# Proteomic analysis of breast tumors confirms the mRNA intrinsic molecular subtypes using different classifiers: a large-scale analysis of fresh frozen tissue samples

**DOI:** 10.1186/s13058-016-0732-2

**Published:** 2016-06-29

**Authors:** Sofia Waldemarson, Emila Kurbasic, Morten Krogh, Paolo Cifani, Tord Berggård, Åke Borg, Peter James

**Affiliations:** Department of Immunotechnology, Lund University, Medicon Village, 223 81 Lund, Sweden; Amber Biosciences AB, Skrivarevägen 9, 226 57 Lund, Sweden; Novo Nordisk A/S, Lund, Sweden; Department of Oncology, Lund University, Medicon Village, 223 81 Lund, Sweden; Turku Centre for Biotechnology, Åbo Akademi University, University of Turku Biocity, Tykistokatu 6, 20520 Turku, Finland

**Keywords:** Breast cancer, Molecular subtyping, Proteomics, Transcriptomics, Mass spectrometry

## Abstract

**Background:**

Breast cancer is a complex and heterogeneous disease that is usually characterized by histological parameters such as tumor size, cellular arrangements/rearrangments, necrosis, nuclear grade and the mitotic index, leading to a set of around twenty subtypes. Together with clinical markers such as hormone receptor status, this classification has considerable prognostic value but there is a large variation in patient response to therapy. Gene expression profiling has provided molecular profiles characteristic of distinct subtypes of breast cancer that reflect the divergent cellular origins and degree of progression.

**Methods:**

Here we present a large-scale proteomic and transcriptomic profiling study of 477 sporadic and hereditary breast cancer tumors with matching mRNA expression analysis. Unsupervised hierarchal clustering was performed and selected proteins from large-scale tandem mass spectrometry (MS/MS) analysis were transferred into a highly multiplexed targeted selected reaction monitoring assay to classify tumors using a hierarchal cluster and support vector machine with leave one out cross-validation.

**Results:**

The subgroups formed upon unsupervised clustering agree very well with groups found at transcriptional level; however, the classifiers (genes or their respective protein products) differ almost entirely between the two datasets. In-depth analysis shows clear differences in pathways unique to each type, which may lie behind their different clinical outcomes. Targeted mass spectrometry analysis and supervised clustering correlate very well with subgroups determined by RNA classification and show convincing agreement with clinical parameters.

**Conclusions:**

This work demonstrates the merits of protein expression profiling for breast cancer stratification. These findings have important implications for the use of genomics and expression analysis for the prediction of protein expression, such as receptor status and drug target expression. The highly multiplexed MS assay is easily implemented in standard clinical chemistry practice, allowing rapid and cheap characterization of tumor tissue suitable for directing the choice of treatment.

**Electronic supplementary material:**

The online version of this article (doi:10.1186/s13058-016-0732-2) contains supplementary material, which is available to authorized users.

## Background

Breast cancer is a heterogeneous disease as seen both at the molecular level and in its clinical presentation and outcome. There is a great need to find parameters to define clinically relevant subgroups. Estrogen receptor (ER) and progesterone (PgR) receptor status divide breast cancer into positive and negative groups, allowing targeted hormone therapies [1, 2]. However, only 60–70 % of ER-positive patients respond to such treatment [3]. This demonstrates the diversity of breast cancer and the need to define the molecular subtypes of the disease.

Comprehensive gene expression profiling has repeatedly confirmed distinct molecular subtypes of breast cancer [4–8]. The five “intrinsic” subtypes luminal A and B, human epidermal growth factor receptor 2 (HER2)-enriched, basal-like and normal-like breast cancer have been shown to be associated with different histological features and clinical outcomes. These have been somewhat controversial but we show here that unsupervised protein analysis supports these broad groupings. Specific genomic alterations have also been associated with some subtypes, further indicating these five subtypes as distinct disease entities [9, 10]. Specific gene expression changes in response to chemotherapy are known to be associated with these subtypes and hence have important prognostic value, such as *p21*^*waf*^, which is strongly associated with the luminal subtypes [11].

The luminal subtypes are generally positive for the ER and/or PgR receptors and can further be subdivided based on the HER2 status and/or proliferation status. The remaining three subgroups are all usually ER-negative. The HER2-enriched tumors are characterized by their expression of ErbB2 (HER2). The basal-like subgroup demonstrates greater genomic instability than other molecular subtypes of breast cancer and has a particularly poor prognosis. Around 80 % of tumors classified as basal-like are also triple-negative (ER/PgR/HER2), which demonstrates an incomplete overlap between molecular subtypes defined by gene expression profiling and the classification through current clinical biomarkers [12]. Many additional markers, especially basal cytokeratins (CK 5/6, CK 17, 18) have been suggested for improved classification of basal-like tumors but there is currently no international consensus. In addition, emerging technologies, such as, for example, somatic copy number alterations on the transcriptome, opens up the possibility of future integration of genomic, epigenomic, and proteomic data to provide additional molecular stratification value [13].

Genetic predisposition can be the cause of breast cancer and germline mutations in the two major breast cancer susceptibility genes *BRCA1* and *BRCA2* confer highly elevated risk of the disease [14]. However, these account for only a fraction of the hereditary cases and furthermore, low-penetrance hereditary genes are being investigated [15, 16]. *BRCA1*-mutated breast tumors typically exhibit features of the basal-like molecular subtype while *BRCA2*-mutated tumors usually are of the luminal subtype [10, 17, 18]. The correlation between DNA copy numbers, gene expression levels, and protein expression levels has been widely studied and discussed, concluding that in most cases DNA copy number, Mrna, and protein levels are not directly correlated [19–21].

Proteome-wide analysis is technically much more challenging than genome-wide measurements because of the dynamic range of protein expression and the plethora of isoforms and post-translational modifications, which leads to the expression of approximately 500,000 protein types from 20,000 genes [22]. The established method for separating proteins is two-dimensional gel electrophoresis (2DE) [23]. This technology coupled with fluorescent labeling (DIGE) allows for large-scale parallel analysis of samples and also resolves protein isoforms and post-translational modified proteins [23]. Over the last decade, a peptide-orientated shotgun mass spectrometry (MS) approach has been developed allowing the identification of tens of thousands of peptides in a sample [24] and the quantification of subsets of proteins of clinical relevance [25]. Cell lines together with isotope labeling provide a powerful model to accurately quantify proteins in depth [26]. Recently, targeted MS has been developed based on selected reaction monitoring (SRM) [27, 28]. This technology targets specific proteins of interest in an assay format avoiding the stochastic sampling problems in shotgun proteomics that complicates the parallel analysis of large sample sets. SRM technology has proven to be robust enough to be suitable for measuring clinical assays [29]. With the addition of isotope-labeled internal standards, good assay accuracy can be achieved and makes assays transferable between different laboratories [30]. However, as for any clinical assays, the SRM assay has to be carefully validated to establish its accuracy and sensitivity (limit of detection, limit of quantification) and a coefficient of variation (CV) of <15 % is a general requirement for clinical application [31]. Such assays also have to be assessed in terms of inter-laboratory transferability [29]. The selection of correct peptides representing each target protein in a specific sample type is critical [32].

In this work we present a large-scale study of breast tumors performed in three parts. Initially 477 sporadic, familial, and hereditary tumors were analyzed at the intact protein level using 2D-DIGE. The mRNA expression profiles of the majority of the tumors were analyzed and the tumors were assigned to molecular subtypes using the classifiers according to Sørlie [6], Hu [7], and the prediction analysis of microarray (PAM50) assay [8]. Unsupervised analysis of these protein data revealed that the most predominant molecular subtypes defined at the gene expression level are also resolved at the protein level. We then carried out an in-depth analysis of sets of pooled tumor material at the peptide level, using a breast cell line as an internal standard and identified 4,255 protein families. Of these, 256 proteins were selected for targeted proteomic profiling using SRM assays: 41 breast cancer tumors, including 17 from an independent dataset, were analyzed and hierarchal clustering of the data revealed a molecular portrait of breast cancer subtypes highly similar to gene expression profiling results. This study demonstrates that abundant proteins that are readily measured with MS in a rapid assay format can define molecular subtypes of value for clinical diagnostics.

## Methods

### Materials

Cy2, Cy3, Cy5 dyes, immobilized pH gradient strips and Pharmalytes were purchased from Serva (Heidelberg, Germany). Acrylamide, urea, Tris, magnesium acetate, DTT, iodoacetamide and the Protein assay kit (Micro Lowry, Peterson’s modification) were bought from Sigma Aldrich (Buchs, Switzerland). Trypsin (sequencing grade modified) was from Promega Corp, Madison, WI, USA).

### Patient and tumor material

Tissue samples (*n* = 477) were collected at Lund University Hospital and anonymized after obtaining informed consent and approval by the Ethics committee (registration numbers LU240-01 and 2009/658). The dataset comprised primary tumors of different histological grade (435), recurrence or second primary tumors [19], lymph nodes [9] and non-malignant tissue [14] from patients predisposed to breast cancer. Out of the lymph node samples, six had a matching primary tumor sample. Out of the recurrences, four tumors had a matching primary tumor sample. There were 215 hereditary samples with 40 samples from patients carrying a *BRCA1* mutation, 15 samples from patients with a *BRCA2* mutation and the rest from patients with a clear hereditary family pattern but unknown mutations (*BRCAx*). The clinical data are presented in Additional file 1: Table S1. The resected samples were snap frozen and stored at –80 °C. A pathologist first examined all samples to obtain representative, viable, and non-necrotic tumor tissue. An appropriate piece of the tumor was excised while keeping the tumor cold. ER and PgR status was determined according to clinical practice and retrieved from medical records. HER2 status was determined using array comparative genomic hybridization (aCGH) as described previously [10, 17]. The tumor was powdered in a Teflon bomb cooled in liquid nitrogen. The sample was divided into two different tubes, one for RNA extraction and the other for protein extraction. RNA extraction was performed as described previously [17].

### Sample Preparation and 2D-DIGE analysis

Lysis buffer (30 μl) containing 8 M urea, 30 mM Tris, 5 mM magnesium acetate and 4 % 3-[(3-Cholamidopropyl)dimethylammonio]-1-propanesulfonate (CHAPS), and with pH 8.5, was added to the homogenized tumor material. The samples were vortexed and cooled on ice for 20 minutes and then centrifuged at 4 °C for 15 minutes and the supernatant was collected. The protein concentration was determined using the Protein Assay Kit (Sigma). The samples were stored at –80 °C. Samples were labeled with Cy3 or Cy5 dyes, respectively, and run on 2D-DIGE and analyzed as described [23].

### Merging of datasets and dye correction

Samples were run in two different batches with different pools in the reference Cy2 channel. Seven gels were run to normalize between the two pools. All samples in the first batch were run with dye swap, i.e., each sample was run once with Cy3 and once with Cy5 on different gels. The duplicates were used to find the systematic dye bias between Cy3 and Cy5 for each spot. As the biological variance was vastly greater than the experimental variance, the rest of the samples were run once. All duplicates were merged and all samples were assembled in a data matrix comprising 473 samples measured in 1750 spots each.

### Data analysis 2D-DIGE

Log_2_ standardized abundances were used. Spots were filtered, for the Sammon map and the hierarchical clustering, by requiring spots to have at most 50 missing values across all 473 samples, and a standard deviation above 0.7. Euclidean distance was used for the Sammon map. For the hierarchical clustering, Pearson-correlation-based distance was used as the similarity measure and average linkage was employed to define cluster-to-cluster distances. The Wilcoxon rank sum test was used for pairwise comparisons between groups. All statistical analysis was performed in R, version 3.1.2 (www.r-project.org). Underlying mRNA data defining the intrinsic subtype have been deposited as part of a larger dataset at the Gene Expression Omnibus at NCBI [GEO:25307].

### Liquid chromatography-tandem mass spectrometry (LC-MS/MS) analysis of breast tumor pools

A subset of female primary tumors with clear classifications that were the same using the Sørlie [6], Hu [7], or the PAM50 [8] gene sets were used for pooling into a normal-like (4 tumors), a luminal A (14 tumors), a luminal B (4 tumors), an HER2 (5 tumors) and a basal group (15 tumors), as detailed in Additional file 2: Table S2. The protein concentration was determined in each pool and 50 μg protein from each tumor pool was further pooled with 50 μg of a stable isotope labeling with amino acids in cell culture (SILAC)-labeled breast cancer cell line (MDA-MB-231) [33].

The proteins were separated by SDS-PAGE as described [34]; each lane was cut into 10 slices and digested with trypsin [30]. Each sample was analyzed on a Thermo LTQ Orbitrap mass spectrometer (ThermoFisher, Bremen, Germany) coupled to an Eksigent 2D NanoLC system (Eksigent technologies, Dublin, CA, USA) as in [25]. Peptides were eluted with a gradient of 5–60 % solvent B over 120 minutes (Buffer A 0.1 % trifluoroascetic acid (TFA)) in water; Buffer B 0.1 % formic acid (FA) in acetonitrile (ACN)). Full-scan MS spectra (m/z 300–2000) were acquired in the LTQ Orbitrap in profile mode, with a resolution of 60,000 at m/z 400. Lock mass was applied. The instrument was operated in data-dependent acquisition mode. The spray voltage was set to 2 kV and the temperature of the heated capillary was 180 °C. The seven most intense ions from the survey scan performed by the Orbitrap were fragmented by collision-induced dissociation in the LTQ (normalized collision energy 35, parent mass selection window 0.7 Da, 30 ms activation time, and minimum signal threshold for MS/MS scans set to 500 counts). All unassigned charge states and singly charged ions were rejected for fragmentation. The dynamic exclusion list was limited to a maximum of 500 masses with a maximum retention time window of 2 minutes and a relative mass window of 10 ppm.

The data were analyzed in the MaxQuant (version 1.2) software suite [35] using the Andromeda search engine with the Swiss-Prot/UniProt release September 2011 [36]. Cysteine carbamidomethylation was set as the fixed modification, while methionine oxidation and protein N-terminus acetylation were added as variable variations. Identifications were filtered with a false discovery rate (FDR) of 0.05 at the peptide and protein levels. Over 4000 protein families and 14,000 proteins were identified when combining the tumor and cell line data (Additional file 3: Table S3). Data from the ten gel slices were merged to create five tumor groups for comparison. The SILAC-labeled cell line pool was used to normalize the data. Proteins of interest were selected for further SRM analysis based on intensity and fold change, and the top 20 % intensity data from each of the five pools were selected. From this subset, proteins with a fold change > ±10 for any pairwise group comparison were selected to give a final list of 256 proteins for further SRM analysis in individual tumor samples (Additional file 4: Table S4). The functional enrichment analysis was carried out with MetaCore™ (Thomson Reuters, version 6.14) and Database for Annotation, Visualization and Integrated Discovery (DAVID) (National Institute of Allergy and Infectious Diseases NIH, version 6.7) [37].

### Targeted SRM analysis

Samples for SRM analysis were prepared by dissolving the protein extracts in Laemmli buffer and then running on a 12 % gel until the sample band was concentrated at the junction of the stacking and running gel. The gel was stained and the band cut out and digested as above. The data identified from the orbitrap analysis described previously was built into the SRM management software Skyline version 1.3 [38] as a spectral library created through the data management platform Proteios version 2.18 [39]. Peptide criteria were set to include peptides 8–25 amino acids long, with carbamidomethylated cysteine residues and excluding ragged ends. One to two proteotypic peptides per target protein that differentiated most between the various groups were selected with an average of four transitions per peptide. The peptides were synthesized (JPT Peptides, Berlin) and SRM transition lists were generated using Skyline software. The SRM measurements were performed on a TSQ Vantage triple-stage quadrupole mass spectrometer (ThermoFisher, San Jose, CA, USA) equipped with a nano-electrospray ion source (ThermoFisher) as described previously [40] and the peptides were eluted with a gradient of 97 % solvent A at 0–5 minutes, 85 % A at 8 minutes, 65 % A at 42 minutes, 10 % A at 45–50 minutes (the peptides and the optimized transitions are given in Additional file 4: Table S4. Forty-one tumor samples were analyzed, each in duplicate (Additional file 5: Table S5). Each sample run was manually inspected using Skyline software at both the sample level and at the peptide level. The dataset was integrated using Anubis automated software version 1.1.6 [41]. All mass spectrometry data, including MS/MS and SRM data have been deposited in the ProteomeXchange Consortium (http://proteomecentral.proteomexchange.org) via the PRIDE partner repository with the dataset identifier PXD000944 and DOI 10.6019/PXD000944.

### Data analysis SRM

Log_2_ standardized abundances were used and duplicate injections were merged. Hierarchical clustering analysis was performed using Qlucore Omics Explorer 2.3 software (Qlucore AB, Lund, Sweden). Multi-group comparison was done using analysis of variance (ANOVA) and complete linkage was used for hierarchal clustering. Supervised classification was performed with a support vector machine. Leave one out cross-validation was employed and only binary classifiers were used with no seed. Every sample was left out once, and the remaining samples were used as the training set. The sample left out was tested on the resulting classifier and a decision value was obtained. Large positive and negative decision values correspond to predictions in the two classes respectively. Varying the threshold decision value between the two classes produces a set of classifiers. Corresponding values of sensitivity and selectivity are plotted as a receiver operating characteristic (ROC) curve. The library e1071 (http://cran.r-project.org/web/packages/e1071/index.html) was used for the support vector machine. Default parameters and a linear kernel were used for the classifier. Only peptides without missing values were used for the classifier and values were logarithmically transformed and normalized. All statistics was performed in R. ROC curves are provided in Additional file 6: Figure S1.

## Results

### Clustering of intrinsic breast cancer subtypes according to protein expression

**Fig. 1:**
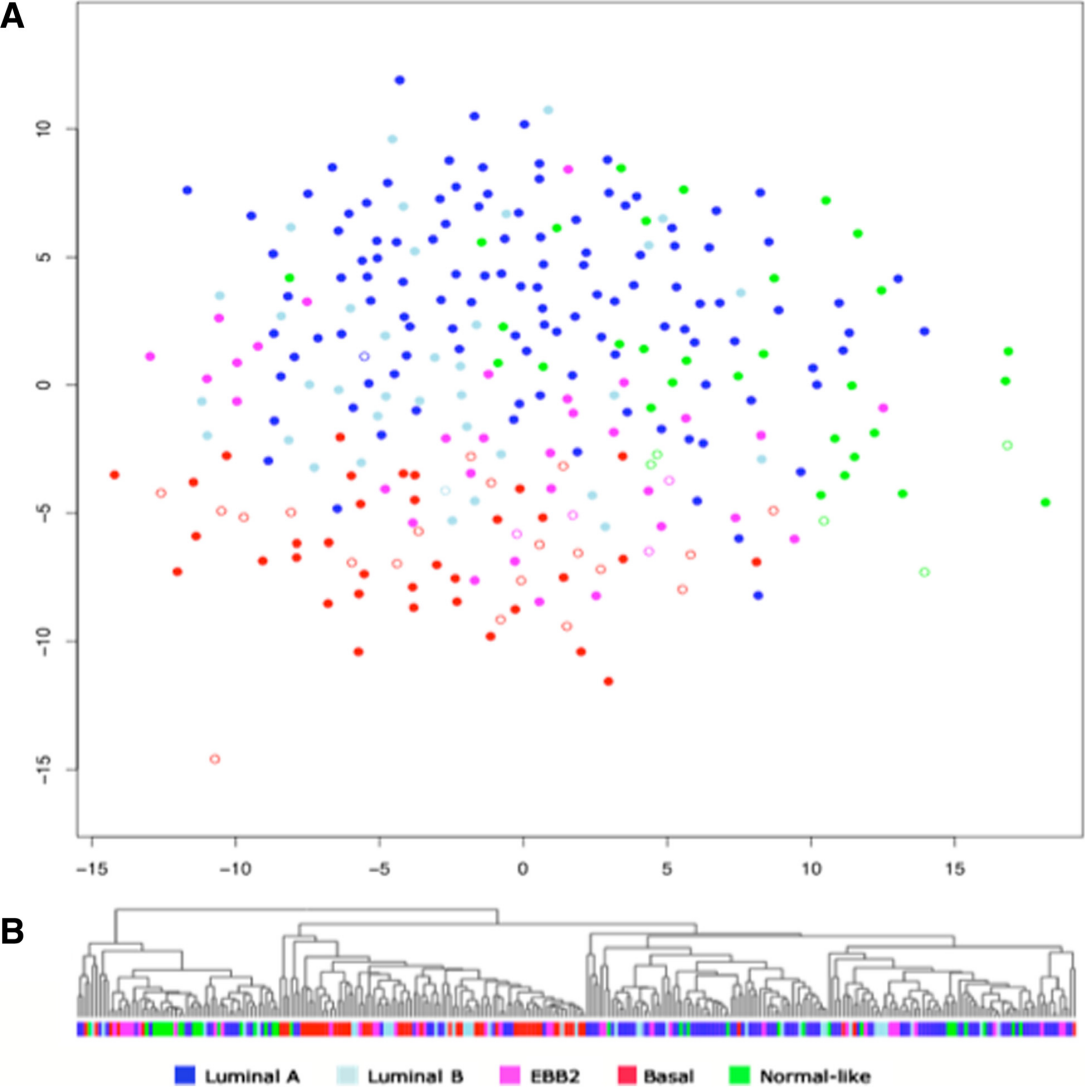
**a** Breast cancer tumors analyzed at protein expression level largely form the same type of clusters as seen at the gene expression level. This is a Sammon map of the tumors using protein expression data from 2D-DIGE with matched mRNA data, color coded according to the Sørlie gene expression classification of the same; luminal A (*dark blue*), luminal B (*light blue*), ERBB2 (*purple*), basal-like (*red*) and normal-like (*green*). Samples from *BRCA1*-mutated patients are indicated as empty rings. **b** Corresponding hierarchal clustering of the same samples using the same data. The basal-like and the luminal A clusters are strikingly homogenous

In total 477 breast tissue samples were successfully analyzed (from a total of over 600 samples) in duplicate using 2D-DIGE, allowing the profiling of several thousand proteins and isoforms/PTMs (post-translational modifications) per sample and the elimination of those with protein degradation. The dataset is described in “Methods” and Additional file 1: Table S1. In parallel, 370 of these tumors were analyzed for gene expression using microarrays [17] and were assigned to the breast cancer subgroups defined by Sørlie/PAM50 or the Hu classifications [6]: the remainder had extensive mRNA degradation and could not be analyzed. Unsupervised analysis of the protein data revealed striking agreement with the gene expression subtyping of the tumors. In Fig. 1, all samples with known gene expression profiles are displayed as a Sammon map (Fig. 1a) and as hierarchal clustering (Fig. 1b). Luminal A and basal-like tumors were most clearly defined by separate clusters. HER2-enriched tumors and normal-like tumors had some degree of clustering, whereas luminal B tumors were more evenly spread across the clusters. Tumors carrying a *BRCA1* mutation (indicated by open rings) fell mostly within the basal-like cluster, in agreement with gene expression results. Strikingly, the *BRCA1*-mutated tumors assigned to other subtypes at a transcriptional level, all fell very close to the basal-like cluster in the Sammon map.

**Fig. 2:**
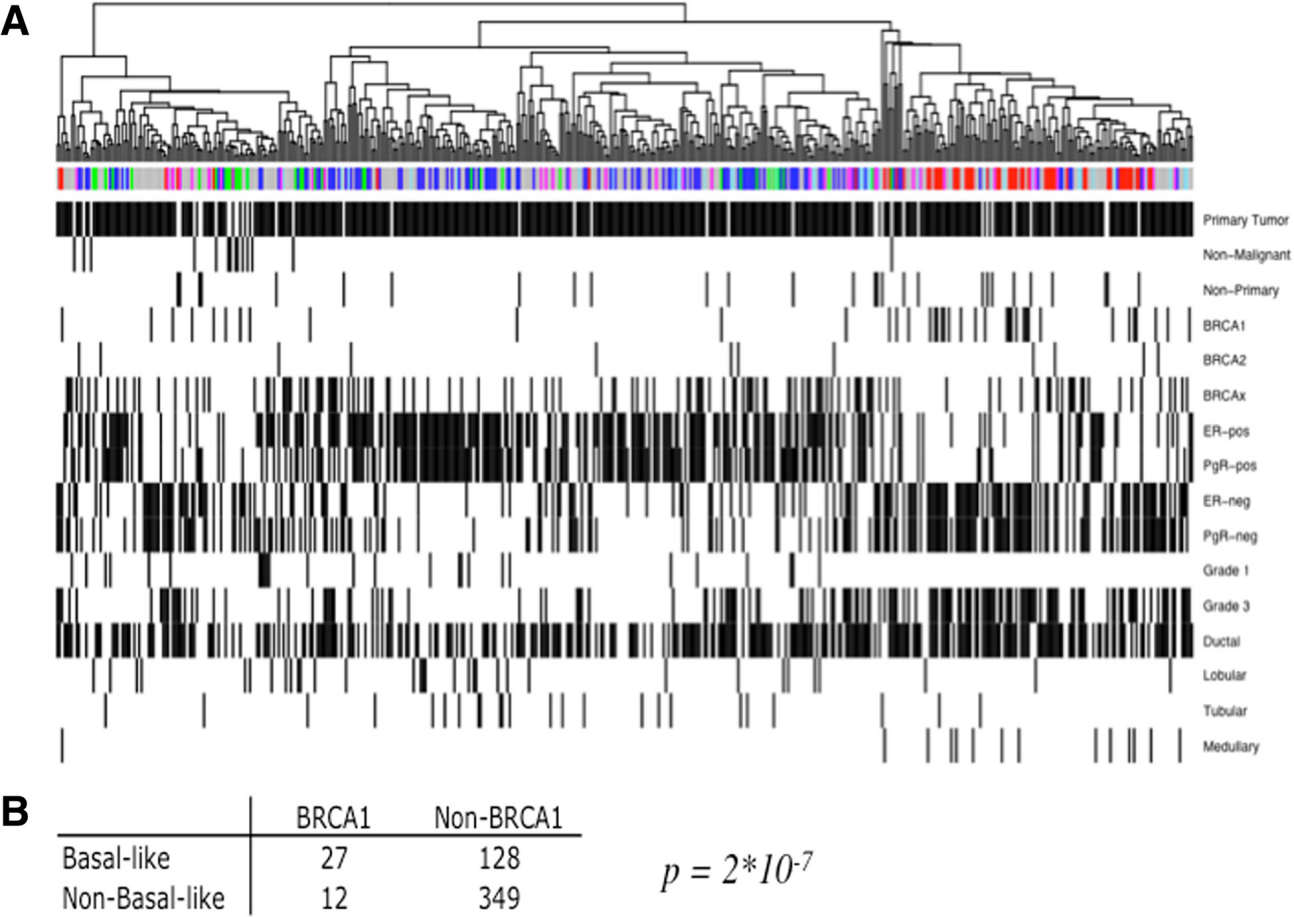
**a** Unsupervised hierarchal clustering of all samples analyzed on 2D-DIGE (fig 2a). Samples previously analyzed at gene expression level are colored according to the Sørlie gene expression classification: luminal A (*dark blue*), luminal B (*light blue*), ERBB2 (purple), basal-like (*red*) and normal-like (*green*). Samples with unknown gene expression are colored *gray*. Clinical parameters are indicated under the cluster where a *black bar* indicates presence of the variable. **b** Distribution of *BRCA1*-mutated tumors in the basal-like cluster compared to the non-basal like cluster Fishers exact test *p* value = 2*10^-7^

Figure 2 shows hierarchical clustering of all tissue included in the 2D-DIGE study including those without a corresponding mRNA analysis. The associated clinical parameters, including tissue type, hereditary mutation status, ER and PgR status, tumor grade, and the histological type of cancer, are indicated below the clusters. Three major branches emerged, one enriched with tumors classified as luminal A and one enriched with basal-like tumors. The normal-like tumors also tended to form a cluster within the third branch and interestingly, the non-malignant samples clustered close to the normal-like tumors, an effect that has also been seen at transcriptional level, which initially named the subgroup [4]. Luminal B and HER2-enriched tumors were more spread across the clusters. Out of the 39 tumors carrying a *BRCA1* mutation, 27 fell within the basal-like cluster, which is a clear overrepresentation (Fig 1b). Out of the 27 *BRCA1*-mutated tumors falling within the basal-like protein cluster, 7 had not been analyzed for gene expression and 3 of these tumors were assigned to other subtypes in the gene expression analysis (HER2 or luminal B). Also most non-primary tumors, i.e., secondary tumors or metastases, were associated with the basal-like cluster. Tumors carrying a *BRCA2* or a *BRCAx* mutation (familial but not *BRCA1* or *BRCA2*) were more evenly spread across the cluster as would be expected, as *BRCA2* tumors usually are associated with the luminal B subtype, which we could not distinguish in this analysis.

The tumors that had not been analyzed for gene expression due to RNA degradation are labeled in gray and are distributed over the entire cluster (Fig. 2). The 2D-PAGE images allowed rapid identification of protein degradation due to the appearance of characteristic low molecular-weight spots corresponding to protein breakdown products. These samples were also eliminated from the analysis (*n* = approximately 130). Sample collection was well-controlled in this study and we therefore conclude that degradation of some samples is due to long storage times in the freezer. The stratification of these tumors could be indicated using the clinical information available for the tumors. ER and PgR status was strongly associated with the different clusters, with the majority of the ER-positive and PgR-positive tumors falling within the cluster dominated by the luminal A tumors and the ER-negative and PgR-negative tumors falling within the basal-like cluster. Also, the majority of the tumors in the basal-like cluster were of grade 3 and all medullary tumors in the dataset were associated with the basal-like cluster.

### Separation of molecular subtypes

**Fig. 3:**
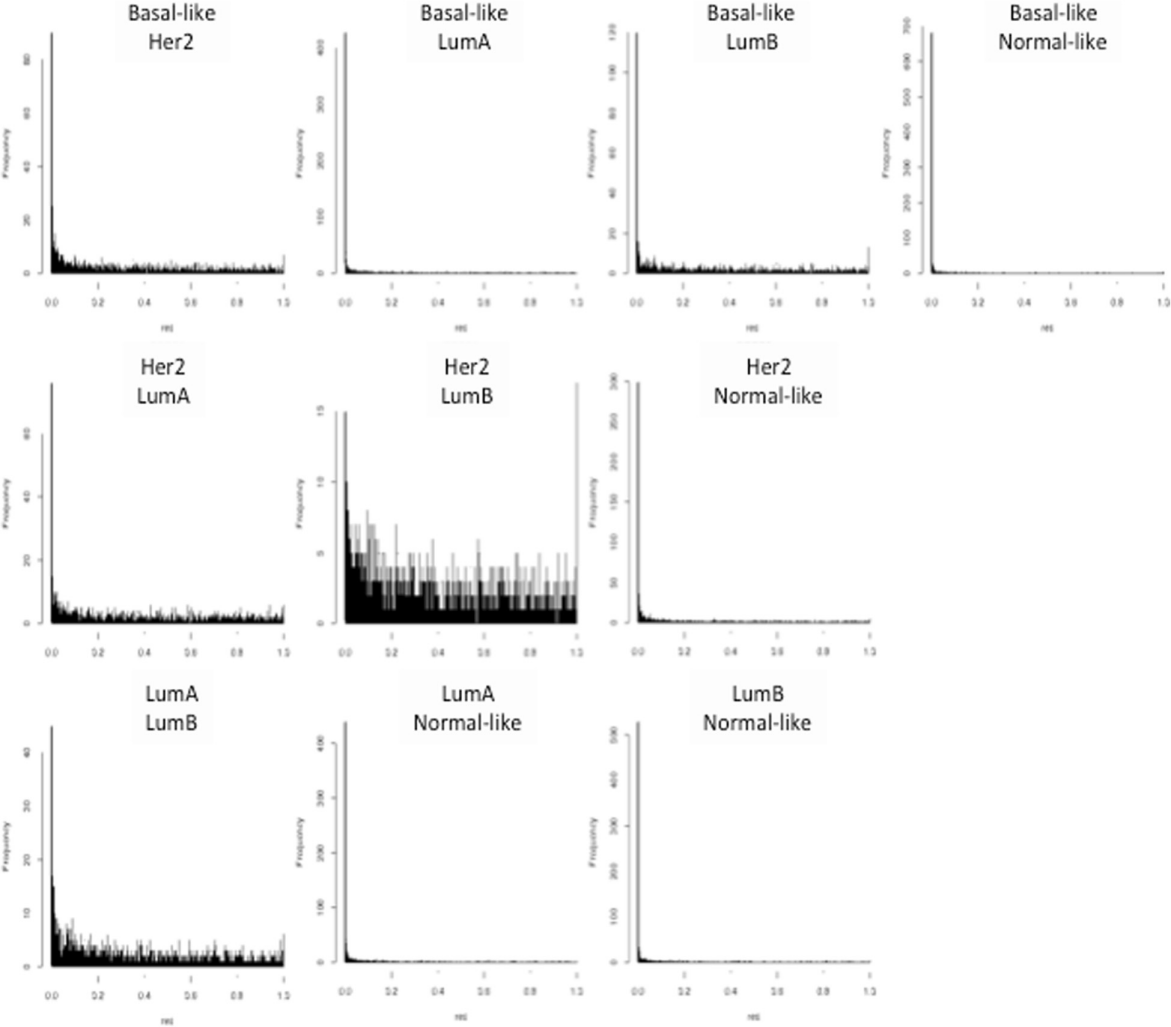
Wilcoxon pairwise comparison of 2D-DIGE data for all tumors grouped according to the Sørlie gene expression classification. The frequency (*y-axis*) of *p* values (*x-axis*, 0–1) for the pairwise comparisons of all five subtypes. All pairwise comparisons demonstrate a clear overrepresentation of *p* values <0.001 except for the human epidermal growth factor receptor 2 (*Her2*) versus luminal B (*LumB*) pairwise comparison

Pairwise Wilcoxon analysis of the subtypes defined at the gene expression level was performed for the set of tumors analyzed for protein expression. This further confirmed the large differences in protein expression for these tumors. Figure 3 shows the distribution of *p* values for all protein spots for each pairwise group comparison. There was a clear overrepresentation of proteins with *p* values below 0.001, indicating that significant subsets of proteins are differentially expressed between the groups. The separation was strong for all pairwise comparisons except for the comparison between the HER2 and luminal B subtypes. The highest number of spots with a *p* value below 0.001 was seen for comparison of the basal-like and luminal A subtypes, with almost 700 protein spots. In summary, unsupervised analysis of the breast tumor proteome demonstrates remarkable similarities with the molecular portraits of breast cancer subtypes and thus, these molecular changes are also reflected at the protein expression level even at the level of abundant proteins represented on 2D gels.

### In-depth protein characterization of molecular subtypes of breast cancer

In order to characterize the tumor groups further and to build a library of breast tumor proteins accessible to mass spectrometric analysis for molecular subtype classification, we performed a comprehensive protein identification analysis. A subset of tumors representing the five molecular subtypes, luminal A, luminal B, HER2, basal-like and normal-like, were selected (Additional file 2: Table S2). The PAM50, Hu and Sørlie gene classifications of all tumors were in concordance. In total 4255 classes of protein representing over 14,000 proteins were identified, which to our knowledge is the largest set of proteins identified from breast cancer tumors (Additional file 3: Table S3).

### Gene expression enrichment and pathway analysis differences in the protein subtypes

An initial overview of the in-depth dataset analyzed using DAVID shows that of the 2734 identifications accepted as Swiss-/UniProt accessions, 768 are epithelial, and 161 are annotated as cancer-associated gene products. The latter proteins are enzymes clustered around drug metabolism, followed by immune system response, DNA repair, cell signaling, and cytoskeletal remodeling. A more thorough investigation using MetaCore™ to compare pathway differences amongst the various intrinsic molecular types showed clear differences.

Both luminal subtypes had strong upregulation of chromatin remodeling including increased expression of BAF53 [SwissProt:O96019] and histone acetyl-transferases indicating nucleosome disassembly. Several proteins in the Toll signaling pathway are also upregulated, probably in response to increased interleukin levels. Several DNA repair proteins RAD50 [SwissProt:Q92878], UBE2V1 [SwissProt:Q13404], and UBE2N [SwissProt:P61088] are increased, while C-Jun [SwissProt:P05412] and MTA1 (metastasis associated protein) [SwissProt:Q13330] are downregulated, leading to a decrease in Bcl-XL-induced apoptosis. The luminal B subtype differs strongly from the luminal A subtype, in that many cytoskeletal remodeling pathways and several clathrins involved with the HER2 receptor and vesicle transport are highly upregulated.

In the basal subtype alpha3, beta1 integrin [SwissProt:P26006 and SwissProt:P05556] are downregulated, which leads to lowering of the activity of focal adhesion kinase (FAK) and hence, many mitogen-activated protein (MAP) kinases (MAP2K4, MAPK14 and MAP2K3 [SwissProt:P45985, SwissProt:Q16539 and SwissProt:P46734]). This is reinforced by downregulation of c-JUN, which lowers PKC, which normally activates FAK. This is supported by downregulation of IKK-beta [SwissProt:O14920], which stops activation of NCOA3, which activates FAK. Poly (ADP-ribose) polymerase 1 (PARP1) [SwissProt:P09874], a protein that in recent years has been extensively studied in breast and ovarian cancer, is upregulated in the basal-like cluster. PARP1 is involved in DNA repair, as is the BRCA1 protein. Targeted PARP1 inhibition has proven especially effective as a treatment for patients carrying a *BRCA1* mutation, and in triple-negative breast cancer (because of the similarities with *BRCA1*-mutated tumors) leading to an inhibition of dual DNA repair pathways leading to cell death [42]. Gene expression of S100-A11 has specifically been reported as upregulated in basal-type breast cancers compared to non-basal types [43], which agrees with our findings and S100-A11 [SwissProt: P31949] has been proposed as a diagnostic marker for breast cancer [44].

The HER2 subtype is defined by upregulation of the Her2 receptor [SwissProt:P04626]. BAF53 [SwissProt:O96019], and BAF60 [SwissProt:P51532] (both part of the CREST-BRG1 complex) are upregulated and this complex may be required for the activation of transcriptional programs associated with oncogene and proto-oncogene mediated growth induction. This is accompanied by downregulation of E3 ubiquitin-protein ligase TRAF2 also known as tumor necrosis factor type 2 receptor-associated protein (TRAF2) [Q12933], tumor necrosis factor receptor type 1-associated DEATH domain protein (TRADD), which usually acts as a tumor suppressor [Q15628], and NF-kappa-B essential modulator (NEMO) [SwissProt:Q9Y6K9], which usually form a complex activating receptor interacting protein (RIP1).

The normal-like subtype has clear upregulation of metabolic pathways, with an increase in tricarboxylic acid (TCA) cycle enzymes, glucose transport, oxidative phosphorylation, and especially the associated nicotinamide adenine dinucleotide (NADH) dehydrogenase subunits. The normal-like subtype also has blood coagulation proteins such as thrombin [SwissProt:P00734], fibrinogen alpha [SwissProt:P02671], Serpine 2 [SwissProt:P07093] and plasmin. Previous studies using highly purified and washed ovarian epithelial cells showed that these proteins were not just due to contaminating blood but that these proteins are either strongly bound to the plasma membrane or have been internalized and can be found in both the gel and shotgun experiments [22].

### Targeted protein assay profiling for tumor classification

**Fig. 4:**
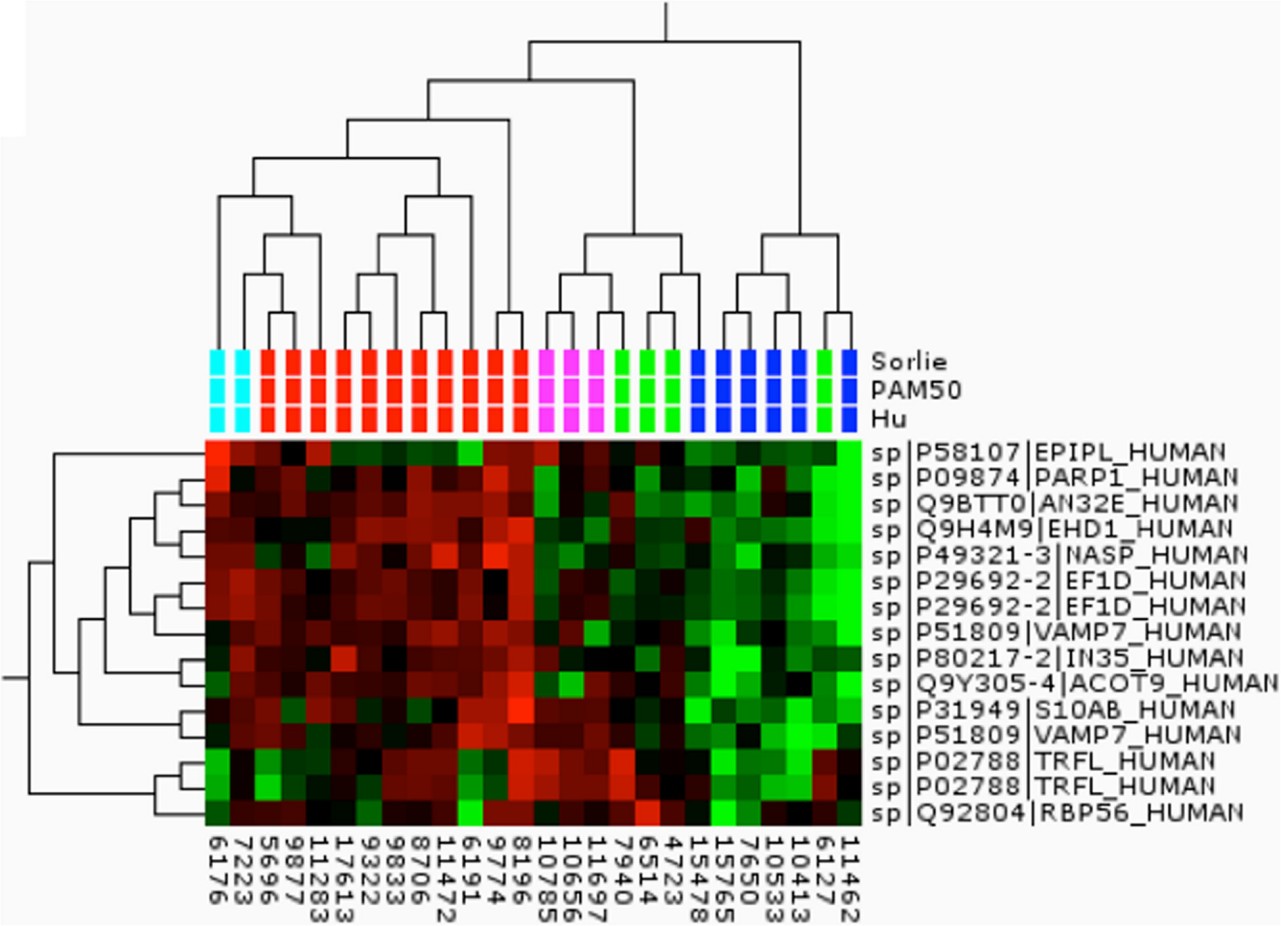
Cluster analysis of the “core set” of tumors using analysis of variance filtering (*p* value 0.01). The tumor classification according to three gene expression profiling methods is indicated below the branching and colored accordingly: *red* basal-like, *magenta* HER2, *green* normal-like, *blue* luminal A, *turquoise* luminal B. Proteins are indicated with the SwissProt ID and short name

Out of the comprehensive set of proteins identified, 256 proteins were chosen for use in an SRM-based subtype classification. The proteins were among the top 20 % most intense in the MS analysis and had the greatest discriminatory power in pairwise group comparisons. A specific MS-based assay, SRM, was established for these proteins, which after refinement, contained 190 proteins represented by 253 peptides (Additional file 4: Table S4): 24 tumors from the original dataset were analyzed in duplicate using the assay, together with 17 tumors from an independent dataset, giving a total of 41 tumors (Additional file 5: Table S5). Of the 41 tumors, 26 were assigned to the same molecular subtype by all the three RNA classifiers. We reasoned that this core set of tumors is representative of the five intrinsic subtypes, as the classification of these was consensual and we used this set for hierarchal clustering using multi-group ANOVA comparison with a *p* value cutoff of 0.01 (Fig. 4). The hierarchal clustering demonstrates remarkably strong similarities to the molecular classification of breast tumors at the gene expression level using the intrinsic gene set [4]. Tumors of luminal and basal origin separated in the first branching followed by further sub branching of tumors assigned to the basal-like, HER2 and normal-like subtypes. The majority of tumors in this analysis clustered with tumors of the same RNA subtype, although there was a slight overlap between the HER2-enriched and normal-like cluster. There were only two tumors classified as luminal B available in this analysis and both clustered with the basal-like tumor.

### Discriminatory power of the SRM signature

**Fig. 5:**
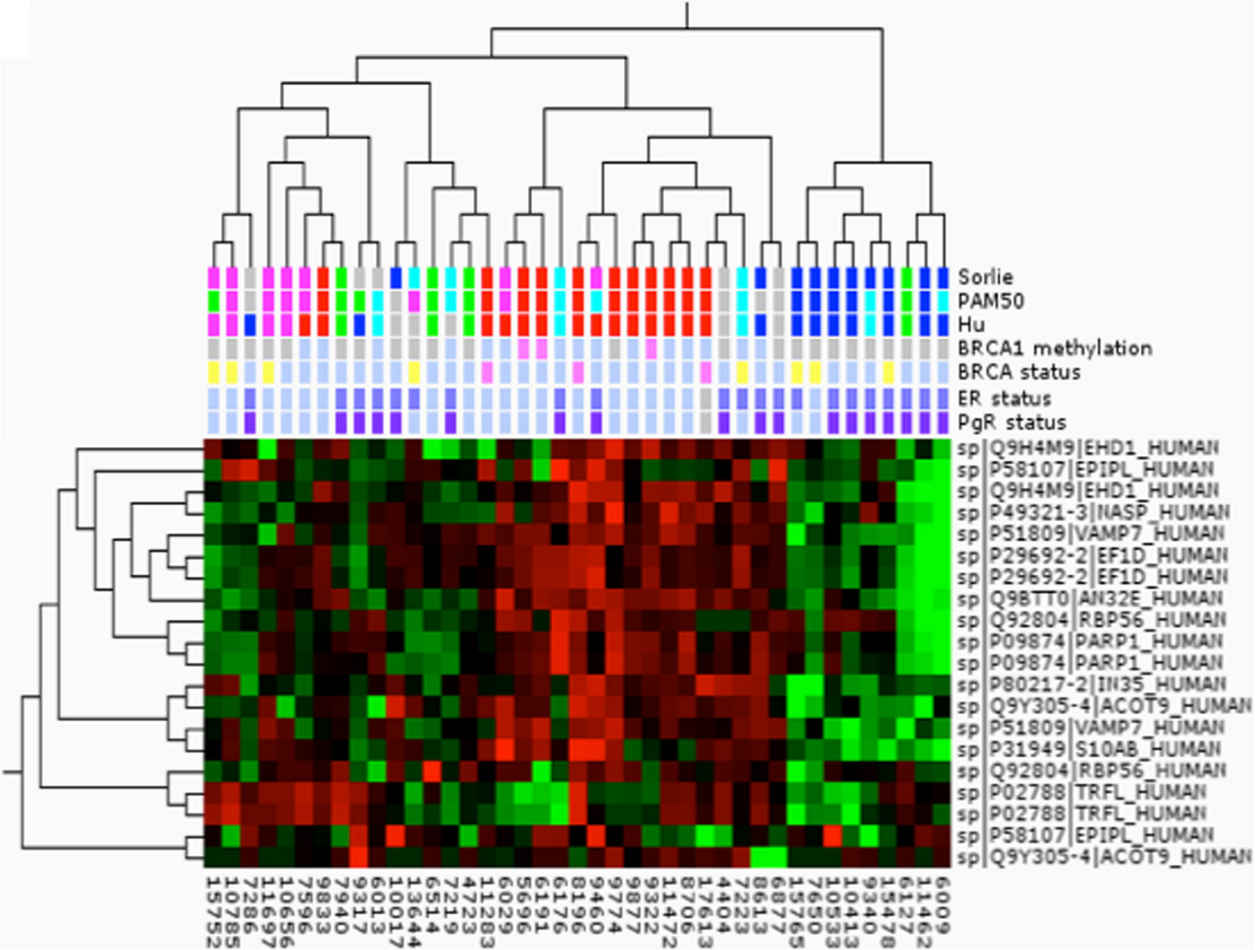
Cluster analysis of all tumors analyzed with the selected reaction monitoring protein assay. The tumor classification according to three gene expression profiling methods is indicated below the branching and colored accordingly: the tumor classification according to three gene expression profiling methods is indicated below the branching and colored accordingly: *red* basal-like, *magenta* human epidermal growth factor receptor 2, *green* normal-like, *blue* luminal A, *turquoise* luminal B, *gray* unknown classification. Clinical parameters including BRCA1 methylation status (*pink* positive), BRCA mutational status (*pink* BRCA1-positive, *yellow* BRCAx-positive), estrogen receptor (*ER*) (*dark blue* positive), and progesterone receptor (*PgR*) (*darker purple* positive) status and overall survival (*green* alive) are indicated below the samples. Proteins are indicated with the SwissProt ID and short name. *PAM50* prediction analysis of microarray

Based on ANOVA, the best discriminators, twelve proteins, were tested using hierarchal clustering of the entire dataset of 41 tumors (Fig. 5). In this total dataset, in addition to tumors with precise classification according to the three gene expression signatures, there were tumors not analyzed at all on gene expression (labeled in gray for all three classifiers) and tumors where the three different classifiers assigned tumors to different subclasses or in some cases, could not classify them at all (labeled in gray for that specific classifier). This dataset also displayed very high similarities to clusters at gene expression level. Luminal A tumors were separated from the remaining tumors of basal origin. Of these tumors, the basal-like subtype formed a clear cluster and HER2 and normal-like tumors formed a separate branch with relatively high separation between these two groups also. Again, the few luminal B tumors were spread across the cluster and it is clear that for proper analysis of this subset more samples would have been needed. Information on the status of *BRCA1* methylation, *BRCA*, ER, and PgR was available: five of the six tumors in this dataset carrying either *BRCA1* methylation (n = 3 tumors) or a *BRCA1* mutation (n = 3 tumors) fell within the basal-like cluster. The rest of the familial (*BRCAx*) tumors were spread across the cluster. Eight out of nine tumors in the luminal A cluster were ER-positive (dark blue) and seven out of nine were PgR-positive (dark purple). The normal-like tumors seem to divide into two separate clusters dominated by ER/PgR-positive or ER/PgR-negative tumors, respectively.

A support vector machine and a leave one out cross-validation approach was used to create ROC curves for all pairwise comparisons. All three different RNA classifiers were tested. Eight out of the ten pairwise comparisons provided good ROC areas with *p* values below 0.05. For four of the pairwise comparisons the PAM50 classification provided the best ROC areas with *p* values below 0.02; ROC areas: basal versus luminal A = 0.95, basal versus normal = 0.83 HER2 versus luminal A = 1, and HER2 versus normal = 0.89. For basal versus luminal B the ROC area was 0.86 and for luminal A versus normal the ROC area was 0.89, using the Hu classifier. Using the Sörlie classifier the best ROC areas were for the basal versus HER2 comparison with a ROC area of 0.75 and a ROC area of 0.84 for the luminal A versus luminal B comparison providing. ROC areas for all classifiers and pairwise comparisons are provided in Additional file 6: Figure S1.

## Discussion

Gene expression profiling of breast cancer for classification into clinically relevant subtypes has become very well-established over the past decade and it is being discussed whether this should be incorporated into clinical practice [12]. Translating these results to protein level has proven difficult as gene expression and protein expression are not directly correlated [19, 21, 45]. We have compared 477 tissue samples at the protein level and 370 at the mRNA level. We used the gene expression data to classify the samples by three different methods, Sørlie [6], Hu [7] and PAM50 [8].

The resultant clustering of protein expression data in this study shows a striking resemblance to subtyping of tumors using gene expression, both with 2D-DIGE technology and a large set of samples, and with the shotgun LC-MS/MS of pooled samples and the targeted SRM analysis with a smaller sample set. However, there is little overlap between the classifiers found with the 2D-DIGE or the LC-MS/MS analysis and the mRNA classifiers found in the three schemes used. This is not surprising for the gel approach because the protein coverage is roughly ten times less than the number of genes analyzed and does not include membrane or membrane-associated proteins such as the hormone receptors and members of the Claudin family. The LC-MS/MS would have been thought to have concordance due to the greater coverage, though this was not the case.

The most clear-cut distinction was seen between the basal-like and the luminal A tumors. The luminal and basal subtypes have repeatedly been identified and validated as the two main classes of breast cancer originating from either the basal or the luminal epithelial cells of the mammary ducts. These two are associated with different ER status, distinctly different gene expression patterns and a significant difference in clinical outcome. Basal-like or triple-negative breast cancer tumors with their particularly poor prognosis would benefit immensely from better prognostic markers [46]. The overlap we see between the HER2 and luminal B (Figs. 1 and 2) groups is also in agreement with what is seen at transcriptional level where these groups have similarities in expression of some of the genes in the gene set defining luminal B tumors. Each of the subtypes has differential enrichment of certain gene sets confirming their definition as functionally different molecular subtypes.

A significant percentage (45 %), of the tumors included in this study was from patients with a family history of breast cancer (the Swedish average is 35 %) with BRCA1 and BRCA12 accounting for 8 % and 3 %, respectively [47]. The rest are considered to comprise a very pathologically heterogeneous group, indicating that these tumors arise from multiple genetic events [48]. Apart from the patients carrying a *BRCA1* mutation, the familial cases in this study were evenly spread across the clusters derived from protein expression analysis (Figs. 2 and 5). Patients carrying germline mutations in *BRCA1* most frequently develop basal-like tumors giving a particularly poor prognosis for these patients [10]. Protein expression analysis also associates the BRCA1 tumors with the basal-like cluster (Figs. 1 and 5). Notably, the BRCA1 tumors not classified as basal-like according to gene expression all fall in or very close to this cluster in the protein expression analysis (Fig. 1). The targeted SRM analysis included only *BRCA1*-mutated tumors classified as basal-like from gene expression. In addition a number of tumors testing positive for methylation of the *BRCA1* gene were included. All of these tumors but one fell within the basal-like cluster (Fig. 5).

In addition to the five intrinsic molecular subtypes described from gene expression analysis, additional subtypes have been reported including a luminal C subtype [49], the molecular apocrine type [50], and a Claudin-low subtype [51]. The protein expression profiling of breast cancer tumors in this study demonstrates a remarkable but not complete overlap with the intrinsic subtypes, but does not demonstrate these other groupings. In the case of the Claudin-low subtype it is probably due to difficulty demonstrating that the expression is low in a shotgun experiment, rather than just absent due to under sampling. In-depth profiling of tumors at the protein expression and post-translational level (especially phosphorylation profiles) will probably allow the definition of further (sub) groups that can be translated into mass spectrometric SRM assays. Such SRM assays allow for rapid large-scale parallel analysis (300 proteins per tissue sample per hour) in a format that is already used clinically for small molecule analysis. Recent studies have demonstrated that this technology can also readily be applied to protein analysis with the required precision and inter-laboratory transferability required [29]. In this work, the peptides were carefully selected and in accordance with established general recommendations [32]. To validate the clinical value and applicability of the assay, parameters such as assay accuracy, reproducibility and laboratory transferability has to be assessed and controlled for. The use of isotopically labeled internal standards in form of proteins or peptides are instrumental in this process, providing detection confidence and means of assessing the previously mentioned parameters [30].

The assay established in this study purposely did not include proteins that are clinically established or proposed markers, though many are breast cancer related proteins, such as the cytokeratin family. Luminal A and B subtypes both express markers of the luminal epithelial layer of normal breast ducts such as keratin 8 and 18 while the basal-like group expresses markers of the basal layer of the normal breast duct such as keratin 5/6. Moreover, ER, PgR, and HER2 could be included to provide more clinical data for the analysis and we have confirmed that we can confidently measure these proteins with targeted MS (unpublished data). In addition, we have recently developed SRM assays that cover all six major DNA repair pathways allowing the response to chemotherapeutic agents to be monitored quantitatively [43]. This would mean a single assay analysis for breast cancer diagnosis and prognosis.

## Conclusions

Disease stratification for improved cancer care and treatment is vital to realize the promise of personalized medicine. The findings in this work are important in that they support and expand the molecular classification markers for breast cancer tumors into major intrinsic subtypes, demonstrating the great overlap of subtypes formed using gene expression and protein expression profiling, though showing the lack of correspondence between the discriminators found by each method. These findings have important implications for the use of genomics and expression analysis for prediction of protein expression, such as receptor status and drug target expression and thus, the utility of protein expression profiling for identifying novel molecular markers. The highly multiplexed assay is easily implemented in standard clinical chemistry practice, allowing rapid and cheap characterization of tumor tissue suitable for directing the choice of treatment.

## Abbreviations

2D-PAGE, two-dimensional polyacrylamide gel electrophoresis; ACN, acetonitrile; ANOVA, analysis of variance; CHAPS, 3-[(3-Cholamidopropyl)dimethylammonio]-1-propanesulfonate; CK, cytokeratin; Cy, cyanine dye label; DAVID, Database for Annotation, Visualization and Integrated Discovery; DIGE, difference gel electrophoresis; ER, estrogen receptor; FA, formic acid; FAK, focal adhesion kinase; FDR, false discovery rate; HER2, human epidermal growth factor receptor 2; LC-MS/MS, liquid chromatography tandem mass spectrometry; MAPK, mitogen-activated protein kinase; PAM50, prediction analysis of microarray; PARP1, Poly (ADP-ribose) polymerase 1; PgR, progesterone receptor; ROC, receiver operating characteristic; SDS, sodium dodecylsulfate; SILAC, stable isotope labeling with amino acids in cell culture; SRM, selected reaction monitoring; TCA, tricarboxylic acid; TFA, trifluoroascetic acid.

## Additional files

Additional file 1: Table S1. The clinical parameters of the tumors used in the 2D-DIGE analysis are given in this table: Sex, tumor type, BRCA type, age at diagnosis, ER status, PgR status, RNA subtype classification according to Hu, Sörlie, and PAM50 and the associated gel number. (PDF 115 kb) Additional file 2: Table S2. The subset of tumors used for the in-depth HPLC-MS/MS analysis. The table gives the sex, tumor type, BRCA typing, consensus RNA classification, BRCA1 promoter methylation state, PgR, and ER status. (PDF 29 kb) Additional file 3: Table S3. The table lists all the proteins identified using MaxQuant, the Protein IDs, protein description, posterior error probability, and confidence score. (PDF 2732 kb) Additional file 4: Table S4. The selected reaction monitoring (SRM) assays set up after refinement, containing 190 proteins represented by 253 peptides. The protein names, peptide sequences, Q1/Q3 transitions, collision energy, and fragment being monitored are given. (PDF 297 kb) Additional file 5: Table S5. The clinical data for the tumors selected for selected reaction monitoring with sample type, BRCA1 methylation status, ER, and PgR status, and the RNA classifications. (PDF 28 kb) Additional file 6: Figure S1. The ROC area curves for all classifiers and pairwise comparisons between each of the three gene classifications (Sörlie, PAM50, and Hu) and SRM-based peptide assays for each of the five subclasses; basal, normal, Her2, luminal A and luminal B. (PDF 178 kb)
