# Supplementary material for: Proteomic analysis of breast tumors confirms the mRNA intrinsic molecular subtypes using different classifiers: a large-scale analysis of fresh frozen tissue samples
Source: Breast Cancer Res. 2016 Jun 29;18:69. doi: 10.1186/s13058-016-0732-2 (PMC4928264; doi:10.1186/s13058-016-0732-2)
Supplement: Additional file 1: Table S1. — The clinical parameters of the tumors used in the 2D-DIGE analysis are given in this table: Sex, tumor type, BRCA type, age at diagnosis, ER status, PgR status, RNA subtype classification according to Hu, Sörlie, and PAM50 and the associated gel number. (PDF 115 kb) [file 13058_2016_732_MOESM1_ESM.pdf]

Cancer No	Patient_sex	HBC sample t	BRCA_Status	HBC family	Sample type	BRCA1 promo	Age at diagno	ER_status	PgR_status	OAS(days)	OAS(event)	Sárlie subtype	Best Hu	Best PAM50	Gel Name
6723	female	brcax		brcax	primary tumor	-	50	er_neg	pgr_neg	1491	dead	-			Spor 1 Cy3
11498	female	brcax		brcax	primary tumor	-	45	er_neg	pgr_neg	1234	dead	-			Spor 1 Cy5
8768	female	brcax		brcax	primary tumor	negative	46	er_neg	pgr_neg	1042	dead	-			Spor 10 Cy5
11302	female	sporadic	sporadic	-	primary tumor	negative	49	er_neg	pgr_pos	4648	alive	Normal-like	Unclassified	Basal	Spor 100 Cy3
10640	female	sporadic		-	recurrence tumor	-	47	er_neg	pgr_neg	4934	alive	-			Spor 100 Cy5
10649	female	sporadic		-	primary tumor	-	50	er_pos	pgr_pos	4933	alive	-			Spor 101 Cy3
10672	female	sporadic	sporadic	-	primary tumor	-	47	er_pos	pgr_pos	2200	dead	Luminal-A	Unclassified	HER2enriched	Spor 101 Cy5
10677	female	sporadic	sporadic	-	primary tumor	-	52	er_pos	pgr_pos	4916	alive	Luminal-B	Unclassified	Basal	Spor 102 Cy3
9833	female	sporadic	sporadic	-	primary tumor	negative	42	er_neg	pgr_neg	1190	dead	Basal	Basal	Basal	Spor 102 Cy5
9460	female	sporadic	sporadic	-	primary tumor	negative	49	er_pos	pgr_pos	1716	dead	ERBB2	Basal	LumB	Spor 103 Cy3
9319	female	sporadic	sporadic	-	primary tumor	negative	40	er_pos	pgr_pos	5511	alive	Luminal-B	HER2enriched	HER2enriched	Spor 103 Cy5
9408	female	sporadic		-	primary tumor	-	44	er_pos	pgr_pos	5476	alive	-			Spor 104 Cy3
9289	female	sporadic	sporadic	-	primary tumor	-	49	er_pos	pgr_pos	5528	alive	Normal-like	Normal	Normal	Spor 104 Cy5
9292	female	sporadic	sporadic	-	primary tumor	negative	44	er_neg	pgr_neg	5525	alive	Basal	Basal	Basal	Spor 105 Cy3
10980	female	sporadic		-	primary tumor	-	43	er_pos	pgr_pos	4772	alive	-			Spor 105 Cy5
11278	female	sporadic	sporadic	-	primary tumor	-	47	er_pos	pgr_pos	4656	alive	Luminal-A	Normal	Normal	Spor 106 Cy3
10847	female	sporadic	sporadic	-	primary tumor	positive	39	er_neg	pgr_neg	4830	alive	Basal	Basal	Basal	Spor 106 Cy5
10830	female	sporadic		-	primary tumor	-	47	er_pos	pgr_pos	4838	alive	-			Spor 107 Cy3
11042	female	sporadic		-	primary tumor	-	50	er_pos	pgr_pos	4748	alive	-			Spor 107 Cy5
11052	female	sporadic		-	primary tumor	-	51	er_pos	pgr_pos	4748	alive	-			Spor 108 Cy3
11183	female	sporadic		-	primary tumor	-	53	er_pos	pgr_neg	4704	alive	-			Spor 108 Cy5
10925	female	sporadic	sporadic	-	primary tumor	negative	33	er_neg	pgr_neg	841	dead	ERBB2	HER2enriched	HER2enriched	Spor 109 Cy3
11882	female	sporadic		-	primary tumor	-	47	er_pos	pgr_pos	4405	alive	-			Spor 109 Cy5
4534	female	brcax		brcax	primary tumor	-	43	er_pos	pgr_pos	7664	alive	-			Spor 11 Cy5
11774	female	sporadic	sporadic	-	primary tumor	negative	50	er_neg	pgr_neg	2004	dead	Luminal-A	Unclassified	Unclassified	Spor 110 Cy3
11335	female	sporadic		-	primary tumor	-	49	er_neg	pgr_neg	4634	alive	-			Spor 110 Cy5
9972	female	sporadic		-	primary tumor	-	85	er_pos	pgr_pos	3095	dead	-			Spor 111 Cy3
10976	female	sporadic	sporadic	-	primary tumor	-	48	er_pos	pgr_pos	4773	alive	Luminal-A	LumB	LumB	Spor 111 Cy5
9894	female	sporadic		-	primary tumor	-	46	er_pos	pgr_pos	5279	alive	-			Spor 112 Cy3
9877	female	sporadic	sporadic	-	primary tumor	negative	46	er_neg	pgr_neg	5287	alive	Basal	Basal	Basal	Spor 112 Cy5
18098	female	sporadic		-	primary tumor	-	28	-	-	1378	alive	-			Spor 113 Cy3
9641	female	sporadic	sporadic	-	primary tumor	-	51	er_neg	pgr_pos	5374	alive	Luminal-A	LumA	LumA	Spor 113 Cy5
8921	female	sporadic	sporadic	-	primary tumor	-	45	er_neg	pgr_neg	5676	alive	Basal	Basal	Basal	Spor 114 Cy3
9718	female	sporadic	sporadic	-	primary tumor	-	50	er_pos	pgr_neg	5341	alive	Luminal-A	Unclassified	LumB	Spor 114 Cy5
11412	female	sporadic	sporadic	-	primary tumor	-	42	er_pos	pgr_pos	4609	alive	Luminal-B	HER2enriched	HER2enriched	Spor 115 Cy3
10786	female	sporadic	sporadic	-	primary tumor	-	43	er_pos	pgr_pos	4860	alive	Luminal-A	LumA	LumA	Spor 115 Cy5
6230	female	sporadic	sporadic	-	primary tumor	-	46	er_pos	pgr_pos	3244	dead	Normal-like	LumA	LumA	Spor 116 Cy3
4602	female	sporadic		-	primary tumor	-	54	er_pos	pgr_neg	7631	alive	-			Spor 116 Cy5
5380	female	sporadic	sporadic	-	primary tumor	-	41	er_pos	pgr_pos	5136	dead	Luminal-A	LumA	LumA	Spor 117 Cy3
6040	female	sporadic	sporadic	-	primary tumor	-	45	er_pos	pgr_pos	3031	dead	Normal-like	Normal	Normal	Spor 117 Cy5
6085	female	sporadic	sporadic	-	primary tumor	negative	46	er_neg	pgr_neg	805	dead	Basal	Basal	Basal	Spor 118 Cy3
5018	female	sporadic		-	primary tumor	-	34	er_pos	pgr_pos	984	dead	-			Spor 118 Cy5
7030	female	sporadic		-	primary tumor	-	47	er_pos	pgr_pos	2601	dead	-			Spor 119 Cy3
6746	female	sporadic	sporadic	-	primary tumor	-	49	er_pos	pgr_pos	6594	alive	Normal-like	Normal	LumA	Spor 119 Cy5
8076	female	brcax		brcax	non_malignant_sample	-	46	er_neg	pgr_pos	-	-	-			Spor 12 Cy3
14321	female	sporadic	sporadic	-	primary tumor	positive	49	er_neg	pgr_neg	3345	alive	Basal	Basal	Basal	Spor 12 Cy5
6747	female	sporadic		-	primary tumor	-	41	er_pos	pgr_pos	6594	alive	-			Spor 120 Cy3
5063	female	sporadic		-	primary tumor	-	51	er_pos	pgr_pos	1642	dead	-			Spor 120 Cy5
5096	female	sporadic		-	primary tumor	-	37	er_pos	pgr_pos	1208	dead	-			Spor 121 Cy3
7036	female	sporadic	sporadic	-	primary tumor	-	45	er_pos	pgr_pos	6456	alive	Luminal-A	LumA	Normal	Spor 121 Cy5
17414	male	brcax		brcax	primary tumor	-	55	-	-	-	-	-			Spor 122 Cy3

8613	female	sporadic	sporadic	-	primary tumor	negative	44	er_pos	pgr_pos		2082	dead	Luminal-A	LumA	Unclassified	Spor 122 Cy5
9544	female	sporadic		-	primary tumor	-	50	er_pos	pgr_pos		5434	alive	-			Spor 123 Cy3
9602	female	sporadic	sporadic	-	primary tumor	positive	48	er_pos	pgr_pos		2387	dead	Luminal-A	LumA	Normal	Spor 123 Cy5
18104	female	sporadic		-	primary tumor	-	63	-	-	-	-		-			Spor 124 Cy3
9469	female	sporadic	sporadic	-	primary tumor	-	52	er_pos	pgr_pos		5446	alive	Luminal-A	Unclassified	Unclassified	Spor 124 Cy5
18100	female	sporadic		-	primary tumor	-	61	-	-		1381	alive	-			Spor 125 Cy3
18091	female	sporadic		-	primary tumor	-	64	-	-		1388	alive	-			Spor 125 Cy5
18088	female	sporadic		-	primary tumor	-	46	-	-		1388	alive	-			Spor 126 Cy3
18092	female	sporadic		-	primary tumor	-	89	-	-		1315	dead	-			Spor 126 Cy5
11822	female	sporadic	sporadic	-	primary tumor	positive	47	er_neg	pgr_neg		4437	alive	Basal	Basal	Basal	Spor 127 Cy3
18105	female	sporadic		-	primary tumor	-	71	-	-		1374	alive	-			Spor 127 Cy5
11348	female	sporadic	sporadic	-	primary tumor	negative	32	er_neg	pgr_pos		1684	dead	Luminal-B	Unclassified	Basal	Spor 128 Cy5
11472	female	sporadic	sporadic	-	primary tumor	negative	41	er_neg	pgr_neg		4580	alive	Basal	Basal	Basal	Spor 129 Cy3
11462	female	sporadic	sporadic	-	primary tumor	-	46	er_pos	pgr_pos		4580	alive	Luminal-A	LumA	LumA	Spor 129 Cy5
11954	female	brcax		brcax	recurrence tumor	-	38	er_neg	pgr_neg		565	dead	-			Spor 13 Cy3
15970	female	brcax		brcax	primary tumor	-	54	er_neg	pgr_neg		1657	dead	-			Spor 13 Cy5
14250	female	sporadic		-	primary tumor	-	52	er_neg	pgr_neg		1349	dead	-			Spor 130 Cy3
9715	female	sporadic		-	primary tumor	-	33	er_neg	pgr_pos		5342	alive	-			Spor 130 Cy5
10921	female	sporadic	sporadic	-	primary tumor	negative	47	er_neg	pgr_neg		4793	alive	ERBB2	HER2enriched	HER2enriched	Spor 131 Cy3
9774	female	sporadic	sporadic	-	primary tumor	-	39	er_neg	pgr_neg		5334	alive	Basal	Basal	Basal	Spor 131 Cy5
10825	female	sporadic	sporadic	-	primary tumor	negative	42	er_pos	pgr_pos		4613	dead	Luminal-A	Unclassified	LumB	Spor 132 Cy3
11059	female	sporadic		-	primary tumor	-	40	er_neg	pgr_pos		4741	alive	-			Spor 132 Cy5
11972	female	sporadic	sporadic	-	primary tumor	negative	32	er_neg	pgr_pos		4375	alive	Luminal-A	LumA	Normal	Spor 133 Cy3
10457	female	sporadic		-	primary tumor	-	50	er_neg	pgr_neg		5014	alive	-			Spor 133 Cy5
10241	female	sporadic		-	primary tumor	-	43	er_neg	pgr_neg		5110	alive	-			Spor 134 Cy3
10365	female	sporadic		-	primary tumor	-	55	er_neg	pgr_neg		5068	alive	-			Spor 134 Cy5
9674	female	sporadic	sporadic	-	primary tumor	-	53	er_pos	pgr_pos		5362	alive	Luminal-A	LumA	LumA	Spor 135 Cy3
10691	female	sporadic		-	primary tumor	-	51	er_neg	pgr_neg		2320	dead	-			Spor 135 Cy5
7144	female	sporadic	sporadic	-	primary tumor	negative	45	er_neg	pgr_neg		173	dead	ERBB2	HER2enriched	HER2enriched	Spor 136 Cy3
14320	female	sporadic		-	primary tumor	-	46	er_neg	pgr_pos		2564	dead	-			Spor 136 Cy5
7802	female	sporadic	sporadic	-	primary tumor	negative	48	er_neg	pgr_neg		6131	alive	ERBB2	Basal	HER2enriched	Spor 137 Cy3
9907	female	sporadic	sporadic	-	primary tumor	-	34	er_pos	pgr_pos		5280	alive	Luminal-A	LumB	LumA	Spor 137 Cy5
9858	female	sporadic	sporadic	-	primary tumor	negative	44	er_neg	pgr_neg		722	dead	ERBB2	Basal	HER2enriched	Spor 138 Cy3
7444	female	sporadic		-	primary tumor	-	43	er_neg	pgr_neg		4222	dead	-			Spor 138 Cy5
10506	female	sporadic	sporadic	-	primary tumor	-	51	er_pos	pgr_pos		4991	alive	Luminal-A	Unclassified	Unclassified	Spor 139 Cy3
14453	female	brcax		brcax	primary tumor	-	48	er_pos	pgr_pos		3283	alive	-			Spor 139 Cy5
12504	female	brcax		brcax	primary tumor	-	40	er_pos	pgr_pos		4166	alive	-			Spor 14 Cy3
5681	female	brcax		brcax	primary tumor	-	27	er_pos	-		7106	alive	-			Spor 14 Cy5
7517	female	sporadic	sporadic	-	primary tumor	-	38	er_pos	pgr_pos		5740	dead	Luminal-A	LumA	LumA	Spor 140 Cy3
5696	female	sporadic	sporadic	-	primary tumor	positive	37	er_neg	pgr_neg		7112	alive	Basal	Basal	Basal	Spor 140 Cy5
10413	female	sporadic	sporadic	-	primary tumor	-	50	er_pos	pgr_pos		5033	alive	Luminal-A	LumA	LumA	Spor 141 Cy3
5114	female	sporadic	sporadic	-	primary tumor	-	30	er_pos	pgr_pos		7379	alive	Luminal-A	LumA	LumA	Spor 141 Cy5
7015	female	sporadic	sporadic	-	primary tumor	-	46	er_pos	pgr_pos		802	dead	Luminal-A	LumA	LumA	Spor 142 Cy3
10549	female	sporadic	sporadic	-	primary tumor	-	53	er_pos	pgr_pos		2708	dead	Luminal-A	LumA	LumA	Spor 142 Cy5
7470	female	sporadic	sporadic	-	primary tumor	negative	48	er_neg	pgr_pos		5340	dead	Normal-like	Normal	Normal	Spor 143 Cy3
16163	female	brcax		brcax	primary tumor	-	74	er_pos	pgr_neg		2548	alive	-			Spor 143 Cy5
11210	female	sporadic	sporadic	-	primary tumor	-	46	er_pos	pgr_pos		3876	dead	Luminal-A	LumA	LumA	Spor 144 Cy3
10147	female	sporadic	sporadic	-	primary tumor	negative	50	er_neg	pgr_neg		5145	alive	Basal	Basal	Normal	Spor 144 Cy5
7800	female	sporadic	sporadic	-	primary tumor	negative	45	er_pos	pgr_pos		6133	alive	Luminal-A	LumB	LumB	Spor 145 Cy3
12720	female	sporadic		-	recurrence tumor	-	46	er_neg	pgr_neg		2396	dead	-			Spor 145 Cy5
5815	female	sporadic	sporadic	-	primary tumor	-	48	er_neg	pgr_neg		995	dead	Basal	Basal	Basal	Spor 146 Cy3
11287	female	sporadic	sporadic	-	primary tumor	-	50	er_pos	pgr_pos		4656	alive	Luminal-A	LumA	LumA	Spor 146 Cy5

7596	female	sporadic	sporadic	-	primary tumor	negative	33	er_neg	pgr_neg	1171	dead	ERBB2	Basal	HER2enriched	Spor 147 Cy3
6176	female	sporadic	sporadic	-	primary tumor	negative	50	er_pos	pgr_pos	6868	alive	Luminal-B	LumB	LumB	Spor 147 Cy5
9846	female	sporadic	sporadic	-	primary tumor	negative	37	er_pos	pgr_pos	2685	dead	Luminal-B	Unclassified	LumB	Spor 148 Cy3
13895	female	sporadic		-	recurrence tumor	-	48	er_neg	pgr_neg	429	dead	-			Spor 148 Cy5
8015	female	sporadic	sporadic	-	primary tumor	-	51	er_pos	pgr_pos	3685	dead	Luminal-A	LumB	LumA	Spor 149 Cy3
7469	female	sporadic		-	primary tumor	-	45	er_pos	pgr_pos	2563	dead	-			Spor 149 Cy5
10395	female	sporadic	sporadic	-	primary tumor	-	52	er_pos	pgr_pos	5043	alive	Luminal-A	LumA	LumA	Spor 15 Cy3
10460	female	sporadic	sporadic	-	primary tumor	-	52	er_pos	pgr_pos	5012	alive	Luminal-A	Unclassified	LumB	Spor 15 Cy5
5300	female	sporadic	sporadic	-	primary tumor	negative	51	er_neg	pgr_neg	7299	alive	ERBB2	HER2enriched	HER2enriched	Spor 150 Cy3
11121	female	sporadic	sporadic	-	primary tumor	negative	46	er_neg	pgr_neg	4725	alive	ERBB2	HER2enriched	HER2enriched	Spor 150 Cy5
7631	female	brca1		brca1	primary tumor	-	48	er_neg	pgr_neg	5796	dead	-			Spor 151 Cy3
4404	female	sporadic	sporadic	-	primary tumor	-	49	er_pos	pgr_pos	2452	dead	Luminal-A	Unclassified	Unclassified	Spor 151 Cy5
6871	female	sporadic	sporadic	-	primary tumor	-	41	er_neg	pgr_neg	732	dead	Basal	Basal	Basal	Spor 152 Cy3
6884	female	brcax		brcax	primary tumor	-	53	er_neg	pgr_neg	6532	alive	-			Spor 152 Cy5
8115	female	sporadic	sporadic	-	primary tumor	-	34	er_pos	pgr_pos	6014	alive	Normal-like	Normal	Unclassified	Spor 153 Cy3
7004	female	sporadic	sporadic	-	primary tumor	negative	40	er_neg	pgr_neg	6469	alive	ERBB2	HER2enriched	HER2enriched	Spor 153 Cy5
5704	female	sporadic	sporadic	-	primary tumor	negative	46	er_neg	pgr_neg	584	dead	ERBB2	Normal	Normal	Spor 154 Cy3
12735	female	brcax		brcax	primary tumor	-	36	er_neg	pgr_neg	4052	alive	-			Spor 154 Cy5
6211	female	sporadic		-	primary tumor	negative	41	er_pos	pgr_neg	2716	dead	-			Spor 155 Cy3
4628	female	sporadic	sporadic	-	primary tumor	negative	52	er_neg	pgr_pos	5643	dead	Luminal-B	HER2enriched	HER2enriched	Spor 155 Cy5
9878	female	sporadic	sporadic	-	primary tumor	positive	46	er_pos	pgr_pos	5287	alive	Luminal-B	Basal	Basal	Spor 156 Cy3
14329	female	sporadic		-	primary tumor	-	56	er_neg	pgr_neg	154	dead	-			Spor 156 Cy5
11950	female	brcax		brcax	primary tumor	negative	56	er_neg	pgr_pos	4384	alive	-			Spor 157 Cy3
14324	female	???		???	primary tumor	-	56	er_pos	pgr_neg	2999	dead	-			Spor 157 Cy5
7194	female	sporadic	sporadic	-	primary tumor	-	38	er_pos	pgr_pos	3015	dead	Luminal-A	LumA	LumB	Spor 158 Cy3
10196	female	sporadic	sporadic	-	primary tumor	negative	81	er_neg	pgr_neg	4378	dead	ERBB2	Basal	Basal	Spor 158 Cy5
9322	female	sporadic	sporadic	-	primary tumor	positive	50	er_neg	pgr_neg	5511	alive	Basal	Basal	Basal	Spor 159 Cy3
10513	female	sporadic	sporadic	-	primary tumor	positive	41	er_neg	pgr_neg	4991	alive	Basal	Basal	Basal	Spor 16 Cy3
10185	female	sporadic	sporadic	-	primary tumor	-	48	er_pos	pgr_pos	5131	alive	Luminal-A	LumA	LumA	Spor 16 Cy5
10162	female	sporadic		-	primary tumor	negative	50	er_neg	pgr_neg	5140	alive	-			Spor 17 Cy3
10206	female	sporadic	sporadic	-	primary tumor	-	52	er_pos	pgr_pos	5123	alive	Normal-like	Normal	Normal	Spor 17 Cy5
10198	female	sporadic	sporadic	-	primary tumor	negative	48	er_pos	pgr_pos	2374	dead	Luminal-A	LumA	LumA	Spor 18 Cy3
10307	female	sporadic	sporadic	-	primary tumor	positive	49	er_neg	pgr_neg	5076	alive	Basal	Basal	Basal	Spor 18 Cy5
10273	female	sporadic		-	primary tumor	-	49	er_neg	pgr_pos	5089	alive	-			Spor 19 Cy3
9759	female	sporadic	sporadic	-	primary tumor	-	48	er_pos	pgr_pos	5336	alive	Normal-like	Normal	Normal	Spor 19 Cy5
12008	female	brcax		brcax	primary tumor	-	46	er_neg	pgr_pos	4360	alive	-			Spor 2 Cy3
8820	female	sporadic		-	primary tumor	-	73	er_pos	pgr_pos	4986	dead	-			Spor 2 Cy5
10339	female	sporadic	sporadic	-	primary tumor	-	53	er_pos	pgr_pos	5070	alive	Luminal-A	LumA	LumA	Spor 20 Cy3
9220	female	sporadic	sporadic	-	primary tumor	negative	36	er_pos	pgr_neg	5544	alive	Luminal-B	LumB	LumB	Spor 20 Cy5
9843	female	sporadic		-	primary tumor	negative	50	er_neg	pgr_neg	5301	alive	-			Spor 21 Cy3
9291	female	sporadic		-	primary tumor	-	39	er_pos	pgr_neg	5523	alive	-			Spor 21 Cy5
9346	female	sporadic	sporadic	-	primary tumor	negative	43	er_neg	pgr_pos	2327	dead	ERBB2	Normal	Normal	Spor 22 Cy3
9317	female	sporadic	sporadic	-	primary tumor	-	47	er_pos	pgr_pos	5518	alive	Luminal-A	LumA	Normal	Spor 22 Cy5
9704	female	sporadic	sporadic	-	primary tumor	-	48	er_pos	pgr_neg	5350	alive	Luminal-A	LumA	LumA	Spor 23 Cy3
6860	female	sporadic		-	primary tumor	-	47	er_pos	pgr_pos	6546	alive	-			Spor 23 Cy5
5089	female	sporadic		-	primary tumor	-	38	er_pos	pgr_pos	7391	alive	-			Spor 24 Cy3
5909	female	sporadic	sporadic	-	primary tumor	negative	48	er_neg	pgr_pos	4660	dead	Luminal-A	LumA	HER2enriched	Spor 24 Cy5
6112	female	sporadic	sporadic	-	primary tumor	-	47	er_pos	pgr_pos	6904	alive	Luminal-A	LumA	LumA	Spor 25 Cy3
6877	female	sporadic	sporadic	-	primary tumor	-	34	er_pos	pgr_pos	5086	dead	Luminal-A	Unclassified	Unclassified	Spor 25 Cy5
6895	female	sporadic	sporadic	-	primary tumor	-	39	er_pos	pgr_pos	2693	dead	Luminal-A	LumA	LumB	Spor 26 Cy3
6532	female	sporadic		-	primary tumor	-	54	er_pos	pgr_pos	563	dead	-			Spor 26 Cy5
6805	female	sporadic	sporadic	-	primary tumor	-	42	er_neg	pgr_pos	6573	alive	Normal-like	Normal	Normal	Spor 27 Cy3

6009	female	sporadic	sporadic	-	primary tumor	-	49	er_pos	pgr_pos		6950	alive	Luminal-A	LumA	LumB	Spor 27 Cy5
7188	female	sporadic	sporadic	-	primary tumor	negative	42	er_neg	pgr_neg		584	dead	Luminal-B	Unclassified	Normal	Spor 28 Cy3
6514	female	sporadic	sporadic	-	primary tumor	-	49	er_neg	pgr_neg		6722	alive	Normal-like	Normal	Normal	Spor 28 Cy5
4570	female	sporadic	sporadic	-	primary tumor	negative	49	er_pos	pgr_pos		852	dead	ERBB2	HER2enriched	HER2enriched	Spor 29 Cy3
5571	female	brcax		brcax	primary tumor	-	39	er_pos	pgr_pos		7148	alive	-			Spor 29 Cy5
18071	female	sporadic		-	primary tumor	-	50	-	-		1398	alive	-			Spor 3 Cy3
18107	female	sporadic		-	primary tumor	-	64	-	-		1382	alive	-			Spor 3 Cy5
6979	female	sporadic	sporadic	-	primary tumor	negative	53	er_neg	pgr_neg		6505	alive	Basal	Basal	Basal	Spor 30 Cy3
6964	female	sporadic		-	primary tumor	-	42	er_neg	pgr_neg		879	dead	-			Spor 30 Cy5
7276	female	sporadic	sporadic	-	primary tumor	-	41	er_pos	pgr_pos		6354	alive	Normal-like	LumA	LumA	Spor 31 Cy3
5570	female	sporadic		-	primary tumor	-	49	er_pos	pgr_pos		1523	dead	-			Spor 31 Cy5
7462	female	sporadic	sporadic	-	primary tumor	negative	42	er_pos	pgr_pos		6260	alive	ERBB2	Unclassified	LumB	Spor 32 Cy3
6627	female	sporadic	sporadic	-	primary tumor	-	43	er_pos	pgr_pos		6649	alive	Normal-like	Normal	Normal	Spor 32 Cy5
8204	female	sporadic	sporadic	-	primary tumor	-	49	er_pos	pgr_neg		5979	alive	Normal-like	Normal	LumA	Spor 33 Cy3
7219	female	sporadic	sporadic	-	primary tumor	negative	46	er_pos	pgr_pos		1097	dead	Luminal-B	Unclassified	LumB	Spor 33 Cy5
7918	female	sporadic		-	primary tumor	-	50	er_pos	pgr_pos		1807	dead	-			Spor 34 Cy3
7278	female	sporadic	sporadic	-	primary tumor	negative	47	er_neg	pgr_neg		6351	alive	ERBB2	HER2enriched	Normal	Spor 34 Cy5
17532	female	brca1		brca1	non_malignant_sample	-	44	-	-	-		-	Normal-like			Spor 35 Cy3
7582	female	sporadic		-	primary tumor	-	52	er_pos	pgr_pos		6231	alive	-			Spor 35 Cy5
17613	female	brca1	brca1	brca1	primary tumor	negative	36	-	-		1743	alive	Basal	Basal	Basal	Spor 36 Cy3
8275	female	sporadic	sporadic	-	primary tumor	negative	42	er_neg	pgr_pos		4122	dead	Luminal-A	LumB	LumA	Spor 36 Cy5
17006	female	sporadic	sporadic	-	primary tumor	negative	64	-	-		1133	dead	Basal	Basal	Basal	Spor 37 Cy5
7940	female	sporadic	sporadic	-	primary tumor	-	53	er_pos	pgr_pos		6084	alive	Normal-like	Normal	Normal	Spor 38 Cy5
9452	female	sporadic	sporadic	-	primary tumor	-	42	er_pos	pgr_pos		2627	dead	Luminal-B	Unclassified	HER2enriched	Spor 39 Cy3
7988	female	sporadic	sporadic	-	primary tumor	-	44	er_pos	pgr_pos		6069	alive	Luminal-A	LumA	LumA	Spor 39 Cy5
18108	female	sporadic		-	primary tumor	-	52	-	-		1382	alive	-			Spor 4 Cy3
11056	male	brca2		brca2	recurrence tumor	-	68	er_neg	pgr_pos		661	dead	-			Spor 4 Cy5
7668	female	sporadic		-	primary tumor	-	52	er_neg	pgr_neg		1391	dead	-			Spor 40 Cy3
17533	female	brca2		brca2	non_malignant_sample	-	35	-	-	-		-	Normal-like			Spor 40 Cy5
9222	female	sporadic	sporadic	-	primary tumor	-	66	er_pos	pgr_neg		5551	alive	Luminal-A	LumB	LumB	Spor 41 Cy3
13057	female	sporadic		-	primary tumor	-	40	er_neg	pgr_pos		1509	dead	-			Spor 41 Cy5
9879	female	sporadic	sporadic	-	primary tumor	-	54	er_pos	pgr_pos		5284	alive	Luminal-A	Normal	Normal	Spor 42 Cy3
8835	female	sporadic		-	primary tumor	-	59	er_neg	pgr_neg		5714	alive	-			Spor 43 Cy3
17755	female	brca2	brca2	brca2	primary tumor	-	48	-	-		1656	alive	Luminal-A	LumB	LumB	Spor 43 Cy5
10227	female	sporadic		-	primary tumor	-	54	er_pos	pgr_neg		5112	alive	-			Spor 44 Cy3
9598	female	sporadic	sporadic	-	primary tumor	-	66	er_pos	pgr_pos		5392	alive	Luminal-A	LumA	LumA	Spor 44 Cy5
8706	female	sporadic	sporadic	-	primary tumor	negative	48	er_neg	pgr_neg		5082	dead	Basal	Basal	Basal	Spor 45 Cy3
10692	female	sporadic	sporadic	-	primary tumor	negative	53	er_neg	pgr_neg		4909	alive	Basal	Basal	Basal	Spor 45 Cy5
8837	female	sporadic	sporadic	-	primary tumor	-	54	er_pos	pgr_pos		3551	dead	Luminal-A	Unclassified	Basal	Spor 46 Cy3
9254	female	sporadic	sporadic	-	primary tumor	-	54	er_pos	pgr_pos		5562	alive	Luminal-A	LumA	LumA	Spor 46 Cy5
9019	female	sporadic	sporadic	-	primary tumor	-	67	er_pos	pgr_pos		5641	alive	Luminal-A	LumA	LumA	Spor 47 Cy3
9340	female	sporadic	sporadic	-	primary tumor	-	52	er_pos	pgr_pos		5501	alive	Luminal-A	LumB	LumB	Spor 47 Cy5
9913	female	sporadic		-	primary tumor	-	37	er_neg	pgr_neg		5272	alive	-			Spor 48 Cy3
12455	female	sporadic		-	primary tumor	-	61	er_neg	pgr_neg		1999	dead	-			Spor 48 Cy5
9047	female	sporadic	sporadic	-	primary tumor	positive	42	er_neg	pgr_neg		5629	alive	Basal	Basal	Basal	Spor 49 Cy3
9362	female	sporadic		-	primary tumor	-	63	er_pos	pgr_pos		5497	alive	-			Spor 49 Cy5
14235	female	sporadic		-	primary tumor	-	64	er_neg	pgr_neg		519	dead	-			Spor 5 Cy3
7747	female	brcax	brcax	brcax	primary tumor	-	35	er_pos	pgr_pos		6167	alive	Luminal-A	LumA	LumA	Spor 5 Cy5
9689	female	sporadic	sporadic	-	primary tumor	-	56	er_pos	pgr_pos		3778	dead	Luminal-B	HER2enriched	HER2enriched	Spor 50 Cy3
9916	female	sporadic	sporadic	-	primary tumor	-	56	er_pos	pgr_pos		5275	alive	Normal-like	Unclassified	Normal	Spor 50 Cy5
10372	female	sporadic		-	primary tumor	-	72	er_pos	pgr_pos		3883	dead	-			Spor 51 Cy3
8869	female	sporadic		-	primary tumor	-	51	er_neg	pgr_neg		5703	alive	-			Spor 51 Cy5

9107	female	sporadic	sporadic	-	primary tumor	-	67	er_pos	pgr_pos	5609	alive	Luminal-A	LumA	Normal	Spor 52 Cy3
9303	female	sporadic		-	primary tumor	-	39	er_pos	pgr_pos	5522	alive	-			Spor 52 Cy5
9949	female	sporadic		-	primary tumor	-	44	er_pos	pgr_pos	5271	alive	-			Spor 53 Cy3
10388	female	sporadic	sporadic	-	primary tumor	-	60	er_pos	pgr_pos	5042	alive	Luminal-A	LumB	Unclassified	Spor 53 Cy5
8883	female	sporadic		-	primary tumor	-	41	er_pos	pgr_pos	5700	alive	-			Spor 54 Cy3
9114	female	sporadic	sporadic	-	primary tumor	-	66	er_pos	pgr_pos	5608	alive	Luminal-A	LumA	LumA	Spor 54 Cy5
9820	female	sporadic		-	primary tumor	-	72	er_pos	pgr_pos	5306	alive	-			Spor 55 Cy3
9952	female	sporadic		-	primary tumor	-	60	er_pos	pgr_pos	5266	alive	-			Spor 55 Cy5
11338	female	sporadic	sporadic	-	primary tumor	negative	45	er_neg	pgr_pos	2957	dead	Luminal-B	Unclassified	LumB	Spor 56 Cy3
13408	female	sporadic	sporadic	-	primary tumor	-	61	er_pos	pgr_neg	3766	alive	Luminal-A	Unclassified	Normal	Spor 56 Cy5
8732	female	sporadic		-	primary tumor	-	59	er_pos	pgr_pos	5756	alive	-			Spor 57 Cy3
8888	female	sporadic	sporadic	-	primary tumor	-	72	er_pos	pgr_pos	2468	dead	Luminal-A	LumB	Normal	Spor 57 Cy5
9152	female	sporadic	sporadic	-	primary tumor	-	45	er_pos	pgr_pos	5586	alive	Luminal-B	HER2enriched	HER2enriched	Spor 58 Cy3
9426	female	sporadic		-	primary tumor	-	69	er_neg	pgr_neg	5376	dead	-			Spor 58 Cy5
10430	female	sporadic	sporadic	-	primary tumor	-	65	er_pos	pgr_pos	5017	alive	Luminal-A	LumA	LumA	Spor 59 Cy3
11370	female	sporadic		-	primary tumor	-	43	er_pos	pgr_pos	4620	alive	-			Spor 59 Cy5
8926	female	brcax		brcax	primary tumor	-	80	er_pos	pgr_pos	5685	alive	-			Spor 6 Cy3
8996	female	brcax		brcax	primary tumor	-	72	er_neg	pgr_neg	633	dead	-			Spor 6 Cy5
8767	female	sporadic	sporadic	-	primary tumor	negative	65	er_pos	pgr_neg	5745	alive	Normal-like	Unclassified	Normal	Spor 60 Cy3
8893	female	sporadic		-	primary tumor	-	67	-	-	5699	alive	-			Spor 60 Cy5
9189	female	sporadic		-	primary tumor	-	62	er_pos	pgr_pos	5567	alive	-			Spor 61 Cy3
9514	female	sporadic		-	primary tumor	-	62	er_neg	pgr_neg	5441	alive	-			Spor 61 Cy5
9865	female	sporadic		-	primary tumor	-	66	er_pos	pgr_pos	5292	alive	-			Spor 62 Cy3
10017	female	sporadic	sporadic	-	primary tumor	-	57	er_pos	pgr_pos	5219	alive	Luminal-A	Unclassified	Unclassified	Spor 62 Cy5
10533	female	sporadic	sporadic	-	primary tumor	-	67	er_pos	pgr_pos	4978	alive	Luminal-A	LumA	LumA	Spor 63 Cy3
8798	female	sporadic	sporadic	-	primary tumor	-	61	er_pos	pgr_pos	5728	alive	Luminal-A	LumA	LumB	Spor 63 Cy5
8950	female	sporadic		-	primary tumor	-	64	er_pos	pgr_pos	5552	dead	-			Spor 64 Cy3
9212	female	sporadic	sporadic	-	primary tumor	-	42	er_pos	pgr_pos	5550	alive	Luminal-A	LumB	LumA	Spor 64 Cy5
9546	female	sporadic	sporadic	-	primary tumor	-	62	er_pos	pgr_neg	2549	dead	Normal-like	Normal	Normal	Spor 65 Cy3
9866	female	sporadic	sporadic	-	primary tumor	-	50	er_pos	pgr_pos	3813	dead	Luminal-A	LumA	LumA	Spor 65 Cy5
10096	female	sporadic		-	primary tumor	-	43	er_pos	pgr_neg	764	dead	-			Spor 66 Cy3
10656	female	sporadic	sporadic	-	primary tumor	-	63	er_neg	pgr_neg	4927	alive	ERBB2	HER2enriched	HER2enriched	Spor 66 Cy5
11305	female	sporadic		-	primary tumor	-	60	er_pos	pgr_pos	1667	dead	-			Spor 67 Cy3
13943	female	sporadic		-	primary tumor	-	49	er_pos	pgr_pos	3516	alive	-			Spor 67 Cy5
16004	female	sporadic		-	primary tumor	-	53	er_pos	pgr_pos	2611	alive	-			Spor 68 Cy3
10065	female	sporadic		-	primary tumor	-	48	-	-	5202	alive	-			Spor 68 Cy5
12078	female	sporadic		-	primary tumor	-	47	er_pos	pgr_pos	4340	alive	-			Spor 69 Cy3
12545	female	sporadic		-	primary tumor	-	56	er_pos	pgr_pos	4157	alive	-			Spor 69 Cy5
11499	female	brcax		brcax	lymph_node	-	45	-	-	1234	dead	-			Spor 7 Cy3
5306	female	sporadic		-	primary tumor	-	39	er_neg	pgr_neg	457	dead	-			Spor 7 Cy5
13327	female	sporadic		-	primary tumor	-	62	er_pos	pgr_pos	3801	alive	-			Spor 70 Cy3
14631	female	sporadic		-	primary tumor	-	51	er_neg	pgr_neg	224	dead	-			Spor 70 Cy5
13833	female	sporadic		-	primary tumor	-	46	er_pos	pgr_pos	3568	alive	-			Spor 71 Cy3
13880	female	sporadic		-	primary tumor	-	51	er_pos	pgr_neg	1785	dead	-			Spor 71 Cy5
14113	female	sporadic		-	primary tumor	-	40	er_pos	pgr_pos	3446	alive	-			Spor 72 Cy3
14256	female	sporadic		-	primary tumor	-	41	er_pos	pgr_pos	3381	alive	-			Spor 72 Cy5
10152	female	sporadic	sporadic	-	primary tumor	-	63	er_pos	pgr_neg	4255	dead	Luminal-A	LumA	Normal	Spor 73 Cy3
9447	female	sporadic		-	primary tumor	-	67	er_pos	pgr_pos	4794	dead	-			Spor 73 Cy5
9612	female	sporadic		-	primary tumor	-	49	er_neg	pgr_neg	1944	dead	-			Spor 74 Cy3
9893	female	sporadic	sporadic	-	primary tumor	-	65	er_pos	pgr_pos	5287	alive	Luminal-A	LumA	LumA	Spor 74 Cy5
9991	female	sporadic		-	primary tumor	-	67	er_pos	pgr_pos	2042	dead	-			Spor 75 Cy3
9665	female	brcax	brcax	brcax	primary tumor	-	56	er_pos	pgr_pos	5364	alive	Luminal-A	LumA	LumA	Spor 75 Cy5

10844	female	sporadic	sporadic	-	primary tumor	-	57	er_pos	pgr_pos	4830	alive	Luminal-A	LumA	LumA	Spor 76 Cy3
12873	female	sporadic	sporadic	-	primary tumor	-	55	er_pos	pgr_neg	998	dead	Luminal-B	LumB	HER2enriched	Spor 76 Cy5
9745	female	sporadic		-	primary tumor	-	68	er_pos	pgr_neg	5334	alive	-			Spor 77 Cy3
11014	female	sporadic		-	primary tumor	-	59	er_pos	pgr_pos	4760	alive	-			Spor 77 Cy5
12984	female	sporadic		-	primary tumor	-	45	er_pos	pgr_pos	3668	dead	-			Spor 78 Cy3
8731	female	sporadic	sporadic	-	primary tumor	-	67	-	-	5753	alive	Normal-like	Normal	Normal	Spor 78 Cy5
9111	female	sporadic		-	primary tumor	-	69	er_pos	pgr_pos	5607	alive	-			Spor 79 Cy3
3193	female	brca2		brca2	primary tumor	-	46	er_neg	pgr_neg	8345	alive	-			Spor 79 Cy5
8665	female	sporadic		-	non_malignant_sample	-	63	er_neg	pgr_neg	5777	alive	-			Spor 8 Cy3
11609	female	sporadic	sporadic	-	primary tumor	negative	33	er_pos	pgr_pos	4528	alive	Luminal-A	LumB	LumB	Spor 8 Cy5
11394	female	???		???	primary tumor	-	34	er_neg	pgr_neg	499	dead	-			Spor 80 Cy3
9002	female	brca1		brca1	primary tumor	-	38	er_neg	pgr_neg	1264	dead	-			Spor 80 Cy5
2654	female	brca1		brca1	primary tumor	-	35	er_neg	pgr_neg	8645	alive	-			Spor 81 Cy3
5905	female	brca1		brca1	primary tumor	-	36	-	-	734	dead	-			Spor 81 Cy5
2318	female	brca1		brca1	primary tumor	-	44	er_pos	pgr_pos	8891	alive	-			Spor 82 Cy3
5447	female	brca1	brca1	brca1	recurrence tumor	-	34	-	-	7217	alive	Basal	Basal	Basal	Spor 82 Cy5
16074	female	brca1		brca1	primary tumor	negative	44	er_neg	pgr_neg	2578	alive	-			Spor 83 Cy3
5851	female	brcax		brcax	primary tumor	-	54	er_pos	pgr_pos	7050	alive	-			Spor 83 Cy5
7119	female	sporadic		-	primary tumor	-	52	er_pos	pgr_neg	3269	dead	-			Spor 84 Cy3
5873	female	brcax	brcax	brcax	primary tumor	-	67	er_pos	pgr_neg	525	dead	Luminal-B	Unclassified	Basal	Spor 84 Cy5
5967	female	brcax		brcax	primary tumor	-	49	er_neg	pgr_neg	6991	alive	-			Spor 85 Cy3
9367	female	brcax	brcax	brcax	primary tumor	-	54	er_pos	pgr_pos	3277	dead	Luminal-B	Unclassified	Unclassified	Spor 85 Cy5
10690	female	brcax	brcax	brcax	primary tumor	-	38	er_pos	pgr_pos	4913	alive	Luminal-A	Unclassified	Unclassified	Spor 86 Cy3
11796	female	brcax		brcax	primary tumor	-	42	er_pos	pgr_pos	4445	alive	-			Spor 86 Cy5
9684	female	brcax	brcax	brcax	primary tumor	-	40	er_neg	pgr_pos	5355	alive	Luminal-A	LumA	LumA	Spor 87 Cy3
12083	female	brcax		brcax	primary tumor	-	37	er_pos	pgr_pos	4333	alive	-			Spor 87 Cy5
10067	female	sporadic		-	primary tumor	-	46	er_neg	pgr_neg	2842	dead	-			Spor 88 Cy3
10081	female	sporadic		-	primary tumor	-	44	er_pos	pgr_pos	5188	alive	-			Spor 88 Cy5
10942	female	sporadic		-	primary tumor	-	44	er_neg	pgr_neg	4781	alive	-			Spor 89 Cy3
14641	female	brcax		brcax	primary tumor	-	34	er_pos	pgr_pos	3212	alive	-			Spor 9 Cy5
9275	female	brcax		brcax	primary tumor	-	56	er_pos	pgr_pos	5595	alive	-			Spor 90 Cy3
14173	female	brcax		brcax	primary tumor	-	64	er_pos	pgr_pos	3425	alive	-			Spor 91 Cy3
9119	female	sporadic		-	primary tumor	-	47	-	-	1131	dead	-			Spor 91 Cy5
10348	female	brcax		brcax	primary tumor	-	71	er_pos	pgr_pos	3891	dead	-			Spor 92 Cy3
12817	female	brcax		brcax	primary tumor	-	49	er_pos	pgr_neg	4031	alive	-			Spor 92 Cy5
14167	female	brcax		brcax	primary tumor	-	41	er_pos	pgr_pos	3425	alive	-			Spor 93 Cy3
15267	female	brcax		brcax	primary tumor	-	46	er_neg	pgr_neg	1724	dead	-			Spor 93 Cy5
8545	female	brcax		brcax	primary tumor	-	47	er_neg	pgr_pos	5825	alive	-			Spor 94 Cy3
9366	female	brcax		brcax	primary tumor	-	36	er_neg	pgr_neg	1813	dead	-			Spor 94 Cy5
10948	female	brcax		brcax	primary tumor	-	75	er_pos	pgr_pos	2174	dead	-			Spor 95 Cy3
7705	female	brcax		brcax	primary tumor	-	61	er_neg	pgr_neg	5145	dead	-			Spor 95 Cy5
10392	female	brcax		brcax	primary tumor	-	40	er_neg	pgr_neg	1989	dead	-			Spor 96 Cy3
6814	female	brca1		brca1	primary tumor	-	26	er_neg	pgr_neg	6564	alive	-			Spor 96 Cy5
8593	female	sporadic		-	primary tumor	-	74	er_pos	pgr_pos	5527	dead	-			Spor 97 Cy3
9327	female	???		???	recurrence_or_2nd_pri	-	34	-	-	3294	dead	-			Spor 97 Cy5
12528	female	brcax		brcax	primary tumor	-	49	er_neg	pgr_neg	2303	dead	-			Spor 98 Cy3
9957	female	brcax		brcax	primary tumor	-	73	er_pos	pgr_pos	5257	alive	-			Spor 98 Cy5
14613	female	brcax		brcax	primary tumor	-	58	er_pos	pgr_pos	3213	alive	-			Spor 99 Cy3
10605	female	brcax		brcax	primary tumor	-	44	er_neg	pgr_neg	328	dead	-			Spor 99 Cy5
6914	female	brcax		brcax	primary tumor	-	29	er_pos	pgr_neg	5553	dead	-			
8363	female	brcax	brcax	brca2	primary tumor	negative	44	er_neg	pgr_neg	1311	dead	Basal	Basal	Basal	
12232	female	brcax	brcax	brcax	primary tumor	-	68	er_pos	pgr_pos	495	dead	Luminal-A	LumB	LumB	

7063	female	brca2	brca2	brca2	primary tumor	negative	70	er_neg	pgr_neg	1868	dead	ERBB2	HER2enriched	HER2enriched
10905	female	brca1	brca1	brca1	primary tumor	negative	51	er_neg	pgr_neg	969	dead	Basal	Basal	Basal
7353	female	brcax	brcax	brcax	primary tumor	-	55	er_pos	pgr_pos	6316	alive	Luminal-A	LumB	LumA
11497	female	brcax	brcax	brcax	primary tumor	-	45	er_neg	pgr_neg	1234	dead	Luminal-B	HER2enriched	HER2enriched
10850	female	brcax	brcax	brcax	primary tumor	-	39	er_pos	pgr_pos	4839	alive	Normal-like	LumA	Normal
11368	female	brcax		brcax	primary tumor	-	50	er_pos	pgr_pos	4627	alive	-		
11900	female	brca2	brca2	brca2	primary tumor	negative	27	er_neg	pgr_pos	4403	alive	Luminal-B	LumB	LumB
14626	female	sporadic		-	primary tumor	-	57	er_neg	pgr_neg	3208	alive	-		
13996	female	brca1	brca1	brca1	lymph_node	negative	52	er_neg	pgr_neg	547	dead	Basal	Basal	Basal
14807	female	brcax	brcax	brcax	primary tumor	-	40	er_pos	pgr_pos	3135	alive	Luminal-A	LumB	LumB
12505	female	brcax	brcax	brcax	primary tumor	-	41	er_neg	pgr_neg	1901	dead	Luminal-B	LumB	HER2enriched
11041	female	sporadic		-	primary tumor	-	55	er_neg	pgr_neg	4748	alive	-		
15526	female	brca2	brca2	brca2	primary tumor	negative	60	er_neg	pgr_neg	2821	alive	Basal	Basal	Basal
17438	female	brcax		brcax	non_malignant_sample	-	45	-	-	-	-	Normal-like		
15016	female	brcax	brcax	brcax	primary tumor	-	67	er_pos	pgr_pos	3059	alive	Luminal-A	LumA	LumA
11130	female	brcax	brcax	brcax	primary tumor	-	38	er_pos	pgr_pos	1731	dead	Luminal-A	LumB	LumB
16779	female	brcax	brcax	brcax	lymph_node	-	66	er_neg	pgr_neg	389	dead	Basal	Basal	Basal
18099	female	sporadic		-	primary tumor	-	52	-	-	1378	alive	-		
9482	female	sporadic	sporadic	-	primary tumor	negative	46	er_pos	pgr_pos	5448	alive	Luminal-B	Unclassified	LumB
5715	female	brcax	brcax	brcax	lymph_node	-	34	er_neg	pgr_neg	3833	dead	ERBB2	HER2enriched	HER2enriched
4631	female	brcax	brcax	brcax	primary tumor	negative	86	er_neg	pgr_neg	210	dead	Basal	Basal	Basal
8196	female	brca1	brca1	brca1	primary tumor	negative	51	er_neg	pgr_neg	2046	dead	Basal	Basal	Basal
12219	female	brcax	brcax	brcax	primary tumor	-	40	er_pos	pgr_pos	4284	alive	Luminal-A	LumA	LumA
13644	female	brcax	brcax	brcax	primary tumor	-	85	er_pos	pgr_neg	926	dead	Luminal-B	Unclassified	HER2enriched
13325	female	brcax	brcax	brcax	primary tumor	-	58	er_pos	pgr_neg	933	dead	Luminal-A	LumB	Unclassified
5714	female	brcax	brcax	brcax	primary tumor	-	34	er_neg	pgr_neg	3833	dead	ERBB2	HER2enriched	HER2enriched
8080	female	brcax		brcax	primary tumor	-	88	er_pos	pgr_pos	1289	dead	-		
8522	female	brcax		brcax	primary tumor	-	47	er_pos	pgr_pos	1386	dead	-		
5799	female	brcax	brcax	brca1	lymph_node	negative	62	er_neg	pgr_neg	1413	dead	ERBB2	HER2enriched	HER2enriched
9252	female	brca1		brca1	primary tumor	negative	36	er_neg	pgr_neg	509	dead	-		
12532	female	brca1	brca1	brca1	lymph_node	negative	33	-	-	499	dead	Basal	Basal	Basal
7513	female	sporadic	sporadic	-	primary tumor	negative	31	er_neg	pgr_neg	6237	alive	Luminal-B	HER2enriched	HER2enriched
11043	female	brcax		brcax	primary tumor	-	28	er_pos	pgr_pos	1997	dead	-		
9549	female	brca1		brca1	primary tumor	-	23	er_neg	pgr_neg	5427	alive	-		
12874	female	brcax		brcax	non_malignant_sample	-	43	-	-	4003	alive	-		
8655	female	sporadic		-	primary tumor	-	42	er_neg	pgr_neg	5796	alive	-		
17080	female	brcax	brcax	brcax	primary tumor	-	35	-	-	2079	alive	Basal	Basal	Basal
6755	female	brcax	brcax	brcax	primary tumor	-	64	er_pos	pgr_pos	2754	dead	Luminal-A	LumB	LumB
11291	female	sporadic	sporadic	-	primary tumor	positive	44	er_neg	pgr_neg	1002	dead	ERBB2	Basal	Basal
8984	female	brcax	brcax	brcax	primary tumor	-	35	er_pos	pgr_neg	1837	dead	ERBB2	Normal	HER2enriched
15243	female	brca2		brca2	primary tumor	-	63	er_pos	pgr_neg	502	dead	-		
16238	female	brca2	brca2	brca2	primary tumor	negative	34	er_pos	pgr_pos	2040	dead	Luminal-A	LumB	LumB
15401	female	brcax	brcax	brcax	primary tumor	-	57	er_pos	pgr_neg	2886	alive	Luminal-A	LumA	LumA
16730	female	brca1	brca1	brca1	primary tumor	negative	50	er_neg	pgr_neg	2297	alive	ERBB2	Basal	Basal
16326	female	brcax	brcax	brcax	primary tumor	negative	50	er_pos	pgr_neg	2472	alive	Luminal-B	Unclassified	Basal
10511	female	brcax	brcax	brca2	primary tumor	-	53	er_pos	pgr_neg	4992	alive	Luminal-A	LumA	LumA
17077	female	brcax	brcax	brcax	primary tumor	-	44	-	-	729	dead	ERBB2	Normal	Normal
16642	female	brca1	brca1	brca1	recurrence_or_2nd_pri	negative	59	er_neg	pgr_neg	1204	dead	ERBB2	Basal	Basal
10463	female	brcax	brcax	brcax	primary tumor	-	50	er_neg	pgr_neg	1997	dead	ERBB2	Basal	Basal
16268	female	brcax	brcax	brcax	primary tumor	-	34	er_pos	pgr_pos	2507	alive	Luminal-A	LumA	LumA
16482	female	brcax		brcax	primary tumor	-	77	er_pos	pgr_pos	2402	alive	-		
12346	female	brcax		brcax	non_malignant_sample	-	63	-	-	4168	alive	-		

16474	female	brcax	brcax	brcax	recurrence tumor	-	54	er_pos	pgr_pos	2410	alive	Luminal-A	LumB	LumB
8572	female	brcax		brcax	lymph_node	-	52	er_neg	pgr_neg	707	dead	-		
17122	female	brcax	brcax	brcax	primary tumor	-	36	-	-	1274	dead	Luminal-A	LumB	LumB
4477	female	brcax		brca1	primary tumor	-	57	er_pos	pgr_neg	55	dead	-		
10776	female	brcax	brcax	brcax	primary tumor	-	55	er_pos	pgr_pos	4867	alive	Luminal-A	Normal	LumA
3007	female	CDKN2A	CDKN2A	CDKN2A	recurrence tumor	-	61	er_neg	pgr_neg	670	dead	ERBB2	HER2enriched	HER2enriched
1843	female	brca1		brca1	primary tumor	-	40	er_pos	pgr_pos	1222	dead	-		
8571	female	brca1	brca1	brca1	primary tumor	negative	46	er_neg	pgr_neg	2870	dead	ERBB2	Basal	Basal
10253	female	brcax	brcax	brcax	primary tumor	negative	49	er_pos	pgr_pos	2745	dead	Normal-like	Normal	Normal
9169	female	brcax	normal	brca1	non_malignant_sample	negative	33	er_neg	pgr_neg	-	-	Normal-like	Basal	Normal
10329	female	brcax	brcax	brcax	primary tumor	-	40	er_pos	pgr_neg	5069	alive	Luminal-B	HER2enriched	HER2enriched
11697	female	brcax	brcax	brcax	primary tumor	-	40	er_neg	pgr_neg	215	dead	ERBB2	HER2enriched	HER2enriched
9278	female	brca1	brca1	brca1	primary tumor	negative	56	er_pos	pgr_pos	5520	dead	Luminal-A	LumA	LumA
16808	female	brcax	brcax	brcax	primary tumor	-	64	er_pos	pgr_neg	2242	alive	Normal-like	Normal	Normal
13414	female	brcax		brcax	recurrence tumor	-	62	er_neg	pgr_pos	1897	dead	-		
15564	female	brcax	brcax	brcax	recurrence tumor	-	46	er_pos	pgr_pos	2802	alive	Luminal-A	LumA	LumA
14315	female	brcax		brcax	recurrence tumor	-	45	er_neg	pgr_neg	395	dead	-		
12054	female	brca1		brca1	primary tumor	negative	38	er_neg	pgr_neg	1405	dead	-		
17405	female	brca1	brca1	brca1	primary tumor	-	28	-	-	1843	alive	Normal-like	Normal	Normal
10359	female	brca1	normal	brca1	non_malignant_sample	-	37	er_neg	pgr_pos	5063	alive	Normal-like	Normal	Normal
17271	female	brcax	brcax	brcax	primary tumor	-	52	-	-	1955	alive	Luminal-A	LumB	LumB
15388	female	brca1		brca1	primary tumor	-	43	er_neg	pgr_neg	455	dead	-		
12727	female	brca1	brca1	brca1	primary tumor	negative	61	er_neg	pgr_neg	1051	dead	Basal	Basal	Basal
10320	female	brcax	brcax	brcax	primary tumor	-	62	er_pos	pgr_neg	2391	dead	Luminal-A	Unclassified	LumA
12364	female	brcax	brcax	brcax	primary tumor	positive	36	er_neg	pgr_neg	4223	alive	Basal	Basal	Basal
14171	female	brca1	brca1	brca1	primary tumor	negative	52	er_pos	pgr_neg	2951	dead	Luminal-B	LumB	LumB
14659	female	brcax	brcax	brcax	primary tumor	-	74	er_pos	pgr_pos	3192	alive	Luminal-A	LumA	LumA
14767	female	brca2	brca2	brca2	primary tumor	negative	57	er_pos	pgr_pos	2194	dead	Luminal-A	LumA	LumA
11247	female	brcax		brcax	primary tumor	-	46	er_pos	pgr_pos	4674	alive	-		
10344	female	brca1		brca1	primary tumor	-	40	er_neg	pgr_neg	489	dead	-		
5241	female	brca1	brca1	brca1	primary tumor	negative	43	er_neg	pgr_neg	5948	dead	Normal-like	Normal	Normal
11778	female	brca1		brca1	non_malignant_sample	-	39	-	-	1840	dead	-		
8601	female	brcax		brcax	primary tumor	-	35	er_pos	pgr_pos	5823	alive	-		
1476	female	brca1		brca1	primary tumor	-	44	er_neg	pgr_neg	4363	dead	-		
15766	female	brcax	brcax	brcax	primary tumor	-	61	er_neg	pgr_neg	1359	dead	Luminal-B	HER2enriched	HER2enriched
6753	female	brcax	brcax	brcax	primary tumor	-	64	er_pos	pgr_pos	3073	dead	Luminal-A	LumB	LumA
17372	female	brcax	brcax	brcax	primary tumor	-	44	-	-	1156	dead	Luminal-B	Unclassified	Unclassified
14317	female	brcax	brcax	brcax	lymph_node	-	62	er_pos	pgr_pos	1567	dead	Luminal-B	HER2enriched	LumB
7178	female	brca1	brca1	brca1	recurrence tumor	negative	44	-	-	43	dead	Basal	Basal	Basal
16804	female	brcax	brcax	brcax	primary tumor	-	35	er_neg	pgr_pos	2241	alive	Normal-like	Unclassified	Unclassified
10865	female	brcax	brcax	brcax	primary tumor	-	61	er_neg	pgr_neg	4816	alive	Luminal-B	HER2enriched	HER2enriched
16481	female	brcax	brcax	brcax	primary tumor	negative	77	er_pos	pgr_pos	2402	alive	Luminal-B	LumB	LumB
7650	female	brcax	brcax	brcax	primary tumor	-	45	er_neg	pgr_neg	6196	alive	Luminal-A	LumA	LumA
10785	female	brcax	brcax	brcax	primary tumor	-	74	er_neg	pgr_neg	808	dead	ERBB2	HER2enriched	HER2enriched
15504	female	brca1	brca1	brca1	primary tumor	negative	73	er_neg	pgr_neg	2828	alive	Basal	Basal	Basal
15478	female	brcax	brcax	brcax	primary tumor	-	57	er_pos	pgr_pos	2842	alive	Luminal-A	LumA	LumA
15752	female	brcax	brcax	brcax	primary tumor	-	50	er_neg	pgr_neg	2732	alive	ERBB2	HER2enriched	Normal
17290	female	brca1	brca1	brca1	primary tumor	negative	53	er_neg	pgr_neg	1931	alive	Basal	Basal	Basal
15143	female	sporadic	sporadic	-	primary tumor	-	68	er_pos	pgr_pos	2976	alive	Luminal-A	LumB	LumB
11101	female	brcax		brcax	primary tumor	-	37	-	-	4732	alive	-		
8648	female	brca2	brca2	brca2	primary tumor	negative	28	er_neg	pgr_neg	733	dead	Luminal-A	LumB	LumB
12530	female	brca1	brca1	brca1	recurrence tumor	-	39	er_neg	pgr_neg	2613	dead	Basal	Basal	Basal

12578	female	brcax	brcax	brcax	primary tumor	-	46	er_neg	pgr_pos		985	dead	Normal-like	Normal	Normal
17179	female	brcax	brcax	brcax	primary tumor	-	40	-	-		2004	alive	Basal	Basal	Basal
17522	female	brcax	brcax	brcax	primary tumor	negative	66	-	-		1777	alive	Basal	Basal	Basal
7720	female	sporadic	sporadic	-	primary tumor	positive	44	er_neg	pgr_neg		6175	alive	Basal	Basal	Basal
12577	female	brcax	brcax	brcax	primary tumor	-	46	er_pos	pgr_pos		985	dead	Luminal-A	LumB	LumB
6605	female	sporadic	sporadic	-	primary tumor	positive	37	er_neg	pgr_neg		6678	alive	Basal	Basal	Basal
10657	female	brcax	brcax	brcax	primary tumor	negative	50	er_pos	pgr_pos		4930	alive	Luminal-A	LumA	LumB
13812	female	brca1	brca1	brca1	primary tumor	negative	47	er_neg	pgr_neg		3584	alive	ERBB2	Basal	Basal
14803	female	brcax	brcax	brcax	primary tumor	-	48	er_pos	pgr_pos		3138	alive	Luminal-A	LumB	LumB
10581	female	brca1	brca1	brca1	primary tumor	negative	59	er_neg	pgr_neg		2162	dead	Basal	Basal	Basal
14616	male	brca2	brca2	brca2	primary tumor	-	85	er_pos	pgr_pos		1041	dead	Luminal-B	LumB	LumB
6023	female	brca1	brca1	brca1	primary tumor	negative	35	er_neg	pgr_neg		2380	dead	Basal	Basal	Basal
7223	female	brcax	brcax	brcax	primary tumor	negative	45	er_pos	pgr_neg		1009	dead	Luminal-B	LumB	LumB
14920	female	brca1		brca1	non_malignant_sample	negative	40	er_neg	pgr_neg	-	-		-		
13714	female	brca1	brca1	brca1	primary tumor	negative	48	er_neg	pgr_neg		406	dead	Basal	Basal	Basal
12994	female	brcax	brcax	brcax	primary tumor	negative	65	er_neg	pgr_neg		3957	alive	ERBB2	Basal	Basal
11577	female	brcax		brcax	recurrence tumor	-	32	er_pos	pgr_pos		4531	alive	-		
12224	female	brca1	brca1	brca1	primary tumor	negative	38	er_neg	pgr_neg		2725	dead	Basal	Basal	Basal
11808	female	brca1	brca1	brca1	primary tumor	negative	38	er_neg	pgr_neg		4444	alive	Basal	Basal	Basal
14510	female	brca1	brca1	brca1	primary tumor	-	40	er_neg	pgr_neg		3264	alive	Basal	Basal	Basal
9053	female	brcax	brcax	brcax	recurrence_or_2nd_pri	-	38	er_neg	pgr_pos		5630	alive	Luminal-A	Normal	LumA
14007	female	brca1		brca1	primary tumor	-	33	er_neg	pgr_neg		3488	alive	-		
16723	female	brcax		brcax	primary tumor	-	43	er_neg	pgr_neg		2296	alive	-		
10683	female	brcax	brcax	brcax	recurrence tumor	-	37	er_pos	pgr_pos		1920	dead	Luminal-A	LumA	Unclassified
9700	female	brcax	brcax	brcax	primary tumor	-	55	er_pos	pgr_pos		4384	dead	Luminal-A	Normal	Normal
10886	female	brcax	brcax	brcax	primary tumor	-	50	er_pos	pgr_pos		2649	dead	Luminal-A	LumB	LumA
11186	female	sporadic	sporadic	-	primary tumor	-	45	er_pos	pgr_pos		4699	alive	Luminal-A	LumA	Normal
12082	female	brcax		brcax	primary tumor	-	39	er_pos	pgr_pos		4335	alive	-		
8228	female	brcax	brcax	brcax	primary tumor	-	39	er_neg	pgr_pos		5971	alive	Normal-like	Normal	Normal
15655	female	brca1	brca1	brca1	primary tumor	negative	28	er_neg	pgr_neg		2765	alive	Basal	Basal	Basal
10360	female	brca1		brca1	non_malignant_sample	-	37	er_neg	pgr_pos		5063	alive	-		
14316	female	brcax	brcax	brcax	primary tumor	negative	62	er_pos	pgr_pos		1567	dead	Luminal-B	Unclassified	HER2enriched
17460	female	brcax	brcax	brcax	primary tumor	-	40	-	-		1805	alive	Luminal-A	LumA	LumA
7592	female	brcax	brcax	brcax	primary tumor	-	44	er_pos	pgr_pos		6217	alive	Luminal-A	Normal	Unclassified
16534	female	brcax	brcax	brcax	primary tumor	-	55	er_pos	pgr_pos	-	-		Luminal-B	LumA	LumA
12314	female	brcax	brcax	brcax	primary tumor	-	35	er_pos	pgr_pos		4242	alive	Luminal-A	LumA	LumA
13091	female	brcax		brcax	primary tumor	-	42	er_pos	pgr_pos		3913	alive	-		
16821	female	brcax	brcax	brcax	primary tumor	-	49	er_pos	pgr_pos		2233	alive	Luminal-A	LumB	LumB
17490	female	brca2		brca2	non_malignant_sample	-	35	-	-	-	-		Normal-like		
13183	female	brcax	brcax	brca2	primary tumor	-	61	er_pos	pgr_neg		3876	alive	Luminal-B	LumB	LumB
9601	female	brcax	brcax	brcax	primary tumor	negative	68	er_pos	pgr_neg		5391	alive	Normal-like	Normal	Normal
14830	female	brcax	brcax	brcax	primary tumor	negative	41	er_neg	pgr_neg		954	dead	Basal	Basal	Basal
16684	female	brcax	brcax	brcax	primary tumor	-	42	er_pos	pgr_pos		2320	alive	Luminal-A	LumA	LumA
16889	female	brcax	brcax	brcax	primary tumor	-	35	er_neg	pgr_pos		1694	dead	ERBB2	Unclassified	LumB
17439	female	brcax		brcax	non_malignant_sample	-	45	-	-	-	-		Normal-like		
13442	female	brcax	brcax	brcax	primary tumor	-	43	er_pos	pgr_neg		3745	alive	Luminal-A	Unclassified	Unclassified
15765	female	brcax	brcax	brcax	primary tumor	-	61	er_pos	pgr_neg		1359	dead	Luminal-A	LumA	LumA
11787	female	brca2	brca2	brca2	primary tumor	negative	54	er_pos	pgr_neg		4447	alive	Luminal-A	LumB	LumB
6150	female	brcax	brcax	brcax	primary tumor	-	55	er_pos	pgr_pos		2576	dead	Luminal-A	LumA	LumA
12237	female	brcax	brcax	brcax	primary tumor	positive	77	er_neg	pgr_neg		86	dead	Basal	Basal	Basal
14090	female	brca1	brca1	brca1	primary tumor	negative	58	er_neg	pgr_neg		3465	alive	Normal-like	Normal	Normal
11736	female	brcax	brcax	brcax	lymph_node	negative	65	er_neg	pgr_neg		4472	alive	Basal	Basal	Basal

10029	female	brca1	brca1	brca1	primary tumor	-	44	er_neg	pgr_neg		486	dead	Basal	Basal	Basal
6874	female	brcax	brcax	brcax	recurrence tumor	-	49	er_neg	pgr_pos		6540	alive	Normal-like	Normal	Normal
9632	female	???		???	primary tumor	-	60	er_pos	pgr_neg		5377	alive	-		
11721	female	brca2		brca2	primary tumor	-	28	er_pos	pgr_neg		439	dead	-		
10697	female	brca1		brca1	primary tumor	-	41	er_neg	pgr_neg		1824	dead	-		
11283	female	brca1	brca1	brca1	primary tumor	negative	29	er_neg	pgr_neg		4656	alive	Basal	Basal	Basal
15050	female	brcax	brcax	brcax	primary tumor	-	71	er_pos	pgr_pos	-	-		Luminal-A	Unclassified	LumB
4594	female	brcax	brcax	brcax	primary tumor	-	70	er_pos	pgr_pos		6513	dead	Luminal-A	LumA	LumA
9153	female	brcax	brcax	brcax	primary tumor	-	46	er_pos	pgr_pos		5586	alive	Luminal-A	LumA	LumA
18090	female	sporadic		-	primary tumor	-	78	-	-		281	dead	-		
