# Supplementary material for: Proteomic analysis of breast tumors confirms the mRNA intrinsic molecular subtypes using different classifiers: a large-scale analysis of fresh frozen tissue samples
Source: Breast Cancer Res. 2016 Jun 29;18:69. doi: 10.1186/s13058-016-0732-2 (PMC4928264; doi:10.1186/s13058-016-0732-2)
Supplement: Additional file 2: Table S2. — The subset of tumors used for the in-depth HPLC-MS/MS analysis. The table gives the sex, tumor type, BRCA typing, consensus RNA classification, BRCA1 promoter methylation state, PgR, and ER status. (PDF 29 kb) [file 13058_2016_732_MOESM2_ESM.pdf]

Cancer No	Patient_sex	HBC sample type	Tumor info ->	Sample type	BRCA1 promotor methylation	ER_status	PgR_status	Expressions data ->	Sörlie subtype classified	Best Hu	Best PAM50
17613	female	brca1	Tumor info ->	primary tumor	negative	-	-	Expressions data ->	Basal	Basal	Basal
17290	female	brca1	Tumor info ->	primary tumor	negative	er_neg	pgr_neg	Expressions data ->	Basal	Basal	Basal
5815	female	sporadic	Tumor info ->	primary tumor	-	er_neg	pgr_neg	Expressions data ->	Basal	Basal	Basal
6871	female	sporadic	Tumor info ->	primary tumor	-	er_neg	pgr_neg	Expressions data ->	Basal	Basal	Basal
12237	female	brcax	Tumor info ->	primary tumor	positive	er_neg	pgr_neg	Expressions data ->	Basal	Basal	Basal
9047	female	sporadic	Tumor info ->	primary tumor	positive	er_neg	pgr_neg	Expressions data ->	Basal	Basal	Basal
6979	female	sporadic	Tumor info ->	primary tumor	negative	er_neg	pgr_neg	Expressions data ->	Basal	Basal	Basal
11822	female	sporadic	Tumor info ->	primary tumor	positive	er_neg	pgr_neg	Expressions data ->	Basal	Basal	Basal
8363	female	brcax	Tumor info ->	primary tumor	negative	er_neg	pgr_neg	Expressions data ->	Basal	Basal	Basal
15526	female	brca2	Tumor info ->	primary tumor	negative	er_neg	pgr_neg	Expressions data ->	Basal	Basal	Basal
4631	female	brcax	Tumor info ->	primary tumor	negative	er_neg	pgr_neg	Expressions data ->	Basal	Basal	Basal
17080	female	brcax	Tumor info ->	primary tumor	-	-	-	Expressions data ->	Basal	Basal	Basal
13714	female	brca1	Tumor info ->	primary tumor	negative	er_neg	pgr_neg	Expressions data ->	Basal	Basal	Basal
12224	female	brca1	Tumor info ->	primary tumor	negative	er_neg	pgr_neg	Expressions data ->	Basal	Basal	Basal
14510	female	brca1	Tumor info ->	primary tumor	-	er_neg	pgr_neg	Expressions data ->	Basal	Basal	Basal
11697	female	brcax	Tumor info ->	primary tumor	-	er_neg	pgr_neg	Expressions data ->	ERBB2	HER2enriched	HER2enriched
10785	female	brcax	Tumor info ->	primary tumor	-	er_neg	pgr_neg	Expressions data ->	ERBB2	HER2enriched	HER2enriched
10656	female	sporadic	Tumor info ->	primary tumor	-	er_neg	pgr_neg	Expressions data ->	ERBB2	HER2enriched	HER2enriched
10925	female	sporadic	Tumor info ->	primary tumor	negative	er_neg	pgr_neg	Expressions data ->	ERBB2	HER2enriched	HER2enriched
10921	female	sporadic	Tumor info ->	primary tumor	negative	er_neg	pgr_neg	Expressions data ->	ERBB2	HER2enriched	HER2enriched
5714	female	brcax	Tumor info ->	primary tumor	-	er_neg	pgr_neg	Expressions data ->	ERBB2	HER2enriched	HER2enriched
16684	female	brcax	Tumor info ->	primary tumor	-	er_pos	pgr_pos	Expressions data ->	Luminal-A	LumA	LumA
15765	female	brcax	Tumor info ->	primary tumor	-	er_pos	pgr_neg	Expressions data ->	Luminal-A	LumA	LumA
7747	female	brcax	Tumor info ->	primary tumor	-	er_pos	pgr_pos	Expressions data ->	Luminal-A	LumA	LumA
10511	female	brcax	Tumor info ->	primary tumor	-	er_pos	pgr_neg	Expressions data ->	Luminal-A	LumA	LumA
16268	female	brcax	Tumor info ->	primary tumor	-	er_pos	pgr_pos	Expressions data ->	Luminal-A	LumA	LumA
6150	female	brcax	Tumor info ->	primary tumor	-	er_pos	pgr_pos	Expressions data ->	Luminal-A	LumA	LumA
10339	female	sporadic	Tumor info ->	primary tumor	-	er_pos	pgr_pos	Expressions data ->	Luminal-A	LumA	LumA
9704	female	sporadic	Tumor info ->	primary tumor	-	er_pos	pgr_neg	Expressions data ->	Luminal-A	LumA	LumA
15401	female	brcax	Tumor info ->	primary tumor	-	er_pos	pgr_neg	Expressions data ->	Luminal-A	LumA	LumA
9153	female	brcax	Tumor info ->	primary tumor	-	er_pos	pgr_pos	Expressions data ->	Luminal-A	LumA	LumA
9665	female	brcax	Tumor info ->	primary tumor	-	er_pos	pgr_pos	Expressions data ->	Luminal-A	LumA	LumA
10549	female	sporadic	Tumor info ->	primary tumor	-	er_pos	pgr_pos	Expressions data ->	Luminal-A	LumA	LumA
10430	female	sporadic	Tumor info ->	primary tumor	-	er_pos	pgr_pos	Expressions data ->	Luminal-A	LumA	LumA
9019	female	sporadic	Tumor info ->	primary tumor	-	er_pos	pgr_pos	Expressions data ->	Luminal-A	LumA	LumA
11900	female	brca2	Tumor info ->	primary tumor	negative	er_neg	pgr_pos	Expressions data ->	Luminal-B	LumB	LumB
16481	female	brcax	Tumor info ->	primary tumor	negative	er_pos	pgr_pos	Expressions data ->	Luminal-B	LumB	LumB
9482	female	sporadic	Tumor info ->	primary tumor	negative	er_pos	pgr_pos	Expressions data ->	Luminal-B	Unclassified	LumB
9846	female	sporadic	Tumor info ->	primary tumor	negative	er_pos	pgr_pos	Expressions data ->	Luminal-B	Unclassified	LumB
9601	female	brcax	Tumor info ->	primary tumor	negative	er_pos	pgr_neg	Expressions data ->	Normal-like	Normal	Normal
14090	female	brca1	Tumor info ->	primary tumor	negative	er_neg	pgr_neg	Expressions data ->	Normal-like	Normal	Normal
6514	female	sporadic	Tumor info ->	primary tumor	-	er_neg	pgr_neg	Expressions data ->	Normal-like	Normal	Normal
16808	female	brcax	Tumor info ->	primary tumor	-	er_pos	pgr_neg	Expressions data ->	Normal-like	Normal	Normal
