# Supplementary material for: Proteomic analysis of breast tumors confirms the mRNA intrinsic molecular subtypes using different classifiers: a large-scale analysis of fresh frozen tissue samples
Source: Breast Cancer Res. 2016 Jun 29;18:69. doi: 10.1186/s13058-016-0732-2 (PMC4928264; doi:10.1186/s13058-016-0732-2)
Supplement: Additional file 3: Table S3. — The table lists all the proteins identified using MaxQuant, the Protein IDs, protein description, posterior error probability, and confidence score. (PDF 2732 kb) [file 13058_2016_732_MOESM3_ESM.pdf]

Majority Protein IDs	Protein Descriptions	PEP	Confidence score
A0AV96	RBM47_HUMAN RNA-binding protein 47 OS=Homo sapiens GN=RBM47 PE=1 SV=2		
A0AV96-2	RBM47_HUMAN Isoform 2 of RNA-binding protein 47 OS=Homo sapiens GN=RBM47		
A0AVF1	TTC26_HUMAN Tetratricopeptide repeat protein 26 OS=Homo sapiens GN=TTC26 PE=2 SV=1		
A0AVK6	E2F8_HUMAN Transcription factor E2F8 OS=Homo sapiens GN=E2F8 PE=1 SV=1		21.98
A0AVT1;A0AVT1-2	UBA6_HUMAN Ubiquitin-like modifier-activating enzyme 6 OS=Homo sapiens GN=UBA6 PE=1 SV=1;>sp|A0AVT1-2|UBA6_HUMAN Iso	3.35E-71	
A0FGR8	ESYT2_HUMAN Extended synaptotagmin-2 OS=Homo sapiens GN=ESYT2 PE=1 SV=1		203.23
A0FGR8-2	ESYT2_HUMAN Isoform 2 of Extended synaptotagmin-2 OS=Homo sapiens GN=ESYT2		255.1
A0FGR8-4	ESYT2_HUMAN Isoform 4 of Extended synaptotagmin-2 OS=Homo sapiens GN=ESYT2		127.29
A0FGR8-5	ESYT2_HUMAN Isoform 5 of Extended synaptotagmin-2 OS=Homo sapiens GN=ESYT2		75.94
A0FGR8-6	ESYT2_HUMAN Isoform 6 of Extended synaptotagmin-2 OS=Homo sapiens GN=ESYT2		203.23
A0M8Q6	LAC7_HUMAN Ig lambda-7 chain C region OS=Homo sapiens GN=IGLC7 PE=1 SV=2		64.41
A0MZ66-3;A0MZ66;A0MZ	3|SHOT1_HUMAN Isoform 3 of Shootin-1 OS=Homo sapiens GN=KIAA1598;>sp|A0MZ66|SHOT1_HUMAN Shootin-1 OS=Homo sapien	2.41E-83	
A1L0T0	ILVBL_HUMAN Acetolactate synthase-like protein OS=Homo sapiens GN=ILVBL PE=1 SV=2		
A1L0T0-2	ILVBL_HUMAN Isoform 2 of Acetolactate synthase-like protein OS=Homo sapiens GN=ILVBL		
A1L0T0;A1L0T0-2	ILVBL_HUMAN Acetolactate synthase-like protein OS=Homo sapiens GN=ILVBL PE=1 SV=2;>sp|A1L0T0-2|ILVBL_HUMAN Isoform 2 o	1.80E-13	
A1X283	SPD2B_HUMAN SH3 and PX domain-containing protein 2B OS=Homo sapiens GN=SH3PXD2B PE=1 SV=3		
A2A2Z9	AN18B_HUMAN Ankyrin repeat domain-containing protein 18B OS=Homo sapiens GN=ANKRD18B PE=2 SV=1		
A2A3N6	PIPSL_HUMAN Putative PIP5K1A and PSMD4-like protein OS=Homo sapiens GN=PIPSL PE=5 SV=1		47.9
A2BFH1	PAL4G_HUMAN Peptidylprolyl cis-trans isomerase A-like 4G OS=Homo sapiens GN=PPIAL4G PE=2 SV=1		39.92
A2RRP1	NBAS_HUMAN Neuroblastoma-amplified sequence OS=Homo sapiens GN=NBAS PE=1 SV=2		
A2RRP1-2	NBAS_HUMAN Isoform 2 of Neuroblastoma-amplified sequence OS=Homo sapiens GN=NBAS		
A2RTX5	SYTC2_HUMAN Probable threonyl-tRNA synthetase 2, cytoplasmic OS=Homo sapiens GN=TARSL2 PE=1 SV=1		97.77
A2RTX5-2	SYTC2_HUMAN Isoform 2 of Probable threonyl-tRNA synthetase 2, cytoplasmic OS=Homo sapiens GN=TARSL2		97.77
A2RTY3	CQ066_HUMAN Uncharacterized protein C17orf66 OS=Homo sapiens GN=C17orf66 PE=2 SV=2		
A2RTY3-2	CQ066_HUMAN Isoform 2 of Uncharacterized protein C17orf66 OS=Homo sapiens GN=C17orf66		
A2RTY3-3	CQ066_HUMAN Isoform 3 of Uncharacterized protein C17orf66 OS=Homo sapiens GN=C17orf66		
A2RTY3-4	CQ066_HUMAN Isoform 4 of Uncharacterized protein C17orf66 OS=Homo sapiens GN=C17orf66		
A2RTY3-5	CQ066_HUMAN Isoform 5 of Uncharacterized protein C17orf66 OS=Homo sapiens GN=C17orf66		
A2RU54	HMX2_HUMAN Homeobox protein HMX2 OS=Homo sapiens GN=HMX2 PE=2 SV=1		
A2RUR9	C144A_HUMAN Coiled-coil domain-containing protein 144A OS=Homo sapiens GN=CCDC144A PE=1 SV=1		
A2RUR9-2	C144A_HUMAN Isoform 2 of Coiled-coil domain-containing protein 144A OS=Homo sapiens GN=CCDC144A		
A2RUR9-3	C144A_HUMAN Isoform 3 of Coiled-coil domain-containing protein 144A OS=Homo sapiens GN=CCDC144A		
A3KMH1;A3KMH1-3;A3KM	K0564_HUMAN Uncharacterized protein KIAA0564 OS=Homo sapiens GN=KIAA0564 PE=1 SV=2;>sp|A3KMH1-3|K0564_HUMAN Isofo	5.56E-33	
A4D0V7	CG058_HUMAN Uncharacterized protein C7orf58 OS=Homo sapiens GN=C7orf58 PE=2 SV=1		
A4D0V7-2	CG058_HUMAN Isoform 2 of Uncharacterized protein C7orf58 OS=Homo sapiens GN=C7orf58		
A4D126	ISPD_HUMAN 2-C-methyl-D-erythritol 4-phosphate cytidylyltransferase-like protein OS=Homo sapiens GN=ISPD PE=3 SV=2		
A4D1E9	GTPBA_HUMAN GTP-binding protein 10 OS=Homo sapiens GN=GTPBP10 PE=1 SV=1		23.38
A4D1E9-2	GTPBA_HUMAN Isoform 2 of GTP-binding protein 10 OS=Homo sapiens GN=GTPBP10		
A4D1E9-3	GTPBA_HUMAN Isoform 3 of GTP-binding protein 10 OS=Homo sapiens GN=GTPBP10		
A4D1E9;C9JNI1;C9J8R7	GTPBA_HUMAN GTP-binding protein 10 OS=Homo sapiens GN=GTPBP10 PE=1 SV=1;>tr|C9JNI1|C9JNI1_HUMAN Uncharacterized pro	2.03E-05	
A4D1F6	LRRD1_HUMAN Leucine-rich repeat and death domain-containing protein 1 OS=Homo sapiens GN=LRRD1 PE=2 SV=2		
A4FU69	EFCB5_HUMAN EF-hand calcium-binding domain-containing protein 5 OS=Homo sapiens GN=EFCAB5 PE=1 SV=2		
A4FU69-2	EFCB5_HUMAN Isoform 2 of EF-hand calcium-binding domain-containing protein 5 OS=Homo sapiens GN=EFCAB5		
A4FU69-3	EFCB5_HUMAN Isoform 3 of EF-hand calcium-binding domain-containing protein 5 OS=Homo sapiens GN=EFCAB5		
A4FU69-4	EFCB5_HUMAN Isoform 4 of EF-hand calcium-binding domain-containing protein 5 OS=Homo sapiens GN=EFCAB5		
A4FU69;A4FU69-3	EFCB5_HUMAN EF-hand calcium-binding domain-containing protein 5 OS=Homo sapiens GN=EFCAB5 PE=1 SV=2;>sp|A4FU69-3|EFCI	4.22E-09	
A4QPH2	PI4P2_HUMAN Putative phosphatidylinositol 4-kinase alpha-like protein P2 OS=Homo sapiens GN=PI4KAP2 PE=5 SV=2		
A4QPH2-2	PI4P2_HUMAN Isoform 2 of Putative phosphatidylinositol 4-kinase alpha-like protein P2 OS=Homo sapiens GN=PI4KAP2		
A4QPH2-3	PI4P2_HUMAN Isoform 3 of Putative phosphatidylinositol 4-kinase alpha-like protein P2 OS=Homo sapiens GN=PI4KAP2		
A4UGR9	XIRP2_HUMAN Xin actin-binding repeat-containing protein 2 OS=Homo sapiens GN=XIRP2 PE=1 SV=2		
A4UGR9-2	XIRP2_HUMAN Isoform 2 of Xin actin-binding repeat-containing protein 2 OS=Homo sapiens GN=XIRP2		
A4UGR9-3	XIRP2_HUMAN Isoform 3 of Xin actin-binding repeat-containing protein 2 OS=Homo sapiens GN=XIRP2		
A5A3E0	POTEF_HUMAN POTE ankyrin domain family member F OS=Homo sapiens GN=POTEF PE=1 SV=2		520.57
A5PLN7	F149A_HUMAN Protein FAM149A OS=Homo sapiens GN=FAM149A PE=2 SV=2		35.09
A5PLN7-2	F149A_HUMAN Isoform 2 of Protein FAM149A OS=Homo sapiens GN=FAM149A		
A5PLN7-3	F149A_HUMAN Isoform 3 of Protein FAM149A OS=Homo sapiens GN=FAM149A		35.09

A5PLN7-4	F149A_HUMAN Isoform 4 of Protein FAM149A OS=Homo sapiens GN=FAM149A		35.09
A5YKK6	CNOT1_HUMAN CCR4-NOT transcription complex subunit 1 OS=Homo sapiens GN=CNOT1 PE=1 SV=2		107.9
A5YKK6-2	CNOT1_HUMAN Isoform 2 of CCR4-NOT transcription complex subunit 1 OS=Homo sapiens GN=CNOT1		107.9
A5YKK6-3	CNOT1_HUMAN Isoform 3 of CCR4-NOT transcription complex subunit 1 OS=Homo sapiens GN=CNOT1		107.9
A5YKK6-4	CNOT1_HUMAN Isoform 4 of CCR4-NOT transcription complex subunit 1 OS=Homo sapiens GN=CNOT1		43.57
A5YKK6;A5YKK6-2;A5YKK	CNOT1_HUMAN CCR4-NOT transcription complex subunit 1 OS=Homo sapiens GN=CNOT1 PE=1 SV=2;>sp|A5YKK6-2|CNOT1_HUMAN	1.68E-40	
A5YM72-2	CRNS1_HUMAN Isoform 2 of Carnosine synthase 1 OS=Homo sapiens GN=CARNS1		
A6H8Y1	BDP1_HUMAN Transcription factor TFIIIB component B'' homolog OS=Homo sapiens GN=BDP1 PE=1 SV=3		44.09
A6H8Y1-2	BDP1_HUMAN Isoform 2 of Transcription factor TFIIIB component B'' homolog OS=Homo sapiens GN=BDP1		44.09
A6H8Y1-3	BDP1_HUMAN Isoform 3 of Transcription factor TFIIIB component B'' homolog OS=Homo sapiens GN=BDP1		38.43
A6H8Y1-4	BDP1_HUMAN Isoform 4 of Transcription factor TFIIIB component B'' homolog OS=Homo sapiens GN=BDP1		38.43
A6H8Y1-5	BDP1_HUMAN Isoform 5 of Transcription factor TFIIIB component B'' homolog OS=Homo sapiens GN=BDP1		37.26
A6H8Y1-6	BDP1_HUMAN Isoform 6 of Transcription factor TFIIIB component B'' homolog OS=Homo sapiens GN=BDP1		44.09
A6H8Y1-7	BDP1_HUMAN Isoform 7 of Transcription factor TFIIIB component B'' homolog OS=Homo sapiens GN=BDP1		44.09
A6H8Y1-8	BDP1_HUMAN Isoform 8 of Transcription factor TFIIIB component B'' homolog OS=Homo sapiens GN=BDP1		5.66
A6NC57	ANR62_HUMAN Ankyrin repeat domain-containing protein 62 OS=Homo sapiens GN=ANKRD62 PE=2 SV=4		
A6NC57-2	ANR62_HUMAN Isoform 2 of Ankyrin repeat domain-containing protein 62 OS=Homo sapiens GN=ANKRD62		
A6NCE7	MP3B2_HUMAN Microtubule-associated proteins 1A/1B light chain 3 beta 2 OS=Homo sapiens GN=MAP1LC3B2 PE=2 SV=1		128.32
A6NCN2	KT81L_HUMAN Keratin-81-like protein OS=Homo sapiens PE=2 SV=3		1.6
A6NCS4	NKX26_HUMAN Homeobox protein Nkx-2.6 OS=Homo sapiens GN=NKX2-6 PE=1 SV=1		
A6NCS6	CB072_HUMAN Uncharacterized protein C2orf72 OS=Homo sapiens GN=C2orf72 PE=2 SV=2		30.11
A6NDG6	PGP_HUMAN Phosphoglycolate phosphatase OS=Homo sapiens GN=PGP PE=1 SV=1	9.24E-28	
A6NDJ8	RB43L_HUMAN Putative Rab-43-like protein ENSP00000330714 OS=Homo sapiens PE=5 SV=3		71.25
A6NDK8	FA35B_HUMAN Protein FAM35B OS=Homo sapiens GN=FAM35B PE=3 SV=3		
A6NDU8	CE051_HUMAN UPF0600 protein C5orf51 OS=Homo sapiens GN=C5orf51 PE=1 SV=1	1.62E-15	
A6NDY0	EPAB2_HUMAN Embryonic polyadenylate-binding protein 2 OS=Homo sapiens GN=PABPN1L PE=2 SV=1		
A6NDY0-2	EPAB2_HUMAN Isoform 2 of Embryonic polyadenylate-binding protein 2 OS=Homo sapiens GN=PABPN1L		
A6NDY0-4	EPAB2_HUMAN Isoform 4 of Embryonic polyadenylate-binding protein 2 OS=Homo sapiens GN=PABPN1L		
A6NDY2	F90AA_HUMAN Putative protein FAM90A10 OS=Homo sapiens GN=FAM90A10 PE=2 SV=1		
A6NE01	F186A_HUMAN Protein FAM186A OS=Homo sapiens GN=FAM186A PE=2 SV=3	3.69E-06	
A6NE21	F90AI_HUMAN Putative protein FAM90A18/FAM90A19 OS=Homo sapiens GN=FAM90A18 PE=1 SV=1		
A6NED2	RCCD1_HUMAN RCC1 domain-containing protein 1 OS=Homo sapiens GN=RCCD1 PE=1 SV=1		
A6NEK1	ARRD5_HUMAN Arrestin domain-containing protein 5 OS=Homo sapiens GN=ARRDC5 PE=2 SV=2		
A6NEW6	F90AG_HUMAN Putative protein FAM90A16/FAM90A17 OS=Homo sapiens GN=FAM90A16 PE=3 SV=1		
A6NGG8	CB071_HUMAN Uncharacterized protein C2orf71 OS=Homo sapiens GN=C2orf71 PE=1 SV=1		26.22
A6NGZ7	U93BL_HUMAN Putative protein unc-93 homolog B1-like protein OS=Homo sapiens PE=5 SV=3		
A6NH08	ZB12L_HUMAN Putative zinc finger and BTB domain-containing protein 12-like protein OS=Homo sapiens PE=5 SV=4		
A6NH11	GLTD2_HUMAN Glycolipid transfer protein domain-containing protein 2 OS=Homo sapiens GN=GLTPD2 PE=1 SV=2		
A6NHG4	DDTL_HUMAN D-dopachrome decarboxylase-like protein OS=Homo sapiens GN=DDTL PE=2 SV=1		66.99
A6NHJ4	ZN860_HUMAN Zinc finger protein 860 OS=Homo sapiens GN=ZNF860 PE=2 SV=3		
A6NHL2	TBAL3_HUMAN Tubulin alpha chain-like 3 OS=Homo sapiens GN=TUBAL3 PE=1 SV=2		214.65
A6NHL2-2	TBAL3_HUMAN Isoform 2 of Tubulin alpha chain-like 3 OS=Homo sapiens GN=TUBAL3		214.65
A6NHL2;A6NHL2-2	TBAL3_HUMAN Tubulin alpha chain-like 3 OS=Homo sapiens GN=TUBAL3 PE=1 SV=2;>sp|A6NHL2-2|TBAL3_HUMAN Isoform 2 of Tub	2.48E-65	
A6NHQ2	FBLL1_HUMAN rRNA/tRNA 2'-O-methyltransferase fibrillarin-like protein 1 OS=Homo sapiens GN=FBLL1 PE=3 SV=1		48.63
A6NHR9	SMHD1_HUMAN Structural maintenance of chromosomes flexible hinge domain-containing protein 1 OS=Homo sapiens GN=SMCHD1 PE=1 SV=2		417.09
A6NHR9-2	SMHD1_HUMAN Isoform 2 of Structural maintenance of chromosomes flexible hinge domain-containing protein 1 OS=Homo sapiens GN=SMCHD1		411
A6NHR9-3	SMHD1_HUMAN Isoform 3 of Structural maintenance of chromosomes flexible hinge domain-containing protein 1 OS=Homo sapiens GN=SMCHD1		198.4
A6NHR9;A6NHR9-2;A6NHI	SMHD1_HUMAN Structural maintenance of chromosomes flexible hinge domain-containing protein 1 OS=Homo sapiens GN=SMCHD1 F	7.01E-119	
A6NHZ5	LR14B_HUMAN Leucine-rich repeat-containing protein 14B OS=Homo sapiens GN=LRRC14B PE=3 SV=3		
A6NI47	POTEM_HUMAN Putative POTE ankyrin domain family member M OS=Homo sapiens GN=POTEM PE=3 SV=2		
A6NI72;P14598;A8MVU1;I	NCF1B_HUMAN Putative neutrophil cytosol factor 1B OS=Homo sapiens GN=NCF1B PE=5 SV=2;>sp|P14598|NCF1_HUMAN Neutrophil	4.82E-06	
A6NIE6	RN3L1_HUMAN RRN3-like protein 1 OS=Homo sapiens PE=2 SV=3		
A6NIJ5	F90AK_HUMAN Putative protein FAM90A20 OS=Homo sapiens GN=FAM90A20 PE=3 SV=1		
A6NIK2	LR10B_HUMAN Leucine-rich repeat-containing protein 10B OS=Homo sapiens GN=LRRC10B PE=4 SV=2		28.31
A6NIL8	YP041_HUMAN Putative CDC37-like protein ENSP00000350273 OS=Homo sapiens PE=5 SV=1		
A6NIZ1	RP1BL_HUMAN Ras-related protein Rap-1b-like protein OS=Homo sapiens PE=2 SV=1		552.9
A6NJJ6	CS067_HUMAN UPF0575 protein C19orf67 OS=Homo sapiens GN=C19orf67 PE=2 SV=2		
A6NJQ4	F90A8_HUMAN Putative protein FAM90A8 OS=Homo sapiens GN=FAM90A8 PE=3 SV=1		
A6NJZ7	RIM3C_HUMAN RIMS-binding protein 3C OS=Homo sapiens GN=RIMBP3C PE=1 SV=2		
A6NK07	IF2BL_HUMAN Eukaryotic translation initiation factor 2 subunit 2-like protein OS=Homo sapiens PE=1 SV=1		624.51
A6NKC0	F90A7_HUMAN Putative protein FAM90A7 OS=Homo sapiens GN=FAM90A7 PE=2 SV=1		

A6NKF1	SAC31_HUMAN SAC3 domain-containing protein 1 OS=Homo sapiens GN=SAC3D1 PE=1 SV=2		
A6NKF2	ARI3C_HUMAN AT-rich interactive domain-containing protein 3C OS=Homo sapiens GN=ARID3C PE=2 SV=1		
A6NKF9	GPHRC_HUMAN Putative Golgi pH regulator C OS=Homo sapiens GN=GPR89C PE=5 SV=2		
A6NKH3	RL37L_HUMAN Putative 60S ribosomal protein L37a-like OS=Homo sapiens GN=RPL37L PE=5 SV=2		24.19
A6NKL6	T200C_HUMAN Transmembrane protein 200C OS=Homo sapiens GN=TMEM200C PE=2 SV=2		
A6NKT7	RGPD3_HUMAN RanBP2-like and GRIP domain-containing protein 3 OS=Homo sapiens GN=RGPD3 PE=2 SV=2		185.39
A6NKZ8	YI016_HUMAN Putative tubulin beta chain-like protein ENSP00000290377 OS=Homo sapiens PE=5 SV=2		543.13
A6NL28	TPM3L_HUMAN Putative tropomyosin alpha-3 chain-like protein OS=Homo sapiens PE=5 SV=2		74.78
A6NL28-2	TPM3L_HUMAN Isoform 2 of Putative tropomyosin alpha-3 chain-like protein OS=Homo sapiens		74.78
A6NL28;A6NL28-2	TPM3L_HUMAN Putative tropomyosin alpha-3 chain-like protein OS=Homo sapiens PE=5 SV=2;>sp|A6NL28-2|TPM3L_HUMAN Isoform	2.84E-52	
A6NLF2	EA3L2_HUMAN RNA polymerase II transcription factor SIII subunit A3-like-2 OS=Homo sapiens GN=TCEB3CL2 PE=3 SV=1		
A6NLP5	TTC36_HUMAN Tetratricopeptide repeat protein 36 OS=Homo sapiens GN=TTC36 PE=2 SV=1		27.44
A6NLX3	SPDE4_HUMAN Speedy protein E4 OS=Homo sapiens GN=SPDYE4 PE=2 SV=2		
A6NM62	LRC53_HUMAN Leucine-rich repeat-containing protein 53 OS=Homo sapiens GN=LRRC53 PE=4 SV=2		
A6NMX2	I4E1B_HUMAN Eukaryotic translation initiation factor 4E type 1B OS=Homo sapiens GN=EIF4E1B PE=2 SV=3		72.69
A6NMY6	AXA2L_HUMAN Putative annexin A2-like protein OS=Homo sapiens GN=ANXA2P2 PE=5 SV=2		2056.54
A6NMZ7;A6NMZ7-2	CO6A6_HUMAN Collagen alpha-6(VI) chain OS=Homo sapiens GN=COL6A6 PE=1 SV=2;>sp|A6NMZ7-2|CO6A6_HUMAN Isoform 2 of (	9.13E-09	
A6NNH2	F90AL_HUMAN Putative protein FAM90A-like OS=Homo sapiens PE=3 SV=2		
A6NNJ1	F90A9_HUMAN Putative protein FAM90A9 OS=Homo sapiens GN=FAM90A9 PE=3 SV=1		
A6NNM3	RIM3B_HUMAN RIMS-binding protein 3B OS=Homo sapiens GN=RIMBP3B PE=2 SV=2		
A6NNW6	ENO4_HUMAN Enolase-like protein ENO4 OS=Homo sapiens GN=ENO4 PE=2 SV=2		26.39
A6NNW6-2	ENO4_HUMAN Isoform 2 of Enolase-like protein ENO4 OS=Homo sapiens GN=ENO4		24.8
A6NNZ2	TBB8B_HUMAN Tubulin beta-8 chain B OS=Homo sapiens PE=1 SV=1		701.95
A6PVS8	LRIQ3_HUMAN Leucine-rich repeat and IQ domain-containing protein 3 OS=Homo sapiens GN=LRRIQ3 PE=2 SV=1		
A6PVS8-2	LRIQ3_HUMAN Isoform 2 of Leucine-rich repeat and IQ domain-containing protein 3 OS=Homo sapiens GN=LRRIQ3		
A6PVS8-3	LRIQ3_HUMAN Isoform 3 of Leucine-rich repeat and IQ domain-containing protein 3 OS=Homo sapiens GN=LRRIQ3		
A6QL64	AN36A_HUMAN Ankyrin repeat domain-containing protein 36A OS=Homo sapiens GN=ANKRD36 PE=2 SV=3		
A6QL64-3	AN36A_HUMAN Isoform 2 of Ankyrin repeat domain-containing protein 36A OS=Homo sapiens GN=ANKRD36		
A7E2Y1	MYH7B_HUMAN Myosin-7B OS=Homo sapiens GN=MYH7B PE=2 SV=3		76.06
A7E2Y1-2	MYH7B_HUMAN Isoform 2 of Myosin-7B OS=Homo sapiens GN=MYH7B		
A7E2Y1-3	MYH7B_HUMAN Isoform 3 of Myosin-7B OS=Homo sapiens GN=MYH7B		
A7E2Y1;A7E2Y1-3	MYH7B_HUMAN Myosin-7B OS=Homo sapiens GN=MYH7B PE=2 SV=3;>sp|A7E2Y1-3|MYH7B_HUMAN Isoform 3 of Myosin-7B OS=Ho	4.66E-06	
A7MCY6	TBKB1_HUMAN TANK-binding kinase 1-binding protein 1 OS=Homo sapiens GN=TBKBP1 PE=1 SV=1		
A7MCY6-2	TBKB1_HUMAN Isoform 2 of TANK-binding kinase 1-binding protein 1 OS=Homo sapiens GN=TBKBP1		
A8K0Z3;Q6VEQ5;C4AMC7	WASH1_HUMAN WAS protein family homolog 1 OS=Homo sapiens GN=WASH1 PE=1 SV=2;>sp|Q6VEQ5|WASH2_HUMAN WAS proteir	2.50E-09	
A8K2U0	A2ML1_HUMAN Alpha-2-macroglobulin-like protein 1 OS=Homo sapiens GN=A2ML1 PE=1 SV=3	0.00016106	
A8MT19	RHN2L_HUMAN Putative rhophilin-2-like protein OS=Homo sapiens PE=5 SV=2		
A8MT69	CENPX_HUMAN Centromere protein X OS=Homo sapiens GN=STRA13 PE=1 SV=1		
A8MT69-2	CENPX_HUMAN Isoform 2 of Centromere protein X OS=Homo sapiens GN=STRA13		
A8MT69-3	CENPX_HUMAN Isoform 3 of Centromere protein X OS=Homo sapiens GN=STRA13		
A8MTJ3	GNAT3_HUMAN Guanine nucleotide-binding protein G(t) subunit alpha-3 OS=Homo sapiens GN=GNAT3 PE=2 SV=2		182.81
A8MUU9	YV023_HUMAN Putative uncharacterized protein ENSP00000383309 OS=Homo sapiens PE=5 SV=3		43.26
A8MW06;P62328	TMSL3_HUMAN Thymosin beta-4-like protein 3 OS=Homo sapiens GN=TMSL3 PE=2 SV=1;>sp|P62328|TYB4_HUMAN Thymosin beta-	3.37E-71	
A8MWA6	F90AM_HUMAN Putative protein FAM90A22 OS=Homo sapiens GN=FAM90A22 PE=5 SV=1		
A8MWD9	RUXGL_HUMAN Small nuclear ribonucleoprotein G-like protein OS=Homo sapiens PE=3 SV=2		290.34
A8MWL7	TM14D_HUMAN Transmembrane protein 14D OS=Homo sapiens GN=TMEM14D PE=3 SV=2		81.8
A8MWS1	KI3P1_HUMAN Putative killer cell immunoglobulin-like receptor like protein KIR3DP1 OS=Homo sapiens GN=KIR3DP1 PE=2 SV=2		
A8MWY0	K132L_HUMAN UPF0577 protein KIAA1324-like OS=Homo sapiens GN=KIAA1324L PE=2 SV=2		
A8MWY0-2	K132L_HUMAN Isoform 2 of UPF0577 protein KIAA1324-like OS=Homo sapiens GN=KIAA1324L		
A8MX19	F90AC_HUMAN Putative protein FAM90A12 OS=Homo sapiens GN=FAM90A12 PE=3 SV=1		
A8MXJ8	F90A5_HUMAN Putative protein FAM90A5 OS=Homo sapiens GN=FAM90A5 PE=3 SV=1		
A8MXV4	NUD19_HUMAN Nucleoside diphosphate-linked moiety X motif 19, mitochondrial OS=Homo sapiens GN=NUDT19 PE=1 SV=1	3.43E-08	
A8MXZ1	F90AN_HUMAN Putative protein FAM90A23 OS=Homo sapiens GN=FAM90A23 PE=5 SV=1		
A8MY62	BLML_HUMAN Putative beta-lactamase-like 1 OS=Homo sapiens GN=LACTBL1 PE=2 SV=2		41.16
A8MYJ9	IMA2L_HUMAN Importin subunit alpha-2-like protein OS=Homo sapiens PE=2 SV=2		
A8MZ26	EFCB9_HUMAN EF-hand calcium-binding domain-containing protein 9 OS=Homo sapiens GN=EFCAB9 PE=4 SV=2		
A8MZ36	EVPLL_HUMAN Envoplakin-like protein OS=Homo sapiens GN=EVPLL PE=2 SV=1		
A9Z1Z3	FR1L4_HUMAN Fer-1-like protein 4 OS=Homo sapiens GN=FER1L4 PE=2 SV=1		
A9Z1Z3-3	FR1L4_HUMAN Isoform 3 of Fer-1-like protein 4 OS=Homo sapiens GN=FER1L4		
A9Z1Z3;A9Z1Z3-3	FR1L4_HUMAN Fer-1-like protein 4 OS=Homo sapiens GN=FER1L4 PE=2 SV=1;>sp|A9Z1Z3-3|FR1L4_HUMAN Isoform 3 of Fer-1-like	9.40E-07	
Accession	Description		

B0I1T2;B0I1T2-4	MYO1G_HUMAN Myosin-Ig OS=Homo sapiens GN=MYO1G PE=1 SV=2;>sp|B0I1T2-4|MYO1G_HUMAN Isoform 4 of Myosin-Ig OS=Hor	1.76E-13	
B2RPK0	HGB1A_HUMAN Putative high mobility group protein B1-like 1 OS=Homo sapiens GN=HMGB1P1 PE=5 SV=1		383.75
B2RTY4	MYO9A_HUMAN Myosin-IXa OS=Homo sapiens GN=MYO9A PE=1 SV=2		
B2RTY4-2	MYO9A_HUMAN Isoform 2 of Myosin-IXa OS=Homo sapiens GN=MYO9A		
B2RTY4-3	MYO9A_HUMAN Isoform 3 of Myosin-IXa OS=Homo sapiens GN=MYO9A		
B2RTY4-4	MYO9A_HUMAN Isoform 4 of Myosin-IXa OS=Homo sapiens GN=MYO9A		
B2RTY4-5	MYO9A_HUMAN Isoform 5 of Myosin-IXa OS=Homo sapiens GN=MYO9A		
B2RU33	POTEC_HUMAN POTE ankyrin domain family member C OS=Homo sapiens GN=POTEC PE=2 SV=2		0.08
B2RU33-2	POTEC_HUMAN Isoform 2 of POTE ankyrin domain family member C OS=Homo sapiens GN=POTEC		0.08
B5MCN3	S14L6_HUMAN Putative SEC14-like protein 6 OS=Homo sapiens GN=SEC14L6 PE=5 SV=1		
B7ZAQ6	GPHRA_HUMAN Golgi pH regulator A OS=Homo sapiens GN=GPR89A PE=1 SV=2		
B7ZAQ6-2	GPHRA_HUMAN Isoform 2 of Golgi pH regulator A OS=Homo sapiens GN=GPR89A		
B7ZAQ6-3	GPHRA_HUMAN Isoform 3 of Golgi pH regulator A OS=Homo sapiens GN=GPR89A		
B9A064	IGLL5_HUMAN Immunoglobulin lambda-like polypeptide 5 OS=Homo sapiens GN=IGLL5 PE=2 SV=2		164.87
C9J798	RAS4B_HUMAN Putative Ras GTPase-activating protein 4B OS=Homo sapiens GN=RASA4B PE=5 SV=2		
C9JLW8	F195B_HUMAN Protein FAM195B OS=Homo sapiens GN=FAM195B PE=1 SV=1		56.78
C9JRZ8	AK1BF_HUMAN Aldo-keto reductase family 1 member B15 OS=Homo sapiens GN=AKR1B15 PE=2 SV=1		46.44
C9JRZ8-2	AK1BF_HUMAN Isoform 2 of Aldo-keto reductase family 1 member B15 OS=Homo sapiens GN=AKR1B15		46.44
O00115	DNS2A_HUMAN Deoxyribonuclease-2-alpha OS=Homo sapiens GN=DNASE2 PE=1 SV=2		31.64
O00116	ADAS_HUMAN Alkyldihydroxyacetonephosphate synthase, peroxisomal OS=Homo sapiens GN=AGPS PE=1 SV=1		326.69
O00139	KIF2A_HUMAN Kinesin-like protein KIF2A OS=Homo sapiens GN=KIF2A PE=1 SV=3		40.02
O00139-1	KIF2A_HUMAN Isoform 1 of Kinesin-like protein KIF2A OS=Homo sapiens GN=KIF2A		40.02
O00139-2	KIF2A_HUMAN Isoform 2 of Kinesin-like protein KIF2A OS=Homo sapiens GN=KIF2A		40.02
O00139-4	KIF2A_HUMAN Isoform 4 of Kinesin-like protein KIF2A OS=Homo sapiens GN=KIF2A		40.02
O00139-4;O00139;D3DW	4|KIF2A_HUMAN Isoform 4 of Kinesin-like protein KIF2A OS=Homo sapiens GN=KIF2A;>sp|O00139|KIF2A_HUMAN Kinesin-like protei	1.75E-14	
O00148	DX39A_HUMAN ATP-dependent RNA helicase DDX39A OS=Homo sapiens GN=DDX39A PE=1 SV=2		486.12
O00151	PDLI1_HUMAN PDZ and LIM domain protein 1 OS=Homo sapiens GN=PDLIM1 PE=1 SV=4		136.38
O00154	BACH_HUMAN Cytosolic acyl coenzyme A thioester hydrolase OS=Homo sapiens GN=ACOT7 PE=1 SV=3		373.8
O00154-2	BACH_HUMAN Isoform 2 of Cytosolic acyl coenzyme A thioester hydrolase OS=Homo sapiens GN=ACOT7		251.26
O00154-3	BACH_HUMAN Isoform 3 of Cytosolic acyl coenzyme A thioester hydrolase OS=Homo sapiens GN=ACOT7		251.26
O00154-4	BACH_HUMAN Isoform 4 of Cytosolic acyl coenzyme A thioester hydrolase OS=Homo sapiens GN=ACOT7		373.8
O00154-4;O00154;B3KQ1	4|BACH_HUMAN Isoform 4 of Cytosolic acyl coenzyme A thioester hydrolase OS=Homo sapiens GN=ACOT7;>sp|O00154|BACH_HUMA	2.52E-122	
O00154-5	BACH_HUMAN Isoform 5 of Cytosolic acyl coenzyme A thioester hydrolase OS=Homo sapiens GN=ACOT7		381.03
O00154-6	BACH_HUMAN Isoform 6 of Cytosolic acyl coenzyme A thioester hydrolase OS=Homo sapiens GN=ACOT7		373.8
O00154;O00154-5;O0015	BACH_HUMAN Cytosolic acyl coenzyme A thioester hydrolase OS=Homo sapiens GN=ACOT7 PE=1 SV=3;>sp|O00154-5|BACH_HUMAI	8.48E-28	
O00159	MYO1C_HUMAN Myosin-Ic OS=Homo sapiens GN=MYO1C PE=1 SV=4		2298.4
O00159-2	MYO1C_HUMAN Isoform 2 of Myosin-Ic OS=Homo sapiens GN=MYO1C		2164.36
O00159-3	MYO1C_HUMAN Isoform 3 of Myosin-Ic OS=Homo sapiens GN=MYO1C		2286.72
O00159-3;O00159;O0015	3|MYO1C_HUMAN Isoform 3 of Myosin-Ic OS=Homo sapiens GN=MYO1C;>sp|O00159|MYO1C_HUMAN Myosin-Ic OS=Homo sapiens G	2.29E-170	
O00159;O00159-3;B7Z3E	MYO1C_HUMAN Myosin-Ic OS=Homo sapiens GN=MYO1C PE=1 SV=4;>sp|O00159-3|MYO1C_HUMAN Isoform 3 of Myosin-Ic OS=Hon	2.65E-200	
O00160	MYO1F_HUMAN Myosin-If OS=Homo sapiens GN=MYO1F PE=1 SV=3	3.73E-36	
O00161	SNP23_HUMAN Synaptosomal-associated protein 23 OS=Homo sapiens GN=SNAP23 PE=1 SV=1		141.07
O00161-2	SNP23_HUMAN Isoform SNAP-23b of Synaptosomal-associated protein 23 OS=Homo sapiens GN=SNAP23		127.33
O00161;O00161-2	SNP23_HUMAN Synaptosomal-associated protein 23 OS=Homo sapiens GN=SNAP23 PE=1 SV=1;>sp|O00161-2|SNP23_HUMAN Isofo	2.88E-33	
O00170	AIP_HUMAN AH receptor-interacting protein OS=Homo sapiens GN=AIP PE=1 SV=2	2.77E-36	
O00178	GTPB1_HUMAN GTP-binding protein 1 OS=Homo sapiens GN=GTPBP1 PE=1 SV=3		26.38
O00182;O00182-2;Q3B8N	LEG9_HUMAN Galectin-9 OS=Homo sapiens GN=LGALS9 PE=1 SV=2;>sp|O00182-2|LEG9_HUMAN Isoform Short of Galectin-9 OS=H	1.03E-15	
O00186	STXB3_HUMAN Syntaxin-binding protein 3 OS=Homo sapiens GN=STXBP3 PE=1 SV=2		248.89
O00193	SMAP_HUMAN Small acidic protein OS=Homo sapiens GN=SMAP PE=1 SV=1	5.22E-12	
O00194	RB27B_HUMAN Ras-related protein Rab-27B OS=Homo sapiens GN=RAB27B PE=1 SV=4		6.47
O00203	AP3B1_HUMAN AP-3 complex subunit beta-1 OS=Homo sapiens GN=AP3B1 PE=1 SV=3	2.21E-15	
O00204;O00204-2	ST2B1_HUMAN Sulfotransferase family cytosolic 2B member 1 OS=Homo sapiens GN=SULT2B1 PE=1 SV=2;>sp|O00204-2|ST2B1_HU	4.62E-25	
O00217	NDUS8_HUMAN NADH dehydrogenase [ubiquinone] iron-sulfur protein 8, mitochondrial OS=Homo sapiens GN=NDUFS8 PE=1 SV=1		144.62
O00220	TR10A_HUMAN Tumor necrosis factor receptor superfamily member 10A OS=Homo sapiens GN=TNFRSF10A PE=1 SV=3		28.84
O00231	PSD11_HUMAN 26S proteasome non-ATPase regulatory subunit 11 OS=Homo sapiens GN=PSMD11 PE=1 SV=3		332.68
O00232	PSD12_HUMAN 26S proteasome non-ATPase regulatory subunit 12 OS=Homo sapiens GN=PSMD12 PE=1 SV=3		223.09
O00233	PSMD9_HUMAN 26S proteasome non-ATPase regulatory subunit 9 OS=Homo sapiens GN=PSMD9 PE=1 SV=3		173
O00233-2	PSMD9_HUMAN Isoform p27-S of 26S proteasome non-ATPase regulatory subunit 9 OS=Homo sapiens GN=PSMD9		173
O00233;O00233-2	PSMD9_HUMAN 26S proteasome non-ATPase regulatory subunit 9 OS=Homo sapiens GN=PSMD9 PE=1 SV=3;>sp|O00233-2|PSMD9_	4.40E-18	
O00258	WRB_HUMAN Tryptophan-rich protein OS=Homo sapiens GN=WRB PE=2 SV=2	2.87E-10	
O00264	PGRC1_HUMAN Membrane-associated progesterone receptor component 1 OS=Homo sapiens GN=PGRMC1 PE=1 SV=3		387.33

O00267;O00267-2	SPT5H_HUMAN Transcription elongation factor SPT5 OS=Homo sapiens GN=SUPT5H PE=1 SV=1;>sp|O00267-2|SPT5H_HUMAN Isofor	2.28E-18	
O00273;O00273-2	DFFA_HUMAN DNA fragmentation factor subunit alpha OS=Homo sapiens GN=DFFA PE=1 SV=1;>sp|O00273-2|DFFA_HUMAN Isoform	3.72E-11	
O00291	HIP1_HUMAN Huntingtin-interacting protein 1 OS=Homo sapiens GN=HIP1 PE=1 SV=5		
O00299	CLIC1_HUMAN Chloride intracellular channel protein 1 OS=Homo sapiens GN=CLIC1 PE=1 SV=4		831.1
O00303	EIF3F_HUMAN Eukaryotic translation initiation factor 3 subunit F OS=Homo sapiens GN=EIF3F PE=1 SV=1		705.47
O00308	WWP2_HUMAN NEDD4-like E3 ubiquitin-protein ligase WWP2 OS=Homo sapiens GN=WWP2 PE=1 SV=2		
O00330	ODPX_HUMAN Pyruvate dehydrogenase protein X component, mitochondrial OS=Homo sapiens GN=PDHX PE=1 SV=3		
O00338	ST1C2_HUMAN Sulfotransferase 1C2 OS=Homo sapiens GN=SULT1C2 PE=1 SV=1		25.05
O00338-2	ST1C2_HUMAN Isoform Long of Sulfotransferase 1C2 OS=Homo sapiens GN=SULT1C2		25.05
O00338-2;O00338	2|ST1C2_HUMAN Isoform Long of Sulfotransferase 1C2 OS=Homo sapiens GN=SULT1C2;>sp|O00338|ST1C2_HUMAN Sulfotransferase	1.89E-05	
O00339;O00339-2;O0033	MATN2_HUMAN Matrilin-2 OS=Homo sapiens GN=MATN2 PE=1 SV=4;>sp|O00339-2|MATN2_HUMAN Isoform 2 of Matrilin-2 OS=Hom	1.68E-06	
O00391;A8K477;A8K4C2;	QSOX1_HUMAN Sulfhydryl oxidase 1 OS=Homo sapiens GN=QSOX1 PE=1 SV=3;>tr|A8K477|A8K477_HUMAN cDNA FLJ78571, highly	6.96E-34	
O00399	DCTN6_HUMAN Dynactin subunit 6 OS=Homo sapiens GN=DCTN6 PE=1 SV=1		81.31
O00400	ACATN_HUMAN Acetyl-coenzyme A transporter 1 OS=Homo sapiens GN=SLC33A1 PE=1 SV=1	1.61E-08	
O00401	WASL_HUMAN Neural Wiskott-Aldrich syndrome protein OS=Homo sapiens GN=WASL PE=1 SV=2	8.46E-10	
O00408	PDE2A_HUMAN cGMP-dependent 3',5'-cyclic phosphodiesterase OS=Homo sapiens GN=PDE2A PE=1 SV=1		
O00410	IPO5_HUMAN Importin-5 OS=Homo sapiens GN=IPO5 PE=1 SV=4		294.95
O00410-2	IPO5_HUMAN Isoform 2 of Importin-5 OS=Homo sapiens GN=IPO5		246.05
O00410-3	IPO5_HUMAN Isoform 3 of Importin-5 OS=Homo sapiens GN=IPO5		294.95
O00410-3;O00410;O0041	3|IPO5_HUMAN Isoform 3 of Importin-5 OS=Homo sapiens GN=IPO5;>sp|O00410|IPO5_HUMAN Importin-5 OS=Homo sapiens GN=I	0	
O00422	SAP18_HUMAN Histone deacetylase complex subunit SAP18 OS=Homo sapiens GN=SAP18 PE=1 SV=1		168.81
O00423	EMAL1_HUMAN Echinoderm microtubule-associated protein-like 1 OS=Homo sapiens GN=EML1 PE=1 SV=3		30.01
O00423-3	EMAL1_HUMAN Isoform 3 of Echinoderm microtubule-associated protein-like 1 OS=Homo sapiens GN=EML1		30.01
O00425	IF2B3_HUMAN Insulin-like growth factor 2 mRNA-binding protein 3 OS=Homo sapiens GN=IGF2BP3 PE=1 SV=2		275.35
O00425-2	IF2B3_HUMAN Isoform 2 of Insulin-like growth factor 2 mRNA-binding protein 3 OS=Homo sapiens GN=IGF2BP3		154.35
O00425;O00425-2	IF2B3_HUMAN Insulin-like growth factor 2 mRNA-binding protein 3 OS=Homo sapiens GN=IGF2BP3 PE=1 SV=2;>sp|O00425-2|IF2B3	2.86E-82	
O00429-3;O00429-6;O00	3|DNM1L_HUMAN Isoform 2 of Dynamin-1-like protein OS=Homo sapiens GN=DNM1L;>sp|O00429-6|DNM1L_HUMAN Isoform 6 of Dy	4.59E-203	
O00429-6;O00429;O0042	6|DNM1L_HUMAN Isoform 6 of Dynamin-1-like protein OS=Homo sapiens GN=DNM1L;>sp|O00429|DNM1L_HUMAN Dynamin-1-like p	2.13E-278	
O00442-2;A8K8K1;O0044	2|RTC1_HUMAN Isoform 2 of RNA 3-terminal phosphate cyclase OS=Homo sapiens GN=RTCD1;>tr|A8K8K1|A8K8K1_HUMAN cDNA FL	7.56E-27	
O00443	P3C2A_HUMAN Phosphatidylinositol-4-phosphate 3-kinase C2 domain-containing subunit alpha OS=Homo sapiens GN=PIK3C2A PE=1 SV=2		
O00445	SYT5_HUMAN Synaptotagmin-5 OS=Homo sapiens GN=SYT5 PE=2 SV=2		2.98
O00458	IFRD1_HUMAN Interferon-related developmental regulator 1 OS=Homo sapiens GN=IFRD1 PE=1 SV=4		
O00459	P85B_HUMAN Phosphatidylinositol 3-kinase regulatory subunit beta OS=Homo sapiens GN=PIK3R2 PE=1 SV=2		32.98
O00461	GOLI4_HUMAN Golgi integral membrane protein 4 OS=Homo sapiens GN=GOLIM4 PE=1 SV=1	1.76E-07	
O00468	AGRIN_HUMAN Agrin OS=Homo sapiens GN=AGRN PE=1 SV=4		
O00469	PLOD2_HUMAN Procollagen-lysine,2-oxoglutarate 5-dioxygenase 2 OS=Homo sapiens GN=PLOD2 PE=1 SV=2		82.15
O00469-2	PLOD2_HUMAN Isoform 2 of Procollagen-lysine,2-oxoglutarate 5-dioxygenase 2 OS=Homo sapiens GN=PLOD2		82.15
O00469-2;O00469	2|PLOD2_HUMAN Isoform 2 of Procollagen-lysine,2-oxoglutarate 5-dioxygenase 2 OS=Homo sapiens GN=PLOD2;>sp|O00469|PLOD2_	2.88E-27	
O00471	EXOC5_HUMAN Exocyst complex component 5 OS=Homo sapiens GN=EXOC5 PE=1 SV=1		31.04
O00472	ELL2_HUMAN RNA polymerase II elongation factor ELL2 OS=Homo sapiens GN=ELL2 PE=1 SV=2		
O00476	NPT4_HUMAN Sodium-dependent phosphate transport protein 4 OS=Homo sapiens GN=SLC17A3 PE=2 SV=2		
O00483	NDUA4_HUMAN NADH dehydrogenase [ubiquinone] 1 alpha subcomplex subunit 4 OS=Homo sapiens GN=NDUFA4 PE=1 SV=1		150.07
O00487	PSDE_HUMAN 26S proteasome non-ATPase regulatory subunit 14 OS=Homo sapiens GN=PSMD14 PE=1 SV=1		264.82
O00488	ZN593_HUMAN Zinc finger protein 593 OS=Homo sapiens GN=ZNF593 PE=1 SV=2		
O00499;O00499-5;O0049	BIN1_HUMAN Myc box-dependent-interacting protein 1 OS=Homo sapiens GN=BIN1 PE=1 SV=1;>sp|O00499-5|BIN1_HUMAN Isoforn	1.25E-08	
O00505	IMA3_HUMAN Importin subunit alpha-3 OS=Homo sapiens GN=KPNA3 PE=1 SV=2		
O00506	STK25_HUMAN Serine/threonine-protein kinase 25 OS=Homo sapiens GN=STK25 PE=1 SV=1		57.95
O00507	USP9Y_HUMAN Probable ubiquitin carboxyl-terminal hydrolase FAF-Y OS=Homo sapiens GN=USP9Y PE=1 SV=2		
O00507-2	USP9Y_HUMAN Isoform Short of Probable ubiquitin carboxyl-terminal hydrolase FAF-Y OS=Homo sapiens GN=USP9Y		
O00507;O00507-2	USP9Y_HUMAN Probable ubiquitin carboxyl-terminal hydrolase FAF-Y OS=Homo sapiens GN=USP9Y PE=1 SV=2;>sp|O00507-2|USP9Y	2.14E-107	
O00515	LAD1_HUMAN Ladinin-1 OS=Homo sapiens GN=LAD1 PE=1 SV=2		
O00534;O00534-2;O0053	VMA5A_HUMAN von Willebrand factor A domain-containing protein 5A OS=Homo sapiens GN=VWA5A PE=2 SV=2;>sp|O00534-2|VMA	8.77E-18	
O00541	PESC_HUMAN Pescadillo homolog OS=Homo sapiens GN=PES1 PE=1 SV=1		89.56
O00541-2	PESC_HUMAN Isoform 2 of Pescadillo homolog OS=Homo sapiens GN=PES1		89.56
O00541;O00541-2	PESC_HUMAN Pescadillo homolog OS=Homo sapiens GN=PES1 PE=1 SV=1;>sp|O00541-2|PESC_HUMAN Isoform 2 of Pescadillo hom	7.10E-20	
O00560	SDCB1_HUMAN Syntenin-1 OS=Homo sapiens GN=SDCBP PE=1 SV=1		
O00560-2	SDCB1_HUMAN Isoform 2 of Syntenin-1 OS=Homo sapiens GN=SDCBP		
O00560-3	SDCB1_HUMAN Isoform 3 of Syntenin-1 OS=Homo sapiens GN=SDCBP		
O00560;O00560-2;O0056	SDCB1_HUMAN Syntenin-1 OS=Homo sapiens GN=SDCBP PE=1 SV=1;>sp|O00560-2|SDCB1_HUMAN Isoform 2 of Syntenin-1 OS=H	2.59E-06	
O00566	MPP10_HUMAN U3 small nucleolar ribonucleoprotein protein MPP10 OS=Homo sapiens GN=MPHOSPH10 PE=1 SV=2		
O00567	NOP56_HUMAN Nucleolar protein 56 OS=Homo sapiens GN=NOP56 PE=1 SV=4		438.61

O00571	DDX3X_HUMAN ATP-dependent RNA helicase DDX3X OS=Homo sapiens GN=DDX3X PE=1 SV=3		824.06
O00584	RNT2_HUMAN Ribonuclease T2 OS=Homo sapiens GN=RNASET2 PE=1 SV=2		31.82
O00592	PODXL_HUMAN Podocalyxin OS=Homo sapiens GN=PODXL PE=1 SV=2		216.6
O00592-2	PODXL_HUMAN Isoform 2 of Podocalyxin OS=Homo sapiens GN=PODXL		216.6
O00592;O00592-2	PODXL_HUMAN Podocalyxin OS=Homo sapiens GN=PODXL PE=1 SV=2;>sp|O00592-2|PODXL_HUMAN Isoform 2 of Podocalyxin OS=I	2.02E-47	
O00622	CYR61_HUMAN Protein CYR61 OS=Homo sapiens GN=CYR61 PE=1 SV=1		358.6
O00625	PIR_HUMAN Pirin OS=Homo sapiens GN=PIR PE=1 SV=1	7.72E-11	
O00629	IMA4_HUMAN Importin subunit alpha-4 OS=Homo sapiens GN=KPNA4 PE=1 SV=1		
O00712	NFIB_HUMAN Nuclear factor 1 B-type OS=Homo sapiens GN=NFIB PE=1 SV=2		
O00712-2	NFIB_HUMAN Isoform 3 of Nuclear factor 1 B-type OS=Homo sapiens GN=NFIB		
O00743-3;O00743;O0074	3|PPP6_HUMAN Isoform 3 of Serine/threonine-protein phosphatase 6 catalytic subunit OS=Homo sapiens GN=PPP6C;>sp|O00743|PPI	1.43E-24	
O00743;O00743-2;O0074	PPP6_HUMAN Serine/threonine-protein phosphatase 6 catalytic subunit OS=Homo sapiens GN=PPP6C PE=1 SV=1;>sp|O00743-2|PPP	5.88E-154	
O00746	NDKM_HUMAN Nucleoside diphosphate kinase, mitochondrial OS=Homo sapiens GN=NME4 PE=1 SV=1	4.70E-09	
O00754	MA2B1_HUMAN Lysosomal alpha-mannosidase OS=Homo sapiens GN=MAN2B1 PE=1 SV=3		182.74
O00757	F16P2_HUMAN Fructose-1,6-bisphosphatase isozyme 2 OS=Homo sapiens GN=FBP2 PE=1 SV=2	4.64E-20	
O00762	UBE2C_HUMAN Ubiquitin-conjugating enzyme E2 C OS=Homo sapiens GN=UBE2C PE=1 SV=1		45.89
O00763;O00763-2	ACACB_HUMAN Acetyl-CoA carboxylase 2 OS=Homo sapiens GN=ACACB PE=1 SV=3;>sp|O00763-2|ACACB_HUMAN Isoform Short of	1.59E-18	
O00764	PDXK_HUMAN Pyridoxal kinase OS=Homo sapiens GN=PDXK PE=1 SV=1		17.69
O00764-2	PDXK_HUMAN Isoform 2 of Pyridoxal kinase OS=Homo sapiens GN=PDXK		17.69
O00764-3	PDXK_HUMAN Isoform 3 of Pyridoxal kinase OS=Homo sapiens GN=PDXK		17.69
O00764;O00764-2;O0076	PDXK_HUMAN Pyridoxal kinase OS=Homo sapiens GN=PDXK PE=1 SV=1;>sp|O00764-2|PDXK_HUMAN Isoform 2 of Pyridoxal kinase	4.98E-215	
O00767	ACOD_HUMAN Acyl-CoA desaturase OS=Homo sapiens GN=SCD PE=1 SV=2		
O14493	CLD4_HUMAN Claudin-4 OS=Homo sapiens GN=CLDN4 PE=1 SV=1		110.55
O14497	ARI1A_HUMAN AT-rich interactive domain-containing protein 1A OS=Homo sapiens GN=ARID1A PE=1 SV=3		120.77
O14497-2	ARI1A_HUMAN Isoform 2 of AT-rich interactive domain-containing protein 1A OS=Homo sapiens GN=ARID1A		68.64
O14497-3	ARI1A_HUMAN Isoform 3 of AT-rich interactive domain-containing protein 1A OS=Homo sapiens GN=ARID1A		120.77
O14497;O14497-2;O1449	ARI1A_HUMAN AT-rich interactive domain-containing protein 1A OS=Homo sapiens GN=ARID1A PE=1 SV=3;>sp|O14497-2|ARI1A_HU	4.35E-05	
O14498	ISLR_HUMAN Immunoglobulin superfamily containing leucine-rich repeat protein OS=Homo sapiens GN=ISLR PE=1 SV=1	1.40E-16	
O14514	BAI1_HUMAN Brain-specific angiogenesis inhibitor 1 OS=Homo sapiens GN=BAI1 PE=1 SV=1	6.60E-06	
O14521	DHSD_HUMAN Succinate dehydrogenase [ubiquinone] cytochrome b small subunit, mitochondrial OS=Homo sapiens GN=SDHD PE=1 SV=1		
O14523	C2C2L_HUMAN C2 domain-containing protein 2-like OS=Homo sapiens GN=C2CD2L PE=1 SV=3		39.52
O14523-2	C2C2L_HUMAN Isoform 2 of C2 domain-containing protein 2-like OS=Homo sapiens GN=C2CD2L		39.52
O14524	T194A_HUMAN Transmembrane protein 194A OS=Homo sapiens GN=TMEM194A PE=1 SV=2		
O14524-2	T194A_HUMAN Isoform 2 of Transmembrane protein 194A OS=Homo sapiens GN=TMEM194A		
O14530	TXND9_HUMAN Thioredoxin domain-containing protein 9 OS=Homo sapiens GN=TXNDC9 PE=1 SV=2	1.90E-10	
O14543	SOCS3_HUMAN Suppressor of cytokine signaling 3 OS=Homo sapiens GN=SOCS3 PE=1 SV=1		19.31
O14548	COX7R_HUMAN Cytochrome c oxidase subunit 7A-related protein, mitochondrial OS=Homo sapiens GN=COX7A2L PE=1 SV=2		40.25
O14556	G3PT_HUMAN Glyceraldehyde-3-phosphate dehydrogenase, testis-specific OS=Homo sapiens GN=GAPDHS PE=1 SV=2		40.53
O14558	HSPB6_HUMAN Heat shock protein beta-6 OS=Homo sapiens GN=HSPB6 PE=1 SV=2	8.40E-45	
O14559	RHG33_HUMAN Rho GTPase-activating protein 33 OS=Homo sapiens GN=ARHGAP33 PE=1 SV=2		
O14559-10	RHG33_HUMAN Isoform 2 of Rho GTPase-activating protein 33 OS=Homo sapiens GN=ARHGAP33		
O14559-11	RHG33_HUMAN Isoform 3 of Rho GTPase-activating protein 33 OS=Homo sapiens GN=ARHGAP33		
O14561	ACPM_HUMAN Acyl carrier protein, mitochondrial OS=Homo sapiens GN=NDUFAB1 PE=1 SV=3		
O14562	UBFD1_HUMAN Ubiquitin domain-containing protein UBFD1 OS=Homo sapiens GN=UBFD1 PE=1 SV=2	4.49E-05	
O14576;O14576-2;O1457	DC1I1_HUMAN Cytoplasmic dynein 1 intermediate chain 1 OS=Homo sapiens GN=DYNC1I1 PE=1 SV=2;>sp|O14576-2|DC1I1_HUMAI	2.33E-08	
O14578	CTRO_HUMAN Citron Rho-interacting kinase OS=Homo sapiens GN=CIT PE=1 SV=2		
O14578-3	CTRO_HUMAN Isoform 3 of Citron Rho-interacting kinase OS=Homo sapiens GN=CIT		
O14578;O14578-3	CTRO_HUMAN Citron Rho-interacting kinase OS=Homo sapiens GN=CIT PE=1 SV=2;>sp|O14578-3|CTRO_HUMAN Isoform 3 of Citror	2.76E-17	
O14579	COPE_HUMAN Coatomer subunit epsilon OS=Homo sapiens GN=COPE PE=1 SV=3		325.08
O14582	TPPC2_HUMAN Trafficking protein particle complex subunit 2 OS=Homo sapiens GN=TRAPPC2 PE=1 SV=3		48.25
O14582-2	TPPC2_HUMAN Isoform 2 of Trafficking protein particle complex subunit 2 OS=Homo sapiens GN=TRAPPC2		48.25
O14602	IF1AY_HUMAN Eukaryotic translation initiation factor 1A, Y-chromosomal OS=Homo sapiens GN=EIF1AY PE=1 SV=4		152.07
O14617-5;O14617;O1461	5|AP3D1_HUMAN Isoform 5 of AP-3 complex subunit delta-1 OS=Homo sapiens GN=AP3D1;>sp|O14617|AP3D1_HUMAN AP-3 comple	6.54E-40	
O14618	CCS_HUMAN Copper chaperone for superoxide dismutase OS=Homo sapiens GN=CCS PE=1 SV=1	7.92E-09	
O14639	ABLM1_HUMAN Actin-binding LIM protein 1 OS=Homo sapiens GN=ABLIM1 PE=1 SV=3		
O14639-2	ABLM1_HUMAN Isoform 2 of Actin-binding LIM protein 1 OS=Homo sapiens GN=ABLIM1		
O14639-3	ABLM1_HUMAN Isoform 3 of Actin-binding LIM protein 1 OS=Homo sapiens GN=ABLIM1		
O14639-4	ABLM1_HUMAN Isoform 4 of Actin-binding LIM protein 1 OS=Homo sapiens GN=ABLIM1		
O14639-5	ABLM1_HUMAN Isoform 5 of Actin-binding LIM protein 1 OS=Homo sapiens GN=ABLIM1		
O14641	DVL2_HUMAN Segment polarity protein dishevelled homolog DVL-2 OS=Homo sapiens GN=DVL2 PE=1 SV=1	4.40E-55	
O14646	CHD1_HUMAN Chromodomain-helicase-DNA-binding protein 1 OS=Homo sapiens GN=CHD1 PE=1 SV=2		103.77

O14646-2	CHD1_HUMAN Isoform 2 of Chromodomain-helicase-DNA-binding protein 1 OS=Homo sapiens GN=CHD1		103.77
O14647	CHD2_HUMAN Chromodomain-helicase-DNA-binding protein 2 OS=Homo sapiens GN=CHD2 PE=1 SV=2		89.56
O14647-2	CHD2_HUMAN Isoform 2 of Chromodomain-helicase-DNA-binding protein 2 OS=Homo sapiens GN=CHD2		89.56
O14656	TOR1A_HUMAN Torsin-1A OS=Homo sapiens GN=TOR1A PE=1 SV=1		43.87
O14657	TOR1B_HUMAN Torsin-1B OS=Homo sapiens GN=TOR1B PE=1 SV=2		80.12
O14662	STX16_HUMAN Syntaxin-16 OS=Homo sapiens GN=STX16 PE=1 SV=3		43.6
O14662-2	STX16_HUMAN Isoform A of Syntaxin-16 OS=Homo sapiens GN=STX16		43.6
O14662-3	STX16_HUMAN Isoform C of Syntaxin-16 OS=Homo sapiens GN=STX16		43.6
O14662-4	STX16_HUMAN Isoform D of Syntaxin-16 OS=Homo sapiens GN=STX16		43.6
O14672	ADA10_HUMAN Disintegrin and metalloproteinase domain-containing protein 10 OS=Homo sapiens GN=ADAM10 PE=1 SV=1		92.7
O14678	ABCD4_HUMAN ATP-binding cassette sub-family D member 4 OS=Homo sapiens GN=ABCD4 PE=2 SV=1		
O14683	P5I11_HUMAN Tumor protein p53-inducible protein 11 OS=Homo sapiens GN=TP53I11 PE=2 SV=2	3.68E-25	
O14684	PTGES_HUMAN Prostaglandin E synthase OS=Homo sapiens GN=PTGES PE=1 SV=2	7.36E-37	
O14686	MLL2_HUMAN Histone-lysine N-methyltransferase MLL2 OS=Homo sapiens GN=MLL2 PE=1 SV=2		40.47
O14686-3	MLL2_HUMAN Isoform 3 of Histone-lysine N-methyltransferase MLL2 OS=Homo sapiens GN=MLL2		40.47
O14715	RGPD8_HUMAN RANBP2-like and GRIP domain-containing protein 8 OS=Homo sapiens GN=RGPD8 PE=1 SV=2		149.46
O14734	ACOT8_HUMAN Acyl-coenzyme A thioesterase 8 OS=Homo sapiens GN=ACOT8 PE=1 SV=1		52.13
O14735	CDIPT_HUMAN CDP-diacylglycerol--inositol 3-phosphatidyltransferase OS=Homo sapiens GN=CDIPT PE=1 SV=1		
O14737	PDCD5_HUMAN Programmed cell death protein 5 OS=Homo sapiens GN=PDCD5 PE=1 SV=3		348.52
O14744	ANM5_HUMAN Protein arginine N-methyltransferase 5 OS=Homo sapiens GN=PRMT5 PE=1 SV=4		70.74
O14745	NHRF1_HUMAN Na(+)/H(+) exchange regulatory cofactor NHE-RF1 OS=Homo sapiens GN=SLC9A3R1 PE=1 SV=4		153.77
O14757	CHK1_HUMAN Serine/threonine-protein kinase Chk1 OS=Homo sapiens GN=CHEK1 PE=1 SV=2		
O14763	TR10B_HUMAN Tumor necrosis factor receptor superfamily member 10B OS=Homo sapiens GN=TNFRSF10B PE=1 SV=2		173.05
O14763-2	TR10B_HUMAN Isoform Short of Tumor necrosis factor receptor superfamily member 10B OS=Homo sapiens GN=TNFRSF10B		173.05
O14772	FPGT_HUMAN Fucose-1-phosphate guanylyltransferase OS=Homo sapiens GN=FPGT PE=1 SV=2		
O14773	TPP1_HUMAN Tripeptidyl-peptidase 1 OS=Homo sapiens GN=TPP1 PE=1 SV=2		262.67
O14773-2	TPP1_HUMAN Isoform 2 of Tripeptidyl-peptidase 1 OS=Homo sapiens GN=TPP1		213.65
O14773;O14773-2	TPP1_HUMAN Tripeptidyl-peptidase 1 OS=Homo sapiens GN=TPP1 PE=1 SV=2;>sp|O14773-2|TPP1_HUMAN Isoform 2 of Tripeptidyl-p	6.60E-39	
O14776	TCRG1_HUMAN Transcription elongation regulator 1 OS=Homo sapiens GN=TCERG1 PE=1 SV=2		275.83
O14776-2	TCRG1_HUMAN Isoform 2 of Transcription elongation regulator 1 OS=Homo sapiens GN=TCERG1		275.83
O14776;O14776-2	TCRG1_HUMAN Transcription elongation regulator 1 OS=Homo sapiens GN=TCERG1 PE=1 SV=2;>sp|O14776-2|TCRG1_HUMAN Isofor	9.96E-42	
O14777	NDC80_HUMAN Kinetochore protein NDC80 homolog OS=Homo sapiens GN=NDC80 PE=1 SV=1	2.10E-52	
O14786	NRP1_HUMAN Neuropilin-1 OS=Homo sapiens GN=NRP1 PE=1 SV=3		200.46
O14786-2	NRP1_HUMAN Isoform 2 of Neuropilin-1 OS=Homo sapiens GN=NRP1		200.46
O14786;O14786-2	NRP1_HUMAN Neuropilin-1 OS=Homo sapiens GN=NRP1 PE=1 SV=3;>sp|O14786-2|NRP1_HUMAN Isoform 2 of Neuropilin-1 OS=Hon	7.00E-05	
O14787	TNPO2_HUMAN Transportin-2 OS=Homo sapiens GN=TNPO2 PE=1 SV=3		
O14787-2	TNPO2_HUMAN Isoform 2 of Transportin-2 OS=Homo sapiens GN=TNPO2		
O14787;O14787-2	TNPO2_HUMAN Transportin-2 OS=Homo sapiens GN=TNPO2 PE=1 SV=3;>sp|O14787-2|TNPO2_HUMAN Isoform 2 of Transportin-2 O	8.19E-45	
O14791-2;O14791	2|APOL1_HUMAN Isoform 2 of Apolipoprotein L1 OS=Homo sapiens GN=APOL1;>sp|O14791|APOL1_HUMAN Apolipoprotein L1 OS=Hc	1.32E-44	
O14795	UN13B_HUMAN Protein unc-13 homolog B OS=Homo sapiens GN=UNC13B PE=1 SV=2		23.13
O14802	RPC1_HUMAN DNA-directed RNA polymerase III subunit RPC1 OS=Homo sapiens GN=POLR3A PE=1 SV=2	1.41E-13	
O14813	PHX2A_HUMAN Paired mesoderm homeobox protein 2A OS=Homo sapiens GN=PHOX2A PE=1 SV=2		
O14818	PSA7_HUMAN Proteasome subunit alpha type-7 OS=Homo sapiens GN=PSMA7 PE=1 SV=1		647.73
O14818-2	PSA7_HUMAN Isoform 2 of Proteasome subunit alpha type-7 OS=Homo sapiens GN=PSMA7		443.57
O14818;O14818-2	PSA7_HUMAN Proteasome subunit alpha type-7 OS=Homo sapiens GN=PSMA7 PE=1 SV=1;>sp|O14818-2|PSA7_HUMAN Isoform 2 of	2.07E-290	
O14828	SCAM3_HUMAN Secretory carrier-associated membrane protein 3 OS=Homo sapiens GN=SCAMP3 PE=1 SV=3		447.04
O14828-2	SCAM3_HUMAN Isoform 2 of Secretory carrier-associated membrane protein 3 OS=Homo sapiens GN=SCAMP3		422.75
O14828;O14828-2	SCAM3_HUMAN Secretory carrier-associated membrane protein 3 OS=Homo sapiens GN=SCAMP3 PE=1 SV=3;>sp|O14828-2|SCAM3_	8.72E-190	
O14841	OPLA_HUMAN 5-oxoprolinase OS=Homo sapiens GN=OPLAH PE=1 SV=3	1.53E-97	
O14879	IFIT3_HUMAN Interferon-induced protein with tetratricopeptide repeats 3 OS=Homo sapiens GN=IFIT3 PE=1 SV=1	1.20E-125	
O14880	MGST3_HUMAN Microsomal glutathione S-transferase 3 OS=Homo sapiens GN=MGST3 PE=1 SV=1		201.24
O14893	GEMI2_HUMAN Survival of motor neuron protein-interacting protein 1 OS=Homo sapiens GN=SIP1 PE=1 SV=1		38.2
O14893-2	GEMI2_HUMAN Isoform 2 of Survival of motor neuron protein-interacting protein 1 OS=Homo sapiens GN=SIP1		38.2
O14893-3	GEMI2_HUMAN Isoform 3 of Survival of motor neuron protein-interacting protein 1 OS=Homo sapiens GN=SIP1		38.2
O14893;O14893-2;O1489	GEMI2_HUMAN Survival of motor neuron protein-interacting protein 1 OS=Homo sapiens GN=SIP1 PE=1 SV=1;>sp|O14893-2|GEMI2_	4.04E-08	
O14896	IRF6_HUMAN Interferon regulatory factor 6 OS=Homo sapiens GN=IRF6 PE=1 SV=1		
O14907	TX1B3_HUMAN Tax1-binding protein 3 OS=Homo sapiens GN=TAX1BP3 PE=1 SV=2	2.87E-30	
O14908	GIPC1_HUMAN PDZ domain-containing protein GIPC1 OS=Homo sapiens GN=GIPC1 PE=1 SV=2		216.54
O14910	LIN7A_HUMAN Protein lin-7 homolog A OS=Homo sapiens GN=LIN7A PE=1 SV=2		254.98
O14920;B4DZ30;B4E0U4;	IKKB_HUMAN Inhibitor of nuclear factor kappa-B kinase subunit beta OS=Homo sapiens GN=IKBKB PE=1 SV=1;>tr|B4DZ30|B4DZ30_	1.20E-06	
O14924	RGS12_HUMAN Regulator of G-protein signaling 12 OS=Homo sapiens GN=RGS12 PE=1 SV=1		31.75

O14924-2	RGS12_HUMAN Isoform 2 of Regulator of G-protein signaling 12 OS=Homo sapiens GN=RGS12		5.86
O14924-3	RGS12_HUMAN Isoform 3 of Regulator of G-protein signaling 12 OS=Homo sapiens GN=RGS12		5.86
O14924-4	RGS12_HUMAN Isoform 4 of Regulator of G-protein signaling 12 OS=Homo sapiens GN=RGS12		31.75
O14929	HAT1_HUMAN Histone acetyltransferase type B catalytic subunit OS=Homo sapiens GN=HAT1 PE=1 SV=1		119.34
O14929-2	HAT1_HUMAN Isoform B of Histone acetyltransferase type B catalytic subunit OS=Homo sapiens GN=HAT1		117.31
O14929;O14929-2	HAT1_HUMAN Histone acetyltransferase type B catalytic subunit OS=Homo sapiens GN=HAT1 PE=1 SV=1;>sp|O14929-2|HAT1_HUMA	8.56E-281	
O14933;O14933-2	UB2L6_HUMAN Ubiquitin/ISG15-conjugating enzyme E2 L6 OS=Homo sapiens GN=UBE2L6 PE=1 SV=4;>sp|O14933-2|UB2L6_HUMAN	4.82E-27	
O14936	CSKP_HUMAN Peripheral plasma membrane protein CASK OS=Homo sapiens GN=CASK PE=1 SV=3		162.88
O14936-2	CSKP_HUMAN Isoform 2 of Peripheral plasma membrane protein CASK OS=Homo sapiens GN=CASK		162.88
O14936-3	CSKP_HUMAN Isoform 3 of Peripheral plasma membrane protein CASK OS=Homo sapiens GN=CASK		162.88
O14936-4	CSKP_HUMAN Isoform 4 of Peripheral plasma membrane protein CASK OS=Homo sapiens GN=CASK		162.88
O14936-5	CSKP_HUMAN Isoform 5 of Peripheral plasma membrane protein CASK OS=Homo sapiens GN=CASK		68.45
O14936-6	CSKP_HUMAN Isoform 6 of Peripheral plasma membrane protein CASK OS=Homo sapiens GN=CASK		162.88
O14936;O14936-2;O1493	CSKP_HUMAN Peripheral plasma membrane protein CASK OS=Homo sapiens GN=CASK PE=1 SV=3;>sp|O14936-2|CSKP_HUMAN Iso	4.27E-07	
O14949	QCR8_HUMAN Cytochrome b-c1 complex subunit 8 OS=Homo sapiens GN=UQCRQ PE=1 SV=4		66.43
O14950	ML12B_HUMAN Myosin regulatory light chain 12B OS=Homo sapiens GN=MYL12B PE=1 SV=2		1326.17
O14964;O14964-2	HGS_HUMAN Hepatocyte growth factor-regulated tyrosine kinase substrate OS=Homo sapiens GN=HGS PE=1 SV=1;>sp|O14964-2|H	2.24E-135	
O14965	STK6_HUMAN Serine/threonine-protein kinase 6 OS=Homo sapiens GN=AURKA PE=1 SV=2		
O14966	RAB7L_HUMAN Ras-related protein Rab-7L1 OS=Homo sapiens GN=RAB7L1 PE=1 SV=1	7.71E-07	
O14967	CLGN_HUMAN Calmegin OS=Homo sapiens GN=CLGN PE=1 SV=1		24.03
O14972	DSCR3_HUMAN Down syndrome critical region protein 3 OS=Homo sapiens GN=DSCR3 PE=1 SV=1		27.74
O14974	MYPT1_HUMAN Protein phosphatase 1 regulatory subunit 12A OS=Homo sapiens GN=PPP1R12A PE=1 SV=1		269.07
O14974-2	MYPT1_HUMAN Isoform 2 of Protein phosphatase 1 regulatory subunit 12A OS=Homo sapiens GN=PPP1R12A		197.71
O14974-3	MYPT1_HUMAN Isoform 3 of Protein phosphatase 1 regulatory subunit 12A OS=Homo sapiens GN=PPP1R12A		237.85
O14974-4	MYPT1_HUMAN Isoform 4 of Protein phosphatase 1 regulatory subunit 12A OS=Homo sapiens GN=PPP1R12A		237.85
O14974;O14974-2;O1497	MYPT1_HUMAN Protein phosphatase 1 regulatory subunit 12A OS=Homo sapiens GN=PPP1R12A PE=1 SV=1;>sp|O14974-2|MYPT1_H	7.28E-32	
O14975	S27A2_HUMAN Very long-chain acyl-CoA synthetase OS=Homo sapiens GN=SLC27A2 PE=1 SV=2		
O14976	GAK_HUMAN Cyclin-G-associated kinase OS=Homo sapiens GN=GAK PE=1 SV=2		27.35
O14978	ZN263_HUMAN Zinc finger protein 263 OS=Homo sapiens GN=ZNF263 PE=2 SV=2		
O14979	HNRDL_HUMAN Heterogeneous nuclear ribonucleoprotein D-like OS=Homo sapiens GN=HNRPDL PE=1 SV=3		266.39
O14979-2	HNRDL_HUMAN Isoform 2 of Heterogeneous nuclear ribonucleoprotein D-like OS=Homo sapiens GN=HNRPDL		246.71
O14979-3	HNRDL_HUMAN Isoform 3 of Heterogeneous nuclear ribonucleoprotein D-like OS=Homo sapiens GN=HNRPDL		246.71
O14979;O14979-2;O1497	HNRDL_HUMAN Heterogeneous nuclear ribonucleoprotein D-like OS=Homo sapiens GN=HNRPDL PE=1 SV=3;>sp|O14979-2|HNRDL_H	4.16E-169	
O14980	XPO1_HUMAN Exportin-1 OS=Homo sapiens GN=XPO1 PE=1 SV=1		489.57
O14981	BTAF1_HUMAN TATA-binding protein-associated factor 172 OS=Homo sapiens GN=BTAF1 PE=1 SV=2	2.46E-17	
O14983	AT2A1_HUMAN Sarcoplasmic/endoplasmic reticulum calcium ATPase 1 OS=Homo sapiens GN=ATP2A1 PE=1 SV=1		541.87
O14983-2	AT2A1_HUMAN Isoform SERCA1A of Sarcoplasmic/endoplasmic reticulum calcium ATPase 1 OS=Homo sapiens GN=ATP2A1		541.87
O14986	PI51B_HUMAN Phosphatidylinositol-4-phosphate 5-kinase type-1 beta OS=Homo sapiens GN=PIP5K1B PE=1 SV=2		
O14986-2	PI51B_HUMAN Isoform 2 of Phosphatidylinositol-4-phosphate 5-kinase type-1 beta OS=Homo sapiens GN=PIP5K1B		
O14994	SYN3_HUMAN Synapsin-3 OS=Homo sapiens GN=SYN3 PE=1 SV=2		
O15020	SPTN2_HUMAN Spectrin beta chain, brain 2 OS=Homo sapiens GN=SPTBN2 PE=1 SV=3		289.66
O15020-2	SPTN2_HUMAN Isoform 2 of Spectrin beta chain, brain 2 OS=Homo sapiens GN=SPTBN2		289.66
O15020;O15020-2	SPTN2_HUMAN Spectrin beta chain, brain 2 OS=Homo sapiens GN=SPTBN2 PE=1 SV=3;>sp|O15020-2|SPTN2_HUMAN Isoform 2 of S	2.88E-89	
O15021;B5MCH9;O15021	MAST4_HUMAN Microtubule-associated serine/threonine-protein kinase 4 OS=Homo sapiens GN=MAST4 PE=1 SV=3;>tr|B5MCH9|B5M	1.98E-07	
O15027	SC16A_HUMAN Protein transport protein Sec16A OS=Homo sapiens GN=SEC16A PE=1 SV=3		30.87
O15027-2	SC16A_HUMAN Isoform 2 of Protein transport protein Sec16A OS=Homo sapiens GN=SEC16A		30.87
O15027-3	SC16A_HUMAN Isoform 3 of Protein transport protein Sec16A OS=Homo sapiens GN=SEC16A		30.87
O15027-4	SC16A_HUMAN Isoform 4 of Protein transport protein Sec16A OS=Homo sapiens GN=SEC16A		30.87
O15027-5	SC16A_HUMAN Isoform 5 of Protein transport protein Sec16A OS=Homo sapiens GN=SEC16A		30.87
O15027-5;O15027;O1502	5|SC16A_HUMAN Isoform 5 of Protein transport protein Sec16A OS=Homo sapiens GN=SEC16A;>sp|O15027|SC16A_HUMAN Protein	2.70E-49	
O15031	PLXB2_HUMAN Plexin-B2 OS=Homo sapiens GN=PLXNB2 PE=1 SV=3		528.49
O15037	KHNYN_HUMAN Protein KHNYN OS=Homo sapiens GN=KHNYN PE=1 SV=3		
O15042	SR140_HUMAN U2 snRNP-associated SURP motif-containing protein OS=Homo sapiens GN=U2SURP PE=1 SV=2		192.53
O15042-2	SR140_HUMAN Isoform 2 of U2 snRNP-associated SURP motif-containing protein OS=Homo sapiens GN=U2SURP		192.53
O15042-3	SR140_HUMAN Isoform 3 of U2 snRNP-associated SURP motif-containing protein OS=Homo sapiens GN=U2SURP		146.84
O15042;O15042-2;O1504	SR140_HUMAN U2 snRNP-associated SURP motif-containing protein OS=Homo sapiens GN=U2SURP PE=1 SV=2;>sp|O15042-2|SR14	3.58E-72	
O15042;O15042-2;O1504	SR140_HUMAN U2-associated protein SR140 OS=Homo sapiens GN=SR140 PE=1 SV=2;>sp|O15042-2|SR140_HUMAN Isoform 2 of U	2.57E-50	
O15056	SYNJ2_HUMAN Synaptojanin-2 OS=Homo sapiens GN=SYNJ2 PE=1 SV=3		33.24
O15056-2	SYNJ2_HUMAN Isoform 2B1 of Synaptojanin-2 OS=Homo sapiens GN=SYNJ2		33.24
O15056-3	SYNJ2_HUMAN Isoform 2A of Synaptojanin-2 OS=Homo sapiens GN=SYNJ2		5.54
O15061	SYNEM_HUMAN Synemin OS=Homo sapiens GN=SYNM PE=1 SV=2		

O15061-2	SYNEM_HUMAN Isoform 2 of Synemin OS=Homo sapiens GN=SYNM		
O15061-3	SYNEM_HUMAN Isoform 3 of Synemin OS=Homo sapiens GN=SYNM		
O15061;O15061-2;C9JIE4	SYNEM_HUMAN Synemin OS=Homo sapiens GN=SYNM PE=1 SV=2;>sp|O15061-2|SYNEM_HUMAN Isoform 2 of Synemin OS=Homo s	1.23E-06	
O15066	KIF3B_HUMAN Kinesin-like protein KIF3B OS=Homo sapiens GN=KIF3B PE=1 SV=1	1.22E-13	
O15067	PUR4_HUMAN Phosphoribosylformylglycinamidine synthase OS=Homo sapiens GN=PFAS PE=1 SV=4	7.02E-102	
O15069	NACAD_HUMAN NAC-alpha domain-containing protein 1 OS=Homo sapiens GN=NACAD PE=2 SV=3		92.85
O15075	DCLK1_HUMAN Serine/threonine-protein kinase DCLK1 OS=Homo sapiens GN=DCLK1 PE=1 SV=2		
O15075-2	DCLK1_HUMAN Isoform 1 of Serine/threonine-protein kinase DCLK1 OS=Homo sapiens GN=DCLK1		
O15075-3	DCLK1_HUMAN Isoform 3 of Serine/threonine-protein kinase DCLK1 OS=Homo sapiens GN=DCLK1		
O15075-4	DCLK1_HUMAN Isoform 4 of Serine/threonine-protein kinase DCLK1 OS=Homo sapiens GN=DCLK1		
O15075;O15075-2	DCLK1_HUMAN Serine/threonine-protein kinase DCLK1 OS=Homo sapiens GN=DCLK1 PE=1 SV=2;>sp|O15075-2|DCLK1_HUMAN Iso	4.37E-14	
O15078;O15078-2	CE290_HUMAN Centrosomal protein of 290 kDa OS=Homo sapiens GN=CEP290 PE=1 SV=2;>sp|O15078-2|CE290_HUMAN Isoform 2	1.42E-11	
O15083	ERC2_HUMAN ERC protein 2 OS=Homo sapiens GN=ERC2 PE=1 SV=3		
O15084-1;O15084-4;O15	1|ANR28_HUMAN Isoform 3 of Serine/threonine-protein phosphatase 6 regulatory ankyrin repeat subunit A OS=Homo sapiens GN=AN	1.38E-10	
O15084;B4DES5;O15084-	ANR28_HUMAN Serine/threonine-protein phosphatase 6 regulatory ankyrin repeat subunit A OS=Homo sapiens GN=ANKRD28 PE=1 S	1.13E-13	
O15085	ARHGB_HUMAN Rho guanine nucleotide exchange factor 11 OS=Homo sapiens GN=ARHGEF11 PE=1 SV=1		
O15091	MRRP3_HUMAN Mitochondrial ribonuclease P protein 3 OS=Homo sapiens GN=KIAA0391 PE=1 SV=2		
O15091-2	MRRP3_HUMAN Isoform 2 of Mitochondrial ribonuclease P protein 3 OS=Homo sapiens GN=KIAA0391		
O15091-4	MRRP3_HUMAN Isoform 4 of Mitochondrial ribonuclease P protein 3 OS=Homo sapiens GN=KIAA0391		
O15091;O15091-2;O1509	MRRP3_HUMAN Mitochondrial ribonuclease P protein 3 OS=Homo sapiens GN=KIAA0391 PE=1 SV=2;>sp|O15091-2|MRRP3_HUMAN I	3.09E-06	
O15118	NPC1_HUMAN Niemann-Pick C1 protein OS=Homo sapiens GN=NPC1 PE=1 SV=2		163.7
O15120	PLCB_HUMAN 1-acyl-sn-glycerol-3-phosphate acyltransferase beta OS=Homo sapiens GN=AGPAT2 PE=1 SV=1		37.92
O15120-2	PLCB_HUMAN Isoform 2 of 1-acyl-sn-glycerol-3-phosphate acyltransferase beta OS=Homo sapiens GN=AGPAT2		37.92
O15126	SCAM1_HUMAN Secretory carrier-associated membrane protein 1 OS=Homo sapiens GN=SCAMP1 PE=1 SV=2		261.71
O15126-2	SCAM1_HUMAN Isoform 2 of Secretory carrier-associated membrane protein 1 OS=Homo sapiens GN=SCAMP1		94.55
O15126;O15126-2	SCAM1_HUMAN Secretory carrier-associated membrane protein 1 OS=Homo sapiens GN=SCAMP1 PE=1 SV=2;>sp|O15126-2|SCAM1_	3.18E-43	
O15127	SCAM2_HUMAN Secretory carrier-associated membrane protein 2 OS=Homo sapiens GN=SCAMP2 PE=1 SV=2		77.45
O15131	IMA5_HUMAN Importin subunit alpha-6 OS=Homo sapiens GN=KPNA5 PE=1 SV=2		
O15143	ARC1B_HUMAN Actin-related protein 2/3 complex subunit 1B OS=Homo sapiens GN=ARPC1B PE=1 SV=3		258.22
O15144	ARPC2_HUMAN Actin-related protein 2/3 complex subunit 2 OS=Homo sapiens GN=ARPC2 PE=1 SV=1		389.77
O15145	ARPC3_HUMAN Actin-related protein 2/3 complex subunit 3 OS=Homo sapiens GN=ARPC3 PE=1 SV=3		426.5
O15155	BET1_HUMAN BET1 homolog OS=Homo sapiens GN=BET1 PE=1 SV=1		111.72
O15160	RPAC1_HUMAN DNA-directed RNA polymerases I and III subunit RPAC1 OS=Homo sapiens GN=POLR1C PE=1 SV=1		123.14
O15160-2	RPAC1_HUMAN Isoform 2 of DNA-directed RNA polymerases I and III subunit RPAC1 OS=Homo sapiens GN=POLR1C		124.12
O15160;O15160-2	RPAC1_HUMAN DNA-directed RNA polymerases I and III subunit RPAC1 OS=Homo sapiens GN=POLR1C PE=1 SV=1;>sp|O15160-2|R	2.17E-13	
O15162	PLS1_HUMAN Phospholipid scramblase 1 OS=Homo sapiens GN=PLSCR1 PE=1 SV=1	4.66E-27	
O15173	PGRC2_HUMAN Membrane-associated progesterone receptor component 2 OS=Homo sapiens GN=PGRMC2 PE=1 SV=1		363.61
O15182	CETN3_HUMAN Centrin-3 OS=Homo sapiens GN=CETN3 PE=2 SV=2		
O15205	UBD_HUMAN Ubiquitin D OS=Homo sapiens GN=UBD PE=1 SV=2		
O15212	PFD6_HUMAN Prefoldin subunit 6 OS=Homo sapiens GN=PFDN6 PE=1 SV=1	2.46E-11	
O15226	NKRF_HUMAN NF-kappa-B-repressing factor OS=Homo sapiens GN=NKRF PE=1 SV=2		
O15230	LAMA5_HUMAN Laminin subunit alpha-5 OS=Homo sapiens GN=LAMA5 PE=1 SV=8		82.38
O15231	ZN185_HUMAN Zinc finger protein 185 OS=Homo sapiens GN=ZNF185 PE=1 SV=3		199.38
O15231-2	ZN185_HUMAN Isoform 2 of Zinc finger protein 185 OS=Homo sapiens GN=ZNF185		154.13
O15231-3	ZN185_HUMAN Isoform 3 of Zinc finger protein 185 OS=Homo sapiens GN=ZNF185		199.38
O15231-3;O15231	3|ZN185_HUMAN Isoform 3 of Zinc finger protein 185 OS=Homo sapiens GN=ZNF185;>sp|O15231|ZN185_HUMAN Zinc finger protei	1.54E-18	
O15235	RT12_HUMAN 28S ribosomal protein S12, mitochondrial OS=Homo sapiens GN=MRPS12 PE=1 SV=1	4.24E-07	
O15240	VGF_HUMAN Neurosecretory protein VGF OS=Homo sapiens GN=VGF PE=1 SV=2		
O15245	S22A1_HUMAN Solute carrier family 22 member 1 OS=Homo sapiens GN=SLC22A1 PE=1 SV=2		
O15247	CLIC2_HUMAN Chloride intracellular channel protein 2 OS=Homo sapiens GN=CLIC2 PE=1 SV=3		57.09
O15254;O15254-2	ACOX3_HUMAN Peroxisomal acyl-coenzyme A oxidase 3 OS=Homo sapiens GN=ACOX3 PE=1 SV=2;>sp|O15254-2|ACOX3_HUMAN Is	1.77E-05	
O15258	RER1_HUMAN Protein RER1 OS=Homo sapiens GN=RER1 PE=1 SV=1		150.01
O15259	NPHP1_HUMAN Nephrocystin-1 OS=Homo sapiens GN=NPHP1 PE=1 SV=1		
O15259-2	NPHP1_HUMAN Isoform 2 of Nephrocystin-1 OS=Homo sapiens GN=NPHP1		
O15259-3	NPHP1_HUMAN Isoform 3 of Nephrocystin-1 OS=Homo sapiens GN=NPHP1		
O15259-4	NPHP1_HUMAN Isoform 4 of Nephrocystin-1 OS=Homo sapiens GN=NPHP1		
O15260	SURF4_HUMAN Surfeit locus protein 4 OS=Homo sapiens GN=SURF4 PE=1 SV=3		371.1
O15260-2	SURF4_HUMAN Isoform 2 of Surfeit locus protein 4 OS=Homo sapiens GN=SURF4		371.1
O15260;B7Z7A8;B7Z8F3;	SURF4_HUMAN Surfeit locus protein 4 OS=Homo sapiens GN=SURF4 PE=1 SV=3;>tr|B7Z7A8|B7Z7A8_HUMAN cDNA FLJ50722, highl	1.57E-09	
O15264	MK13_HUMAN Mitogen-activated protein kinase 13 OS=Homo sapiens GN=MAPK13 PE=1 SV=1		50.95
O15269	SPTC1_HUMAN Serine palmitoyltransferase 1 OS=Homo sapiens GN=SPTLC1 PE=1 SV=1	2.86E-42	

O15270	SPTC2_HUMAN Serine palmitoyltransferase 2 OS=Homo sapiens GN=SPTLC2 PE=1 SV=1	1.71E-31	
O15294	OGT1_HUMAN UDP-N-acetylglucosamine--peptide N-acetylglucosaminyltransferase 110 kDa subunit OS=Homo sapiens GN=OGT PE=1 SV=3		
O15294-2	OGT1_HUMAN Isoform 2 of UDP-N-acetylglucosamine--peptide N-acetylglucosaminyltransferase 110 kDa subunit OS=Homo sapiens GN=OGT		
O15294-3	OGT1_HUMAN Isoform 1 of UDP-N-acetylglucosamine--peptide N-acetylglucosaminyltransferase 110 kDa subunit OS=Homo sapiens GN=OGT		
O15294-4	OGT1_HUMAN Isoform 4 of UDP-N-acetylglucosamine--peptide N-acetylglucosaminyltransferase 110 kDa subunit OS=Homo sapiens GN=OGT		
O15294;O15294-3;O1529	OGT1_HUMAN UDP-N-acetylglucosamine--peptide N-acetylglucosaminyltransferase 110 kDa subunit OS=Homo sapiens GN=OGT PE=1	8.63E-31	
O15296;O15296-4;O1529	LX15B_HUMAN Arachidonate 15-lipoxygenase B OS=Homo sapiens GN=ALOX15B PE=2 SV=3;>sp|O15296-4|LX15B_HUMAN Isoform	1.08E-16	
O15305	PMM2_HUMAN Phosphomannomutase 2 OS=Homo sapiens GN=PMM2 PE=1 SV=1	1.80E-24	
O15321	TM9S1_HUMAN Transmembrane 9 superfamily member 1 OS=Homo sapiens GN=TM9SF1 PE=2 SV=2		
O15327	INP4B_HUMAN Type II inositol-3,4-bisphosphate 4-phosphatase OS=Homo sapiens GN=INPP4B PE=2 SV=4	1.65E-34	
O15344;O15344-2	TRI18_HUMAN Midline-1 OS=Homo sapiens GN=MID1 PE=1 SV=1;>sp|O15344-2|TRI18_HUMAN Isoform 2 of Midline-1 OS=Homo sa	1.84E-11	
O15347	HMGB3_HUMAN High mobility group protein B3 OS=Homo sapiens GN=HMGB3 PE=1 SV=4		313.54
O15355	PPM1G_HUMAN Protein phosphatase 1G OS=Homo sapiens GN=PPM1G PE=1 SV=1		292.57
O15357	SHIP2_HUMAN Phosphatidylinositol-3,4,5-trisphosphate 5-phosphatase 2 OS=Homo sapiens GN=INPPL1 PE=1 SV=2		
O15357-2	SHIP2_HUMAN Isoform 2 of Phosphatidylinositol-3,4,5-trisphosphate 5-phosphatase 2 OS=Homo sapiens GN=INPPL1		
O15357;O15357-2	SHIP2_HUMAN Phosphatidylinositol-3,4,5-trisphosphate 5-phosphatase 2 OS=Homo sapiens GN=INPPL1 PE=1 SV=2;>sp|O15357-2|S	3.82E-14	
O15371	EIF3D_HUMAN Eukaryotic translation initiation factor 3 subunit D OS=Homo sapiens GN=EIF3D PE=1 SV=1		163.53
O15372	EIF3H_HUMAN Eukaryotic translation initiation factor 3 subunit H OS=Homo sapiens GN=EIF3H PE=1 SV=1		560.26
O15379	HDAC3_HUMAN Histone deacetylase 3 OS=Homo sapiens GN=HDAC3 PE=1 SV=2		
O15379-2	HDAC3_HUMAN Isoform 2 of Histone deacetylase 3 OS=Homo sapiens GN=HDAC3		
O15381	NVL_HUMAN Nuclear valosin-containing protein-like OS=Homo sapiens GN=NVL PE=1 SV=1		
O15381-2	NVL_HUMAN Isoform 2 of Nuclear valosin-containing protein-like OS=Homo sapiens GN=NVL		
O15381-3	NVL_HUMAN Isoform 3 of Nuclear valosin-containing protein-like OS=Homo sapiens GN=NVL		
O15381;O15381-2;O1538	NVL_HUMAN Nuclear valosin-containing protein-like OS=Homo sapiens GN=NVL PE=1 SV=1;>sp|O15381-2|NVL_HUMAN Isoform 2 of	3.00E-27	
O15382	BCAT2_HUMAN Branched-chain-amino-acid aminotransferase, mitochondrial OS=Homo sapiens GN=BCAT2 PE=1 SV=2		141.48
O15382-2	BCAT2_HUMAN Isoform B of Branched-chain-amino-acid aminotransferase, mitochondrial OS=Homo sapiens GN=BCAT2		109.74
O15382;O15382-2	BCAT2_HUMAN Branched-chain-amino-acid aminotransferase, mitochondrial OS=Homo sapiens GN=BCAT2 PE=1 SV=2;>sp|O15382-2	1.62E-08	
O15391	TYY2_HUMAN Transcription factor YY2 OS=Homo sapiens GN=YY2 PE=2 SV=1		
O15397	IPO8_HUMAN Importin-8 OS=Homo sapiens GN=IPO8 PE=1 SV=2	3.94E-12	
O15399	NMDE4_HUMAN Glutamate [NMDA] receptor subunit epsilon-4 OS=Homo sapiens GN=GRIN2D PE=1 SV=2		31.96
O15400	STX7_HUMAN Syntaxin-7 OS=Homo sapiens GN=STX7 PE=1 SV=4		383.92
O15400-2	STX7_HUMAN Isoform 2 of Syntaxin-7 OS=Homo sapiens GN=STX7		383.92
O15400;O15400-2	STX7_HUMAN Syntaxin-7 OS=Homo sapiens GN=STX7 PE=1 SV=4;>sp|O15400-2|STX7_HUMAN Isoform 2 of Syntaxin-7 OS=Homo s	7.60E-11	
O15417	TNC18_HUMAN Trinucleotide repeat-containing gene 18 protein OS=Homo sapiens GN=TNRC18 PE=1 SV=3		
O15417-2	TNC18_HUMAN Isoform 2 of Trinucleotide repeat-containing gene 18 protein OS=Homo sapiens GN=TNRC18		
O15427	MOT4_HUMAN Monocarboxylate transporter 4 OS=Homo sapiens GN=SLC16A3 PE=1 SV=1		341.21
O15439	MRP4_HUMAN Multidrug resistance-associated protein 4 OS=Homo sapiens GN=ABCC4 PE=1 SV=3		93.54
O15439-2	MRP4_HUMAN Isoform 2 of Multidrug resistance-associated protein 4 OS=Homo sapiens GN=ABCC4		93.54
O15440	MRP5_HUMAN Multidrug resistance-associated protein 5 OS=Homo sapiens GN=ABCC5 PE=1 SV=2		
O15446-2;A8K818;O1544	2|RPA34_HUMAN Isoform 2 of DNA-directed RNA polymerase I subunit RPA34 OS=Homo sapiens GN=CD3EAP;>tr|A8K818|A8K818_H	3.39E-10	
O15455	TLR3_HUMAN Toll-like receptor 3 OS=Homo sapiens GN=TLR3 PE=1 SV=1		
O15460	P4HA2_HUMAN Prolyl 4-hydroxylase subunit alpha-2 OS=Homo sapiens GN=P4HA2 PE=1 SV=1		25.63
O15460-2	P4HA2_HUMAN Isoform IIa of Prolyl 4-hydroxylase subunit alpha-2 OS=Homo sapiens GN=P4HA2		25.63
O15460;O15460-2	P4HA2_HUMAN Prolyl 4-hydroxylase subunit alpha-2 OS=Homo sapiens GN=P4HA2 PE=1 SV=1;>sp|O15460-2|P4HA2_HUMAN Isofor	1.92E-13	
O15479	MAGB2_HUMAN Melanoma-associated antigen B2 OS=Homo sapiens GN=MAGEB2 PE=1 SV=3	8.48E-19	
O15480	MAGB3_HUMAN Melanoma-associated antigen B3 OS=Homo sapiens GN=MAGEB3 PE=2 SV=2		26.3
O15484	CAN5_HUMAN Calpain-5 OS=Homo sapiens GN=CAPN5 PE=1 SV=2		
O15498	YKT6_HUMAN Synaptobrevin homolog YKT6 OS=Homo sapiens GN=YKT6 PE=1 SV=1		18.58
O15499	GSC2_HUMAN Homeobox protein goosecoid-2 OS=Homo sapiens GN=GSC2 PE=2 SV=1		
O15504	NUPL2_HUMAN Nucleoporin-like protein 2 OS=Homo sapiens GN=NUPL2 PE=1 SV=1		
O15504-3	NUPL2_HUMAN Isoform 3 of Nucleoporin-like protein 2 OS=Homo sapiens GN=NUPL2		
O15511	ARPC5_HUMAN Actin-related protein 2/3 complex subunit 5 OS=Homo sapiens GN=ARPC5 PE=1 SV=3		136.11
O15511-2	ARPC5_HUMAN Isoform 2 of Actin-related protein 2/3 complex subunit 5 OS=Homo sapiens GN=ARPC5		91.52
O15511;B1ALC0;O15511-	ARPC5_HUMAN Actin-related protein 2/3 complex subunit 5 OS=Homo sapiens GN=ARPC5 PE=1 SV=3;>tr|B1ALC0|B1ALC0_HUMAN A	3.93E-18	
O15514	RPB4_HUMAN DNA-directed RNA polymerase II subunit RPB4 OS=Homo sapiens GN=POLR2D PE=1 SV=1		62.23
O15523	DDX3Y_HUMAN ATP-dependent RNA helicase DDX3Y OS=Homo sapiens GN=DDX3Y PE=1 SV=2		501.99
O15525	MAFG_HUMAN Transcription factor MafG OS=Homo sapiens GN=MAFG PE=1 SV=1		
O15530	PDPK1_HUMAN 3-phosphoinositide-dependent protein kinase 1 OS=Homo sapiens GN=PDPK1 PE=1 SV=1		
O15530-2	PDPK1_HUMAN Isoform 2 of 3-phosphoinositide-dependent protein kinase 1 OS=Homo sapiens GN=PDPK1		
O15530-3	PDPK1_HUMAN Isoform 3 of 3-phosphoinositide-dependent protein kinase 1 OS=Homo sapiens GN=PDPK1		
O15533	TPSN_HUMAN Tapasin OS=Homo sapiens GN=TAPBP PE=1 SV=1		26.6

O15533-2	TPSN_HUMAN Isoform 2 of Tapasin OS=Homo sapiens GN=TAPBP		26.6
O15533-3	TPSN_HUMAN Isoform 3 of Tapasin OS=Homo sapiens GN=TAPBP		26.6
O15533-3;A2AB90;A2ABB	3|TPSN_HUMAN Isoform 3 of Tapasin OS=Homo sapiens GN=TAPBP;>tr|A2AB90|A2AB90_HUMAN TAP binding protein (Tapasin) OS=H	4.85E-05	
O15540	FABP7_HUMAN Fatty acid-binding protein, brain OS=Homo sapiens GN=FABP7 PE=1 SV=3	9.80E-194	
O15541	R113A_HUMAN RING finger protein 113A OS=Homo sapiens GN=RNF113A PE=1 SV=1	1.77E-10	
O15551	CLD3_HUMAN Claudin-3 OS=Homo sapiens GN=CLDN3 PE=1 SV=1		79.62
O43143	DHX15_HUMAN Putative pre-splicing factor ATP-dependent RNA helicase DHX15 OS=Homo sapiens GN=DHX15 PE=1 SV=2		609.4
O43143;B4E0S6	DHX15_HUMAN Putative pre-mRNA-splicing factor ATP-dependent RNA helicase DHX15 OS=Homo sapiens GN=DHX15 PE=1 SV=2;>tr	1.27E-193	
O43148	MCES_HUMAN mRNA cap guanine-N7 methyltransferase OS=Homo sapiens GN=RNMT PE=1 SV=1		66.87
O43148-2	MCES_HUMAN Isoform 2 of mRNA cap guanine-N7 methyltransferase OS=Homo sapiens GN=RNMT		66.87
O43148-2;O43148	2|MCES_HUMAN Isoform 2 of mRNA cap guanine-N7 methyltransferase OS=Homo sapiens GN=RNMT;>sp|O43148|MCES_HUMAN mR	6.79E-13	
O43149	ZZEF1_HUMAN Zinc finger ZZ-type and EF-hand domain-containing protein 1 OS=Homo sapiens GN=ZZEF1 PE=1 SV=6		49.33
O43149-2	ZZEF1_HUMAN Isoform 2 of Zinc finger ZZ-type and EF-hand domain-containing protein 1 OS=Homo sapiens GN=ZZEF1		20.6
O43149-3	ZZEF1_HUMAN Isoform 3 of Zinc finger ZZ-type and EF-hand domain-containing protein 1 OS=Homo sapiens GN=ZZEF1		2.94
O43150	ASAP2_HUMAN Arf-GAP with SH3 domain, ANK repeat and PH domain-containing protein 2 OS=Homo sapiens GN=ASAP2 PE=1 SV=3		40.04
O43150-2	ASAP2_HUMAN Isoform 2 of Arf-GAP with SH3 domain, ANK repeat and PH domain-containing protein 2 OS=Homo sapiens GN=ASAP2		40.04
O43151	TET3_HUMAN Methylcytosine dioxygenase TET3 OS=Homo sapiens GN=TET3 PE=2 SV=3		
O43151-2	TET3_HUMAN Isoform 2 of Methylcytosine dioxygenase TET3 OS=Homo sapiens GN=TET3		
O43151-3	TET3_HUMAN Isoform 3 of Methylcytosine dioxygenase TET3 OS=Homo sapiens GN=TET3		
O43156	TTI1_HUMAN TEL2-interacting protein 1 homolog OS=Homo sapiens GN=TTI1 PE=1 SV=3	4.66E-05	
O43169	CYB5B_HUMAN Cytochrome b5 type B OS=Homo sapiens GN=CYB5B PE=1 SV=2		301.24
O43172	PRP4_HUMAN U4/U6 small nuclear ribonucleoprotein Prp4 OS=Homo sapiens GN=PRPF4 PE=1 SV=2		81.75
O43172-2	PRP4_HUMAN Isoform 2 of U4/U6 small nuclear ribonucleoprotein Prp4 OS=Homo sapiens GN=PRPF4		81.75
O43172;O43172-2	PRP4_HUMAN U4/U6 small nuclear ribonucleoprotein Prp4 OS=Homo sapiens GN=PRPF4 PE=1 SV=2;>sp|O43172-2|PRP4_HUMAN Isc	3.65E-29	
O43175	SERA_HUMAN D-3-phosphoglycerate dehydrogenase OS=Homo sapiens GN=PHGDH PE=1 SV=4		
O43181	NDUS4_HUMAN NADH dehydrogenase [ubiquinone] iron-sulfur protein 4, mitochondrial OS=Homo sapiens GN=NDUFS4 PE=1 SV=1		
O43236	SEPT4_HUMAN Septin-4 OS=Homo sapiens GN=SEPT4 PE=1 SV=1		22.04
O43236-2	SEPT4_HUMAN Isoform 2 of Septin-4 OS=Homo sapiens GN=SEPT4		22.04
O43236-3	SEPT4_HUMAN Isoform 3 of Septin-4 OS=Homo sapiens GN=SEPT4		22.04
O43236-4	SEPT4_HUMAN Isoform 4 of Septin-4 OS=Homo sapiens GN=SEPT4		22.04
O43236-5	SEPT4_HUMAN Isoform 5 of Septin-4 OS=Homo sapiens GN=SEPT4		21.59
O43236-6	SEPT4_HUMAN Isoform ARTS of Septin-4 OS=Homo sapiens GN=SEPT4		0.45
O43237	DC1L2_HUMAN Cytoplasmic dynein 1 light intermediate chain 2 OS=Homo sapiens GN=DYNC1LI2 PE=1 SV=1		281.75
O43242	PSMD3_HUMAN 26S proteasome non-ATPase regulatory subunit 3 OS=Homo sapiens GN=PSMD3 PE=1 SV=2		1077.91
O43252	PAPS1_HUMAN Bifunctional 3-phosphoadenosine 5-phosphosulfate synthase 1 OS=Homo sapiens GN=PAPSS1 PE=1 SV=2	7.77E-45	
O43264	ZW10_HUMAN Centromere/kinetochore protein zw10 homolog OS=Homo sapiens GN=ZW10 PE=1 SV=3	8.08E-82	
O43272	PROD_HUMAN Proline dehydrogenase 1, mitochondrial OS=Homo sapiens GN=PRODH PE=1 SV=3		
O43272-1	PROD_HUMAN Isoform 3 of Proline dehydrogenase 1, mitochondrial OS=Homo sapiens GN=PRODH		
O43272-2	PROD_HUMAN Isoform 2 of Proline dehydrogenase 1, mitochondrial OS=Homo sapiens GN=PRODH		
O43278;O43278-2	SPIT1_HUMAN Kunitz-type protease inhibitor 1 OS=Homo sapiens GN=SPINT1 PE=1 SV=2;>sp|O43278-2|SPIT1_HUMAN Isoform 2 o	1.06E-11	
O43286	B4GT5_HUMAN Beta-1,4-galactosyltransferase 5 OS=Homo sapiens GN=B4GALT5 PE=2 SV=1		41.38
O43290	SNUT1_HUMAN U4/U6.U5 tri-snRNP-associated protein 1 OS=Homo sapiens GN=SART1 PE=1 SV=1		168.59
O43291	SPIT2_HUMAN Kunitz-type protease inhibitor 2 OS=Homo sapiens GN=SPINT2 PE=1 SV=2		
O43294;O43294-2	TGFI1_HUMAN Transforming growth factor beta-1-induced transcript 1 protein OS=Homo sapiens GN=TGFB1I1 PE=1 SV=2;>sp|O432	1.97E-26	
O43299	K0415_HUMAN Protein KIAA0415 OS=Homo sapiens GN=KIAA0415 PE=1 SV=2		20.62
O43301	HS12A_HUMAN Heat shock 70 kDa protein 12A OS=Homo sapiens GN=HSPA12A PE=1 SV=2	2.92E-10	
O43303	CP110_HUMAN Centriolar coiled-coil protein of 110 kDa OS=Homo sapiens GN=CCP110 PE=1 SV=3		
O43303-2	CP110_HUMAN Isoform 2 of Centriolar coiled-coil protein of 110 kDa OS=Homo sapiens GN=CCP110		
O43313	ATMIN_HUMAN ATM interactor OS=Homo sapiens GN=ATMIN PE=1 SV=2		23.29
O43313-2	ATMIN_HUMAN Isoform 2 of ATM interactor OS=Homo sapiens GN=ATMIN		21.37
O43314	VIP2_HUMAN Inositol hexakisphosphate and diphosphoinositol-pentakisphosphate kinase 2 OS=Homo sapiens GN=PPIP5K2 PE=1 SV=3		46.8
O43314-2	VIP2_HUMAN Isoform 2 of Inositol hexakisphosphate and diphosphoinositol-pentakisphosphate kinase 2 OS=Homo sapiens GN=PPIP5K2		46.8
O43324	MCA3_HUMAN Eukaryotic translation elongation factor 1 epsilon-1 OS=Homo sapiens GN=EEF1E1 PE=1 SV=1		137.22
O43325	LYRM1_HUMAN LYR motif-containing protein 1 OS=Homo sapiens GN=LYRM1 PE=2 SV=1		
O43374	RASL2_HUMAN Ras GTPase-activating protein 4 OS=Homo sapiens GN=RASA4 PE=2 SV=2		
O43374-2	RASL2_HUMAN Isoform 2 of Ras GTPase-activating protein 4 OS=Homo sapiens GN=RASA4		
O43390	HNRPR_HUMAN Heterogeneous nuclear ribonucleoprotein R OS=Homo sapiens GN=HNRNPR PE=1 SV=1		722.1
O43390-2	HNRPR_HUMAN Isoform 2 of Heterogeneous nuclear ribonucleoprotein R OS=Homo sapiens GN=HNRNPR		682.83
O43390;O43390-2	HNRPR_HUMAN Heterogeneous nuclear ribonucleoprotein R OS=Homo sapiens GN=HNRNPR PE=1 SV=1;>sp|O43390-2|HNRPR_HUM/	0	
O43395	PRPF3_HUMAN U4/U6 small nuclear ribonucleoprotein Prp3 OS=Homo sapiens GN=PRPF3 PE=1 SV=2		146.84
O43396	TXNL1_HUMAN Thioredoxin-like protein 1 OS=Homo sapiens GN=TXNL1 PE=1 SV=3	3.42E-206	

O43399	TPD54_HUMAN Tumor protein D54 OS=Homo sapiens GN=TPD52L2 PE=1 SV=2		463.42
O43399-2	TPD54_HUMAN Isoform 2 of Tumor protein D54 OS=Homo sapiens GN=TPD52L2		392.6
O43399-3	TPD54_HUMAN Isoform 3 of Tumor protein D54 OS=Homo sapiens GN=TPD52L2		392.6
O43399-3;O43399-4;O43:	3|TPD54_HUMAN Isoform 3 of Tumor protein D54 OS=Homo sapiens GN=TPD52L2;>sp|O43399-4|TPD54_HUMAN Isoform 4 of Tumor	2.76E-162	
O43399-4	TPD54_HUMAN Isoform 4 of Tumor protein D54 OS=Homo sapiens GN=TPD52L2		392.6
O43402	CX4NB_HUMAN Neighbor of COX4 OS=Homo sapiens GN=COX4NB PE=1 SV=1		154.76
O43414;B4DN03;C9JA75;(	ERI3_HUMAN ERI1 exoribonuclease 3 OS=Homo sapiens GN=ERI3 PE=1 SV=2;>tr|B4DN03|B4DN03_HUMAN Prion protein interacting	3.02E-15	
O43423	AN32C_HUMAN Acidic leucine-rich nuclear phosphoprotein 32 family member C OS=Homo sapiens GN=ANP32C PE=2 SV=1		116.02
O43427;O43427-2	FIBP_HUMAN Acidic fibroblast growth factor intracellular-binding protein OS=Homo sapiens GN=FIBP PE=1 SV=3;>sp|O43427-2|FIBP	5.94E-06	
O43432	IF4G3_HUMAN Eukaryotic translation initiation factor 4 gamma 3 OS=Homo sapiens GN=EIF4G3 PE=1 SV=2		176.18
O43432-2	IF4G3_HUMAN Isoform 2 of Eukaryotic translation initiation factor 4 gamma 3 OS=Homo sapiens GN=EIF4G3		5.57
O43447	PPIH_HUMAN Peptidyl-prolyl cis-trans isomerase H OS=Homo sapiens GN=PPIH PE=1 SV=1		136.69
O43448	KCAB3_HUMAN Voltage-gated potassium channel subunit beta-3 OS=Homo sapiens GN=KCNAB3 PE=1 SV=2		
O43464	HTRA2_HUMAN Serine protease HTRA2, mitochondrial OS=Homo sapiens GN=HTRA2 PE=1 SV=2		56.21
O43464-2	HTRA2_HUMAN Isoform 2 of Serine protease HTRA2, mitochondrial OS=Homo sapiens GN=HTRA2		43.72
O43464-3	HTRA2_HUMAN Isoform 3 of Serine protease HTRA2, mitochondrial OS=Homo sapiens GN=HTRA2		56.21
O43464-4	HTRA2_HUMAN Isoform 4 of Serine protease HTRA2, mitochondrial OS=Homo sapiens GN=HTRA2		56.21
O43464;A8K7G2;O43464-	HTRA2_HUMAN Serine protease HTRA2, mitochondrial OS=Homo sapiens GN=HTRA2 PE=1 SV=2;>tr|A8K7G2|A8K7G2_HUMAN cDNA	5.47E-13	
O43488	ARK72_HUMAN Aflatoxin B1 aldehyde reductase member 2 OS=Homo sapiens GN=AKR7A2 PE=1 SV=3		96.51
O43490;O43490-2;O4349	PROM1_HUMAN Prominin-1 OS=Homo sapiens GN=PROM1 PE=1 SV=1;>sp|O43490-2|PROM1_HUMAN Isoform 2 of Prominin-1 OS=H	1.48E-67	
O43491	E41L2_HUMAN Band 4.1-like protein 2 OS=Homo sapiens GN=EPB41L2 PE=1 SV=1		260.43
O43493	TGON2_HUMAN Trans-Golgi network integral membrane protein 2 OS=Homo sapiens GN=TGOLN2 PE=1 SV=2		210
O43493-2	TGON2_HUMAN Isoform TGN46 of Trans-Golgi network integral membrane protein 2 OS=Homo sapiens GN=TGOLN2		210
O43493-3	TGON2_HUMAN Isoform TGN48 of Trans-Golgi network integral membrane protein 2 OS=Homo sapiens GN=TGOLN2		210
O43493-4	TGON2_HUMAN Isoform 4 of Trans-Golgi network integral membrane protein 2 OS=Homo sapiens GN=TGOLN2		183.21
O43493-5	TGON2_HUMAN Isoform 5 of Trans-Golgi network integral membrane protein 2 OS=Homo sapiens GN=TGOLN2		210
O43493-6	TGON2_HUMAN Isoform 6 of Trans-Golgi network integral membrane protein 2 OS=Homo sapiens GN=TGOLN2		79.52
O43493;O43493-3;O4349	TGON2_HUMAN Trans-Golgi network integral membrane protein 2 OS=Homo sapiens GN=TGOLN2 PE=1 SV=2;>sp|O43493-3|TGON2	7.54E-151	
O43497	CAC1G_HUMAN Voltage-dependent T-type calcium channel subunit alpha-1G OS=Homo sapiens GN=CACNA1G PE=2 SV=3		
O43497-10	CAC1G_HUMAN Isoform 10 of Voltage-dependent T-type calcium channel subunit alpha-1G OS=Homo sapiens GN=CACNA1G		
O43497-11	CAC1G_HUMAN Isoform 11 of Voltage-dependent T-type calcium channel subunit alpha-1G OS=Homo sapiens GN=CACNA1G		
O43497-12	CAC1G_HUMAN Isoform 12 of Voltage-dependent T-type calcium channel subunit alpha-1G OS=Homo sapiens GN=CACNA1G		
O43497-13	CAC1G_HUMAN Isoform 13 of Voltage-dependent T-type calcium channel subunit alpha-1G OS=Homo sapiens GN=CACNA1G		
O43497-2	CAC1G_HUMAN Isoform 1 of Voltage-dependent T-type calcium channel subunit alpha-1G OS=Homo sapiens GN=CACNA1G		
O43497-3	CAC1G_HUMAN Isoform 2 of Voltage-dependent T-type calcium channel subunit alpha-1G OS=Homo sapiens GN=CACNA1G		
O43497-4	CAC1G_HUMAN Isoform 3 of Voltage-dependent T-type calcium channel subunit alpha-1G OS=Homo sapiens GN=CACNA1G		
O43497-5	CAC1G_HUMAN Isoform 4 of Voltage-dependent T-type calcium channel subunit alpha-1G OS=Homo sapiens GN=CACNA1G		
O43497-6	CAC1G_HUMAN Isoform 6 of Voltage-dependent T-type calcium channel subunit alpha-1G OS=Homo sapiens GN=CACNA1G		
O43497-7	CAC1G_HUMAN Isoform 7 of Voltage-dependent T-type calcium channel subunit alpha-1G OS=Homo sapiens GN=CACNA1G		
O43497-8	CAC1G_HUMAN Isoform 8 of Voltage-dependent T-type calcium channel subunit alpha-1G OS=Homo sapiens GN=CACNA1G		
O43497-9	CAC1G_HUMAN Isoform 9 of Voltage-dependent T-type calcium channel subunit alpha-1G OS=Homo sapiens GN=CACNA1G		
O43504	HBXIP_HUMAN Hepatitis B virus X-interacting protein OS=Homo sapiens GN=HBXIP PE=1 SV=1	1.28E-09	
O43520	AT8B1_HUMAN Probable phospholipid-transporting ATPase IC OS=Homo sapiens GN=ATP8B1 PE=1 SV=3		27.33
O43548	TGM5_HUMAN Protein-glutamine gamma-glutamyltransferase 5 OS=Homo sapiens GN=TGM5 PE=1 SV=4		33.68
O43548-2	TGM5_HUMAN Isoform Short of Protein-glutamine gamma-glutamyltransferase 5 OS=Homo sapiens GN=TGM5		33.68
O43570	CAH12_HUMAN Carbonic anhydrase 12 OS=Homo sapiens GN=CA12 PE=1 SV=1		
O43570-2	CAH12_HUMAN Isoform 2 of Carbonic anhydrase 12 OS=Homo sapiens GN=CA12		
O43570;O43570-2	CAH12_HUMAN Carbonic anhydrase 12 OS=Homo sapiens GN=CA12 PE=1 SV=1;>sp|O43570-2|CAH12_HUMAN Isoform 2 of Carboni	1.23E-08	
O43583	DENR_HUMAN Density-regulated protein OS=Homo sapiens GN=DENR PE=1 SV=2		59.37
O43592	XPOT_HUMAN Exportin-T OS=Homo sapiens GN=XPOT PE=1 SV=2	0	
O43598	RCL_HUMAN Deoxyribonucleoside 5-monophosphate N-glycosidase OS=Homo sapiens GN=RCL PE=1 SV=1	5.96E-19	
O43615	TIM44_HUMAN Mitochondrial import inner membrane translocase subunit TIM44 OS=Homo sapiens GN=TIMM44 PE=1 SV=2		290.29
O43617	TPPC3_HUMAN Trafficking protein particle complex subunit 3 OS=Homo sapiens GN=TRAPPC3 PE=1 SV=1		26.27
O43633	CHM2A_HUMAN Charged multivesicular body protein 2a OS=Homo sapiens GN=CHMP2A PE=1 SV=1	8.86E-09	
O43660	PLRG1_HUMAN Pleiotropic regulator 1 OS=Homo sapiens GN=PLRG1 PE=1 SV=1		38.54
O43660-2	PLRG1_HUMAN Isoform 2 of Pleiotropic regulator 1 OS=Homo sapiens GN=PLRG1		38.54
O43660;O43660-2	PLRG1_HUMAN Pleiotropic regulator 1 OS=Homo sapiens GN=PLRG1 PE=1 SV=1;>sp|O43660-2|PLRG1_HUMAN Isoform 2 of Pleiotro	1.07E-08	
O43663	PRC1_HUMAN Protein regulator of cytokinesis 1 OS=Homo sapiens GN=PRC1 PE=1 SV=2		
O43663-2	PRC1_HUMAN Isoform 2 of Protein regulator of cytokinesis 1 OS=Homo sapiens GN=PRC1		
O43663-3	PRC1_HUMAN Isoform 3 of Protein regulator of cytokinesis 1 OS=Homo sapiens GN=PRC1		
O43665	RGS10_HUMAN Regulator of G-protein signaling 10 OS=Homo sapiens GN=RGS10 PE=1 SV=2		168.38

O43665-2	RGS10_HUMAN Isoform 2 of Regulator of G-protein signaling 10 OS=Homo sapiens GN=RGS10		168.38
O43665-3	RGS10_HUMAN Isoform 3 of Regulator of G-protein signaling 10 OS=Homo sapiens GN=RGS10		200.76
O43665-3;O43665;O4366	3|RGS10_HUMAN Isoform 3 of Regulator of G-protein signaling 10 OS=Homo sapiens GN=RGS10;>sp|O43665|RGS10_HUMAN Regula	4.35E-69	
O43670	ZN207_HUMAN Zinc finger protein 207 OS=Homo sapiens GN=ZNF207 PE=1 SV=1		37.22
O43670-2	ZN207_HUMAN Isoform 2 of Zinc finger protein 207 OS=Homo sapiens GN=ZNF207		37.22
O43670-3	ZN207_HUMAN Isoform 3 of Zinc finger protein 207 OS=Homo sapiens GN=ZNF207		
O43670;O43670-2	ZN207_HUMAN Zinc finger protein 207 OS=Homo sapiens GN=ZNF207 PE=1 SV=1;>sp|O43670-2|ZN207_HUMAN Isoform 2 of Zinc f	1.04E-65	
O43674	NDUB5_HUMAN NADH dehydrogenase [ubiquinone] 1 beta subcomplex subunit 5, mitochondrial OS=Homo sapiens GN=NDUFB5 PE=1	2.07E-12	
O43676	NDUB3_HUMAN NADH dehydrogenase [ubiquinone] 1 beta subcomplex subunit 3 OS=Homo sapiens GN=NDUFB3 PE=1 SV=3		59.4
O43678	NDUA2_HUMAN NADH dehydrogenase [ubiquinone] 1 alpha subcomplex subunit 2 OS=Homo sapiens GN=NDUFA2 PE=1 SV=3		
O43681	ASNA_HUMAN ATPase ASNA1 OS=Homo sapiens GN=ASNA1 PE=1 SV=2		76.42
O43683	BUB1_HUMAN Mitotic checkpoint serine/threonine-protein kinase BUB1 OS=Homo sapiens GN=BUB1 PE=1 SV=1	4.13E-20	
O43684	BUB3_HUMAN Mitotic checkpoint protein BUB3 OS=Homo sapiens GN=BUB3 PE=1 SV=1		253.53
O43684-2	BUB3_HUMAN Isoform 2 of Mitotic checkpoint protein BUB3 OS=Homo sapiens GN=BUB3		253.53
O43684;O43684-2	BUB3_HUMAN Mitotic checkpoint protein BUB3 OS=Homo sapiens GN=BUB3 PE=1 SV=1;>sp|O43684-2|BUB3_HUMAN Isoform 2 of M	2.15E-110	
O43688	LPP2_HUMAN Lipid phosphate phosphohydrolase 2 OS=Homo sapiens GN=PPAP2C PE=1 SV=1		44.73
O43688-2	LPP2_HUMAN Isoform 2 of Lipid phosphate phosphohydrolase 2 OS=Homo sapiens GN=PPAP2C		44.73
O43688-2;O43688	2|LPP2_HUMAN Isoform 2 of Lipid phosphate phosphohydrolase 2 OS=Homo sapiens GN=PPAP2C;>sp|O43688|LPP2_HUMAN Lipid pho	2.07E-06	
O43707	ACTN4_HUMAN Alpha-actinin-4 OS=Homo sapiens GN=ACTN4 PE=1 SV=2		3415.39
O43708;O43708-2	MAAI_HUMAN Maleylacetoacetate isomerase OS=Homo sapiens GN=GSTZ1 PE=1 SV=3;>sp|O43708-2|MAAI_HUMAN Isoform 2 of Ma	9.89E-08	
O43709	WBS22_HUMAN Uncharacterized methyltransferase WBSCR22 OS=Homo sapiens GN=WBSCR22 PE=1 SV=2		36.22
O43709-2	WBS22_HUMAN Isoform 2 of Uncharacterized methyltransferase WBSCR22 OS=Homo sapiens GN=WBSCR22		36.22
O43711	TLX3_HUMAN T-cell leukemia homeobox protein 3 OS=Homo sapiens GN=TLX3 PE=1 SV=3		
O43719	HTSF1_HUMAN HIV Tat-specific factor 1 OS=Homo sapiens GN=HTATSF1 PE=1 SV=1		
O43731	ERD23_HUMAN ER lumen protein retaining receptor 3 OS=Homo sapiens GN=KDELR3 PE=2 SV=1		28.17
O43731-2	ERD23_HUMAN Isoform 2 of ER lumen protein retaining receptor 3 OS=Homo sapiens GN=KDELR3		28.17
O43747	AP1G1_HUMAN AP-1 complex subunit gamma-1 OS=Homo sapiens GN=AP1G1 PE=1 SV=5		31.27
O43747-2	AP1G1_HUMAN Isoform 2 of AP-1 complex subunit gamma-1 OS=Homo sapiens GN=AP1G1		31.27
O43747-2;O43747	2|AP1G1_HUMAN Isoform 2 of AP-1 complex subunit gamma-1 OS=Homo sapiens GN=AP1G1;>sp|O43747|AP1G1_HUMAN AP-1 com	5.64E-74	
O43752	STX6_HUMAN Syntaxin-6 OS=Homo sapiens GN=STX6 PE=1 SV=1		116.34
O43760	SNG2_HUMAN Synaptogyrin-2 OS=Homo sapiens GN=SYNGR2 PE=1 SV=1		214.72
O43761	SNG3_HUMAN Synaptogyrin-3 OS=Homo sapiens GN=SYNGR3 PE=2 SV=2	6.39E-09	
O43763	TLX2_HUMAN T-cell leukemia homeobox protein 2 OS=Homo sapiens GN=TLX2 PE=1 SV=2		
O43765	SGTA_HUMAN Small glutamine-rich tetratricopeptide repeat-containing protein alpha OS=Homo sapiens GN=SGTA PE=1 SV=1		104.07
O43768-3;O43768-7;O437	3|ENSA_HUMAN Isoform 3 of Alpha-endosulfine OS=Homo sapiens GN=ENSA;>sp|O43768-7|ENSA_HUMAN Isoform 7 of Alpha-endos	4.68E-06	
O43768-4;O43768-3;O437	4|ENSA_HUMAN Isoform 4 of Alpha-endosulfine OS=Homo sapiens GN=ENSA;>sp|O43768-3|ENSA_HUMAN Isoform 3 of Alpha-endos	8.38E-67	
O43772	MCAT_HUMAN Mitochondrial carnitine/acylcarnitine carrier protein OS=Homo sapiens GN=SLC25A20 PE=1 SV=1		
O43776	SYNC_HUMAN Asparaginyl-tRNA synthetase, cytoplasmic OS=Homo sapiens GN=NARS PE=1 SV=1		237.57
O43790	KRT86_HUMAN Keratin, type II cuticular Hb6 OS=Homo sapiens GN=KRT86 PE=1 SV=1		81.03
O43795	MYO1B_HUMAN Myosin-Ib OS=Homo sapiens GN=MYO1B PE=1 SV=3		624.13
O43795-2	MYO1B_HUMAN Isoform 2 of Myosin-Ib OS=Homo sapiens GN=MYO1B		624.13
O43795;O43795-2	MYO1B_HUMAN Myosin-Ib OS=Homo sapiens GN=MYO1B PE=2 SV=3;>sp|O43795-2|MYO1B_HUMAN Isoform 2 of Myosin-Ib OS=Hor	4.89E-50	
O43805	SSNA1_HUMAN Sjoegren syndrome nuclear autoantigen 1 OS=Homo sapiens GN=SSNA1 PE=1 SV=2	2.53E-09	
O43809	CPSF5_HUMAN Cleavage and polyadenylation specificity factor subunit 5 OS=Homo sapiens GN=NUDT21 PE=1 SV=1		586.94
O43813	LANC1_HUMAN LanC-like protein 1 OS=Homo sapiens GN=LANCL1 PE=1 SV=1	1.96E-07	
O43815;O43815-2	STRN_HUMAN Striatin OS=Homo sapiens GN=STRN PE=1 SV=4;>sp|O43815-2|STRN_HUMAN Isoform 2 of Striatin OS=Homo sapiens	2.31E-15	
O43818	U3IP2_HUMAN U3 small nucleolar RNA-interacting protein 2 OS=Homo sapiens GN=RRP9 PE=1 SV=1		
O43823	AKAP8_HUMAN A-kinase anchor protein 8 OS=Homo sapiens GN=AKAP8 PE=1 SV=1		
O43829	ZF161_HUMAN Zinc finger protein 161 homolog OS=Homo sapiens GN=ZFP161 PE=2 SV=2		
O43837	IDH3B_HUMAN Isocitrate dehydrogenase [NAD] subunit beta, mitochondrial OS=Homo sapiens GN=IDH3B PE=1 SV=2		208.08
O43837-2	IDH3B_HUMAN Isoform A of Isocitrate dehydrogenase [NAD] subunit beta, mitochondrial OS=Homo sapiens GN=IDH3B		172.16
O43837-3	IDH3B_HUMAN Isoform C of Isocitrate dehydrogenase [NAD] subunit beta, mitochondrial OS=Homo sapiens GN=IDH3B		98.71
O43837;O43837-2	IDH3B_HUMAN Isocitrate dehydrogenase [NAD] subunit beta, mitochondrial OS=Homo sapiens GN=IDH3B PE=1 SV=2;>sp|O43837-2	1.13E-91	
O43847-2;O43847	2|NRDC_HUMAN Isoform 2 of Nardilysin OS=Homo sapiens GN=NRD1;>sp|O43847|NRDC_HUMAN Nardilysin OS=Homo sapiens GN=	1.16E-14	
O43852	CALU_HUMAN Calumenin OS=Homo sapiens GN=CALU PE=1 SV=2		1012.32
O43852-2	CALU_HUMAN Isoform 2 of Calumenin OS=Homo sapiens GN=CALU		989.13
O43852;O43852-2	CALU_HUMAN Calumenin OS=Homo sapiens GN=CALU PE=1 SV=2;>sp|O43852-2|CALU_HUMAN Isoform 2 of Calumenin OS=Homo s	4.00E-112	
O43865;O43865-2	SAHH2_HUMAN Putative adenosylhomocysteinase 2 OS=Homo sapiens GN=AHCYL1 PE=1 SV=2;>sp|O43865-2|SAHH2_HUMAN Isofo	1.85E-26	
O43866	CD5L_HUMAN CD5 antigen-like OS=Homo sapiens GN=CD5L PE=1 SV=1	2.96E-50	
O43869	OR2T1_HUMAN Olfactory receptor 2T1 OS=Homo sapiens GN=OR2T1 PE=2 SV=3		
O43896	KIF1C_HUMAN Kinesin-like protein KIF1C OS=Homo sapiens GN=KIF1C PE=1 SV=3		

O43913	ORC5_HUMAN Origin recognition complex subunit 5 OS=Homo sapiens GN=ORC5 PE=1 SV=1		
O43914;O43914-2	TYOBP_HUMAN TYRO protein tyrosine kinase-binding protein OS=Homo sapiens GN=TYROBP PE=1 SV=1;>sp|O43914-2|TYOBP_HUM	1.23E-29	
O43920	NDUS5_HUMAN NADH dehydrogenase [ubiquinone] iron-sulfur protein 5 OS=Homo sapiens GN=NDUFS5 PE=1 SV=3		232.98
O43924	PDE6D_HUMAN Retinal rod rhodopsin-sensitive cGMP 3,5-cyclic phosphodiesterase subunit delta OS=Homo sapiens GN=PDE6D PE=1	9.39E-05	
O43929	ORC4_HUMAN Origin recognition complex subunit 4 OS=Homo sapiens GN=ORC4 PE=1 SV=2		32.73
O60216	RAD21_HUMAN Double-strand-break repair protein rad21 homolog OS=Homo sapiens GN=RAD21 PE=1 SV=2		
O60218	AK1BA_HUMAN Aldo-keto reductase family 1 member B10 OS=Homo sapiens GN=AKR1B10 PE=1 SV=2	0.00013979	
O60220	TIM8A_HUMAN Mitochondrial import inner membrane translocase subunit Tim8 A OS=Homo sapiens GN=TIMM8A PE=1 SV=1		38.91
O60231	DHX16_HUMAN Putative pre-splicing factor ATP-dependent RNA helicase DHX16 OS=Homo sapiens GN=DHX16 PE=1 SV=2		158.56
O60232	SSA27_HUMAN Sjoegren syndrome/scleroderma autoantigen 1 OS=Homo sapiens GN=SSSCA1 PE=1 SV=1	2.56E-38	
O60234	GMFG_HUMAN Glia maturation factor gamma OS=Homo sapiens GN=GMFG PE=1 SV=1	7.84E-21	
O60238	BNI3L_HUMAN BCL2/adenovirus E1B 19 kDa protein-interacting protein 3-like OS=Homo sapiens GN=BNIP3L PE=1 SV=1		
O60239;B2R7Y8;B3KQW6	3BP5_HUMAN SH3 domain-binding protein 5 OS=Homo sapiens GN=SH3BP5 PE=1 SV=2;>tr|B2R7Y8|B2R7Y8_HUMAN cDNA, FLJ9366	5.36E-06	
O60240	PLIN1_HUMAN Perilipin-1 OS=Homo sapiens GN=PLIN1 PE=2 SV=2	1.18E-291	
O60244	MED14_HUMAN Mediator of RNA polymerase II transcription subunit 14 OS=Homo sapiens GN=MED14 PE=1 SV=2		
O60256	KPRB_HUMAN Phosphoribosyl pyrophosphate synthase-associated protein 2 OS=Homo sapiens GN=PRPSAP2 PE=1 SV=1	1.03E-15	
O60264	SMCA5_HUMAN SWI/SNF-related matrix-associated actin-dependent regulator of chromatin subfamily A member 5 OS=Homo sapiens GN=SMARCA5		194.93
O60271	JIP4_HUMAN C-Jun-amino-terminal kinase-interacting protein 4 OS=Homo sapiens GN=SPAG9 PE=1 SV=4		
O60271-2	JIP4_HUMAN Isoform 2 of C-Jun-amino-terminal kinase-interacting protein 4 OS=Homo sapiens GN=SPAG9		
O60271-3	JIP4_HUMAN Isoform 3 of C-Jun-amino-terminal kinase-interacting protein 4 OS=Homo sapiens GN=SPAG9		
O60271-4	JIP4_HUMAN Isoform 4 of C-Jun-amino-terminal kinase-interacting protein 4 OS=Homo sapiens GN=SPAG9		
O60271-4;O60271;O6027	4|JIP4_HUMAN Isoform 4 of C-Jun-amino-terminal kinase-interacting protein 4 OS=Homo sapiens GN=SPAG9;>sp|O60271|JIP4_HUM	1.38E-78	
O60271-5	JIP4_HUMAN Isoform 5 of C-Jun-amino-terminal kinase-interacting protein 4 OS=Homo sapiens GN=SPAG9		
O60271-6	JIP4_HUMAN Isoform 6 of C-Jun-amino-terminal kinase-interacting protein 4 OS=Homo sapiens GN=SPAG9		
O60271-7	JIP4_HUMAN Isoform 7 of C-Jun-amino-terminal kinase-interacting protein 4 OS=Homo sapiens GN=SPAG9		
O60271-7;O60271-3;O60	7|JIP4_HUMAN Isoform 7 of C-Jun-amino-terminal kinase-interacting protein 4 OS=Homo sapiens GN=SPAG9;>sp|O60271-3|JIP4_HU	2.28E-70	
O60271-8	JIP4_HUMAN Isoform 8 of C-Jun-amino-terminal kinase-interacting protein 4 OS=Homo sapiens GN=SPAG9		
O60271;O60271-2;O6027	JIP4_HUMAN C-Jun-amino-terminal kinase-interacting protein 4 OS=Homo sapiens GN=SPAG9 PE=1 SV=4;>sp|O60271-2|JIP4_HUM	1.45E-83	
O60281	ZN292_HUMAN Zinc finger protein 292 OS=Homo sapiens GN=ZNF292 PE=1 SV=3		29.82
O60281-2	ZN292_HUMAN Isoform 2 of Zinc finger protein 292 OS=Homo sapiens GN=ZNF292		29.82
O60281;O60281-2	ZN292_HUMAN Zinc finger protein 292 OS=Homo sapiens GN=ZNF292 PE=1 SV=3;>sp|O60281-2|ZN292_HUMAN Isoform 2 of Zinc f	1.94E-08	
O60282	KIF5C_HUMAN Kinesin heavy chain isoform 5C OS=Homo sapiens GN=KIF5C PE=1 SV=1		
O60282-2	KIF5C_HUMAN Isoform 2 of Kinesin heavy chain isoform 5C OS=Homo sapiens GN=KIF5C		
O60282;O60282-2	KIF5C_HUMAN Kinesin heavy chain isoform 5C OS=Homo sapiens GN=KIF5C PE=1 SV=1;>sp|O60282-2|KIF5C_HUMAN Isoform 2 of	4.08E-143	
O60287	NPA1P_HUMAN Nucleolar pre-ribosomal-associated protein 1 OS=Homo sapiens GN=URB1 PE=1 SV=4		87.16
O60290	ZN862_HUMAN Zinc finger protein 862 OS=Homo sapiens GN=ZNF862 PE=2 SV=2		23.48
O60290-2	ZN862_HUMAN Isoform 2 of Zinc finger protein 862 OS=Homo sapiens GN=ZNF862		19.1
O60293	ZC3H1_HUMAN Zinc finger C3H1 domain-containing protein OS=Homo sapiens GN=ZFC3H1 PE=1 SV=3		33.44
O60293-2	ZC3H1_HUMAN Isoform 2 of Zinc finger C3H1 domain-containing protein OS=Homo sapiens GN=ZFC3H1		33.44
O60299	PRIP1_HUMAN ProSAP-interacting protein 1 OS=Homo sapiens GN=PROSAPIP1 PE=2 SV=1		
O60299-2	PRIP1_HUMAN Isoform 2 of ProSAP-interacting protein 1 OS=Homo sapiens GN=PROSAPIP1		
O60306	AQR_HUMAN Intron-binding protein aquarius OS=Homo sapiens GN=AQR PE=1 SV=4		
O60308	CE104_HUMAN Centrosomal protein of 104 kDa OS=Homo sapiens GN=CEP104 PE=1 SV=1		
O60313	OPA1_HUMAN Dynamin-like 120 kDa protein, mitochondrial OS=Homo sapiens GN=OPA1 PE=1 SV=3		29.56
O60313-2	OPA1_HUMAN Isoform 2 of Dynamin-like 120 kDa protein, mitochondrial OS=Homo sapiens GN=OPA1		29.56
O60313-2;D3DNW4;O603	2|OPA1_HUMAN Isoform 2 of Dynamin-like 120 kDa protein, mitochondrial OS=Homo sapiens GN=OPA1;>tr|D3DNW4|D3DNW4_HUM	2.96E-50	
O60315	ZEB2_HUMAN Zinc finger E-box-binding homeobox 2 OS=Homo sapiens GN=ZEB2 PE=1 SV=1		
O60331	PI51C_HUMAN Phosphatidylinositol-4-phosphate 5-kinase type-1 gamma OS=Homo sapiens GN=PIP5K1C PE=1 SV=2		4.96
O60333-4;O60333;O6033	4|KIF1B_HUMAN Isoform 4 of Kinesin-like protein KIF1B OS=Homo sapiens GN=KIF1B;>sp|O60333|KIF1B_HUMAN Kinesin-like protei	1.37E-05	
O60341	KDM1A_HUMAN Lysine-specific histone demethylase 1A OS=Homo sapiens GN=KDM1A PE=1 SV=2		
O60341-2	KDM1A_HUMAN Isoform 2 of Lysine-specific histone demethylase 1A OS=Homo sapiens GN=KDM1A		
O60341-2;O60341	2|KDM1A_HUMAN Isoform 2 of Lysine-specific histone demethylase 1A OS=Homo sapiens GN=KDM1A;>sp|O60341|KDM1A_HUMAN L	1.49E-49	
O60343;O60343-3;O6034	TBCD4_HUMAN TBC1 domain family member 4 OS=Homo sapiens GN=TBC1D4 PE=1 SV=2;>sp|O60343-3|TBCD4_HUMAN Isoform 3	3.25E-07	
O60344	ECE2_HUMAN Endothelin-converting enzyme 2 OS=Homo sapiens GN=ECE2 PE=1 SV=4		3.71
O60344-2	ECE2_HUMAN Isoform B of Endothelin-converting enzyme 2 OS=Homo sapiens GN=ECE2		3.71
O60344-3	ECE2_HUMAN Isoform C of Endothelin-converting enzyme 2 OS=Homo sapiens GN=ECE2		3.71
O60344-5	ECE2_HUMAN Isoform 2C of Endothelin-converting enzyme 2 OS=Homo sapiens GN=ECE2		20.65
O60361	NDK8_HUMAN Putative nucleoside diphosphate kinase OS=Homo sapiens GN=NME2P1 PE=5 SV=1		599.59
O60427	FADS1_HUMAN Fatty acid desaturase 1 OS=Homo sapiens GN=FADS1 PE=1 SV=1	5.17E-07	
O60437	PEPL_HUMAN Periplakin OS=Homo sapiens GN=PPL PE=1 SV=4		
O60443	DFNA5_HUMAN Non-syndromic hearing impairment protein 5 OS=Homo sapiens GN=DFNA5 PE=1 SV=2	2.42E-09	

O60447	EVI5_HUMAN Ecotropic viral integration site 5 protein homolog OS=Homo sapiens GN=EVI5 PE=1 SV=3		
O60449	LY75_HUMAN Lymphocyte antigen 75 OS=Homo sapiens GN=LY75 PE=1 SV=3		
O60449-2	LY75_HUMAN Isoform 2 of Lymphocyte antigen 75 OS=Homo sapiens GN=LY75		
O60449-3	LY75_HUMAN Isoform 3 of Lymphocyte antigen 75 OS=Homo sapiens GN=LY75		
O60469	DSCAM_HUMAN Down syndrome cell adhesion molecule OS=Homo sapiens GN=DSCAM PE=1 SV=2		
O60469-2	DSCAM_HUMAN Isoform Short of Down syndrome cell adhesion molecule OS=Homo sapiens GN=DSCAM		
O60481	ZIC3_HUMAN Zinc finger protein ZIC 3 OS=Homo sapiens GN=ZIC3 PE=1 SV=1		32.53
O60486	PLXC1_HUMAN Plexin-C1 OS=Homo sapiens GN=PLXNC1 PE=1 SV=1		48.06
O60488	ACSL4_HUMAN Long-chain-fatty-acid--CoA ligase 4 OS=Homo sapiens GN=ACSL4 PE=1 SV=2		392.68
O60488-2	ACSL4_HUMAN Isoform Short of Long-chain-fatty-acid--CoA ligase 4 OS=Homo sapiens GN=ACSL4		392.68
O60488;O60488-2	ACSL4_HUMAN Long-chain-fatty-acid--CoA ligase 4 OS=Homo sapiens GN=ACSL4 PE=1 SV=2;>sp|O60488-2|ACSL4_HUMAN Isoform	4.11E-45	
O60493	SNX3_HUMAN Sorting nexin-3 OS=Homo sapiens GN=SNX3 PE=1 SV=3		191.34
O60493-2	SNX3_HUMAN Isoform 2 of Sorting nexin-3 OS=Homo sapiens GN=SNX3		86.75
O60493-3	SNX3_HUMAN Isoform 3 of Sorting nexin-3 OS=Homo sapiens GN=SNX3		123.07
O60493-4	SNX3_HUMAN Isoform 4 of Sorting nexin-3 OS=Homo sapiens GN=SNX3		116.8
O60493;O60493-4;O6049	SNX3_HUMAN Sorting nexin-3 OS=Homo sapiens GN=SNX3 PE=1 SV=3;>sp|O60493-4|SNX3_HUMAN Isoform 4 of Sorting nexin-3 O	8.56E-16	
O60494	CUBN_HUMAN Cubilin OS=Homo sapiens GN=CUBN PE=1 SV=5		31.25
O60496	DOK2_HUMAN Docking protein 2 OS=Homo sapiens GN=DOK2 PE=1 SV=2	6.03E-05	
O60499	STX10_HUMAN Syntaxin-10 OS=Homo sapiens GN=STX10 PE=1 SV=1		77.29
O60499-2	STX10_HUMAN Isoform 2 of Syntaxin-10 OS=Homo sapiens GN=STX10		67.87
O60499;O60499-2	STX10_HUMAN Syntaxin-10 OS=Homo sapiens GN=STX10 PE=1 SV=1;>sp|O60499-2|STX10_HUMAN Isoform 2 of Syntaxin-10 OS=I	0.00019025	
O60502;O60502-2	NCOAT_HUMAN Bifunctional protein NCOAT OS=Homo sapiens GN=MGEA5 PE=1 SV=2;>sp|O60502-2|NCOAT_HUMAN Isoform 2 of B	4.25E-33	
O60506	HNRPQ_HUMAN Heterogeneous nuclear ribonucleoprotein Q OS=Homo sapiens GN=SYNCRIP PE=1 SV=2		981.04
O60506-2	HNRPQ_HUMAN Isoform 2 of Heterogeneous nuclear ribonucleoprotein Q OS=Homo sapiens GN=SYNCRIP		981.04
O60506-3	HNRPQ_HUMAN Isoform 3 of Heterogeneous nuclear ribonucleoprotein Q OS=Homo sapiens GN=SYNCRIP		1016.23
O60506-3;O60506-4;O60!	3|HNRPQ_HUMAN Isoform 3 of Heterogeneous nuclear ribonucleoprotein Q OS=Homo sapiens GN=SYNCRIP;>sp|O60506-4|HNRPQ_H	0	
O60506-4	HNRPQ_HUMAN Isoform 4 of Heterogeneous nuclear ribonucleoprotein Q OS=Homo sapiens GN=SYNCRIP		1016.23
O60506-5	HNRPQ_HUMAN Isoform 5 of Heterogeneous nuclear ribonucleoprotein Q OS=Homo sapiens GN=SYNCRIP		713.07
O60506;O60506-2	HNRPQ_HUMAN Heterogeneous nuclear ribonucleoprotein Q OS=Homo sapiens GN=SYNCRIP PE=1 SV=2;>sp|O60506-2|HNRPQ_HUM	0	
O60508	PRP17_HUMAN Pre-processing factor 17 OS=Homo sapiens GN=CDC40 PE=1 SV=1		
O60518	RNBP6_HUMAN Ran-binding protein 6 OS=Homo sapiens GN=RANBP6 PE=1 SV=2		88.23
O60524	NEMF_HUMAN Nuclear export mediator factor NEMF OS=Homo sapiens GN=NEMF PE=1 SV=4		
O60524-2	NEMF_HUMAN Isoform 2 of Nuclear export mediator factor NEMF OS=Homo sapiens GN=NEMF		
O60524-3	NEMF_HUMAN Isoform 3 of Nuclear export mediator factor NEMF OS=Homo sapiens GN=NEMF		
O60524-4	NEMF_HUMAN Isoform 4 of Nuclear export mediator factor NEMF OS=Homo sapiens GN=NEMF		
O60524-5	NEMF_HUMAN Isoform 5 of Nuclear export mediator factor NEMF OS=Homo sapiens GN=NEMF		
O60524;O60524-3;O6052	NEMF_HUMAN Nuclear export mediator factor NEMF OS=Homo sapiens GN=NEMF PE=1 SV=4;>sp|O60524-3|NEMF_HUMAN Isoform 3	1.09E-14	
O60547	GMDS_HUMAN GDP-mannose 4,6 dehydratase OS=Homo sapiens GN=GMDS PE=1 SV=1	2.97E-17	
O60551	NMT2_HUMAN Glycylpeptide N-tetradecanoyltransferase 2 OS=Homo sapiens GN=NMT2 PE=1 SV=1		164.8
O60563	CCNT1_HUMAN Cyclin-T1 OS=Homo sapiens GN=CCNT1 PE=1 SV=1	4.94E-10	
O60566	BUB1B_HUMAN Mitotic checkpoint serine/threonine-protein kinase BUB1 beta OS=Homo sapiens GN=BUB1B PE=1 SV=3		
O60566-2	BUB1B_HUMAN Isoform 2 of Mitotic checkpoint serine/threonine-protein kinase BUB1 beta OS=Homo sapiens GN=BUB1B		
O60566-3	BUB1B_HUMAN Isoform 3 of Mitotic checkpoint serine/threonine-protein kinase BUB1 beta OS=Homo sapiens GN=BUB1B		
O60566-3;O60566;O6056	3|BUB1B_HUMAN Isoform 3 of Mitotic checkpoint serine/threonine-protein kinase BUB1 beta OS=Homo sapiens GN=BUB1B;>sp|O605	1.40E-09	
O60568	PLOD3_HUMAN Procollagen-lysine,2-oxoglutarate 5-dioxygenase 3 OS=Homo sapiens GN=PLOD3 PE=1 SV=1		555.24
O60573	IF4E2_HUMAN Eukaryotic translation initiation factor 4E type 2 OS=Homo sapiens GN=EIF4E2 PE=1 SV=1		58.98
O60610	DIAP1_HUMAN Protein diaphanous homolog 1 OS=Homo sapiens GN=DIAPH1 PE=1 SV=2		
O60610-2	DIAP1_HUMAN Isoform 2 of Protein diaphanous homolog 1 OS=Homo sapiens GN=DIAPH1		
O60610;O60610-2	DIAP1_HUMAN Protein diaphanous homolog 1 OS=Homo sapiens GN=DIAPH1 PE=1 SV=2;>sp|O60610-2|DIAP1_HUMAN Isoform 2 of	0	
O60613	SEP15_HUMAN 15 kDa selenoprotein OS=Homo sapiens GN=SEP15 PE=1 SV=3		122.67
O60641	AP180_HUMAN Clathrin coat assembly protein AP180 OS=Homo sapiens GN=SNAP91 PE=1 SV=2		85.45
O60641-2	AP180_HUMAN Isoform 2 of Clathrin coat assembly protein AP180 OS=Homo sapiens GN=SNAP91		0.75
O60641-3	AP180_HUMAN Isoform 3 of Clathrin coat assembly protein AP180 OS=Homo sapiens GN=SNAP91		85.45
O60645	EXOC3_HUMAN Exocyst complex component 3 OS=Homo sapiens GN=EXOC3 PE=1 SV=2		46.93
O60645-2	EXOC3_HUMAN Isoform 2 of Exocyst complex component 3 OS=Homo sapiens GN=EXOC3		46.93
O60645;O60645-2	EXOC3_HUMAN Exocyst complex component 3 OS=Homo sapiens GN=EXOC3 PE=1 SV=2;>sp|O60645-2|EXOC3_HUMAN Isoform 2 o	5.13E-09	
O60664	PLIN3_HUMAN Perilipin-3 OS=Homo sapiens GN=PLIN3 PE=1 SV=3		144.63
O60664-2	PLIN3_HUMAN Isoform A of Perilipin-3 OS=Homo sapiens GN=PLIN3		18.78
O60664-3	PLIN3_HUMAN Isoform 3 of Perilipin-3 OS=Homo sapiens GN=PLIN3		144.63
O60664;O60664-3	PLIN3_HUMAN Perilipin-3 OS=Homo sapiens GN=PLIN3 PE=1 SV=3;>sp|O60664-3|PLIN3_HUMAN Isoform 3 of Perilipin-3 OS=Homo	0	
O60667	FAIM3_HUMAN Fas apoptotic inhibitory molecule 3 OS=Homo sapiens GN=FAIM3 PE=2 SV=1		

O60669	MOT2_HUMAN Monocarboxylate transporter 2 OS=Homo sapiens GN=SLC16A7 PE=2 SV=2		
O60675	MAFK_HUMAN Transcription factor MafK OS=Homo sapiens GN=MAFK PE=1 SV=1		
O60684	IMA7_HUMAN Importin subunit alpha-7 OS=Homo sapiens GN=KPNA6 PE=1 SV=1		
O60701	UGDH_HUMAN UDP-glucose 6-dehydrogenase OS=Homo sapiens GN=UGDH PE=1 SV=1		
O60711	LPXN_HUMAN Leupaxin OS=Homo sapiens GN=LPXN PE=1 SV=1	1.08E-10	
O60716	CTND1_HUMAN Catenin delta-1 OS=Homo sapiens GN=CTNND1 PE=1 SV=1		
O60716-10	CTND1_HUMAN Isoform 2AB of Catenin delta-1 OS=Homo sapiens GN=CTNND1		
O60716-11	CTND1_HUMAN Isoform 2AC of Catenin delta-1 OS=Homo sapiens GN=CTNND1		
O60716-12	CTND1_HUMAN Isoform 2BC of Catenin delta-1 OS=Homo sapiens GN=CTNND1		
O60716-13	CTND1_HUMAN Isoform 2A of Catenin delta-1 OS=Homo sapiens GN=CTNND1		
O60716-14	CTND1_HUMAN Isoform 2B of Catenin delta-1 OS=Homo sapiens GN=CTNND1		
O60716-15	CTND1_HUMAN Isoform 2C of Catenin delta-1 OS=Homo sapiens GN=CTNND1		
O60716-16	CTND1_HUMAN Isoform 2 of Catenin delta-1 OS=Homo sapiens GN=CTNND1		
O60716-17	CTND1_HUMAN Isoform 3ABC of Catenin delta-1 OS=Homo sapiens GN=CTNND1		
O60716-18	CTND1_HUMAN Isoform 3AB of Catenin delta-1 OS=Homo sapiens GN=CTNND1		
O60716-19	CTND1_HUMAN Isoform 3AC of Catenin delta-1 OS=Homo sapiens GN=CTNND1		
O60716-2	CTND1_HUMAN Isoform 1AB of Catenin delta-1 OS=Homo sapiens GN=CTNND1		
O60716-20	CTND1_HUMAN Isoform 3BC of Catenin delta-1 OS=Homo sapiens GN=CTNND1		
O60716-21	CTND1_HUMAN Isoform 3A of Catenin delta-1 OS=Homo sapiens GN=CTNND1		
O60716-22	CTND1_HUMAN Isoform 3B of Catenin delta-1 OS=Homo sapiens GN=CTNND1		
O60716-23	CTND1_HUMAN Isoform 3C of Catenin delta-1 OS=Homo sapiens GN=CTNND1		
O60716-24	CTND1_HUMAN Isoform 3 of Catenin delta-1 OS=Homo sapiens GN=CTNND1		
O60716-25	CTND1_HUMAN Isoform 4ABC of Catenin delta-1 OS=Homo sapiens GN=CTNND1		
O60716-26	CTND1_HUMAN Isoform 4AB of Catenin delta-1 OS=Homo sapiens GN=CTNND1		
O60716-27	CTND1_HUMAN Isoform 4AC of Catenin delta-1 OS=Homo sapiens GN=CTNND1		
O60716-28	CTND1_HUMAN Isoform 4BC of Catenin delta-1 OS=Homo sapiens GN=CTNND1		
O60716-29	CTND1_HUMAN Isoform 4A of Catenin delta-1 OS=Homo sapiens GN=CTNND1		
O60716-3	CTND1_HUMAN Isoform 1AC of Catenin delta-1 OS=Homo sapiens GN=CTNND1		
O60716-3;O60716-5;O60	3|CTND1_HUMAN Isoform 1AC of Catenin delta-1 OS=Homo sapiens GN=CTNND1;>sp|O60716-5|CTND1_HUMAN Isoform 1A of Cate	2.00E-108	
O60716-30	CTND1_HUMAN Isoform 4B of Catenin delta-1 OS=Homo sapiens GN=CTNND1		
O60716-31	CTND1_HUMAN Isoform 4C of Catenin delta-1 OS=Homo sapiens GN=CTNND1		
O60716-32	CTND1_HUMAN Isoform 4 of Catenin delta-1 OS=Homo sapiens GN=CTNND1		
O60716-4	CTND1_HUMAN Isoform 1BC of Catenin delta-1 OS=Homo sapiens GN=CTNND1		
O60716-5	CTND1_HUMAN Isoform 1A of Catenin delta-1 OS=Homo sapiens GN=CTNND1		
O60716-6	CTND1_HUMAN Isoform 1B of Catenin delta-1 OS=Homo sapiens GN=CTNND1		
O60716-7	CTND1_HUMAN Isoform 1C of Catenin delta-1 OS=Homo sapiens GN=CTNND1		
O60716-8	CTND1_HUMAN Isoform 1 of Catenin delta-1 OS=Homo sapiens GN=CTNND1		
O60716-9	CTND1_HUMAN Isoform 2ABC of Catenin delta-1 OS=Homo sapiens GN=CTNND1		
O60716;O60716-2;C9JKX	CTND1_HUMAN Catenin delta-1 OS=Homo sapiens GN=CTNND1 PE=1 SV=1;>sp|O60716-2|CTND1_HUMAN Isoform 1AB of Catenin d	1.51E-15	
O60739	EIF1B_HUMAN Eukaryotic translation initiation factor 1b OS=Homo sapiens GN=EIF1B PE=1 SV=2		135.44
O60741	HCN1_HUMAN Potassium/sodium hyperpolarization-activated cyclic nucleotide-gated channel 1 OS=Homo sapiens GN=HCN1 PE=2 SV=3		
O60749	SNX2_HUMAN Sorting nexin-2 OS=Homo sapiens GN=SNX2 PE=1 SV=2	1.31E-16	
O60762	DPM1_HUMAN Dolichol-phosphate mannosyltransferase OS=Homo sapiens GN=DPM1 PE=1 SV=1		281.4
O60763	USO1_HUMAN General vesicular transport factor p115 OS=Homo sapiens GN=USO1 PE=1 SV=2		77.57
O60763-2	USO1_HUMAN Isoform 2 of General vesicular transport factor p115 OS=Homo sapiens GN=USO1		77.57
O60763-2;O60763	2|USO1_HUMAN Isoform 2 of General vesicular transport factor p115 OS=Homo sapiens GN=USO1;>sp|O60763|USO1_HUMAN Gener	3.60E-133	
O60763;O60763-2	USO1_HUMAN General vesicular transport factor p115 OS=Homo sapiens GN=USO1 PE=1 SV=2;>sp|O60763-2|USO1_HUMAN Isofor	1.54E-46	
O60783	RT14_HUMAN 28S ribosomal protein S14, mitochondrial OS=Homo sapiens GN=MRPS14 PE=1 SV=1		113.99
O60784-2;O60784;O6078	2|TOM1_HUMAN Isoform 2 of Target of Myb protein 1 OS=Homo sapiens GN=TOM1;>sp|O60784|TOM1_HUMAN Target of Myb protein	1.99E-07	
O60812	HNRCL_HUMAN Heterogeneous nuclear ribonucleoprotein C-like 1 OS=Homo sapiens GN=HNRNPCL1 PE=1 SV=1		277.96
O60814	H2B1K_HUMAN Histone H2B type 1-K OS=Homo sapiens GN=HIST1H2BK PE=1 SV=3		705.5
O60825;B0FLL2;O60825-	F262_HUMAN 6-phosphofructo-2-kinase/fructose-2,6-biphosphatase 2 OS=Homo sapiens GN=PFKFB2 PE=1 SV=2;>tr|B0FLL2|B0FLL	5.14E-12	
O60826	CCD22_HUMAN Coiled-coil domain-containing protein 22 OS=Homo sapiens GN=CCDC22 PE=1 SV=1	7.99E-10	
O60828	PQBP1_HUMAN Polyglutamine-binding protein 1 OS=Homo sapiens GN=PQBP1 PE=1 SV=1		73.59
O60828-10	PQBP1_HUMAN Isoform 10 of Polyglutamine-binding protein 1 OS=Homo sapiens GN=PQBP1		28.66
O60828-2	PQBP1_HUMAN Isoform 2 of Polyglutamine-binding protein 1 OS=Homo sapiens GN=PQBP1		73.59
O60828-3	PQBP1_HUMAN Isoform 3 of Polyglutamine-binding protein 1 OS=Homo sapiens GN=PQBP1		73.59
O60828-4	PQBP1_HUMAN Isoform 4 of Polyglutamine-binding protein 1 OS=Homo sapiens GN=PQBP1		28.66
O60828-5	PQBP1_HUMAN Isoform 5 of Polyglutamine-binding protein 1 OS=Homo sapiens GN=PQBP1		73.59
O60828-6	PQBP1_HUMAN Isoform 6 of Polyglutamine-binding protein 1 OS=Homo sapiens GN=PQBP1		28.66
O60828-7	PQBP1_HUMAN Isoform 7 of Polyglutamine-binding protein 1 OS=Homo sapiens GN=PQBP1		32.03

O60828-8	PQBP1_HUMAN Isoform 8 of Polyglutamine-binding protein 1 OS=Homo sapiens GN=PQBP1		28.66
O60828-9	PQBP1_HUMAN Isoform 9 of Polyglutamine-binding protein 1 OS=Homo sapiens GN=PQBP1		28.66
O60828;O60828-2;O6082	PQBP1_HUMAN Polyglutamine-binding protein 1 OS=Homo sapiens GN=PQBP1 PE=1 SV=1;>sp|O60828-2|PQBP1_HUMAN Isoform 2 (	1.13E-06	
O60830	TI17B_HUMAN Mitochondrial import inner membrane translocase subunit Tim17-B OS=Homo sapiens GN=TIMM17B PE=1 SV=1	1.71E-15	
O60831	PRAF2_HUMAN PRA1 family protein 2 OS=Homo sapiens GN=PRAF2 PE=1 SV=1		112.56
O60832	DKC1_HUMAN H/ACA ribonucleoprotein complex subunit 4 OS=Homo sapiens GN=DKC1 PE=1 SV=3		401.32
O60840	CAC1F_HUMAN Voltage-dependent L-type calcium channel subunit alpha-1F OS=Homo sapiens GN=CACNA1F PE=1 SV=2		
O60840-2	CAC1F_HUMAN Isoform 2 of Voltage-dependent L-type calcium channel subunit alpha-1F OS=Homo sapiens GN=CACNA1F		
O60841	IF2P_HUMAN Eukaryotic translation initiation factor 5B OS=Homo sapiens GN=EIF5B PE=1 SV=4		257.27
O60869	EDF1_HUMAN Endothelial differentiation-related factor 1 OS=Homo sapiens GN=EDF1 PE=1 SV=1		292.23
O60869-2	EDF1_HUMAN Isoform 2 of Endothelial differentiation-related factor 1 OS=Homo sapiens GN=EDF1		275.94
O60869;O60869-2	EDF1_HUMAN Endothelial differentiation-related factor 1 OS=Homo sapiens GN=EDF1 PE=1 SV=1;>sp|O60869-2|EDF1_HUMAN Isofo	1.67E-73	
O60870	KIN17_HUMAN DNA/RNA-binding protein KIN17 OS=Homo sapiens GN=KIN PE=1 SV=2		
O60879-2;O60879;O6087	2|DIAP2_HUMAN Isoform 2 of Protein diaphanous homolog 2 OS=Homo sapiens GN=DIAPH2;>sp|O60879|DIAP2_HUMAN Protein diap	1.41E-17	
O60880;O60880-4	SH21A_HUMAN SH2 domain-containing protein 1A OS=Homo sapiens GN=SH2D1A PE=1 SV=1;>sp|O60880-4|SH21A_HUMAN Isofor	0.00021694	
O60884	DNJA2_HUMAN DnaJ homolog subfamily A member 2 OS=Homo sapiens GN=DNAJA2 PE=1 SV=1	4.25E-80	
O60885	BRD4_HUMAN Bromodomain-containing protein 4 OS=Homo sapiens GN=BRD4 PE=1 SV=2		84.52
O60885-2	BRD4_HUMAN Isoform 2 of Bromodomain-containing protein 4 OS=Homo sapiens GN=BRD4		71.23
O60885;Q15059;O60885-	BRD4_HUMAN Bromodomain-containing protein 4 OS=Homo sapiens GN=BRD4 PE=1 SV=2;>sp|Q15059|BRD3_HUMAN Bromodomair	4.22E-09	
O60888	CUTA_HUMAN Protein CutA OS=Homo sapiens GN=CUTA PE=1 SV=2		125.11
O60888-2	CUTA_HUMAN Isoform A of Protein CutA OS=Homo sapiens GN=CUTA		125.11
O60888-2;O60888;O6088	2|CUTA_HUMAN Isoform A of Protein CutA OS=Homo sapiens GN=CUTA;>sp|O60888|CUTA_HUMAN Protein CutA OS=Homo sapiens C	1.16E-25	
O60888-3	CUTA_HUMAN Isoform C of Protein CutA OS=Homo sapiens GN=CUTA		125.11
O60890	OPHN1_HUMAN Oligophrenin-1 OS=Homo sapiens GN=OPHN1 PE=2 SV=1		35.36
O60907	TBL1X_HUMAN F-box-like/WD repeat-containing protein TBL1X OS=Homo sapiens GN=TBL1X PE=1 SV=3		22.51
O60907-2	TBL1X_HUMAN Isoform 2 of F-box-like/WD repeat-containing protein TBL1X OS=Homo sapiens GN=TBL1X		22.51
O60925	PFD1_HUMAN Prefoldin subunit 1 OS=Homo sapiens GN=PFDN1 PE=1 SV=2		63.24
O60927	PP1RB_HUMAN Protein phosphatase 1 regulatory subunit 11 OS=Homo sapiens GN=PPP1R11 PE=1 SV=1	6.75E-05	
O60934	NBN_HUMAN Nibrin OS=Homo sapiens GN=NBN PE=1 SV=1	9.38E-12	
O60936;O60936-2	NOL3_HUMAN Nucleolar protein 3 OS=Homo sapiens GN=NOL3 PE=1 SV=1;>sp|O60936-2|NOL3_HUMAN Isoform 2 of Nucleolar prot	5.90E-10	
O60938	KERA_HUMAN Keratocan OS=Homo sapiens GN=KERA PE=1 SV=1		
O60942;O60942-2;O6094	MCE1_HUMAN mRNA-capping enzyme OS=Homo sapiens GN=RNGTT PE=1 SV=1;>sp|O60942-2|MCE1_HUMAN Isoform 2 of mRNA-ca	5.36E-09	
O71037	ENK3_HUMAN HERV-K_19q12 provirus ancestral Env polyprotein OS=Homo sapiens PE=1 SV=2		
O75027-2;O75027	2|ABCB7_HUMAN Isoform 2 of ATP-binding cassette sub-family B member 7, mitochondrial OS=Homo sapiens GN=ABCB7;>sp|O7502	4.20E-10	
O75038	PLCH2_HUMAN 1-phosphatidylinositol-4,5-bisphosphate phosphodiesterase eta-2 OS=Homo sapiens GN=PLCH2 PE=2 SV=3		
O75038-3	PLCH2_HUMAN Isoform 3 of 1-phosphatidylinositol-4,5-bisphosphate phosphodiesterase eta-2 OS=Homo sapiens GN=PLCH2		
O75038-4	PLCH2_HUMAN Isoform 4 of 1-phosphatidylinositol-4,5-bisphosphate phosphodiesterase eta-2 OS=Homo sapiens GN=PLCH2		
O75038-5	PLCH2_HUMAN Isoform 5 of 1-phosphatidylinositol-4,5-bisphosphate phosphodiesterase eta-2 OS=Homo sapiens GN=PLCH2		
O75044	FNBP2_HUMAN SLIT-ROBO Rho GTPase-activating protein 2 OS=Homo sapiens GN=SRGAP2 PE=1 SV=2	8.03E-58	
O75063	XYLK_HUMAN Glycosaminoglycan xylosylkinase OS=Homo sapiens GN=FAM20B PE=1 SV=1		
O75069	TMCC2_HUMAN Transmembrane and coiled-coil domains protein 2 OS=Homo sapiens GN=TMCC2 PE=1 SV=3		
O75083	WDR1_HUMAN WD repeat-containing protein 1 OS=Homo sapiens GN=WDR1 PE=1 SV=4		334.7
O75083-3	WDR1_HUMAN Isoform 2 of WD repeat-containing protein 1 OS=Homo sapiens GN=WDR1		134.37
O75083;O75083-3	WDR1_HUMAN WD repeat-containing protein 1 OS=Homo sapiens GN=WDR1 PE=1 SV=4;>sp|O75083-3|WDR1_HUMAN Isoform 2 of	0	
O75084	FZD7_HUMAN Frizzled-7 OS=Homo sapiens GN=FZD7 PE=1 SV=2		74.7
O75116	ROCK2_HUMAN Rho-associated protein kinase 2 OS=Homo sapiens GN=ROCK2 PE=1 SV=4		53.3
O75122	CLAP2_HUMAN CLIP-associating protein 2 OS=Homo sapiens GN=CLASP2 PE=1 SV=2	1.19E-87	
O75131	CPNE3_HUMAN Copine-3 OS=Homo sapiens GN=CPNE3 PE=1 SV=1		23.37
O75146	HIP1R_HUMAN Huntingtin-interacting protein 1-related protein OS=Homo sapiens GN=HIP1R PE=1 SV=2		261.27
O75147	OBSL1_HUMAN Obscurin-like protein 1 OS=Homo sapiens GN=OBSL1 PE=1 SV=4		
O75147-1	OBSL1_HUMAN Isoform 3 of Obscurin-like protein 1 OS=Homo sapiens GN=OBSL1		
O75147-2	OBSL1_HUMAN Isoform 2 of Obscurin-like protein 1 OS=Homo sapiens GN=OBSL1		
O75150	BRE1B_HUMAN E3 ubiquitin-protein ligase BRE1B OS=Homo sapiens GN=RNF40 PE=1 SV=4		
O75150-3	BRE1B_HUMAN Isoform 3 of E3 ubiquitin-protein ligase BRE1B OS=Homo sapiens GN=RNF40		
O75150-4	BRE1B_HUMAN Isoform 4 of E3 ubiquitin-protein ligase BRE1B OS=Homo sapiens GN=RNF40		
O75150;A8K6K1;O75150-	BRE1B_HUMAN E3 ubiquitin-protein ligase BRE1B OS=Homo sapiens GN=RNF40 PE=1 SV=4;>tr|A8K6K1|A8K6K1_HUMAN cDNA FLJ7	4.40E-06	
O75151	PHF2_HUMAN Lysine-specific demethylase PHF2 OS=Homo sapiens GN=PHF2 PE=1 SV=4		
O75152	ZC11A_HUMAN Zinc finger CCCH domain-containing protein 11A OS=Homo sapiens GN=ZC3H11A PE=1 SV=3		
O75153	K0664_HUMAN Protein KIAA0664 OS=Homo sapiens GN=KIAA0664 PE=1 SV=2	5.65E-29	
O75155	CAND2_HUMAN Cullin-associated NEDD8-dissociated protein 2 OS=Homo sapiens GN=CAND2 PE=1 SV=3		0.36
O75155-2	CAND2_HUMAN Isoform 2 of Cullin-associated NEDD8-dissociated protein 2 OS=Homo sapiens GN=CAND2		0.36

<table>
<tr><td>O75157;O75157-2</td><td>T22D2_HUMAN TSC22 domain family protein 2 OS=Homo sapiens GN=TSC22D2 PE=1 SV=3;>sp|O75157-2|T22D2_HUMAN Isoform 2</td><td>2.66E-05</td><td></td></tr>
<tr><td>O75165</td><td>DJC13_HUMAN DnaJ homolog subfamily C member 13 OS=Homo sapiens GN=DNAJC13 PE=1 SV=5</td><td></td><td>107.05</td></tr>
<tr><td>O75170</td><td>PP6R2_HUMAN Serine/threonine-protein phosphatase 6 regulatory subunit 2 OS=Homo sapiens GN=PPP6R2 PE=1 SV=2</td><td></td><td></td></tr>
<tr><td>O75170-2</td><td>PP6R2_HUMAN Isoform 2 of Serine/threonine-protein phosphatase 6 regulatory subunit 2 OS=Homo sapiens GN=PPP6R2</td><td></td><td></td></tr>
<tr><td>O75170-3</td><td>PP6R2_HUMAN Isoform 3 of Serine/threonine-protein phosphatase 6 regulatory subunit 2 OS=Homo sapiens GN=PPP6R2</td><td></td><td></td></tr>
<tr><td>O75170-4</td><td>PP6R2_HUMAN Isoform 4 of Serine/threonine-protein phosphatase 6 regulatory subunit 2 OS=Homo sapiens GN=PPP6R2</td><td></td><td></td></tr>
<tr><td>O75170-5</td><td>PP6R2_HUMAN Isoform 5 of Serine/threonine-protein phosphatase 6 regulatory subunit 2 OS=Homo sapiens GN=PPP6R2</td><td></td><td></td></tr>
<tr><td>O75170-6</td><td>PP6R2_HUMAN Isoform 6 of Serine/threonine-protein phosphatase 6 regulatory subunit 2 OS=Homo sapiens GN=PPP6R2</td><td></td><td></td></tr>
<tr><td>O75170;O75170-5;O7517</td><td>PP6R2_HUMAN Serine/threonine-protein phosphatase 6 regulatory subunit 2 OS=Homo sapiens GN=PPP6R2 PE=1 SV=2;>sp|O75170</td><td>5.59E-05</td><td></td></tr>
<tr><td>O75175;B7Z6J7;O75175-2</td><td>CNOT3_HUMAN CCR4-NOT transcription complex subunit 3 OS=Homo sapiens GN=CNOT3 PE=1 SV=1;>tr|B7Z6J7|B7Z6J7_HUMAN cD</td><td>3.87E-09</td><td></td></tr>
<tr><td>O75179;O75179-2;B4DTP</td><td>ANR17_HUMAN Ankyrin repeat domain-containing protein 17 OS=Homo sapiens GN=ANKRD17 PE=1 SV=3;>sp|O75179-2|ANR17_HU</td><td>3.82E-76</td><td></td></tr>
<tr><td>O75182;O75182-2;B7Z39</td><td>SIN3B_HUMAN Paired amphipathic helix protein Sin3b OS=Homo sapiens GN=SIN3B PE=1 SV=2;>sp|O75182-2|SIN3B_HUMAN Isofo</td><td>5.08E-06</td><td></td></tr>
<tr><td>O75190</td><td>DNJB6_HUMAN DnaJ homolog subfamily B member 6 OS=Homo sapiens GN=DNAJB6 PE=1 SV=2</td><td></td><td>82.28</td></tr>
<tr><td>O75190-2</td><td>DNJB6_HUMAN Isoform B of DnaJ homolog subfamily B member 6 OS=Homo sapiens GN=DNAJB6</td><td></td><td></td></tr>
<tr><td>O75190-3</td><td>DNJB6_HUMAN Isoform C of DnaJ homolog subfamily B member 6 OS=Homo sapiens GN=DNAJB6</td><td></td><td>42.9</td></tr>
<tr><td>O75190;O75190-3;O7519</td><td>DNJB6_HUMAN DnaJ homolog subfamily B member 6 OS=Homo sapiens GN=DNAJB6 PE=1 SV=2;>sp|O75190-3|DNJB6_HUMAN Isof</td><td>7.21E-21</td><td></td></tr>
<tr><td>O75208</td><td>COQ9_HUMAN Ubiquinone biosynthesis protein COQ9, mitochondrial OS=Homo sapiens GN=COQ9 PE=1 SV=1</td><td>4.15E-07</td><td></td></tr>
<tr><td>O75223</td><td>GGCT_HUMAN Gamma-glutamylcyclotransferase OS=Homo sapiens GN=GGCT PE=1 SV=1</td><td>6.44E-11</td><td></td></tr>
<tr><td>O75251</td><td>NDUS7_HUMAN NADH dehydrogenase [ubiquinone] iron-sulfur protein 7, mitochondrial OS=Homo sapiens GN=NDUFS7 PE=1 SV=3</td><td></td><td>80.53</td></tr>
<tr><td>O75306</td><td>NDUS2_HUMAN NADH dehydrogenase [ubiquinone] iron-sulfur protein 2, mitochondrial OS=Homo sapiens GN=NDUFS2 PE=1 SV=2</td><td></td><td>81.24</td></tr>
<tr><td>O75312</td><td>ZPR1_HUMAN Zinc finger protein ZPR1 OS=Homo sapiens GN=ZNF259 PE=1 SV=1</td><td>3.12E-22</td><td></td></tr>
<tr><td>O75317</td><td>UBP12_HUMAN Ubiquitin carboxyl-terminal hydrolase 12 OS=Homo sapiens GN=USP12 PE=1 SV=2</td><td></td><td></td></tr>
<tr><td>O75323</td><td>NIPS2_HUMAN Protein NipSnap homolog 2 OS=Homo sapiens GN=GBAS PE=1 SV=1</td><td></td><td></td></tr>
<tr><td>O75339</td><td>CILP1_HUMAN Cartilage intermediate layer protein 1 OS=Homo sapiens GN=CILP PE=1 SV=4</td><td>2.18E-13</td><td></td></tr>
<tr><td>O75340</td><td>PDCD6_HUMAN Programmed cell death protein 6 OS=Homo sapiens GN=PDCD6 PE=1 SV=1</td><td></td><td>161.77</td></tr>
<tr><td>O75347</td><td>TBCA_HUMAN Tubulin-specific chaperone A OS=Homo sapiens GN=TBCA PE=1 SV=3</td><td></td><td>218.7</td></tr>
<tr><td>O75348</td><td>VATG1_HUMAN V-type proton ATPase subunit G 1 OS=Homo sapiens GN=ATP6V1G1 PE=1 SV=3</td><td></td><td>86.84</td></tr>
<tr><td>O75351</td><td>VPS4B_HUMAN Vacuolar protein sorting-associated protein 4B OS=Homo sapiens GN=VPS4B PE=1 SV=2</td><td>3.71E-60</td><td></td></tr>
<tr><td>O75352</td><td>MPU1_HUMAN Mannose-P-dolichol utilization defect 1 protein OS=Homo sapiens GN=MPDU1 PE=1 SV=2</td><td></td><td>81.25</td></tr>
<tr><td>O75354</td><td>ENTP6_HUMAN Ectonucleoside triphosphate diphosphohydrolase 6 OS=Homo sapiens GN=ENTPD6 PE=1 SV=3</td><td></td><td>35.54</td></tr>
<tr><td>O75354-2</td><td>ENTP6_HUMAN Isoform 2 of Ectonucleoside triphosphate diphosphohydrolase 6 OS=Homo sapiens GN=ENTPD6</td><td></td><td>35.54</td></tr>
<tr><td>O75362</td><td>ZN217_HUMAN Zinc finger protein 217 OS=Homo sapiens GN=ZNF217 PE=1 SV=1</td><td></td><td></td></tr>
<tr><td>O75363-2;O75363</td><td>2|BCAS1_HUMAN Isoform 2 of Breast carcinoma-amplified sequence 1 OS=Homo sapiens GN=BCAS1;>sp|O75363|BCAS1_HUMAN Br</td><td>0</td><td></td></tr>
<tr><td>O75366</td><td>AVIL_HUMAN Advillin OS=Homo sapiens GN=AVIL PE=1 SV=3</td><td></td><td></td></tr>
<tr><td>O75366-2</td><td>AVIL_HUMAN Isoform 2 of Advillin OS=Homo sapiens GN=AVIL</td><td></td><td></td></tr>
<tr><td>O75367</td><td>H2AY_HUMAN Core histone macro-H2A.1 OS=Homo sapiens GN=H2AFY PE=1 SV=4</td><td></td><td>323.46</td></tr>
<tr><td>O75367-2</td><td>H2AY_HUMAN Isoform 1 of Core histone macro-H2A.1 OS=Homo sapiens GN=H2AFY</td><td></td><td>323.46</td></tr>
<tr><td>O75367-3</td><td>H2AY_HUMAN Isoform 3 of Core histone macro-H2A.1 OS=Homo sapiens GN=H2AFY</td><td></td><td>323.46</td></tr>
<tr><td>O75367;O75367-3;O7536</td><td>H2AY_HUMAN Core histone macro-H2A.1 OS=Homo sapiens GN=H2AFY PE=1 SV=4;>sp|O75367-3|H2AY_HUMAN Isoform 3 of Core h</td><td>0</td><td></td></tr>
<tr><td>O75368</td><td>SH3L1_HUMAN SH3 domain-binding glutamic acid-rich-like protein OS=Homo sapiens GN=SH3BGRL PE=1 SV=1</td><td></td><td>71.1</td></tr>
<tr><td>O75369</td><td>FLNB_HUMAN Filamin-B OS=Homo sapiens GN=FLNB PE=1 SV=2</td><td></td><td>5627.78</td></tr>
<tr><td>O75369-2</td><td>FLNB_HUMAN Isoform 2 of Filamin-B OS=Homo sapiens GN=FLNB</td><td></td><td>5627.78</td></tr>
<tr><td>O75369-3</td><td>FLNB_HUMAN Isoform 3 of Filamin-B OS=Homo sapiens GN=FLNB</td><td></td><td>5381.87</td></tr>
<tr><td>O75369-4</td><td>FLNB_HUMAN Isoform 4 of Filamin-B OS=Homo sapiens GN=FLNB</td><td></td><td>4463.6</td></tr>
<tr><td>O75369-5</td><td>FLNB_HUMAN Isoform 5 of Filamin-B OS=Homo sapiens GN=FLNB</td><td></td><td>4463.6</td></tr>
<tr><td>O75369-6</td><td>FLNB_HUMAN Isoform 6 of Filamin-B OS=Homo sapiens GN=FLNB</td><td></td><td>5381.87</td></tr>
<tr><td>O75369-7</td><td>FLNB_HUMAN Isoform 7 of Filamin-B OS=Homo sapiens GN=FLNB</td><td></td><td>4902.93</td></tr>
<tr><td>O75369;O75369-2;O7536</td><td>FLNB_HUMAN Filamin-B OS=Homo sapiens GN=FLNB PE=1 SV=2;>sp|O75369-2|FLNB_HUMAN Isoform 2 of Filamin-B OS=Homo sap</td><td>0</td><td></td></tr>
<tr><td>O75376</td><td>NCOR1_HUMAN Nuclear receptor corepressor 1 OS=Homo sapiens GN=NCOR1 PE=1 SV=2</td><td></td><td>66.35</td></tr>
<tr><td>O75376-2</td><td>NCOR1_HUMAN Isoform 2 of Nuclear receptor corepressor 1 OS=Homo sapiens GN=NCOR1</td><td></td><td>66.35</td></tr>
<tr><td>O75376;O75376-2</td><td>NCOR1_HUMAN Nuclear receptor corepressor 1 OS=Homo sapiens GN=NCOR1 PE=1 SV=2;>sp|O75376-2|NCOR1_HUMAN Isoform 2</td><td>1.86E-08</td><td></td></tr>
<tr><td>O75379</td><td>VAMP4_HUMAN Vesicle-associated membrane protein 4 OS=Homo sapiens GN=VAMP4 PE=1 SV=2</td><td></td><td>21.96</td></tr>
<tr><td>O75379-2</td><td>VAMP4_HUMAN Isoform 2 of Vesicle-associated membrane protein 4 OS=Homo sapiens GN=VAMP4</td><td></td><td>21.96</td></tr>
<tr><td>O75379;O75379-2</td><td>VAMP4_HUMAN Vesicle-associated membrane protein 4 OS=Homo sapiens GN=VAMP4 PE=1 SV=2;>sp|O75379-2|VAMP4_HUMAN Isc</td><td>1.04E-09</td><td></td></tr>
<tr><td>O75380</td><td>NDUS6_HUMAN NADH dehydrogenase [ubiquinone] iron-sulfur protein 6, mitochondrial OS=Homo sapiens GN=NDUFS6 PE=1 SV=1</td><td></td><td>92.52</td></tr>
<tr><td>O75381</td><td>PEX14_HUMAN Peroxisomal membrane protein PEX14 OS=Homo sapiens GN=PEX14 PE=1 SV=1</td><td></td><td>38.58</td></tr>
<tr><td>O75381-2</td><td>PEX14_HUMAN Isoform 2 of Peroxisomal membrane protein PEX14 OS=Homo sapiens GN=PEX14</td><td></td><td>38.58</td></tr>
<tr><td>O75381;O75381-2</td><td>PEX14_HUMAN Peroxisomal membrane protein PEX14 OS=Homo sapiens GN=PEX14 PE=1 SV=1;>sp|O75381-2|PEX14_HUMAN Isofo</td><td>2.36E-15</td><td></td></tr>
<tr><td>O75390</td><td>CISY_HUMAN Citrate synthase, mitochondrial OS=Homo sapiens GN=CS PE=1 SV=2</td><td></td><td>302.73</td></tr>
<tr><td>O75391</td><td>SPAG7_HUMAN Sperm-associated antigen 7 OS=Homo sapiens GN=SPAG7 PE=1 SV=2</td><td>3.62E-06</td><td></td></tr>
<tr><td>O75394</td><td>RM33_HUMAN 39S ribosomal protein L33, mitochondrial OS=Homo sapiens GN=MRPL33 PE=1 SV=1</td><td></td><td>28.61</td></tr>
</table>

O75396	SC22B_HUMAN Vesicle-trafficking protein SEC22b OS=Homo sapiens GN=SEC22B PE=1 SV=4		391.75
O75400	PR40A_HUMAN Pre-processing factor 40 homolog A OS=Homo sapiens GN=PRPF40A PE=1 SV=2		244.33
O75400-2	PR40A_HUMAN Isoform 2 of Pre-processing factor 40 homolog A OS=Homo sapiens GN=PRPF40A		244.33
O75400-3	PR40A_HUMAN Isoform 3 of Pre-processing factor 40 homolog A OS=Homo sapiens GN=PRPF40A		260.58
O75400;O75400-2;O7540	PR40A_HUMAN Pre-mRNA-processing factor 40 homolog A OS=Homo sapiens GN=PRPF40A PE=1 SV=2;>sp|O75400-2|PR40A_HUMA	1.40E-200	
O75410	TACC1_HUMAN Transforming acidic coiled-coil-containing protein 1 OS=Homo sapiens GN=TACC1 PE=1 SV=2		
O75410-2	TACC1_HUMAN Isoform 2 of Transforming acidic coiled-coil-containing protein 1 OS=Homo sapiens GN=TACC1		
O75410-2;D3DSX6;O7541	2|TACC1_HUMAN Isoform 2 of Transforming acidic coiled-coil-containing protein 1 OS=Homo sapiens GN=TACC1;>tr|D3DSX6|D3DSX	4.83E-10	
O75410-3	TACC1_HUMAN Isoform 3 of Transforming acidic coiled-coil-containing protein 1 OS=Homo sapiens GN=TACC1		
O75410-4	TACC1_HUMAN Isoform 4 of Transforming acidic coiled-coil-containing protein 1 OS=Homo sapiens GN=TACC1		
O75410-5	TACC1_HUMAN Isoform 5 of Transforming acidic coiled-coil-containing protein 1 OS=Homo sapiens GN=TACC1		
O75410-6	TACC1_HUMAN Isoform 6 of Transforming acidic coiled-coil-containing protein 1 OS=Homo sapiens GN=TACC1		
O75410-7	TACC1_HUMAN Isoform 7 of Transforming acidic coiled-coil-containing protein 1 OS=Homo sapiens GN=TACC1		
O75410-8	TACC1_HUMAN Isoform 8 of Transforming acidic coiled-coil-containing protein 1 OS=Homo sapiens GN=TACC1		
O75414	NDK6_HUMAN Nucleoside diphosphate kinase 6 OS=Homo sapiens GN=NME6 PE=1 SV=3	9.47E-05	
O75417	DPOLQ_HUMAN DNA polymerase theta OS=Homo sapiens GN=POLQ PE=1 SV=2		31.37
O75417-2	DPOLQ_HUMAN Isoform 2 of DNA polymerase theta OS=Homo sapiens GN=POLQ		15.4
O75420	PERQ1_HUMAN PERQ amino acid-rich with GYF domain-containing protein 1 OS=Homo sapiens GN=GIGYF1 PE=1 SV=2		1.89
O75431	MTX2_HUMAN Metaxin-2 OS=Homo sapiens GN=MTX2 PE=1 SV=1		64.97
O75436	VP26A_HUMAN Vacuolar protein sorting-associated protein 26A OS=Homo sapiens GN=VPS26A PE=1 SV=2		62.85
O75438	NDUB1_HUMAN NADH dehydrogenase [ubiquinone] 1 beta subcomplex subunit 1 OS=Homo sapiens GN=NDUFB1 PE=1 SV=1		
O75439	MPPB_HUMAN Mitochondrial-processing peptidase subunit beta OS=Homo sapiens GN=PMPCB PE=1 SV=2		65.82
O75443	TECTA_HUMAN Alpha-tectorin OS=Homo sapiens GN=TECTA PE=1 SV=3		
O75446	SAP30_HUMAN Histone deacetylase complex subunit SAP30 OS=Homo sapiens GN=SAP30 PE=1 SV=1		
O75448	MED24_HUMAN Mediator of RNA polymerase II transcription subunit 24 OS=Homo sapiens GN=MED24 PE=1 SV=1		
O75448-2	MED24_HUMAN Isoform 2 of Mediator of RNA polymerase II transcription subunit 24 OS=Homo sapiens GN=MED24		
O75461	E2F6_HUMAN Transcription factor E2F6 OS=Homo sapiens GN=E2F6 PE=1 SV=1		
O75475	PSIP1_HUMAN PC4 and SFRS1-interacting protein OS=Homo sapiens GN=PSIP1 PE=1 SV=1		168.44
O75475-2	PSIP1_HUMAN Isoform 2 of PC4 and SFRS1-interacting protein OS=Homo sapiens GN=PSIP1		57.97
O75475;O75475-2	PSIP1_HUMAN PC4 and SFRS1-interacting protein OS=Homo sapiens GN=PSIP1 PE=1 SV=1;>sp|O75475-2|PSIP1_HUMAN Isoform 2	0	
O75477	ERLN1_HUMAN Erlin-1 OS=Homo sapiens GN=ERLIN1 PE=1 SV=1		527.37
O75478	TAD2A_HUMAN Transcriptional adapter 2-alpha OS=Homo sapiens GN=TADA2A PE=1 SV=3		
O75487	GPC4_HUMAN Glypican-4 OS=Homo sapiens GN=GPC4 PE=1 SV=4		
O75489	NDUS3_HUMAN NADH dehydrogenase [ubiquinone] iron-sulfur protein 3, mitochondrial OS=Homo sapiens GN=NDUFS3 PE=1 SV=1		431.12
O75494	SRS10_HUMAN Serine/arginine-rich splicing factor 10 OS=Homo sapiens GN=SRSF10 PE=1 SV=1		108.41
O75494-2	SRS10_HUMAN Isoform 2 of Serine/arginine-rich splicing factor 10 OS=Homo sapiens GN=SRSF10		108.41
O75494-3	SRS10_HUMAN Isoform 3 of Serine/arginine-rich splicing factor 10 OS=Homo sapiens GN=SRSF10		108.41
O75494-4	SRS10_HUMAN Isoform 4 of Serine/arginine-rich splicing factor 10 OS=Homo sapiens GN=SRSF10		108.41
O75494;O75494-2;O7549	SRS10_HUMAN Serine/arginine-rich splicing factor 10 OS=Homo sapiens GN=SRSF10 PE=1 SV=1;>sp|O75494-2|SRS10_HUMAN Isof	6.97E-08	
O75509	TNR21_HUMAN Tumor necrosis factor receptor superfamily member 21 OS=Homo sapiens GN=TNFRSF21 PE=1 SV=1		32.13
O75521	ECI2_HUMAN Enoyl-CoA delta isomerase 2, mitochondrial OS=Homo sapiens GN=ECI2 PE=1 SV=4		315.77
O75521-2	ECI2_HUMAN Isoform 2 of Enoyl-CoA delta isomerase 2, mitochondrial OS=Homo sapiens GN=ECI2		311.55
O75521;O75521-2	ECI2_HUMAN Enoyl-CoA delta isomerase 2, mitochondrial OS=Homo sapiens GN=ECI2 PE=1 SV=4;>sp|O75521-2|ECI2_HUMAN Isofo	2.79E-48	
O75521;Q53GC8;Q53HG3	PECI_HUMAN Peroxisomal 3,2-trans-enoyl-CoA isomerase OS=Homo sapiens GN=PECI PE=1 SV=4;>tr|Q53GC8|Q53GC8_HUMAN Per	2.30E-68	
O75525	KHDR3_HUMAN KH domain-containing, RNA-binding, signal transduction-associated protein 3 OS=Homo sapiens GN=KHDRBS3 PE=1 SV=1		
O75525-2	KHDR3_HUMAN Isoform 2 of KH domain-containing, RNA-binding, signal transduction-associated protein 3 OS=Homo sapiens GN=KHDRBS3		
O75526	HNRGT_HUMAN RNA-binding motif protein, X-linked-like-2 OS=Homo sapiens GN=RBMXL2 PE=1 SV=3		94.97
O75528	TADA3_HUMAN Transcriptional adapter 3 OS=Homo sapiens GN=TADA3 PE=1 SV=1		
O75528-2	TADA3_HUMAN Isoform 2 of Transcriptional adapter 3 OS=Homo sapiens GN=TADA3		
O75530	EED_HUMAN Polycomb protein EED OS=Homo sapiens GN=EED PE=1 SV=2		
O75530-2	EED_HUMAN Isoform 2 of Polycomb protein EED OS=Homo sapiens GN=EED		
O75530-3	EED_HUMAN Isoform 3 of Polycomb protein EED OS=Homo sapiens GN=EED		
O75531	BAF_HUMAN Barrier-to-autointegration factor OS=Homo sapiens GN=BANF1 PE=1 SV=1		386.57
O75533	SF3B1_HUMAN Splicing factor 3B subunit 1 OS=Homo sapiens GN=SF3B1 PE=1 SV=3		718.49
O75534	CSDE1_HUMAN Cold shock domain-containing protein E1 OS=Homo sapiens GN=CSDE1 PE=1 SV=2		
O75534-2	CSDE1_HUMAN Isoform Short of Cold shock domain-containing protein E1 OS=Homo sapiens GN=CSDE1		
O75534;O75534-2	CSDE1_HUMAN Cold shock domain-containing protein E1 OS=Homo sapiens GN=CSDE1 PE=1 SV=2;>sp|O75534-2|CSDE1_HUMAN I	4.27E-64	
O75569	PRKRA_HUMAN Interferon-inducible double stranded RNA-dependent protein kinase activator A OS=Homo sapiens GN=PRKRA PE=1 SV=1		99.69
O75569-2	PRKRA_HUMAN Isoform 2 of Interferon-inducible double stranded RNA-dependent protein kinase activator A OS=Homo sapiens GN=PRKRA		99.69
O75569-3	PRKRA_HUMAN Isoform 3 of Interferon-inducible double stranded RNA-dependent protein kinase activator A OS=Homo sapiens GN=PRKRA		99.69
O75569;O75569-2;O7556	PRKRA_HUMAN Interferon-inducible double stranded RNA-dependent protein kinase activator A OS=Homo sapiens GN=PRKRA PE=1 S	2.41E-11	

O75578	ITA10_HUMAN Integrin alpha-10 OS=Homo sapiens GN=ITGA10 PE=2 SV=2		
O75581	LRP6_HUMAN Low-density lipoprotein receptor-related protein 6 OS=Homo sapiens GN=LRP6 PE=1 SV=2		28.8
O75586	MED6_HUMAN Mediator of RNA polymerase II transcription subunit 6 OS=Homo sapiens GN=MED6 PE=1 SV=2		
O75592	MYCB2_HUMAN Probable E3 ubiquitin-protein ligase MYCBP2 OS=Homo sapiens GN=MYCBP2 PE=1 SV=3		76.79
O75592-2	MYCB2_HUMAN Isoform 2 of Probable E3 ubiquitin-protein ligase MYCBP2 OS=Homo sapiens GN=MYCBP2		76.79
O75596	CLC3A_HUMAN C-type lectin domain family 3 member A OS=Homo sapiens GN=CLEC3A PE=1 SV=1	0.00020953	
O75607	NPM3_HUMAN Nucleoplasmin-3 OS=Homo sapiens GN=NPM3 PE=1 SV=3		330.64
O75608	LYPA1_HUMAN Acyl-protein thioesterase 1 OS=Homo sapiens GN=LYPLA1 PE=1 SV=1		86.37
O75608-2	LYPA1_HUMAN Isoform 2 of Acyl-protein thioesterase 1 OS=Homo sapiens GN=LYPLA1		86.37
O75608;O75608-2	LYPA1_HUMAN Acyl-protein thioesterase 1 OS=Homo sapiens GN=LYPLA1 PE=1 SV=1;>sp|O75608-2|LYPA1_HUMAN Isoform 2 of Acy	1.35E-67	
O75628	REM1_HUMAN GTP-binding protein REM 1 OS=Homo sapiens GN=REM1 PE=1 SV=2		
O75643	U520_HUMAN U5 small nuclear ribonucleoprotein 200 kDa helicase OS=Homo sapiens GN=SNRNP200 PE=1 SV=2		2604.04
O75643-2	U520_HUMAN Isoform 2 of U5 small nuclear ribonucleoprotein 200 kDa helicase OS=Homo sapiens GN=SNRNP200		625.58
O75643;A4FU77;C9JAQ9;(	U520_HUMAN U5 small nuclear ribonucleoprotein 200 kDa helicase OS=Homo sapiens GN=SNRNP200 PE=1 SV=2;>tr|A4FU77|A4FU7	1.16E-109	
O75663;O75663-2	TIPRL_HUMAN TIP41-like protein OS=Homo sapiens GN=TIPRL PE=1 SV=2;>sp|O75663-2|TIPRL_HUMAN Isoform 2 of TIP41-like pro	5.03E-40	
O75665	OFD1_HUMAN Oral-facial-digital syndrome 1 protein OS=Homo sapiens GN=OFD1 PE=1 SV=1		35.83
O75665-2	OFD1_HUMAN Isoform 2 of Oral-facial-digital syndrome 1 protein OS=Homo sapiens GN=OFD1		33.75
O75665-3	OFD1_HUMAN Isoform 3 of Oral-facial-digital syndrome 1 protein OS=Homo sapiens GN=OFD1		35.83
O75676;O75676-2	KS6A4_HUMAN Ribosomal protein S6 kinase alpha-4 OS=Homo sapiens GN=RPS6KA4 PE=1 SV=1;>sp|O75676-2|KS6A4_HUMAN Iso	4.94E-15	
O75678	RFPL2_HUMAN Ret finger protein-like 2 OS=Homo sapiens GN=RFPL2 PE=2 SV=3		
O75683	SURF6_HUMAN Surfeit locus protein 6 OS=Homo sapiens GN=SURF6 PE=1 SV=3		
O75688;O75688-2;O7568	PPM1B_HUMAN Protein phosphatase 1B OS=Homo sapiens GN=PPM1B PE=1 SV=1;>sp|O75688-2|PPM1B_HUMAN Isoform Beta-2 of	2.52E-17	
O75691	UTP20_HUMAN Small subunit processome component 20 homolog OS=Homo sapiens GN=UTP20 PE=1 SV=3		71.72
O75694	NU155_HUMAN Nuclear pore complex protein Nup155 OS=Homo sapiens GN=NUP155 PE=1 SV=1		32.63
O75694-2	NU155_HUMAN Isoform 2 of Nuclear pore complex protein Nup155 OS=Homo sapiens GN=NUP155		32.63
O75694;B4DLT2;O75694-:	NU155_HUMAN Nuclear pore complex protein Nup155 OS=Homo sapiens GN=NUP155 PE=1 SV=1;>tr|B4DLT2|B4DLT2_HUMAN cDNA	1.41E-81	
O75695	XRP2_HUMAN Protein XRP2 OS=Homo sapiens GN=RP2 PE=1 SV=4		178.97
O75698	HUG1_HUMAN Putative uncharacterized protein HUG-1 OS=Homo sapiens GN=HUG1 PE=5 SV=2		
O75717	WDHD1_HUMAN WD repeat and HMG-box DNA-binding protein 1 OS=Homo sapiens GN=WDHD1 PE=1 SV=1		57.49
O75718	CRTAP_HUMAN Cartilage-associated protein OS=Homo sapiens GN=CRTAP PE=1 SV=1		211.53
O75746	CMC1_HUMAN Calcium-binding mitochondrial carrier protein Aralar1 OS=Homo sapiens GN=SLC25A12 PE=1 SV=2		
O75747	P3C2G_HUMAN Phosphatidylinositol-4-phosphate 3-kinase C2 domain-containing subunit gamma OS=Homo sapiens GN=PIK3C2G PE=1 SV=3		30.21
O75787	RENR_HUMAN Renin receptor OS=Homo sapiens GN=ATP6AP2 PE=1 SV=2		
O75792	RNH2A_HUMAN Ribonuclease H2 subunit A OS=Homo sapiens GN=RNASEH2A PE=1 SV=2	8.23E-66	
O75794	CD123_HUMAN Cell division cycle protein 123 homolog OS=Homo sapiens GN=CDC123 PE=1 SV=1		23.33
O75815	BCAR3_HUMAN Breast cancer anti-estrogen resistance protein 3 OS=Homo sapiens GN=BCAR3 PE=1 SV=1		
O75815-2	BCAR3_HUMAN Isoform 2 of Breast cancer anti-estrogen resistance protein 3 OS=Homo sapiens GN=BCAR3		
O75817	POP7_HUMAN Ribonuclease P protein subunit p20 OS=Homo sapiens GN=POP7 PE=1 SV=2		82.81
O75818;O75818-2	RPP40_HUMAN Ribonuclease P protein subunit p40 OS=Homo sapiens GN=RPP40 PE=1 SV=3;>sp|O75818-2|RPP40_HUMAN Isoform	1.34E-12	
O75821	EIF3G_HUMAN Eukaryotic translation initiation factor 3 subunit G OS=Homo sapiens GN=EIF3G PE=1 SV=2		223.93
O75822	EIF3J_HUMAN Eukaryotic translation initiation factor 3 subunit J OS=Homo sapiens GN=EIF3J PE=1 SV=2		184.93
O75828	CBR3_HUMAN Carbonyl reductase [NADPH] 3 OS=Homo sapiens GN=CBR3 PE=1 SV=3	7.45E-100	
O75832	PSD10_HUMAN 26S proteasome non-ATPase regulatory subunit 10 OS=Homo sapiens GN=PSMD10 PE=1 SV=1	5.53E-36	
O75843	AP1G2_HUMAN AP-1 complex subunit gamma-like 2 OS=Homo sapiens GN=AP1G2 PE=1 SV=1		
O75844	FACE1_HUMAN CAAX prenyl protease 1 homolog OS=Homo sapiens GN=ZMPSTE24 PE=1 SV=2		91.59
O75874	IDHC_HUMAN Isocitrate dehydrogenase [NADP] cytoplasmic OS=Homo sapiens GN=IDH1 PE=1 SV=2		
O75880	SCO1_HUMAN Protein SCO1 homolog, mitochondrial OS=Homo sapiens GN=SCO1 PE=1 SV=1		93.86
O75886;O75886-2	STAM2_HUMAN Signal transducing adapter molecule 2 OS=Homo sapiens GN=STAM2 PE=1 SV=1;>sp|O75886-2|STAM2_HUMAN Isof	7.16E-14	
O75891	AL1L1_HUMAN Aldehyde dehydrogenase family 1 member L1 OS=Homo sapiens GN=ALDH1L1 PE=1 SV=2	1.42E-14	
O75896	TUSC2_HUMAN Tumor suppressor candidate 2 OS=Homo sapiens GN=TUSC2 PE=1 SV=3	0.00024521	
O75909-4;O75909;O7590	4|CCNK_HUMAN Isoform 4 of Cyclin-K OS=Homo sapiens GN=CCNK;>sp|O75909|CCNK_HUMAN Cyclin-K OS=Homo sapiens GN=CCN	1.10E-16	
O75911	DHRS3_HUMAN Short-chain dehydrogenase/reductase 3 OS=Homo sapiens GN=DHRS3 PE=1 SV=2	3.63E-13	
O75915	PRAF3_HUMAN PRA1 family protein 3 OS=Homo sapiens GN=ARL6IP5 PE=1 SV=1		18.55
O75923	DYSF_HUMAN Dysferlin OS=Homo sapiens GN=DYSF PE=1 SV=1		225.89
O75923-10	DYSF_HUMAN Isoform 10 of Dysferlin OS=Homo sapiens GN=DYSF		225.89
O75923-11	DYSF_HUMAN Isoform 11 of Dysferlin OS=Homo sapiens GN=DYSF		225.89
O75923-12	DYSF_HUMAN Isoform 12 of Dysferlin OS=Homo sapiens GN=DYSF		225.89
O75923-13	DYSF_HUMAN Isoform 13 of Dysferlin OS=Homo sapiens GN=DYSF		225.89
O75923-13;O75923-7;O7!	13|DYSF_HUMAN Isoform 13 of Dysferlin OS=Homo sapiens GN=DYSF;>sp|O75923-7|DYSF_HUMAN Isoform 7 of Dysferlin OS=Homc	4.96E-19	
O75923-14	DYSF_HUMAN Isoform 14 of Dysferlin OS=Homo sapiens GN=DYSF		225.89
O75923-15	DYSF_HUMAN Isoform 15 of Dysferlin OS=Homo sapiens GN=DYSF		225.73

O75923-2	DYSF_HUMAN Isoform 2 of Dysferlin OS=Homo sapiens GN=DYSF		225.89
O75923-3	DYSF_HUMAN Isoform 3 of Dysferlin OS=Homo sapiens GN=DYSF		225.89
O75923-4	DYSF_HUMAN Isoform 4 of Dysferlin OS=Homo sapiens GN=DYSF		225.89
O75923-5	DYSF_HUMAN Isoform 5 of Dysferlin OS=Homo sapiens GN=DYSF		225.89
O75923-6	DYSF_HUMAN Isoform 6 of Dysferlin OS=Homo sapiens GN=DYSF		225.89
O75923-7	DYSF_HUMAN Isoform 7 of Dysferlin OS=Homo sapiens GN=DYSF		225.89
O75923-8	DYSF_HUMAN Isoform 8 of Dysferlin OS=Homo sapiens GN=DYSF		225.89
O75923-9	DYSF_HUMAN Isoform 9 of Dysferlin OS=Homo sapiens GN=DYSF		225.89
O75934	SPF27_HUMAN Pre-splicing factor SPF27 OS=Homo sapiens GN=BCAS2 PE=1 SV=1		295.85
O75935;O75935-3;O7593	DCTN3_HUMAN Dynactin subunit 3 OS=Homo sapiens GN=DCTN3 PE=1 SV=1;>sp|O75935-3|DCTN3_HUMAN Isoform 3 of Dynactin s	2.98E-18	
O75937	DNJC8_HUMAN DnaJ homolog subfamily C member 8 OS=Homo sapiens GN=DNAJC8 PE=1 SV=2		167.3
O75940	SPF30_HUMAN Survival of motor neuron-related-splicing factor 30 OS=Homo sapiens GN=SMNDC1 PE=1 SV=1		
O75947	ATP5H_HUMAN ATP synthase subunit d, mitochondrial OS=Homo sapiens GN=ATP5H PE=1 SV=3		377.77
O75947-2	ATP5H_HUMAN Isoform 2 of ATP synthase subunit d, mitochondrial OS=Homo sapiens GN=ATP5H		377.77
O75947;O75947-2	ATP5H_HUMAN ATP synthase subunit d, mitochondrial OS=Homo sapiens GN=ATP5H PE=1 SV=3;>sp|O75947-2|ATP5H_HUMAN Isofo	4.58E-172	
O75955	FLOT1_HUMAN Flotillin-1 OS=Homo sapiens GN=FLOT1 PE=1 SV=3		813.86
O75962	TRIO_HUMAN Triple functional domain protein OS=Homo sapiens GN=TRIO PE=1 SV=2		47.1
O75962-2	TRIO_HUMAN Isoform 2 of Triple functional domain protein OS=Homo sapiens GN=TRIO		47.1
O75962-3	TRIO_HUMAN Isoform 3 of Triple functional domain protein OS=Homo sapiens GN=TRIO		
O75962-4	TRIO_HUMAN Isoform 4 of Triple functional domain protein OS=Homo sapiens GN=TRIO		41.57
O75962-5	TRIO_HUMAN Isoform 5 of Triple functional domain protein OS=Homo sapiens GN=TRIO		47.1
O75962;O75962-2;O7596	TRIO_HUMAN Triple functional domain protein OS=Homo sapiens GN=TRIO PE=1 SV=2;>sp|O75962-2|TRIO_HUMAN Isoform 2 of Tri	9.10E-35	
O75964	ATP5L_HUMAN ATP synthase subunit g, mitochondrial OS=Homo sapiens GN=ATP5L PE=1 SV=3		226.81
O75976	CBPD_HUMAN Carboxypeptidase D OS=Homo sapiens GN=CPD PE=1 SV=2		237.86
O76003	GLRX3_HUMAN Glutaredoxin-3 OS=Homo sapiens GN=GLRX3 PE=1 SV=2		
O76009	KT33A_HUMAN Keratin, type I cuticular Ha3-I OS=Homo sapiens GN=KRT33A PE=1 SV=2		17.3
O76011	KRT34_HUMAN Keratin, type I cuticular Ha4 OS=Homo sapiens GN=KRT34 PE=1 SV=2		
O76013	KRT36_HUMAN Keratin, type I cuticular Ha6 OS=Homo sapiens GN=KRT36 PE=1 SV=1		65.9
O76013-2	KRT36_HUMAN Isoform 2 of Keratin, type I cuticular Ha6 OS=Homo sapiens GN=KRT36		65.9
O76014	KRT37_HUMAN Keratin, type I cuticular Ha7 OS=Homo sapiens GN=KRT37 PE=2 SV=3		76.5
O76015	KRT38_HUMAN Keratin, type I cuticular Ha8 OS=Homo sapiens GN=KRT38 PE=2 SV=3		85.88
O76021	RL1D1_HUMAN Ribosomal L1 domain-containing protein 1 OS=Homo sapiens GN=RSL1D1 PE=1 SV=3		166.24
O76024	WFS1_HUMAN Wolframin OS=Homo sapiens GN=WFS1 PE=1 SV=2		28.94
O76031	CLPX_HUMAN ATP-dependent Clp protease ATP-binding subunit clpX-like, mitochondrial OS=Homo sapiens GN=CLPX PE=1 SV=2		88.42
O76041	NEBL_HUMAN Nebulette OS=Homo sapiens GN=NEBL PE=2 SV=1	6.69E-11	
O76054	S14L2_HUMAN SEC14-like protein 2 OS=Homo sapiens GN=SEC14L2 PE=1 SV=1	1.46E-20	
O76061	STC2_HUMAN Stanniocalcin-2 OS=Homo sapiens GN=STC2 PE=1 SV=1	1.91E-05	
O76062	ERG24_HUMAN Delta(14)-sterol reductase OS=Homo sapiens GN=TM7SF2 PE=2 SV=3		
O76062-2	ERG24_HUMAN Isoform 2 of Delta(14)-sterol reductase OS=Homo sapiens GN=TM7SF2		
O76070	SYUG_HUMAN Gamma-synuclein OS=Homo sapiens GN=SNCG PE=1 SV=2	2.72E-152	
O76071	CIAO1_HUMAN Probable cytosolic iron-sulfur protein assembly protein CIAO1 OS=Homo sapiens GN=CIAO1 PE=1 SV=1	3.36E-12	
O76083-5	PDE9A_HUMAN Isoform PDE9A5 of High affinity cGMP-specific 3',5'-cyclic phosphodiesterase 9A OS=Homo sapiens GN=PDE9A		21.53
O76094	SRP72_HUMAN Signal recognition particle 72 kDa protein OS=Homo sapiens GN=SRP72 PE=1 SV=3		315.04
O76095	JTB_HUMAN Protein JTB OS=Homo sapiens GN=JTB PE=1 SV=1		32.3
O76095-2	JTB_HUMAN Isoform 2 of Protein JTB OS=Homo sapiens GN=JTB		32.3
O94760	DDAH1_HUMAN N(G),N(G)-dimethylarginine dimethylaminohydrolase 1 OS=Homo sapiens GN=DDAH1 PE=1 SV=3		52.6
O94763	RMP_HUMAN Unconventional prefoldin RPB5 interactor OS=Homo sapiens GN=RMP PE=1 SV=3		41.16
O94763-2	RMP_HUMAN Isoform 2 of Unconventional prefoldin RPB5 interactor OS=Homo sapiens GN=RMP		41.16
O94763;O94763-2	RMP_HUMAN Unconventional prefoldin RPB5 interactor OS=Homo sapiens GN=RMP PE=1 SV=3;>sp|O94763-2|RMP_HUMAN Isoform	5.66E-05	
O94766	B3GA3_HUMAN Galactosylgalactosylxylosylprotein 3-beta-glucuronosyltransferase 3 OS=Homo sapiens GN=B3GAT3 PE=1 SV=2		28.61
O94776	MTA2_HUMAN Metastasis-associated protein MTA2 OS=Homo sapiens GN=MTA2 PE=1 SV=1		26.65
O94788	AL1A2_HUMAN Retinal dehydrogenase 2 OS=Homo sapiens GN=ALDH1A2 PE=1 SV=3		
O94788-2	AL1A2_HUMAN Isoform 2 of Retinal dehydrogenase 2 OS=Homo sapiens GN=ALDH1A2		
O94788;O94788-2	AL1A2_HUMAN Retinal dehydrogenase 2 OS=Homo sapiens GN=ALDH1A2 PE=1 SV=3;>sp|O94788-2|AL1A2_HUMAN Isoform 2 of Re	3.02E-11	
O94804	STK10_HUMAN Serine/threonine-protein kinase 10 OS=Homo sapiens GN=STK10 PE=1 SV=1		
O94805	ACL6B_HUMAN Actin-like protein 6B OS=Homo sapiens GN=ACTL6B PE=1 SV=1		
O94806-2	KPCD3_HUMAN Isoform 2 of Serine/threonine-protein kinase D3 OS=Homo sapiens GN=PRKD3		
O94822	LTN1_HUMAN E3 ubiquitin-protein ligase listerin OS=Homo sapiens GN=LTN1 PE=1 SV=6	3.14E-13	
O94823	AT10B_HUMAN Probable phospholipid-transporting ATPase VB OS=Homo sapiens GN=ATP10B PE=2 SV=2		
O94823-2	AT10B_HUMAN Isoform B of Probable phospholipid-transporting ATPase VB OS=Homo sapiens GN=ATP10B		
O94823-3	AT10B_HUMAN Isoform C of Probable phospholipid-transporting ATPase VB OS=Homo sapiens GN=ATP10B		

O94826	TOM70_HUMAN Mitochondrial import receptor subunit TOM70 OS=Homo sapiens GN=TOMM70A PE=1 SV=1		317.14
O94832	MYO1D_HUMAN Myosin-Id OS=Homo sapiens GN=MYO1D PE=1 SV=2		
O94842;B4DPY8;B4DSM0;	TOX4_HUMAN TOX high mobility group box family member 4 OS=Homo sapiens GN=TOX4 PE=1 SV=1;>tr|B4DPY8|B4DPY8_HUMAN c	1.38E-06	
O94851;O94851-2	MICA2_HUMAN Protein MICAL-2 OS=Homo sapiens GN=MICAL2 PE=1 SV=1;>sp|O94851-2|MICA2_HUMAN Isoform 2 of Protein MICA	1.39E-21	
O94855-2;A8K6V0;O9485	2|SC24D_HUMAN Isoform 2 of Protein transport protein Sec24D OS=Homo sapiens GN=SEC24D;>tr|A8K6V0|A8K6V0_HUMAN cDNA F	2.70E-71	
O94874;O94874-2	UFL1_HUMAN E3 UFM1-protein ligase 1 OS=Homo sapiens GN=KIAA0776 PE=1 SV=2;>sp|O94874-2|UFL1_HUMAN Isoform 2 of E3 U	3.03E-23	
O94875-6	SRBS2_HUMAN Isoform 6 of Sorbin and SH3 domain-containing protein 2 OS=Homo sapiens GN=SORBS2		
O94880	PHF14_HUMAN PHD finger protein 14 OS=Homo sapiens GN=PHF14 PE=1 SV=2		
O94880-2	PHF14_HUMAN Isoform 2 of PHD finger protein 14 OS=Homo sapiens GN=PHF14		
O94886	TM63A_HUMAN Transmembrane protein 63A OS=Homo sapiens GN=TMEM63A PE=1 SV=3	1.28E-09	
O94888	UBXN7_HUMAN UBX domain-containing protein 7 OS=Homo sapiens GN=UBXN7 PE=1 SV=2		92.14
O94901	SUN1_HUMAN SUN domain-containing protein 1 OS=Homo sapiens GN=SUN1 PE=1 SV=3		
O94901-5	SUN1_HUMAN Isoform 5 of SUN domain-containing protein 1 OS=Homo sapiens GN=SUN1		
O94903	PROSC_HUMAN Proline synthase co-transcribed bacterial homolog protein OS=Homo sapiens GN=PROSC PE=1 SV=1		54.46
O94905	ERLN2_HUMAN Erlin-2 OS=Homo sapiens GN=ERLIN2 PE=1 SV=1		430.74
O94905-2	ERLN2_HUMAN Isoform 2 of Erlin-2 OS=Homo sapiens GN=ERLIN2		181.71
O94905-3	ERLN2_HUMAN Isoform 3 of Erlin-2 OS=Homo sapiens GN=ERLIN2		181.71
O94906	PRP6_HUMAN Pre-processing factor 6 OS=Homo sapiens GN=PRPF6 PE=1 SV=1		387.61
O94915	FRYL_HUMAN Protein furry homolog-like OS=Homo sapiens GN=FRYL PE=1 SV=2	3.91E-08	
O94919	ENDD1_HUMAN Endonuclease domain-containing 1 protein OS=Homo sapiens GN=ENDOD1 PE=1 SV=2		70.67
O94921	CDK14_HUMAN Cyclin-dependent kinase 14 OS=Homo sapiens GN=CDK14 PE=1 SV=3		48.83
O94921-2	CDK14_HUMAN Isoform 2 of Cyclin-dependent kinase 14 OS=Homo sapiens GN=CDK14		48.83
O94921-3	CDK14_HUMAN Isoform 3 of Cyclin-dependent kinase 14 OS=Homo sapiens GN=CDK14		48.83
O94925	GLSK_HUMAN Glutaminase kidney isoform, mitochondrial OS=Homo sapiens GN=GLS PE=1 SV=1		118.65
O94925-2	GLSK_HUMAN Isoform 2 of Glutaminase kidney isoform, mitochondrial OS=Homo sapiens GN=GLS		58.93
O94925-3	GLSK_HUMAN Isoform 3 of Glutaminase kidney isoform, mitochondrial OS=Homo sapiens GN=GLS		118.65
O94925-3;A8K132;O9492	3|GLSK_HUMAN Isoform 3 of Glutaminase kidney isoform, mitochondrial OS=Homo sapiens GN=GLS;>tr|A8K132|A8K132_HUMAN cD	1.44E-208	
O94964-2;O94964;O9496	2|K0889_HUMAN Isoform 2 of Uncharacterized protein KIAA0889 OS=Homo sapiens GN=KIAA0889;>sp|O94964|K0889_HUMAN Unch	1.83E-34	
O94973	AP2A2_HUMAN AP-2 complex subunit alpha-2 OS=Homo sapiens GN=AP2A2 PE=1 SV=2		424.85
O94973-2	AP2A2_HUMAN Isoform 2 of AP-2 complex subunit alpha-2 OS=Homo sapiens GN=AP2A2		424.85
O94973-2;O94973;B7Z5S	2|AP2A2_HUMAN Isoform 2 of AP-2 complex subunit alpha-2 OS=Homo sapiens GN=AP2A2;>sp|O94973|AP2A2_HUMAN AP-2 comple	1.24E-101	
O94973-3	AP2A2_HUMAN Isoform 3 of AP-2 complex subunit alpha-2 OS=Homo sapiens GN=AP2A2		336.68
O94979	SC31A_HUMAN Protein transport protein Sec31A OS=Homo sapiens GN=SEC31A PE=1 SV=3		
O94979-2	SC31A_HUMAN Isoform 2 of Protein transport protein Sec31A OS=Homo sapiens GN=SEC31A		
O94979-3	SC31A_HUMAN Isoform 3 of Protein transport protein Sec31A OS=Homo sapiens GN=SEC31A		
O94979-4	SC31A_HUMAN Isoform 4 of Protein transport protein Sec31A OS=Homo sapiens GN=SEC31A		
O94979-5	SC31A_HUMAN Isoform 5 of Protein transport protein Sec31A OS=Homo sapiens GN=SEC31A		
O94979-6	SC31A_HUMAN Isoform 6 of Protein transport protein Sec31A OS=Homo sapiens GN=SEC31A		
O94979-7	SC31A_HUMAN Isoform 7 of Protein transport protein Sec31A OS=Homo sapiens GN=SEC31A		
O94979-8	SC31A_HUMAN Isoform 8 of Protein transport protein Sec31A OS=Homo sapiens GN=SEC31A		
O94979-8;O94979;O9497	8|SC31A_HUMAN Isoform 8 of Protein transport protein Sec31A OS=Homo sapiens GN=SEC31A;>sp|O94979|SC31A_HUMAN Protein	2.37E-46	
O94992	HEXI1_HUMAN Protein HEXIM1 OS=Homo sapiens GN=HEXIM1 PE=1 SV=1	4.58E-18	
O95049	ZO3_HUMAN Tight junction protein ZO-3 OS=Homo sapiens GN=TJP3 PE=1 SV=2		
O95049-2	ZO3_HUMAN Isoform 2 of Tight junction protein ZO-3 OS=Homo sapiens GN=TJP3		
O95049-3	ZO3_HUMAN Isoform 3 of Tight junction protein ZO-3 OS=Homo sapiens GN=TJP3		
O95059	RPP14_HUMAN Ribonuclease P protein subunit p14 OS=Homo sapiens GN=RPP14 PE=1 SV=3		194.46
O95070	YIF1A_HUMAN Protein YIF1A OS=Homo sapiens GN=YIF1A PE=1 SV=2		113.65
O95071	UBR5_HUMAN E3 ubiquitin-protein ligase UBR5 OS=Homo sapiens GN=UBR5 PE=1 SV=2		244.05
O95073	FSBP_HUMAN Fibrinogen silencer-binding protein OS=Homo sapiens GN=FSBP PE=1 SV=1		
O95073-2	FSBP_HUMAN Isoform 2 of Fibrinogen silencer-binding protein OS=Homo sapiens GN=FSBP		
O95076	ALX3_HUMAN Homeobox protein aristaless-like 3 OS=Homo sapiens GN=ALX3 PE=1 SV=2		
O95096	NKX22_HUMAN Homeobox protein Nkx-2.2 OS=Homo sapiens GN=NKX2-2 PE=2 SV=1		
O95104	SFR15_HUMAN Splicing factor, arginine/serine-rich 15 OS=Homo sapiens GN=SCAF4 PE=1 SV=3		64.58
O95104-2	SFR15_HUMAN Isoform 2 of Splicing factor, arginine/serine-rich 15 OS=Homo sapiens GN=SCAF4		64.58
O95104;O95104-2	SFR15_HUMAN Splicing factor, arginine/serine-rich 15 OS=Homo sapiens GN=SCAF4 PE=1 SV=3;>sp|O95104-2|SFR15_HUMAN Isofo	1.49E-15	
O95139	NDUB6_HUMAN NADH dehydrogenase [ubiquinone] 1 beta subcomplex subunit 6 OS=Homo sapiens GN=NDUFB6 PE=1 SV=3		89.32
O95140	MFN2_HUMAN Mitofusin-2 OS=Homo sapiens GN=MFN2 PE=1 SV=3		
O95140-2	MFN2_HUMAN Isoform 2 of Mitofusin-2 OS=Homo sapiens GN=MFN2		
O95154	ARK73_HUMAN Aflatoxin B1 aldehyde reductase member 3 OS=Homo sapiens GN=AKR7A3 PE=1 SV=2	1.33E-110	
O95155;A8K8S9;O95155-	UBE4B_HUMAN Ubiquitin conjugation factor E4 B OS=Homo sapiens GN=UBE4B PE=1 SV=1;>tr|A8K8S9|A8K8S9_HUMAN cDNA FLJ78	4.54E-07	
O95159	ZFPL1_HUMAN Zinc finger protein-like 1 OS=Homo sapiens GN=ZFPL1 PE=1 SV=2		86.07

O95163	ELP1_HUMAN Elongator complex protein 1 OS=Homo sapiens GN=IKBKAP PE=1 SV=3	6.51E-43	
O95168	NDUB4_HUMAN NADH dehydrogenase [ubiquinone] 1 beta subcomplex subunit 4 OS=Homo sapiens GN=NDUFB4 PE=1 SV=3		
O95169	NDUB8_HUMAN NADH dehydrogenase [ubiquinone] 1 beta subcomplex subunit 8, mitochondrial OS=Homo sapiens GN=NDUFB8 PE=1 SV=1		70.25
O95170	CDRT1_HUMAN CMT1A duplicated region transcript 1 protein OS=Homo sapiens GN=CDRT1 PE=2 SV=3		
O95170-2	CDRT1_HUMAN Isoform 2 of CMT1A duplicated region transcript 1 protein OS=Homo sapiens GN=CDRT1		
O95171	SCEL_HUMAN Sciellin OS=Homo sapiens GN=SCEL PE=1 SV=2		
O95171-2	SCEL_HUMAN Isoform 2 of Sciellin OS=Homo sapiens GN=SCEL		
O95182	NDUA7_HUMAN NADH dehydrogenase [ubiquinone] 1 alpha subcomplex subunit 7 OS=Homo sapiens GN=NDUFA7 PE=1 SV=3		132.91
O95197	RTN3_HUMAN Reticulon-3 OS=Homo sapiens GN=RTN3 PE=1 SV=2		145.87
O95197-2	RTN3_HUMAN Isoform 2 of Reticulon-3 OS=Homo sapiens GN=RTN3		145.87
O95197-3	RTN3_HUMAN Isoform 3 of Reticulon-3 OS=Homo sapiens GN=RTN3		148.57
O95197-4	RTN3_HUMAN Isoform 4 of Reticulon-3 OS=Homo sapiens GN=RTN3		134.67
O95197-5	RTN3_HUMAN Isoform 5 of Reticulon-3 OS=Homo sapiens GN=RTN3		53.44
O95197-5;O95197-3;B7Z	5|RTN3_HUMAN Isoform 5 of Reticulon-3 OS=Homo sapiens GN=RTN3;>sp|O95197-3|RTN3_HUMAN Isoform 3 of Reticulon-3 OS=Ho	3.84E-14	
O95197;O95197-2;O9519	RTN3_HUMAN Reticulon-3 OS=Homo sapiens GN=RTN3 PE=1 SV=2;>sp|O95197-2|RTN3_HUMAN Isoform 2 of Reticulon-3 OS=Homo	3.22E-15	
O95202	LETM1_HUMAN LETM1 and EF-hand domain-containing protein 1, mitochondrial OS=Homo sapiens GN=LETM1 PE=1 SV=1		465.46
O95202-2	LETM1_HUMAN Isoform 2 of LETM1 and EF-hand domain-containing protein 1, mitochondrial OS=Homo sapiens GN=LETM1		151.42
O95202-3	LETM1_HUMAN Isoform 3 of LETM1 and EF-hand domain-containing protein 1, mitochondrial OS=Homo sapiens GN=LETM1		151.42
O95202;D3DVQ1	LETM1_HUMAN LETM1 and EF-hand domain-containing protein 1, mitochondrial OS=Homo sapiens GN=LETM1 PE=1 SV=1;>tr|D3DV	3.29E-195	
O95208	EPN2_HUMAN Epsin-2 OS=Homo sapiens GN=EPN2 PE=1 SV=3		32.77
O95208-2	EPN2_HUMAN Isoform 2 of Epsin-2 OS=Homo sapiens GN=EPN2		32.77
O95208-3	EPN2_HUMAN Isoform 3 of Epsin-2 OS=Homo sapiens GN=EPN2		20.66
O95214	LERL1_HUMAN Leptin receptor overlapping transcript-like 1 OS=Homo sapiens GN=LEPROTL1 PE=2 SV=2		34.68
O95218	ZRAB2_HUMAN Zinc finger Ran-binding domain-containing protein 2 OS=Homo sapiens GN=ZRANB2 PE=1 SV=2		
O95218-2	ZRAB2_HUMAN Isoform 2 of Zinc finger Ran-binding domain-containing protein 2 OS=Homo sapiens GN=ZRANB2		
O95218;O95218-2	ZRAB2_HUMAN Zinc finger Ran-binding domain-containing protein 2 OS=Homo sapiens GN=ZRANB2 PE=1 SV=2;>sp|O95218-2|ZRA	8.85E-58	
O95219	SNX4_HUMAN Sorting nexin-4 OS=Homo sapiens GN=SNX4 PE=1 SV=1		64.61
O95229	ZWINT_HUMAN ZW10 interactor OS=Homo sapiens GN=ZWINT PE=1 SV=2	4.11E-07	
O95231	VENTX_HUMAN Homeobox protein VENTX OS=Homo sapiens GN=VENTX PE=2 SV=1		
O95232	LC7L3_HUMAN Luc7-like protein 3 OS=Homo sapiens GN=LUC7L3 PE=1 SV=2		142.5
O95232-2	LC7L3_HUMAN Isoform 2 of Luc7-like protein 3 OS=Homo sapiens GN=LUC7L3		
O95236;O95236-2;O9523	APOL3_HUMAN Apolipoprotein L3 OS=Homo sapiens GN=APOL3 PE=2 SV=3;>sp|O95236-2|APOL3_HUMAN Isoform 2 of Apolipoprote	7.32E-21	
O95239	KIF4A_HUMAN Chromosome-associated kinesin KIF4A OS=Homo sapiens GN=KIF4A PE=1 SV=3		46.17
O95239-2	KIF4A_HUMAN Isoform 2 of Chromosome-associated kinesin KIF4A OS=Homo sapiens GN=KIF4A		
O95239;O95239-2	KIF4A_HUMAN Chromosome-associated kinesin KIF4A OS=Homo sapiens GN=KIF4A PE=1 SV=3;>sp|O95239-2|KIF4A_HUMAN Isofor	3.33E-34	
O95249	GOSR1_HUMAN Golgi SNAP receptor complex member 1 OS=Homo sapiens GN=GOSR1 PE=1 SV=1		135.36
O95251	MYST2_HUMAN Histone acetyltransferase MYST2 OS=Homo sapiens GN=MYST2 PE=1 SV=1		
O95255	MRP6_HUMAN Multidrug resistance-associated protein 6 OS=Homo sapiens GN=ABCC6 PE=1 SV=2		
O95259	KCNH1_HUMAN Potassium voltage-gated channel subfamily H member 1 OS=Homo sapiens GN=KCNH1 PE=1 SV=1		1.89
O95259-2	KCNH1_HUMAN Isoform 1 of Potassium voltage-gated channel subfamily H member 1 OS=Homo sapiens GN=KCNH1		1.89
O95273;O95273-2	CCDB1_HUMAN Cyclin-D1-binding protein 1 OS=Homo sapiens GN=CCNDBP1 PE=1 SV=2;>sp|O95273-2|CCDB1_HUMAN Isoform 2 o	1.97E-06	
O95292	VAPB_HUMAN Vesicle-associated membrane protein-associated protein B/C OS=Homo sapiens GN=VAPB PE=1 SV=3		304.38
O95292-2	VAPB_HUMAN Isoform 2 of Vesicle-associated membrane protein-associated protein B/C OS=Homo sapiens GN=VAPB		120.89
O95292;Q53XM7;B3KVU7	VAPB_HUMAN Vesicle-associated membrane protein-associated protein B/C OS=Homo sapiens GN=VAPB PE=1 SV=3;>tr|Q53XM7|Q5	1.66E-52	
O95294;O95294-2	RASL1_HUMAN RasGAP-activating-like protein 1 OS=Homo sapiens GN=RASAL1 PE=1 SV=3;>sp|O95294-2|RASL1_HUMAN Isoform 2	3.87E-15	
O95297	MPZL1_HUMAN Myelin protein zero-like protein 1 OS=Homo sapiens GN=MPZL1 PE=1 SV=1		79.89
O95297-2	MPZL1_HUMAN Isoform 2 of Myelin protein zero-like protein 1 OS=Homo sapiens GN=MPZL1		79.89
O95297-3	MPZL1_HUMAN Isoform 3 of Myelin protein zero-like protein 1 OS=Homo sapiens GN=MPZL1		52.65
O95297-3;Q9UEL6	3|MPZL1_HUMAN Isoform 3 of Myelin protein zero-like protein 1 OS=Homo sapiens GN=MPZL1;>tr|Q9UEL6|Q9UEL6_HUMAN Putative	5.18E-05	
O95297-4	MPZL1_HUMAN Isoform 4 of Myelin protein zero-like protein 1 OS=Homo sapiens GN=MPZL1		79.89
O95297;O95297-2;O9529	MPZL1_HUMAN Myelin protein zero-like protein 1 OS=Homo sapiens GN=MPZL1 PE=1 SV=1;>sp|O95297-2|MPZL1_HUMAN Isoform 2	8.26E-43	
O95298	NDUC2_HUMAN NADH dehydrogenase [ubiquinone] 1 subunit C2 OS=Homo sapiens GN=NDUFC2 PE=1 SV=1		
O95299	NDUAA_HUMAN NADH dehydrogenase [ubiquinone] 1 alpha subcomplex subunit 10, mitochondrial OS=Homo sapiens GN=NDUFA10 PE=1 SV=1		139.09
O95302	FKBP9_HUMAN Peptidyl-prolyl cis-trans isomerase FKBP9 OS=Homo sapiens GN=FKBP9 PE=1 SV=2		
O95319	CELF2_HUMAN CUGBP Elav-like family member 2 OS=Homo sapiens GN=CELF2 PE=1 SV=1		27.76
O95319-2	CELF2_HUMAN Isoform 2 of CUGBP Elav-like family member 2 OS=Homo sapiens GN=CELF2		27.76
O95319-3	CELF2_HUMAN Isoform 3 of CUGBP Elav-like family member 2 OS=Homo sapiens GN=CELF2		27.76
O95319-3;O95319-4;O95	3|CELF2_HUMAN Isoform 3 of CUGBP Elav-like family member 2 OS=Homo sapiens GN=CELF2;>sp|O95319-4|CELF2_HUMAN Isoform	4.99E-26	
O95319-4	CELF2_HUMAN Isoform 4 of CUGBP Elav-like family member 2 OS=Homo sapiens GN=CELF2		27.76
O95319-5	CELF2_HUMAN Isoform 5 of CUGBP Elav-like family member 2 OS=Homo sapiens GN=CELF2		27.76
O95336	6PGL_HUMAN 6-phosphogluconolactonase OS=Homo sapiens GN=PGLS PE=1 SV=2		189.19

O95340-2;O95340	2|PAPS2_HUMAN Isoform B of Bifunctional 3-phosphoadenosine 5-phosphosulfate synthase 2 OS=Homo sapiens GN=PAPSS2;>sp|O9!	3.40E-45	
O95342	ABCBB_HUMAN Bile salt export pump OS=Homo sapiens GN=ABCB11 PE=1 SV=2		
O95347;O95347-2	SMC2_HUMAN Structural maintenance of chromosomes protein 2 OS=Homo sapiens GN=SMC2 PE=1 SV=2;>sp|O95347-2|SMC2_HUI	5.96E-117	
O95352;O95352-2	ATG7_HUMAN Ubiquitin-like modifier-activating enzyme ATG7 OS=Homo sapiens GN=ATG7 PE=1 SV=1;>sp|O95352-2|ATG7_HUMAN	8.77E-13	
O95359;O95359-3	TACC2_HUMAN Transforming acidic coiled-coil-containing protein 2 OS=Homo sapiens GN=TACC2 PE=1 SV=3;>sp|O95359-3|TACC2_	3.76E-181	
O95365	ZBT7A_HUMAN Zinc finger and BTB domain-containing protein 7A OS=Homo sapiens GN=ZBTB7A PE=1 SV=1		
O95372	LYPA2_HUMAN Acyl-protein thioesterase 2 OS=Homo sapiens GN=LYPLA2 PE=1 SV=1		39.88
O95373	IPO7_HUMAN Importin-7 OS=Homo sapiens GN=IPO7 PE=1 SV=1		233.61
O95379	TFIP8_HUMAN Tumor necrosis factor alpha-induced protein 8 OS=Homo sapiens GN=TNFAIP8 PE=1 SV=1		40.44
O95379-2	TFIP8_HUMAN Isoform 2 of Tumor necrosis factor alpha-induced protein 8 OS=Homo sapiens GN=TNFAIP8		40.44
O95379-3	TFIP8_HUMAN Isoform 3 of Tumor necrosis factor alpha-induced protein 8 OS=Homo sapiens GN=TNFAIP8		40.44
O95379;O95379-2;O9537	TFIP8_HUMAN Tumor necrosis factor alpha-induced protein 8 OS=Homo sapiens GN=TNFAIP8 PE=2 SV=1;>sp|O95379-2|TFIP8_HUM.	9.64E-08	
O95391	SLU7_HUMAN Pre-splicing factor SLU7 OS=Homo sapiens GN=SLU7 PE=1 SV=2		29.3
O95394	AGM1_HUMAN Phosphoacetylglucosamine mutase OS=Homo sapiens GN=PGM3 PE=1 SV=1		59.35
O95395	GCNT3_HUMAN Beta-1,3-galactosyl-O-glycosyl-glycoprotein beta-1,6-N-acetylglucosaminyltransferase 3 OS=Homo sapiens GN=GCNT3 PE=2 SV=1		
O95396	MOCS3_HUMAN Adenylyltransferase and sulfurtransferase MOCS3 OS=Homo sapiens GN=MOCS3 PE=1 SV=1	2.27E-23	
O95399	UTS2_HUMAN Urotensin-2 OS=Homo sapiens GN=UTS2 PE=1 SV=1		42.23
O95399-2	UTS2_HUMAN Isoform 2 of Urotensin-2 OS=Homo sapiens GN=UTS2		42.23
O95400	CD2B2_HUMAN CD2 antigen cytoplasmic tail-binding protein 2 OS=Homo sapiens GN=CD2BP2 PE=1 SV=1		
O95425	SVIL_HUMAN Supervillin OS=Homo sapiens GN=SVIL PE=1 SV=2		357.73
O95425-2	SVIL_HUMAN Isoform 2 of Supervillin OS=Homo sapiens GN=SVIL		335.71
O95425;O95425-2	SVIL_HUMAN Supervillin OS=Homo sapiens GN=SVIL PE=1 SV=2;>sp|O95425-2|SVIL_HUMAN Isoform 2 of Supervillin OS=Homo sa|	5.97E-18	
O95428	PPN_HUMAN Papilin OS=Homo sapiens GN=PAPLN PE=2 SV=4		
O95428-4	PPN_HUMAN Isoform 4 of Papilin OS=Homo sapiens GN=PAPLN		
O95428-5	PPN_HUMAN Isoform 5 of Papilin OS=Homo sapiens GN=PAPLN		
O95428-6	PPN_HUMAN Isoform 6 of Papilin OS=Homo sapiens GN=PAPLN		
O95433	AHSA1_HUMAN Activator of 90 kDa heat shock protein ATPase homolog 1 OS=Homo sapiens GN=AHSA1 PE=1 SV=1		70.36
O95445	APOM_HUMAN Apolipoprotein M OS=Homo sapiens GN=APOM PE=1 SV=2	2.07E-06	
O95450	ATS2_HUMAN A disintegrin and metalloproteinase with thrombospondin motifs 2 OS=Homo sapiens GN=ADAMTS2 PE=2 SV=2		
O95456	PSMG1_HUMAN Proteasome assembly chaperone 1 OS=Homo sapiens GN=PSMG1 PE=1 SV=1		39.96
O95456-2	PSMG1_HUMAN Isoform 2 of Proteasome assembly chaperone 1 OS=Homo sapiens GN=PSMG1		39.96
O95456-2;O95456	2|PSMG1_HUMAN Isoform 2 of Proteasome assembly chaperone 1 OS=Homo sapiens GN=PSMG1;>sp|O95456|PSMG1_HUMAN Protea	2.20E-11	
O95456;O95456-2	PSMG1_HUMAN Proteasome assembly chaperone 1 OS=Homo sapiens GN=PSMG1 PE=1 SV=1;>sp|O95456-2|PSMG1_HUMAN Isoforr	6.79E-18	
O95466	FMNL_HUMAN Formin-like protein 1 OS=Homo sapiens GN=FMNL1 PE=1 SV=3		242.14
O95466-2	FMNL_HUMAN Isoform 2 of Formin-like protein 1 OS=Homo sapiens GN=FMNL1		242.14
O95466-2;O95466	2|FMNL_HUMAN Isoform 2 of Formin-like protein 1 OS=Homo sapiens GN=FMNL1;>sp|O95466|FMNL_HUMAN Formin-like protein 1 O	2.29E-44	
O95470	SGPL1_HUMAN Sphingosine-1-phosphate lyase 1 OS=Homo sapiens GN=SGPL1 PE=1 SV=3		65.76
O95477	ABCA1_HUMAN ATP-binding cassette sub-family A member 1 OS=Homo sapiens GN=ABCA1 PE=1 SV=3		74.93
O95478	NSA2_HUMAN Ribosome biogenesis protein NSA2 homolog OS=Homo sapiens GN=NSA2 PE=1 SV=1		
O95479	G6PE_HUMAN GDH/6PGL endoplasmic bifunctional protein OS=Homo sapiens GN=H6PD PE=1 SV=2	3.43E-27	
O95484	CLD9_HUMAN Claudin-9 OS=Homo sapiens GN=CLDN9 PE=1 SV=1		79.62
O95486	SC24A_HUMAN Protein transport protein Sec24A OS=Homo sapiens GN=SEC24A PE=1 SV=2		
O95487	SC24B_HUMAN Protein transport protein Sec24B OS=Homo sapiens GN=SEC24B PE=1 SV=2		
O95487-2	SC24B_HUMAN Isoform 2 of Protein transport protein Sec24B OS=Homo sapiens GN=SEC24B		
O95487;O95487-2	SC24B_HUMAN Protein transport protein Sec24B OS=Homo sapiens GN=SEC24B PE=1 SV=2;>sp|O95487-2|SC24B_HUMAN Isoform	7.26E-38	
O95503	CBX6_HUMAN Chromobox protein homolog 6 OS=Homo sapiens GN=CBX6 PE=1 SV=1		
O95551-2;O95551;O9555	2|TYDP2_HUMAN Isoform 2 of Tyrosyl-DNA phosphodiesterase 2 OS=Homo sapiens GN=TDP2;>sp|O95551|TYDP2_HUMAN Tyrosyl-DI	1.73E-12	
O95563	BR44_HUMAN Brain protein 44 OS=Homo sapiens GN=BRP44 PE=1 SV=1		71.69
O95571	ETHE1_HUMAN Protein ETHE1, mitochondrial OS=Homo sapiens GN=ETHE1 PE=1 SV=2		66.53
O95573	ACSL3_HUMAN Long-chain-fatty-acid--CoA ligase 3 OS=Homo sapiens GN=ACSL3 PE=1 SV=3		269.47
O95602	RPA1_HUMAN DNA-directed RNA polymerase I subunit RPA1 OS=Homo sapiens GN=POLR1A PE=1 SV=2		79
O95613	PCNT_HUMAN Pericentrin OS=Homo sapiens GN=PCNT PE=1 SV=4		
O95613-2	PCNT_HUMAN Isoform 2 of Pericentrin OS=Homo sapiens GN=PCNT		
O95626	AN32D_HUMAN Acidic leucine-rich nuclear phosphoprotein 32 family member D OS=Homo sapiens GN=ANP32D PE=1 SV=2		75.91
O95630	STABP_HUMAN STAM-binding protein OS=Homo sapiens GN=STAMBP PE=1 SV=1	8.89E-10	
O95639	CPSF4_HUMAN Cleavage and polyadenylation specificity factor subunit 4 OS=Homo sapiens GN=CPSF4 PE=1 SV=1		
O95639-2	CPSF4_HUMAN Isoform 2 of Cleavage and polyadenylation specificity factor subunit 4 OS=Homo sapiens GN=CPSF4		
O95639-3	CPSF4_HUMAN Isoform 3 of Cleavage and polyadenylation specificity factor subunit 4 OS=Homo sapiens GN=CPSF4		
O95639;O95639-2;O9563	CPSF4_HUMAN Cleavage and polyadenylation specificity factor subunit 4 OS=Homo sapiens GN=CPSF4 PE=1 SV=1;>sp|O95639-2|CP	3.59E-09	
O95671;O95671-2	ASML_HUMAN N-acetylserotonin O-methyltransferase-like protein OS=Homo sapiens GN=ASMTL PE=1 SV=3;>sp|O95671-2|ASML_HU	1.35E-10	
O95674	CDS2_HUMAN Phosphatidate cytidylyltransferase 2 OS=Homo sapiens GN=CDS2 PE=1 SV=1	1.33E-06	

O95678	K2C75_HUMAN Keratin, type II cytoskeletal 75 OS=Homo sapiens GN=KRT75 PE=1 SV=2		526.5
O95684;O95684-2	FR1OP_HUMAN FGFR1 oncogene partner OS=Homo sapiens GN=FGFR1OP PE=1 SV=1;>sp|O95684-2|FR1OP_HUMAN Isoform 2 of FG	2.95E-09	
O95707	RPP29_HUMAN Ribonuclease P protein subunit p29 OS=Homo sapiens GN=POP4 PE=1 SV=2		124.59
O95714	HERC2_HUMAN E3 ubiquitin-protein ligase HERC2 OS=Homo sapiens GN=HERC2 PE=1 SV=2		76.48
O95716	RAB3D_HUMAN Ras-related protein Rab-3D OS=Homo sapiens GN=RAB3D PE=1 SV=1		134.86
O95718	ERR2_HUMAN Steroid hormone receptor ERR2 OS=Homo sapiens GN=ESRRB PE=1 SV=1		28.97
O95721	SNP29_HUMAN Synaptosomal-associated protein 29 OS=Homo sapiens GN=SNAP29 PE=1 SV=1		408.8
O95741	CPNE6_HUMAN Copine-6 OS=Homo sapiens GN=CPNE6 PE=1 SV=3		27.17
O95747	OXSR1_HUMAN Serine/threonine-protein kinase OSR1 OS=Homo sapiens GN=OXSR1 PE=1 SV=1		42.04
O95749	GGPPS_HUMAN Geranylgeranyl pyrophosphate synthase OS=Homo sapiens GN=GGPS1 PE=1 SV=1	1.87E-12	
O95757	HS74L_HUMAN Heat shock 70 kDa protein 4L OS=Homo sapiens GN=HSPA4L PE=1 SV=3		56.58
O95758	ROD1_HUMAN Regulator of differentiation 1 OS=Homo sapiens GN=ROD1 PE=1 SV=2		61.1
O95758-1	ROD1_HUMAN Isoform 1 of Regulator of differentiation 1 OS=Homo sapiens GN=ROD1		61.1
O95758-2	ROD1_HUMAN Isoform 2 of Regulator of differentiation 1 OS=Homo sapiens GN=ROD1		61.1
O95758-4	ROD1_HUMAN Isoform 4 of Regulator of differentiation 1 OS=Homo sapiens GN=ROD1		61.1
O95758-4;O95758-5;O957	4|ROD1_HUMAN Isoform 4 of Regulator of differentiation 1 OS=Homo sapiens GN=ROD1;>sp|O95758-5|ROD1_HUMAN Isoform 5 of R	5.70E-15	
O95758-5	ROD1_HUMAN Isoform 5 of Regulator of differentiation 1 OS=Homo sapiens GN=ROD1		61.1
O95759;Q0IIM8;O95759-2	TBCD8_HUMAN TBC1 domain family member 8 OS=Homo sapiens GN=TBC1D8 PE=1 SV=3;>sp|Q0IIM8|TBC8B_HUMAN TBC1 domain	3.22E-06	
O95777	NAA38_HUMAN N-alpha-acetyltransferase 38, NatC auxiliary subunit OS=Homo sapiens GN=NAA38 PE=1 SV=3		327.67
O95782	AP2A1_HUMAN AP-2 complex subunit alpha-1 OS=Homo sapiens GN=AP2A1 PE=1 SV=3		1500.03
O95782-2	AP2A1_HUMAN Isoform B of AP-2 complex subunit alpha-1 OS=Homo sapiens GN=AP2A1		1500.03
O95782;O95782-2	AP2A1_HUMAN AP-2 complex subunit alpha-1 OS=Homo sapiens GN=AP2A1 PE=1 SV=3;>sp|O95782-2|AP2A1_HUMAN Isoform B of	1.55E-31	
O95786	DDX58_HUMAN Probable ATP-dependent RNA helicase DDX58 OS=Homo sapiens GN=DDX58 PE=1 SV=2		23.3
O95786-2	DDX58_HUMAN Isoform 2 of Probable ATP-dependent RNA helicase DDX58 OS=Homo sapiens GN=DDX58		23.3
O95786;O95786-2	DDX58_HUMAN Probable ATP-dependent RNA helicase DDX58 OS=Homo sapiens GN=DDX58 PE=1 SV=2;>sp|O95786-2|DDX58_HUM	6.73E-100	
O95793	STAU1_HUMAN Double-stranded RNA-binding protein Staufen homolog 1 OS=Homo sapiens GN=STAU1 PE=1 SV=2		567.97
O95793-2	STAU1_HUMAN Isoform Short of Double-stranded RNA-binding protein Staufen homolog 1 OS=Homo sapiens GN=STAU1		567.97
O95793;O95793-2	STAU1_HUMAN Double-stranded RNA-binding protein Staufen homolog 1 OS=Homo sapiens GN=STAU1 PE=1 SV=2;>sp|O95793-2|ST	2.73E-60	
O95801	TTC4_HUMAN Tetratricopeptide repeat protein 4 OS=Homo sapiens GN=TTC4 PE=1 SV=3	3.68E-25	
O95810	SDPR_HUMAN Serum deprivation-response protein OS=Homo sapiens GN=SDPR PE=1 SV=3		367.08
O95816	BAG2_HUMAN BAG family molecular chaperone regulator 2 OS=Homo sapiens GN=BAG2 PE=1 SV=1		523.79
O95817	BAG3_HUMAN BAG family molecular chaperone regulator 3 OS=Homo sapiens GN=BAG3 PE=1 SV=3		27.81
O95819	M4K4_HUMAN Mitogen-activated protein kinase kinase kinase kinase 4 OS=Homo sapiens GN=MAP4K4 PE=1 SV=2		128.57
O95819-2	M4K4_HUMAN Isoform 2 of Mitogen-activated protein kinase kinase kinase kinase 4 OS=Homo sapiens GN=MAP4K4		128.57
O95819-3	M4K4_HUMAN Isoform 3 of Mitogen-activated protein kinase kinase kinase kinase 4 OS=Homo sapiens GN=MAP4K4		128.57
O95819-4	M4K4_HUMAN Isoform 4 of Mitogen-activated protein kinase kinase kinase kinase 4 OS=Homo sapiens GN=MAP4K4		128.57
O95819-5	M4K4_HUMAN Isoform 5 of Mitogen-activated protein kinase kinase kinase kinase 4 OS=Homo sapiens GN=MAP4K4		128.57
O95825	QORL1_HUMAN Quinone oxidoreductase-like protein 1 OS=Homo sapiens GN=CRYZL1 PE=1 SV=2	2.85E-11	
O95831	AIFM1_HUMAN Apoptosis-inducing factor 1, mitochondrial OS=Homo sapiens GN=AIFM1 PE=1 SV=1		390.02
O95831-2	AIFM1_HUMAN Isoform 2 of Apoptosis-inducing factor 1, mitochondrial OS=Homo sapiens GN=AIFM1		157.59
O95831-3	AIFM1_HUMAN Isoform 3 of Apoptosis-inducing factor 1, mitochondrial OS=Homo sapiens GN=AIFM1		384.65
O95831;D3DTE9;O95831-	AIFM1_HUMAN Apoptosis-inducing factor 1, mitochondrial OS=Homo sapiens GN=AIFM1 PE=1 SV=1;>tr|D3DTE9|D3DTE9_HUMAN Pr	4.41E-135	
O95832	CLD1_HUMAN Claudin-1 OS=Homo sapiens GN=CLDN1 PE=1 SV=1	3.29E-10	
O95833	CLIC3_HUMAN Chloride intracellular channel protein 3 OS=Homo sapiens GN=CLIC3 PE=1 SV=2	6.79E-19	
O95837	GNA14_HUMAN Guanine nucleotide-binding protein subunit alpha-14 OS=Homo sapiens GN=GNA14 PE=2 SV=1		172.18
O95838	GLP2R_HUMAN Glucagon-like peptide 2 receptor OS=Homo sapiens GN=GLP2R PE=1 SV=1		23.71
O95861-2;C9JI50;D3DTA9	2|BPNT1_HUMAN Isoform 2 of 3(2),5-bisphosphate nucleotidase 1 OS=Homo sapiens GN=BPNT1;>tr|C9JI50|C9JI50_HUMAN Unchara	1.89E-180	
O95864	FADS2_HUMAN Fatty acid desaturase 2 OS=Homo sapiens GN=FADS2 PE=1 SV=1		63.13
O95864-2	FADS2_HUMAN Isoform 2 of Fatty acid desaturase 2 OS=Homo sapiens GN=FADS2		45.92
O95864-3	FADS2_HUMAN Isoform 3 of Fatty acid desaturase 2 OS=Homo sapiens GN=FADS2		17.21
O95864;O95864-2;O9586	FADS2_HUMAN Fatty acid desaturase 2 OS=Homo sapiens GN=FADS2 PE=1 SV=1;>sp|O95864-2|FADS2_HUMAN Isoform 2 of Fatty a	9.98E-27	
O95865	DDAH2_HUMAN N(G),N(G)-dimethylarginine dimethylaminohydrolase 2 OS=Homo sapiens GN=DDAH2 PE=1 SV=1		106.98
O95876	FRITZ_HUMAN WD repeat-containing and planar cell polarity effector protein fritz homolog OS=Homo sapiens GN=WDPCP PE=1 SV=2		
O95876-2	FRITZ_HUMAN Isoform 2 of WD repeat-containing and planar cell polarity effector protein fritz homolog OS=Homo sapiens GN=WDPCP		
O95876-3	FRITZ_HUMAN Isoform 3 of WD repeat-containing and planar cell polarity effector protein fritz homolog OS=Homo sapiens GN=WDPCP		
O95881	TXD12_HUMAN Thioredoxin domain-containing protein 12 OS=Homo sapiens GN=TXNDC12 PE=1 SV=1		260.8
O95905	SGT1_HUMAN Protein SGT1 OS=Homo sapiens GN=ECD PE=1 SV=1	2.70E-08	
O95926	SYF2_HUMAN Pre-splicing factor SYF2 OS=Homo sapiens GN=SYF2 PE=1 SV=1		
O95967	FBLN4_HUMAN EGF-containing fibulin-like extracellular matrix protein 2 OS=Homo sapiens GN=EFEMP2 PE=1 SV=3	0.00013111	
O95969	SG1D2_HUMAN Secretoglobin family 1D member 2 OS=Homo sapiens GN=SCGB1D2 PE=2 SV=1	2.76E-08	
O95983	MBD3_HUMAN Methyl-CpG-binding domain protein 3 OS=Homo sapiens GN=MBD3 PE=1 SV=1		77.26

O95983-2	MBD3_HUMAN Isoform 2 of Methyl-CpG-binding domain protein 3 OS=Homo sapiens GN=MBD3		77.26
O95983;O95983-2	MBD3_HUMAN Methyl-CpG-binding domain protein 3 OS=Homo sapiens GN=MBD3 PE=1 SV=1;>sp|O95983-2|MBD3_HUMAN Isoform	8.15E-10	
O95989	NUDT3_HUMAN Diphosphoinositol polyphosphate phosphohydrolase 1 OS=Homo sapiens GN=NUDT3 PE=1 SV=1	1.47E-05	
O95994	AGR2_HUMAN Anterior gradient protein 2 homolog OS=Homo sapiens GN=AGR2 PE=1 SV=1		23.53
O95999	BCL10_HUMAN B-cell lymphoma/leukemia 10 OS=Homo sapiens GN=BCL10 PE=1 SV=1	6.11E-16	
O96000	NDUBA_HUMAN NADH dehydrogenase [ubiquinone] 1 beta subcomplex subunit 10 OS=Homo sapiens GN=NDUFB10 PE=1 SV=3		248.59
O96005	CLPT1_HUMAN Cleft lip and palate transmembrane protein 1 OS=Homo sapiens GN=CLPTM1 PE=1 SV=1		38.78
O96005-2	CLPT1_HUMAN Isoform 2 of Cleft lip and palate transmembrane protein 1 OS=Homo sapiens GN=CLPTM1		38.78
O96005-2;O96005	2|CLPT1_HUMAN Isoform 2 of Cleft lip and palate transmembrane protein 1 OS=Homo sapiens GN=CLPTM1;>sp|O96005|CLPT1_HUM	3.89E-05	
O96007	MOC2B_HUMAN Molybdopterin synthase catalytic subunit OS=Homo sapiens GN=MOCS2 PE=1 SV=1		24.88
O96008	TOM40_HUMAN Mitochondrial import receptor subunit TOM40 homolog OS=Homo sapiens GN=TOMM40 PE=1 SV=1		301.26
O96008-2	TOM40_HUMAN Isoform 2 of Mitochondrial import receptor subunit TOM40 homolog OS=Homo sapiens GN=TOMM40		239.64
O96008;O96008-2	TOM40_HUMAN Mitochondrial import receptor subunit TOM40 homolog OS=Homo sapiens GN=TOMM40 PE=1 SV=1;>sp|O96008-2|T(	1.93E-209	
O96011	PX11B_HUMAN Peroxisomal membrane protein 11B OS=Homo sapiens GN=PEX11B PE=1 SV=1		
O96013-2;O96013;O9601	2|PAK4_HUMAN Isoform 2 of Serine/threonine-protein kinase PAK 4 OS=Homo sapiens GN=PAK4;>sp|O96013|PAK4_HUMAN Serine/t	7.90E-11	
O96013;A8K724;O96013-	PAK4_HUMAN Serine/threonine-protein kinase PAK 4 OS=Homo sapiens GN=PAK4 PE=1 SV=1;>tr|A8K724|A8K724_HUMAN cDNA FL	6.72E-11	
O96019	ACL6A_HUMAN Actin-like protein 6A OS=Homo sapiens GN=ACTL6A PE=1 SV=1		102.65
O96019-2	ACL6A_HUMAN Isoform 2 of Actin-like protein 6A OS=Homo sapiens GN=ACTL6A		102.65
O96019;O96019-2	ACL6A_HUMAN Actin-like protein 6A OS=Homo sapiens GN=ACTL6A PE=1 SV=1;>sp|O96019-2|ACL6A_HUMAN Isoform 2 of Actin-like	3.48E-86	
P00156	CYB_HUMAN Cytochrome b OS=Homo sapiens GN=MT-CYB PE=1 SV=1		
P00167	CYB5_HUMAN Cytochrome b5 OS=Homo sapiens GN=CYB5A PE=1 SV=2		
P00167-2	CYB5_HUMAN Isoform 2 of Cytochrome b5 OS=Homo sapiens GN=CYB5A		
P00167;P00167-2	CYB5_HUMAN Cytochrome b5 OS=Homo sapiens GN=CYB5A PE=1 SV=2;>sp|P00167-2|CYB5_HUMAN Isoform 2 of Cytochrome b5 O	9.55E-31	
P00325;A8MYN5;B4DVC3;	ADH1B_HUMAN Alcohol dehydrogenase 1B OS=Homo sapiens GN=ADH1B PE=1 SV=2;>tr|A8MYN5|A8MYN5_HUMAN Uncharacterized	1.04E-244	
P00338	LDHA_HUMAN L-lactate dehydrogenase A chain OS=Homo sapiens GN=LDHA PE=1 SV=2		1382.58
P00338-2	LDHA_HUMAN Isoform 2 of L-lactate dehydrogenase A chain OS=Homo sapiens GN=LDHA		1165.53
P00338;P00338-2	LDHA_HUMAN L-lactate dehydrogenase A chain OS=Homo sapiens GN=LDHA PE=1 SV=2;>sp|P00338-2|LDHA_HUMAN Isoform 2 of L	0	
P00352	AL1A1_HUMAN Retinal dehydrogenase 1 OS=Homo sapiens GN=ALDH1A1 PE=1 SV=2		78.41
P00367	DHE3_HUMAN Glutamate dehydrogenase 1, mitochondrial OS=Homo sapiens GN=GLUD1 PE=1 SV=2		859.77
P00374	DYR_HUMAN Dihydrofolate reductase OS=Homo sapiens GN=DHFR PE=1 SV=2	2.74E-31	
P00387	NB5R3_HUMAN NADH-cytochrome b5 reductase 3 OS=Homo sapiens GN=CYB5R3 PE=1 SV=3		610.07
P00387-2	NB5R3_HUMAN Isoform 2 of NADH-cytochrome b5 reductase 3 OS=Homo sapiens GN=CYB5R3		610.07
P00387;P00387-2	NB5R3_HUMAN NADH-cytochrome b5 reductase 3 OS=Homo sapiens GN=CYB5R3 PE=1 SV=3;>sp|P00387-2|NB5R3_HUMAN Isoform	1.58E-22	
P00390;P00390-2	GSHR_HUMAN Glutathione reductase, mitochondrial OS=Homo sapiens GN=GSR PE=1 SV=2;>sp|P00390-2|GSHR_HUMAN Isoform C	8.22E-51	
P00395	COX1_HUMAN Cytochrome c oxidase subunit 1 OS=Homo sapiens GN=MT-CO1 PE=1 SV=1	2.06E-14	
P00403	COX2_HUMAN Cytochrome c oxidase subunit 2 OS=Homo sapiens GN=MT-CO2 PE=1 SV=1		449.9
P00441	SODC_HUMAN Superoxide dismutase [Cu-Zn] OS=Homo sapiens GN=SOD1 PE=1 SV=2		216.39
P00450	CERU_HUMAN Ceruloplasmin OS=Homo sapiens GN=CP PE=1 SV=1		188.2
P00488	F13A_HUMAN Coagulation factor XIII A chain OS=Homo sapiens GN=F13A1 PE=1 SV=4	6.15E-50	
P00491	PNPH_HUMAN Purine nucleoside phosphorylase OS=Homo sapiens GN=PNP PE=1 SV=2		313.82
P00492	HPRT_HUMAN Hypoxanthine-guanine phosphoribosyltransferase OS=Homo sapiens GN=HPRT1 PE=1 SV=2		178.89
P00505	AATM_HUMAN Aspartate aminotransferase, mitochondrial OS=Homo sapiens GN=GOT2 PE=1 SV=3		1073.24
P00533	EGFR_HUMAN Epidermal growth factor receptor OS=Homo sapiens GN=EGFR PE=1 SV=2		684.32
P00533-2	EGFR_HUMAN Isoform 2 of Epidermal growth factor receptor OS=Homo sapiens GN=EGFR		389.54
P00533-3	EGFR_HUMAN Isoform 3 of Epidermal growth factor receptor OS=Homo sapiens GN=EGFR		444.83
P00533-4	EGFR_HUMAN Isoform 4 of Epidermal growth factor receptor OS=Homo sapiens GN=EGFR		444.83
P00533;P00533-3;P00533	EGFR_HUMAN Epidermal growth factor receptor OS=Homo sapiens GN=EGFR PE=1 SV=2;>sp|P00533-3|EGFR_HUMAN Isoform 3 of E	8.04E-172	
P00558	PGK1_HUMAN Phosphoglycerate kinase 1 OS=Homo sapiens GN=PGK1 PE=1 SV=3		1385.17
P00568	KAD1_HUMAN Adenylate kinase isoenzyme 1 OS=Homo sapiens GN=AK1 PE=1 SV=3		119.49
P00734	THRB_HUMAN Prothrombin OS=Homo sapiens GN=F2 PE=1 SV=2	0	
P00736	C1R_HUMAN Complement C1r subcomponent OS=Homo sapiens GN=C1R PE=1 SV=2	2.52E-96	
P00738	HPT_HUMAN Haptoglobin OS=Homo sapiens GN=HP PE=1 SV=1		469.73
P00739	HPTR_HUMAN Haptoglobin-related protein OS=Homo sapiens GN=HPR PE=1 SV=2		282.05
P00739-2	HPTR_HUMAN Isoform 2 of Haptoglobin-related protein OS=Homo sapiens GN=HPR		268.1
P00739-2;P00739	2|HPTR_HUMAN Isoform 2 of Haptoglobin-related protein OS=Homo sapiens GN=HPR;>sp|P00739|HPTR_HUMAN Haptoglobin-related	6.98E-290	
P00742	FA10_HUMAN Coagulation factor X OS=Homo sapiens GN=F10 PE=1 SV=2	8.97E-14	
P00746	CFAD_HUMAN Complement factor D OS=Homo sapiens GN=CFD PE=1 SV=5	2.26E-11	
P00747	PLMN_HUMAN Plasminogen OS=Homo sapiens GN=PLG PE=1 SV=2		138.3
P00748	FA12_HUMAN Coagulation factor XII OS=Homo sapiens GN=F12 PE=1 SV=3	2.94E-83	
P00749	UROK_HUMAN Urokinase-type plasminogen activator OS=Homo sapiens GN=PLAU PE=1 SV=2		119.46
P00749-2	UROK_HUMAN Isoform 2 of Urokinase-type plasminogen activator OS=Homo sapiens GN=PLAU		119.46

P00751	CFAB_HUMAN Complement factor B OS=Homo sapiens GN=CFB PE=1 SV=2		221.22
P00751-2	CFAB_HUMAN Isoform 2 of Complement factor B OS=Homo sapiens GN=CFB		79.42
P00813	ADA_HUMAN Adenosine deaminase OS=Homo sapiens GN=ADA PE=1 SV=3	3.30E-10	
P00846	ATP6_HUMAN ATP synthase subunit a OS=Homo sapiens GN=MT-ATP6 PE=1 SV=1		63.05
P00915	CAH1_HUMAN Carbonic anhydrase 1 OS=Homo sapiens GN=CA1 PE=1 SV=2		179.57
P00918	CAH2_HUMAN Carbonic anhydrase 2 OS=Homo sapiens GN=CA2 PE=1 SV=2		43.23
P00966	ASSY_HUMAN Argininosuccinate synthase OS=Homo sapiens GN=ASS1 PE=1 SV=2	7.98E-236	
P00973-3;P00973;P00973	3|OAS1_HUMAN Isoform p48 of 2-5-oligoadenylate synthase 1 OS=Homo sapiens GN=OAS1;>sp|P00973|OAS1_HUMAN 2-5-oligoade	4.97E-08	
P01008	ANT3_HUMAN Antithrombin-III OS=Homo sapiens GN=SERPINC1 PE=1 SV=1		137.91
P01009	A1AT_HUMAN Alpha-1-antitrypsin OS=Homo sapiens GN=SERPINA1 PE=1 SV=3		562.84
P01009-2	A1AT_HUMAN Isoform 2 of Alpha-1-antitrypsin OS=Homo sapiens GN=SERPINA1		515.76
P01009-3	A1AT_HUMAN Isoform 3 of Alpha-1-antitrypsin OS=Homo sapiens GN=SERPINA1		417.52
P01009;P01009-2;P01009	A1AT_HUMAN Alpha-1-antitrypsin OS=Homo sapiens GN=SERPINA1 PE=1 SV=3;>sp|P01009-2|A1AT_HUMAN Isoform 2 of Alpha-1-ar	0	
P01011	AACT_HUMAN Alpha-1-antichymotrypsin OS=Homo sapiens GN=SERPINA3 PE=1 SV=2		156.84
P01011-2	AACT_HUMAN Isoform 2 of Alpha-1-antichymotrypsin OS=Homo sapiens GN=SERPINA3		23.72
P01019	ANGT_HUMAN Angiotensinogen OS=Homo sapiens GN=AGT PE=1 SV=1		137.5
P01023	A2MG_HUMAN Alpha-2-macroglobulin OS=Homo sapiens GN=A2M PE=1 SV=3		1505.35
P01024	CO3_HUMAN Complement C3 OS=Homo sapiens GN=C3 PE=1 SV=2		971.81
P01031	CO5_HUMAN Complement C5 OS=Homo sapiens GN=C5 PE=1 SV=4		
P01033	TIMP1_HUMAN Metalloproteinase inhibitor 1 OS=Homo sapiens GN=TIMP1 PE=1 SV=1		53.19
P01034	CYTC_HUMAN Cystatin-C OS=Homo sapiens GN=CST3 PE=1 SV=1		120.36
P01036	CYTS_HUMAN Cystatin-S OS=Homo sapiens GN=CST4 PE=1 SV=3		165.92
P01037	CYTN_HUMAN Cystatin-SN OS=Homo sapiens GN=CST1 PE=1 SV=3		160.37
P01040	CYTA_HUMAN Cystatin-A OS=Homo sapiens GN=CSTA PE=1 SV=1	8.96E-13	
P01042	KNG1_HUMAN Kininogen-1 OS=Homo sapiens GN=KNG1 PE=1 SV=2		73.85
P01042-2	KNG1_HUMAN Isoform LMW of Kininogen-1 OS=Homo sapiens GN=KNG1		142.48
P01106	MYC_HUMAN Myc proto-oncogene protein OS=Homo sapiens GN=MYC PE=1 SV=1		
P01106-2	MYC_HUMAN Isoform 2 of Myc proto-oncogene protein OS=Homo sapiens GN=MYC		
P01111	RASN_HUMAN GTPase NRas OS=Homo sapiens GN=NRAS PE=1 SV=1		342.05
P01112	RASH_HUMAN GTPase HRas OS=Homo sapiens GN=HRAS PE=1 SV=1		383.61
P01112-2	RASH_HUMAN Isoform 2 of GTPase HRas OS=Homo sapiens GN=HRAS		300.81
P01112;P01112-2	RASH_HUMAN GTPase HRas OS=Homo sapiens GN=HRAS PE=1 SV=1;>sp|P01112-2|RASH_HUMAN Isoform 2 of GTPase HRas OS=H	1.72E-40	
P01116	RASK_HUMAN GTPase KRas OS=Homo sapiens GN=KRAS PE=1 SV=1		299.68
P01116-2	RASK_HUMAN Isoform 2B of GTPase KRas OS=Homo sapiens GN=KRAS		338.66
P01116-2;P01116	2|RASK_HUMAN Isoform 2B of GTPase KRas OS=Homo sapiens GN=KRAS;>sp|P01116|RASK_HUMAN GTPase KRas OS=Homo sapiens	2.34E-33	
P01130	LDLR_HUMAN Low-density lipoprotein receptor OS=Homo sapiens GN=LDLR PE=1 SV=1		113.36
P01213	PDYN_HUMAN Proenkephalin-B OS=Homo sapiens GN=PDYN PE=1 SV=1		
P01344	IGF2_HUMAN Insulin-like growth factor II OS=Homo sapiens GN=IGF2 PE=1 SV=1		14.45
P01344-2	IGF2_HUMAN Isoform 2 of Insulin-like growth factor II OS=Homo sapiens GN=IGF2		14.45
P01591	IGJ_HUMAN Immunoglobulin J chain OS=Homo sapiens GN=IGJ PE=1 SV=4	5.13E-18	
P01594	KV102_HUMAN Ig kappa chain V-I region AU OS=Homo sapiens PE=1 SV=1	4.82E-193	
P01596	KV104_HUMAN Ig kappa chain V-I region CAR OS=Homo sapiens PE=1 SV=1		75.44
P01598	KV106_HUMAN Ig kappa chain V-I region EU OS=Homo sapiens PE=1 SV=1		75.44
P01602	KV110_HUMAN Ig kappa chain V-I region HK102 (Fragment) OS=Homo sapiens GN=IGKV1-5 PE=4 SV=1	4.64E-08	
P01608;P01593;P01599;P	KV116_HUMAN Ig kappa chain V-I region Roy OS=Homo sapiens PE=1 SV=1;>sp|P01593|KV101_HUMAN Ig kappa chain V-I region A	1.45E-208	
P01609	KV117_HUMAN Ig kappa chain V-I region Scw OS=Homo sapiens PE=1 SV=1	6.57E-192	
P01611	KV119_HUMAN Ig kappa chain V-I region Wes OS=Homo sapiens PE=1 SV=1	7.86E-112	
P01612	KV120_HUMAN Ig kappa chain V-I region Mev OS=Homo sapiens PE=1 SV=1	1.93E-54	
P01613	KV121_HUMAN Ig kappa chain V-I region Ni OS=Homo sapiens PE=1 SV=1		38.14
P01619	KV301_HUMAN Ig kappa chain V-III region B6 OS=Homo sapiens PE=1 SV=1		58.74
P01620	KV302_HUMAN Ig kappa chain V-III region SIE OS=Homo sapiens PE=1 SV=1		56.99
P01621	KV303_HUMAN Ig kappa chain V-III region NG9 (Fragment) OS=Homo sapiens PE=1 SV=1	2.13E-19	
P01622	KV304_HUMAN Ig kappa chain V-III region Ti OS=Homo sapiens PE=1 SV=1		56.99
P01623	KV305_HUMAN Ig kappa chain V-III region WOL OS=Homo sapiens PE=1 SV=1		56.99
P01625	KV402_HUMAN Ig kappa chain V-IV region Len OS=Homo sapiens PE=1 SV=2	9.21E-175	
P01700;P04208	LV102_HUMAN Ig lambda chain V-I region HA OS=Homo sapiens PE=1 SV=1;>sp|P04208|LV106_HUMAN Ig lambda chain V-I region	9.08E-26	
P01714	LV301_HUMAN Ig lambda chain V-III region SH OS=Homo sapiens PE=1 SV=1	1.11E-121	
P01717	LV403_HUMAN Ig lambda chain V-IV region Hil OS=Homo sapiens PE=1 SV=1	1.31E-86	
P01743	HV102_HUMAN Ig heavy chain V-I region HG3 OS=Homo sapiens PE=4 SV=1	3.03E-29	
P01763	HV302_HUMAN Ig heavy chain V-III region WEA OS=Homo sapiens PE=1 SV=1		
P01764	HV303_HUMAN Ig heavy chain V-III region VH26 OS=Homo sapiens PE=1 SV=1	5.20E-136	

P01765;P01774;P01776	HV304_HUMAN Ig heavy chain V-III region TIL OS=Homo sapiens PE=1 SV=1;>sp|P01774|HV313_HUMAN Ig heavy chain V-III regior	1.24E-245	
P01766;P01777;P01767;P	HV305_HUMAN Ig heavy chain V-III region BRO OS=Homo sapiens PE=1 SV=1;>sp|P01777|HV316_HUMAN Ig heavy chain V-III regi	9.04E-285	
P01771	HV310_HUMAN Ig heavy chain V-III region HIL OS=Homo sapiens PE=1 SV=1	1.96E-05	
P01779	HV318_HUMAN Ig heavy chain V-III region TUR OS=Homo sapiens PE=1 SV=1	2.19E-130	
P01781	HV320_HUMAN Ig heavy chain V-III region GAL OS=Homo sapiens PE=1 SV=1	1.88E-33	
P01833	PIGR_HUMAN Polymeric immunoglobulin receptor OS=Homo sapiens GN=PIGR PE=1 SV=4	2.72E-137	
P01834	IGKC_HUMAN Ig kappa chain C region OS=Homo sapiens GN=IGKC PE=1 SV=1		144.54
P01857	IGHG1_HUMAN Ig gamma-1 chain C region OS=Homo sapiens GN=IGHG1 PE=1 SV=1		352.81
P01859	IGHG2_HUMAN Ig gamma-2 chain C region OS=Homo sapiens GN=IGHG2 PE=1 SV=2		74.57
P01860	IGHG3_HUMAN Ig gamma-3 chain C region OS=Homo sapiens GN=IGHG3 PE=1 SV=2		256.42
P01861	IGHG4_HUMAN Ig gamma-4 chain C region OS=Homo sapiens GN=IGHG4 PE=1 SV=1		231.36
P01871	IGHM_HUMAN Ig mu chain C region OS=Homo sapiens GN=IGHM PE=1 SV=3		385.13
P01871-2	IGHM_HUMAN Isoform 2 of Ig mu chain C region OS=Homo sapiens GN=IGHM		385.13
P01871-2;P01871	2|IGHM_HUMAN Isoform 2 of Ig mu chain C region OS=Homo sapiens GN=IGHM;>sp|P01871|IGHM_HUMAN Ig mu chain C region OS	0	
P01876	IGHA1_HUMAN Ig alpha-1 chain C region OS=Homo sapiens GN=IGHA1 PE=1 SV=2		
P01877	IGHA2_HUMAN Ig alpha-2 chain C region OS=Homo sapiens GN=IGHA2 PE=1 SV=3		
P01880-2;P01880	2|IGHD_HUMAN Isoform 2 of Ig delta chain C region OS=Homo sapiens GN=IGHD;>sp|P01880|IGHD_HUMAN Ig delta chain C region	3.53E-17	
P01889	1B07_HUMAN HLA class I histocompatibility antigen, B-7 alpha chain OS=Homo sapiens GN=HLA-B PE=1 SV=3		430.5
P01891	1A68_HUMAN HLA class I histocompatibility antigen, A-68 alpha chain OS=Homo sapiens GN=HLA-A PE=1 SV=4		817.83
P01892	1A02_HUMAN HLA class I histocompatibility antigen, A-2 alpha chain OS=Homo sapiens GN=HLA-A PE=1 SV=1		1465.4
P01893	HLAH_HUMAN Putative HLA class I histocompatibility antigen, alpha chain H OS=Homo sapiens GN=HLA-H PE=5 SV=3		164.13
P01903	DRA_HUMAN HLA class II histocompatibility antigen, DR alpha chain OS=Homo sapiens GN=HLA-DRA PE=1 SV=1	0	
P01906	DQA2_HUMAN HLA class II histocompatibility antigen, DQ alpha 2 chain OS=Homo sapiens GN=HLA-DQA2 PE=2 SV=2	1.79E-37	
P01909	DQA1_HUMAN HLA class II histocompatibility antigen, DQ alpha 1 chain OS=Homo sapiens GN=HLA-DQA1 PE=1 SV=1	1.31E-61	
P01911	2B1F_HUMAN HLA class II histocompatibility antigen, DRB1-15 beta chain OS=Homo sapiens GN=HLA-DRB1 PE=1 SV=2	1.40E-154	
P01912	2B13_HUMAN HLA class II histocompatibility antigen, DRB1-3 chain OS=Homo sapiens GN=HLA-DRB1 PE=1 SV=2		0.15
P01920	DQB1_HUMAN HLA class II histocompatibility antigen, DQ beta 1 chain OS=Homo sapiens GN=HLA-DQB1 PE=1 SV=2	1.04E-123	
P02008	HBAZ_HUMAN Hemoglobin subunit zeta OS=Homo sapiens GN=HBZ PE=1 SV=2		5.44
P02042	HBD_HUMAN Hemoglobin subunit delta OS=Homo sapiens GN=HBD PE=1 SV=2		761.15
P02100	HBE_HUMAN Hemoglobin subunit epsilon OS=Homo sapiens GN=HBE1 PE=1 SV=2		78.42
P02144	MYG_HUMAN Myoglobin OS=Homo sapiens GN=MB PE=1 SV=2	3.70E-12	
P02452	CO1A1_HUMAN Collagen alpha-1(I) chain OS=Homo sapiens GN=COL1A1 PE=1 SV=5		40.2
P02461	CO3A1_HUMAN Collagen alpha-1(III) chain OS=Homo sapiens GN=COL3A1 PE=1 SV=4		39.77
P02461-2	CO3A1_HUMAN Isoform 2 of Collagen alpha-1(III) chain OS=Homo sapiens GN=COL3A1		2.6
P02462;P02462-2	CO4A1_HUMAN Collagen alpha-1(IV) chain OS=Homo sapiens GN=COL4A1 PE=1 SV=3;>sp|P02462-2|CO4A1_HUMAN Isoform 2 of C	8.82E-204	
P02511	CRYAB_HUMAN Alpha-crystallin B chain OS=Homo sapiens GN=CRYAB PE=1 SV=2	1.24E-85	
P02533	K1C14_HUMAN Keratin, type I cytoskeletal 14 OS=Homo sapiens GN=KRT14 PE=1 SV=4		1018.81
P02538	K2C6A_HUMAN Keratin, type II cytoskeletal 6A OS=Homo sapiens GN=KRT6A PE=1 SV=3		1074.76
P02545	LMNA_HUMAN Prelamin-A/C OS=Homo sapiens GN=LMNA PE=1 SV=1		1879.38
P02545-2	LMNA_HUMAN Isoform C of Prelamin-A/C OS=Homo sapiens GN=LMNA		1838.8
P02545-3	LMNA_HUMAN Isoform ADelta10 of Prelamin-A/C OS=Homo sapiens GN=LMNA		1879.38
P02545;P02545-2;P02545	LMNA_HUMAN Prelamin-A/C OS=Homo sapiens GN=LMNA PE=1 SV=1;>sp|P02545-2|LMNA_HUMAN Isoform C of Prelamin-A/C OS=H	1.64E-222	
P02549;P02549-2	SPTA1_HUMAN Spectrin alpha chain, erythrocyte OS=Homo sapiens GN=SPTA1 PE=1 SV=5;>sp|P02549-2|SPTA1_HUMAN Isoform 2 (	9.87E-11	
P02585	TNNC2_HUMAN Troponin C, skeletal muscle OS=Homo sapiens GN=TNNC2 PE=1 SV=2		
P02647	APOA1_HUMAN Apolipoprotein A-I OS=Homo sapiens GN=APOA1 PE=1 SV=1		407.94
P02649	APOE_HUMAN Apolipoprotein E OS=Homo sapiens GN=APOE PE=1 SV=1		
P02652	APOA2_HUMAN Apolipoprotein A-II OS=Homo sapiens GN=APOA2 PE=1 SV=1		189
P02656	APOC3_HUMAN Apolipoprotein C-III OS=Homo sapiens GN=APOC3 PE=1 SV=1		45.9
P02671	FIBA_HUMAN Fibrinogen alpha chain OS=Homo sapiens GN=FGA PE=1 SV=2		414.69
P02671-2	FIBA_HUMAN Isoform 2 of Fibrinogen alpha chain OS=Homo sapiens GN=FGA		414.69
P02671;P02671-2	FIBA_HUMAN Fibrinogen alpha chain OS=Homo sapiens GN=FGA PE=1 SV=2;>sp|P02671-2|FIBA_HUMAN Isoform 2 of Fibrinogen alp	0	
P02675	FIBB_HUMAN Fibrinogen beta chain OS=Homo sapiens GN=FGB PE=1 SV=2		438.39
P02679	FIBG_HUMAN Fibrinogen gamma chain OS=Homo sapiens GN=FGG PE=1 SV=3		322.86
P02679-2	FIBG_HUMAN Isoform Gamma-A of Fibrinogen gamma chain OS=Homo sapiens GN=FGG		322.86
P02679;P02679-2	FIBG_HUMAN Fibrinogen gamma chain OS=Homo sapiens GN=FGG PE=1 SV=3;>sp|P02679-2|FIBG_HUMAN Isoform Gamma-A of Fit	1.20E-09	
P02686	MBP_HUMAN Myelin basic protein OS=Homo sapiens GN=MBP PE=1 SV=3		41.64
P02686-2	MBP_HUMAN Isoform 2 of Myelin basic protein OS=Homo sapiens GN=MBP		40.35
P02686-3	MBP_HUMAN Isoform 3 of Myelin basic protein OS=Homo sapiens GN=MBP		41.64
P02686-4	MBP_HUMAN Isoform 4 of Myelin basic protein OS=Homo sapiens GN=MBP		41.64
P02686-5	MBP_HUMAN Isoform 5 of Myelin basic protein OS=Homo sapiens GN=MBP		41.64
P02686-6	MBP_HUMAN Isoform 6 of Myelin basic protein OS=Homo sapiens GN=MBP		41.64

P02686-7	MBP_HUMAN Isoform 7 of Myelin basic protein OS=Homo sapiens GN=MBP		40.35
P02686;A6NP45;B7Z3Y6;	MBP_HUMAN Myelin basic protein OS=Homo sapiens GN=MBP PE=1 SV=3;>tr|A6NP45|A6NP45_HUMAN Uncharacterized protein OS=	9.49E-06	
P02730	B3AT_HUMAN Band 3 anion transport protein OS=Homo sapiens GN=SLC4A1 PE=1 SV=3	2.47E-15	
P02735	SAA_HUMAN Serum amyloid A protein OS=Homo sapiens GN=SAA1 PE=1 SV=2		39.01
P02743	SAMP_HUMAN Serum amyloid P-component OS=Homo sapiens GN=APCS PE=1 SV=2		
P02745	C1QA_HUMAN Complement C1q subcomponent subunit A OS=Homo sapiens GN=C1QA PE=1 SV=2	1.88E-05	
P02746	C1QB_HUMAN Complement C1q subcomponent subunit B OS=Homo sapiens GN=C1QB PE=1 SV=3	4.13E-102	
P02747	C1QC_HUMAN Complement C1q subcomponent subunit C OS=Homo sapiens GN=C1QC PE=1 SV=3		76.81
P02748	CO9_HUMAN Complement component C9 OS=Homo sapiens GN=C9 PE=1 SV=2		36.78
P02749	APOH_HUMAN Beta-2-glycoprotein 1 OS=Homo sapiens GN=APOH PE=1 SV=3		81.82
P02750	A2GL_HUMAN Leucine-rich alpha-2-glycoprotein OS=Homo sapiens GN=LRG1 PE=1 SV=2	3.11E-77	
P02751	FINC_HUMAN Fibronectin OS=Homo sapiens GN=FN1 PE=1 SV=4		117.63
P02751-10	FINC_HUMAN Isoform 10 of Fibronectin OS=Homo sapiens GN=FN1		117.63
P02751-11	FINC_HUMAN Isoform 11 of Fibronectin OS=Homo sapiens GN=FN1		79.03
P02751-12	FINC_HUMAN Isoform 12 of Fibronectin OS=Homo sapiens GN=FN1		77.83
P02751-13	FINC_HUMAN Isoform 13 of Fibronectin OS=Homo sapiens GN=FN1		117.63
P02751-14	FINC_HUMAN Isoform 14 of Fibronectin OS=Homo sapiens GN=FN1		117.63
P02751-15	FINC_HUMAN Isoform 15 of Fibronectin OS=Homo sapiens GN=FN1		117.63
P02751-15;P02751-7;P02	15|FINC_HUMAN Isoform 15 of Fibronectin OS=Homo sapiens GN=FN1;>sp|P02751-7|FINC_HUMAN Isoform 7 of Fibronectin OS=Ho	1.20E-98	
P02751-3	FINC_HUMAN Isoform 3 of Fibronectin OS=Homo sapiens GN=FN1		117.63
P02751-4	FINC_HUMAN Isoform 4 of Fibronectin OS=Homo sapiens GN=FN1		117.63
P02751-5	FINC_HUMAN Isoform 5 of Fibronectin OS=Homo sapiens GN=FN1		117.63
P02751-6	FINC_HUMAN Isoform 6 of Fibronectin OS=Homo sapiens GN=FN1		117.63
P02751-7	FINC_HUMAN Isoform 7 of Fibronectin OS=Homo sapiens GN=FN1		117.63
P02751-8	FINC_HUMAN Isoform 8 of Fibronectin OS=Homo sapiens GN=FN1		117.63
P02751-9	FINC_HUMAN Isoform 9 of Fibronectin OS=Homo sapiens GN=FN1		117.63
P02751;P02751-3;P02751	FINC_HUMAN Fibronectin OS=Homo sapiens GN=FN1 PE=1 SV=4;>sp|P02751-3|FINC_HUMAN Isoform 3 of Fibronectin OS=Homo sa	0	
P02753	RET4_HUMAN Retinol-binding protein 4 OS=Homo sapiens GN=RBP4 PE=1 SV=3		65.86
P02760	AMBP_HUMAN Protein AMBP OS=Homo sapiens GN=AMBP PE=1 SV=1		59.37
P02763	A1AG1_HUMAN Alpha-1-acid glycoprotein 1 OS=Homo sapiens GN=ORM1 PE=1 SV=1		40.8
P02765	FETUA_HUMAN Alpha-2-HS-glycoprotein OS=Homo sapiens GN=AHSG PE=1 SV=1		185.05
P02766	TTHY_HUMAN Transthyretin OS=Homo sapiens GN=TTR PE=1 SV=1		131.53
P02768	ALBU_HUMAN Serum albumin OS=Homo sapiens GN=ALB PE=1 SV=2		3421.59
P02768-2	ALBU_HUMAN Isoform 2 of Serum albumin OS=Homo sapiens GN=ALB		2194.61
P02774	VTDB_HUMAN Vitamin D-binding protein OS=Homo sapiens GN=GC PE=1 SV=1		225.87
P02774-2	VTDB_HUMAN Isoform 2 of Vitamin D-binding protein OS=Homo sapiens GN=GC		225.87
P02774;P02774-2	VTDB_HUMAN Vitamin D-binding protein OS=Homo sapiens GN=GC PE=1 SV=1;>sp|P02774-2|VTDB_HUMAN Isoform 2 of Vitamin D	0	
P02786	TFR1_HUMAN Transferrin receptor protein 1 OS=Homo sapiens GN=TFRC PE=1 SV=2		1251.05
P02787	TRFE_HUMAN Serotransferrin OS=Homo sapiens GN=TF PE=1 SV=3		1054.12
P02788	TRFL_HUMAN Lactotransferrin OS=Homo sapiens GN=LTF PE=1 SV=6	0	
P02790	HEMO_HUMAN Hemopexin OS=Homo sapiens GN=HPX PE=1 SV=2		47.26
P02792	FRIL_HUMAN Ferritin light chain OS=Homo sapiens GN=FTL PE=1 SV=2		219.36
P02794	FRIH_HUMAN Ferritin heavy chain OS=Homo sapiens GN=FTH1 PE=1 SV=2		481.82
P03372;P03372-2;P03372	ESR1_HUMAN Estrogen receptor OS=Homo sapiens GN=ESR1 PE=1 SV=2;>sp|P03372-2|ESR1_HUMAN Isoform 2 of Estrogen recept	0.0001966	
P03886	NU1M_HUMAN NADH-ubiquinone oxidoreductase chain 1 OS=Homo sapiens GN=MT-ND1 PE=1 SV=1		
P03897	NU3M_HUMAN NADH-ubiquinone oxidoreductase chain 3 OS=Homo sapiens GN=MT-ND3 PE=1 SV=1		
P03905	NU4M_HUMAN NADH-ubiquinone oxidoreductase chain 4 OS=Homo sapiens GN=MT-ND4 PE=1 SV=1		
P03950	ANGI_HUMAN Angiogenin OS=Homo sapiens GN=ANG PE=1 SV=1	9.84E-09	
P03951	FA11_HUMAN Coagulation factor XI OS=Homo sapiens GN=F11 PE=1 SV=1		
P03951-2	FA11_HUMAN Isoform 2 of Coagulation factor XI OS=Homo sapiens GN=F11		
P03952	KLKB1_HUMAN Plasma kallikrein OS=Homo sapiens GN=KLKB1 PE=1 SV=1	3.08E-33	
P03989	1B27_HUMAN HLA class I histocompatibility antigen, B-27 alpha chain OS=Homo sapiens GN=HLA-B PE=1 SV=2		469.29
P04003	C4BPA_HUMAN C4b-binding protein alpha chain OS=Homo sapiens GN=C4BPA PE=1 SV=2	5.40E-154	
P04004	VTNC_HUMAN Vitronectin OS=Homo sapiens GN=VTN PE=1 SV=1	7.98E-54	
P04035	HMDH_HUMAN 3-hydroxy-3-methylglutaryl-coenzyme A reductase OS=Homo sapiens GN=HMGCR PE=1 SV=1		
P04035-2	HMDH_HUMAN Isoform 2 of 3-hydroxy-3-methylglutaryl-coenzyme A reductase OS=Homo sapiens GN=HMGCR		
P04040	CATA_HUMAN Catalase OS=Homo sapiens GN=CAT PE=1 SV=3		182.34
P04062	GLCM_HUMAN Glucosylceramidase OS=Homo sapiens GN=GBA PE=1 SV=3		
P04062-2	GLCM_HUMAN Isoform Short of Glucosylceramidase OS=Homo sapiens GN=GBA		
P04062-3	GLCM_HUMAN Isoform 3 of Glucosylceramidase OS=Homo sapiens GN=GBA		
P04062;P04062-2	GLCM_HUMAN Glucosylceramidase OS=Homo sapiens GN=GBA PE=1 SV=3;>sp|P04062-2|GLCM_HUMAN Isoform Short of Glucosylce	2.06E-05	

P04075	ALDOA_HUMAN Fructose-bisphosphate aldolase A OS=Homo sapiens GN=ALDOA PE=1 SV=2		1034.27
P04080	CYTB_HUMAN Cystatin-B OS=Homo sapiens GN=CSTB PE=1 SV=2		154.42
P04083	ANXA1_HUMAN Annexin A1 OS=Homo sapiens GN=ANXA1 PE=1 SV=2		1065.67
P04114	APOB_HUMAN Apolipoprotein B-100 OS=Homo sapiens GN=APOB PE=1 SV=2		579.28
P04150-3;B6ZGU6;D3DQF	3|GCR_HUMAN Isoform Alpha-2 of Glucocorticoid receptor OS=Homo sapiens GN=NR3C1;>tr|B6ZGU6|B6ZGU6_HUMAN Glucocorticoi	5.49E-05	
P04155	TFF1_HUMAN Trefoil factor 1 OS=Homo sapiens GN=TFF1 PE=1 SV=1		
P04156	PRIO_HUMAN Major prion protein OS=Homo sapiens GN=PRNP PE=1 SV=1		207.48
P04156-2	PRIO_HUMAN Isoform 2 of Major prion protein OS=Homo sapiens GN=PRNP		207.48
P04156;P04156-2	PRIO_HUMAN Major prion protein OS=Homo sapiens GN=PRNP PE=1 SV=1;>sp|P04156-2|PRIO_HUMAN Isoform 2 of Major prion pro	1.57E-59	
P04179	SODM_HUMAN Superoxide dismutase [Mn], mitochondrial OS=Homo sapiens GN=SOD2 PE=1 SV=2		345.74
P04181	OAT_HUMAN Ornithine aminotransferase, mitochondrial OS=Homo sapiens GN=OAT PE=1 SV=1		314.67
P04183	KITH_HUMAN Thymidine kinase, cytosolic OS=Homo sapiens GN=TK1 PE=1 SV=2	1.67E-47	
P04196	HRG_HUMAN Histidine-rich glycoprotein OS=Homo sapiens GN=HRG PE=1 SV=1		75.07
P04206	KV307_HUMAN Ig kappa chain V-III region GOL OS=Homo sapiens PE=1 SV=1		56.99
P04207;P04434;P01624;P	KV308_HUMAN Ig kappa chain V-III region CLL OS=Homo sapiens PE=1 SV=2;>sp|P04434|KV310_HUMAN Ig kappa chain V-III regio	5.26E-10	
P04216	THY1_HUMAN Thy-1 membrane glycoprotein OS=Homo sapiens GN=THY1 PE=1 SV=2	2.78E-93	
P04217	A1BG_HUMAN Alpha-1B-glycoprotein OS=Homo sapiens GN=A1BG PE=1 SV=4		112.24
P04217-2	A1BG_HUMAN Isoform 2 of Alpha-1B-glycoprotein OS=Homo sapiens GN=A1BG		26.53
P04217;P04217-2	A1BG_HUMAN Alpha-1B-glycoprotein OS=Homo sapiens GN=A1BG PE=1 SV=4;>sp|P04217-2|A1BG_HUMAN Isoform 2 of Alpha-1B-g	0	
P04220	MUCB_HUMAN Ig mu heavy chain disease protein OS=Homo sapiens PE=1 SV=1		309.78
P04222	1C03_HUMAN HLA class I histocompatibility antigen, Cw-3 alpha chain OS=Homo sapiens GN=HLA-C PE=1 SV=2		323.82
P04229	2B11_HUMAN HLA class II histocompatibility antigen, DRB1-1 beta chain OS=Homo sapiens GN=HLA-DRB1 PE=1 SV=2		29.42
P04233	HG2A_HUMAN HLA class II histocompatibility antigen gamma chain OS=Homo sapiens GN=CD74 PE=1 SV=3		121.89
P04233-2	HG2A_HUMAN Isoform 2 of HLA class II histocompatibility antigen gamma chain OS=Homo sapiens GN=CD74		121.89
P04233-2;P04233	2|HG2A_HUMAN Isoform 2 of HLA class II histocompatibility antigen gamma chain OS=Homo sapiens GN=CD74;>sp|P04233|HG2A_H	1.05E-111	
P04233-3	HG2A_HUMAN Isoform 3 of HLA class II histocompatibility antigen gamma chain OS=Homo sapiens GN=CD74		84.75
P04233;P04233-2	HG2A_HUMAN HLA class II histocompatibility antigen gamma chain OS=Homo sapiens GN=CD74 PE=1 SV=3;>sp|P04233-2|HG2A_HU	2.10E-10	
P04259	K2C6B_HUMAN Keratin, type II cytoskeletal 6B OS=Homo sapiens GN=KRT6B PE=1 SV=5		989.24
P04264	K2C1_HUMAN Keratin, type II cytoskeletal 1 OS=Homo sapiens GN=KRT1 PE=1 SV=6		2663.38
P04275	VWF_HUMAN von Willebrand factor OS=Homo sapiens GN=VWF PE=1 SV=4	2.32E-48	
P04278;P04278-2;B0FWH	SHBG_HUMAN Sex hormone-binding globulin OS=Homo sapiens GN=SHBG PE=1 SV=2;>sp|P04278-2|SHBG_HUMAN Isoform 2 of Se	1.22E-08	
P04350	TBB4_HUMAN Tubulin beta-4 chain OS=Homo sapiens GN=TUBB4 PE=1 SV=2		1886.18
P04406	G3P_HUMAN Glyceraldehyde-3-phosphate dehydrogenase OS=Homo sapiens GN=GAPDH PE=1 SV=3		2232.02
P04424	ARLY_HUMAN Argininosuccinate lyase OS=Homo sapiens GN=ASL PE=1 SV=4	8.64E-32	
P04430	KV122_HUMAN Ig kappa chain V-I region BAN OS=Homo sapiens PE=1 SV=1	1.21E-149	
P04433	KV309_HUMAN Ig kappa chain V-III region VG (Fragment) OS=Homo sapiens PE=1 SV=1		44.82
P04439	1A03_HUMAN HLA class I histocompatibility antigen, A-3 alpha chain OS=Homo sapiens GN=HLA-A PE=1 SV=2		504.96
P04440	DPB1_HUMAN HLA class II histocompatibility antigen, DP beta 1 chain OS=Homo sapiens GN=HLA-DPB1 PE=1 SV=1	4.23E-46	
P04626	ERBB2_HUMAN Receptor tyrosine-protein kinase erbB-2 OS=Homo sapiens GN=ERBB2 PE=1 SV=1		85.48
P04626-2	ERBB2_HUMAN Isoform 2 of Receptor tyrosine-protein kinase erbB-2 OS=Homo sapiens GN=ERBB2		83.09
P04626-3	ERBB2_HUMAN Isoform 3 of Receptor tyrosine-protein kinase erbB-2 OS=Homo sapiens GN=ERBB2		83.09
P04626-4	ERBB2_HUMAN Isoform 4 of Receptor tyrosine-protein kinase erbB-2 OS=Homo sapiens GN=ERBB2		85.48
P04626;P04626-4;B4DTR1	ERBB2_HUMAN Receptor tyrosine-protein kinase erbB-2 OS=Homo sapiens GN=ERBB2 PE=1 SV=1;>sp|P04626-4|ERBB2_HUMAN Iso	7.26E-32	
P04632	CPNS1_HUMAN Calpain small subunit 1 OS=Homo sapiens GN=CAPNS1 PE=1 SV=1		604.95
P04637	P53_HUMAN Cellular tumor antigen p53 OS=Homo sapiens GN=TP53 PE=1 SV=4		83.7
P04637-2	P53_HUMAN Isoform 2 of Cellular tumor antigen p53 OS=Homo sapiens GN=TP53		83.7
P04637-3	P53_HUMAN Isoform 3 of Cellular tumor antigen p53 OS=Homo sapiens GN=TP53		83.7
P04637-4	P53_HUMAN Isoform 4 of Cellular tumor antigen p53 OS=Homo sapiens GN=TP53		83.7
P04637-5	P53_HUMAN Isoform 5 of Cellular tumor antigen p53 OS=Homo sapiens GN=TP53		83.7
P04637-6	P53_HUMAN Isoform 6 of Cellular tumor antigen p53 OS=Homo sapiens GN=TP53		83.7
P04637-7	P53_HUMAN Isoform 7 of Cellular tumor antigen p53 OS=Homo sapiens GN=TP53		37.37
P04637-8	P53_HUMAN Isoform 8 of Cellular tumor antigen p53 OS=Homo sapiens GN=TP53		37.37
P04637-9	P53_HUMAN Isoform 9 of Cellular tumor antigen p53 OS=Homo sapiens GN=TP53		37.37
P04637;P04637-4;P04637	P53_HUMAN Cellular tumor antigen p53 OS=Homo sapiens GN=TP53 PE=1 SV=4;>sp|P04637-4|P53_HUMAN Isoform 4 of Cellular tu	2.45E-48	
P04745	AMY1_HUMAN Alpha-amylase 1 OS=Homo sapiens GN=AMY1A PE=1 SV=2	2.71E-05	
P04792	HSPB1_HUMAN Heat shock protein beta-1 OS=Homo sapiens GN=HSPB1 PE=1 SV=2		589.72
P04818	TYSY_HUMAN Thymidylate synthase OS=Homo sapiens GN=TYMS PE=1 SV=3	3.48E-30	
P04839	CY24B_HUMAN Cytochrome b-245 heavy chain OS=Homo sapiens GN=CYBB PE=1 SV=2	2.78E-64	
P04843	RPN1_HUMAN Dolichyl-diphosphooligosaccharide--protein glycosyltransferase subunit 1 OS=Homo sapiens GN=RPN1 PE=1 SV=1		1012.05
P04844	RPN2_HUMAN Dolichyl-diphosphooligosaccharide--protein glycosyltransferase subunit 2 OS=Homo sapiens GN=RPN2 PE=1 SV=3		1046.16
P04899	GNAI2_HUMAN Guanine nucleotide-binding protein G(i) subunit alpha-2 OS=Homo sapiens GN=GNAI2 PE=1 SV=3		1171.68

P04899-2	GNAI2_HUMAN Isoform 2 of Guanine nucleotide-binding protein G(i) subunit alpha-2 OS=Homo sapiens GN=GNAI2		1099
P04899;P04899-2	GNAI2_HUMAN Guanine nucleotide-binding protein G(i) subunit alpha-2 OS=Homo sapiens GN=GNAI2 PE=1 SV=3;>sp|P04899-2|GN.	8.09E-190	
P04908	H2A1B_HUMAN Histone H2A type 1-B/E OS=Homo sapiens GN=HIST1H2AB PE=1 SV=2		212.14
P05023	AT1A1_HUMAN Sodium/potassium-transporting ATPase subunit alpha-1 OS=Homo sapiens GN=ATP1A1 PE=1 SV=1		2674.16
P05023-2	AT1A1_HUMAN Isoform Short of Sodium/potassium-transporting ATPase subunit alpha-1 OS=Homo sapiens GN=ATP1A1		1594.29
P05023;P05023-2	AT1A1_HUMAN Sodium/potassium-transporting ATPase subunit alpha-1 OS=Homo sapiens GN=ATP1A1 PE=1 SV=1;>sp|P05023-2|AT	0	
P05026	AT1B1_HUMAN Sodium/potassium-transporting ATPase subunit beta-1 OS=Homo sapiens GN=ATP1B1 PE=1 SV=1		493.48
P05026-2	AT1B1_HUMAN Isoform 2 of Sodium/potassium-transporting ATPase subunit beta-1 OS=Homo sapiens GN=ATP1B1		493.48
P05026;P05026-2	AT1B1_HUMAN Sodium/potassium-transporting ATPase subunit beta-1 OS=Homo sapiens GN=ATP1B1 PE=1 SV=1;>sp|P05026-2|AT1	4.84E-65	
P05060	SCG1_HUMAN Secretogranin-1 OS=Homo sapiens GN=CHGB PE=1 SV=2	3.44E-31	
P05067	A4_HUMAN Amyloid beta A4 protein OS=Homo sapiens GN=APP PE=1 SV=3		28.16
P05067-10	A4_HUMAN Isoform APP639 of Amyloid beta A4 protein OS=Homo sapiens GN=APP		24.99
P05067-2	A4_HUMAN Isoform APP305 of Amyloid beta A4 protein OS=Homo sapiens GN=APP		0.64
P05067-3	A4_HUMAN Isoform L-APP677 of Amyloid beta A4 protein OS=Homo sapiens GN=APP		25.63
P05067-4	A4_HUMAN Isoform APP695 of Amyloid beta A4 protein OS=Homo sapiens GN=APP		25.63
P05067-5	A4_HUMAN Isoform L-APP696 of Amyloid beta A4 protein OS=Homo sapiens GN=APP		28.16
P05067-6	A4_HUMAN Isoform APP714 of Amyloid beta A4 protein OS=Homo sapiens GN=APP		28.16
P05067-7	A4_HUMAN Isoform L-APP733 of Amyloid beta A4 protein OS=Homo sapiens GN=APP		25.63
P05067-8	A4_HUMAN Isoform APP751 of Amyloid beta A4 protein OS=Homo sapiens GN=APP		25.63
P05067-9	A4_HUMAN Isoform L-APP752 of Amyloid beta A4 protein OS=Homo sapiens GN=APP		28.16
P05067;P05067-8;P05067	A4_HUMAN Amyloid beta A4 protein OS=Homo sapiens GN=APP PE=1 SV=3;>sp|P05067-8|A4_HUMAN Isoform APP751 of Amyloid be	2.28E-09	
P05089	ARGI1_HUMAN Arginase-1 OS=Homo sapiens GN=ARG1 PE=1 SV=2		27.13
P05089-2	ARGI1_HUMAN Isoform 2 of Arginase-1 OS=Homo sapiens GN=ARG1		26.08
P05089-3	ARGI1_HUMAN Isoform 3 of Arginase-1 OS=Homo sapiens GN=ARG1		27.13
P05090	APOD_HUMAN Apolipoprotein D OS=Homo sapiens GN=APOD PE=1 SV=1	1.27E-191	
P05091	ALDH2_HUMAN Aldehyde dehydrogenase, mitochondrial OS=Homo sapiens GN=ALDH2 PE=1 SV=2		283.54
P05107	ITB2_HUMAN Integrin beta-2 OS=Homo sapiens GN=ITGB2 PE=1 SV=2		
P05109	S10A8_HUMAN Protein S100-A8 OS=Homo sapiens GN=S100A8 PE=1 SV=1		39.43
P05114	HMGN1_HUMAN Non-histone chromosomal protein HMG-14 OS=Homo sapiens GN=HMGN1 PE=1 SV=3		68.17
P05120	PAI2_HUMAN Plasminogen activator inhibitor 2 OS=Homo sapiens GN=SERPINB2 PE=1 SV=2	0	
P05121	PAI1_HUMAN Plasminogen activator inhibitor 1 OS=Homo sapiens GN=SERPINE1 PE=1 SV=1		216.61
P05129	KPCG_HUMAN Protein kinase C gamma type OS=Homo sapiens GN=PRKCG PE=1 SV=3		
P05141	ADT2_HUMAN ADP/ATP translocase 2 OS=Homo sapiens GN=SLC25A5 PE=1 SV=7		1027.86
P05155	IC1_HUMAN Plasma protease C1 inhibitor OS=Homo sapiens GN=SERPING1 PE=1 SV=2	3.85E-122	
P05156	CFAI_HUMAN Complement factor I OS=Homo sapiens GN=CFI PE=1 SV=2	3.53E-51	
P05161	ISG15_HUMAN Ubiquitin-like protein ISG15 OS=Homo sapiens GN=ISG15 PE=1 SV=5		31.67
P05164	PERM_HUMAN Myeloperoxidase OS=Homo sapiens GN=MPO PE=1 SV=1		
P05164-2	PERM_HUMAN Isoform H14 of Myeloperoxidase OS=Homo sapiens GN=MPO		
P05164-3	PERM_HUMAN Isoform H7 of Myeloperoxidase OS=Homo sapiens GN=MPO		
P05164-3;P05164;P05164	3|PERM_HUMAN Isoform H7 of Myeloperoxidase OS=Homo sapiens GN=MPO;>sp|P05164|PERM_HUMAN Myeloperoxidase OS=Homo s	1.05E-23	
P05165;P05165-2	PCCA_HUMAN Propionyl-CoA carboxylase alpha chain, mitochondrial OS=Homo sapiens GN=PCCA PE=1 SV=4;>sp|P05165-2|PCCA_H	2.60E-19	
P05166	PCCB_HUMAN Propionyl-CoA carboxylase beta chain, mitochondrial OS=Homo sapiens GN=PCCB PE=1 SV=3	1.50E-06	
P05186	PPBT_HUMAN Alkaline phosphatase, tissue-nonspecific isozyme OS=Homo sapiens GN=ALPL PE=1 SV=4	3.17E-15	
P05198	IF2A_HUMAN Eukaryotic translation initiation factor 2 subunit 1 OS=Homo sapiens GN=EIF2S1 PE=1 SV=3		953.55
P05204	HMGN2_HUMAN Non-histone chromosomal protein HMG-17 OS=Homo sapiens GN=HMGN2 PE=1 SV=3		36.91
P05231	IL6_HUMAN Interleukin-6 OS=Homo sapiens GN=IL6 PE=1 SV=1		185.28
P05305	EDN1_HUMAN Endothelin-1 OS=Homo sapiens GN=EDN1 PE=1 SV=1		
P05362	ICAM1_HUMAN Intercellular adhesion molecule 1 OS=Homo sapiens GN=ICAM1 PE=1 SV=2		608.87
P05386	RLA1_HUMAN 60S acidic ribosomal protein P1 OS=Homo sapiens GN=RPLP1 PE=1 SV=1		266.41
P05387	RLA2_HUMAN 60S acidic ribosomal protein P2 OS=Homo sapiens GN=RPLP2 PE=1 SV=1		756.97
P05388	RLA0_HUMAN 60S acidic ribosomal protein P0 OS=Homo sapiens GN=RPLP0 PE=1 SV=1		922.15
P05412	JUN_HUMAN Transcription factor AP-1 OS=Homo sapiens GN=JUN PE=1 SV=2	0.00021084	
P05413	FABPH_HUMAN Fatty acid-binding protein, heart OS=Homo sapiens GN=FABP3 PE=1 SV=4	7.50E-15	
P05452	TETN_HUMAN Tetranectin OS=Homo sapiens GN=CLEC3B PE=1 SV=3		
P05455	LA_HUMAN Lupus La protein OS=Homo sapiens GN=SSB PE=1 SV=2		668.44
P05534	1A24_HUMAN HLA class I histocompatibility antigen, A-24 alpha chain OS=Homo sapiens GN=HLA-A PE=1 SV=2		596.6
P05546	HEP2_HUMAN Heparin cofactor 2 OS=Homo sapiens GN=SERPIND1 PE=1 SV=3	5.91E-65	
P05549	AP2A_HUMAN Transcription factor AP-2-alpha OS=Homo sapiens GN=TFAP2A PE=1 SV=1		
P05549-2	AP2A_HUMAN Isoform 4 of Transcription factor AP-2-alpha OS=Homo sapiens GN=TFAP2A		
P05556	ITB1_HUMAN Integrin beta-1 OS=Homo sapiens GN=ITGB1 PE=1 SV=2		1647.83
P05556-2	ITB1_HUMAN Isoform Beta-1B of Integrin beta-1 OS=Homo sapiens GN=ITGB1		1547.11

P05556-3	ITB1_HUMAN Isoform Beta-1C of Integrin beta-1 OS=Homo sapiens GN=ITGB1		1547.11
P05556-3;P05556-4;P055	3|ITB1_HUMAN Isoform Beta-1C of Integrin beta-1 OS=Homo sapiens GN=ITGB1;>sp|P05556-4|ITB1_HUMAN Isoform Beta-1C-2 of I	1.58E-13	
P05556-4	ITB1_HUMAN Isoform Beta-1C-2 of Integrin beta-1 OS=Homo sapiens GN=ITGB1		1547.11
P05556-5	ITB1_HUMAN Isoform Beta-1D of Integrin beta-1 OS=Homo sapiens GN=ITGB1		1547.11
P05556;P05556-3;P05556	ITB1_HUMAN Integrin beta-1 OS=Homo sapiens GN=ITGB1 PE=1 SV=2;>sp|P05556-3|ITB1_HUMAN Isoform Beta-1C of Integrin beta	0	
P05771-2;P05771	2|KPCB_HUMAN Isoform Beta-II of Protein kinase C beta type OS=Homo sapiens GN=PRKCB;>sp|P05771|KPCB_HUMAN Protein kinas	7.89E-17	
P05783	K1C18_HUMAN Keratin, type I cytoskeletal 18 OS=Homo sapiens GN=KRT18 PE=1 SV=2		1087.06
P05787	K2C8_HUMAN Keratin, type II cytoskeletal 8 OS=Homo sapiens GN=KRT8 PE=1 SV=7		1682.12
P05976	MYL1_HUMAN Myosin light chain 1/3, skeletal muscle isoform OS=Homo sapiens GN=MYL1 PE=1 SV=3		113.01
P05976-2	MYL1_HUMAN Isoform MLC3 of Myosin light chain 1/3, skeletal muscle isoform OS=Homo sapiens GN=MYL1		113.01
P05997	CO5A2_HUMAN Collagen alpha-2(V) chain OS=Homo sapiens GN=COL5A2 PE=1 SV=3	1.12E-11	
P06132	DCUP_HUMAN Uroporphyrinogen decarboxylase OS=Homo sapiens GN=UROD PE=1 SV=2		50.53
P06133	UD2B4_HUMAN UDP-glucuronosyltransferase 2B4 OS=Homo sapiens GN=UGT2B4 PE=1 SV=2		
P06239	LCK_HUMAN Tyrosine-protein kinase Lck OS=Homo sapiens GN=LCK PE=1 SV=6		62.5
P06239-2	LCK_HUMAN Isoform Short of Tyrosine-protein kinase Lck OS=Homo sapiens GN=LCK		13.74
P06239-3	LCK_HUMAN Isoform 3 of Tyrosine-protein kinase Lck OS=Homo sapiens GN=LCK		62.5
P06241	FYN_HUMAN Tyrosine-protein kinase Fyn OS=Homo sapiens GN=FYN PE=1 SV=3		108.67
P06241-2	FYN_HUMAN Isoform 2 of Tyrosine-protein kinase Fyn OS=Homo sapiens GN=FYN		108.67
P06241-3	FYN_HUMAN Isoform 3 of Tyrosine-protein kinase Fyn OS=Homo sapiens GN=FYN		108.67
P06276	CHLE_HUMAN Cholinesterase OS=Homo sapiens GN=BCHE PE=1 SV=1		
P06280	AGAL_HUMAN Alpha-galactosidase A OS=Homo sapiens GN=GLA PE=1 SV=1	2.15E-28	
P06309;P01617;P06310;P	KV205_HUMAN Ig kappa chain V-II region GM607 (Fragment) OS=Homo sapiens PE=4 SV=1;>sp|P01617|KV204_HUMAN Ig kappa ch	9.06E-48	
P06313;P06312;P06314;C	KV403_HUMAN Ig kappa chain V-IV region JI OS=Homo sapiens PE=4 SV=1;>sp|P06312|KV401_HUMAN Ig kappa chain V-IV region (	4.50E-70	
P06316;P01702	LV107_HUMAN Ig lambda chain V-I region BL2 OS=Homo sapiens PE=2 SV=1;>sp|P01702|LV104_HUMAN Ig lambda chain V-I region	0.00024023	
P06331;P01825	HV209_HUMAN Ig heavy chain V-II region ARH-77 OS=Homo sapiens PE=4 SV=1;>sp|P01825|HV207_HUMAN Ig heavy chain V-II reg	1.55E-55	
P06396	GELS_HUMAN Gelsolin OS=Homo sapiens GN=GSN PE=1 SV=1		71.28
P06396-2	GELS_HUMAN Isoform 2 of Gelsolin OS=Homo sapiens GN=GSN		71.28
P06396;Q5T0I2	GELS_HUMAN Gelsolin OS=Homo sapiens GN=GSN PE=1 SV=1;>tr|Q5T0I2|Q5T0I2_HUMAN Gelsolin (Amyloidosis, Finnish type) OS=	0	
P06400	RB_HUMAN Retinoblastoma-associated protein OS=Homo sapiens GN=RB1 PE=1 SV=2		
P06401;P06401-2	PRGR_HUMAN Progesterone receptor OS=Homo sapiens GN=PGR PE=1 SV=4;>sp|P06401-2|PRGR_HUMAN Isoform A of Progesterone	2.90E-06	
P06454	PTMA_HUMAN Prothymosin alpha OS=Homo sapiens GN=PTMA PE=1 SV=2		
P06454-2	PTMA_HUMAN Isoform 2 of Prothymosin alpha OS=Homo sapiens GN=PTMA		
P06454;P06454-2	PTMA_HUMAN Prothymosin alpha OS=Homo sapiens GN=PTMA PE=1 SV=2;>sp|P06454-2|PTMA_HUMAN Isoform 2 of Prothymosin al	2.03E-53	
P06493	CDK1_HUMAN Cyclin-dependent kinase 1 OS=Homo sapiens GN=CDK1 PE=1 SV=3		394.17
P06493-2	CDK1_HUMAN Isoform 2 of Cyclin-dependent kinase 1 OS=Homo sapiens GN=CDK1		259.86
P06493;P06493-2	CDK1_HUMAN Cyclin-dependent kinase 1 OS=Homo sapiens GN=CDK1 PE=1 SV=3;>sp|P06493-2|CDK1_HUMAN Isoform 2 of Cyclin-	1.26E-90	
P06576	ATPB_HUMAN ATP synthase subunit beta, mitochondrial OS=Homo sapiens GN=ATP5B PE=1 SV=3		1764.07
P06681	CO2_HUMAN Complement C2 OS=Homo sapiens GN=C2 PE=1 SV=2	7.33E-06	
P06702	S10A9_HUMAN Protein S100-A9 OS=Homo sapiens GN=S100A9 PE=1 SV=1		
P06703	S10A6_HUMAN Protein S100-A6 OS=Homo sapiens GN=S100A6 PE=1 SV=1		286.8
P06727	APOA4_HUMAN Apolipoprotein A-IV OS=Homo sapiens GN=APOA4 PE=1 SV=3		106.68
P06730	IF4E_HUMAN Eukaryotic translation initiation factor 4E OS=Homo sapiens GN=EIF4E PE=1 SV=2		150.03
P06733	ENOA_HUMAN Alpha-enolase OS=Homo sapiens GN=ENO1 PE=1 SV=2		2024.09
P06733-2	ENOA_HUMAN Isoform MBP-1 of Alpha-enolase OS=Homo sapiens GN=ENO1		1607.74
P06733;P06733-2	ENOA_HUMAN Alpha-enolase OS=Homo sapiens GN=ENO1 PE=1 SV=2;>sp|P06733-2|ENOA_HUMAN Isoform MBP-1 of Alpha-enolase	0	
P06734;P06734-2	FCER2_HUMAN Low affinity immunoglobulin epsilon Fc receptor OS=Homo sapiens GN=FCER2 PE=1 SV=1;>sp|P06734-2|FCER2_HUN	9.63E-19	
P06737	PYGL_HUMAN Glycogen phosphorylase, liver form OS=Homo sapiens GN=PYGL PE=1 SV=4		83.09
P06744	G6PI_HUMAN Glucose-6-phosphate isomerase OS=Homo sapiens GN=GPI PE=1 SV=4		673.22
P06746;Q53EV2;B7Z1W5	DPOLB_HUMAN DNA polymerase beta OS=Homo sapiens GN=POLB PE=1 SV=3;>tr|Q53EV2|Q53EV2_HUMAN Polymerase (DNA direct	1.95E-07	
P06748	NPM_HUMAN Nucleophosmin OS=Homo sapiens GN=NPM1 PE=1 SV=2		1128.21
P06748-2	NPM_HUMAN Isoform 2 of Nucleophosmin OS=Homo sapiens GN=NPM1		1128.21
P06748;P06748-2	NPM_HUMAN Nucleophosmin OS=Homo sapiens GN=NPM1 PE=1 SV=2;>sp|P06748-2|NPM_HUMAN Isoform 2 of Nucleophosmin OS=	0	
P06753	TPM3_HUMAN Tropomyosin alpha-3 chain OS=Homo sapiens GN=TPM3 PE=1 SV=1		398.84
P06753-2	TPM3_HUMAN Isoform 2 of Tropomyosin alpha-3 chain OS=Homo sapiens GN=TPM3		621.22
P06753-2;P06753-3	2|TPM3_HUMAN Isoform 2 of Tropomyosin alpha-3 chain OS=Homo sapiens GN=TPM3;>sp|P06753-3|TPM3_HUMAN Isoform 3 of Trop	0	
P06753-3	TPM3_HUMAN Isoform 3 of Tropomyosin alpha-3 chain OS=Homo sapiens GN=TPM3		555.83
P06756	ITAV_HUMAN Integrin alpha-V OS=Homo sapiens GN=ITGAV PE=1 SV=2		125.37
P06756-2	ITAV_HUMAN Isoform 2 of Integrin alpha-V OS=Homo sapiens GN=ITGAV		125.37
P06756;P06756-2	ITAV_HUMAN Integrin alpha-V OS=Homo sapiens GN=ITGAV PE=1 SV=2;>sp|P06756-2|ITAV_HUMAN Isoform 2 of Integrin alpha-V C	6.16E-78	
P06865	HEXA_HUMAN Beta-hexosaminidase subunit alpha OS=Homo sapiens GN=HEXA PE=1 SV=2		
P06899	H2B1J_HUMAN Histone H2B type 1-J OS=Homo sapiens GN=HIST1H2BJ PE=1 SV=3		729.36

P07093;P07093-2	GDN_HUMAN Glia-derived nexin OS=Homo sapiens GN=SERPINE2 PE=1 SV=1;>sp|P07093-2|GDN_HUMAN Isoform 2 of Glia-derived	4.61E-17	
P07099	HYEP_HUMAN Epoxide hydrolase 1 OS=Homo sapiens GN=EPHX1 PE=1 SV=1		149.84
P07108	ACBP_HUMAN Acyl-CoA-binding protein OS=Homo sapiens GN=DBI PE=1 SV=2		76.14
P07108-2	ACBP_HUMAN Isoform 2 of Acyl-CoA-binding protein OS=Homo sapiens GN=DBI		76.14
P07108-2;P07108-3;P071	2|ACBP_HUMAN Isoform 2 of Acyl-CoA-binding protein OS=Homo sapiens GN=DBI;>sp|P07108-3|ACBP_HUMAN Isoform 3 of Acyl-Co	9.71E-36	
P07108-3	ACBP_HUMAN Isoform 3 of Acyl-CoA-binding protein OS=Homo sapiens GN=DBI		76.14
P07108;P07108-2;P07108	ACBP_HUMAN Acyl-CoA-binding protein OS=Homo sapiens GN=DBI PE=1 SV=2;>sp|P07108-2|ACBP_HUMAN Isoform 2 of Acyl-CoA-b	4.94E-20	
P07195	LDHB_HUMAN L-lactate dehydrogenase B chain OS=Homo sapiens GN=LDHB PE=1 SV=2		725.87
P07196	NFL_HUMAN Neurofilament light polypeptide OS=Homo sapiens GN=NEFL PE=1 SV=3		103.13
P07197	NFM_HUMAN Neurofilament medium polypeptide OS=Homo sapiens GN=NEFM PE=1 SV=3		97.16
P07203	GPX1_HUMAN Glutathione peroxidase 1 OS=Homo sapiens GN=GPX1 PE=1 SV=4		240.85
P07205	PGK2_HUMAN Phosphoglycerate kinase 2 OS=Homo sapiens GN=PGK2 PE=1 SV=3		242.74
P07237	PDIA1_HUMAN Protein disulfide-isomerase OS=Homo sapiens GN=P4HB PE=1 SV=3		1878.31
P07305	H10_HUMAN Histone H1.0 OS=Homo sapiens GN=H1F0 PE=1 SV=3		258.54
P07311	ACYP1_HUMAN Acylphosphatase-1 OS=Homo sapiens GN=ACYP1 PE=1 SV=2		48.79
P07339	CATD_HUMAN Cathepsin D OS=Homo sapiens GN=CTSD PE=1 SV=1		774.29
P07355	ANXA2_HUMAN Annexin A2 OS=Homo sapiens GN=ANXA2 PE=1 SV=2		2941.65
P07355-2	ANXA2_HUMAN Isoform 2 of Annexin A2 OS=Homo sapiens GN=ANXA2		2912.28
P07355-2;P07355;A6NMY	2|ANXA2_HUMAN Isoform 2 of Annexin A2 OS=Homo sapiens GN=ANXA2;>sp|P07355|ANXA2_HUMAN Annexin A2 OS=Homo sapiens	2.83E-269	
P07357	CO8A_HUMAN Complement component C8 alpha chain OS=Homo sapiens GN=C8A PE=1 SV=2	8.16E-100	
P07358	CO8B_HUMAN Complement component C8 beta chain OS=Homo sapiens GN=C8B PE=1 SV=3	6.48E-13	
P07360	CO8G_HUMAN Complement component C8 gamma chain OS=Homo sapiens GN=C8G PE=1 SV=3	3.55E-37	
P07384	CAN1_HUMAN Calpain-1 catalytic subunit OS=Homo sapiens GN=CAPN1 PE=1 SV=1		
P07437	TBB5_HUMAN Tubulin beta chain OS=Homo sapiens GN=TUBB PE=1 SV=2		2474.18
P07476	INVO_HUMAN Involucrin OS=Homo sapiens GN=IVL PE=1 SV=2		
P07477	TRY1_HUMAN Trypsin-1 OS=Homo sapiens GN=PRSS1 PE=1 SV=1		
P07478	TRY2_HUMAN Trypsin-2 OS=Homo sapiens GN=PRSS2 PE=1 SV=1		
P07498	CASK_HUMAN Kappa-casein OS=Homo sapiens GN=CSN3 PE=1 SV=3	1.12E-05	
P07585;P07585-4	PGS2_HUMAN Decorin OS=Homo sapiens GN=DCN PE=1 SV=1;>sp|P07585-4|PGS2_HUMAN Isoform D of Decorin OS=Homo sapiens	0	
P07602	SAP_HUMAN Proactivator polypeptide OS=Homo sapiens GN=PSAP PE=1 SV=2		484.31
P07602-2	SAP_HUMAN Isoform Sap-mu-6 of Proactivator polypeptide OS=Homo sapiens GN=PSAP		484.31
P07602-3	SAP_HUMAN Isoform Sap-mu-9 of Proactivator polypeptide OS=Homo sapiens GN=PSAP		484.31
P07602-3;P07602-2;P076	3|SAP_HUMAN Isoform Sap-mu-9 of Proactivator polypeptide OS=Homo sapiens GN=PSAP;>sp|P07602-2|SAP_HUMAN Isoform Sap-r	6.84E-58	
P07602;P07602-3;P07602	SAP_HUMAN Proactivator polypeptide OS=Homo sapiens GN=PSAP PE=1 SV=2;>sp|P07602-3|SAP_HUMAN Isoform Sap-mu-9 of Proa	1.79E-154	
P07686	HEXB_HUMAN Beta-hexosaminidase subunit beta OS=Homo sapiens GN=HEXB PE=1 SV=3		270.85
P07711	CATL1_HUMAN Cathepsin L1 OS=Homo sapiens GN=CTSL1 PE=1 SV=2	2.23E-08	
P07737	PROF1_HUMAN Profilin-1 OS=Homo sapiens GN=PFN1 PE=1 SV=2		1084.54
P07738	PMGE_HUMAN Bisphosphoglycerate mutase OS=Homo sapiens GN=BPGM PE=1 SV=2	1.97E-30	
P07741	APT_HUMAN Adenine phosphoribosyltransferase OS=Homo sapiens GN=APRT PE=1 SV=2		243.17
P07814	SYEP_HUMAN Bifunctional aminoacyl-tRNA synthetase OS=Homo sapiens GN=EPRS PE=1 SV=5		1232.26
P07858	CATB_HUMAN Cathepsin B OS=Homo sapiens GN=CTSB PE=1 SV=3		271.52
P07864	LDHC_HUMAN L-lactate dehydrogenase C chain OS=Homo sapiens GN=LDHC PE=2 SV=4		134.46
P07900	HS90A_HUMAN Heat shock protein HSP 90-alpha OS=Homo sapiens GN=HSP90AA1 PE=1 SV=5		2001.57
P07900-2	HS90A_HUMAN Isoform 2 of Heat shock protein HSP 90-alpha OS=Homo sapiens GN=HSP90AA1		2008.35
P07900-2;P07900	2|HS90A_HUMAN Isoform 2 of Heat shock protein HSP 90-alpha OS=Homo sapiens GN=HSP90AA1;>sp|P07900|HS90A_HUMAN Heat	0	
P07910	HNRPC_HUMAN Heterogeneous nuclear ribonucleoproteins C1/C2 OS=Homo sapiens GN=HNRNPC PE=1 SV=4		561.45
P07910-2	HNRPC_HUMAN Isoform C1 of Heterogeneous nuclear ribonucleoproteins C1/C2 OS=Homo sapiens GN=HNRNPC		681.22
P07910-3	HNRPC_HUMAN Isoform 3 of Heterogeneous nuclear ribonucleoproteins C1/C2 OS=Homo sapiens GN=HNRNPC		250.05
P07910-4	HNRPC_HUMAN Isoform 4 of Heterogeneous nuclear ribonucleoproteins C1/C2 OS=Homo sapiens GN=HNRNPC		655.48
P07910;P07910-2;P07910	HNRPC_HUMAN Heterogeneous nuclear ribonucleoproteins C1/C2 OS=Homo sapiens GN=HNRNPC PE=1 SV=4;>sp|P07910-2|HNRPC_	1.60E-64	
P07919	QCR6_HUMAN Cytochrome b-c1 complex subunit 6, mitochondrial OS=Homo sapiens GN=UQCRH PE=1 SV=2		78.96
P07942	LAMB1_HUMAN Laminin subunit beta-1 OS=Homo sapiens GN=LAMB1 PE=1 SV=2	2.35E-207	
P07947	YES_HUMAN Tyrosine-protein kinase Yes OS=Homo sapiens GN=YES1 PE=1 SV=3		109.1
P07948	LYN_HUMAN Tyrosine-protein kinase Lyn OS=Homo sapiens GN=LYN PE=1 SV=3		150.52
P07948-2	LYN_HUMAN Isoform LYN B of Tyrosine-protein kinase Lyn OS=Homo sapiens GN=LYN		150.52
P07948;P07948-2	LYN_HUMAN Tyrosine-protein kinase Lyn OS=Homo sapiens GN=LYN PE=1 SV=3;>sp|P07948-2|LYN_HUMAN Isoform LYN B of Tyrosin	3.86E-14	
P07949	RET_HUMAN Proto-oncogene tyrosine-protein kinase receptor Ret OS=Homo sapiens GN=RET PE=1 SV=3		
P07949-2	RET_HUMAN Isoform 2 of Proto-oncogene tyrosine-protein kinase receptor Ret OS=Homo sapiens GN=RET		
P07951	TPM2_HUMAN Tropomyosin beta chain OS=Homo sapiens GN=TPM2 PE=1 SV=1		267.27
P07951-2	TPM2_HUMAN Isoform 2 of Tropomyosin beta chain OS=Homo sapiens GN=TPM2		267.27
P07951-3	TPM2_HUMAN Isoform 3 of Tropomyosin beta chain OS=Homo sapiens GN=TPM2		257.43

P07954	FUMH_HUMAN Fumarate hydratase, mitochondrial OS=Homo sapiens GN=FH PE=1 SV=3		327.75
P07954-2	FUMH_HUMAN Isoform Cytoplasmic of Fumarate hydratase, mitochondrial OS=Homo sapiens GN=FH		327.75
P07954;P07954-2	FUMH_HUMAN Fumarate hydratase, mitochondrial OS=Homo sapiens GN=FH PE=1 SV=3;>sp|P07954-2|FUMH_HUMAN Isoform Cytop	0	
P07996	TSP1_HUMAN Thrombospondin-1 OS=Homo sapiens GN=THBS1 PE=1 SV=2		357.25
P08069	IGF1R_HUMAN Insulin-like growth factor 1 receptor OS=Homo sapiens GN=IGF1R PE=1 SV=1		
P08107	HSP71_HUMAN Heat shock 70 kDa protein 1A/1B OS=Homo sapiens GN=HSPA1A PE=1 SV=5		662.66
P08123	CO1A2_HUMAN Collagen alpha-2(I) chain OS=Homo sapiens GN=COL1A2 PE=1 SV=7		37.78
P08133	ANXA6_HUMAN Annexin A6 OS=Homo sapiens GN=ANXA6 PE=1 SV=3		1772.32
P08134	RHOC_HUMAN Rho-related GTP-binding protein RhoC OS=Homo sapiens GN=RHOC PE=1 SV=1		803.66
P08174	DAF_HUMAN Complement decay-accelerating factor OS=Homo sapiens GN=CD55 PE=1 SV=4		134.17
P08174-2	DAF_HUMAN Isoform 1 of Complement decay-accelerating factor OS=Homo sapiens GN=CD55		134.17
P08174-2;P08174	2|DAF_HUMAN Isoform 1 of Complement decay-accelerating factor OS=Homo sapiens GN=CD55;>sp|P08174|DAF_HUMAN Compleme	2.58E-15	
P08185	CBG_HUMAN Corticosteroid-binding globulin OS=Homo sapiens GN=SERPINA6 PE=1 SV=1		87.92
P08195	4F2_HUMAN 4F2 cell-surface antigen heavy chain OS=Homo sapiens GN=SLC3A2 PE=1 SV=3		1433.53
P08195-2	4F2_HUMAN Isoform 2 of 4F2 cell-surface antigen heavy chain OS=Homo sapiens GN=SLC3A2		1433.53
P08195-3	4F2_HUMAN Isoform 3 of 4F2 cell-surface antigen heavy chain OS=Homo sapiens GN=SLC3A2		1433.53
P08195-4	4F2_HUMAN Isoform 4 of 4F2 cell-surface antigen heavy chain OS=Homo sapiens GN=SLC3A2		1433.53
P08195-4;P08195;P08195	4|4F2_HUMAN Isoform 4 of 4F2 cell-surface antigen heavy chain OS=Homo sapiens GN=SLC3A2;>sp|P08195|4F2_HUMAN 4F2 cell-su	0	
P08236;B4E1F6;B2R6X2;F	BGLR_HUMAN Beta-glucuronidase OS=Homo sapiens GN=GUSB PE=1 SV=2;>tr|B4E1F6|B4E1F6_HUMAN cDNA FLJ53268, highly simi	2.11E-46	
P08237;P08237-2	K6PF_HUMAN 6-phosphofructokinase, muscle type OS=Homo sapiens GN=PFKM PE=1 SV=2;>sp|P08237-2|K6PF_HUMAN Isoform 2 c	1.92E-67	
P08238	HS90B_HUMAN Heat shock protein HSP 90-beta OS=Homo sapiens GN=HSP90AB1 PE=1 SV=4		2366.92
P08240	SRPR_HUMAN Signal recognition particle receptor subunit alpha OS=Homo sapiens GN=SRPR PE=1 SV=2		114.24
P08243	ASNS_HUMAN Asparagine synthetase [glutamine-hydrolyzing] OS=Homo sapiens GN=ASNS PE=1 SV=4		23.35
P08246	ELNE_HUMAN Neutrophil elastase OS=Homo sapiens GN=ELANE PE=1 SV=1	5.74E-15	
P08253	MMP2_HUMAN 72 kDa type IV collagenase OS=Homo sapiens GN=MMP2 PE=1 SV=2	4.88E-46	
P08254	MMP3_HUMAN Stromelysin-1 OS=Homo sapiens GN=MMP3 PE=1 SV=2		
P08263;P09210;Q16772;(	GSTA1_HUMAN Glutathione S-transferase A1 OS=Homo sapiens GN=GSTA1 PE=1 SV=3;>sp|P09210|GSTA2_HUMAN Glutathione S-tr	1.91E-17	
P08294	SODE_HUMAN Extracellular superoxide dismutase [Cu-Zn] OS=Homo sapiens GN=SOD3 PE=1 SV=2	4.12E-69	
P08311	CATG_HUMAN Cathepsin G OS=Homo sapiens GN=CTSG PE=1 SV=2	1.42E-20	
P08397	HEM3_HUMAN Porphobilinogen deaminase OS=Homo sapiens GN=HMBS PE=1 SV=2		
P08397-2	HEM3_HUMAN Isoform 2 of Porphobilinogen deaminase OS=Homo sapiens GN=HMBS		
P08397;P08397-2	HEM3_HUMAN Porphobilinogen deaminase OS=Homo sapiens GN=HMBS PE=1 SV=2;>sp|P08397-2|HEM3_HUMAN Isoform 2 of Porph	1.41E-39	
P08493	MGP_HUMAN Matrix Gla protein OS=Homo sapiens GN=MGP PE=1 SV=2	5.18E-39	
P08519	APOA_HUMAN Apolipoprotein(a) OS=Homo sapiens GN=LPA PE=1 SV=1	0.00011439	
P08559	ODPA_HUMAN Pyruvate dehydrogenase E1 component subunit alpha, somatic form, mitochondrial OS=Homo sapiens GN=PDHA1 PE=1 SV=3		239.94
P08567	PLEK_HUMAN Pleckstrin OS=Homo sapiens GN=PLEK PE=1 SV=3	6.71E-271	
P08571	CD14_HUMAN Monocyte differentiation antigen CD14 OS=Homo sapiens GN=CD14 PE=1 SV=2	3.05E-146	
P08572	CO4A2_HUMAN Collagen alpha-2(IV) chain OS=Homo sapiens GN=COL4A2 PE=1 SV=4	1.38E-162	
P08574	CY1_HUMAN Cytochrome c1, heme protein, mitochondrial OS=Homo sapiens GN=CYC1 PE=1 SV=3		288.64
P08575;P08575-2	PTPRC_HUMAN Receptor-type tyrosine-protein phosphatase C OS=Homo sapiens GN=PTPRC PE=1 SV=2;>sp|P08575-2|PTPRC_HUMA	4.62E-15	
P08579	RU2B_HUMAN U2 small nuclear ribonucleoprotein B'' OS=Homo sapiens GN=SNRPB2 PE=1 SV=1		153.72
P08581-2;A1L467;P08581	2|MET_HUMAN Isoform 2 of Hepatocyte growth factor receptor OS=Homo sapiens GN=MET;>tr|A1L467|A1L467_HUMAN Met proto-on	8.86E-30	
P08582	TRFM_HUMAN Melanotransferrin OS=Homo sapiens GN=MFI2 PE=1 SV=2		117.89
P08582-2	TRFM_HUMAN Isoform 2 of Melanotransferrin OS=Homo sapiens GN=MFI2		64.97
P08590	MYL3_HUMAN Myosin light chain 3 OS=Homo sapiens GN=MYL3 PE=1 SV=3		114.73
P08603	CFAH_HUMAN Complement factor H OS=Homo sapiens GN=CFH PE=1 SV=4	0	
P08621	RU17_HUMAN U1 small nuclear ribonucleoprotein 70 kDa OS=Homo sapiens GN=SNRNP70 PE=1 SV=2		160.09
P08621-2	RU17_HUMAN Isoform 2 of U1 small nuclear ribonucleoprotein 70 kDa OS=Homo sapiens GN=SNRNP70		160.09
P08621-3	RU17_HUMAN Isoform 3 of U1 small nuclear ribonucleoprotein 70 kDa OS=Homo sapiens GN=SNRNP70		160.09
P08621-4	RU17_HUMAN Isoform 4 of U1 small nuclear ribonucleoprotein 70 kDa OS=Homo sapiens GN=SNRNP70		
P08621;A8KAQ5;P08621-:	RU17_HUMAN U1 small nuclear ribonucleoprotein 70 kDa OS=Homo sapiens GN=SNRNP70 PE=1 SV=2;>tr|A8KAQ5|A8KAQ5_HUMAN	1.12E-179	
P08631	HCK_HUMAN Tyrosine-protein kinase HCK OS=Homo sapiens GN=HCK PE=1 SV=5		145.17
P08631-2	HCK_HUMAN Isoform p59-HCK of Tyrosine-protein kinase HCK OS=Homo sapiens GN=HCK		145.17
P08631;P08631-2	HCK_HUMAN Tyrosine-protein kinase HCK OS=Homo sapiens GN=HCK PE=1 SV=5;>sp|P08631-2|HCK_HUMAN Isoform p59-HCK of T	5.27E-11	
P08637	FCG3A_HUMAN Low affinity immunoglobulin gamma Fc region receptor III-A OS=Homo sapiens GN=FCGR3A PE=1 SV=2	1.29E-05	
P08648	ITA5_HUMAN Integrin alpha-5 OS=Homo sapiens GN=ITGA5 PE=1 SV=2		79.53
P08651	NFIC_HUMAN Nuclear factor 1 C-type OS=Homo sapiens GN=NFIC PE=1 SV=2		
P08651-2	NFIC_HUMAN Isoform 1 of Nuclear factor 1 C-type OS=Homo sapiens GN=NFIC		
P08651-3	NFIC_HUMAN Isoform 2 of Nuclear factor 1 C-type OS=Homo sapiens GN=NFIC		
P08651-4	NFIC_HUMAN Isoform 3 of Nuclear factor 1 C-type OS=Homo sapiens GN=NFIC		
P08651-5	NFIC_HUMAN Isoform 5 of Nuclear factor 1 C-type OS=Homo sapiens GN=NFIC		

P08670	VIME_HUMAN Vimentin OS=Homo sapiens GN=VIM PE=1 SV=4		3540.48
P08697	A2AP_HUMAN Alpha-2-antiplasmin OS=Homo sapiens GN=SERPINF2 PE=1 SV=3		55.18
P08708	RS17_HUMAN 40S ribosomal protein S17 OS=Homo sapiens GN=RPS17 PE=1 SV=2		956.1
P08727	K1C19_HUMAN Keratin, type I cytoskeletal 19 OS=Homo sapiens GN=KRT19 PE=1 SV=4		1390.5
P08729	K2C7_HUMAN Keratin, type II cytoskeletal 7 OS=Homo sapiens GN=KRT7 PE=1 SV=5		973.33
P08754	GNAI3_HUMAN Guanine nucleotide-binding protein G(k) subunit alpha OS=Homo sapiens GN=GNAI3 PE=1 SV=3		500.09
P08758	ANXA5_HUMAN Annexin A5 OS=Homo sapiens GN=ANXA5 PE=1 SV=2		1060.51
P08779	K1C16_HUMAN Keratin, type I cytoskeletal 16 OS=Homo sapiens GN=KRT16 PE=1 SV=4		836.04
P08833	IBP1_HUMAN Insulin-like growth factor-binding protein 1 OS=Homo sapiens GN=IGFBP1 PE=1 SV=1		30.29
P08842	STS_HUMAN Steryl-sulfatase OS=Homo sapiens GN=STS PE=1 SV=2		
P08865	RSSA_HUMAN 40S ribosomal protein SA OS=Homo sapiens GN=RPSA PE=1 SV=4		903.89
P08912	ACM5_HUMAN Muscarinic acetylcholine receptor M5 OS=Homo sapiens GN=CHRM5 PE=2 SV=2		32.52
P08922	ROS_HUMAN Proto-oncogene tyrosine-protein kinase ROS OS=Homo sapiens GN=ROS1 PE=2 SV=3		
P08962	CD63_HUMAN CD63 antigen OS=Homo sapiens GN=CD63 PE=1 SV=2		
P09001	RM03_HUMAN 39S ribosomal protein L3, mitochondrial OS=Homo sapiens GN=MRPL3 PE=1 SV=1		
P09012	SNRPA_HUMAN U1 small nuclear ribonucleoprotein A OS=Homo sapiens GN=SNRPA PE=1 SV=3		111.35
P09104	ENOG_HUMAN Gamma-enolase OS=Homo sapiens GN=ENO2 PE=1 SV=3		358.26
P09110	THIK_HUMAN 3-ketoacyl-CoA thiolase, peroxisomal OS=Homo sapiens GN=ACAA1 PE=1 SV=2		47.74
P09131	P3_HUMAN P3 protein OS=Homo sapiens GN=SLC10A3 PE=2 SV=1		
P09132	SRP19_HUMAN Signal recognition particle 19 kDa protein OS=Homo sapiens GN=SRP19 PE=1 SV=3		329.53
P09211	GSTP1_HUMAN Glutathione S-transferase P OS=Homo sapiens GN=GSTP1 PE=1 SV=2		839.4
P09234	RU1C_HUMAN U1 small nuclear ribonucleoprotein C OS=Homo sapiens GN=SNRPC PE=1 SV=1		242.45
P09327	VILI_HUMAN Villin-1 OS=Homo sapiens GN=VIL1 PE=1 SV=4		
P09382	LEG1_HUMAN Galectin-1 OS=Homo sapiens GN=LGALS1 PE=1 SV=2		935.43
P09417	DHPR_HUMAN Dihydropteridine reductase OS=Homo sapiens GN=QDPR PE=1 SV=2	1.86E-249	
P09429	HMGB1_HUMAN High mobility group protein B1 OS=Homo sapiens GN=HMGB1 PE=1 SV=3		649.92
P09455	RET1_HUMAN Retinol-binding protein 1 OS=Homo sapiens GN=RBP1 PE=1 SV=2	4.13E-128	
P09467	F16P1_HUMAN Fructose-1,6-bisphosphatase 1 OS=Homo sapiens GN=FBP1 PE=1 SV=5		
P09471	GNAO_HUMAN Guanine nucleotide-binding protein G(o) subunit alpha OS=Homo sapiens GN=GNAO1 PE=1 SV=4		160.28
P09471-2	GNAO_HUMAN Isoform Alpha-2 of Guanine nucleotide-binding protein G(o) subunit alpha OS=Homo sapiens GN=GNAO1		211.04
P09486	SPRC_HUMAN SPARC OS=Homo sapiens GN=SPARC PE=1 SV=1	1.08E-10	
P09488;P09488-2	GSTM1_HUMAN Glutathione S-transferase Mu 1 OS=Homo sapiens GN=GSTM1 PE=1 SV=3;>sp|P09488-2|GSTM1_HUMAN Isoform 2	9.33E-96	
P09493	TPM1_HUMAN Tropomyosin alpha-1 chain OS=Homo sapiens GN=TPM1 PE=1 SV=2		198
P09493-2	TPM1_HUMAN Isoform 2 of Tropomyosin alpha-1 chain OS=Homo sapiens GN=TPM1		192.8
P09493-3	TPM1_HUMAN Isoform 3 of Tropomyosin alpha-1 chain OS=Homo sapiens GN=TPM1		211.33
P09493-3;P09493-4	3|TPM1_HUMAN Isoform 3 of Tropomyosin alpha-1 chain OS=Homo sapiens GN=TPM1;>sp|P09493-4|TPM1_HUMAN Isoform 4 of Trop	7.35E-32	
P09493-4	TPM1_HUMAN Isoform 4 of Tropomyosin alpha-1 chain OS=Homo sapiens GN=TPM1		211.33
P09493-5	TPM1_HUMAN Isoform 5 of Tropomyosin alpha-1 chain OS=Homo sapiens GN=TPM1		252.84
P09493-6	TPM1_HUMAN Isoform 6 of Tropomyosin alpha-1 chain OS=Homo sapiens GN=TPM1		192.49
P09496	CLCA_HUMAN Clathrin light chain A OS=Homo sapiens GN=CLTA PE=1 SV=1		251.86
P09496-2	CLCA_HUMAN Isoform Non-brain of Clathrin light chain A OS=Homo sapiens GN=CLTA		363.31
P09496-3	CLCA_HUMAN Isoform 3 of Clathrin light chain A OS=Homo sapiens GN=CLTA		251.86
P09496;P09496-3;B4DIN1	CLCA_HUMAN Clathrin light chain A OS=Homo sapiens GN=CLTA PE=1 SV=1;>sp|P09496-3|CLCA_HUMAN Isoform 3 of Clathrin light	8.03E-12	
P09497	CLCB_HUMAN Clathrin light chain B OS=Homo sapiens GN=CLTB PE=1 SV=1		216.98
P09497-2	CLCB_HUMAN Isoform Non-brain of Clathrin light chain B OS=Homo sapiens GN=CLTB		224.34
P09497;P09497-2	CLCB_HUMAN Clathrin light chain B OS=Homo sapiens GN=CLTB PE=1 SV=1;>sp|P09497-2|CLCB_HUMAN Isoform Non-brain of Clath	1.23E-14	
P09525	ANXA4_HUMAN Annexin A4 OS=Homo sapiens GN=ANXA4 PE=1 SV=4		99.84
P09543	CN37_HUMAN 2',3'-cyclic-nucleotide 3'-phosphodiesterase OS=Homo sapiens GN=CNP PE=1 SV=2		658.5
P09543-2	CN37_HUMAN Isoform CNPI of 2',3'-cyclic-nucleotide 3'-phosphodiesterase OS=Homo sapiens GN=CNP		658.5
P09543;P09543-2	CN37_HUMAN 2,3-cyclic-nucleotide 3-phosphodiesterase OS=Homo sapiens GN=CNP PE=1 SV=2;>sp|P09543-2|CN37_HUMAN Isofor	1.42E-24	
P09601	HMOX1_HUMAN Heme oxygenase 1 OS=Homo sapiens GN=HMOX1 PE=1 SV=1	1.55E-05	
P09622	DLDH_HUMAN Dihydrolipoyl dehydrogenase, mitochondrial OS=Homo sapiens GN=DLD PE=1 SV=2		668.33
P09651	ROA1_HUMAN Heterogeneous nuclear ribonucleoprotein A1 OS=Homo sapiens GN=HNRNPA1 PE=1 SV=5		1287.51
P09651-2	ROA1_HUMAN Isoform A1-A of Heterogeneous nuclear ribonucleoprotein A1 OS=Homo sapiens GN=HNRNPA1		1287.51
P09651-3	ROA1_HUMAN Isoform 2 of Heterogeneous nuclear ribonucleoprotein A1 OS=Homo sapiens GN=HNRNPA1		1271.87
P09651;P09651-2;P09651	ROA1_HUMAN Heterogeneous nuclear ribonucleoprotein A1 OS=Homo sapiens GN=HNRNPA1 PE=1 SV=5;>sp|P09651-2|ROA1_HUMA	9.12E-280	
P09661	RU2A_HUMAN U2 small nuclear ribonucleoprotein A' OS=Homo sapiens GN=SNRPA1 PE=1 SV=2		421.3
P09668	CATH_HUMAN Pro-cathepsin H OS=Homo sapiens GN=CTSH PE=1 SV=4		
P09668;Q6IBC3;Q96NY6;(	CATH_HUMAN Cathepsin H OS=Homo sapiens GN=CTSH PE=1 SV=4;>tr|Q6IBC3|Q6IBC3_HUMAN CTSH protein OS=Homo sapiens G	2.40E-24	
P09669	COX6C_HUMAN Cytochrome c oxidase subunit 6C OS=Homo sapiens GN=COX6C PE=1 SV=2		61.09
P09758	TACD2_HUMAN Tumor-associated calcium signal transducer 2 OS=Homo sapiens GN=TACSTD2 PE=1 SV=3		208.25

P09769	FGR_HUMAN Tyrosine-protein kinase Fgr OS=Homo sapiens GN=FGR PE=1 SV=2		55.19
P09871	C1S_HUMAN Complement C1s subcomponent OS=Homo sapiens GN=C1S PE=1 SV=1	4.07E-79	
P09874	PARP1_HUMAN Poly [ADP-ribose] polymerase 1 OS=Homo sapiens GN=PARP1 PE=1 SV=4		1202.04
P09884	DPOLA_HUMAN DNA polymerase alpha catalytic subunit OS=Homo sapiens GN=POLA1 PE=1 SV=2	6.21E-09	
P09913	IFIT2_HUMAN Interferon-induced protein with tetratricopeptide repeats 2 OS=Homo sapiens GN=IFIT2 PE=1 SV=1	2.29E-43	
P09914	IFIT1_HUMAN Interferon-induced protein with tetratricopeptide repeats 1 OS=Homo sapiens GN=IFIT1 PE=1 SV=2	1.81E-134	
P09917	LOX5_HUMAN Arachidonate 5-lipoxygenase OS=Homo sapiens GN=ALOX5 PE=1 SV=2	3.38E-21	
P09936	UCHL1_HUMAN Ubiquitin carboxyl-terminal hydrolase isozyme L1 OS=Homo sapiens GN=UCHL1 PE=1 SV=2	9.78E-190	
P09960	LKHA4_HUMAN Leukotriene A-4 hydrolase OS=Homo sapiens GN=LTA4H PE=1 SV=2		33.81
P09960-2	LKHA4_HUMAN Isoform 2 of Leukotriene A-4 hydrolase OS=Homo sapiens GN=LTA4H		33.81
P09960-3	LKHA4_HUMAN Isoform 3 of Leukotriene A-4 hydrolase OS=Homo sapiens GN=LTA4H		33.81
P09960;B4DVZ8;P09960-[illegible]	LKHA4_HUMAN Leukotriene A-4 hydrolase OS=Homo sapiens GN=LTA4H PE=1 SV=2;>tr|B4DVZ8|B4DVZ8_HUMAN cDNA FLJ52432, H	0	
P09972	ALDOC_HUMAN Fructose-bisphosphate aldolase C OS=Homo sapiens GN=ALDOC PE=1 SV=2		57.85
P0C0L4	CO4A_HUMAN Complement C4-A OS=Homo sapiens GN=C4A PE=1 SV=1		445.87
P0C0L5	CO4B_HUMAN Complement C4-B OS=Homo sapiens GN=C4B PE=1 SV=1		445.87
P0C0S5	H2AZ_HUMAN Histone H2A.Z OS=Homo sapiens GN=H2AFZ PE=1 SV=2		250.74
P0C0S8	H2A1_HUMAN Histone H2A type 1 OS=Homo sapiens GN=HIST1H2AG PE=1 SV=2		288.83
P0C221	CN038_HUMAN Uncharacterized protein C14orf38 OS=Homo sapiens GN=C14orf38 PE=2 SV=1		
P0C6E5	HMG3M_HUMAN Putative high mobility group protein B3-like protein OS=Homo sapiens PE=5 SV=1		105.96
P0C7P0	CISD3_HUMAN CDGSH iron-sulfur domain-containing protein 3, mitochondrial OS=Homo sapiens GN=CISD3 PE=1 SV=1	1.59E-08	
P0C7P4	UCRIL_HUMAN Putative cytochrome b-c1 complex subunit Rieske-like protein 1 OS=Homo sapiens GN=UQCRFS1P1 PE=5 SV=1		270.07
P0C7V4	F90AJ_HUMAN Putative protein FAM90A15 OS=Homo sapiens GN=FAM90A15 PE=3 SV=1		
P0C7W8	F90AD_HUMAN Putative protein FAM90A13 OS=Homo sapiens GN=FAM90A13 PE=5 SV=1		
P0C7W9	F90AE_HUMAN Putative protein FAM90A14 OS=Homo sapiens GN=FAM90A14 PE=3 SV=1		
P0C7X0	F90AO_HUMAN Putative protein FAM90A24 OS=Homo sapiens GN=FAM90A24P PE=5 SV=1		
P0C7X5	ZN806_HUMAN Zinc finger protein 806 OS=Homo sapiens GN=ZNF806 PE=2 SV=1		
P0CB38	PAB4L_HUMAN Polyadenylate-binding protein 4-like OS=Homo sapiens GN=PABPC4L PE=2 SV=1		149.56
P0CB43;Q9BTY7	F203B_HUMAN Protein FAM203B OS=Homo sapiens GN=FAM203B PE=3 SV=1;>sp|Q9BTY7|F203A_HUMAN Protein FAM203A OS=Hon	0.00012735	
P0CF74	LAC6_HUMAN Ig lambda-6 chain C region OS=Homo sapiens GN=IGLC6 PE=4 SV=1		158.78
P0CG04	LAC1_HUMAN Ig lambda-1 chain C regions OS=Homo sapiens GN=IGLC1 PE=1 SV=1		164.87
P0CG05	LAC2_HUMAN Ig lambda-2 chain C regions OS=Homo sapiens GN=IGLC2 PE=1 SV=1		179.92
P0CG06	LAC3_HUMAN Ig lambda-3 chain C regions OS=Homo sapiens GN=IGLC3 PE=1 SV=1		179.92
P0CG08	GPHRB_HUMAN Golgi pH regulator B OS=Homo sapiens GN=GPR89B PE=1 SV=1		
P0CG22	DR4L1_HUMAN Putative dehydrogenase/reductase SDR family member 4-like 2 OS=Homo sapiens GN=DHRS4L1 PE=5 SV=1		43.71
P0CG29	GST2_HUMAN Glutathione S-transferase theta-2 OS=Homo sapiens GN=GSTT2 PE=1 SV=1		20.96
P0CG30	GSTT2_HUMAN Glutathione S-transferase theta-2B OS=Homo sapiens GN=GSTT2B PE=1 SV=1		20.96
P0CG31	Z286B_HUMAN Putative zinc finger protein 286B OS=Homo sapiens GN=ZNF286B PE=5 SV=1		20.45
P0CG38	POTEI_HUMAN POTE ankyrin domain family member I OS=Homo sapiens GN=POTEI PE=3 SV=1		427.71
P0CG39	POTEJ_HUMAN POTE ankyrin domain family member J OS=Homo sapiens GN=POTEJ PE=3 SV=1		365.22
P0CG47	UBB_HUMAN Polyubiquitin-B OS=Homo sapiens GN=UBB PE=1 SV=1		465.4
P0CG48	UBC_HUMAN Polyubiquitin-C OS=Homo sapiens GN=UBC PE=1 SV=2		465.4
P0CL83	ST3L1_HUMAN STAG3-like protein 1 OS=Homo sapiens GN=STAG3L1 PE=3 SV=1		
P0CW22	RS17L_HUMAN 40S ribosomal protein S17-like OS=Homo sapiens GN=RPS17L PE=3 SV=1		956.1
P10071	GLI3_HUMAN Transcriptional activator GLI3 OS=Homo sapiens GN=GLI3 PE=1 SV=6		
P10109	ADX_HUMAN Adrenodoxin, mitochondrial OS=Homo sapiens GN=FDX1 PE=1 SV=1		65.92
P10114	RAP2A_HUMAN Ras-related protein Rap-2a OS=Homo sapiens GN=RAP2A PE=1 SV=1		35.77
P10145	IL8_HUMAN Interleukin-8 OS=Homo sapiens GN=IL8 PE=1 SV=1		45.51
P10145-2	IL8_HUMAN Isoform 2 of Interleukin-8 OS=Homo sapiens GN=IL8		45.51
P10155	RO60_HUMAN 60 kDa SS-A/Ro ribonucleoprotein OS=Homo sapiens GN=TROVE2 PE=1 SV=2		43.16
P10244	MYBB_HUMAN Myb-related protein B OS=Homo sapiens GN=MYBL2 PE=1 SV=1		
P10253	LYAG_HUMAN Lysosomal alpha-glucosidase OS=Homo sapiens GN=GAA PE=1 SV=4		236.08
P10301	RRAS_HUMAN Ras-related protein R-Ras OS=Homo sapiens GN=RRAS PE=1 SV=1		411.73
P10314	1A32_HUMAN HLA class I histocompatibility antigen, A-32 alpha chain OS=Homo sapiens GN=HLA-A PE=2 SV=2		657.24
P10316	1A69_HUMAN HLA class I histocompatibility antigen, A-69 alpha chain OS=Homo sapiens GN=HLA-A PE=1 SV=2		1276.24
P10319	1B58_HUMAN HLA class I histocompatibility antigen, B-58 alpha chain OS=Homo sapiens GN=HLA-B PE=2 SV=1		573.47
P10321	1C07_HUMAN HLA class I histocompatibility antigen, Cw-7 alpha chain OS=Homo sapiens GN=HLA-C PE=1 SV=3		410.17
P10398	ARAF_HUMAN Serine/threonine-protein kinase A-Raf OS=Homo sapiens GN=ARAF PE=1 SV=2	3.72E-14	
P10412	H14_HUMAN Histone H1.4 OS=Homo sapiens GN=HIST1H1E PE=1 SV=2		521.36
P10415;P10415-2	BCL2_HUMAN Apoptosis regulator Bcl-2 OS=Homo sapiens GN=BCL2 PE=1 SV=2;>sp|P10415-2|BCL2_HUMAN Isoform Beta of Apopt	1.20E-66	
P10515	ODP2_HUMAN Dihydrolipoyllysine-residue acetyltransferase component of pyruvate dehydrogenase complex, mitochondrial OS=Homo sapiens GN=D		293.73
P10586	PTPRF_HUMAN Receptor-type tyrosine-protein phosphatase F OS=Homo sapiens GN=PTPRF PE=1 SV=2		94.94

P10586-2	PTPRF_HUMAN Isoform 2 of Receptor-type tyrosine-protein phosphatase F OS=Homo sapiens GN=PTPRF		94.94
P10586;P10586-2	PTPRF_HUMAN Receptor-type tyrosine-protein phosphatase F OS=Homo sapiens GN=PTPRF PE=1 SV=2;>sp|P10586-2|PTPRF_HUMAN	3.95E-29	
P10588	NR2F6_HUMAN Nuclear receptor subfamily 2 group F member 6 OS=Homo sapiens GN=NR2F6 PE=1 SV=2		45.93
P10589	COT1_HUMAN COUP transcription factor 1 OS=Homo sapiens GN=NR2F1 PE=1 SV=1		57.79
P10599	THIO_HUMAN Thioredoxin OS=Homo sapiens GN=TXN PE=1 SV=3		293.43
P10606	COX5B_HUMAN Cytochrome c oxidase subunit 5B, mitochondrial OS=Homo sapiens GN=COX5B PE=1 SV=2		188.8
P10619	PPGB_HUMAN Lysosomal protective protein OS=Homo sapiens GN=CTSA PE=1 SV=2		147.01
P10620	MGST1_HUMAN Microsomal glutathione S-transferase 1 OS=Homo sapiens GN=MGST1 PE=1 SV=1		152.65
P10643	CO7_HUMAN Complement component C7 OS=Homo sapiens GN=C7 PE=1 SV=2	4.83E-08	
P10644	KAP0_HUMAN cAMP-dependent protein kinase type I-alpha regulatory subunit OS=Homo sapiens GN=PRKAR1A PE=1 SV=1		65.4
P10768	ESTD_HUMAN S-formylglutathione hydrolase OS=Homo sapiens GN=ESD PE=1 SV=2		79.56
P10809	CH60_HUMAN 60 kDa heat shock protein, mitochondrial OS=Homo sapiens GN=HSPD1 PE=1 SV=2		3324.19
P10827	THA_HUMAN Thyroid hormone receptor alpha OS=Homo sapiens GN=THRA PE=1 SV=1		
P10827-2	THA_HUMAN Isoform Alpha-1 of Thyroid hormone receptor alpha OS=Homo sapiens GN=THRA		
P10827-3	THA_HUMAN Isoform Alpha-3 of Thyroid hormone receptor alpha OS=Homo sapiens GN=THRA		
P10827-4	THA_HUMAN Isoform Alpha-4 of Thyroid hormone receptor alpha OS=Homo sapiens GN=THRA		
P10828	THB_HUMAN Thyroid hormone receptor beta OS=Homo sapiens GN=THRB PE=1 SV=2		
P10828-2	THB_HUMAN Isoform Beta-2 of Thyroid hormone receptor beta OS=Homo sapiens GN=THRB		
P10909	CLUS_HUMAN Clusterin OS=Homo sapiens GN=CLU PE=1 SV=1		23.1
P10909-2	CLUS_HUMAN Isoform 2 of Clusterin OS=Homo sapiens GN=CLU		23.1
P10909-2;P10909-5;P109	2|CLUS_HUMAN Isoform 2 of Clusterin OS=Homo sapiens GN=CLU;>sp|P10909-5|CLUS_HUMAN Isoform 5 of Clusterin OS=Homo sap	4.65E-303	
P10909-3	CLUS_HUMAN Isoform 3 of Clusterin OS=Homo sapiens GN=CLU		1.79
P10909-4	CLUS_HUMAN Isoform 4 of Clusterin OS=Homo sapiens GN=CLU		23.1
P10909-5	CLUS_HUMAN Isoform 5 of Clusterin OS=Homo sapiens GN=CLU		23.1
P10915	HPLN1_HUMAN Hyaluronan and proteoglycan link protein 1 OS=Homo sapiens GN=HAPLN1 PE=2 SV=2		
P11021	GRP78_HUMAN 78 kDa glucose-regulated protein OS=Homo sapiens GN=HSPA5 PE=1 SV=2		3221.8
P11047	LAMC1_HUMAN Laminin subunit gamma-1 OS=Homo sapiens GN=LAMC1 PE=1 SV=3		
P11055	MYH3_HUMAN Myosin-3 OS=Homo sapiens GN=MYH3 PE=1 SV=3		68.2
P11086	PNMT_HUMAN Phenylethanolamine N-methyltransferase OS=Homo sapiens GN=PNMT PE=1 SV=1	1.07E-21	
P11117;B7Z6L8;B7Z552;E	PPAL_HUMAN Lysosomal acid phosphatase OS=Homo sapiens GN=ACP2 PE=1 SV=3;>tr|B7Z6L8|B7Z6L8_HUMAN cDNA FLJ53923, hig	6.40E-06	
P11137	MAP2_HUMAN Microtubule-associated protein 2 OS=Homo sapiens GN=MAP2 PE=1 SV=4		
P11137-2	MAP2_HUMAN Isoform 2 of Microtubule-associated protein 2 OS=Homo sapiens GN=MAP2		
P11137-3	MAP2_HUMAN Isoform 3 of Microtubule-associated protein 2 OS=Homo sapiens GN=MAP2		
P11142	HSP7C_HUMAN Heat shock cognate 71 kDa protein OS=Homo sapiens GN=HSPA8 PE=1 SV=1		1999.48
P11142-2	HSP7C_HUMAN Isoform 2 of Heat shock cognate 71 kDa protein OS=Homo sapiens GN=HSPA8		1810.25
P11142;P11142-2	HSP7C_HUMAN Heat shock cognate 71 kDa protein OS=Homo sapiens GN=HSPA8 PE=1 SV=1;>sp|P11142-2|HSP7C_HUMAN Isoform	0	
P11166	GTR1_HUMAN Solute carrier family 2, facilitated glucose transporter member 1 OS=Homo sapiens GN=SLC2A1 PE=1 SV=2		420.75
P11171	41_HUMAN Protein 4.1 OS=Homo sapiens GN=EPB41 PE=1 SV=4		
P11171-2	41_HUMAN Isoform 2 of Protein 4.1 OS=Homo sapiens GN=EPB41		
P11171-3	41_HUMAN Isoform 3 of Protein 4.1 OS=Homo sapiens GN=EPB41		
P11171-4	41_HUMAN Isoform 4 of Protein 4.1 OS=Homo sapiens GN=EPB41		
P11171-5	41_HUMAN Isoform 5 of Protein 4.1 OS=Homo sapiens GN=EPB41		
P11171-6	41_HUMAN Isoform 6 of Protein 4.1 OS=Homo sapiens GN=EPB41		
P11171-7	41_HUMAN Isoform 7 of Protein 4.1 OS=Homo sapiens GN=EPB41		
P11172;P11172-2	UMPS_HUMAN Uridine 5-monophosphate synthase OS=Homo sapiens GN=UMPS PE=1 SV=1;>sp|P11172-2|UMPS_HUMAN Isoform 2	6.63E-108	
P11177	ODPB_HUMAN Pyruvate dehydrogenase E1 component subunit beta, mitochondrial OS=Homo sapiens GN=PDHB PE=1 SV=3		664.04
P11177-2	ODPB_HUMAN Isoform 2 of Pyruvate dehydrogenase E1 component subunit beta, mitochondrial OS=Homo sapiens GN=PDHB		664.04
P11177;B4DDD7;P11177-	ODPB_HUMAN Pyruvate dehydrogenase E1 component subunit beta, mitochondrial OS=Homo sapiens GN=PDHB PE=1 SV=3;>tr|B4D	5.91E-84	
P11182	ODB2_HUMAN Lipoamide acyltransferase component of branched-chain alpha-keto acid dehydrogenase complex, mitochondrial OS=Homo sapiens GN=DBT PE=1 SV=3		
P11215	ITAM_HUMAN Integrin alpha-M OS=Homo sapiens GN=ITGAM PE=1 SV=2	2.15E-08	
P11216	PYGB_HUMAN Glycogen phosphorylase, brain form OS=Homo sapiens GN=PYGB PE=1 SV=5		210.34
P11217	PYGM_HUMAN Glycogen phosphorylase, muscle form OS=Homo sapiens GN=PYGM PE=1 SV=6		25.37
P11233	RALA_HUMAN Ras-related protein Ral-A OS=Homo sapiens GN=RALA PE=1 SV=1		431.26
P11234	RALB_HUMAN Ras-related protein Ral-B OS=Homo sapiens GN=RALB PE=1 SV=1		597.62
P11274	BCR_HUMAN Breakpoint cluster region protein OS=Homo sapiens GN=BCR PE=1 SV=2		
P11274-2	BCR_HUMAN Isoform 2 of Breakpoint cluster region protein OS=Homo sapiens GN=BCR		
P11274;P11274-2;Q1284	BCR_HUMAN Breakpoint cluster region protein OS=Homo sapiens GN=BCR PE=1 SV=2;>sp|P11274-2|BCR_HUMAN Isoform 2 of Brea	6.45E-05	
P11277	SPTB1_HUMAN Spectrin beta chain, erythrocyte OS=Homo sapiens GN=SPTB PE=1 SV=5		159.61
P11277-2	SPTB1_HUMAN Isoform 2 of Spectrin beta chain, erythrocyte OS=Homo sapiens GN=SPTB		159.61
P11277-2;P11277	2|SPTB1_HUMAN Isoform 2 of Spectrin beta chain, erythrocyte OS=Homo sapiens GN=SPTB;>sp|P11277|SPTB1_HUMAN Spectrin bet	4.46E-26	
P11277-3	SPTB1_HUMAN Isoform 3 of Spectrin beta chain, erythrocyte OS=Homo sapiens GN=SPTB		159.61

P11279	LAMP1_HUMAN Lysosome-associated membrane glycoprotein 1 OS=Homo sapiens GN=LAMP1 PE=1 SV=3		236.99
P11310-2;P11310	2|ACADM_HUMAN Isoform 2 of Medium-chain specific acyl-CoA dehydrogenase, mitochondrial OS=Homo sapiens GN=ACADM;>sp|P1:	2.32E-218	
P11362	FGFR1_HUMAN Basic fibroblast growth factor receptor 1 OS=Homo sapiens GN=FGFR1 PE=1 SV=3		71.31
P11362-10	FGFR1_HUMAN Isoform 7 of Basic fibroblast growth factor receptor 1 OS=Homo sapiens GN=FGFR1		15.24
P11362-11	FGFR1_HUMAN Isoform 9 of Basic fibroblast growth factor receptor 1 OS=Homo sapiens GN=FGFR1		15.24
P11362-12	FGFR1_HUMAN Isoform 11 of Basic fibroblast growth factor receptor 1 OS=Homo sapiens GN=FGFR1		7.46
P11362-13	FGFR1_HUMAN Isoform 13 of Basic fibroblast growth factor receptor 1 OS=Homo sapiens GN=FGFR1		7.46
P11362-14	FGFR1_HUMAN Isoform 15 of Basic fibroblast growth factor receptor 1 OS=Homo sapiens GN=FGFR1		59.4
P11362-15	FGFR1_HUMAN Isoform 16 of Basic fibroblast growth factor receptor 1 OS=Homo sapiens GN=FGFR1		4.13
P11362-16	FGFR1_HUMAN Isoform 17 of Basic fibroblast growth factor receptor 1 OS=Homo sapiens GN=FGFR1		7.78
P11362-19	FGFR1_HUMAN Isoform 19 of Basic fibroblast growth factor receptor 1 OS=Homo sapiens GN=FGFR1		64.15
P11362-2	FGFR1_HUMAN Isoform 4 of Basic fibroblast growth factor receptor 1 OS=Homo sapiens GN=FGFR1		71.31
P11362-3	FGFR1_HUMAN Isoform 6 of Basic fibroblast growth factor receptor 1 OS=Homo sapiens GN=FGFR1		67.18
P11362-4	FGFR1_HUMAN Isoform 8 of Basic fibroblast growth factor receptor 1 OS=Homo sapiens GN=FGFR1		67.18
P11362-5	FGFR1_HUMAN Isoform 10 of Basic fibroblast growth factor receptor 1 OS=Homo sapiens GN=FGFR1		59.4
P11362-6	FGFR1_HUMAN Isoform 12 of Basic fibroblast growth factor receptor 1 OS=Homo sapiens GN=FGFR1		59.4
P11362-7	FGFR1_HUMAN Isoform 14 of Basic fibroblast growth factor receptor 1 OS=Homo sapiens GN=FGFR1		63.53
P11362-8	FGFR1_HUMAN Isoform 2 of Basic fibroblast growth factor receptor 1 OS=Homo sapiens GN=FGFR1		19.37
P11362-9	FGFR1_HUMAN Isoform 5 of Basic fibroblast growth factor receptor 1 OS=Homo sapiens GN=FGFR1		19.37
P11387	TOP1_HUMAN DNA topoisomerase 1 OS=Homo sapiens GN=TOP1 PE=1 SV=2		546.53
P11388	TOP2A_HUMAN DNA topoisomerase 2-alpha OS=Homo sapiens GN=TOP2A PE=1 SV=3		820.38
P11388-2	TOP2A_HUMAN Isoform 2 of DNA topoisomerase 2-alpha OS=Homo sapiens GN=TOP2A		820.38
P11388-3	TOP2A_HUMAN Isoform 3 of DNA topoisomerase 2-alpha OS=Homo sapiens GN=TOP2A		820.38
P11388-4	TOP2A_HUMAN Isoform 4 of DNA topoisomerase 2-alpha OS=Homo sapiens GN=TOP2A		820.38
P11388-4;P11388-3;P113	4|TOP2A_HUMAN Isoform 4 of DNA topoisomerase 2-alpha OS=Homo sapiens GN=TOP2A;>sp|P11388-3|TOP2A_HUMAN Isoform 3 of	0	
P11413	G6PD_HUMAN Glucose-6-phosphate 1-dehydrogenase OS=Homo sapiens GN=G6PD PE=1 SV=4		167.14
P11413-2	G6PD_HUMAN Isoform Long of Glucose-6-phosphate 1-dehydrogenase OS=Homo sapiens GN=G6PD		167.14
P11413-2;P11413-3;A8K8	2|G6PD_HUMAN Isoform Long of Glucose-6-phosphate 1-dehydrogenase OS=Homo sapiens GN=G6PD;>sp|P11413-3|G6PD_HUMAN I	6.23E-142	
P11413-3	G6PD_HUMAN Isoform 3 of Glucose-6-phosphate 1-dehydrogenase OS=Homo sapiens GN=G6PD		167.14
P11441	UBL4A_HUMAN Ubiquitin-like protein 4A OS=Homo sapiens GN=UBL4A PE=1 SV=1		44.51
P11473	VDR_HUMAN Vitamin D3 receptor OS=Homo sapiens GN=VDR PE=1 SV=1		
P11474	ERR1_HUMAN Steroid hormone receptor ERR1 OS=Homo sapiens GN=ESRRA PE=1 SV=3		
P11474-2	ERR1_HUMAN Isoform 2 of Steroid hormone receptor ERR1 OS=Homo sapiens GN=ESRRA		
P11488	GNAT1_HUMAN Guanine nucleotide-binding protein G(t) subunit alpha-1 OS=Homo sapiens GN=GNAT1 PE=1 SV=5		175.53
P11498	PYC_HUMAN Pyruvate carboxylase, mitochondrial OS=Homo sapiens GN=PC PE=1 SV=2		77.41
P11509	CP2A6_HUMAN Cytochrome P450 2A6 OS=Homo sapiens GN=CYP2A6 PE=1 SV=3		
P11511	CP19A_HUMAN Cytochrome P450 19A1 OS=Homo sapiens GN=CYP19A1 PE=1 SV=3		
P11532	DMD_HUMAN Dystrophin OS=Homo sapiens GN=DMD PE=1 SV=3		90.26
P11532-2	DMD_HUMAN Isoform 1 of Dystrophin OS=Homo sapiens GN=DMD		57.96
P11532-3	DMD_HUMAN Isoform 2 of Dystrophin OS=Homo sapiens GN=DMD		57.96
P11532-4	DMD_HUMAN Isoform 3 of Dystrophin OS=Homo sapiens GN=DMD		90.26
P11532;P11532-4;P11532	DMD_HUMAN Dystrophin OS=Homo sapiens GN=DMD PE=1 SV=3;>sp|P11532-4|DMD_HUMAN Isoform 3 of Dystrophin OS=Homo sa	2.07E-09	
P11586	C1TC_HUMAN C-1-tetrahydrofolate synthase, cytoplasmic OS=Homo sapiens GN=MTHFD1 PE=1 SV=3		244.59
P11678	PERE_HUMAN Eosinophil peroxidase OS=Homo sapiens GN=EPX PE=1 SV=2	3.29E-05	
P11712	CP2C9_HUMAN Cytochrome P450 2C9 OS=Homo sapiens GN=CYP2C9 PE=1 SV=3		
P11717	MPRI_HUMAN Cation-independent mannose-6-phosphate receptor OS=Homo sapiens GN=IGF2R PE=1 SV=3		912.79
P11766	ADHX_HUMAN Alcohol dehydrogenase class-3 OS=Homo sapiens GN=ADH5 PE=1 SV=4		43.06
P11802	CDK4_HUMAN Cyclin-dependent kinase 4 OS=Homo sapiens GN=CDK4 PE=1 SV=2		49.63
P11836	CD20_HUMAN B-lymphocyte antigen CD20 OS=Homo sapiens GN=MS4A1 PE=1 SV=1	2.27E-179	
P11908	PRPS2_HUMAN Ribose-phosphate pyrophosphokinase 2 OS=Homo sapiens GN=PRPS2 PE=1 SV=2		27.09
P11908-2	PRPS2_HUMAN Isoform 2 of Ribose-phosphate pyrophosphokinase 2 OS=Homo sapiens GN=PRPS2		27.09
P11908-2;B2R860;P11908	2|PRPS2_HUMAN Isoform 2 of Ribose-phosphate pyrophosphokinase 2 OS=Homo sapiens GN=PRPS2;>tr|B2R860|B2R860_HUMAN Ri	2.39E-32	
P11926	DCOR_HUMAN Ornithine decarboxylase OS=Homo sapiens GN=ODC1 PE=1 SV=2		
P11940	PABP1_HUMAN Polyadenylate-binding protein 1 OS=Homo sapiens GN=PABPC1 PE=1 SV=2		1822.41
P11940-2	PABP1_HUMAN Isoform 2 of Polyadenylate-binding protein 1 OS=Homo sapiens GN=PABPC1		1822.41
P11940;B4DZW4;P11940-	PABP1_HUMAN Polyadenylate-binding protein 1 OS=Homo sapiens GN=PABPC1 PE=1 SV=2;>tr|B4DZW4|B4DZW4_HUMAN cDNA FLJ5	0	
P12004	PCNA_HUMAN Proliferating cell nuclear antigen OS=Homo sapiens GN=PCNA PE=1 SV=1		533.14
P12035	K2C3_HUMAN Keratin, type II cytoskeletal 3 OS=Homo sapiens GN=KRT3 PE=1 SV=3		340.41
P12036	NFH_HUMAN Neurofilament heavy polypeptide OS=Homo sapiens GN=NEFH PE=1 SV=4		214.44
P12036-2	NFH_HUMAN Isoform 2 of Neurofilament heavy polypeptide OS=Homo sapiens GN=NEFH		206.02
P12036;P12036-2	NFH_HUMAN Neurofilament heavy polypeptide OS=Homo sapiens GN=NEFH PE=1 SV=4;>sp|P12036-2|NFH_HUMAN Isoform 2 of Neu	2.86E-30	

P12081	SYHC_HUMAN Histidyl-tRNA synthetase, cytoplasmic OS=Homo sapiens GN=HARS PE=1 SV=2		328.67
P12107-2;P12107;P12107	2|COBA1_HUMAN Isoform B of Collagen alpha-1(XI) chain OS=Homo sapiens GN=COL11A1;>sp|P12107|COBA1_HUMAN Collagen alp	2.14E-06	
P12109	CO6A1_HUMAN Collagen alpha-1(VI) chain OS=Homo sapiens GN=COL6A1 PE=1 SV=3	0	
P12110	CO6A2_HUMAN Collagen alpha-2(VI) chain OS=Homo sapiens GN=COL6A2 PE=1 SV=4		
P12110-2	CO6A2_HUMAN Isoform 2C2A of Collagen alpha-2(VI) chain OS=Homo sapiens GN=COL6A2		
P12110-3	CO6A2_HUMAN Isoform 2C2A' of Collagen alpha-2(VI) chain OS=Homo sapiens GN=COL6A2		
P12110;P12110-2;P12110	CO6A2_HUMAN Collagen alpha-2(VI) chain OS=Homo sapiens GN=COL6A2 PE=1 SV=4;>sp|P12110-2|CO6A2_HUMAN Isoform 2C2A	0	
P12111;P12111-2	CO6A3_HUMAN Collagen alpha-3(VI) chain OS=Homo sapiens GN=COL6A3 PE=1 SV=5;>sp|P12111-2|CO6A3_HUMAN Isoform 2 of C	0	
P12235	ADT1_HUMAN ADP/ATP translocase 1 OS=Homo sapiens GN=SLC25A4 PE=1 SV=4		979.22
P12236	ADT3_HUMAN ADP/ATP translocase 3 OS=Homo sapiens GN=SLC25A6 PE=1 SV=4		1085.95
P12259	FA5_HUMAN Coagulation factor V OS=Homo sapiens GN=F5 PE=1 SV=4		
P12268	IMDH2_HUMAN Inosine-5'-monophosphate dehydrogenase 2 OS=Homo sapiens GN=IMPDH2 PE=1 SV=2		205.66
P12270	TPR_HUMAN Nucleoprotein TPR OS=Homo sapiens GN=TPR PE=1 SV=3		1149.68
P12273	PIP_HUMAN Prolactin-inducible protein OS=Homo sapiens GN=PIP PE=1 SV=1		
P12277	KCRB_HUMAN Creatine kinase B-type OS=Homo sapiens GN=CKB PE=1 SV=1	2.57E-23	
P12429	ANXA3_HUMAN Annexin A3 OS=Homo sapiens GN=ANXA3 PE=1 SV=3		357.49
P12524	MYCL1_HUMAN Protein L-Myc-1 OS=Homo sapiens GN=MYCL1 PE=1 SV=2		
P12525	MYCL2_HUMAN Protein L-Myc-2 OS=Homo sapiens GN=MYCL2 PE=2 SV=1		
P12532	KCRU_HUMAN Creatine kinase U-type, mitochondrial OS=Homo sapiens GN=CKMT1A PE=1 SV=1		
P12532-2	KCRU_HUMAN Isoform 2 of Creatine kinase U-type, mitochondrial OS=Homo sapiens GN=CKMT1A		
P12532;P12532-2	KCRU_HUMAN Creatine kinase U-type, mitochondrial OS=Homo sapiens GN=CKMT1A PE=1 SV=1;>sp|P12532-2|KCRU_HUMAN Isofor	4.90E-94	
P12643	BMP2_HUMAN Bone morphogenetic protein 2 OS=Homo sapiens GN=BMP2 PE=1 SV=1		
P12694	ODBA_HUMAN 2-oxoisovalerate dehydrogenase subunit alpha, mitochondrial OS=Homo sapiens GN=BCKDHA PE=1 SV=2	2.71E-19	
P12757	SKIL_HUMAN Ski-like protein OS=Homo sapiens GN=SKIL PE=1 SV=2		
P12757-2	SKIL_HUMAN Isoform SNOA of Ski-like protein OS=Homo sapiens GN=SKIL		
P12757-3	SKIL_HUMAN Isoform SNON2 of Ski-like protein OS=Homo sapiens GN=SKIL		
P12757-4	SKIL_HUMAN Isoform SNOI of Ski-like protein OS=Homo sapiens GN=SKIL		
P12757-5	SKIL_HUMAN Isoform 5 of Ski-like protein OS=Homo sapiens GN=SKIL		
P12814	ACTN1_HUMAN Alpha-actinin-1 OS=Homo sapiens GN=ACTN1 PE=1 SV=2		2498.27
P12814-2	ACTN1_HUMAN Isoform 2 of Alpha-actinin-1 OS=Homo sapiens GN=ACTN1		2472.44
P12814;P12814-2	ACTN1_HUMAN Alpha-actinin-1 OS=Homo sapiens GN=ACTN1 PE=1 SV=2;>sp|P12814-2|ACTN1_HUMAN Isoform 2 of Alpha-actinin-1	0	
P12829	MYL4_HUMAN Myosin light chain 4 OS=Homo sapiens GN=MYL4 PE=1 SV=3		
P12830	CADH1_HUMAN Cadherin-1 OS=Homo sapiens GN=CDH1 PE=1 SV=3		
P12882	MYH1_HUMAN Myosin-1 OS=Homo sapiens GN=MYH1 PE=1 SV=3		113.13
P12883	MYH7_HUMAN Myosin-7 OS=Homo sapiens GN=MYH7 PE=1 SV=5		76.64
P12931	SRC_HUMAN Proto-oncogene tyrosine-protein kinase Src OS=Homo sapiens GN=SRC PE=1 SV=3		43.99
P12931-2	SRC_HUMAN Isoform 2 of Proto-oncogene tyrosine-protein kinase Src OS=Homo sapiens GN=SRC		43.99
P12931-2;E1P5V4;P12931	2|SRC_HUMAN Isoform 2 of Proto-oncogene tyrosine-protein kinase Src OS=Homo sapiens GN=SRC;>tr|E1P5V4|E1P5V4_HUMAN V-s	5.69E-13	
P12955	PEPD_HUMAN Xaa-Pro dipeptidase OS=Homo sapiens GN=PEPD PE=1 SV=3		109.04
P12956	XRCC6_HUMAN X-ray repair cross-complementing protein 6 OS=Homo sapiens GN=XRCC6 PE=1 SV=2		1236.74
P13010	XRCC5_HUMAN X-ray repair cross-complementing protein 5 OS=Homo sapiens GN=XRCC5 PE=1 SV=3		2210.31
P13051	UNG_HUMAN Uracil-DNA glycosylase OS=Homo sapiens GN=UNG PE=1 SV=2		22.56
P13051-2	UNG_HUMAN Isoform 1 of Uracil-DNA glycosylase OS=Homo sapiens GN=UNG		22.56
P13073	COX41_HUMAN Cytochrome c oxidase subunit 4 isoform 1, mitochondrial OS=Homo sapiens GN=COX4I1 PE=1 SV=1		350.18
P13164	IFM1_HUMAN Interferon-induced transmembrane protein 1 OS=Homo sapiens GN=IFITM1 PE=1 SV=3		202.65
P13284	GILT_HUMAN Gamma-interferon-inducible lysosomal thiol reductase OS=Homo sapiens GN=IFI30 PE=1 SV=3	3.23E-43	
P13473	LAMP2_HUMAN Lysosome-associated membrane glycoprotein 2 OS=Homo sapiens GN=LAMP2 PE=1 SV=2		159.41
P13473-2	LAMP2_HUMAN Isoform LAMP-2B of Lysosome-associated membrane glycoprotein 2 OS=Homo sapiens GN=LAMP2		159.41
P13473;P13473-2	LAMP2_HUMAN Lysosome-associated membrane glycoprotein 2 OS=Homo sapiens GN=LAMP2 PE=1 SV=2;>sp|P13473-2|LAMP2_HUM	2.21E-09	
P13489	RINI_HUMAN Ribonuclease inhibitor OS=Homo sapiens GN=RNH1 PE=1 SV=2	9.72E-164	
P13498	CY24A_HUMAN Cytochrome b-245 light chain OS=Homo sapiens GN=CYBA PE=1 SV=3		58.15
P13521	SCG2_HUMAN Secretogranin-2 OS=Homo sapiens GN=SCG2 PE=1 SV=2		
P13533	MYH6_HUMAN Myosin-6 OS=Homo sapiens GN=MYH6 PE=1 SV=5		68.13
P13535	MYH8_HUMAN Myosin-8 OS=Homo sapiens GN=MYH8 PE=1 SV=3		121.32
P13584	CP4B1_HUMAN Cytochrome P450 4B1 OS=Homo sapiens GN=CYP4B1 PE=1 SV=2		
P13584-2	CP4B1_HUMAN Isoform 2 of Cytochrome P450 4B1 OS=Homo sapiens GN=CYP4B1		
P13611;P13611-5;P13611	CSPG2_HUMAN Versican core protein OS=Homo sapiens GN=VCAN PE=1 SV=3;>sp|P13611-5|CSPG2_HUMAN Isoform Vint of Versica	4.01E-195	
P13637	AT1A3_HUMAN Sodium/potassium-transporting ATPase subunit alpha-3 OS=Homo sapiens GN=ATP1A3 PE=1 SV=3		1392.63
P13639	EF2_HUMAN Elongation factor 2 OS=Homo sapiens GN=EEF2 PE=1 SV=4		1772.06
P13645	K1C10_HUMAN Keratin, type I cytoskeletal 10 OS=Homo sapiens GN=KRT10 PE=1 SV=6		2613.94
P13646	K1C13_HUMAN Keratin, type I cytoskeletal 13 OS=Homo sapiens GN=KRT13 PE=1 SV=4		513.67

P13646-2	K1C13_HUMAN Isoform 2 of Keratin, type I cytoskeletal 13 OS=Homo sapiens GN=KRT13		451.64
P13646-3	K1C13_HUMAN Isoform 3 of Keratin, type I cytoskeletal 13 OS=Homo sapiens GN=KRT13		511.94
P13647	K2C5_HUMAN Keratin, type II cytoskeletal 5 OS=Homo sapiens GN=KRT5 PE=1 SV=3		1184.7
P13667	PDIA4_HUMAN Protein disulfide-isomerase A4 OS=Homo sapiens GN=PDIA4 PE=1 SV=2		1819.04
P13671	CO6_HUMAN Complement component C6 OS=Homo sapiens GN=C6 PE=1 SV=3		
P13674	P4HA1_HUMAN Prolyl 4-hydroxylase subunit alpha-1 OS=Homo sapiens GN=P4HA1 PE=1 SV=2		506.63
P13674-2	P4HA1_HUMAN Isoform 2 of Prolyl 4-hydroxylase subunit alpha-1 OS=Homo sapiens GN=P4HA1		512.59
P13674-2;P13674	2|P4HA1_HUMAN Isoform 2 of Prolyl 4-hydroxylase subunit alpha-1 OS=Homo sapiens GN=P4HA1;>sp|P13674|P4HA1_HUMAN Prolyl	1.16E-82	
P13674;P13674-2	P4HA1_HUMAN Prolyl 4-hydroxylase subunit alpha-1 OS=Homo sapiens GN=P4HA1 PE=1 SV=2;>sp|P13674-2|P4HA1_HUMAN Isofori	0	
P13693	TCTP_HUMAN Translationally-controlled tumor protein OS=Homo sapiens GN=TPT1 PE=1 SV=1		442.94
P13716-2;P13716	2|HEM2_HUMAN Isoform 2 of Delta-aminolevulinic acid dehydratase OS=Homo sapiens GN=ALAD;>sp|P13716|HEM2_HUMAN Delta-a	3.63E-10	
P13716;B7Z3I9;P13716-2	HEM2_HUMAN Delta-aminolevulinic acid dehydratase OS=Homo sapiens GN=ALAD PE=1 SV=1;>tr|B7Z3I9|B7Z3I9_HUMAN Delta-am	2.83E-19	
P13726	TF_HUMAN Tissue factor OS=Homo sapiens GN=F3 PE=1 SV=1		220.43
P13746	1A11_HUMAN HLA class I histocompatibility antigen, A-11 alpha chain OS=Homo sapiens GN=HLA-A PE=1 SV=1		443.15
P13746-2	1A11_HUMAN Isoform 2 of HLA class I histocompatibility antigen, A-11 alpha chain OS=Homo sapiens GN=HLA-A		443.15
P13747	HLAE_HUMAN HLA class I histocompatibility antigen, alpha chain E OS=Homo sapiens GN=HLA-E PE=1 SV=3		112.87
P13760	2B14_HUMAN HLA class II histocompatibility antigen, DRB1-4 beta chain OS=Homo sapiens GN=HLA-DRB1 PE=1 SV=1		29.42
P13761	2B17_HUMAN HLA class II histocompatibility antigen, DRB1-7 beta chain OS=Homo sapiens GN=HLA-DRB1 PE=1 SV=1		29.42
P13762	DRB4_HUMAN HLA class II histocompatibility antigen, DR beta 4 chain OS=Homo sapiens GN=HLA-DRB4 PE=1 SV=2	0	
P13796	PLSL_HUMAN Plastin-2 OS=Homo sapiens GN=LCP1 PE=1 SV=6		31.36
P13797	PLST_HUMAN Plastin-3 OS=Homo sapiens GN=PLS3 PE=1 SV=4		158.55
P13798	ACPH_HUMAN Acylamino-acid-releasing enzyme OS=Homo sapiens GN=APEH PE=1 SV=4	2.86E-18	
P13804	ETFA_HUMAN Electron transfer flavoprotein subunit alpha, mitochondrial OS=Homo sapiens GN=ETFA PE=1 SV=1		616.04
P13807	GYS1_HUMAN Glycogen [starch] synthase, muscle OS=Homo sapiens GN=GYS1 PE=1 SV=2	1.17E-12	
P13861	KAP2_HUMAN cAMP-dependent protein kinase type II-alpha regulatory subunit OS=Homo sapiens GN=PRKAR2A PE=1 SV=2		40.92
P13928	ANXA8_HUMAN Annexin A8 OS=Homo sapiens GN=ANXA8 PE=1 SV=3		
P13929	ENOB_HUMAN Beta-enolase OS=Homo sapiens GN=ENO3 PE=1 SV=4		452.13
P13929-2	ENOB_HUMAN Isoform 2 of Beta-enolase OS=Homo sapiens GN=ENO3		452.13
P13929-3	ENOB_HUMAN Isoform 3 of Beta-enolase OS=Homo sapiens GN=ENO3		452.13
P13929;P13929-2;P13929	ENOB_HUMAN Beta-enolase OS=Homo sapiens GN=ENO3 PE=1 SV=4;>sp|P13929-2|ENOB_HUMAN Isoform 2 of Beta-enolase OS=H	1.62E-251	
P13984	T2FB_HUMAN General transcription factor IIF subunit 2 OS=Homo sapiens GN=GTF2F2 PE=1 SV=2		179.11
P13987	CD59_HUMAN CD59 glycoprotein OS=Homo sapiens GN=CD59 PE=1 SV=1		227.86
P13995	MTDC_HUMAN Bifunctional methylenetetrahydrofolate dehydrogenase/cyclohydrolase, mitochondrial OS=Homo sapiens GN=MTHFD2 PE=1 SV=2		
P14060	3BHS1_HUMAN 3 beta-hydroxysteroid dehydrogenase/Delta 5-->4-isomerase type 1 OS=Homo sapiens GN=HSD3B1 PE=1 SV=2		22.67
P14136	GFAP_HUMAN Glial fibrillary acidic protein OS=Homo sapiens GN=GFAP PE=1 SV=1		90.26
P14136-2	GFAP_HUMAN Isoform 2 of Glial fibrillary acidic protein OS=Homo sapiens GN=GFAP		90.26
P14136-3	GFAP_HUMAN Isoform 3 of Glial fibrillary acidic protein OS=Homo sapiens GN=GFAP		90.26
P14174	MIF_HUMAN Macrophage migration inhibitory factor OS=Homo sapiens GN=MIF PE=1 SV=4		231.01
P14209	CD99_HUMAN CD99 antigen OS=Homo sapiens GN=CD99 PE=1 SV=1		50.38
P14314	GLU2B_HUMAN Glucosidase 2 subunit beta OS=Homo sapiens GN=PRKCSH PE=1 SV=2		903.23
P14317	HCLS1_HUMAN Hematopoietic lineage cell-specific protein OS=Homo sapiens GN=HCLS1 PE=1 SV=3	8.38E-167	
P14324	FPPS_HUMAN Farnesyl pyrophosphate synthase OS=Homo sapiens GN=FDPS PE=1 SV=4		111.87
P14384	CBPM_HUMAN Carboxypeptidase M OS=Homo sapiens GN=CPM PE=1 SV=2		
P14406	CX7A2_HUMAN Cytochrome c oxidase subunit 7A2, mitochondrial OS=Homo sapiens GN=COX7A2 PE=1 SV=1		52.89
P14410	SUIS_HUMAN Sucrase-isomaltase, intestinal OS=Homo sapiens GN=SI PE=1 SV=6		
P14543;P14543-2	NID1_HUMAN Nidogen-1 OS=Homo sapiens GN=NID1 PE=1 SV=3;>sp|P14543-2|NID1_HUMAN Isoform 2 of Nidogen-1 OS=Homo sa	5.11E-160	
P14550	AK1A1_HUMAN Alcohol dehydrogenase [NADP+] OS=Homo sapiens GN=AKR1A1 PE=1 SV=3		43.59
P14616	INSRR_HUMAN Insulin receptor-related protein OS=Homo sapiens GN=INSRR PE=2 SV=2		
P14618	KPYM_HUMAN Pyruvate kinase isozymes M1/M2 OS=Homo sapiens GN=PKM2 PE=1 SV=4		2072.91
P14618-2	KPYM_HUMAN Isoform M1 of Pyruvate kinase isozymes M1/M2 OS=Homo sapiens GN=PKM2		1788.15
P14618-2;B4DRT3;Q504U	2|KPYM_HUMAN Isoform M1 of Pyruvate kinase isozymes M1/M2 OS=Homo sapiens GN=PKM2;>tr|B4DRT3|B4DRT3_HUMAN Pyruvate	0	
P14618;B4DUU6;B4DNK4;	KPYM_HUMAN Pyruvate kinase isozymes M1/M2 OS=Homo sapiens GN=PKM2 PE=1 SV=4;>tr|B4DUU6|B4DUU6_HUMAN Pyruvate kin	0	
P14621	ACYP2_HUMAN Acylphosphatase-2 OS=Homo sapiens GN=ACYP2 PE=1 SV=2	2.14E-41	
P14625	ENPL_HUMAN Endoplasmin OS=Homo sapiens GN=HSP90B1 PE=1 SV=1		2600.82
P14635	CCNB1_HUMAN G2/mitotic-specific cyclin-B1 OS=Homo sapiens GN=CCNB1 PE=1 SV=1		
P14649	MYL6B_HUMAN Myosin light chain 6B OS=Homo sapiens GN=MYL6B PE=1 SV=1		195.19
P14678	RSMB_HUMAN Small nuclear ribonucleoprotein-associated proteins B and B' OS=Homo sapiens GN=SNRPB PE=1 SV=2		494.76
P14678-2	RSMB_HUMAN Isoform SM-B of Small nuclear ribonucleoprotein-associated proteins B and B' OS=Homo sapiens GN=SNRPB		495.35
P14678-3	RSMB_HUMAN Isoform SM-B1 of Small nuclear ribonucleoprotein-associated proteins B and B' OS=Homo sapiens GN=SNRPB		494.76
P14678-3;A8MT02;Q1518	3|RSMB_HUMAN Isoform SM-B1 of Small nuclear ribonucleoprotein-associated proteins B and B OS=Homo sapiens GN=SNRPB;>tr|A8	9.50E-40	
P14735	IDE_HUMAN Insulin-degrading enzyme OS=Homo sapiens GN=IDE PE=1 SV=4		

P14780	MMP9_HUMAN Matrix metalloproteinase-9 OS=Homo sapiens GN=MMP9 PE=1 SV=3	2.50E-37	
P14854	CX6B1_HUMAN Cytochrome c oxidase subunit 6B1 OS=Homo sapiens GN=COX6B1 PE=1 SV=2		146.85
P14859-2;P14859;P14859	2|PO2F1_HUMAN Isoform 2 of POU domain, class 2, transcription factor 1 OS=Homo sapiens GN=POU2F1;>sp|P14859|PO2F1_HUMAN	2.37E-12	
P14866	HNRPL_HUMAN Heterogeneous nuclear ribonucleoprotein L OS=Homo sapiens GN=HNRNPL PE=1 SV=2		530.51
P14868	SYDC_HUMAN Aspartyl-tRNA synthetase, cytoplasmic OS=Homo sapiens GN=DARS PE=1 SV=2		831.82
P14921;P14921-2	ETS1_HUMAN Protein C-ets-1 OS=Homo sapiens GN=ETS1 PE=1 SV=1;>sp|P14921-2|ETS1_HUMAN Isoform c-ETS-1B of Protein C-et	8.78E-14	
P14923	PLAK_HUMAN Junction plakoglobin OS=Homo sapiens GN=JUP PE=1 SV=3		69.84
P14927	QCR7_HUMAN Cytochrome b-c1 complex subunit 7 OS=Homo sapiens GN=UQCRB PE=1 SV=2		61.15
P15086	CBPB1_HUMAN Carboxypeptidase B OS=Homo sapiens GN=CPB1 PE=1 SV=4	0	
P15088	CBPA3_HUMAN Mast cell carboxypeptidase A OS=Homo sapiens GN=CPA3 PE=1 SV=2	1.48E-48	
P15090	FABP4_HUMAN Fatty acid-binding protein, adipocyte OS=Homo sapiens GN=FABP4 PE=1 SV=3	1.15E-145	
P15104	GLNA_HUMAN Glutamine synthetase OS=Homo sapiens GN=GLUL PE=1 SV=4	1.48E-112	
P15121	ALDR_HUMAN Aldose reductase OS=Homo sapiens GN=AKR1B1 PE=1 SV=3		241.51
P15144	AMPN_HUMAN Aminopeptidase N OS=Homo sapiens GN=ANPEP PE=1 SV=4	9.94E-152	
P15151	PVR_HUMAN Poliovirus receptor OS=Homo sapiens GN=PVR PE=1 SV=2		157.37
P15151-2	PVR_HUMAN Isoform Beta of Poliovirus receptor OS=Homo sapiens GN=PVR		157.37
P15151-3	PVR_HUMAN Isoform Gamma of Poliovirus receptor OS=Homo sapiens GN=PVR		157.37
P15151-4	PVR_HUMAN Isoform Delta of Poliovirus receptor OS=Homo sapiens GN=PVR		157.37
P15151;P15151-2;P15151	PVR_HUMAN Poliovirus receptor OS=Homo sapiens GN=PVR PE=1 SV=2;>sp|P15151-2|PVR_HUMAN Isoform Beta of Poliovirus recept	1.23E-31	
P15153	RAC2_HUMAN Ras-related C3 botulinum toxin substrate 2 OS=Homo sapiens GN=RAC2 PE=1 SV=1		347.8
P15170	ERF3A_HUMAN Eukaryotic peptide chain release factor GTP-binding subunit ERF3A OS=Homo sapiens GN=GSPT1 PE=1 SV=1	1.85E-47	
P15259	PGAM2_HUMAN Phosphoglycerate mutase 2 OS=Homo sapiens GN=PGAM2 PE=1 SV=3		218.7
P15289	ARSA_HUMAN Arylsulfatase A OS=Homo sapiens GN=ARSA PE=1 SV=3	7.78E-17	
P15291	B4GT1_HUMAN Beta-1,4-galactosyltransferase 1 OS=Homo sapiens GN=B4GALT1 PE=1 SV=5		45.33
P15291-2	B4GT1_HUMAN Isoform Short of Beta-1,4-galactosyltransferase 1 OS=Homo sapiens GN=B4GALT1		45.88
P15311	EZRI_HUMAN Ezrin OS=Homo sapiens GN=EZR PE=1 SV=4		2489.77
P15374	UCHL3_HUMAN Ubiquitin carboxyl-terminal hydrolase isozyme L3 OS=Homo sapiens GN=UCHL3 PE=1 SV=1	2.35E-48	
P15407	FOSL1_HUMAN Fos-related antigen 1 OS=Homo sapiens GN=FOSL1 PE=1 SV=1		81.31
P15408	FOSL2_HUMAN Fos-related antigen 2 OS=Homo sapiens GN=FOSL2 PE=1 SV=1		
P15408-2	FOSL2_HUMAN Isoform 2 of Fos-related antigen 2 OS=Homo sapiens GN=FOSL2		
P15498	VAV_HUMAN Proto-oncogene vav OS=Homo sapiens GN=VAV1 PE=1 SV=4	9.20E-13	
P15509	CSF2R_HUMAN Granulocyte-macrophage colony-stimulating factor receptor subunit alpha OS=Homo sapiens GN=CSF2RA PE=1 SV=1		46.46
P15509-2	CSF2R_HUMAN Isoform 2 of Granulocyte-macrophage colony-stimulating factor receptor subunit alpha OS=Homo sapiens GN=CSF2RA		46.46
P15509-2;P15509;P15509	2|CSF2R_HUMAN Isoform 2 of Granulocyte-macrophage colony-stimulating factor receptor subunit alpha OS=Homo sapiens GN=CSF2	6.84E-06	
P15509-3	CSF2R_HUMAN Isoform 3 of Granulocyte-macrophage colony-stimulating factor receptor subunit alpha OS=Homo sapiens GN=CSF2RA		46.46
P15509-4	CSF2R_HUMAN Isoform 4 of Granulocyte-macrophage colony-stimulating factor receptor subunit alpha OS=Homo sapiens GN=CSF2RA		46.46
P15509-5	CSF2R_HUMAN Isoform 5 of Granulocyte-macrophage colony-stimulating factor receptor subunit alpha OS=Homo sapiens GN=CSF2RA		46.46
P15509-6	CSF2R_HUMAN Isoform 6 of Granulocyte-macrophage colony-stimulating factor receptor subunit alpha OS=Homo sapiens GN=CSF2RA		37.92
P15529	MCP_HUMAN Membrane cofactor protein OS=Homo sapiens GN=CD46 PE=1 SV=3		193.07
P15529-10	MCP_HUMAN Isoform 2 of Membrane cofactor protein OS=Homo sapiens GN=CD46		193.07
P15529-11	MCP_HUMAN Isoform C of Membrane cofactor protein OS=Homo sapiens GN=CD46		193.07
P15529-12	MCP_HUMAN Isoform E of Membrane cofactor protein OS=Homo sapiens GN=CD46		193.07
P15529-13	MCP_HUMAN Isoform G of Membrane cofactor protein OS=Homo sapiens GN=CD46		193.07
P15529-14	MCP_HUMAN Isoform I of Membrane cofactor protein OS=Homo sapiens GN=CD46		193.07
P15529-15	MCP_HUMAN Isoform K of Membrane cofactor protein OS=Homo sapiens GN=CD46		193.07
P15529-16	MCP_HUMAN Isoform 3 of Membrane cofactor protein OS=Homo sapiens GN=CD46		188.44
P15529-2	MCP_HUMAN Isoform B of Membrane cofactor protein OS=Homo sapiens GN=CD46		210.2
P15529-2;P15529-5;P155	2|MCP_HUMAN Isoform B of Membrane cofactor protein OS=Homo sapiens GN=CD46;>sp|P15529-5|MCP_HUMAN Isoform H of Memb	5.18E-10	
P15529-3	MCP_HUMAN Isoform D of Membrane cofactor protein OS=Homo sapiens GN=CD46		210.2
P15529-4	MCP_HUMAN Isoform F of Membrane cofactor protein OS=Homo sapiens GN=CD46		210.2
P15529-5	MCP_HUMAN Isoform H of Membrane cofactor protein OS=Homo sapiens GN=CD46		210.2
P15529-6	MCP_HUMAN Isoform J of Membrane cofactor protein OS=Homo sapiens GN=CD46		210.2
P15529-7	MCP_HUMAN Isoform L of Membrane cofactor protein OS=Homo sapiens GN=CD46		210.2
P15529-8	MCP_HUMAN Isoform M of Membrane cofactor protein OS=Homo sapiens GN=CD46		178.12
P15529-9	MCP_HUMAN Isoform N of Membrane cofactor protein OS=Homo sapiens GN=CD46		178.12
P15531	NDKA_HUMAN Nucleoside diphosphate kinase A OS=Homo sapiens GN=NME1 PE=1 SV=1		640.93
P15531-2	NDKA_HUMAN Isoform 2 of Nucleoside diphosphate kinase A OS=Homo sapiens GN=NME1		640.93
P15531-2;P15531	2|NDKA_HUMAN Isoform 2 of Nucleoside diphosphate kinase A OS=Homo sapiens GN=NME1;>sp|P15531|NDKA_HUMAN Nucleoside c	1.17E-155	
P15538	C11B1_HUMAN Cytochrome P450 11B1, mitochondrial OS=Homo sapiens GN=CYP11B1 PE=1 SV=5		
P15559	NQO1_HUMAN NAD(P)H dehydrogenase [quinone] 1 OS=Homo sapiens GN=NQO1 PE=1 SV=1	7.33E-74	
P15586	GNS_HUMAN N-acetylglucosamine-6-sulfatase OS=Homo sapiens GN=GNS PE=1 SV=3		116.73

P15692	VEGFA_HUMAN Vascular endothelial growth factor A OS=Homo sapiens GN=VEGFA PE=1 SV=2		18.83
P15692-10	VEGFA_HUMAN Isoform VEGF111 of Vascular endothelial growth factor A OS=Homo sapiens GN=VEGFA		18.83
P15692-11	VEGFA_HUMAN Isoform L-VEGF165 of Vascular endothelial growth factor A OS=Homo sapiens GN=VEGFA		20.62
P15692-12	VEGFA_HUMAN Isoform L-VEGF121 of Vascular endothelial growth factor A OS=Homo sapiens GN=VEGFA		20.62
P15692-13	VEGFA_HUMAN Isoform L-VEGF189 of Vascular endothelial growth factor A OS=Homo sapiens GN=VEGFA		20.62
P15692-14	VEGFA_HUMAN Isoform L-VEGF206 of Vascular endothelial growth factor A OS=Homo sapiens GN=VEGFA		20.62
P15692-2	VEGFA_HUMAN Isoform VEGF189 of Vascular endothelial growth factor A OS=Homo sapiens GN=VEGFA		18.83
P15692-3	VEGFA_HUMAN Isoform VEGF183 of Vascular endothelial growth factor A OS=Homo sapiens GN=VEGFA		18.83
P15692-4	VEGFA_HUMAN Isoform VEGF165 of Vascular endothelial growth factor A OS=Homo sapiens GN=VEGFA		18.83
P15692-5	VEGFA_HUMAN Isoform VEGF148 of Vascular endothelial growth factor A OS=Homo sapiens GN=VEGFA		18.83
P15692-6	VEGFA_HUMAN Isoform VEGF145 of Vascular endothelial growth factor A OS=Homo sapiens GN=VEGFA		18.83
P15692-8	VEGFA_HUMAN Isoform VEGF165B of Vascular endothelial growth factor A OS=Homo sapiens GN=VEGFA		18.83
P15692-9	VEGFA_HUMAN Isoform VEGF121 of Vascular endothelial growth factor A OS=Homo sapiens GN=VEGFA		18.83
P15814	IGLL1_HUMAN Immunoglobulin lambda-like polypeptide 1 OS=Homo sapiens GN=IGLL1 PE=1 SV=1	8.52E-08	
P15822	ZEP1_HUMAN Zinc finger protein 40 OS=Homo sapiens GN=HIVEP1 PE=1 SV=3		
P15880	RS2_HUMAN 40S ribosomal protein S2 OS=Homo sapiens GN=RPS2 PE=1 SV=2		1107.64
P15918	RAG1_HUMAN V(D)J recombination-activating protein 1 OS=Homo sapiens GN=RAG1 PE=1 SV=2		
P15923	TFE2_HUMAN Transcription factor E2-alpha OS=Homo sapiens GN=TCF3 PE=1 SV=1		
P15923-2	TFE2_HUMAN Isoform E47 of Transcription factor E2-alpha OS=Homo sapiens GN=TCF3		
P15924	DESP_HUMAN Desmoplakin OS=Homo sapiens GN=DSP PE=1 SV=3		156.18
P15924-2	DESP_HUMAN Isoform DPII of Desmoplakin OS=Homo sapiens GN=DSP		113.8
P15924;D7RX09;Q4LE79;I	DESP_HUMAN Desmoplakin OS=Homo sapiens GN=DSP PE=1 SV=3;>tr|D7RX09|D7RX09_HUMAN Desmoplakin Ia OS=Homo sapiens	0	
P15927	RFA2_HUMAN Replication protein A 32 kDa subunit OS=Homo sapiens GN=RPA2 PE=1 SV=1		267.91
P15927-2	RFA2_HUMAN Isoform 2 of Replication protein A 32 kDa subunit OS=Homo sapiens GN=RPA2		267.91
P15927-3	RFA2_HUMAN Isoform 3 of Replication protein A 32 kDa subunit OS=Homo sapiens GN=RPA2		267.91
P15927-3;P15927-2;B2R7	3|RFA2_HUMAN Isoform 3 of Replication protein A 32 kDa subunit OS=Homo sapiens GN=RPA2;>sp|P15927-2|RFA2_HUMAN Isoform	4.37E-23	
P15941	MUC1_HUMAN Mucin-1 OS=Homo sapiens GN=MUC1 PE=1 SV=3		
P15941-10	MUC1_HUMAN Isoform 10 of Mucin-1 OS=Homo sapiens GN=MUC1		
P15941-2	MUC1_HUMAN Isoform 2 of Mucin-1 OS=Homo sapiens GN=MUC1		
P15941-2;P15941;P15941	2|MUC1_HUMAN Isoform 2 of Mucin-1 OS=Homo sapiens GN=MUC1;>sp|P15941|MUC1_HUMAN Mucin-1 OS=Homo sapiens GN=MUC	2.35E-105	
P15941-3	MUC1_HUMAN Isoform 3 of Mucin-1 OS=Homo sapiens GN=MUC1		
P15941-4	MUC1_HUMAN Isoform 4 of Mucin-1 OS=Homo sapiens GN=MUC1		
P15941-6	MUC1_HUMAN Isoform 6 of Mucin-1 OS=Homo sapiens GN=MUC1		
P15941-7	MUC1_HUMAN Isoform 7 of Mucin-1 OS=Homo sapiens GN=MUC1		
P15941-8	MUC1_HUMAN Isoform 8 of Mucin-1 OS=Homo sapiens GN=MUC1		
P15941-9	MUC1_HUMAN Isoform 9 of Mucin-1 OS=Homo sapiens GN=MUC1		
P15954	COX7C_HUMAN Cytochrome c oxidase subunit 7C, mitochondrial OS=Homo sapiens GN=COX7C PE=1 SV=1		
P16035	TIMP2_HUMAN Metalloproteinase inhibitor 2 OS=Homo sapiens GN=TIMP2 PE=1 SV=2		63.67
P16066	ANPRA_HUMAN Atrial natriuretic peptide receptor 1 OS=Homo sapiens GN=NPR1 PE=1 SV=1		
P16070	CD44_HUMAN CD44 antigen OS=Homo sapiens GN=CD44 PE=1 SV=3		788.23
P16070-10	CD44_HUMAN Isoform 10 of CD44 antigen OS=Homo sapiens GN=CD44		788.23
P16070-11	CD44_HUMAN Isoform 11 of CD44 antigen OS=Homo sapiens GN=CD44		788.23
P16070-12	CD44_HUMAN Isoform 12 of CD44 antigen OS=Homo sapiens GN=CD44		788.23
P16070-13	CD44_HUMAN Isoform 13 of CD44 antigen OS=Homo sapiens GN=CD44		788.23
P16070-14	CD44_HUMAN Isoform 14 of CD44 antigen OS=Homo sapiens GN=CD44		788.51
P16070-15	CD44_HUMAN Isoform 15 of CD44 antigen OS=Homo sapiens GN=CD44		246.83
P16070-16	CD44_HUMAN Isoform 16 of CD44 antigen OS=Homo sapiens GN=CD44		788.23
P16070-17	CD44_HUMAN Isoform 17 of CD44 antigen OS=Homo sapiens GN=CD44		788.23
P16070-3	CD44_HUMAN Isoform 3 of CD44 antigen OS=Homo sapiens GN=CD44		788.23
P16070-4	CD44_HUMAN Isoform 4 of CD44 antigen OS=Homo sapiens GN=CD44		788.23
P16070-5	CD44_HUMAN Isoform 5 of CD44 antigen OS=Homo sapiens GN=CD44		788.23
P16070-6	CD44_HUMAN Isoform 6 of CD44 antigen OS=Homo sapiens GN=CD44		788.23
P16070-7	CD44_HUMAN Isoform 7 of CD44 antigen OS=Homo sapiens GN=CD44		788.23
P16070-8	CD44_HUMAN Isoform 8 of CD44 antigen OS=Homo sapiens GN=CD44		788.23
P16070-9	CD44_HUMAN Isoform 9 of CD44 antigen OS=Homo sapiens GN=CD44		246.83
P16070;P16070-5;P16070	CD44_HUMAN CD44 antigen OS=Homo sapiens GN=CD44 PE=1 SV=3;>sp|P16070-5|CD44_HUMAN Isoform 5 of CD44 antigen OS=H	5.47E-220	
P16083	NQO2_HUMAN Ribosyldihydronicotinamide dehydrogenase [quinone] OS=Homo sapiens GN=NQO2 PE=1 SV=5	2.49E-63	
P16104	H2AX_HUMAN Histone H2A.x OS=Homo sapiens GN=H2AFX PE=1 SV=2		145.19
P16144	ITB4_HUMAN Integrin beta-4 OS=Homo sapiens GN=ITGB4 PE=1 SV=5		215.73
P16144-2	ITB4_HUMAN Isoform Beta-4A of Integrin beta-4 OS=Homo sapiens GN=ITGB4		215.73
P16144-3	ITB4_HUMAN Isoform Beta-4B of Integrin beta-4 OS=Homo sapiens GN=ITGB4		215.73

P16144-4	ITB4_HUMAN Isoform Beta-4D of Integrin beta-4 OS=Homo sapiens GN=ITGB4		215.73
P16144-5	ITB4_HUMAN Isoform Beta-4E of Integrin beta-4 OS=Homo sapiens GN=ITGB4		19.35
P16144;P16144-3;A0AVL6	ITB4_HUMAN Integrin beta-4 OS=Homo sapiens GN=ITGB4 PE=1 SV=5;>sp|P16144-3|ITB4_HUMAN Isoform Beta-4B of Integrin beta	3.98E-48	
P16150	LEUK_HUMAN Leukosialin OS=Homo sapiens GN=SPN PE=1 SV=1		23.51
P16152	CBR1_HUMAN Carbonyl reductase [NADPH] 1 OS=Homo sapiens GN=CBR1 PE=1 SV=3		160.5
P16188	1A30_HUMAN HLA class I histocompatibility antigen, A-30 alpha chain OS=Homo sapiens GN=HLA-A PE=1 SV=2		521.97
P16189	1A31_HUMAN HLA class I histocompatibility antigen, A-31 alpha chain OS=Homo sapiens GN=HLA-A PE=2 SV=2		599.27
P16190	1A33_HUMAN HLA class I histocompatibility antigen, A-33 alpha chain OS=Homo sapiens GN=HLA-A PE=1 SV=3		541.14
P16219	ACADS_HUMAN Short-chain specific acyl-CoA dehydrogenase, mitochondrial OS=Homo sapiens GN=ACADS PE=1 SV=1	3.28E-08	
P16220	CREB1_HUMAN Cyclic AMP-responsive element-binding protein 1 OS=Homo sapiens GN=CREB1 PE=1 SV=2		18.55
P16220-2	CREB1_HUMAN Isoform CREB-B of Cyclic AMP-responsive element-binding protein 1 OS=Homo sapiens GN=CREB1		18.55
P16260	GDC_HUMAN Graves disease carrier protein OS=Homo sapiens GN=SLC25A16 PE=2 SV=3		
P16278	BGAL_HUMAN Beta-galactosidase OS=Homo sapiens GN=GLB1 PE=1 SV=2		76.49
P16278-2	BGAL_HUMAN Isoform 2 of Beta-galactosidase OS=Homo sapiens GN=GLB1		56.09
P16278-3	BGAL_HUMAN Isoform 3 of Beta-galactosidase OS=Homo sapiens GN=GLB1		76.49
P16278;P16278-3;P16278	BGAL_HUMAN Beta-galactosidase OS=Homo sapiens GN=GLB1 PE=1 SV=2;>sp|P16278-3|BGAL_HUMAN Isoform 3 of Beta-galactosic	1.20E-23	
P16284;P16284-6;P16284	PECA1_HUMAN Platelet endothelial cell adhesion molecule OS=Homo sapiens GN=PECAM1 PE=1 SV=1;>sp|P16284-6|PECA1_HUMAN	6.62E-06	
P16298	PP2BB_HUMAN Serine/threonine-protein phosphatase 2B catalytic subunit beta isoform OS=Homo sapiens GN=PPP3CB PE=1 SV=2		64.47
P16298-2	PP2BB_HUMAN Isoform 2 of Serine/threonine-protein phosphatase 2B catalytic subunit beta isoform OS=Homo sapiens GN=PPP3CB		64.47
P16298-3	PP2BB_HUMAN Isoform 3 of Serine/threonine-protein phosphatase 2B catalytic subunit beta isoform OS=Homo sapiens GN=PPP3CB		64.47
P16333	NCK1_HUMAN Cytoplasmic protein NCK1 OS=Homo sapiens GN=NCK1 PE=1 SV=1	6.72E-10	
P16383	GCF_HUMAN GC-rich sequence DNA-binding factor OS=Homo sapiens GN=TCF9 PE=1 SV=2		25.75
P16383-2	GCF_HUMAN Isoform 2 of GC-rich sequence DNA-binding factor OS=Homo sapiens GN=TCF9		25.75
P16401	H15_HUMAN Histone H1.5 OS=Homo sapiens GN=HIST1H1B PE=1 SV=3		20.3
P16402	H13_HUMAN Histone H1.3 OS=Homo sapiens GN=HIST1H1D PE=1 SV=2		488.18
P16403	H12_HUMAN Histone H1.2 OS=Homo sapiens GN=HIST1H1C PE=1 SV=2		515.77
P16422	EPCAM_HUMAN Epithelial cell adhesion molecule OS=Homo sapiens GN=EPCAM PE=1 SV=2		69.59
P16435	NCPR_HUMAN NADPH--cytochrome P450 reductase OS=Homo sapiens GN=POR PE=1 SV=2		53.08
P16444	DPEP1_HUMAN Dipeptidase 1 OS=Homo sapiens GN=DPEP1 PE=1 SV=3		
P16455	MGMT_HUMAN Methylated-DNA--protein-cysteine methyltransferase OS=Homo sapiens GN=MGMT PE=1 SV=1		
P16471	PRLR_HUMAN Prolactin receptor OS=Homo sapiens GN=PRLR PE=1 SV=1		29.66
P16471-2	PRLR_HUMAN Isoform 2 of Prolactin receptor OS=Homo sapiens GN=PRLR		29.66
P16499	PDE6A_HUMAN Rod cGMP-specific 3,5-cyclic phosphodiesterase subunit alpha OS=Homo sapiens GN=PDE6A PE=1 SV=4	1.14E-09	
P16520	GBB3_HUMAN Guanine nucleotide-binding protein G(I)/G(S)/G(T) subunit beta-3 OS=Homo sapiens GN=GNB3 PE=1 SV=1		86.07
P16615	AT2A2_HUMAN Sarcoplasmic/endoplasmic reticulum calcium ATPase 2 OS=Homo sapiens GN=ATP2A2 PE=1 SV=1		1380.67
P16615-2	AT2A2_HUMAN Isoform 2 of Sarcoplasmic/endoplasmic reticulum calcium ATPase 2 OS=Homo sapiens GN=ATP2A2		1363.36
P16615-3	AT2A2_HUMAN Isoform 3 of Sarcoplasmic/endoplasmic reticulum calcium ATPase 2 OS=Homo sapiens GN=ATP2A2		1364.05
P16615-4	AT2A2_HUMAN Isoform 4 of Sarcoplasmic/endoplasmic reticulum calcium ATPase 2 OS=Homo sapiens GN=ATP2A2		1291.34
P16615-5	AT2A2_HUMAN Isoform 5 of Sarcoplasmic/endoplasmic reticulum calcium ATPase 2 OS=Homo sapiens GN=ATP2A2		1363.36
P16615;P16615-3;P16615	AT2A2_HUMAN Sarcoplasmic/endoplasmic reticulum calcium ATPase 2 OS=Homo sapiens GN=ATP2A2 PE=1 SV=1;>sp|P16615-3|AT2	1.17E-133	
P16671	CD36_HUMAN Platelet glycoprotein 4 OS=Homo sapiens GN=CD36 PE=1 SV=2	0	
P16885	PLCG2_HUMAN 1-phosphatidylinositol-4,5-bisphosphate phosphodiesterase gamma-2 OS=Homo sapiens GN=PLCG2 PE=1 SV=4	0	
P16930	FAAA_HUMAN Fumarylacetoacetase OS=Homo sapiens GN=FAH PE=1 SV=2	8.31E-18	
P16949	STMN1_HUMAN Stathmin OS=Homo sapiens GN=STMN1 PE=1 SV=3		370.21
P16949-2	STMN1_HUMAN Isoform 2 of Stathmin OS=Homo sapiens GN=STMN1		343.99
P16949;P16949-2	STMN1_HUMAN Stathmin OS=Homo sapiens GN=STMN1 PE=1 SV=3;>sp|P16949-2|STMN1_HUMAN Isoform 2 of Stathmin OS=Homo	6.49E-107	
P16989	DBPA_HUMAN DNA-binding protein A OS=Homo sapiens GN=CSDA PE=1 SV=4		448.66
P16989-2	DBPA_HUMAN Isoform 2 of DNA-binding protein A OS=Homo sapiens GN=CSDA		386.46
P16989-3	DBPA_HUMAN Isoform 3 of DNA-binding protein A OS=Homo sapiens GN=CSDA		391.99
P16989;P16989-3;P16989	DBPA_HUMAN DNA-binding protein A OS=Homo sapiens GN=CSDA PE=1 SV=4;>sp|P16989-3|DBPA_HUMAN Isoform 3 of DNA-bindin	6.28E-261	
P17014	ZNF12_HUMAN Zinc finger protein 12 OS=Homo sapiens GN=ZNF12 PE=2 SV=3		
P17014-2	ZNF12_HUMAN Isoform 2 of Zinc finger protein 12 OS=Homo sapiens GN=ZNF12		
P17014-4	ZNF12_HUMAN Isoform 4 of Zinc finger protein 12 OS=Homo sapiens GN=ZNF12		
P17014-5	ZNF12_HUMAN Isoform 5 of Zinc finger protein 12 OS=Homo sapiens GN=ZNF12		
P17050	NAGAB_HUMAN Alpha-N-acetylgalactosaminidase OS=Homo sapiens GN=NAGA PE=1 SV=2	3.89E-08	
P17066	HSP76_HUMAN Heat shock 70 kDa protein 6 OS=Homo sapiens GN=HSPA6 PE=1 SV=2		327.72
P17081	RHOQ_HUMAN Rho-related GTP-binding protein RhoQ OS=Homo sapiens GN=RHOQ PE=1 SV=2		59.44
P17096	HMGA1_HUMAN High mobility group protein HMG-I/HMG-Y OS=Homo sapiens GN=HMGA1 PE=1 SV=3		106.31
P17096-2	HMGA1_HUMAN Isoform HMG-Y of High mobility group protein HMG-I/HMG-Y OS=Homo sapiens GN=HMGA1		22.07
P17096-3	HMGA1_HUMAN Isoform HMG-R of High mobility group protein HMG-I/HMG-Y OS=Homo sapiens GN=HMGA1		106.31
P17096-3;P17096	3|HMGA1_HUMAN Isoform HMG-R of High mobility group protein HMG-I/HMG-Y OS=Homo sapiens GN=HMGA1;>sp|P17096|HMGA1_I	2.22E-57	

P17096;P17096-3	HMGA1_HUMAN High mobility group protein HMG-I/HMG-Y OS=Homo sapiens GN=HMGA1 PE=1 SV=3;>sp|P17096-3|HMGA1_HUMAN	3.92E-37	
P17152	TMM11_HUMAN Transmembrane protein 11, mitochondrial OS=Homo sapiens GN=TMEM11 PE=1 SV=1	4.01E-06	
P17174	AATC_HUMAN Aspartate aminotransferase, cytoplasmic OS=Homo sapiens GN=GOT1 PE=1 SV=3		73.91
P17252	KPCA_HUMAN Protein kinase C alpha type OS=Homo sapiens GN=PRKCA PE=1 SV=4	7.71E-16	
P17301	ITA2_HUMAN Integrin alpha-2 OS=Homo sapiens GN=ITGA2 PE=1 SV=1		1035.99
P17480	UBF1_HUMAN Nucleolar transcription factor 1 OS=Homo sapiens GN=UBTF PE=1 SV=1		
P17480-2	UBF1_HUMAN Isoform UBF2 of Nucleolar transcription factor 1 OS=Homo sapiens GN=UBTF		
P17480;P17480-2	UBF1_HUMAN Nucleolar transcription factor 1 OS=Homo sapiens GN=UBTF PE=1 SV=1;>sp|P17480-2|UBF1_HUMAN Isoform UBF2 of	2.14E-30	
P17540	KCRS_HUMAN Creatine kinase S-type, mitochondrial OS=Homo sapiens GN=CKMT2 PE=1 SV=2		
P17542	TAL1_HUMAN T-cell acute lymphocytic leukemia protein 1 OS=Homo sapiens GN=TAL1 PE=1 SV=2		
P17542-2	TAL1_HUMAN Isoform PP39-TAL1 of T-cell acute lymphocytic leukemia protein 1 OS=Homo sapiens GN=TAL1		
P17542-3	TAL1_HUMAN Isoform PP22-TAL1 of T-cell acute lymphocytic leukemia protein 1 OS=Homo sapiens GN=TAL1		
P17568	NDUB7_HUMAN NADH dehydrogenase [ubiquinone] 1 beta subcomplex subunit 7 OS=Homo sapiens GN=NDUFB7 PE=1 SV=4	6.53E-08	
P17612	KAPCA_HUMAN cAMP-dependent protein kinase catalytic subunit alpha OS=Homo sapiens GN=PRKACA PE=1 SV=2		115.92
P17612-2	KAPCA_HUMAN Isoform 2 of cAMP-dependent protein kinase catalytic subunit alpha OS=Homo sapiens GN=PRKACA		115.92
P17612;A8K8B9;P17612-2	KAPCA_HUMAN cAMP-dependent protein kinase catalytic subunit alpha OS=Homo sapiens GN=PRKACA PE=1 SV=2;>tr|A8K8B9|A8K8	9.93E-58	
P17655	CAN2_HUMAN Calpain-2 catalytic subunit OS=Homo sapiens GN=CAPN2 PE=1 SV=6		167.37
P17661	DESM_HUMAN Desmin OS=Homo sapiens GN=DES PE=1 SV=3		336.53
P17693	HLAG_HUMAN HLA class I histocompatibility antigen, alpha chain G OS=Homo sapiens GN=HLA-G PE=1 SV=1		37.17
P17706;Q59F91;D3DUJ3	PTN2_HUMAN Tyrosine-protein phosphatase non-receptor type 2 OS=Homo sapiens GN=PTPN2 PE=1 SV=2;>tr|Q59F91|Q59F91_HUM	5.37E-05	
P17812	PYRG1_HUMAN CTP synthase 1 OS=Homo sapiens GN=CTPS PE=1 SV=2		68.25
P17813;P17813-2	EGLN_HUMAN Endoglin OS=Homo sapiens GN=ENG PE=1 SV=2;>sp|P17813-2|EGLN_HUMAN Isoform Short of Endoglin OS=Homo sa	5.47E-14	
P17844	DDX5_HUMAN Probable ATP-dependent RNA helicase DDX5 OS=Homo sapiens GN=DDX5 PE=1 SV=1		324.56
P17858	K6PL_HUMAN 6-phosphofructokinase, liver type OS=Homo sapiens GN=PFKL PE=1 SV=6		120.28
P17858-2	K6PL_HUMAN Isoform 2 of 6-phosphofructokinase, liver type OS=Homo sapiens GN=PFKL		55.7
P17858-2;P17858	2|K6PL_HUMAN Isoform 2 of 6-phosphofructokinase, liver type OS=Homo sapiens GN=PFKL;>sp|P17858|K6PL_HUMAN 6-phosphofru	0	
P17858;P17858-2	K6PL_HUMAN 6-phosphofructokinase, liver type OS=Homo sapiens GN=PFKL PE=1 SV=6;>sp|P17858-2|K6PL_HUMAN Isoform 2 of 6-	9.35E-291	
P17900	SAP3_HUMAN Ganglioside GM2 activator OS=Homo sapiens GN=GM2A PE=1 SV=4		158.92
P17931	LEG3_HUMAN Galectin-3 OS=Homo sapiens GN=LGALS3 PE=1 SV=5		687.47
P17980	PRS6A_HUMAN 26S protease regulatory subunit 6A OS=Homo sapiens GN=PSMC3 PE=1 SV=3		527.95
P17987	TCPA_HUMAN T-complex protein 1 subunit alpha OS=Homo sapiens GN=TCP1 PE=1 SV=1		485.11
P18031	PTN1_HUMAN Tyrosine-protein phosphatase non-receptor type 1 OS=Homo sapiens GN=PTPN1 PE=1 SV=1		427.83
P18074	ERCC2_HUMAN TFIIH basal transcription factor complex helicase XPD subunit OS=Homo sapiens GN=ERCC2 PE=1 SV=1		119.44
P18077	RL35A_HUMAN 60S ribosomal protein L35a OS=Homo sapiens GN=RPL35A PE=1 SV=2		393.9
P18084	ITB5_HUMAN Integrin beta-5 OS=Homo sapiens GN=ITGB5 PE=1 SV=1		83.05
P18085	ARF4_HUMAN ADP-ribosylation factor 4 OS=Homo sapiens GN=ARF4 PE=1 SV=3		598.8
P18124	RL7_HUMAN 60S ribosomal protein L7 OS=Homo sapiens GN=RPL7 PE=1 SV=1		1321.48
P18135;P18136	KV312_HUMAN Ig kappa chain V-III region HAH OS=Homo sapiens PE=2 SV=1;>sp|P18136|KV313_HUMAN Ig kappa chain V-III regi	4.81E-62	
P18206	VINC_HUMAN Vinculin OS=Homo sapiens GN=VCL PE=1 SV=4		600.82
P18206-2	VINC_HUMAN Isoform 1 of Vinculin OS=Homo sapiens GN=VCL		600.82
P18206;P18206-2	VINC_HUMAN Vinculin OS=Homo sapiens GN=VCL PE=1 SV=4;>sp|P18206-2|VINC_HUMAN Isoform 1 of Vinculin OS=Homo sapiens	2.21E-22	
P18428	LBP_HUMAN Lipopolysaccharide-binding protein OS=Homo sapiens GN=LBP PE=1 SV=3	6.58E-12	
P18440	ARY1_HUMAN Arylamine N-acetyltransferase 1 OS=Homo sapiens GN=NAT1 PE=1 SV=2	1.84E-205	
P18462	1A25_HUMAN HLA class I histocompatibility antigen, A-25 alpha chain OS=Homo sapiens GN=HLA-A PE=2 SV=1		587.75
P18463	1B37_HUMAN HLA class I histocompatibility antigen, B-37 alpha chain OS=Homo sapiens GN=HLA-B PE=2 SV=1		441.04
P18464	1B51_HUMAN HLA class I histocompatibility antigen, B-51 alpha chain OS=Homo sapiens GN=HLA-B PE=1 SV=1		413.81
P18465	1B57_HUMAN HLA class I histocompatibility antigen, B-57 alpha chain OS=Homo sapiens GN=HLA-B PE=1 SV=1		525.42
P18564	ITB6_HUMAN Integrin beta-6 OS=Homo sapiens GN=ITGB6 PE=1 SV=2	4.59E-06	
P18583	SON_HUMAN Protein SON OS=Homo sapiens GN=SON PE=1 SV=4		
P18583-10	SON_HUMAN Isoform J of Protein SON OS=Homo sapiens GN=SON		
P18583-2	SON_HUMAN Isoform A of Protein SON OS=Homo sapiens GN=SON		
P18583-3	SON_HUMAN Isoform B of Protein SON OS=Homo sapiens GN=SON		
P18583-4	SON_HUMAN Isoform C of Protein SON OS=Homo sapiens GN=SON		
P18583-5	SON_HUMAN Isoform D of Protein SON OS=Homo sapiens GN=SON		
P18583-6	SON_HUMAN Isoform E of Protein SON OS=Homo sapiens GN=SON		
P18583-7	SON_HUMAN Isoform G of Protein SON OS=Homo sapiens GN=SON		
P18583-8	SON_HUMAN Isoform H of Protein SON OS=Homo sapiens GN=SON		
P18583-9	SON_HUMAN Isoform I of Protein SON OS=Homo sapiens GN=SON		
P18583-9;P18583-5;P185	9|SON_HUMAN Isoform I of Protein SON OS=Homo sapiens GN=SON;>sp|P18583-5|SON_HUMAN Isoform D of Protein SON OS=Hom	1.35E-08	
P18615	NELFE_HUMAN Negative elongation factor E OS=Homo sapiens GN=RDBP PE=1 SV=3	5.48E-63	
P18621	RL17_HUMAN 60S ribosomal protein L17 OS=Homo sapiens GN=RPL17 PE=1 SV=3		897.6

P18669	PGAM1_HUMAN Phosphoglycerate mutase 1 OS=Homo sapiens GN=PGAM1 PE=1 SV=2		798.17
P18754	RCC1_HUMAN Regulator of chromosome condensation OS=Homo sapiens GN=RCC1 PE=1 SV=1		299.74
P18754-2	RCC1_HUMAN Isoform 2 of Regulator of chromosome condensation OS=Homo sapiens GN=RCC1		299.74
P18754-2;P18754	2|RCC1_HUMAN Isoform 2 of Regulator of chromosome condensation OS=Homo sapiens GN=RCC1;>sp|P18754|RCC1_HUMAN Regula	4.79E-29	
P18827	SDC1_HUMAN Syndecan-1 OS=Homo sapiens GN=SDC1 PE=1 SV=3		
P18846	ATF1_HUMAN Cyclic AMP-dependent transcription factor ATF-1 OS=Homo sapiens GN=ATF1 PE=1 SV=2		18.55
P18858	DNLI1_HUMAN DNA ligase 1 OS=Homo sapiens GN=LIG1 PE=1 SV=1		46.73
P18859	ATP5J_HUMAN ATP synthase-coupling factor 6, mitochondrial OS=Homo sapiens GN=ATP5J PE=1 SV=1		22.86
P18887	XRCC1_HUMAN DNA repair protein XRCC1 OS=Homo sapiens GN=XRCC1 PE=1 SV=2		
P19012	K1C15_HUMAN Keratin, type I cytoskeletal 15 OS=Homo sapiens GN=KRT15 PE=1 SV=3		502.08
P19013	K2C4_HUMAN Keratin, type II cytoskeletal 4 OS=Homo sapiens GN=KRT4 PE=1 SV=4		364.8
P19022	CADH2_HUMAN Cadherin-2 OS=Homo sapiens GN=CDH2 PE=1 SV=4		
P19086	GNAZ_HUMAN Guanine nucleotide-binding protein G(z) subunit alpha OS=Homo sapiens GN=GNAZ PE=2 SV=3		1.77
P19087	GNAT2_HUMAN Guanine nucleotide-binding protein G(t) subunit alpha-2 OS=Homo sapiens GN=GNAT2 PE=1 SV=4		178.57
P19099	C11B2_HUMAN Cytochrome P450 11B2, mitochondrial OS=Homo sapiens GN=CYP11B2 PE=1 SV=3		
P19105	ML12A_HUMAN Myosin regulatory light chain 12A OS=Homo sapiens GN=MYL12A PE=1 SV=2		1287.85
P19174	PLCG1_HUMAN 1-phosphatidylinositol-4,5-bisphosphate phosphodiesterase gamma-1 OS=Homo sapiens GN=PLCG1 PE=1 SV=1		
P19174-2	PLCG1_HUMAN Isoform 2 of 1-phosphatidylinositol-4,5-bisphosphate phosphodiesterase gamma-1 OS=Homo sapiens GN=PLCG1		
P19174-2;P19174	2|PLCG1_HUMAN Isoform 2 of 1-phosphatidylinositol-4,5-bisphosphate phosphodiesterase gamma-1 OS=Homo sapiens GN=PLCG1;>s	2.01E-63	
P19256	LFA3_HUMAN Lymphocyte function-associated antigen 3 OS=Homo sapiens GN=CD58 PE=1 SV=1		
P19256-2	LFA3_HUMAN Isoform 2 of Lymphocyte function-associated antigen 3 OS=Homo sapiens GN=CD58		
P19256-3	LFA3_HUMAN Isoform 3 of Lymphocyte function-associated antigen 3 OS=Homo sapiens GN=CD58		
P19338	NUCL_HUMAN Nucleolin OS=Homo sapiens GN=NCL PE=1 SV=3		1438.17
P19367	HXK1_HUMAN Hexokinase-1 OS=Homo sapiens GN=HK1 PE=1 SV=3		792.34
P19367-2	HXK1_HUMAN Isoform 2 of Hexokinase-1 OS=Homo sapiens GN=HK1		792.34
P19367-3	HXK1_HUMAN Isoform 3 of Hexokinase-1 OS=Homo sapiens GN=HK1		792.34
P19367-3;P19367;P19367	3|HXK1_HUMAN Isoform 3 of Hexokinase-1 OS=Homo sapiens GN=HK1;>sp|P19367|HXK1_HUMAN Hexokinase-1 OS=Homo sapiens	1.67E-115	
P19367-4	HXK1_HUMAN Isoform 4 of Hexokinase-1 OS=Homo sapiens GN=HK1		792.34
P19367;P19367-3;P19367	HXK1_HUMAN Hexokinase-1 OS=Homo sapiens GN=HK1 PE=1 SV=3;>sp|P19367-3|HXK1_HUMAN Isoform 3 of Hexokinase-1 OS=Hc	1.03E-92	
P19387	RPB3_HUMAN DNA-directed RNA polymerase II subunit RPB3 OS=Homo sapiens GN=POLR2C PE=1 SV=2		
P19388	RPAB1_HUMAN DNA-directed RNA polymerases I, II, and III subunit RPABC1 OS=Homo sapiens GN=POLR2E PE=1 SV=4		156.26
P19404	NDUV2_HUMAN NADH dehydrogenase [ubiquinone] flavoprotein 2, mitochondrial OS=Homo sapiens GN=NDUFV2 PE=1 SV=2		191.72
P19447	ERCC3_HUMAN TFIIH basal transcription factor complex helicase XPB subunit OS=Homo sapiens GN=ERCC3 PE=1 SV=1		
P19474;P19474-2	RO52_HUMAN E3 ubiquitin-protein ligase TRIM21 OS=Homo sapiens GN=TRIM21 PE=1 SV=1;>sp|P19474-2|RO52_HUMAN Isoform 2	1.46E-07	
P19525	E2AK2_HUMAN Interferon-induced, double-stranded RNA-activated protein kinase OS=Homo sapiens GN=EIF2AK2 PE=1 SV=2		75.46
P19623	SPEE_HUMAN Spermidine synthase OS=Homo sapiens GN=SRM PE=1 SV=1		76.03
P19652	A1AG2_HUMAN Alpha-1-acid glycoprotein 2 OS=Homo sapiens GN=ORM2 PE=1 SV=2		79.36
P19784	CSK22_HUMAN Casein kinase II subunit alpha' OS=Homo sapiens GN=CSNK2A2 PE=1 SV=1		
P19793	RXRA_HUMAN Retinoic acid receptor RXR-alpha OS=Homo sapiens GN=RXRA PE=1 SV=1		
P19823	ITIH2_HUMAN Inter-alpha-trypsin inhibitor heavy chain H2 OS=Homo sapiens GN=ITIH2 PE=1 SV=2		140.64
P19827	ITIH1_HUMAN Inter-alpha-trypsin inhibitor heavy chain H1 OS=Homo sapiens GN=ITIH1 PE=1 SV=3		54.91
P19971	TYPH_HUMAN Thymidine phosphorylase OS=Homo sapiens GN=TYMP PE=1 SV=2	0	
P20020	AT2B1_HUMAN Plasma membrane calcium-transporting ATPase 1 OS=Homo sapiens GN=ATP2B1 PE=1 SV=3		1172.52
P20020-2	AT2B1_HUMAN Isoform A of Plasma membrane calcium-transporting ATPase 1 OS=Homo sapiens GN=ATP2B1		1101.42
P20020-3	AT2B1_HUMAN Isoform B of Plasma membrane calcium-transporting ATPase 1 OS=Homo sapiens GN=ATP2B1		1177.92
P20020-4	AT2B1_HUMAN Isoform C of Plasma membrane calcium-transporting ATPase 1 OS=Homo sapiens GN=ATP2B1		1172.52
P20020-5	AT2B1_HUMAN Isoform E of Plasma membrane calcium-transporting ATPase 1 OS=Homo sapiens GN=ATP2B1		1101.42
P20020-6	AT2B1_HUMAN Isoform K of Plasma membrane calcium-transporting ATPase 1 OS=Homo sapiens GN=ATP2B1		1177.92
P20020;P20020-4;P20020	AT2B1_HUMAN Plasma membrane calcium-transporting ATPase 1 OS=Homo sapiens GN=ATP2B1 PE=1 SV=3;>sp|P20020-4|AT2B1_H	1.92E-100	
P20036	DPA1_HUMAN HLA class II histocompatibility antigen, DP alpha 1 chain OS=Homo sapiens GN=HLA-DPA1 PE=1 SV=1	3.11E-38	
P20039	2B1B_HUMAN HLA class II histocompatibility antigen, DRB1-11 beta chain OS=Homo sapiens GN=HLA-DRB1 PE=1 SV=1		29.42
P20042	IF2B_HUMAN Eukaryotic translation initiation factor 2 subunit 2 OS=Homo sapiens GN=EIF2S2 PE=1 SV=2		689.37
P20073	ANXA7_HUMAN Annexin A7 OS=Homo sapiens GN=ANXA7 PE=1 SV=3		
P20073-2	ANXA7_HUMAN Isoform 2 of Annexin A7 OS=Homo sapiens GN=ANXA7		
P20073;P20073-2	ANXA7_HUMAN Annexin A7 OS=Homo sapiens GN=ANXA7 PE=1 SV=3;>sp|P20073-2|ANXA7_HUMAN Isoform 2 of Annexin A7 OS=H	5.48E-166	
P20132	SDHL_HUMAN L-serine dehydratase/L-threonine deaminase OS=Homo sapiens GN=SDS PE=1 SV=2	9.47E-05	
P20226	TBP_HUMAN TATA-box-binding protein OS=Homo sapiens GN=TBP PE=1 SV=2		
P20231;Q15661;Q15661-:	TRYB2_HUMAN Tryptase beta-2 OS=Homo sapiens GN=TPSB2 PE=1 SV=2;>sp|Q15661|TRYB1_HUMAN Tryptase alpha/beta-1 OS=Hc	8.16E-185	
P20290	BTF3_HUMAN Transcription factor BTF3 OS=Homo sapiens GN=BTF3 PE=1 SV=1		406.47
P20290-2	BTF3_HUMAN Isoform 2 of Transcription factor BTF3 OS=Homo sapiens GN=BTF3		442.51
P20290;P20290-2	BTF3_HUMAN Transcription factor BTF3 OS=Homo sapiens GN=BTF3 PE=1 SV=1;>sp|P20290-2|BTF3_HUMAN Isoform 2 of Transcripti	2.24E-138	

P20292	AL5AP_HUMAN Arachidonate 5-lipoxygenase-activating protein OS=Homo sapiens GN=ALOX5AP PE=1 SV=2	6.37E-10	
P20336	RAB3A_HUMAN Ras-related protein Rab-3A OS=Homo sapiens GN=RAB3A PE=1 SV=1		90.26
P20337	RAB3B_HUMAN Ras-related protein Rab-3B OS=Homo sapiens GN=RAB3B PE=1 SV=2		90.26
P20338	RAB4A_HUMAN Ras-related protein Rab-4A OS=Homo sapiens GN=RAB4A PE=1 SV=2		98.54
P20339	RAB5A_HUMAN Ras-related protein Rab-5A OS=Homo sapiens GN=RAB5A PE=1 SV=2		712.12
P20340	RAB6A_HUMAN Ras-related protein Rab-6A OS=Homo sapiens GN=RAB6A PE=1 SV=3		396.63
P20340-2	RAB6A_HUMAN Isoform 2 of Ras-related protein Rab-6A OS=Homo sapiens GN=RAB6A		467.87
P20340-2;Q6FGX3;P2034(	2|RAB6A_HUMAN Isoform 2 of Ras-related protein Rab-6A OS=Homo sapiens GN=RAB6A;>tr|Q6FGX3|Q6FGX3_HUMAN RAB6A protei	7.77E-55	
P20340;P20340-2;Q9NRW	RAB6A_HUMAN Ras-related protein Rab-6A OS=Homo sapiens GN=RAB6A PE=1 SV=3;>sp|P20340-2|RAB6A_HUMAN Isoform 2 of Ra	4.72E-125	
P20585	MSH3_HUMAN DNA mismatch repair protein Msh3 OS=Homo sapiens GN=MSH3 PE=1 SV=4		60.9
P20591	MX1_HUMAN Interferon-induced GTP-binding protein Mx1 OS=Homo sapiens GN=MX1 PE=1 SV=4	7.57E-284	
P20592	MX2_HUMAN Interferon-induced GTP-binding protein Mx2 OS=Homo sapiens GN=MX2 PE=1 SV=1	1.12E-51	
P20618	PSB1_HUMAN Proteasome subunit beta type-1 OS=Homo sapiens GN=PSMB1 PE=1 SV=2		861.88
P20645	MPRD_HUMAN Cation-dependent mannose-6-phosphate receptor OS=Homo sapiens GN=M6PR PE=1 SV=1	1.07E-91	
P20648	ATP4A_HUMAN Potassium-transporting ATPase alpha chain 1 OS=Homo sapiens GN=ATP4A PE=2 SV=5		333.64
P20671	H2A1D_HUMAN Histone H2A type 1-D OS=Homo sapiens GN=HIST1H2AD PE=1 SV=2		288.83
P20674	COX5A_HUMAN Cytochrome c oxidase subunit 5A, mitochondrial OS=Homo sapiens GN=COX5A PE=1 SV=2		254.5
P20700	LMNB1_HUMAN Lamin-B1 OS=Homo sapiens GN=LMNB1 PE=1 SV=2		270.35
P20701-2;P20701	2|ITAL_HUMAN Isoform 2 of Integrin alpha-L OS=Homo sapiens GN=ITGAL;>sp|P20701|ITAL_HUMAN Integrin alpha-L OS=Homo sap	1.49E-06	
P20742	PZP_HUMAN Pregnancy zone protein OS=Homo sapiens GN=PZP PE=1 SV=4		296.87
P20742-2	PZP_HUMAN Isoform 2 of Pregnancy zone protein OS=Homo sapiens GN=PZP		296.08
P20742;P20742-2	PZP_HUMAN Pregnancy zone protein OS=Homo sapiens GN=PZP PE=1 SV=4;>sp|P20742-2|PZP_HUMAN Isoform 2 of Pregnancy zon(	2.97E-218	
P20774	MIME_HUMAN Mimecan OS=Homo sapiens GN=OGN PE=1 SV=1	2.84E-294	
P20794	MAK_HUMAN Serine/threonine-protein kinase MAK OS=Homo sapiens GN=MAK PE=2 SV=2		60.33
P20810	ICAL_HUMAN Calpastatin OS=Homo sapiens GN=CAST PE=1 SV=4		861.31
P20810-2	ICAL_HUMAN Isoform 2 of Calpastatin OS=Homo sapiens GN=CAST		813.8
P20810-3	ICAL_HUMAN Isoform 3 of Calpastatin OS=Homo sapiens GN=CAST		721.08
P20810-4	ICAL_HUMAN Isoform 4 of Calpastatin OS=Homo sapiens GN=CAST		849.77
P20810-5	ICAL_HUMAN Isoform 5 of Calpastatin OS=Homo sapiens GN=CAST		813.8
P20810-6	ICAL_HUMAN Isoform 6 of Calpastatin OS=Homo sapiens GN=CAST		861.31
P20810-6;P20810-5;P208	6|ICAL_HUMAN Isoform 6 of Calpastatin OS=Homo sapiens GN=CAST;>sp|P20810-5|ICAL_HUMAN Isoform 5 of Calpastatin OS=Hom(	5.29E-186	
P20810-7	ICAL_HUMAN Isoform 7 of Calpastatin OS=Homo sapiens GN=CAST		845.78
P20839-3;P20839;P20839	3|IMDH1_HUMAN Isoform 3 of Inosine-5-monophosphate dehydrogenase 1 OS=Homo sapiens GN=IMPDH1;>sp|P20839|IMDH1_HUM	4.71E-10	
P20853	CP2A7_HUMAN Cytochrome P450 2A7 OS=Homo sapiens GN=CYP2A7 PE=1 SV=2		
P20908	CO5A1_HUMAN Collagen alpha-1(V) chain OS=Homo sapiens GN=COL5A1 PE=1 SV=3		
P20929	NEBU_HUMAN Nebulin OS=Homo sapiens GN=NEB PE=1 SV=4		72.57
P20930	FILA_HUMAN Filaggrin OS=Homo sapiens GN=FLG PE=1 SV=3		42.21
P20933	ASPG_HUMAN N(4)-(beta-N-acetylglucosaminyl)-L-asparaginase OS=Homo sapiens GN=AGA PE=1 SV=2		48.85
P20936;B4DTX4;B4DTL2;I	RASA1_HUMAN Ras GTPase-activating protein 1 OS=Homo sapiens GN=RASA1 PE=1 SV=1;>tr|B4DTX4|B4DTX4_HUMAN cDNA FLJ59	6.77E-47	
P20962	PTMS_HUMAN Parathymosin OS=Homo sapiens GN=PTMS PE=1 SV=2		
P21108	PRPS3_HUMAN Ribose-phosphate pyrophosphokinase 3 OS=Homo sapiens GN=PRPS1L1 PE=1 SV=2		39.14
P21127	CD11B_HUMAN Cyclin-dependent kinase 11B OS=Homo sapiens GN=CDK11B PE=1 SV=3		85.59
P21127-10	CD11B_HUMAN Isoform SV11 of Cyclin-dependent kinase 11B OS=Homo sapiens GN=CDK11B		68.47
P21127-12	CD11B_HUMAN Isoform 7 of Cyclin-dependent kinase 11B OS=Homo sapiens GN=CDK11B		68.47
P21127-2	CD11B_HUMAN Isoform SV1 of Cyclin-dependent kinase 11B OS=Homo sapiens GN=CDK11B		85.59
P21127-3	CD11B_HUMAN Isoform 2 of Cyclin-dependent kinase 11B OS=Homo sapiens GN=CDK11B		74.27
P21127-4	CD11B_HUMAN Isoform 3 of Cyclin-dependent kinase 11B OS=Homo sapiens GN=CDK11B		68.47
P21127-5	CD11B_HUMAN Isoform SV4 of Cyclin-dependent kinase 11B OS=Homo sapiens GN=CDK11B		68.47
P21127-6	CD11B_HUMAN Isoform SV5 of Cyclin-dependent kinase 11B OS=Homo sapiens GN=CDK11B		74.27
P21127-8	CD11B_HUMAN Isoform 8 of Cyclin-dependent kinase 11B OS=Homo sapiens GN=CDK11B		74.27
P21127-9	CD11B_HUMAN Isoform SV10 of Cyclin-dependent kinase 11B OS=Homo sapiens GN=CDK11B		85.59
P21127;Q9UQ88;P21127-:	CD11B_HUMAN Cyclin-dependent kinase 11B OS=Homo sapiens GN=CDK11B PE=1 SV=3;>sp|Q9UQ88|CD11A_HUMAN Cyclin-depen(	1.86E-37	
P21266	GSTM3_HUMAN Glutathione S-transferase Mu 3 OS=Homo sapiens GN=GSTM3 PE=1 SV=3		139.86
P21281	VATB2_HUMAN V-type proton ATPase subunit B, brain isoform OS=Homo sapiens GN=ATP6V1B2 PE=1 SV=3	1.55E-97	
P21283	VATC1_HUMAN V-type proton ATPase subunit C 1 OS=Homo sapiens GN=ATP6V1C1 PE=1 SV=4		
P21291	CSRP1_HUMAN Cysteine and glycine-rich protein 1 OS=Homo sapiens GN=CSRP1 PE=1 SV=3		401.5
P21333	FLNA_HUMAN Filamin-A OS=Homo sapiens GN=FLNA PE=1 SV=4		5069.2
P21333-2	FLNA_HUMAN Isoform 2 of Filamin-A OS=Homo sapiens GN=FLNA		5069.2
P21333;P21333-2	FLNA_HUMAN Filamin-A OS=Homo sapiens GN=FLNA PE=1 SV=4;>sp|P21333-2|FLNA_HUMAN Isoform 2 of Filamin-A OS=Homo sapi	0	
P21397	AOFA_HUMAN Amine oxidase [flavin-containing] A OS=Homo sapiens GN=MAOA PE=1 SV=1		
P21399	ACOC_HUMAN Cytoplasmic aconitate hydratase OS=Homo sapiens GN=ACO1 PE=1 SV=3	1.35E-47	

P21579	SYT1_HUMAN Synaptotagmin-1 OS=Homo sapiens GN=SYT1 PE=1 SV=1		20.43
P21580	TNAP3_HUMAN Tumor necrosis factor alpha-induced protein 3 OS=Homo sapiens GN=TNFAIP3 PE=1 SV=1		
P21589	5NTD_HUMAN 5'-nucleotidase OS=Homo sapiens GN=NT5E PE=1 SV=1		1228.05
P21675	TAF1_HUMAN Transcription initiation factor TFIID subunit 1 OS=Homo sapiens GN=TAF1 PE=1 SV=2		
P21675-2	TAF1_HUMAN Isoform 2 of Transcription initiation factor TFIID subunit 1 OS=Homo sapiens GN=TAF1		
P21675-3	TAF1_HUMAN Isoform 3 of Transcription initiation factor TFIID subunit 1 OS=Homo sapiens GN=TAF1		
P21675-4	TAF1_HUMAN Isoform 4 of Transcription initiation factor TFIID subunit 1 OS=Homo sapiens GN=TAF1		
P21675-4;P21675-3;P216	4|TAF1_HUMAN Isoform 4 of Transcription initiation factor TFIID subunit 1 OS=Homo sapiens GN=TAF1;>sp|P21675-3|TAF1_HUMAN I	7.93E-08	
P21695	GPDA_HUMAN Glycerol-3-phosphate dehydrogenase [NAD+], cytoplasmic OS=Homo sapiens GN=GPD1 PE=1 SV=4	7.21E-274	
P21741	MK_HUMAN Midkine OS=Homo sapiens GN=MDK PE=1 SV=1	3.48E-08	
P21796	VDAC1_HUMAN Voltage-dependent anion-selective channel protein 1 OS=Homo sapiens GN=VDAC1 PE=1 SV=2		986.53
P21802	FGFR2_HUMAN Fibroblast growth factor receptor 2 OS=Homo sapiens GN=FGFR2 PE=1 SV=1		65.01
P21802-10	FGFR2_HUMAN Isoform 10 of Fibroblast growth factor receptor 2 OS=Homo sapiens GN=FGFR2		65.01
P21802-11	FGFR2_HUMAN Isoform 11 of Fibroblast growth factor receptor 2 OS=Homo sapiens GN=FGFR2		65.01
P21802-12	FGFR2_HUMAN Isoform 12 of Fibroblast growth factor receptor 2 OS=Homo sapiens GN=FGFR2		65.01
P21802-13	FGFR2_HUMAN Isoform 13 of Fibroblast growth factor receptor 2 OS=Homo sapiens GN=FGFR2		65.72
P21802-14	FGFR2_HUMAN Isoform 14 of Fibroblast growth factor receptor 2 OS=Homo sapiens GN=FGFR2		8.08
P21802-15	FGFR2_HUMAN Isoform 15 of Fibroblast growth factor receptor 2 OS=Homo sapiens GN=FGFR2		64.91
P21802-16	FGFR2_HUMAN Isoform 16 of Fibroblast growth factor receptor 2 OS=Homo sapiens GN=FGFR2		65.01
P21802-17	FGFR2_HUMAN Isoform 17 of Fibroblast growth factor receptor 2 OS=Homo sapiens GN=FGFR2		65.01
P21802-18	FGFR2_HUMAN Isoform 18 of Fibroblast growth factor receptor 2 OS=Homo sapiens GN=FGFR2		65.01
P21802-19	FGFR2_HUMAN Isoform 19 of Fibroblast growth factor receptor 2 OS=Homo sapiens GN=FGFR2		8.08
P21802-2	FGFR2_HUMAN Isoform 2 of Fibroblast growth factor receptor 2 OS=Homo sapiens GN=FGFR2		65.01
P21802-20	FGFR2_HUMAN Isoform 20 of Fibroblast growth factor receptor 2 OS=Homo sapiens GN=FGFR2		56.93
P21802-3	FGFR2_HUMAN Isoform 3 of Fibroblast growth factor receptor 2 OS=Homo sapiens GN=FGFR2		65.01
P21802-4	FGFR2_HUMAN Isoform 4 of Fibroblast growth factor receptor 2 OS=Homo sapiens GN=FGFR2		56.93
P21802-5	FGFR2_HUMAN Isoform 5 of Fibroblast growth factor receptor 2 OS=Homo sapiens GN=FGFR2		65.01
P21802-6	FGFR2_HUMAN Isoform 6 of Fibroblast growth factor receptor 2 OS=Homo sapiens GN=FGFR2		65.01
P21802-7	FGFR2_HUMAN Isoform 7 of Fibroblast growth factor receptor 2 OS=Homo sapiens GN=FGFR2		73.08
P21802-8	FGFR2_HUMAN Isoform 8 of Fibroblast growth factor receptor 2 OS=Homo sapiens GN=FGFR2		65.01
P21802-9	FGFR2_HUMAN Isoform 9 of Fibroblast growth factor receptor 2 OS=Homo sapiens GN=FGFR2		65.01
P21810	PGS1_HUMAN Biglycan OS=Homo sapiens GN=BGN PE=1 SV=2		17.73
P21817	RYR1_HUMAN Ryanodine receptor 1 OS=Homo sapiens GN=RYR1 PE=1 SV=3		
P21817-2	RYR1_HUMAN Isoform 2 of Ryanodine receptor 1 OS=Homo sapiens GN=RYR1		
P21817-3	RYR1_HUMAN Isoform 3 of Ryanodine receptor 1 OS=Homo sapiens GN=RYR1		
P21912	DHSB_HUMAN Succinate dehydrogenase [ubiquinone] iron-sulfur subunit, mitochondrial OS=Homo sapiens GN=SDHB PE=1 SV=3		413.68
P21926	CD9_HUMAN CD9 antigen OS=Homo sapiens GN=CD9 PE=1 SV=4		122.15
P21953	ODBB_HUMAN 2-oxoisovalerate dehydrogenase subunit beta, mitochondrial OS=Homo sapiens GN=BCKDHB PE=1 SV=2	7.70E-10	
P21964	COMT_HUMAN Catechol O-methyltransferase OS=Homo sapiens GN=COMT PE=1 SV=2		214.95
P21964-2	COMT_HUMAN Isoform Soluble of Catechol O-methyltransferase OS=Homo sapiens GN=COMT		214.95
P21964;P21964-2	COMT_HUMAN Catechol O-methyltransferase OS=Homo sapiens GN=COMT PE=1 SV=2;>sp|P21964-2|COMT_HUMAN Isoform Soluble	2.41E-60	
P21980	TGM2_HUMAN Protein-glutamine gamma-glutamyltransferase 2 OS=Homo sapiens GN=TGM2 PE=1 SV=2		397.27
P21980-2	TGM2_HUMAN Isoform 2 of Protein-glutamine gamma-glutamyltransferase 2 OS=Homo sapiens GN=TGM2		196.44
P21980-3	TGM2_HUMAN Isoform 3 of Protein-glutamine gamma-glutamyltransferase 2 OS=Homo sapiens GN=TGM2		111.89
P21980;P21980-2	TGM2_HUMAN Protein-glutamine gamma-glutamyltransferase 2 OS=Homo sapiens GN=TGM2 PE=1 SV=2;>sp|P21980-2|TGM2_HUMA	3.58E-292	
P22033	MUTA_HUMAN Methylmalonyl-CoA mutase, mitochondrial OS=Homo sapiens GN=MUT PE=1 SV=4	2.33E-09	
P22059	OSBP1_HUMAN Oxysterol-binding protein 1 OS=Homo sapiens GN=OSBP PE=1 SV=1	3.43E-17	
P22061	PIMT_HUMAN Protein-L-isoaspartate(D-aspartate) O-methyltransferase OS=Homo sapiens GN=PCMT1 PE=1 SV=4		122.42
P22061-2	PIMT_HUMAN Isoform 2 of Protein-L-isoaspartate(D-aspartate) O-methyltransferase OS=Homo sapiens GN=PCMT1		122.42
P22061-2;P22061	2|PIMT_HUMAN Isoform 2 of Protein-L-isoaspartate(D-aspartate) O-methyltransferase OS=Homo sapiens GN=PCMT1;>sp|P22061|PIM	9.25E-43	
P22087	FBRL_HUMAN rRNA 2'-O-methyltransferase fibrillarin OS=Homo sapiens GN=FBL PE=1 SV=2		177.42
P22087;Q96BS4;B4DLD4	FBRL_HUMAN rRNA 2-O-methyltransferase fibrillarin OS=Homo sapiens GN=FBL PE=1 SV=2;>tr|Q96BS4|Q96BS4_HUMAN FBL protei	4.93E-46	
P22090	RS4Y1_HUMAN 40S ribosomal protein S4, Y isoform 1 OS=Homo sapiens GN=RPS4Y1 PE=2 SV=2		309.47
P22102	PUR2_HUMAN Trifunctional purine biosynthetic protein adenosine-3 OS=Homo sapiens GN=GART PE=1 SV=1		63.36
P22105;P22105-3	TENX_HUMAN Tenascin-X OS=Homo sapiens GN=TNXB PE=1 SV=3;>sp|P22105-3|TENX_HUMAN Isoform 3 of Tenascin-X OS=Homo s	3.18E-88	
P22223	CADH3_HUMAN Cadherin-3 OS=Homo sapiens GN=CDH3 PE=1 SV=2	2.06E-35	
P22234	PUR6_HUMAN Multifunctional protein ADE2 OS=Homo sapiens GN=PAICS PE=1 SV=3		157.64
P22234-2	PUR6_HUMAN Isoform 2 of Multifunctional protein ADE2 OS=Homo sapiens GN=PAICS		104.43
P22234;P22234-2	PUR6_HUMAN Multifunctional protein ADE2 OS=Homo sapiens GN=PAICS PE=1 SV=3;>sp|P22234-2|PUR6_HUMAN Isoform 2 of Multi	0	
P22307	NLTP_HUMAN Non-specific lipid-transfer protein OS=Homo sapiens GN=SCP2 PE=1 SV=2		450.56
P22307-2	NLTP_HUMAN Isoform SCP2 of Non-specific lipid-transfer protein OS=Homo sapiens GN=SCP2		400.31

P22307;P22307-2	NLTP_HUMAN Non-specific lipid-transfer protein OS=Homo sapiens GN=SCP2 PE=1 SV=2;>sp|P22307-2|NLTP_HUMAN Isoform SCP2	9.21E-126	
P22314	UBA1_HUMAN Ubiquitin-like modifier-activating enzyme 1 OS=Homo sapiens GN=UBA1 PE=1 SV=3		1136.12
P22352	GPX3_HUMAN Glutathione peroxidase 3 OS=Homo sapiens GN=GPX3 PE=1 SV=2	4.03E-20	
P22392	NDKB_HUMAN Nucleoside diphosphate kinase B OS=Homo sapiens GN=NME2 PE=1 SV=1		813.02
P22392-2	NDKB_HUMAN Isoform 3 of Nucleoside diphosphate kinase B OS=Homo sapiens GN=NME2		996.81
P22392-2;P22392	2|NDKB_HUMAN Isoform 3 of Nucleoside diphosphate kinase B OS=Homo sapiens GN=NME2;>sp|P22392|NDKB_HUMAN Nucleoside c	1.62E-275	
P22413	ENPP1_HUMAN Ectonucleotide pyrophosphatase/phosphodiesterase family member 1 OS=Homo sapiens GN=ENPP1 PE=1 SV=2		692.53
P22455	FGFR4_HUMAN Fibroblast growth factor receptor 4 OS=Homo sapiens GN=FGFR4 PE=1 SV=2		84.48
P22455-2	FGFR4_HUMAN Isoform 2 of Fibroblast growth factor receptor 4 OS=Homo sapiens GN=FGFR4		84.48
P22492	H1T_HUMAN Histone H1t OS=Homo sapiens GN=HIST1H1T PE=1 SV=4		160.05
P22528	SPR1B_HUMAN Cornifin-B OS=Homo sapiens GN=SPRR1B PE=1 SV=2		
P22531	SPR2E_HUMAN Small proline-rich protein 2E OS=Homo sapiens GN=SPRR2E PE=2 SV=2		23.4
P22532	SPR2D_HUMAN Small proline-rich protein 2D OS=Homo sapiens GN=SPRR2D PE=2 SV=2		23.4
P22570	ADRO_HUMAN NADPH:adrenodoxin oxidoreductase, mitochondrial OS=Homo sapiens GN=FDXR PE=1 SV=3		
P22570-2	ADRO_HUMAN Isoform Long of NADPH:adrenodoxin oxidoreductase, mitochondrial OS=Homo sapiens GN=FDXR		
P22570-2;P22570	2|ADRO_HUMAN Isoform Long of NADPH:adrenodoxin oxidoreductase, mitochondrial OS=Homo sapiens GN=FDXR;>sp|P22570|ADRO	3.09E-16	
P22570;P22570-2	ADRO_HUMAN NADPH:adrenodoxin oxidoreductase, mitochondrial OS=Homo sapiens GN=FDXR PE=1 SV=3;>sp|P22570-2|ADRO_HU	4.43E-107	
P22607	FGFR3_HUMAN Fibroblast growth factor receptor 3 OS=Homo sapiens GN=FGFR3 PE=1 SV=1		49.3
P22607-2	FGFR3_HUMAN Isoform 2 of Fibroblast growth factor receptor 3 OS=Homo sapiens GN=FGFR3		49.3
P22607-3	FGFR3_HUMAN Isoform 3 of Fibroblast growth factor receptor 3 OS=Homo sapiens GN=FGFR3		49.3
P22607-4	FGFR3_HUMAN Isoform 4 of Fibroblast growth factor receptor 3 OS=Homo sapiens GN=FGFR3		49.3
P22612	KAPCG_HUMAN cAMP-dependent protein kinase catalytic subunit gamma OS=Homo sapiens GN=PRKACG PE=1 SV=3		19.63
P22626	ROA2_HUMAN Heterogeneous nuclear ribonucleoproteins A2/B1 OS=Homo sapiens GN=HNRNPA2B1 PE=1 SV=2		1151.26
P22626-2	ROA2_HUMAN Isoform A2 of Heterogeneous nuclear ribonucleoproteins A2/B1 OS=Homo sapiens GN=HNRNPA2B1		1146.02
P22626;P22626-2	ROA2_HUMAN Heterogeneous nuclear ribonucleoproteins A2/B1 OS=Homo sapiens GN=HNRNPA2B1 PE=1 SV=2;>sp|P22626-2|ROA2	0	
P22670	RFX1_HUMAN MHC class II regulatory factor RFX1 OS=Homo sapiens GN=RFX1 PE=1 SV=2		
P22676	CALB2_HUMAN Calretinin OS=Homo sapiens GN=CALB2 PE=1 SV=2		32.25
P22681	CBL_HUMAN E3 ubiquitin-protein ligase CBL OS=Homo sapiens GN=CBL PE=1 SV=2	2.98E-09	
P22692	IBP4_HUMAN Insulin-like growth factor-binding protein 4 OS=Homo sapiens GN=IGFBP4 PE=1 SV=2		159.78
P22694	KAPCB_HUMAN cAMP-dependent protein kinase catalytic subunit beta OS=Homo sapiens GN=PRKACB PE=1 SV=2		73.46
P22694-2	KAPCB_HUMAN Isoform 2 of cAMP-dependent protein kinase catalytic subunit beta OS=Homo sapiens GN=PRKACB		73.46
P22694-2;P22694-6;P226	2|KAPCB_HUMAN Isoform 2 of cAMP-dependent protein kinase catalytic subunit beta OS=Homo sapiens GN=PRKACB;>sp|P22694-6|K	1.90E-32	
P22694-3	KAPCB_HUMAN Isoform 3 of cAMP-dependent protein kinase catalytic subunit beta OS=Homo sapiens GN=PRKACB		73.46
P22694-4	KAPCB_HUMAN Isoform 4 of cAMP-dependent protein kinase catalytic subunit beta OS=Homo sapiens GN=PRKACB		73.46
P22694-5	KAPCB_HUMAN Isoform 5 of cAMP-dependent protein kinase catalytic subunit beta OS=Homo sapiens GN=PRKACB		73.46
P22694-6	KAPCB_HUMAN Isoform 6 of cAMP-dependent protein kinase catalytic subunit beta OS=Homo sapiens GN=PRKACB		73.46
P22694-7	KAPCB_HUMAN Isoform 7 of cAMP-dependent protein kinase catalytic subunit beta OS=Homo sapiens GN=PRKACB		73.46
P22694-8	KAPCB_HUMAN Isoform 8 of cAMP-dependent protein kinase catalytic subunit beta OS=Homo sapiens GN=PRKACB		18.41
P22695	QCR2_HUMAN Cytochrome b-c1 complex subunit 2, mitochondrial OS=Homo sapiens GN=UQCRC2 PE=1 SV=3		446.83
P22792	CPN2_HUMAN Carboxypeptidase N subunit 2 OS=Homo sapiens GN=CPN2 PE=1 SV=3	1.02E-05	
P22830-2;P22830	2|HEMH_HUMAN Isoform 2 of Ferrochelatase, mitochondrial OS=Homo sapiens GN=FECH;>sp|P22830|HEMH_HUMAN Ferrochelatase,	3.37E-05	
P22897	MRC1_HUMAN Macrophage mannose receptor 1 OS=Homo sapiens GN=MRC1 PE=1 SV=1	2.89E-09	
P23141	EST1_HUMAN Liver carboxylesterase 1 OS=Homo sapiens GN=CES1 PE=1 SV=2		
P23141-2	EST1_HUMAN Isoform 2 of Liver carboxylesterase 1 OS=Homo sapiens GN=CES1		
P23141-2;P23141	2|EST1_HUMAN Isoform 2 of Liver carboxylesterase 1 OS=Homo sapiens GN=CES1;>sp|P23141|EST1_HUMAN Liver carboxylesterase	5.79E-99	
P23142-4	4|FBLN1_HUMAN Isoform C of Fibulin-1 OS=Homo sapiens GN=FBLN1	0	
P23142;P23142-3;P23142	FBLN1_HUMAN Fibulin-1 OS=Homo sapiens GN=FBLN1 PE=1 SV=4;>sp|P23142-3|FBLN1_HUMAN Isoform B of Fibulin-1 OS=Homo sa	0	
P23193	TCEA1_HUMAN Transcription elongation factor A protein 1 OS=Homo sapiens GN=TCEA1 PE=1 SV=2		61.58
P23193-2	TCEA1_HUMAN Isoform 2 of Transcription elongation factor A protein 1 OS=Homo sapiens GN=TCEA1		78.33
P23193;B7Z4W0;P23193-	TCEA1_HUMAN Transcription elongation factor A protein 1 OS=Homo sapiens GN=TCEA1 PE=1 SV=2;>tr|B7Z4W0|B7Z4W0_HUMAN c	3.40E-76	
P23229	ITA6_HUMAN Integrin alpha-6 OS=Homo sapiens GN=ITGA6 PE=1 SV=4		799.77
P23229-2	ITA6_HUMAN Isoform Alpha-6X1A of Integrin alpha-6 OS=Homo sapiens GN=ITGA6		812.95
P23229-3	ITA6_HUMAN Isoform Alpha-6X1B of Integrin alpha-6 OS=Homo sapiens GN=ITGA6		799.77
P23229-4	ITA6_HUMAN Isoform Alpha-6X2A of Integrin alpha-6 OS=Homo sapiens GN=ITGA6		812.95
P23229-5	ITA6_HUMAN Isoform Alpha-6X2B of Integrin alpha-6 OS=Homo sapiens GN=ITGA6		799.77
P23229-6	ITA6_HUMAN Isoform Alpha-6X1X2A of Integrin alpha-6 OS=Homo sapiens GN=ITGA6		812.95
P23229-6;P23229-9;P232	6|ITA6_HUMAN Isoform Alpha-6X1X2A of Integrin alpha-6 OS=Homo sapiens GN=ITGA6;>sp|P23229-9|ITA6_HUMAN Isoform 9 of In	0	
P23229-7	ITA6_HUMAN Isoform 7 of Integrin alpha-6 OS=Homo sapiens GN=ITGA6		707.09
P23229-9	ITA6_HUMAN Isoform 9 of Integrin alpha-6 OS=Homo sapiens GN=ITGA6		812.95
P23229;P23229-3;P23229	ITA6_HUMAN Integrin alpha-6 OS=Homo sapiens GN=ITGA6 PE=1 SV=4;>sp|P23229-3|ITA6_HUMAN Isoform Alpha-6X1B of Integrin	0	
P23246	SFPQ_HUMAN Splicing factor, proline- and glutamine-rich OS=Homo sapiens GN=SFPQ PE=1 SV=2		1126.52

P23246-2	SFPQ_HUMAN Isoform Short of Splicing factor, proline- and glutamine-rich OS=Homo sapiens GN=SFPQ		1012.19
P23246;P23246-2	SFPQ_HUMAN Splicing factor, proline- and glutamine-rich OS=Homo sapiens GN=SFPQ PE=1 SV=2;>sp|P23246-2|SFPQ_HUMAN Isofo	0	
P23258	TBG1_HUMAN Tubulin gamma-1 chain OS=Homo sapiens GN=TUBG1 PE=1 SV=2		30.66
P23284	PPIB_HUMAN Peptidyl-prolyl cis-trans isomerase B OS=Homo sapiens GN=PPIB PE=1 SV=2		1137.2
P23297	S10A1_HUMAN Protein S100-A1 OS=Homo sapiens GN=S100A1 PE=1 SV=2	2.79E-64	
P23368	MAOM_HUMAN NAD-dependent malic enzyme, mitochondrial OS=Homo sapiens GN=ME2 PE=1 SV=1		104.88
P23378	GCSP_HUMAN Glycine dehydrogenase [decarboxylating], mitochondrial OS=Homo sapiens GN=GLDC PE=1 SV=2	2.16E-14	
P23381	SYWC_HUMAN Tryptophanyl-tRNA synthetase, cytoplasmic OS=Homo sapiens GN=WARS PE=1 SV=2		73.99
P23381-2	SYWC_HUMAN Isoform 2 of Tryptophanyl-tRNA synthetase, cytoplasmic OS=Homo sapiens GN=WARS		73.99
P23381;P23381-2	SYWC_HUMAN Tryptophanyl-tRNA synthetase, cytoplasmic OS=Homo sapiens GN=WARS PE=1 SV=2;>sp|P23381-2|SYWC_HUMAN Is	0	
P23396	RS3_HUMAN 40S ribosomal protein S3 OS=Homo sapiens GN=RPS3 PE=1 SV=2		1481.05
P23434	GCSH_HUMAN Glycine cleavage system H protein, mitochondrial OS=Homo sapiens GN=GCSH PE=1 SV=2		
P23443;P23443-2	KS6B1_HUMAN Ribosomal protein S6 kinase beta-1 OS=Homo sapiens GN=RPS6KB1 PE=1 SV=2;>sp|P23443-2|KS6B1_HUMAN Isofo	2.88E-31	
P23458	JAK1_HUMAN Tyrosine-protein kinase JAK1 OS=Homo sapiens GN=JAK1 PE=1 SV=2		71.51
P23468;P23468-2	PTPRD_HUMAN Receptor-type tyrosine-protein phosphatase delta OS=Homo sapiens GN=PTPRD PE=1 SV=2;>sp|P23468-2|PTPRD_H	4.02E-11	
P23470;P23470-2	PTPRG_HUMAN Receptor-type tyrosine-protein phosphatase gamma OS=Homo sapiens GN=PTPRG PE=1 SV=4;>sp|P23470-2|PTPRG_	3.85E-06	
P23497	SP100_HUMAN Nuclear autoantigen Sp-100 OS=Homo sapiens GN=SP100 PE=1 SV=3		75.36
P23497-2	SP100_HUMAN Isoform Sp100-A of Nuclear autoantigen Sp-100 OS=Homo sapiens GN=SP100		53.24
P23497-3	SP100_HUMAN Isoform Sp100-B of Nuclear autoantigen Sp-100 OS=Homo sapiens GN=SP100		53.24
P23497-4	SP100_HUMAN Isoform Sp100-C of Nuclear autoantigen Sp-100 OS=Homo sapiens GN=SP100		53.4
P23497-5	SP100_HUMAN Isoform SpAlt-C of Nuclear autoantigen Sp-100 OS=Homo sapiens GN=SP100		53.24
P23497;P23497-4;P23497	SP100_HUMAN Nuclear autoantigen Sp-100 OS=Homo sapiens GN=SP100 PE=1 SV=3;>sp|P23497-4|SP100_HUMAN Isoform Sp100-	7.08E-20	
P23511	NFYA_HUMAN Nuclear transcription factor Y subunit alpha OS=Homo sapiens GN=NFYA PE=1 SV=2		67.3
P23511-2	NFYA_HUMAN Isoform Short of Nuclear transcription factor Y subunit alpha OS=Homo sapiens GN=NFYA		67.3
P23511;P23511-2	NFYA_HUMAN Nuclear transcription factor Y subunit alpha OS=Homo sapiens GN=NFYA PE=1 SV=2;>sp|P23511-2|NFYA_HUMAN Isof	5.58E-08	
P23526	SAHH_HUMAN Adenosylhomocysteinase OS=Homo sapiens GN=AHCY PE=1 SV=4		353.82
P23527	H2B1O_HUMAN Histone H2B type 1-O OS=Homo sapiens GN=HIST1H2BO PE=1 SV=3		732.38
P23528	COF1_HUMAN Cofilin-1 OS=Homo sapiens GN=CFL1 PE=1 SV=3		922.05
P23588	IF4B_HUMAN Eukaryotic translation initiation factor 4B OS=Homo sapiens GN=EIF4B PE=1 SV=2		266.91
P23634	AT2B4_HUMAN Plasma membrane calcium-transporting ATPase 4 OS=Homo sapiens GN=ATP2B4 PE=1 SV=2		754.45
P23634-2	AT2B4_HUMAN Isoform XA of Plasma membrane calcium-transporting ATPase 4 OS=Homo sapiens GN=ATP2B4		754.45
P23634-3	AT2B4_HUMAN Isoform ZA of Plasma membrane calcium-transporting ATPase 4 OS=Homo sapiens GN=ATP2B4		754.45
P23634-4	AT2B4_HUMAN Isoform XK of Plasma membrane calcium-transporting ATPase 4 OS=Homo sapiens GN=ATP2B4		754.45
P23634-5	AT2B4_HUMAN Isoform ZK of Plasma membrane calcium-transporting ATPase 4 OS=Homo sapiens GN=ATP2B4		754.45
P23634-6	AT2B4_HUMAN Isoform XB of Plasma membrane calcium-transporting ATPase 4 OS=Homo sapiens GN=ATP2B4		754.45
P23634-7	AT2B4_HUMAN Isoform ZB of Plasma membrane calcium-transporting ATPase 4 OS=Homo sapiens GN=ATP2B4		754.45
P23634-8	AT2B4_HUMAN Isoform ZD of Plasma membrane calcium-transporting ATPase 4 OS=Homo sapiens GN=ATP2B4		754.45
P23634;P23634-8;P23634	AT2B4_HUMAN Plasma membrane calcium-transporting ATPase 4 OS=Homo sapiens GN=ATP2B4 PE=1 SV=2;>sp|P23634-8|AT2B4_H	9.90E-158	
P23743	DGKA_HUMAN Diacylglycerol kinase alpha OS=Homo sapiens GN=DGKA PE=1 SV=3		23.9
P23760	PAX3_HUMAN Paired box protein Pax-3 OS=Homo sapiens GN=PAX3 PE=1 SV=2		
P23760-2	PAX3_HUMAN Isoform Pax3A of Paired box protein Pax-3 OS=Homo sapiens GN=PAX3		
P23760-3	PAX3_HUMAN Isoform Pax3B of Paired box protein Pax-3 OS=Homo sapiens GN=PAX3		
P23763	VAMP1_HUMAN Vesicle-associated membrane protein 1 OS=Homo sapiens GN=VAMP1 PE=1 SV=1		52.49
P23763-2	VAMP1_HUMAN Isoform 3 of Vesicle-associated membrane protein 1 OS=Homo sapiens GN=VAMP1		52.49
P23763-3	VAMP1_HUMAN Isoform 2 of Vesicle-associated membrane protein 1 OS=Homo sapiens GN=VAMP1		52.49
P23771-2;P23771	2|GATA3_HUMAN Isoform 2 of Trans-acting T-cell-specific transcription factor GATA-3 OS=Homo sapiens GN=GATA3;>sp|P23771|GATA	1.11E-69	
P23786	CPT2_HUMAN Carnitine O-palmitoyltransferase 2, mitochondrial OS=Homo sapiens GN=CPT2 PE=1 SV=2		
P23919	KTHY_HUMAN Thymidylate kinase OS=Homo sapiens GN=DTYMK PE=1 SV=4		190.13
P23921	RIR1_HUMAN Ribonucleoside-diphosphate reductase large subunit OS=Homo sapiens GN=RRM1 PE=1 SV=1	1.60E-90	
P23942	PRPH2_HUMAN Peripherin-2 OS=Homo sapiens GN=PRPH2 PE=1 SV=1		
P23946	CMA1_HUMAN Chymase OS=Homo sapiens GN=CMA1 PE=1 SV=1	2.38E-24	
P24043	LAMA2_HUMAN Laminin subunit alpha-2 OS=Homo sapiens GN=LAMA2 PE=1 SV=4		
P24046	GBRR1_HUMAN Gamma-aminobutyric acid receptor subunit rho-1 OS=Homo sapiens GN=GABRR1 PE=2 SV=2		
P24046-2	GBRR1_HUMAN Isoform 2 of Gamma-aminobutyric acid receptor subunit rho-1 OS=Homo sapiens GN=GABRR1		
P24298	ALAT1_HUMAN Alanine aminotransferase 1 OS=Homo sapiens GN=GPT PE=1 SV=3		
P24385	CCND1_HUMAN G1/S-specific cyclin-D1 OS=Homo sapiens GN=CCND1 PE=1 SV=1	3.58E-39	
P24386;P26374	RAE1_HUMAN Rab proteins geranylgeranyltransferase component A 1 OS=Homo sapiens GN=CHM PE=1 SV=3;>sp|P26374|RAE2_HU	1.24E-10	
P24390	ERD21_HUMAN ER lumen protein retaining receptor 1 OS=Homo sapiens GN=KDELR1 PE=1 SV=1		77.78
P24468	COT2_HUMAN COUP transcription factor 2 OS=Homo sapiens GN=NR2F2 PE=1 SV=1		57.79
P24534	EF1B_HUMAN Elongation factor 1-beta OS=Homo sapiens GN=EEF1B2 PE=1 SV=3		593.61
P24539	AT5F1_HUMAN ATP synthase subunit b, mitochondrial OS=Homo sapiens GN=ATP5F1 PE=1 SV=2		206.02

P24588	AKAP5_HUMAN A-kinase anchor protein 5 OS=Homo sapiens GN=AKAP5 PE=1 SV=3		
P24592	IBP6_HUMAN Insulin-like growth factor-binding protein 6 OS=Homo sapiens GN=IGFBP6 PE=1 SV=1		27.65
P24593	IBP5_HUMAN Insulin-like growth factor-binding protein 5 OS=Homo sapiens GN=IGFBP5 PE=1 SV=1	1.82E-05	
P24666	PPAC_HUMAN Low molecular weight phosphotyrosine protein phosphatase OS=Homo sapiens GN=ACP1 PE=1 SV=3		
P24666-2	PPAC_HUMAN Isoform 2 of Low molecular weight phosphotyrosine protein phosphatase OS=Homo sapiens GN=ACP1		
P24666-2;D3YTI2	2|PPAC_HUMAN Isoform 2 of Low molecular weight phosphotyrosine protein phosphatase OS=Homo sapiens GN=ACP1;>tr|D3YTI2|D3	2.77E-36	
P24666-3	PPAC_HUMAN Isoform 3 of Low molecular weight phosphotyrosine protein phosphatase OS=Homo sapiens GN=ACP1		
P24666;P24666-2;P24666	PPAC_HUMAN Low molecular weight phosphotyrosine protein phosphatase OS=Homo sapiens GN=ACP1 PE=1 SV=3;>sp|P24666-2|PF	3.20E-32	
P24752	THIL_HUMAN Acetyl-CoA acetyltransferase, mitochondrial OS=Homo sapiens GN=ACAT1 PE=1 SV=1		631.48
P24821-4;P24821-2	4|TENA_HUMAN Isoform 4 of Tenascin OS=Homo sapiens GN=TNC;>sp|P24821-2|TENA_HUMAN Isoform 2 of Tenascin OS=Homo sap	0	
P24821-5	5|TENA_HUMAN Isoform 5 of Tenascin OS=Homo sapiens GN=TNC	0	
P24821;P24821-3	TENA_HUMAN Tenascin OS=Homo sapiens GN=TNC PE=1 SV=3;>sp|P24821-3|TENA_HUMAN Isoform 3 of Tenascin OS=Homo sapien	0	
P24844	MYL9_HUMAN Myosin regulatory light polypeptide 9 OS=Homo sapiens GN=MYL9 PE=1 SV=4		810.15
P24863	CCNC_HUMAN Cyclin-C OS=Homo sapiens GN=CCNC PE=1 SV=2		
P24928	RPB1_HUMAN DNA-directed RNA polymerase II subunit RPB1 OS=Homo sapiens GN=POLR2A PE=1 SV=2		229.17
P24941	CDK2_HUMAN Cyclin-dependent kinase 2 OS=Homo sapiens GN=CDK2 PE=1 SV=2		180.97
P24941;B4DDL9	CDK2_HUMAN Cell division protein kinase 2 OS=Homo sapiens GN=CDK2 PE=1 SV=2;>tr|B4DDL9|B4DDL9_HUMAN cDNA FLJ54979,	1.39E-14	
P24941;Q00526	CDK2_HUMAN Cyclin-dependent kinase 2 OS=Homo sapiens GN=CDK2 PE=1 SV=2;>sp|Q00526|CDK3_HUMAN Cyclin-dependent kina	1.42E-52	
P25098;P35626	ARBK1_HUMAN Beta-adrenergic receptor kinase 1 OS=Homo sapiens GN=ADRBK1 PE=1 SV=2;>sp|P35626|ARBK2_HUMAN Beta-adre	1.15E-18	
P25205	MCM3_HUMAN DNA replication licensing factor MCM3 OS=Homo sapiens GN=MCM3 PE=1 SV=3		462.86
P25311	ZA2G_HUMAN Zinc-alpha-2-glycoprotein OS=Homo sapiens GN=AZGP1 PE=1 SV=2	7.37E-218	
P25325	THTM_HUMAN 3-mercaptopyruvate sulfurtransferase OS=Homo sapiens GN=MPST PE=1 SV=3		54.66
P25398	RS12_HUMAN 40S ribosomal protein S12 OS=Homo sapiens GN=RPS12 PE=1 SV=3		497.97
P25445;P25445-6	TNR6_HUMAN Tumor necrosis factor receptor superfamily member 6 OS=Homo sapiens GN=FAS PE=1 SV=1;>sp|P25445-6|TNR6_HU	4.05E-09	
P25490	TYY1_HUMAN Transcriptional repressor protein YY1 OS=Homo sapiens GN=YY1 PE=1 SV=2		
P25685	DNJB1_HUMAN DnaJ homolog subfamily B member 1 OS=Homo sapiens GN=DNAJB1 PE=1 SV=4		180.32
P25686	DNJB2_HUMAN DnaJ homolog subfamily B member 2 OS=Homo sapiens GN=DNAJB2 PE=2 SV=3		
P25686-2	DNJB2_HUMAN Isoform 2 of DnaJ homolog subfamily B member 2 OS=Homo sapiens GN=DNAJB2		
P25705	ATPA_HUMAN ATP synthase subunit alpha, mitochondrial OS=Homo sapiens GN=ATP5A1 PE=1 SV=1		1153.74
P25774	CATS_HUMAN Cathepsin S OS=Homo sapiens GN=CTSS PE=1 SV=3		48.17
P25786	PSA1_HUMAN Proteasome subunit alpha type-1 OS=Homo sapiens GN=PSMA1 PE=1 SV=1		689.28
P25786-2	PSA1_HUMAN Isoform Long of Proteasome subunit alpha type-1 OS=Homo sapiens GN=PSMA1		689.28
P25786-2;P25786	2|PSA1_HUMAN Isoform Long of Proteasome subunit alpha type-1 OS=Homo sapiens GN=PSMA1;>sp|P25786|PSA1_HUMAN Proteasc	5.96E-178	
P25787	PSA2_HUMAN Proteasome subunit alpha type-2 OS=Homo sapiens GN=PSMA2 PE=1 SV=2		672.83
P25788	PSA3_HUMAN Proteasome subunit alpha type-3 OS=Homo sapiens GN=PSMA3 PE=1 SV=2		360.51
P25788-2	PSA3_HUMAN Isoform 2 of Proteasome subunit alpha type-3 OS=Homo sapiens GN=PSMA3		360.51
P25788;P25788-2	PSA3_HUMAN Proteasome subunit alpha type-3 OS=Homo sapiens GN=PSMA3 PE=1 SV=2;>sp|P25788-2|PSA3_HUMAN Isoform 2 of	2.79E-147	
P25789	PSA4_HUMAN Proteasome subunit alpha type-4 OS=Homo sapiens GN=PSMA4 PE=1 SV=1		446.36
P25815	S100P_HUMAN Protein S100-P OS=Homo sapiens GN=S100P PE=1 SV=2		
P25940	CO5A3_HUMAN Collagen alpha-3(V) chain OS=Homo sapiens GN=COL5A3 PE=1 SV=3		28.1
P26006	ITA3_HUMAN Integrin alpha-3 OS=Homo sapiens GN=ITGA3 PE=1 SV=4		952.6
P26006-1;P26006	1|ITA3_HUMAN Isoform 2 of Integrin alpha-3 OS=Homo sapiens GN=ITGA3;>sp|P26006|ITA3_HUMAN Integrin alpha-3 OS=Homo sa	1.29E-219	
P26006-2	ITA3_HUMAN Isoform Alpha-3A of Integrin alpha-3 OS=Homo sapiens GN=ITGA3		952.6
P26022	PTX3_HUMAN Pentraxin-related protein PTX3 OS=Homo sapiens GN=PTX3 PE=1 SV=3		33.85
P26038	MOES_HUMAN Moesin OS=Homo sapiens GN=MSN PE=1 SV=3		3902.81
P26196	DDX6_HUMAN Probable ATP-dependent RNA helicase DDX6 OS=Homo sapiens GN=DDX6 PE=1 SV=2		275.69
P26232	CTNA2_HUMAN Catenin alpha-2 OS=Homo sapiens GN=CTNNA2 PE=1 SV=5		32.97
P26232-2	CTNA2_HUMAN Isoform 2 of Catenin alpha-2 OS=Homo sapiens GN=CTNNA2		32.97
P26232-3	CTNA2_HUMAN Isoform 3 of Catenin alpha-2 OS=Homo sapiens GN=CTNNA2		32.97
P26232-4	CTNA2_HUMAN Isoform 4 of Catenin alpha-2 OS=Homo sapiens GN=CTNNA2		10.63
P26232-5	CTNA2_HUMAN Isoform 5 of Catenin alpha-2 OS=Homo sapiens GN=CTNNA2		39.42
P26232-6	CTNA2_HUMAN Isoform 6 of Catenin alpha-2 OS=Homo sapiens GN=CTNNA2		10.63
P26232;P26232-5;P26232	CTNA2_HUMAN Catenin alpha-2 OS=Homo sapiens GN=CTNNA2 PE=1 SV=5;>sp|P26232-5|CTNA2_HUMAN Isoform 5 of Catenin alph	3.17E-21	
P26358	DNMT1_HUMAN DNA (cytosine-5)-methyltransferase 1 OS=Homo sapiens GN=DNMT1 PE=1 SV=2		736.05
P26358-2	DNMT1_HUMAN Isoform 2 of DNA (cytosine-5)-methyltransferase 1 OS=Homo sapiens GN=DNMT1		723.05
P26358-2;P26358;P26358	2|DNMT1_HUMAN Isoform 2 of DNA (cytosine-5)-methyltransferase 1 OS=Homo sapiens GN=DNMT1;>sp|P26358|DNMT1_HUMAN DN	2.63E-118	
P26358-3	DNMT1_HUMAN Isoform 3 of DNA (cytosine-5)-methyltransferase 1 OS=Homo sapiens GN=DNMT1		516.3
P26368	U2AF2_HUMAN Splicing factor U2AF 65 kDa subunit OS=Homo sapiens GN=U2AF2 PE=1 SV=4		355.81
P26368-2	U2AF2_HUMAN Isoform 2 of Splicing factor U2AF 65 kDa subunit OS=Homo sapiens GN=U2AF2		355.81
P26368;P26368-2	U2AF2_HUMAN Splicing factor U2AF 65 kDa subunit OS=Homo sapiens GN=U2AF2 PE=1 SV=4;>sp|P26368-2|U2AF2_HUMAN Isoform	0	
P26373	RL13_HUMAN 60S ribosomal protein L13 OS=Homo sapiens GN=RPL13 PE=1 SV=4		663.06

P26378	ELAV4_HUMAN ELAV-like protein 4 OS=Homo sapiens GN=ELAVL4 PE=1 SV=2		
P26378-2	ELAV4_HUMAN Isoform 2 of ELAV-like protein 4 OS=Homo sapiens GN=ELAVL4		
P26378-3	ELAV4_HUMAN Isoform 3 of ELAV-like protein 4 OS=Homo sapiens GN=ELAVL4		
P26378-4	ELAV4_HUMAN Isoform 4 of ELAV-like protein 4 OS=Homo sapiens GN=ELAVL4		
P26439	3BHS2_HUMAN 3 beta-hydroxysteroid dehydrogenase/Delta 5-->4-isomerase type 2 OS=Homo sapiens GN=HSD3B2 PE=1 SV=2		5.47
P26440	IVD_HUMAN Isovaleryl-CoA dehydrogenase, mitochondrial OS=Homo sapiens GN=IVD PE=1 SV=1		62.71
P26447	S10A4_HUMAN Protein S100-A4 OS=Homo sapiens GN=S100A4 PE=1 SV=1		71.81
P26583	HMGB2_HUMAN High mobility group protein B2 OS=Homo sapiens GN=HMGB2 PE=1 SV=2		669.74
P26599	PTBP1_HUMAN Polypyrimidine tract-binding protein 1 OS=Homo sapiens GN=PTBP1 PE=1 SV=1		1060.03
P26599-2	PTBP1_HUMAN Isoform 2 of Polypyrimidine tract-binding protein 1 OS=Homo sapiens GN=PTBP1		1060.03
P26599-2;P26599	2|PTBP1_HUMAN Isoform 2 of Polypyrimidine tract-binding protein 1 OS=Homo sapiens GN=PTBP1;>sp|P26599|PTBP1_HUMAN Polyp	0	
P26639	SYTC_HUMAN Threonyl-tRNA synthetase, cytoplasmic OS=Homo sapiens GN=TARS PE=1 SV=3		670.45
P26640	SYVC_HUMAN Valyl-tRNA synthetase OS=Homo sapiens GN=VARS PE=1 SV=4		888.51
P26641	EF1G_HUMAN Elongation factor 1-gamma OS=Homo sapiens GN=EEF1G PE=1 SV=3		1100.96
P26885	FKBP2_HUMAN Peptidyl-prolyl cis-trans isomerase FKBP2 OS=Homo sapiens GN=FKBP2 PE=1 SV=2		227.42
P27105	STOM_HUMAN Erythrocyte band 7 integral membrane protein OS=Homo sapiens GN=STOM PE=1 SV=3		125.83
P27144	KAD4_HUMAN Adenylate kinase isoenzyme 4, mitochondrial OS=Homo sapiens GN=AK4 PE=1 SV=1		492.91
P27169	PON1_HUMAN Serum paraoxonase/arylesterase 1 OS=Homo sapiens GN=PON1 PE=1 SV=3	1.01E-15	
P27216-2;P27216	2|ANX13_HUMAN Isoform B of Annexin A13 OS=Homo sapiens GN=ANXA13;>sp|P27216|ANX13_HUMAN Annexin A13 OS=Homo sap	7.30E-08	
P27338	AOFB_HUMAN Amine oxidase [flavin-containing] B OS=Homo sapiens GN=MAOB PE=1 SV=3	2.01E-62	
P27348	1433T_HUMAN 14-3-3 protein theta OS=Homo sapiens GN=YWHAQ PE=1 SV=1		1143.1
P27352	IF_HUMAN Gastric intrinsic factor OS=Homo sapiens GN=GIF PE=1 SV=2		22.14
P27352-2	IF_HUMAN Isoform 2 of Gastric intrinsic factor OS=Homo sapiens GN=GIF		22.14
P27361	MK03_HUMAN Mitogen-activated protein kinase 3 OS=Homo sapiens GN=MAPK3 PE=1 SV=4		27.93
P27449	VATL_HUMAN V-type proton ATPase 16 kDa proteolipid subunit OS=Homo sapiens GN=ATP6V0C PE=1 SV=1		56.88
P27482	CALL3_HUMAN Calmodulin-like protein 3 OS=Homo sapiens GN=CALML3 PE=1 SV=2		14.07
P27635	RL10_HUMAN 60S ribosomal protein L10 OS=Homo sapiens GN=RPL10 PE=1 SV=4		1138.91
P27658	CO8A1_HUMAN Collagen alpha-1(VIII) chain OS=Homo sapiens GN=COL8A1 PE=1 SV=2		97.02
P27694	RFA1_HUMAN Replication protein A 70 kDa DNA-binding subunit OS=Homo sapiens GN=RPA1 PE=1 SV=2		326.3
P27695	APEX1_HUMAN DNA-(apurinic or apyrimidinic site) lyase OS=Homo sapiens GN=APEX1 PE=1 SV=2		623.31
P27701	CD82_HUMAN CD82 antigen OS=Homo sapiens GN=CD82 PE=1 SV=1		186.28
P27707	DCK_HUMAN Deoxycytidine kinase OS=Homo sapiens GN=DCK PE=1 SV=1	5.35E-36	
P27708	PYR1_HUMAN CAD protein OS=Homo sapiens GN=CAD PE=1 SV=3		116.29
P27797	CALR_HUMAN Calreticulin OS=Homo sapiens GN=CALR PE=1 SV=1		1143.12
P27816	MAP4_HUMAN Microtubule-associated protein 4 OS=Homo sapiens GN=MAP4 PE=1 SV=3		1481.49
P27816-2	MAP4_HUMAN Isoform 2 of Microtubule-associated protein 4 OS=Homo sapiens GN=MAP4		1283.22
P27816-4	MAP4_HUMAN Isoform 4 of Microtubule-associated protein 4 OS=Homo sapiens GN=MAP4		552.76
P27816-5	MAP4_HUMAN Isoform 5 of Microtubule-associated protein 4 OS=Homo sapiens GN=MAP4		774.5
P27816-6	MAP4_HUMAN Isoform 6 of Microtubule-associated protein 4 OS=Homo sapiens GN=MAP4		1481.49
P27816;P27816-6;P27816	MAP4_HUMAN Microtubule-associated protein 4 OS=Homo sapiens GN=MAP4 PE=1 SV=3;>sp|P27816-6|MAP4_HUMAN Isoform 6 of M	0	
P27824	CALX_HUMAN Calnexin OS=Homo sapiens GN=CANX PE=1 SV=2		1487.72
P27986-4;P27986	4|P85A_HUMAN Isoform 4 of Phosphatidylinositol 3-kinase regulatory subunit alpha OS=Homo sapiens GN=PIK3R1;>sp|P27986|P85A	1.54E-13	
P28062	PSB8_HUMAN Proteasome subunit beta type-8 OS=Homo sapiens GN=PSMB8 PE=1 SV=3		278.16
P28062-2	PSB8_HUMAN Isoform 2 of Proteasome subunit beta type-8 OS=Homo sapiens GN=PSMB8		282.78
P28062;P28062-2	PSB8_HUMAN Proteasome subunit beta type-8 OS=Homo sapiens GN=PSMB8 PE=1 SV=3;>sp|P28062-2|PSB8_HUMAN Isoform 2 of I	3.03E-55	
P28065	PSB9_HUMAN Proteasome subunit beta type-9 OS=Homo sapiens GN=PSMB9 PE=1 SV=2		121.49
P28065-2	PSB9_HUMAN Isoform LMP2.S of Proteasome subunit beta type-9 OS=Homo sapiens GN=PSMB9		121.49
P28065;P28065-2	PSB9_HUMAN Proteasome subunit beta type-9 OS=Homo sapiens GN=PSMB9 PE=1 SV=2;>sp|P28065-2|PSB9_HUMAN Isoform LMP2	7.46E-24	
P28066	PSA5_HUMAN Proteasome subunit alpha type-5 OS=Homo sapiens GN=PSMA5 PE=1 SV=3		467.32
P28068	DMB_HUMAN HLA class II histocompatibility antigen, DM beta chain OS=Homo sapiens GN=HLA-DMB PE=1 SV=1	1.42E-08	
P28070	PSB4_HUMAN Proteasome subunit beta type-4 OS=Homo sapiens GN=PSMB4 PE=1 SV=4		580.29
P28072	PSB6_HUMAN Proteasome subunit beta type-6 OS=Homo sapiens GN=PSMB6 PE=1 SV=4		304.32
P28074	PSB5_HUMAN Proteasome subunit beta type-5 OS=Homo sapiens GN=PSMB5 PE=1 SV=3		916.85
P28074-2	PSB5_HUMAN Isoform 2 of Proteasome subunit beta type-5 OS=Homo sapiens GN=PSMB5		407.13
P28074;P28074-2	PSB5_HUMAN Proteasome subunit beta type-5 OS=Homo sapiens GN=PSMB5 PE=1 SV=3;>sp|P28074-2|PSB5_HUMAN Isoform 2 of I	1.56E-70	
P28161	GSTM2_HUMAN Glutathione S-transferase Mu 2 OS=Homo sapiens GN=GSTM2 PE=1 SV=2	9.26E-86	
P28288	ABCD3_HUMAN ATP-binding cassette sub-family D member 3 OS=Homo sapiens GN=ABCD3 PE=1 SV=1		165.35
P28288-2	ABCD3_HUMAN Isoform 2 of ATP-binding cassette sub-family D member 3 OS=Homo sapiens GN=ABCD3		165.35
P28288-3	ABCD3_HUMAN Isoform 3 of ATP-binding cassette sub-family D member 3 OS=Homo sapiens GN=ABCD3		108.28
P28288;P28288-2	ABCD3_HUMAN ATP-binding cassette sub-family D member 3 OS=Homo sapiens GN=ABCD3 PE=1 SV=1;>sp|P28288-2|ABCD3_HUM/	1.39E-14	
P28290	SSFA2_HUMAN Sperm-specific antigen 2 OS=Homo sapiens GN=SSFA2 PE=1 SV=3		27.58

P28290-2	SSFA2_HUMAN Isoform 2 of Sperm-specific antigen 2 OS=Homo sapiens GN=SSFA2		27.58
P28331	NDUS1_HUMAN NADH-ubiquinone oxidoreductase 75 kDa subunit, mitochondrial OS=Homo sapiens GN=NDUFS1 PE=1 SV=3		231.28
P28332-2;P28332;Q8IUN7	2|ADH6_HUMAN Isoform 2 of Alcohol dehydrogenase 6 OS=Homo sapiens GN=ADH6;>sp|P28332|ADH6_HUMAN Alcohol dehydrogena	6.97E-05	
P28340	DPOD1_HUMAN DNA polymerase delta catalytic subunit OS=Homo sapiens GN=POLD1 PE=1 SV=2		93.24
P28347	TEAD1_HUMAN Transcriptional enhancer factor TEF-1 OS=Homo sapiens GN=TEAD1 PE=1 SV=2		22.7
P28360	MSX1_HUMAN Homeobox protein MSX-1 OS=Homo sapiens GN=MSX1 PE=1 SV=2		
P28370	SMCA1_HUMAN Probable global transcription activator SNF2L1 OS=Homo sapiens GN=SMARCA1 PE=1 SV=2		147.73
P28370-2	SMCA1_HUMAN Isoform 2 of Probable global transcription activator SNF2L1 OS=Homo sapiens GN=SMARCA1		147.73
P28370;P28370-2	SMCA1_HUMAN Probable global transcription activator SNF2L1 OS=Homo sapiens GN=SMARCA1 PE=1 SV=2;>sp|P28370-2|SMCA1_H	5.49E-36	
P28476	GBRR2_HUMAN Gamma-aminobutyric acid receptor subunit rho-2 OS=Homo sapiens GN=GABRR2 PE=2 SV=4		
P28482	MK01_HUMAN Mitogen-activated protein kinase 1 OS=Homo sapiens GN=MAPK1 PE=1 SV=3		50.06
P28676	GRAN_HUMAN Grancalcin OS=Homo sapiens GN=GCA PE=1 SV=2	3.69E-18	
P28749	RBL1_HUMAN Retinoblastoma-like protein 1 OS=Homo sapiens GN=RBL1 PE=1 SV=3		
P28749-2	RBL1_HUMAN Isoform 2 of Retinoblastoma-like protein 1 OS=Homo sapiens GN=RBL1		
P28799	GRN_HUMAN Granulins OS=Homo sapiens GN=GRN PE=1 SV=2		177.41
P28799-2	GRN_HUMAN Isoform 2 of Granulins OS=Homo sapiens GN=GRN		136.92
P28838	AMPL_HUMAN Cytosol aminopeptidase OS=Homo sapiens GN=LAP3 PE=1 SV=3		194.99
P28838-2	AMPL_HUMAN Isoform 2 of Cytosol aminopeptidase OS=Homo sapiens GN=LAP3		194.99
P28838;P28838-2	AMPL_HUMAN Cytosol aminopeptidase OS=Homo sapiens GN=LAP3 PE=1 SV=3;>sp|P28838-2|AMPL_HUMAN Isoform 2 of Cytosol an	0	
P28907	CD38_HUMAN ADP-ribosyl cyclase 1 OS=Homo sapiens GN=CD38 PE=1 SV=2	1.64E-06	
P29034	S10A2_HUMAN Protein S100-A2 OS=Homo sapiens GN=S100A2 PE=1 SV=3	1.92E-08	
P29083	T2EA_HUMAN General transcription factor IIE subunit 1 OS=Homo sapiens GN=GTF2E1 PE=1 SV=2	2.16E-07	
P29084	T2EB_HUMAN Transcription initiation factor IIE subunit beta OS=Homo sapiens GN=GTF2E2 PE=1 SV=1	3.70E-06	
P29120	NEC1_HUMAN Neuroendocrine convertase 1 OS=Homo sapiens GN=PCSK1 PE=1 SV=2		33.17
P29144	TPP2_HUMAN Tripeptidyl-peptidase 2 OS=Homo sapiens GN=TPP2 PE=1 SV=4		39.84
P29218	IMPA1_HUMAN Inositol monophosphatase 1 OS=Homo sapiens GN=IMPA1 PE=1 SV=1		
P29279	CTGF_HUMAN Connective tissue growth factor OS=Homo sapiens GN=CTGF PE=1 SV=2		142.73
P29279-2	CTGF_HUMAN Isoform 2 of Connective tissue growth factor OS=Homo sapiens GN=CTGF		101.55
P29279;P29279-2	CTGF_HUMAN Connective tissue growth factor OS=Homo sapiens GN=CTGF PE=1 SV=2;>sp|P29279-2|CTGF_HUMAN Isoform 2 of Co	1.95E-07	
P29317	EPHA2_HUMAN Ephrin type-A receptor 2 OS=Homo sapiens GN=EPHA2 PE=1 SV=2		867.41
P29320	EPHA3_HUMAN Ephrin type-A receptor 3 OS=Homo sapiens GN=EPHA3 PE=1 SV=2		43.02
P29320-2	EPHA3_HUMAN Isoform 2 of Ephrin type-A receptor 3 OS=Homo sapiens GN=EPHA3		3.41
P29322	EPHA8_HUMAN Ephrin type-A receptor 8 OS=Homo sapiens GN=EPHA8 PE=1 SV=2		41.17
P29323	EPHB2_HUMAN Ephrin type-B receptor 2 OS=Homo sapiens GN=EPHB2 PE=1 SV=5		93.96
P29323-2	EPHB2_HUMAN Isoform 2 of Ephrin type-B receptor 2 OS=Homo sapiens GN=EPHB2		93.96
P29323-3	EPHB2_HUMAN Isoform 3 of Ephrin type-B receptor 2 OS=Homo sapiens GN=EPHB2		93.96
P29323;P29323-3;P29323	EPHB2_HUMAN Ephrin type-B receptor 2 OS=Homo sapiens GN=EPHB2 PE=1 SV=5;>sp|P29323-3|EPHB2_HUMAN Isoform 3 of Ephri	2.13E-52	
P29350-3;P29350;P29350	3|PTN6_HUMAN Isoform 2 of Tyrosine-protein phosphatase non-receptor type 6 OS=Homo sapiens GN=PTPN6;>sp|P29350|PTN6_HU	0	
P29353-6;P29353;B4DL02	6|SHC1_HUMAN Isoform 6 of SHC-transforming protein 1 OS=Homo sapiens GN=SHC1;>sp|P29353|SHC1_HUMAN SHC-transforming	2.52E-09	
P29372	3MG_HUMAN DNA-3-methyladenine glycosylase OS=Homo sapiens GN=MPG PE=1 SV=3		43.2
P29372-2	3MG_HUMAN Isoform 2 of DNA-3-methyladenine glycosylase OS=Homo sapiens GN=MPG		43.2
P29373	RABP2_HUMAN Cellular retinoic acid-binding protein 2 OS=Homo sapiens GN=CRABP2 PE=1 SV=2		
P29401	TKT_HUMAN Transketolase OS=Homo sapiens GN=TKT PE=1 SV=3		809.29
P29466;P29466-2;P29466	CASP1_HUMAN Caspase-1 OS=Homo sapiens GN=CASP1 PE=1 SV=1;>sp|P29466-2|CASP1_HUMAN Isoform Beta of Caspase-1 OS=H	4.11E-108	
P29508	SPB3_HUMAN Serpin B3 OS=Homo sapiens GN=SERPINB3 PE=1 SV=2		
P29508-2	SPB3_HUMAN Isoform 2 of Serpin B3 OS=Homo sapiens GN=SERPINB3		
P29536	LMOD1_HUMAN Leiomodin-1 OS=Homo sapiens GN=LMOD1 PE=1 SV=3		
P29536-2	LMOD1_HUMAN Isoform 2 of Leiomodin-1 OS=Homo sapiens GN=LMOD1		
P29558	RBMS1_HUMAN RNA-binding motif, single-stranded-interacting protein 1 OS=Homo sapiens GN=RBMS1 PE=1 SV=3		55.15
P29558-2	RBMS1_HUMAN Isoform 2 of RNA-binding motif, single-stranded-interacting protein 1 OS=Homo sapiens GN=RBMS1		55.15
P29590	PML_HUMAN Protein PML OS=Homo sapiens GN=PML PE=1 SV=3		
P29590-10	PML_HUMAN Isoform PML-7 of Protein PML OS=Homo sapiens GN=PML		
P29590-11	PML_HUMAN Isoform PML-11 of Protein PML OS=Homo sapiens GN=PML		
P29590-12	PML_HUMAN Isoform PML-12 of Protein PML OS=Homo sapiens GN=PML		
P29590-13	PML_HUMAN Isoform PML-13 of Protein PML OS=Homo sapiens GN=PML		
P29590-14	PML_HUMAN Isoform PML-14 of Protein PML OS=Homo sapiens GN=PML		
P29590-2	PML_HUMAN Isoform PML-5 of Protein PML OS=Homo sapiens GN=PML		
P29590-3	PML_HUMAN Isoform PML-8 of Protein PML OS=Homo sapiens GN=PML		
P29590-4	PML_HUMAN Isoform PML-6 of Protein PML OS=Homo sapiens GN=PML		
P29590-5	PML_HUMAN Isoform PML-4 of Protein PML OS=Homo sapiens GN=PML		
P29590-6	PML_HUMAN Isoform PML-9 of Protein PML OS=Homo sapiens GN=PML		

P29590-7	PML_HUMAN Isoform PML-10 of Protein PML OS=Homo sapiens GN=PML		
P29590-8	PML_HUMAN Isoform PML-2 of Protein PML OS=Homo sapiens GN=PML		
P29590-9	PML_HUMAN Isoform PML-3 of Protein PML OS=Homo sapiens GN=PML		
P29590;P29590-7;P29590	PML_HUMAN Protein PML OS=Homo sapiens GN=PML PE=1 SV=3;>sp|P29590-7|PML_HUMAN Isoform PML-10 of Protein PML OS=Hor	2.08E-155	
P29590;P29590-7;Q59FP	PML_HUMAN Probable transcription factor PML OS=Homo sapiens GN=PML PE=1 SV=3;>sp|P29590-7|PML_HUMAN Isoform PML-5 of	5.47E-114	
P29597	TYK2_HUMAN Non-receptor tyrosine-protein kinase TYK2 OS=Homo sapiens GN=TYK2 PE=1 SV=3		
P29622	KAIN_HUMAN Kallistatin OS=Homo sapiens GN=SERPINA4 PE=1 SV=3		21.82
P29692	EF1D_HUMAN Elongation factor 1-delta OS=Homo sapiens GN=EEF1D PE=1 SV=5		919.26
P29692-2	EF1D_HUMAN Isoform 2 of Elongation factor 1-delta OS=Homo sapiens GN=EEF1D		920.19
P29692-2;P29692	2|EF1D_HUMAN Isoform 2 of Elongation factor 1-delta OS=Homo sapiens GN=EEF1D;>sp|P29692|EF1D_HUMAN Elongation factor 1-c	0	
P29728;P29728-2	OAS2_HUMAN 2-5-oligoadenylate synthase 2 OS=Homo sapiens GN=OAS2 PE=1 SV=3;>sp|P29728-2|OAS2_HUMAN Isoform p69 of	6.62E-190	
P29762	RABP1_HUMAN Cellular retinoic acid-binding protein 1 OS=Homo sapiens GN=CRABP1 PE=1 SV=2		
P29803	ODPAT_HUMAN Pyruvate dehydrogenase E1 component subunit alpha, testis-specific form, mitochondrial OS=Homo sapiens GN=PDHA2 PE=1 SV=1		122.06
P29966	MARCS_HUMAN Myristoylated alanine-rich C-kinase substrate OS=Homo sapiens GN=MARCKS PE=1 SV=4		397.04
P29972	AQP1_HUMAN Aquaporin-1 OS=Homo sapiens GN=AQP1 PE=1 SV=3	5.37E-27	
P29992	GNA11_HUMAN Guanine nucleotide-binding protein subunit alpha-11 OS=Homo sapiens GN=GNA11 PE=1 SV=2		327.68
P30038	AL4A1_HUMAN Delta-1-pyrroline-5-carboxylate dehydrogenase, mitochondrial OS=Homo sapiens GN=ALDH4A1 PE=1 SV=3		
P30040	ERP29_HUMAN Endoplasmic reticulum resident protein 29 OS=Homo sapiens GN=ERP29 PE=1 SV=4		784.78
P30041	PRDX6_HUMAN Peroxiredoxin-6 OS=Homo sapiens GN=PRDX6 PE=1 SV=3		920.43
P30042	ES1_HUMAN ES1 protein homolog, mitochondrial OS=Homo sapiens GN=C21orf33 PE=1 SV=3		105.48
P30042-2	ES1_HUMAN Isoform Short of ES1 protein homolog, mitochondrial OS=Homo sapiens GN=C21orf33		105.48
P30042;P30042-2	ES1_HUMAN ES1 protein homolog, mitochondrial OS=Homo sapiens GN=C21orf33 PE=1 SV=3;>sp|P30042-2|ES1_HUMAN Isoform S	3.37E-56	
P30043	BLVRB_HUMAN Flavin reductase OS=Homo sapiens GN=BLVRB PE=1 SV=3		200.41
P30044	PRDX5_HUMAN Peroxiredoxin-5, mitochondrial OS=Homo sapiens GN=PRDX5 PE=1 SV=4		381.07
P30044-2	PRDX5_HUMAN Isoform Cytoplasmic+peroxisomal of Peroxiredoxin-5, mitochondrial OS=Homo sapiens GN=PRDX5		386.82
P30044;P30044-2	PRDX5_HUMAN Peroxiredoxin-5, mitochondrial OS=Homo sapiens GN=PRDX5 PE=1 SV=4;>sp|P30044-2|PRDX5_HUMAN Isoform Cyt	6.99E-167	
P30046	DOPD_HUMAN D-dopachrome decarboxylase OS=Homo sapiens GN=DDT PE=1 SV=3		111.07
P30047	GFRP_HUMAN GTP cyclohydrolase 1 feedback regulatory protein OS=Homo sapiens GN=GCHFR PE=1 SV=3	1.53E-08	
P30048	PRDX3_HUMAN Thioredoxin-dependent peroxide reductase, mitochondrial OS=Homo sapiens GN=PRDX3 PE=1 SV=3		371.09
P30049	ATPD_HUMAN ATP synthase subunit delta, mitochondrial OS=Homo sapiens GN=ATP5D PE=1 SV=2		163.67
P30050	RL12_HUMAN 60S ribosomal protein L12 OS=Homo sapiens GN=RPL12 PE=1 SV=1		646.06
P30050-2	RL12_HUMAN Isoform 2 of 60S ribosomal protein L12 OS=Homo sapiens GN=RPL12		491.49
P30050;P30050-2	RL12_HUMAN 60S ribosomal protein L12 OS=Homo sapiens GN=RPL12 PE=1 SV=1;>sp|P30050-2|RL12_HUMAN Isoform 2 of 60S rib	0	
P30084	ECHM_HUMAN Enoyl-CoA hydratase, mitochondrial OS=Homo sapiens GN=ECHS1 PE=1 SV=4		340.72
P30085	KCY_HUMAN UMP-CMP kinase OS=Homo sapiens GN=CMPK1 PE=1 SV=3		348.69
P30086	PEBP1_HUMAN Phosphatidylethanolamine-binding protein 1 OS=Homo sapiens GN=PEBP1 PE=1 SV=3		549.13
P30101	PDIA3_HUMAN Protein disulfide-isomerase A3 OS=Homo sapiens GN=PDIA3 PE=1 SV=4		1746.83
P30153	2AAA_HUMAN Serine/threonine-protein phosphatase 2A 65 kDa regulatory subunit A alpha isoform OS=Homo sapiens GN=PPP2R1A PE=1 SV=4		465.55
P30154	2AAB_HUMAN Serine/threonine-protein phosphatase 2A 65 kDa regulatory subunit A beta isoform OS=Homo sapiens GN=PPP2R1B PE=1 SV=3		75.54
P30154-2	2AAB_HUMAN Isoform 2 of Serine/threonine-protein phosphatase 2A 65 kDa regulatory subunit A beta isoform OS=Homo sapiens GN=PPP2R1B		75.54
P30154-2;A8MY67;P3015	2|2AAB_HUMAN Isoform 2 of Serine/threonine-protein phosphatase 2A 65 kDa regulatory subunit A beta isoform OS=Homo sapiens G	5.36E-101	
P30154;P30154-2	2AAB_HUMAN Serine/threonine-protein phosphatase 2A 65 kDa regulatory subunit A beta isoform OS=Homo sapiens GN=PPP2R1B PE	1.17E-103	
P30260	CDC27_HUMAN Cell division cycle protein 27 homolog OS=Homo sapiens GN=CDC27 PE=1 SV=2		
P30273	FCERG_HUMAN High affinity immunoglobulin epsilon receptor subunit gamma OS=Homo sapiens GN=FCER1G PE=1 SV=1	1.87E-24	
P30405	PPIF_HUMAN Peptidyl-prolyl cis-trans isomerase F, mitochondrial OS=Homo sapiens GN=PPIF PE=1 SV=1		113.65
P30408	T4S1_HUMAN Transmembrane 4 L6 family member 1 OS=Homo sapiens GN=TM4SF1 PE=1 SV=1		57.86
P30419	NMT1_HUMAN Glycylpeptide N-tetradecanoyltransferase 1 OS=Homo sapiens GN=NMT1 PE=1 SV=2		406.4
P30419-2	NMT1_HUMAN Isoform Short of Glycylpeptide N-tetradecanoyltransferase 1 OS=Homo sapiens GN=NMT1		322.44
P30419;P30419-2	NMT1_HUMAN Glycylpeptide N-tetradecanoyltransferase 1 OS=Homo sapiens GN=NMT1 PE=1 SV=2;>sp|P30419-2|NMT1_HUMAN Isc	4.64E-122	
P30443	1A01_HUMAN HLA class I histocompatibility antigen, A-1 alpha chain OS=Homo sapiens GN=HLA-A PE=1 SV=1		436.03
P30447	1A23_HUMAN HLA class I histocompatibility antigen, A-23 alpha chain OS=Homo sapiens GN=HLA-A PE=1 SV=1		596.69
P30450	1A26_HUMAN HLA class I histocompatibility antigen, A-26 alpha chain OS=Homo sapiens GN=HLA-A PE=1 SV=2		587.75
P30453	1A34_HUMAN HLA class I histocompatibility antigen, A-34 alpha chain OS=Homo sapiens GN=HLA-A PE=1 SV=1		663.45
P30455	1A36_HUMAN HLA class I histocompatibility antigen, A-36 alpha chain OS=Homo sapiens GN=HLA-A PE=1 SV=1		436.03
P30456	1A43_HUMAN HLA class I histocompatibility antigen, A-43 alpha chain OS=Homo sapiens GN=HLA-A PE=2 SV=1		587.75
P30457	1A66_HUMAN HLA class I histocompatibility antigen, A-66 alpha chain OS=Homo sapiens GN=HLA-A PE=1 SV=1		587.75
P30459	1A74_HUMAN HLA class I histocompatibility antigen, A-74 alpha chain OS=Homo sapiens GN=HLA-A PE=1 SV=1		657.24
P30460	1B08_HUMAN HLA class I histocompatibility antigen, B-8 alpha chain OS=Homo sapiens GN=HLA-B PE=1 SV=1		500.32
P30461	1B13_HUMAN HLA class I histocompatibility antigen, B-13 alpha chain OS=Homo sapiens GN=HLA-B PE=2 SV=1		450.59
P30462	1B14_HUMAN HLA class I histocompatibility antigen, B-14 alpha chain OS=Homo sapiens GN=HLA-B PE=1 SV=1		332.41
P30464	1B15_HUMAN HLA class I histocompatibility antigen, B-15 alpha chain OS=Homo sapiens GN=HLA-B PE=1 SV=2		517.72

P30466	1B18_HUMAN HLA class I histocompatibility antigen, B-18 alpha chain OS=Homo sapiens GN=HLA-B PE=1 SV=1		420.29
P30475	1B39_HUMAN HLA class I histocompatibility antigen, B-39 alpha chain OS=Homo sapiens GN=HLA-B PE=2 SV=1		382.83
P30479	1B41_HUMAN HLA class I histocompatibility antigen, B-41 alpha chain OS=Homo sapiens GN=HLA-B PE=1 SV=1		684.43
P30480	1B42_HUMAN HLA class I histocompatibility antigen, B-42 alpha chain OS=Homo sapiens GN=HLA-B PE=1 SV=1		498.09
P30481	1B44_HUMAN HLA class I histocompatibility antigen, B-44 alpha chain OS=Homo sapiens GN=HLA-B PE=1 SV=1		468.99
P30483	1B45_HUMAN HLA class I histocompatibility antigen, B-45 alpha chain OS=Homo sapiens GN=HLA-B PE=2 SV=1		433.19
P30484	1B46_HUMAN HLA class I histocompatibility antigen, B-46 alpha chain OS=Homo sapiens GN=HLA-B PE=2 SV=1		491.88
P30485	1B47_HUMAN HLA class I histocompatibility antigen, B-47 alpha chain OS=Homo sapiens GN=HLA-B PE=2 SV=1		509.17
P30486	1B48_HUMAN HLA class I histocompatibility antigen, B-48 alpha chain OS=Homo sapiens GN=HLA-B PE=1 SV=1		389.3
P30487	1B49_HUMAN HLA class I histocompatibility antigen, B-49 alpha chain OS=Homo sapiens GN=HLA-B PE=2 SV=2		433.19
P30488	1B50_HUMAN HLA class I histocompatibility antigen, B-50 alpha chain OS=Homo sapiens GN=HLA-B PE=2 SV=1		433.19
P30490	1B52_HUMAN HLA class I histocompatibility antigen, B-52 alpha chain OS=Homo sapiens GN=HLA-B PE=2 SV=1		439.65
P30491	1B53_HUMAN HLA class I histocompatibility antigen, B-53 alpha chain OS=Homo sapiens GN=HLA-B PE=1 SV=1		471.94
P30492	1B54_HUMAN HLA class I histocompatibility antigen, B-54 alpha chain OS=Homo sapiens GN=HLA-B PE=2 SV=1		499.59
P30493	1B55_HUMAN HLA class I histocompatibility antigen, B-55 alpha chain OS=Homo sapiens GN=HLA-B PE=1 SV=1		499.59
P30495	1B56_HUMAN HLA class I histocompatibility antigen, B-56 alpha chain OS=Homo sapiens GN=HLA-B PE=2 SV=1		423.89
P30498	1B78_HUMAN HLA class I histocompatibility antigen, B-78 alpha chain OS=Homo sapiens GN=HLA-B PE=2 SV=1		416.04
P30499	1C01_HUMAN HLA class I histocompatibility antigen, Cw-1 alpha chain OS=Homo sapiens GN=HLA-C PE=2 SV=1		326.4
P30501	1C02_HUMAN HLA class I histocompatibility antigen, Cw-2 alpha chain OS=Homo sapiens GN=HLA-C PE=1 SV=1		358.48
P30504	1C04_HUMAN HLA class I histocompatibility antigen, Cw-4 alpha chain OS=Homo sapiens GN=HLA-C PE=1 SV=1		465.81
P30505	1C08_HUMAN HLA class I histocompatibility antigen, Cw-8 alpha chain OS=Homo sapiens GN=HLA-C PE=2 SV=1		373.69
P30508	1C12_HUMAN HLA class I histocompatibility antigen, Cw-12 alpha chain OS=Homo sapiens GN=HLA-C PE=1 SV=2		562.7
P30510	1C14_HUMAN HLA class I histocompatibility antigen, Cw-14 alpha chain OS=Homo sapiens GN=HLA-C PE=2 SV=2		465.81
P30511	HLAF_HUMAN HLA class I histocompatibility antigen, alpha chain F OS=Homo sapiens GN=HLA-F PE=2 SV=3		71.99
P30511-2	HLAF_HUMAN Isoform 2 of HLA class I histocompatibility antigen, alpha chain F OS=Homo sapiens GN=HLA-F		58.9
P30511-3	HLAF_HUMAN Isoform 3 of HLA class I histocompatibility antigen, alpha chain F OS=Homo sapiens GN=HLA-F		71.99
P30511-3;P30511	3|HLAF_HUMAN Isoform 3 of HLA class I histocompatibility antigen, alpha chain F OS=Homo sapiens GN=HLA-F;>sp|P30511|HLAF_HU	4.51E-16	
P30512	1A29_HUMAN HLA class I histocompatibility antigen, A-29 alpha chain OS=Homo sapiens GN=HLA-A PE=2 SV=2		595.43
P30519	HMOX2_HUMAN Heme oxygenase 2 OS=Homo sapiens GN=HMOX2 PE=1 SV=2		191.66
P30520	PURA2_HUMAN Adenylosuccinate synthetase isozyme 2 OS=Homo sapiens GN=ADSS PE=1 SV=3	6.95E-86	
P30530	UFO_HUMAN Tyrosine-protein kinase receptor UFO OS=Homo sapiens GN=AXL PE=1 SV=3		
P30530-2	UFO_HUMAN Isoform Short of Tyrosine-protein kinase receptor UFO OS=Homo sapiens GN=AXL		
P30530;P30530-2	UFO_HUMAN Tyrosine-protein kinase receptor UFO OS=Homo sapiens GN=AXL PE=1 SV=3;>sp|P30530-2|UFO_HUMAN Isoform Short	2.26E-110	
P30533	AMRP_HUMAN Alpha-2-macroglobulin receptor-associated protein OS=Homo sapiens GN=LRPAP1 PE=1 SV=1		153.14
P30536	TSPOA_HUMAN Translocator protein OS=Homo sapiens GN=TSPO PE=1 SV=3		61.31
P30566	PUR8_HUMAN Adenylosuccinate lyase OS=Homo sapiens GN=ADSL PE=1 SV=2		91.48
P30566-2	PUR8_HUMAN Isoform 2 of Adenylosuccinate lyase OS=Homo sapiens GN=ADSL		46.94
P30566;P30566-2	PUR8_HUMAN Adenylosuccinate lyase OS=Homo sapiens GN=ADSL PE=1 SV=2;>sp|P30566-2|PUR8_HUMAN Isoform 2 of Adenylosuc	5.83E-149	
P30613	KPYR_HUMAN Pyruvate kinase isozymes R/L OS=Homo sapiens GN=PKLR PE=1 SV=2		59.25
P30613-2	KPYR_HUMAN Isoform L-type of Pyruvate kinase isozymes R/L OS=Homo sapiens GN=PKLR		59.25
P30613;P30613-2	KPYR_HUMAN Pyruvate kinase isozymes R/L OS=Homo sapiens GN=PKLR PE=1 SV=2;>sp|P30613-2|KPYR_HUMAN Isoform L-type of	4.65E-09	
P30622	CLIP1_HUMAN CAP-Gly domain-containing linker protein 1 OS=Homo sapiens GN=CLIP1 PE=1 SV=2		
P30622-1	CLIP1_HUMAN Isoform 2 of CAP-Gly domain-containing linker protein 1 OS=Homo sapiens GN=CLIP1		
P30622-2	CLIP1_HUMAN Isoform 3 of CAP-Gly domain-containing linker protein 1 OS=Homo sapiens GN=CLIP1		
P30622;P30622-1;P30622	CLIP1_HUMAN CAP-Gly domain-containing linker protein 1 OS=Homo sapiens GN=CLIP1 PE=1 SV=2;>sp|P30622-1|CLIP1_HUMAN Is	7.19E-38	
P30626	SORCN_HUMAN Sorcin OS=Homo sapiens GN=SRI PE=1 SV=1		127.64
P30685	1B35_HUMAN HLA class I histocompatibility antigen, B-35 alpha chain OS=Homo sapiens GN=HLA-B PE=1 SV=1		475.37
P30711	GSTT1_HUMAN Glutathione S-transferase theta-1 OS=Homo sapiens GN=GSTT1 PE=1 SV=4	4.32E-30	
P30740	ILEU_HUMAN Leukocyte elastase inhibitor OS=Homo sapiens GN=SERPINB1 PE=1 SV=1		71.64
P30825	CTR1_HUMAN High affinity cationic amino acid transporter 1 OS=Homo sapiens GN=SLC7A1 PE=1 SV=1		213.98
P30837	AL1B1_HUMAN Aldehyde dehydrogenase X, mitochondrial OS=Homo sapiens GN=ALDH1B1 PE=1 SV=3		206.27
P30876	RPB2_HUMAN DNA-directed RNA polymerase II subunit RPB2 OS=Homo sapiens GN=POLR2B PE=1 SV=1		18.79
P31025	LCN1_HUMAN Lipocalin-1 OS=Homo sapiens GN=LCN1 PE=1 SV=1		
P31040	DHSA_HUMAN Succinate dehydrogenase [ubiquinone] flavoprotein subunit, mitochondrial OS=Homo sapiens GN=SDHA PE=1 SV=2		578.12
P31146	COR1A_HUMAN Coronin-1A OS=Homo sapiens GN=CORO1A PE=1 SV=4		
P31150	GDIA_HUMAN Rab GDP dissociation inhibitor alpha OS=Homo sapiens GN=GDI1 PE=1 SV=2		317.81
P31151	S10A7_HUMAN Protein S100-A7 OS=Homo sapiens GN=S100A7 PE=1 SV=4		
P31152	MK04_HUMAN Mitogen-activated protein kinase 4 OS=Homo sapiens GN=MAPK4 PE=1 SV=2		27.93
P31153	METK2_HUMAN S-adenosylmethionine synthase isoform type-2 OS=Homo sapiens GN=MAT2A PE=1 SV=1		101.15
P31323	KAP3_HUMAN cAMP-dependent protein kinase type II-beta regulatory subunit OS=Homo sapiens GN=PRKAR2B PE=1 SV=3	4.07E-17	
P31327	CPSM_HUMAN Carbamoyl-phosphate synthase [ammonia], mitochondrial OS=Homo sapiens GN=CPS1 PE=1 SV=2		44.62

P31327-2	CPSM_HUMAN Isoform 2 of Carbamoyl-phosphate synthase [ammonia], mitochondrial OS=Homo sapiens GN=CPS1		8.77
P31327;P31327-2	CPSM_HUMAN Carbamoyl-phosphate synthase [ammonia], mitochondrial OS=Homo sapiens GN=CPS1 PE=1 SV=2;>sp|P31327-2|CPS	0.00019025	
P31350	RIR2_HUMAN Ribonucleoside-diphosphate reductase subunit M2 OS=Homo sapiens GN=RRM2 PE=1 SV=1	3.71E-70	
P31431	SDC4_HUMAN Syndecan-4 OS=Homo sapiens GN=SDC4 PE=1 SV=2		26.89
P31483	TIA1_HUMAN Nucleolysin TIA-1 isoform p40 OS=Homo sapiens GN=TIA1 PE=1 SV=3		97.03
P31483-2	TIA1_HUMAN Isoform Short of Nucleolysin TIA-1 isoform p40 OS=Homo sapiens GN=TIA1		97.03
P31483-2;C9JTN7;Q59G9	2|TIA1_HUMAN Isoform Short of Nucleolysin TIA-1 isoform p40 OS=Homo sapiens GN=TIA1;>tr|C9JTN7|C9JTN7_HUMAN Uncharacte	7.34E-18	
P31483;P31483-2	TIA1_HUMAN Nucleolysin TIA-1 isoform p40 OS=Homo sapiens GN=TIA1 PE=1 SV=3;>sp|P31483-2|TIA1_HUMAN Isoform Short of N	3.87E-28	
P31629	ZEP2_HUMAN Transcription factor HIVEP2 OS=Homo sapiens GN=HIVEP2 PE=1 SV=2		
P31689	DNJA1_HUMAN DnaJ homolog subfamily A member 1 OS=Homo sapiens GN=DNAJA1 PE=1 SV=2		96.9
P31749	AKT1_HUMAN RAC-alpha serine/threonine-protein kinase OS=Homo sapiens GN=AKT1 PE=1 SV=2	4.39E-45	
P31751	AKT2_HUMAN RAC-beta serine/threonine-protein kinase OS=Homo sapiens GN=AKT2 PE=1 SV=2	2.43E-31	
P31930	QCR1_HUMAN Cytochrome b-c1 complex subunit 1, mitochondrial OS=Homo sapiens GN=UQCRC1 PE=1 SV=3		269.4
P31937	3HIDH_HUMAN 3-hydroxyisobutyrate dehydrogenase, mitochondrial OS=Homo sapiens GN=HIBADH PE=1 SV=2		242.67
P31939	PUR9_HUMAN Bifunctional purine biosynthesis protein PURH OS=Homo sapiens GN=ATIC PE=1 SV=3		
P31941	ABC3A_HUMAN Probable DNA dC->dU-editing enzyme APOBEC-3A OS=Homo sapiens GN=APOBEC3A PE=1 SV=3		
P31942	HNRH3_HUMAN Heterogeneous nuclear ribonucleoprotein H3 OS=Homo sapiens GN=HNRNPH3 PE=1 SV=2		369.07
P31942-2	HNRH3_HUMAN Isoform 2 of Heterogeneous nuclear ribonucleoprotein H3 OS=Homo sapiens GN=HNRNPH3		369.07
P31942-3	HNRH3_HUMAN Isoform 3 of Heterogeneous nuclear ribonucleoprotein H3 OS=Homo sapiens GN=HNRNPH3		351.89
P31942-4	HNRH3_HUMAN Isoform 4 of Heterogeneous nuclear ribonucleoprotein H3 OS=Homo sapiens GN=HNRNPH3		215.05
P31942-5	HNRH3_HUMAN Isoform 5 of Heterogeneous nuclear ribonucleoprotein H3 OS=Homo sapiens GN=HNRNPH3		171.55
P31942-6	HNRH3_HUMAN Isoform 6 of Heterogeneous nuclear ribonucleoprotein H3 OS=Homo sapiens GN=HNRNPH3		171.55
P31942;P31942-2;P31942	HNRH3_HUMAN Heterogeneous nuclear ribonucleoprotein H3 OS=Homo sapiens GN=HNRNPH3 PE=1 SV=2;>sp|P31942-2|HNRH3_HU	1.43E-55	
P31943	HNRH1_HUMAN Heterogeneous nuclear ribonucleoprotein H OS=Homo sapiens GN=HNRNPH1 PE=1 SV=4		451.95
P31946	1433B_HUMAN 14-3-3 protein beta/alpha OS=Homo sapiens GN=YWHAB PE=1 SV=3		1433.97
P31946-2	1433B_HUMAN Isoform Short of 14-3-3 protein beta/alpha OS=Homo sapiens GN=YWHAB		1455.94
P31947	1433S_HUMAN 14-3-3 protein sigma OS=Homo sapiens GN=SFN PE=1 SV=1		510.78
P31947-2	1433S_HUMAN Isoform 2 of 14-3-3 protein sigma OS=Homo sapiens GN=SFN		394.32
P31947;P31947-2	1433S_HUMAN 14-3-3 protein sigma OS=Homo sapiens GN=SFN PE=1 SV=1;>sp|P31947-2|1433S_HUMAN Isoform 2 of 14-3-3 prot	4.32E-25	
P31948	STIP1_HUMAN Stress-induced-phosphoprotein 1 OS=Homo sapiens GN=STIP1 PE=1 SV=1		905.88
P31949	S10AB_HUMAN Protein S100-A11 OS=Homo sapiens GN=S100A11 PE=1 SV=2		188.97
P32004	L1CAM_HUMAN Neural cell adhesion molecule L1 OS=Homo sapiens GN=L1CAM PE=1 SV=2		113.6
P32004-2	L1CAM_HUMAN Isoform 2 of Neural cell adhesion molecule L1 OS=Homo sapiens GN=L1CAM		113.6
P32004;P32004-2	L1CAM_HUMAN Neural cell adhesion molecule L1 OS=Homo sapiens GN=L1CAM PE=1 SV=2;>sp|P32004-2|L1CAM_HUMAN Isoform 2	5.77E-30	
P32119	PRDX2_HUMAN Peroxiredoxin-2 OS=Homo sapiens GN=PRDX2 PE=1 SV=5		509.81
P32238	CCKAR_HUMAN Cholecystokinin receptor type A OS=Homo sapiens GN=CCKAR PE=1 SV=1		
P32247	BRS3_HUMAN Bombesin receptor subtype-3 OS=Homo sapiens GN=BRS3 PE=1 SV=1		
P32297	ACHA3_HUMAN Neuronal acetylcholine receptor subunit alpha-3 OS=Homo sapiens GN=CHRNA3 PE=1 SV=4		
P32297-1	ACHA3_HUMAN Isoform 1 of Neuronal acetylcholine receptor subunit alpha-3 OS=Homo sapiens GN=CHRNA3		
P32297-3	ACHA3_HUMAN Isoform 3 of Neuronal acetylcholine receptor subunit alpha-3 OS=Homo sapiens GN=CHRNA3		
P32321	DCTD_HUMAN Deoxycytidylate deaminase OS=Homo sapiens GN=DCTD PE=1 SV=2		44.65
P32321-2	DCTD_HUMAN Isoform 2 of Deoxycytidylate deaminase OS=Homo sapiens GN=DCTD		44.65
P32321-2;P32321	2|DCTD_HUMAN Isoform 2 of Deoxycytidylate deaminase OS=Homo sapiens GN=DCTD;>sp|P32321|DCTD_HUMAN Deoxycytidylate d	1.51E-07	
P32322	P5CR1_HUMAN Pyrroline-5-carboxylate reductase 1, mitochondrial OS=Homo sapiens GN=PYCR1 PE=1 SV=2		736.29
P32418	NAC1_HUMAN Sodium/calcium exchanger 1 OS=Homo sapiens GN=SLC8A1 PE=1 SV=3		43.13
P32418-2	NAC1_HUMAN Isoform 3 of Sodium/calcium exchanger 1 OS=Homo sapiens GN=SLC8A1		43.13
P32418-3	NAC1_HUMAN Isoform 7 of Sodium/calcium exchanger 1 OS=Homo sapiens GN=SLC8A1		43.13
P32418-4	NAC1_HUMAN Isoform 10 of Sodium/calcium exchanger 1 OS=Homo sapiens GN=SLC8A1		43.13
P32455	GBP1_HUMAN Interferon-induced guanylate-binding protein 1 OS=Homo sapiens GN=GBP1 PE=1 SV=2		68.82
P32456	GBP2_HUMAN Interferon-induced guanylate-binding protein 2 OS=Homo sapiens GN=GBP2 PE=2 SV=3		
P32519	ELF1_HUMAN ETS-related transcription factor Elf-1 OS=Homo sapiens GN=ELF1 PE=1 SV=2	9.15E-09	
P32780	TF2H1_HUMAN General transcription factor IIH subunit 1 OS=Homo sapiens GN=GTF2H1 PE=1 SV=1		71.23
P32929;B4E1R2;P32929-2	CGL_HUMAN Cystathionine gamma-lyase OS=Homo sapiens GN=CTH PE=1 SV=3;>tr|B4E1R2|B4E1R2_HUMAN cDNA FLJ50357, highl	6.59E-20	
P32969	RL9_HUMAN 60S ribosomal protein L9 OS=Homo sapiens GN=RPL9 PE=1 SV=1		866.94
P32970	CD70_HUMAN CD70 antigen OS=Homo sapiens GN=CD70 PE=1 SV=2	1.80E-64	
P33121;P33121-2	ACSL1_HUMAN Long-chain-fatty-acid--CoA ligase 1 OS=Homo sapiens GN=ACSL1 PE=1 SV=1;>sp|P33121-2|ACSL1_HUMAN Isoform	3.13E-09	
P33176	KINH_HUMAN Kinesin-1 heavy chain OS=Homo sapiens GN=KIF5B PE=1 SV=1		69.58
P33240	CSTF2_HUMAN Cleavage stimulation factor subunit 2 OS=Homo sapiens GN=CSTF2 PE=1 SV=1		104.03
P33240-2	CSTF2_HUMAN Isoform 2 of Cleavage stimulation factor subunit 2 OS=Homo sapiens GN=CSTF2		104.03
P33240;P33240-2;Q9H0L4	CSTF2_HUMAN Cleavage stimulation factor subunit 2 OS=Homo sapiens GN=CSTF2 PE=1 SV=1;>sp|P33240-2|CSTF2_HUMAN Isoforr	1.23E-10	
P33241	LSP1_HUMAN Lymphocyte-specific protein 1 OS=Homo sapiens GN=LSP1 PE=1 SV=1	1.04E-67	

P33260	CP2CI_HUMAN Cytochrome P450 2C18 OS=Homo sapiens GN=CYP2C18 PE=1 SV=3		
P33261	CP2CJ_HUMAN Cytochrome P450 2C19 OS=Homo sapiens GN=CYP2C19 PE=1 SV=3		
P33316	DUT_HUMAN Deoxyuridine 5'-triphosphate nucleotidohydrolase, mitochondrial OS=Homo sapiens GN=DUT PE=1 SV=3		306.32
P33316-2	DUT_HUMAN Isoform 2 of Deoxyuridine 5'-triphosphate nucleotidohydrolase, mitochondrial OS=Homo sapiens GN=DUT		306.32
P33316-3	DUT_HUMAN Isoform 3 of Deoxyuridine 5'-triphosphate nucleotidohydrolase, mitochondrial OS=Homo sapiens GN=DUT		306.32
P33316;P33316-2	DUT_HUMAN Deoxyuridine 5-triphosphate nucleotidohydrolase, mitochondrial OS=Homo sapiens GN=DUT PE=1 SV=4;>sp|P33316-2|	3.09E-36	
P33527	MRP1_HUMAN Multidrug resistance-associated protein 1 OS=Homo sapiens GN=ABCC1 PE=1 SV=3		138.03
P33527-2	MRP1_HUMAN Isoform 2 of Multidrug resistance-associated protein 1 OS=Homo sapiens GN=ABCC1		138.03
P33527-3	MRP1_HUMAN Isoform 3 of Multidrug resistance-associated protein 1 OS=Homo sapiens GN=ABCC1		138.03
P33527-4	MRP1_HUMAN Isoform 4 of Multidrug resistance-associated protein 1 OS=Homo sapiens GN=ABCC1		138.03
P33527-5	MRP1_HUMAN Isoform 5 of Multidrug resistance-associated protein 1 OS=Homo sapiens GN=ABCC1		138.03
P33527-6	MRP1_HUMAN Isoform 6 of Multidrug resistance-associated protein 1 OS=Homo sapiens GN=ABCC1		138.03
P33527-7	MRP1_HUMAN Isoform 7 of Multidrug resistance-associated protein 1 OS=Homo sapiens GN=ABCC1		138.03
P33527-8	MRP1_HUMAN Isoform 8 of Multidrug resistance-associated protein 1 OS=Homo sapiens GN=ABCC1		138.03
P33527-9	MRP1_HUMAN Isoform 9 of Multidrug resistance-associated protein 1 OS=Homo sapiens GN=ABCC1		138.03
P33527-9;P33527;P33527	9|MRP1_HUMAN Isoform 9 of Multidrug resistance-associated protein 1 OS=Homo sapiens GN=ABCC1;>sp|P33527|MRP1_HUMAN Mu	2.58E-12	
P33552	CKS2_HUMAN Cyclin-dependent kinases regulatory subunit 2 OS=Homo sapiens GN=CKS2 PE=1 SV=1		69.55
P33764	S10A3_HUMAN Protein S100-A3 OS=Homo sapiens GN=S100A3 PE=1 SV=1		
P33778	H2B1B_HUMAN Histone H2B type 1-B OS=Homo sapiens GN=HIST1H2BB PE=1 SV=2		729.71
P33897	ABCD1_HUMAN ATP-binding cassette sub-family D member 1 OS=Homo sapiens GN=ABCD1 PE=1 SV=2	6.13E-13	
P33908	MA1A1_HUMAN Mannosyl-oligosaccharide 1,2-alpha-mannosidase IA OS=Homo sapiens GN=MAN1A1 PE=1 SV=3		
P33947	ERD22_HUMAN ER lumen protein retaining receptor 2 OS=Homo sapiens GN=KDELR2 PE=1 SV=1		109.75
P33947-2	ERD22_HUMAN Isoform 2 of ER lumen protein retaining receptor 2 OS=Homo sapiens GN=KDELR2		81.58
P33991	MCM4_HUMAN DNA replication licensing factor MCM4 OS=Homo sapiens GN=MCM4 PE=1 SV=5		374.43
P33992	MCM5_HUMAN DNA replication licensing factor MCM5 OS=Homo sapiens GN=MCM5 PE=1 SV=5		88.33
P33993	MCM7_HUMAN DNA replication licensing factor MCM7 OS=Homo sapiens GN=MCM7 PE=1 SV=4		438
P33993-2	MCM7_HUMAN Isoform 2 of DNA replication licensing factor MCM7 OS=Homo sapiens GN=MCM7		198.91
P33993;P33993-2	MCM7_HUMAN DNA replication licensing factor MCM7 OS=Homo sapiens GN=MCM7 PE=1 SV=4;>sp|P33993-2|MCM7_HUMAN Isoforn	8.09E-100	
P34096	RNAS4_HUMAN Ribonuclease 4 OS=Homo sapiens GN=RNASE4 PE=1 SV=3	0.00022164	
P34896	GLYC_HUMAN Serine hydroxymethyltransferase, cytosolic OS=Homo sapiens GN=SHMT1 PE=1 SV=1		
P34896-2	GLYC_HUMAN Isoform 2 of Serine hydroxymethyltransferase, cytosolic OS=Homo sapiens GN=SHMT1		
P34896-3	GLYC_HUMAN Isoform 3 of Serine hydroxymethyltransferase, cytosolic OS=Homo sapiens GN=SHMT1		
P34896;P34896-2;P34896	GLYC_HUMAN Serine hydroxymethyltransferase, cytosolic OS=Homo sapiens GN=SHMT1 PE=1 SV=1;>sp|P34896-2|GLYC_HUMAN Isc	4.31E-82	
P34897	GLYM_HUMAN Serine hydroxymethyltransferase, mitochondrial OS=Homo sapiens GN=SHMT2 PE=1 SV=3		697.1
P34931	HS71L_HUMAN Heat shock 70 kDa protein 1-like OS=Homo sapiens GN=HSPA1L PE=1 SV=2		654.42
P34932	HSP74_HUMAN Heat shock 70 kDa protein 4 OS=Homo sapiens GN=HSPA4 PE=1 SV=4		423.75
P34949;P34949-2	MPI_HUMAN Mannose-6-phosphate isomerase OS=Homo sapiens GN=MPI PE=1 SV=2;>sp|P34949-2|MPI_HUMAN Isoform 2 of Manno	8.85E-18	
P35030	TRY3_HUMAN Trypsin-3 OS=Homo sapiens GN=PRSS3 PE=1 SV=2		57.19
P35030-2	TRY3_HUMAN Isoform B of Trypsin-3 OS=Homo sapiens GN=PRSS3		55.93
P35030-3	TRY3_HUMAN Isoform C of Trypsin-3 OS=Homo sapiens GN=PRSS3		55.93
P35030;P35030-2;P35030	TRY3_HUMAN Trypsin-3 OS=Homo sapiens GN=PRSS3 PE=1 SV=2;>sp|P35030-2|TRY3_HUMAN Isoform B of Trypsin-3 OS=Homo sap	2.32E-59	
P35052	GPC1_HUMAN Glypican-1 OS=Homo sapiens GN=GPC1 PE=1 SV=2	5.32E-16	
P35080	PROF2_HUMAN Profilin-2 OS=Homo sapiens GN=PFN2 PE=1 SV=3	5.46E-63	
P35080-2	PROF2_HUMAN Isoform IIb of Profilin-2 OS=Homo sapiens GN=PFN2		
P35219	CAH8_HUMAN Carbonic anhydrase-related protein OS=Homo sapiens GN=CA8 PE=1 SV=3	5.19E-06	
P35221	CTNA1_HUMAN Catenin alpha-1 OS=Homo sapiens GN=CTNNA1 PE=1 SV=1		226.37
P35221-2	CTNA1_HUMAN Isoform 2 of Catenin alpha-1 OS=Homo sapiens GN=CTNNA1		189.16
P35221-2;P35221	2|CTNA1_HUMAN Isoform 2 of Catenin alpha-1 OS=Homo sapiens GN=CTNNA1;>sp|P35221|CTNA1_HUMAN Catenin alpha-1 OS=Hor	0	
P35221;B4E2G8;P35221-2	CTNA1_HUMAN Catenin alpha-1 OS=Homo sapiens GN=CTNNA1 PE=1 SV=1;>tr|B4E2G8|B4E2G8_HUMAN cDNA FLJ54047, highly sin	6.17E-224	
P35222	CTNB1_HUMAN Catenin beta-1 OS=Homo sapiens GN=CTNNB1 PE=1 SV=1		86.14
P35222-2	CTNB1_HUMAN Isoform 2 of Catenin beta-1 OS=Homo sapiens GN=CTNNB1		
P35228	NOS2_HUMAN Nitric oxide synthase, inducible OS=Homo sapiens GN=NOS2 PE=1 SV=2		
P35228-2	NOS2_HUMAN Isoform 2 of Nitric oxide synthase, inducible OS=Homo sapiens GN=NOS2		
P35228;P35228-2	NOS2_HUMAN Nitric oxide synthase, inducible OS=Homo sapiens GN=NOS2 PE=1 SV=2;>sp|P35228-2|NOS2_HUMAN Isoform 2 of N	1.87E-08	
P35232	PHB_HUMAN Prohibitin OS=Homo sapiens GN=PHB PE=1 SV=1		807.14
P35237	SPB6_HUMAN Serpin B6 OS=Homo sapiens GN=SERPINB6 PE=1 SV=3		125.77
P35241	RADI_HUMAN Radixin OS=Homo sapiens GN=RDX PE=1 SV=1		2170.24
P35244	RFA3_HUMAN Replication protein A 14 kDa subunit OS=Homo sapiens GN=RPA3 PE=1 SV=1		549.94
P35249	RFC4_HUMAN Replication factor C subunit 4 OS=Homo sapiens GN=RFC4 PE=1 SV=2		62.69
P35250	RFC2_HUMAN Replication factor C subunit 2 OS=Homo sapiens GN=RFC2 PE=1 SV=3		183.3
P35250-2	RFC2_HUMAN Isoform 2 of Replication factor C subunit 2 OS=Homo sapiens GN=RFC2		183.3

P35250;P35250-2	RFC2_HUMAN Replication factor C subunit 2 OS=Homo sapiens GN=RFC2 PE=1 SV=3;>sp|P35250-2|RFC2_HUMAN Isoform 2 of Repli	1.85E-11	
P35251	RFC1_HUMAN Replication factor C subunit 1 OS=Homo sapiens GN=RFC1 PE=1 SV=4		150.03
P35251-2	RFC1_HUMAN Isoform 2 of Replication factor C subunit 1 OS=Homo sapiens GN=RFC1		150.03
P35251;P35251-2	RFC1_HUMAN Replication factor C subunit 1 OS=Homo sapiens GN=RFC1 PE=1 SV=4;>sp|P35251-2|RFC1_HUMAN Isoform 2 of Repli	1.32E-43	
P35268	RL22_HUMAN 60S ribosomal protein L22 OS=Homo sapiens GN=RPL22 PE=1 SV=2		455.96
P35269	T2FA_HUMAN General transcription factor IIF subunit 1 OS=Homo sapiens GN=GTF2F1 PE=1 SV=2		66.52
P35270	SPRE_HUMAN Sepiapterin reductase OS=Homo sapiens GN=SPR PE=1 SV=1		192.31
P35321	SPR1A_HUMAN Cornifin-A OS=Homo sapiens GN=SPRR1A PE=1 SV=2		
P35325	SPR2B_HUMAN Small proline-rich protein 2B OS=Homo sapiens GN=SPRR2B PE=2 SV=1		23.4
P35326	SPR2A_HUMAN Small proline-rich protein 2A OS=Homo sapiens GN=SPRR2A PE=2 SV=1		23.4
P35348	ADA1A_HUMAN Alpha-1A adrenergic receptor OS=Homo sapiens GN=ADRA1A PE=2 SV=2		
P35348-2	ADA1A_HUMAN Isoform 2 of Alpha-1A adrenergic receptor OS=Homo sapiens GN=ADRA1A		
P35348-3	ADA1A_HUMAN Isoform 3 of Alpha-1A adrenergic receptor OS=Homo sapiens GN=ADRA1A		
P35348-4	ADA1A_HUMAN Isoform 4 of Alpha-1A adrenergic receptor OS=Homo sapiens GN=ADRA1A		
P35348-5	ADA1A_HUMAN Isoform 5 of Alpha-1A adrenergic receptor OS=Homo sapiens GN=ADRA1A		
P35348-6	ADA1A_HUMAN Isoform 6 of Alpha-1A adrenergic receptor OS=Homo sapiens GN=ADRA1A		
P35348-7	ADA1A_HUMAN Isoform 7 of Alpha-1A adrenergic receptor OS=Homo sapiens GN=ADRA1A		
P35348-8	ADA1A_HUMAN Isoform 8 of Alpha-1A adrenergic receptor OS=Homo sapiens GN=ADRA1A		
P35348-9	ADA1A_HUMAN Isoform 9 of Alpha-1A adrenergic receptor OS=Homo sapiens GN=ADRA1A		
P35354	PGH2_HUMAN Prostaglandin G/H synthase 2 OS=Homo sapiens GN=PTGS2 PE=1 SV=2	7.19E-08	
P35410	MAS1L_HUMAN Mas-related G-protein coupled receptor MRG OS=Homo sapiens GN=MAS1L PE=2 SV=1		24.84
P35442	TSP2_HUMAN Thrombospondin-2 OS=Homo sapiens GN=THBS2 PE=1 SV=2	0	
P35498	SCN1A_HUMAN Sodium channel protein type 1 subunit alpha OS=Homo sapiens GN=SCN1A PE=1 SV=2		
P35498-2	SCN1A_HUMAN Isoform 2 of Sodium channel protein type 1 subunit alpha OS=Homo sapiens GN=SCN1A		
P35499	SCN4A_HUMAN Sodium channel protein type 4 subunit alpha OS=Homo sapiens GN=SCN4A PE=1 SV=4		
P35520-2;D3DSK4;P3552	2|CBS_HUMAN Isoform 2 of Cystathionine beta-synthase OS=Homo sapiens GN=CBS;>tr|D3DSK4|D3DSK4_HUMAN Cystathionine-be	4.03E-05	
P35527	K1C9_HUMAN Keratin, type I cytoskeletal 9 OS=Homo sapiens GN=KRT9 PE=1 SV=3		2081.63
P35542	SAA4_HUMAN Serum amyloid A-4 protein OS=Homo sapiens GN=SAA4 PE=1 SV=2	4.39E-45	
P35548	MSX2_HUMAN Homeobox protein MSX-2 OS=Homo sapiens GN=MSX2 PE=1 SV=3		
P35555	FBN1_HUMAN Fibrillin-1 OS=Homo sapiens GN=FBN1 PE=1 SV=3	3.31E-201	
P35573;P35573-3;P35573	GDE_HUMAN Glycogen debranching enzyme OS=Homo sapiens GN=AGL PE=1 SV=3;>sp|P35573-3|GDE_HUMAN Isoform 6 of Glycog	0	
P35579	MYH9_HUMAN Myosin-9 OS=Homo sapiens GN=MYH9 PE=1 SV=4		7844.73
P35579-2	MYH9_HUMAN Isoform 2 of Myosin-9 OS=Homo sapiens GN=MYH9		5228.19
P35579;P35579-2	MYH9_HUMAN Myosin-9 OS=Homo sapiens GN=MYH9 PE=1 SV=4;>sp|P35579-2|MYH9_HUMAN Isoform 2 of Myosin-9 OS=Homo sap	0	
P35580	MYH10_HUMAN Myosin-10 OS=Homo sapiens GN=MYH10 PE=1 SV=3		1282.66
P35580-2	MYH10_HUMAN Isoform 2 of Myosin-10 OS=Homo sapiens GN=MYH10		1282.66
P35580-3	MYH10_HUMAN Isoform 3 of Myosin-10 OS=Homo sapiens GN=MYH10		1282.66
P35580-3;P35580-2;P355	3|MYH10_HUMAN Isoform 3 of Myosin-10 OS=Homo sapiens GN=MYH10;>sp|P35580-2|MYH10_HUMAN Isoform 2 of Myosin-10 OS=I	0	
P35606	COPB2_HUMAN Coatomer subunit beta' OS=Homo sapiens GN=COPB2 PE=1 SV=2		76.92
P35609	ACTN2_HUMAN Alpha-actinin-2 OS=Homo sapiens GN=ACTN2 PE=1 SV=1		916.96
P35610	SOAT1_HUMAN Sterol O-acyltransferase 1 OS=Homo sapiens GN=SOAT1 PE=1 SV=3		50.63
P35611	ADDA_HUMAN Alpha-adducin OS=Homo sapiens GN=ADD1 PE=1 SV=2		47.14
P35611-2	ADDA_HUMAN Isoform 2 of Alpha-adducin OS=Homo sapiens GN=ADD1		47.14
P35611-3	ADDA_HUMAN Isoform 3 of Alpha-adducin OS=Homo sapiens GN=ADD1		47.14
P35611-3;P35611;P35611	3|ADDA_HUMAN Isoform 3 of Alpha-adducin OS=Homo sapiens GN=ADD1;>sp|P35611|ADDA_HUMAN Alpha-adducin OS=Homo sapie	1.52E-17	
P35613	BASI_HUMAN Basigin OS=Homo sapiens GN=BSG PE=1 SV=2		631.92
P35613-2	BASI_HUMAN Isoform 2 of Basigin OS=Homo sapiens GN=BSG		631.92
P35613;P35613-2	BASI_HUMAN Basigin OS=Homo sapiens GN=BSG PE=1 SV=2;>sp|P35613-2|BASI_HUMAN Isoform 2 of Basigin OS=Homo sapiens G	5.71E-68	
P35625	TIMP3_HUMAN Metalloproteinase inhibitor 3 OS=Homo sapiens GN=TIMP3 PE=1 SV=2		51.53
P35637	FUS_HUMAN RNA-binding protein FUS OS=Homo sapiens GN=FUS PE=1 SV=1		106.82
P35637-2	FUS_HUMAN Isoform Short of RNA-binding protein FUS OS=Homo sapiens GN=FUS		106.82
P35637;P35637-2	FUS_HUMAN RNA-binding protein FUS OS=Homo sapiens GN=FUS PE=1 SV=1;>sp|P35637-2|FUS_HUMAN Isoform Short of RNA-binc	3.40E-300	
P35658	NU214_HUMAN Nuclear pore complex protein Nup214 OS=Homo sapiens GN=NUP214 PE=1 SV=2		78.13
P35658-2	NU214_HUMAN Isoform 2 of Nuclear pore complex protein Nup214 OS=Homo sapiens GN=NUP214		78.13
P35658-3	NU214_HUMAN Isoform 3 of Nuclear pore complex protein Nup214 OS=Homo sapiens GN=NUP214		78.13
P35658-3;P35658;P35658	3|NU214_HUMAN Isoform 3 of Nuclear pore complex protein Nup214 OS=Homo sapiens GN=NUP214;>sp|P35658|NU214_HUMAN Nu	5.54E-53	
P35658-4	NU214_HUMAN Isoform 4 of Nuclear pore complex protein Nup214 OS=Homo sapiens GN=NUP214		78.13
P35658-5	NU214_HUMAN Isoform 5 of Nuclear pore complex protein Nup214 OS=Homo sapiens GN=NUP214		78.98
P35658-5;P35658-3;P356	5|NU214_HUMAN Isoform 5 of Nuclear pore complex protein Nup214 OS=Homo sapiens GN=NUP214;>sp|P35658-3|NU214_HUMAN I	9.51E-11	
P35659	DEK_HUMAN Protein DEK OS=Homo sapiens GN=DEK PE=1 SV=1		136.33
P35749	MYH11_HUMAN Myosin-11 OS=Homo sapiens GN=MYH11 PE=1 SV=3		994.36

P35754	GLRX1_HUMAN Glutaredoxin-1 OS=Homo sapiens GN=GLRX PE=1 SV=2		26.23
P35790;P35790-2	CHKA_HUMAN Choline kinase alpha OS=Homo sapiens GN=CHKA PE=1 SV=3;>sp|P35790-2|CHKA_HUMAN Isoform 2 of Choline kina	6.29E-08	
P35813;O75688;O75688-:	PPM1A_HUMAN Protein phosphatase 1A OS=Homo sapiens GN=PPM1A PE=1 SV=1;>sp|O75688|PPM1B_HUMAN Protein phosphatase	2.58E-09	
P35858	ALS_HUMAN Insulin-like growth factor-binding protein complex acid labile subunit OS=Homo sapiens GN=IGFALS PE=1 SV=1		33.5
P35900	K1C20_HUMAN Keratin, type I cytoskeletal 20 OS=Homo sapiens GN=KRT20 PE=1 SV=1		71.33
P35908	K22E_HUMAN Keratin, type II cytoskeletal 2 epidermal OS=Homo sapiens GN=KRT2 PE=1 SV=2		2115.08
P35914	HMGCL_HUMAN Hydroxymethylglutaryl-CoA lyase, mitochondrial OS=Homo sapiens GN=HMGCL PE=1 SV=2		
P35998	PRS7_HUMAN 26S protease regulatory subunit 7 OS=Homo sapiens GN=PSMC2 PE=1 SV=3		483.35
P36021	MOT8_HUMAN Monocarboxylate transporter 8 OS=Homo sapiens GN=SLC16A2 PE=1 SV=2	1.73E-15	
P36222	CH3L1_HUMAN Chitinase-3-like protein 1 OS=Homo sapiens GN=CHI3L1 PE=1 SV=2	1.05E-41	
P36269;P36269-2	GGT5_HUMAN Gamma-glutamyltransferase 5 OS=Homo sapiens GN=GGT5 PE=1 SV=2;>sp|P36269-2|GGT5_HUMAN Isoform 2 of Ga	1.71E-24	
P36404	ARL2_HUMAN ADP-ribosylation factor-like protein 2 OS=Homo sapiens GN=ARL2 PE=1 SV=4		54.94
P36405	ARL3_HUMAN ADP-ribosylation factor-like protein 3 OS=Homo sapiens GN=ARL3 PE=1 SV=2	1.98E-19	
P36406	TRI23_HUMAN E3 ubiquitin-protein ligase TRIM23 OS=Homo sapiens GN=TRIM23 PE=1 SV=1		62.44
P36406-2	TRI23_HUMAN Isoform Beta of E3 ubiquitin-protein ligase TRIM23 OS=Homo sapiens GN=TRIM23		62.44
P36406-3	TRI23_HUMAN Isoform Gamma of E3 ubiquitin-protein ligase TRIM23 OS=Homo sapiens GN=TRIM23		62.44
P36406;P36406-2;P36406	TRI23_HUMAN E3 ubiquitin-protein ligase TRIM23 OS=Homo sapiens GN=TRIM23 PE=1 SV=1;>sp|P36406-2|TRI23_HUMAN Isoform	2.04E-05	
P36507	MP2K2_HUMAN Dual specificity mitogen-activated protein kinase kinase 2 OS=Homo sapiens GN=MAP2K2 PE=1 SV=1	1.15E-34	
P36542	ATPG_HUMAN ATP synthase subunit gamma, mitochondrial OS=Homo sapiens GN=ATP5C1 PE=1 SV=1		257.15
P36542-2	ATPG_HUMAN Isoform Heart of ATP synthase subunit gamma, mitochondrial OS=Homo sapiens GN=ATP5C1		257.15
P36542;P36542-2	ATPG_HUMAN ATP synthase subunit gamma, mitochondrial OS=Homo sapiens GN=ATP5C1 PE=1 SV=1;>sp|P36542-2|ATPG_HUMAN I	3.92E-116	
P36543	VATE1_HUMAN V-type proton ATPase subunit E 1 OS=Homo sapiens GN=ATP6V1E1 PE=1 SV=1		88.78
P36551	HEM6_HUMAN Coproporphyrinogen-III oxidase, mitochondrial OS=Homo sapiens GN=CPOX PE=1 SV=3		490.66
P36578	RL4_HUMAN 60S ribosomal protein L4 OS=Homo sapiens GN=RPL4 PE=1 SV=5		1425.93
P36639;P36639-2;P36639	8ODP_HUMAN 7,8-dihydro-8-oxoguanine triphosphatase OS=Homo sapiens GN=NUDT1 PE=1 SV=3;>sp|P36639-2|8ODP_HUMAN Iso	2.00E-11	
P36776	LONM_HUMAN Lon protease homolog, mitochondrial OS=Homo sapiens GN=LONP1 PE=1 SV=2		388.39
P36871	PGM1_HUMAN Phosphoglucomutase-1 OS=Homo sapiens GN=PGM1 PE=1 SV=3		109.93
P36871-2	PGM1_HUMAN Isoform 2 of Phosphoglucomutase-1 OS=Homo sapiens GN=PGM1		80.69
P36871-2;P36871	2|PGM1_HUMAN Isoform 2 of Phosphoglucomutase-1 OS=Homo sapiens GN=PGM1;>sp|P36871|PGM1_HUMAN Phosphoglucomutase-	3.55E-126	
P36871;P36871-2	PGM1_HUMAN Phosphoglucomutase-1 OS=Homo sapiens GN=PGM1 PE=1 SV=3;>sp|P36871-2|PGM1_HUMAN Isoform 2 of Phosphog	1.98E-30	
P36873	PP1G_HUMAN Serine/threonine-protein phosphatase PP1-gamma catalytic subunit OS=Homo sapiens GN=PPP1CC PE=1 SV=1		811.54
P36873-2	PP1G_HUMAN Isoform Gamma-2 of Serine/threonine-protein phosphatase PP1-gamma catalytic subunit OS=Homo sapiens GN=PPP1CC		814.1
P36873-2;P36873	2|PP1G_HUMAN Isoform Gamma-2 of Serine/threonine-protein phosphatase PP1-gamma catalytic subunit OS=Homo sapiens GN=PPP	0	
P36897-2;P36897;P36897	2|TGFR1_HUMAN Isoform 2 of TGF-beta receptor type-1 OS=Homo sapiens GN=TGFBR1;>sp|P36897|TGFR1_HUMAN TGF-beta recept	1.01E-13	
P36915;B4DYK6;Q0EFC6;	GNL1_HUMAN Guanine nucleotide-binding protein-like 1 OS=Homo sapiens GN=GNL1 PE=1 SV=2;>tr|B4DYK6|B4DYK6_HUMAN cDNA	2.57E-11	
P36952	SPB5_HUMAN Serpin B5 OS=Homo sapiens GN=SERPINB5 PE=1 SV=2	0	
P36954	RPB9_HUMAN DNA-directed RNA polymerase II subunit RPB9 OS=Homo sapiens GN=POLR2I PE=1 SV=1		18.86
P36955	PEDF_HUMAN Pigment epithelium-derived factor OS=Homo sapiens GN=SERPINF1 PE=1 SV=4	0	
P36957	ODO2_HUMAN Dihydrolipoyllysine-residue succinyltransferase component of 2-oxoglutarate dehydrogenase complex, mitochondrial OS=Homo sapier		271.8
P36959	GMPR1_HUMAN GMP reductase 1 OS=Homo sapiens GN=GMPR PE=1 SV=1		
P36969	GPX4_HUMAN Phospholipid hydroperoxide glutathione peroxidase, mitochondrial OS=Homo sapiens GN=GPX4 PE=1 SV=3		60.28
P36969-2	GPX4_HUMAN Isoform Cytoplasmic of Phospholipid hydroperoxide glutathione peroxidase, mitochondrial OS=Homo sapiens GN=GPX4		54.33
P36969;P36969-2	GPX4_HUMAN Phospholipid hydroperoxide glutathione peroxidase, mitochondrial OS=Homo sapiens GN=GPX4 PE=1 SV=3;>sp|P3696	2.51E-22	
P36980;P36980-2	FHR2_HUMAN Complement factor H-related protein 2 OS=Homo sapiens GN=CFHR2 PE=1 SV=1;>sp|P36980-2|FHR2_HUMAN Isoforr	5.83E-32	
P37108	SRP14_HUMAN Signal recognition particle 14 kDa protein OS=Homo sapiens GN=SRP14 PE=1 SV=2		492.74
P37198	NUP62_HUMAN Nuclear pore glycoprotein p62 OS=Homo sapiens GN=NUP62 PE=1 SV=3		
P37235	HPCL1_HUMAN Hippocalcin-like protein 1 OS=Homo sapiens GN=HPCAL1 PE=1 SV=3		526.8
P37268	FDFT_HUMAN Squalene synthase OS=Homo sapiens GN=FDFT1 PE=1 SV=1	3.69E-82	
P37275	ZEB1_HUMAN Zinc finger E-box-binding homeobox 1 OS=Homo sapiens GN=ZEB1 PE=1 SV=2		59.21
P37802	TAGL2_HUMAN Transgelin-2 OS=Homo sapiens GN=TAGLN2 PE=1 SV=3		1137.85
P37837	TALDO_HUMAN Transaldolase OS=Homo sapiens GN=TALDO1 PE=1 SV=2		303.09
P38117	ETFB_HUMAN Electron transfer flavoprotein subunit beta OS=Homo sapiens GN=ETFB PE=1 SV=3		162.55
P38117-2	ETFB_HUMAN Isoform 2 of Electron transfer flavoprotein subunit beta OS=Homo sapiens GN=ETFB		163.98
P38117-2;P38117	2|ETFB_HUMAN Isoform 2 of Electron transfer flavoprotein subunit beta OS=Homo sapiens GN=ETFB;>sp|P38117|ETFB_HUMAN Elect	4.92E-55	
P38117;P38117-2	ETFB_HUMAN Electron transfer flavoprotein subunit beta OS=Homo sapiens GN=ETFB PE=1 SV=3;>sp|P38117-2|ETFB_HUMAN Isofor	3.27E-64	
P38159	HNRPG_HUMAN Heterogeneous nuclear ribonucleoprotein G OS=Homo sapiens GN=RBMX PE=1 SV=3		332.85
P38405	GNAL_HUMAN Guanine nucleotide-binding protein G(olf) subunit alpha OS=Homo sapiens GN=GNAL PE=1 SV=1		117.15
P38432	COIL_HUMAN Coilin OS=Homo sapiens GN=COIL PE=1 SV=1		27.51
P38571;P38571-2	LICH_HUMAN Lysosomal acid lipase/cholesteryl ester hydrolase OS=Homo sapiens GN=LIPA PE=1 SV=2;>sp|P38571-2|LICH_HUMAN	7.73E-06	
P38606	VATA_HUMAN V-type proton ATPase catalytic subunit A OS=Homo sapiens GN=ATP6V1A PE=1 SV=2		
P38646	GRP75_HUMAN Stress-70 protein, mitochondrial OS=Homo sapiens GN=HSPA9 PE=1 SV=2		1677.46

P38919	IF4A3_HUMAN Eukaryotic initiation factor 4A-III OS=Homo sapiens GN=EIF4A3 PE=1 SV=4		218.06
P39019	RS19_HUMAN 40S ribosomal protein S19 OS=Homo sapiens GN=RPS19 PE=1 SV=2		590.92
P39023	RL3_HUMAN 60S ribosomal protein L3 OS=Homo sapiens GN=RPL3 PE=1 SV=2		1073.93
P39059	COFA1_HUMAN Collagen alpha-1(XV) chain OS=Homo sapiens GN=COL15A1 PE=1 SV=2		25.51
P39060;P39060-1;D3DSM	COIA1_HUMAN Collagen alpha-1(XVIII) chain OS=Homo sapiens GN=COL18A1 PE=1 SV=5;>sp|P39060-1|COIA1_HUMAN Isoform 2 c	3.62E-130	
P39210	MPV17_HUMAN Protein Mpv17 OS=Homo sapiens GN=MPV17 PE=1 SV=1	4.75E-08	
P39656	OST48_HUMAN Dolichyl-diphosphooligosaccharide--protein glycosyltransferase 48 kDa subunit OS=Homo sapiens GN=DDOST PE=1 SV=4		299.02
P39687	AN32A_HUMAN Acidic leucine-rich nuclear phosphoprotein 32 family member A OS=Homo sapiens GN=ANP32A PE=1 SV=1		547.31
P39748	FEN1_HUMAN Flap endonuclease 1 OS=Homo sapiens GN=FEN1 PE=1 SV=1		242.56
P39880	CUX1_HUMAN Homeobox protein cut-like 1 OS=Homo sapiens GN=CUX1 PE=1 SV=3		74.1
P39880-2	CUX1_HUMAN Isoform 2 of Homeobox protein cut-like 1 OS=Homo sapiens GN=CUX1		74.1
P39880-3	CUX1_HUMAN Isoform 3 of Homeobox protein cut-like 1 OS=Homo sapiens GN=CUX1		67.73
P39880-3;P39880;P39880	3|CUX1_HUMAN Isoform 3 of Homeobox protein cut-like 1 OS=Homo sapiens GN=CUX1;>sp|P39880|CUX1_HUMAN Homeobox protei	5.15E-13	
P39880-4	CUX1_HUMAN Isoform 5 of Homeobox protein cut-like 1 OS=Homo sapiens GN=CUX1		74.1
P39880-5	CUX1_HUMAN Isoform 6 of Homeobox protein cut-like 1 OS=Homo sapiens GN=CUX1		74.1
P39880-6	CUX1_HUMAN Isoform 7 of Homeobox protein cut-like 1 OS=Homo sapiens GN=CUX1		74.1
P40121	CAPG_HUMAN Macrophage-capping protein OS=Homo sapiens GN=CAPG PE=1 SV=2		324.95
P40123	CAP2_HUMAN Adenylyl cyclase-associated protein 2 OS=Homo sapiens GN=CAP2 PE=1 SV=1	1.58E-43	
P40199	CEAM6_HUMAN Carcinoembryonic antigen-related cell adhesion molecule 6 OS=Homo sapiens GN=CEACAM6 PE=1 SV=3	1.11E-67	
P40222	TXLNA_HUMAN Alpha-taxilin OS=Homo sapiens GN=TXLNA PE=1 SV=3		248.95
P40227	TCPZ_HUMAN T-complex protein 1 subunit zeta OS=Homo sapiens GN=CCT6A PE=1 SV=3		387.13
P40259	CD79B_HUMAN B-cell antigen receptor complex-associated protein beta chain OS=Homo sapiens GN=CD79B PE=1 SV=1	1.95E-54	
P40261	NNMT_HUMAN Nicotinamide N-methyltransferase OS=Homo sapiens GN=NNMT PE=1 SV=1		83.8
P40306	PSB10_HUMAN Proteasome subunit beta type-10 OS=Homo sapiens GN=PSMB10 PE=1 SV=1	1.44E-11	
P40394	ADH7_HUMAN Alcohol dehydrogenase class 4 mu/sigma chain OS=Homo sapiens GN=ADH7 PE=1 SV=2		17.58
P40429	RL13A_HUMAN 60S ribosomal protein L13a OS=Homo sapiens GN=RPL13A PE=1 SV=2		482.22
P40616	ARL1_HUMAN ADP-ribosylation factor-like protein 1 OS=Homo sapiens GN=ARL1 PE=1 SV=1		317.64
P40692	MLH1_HUMAN DNA mismatch repair protein Mlh1 OS=Homo sapiens GN=MLH1 PE=1 SV=1	8.00E-09	
P40763;P40763-2	STAT3_HUMAN Signal transducer and activator of transcription 3 OS=Homo sapiens GN=STAT3 PE=1 SV=2;>sp|P40763-2|STAT3_HUI	8.57E-09	
P40855;P40855-5	PEX19_HUMAN Peroxisomal biogenesis factor 19 OS=Homo sapiens GN=PEX19 PE=1 SV=1;>sp|P40855-5|PEX19_HUMAN Isoform 5 c	5.41E-29	
P40925	MDHC_HUMAN Malate dehydrogenase, cytoplasmic OS=Homo sapiens GN=MDH1 PE=1 SV=4		318.16
P40926	MDHM_HUMAN Malate dehydrogenase, mitochondrial OS=Homo sapiens GN=MDH2 PE=1 SV=3		1243.89
P40937	RFC5_HUMAN Replication factor C subunit 5 OS=Homo sapiens GN=RFC5 PE=1 SV=1		367.95
P40938	RFC3_HUMAN Replication factor C subunit 3 OS=Homo sapiens GN=RFC3 PE=1 SV=2		192.59
P40939	ECHA_HUMAN Trifunctional enzyme subunit alpha, mitochondrial OS=Homo sapiens GN=HADHA PE=1 SV=2		822.97
P41091	IF2G_HUMAN Eukaryotic translation initiation factor 2 subunit 3 OS=Homo sapiens GN=EIF2S3 PE=1 SV=3		756.97
P41161	ETV5_HUMAN ETS translocation variant 5 OS=Homo sapiens GN=ETV5 PE=1 SV=1		31.97
P41180	CASR_HUMAN Extracellular calcium-sensing receptor OS=Homo sapiens GN=CASR PE=1 SV=2		
P41180-2	CASR_HUMAN Isoform 2 of Extracellular calcium-sensing receptor OS=Homo sapiens GN=CASR		
P41208	CETN2_HUMAN Centrin-2 OS=Homo sapiens GN=CETN2 PE=1 SV=1		
P41212	ETV6_HUMAN Transcription factor ETV6 OS=Homo sapiens GN=ETV6 PE=1 SV=1		
P41214	EIF2D_HUMAN Eukaryotic translation initiation factor 2D OS=Homo sapiens GN=EIF2D PE=1 SV=3	5.01E-05	
P41214;Q5SY38;Q59FU5	LIGA_HUMAN Ligatin OS=Homo sapiens GN=LGTN PE=1 SV=3;>tr|Q5SY38|Q5SY38_HUMAN Ligatin (Fragment) OS=Homo sapiens G	5.33E-06	
P41218	MNDA_HUMAN Myeloid cell nuclear differentiation antigen OS=Homo sapiens GN=MNDA PE=1 SV=1	1.49E-126	
P41219	PERI_HUMAN Peripherin OS=Homo sapiens GN=PRPH PE=1 SV=2		350.38
P41219-2	PERI_HUMAN Isoform 2 of Peripherin OS=Homo sapiens GN=PRPH		349.76
P41219-2;P41219	2|PERI_HUMAN Isoform 2 of Peripherin OS=Homo sapiens GN=PRPH;>sp|P41219|PERI_HUMAN Peripherin OS=Homo sapiens GN=PR	1.85E-170	
P41223	BUD31_HUMAN Protein BUD31 homolog OS=Homo sapiens GN=BUD31 PE=1 SV=2		78.33
P41226	UBA7_HUMAN Ubiquitin-like modifier-activating enzyme 7 OS=Homo sapiens GN=UBA7 PE=1 SV=2	1.72E-38	
P41227	NAA10_HUMAN N-alpha-acetyltransferase 10, NatA catalytic subunit OS=Homo sapiens GN=NAA10 PE=1 SV=1		43.15
P41229	KDM5C_HUMAN Lysine-specific demethylase 5C OS=Homo sapiens GN=KDM5C PE=1 SV=2		33.14
P41229-2	KDM5C_HUMAN Isoform 2 of Lysine-specific demethylase 5C OS=Homo sapiens GN=KDM5C		33.14
P41229-3	KDM5C_HUMAN Isoform 3 of Lysine-specific demethylase 5C OS=Homo sapiens GN=KDM5C		33.14
P41236;Q6NXS1	IPP2_HUMAN Protein phosphatase inhibitor 2 OS=Homo sapiens GN=PPP1R2 PE=1 SV=2;>sp|Q6NXS1|IPP2M_HUMAN Putative protei	1.31E-10	
P41240	CSK_HUMAN Tyrosine-protein kinase CSK OS=Homo sapiens GN=CSK PE=1 SV=1	1.05E-88	
P41247	PLPL4_HUMAN Patatin-like phospholipase domain-containing protein 4 OS=Homo sapiens GN=PNPLA4 PE=1 SV=3		
P41250	SYG_HUMAN Glycyl-tRNA synthetase OS=Homo sapiens GN=GARS PE=1 SV=3		355.95
P41252	SYIC_HUMAN Isoleucyl-tRNA synthetase, cytoplasmic OS=Homo sapiens GN=IARS PE=1 SV=2		839.64
P41567	EIF1_HUMAN Eukaryotic translation initiation factor 1 OS=Homo sapiens GN=EIF1 PE=1 SV=1		267.62
P41743	KPCI_HUMAN Protein kinase C iota type OS=Homo sapiens GN=PRKCI PE=1 SV=2		16.43
P42025	ACTY_HUMAN Beta-centractin OS=Homo sapiens GN=ACTR1B PE=1 SV=1		123.68

P42126	ECI1_HUMAN Enoyl-CoA delta isomerase 1, mitochondrial OS=Homo sapiens GN=ECI1 PE=1 SV=1		199.89
P42126-2	ECI1_HUMAN Isoform 2 of Enoyl-CoA delta isomerase 1, mitochondrial OS=Homo sapiens GN=ECI1		134.26
P42126;P42126-2	ECI1_HUMAN Enoyl-CoA delta isomerase 1, mitochondrial OS=Homo sapiens GN=ECI1 PE=1 SV=1;>sp|P42126-2|ECI1_HUMAN Isofo	4.43E-283	
P42126;P42126-2;Q96DC	D3D2_HUMAN 3,2-trans-enoyl-CoA isomerase, mitochondrial OS=Homo sapiens GN=DCI PE=1 SV=1;>sp|P42126-2|D3D2_HUMAN Is	1.65E-89	
P42166	LAP2A_HUMAN Lamina-associated polypeptide 2, isoform alpha OS=Homo sapiens GN=TMPO PE=1 SV=2		731.87
P42167	LAP2B_HUMAN Lamina-associated polypeptide 2, isoforms beta/gamma OS=Homo sapiens GN=TMPO PE=1 SV=2		377.37
P42167-2	LAP2B_HUMAN Isoform Gamma of Lamina-associated polypeptide 2, isoforms beta/gamma OS=Homo sapiens GN=TMPO		377.37
P42167;P42167-2	LAP2B_HUMAN Lamina-associated polypeptide 2, isoforms beta/gamma OS=Homo sapiens GN=TMPO PE=1 SV=2;>sp|P42167-2|LAP2	1.07E-271	
P42224;P42224-2	STAT1_HUMAN Signal transducer and activator of transcription 1-alpha/beta OS=Homo sapiens GN=STAT1 PE=1 SV=2;>sp|P42224-2	9.56E-23	
P42226	STAT6_HUMAN Signal transducer and activator of transcription 6 OS=Homo sapiens GN=STAT6 PE=1 SV=1	1.84E-12	
P42229	STA5A_HUMAN Signal transducer and activator of transcription 5A OS=Homo sapiens GN=STAT5A PE=1 SV=1	3.96E-25	
P42285	SK2L2_HUMAN Superkiller viralicidic activity 2-like 2 OS=Homo sapiens GN=SKIV2L2 PE=1 SV=3		67.08
P42330;P17516	AK1C3_HUMAN Aldo-keto reductase family 1 member C3 OS=Homo sapiens GN=AKR1C3 PE=1 SV=4;>sp|P17516|AK1C4_HUMAN Alc	5.55E-157	
P42345	MTOR_HUMAN Serine/threonine-protein kinase mTOR OS=Homo sapiens GN=MTOR PE=1 SV=1	1.01E-09	
P42356	PI4KA_HUMAN Phosphatidylinositol 4-kinase alpha OS=Homo sapiens GN=PI4KA PE=1 SV=3		
P42356-2	PI4KA_HUMAN Isoform 2 of Phosphatidylinositol 4-kinase alpha OS=Homo sapiens GN=PI4KA		
P42566	EPS15_HUMAN Epidermal growth factor receptor substrate 15 OS=Homo sapiens GN=EPS15 PE=1 SV=2	6.04E-42	
P42574	CASP3_HUMAN Caspase-3 OS=Homo sapiens GN=CASP3 PE=1 SV=2	3.40E-70	
P42677	RS27_HUMAN 40S ribosomal protein S27 OS=Homo sapiens GN=RPS27 PE=1 SV=3		372.36
P42685	FRK_HUMAN Tyrosine-protein kinase FRK OS=Homo sapiens GN=FRK PE=1 SV=1	3.17E-05	
P42694	HELZ_HUMAN Probable helicase with zinc finger domain OS=Homo sapiens GN=HELZ PE=1 SV=2		
P42695	CNDD3_HUMAN Condensin-2 complex subunit D3 OS=Homo sapiens GN=NCAPD3 PE=1 SV=2	2.40E-34	
P42696	RBM34_HUMAN RNA-binding protein 34 OS=Homo sapiens GN=RBM34 PE=1 SV=2		22.91
P42696-2	RBM34_HUMAN Isoform 2 of RNA-binding protein 34 OS=Homo sapiens GN=RBM34		22.91
P42704	LPPRC_HUMAN Leucine-rich PPR motif-containing protein, mitochondrial OS=Homo sapiens GN=LRPPRC PE=1 SV=3		2479.11
P42765	THIM_HUMAN 3-ketoacyl-CoA thiolase, mitochondrial OS=Homo sapiens GN=ACAA2 PE=1 SV=2		
P42766	RL35_HUMAN 60S ribosomal protein L35 OS=Homo sapiens GN=RPL35 PE=1 SV=2		238.78
P42768	WASP_HUMAN Wiskott-Aldrich syndrome protein OS=Homo sapiens GN=WAS PE=1 SV=4	2.20E-23	
P42771;P42771-2	CD2A1_HUMAN Cyclin-dependent kinase inhibitor 2A, isoforms 1/2/3 OS=Homo sapiens GN=CDKN2A PE=1 SV=2;>sp|P42771-2|CD2	1.66E-14	
P42785	PCP_HUMAN Lysosomal Pro-X carboxypeptidase OS=Homo sapiens GN=PRCP PE=1 SV=1		
P42858	HD_HUMAN Huntingtin OS=Homo sapiens GN=HTT PE=1 SV=2		
P42892	ECE1_HUMAN Endothelin-converting enzyme 1 OS=Homo sapiens GN=ECE1 PE=1 SV=2		434.96
P42892-2	ECE1_HUMAN Isoform A of Endothelin-converting enzyme 1 OS=Homo sapiens GN=ECE1		434.96
P42892-3	ECE1_HUMAN Isoform C of Endothelin-converting enzyme 1 OS=Homo sapiens GN=ECE1		434.96
P42892-4	ECE1_HUMAN Isoform D of Endothelin-converting enzyme 1 OS=Homo sapiens GN=ECE1		434.96
P42892;P42892-4;P42892	ECE1_HUMAN Endothelin-converting enzyme 1 OS=Homo sapiens GN=ECE1 PE=1 SV=2;>sp|P42892-4|ECE1_HUMAN Isoform D of En	1.36E-17	
P43003	EAA1_HUMAN Excitatory amino acid transporter 1 OS=Homo sapiens GN=SLC1A3 PE=1 SV=1	0.00013158	
P43007	SATT_HUMAN Neutral amino acid transporter A OS=Homo sapiens GN=SLC1A4 PE=1 SV=1		
P43026	GDF5_HUMAN Growth/differentiation factor 5 OS=Homo sapiens GN=GDF5 PE=1 SV=3		
P43034	LIS1_HUMAN Platelet-activating factor acetylhydrolase IB subunit alpha OS=Homo sapiens GN=PAFAH1B1 PE=1 SV=2		48.16
P43034-2	LIS1_HUMAN Isoform 2 of Platelet-activating factor acetylhydrolase IB subunit alpha OS=Homo sapiens GN=PAFAH1B1		5.8
P43121	MUC18_HUMAN Cell surface glycoprotein MUC18 OS=Homo sapiens GN=MCAM PE=1 SV=2		134.7
P43121-2	MUC18_HUMAN Isoform 2 of Cell surface glycoprotein MUC18 OS=Homo sapiens GN=MCAM		44.8
P43121;P43121-2	MUC18_HUMAN Cell surface glycoprotein MUC18 OS=Homo sapiens GN=MCAM PE=1 SV=2;>sp|P43121-2|MUC18_HUMAN Isoform 2	1.29E-11	
P43155	CACP_HUMAN Carnitine O-acetyltransferase OS=Homo sapiens GN=CRAT PE=1 SV=5		33.58
P43155-2	CACP_HUMAN Isoform 2 of Carnitine O-acetyltransferase OS=Homo sapiens GN=CRAT		33.58
P43155-3	CACP_HUMAN Isoform 3 of Carnitine O-acetyltransferase OS=Homo sapiens GN=CRAT		27.84
P43155;P43155-2;C9JBD1	CACP_HUMAN Carnitine O-acetyltransferase OS=Homo sapiens GN=CRAT PE=1 SV=5;>sp|P43155-2|CACP_HUMAN Isoform 2 of Carn	1.09E-81	
P43243	MATR3_HUMAN Matrin-3 OS=Homo sapiens GN=MATR3 PE=1 SV=2		555.47
P43246	MSH2_HUMAN DNA mismatch repair protein Msh2 OS=Homo sapiens GN=MSH2 PE=1 SV=1		304.41
P43268	ETV4_HUMAN ETS translocation variant 4 OS=Homo sapiens GN=ETV4 PE=1 SV=3		29.69
P43304	GPDM_HUMAN Glycerol-3-phosphate dehydrogenase, mitochondrial OS=Homo sapiens GN=GPD2 PE=1 SV=3		95
P43304-2	GPDM_HUMAN Isoform 2 of Glycerol-3-phosphate dehydrogenase, mitochondrial OS=Homo sapiens GN=GPD2		91.35
P43304;P43304-2	GPDM_HUMAN Glycerol-3-phosphate dehydrogenase, mitochondrial OS=Homo sapiens GN=GPD2 PE=1 SV=3;>sp|P43304-2|GPDM_H	8.84E-40	
P43307	SSRA_HUMAN Translocon-associated protein subunit alpha OS=Homo sapiens GN=SSR1 PE=1 SV=3		154.31
P43307-2	SSRA_HUMAN Isoform 2 of Translocon-associated protein subunit alpha OS=Homo sapiens GN=SSR1		116.46
P43307;P43307-2	SSRA_HUMAN Translocon-associated protein subunit alpha OS=Homo sapiens GN=SSR1 PE=1 SV=3;>sp|P43307-2|SSRA_HUMAN Isc	5.12E-56	
P43378	PTN9_HUMAN Tyrosine-protein phosphatase non-receptor type 9 OS=Homo sapiens GN=PTPN9 PE=1 SV=1		
P43405;P43405-2	KSYK_HUMAN Tyrosine-protein kinase SYK OS=Homo sapiens GN=SYK PE=1 SV=1;>sp|P43405-2|KSYK_HUMAN Isoform Short of Tyr	1.14E-142	
P43487	RANG_HUMAN Ran-specific GTPase-activating protein OS=Homo sapiens GN=RANBP1 PE=1 SV=1		164.4
P43490	NAMPT_HUMAN Nicotinamide phosphoribosyltransferase OS=Homo sapiens GN=NAMPT PE=1 SV=1		564.06

P43626	KI2L1_HUMAN Killer cell immunoglobulin-like receptor 2DL1 OS=Homo sapiens GN=KIR2DL1 PE=1 SV=1		
P43627	KI2L2_HUMAN Killer cell immunoglobulin-like receptor 2DL2 OS=Homo sapiens GN=KIR2DL2 PE=1 SV=1		
P43628	KI2L3_HUMAN Killer cell immunoglobulin-like receptor 2DL3 OS=Homo sapiens GN=KIR2DL3 PE=1 SV=1		
P43628-2	KI2L3_HUMAN Isoform 2 of Killer cell immunoglobulin-like receptor 2DL3 OS=Homo sapiens GN=KIR2DL3		
P43629	KI3L1_HUMAN Killer cell immunoglobulin-like receptor 3DL1 OS=Homo sapiens GN=KIR3DL1 PE=2 SV=1		
P43631	KI2S2_HUMAN Killer cell immunoglobulin-like receptor 2DS2 OS=Homo sapiens GN=KIR2DS2 PE=1 SV=2		
P43652	AFAM_HUMAN Afamin OS=Homo sapiens GN=AFM PE=1 SV=1		31.34
P43686	PRS6B_HUMAN 26S protease regulatory subunit 6B OS=Homo sapiens GN=PSMC4 PE=1 SV=2		286.19
P43686-2	PRS6B_HUMAN Isoform 2 of 26S protease regulatory subunit 6B OS=Homo sapiens GN=PSMC4		286.19
P43686;P43686-2	PRS6B_HUMAN 26S protease regulatory subunit 6B OS=Homo sapiens GN=PSMC4 PE=1 SV=2;>sp|P43686-2|PRS6B_HUMAN Isoform	1.19E-255	
P43897	EFTS_HUMAN Elongation factor Ts, mitochondrial OS=Homo sapiens GN=TSFM PE=1 SV=2		
P43897-2	EFTS_HUMAN Isoform 2 of Elongation factor Ts, mitochondrial OS=Homo sapiens GN=TSFM		
P43897-2;P43897	2|EFTS_HUMAN Isoform 2 of Elongation factor Ts, mitochondrial OS=Homo sapiens GN=TSFM;>sp|P43897|EFTS_HUMAN Elongation f	4.45E-46	
P45877	PPIC_HUMAN Peptidyl-prolyl cis-trans isomerase C OS=Homo sapiens GN=PPIC PE=1 SV=1		49.08
P45880	VDAC2_HUMAN Voltage-dependent anion-selective channel protein 2 OS=Homo sapiens GN=VDAC2 PE=1 SV=2		586.15
P45880-1	VDAC2_HUMAN Isoform 1 of Voltage-dependent anion-selective channel protein 2 OS=Homo sapiens GN=VDAC2		586.15
P45880-1;P45880;P45880	1|VDAC2_HUMAN Isoform 1 of Voltage-dependent anion-selective channel protein 2 OS=Homo sapiens GN=VDAC2;>sp|P45880|VDAC	7.34E-25	
P45880-2	VDAC2_HUMAN Isoform 2 of Voltage-dependent anion-selective channel protein 2 OS=Homo sapiens GN=VDAC2		586.15
P45880;P45880-1;P45880	VDAC2_HUMAN Voltage-dependent anion-selective channel protein 2 OS=Homo sapiens GN=VDAC2 PE=1 SV=2;>sp|P45880-1|VDAC	0	
P45954	ACDSB_HUMAN Short/branched chain specific acyl-CoA dehydrogenase, mitochondrial OS=Homo sapiens GN=ACADSB PE=1 SV=1	7.08E-115	
P45973	CBX5_HUMAN Chromobox protein homolog 5 OS=Homo sapiens GN=CBX5 PE=1 SV=1		43.49
P45974	UBP5_HUMAN Ubiquitin carboxyl-terminal hydrolase 5 OS=Homo sapiens GN=USP5 PE=1 SV=2		
P45974-2	UBP5_HUMAN Isoform Short of Ubiquitin carboxyl-terminal hydrolase 5 OS=Homo sapiens GN=USP5		
P45974-2;P45974	2|UBP5_HUMAN Isoform Short of Ubiquitin carboxyl-terminal hydrolase 5 OS=Homo sapiens GN=USP5;>sp|P45974|UBP5_HUMAN Ub	0	
P45974;P45974-2	UBP5_HUMAN Ubiquitin carboxyl-terminal hydrolase 5 OS=Homo sapiens GN=USP5 PE=1 SV=2;>sp|P45974-2|UBP5_HUMAN Isoform	2.99E-166	
P45983	MK08_HUMAN Mitogen-activated protein kinase 8 OS=Homo sapiens GN=MAPK8 PE=1 SV=2		41.33
P45983-2	MK08_HUMAN Isoform 1 of Mitogen-activated protein kinase 8 OS=Homo sapiens GN=MAPK8		41.33
P45983-3	MK08_HUMAN Isoform 3 of Mitogen-activated protein kinase 8 OS=Homo sapiens GN=MAPK8		41.33
P45983-4	MK08_HUMAN Isoform 4 of Mitogen-activated protein kinase 8 OS=Homo sapiens GN=MAPK8		41.33
P45984	MK09_HUMAN Mitogen-activated protein kinase 9 OS=Homo sapiens GN=MAPK9 PE=1 SV=2		45.31
P45984-2	MK09_HUMAN Isoform Alpha-1 of Mitogen-activated protein kinase 9 OS=Homo sapiens GN=MAPK9		45.31
P45984-3	MK09_HUMAN Isoform Beta-1 of Mitogen-activated protein kinase 9 OS=Homo sapiens GN=MAPK9		45.31
P45984-4	MK09_HUMAN Isoform Beta-2 of Mitogen-activated protein kinase 9 OS=Homo sapiens GN=MAPK9		45.31
P45985-2;D3DTS5;P4598	2|MP2K4_HUMAN Isoform 2 of Dual specificity mitogen-activated protein kinase kinase 4 OS=Homo sapiens GN=MAP2K4;>tr|D3DTS5	1.98E-80	
P46013	KI67_HUMAN Antigen KI-67 OS=Homo sapiens GN=MKI67 PE=1 SV=2		991.32
P46013-2	KI67_HUMAN Isoform Short of Antigen KI-67 OS=Homo sapiens GN=MKI67		944.96
P46013;P46013-2	KI67_HUMAN Antigen KI-67 OS=Homo sapiens GN=MKI67 PE=1 SV=2;>sp|P46013-2|KI67_HUMAN Isoform Short of Antigen KI-67 C	6.43E-82	
P46019	KPB2_HUMAN Phosphorylase b kinase regulatory subunit alpha, liver isoform OS=Homo sapiens GN=PHKA2 PE=1 SV=1		
P46020	KPB1_HUMAN Phosphorylase b kinase regulatory subunit alpha, skeletal muscle isoform OS=Homo sapiens GN=PHKA1 PE=1 SV=2		82.15
P46020-2	KPB1_HUMAN Isoform 2 of Phosphorylase b kinase regulatory subunit alpha, skeletal muscle isoform OS=Homo sapiens GN=PHKA1		82.15
P46060	RAGP1_HUMAN Ran GTPase-activating protein 1 OS=Homo sapiens GN=RANGAP1 PE=1 SV=1		190.58
P46063	RECQ1_HUMAN ATP-dependent DNA helicase Q1 OS=Homo sapiens GN=RECQL PE=1 SV=3		42.59
P46087	NOP2_HUMAN Putative ribosomal RNA methyltransferase NOP2 OS=Homo sapiens GN=NOP2 PE=1 SV=2		141.64
P46087-2	NOP2_HUMAN Isoform 2 of Putative ribosomal RNA methyltransferase NOP2 OS=Homo sapiens GN=NOP2		141.64
P46087;P46087-2	NOP2_HUMAN Putative ribosomal RNA methyltransferase NOP2 OS=Homo sapiens GN=NOP2 PE=1 SV=2;>sp|P46087-2|NOP2_HUMA	5.65E-219	
P46100	ATRX_HUMAN Transcriptional regulator ATRX OS=Homo sapiens GN=ATRX PE=1 SV=5		
P46100-2	ATRX_HUMAN Isoform 1 of Transcriptional regulator ATRX OS=Homo sapiens GN=ATRX		
P46100-3	ATRX_HUMAN Isoform 2 of Transcriptional regulator ATRX OS=Homo sapiens GN=ATRX		
P46100-4	ATRX_HUMAN Isoform 3 of Transcriptional regulator ATRX OS=Homo sapiens GN=ATRX		
P46100-5	ATRX_HUMAN Isoform 5 of Transcriptional regulator ATRX OS=Homo sapiens GN=ATRX		
P46100-6	ATRX_HUMAN Isoform 6 of Transcriptional regulator ATRX OS=Homo sapiens GN=ATRX		
P46100;P46100-4;P46100	ATRX_HUMAN Transcriptional regulator ATRX OS=Homo sapiens GN=ATRX PE=1 SV=5;>sp|P46100-4|ATRX_HUMAN Isoform 3 of Trar	4.70E-10	
P46108	CRK_HUMAN Adapter molecule crk OS=Homo sapiens GN=CRK PE=1 SV=2		42.77
P46108-2	CRK_HUMAN Isoform Crk-I of Adapter molecule crk OS=Homo sapiens GN=CRK		42.77
P46108;P46108-2	CRK_HUMAN Adapter molecule crk OS=Homo sapiens GN=CRK PE=1 SV=2;>sp|P46108-2|CRK_HUMAN Isoform Crk-I of Adapter mol	2.26E-44	
P46109	CRKL_HUMAN Crk-like protein OS=Homo sapiens GN=CRKL PE=1 SV=1	8.77E-31	
P46199	IF2M_HUMAN Translation initiation factor IF-2, mitochondrial OS=Homo sapiens GN=MTIF2 PE=1 SV=2	8.45E-15	
P46379	BAG6_HUMAN Large proline-rich protein BAG6 OS=Homo sapiens GN=BAG6 PE=1 SV=2		191.67
P46379-2	BAG6_HUMAN Isoform 2 of Large proline-rich protein BAG6 OS=Homo sapiens GN=BAG6		191.67
P46379-3	BAG6_HUMAN Isoform 3 of Large proline-rich protein BAG6 OS=Homo sapiens GN=BAG6		191.67
P46379-3;P46379;B0UX8	3|BAT3_HUMAN Isoform 3 of Large proline-rich protein BAT3 OS=Homo sapiens GN=BAT3;>sp|P46379|BAT3_HUMAN Large proline-ri	2.98E-22	

P46379-3;P46379;P46379	3|BAG6_HUMAN Isoform 3 of Large proline-rich protein BAG6 OS=Homo sapiens GN=BAG6;>sp|P46379|BAG6_HUMAN Large proline-	2.86E-28	
P46459	NSF_HUMAN Vesicle-fusing ATPase OS=Homo sapiens GN=NSF PE=1 SV=3		52.77
P46531	NOTC1_HUMAN Neurogenic locus notch homolog protein 1 OS=Homo sapiens GN=NOTCH1 PE=1 SV=4		7.52
P46734-3;C9JI18;P46734;	3|MP2K3_HUMAN Isoform 2 of Dual specificity mitogen-activated protein kinase kinase 3 OS=Homo sapiens GN=MAP2K3;>tr|C9JI18|	2.23E-35	
P46736;P46736-3;P46736	BRCC3_HUMAN Lys-63-specific deubiquitinase BRCC36 OS=Homo sapiens GN=BRCC3 PE=1 SV=2;>sp|P46736-3|BRCC3_HUMAN Isof	2.15E-05	
P46776	RL27A_HUMAN 60S ribosomal protein L27a OS=Homo sapiens GN=RPL27A PE=1 SV=2		270.78
P46777	RL5_HUMAN 60S ribosomal protein L5 OS=Homo sapiens GN=RPL5 PE=1 SV=3		1740.68
P46778	RL21_HUMAN 60S ribosomal protein L21 OS=Homo sapiens GN=RPL21 PE=1 SV=2		671.68
P46779	RL28_HUMAN 60S ribosomal protein L28 OS=Homo sapiens GN=RPL28 PE=1 SV=3		403.57
P46781	RS9_HUMAN 40S ribosomal protein S9 OS=Homo sapiens GN=RPS9 PE=1 SV=3		840.85
P46782	RS5_HUMAN 40S ribosomal protein S5 OS=Homo sapiens GN=RPS5 PE=1 SV=4		808.8
P46783	RS10_HUMAN 40S ribosomal protein S10 OS=Homo sapiens GN=RPS10 PE=1 SV=1		519.22
P46821	MAP1B_HUMAN Microtubule-associated protein 1B OS=Homo sapiens GN=MAP1B PE=1 SV=2		1615.97
P46926	GNPI1_HUMAN Glucosamine-6-phosphate isomerase 1 OS=Homo sapiens GN=GNPDA1 PE=1 SV=1	8.11E-22	
P46934-4;P46934;P46934	4|NEDD4_HUMAN Isoform 4 of E3 ubiquitin-protein ligase NEDD4 OS=Homo sapiens GN=NEDD4;>sp|P46934|NEDD4_HUMAN E3 ubi	2.58E-98	
P46937	YAP1_HUMAN Yorkie homolog OS=Homo sapiens GN=YAP1 PE=1 SV=2		68.37
P46937-2	YAP1_HUMAN Isoform 2 of Yorkie homolog OS=Homo sapiens GN=YAP1		68.37
P46937-3	YAP1_HUMAN Isoform 3 of Yorkie homolog OS=Homo sapiens GN=YAP1		68.37
P46937;P46937-2;P46937	YAP1_HUMAN Yorkie homolog OS=Homo sapiens GN=YAP1 PE=1 SV=2;>sp|P46937-2|YAP1_HUMAN Isoform 2 of Yorkie homolog OS=	1.18E-11	
P46939	UTRO_HUMAN Utrophin OS=Homo sapiens GN=UTRN PE=1 SV=2		1475.41
P46940	IQGA1_HUMAN Ras GTPase-activating-like protein IQGAP1 OS=Homo sapiens GN=IQGAP1 PE=1 SV=1		3781.81
P46976;P46976-2;P46976	GLYG_HUMAN Glycogenin-1 OS=Homo sapiens GN=GYG1 PE=1 SV=4;>sp|P46976-2|GLYG_HUMAN Isoform GN-1 of Glycogenin-1 OS	9.26E-11	
P46977	STT3A_HUMAN Dolichyl-diphosphooligosaccharide--protein glycosyltransferase subunit STT3A OS=Homo sapiens GN=STT3A PE=1 SV=2		269.77
P47224	MSS4_HUMAN Guanine nucleotide exchange factor MSS4 OS=Homo sapiens GN=RABIF PE=1 SV=2	4.49E-13	
P47736	RPGP1_HUMAN Rap1 GTPase-activating protein 1 OS=Homo sapiens GN=RAP1GAP PE=1 SV=2		29.08
P47736-2	RPGP1_HUMAN Isoform 2 of Rap1 GTPase-activating protein 1 OS=Homo sapiens GN=RAP1GAP		29.08
P47736-3	RPGP1_HUMAN Isoform 3 of Rap1 GTPase-activating protein 1 OS=Homo sapiens GN=RAP1GAP		28.94
P47755	CAZA2_HUMAN F-actin-capping protein subunit alpha-2 OS=Homo sapiens GN=CAPZA2 PE=1 SV=3		146.35
P47756	CAPZB_HUMAN F-actin-capping protein subunit beta OS=Homo sapiens GN=CAPZB PE=1 SV=4		529.11
P47756-2	CAPZB_HUMAN Isoform 2 of F-actin-capping protein subunit beta OS=Homo sapiens GN=CAPZB		625.99
P47756-2;P47756	2|CAPZB_HUMAN Isoform 2 of F-actin-capping protein subunit beta OS=Homo sapiens GN=CAPZB;>sp|P47756|CAPZB_HUMAN F-acti	6.35E-68	
P47756;P47756-2	CAPZB_HUMAN F-actin-capping protein subunit beta OS=Homo sapiens GN=CAPZB PE=1 SV=4;>sp|P47756-2|CAPZB_HUMAN Isofori	2.03E-63	
P47813	IF1AX_HUMAN Eukaryotic translation initiation factor 1A, X-chromosomal OS=Homo sapiens GN=EIF1AX PE=1 SV=2		180.66
P47895	AL1A3_HUMAN Aldehyde dehydrogenase family 1 member A3 OS=Homo sapiens GN=ALDH1A3 PE=1 SV=2		20.89
P47897	SYQ_HUMAN Glutaminyl-tRNA synthetase OS=Homo sapiens GN=QARS PE=1 SV=1		645.34
P47898	5HT5A_HUMAN 5-hydroxytryptamine receptor 5A OS=Homo sapiens GN=HTR5A PE=1 SV=1		
P47914	RL29_HUMAN 60S ribosomal protein L29 OS=Homo sapiens GN=RPL29 PE=1 SV=2		132.01
P47929	LEG7_HUMAN Galectin-7 OS=Homo sapiens GN=LGALS7 PE=1 SV=2		
P47985	UCRI_HUMAN Cytochrome b-c1 complex subunit Rieske, mitochondrial OS=Homo sapiens GN=UQCRFS1 PE=1 SV=2		361.62
P47989	XDH_HUMAN Xanthine dehydrogenase/oxidase OS=Homo sapiens GN=XDH PE=1 SV=4	1.21E-08	
P48047	ATPO_HUMAN ATP synthase subunit O, mitochondrial OS=Homo sapiens GN=ATP5O PE=1 SV=1		654.16
P48058	GRIA4_HUMAN Glutamate receptor 4 OS=Homo sapiens GN=GRIA4 PE=2 SV=2		
P48059	LIMS1_HUMAN LIM and senescent cell antigen-like-containing domain protein 1 OS=Homo sapiens GN=LIMS1 PE=1 SV=4		189.42
P48060	GLIP1_HUMAN Glioma pathogenesis-related protein 1 OS=Homo sapiens GN=GLIPR1 PE=1 SV=3		39.69
P48147	PPCE_HUMAN Prolyl endopeptidase OS=Homo sapiens GN=PREP PE=1 SV=2	6.81E-08	
P48163	MAOX_HUMAN NADP-dependent malic enzyme OS=Homo sapiens GN=ME1 PE=1 SV=1	6.43E-13	
P48426	PI42A_HUMAN Phosphatidylinositol-5-phosphate 4-kinase type-2 alpha OS=Homo sapiens GN=PIP4K2A PE=1 SV=2	8.70E-23	
P48443	RXRG_HUMAN Retinoic acid receptor RXR-gamma OS=Homo sapiens GN=RXRG PE=1 SV=1		
P48444	COPD_HUMAN Coatomer subunit delta OS=Homo sapiens GN=ARCN1 PE=1 SV=1		492.34
P48449	ERG7_HUMAN Lanosterol synthase OS=Homo sapiens GN=LSS PE=1 SV=1		223.8
P48454	PP2BC_HUMAN Serine/threonine-protein phosphatase 2B catalytic subunit gamma isoform OS=Homo sapiens GN=PPP3CC PE=1 SV=3		
P48454-2	PP2BC_HUMAN Isoform 2 of Serine/threonine-protein phosphatase 2B catalytic subunit gamma isoform OS=Homo sapiens GN=PPP3CC		
P48507	GSH0_HUMAN Glutamate--cysteine ligase regulatory subunit OS=Homo sapiens GN=GCLM PE=1 SV=1	0.0001736	
P48509	CD151_HUMAN CD151 antigen OS=Homo sapiens GN=CD151 PE=1 SV=3		124.2
P48552	NRIP1_HUMAN Nuclear receptor-interacting protein 1 OS=Homo sapiens GN=NRIP1 PE=1 SV=2		
P48556	PSMD8_HUMAN 26S proteasome non-ATPase regulatory subunit 8 OS=Homo sapiens GN=PSMD8 PE=1 SV=2		242.53
P48594	SPB4_HUMAN Serpin B4 OS=Homo sapiens GN=SERPINB4 PE=1 SV=2		
P48595	SPB10_HUMAN Serpin B10 OS=Homo sapiens GN=SERPINB10 PE=1 SV=1	4.38E-25	
P48634	PRC2A_HUMAN Protein PRRC2A OS=Homo sapiens GN=PRRC2A PE=1 SV=3		519.89
P48634-2	PRC2A_HUMAN Isoform 2 of Protein PRRC2A OS=Homo sapiens GN=PRRC2A		462.28
P48634-3	PRC2A_HUMAN Isoform 3 of Protein PRRC2A OS=Homo sapiens GN=PRRC2A		496.01

P48634-4	PRC2A_HUMAN Isoform 4 of Protein PRRC2A OS=Homo sapiens GN=PRRC2A		369.16
P48634;P48634-3;P48634	PRC2A_HUMAN Protein PRRC2A OS=Homo sapiens GN=PRRC2A PE=1 SV=3;>sp|P48634-3|PRC2A_HUMAN Isoform 3 of Protein PRRC	8.33E-22	
P48637	GSHB_HUMAN Glutathione synthetase OS=Homo sapiens GN=GSS PE=1 SV=1		40.33
P48643	TCPE_HUMAN T-complex protein 1 subunit epsilon OS=Homo sapiens GN=CCT5 PE=1 SV=1		550.62
P48651	PTSS1_HUMAN Phosphatidylserine synthase 1 OS=Homo sapiens GN=PTDSS1 PE=1 SV=1		103.86
P48664	EAA4_HUMAN Excitatory amino acid transporter 4 OS=Homo sapiens GN=SLC1A6 PE=2 SV=1		40.4
P48668	K2C6C_HUMAN Keratin, type II cytoskeletal 6C OS=Homo sapiens GN=KRT6C PE=1 SV=3		1061.4
P48681	NEST_HUMAN Nestin OS=Homo sapiens GN=NES PE=1 SV=2	2.17E-38	
P48729	KC1A_HUMAN Casein kinase I isoform alpha OS=Homo sapiens GN=CSNK1A1 PE=1 SV=2		65.77
P48729-2	KC1A_HUMAN Isoform 2 of Casein kinase I isoform alpha OS=Homo sapiens GN=CSNK1A1		65.77
P48729-2;P48729	2|KC1A_HUMAN Isoform 2 of Casein kinase I isoform alpha OS=Homo sapiens GN=CSNK1A1;>sp|P48729|KC1A_HUMAN Casein kinas	7.35E-41	
P48735	IDHP_HUMAN Isocitrate dehydrogenase [NADP], mitochondrial OS=Homo sapiens GN=IDH2 PE=1 SV=2		
P48739-2;P48739	2|PIPNB_HUMAN Isoform 2 of Phosphatidylinositol transfer protein beta isoform OS=Homo sapiens GN=PITPNB;>sp|P48739|PIPNB_H	9.72E-62	
P48741	HSP77_HUMAN Putative heat shock 70 kDa protein 7 OS=Homo sapiens GN=HSPA7 PE=5 SV=2		267.36
P48960	CD97_HUMAN CD97 antigen OS=Homo sapiens GN=CD97 PE=1 SV=4		1002.34
P48960-2	CD97_HUMAN Isoform 2 of CD97 antigen OS=Homo sapiens GN=CD97		1002.34
P48960-3	CD97_HUMAN Isoform 3 of CD97 antigen OS=Homo sapiens GN=CD97		1002.34
P48960;P48960-3;B4DTS(	CD97_HUMAN CD97 antigen OS=Homo sapiens GN=CD97 PE=1 SV=4;>sp|P48960-3|CD97_HUMAN Isoform 3 of CD97 antigen OS=H	7.45E-249	
P48995	TRPC1_HUMAN Short transient receptor potential channel 1 OS=Homo sapiens GN=TRPC1 PE=1 SV=1		
P48995-2	TRPC1_HUMAN Isoform Short of Short transient receptor potential channel 1 OS=Homo sapiens GN=TRPC1		
P49005	DPOD2_HUMAN DNA polymerase delta subunit 2 OS=Homo sapiens GN=POLD2 PE=1 SV=1	4.83E-07	
P49006	MRP_HUMAN MARCKS-related protein OS=Homo sapiens GN=MARCKSL1 PE=1 SV=2		107.17
P49023-3;B7ZMB4;P4902	3|PAXI_HUMAN Isoform Gamma of Paxillin OS=Homo sapiens GN=PXN;>tr|B7ZMB4|B7ZMB4_HUMAN Paxillin OS=Homo sapiens GN=	6.55E-230	
P49137	MAPK2_HUMAN MAP kinase-activated protein kinase 2 OS=Homo sapiens GN=MAPKAPK2 PE=1 SV=1	4.92E-13	
P49189	AL9A1_HUMAN 4-trimethylaminobutyraldehyde dehydrogenase OS=Homo sapiens GN=ALDH9A1 PE=1 SV=3		87.15
P49190	PTH2R_HUMAN Parathyroid hormone 2 receptor OS=Homo sapiens GN=PTH2R PE=1 SV=1		
P49207	RL34_HUMAN 60S ribosomal protein L34 OS=Homo sapiens GN=RPL34 PE=1 SV=3		246.96
P49221	TGM4_HUMAN Protein-glutamine gamma-glutamyltransferase 4 OS=Homo sapiens GN=TGM4 PE=1 SV=2	1.16E-11	
P49247	RPIA_HUMAN Ribose-5-phosphate isomerase OS=Homo sapiens GN=RPIA PE=1 SV=3	9.95E-21	
P49257	LMAN1_HUMAN Protein ERGIC-53 OS=Homo sapiens GN=LMAN1 PE=1 SV=2		126.61
P49321	NASP_HUMAN Nuclear autoantigenic sperm protein OS=Homo sapiens GN=NASP PE=1 SV=2		456.23
P49321-2	NASP_HUMAN Isoform 2 of Nuclear autoantigenic sperm protein OS=Homo sapiens GN=NASP		279.93
P49321-3	NASP_HUMAN Isoform 3 of Nuclear autoantigenic sperm protein OS=Homo sapiens GN=NASP		456.23
P49321-3;D3DQ07;P4932	3|NASP_HUMAN Isoform 3 of Nuclear autoantigenic sperm protein OS=Homo sapiens GN=NASP;>tr|D3DQ07|D3DQ07_HUMAN Nuclea	9.91E-257	
P49327	FAS_HUMAN Fatty acid synthase OS=Homo sapiens GN=FASN PE=1 SV=3		682.94
P49354;P49354-2	FNTA_HUMAN Protein farnesyltransferase/geranylgeranyltransferase type-1 subunit alpha OS=Homo sapiens GN=FNTA PE=1 SV=1;>s	4.81E-18	
P49366;P49366-2	DHYS_HUMAN Deoxyhypusine synthase OS=Homo sapiens GN=DHPS PE=1 SV=1;>sp|P49366-2|DHYS_HUMAN Isoform Short of Deo	1.94E-21	
P49368	TCPG_HUMAN T-complex protein 1 subunit gamma OS=Homo sapiens GN=CCT3 PE=1 SV=4		755.89
P49406	RM19_HUMAN 39S ribosomal protein L19, mitochondrial OS=Homo sapiens GN=MRPL19 PE=1 SV=2		105.17
P49407;P49407-2	ARRB1_HUMAN Beta-arrestin-1 OS=Homo sapiens GN=ARRB1 PE=1 SV=2;>sp|P49407-2|ARRB1_HUMAN Isoform 1B of Beta-arrestin	1.74E-09	
P49411	EFTU_HUMAN Elongation factor Tu, mitochondrial OS=Homo sapiens GN=TUFM PE=1 SV=2		744.25
P49419	AL7A1_HUMAN Alpha-aminoadipic semialdehyde dehydrogenase OS=Homo sapiens GN=ALDH7A1 PE=1 SV=5		34.24
P49419-2	AL7A1_HUMAN Isoform 2 of Alpha-aminoadipic semialdehyde dehydrogenase OS=Homo sapiens GN=ALDH7A1		34.24
P49419-3	AL7A1_HUMAN Isoform 3 of Alpha-aminoadipic semialdehyde dehydrogenase OS=Homo sapiens GN=ALDH7A1		34.24
P49419;P49419-2	AL7A1_HUMAN Alpha-aminoadipic semialdehyde dehydrogenase OS=Homo sapiens GN=ALDH7A1 PE=1 SV=5;>sp|P49419-2|AL7A1_	2.47E-130	
P49441	INPP_HUMAN Inositol polyphosphate 1-phosphatase OS=Homo sapiens GN=INPP1 PE=1 SV=1	2.14E-06	
P49447	CY561_HUMAN Cytochrome b561 OS=Homo sapiens GN=CYB561 PE=2 SV=2	8.21E-10	
P49448	DHE4_HUMAN Glutamate dehydrogenase 2, mitochondrial OS=Homo sapiens GN=GLUD2 PE=1 SV=2		608.66
P49454	CENPF_HUMAN Centromere protein F OS=Homo sapiens GN=CENPF PE=1 SV=2		
P49458	SRP09_HUMAN Signal recognition particle 9 kDa protein OS=Homo sapiens GN=SRP9 PE=1 SV=2		252.18
P49458-2	SRP09_HUMAN Isoform 2 of Signal recognition particle 9 kDa protein OS=Homo sapiens GN=SRP9		122.59
P49458;P49458-2	SRP09_HUMAN Signal recognition particle 9 kDa protein OS=Homo sapiens GN=SRP9 PE=1 SV=2;>sp|P49458-2|SRP09_HUMAN Isofo	2.56E-25	
P49459	UBE2A_HUMAN Ubiquitin-conjugating enzyme E2 A OS=Homo sapiens GN=UBE2A PE=1 SV=2		20.35
P49585	PCY1A_HUMAN Choline-phosphate cytidylyltransferase A OS=Homo sapiens GN=PCYT1A PE=1 SV=2		135.3
P49588	SYAC_HUMAN Alanyl-tRNA synthetase, cytoplasmic OS=Homo sapiens GN=AARS PE=1 SV=2	0	
P49589	SYCC_HUMAN Cysteinyl-tRNA synthetase, cytoplasmic OS=Homo sapiens GN=CARS PE=1 SV=3		202.47
P49589-2	SYCC_HUMAN Isoform 2 of Cysteinyl-tRNA synthetase, cytoplasmic OS=Homo sapiens GN=CARS		202.47
P49589;P49589-2	SYCC_HUMAN Cysteinyl-tRNA synthetase, cytoplasmic OS=Homo sapiens GN=CARS PE=1 SV=3;>sp|P49589-2|SYCC_HUMAN Isoforn	9.22E-110	
P49590	SYHM_HUMAN Probable histidyl-tRNA synthetase, mitochondrial OS=Homo sapiens GN=HARS2 PE=1 SV=1		129.88
P49591	SYSC_HUMAN Seryl-tRNA synthetase, cytoplasmic OS=Homo sapiens GN=SARS PE=1 SV=3		147.23
P49593	PPM1F_HUMAN Protein phosphatase 1F OS=Homo sapiens GN=PPM1F PE=1 SV=3	7.58E-07	

P49642	PRI1_HUMAN DNA primase small subunit OS=Homo sapiens GN=PRIM1 PE=1 SV=1	1.81E-11	
P49643	PRI2_HUMAN DNA primase large subunit OS=Homo sapiens GN=PRIM2 PE=1 SV=2		33.72
P49674;Q53EZ1;Q5U045;	KC1E_HUMAN Casein kinase I isoform epsilon OS=Homo sapiens GN=CSNK1E PE=1 SV=1;>tr|Q53EZ1|Q53EZ1_HUMAN Casein kinas	7.87E-07	
P49703	ARL4D_HUMAN ADP-ribosylation factor-like protein 4D OS=Homo sapiens GN=ARL4D PE=1 SV=2		
P49711	CTCF_HUMAN Transcriptional repressor CTCF OS=Homo sapiens GN=CTCF PE=1 SV=1		
P49720	PSB3_HUMAN Proteasome subunit beta type-3 OS=Homo sapiens GN=PSMB3 PE=1 SV=2		620.12
P49721	PSB2_HUMAN Proteasome subunit beta type-2 OS=Homo sapiens GN=PSMB2 PE=1 SV=1		761.13
P49736	MCM2_HUMAN DNA replication licensing factor MCM2 OS=Homo sapiens GN=MCM2 PE=1 SV=4		456.19
P49747	COMP_HUMAN Cartilage oligomeric matrix protein OS=Homo sapiens GN=COMP PE=1 SV=2	2.37E-246	
P49748	ACADV_HUMAN Very long-chain specific acyl-CoA dehydrogenase, mitochondrial OS=Homo sapiens GN=ACADVL PE=1 SV=1		364.95
P49748-2	ACADV_HUMAN Isoform 2 of Very long-chain specific acyl-CoA dehydrogenase, mitochondrial OS=Homo sapiens GN=ACADVL		364.95
P49748;P49748-2	ACADV_HUMAN Very long-chain specific acyl-CoA dehydrogenase, mitochondrial OS=Homo sapiens GN=ACADVL PE=1 SV=1;>sp|P49	1.26E-131	
P49750	YLPM1_HUMAN YLP motif-containing protein 1 OS=Homo sapiens GN=YLPM1 PE=1 SV=3		148.9
P49750-3	YLPM1_HUMAN Isoform 3 of YLP motif-containing protein 1 OS=Homo sapiens GN=YLPM1		103.71
P49750-4	YLPM1_HUMAN Isoform 4 of YLP motif-containing protein 1 OS=Homo sapiens GN=YLPM1		148.9
P49750-4;P49750;P49750	4|YLPM1_HUMAN Isoform 4 of YLP motif-containing protein 1 OS=Homo sapiens GN=YLPM1;>sp|P49750|YLPM1_HUMAN YLP motif-co	3.63E-09	
P49753;B4DV16;Q86TX2;	ACOT2_HUMAN Acyl-coenzyme A thioesterase 2, mitochondrial OS=Homo sapiens GN=ACOT2 PE=1 SV=6;>tr|B4DV16|B4DV16_HUM	6.11E-61	
P49755	TMEDA_HUMAN Transmembrane emp24 domain-containing protein 10 OS=Homo sapiens GN=TMED10 PE=1 SV=2		728.29
P49756	RBM25_HUMAN RNA-binding protein 25 OS=Homo sapiens GN=RBM25 PE=1 SV=3		267.64
P49756-2	RBM25_HUMAN Isoform 2 of RNA-binding protein 25 OS=Homo sapiens GN=RBM25		82.52
P49756-3	RBM25_HUMAN Isoform 3 of RNA-binding protein 25 OS=Homo sapiens GN=RBM25		82.52
P49756-4	RBM25_HUMAN Isoform 4 of RNA-binding protein 25 OS=Homo sapiens GN=RBM25		
P49757;B2RCI6;P49757-3	NUMB_HUMAN Protein numb homolog OS=Homo sapiens GN=NUMB PE=1 SV=2;>tr|B2RCI6|B2RCI6_HUMAN cDNA, FLJ96094, highly	1.44E-06	
P49760	CLK2_HUMAN Dual specificity protein kinase CLK2 OS=Homo sapiens GN=CLK2 PE=1 SV=1		
P49760-2	CLK2_HUMAN Isoform 2 of Dual specificity protein kinase CLK2 OS=Homo sapiens GN=CLK2		
P49760-3	CLK2_HUMAN Isoform 3 of Dual specificity protein kinase CLK2 OS=Homo sapiens GN=CLK2		
P49768	PSN1_HUMAN Presenilin-1 OS=Homo sapiens GN=PSEN1 PE=1 SV=1		34.82
P49768-2	PSN1_HUMAN Isoform 2 of Presenilin-1 OS=Homo sapiens GN=PSEN1		34.82
P49768-3	PSN1_HUMAN Isoform 3 of Presenilin-1 OS=Homo sapiens GN=PSEN1		34.82
P49768-5	PSN1_HUMAN Isoform 5 of Presenilin-1 OS=Homo sapiens GN=PSEN1		34.82
P49768-6	PSN1_HUMAN Isoform 6 of Presenilin-1 OS=Homo sapiens GN=PSEN1		34.82
P49770	EI2BB_HUMAN Translation initiation factor eIF-2B subunit beta OS=Homo sapiens GN=EIF2B2 PE=1 SV=3	3.34E-21	
P49773	HINT1_HUMAN Histidine triad nucleotide-binding protein 1 OS=Homo sapiens GN=HINT1 PE=1 SV=2		471.23
P49790	NU153_HUMAN Nuclear pore complex protein Nup153 OS=Homo sapiens GN=NUP153 PE=1 SV=2		
P49792	RBP2_HUMAN E3 SUMO-protein ligase RanBP2 OS=Homo sapiens GN=RANBP2 PE=1 SV=2		888.42
P49798	RGS4_HUMAN Regulator of G-protein signaling 4 OS=Homo sapiens GN=RGS4 PE=1 SV=1		31.61
P49810	PSN2_HUMAN Presenilin-2 OS=Homo sapiens GN=PSEN2 PE=1 SV=1		34.82
P49821	NDUV1_HUMAN NADH dehydrogenase [ubiquinone] flavoprotein 1, mitochondrial OS=Homo sapiens GN=NDUFV1 PE=1 SV=4		129.15
P49821-2	NDUV1_HUMAN Isoform 2 of NADH dehydrogenase [ubiquinone] flavoprotein 1, mitochondrial OS=Homo sapiens GN=NDUFV1		129.15
P49821;P49821-2	NDUV1_HUMAN NADH dehydrogenase [ubiquinone] flavoprotein 1, mitochondrial OS=Homo sapiens GN=NDUFV1 PE=1 SV=4;>sp|P4	2.62E-25	
P49841-2;P49841	2|GSK3B_HUMAN Isoform 2 of Glycogen synthase kinase-3 beta OS=Homo sapiens GN=GSK3B;>sp|P49841|GSK3B_HUMAN Glycoge	1.32E-10	
P49863	GRAK_HUMAN Granzyme K OS=Homo sapiens GN=GZMK PE=1 SV=1		14.6
P49902	5NTC_HUMAN Cytosolic purine 5-nucleotidase OS=Homo sapiens GN=NT5C2 PE=1 SV=1	3.64E-31	
P49903	SPS1_HUMAN Selenide, water dikinase 1 OS=Homo sapiens GN=SEPHS1 PE=1 SV=2		155.28
P49913	CAMP_HUMAN Cathelicidin antimicrobial peptide OS=Homo sapiens GN=CAMP PE=1 SV=1	9.68E-08	
P49914	MTHFS_HUMAN 5-formyltetrahydrofolate cyclo-ligase OS=Homo sapiens GN=MTHFS PE=1 SV=2	1.55E-10	
P49915	GUAA_HUMAN GMP synthase [glutamine-hydrolyzing] OS=Homo sapiens GN=GMPS PE=1 SV=1		90.9
P49916	DNLI3_HUMAN DNA ligase 3 OS=Homo sapiens GN=LIG3 PE=1 SV=2		233.61
P49916-2	DNLI3_HUMAN Isoform Beta of DNA ligase 3 OS=Homo sapiens GN=LIG3		188.53
P49916;P49916-2	DNLI3_HUMAN DNA ligase 3 OS=Homo sapiens GN=LIG3 PE=1 SV=2;>sp|P49916-2|DNLI3_HUMAN Isoform Beta of DNA ligase 3 OS	8.13E-12	
P49917	DNLI4_HUMAN DNA ligase 4 OS=Homo sapiens GN=LIG4 PE=1 SV=2		41.37
P49959	MRE11_HUMAN Double-strand break repair protein MRE11A OS=Homo sapiens GN=MRE11A PE=1 SV=3		55.06
P49959-2	MRE11_HUMAN Isoform 2 of Double-strand break repair protein MRE11A OS=Homo sapiens GN=MRE11A		55.06
P49959;P49959-2	MRE11_HUMAN Double-strand break repair protein MRE11A OS=Homo sapiens GN=MRE11A PE=1 SV=3;>sp|P49959-2|MRE11_HUMA	4.64E-76	
P49961	ENTP1_HUMAN Ectonucleoside triphosphate diphosphohydrolase 1 OS=Homo sapiens GN=ENTPD1 PE=1 SV=1		
P49961-2	ENTP1_HUMAN Isoform Placental I of Ectonucleoside triphosphate diphosphohydrolase 1 OS=Homo sapiens GN=ENTPD1		
P50135	HNMT_HUMAN Histamine N-methyltransferase OS=Homo sapiens GN=HNMT PE=1 SV=1		
P50148	GNAQ_HUMAN Guanine nucleotide-binding protein G(q) subunit alpha OS=Homo sapiens GN=GNAQ PE=1 SV=4		198.61
P50151	GBG10_HUMAN Guanine nucleotide-binding protein G(I)/G(S)/G(O) subunit gamma-10 OS=Homo sapiens GN=GNG10 PE=1 SV=1		
P50213	IDH3A_HUMAN Isocitrate dehydrogenase [NAD] subunit alpha, mitochondrial OS=Homo sapiens GN=IDH3A PE=1 SV=1		348.61
P50213-2	IDH3A_HUMAN Isoform 2 of Isocitrate dehydrogenase [NAD] subunit alpha, mitochondrial OS=Homo sapiens GN=IDH3A		225.06

P50213;P50213-2	IDH3A_HUMAN Isocitrate dehydrogenase [NAD] subunit alpha, mitochondrial OS=Homo sapiens GN=IDH3A PE=1 SV=1;>sp|P50213-	5.08E-65	
P50224;P50225-2	ST1A3_HUMAN Sulfotransferase 1A3/1A4 OS=Homo sapiens GN=SULT1A3 PE=1 SV=1;>sp|P50225-2|ST1A1_HUMAN Isoform 2 of Su	9.53E-13	
P50225;P50226	ST1A1_HUMAN Sulfotransferase 1A1 OS=Homo sapiens GN=SULT1A1 PE=1 SV=3;>sp|P50226|ST1A2_HUMAN Sulfotransferase 1A2 (	2.86E-11	
P50226;P50225	ST1A2_HUMAN Sulfotransferase 1A2 OS=Homo sapiens GN=SULT1A2 PE=1 SV=2;>sp|P50225|ST1A1_HUMAN Sulfotransferase 1A1 (	2.18E-12	
P50238	CRIP1_HUMAN Cysteine-rich protein 1 OS=Homo sapiens GN=CRIP1 PE=1 SV=3	8.10E-09	
P50281	MMP14_HUMAN Matrix metalloproteinase-14 OS=Homo sapiens GN=MMP14 PE=1 SV=3		268.2
P50395	GDIB_HUMAN Rab GDP dissociation inhibitor beta OS=Homo sapiens GN=GDI2 PE=1 SV=2		654.22
P50402	EMD_HUMAN Emerin OS=Homo sapiens GN=EMD PE=1 SV=1		121.96
P50416	CPT1A_HUMAN Carnitine O-palmitoyltransferase 1, liver isoform OS=Homo sapiens GN=CPT1A PE=1 SV=2		
P50416-2	CPT1A_HUMAN Isoform 2 of Carnitine O-palmitoyltransferase 1, liver isoform OS=Homo sapiens GN=CPT1A		
P50416;P50416-2	CPT1A_HUMAN Carnitine O-palmitoyltransferase 1, liver isoform OS=Homo sapiens GN=CPT1A PE=1 SV=2;>sp|P50416-2|CPT1A_HUI	0.00017048	
P50440;P50440-2;P50440	GATM_HUMAN Glycine amidinotransferase, mitochondrial OS=Homo sapiens GN=GATM PE=1 SV=1;>sp|P50440-2|GATM_HUMAN Isof	5.23E-78	
P50452	SPB8_HUMAN Serpin B8 OS=Homo sapiens GN=SERPINB8 PE=1 SV=2		43.62
P50453	SPB9_HUMAN Serpin B9 OS=Homo sapiens GN=SERPINB9 PE=1 SV=1		29.52
P50454	SERPH_HUMAN Serpin H1 OS=Homo sapiens GN=SERPINH1 PE=1 SV=2		1537.96
P50479	PDLI4_HUMAN PDZ and LIM domain protein 4 OS=Homo sapiens GN=PDLIM4 PE=1 SV=2	3.15E-11	
P50502	F10A1_HUMAN Hsc70-interacting protein OS=Homo sapiens GN=ST13 PE=1 SV=2		123.18
P50542	PEX5_HUMAN Peroxisomal targeting signal 1 receptor OS=Homo sapiens GN=PEX5 PE=1 SV=3		
P50542-2	PEX5_HUMAN Isoform 2 of Peroxisomal targeting signal 1 receptor OS=Homo sapiens GN=PEX5		
P50542-3	PEX5_HUMAN Isoform 3 of Peroxisomal targeting signal 1 receptor OS=Homo sapiens GN=PEX5		
P50549	ETV1_HUMAN ETS translocation variant 1 OS=Homo sapiens GN=ETV1 PE=1 SV=2		29.69
P50549-2	ETV1_HUMAN Isoform 2 of ETS translocation variant 1 OS=Homo sapiens GN=ETV1		29.69
P50552	VASP_HUMAN Vasodilator-stimulated phosphoprotein OS=Homo sapiens GN=VASP PE=1 SV=3		219.45
P50570	DYN2_HUMAN Dynamin-2 OS=Homo sapiens GN=DNM2 PE=1 SV=2		269.41
P50570-2	DYN2_HUMAN Isoform 2 of Dynamin-2 OS=Homo sapiens GN=DNM2		269.41
P50570;P50570-2	DYN2_HUMAN Dynamin-2 OS=Homo sapiens GN=DNM2 PE=1 SV=2;>sp|P50570-2|DYN2_HUMAN Isoform 2 of Dynamin-2 OS=Homc	6.27E-102	
P50579	AMPM2_HUMAN Methionine aminopeptidase 2 OS=Homo sapiens GN=METAP2 PE=1 SV=1		27.7
P50583	AP4A_HUMAN Bis(5'-nucleosyl)-tetraphosphatase [asymmetrical] OS=Homo sapiens GN=NUDT2 PE=1 SV=3		
P50613	CDK7_HUMAN Cyclin-dependent kinase 7 OS=Homo sapiens GN=CDK7 PE=1 SV=1		147.03
P50613;D6RAD4;D6R9G1;	CDK7_HUMAN Cell division protein kinase 7 OS=Homo sapiens GN=CDK7 PE=1 SV=1;>tr|D6RAD4|D6RAD4_HUMAN Uncharacterized	1.02E-23	
P50748	KNTC1_HUMAN Kinetochore-associated protein 1 OS=Homo sapiens GN=KNTC1 PE=1 SV=1	2.99E-11	
P50750	CDK9_HUMAN Cyclin-dependent kinase 9 OS=Homo sapiens GN=CDK9 PE=1 SV=3		133.41
P50750-2	CDK9_HUMAN Isoform 2 of Cyclin-dependent kinase 9 OS=Homo sapiens GN=CDK9		133.41
P50750-2;B2R9L6;B5BU5	2|CDK9_HUMAN Isoform 2 of Cell division protein kinase 9 OS=Homo sapiens GN=CDK9;>tr|B2R9L6|B2R9L6_HUMAN cDNA, FLJ9445	7.36E-14	
P50750-2;P50750	2|CDK9_HUMAN Isoform 2 of Cyclin-dependent kinase 9 OS=Homo sapiens GN=CDK9;>sp|P50750|CDK9_HUMAN Cyclin-dependent k	4.29E-14	
P50851	LRBA_HUMAN Lipopolysaccharide-responsive and beige-like anchor protein OS=Homo sapiens GN=LRBA PE=1 SV=4		
P50851-2	LRBA_HUMAN Isoform 2 of Lipopolysaccharide-responsive and beige-like anchor protein OS=Homo sapiens GN=LRBA		
P50851-2;P50851	2|LRBA_HUMAN Isoform 2 of Lipopolysaccharide-responsive and beige-like anchor protein OS=Homo sapiens GN=LRBA;>sp|P50851|L	1.57E-23	
P50895	BCAM_HUMAN Basal cell adhesion molecule OS=Homo sapiens GN=BCAM PE=1 SV=2		
P50897	PPT1_HUMAN Palmitoyl-protein thioesterase 1 OS=Homo sapiens GN=PPT1 PE=1 SV=1		195.43
P50914	RL14_HUMAN 60S ribosomal protein L14 OS=Homo sapiens GN=RPL14 PE=1 SV=4		475.66
P50990	TCPQ_HUMAN T-complex protein 1 subunit theta OS=Homo sapiens GN=CCT8 PE=1 SV=4		541.15
P50991	TCPD_HUMAN T-complex protein 1 subunit delta OS=Homo sapiens GN=CCT4 PE=1 SV=4		644.37
P50993	AT1A2_HUMAN Sodium/potassium-transporting ATPase subunit alpha-2 OS=Homo sapiens GN=ATP1A2 PE=1 SV=1		1268.58
P50995	ANX11_HUMAN Annexin A11 OS=Homo sapiens GN=ANXA11 PE=1 SV=1		60.75
P51003	PAPOA_HUMAN Poly(A) polymerase alpha OS=Homo sapiens GN=PAPOLA PE=1 SV=4	2.51E-12	
P51114	FXR1_HUMAN Fragile X mental retardation syndrome-related protein 1 OS=Homo sapiens GN=FXR1 PE=1 SV=3		442.55
P51114-2	FXR1_HUMAN Isoform 2 of Fragile X mental retardation syndrome-related protein 1 OS=Homo sapiens GN=FXR1		365.18
P51114-3	FXR1_HUMAN Isoform 3 of Fragile X mental retardation syndrome-related protein 1 OS=Homo sapiens GN=FXR1		333.08
P51114;B4DXZ6;P51114-2	FXR1_HUMAN Fragile X mental retardation syndrome-related protein 1 OS=Homo sapiens GN=FXR1 PE=1 SV=3;>tr|B4DXZ6|B4DXZ6	1.94E-113	
P51116	FXR2_HUMAN Fragile X mental retardation syndrome-related protein 2 OS=Homo sapiens GN=FXR2 PE=1 SV=2		135.81
P51148	RAB5C_HUMAN Ras-related protein Rab-5C OS=Homo sapiens GN=RAB5C PE=1 SV=2		896.67
P51149	RAB7A_HUMAN Ras-related protein Rab-7a OS=Homo sapiens GN=RAB7A PE=1 SV=1		515.7
P51151	RAB9A_HUMAN Ras-related protein Rab-9A OS=Homo sapiens GN=RAB9A PE=1 SV=1		74.68
P51153	RAB13_HUMAN Ras-related protein Rab-13 OS=Homo sapiens GN=RAB13 PE=1 SV=1		288.56
P51157	RAB28_HUMAN Ras-related protein Rab-28 OS=Homo sapiens GN=RAB28 PE=1 SV=2		46.29
P51157-2	RAB28_HUMAN Isoform L of Ras-related protein Rab-28 OS=Homo sapiens GN=RAB28		46.29
P51157;P51157-2;D6RHF	RAB28_HUMAN Ras-related protein Rab-28 OS=Homo sapiens GN=RAB28 PE=1 SV=2;>sp|P51157-2|RAB28_HUMAN Isoform L of Ras	3.89E-06	
P51159	RB27A_HUMAN Ras-related protein Rab-27A OS=Homo sapiens GN=RAB27A PE=1 SV=3		122.6
P51159-2	RB27A_HUMAN Isoform Short of Ras-related protein Rab-27A OS=Homo sapiens GN=RAB27A		105.77
P51159;P51159-2	RB27A_HUMAN Ras-related protein Rab-27A OS=Homo sapiens GN=RAB27A PE=1 SV=3;>sp|P51159-2|RB27A_HUMAN Isoform Shor	2.11E-10	

P51398	RT29_HUMAN 28S ribosomal protein S29, mitochondrial OS=Homo sapiens GN=DAP3 PE=1 SV=1		403.6
P51451	BLK_HUMAN Tyrosine-protein kinase Blk OS=Homo sapiens GN=BLK PE=1 SV=3		65.4
P51452	DUS3_HUMAN Dual specificity protein phosphatase 3 OS=Homo sapiens GN=DUSP3 PE=1 SV=1		93.16
P51511	MMP15_HUMAN Matrix metalloproteinase-15 OS=Homo sapiens GN=MMP15 PE=1 SV=1		10.09
P51531	SMCA2_HUMAN Probable global transcription activator SNF2L2 OS=Homo sapiens GN=SMARCA2 PE=1 SV=2		35.68
P51531-2	SMCA2_HUMAN Isoform Short of Probable global transcription activator SNF2L2 OS=Homo sapiens GN=SMARCA2		35.68
P51531;P51531-2	SMCA2_HUMAN Probable global transcription activator SNF2L2 OS=Homo sapiens GN=SMARCA2 PE=1 SV=2;>sp|P51531-2|SMCA2_H	2.33E-31	
P51532	SMCA4_HUMAN Transcription activator BRG1 OS=Homo sapiens GN=SMARCA4 PE=1 SV=2		152.47
P51553	IDH3G_HUMAN Isocitrate dehydrogenase [NAD] subunit gamma, mitochondrial OS=Homo sapiens GN=IDH3G PE=1 SV=1		
P51570	GALK1_HUMAN Galactokinase OS=Homo sapiens GN=GALK1 PE=1 SV=1	2.34E-24	
P51571	SSRD_HUMAN Translocon-associated protein subunit delta OS=Homo sapiens GN=SSR4 PE=1 SV=1		339.89
P51572	BAP31_HUMAN B-cell receptor-associated protein 31 OS=Homo sapiens GN=BCAP31 PE=1 SV=3		979.89
P51580	TPMT_HUMAN Thiopurine S-methyltransferase OS=Homo sapiens GN=TPMT PE=1 SV=1	9.21E-10	
P51587	BRCA2_HUMAN Breast cancer type 2 susceptibility protein OS=Homo sapiens GN=BRCA2 PE=1 SV=2		
P51606;A6NKZ2;P51606-2	RENBP_HUMAN N-acylglucosamine 2-epimerase OS=Homo sapiens GN=RENBP PE=1 SV=2;>tr|A6NKZ2|A6NKZ2_HUMAN Uncharacter	6.36E-18	
P51608	MECP2_HUMAN Methyl-CpG-binding protein 2 OS=Homo sapiens GN=MECP2 PE=1 SV=1		
P51608-2	MECP2_HUMAN Isoform B of Methyl-CpG-binding protein 2 OS=Homo sapiens GN=MECP2		
P51608-2;A8K079;C6KG4	2|MECP2_HUMAN Isoform B of Methyl-CpG-binding protein 2 OS=Homo sapiens GN=MECP2;>tr|A8K079|A8K079_HUMAN cDNA FLJ75	4.50E-10	
P51610	HCFC1_HUMAN Host cell factor 1 OS=Homo sapiens GN=HCFC1 PE=1 SV=2		124.31
P51610-2	HCFC1_HUMAN Isoform 2 of Host cell factor 1 OS=Homo sapiens GN=HCFC1		124.31
P51610-3	HCFC1_HUMAN Isoform 3 of Host cell factor 1 OS=Homo sapiens GN=HCFC1		14.89
P51610;P51610-2	HCFC1_HUMAN Host cell factor 1 OS=Homo sapiens GN=HCFC1 PE=1 SV=2;>sp|P51610-2|HCFC1_HUMAN Isoform 2 of Host cell fact	3.59E-121	
P51636	CAV2_HUMAN Caveolin-2 OS=Homo sapiens GN=CAV2 PE=1 SV=2		134.32
P51636-2	CAV2_HUMAN Isoform Beta of Caveolin-2 OS=Homo sapiens GN=CAV2		134.32
P51636-3	CAV2_HUMAN Isoform C of Caveolin-2 OS=Homo sapiens GN=CAV2		31.55
P51636;Q53X57;P51636-2	CAV2_HUMAN Caveolin-2 OS=Homo sapiens GN=CAV2 PE=1 SV=2;>tr|Q53X57|Q53X57_HUMAN Caveolin (Fragment) OS=Homo sap	1.73E-05	
P51648-2;P51648	2|AL3A2_HUMAN Isoform 2 of Fatty aldehyde dehydrogenase OS=Homo sapiens GN=ALDH3A2;>sp|P51648|AL3A2_HUMAN Fatty alde	2.95E-09	
P51649	SSDH_HUMAN Succinate-semialdehyde dehydrogenase, mitochondrial OS=Homo sapiens GN=ALDH5A1 PE=1 SV=2		
P51659	DHB4_HUMAN Peroxisomal multifunctional enzyme type 2 OS=Homo sapiens GN=HSD17B4 PE=1 SV=3		415.91
P51665	PSD7_HUMAN 26S proteasome non-ATPase regulatory subunit 7 OS=Homo sapiens GN=PSMD7 PE=1 SV=2		695.8
P51687	SUOX_HUMAN Sulfite oxidase, mitochondrial OS=Homo sapiens GN=SUOX PE=1 SV=2	1.13E-09	
P51692	STA5B_HUMAN Signal transducer and activator of transcription 5B OS=Homo sapiens GN=STAT5B PE=1 SV=2	9.68E-10	
P51784	UBP11_HUMAN Ubiquitin carboxyl-terminal hydrolase 11 OS=Homo sapiens GN=USP11 PE=1 SV=3	1.41E-15	
P51787	KCNQ1_HUMAN Potassium voltage-gated channel subfamily KQT member 1 OS=Homo sapiens GN=KCNQ1 PE=1 SV=3		
P51787-2	KCNQ1_HUMAN Isoform 2 of Potassium voltage-gated channel subfamily KQT member 1 OS=Homo sapiens GN=KCNQ1		
P51805	PLXA3_HUMAN Plexin-A3 OS=Homo sapiens GN=PLXNA3 PE=1 SV=2		
P51808	DYLT3_HUMAN Dynein light chain Tctex-type 3 OS=Homo sapiens GN=DYNLT3 PE=1 SV=1	8.65E-35	
P51809	VAMP7_HUMAN Vesicle-associated membrane protein 7 OS=Homo sapiens GN=VAMP7 PE=1 SV=3		198.23
P51809-2	VAMP7_HUMAN Isoform 2 of Vesicle-associated membrane protein 7 OS=Homo sapiens GN=VAMP7		157.47
P51809-2;P51809;P51809	2|VAMP7_HUMAN Isoform 2 of Vesicle-associated membrane protein 7 OS=Homo sapiens GN=VAMP7;>sp|P51809|VAMP7_HUMAN Ve	5.50E-67	
P51809-3	VAMP7_HUMAN Isoform 3 of Vesicle-associated membrane protein 7 OS=Homo sapiens GN=VAMP7		198.23
P51809;P51809-3;P51809	VAMP7_HUMAN Vesicle-associated membrane protein 7 OS=Homo sapiens GN=VAMP7 PE=1 SV=3;>sp|P51809-3|VAMP7_HUMAN Iso	1.18E-24	
P51812	KS6A3_HUMAN Ribosomal protein S6 kinase alpha-3 OS=Homo sapiens GN=RPS6KA3 PE=1 SV=1	2.14E-34	
P51814	ZNF41_HUMAN Zinc finger protein 41 OS=Homo sapiens GN=ZNF41 PE=1 SV=2		
P51814-2	ZNF41_HUMAN Isoform 2 of Zinc finger protein 41 OS=Homo sapiens GN=ZNF41		
P51814-3	ZNF41_HUMAN Isoform 3 of Zinc finger protein 41 OS=Homo sapiens GN=ZNF41		
P51814-4	ZNF41_HUMAN Isoform 4 of Zinc finger protein 41 OS=Homo sapiens GN=ZNF41		
P51814-5	ZNF41_HUMAN Isoform 5 of Zinc finger protein 41 OS=Homo sapiens GN=ZNF41		
P51814-6	ZNF41_HUMAN Isoform 6 of Zinc finger protein 41 OS=Homo sapiens GN=ZNF41		
P51814-7	ZNF41_HUMAN Isoform 7 of Zinc finger protein 41 OS=Homo sapiens GN=ZNF41		
P51814-8	ZNF41_HUMAN Isoform 8 of Zinc finger protein 41 OS=Homo sapiens GN=ZNF41		
P51825	AFF1_HUMAN AF4/FMR2 family member 1 OS=Homo sapiens GN=AFF1 PE=1 SV=1		23.73
P51841	GUC2F_HUMAN Retinal guanylyl cyclase 2 OS=Homo sapiens GN=GUCY2F PE=2 SV=2		
P51858	HDGF_HUMAN Hepatoma-derived growth factor OS=Homo sapiens GN=HDGF PE=1 SV=1		352.29
P51878	CASP5_HUMAN Caspase-5 OS=Homo sapiens GN=CASP5 PE=1 SV=3		
P51878-2	CASP5_HUMAN Isoform 2 of Caspase-5 OS=Homo sapiens GN=CASP5		
P51878-4	CASP5_HUMAN Isoform 4 of Caspase-5 OS=Homo sapiens GN=CASP5		
P51878-5	CASP5_HUMAN Isoform 5 of Caspase-5 OS=Homo sapiens GN=CASP5		
P51878-6	CASP5_HUMAN Isoform 6 of Caspase-5 OS=Homo sapiens GN=CASP5		
P51884	LUM_HUMAN Lumican OS=Homo sapiens GN=LUM PE=1 SV=2	0	
P51888	PRELP_HUMAN Prolargin OS=Homo sapiens GN=PRELP PE=1 SV=1	0	

P51911	CNN1_HUMAN Calponin-1 OS=Homo sapiens GN=CNN1 PE=1 SV=2		7.03
P51946	CCNH_HUMAN Cyclin-H OS=Homo sapiens GN=CCNH PE=1 SV=1		118
P51948	MAT1_HUMAN CDK-activating kinase assembly factor MAT1 OS=Homo sapiens GN=MNAT1 PE=1 SV=1		53.44
P51965;A6NDQ7;C9J2P0;(	UB2E1_HUMAN Ubiquitin-conjugating enzyme E2 E1 OS=Homo sapiens GN=UBE2E1 PE=1 SV=1;>tr|A6NDQ7|A6NDQ7_HUMAN Ubiqu	1.23E-16	
P51970	NDUA8_HUMAN NADH dehydrogenase [ubiquinone] 1 alpha subcomplex subunit 8 OS=Homo sapiens GN=NDUFA8 PE=1 SV=3		138.33
P51991	ROA3_HUMAN Heterogeneous nuclear ribonucleoprotein A3 OS=Homo sapiens GN=HNRNPA3 PE=1 SV=2		451.94
P51991-2	ROA3_HUMAN Isoform 2 of Heterogeneous nuclear ribonucleoprotein A3 OS=Homo sapiens GN=HNRNPA3		435.52
P51991;P51991-2	ROA3_HUMAN Heterogeneous nuclear ribonucleoprotein A3 OS=Homo sapiens GN=HNRNPA3 PE=1 SV=2;>sp|P51991-2|ROA3_HUMA	7.88E-225	
P52209	6PGD_HUMAN 6-phosphogluconate dehydrogenase, decarboxylating OS=Homo sapiens GN=PGD PE=1 SV=3		103.13
P52272	HNRPM_HUMAN Heterogeneous nuclear ribonucleoprotein M OS=Homo sapiens GN=HNRNPM PE=1 SV=3		1154.91
P52272-2	HNRPM_HUMAN Isoform 2 of Heterogeneous nuclear ribonucleoprotein M OS=Homo sapiens GN=HNRNPM		1154.91
P52272;P52272-2	HNRPM_HUMAN Heterogeneous nuclear ribonucleoprotein M OS=Homo sapiens GN=HNRNPM PE=1 SV=3;>sp|P52272-2|HNRPM_HUM	0	
P52292	IMA2_HUMAN Importin subunit alpha-2 OS=Homo sapiens GN=KPNA2 PE=1 SV=1		
P52294	IMA1_HUMAN Importin subunit alpha-1 OS=Homo sapiens GN=KPNA1 PE=1 SV=3		
P52298	NCBP2_HUMAN Nuclear cap-binding protein subunit 2 OS=Homo sapiens GN=NCBP2 PE=1 SV=1		163.14
P52298-2	NCBP2_HUMAN Isoform 2 of Nuclear cap-binding protein subunit 2 OS=Homo sapiens GN=NCBP2		89.45
P52298;P52298-2	NCBP2_HUMAN Nuclear cap-binding protein subunit 2 OS=Homo sapiens GN=NCBP2 PE=1 SV=1;>sp|P52298-2|NCBP2_HUMAN Isofo	1.46E-05	
P52306	GDS1_HUMAN Rap1 GTPase-GDP dissociation stimulator 1 OS=Homo sapiens GN=RAP1GDS1 PE=1 SV=3		
P52306-2	GDS1_HUMAN Isoform 2 of Rap1 GTPase-GDP dissociation stimulator 1 OS=Homo sapiens GN=RAP1GDS1		
P52306;P52306-2	GDS1_HUMAN Rap1 GTPase-GDP dissociation stimulator 1 OS=Homo sapiens GN=RAP1GDS1 PE=1 SV=3;>sp|P52306-2|GDS1_HUM/	2.84E-24	
P52333	JAK3_HUMAN Tyrosine-protein kinase JAK3 OS=Homo sapiens GN=JAK3 PE=1 SV=2		
P52333-2	JAK3_HUMAN Isoform 1 of Tyrosine-protein kinase JAK3 OS=Homo sapiens GN=JAK3		
P52434	RPAB3_HUMAN DNA-directed RNA polymerases I, II, and III subunit RPABC3 OS=Homo sapiens GN=POLR2H PE=1 SV=4		193.17
P52435	RPB11_HUMAN DNA-directed RNA polymerase II subunit RPB11-a OS=Homo sapiens GN=POLR2J PE=1 SV=1		30.42
P52565	GDIR1_HUMAN Rho GDP-dissociation inhibitor 1 OS=Homo sapiens GN=ARHGDIA PE=1 SV=3		252.34
P52566	GDIR2_HUMAN Rho GDP-dissociation inhibitor 2 OS=Homo sapiens GN=ARHGDIB PE=1 SV=3		131.94
P52569	CTR2_HUMAN Low affinity cationic amino acid transporter 2 OS=Homo sapiens GN=SLC7A2 PE=1 SV=2		45.99
P52569-2	CTR2_HUMAN Isoform 2 of Low affinity cationic amino acid transporter 2 OS=Homo sapiens GN=SLC7A2		
P52569-3	CTR2_HUMAN Isoform 3 of Low affinity cationic amino acid transporter 2 OS=Homo sapiens GN=SLC7A2		45.99
P52594	AGFG1_HUMAN Arf-GAP domain and FG repeats-containing protein 1 OS=Homo sapiens GN=AGFG1 PE=1 SV=2		67.97
P52594-2	AGFG1_HUMAN Isoform 2 of Arf-GAP domain and FG repeats-containing protein 1 OS=Homo sapiens GN=AGFG1		67.97
P52594-3	AGFG1_HUMAN Isoform 3 of Arf-GAP domain and FG repeats-containing protein 1 OS=Homo sapiens GN=AGFG1		67.97
P52594;P52594-3;P52594	AGFG1_HUMAN Arf-GAP domain and FG repeats-containing protein 1 OS=Homo sapiens GN=AGFG1 PE=1 SV=2;>sp|P52594-3|AGFG	1.46E-09	
P52597	HNRPF_HUMAN Heterogeneous nuclear ribonucleoprotein F OS=Homo sapiens GN=HNRNPF PE=1 SV=3		409.4
P52630;B4DLC7;P52630-2	STAT2_HUMAN Signal transducer and activator of transcription 2 OS=Homo sapiens GN=STAT2 PE=1 SV=1;>tr|B4DLC7|B4DLC7_HUM	7.17E-19	
P52655	TF2AA_HUMAN Transcription initiation factor IIA subunit 1 OS=Homo sapiens GN=GTF2A1 PE=1 SV=1	4.33E-05	
P52657	T2AG_HUMAN Transcription initiation factor IIA subunit 2 OS=Homo sapiens GN=GTF2A2 PE=1 SV=1		144.55
P52701	MSH6_HUMAN DNA mismatch repair protein Msh6 OS=Homo sapiens GN=MSH6 PE=1 SV=2		336.57
P52701-2	MSH6_HUMAN Isoform GTBP-alt of DNA mismatch repair protein Msh6 OS=Homo sapiens GN=MSH6		311.63
P52701;B4DF41;B4E3I4;P	MSH6_HUMAN DNA mismatch repair protein Msh6 OS=Homo sapiens GN=MSH6 PE=1 SV=2;>tr|B4DF41|B4DF41_HUMAN cDNA FLJ5	3.37E-35	
P52732	KIF11_HUMAN Kinesin-like protein KIF11 OS=Homo sapiens GN=KIF11 PE=1 SV=2		
P52735-2;P52735-3;P527	2|VAV2_HUMAN Isoform 2 of Guanine nucleotide exchange factor VAV2 OS=Homo sapiens GN=VAV2;>sp|P52735-3|VAV2_HUMAN Isc	1.53E-24	
P52735;P52735-2;P52735	VAV2_HUMAN Guanine nucleotide exchange factor VAV2 OS=Homo sapiens GN=VAV2 PE=1 SV=2;>sp|P52735-2|VAV2_HUMAN Isofor	7.97E-07	
P52756	RBM5_HUMAN RNA-binding protein 5 OS=Homo sapiens GN=RBM5 PE=1 SV=2	3.85E-15	
P52758	UK114_HUMAN Ribonuclease UK114 OS=Homo sapiens GN=HRSP12 PE=1 SV=1		29.31
P52788	SPSY_HUMAN Spermine synthase OS=Homo sapiens GN=SMS PE=1 SV=2		35.14
P52789	HXK2_HUMAN Hexokinase-2 OS=Homo sapiens GN=HK2 PE=1 SV=2		163.76
P52790	HXK3_HUMAN Hexokinase-3 OS=Homo sapiens GN=HK3 PE=1 SV=2		73.99
P52815	RM12_HUMAN 39S ribosomal protein L12, mitochondrial OS=Homo sapiens GN=MRPL12 PE=1 SV=2		168.02
P52888	THOP1_HUMAN Thimet oligopeptidase OS=Homo sapiens GN=THOP1 PE=1 SV=2		34.22
P52895	AK1C2_HUMAN Aldo-keto reductase family 1 member C2 OS=Homo sapiens GN=AKR1C2 PE=1 SV=3	6.69E-75	
P52907	CAZA1_HUMAN F-actin-capping protein subunit alpha-1 OS=Homo sapiens GN=CAPZA1 PE=1 SV=3		311.51
P52943	CRIP2_HUMAN Cysteine-rich protein 2 OS=Homo sapiens GN=CRIP2 PE=1 SV=1		177.27
P52948	NUP98_HUMAN Nuclear pore complex protein Nup98-Nup96 OS=Homo sapiens GN=NUP98 PE=1 SV=4		
P52948-2	NUP98_HUMAN Isoform 2 of Nuclear pore complex protein Nup98-Nup96 OS=Homo sapiens GN=NUP98		
P52948-3	NUP98_HUMAN Isoform 3 of Nuclear pore complex protein Nup98-Nup96 OS=Homo sapiens GN=NUP98		
P52948-4	NUP98_HUMAN Isoform 4 of Nuclear pore complex protein Nup98-Nup96 OS=Homo sapiens GN=NUP98		
P52948-5	NUP98_HUMAN Isoform 5 of Nuclear pore complex protein Nup98-Nup96 OS=Homo sapiens GN=NUP98		
P52948-6	NUP98_HUMAN Isoform 6 of Nuclear pore complex protein Nup98-Nup96 OS=Homo sapiens GN=NUP98		
P52948;P52948-5;P52948	NUP98_HUMAN Nuclear pore complex protein Nup98-Nup96 OS=Homo sapiens GN=NUP98 PE=1 SV=4;>sp|P52948-5|NUP98_HUMAN	5.12E-23	
P53004	BIEA_HUMAN Biliverdin reductase A OS=Homo sapiens GN=BLVRA PE=1 SV=2		129.83

P53007	TXTP_HUMAN Tricarboxylate transport protein, mitochondrial OS=Homo sapiens GN=SLC25A1 PE=1 SV=2		309.63
P53041	PPP5_HUMAN Serine/threonine-protein phosphatase 5 OS=Homo sapiens GN=PPP5C PE=1 SV=1	4.48E-41	
P53365	ARFP2_HUMAN Arfaptin-2 OS=Homo sapiens GN=ARFIP2 PE=1 SV=1	1.89E-10	
P53367	ARFP1_HUMAN Arfaptin-1 OS=Homo sapiens GN=ARFIP1 PE=1 SV=2		
P53367-2	ARFP1_HUMAN Isoform A of Arfaptin-1 OS=Homo sapiens GN=ARFIP1		
P53367-2;P53367	2|ARFP1_HUMAN Isoform A of Arfaptin-1 OS=Homo sapiens GN=ARFIP1;>sp|P53367|ARFP1_HUMAN Arfaptin-1 OS=Homo sapiens GI	1.57E-21	
P53367;P53367-2	ARFP1_HUMAN Arfaptin-1 OS=Homo sapiens GN=ARFIP1 PE=1 SV=2;>sp|P53367-2|ARFP1_HUMAN Isoform A of Arfaptin-1 OS=Hom	1.44E-44	
P53384;P53384-2	NUBP1_HUMAN Cytosolic Fe-S cluster assembly factor NUBP1 OS=Homo sapiens GN=NUBP1 PE=1 SV=2;>sp|P53384-2|NUBP1_HUM/	3.10E-12	
P53396	ACLY_HUMAN ATP-citrate synthase OS=Homo sapiens GN=ACLY PE=1 SV=3		561.59
P53582	AMPM1_HUMAN Methionine aminopeptidase 1 OS=Homo sapiens GN=METAP1 PE=1 SV=2		
P53597	SUCA_HUMAN Succinyl-CoA ligase [GDP-forming] subunit alpha, mitochondrial OS=Homo sapiens GN=SUCLG1 PE=1 SV=4		143.23
P53602	MVD1_HUMAN Diphosphomevalonate decarboxylase OS=Homo sapiens GN=MVD PE=1 SV=1	7.05E-17	
P53611	PGTB2_HUMAN Geranylgeranyl transferase type-2 subunit beta OS=Homo sapiens GN=RABGGTB PE=1 SV=2	3.85E-15	
P53618	COPB_HUMAN Coatomer subunit beta OS=Homo sapiens GN=COPB1 PE=1 SV=3		227.45
P53621	COPA_HUMAN Coatomer subunit alpha OS=Homo sapiens GN=COPA PE=1 SV=2		398.2
P53621-2	COPA_HUMAN Isoform 2 of Coatomer subunit alpha OS=Homo sapiens GN=COPA		398.2
P53621-2;P53621	2|COPA_HUMAN Isoform 2 of Coatomer subunit alpha OS=Homo sapiens GN=COPA;>sp|P53621|COPA_HUMAN Coatomer subunit alpl	1.24E-231	
P53634	CATC_HUMAN Dipeptidyl peptidase 1 OS=Homo sapiens GN=CTSC PE=1 SV=2		278.82
P53634-2	CATC_HUMAN Isoform 2 of Dipeptidyl peptidase 1 OS=Homo sapiens GN=CTSC		77.26
P53634;B4DJQ8	CATC_HUMAN Dipeptidyl peptidase 1 OS=Homo sapiens GN=CTSC PE=1 SV=2;>tr|B4DJQ8|B4DJQ8_HUMAN cDNA FLJ55694, highly s	1.36E-20	
P53667	LIMK1_HUMAN LIM domain kinase 1 OS=Homo sapiens GN=LIMK1 PE=1 SV=3		
P53667-2	LIMK1_HUMAN Isoform 2 of LIM domain kinase 1 OS=Homo sapiens GN=LIMK1		
P53667-3	LIMK1_HUMAN Isoform 3 of LIM domain kinase 1 OS=Homo sapiens GN=LIMK1		
P53675	CLH2_HUMAN Clathrin heavy chain 2 OS=Homo sapiens GN=CLTCL1 PE=1 SV=2		1384.51
P53675-2	CLH2_HUMAN Isoform 2 of Clathrin heavy chain 2 OS=Homo sapiens GN=CLTCL1		1384.51
P53680	AP2S1_HUMAN AP-2 complex subunit sigma OS=Homo sapiens GN=AP2S1 PE=1 SV=2		57.99
P53680-2	AP2S1_HUMAN Isoform 2 of AP-2 complex subunit sigma OS=Homo sapiens GN=AP2S1		27.47
P53680;P53680-2	AP2S1_HUMAN AP-2 complex subunit sigma OS=Homo sapiens GN=AP2S1 PE=1 SV=2;>sp|P53680-2|AP2S1_HUMAN Isoform 2 of AI	3.42E-12	
P53701	CCHL_HUMAN Cytochrome c-type heme lyase OS=Homo sapiens GN=HCCS PE=1 SV=1		
P53778	MK12_HUMAN Mitogen-activated protein kinase 12 OS=Homo sapiens GN=MAPK12 PE=1 SV=3		43.93
P53779	MK10_HUMAN Mitogen-activated protein kinase 10 OS=Homo sapiens GN=MAPK10 PE=1 SV=2		41.33
P53779-2	MK10_HUMAN Isoform Alpha-1 of Mitogen-activated protein kinase 10 OS=Homo sapiens GN=MAPK10		41.33
P53779;A8MTD8;P53779-:	MK10_HUMAN Mitogen-activated protein kinase 10 OS=Homo sapiens GN=MAPK10 PE=1 SV=2;>tr|A8MTD8|A8MTD8_HUMAN Unchar	1.35E-06	
P53801	PTTG_HUMAN Pituitary tumor-transforming gene 1 protein-interacting protein OS=Homo sapiens GN=PTTG1IP PE=1 SV=1		94.88
P53803	RPAB4_HUMAN DNA-directed RNA polymerases I, II, and III subunit RPABC4 OS=Homo sapiens GN=POLR2K PE=2 SV=1		
P53814	SMTN_HUMAN Smoothelin OS=Homo sapiens GN=SMTN PE=1 SV=7		24.71
P53814-2	SMTN_HUMAN Isoform A of Smoothelin OS=Homo sapiens GN=SMTN		24.71
P53814-5	SMTN_HUMAN Isoform B2 of Smoothelin OS=Homo sapiens GN=SMTN		24.71
P53814-6	SMTN_HUMAN Isoform B3 of Smoothelin OS=Homo sapiens GN=SMTN		24.71
P53985	MOT1_HUMAN Monocarboxylate transporter 1 OS=Homo sapiens GN=SLC16A1 PE=1 SV=3	2.56E-14	
P53990-4;P53990;P53990	4|IST1_HUMAN Isoform 4 of IST1 homolog OS=Homo sapiens GN=KIAA0174;>sp|P53990|IST1_HUMAN IST1 homolog OS=Homo sap	1.79E-15	
P53992	SC24C_HUMAN Protein transport protein Sec24C OS=Homo sapiens GN=SEC24C PE=1 SV=3		
P53999	TCP4_HUMAN Activated RNA polymerase II transcriptional coactivator p15 OS=Homo sapiens GN=SUB1 PE=1 SV=3		272.55
P54105	ICLN_HUMAN Methylosome subunit pICln OS=Homo sapiens GN=CLNS1A PE=1 SV=1	1.64E-97	
P54132	BLM_HUMAN Bloom syndrome protein OS=Homo sapiens GN=BLM PE=1 SV=1		
P54136	SYRC_HUMAN Arginyl-tRNA synthetase, cytoplasmic OS=Homo sapiens GN=RARS PE=1 SV=2		1114.41
P54136-2	SYRC_HUMAN Isoform Monomeric of Arginyl-tRNA synthetase, cytoplasmic OS=Homo sapiens GN=RARS		1057.53
P54136;P54136-2	SYRC_HUMAN Arginyl-tRNA synthetase, cytoplasmic OS=Homo sapiens GN=RARS PE=1 SV=2;>sp|P54136-2|SYRC_HUMAN Isoform I	0	
P54277	PMS1_HUMAN PMS1 protein homolog 1 OS=Homo sapiens GN=PMS1 PE=1 SV=1		
P54289	CA2D1_HUMAN Voltage-dependent calcium channel subunit alpha-2/delta-1 OS=Homo sapiens GN=CACNA2D1 PE=1 SV=3		
P54289-2	CA2D1_HUMAN Isoform 2 of Voltage-dependent calcium channel subunit alpha-2/delta-1 OS=Homo sapiens GN=CACNA2D1		
P54289-3	CA2D1_HUMAN Isoform 3 of Voltage-dependent calcium channel subunit alpha-2/delta-1 OS=Homo sapiens GN=CACNA2D1		
P54289-4	CA2D1_HUMAN Isoform 4 of Voltage-dependent calcium channel subunit alpha-2/delta-1 OS=Homo sapiens GN=CACNA2D1		
P54289-5	CA2D1_HUMAN Isoform 5 of Voltage-dependent calcium channel subunit alpha-2/delta-1 OS=Homo sapiens GN=CACNA2D1		
P54296	MYOM2_HUMAN Myomesin-2 OS=Homo sapiens GN=MYOM2 PE=2 SV=2	7.14E-11	
P54577	SYYC_HUMAN Tyrosyl-tRNA synthetase, cytoplasmic OS=Homo sapiens GN=YARS PE=1 SV=4		489.1
P54578	UBP14_HUMAN Ubiquitin carboxyl-terminal hydrolase 14 OS=Homo sapiens GN=USP14 PE=1 SV=3		74.34
P54619	AAKG1_HUMAN 5-AMP-activated protein kinase subunit gamma-1 OS=Homo sapiens GN=PRKAG1 PE=1 SV=1	1.92E-07	
P54652	HSP72_HUMAN Heat shock-related 70 kDa protein 2 OS=Homo sapiens GN=HSPA2 PE=1 SV=1		697.05
P54687	BCAT1_HUMAN Branched-chain-amino-acid aminotransferase, cytosolic OS=Homo sapiens GN=BCAT1 PE=1 SV=3	2.71E-158	
P54707	AT12A_HUMAN Potassium-transporting ATPase alpha chain 2 OS=Homo sapiens GN=ATP12A PE=2 SV=3		289.58

P54707-2	AT12A_HUMAN Isoform 2 of Potassium-transporting ATPase alpha chain 2 OS=Homo sapiens GN=ATP12A		289.58
P54709	AT1B3_HUMAN Sodium/potassium-transporting ATPase subunit beta-3 OS=Homo sapiens GN=ATP1B3 PE=1 SV=1		348.91
P54725	RD23A_HUMAN UV excision repair protein RAD23 homolog A OS=Homo sapiens GN=RAD23A PE=1 SV=1	7.49E-62	
P54727	RD23B_HUMAN UV excision repair protein RAD23 homolog B OS=Homo sapiens GN=RAD23B PE=1 SV=1		
P54753	EPHB3_HUMAN Ephrin type-B receptor 3 OS=Homo sapiens GN=EPHB3 PE=1 SV=2		92.5
P54756	EPHA5_HUMAN Ephrin type-A receptor 5 OS=Homo sapiens GN=EPHA5 PE=1 SV=3		40.71
P54756-2	EPHA5_HUMAN Isoform 2 of Ephrin type-A receptor 5 OS=Homo sapiens GN=EPHA5		40.71
P54756-3	EPHA5_HUMAN Isoform 3 of Ephrin type-A receptor 5 OS=Homo sapiens GN=EPHA5		40.71
P54760	EPHB4_HUMAN Ephrin type-B receptor 4 OS=Homo sapiens GN=EPHB4 PE=1 SV=2		102.7
P54762	EPHB1_HUMAN Ephrin type-B receptor 1 OS=Homo sapiens GN=EPHB1 PE=1 SV=1		103.85
P54764	EPHA4_HUMAN Ephrin type-A receptor 4 OS=Homo sapiens GN=EPHA4 PE=1 SV=1		49.98
P54802	ANAG_HUMAN Alpha-N-acetylglucosaminidase OS=Homo sapiens GN=NAGLU PE=1 SV=2	5.34E-06	
P54819	KAD2_HUMAN Adenylate kinase 2, mitochondrial OS=Homo sapiens GN=AK2 PE=1 SV=2		782.41
P54819-2	KAD2_HUMAN Isoform 2 of Adenylate kinase 2, mitochondrial OS=Homo sapiens GN=AK2		782.41
P54819-3	KAD2_HUMAN Isoform 3 of Adenylate kinase 2, mitochondrial OS=Homo sapiens GN=AK2		706.14
P54819-4	KAD2_HUMAN Isoform 4 of Adenylate kinase 2, mitochondrial OS=Homo sapiens GN=AK2		584.22
P54819-5	KAD2_HUMAN Isoform 5 of Adenylate kinase 2, mitochondrial OS=Homo sapiens GN=AK2		723.99
P54819-6	KAD2_HUMAN Isoform 6 of Adenylate kinase 2, mitochondrial OS=Homo sapiens GN=AK2		665.53
P54819;P54819-2;P54819	KAD2_HUMAN Adenylate kinase 2, mitochondrial OS=Homo sapiens GN=AK2 PE=1 SV=2;>sp|P54819-2|KAD2_HUMAN Isoform 2 of A	3.26E-86	
P54821	PRRX1_HUMAN Paired mesoderm homeobox protein 1 OS=Homo sapiens GN=PRRX1 PE=2 SV=2		
P54821-2	PRRX1_HUMAN Isoform PMX1-A of Paired mesoderm homeobox protein 1 OS=Homo sapiens GN=PRRX1		
P54852	EMP3_HUMAN Epithelial membrane protein 3 OS=Homo sapiens GN=EMP3 PE=2 SV=1		24.13
P54868	HMCS2_HUMAN Hydroxymethylglutaryl-CoA synthase, mitochondrial OS=Homo sapiens GN=HMGCS2 PE=1 SV=1		
P54886	P5CS_HUMAN Delta-1-pyrroline-5-carboxylate synthase OS=Homo sapiens GN=ALDH18A1 PE=1 SV=2		557.83
P54886-2	P5CS_HUMAN Isoform Short of Delta-1-pyrroline-5-carboxylate synthase OS=Homo sapiens GN=ALDH18A1		557.16
P54886;P54886-2	P5CS_HUMAN Delta-1-pyrroline-5-carboxylate synthase OS=Homo sapiens GN=ALDH18A1 PE=1 SV=2;>sp|P54886-2|P5CS_HUMAN I	7.17E-133	
P54920	SNAA_HUMAN Alpha-soluble NSF attachment protein OS=Homo sapiens GN=NAPA PE=1 SV=3		45.35
P55010	IF5_HUMAN Eukaryotic translation initiation factor 5 OS=Homo sapiens GN=EIF5 PE=1 SV=2		224.63
P55011	S12A2_HUMAN Solute carrier family 12 member 2 OS=Homo sapiens GN=SLC12A2 PE=1 SV=1		
P55011-3	S12A2_HUMAN Isoform 2 of Solute carrier family 12 member 2 OS=Homo sapiens GN=SLC12A2		
P55011;P55011-3	S12A2_HUMAN Solute carrier family 12 member 2 OS=Homo sapiens GN=SLC12A2 PE=1 SV=1;>sp|P55011-3|S12A2_HUMAN Isoform	4.28E-05	
P55036	PSMD4_HUMAN 26S proteasome non-ATPase regulatory subunit 4 OS=Homo sapiens GN=PSMD4 PE=1 SV=1		156.35
P55036-2	PSMD4_HUMAN Isoform Rpn10E of 26S proteasome non-ATPase regulatory subunit 4 OS=Homo sapiens GN=PSMD4		91.38
P55036;P55036-2	PSMD4_HUMAN 26S proteasome non-ATPase regulatory subunit 4 OS=Homo sapiens GN=PSMD4 PE=1 SV=1;>sp|P55036-2|PSMD4_	6.27E-41	
P55039	DRG2_HUMAN Developmentally-regulated GTP-binding protein 2 OS=Homo sapiens GN=DRG2 PE=1 SV=1	2.89E-15	
P55058;P55058-2	PLTP_HUMAN Phospholipid transfer protein OS=Homo sapiens GN=PLTP PE=1 SV=1;>sp|P55058-2|PLTP_HUMAN Isoform 2 of Phosph	6.26E-17	
P55060	XPO2_HUMAN Exportin-2 OS=Homo sapiens GN=CSE1L PE=1 SV=3		469.45
P55060-2	XPO2_HUMAN Isoform 2 of Exportin-2 OS=Homo sapiens GN=CSE1L		57.18
P55060-3	XPO2_HUMAN Isoform 3 of Exportin-2 OS=Homo sapiens GN=CSE1L		469.45
P55060;P55060-3	XPO2_HUMAN Exportin-2 OS=Homo sapiens GN=CSE1L PE=1 SV=3;>sp|P55060-3|XPO2_HUMAN Isoform 3 of Exportin-2 OS=Homo	0	
P55061	BI1_HUMAN Bax inhibitor 1 OS=Homo sapiens GN=TMBIM6 PE=1 SV=2		
P55072	TERA_HUMAN Transitional endoplasmic reticulum ATPase OS=Homo sapiens GN=VCP PE=1 SV=4		1995.04
P55081	MFAP1_HUMAN Microfibrillar-associated protein 1 OS=Homo sapiens GN=MFAP1 PE=1 SV=2		
P55083	MFAP4_HUMAN Microfibril-associated glycoprotein 4 OS=Homo sapiens GN=MFAP4 PE=1 SV=2	1.32E-35	
P55084	ECHB_HUMAN Trifunctional enzyme subunit beta, mitochondrial OS=Homo sapiens GN=HADHB PE=1 SV=3		397.65
P55145	MANF_HUMAN Mesencephalic astrocyte-derived neurotrophic factor OS=Homo sapiens GN=MANF PE=1 SV=3		800.32
P55160	NCKPL_HUMAN Nck-associated protein 1-like OS=Homo sapiens GN=NCKAP1L PE=1 SV=3	7.74E-11	
P55196	AFAD_HUMAN Afadin OS=Homo sapiens GN=MLLT4 PE=1 SV=3		140.27
P55196-1	AFAD_HUMAN Isoform 2 of Afadin OS=Homo sapiens GN=MLLT4		136.87
P55196-1;P55196-5;P551	1|AFAD_HUMAN Isoform 2 of Afadin OS=Homo sapiens GN=MLLT4;>sp|P55196-5|AFAD_HUMAN Isoform 5 of Afadin OS=Homo sapier	9.19E-77	
P55196-2	AFAD_HUMAN Isoform 1 of Afadin OS=Homo sapiens GN=MLLT4		136.87
P55196-3	AFAD_HUMAN Isoform 3 of Afadin OS=Homo sapiens GN=MLLT4		136.87
P55196-5	AFAD_HUMAN Isoform 5 of Afadin OS=Homo sapiens GN=MLLT4		136.87
P55196-5;A8MQ02;P5519	5|AFAD_HUMAN Isoform 5 of Afadin OS=Homo sapiens GN=MLLT4;>tr|A8MQ02|A8MQ02_HUMAN Uncharacterized protein OS=Homo	2.37E-06	
P55196-6	AFAD_HUMAN Isoform 6 of Afadin OS=Homo sapiens GN=MLLT4		140.27
P55197	AF10_HUMAN Protein AF-10 OS=Homo sapiens GN=MLLT10 PE=1 SV=1		
P55209	NP1L1_HUMAN Nucleosome assembly protein 1-like 1 OS=Homo sapiens GN=NAP1L1 PE=1 SV=1		149.53
P55210	CASP7_HUMAN Caspase-7 OS=Homo sapiens GN=CASP7 PE=1 SV=1		5.6
P55210-2	CASP7_HUMAN Isoform Beta of Caspase-7 OS=Homo sapiens GN=CASP7		67.81
P55210-3	CASP7_HUMAN Isoform Alpha' of Caspase-7 OS=Homo sapiens GN=CASP7		5.6
P55212	CASP6_HUMAN Caspase-6 OS=Homo sapiens GN=CASP6 PE=1 SV=2	9.02E-16	

P55263	ADK_HUMAN Adenosine kinase OS=Homo sapiens GN=ADK PE=1 SV=2		51.93
P55263-2	ADK_HUMAN Isoform Short of Adenosine kinase OS=Homo sapiens GN=ADK		51.93
P55263;P55263-2	ADK_HUMAN Adenosine kinase OS=Homo sapiens GN=ADK PE=1 SV=2;>sp|P55263-2|ADK_HUMAN Isoform Short of Adenosine kina:	1.03E-24	
P55265	DSRAD_HUMAN Double-stranded RNA-specific adenosine deaminase OS=Homo sapiens GN=ADAR PE=1 SV=4		60.97
P55265-2	DSRAD_HUMAN Isoform 2 of Double-stranded RNA-specific adenosine deaminase OS=Homo sapiens GN=ADAR		60.97
P55265-3	DSRAD_HUMAN Isoform 3 of Double-stranded RNA-specific adenosine deaminase OS=Homo sapiens GN=ADAR		60.97
P55265-4	DSRAD_HUMAN Isoform 4 of Double-stranded RNA-specific adenosine deaminase OS=Homo sapiens GN=ADAR		60.97
P55265-4;P55265;P55265	4|DSRAD_HUMAN Isoform 4 of Double-stranded RNA-specific adenosine deaminase OS=Homo sapiens GN=ADAR;>sp|P55265|DSRAD	6.43E-255	
P55265-5	DSRAD_HUMAN Isoform 5 of Double-stranded RNA-specific adenosine deaminase OS=Homo sapiens GN=ADAR		60.97
P55268	LAMB2_HUMAN Laminin subunit beta-2 OS=Homo sapiens GN=LAMB2 PE=1 SV=2	1.09E-174	
P55287;P55287-2	CAD11_HUMAN Cadherin-11 OS=Homo sapiens GN=CDH11 PE=1 SV=2;>sp|P55287-2|CAD11_HUMAN Isoform 2 of Cadherin-11 OS=	6.68E-05	
P55317	FOXA1_HUMAN Hepatocyte nuclear factor 3-alpha OS=Homo sapiens GN=FOXA1 PE=1 SV=2		
P55318	FOXA3_HUMAN Hepatocyte nuclear factor 3-gamma OS=Homo sapiens GN=FOXA3 PE=1 SV=2		
P55327-3;D0UFD1;D0UFD	3|TPD52_HUMAN Isoform 3 of Tumor protein D52 OS=Homo sapiens GN=TPD52;>tr|D0UFD1|D0UFD1_HUMAN Prostate leucine zippe	2.01E-123	
P55735	SEC13_HUMAN Protein SEC13 homolog OS=Homo sapiens GN=SEC13 PE=1 SV=3	3.58E-196	
P55769	NH2L1_HUMAN NHP2-like protein 1 OS=Homo sapiens GN=NHP2L1 PE=1 SV=3		101.79
P55786	PSA_HUMAN Puromycin-sensitive aminopeptidase OS=Homo sapiens GN=NPEPPS PE=1 SV=2		72.31
P55795	HNRH2_HUMAN Heterogeneous nuclear ribonucleoprotein H2 OS=Homo sapiens GN=HNRNPH2 PE=1 SV=1		416.42
P55809	SCOT1_HUMAN Succinyl-CoA:3-ketoacid-coenzyme A transferase 1, mitochondrial OS=Homo sapiens GN=OXCT1 PE=1 SV=1		
P55822	SH3BG_HUMAN SH3 domain-binding glutamic acid-rich protein OS=Homo sapiens GN=SH3BGR PE=1 SV=3	3.59E-05	
P55854	SUMO3_HUMAN Small ubiquitin-related modifier 3 OS=Homo sapiens GN=SUMO3 PE=1 SV=2		110.85
P55884	EIF3B_HUMAN Eukaryotic translation initiation factor 3 subunit B OS=Homo sapiens GN=EIF3B PE=1 SV=3		590.37
P55884-2	EIF3B_HUMAN Isoform 2 of Eukaryotic translation initiation factor 3 subunit B OS=Homo sapiens GN=EIF3B		590.37
P55884-2;P55884	2|EIF3B_HUMAN Isoform 2 of Eukaryotic translation initiation factor 3 subunit B OS=Homo sapiens GN=EIF3B;>sp|P55884|EIF3B_HU	4.47E-182	
P55899	FCGRN_HUMAN IgG receptor FcRn large subunit p51 OS=Homo sapiens GN=FCGRT PE=1 SV=1		24.98
P55957	BID_HUMAN BH3-interacting domain death agonist OS=Homo sapiens GN=BID PE=1 SV=1		163.03
P55957-2	BID_HUMAN Isoform 2 of BH3-interacting domain death agonist OS=Homo sapiens GN=BID		163.03
P55957-2;P55957	2|BID_HUMAN Isoform 2 of BH3-interacting domain death agonist OS=Homo sapiens GN=BID;>sp|P55957|BID_HUMAN BH3-interacti	3.89E-21	
P55957-4	BID_HUMAN Isoform 4 of BH3-interacting domain death agonist OS=Homo sapiens GN=BID		64.37
P56134	ATPK_HUMAN ATP synthase subunit f, mitochondrial OS=Homo sapiens GN=ATP5J2 PE=1 SV=3		82.43
P56134-2	ATPK_HUMAN Isoform 2 of ATP synthase subunit f, mitochondrial OS=Homo sapiens GN=ATP5J2		82.43
P56134;P56134-2	ATPK_HUMAN ATP synthase subunit f, mitochondrial OS=Homo sapiens GN=ATP5J2 PE=1 SV=3;>sp|P56134-2|ATPK_HUMAN Isoform	5.98E-20	
P56159	GFRA1_HUMAN GDNF family receptor alpha-1 OS=Homo sapiens GN=GFRA1 PE=2 SV=2		
P56159-2	GFRA1_HUMAN Isoform 2 of GDNF family receptor alpha-1 OS=Homo sapiens GN=GFRA1		
P56159-2;P56159	2|GFRA1_HUMAN Isoform 2 of GDNF family receptor alpha-1 OS=Homo sapiens GN=GFRA1;>sp|P56159|GFRA1_HUMAN GDNF family	2.09E-221	
P56181	NDUV3_HUMAN NADH dehydrogenase [ubiquinone] flavoprotein 3, mitochondrial OS=Homo sapiens GN=NDUFV3 PE=1 SV=2		32.95
P56181-2	NDUV3_HUMAN Isoform 2 of NADH dehydrogenase [ubiquinone] flavoprotein 3, mitochondrial OS=Homo sapiens GN=NDUFV3		31.12
P56182	RRP1_HUMAN Ribosomal RNA processing protein 1 homolog A OS=Homo sapiens GN=RRP1 PE=1 SV=1		
P56192	SYMC_HUMAN Methionyl-tRNA synthetase, cytoplasmic OS=Homo sapiens GN=MARS PE=1 SV=2		442.18
P56199	ITA1_HUMAN Integrin alpha-1 OS=Homo sapiens GN=ITGA1 PE=1 SV=2	2.43E-10	
P56211;P56211-2	ARP19_HUMAN cAMP-regulated phosphoprotein 19 OS=Homo sapiens GN=ARPP19 PE=1 SV=2;>sp|P56211-2|ARP19_HUMAN Isoforn	9.88E-09	
P56270	MAZ_HUMAN Myc-associated zinc finger protein OS=Homo sapiens GN=MAZ PE=1 SV=1		
P56279	TCL1A_HUMAN T-cell leukemia/lymphoma protein 1A OS=Homo sapiens GN=TCL1A PE=1 SV=1	4.46E-67	
P56377	AP1S2_HUMAN AP-1 complex subunit sigma-2 OS=Homo sapiens GN=AP1S2 PE=1 SV=1		
P56378	68MP_HUMAN 6.8 kDa mitochondrial proteolipid OS=Homo sapiens GN=MP68 PE=1 SV=1		29.91
P56381	ATP5E_HUMAN ATP synthase subunit epsilon, mitochondrial OS=Homo sapiens GN=ATP5E PE=1 SV=2		
P56385	ATP5I_HUMAN ATP synthase subunit e, mitochondrial OS=Homo sapiens GN=ATP5I PE=1 SV=2		43.4
P56524	HDAC4_HUMAN Histone deacetylase 4 OS=Homo sapiens GN=HDAC4 PE=1 SV=3		
P56537	IF6_HUMAN Eukaryotic translation initiation factor 6 OS=Homo sapiens GN=EIF6 PE=1 SV=1		548.09
P56539	CAV3_HUMAN Caveolin-3 OS=Homo sapiens GN=CAV3 PE=1 SV=1		57.05
P56545	CTBP2_HUMAN C-terminal-binding protein 2 OS=Homo sapiens GN=CTBP2 PE=1 SV=1		
P56545-2	CTBP2_HUMAN Isoform 2 of C-terminal-binding protein 2 OS=Homo sapiens GN=CTBP2		
P56545-2;P56545	2|CTBP2_HUMAN Isoform 2 of C-terminal-binding protein 2 OS=Homo sapiens GN=CTBP2;>sp|P56545|CTBP2_HUMAN C-terminal-bin	2.92E-22	
P56556	NDUA6_HUMAN NADH dehydrogenase [ubiquinone] 1 alpha subcomplex subunit 6 OS=Homo sapiens GN=NDUFA6 PE=1 SV=3		54.91
P56747	CLD6_HUMAN Claudin-6 OS=Homo sapiens GN=CLDN6 PE=1 SV=2		79.62
P56962	STX17_HUMAN Syntaxin-17 OS=Homo sapiens GN=STX17 PE=1 SV=2	6.84E-06	
P57053	H2BFS_HUMAN Histone H2B type F-S OS=Homo sapiens GN=H2BFS PE=1 SV=2		700.06
P57059	SIK1_HUMAN Serine/threonine-protein kinase SIK1 OS=Homo sapiens GN=SIK1 PE=1 SV=2		18.52
P57081	WDR4_HUMAN tRNA (guanine-N(7)-)-methyltransferase subunit WDR4 OS=Homo sapiens GN=WDR4 PE=1 SV=2		
P57081-2	WDR4_HUMAN Isoform 2 of tRNA (guanine-N(7)-)-methyltransferase subunit WDR4 OS=Homo sapiens GN=WDR4		
P57081-3	WDR4_HUMAN Isoform 3 of tRNA (guanine-N(7)-)-methyltransferase subunit WDR4 OS=Homo sapiens GN=WDR4		

P57088	TMM33_HUMAN Transmembrane protein 33 OS=Homo sapiens GN=TMEM33 PE=1 SV=2		255.42
P57105	SYJ2B_HUMAN Synaptojanin-2-binding protein OS=Homo sapiens GN=SYNJ2BP PE=1 SV=2		37.11
P57678	GEMI4_HUMAN Component of gems 4 OS=Homo sapiens GN=GEMIN4 PE=1 SV=2	8.55E-11	
P57721	PCBP3_HUMAN Poly(rC)-binding protein 3 OS=Homo sapiens GN=PCBP3 PE=1 SV=2		116.51
P57721-2	PCBP3_HUMAN Isoform 2 of Poly(rC)-binding protein 3 OS=Homo sapiens GN=PCBP3		116.51
P57721-3	PCBP3_HUMAN Isoform 3 of Poly(rC)-binding protein 3 OS=Homo sapiens GN=PCBP3		116.51
P57721-4	PCBP3_HUMAN Isoform 4 of Poly(rC)-binding protein 3 OS=Homo sapiens GN=PCBP3		116.51
P57721-5	PCBP3_HUMAN Isoform 5 of Poly(rC)-binding protein 3 OS=Homo sapiens GN=PCBP3		116.51
P57721;P57721-4;P57721	PCBP3_HUMAN Poly(rC)-binding protein 3 OS=Homo sapiens GN=PCBP3 PE=1 SV=2;>sp|P57721-4|PCBP3_HUMAN Isoform 4 of Poly(	3.37E-84	
P57723	PCBP4_HUMAN Poly(rC)-binding protein 4 OS=Homo sapiens GN=PCBP4 PE=2 SV=1		7.97
P57723-2	PCBP4_HUMAN Isoform 2 of Poly(rC)-binding protein 4 OS=Homo sapiens GN=PCBP4		7.97
P57735	RAB25_HUMAN Ras-related protein Rab-25 OS=Homo sapiens GN=RAB25 PE=1 SV=2		67.49
P57737;B4DFD6;B4DKU9;	CORO7_HUMAN Coronin-7 OS=Homo sapiens GN=CORO7 PE=1 SV=2;>tr|B4DFD6|B4DFD6_HUMAN cDNA FLJ54091, highly similar tc	5.31E-29	
P57740	NU107_HUMAN Nuclear pore complex protein Nup107 OS=Homo sapiens GN=NUP107 PE=1 SV=1		
P57764	GSDMD_HUMAN Gasdermin-D OS=Homo sapiens GN=GSDMD PE=1 SV=1	4.05E-07	
P57772	SELB_HUMAN Selenocysteine-specific elongation factor OS=Homo sapiens GN=EEFSEC PE=1 SV=4	4.22E-14	
P57796	CABP4_HUMAN Calcium-binding protein 4 OS=Homo sapiens GN=CABP4 PE=1 SV=2		
P57796-2	CABP4_HUMAN Isoform 2 of Calcium-binding protein 4 OS=Homo sapiens GN=CABP4		
P58107	EPIPL_HUMAN Epiplakin OS=Homo sapiens GN=EPPK1 PE=1 SV=2		399.76
P58304	VSX2_HUMAN Visual system homeobox 2 OS=Homo sapiens GN=VSX2 PE=1 SV=1		
P58335	ANTR2_HUMAN Anthrax toxin receptor 2 OS=Homo sapiens GN=ANTXR2 PE=1 SV=5		22.7
P58335-2	ANTR2_HUMAN Isoform 2 of Anthrax toxin receptor 2 OS=Homo sapiens GN=ANTXR2		22.7
P58335-3	ANTR2_HUMAN Isoform 3 of Anthrax toxin receptor 2 OS=Homo sapiens GN=ANTXR2		2.83
P58335-4	ANTR2_HUMAN Isoform 4 of Anthrax toxin receptor 2 OS=Homo sapiens GN=ANTXR2		22.7
P58335;P58335-4;A4FUA5	ANTR2_HUMAN Anthrax toxin receptor 2 OS=Homo sapiens GN=ANTXR2 PE=1 SV=5;>sp|P58335-4|ANTR2_HUMAN Isoform 4 of Antl	6.29E-05	
P58546	MTPN_HUMAN Myotrophin OS=Homo sapiens GN=MTPN PE=1 SV=2		273.83
P58876	H2B1D_HUMAN Histone H2B type 1-D OS=Homo sapiens GN=HIST1H2BD PE=1 SV=2		705.5
P59045;P59045-2;P59045	NAL11_HUMAN NACHT, LRR and PYD domains-containing protein 11 OS=Homo sapiens GN=NLRP11 PE=2 SV=2;>sp|P59045-2|NAL1	2.83E-10	
P59074	CG301_HUMAN Putative protein CGI-301 OS=Homo sapiens GN=CGI-301 PE=5 SV=1		
P59190	RAB15_HUMAN Ras-related protein Rab-15 OS=Homo sapiens GN=RAB15 PE=1 SV=1		158.68
P59190-2	RAB15_HUMAN Isoform 2 of Ras-related protein Rab-15 OS=Homo sapiens GN=RAB15		143.66
P59665;P59666	DEF1_HUMAN Neutrophil defensin 1 OS=Homo sapiens GN=DEFA1 PE=1 SV=1;>sp|P59666|DEF3_HUMAN Neutrophil defensin 3 OS=	6.64E-17	
P59780	AP3S2_HUMAN AP-3 complex subunit sigma-2 OS=Homo sapiens GN=AP3S2 PE=2 SV=1	3.06E-10	
P59998	ARPC4_HUMAN Actin-related protein 2/3 complex subunit 4 OS=Homo sapiens GN=ARPC4 PE=1 SV=3		345.63
P60033	CD81_HUMAN CD81 antigen OS=Homo sapiens GN=CD81 PE=1 SV=1		39.17
P60059	SC61G_HUMAN Protein transport protein Sec61 subunit gamma OS=Homo sapiens GN=SEC61G PE=2 SV=1		13.95
P60174	TPIS_HUMAN Triosephosphate isomerase OS=Homo sapiens GN=TPI1 PE=1 SV=2		1372.48
P60174-2	TPIS_HUMAN Isoform 2 of Triosephosphate isomerase OS=Homo sapiens GN=TPI1		1018.64
P60174;P60174-2	TPIS_HUMAN Triosephosphate isomerase OS=Homo sapiens GN=TPI1 PE=1 SV=2;>sp|P60174-2|TPIS_HUMAN Isoform 2 of Triosepho	0	
P60228	EIF3E_HUMAN Eukaryotic translation initiation factor 3 subunit E OS=Homo sapiens GN=EIF3E PE=1 SV=1		462.9
P60468	SC61B_HUMAN Protein transport protein Sec61 subunit beta OS=Homo sapiens GN=SEC61B PE=1 SV=2		195.48
P60510	PP4C_HUMAN Serine/threonine-protein phosphatase 4 catalytic subunit OS=Homo sapiens GN=PPP4C PE=1 SV=1		143.36
P60520	GBRL2_HUMAN Gamma-aminobutyric acid receptor-associated protein-like 2 OS=Homo sapiens GN=GABARAPL2 PE=1 SV=1		
P60602	ROMO1_HUMAN Reactive oxygen species modulator 1 OS=Homo sapiens GN=ROMO1 PE=1 SV=1		31.07
P60602-2	ROMO1_HUMAN Isoform 2 of Reactive oxygen species modulator 1 OS=Homo sapiens GN=ROMO1		31.07
P60604	UB2G2_HUMAN Ubiquitin-conjugating enzyme E2 G2 OS=Homo sapiens GN=UBE2G2 PE=1 SV=1	1.64E-08	
P60660	MYL6_HUMAN Myosin light polypeptide 6 OS=Homo sapiens GN=MYL6 PE=1 SV=2		774.11
P60660-2	MYL6_HUMAN Isoform Smooth muscle of Myosin light polypeptide 6 OS=Homo sapiens GN=MYL6		840.94
P60660;P60660-2	MYL6_HUMAN Myosin light polypeptide 6 OS=Homo sapiens GN=MYL6 PE=1 SV=2;>sp|P60660-2|MYL6_HUMAN Isoform Smooth mus	1.98E-158	
P60709	ACTB_HUMAN Actin, cytoplasmic 1 OS=Homo sapiens GN=ACTB PE=1 SV=1		2352.17
P60763	RAC3_HUMAN Ras-related C3 botulinum toxin substrate 3 OS=Homo sapiens GN=RAC3 PE=1 SV=1		241.21
P60842	IF4A1_HUMAN Eukaryotic initiation factor 4A-I OS=Homo sapiens GN=EIF4A1 PE=1 SV=1		1103.33
P60866	RS20_HUMAN 40S ribosomal protein S20 OS=Homo sapiens GN=RPS20 PE=1 SV=1		334.69
P60891	PRPS1_HUMAN Ribose-phosphate pyrophosphokinase 1 OS=Homo sapiens GN=PRPS1 PE=1 SV=2		39.32
P60900	PSA6_HUMAN Proteasome subunit alpha type-6 OS=Homo sapiens GN=PSMA6 PE=1 SV=1		675.45
P60903	S10AA_HUMAN Protein S100-A10 OS=Homo sapiens GN=S100A10 PE=1 SV=2		424.1
P60953	CDC42_HUMAN Cell division control protein 42 homolog OS=Homo sapiens GN=CDC42 PE=1 SV=2		382.73
P60953-1	CDC42_HUMAN Isoform 1 of Cell division control protein 42 homolog OS=Homo sapiens GN=CDC42		216.04
P60953;P60953-1	CDC42_HUMAN Cell division control protein 42 homolog OS=Homo sapiens GN=CDC42 PE=1 SV=2;>sp|P60953-1|CDC42_HUMAN Isc	3.90E-81	
P60981	DEST_HUMAN Destrin OS=Homo sapiens GN=DSTN PE=1 SV=3		150.71
P60983	GMFB_HUMAN Glia maturation factor beta OS=Homo sapiens GN=GMFB PE=1 SV=2		38.53

P61006	RAB8A_HUMAN Ras-related protein Rab-8A OS=Homo sapiens GN=RAB8A PE=1 SV=1		648.89
P61009	SPCS3_HUMAN Signal peptidase complex subunit 3 OS=Homo sapiens GN=SPCS3 PE=1 SV=1		152.05
P61011	SRP54_HUMAN Signal recognition particle 54 kDa protein OS=Homo sapiens GN=SRP54 PE=1 SV=1		245.3
P61018	RAB4B_HUMAN Ras-related protein Rab-4B OS=Homo sapiens GN=RAB4B PE=1 SV=1		103.32
P61018-2	RAB4B_HUMAN Isoform 2 of Ras-related protein Rab-4B OS=Homo sapiens GN=RAB4B		103.32
P61018-2;P61018;Q6PIK3	2|RAB4B_HUMAN Isoform 2 of Ras-related protein Rab-4B OS=Homo sapiens GN=RAB4B;>sp|P61018|RAB4B_HUMAN Ras-related pro	2.33E-07	
P61018;P61018-2	RAB4B_HUMAN Ras-related protein Rab-4B OS=Homo sapiens GN=RAB4B PE=1 SV=1;>sp|P61018-2|RAB4B_HUMAN Isoform 2 of Ra	3.12E-07	
P61019	RAB2A_HUMAN Ras-related protein Rab-2A OS=Homo sapiens GN=RAB2A PE=1 SV=1		637.4
P61020	RAB5B_HUMAN Ras-related protein Rab-5B OS=Homo sapiens GN=RAB5B PE=1 SV=1		490.89
P61024	CKS1_HUMAN Cyclin-dependent kinases regulatory subunit 1 OS=Homo sapiens GN=CKS1B PE=1 SV=1		65.91
P61026	RAB10_HUMAN Ras-related protein Rab-10 OS=Homo sapiens GN=RAB10 PE=1 SV=1		535.49
P61073-2;P61073	2|CXCR4_HUMAN Isoform 2 of C-X-C chemokine receptor type 4 OS=Homo sapiens GN=CXCR4;>sp|P61073|CXCR4_HUMAN C-X-C ch	3.27E-15	
P61077	UB2D3_HUMAN Ubiquitin-conjugating enzyme E2 D3 OS=Homo sapiens GN=UBE2D3 PE=1 SV=1		28.74
P61077-2	UB2D3_HUMAN Isoform 2 of Ubiquitin-conjugating enzyme E2 D3 OS=Homo sapiens GN=UBE2D3		28.74
P61077-2;P61077;P61077	2|UB2D3_HUMAN Isoform 2 of Ubiquitin-conjugating enzyme E2 D3 OS=Homo sapiens GN=UBE2D3;>sp|P61077|UB2D3_HUMAN Ubi	5.30E-18	
P61077-3	UB2D3_HUMAN Isoform 3 of Ubiquitin-conjugating enzyme E2 D3 OS=Homo sapiens GN=UBE2D3		28.74
P61077-3;P61077-2;D3D0	3|UB2D3_HUMAN Isoform 3 of Ubiquitin-conjugating enzyme E2 D3 OS=Homo sapiens GN=UBE2D3;>sp|P61077-2|UB2D3_HUMAN Is	2.28E-11	
P61081	UBC12_HUMAN NEDD8-conjugating enzyme Ubc12 OS=Homo sapiens GN=UBE2M PE=1 SV=1		153.22
P61086	UBE2K_HUMAN Ubiquitin-conjugating enzyme E2 K OS=Homo sapiens GN=UBE2K PE=1 SV=3		159.75
P61086-2	UBE2K_HUMAN Isoform 2 of Ubiquitin-conjugating enzyme E2 K OS=Homo sapiens GN=UBE2K		63.06
P61086;B4DIZ2;C9JGP1;D	UBE2K_HUMAN Ubiquitin-conjugating enzyme E2 K OS=Homo sapiens GN=UBE2K PE=1 SV=3;>tr|B4DIZ2|B4DIZ2_HUMAN cDNA FLJ	9.89E-31	
P61088	UBE2N_HUMAN Ubiquitin-conjugating enzyme E2 N OS=Homo sapiens GN=UBE2N PE=1 SV=1		351.72
P61106	RAB14_HUMAN Ras-related protein Rab-14 OS=Homo sapiens GN=RAB14 PE=1 SV=4		601.41
P61158	ARP3_HUMAN Actin-related protein 3 OS=Homo sapiens GN=ACTR3 PE=1 SV=3		402.51
P61160	ARP2_HUMAN Actin-related protein 2 OS=Homo sapiens GN=ACTR2 PE=1 SV=1		426.29
P61163	ACTZ_HUMAN Alpha-centractin OS=Homo sapiens GN=ACTR1A PE=1 SV=1		267.5
P61201	CSN2_HUMAN COP9 signalosome complex subunit 2 OS=Homo sapiens GN=COPS2 PE=1 SV=1		83.64
P61201-2	CSN2_HUMAN Isoform 2 of COP9 signalosome complex subunit 2 OS=Homo sapiens GN=COPS2		83.64
P61201-2;P61201	2|CSN2_HUMAN Isoform 2 of COP9 signalosome complex subunit 2 OS=Homo sapiens GN=COPS2;>sp|P61201|CSN2_HUMAN COP9 s	8.36E-51	
P61204	ARF3_HUMAN ADP-ribosylation factor 3 OS=Homo sapiens GN=ARF3 PE=1 SV=2		848.68
P61218	RPAB2_HUMAN DNA-directed RNA polymerases I, II, and III subunit RPABC2 OS=Homo sapiens GN=POLR2F PE=1 SV=1		26.99
P61221	ABCE1_HUMAN ATP-binding cassette sub-family E member 1 OS=Homo sapiens GN=ABCE1 PE=1 SV=1		621.03
P61224	RAP1B_HUMAN Ras-related protein Rap-1b OS=Homo sapiens GN=RAP1B PE=1 SV=1		648.23
P61225	RAP2B_HUMAN Ras-related protein Rap-2b OS=Homo sapiens GN=RAP2B PE=1 SV=1		271.79
P61244	MAX_HUMAN Protein max OS=Homo sapiens GN=MAX PE=1 SV=1		43.65
P61244-2	MAX_HUMAN Isoform 2 of Protein max OS=Homo sapiens GN=MAX		43.65
P61244-3	MAX_HUMAN Isoform 3 of Protein max OS=Homo sapiens GN=MAX		43.65
P61247	RS3A_HUMAN 40S ribosomal protein S3a OS=Homo sapiens GN=RPS3A PE=1 SV=2		1582.55
P61254	RL26_HUMAN 60S ribosomal protein L26 OS=Homo sapiens GN=RPL26 PE=1 SV=1		661.96
P61289	PSME3_HUMAN Proteasome activator complex subunit 3 OS=Homo sapiens GN=PSME3 PE=1 SV=1		711.34
P61289-2	PSME3_HUMAN Isoform 2 of Proteasome activator complex subunit 3 OS=Homo sapiens GN=PSME3		711.34
P61289-2;P61289	2|PSME3_HUMAN Isoform 2 of Proteasome activator complex subunit 3 OS=Homo sapiens GN=PSME3;>sp|P61289|PSME3_HUMAN Pr	1.39E-83	
P61296	HAND2_HUMAN Heart- and neural crest derivatives-expressed protein 2 OS=Homo sapiens GN=HAND2 PE=1 SV=1		
P61313	RL15_HUMAN 60S ribosomal protein L15 OS=Homo sapiens GN=RPL15 PE=1 SV=2		576.72
P61326	MGN_HUMAN Protein mago nashi homolog OS=Homo sapiens GN=MAGOH PE=1 SV=1		300.21
P61353	RL27_HUMAN 60S ribosomal protein L27 OS=Homo sapiens GN=RPL27 PE=1 SV=2		374.34
P61421	VA0D1_HUMAN V-type proton ATPase subunit d 1 OS=Homo sapiens GN=ATP6V0D1 PE=1 SV=1		
P61457	PHS_HUMAN Pterin-4-alpha-carbinolamine dehydratase OS=Homo sapiens GN=PCBD1 PE=1 SV=2		102.33
P61513	RL37A_HUMAN 60S ribosomal protein L37a OS=Homo sapiens GN=RPL37A PE=1 SV=2		317.52
P61565	ENK1_HUMAN HERV-K_12q14.1 provirus ancestral Env polyprotein OS=Homo sapiens PE=1 SV=1		
P61566	ENK11_HUMAN HERV-K_22q11.21 provirus ancestral Env polyprotein OS=Homo sapiens PE=2 SV=1		
P61567	ENK12_HUMAN HERV-K_1q22 provirus ancestral Env polyprotein OS=Homo sapiens PE=2 SV=1		
P61570	ENK17_HUMAN HERV-K_11q22.1 provirus ancestral Env polyprotein OS=Homo sapiens PE=3 SV=1		
P61571	REC1_HUMAN HERV-K_12q14.1 provirus Rec protein OS=Homo sapiens PE=1 SV=1		
P61586	RHOA_HUMAN Transforming protein RhoA OS=Homo sapiens GN=RHOA PE=1 SV=1		987.22
P61599	NAA20_HUMAN N-alpha-acetyltransferase 20, NatB catalytic subunit OS=Homo sapiens GN=NAA20 PE=1 SV=1	9.44E-07	
P61601	NCALD_HUMAN Neurocalcin-delta OS=Homo sapiens GN=NCALD PE=2 SV=2		204.25
P61604	CH10_HUMAN 10 kDa heat shock protein, mitochondrial OS=Homo sapiens GN=HSPE1 PE=1 SV=2		530.55
P61619	S61A1_HUMAN Protein transport protein Sec61 subunit alpha isoform 1 OS=Homo sapiens GN=SEC61A1 PE=1 SV=2		259.03
P61619-3	S61A1_HUMAN Isoform 3 of Protein transport protein Sec61 subunit alpha isoform 1 OS=Homo sapiens GN=SEC61A1		120.6
P61619;P61619-3	S61A1_HUMAN Protein transport protein Sec61 subunit alpha isoform 1 OS=Homo sapiens GN=SEC61A1 PE=1 SV=2;>sp|P61619-3|S	2.47E-36	

P61626	LYSC_HUMAN Lysozyme C OS=Homo sapiens GN=LYZ PE=1 SV=1	6.60E-199	
P61758	PFD3_HUMAN Prefoldin subunit 3 OS=Homo sapiens GN=VBP1 PE=1 SV=3		313.67
P61764	STXB1_HUMAN Syntaxin-binding protein 1 OS=Homo sapiens GN=STXBP1 PE=1 SV=1		25.19
P61764-2	STXB1_HUMAN Isoform 2 of Syntaxin-binding protein 1 OS=Homo sapiens GN=STXBP1		25.19
P61764-2;P61764	2|STXB1_HUMAN Isoform 2 of Syntaxin-binding protein 1 OS=Homo sapiens GN=STXBP1;>sp|P61764|STXB1_HUMAN Syntaxin-bindi	4.82E-46	
P61769	B2MG_HUMAN Beta-2-microglobulin OS=Homo sapiens GN=B2M PE=1 SV=1		208.37
P61803	DAD1_HUMAN Dolichyl-diphosphooligosaccharide--protein glycosyltransferase subunit DAD1 OS=Homo sapiens GN=DAD1 PE=1 SV=3		192.58
P61812-2;B2R7T2;P61812	2|TGFB2_HUMAN Isoform B of Transforming growth factor beta-2 OS=Homo sapiens GN=TGFB2;>tr|B2R7T2|B2R7T2_HUMAN cDNA, I	6.53E-23	
P61916	NPC2_HUMAN Epididymal secretory protein E1 OS=Homo sapiens GN=NPC2 PE=1 SV=1		264.4
P61923	COPZ1_HUMAN Coatomer subunit zeta-1 OS=Homo sapiens GN=COPZ1 PE=1 SV=1		187.87
P61927	RL37_HUMAN 60S ribosomal protein L37 OS=Homo sapiens GN=RPL37 PE=1 SV=2	1.71E-06	
P61956	SUMO2_HUMAN Small ubiquitin-related modifier 2 OS=Homo sapiens GN=SUMO2 PE=1 SV=2		64.64
P61960	UFM1_HUMAN Ubiquitin-fold modifier 1 OS=Homo sapiens GN=UFM1 PE=1 SV=1		33.51
P61962	DCAF7_HUMAN DDB1- and CUL4-associated factor 7 OS=Homo sapiens GN=DCAF7 PE=1 SV=1		
P61964	WDR5_HUMAN WD repeat-containing protein 5 OS=Homo sapiens GN=WDR5 PE=1 SV=1		209.21
P61966	AP1S1_HUMAN AP-1 complex subunit sigma-1A OS=Homo sapiens GN=AP1S1 PE=1 SV=1		
P61966-2	AP1S1_HUMAN Isoform 2 of AP-1 complex subunit sigma-1A OS=Homo sapiens GN=AP1S1		
P61966;P61966-2	AP1S1_HUMAN AP-1 complex subunit sigma-1A OS=Homo sapiens GN=AP1S1 PE=1 SV=1;>sp|P61966-2|AP1S1_HUMAN Isoform 2 o	1.70E-34	
P61970	NTF2_HUMAN Nuclear transport factor 2 OS=Homo sapiens GN=NUTF2 PE=1 SV=1		189.29
P61978	HNRPK_HUMAN Heterogeneous nuclear ribonucleoprotein K OS=Homo sapiens GN=HNRNPK PE=1 SV=1		1276.79
P61978-2	HNRPK_HUMAN Isoform 2 of Heterogeneous nuclear ribonucleoprotein K OS=Homo sapiens GN=HNRNPK		1276.79
P61978-2;P61978	2|HNRPK_HUMAN Isoform 2 of Heterogeneous nuclear ribonucleoprotein K OS=Homo sapiens GN=HNRNPK;>sp|P61978|HNRPK_HUM.	0	
P61978-3	HNRPK_HUMAN Isoform 3 of Heterogeneous nuclear ribonucleoprotein K OS=Homo sapiens GN=HNRNPK		1196.33
P61978-3;B4DUQ1;Q5T6V	3|HNRPK_HUMAN Isoform 3 of Heterogeneous nuclear ribonucleoprotein K OS=Homo sapiens GN=HNRNPK;>tr|B4DUQ1|B4DUQ1_HU	0	
P61981	1433G_HUMAN 14-3-3 protein gamma OS=Homo sapiens GN=YWHAG PE=1 SV=2		829.73
P62068	UBP46_HUMAN Ubiquitin carboxyl-terminal hydrolase 46 OS=Homo sapiens GN=USP46 PE=1 SV=1		
P62068-2	UBP46_HUMAN Isoform 2 of Ubiquitin carboxyl-terminal hydrolase 46 OS=Homo sapiens GN=USP46		
P62068-3	UBP46_HUMAN Isoform 3 of Ubiquitin carboxyl-terminal hydrolase 46 OS=Homo sapiens GN=USP46		
P62068-4	UBP46_HUMAN Isoform 4 of Ubiquitin carboxyl-terminal hydrolase 46 OS=Homo sapiens GN=USP46		
P62070	RRAS2_HUMAN Ras-related protein R-Ras2 OS=Homo sapiens GN=RRAS2 PE=1 SV=1		485.94
P62072	TIM10_HUMAN Mitochondrial import inner membrane translocase subunit Tim10 OS=Homo sapiens GN=TIMM10 PE=1 SV=1		142.36
P62081	RS7_HUMAN 40S ribosomal protein S7 OS=Homo sapiens GN=RPS7 PE=1 SV=1		903.13
P62136	PP1A_HUMAN Serine/threonine-protein phosphatase PP1-alpha catalytic subunit OS=Homo sapiens GN=PPP1CA PE=1 SV=1		835.58
P62140	PP1B_HUMAN Serine/threonine-protein phosphatase PP1-beta catalytic subunit OS=Homo sapiens GN=PPP1CB PE=1 SV=3		820.69
P62158	CALM_HUMAN Calmodulin OS=Homo sapiens GN=CALM1 PE=1 SV=2		715.55
P62166	NCS1_HUMAN Neuronal calcium sensor 1 OS=Homo sapiens GN=NCS1 PE=1 SV=2		26.14
P62191	PRS4_HUMAN 26S protease regulatory subunit 4 OS=Homo sapiens GN=PSMC1 PE=1 SV=1		485.56
P62195	PRS8_HUMAN 26S protease regulatory subunit 8 OS=Homo sapiens GN=PSMC5 PE=1 SV=1		478.44
P62241	RS8_HUMAN 40S ribosomal protein S8 OS=Homo sapiens GN=RPS8 PE=1 SV=2		778.23
P62244	RS15A_HUMAN 40S ribosomal protein S15a OS=Homo sapiens GN=RPS15A PE=1 SV=2		533.45
P62249	RS16_HUMAN 40S ribosomal protein S16 OS=Homo sapiens GN=RPS16 PE=1 SV=2		680.96
P62256	UBE2H_HUMAN Ubiquitin-conjugating enzyme E2 H OS=Homo sapiens GN=UBE2H PE=1 SV=1	1.49E-17	
P62258	1433E_HUMAN 14-3-3 protein epsilon OS=Homo sapiens GN=YWHAE PE=1 SV=1		1579.84
P62258-2	1433E_HUMAN Isoform SV of 14-3-3 protein epsilon OS=Homo sapiens GN=YWHAE		1423.75
P62258;P62258-2	1433E_HUMAN 14-3-3 protein epsilon OS=Homo sapiens GN=YWHAE PE=1 SV=1;>sp|P62258-2|1433E_HUMAN Isoform SV of 14-3-3	0	
P62263	RS14_HUMAN 40S ribosomal protein S14 OS=Homo sapiens GN=RPS14 PE=1 SV=3		643.14
P62266	RS23_HUMAN 40S ribosomal protein S23 OS=Homo sapiens GN=RPS23 PE=1 SV=3		271.93
P62269	RS18_HUMAN 40S ribosomal protein S18 OS=Homo sapiens GN=RPS18 PE=1 SV=3		827.24
P62273	RS29_HUMAN 40S ribosomal protein S29 OS=Homo sapiens GN=RPS29 PE=1 SV=2		202.05
P62277	RS13_HUMAN 40S ribosomal protein S13 OS=Homo sapiens GN=RPS13 PE=1 SV=2		564.61
P62280	RS11_HUMAN 40S ribosomal protein S11 OS=Homo sapiens GN=RPS11 PE=1 SV=3		612.88
P62304	RUXE_HUMAN Small nuclear ribonucleoprotein E OS=Homo sapiens GN=SNRPE PE=1 SV=1		569.46
P62306	RUXF_HUMAN Small nuclear ribonucleoprotein F OS=Homo sapiens GN=SNRPF PE=1 SV=1		420.41
P62308	RUXG_HUMAN Small nuclear ribonucleoprotein G OS=Homo sapiens GN=SNRPG PE=1 SV=1		316.18
P62310	LSM3_HUMAN U6 snRNA-associated Sm-like protein LSm3 OS=Homo sapiens GN=LSM3 PE=1 SV=2		
P62312	LSM6_HUMAN U6 snRNA-associated Sm-like protein LSm6 OS=Homo sapiens GN=LSM6 PE=1 SV=1		197.3
P62314	SMD1_HUMAN Small nuclear ribonucleoprotein Sm D1 OS=Homo sapiens GN=SNRPD1 PE=1 SV=1		445.02
P62316	SMD2_HUMAN Small nuclear ribonucleoprotein Sm D2 OS=Homo sapiens GN=SNRPD2 PE=1 SV=1		539.22
P62318	SMD3_HUMAN Small nuclear ribonucleoprotein Sm D3 OS=Homo sapiens GN=SNRPD3 PE=1 SV=1		306.06
P62330	ARF6_HUMAN ADP-ribosylation factor 6 OS=Homo sapiens GN=ARF6 PE=1 SV=2		454.95
P62333	PRS10_HUMAN 26S protease regulatory subunit 10B OS=Homo sapiens GN=PSMC6 PE=1 SV=1		330.86

P62341	SELT_HUMAN Selenoprotein T OS=Homo sapiens GN=SELT PE=2 SV=2	1.76E-05	
P62380	TBPL1_HUMAN TATA box-binding protein-like protein 1 OS=Homo sapiens GN=TBPL1 PE=1 SV=1		45.16
P62424	RL7A_HUMAN 60S ribosomal protein L7a OS=Homo sapiens GN=RPL7A PE=1 SV=2		1211.55
P62487	RPB7_HUMAN DNA-directed RNA polymerase II subunit RPB7 OS=Homo sapiens GN=POLR2G PE=1 SV=1		111.71
P62491	RB11A_HUMAN Ras-related protein Rab-11A OS=Homo sapiens GN=RAB11A PE=1 SV=3		425.5
P62495	ERF1_HUMAN Eukaryotic peptide chain release factor subunit 1 OS=Homo sapiens GN=ETF1 PE=1 SV=3		232.99
P62633	CNBP_HUMAN Cellular nucleic acid-binding protein OS=Homo sapiens GN=CNBP PE=1 SV=1		183.38
P62633-2	CNBP_HUMAN Isoform 2 of Cellular nucleic acid-binding protein OS=Homo sapiens GN=CNBP		183.38
P62633-3	CNBP_HUMAN Isoform 3 of Cellular nucleic acid-binding protein OS=Homo sapiens GN=CNBP		122.76
P62633;P62633-2;P62633	CNBP_HUMAN Cellular nucleic acid-binding protein OS=Homo sapiens GN=CNBP PE=1 SV=1;>sp|P62633-2|CNBP_HUMAN Isoform 2 c	2.25E-50	
P62699	YPEL5_HUMAN Protein yippee-like 5 OS=Homo sapiens GN=YPEL5 PE=2 SV=1	6.45E-17	
P62701	RS4X_HUMAN 40S ribosomal protein S4, X isoform OS=Homo sapiens GN=RPS4X PE=1 SV=2		1108.72
P62714	PP2AB_HUMAN Serine/threonine-protein phosphatase 2A catalytic subunit beta isoform OS=Homo sapiens GN=PPP2CB PE=1 SV=1		172.98
P62736	ACTA_HUMAN Actin, aortic smooth muscle OS=Homo sapiens GN=ACTA2 PE=1 SV=1		1109.31
P62745	RHOB_HUMAN Rho-related GTP-binding protein RhoB OS=Homo sapiens GN=RHOB PE=1 SV=1		172.97
P62750	RL23A_HUMAN 60S ribosomal protein L23a OS=Homo sapiens GN=RPL23A PE=1 SV=1		648.54
P62753	RS6_HUMAN 40S ribosomal protein S6 OS=Homo sapiens GN=RPS6 PE=1 SV=1		607.04
P62805	H4_HUMAN Histone H4 OS=Homo sapiens GN=HIST1H4A PE=1 SV=2		895.29
P62807	H2B1C_HUMAN Histone H2B type 1-C/E/F/G/I OS=Homo sapiens GN=HIST1H2BC PE=1 SV=4		705.5
P62820	RAB1A_HUMAN Ras-related protein Rab-1A OS=Homo sapiens GN=RAB1A PE=1 SV=3		1001.3
P62820-2	RAB1A_HUMAN Isoform 2 of Ras-related protein Rab-1A OS=Homo sapiens GN=RAB1A		734.32
P62820-3	RAB1A_HUMAN Isoform 3 of Ras-related protein Rab-1A OS=Homo sapiens GN=RAB1A		739.85
P62820;P62820-2;P62820	RAB1A_HUMAN Ras-related protein Rab-1A OS=Homo sapiens GN=RAB1A PE=1 SV=3;>sp|P62820-2|RAB1A_HUMAN Isoform 2 of Ra	4.61E-255	
P62826	RAN_HUMAN GTP-binding nuclear protein Ran OS=Homo sapiens GN=RAN PE=1 SV=3		535.51
P62829	RL23_HUMAN 60S ribosomal protein L23 OS=Homo sapiens GN=RPL23 PE=1 SV=1		789.04
P62834	RAP1A_HUMAN Ras-related protein Rap-1A OS=Homo sapiens GN=RAP1A PE=1 SV=1		493.61
P62837	UB2D2_HUMAN Ubiquitin-conjugating enzyme E2 D2 OS=Homo sapiens GN=UBE2D2 PE=1 SV=1		28.74
P62841	RS15_HUMAN 40S ribosomal protein S15 OS=Homo sapiens GN=RPS15 PE=1 SV=2		889.24
P62847	RS24_HUMAN 40S ribosomal protein S24 OS=Homo sapiens GN=RPS24 PE=1 SV=1		392.93
P62847-2	RS24_HUMAN Isoform 2 of 40S ribosomal protein S24 OS=Homo sapiens GN=RPS24		392.93
P62847-3	RS24_HUMAN Isoform 3 of 40S ribosomal protein S24 OS=Homo sapiens GN=RPS24		392.93
P62847;P62847-3;P62847	RS24_HUMAN 40S ribosomal protein S24 OS=Homo sapiens GN=RPS24 PE=1 SV=1;>sp|P62847-3|RS24_HUMAN Isoform 3 of 40S ri	1.21E-96	
P62851	RS25_HUMAN 40S ribosomal protein S25 OS=Homo sapiens GN=RPS25 PE=1 SV=1		288.38
P62854	RS26_HUMAN 40S ribosomal protein S26 OS=Homo sapiens GN=RPS26 PE=1 SV=3		309.45
P62857	RS28_HUMAN 40S ribosomal protein S28 OS=Homo sapiens GN=RPS28 PE=1 SV=1		170.27
P62861	RS30_HUMAN 40S ribosomal protein S30 OS=Homo sapiens GN=FAU PE=1 SV=1		120.85
P62873	GBB1_HUMAN Guanine nucleotide-binding protein G(I)/G(S)/G(T) subunit beta-1 OS=Homo sapiens GN=GNB1 PE=1 SV=3		528.59
P62875	RPAB5_HUMAN DNA-directed RNA polymerases I, II, and III subunit RPABC5 OS=Homo sapiens GN=POLR2L PE=1 SV=1		101.67
P62877	RBX1_HUMAN E3 ubiquitin-protein ligase RBX1 OS=Homo sapiens GN=RBX1 PE=1 SV=1		41.12
P62879	GBB2_HUMAN Guanine nucleotide-binding protein G(I)/G(S)/G(T) subunit beta-2 OS=Homo sapiens GN=GNB2 PE=1 SV=3		868.57
P62888	RL30_HUMAN 60S ribosomal protein L30 OS=Homo sapiens GN=RPL30 PE=1 SV=2		735.19
P62891;Q59GN2	RL39_HUMAN 60S ribosomal protein L39 OS=Homo sapiens GN=RPL39 PE=2 SV=2;>sp|Q59GN2|R39L5_HUMAN Putative 60S ribosor	2.91E-06	
P62899	RL31_HUMAN 60S ribosomal protein L31 OS=Homo sapiens GN=RPL31 PE=1 SV=1		305.62
P62906	RL10A_HUMAN 60S ribosomal protein L10a OS=Homo sapiens GN=RPL10A PE=1 SV=2		957.27
P62910	RL32_HUMAN 60S ribosomal protein L32 OS=Homo sapiens GN=RPL32 PE=1 SV=2		445.24
P62913	RL11_HUMAN 60S ribosomal protein L11 OS=Homo sapiens GN=RPL11 PE=1 SV=2		275.6
P62913-2	RL11_HUMAN Isoform 2 of 60S ribosomal protein L11 OS=Homo sapiens GN=RPL11		275.6
P62913-2;P62913	2|RL11_HUMAN Isoform 2 of 60S ribosomal protein L11 OS=Homo sapiens GN=RPL11;>sp|P62913|RL11_HUMAN 60S ribosomal prote	7.20E-177	
P62913;P62913-2	RL11_HUMAN 60S ribosomal protein L11 OS=Homo sapiens GN=RPL11 PE=1 SV=2;>sp|P62913-2|RL11_HUMAN Isoform 2 of 60S rib	8.18E-105	
P62917	RL8_HUMAN 60S ribosomal protein L8 OS=Homo sapiens GN=RPL8 PE=1 SV=2		652.19
P62937	PPIA_HUMAN Peptidyl-prolyl cis-trans isomerase A OS=Homo sapiens GN=PPIA PE=1 SV=2		1366.76
P62942	FKB1A_HUMAN Peptidyl-prolyl cis-trans isomerase FKBP1A OS=Homo sapiens GN=FKBP1A PE=1 SV=2		202.14
P62979	RS27A_HUMAN Ubiquitin-40S ribosomal protein S27a OS=Homo sapiens GN=RPS27A PE=1 SV=2		548.86
P62987	RL40_HUMAN Ubiquitin-60S ribosomal protein L40 OS=Homo sapiens GN=UBA52 PE=1 SV=2		469.82
P62993	GRB2_HUMAN Growth factor receptor-bound protein 2 OS=Homo sapiens GN=GRB2 PE=1 SV=1		203.67
P62993-2	GRB2_HUMAN Isoform GRB3-3 of Growth factor receptor-bound protein 2 OS=Homo sapiens GN=GRB2		167.14
P62993;P62993-2	GRB2_HUMAN Growth factor receptor-bound protein 2 OS=Homo sapiens GN=GRB2 PE=1 SV=1;>sp|P62993-2|GRB2_HUMAN Isoforr	1.02E-63	
P62995	TRA2B_HUMAN Transformer-2 protein homolog beta OS=Homo sapiens GN=TRA2B PE=1 SV=1		51.06
P62995-3	TRA2B_HUMAN Isoform 3 of Transformer-2 protein homolog beta OS=Homo sapiens GN=TRA2B		50.67
P62995;P62995-3	TRA2B_HUMAN Transformer-2 protein homolog beta OS=Homo sapiens GN=TRA2B PE=1 SV=1;>sp|P62995-3|TRA2B_HUMAN Isoforr	4.64E-52	
P63000	RAC1_HUMAN Ras-related C3 botulinum toxin substrate 1 OS=Homo sapiens GN=RAC1 PE=1 SV=1		486.08

P63000-2	RAC1_HUMAN Isoform B of Ras-related C3 botulinum toxin substrate 1 OS=Homo sapiens GN=RAC1		461.82
P63000-2;P63000	2|RAC1_HUMAN Isoform B of Ras-related C3 botulinum toxin substrate 1 OS=Homo sapiens GN=RAC1;>sp|P63000|RAC1_HUMAN Ras	2.24E-69	
P63010	AP2B1_HUMAN AP-2 complex subunit beta OS=Homo sapiens GN=AP2B1 PE=1 SV=1		1625.5
P63010-2	AP2B1_HUMAN Isoform 2 of AP-2 complex subunit beta OS=Homo sapiens GN=AP2B1		1625.5
P63010-2;P63010	2|AP2B1_HUMAN Isoform 2 of AP-2 complex subunit beta OS=Homo sapiens GN=AP2B1;>sp|P63010|AP2B1_HUMAN AP-2 complex su	2.04E-166	
P63027	VAMP2_HUMAN Vesicle-associated membrane protein 2 OS=Homo sapiens GN=VAMP2 PE=1 SV=3		363.18
P63092	GNAS2_HUMAN Guanine nucleotide-binding protein G(s) subunit alpha isoforms short OS=Homo sapiens GN=GNAS PE=1 SV=1		592.86
P63092-2	GNAS2_HUMAN Isoform Gnas-2 of Guanine nucleotide-binding protein G(s) subunit alpha isoforms short OS=Homo sapiens GN=GNAS		592.86
P63092-3	GNAS2_HUMAN Isoform 3 of Guanine nucleotide-binding protein G(s) subunit alpha isoforms short OS=Homo sapiens GN=GNAS		592.86
P63096	GNAI1_HUMAN Guanine nucleotide-binding protein G(i) subunit alpha-1 OS=Homo sapiens GN=GNAI1 PE=1 SV=2		327.66
P63098	CANB1_HUMAN Calcineurin subunit B type 1 OS=Homo sapiens GN=PPP3R1 PE=1 SV=2		101.98
P63104	1433Z_HUMAN 14-3-3 protein zeta/delta OS=Homo sapiens GN=YWHAZ PE=1 SV=1		1484.68
P63135	POK12_HUMAN HERV-K_1q22 provirus ancestral Pol protein OS=Homo sapiens PE=3 SV=1		
P63151	2ABA_HUMAN Serine/threonine-protein phosphatase 2A 55 kDa regulatory subunit B alpha isoform OS=Homo sapiens GN=PPP2R2A PE=1 SV=1		66.3
P63162	RSMN_HUMAN Small nuclear ribonucleoprotein-associated protein N OS=Homo sapiens GN=SNRPN PE=1 SV=1		415.68
P63165	SUMO1_HUMAN Small ubiquitin-related modifier 1 OS=Homo sapiens GN=SUMO1 PE=1 SV=1		61.72
P63167	DYL1_HUMAN Dynein light chain 1, cytoplasmic OS=Homo sapiens GN=DYNLL1 PE=1 SV=1		283
P63172	DYLT1_HUMAN Dynein light chain Tctex-type 1 OS=Homo sapiens GN=DYNLT1 PE=1 SV=1		132.61
P63173	RL38_HUMAN 60S ribosomal protein L38 OS=Homo sapiens GN=RPL38 PE=1 SV=2		427.88
P63208	SKP1_HUMAN S-phase kinase-associated protein 1 OS=Homo sapiens GN=SKP1 PE=1 SV=2		422.82
P63208-2	SKP1_HUMAN Isoform 2 of S-phase kinase-associated protein 1 OS=Homo sapiens GN=SKP1		350.64
P63208;P63208-2	SKP1_HUMAN S-phase kinase-associated protein 1 OS=Homo sapiens GN=SKP1 PE=1 SV=2;>sp|P63208-2|SKP1_HUMAN Isoform 2 c	2.39E-43	
P63218	GBG5_HUMAN Guanine nucleotide-binding protein G(I)/G(S)/G(O) subunit gamma-5 OS=Homo sapiens GN=GNG5 PE=1 SV=3		31.66
P63220	RS21_HUMAN 40S ribosomal protein S21 OS=Homo sapiens GN=RPS21 PE=1 SV=1		476.88
P63241	IF5A1_HUMAN Eukaryotic translation initiation factor 5A-1 OS=Homo sapiens GN=EIF5A PE=1 SV=2		822.63
P63241-2	IF5A1_HUMAN Isoform 2 of Eukaryotic translation initiation factor 5A-1 OS=Homo sapiens GN=EIF5A		822.63
P63241-2;D3DTP2;P6324	2|IF5A1_HUMAN Isoform 2 of Eukaryotic translation initiation factor 5A-1 OS=Homo sapiens GN=EIF5A;>tr|D3DTP2|D3DTP2_HUMAN	4.61E-218	
P63244	GBLP_HUMAN Guanine nucleotide-binding protein subunit beta-2-like 1 OS=Homo sapiens GN=GNB2L1 PE=1 SV=3		1369.53
P63261	ACTG_HUMAN Actin, cytoplasmic 2 OS=Homo sapiens GN=ACTG1 PE=1 SV=1		2352.17
P63267	ACTH_HUMAN Actin, gamma-enteric smooth muscle OS=Homo sapiens GN=ACTG2 PE=1 SV=1		1105.01
P63272	SPT4H_HUMAN Transcription elongation factor SPT4 OS=Homo sapiens GN=SUPT4H1 PE=1 SV=1		85.98
P63279	UBC9_HUMAN SUMO-conjugating enzyme UBC9 OS=Homo sapiens GN=UBE2I PE=1 SV=1		232.84
P63313	TYB10_HUMAN Thymosin beta-10 OS=Homo sapiens GN=TMSB10 PE=1 SV=2	7.62E-11	
P67775	PP2AA_HUMAN Serine/threonine-protein phosphatase 2A catalytic subunit alpha isoform OS=Homo sapiens GN=PPP2CA PE=1 SV=1		198.87
P67809	YBOX1_HUMAN Nuclease-sensitive element-binding protein 1 OS=Homo sapiens GN=YBX1 PE=1 SV=3		835.99
P67812	SC11A_HUMAN Signal peptidase complex catalytic subunit SEC11A OS=Homo sapiens GN=SEC11A PE=1 SV=1		135.69
P67870	CSK2B_HUMAN Casein kinase II subunit beta OS=Homo sapiens GN=CSNK2B PE=1 SV=1		157.41
P67936	TPM4_HUMAN Tropomyosin alpha-4 chain OS=Homo sapiens GN=TPM4 PE=1 SV=3		663.62
P67936-2	TPM4_HUMAN Isoform 2 of Tropomyosin alpha-4 chain OS=Homo sapiens GN=TPM4		386.92
P67936;B4DVY2;B4DTB1	TPM4_HUMAN Tropomyosin alpha-4 chain OS=Homo sapiens GN=TPM4 PE=1 SV=3;>tr|B4DVY2|B4DVY2_HUMAN cDNA FLJ54184, hig	1.23E-188	
P68032	ACTC_HUMAN Actin, alpha cardiac muscle 1 OS=Homo sapiens GN=ACTC1 PE=1 SV=1		1210.38
P68036	UB2L3_HUMAN Ubiquitin-conjugating enzyme E2 L3 OS=Homo sapiens GN=UBE2L3 PE=1 SV=1		109.42
P68104	EF1A1_HUMAN Elongation factor 1-alpha 1 OS=Homo sapiens GN=EEF1A1 PE=1 SV=1		1752.46
P68133	ACTS_HUMAN Actin, alpha skeletal muscle OS=Homo sapiens GN=ACTA1 PE=1 SV=1		1198.17
P68363	TBA1B_HUMAN Tubulin alpha-1B chain OS=Homo sapiens GN=TUBA1B PE=1 SV=1		1294.17
P68366	TBA4A_HUMAN Tubulin alpha-4A chain OS=Homo sapiens GN=TUBA4A PE=1 SV=1		1094.51
P68371	TBB2C_HUMAN Tubulin beta-2C chain OS=Homo sapiens GN=TUBB2C PE=1 SV=1		2376.63
P68400	CSK21_HUMAN Casein kinase II subunit alpha OS=Homo sapiens GN=CSNK2A1 PE=1 SV=1		290.34
P68402	PA1B2_HUMAN Platelet-activating factor acetylhydrolase IB subunit beta OS=Homo sapiens GN=PAFAH1B2 PE=1 SV=1		112.51
P68431	H31_HUMAN Histone H3.1 OS=Homo sapiens GN=HIST1H3A PE=1 SV=2		216.15
P68871	HBB_HUMAN Hemoglobin subunit beta OS=Homo sapiens GN=HBB PE=1 SV=2		1045.75
P69849	NOMO3_HUMAN Nodal modulator 3 OS=Homo sapiens GN=NOMO3 PE=2 SV=2		174.04
P69891	HBG1_HUMAN Hemoglobin subunit gamma-1 OS=Homo sapiens GN=HBG1 PE=1 SV=2		66.95
P69892	HBG2_HUMAN Hemoglobin subunit gamma-2 OS=Homo sapiens GN=HBG2 PE=1 SV=2		66.95
P69905	HBA_HUMAN Hemoglobin subunit alpha OS=Homo sapiens GN=HBA1 PE=1 SV=2		484.03
P78312	F193A_HUMAN Protein FAM193A OS=Homo sapiens GN=FAM193A PE=1 SV=2		
P78312-2	F193A_HUMAN Isoform 2 of Protein FAM193A OS=Homo sapiens GN=FAM193A		
P78312-4	F193A_HUMAN Isoform 4 of Protein FAM193A OS=Homo sapiens GN=FAM193A		
P78316;P78316-2	NOP14_HUMAN Nucleolar protein 14 OS=Homo sapiens GN=NOP14 PE=1 SV=3;>sp|P78316-2|NOP14_HUMAN Isoform 2 of Nucleolar	3.40E-11	
P78318	IGBP1_HUMAN Immunoglobulin-binding protein 1 OS=Homo sapiens GN=IGBP1 PE=1 SV=1	1.35E-14	
P78324-2;P78324;P78324	2|SHPS1_HUMAN Isoform 2 of Tyrosine-protein phosphatase non-receptor type substrate 1 OS=Homo sapiens GN=SIRPA;>sp|P7832	7.68E-15	

P78330	SERB_HUMAN Phosphoserine phosphatase OS=Homo sapiens GN=PSPH PE=1 SV=2		29.78
P78332	RBM6_HUMAN RNA-binding protein 6 OS=Homo sapiens GN=RBM6 PE=1 SV=5		
P78344	IF4G2_HUMAN Eukaryotic translation initiation factor 4 gamma 2 OS=Homo sapiens GN=EIF4G2 PE=1 SV=1		340.02
P78344-2	IF4G2_HUMAN Isoform 2 of Eukaryotic translation initiation factor 4 gamma 2 OS=Homo sapiens GN=EIF4G2		340.02
P78344;P78344-2	IF4G2_HUMAN Eukaryotic translation initiation factor 4 gamma 2 OS=Homo sapiens GN=EIF4G2 PE=1 SV=1;>sp|P78344-2|IF4G2_HU	1.82E-93	
P78345	RPP38_HUMAN Ribonuclease P protein subunit p38 OS=Homo sapiens GN=RPP38 PE=1 SV=2		
P78346	RPP30_HUMAN Ribonuclease P protein subunit p30 OS=Homo sapiens GN=RPP30 PE=1 SV=1		314.86
P78347	GTF2I_HUMAN General transcription factor II-I OS=Homo sapiens GN=GTF2I PE=1 SV=2		448.18
P78347-2	GTF2I_HUMAN Isoform 2 of General transcription factor II-I OS=Homo sapiens GN=GTF2I		448.18
P78347-3	GTF2I_HUMAN Isoform 3 of General transcription factor II-I OS=Homo sapiens GN=GTF2I		448.18
P78347-4	GTF2I_HUMAN Isoform 4 of General transcription factor II-I OS=Homo sapiens GN=GTF2I		448.18
P78347;B4DH52;A8K9W7;	GTF2I_HUMAN General transcription factor II-I OS=Homo sapiens GN=GTF2I PE=1 SV=2;>tr|B4DH52|B4DH52_HUMAN cDNA FLJ559	5.36E-44	
P78352	DLG4_HUMAN Disks large homolog 4 OS=Homo sapiens GN=DLG4 PE=1 SV=3		
P78352-2	DLG4_HUMAN Isoform 2 of Disks large homolog 4 OS=Homo sapiens GN=DLG4		
P78356	PI42B_HUMAN Phosphatidylinositol-5-phosphate 4-kinase type-2 beta OS=Homo sapiens GN=PIP4K2B PE=1 SV=1	5.92E-48	
P78362-2;P78362	2|SRPK2_HUMAN Isoform 2 of Serine/threonine-protein kinase SRPK2 OS=Homo sapiens GN=SRPK2;>sp|P78362|SRPK2_HUMAN Ser	1.27E-24	
P78371	TCPB_HUMAN T-complex protein 1 subunit beta OS=Homo sapiens GN=CCT2 PE=1 SV=4		682.5
P78383	S35B1_HUMAN Solute carrier family 35 member B1 OS=Homo sapiens GN=SLC35B1 PE=2 SV=1		
P78385	KRT83_HUMAN Keratin, type II cuticular Hb3 OS=Homo sapiens GN=KRT83 PE=1 SV=2		45
P78386	KRT85_HUMAN Keratin, type II cuticular Hb5 OS=Homo sapiens GN=KRT85 PE=1 SV=1		43.4
P78406	RAE1L_HUMAN mRNA export factor OS=Homo sapiens GN=RAE1 PE=1 SV=1		22.05
P78417	GSTO1_HUMAN Glutathione S-transferase omega-1 OS=Homo sapiens GN=GSTO1 PE=1 SV=2		396.5
P78423	X3CL1_HUMAN Fractalkine OS=Homo sapiens GN=CX3CL1 PE=1 SV=1		
P78424	PO6F2_HUMAN POU domain, class 6, transcription factor 2 OS=Homo sapiens GN=POU6F2 PE=1 SV=3		
P78424-2	PO6F2_HUMAN Isoform 2 of POU domain, class 6, transcription factor 2 OS=Homo sapiens GN=POU6F2		
P78527	PRKDC_HUMAN DNA-dependent protein kinase catalytic subunit OS=Homo sapiens GN=PRKDC PE=1 SV=3		14506.87
P78527-2	PRKDC_HUMAN Isoform 2 of DNA-dependent protein kinase catalytic subunit OS=Homo sapiens GN=PRKDC		14257.34
P78527;P78527-2	PRKDC_HUMAN DNA-dependent protein kinase catalytic subunit OS=Homo sapiens GN=PRKDC PE=1 SV=3;>sp|P78527-2|PRKDC_HU	0	
P78536	ADA17_HUMAN Disintegrin and metalloproteinase domain-containing protein 17 OS=Homo sapiens GN=ADAM17 PE=1 SV=1		
P78536-2	ADA17_HUMAN Isoform B of Disintegrin and metalloproteinase domain-containing protein 17 OS=Homo sapiens GN=ADAM17		
P78537	BL1S1_HUMAN Biogenesis of lysosome-related organelles complex 1 subunit 1 OS=Homo sapiens GN=BLOC1S1 PE=1 SV=2		51.97
P78537-2	BL1S1_HUMAN Isoform 2 of Biogenesis of lysosome-related organelles complex 1 subunit 1 OS=Homo sapiens GN=BLOC1S1		51.97
P78537;P78537-2	BL1S1_HUMAN Biogenesis of lysosome-related organelles complex 1 subunit 1 OS=Homo sapiens GN=BLOC1S1 PE=1 SV=2;>sp|P785	6.21E-07	
P78559	MAP1A_HUMAN Microtubule-associated protein 1A OS=Homo sapiens GN=MAP1A PE=1 SV=6		144.07
P78559-2	MAP1A_HUMAN Isoform 2 of Microtubule-associated protein 1A OS=Homo sapiens GN=MAP1A		144.07
P78559-2;P78559	2|MAP1A_HUMAN Isoform 2 of Microtubule-associated protein 1A OS=Homo sapiens GN=MAP1A;>sp|P78559|MAP1A_HUMAN Microtul	7.42E-09	
P79483	DRB3_HUMAN HLA class II histocompatibility antigen, DR beta 3 chain OS=Homo sapiens GN=HLA-DRB3 PE=1 SV=1		29.42
P80108	PHLD_HUMAN Phosphatidylinositol-glycan-specific phospholipase D OS=Homo sapiens GN=GPLD1 PE=1 SV=3		36.67
P80188	NGAL_HUMAN Neutrophil gelatinase-associated lipocalin OS=Homo sapiens GN=LCN2 PE=1 SV=2		26.65
P80188-2	NGAL_HUMAN Isoform 2 of Neutrophil gelatinase-associated lipocalin OS=Homo sapiens GN=LCN2		26.65
P80188;P80188-2	NGAL_HUMAN Neutrophil gelatinase-associated lipocalin OS=Homo sapiens GN=LCN2 PE=1 SV=2;>sp|P80188-2|NGAL_HUMAN Isofor	6.01E-146	
P80217-2;P80217	2|IN35_HUMAN Isoform 2 of Interferon-induced 35 kDa protein OS=Homo sapiens GN=IFI35;>sp|P80217|IN35_HUMAN Interferon-in	4.51E-15	
P80294	MT1H_HUMAN Metallothionein-1H OS=Homo sapiens GN=MT1H PE=1 SV=1	9.09E-09	
P80303	NUCB2_HUMAN Nucleobindin-2 OS=Homo sapiens GN=NUCB2 PE=1 SV=2		519.18
P80303-2	NUCB2_HUMAN Isoform 2 of Nucleobindin-2 OS=Homo sapiens GN=NUCB2		519.18
P80303;P80303-2	NUCB2_HUMAN Nucleobindin-2 OS=Homo sapiens GN=NUCB2 PE=1 SV=2;>sp|P80303-2|NUCB2_HUMAN Isoform 2 of Nucleobindin-2	4.95E-15	
P80370	DLK1_HUMAN Protein delta homolog 1 OS=Homo sapiens GN=DLK1 PE=1 SV=3		
P80370-2	DLK1_HUMAN Isoform Short of Protein delta homolog 1 OS=Homo sapiens GN=DLK1		
P80404	GABT_HUMAN 4-aminobutyrate aminotransferase, mitochondrial OS=Homo sapiens GN=ABAT PE=1 SV=3		
P80723	BASP1_HUMAN Brain acid soluble protein 1 OS=Homo sapiens GN=BASP1 PE=1 SV=2		846.85
P80723-2	BASP1_HUMAN Isoform 2 of Brain acid soluble protein 1 OS=Homo sapiens GN=BASP1		556.65
P80723;P80723-2	BASP1_HUMAN Brain acid soluble protein 1 OS=Homo sapiens GN=BASP1 PE=1 SV=2;>sp|P80723-2|BASP1_HUMAN Isoform 2 of Bra	9.46E-14	
P80748	LV302_HUMAN Ig lambda chain V-III region LOI OS=Homo sapiens PE=1 SV=1	6.31E-88	
P81605	DCD_HUMAN Dermcidin OS=Homo sapiens GN=DCD PE=1 SV=2		138.83
P82094	TMF1_HUMAN TATA element modulatory factor OS=Homo sapiens GN=TMF1 PE=1 SV=2		
P82094-2	TMF1_HUMAN Isoform 2 of TATA element modulatory factor OS=Homo sapiens GN=TMF1		
P82094-2;P82094	2|TMF1_HUMAN Isoform 2 of TATA element modulatory factor OS=Homo sapiens GN=TMF1;>sp|P82094|TMF1_HUMAN TATA element	1.27E-34	
P82650	RT22_HUMAN 28S ribosomal protein S22, mitochondrial OS=Homo sapiens GN=MRPS22 PE=1 SV=1		200.83
P82663	RT25_HUMAN 28S ribosomal protein S25, mitochondrial OS=Homo sapiens GN=MRPS25 PE=1 SV=1		86.51
P82664	RT10_HUMAN 28S ribosomal protein S10, mitochondrial OS=Homo sapiens GN=MRPS10 PE=1 SV=2		34.76
P82673	RT35_HUMAN 28S ribosomal protein S35, mitochondrial OS=Homo sapiens GN=MRPS35 PE=1 SV=1		156.75

P82673-2	RT35_HUMAN Isoform 2 of 28S ribosomal protein S35, mitochondrial OS=Homo sapiens GN=MRPS35		126.15
P82673;C9JQV1;P82673-2	RT35_HUMAN 28S ribosomal protein S35, mitochondrial OS=Homo sapiens GN=MRPS35 PE=1 SV=1;>tr|C9JQV1|C9JQV1_HUMAN Un	2.31E-21	
P82675	RT05_HUMAN 28S ribosomal protein S5, mitochondrial OS=Homo sapiens GN=MRPS5 PE=1 SV=2		182.84
P82675-2	RT05_HUMAN Isoform 2 of 28S ribosomal protein S5, mitochondrial OS=Homo sapiens GN=MRPS5		139.95
P82675;B4DIW8;P82675-2	RT05_HUMAN 28S ribosomal protein S5, mitochondrial OS=Homo sapiens GN=MRPS5 PE=1 SV=2;>tr|B4DIW8|B4DIW8_HUMAN cDNA	2.63E-25	
P82909	RT36_HUMAN 28S ribosomal protein S36, mitochondrial OS=Homo sapiens GN=MRPS36 PE=1 SV=2		133.49
P82912	RT11_HUMAN 28S ribosomal protein S11, mitochondrial OS=Homo sapiens GN=MRPS11 PE=1 SV=2		76.37
P82912-2	RT11_HUMAN Isoform 2 of 28S ribosomal protein S11, mitochondrial OS=Homo sapiens GN=MRPS11		76.37
P82912-3	RT11_HUMAN Isoform 3 of 28S ribosomal protein S11, mitochondrial OS=Homo sapiens GN=MRPS11		29.36
P82912;P82912-2	RT11_HUMAN 28S ribosomal protein S11, mitochondrial OS=Homo sapiens GN=MRPS11 PE=1 SV=2;>sp|P82912-2|RT11_HUMAN Iso	1.94E-10	
P82914	RT15_HUMAN 28S ribosomal protein S15, mitochondrial OS=Homo sapiens GN=MRPS15 PE=1 SV=1		171.62
P82921	RT21_HUMAN 28S ribosomal protein S21, mitochondrial OS=Homo sapiens GN=MRPS21 PE=1 SV=2		
P82930	RT34_HUMAN 28S ribosomal protein S34, mitochondrial OS=Homo sapiens GN=MRPS34 PE=1 SV=2		180.86
P82932	RT06_HUMAN 28S ribosomal protein S6, mitochondrial OS=Homo sapiens GN=MRPS6 PE=1 SV=3		76.91
P82933	RT09_HUMAN 28S ribosomal protein S9, mitochondrial OS=Homo sapiens GN=MRPS9 PE=1 SV=2		138.26
P82979	SARNP_HUMAN SAP domain-containing ribonucleoprotein OS=Homo sapiens GN=SARNP PE=1 SV=3		255.93
P82980	RET5_HUMAN Retinol-binding protein 5 OS=Homo sapiens GN=RBP5 PE=1 SV=3	0.00016687	
P83111	LACTB_HUMAN Serine beta-lactamase-like protein LACTB, mitochondrial OS=Homo sapiens GN=LACTB PE=1 SV=2		
P83111-2	LACTB_HUMAN Isoform 2 of Serine beta-lactamase-like protein LACTB, mitochondrial OS=Homo sapiens GN=LACTB		
P83436	COG7_HUMAN Conserved oligomeric Golgi complex subunit 7 OS=Homo sapiens GN=COG7 PE=1 SV=1	1.27E-05	
P83731	RL24_HUMAN 60S ribosomal protein L24 OS=Homo sapiens GN=RPL24 PE=1 SV=1		545.14
P83876	TXN4A_HUMAN Thioredoxin-like protein 4A OS=Homo sapiens GN=TXNL4A PE=1 SV=1		266.34
P83881	RL36A_HUMAN 60S ribosomal protein L36a OS=Homo sapiens GN=RPL36A PE=1 SV=2		145.77
P83916	CBX1_HUMAN Chromobox protein homolog 1 OS=Homo sapiens GN=CBX1 PE=1 SV=1		743.83
P84022;Q15796;Q15796-2	SMAD3_HUMAN Mothers against decapentaplegic homolog 3 OS=Homo sapiens GN=SMAD3 PE=1 SV=1;>sp|Q15796|SMAD2_HUMAN	1.90E-17	
P84074	HPCA_HUMAN Neuron-specific calcium-binding protein hippocalcin OS=Homo sapiens GN=HPCA PE=1 SV=2		198.98
P84077	ARF1_HUMAN ADP-ribosylation factor 1 OS=Homo sapiens GN=ARF1 PE=1 SV=2		857.87
P84085	ARF5_HUMAN ADP-ribosylation factor 5 OS=Homo sapiens GN=ARF5 PE=1 SV=2		417.44
P84090	ERH_HUMAN Enhancer of rudimentary homolog OS=Homo sapiens GN=ERH PE=1 SV=1		142.95
P84095	RHOG_HUMAN Rho-related GTP-binding protein RhoG OS=Homo sapiens GN=RHOG PE=1 SV=1		398.48
P84098	RL19_HUMAN 60S ribosomal protein L19 OS=Homo sapiens GN=RPL19 PE=1 SV=1		430.13
P84103	SRSF3_HUMAN Serine/arginine-rich splicing factor 3 OS=Homo sapiens GN=SRSF3 PE=1 SV=1		289.26
P84157	MXRA7_HUMAN Matrix-remodeling-associated protein 7 OS=Homo sapiens GN=MXRA7 PE=1 SV=1		
P84157-2	MXRA7_HUMAN Isoform 2 of Matrix-remodeling-associated protein 7 OS=Homo sapiens GN=MXRA7		
P84157-2;P84157;P84157	2|MXRA7_HUMAN Isoform 2 of Matrix-remodeling-associated protein 7 OS=Homo sapiens GN=MXRA7;>sp|P84157|MXRA7_HUMAN M	1.79E-100	
P84157-3	MXRA7_HUMAN Isoform 3 of Matrix-remodeling-associated protein 7 OS=Homo sapiens GN=MXRA7		
P84157;P84157-3;P84157	MXRA7_HUMAN Matrix-remodeling-associated protein 7 OS=Homo sapiens GN=MXRA7 PE=1 SV=1;>sp|P84157-3|MXRA7_HUMAN Iso	4.60E-13	
P84243	H33_HUMAN Histone H3.3 OS=Homo sapiens GN=H3F3A PE=1 SV=2		180.26
P84550;P84550-3;P84550	SKOR1_HUMAN SKI family transcriptional corepressor 1 OS=Homo sapiens GN=SKOR1 PE=1 SV=1;>sp|P84550-3|SKOR1_HUMAN Iso	8.17E-06	
P85037;B4DY33;A4D1Z4;	FOXK1_HUMAN Forkhead box protein K1 OS=Homo sapiens GN=FOXK1 PE=1 SV=1;>tr|B4DY33|B4DY33_HUMAN cDNA FLJ55600, hig	9.14E-28	
P85299	PRR5_HUMAN Proline-rich protein 5 OS=Homo sapiens GN=PRR5 PE=1 SV=1		
P85299-2	PRR5_HUMAN Isoform 2 of Proline-rich protein 5 OS=Homo sapiens GN=PRR5		
P85299-3	PRR5_HUMAN Isoform 3 of Proline-rich protein 5 OS=Homo sapiens GN=PRR5		
P85299-4	PRR5_HUMAN Isoform 4 of Proline-rich protein 5 OS=Homo sapiens GN=PRR5		
P86790;P86791	CCZ1L_HUMAN Vacuolar fusion protein CCZ1 homolog-like OS=Homo sapiens GN=C7orf28B PE=1 SV=1;>sp|P86791|CCZ1_HUMAN V	6.41E-10	
P98095-2;P98095	2|FBLN2_HUMAN Isoform 2 of Fibulin-2 OS=Homo sapiens GN=FBLN2;>sp|P98095|FBLN2_HUMAN Fibulin-2 OS=Homo sapiens GN=F	1.05E-206	
P98160	PGBM_HUMAN Basement membrane-specific heparan sulfate proteoglycan core protein OS=Homo sapiens GN=HSPG2 PE=1 SV=4	0	
P98161	PKD1_HUMAN Polycystin-1 OS=Homo sapiens GN=PKD1 PE=1 SV=3		28.88
P98161-2	PKD1_HUMAN Isoform 2 of Polycystin-1 OS=Homo sapiens GN=PKD1		28.88
P98161-3	PKD1_HUMAN Isoform 3 of Polycystin-1 OS=Homo sapiens GN=PKD1		28.88
P98164	LRP2_HUMAN Low-density lipoprotein receptor-related protein 2 OS=Homo sapiens GN=LRP2 PE=1 SV=3		
P98171	RHG04_HUMAN Rho GTPase-activating protein 4 OS=Homo sapiens GN=ARHGAP4 PE=1 SV=2	6.92E-20	
P98173	FAM3A_HUMAN Protein FAM3A OS=Homo sapiens GN=FAM3A PE=1 SV=2		35.06
P98175	RBM10_HUMAN RNA-binding protein 10 OS=Homo sapiens GN=RBM10 PE=1 SV=3		46.24
P98175-2	RBM10_HUMAN Isoform 2 of RNA-binding protein 10 OS=Homo sapiens GN=RBM10		46.24
P98175-3	RBM10_HUMAN Isoform 3 of RNA-binding protein 10 OS=Homo sapiens GN=RBM10		46.24
P98175-4	RBM10_HUMAN Isoform 4 of RNA-binding protein 10 OS=Homo sapiens GN=RBM10		46.24
P98177	FOXO4_HUMAN Forkhead box protein O4 OS=Homo sapiens GN=FOXO4 PE=1 SV=5		21.17
P98177-2	FOXO4_HUMAN Isoform Zeta of Forkhead box protein O4 OS=Homo sapiens GN=FOXO4		21.17
P98179	RBM3_HUMAN Putative RNA-binding protein 3 OS=Homo sapiens GN=RBM3 PE=1 SV=1		534.76
P99999	CYC_HUMAN Cytochrome c OS=Homo sapiens GN=CYCS PE=1 SV=2		393.93

Q00005	2ABB_HUMAN Serine/threonine-protein phosphatase 2A 55 kDa regulatory subunit B beta isoform OS=Homo sapiens GN=PPP2R2B PE=1 SV=1		64.4
Q00005-2	2ABB_HUMAN Isoform 2 of Serine/threonine-protein phosphatase 2A 55 kDa regulatory subunit B beta isoform OS=Homo sapiens GN=PPP2R2B		64.4
Q00005-3	2ABB_HUMAN Isoform 3 of Serine/threonine-protein phosphatase 2A 55 kDa regulatory subunit B beta isoform OS=Homo sapiens GN=PPP2R2B		64.4
Q00005-4	2ABB_HUMAN Isoform 4 of Serine/threonine-protein phosphatase 2A 55 kDa regulatory subunit B beta isoform OS=Homo sapiens GN=PPP2R2B		64.4
Q00005-5	2ABB_HUMAN Isoform 5 of Serine/threonine-protein phosphatase 2A 55 kDa regulatory subunit B beta isoform OS=Homo sapiens GN=PPP2R2B		64.4
Q00005-5;Q00005-4;Q66I	5|2ABB_HUMAN Isoform 5 of Serine/threonine-protein phosphatase 2A 55 kDa regulatory subunit B beta isoform OS=Homo sapiens G	1.73E-45	
Q00059	TFAM_HUMAN Transcription factor A, mitochondrial OS=Homo sapiens GN=TFAM PE=1 SV=1		463.54
Q00169	PIPNA_HUMAN Phosphatidylinositol transfer protein alpha isoform OS=Homo sapiens GN=PITPNA PE=1 SV=2	4.26E-08	
Q00266	METK1_HUMAN S-adenosylmethionine synthase isoform type-1 OS=Homo sapiens GN=MAT1A PE=1 SV=2		37.34
Q00325	MPCP_HUMAN Phosphate carrier protein, mitochondrial OS=Homo sapiens GN=SLC25A3 PE=1 SV=2		959.16
Q00325-2	MPCP_HUMAN Isoform B of Phosphate carrier protein, mitochondrial OS=Homo sapiens GN=SLC25A3		1020.63
Q00325-2;Q00325	2|MPCP_HUMAN Isoform B of Phosphate carrier protein, mitochondrial OS=Homo sapiens GN=SLC25A3;>sp|Q00325|MPCP_HUMAN Pl	0	
Q00325;Q00325-2	MPCP_HUMAN Phosphate carrier protein, mitochondrial OS=Homo sapiens GN=SLC25A3 PE=1 SV=2;>sp|Q00325-2|MPCP_HUMAN Isc	9.06E-65	
Q00341	VIGLN_HUMAN Vigilin OS=Homo sapiens GN=HDLBP PE=1 SV=2		831.84
Q00403	TF2B_HUMAN Transcription initiation factor IIB OS=Homo sapiens GN=GTF2B PE=1 SV=1	3.42E-09	
Q00526	CDK3_HUMAN Cyclin-dependent kinase 3 OS=Homo sapiens GN=CDK3 PE=1 SV=1		143.28
Q00532	CDKL1_HUMAN Cyclin-dependent kinase-like 1 OS=Homo sapiens GN=CDKL1 PE=2 SV=5		29.48
Q00532-2	CDKL1_HUMAN Isoform 2 of Cyclin-dependent kinase-like 1 OS=Homo sapiens GN=CDKL1		50.82
Q00534	CDK6_HUMAN Cyclin-dependent kinase 6 OS=Homo sapiens GN=CDK6 PE=1 SV=1		53.46
Q00535	CDK5_HUMAN Cyclin-dependent kinase 5 OS=Homo sapiens GN=CDK5 PE=1 SV=3		51.25
Q00535;Q6IAW3;A1XKG3	CDK5_HUMAN Cell division protein kinase 5 OS=Homo sapiens GN=CDK5 PE=1 SV=3;>tr|Q6IAW3|Q6IAW3_HUMAN CDK5 protein OS	6.41E-17	
Q00536	CDK16_HUMAN Cyclin-dependent kinase 16 OS=Homo sapiens GN=CDK16 PE=1 SV=1		54.92
Q00537	CDK17_HUMAN Cyclin-dependent kinase 17 OS=Homo sapiens GN=CDK17 PE=1 SV=2		53.66
Q00577	PURA_HUMAN Transcriptional activator protein Pur-alpha OS=Homo sapiens GN=PURA PE=1 SV=2		125.45
Q00610	CLH1_HUMAN Clathrin heavy chain 1 OS=Homo sapiens GN=CLTC PE=1 SV=5		5321.31
Q00610-2	CLH1_HUMAN Isoform 2 of Clathrin heavy chain 1 OS=Homo sapiens GN=CLTC		5321.31
Q00610;Q00610-2	CLH1_HUMAN Clathrin heavy chain 1 OS=Homo sapiens GN=CLTC PE=1 SV=5;>sp|Q00610-2|CLH1_HUMAN Isoform 2 of Clathrin hea	0	
Q00613;Q00613-2	HSF1_HUMAN Heat shock factor protein 1 OS=Homo sapiens GN=HSF1 PE=1 SV=1;>sp|Q00613-2|HSF1_HUMAN Isoform Short of He	6.65E-14	
Q00653;Q00653-4	NFKB2_HUMAN Nuclear factor NF-kappa-B p100 subunit OS=Homo sapiens GN=NFKB2 PE=1 SV=4;>sp|Q00653-4|NFKB2_HUMAN Isc	2.37E-16	
Q00688	FKBP3_HUMAN Peptidyl-prolyl cis-trans isomerase FKBP3 OS=Homo sapiens GN=FKBP3 PE=1 SV=1		631.08
Q00722	PLCB2_HUMAN 1-phosphatidylinositol-4,5-bisphosphate phosphodiesterase beta-2 OS=Homo sapiens GN=PLCB2 PE=1 SV=2		
Q00722-2	PLCB2_HUMAN Isoform 2 of 1-phosphatidylinositol-4,5-bisphosphate phosphodiesterase beta-2 OS=Homo sapiens GN=PLCB2		
Q00765	REEP5_HUMAN Receptor expression-enhancing protein 5 OS=Homo sapiens GN=REEP5 PE=1 SV=3		167.36
Q00796	DHSO_HUMAN Sorbitol dehydrogenase OS=Homo sapiens GN=SORD PE=1 SV=4		61.15
Q00839	HNRPU_HUMAN Heterogeneous nuclear ribonucleoprotein U OS=Homo sapiens GN=HNRNPU PE=1 SV=6		1225.18
Q00839-2	HNRPU_HUMAN Isoform Short of Heterogeneous nuclear ribonucleoprotein U OS=Homo sapiens GN=HNRNPU		1165.2
Q00839-2;B4DLR3	2|HNRPU_HUMAN Isoform Short of Heterogeneous nuclear ribonucleoprotein U OS=Homo sapiens GN=HNRNPU;>tr|B4DLR3|B4DLR3_	0	
Q00839;B3KX72;Q5RI17;(	HNRPU_HUMAN Heterogeneous nuclear ribonucleoprotein U OS=Homo sapiens GN=HNRNPU PE=1 SV=6;>tr|B3KX72|B3KX72_HUMAN	0	
Q008S8	ECT2L_HUMAN Epithelial cell-transforming sequence 2 oncogene-like OS=Homo sapiens GN=ECT2L PE=2 SV=2	2.57E-05	
Q00973	B4GN1_HUMAN Beta-1,4 N-acetylgalactosaminyltransferase 1 OS=Homo sapiens GN=B4GALNT1 PE=1 SV=2		
Q00978	IRF9_HUMAN Interferon regulatory factor 9 OS=Homo sapiens GN=IRF9 PE=1 SV=1	1.09E-06	
Q01064	PDE1B_HUMAN Calcium/calmodulin-dependent 3',5'-cyclic nucleotide phosphodiesterase 1B OS=Homo sapiens GN=PDE1B PE=1 SV=2		31.06
Q01064-2	PDE1B_HUMAN Isoform PDE1B2 of Calcium/calmodulin-dependent 3',5'-cyclic nucleotide phosphodiesterase 1B OS=Homo sapiens GN=PDE1B		31.06
Q01081	U2AF1_HUMAN Splicing factor U2AF 35 kDa subunit OS=Homo sapiens GN=U2AF1 PE=1 SV=3		362.42
Q01082	SPTB2_HUMAN Spectrin beta chain, brain 1 OS=Homo sapiens GN=SPTBN1 PE=1 SV=2		5364.1
Q01082-2	SPTB2_HUMAN Isoform Short of Spectrin beta chain, brain 1 OS=Homo sapiens GN=SPTBN1		4959.09
Q01082-3	SPTB2_HUMAN Isoform 2 of Spectrin beta chain, brain 1 OS=Homo sapiens GN=SPTBN1		4949.69
Q01082;Q01082-2	SPTB2_HUMAN Spectrin beta chain, brain 1 OS=Homo sapiens GN=SPTBN1 PE=1 SV=2;>sp|Q01082-2|SPTB2_HUMAN Isoform Short	0	
Q01085	TIAR_HUMAN Nucleolysin TIAR OS=Homo sapiens GN=TIAL1 PE=1 SV=1		106.46
Q01105	SET_HUMAN Protein SET OS=Homo sapiens GN=SET PE=1 SV=3		539.66
Q01105-2	SET_HUMAN Isoform 2 of Protein SET OS=Homo sapiens GN=SET		515.14
Q01105-2;Q01105	2|SET_HUMAN Isoform 2 of Protein SET OS=Homo sapiens GN=SET;>sp|Q01105|SET_HUMAN Protein SET OS=Homo sapiens GN=SE	3.16E-84	
Q01130	SRSF2_HUMAN Serine/arginine-rich splicing factor 2 OS=Homo sapiens GN=SRSF2 PE=1 SV=4		432.78
Q01196	RUNX1_HUMAN Runt-related transcription factor 1 OS=Homo sapiens GN=RUNX1 PE=1 SV=3		
Q01196-10	RUNX1_HUMAN Isoform AML-1I of Runt-related transcription factor 1 OS=Homo sapiens GN=RUNX1		
Q01196-11	RUNX1_HUMAN Isoform AML-1L of Runt-related transcription factor 1 OS=Homo sapiens GN=RUNX1		
Q01196-2	RUNX1_HUMAN Isoform AML-1A of Runt-related transcription factor 1 OS=Homo sapiens GN=RUNX1		
Q01196-3	RUNX1_HUMAN Isoform AML-1C of Runt-related transcription factor 1 OS=Homo sapiens GN=RUNX1		
Q01196-4	RUNX1_HUMAN Isoform AML-1E of Runt-related transcription factor 1 OS=Homo sapiens GN=RUNX1		
Q01196-5	RUNX1_HUMAN Isoform AML-1FA of Runt-related transcription factor 1 OS=Homo sapiens GN=RUNX1		
Q01196-6	RUNX1_HUMAN Isoform AML-1FB of Runt-related transcription factor 1 OS=Homo sapiens GN=RUNX1		

Q01196-7	RUNX1_HUMAN Isoform AML-1FC of Runt-related transcription factor 1 OS=Homo sapiens GN=RUNX1		
Q01196-8	RUNX1_HUMAN Isoform AML-1G of Runt-related transcription factor 1 OS=Homo sapiens GN=RUNX1		
Q01196-9	RUNX1_HUMAN Isoform AML-1H of Runt-related transcription factor 1 OS=Homo sapiens GN=RUNX1		
Q01432;Q01432-3	AMPD3_HUMAN AMP deaminase 3 OS=Homo sapiens GN=AMPD3 PE=1 SV=1;>sp|Q01432-3|AMPD3_HUMAN Isoform 1C of AMP dear	1.55E-07	
Q01433;Q01433-2;Q0143	AMPD2_HUMAN AMP deaminase 2 OS=Homo sapiens GN=AMPD2 PE=1 SV=2;>sp|Q01433-2|AMPD2_HUMAN Isoform Ex1A-2-3 of AM	1.95E-14	
Q01469	FABP5_HUMAN Fatty acid-binding protein, epidermal OS=Homo sapiens GN=FABP5 PE=1 SV=3		50.99
Q01484	ANK2_HUMAN Ankyrin-2 OS=Homo sapiens GN=ANK2 PE=1 SV=3		
Q01484-2	ANK2_HUMAN Isoform 2 of Ankyrin-2 OS=Homo sapiens GN=ANK2		
Q01484-4	ANK2_HUMAN Isoform 3 of Ankyrin-2 OS=Homo sapiens GN=ANK2		
Q01484-4;Q01484;Q0148	4|ANK2_HUMAN Isoform 3 of Ankyrin-2 OS=Homo sapiens GN=ANK2;>sp|Q01484|ANK2_HUMAN Ankyrin-2 OS=Homo sapiens GN=A	1.18E-10	
Q01484-5	ANK2_HUMAN Isoform 4 of Ankyrin-2 OS=Homo sapiens GN=ANK2		
Q01518	CAP1_HUMAN Adenylyl cyclase-associated protein 1 OS=Homo sapiens GN=CAP1 PE=1 SV=5		396.72
Q01518-2	CAP1_HUMAN Isoform 2 of Adenylyl cyclase-associated protein 1 OS=Homo sapiens GN=CAP1		300.33
Q01518;Q01518-2	CAP1_HUMAN Adenylyl cyclase-associated protein 1 OS=Homo sapiens GN=CAP1 PE=1 SV=5;>sp|Q01518-2|CAP1_HUMAN Isoform 2	0	
Q01546	K22O_HUMAN Keratin, type II cytoskeletal 2 oral OS=Homo sapiens GN=KRT76 PE=1 SV=2		431.38
Q01581	HMCS1_HUMAN Hydroxymethylglutaryl-CoA synthase, cytoplasmic OS=Homo sapiens GN=HMGCS1 PE=1 SV=2	2.55E-88	
Q01628	IFM3_HUMAN Interferon-induced transmembrane protein 3 OS=Homo sapiens GN=IFITM3 PE=1 SV=2		202.65
Q01629	IFM2_HUMAN Interferon-induced transmembrane protein 2 OS=Homo sapiens GN=IFITM2 PE=1 SV=2		202.65
Q01650	LAT1_HUMAN Large neutral amino acids transporter small subunit 1 OS=Homo sapiens GN=SLC7A5 PE=1 SV=2		331.76
Q01658	NC2B_HUMAN Protein Dr1 OS=Homo sapiens GN=DR1 PE=1 SV=1		108.48
Q01780	EXOSX_HUMAN Exosome component 10 OS=Homo sapiens GN=EXOSC10 PE=1 SV=2		66.23
Q01780-2	EXOSX_HUMAN Isoform 2 of Exosome component 10 OS=Homo sapiens GN=EXOSC10		66.23
Q01780;Q01780-2	EXOSX_HUMAN Exosome component 10 OS=Homo sapiens GN=EXOSC10 PE=1 SV=2;>sp|Q01780-2|EXOSX_HUMAN Isoform 2 of Ex	2.00E-17	
Q01804	OTUD4_HUMAN OTU domain-containing protein 4 OS=Homo sapiens GN=OTUD4 PE=1 SV=3		39.12
Q01804-2	OTUD4_HUMAN Isoform 2 of OTU domain-containing protein 4 OS=Homo sapiens GN=OTUD4		38.47
Q01804-3	OTUD4_HUMAN Isoform 3 of OTU domain-containing protein 4 OS=Homo sapiens GN=OTUD4		0.65
Q01804;Q01804-3;Q7RTX	OTUD4_HUMAN OTU domain-containing protein 4 OS=Homo sapiens GN=OTUD4 PE=1 SV=3;>sp|Q01804-3|OTUD4_HUMAN Isoform	2.41E-08	
Q01813	K6PP_HUMAN 6-phosphofructokinase type C OS=Homo sapiens GN=PFKP PE=1 SV=2		370.72
Q01814	AT2B2_HUMAN Plasma membrane calcium-transporting ATPase 2 OS=Homo sapiens GN=ATP2B2 PE=1 SV=2		368.6
Q01814-2	AT2B2_HUMAN Isoform WA of Plasma membrane calcium-transporting ATPase 2 OS=Homo sapiens GN=ATP2B2		316.94
Q01814-3	AT2B2_HUMAN Isoform YA of Plasma membrane calcium-transporting ATPase 2 OS=Homo sapiens GN=ATP2B2		316.94
Q01814-4	AT2B2_HUMAN Isoform ZA of Plasma membrane calcium-transporting ATPase 2 OS=Homo sapiens GN=ATP2B2		316.94
Q01814-5	AT2B2_HUMAN Isoform YB of Plasma membrane calcium-transporting ATPase 2 OS=Homo sapiens GN=ATP2B2		368.6
Q01814-6	AT2B2_HUMAN Isoform ZB of Plasma membrane calcium-transporting ATPase 2 OS=Homo sapiens GN=ATP2B2		368.6
Q01814-7	AT2B2_HUMAN Isoform XA of Plasma membrane calcium-transporting ATPase 2 OS=Homo sapiens GN=ATP2B2		316.94
Q01814-8	AT2B2_HUMAN Isoform XB of Plasma membrane calcium-transporting ATPase 2 OS=Homo sapiens GN=ATP2B2		368.6
Q01814;Q01814-5;Q0181	AT2B2_HUMAN Plasma membrane calcium-transporting ATPase 2 OS=Homo sapiens GN=ATP2B2 PE=1 SV=2;>sp|Q01814-5|AT2B2_H	1.46E-54	
Q01844	EWS_HUMAN RNA-binding protein EWS OS=Homo sapiens GN=EWSR1 PE=1 SV=1		40.35
Q01844-2	EWS_HUMAN Isoform EWS-B of RNA-binding protein EWS OS=Homo sapiens GN=EWSR1		40.35
Q01844;Q01844-2	EWS_HUMAN RNA-binding protein EWS OS=Homo sapiens GN=EWSR1 PE=1 SV=1;>sp|Q01844-2|EWS_HUMAN Isoform EWS-B of RN	1.17E-90	
Q01968	OCRL_HUMAN Inositol polyphosphate 5-phosphatase OCRL-1 OS=Homo sapiens GN=OCRL PE=1 SV=3		33.65
Q01968-2	OCRL_HUMAN Isoform B of Inositol polyphosphate 5-phosphatase OCRL-1 OS=Homo sapiens GN=OCRL		33.65
Q01970	PLCB3_HUMAN 1-phosphatidylinositol-4,5-bisphosphate phosphodiesterase beta-3 OS=Homo sapiens GN=PLCB3 PE=1 SV=2		147.63
Q01973	ROR1_HUMAN Tyrosine-protein kinase transmembrane receptor ROR1 OS=Homo sapiens GN=ROR1 PE=2 SV=2		32.09
Q01973-2	ROR1_HUMAN Isoform Short of Tyrosine-protein kinase transmembrane receptor ROR1 OS=Homo sapiens GN=ROR1		16.32
Q01995	TAGL_HUMAN Transgelin OS=Homo sapiens GN=TAGLN PE=1 SV=4	0	
Q02040	AK17A_HUMAN A-kinase anchor protein 17A OS=Homo sapiens GN=AKAP17A PE=1 SV=2		
Q02040-2	AK17A_HUMAN Isoform 2 of A-kinase anchor protein 17A OS=Homo sapiens GN=AKAP17A		
Q02040-3	AK17A_HUMAN Isoform 3 of A-kinase anchor protein 17A OS=Homo sapiens GN=AKAP17A		
Q02040;Q02040-3;Q0204	AK17A_HUMAN A-kinase anchor protein 17A OS=Homo sapiens GN=AKAP17A PE=1 SV=2;>sp|Q02040-3|AK17A_HUMAN Isoform 3 o	6.42E-116	
Q02083;Q02083-2;B4DVL	NAAA_HUMAN N-acylethanolamine-hydrolyzing acid amidase OS=Homo sapiens GN=NAAA PE=1 SV=3;>sp|Q02083-2|NAAA_HUMAN	7.59E-08	
Q02127	PYRD_HUMAN Dihydroorotate dehydrogenase, mitochondrial OS=Homo sapiens GN=DHODH PE=1 SV=3	5.53E-24	
Q02156	KPCE_HUMAN Protein kinase C epsilon type OS=Homo sapiens GN=PRKCE PE=1 SV=1		
Q02218	ODO1_HUMAN 2-oxoglutarate dehydrogenase, mitochondrial OS=Homo sapiens GN=OGDH PE=1 SV=3		133.85
Q02224	CENPE_HUMAN Centromere-associated protein E OS=Homo sapiens GN=CENPE PE=1 SV=2		
Q02224-2	CENPE_HUMAN Isoform 2 of Centromere-associated protein E OS=Homo sapiens GN=CENPE		
Q02224-3	CENPE_HUMAN Isoform 3 of Centromere-associated protein E OS=Homo sapiens GN=CENPE		
Q02224;Q02224-2;Q0222	CENPE_HUMAN Centromere-associated protein E OS=Homo sapiens GN=CENPE PE=1 SV=2;>sp|Q02224-2|CENPE_HUMAN Isoform 2	1.11E-08	
Q02241	KIF23_HUMAN Kinesin-like protein KIF23 OS=Homo sapiens GN=KIF23 PE=1 SV=3		95.09
Q02241-2	KIF23_HUMAN Isoform 2 of Kinesin-like protein KIF23 OS=Homo sapiens GN=KIF23		95.09
Q02241;Q02241-2	KIF23_HUMAN Kinesin-like protein KIF23 OS=Homo sapiens GN=KIF23 PE=1 SV=3;>sp|Q02241-2|KIF23_HUMAN Isoform 2 of Kinesi	1.98E-20	

Q02246	CNTN2_HUMAN Contactin-2 OS=Homo sapiens GN=CNTN2 PE=1 SV=1		
Q02252	MMSA_HUMAN Methylmalonate-semialdehyde dehydrogenase [acylating], mitochondrial OS=Homo sapiens GN=ALDH6A1 PE=1 SV=2		
Q02338	BDH_HUMAN D-beta-hydroxybutyrate dehydrogenase, mitochondrial OS=Homo sapiens GN=BDH1 PE=1 SV=3		174.09
Q02388;Q02388-2	CO7A1_HUMAN Collagen alpha-1(VII) chain OS=Homo sapiens GN=COL7A1 PE=1 SV=2;>sp|Q02388-2|CO7A1_HUMAN Isoform 2 of	1.32E-10	
Q02413	DSG1_HUMAN Desmoglein-1 OS=Homo sapiens GN=DSG1 PE=1 SV=2		
Q02487	DSC2_HUMAN Desmocollin-2 OS=Homo sapiens GN=DSC2 PE=1 SV=1		
Q02487-2	DSC2_HUMAN Isoform 2B of Desmocollin-2 OS=Homo sapiens GN=DSC2		
Q02539	H11_HUMAN Histone H1.1 OS=Homo sapiens GN=HIST1H1A PE=1 SV=3		175
Q02543	RL18A_HUMAN 60S ribosomal protein L18a OS=Homo sapiens GN=RPL18A PE=1 SV=2		583.5
Q02750;Q02750-2	MP2K1_HUMAN Dual specificity mitogen-activated protein kinase kinase 1 OS=Homo sapiens GN=MAP2K1 PE=1 SV=2;>sp|Q02750-2	2.40E-08	
Q02779	M3K10_HUMAN Mitogen-activated protein kinase kinase kinase 10 OS=Homo sapiens GN=MAP3K10 PE=1 SV=3		
Q02790	FKBP4_HUMAN Peptidyl-prolyl cis-trans isomerase FKBP4 OS=Homo sapiens GN=FKBP4 PE=1 SV=3		110.71
Q02809	PLOD1_HUMAN Procollagen-lysine,2-oxoglutarate 5-dioxygenase 1 OS=Homo sapiens GN=PLOD1 PE=1 SV=2		542.39
Q02818	NUCB1_HUMAN Nucleobindin-1 OS=Homo sapiens GN=NUCB1 PE=1 SV=4		333.14
Q02846	GUC2D_HUMAN Retinal guanylyl cyclase 1 OS=Homo sapiens GN=GUCY2D PE=1 SV=2		
Q02878	RL6_HUMAN 60S ribosomal protein L6 OS=Homo sapiens GN=RPL6 PE=1 SV=3		845.68
Q02880	TOP2B_HUMAN DNA topoisomerase 2-beta OS=Homo sapiens GN=TOP2B PE=1 SV=3		760.91
Q02880-2	TOP2B_HUMAN Isoform Beta-1 of DNA topoisomerase 2-beta OS=Homo sapiens GN=TOP2B		759.6
Q02880;Q02880-2	TOP2B_HUMAN DNA topoisomerase 2-beta OS=Homo sapiens GN=TOP2B PE=1 SV=3;>sp|Q02880-2|TOP2B_HUMAN Isoform Beta-1	0	
Q02930	CREB5_HUMAN Cyclic AMP-responsive element-binding protein 5 OS=Homo sapiens GN=CREB5 PE=1 SV=3		32.95
Q02930-2	CREB5_HUMAN Isoform 2 of Cyclic AMP-responsive element-binding protein 5 OS=Homo sapiens GN=CREB5		32.95
Q02930-3	CREB5_HUMAN Isoform 3 of Cyclic AMP-responsive element-binding protein 5 OS=Homo sapiens GN=CREB5		32.95
Q02930-4	CREB5_HUMAN Isoform 4 of Cyclic AMP-responsive element-binding protein 5 OS=Homo sapiens GN=CREB5		32.95
Q02952;A6NEC7;Q02952-	AKA12_HUMAN A-kinase anchor protein 12 OS=Homo sapiens GN=AKAP12 PE=1 SV=4;>tr|A6NEC7|A6NEC7_HUMAN Uncharacterizec	1.41E-47	
Q02978	M2OM_HUMAN Mitochondrial 2-oxoglutarate/malate carrier protein OS=Homo sapiens GN=SLC25A11 PE=1 SV=3		250.13
Q03001	DYST_HUMAN Dystonin OS=Homo sapiens GN=DST PE=1 SV=4		229.79
Q03001-10	DYST_HUMAN Isoform 5 of Dystonin OS=Homo sapiens GN=DST		1.02
Q03001-11;Q03001;Q5TB	11|DYST_HUMAN Isoform 6 of Dystonin OS=Homo sapiens GN=DST;>sp|Q03001|DYST_HUMAN Dystonin OS=Homo sapiens GN=DST	0	
Q03001-13	DYST_HUMAN Isoform 8 of Dystonin OS=Homo sapiens GN=DST		55.13
Q03001-3	DYST_HUMAN Isoform 3 of Dystonin OS=Homo sapiens GN=DST		28.65
Q03001-8	DYST_HUMAN Isoform 2 of Dystonin OS=Homo sapiens GN=DST		175.29
Q03001-9	DYST_HUMAN Isoform 4 of Dystonin OS=Homo sapiens GN=DST		21.36
Q03001;Q03001-8	DYST_HUMAN Dystonin OS=Homo sapiens GN=DST PE=1 SV=4;>sp|Q03001-8|DYST_HUMAN Isoform 2 of Dystonin OS=Homo sapie	0	
Q03013;Q03013-2	GSTM4_HUMAN Glutathione S-transferase Mu 4 OS=Homo sapiens GN=GSTM4 PE=1 SV=3;>sp|Q03013-2|GSTM4_HUMAN Isoform 2	6.92E-84	
Q03014	HHEX_HUMAN Hematopoietically-expressed homeobox protein HHEX OS=Homo sapiens GN=HHEX PE=1 SV=1		
Q03060-21	CREM_HUMAN Isoform 18 of cAMP-responsive element modulator OS=Homo sapiens GN=CREM		18.55
Q03060-6	CREM_HUMAN Isoform 3 of cAMP-responsive element modulator OS=Homo sapiens GN=CREM		18.55
Q03060-8	CREM_HUMAN Isoform 6 of cAMP-responsive element modulator OS=Homo sapiens GN=CREM		18.55
Q03060-9	CREM_HUMAN Isoform 7 of cAMP-responsive element modulator OS=Homo sapiens GN=CREM		18.55
Q03112	EVI1_HUMAN MDS1 and EVI1 complex locus protein EVI1 OS=Homo sapiens GN=MECOM PE=1 SV=2		
Q03112-3	EVI1_HUMAN Isoform 2 of MDS1 and EVI1 complex locus protein EVI1 OS=Homo sapiens GN=MECOM		
Q03112-5	EVI1_HUMAN Isoform 5 of MDS1 and EVI1 complex locus protein EVI1 OS=Homo sapiens GN=MECOM		
Q03112-6	EVI1_HUMAN Isoform 6 of MDS1 and EVI1 complex locus protein EVI1 OS=Homo sapiens GN=MECOM		
Q03113	GNA12_HUMAN Guanine nucleotide-binding protein subunit alpha-12 OS=Homo sapiens GN=GNA12 PE=1 SV=4		85.18
Q03135	CAV1_HUMAN Caveolin-1 OS=Homo sapiens GN=CAV1 PE=1 SV=4		625.56
Q03135-2	CAV1_HUMAN Isoform Beta of Caveolin-1 OS=Homo sapiens GN=CAV1		374.65
Q03135;Q03135-2	CAV1_HUMAN Caveolin-1 OS=Homo sapiens GN=CAV1 PE=1 SV=4;>sp|Q03135-2|CAV1_HUMAN Isoform Beta of Caveolin-1 OS=Hon	2.39E-47	
Q03154	ACY1_HUMAN Aminoacylase-1 OS=Homo sapiens GN=ACY1 PE=1 SV=1	5.25E-09	
Q03164	MLL1_HUMAN Histone-lysine N-methyltransferase MLL OS=Homo sapiens GN=MLL PE=1 SV=5		65.5
Q03164-2	MLL1_HUMAN Isoform 14P-18B of Histone-lysine N-methyltransferase MLL OS=Homo sapiens GN=MLL		65.5
Q03164;Q03164-2	MLL1_HUMAN Histone-lysine N-methyltransferase MLL OS=Homo sapiens GN=MLL PE=1 SV=5;>sp|Q03164-2|MLL1_HUMAN Isoform	6.74E-18	
Q03169	TNAP2_HUMAN Tumor necrosis factor alpha-induced protein 2 OS=Homo sapiens GN=TNFAIP2 PE=1 SV=2		113.17
Q03188	CENPC_HUMAN Centromere protein C 1 OS=Homo sapiens GN=CENPC1 PE=1 SV=2		
Q03252	LMNB2_HUMAN Lamin-B2 OS=Homo sapiens GN=LMNB2 PE=1 SV=3		115.85
Q03405	UPAR_HUMAN Urokinase plasminogen activator surface receptor OS=Homo sapiens GN=PLAUR PE=1 SV=1		228.52
Q03405-2	UPAR_HUMAN Isoform 2 of Urokinase plasminogen activator surface receptor OS=Homo sapiens GN=PLAUR		193
Q03405-3	UPAR_HUMAN Isoform 3 of Urokinase plasminogen activator surface receptor OS=Homo sapiens GN=PLAUR		201.23
Q03405;Q03405-2;Q9UPI	UPAR_HUMAN Urokinase plasminogen activator surface receptor OS=Homo sapiens GN=PLAUR PE=1 SV=1;>sp|Q03405-2|UPAR_HUN	9.55E-07	
Q03426	KIME_HUMAN Mevalonate kinase OS=Homo sapiens GN=MVK PE=1 SV=1	2.54E-12	
Q03518	TAP1_HUMAN Antigen peptide transporter 1 OS=Homo sapiens GN=TAP1 PE=1 SV=2		76.38
Q03519	TAP2_HUMAN Antigen peptide transporter 2 OS=Homo sapiens GN=TAP2 PE=1 SV=1		121.89

Q03519-2	TAP2_HUMAN Isoform 2 of Antigen peptide transporter 2 OS=Homo sapiens GN=TAP2		121.89
Q03519;Q03519-2	TAP2_HUMAN Antigen peptide transporter 2 OS=Homo sapiens GN=TAP2 PE=1 SV=1;>sp|Q03519-2|TAP2_HUMAN Isoform 2 of Antig	4.17E-18	
Q03591	FHR1_HUMAN Complement factor H-related protein 1 OS=Homo sapiens GN=CFHR1 PE=1 SV=2	1.99E-72	
Q03701	CEBPZ_HUMAN CCAAT/enhancer-binding protein zeta OS=Homo sapiens GN=CEBPZ PE=1 SV=3		
Q03938	ZNF90_HUMAN Zinc finger protein 90 OS=Homo sapiens GN=ZNF90 PE=2 SV=3		
Q03989	ARI5A_HUMAN AT-rich interactive domain-containing protein 5A OS=Homo sapiens GN=ARID5A PE=2 SV=2		
Q04118	PRB3_HUMAN Basic salivary proline-rich protein 3 OS=Homo sapiens GN=PRB3 PE=1 SV=2	2.10E-09	
Q04206-3	TF65_HUMAN Isoform 3 of Transcription factor p65 OS=Homo sapiens GN=RELA		
Q04206;Q04206-4;Q0420	TF65_HUMAN Transcription factor p65 OS=Homo sapiens GN=RELA PE=1 SV=2;>sp|Q04206-4|TF65_HUMAN Isoform 4 of Transcriptio	5.15E-73	
Q04323	UBXN1_HUMAN UBX domain-containing protein 1 OS=Homo sapiens GN=UBXN1 PE=1 SV=2		64.64
Q04323-2	UBXN1_HUMAN Isoform 2 of UBX domain-containing protein 1 OS=Homo sapiens GN=UBXN1		64.64
Q04323-2;Q04323	2|UBXN1_HUMAN Isoform 2 of UBX domain-containing protein 1 OS=Homo sapiens GN=UBXN1;>sp|Q04323|UBXN1_HUMAN UBX dor	1.01E-05	
Q04446	GLGB_HUMAN 1,4-alpha-glucan-branching enzyme OS=Homo sapiens GN=GBE1 PE=1 SV=2		32.73
Q04609	FOLH1_HUMAN Glutamate carboxypeptidase 2 OS=Homo sapiens GN=FOLH1 PE=1 SV=1		
Q04609-3	FOLH1_HUMAN Isoform PSMA-3 of Glutamate carboxypeptidase 2 OS=Homo sapiens GN=FOLH1		
Q04609-6	FOLH1_HUMAN Isoform PSMA' of Glutamate carboxypeptidase 2 OS=Homo sapiens GN=FOLH1		
Q04609-7	FOLH1_HUMAN Isoform PSMA-7 of Glutamate carboxypeptidase 2 OS=Homo sapiens GN=FOLH1		
Q04609-8	FOLH1_HUMAN Isoform PSMA-8 of Glutamate carboxypeptidase 2 OS=Homo sapiens GN=FOLH1		
Q04609-9	FOLH1_HUMAN Isoform PSMA-9 of Glutamate carboxypeptidase 2 OS=Homo sapiens GN=FOLH1		
Q04637	IF4G1_HUMAN Eukaryotic translation initiation factor 4 gamma 1 OS=Homo sapiens GN=EIF4G1 PE=1 SV=4		911.25
Q04637-3	IF4G1_HUMAN Isoform B of Eukaryotic translation initiation factor 4 gamma 1 OS=Homo sapiens GN=EIF4G1		911.25
Q04637-4	IF4G1_HUMAN Isoform C of Eukaryotic translation initiation factor 4 gamma 1 OS=Homo sapiens GN=EIF4G1		911.25
Q04637-5	IF4G1_HUMAN Isoform D of Eukaryotic translation initiation factor 4 gamma 1 OS=Homo sapiens GN=EIF4G1		911.25
Q04637-6	IF4G1_HUMAN Isoform E of Eukaryotic translation initiation factor 4 gamma 1 OS=Homo sapiens GN=EIF4G1		911.25
Q04637;Q04637-3;Q0463	IF4G1_HUMAN Eukaryotic translation initiation factor 4 gamma 1 OS=Homo sapiens GN=EIF4G1 PE=1 SV=4;>sp|Q04637-3|IF4G1_H	9.40E-218	
Q04695	K1C17_HUMAN Keratin, type I cytoskeletal 17 OS=Homo sapiens GN=KRT17 PE=1 SV=2		539.66
Q04721	NOTC2_HUMAN Neurogenic locus notch homolog protein 2 OS=Homo sapiens GN=NOTCH2 PE=1 SV=3		29.81
Q04724	TLE1_HUMAN Transducin-like enhancer protein 1 OS=Homo sapiens GN=TLE1 PE=1 SV=2		50.22
Q04725	TLE2_HUMAN Transducin-like enhancer protein 2 OS=Homo sapiens GN=TLE2 PE=1 SV=2		50.15
Q04726;Q04726-4;Q0472	TLE3_HUMAN Transducin-like enhancer protein 3 OS=Homo sapiens GN=TLE3 PE=1 SV=2;>sp|Q04726-4|TLE3_HUMAN Isoform 4 of	3.40E-07	
Q04727	TLE4_HUMAN Transducin-like enhancer protein 4 OS=Homo sapiens GN=TLE4 PE=1 SV=3		37.54
Q04727-2	TLE4_HUMAN Isoform 2 of Transducin-like enhancer protein 4 OS=Homo sapiens GN=TLE4		37.54
Q04727-3	TLE4_HUMAN Isoform 3 of Transducin-like enhancer protein 4 OS=Homo sapiens GN=TLE4		37.54
Q04760	LGUL_HUMAN Lactoylglutathione lyase OS=Homo sapiens GN=GLO1 PE=1 SV=4		687.24
Q04760-2	LGUL_HUMAN Isoform 2 of Lactoylglutathione lyase OS=Homo sapiens GN=GLO1		687.24
Q04760;Q04760-2	LGUL_HUMAN Lactoylglutathione lyase OS=Homo sapiens GN=GLO1 PE=1 SV=4;>sp|Q04760-2|LGUL_HUMAN Isoform 2 of Lactoylglu	6.03E-169	
Q04826	1B40_HUMAN HLA class I histocompatibility antigen, B-40 alpha chain OS=Homo sapiens GN=HLA-B PE=1 SV=1		659.47
Q04828	AK1C1_HUMAN Aldo-keto reductase family 1 member C1 OS=Homo sapiens GN=AKR1C1 PE=1 SV=1	1.46E-207	
Q04837	SSBP_HUMAN Single-stranded DNA-binding protein, mitochondrial OS=Homo sapiens GN=SSBP1 PE=1 SV=1		664.52
Q04917	1433F_HUMAN 14-3-3 protein eta OS=Homo sapiens GN=YWHAH PE=1 SV=4		680.6
Q04941	PLP2_HUMAN Proteolipid protein 2 OS=Homo sapiens GN=PLP2 PE=1 SV=1		156.14
Q04941-2	PLP2_HUMAN Isoform 2 of Proteolipid protein 2 OS=Homo sapiens GN=PLP2		78.39
Q04941;Q04941-2	PLP2_HUMAN Proteolipid protein 2 OS=Homo sapiens GN=PLP2 PE=1 SV=1;>sp|Q04941-2|PLP2_HUMAN Isoform 2 of Proteolipid prot	7.53E-10	
Q05048	CSTF1_HUMAN Cleavage stimulation factor subunit 1 OS=Homo sapiens GN=CSTF1 PE=1 SV=1		
Q05086;Q05086-3;Q0508	UBE3A_HUMAN Ubiquitin-protein ligase E3A OS=Homo sapiens GN=UBE3A PE=1 SV=4;>sp|Q05086-3|UBE3A_HUMAN Isoform III of	3.16E-06	
Q05193	DYN1_HUMAN Dynamin-1 OS=Homo sapiens GN=DNM1 PE=1 SV=2		109.34
Q05193-2	DYN1_HUMAN Isoform 2 of Dynamin-1 OS=Homo sapiens GN=DNM1		111.04
Q05193-3	DYN1_HUMAN Isoform 3 of Dynamin-1 OS=Homo sapiens GN=DNM1		109.34
Q05193-4	DYN1_HUMAN Isoform 4 of Dynamin-1 OS=Homo sapiens GN=DNM1		109.34
Q05193-5	DYN1_HUMAN Isoform 5 of Dynamin-1 OS=Homo sapiens GN=DNM1		111.04
Q05209	PTN12_HUMAN Tyrosine-protein phosphatase non-receptor type 12 OS=Homo sapiens GN=PTPN12 PE=1 SV=3	1.34E-148	
Q05397	FAK1_HUMAN Focal adhesion kinase 1 OS=Homo sapiens GN=PTK2 PE=1 SV=2		37
Q05397-2	FAK1_HUMAN Isoform 2 of Focal adhesion kinase 1 OS=Homo sapiens GN=PTK2		32.96
Q05397-3	FAK1_HUMAN Isoform 3 of Focal adhesion kinase 1 OS=Homo sapiens GN=PTK2		
Q05397-4	FAK1_HUMAN Isoform 4 of Focal adhesion kinase 1 OS=Homo sapiens GN=PTK2		1.45
Q05397;Q05397-2;Q0539	FAK1_HUMAN Focal adhesion kinase 1 OS=Homo sapiens GN=PTK2 PE=1 SV=2;>sp|Q05397-2|FAK1_HUMAN Isoform 2 of Focal adhe	1.07E-46	
Q05469;Q05469-2	LIPS_HUMAN Hormone-sensitive lipase OS=Homo sapiens GN=LIPE PE=1 SV=4;>sp|Q05469-2|LIPS_HUMAN Isoform 2 of Hormone-s	6.15E-13	
Q05519	SRS11_HUMAN Serine/arginine-rich splicing factor 11 OS=Homo sapiens GN=SRSF11 PE=1 SV=1		146.5
Q05639	EF1A2_HUMAN Elongation factor 1-alpha 2 OS=Homo sapiens GN=EEF1A2 PE=1 SV=1		813.55
Q05655	KPCD_HUMAN Protein kinase C delta type OS=Homo sapiens GN=PRKCD PE=1 SV=2	2.04E-33	
Q05682	CALD1_HUMAN Caldesmon OS=Homo sapiens GN=CALD1 PE=1 SV=3		271.12

Q05682-2	CALD1_HUMAN Isoform 2 of Caldesmon OS=Homo sapiens GN=CALD1		256.1
Q05682-3	CALD1_HUMAN Isoform 3 of Caldesmon OS=Homo sapiens GN=CALD1		255.89
Q05682-4	CALD1_HUMAN Isoform 4 of Caldesmon OS=Homo sapiens GN=CALD1		256.1
Q05682-4;Q05682-5;Q05(	4|CALD1_HUMAN Isoform 4 of Caldesmon OS=Homo sapiens GN=CALD1;>sp|Q05682-5|CALD1_HUMAN Isoform 5 of Caldesmon OS=	1.51E-216	
Q05682-5	CALD1_HUMAN Isoform 5 of Caldesmon OS=Homo sapiens GN=CALD1		255.89
Q05682-5;Q05682-3;B7Z(	5|CALD1_HUMAN Isoform 5 of Caldesmon OS=Homo sapiens GN=CALD1;>sp|Q05682-3|CALD1_HUMAN Isoform 3 of Caldesmon OS=	0	
Q05707;Q05707-2;Q0570	COEA1_HUMAN Collagen alpha-1(XIV) chain OS=Homo sapiens GN=COL14A1 PE=1 SV=3;>sp|Q05707-2|COEA1_HUMAN Isoform 2 o	0	
Q05BV3	EMAL5_HUMAN Echinoderm microtubule-associated protein-like 5 OS=Homo sapiens GN=EML5 PE=2 SV=3		
Q05BV3-2	EMAL5_HUMAN Isoform 2 of Echinoderm microtubule-associated protein-like 5 OS=Homo sapiens GN=EML5		
Q05BV3-4	EMAL5_HUMAN Isoform 4 of Echinoderm microtubule-associated protein-like 5 OS=Homo sapiens GN=EML5		
Q05BV3-5	EMAL5_HUMAN Isoform 5 of Echinoderm microtubule-associated protein-like 5 OS=Homo sapiens GN=EML5		
Q05DH4	F16A1_HUMAN Protein FAM160A1 OS=Homo sapiens GN=FAM160A1 PE=2 SV=2		
Q06124-2;Q06124;Q0612	2|PTN11_HUMAN Isoform 2 of Tyrosine-protein phosphatase non-receptor type 11 OS=Homo sapiens GN=PTPN11;>sp|Q06124|PTN11	0	
Q06124;Q06124-2;Q0612	PTN11_HUMAN Tyrosine-protein phosphatase non-receptor type 11 OS=Homo sapiens GN=PTPN11 PE=1 SV=2;>sp|Q06124-2|PTN11	3.93E-14	
Q06136	KDSR_HUMAN 3-ketodihydrosphingosine reductase OS=Homo sapiens GN=KDSR PE=1 SV=1	4.55E-10	
Q06187	BTK_HUMAN Tyrosine-protein kinase BTK OS=Homo sapiens GN=BTK PE=1 SV=3		47.27
Q06190	P2R3A_HUMAN Serine/threonine-protein phosphatase 2A regulatory subunit B'' subunit alpha OS=Homo sapiens GN=PPP2R3A PE=1 SV=1		
Q06190-2	P2R3A_HUMAN Isoform PR72 of Serine/threonine-protein phosphatase 2A regulatory subunit B'' subunit alpha OS=Homo sapiens GN=PPP2R3A		
Q06203	PUR1_HUMAN Amidophosphoribosyltransferase OS=Homo sapiens GN=PPAT PE=1 SV=1	2.38E-26	
Q06210;Q06210-2	GFPT1_HUMAN Glucosamine--fructose-6-phosphate aminotransferase [isomerizing] 1 OS=Homo sapiens GN=GFPT1 PE=1 SV=3;>sp|(	3.49E-98	
Q06265	EXOS9_HUMAN Exosome complex component RRP45 OS=Homo sapiens GN=EXOSC9 PE=1 SV=3		110.1
Q06265-2	EXOS9_HUMAN Isoform 2 of Exosome complex component RRP45 OS=Homo sapiens GN=EXOSC9		110.1
Q06265-2;A5PLM5;Q0626	2|EXOS9_HUMAN Isoform 2 of Exosome complex exonuclease RRP45 OS=Homo sapiens GN=EXOSC9;>tr|A5PLM5|A5PLM5_HUMAN E	4.09E-51	
Q06265-3	EXOS9_HUMAN Isoform 3 of Exosome complex component RRP45 OS=Homo sapiens GN=EXOSC9		110.1
Q06265-4	EXOS9_HUMAN Isoform 4 of Exosome complex component RRP45 OS=Homo sapiens GN=EXOSC9		110.1
Q06323	PSME1_HUMAN Proteasome activator complex subunit 1 OS=Homo sapiens GN=PSME1 PE=1 SV=1		790.34
Q06330;B4DY22;Q06330-	SUH_HUMAN Recombining binding protein suppressor of hairless OS=Homo sapiens GN=RBPJ PE=1 SV=2;>tr|B4DY22|B4DY22_HUMA	2.10E-12	
Q06481	APLP2_HUMAN Amyloid-like protein 2 OS=Homo sapiens GN=APLP2 PE=1 SV=2		62.8
Q06481-2	APLP2_HUMAN Isoform 2 of Amyloid-like protein 2 OS=Homo sapiens GN=APLP2		62.8
Q06481-3	APLP2_HUMAN Isoform 3 of Amyloid-like protein 2 OS=Homo sapiens GN=APLP2		62.8
Q06481-4	APLP2_HUMAN Isoform 4 of Amyloid-like protein 2 OS=Homo sapiens GN=APLP2		62.8
Q06481-5	APLP2_HUMAN Isoform 5 of Amyloid-like protein 2 OS=Homo sapiens GN=APLP2		62.8
Q06481;Q06481-3;Q0648	APLP2_HUMAN Amyloid-like protein 2 OS=Homo sapiens GN=APLP2 PE=1 SV=2;>sp|Q06481-3|APLP2_HUMAN Isoform 3 of Amyloid-	4.27E-11	
Q06587	RING1_HUMAN E3 ubiquitin-protein ligase RING1 OS=Homo sapiens GN=RING1 PE=1 SV=2		29.96
Q06587-2	RING1_HUMAN Isoform 2 of E3 ubiquitin-protein ligase RING1 OS=Homo sapiens GN=RING1		29.96
Q06587;Q06587-2	RING1_HUMAN E3 ubiquitin-protein ligase RING1 OS=Homo sapiens GN=RING1 PE=1 SV=2;>sp|Q06587-2|RING1_HUMAN Isoform 2	1.36E-11	
Q06732	ZN33B_HUMAN Zinc finger protein 33B OS=Homo sapiens GN=ZNF33B PE=2 SV=2	0.00019949	
Q06787	FMR1_HUMAN Fragile X mental retardation 1 protein OS=Homo sapiens GN=FMR1 PE=1 SV=1		91.47
Q06787-2	FMR1_HUMAN Isoform 1 of Fragile X mental retardation 1 protein OS=Homo sapiens GN=FMR1		94.63
Q06787-3	FMR1_HUMAN Isoform 2 of Fragile X mental retardation 1 protein OS=Homo sapiens GN=FMR1		91.47
Q06787-4	FMR1_HUMAN Isoform 3 of Fragile X mental retardation 1 protein OS=Homo sapiens GN=FMR1		94.63
Q06787-5	FMR1_HUMAN Isoform 4 of Fragile X mental retardation 1 protein OS=Homo sapiens GN=FMR1		91.47
Q06787-6	FMR1_HUMAN Isoform 5 of Fragile X mental retardation 1 protein OS=Homo sapiens GN=FMR1		94.63
Q06787-7	FMR1_HUMAN Isoform 7 of Fragile X mental retardation 1 protein OS=Homo sapiens GN=FMR1		94.63
Q06787-8	FMR1_HUMAN Isoform 8 of Fragile X mental retardation 1 protein OS=Homo sapiens GN=FMR1		91.47
Q06787;Q06787-3;Q0678	FMR1_HUMAN Fragile X mental retardation 1 protein OS=Homo sapiens GN=FMR1 PE=1 SV=1;>sp|Q06787-3|FMR1_HUMAN Isoform	7.40E-13	
Q06828	FMOD_HUMAN Fibromodulin OS=Homo sapiens GN=FMOD PE=1 SV=2	1.57E-64	
Q06830	PRDX1_HUMAN Peroxiredoxin-1 OS=Homo sapiens GN=PRDX1 PE=1 SV=1		921.03
Q07000	1C15_HUMAN HLA class I histocompatibility antigen, Cw-15 alpha chain OS=Homo sapiens GN=HLA-C PE=1 SV=1		458.03
Q07002	CDK18_HUMAN Cyclin-dependent kinase 18 OS=Homo sapiens GN=CDK18 PE=1 SV=3		97.41
Q07002-2	CDK18_HUMAN Isoform 2 of Cyclin-dependent kinase 18 OS=Homo sapiens GN=CDK18		97.41
Q07002-3	CDK18_HUMAN Isoform 3 of Cyclin-dependent kinase 18 OS=Homo sapiens GN=CDK18		97.41
Q07002-3;Q07002-2;Q070	3|CDK18_HUMAN Isoform 3 of Cyclin-dependent kinase 18 OS=Homo sapiens GN=CDK18;>sp|Q07002-2|CDK18_HUMAN Isoform 2 o	1.37E-07	
Q07020	RL18_HUMAN 60S ribosomal protein L18 OS=Homo sapiens GN=RPL18 PE=1 SV=2		556.48
Q07021	C1QBP_HUMAN Complement component 1 Q subcomponent-binding protein, mitochondrial OS=Homo sapiens GN=C1QBP PE=1 SV=1		500.12
Q07065	CKAP4_HUMAN Cytoskeleton-associated protein 4 OS=Homo sapiens GN=CKAP4 PE=1 SV=2		1832.11
Q07157	ZO1_HUMAN Tight junction protein ZO-1 OS=Homo sapiens GN=TJP1 PE=1 SV=3		101.38
Q07157-2	ZO1_HUMAN Isoform Short of Tight junction protein ZO-1 OS=Homo sapiens GN=TJP1		101.38
Q07157;Q07157-2	ZO1_HUMAN Tight junction protein ZO-1 OS=Homo sapiens GN=TJP1 PE=1 SV=3;>sp|Q07157-2|ZO1_HUMAN Isoform Short of Tight	1.12E-59	
Q07283	TRHY_HUMAN Trichohyalin OS=Homo sapiens GN=TCHH PE=1 SV=2		
Q07507	DERM_HUMAN Dermatopontin OS=Homo sapiens GN=DPT PE=2 SV=2	5.71E-112	

Q07666	KHDR1_HUMAN KH domain-containing, RNA-binding, signal transduction-associated protein 1 OS=Homo sapiens GN=KHDRBS1 PE=1 SV=1		539.52
Q07666-2	KHDR1_HUMAN Isoform 2 of KH domain-containing, RNA-binding, signal transduction-associated protein 1 OS=Homo sapiens GN=KHDRBS1		539.52
Q07666-3	KHDR1_HUMAN Isoform 3 of KH domain-containing, RNA-binding, signal transduction-associated protein 1 OS=Homo sapiens GN=KHDRBS1		477.27
Q07666;Q07666-2;Q0766	KHDR1_HUMAN KH domain-containing, RNA-binding, signal transduction-associated protein 1 OS=Homo sapiens GN=KHDRBS1 PE=1	3.16E-222	
Q07812	BAX_HUMAN Apoptosis regulator BAX OS=Homo sapiens GN=BAX PE=1 SV=1		384.58
Q07812-2	BAX_HUMAN Isoform Beta of Apoptosis regulator BAX OS=Homo sapiens GN=BAX		384.58
Q07812-2;Q07812;Q0781	2|BAX_HUMAN Isoform Beta of Apoptosis regulator BAX OS=Homo sapiens GN=BAX;>sp|Q07812|BAX_HUMAN Apoptosis regulator BA	5.07E-169	
Q07812-4	BAX_HUMAN Isoform Delta of Apoptosis regulator BAX OS=Homo sapiens GN=BAX		110.84
Q07812-5	BAX_HUMAN Isoform Epsilon of Apoptosis regulator BAX OS=Homo sapiens GN=BAX		336.02
Q07812-6	BAX_HUMAN Isoform Zeta of Apoptosis regulator BAX OS=Homo sapiens GN=BAX		210.59
Q07812-7	BAX_HUMAN Isoform Psi of Apoptosis regulator BAX OS=Homo sapiens GN=BAX		342.42
Q07812-8	BAX_HUMAN Isoform Sigma of Apoptosis regulator BAX OS=Homo sapiens GN=BAX		384.58
Q07817	B2CL1_HUMAN Bcl-2-like protein 1 OS=Homo sapiens GN=BCL2L1 PE=1 SV=1		
Q07817-2	B2CL1_HUMAN Isoform Bcl-X(S) of Bcl-2-like protein 1 OS=Homo sapiens GN=BCL2L1		
Q07817-3	B2CL1_HUMAN Isoform Bcl-X(beta) of Bcl-2-like protein 1 OS=Homo sapiens GN=BCL2L1		
Q07864	DPOE1_HUMAN DNA polymerase epsilon catalytic subunit A OS=Homo sapiens GN=POLE PE=1 SV=5		35.24
Q07866-4;Q07866-6;Q07	4|KLC1_HUMAN Isoform J of Kinesin light chain 1 OS=Homo sapiens GN=KLC1;>sp|Q07866-6|KLC1_HUMAN Isoform N of Kinesin ligh	1.42E-156	
Q07866-9;Q07866-4;Q07	9|KLC1_HUMAN Isoform I of Kinesin light chain 1 OS=Homo sapiens GN=KLC1;>sp|Q07866-4|KLC1_HUMAN Isoform J of Kinesin light	2.17E-45	
Q07889	SOS1_HUMAN Son of sevenless homolog 1 OS=Homo sapiens GN=SOS1 PE=1 SV=1		30.67
Q07912	ACK1_HUMAN Activated CDC42 kinase 1 OS=Homo sapiens GN=TNK2 PE=1 SV=3		21.14
Q07912-2	ACK1_HUMAN Isoform 2 of Activated CDC42 kinase 1 OS=Homo sapiens GN=TNK2		21.14
Q07912-3	ACK1_HUMAN Isoform 3 of Activated CDC42 kinase 1 OS=Homo sapiens GN=TNK2		21.14
Q07954	LRP1_HUMAN Prolow-density lipoprotein receptor-related protein 1 OS=Homo sapiens GN=LRP1 PE=1 SV=2		61.66
Q07955	SRSF1_HUMAN Serine/arginine-rich splicing factor 1 OS=Homo sapiens GN=SRSF1 PE=1 SV=2		543.95
Q07955-2	SRSF1_HUMAN Isoform ASF-2 of Serine/arginine-rich splicing factor 1 OS=Homo sapiens GN=SRSF1		529.49
Q07955-3	SRSF1_HUMAN Isoform ASF-3 of Serine/arginine-rich splicing factor 1 OS=Homo sapiens GN=SRSF1		529.49
Q07955;Q07955-2;Q0795	SRSF1_HUMAN Serine/arginine-rich splicing factor 1 OS=Homo sapiens GN=SRSF1 PE=1 SV=2;>sp|Q07955-2|SRSF1_HUMAN Isoforr	5.04E-58	
Q07960	RHG01_HUMAN Rho GTPase-activating protein 1 OS=Homo sapiens GN=ARHGAP1 PE=1 SV=1	1.26E-37	
Q08043	ACTN3_HUMAN Alpha-actinin-3 OS=Homo sapiens GN=ACTN3 PE=1 SV=2		562.01
Q08117	AES_HUMAN Amino-terminal enhancer of split OS=Homo sapiens GN=AES PE=1 SV=4		20.05
Q08170	SRSF4_HUMAN Serine/arginine-rich splicing factor 4 OS=Homo sapiens GN=SRSF4 PE=1 SV=2		67.71
Q08209	PP2BA_HUMAN Serine/threonine-protein phosphatase 2B catalytic subunit alpha isoform OS=Homo sapiens GN=PPP3CA PE=1 SV=1		128.96
Q08209-2	PP2BA_HUMAN Isoform 2 of Serine/threonine-protein phosphatase 2B catalytic subunit alpha isoform OS=Homo sapiens GN=PPP3CA		128.96
Q08209;Q08209-2;P1629	PP2BA_HUMAN Serine/threonine-protein phosphatase 2B catalytic subunit alpha isoform OS=Homo sapiens GN=PPP3CA PE=1 SV=1;>	2.25E-18	
Q08211	DHX9_HUMAN ATP-dependent RNA helicase A OS=Homo sapiens GN=DHX9 PE=1 SV=4		1586.43
Q08257	QOR_HUMAN Quinone oxidoreductase OS=Homo sapiens GN=CRYZ PE=1 SV=1	4.09E-11	
Q08345	DDR1_HUMAN Epithelial discoidin domain-containing receptor 1 OS=Homo sapiens GN=DDR1 PE=1 SV=1		
Q08345-2	DDR1_HUMAN Isoform 2 of Epithelial discoidin domain-containing receptor 1 OS=Homo sapiens GN=DDR1		
Q08345-5	DDR1_HUMAN Isoform 4 of Epithelial discoidin domain-containing receptor 1 OS=Homo sapiens GN=DDR1		
Q08357	S20A2_HUMAN Sodium-dependent phosphate transporter 2 OS=Homo sapiens GN=SLC20A2 PE=1 SV=1		39.28
Q08378	GOGA3_HUMAN Golgin subfamily A member 3 OS=Homo sapiens GN=GOLGA3 PE=1 SV=2		26.72
Q08378-2	GOGA3_HUMAN Isoform 2 of Golgin subfamily A member 3 OS=Homo sapiens GN=GOLGA3		26.72
Q08378-4	GOGA3_HUMAN Isoform 3 of Golgin subfamily A member 3 OS=Homo sapiens GN=GOLGA3		23.88
Q08378;Q08378-2;Q0837	GOGA3_HUMAN Golgin subfamily A member 3 OS=Homo sapiens GN=GOLGA3 PE=1 SV=2;>sp|Q08378-2|GOGA3_HUMAN Isoform 2	5.08E-08	
Q08379	GOGA2_HUMAN Golgin subfamily A member 2 OS=Homo sapiens GN=GOLGA2 PE=1 SV=3		
Q08379-2	GOGA2_HUMAN Isoform 2 of Golgin subfamily A member 2 OS=Homo sapiens GN=GOLGA2		
Q08379;B7ZC06	GOGA2_HUMAN Golgin subfamily A member 2 OS=Homo sapiens GN=GOLGA2 PE=1 SV=3;>tr|B7ZC06|B7ZC06_HUMAN Golgi autoar	7.64E-19	
Q08380	LG3BP_HUMAN Galectin-3-binding protein OS=Homo sapiens GN=LGALS3BP PE=1 SV=1		94.59
Q08431;B3KTQ2;Q08431-	MFGM_HUMAN Lactadherin OS=Homo sapiens GN=MFGE8 PE=1 SV=2;>tr|B3KTQ2|B3KTQ2_HUMAN cDNA FLJ38589 fis, clone HCHOI	1.33E-18	
Q08462	ADCY2_HUMAN Adenylate cyclase type 2 OS=Homo sapiens GN=ADCY2 PE=1 SV=5		36.25
Q08499	PDE4D_HUMAN cAMP-specific 3',5'-cyclic phosphodiesterase 4D OS=Homo sapiens GN=PDE4D PE=1 SV=2		16.05
Q08499-10	PDE4D_HUMAN Isoform 9 of cAMP-specific 3',5'-cyclic phosphodiesterase 4D OS=Homo sapiens GN=PDE4D		14.71
Q08499-11	PDE4D_HUMAN Isoform 7 of cAMP-specific 3',5'-cyclic phosphodiesterase 4D OS=Homo sapiens GN=PDE4D		13.38
Q08499-12	PDE4D_HUMAN Isoform 12 of cAMP-specific 3',5'-cyclic phosphodiesterase 4D OS=Homo sapiens GN=PDE4D		49.73
Q08499-2	PDE4D_HUMAN Isoform 3 of cAMP-specific 3',5'-cyclic phosphodiesterase 4D OS=Homo sapiens GN=PDE4D		16.05
Q08499-3	PDE4D_HUMAN Isoform 10 of cAMP-specific 3',5'-cyclic phosphodiesterase 4D OS=Homo sapiens GN=PDE4D		16.05
Q08499-4	PDE4D_HUMAN Isoform 1 of cAMP-specific 3',5'-cyclic phosphodiesterase 4D OS=Homo sapiens GN=PDE4D		7.45
Q08499-5	PDE4D_HUMAN Isoform 2 of cAMP-specific 3',5'-cyclic phosphodiesterase 4D OS=Homo sapiens GN=PDE4D		16.05
Q08499-6	PDE4D_HUMAN Isoform 5 of cAMP-specific 3',5'-cyclic phosphodiesterase 4D OS=Homo sapiens GN=PDE4D		65.78
Q08499-7	PDE4D_HUMAN Isoform N3 of cAMP-specific 3',5'-cyclic phosphodiesterase 4D OS=Homo sapiens GN=PDE4D		49.73
Q08499-8	PDE4D_HUMAN Isoform 6 of cAMP-specific 3',5'-cyclic phosphodiesterase 4D OS=Homo sapiens GN=PDE4D		16.05

Q08499-9	PDE4D_HUMAN Isoform 8 of cAMP-specific 3',5'-cyclic phosphodiesterase 4D OS=Homo sapiens GN=PDE4D		16.05
Q08554	DSC1_HUMAN Desmocollin-1 OS=Homo sapiens GN=DSC1 PE=1 SV=2		30.92
Q08554-2	DSC1_HUMAN Isoform 1B of Desmocollin-1 OS=Homo sapiens GN=DSC1		30.92
Q08623	HDHD1_HUMAN Pseudouridine-5-monophosphatase OS=Homo sapiens GN=HDHD1 PE=1 SV=3	3.94E-11	
Q08623;B7Z6Q3	HDHD1_HUMAN Haloacid dehalogenase-like hydrolase domain-containing protein 1 OS=Homo sapiens GN=HDHD1 PE=1 SV=3;>tr|B7	9.03E-11	
Q08708	CLM6_HUMAN CMRF35-like molecule 6 OS=Homo sapiens GN=CD300C PE=2 SV=1		37.31
Q08722	CD47_HUMAN Leukocyte surface antigen CD47 OS=Homo sapiens GN=CD47 PE=1 SV=1		84.38
Q08722-2	CD47_HUMAN Isoform OA3-293 of Leukocyte surface antigen CD47 OS=Homo sapiens GN=CD47		84.38
Q08722-3	CD47_HUMAN Isoform OA3-305 of Leukocyte surface antigen CD47 OS=Homo sapiens GN=CD47		84.38
Q08722-4	CD47_HUMAN Isoform OA3-312 of Leukocyte surface antigen CD47 OS=Homo sapiens GN=CD47		84.38
Q08722;Q08722-4;Q0872	CD47_HUMAN Leukocyte surface antigen CD47 OS=Homo sapiens GN=CD47 PE=1 SV=1;>sp|Q08722-4|CD47_HUMAN Isoform OA3-	8.18E-11	
Q08752	PPID_HUMAN Peptidyl-prolyl cis-trans isomerase D OS=Homo sapiens GN=PPID PE=1 SV=3	2.62E-22	
Q08945	SSRP1_HUMAN FACT complex subunit SSRP1 OS=Homo sapiens GN=SSRP1 PE=1 SV=1		507.72
Q08999	RBL2_HUMAN Retinoblastoma-like protein 2 OS=Homo sapiens GN=RBL2 PE=1 SV=3		
Q08AF3	SLFN5_HUMAN Schlafen family member 5 OS=Homo sapiens GN=SLFN5 PE=1 SV=1		
Q08AF3-2	SLFN5_HUMAN Isoform 2 of Schlafen family member 5 OS=Homo sapiens GN=SLFN5		
Q08AF3;Q08AF3-2	SLFN5_HUMAN Schlafen family member 5 OS=Homo sapiens GN=SLFN5 PE=1 SV=1;>sp|Q08AF3-2|SLFN5_HUMAN Isoform 2 of Schl	1.52E-29	
Q08AH1	ACSM1_HUMAN Acyl-coenzyme A synthetase ACSM1, mitochondrial OS=Homo sapiens GN=ACSM1 PE=2 SV=1		
Q08AH1-2	ACSM1_HUMAN Isoform 2 of Acyl-coenzyme A synthetase ACSM1, mitochondrial OS=Homo sapiens GN=ACSM1		
Q08AM6	VAC14_HUMAN Protein VAC14 homolog OS=Homo sapiens GN=VAC14 PE=1 SV=1	9.79E-22	
Q08J23	NSUN2_HUMAN tRNA (cytosine(34)-C(5))-methyltransferase OS=Homo sapiens GN=NSUN2 PE=1 SV=2		380.53
Q08J23;B4DQW2;A8K529	NSUN2_HUMAN tRNA (cytosine-5-)-methyltransferase NSUN2 OS=Homo sapiens GN=NSUN2 PE=1 SV=2;>tr|B4DQW2|B4DQW2_HUN	8.21E-84	
Q09019	DMWD_HUMAN Dystrophia myotonica WD repeat-containing protein OS=Homo sapiens GN=DMWD PE=1 SV=3		
Q09028	RBBP4_HUMAN Histone-binding protein RBBP4 OS=Homo sapiens GN=RBBP4 PE=1 SV=3		102.89
Q09028-2	RBBP4_HUMAN Isoform 2 of Histone-binding protein RBBP4 OS=Homo sapiens GN=RBBP4		102.89
Q09028-3	RBBP4_HUMAN Isoform 3 of Histone-binding protein RBBP4 OS=Homo sapiens GN=RBBP4		102.89
Q09028-4	RBBP4_HUMAN Isoform 4 of Histone-binding protein RBBP4 OS=Homo sapiens GN=RBBP4		102.89
Q09028;Q09028-2;Q0902	RBBP4_HUMAN Histone-binding protein RBBP4 OS=Homo sapiens GN=RBBP4 PE=1 SV=3;>sp|Q09028-2|RBBP4_HUMAN Isoform 2 of	5.68E-103	
Q09160	1A80_HUMAN HLA class I histocompatibility antigen, A-80 alpha chain OS=Homo sapiens GN=HLA-A PE=2 SV=1		246.13
Q09161	NCBP1_HUMAN Nuclear cap-binding protein subunit 1 OS=Homo sapiens GN=NCBP1 PE=1 SV=1		329.41
Q09472	EP300_HUMAN Histone acetyltransferase p300 OS=Homo sapiens GN=EP300 PE=1 SV=2		59.72
Q09666	AHNK_HUMAN Neuroblast differentiation-associated protein AHNAK OS=Homo sapiens GN=AHNAK PE=1 SV=2		24526.76
Q09MP3	R51A2_HUMAN RAD51-associated protein 2 OS=Homo sapiens GN=RAD51AP2 PE=1 SV=1		
Q0JRZ9;B4DHK0;Q0JRZ9-	FCHO2_HUMAN FCH domain only protein 2 OS=Homo sapiens GN=FCHO2 PE=1 SV=2;>tr|B4DHK0|B4DHK0_HUMAN cDNA FLJ60538,	3.54E-09	
Q0VAA2	CN16B_HUMAN Uncharacterized protein C14orf166B OS=Homo sapiens GN=C14orf166B PE=2 SV=2	6.16E-06	
Q0VD83	APOBR_HUMAN Apolipoprotein B receptor OS=Homo sapiens GN=APOBR PE=1 SV=1		
Q0VD83-3	APOBR_HUMAN Isoform 3 of Apolipoprotein B receptor OS=Homo sapiens GN=APOBR		
Q0VDD8	DYH14_HUMAN Dynein heavy chain 14, axonemal OS=Homo sapiens GN=DNAH14 PE=2 SV=3		
Q0VDD8-2	DYH14_HUMAN Isoform 2 of Dynein heavy chain 14, axonemal OS=Homo sapiens GN=DNAH14		
Q0VDD8-3	DYH14_HUMAN Isoform 3 of Dynein heavy chain 14, axonemal OS=Homo sapiens GN=DNAH14		
Q0VDD8-4	DYH14_HUMAN Isoform 4 of Dynein heavy chain 14, axonemal OS=Homo sapiens GN=DNAH14		
Q0VDD8-4;Q0VDD8	4|DYH14_HUMAN Isoform 4 of Dynein heavy chain 14, axonemal OS=Homo sapiens GN=DNAH14;>sp|Q0VDD8|DYH14_HUMAN Dyne	6.57E-12	
Q0VDF9	HSP7E_HUMAN Heat shock 70 kDa protein 14 OS=Homo sapiens GN=HSPA14 PE=1 SV=1	1.64E-10	
Q0VF96	CGNL1_HUMAN Cingulin-like protein 1 OS=Homo sapiens GN=CGNL1 PE=1 SV=2		
Q0VF96-2	CGNL1_HUMAN Isoform 2 of Cingulin-like protein 1 OS=Homo sapiens GN=CGNL1		
Q0VGL1	CG059_HUMAN UPF0539 protein C7orf59 OS=Homo sapiens GN=C7orf59 PE=1 SV=1		
Q0ZGT2	NEXN_HUMAN Nexilin OS=Homo sapiens GN=NEXN PE=1 SV=1		59.98
Q0ZGT2-2	NEXN_HUMAN Isoform 2 of Nexilin OS=Homo sapiens GN=NEXN		59.98
Q0ZGT2-3	NEXN_HUMAN Isoform 3 of Nexilin OS=Homo sapiens GN=NEXN		56.33
Q0ZGT2;Q0ZGT2-2;Q0ZG	NEXN_HUMAN Nexilin OS=Homo sapiens GN=NEXN PE=1 SV=1;>sp|Q0ZGT2-2|NEXN_HUMAN Isoform 2 of Nexilin OS=Homo sapiens	2.58E-05	
Q10471	GALT2_HUMAN Polypeptide N-acetylgalactosaminyltransferase 2 OS=Homo sapiens GN=GALNT2 PE=1 SV=1		67.91
Q10472	GALT1_HUMAN Polypeptide N-acetylgalactosaminyltransferase 1 OS=Homo sapiens GN=GALNT1 PE=1 SV=1		20.79
Q10567	AP1B1_HUMAN AP-1 complex subunit beta-1 OS=Homo sapiens GN=AP1B1 PE=1 SV=2		696.58
Q10567-2	AP1B1_HUMAN Isoform B of AP-1 complex subunit beta-1 OS=Homo sapiens GN=AP1B1		696.58
Q10567-3	AP1B1_HUMAN Isoform C of AP-1 complex subunit beta-1 OS=Homo sapiens GN=AP1B1		696.58
Q10567-3;Q10567;Q1056	3|AP1B1_HUMAN Isoform C of AP-1 complex subunit beta-1 OS=Homo sapiens GN=AP1B1;>sp|Q10567|AP1B1_HUMAN AP-1 complex	0	
Q10567;Q10567-2;Q1056	AP1B1_HUMAN AP-1 complex subunit beta-1 OS=Homo sapiens GN=AP1B1 PE=1 SV=2;>sp|Q10567-2|AP1B1_HUMAN Isoform B of A	5.17E-118	
Q10570	CPSF1_HUMAN Cleavage and polyadenylation specificity factor subunit 1 OS=Homo sapiens GN=CPSF1 PE=1 SV=2		31.57
Q10588	BST1_HUMAN ADP-ribosyl cyclase 2 OS=Homo sapiens GN=BST1 PE=1 SV=2		
Q10589	BST2_HUMAN Bone marrow stromal antigen 2 OS=Homo sapiens GN=BST2 PE=1 SV=1		150.94
Q10713	MPPA_HUMAN Mitochondrial-processing peptidase subunit alpha OS=Homo sapiens GN=PMPCA PE=1 SV=2		33.59

Q12756	KIF1A_HUMAN Kinesin-like protein KIF1A OS=Homo sapiens GN=KIF1A PE=2 SV=2		
Q12756-2	KIF1A_HUMAN Isoform 2 of Kinesin-like protein KIF1A OS=Homo sapiens GN=KIF1A		
Q12765	SCRN1_HUMAN Secernin-1 OS=Homo sapiens GN=SCRN1 PE=1 SV=2	1.91E-11	
Q12768	STRUM_HUMAN WASH complex subunit strumpellin OS=Homo sapiens GN=KIAA0196 PE=1 SV=1	1.26E-11	
Q12769	NU160_HUMAN Nuclear pore complex protein Nup160 OS=Homo sapiens GN=NUP160 PE=1 SV=3		
Q12769-3	NU160_HUMAN Isoform 3 of Nuclear pore complex protein Nup160 OS=Homo sapiens GN=NUP160		
Q12769;C9JIP0	NU160_HUMAN Nuclear pore complex protein Nup160 OS=Homo sapiens GN=NUP160 PE=1 SV=3;>tr|C9JIP0|C9JIP0_HUMAN Unchar	2.27E-07	
Q12788	TBL3_HUMAN Transducin beta-like protein 3 OS=Homo sapiens GN=TBL3 PE=1 SV=2		
Q12789	TF3C1_HUMAN General transcription factor 3C polypeptide 1 OS=Homo sapiens GN=GTF3C1 PE=1 SV=4		
Q12789-3	TF3C1_HUMAN Isoform 2 of General transcription factor 3C polypeptide 1 OS=Homo sapiens GN=GTF3C1		
Q12789;Q12789-3	TF3C1_HUMAN General transcription factor 3C polypeptide 1 OS=Homo sapiens GN=GTF3C1 PE=1 SV=4;>sp|Q12789-3|TF3C1_HUM/	2.19E-22	
Q12791	KCMA1_HUMAN Calcium-activated potassium channel subunit alpha-1 OS=Homo sapiens GN=KCNMA1 PE=1 SV=2		
Q12791-2	KCMA1_HUMAN Isoform 2 of Calcium-activated potassium channel subunit alpha-1 OS=Homo sapiens GN=KCNMA1		
Q12791-3	KCMA1_HUMAN Isoform 3 of Calcium-activated potassium channel subunit alpha-1 OS=Homo sapiens GN=KCNMA1		
Q12791-4	KCMA1_HUMAN Isoform 4 of Calcium-activated potassium channel subunit alpha-1 OS=Homo sapiens GN=KCNMA1		
Q12791-5	KCMA1_HUMAN Isoform 5 of Calcium-activated potassium channel subunit alpha-1 OS=Homo sapiens GN=KCNMA1		
Q12791-7	KCMA1_HUMAN Isoform 7 of Calcium-activated potassium channel subunit alpha-1 OS=Homo sapiens GN=KCNMA1		
Q12792	TWF1_HUMAN Twinfilin-1 OS=Homo sapiens GN=TWF1 PE=1 SV=3		18.97
Q12792-3	TWF1_HUMAN Isoform 3 of Twinfilin-1 OS=Homo sapiens GN=TWF1		18.97
Q12792-4	TWF1_HUMAN Isoform 4 of Twinfilin-1 OS=Homo sapiens GN=TWF1		18.97
Q12792;Q12792-3;Q1279	TWF1_HUMAN Twinfilin-1 OS=Homo sapiens GN=TWF1 PE=1 SV=3;>sp|Q12792-3|TWF1_HUMAN Isoform 3 of Twinfilin-1 OS=Homo s	1.85E-81	
Q12797	ASPH_HUMAN Aspartyl/asparaginyl beta-hydroxylase OS=Homo sapiens GN=ASPH PE=1 SV=3		
Q12797-2	ASPH_HUMAN Isoform Junctate of Aspartyl/asparaginyl beta-hydroxylase OS=Homo sapiens GN=ASPH		
Q12797-3	ASPH_HUMAN Isoform Junctin-1 of Aspartyl/asparaginyl beta-hydroxylase OS=Homo sapiens GN=ASPH		41.77
Q12797-4	ASPH_HUMAN Isoform Junctin-2 of Aspartyl/asparaginyl beta-hydroxylase OS=Homo sapiens GN=ASPH		41.77
Q12797;B4E2K4	ASPH_HUMAN Aspartyl/asparaginyl beta-hydroxylase OS=Homo sapiens GN=ASPH PE=1 SV=3;>tr|B4E2K4|B4E2K4_HUMAN cDNA FL	3.97E-104	
Q12799	TCP10_HUMAN T-complex protein 10A homolog OS=Homo sapiens GN=TCP10 PE=2 SV=3		35.33
Q12799-2	TCP10_HUMAN Isoform 2 of T-complex protein 10A homolog OS=Homo sapiens GN=TCP10		35.33
Q12800;Q12800-4;Q1280	TFCP2_HUMAN Alpha-globin transcription factor CP2 OS=Homo sapiens GN=TFCP2 PE=1 SV=2;>sp|Q12800-4|TFCP2_HUMAN Isoforn	2.07E-92	
Q12802	AKP13_HUMAN A-kinase anchor protein 13 OS=Homo sapiens GN=AKAP13 PE=1 SV=2		
Q12802-2	AKP13_HUMAN Isoform 2 of A-kinase anchor protein 13 OS=Homo sapiens GN=AKAP13		
Q12802-3	AKP13_HUMAN Isoform 3 of A-kinase anchor protein 13 OS=Homo sapiens GN=AKAP13		
Q12802-4	AKP13_HUMAN Isoform 4 of A-kinase anchor protein 13 OS=Homo sapiens GN=AKAP13		
Q12802-6	AKP13_HUMAN Isoform 6 of A-kinase anchor protein 13 OS=Homo sapiens GN=AKAP13		
Q12802-7	AKP13_HUMAN Isoform 7 of A-kinase anchor protein 13 OS=Homo sapiens GN=AKAP13		
Q12805;Q12805-3;Q1280	FBLN3_HUMAN EGF-containing fibulin-like extracellular matrix protein 1 OS=Homo sapiens GN=EFEMP1 PE=1 SV=2;>sp|Q12805-3|FI	1.97E-72	
Q12816	TROP_HUMAN Trophinin OS=Homo sapiens GN=TRO PE=1 SV=3		21.92
Q12824	SNF5_HUMAN SWI/SNF-related matrix-associated actin-dependent regulator of chromatin subfamily B member 1 OS=Homo sapiens GN=SMARCB1 PE=1 SV=2		
Q12824-2	SNF5_HUMAN Isoform B of SWI/SNF-related matrix-associated actin-dependent regulator of chromatin subfamily B member 1 OS=Homo sapiens GN=SMARCB1		
Q12824;Q12824-2	SNF5_HUMAN SWI/SNF-related matrix-associated actin-dependent regulator of chromatin subfamily B member 1 OS=Homo sapiens G	2.39E-11	
Q12829	RB40B_HUMAN Ras-related protein Rab-40B OS=Homo sapiens GN=RAB40B PE=2 SV=1		
Q12830	BPTF_HUMAN Nucleosome-remodeling factor subunit BPTF OS=Homo sapiens GN=BPTF PE=1 SV=3		
Q12830-2	BPTF_HUMAN Isoform 2 of Nucleosome-remodeling factor subunit BPTF OS=Homo sapiens GN=BPTF		
Q12830-4	BPTF_HUMAN Isoform 4 of Nucleosome-remodeling factor subunit BPTF OS=Homo sapiens GN=BPTF		
Q12830;Q12830-2;Q1283	BPTF_HUMAN Nucleosome-remodeling factor subunit BPTF OS=Homo sapiens GN=BPTF PE=1 SV=3;>sp|Q12830-2|BPTF_HUMAN Isof	4.10E-24	
Q12834	CDC20_HUMAN Cell division cycle protein 20 homolog OS=Homo sapiens GN=CDC20 PE=1 SV=2	1.21E-05	
Q12840	KIF5A_HUMAN Kinesin heavy chain isoform 5A OS=Homo sapiens GN=KIF5A PE=1 SV=2		39.76
Q12841	FSTL1_HUMAN Follistatin-related protein 1 OS=Homo sapiens GN=FSTL1 PE=1 SV=1		27.56
Q12846	STX4_HUMAN Syntaxin-4 OS=Homo sapiens GN=STX4 PE=1 SV=2		112.8
Q12849	GRSF1_HUMAN G-rich sequence factor 1 OS=Homo sapiens GN=GRSF1 PE=1 SV=3		
Q12857	NFIA_HUMAN Nuclear factor 1 A-type OS=Homo sapiens GN=NFIA PE=1 SV=2		
Q12857-2	NFIA_HUMAN Isoform 2 of Nuclear factor 1 A-type OS=Homo sapiens GN=NFIA		
Q12873	CHD3_HUMAN Chromodomain-helicase-DNA-binding protein 3 OS=Homo sapiens GN=CHD3 PE=1 SV=3		687.51
Q12873-2	CHD3_HUMAN Isoform 2 of Chromodomain-helicase-DNA-binding protein 3 OS=Homo sapiens GN=CHD3		687.51
Q12873;D3DTQ9;Q12873-	CHD3_HUMAN Chromodomain-helicase-DNA-binding protein 3 OS=Homo sapiens GN=CHD3 PE=1 SV=3;>tr|D3DTQ9|D3DTQ9_HUMA	4.02E-16	
Q12874	SF3A3_HUMAN Splicing factor 3A subunit 3 OS=Homo sapiens GN=SF3A3 PE=1 SV=1		295.71
Q12882	DPYD_HUMAN Dihydropyrimidine dehydrogenase [NADP+] OS=Homo sapiens GN=DPYD PE=1 SV=2		
Q12884	SEPR_HUMAN Seprase OS=Homo sapiens GN=FAP PE=1 SV=5	7.99E-06	
Q12888	TP53B_HUMAN Tumor suppressor p53-binding protein 1 OS=Homo sapiens GN=TP53BP1 PE=1 SV=2		
Q12888-2	TP53B_HUMAN Isoform 2 of Tumor suppressor p53-binding protein 1 OS=Homo sapiens GN=TP53BP1		
Q12888-2;Q12888	2|TP53B_HUMAN Isoform 2 of Tumor suppressor p53-binding protein 1 OS=Homo sapiens GN=TP53BP1;>sp|Q12888|TP53B_HUMAN	7.65E-06	

<table>
<tr><td>Q12904</td><td>AIMP1_HUMAN Aminoacyl tRNA synthase complex-interacting multifunctional protein 1 OS=Homo sapiens GN=AIMP1 PE=1 SV=2</td><td></td><td>621.48</td></tr>
<tr><td>Q12905</td><td>ILF2_HUMAN Interleukin enhancer-binding factor 2 OS=Homo sapiens GN=ILF2 PE=1 SV=2</td><td></td><td>650.11</td></tr>
<tr><td>Q12906</td><td>ILF3_HUMAN Interleukin enhancer-binding factor 3 OS=Homo sapiens GN=ILF3 PE=1 SV=3</td><td></td><td>1082.52</td></tr>
<tr><td>Q12906-2</td><td>ILF3_HUMAN Isoform 2 of Interleukin enhancer-binding factor 3 OS=Homo sapiens GN=ILF3</td><td></td><td>1082.52</td></tr>
<tr><td>Q12906-3</td><td>ILF3_HUMAN Isoform 3 of Interleukin enhancer-binding factor 3 OS=Homo sapiens GN=ILF3</td><td></td><td>1082.52</td></tr>
<tr><td>Q12906-4</td><td>ILF3_HUMAN Isoform 4 of Interleukin enhancer-binding factor 3 OS=Homo sapiens GN=ILF3</td><td></td><td>1082.52</td></tr>
<tr><td>Q12906-5</td><td>ILF3_HUMAN Isoform 5 of Interleukin enhancer-binding factor 3 OS=Homo sapiens GN=ILF3</td><td></td><td>1082.52</td></tr>
<tr><td>Q12906-6</td><td>ILF3_HUMAN Isoform 6 of Interleukin enhancer-binding factor 3 OS=Homo sapiens GN=ILF3</td><td></td><td>1082.52</td></tr>
<tr><td>Q12906-6;Q12906-4</td><td>6|ILF3_HUMAN Isoform 6 of Interleukin enhancer-binding factor 3 OS=Homo sapiens GN=ILF3;>sp|Q12906-4|ILF3_HUMAN Isoform 4</td><td>1.82E-187</td><td></td></tr>
<tr><td>Q12906;C9JFV5;Q12906-</td><td>ILF3_HUMAN Interleukin enhancer-binding factor 3 OS=Homo sapiens GN=ILF3 PE=1 SV=3;>tr|C9JFV5|C9JFV5_HUMAN Uncharacteri</td><td>2.31E-184</td><td></td></tr>
<tr><td>Q12907</td><td>LMAN2_HUMAN Vesicular integral-membrane protein VIP36 OS=Homo sapiens GN=LMAN2 PE=1 SV=1</td><td></td><td>128.08</td></tr>
<tr><td>Q12912</td><td>LRMP_HUMAN Lymphoid-restricted membrane protein OS=Homo sapiens GN=LRMP PE=1 SV=3</td><td></td><td>65.16</td></tr>
<tr><td>Q12912-2</td><td>LRMP_HUMAN Isoform 2 of Lymphoid-restricted membrane protein OS=Homo sapiens GN=LRMP</td><td></td><td>65.16</td></tr>
<tr><td>Q12912;Q12912-2</td><td>LRMP_HUMAN Lymphoid-restricted membrane protein OS=Homo sapiens GN=LRMP PE=1 SV=3;>sp|Q12912-2|LRMP_HUMAN Isoform</td><td>1.01E-103</td><td></td></tr>
<tr><td>Q12923-4;Q12923;Q1292</td><td>4|PTN13_HUMAN Isoform 4 of Tyrosine-protein phosphatase non-receptor type 13 OS=Homo sapiens GN=PTPN13;>sp|Q12923|PTN1</td><td>1.51E-05</td><td></td></tr>
<tr><td>Q12926</td><td>ELAV2_HUMAN ELAV-like protein 2 OS=Homo sapiens GN=ELAVL2 PE=1 SV=2</td><td></td><td></td></tr>
<tr><td>Q12926-2</td><td>ELAV2_HUMAN Isoform 2 of ELAV-like protein 2 OS=Homo sapiens GN=ELAVL2</td><td></td><td></td></tr>
<tr><td>Q12929</td><td>EPS8_HUMAN Epidermal growth factor receptor kinase substrate 8 OS=Homo sapiens GN=EPS8 PE=1 SV=1</td><td></td><td></td></tr>
<tr><td>Q12931</td><td>TRAP1_HUMAN Heat shock protein 75 kDa, mitochondrial OS=Homo sapiens GN=TRAP1 PE=1 SV=3</td><td></td><td>541.98</td></tr>
<tr><td>Q12933;Q12933-3;Q1293</td><td>TRAF2_HUMAN TNF receptor-associated factor 2 OS=Homo sapiens GN=TRAF2 PE=1 SV=2;>sp|Q12933-3|TRAF2_HUMAN Isoform 3</td><td>5.98E-08</td><td></td></tr>
<tr><td>Q12955</td><td>ANK3_HUMAN Ankyrin-3 OS=Homo sapiens GN=ANK3 PE=1 SV=3</td><td></td><td></td></tr>
<tr><td>Q12959</td><td>DLG1_HUMAN Disks large homolog 1 OS=Homo sapiens GN=DLG1 PE=1 SV=2</td><td></td><td>145.84</td></tr>
<tr><td>Q12959-2</td><td>DLG1_HUMAN Isoform 2 of Disks large homolog 1 OS=Homo sapiens GN=DLG1</td><td></td><td>145.84</td></tr>
<tr><td>Q12959-2;Q12959-7;Q12</td><td>2|DLG1_HUMAN Isoform 2 of Disks large homolog 1 OS=Homo sapiens GN=DLG1;>sp|Q12959-7|DLG1_HUMAN Isoform 7 of Disks la</td><td>5.03E-95</td><td></td></tr>
<tr><td>Q12959-3</td><td>DLG1_HUMAN Isoform 3 of Disks large homolog 1 OS=Homo sapiens GN=DLG1</td><td></td><td>145.84</td></tr>
<tr><td>Q12959-4</td><td>DLG1_HUMAN Isoform 4 of Disks large homolog 1 OS=Homo sapiens GN=DLG1</td><td></td><td>145.84</td></tr>
<tr><td>Q12959-5</td><td>DLG1_HUMAN Isoform 5 of Disks large homolog 1 OS=Homo sapiens GN=DLG1</td><td></td><td>145.84</td></tr>
<tr><td>Q12959-6</td><td>DLG1_HUMAN Isoform 6 of Disks large homolog 1 OS=Homo sapiens GN=DLG1</td><td></td><td>145.84</td></tr>
<tr><td>Q12959-7</td><td>DLG1_HUMAN Isoform 7 of Disks large homolog 1 OS=Homo sapiens GN=DLG1</td><td></td><td>145.84</td></tr>
<tr><td>Q12965</td><td>MYO1E_HUMAN Myosin-Ie OS=Homo sapiens GN=MYO1E PE=1 SV=2</td><td></td><td></td></tr>
<tr><td>Q12968</td><td>NFAC3_HUMAN Nuclear factor of activated T-cells, cytoplasmic 3 OS=Homo sapiens GN=NFATC3 PE=1 SV=1</td><td></td><td>21.61</td></tr>
<tr><td>Q12968-2</td><td>NFAC3_HUMAN Isoform 2 of Nuclear factor of activated T-cells, cytoplasmic 3 OS=Homo sapiens GN=NFATC3</td><td></td><td>21.61</td></tr>
<tr><td>Q12968-3</td><td>NFAC3_HUMAN Isoform 3 of Nuclear factor of activated T-cells, cytoplasmic 3 OS=Homo sapiens GN=NFATC3</td><td></td><td>21.61</td></tr>
<tr><td>Q12968-4</td><td>NFAC3_HUMAN Isoform 4 of Nuclear factor of activated T-cells, cytoplasmic 3 OS=Homo sapiens GN=NFATC3</td><td></td><td>21.61</td></tr>
<tr><td>Q12968-5</td><td>NFAC3_HUMAN Isoform 5 of Nuclear factor of activated T-cells, cytoplasmic 3 OS=Homo sapiens GN=NFATC3</td><td></td><td>21.61</td></tr>
<tr><td>Q12968-6</td><td>NFAC3_HUMAN Isoform 6 of Nuclear factor of activated T-cells, cytoplasmic 3 OS=Homo sapiens GN=NFATC3</td><td></td><td>21.61</td></tr>
<tr><td>Q12972</td><td>PP1R8_HUMAN Nuclear inhibitor of protein phosphatase 1 OS=Homo sapiens GN=PPP1R8 PE=1 SV=2</td><td></td><td>74.89</td></tr>
<tr><td>Q12972-2</td><td>PP1R8_HUMAN Isoform Beta of Nuclear inhibitor of protein phosphatase 1 OS=Homo sapiens GN=PPP1R8</td><td></td><td>74.62</td></tr>
<tr><td>Q12972;Q12972-2</td><td>PP1R8_HUMAN Nuclear inhibitor of protein phosphatase 1 OS=Homo sapiens GN=PPP1R8 PE=1 SV=2;>sp|Q12972-2|PP1R8_HUMAN</td><td>6.43E-05</td><td></td></tr>
<tr><td>Q12974</td><td>TP4A2_HUMAN Protein tyrosine phosphatase type IVA 2 OS=Homo sapiens GN=PTP4A2 PE=1 SV=1</td><td></td><td>40.26</td></tr>
<tr><td>Q12979</td><td>ABR_HUMAN Active breakpoint cluster region-related protein OS=Homo sapiens GN=ABR PE=1 SV=1</td><td></td><td></td></tr>
<tr><td>Q12979-2</td><td>ABR_HUMAN Isoform Short of Active breakpoint cluster region-related protein OS=Homo sapiens GN=ABR</td><td></td><td></td></tr>
<tr><td>Q12979-2;Q12979</td><td>2|ABR_HUMAN Isoform Short of Active breakpoint cluster region-related protein OS=Homo sapiens GN=ABR;>sp|Q12979|ABR_HUMA</td><td>1.60E-25</td><td></td></tr>
<tr><td>Q12980</td><td>NPRL3_HUMAN Nitrogen permease regulator 3-like protein OS=Homo sapiens GN=NPRL3 PE=1 SV=1</td><td></td><td>22.37</td></tr>
<tr><td>Q12983</td><td>BNIP3_HUMAN BCL2/adenovirus E1B 19 kDa protein-interacting protein 3 OS=Homo sapiens GN=BNIP3 PE=1 SV=2</td><td></td><td></td></tr>
<tr><td>Q12996</td><td>CSTF3_HUMAN Cleavage stimulation factor subunit 3 OS=Homo sapiens GN=CSTF3 PE=1 SV=1</td><td></td><td>472.93</td></tr>
<tr><td>Q13011</td><td>ECH1_HUMAN Delta(3,5)-Delta(2,4)-dienoyl-CoA isomerase, mitochondrial OS=Homo sapiens GN=ECH1 PE=1 SV=2</td><td></td><td>209.55</td></tr>
<tr><td>Q13017;Q13017-2</td><td>RHG05_HUMAN Rho GTPase-activating protein 5 OS=Homo sapiens GN=ARHGAP5 PE=1 SV=2;>sp|Q13017-2|RHG05_HUMAN Isoform</td><td>5.08E-15</td><td></td></tr>
<tr><td>Q13029</td><td>PRDM2_HUMAN PR domain zinc finger protein 2 OS=Homo sapiens GN=PRDM2 PE=1 SV=3</td><td></td><td>33.62</td></tr>
<tr><td>Q13029-2</td><td>PRDM2_HUMAN Isoform 2 of PR domain zinc finger protein 2 OS=Homo sapiens GN=PRDM2</td><td></td><td>33.62</td></tr>
<tr><td>Q13029-3</td><td>PRDM2_HUMAN Isoform 3 of PR domain zinc finger protein 2 OS=Homo sapiens GN=PRDM2</td><td></td><td>4.79</td></tr>
<tr><td>Q13033;Q13033-2</td><td>STRN3_HUMAN Striatin-3 OS=Homo sapiens GN=STRN3 PE=1 SV=3;>sp|Q13033-2|STRN3_HUMAN Isoform Alpha of Striatin-3 OS=</td><td>1.68E-10</td><td></td></tr>
<tr><td>Q13042</td><td>CDC16_HUMAN Cell division cycle protein 16 homolog OS=Homo sapiens GN=CDC16 PE=1 SV=2</td><td></td><td></td></tr>
<tr><td>Q13042-2</td><td>CDC16_HUMAN Isoform 2 of Cell division cycle protein 16 homolog OS=Homo sapiens GN=CDC16</td><td></td><td></td></tr>
<tr><td>Q13042-3</td><td>CDC16_HUMAN Isoform 3 of Cell division cycle protein 16 homolog OS=Homo sapiens GN=CDC16</td><td></td><td></td></tr>
<tr><td>Q13043;Q13043-2</td><td>STK4_HUMAN Serine/threonine-protein kinase 4 OS=Homo sapiens GN=STK4 PE=1 SV=2;>sp|Q13043-2|STK4_HUMAN Isoform 2 of</td><td>6.06E-51</td><td></td></tr>
<tr><td>Q13045</td><td>FLII_HUMAN Protein flightless-1 homolog OS=Homo sapiens GN=FLII PE=1 SV=2</td><td>7.89E-34</td><td></td></tr>
<tr><td>Q13049</td><td>TRI32_HUMAN E3 ubiquitin-protein ligase TRIM32 OS=Homo sapiens GN=TRIM32 PE=1 SV=2</td><td>6.64E-07</td><td></td></tr>
<tr><td>Q13057</td><td>COASY_HUMAN Bifunctional coenzyme A synthase OS=Homo sapiens GN=COASY PE=1 SV=4</td><td></td><td></td></tr>
<tr><td>Q13057-2</td><td>COASY_HUMAN Isoform 2 of Bifunctional coenzyme A synthase OS=Homo sapiens GN=COASY</td><td></td><td></td></tr>
<tr><td>Q13057-2;Q13057</td><td>2|COASY_HUMAN Isoform 2 of Bifunctional coenzyme A synthase OS=Homo sapiens GN=COASY;>sp|Q13057|COASY_HUMAN Bifuncti</td><td>6.86E-34</td><td></td></tr>
</table>

Q13084	RM28_HUMAN 39S ribosomal protein L28, mitochondrial OS=Homo sapiens GN=MRPL28 PE=1 SV=4		138.84
Q13085	ACACA_HUMAN Acetyl-CoA carboxylase 1 OS=Homo sapiens GN=ACACA PE=1 SV=2		
Q13085-2	ACACA_HUMAN Isoform 2 of Acetyl-CoA carboxylase 1 OS=Homo sapiens GN=ACACA		
Q13085-3	ACACA_HUMAN Isoform 3 of Acetyl-CoA carboxylase 1 OS=Homo sapiens GN=ACACA		
Q13085-4	ACACA_HUMAN Isoform 4 of Acetyl-CoA carboxylase 1 OS=Homo sapiens GN=ACACA		
Q13085-4;Q13085;Q1308	4|ACACA_HUMAN Isoform 4 of Acetyl-CoA carboxylase 1 OS=Homo sapiens GN=ACACA;>sp|Q13085|ACACA_HUMAN Acetyl-CoA carb	3.19E-31	
Q13087;Q13087-2	PDIA2_HUMAN Protein disulfide-isomerase A2 OS=Homo sapiens GN=PDIA2 PE=1 SV=2;>sp|Q13087-2|PDIA2_HUMAN Isoform 2 of I	6.93E-29	
Q13098	CSN1_HUMAN COP9 signalosome complex subunit 1 OS=Homo sapiens GN=GPS1 PE=1 SV=4		
Q13098-5	CSN1_HUMAN Isoform 4 of COP9 signalosome complex subunit 1 OS=Homo sapiens GN=GPS1		
Q13098-6	CSN1_HUMAN Isoform 3 of COP9 signalosome complex subunit 1 OS=Homo sapiens GN=GPS1		
Q13098-6;Q13098-7;Q13(	6|CSN1_HUMAN Isoform 3 of COP9 signalosome complex subunit 1 OS=Homo sapiens GN=GPS1;>sp|Q13098-7|CSN1_HUMAN Isofor	7.79E-10	
Q13098-7	CSN1_HUMAN Isoform 2 of COP9 signalosome complex subunit 1 OS=Homo sapiens GN=GPS1		
Q13098-7;Q13098;Q1309	7|CSN1_HUMAN Isoform 2 of COP9 signalosome complex subunit 1 OS=Homo sapiens GN=GPS1;>sp|Q13098|CSN1_HUMAN COP9 si	2.47E-15	
Q13107;Q13107-2	UBP4_HUMAN Ubiquitin carboxyl-terminal hydrolase 4 OS=Homo sapiens GN=USP4 PE=1 SV=3;>sp|Q13107-2|UBP4_HUMAN Isoform	1.39E-07	
Q13123	RED_HUMAN Protein Red OS=Homo sapiens GN=IK PE=1 SV=3		72.9
Q13126	MTAP_HUMAN S-methyl-5-thioadenosine phosphorylase OS=Homo sapiens GN=MTAP PE=1 SV=2	1.25E-75	
Q13131-2;Q13131	2|AAPK1_HUMAN Isoform 2 of 5-AMP-activated protein kinase catalytic subunit alpha-1 OS=Homo sapiens GN=PRKAA1;>sp|Q13131|,	3.46E-40	
Q13136	LIPA1_HUMAN Liprin-alpha-1 OS=Homo sapiens GN=PPFIA1 PE=1 SV=1		59.43
Q13136-2	LIPA1_HUMAN Isoform 2 of Liprin-alpha-1 OS=Homo sapiens GN=PPFIA1		59.43
Q13136;Q13136-2	LIPA1_HUMAN Liprin-alpha-1 OS=Homo sapiens GN=PPFIA1 PE=1 SV=1;>sp|Q13136-2|LIPA1_HUMAN Isoform 2 of Liprin-alpha-1 OS	0.00013768	
Q13137	CACO2_HUMAN Calcium-binding and coiled-coil domain-containing protein 2 OS=Homo sapiens GN=CALCOCO2 PE=1 SV=1	3.67E-06	
Q13144	EI2BE_HUMAN Translation initiation factor eIF-2B subunit epsilon OS=Homo sapiens GN=EIF2B5 PE=1 SV=3		61.81
Q13148	TADBP_HUMAN TAR DNA-binding protein 43 OS=Homo sapiens GN=TARDBP PE=1 SV=1		262.56
Q13148-2	TADBP_HUMAN Isoform 2 of TAR DNA-binding protein 43 OS=Homo sapiens GN=TARDBP		262.56
Q13148-2;E2PU12;Q1314	2|TADBP_HUMAN Isoform 2 of TAR DNA-binding protein 43 OS=Homo sapiens GN=TARDBP;>tr|E2PU12|E2PU12_HUMAN TAR DNA bir	1.76E-95	
Q13148-3	TADBP_HUMAN Isoform 3 of TAR DNA-binding protein 43 OS=Homo sapiens GN=TARDBP		74.33
Q13151	ROA0_HUMAN Heterogeneous nuclear ribonucleoprotein A0 OS=Homo sapiens GN=HNRNPA0 PE=1 SV=1		390.62
Q13153-2;Q13153	2|PAK1_HUMAN Isoform 2 of Serine/threonine-protein kinase PAK 1 OS=Homo sapiens GN=PAK1;>sp|Q13153|PAK1_HUMAN Serine/t	3.31E-17	
Q13155	AIMP2_HUMAN Aminoacyl tRNA synthase complex-interacting multifunctional protein 2 OS=Homo sapiens GN=AIMP2 PE=1 SV=2		192.96
Q13158	FADD_HUMAN Protein FADD OS=Homo sapiens GN=FADD PE=1 SV=1	6.29E-62	
Q13162	PRDX4_HUMAN Peroxiredoxin-4 OS=Homo sapiens GN=PRDX4 PE=1 SV=1		785.69
Q13164	MK07_HUMAN Mitogen-activated protein kinase 7 OS=Homo sapiens GN=MAPK7 PE=1 SV=2		
Q13164-2	MK07_HUMAN Isoform 2 of Mitogen-activated protein kinase 7 OS=Homo sapiens GN=MAPK7		
Q13164-3	MK07_HUMAN Isoform 3 of Mitogen-activated protein kinase 7 OS=Homo sapiens GN=MAPK7		
Q13177	PAK2_HUMAN Serine/threonine-protein kinase PAK 2 OS=Homo sapiens GN=PAK2 PE=1 SV=3	1.20E-156	
Q13185	CBX3_HUMAN Chromobox protein homolog 3 OS=Homo sapiens GN=CBX3 PE=1 SV=4		806.67
Q13188	STK3_HUMAN Serine/threonine-protein kinase 3 OS=Homo sapiens GN=STK3 PE=1 SV=2	6.58E-36	
Q13190	STX5_HUMAN Syntaxin-5 OS=Homo sapiens GN=STX5 PE=1 SV=2		108.11
Q13190-2	STX5_HUMAN Isoform 2 of Syntaxin-5 OS=Homo sapiens GN=STX5		108.11
Q13190-3	STX5_HUMAN Isoform 3 of Syntaxin-5 OS=Homo sapiens GN=STX5		108.11
Q13190;Q13190-2;Q1319	STX5_HUMAN Syntaxin-5 OS=Homo sapiens GN=STX5 PE=1 SV=2;>sp|Q13190-2|STX5_HUMAN Isoform 2 of Syntaxin-5 OS=Homo s	4.46E-14	
Q13200	PSMD2_HUMAN 26S proteasome non-ATPase regulatory subunit 2 OS=Homo sapiens GN=PSMD2 PE=1 SV=3		634.07
Q13206	DDX10_HUMAN Probable ATP-dependent RNA helicase DDX10 OS=Homo sapiens GN=DDX10 PE=1 SV=2		
Q13216	ERCC8_HUMAN DNA excision repair protein ERCC-8 OS=Homo sapiens GN=ERCC8 PE=1 SV=1		
Q13216-2	ERCC8_HUMAN Isoform 2 of DNA excision repair protein ERCC-8 OS=Homo sapiens GN=ERCC8		
Q13217	DNJC3_HUMAN DnaJ homolog subfamily C member 3 OS=Homo sapiens GN=DNAJC3 PE=1 SV=1		160.47
Q13228	SBP1_HUMAN Selenium-binding protein 1 OS=Homo sapiens GN=SELENBP1 PE=1 SV=2		
Q13228-2	SBP1_HUMAN Isoform 2 of Selenium-binding protein 1 OS=Homo sapiens GN=SELENBP1		
Q13228;B4E1F3;Q13228-:	SBP1_HUMAN Selenium-binding protein 1 OS=Homo sapiens GN=SELENBP1 PE=1 SV=2;>tr|B4E1F3|B4E1F3_HUMAN cDNA FLJ61035	0	
Q13232	NDK3_HUMAN Nucleoside diphosphate kinase 3 OS=Homo sapiens GN=NME3 PE=1 SV=2	3.91E-53	
Q13239	SLAP1_HUMAN Src-like-adapter OS=Homo sapiens GN=SLA PE=1 SV=3		
Q13242	SRSF9_HUMAN Serine/arginine-rich splicing factor 9 OS=Homo sapiens GN=SRSF9 PE=1 SV=1		476.52
Q13243	SRSF5_HUMAN Serine/arginine-rich splicing factor 5 OS=Homo sapiens GN=SRSF5 PE=1 SV=1		179.67
Q13243-2	SRSF5_HUMAN Isoform SRP40-2 of Serine/arginine-rich splicing factor 5 OS=Homo sapiens GN=SRSF5		96.14
Q13243-3	SRSF5_HUMAN Isoform SRP40-4 of Serine/arginine-rich splicing factor 5 OS=Homo sapiens GN=SRSF5		83.53
Q13243;Q13243-3	SRSF5_HUMAN Serine/arginine-rich splicing factor 5 OS=Homo sapiens GN=SRSF5 PE=1 SV=1;>sp|Q13243-3|SRSF5_HUMAN Isoforı	5.83E-24	
Q13247	SRSF6_HUMAN Serine/arginine-rich splicing factor 6 OS=Homo sapiens GN=SRSF6 PE=1 SV=2		69.99
Q13247-2	SRSF6_HUMAN Isoform SRP55-2 of Serine/arginine-rich splicing factor 6 OS=Homo sapiens GN=SRSF6		28.9
Q13247-3	SRSF6_HUMAN Isoform SRP55-3 of Serine/arginine-rich splicing factor 6 OS=Homo sapiens GN=SRSF6		69.99
Q13247;Q13247-3	SRSF6_HUMAN Serine/arginine-rich splicing factor 6 OS=Homo sapiens GN=SRSF6 PE=1 SV=2;>sp|Q13247-3|SRSF6_HUMAN Isoforı	7.14E-38	
Q13255	GRM1_HUMAN Metabotropic glutamate receptor 1 OS=Homo sapiens GN=GRM1 PE=1 SV=3		

Q13255-2	GRM1_HUMAN Isoform Beta of Metabotropic glutamate receptor 1 OS=Homo sapiens GN=GRM1		
Q13257	MD2L1_HUMAN Mitotic spindle assembly checkpoint protein MAD2A OS=Homo sapiens GN=MAD2L1 PE=1 SV=1		111.16
Q13263	TIF1B_HUMAN Transcription intermediary factor 1-beta OS=Homo sapiens GN=TRIM28 PE=1 SV=5		319.3
Q13263-2	TIF1B_HUMAN Isoform 2 of Transcription intermediary factor 1-beta OS=Homo sapiens GN=TRIM28		316.38
Q13263;Q13263-2	TIF1B_HUMAN Transcription intermediary factor 1-beta OS=Homo sapiens GN=TRIM28 PE=1 SV=5;>sp|Q13263-2|TIF1B_HUMAN Iso	0	
Q13268;Q13268-2	DHRS2_HUMAN Dehydrogenase/reductase SDR family member 2 OS=Homo sapiens GN=DHRS2 PE=1 SV=3;>sp|Q13268-2|DHRS2_H	2.77E-21	
Q13277	STX3_HUMAN Syntaxin-3 OS=Homo sapiens GN=STX3 PE=1 SV=3		88.18
Q13277-2	STX3_HUMAN Isoform B of Syntaxin-3 OS=Homo sapiens GN=STX3		88.18
Q13277;Q13277-2	STX3_HUMAN Syntaxin-3 OS=Homo sapiens GN=STX3 PE=1 SV=3;>sp|Q13277-2|STX3_HUMAN Isoform B of Syntaxin-3 OS=Homo	5.60E-13	
Q13283	G3BP1_HUMAN Ras GTPase-activating protein-binding protein 1 OS=Homo sapiens GN=G3BP1 PE=1 SV=1		923.75
Q13287	NMI_HUMAN N-myc-interactor OS=Homo sapiens GN=NMI PE=1 SV=2	8.68E-07	
Q13303	KCAB2_HUMAN Voltage-gated potassium channel subunit beta-2 OS=Homo sapiens GN=KCNAB2 PE=1 SV=2		
Q13303-2	KCAB2_HUMAN Isoform 2 of Voltage-gated potassium channel subunit beta-2 OS=Homo sapiens GN=KCNAB2		
Q13303-3	KCAB2_HUMAN Isoform 3 of Voltage-gated potassium channel subunit beta-2 OS=Homo sapiens GN=KCNAB2		
Q13303-3;Q13303;Q1330	3|KCAB2_HUMAN Isoform 3 of Voltage-gated potassium channel subunit beta-2 OS=Homo sapiens GN=KCNAB2;>sp|Q13303|KCAB2_	2.64E-15	
Q13308	PTK7_HUMAN Inactive tyrosine-protein kinase 7 OS=Homo sapiens GN=PTK7 PE=1 SV=2		143.56
Q13308-2	PTK7_HUMAN Isoform 2 of Inactive tyrosine-protein kinase 7 OS=Homo sapiens GN=PTK7		143.56
Q13308-3	PTK7_HUMAN Isoform 3 of Inactive tyrosine-protein kinase 7 OS=Homo sapiens GN=PTK7		134.76
Q13308-4	PTK7_HUMAN Isoform 4 of Inactive tyrosine-protein kinase 7 OS=Homo sapiens GN=PTK7		137.76
Q13308-5	PTK7_HUMAN Isoform 5 of Inactive tyrosine-protein kinase 7 OS=Homo sapiens GN=PTK7		104.05
Q13308;Q13308-2;Q1330	PTK7_HUMAN Inactive tyrosine-protein kinase 7 OS=Homo sapiens GN=PTK7 PE=1 SV=2;>sp|Q13308-2|PTK7_HUMAN Isoform 2 of I	6.72E-32	
Q13310	PABP4_HUMAN Polyadenylate-binding protein 4 OS=Homo sapiens GN=PABPC4 PE=1 SV=1		1343.92
Q13310-2	PABP4_HUMAN Isoform 2 of Polyadenylate-binding protein 4 OS=Homo sapiens GN=PABPC4		1374.28
Q13310-2;Q13310	2|PABP4_HUMAN Isoform 2 of Polyadenylate-binding protein 4 OS=Homo sapiens GN=PABPC4;>sp|Q13310|PABP4_HUMAN Polyadeny	0	
Q13310;Q13310-2	PABP4_HUMAN Polyadenylate-binding protein 4 OS=Homo sapiens GN=PABPC4 PE=1 SV=1;>sp|Q13310-2|PABP4_HUMAN Isoform 2	0	
Q13315	ATM_HUMAN Serine-protein kinase ATM OS=Homo sapiens GN=ATM PE=1 SV=3		188.5
Q13325	IFIT5_HUMAN Interferon-induced protein with tetratricopeptide repeats 5 OS=Homo sapiens GN=IFIT5 PE=1 SV=1		27.96
Q13330	MTA1_HUMAN Metastasis-associated protein MTA1 OS=Homo sapiens GN=MTA1 PE=1 SV=2		
Q13330-2	MTA1_HUMAN Isoform Short of Metastasis-associated protein MTA1 OS=Homo sapiens GN=MTA1		
Q13330;Q13330-2	MTA1_HUMAN Metastasis-associated protein MTA1 OS=Homo sapiens GN=MTA1 PE=1 SV=2;>sp|Q13330-2|MTA1_HUMAN Isoform Sh	2.98E-17	
Q13332	PTPRS_HUMAN Receptor-type tyrosine-protein phosphatase S OS=Homo sapiens GN=PTPRS PE=1 SV=3		
Q13332-2	PTPRS_HUMAN Isoform PTPS-MEA of Receptor-type tyrosine-protein phosphatase S OS=Homo sapiens GN=PTPRS		
Q13332-3	PTPRS_HUMAN Isoform PTPS-MEB of Receptor-type tyrosine-protein phosphatase S OS=Homo sapiens GN=PTPRS		
Q13332-4	PTPRS_HUMAN Isoform PTPS-MEC of Receptor-type tyrosine-protein phosphatase S OS=Homo sapiens GN=PTPRS		
Q13332-5	PTPRS_HUMAN Isoform PTPS-F4-7 of Receptor-type tyrosine-protein phosphatase S OS=Homo sapiens GN=PTPRS		
Q13332-6	PTPRS_HUMAN Isoform 2 of Receptor-type tyrosine-protein phosphatase S OS=Homo sapiens GN=PTPRS		
Q13332-7	PTPRS_HUMAN Isoform 3 of Receptor-type tyrosine-protein phosphatase S OS=Homo sapiens GN=PTPRS		
Q13342	LY10_HUMAN Nuclear body protein SP140 OS=Homo sapiens GN=SP140 PE=2 SV=2		0.25
Q13342-3	LY10_HUMAN Isoform Sp140 of Nuclear body protein SP140 OS=Homo sapiens GN=SP140		0.25
Q13347	EIF3I_HUMAN Eukaryotic translation initiation factor 3 subunit I OS=Homo sapiens GN=EIF3I PE=1 SV=1		494.28
Q13362	2A5G_HUMAN Serine/threonine-protein phosphatase 2A 56 kDa regulatory subunit gamma isoform OS=Homo sapiens GN=PPP2R5C PE=1 SV=3		25.65
Q13362-2	2A5G_HUMAN Isoform Gamma-1 of Serine/threonine-protein phosphatase 2A 56 kDa regulatory subunit gamma isoform OS=Homo sapiens GN=PPP		25.65
Q13362-3	2A5G_HUMAN Isoform Gamma-2 of Serine/threonine-protein phosphatase 2A 56 kDa regulatory subunit gamma isoform OS=Homo sapiens GN=PPP		25.65
Q13362;Q13362-3;Q1336	2A5G_HUMAN Serine/threonine-protein phosphatase 2A 56 kDa regulatory subunit gamma isoform OS=Homo sapiens GN=PPP2R5C P	8.81E-84	
Q13363	CTBP1_HUMAN C-terminal-binding protein 1 OS=Homo sapiens GN=CTBP1 PE=1 SV=2		147.73
Q13371;Q4VXB6;Q4VXB5	PHLP_HUMAN Phosducin-like protein OS=Homo sapiens GN=PDCL PE=1 SV=3;>tr|Q4VXB6|Q4VXB6_HUMAN Phosducin-like OS=Hom	1.96E-07	
Q13395	TARB1_HUMAN Probable methyltransferase TARBP1 OS=Homo sapiens GN=TARBP1 PE=1 SV=1		
Q13404	UB2V1_HUMAN Ubiquitin-conjugating enzyme E2 variant 1 OS=Homo sapiens GN=UBE2V1 PE=1 SV=2		206.5
Q13404-1	UB2V1_HUMAN Isoform 1 of Ubiquitin-conjugating enzyme E2 variant 1 OS=Homo sapiens GN=UBE2V1		206.5
Q13404-1;Q13404-2;Q13	1|UB2V1_HUMAN Isoform 1 of Ubiquitin-conjugating enzyme E2 variant 1 OS=Homo sapiens GN=UBE2V1;>sp|Q13404-2|UB2V1_HUN	1.70E-22	
Q13404-2	UB2V1_HUMAN Isoform 2 of Ubiquitin-conjugating enzyme E2 variant 1 OS=Homo sapiens GN=UBE2V1		206.5
Q13404-6	UB2V1_HUMAN Isoform 4 of Ubiquitin-conjugating enzyme E2 variant 1 OS=Homo sapiens GN=UBE2V1		92.87
Q13404-7	UB2V1_HUMAN Isoform 5 of Ubiquitin-conjugating enzyme E2 variant 1 OS=Homo sapiens GN=UBE2V1		206.5
Q13404;Q13404-1;Q1340	UB2V1_HUMAN Ubiquitin-conjugating enzyme E2 variant 1 OS=Homo sapiens GN=UBE2V1 PE=1 SV=2;>sp|Q13404-1|UB2V1_HUMAI	7.63E-137	
Q13405	RM49_HUMAN 39S ribosomal protein L49, mitochondrial OS=Homo sapiens GN=MRPL49 PE=1 SV=1		172.72
Q13409	DC1I2_HUMAN Cytoplasmic dynein 1 intermediate chain 2 OS=Homo sapiens GN=DYNC1I2 PE=1 SV=3		
Q13409-2	DC1I2_HUMAN Isoform 2B of Cytoplasmic dynein 1 intermediate chain 2 OS=Homo sapiens GN=DYNC1I2		
Q13409-3	DC1I2_HUMAN Isoform 2C of Cytoplasmic dynein 1 intermediate chain 2 OS=Homo sapiens GN=DYNC1I2		
Q13409-5	DC1I2_HUMAN Isoform 2E of Cytoplasmic dynein 1 intermediate chain 2 OS=Homo sapiens GN=DYNC1I2		
Q13409-6	DC1I2_HUMAN Isoform 2F of Cytoplasmic dynein 1 intermediate chain 2 OS=Homo sapiens GN=DYNC1I2		
Q13409;Q13409-5;Q1340	DC1I2_HUMAN Cytoplasmic dynein 1 intermediate chain 2 OS=Homo sapiens GN=DYNC1I2 PE=1 SV=3;>sp|Q13409-5|DC1I2_HUMAI	8.68E-64	

Q13416	ORC2_HUMAN Origin recognition complex subunit 2 OS=Homo sapiens GN=ORC2 PE=1 SV=2		42.81
Q13418	ILK_HUMAN Integrin-linked protein kinase OS=Homo sapiens GN=ILK PE=1 SV=2		181.89
Q13422;Q13422-7;Q1342	IKZF1_HUMAN DNA-binding protein Ikaros OS=Homo sapiens GN=IKZF1 PE=1 SV=1;>sp|Q13422-7|IKZF1_HUMAN Isoform Ik7 of DN	1.33E-16	
Q13423	NNTM_HUMAN NAD(P) transhydrogenase, mitochondrial OS=Homo sapiens GN=NNT PE=1 SV=3		83.99
Q13425	SNTB2_HUMAN Beta-2-syntrophin OS=Homo sapiens GN=SNTB2 PE=1 SV=1		63.76
Q13425-2	SNTB2_HUMAN Isoform 2 of Beta-2-syntrophin OS=Homo sapiens GN=SNTB2		60.37
Q13426;Q13426-2;Q1342	XRCC4_HUMAN DNA repair protein XRCC4 OS=Homo sapiens GN=XRCC4 PE=1 SV=2;>sp|Q13426-2|XRCC4_HUMAN Isoform 2 of DN	1.68E-06	
Q13427	PPIG_HUMAN Peptidyl-prolyl cis-trans isomerase G OS=Homo sapiens GN=PPIG PE=1 SV=2		21.75
Q13428	TCOF_HUMAN Treacle protein OS=Homo sapiens GN=TCOF1 PE=1 SV=3		1022.86
Q13428-2	TCOF_HUMAN Isoform 2 of Treacle protein OS=Homo sapiens GN=TCOF1		989.46
Q13428-3	TCOF_HUMAN Isoform 3 of Treacle protein OS=Homo sapiens GN=TCOF1		1029.1
Q13428-4	TCOF_HUMAN Isoform 4 of Treacle protein OS=Homo sapiens GN=TCOF1		1022.86
Q13428-4;Q13428-3;Q13	4|TCOF_HUMAN Isoform 4 of Treacle protein OS=Homo sapiens GN=TCOF1;>sp|Q13428-3|TCOF_HUMAN Isoform 3 of Treacle protein	2.31E-44	
Q13428-5	TCOF_HUMAN Isoform 5 of Treacle protein OS=Homo sapiens GN=TCOF1		551.65
Q13428-6	TCOF_HUMAN Isoform 6 of Treacle protein OS=Homo sapiens GN=TCOF1		927.79
Q13428-7	TCOF_HUMAN Isoform 7 of Treacle protein OS=Homo sapiens GN=TCOF1		934.03
Q13428-8	TCOF_HUMAN Isoform 8 of Treacle protein OS=Homo sapiens GN=TCOF1		995.7
Q13433;Q13433-2	S39A6_HUMAN Zinc transporter ZIP6 OS=Homo sapiens GN=SLC39A6 PE=1 SV=3;>sp|Q13433-2|S39A6_HUMAN Isoform 2 of Zinc tr	1.32E-31	
Q13435	SF3B2_HUMAN Splicing factor 3B subunit 2 OS=Homo sapiens GN=SF3B2 PE=1 SV=2		394.48
Q13439	GOGA4_HUMAN Golgin subfamily A member 4 OS=Homo sapiens GN=GOLGA4 PE=1 SV=1		
Q13439-3	GOGA4_HUMAN Isoform 3 of Golgin subfamily A member 4 OS=Homo sapiens GN=GOLGA4		
Q13439-4	GOGA4_HUMAN Isoform 4 of Golgin subfamily A member 4 OS=Homo sapiens GN=GOLGA4		
Q13439;Q13439-4;Q1343	GOGA4_HUMAN Golgin subfamily A member 4 OS=Homo sapiens GN=GOLGA4 PE=1 SV=1;>sp|Q13439-4|GOGA4_HUMAN Isoform 4	3.87E-40	
Q13442	HAP28_HUMAN 28 kDa heat- and acid-stable phosphoprotein OS=Homo sapiens GN=PDAP1 PE=1 SV=1		430.4
Q13443	ADAM9_HUMAN Disintegrin and metalloproteinase domain-containing protein 9 OS=Homo sapiens GN=ADAM9 PE=1 SV=1		110.67
Q13443-2	ADAM9_HUMAN Isoform 2 of Disintegrin and metalloproteinase domain-containing protein 9 OS=Homo sapiens GN=ADAM9		75.02
Q13443;C9J643;A0AVL1;C	ADAM9_HUMAN Disintegrin and metalloproteinase domain-containing protein 9 OS=Homo sapiens GN=ADAM9 PE=1 SV=1;>tr|C9J64	1.20E-10	
Q13445	TMED1_HUMAN Transmembrane emp24 domain-containing protein 1 OS=Homo sapiens GN=TMED1 PE=1 SV=1		252.07
Q13451	FKBP5_HUMAN Peptidyl-prolyl cis-trans isomerase FKBP5 OS=Homo sapiens GN=FKBP5 PE=1 SV=2		205.32
Q13454;Q13454-2	TUSC3_HUMAN Tumor suppressor candidate 3 OS=Homo sapiens GN=TUSC3 PE=2 SV=1;>sp|Q13454-2|TUSC3_HUMAN Isoform 2 of	0.00014873	
Q13459;Q13459-2	MYO9B_HUMAN Myosin-IXb OS=Homo sapiens GN=MYO9B PE=1 SV=3;>sp|Q13459-2|MYO9B_HUMAN Isoform Short of Myosin-IXb O	6.99E-15	
Q13464	ROCK1_HUMAN Rho-associated protein kinase 1 OS=Homo sapiens GN=ROCK1 PE=1 SV=1		
Q13465	MDS1_HUMAN MDS1 and EVI1 complex locus protein MDS1 OS=Homo sapiens GN=MECOM PE=2 SV=1		
Q13472	TOP3A_HUMAN DNA topoisomerase 3-alpha OS=Homo sapiens GN=TOP3A PE=1 SV=1		
Q13472-2	TOP3A_HUMAN Isoform Short of DNA topoisomerase 3-alpha OS=Homo sapiens GN=TOP3A		
Q13492	PICAL_HUMAN Phosphatidylinositol-binding clathrin assembly protein OS=Homo sapiens GN=PICALM PE=1 SV=2		522.33
Q13492-2	PICAL_HUMAN Isoform 2 of Phosphatidylinositol-binding clathrin assembly protein OS=Homo sapiens GN=PICALM		522.33
Q13492-3	PICAL_HUMAN Isoform 3 of Phosphatidylinositol-binding clathrin assembly protein OS=Homo sapiens GN=PICALM		522.33
Q13492;Q13492-2;Q1349	PICAL_HUMAN Phosphatidylinositol-binding clathrin assembly protein OS=Homo sapiens GN=PICALM PE=1 SV=2;>sp|Q13492-2|PICA	8.67E-85	
Q13501	SQSTM_HUMAN Sequestosome-1 OS=Homo sapiens GN=SQSTM1 PE=1 SV=1		67.74
Q13501-2	SQSTM_HUMAN Isoform 2 of Sequestosome-1 OS=Homo sapiens GN=SQSTM1		67.74
Q13501;Q13501-2	SQSTM_HUMAN Sequestosome-1 OS=Homo sapiens GN=SQSTM1 PE=1 SV=1;>sp|Q13501-2|SQSTM_HUMAN Isoform 2 of Sequestos	1.51E-20	
Q13503	MED21_HUMAN Mediator of RNA polymerase II transcription subunit 21 OS=Homo sapiens GN=MED21 PE=1 SV=1	8.82E-35	
Q13505	MTX1_HUMAN Metaxin-1 OS=Homo sapiens GN=MTX1 PE=1 SV=2		
Q13505-2	MTX1_HUMAN Isoform 2 of Metaxin-1 OS=Homo sapiens GN=MTX1		
Q13505-3	MTX1_HUMAN Isoform 3 of Metaxin-1 OS=Homo sapiens GN=MTX1		
Q13505;Q13505-2;Q1350	MTX1_HUMAN Metaxin-1 OS=Homo sapiens GN=MTX1 PE=1 SV=2;>sp|Q13505-2|MTX1_HUMAN Isoform 2 of Metaxin-1 OS=Homo s	5.37E-09	
Q13507	TRPC3_HUMAN Short transient receptor potential channel 3 OS=Homo sapiens GN=TRPC3 PE=1 SV=3		29.65
Q13509	TBB3_HUMAN Tubulin beta-3 chain OS=Homo sapiens GN=TUBB3 PE=1 SV=2		1440.15
Q13510	ASAH1_HUMAN Acid ceramidase OS=Homo sapiens GN=ASAH1 PE=1 SV=5		
Q13510-2	ASAH1_HUMAN Isoform 2 of Acid ceramidase OS=Homo sapiens GN=ASAH1		
Q13510-2;Q13510	2|ASAH1_HUMAN Isoform 2 of Acid ceramidase OS=Homo sapiens GN=ASAH1;>sp|Q13510|ASAH1_HUMAN Acid ceramidase OS=Hon	9.89E-14	
Q13523	PRP4B_HUMAN Serine/threonine-protein kinase PRP4 homolog OS=Homo sapiens GN=PRPF4B PE=1 SV=3		
Q13526	PIN1_HUMAN Peptidyl-prolyl cis-trans isomerase NIMA-interacting 1 OS=Homo sapiens GN=PIN1 PE=1 SV=1		163.2
Q13535	ATR_HUMAN Serine/threonine-protein kinase ATR OS=Homo sapiens GN=ATR PE=1 SV=3		
Q13535-2	ATR_HUMAN Isoform 2 of Serine/threonine-protein kinase ATR OS=Homo sapiens GN=ATR		
Q13535-3	ATR_HUMAN Isoform 3 of Serine/threonine-protein kinase ATR OS=Homo sapiens GN=ATR		
Q13546	RIPK1_HUMAN Receptor-interacting serine/threonine-protein kinase 1 OS=Homo sapiens GN=RIPK1 PE=1 SV=3	2.30E-08	
Q13547	HDAC1_HUMAN Histone deacetylase 1 OS=Homo sapiens GN=HDAC1 PE=1 SV=1		166.81
Q13554-2	KCC2B_HUMAN Isoform 1 of Calcium/calmodulin-dependent protein kinase type II subunit beta OS=Homo sapiens GN=CAMK2B		5.71
Q13554-4	KCC2B_HUMAN Isoform 3 of Calcium/calmodulin-dependent protein kinase type II subunit beta OS=Homo sapiens GN=CAMK2B		5.71

Q13554-5	KCC2B_HUMAN Isoform 5 of Calcium/calmodulin-dependent protein kinase type II subunit beta OS=Homo sapiens GN=CAMK2B		5.71
Q13554-6	KCC2B_HUMAN Isoform 6 of Calcium/calmodulin-dependent protein kinase type II subunit beta OS=Homo sapiens GN=CAMK2B		5.71
Q13554-7	KCC2B_HUMAN Isoform 7 of Calcium/calmodulin-dependent protein kinase type II subunit beta OS=Homo sapiens GN=CAMK2B		5.71
Q13554-8	KCC2B_HUMAN Isoform 8 of Calcium/calmodulin-dependent protein kinase type II subunit beta OS=Homo sapiens GN=CAMK2B		5.71
Q13555	KCC2G_HUMAN Calcium/calmodulin-dependent protein kinase type II subunit gamma OS=Homo sapiens GN=CAMK2G PE=1 SV=3		5.71
Q13555-2	KCC2G_HUMAN Isoform 2 of Calcium/calmodulin-dependent protein kinase type II subunit gamma OS=Homo sapiens GN=CAMK2G		5.71
Q13555-3	KCC2G_HUMAN Isoform 3 of Calcium/calmodulin-dependent protein kinase type II subunit gamma OS=Homo sapiens GN=CAMK2G		5.71
Q13555-4	KCC2G_HUMAN Isoform 4 of Calcium/calmodulin-dependent protein kinase type II subunit gamma OS=Homo sapiens GN=CAMK2G		5.71
Q13555-5	KCC2G_HUMAN Isoform 5 of Calcium/calmodulin-dependent protein kinase type II subunit gamma OS=Homo sapiens GN=CAMK2G		5.71
Q13555-6	KCC2G_HUMAN Isoform 6 of Calcium/calmodulin-dependent protein kinase type II subunit gamma OS=Homo sapiens GN=CAMK2G		5.71
Q13555-6;Q13555;Q1355	6|KCC2G_HUMAN Isoform 6 of Calcium/calmodulin-dependent protein kinase type II subunit gamma OS=Homo sapiens GN=CAMK2G;	6.67E-22	
Q13555-7	KCC2G_HUMAN Isoform 7 of Calcium/calmodulin-dependent protein kinase type II subunit gamma OS=Homo sapiens GN=CAMK2G		5.71
Q13555-8	KCC2G_HUMAN Isoform 8 of Calcium/calmodulin-dependent protein kinase type II subunit gamma OS=Homo sapiens GN=CAMK2G		5.71
Q13555-9	KCC2G_HUMAN Isoform 9 of Calcium/calmodulin-dependent protein kinase type II subunit gamma OS=Homo sapiens GN=CAMK2G		5.71
Q13557	KCC2D_HUMAN Calcium/calmodulin-dependent protein kinase type II subunit delta OS=Homo sapiens GN=CAMK2D PE=1 SV=3		51.28
Q13557-10	KCC2D_HUMAN Isoform Delta 10 of Calcium/calmodulin-dependent protein kinase type II subunit delta OS=Homo sapiens GN=CAMK2D		51.28
Q13557-11	KCC2D_HUMAN Isoform Delta 11 of Calcium/calmodulin-dependent protein kinase type II subunit delta OS=Homo sapiens GN=CAMK2D		51.28
Q13557-11;Q13557-4;Q1	11|KCC2D_HUMAN Isoform Delta 11 of Calcium/calmodulin-dependent protein kinase type II subunit delta OS=Homo sapiens GN=CA	6.25E-137	
Q13557-3	KCC2D_HUMAN Isoform Delta 3 of Calcium/calmodulin-dependent protein kinase type II subunit delta OS=Homo sapiens GN=CAMK2D		51.28
Q13557-4	KCC2D_HUMAN Isoform Delta 4 of Calcium/calmodulin-dependent protein kinase type II subunit delta OS=Homo sapiens GN=CAMK2D		51.28
Q13557-5	KCC2D_HUMAN Isoform Delta 8 of Calcium/calmodulin-dependent protein kinase type II subunit delta OS=Homo sapiens GN=CAMK2D		51.28
Q13557-6	KCC2D_HUMAN Isoform Delta 9 of Calcium/calmodulin-dependent protein kinase type II subunit delta OS=Homo sapiens GN=CAMK2D		51.28
Q13557-8	KCC2D_HUMAN Isoform Delta 6 of Calcium/calmodulin-dependent protein kinase type II subunit delta OS=Homo sapiens GN=CAMK2D		51.28
Q13557-9	KCC2D_HUMAN Isoform Delta 7 of Calcium/calmodulin-dependent protein kinase type II subunit delta OS=Homo sapiens GN=CAMK2D		51.28
Q13561	DCTN2_HUMAN Dynactin subunit 2 OS=Homo sapiens GN=DCTN2 PE=1 SV=4		99.53
Q13561-2	DCTN2_HUMAN Isoform 2 of Dynactin subunit 2 OS=Homo sapiens GN=DCTN2		99.53
Q13561-2;Q13561-3;Q13	2|DCTN2_HUMAN Isoform 2 of Dynactin subunit 2 OS=Homo sapiens GN=DCTN2;>sp|Q13561-3|DCTN2_HUMAN Isoform 3 of Dynact	6.05E-53	
Q13561-3	DCTN2_HUMAN Isoform 3 of Dynactin subunit 2 OS=Homo sapiens GN=DCTN2		99.53
Q13564	ULA1_HUMAN NEDD8-activating enzyme E1 regulatory subunit OS=Homo sapiens GN=NAE1 PE=1 SV=1	1.42E-25	
Q13573	SNW1_HUMAN SNW domain-containing protein 1 OS=Homo sapiens GN=SNW1 PE=1 SV=1		
Q13576	IQGA2_HUMAN Ras GTPase-activating-like protein IQGAP2 OS=Homo sapiens GN=IQGAP2 PE=1 SV=4		172.66
Q13576-2	IQGA2_HUMAN Isoform 2 of Ras GTPase-activating-like protein IQGAP2 OS=Homo sapiens GN=IQGAP2		133.17
Q13576;B7Z7U6;D6R939	IQGA2_HUMAN Ras GTPase-activating-like protein IQGAP2 OS=Homo sapiens GN=IQGAP2 PE=1 SV=4;>tr|B7Z7U6|B7Z7U6_HUMAN	2.00E-73	
Q13586	STIM1_HUMAN Stromal interaction molecule 1 OS=Homo sapiens GN=STIM1 PE=1 SV=3		51.16
Q13591	SEM5A_HUMAN Semaphorin-5A OS=Homo sapiens GN=SEMA5A PE=1 SV=3		
Q13595	TRA2A_HUMAN Transformer-2 protein homolog alpha OS=Homo sapiens GN=TRA2A PE=1 SV=1		
Q13595-2	TRA2A_HUMAN Isoform Short of Transformer-2 protein homolog alpha OS=Homo sapiens GN=TRA2A		
Q13595;Q13595-2	TRA2A_HUMAN Transformer-2 protein homolog alpha OS=Homo sapiens GN=TRA2A PE=1 SV=1;>sp|Q13595-2|TRA2A_HUMAN Isofo	5.58E-24	
Q13596;Q13596-2	SNX1_HUMAN Sorting nexin-1 OS=Homo sapiens GN=SNX1 PE=1 SV=3;>sp|Q13596-2|SNX1_HUMAN Isoform 1A of Sorting nexin-1	2.83E-13	
Q13601	KRR1_HUMAN KRR1 small subunit processome component homolog OS=Homo sapiens GN=KRR1 PE=1 SV=4		33.18
Q13608	PEX6_HUMAN Peroxisome assembly factor 2 OS=Homo sapiens GN=PEX6 PE=1 SV=2		
Q13610	PWP1_HUMAN Periodic tryptophan protein 1 homolog OS=Homo sapiens GN=PWP1 PE=1 SV=1	2.27E-05	
Q13614	MTMR2_HUMAN Myotubularin-related protein 2 OS=Homo sapiens GN=MTMR2 PE=1 SV=4		
Q13616	CUL1_HUMAN Cullin-1 OS=Homo sapiens GN=CUL1 PE=1 SV=2		82.43
Q13617	CUL2_HUMAN Cullin-2 OS=Homo sapiens GN=CUL2 PE=1 SV=2		38.79
Q13618	CUL3_HUMAN Cullin-3 OS=Homo sapiens GN=CUL3 PE=1 SV=2		41.1
Q13618-2	CUL3_HUMAN Isoform 2 of Cullin-3 OS=Homo sapiens GN=CUL3		41.1
Q13618-3	CUL3_HUMAN Isoform 3 of Cullin-3 OS=Homo sapiens GN=CUL3		6.06
Q13618;Q13618-2;Q1361	CUL3_HUMAN Cullin-3 OS=Homo sapiens GN=CUL3 PE=1 SV=2;>sp|Q13618-2|CUL3_HUMAN Isoform 2 of Cullin-3 OS=Homo sapien	1.11E-35	
Q13619	CUL4A_HUMAN Cullin-4A OS=Homo sapiens GN=CUL4A PE=1 SV=3		159.84
Q13619-2	CUL4A_HUMAN Isoform 2 of Cullin-4A OS=Homo sapiens GN=CUL4A		159.84
Q13619;A8MSH7;B2RBV7	CUL4A_HUMAN Cullin-4A OS=Homo sapiens GN=CUL4A PE=1 SV=3;>tr|A8MSH7|A8MSH7_HUMAN Uncharacterized protein OS=Hom	8.22E-28	
Q13620	CUL4B_HUMAN Cullin-4B OS=Homo sapiens GN=CUL4B PE=1 SV=4		328.42
Q13620-1	CUL4B_HUMAN Isoform 2 of Cullin-4B OS=Homo sapiens GN=CUL4B		328.42
Q13620-3	CUL4B_HUMAN Isoform 3 of Cullin-4B OS=Homo sapiens GN=CUL4B		267.8
Q13620;E0CX08;Q13620-	CUL4B_HUMAN Cullin-4B OS=Homo sapiens GN=CUL4B PE=1 SV=4;>tr|E0CX08|E0CX08_HUMAN Cullin 4B, isoform CRA_e OS=Hom	7.91E-195	
Q13621	S12A1_HUMAN Solute carrier family 12 member 1 OS=Homo sapiens GN=SLC12A1 PE=1 SV=2		
Q13621-3	S12A1_HUMAN Isoform F of Solute carrier family 12 member 1 OS=Homo sapiens GN=SLC12A1		
Q13630	FCL_HUMAN GDP-L-fucose synthase OS=Homo sapiens GN=TSTA3 PE=1 SV=1	2.20E-30	
Q13636	RAB31_HUMAN Ras-related protein Rab-31 OS=Homo sapiens GN=RAB31 PE=1 SV=1		145.83
Q13637	RAB32_HUMAN Ras-related protein Rab-32 OS=Homo sapiens GN=RAB32 PE=1 SV=3		41.99

Q13641	TPBG_HUMAN Trophoblast glycoprotein OS=Homo sapiens GN=TPBG PE=1 SV=1		88.02
Q13642-1;Q13642;Q1364	1|FHL1_HUMAN Isoform 1 of Four and a half LIM domains protein 1 OS=Homo sapiens GN=FHL1;>sp|Q13642|FHL1_HUMAN Four and	1.36E-234	
Q13643	FHL3_HUMAN Four and a half LIM domains protein 3 OS=Homo sapiens GN=FHL3 PE=1 SV=4	4.51E-15	
Q13698	CAC1S_HUMAN Voltage-dependent L-type calcium channel subunit alpha-1S OS=Homo sapiens GN=CACNA1S PE=1 SV=4		
Q13724	MOGS_HUMAN Mannosyl-oligosaccharide glucosidase OS=Homo sapiens GN=MOGS PE=1 SV=5		326.87
Q13733	AT1A4_HUMAN Sodium/potassium-transporting ATPase subunit alpha-4 OS=Homo sapiens GN=ATP1A4 PE=1 SV=3		783.01
Q13733-2	AT1A4_HUMAN Isoform 2 of Sodium/potassium-transporting ATPase subunit alpha-4 OS=Homo sapiens GN=ATP1A4		
Q13740	CD166_HUMAN CD166 antigen OS=Homo sapiens GN=ALCAM PE=1 SV=2		429.49
Q13740-2	CD166_HUMAN Isoform 2 of CD166 antigen OS=Homo sapiens GN=ALCAM		429.49
Q13740;B4DTU0;Q13740-	CD166_HUMAN CD166 antigen OS=Homo sapiens GN=ALCAM PE=1 SV=2;>tr|B4DTU0|B4DTU0_HUMAN cDNA FLJ59249, highly simila	1.31E-145	
Q13748	TBA3C_HUMAN Tubulin alpha-3C/D chain OS=Homo sapiens GN=TUBA3C PE=1 SV=3		968.57
Q13748-2	TBA3C_HUMAN Isoform 2 of Tubulin alpha-3C/D chain OS=Homo sapiens GN=TUBA3C		854.87
Q13751	LAMB3_HUMAN Laminin subunit beta-3 OS=Homo sapiens GN=LAMB3 PE=1 SV=1	2.39E-15	
Q13753;Q13753-2	LAMC2_HUMAN Laminin subunit gamma-2 OS=Homo sapiens GN=LAMC2 PE=1 SV=2;>sp|Q13753-2|LAMC2_HUMAN Isoform Short o	1.42E-12	
Q13761	RUNX3_HUMAN Runt-related transcription factor 3 OS=Homo sapiens GN=RUNX3 PE=1 SV=2		
Q13761-2	RUNX3_HUMAN Isoform 2 of Runt-related transcription factor 3 OS=Homo sapiens GN=RUNX3		
Q13765	NACA_HUMAN Nascent polypeptide-associated complex subunit alpha OS=Homo sapiens GN=NACA PE=1 SV=1		624.48
Q13769	THOC5_HUMAN THO complex subunit 5 homolog OS=Homo sapiens GN=THOC5 PE=1 SV=2	6.99E-08	
Q13772	NCOA4_HUMAN Nuclear receptor coactivator 4 OS=Homo sapiens GN=NCOA4 PE=1 SV=1		20.8
Q13772-2	NCOA4_HUMAN Isoform Beta of Nuclear receptor coactivator 4 OS=Homo sapiens GN=NCOA4		0.06
Q13795	ARFRP_HUMAN ADP-ribosylation factor-related protein 1 OS=Homo sapiens GN=ARFRP1 PE=1 SV=1	2.88E-07	
Q13813	SPTA2_HUMAN Spectrin alpha chain, brain OS=Homo sapiens GN=SPTAN1 PE=1 SV=3		6463.59
Q13813-2	SPTA2_HUMAN Isoform 2 of Spectrin alpha chain, brain OS=Homo sapiens GN=SPTAN1		6425.56
Q13813-3	SPTA2_HUMAN Isoform 3 of Spectrin alpha chain, brain OS=Homo sapiens GN=SPTAN1		6448.78
Q13813;Q13813-2;Q1381	SPTA2_HUMAN Spectrin alpha chain, brain OS=Homo sapiens GN=SPTAN1 PE=1 SV=3;>sp|Q13813-2|SPTA2_HUMAN Isoform 2 of Sp	0	
Q13822	ENPP2_HUMAN Ectonucleotide pyrophosphatase/phosphodiesterase family member 2 OS=Homo sapiens GN=ENPP2 PE=1 SV=3		43.12
Q13822-2	ENPP2_HUMAN Isoform 2 of Ectonucleotide pyrophosphatase/phosphodiesterase family member 2 OS=Homo sapiens GN=ENPP2		43.12
Q13822-3	ENPP2_HUMAN Isoform 3 of Ectonucleotide pyrophosphatase/phosphodiesterase family member 2 OS=Homo sapiens GN=ENPP2		43.12
Q13823	NOG2_HUMAN Nucleolar GTP-binding protein 2 OS=Homo sapiens GN=GNL2 PE=1 SV=1		
Q13835	PKP1_HUMAN Plakophilin-1 OS=Homo sapiens GN=PKP1 PE=1 SV=2		
Q13835-2	PKP1_HUMAN Isoform 1 of Plakophilin-1 OS=Homo sapiens GN=PKP1		
Q13838	DX39B_HUMAN Spliceosome RNA helicase DDX39B OS=Homo sapiens GN=DDX39B PE=1 SV=1		534.61
Q13838-2	DX39B_HUMAN Isoform 2 of Spliceosome RNA helicase DDX39B OS=Homo sapiens GN=DDX39B		534.73
Q13838-2;Q13838	2|DX39B_HUMAN Isoform 2 of Spliceosome RNA helicase DDX39B OS=Homo sapiens GN=DDX39B;>sp|Q13838|DX39B_HUMAN Splic	0	
Q13838-2;Q13838;Q5STA	2|UAP56_HUMAN Isoform 2 of Spliceosome RNA helicase BAT1 OS=Homo sapiens GN=BAT1;>sp|Q13838|UAP56_HUMAN Spliceosome	1.07E-207	
Q13867	BLMH_HUMAN Bleomycin hydrolase OS=Homo sapiens GN=BLMH PE=1 SV=1		45.42
Q13868	EXOS2_HUMAN Exosome complex component RRP4 OS=Homo sapiens GN=EXOSC2 PE=1 SV=2		333.96
Q13868;A3KFL5;A3KFL2;E	EXOS2_HUMAN Exosome complex exonuclease RRP4 OS=Homo sapiens GN=EXOSC2 PE=1 SV=2;>tr|A3KFL5|A3KFL5_HUMAN Exosor	6.31E-50	
Q13882	PTK6_HUMAN Protein-tyrosine kinase 6 OS=Homo sapiens GN=PTK6 PE=1 SV=1		49.04
Q13885	TBB2A_HUMAN Tubulin beta-2A chain OS=Homo sapiens GN=TUBB2A PE=1 SV=1		2168.12
Q13888	TF2H2_HUMAN General transcription factor IIH subunit 2 OS=Homo sapiens GN=GTF2H2 PE=1 SV=1		36.59
Q13889	TF2H3_HUMAN General transcription factor IIH subunit 3 OS=Homo sapiens GN=GTF2H3 PE=1 SV=2		61.34
Q13895	BYST_HUMAN Bystin OS=Homo sapiens GN=BYSL PE=1 SV=3		110.48
Q13907-2;Q13907	2|IDI1_HUMAN Isoform 2 of Isopentenyl-diphosphate Delta-isomerase 1 OS=Homo sapiens GN=IDI1;>sp|Q13907|IDI1_HUMAN Isope	5.94E-58	
Q13938	CAYP1_HUMAN Calcyphosin OS=Homo sapiens GN=CAPS PE=1 SV=1	1.90E-62	
Q13948	CASP_HUMAN Protein CASP OS=Homo sapiens GN=CUX1 PE=1 SV=2		93.09
Q13948-2	CASP_HUMAN Isoform 8 of Protein CASP OS=Homo sapiens GN=CUX1		93.09
Q13948;Q13948-2;P3988(	CASP_HUMAN Protein CASP OS=Homo sapiens GN=CUX1 PE=1 SV=2;>sp|Q13948-2|CASP_HUMAN Isoform 8 of Protein CASP OS=Ho	8.89E-11	
Q13950	RUNX2_HUMAN Runt-related transcription factor 2 OS=Homo sapiens GN=RUNX2 PE=1 SV=2		
Q13950-2	RUNX2_HUMAN Isoform 2 of Runt-related transcription factor 2 OS=Homo sapiens GN=RUNX2		
Q13950-3	RUNX2_HUMAN Isoform 3 of Runt-related transcription factor 2 OS=Homo sapiens GN=RUNX2		
Q13951	PEBB_HUMAN Core-binding factor subunit beta OS=Homo sapiens GN=CBFB PE=1 SV=2		313.88
Q13951-2	PEBB_HUMAN Isoform 2 of Core-binding factor subunit beta OS=Homo sapiens GN=CBFB		339.78
Q13951;A8K719;Q13951-	PEBB_HUMAN Core-binding factor subunit beta OS=Homo sapiens GN=CBFB PE=1 SV=2;>tr|A8K719|A8K719_HUMAN cDNA FLJ7641	3.86E-26	
Q13952	NFYC_HUMAN Nuclear transcription factor Y subunit gamma OS=Homo sapiens GN=NFYC PE=1 SV=3		39.26
Q13952-2	NFYC_HUMAN Isoform 1 of Nuclear transcription factor Y subunit gamma OS=Homo sapiens GN=NFYC		39.26
Q13952-3	NFYC_HUMAN Isoform 2 of Nuclear transcription factor Y subunit gamma OS=Homo sapiens GN=NFYC		39.26
Q13976	KGP1_HUMAN cGMP-dependent protein kinase 1 OS=Homo sapiens GN=PRKG1 PE=1 SV=3		30.71
Q13976-2	KGP1_HUMAN Isoform Beta of cGMP-dependent protein kinase 1 OS=Homo sapiens GN=PRKG1		25.19
Q14004	CDK13_HUMAN Cyclin-dependent kinase 13 OS=Homo sapiens GN=CDK13 PE=1 SV=2		109.05
Q14004-2	CDK13_HUMAN Isoform 2 of Cyclin-dependent kinase 13 OS=Homo sapiens GN=CDK13		107.37

Q14004;Q14004-2;Q9NYV	CDK13_HUMAN Cyclin-dependent kinase 13 OS=Homo sapiens GN=CDK13 PE=1 SV=2;>sp|Q14004-2|CDK13_HUMAN Isoform 2 of C	1.43E-13	
Q14005;Q14005-2;B7WNI	IL16_HUMAN Pro-interleukin-16 OS=Homo sapiens GN=IL16 PE=1 SV=4;>sp|Q14005-2|IL16_HUMAN Isoform 2 of Pro-interleukin-16	3.25E-22	
Q14008	CKAP5_HUMAN Cytoskeleton-associated protein 5 OS=Homo sapiens GN=CKAP5 PE=1 SV=3		815.65
Q14008-2	CKAP5_HUMAN Isoform 2 of Cytoskeleton-associated protein 5 OS=Homo sapiens GN=CKAP5		809.43
Q14008-3	CKAP5_HUMAN Isoform 3 of Cytoskeleton-associated protein 5 OS=Homo sapiens GN=CKAP5		815.65
Q14008-3;Q14008;Q1400	3|CKAP5_HUMAN Isoform 3 of Cytoskeleton-associated protein 5 OS=Homo sapiens GN=CKAP5;>sp|Q14008|CKAP5_HUMAN Cytoske	7.80E-97	
Q14011	CIRBP_HUMAN Cold-inducible RNA-binding protein OS=Homo sapiens GN=CIRBP PE=1 SV=1		350.39
Q14012	KCC1A_HUMAN Calcium/calmodulin-dependent protein kinase type 1 OS=Homo sapiens GN=CAMK1 PE=1 SV=1	3.44E-09	
Q14019	COTL1_HUMAN Coactosin-like protein OS=Homo sapiens GN=COTL1 PE=1 SV=3		193.13
Q14028	CNGB1_HUMAN Cyclic nucleotide-gated cation channel beta-1 OS=Homo sapiens GN=CNGB1 PE=1 SV=2		53.62
Q14028-2	CNGB1_HUMAN Isoform RCNC2A of Cyclic nucleotide-gated cation channel beta-1 OS=Homo sapiens GN=CNGB1		51.28
Q14061	COX17_HUMAN Cytochrome c oxidase copper chaperone OS=Homo sapiens GN=COX17 PE=1 SV=2		57.41
Q14088	RB33A_HUMAN Ras-related protein Rab-33A OS=Homo sapiens GN=RAB33A PE=1 SV=2		3.92
Q14103	HNRPD_HUMAN Heterogeneous nuclear ribonucleoprotein D0 OS=Homo sapiens GN=HNRNPD PE=1 SV=1		734.86
Q14103-2	HNRPD_HUMAN Isoform 2 of Heterogeneous nuclear ribonucleoprotein D0 OS=Homo sapiens GN=HNRNPD		592.3
Q14103-2;Q14103-4	2|HNRPD_HUMAN Isoform 2 of Heterogeneous nuclear ribonucleoprotein D0 OS=Homo sapiens GN=HNRNPD;>sp|Q14103-4|HNRPD_I	0	
Q14103-3	HNRPD_HUMAN Isoform 3 of Heterogeneous nuclear ribonucleoprotein D0 OS=Homo sapiens GN=HNRNPD		734.86
Q14103-4	HNRPD_HUMAN Isoform 4 of Heterogeneous nuclear ribonucleoprotein D0 OS=Homo sapiens GN=HNRNPD		592.3
Q14103-4;B9ZVU1;Q1410	4|HNRPD_HUMAN Isoform 4 of Heterogeneous nuclear ribonucleoprotein D0 OS=Homo sapiens GN=HNRNPD;>tr|B9ZVU1|B9ZVU1_HU	1.27E-206	
Q14103;Q14103-2;Q1410	HNRPD_HUMAN Heterogeneous nuclear ribonucleoprotein D0 OS=Homo sapiens GN=HNRNPD PE=1 SV=1;>sp|Q14103-2|HNRPD_HUI	9.45E-198	
Q14108	SCRB2_HUMAN Lysosome membrane protein 2 OS=Homo sapiens GN=SCARB2 PE=1 SV=2		30.09
Q14112;Q14112-2	NID2_HUMAN Nidogen-2 OS=Homo sapiens GN=NID2 PE=1 SV=3;>sp|Q14112-2|NID2_HUMAN Isoform 2 of Nidogen-2 OS=Homo sa	5.53E-133	
Q14116	IL18_HUMAN Interleukin-18 OS=Homo sapiens GN=IL18 PE=1 SV=1	1.02E-06	
Q14118	DAG1_HUMAN Dystroglycan OS=Homo sapiens GN=DAG1 PE=1 SV=2		139.98
Q14119	VEZF1_HUMAN Vascular endothelial zinc finger 1 OS=Homo sapiens GN=VEZF1 PE=1 SV=2		
Q14126	DSG2_HUMAN Desmoglein-2 OS=Homo sapiens GN=DSG2 PE=1 SV=2	4.17E-37	
Q14134;Q14134-2	TRI29_HUMAN Tripartite motif-containing protein 29 OS=Homo sapiens GN=TRIM29 PE=1 SV=2;>sp|Q14134-2|TRI29_HUMAN Isofor	4.02E-26	
Q14137	BOP1_HUMAN Ribosome biogenesis protein BOP1 OS=Homo sapiens GN=BOP1 PE=1 SV=2		
Q14139	UBE4A_HUMAN Ubiquitin conjugation factor E4 A OS=Homo sapiens GN=UBE4A PE=1 SV=2		
Q14139-2	UBE4A_HUMAN Isoform 2 of Ubiquitin conjugation factor E4 A OS=Homo sapiens GN=UBE4A		
Q14139-2;Q14139	2|UBE4A_HUMAN Isoform 2 of Ubiquitin conjugation factor E4 A OS=Homo sapiens GN=UBE4A;>sp|Q14139|UBE4A_HUMAN Ubiquitin	2.50E-12	
Q14141	SEPT6_HUMAN Septin-6 OS=Homo sapiens GN=SEPT6 PE=1 SV=4		319.44
Q14141-2	SEPT6_HUMAN Isoform I of Septin-6 OS=Homo sapiens GN=SEPT6		319.44
Q14141-3	SEPT6_HUMAN Isoform IV of Septin-6 OS=Homo sapiens GN=SEPT6		162.53
Q14141-4	SEPT6_HUMAN Isoform V of Septin-6 OS=Homo sapiens GN=SEPT6		319.44
Q14141;Q14141-4;Q1414	SEPT6_HUMAN Septin-6 OS=Homo sapiens GN=SEPT6 PE=1 SV=4;>sp|Q14141-4|SEPT6_HUMAN Isoform V of Septin-6 OS=Homo sa	0	
Q14145	KEAP1_HUMAN Kelch-like ECH-associated protein 1 OS=Homo sapiens GN=KEAP1 PE=1 SV=2		
Q14146	URB2_HUMAN Unhealthy ribosome biogenesis protein 2 homolog OS=Homo sapiens GN=URB2 PE=1 SV=2		
Q14149;Q4VBZ9;A2RU29;	MORC3_HUMAN MORC family CW-type zinc finger protein 3 OS=Homo sapiens GN=MORC3 PE=1 SV=3;>tr|Q4VBZ9|Q4VBZ9_HUMAN	5.74E-06	
Q14151	SAFB2_HUMAN Scaffold attachment factor B2 OS=Homo sapiens GN=SAFB2 PE=1 SV=1		
Q14152	EIF3A_HUMAN Eukaryotic translation initiation factor 3 subunit A OS=Homo sapiens GN=EIF3A PE=1 SV=1		920.67
Q14155;Q14155-3;Q1415	ARHG7_HUMAN Rho guanine nucleotide exchange factor 7 OS=Homo sapiens GN=ARHGEF7 PE=1 SV=2;>sp|Q14155-3|ARHG7_HUM/	2.14E-21	
Q14156	EFR3A_HUMAN Protein EFR3 homolog A OS=Homo sapiens GN=EFR3A PE=1 SV=2		28.89
Q14156-2	EFR3A_HUMAN Isoform 2 of Protein EFR3 homolog A OS=Homo sapiens GN=EFR3A		28.89
Q14156-3	EFR3A_HUMAN Isoform 3 of Protein EFR3 homolog A OS=Homo sapiens GN=EFR3A		28.89
Q14157	UBP2L_HUMAN Ubiquitin-associated protein 2-like OS=Homo sapiens GN=UBAP2L PE=1 SV=2		327.29
Q14157-1	UBP2L_HUMAN Isoform 2 of Ubiquitin-associated protein 2-like OS=Homo sapiens GN=UBAP2L		327.29
Q14157-3	UBP2L_HUMAN Isoform 3 of Ubiquitin-associated protein 2-like OS=Homo sapiens GN=UBAP2L		327.29
Q14157-4	UBP2L_HUMAN Isoform 4 of Ubiquitin-associated protein 2-like OS=Homo sapiens GN=UBAP2L		327.29
Q14157;B4DYY5;Q14157-	UBP2L_HUMAN Ubiquitin-associated protein 2-like OS=Homo sapiens GN=UBAP2L PE=1 SV=2;>tr|B4DYY5|B4DYY5_HUMAN cDNA FLJ	1.06E-133	
Q14160	SCRIB_HUMAN Protein scribble homolog OS=Homo sapiens GN=SCRIB PE=1 SV=4		26.26
Q14160-2	SCRIB_HUMAN Isoform 2 of Protein scribble homolog OS=Homo sapiens GN=SCRIB		26.26
Q14160-3	SCRIB_HUMAN Isoform 3 of Protein scribble homolog OS=Homo sapiens GN=SCRIB		26.26
Q14160-3;Q14160	3|SCRIB_HUMAN Isoform 3 of Protein scribble homolog OS=Homo sapiens GN=SCRIB;>sp|Q14160|SCRIB_HUMAN Protein scribble hc	1.08E-46	
Q14161	GIT2_HUMAN ARF GTPase-activating protein GIT2 OS=Homo sapiens GN=GIT2 PE=1 SV=2		31.31
Q14161-10	GIT2_HUMAN Isoform 10 of ARF GTPase-activating protein GIT2 OS=Homo sapiens GN=GIT2		27.72
Q14161-11	GIT2_HUMAN Isoform 11 of ARF GTPase-activating protein GIT2 OS=Homo sapiens GN=GIT2		27.72
Q14161-3	GIT2_HUMAN Isoform 3 of ARF GTPase-activating protein GIT2 OS=Homo sapiens GN=GIT2		31.31
Q14161-4	GIT2_HUMAN Isoform 4 of ARF GTPase-activating protein GIT2 OS=Homo sapiens GN=GIT2		31.31
Q14161-5	GIT2_HUMAN Isoform 5 of ARF GTPase-activating protein GIT2 OS=Homo sapiens GN=GIT2		27.72
Q14161-6	GIT2_HUMAN Isoform 6 of ARF GTPase-activating protein GIT2 OS=Homo sapiens GN=GIT2		27.72

Q14161-7	GIT2_HUMAN Isoform 7 of ARF GTPase-activating protein GIT2 OS=Homo sapiens GN=GIT2		27.72
Q14161-8	GIT2_HUMAN Isoform 8 of ARF GTPase-activating protein GIT2 OS=Homo sapiens GN=GIT2		27.72
Q14161-9	GIT2_HUMAN Isoform 9 of ARF GTPase-activating protein GIT2 OS=Homo sapiens GN=GIT2		27.72
Q14165	MLEC_HUMAN Malectin OS=Homo sapiens GN=MLEC PE=1 SV=1		328.17
Q14166	TTL12_HUMAN Tubulin--tyrosine ligase-like protein 12 OS=Homo sapiens GN=TTLL12 PE=1 SV=2		80.85
Q14191	WRN_HUMAN Werner syndrome ATP-dependent helicase OS=Homo sapiens GN=WRN PE=1 SV=2		25.02
Q14192	FHL2_HUMAN Four and a half LIM domains protein 2 OS=Homo sapiens GN=FHL2 PE=1 SV=3	1.29E-73	
Q14195	DPYL3_HUMAN Dihydropyrimidinase-related protein 3 OS=Homo sapiens GN=DPYSL3 PE=1 SV=1	0	
Q14197	ICT1_HUMAN Peptidyl-tRNA hydrolase ICT1, mitochondrial OS=Homo sapiens GN=ICT1 PE=1 SV=1		109.1
Q14202	ZMYM3_HUMAN Zinc finger MYM-type protein 3 OS=Homo sapiens GN=ZMYM3 PE=1 SV=2		
Q14202-2	ZMYM3_HUMAN Isoform 2 of Zinc finger MYM-type protein 3 OS=Homo sapiens GN=ZMYM3		
Q14203	DCTN1_HUMAN Dynactin subunit 1 OS=Homo sapiens GN=DCTN1 PE=1 SV=3		
Q14203-2	DCTN1_HUMAN Isoform p135 of Dynactin subunit 1 OS=Homo sapiens GN=DCTN1		
Q14203;Q14203-2	DCTN1_HUMAN Dynactin subunit 1 OS=Homo sapiens GN=DCTN1 PE=1 SV=3;>sp|Q14203-2|DCTN1_HUMAN Isoform p135 of Dynac	3.79E-19	
Q14204	DYHC1_HUMAN Cytoplasmic dynein 1 heavy chain 1 OS=Homo sapiens GN=DYNC1H1 PE=1 SV=5		5322.31
Q14232	EI2BA_HUMAN Translation initiation factor eIF-2B subunit alpha OS=Homo sapiens GN=EIF2B1 PE=1 SV=1		188.52
Q14240	IF4A2_HUMAN Eukaryotic initiation factor 4A-II OS=Homo sapiens GN=EIF4A2 PE=1 SV=2		744.4
Q14240-2	IF4A2_HUMAN Isoform 2 of Eukaryotic initiation factor 4A-II OS=Homo sapiens GN=EIF4A2		744.4
Q14240-2;D3DNU9;Q142	2|IF4A2_HUMAN Isoform 2 of Eukaryotic initiation factor 4A-II OS=Homo sapiens GN=EIF4A2;>tr|D3DNU9|D3DNU9_HUMAN Eukaryo	1.58E-187	
Q14244;Q14244-2;Q1424	MAP7_HUMAN Ensconsin OS=Homo sapiens GN=MAP7 PE=1 SV=1;>sp|Q14244-2|MAP7_HUMAN Isoform 2 of Ensconsin OS=Homo sa	1.61E-12	
Q14247	SRC8_HUMAN Src substrate cortactin OS=Homo sapiens GN=CTTN PE=1 SV=2	0	
Q14254	FLOT2_HUMAN Flotillin-2 OS=Homo sapiens GN=FLOT2 PE=1 SV=2		326.25
Q14257	RCN2_HUMAN Reticulocalbin-2 OS=Homo sapiens GN=RCN2 PE=1 SV=1		
Q14258	TRI25_HUMAN E3 ubiquitin/ISG15 ligase TRIM25 OS=Homo sapiens GN=TRIM25 PE=1 SV=2		704.42
Q14314	FGL2_HUMAN Fibroleukin OS=Homo sapiens GN=FGL2 PE=1 SV=1	2.04E-05	
Q14315	FLNC_HUMAN Filamin-C OS=Homo sapiens GN=FLNC PE=1 SV=3		2605.79
Q14315-2	FLNC_HUMAN Isoform 2 of Filamin-C OS=Homo sapiens GN=FLNC		2605.79
Q14315;Q14315-2	FLNC_HUMAN Filamin-C OS=Homo sapiens GN=FLNC PE=1 SV=3;>sp|Q14315-2|FLNC_HUMAN Isoform 2 of Filamin-C OS=Homo sap	0	
Q14318	FKBP8_HUMAN Peptidyl-prolyl cis-trans isomerase FKBP8 OS=Homo sapiens GN=FKBP8 PE=1 SV=2		23.78
Q14318-2	FKBP8_HUMAN Isoform 2 of Peptidyl-prolyl cis-trans isomerase FKBP8 OS=Homo sapiens GN=FKBP8		23.78
Q14318-2;Q14318	2|FKBP8_HUMAN Isoform 2 of Peptidyl-prolyl cis-trans isomerase FKBP8 OS=Homo sapiens GN=FKBP8;>sp|Q14318|FKBP8_HUMAN P	3.88E-127	
Q14320	FA50A_HUMAN Protein FAM50A OS=Homo sapiens GN=FAM50A PE=1 SV=2		27.88
Q14331	FRG1_HUMAN Protein FRG1 OS=Homo sapiens GN=FRG1 PE=1 SV=1		70.74
Q14332	FZD2_HUMAN Frizzled-2 OS=Homo sapiens GN=FZD2 PE=2 SV=1		48.55
Q14344	GNA13_HUMAN Guanine nucleotide-binding protein subunit alpha-13 OS=Homo sapiens GN=GNA13 PE=1 SV=2		364.59
Q14353	GAMT_HUMAN Guanidinoacetate N-methyltransferase OS=Homo sapiens GN=GAMT PE=1 SV=1		25.84
Q14376	GALE_HUMAN UDP-glucose 4-epimerase OS=Homo sapiens GN=GALE PE=1 SV=2	1.31E-44	
Q14435	GALT3_HUMAN Polypeptide N-acetylgalactosaminyltransferase 3 OS=Homo sapiens GN=GALNT3 PE=2 SV=2	4.42E-07	
Q14439	GP176_HUMAN Probable G-protein coupled receptor 176 OS=Homo sapiens GN=GPR176 PE=2 SV=1		
Q14444	CAPR1_HUMAN Caprin-1 OS=Homo sapiens GN=CAPRIN1 PE=1 SV=2		341.9
Q14444-2	CAPR1_HUMAN Isoform 2 of Caprin-1 OS=Homo sapiens GN=CAPRIN1		341.9
Q14444;Q14444-2	CAPR1_HUMAN Caprin-1 OS=Homo sapiens GN=CAPRIN1 PE=1 SV=2;>sp|Q14444-2|CAPR1_HUMAN Isoform 2 of Caprin-1 OS=Hom	5.26E-175	
Q14451	GRB7_HUMAN Growth factor receptor-bound protein 7 OS=Homo sapiens GN=GRB7 PE=1 SV=2		
Q14451-2	GRB7_HUMAN Isoform 2 of Growth factor receptor-bound protein 7 OS=Homo sapiens GN=GRB7		
Q14451-3;Q14451;Q1445	3|GRB7_HUMAN Isoform 3 of Growth factor receptor-bound protein 7 OS=Homo sapiens GN=GRB7;>sp|Q14451|GRB7_HUMAN Growt	4.22E-33	
Q14457	BECN1_HUMAN Beclin-1 OS=Homo sapiens GN=BECN1 PE=1 SV=2		34.86
Q14498	RBM39_HUMAN RNA-binding protein 39 OS=Homo sapiens GN=RBM39 PE=1 SV=2		161.24
Q14498-2	RBM39_HUMAN Isoform 2 of RNA-binding protein 39 OS=Homo sapiens GN=RBM39		161.24
Q14498;Q14498-2	RBM39_HUMAN RNA-binding protein 39 OS=Homo sapiens GN=RBM39 PE=1 SV=2;>sp|Q14498-2|RBM39_HUMAN Isoform 2 of RNA-	4.75E-72	
Q14508;Q14508-3;Q1450	WFDC2_HUMAN WAP four-disulfide core domain protein 2 OS=Homo sapiens GN=WFDC2 PE=1 SV=2;>sp|Q14508-3|WFDC2_HUMAN	3.35E-05	
Q14517	FAT1_HUMAN Protocadherin Fat 1 OS=Homo sapiens GN=FAT1 PE=1 SV=2		
Q14520	HABP2_HUMAN Hyaluronan-binding protein 2 OS=Homo sapiens GN=HABP2 PE=1 SV=1	1.68E-05	
Q14525	KT33B_HUMAN Keratin, type I cuticular Ha3-II OS=Homo sapiens GN=KRT33B PE=1 SV=3		73.45
Q14527	HLTF_HUMAN Helicase-like transcription factor OS=Homo sapiens GN=HLTF PE=1 SV=2		49.45
Q14527-2	HLTF_HUMAN Isoform 2 of Helicase-like transcription factor OS=Homo sapiens GN=HLTF		49.45
Q14532	K1H2_HUMAN Keratin, type I cuticular Ha2 OS=Homo sapiens GN=KRT32 PE=1 SV=3		59.53
Q14533	KRT81_HUMAN Keratin, type II cuticular Hb1 OS=Homo sapiens GN=KRT81 PE=1 SV=3		81.03
Q14534	ERG1_HUMAN Squalene monooxygenase OS=Homo sapiens GN=SQLE PE=2 SV=3		
Q14554	PDIA5_HUMAN Protein disulfide-isomerase A5 OS=Homo sapiens GN=PDIA5 PE=1 SV=1	9.32E-07	
Q14558	KPRA_HUMAN Phosphoribosyl pyrophosphate synthase-associated protein 1 OS=Homo sapiens GN=PRPSAP1 PE=1 SV=2		77.11
Q14558-2	KPRA_HUMAN Isoform 2 of Phosphoribosyl pyrophosphate synthase-associated protein 1 OS=Homo sapiens GN=PRPSAP1		77.11

Q14558-2;Q14558	2|KPRA_HUMAN Isoform 2 of Phosphoribosyl pyrophosphate synthase-associated protein 1 OS=Homo sapiens GN=PRPSAP1;>sp|Q14!	2.49E-20	
Q14562	DHX8_HUMAN ATP-dependent RNA helicase DHX8 OS=Homo sapiens GN=DHX8 PE=1 SV=1		
Q14566	MCM6_HUMAN DNA replication licensing factor MCM6 OS=Homo sapiens GN=MCM6 PE=1 SV=1		430.87
Q14568	HS902_HUMAN Putative heat shock protein HSP 90-alpha A2 OS=Homo sapiens GN=HSP90AA2 PE=1 SV=2		219.6
Q14571	ITPR2_HUMAN Inositol 1,4,5-trisphosphate receptor type 2 OS=Homo sapiens GN=ITPR2 PE=1 SV=2		53.43
Q14571-2	ITPR2_HUMAN Isoform Short of Inositol 1,4,5-trisphosphate receptor type 2 OS=Homo sapiens GN=ITPR2		6.82
Q14573	ITPR3_HUMAN Inositol 1,4,5-trisphosphate receptor type 3 OS=Homo sapiens GN=ITPR3 PE=1 SV=2		434.54
Q14574	DSC3_HUMAN Desmocollin-3 OS=Homo sapiens GN=DSC3 PE=1 SV=3		
Q14574-2	DSC3_HUMAN Isoform 3B of Desmocollin-3 OS=Homo sapiens GN=DSC3		
Q14576-2	ELAV3_HUMAN Isoform 2 of ELAV-like protein 3 OS=Homo sapiens GN=ELAVL3		
Q14588	ZN234_HUMAN Zinc finger protein 234 OS=Homo sapiens GN=ZNF234 PE=2 SV=3		39.48
Q14624	ITIH4_HUMAN Inter-alpha-trypsin inhibitor heavy chain H4 OS=Homo sapiens GN=ITIH4 PE=1 SV=4		74.38
Q14624-2	ITIH4_HUMAN Isoform 2 of Inter-alpha-trypsin inhibitor heavy chain H4 OS=Homo sapiens GN=ITIH4		74.38
Q14624-2;Q14624	2|ITIH4_HUMAN Isoform 2 of Inter-alpha-trypsin inhibitor heavy chain H4 OS=Homo sapiens GN=ITIH4;>sp|Q14624|ITIH4_HUMAN I	3.33E-190	
Q14642	I5P1_HUMAN Type I inositol-1,4,5-trisphosphate 5-phosphatase OS=Homo sapiens GN=INPP5A PE=2 SV=1		
Q14643	ITPR1_HUMAN Inositol 1,4,5-trisphosphate receptor type 1 OS=Homo sapiens GN=ITPR1 PE=1 SV=2		62.94
Q14643-2	ITPR1_HUMAN Isoform 2 of Inositol 1,4,5-trisphosphate receptor type 1 OS=Homo sapiens GN=ITPR1		62.94
Q14643-3	ITPR1_HUMAN Isoform 3 of Inositol 1,4,5-trisphosphate receptor type 1 OS=Homo sapiens GN=ITPR1		60.59
Q14643-4	ITPR1_HUMAN Isoform 4 of Inositol 1,4,5-trisphosphate receptor type 1 OS=Homo sapiens GN=ITPR1		60.59
Q14643-5	ITPR1_HUMAN Isoform 5 of Inositol 1,4,5-trisphosphate receptor type 1 OS=Homo sapiens GN=ITPR1		60.59
Q14643-6	ITPR1_HUMAN Isoform 6 of Inositol 1,4,5-trisphosphate receptor type 1 OS=Homo sapiens GN=ITPR1		62.94
Q14643-7	ITPR1_HUMAN Isoform 7 of Inositol 1,4,5-trisphosphate receptor type 1 OS=Homo sapiens GN=ITPR1		62.94
Q14643-8	ITPR1_HUMAN Isoform 8 of Inositol 1,4,5-trisphosphate receptor type 1 OS=Homo sapiens GN=ITPR1		60.59
Q14651	PLSI_HUMAN Plastin-1 OS=Homo sapiens GN=PLS1 PE=1 SV=2		31.74
Q14653	IRF3_HUMAN Interferon regulatory factor 3 OS=Homo sapiens GN=IRF3 PE=1 SV=1	3.85E-20	
Q14657	LAGE3_HUMAN L antigen family member 3 OS=Homo sapiens GN=LAGE3 PE=1 SV=2		145.3
Q14667	K0100_HUMAN UPF0378 protein KIAA0100 OS=Homo sapiens GN=KIAA0100 PE=1 SV=3		
Q14667-2	K0100_HUMAN Isoform 2 of UPF0378 protein KIAA0100 OS=Homo sapiens GN=KIAA0100		
Q14667-4	K0100_HUMAN Isoform 4 of UPF0378 protein KIAA0100 OS=Homo sapiens GN=KIAA0100		
Q14669	TRIPC_HUMAN Probable E3 ubiquitin-protein ligase TRIP12 OS=Homo sapiens GN=TRIP12 PE=1 SV=1		154.14
Q14671;Q14671-2	PUM1_HUMAN Pumilio homolog 1 OS=Homo sapiens GN=PUM1 PE=1 SV=3;>sp|Q14671-2|PUM1_HUMAN Isoform 2 of Pumilio homol	6.71E-23	
Q14676	MDC1_HUMAN Mediator of DNA damage checkpoint protein 1 OS=Homo sapiens GN=MDC1 PE=1 SV=3		352.35
Q14676-2	MDC1_HUMAN Isoform 2 of Mediator of DNA damage checkpoint protein 1 OS=Homo sapiens GN=MDC1		352.35
Q14676-3	MDC1_HUMAN Isoform 3 of Mediator of DNA damage checkpoint protein 1 OS=Homo sapiens GN=MDC1		336.08
Q14676-4	MDC1_HUMAN Isoform 4 of Mediator of DNA damage checkpoint protein 1 OS=Homo sapiens GN=MDC1		283.13
Q14677	EPN4_HUMAN Clathrin interactor 1 OS=Homo sapiens GN=CLINT1 PE=1 SV=1		23.44
Q14677-2	EPN4_HUMAN Isoform 2 of Clathrin interactor 1 OS=Homo sapiens GN=CLINT1		23.44
Q14677;Q14677-2	EPN4_HUMAN Clathrin interactor 1 OS=Homo sapiens GN=CLINT1 PE=1 SV=1;>sp|Q14677-2|EPN4_HUMAN Isoform 2 of Clathrin inte	1.36E-108	
Q14680	MELK_HUMAN Maternal embryonic leucine zipper kinase OS=Homo sapiens GN=MELK PE=1 SV=3		
Q14683	SMC1A_HUMAN Structural maintenance of chromosomes protein 1A OS=Homo sapiens GN=SMC1A PE=1 SV=2		333.95
Q14684	RRP1B_HUMAN Ribosomal RNA processing protein 1 homolog B OS=Homo sapiens GN=RRP1B PE=1 SV=3		
Q14684-2	RRP1B_HUMAN Isoform 2 of Ribosomal RNA processing protein 1 homolog B OS=Homo sapiens GN=RRP1B		
Q14684;C9JHZ3;Q14684-	RRP1B_HUMAN Ribosomal RNA processing protein 1 homolog B OS=Homo sapiens GN=RRP1B PE=1 SV=3;>tr|C9JHZ3|C9JHZ3_HUM/	2.50E-10	
Q14690	RRP5_HUMAN Protein RRP5 homolog OS=Homo sapiens GN=PDCD11 PE=1 SV=3		75.83
Q14691	PSF1_HUMAN DNA replication complex GINS protein PSF1 OS=Homo sapiens GN=GINS1 PE=1 SV=1	2.59E-15	
Q14692	BMS1_HUMAN Ribosome biogenesis protein BMS1 homolog OS=Homo sapiens GN=BMS1 PE=1 SV=1		
Q14694	UBP10_HUMAN Ubiquitin carboxyl-terminal hydrolase 10 OS=Homo sapiens GN=USP10 PE=1 SV=2		
Q14694-2	UBP10_HUMAN Isoform 2 of Ubiquitin carboxyl-terminal hydrolase 10 OS=Homo sapiens GN=USP10		
Q14694-2;Q14694-3;Q14(	2|UBP10_HUMAN Isoform 2 of Ubiquitin carboxyl-terminal hydrolase 10 OS=Homo sapiens GN=USP10;>sp|Q14694-3|UBP10_HUMAN	4.18E-65	
Q14694-3	UBP10_HUMAN Isoform 3 of Ubiquitin carboxyl-terminal hydrolase 10 OS=Homo sapiens GN=USP10		
Q14696	MESD_HUMAN LDLR chaperone MESD OS=Homo sapiens GN=MESDC2 PE=1 SV=2		258.1
Q14697	GANAB_HUMAN Neutral alpha-glucosidase AB OS=Homo sapiens GN=GANAB PE=1 SV=3		2320.54
Q14697-2	GANAB_HUMAN Isoform 2 of Neutral alpha-glucosidase AB OS=Homo sapiens GN=GANAB		2274.96
Q14697-2;Q14697	2|GANAB_HUMAN Isoform 2 of Neutral alpha-glucosidase AB OS=Homo sapiens GN=GANAB;>sp|Q14697|GANAB_HUMAN Neutral alpl	0	
Q14697-3	GANAB_HUMAN Isoform 3 of Neutral alpha-glucosidase AB OS=Homo sapiens GN=GANAB		43.34
Q14697;Q14697-2	GANAB_HUMAN Neutral alpha-glucosidase AB OS=Homo sapiens GN=GANAB PE=1 SV=3;>sp|Q14697-2|GANAB_HUMAN Isoform 2 of	0	
Q14699	RFTN1_HUMAN Raftlin OS=Homo sapiens GN=RFTN1 PE=1 SV=4		93.84
Q14722	KCAB1_HUMAN Voltage-gated potassium channel subunit beta-1 OS=Homo sapiens GN=KCNAB1 PE=2 SV=1		
Q14722-3	KCAB1_HUMAN Isoform KvB1.2 of Voltage-gated potassium channel subunit beta-1 OS=Homo sapiens GN=KCNAB1		
Q14728	MFS10_HUMAN Major facilitator superfamily domain-containing protein 10 OS=Homo sapiens GN=MFSD10 PE=2 SV=1		43.01
Q14738	2A5D_HUMAN Serine/threonine-protein phosphatase 2A 56 kDa regulatory subunit delta isoform OS=Homo sapiens GN=PPP2R5D PE=1 SV=1		70.04

Q14738-2	2A5D_HUMAN Isoform Delta-2 of Serine/threonine-protein phosphatase 2A 56 kDa regulatory subunit delta isoform OS=Homo sapiens GN=PPP2R5D		70.04
Q14738-3	2A5D_HUMAN Isoform Delta-3 of Serine/threonine-protein phosphatase 2A 56 kDa regulatory subunit delta isoform OS=Homo sapiens GN=PPP2R5D		70.04
Q14738;Q14738-2;Q1473	2A5D_HUMAN Serine/threonine-protein phosphatase 2A 56 kDa regulatory subunit delta isoform OS=Homo sapiens GN=PPP2R5D PE=	1.63E-93	
Q14739	LBR_HUMAN Lamin-B receptor OS=Homo sapiens GN=LBR PE=1 SV=2		105.81
Q14746	COG2_HUMAN Conserved oligomeric Golgi complex subunit 2 OS=Homo sapiens GN=COG2 PE=1 SV=1	5.86E-05	
Q14764	MVP_HUMAN Major vault protein OS=Homo sapiens GN=MVP PE=1 SV=4		611.54
Q14767	LTBP2_HUMAN Latent-transforming growth factor beta-binding protein 2 OS=Homo sapiens GN=LTBP2 PE=1 SV=3	4.98E-104	
Q14789	GOGB1_HUMAN Golgin subfamily B member 1 OS=Homo sapiens GN=GOLGB1 PE=1 SV=2		148.47
Q147U4	L37B2_HUMAN Putative LRRC37B-like protein 2 OS=Homo sapiens GN=LRRC37BP1 PE=5 SV=2		
Q14802	FXYD3_HUMAN FXYD domain-containing ion transport regulator 3 OS=Homo sapiens GN=FXYD3 PE=2 SV=1		
Q14802-2	FXYD3_HUMAN Isoform 2 of FXYD domain-containing ion transport regulator 3 OS=Homo sapiens GN=FXYD3		
Q14802-2;Q14802	2|FXYD3_HUMAN Isoform 2 of FXYD domain-containing ion transport regulator 3 OS=Homo sapiens GN=FXYD3;>sp|Q14802|FXYD3_I	4.20E-05	
Q14807	KIF22_HUMAN Kinesin-like protein KIF22 OS=Homo sapiens GN=KIF22 PE=1 SV=5		
Q14832	GRM3_HUMAN Metabotropic glutamate receptor 3 OS=Homo sapiens GN=GRM3 PE=2 SV=2		
Q14839	CHD4_HUMAN Chromodomain-helicase-DNA-binding protein 4 OS=Homo sapiens GN=CHD4 PE=1 SV=2		1602.57
Q14839-2	CHD4_HUMAN Isoform 2 of Chromodomain-helicase-DNA-binding protein 4 OS=Homo sapiens GN=CHD4		1602.57
Q14839-2;Q14839	2|CHD4_HUMAN Isoform 2 of Chromodomain-helicase-DNA-binding protein 4 OS=Homo sapiens GN=CHD4;>sp|Q14839|CHD4_HUMA	0	
Q14847	LASP1_HUMAN LIM and SH3 domain protein 1 OS=Homo sapiens GN=LASP1 PE=1 SV=2		689.49
Q14847-2	LASP1_HUMAN Isoform 2 of LIM and SH3 domain protein 1 OS=Homo sapiens GN=LASP1		651.44
Q14847;Q14847-2	LASP1_HUMAN LIM and SH3 domain protein 1 OS=Homo sapiens GN=LASP1 PE=1 SV=2;>sp|Q14847-2|LASP1_HUMAN Isoform 2 of	2.93E-11	
Q14849	STAR3_HUMAN StAR-related lipid transfer protein 3 OS=Homo sapiens GN=STARD3 PE=1 SV=2		
Q14914	PTGR1_HUMAN Prostaglandin reductase 1 OS=Homo sapiens GN=PTGR1 PE=1 SV=2		86.34
Q14919	NC2A_HUMAN Dr1-associated corepressor OS=Homo sapiens GN=DRAP1 PE=1 SV=3		118.57
Q14919-2	NC2A_HUMAN Isoform 2 of Dr1-associated corepressor OS=Homo sapiens GN=DRAP1		118.57
Q14919-2;Q14919	2|NC2A_HUMAN Isoform 2 of Dr1-associated corepressor OS=Homo sapiens GN=DRAP1;>sp|Q14919|NC2A_HUMAN Dr1-associated c	2.61E-23	
Q14938	NFIX_HUMAN Nuclear factor 1 X-type OS=Homo sapiens GN=NFIX PE=1 SV=2		26.6
Q14938-2	NFIX_HUMAN Isoform 2 of Nuclear factor 1 X-type OS=Homo sapiens GN=NFIX		26.6
Q14938-3	NFIX_HUMAN Isoform 3 of Nuclear factor 1 X-type OS=Homo sapiens GN=NFIX		26.6
Q14938-4	NFIX_HUMAN Isoform 4 of Nuclear factor 1 X-type OS=Homo sapiens GN=NFIX		26.6
Q14938-5	NFIX_HUMAN Isoform 5 of Nuclear factor 1 X-type OS=Homo sapiens GN=NFIX		26.6
Q14943	KI3S1_HUMAN Killer cell immunoglobulin-like receptor 3DS1 OS=Homo sapiens GN=KIR3DS1 PE=2 SV=1		
Q14953	KI2S5_HUMAN Killer cell immunoglobulin-like receptor 2DS5 OS=Homo sapiens GN=KIR2DS5 PE=2 SV=1		
Q14954	KI2S1_HUMAN Killer cell immunoglobulin-like receptor 2DS1 OS=Homo sapiens GN=KIR2DS1 PE=2 SV=1		
Q14956;Q14956-2	GPNMB_HUMAN Transmembrane glycoprotein NMB OS=Homo sapiens GN=GPNMB PE=1 SV=2;>sp|Q14956-2|GPNMB_HUMAN Isoforr	2.06E-75	
Q14964	RB39A_HUMAN Ras-related protein Rab-39A OS=Homo sapiens GN=RAB39 PE=2 SV=2		61.85
Q14966	ZN638_HUMAN Zinc finger protein 638 OS=Homo sapiens GN=ZNF638 PE=1 SV=2		60.64
Q14966-2	ZN638_HUMAN Isoform 2 of Zinc finger protein 638 OS=Homo sapiens GN=ZNF638		1.6
Q14966-3	ZN638_HUMAN Isoform 3 of Zinc finger protein 638 OS=Homo sapiens GN=ZNF638		40.19
Q14966-4	ZN638_HUMAN Isoform 4 of Zinc finger protein 638 OS=Homo sapiens GN=ZNF638		5.69
Q14966-5	ZN638_HUMAN Isoform 5 of Zinc finger protein 638 OS=Homo sapiens GN=ZNF638		59.08
Q14966;Q14966-5	ZN638_HUMAN Zinc finger protein 638 OS=Homo sapiens GN=ZNF638 PE=1 SV=2;>sp|Q14966-5|ZN638_HUMAN Isoform 5 of Zinc f	2.01E-13	
Q14974	IMB1_HUMAN Importin subunit beta-1 OS=Homo sapiens GN=KPNB1 PE=1 SV=2		754.68
Q14978	NOLC1_HUMAN Nucleolar and coiled-body phosphoprotein 1 OS=Homo sapiens GN=NOLC1 PE=1 SV=2		235.45
Q14978-2	NOLC1_HUMAN Isoform Beta of Nucleolar and coiled-body phosphoprotein 1 OS=Homo sapiens GN=NOLC1		242.82
Q14978-2;B2RAU8;Q1497	2|NOLC1_HUMAN Isoform Beta of Nucleolar and coiled-body phosphoprotein 1 OS=Homo sapiens GN=NOLC1;>tr|B2RAU8|B2RAU8_H	3.77E-40	
Q14978-3	NOLC1_HUMAN Isoform 3 of Nucleolar and coiled-body phosphoprotein 1 OS=Homo sapiens GN=NOLC1		122.12
Q14980	NUMA1_HUMAN Nuclear mitotic apparatus protein 1 OS=Homo sapiens GN=NUMA1 PE=1 SV=2		925.1
Q14980-2	NUMA1_HUMAN Isoform 2 of Nuclear mitotic apparatus protein 1 OS=Homo sapiens GN=NUMA1		933.86
Q14980-2;Q14980	2|NUMA1_HUMAN Isoform 2 of Nuclear mitotic apparatus protein 1 OS=Homo sapiens GN=NUMA1;>sp|Q14980|NUMA1_HUMAN Nucl	0	
Q14980;Q14980-2	NUMA1_HUMAN Nuclear mitotic apparatus protein 1 OS=Homo sapiens GN=NUMA1 PE=1 SV=2;>sp|Q14980-2|NUMA1_HUMAN Isofor	0	
Q14994	NR1I3_HUMAN Nuclear receptor subfamily 1 group I member 3 OS=Homo sapiens GN=NR1I3 PE=1 SV=2		
Q14994-2	NR1I3_HUMAN Isoform 2 of Nuclear receptor subfamily 1 group I member 3 OS=Homo sapiens GN=NR1I3		
Q14995	NR1D2_HUMAN Nuclear receptor subfamily 1 group D member 2 OS=Homo sapiens GN=NR1D2 PE=1 SV=3	6.24E-05	
Q14997	PSME4_HUMAN Proteasome activator complex subunit 4 OS=Homo sapiens GN=PSME4 PE=1 SV=2	2.42E-08	
Q149N8	SHPRH_HUMAN E3 ubiquitin-protein ligase SHPRH OS=Homo sapiens GN=SHPRH PE=1 SV=2		32.18
Q149N8-2	SHPRH_HUMAN Isoform 2 of E3 ubiquitin-protein ligase SHPRH OS=Homo sapiens GN=SHPRH		13.71
Q149N8-4	SHPRH_HUMAN Isoform 3 of E3 ubiquitin-protein ligase SHPRH OS=Homo sapiens GN=SHPRH		31.58
Q149N8-5	SHPRH_HUMAN Isoform 4 of E3 ubiquitin-protein ligase SHPRH OS=Homo sapiens GN=SHPRH		0.96
Q14BN4;Q14BN4-3;Q14BI	SLMAP_HUMAN Sarcolemmal membrane-associated protein OS=Homo sapiens GN=SLMAP PE=1 SV=1;>sp|Q14BN4-3|SLMAP_HUMAN	6.04E-30	
Q14C86-6;Q14C86;Q14C8	6|GAPD1_HUMAN Isoform 6 of GTPase-activating protein and VPS9 domain-containing protein 1 OS=Homo sapiens GN=GAPVD1;>sp|	8.34E-13	
Q14C87	T132D_HUMAN Transmembrane protein 132D OS=Homo sapiens GN=TMEM132D PE=2 SV=1		

Q14CN4	K2C72_HUMAN Keratin, type II cytoskeletal 72 OS=Homo sapiens GN=KRT72 PE=1 SV=2		280.69
Q14CN4-2	K2C72_HUMAN Isoform 2 of Keratin, type II cytoskeletal 72 OS=Homo sapiens GN=KRT72		280.69
Q14CX7	NAA25_HUMAN N-alpha-acetyltransferase 25, NatB auxiliary subunit OS=Homo sapiens GN=NAA25 PE=1 SV=1		57.11
Q14CX7-2	NAA25_HUMAN Isoform 2 of N-alpha-acetyltransferase 25, NatB auxiliary subunit OS=Homo sapiens GN=NAA25		57.11
Q14CX7;Q14CX7-2	NAA25_HUMAN N-alpha-acetyltransferase 25, NatB auxiliary subunit OS=Homo sapiens GN=NAA25 PE=1 SV=1;>sp|Q14CX7-2|NAA2	8.87E-12	
Q15003	CND2_HUMAN Condensin complex subunit 2 OS=Homo sapiens GN=NCAPH PE=1 SV=3	1.76E-14	
Q15004	PAF_HUMAN PCNA-associated factor OS=Homo sapiens GN=PAF PE=1 SV=1		
Q15005	SPCS2_HUMAN Signal peptidase complex subunit 2 OS=Homo sapiens GN=SPCS2 PE=1 SV=3		258.47
Q15006	TTC35_HUMAN Tetratricopeptide repeat protein 35 OS=Homo sapiens GN=TTC35 PE=1 SV=1		215.2
Q15007	FL2D_HUMAN Pre-splicing regulator WTAP OS=Homo sapiens GN=WTAP PE=1 SV=2		
Q15007-2	FL2D_HUMAN Isoform 2 of Pre-splicing regulator WTAP OS=Homo sapiens GN=WTAP		
Q15007;Q15007-2	FL2D_HUMAN Pre-mRNA-splicing regulator WTAP OS=Homo sapiens GN=WTAP PE=1 SV=2;>sp|Q15007-2|FL2D_HUMAN Isoform 2 of	2.64E-70	
Q15008	PSMD6_HUMAN 26S proteasome non-ATPase regulatory subunit 6 OS=Homo sapiens GN=PSMD6 PE=1 SV=1		233.55
Q15011	HERP1_HUMAN Homocysteine-responsive endoplasmic reticulum-resident ubiquitin-like domain member 1 protein OS=Homo sapiens GN=HERPUD1 I		27.87
Q15011-2	HERP1_HUMAN Isoform 2 of Homocysteine-responsive endoplasmic reticulum-resident ubiquitin-like domain member 1 protein OS=Homo sapiens GN		27.87
Q15011-3	HERP1_HUMAN Isoform 3 of Homocysteine-responsive endoplasmic reticulum-resident ubiquitin-like domain member 1 protein OS=Homo sapiens GN		27.87
Q15014	MO4L2_HUMAN Mortality factor 4-like protein 2 OS=Homo sapiens GN=MORF4L2 PE=1 SV=1		109.44
Q15018	F175B_HUMAN BRISC complex subunit Abro1 OS=Homo sapiens GN=FAM175B PE=1 SV=2	2.58E-06	
Q15019	SEPT2_HUMAN Septin-2 OS=Homo sapiens GN=SEPT2 PE=1 SV=1		813.81
Q15019-2	SEPT2_HUMAN Isoform 2 of Septin-2 OS=Homo sapiens GN=SEPT2		832.4
Q15019-2;Q15019	2|SEPT2_HUMAN Isoform 2 of Septin-2 OS=Homo sapiens GN=SEPT2;>sp|Q15019|SEPT2_HUMAN Septin-2 OS=Homo sapiens GN=S	5.15E-300	
Q15020	SART3_HUMAN Squamous cell carcinoma antigen recognized by T-cells 3 OS=Homo sapiens GN=SART3 PE=1 SV=1		238.98
Q15020-2	SART3_HUMAN Isoform 2 of Squamous cell carcinoma antigen recognized by T-cells 3 OS=Homo sapiens GN=SART3		40.91
Q15020;B7ZKM0;B4DYA2	SART3_HUMAN Squamous cell carcinoma antigen recognized by T-cells 3 OS=Homo sapiens GN=SART3 PE=1 SV=1;>tr|B7ZKM0|B7Z	5.84E-79	
Q15021	CND1_HUMAN Condensin complex subunit 1 OS=Homo sapiens GN=NCAPD2 PE=1 SV=3		40.64
Q15024	EXOS7_HUMAN Exosome complex component RRP42 OS=Homo sapiens GN=EXOSC7 PE=1 SV=3		35.52
Q15024;B2RDZ9;B4DIU0	EXOS7_HUMAN Exosome complex exonuclease RRP42 OS=Homo sapiens GN=EXOSC7 PE=1 SV=2;>tr|B2RDZ9|B2RDZ9_HUMAN cDN	2.25E-33	
Q15025	TNIP1_HUMAN TNFAIP3-interacting protein 1 OS=Homo sapiens GN=TNIP1 PE=1 SV=2		38.43
Q15025-2	TNIP1_HUMAN Isoform 2 of TNFAIP3-interacting protein 1 OS=Homo sapiens GN=TNIP1		38.43
Q15029	U5S1_HUMAN 116 kDa U5 small nuclear ribonucleoprotein component OS=Homo sapiens GN=EFTUD2 PE=1 SV=1		696.9
Q15031	SYLM_HUMAN Probable leucyl-tRNA synthetase, mitochondrial OS=Homo sapiens GN=LARS2 PE=1 SV=2	1.65E-12	
Q15032	R3HD1_HUMAN R3H domain-containing protein 1 OS=Homo sapiens GN=R3HDM1 PE=1 SV=3		
Q15032-2	R3HD1_HUMAN Isoform 2 of R3H domain-containing protein 1 OS=Homo sapiens GN=R3HDM1		
Q15035	TRAM2_HUMAN Translocating chain-associated membrane protein 2 OS=Homo sapiens GN=TRAM2 PE=1 SV=1	1.31E-05	
Q15040	JOS1_HUMAN Josephin-1 OS=Homo sapiens GN=JOSD1 PE=1 SV=1		
Q15041	AR6P1_HUMAN ADP-ribosylation factor-like protein 6-interacting protein 1 OS=Homo sapiens GN=ARL6IP1 PE=1 SV=2		50.78
Q15042	RB3GP_HUMAN Rab3 GTPase-activating protein catalytic subunit OS=Homo sapiens GN=RAB3GAP1 PE=1 SV=3	3.43E-10	
Q15046	SYK_HUMAN Lysyl-tRNA synthetase OS=Homo sapiens GN=KARS PE=1 SV=3		718.18
Q15046-2	SYK_HUMAN Isoform Mitochondrial of Lysyl-tRNA synthetase OS=Homo sapiens GN=KARS		711.72
Q15046;D3DUK4;Q15046-	SYK_HUMAN Lysyl-tRNA synthetase OS=Homo sapiens GN=KARS PE=1 SV=3;>tr|D3DUK4|D3DUK4_HUMAN Lysyl-tRNA synthetase O	9.01E-265	
Q15050	RRS1_HUMAN Ribosome biogenesis regulatory protein homolog OS=Homo sapiens GN=RRS1 PE=1 SV=2		
Q15052	ARHG6_HUMAN Rho guanine nucleotide exchange factor 6 OS=Homo sapiens GN=ARHGEF6 PE=1 SV=2		
Q15052-2	ARHG6_HUMAN Isoform 2 of Rho guanine nucleotide exchange factor 6 OS=Homo sapiens GN=ARHGEF6		
Q15054	DPOD3_HUMAN DNA polymerase delta subunit 3 OS=Homo sapiens GN=POLD3 PE=1 SV=2		
Q15056	IF4H_HUMAN Eukaryotic translation initiation factor 4H OS=Homo sapiens GN=EIF4H PE=1 SV=5		299.06
Q15056-2	IF4H_HUMAN Isoform Short of Eukaryotic translation initiation factor 4H OS=Homo sapiens GN=EIF4H		299.06
Q15056;Q15056-2	IF4H_HUMAN Eukaryotic translation initiation factor 4H OS=Homo sapiens GN=EIF4H PE=1 SV=5;>sp|Q15056-2|IF4H_HUMAN Isofor	3.82E-129	
Q15057	ACAP2_HUMAN Arf-GAP with coiled-coil, ANK repeat and PH domain-containing protein 2 OS=Homo sapiens GN=ACAP2 PE=1 SV=3	4.31E-06	
Q15058	KIF14_HUMAN Kinesin-like protein KIF14 OS=Homo sapiens GN=KIF14 PE=1 SV=1	5.66E-06	
Q15059	BRD3_HUMAN Bromodomain-containing protein 3 OS=Homo sapiens GN=BRD3 PE=1 SV=1		
Q15059-2	BRD3_HUMAN Isoform 2 of Bromodomain-containing protein 3 OS=Homo sapiens GN=BRD3		
Q15061	WDR43_HUMAN WD repeat-containing protein 43 OS=Homo sapiens GN=WDR43 PE=1 SV=3		21.1
Q15063	POSTN_HUMAN Periostin OS=Homo sapiens GN=POSTN PE=1 SV=2	0	
Q15063-2	2|POSTN_HUMAN Isoform 2 of Periostin OS=Homo sapiens GN=POSTN	0	
Q15063-3	3|POSTN_HUMAN Isoform 3 of Periostin OS=Homo sapiens GN=POSTN	0	
Q15067;Q15067-2	ACOX1_HUMAN Peroxisomal acyl-coenzyme A oxidase 1 OS=Homo sapiens GN=ACOX1 PE=1 SV=3;>sp|Q15067-2|ACOX1_HUMAN Is	8.90E-15	
Q15070	OXA1L_HUMAN Mitochondrial inner membrane protein OXA1L OS=Homo sapiens GN=OXA1L PE=1 SV=3		45.67
Q15070-2	OXA1L_HUMAN Isoform 2 of Mitochondrial inner membrane protein OXA1L OS=Homo sapiens GN=OXA1L		45.67
Q15070-3	OXA1L_HUMAN Isoform 3 of Mitochondrial inner membrane protein OXA1L OS=Homo sapiens GN=OXA1L		45.67
Q15070;Q15070-2;Q1507	OXA1L_HUMAN Mitochondrial inner membrane protein OXA1L OS=Homo sapiens GN=OXA1L PE=1 SV=3;>sp|Q15070-2|OXA1L_HUM/	5.24E-10	
Q15075	EEA1_HUMAN Early endosome antigen 1 OS=Homo sapiens GN=EEA1 PE=1 SV=2		437.89

Q15084	PDIA6_HUMAN Protein disulfide-isomerase A6 OS=Homo sapiens GN=PDIA6 PE=1 SV=1		894.82
Q15084-2	PDIA6_HUMAN Isoform 2 of Protein disulfide-isomerase A6 OS=Homo sapiens GN=PDIA6		894.82
Q15084-2;B3KY95;B5MBV	2|PDIA6_HUMAN Isoform 2 of Protein disulfide-isomerase A6 OS=Homo sapiens GN=PDIA6;>tr|B3KY95|B3KY95_HUMAN cDNA FLJ16	0	
Q15102	PA1B3_HUMAN Platelet-activating factor acetylhydrolase IB subunit gamma OS=Homo sapiens GN=PAFAH1B3 PE=1 SV=1		114.53
Q15109	RAGE_HUMAN Advanced glycosylation end product-specific receptor OS=Homo sapiens GN=AGER PE=1 SV=1		
Q15109-2	RAGE_HUMAN Isoform 2 of Advanced glycosylation end product-specific receptor OS=Homo sapiens GN=AGER		
Q15113	PCOC1_HUMAN Procollagen C-endopeptidase enhancer 1 OS=Homo sapiens GN=PCOLCE PE=1 SV=2	8.93E-50	
Q15119	PDK2_HUMAN [Pyruvate dehydrogenase [lipoamide]] kinase isozyme 2, mitochondrial OS=Homo sapiens GN=PDK2 PE=1 SV=2		
Q15121	PEA15_HUMAN Astrocytic phosphoprotein PEA-15 OS=Homo sapiens GN=PEA15 PE=1 SV=2		243.78
Q15124	PGM5_HUMAN Phosphoglucomutase-like protein 5 OS=Homo sapiens GN=PGM5 PE=1 SV=2		
Q15124-2	PGM5_HUMAN Isoform 2 of Phosphoglucomutase-like protein 5 OS=Homo sapiens GN=PGM5		
Q15124;Q15124-2	PGM5_HUMAN Phosphoglucomutase-like protein 5 OS=Homo sapiens GN=PGM5 PE=1 SV=2;>sp|Q15124-2|PGM5_HUMAN Isoform 2	4.85E-21	
Q15125	EBP_HUMAN 3-beta-hydroxysteroid-Delta(8),Delta(7)-isomerase OS=Homo sapiens GN=EBP PE=1 SV=3		53.13
Q15126	PMVK_HUMAN Phosphomevalonate kinase OS=Homo sapiens GN=PMVK PE=1 SV=3	7.66E-18	
Q15139	KPCD1_HUMAN Serine/threonine-protein kinase D1 OS=Homo sapiens GN=PRKD1 PE=1 SV=2		
Q15149	PLEC_HUMAN Plectin OS=Homo sapiens GN=PLEC PE=1 SV=3		11556.57
Q15149-2	PLEC_HUMAN Isoform 2 of Plectin OS=Homo sapiens GN=PLEC		11519.68
Q15149-3	PLEC_HUMAN Isoform 3 of Plectin OS=Homo sapiens GN=PLEC		11588.01
Q15149-4	PLEC_HUMAN Isoform 4 of Plectin OS=Homo sapiens GN=PLEC		11633.37
Q15149-5	PLEC_HUMAN Isoform 5 of Plectin OS=Homo sapiens GN=PLEC		11519.68
Q15149-6	PLEC_HUMAN Isoform 6 of Plectin OS=Homo sapiens GN=PLEC		11519.68
Q15149-7	PLEC_HUMAN Isoform 7 of Plectin OS=Homo sapiens GN=PLEC		11519.68
Q15149-8	PLEC_HUMAN Isoform 8 of Plectin OS=Homo sapiens GN=PLEC		11519.68
Q15149-9	PLEC_HUMAN Isoform 9 of Plectin OS=Homo sapiens GN=PLEC		11519.68
Q15149;Q15149-2;Q1514	PLEC_HUMAN Plectin OS=Homo sapiens GN=PLEC PE=1 SV=3;>sp|Q15149-2|PLEC_HUMAN Isoform 2 of Plectin OS=Homo sapiens G	0	
Q15154;Q15154-2	PCM1_HUMAN Pericentriolar material 1 protein OS=Homo sapiens GN=PCM1 PE=1 SV=4;>sp|Q15154-2|PCM1_HUMAN Isoform 2 of P	8.29E-20	
Q15155	NOMO1_HUMAN Nodal modulator 1 OS=Homo sapiens GN=NOMO1 PE=1 SV=5		204.65
Q15165;Q15165-1;Q1516	PON2_HUMAN Serum paraoxonase/arylesterase 2 OS=Homo sapiens GN=PON2 PE=1 SV=3;>sp|Q15165-1|PON2_HUMAN Isoform 1 c	8.64E-24	
Q15173	2A5B_HUMAN Serine/threonine-protein phosphatase 2A 56 kDa regulatory subunit beta isoform OS=Homo sapiens GN=PPP2R5B PE=1 SV=1		
Q15173-2	2A5B_HUMAN Isoform Beta-2 of Serine/threonine-protein phosphatase 2A 56 kDa regulatory subunit beta isoform OS=Homo sapiens GN=PPP2R5B		
Q15181	IPYR_HUMAN Inorganic pyrophosphatase OS=Homo sapiens GN=PPA1 PE=1 SV=2		138.66
Q15185	TEBP_HUMAN Prostaglandin E synthase 3 OS=Homo sapiens GN=PTGES3 PE=1 SV=1		191.08
Q15208	STK38_HUMAN Serine/threonine-protein kinase 38 OS=Homo sapiens GN=STK38 PE=1 SV=1	1.38E-18	
Q15233	NONO_HUMAN Non-POU domain-containing octamer-binding protein OS=Homo sapiens GN=NONO PE=1 SV=4		1092.91
Q15257	PTPA_HUMAN Serine/threonine-protein phosphatase 2A activator OS=Homo sapiens GN=PPP2R4 PE=1 SV=3		89.83
Q15257-2	PTPA_HUMAN Isoform 1 of Serine/threonine-protein phosphatase 2A activator OS=Homo sapiens GN=PPP2R4		89.83
Q15257-3	PTPA_HUMAN Isoform 3 of Serine/threonine-protein phosphatase 2A activator OS=Homo sapiens GN=PPP2R4		89.83
Q15257-4	PTPA_HUMAN Isoform 4 of Serine/threonine-protein phosphatase 2A activator OS=Homo sapiens GN=PPP2R4		11.27
Q15257;A6PVN5;Q15257-	PTPA_HUMAN Serine/threonine-protein phosphatase 2A activator OS=Homo sapiens GN=PPP2R4 PE=1 SV=3;>tr|A6PVN5|A6PVN5_HU	8.54E-40	
Q15269	PWP2_HUMAN Periodic tryptophan protein 2 homolog OS=Homo sapiens GN=PWP2 PE=1 SV=2		
Q15274	NADC_HUMAN Nicotinate-nucleotide pyrophosphorylase [carboxylating] OS=Homo sapiens GN=QPRT PE=1 SV=3	2.63E-12	
Q15276	RABE1_HUMAN Rab GTPase-binding effector protein 1 OS=Homo sapiens GN=RABEP1 PE=1 SV=2		
Q15276-2	RABE1_HUMAN Isoform 2 of Rab GTPase-binding effector protein 1 OS=Homo sapiens GN=RABEP1		
Q15276;Q15276-2	RABE1_HUMAN Rab GTPase-binding effector protein 1 OS=Homo sapiens GN=RABEP1 PE=1 SV=2;>sp|Q15276-2|RABE1_HUMAN Isof	2.97E-18	
Q15286	RAB35_HUMAN Ras-related protein Rab-35 OS=Homo sapiens GN=RAB35 PE=1 SV=1		441.18
Q15287	RNPS1_HUMAN RNA-binding protein with serine-rich domain 1 OS=Homo sapiens GN=RNPS1 PE=1 SV=1		
Q15287-2	RNPS1_HUMAN Isoform 2 of RNA-binding protein with serine-rich domain 1 OS=Homo sapiens GN=RNPS1		
Q15287-3	RNPS1_HUMAN Isoform 3 of RNA-binding protein with serine-rich domain 1 OS=Homo sapiens GN=RNPS1		
Q15287;Q15287-2;Q1528	RNPS1_HUMAN RNA-binding protein with serine-rich domain 1 OS=Homo sapiens GN=RNPS1 PE=1 SV=1;>sp|Q15287-2|RNPS1_HUM	1.90E-122	
Q15291	RBBP5_HUMAN Retinoblastoma-binding protein 5 OS=Homo sapiens GN=RBBP5 PE=1 SV=2		38
Q15291-2	RBBP5_HUMAN Isoform 2 of Retinoblastoma-binding protein 5 OS=Homo sapiens GN=RBBP5		38
Q15291;Q15291-2	RBBP5_HUMAN Retinoblastoma-binding protein 5 OS=Homo sapiens GN=RBBP5 PE=1 SV=2;>sp|Q15291-2|RBBP5_HUMAN Isoform 2	1.12E-09	
Q15293	RCN1_HUMAN Reticulocalbin-1 OS=Homo sapiens GN=RCN1 PE=1 SV=1		775.01
Q15303	ERBB4_HUMAN Receptor tyrosine-protein kinase erbB-4 OS=Homo sapiens GN=ERBB4 PE=1 SV=1		84.23
Q15303-2	ERBB4_HUMAN Isoform JM-B CYT-1 of Receptor tyrosine-protein kinase erbB-4 OS=Homo sapiens GN=ERBB4		84.23
Q15303-3	ERBB4_HUMAN Isoform JM-A CYT-2 of Receptor tyrosine-protein kinase erbB-4 OS=Homo sapiens GN=ERBB4		84.23
Q15303-4	ERBB4_HUMAN Isoform JM-B CYT-2 of Receptor tyrosine-protein kinase erbB-4 OS=Homo sapiens GN=ERBB4		84.23
Q15306;Q15306-2	IRF4_HUMAN Interferon regulatory factor 4 OS=Homo sapiens GN=IRF4 PE=1 SV=1;>sp|Q15306-2|IRF4_HUMAN Isoform 2 of Interfe	1.47E-16	
Q15323	K1H1_HUMAN Keratin, type I cuticular Ha1 OS=Homo sapiens GN=KRT31 PE=1 SV=3		62.43
Q15327	ANKR1_HUMAN Ankyrin repeat domain-containing protein 1 OS=Homo sapiens GN=ANKRD1 PE=1 SV=2		
Q15363	TMED2_HUMAN Transmembrane emp24 domain-containing protein 2 OS=Homo sapiens GN=TMED2 PE=1 SV=1		289.71

Q15365	PCBP1_HUMAN Poly(rC)-binding protein 1 OS=Homo sapiens GN=PCBP1 PE=1 SV=2		325.69
Q15366	PCBP2_HUMAN Poly(rC)-binding protein 2 OS=Homo sapiens GN=PCBP2 PE=1 SV=1		394.9
Q15369	ELOC_HUMAN Transcription elongation factor B polypeptide 1 OS=Homo sapiens GN=TCEB1 PE=1 SV=1		279.31
Q15370	ELOB_HUMAN Transcription elongation factor B polypeptide 2 OS=Homo sapiens GN=TCEB2 PE=1 SV=1		165.67
Q15375	EPHA7_HUMAN Ephrin type-A receptor 7 OS=Homo sapiens GN=EPHA7 PE=1 SV=3		89.59
Q15375-2	EPHA7_HUMAN Isoform 2 of Ephrin type-A receptor 7 OS=Homo sapiens GN=EPHA7		89.59
Q15375;Q15375-2	EPHA7_HUMAN Ephrin type-A receptor 7 OS=Homo sapiens GN=EPHA7 PE=1 SV=3;>sp|Q15375-2|EPHA7_HUMAN Isoform 2 of Ephri	2.57E-09	
Q15382	RHEB_HUMAN GTP-binding protein Rheb OS=Homo sapiens GN=RHEB PE=1 SV=1		188.06
Q15386;B4DHJ9	UBE3C_HUMAN Ubiquitin-protein ligase E3C OS=Homo sapiens GN=UBE3C PE=1 SV=3;>tr|B4DHJ9|B4DHJ9_HUMAN cDNA FLJ61262,	4.83E-10	
Q15388	TOM20_HUMAN Mitochondrial import receptor subunit TOM20 homolog OS=Homo sapiens GN=TOMM20 PE=1 SV=1		172.31
Q15392	DHC24_HUMAN Delta(24)-sterol reductase OS=Homo sapiens GN=DHCR24 PE=1 SV=2		
Q15393	SF3B3_HUMAN Splicing factor 3B subunit 3 OS=Homo sapiens GN=SF3B3 PE=1 SV=4		671.38
Q15393-2	SF3B3_HUMAN Isoform 2 of Splicing factor 3B subunit 3 OS=Homo sapiens GN=SF3B3		128.18
Q15393-3	SF3B3_HUMAN Isoform 3 of Splicing factor 3B subunit 3 OS=Homo sapiens GN=SF3B3		354.7
Q15397	K0020_HUMAN Pumilio domain-containing protein KIAA0020 OS=Homo sapiens GN=KIAA0020 PE=1 SV=3		93.34
Q15398;Q15398-1	DLGP5_HUMAN Disks large-associated protein 5 OS=Homo sapiens GN=DLGAP5 PE=1 SV=2;>sp|Q15398-1|DLGP5_HUMAN Isoform 2	2.17E-69	
Q15404	RSU1_HUMAN Ras suppressor protein 1 OS=Homo sapiens GN=RSU1 PE=1 SV=3		254.57
Q15417	CNN3_HUMAN Calponin-3 OS=Homo sapiens GN=CNN3 PE=1 SV=1		553.62
Q15418-2;Q15418;Q1541	2|KS6A1_HUMAN Isoform 2 of Ribosomal protein S6 kinase alpha-1 OS=Homo sapiens GN=RPS6KA1;>sp|Q15418|KS6A1_HUMAN Rib	5.14E-66	
Q15424	SAFB1_HUMAN Scaffold attachment factor B1 OS=Homo sapiens GN=SAFB PE=1 SV=4		
Q15427	SF3B4_HUMAN Splicing factor 3B subunit 4 OS=Homo sapiens GN=SF3B4 PE=1 SV=1		172.76
Q15428	SF3A2_HUMAN Splicing factor 3A subunit 2 OS=Homo sapiens GN=SF3A2 PE=1 SV=2		117.44
Q15434	RBMS2_HUMAN RNA-binding motif, single-stranded-interacting protein 2 OS=Homo sapiens GN=RBMS2 PE=1 SV=1		19.33
Q15435	PP1R7_HUMAN Protein phosphatase 1 regulatory subunit 7 OS=Homo sapiens GN=PPP1R7 PE=1 SV=1		74.87
Q15435-2	PP1R7_HUMAN Isoform 2 of Protein phosphatase 1 regulatory subunit 7 OS=Homo sapiens GN=PPP1R7		74.87
Q15435-3	PP1R7_HUMAN Isoform 3 of Protein phosphatase 1 regulatory subunit 7 OS=Homo sapiens GN=PPP1R7		69.81
Q15435-4	PP1R7_HUMAN Isoform 4 of Protein phosphatase 1 regulatory subunit 7 OS=Homo sapiens GN=PPP1R7		69.81
Q15435;Q15435-2;C9J17	PP1R7_HUMAN Protein phosphatase 1 regulatory subunit 7 OS=Homo sapiens GN=PPP1R7 PE=1 SV=1;>sp|Q15435-2|PP1R7_HUMAN	2.50E-116	
Q15436	SC23A_HUMAN Protein transport protein Sec23A OS=Homo sapiens GN=SEC23A PE=1 SV=2		165.87
Q15437	SC23B_HUMAN Protein transport protein Sec23B OS=Homo sapiens GN=SEC23B PE=1 SV=2		51.31
Q15459	SF3A1_HUMAN Splicing factor 3A subunit 1 OS=Homo sapiens GN=SF3A1 PE=1 SV=1		411.48
Q15477	SKIV2_HUMAN Helicase SKI2W OS=Homo sapiens GN=SKIV2L PE=1 SV=3	2.35E-05	
Q15493	RGN_HUMAN Regucalcin OS=Homo sapiens GN=RGN PE=1 SV=1	3.94E-48	
Q15526	SURF1_HUMAN Surfeit locus protein 1 OS=Homo sapiens GN=SURF1 PE=1 SV=1		
Q15526-2	SURF1_HUMAN Isoform 2 of Surfeit locus protein 1 OS=Homo sapiens GN=SURF1		
Q15528	MED22_HUMAN Mediator of RNA polymerase II transcription subunit 22 OS=Homo sapiens GN=MED22 PE=1 SV=2		
Q15528-2	MED22_HUMAN Isoform Surf5A of Mediator of RNA polymerase II transcription subunit 22 OS=Homo sapiens GN=MED22		
Q15542	TAF5_HUMAN Transcription initiation factor TFIID subunit 5 OS=Homo sapiens GN=TAF5 PE=1 SV=3		
Q15542-2	TAF5_HUMAN Isoform Short of Transcription initiation factor TFIID subunit 5 OS=Homo sapiens GN=TAF5		
Q15545	TAF7_HUMAN Transcription initiation factor TFIID subunit 7 OS=Homo sapiens GN=TAF7 PE=1 SV=1	4.01E-05	
Q15555;B7Z2L3;B4DJV4;I	MARE2_HUMAN Microtubule-associated protein RP/EB family member 2 OS=Homo sapiens GN=MAPRE2 PE=1 SV=1;>tr|B7Z2L3|B7Z2	2.09E-11	
Q15560	TCEA2_HUMAN Transcription elongation factor A protein 2 OS=Homo sapiens GN=TCEA2 PE=1 SV=1		
Q15582	BGH3_HUMAN Transforming growth factor-beta-induced protein ig-h3 OS=Homo sapiens GN=TGFBI PE=1 SV=1	2.99E-217	
Q15599	NHRF2_HUMAN Na(+)/H(+) exchange regulatory cofactor NHE-RF2 OS=Homo sapiens GN=SLC9A3R2 PE=1 SV=2		540.43
Q15599-2	NHRF2_HUMAN Isoform 2 of Na(+)/H(+) exchange regulatory cofactor NHE-RF2 OS=Homo sapiens GN=SLC9A3R2		516.87
Q15599;Q15599-2	NHRF2_HUMAN Na(+)/H(+) exchange regulatory cofactor NHE-RF2 OS=Homo sapiens GN=SLC9A3R2 PE=1 SV=2;>sp|Q15599-2|NHI	2.11E-16	
Q155Q3	DIXC1_HUMAN Dixin OS=Homo sapiens GN=DIXDC1 PE=1 SV=2		34.06
Q155Q3-2	DIXC1_HUMAN Isoform 2 of Dixin OS=Homo sapiens GN=DIXDC1		14.83
Q155Q3-3	DIXC1_HUMAN Isoform 3 of Dixin OS=Homo sapiens GN=DIXDC1		14.83
Q155Q3-4	DIXC1_HUMAN Isoform 4 of Dixin OS=Homo sapiens GN=DIXDC1		19.23
Q15628	TRADD_HUMAN Tumor necrosis factor receptor type 1-associated DEATH domain protein OS=Homo sapiens GN=TRADD PE=1 SV=2	1.48E-09	
Q15629	TRAM1_HUMAN Translocating chain-associated membrane protein 1 OS=Homo sapiens GN=TRAM1 PE=1 SV=3		41.36
Q15631	TSN_HUMAN Translin OS=Homo sapiens GN=TSN PE=1 SV=1		91.9
Q15637	SF01_HUMAN Splicing factor 1 OS=Homo sapiens GN=SF1 PE=1 SV=4		412.9
Q15637-2	SF01_HUMAN Isoform 2 of Splicing factor 1 OS=Homo sapiens GN=SF1		412.9
Q15637-3	SF01_HUMAN Isoform 3 of Splicing factor 1 OS=Homo sapiens GN=SF1		412.9
Q15637-4	SF01_HUMAN Isoform 4 of Splicing factor 1 OS=Homo sapiens GN=SF1		412.9
Q15637-5	SF01_HUMAN Isoform 5 of Splicing factor 1 OS=Homo sapiens GN=SF1		418.48
Q15637-6	SF01_HUMAN Isoform 6 of Splicing factor 1 OS=Homo sapiens GN=SF1		412.9
Q15637;Q15637-2;B4DJU	SF01_HUMAN Splicing factor 1 OS=Homo sapiens GN=SF1 PE=1 SV=4;>sp|Q15637-2|SF01_HUMAN Isoform 2 of Splicing factor 1 OS	9.03E-51	
Q15642	CIP4_HUMAN Cdc42-interacting protein 4 OS=Homo sapiens GN=TRIP10 PE=1 SV=3		80.13

Q15642-2	CIP4_HUMAN Isoform 2 of Cdc42-interacting protein 4 OS=Homo sapiens GN=TRIP10		80.13
Q15642-3	CIP4_HUMAN Isoform 3 of Cdc42-interacting protein 4 OS=Homo sapiens GN=TRIP10		80.13
Q15642-4	CIP4_HUMAN Isoform 4 of Cdc42-interacting protein 4 OS=Homo sapiens GN=TRIP10		80.13
Q15642-5	CIP4_HUMAN Isoform 5 of Cdc42-interacting protein 4 OS=Homo sapiens GN=TRIP10		46.86
Q15642;Q15642-2;Q1564	CIP4_HUMAN Cdc42-interacting protein 4 OS=Homo sapiens GN=TRIP10 PE=1 SV=3;>sp|Q15642-2|CIP4_HUMAN Isoform 2 of Cdc42	9.45E-129	
Q15643	TRIPB_HUMAN Thyroid receptor-interacting protein 11 OS=Homo sapiens GN=TRIP11 PE=1 SV=3		
Q15645	PCH2_HUMAN Pachytene checkpoint protein 2 homolog OS=Homo sapiens GN=TRIP13 PE=1 SV=2		
Q15645-2	PCH2_HUMAN Isoform 2 of Pachytene checkpoint protein 2 homolog OS=Homo sapiens GN=TRIP13		
Q15648	MED1_HUMAN Mediator of RNA polymerase II transcription subunit 1 OS=Homo sapiens GN=MED1 PE=1 SV=4		
Q15648-3	MED1_HUMAN Isoform 2 of Mediator of RNA polymerase II transcription subunit 1 OS=Homo sapiens GN=MED1		
Q15648;B2RAG9;B4DSK7	MED1_HUMAN Mediator of RNA polymerase II transcription subunit 1 OS=Homo sapiens GN=MED1 PE=1 SV=4;>tr|B2RAG9|B2RAG9_	2.75E-11	
Q15650	TRIP4_HUMAN Activating signal cointegrator 1 OS=Homo sapiens GN=TRIP4 PE=1 SV=4		
Q15651;P05204;Q15651-:	HMGN3_HUMAN High mobility group nucleosome-binding domain-containing protein 3 OS=Homo sapiens GN=HMGN3 PE=1 SV=2;>sp	3.31E-12	
Q15654	TRIP6_HUMAN Thyroid receptor-interacting protein 6 OS=Homo sapiens GN=TRIP6 PE=1 SV=3	5.97E-11	
Q15678	PTN14_HUMAN Tyrosine-protein phosphatase non-receptor type 14 OS=Homo sapiens GN=PTPN14 PE=1 SV=2		43.95
Q15691	MARE1_HUMAN Microtubule-associated protein RP/EB family member 1 OS=Homo sapiens GN=MAPRE1 PE=1 SV=3		318.93
Q15695	U2AFL_HUMAN U2 small nuclear ribonucleoprotein auxiliary factor 35 kDa subunit-related protein 1 OS=Homo sapiens GN=ZRSR1 PE=2 SV=2		
Q15699	ALX1_HUMAN ALX homeobox protein 1 OS=Homo sapiens GN=ALX1 PE=1 SV=2		
Q15714-2;Q15714;Q1571	2|T22D1_HUMAN Isoform 2 of TSC22 domain family protein 1 OS=Homo sapiens GN=TSC22D1;>sp|Q15714|T22D1_HUMAN TSC22 d	2.18E-60	
Q15717	ELAV1_HUMAN ELAV-like protein 1 OS=Homo sapiens GN=ELAVL1 PE=1 SV=2		626.96
Q15738	NSDHL_HUMAN Sterol-4-alpha-carboxylate 3-dehydrogenase, decarboxylating OS=Homo sapiens GN=NSDHL PE=1 SV=2		195.24
Q15746	MYLK_HUMAN Myosin light chain kinase, smooth muscle OS=Homo sapiens GN=MYLK PE=1 SV=4		46.14
Q15746-2	MYLK_HUMAN Isoform 2 of Myosin light chain kinase, smooth muscle OS=Homo sapiens GN=MYLK		44.95
Q15746-3	MYLK_HUMAN Isoform 3A of Myosin light chain kinase, smooth muscle OS=Homo sapiens GN=MYLK		46.14
Q15746-4	MYLK_HUMAN Isoform 3B of Myosin light chain kinase, smooth muscle OS=Homo sapiens GN=MYLK		44.95
Q15746-5	MYLK_HUMAN Isoform 4 of Myosin light chain kinase, smooth muscle OS=Homo sapiens GN=MYLK		46.14
Q15746-6	MYLK_HUMAN Isoform Del-1790 of Myosin light chain kinase, smooth muscle OS=Homo sapiens GN=MYLK		46.14
Q15746-7	MYLK_HUMAN Isoform 5 of Myosin light chain kinase, smooth muscle OS=Homo sapiens GN=MYLK		44.95
Q15746;Q15746-6;Q1574	MYLK_HUMAN Myosin light chain kinase, smooth muscle OS=Homo sapiens GN=MYLK PE=1 SV=4;>sp|Q15746-6|MYLK_HUMAN Isofo	1.97E-20	
Q15750	TAB1_HUMAN TGF-beta-activated kinase 1 and MAP3K7-binding protein 1 OS=Homo sapiens GN=TAB1 PE=1 SV=1	0.00016412	
Q15751	HERC1_HUMAN Probable E3 ubiquitin-protein ligase HERC1 OS=Homo sapiens GN=HERC1 PE=1 SV=2		
Q15758	AAAT_HUMAN Neutral amino acid transporter B(0) OS=Homo sapiens GN=SLC1A5 PE=1 SV=2		574.24
Q15759	MK11_HUMAN Mitogen-activated protein kinase 11 OS=Homo sapiens GN=MAPK11 PE=1 SV=2		47.34
Q15760	GPR19_HUMAN Probable G-protein coupled receptor 19 OS=Homo sapiens GN=GPR19 PE=2 SV=2		
Q15771	RAB30_HUMAN Ras-related protein Rab-30 OS=Homo sapiens GN=RAB30 PE=1 SV=2		61.85
Q15785	TOM34_HUMAN Mitochondrial import receptor subunit TOM34 OS=Homo sapiens GN=TOMM34 PE=1 SV=2		214.04
Q15796;Q15796-2;O1519	SMAD2_HUMAN Mothers against decapentaplegic homolog 2 OS=Homo sapiens GN=SMAD2 PE=1 SV=1;>sp|Q15796-2|SMAD2_HUM/	1.74E-07	
Q15800	ERG25_HUMAN C-4 methylsterol oxidase OS=Homo sapiens GN=SC4MOL PE=1 SV=1		46.51
Q15811;Q15811-4;Q1581	ITSN1_HUMAN Intersectin-1 OS=Homo sapiens GN=ITSN1 PE=1 SV=3;>sp|Q15811-4|ITSN1_HUMAN Isoform 4 of Intersectin-1 OS=	1.73E-06	
Q15813	TBCE_HUMAN Tubulin-specific chaperone E OS=Homo sapiens GN=TBCE PE=1 SV=1	7.33E-20	
Q15814	TBCC_HUMAN Tubulin-specific chaperone C OS=Homo sapiens GN=TBCC PE=1 SV=2	1.58E-49	
Q15819	UB2V2_HUMAN Ubiquitin-conjugating enzyme E2 variant 2 OS=Homo sapiens GN=UBE2V2 PE=1 SV=4		239.46
Q15828	CYTM_HUMAN Cystatin-M OS=Homo sapiens GN=CST6 PE=1 SV=1	2.72E-11	
Q15833	STXB2_HUMAN Syntaxin-binding protein 2 OS=Homo sapiens GN=STXBP2 PE=1 SV=2		59.12
Q15833-2	STXB2_HUMAN Isoform 2 of Syntaxin-binding protein 2 OS=Homo sapiens GN=STXBP2		59.12
Q15833;Q15833-2	STXB2_HUMAN Syntaxin-binding protein 2 OS=Homo sapiens GN=STXBP2 PE=1 SV=2;>sp|Q15833-2|STXB2_HUMAN Isoform 2 of Sy	3.07E-17	
Q15836	VAMP3_HUMAN Vesicle-associated membrane protein 3 OS=Homo sapiens GN=VAMP3 PE=1 SV=3		310.33
Q15843	NEDD8_HUMAN NEDD8 OS=Homo sapiens GN=NEDD8 PE=1 SV=1		283.73
Q15847	APM2_HUMAN Adipose most abundant gene transcript 2 protein OS=Homo sapiens GN=APM2 PE=1 SV=1	8.73E-28	
Q15904	VAS1_HUMAN V-type proton ATPase subunit S1 OS=Homo sapiens GN=ATP6AP1 PE=1 SV=2		
Q15907	RB11B_HUMAN Ras-related protein Rab-11B OS=Homo sapiens GN=RAB11B PE=1 SV=4		612.85
Q15910	EZH2_HUMAN Histone-lysine N-methyltransferase EZH2 OS=Homo sapiens GN=EZH2 PE=1 SV=2		
Q15910-2	EZH2_HUMAN Isoform 2 of Histone-lysine N-methyltransferase EZH2 OS=Homo sapiens GN=EZH2		
Q15910-3	EZH2_HUMAN Isoform 3 of Histone-lysine N-methyltransferase EZH2 OS=Homo sapiens GN=EZH2		
Q15910-4	EZH2_HUMAN Isoform 4 of Histone-lysine N-methyltransferase EZH2 OS=Homo sapiens GN=EZH2		
Q15910-5	EZH2_HUMAN Isoform 5 of Histone-lysine N-methyltransferase EZH2 OS=Homo sapiens GN=EZH2		
Q15911	ZFHX3_HUMAN Zinc finger homeobox protein 3 OS=Homo sapiens GN=ZFHX3 PE=1 SV=2		52.77
Q15911-2	ZFHX3_HUMAN Isoform B of Zinc finger homeobox protein 3 OS=Homo sapiens GN=ZFHX3		28.05
Q15915	ZIC1_HUMAN Zinc finger protein ZIC 1 OS=Homo sapiens GN=ZIC1 PE=2 SV=2		7.49
Q15942	ZYX_HUMAN Zyxin OS=Homo sapiens GN=ZYX PE=1 SV=1		323.32
Q16134	ETFD_HUMAN Electron transfer flavoprotein-ubiquinone oxidoreductase, mitochondrial OS=Homo sapiens GN=ETFDH PE=1 SV=2	1.89E-29	

Q16181	SEPT7_HUMAN Septin-7 OS=Homo sapiens GN=SEPT7 PE=1 SV=2		680.59
Q16181-2	SEPT7_HUMAN Isoform 2 of Septin-7 OS=Homo sapiens GN=SEPT7		680.59
Q16181;Q16181-2	SEPT7_HUMAN Septin-7 OS=Homo sapiens GN=SEPT7 PE=1 SV=2;>sp|Q16181-2|SEPT7_HUMAN Isoform 2 of Septin-7 OS=Homo sa	7.12E-146	
Q16186	ADRM1_HUMAN Proteasomal ubiquitin receptor ADRM1 OS=Homo sapiens GN=ADRM1 PE=1 SV=2		102.19
Q16204	CCDC6_HUMAN Coiled-coil domain-containing protein 6 OS=Homo sapiens GN=CCDC6 PE=1 SV=2	3.28E-30	
Q16222;Q16222-3;Q1622	UAP1_HUMAN UDP-N-acetylhexosamine pyrophosphorylase OS=Homo sapiens GN=UAP1 PE=1 SV=3;>sp|Q16222-3|UAP1_HUMAN Is	1.54E-278	
Q16270	IBP7_HUMAN Insulin-like growth factor-binding protein 7 OS=Homo sapiens GN=IGFBP7 PE=1 SV=1		356.68
Q16280	CNGA2_HUMAN Cyclic nucleotide-gated olfactory channel OS=Homo sapiens GN=CNGA2 PE=2 SV=2		23.99
Q16322	KCA10_HUMAN Potassium voltage-gated channel subfamily A member 10 OS=Homo sapiens GN=KCNA10 PE=1 SV=2		
Q16352	AINX_HUMAN Alpha-internexin OS=Homo sapiens GN=INA PE=1 SV=2		73.44
Q16363;Q16363-2	LAMA4_HUMAN Laminin subunit alpha-4 OS=Homo sapiens GN=LAMA4 PE=1 SV=4;>sp|Q16363-2|LAMA4_HUMAN Isoform 2 of Lami	0	
Q16394	EXT1_HUMAN Exostosin-1 OS=Homo sapiens GN=EXT1 PE=1 SV=2		
Q16401	PSMD5_HUMAN 26S proteasome non-ATPase regulatory subunit 5 OS=Homo sapiens GN=PSMD5 PE=1 SV=3		192.96
Q16512	PKN1_HUMAN Serine/threonine-protein kinase N1 OS=Homo sapiens GN=PKN1 PE=1 SV=2		30.51
Q16512-2	PKN1_HUMAN Isoform 2 of Serine/threonine-protein kinase N1 OS=Homo sapiens GN=PKN1		30.51
Q16512-2;Q16512	2|PKN1_HUMAN Isoform 2 of Serine/threonine-protein kinase N1 OS=Homo sapiens GN=PKN1;>sp|Q16512|PKN1_HUMAN Serine/thre	8.83E-15	
Q16512-3	PKN1_HUMAN Isoform 3 of Serine/threonine-protein kinase N1 OS=Homo sapiens GN=PKN1		30.51
Q16512;Q16512-2;Q1651	PKN1_HUMAN Serine/threonine-protein kinase N1 OS=Homo sapiens GN=PKN1 PE=1 SV=2;>sp|Q16512-2|PKN1_HUMAN Isoform 2 c	1.84E-10	
Q16513	PKN2_HUMAN Serine/threonine-protein kinase N2 OS=Homo sapiens GN=PKN2 PE=1 SV=1		50.71
Q16527	CSRP2_HUMAN Cysteine and glycine-rich protein 2 OS=Homo sapiens GN=CSRP2 PE=1 SV=3		48.9
Q16531	DDB1_HUMAN DNA damage-binding protein 1 OS=Homo sapiens GN=DDB1 PE=1 SV=1		359.43
Q16533	SNPC1_HUMAN snRNA-activating protein complex subunit 1 OS=Homo sapiens GN=SNAPC1 PE=1 SV=1	1.19E-08	
Q16537	2A5E_HUMAN Serine/threonine-protein phosphatase 2A 56 kDa regulatory subunit epsilon isoform OS=Homo sapiens GN=PPP2R5E PE=1 SV=1		
Q16539	MK14_HUMAN Mitogen-activated protein kinase 14 OS=Homo sapiens GN=MAPK14 PE=1 SV=3		44.31
Q16539-2	MK14_HUMAN Isoform CSBP1 of Mitogen-activated protein kinase 14 OS=Homo sapiens GN=MAPK14		44.31
Q16539-3	MK14_HUMAN Isoform Mxi2 of Mitogen-activated protein kinase 14 OS=Homo sapiens GN=MAPK14		44.31
Q16539-4	MK14_HUMAN Isoform Exip of Mitogen-activated protein kinase 14 OS=Homo sapiens GN=MAPK14		44.31
Q16539;Q16539-2;B4E0K	MK14_HUMAN Mitogen-activated protein kinase 14 OS=Homo sapiens GN=MAPK14 PE=1 SV=3;>sp|Q16539-2|MK14_HUMAN Isoform	4.89E-136	
Q16540	RM23_HUMAN 39S ribosomal protein L23, mitochondrial OS=Homo sapiens GN=MRPL23 PE=1 SV=1		133.91
Q16543	CDC37_HUMAN Hsp90 co-chaperone Cdc37 OS=Homo sapiens GN=CDC37 PE=1 SV=1		86.26
Q16555	DPYL2_HUMAN Dihydropyrimidinase-related protein 2 OS=Homo sapiens GN=DPYSL2 PE=1 SV=1	0	
Q16563	SYPL1_HUMAN Synaptophysin-like protein 1 OS=Homo sapiens GN=SYPL1 PE=1 SV=1		76.28
Q16563-2	SYPL1_HUMAN Isoform 2 of Synaptophysin-like protein 1 OS=Homo sapiens GN=SYPL1		76.28
Q16563;Q16563-2	SYPL1_HUMAN Synaptophysin-like protein 1 OS=Homo sapiens GN=SYPL1 PE=1 SV=1;>sp|Q16563-2|SYPL1_HUMAN Isoform 2 of Sy	1.50E-36	
Q16566	KCC4_HUMAN Calcium/calmodulin-dependent protein kinase type IV OS=Homo sapiens GN=CAMK4 PE=1 SV=1		
Q16568	CART_HUMAN Cocaine- and amphetamine-regulated transcript protein OS=Homo sapiens GN=CARTPT PE=1 SV=1	1.60E-11	
Q16576	RBBP7_HUMAN Histone-binding protein RBBP7 OS=Homo sapiens GN=RBBP7 PE=1 SV=1		126.52
Q16594	TAF9_HUMAN Transcription initiation factor TFIID subunit 9 OS=Homo sapiens GN=TAF9 PE=1 SV=1		91.13
Q16595	FRDA_HUMAN Frataxin, mitochondrial OS=Homo sapiens GN=FXN PE=1 SV=2		41.04
Q16595-2	FRDA_HUMAN Isoform 2 of Frataxin, mitochondrial OS=Homo sapiens GN=FXN		42.81
Q16595;A8MXJ6;C9JJ89;C	FRDA_HUMAN Frataxin, mitochondrial OS=Homo sapiens GN=FXN PE=1 SV=2;>tr|A8MXJ6|A8MXJ6_HUMAN Uncharacterized protein (	1.75E-10	
Q16610-3	ECM1_HUMAN Isoform 3 of Extracellular matrix protein 1 OS=Homo sapiens GN=ECM1		
Q16610-4;Q16610;C8CHS	4|ECM1_HUMAN Isoform 4 of Extracellular matrix protein 1 OS=Homo sapiens GN=ECM1;>sp|Q16610|ECM1_HUMAN Extracellular ma	2.86E-11	
Q16611	BAK_HUMAN Bcl-2 homologous antagonist/killer OS=Homo sapiens GN=BAK1 PE=1 SV=1		
Q16625	OCLN_HUMAN Occludin OS=Homo sapiens GN=OCLN PE=1 SV=1		
Q16626	MEA1_HUMAN Male-enhanced antigen 1 OS=Homo sapiens GN=MEA1 PE=1 SV=2	4.25E-37	
Q16629	SRSF7_HUMAN Serine/arginine-rich splicing factor 7 OS=Homo sapiens GN=SRSF7 PE=1 SV=1		295.38
Q16629-2	SRSF7_HUMAN Isoform 2 of Serine/arginine-rich splicing factor 7 OS=Homo sapiens GN=SRSF7		295.38
Q16629-3	SRSF7_HUMAN Isoform 3 of Serine/arginine-rich splicing factor 7 OS=Homo sapiens GN=SRSF7		295.38
Q16629;Q16629-2;Q1662	SRSF7_HUMAN Serine/arginine-rich splicing factor 7 OS=Homo sapiens GN=SRSF7 PE=1 SV=1;>sp|Q16629-2|SRSF7_HUMAN Isoforr	7.25E-71	
Q16630	CPSF6_HUMAN Cleavage and polyadenylation specificity factor subunit 6 OS=Homo sapiens GN=CPSF6 PE=1 SV=2		265.7
Q16630-2	CPSF6_HUMAN Isoform 2 of Cleavage and polyadenylation specificity factor subunit 6 OS=Homo sapiens GN=CPSF6		265.7
Q16630-2;Q16630;C9JGC	2|CPSF6_HUMAN Isoform 2 of Cleavage and polyadenylation specificity factor subunit 6 OS=Homo sapiens GN=CPSF6;>sp|Q16630|Cl	0	
Q16630-3	CPSF6_HUMAN Isoform 3 of Cleavage and polyadenylation specificity factor subunit 6 OS=Homo sapiens GN=CPSF6		263.6
Q16637;Q16637-3;Q1663	SMN_HUMAN Survival motor neuron protein OS=Homo sapiens GN=SMN1 PE=1 SV=1;>sp|Q16637-3|SMN_HUMAN Isoform SMN-delt	2.06E-14	
Q16643	DREB_HUMAN Drebrin OS=Homo sapiens GN=DBN1 PE=1 SV=4		
Q16643-2	DREB_HUMAN Isoform 2 of Drebrin OS=Homo sapiens GN=DBN1		
Q16643-2;Q16643	2|DREB_HUMAN Isoform 2 of Drebrin OS=Homo sapiens GN=DBN1;>sp|Q16643|DREB_HUMAN Drebrin OS=Homo sapiens GN=DBN1	7.07E-86	
Q16643;Q16643-2	DREB_HUMAN Drebrin OS=Homo sapiens GN=DBN1 PE=1 SV=4;>sp|Q16643-2|DREB_HUMAN Isoform 2 of Drebrin OS=Homo sapier	2.25E-25	
Q16647	PTGIS_HUMAN Prostacyclin synthase OS=Homo sapiens GN=PTGIS PE=1 SV=1	8.69E-20	
Q16650	TBR1_HUMAN T-box brain protein 1 OS=Homo sapiens GN=TBR1 PE=1 SV=1		

Q16651	PRSS8_HUMAN Prostasin OS=Homo sapiens GN=PRSS8 PE=1 SV=1	1.39E-12	
Q16656	NRF1_HUMAN Nuclear respiratory factor 1 OS=Homo sapiens GN=NRF1 PE=1 SV=1		
Q16656-2	NRF1_HUMAN Isoform Short of Nuclear respiratory factor 1 OS=Homo sapiens GN=NRF1		
Q16658	FSCN1_HUMAN Fascin OS=Homo sapiens GN=FSCN1 PE=1 SV=3	4.18E-29	
Q16659	MK06_HUMAN Mitogen-activated protein kinase 6 OS=Homo sapiens GN=MAPK6 PE=1 SV=1		27.93
Q16661	GUC2B_HUMAN Guanylate cyclase activator 2B OS=Homo sapiens GN=GUCA2B PE=1 SV=1		
Q16666	IF16_HUMAN Gamma-interferon-inducible protein 16 OS=Homo sapiens GN=IFI16 PE=1 SV=3		342.24
Q16666-2	IF16_HUMAN Isoform 2 of Gamma-interferon-inducible protein 16 OS=Homo sapiens GN=IFI16		342.24
Q16666-3	IF16_HUMAN Isoform 3 of Gamma-interferon-inducible protein 16 OS=Homo sapiens GN=IFI16		342.24
Q16666-5	IF16_HUMAN Isoform 4 of Gamma-interferon-inducible protein 16 OS=Homo sapiens GN=IFI16		313.86
Q16666;D3DUZ3;Q16666-	IF16_HUMAN Gamma-interferon-inducible protein 16 OS=Homo sapiens GN=IFI16 PE=1 SV=3;>tr|D3DUZ3|D3DUZ3_HUMAN Interfer	1.12E-29	
Q16695	H31T_HUMAN Histone H3.1t OS=Homo sapiens GN=HIST3H3 PE=1 SV=3		180.26
Q16696	CP2AD_HUMAN Cytochrome P450 2A13 OS=Homo sapiens GN=CYP2A13 PE=1 SV=3		
Q16698	DECR_HUMAN 2,4-dienoyl-CoA reductase, mitochondrial OS=Homo sapiens GN=DECR1 PE=1 SV=1		312.75
Q16706	MA2A1_HUMAN Alpha-mannosidase 2 OS=Homo sapiens GN=MAN2A1 PE=1 SV=2		37.78
Q16718	NDUA5_HUMAN NADH dehydrogenase [ubiquinone] 1 alpha subcomplex subunit 5 OS=Homo sapiens GN=NDUFA5 PE=1 SV=3		125.88
Q16719	KYNU_HUMAN Kynureninase OS=Homo sapiens GN=KYNU PE=1 SV=1		
Q16720	AT2B3_HUMAN Plasma membrane calcium-transporting ATPase 3 OS=Homo sapiens GN=ATP2B3 PE=1 SV=3		465.41
Q16720-2	AT2B3_HUMAN Isoform XA of Plasma membrane calcium-transporting ATPase 3 OS=Homo sapiens GN=ATP2B3		460.01
Q16720-3	AT2B3_HUMAN Isoform ZA of Plasma membrane calcium-transporting ATPase 3 OS=Homo sapiens GN=ATP2B3		460.01
Q16720-4	AT2B3_HUMAN Isoform ZB of Plasma membrane calcium-transporting ATPase 3 OS=Homo sapiens GN=ATP2B3		465.41
Q16720-5	AT2B3_HUMAN Isoform XE of Plasma membrane calcium-transporting ATPase 3 OS=Homo sapiens GN=ATP2B3		460.01
Q16720-6	AT2B3_HUMAN Isoform ZE of Plasma membrane calcium-transporting ATPase 3 OS=Homo sapiens GN=ATP2B3		460.01
Q16720-7	AT2B3_HUMAN Isoform XG of Plasma membrane calcium-transporting ATPase 3 OS=Homo sapiens GN=ATP2B3		465.22
Q16720-8	AT2B3_HUMAN Isoform ZG of Plasma membrane calcium-transporting ATPase 3 OS=Homo sapiens GN=ATP2B3		465.22
Q16720;Q16720-4;Q1672	AT2B3_HUMAN Plasma membrane calcium-transporting ATPase 3 OS=Homo sapiens GN=ATP2B3 PE=1 SV=3;>sp|Q16720-4|AT2B3_H	4.06E-54	
Q16739	CEGT_HUMAN Ceramide glucosyltransferase OS=Homo sapiens GN=UGCG PE=1 SV=1		86.99
Q16740	CLPP_HUMAN Putative ATP-dependent Clp protease proteolytic subunit, mitochondrial OS=Homo sapiens GN=CLPP PE=1 SV=1		213.19
Q16762	THTR_HUMAN Thiosulfate sulfurtransferase OS=Homo sapiens GN=TST PE=1 SV=4		60.52
Q16763	UBE2S_HUMAN Ubiquitin-conjugating enzyme E2 S OS=Homo sapiens GN=UBE2S PE=1 SV=2	1.70E-17	
Q16774	KGUA_HUMAN Guanylate kinase OS=Homo sapiens GN=GUK1 PE=1 SV=2	1.40E-14	
Q16775	GLO2_HUMAN Hydroxyacylglutathione hydrolase, mitochondrial OS=Homo sapiens GN=HAGH PE=1 SV=2		32.94
Q16775-2	GLO2_HUMAN Isoform 2 of Hydroxyacylglutathione hydrolase, mitochondrial OS=Homo sapiens GN=HAGH		32.94
Q16775;B4DT01;Q16775-	GLO2_HUMAN Hydroxyacylglutathione hydrolase, mitochondrial OS=Homo sapiens GN=HAGH PE=1 SV=2;>tr|B4DT01|B4DT01_HUMA	7.43E-18	
Q16777	H2A2C_HUMAN Histone H2A type 2-C OS=Homo sapiens GN=HIST2H2AC PE=1 SV=4		288.83
Q16778	H2B2E_HUMAN Histone H2B type 2-E OS=Homo sapiens GN=HIST2H2BE PE=1 SV=3		729.36
Q16787;Q16787-1	LAMA3_HUMAN Laminin subunit alpha-3 OS=Homo sapiens GN=LAMA3 PE=1 SV=2;>sp|Q16787-1|LAMA3_HUMAN Isoform 1 of Lami	1.38E-17	
Q16795	NDUA9_HUMAN NADH dehydrogenase [ubiquinone] 1 alpha subcomplex subunit 9, mitochondrial OS=Homo sapiens GN=NDUFA9 PE=1 SV=2		161.52
Q16799	RTN1_HUMAN Reticulon-1 OS=Homo sapiens GN=RTN1 PE=1 SV=1		38.7
Q16799-2	RTN1_HUMAN Isoform RTN1-B of Reticulon-1 OS=Homo sapiens GN=RTN1		38.7
Q16799-3	RTN1_HUMAN Isoform RTN1-C of Reticulon-1 OS=Homo sapiens GN=RTN1		38.7
Q16799;Q16799-2;Q1679	RTN1_HUMAN Reticulon-1 OS=Homo sapiens GN=RTN1 PE=1 SV=1;>sp|Q16799-2|RTN1_HUMAN Isoform RTN1-B of Reticulon-1 OS=	1.64E-06	
Q16816	PHKG1_HUMAN Phosphorylase b kinase gamma catalytic chain, skeletal muscle isoform OS=Homo sapiens GN=PHKG1 PE=2 SV=3		20.47
Q16820	MEP1B_HUMAN Meprin A subunit beta OS=Homo sapiens GN=MEP1B PE=1 SV=3		
Q16822	PCKGM_HUMAN Phosphoenolpyruvate carboxykinase [GTP], mitochondrial OS=Homo sapiens GN=PCK2 PE=1 SV=3		429.01
Q16822-2	PCKGM_HUMAN Isoform 2 of Phosphoenolpyruvate carboxykinase [GTP], mitochondrial OS=Homo sapiens GN=PCK2		210.18
Q16822;Q16822-2	PCKGM_HUMAN Phosphoenolpyruvate carboxykinase [GTP], mitochondrial OS=Homo sapiens GN=PCK2 PE=1 SV=3;>sp|Q16822-2|P(	1.90E-219	
Q16825	PTN21_HUMAN Tyrosine-protein phosphatase non-receptor type 21 OS=Homo sapiens GN=PTPN21 PE=1 SV=2		2.47
Q16831	UPP1_HUMAN Uridine phosphorylase 1 OS=Homo sapiens GN=UPP1 PE=1 SV=1	7.90E-11	
Q16832	DDR2_HUMAN Discoidin domain-containing receptor 2 OS=Homo sapiens GN=DDR2 PE=1 SV=2		
Q16836	HCDH_HUMAN Hydroxyacyl-coenzyme A dehydrogenase, mitochondrial OS=Homo sapiens GN=HADH PE=1 SV=3		345.25
Q16836-2	HCDH_HUMAN Isoform 2 of Hydroxyacyl-coenzyme A dehydrogenase, mitochondrial OS=Homo sapiens GN=HADH		345.25
Q16836-2;B3KTT6;B2RB0	2|HCDH_HUMAN Isoform 2 of Hydroxyacyl-coenzyme A dehydrogenase, mitochondrial OS=Homo sapiens GN=HADH;>tr|B3KTT6|B3K	2.50E-76	
Q16850	CP51A_HUMAN Lanosterol 14-alpha demethylase OS=Homo sapiens GN=CYP51A1 PE=1 SV=3		63.12
Q16850-2	CP51A_HUMAN Isoform 2 of Lanosterol 14-alpha demethylase OS=Homo sapiens GN=CYP51A1		63.12
Q16850;B3KRC6;Q05D40;	CP51A_HUMAN Lanosterol 14-alpha demethylase OS=Homo sapiens GN=CYP51A1 PE=1 SV=3;>tr|B3KRC6|B3KRC6_HUMAN cDNA FL	1.88E-27	
Q16851;Q16851-2	UGPA_HUMAN UTP--glucose-1-phosphate uridylyltransferase OS=Homo sapiens GN=UGP2 PE=1 SV=5;>sp|Q16851-2|UGPA_HUMAN	9.94E-56	
Q16853	AOC3_HUMAN Membrane primary amine oxidase OS=Homo sapiens GN=AOC3 PE=1 SV=3	4.02E-09	
Q16864	VATF_HUMAN V-type proton ATPase subunit F OS=Homo sapiens GN=ATP6V1F PE=1 SV=2		
Q16875	F263_HUMAN 6-phosphofructo-2-kinase/fructose-2,6-biphosphatase 3 OS=Homo sapiens GN=PFKFB3 PE=1 SV=1		
Q16875-2	F263_HUMAN Isoform 2 of 6-phosphofructo-2-kinase/fructose-2,6-biphosphatase 3 OS=Homo sapiens GN=PFKFB3		

Q16877	F264_HUMAN 6-phosphofructo-2-kinase/fructose-2,6-biphosphatase 4 OS=Homo sapiens GN=PFKFB4 PE=2 SV=6		
Q16881	TRXR1_HUMAN Thioredoxin reductase 1, cytoplasmic OS=Homo sapiens GN=TXNRD1 PE=1 SV=3		66.12
Q16881-2	TRXR1_HUMAN Isoform 2 of Thioredoxin reductase 1, cytoplasmic OS=Homo sapiens GN=TXNRD1		66.12
Q16881-3	TRXR1_HUMAN Isoform 3 of Thioredoxin reductase 1, cytoplasmic OS=Homo sapiens GN=TXNRD1		66.12
Q16881-4	TRXR1_HUMAN Isoform 4 of Thioredoxin reductase 1, cytoplasmic OS=Homo sapiens GN=TXNRD1		66.12
Q16881-5	TRXR1_HUMAN Isoform 5 of Thioredoxin reductase 1, cytoplasmic OS=Homo sapiens GN=TXNRD1		66.12
Q16881-6	TRXR1_HUMAN Isoform 6 of Thioredoxin reductase 1, cytoplasmic OS=Homo sapiens GN=TXNRD1		66.12
Q16881;B7Z904;Q16881-	TRXR1_HUMAN Thioredoxin reductase 1, cytoplasmic OS=Homo sapiens GN=TXNRD1 PE=1 SV=3;>tr|B7Z904|B7Z904_HUMAN Thior	0	
Q16890;Q16890-2;Q1689	TPD53_HUMAN Tumor protein D53 OS=Homo sapiens GN=TPD52L1 PE=1 SV=1;>sp|Q16890-2|TPD53_HUMAN Isoform 2 of Tumor pr	7.09E-31	
Q16891	IMMT_HUMAN Mitochondrial inner membrane protein OS=Homo sapiens GN=IMMT PE=1 SV=1		596.96
Q16891-2	IMMT_HUMAN Isoform 2 of Mitochondrial inner membrane protein OS=Homo sapiens GN=IMMT		599.95
Q16891-3	IMMT_HUMAN Isoform 3 of Mitochondrial inner membrane protein OS=Homo sapiens GN=IMMT		560.72
Q16891;Q16891-2;Q1689	IMMT_HUMAN Mitochondrial inner membrane protein OS=Homo sapiens GN=IMMT PE=1 SV=1;>sp|Q16891-2|IMMT_HUMAN Isoform	0	
Q17R89	RHG44_HUMAN Rho GTPase-activating protein 44 OS=Homo sapiens GN=ARHGAP44 PE=1 SV=1		
Q17R98	ZN827_HUMAN Zinc finger protein 827 OS=Homo sapiens GN=ZNF827 PE=2 SV=1		
Q17R98-2	ZN827_HUMAN Isoform 2 of Zinc finger protein 827 OS=Homo sapiens GN=ZNF827		
Q17R98-3	ZN827_HUMAN Isoform 3 of Zinc finger protein 827 OS=Homo sapiens GN=ZNF827		
Q17RW2	COOA1_HUMAN Collagen alpha-1(XXIV) chain OS=Homo sapiens GN=COL24A1 PE=2 SV=2		
Q17RW2-2	COOA1_HUMAN Isoform 2 of Collagen alpha-1(XXIV) chain OS=Homo sapiens GN=COL24A1		
Q1ED39	CP088_HUMAN Protein C16orf88 OS=Homo sapiens GN=C16orf88 PE=1 SV=1		
Q1HG43	DOXA1_HUMAN Dual oxidase maturation factor 1 OS=Homo sapiens GN=DUOXA1 PE=1 SV=1		
Q1HG43-2	DOXA1_HUMAN Isoform 2 of Dual oxidase maturation factor 1 OS=Homo sapiens GN=DUOXA1		
Q1HG43-3	DOXA1_HUMAN Isoform 3 of Dual oxidase maturation factor 1 OS=Homo sapiens GN=DUOXA1		
Q1KMD3	HNRL2_HUMAN Heterogeneous nuclear ribonucleoprotein U-like protein 2 OS=Homo sapiens GN=HNRNPUL2 PE=1 SV=1		334.89
Q1MX18	INSC_HUMAN Protein inscuteable homolog OS=Homo sapiens GN=INSC PE=1 SV=1		
Q1MX18-2	INSC_HUMAN Isoform 2 of Protein inscuteable homolog OS=Homo sapiens GN=INSC		
Q1MX18-4	INSC_HUMAN Isoform 4 of Protein inscuteable homolog OS=Homo sapiens GN=INSC		
Q1RN00	YC018_HUMAN Putative uncharacterized protein LOC151760 OS=Homo sapiens PE=2 SV=1		
Q24JP5	T132A_HUMAN Transmembrane protein 132A OS=Homo sapiens GN=TMEM132A PE=2 SV=1		
Q24JP5-2	T132A_HUMAN Isoform 2 of Transmembrane protein 132A OS=Homo sapiens GN=TMEM132A		
Q24JP5-3	T132A_HUMAN Isoform 3 of Transmembrane protein 132A OS=Homo sapiens GN=TMEM132A		
Q24JP5-4	T132A_HUMAN Isoform 4 of Transmembrane protein 132A OS=Homo sapiens GN=TMEM132A		
Q27J81	INF2_HUMAN Inverted formin-2 OS=Homo sapiens GN=INF2 PE=1 SV=2		39.34
Q27J81-2	INF2_HUMAN Isoform 2 of Inverted formin-2 OS=Homo sapiens GN=INF2		39.34
Q27J81-3	INF2_HUMAN Isoform 3 of Inverted formin-2 OS=Homo sapiens GN=INF2		35.61
Q27J81;Q27J81-2	INF2_HUMAN Inverted formin-2 OS=Homo sapiens GN=INF2 PE=1 SV=2;>sp|Q27J81-2|INF2_HUMAN Isoform 2 of Inverted formin-2	1.01E-48	
Q29718	1B82_HUMAN HLA class I histocompatibility antigen, B-82 alpha chain OS=Homo sapiens GN=HLA-B PE=2 SV=1		314.84
Q29836	1B67_HUMAN HLA class I histocompatibility antigen, B-67 alpha chain OS=Homo sapiens GN=HLA-B PE=1 SV=1		382.83
Q29865	1C18_HUMAN HLA class I histocompatibility antigen, Cw-18 alpha chain OS=Homo sapiens GN=HLA-C PE=2 SV=1		357.73
Q29940	1B59_HUMAN HLA class I histocompatibility antigen, B-59 alpha chain OS=Homo sapiens GN=HLA-B PE=2 SV=1		499.59
Q29960	1C16_HUMAN HLA class I histocompatibility antigen, Cw-16 alpha chain OS=Homo sapiens GN=HLA-C PE=2 SV=1		407.43
Q29960-2	1C16_HUMAN Isoform 2 of HLA class I histocompatibility antigen, Cw-16 alpha chain OS=Homo sapiens GN=HLA-C		407.43
Q29963	1C06_HUMAN HLA class I histocompatibility antigen, Cw-6 alpha chain OS=Homo sapiens GN=HLA-C PE=1 SV=2		417.3
Q29974	2B1G_HUMAN HLA class II histocompatibility antigen, DRB1-16 beta chain OS=Homo sapiens GN=HLA-DRB1 PE=1 SV=1		29.42
Q29980	MICB_HUMAN MHC class I polypeptide-related sequence B OS=Homo sapiens GN=MICB PE=1 SV=1		
Q29RF7	PDS5A_HUMAN Sister chromatid cohesion protein PDS5 homolog A OS=Homo sapiens GN=PDS5A PE=1 SV=1		224.81
Q29RF7-3	PDS5A_HUMAN Isoform 2 of Sister chromatid cohesion protein PDS5 homolog A OS=Homo sapiens GN=PDS5A		55.9
Q29RF7;Q29RF7-3	PDS5A_HUMAN Sister chromatid cohesion protein PDS5 homolog A OS=Homo sapiens GN=PDS5A PE=1 SV=1;>sp|Q29RF7-3|PDS5A_	3.93E-116	
Q2KHT3	CL16A_HUMAN Protein CLEC16A OS=Homo sapiens GN=CLEC16A PE=2 SV=2		
Q2KHT3-2	CL16A_HUMAN Isoform 2 of Protein CLEC16A OS=Homo sapiens GN=CLEC16A		
Q2KHT3;Q2KHT3-2	CL16A_HUMAN Protein CLEC16A OS=Homo sapiens GN=CLEC16A PE=2 SV=2;>sp|Q2KHT3-2|CL16A_HUMAN Isoform 2 of Protein CLI	5.86E-06	
Q2KHT4	GSG1_HUMAN Germ cell-specific gene 1 protein OS=Homo sapiens GN=GSG1 PE=2 SV=2		
Q2KHT4-2	GSG1_HUMAN Isoform 2 of Germ cell-specific gene 1 protein OS=Homo sapiens GN=GSG1		
Q2KHT4-3	GSG1_HUMAN Isoform 3 of Germ cell-specific gene 1 protein OS=Homo sapiens GN=GSG1		
Q2KHT4-4	GSG1_HUMAN Isoform 4 of Germ cell-specific gene 1 protein OS=Homo sapiens GN=GSG1		
Q2KHT4-5	GSG1_HUMAN Isoform 5 of Germ cell-specific gene 1 protein OS=Homo sapiens GN=GSG1		
Q2KHT4-6	GSG1_HUMAN Isoform 6 of Germ cell-specific gene 1 protein OS=Homo sapiens GN=GSG1		
Q2KHT4-7	GSG1_HUMAN Isoform 7 of Germ cell-specific gene 1 protein OS=Homo sapiens GN=GSG1		
Q2KHT4-8	GSG1_HUMAN Isoform 8 of Germ cell-specific gene 1 protein OS=Homo sapiens GN=GSG1		
Q2LD37;Q2LD37-4;C9JN5	K1109_HUMAN Uncharacterized protein KIAA1109 OS=Homo sapiens GN=KIAA1109 PE=1 SV=2;>sp|Q2LD37-4|K1109_HUMAN Isofo	1.78E-07	
Q2M1K9	ZN423_HUMAN Zinc finger protein 423 OS=Homo sapiens GN=ZNF423 PE=1 SV=1		20.89

Q2M238	RRN3L_HUMAN Putative RRN3-like protein FLJ77916 OS=Homo sapiens PE=5 SV=1		
Q2M243	CCD27_HUMAN Coiled-coil domain-containing protein 27 OS=Homo sapiens GN=CCDC27 PE=2 SV=2		
Q2M296	MTHSD_HUMAN Methenyltetrahydrofolate synthase domain-containing protein OS=Homo sapiens GN=MTHFSD PE=1 SV=2		
Q2M296-2	MTHSD_HUMAN Isoform 2 of Methenyltetrahydrofolate synthase domain-containing protein OS=Homo sapiens GN=MTHFSD		
Q2M2I5	K1C24_HUMAN Keratin, type I cytoskeletal 24 OS=Homo sapiens GN=KRT24 PE=1 SV=1		139.63
Q2M2I8;B7ZLC4;Q2M2I8-	AAK1_HUMAN AP2-associated protein kinase 1 OS=Homo sapiens GN=AAK1 PE=1 SV=3;>tr|B7ZLC4|B7ZLC4_HUMAN AAK1 protein C	3.31E-06	
Q2M329	CCD96_HUMAN Coiled-coil domain-containing protein 96 OS=Homo sapiens GN=CCDC96 PE=2 SV=2		
Q2M389	WAHS7_HUMAN WASH complex subunit 7 OS=Homo sapiens GN=KIAA1033 PE=1 SV=2		
Q2M389-2	WAHS7_HUMAN Isoform 2 of WASH complex subunit 7 OS=Homo sapiens GN=KIAA1033		
Q2M389;Q2M389-2	WAHS7_HUMAN WASH complex subunit 7 OS=Homo sapiens GN=KIAA1033 PE=1 SV=2;>sp|Q2M389-2|WAHS7_HUMAN Isoform 2 of	2.23E-11	
Q2M3C7	SPKAP_HUMAN A-kinase anchor protein SPHKAP OS=Homo sapiens GN=SPHKAP PE=1 SV=1		
Q2M3C7-2	SPKAP_HUMAN Isoform 2 of A-kinase anchor protein SPHKAP OS=Homo sapiens GN=SPHKAP		
Q2M3X9	ZN674_HUMAN Zinc finger protein 674 OS=Homo sapiens GN=ZNF674 PE=1 SV=1		27.01
Q2NKQ1	SGSM1_HUMAN Small G protein signaling modulator 1 OS=Homo sapiens GN=SGSM1 PE=1 SV=2		
Q2NKQ1-3	SGSM1_HUMAN Isoform 2 of Small G protein signaling modulator 1 OS=Homo sapiens GN=SGSM1		
Q2NKQ1-4	SGSM1_HUMAN Isoform 3 of Small G protein signaling modulator 1 OS=Homo sapiens GN=SGSM1		
Q2NKX8	ERC6L_HUMAN DNA excision repair protein ERCC-6-like OS=Homo sapiens GN=ERCC6L PE=1 SV=1	5.31E-07	
Q2NL67	PARP6_HUMAN Poly [ADP-ribose] polymerase 6 OS=Homo sapiens GN=PARP6 PE=2 SV=1		
Q2NL67-2	PARP6_HUMAN Isoform 2 of Poly [ADP-ribose] polymerase 6 OS=Homo sapiens GN=PARP6		
Q2NL67-3	PARP6_HUMAN Isoform 3 of Poly [ADP-ribose] polymerase 6 OS=Homo sapiens GN=PARP6		
Q2NL82	TSR1_HUMAN Pre-rRNA-processing protein TSR1 homolog OS=Homo sapiens GN=TSR1 PE=1 SV=1		212.82
Q2Q1W2	LIN41_HUMAN Tripartite motif-containing protein 71 OS=Homo sapiens GN=TRIM71 PE=2 SV=1		
Q2T9F4	IN4L2_HUMAN Integrator complex subunit 4-like protein 2 OS=Homo sapiens GN=INTS4L2 PE=2 SV=1		
Q2TAA2	IAH1_HUMAN Isoamyl acetate-hydrolyzing esterase 1 homolog OS=Homo sapiens GN=IAH1 PE=1 SV=1		55.99
Q2TAL8	QRIC1_HUMAN Glutamine-rich protein 1 OS=Homo sapiens GN=QRICH1 PE=1 SV=1	1.20E-17	
Q2TAY7	SMU1_HUMAN WD40 repeat-containing protein SMU1 OS=Homo sapiens GN=SMU1 PE=1 SV=2		227.67
Q2TB90	HKDC1_HUMAN Putative hexokinase HKDC1 OS=Homo sapiens GN=HKDC1 PE=2 SV=3		99.24
Q2TB90-2	HKDC1_HUMAN Isoform 2 of Putative hexokinase HKDC1 OS=Homo sapiens GN=HKDC1		7.7
Q2TB90-3	HKDC1_HUMAN Isoform 3 of Putative hexokinase HKDC1 OS=Homo sapiens GN=HKDC1		7.7
Q2TBA0;Q2TBA0-2	KBTB5_HUMAN Kelch repeat and BTB domain-containing protein 5 OS=Homo sapiens GN=KBTBD5 PE=2 SV=2;>sp|Q2TBA0-2|KBTB5_	8.00E-06	
Q2TBF2	WSCD2_HUMAN WSC domain-containing protein 2 OS=Homo sapiens GN=WSCD2 PE=2 SV=2		
Q2TBF2-2	WSCD2_HUMAN Isoform 2 of WSC domain-containing protein 2 OS=Homo sapiens GN=WSCD2		
Q2VIQ3	KIF4B_HUMAN Chromosome-associated kinesin KIF4B OS=Homo sapiens GN=KIF4B PE=1 SV=2		
Q2VIR3	IF2GL_HUMAN Eukaryotic translation initiation factor 2 subunit 3-like protein OS=Homo sapiens GN=EIF2S3L PE=1 SV=2		543.38
Q2VIR3-2	IF2GL_HUMAN Isoform 2 of Eukaryotic translation initiation factor 2 subunit 3-like protein OS=Homo sapiens GN=EIF2S3L		477.71
Q30134	2B18_HUMAN HLA class II histocompatibility antigen, DRB1-8 beta chain OS=Homo sapiens GN=HLA-DRB1 PE=1 SV=2		29.27
Q30154	DRB5_HUMAN HLA class II histocompatibility antigen, DR beta 5 chain OS=Homo sapiens GN=HLA-DRB5 PE=1 SV=1		29.27
Q30167	2B1A_HUMAN HLA class II histocompatibility antigen, DRB1-10 beta chain OS=Homo sapiens GN=HLA-DRB1 PE=1 SV=2		29.42
Q30201	HFE_HUMAN Hereditary hemochromatosis protein OS=Homo sapiens GN=HFE PE=1 SV=1		
Q30201-2	HFE_HUMAN Isoform 2 of Hereditary hemochromatosis protein OS=Homo sapiens GN=HFE		
Q30201-5	HFE_HUMAN Isoform 5 of Hereditary hemochromatosis protein OS=Homo sapiens GN=HFE		
Q30201-6	HFE_HUMAN Isoform 6 of Hereditary hemochromatosis protein OS=Homo sapiens GN=HFE		
Q30201-7	HFE_HUMAN Isoform 7 of Hereditary hemochromatosis protein OS=Homo sapiens GN=HFE		
Q30201-8	HFE_HUMAN Isoform 8 of Hereditary hemochromatosis protein OS=Homo sapiens GN=HFE		
Q31610	1B81_HUMAN HLA class I histocompatibility antigen, B-81 alpha chain OS=Homo sapiens GN=HLA-B PE=1 SV=1		363.46
Q31612	1B73_HUMAN HLA class I histocompatibility antigen, B-73 alpha chain OS=Homo sapiens GN=HLA-B PE=1 SV=1		368.35
Q32M45	ANO4_HUMAN Anoctamin-4 OS=Homo sapiens GN=ANO4 PE=2 SV=1		
Q32M45-2	ANO4_HUMAN Isoform 2 of Anoctamin-4 OS=Homo sapiens GN=ANO4		
Q32M84-2	BTBDG_HUMAN Isoform 2 of BTB/POZ domain-containing protein 16 OS=Homo sapiens GN=BTBD16		
Q32MK0	MYLK3_HUMAN Putative myosin light chain kinase 3 OS=Homo sapiens GN=MYLK3 PE=2 SV=3		44.43
Q32MZ4	LRRF1_HUMAN Leucine-rich repeat flightless-interacting protein 1 OS=Homo sapiens GN=LRRFIP1 PE=1 SV=2		94.2
Q32MZ4-2	LRRF1_HUMAN Isoform 2 of Leucine-rich repeat flightless-interacting protein 1 OS=Homo sapiens GN=LRRFIP1		92.4
Q32MZ4-2;Q32MZ4-3;Q32	2|LRRF1_HUMAN Isoform 2 of Leucine-rich repeat flightless-interacting protein 1 OS=Homo sapiens GN=LRRFIP1;>sp|Q32MZ4-3|LRR	3.13E-256	
Q32MZ4-3	LRRF1_HUMAN Isoform 3 of Leucine-rich repeat flightless-interacting protein 1 OS=Homo sapiens GN=LRRFIP1		83.33
Q32MZ4;Q32MZ4-2;Q32M	LRRF1_HUMAN Leucine-rich repeat flightless-interacting protein 1 OS=Homo sapiens GN=LRRFIP1 PE=1 SV=2;>sp|Q32MZ4-2|LRRF1_	1.62E-162	
Q32NB8	PGPS1_HUMAN CDP-diacylglycerol--glycerol-3-phosphate 3-phosphatidyltransferase, mitochondrial OS=Homo sapiens GN=PGS1 PE=2 SV=1		
Q32P28	P3H1_HUMAN Prolyl 3-hydroxylase 1 OS=Homo sapiens GN=LEPRE1 PE=1 SV=2		394.67
Q32P28-2	P3H1_HUMAN Isoform 2 of Prolyl 3-hydroxylase 1 OS=Homo sapiens GN=LEPRE1		167.23
Q32P28-3	P3H1_HUMAN Isoform 3 of Prolyl 3-hydroxylase 1 OS=Homo sapiens GN=LEPRE1		389.53
Q32P28;Q32P28-3	P3H1_HUMAN Prolyl 3-hydroxylase 1 OS=Homo sapiens GN=LEPRE1 PE=1 SV=2;>sp|Q32P28-3|P3H1_HUMAN Isoform 3 of Prolyl 3-h	3.10E-121	
Q32P51	RA1L2_HUMAN Heterogeneous nuclear ribonucleoprotein A1-like 2 OS=Homo sapiens GN=HNRNPA1L2 PE=2 SV=2		859.38

Q38SD2	LRRK1_HUMAN Leucine-rich repeat serine/threonine-protein kinase 1 OS=Homo sapiens GN=LRRK1 PE=1 SV=3	4.18E-14	
Q3B726	RPA43_HUMAN DNA-directed RNA polymerase I subunit RPA43 OS=Homo sapiens GN=TWISTNB PE=1 SV=1		
Q3B7T1	EDRF1_HUMAN Erythroid differentiation-related factor 1 OS=Homo sapiens GN=EDRF1 PE=1 SV=1		
Q3B7T1-3	EDRF1_HUMAN Isoform 2 of Erythroid differentiation-related factor 1 OS=Homo sapiens GN=EDRF1		
Q3B7T1-4	EDRF1_HUMAN Isoform 3 of Erythroid differentiation-related factor 1 OS=Homo sapiens GN=EDRF1		
Q3B7T1-5	EDRF1_HUMAN Isoform 4 of Erythroid differentiation-related factor 1 OS=Homo sapiens GN=EDRF1		
Q3B8N5	PROX2_HUMAN Prospero homeobox protein 2 OS=Homo sapiens GN=PROX2 PE=2 SV=3		
Q3B8N5-2	PROX2_HUMAN Isoform 2 of Prospero homeobox protein 2 OS=Homo sapiens GN=PROX2		
Q3C1V8	BSH_HUMAN Brain-specific homeobox protein homolog OS=Homo sapiens GN=BSX PE=2 SV=2		
Q3KNS1	PTHD3_HUMAN Patched domain-containing protein 3 OS=Homo sapiens GN=PTCHD3 PE=1 SV=2		
Q3KNS6	ZN829_HUMAN Zinc finger protein 829 OS=Homo sapiens GN=ZNF829 PE=2 SV=1		
Q3KNS6-2	ZN829_HUMAN Isoform 2 of Zinc finger protein 829 OS=Homo sapiens GN=ZNF829		
Q3KQU3	MA7D1_HUMAN MAP7 domain-containing protein 1 OS=Homo sapiens GN=MAP7D1 PE=1 SV=1		38.4
Q3KQU3-2	MA7D1_HUMAN Isoform 2 of MAP7 domain-containing protein 1 OS=Homo sapiens GN=MAP7D1		38.4
Q3KQU3-3	MA7D1_HUMAN Isoform 3 of MAP7 domain-containing protein 1 OS=Homo sapiens GN=MAP7D1		32.83
Q3KQU3-4	MA7D1_HUMAN Isoform 4 of MAP7 domain-containing protein 1 OS=Homo sapiens GN=MAP7D1		38.4
Q3KQU3;D3DPS3;D3DPS4	MA7D1_HUMAN MAP7 domain-containing protein 1 OS=Homo sapiens GN=MAP7D1 PE=1 SV=1;>tr|D3DPS3|D3DPS3_HUMAN Argininn	5.68E-23	
Q3KQV9	UAP1L_HUMAN UDP-N-acetylhexosamine pyrophosphorylase-like protein 1 OS=Homo sapiens GN=UAP1L1 PE=2 SV=2	2.92E-12	
Q3KR37	GRM1B_HUMAN GRAM domain-containing protein 1B OS=Homo sapiens GN=GRAMD1B PE=1 SV=1		
Q3L8U1	CHD9_HUMAN Chromodomain-helicase-DNA-binding protein 9 OS=Homo sapiens GN=CHD9 PE=1 SV=2		81.71
Q3L8U1-2	CHD9_HUMAN Isoform 2 of Chromodomain-helicase-DNA-binding protein 9 OS=Homo sapiens GN=CHD9		81.71
Q3L8U1-3	CHD9_HUMAN Isoform 3 of Chromodomain-helicase-DNA-binding protein 9 OS=Homo sapiens GN=CHD9		81.71
Q3LXA3	DHAK_HUMAN Bifunctional ATP-dependent dihydroxyacetone kinase/FAD-AMP lyase (cyclizing) OS=Homo sapiens GN=DAK PE=1 SV=	7.52E-119	
Q3MHD2	LSM12_HUMAN Protein LSM12 homolog OS=Homo sapiens GN=LSM12 PE=1 SV=2		131.39
Q3MHD2-2	LSM12_HUMAN Isoform 2 of Protein LSM12 homolog OS=Homo sapiens GN=LSM12		131.39
Q3MHD2-2;Q3MHD2	2|LSM12_HUMAN Isoform 2 of Protein LSM12 homolog OS=Homo sapiens GN=LSM12;>sp|Q3MHD2|LSM12_HUMAN Protein LSM12 hc	4.13E-21	
Q3MIR4	CC50B_HUMAN Cell cycle control protein 50B OS=Homo sapiens GN=TMEM30B PE=2 SV=1		
Q3MJ40	C144B_HUMAN Coiled-coil domain-containing protein 144B OS=Homo sapiens GN=CCDC144B PE=2 SV=1		
Q3MJ40-2	C144B_HUMAN Isoform 2 of Coiled-coil domain-containing protein 144B OS=Homo sapiens GN=CCDC144B		
Q3SXM5	HSDL1_HUMAN Inactive hydroxysteroid dehydrogenase-like protein 1 OS=Homo sapiens GN=HSDL1 PE=1 SV=3	0.00022061	
Q3SY69	AL1L2_HUMAN Aldehyde dehydrogenase family 1 member L2, mitochondrial OS=Homo sapiens GN=ALDH1L2 PE=1 SV=2	1.67E-11	
Q3SY84	K2C71_HUMAN Keratin, type II cytoskeletal 71 OS=Homo sapiens GN=KRT71 PE=1 SV=3		246.28
Q3SY89	EA3L1_HUMAN RNA polymerase II transcription factor SIII subunit A3-like-1 OS=Homo sapiens GN=TCEB3CL PE=2 SV=1		
Q3SYG4	PTHB1_HUMAN Protein PTHB1 OS=Homo sapiens GN=BBS9 PE=1 SV=1		
Q3SYG4-2	PTHB1_HUMAN Isoform 2 of Protein PTHB1 OS=Homo sapiens GN=BBS9		
Q3SYG4-3	PTHB1_HUMAN Isoform 3 of Protein PTHB1 OS=Homo sapiens GN=BBS9		
Q3SYG4-4	PTHB1_HUMAN Isoform 4 of Protein PTHB1 OS=Homo sapiens GN=BBS9		
Q3SYG4-5	PTHB1_HUMAN Isoform 5 of Protein PTHB1 OS=Homo sapiens GN=BBS9		
Q3SYG4-6	PTHB1_HUMAN Isoform 6 of Protein PTHB1 OS=Homo sapiens GN=BBS9		
Q3T8J9	GON4L_HUMAN GON-4-like protein OS=Homo sapiens GN=GON4L PE=1 SV=1		
Q3T8J9-2	GON4L_HUMAN Isoform 2 of GON-4-like protein OS=Homo sapiens GN=GON4L		
Q3T8J9-3	GON4L_HUMAN Isoform 3 of GON-4-like protein OS=Homo sapiens GN=GON4L		
Q3T906	GNPTA_HUMAN N-acetylglucosamine-1-phosphotransferase subunits alpha/beta OS=Homo sapiens GN=GNPTAB PE=1 SV=1		
Q3T906-2	GNPTA_HUMAN Isoform 2 of N-acetylglucosamine-1-phosphotransferase subunits alpha/beta OS=Homo sapiens GN=GNPTAB		
Q3V6T2	GRDN_HUMAN Girdin OS=Homo sapiens GN=CCDC88A PE=1 SV=2		
Q3V6T2-2	GRDN_HUMAN Isoform 2 of Girdin OS=Homo sapiens GN=CCDC88A		
Q3V6T2-3	GRDN_HUMAN Isoform 3 of Girdin OS=Homo sapiens GN=CCDC88A		
Q3V6T2-4	GRDN_HUMAN Isoform 4 of Girdin OS=Homo sapiens GN=CCDC88A		
Q3V6T2;Q3V6T2-2;Q3V6T	GRDN_HUMAN Girdin OS=Homo sapiens GN=CCDC88A PE=1 SV=2;>sp|Q3V6T2-2|GRDN_HUMAN Isoform 2 of Girdin OS=Homo sapi	8.54E-15	
Q3YEC7-2;Q3YEC7;Q3YEC	2|PARF_HUMAN Isoform 2 of Putative GTP-binding protein Parf OS=Homo sapiens GN=PARF;>sp|Q3YEC7|PARF_HUMAN Putative GTP-	1.68E-66	
Q3ZAQ7	VMA21_HUMAN Vacuolar ATPase assembly integral membrane protein VMA21 OS=Homo sapiens GN=VMA21 PE=1 SV=1		
Q3ZAQ7-2	VMA21_HUMAN Isoform 2 of Vacuolar ATPase assembly integral membrane protein VMA21 OS=Homo sapiens GN=VMA21		
Q3ZAQ7-2;Q3ZAQ7	2|VMA21_HUMAN Isoform 2 of Vacuolar ATPase assembly integral membrane protein VMA21 OS=Homo sapiens GN=VMA21;>sp|Q3Z	3.67E-38	
Q3ZAQ7;Q3ZAQ7-2	VMA21_HUMAN Vacuolar ATPase assembly integral membrane protein VMA21 OS=Homo sapiens GN=VMA21 PE=1 SV=1;>sp|Q3ZAQ	1.80E-15	
Q3ZCM7	TBB8_HUMAN Tubulin beta-8 chain OS=Homo sapiens GN=TUBB8 PE=1 SV=2		844.07
Q3ZCQ8	TIM50_HUMAN Mitochondrial import inner membrane translocase subunit TIM50 OS=Homo sapiens GN=TIMM50 PE=1 SV=2		146.16
Q3ZCQ8-2	TIM50_HUMAN Isoform 2 of Mitochondrial import inner membrane translocase subunit TIM50 OS=Homo sapiens GN=TIMM50		146.16
Q3ZCQ8-2;Q3ZCQ8	2|TIM50_HUMAN Isoform 2 of Mitochondrial import inner membrane translocase subunit TIM50 OS=Homo sapiens GN=TIMM50;>sp|C	4.61E-18	
Q460N5;Q460N5-1;Q460N	PAR14_HUMAN Poly [ADP-ribose] polymerase 14 OS=Homo sapiens GN=PARP14 PE=1 SV=3;>sp|Q460N5-1|PAR14_HUMAN Isoform	1.43E-31	
Q494R4	CC153_HUMAN Coiled-coil domain-containing protein 153 OS=Homo sapiens GN=CCDC153 PE=2 SV=2		
Q494R4-2	CC153_HUMAN Isoform 2 of Coiled-coil domain-containing protein 153 OS=Homo sapiens GN=CCDC153		

Q495T6	MMEL1_HUMAN Membrane metallo-endopeptidase-like 1 OS=Homo sapiens GN=MMEL1 PE=1 SV=2		
Q495T6-2	MMEL1_HUMAN Isoform 2 of Membrane metallo-endopeptidase-like 1 OS=Homo sapiens GN=MMEL1		
Q495T6-3	MMEL1_HUMAN Isoform 3 of Membrane metallo-endopeptidase-like 1 OS=Homo sapiens GN=MMEL1		
Q495X7	TRI60_HUMAN Tripartite motif-containing protein 60 OS=Homo sapiens GN=TRIM60 PE=2 SV=2		
Q496J9	SV2C_HUMAN Synaptic vesicle glycoprotein 2C OS=Homo sapiens GN=SV2C PE=2 SV=1		27.7
Q496Y0	LONF3_HUMAN LON peptidase N-terminal domain and RING finger protein 3 OS=Homo sapiens GN=LONRF3 PE=1 SV=1		
Q496Y0-2	LONF3_HUMAN Isoform 2 of LON peptidase N-terminal domain and RING finger protein 3 OS=Homo sapiens GN=LONRF3		
Q496Y0-3	LONF3_HUMAN Isoform 3 of LON peptidase N-terminal domain and RING finger protein 3 OS=Homo sapiens GN=LONRF3		
Q49A26	GLYR1_HUMAN Putative oxidoreductase GLYR1 OS=Homo sapiens GN=GLYR1 PE=1 SV=3		
Q49A26-2	GLYR1_HUMAN Isoform 2 of Putative oxidoreductase GLYR1 OS=Homo sapiens GN=GLYR1		
Q49A26-3	GLYR1_HUMAN Isoform 3 of Putative oxidoreductase GLYR1 OS=Homo sapiens GN=GLYR1		
Q49A26-4	GLYR1_HUMAN Isoform 4 of Putative oxidoreductase GLYR1 OS=Homo sapiens GN=GLYR1		
Q49A26-5	GLYR1_HUMAN Isoform 5 of Putative oxidoreductase GLYR1 OS=Homo sapiens GN=GLYR1		
Q49A26;Q49A26-3;Q49A2	GLYR1_HUMAN Putative oxidoreductase GLYR1 OS=Homo sapiens GN=GLYR1 PE=1 SV=3;>sp|Q49A26-3|GLYR1_HUMAN Isoform 3 of	4.02E-05	
Q49AJ0	F135B_HUMAN Protein FAM135B OS=Homo sapiens GN=FAM135B PE=2 SV=2		
Q49AJ0-2	F135B_HUMAN Isoform 2 of Protein FAM135B OS=Homo sapiens GN=FAM135B		
Q49AJ0-3	F135B_HUMAN Isoform 3 of Protein FAM135B OS=Homo sapiens GN=FAM135B		
Q49AJ0-4	F135B_HUMAN Isoform 4 of Protein FAM135B OS=Homo sapiens GN=FAM135B		
Q49MG5	MAP9_HUMAN Microtubule-associated protein 9 OS=Homo sapiens GN=MAP9 PE=1 SV=3		
Q4AC94	C2CD3_HUMAN C2 domain-containing protein 3 OS=Homo sapiens GN=C2CD3 PE=1 SV=4		59.42
Q4AC94-1	C2CD3_HUMAN Isoform 1 of C2 domain-containing protein 3 OS=Homo sapiens GN=C2CD3		48.61
Q4AC94-2	C2CD3_HUMAN Isoform 2 of C2 domain-containing protein 3 OS=Homo sapiens GN=C2CD3		40.52
Q4AC94-3	C2CD3_HUMAN Isoform 3 of C2 domain-containing protein 3 OS=Homo sapiens GN=C2CD3		48.61
Q4AC94-4	C2CD3_HUMAN Isoform 4 of C2 domain-containing protein 3 OS=Homo sapiens GN=C2CD3		40.52
Q4ADV7	RIC1_HUMAN Protein RIC1 homolog OS=Homo sapiens GN=KIAA1432 PE=1 SV=2		
Q4ADV7-2	RIC1_HUMAN Isoform 2 of Protein RIC1 homolog OS=Homo sapiens GN=KIAA1432		
Q4FZB7	SV421_HUMAN Histone-lysine N-methyltransferase SUV420H1 OS=Homo sapiens GN=SUV420H1 PE=1 SV=4		
Q4FZB7-2	SV421_HUMAN Isoform 2 of Histone-lysine N-methyltransferase SUV420H1 OS=Homo sapiens GN=SUV420H1		
Q4FZB7-4	SV421_HUMAN Isoform 3 of Histone-lysine N-methyltransferase SUV420H1 OS=Homo sapiens GN=SUV420H1		
Q4G0F5	VP26B_HUMAN Vacuolar protein sorting-associated protein 26B OS=Homo sapiens GN=VPS26B PE=1 SV=2		49.33
Q4G0J3	LARP7_HUMAN La-related protein 7 OS=Homo sapiens GN=LARP7 PE=1 SV=1	2.61E-16	
Q4G0N4	CE033_HUMAN UPF0465 protein C5orf33 OS=Homo sapiens GN=C5orf33 PE=1 SV=2		
Q4G0N4-2	CE033_HUMAN Isoform 2 of UPF0465 protein C5orf33 OS=Homo sapiens GN=C5orf33		
Q4G0N4-3	CE033_HUMAN Isoform 3 of UPF0465 protein C5orf33 OS=Homo sapiens GN=C5orf33		
Q4G0N4;Q4G0N4-2;B7Z8'	CE033_HUMAN UPF0465 protein C5orf33 OS=Homo sapiens GN=C5orf33 PE=1 SV=2;>sp|Q4G0N4-2|CE033_HUMAN Isoform 2 of UP	4.55E-58	
Q4G0N4;Q4G0N4-2;Q4G0	NAKD1_HUMAN NAD kinase domain-containing protein 1 OS=Homo sapiens GN=NADKD1 PE=1 SV=2;>sp|Q4G0N4-2|NAKD1_HUMAN	1.74E-41	
Q4G0X4	KCD21_HUMAN BTB/POZ domain-containing protein KCTD21 OS=Homo sapiens GN=KCTD21 PE=2 SV=1	0.00015348	
Q4G0X9	CCD40_HUMAN Coiled-coil domain-containing protein 40 OS=Homo sapiens GN=CCDC40 PE=2 SV=2		
Q4G0X9-2	CCD40_HUMAN Isoform 2 of Coiled-coil domain-containing protein 40 OS=Homo sapiens GN=CCDC40		
Q4G0X9-3	CCD40_HUMAN Isoform 3 of Coiled-coil domain-containing protein 40 OS=Homo sapiens GN=CCDC40		
Q4G0X9-4	CCD40_HUMAN Isoform 4 of Coiled-coil domain-containing protein 40 OS=Homo sapiens GN=CCDC40		
Q4G0Z9-4	CH045_HUMAN Isoform 4 of Uncharacterized protein C8orf45 OS=Homo sapiens GN=C8orf45		
Q4G148	GXLT1_HUMAN Glucoside xylosyltransferase 1 OS=Homo sapiens GN=GXYLT1 PE=1 SV=2		
Q4G148-2	GXLT1_HUMAN Isoform 2 of Glucoside xylosyltransferase 1 OS=Homo sapiens GN=GXYLT1		
Q4G176	ACSF3_HUMAN Acyl-CoA synthetase family member 3, mitochondrial OS=Homo sapiens GN=ACSF3 PE=1 SV=3		
Q4G176-2	ACSF3_HUMAN Isoform 2 of Acyl-CoA synthetase family member 3, mitochondrial OS=Homo sapiens GN=ACSF3		
Q4G176;Q4G176-2	ACSF3_HUMAN Acyl-CoA synthetase family member 3, mitochondrial OS=Homo sapiens GN=ACSF3 PE=1 SV=3;>sp|Q4G176-2|ACSF	4.68E-28	
Q4G1C9	GRPL2_HUMAN GLIPR1-like protein 2 OS=Homo sapiens GN=GLIPR1L2 PE=2 SV=2		
Q4G1C9-2	GRPL2_HUMAN Isoform 2 of GLIPR1-like protein 2 OS=Homo sapiens GN=GLIPR1L2		
Q4G1C9-3	GRPL2_HUMAN Isoform 3 of GLIPR1-like protein 2 OS=Homo sapiens GN=GLIPR1L2		
Q4G1C9-4	GRPL2_HUMAN Isoform 4 of GLIPR1-like protein 2 OS=Homo sapiens GN=GLIPR1L2		
Q4G1C9-5	GRPL2_HUMAN Isoform 5 of GLIPR1-like protein 2 OS=Homo sapiens GN=GLIPR1L2		
Q4G1C9-6	GRPL2_HUMAN Isoform 6 of GLIPR1-like protein 2 OS=Homo sapiens GN=GLIPR1L2		
Q4KMQ2	ANO6_HUMAN Anoctamin-6 OS=Homo sapiens GN=ANO6 PE=1 SV=2		30.65
Q4KWH8	PLCH1_HUMAN 1-phosphatidylinositol-4,5-bisphosphate phosphodiesterase eta-1 OS=Homo sapiens GN=PLCH1 PE=1 SV=1		95.9
Q4KWH8-2	PLCH1_HUMAN Isoform 2 of 1-phosphatidylinositol-4,5-bisphosphate phosphodiesterase eta-1 OS=Homo sapiens GN=PLCH1		84.41
Q4KWH8-3	PLCH1_HUMAN Isoform 3 of 1-phosphatidylinositol-4,5-bisphosphate phosphodiesterase eta-1 OS=Homo sapiens GN=PLCH1		87.23
Q4KWH8-4	PLCH1_HUMAN Isoform 4 of 1-phosphatidylinositol-4,5-bisphosphate phosphodiesterase eta-1 OS=Homo sapiens GN=PLCH1		89.15
Q4KWH8;Q4KWH8-2;Q4K'	PLCH1_HUMAN 1-phosphatidylinositol-4,5-bisphosphate phosphodiesterase eta-1 OS=Homo sapiens GN=PLCH1 PE=1 SV=1;>sp|Q4K	2.83E-07	
Q4L180	FIL1L_HUMAN Filamin A-interacting protein 1-like OS=Homo sapiens GN=FILIP1L PE=1 SV=2		
Q4L180-2	FIL1L_HUMAN Isoform 2 of Filamin A-interacting protein 1-like OS=Homo sapiens GN=FILIP1L		

Q4L180-3	FIL1L_HUMAN Isoform 3 of Filamin A-interacting protein 1-like OS=Homo sapiens GN=FILIP1L		
Q4L180-4	FIL1L_HUMAN Isoform 4 of Filamin A-interacting protein 1-like OS=Homo sapiens GN=FILIP1L		
Q4L180-5	FIL1L_HUMAN Isoform 5 of Filamin A-interacting protein 1-like OS=Homo sapiens GN=FILIP1L		
Q4L180-6	FIL1L_HUMAN Isoform 6 of Filamin A-interacting protein 1-like OS=Homo sapiens GN=FILIP1L		
Q4L180-7	FIL1L_HUMAN Isoform 7 of Filamin A-interacting protein 1-like OS=Homo sapiens GN=FILIP1L		
Q4L235	ACSF4_HUMAN Acyl-CoA synthetase family member 4 OS=Homo sapiens GN=AASDH PE=2 SV=3		42.01
Q4L235-2	ACSF4_HUMAN Isoform 2 of Acyl-CoA synthetase family member 4 OS=Homo sapiens GN=AASDH		42.01
Q4L235-3	ACSF4_HUMAN Isoform 3 of Acyl-CoA synthetase family member 4 OS=Homo sapiens GN=AASDH		42.01
Q4L235-4	ACSF4_HUMAN Isoform 4 of Acyl-CoA synthetase family member 4 OS=Homo sapiens GN=AASDH		42.01
Q4LDE5	SVEP1_HUMAN Sushi, von Willebrand factor type A, EGF and pentraxin domain-containing protein 1 OS=Homo sapiens GN=SVEP1 PE=1 SV=3		18.52
Q4LDE5-3	SVEP1_HUMAN Isoform 3 of Sushi, von Willebrand factor type A, EGF and pentraxin domain-containing protein 1 OS=Homo sapiens GN=SVEP1		18.52
Q4LEZ3	AARD_HUMAN Alanine and arginine-rich domain-containing protein OS=Homo sapiens GN=AARD PE=2 SV=1		31.98
Q4UJ75	A20A4_HUMAN Ankyrin repeat domain-containing protein 20A4 OS=Homo sapiens GN=ANKRD20A4 PE=2 SV=1		
Q4V328;Q4V328-4;Q4V32	GRAP1_HUMAN GRIP1-associated protein 1 OS=Homo sapiens GN=GRIPAP1 PE=1 SV=1;>sp|Q4V328-4|GRAP1_HUMAN Isoform 4 of	2.64E-18	
Q4VC05	BCL7A_HUMAN B-cell CLL/lymphoma 7 protein family member A OS=Homo sapiens GN=BCL7A PE=1 SV=1		
Q4VC05-2	BCL7A_HUMAN Isoform 2 of B-cell CLL/lymphoma 7 protein family member A OS=Homo sapiens GN=BCL7A		
Q4VC31	CCD58_HUMAN Coiled-coil domain-containing protein 58 OS=Homo sapiens GN=CCDC58 PE=1 SV=1		171.86
Q4VXU2	PAP1L_HUMAN Polyadenylate-binding protein 1-like OS=Homo sapiens GN=PABPC1L PE=2 SV=1		342.78
Q4ZG55-2	GREB1_HUMAN Isoform 2 of Protein GREB1 OS=Homo sapiens GN=GREB1		1.34
Q4ZG55-3	GREB1_HUMAN Isoform 3 of Protein GREB1 OS=Homo sapiens GN=GREB1		1.34
Q4ZHG4	FNDC1_HUMAN Fibronectin type III domain-containing protein 1 OS=Homo sapiens GN=FNDC1 PE=1 SV=4		
Q4ZHG4-2	FNDC1_HUMAN Isoform 2 of Fibronectin type III domain-containing protein 1 OS=Homo sapiens GN=FNDC1		
Q504Q3	PAN2_HUMAN PAB-dependent poly(A)-specific ribonuclease subunit 2 OS=Homo sapiens GN=PAN2 PE=1 SV=3		
Q504Q3-2	PAN2_HUMAN Isoform 2 of PAB-dependent poly(A)-specific ribonuclease subunit 2 OS=Homo sapiens GN=PAN2		
Q504Q3-3	PAN2_HUMAN Isoform 3 of PAB-dependent poly(A)-specific ribonuclease subunit 2 OS=Homo sapiens GN=PAN2		
Q52LJ0	FA98B_HUMAN Protein FAM98B OS=Homo sapiens GN=FAM98B PE=1 SV=1		83.28
Q52LW3	RHG29_HUMAN Rho GTPase-activating protein 29 OS=Homo sapiens GN=ARHGAP29 PE=1 SV=2		
Q52LW3-2	RHG29_HUMAN Isoform 2 of Rho GTPase-activating protein 29 OS=Homo sapiens GN=ARHGAP29		
Q52LW3;Q52LW3-2	RHG29_HUMAN Rho GTPase-activating protein 29 OS=Homo sapiens GN=ARHGAP29 PE=1 SV=2;>sp|Q52LW3-2|RHG29_HUMAN Isof	1.29E-43	
Q52M75	CE027_HUMAN Putative uncharacterized protein C5orf27 OS=Homo sapiens GN=C5orf27 PE=2 SV=2		23.03
Q53EL6	PDCD4_HUMAN Programmed cell death protein 4 OS=Homo sapiens GN=PDCD4 PE=1 SV=2	0	
Q53EP0;Q53EP0-2	FND3B_HUMAN Fibronectin type III domain-containing protein 3B OS=Homo sapiens GN=FNDC3B PE=1 SV=2;>sp|Q53EP0-2|FND3B_	1.12E-05	
Q53EZ4;Q53EZ4-2	CEP55_HUMAN Centrosomal protein of 55 kDa OS=Homo sapiens GN=CEP55 PE=1 SV=3;>sp|Q53EZ4-2|CEP55_HUMAN Isoform 2 of	1.22E-51	
Q53F19	CQ085_HUMAN Uncharacterized protein C17orf85 OS=Homo sapiens GN=C17orf85 PE=1 SV=2		
Q53F19-2	CQ085_HUMAN Isoform 2 of Uncharacterized protein C17orf85 OS=Homo sapiens GN=C17orf85		
Q53F19;Q53F19-2	CQ085_HUMAN Uncharacterized protein C17orf85 OS=Homo sapiens GN=C17orf85 PE=1 SV=2;>sp|Q53F19-2|CQ085_HUMAN Isofor	0.00024906	
Q53FA7;Q53FA7-2	QORX_HUMAN Quinone oxidoreductase PIG3 OS=Homo sapiens GN=TP53I3 PE=1 SV=2;>sp|Q53FA7-2|QORX_HUMAN Isoform 2 of Q	5.15E-14	
Q53FE4	CD017_HUMAN Uncharacterized protein C4orf17 OS=Homo sapiens GN=C4orf17 PE=2 SV=3		
Q53FT3	CK073_HUMAN Uncharacterized protein C11orf73 OS=Homo sapiens GN=C11orf73 PE=1 SV=2	7.16E-18	
Q53FV1	ORML2_HUMAN ORM1-like protein 2 OS=Homo sapiens GN=ORMDL2 PE=2 SV=2		
Q53GA4	PHLA2_HUMAN Pleckstrin homology-like domain family A member 2 OS=Homo sapiens GN=PHLDA2 PE=1 SV=2		34.57
Q53GD3	CTL4_HUMAN Choline transporter-like protein 4 OS=Homo sapiens GN=SLC44A4 PE=2 SV=2		
Q53GD3-2	CTL4_HUMAN Isoform 2 of Choline transporter-like protein 4 OS=Homo sapiens GN=SLC44A4		
Q53GD3;B4DU94;A2BED4	CTL4_HUMAN Choline transporter-like protein 4 OS=Homo sapiens GN=SLC44A4 PE=2 SV=2;>tr|B4DU94|B4DU94_HUMAN cDNA FLJ	4.67E-07	
Q53GG5;Q53GG5-2;Q53G	PDLI3_HUMAN PDZ and LIM domain protein 3 OS=Homo sapiens GN=PDLIM3 PE=2 SV=1;>sp|Q53GG5-2|PDLI3_HUMAN Isoform 2 o	1.98E-27	
Q53GL7	PAR10_HUMAN Poly [ADP-ribose] polymerase 10 OS=Homo sapiens GN=PARP10 PE=1 SV=2		
Q53GQ0	DHB12_HUMAN Estradiol 17-beta-dehydrogenase 12 OS=Homo sapiens GN=HSD17B12 PE=1 SV=2		726.21
Q53GS7	GLE1_HUMAN Nucleoporin GLE1 OS=Homo sapiens GN=GLE1 PE=1 SV=2		
Q53GS7-2	GLE1_HUMAN Isoform 2 of Nucleoporin GLE1 OS=Homo sapiens GN=GLE1		
Q53GS9	SNUT2_HUMAN U4/U6.U5 tri-snRNP-associated protein 2 OS=Homo sapiens GN=USP39 PE=1 SV=2		366.81
Q53H12	AGK_HUMAN Acylglycerol kinase, mitochondrial OS=Homo sapiens GN=AGK PE=1 SV=2		112.35
Q53H47	SETMR_HUMAN Histone-lysine N-methyltransferase SETMAR OS=Homo sapiens GN=SETMAR PE=1 SV=1		
Q53H47-2	SETMR_HUMAN Isoform 2 of Histone-lysine N-methyltransferase SETMAR OS=Homo sapiens GN=SETMAR		
Q53H82	LACB2_HUMAN Beta-lactamase-like protein 2 OS=Homo sapiens GN=LACTB2 PE=1 SV=2		88.58
Q53H96	P5CR3_HUMAN Pyrroline-5-carboxylate reductase 3 OS=Homo sapiens GN=PYCRL PE=1 SV=2	7.39E-05	
Q53HL2	BOREA_HUMAN Borealin OS=Homo sapiens GN=CDCA8 PE=1 SV=2		
Q53S58	TM177_HUMAN Transmembrane protein 177 OS=Homo sapiens GN=TMEM177 PE=2 SV=1		
Q53TN4	CYBR1_HUMAN Cytochrome b reductase 1 OS=Homo sapiens GN=CYBRD1 PE=1 SV=1		75.23
Q562E7	WDR81_HUMAN WD repeat-containing protein 81 OS=Homo sapiens GN=WDR81 PE=2 SV=1		
Q562R1	ACTBL_HUMAN Beta-actin-like protein 2 OS=Homo sapiens GN=ACTBL2 PE=1 SV=2		562.21
Q567U6	CCD93_HUMAN Coiled-coil domain-containing protein 93 OS=Homo sapiens GN=CCDC93 PE=1 SV=2		53.66

Q56NI9	ESCO2_HUMAN N-acetyltransferase ESCO2 OS=Homo sapiens GN=ESCO2 PE=1 SV=1		34.77
Q56UQ5	TPT1L_HUMAN TPT1-like protein OS=Homo sapiens PE=1 SV=1		120.36
Q56VL3	OCAD2_HUMAN OCIA domain-containing protein 2 OS=Homo sapiens GN=OCIAD2 PE=1 SV=1		230.1
Q56VL3-2	OCAD2_HUMAN Isoform 2 of OCIA domain-containing protein 2 OS=Homo sapiens GN=OCIAD2		28.83
Q56VL3;Q56VL3-2	OCAD2_HUMAN OCIA domain-containing protein 2 OS=Homo sapiens GN=OCIAD2 PE=1 SV=1;>sp|Q56VL3-2|OCAD2_HUMAN Isofor	8.78E-17	
Q58EX2	SDK2_HUMAN Protein sidekick-2 OS=Homo sapiens GN=SDK2 PE=1 SV=3		
Q58EX2-2	SDK2_HUMAN Isoform 2 of Protein sidekick-2 OS=Homo sapiens GN=SDK2		
Q58EX2-3	SDK2_HUMAN Isoform 3 of Protein sidekick-2 OS=Homo sapiens GN=SDK2		
Q58EX2-4	SDK2_HUMAN Isoform 4 of Protein sidekick-2 OS=Homo sapiens GN=SDK2		
Q58FF3	ENPLL_HUMAN Putative endoplasmin-like protein OS=Homo sapiens GN=HSP90B2P PE=5 SV=1		213.64
Q58FF6	H90B4_HUMAN Putative heat shock protein HSP 90-beta 4 OS=Homo sapiens GN=HSP90AB4P PE=5 SV=1		233.79
Q58FF7	H90B3_HUMAN Putative heat shock protein HSP 90-beta-3 OS=Homo sapiens GN=HSP90AB3P PE=5 SV=1		751.7
Q58FF8	H90B2_HUMAN Putative heat shock protein HSP 90-beta 2 OS=Homo sapiens GN=HSP90AB2P PE=1 SV=2		491.4
Q58FG0	HS905_HUMAN Putative heat shock protein HSP 90-alpha A5 OS=Homo sapiens GN=HSP90AA5P PE=1 SV=1		281.74
Q58FG1	HS904_HUMAN Putative heat shock protein HSP 90-alpha A4 OS=Homo sapiens GN=HSP90AA4P PE=5 SV=1		219
Q59H18	TNI3K_HUMAN Serine/threonine-protein kinase TNNI3K OS=Homo sapiens GN=TNNI3K PE=1 SV=3		
Q59H18-1	TNI3K_HUMAN Isoform 1 of Serine/threonine-protein kinase TNNI3K OS=Homo sapiens GN=TNNI3K		
Q59H18-3	TNI3K_HUMAN Isoform 3 of Serine/threonine-protein kinase TNNI3K OS=Homo sapiens GN=TNNI3K		
Q59H18-4	TNI3K_HUMAN Isoform 4 of Serine/threonine-protein kinase TNNI3K OS=Homo sapiens GN=TNNI3K		
Q5BJD5	TM41B_HUMAN Transmembrane protein 41B OS=Homo sapiens GN=TMEM41B PE=1 SV=1		40.86
Q5BJD5-2	TM41B_HUMAN Isoform 2 of Transmembrane protein 41B OS=Homo sapiens GN=TMEM41B		39.13
Q5BJF2	TMM97_HUMAN Transmembrane protein 97 OS=Homo sapiens GN=TMEM97 PE=1 SV=1		
Q5BJH7	YIF1B_HUMAN Protein YIF1B OS=Homo sapiens GN=YIF1B PE=1 SV=1		66.06
Q5BJH7-2	YIF1B_HUMAN Isoform 2 of Protein YIF1B OS=Homo sapiens GN=YIF1B		66.06
Q5BJH7-3	YIF1B_HUMAN Isoform 3 of Protein YIF1B OS=Homo sapiens GN=YIF1B		66.06
Q5BJH7-4	YIF1B_HUMAN Isoform 4 of Protein YIF1B OS=Homo sapiens GN=YIF1B		2.48
Q5BJH7-5	YIF1B_HUMAN Isoform 5 of Protein YIF1B OS=Homo sapiens GN=YIF1B		2.48
Q5BKU9	CQ090_HUMAN Uncharacterized protein C17orf90 OS=Homo sapiens GN=C17orf90 PE=2 SV=1	5.65E-11	
Q5BKZ1	ZN326_HUMAN Zinc finger protein 326 OS=Homo sapiens GN=ZNF326 PE=1 SV=2		
Q5C9Z4	NOM1_HUMAN Nucleolar MIF4G domain-containing protein 1 OS=Homo sapiens GN=NOM1 PE=1 SV=1		
Q5CZ79	AN20B_HUMAN Ankyrin repeat domain-containing protein 20B OS=Homo sapiens GN=ANKRD20A8P PE=2 SV=2		
Q5CZ79-2	AN20B_HUMAN Isoform 2 of Ankyrin repeat domain-containing protein 20B OS=Homo sapiens GN=ANKRD20A8P		
Q5CZC0	FSIP2_HUMAN Fibrous sheath-interacting protein 2 OS=Homo sapiens GN=FSIP2 PE=1 SV=4		66
Q5CZC0-2	FSIP2_HUMAN Isoform 2 of Fibrous sheath-interacting protein 2 OS=Homo sapiens GN=FSIP2		2.03
Q5D0E6	DALD3_HUMAN DALR anticodon-binding domain-containing protein 3 OS=Homo sapiens GN=DALRD3 PE=2 SV=2		
Q5D0E6-2	DALD3_HUMAN Isoform 2 of DALR anticodon-binding domain-containing protein 3 OS=Homo sapiens GN=DALRD3		
Q5D0E6-3	DALD3_HUMAN Isoform 3 of DALR anticodon-binding domain-containing protein 3 OS=Homo sapiens GN=DALRD3		
Q5D0E6-4	DALD3_HUMAN Isoform 4 of DALR anticodon-binding domain-containing protein 3 OS=Homo sapiens GN=DALRD3		
Q5D862	FILA2_HUMAN Filaggrin-2 OS=Homo sapiens GN=FLG2 PE=1 SV=1		66.77
Q5EBL8	PDZ11_HUMAN PDZ domain-containing protein 11 OS=Homo sapiens GN=PDZD11 PE=1 SV=2		134.53
Q5EBL8-2	PDZ11_HUMAN Isoform 2 of PDZ domain-containing protein 11 OS=Homo sapiens GN=PDZD11		134.53
Q5EBL8-2;D3DVU3;Q5EBL	2|PDZ11_HUMAN Isoform 2 of PDZ domain-containing protein 11 OS=Homo sapiens GN=PDZD11;>tr|D3DVU3|D3DVU3_HUMAN PDZ	3.70E-45	
Q5EBM0;Q5EBM0-4;Q5EB	CMPK2_HUMAN UMP-CMP kinase 2, mitochondrial OS=Homo sapiens GN=CMPK2 PE=1 SV=3;>sp|Q5EBM0-4|CMPK2_HUMAN Isoform	4.64E-68	
Q5FWF4	ZRAB3_HUMAN Zinc finger Ran-binding domain-containing protein 3 OS=Homo sapiens GN=ZRANB3 PE=2 SV=2		
Q5FWF4-2	ZRAB3_HUMAN Isoform 2 of Zinc finger Ran-binding domain-containing protein 3 OS=Homo sapiens GN=ZRANB3		
Q5FWF4-3	ZRAB3_HUMAN Isoform 3 of Zinc finger Ran-binding domain-containing protein 3 OS=Homo sapiens GN=ZRANB3		
Q5GJ75	TP8L3_HUMAN Tumor necrosis factor alpha-induced protein 8-like protein 3 OS=Homo sapiens GN=TNFAIP8L3 PE=2 SV=1		29.43
Q5GLZ8;Q5GLZ8-2;Q5GLZ	HERC4_HUMAN Probable E3 ubiquitin-protein ligase HERC4 OS=Homo sapiens GN=HERC4 PE=1 SV=1;>sp|Q5GLZ8-2|HERC4_HUMAN	1.63E-20	
Q5H9M0	MUML1_HUMAN PWWP domain-containing protein MUM1L1 OS=Homo sapiens GN=MUM1L1 PE=2 SV=1		
Q5H9R7-5;Q5H9R7;Q5H9I	5|PP6R3_HUMAN Isoform 5 of Serine/threonine-protein phosphatase 6 regulatory subunit 3 OS=Homo sapiens GN=PPP6R3;>sp|Q5H9	1.61E-11	
Q5H9U9	DDX6L_HUMAN Probable ATP-dependent RNA helicase DDX60-like OS=Homo sapiens GN=DDX60L PE=2 SV=2		36.54
Q5H9U9-2	DDX6L_HUMAN Isoform 2 of Probable ATP-dependent RNA helicase DDX60-like OS=Homo sapiens GN=DDX60L		33.06
Q5HYA8	MKS3_HUMAN Meckelin OS=Homo sapiens GN=TMEM67 PE=1 SV=2		29.14
Q5HYC2	K2026_HUMAN Uncharacterized protein KIAA2026 OS=Homo sapiens GN=KIAA2026 PE=2 SV=2		
Q5HYC2-2	K2026_HUMAN Isoform 2 of Uncharacterized protein KIAA2026 OS=Homo sapiens GN=KIAA2026		
Q5HYC2;Q5HYC2-2	K2026_HUMAN Uncharacterized protein KIAA2026 OS=Homo sapiens GN=KIAA2026 PE=2 SV=2;>sp|Q5HYC2-2|K2026_HUMAN Isofo	2.73E-10	
Q5HYI8	RABL3_HUMAN Rab-like protein 3 OS=Homo sapiens GN=RABL3 PE=1 SV=1		93.59
Q5HYK3;B2RDU9;B3GK62	COQ5_HUMAN Ubiquinone biosynthesis methyltransferase COQ5, mitochondrial OS=Homo sapiens GN=COQ5 PE=1 SV=2;>tr|B2RDU9	1.32E-07	
Q5HYK3;Q5HYK3-2	COQ5_HUMAN 2-methoxy-6-polyprenyl-1,4-benzoquinol methylase, mitochondrial OS=Homo sapiens GN=COQ5 PE=1 SV=2;>sp|Q5H	5.29E-06	
Q5J8M3	TMM85_HUMAN Transmembrane protein 85 OS=Homo sapiens GN=TMEM85 PE=1 SV=2		75.84
Q5J8M3-2	TMM85_HUMAN Isoform 2 of Transmembrane protein 85 OS=Homo sapiens GN=TMEM85		75.84

Q5J8M3-3	TMM85_HUMAN Isoform 3 of Transmembrane protein 85 OS=Homo sapiens GN=TMEM85		75.84
Q5J8M3;Q5J8M3-2;Q5J8M	TMM85_HUMAN Transmembrane protein 85 OS=Homo sapiens GN=TMEM85 PE=1 SV=2;>sp|Q5J8M3-2|TMM85_HUMAN Isoform 2 of	2.93E-34	
Q5JNZ3	ZN311_HUMAN Zinc finger protein 311 OS=Homo sapiens GN=ZNF311 PE=1 SV=2		
Q5JNZ5	RS26L_HUMAN Putative 40S ribosomal protein S26-like 1 OS=Homo sapiens GN=RPS26P11 PE=5 SV=1		227.99
Q5JPE7	NOMO2_HUMAN Nodal modulator 2 OS=Homo sapiens GN=NOMO2 PE=1 SV=1		174.04
Q5JPE7-2	NOMO2_HUMAN Isoform 2 of Nodal modulator 2 OS=Homo sapiens GN=NOMO2		174.04
Q5JPE7;P69849;Q15155;(	NOMO2_HUMAN Nodal modulator 2 OS=Homo sapiens GN=NOMO2 PE=1 SV=1;>sp|P69849|NOMO3_HUMAN Nodal modulator 3 OS=	3.05E-20	
Q5JPF3	AN36C_HUMAN Ankyrin repeat domain-containing protein 36C OS=Homo sapiens PE=2 SV=3		
Q5JPF3-2	AN36C_HUMAN Isoform 2 of Ankyrin repeat domain-containing protein 36C OS=Homo sapiens		
Q5JPH6	SYEM_HUMAN Probable glutamyl-tRNA synthetase, mitochondrial OS=Homo sapiens GN=EARS2 PE=1 SV=2	1.17E-08	
Q5JQF8	PAP1M_HUMAN Polyadenylate-binding protein 1-like 2 OS=Homo sapiens GN=PABPC1L2A PE=1 SV=1		160.94
Q5JR59	MTUS2_HUMAN Microtubule-associated tumor suppressor candidate 2 OS=Homo sapiens GN=MTUS2 PE=1 SV=3		
Q5JR59-3	MTUS2_HUMAN Isoform 2 of Microtubule-associated tumor suppressor candidate 2 OS=Homo sapiens GN=MTUS2		
Q5JR59-4	MTUS2_HUMAN Isoform 3 of Microtubule-associated tumor suppressor candidate 2 OS=Homo sapiens GN=MTUS2		
Q5JRA6	MIA3_HUMAN Melanoma inhibitory activity protein 3 OS=Homo sapiens GN=MIA3 PE=1 SV=1		56.89
Q5JRA6-2	MIA3_HUMAN Isoform 2 of Melanoma inhibitory activity protein 3 OS=Homo sapiens GN=MIA3		56.89
Q5JRA6-3	MIA3_HUMAN Isoform 3 of Melanoma inhibitory activity protein 3 OS=Homo sapiens GN=MIA3		9.54
Q5JRA6;Q5JRA6-2	MIA3_HUMAN Melanoma inhibitory activity protein 3 OS=Homo sapiens GN=MIA3 PE=1 SV=1;>sp|Q5JRA6-2|MIA3_HUMAN Isoform 2	4.80E-23	
Q5JRX3	PREP_HUMAN Presequence protease, mitochondrial OS=Homo sapiens GN=PITRM1 PE=1 SV=2		70.83
Q5JRX3-2	PREP_HUMAN Isoform 2 of Presequence protease, mitochondrial OS=Homo sapiens GN=PITRM1		70.83
Q5JRX3-2;Q5JRX3	2|PREP_HUMAN Isoform 2 of Presequence protease, mitochondrial OS=Homo sapiens GN=PITRM1;>sp|Q5JRX3|PREP_HUMAN Presequ	8.13E-19	
Q5JS54	PSMG4_HUMAN Proteasome assembly chaperone 4 OS=Homo sapiens GN=PSMG4 PE=2 SV=2	2.58E-06	
Q5JSH3;Q5JSH3-2	WDR44_HUMAN WD repeat-containing protein 44 OS=Homo sapiens GN=WDR44 PE=1 SV=1;>sp|Q5JSH3-2|WDR44_HUMAN Isoform	1.87E-22	
Q5JSL3	DOC11_HUMAN Dedicator of cytokinesis protein 11 OS=Homo sapiens GN=DOCK11 PE=1 SV=2		
Q5JSP0;Q5JSP0-2	FGD3_HUMAN FYVE, RhoGEF and PH domain-containing protein 3 OS=Homo sapiens GN=FGD3 PE=1 SV=1;>sp|Q5JSP0-2|FGD3_HUI	1.91E-06	
Q5JTD0	TJAP1_HUMAN Tight junction-associated protein 1 OS=Homo sapiens GN=TJAP1 PE=1 SV=1		
Q5JTD0-2	TJAP1_HUMAN Isoform 2 of Tight junction-associated protein 1 OS=Homo sapiens GN=TJAP1		
Q5JTD0-3	TJAP1_HUMAN Isoform 3 of Tight junction-associated protein 1 OS=Homo sapiens GN=TJAP1		
Q5JTD0-4	TJAP1_HUMAN Isoform 4 of Tight junction-associated protein 1 OS=Homo sapiens GN=TJAP1		
Q5JTD0;Q5JTD0-2;Q5JTD(	TJAP1_HUMAN Tight junction-associated protein 1 OS=Homo sapiens GN=TJAP1 PE=1 SV=1;>sp|Q5JTD0-2|TJAP1_HUMAN Isoform 2	1.39E-13	
Q5JTH9	RRP12_HUMAN RRP12-like protein OS=Homo sapiens GN=RRP12 PE=1 SV=2		59.03
Q5JTH9-2	RRP12_HUMAN Isoform 2 of RRP12-like protein OS=Homo sapiens GN=RRP12		59.03
Q5JTH9;Q5JTH9-2	RRP12_HUMAN RRP12-like protein OS=Homo sapiens GN=RRP12 PE=1 SV=2;>sp|Q5JTH9-2|RRP12_HUMAN Isoform 2 of RRP12-like	5.33E-202	
Q5JTJ3	CA031_HUMAN Uncharacterized protein C1orf31 OS=Homo sapiens GN=C1orf31 PE=2 SV=1		48.06
Q5JTJ3-2	CA031_HUMAN Isoform 2 of Uncharacterized protein C1orf31 OS=Homo sapiens GN=C1orf31		48.06
Q5JTJ3-2;Q5JTJ3;Q5JTJ3-	2|CA031_HUMAN Isoform 2 of Uncharacterized protein C1orf31 OS=Homo sapiens GN=C1orf31;>sp|Q5JTJ3|CA031_HUMAN Uncharac	1.08E-45	
Q5JTJ3-3	CA031_HUMAN Isoform 3 of Uncharacterized protein C1orf31 OS=Homo sapiens GN=C1orf31		48.06
Q5JTV8	TOIP1_HUMAN Torsin-1A-interacting protein 1 OS=Homo sapiens GN=TOR1AIP1 PE=1 SV=2		
Q5JTV8-2	TOIP1_HUMAN Isoform 2 of Torsin-1A-interacting protein 1 OS=Homo sapiens GN=TOR1AIP1		
Q5JTV8;Q5JTV8-2	TOIP1_HUMAN Torsin-1A-interacting protein 1 OS=Homo sapiens GN=TOR1AIP1 PE=1 SV=2;>sp|Q5JTV8-2|TOIP1_HUMAN Isoform 2	1.90E-64	
Q5JTZ9	SYAM_HUMAN Alanyl-tRNA synthetase, mitochondrial OS=Homo sapiens GN=AARS2 PE=1 SV=1		39.32
Q5JU00	TCTE1_HUMAN T-complex-associated testis-expressed protein 1 OS=Homo sapiens GN=TCTE1 PE=2 SV=1		20.6
Q5JU69	TOR2A_HUMAN Torsin-2A OS=Homo sapiens GN=TOR2A PE=2 SV=1		23.56
Q5JUX0	SPIN3_HUMAN Spindlin-3 OS=Homo sapiens GN=SPIN3 PE=2 SV=1		38.06
Q5JV73	FRPD3_HUMAN FERM and PDZ domain-containing protein 3 OS=Homo sapiens GN=FRMPD3 PE=2 SV=2		42.76
Q5JVF3	PCID2_HUMAN PCI domain-containing protein 2 OS=Homo sapiens GN=PCID2 PE=1 SV=2		21.93
Q5JVF3-2	PCID2_HUMAN Isoform 2 of PCI domain-containing protein 2 OS=Homo sapiens GN=PCID2		21.93
Q5JVF3-3	PCID2_HUMAN Isoform 3 of PCI domain-containing protein 2 OS=Homo sapiens GN=PCID2		21.93
Q5JVF3-4	PCID2_HUMAN Isoform 4 of PCI domain-containing protein 2 OS=Homo sapiens GN=PCID2		21.93
Q5JVF3-4;C9J439;A6NM5:	4|PCID2_HUMAN Isoform 4 of PCI domain-containing protein 2 OS=Homo sapiens GN=PCID2;>tr|C9J439|C9J439_HUMAN Uncharacte	4.80E-09	
Q5JWF2	GNAS1_HUMAN Guanine nucleotide-binding protein G(s) subunit alpha isoforms XLas OS=Homo sapiens GN=GNAS PE=1 SV=2		598.18
Q5JWF2-2	GNAS1_HUMAN Isoform XLas-2 of Guanine nucleotide-binding protein G(s) subunit alpha isoforms XLas OS=Homo sapiens GN=GNAS		598.18
Q5JWF2-3	GNAS1_HUMAN Isoform XLas-3 of Guanine nucleotide-binding protein G(s) subunit alpha isoforms XLas OS=Homo sapiens GN=GNAS		4.85
Q5JWF2;Q5JWF2-2;P6309	GNAS1_HUMAN Guanine nucleotide-binding protein G(s) subunit alpha isoforms XLas OS=Homo sapiens GN=GNAS PE=1 SV=2;>sp|(	1.42E-34	
Q5JWF8	CT134_HUMAN Putative uncharacterized actin family protein C20orf134 OS=Homo sapiens GN=C20orf134 PE=2 SV=1		17.81
Q5JWR5	DOP1_HUMAN Protein dopey-1 OS=Homo sapiens GN=DOPEY1 PE=1 SV=1		
Q5JXB2	UE2NL_HUMAN Putative ubiquitin-conjugating enzyme E2 N-like OS=Homo sapiens GN=UBE2NL PE=5 SV=1		202.01
Q5JXC2	MIIP_HUMAN Migration and invasion-inhibitory protein OS=Homo sapiens GN=MIIP PE=1 SV=3		22.78
Q5JXC2-2	MIIP_HUMAN Isoform 2 of Migration and invasion-inhibitory protein OS=Homo sapiens GN=MIIP		22.78
Q5JYT7	K1755_HUMAN Uncharacterized protein KIAA1755 OS=Homo sapiens GN=KIAA1755 PE=2 SV=2		
Q5K4L6;Q5K4L6-2;Q5K4L	S27A3_HUMAN Long-chain fatty acid transport protein 3 OS=Homo sapiens GN=SLC27A3 PE=2 SV=3;>sp|Q5K4L6-2|S27A3_HUMAN	1.83E-27	

Q5K651	SAMD9_HUMAN Sterile alpha motif domain-containing protein 9 OS=Homo sapiens GN=SAMD9 PE=1 SV=1	9.85E-11	
Q5M775	CYTSB_HUMAN Cytospin-B OS=Homo sapiens GN=SPECC1 PE=1 SV=1		31.72
Q5M775-2	CYTSB_HUMAN Isoform 2 of Cytospin-B OS=Homo sapiens GN=SPECC1		8.69
Q5M775-3	CYTSB_HUMAN Isoform 3 of Cytospin-B OS=Homo sapiens GN=SPECC1		8.69
Q5M775-4	CYTSB_HUMAN Isoform 4 of Cytospin-B OS=Homo sapiens GN=SPECC1		31.72
Q5M775-5	CYTSB_HUMAN Isoform 5 of Cytospin-B OS=Homo sapiens GN=SPECC1		8.69
Q5M775;A8MV89;B4E2A4	CYTSB_HUMAN Cytospin-B OS=Homo sapiens GN=CYTSB PE=1 SV=1;>tr|A8MV89|A8MV89_HUMAN Uncharacterized protein OS=Hon	5.54E-07	
Q5M9N0	CD158_HUMAN Coiled-coil domain-containing protein 158 OS=Homo sapiens GN=CCDC158 PE=2 SV=2		30.85
Q5M9N0-2	CD158_HUMAN Isoform 2 of Coiled-coil domain-containing protein 158 OS=Homo sapiens GN=CCDC158		7.57
Q5M9N0-3	CD158_HUMAN Isoform 3 of Coiled-coil domain-containing protein 158 OS=Homo sapiens GN=CCDC158		7.57
Q5M9N0;Q5M9N0-2;Q5M9	CD158_HUMAN Coiled-coil domain-containing protein 158 OS=Homo sapiens GN=CCDC158 PE=2 SV=2;>sp|Q5M9N0-2|CD158_HUMA	1.14E-06	
Q5MCW4	ZN569_HUMAN Zinc finger protein 569 OS=Homo sapiens GN=ZNF569 PE=2 SV=1		
Q5MIZ7	P4R3B_HUMAN Serine/threonine-protein phosphatase 4 regulatory subunit 3B OS=Homo sapiens GN=SMEK2 PE=1 SV=2		0.63
Q5MIZ7-2	P4R3B_HUMAN Isoform 2 of Serine/threonine-protein phosphatase 4 regulatory subunit 3B OS=Homo sapiens GN=SMEK2		0.63
Q5MIZ7-3	P4R3B_HUMAN Isoform 3 of Serine/threonine-protein phosphatase 4 regulatory subunit 3B OS=Homo sapiens GN=SMEK2		0.63
Q5MIZ7;Q5MIZ7-2;Q5MIZ	P4R3B_HUMAN Serine/threonine-protein phosphatase 4 regulatory subunit 3B OS=Homo sapiens GN=SMEK2 PE=1 SV=2;>sp|Q5MIZ	7.53E-13	
Q5PRF9	SMAG2_HUMAN Protein Smaug homolog 2 OS=Homo sapiens GN=SAMD4B PE=1 SV=1	3.69E-23	
Q5PSV4	BRM1L_HUMAN Breast cancer metastasis-suppressor 1-like protein OS=Homo sapiens GN=BRMS1L PE=1 SV=2		
Q5QJE6	TDIF2_HUMAN Deoxynucleotidyltransferase terminal-interacting protein 2 OS=Homo sapiens GN=DNTTIP2 PE=1 SV=2		
Q5QNW6	H2B2F_HUMAN Histone H2B type 2-F OS=Homo sapiens GN=HIST2H2BF PE=1 SV=3		708.52
Q5QP74	CJ115_HUMAN Putative uncharacterized protein C10orf115 OS=Homo sapiens GN=C10orf115 PE=2 SV=2		
Q5QP82	DCA10_HUMAN DDB1- and CUL4-associated factor 10 OS=Homo sapiens GN=DCAF10 PE=1 SV=1		
Q5QP82-2	DCA10_HUMAN Isoform 2 of DDB1- and CUL4-associated factor 10 OS=Homo sapiens GN=DCAF10		
Q5R3I4	TTC38_HUMAN Tetratricopeptide repeat protein 38 OS=Homo sapiens GN=TTC38 PE=1 SV=1	9.14E-32	
Q5RHP9	CA173_HUMAN Uncharacterized protein C1orf173 OS=Homo sapiens GN=C1orf173 PE=2 SV=1		
Q5RHP9-2	CA173_HUMAN Isoform 2 of Uncharacterized protein C1orf173 OS=Homo sapiens GN=C1orf173		
Q5RHP9-3	CA173_HUMAN Isoform 3 of Uncharacterized protein C1orf173 OS=Homo sapiens GN=C1orf173		
Q5RI15	FA36A_HUMAN Protein FAM36A OS=Homo sapiens GN=FAM36A PE=1 SV=2		88.13
Q5RI15-2	FA36A_HUMAN Isoform 2 of Protein FAM36A OS=Homo sapiens GN=FAM36A		88.13
Q5RI15-2;B3KM21;Q5RI1	2|FA36A_HUMAN Isoform 2 of Protein FAM36A OS=Homo sapiens GN=FAM36A;>tr|B3KM21|B3KM21_HUMAN Family with sequence si	7.49E-16	
Q5RKV6	EXOS6_HUMAN Exosome complex component MTR3 OS=Homo sapiens GN=EXOSC6 PE=1 SV=1		237.69
Q5SNT6	FA21B_HUMAN WASH complex subunit FAM21B OS=Homo sapiens GN=FAM21B PE=1 SV=2		35.35
Q5SQ80	A20A2_HUMAN Ankyrin repeat domain-containing protein 20A2 OS=Homo sapiens GN=ANKRD20A2 PE=2 SV=1		
Q5SQS7	SH24B_HUMAN SH2 domain-containing protein 4B OS=Homo sapiens GN=SH2D4B PE=2 SV=1		28.25
Q5SQS7-2	SH24B_HUMAN Isoform 2 of SH2 domain-containing protein 4B OS=Homo sapiens GN=SH2D4B		28.25
Q5SQS7-3	SH24B_HUMAN Isoform 3 of SH2 domain-containing protein 4B OS=Homo sapiens GN=SH2D4B		28.25
Q5SRD0	FA21D_HUMAN Putative WASH complex subunit FAM21 OS=Homo sapiens GN=FAM21D PE=3 SV=2		31.89
Q5SRD1;O14925	TI23B_HUMAN Putative mitochondrial import inner membrane translocase subunit Tim23B OS=Homo sapiens GN=TIMM23B PE=5 SV=	1.48E-58	
Q5SRE5	NU188_HUMAN Nucleoporin NUP188 homolog OS=Homo sapiens GN=NUP188 PE=1 SV=1		
Q5SRE5-2	NU188_HUMAN Isoform 2 of Nucleoporin NUP188 homolog OS=Homo sapiens GN=NUP188		
Q5SRE5;Q5SRE5-2	NU188_HUMAN Nucleoporin NUP188 homolog OS=Homo sapiens GN=NUP188 PE=1 SV=1;>sp|Q5SRE5-2|NU188_HUMAN Isoform 2 c	5.92E-18	
Q5SRE7-3;Q5SRE7;B3KN1	3|PHYD1_HUMAN Isoform 3 of Phytanoyl-CoA dioxygenase domain-containing protein 1 OS=Homo sapiens GN=PHYHD1;>sp|Q5SRE7	5.66E-11	
Q5SRE7;Q5SRE7-2	PHYD1_HUMAN Phytanoyl-CoA dioxygenase domain-containing protein 1 OS=Homo sapiens GN=PHYHD1 PE=1 SV=2;>sp|Q5SRE7-2|	7.63E-22	
Q5SSJ5	HP1B3_HUMAN Heterochromatin protein 1-binding protein 3 OS=Homo sapiens GN=HP1BP3 PE=1 SV=1		337.56
Q5SSJ5-2	HP1B3_HUMAN Isoform 2 of Heterochromatin protein 1-binding protein 3 OS=Homo sapiens GN=HP1BP3		278.86
Q5SSJ5-3	HP1B3_HUMAN Isoform 3 of Heterochromatin protein 1-binding protein 3 OS=Homo sapiens GN=HP1BP3		278.86
Q5SSJ5-5	HP1B3_HUMAN Isoform 4 of Heterochromatin protein 1-binding protein 3 OS=Homo sapiens GN=HP1BP3		58.7
Q5SSJ5;Q5SSJ5-2;Q5SSJ5	HP1B3_HUMAN Heterochromatin protein 1-binding protein 3 OS=Homo sapiens GN=HP1BP3 PE=1 SV=1;>sp|Q5SSJ5-2|HP1B3_HUMA	1.36E-103	
Q5ST30	SYVM_HUMAN Valyl-tRNA synthetase, mitochondrial OS=Homo sapiens GN=VARS2 PE=1 SV=2		
Q5ST30-2	SYVM_HUMAN Isoform 2 of Valyl-tRNA synthetase, mitochondrial OS=Homo sapiens GN=VARS2		
Q5SW79	CE170_HUMAN Centrosomal protein of 170 kDa OS=Homo sapiens GN=CEP170 PE=1 SV=1		107.16
Q5SW79-2	CE170_HUMAN Isoform 2 of Centrosomal protein of 170 kDa OS=Homo sapiens GN=CEP170		107.16
Q5SW79-3	CE170_HUMAN Isoform 3 of Centrosomal protein of 170 kDa OS=Homo sapiens GN=CEP170		107.16
Q5SW79;B2RTS4;Q5SW79	CE170_HUMAN Centrosomal protein of 170 kDa OS=Homo sapiens GN=CEP170 PE=1 SV=1;>tr|B2RTS4|B2RTS4_HUMAN Centrosoma	2.20E-34	
Q5SW96	ARH_HUMAN Low density lipoprotein receptor adapter protein 1 OS=Homo sapiens GN=LDLRAP1 PE=1 SV=3	1.05E-09	
Q5SXM8	DNLZ_HUMAN DNL-type zinc finger protein OS=Homo sapiens GN=DNLZ PE=2 SV=1		
Q5SY16	NOL9_HUMAN Polynucleotide 5-hydroxyl-kinase NOL9 OS=Homo sapiens GN=NOL9 PE=1 SV=1	6.12E-22	
Q5SYB0	FRPD1_HUMAN FERM and PDZ domain-containing protein 1 OS=Homo sapiens GN=FRMPD1 PE=1 SV=1		
Q5SYC1	CLVS2_HUMAN Clavesin-2 OS=Homo sapiens GN=CLVS2 PE=2 SV=1		26.97
Q5SYC1-2	CLVS2_HUMAN Isoform 2 of Clavesin-2 OS=Homo sapiens GN=CLVS2		22.06
Q5SYE7	NHSL1_HUMAN NHS-like protein 1 OS=Homo sapiens GN=NHSL1 PE=1 SV=2		20.76

Q5SYE7-2	NHSL1_HUMAN Isoform 2 of NHS-like protein 1 OS=Homo sapiens GN=NHSL1		24.92
Q5SZK8	FREM2_HUMAN FRAS1-related extracellular matrix protein 2 OS=Homo sapiens GN=FREM2 PE=1 SV=2		
Q5SZK8-2	FREM2_HUMAN Isoform 2 of FRAS1-related extracellular matrix protein 2 OS=Homo sapiens GN=FREM2		
Q5SZQ8	CELF3_HUMAN CUGBP Elav-like family member 3 OS=Homo sapiens GN=CELF3 PE=1 SV=1		
Q5SZQ8-2	CELF3_HUMAN Isoform 2 of CUGBP Elav-like family member 3 OS=Homo sapiens GN=CELF3		
Q5SZQ8-3	CELF3_HUMAN Isoform 3 of CUGBP Elav-like family member 3 OS=Homo sapiens GN=CELF3		
Q5T013	HYI_HUMAN Putative hydroxypyruvate isomerase OS=Homo sapiens GN=HYI PE=2 SV=2		28.73
Q5T013-2	HYI_HUMAN Isoform 2 of Putative hydroxypyruvate isomerase OS=Homo sapiens GN=HYI		28.73
Q5T013-3	HYI_HUMAN Isoform 3 of Putative hydroxypyruvate isomerase OS=Homo sapiens GN=HYI		28.73
Q5T013-4	HYI_HUMAN Isoform 4 of Putative hydroxypyruvate isomerase OS=Homo sapiens GN=HYI		28.73
Q5T013;Q5T013-4;Q5T01	HYI_HUMAN Putative hydroxypyruvate isomerase OS=Homo sapiens GN=HYI PE=2 SV=2;>sp|Q5T013-4|HYI_HUMAN Isoform 4 of Pu	4.33E-24	
Q5T035	CI129_HUMAN Putative uncharacterized protein C9orf129 OS=Homo sapiens GN=C9orf129 PE=1 SV=1		7.25
Q5T0N5;Q5T0N5-5;Q5T0N	FBP1L_HUMAN Formin-binding protein 1-like OS=Homo sapiens GN=FNBP1L PE=1 SV=2;>sp|Q5T0N5-5|FBP1L_HUMAN Isoform 5 of I	2.98E-07	
Q5T0U0	CC122_HUMAN Coiled-coil domain-containing protein 122 OS=Homo sapiens GN=CCDC122 PE=2 SV=1		
Q5T0U0-2	CC122_HUMAN Isoform 2 of Coiled-coil domain-containing protein 122 OS=Homo sapiens GN=CCDC122		
Q5T160	SYRM_HUMAN Probable arginyl-tRNA synthetase, mitochondrial OS=Homo sapiens GN=RARS2 PE=1 SV=1		
Q5T1C6	THEM4_HUMAN Thioesterase superfamily member 4 OS=Homo sapiens GN=THEM4 PE=1 SV=1	2.23E-06	
Q5T1J5	CHCH9_HUMAN Putative coiled-coil-helix-coiled-coil-helix domain-containing protein CHCHD2P9, mitochondrial OS=Homo sapiens GN=CHCHD2P9 PE		40.68
Q5T1N1	AKND1_HUMAN Protein AKNAD1 OS=Homo sapiens GN=AKNAD1 PE=2 SV=3		
Q5T1N1-2	AKND1_HUMAN Isoform 2 of Protein AKNAD1 OS=Homo sapiens GN=AKNAD1		
Q5T1N1-3	AKND1_HUMAN Isoform 3 of Protein AKNAD1 OS=Homo sapiens GN=AKNAD1		
Q5T1N1-4	AKND1_HUMAN Isoform 4 of Protein AKNAD1 OS=Homo sapiens GN=AKNAD1		
Q5T1V6-2	DDX59_HUMAN Isoform 2 of Probable ATP-dependent RNA helicase DDX59 OS=Homo sapiens GN=DDX59		
Q5T200	ZC3HD_HUMAN Zinc finger CCCH domain-containing protein 13 OS=Homo sapiens GN=ZC3H13 PE=1 SV=1		
Q5T200-2	ZC3HD_HUMAN Isoform 2 of Zinc finger CCCH domain-containing protein 13 OS=Homo sapiens GN=ZC3H13		
Q5T200;Q5T200-2	ZC3HD_HUMAN Zinc finger CCCH domain-containing protein 13 OS=Homo sapiens GN=ZC3H13 PE=1 SV=1;>sp|Q5T200-2|ZC3HD_H	2.36E-11	
Q5T280	CI114_HUMAN Uncharacterized protein C9orf114 OS=Homo sapiens GN=C9orf114 PE=1 SV=3		
Q5T2E6;Q5T2E6-2;B3KUU	CJ076_HUMAN UPF0668 protein C10orf76 OS=Homo sapiens GN=C10orf76 PE=2 SV=1;>sp|Q5T2E6-2|CJ076_HUMAN Isoform 2 of U	1.40E-05	
Q5T2N8	ATD3C_HUMAN ATPase family AAA domain-containing protein 3C OS=Homo sapiens GN=ATAD3C PE=1 SV=2		255.81
Q5T2P8	AXA81_HUMAN Annexin A8-like protein 1 OS=Homo sapiens GN=ANXA8L1 PE=2 SV=1		
Q5T2S8	ARMC4_HUMAN Armadillo repeat-containing protein 4 OS=Homo sapiens GN=ARMC4 PE=2 SV=1		
Q5T2T1	MPP7_HUMAN MAGUK p55 subfamily member 7 OS=Homo sapiens GN=MPP7 PE=1 SV=1		
Q5T3F8	TM63B_HUMAN Transmembrane protein 63B OS=Homo sapiens GN=TMEM63B PE=1 SV=1		
Q5T3F8-3	TM63B_HUMAN Isoform 3 of Transmembrane protein 63B OS=Homo sapiens GN=TMEM63B		
Q5T440	CAF17_HUMAN Putative transferase CAF17, mitochondrial OS=Homo sapiens GN=IBA57 PE=1 SV=1	7.89E-28	
Q5T4S7-2;Q5T4S7;Q5T4S	2|UBR4_HUMAN Isoform 2 of E3 ubiquitin-protein ligase UBR4 OS=Homo sapiens GN=UBR4;>sp|Q5T4S7|UBR4_HUMAN E3 ubiquitin-	3.17E-94	
Q5T5J6	SWT1_HUMAN Transcriptional protein SWT1 OS=Homo sapiens GN=SWT1 PE=2 SV=1		
Q5T5P2	SKT_HUMAN Sickle tail protein homolog OS=Homo sapiens GN=SKT PE=1 SV=2		73.89
Q5T5P2-2	SKT_HUMAN Isoform 2 of Sickle tail protein homolog OS=Homo sapiens GN=SKT		71.79
Q5T5P2-3	SKT_HUMAN Isoform 3 of Sickle tail protein homolog OS=Homo sapiens GN=SKT		75.9
Q5T5P2-4	SKT_HUMAN Isoform 4 of Sickle tail protein homolog OS=Homo sapiens GN=SKT		73.8
Q5T5P2-6	SKT_HUMAN Isoform 6 of Sickle tail protein homolog OS=Homo sapiens GN=SKT		69.37
Q5T5P2-7	SKT_HUMAN Isoform 5 of Sickle tail protein homolog OS=Homo sapiens GN=SKT		70.74
Q5T5P2-8	SKT_HUMAN Isoform 7 of Sickle tail protein homolog OS=Homo sapiens GN=SKT		69.37
Q5T5S1	K1984_HUMAN Uncharacterized coiled-coil domain-containing protein KIAA1984 OS=Homo sapiens GN=KIAA1984 PE=2 SV=3		
Q5T5S1-2	K1984_HUMAN Isoform 2 of Uncharacterized coiled-coil domain-containing protein KIAA1984 OS=Homo sapiens GN=KIAA1984		
Q5T5S1-3	K1984_HUMAN Isoform 3 of Uncharacterized coiled-coil domain-containing protein KIAA1984 OS=Homo sapiens GN=KIAA1984		
Q5T5S1-4	K1984_HUMAN Isoform 4 of Uncharacterized coiled-coil domain-containing protein KIAA1984 OS=Homo sapiens GN=KIAA1984		
Q5T619	ZN648_HUMAN Zinc finger protein 648 OS=Homo sapiens GN=ZNF648 PE=1 SV=1	3.70E-05	
Q5T653	RM02_HUMAN 39S ribosomal protein L2, mitochondrial OS=Homo sapiens GN=MRPL2 PE=1 SV=2	1.71E-30	
Q5T655	CC147_HUMAN Coiled-coil domain-containing protein 147 OS=Homo sapiens GN=CCDC147 PE=2 SV=1		
Q5T6C5	AT7L2_HUMAN Ataxin-7-like protein 2 OS=Homo sapiens GN=ATXN7L2 PE=2 SV=1		30.18
Q5T6F2	UBAP2_HUMAN Ubiquitin-associated protein 2 OS=Homo sapiens GN=UBAP2 PE=1 SV=1		126.05
Q5T6V5	CI064_HUMAN UPF0553 protein C9orf64 OS=Homo sapiens GN=C9orf64 PE=1 SV=1	2.61E-49	
Q5T749	KPRP_HUMAN Keratinocyte proline-rich protein OS=Homo sapiens GN=KPRP PE=1 SV=1		
Q5T750	XP32_HUMAN Skin-specific protein 32 OS=Homo sapiens GN=XP32 PE=1 SV=1		
Q5T764	IFT1B_HUMAN Interferon-induced protein with tetratricopeptide repeats 1B OS=Homo sapiens GN=IFIT1B PE=2 SV=1	7.81E-17	
Q5T7B8	KIF24_HUMAN Kinesin-like protein KIF24 OS=Homo sapiens GN=KIF24 PE=1 SV=2		
Q5T7B8-2	KIF24_HUMAN Isoform 2 of Kinesin-like protein KIF24 OS=Homo sapiens GN=KIF24		
Q5T7B8-3	KIF24_HUMAN Isoform 3 of Kinesin-like protein KIF24 OS=Homo sapiens GN=KIF24		
Q5T7B8-4	KIF24_HUMAN Isoform 4 of Kinesin-like protein KIF24 OS=Homo sapiens GN=KIF24		

Q5T7V8	GORAB_HUMAN RAB6-interacting golgin OS=Homo sapiens GN=GORAB PE=1 SV=1		
Q5T7V8-2	GORAB_HUMAN Isoform 2 of RAB6-interacting golgin OS=Homo sapiens GN=GORAB		
Q5T8D3;Q5T8D3-3;B7Z2F	ACBD5_HUMAN Acyl-CoA-binding domain-containing protein 5 OS=Homo sapiens GN=ACBD5 PE=1 SV=1;>sp|Q5T8D3-3|ACBD5_HUI	6.60E-08	
Q5T8P6	RBM26_HUMAN RNA-binding protein 26 OS=Homo sapiens GN=RBM26 PE=1 SV=3		131.43
Q5T8P6-2	RBM26_HUMAN Isoform 2 of RNA-binding protein 26 OS=Homo sapiens GN=RBM26		131.43
Q5T8P6-3	RBM26_HUMAN Isoform 3 of RNA-binding protein 26 OS=Homo sapiens GN=RBM26		84.5
Q5T8P6-4	RBM26_HUMAN Isoform 4 of RNA-binding protein 26 OS=Homo sapiens GN=RBM26		70.4
Q5T8P6-5	RBM26_HUMAN Isoform 5 of RNA-binding protein 26 OS=Homo sapiens GN=RBM26		70.4
Q5T8P6-6	RBM26_HUMAN Isoform 6 of RNA-binding protein 26 OS=Homo sapiens GN=RBM26		14.1
Q5T8P6;Q5T8P6-2;Q5T8P	RBM26_HUMAN RNA-binding protein 26 OS=Homo sapiens GN=RBM26 PE=1 SV=3;>sp|Q5T8P6-2|RBM26_HUMAN Isoform 2 of RNA-I	4.90E-20	
Q5T9A4	ATD3B_HUMAN ATPase family AAA domain-containing protein 3B OS=Homo sapiens GN=ATAD3B PE=1 SV=1		381.7
Q5T9A4-2	ATD3B_HUMAN Isoform 2 of ATPase family AAA domain-containing protein 3B OS=Homo sapiens GN=ATAD3B		94.91
Q5T9A4-3	ATD3B_HUMAN Isoform 3 of ATPase family AAA domain-containing protein 3B OS=Homo sapiens GN=ATAD3B		249.68
Q5T9A4;Q5T9A4-3	ATD3B_HUMAN ATPase family AAA domain-containing protein 3B OS=Homo sapiens GN=ATAD3B PE=1 SV=1;>sp|Q5T9A4-3|ATD3B_I	9.46E-44	
Q5T9S5	CCD18_HUMAN Coiled-coil domain-containing protein 18 OS=Homo sapiens GN=CCDC18 PE=1 SV=1		
Q5T9S5-2	CCD18_HUMAN Isoform 2 of Coiled-coil domain-containing protein 18 OS=Homo sapiens GN=CCDC18		
Q5TA45	INT11_HUMAN Integrator complex subunit 11 OS=Homo sapiens GN=CPSF3L PE=1 SV=2		
Q5TA45-2	INT11_HUMAN Isoform 2 of Integrator complex subunit 11 OS=Homo sapiens GN=CPSF3L		
Q5TA45-3	INT11_HUMAN Isoform 3 of Integrator complex subunit 11 OS=Homo sapiens GN=CPSF3L		
Q5TAH2	S9A11_HUMAN Sodium/hydrogen exchanger 11 OS=Homo sapiens GN=SLC9A11 PE=2 SV=1		
Q5TAP6	UT14C_HUMAN U3 small nucleolar RNA-associated protein 14 homolog C OS=Homo sapiens GN=UTP14C PE=1 SV=1		
Q5TAQ9;Q5TAQ9-2	DCAF8_HUMAN DDB1- and CUL4-associated factor 8 OS=Homo sapiens GN=DCAF8 PE=1 SV=1;>sp|Q5TAQ9-2|DCAF8_HUMAN Isofor	8.93E-09	
Q5TAT6	CODA1_HUMAN Collagen alpha-1(XIII) chain OS=Homo sapiens GN=COL13A1 PE=1 SV=1		35.3
Q5TAT6-2	CODA1_HUMAN Isoform 2 of Collagen alpha-1(XIII) chain OS=Homo sapiens GN=COL13A1		35.3
Q5TAT6-3	CODA1_HUMAN Isoform 3 of Collagen alpha-1(XIII) chain OS=Homo sapiens GN=COL13A1		35.3
Q5TAT6-4	CODA1_HUMAN Isoform 4 of Collagen alpha-1(XIII) chain OS=Homo sapiens GN=COL13A1		35.3
Q5TAT6-5	CODA1_HUMAN Isoform 5 of Collagen alpha-1(XIII) chain OS=Homo sapiens GN=COL13A1		35.3
Q5TAT6-6	CODA1_HUMAN Isoform 6 of Collagen alpha-1(XIII) chain OS=Homo sapiens GN=COL13A1		35.3
Q5TAT6-7	CODA1_HUMAN Isoform 7 of Collagen alpha-1(XIII) chain OS=Homo sapiens GN=COL13A1		35.3
Q5TAT6-8	CODA1_HUMAN Isoform 8 of Collagen alpha-1(XIII) chain OS=Homo sapiens GN=COL13A1		35.3
Q5TAT6-9	CODA1_HUMAN Isoform 9 of Collagen alpha-1(XIII) chain OS=Homo sapiens GN=COL13A1		35.3
Q5TAT6;Q5TAT6-6;Q5TAT	CODA1_HUMAN Collagen alpha-1(XIII) chain OS=Homo sapiens GN=COL13A1 PE=1 SV=1;>sp|Q5TAT6-6|CODA1_HUMAN Isoform 6	1.71E-12	
Q5TAX3	TUT4_HUMAN Terminal uridylyltransferase 4 OS=Homo sapiens GN=ZCCHC11 PE=1 SV=3		
Q5TAX3-2	TUT4_HUMAN Isoform 2 of Terminal uridylyltransferase 4 OS=Homo sapiens GN=ZCCHC11		
Q5TB30	DEP1A_HUMAN DEP domain-containing protein 1A OS=Homo sapiens GN=DEPDC1 PE=1 SV=2		13.36
Q5TB30-2	DEP1A_HUMAN Isoform 2 of DEP domain-containing protein 1A OS=Homo sapiens GN=DEPDC1		13.36
Q5TBB1	RNH2B_HUMAN Ribonuclease H2 subunit B OS=Homo sapiens GN=RNASEH2B PE=1 SV=1		33.55
Q5TC82	RC3H1_HUMAN Roquin OS=Homo sapiens GN=RC3H1 PE=1 SV=1		19.48
Q5TC82-2	RC3H1_HUMAN Isoform 2 of Roquin OS=Homo sapiens GN=RC3H1		19.48
Q5TDH0-3;Q5TDH0;Q5TD	3|DDI2_HUMAN Isoform 3 of Protein DDI1 homolog 2 OS=Homo sapiens GN=DDI2;>sp|Q5TDH0|DDI2_HUMAN Protein DDI1 homolog	6.30E-08	
Q5TEZ5	CF163_HUMAN Uncharacterized protein C6orf163 OS=Homo sapiens GN=C6orf163 PE=1 SV=2		
Q5TF21	CF174_HUMAN Uncharacterized protein C6orf174 OS=Homo sapiens GN=C6orf174 PE=2 SV=1		41.66
Q5TFE4	NT5D1_HUMAN 5-nucleotidase domain-containing protein 1 OS=Homo sapiens GN=NT5DC1 PE=1 SV=1	1.39E-49	
Q5TGZ0	CA151_HUMAN UPF0327 protein C1orf151 OS=Homo sapiens GN=C1orf151 PE=1 SV=1		56.63
Q5TH69	BIG3_HUMAN Brefeldin A-inhibited guanine nucleotide-exchange protein 3 OS=Homo sapiens GN=ARFGEF3 PE=1 SV=3		
Q5THJ4	VP13D_HUMAN Vacuolar protein sorting-associated protein 13D OS=Homo sapiens GN=VPS13D PE=1 SV=1		
Q5THJ4-2	VP13D_HUMAN Isoform 2 of Vacuolar protein sorting-associated protein 13D OS=Homo sapiens GN=VPS13D		
Q5THJ4;Q5THJ4-2	VP13D_HUMAN Vacuolar protein sorting-associated protein 13D OS=Homo sapiens GN=VPS13D PE=1 SV=1;>sp|Q5THJ4-2|VP13D_HI	1.61E-09	
Q5TID7	CA114_HUMAN Uncharacterized protein C1orf114 OS=Homo sapiens GN=C1orf114 PE=2 SV=1		28.93
Q5TID7-2	CA114_HUMAN Isoform 2 of Uncharacterized protein C1orf114 OS=Homo sapiens GN=C1orf114		28.93
Q5TID7-3	CA114_HUMAN Isoform 3 of Uncharacterized protein C1orf114 OS=Homo sapiens GN=C1orf114		28.93
Q5TYW2	A20A1_HUMAN Ankyrin repeat domain-containing protein 20A1 OS=Homo sapiens GN=ANKRD20A1 PE=1 SV=1		
Q5TZA2	CROCC_HUMAN Rootletin OS=Homo sapiens GN=CROCC PE=1 SV=1		220.61
Q5TZA2-2	CROCC_HUMAN Isoform 2 of Rootletin OS=Homo sapiens GN=CROCC		169.14
Q5TZA2;Q5TZA2-2	CROCC_HUMAN Rootletin OS=Homo sapiens GN=CROCC PE=1 SV=1;>sp|Q5TZA2-2|CROCC_HUMAN Isoform 2 of Rootletin OS=Hom	2.87E-06	
Q5U3C3	TM164_HUMAN Transmembrane protein 164 OS=Homo sapiens GN=TMEM164 PE=1 SV=1		
Q5U5X0	LYRM7_HUMAN LYR motif-containing protein 7 OS=Homo sapiens GN=LYRM7 PE=1 SV=1		
Q5UIP0	RIF1_HUMAN Telomere-associated protein RIF1 OS=Homo sapiens GN=RIF1 PE=1 SV=2		240.03
Q5UIP0-2	RIF1_HUMAN Isoform 2 of Telomere-associated protein RIF1 OS=Homo sapiens GN=RIF1		221.58
Q5UIP0;Q5UIP0-2	RIF1_HUMAN Telomere-associated protein RIF1 OS=Homo sapiens GN=RIF1 PE=1 SV=2;>sp|Q5UIP0-2|RIF1_HUMAN Isoform 2 of Te	9.00E-12	
Q5VIR6	VPS53_HUMAN Vacuolar protein sorting-associated protein 53 homolog OS=Homo sapiens GN=VPS53 PE=1 SV=1		19.75

Q5VIR6-2	VPS53_HUMAN Isoform 2 of Vacuolar protein sorting-associated protein 53 homolog OS=Homo sapiens GN=VPS53		19.75
Q5VIR6-3	VPS53_HUMAN Isoform 3 of Vacuolar protein sorting-associated protein 53 homolog OS=Homo sapiens GN=VPS53		19.75
Q5VIR6;Q5VIR6-3;Q5VIR6	VPS53_HUMAN Vacuolar protein sorting-associated protein 53 homolog OS=Homo sapiens GN=VPS53 PE=1 SV=1;>sp|Q5VIR6-3|VPS	8.29E-13	
Q5VST9	OBSCN_HUMAN Obscurin OS=Homo sapiens GN=OBSCN PE=1 SV=3		100.34
Q5VST9-2	OBSCN_HUMAN Isoform 2 of Obscurin OS=Homo sapiens GN=OBSCN		100.34
Q5VST9-3	OBSCN_HUMAN Isoform 3 of Obscurin OS=Homo sapiens GN=OBSCN		91.17
Q5VST9-5	OBSCN_HUMAN Isoform 4 of Obscurin OS=Homo sapiens GN=OBSCN		68.47
Q5VST9-6	OBSCN_HUMAN Isoform 5 of Obscurin OS=Homo sapiens GN=OBSCN		100.34
Q5VT06	CE350_HUMAN Centrosome-associated protein 350 OS=Homo sapiens GN=CEP350 PE=1 SV=1		
Q5VT25	MRCKA_HUMAN Serine/threonine-protein kinase MRCK alpha OS=Homo sapiens GN=CDC42BPA PE=1 SV=1		
Q5VT25-2	MRCKA_HUMAN Isoform 2 of Serine/threonine-protein kinase MRCK alpha OS=Homo sapiens GN=CDC42BPA		
Q5VT25-3	MRCKA_HUMAN Isoform 3 of Serine/threonine-protein kinase MRCK alpha OS=Homo sapiens GN=CDC42BPA		
Q5VT25-4	MRCKA_HUMAN Isoform 4 of Serine/threonine-protein kinase MRCK alpha OS=Homo sapiens GN=CDC42BPA		
Q5VT25-5	MRCKA_HUMAN Isoform 5 of Serine/threonine-protein kinase MRCK alpha OS=Homo sapiens GN=CDC42BPA		
Q5VT25-6	MRCKA_HUMAN Isoform 6 of Serine/threonine-protein kinase MRCK alpha OS=Homo sapiens GN=CDC42BPA		
Q5VT25-6;Q5VT25-2;Q5V	6|MRCKA_HUMAN Isoform 6 of Serine/threonine-protein kinase MRCK alpha OS=Homo sapiens GN=CDC42BPA;>sp|Q5VT25-2|MRCKA	2.56E-08	
Q5VT40	FA78B_HUMAN Protein FAM78B OS=Homo sapiens GN=FAM78B PE=2 SV=1		
Q5VT52	RPRD2_HUMAN Regulation of nuclear pre domain-containing protein 2 OS=Homo sapiens GN=RPRD2 PE=1 SV=1		
Q5VT52-2	RPRD2_HUMAN Isoform 2 of Regulation of nuclear pre domain-containing protein 2 OS=Homo sapiens GN=RPRD2		
Q5VT52-3	RPRD2_HUMAN Isoform 3 of Regulation of nuclear pre domain-containing protein 2 OS=Homo sapiens GN=RPRD2		
Q5VT52-4	RPRD2_HUMAN Isoform 4 of Regulation of nuclear pre domain-containing protein 2 OS=Homo sapiens GN=RPRD2		
Q5VT52;Q5VT52-2;Q5VT5	RPRD2_HUMAN Regulation of nuclear pre-mRNA domain-containing protein 2 OS=Homo sapiens GN=RPRD2 PE=1 SV=1;>sp|Q5VT52	8.18E-08	
Q5VT79	AXA82_HUMAN Annexin A8-like protein 2 OS=Homo sapiens GN=ANXA8L2 PE=2 SV=1		
Q5VT79-2	AXA82_HUMAN Isoform 2 of Annexin A8-like protein 2 OS=Homo sapiens GN=ANXA8L2		
Q5VT79;P13928;Q5T2P8;(	AXA82_HUMAN Annexin A8-like protein 2 OS=Homo sapiens GN=ANXA8L2 PE=2 SV=1;>sp|P13928|ANXA8_HUMAN Annexin A8 OS=I	5.53E-97	
Q5VTE0	EF1A3_HUMAN Putative elongation factor 1-alpha-like 3 OS=Homo sapiens GN=EEF1A1P5 PE=5 SV=1		1752.46
Q5VTH9	WDR78_HUMAN WD repeat-containing protein 78 OS=Homo sapiens GN=WDR78 PE=2 SV=1		21.96
Q5VTH9-3	WDR78_HUMAN Isoform 3 of WD repeat-containing protein 78 OS=Homo sapiens GN=WDR78		21.23
Q5VTL8	PR38B_HUMAN Pre-splicing factor 38B OS=Homo sapiens GN=PRPF38B PE=1 SV=1		142.55
Q5VTL8-2	PR38B_HUMAN Isoform 2 of Pre-splicing factor 38B OS=Homo sapiens GN=PRPF38B		107
Q5VTL8;Q5VTL8-2	PR38B_HUMAN Pre-mRNA-splicing factor 38B OS=Homo sapiens GN=PRPF38B PE=1 SV=1;>sp|Q5VTL8-2|PR38B_HUMAN Isoform 2 c	2.30E-16	
Q5VTR2	BRE1A_HUMAN E3 ubiquitin-protein ligase BRE1A OS=Homo sapiens GN=RNF20 PE=1 SV=2		93.29
Q5VTU8	AT5EL_HUMAN ATP synthase subunit epsilon-like protein, mitochondrial OS=Homo sapiens GN=ATP5EP2 PE=1 SV=1		
Q5VU43	MYOME_HUMAN Myomegalin OS=Homo sapiens GN=PDE4DIP PE=1 SV=1		36
Q5VU43-10	MYOME_HUMAN Isoform 10 of Myomegalin OS=Homo sapiens GN=PDE4DIP		4.54
Q5VU43-11	MYOME_HUMAN Isoform 11 of Myomegalin OS=Homo sapiens GN=PDE4DIP		4.54
Q5VU43-12	MYOME_HUMAN Isoform 12 of Myomegalin OS=Homo sapiens GN=PDE4DIP		4.54
Q5VU43-2	MYOME_HUMAN Isoform 2 of Myomegalin OS=Homo sapiens GN=PDE4DIP		1.76
Q5VU43-3	MYOME_HUMAN Isoform 3 of Myomegalin OS=Homo sapiens GN=PDE4DIP		34.96
Q5VU43-4	MYOME_HUMAN Isoform 4 of Myomegalin OS=Homo sapiens GN=PDE4DIP		36
Q5VU43-5	MYOME_HUMAN Isoform 5 of Myomegalin OS=Homo sapiens GN=PDE4DIP		29.7
Q5VU43-6	MYOME_HUMAN Isoform 6 of Myomegalin OS=Homo sapiens GN=PDE4DIP		6.3
Q5VU43-7	MYOME_HUMAN Isoform 7 of Myomegalin OS=Homo sapiens GN=PDE4DIP		6.3
Q5VU43-8	MYOME_HUMAN Isoform 8 of Myomegalin OS=Homo sapiens GN=PDE4DIP		4.54
Q5VU43-9	MYOME_HUMAN Isoform 9 of Myomegalin OS=Homo sapiens GN=PDE4DIP		4.54
Q5VU97	CAHD1_HUMAN VWFA and cache domain-containing protein 1 OS=Homo sapiens GN=CACHD1 PE=1 SV=2		
Q5VU97-2	CAHD1_HUMAN Isoform 2 of VWFA and cache domain-containing protein 1 OS=Homo sapiens GN=CACHD1		
Q5VU97;Q5VU97-2	CAHD1_HUMAN VWFA and cache domain-containing protein 1 OS=Homo sapiens GN=CACHD1 PE=1 SV=2;>sp|Q5VU97-2|CAHD1_HU	4.16E-06	
Q5VUR7	A20A3_HUMAN Ankyrin repeat domain-containing protein 20A3 OS=Homo sapiens GN=ANKRD20A3 PE=4 SV=1		
Q5VV42;Q5VV42-2	CDKAL_HUMAN CDK5 regulatory subunit-associated protein 1-like 1 OS=Homo sapiens GN=CDKAL1 PE=1 SV=1;>sp|Q5VV42-2|CDKA	9.94E-10	
Q5VV67	PPRC1_HUMAN Peroxisome proliferator-activated receptor gamma coactivator-related protein 1 OS=Homo sapiens GN=PPRC1 PE=1 SV=1		51.32
Q5VV67-2	PPRC1_HUMAN Isoform 2 of Peroxisome proliferator-activated receptor gamma coactivator-related protein 1 OS=Homo sapiens GN=PPRC1		51.32
Q5VVM6;Q5VVM6-2	CCD30_HUMAN Coiled-coil domain-containing protein 30 OS=Homo sapiens GN=CCDC30 PE=2 SV=1;>sp|Q5VVM6-2|CCD30_HUMAN	1.27E-11	
Q5VW32	BROX_HUMAN BRO1 domain-containing protein BROX OS=Homo sapiens GN=BROX PE=1 SV=1	9.11E-27	
Q5VW36	K1797_HUMAN Uncharacterized protein KIAA1797 OS=Homo sapiens GN=KIAA1797 PE=1 SV=1	3.44E-12	
Q5VW38;Q5VW38-2;Q5VV	GP107_HUMAN Protein GPR107 OS=Homo sapiens GN=GPR107 PE=2 SV=1;>sp|Q5VW38-2|GP107_HUMAN Isoform 2 of Protein GPR	2.01E-09	
Q5VWI1	TCRGL_HUMAN Transcription elongation regulator 1-like protein OS=Homo sapiens GN=TCERG1L PE=2 SV=2		14.56
Q5VWQ8;Q5VWQ8-2;Q96I	DAB2P_HUMAN Disabled homolog 2-interacting protein OS=Homo sapiens GN=DAB2IP PE=1 SV=2;>sp|Q5VWQ8-2|DAB2P_HUMAN Is	4.26E-06	
Q5VWX1	KHDR2_HUMAN KH domain-containing, RNA-binding, signal transduction-associated protein 2 OS=Homo sapiens GN=KHDRBS2 PE=1 SV=1		
Q5VWZ2;Q5VWZ2-2	LYPL1_HUMAN Lysophospholipase-like protein 1 OS=Homo sapiens GN=LYPLAL1 PE=1 SV=3;>sp|Q5VWZ2-2|LYPL1_HUMAN Isoform 2	1.59E-08	
Q5VXD3	SAM13_HUMAN Sterile alpha motif domain-containing protein 13 OS=Homo sapiens GN=SAMD13 PE=2 SV=1		

Q5VXD3-2	SAM13_HUMAN Isoform 2 of Sterile alpha motif domain-containing protein 13 OS=Homo sapiens GN=SAMD13		
Q5VXD3-3	SAM13_HUMAN Isoform 3 of Sterile alpha motif domain-containing protein 13 OS=Homo sapiens GN=SAMD13		
Q5VYJ4	RUEL1_HUMAN Putative small nuclear ribonucleoprotein polypeptide E-like protein 1 OS=Homo sapiens GN=SNRPEL1 PE=5 SV=1		186.64
Q5VYJ5	CJ112_HUMAN MAM and LDL-receptor class A domain-containing protein C10orf112 OS=Homo sapiens GN=C10orf112 PE=2 SV=3		
Q5VYK3	ECM29_HUMAN Proteasome-associated protein ECM29 homolog OS=Homo sapiens GN=ECM29 PE=1 SV=2		311.92
Q5VYS8	TUT7_HUMAN Terminal uridylyltransferase 7 OS=Homo sapiens GN=ZCCHC6 PE=1 SV=1		
Q5VYS8-2	TUT7_HUMAN Isoform 2 of Terminal uridylyltransferase 7 OS=Homo sapiens GN=ZCCHC6		
Q5VYS8-4	TUT7_HUMAN Isoform 4 of Terminal uridylyltransferase 7 OS=Homo sapiens GN=ZCCHC6		
Q5VYS8-6	TUT7_HUMAN Isoform 6 of Terminal uridylyltransferase 7 OS=Homo sapiens GN=ZCCHC6		
Q5VYS8;Q5VYS8-6;Q5VYS	TUT7_HUMAN Terminal uridylyltransferase 7 OS=Homo sapiens GN=ZCCHC6 PE=1 SV=1;>sp|Q5VYS8-6|TUT7_HUMAN Isoform 6 of T	3.24E-05	
Q5VYY1	ANR22_HUMAN Ankyrin repeat domain-containing protein 22 OS=Homo sapiens GN=ANKRD22 PE=2 SV=1	2.56E-09	
Q5VZ03	NXNL2_HUMAN Nucleoredoxin-like protein 2 OS=Homo sapiens GN=NXNL2 PE=2 SV=1		
Q5VZ03-3	NXNL2_HUMAN Isoform 2 of Nucleoredoxin-like protein 2 OS=Homo sapiens GN=NXNL2		
Q5VZF2	MBNL2_HUMAN Muscleblind-like protein 2 OS=Homo sapiens GN=MBNL2 PE=1 SV=2		
Q5VZF2-2	MBNL2_HUMAN Isoform 2 of Muscleblind-like protein 2 OS=Homo sapiens GN=MBNL2		
Q5VZF2-3	MBNL2_HUMAN Isoform 3 of Muscleblind-like protein 2 OS=Homo sapiens GN=MBNL2		
Q5VZF2;Q5VZF2-2;Q5VZF	MBNL2_HUMAN Muscleblind-like protein 2 OS=Homo sapiens GN=MBNL2 PE=1 SV=2;>sp|Q5VZF2-2|MBNL2_HUMAN Isoform 2 of Mu	8.55E-11	
Q5VZK9	LR16A_HUMAN Leucine-rich repeat-containing protein 16A OS=Homo sapiens GN=LRRC16A PE=1 SV=1		
Q5VZK9-2	LR16A_HUMAN Isoform 2 of Leucine-rich repeat-containing protein 16A OS=Homo sapiens GN=LRRC16A		
Q5VZK9-3	LR16A_HUMAN Isoform 3 of Leucine-rich repeat-containing protein 16A OS=Homo sapiens GN=LRRC16A		
Q5W0B1	RN219_HUMAN RING finger protein 219 OS=Homo sapiens GN=RNF219 PE=1 SV=1		
Q5W111	SPRY7_HUMAN SPRY domain-containing protein 7 OS=Homo sapiens GN=SPRYD7 PE=1 SV=2		71.12
Q5W111-2	SPRY7_HUMAN Isoform 2 of SPRY domain-containing protein 7 OS=Homo sapiens GN=SPRYD7		71.12
Q5W111;Q5W111-2	SPRY7_HUMAN SPRY domain-containing protein 7 OS=Homo sapiens GN=SPRYD7 PE=1 SV=2;>sp|Q5W111-2|SPRY7_HUMAN Isoforn	5.49E-17	
Q5XKE5	K2C79_HUMAN Keratin, type II cytoskeletal 79 OS=Homo sapiens GN=KRT79 PE=1 SV=2		415.11
Q5XKP0	QIL1_HUMAN Protein QIL1 OS=Homo sapiens GN=QIL1 PE=1 SV=1		107.22
Q5XKR4	OTP_HUMAN Homeobox protein orthopedia OS=Homo sapiens GN=OTP PE=1 SV=1		
Q5XPI4	RN123_HUMAN E3 ubiquitin-protein ligase RNF123 OS=Homo sapiens GN=RNF123 PE=1 SV=1		
Q5XPI4-2	RN123_HUMAN Isoform 2 of E3 ubiquitin-protein ligase RNF123 OS=Homo sapiens GN=RNF123		
Q5XX13	FBW10_HUMAN F-box/WD repeat-containing protein 10 OS=Homo sapiens GN=FBXW10 PE=2 SV=2		
Q5XX13-2	FBW10_HUMAN Isoform 2 of F-box/WD repeat-containing protein 10 OS=Homo sapiens GN=FBXW10		
Q5XX13-3	FBW10_HUMAN Isoform 3 of F-box/WD repeat-containing protein 10 OS=Homo sapiens GN=FBXW10		
Q5XX13-4	FBW10_HUMAN Isoform 4 of F-box/WD repeat-containing protein 10 OS=Homo sapiens GN=FBXW10		
Q5Y7A7	2B1D_HUMAN HLA class II histocompatibility antigen, DRB1-13 beta chain OS=Homo sapiens GN=HLA-DRB1 PE=1 SV=1		0.15
Q5ZPR3	CD276_HUMAN CD276 antigen OS=Homo sapiens GN=CD276 PE=1 SV=1		
Q5ZPR3-2	CD276_HUMAN Isoform 2 of CD276 antigen OS=Homo sapiens GN=CD276		
Q5ZPR3-3	CD276_HUMAN Isoform 3 of CD276 antigen OS=Homo sapiens GN=CD276		
Q5ZPR3-4	CD276_HUMAN Isoform 4 of CD276 antigen OS=Homo sapiens GN=CD276		
Q63HN8	RN213_HUMAN RING finger protein 213 OS=Homo sapiens GN=RNF213 PE=1 SV=2		132.26
Q63HN8-2	RN213_HUMAN Isoform 2 of RING finger protein 213 OS=Homo sapiens GN=RNF213		17.09
Q63HR2	TENC1_HUMAN Tensin-like C1 domain-containing phosphatase OS=Homo sapiens GN=TENC1 PE=1 SV=2		
Q63HR2-2	TENC1_HUMAN Isoform 2 of Tensin-like C1 domain-containing phosphatase OS=Homo sapiens GN=TENC1		
Q63HR2-4	TENC1_HUMAN Isoform 4 of Tensin-like C1 domain-containing phosphatase OS=Homo sapiens GN=TENC1		
Q63HR2-4;A7E2A6;Q63HF	4|TENC1_HUMAN Isoform 4 of Tensin-like C1 domain-containing phosphatase OS=Homo sapiens GN=TENC1;>tr|A7E2A6|A7E2A6_HU	4.53E-07	
Q63HR2-5	TENC1_HUMAN Isoform 5 of Tensin-like C1 domain-containing phosphatase OS=Homo sapiens GN=TENC1		
Q63HR2-6	TENC1_HUMAN Isoform 6 of Tensin-like C1 domain-containing phosphatase OS=Homo sapiens GN=TENC1		
Q63ZE4	S22AA_HUMAN Solute carrier family 22 member 10 OS=Homo sapiens GN=SLC22A10 PE=2 SV=2		24.93
Q63ZY3	KANK2_HUMAN KN motif and ankyrin repeat domain-containing protein 2 OS=Homo sapiens GN=KANK2 PE=1 SV=1		
Q63ZY3-2	KANK2_HUMAN Isoform 2 of KN motif and ankyrin repeat domain-containing protein 2 OS=Homo sapiens GN=KANK2		
Q63ZY3-2;Q63ZY3;Q63ZY	2|KANK2_HUMAN Isoform 2 of KN motif and ankyrin repeat domain-containing protein 2 OS=Homo sapiens GN=KANK2;>sp|Q63ZY3|	4.45E-11	
Q63ZY3-3	KANK2_HUMAN Isoform 3 of KN motif and ankyrin repeat domain-containing protein 2 OS=Homo sapiens GN=KANK2		
Q63ZY6	NSN5C_HUMAN Putative methyltransferase NSUN5C OS=Homo sapiens GN=NSUN5P2 PE=5 SV=2		3.39
Q63ZY6-4	NSN5C_HUMAN Isoform 3 of Putative methyltransferase NSUN5C OS=Homo sapiens GN=NSUN5P2		3.39
Q63ZY6-5	NSN5C_HUMAN Isoform 4 of Putative methyltransferase NSUN5C OS=Homo sapiens GN=NSUN5P2		3.39
Q641Q2	FA21A_HUMAN WASH complex subunit FAM21A OS=Homo sapiens GN=FAM21A PE=1 SV=3		35.35
Q641Q2-2	FA21A_HUMAN Isoform 2 of WASH complex subunit FAM21A OS=Homo sapiens GN=FAM21A		35.35
Q641Q2;B7ZME8;Q641Q2	FA21A_HUMAN WASH complex subunit FAM21A OS=Homo sapiens GN=FAM21A PE=1 SV=3;>tr|B7ZME8|B7ZME8_HUMAN FAM21A pr	4.36E-09	
Q658T7	F90A2_HUMAN Putative protein FAM90A2 OS=Homo sapiens GN=FAM90A2P PE=5 SV=2		
Q658T7-2	F90A2_HUMAN Isoform 2 of Putative protein FAM90A2 OS=Homo sapiens GN=FAM90A2P		
Q658Y4	F91A1_HUMAN Protein FAM91A1 OS=Homo sapiens GN=FAM91A1 PE=1 SV=3		52.8
Q659C4	LAR1B_HUMAN La-related protein 1B OS=Homo sapiens GN=LARP1B PE=1 SV=2		96.24

Q659C4-2	LAR1B_HUMAN Isoform 2 of La-related protein 1B OS=Homo sapiens GN=LARP1B		94.75
Q659C4-3	LAR1B_HUMAN Isoform 3 of La-related protein 1B OS=Homo sapiens GN=LARP1B		0.34
Q659C4-4	LAR1B_HUMAN Isoform 4 of La-related protein 1B OS=Homo sapiens GN=LARP1B		
Q659C4-5	LAR1B_HUMAN Isoform 5 of La-related protein 1B OS=Homo sapiens GN=LARP1B		94.75
Q659C4-6	LAR1B_HUMAN Isoform 6 of La-related protein 1B OS=Homo sapiens GN=LARP1B		94.75
Q659C4-7	LAR1B_HUMAN Isoform 7 of La-related protein 1B OS=Homo sapiens GN=LARP1B		94.75
Q659C4-9	LAR1B_HUMAN Isoform 9 of La-related protein 1B OS=Homo sapiens GN=LARP1B		34.3
Q66K14	TBC9B_HUMAN TBC1 domain family member 9B OS=Homo sapiens GN=TBC1D9B PE=1 SV=3		25.06
Q66K14-2	TBC9B_HUMAN Isoform 2 of TBC1 domain family member 9B OS=Homo sapiens GN=TBC1D9B		25.06
Q66K14;Q66K14-2	TBC9B_HUMAN TBC1 domain family member 9B OS=Homo sapiens GN=TBC1D9B PE=1 SV=3;>sp|Q66K14-2|TBC9B_HUMAN Isoform	2.78E-12	
Q66K66	TM198_HUMAN Transmembrane protein 198 OS=Homo sapiens GN=TMEM198 PE=2 SV=1		
Q66K74	MAP1S_HUMAN Microtubule-associated protein 1S OS=Homo sapiens GN=MAP1S PE=1 SV=2		57.79
Q66K79	CBPZ_HUMAN Carboxypeptidase Z OS=Homo sapiens GN=CPZ PE=1 SV=2		
Q66K79-2	CBPZ_HUMAN Isoform 2 of Carboxypeptidase Z OS=Homo sapiens GN=CPZ		
Q66K79-3	CBPZ_HUMAN Isoform 3 of Carboxypeptidase Z OS=Homo sapiens GN=CPZ		
Q66LE6	2ABD_HUMAN Serine/threonine-protein phosphatase 2A 55 kDa regulatory subunit B delta isoform OS=Homo sapiens GN=PPP2R2D PE=1 SV=1		63.98
Q66PJ3-2;Q66PJ3;Q66PJ3	2|AR6P4_HUMAN Isoform 2 of ADP-ribosylation factor-like protein 6-interacting protein 4 OS=Homo sapiens GN=ARL6IP4;>sp|Q66PJ3	1.78E-16	
Q66PJ3;Q66PJ3-2;Q66PJ3	AR6P4_HUMAN ADP-ribosylation factor-like protein 6-interacting protein 4 OS=Homo sapiens GN=ARL6IP4 PE=1 SV=1;>sp|Q66PJ3-2	3.25E-59	
Q68CJ6	SLIP_HUMAN GTPase SLIP-GC OS=Homo sapiens GN=C8orf80 PE=1 SV=3		
Q68CP9	ARID2_HUMAN AT-rich interactive domain-containing protein 2 OS=Homo sapiens GN=ARID2 PE=1 SV=2		
Q68CP9-2	ARID2_HUMAN Isoform 2 of AT-rich interactive domain-containing protein 2 OS=Homo sapiens GN=ARID2		
Q68CP9-3	ARID2_HUMAN Isoform 3 of AT-rich interactive domain-containing protein 2 OS=Homo sapiens GN=ARID2		
Q68CP9;Q68CP9-3	ARID2_HUMAN AT-rich interactive domain-containing protein 2 OS=Homo sapiens GN=ARID2 PE=1 SV=2;>sp|Q68CP9-3|ARID2_HUM	7.61E-05	
Q68CR1	SE1L3_HUMAN Protein sel-1 homolog 3 OS=Homo sapiens GN=SEL1L3 PE=1 SV=2		19.23
Q68CR1-2	SE1L3_HUMAN Isoform 2 of Protein sel-1 homolog 3 OS=Homo sapiens GN=SEL1L3		25.54
Q68CR1-3	SE1L3_HUMAN Isoform 3 of Protein sel-1 homolog 3 OS=Homo sapiens GN=SEL1L3		17.6
Q68CZ2;Q59GW4;C9JHU5	TENS3_HUMAN Tensin-3 OS=Homo sapiens GN=TNS3 PE=1 SV=2;>tr|Q59GW4|Q59GW4_HUMAN Tensin 3 variant (Fragment) OS=Ho	3.39E-42	
Q68D06	SLN13_HUMAN Schlafen family member 13 OS=Homo sapiens GN=SLFN13 PE=2 SV=1		
Q68D06-2	SLN13_HUMAN Isoform 2 of Schlafen family member 13 OS=Homo sapiens GN=SLFN13		
Q68D10	SPT2_HUMAN Protein SPT2 homolog OS=Homo sapiens GN=SPTY2D1 PE=1 SV=3		
Q68D10-2	SPT2_HUMAN Isoform 2 of Protein SPT2 homolog OS=Homo sapiens GN=SPTY2D1		
Q68D10-3	SPT2_HUMAN Isoform 3 of Protein SPT2 homolog OS=Homo sapiens GN=SPTY2D1		
Q68DA7	FMN1_HUMAN Formin-1 OS=Homo sapiens GN=FMN1 PE=1 SV=3		
Q68DA7-2	FMN1_HUMAN Isoform 2 of Formin-1 OS=Homo sapiens GN=FMN1		
Q68DA7-3	FMN1_HUMAN Isoform 3 of Formin-1 OS=Homo sapiens GN=FMN1		
Q68DA7-5	FMN1_HUMAN Isoform 5 of Formin-1 OS=Homo sapiens GN=FMN1		
Q68DK2	ZFY26_HUMAN Zinc finger FYVE domain-containing protein 26 OS=Homo sapiens GN=ZFYVE26 PE=1 SV=3		
Q68DK2-2	ZFY26_HUMAN Isoform 2 of Zinc finger FYVE domain-containing protein 26 OS=Homo sapiens GN=ZFYVE26		
Q68DK2-3	ZFY26_HUMAN Isoform 3 of Zinc finger FYVE domain-containing protein 26 OS=Homo sapiens GN=ZFYVE26		
Q68DK2-4	ZFY26_HUMAN Isoform 4 of Zinc finger FYVE domain-containing protein 26 OS=Homo sapiens GN=ZFYVE26		
Q68DN6	RGPD1_HUMAN RANBP2-like and GRIP domain-containing protein 1/2 OS=Homo sapiens GN=RGPD1 PE=1 SV=2		155.73
Q68DN6-2	RGPD1_HUMAN Isoform 2 of RANBP2-like and GRIP domain-containing protein 1/2 OS=Homo sapiens GN=RGPD1		87.62
Q68E01	INT3_HUMAN Integrator complex subunit 3 OS=Homo sapiens GN=INTS3 PE=1 SV=1		141.53
Q68E01-2	INT3_HUMAN Isoform 2 of Integrator complex subunit 3 OS=Homo sapiens GN=INTS3		141.53
Q68E01-3	INT3_HUMAN Isoform 3 of Integrator complex subunit 3 OS=Homo sapiens GN=INTS3		141.53
Q68E01-4	INT3_HUMAN Isoform 4 of Integrator complex subunit 3 OS=Homo sapiens GN=INTS3		89.92
Q68E01;Q68E01-2;Q68E0	INT3_HUMAN Integrator complex subunit 3 OS=Homo sapiens GN=INTS3 PE=1 SV=1;>sp|Q68E01-2|INT3_HUMAN Isoform 2 of Inte	6.93E-13	
Q68EM7-4	RHG17_HUMAN Isoform 4 of Rho GTPase-activating protein 17 OS=Homo sapiens GN=ARHGAP17		
Q68EM7-5	RHG17_HUMAN Isoform 5 of Rho GTPase-activating protein 17 OS=Homo sapiens GN=ARHGAP17		
Q68EM7-6	RHG17_HUMAN Isoform 6 of Rho GTPase-activating protein 17 OS=Homo sapiens GN=ARHGAP17		
Q68EM7;Q68EM7-5;C9JE6	RHG17_HUMAN Rho GTPase-activating protein 17 OS=Homo sapiens GN=ARHGAP17 PE=1 SV=1;>sp|Q68EM7-5|RHG17_HUMAN Isof	9.84E-21	
Q69384	ENK2_HUMAN HERV-K_7p22.1 provirus ancestral Env polyprotein OS=Homo sapiens GN=ERVK6 PE=1 SV=1		
Q69YL0	YC029_HUMAN Uncharacterized protein DKFZp762I1415 OS=Homo sapiens PE=4 SV=1		
Q69YN4	VIR_HUMAN Protein virilizer homolog OS=Homo sapiens GN=KIAA1429 PE=1 SV=2		354.15
Q69YN4-2	VIR_HUMAN Isoform 2 of Protein virilizer homolog OS=Homo sapiens GN=KIAA1429		268.72
Q69YN4-3	VIR_HUMAN Isoform 3 of Protein virilizer homolog OS=Homo sapiens GN=KIAA1429		354.15
Q69YN4-4	VIR_HUMAN Isoform 4 of Protein virilizer homolog OS=Homo sapiens GN=KIAA1429		176.5
Q69YN4;Q69YN4-3;Q69YN	VIR_HUMAN Protein virilizer homolog OS=Homo sapiens GN=KIAA1429 PE=1 SV=2;>sp|Q69YN4-3|VIR_HUMAN Isoform 3 of Protein	5.98E-14	
Q69YQ0	CYTSA_HUMAN Cytospin-A OS=Homo sapiens GN=SPECC1L PE=1 SV=2	5.77E-10	
Q6A162	K1C40_HUMAN Keratin, type I cytoskeletal 40 OS=Homo sapiens GN=KRT40 PE=1 SV=2		7.18
Q6A1A2	PDPK2_HUMAN Putative 3-phosphoinositide-dependent protein kinase 2 OS=Homo sapiens GN=PDPK2 PE=5 SV=1		

Q6AI08	HEAT6_HUMAN HEAT repeat-containing protein 6 OS=Homo sapiens GN=HEATR6 PE=1 SV=1		
Q6AWC2	WWC2_HUMAN Protein WWC2 OS=Homo sapiens GN=WWC2 PE=1 SV=2		
Q6AWC2-2	WWC2_HUMAN Isoform 2 of Protein WWC2 OS=Homo sapiens GN=WWC2		
Q6AWC2-3	WWC2_HUMAN Isoform 3 of Protein WWC2 OS=Homo sapiens GN=WWC2		
Q6AWC2-4	WWC2_HUMAN Isoform 4 of Protein WWC2 OS=Homo sapiens GN=WWC2		
Q6AWC2-5	WWC2_HUMAN Isoform 5 of Protein WWC2 OS=Homo sapiens GN=WWC2		
Q6AWC2-6	WWC2_HUMAN Isoform 6 of Protein WWC2 OS=Homo sapiens GN=WWC2		
Q6AWC2-7	WWC2_HUMAN Isoform 7 of Protein WWC2 OS=Homo sapiens GN=WWC2		
Q6BCY4;Q6BCY4-2	NB5R2_HUMAN NADH-cytochrome b5 reductase 2 OS=Homo sapiens GN=CYB5R2 PE=1 SV=1;>sp|Q6BCY4-2|NB5R2_HUMAN Isoforn	4.22E-08	
Q6DD88	ATLA3_HUMAN Atlastin-3 OS=Homo sapiens GN=ATL3 PE=1 SV=1		560.87
Q6DKI1	RL7L_HUMAN 60S ribosomal protein L7-like 1 OS=Homo sapiens GN=RPL7L1 PE=1 SV=1		177.7
Q6DKJ4;Q6DKJ4-2	NXN_HUMAN Nucleoredoxin OS=Homo sapiens GN=NXN PE=1 SV=2;>sp|Q6DKJ4-2|NXN_HUMAN Isoform 2 of Nucleoredoxin OS=Ho	1.59E-09	
Q6DKK2	TTC19_HUMAN Tetratricopeptide repeat protein 19, mitochondrial OS=Homo sapiens GN=TTC19 PE=1 SV=4	7.86E-06	
Q6DN03	H2B2C_HUMAN Putative histone H2B type 2-C OS=Homo sapiens GN=HIST2H2BC PE=5 SV=3		179.62
Q6DN12	MCTP2_HUMAN Multiple C2 and transmembrane domain-containing protein 2 OS=Homo sapiens GN=MCTP2 PE=1 SV=3		
Q6DN12-2	MCTP2_HUMAN Isoform 2 of Multiple C2 and transmembrane domain-containing protein 2 OS=Homo sapiens GN=MCTP2		
Q6DN12-3	MCTP2_HUMAN Isoform 3 of Multiple C2 and transmembrane domain-containing protein 2 OS=Homo sapiens GN=MCTP2		
Q6DN12-4	MCTP2_HUMAN Isoform 4 of Multiple C2 and transmembrane domain-containing protein 2 OS=Homo sapiens GN=MCTP2		
Q6DN90	IQEC1_HUMAN IQ motif and SEC7 domain-containing protein 1 OS=Homo sapiens GN=IQSEC1 PE=1 SV=1		27.18
Q6DN90-2	IQEC1_HUMAN Isoform 2 of IQ motif and SEC7 domain-containing protein 1 OS=Homo sapiens GN=IQSEC1		20.91
Q6DN90;Q6DN90-2	IQEC1_HUMAN IQ motif and SEC7 domain-containing protein 1 OS=Homo sapiens GN=IQSEC1 PE=1 SV=1;>sp|Q6DN90-2|IQEC1_HU	7.24E-14	
Q6DRA6	H2B2D_HUMAN Putative histone H2B type 2-D OS=Homo sapiens GN=HIST2H2BD PE=5 SV=3		182.24
Q6EEV4	GL1AD_HUMAN Protein GRINL1A, isoforms 4/5 OS=Homo sapiens GN=GRINL1A PE=2 SV=1		
Q6EEV4-2	GL1AD_HUMAN Isoform 5 of Protein GRINL1A, isoforms 4/5 OS=Homo sapiens GN=GRINL1A		
Q6EEV6	SUMO4_HUMAN Small ubiquitin-related modifier 4 OS=Homo sapiens GN=SUMO4 PE=1 SV=2		67.61
Q6EKJ0	GTD2B_HUMAN General transcription factor II-I repeat domain-containing protein 2B OS=Homo sapiens GN=GTF2IRD2B PE=1 SV=1		
Q6EKJ0-2	GTD2B_HUMAN Isoform 2 of General transcription factor II-I repeat domain-containing protein 2B OS=Homo sapiens GN=GTF2IRD2B		
Q6EMB2-3	TTLL5_HUMAN Isoform 3 of Tubulin polyglutamylase TTLL5 OS=Homo sapiens GN=TTLL5		
Q6FHJ7	SFRP4_HUMAN Secreted frizzled-related protein 4 OS=Homo sapiens GN=SFRP4 PE=1 SV=2	8.79E-13	
Q6FI13	H2A2A_HUMAN Histone H2A type 2-A OS=Homo sapiens GN=HIST2H2AA3 PE=1 SV=3		288.83
Q6FI81;Q6FI81-3	CPIN1_HUMAN Anamorsin OS=Homo sapiens GN=CIAPIN1 PE=1 SV=2;>sp|Q6FI81-3|CPIN1_HUMAN Isoform 3 of Anamorsin OS=Ho	5.98E-19	
Q6GMV2	SMYD5_HUMAN SET and MYND domain-containing protein 5 OS=Homo sapiens GN=SMYD5 PE=1 SV=2	1.26E-09	
Q6GMV3	PTRD1_HUMAN Putative peptidyl-tRNA hydrolase PTRHD1 OS=Homo sapiens GN=PTRHD1 PE=1 SV=1	1.16E-09	
Q6I9Y2	THOC7_HUMAN THO complex subunit 7 homolog OS=Homo sapiens GN=THOC7 PE=1 SV=3		88.06
Q6IA69	NADE_HUMAN Glutamine-dependent NAD(+) synthetase OS=Homo sapiens GN=NADSYN1 PE=1 SV=3	3.32E-14	
Q6IAA8	LTOR1_HUMAN Ragulator complex protein LAMTOR1 OS=Homo sapiens GN=LAMTOR1 PE=1 SV=2		108.59
Q6IAA8;B4DUK2	PDRO_HUMAN Ragulator complex protein PDRO OS=Homo sapiens GN=PDRO PE=1 SV=2;>tr|B4DUK2|B4DUK2_HUMAN cDNA FLJ513	3.42E-59	
Q6IAN0	DRS7B_HUMAN Dehydrogenase/reductase SDR family member 7B OS=Homo sapiens GN=DHRS7B PE=1 SV=2		185.01
Q6IB77	GLYAT_HUMAN Glycine N-acyltransferase OS=Homo sapiens GN=GLYAT PE=1 SV=3		
Q6IB77-2	GLYAT_HUMAN Isoform 2 of Glycine N-acyltransferase OS=Homo sapiens GN=GLYAT		
Q6IBS0	TWF2_HUMAN Twinfilin-2 OS=Homo sapiens GN=TWF2 PE=1 SV=2		135.85
Q6IEE8-2	SN12L_HUMAN Isoform 2 of Schlafen family member 12-like OS=Homo sapiens GN=SLFN12L		
Q6IN85	P4R3A_HUMAN Serine/threonine-protein phosphatase 4 regulatory subunit 3A OS=Homo sapiens GN=SMEK1 PE=1 SV=1		34.84
Q6IN85-2	P4R3A_HUMAN Isoform 2 of Serine/threonine-protein phosphatase 4 regulatory subunit 3A OS=Homo sapiens GN=SMEK1		34.84
Q6IN85-4	P4R3A_HUMAN Isoform 4 of Serine/threonine-protein phosphatase 4 regulatory subunit 3A OS=Homo sapiens GN=SMEK1		34.84
Q6IN85-5	P4R3A_HUMAN Isoform 5 of Serine/threonine-protein phosphatase 4 regulatory subunit 3A OS=Homo sapiens GN=SMEK1		32.38
Q6IN85;Q6IN85-2;Q6IN85	P4R3A_HUMAN Serine/threonine-protein phosphatase 4 regulatory subunit 3A OS=Homo sapiens GN=SMEK1 PE=1 SV=1;>sp|Q6IN85	1.99E-11	
Q6IPM2	IQCE_HUMAN IQ domain-containing protein E OS=Homo sapiens GN=IQCE PE=1 SV=2		24.76
Q6IPM2-2	IQCE_HUMAN Isoform 2 of IQ domain-containing protein E OS=Homo sapiens GN=IQCE		28.21
Q6IPM2-3	IQCE_HUMAN Isoform 3 of IQ domain-containing protein E OS=Homo sapiens GN=IQCE		24.76
Q6IPM2-4	IQCE_HUMAN Isoform 4 of IQ domain-containing protein E OS=Homo sapiens GN=IQCE		24.76
Q6IPW1-2	CK071_HUMAN Isoform 2 of Uncharacterized protein C11orf71 OS=Homo sapiens GN=C11orf71		
Q6IQ22	RAB12_HUMAN Ras-related protein Rab-12 OS=Homo sapiens GN=RAB12 PE=1 SV=3		161.75
Q6IQ26	DEN5A_HUMAN DENN domain-containing protein 5A OS=Homo sapiens GN=DENND5A PE=1 SV=2		
Q6IS14	IF5AL_HUMAN Eukaryotic translation initiation factor 5A-1-like OS=Homo sapiens GN=EIF5AL1 PE=1 SV=2		430.67
Q6ISB3	GRHL2_HUMAN Grainyhead-like protein 2 homolog OS=Homo sapiens GN=GRHL2 PE=1 SV=1		
Q6ISB3-2	GRHL2_HUMAN Isoform 2 of Grainyhead-like protein 2 homolog OS=Homo sapiens GN=GRHL2		
Q6JBY9	CPZIP_HUMAN CapZ-interacting protein OS=Homo sapiens GN=RCSD1 PE=1 SV=1	1.03E-14	
Q6JEL2	KLH10_HUMAN Kelch-like protein 10 OS=Homo sapiens GN=KLHL10 PE=2 SV=1		
Q6JQN1	ACD10_HUMAN Acyl-CoA dehydrogenase family member 10 OS=Homo sapiens GN=ACAD10 PE=2 SV=1		
Q6JQN1-2	ACD10_HUMAN Isoform 2 of Acyl-CoA dehydrogenase family member 10 OS=Homo sapiens GN=ACAD10		

Q6JQN1-4	ACD10_HUMAN Isoform 4 of Acyl-CoA dehydrogenase family member 10 OS=Homo sapiens GN=ACAD10		
Q6K0P9	IFIX_HUMAN Pyrin and HIN domain-containing protein 1 OS=Homo sapiens GN=PYHIN1 PE=1 SV=1		70.07
Q6K0P9-2	IFIX_HUMAN Isoform 2 of Pyrin and HIN domain-containing protein 1 OS=Homo sapiens GN=PYHIN1		70.07
Q6K0P9-3	IFIX_HUMAN Isoform 3 of Pyrin and HIN domain-containing protein 1 OS=Homo sapiens GN=PYHIN1		70.07
Q6K0P9-4	IFIX_HUMAN Isoform 4 of Pyrin and HIN domain-containing protein 1 OS=Homo sapiens GN=PYHIN1		70.07
Q6K0P9-5	IFIX_HUMAN Isoform 5 of Pyrin and HIN domain-containing protein 1 OS=Homo sapiens GN=PYHIN1		68.46
Q6K0P9-6	IFIX_HUMAN Isoform 6 of Pyrin and HIN domain-containing protein 1 OS=Homo sapiens GN=PYHIN1		68.46
Q6K0P9;Q6K0P9-2;Q6K0P	IFIX_HUMAN Pyrin and HIN domain-containing protein 1 OS=Homo sapiens GN=PYHIN1 PE=1 SV=1;>sp|Q6K0P9-2|IFIX_HUMAN Isof	1.41E-10	
Q6KB66	K2C80_HUMAN Keratin, type II cytoskeletal 80 OS=Homo sapiens GN=KRT80 PE=1 SV=2		103.34
Q6KB66-2	K2C80_HUMAN Isoform 2 of Keratin, type II cytoskeletal 80 OS=Homo sapiens GN=KRT80		103.34
Q6KB66-3	K2C80_HUMAN Isoform 3 of Keratin, type II cytoskeletal 80 OS=Homo sapiens GN=KRT80		103.34
Q6KC79	NIPBL_HUMAN Nipped-B-like protein OS=Homo sapiens GN=NIPBL PE=1 SV=2		219.4
Q6KC79-2	NIPBL_HUMAN Isoform 2 of Nipped-B-like protein OS=Homo sapiens GN=NIPBL		219.4
Q6KC79-3	NIPBL_HUMAN Isoform 3 of Nipped-B-like protein OS=Homo sapiens GN=NIPBL		18.86
Q6KC79;Q6KC79-2	NIPBL_HUMAN Nipped-B-like protein OS=Homo sapiens GN=NIPBL PE=1 SV=2;>sp|Q6KC79-2|NIPBL_HUMAN Isoform 2 of Nipped-B-	2.05E-08	
Q6KCM7	SCMC2_HUMAN Calcium-binding mitochondrial carrier protein SCaMC-2 OS=Homo sapiens GN=SLC25A25 PE=1 SV=1		2.02
Q6KCM7-2	SCMC2_HUMAN Isoform 2 of Calcium-binding mitochondrial carrier protein SCaMC-2 OS=Homo sapiens GN=SLC25A25		2.02
Q6KCM7-4	SCMC2_HUMAN Isoform 4 of Calcium-binding mitochondrial carrier protein SCaMC-2 OS=Homo sapiens GN=SLC25A25		2.02
Q6KCM7-6	SCMC2_HUMAN Isoform 6 of Calcium-binding mitochondrial carrier protein SCaMC-2 OS=Homo sapiens GN=SLC25A25		2.02
Q6L8Q7;Q6L8Q7-2	PDE12_HUMAN 2,5-phosphodiesterase 12 OS=Homo sapiens GN=PDE12 PE=1 SV=2;>sp|Q6L8Q7-2|PDE12_HUMAN Isoform 2 of 2,5-	2.04E-56	
Q6MZM0	HPHL1_HUMAN Hephaestin-like protein 1 OS=Homo sapiens GN=HEPHL1 PE=2 SV=2		
Q6N021	TET2_HUMAN Methylcytosine dioxygenase TET2 OS=Homo sapiens GN=TET2 PE=1 SV=3		24.29
Q6NSI1	AR26L_HUMAN Putative ankyrin repeat domain-containing protein 26-like protein OS=Homo sapiens GN=ANKRD26P1 PE=5 SV=2		
Q6NSI8	K1841_HUMAN Uncharacterized protein KIAA1841 OS=Homo sapiens GN=KIAA1841 PE=2 SV=2		
Q6NSI8-2	K1841_HUMAN Isoform 2 of Uncharacterized protein KIAA1841 OS=Homo sapiens GN=KIAA1841		
Q6NSI8-3	K1841_HUMAN Isoform 3 of Uncharacterized protein KIAA1841 OS=Homo sapiens GN=KIAA1841		
Q6NSJ5	LRC8E_HUMAN Leucine-rich repeat-containing protein 8E OS=Homo sapiens GN=LRRC8E PE=2 SV=2	2.38E-08	
Q6NSX1	CCD70_HUMAN Coiled-coil domain-containing protein 70 OS=Homo sapiens GN=CCDC70 PE=2 SV=1		
Q6NSZ9	ZN498_HUMAN Zinc finger protein 498 OS=Homo sapiens GN=ZNF498 PE=2 SV=3		
Q6NSZ9-2	ZN498_HUMAN Isoform 2 of Zinc finger protein 498 OS=Homo sapiens GN=ZNF498		
Q6NSZ9-4	ZN498_HUMAN Isoform 4 of Zinc finger protein 498 OS=Homo sapiens GN=ZNF498		
Q6NTF9	RHBD2_HUMAN Rhomboid domain-containing protein 2 OS=Homo sapiens GN=RHBDD2 PE=2 SV=2	4.34E-09	
Q6NUI2	GPAT2_HUMAN Glycerol-3-phosphate acyltransferase 2, mitochondrial OS=Homo sapiens GN=GPAT2 PE=2 SV=2		36.72
Q6NUI2-3	GPAT2_HUMAN Isoform 2 of Glycerol-3-phosphate acyltransferase 2, mitochondrial OS=Homo sapiens GN=GPAT2		10.38
Q6NUI2-4	GPAT2_HUMAN Isoform 3 of Glycerol-3-phosphate acyltransferase 2, mitochondrial OS=Homo sapiens GN=GPAT2		36.72
Q6NUI2-5	GPAT2_HUMAN Isoform 4 of Glycerol-3-phosphate acyltransferase 2, mitochondrial OS=Homo sapiens GN=GPAT2		27.5
Q6NUK1	SCMC1_HUMAN Calcium-binding mitochondrial carrier protein SCaMC-1 OS=Homo sapiens GN=SLC25A24 PE=1 SV=2		355.01
Q6NUK1-2	SCMC1_HUMAN Isoform 2 of Calcium-binding mitochondrial carrier protein SCaMC-1 OS=Homo sapiens GN=SLC25A24		282.34
Q6NUK1;Q6NUK1-2	SCMC1_HUMAN Calcium-binding mitochondrial carrier protein SCaMC-1 OS=Homo sapiens GN=SLC25A24 PE=1 SV=2;>sp|Q6NUK1-2	5.82E-107	
Q6NUM9	RETST_HUMAN All-trans-retinol 13,14-reductase OS=Homo sapiens GN=RETSAT PE=2 SV=2		32.79
Q6NUM9-2	RETST_HUMAN Isoform 2 of All-trans-retinol 13,14-reductase OS=Homo sapiens GN=RETSAT		32.79
Q6NUM9;Q6NUM9-2	RETST_HUMAN All-trans-retinol 13,14-reductase OS=Homo sapiens GN=RETSAT PE=2 SV=2;>sp|Q6NUM9-2|RETST_HUMAN Isoform	1.10E-12	
Q6NUQ4	TM214_HUMAN Transmembrane protein 214 OS=Homo sapiens GN=TMEM214 PE=1 SV=2		
Q6NUQ4-2	TM214_HUMAN Isoform 2 of Transmembrane protein 214 OS=Homo sapiens GN=TMEM214		
Q6NUQ4;Q6NUQ4-2	TM214_HUMAN Transmembrane protein 214 OS=Homo sapiens GN=TMEM214 PE=1 SV=2;>sp|Q6NUQ4-2|TM214_HUMAN Isoform 2	7.40E-13	
Q6NVV1	R13AX_HUMAN Putative 60S ribosomal protein L13a-like MGC87657 OS=Homo sapiens PE=5 SV=1		264.98
Q6NVY1	HIBCH_HUMAN 3-hydroxyisobutyryl-CoA hydrolase, mitochondrial OS=Homo sapiens GN=HIBCH PE=1 SV=2		61.44
Q6NVY1-2	HIBCH_HUMAN Isoform 2 of 3-hydroxyisobutyryl-CoA hydrolase, mitochondrial OS=Homo sapiens GN=HIBCH		60.48
Q6NVY1;Q6NVY1-2	HIBCH_HUMAN 3-hydroxyisobutyryl-CoA hydrolase, mitochondrial OS=Homo sapiens GN=HIBCH PE=1 SV=2;>sp|Q6NVY1-2|HIBCH_I	6.27E-40	
Q6NXE6;Q6NXE6-2	ARMC6_HUMAN Armadillo repeat-containing protein 6 OS=Homo sapiens GN=ARMC6 PE=1 SV=2;>sp|Q6NXE6-2|ARMC6_HUMAN Isof	3.05E-115	
Q6NXG1	ESRP1_HUMAN Epithelial splicing regulatory protein 1 OS=Homo sapiens GN=ESRP1 PE=1 SV=2		37.7
Q6NXG1-2	ESRP1_HUMAN Isoform 2 of Epithelial splicing regulatory protein 1 OS=Homo sapiens GN=ESRP1		37.7
Q6NXG1-3	ESRP1_HUMAN Isoform 3 of Epithelial splicing regulatory protein 1 OS=Homo sapiens GN=ESRP1		37.7
Q6NXG1-4	ESRP1_HUMAN Isoform 4 of Epithelial splicing regulatory protein 1 OS=Homo sapiens GN=ESRP1		37.7
Q6NXG1;Q6NXG1-3;Q6NX	ESRP1_HUMAN Epithelial splicing regulatory protein 1 OS=Homo sapiens GN=ESRP1 PE=1 SV=2;>sp|Q6NXG1-3|ESRP1_HUMAN Isofo	4.59E-13	
Q6NXR0	IIGP5_HUMAN Interferon-inducible GTPase 5 OS=Homo sapiens GN=IRGC PE=2 SV=1		29.26
Q6NXT2	H3C_HUMAN Histone H3.3C OS=Homo sapiens GN=H3F3C PE=1 SV=3		108.52
Q6NXT6	TAPT1_HUMAN Transmembrane anterior posterior transformation protein 1 homolog OS=Homo sapiens GN=TAPT1 PE=1 SV=1		
Q6NXT6-2	TAPT1_HUMAN Isoform 2 of Transmembrane anterior posterior transformation protein 1 homolog OS=Homo sapiens GN=TAPT1		
Q6NY19	KANK3_HUMAN KN motif and ankyrin repeat domain-containing protein 3 OS=Homo sapiens GN=KANK3 PE=2 SV=1		
Q6NY19-2	KANK3_HUMAN Isoform 2 of KN motif and ankyrin repeat domain-containing protein 3 OS=Homo sapiens GN=KANK3		

Q6NYC1-3;Q6NYC1;Q6NY(	3|JMJD6_HUMAN Isoform 3 of Bifunctional arginine demethylase and lysyl-hydroxylase JMJD6 OS=Homo sapiens GN=JMJD6;>sp|Q6N	2.07E-09	
Q6NYC8;Q6NYC8-2	PHTNS_HUMAN Phostensin OS=Homo sapiens GN=KIAA1949 PE=1 SV=1;>sp|Q6NYC8-2|PHTNS_HUMAN Isoform 2 of Phostensin OS=	2.05E-117	
Q6NZ67	MZT2B_HUMAN Mitotic-spindle organizing protein 2B OS=Homo sapiens GN=MZT2B PE=1 SV=1		59.39
Q6NZI2	PTRF_HUMAN Polymerase I and transcript release factor OS=Homo sapiens GN=PTRF PE=1 SV=1		1132.18
Q6NZI2-2	PTRF_HUMAN Isoform 2 of Polymerase I and transcript release factor OS=Homo sapiens GN=PTRF		751.67
Q6NZI2-3	PTRF_HUMAN Isoform 3 of Polymerase I and transcript release factor OS=Homo sapiens GN=PTRF		184.72
Q6NZI2;B4DNU9;B4DPZ5;	PTRF_HUMAN Polymerase I and transcript release factor OS=Homo sapiens GN=PTRF PE=1 SV=1;>tr|B4DNU9|B4DNU9_HUMAN cDN/	2.76E-283	
Q6NZY4-2	ZCHC8_HUMAN Isoform 2 of Zinc finger CCHC domain-containing protein 8 OS=Homo sapiens GN=ZCCHC8		
Q6NZY4;Q6NZY4-2	ZCHC8_HUMAN Zinc finger CCHC domain-containing protein 8 OS=Homo sapiens GN=ZCCHC8 PE=1 SV=2;>sp|Q6NZY4-2|ZCHC8_HU	1.35E-29	
Q6P158	DHX57_HUMAN Putative ATP-dependent RNA helicase DHX57 OS=Homo sapiens GN=DHX57 PE=1 SV=2		18.65
Q6P158-2	DHX57_HUMAN Isoform 2 of Putative ATP-dependent RNA helicase DHX57 OS=Homo sapiens GN=DHX57		
Q6P158-3	DHX57_HUMAN Isoform 3 of Putative ATP-dependent RNA helicase DHX57 OS=Homo sapiens GN=DHX57		
Q6P161	RM54_HUMAN 39S ribosomal protein L54, mitochondrial OS=Homo sapiens GN=MRPL54 PE=1 SV=1		44.03
Q6P179;Q6P179-3	ERAP2_HUMAN Endoplasmic reticulum aminopeptidase 2 OS=Homo sapiens GN=ERAP2 PE=1 SV=2;>sp|Q6P179-3|ERAP2_HUMAN Is	4.29E-111	
Q6P1A2	MBOA5_HUMAN Lysophospholipid acyltransferase 5 OS=Homo sapiens GN=LPCAT3 PE=1 SV=1	8.60E-09	
Q6P1J6	PLB1_HUMAN Phospholipase B1, membrane-associated OS=Homo sapiens GN=PLB1 PE=1 SV=3		
Q6P1J6-3	PLB1_HUMAN Isoform 3 of Phospholipase B1, membrane-associated OS=Homo sapiens GN=PLB1		
Q6P1J6-4	PLB1_HUMAN Isoform 4 of Phospholipase B1, membrane-associated OS=Homo sapiens GN=PLB1		
Q6P1J6-5	PLB1_HUMAN Isoform 5 of Phospholipase B1, membrane-associated OS=Homo sapiens GN=PLB1		
Q6P1J9	CDC73_HUMAN Parafibromin OS=Homo sapiens GN=CDC73 PE=1 SV=1		70.02
Q6P1K8	T2H2L_HUMAN General transcription factor IIH subunit 2-like protein OS=Homo sapiens GN=GTF2H2C PE=2 SV=1		36.59
Q6P1L8	RM14_HUMAN 39S ribosomal protein L14, mitochondrial OS=Homo sapiens GN=MRPL14 PE=1 SV=1		258.37
Q6P1M0	S27A4_HUMAN Long-chain fatty acid transport protein 4 OS=Homo sapiens GN=SLC27A4 PE=1 SV=1		
Q6P1M3	L2GL2_HUMAN Lethal(2) giant larvae protein homolog 2 OS=Homo sapiens GN=LLGL2 PE=1 SV=2		
Q6P1M3-2	L2GL2_HUMAN Isoform A of Lethal(2) giant larvae protein homolog 2 OS=Homo sapiens GN=LLGL2		
Q6P1M3-3	L2GL2_HUMAN Isoform B of Lethal(2) giant larvae protein homolog 2 OS=Homo sapiens GN=LLGL2		
Q6P1M3;Q6P1M3-2	L2GL2_HUMAN Lethal(2) giant larvae protein homolog 2 OS=Homo sapiens GN=LLGL2 PE=1 SV=2;>sp|Q6P1M3-2|L2GL2_HUMAN Isc	1.86E-127	
Q6P1N0;Q6P1N0-2	C2D1A_HUMAN Coiled-coil and C2 domain-containing protein 1A OS=Homo sapiens GN=CC2D1A PE=1 SV=1;>sp|Q6P1N0-2|C2D1A_	3.12E-14	
Q6P1N9;Q6P1N9-2	TATD1_HUMAN Putative deoxyribonuclease TATDN1 OS=Homo sapiens GN=TATDN1 PE=1 SV=2;>sp|Q6P1N9-2|TATD1_HUMAN Isofor	5.17E-09	
Q6P1Q9;Q6P1Q9-2	MTL2B_HUMAN Methyltransferase-like protein 2B OS=Homo sapiens GN=METTL2B PE=1 SV=3;>sp|Q6P1Q9-2|MTL2B_HUMAN Isoforr	7.12E-20	
Q6P1R4	DUS1L_HUMAN tRNA-dihydrouridine synthase 1-like OS=Homo sapiens GN=DUS1L PE=1 SV=1		
Q6P1X5	TAF2_HUMAN Transcription initiation factor TFIID subunit 2 OS=Homo sapiens GN=TAF2 PE=1 SV=3		
Q6P1X6	CH082_HUMAN UPF0598 protein C8orf82 OS=Homo sapiens GN=C8orf82 PE=1 SV=2		
Q6P1X6-2	CH082_HUMAN Isoform 2 of UPF0598 protein C8orf82 OS=Homo sapiens GN=C8orf82		
Q6P280	ZN529_HUMAN Zinc finger protein 529 OS=Homo sapiens GN=ZNF529 PE=2 SV=1		34.45
Q6P280-2	ZN529_HUMAN Isoform 2 of Zinc finger protein 529 OS=Homo sapiens GN=ZNF529		34.45
Q6P2C8;Q6P2C8-2;B4DPP	MED27_HUMAN Mediator of RNA polymerase II transcription subunit 27 OS=Homo sapiens GN=MED27 PE=1 SV=1;>sp|Q6P2C8-2|ME	6.43E-05	
Q6P2E9;Q6P2E9-2	EDC4_HUMAN Enhancer of mRNA-decapping protein 4 OS=Homo sapiens GN=EDC4 PE=1 SV=1;>sp|Q6P2E9-2|EDC4_HUMAN Isoforr	6.81E-24	
Q6P2H3	CEP85_HUMAN Centrosomal protein of 85 kDa OS=Homo sapiens GN=CEP85 PE=1 SV=1		
Q6P2H3-2	CEP85_HUMAN Isoform 2 of Centrosomal protein of 85 kDa OS=Homo sapiens GN=CEP85		
Q6P2H3-3	CEP85_HUMAN Isoform 3 of Centrosomal protein of 85 kDa OS=Homo sapiens GN=CEP85		
Q6P2H8	TMM53_HUMAN Transmembrane protein 53 OS=Homo sapiens GN=TMEM53 PE=2 SV=1		
Q6P2H8-2	TMM53_HUMAN Isoform 2 of Transmembrane protein 53 OS=Homo sapiens GN=TMEM53		
Q6P2I3;Q96GK7	FAH2B_HUMAN Fumarylacetoacetate hydrolase domain-containing protein 2B OS=Homo sapiens GN=FAHD2B PE=1 SV=1;>sp|Q96GK	4.79E-17	
Q6P2Q9	PRP8_HUMAN Pre-processing-splicing factor 8 OS=Homo sapiens GN=PRPF8 PE=1 SV=2		3517.23
Q6P2Q9;B4DK16	PRP8_HUMAN Pre-mRNA-processing-splicing factor 8 OS=Homo sapiens GN=PRPF8 PE=1 SV=2;>tr|B4DK16|B4DK16_HUMAN cDNA F	2.37E-64	
Q6P3R8	NEK5_HUMAN Serine/threonine-protein kinase Nek5 OS=Homo sapiens GN=NEK5 PE=1 SV=1		
Q6P3X3	TTC27_HUMAN Tetratricopeptide repeat protein 27 OS=Homo sapiens GN=TTC27 PE=1 SV=1		
Q6P4A8	PLBL1_HUMAN Phospholipase B-like 1 OS=Homo sapiens GN=PLBD1 PE=1 SV=2	1.10E-10	
Q6P4A8;A8K4E9	PLBL1_HUMAN Putative phospholipase B-like 1 OS=Homo sapiens GN=PLBD1 PE=1 SV=2;>tr|A8K4E9|A8K4E9_HUMAN cDNA FLJ771(	3.04E-11	
Q6P4I2	WDR73_HUMAN WD repeat-containing protein 73 OS=Homo sapiens GN=WDR73 PE=1 SV=1	3.12E-11	
Q6P4R8-2;Q6P4R8;Q6P4R	2|NFRKB_HUMAN Isoform 2 of Nuclear factor related to kappa-B-binding protein OS=Homo sapiens GN=NFRKB;>sp|Q6P4R8|NFRKB_	7.94E-10	
Q6P575	YV019_HUMAN Putative uncharacterized protein LOC91316 OS=Homo sapiens PE=5 SV=2		
Q6P582	MZT2A_HUMAN Mitotic-spindle organizing protein 2A OS=Homo sapiens GN=MZT2A PE=1 SV=2		9.6
Q6P587	FAHD1_HUMAN Fumarylacetoacetate hydrolase domain-containing protein 1 OS=Homo sapiens GN=FAHD1 PE=1 SV=2		
Q6P587-2	FAHD1_HUMAN Isoform 2 of Fumarylacetoacetate hydrolase domain-containing protein 1 OS=Homo sapiens GN=FAHD1		
Q6P587-2;Q6P587	2|FAHD1_HUMAN Isoform 2 of Fumarylacetoacetate hydrolase domain-containing protein 1 OS=Homo sapiens GN=FAHD1;>sp|Q6P58	8.08E-31	
Q6P589	TP8L2_HUMAN Tumor necrosis factor alpha-induced protein 8-like protein 2 OS=Homo sapiens GN=TNFAIP8L2 PE=1 SV=1		
Q6P5R6	RL22L_HUMAN 60S ribosomal protein L22-like 1 OS=Homo sapiens GN=RPL22L1 PE=1 SV=2		20.47
Q6P5Z2	PKN3_HUMAN Serine/threonine-protein kinase N3 OS=Homo sapiens GN=PKN3 PE=1 SV=1		
Q6P656	CO026_HUMAN Uncharacterized protein C15orf26 OS=Homo sapiens GN=C15orf26 PE=2 SV=1		23.84

Q6P656-2	CO026_HUMAN Isoform 2 of Uncharacterized protein C15orf26 OS=Homo sapiens GN=C15orf26		23.84
Q6P6C2	ALKB5_HUMAN Probable alpha-ketoglutarate-dependent dioxygenase ABH5 OS=Homo sapiens GN=ALKBH5 PE=1 SV=1		
Q6P6C2-2	ALKB5_HUMAN Isoform 2 of Probable alpha-ketoglutarate-dependent dioxygenase ABH5 OS=Homo sapiens GN=ALKBH5		
Q6P6C2;D3DXC6;Q6P6C2-	ALKB5_HUMAN Probable alpha-ketoglutarate-dependent dioxygenase ABH5 OS=Homo sapiens GN=ALKBH5 PE=1 SV=1;>tr|D3DXC6|	1.51E-12	
Q6P996	PDXD1_HUMAN Pyridoxal-dependent decarboxylase domain-containing protein 1 OS=Homo sapiens GN=PDXDC1 PE=1 SV=2	1.89E-211	
Q6P9B6	K1609_HUMAN TLD domain-containing protein KIAA1609 OS=Homo sapiens GN=KIAA1609 PE=1 SV=2		27.49
Q6P9B9	INT5_HUMAN Integrator complex subunit 5 OS=Homo sapiens GN=INTS5 PE=1 SV=1		
Q6PCB7	S27A1_HUMAN Long-chain fatty acid transport protein 1 OS=Homo sapiens GN=SLC27A1 PE=2 SV=1		33.05
Q6PCE3	PGM2L_HUMAN Glucose 1,6-bisphosphate synthase OS=Homo sapiens GN=PGM2L1 PE=1 SV=3	1.77E-07	
Q6PD62	CTR9_HUMAN RNA polymerase-associated protein CTR9 homolog OS=Homo sapiens GN=CTR9 PE=1 SV=1		64.82
Q6PD74	AAGAB_HUMAN Alpha- and gamma-adaptin-binding protein p34 OS=Homo sapiens GN=AAGAB PE=1 SV=1	2.29E-07	
Q6PEY2	TBA3E_HUMAN Tubulin alpha-3E chain OS=Homo sapiens GN=TUBA3E PE=1 SV=2		861.23
Q6PGP7	TTC37_HUMAN Tetratricopeptide repeat protein 37 OS=Homo sapiens GN=TTC37 PE=1 SV=1	2.13E-17	
Q6PI48	SYDM_HUMAN Aspartyl-tRNA synthetase, mitochondrial OS=Homo sapiens GN=DARS2 PE=1 SV=1		
Q6PI78	TMM65_HUMAN Transmembrane protein 65 OS=Homo sapiens GN=TMEM65 PE=1 SV=2	5.80E-13	
Q6PII5	HAGHL_HUMAN Hydroxyacylglutathione hydrolase-like protein OS=Homo sapiens GN=HAGHL PE=2 SV=1		27.16
Q6PII5-2	HAGHL_HUMAN Isoform 2 of Hydroxyacylglutathione hydrolase-like protein OS=Homo sapiens GN=HAGHL		
Q6PIU2	NCEH1_HUMAN Neutral cholesterol ester hydrolase 1 OS=Homo sapiens GN=NCEH1 PE=1 SV=3		390.53
Q6PIU2-2	NCEH1_HUMAN Isoform 2 of Neutral cholesterol ester hydrolase 1 OS=Homo sapiens GN=NCEH1		390.53
Q6PIU2-2;Q6PIU2;Q6PIU2	2|NCEH1_HUMAN Isoform 2 of Neutral cholesterol ester hydrolase 1 OS=Homo sapiens GN=NCEH1;>sp|Q6PIU2|NCEH1_HUMAN Neut	1.26E-45	
Q6PIU2-3	NCEH1_HUMAN Isoform 3 of Neutral cholesterol ester hydrolase 1 OS=Homo sapiens GN=NCEH1		279.43
Q6PIW4	FIGL1_HUMAN Fidgetin-like protein 1 OS=Homo sapiens GN=FIGNL1 PE=1 SV=2		
Q6PIW4-2	FIGL1_HUMAN Isoform 2 of Fidgetin-like protein 1 OS=Homo sapiens GN=FIGNL1		
Q6PIY5	CA228_HUMAN Uncharacterized protein C1orf228 OS=Homo sapiens GN=C1orf228 PE=2 SV=2		
Q6PIY5-2	CA228_HUMAN Isoform 2 of Uncharacterized protein C1orf228 OS=Homo sapiens GN=C1orf228		
Q6PJG6	BRAT1_HUMAN BRCA1-associated ATM activator 1 OS=Homo sapiens GN=BRAT1 PE=1 SV=2	2.12E-18	
Q6PJG6-2	BRAT1_HUMAN Isoform 2 of BRCA1-associated ATM activator 1 OS=Homo sapiens GN=BRAT1		9.45
Q6PJG6;Q6PJG6-2	BRAT1_HUMAN BRCA1-associated ATM activator 1 OS=Homo sapiens GN=BRAT1 PE=1 SV=2;>sp|Q6PJG6-2|BRAT1_HUMAN Isoform :	2.05E-14	
Q6PJP8	DCR1A_HUMAN DNA cross-link repair 1A protein OS=Homo sapiens GN=DCLRE1A PE=1 SV=3		
Q6PJT7	ZC3HE_HUMAN Zinc finger CCCH domain-containing protein 14 OS=Homo sapiens GN=ZC3H14 PE=1 SV=1		
Q6PJT7-2	ZC3HE_HUMAN Isoform 2 of Zinc finger CCCH domain-containing protein 14 OS=Homo sapiens GN=ZC3H14		
Q6PJT7-3	ZC3HE_HUMAN Isoform 3 of Zinc finger CCCH domain-containing protein 14 OS=Homo sapiens GN=ZC3H14		
Q6PJT7-4	ZC3HE_HUMAN Isoform 4 of Zinc finger CCCH domain-containing protein 14 OS=Homo sapiens GN=ZC3H14		
Q6PJT7-5	ZC3HE_HUMAN Isoform 5 of Zinc finger CCCH domain-containing protein 14 OS=Homo sapiens GN=ZC3H14		
Q6PK04	CC137_HUMAN Coiled-coil domain-containing protein 137 OS=Homo sapiens GN=CCDC137 PE=1 SV=1		26.6
Q6PK81	ZN773_HUMAN Zinc finger protein 773 OS=Homo sapiens GN=ZNF773 PE=2 SV=1		
Q6PK81-2	ZN773_HUMAN Isoform 2 of Zinc finger protein 773 OS=Homo sapiens GN=ZNF773		
Q6PKG0	LARP1_HUMAN La-related protein 1 OS=Homo sapiens GN=LARP1 PE=1 SV=2		277.28
Q6PKG0-3	LARP1_HUMAN Isoform 2 of La-related protein 1 OS=Homo sapiens GN=LARP1		226.81
Q6PKG0;Q6PKG0-3	LARP1_HUMAN La-related protein 1 OS=Homo sapiens GN=LARP1 PE=1 SV=2;>sp|Q6PKG0-3|LARP1_HUMAN Isoform 2 of La-related	7.64E-48	
Q6PKX4	DOK6_HUMAN Docking protein 6 OS=Homo sapiens GN=DOK6 PE=1 SV=1		
Q6PL18	ATAD2_HUMAN ATPase family AAA domain-containing protein 2 OS=Homo sapiens GN=ATAD2 PE=1 SV=1		
Q6PL18-2	ATAD2_HUMAN Isoform 2 of ATPase family AAA domain-containing protein 2 OS=Homo sapiens GN=ATAD2		
Q6Q0C0	TRAF7_HUMAN E3 ubiquitin-protein ligase TRAF7 OS=Homo sapiens GN=TRAF7 PE=1 SV=1		
Q6Q0C0-2	TRAF7_HUMAN Isoform 2 of E3 ubiquitin-protein ligase TRAF7 OS=Homo sapiens GN=TRAF7		
Q6Q759	SPG17_HUMAN Sperm-associated antigen 17 OS=Homo sapiens GN=SPAG17 PE=1 SV=1		
Q6QEF8	CORO6_HUMAN Coronin-6 OS=Homo sapiens GN=CORO6 PE=1 SV=2		79.56
Q6QEF8-2	CORO6_HUMAN Isoform 2 of Coronin-6 OS=Homo sapiens GN=CORO6		79.56
Q6QEF8-3	CORO6_HUMAN Isoform 3 of Coronin-6 OS=Homo sapiens GN=CORO6		79.56
Q6QEF8-4	CORO6_HUMAN Isoform 4 of Coronin-6 OS=Homo sapiens GN=CORO6		79.56
Q6QEF8-5	CORO6_HUMAN Isoform 5 of Coronin-6 OS=Homo sapiens GN=CORO6		79.56
Q6QEF8;Q6QEF8-5	CORO6_HUMAN Coronin-6 OS=Homo sapiens GN=CORO6 PE=1 SV=2;>sp|Q6QEF8-5|CORO6_HUMAN Isoform 5 of Coronin-6 OS=Ho	1.35E-08	
Q6R327-3;Q6R327	3|RICTR_HUMAN Isoform 3 of Rapamycin-insensitive companion of mTOR OS=Homo sapiens GN=RICTOR;>sp|Q6R327|RICTR_HUMAI	1.96E-06	
Q6RW13	ATRAP_HUMAN Type-1 angiotensin II receptor-associated protein OS=Homo sapiens GN=AGTRAP PE=1 SV=1		25.38
Q6RW13-2	ATRAP_HUMAN Isoform 2 of Type-1 angiotensin II receptor-associated protein OS=Homo sapiens GN=AGTRAP		25.38
Q6RW13-3	ATRAP_HUMAN Isoform 3 of Type-1 angiotensin II receptor-associated protein OS=Homo sapiens GN=AGTRAP		25.38
Q6RW13-4	ATRAP_HUMAN Isoform 4 of Type-1 angiotensin II receptor-associated protein OS=Homo sapiens GN=AGTRAP		25.38
Q6RW13-5	ATRAP_HUMAN Isoform 5 of Type-1 angiotensin II receptor-associated protein OS=Homo sapiens GN=AGTRAP		25.38
Q6S545	POTEH_HUMAN POTE ankyrin domain family member H OS=Homo sapiens GN=POTEH PE=2 SV=3		
Q6S5H4	POTEB_HUMAN POTE ankyrin domain family member B OS=Homo sapiens GN=POTEB PE=2 SV=1		0.08
Q6S5H4-2	POTEB_HUMAN Isoform 2 of POTE ankyrin domain family member B OS=Homo sapiens GN=POTEB		0.08

Q6S5H4-3	POTEB_HUMAN Isoform 3 of POTE ankyrin domain family member B OS=Homo sapiens GN=POTEB		0.08
Q6S5H5	POTEG_HUMAN POTE ankyrin domain family member G OS=Homo sapiens GN=POTEG PE=2 SV=5		
Q6S5H5-2	POTEG_HUMAN Isoform 2 of POTE ankyrin domain family member G OS=Homo sapiens GN=POTEG		
Q6S8J3	POTEE_HUMAN POTE ankyrin domain family member E OS=Homo sapiens GN=POTEE PE=1 SV=3		937.56
Q6S8J3-2	POTEE_HUMAN Isoform 2 of POTE ankyrin domain family member E OS=Homo sapiens GN=POTEE		24.09
Q6S8J3-3	POTEE_HUMAN Isoform 3 of POTE ankyrin domain family member E OS=Homo sapiens GN=POTEE		24.09
Q6S8J3;A5A3E0;P0CG38;I	POTEE_HUMAN POTE ankyrin domain family member E OS=Homo sapiens GN=POTEE PE=1 SV=3;>sp|A5A3E0|POTEF_HUMAN POTE a	0	
Q6SA08	TSSK4_HUMAN Testis-specific serine/threonine-protein kinase 4 OS=Homo sapiens GN=TSSK4 PE=1 SV=1		
Q6SA08-2	TSSK4_HUMAN Isoform 2 of Testis-specific serine/threonine-protein kinase 4 OS=Homo sapiens GN=TSSK4		
Q6SA08-3	TSSK4_HUMAN Isoform 3 of Testis-specific serine/threonine-protein kinase 4 OS=Homo sapiens GN=TSSK4		
Q6STE5	SMRD3_HUMAN SWI/SNF-related matrix-associated actin-dependent regulator of chromatin subfamily D member 3 OS=Homo sapiens GN=SMARCD3 PE=1 SV=1		
Q6STE5-2	SMRD3_HUMAN Isoform 2 of SWI/SNF-related matrix-associated actin-dependent regulator of chromatin subfamily D member 3 OS=Homo sapiens GN=SMARCD3		
Q6UB35	C1TM_HUMAN Monofunctional C1-tetrahydrofolate synthase, mitochondrial OS=Homo sapiens GN=MTHFD1L PE=1 SV=1		48.45
Q6UB35-2	C1TM_HUMAN Isoform 2 of Monofunctional C1-tetrahydrofolate synthase, mitochondrial OS=Homo sapiens GN=MTHFD1L		2.13
Q6UB98	ANR12_HUMAN Ankyrin repeat domain-containing protein 12 OS=Homo sapiens GN=ANKRD12 PE=1 SV=3		
Q6UB98-2	ANR12_HUMAN Isoform 2 of Ankyrin repeat domain-containing protein 12 OS=Homo sapiens GN=ANKRD12		
Q6UB99	ANR11_HUMAN Ankyrin repeat domain-containing protein 11 OS=Homo sapiens GN=ANKRD11 PE=1 SV=3		40.64
Q6UN15	FIP1_HUMAN Pre 3'-end-processing factor FIP1 OS=Homo sapiens GN=FIP1L1 PE=1 SV=1		
Q6UN15-3	FIP1_HUMAN Isoform 3 of Pre 3'-end-processing factor FIP1 OS=Homo sapiens GN=FIP1L1		
Q6UN15-4	FIP1_HUMAN Isoform 4 of Pre 3'-end-processing factor FIP1 OS=Homo sapiens GN=FIP1L1		
Q6UN15;Q6UN15-3;B4DIF	FIP1_HUMAN Pre-mRNA 3-end-processing factor FIP1 OS=Homo sapiens GN=FIP1L1 PE=1 SV=1;>sp|Q6UN15-3|FIP1_HUMAN Isoforr	1.58E-98	
Q6UVK1	CSPG4_HUMAN Chondroitin sulfate proteoglycan 4 OS=Homo sapiens GN=CSPG4 PE=1 SV=2		37.18
Q6UVY6	MOXD1_HUMAN DBH-like monooxygenase protein 1 OS=Homo sapiens GN=MOXD1 PE=2 SV=1		18.11
Q6UVY6-2	MOXD1_HUMAN Isoform 2 of DBH-like monooxygenase protein 1 OS=Homo sapiens GN=MOXD1		18.11
Q6UW49	SPESP_HUMAN Sperm equatorial segment protein 1 OS=Homo sapiens GN=SPESP1 PE=1 SV=2		
Q6UW63	KDEL1_HUMAN KDEL motif-containing protein 1 OS=Homo sapiens GN=KDELC1 PE=1 SV=1		
Q6UW68	TM205_HUMAN Transmembrane protein 205 OS=Homo sapiens GN=TMEM205 PE=1 SV=1		28.35
Q6UW78	CK083_HUMAN UPF0723 protein C11orf83 OS=Homo sapiens GN=C11orf83 PE=1 SV=2		18.15
Q6UWE0	LRSM1_HUMAN E3 ubiquitin-protein ligase LRSAM1 OS=Homo sapiens GN=LRSAM1 PE=1 SV=1		
Q6UWE0-2	LRSM1_HUMAN Isoform 2 of E3 ubiquitin-protein ligase LRSAM1 OS=Homo sapiens GN=LRSAM1		
Q6UWE0-3	LRSM1_HUMAN Isoform 3 of E3 ubiquitin-protein ligase LRSAM1 OS=Homo sapiens GN=LRSAM1		
Q6UWE0;Q6UWE0-2	LRSM1_HUMAN E3 ubiquitin-protein ligase LRSAM1 OS=Homo sapiens GN=LRSAM1 PE=1 SV=1;>sp|Q6UWE0-2|LRSM1_HUMAN Isofo	3.17E-32	
Q6UWP2;Q6UWP2-2	DHR11_HUMAN Dehydrogenase/reductase SDR family member 11 OS=Homo sapiens GN=DHRS11 PE=1 SV=1;>sp|Q6UWP2-2|DHR1	1.11E-07	
Q6UWR7	ENPP6_HUMAN Ectonucleotide pyrophosphatase/phosphodiesterase family member 6 OS=Homo sapiens GN=ENPP6 PE=2 SV=2		
Q6UWY5	OLFL1_HUMAN Olfactomedin-like protein 1 OS=Homo sapiens GN=OLFML1 PE=1 SV=2	4.44E-58	
Q6UWZ7	F175A_HUMAN BRCA1-A complex subunit Abraxas OS=Homo sapiens GN=FAM175A PE=1 SV=2		
Q6UX04;Q6UX04-2	CWC27_HUMAN Peptidyl-prolyl cis-trans isomerase CWC27 homolog OS=Homo sapiens GN=CWC27 PE=1 SV=1;>sp|Q6UX04-2|CWC2	4.18E-12	
Q6UX07;Q6UX07-2	DHR13_HUMAN Dehydrogenase/reductase SDR family member 13 OS=Homo sapiens GN=DHRS13 PE=1 SV=1;>sp|Q6UX07-2|DHR1	1.43E-26	
Q6UX27-2	VSTM1_HUMAN Isoform 2 of V-set and transmembrane domain-containing protein 1 OS=Homo sapiens GN=VSTM1		
Q6UX71;B4E367;B7WP84;	PXDC2_HUMAN Plexin domain-containing protein 2 OS=Homo sapiens GN=PLXDC2 PE=1 SV=1;>tr|B4E367|B4E367_HUMAN cDNA FL	4.39E-08	
Q6UXG2;Q6UXG2-3;Q6UX	K1324_HUMAN UPF0577 protein KIAA1324 OS=Homo sapiens GN=KIAA1324 PE=2 SV=2;>sp|Q6UXG2-3|K1324_HUMAN Isoform 3 o	2.22E-23	
Q6UXH1	CREL2_HUMAN Cysteine-rich with EGF-like domain protein 2 OS=Homo sapiens GN=CRELD2 PE=1 SV=1		44.22
Q6UXH1-2	CREL2_HUMAN Isoform 2 of Cysteine-rich with EGF-like domain protein 2 OS=Homo sapiens GN=CRELD2		13.65
Q6UXH1-3	CREL2_HUMAN Isoform 3 of Cysteine-rich with EGF-like domain protein 2 OS=Homo sapiens GN=CRELD2		13.65
Q6UXH1-4	CREL2_HUMAN Isoform 4 of Cysteine-rich with EGF-like domain protein 2 OS=Homo sapiens GN=CRELD2		44.22
Q6UXH1-5	CREL2_HUMAN Isoform 5 of Cysteine-rich with EGF-like domain protein 2 OS=Homo sapiens GN=CRELD2		44.22
Q6UXH1-5;A5GZA6;Q6UX	5|CREL2_HUMAN Isoform 5 of Cysteine-rich with EGF-like domain protein 2 OS=Homo sapiens GN=CRELD2;>tr|A5GZA6|A5GZA6_HU	4.40E-05	
Q6UXH1-6	CREL2_HUMAN Isoform 6 of Cysteine-rich with EGF-like domain protein 2 OS=Homo sapiens GN=CRELD2		44.22
Q6UXI9	NPNT_HUMAN Nephronectin OS=Homo sapiens GN=NPNT PE=2 SV=3		21.67
Q6UXI9-2	NPNT_HUMAN Isoform 2 of Nephronectin OS=Homo sapiens GN=NPNT		21.67
Q6UXN9	WDR82_HUMAN WD repeat-containing protein 82 OS=Homo sapiens GN=WDR82 PE=1 SV=1		223.56
Q6UXV4	APOOL_HUMAN Apolipoprotein O-like OS=Homo sapiens GN=APOOL PE=1 SV=1	6.11E-10	
Q6UY01	LRC31_HUMAN Leucine-rich repeat-containing protein 31 OS=Homo sapiens GN=LRRC31 PE=2 SV=1		
Q6UY01-2	LRC31_HUMAN Isoform 2 of Leucine-rich repeat-containing protein 31 OS=Homo sapiens GN=LRRC31		
Q6UY01-3	LRC31_HUMAN Isoform 3 of Leucine-rich repeat-containing protein 31 OS=Homo sapiens GN=LRRC31		
Q6VUC0	AP2E_HUMAN Transcription factor AP-2-epsilon OS=Homo sapiens GN=TFAP2E PE=2 SV=1		
Q6VY07	PACS1_HUMAN Phosphofurin acidic cluster sorting protein 1 OS=Homo sapiens GN=PACS1 PE=1 SV=2		28.44
Q6VY07-2	PACS1_HUMAN Isoform 2 of Phosphofurin acidic cluster sorting protein 1 OS=Homo sapiens GN=PACS1		28.44
Q6VY07-2;Q6VY07	2|PACS1_HUMAN Isoform 2 of Phosphofurin acidic cluster sorting protein 1 OS=Homo sapiens GN=PACS1;>sp|Q6VY07|PACS1_HUMAI	2.70E-23	
Q6VY07;Q6VY07-2	PACS1_HUMAN Phosphofurin acidic cluster sorting protein 1 OS=Homo sapiens GN=PACS1 PE=1 SV=2;>sp|Q6VY07-2|PACS1_HUMAN	2.03E-22	
Q6WCQ1-2;B9EGI2;Q6WC	2|MPRIP_HUMAN Isoform 2 of Myosin phosphatase Rho-interacting protein OS=Homo sapiens GN=MPRIP;>tr|B9EGI2|B9EGI2_HUMAN	2.95E-19	

Q6WKZ4	RFIP1_HUMAN Rab11 family-interacting protein 1 OS=Homo sapiens GN=RAB11FIP1 PE=1 SV=2		159.03
Q6WKZ4-1	RFIP1_HUMAN Isoform 3 of Rab11 family-interacting protein 1 OS=Homo sapiens GN=RAB11FIP1		81.3
Q6WKZ4-2	RFIP1_HUMAN Isoform 4 of Rab11 family-interacting protein 1 OS=Homo sapiens GN=RAB11FIP1		72.68
Q6WKZ4-3	RFIP1_HUMAN Isoform 2 of Rab11 family-interacting protein 1 OS=Homo sapiens GN=RAB11FIP1		152.02
Q6WKZ4-5	RFIP1_HUMAN Isoform 5 of Rab11 family-interacting protein 1 OS=Homo sapiens GN=RAB11FIP1		5.05
Q6WKZ4;Q307T1;Q6WKZ	RFIP1_HUMAN Rab11 family-interacting protein 1 OS=Homo sapiens GN=RAB11FIP1 PE=1 SV=2;>tr|Q307T1|Q307T1_HUMAN RAB11	4.07E-17	
Q6WRX3	ZY11A_HUMAN Protein zyg-11 homolog A OS=Homo sapiens GN=ZYG11A PE=2 SV=3		
Q6WRX3-2	ZY11A_HUMAN Isoform 2 of Protein zyg-11 homolog A OS=Homo sapiens GN=ZYG11A		
Q6XE24	RBMS3_HUMAN RNA-binding motif, single-stranded-interacting protein 3 OS=Homo sapiens GN=RBMS3 PE=1 SV=1		58.06
Q6XE24-2	RBMS3_HUMAN Isoform 2 of RNA-binding motif, single-stranded-interacting protein 3 OS=Homo sapiens GN=RBMS3		58.06
Q6XE24-3	RBMS3_HUMAN Isoform 3 of RNA-binding motif, single-stranded-interacting protein 3 OS=Homo sapiens GN=RBMS3		58.06
Q6XE24-4	RBMS3_HUMAN Isoform 4 of RNA-binding motif, single-stranded-interacting protein 3 OS=Homo sapiens GN=RBMS3		58.06
Q6XE24;Q6XE24-2;Q6XE2	RBMS3_HUMAN RNA-binding motif, single-stranded-interacting protein 3 OS=Homo sapiens GN=RBMS3 PE=1 SV=1;>sp|Q6XE24-2|R	1.16E-05	
Q6XQN6;Q6XQN6-3;Q6XQ	PNCB_HUMAN Nicotinate phosphoribosyltransferase OS=Homo sapiens GN=NAPRT1 PE=1 SV=2;>sp|Q6XQN6-3|PNCB_HUMAN Isofor	2.07E-35	
Q6XZF7	DNMBP_HUMAN Dynamin-binding protein OS=Homo sapiens GN=DNMBP PE=1 SV=1		29.6
Q6XZF7-2	DNMBP_HUMAN Isoform 2 of Dynamin-binding protein OS=Homo sapiens GN=DNMBP		29.6
Q6XZF7;Q6XZF7-2	DNMBP_HUMAN Dynamin-binding protein OS=Homo sapiens GN=DNMBP PE=1 SV=1;>sp|Q6XZF7-2|DNMBP_HUMAN Isoform 2 of Dy	9.55E-78	
Q6Y1H2	HACD2_HUMAN 3-hydroxyacyl-CoA dehydratase 2 OS=Homo sapiens GN=PTPLB PE=1 SV=1	1.21E-08	
Q6Y7W6-3;A6H8W4;Q6Y7	3|PERQ2_HUMAN Isoform 2 of PERQ amino acid-rich with GYF domain-containing protein 2 OS=Homo sapiens GN=GIGYF2;>tr|A6H8V	4.04E-44	
Q6YHK3	CD109_HUMAN CD109 antigen OS=Homo sapiens GN=CD109 PE=1 SV=2		
Q6YHK3-2	CD109_HUMAN Isoform 2 of CD109 antigen OS=Homo sapiens GN=CD109		
Q6YHK3-3	CD109_HUMAN Isoform 3 of CD109 antigen OS=Homo sapiens GN=CD109		
Q6YHK3-4	CD109_HUMAN Isoform 4 of CD109 antigen OS=Homo sapiens GN=CD109		
Q6YHK3;Q6YHK3-4;Q6YH	CD109_HUMAN CD109 antigen OS=Homo sapiens GN=CD109 PE=1 SV=2;>sp|Q6YHK3-4|CD109_HUMAN Isoform 4 of CD109 antiger	4.93E-12	
Q6YHU6	THADA_HUMAN Thyroid adenoma-associated protein OS=Homo sapiens GN=THADA PE=1 SV=1		
Q6YHU6-2	THADA_HUMAN Isoform 2 of Thyroid adenoma-associated protein OS=Homo sapiens GN=THADA		
Q6YHU6-3	THADA_HUMAN Isoform 3 of Thyroid adenoma-associated protein OS=Homo sapiens GN=THADA		
Q6YHU6-4	THADA_HUMAN Isoform 4 of Thyroid adenoma-associated protein OS=Homo sapiens GN=THADA		
Q6YHU6-5	THADA_HUMAN Isoform 5 of Thyroid adenoma-associated protein OS=Homo sapiens GN=THADA		
Q6YN16	HSDL2_HUMAN Hydroxysteroid dehydrogenase-like protein 2 OS=Homo sapiens GN=HSDL2 PE=1 SV=1		193.11
Q6YN16-2	HSDL2_HUMAN Isoform 2 of Hydroxysteroid dehydrogenase-like protein 2 OS=Homo sapiens GN=HSDL2		162.76
Q6YN16;B2R923;Q6YN16-	HSDL2_HUMAN Hydroxysteroid dehydrogenase-like protein 2 OS=Homo sapiens GN=HSDL2 PE=1 SV=1;>tr|B2R923|B2R923_HUMAN	2.68E-240	
Q6YP21	KAT3_HUMAN Kynurenine--oxoglutarate transaminase 3 OS=Homo sapiens GN=CCBL2 PE=1 SV=1	5.20E-35	
Q6ZMC9	SIG15_HUMAN Sialic acid-binding Ig-like lectin 15 OS=Homo sapiens GN=SIGLEC15 PE=1 SV=1		
Q6ZMI0	KLRAQ_HUMAN KLRAQ motif-containing protein 1 OS=Homo sapiens GN=KLRAQ1 PE=1 SV=1		34.36
Q6ZMI0-2	KLRAQ_HUMAN Isoform 2 of KLRAQ motif-containing protein 1 OS=Homo sapiens GN=KLRAQ1		34.36
Q6ZMI0-3	KLRAQ_HUMAN Isoform 3 of KLRAQ motif-containing protein 1 OS=Homo sapiens GN=KLRAQ1		34.36
Q6ZMI0-4	KLRAQ_HUMAN Isoform 4 of KLRAQ motif-containing protein 1 OS=Homo sapiens GN=KLRAQ1		34.36
Q6ZMI0;Q6ZMI0-2;B7ZKY	KLRAQ_HUMAN KLRAQ motif-containing protein 1 OS=Homo sapiens GN=KLRAQ1 PE=1 SV=1;>sp|Q6ZMI0-2|KLRAQ_HUMAN Isoform	2.22E-10	
Q6ZMK1;Q6ZMK1-2	CYHR1_HUMAN Cysteine and histidine-rich protein 1 OS=Homo sapiens GN=CYHR1 PE=2 SV=2;>sp|Q6ZMK1-2|CYHR1_HUMAN Isofor	3.91E-13	
Q6ZMR3	LDH6A_HUMAN L-lactate dehydrogenase A-like 6A OS=Homo sapiens GN=LDHAL6A PE=2 SV=1		106.15
Q6ZMU5;Q6ZMU5-2	TRI72_HUMAN Tripartite motif-containing protein 72 OS=Homo sapiens GN=TRIM72 PE=1 SV=2;>sp|Q6ZMU5-2|TRI72_HUMAN Isofo	3.64E-63	
Q6ZMV9-2	KIF6_HUMAN Isoform 2 of Kinesin-like protein KIF6 OS=Homo sapiens GN=KIF6		
Q6ZMV9-4	KIF6_HUMAN Isoform 4 of Kinesin-like protein KIF6 OS=Homo sapiens GN=KIF6		
Q6ZMW3	EMAL6_HUMAN Echinoderm microtubule-associated protein-like 6 OS=Homo sapiens GN=EML6 PE=2 SV=2		
Q6ZMW3-2	EMAL6_HUMAN Isoform 2 of Echinoderm microtubule-associated protein-like 6 OS=Homo sapiens GN=EML6		
Q6ZMZ3	SYNE3_HUMAN Nesprin-3 OS=Homo sapiens GN=C14orf49 PE=1 SV=2		
Q6ZMZ3-2	SYNE3_HUMAN Isoform 2 of Nesprin-3 OS=Homo sapiens GN=C14orf49		
Q6ZMZ3-3	SYNE3_HUMAN Isoform 3 of Nesprin-3 OS=Homo sapiens GN=C14orf49		
Q6ZN17	LN28B_HUMAN Protein lin-28 homolog B OS=Homo sapiens GN=LIN28B PE=1 SV=1		
Q6ZN17-2	LN28B_HUMAN Isoform 2 of Protein lin-28 homolog B OS=Homo sapiens GN=LIN28B		
Q6ZNG1	ZN600_HUMAN Zinc finger protein 600 OS=Homo sapiens GN=ZNF600 PE=1 SV=2		
Q6ZNJ1;Q6ZNJ1-2;Q6ZNJ	NBEL2_HUMAN Neurobeachin-like protein 2 OS=Homo sapiens GN=NBEAL2 PE=1 SV=2;>sp|Q6ZNJ1-2|NBEL2_HUMAN Isoform 2 of N	3.93E-10	
Q6ZR08	DYH12_HUMAN Dynein heavy chain 12, axonemal OS=Homo sapiens GN=DNAH12 PE=1 SV=2		
Q6ZR08-2	DYH12_HUMAN Isoform 2 of Dynein heavy chain 12, axonemal OS=Homo sapiens GN=DNAH12		
Q6ZR08-3	DYH12_HUMAN Isoform 3 of Dynein heavy chain 12, axonemal OS=Homo sapiens GN=DNAH12		
Q6ZR08-4	DYH12_HUMAN Isoform 4 of Dynein heavy chain 12, axonemal OS=Homo sapiens GN=DNAH12		
Q6ZRF8	RN207_HUMAN RING finger protein 207 OS=Homo sapiens GN=RNF207 PE=1 SV=2		30.97
Q6ZRP7	QSOX2_HUMAN Sulfhydryl oxidase 2 OS=Homo sapiens GN=QSOX2 PE=1 SV=3		111.54
Q6ZRQ5	MMS22_HUMAN Protein MMS22-like OS=Homo sapiens GN=MMS22L PE=1 SV=3		
Q6ZRS2	SRCAP_HUMAN Helicase SRCAP OS=Homo sapiens GN=SRCAP PE=1 SV=3		

Q6ZRS2-2	SRCAP_HUMAN Isoform 2 of Helicase SRCAP OS=Homo sapiens GN=SRCAP		
Q6ZRS2-3	SRCAP_HUMAN Isoform 3 of Helicase SRCAP OS=Homo sapiens GN=SRCAP		
Q6ZS17;Q6ZS17-2	FA65A_HUMAN Protein FAM65A OS=Homo sapiens GN=FAM65A PE=1 SV=1;>sp|Q6ZS17-2|FA65A_HUMAN Isoform 2 of Protein FAM6	1.19E-95	
Q6ZS30	NBEL1_HUMAN Neurobeachin-like protein 1 OS=Homo sapiens GN=NBEAL1 PE=2 SV=3		27.64
Q6ZS30-1	NBEL1_HUMAN Isoform 1 of Neurobeachin-like protein 1 OS=Homo sapiens GN=NBEAL1		27.64
Q6ZSJ8	CA122_HUMAN Uncharacterized protein C1orf122 OS=Homo sapiens GN=C1orf122 PE=2 SV=2		22.34
Q6ZSR9	YJ005_HUMAN Uncharacterized protein FLJ45252 OS=Homo sapiens PE=1 SV=2		17.69
Q6ZSZ5	ARHGI_HUMAN Rho guanine nucleotide exchange factor 18 OS=Homo sapiens GN=ARHGEF18 PE=1 SV=3		
Q6ZSZ5-2	ARHGI_HUMAN Isoform 2 of Rho guanine nucleotide exchange factor 18 OS=Homo sapiens GN=ARHGEF18		
Q6ZSZ5-3	ARHGI_HUMAN Isoform 3 of Rho guanine nucleotide exchange factor 18 OS=Homo sapiens GN=ARHGEF18		
Q6ZSZ5;A8MV62;Q6ZSZ5	ARHGI_HUMAN Rho guanine nucleotide exchange factor 18 OS=Homo sapiens GN=ARHGEF18 PE=1 SV=3;>tr|A8MV62|A8MV62_HUM	2.14E-26	
Q6ZT21	TMPPE_HUMAN Transmembrane protein with metallophosphoesterase domain OS=Homo sapiens GN=TMPPE PE=2 SV=2		27.9
Q6ZT21-2	TMPPE_HUMAN Isoform 2 of Transmembrane protein with metallophosphoesterase domain OS=Homo sapiens GN=TMPPE		27.9
Q6ZTR5	CX022_HUMAN Uncharacterized protein CXorf22 OS=Homo sapiens GN=CXorf22 PE=2 SV=3		25.45
Q6ZTR5-2	CX022_HUMAN Isoform 2 of Uncharacterized protein CXorf22 OS=Homo sapiens GN=CXorf22		25.45
Q6ZTW0	TPGS1_HUMAN Tubulin polyglutamylase complex subunit 1 OS=Homo sapiens GN=C19orf20 PE=2 SV=2		
Q6ZTW0-2	TPGS1_HUMAN Isoform 2 of Tubulin polyglutamylase complex subunit 1 OS=Homo sapiens GN=C19orf20		
Q6ZU11-2	YD002_HUMAN Isoform 2 of Uncharacterized protein FLJ44066 OS=Homo sapiens		
Q6ZU15	SEP14_HUMAN Septin-14 OS=Homo sapiens GN=SEPT14 PE=1 SV=2		55.93
Q6ZUB1	CI079_HUMAN FAM75-like protein C9orf79 OS=Homo sapiens GN=C9orf79 PE=2 SV=2		
Q6ZUI0	TPRG1_HUMAN Tumor protein p63-regulated gene 1 protein OS=Homo sapiens GN=TPRG1 PE=2 SV=1	0.00024023	
Q6ZUJ8;Q6ZUJ8-2	BCAP_HUMAN Phosphoinositide 3-kinase adapter protein 1 OS=Homo sapiens GN=PIK3AP1 PE=1 SV=2;>sp|Q6ZUJ8-2|BCAP_HUMAN	1.57E-12	
Q6ZUL3	CH086_HUMAN Uncharacterized protein C8orf86 OS=Homo sapiens GN=C8orf86 PE=2 SV=1		
Q6ZUL3-2	CH086_HUMAN Isoform 2 of Uncharacterized protein C8orf86 OS=Homo sapiens GN=C8orf86		
Q6ZUT6	CO052_HUMAN Uncharacterized protein C15orf52 OS=Homo sapiens GN=C15orf52 PE=1 SV=1		69.21
Q6ZUT6-2	CO052_HUMAN Isoform 2 of Uncharacterized protein C15orf52 OS=Homo sapiens GN=C15orf52		13.47
Q6ZUT6-3	CO052_HUMAN Isoform 3 of Uncharacterized protein C15orf52 OS=Homo sapiens GN=C15orf52		27.61
Q6ZUT6-4	CO052_HUMAN Isoform 4 of Uncharacterized protein C15orf52 OS=Homo sapiens GN=C15orf52		41.08
Q6ZUT6;Q6ZUT6-4;Q6ZU	CO052_HUMAN Uncharacterized protein C15orf52 OS=Homo sapiens GN=C15orf52 PE=1 SV=1;>sp|Q6ZUT6-4|CO052_HUMAN Isofor	2.42E-10	
Q6ZUT9	DEN5B_HUMAN DENN domain-containing protein 5B OS=Homo sapiens GN=DENND5B PE=1 SV=2		
Q6ZUT9-2	DEN5B_HUMAN Isoform 2 of DENN domain-containing protein 5B OS=Homo sapiens GN=DENND5B		
Q6ZUT9-3	DEN5B_HUMAN Isoform 3 of DENN domain-containing protein 5B OS=Homo sapiens GN=DENND5B		
Q6ZUT9-4	DEN5B_HUMAN Isoform 4 of DENN domain-containing protein 5B OS=Homo sapiens GN=DENND5B		
Q6ZV73;Q6ZV73-2;A4FVC	FGD6_HUMAN FYVE, RhoGEF and PH domain-containing protein 6 OS=Homo sapiens GN=FGD6 PE=1 SV=2;>sp|Q6ZV73-2|FGD6_HU	2.21E-06	
Q6ZVC0	CG051_HUMAN Uncharacterized protein C7orf51 OS=Homo sapiens GN=C7orf51 PE=1 SV=1		
Q6ZVC0-2	CG051_HUMAN Isoform 2 of Uncharacterized protein C7orf51 OS=Homo sapiens GN=C7orf51		
Q6ZVD8	PHLP2_HUMAN PH domain leucine-rich repeat-containing protein phosphatase 2 OS=Homo sapiens GN=PHLPP2 PE=1 SV=3		
Q6ZVD8-2	PHLP2_HUMAN Isoform 2 of PH domain leucine-rich repeat-containing protein phosphatase 2 OS=Homo sapiens GN=PHLPP2		
Q6ZVD8-3	PHLP2_HUMAN Isoform 3 of PH domain leucine-rich repeat-containing protein phosphatase 2 OS=Homo sapiens GN=PHLPP2		
Q6ZVF9	GRIN3_HUMAN G protein-regulated inducer of neurite outgrowth 3 OS=Homo sapiens GN=GPRIN3 PE=2 SV=2		
Q6ZW49	PAXI1_HUMAN PAX-interacting protein 1 OS=Homo sapiens GN=PAXIP1 PE=1 SV=2		19.65
Q6ZW49-1	PAXI1_HUMAN Isoform 2 of PAX-interacting protein 1 OS=Homo sapiens GN=PAXIP1		19.65
Q6ZW49-2	PAXI1_HUMAN Isoform 3 of PAX-interacting protein 1 OS=Homo sapiens GN=PAXIP1		19.65
Q6ZW49-3	PAXI1_HUMAN Isoform 4 of PAX-interacting protein 1 OS=Homo sapiens GN=PAXIP1		19.65
Q6ZW49-4	PAXI1_HUMAN Isoform 5 of PAX-interacting protein 1 OS=Homo sapiens GN=PAXIP1		19.65
Q6ZXV5	TMTC3_HUMAN Transmembrane and TPR repeat-containing protein 3 OS=Homo sapiens GN=TMTC3 PE=1 SV=2		
Q6ZXV5-2	TMTC3_HUMAN Isoform 2 of Transmembrane and TPR repeat-containing protein 3 OS=Homo sapiens GN=TMTC3		
Q702N8	XIRP1_HUMAN Xin actin-binding repeat-containing protein 1 OS=Homo sapiens GN=XIRP1 PE=1 SV=1		
Q702N8-2	XIRP1_HUMAN Isoform B of Xin actin-binding repeat-containing protein 1 OS=Homo sapiens GN=XIRP1		
Q702N8;Q702N8-2	XIRP1_HUMAN Xin actin-binding repeat-containing protein 1 OS=Homo sapiens GN=XIRP1 PE=1 SV=1;>sp|Q702N8-2|XIRP1_HUMAN	3.96E-11	
Q709C8;Q709C8-3;Q709C	VP13C_HUMAN Vacuolar protein sorting-associated protein 13C OS=Homo sapiens GN=VPS13C PE=1 SV=1;>sp|Q709C8-3|VP13C_HU	4.68E-11	
Q70E73	RAPH1_HUMAN Ras-associated and pleckstrin homology domains-containing protein 1 OS=Homo sapiens GN=RAPH1 PE=1 SV=3		
Q70E73-2	RAPH1_HUMAN Isoform RMO1 of Ras-associated and pleckstrin homology domains-containing protein 1 OS=Homo sapiens GN=RAPH1		
Q70E73-3	RAPH1_HUMAN Isoform RMO1a of Ras-associated and pleckstrin homology domains-containing protein 1 OS=Homo sapiens GN=RAPH1		
Q70E73-4	RAPH1_HUMAN Isoform RMO1b of Ras-associated and pleckstrin homology domains-containing protein 1 OS=Homo sapiens GN=RAPH1		27.69
Q70E73-5	RAPH1_HUMAN Isoform RMO1c of Ras-associated and pleckstrin homology domains-containing protein 1 OS=Homo sapiens GN=RAPH1		
Q70E73-6	RAPH1_HUMAN Isoform RMO1ab of Ras-associated and pleckstrin homology domains-containing protein 1 OS=Homo sapiens GN=RAPH1		27.69
Q70E73-6;Q70E73-9;Q70	6|RAPH1_HUMAN Isoform RMO1ab of Ras-associated and pleckstrin homology domains-containing protein 1 OS=Homo sapiens GN=R	1.48E-16	
Q70E73-7	RAPH1_HUMAN Isoform RMO1ac of Ras-associated and pleckstrin homology domains-containing protein 1 OS=Homo sapiens GN=RAPH1		
Q70E73-8	RAPH1_HUMAN Isoform RMO1bc of Ras-associated and pleckstrin homology domains-containing protein 1 OS=Homo sapiens GN=RAPH1		27.69
Q70E73-9	RAPH1_HUMAN Isoform RMO1abc of Ras-associated and pleckstrin homology domains-containing protein 1 OS=Homo sapiens GN=RAPH1		27.69

Q70E73;Q70E73-2;Q70E7	RAPH1_HUMAN Ras-associated and pleckstrin homology domains-containing protein 1 OS=Homo sapiens GN=RAPH1 PE=1 SV=3;>sp	1.11E-32	
Q70J99	UN13D_HUMAN Protein unc-13 homolog D OS=Homo sapiens GN=UNC13D PE=1 SV=1		44.2
Q70J99-2	UN13D_HUMAN Isoform 2 of Protein unc-13 homolog D OS=Homo sapiens GN=UNC13D		44.2
Q70J99-3	UN13D_HUMAN Isoform 3 of Protein unc-13 homolog D OS=Homo sapiens GN=UNC13D		44.2
Q70J99-3;Q70J99	3|UN13D_HUMAN Isoform 3 of Protein unc-13 homolog D OS=Homo sapiens GN=UNC13D;>sp|Q70J99|UN13D_HUMAN Protein unc-1	2.06E-107	
Q70UQ0	IKIP_HUMAN Inhibitor of nuclear factor kappa-B kinase-interacting protein OS=Homo sapiens GN=IKBIP PE=1 SV=1		552.45
Q70UQ0-2	IKIP_HUMAN Isoform 2 of Inhibitor of nuclear factor kappa-B kinase-interacting protein OS=Homo sapiens GN=IKBIP		406.12
Q70UQ0-3	IKIP_HUMAN Isoform 3 of Inhibitor of nuclear factor kappa-B kinase-interacting protein OS=Homo sapiens GN=IKBIP		23.91
Q70UQ0-4	IKIP_HUMAN Isoform 4 of Inhibitor of nuclear factor kappa-B kinase-interacting protein OS=Homo sapiens GN=IKBIP		680.44
Q70UQ0;Q3B7K3;Q70UQ0	IKIP_HUMAN Inhibitor of nuclear factor kappa-B kinase-interacting protein OS=Homo sapiens GN=IKBIP PE=1 SV=1;>tr|Q3B7K3|Q3E	1.10E-152	
Q70Z53	F10C1_HUMAN Protein FRA10AC1 OS=Homo sapiens GN=FRA10AC1 PE=1 SV=3		
Q70Z53-2	F10C1_HUMAN Isoform 2 of Protein FRA10AC1 OS=Homo sapiens GN=FRA10AC1		
Q70Z53-3	F10C1_HUMAN Isoform 3 of Protein FRA10AC1 OS=Homo sapiens GN=FRA10AC1		
Q70Z53-4	F10C1_HUMAN Isoform 4 of Protein FRA10AC1 OS=Homo sapiens GN=FRA10AC1		
Q70Z53-5	F10C1_HUMAN Isoform 5 of Protein FRA10AC1 OS=Homo sapiens GN=FRA10AC1		
Q712K3	UB2R2_HUMAN Ubiquitin-conjugating enzyme E2 R2 OS=Homo sapiens GN=UBE2R2 PE=1 SV=1	3.03E-07	
Q71DI3	H32_HUMAN Histone H3.2 OS=Homo sapiens GN=HIST2H3A PE=1 SV=3		180.26
Q71F56	MD13L_HUMAN Mediator of RNA polymerase II transcription subunit 13-like OS=Homo sapiens GN=MED13L PE=1 SV=1		30.39
Q71RC2	LARP4_HUMAN La-related protein 4 OS=Homo sapiens GN=LARP4 PE=1 SV=3		132.01
Q71RC2-2	LARP4_HUMAN Isoform 2 of La-related protein 4 OS=Homo sapiens GN=LARP4		87.53
Q71RC2-3	LARP4_HUMAN Isoform 3 of La-related protein 4 OS=Homo sapiens GN=LARP4		132.01
Q71RC2-4	LARP4_HUMAN Isoform 4 of La-related protein 4 OS=Homo sapiens GN=LARP4		132.01
Q71RC2-4;Q71RC2;Q71R(	4|LARP4_HUMAN Isoform 4 of La-related protein 4 OS=Homo sapiens GN=LARP4;>sp|Q71RC2|LARP4_HUMAN La-related protein 4 OS	3.73E-23	
Q71RS6	NCKX5_HUMAN Sodium/potassium/calcium exchanger 5 OS=Homo sapiens GN=SLC24A5 PE=1 SV=1		25
Q71U36	TBA1A_HUMAN Tubulin alpha-1A chain OS=Homo sapiens GN=TUBA1A PE=1 SV=1		1221.3
Q71UI9	H2AV_HUMAN Histone H2A.V OS=Homo sapiens GN=H2AFV PE=1 SV=3		250.74
Q71UM5	RS27L_HUMAN 40S ribosomal protein S27-like OS=Homo sapiens GN=RPS27L PE=1 SV=3		306.4
Q75LS8	FKB9L_HUMAN Putative FK506-binding protein 9-like protein OS=Homo sapiens GN=FKBP9L PE=5 SV=1		
Q75QN2	INT8_HUMAN Integrator complex subunit 8 OS=Homo sapiens GN=INTS8 PE=1 SV=1		
Q75QN2-2	INT8_HUMAN Isoform 2 of Integrator complex subunit 8 OS=Homo sapiens GN=INTS8		
Q75V66	ANO5_HUMAN Anoctamin-5 OS=Homo sapiens GN=ANO5 PE=1 SV=1		
Q76FK4	NOL8_HUMAN Nucleolar protein 8 OS=Homo sapiens GN=NOL8 PE=1 SV=1		
Q76FK4-2	NOL8_HUMAN Isoform 2 of Nucleolar protein 8 OS=Homo sapiens GN=NOL8		
Q76FK4-3	NOL8_HUMAN Isoform 3 of Nucleolar protein 8 OS=Homo sapiens GN=NOL8		
Q76FK4-4	NOL8_HUMAN Isoform 4 of Nucleolar protein 8 OS=Homo sapiens GN=NOL8		
Q76KD6	SPERI_HUMAN Speriolin OS=Homo sapiens GN=SPATC1 PE=2 SV=2	1.09E-08	
Q76M96	CCD80_HUMAN Coiled-coil domain-containing protein 80 OS=Homo sapiens GN=CCDC80 PE=1 SV=1		
Q76M96-2	CCD80_HUMAN Isoform 2 of Coiled-coil domain-containing protein 80 OS=Homo sapiens GN=CCDC80		
Q7KYR7	BT2A1_HUMAN Butyrophilin subfamily 2 member A1 OS=Homo sapiens GN=BTN2A1 PE=1 SV=3		54.17
Q7KYR7-1	BT2A1_HUMAN Isoform 2 of Butyrophilin subfamily 2 member A1 OS=Homo sapiens GN=BTN2A1		50.26
Q7KYR7-3	BT2A1_HUMAN Isoform 3 of Butyrophilin subfamily 2 member A1 OS=Homo sapiens GN=BTN2A1		54.17
Q7KZ85	SPT6H_HUMAN Transcription elongation factor SPT6 OS=Homo sapiens GN=SUPT6H PE=1 SV=2		109.68
Q7KZ85-2	SPT6H_HUMAN Isoform 2 of Transcription elongation factor SPT6 OS=Homo sapiens GN=SUPT6H		34.05
Q7KZ85-3	SPT6H_HUMAN Isoform 3 of Transcription elongation factor SPT6 OS=Homo sapiens GN=SUPT6H		
Q7KZF4	SND1_HUMAN Staphylococcal nuclease domain-containing protein 1 OS=Homo sapiens GN=SND1 PE=1 SV=1		1697.89
Q7KZI7;Q7KZI7-8;Q7KZI7	MARK2_HUMAN Serine/threonine-protein kinase MARK2 OS=Homo sapiens GN=MARK2 PE=1 SV=2;>sp|Q7KZI7-8|MARK2_HUMAN Is	5.27E-14	
Q7L014	DDX46_HUMAN Probable ATP-dependent RNA helicase DDX46 OS=Homo sapiens GN=DDX46 PE=1 SV=2		363.92
Q7L0Y3	MRRP1_HUMAN Mitochondrial ribonuclease P protein 1 OS=Homo sapiens GN=RG9MTD1 PE=1 SV=2		
Q7L1Q6	BZW1_HUMAN Basic leucine zipper and W2 domain-containing protein 1 OS=Homo sapiens GN=BZW1 PE=1 SV=1		572.82
Q7L1Q6-2	BZW1_HUMAN Isoform 2 of Basic leucine zipper and W2 domain-containing protein 1 OS=Homo sapiens GN=BZW1		543.17
Q7L1Q6;Q7L1Q6-2	BZW1_HUMAN Basic leucine zipper and W2 domain-containing protein 1 OS=Homo sapiens GN=BZW1 PE=1 SV=1;>sp|Q7L1Q6-2|BZ	2.39E-83	
Q7L273	KCTD9_HUMAN BTB/POZ domain-containing protein KCTD9 OS=Homo sapiens GN=KCTD9 PE=2 SV=1	3.52E-09	
Q7L2E3	DHX30_HUMAN Putative ATP-dependent RNA helicase DHX30 OS=Homo sapiens GN=DHX30 PE=1 SV=1		156.38
Q7L2E3-2	DHX30_HUMAN Isoform 2 of Putative ATP-dependent RNA helicase DHX30 OS=Homo sapiens GN=DHX30		156.38
Q7L2E3-2;Q7L2E3;C9JC37	2|DHX30_HUMAN Isoform 2 of Putative ATP-dependent RNA helicase DHX30 OS=Homo sapiens GN=DHX30;>sp|Q7L2E3|DHX30_HUM	1.32E-17	
Q7L2E3-3	DHX30_HUMAN Isoform 3 of Putative ATP-dependent RNA helicase DHX30 OS=Homo sapiens GN=DHX30		156.38
Q7L2H7	EIF3M_HUMAN Eukaryotic translation initiation factor 3 subunit M OS=Homo sapiens GN=EIF3M PE=1 SV=1		182.29
Q7L2J0	MEPCE_HUMAN 7SK snRNA methylphosphate capping enzyme OS=Homo sapiens GN=MEPCE PE=1 SV=1		25.25
Q7L2R6	ZN765_HUMAN Zinc finger protein 765 OS=Homo sapiens GN=ZNF765 PE=2 SV=2	6.24E-19	
Q7L4I2;Q7L4I2-2;E1B6W3	RSRC2_HUMAN Arginine/serine-rich coiled-coil protein 2 OS=Homo sapiens GN=RSRC2 PE=1 SV=1;>sp|Q7L4I2-2|RSRC2_HUMAN Iso	3.80E-49	
Q7L4S7	ARMX6_HUMAN Protein ARMCX6 OS=Homo sapiens GN=ARMCX6 PE=1 SV=1		

Q7L513-9;Q7L513-2;Q7L5	9|FCRLA_HUMAN Isoform 9 of Fc receptor-like A OS=Homo sapiens GN=FCRLA;>sp|Q7L513-2|FCRLA_HUMAN Isoform 2 of Fc recepto	1.36E-121	
Q7L576	CYFP1_HUMAN Cytoplasmic FMR1-interacting protein 1 OS=Homo sapiens GN=CYFIP1 PE=1 SV=1		257.43
Q7L576-2	CYFP1_HUMAN Isoform 2 of Cytoplasmic FMR1-interacting protein 1 OS=Homo sapiens GN=CYFIP1		97.79
Q7L576-3	CYFP1_HUMAN Isoform 3 of Cytoplasmic FMR1-interacting protein 1 OS=Homo sapiens GN=CYFIP1		73.61
Q7L576;Q7L576-2	CYFP1_HUMAN Cytoplasmic FMR1-interacting protein 1 OS=Homo sapiens GN=CYFIP1 PE=1 SV=1;>sp|Q7L576-2|CYFP1_HUMAN Isof	9.07E-98	
Q7L591;Q7L591-3;Q7L59	DOK3_HUMAN Docking protein 3 OS=Homo sapiens GN=DOK3 PE=1 SV=2;>sp|Q7L591-3|DOK3_HUMAN Isoform 3 of Docking protei	2.94E-33	
Q7L5D6	GET4_HUMAN Golgi to ER traffic protein 4 homolog OS=Homo sapiens GN=GET4 PE=1 SV=1		
Q7L5D6-2	GET4_HUMAN Isoform 2 of Golgi to ER traffic protein 4 homolog OS=Homo sapiens GN=GET4		
Q7L5D6;Q7L5D6-2	GET4_HUMAN Golgi to ER traffic protein 4 homolog OS=Homo sapiens GN=GET4 PE=1 SV=1;>sp|Q7L5D6-2|GET4_HUMAN Isoform 2	8.68E-82	
Q7L5L3	GDPD3_HUMAN Glycerophosphodiester phosphodiesterase domain-containing protein 3 OS=Homo sapiens GN=GDPD3 PE=2 SV=3		
Q7L5L3-1	GDPD3_HUMAN Isoform 2 of Glycerophosphodiester phosphodiesterase domain-containing protein 3 OS=Homo sapiens GN=GDPD3		
Q7L5L3;Q7L5L3-1	GDPD3_HUMAN Glycerophosphodiester phosphodiesterase domain-containing protein 3 OS=Homo sapiens GN=GDPD3 PE=2 SV=3;>s	5.73E-17	
Q7L5N1	CSN6_HUMAN COP9 signalosome complex subunit 6 OS=Homo sapiens GN=COPS6 PE=1 SV=1		64.37
Q7L5Y1	ENOF1_HUMAN Mitochondrial enolase superfamily member 1 OS=Homo sapiens GN=ENOSF1 PE=1 SV=1		24
Q7L5Y1-2	ENOF1_HUMAN Isoform 2 of Mitochondrial enolase superfamily member 1 OS=Homo sapiens GN=ENOSF1		24
Q7L5Y9;Q7L5Y9-2;Q7L5Y	MAEA_HUMAN Macrophage erythroblast attacher OS=Homo sapiens GN=MAEA PE=1 SV=1;>sp|Q7L5Y9-2|MAEA_HUMAN Isoform 2 of	4.93E-06	
Q7L775	EPMIP_HUMAN EPM2A-interacting protein 1 OS=Homo sapiens GN=EPM2AIP1 PE=1 SV=1		30.62
Q7L7L0	H2A3_HUMAN Histone H2A type 3 OS=Homo sapiens GN=HIST3H2A PE=1 SV=3		212.14
Q7L7V1	DHX32_HUMAN Putative pre-splicing factor ATP-dependent RNA helicase DHX32 OS=Homo sapiens GN=DHX32 PE=1 SV=1		0.66
Q7L7X3;Q9H2K8	TAOK1_HUMAN Serine/threonine-protein kinase TAO1 OS=Homo sapiens GN=TAOK1 PE=1 SV=1;>sp|Q9H2K8|TAOK3_HUMAN Serine	2.34E-05	
Q7L804	RFIP2_HUMAN Rab11 family-interacting protein 2 OS=Homo sapiens GN=RAB11FIP2 PE=1 SV=1		53.26
Q7L8L6;B4DWZ8	FAKD5_HUMAN FAST kinase domain-containing protein 5 OS=Homo sapiens GN=FASTKD5 PE=1 SV=1;>tr|B4DWZ8|B4DWZ8_HUMAN	4.40E-06	
Q7L9L4;Q9H8S9;Q9H8S9-	MOL1A_HUMAN Mps one binder kinase activator-like 1A OS=Homo sapiens GN=MOBKL1A PE=1 SV=3;>sp|Q9H8S9|MOL1B_HUMAN M	3.15E-16	
Q7LBC6	KDM3B_HUMAN Lysine-specific demethylase 3B OS=Homo sapiens GN=KDM3B PE=1 SV=2		104.29
Q7LBC6-2	KDM3B_HUMAN Isoform 2 of Lysine-specific demethylase 3B OS=Homo sapiens GN=KDM3B		55.79
Q7LBC6-3	KDM3B_HUMAN Isoform 3 of Lysine-specific demethylase 3B OS=Homo sapiens GN=KDM3B		13.36
Q7LBR1	CHM1B_HUMAN Charged multivesicular body protein 1b OS=Homo sapiens GN=CHMP1B PE=1 SV=1	4.11E-05	
Q7LFX5	CHSTF_HUMAN Carbohydrate sulfotransferase 15 OS=Homo sapiens GN=CHST15 PE=1 SV=1		
Q7LG56;Q7LG56-2	RIR2B_HUMAN Ribonucleoside-diphosphate reductase subunit M2 B OS=Homo sapiens GN=RRM2B PE=1 SV=1;>sp|Q7LG56-2|RIR2B	2.29E-45	
Q7RTM1	OTOP1_HUMAN Otopetrin-1 OS=Homo sapiens GN=OTOP1 PE=2 SV=1		
Q7RTS1	BHA15_HUMAN Class A basic helix-loop-helix protein 15 OS=Homo sapiens GN=BHLHA15 PE=1 SV=1		
Q7RTS7	K2C74_HUMAN Keratin, type II cytoskeletal 74 OS=Homo sapiens GN=KRT74 PE=1 SV=2		310.41
Q7RTT6	SSX6_HUMAN Putative protein SSX6 OS=Homo sapiens GN=SSX6 PE=5 SV=1		
Q7RTV0	PHF5A_HUMAN PHD finger-like domain-containing protein 5A OS=Homo sapiens GN=PHF5A PE=1 SV=1		177.25
Q7RTV5	CI021_HUMAN UPF0308 protein C9orf21 OS=Homo sapiens GN=C9orf21 PE=2 SV=1	6.25E-05	
Q7RTX8	HIN1L_HUMAN Putative HIN1-like protein OS=Homo sapiens GN=HIN1L PE=5 SV=1		38.47
Q7Z2K6	ERMP1_HUMAN Endoplasmic reticulum metallopeptidase 1 OS=Homo sapiens GN=ERMP1 PE=1 SV=2		
Q7Z2T5	TRM1L_HUMAN TRMT1-like protein OS=Homo sapiens GN=TRMT1L PE=1 SV=2		96.32
Q7Z2T5-2	TRM1L_HUMAN Isoform 2 of TRMT1-like protein OS=Homo sapiens GN=TRMT1L		20.23
Q7Z2T5;Q7Z2T5-2	TRM1L_HUMAN TRMT1-like protein OS=Homo sapiens GN=TRMT1L PE=1 SV=2;>sp|Q7Z2T5-2|TRM1L_HUMAN Isoform 2 of TRMT1-lik	3.72E-20	
Q7Z2W4	ZCCHV_HUMAN Zinc finger CCCH-type antiviral protein 1 OS=Homo sapiens GN=ZC3HAV1 PE=1 SV=3		62.71
Q7Z2W4-2	ZCCHV_HUMAN Isoform 2 of Zinc finger CCCH-type antiviral protein 1 OS=Homo sapiens GN=ZC3HAV1		62.43
Q7Z2W4-3	ZCCHV_HUMAN Isoform 3 of Zinc finger CCCH-type antiviral protein 1 OS=Homo sapiens GN=ZC3HAV1		78.39
Q7Z2W4-4	ZCCHV_HUMAN Isoform 4 of Zinc finger CCCH-type antiviral protein 1 OS=Homo sapiens GN=ZC3HAV1		0.28
Q7Z2W4;Q7Z2W4-2	ZCCHV_HUMAN Zinc finger CCCH-type antiviral protein 1 OS=Homo sapiens GN=ZC3HAV1 PE=1 SV=3;>sp|Q7Z2W4-2|ZCCHV_HUMA	6.36E-84	
Q7Z2W9	RM21_HUMAN 39S ribosomal protein L21, mitochondrial OS=Homo sapiens GN=MRPL21 PE=1 SV=2	6.13E-19	
Q7Z2Y8	GVIN1_HUMAN Interferon-induced very large GTPase 1 OS=Homo sapiens GN=GVINP1 PE=2 SV=2		
Q7Z2Z2	ETUD1_HUMAN Elongation factor Tu GTP-binding domain-containing protein 1 OS=Homo sapiens GN=EFTUD1 PE=1 SV=2		
Q7Z2Z2-2	ETUD1_HUMAN Isoform 2 of Elongation factor Tu GTP-binding domain-containing protein 1 OS=Homo sapiens GN=EFTUD1		
Q7Z2Z2-3	ETUD1_HUMAN Isoform 3 of Elongation factor Tu GTP-binding domain-containing protein 1 OS=Homo sapiens GN=EFTUD1		
Q7Z2Z2;Q7Z2Z2-2	ETUD1_HUMAN Elongation factor Tu GTP-binding domain-containing protein 1 OS=Homo sapiens GN=EFTUD1 PE=1 SV=2;>sp|Q7Z2Z	2.16E-20	
Q7Z333	SETX_HUMAN Probable helicase senataxin OS=Homo sapiens GN=SETX PE=1 SV=4		49.02
Q7Z333-3	SETX_HUMAN Isoform 3 of Probable helicase senataxin OS=Homo sapiens GN=SETX		45.11
Q7Z333-4	SETX_HUMAN Isoform 4 of Probable helicase senataxin OS=Homo sapiens GN=SETX		49.02
Q7Z3B4	NUP54_HUMAN Nucleoporin p54 OS=Homo sapiens GN=NUP54 PE=1 SV=2		
Q7Z3B4-2	NUP54_HUMAN Isoform 2 of Nucleoporin p54 OS=Homo sapiens GN=NUP54		
Q7Z3B4;Q7Z3B4-2	NUP54_HUMAN Nucleoporin p54 OS=Homo sapiens GN=NUP54 PE=1 SV=2;>sp|Q7Z3B4-2|NUP54_HUMAN Isoform 2 of Nucleoporin p	6.22E-47	
Q7Z3E2	CJ118_HUMAN Uncharacterized protein C10orf118 OS=Homo sapiens GN=C10orf118 PE=2 SV=2	2.97E-08	
Q7Z3E5	ARMC9_HUMAN LisH domain-containing protein ARMC9 OS=Homo sapiens GN=ARMC9 PE=2 SV=2		
Q7Z3E5-2	ARMC9_HUMAN Isoform 2 of LisH domain-containing protein ARMC9 OS=Homo sapiens GN=ARMC9		
Q7Z3E5;A6NIB1;Q7Z3E5-	ARMC9_HUMAN LisH domain-containing protein ARMC9 OS=Homo sapiens GN=ARMC9 PE=2 SV=2;>tr|A6NIB1|A6NIB1_HUMAN Unch	8.22E-39	

Q7Z3J3	RGPD4_HUMAN RanBP2-like and GRIP domain-containing protein 4 OS=Homo sapiens GN=RGPD4 PE=2 SV=3		182.51
Q7Z3K3	POGZ_HUMAN Pogo transposable element with ZNF domain OS=Homo sapiens GN=POGZ PE=1 SV=2		
Q7Z3K3-2	POGZ_HUMAN Isoform 2 of Pogo transposable element with ZNF domain OS=Homo sapiens GN=POGZ		
Q7Z3K3-3	POGZ_HUMAN Isoform 3 of Pogo transposable element with ZNF domain OS=Homo sapiens GN=POGZ		
Q7Z3K3-4	POGZ_HUMAN Isoform 4 of Pogo transposable element with ZNF domain OS=Homo sapiens GN=POGZ		
Q7Z3K3-5	POGZ_HUMAN Isoform 5 of Pogo transposable element with ZNF domain OS=Homo sapiens GN=POGZ		
Q7Z3K3;Q7Z3K3-3;Q7Z3K	POGZ_HUMAN Pogo transposable element with ZNF domain OS=Homo sapiens GN=POGZ PE=1 SV=2;>sp|Q7Z3K3-3|POGZ_HUMAN I	8.56E-26	
Q7Z3K6-3	MIER3_HUMAN Isoform 3 of Mesoderm induction early response protein 3 OS=Homo sapiens GN=MIER3		
Q7Z3T8	ZFY16_HUMAN Zinc finger FYVE domain-containing protein 16 OS=Homo sapiens GN=ZFYVE16 PE=1 SV=3		
Q7Z3T8-3	ZFY16_HUMAN Isoform 2 of Zinc finger FYVE domain-containing protein 16 OS=Homo sapiens GN=ZFYVE16		
Q7Z3Y7	K1C28_HUMAN Keratin, type I cytoskeletal 28 OS=Homo sapiens GN=KRT28 PE=1 SV=2		253
Q7Z3Y8	K1C27_HUMAN Keratin, type I cytoskeletal 27 OS=Homo sapiens GN=KRT27 PE=1 SV=2		196.25
Q7Z3Y9	K1C26_HUMAN Keratin, type I cytoskeletal 26 OS=Homo sapiens GN=KRT26 PE=1 SV=2		132.43
Q7Z3Z0	K1C25_HUMAN Keratin, type I cytoskeletal 25 OS=Homo sapiens GN=KRT25 PE=1 SV=1		207.09
Q7Z401	MYCPP_HUMAN C-myc promoter-binding protein OS=Homo sapiens GN=DENND4A PE=1 SV=2		
Q7Z403	TMC6_HUMAN Transmembrane channel-like protein 6 OS=Homo sapiens GN=TMC6 PE=1 SV=2		22.31
Q7Z403-2	TMC6_HUMAN Isoform 2 of Transmembrane channel-like protein 6 OS=Homo sapiens GN=TMC6		7.4
Q7Z403-3	TMC6_HUMAN Isoform 3 of Transmembrane channel-like protein 6 OS=Homo sapiens GN=TMC6		14.91
Q7Z406	MYH14_HUMAN Myosin-14 OS=Homo sapiens GN=MYH14 PE=1 SV=2		588.71
Q7Z406-2	MYH14_HUMAN Isoform 2 of Myosin-14 OS=Homo sapiens GN=MYH14		588.71
Q7Z406-2;Q7Z406-6;Q7Z	2|MYH14_HUMAN Isoform 2 of Myosin-14 OS=Homo sapiens GN=MYH14;>sp|Q7Z406-6|MYH14_HUMAN Isoform 6 of Myosin-14 OS=	7.44E-157	
Q7Z406-4	MYH14_HUMAN Isoform 4 of Myosin-14 OS=Homo sapiens GN=MYH14		426.16
Q7Z406-5	MYH14_HUMAN Isoform 5 of Myosin-14 OS=Homo sapiens GN=MYH14		434.45
Q7Z406-6	MYH14_HUMAN Isoform 6 of Myosin-14 OS=Homo sapiens GN=MYH14		588.71
Q7Z417	NUFP2_HUMAN Nuclear fragile X mental retardation-interacting protein 2 OS=Homo sapiens GN=NUFIP2 PE=1 SV=1	1.08E-14	
Q7Z418	KCNKI_HUMAN Potassium channel subfamily K member 18 OS=Homo sapiens GN=KCNK18 PE=1 SV=1		
Q7Z434	MAVS_HUMAN Mitochondrial antiviral-signaling protein OS=Homo sapiens GN=MAVS PE=1 SV=2		136.91
Q7Z434-2	MAVS_HUMAN Isoform 2 of Mitochondrial antiviral-signaling protein OS=Homo sapiens GN=MAVS		14.35
Q7Z434;A8K6X0	MAVS_HUMAN Mitochondrial antiviral-signaling protein OS=Homo sapiens GN=MAVS PE=1 SV=2;>tr|A8K6X0|A8K6X0_HUMAN cDNA	5.33E-84	
Q7Z460	CLAP1_HUMAN CLIP-associating protein 1 OS=Homo sapiens GN=CLASP1 PE=1 SV=1		174.16
Q7Z460-2	CLAP1_HUMAN Isoform 2 of CLIP-associating protein 1 OS=Homo sapiens GN=CLASP1		162.72
Q7Z460-3	CLAP1_HUMAN Isoform 3 of CLIP-associating protein 1 OS=Homo sapiens GN=CLASP1		162.72
Q7Z460;Q7Z460-3;A2RU2	CLAP1_HUMAN CLIP-associating protein 1 OS=Homo sapiens GN=CLASP1 PE=1 SV=1;>sp|Q7Z460-3|CLAP1_HUMAN Isoform 3 of CL	1.19E-26	
Q7Z478	DHX29_HUMAN ATP-dependent RNA helicase DHX29 OS=Homo sapiens GN=DHX29 PE=1 SV=2		590.99
Q7Z4G1-2;Q7Z4G1	2|COMD6_HUMAN Isoform 2 of COMM domain-containing protein 6 OS=Homo sapiens GN=COMMD6;>sp|Q7Z4G1|COMD6_HUMAN C	5.22E-07	
Q7Z4H3;Q7Z4H3-2;Q7Z4	HDDC2_HUMAN HD domain-containing protein 2 OS=Homo sapiens GN=HDDC2 PE=1 SV=1;>sp|Q7Z4H3-2|HDDC2_HUMAN Isoform	6.74E-13	
Q7Z4H8	KDEL2_HUMAN KDEL motif-containing protein 2 OS=Homo sapiens GN=KDELC2 PE=1 SV=2		158.23
Q7Z4H8-2	KDEL2_HUMAN Isoform 2 of KDEL motif-containing protein 2 OS=Homo sapiens GN=KDELC2		99.75
Q7Z4H8-3	KDEL2_HUMAN Isoform 3 of KDEL motif-containing protein 2 OS=Homo sapiens GN=KDELC2		99.75
Q7Z4H8;Q7Z4H8-2;B2R8	KDEL2_HUMAN KDEL motif-containing protein 2 OS=Homo sapiens GN=KDELC2 PE=1 SV=2;>sp|Q7Z4H8-2|KDEL2_HUMAN Isoform	1.43E-34	
Q7Z4I7-2;Q7Z4I7;B3KNZ	2|LIMS2_HUMAN Isoform 2 of LIM and senescent cell antigen-like-containing domain protein 2 OS=Homo sapiens GN=LIMS2;>sp|Q7	1.25E-34	
Q7Z4Q2	HEAT3_HUMAN HEAT repeat-containing protein 3 OS=Homo sapiens GN=HEATR3 PE=1 SV=2		
Q7Z4Q2-2	HEAT3_HUMAN Isoform 2 of HEAT repeat-containing protein 3 OS=Homo sapiens GN=HEATR3		
Q7Z4Q2-3	HEAT3_HUMAN Isoform 3 of HEAT repeat-containing protein 3 OS=Homo sapiens GN=HEATR3		
Q7Z4Q2;Q7Z4Q2-2	HEAT3_HUMAN HEAT repeat-containing protein 3 OS=Homo sapiens GN=HEATR3 PE=1 SV=2;>sp|Q7Z4Q2-2|HEAT3_HUMAN Isoform	1.72E-06	
Q7Z4R8	CF120_HUMAN UPF0669 protein C6orf120 OS=Homo sapiens GN=C6orf120 PE=1 SV=1		31.3
Q7Z4S6	KI21A_HUMAN Kinesin-like protein KIF21A OS=Homo sapiens GN=KIF21A PE=1 SV=2		42.89
Q7Z4S6-2	KI21A_HUMAN Isoform 2 of Kinesin-like protein KIF21A OS=Homo sapiens GN=KIF21A		42.89
Q7Z4S6-3	KI21A_HUMAN Isoform 3 of Kinesin-like protein KIF21A OS=Homo sapiens GN=KIF21A		42.89
Q7Z4S6-4	KI21A_HUMAN Isoform 4 of Kinesin-like protein KIF21A OS=Homo sapiens GN=KIF21A		42.89
Q7Z4V5	HDGR2_HUMAN Hepatoma-derived growth factor-related protein 2 OS=Homo sapiens GN=HDGFRP2 PE=1 SV=1		
Q7Z4V5-2	HDGR2_HUMAN Isoform 2 of Hepatoma-derived growth factor-related protein 2 OS=Homo sapiens GN=HDGFRP2		
Q7Z4V5;Q7Z4V5-2	HDGR2_HUMAN Hepatoma-derived growth factor-related protein 2 OS=Homo sapiens GN=HDGFRP2 PE=1 SV=1;>sp|Q7Z4V5-2|HDG	9.64E-18	
Q7Z4W1	DCXR_HUMAN L-xylulose reductase OS=Homo sapiens GN=DCXR PE=1 SV=2		59.29
Q7Z5G4	GOGA7_HUMAN Golgin subfamily A member 7 OS=Homo sapiens GN=GOLGA7 PE=1 SV=2		247.67
Q7Z5K2	WAPL_HUMAN Wings apart-like protein homolog OS=Homo sapiens GN=WAPAL PE=1 SV=1		52.89
Q7Z5K2-2	WAPL_HUMAN Isoform 2 of Wings apart-like protein homolog OS=Homo sapiens GN=WAPAL		52.89
Q7Z5K2-3	WAPL_HUMAN Isoform 3 of Wings apart-like protein homolog OS=Homo sapiens GN=WAPAL		52.89
Q7Z5K2-3;Q7Z5K2-2;Q7Z	3|WAPL_HUMAN Isoform 3 of Wings apart-like protein homolog OS=Homo sapiens GN=WAPAL;>sp|Q7Z5K2-2|WAPL_HUMAN Isoform	1.56E-16	
Q7Z5L7-3;Q7Z5L7-2;Q7Z	3|PODN_HUMAN Isoform 3 of Podocan OS=Homo sapiens GN=PODN;>sp|Q7Z5L7-2|PODN_HUMAN Isoform 2 of Podocan OS=Homo s	3.31E-24	
Q7Z5L9	I2BP2_HUMAN Interferon regulatory factor 2-binding protein 2 OS=Homo sapiens GN=IRF2BP2 PE=1 SV=2		

Q7Z5L9-2	I2BP2_HUMAN Isoform 2 of Interferon regulatory factor 2-binding protein 2 OS=Homo sapiens GN=IRF2BP2		
Q7Z5L9-3	I2BP2_HUMAN Isoform 3 of Interferon regulatory factor 2-binding protein 2 OS=Homo sapiens GN=IRF2BP2		
Q7Z5L9;Q7Z5L9-2	I2BP2_HUMAN Interferon regulatory factor 2-binding protein 2 OS=Homo sapiens GN=IRF2BP2 PE=1 SV=2;>sp|Q7Z5L9-2|I2BP2_HU	1.47E-29	
Q7Z5N4	SDK1_HUMAN Protein sidekick-1 OS=Homo sapiens GN=SDK1 PE=1 SV=3		
Q7Z5N4-3	SDK1_HUMAN Isoform 3 of Protein sidekick-1 OS=Homo sapiens GN=SDK1		
Q7Z5P9	MUC19_HUMAN Mucin-19 OS=Homo sapiens GN=MUC19 PE=1 SV=2		
Q7Z5P9-2	MUC19_HUMAN Isoform 2 of Mucin-19 OS=Homo sapiens GN=MUC19		
Q7Z5R6	AB1IP_HUMAN Amyloid beta A4 precursor protein-binding family B member 1-interacting protein OS=Homo sapiens GN=APBB1IP PE=	1.43E-22	
Q7Z695	ADCK2_HUMAN Uncharacterized aarF domain-containing protein kinase 2 OS=Homo sapiens GN=ADCK2 PE=2 SV=1		
Q7Z6J4-2	2|FGD2_HUMAN Isoform 2 of FYVE, RhoGEF and PH domain-containing protein 2 OS=Homo sapiens GN=FGD2	8.57E-05	
Q7Z6J6	FRMD5_HUMAN FERM domain-containing protein 5 OS=Homo sapiens GN=FRMD5 PE=2 SV=1		
Q7Z6J6-2	FRMD5_HUMAN Isoform 2 of FERM domain-containing protein 5 OS=Homo sapiens GN=FRMD5		
Q7Z6K1	THAP5_HUMAN THAP domain-containing protein 5 OS=Homo sapiens GN=THAP5 PE=1 SV=2		
Q7Z6M2	FBX33_HUMAN F-box only protein 33 OS=Homo sapiens GN=FBXO33 PE=2 SV=1		
Q7Z6M4	MTER2_HUMAN mTERF domain-containing protein 2 OS=Homo sapiens GN=MTERFD2 PE=1 SV=3		
Q7Z6R9	AP2D_HUMAN Transcription factor AP-2-delta OS=Homo sapiens GN=TFAP2D PE=2 SV=1		
Q7Z6Z7	HUWE1_HUMAN E3 ubiquitin-protein ligase HUWE1 OS=Homo sapiens GN=HUWE1 PE=1 SV=3		1015.88
Q7Z6Z7-2	HUWE1_HUMAN Isoform 2 of E3 ubiquitin-protein ligase HUWE1 OS=Homo sapiens GN=HUWE1		1015.88
Q7Z6Z7-3	HUWE1_HUMAN Isoform 3 of E3 ubiquitin-protein ligase HUWE1 OS=Homo sapiens GN=HUWE1		1015.88
Q7Z6Z7;Q7Z6Z7-3;Q7Z6Z	HUWE1_HUMAN E3 ubiquitin-protein ligase HUWE1 OS=Homo sapiens GN=HUWE1 PE=1 SV=3;>sp|Q7Z6Z7-3|HUWE1_HUMAN Isofor	1.28E-83	
Q7Z739	YTHD3_HUMAN YTH domain family protein 3 OS=Homo sapiens GN=YTHDF3 PE=1 SV=1		171.7
Q7Z794	K2C1B_HUMAN Keratin, type II cytoskeletal 1b OS=Homo sapiens GN=KRT77 PE=1 SV=3		271.81
Q7Z7A4;Q7Z7A4-4;Q7Z7A	PXK_HUMAN PX domain-containing protein kinase-like protein OS=Homo sapiens GN=PXK PE=1 SV=1;>sp|Q7Z7A4-4|PXK_HUMAN Is	2.40E-25	
Q7Z7B0	FLIP1_HUMAN Filamin-A-interacting protein 1 OS=Homo sapiens GN=FILIP1 PE=1 SV=1		39.89
Q7Z7B0-2	FLIP1_HUMAN Isoform 2 of Filamin-A-interacting protein 1 OS=Homo sapiens GN=FILIP1		38.56
Q7Z7B0-3	FLIP1_HUMAN Isoform 3 of Filamin-A-interacting protein 1 OS=Homo sapiens GN=FILIP1		38.51
Q7Z7D3	VTCN1_HUMAN V-set domain-containing T-cell activation inhibitor 1 OS=Homo sapiens GN=VTCN1 PE=1 SV=1		
Q7Z7D3-2	VTCN1_HUMAN Isoform 2 of V-set domain-containing T-cell activation inhibitor 1 OS=Homo sapiens GN=VTCN1		
Q7Z7E8;Q7Z7E8-2	UB2Q1_HUMAN Ubiquitin-conjugating enzyme E2 Q1 OS=Homo sapiens GN=UBE2Q1 PE=1 SV=1;>sp|Q7Z7E8-2|UB2Q1_HUMAN Isof	3.65E-12	
Q7Z7G0-4	TARSH_HUMAN Isoform 4 of Target of Nesh-SH3 OS=Homo sapiens GN=ABI3BP		
Q7Z7G0;Q7Z7G0-4;Q7Z7G	TARSH_HUMAN Target of Nesh-SH3 OS=Homo sapiens GN=ABI3BP PE=1 SV=1;>sp|Q7Z7G0-4|TARSH_HUMAN Isoform 4 of Target of	1.56E-12	
Q7Z7G8	VP13B_HUMAN Vacuolar protein sorting-associated protein 13B OS=Homo sapiens GN=VPS13B PE=1 SV=2		
Q7Z7G8-2	VP13B_HUMAN Isoform 2 of Vacuolar protein sorting-associated protein 13B OS=Homo sapiens GN=VPS13B		
Q7Z7G8-3	VP13B_HUMAN Isoform 3 of Vacuolar protein sorting-associated protein 13B OS=Homo sapiens GN=VPS13B		
Q7Z7G8-4	VP13B_HUMAN Isoform 4 of Vacuolar protein sorting-associated protein 13B OS=Homo sapiens GN=VPS13B		
Q7Z7G8-6	VP13B_HUMAN Isoform 6 of Vacuolar protein sorting-associated protein 13B OS=Homo sapiens GN=VPS13B		
Q7Z7H5	TMED4_HUMAN Transmembrane emp24 domain-containing protein 4 OS=Homo sapiens GN=TMED4 PE=1 SV=1		351.19
Q7Z7H5-2	TMED4_HUMAN Isoform 2 of Transmembrane emp24 domain-containing protein 4 OS=Homo sapiens GN=TMED4		351.19
Q7Z7H5-3	TMED4_HUMAN Isoform 3 of Transmembrane emp24 domain-containing protein 4 OS=Homo sapiens GN=TMED4		351.19
Q7Z7H5;C9JUN6;Q7Z7H5-	TMED4_HUMAN Transmembrane emp24 domain-containing protein 4 OS=Homo sapiens GN=TMED4 PE=1 SV=1;>tr|C9JUN6|C9JUN6_	6.46E-19	
Q7Z7H8	RM10_HUMAN 39S ribosomal protein L10, mitochondrial OS=Homo sapiens GN=MRPL10 PE=1 SV=3		
Q7Z7K0	COXAM_HUMAN COX assembly mitochondrial protein homolog OS=Homo sapiens GN=CMC1 PE=1 SV=1		43.72
Q7Z7K6	CENPV_HUMAN Centromere protein V OS=Homo sapiens GN=CENPV PE=1 SV=1		
Q7Z7K6-2	CENPV_HUMAN Isoform 2 of Centromere protein V OS=Homo sapiens GN=CENPV		
Q7Z7K6-3	CENPV_HUMAN Isoform 3 of Centromere protein V OS=Homo sapiens GN=CENPV		
Q7Z7L1	SLN11_HUMAN Schlafen family member 11 OS=Homo sapiens GN=SLFN11 PE=1 SV=2		42.45
Q86SE5	RALYL_HUMAN RNA-binding Raly-like protein OS=Homo sapiens GN=RALYL PE=1 SV=2		72.7
Q86SE5-2	RALYL_HUMAN Isoform 2 of RNA-binding Raly-like protein OS=Homo sapiens GN=RALYL		2.32
Q86SF2	GALT7_HUMAN N-acetylgalactosaminyltransferase 7 OS=Homo sapiens GN=GALNT7 PE=1 SV=1		21.59
Q86SQ0	PHLB2_HUMAN Pleckstrin homology-like domain family B member 2 OS=Homo sapiens GN=PHLDB2 PE=1 SV=2		92.39
Q86SQ0-2	PHLB2_HUMAN Isoform 2 of Pleckstrin homology-like domain family B member 2 OS=Homo sapiens GN=PHLDB2		92.39
Q86SQ0-3	PHLB2_HUMAN Isoform 3 of Pleckstrin homology-like domain family B member 2 OS=Homo sapiens GN=PHLDB2		92.39
Q86SQ0;Q86SQ0-3;Q86S(	PHLB2_HUMAN Pleckstrin homology-like domain family B member 2 OS=Homo sapiens GN=PHLDB2 PE=1 SV=2;>sp|Q86SQ0-3|PHLE	8.95E-08	
Q86SS6	SYT9_HUMAN Synaptotagmin-9 OS=Homo sapiens GN=SYT9 PE=2 SV=1		
Q86SX6	GLRX5_HUMAN Glutaredoxin-related protein 5, mitochondrial OS=Homo sapiens GN=GLRX5 PE=1 SV=2		130.73
Q86SZ2	TPC6B_HUMAN Trafficking protein particle complex subunit 6B OS=Homo sapiens GN=TRAPPC6B PE=1 SV=1		51.37
Q86SZ2-2	TPC6B_HUMAN Isoform 2 of Trafficking protein particle complex subunit 6B OS=Homo sapiens GN=TRAPPC6B		51.37
Q86SZ2;B4DFZ8;Q86SZ2-	TPC6B_HUMAN Trafficking protein particle complex subunit 6B OS=Homo sapiens GN=TRAPPC6B PE=1 SV=1;>tr|B4DFZ8|B4DFZ8_HU	1.07E-26	
Q86T23	CROL1_HUMAN Putative ciliary rootlet coiled-coil protein-like 1 protein OS=Homo sapiens GN=CROCCP2 PE=5 SV=1		1.66
Q86T90	K1328_HUMAN Uncharacterized protein KIAA1328 OS=Homo sapiens GN=KIAA1328 PE=1 SV=2		
Q86T90-2	K1328_HUMAN Isoform 2 of Uncharacterized protein KIAA1328 OS=Homo sapiens GN=KIAA1328		

Q86T90-3	K1328_HUMAN Isoform 3 of Uncharacterized protein KIAA1328 OS=Homo sapiens GN=KIAA1328		
Q86T90-4	K1328_HUMAN Isoform 4 of Uncharacterized protein KIAA1328 OS=Homo sapiens GN=KIAA1328		
Q86TB9	PATL1_HUMAN Protein PAT1 homolog 1 OS=Homo sapiens GN=PATL1 PE=1 SV=2		95.24
Q86TB9-2	PATL1_HUMAN Isoform 2 of Protein PAT1 homolog 1 OS=Homo sapiens GN=PATL1		95.24
Q86TB9-4	PATL1_HUMAN Isoform 4 of Protein PAT1 homolog 1 OS=Homo sapiens GN=PATL1		95.24
Q86TB9;B3KXT9;Q86TB9-	PATL1_HUMAN Protein PAT1 homolog 1 OS=Homo sapiens GN=PATL1 PE=1 SV=2;>tr|B3KXT9|B3KXT9_HUMAN cDNA FLJ46053 fis, cl	4.97E-25	
Q86TC9;Q86TC9-2	MYPN_HUMAN Myopalladin OS=Homo sapiens GN=MYPN PE=1 SV=2;>sp|Q86TC9-2|MYPN_HUMAN Isoform 2 of Myopalladin OS=Hon	7.42E-15	
Q86TG7;B4DSP0;Q86TG7-	PEG10_HUMAN Retrotransposon-derived protein PEG10 OS=Homo sapiens GN=PEG10 PE=1 SV=2;>tr|B4DSP0|B4DSP0_HUMAN HCG	4.14E-42	
Q86TI0	TBCD1_HUMAN TBC1 domain family member 1 OS=Homo sapiens GN=TBC1D1 PE=1 SV=2	1.01E-18	
Q86TI2-2;Q1ZZB8;Q86TI2	2|DPP9_HUMAN Isoform 2 of Dipeptidyl peptidase 9 OS=Homo sapiens GN=DPP9;>tr|Q1ZZB8|Q1ZZB8_HUMAN Dipeptidyl peptidase	4.92E-10	
Q86TM6	SYVN1_HUMAN E3 ubiquitin-protein ligase synoviolin OS=Homo sapiens GN=SYVN1 PE=1 SV=2		
Q86TM6-2	SYVN1_HUMAN Isoform 2 of E3 ubiquitin-protein ligase synoviolin OS=Homo sapiens GN=SYVN1		
Q86TM6-3	SYVN1_HUMAN Isoform 3 of E3 ubiquitin-protein ligase synoviolin OS=Homo sapiens GN=SYVN1		
Q86TU7	SETD3_HUMAN Histone-lysine N-methyltransferase setd3 OS=Homo sapiens GN=SETD3 PE=1 SV=1		56.27
Q86TU7-2	SETD3_HUMAN Isoform 2 of Histone-lysine N-methyltransferase setd3 OS=Homo sapiens GN=SETD3		43.79
Q86TU7-3	SETD3_HUMAN Isoform 3 of Histone-lysine N-methyltransferase setd3 OS=Homo sapiens GN=SETD3		43.79
Q86TU7;A5PLP0;A0PJU3;(	SETD3_HUMAN SET domain-containing protein 3 OS=Homo sapiens GN=SETD3 PE=1 SV=1;>tr|A5PLP0|A5PLP0_HUMAN SET domain	8.56E-75	
Q86TU7;Q86TU7-2;Q86TU	SETD3_HUMAN Histone-lysine N-methyltransferase setd3 OS=Homo sapiens GN=SETD3 PE=1 SV=1;>sp|Q86TU7-2|SETD3_HUMAN I	7.51E-18	
Q86U06	RBM23_HUMAN Probable RNA-binding protein 23 OS=Homo sapiens GN=RBM23 PE=1 SV=1		12.59
Q86U06-2	RBM23_HUMAN Isoform 2 of Probable RNA-binding protein 23 OS=Homo sapiens GN=RBM23		12.59
Q86U06-3	RBM23_HUMAN Isoform 3 of Probable RNA-binding protein 23 OS=Homo sapiens GN=RBM23		12.59
Q86U06-4	RBM23_HUMAN Isoform 4 of Probable RNA-binding protein 23 OS=Homo sapiens GN=RBM23		12.59
Q86U06-5	RBM23_HUMAN Isoform 5 of Probable RNA-binding protein 23 OS=Homo sapiens GN=RBM23		12.59
Q86U28	ISCA2_HUMAN Iron-sulfur cluster assembly 2 homolog, mitochondrial OS=Homo sapiens GN=ISCA2 PE=1 SV=2		22.63
Q86U38	CN021_HUMAN Pumilio domain-containing protein C14orf21 OS=Homo sapiens GN=C14orf21 PE=1 SV=1		
Q86U42	PABP2_HUMAN Polyadenylate-binding protein 2 OS=Homo sapiens GN=PABPN1 PE=1 SV=3		46.52
Q86U42-2	PABP2_HUMAN Isoform 2 of Polyadenylate-binding protein 2 OS=Homo sapiens GN=PABPN1		46.52
Q86U42;Q86U42-2	PABP2_HUMAN Polyadenylate-binding protein 2 OS=Homo sapiens GN=PABPN1 PE=1 SV=3;>sp|Q86U42-2|PABP2_HUMAN Isoform 2	1.88E-20	
Q86U86	PB1_HUMAN Protein polybromo-1 OS=Homo sapiens GN=PBRM1 PE=1 SV=1		
Q86U86-2	PB1_HUMAN Isoform 2 of Protein polybromo-1 OS=Homo sapiens GN=PBRM1		
Q86U86-3	PB1_HUMAN Isoform 3 of Protein polybromo-1 OS=Homo sapiens GN=PBRM1		
Q86U86-4	PB1_HUMAN Isoform 4 of Protein polybromo-1 OS=Homo sapiens GN=PBRM1		
Q86U86-5	PB1_HUMAN Isoform 5 of Protein polybromo-1 OS=Homo sapiens GN=PBRM1		
Q86U86-6	PB1_HUMAN Isoform 6 of Protein polybromo-1 OS=Homo sapiens GN=PBRM1		
Q86U86-7	PB1_HUMAN Isoform 7 of Protein polybromo-1 OS=Homo sapiens GN=PBRM1		
Q86U86-8	PB1_HUMAN Isoform 8 of Protein polybromo-1 OS=Homo sapiens GN=PBRM1		
Q86U86-9	PB1_HUMAN Isoform 9 of Protein polybromo-1 OS=Homo sapiens GN=PBRM1		
Q86U86;Q86U86-8;Q86U8	PB1_HUMAN Protein polybromo-1 OS=Homo sapiens GN=PBRM1 PE=1 SV=1;>sp|Q86U86-8|PB1_HUMAN Isoform 8 of Protein polybr	6.42E-13	
Q86U90	YRDC_HUMAN YrdC domain-containing protein, mitochondrial OS=Homo sapiens GN=YRDC PE=1 SV=1		
Q86UC2	RSPH3_HUMAN Radial spoke head protein 3 homolog OS=Homo sapiens GN=RSPH3 PE=2 SV=1		
Q86UC2-2	RSPH3_HUMAN Isoform 2 of Radial spoke head protein 3 homolog OS=Homo sapiens GN=RSPH3		
Q86UE3	ZN546_HUMAN Zinc finger protein 546 OS=Homo sapiens GN=ZNF546 PE=2 SV=2		
Q86UE4	LYRIC_HUMAN Protein LYRIC OS=Homo sapiens GN=MTDH PE=1 SV=2		367.28
Q86UE8	TLK2_HUMAN Serine/threonine-protein kinase tousled-like 2 OS=Homo sapiens GN=TLK2 PE=1 SV=2		44.82
Q86UE8-2	TLK2_HUMAN Isoform 2 of Serine/threonine-protein kinase tousled-like 2 OS=Homo sapiens GN=TLK2		44.82
Q86UE8-3	TLK2_HUMAN Isoform 3 of Serine/threonine-protein kinase tousled-like 2 OS=Homo sapiens GN=TLK2		44.82
Q86UE8;Q86UE8-2;Q86UE	TLK2_HUMAN Serine/threonine-protein kinase tousled-like 2 OS=Homo sapiens GN=TLK2 PE=1 SV=2;>sp|Q86UE8-2|TLK2_HUMAN Is	8.08E-13	
Q86UF1	TSN33_HUMAN Tetraspanin-33 OS=Homo sapiens GN=TSPAN33 PE=2 SV=1		
Q86UK7	ZN598_HUMAN Zinc finger protein 598 OS=Homo sapiens GN=ZNF598 PE=1 SV=1		48.54
Q86UK7-2	ZN598_HUMAN Isoform 2 of Zinc finger protein 598 OS=Homo sapiens GN=ZNF598		48.54
Q86UK7-3	ZN598_HUMAN Isoform 3 of Zinc finger protein 598 OS=Homo sapiens GN=ZNF598		48.54
Q86UP2	KTN1_HUMAN Kinectin OS=Homo sapiens GN=KTN1 PE=1 SV=1		1575.99
Q86UP2-2	KTN1_HUMAN Isoform 2 of Kinectin OS=Homo sapiens GN=KTN1		1526.57
Q86UP2;B4DZ88;Q86UP2-	KTN1_HUMAN Kinectin OS=Homo sapiens GN=KTN1 PE=1 SV=1;>tr|B4DZ88|B4DZ88_HUMAN KTN1 protein OS=Homo sapiens GN=K	0	
Q86UP8-2	GTD2A_HUMAN Isoform 2 of General transcription factor II-I repeat domain-containing protein 2A OS=Homo sapiens GN=GTF2IRD2		
Q86UP8-3	GTD2A_HUMAN Isoform 3 of General transcription factor II-I repeat domain-containing protein 2A OS=Homo sapiens GN=GTF2IRD2		
Q86UP8-4	GTD2A_HUMAN Isoform 4 of General transcription factor II-I repeat domain-containing protein 2A OS=Homo sapiens GN=GTF2IRD2		
Q86UP8-5	GTD2A_HUMAN Isoform 5 of General transcription factor II-I repeat domain-containing protein 2A OS=Homo sapiens GN=GTF2IRD2		
Q86UQ0	ZN589_HUMAN Zinc finger protein 589 OS=Homo sapiens GN=ZNF589 PE=1 SV=1		
Q86UQ0-2	ZN589_HUMAN Isoform 2 of Zinc finger protein 589 OS=Homo sapiens GN=ZNF589		
Q86UQ0-3	ZN589_HUMAN Isoform 3 of Zinc finger protein 589 OS=Homo sapiens GN=ZNF589		

Q86UQ4	ABCAD_HUMAN ATP-binding cassette sub-family A member 13 OS=Homo sapiens GN=ABCA13 PE=2 SV=3		110.3
Q86UQ4-2	ABCAD_HUMAN Isoform 2 of ATP-binding cassette sub-family A member 13 OS=Homo sapiens GN=ABCA13		
Q86UQ4-3	ABCAD_HUMAN Isoform 3 of ATP-binding cassette sub-family A member 13 OS=Homo sapiens GN=ABCA13		12.5
Q86UQ4;Q86UQ4-3	ABCAD_HUMAN ATP-binding cassette sub-family A member 13 OS=Homo sapiens GN=ABCA13 PE=2 SV=3;>sp|Q86UQ4-3|ABCAD_HU	8.93E-23	
Q86UQ8	NFE4_HUMAN Transcription factor NF-E4 OS=Homo sapiens GN=NFE4 PE=1 SV=1		
Q86UQ8-2	NFE4_HUMAN Isoform 2 of Transcription factor NF-E4 OS=Homo sapiens GN=NFE4		
Q86UR5	RIMS1_HUMAN Regulating synaptic membrane exocytosis protein 1 OS=Homo sapiens GN=RIMS1 PE=1 SV=1		
Q86UT8	CCD84_HUMAN Coiled-coil domain-containing protein 84 OS=Homo sapiens GN=CCDC84 PE=2 SV=1		
Q86UV5	UBP48_HUMAN Ubiquitin carboxyl-terminal hydrolase 48 OS=Homo sapiens GN=USP48 PE=1 SV=1		68.5
Q86UV5-2	UBP48_HUMAN Isoform 2 of Ubiquitin carboxyl-terminal hydrolase 48 OS=Homo sapiens GN=USP48		68.5
Q86UV5-3	UBP48_HUMAN Isoform 3 of Ubiquitin carboxyl-terminal hydrolase 48 OS=Homo sapiens GN=USP48		68.5
Q86UV5;Q86UV5-2;Q86U\	UBP48_HUMAN Ubiquitin carboxyl-terminal hydrolase 48 OS=Homo sapiens GN=USP48 PE=1 SV=1;>sp|Q86UV5-2|UBP48_HUMAN Is	2.99E-21	
Q86UX7;Q86UX7-2	URP2_HUMAN Fermitin family homolog 3 OS=Homo sapiens GN=FERMT3 PE=1 SV=1;>sp|Q86UX7-2|URP2_HUMAN Isoform 2 of Ferm	1.99E-97	
Q86UY8;Q86UY8-2	NT5D3_HUMAN 5-nucleotidase domain-containing protein 3 OS=Homo sapiens GN=NT5DC3 PE=2 SV=1;>sp|Q86UY8-2|NT5D3_HUM/	1.62E-19	
Q86V15	CASZ1_HUMAN Zinc finger protein castor homolog 1 OS=Homo sapiens GN=CASZ1 PE=2 SV=4		28.06
Q86V15-2	CASZ1_HUMAN Isoform 2 of Zinc finger protein castor homolog 1 OS=Homo sapiens GN=CASZ1		28.06
Q86V20	FA35A_HUMAN Protein FAM35A OS=Homo sapiens GN=FAM35A PE=1 SV=1		
Q86V20-2	FA35A_HUMAN Isoform 2 of Protein FAM35A OS=Homo sapiens GN=FAM35A		
Q86V21;Q86V21-2;Q86V2	AACS_HUMAN Acetoacetyl-CoA synthetase OS=Homo sapiens GN=AACS PE=1 SV=1;>sp|Q86V21-2|AACS_HUMAN Isoform 2 of Aceto	7.14E-17	
Q86V48	LUZP1_HUMAN Leucine zipper protein 1 OS=Homo sapiens GN=LUZP1 PE=1 SV=2		575.02
Q86V48-2	LUZP1_HUMAN Isoform 2 of Leucine zipper protein 1 OS=Homo sapiens GN=LUZP1		534.59
Q86V48-3	LUZP1_HUMAN Isoform 3 of Leucine zipper protein 1 OS=Homo sapiens GN=LUZP1		534.59
Q86V48;Q86V48-3;Q86V4	LUZP1_HUMAN Leucine zipper protein 1 OS=Homo sapiens GN=LUZP1 PE=1 SV=2;>sp|Q86V48-3|LUZP1_HUMAN Isoform 3 of Leucin	8.93E-23	
Q86V81	THOC4_HUMAN THO complex subunit 4 OS=Homo sapiens GN=THOC4 PE=1 SV=3		290.48
Q86V88;Q86V88-2;Q86V8	MGDP1_HUMAN Magnesium-dependent phosphatase 1 OS=Homo sapiens GN=MDP1 PE=1 SV=1;>sp|Q86V88-2|MGDP1_HUMAN Isofo	7.09E-10	
Q86VB7-2;Q86VB7;Q86VE	2|C163A_HUMAN Isoform 2 of Scavenger receptor cysteine-rich type 1 protein M130 OS=Homo sapiens GN=CD163;>sp|Q86VB7|C16	7.47E-154	
Q86VD1	MORC1_HUMAN MORC family CW-type zinc finger protein 1 OS=Homo sapiens GN=MORC1 PE=2 SV=2		
Q86VI3	IQGA3_HUMAN Ras GTPase-activating-like protein IQGAP3 OS=Homo sapiens GN=IQGAP3 PE=1 SV=2		281.47
Q86VM9	ZCH18_HUMAN Zinc finger CCCH domain-containing protein 18 OS=Homo sapiens GN=ZC3H18 PE=1 SV=2		
Q86VM9-2	ZCH18_HUMAN Isoform 2 of Zinc finger CCCH domain-containing protein 18 OS=Homo sapiens GN=ZC3H18		
Q86VM9;Q86VM9-2	ZCH18_HUMAN Zinc finger CCCH domain-containing protein 18 OS=Homo sapiens GN=ZC3H18 PE=1 SV=2;>sp|Q86VM9-2|ZCH18_H	1.67E-23	
Q86VP6	CAND1_HUMAN Cullin-associated NEDD8-dissociated protein 1 OS=Homo sapiens GN=CAND1 PE=1 SV=2		474.18
Q86VP6-2	CAND1_HUMAN Isoform 2 of Cullin-associated NEDD8-dissociated protein 1 OS=Homo sapiens GN=CAND1		435.96
Q86VP6-3	CAND1_HUMAN Isoform 3 of Cullin-associated NEDD8-dissociated protein 1 OS=Homo sapiens GN=CAND1		83.41
Q86VP6;Q86VP6-2	CAND1_HUMAN Cullin-associated NEDD8-dissociated protein 1 OS=Homo sapiens GN=CAND1 PE=1 SV=2;>sp|Q86VP6-2|CAND1_HUI	0	
Q86VR2	F134C_HUMAN Protein FAM134C OS=Homo sapiens GN=FAM134C PE=1 SV=1		
Q86VS8	HOOK3_HUMAN Protein Hook homolog 3 OS=Homo sapiens GN=HOOK3 PE=1 SV=2	8.28E-14	
Q86VV8	RTTN_HUMAN Rotatin OS=Homo sapiens GN=RTTN PE=1 SV=3		41.81
Q86VV8-2	RTTN_HUMAN Isoform 2 of Rotatin OS=Homo sapiens GN=RTTN		20.72
Q86VV8-3	RTTN_HUMAN Isoform 3 of Rotatin OS=Homo sapiens GN=RTTN		41.81
Q86VV8-4	RTTN_HUMAN Isoform 4 of Rotatin OS=Homo sapiens GN=RTTN		21.9
Q86VV8-5	RTTN_HUMAN Isoform 5 of Rotatin OS=Homo sapiens GN=RTTN		26.54
Q86VW0	SESD1_HUMAN SEC14 domain and spectrin repeat-containing protein 1 OS=Homo sapiens GN=SESTD1 PE=1 SV=2		
Q86VX2	COMD7_HUMAN COMM domain-containing protein 7 OS=Homo sapiens GN=COMMD7 PE=1 SV=2		38.38
Q86VX2-2	COMD7_HUMAN Isoform 2 of COMM domain-containing protein 7 OS=Homo sapiens GN=COMMD7		38.38
Q86VX2;Q86VX2-2	COMD7_HUMAN COMM domain-containing protein 7 OS=Homo sapiens GN=COMMD7 PE=1 SV=2;>sp|Q86VX2-2|COMD7_HUMAN Iso	2.12E-05	
Q86VY4	TSYL5_HUMAN Testis-specific Y-encoded-like protein 5 OS=Homo sapiens GN=TSPYL5 PE=1 SV=2		
Q86W24	NAL14_HUMAN NACHT, LRR and PYD domains-containing protein 14 OS=Homo sapiens GN=NLRP14 PE=2 SV=1		
Q86W25	NAL13_HUMAN NACHT, LRR and PYD domains-containing protein 13 OS=Homo sapiens GN=NLRP13 PE=1 SV=2		26.16
Q86W26	NAL10_HUMAN NACHT, LRR and PYD domains-containing protein 10 OS=Homo sapiens GN=NLRP10 PE=1 SV=1		
Q86W34	AMZ2_HUMAN Archaemetzincin-2 OS=Homo sapiens GN=AMZ2 PE=1 SV=2		
Q86W42	THOC6_HUMAN THO complex subunit 6 homolog OS=Homo sapiens GN=THOC6 PE=1 SV=1		59.73
Q86W42-2	THOC6_HUMAN Isoform 2 of THO complex subunit 6 homolog OS=Homo sapiens GN=THOC6		59.73
Q86W42-3	THOC6_HUMAN Isoform 3 of THO complex subunit 6 homolog OS=Homo sapiens GN=THOC6		59.73
Q86W42;Q86W42-2;Q86V	THOC6_HUMAN THO complex subunit 6 homolog OS=Homo sapiens GN=THOC6 PE=1 SV=1;>sp|Q86W42-2|THOC6_HUMAN Isoform	2.28E-08	
Q86W56;Q86W56-2;Q86V	PARG_HUMAN Poly(ADP-ribose) glycohydrolase OS=Homo sapiens GN=PARG PE=1 SV=1;>sp|Q86W56-2|PARG_HUMAN Isoform 2 of	1.49E-08	
Q86W92;Q86W92-2;Q86V	LIPB1_HUMAN Liprin-beta-1 OS=Homo sapiens GN=PPFIBP1 PE=1 SV=2;>sp|Q86W92-2|LIPB1_HUMAN Isoform 2 of Liprin-beta-1 OS	8.09E-05	
Q86WA6	BPHL_HUMAN Valacyclovir hydrolase OS=Homo sapiens GN=BPHL PE=1 SV=1		76.3
Q86WA6-2	BPHL_HUMAN Isoform 2 of Valacyclovir hydrolase OS=Homo sapiens GN=BPHL		76.3
Q86WA6;Q86WA6-2	BPHL_HUMAN Valacyclovir hydrolase OS=Homo sapiens GN=BPHL PE=1 SV=1;>sp|Q86WA6-2|BPHL_HUMAN Isoform 2 of Valacyclovi	1.53E-06	
Q86WB0;Q86WB0-2;Q86V	NIPA_HUMAN Nuclear-interacting partner of ALK OS=Homo sapiens GN=ZC3HC1 PE=1 SV=1;>sp|Q86WB0-2|NIPA_HUMAN Isoform 2	2.49E-05	

Q86WG5	MTMRD_HUMAN Myotubularin-related protein 13 OS=Homo sapiens GN=SBF2 PE=1 SV=1		19.32
Q86WG5-3	MTMRD_HUMAN Isoform 3 of Myotubularin-related protein 13 OS=Homo sapiens GN=SBF2		19.32
Q86WI3	NLRC5_HUMAN Protein NLRC5 OS=Homo sapiens GN=NLRC5 PE=1 SV=3		
Q86WI3-2	NLRC5_HUMAN Isoform 2 of Protein NLRC5 OS=Homo sapiens GN=NLRC5		
Q86WI3-3	NLRC5_HUMAN Isoform 3 of Protein NLRC5 OS=Homo sapiens GN=NLRC5		
Q86WI3-4	NLRC5_HUMAN Isoform 4 of Protein NLRC5 OS=Homo sapiens GN=NLRC5		
Q86WI3-5	NLRC5_HUMAN Isoform 5 of Protein NLRC5 OS=Homo sapiens GN=NLRC5		
Q86WI3-6	NLRC5_HUMAN Isoform 6 of Protein NLRC5 OS=Homo sapiens GN=NLRC5		
Q86WQ0	NR2CA_HUMAN Nuclear receptor 2C2-associated protein OS=Homo sapiens GN=NR2C2AP PE=1 SV=1	1.38E-17	
Q86WR0	CCD25_HUMAN Coiled-coil domain-containing protein 25 OS=Homo sapiens GN=CCDC25 PE=1 SV=2	6.90E-05	
Q86WR7	CJ047_HUMAN Uncharacterized protein C10orf47 OS=Homo sapiens GN=C10orf47 PE=1 SV=2	2.47E-10	
Q86WV6	TM173_HUMAN Transmembrane protein 173 OS=Homo sapiens GN=TMEM173 PE=1 SV=1	8.56E-07	
Q86WX3	AROS_HUMAN Active regulator of SIRT1 OS=Homo sapiens GN=RPS19BP1 PE=1 SV=1		41.43
Q86X29-2	LSR_HUMAN Isoform 2 of Lipolysis-stimulated lipoprotein receptor OS=Homo sapiens GN=LSR		
Q86X55;Q86X55-1;Q86X5	CARM1_HUMAN Histone-arginine methyltransferase CARM1 OS=Homo sapiens GN=CARM1 PE=1 SV=3;>sp|Q86X55-1|CARM1_HUMAI	1.25E-09	
Q86X67	NUD13_HUMAN Nucleoside diphosphate-linked moiety X motif 13 OS=Homo sapiens GN=NUDT13 PE=2 SV=3		21.17
Q86X67-2	NUD13_HUMAN Isoform 2 of Nucleoside diphosphate-linked moiety X motif 13 OS=Homo sapiens GN=NUDT13		21.17
Q86X67;Q86X67-2	NUD13_HUMAN Nucleoside diphosphate-linked moiety X motif 13 OS=Homo sapiens GN=NUDT13 PE=2 SV=3;>sp|Q86X67-2|NUD13_	2.32E-05	
Q86X76	NIT1_HUMAN Nitrilase homolog 1 OS=Homo sapiens GN=NIT1 PE=1 SV=2		23.72
Q86X76-2	NIT1_HUMAN Isoform 1 of Nitrilase homolog 1 OS=Homo sapiens GN=NIT1		23.72
Q86X76-3	NIT1_HUMAN Isoform 4 of Nitrilase homolog 1 OS=Homo sapiens GN=NIT1		23.72
Q86X76-3;B2R8D1;Q86X7	3|NIT1_HUMAN Isoform 4 of Nitrilase homolog 1 OS=Homo sapiens GN=NIT1;>tr|B2R8D1|B2R8D1_HUMAN cDNA, FLJ93841, highly s	4.02E-16	
Q86X76-4	NIT1_HUMAN Isoform 5 of Nitrilase homolog 1 OS=Homo sapiens GN=NIT1		28.97
Q86X83	COMD2_HUMAN COMM domain-containing protein 2 OS=Homo sapiens GN=COMMD2 PE=1 SV=2		
Q86XA9;Q86XA9-2	HTR5A_HUMAN HEAT repeat-containing protein 5A OS=Homo sapiens GN=HEATR5A PE=1 SV=2;>sp|Q86XA9-2|HTR5A_HUMAN Isofo	3.38E-07	
Q86XD8	ANUB1_HUMAN AN1-type zinc finger and ubiquitin domain-containing protein 1 OS=Homo sapiens GN=ANUBL1 PE=2 SV=2		
Q86XF0	DYRL1_HUMAN Dihydrofolate reductase-like protein 1 OS=Homo sapiens GN=DHFRL1 PE=2 SV=1		
Q86XH1;Q86XH1-2	IQCA1_HUMAN IQ and AAA domain-containing protein 1 OS=Homo sapiens GN=IQCA1 PE=2 SV=1;>sp|Q86XH1-2|IQCA1_HUMAN Isc	3.33E-05	
Q86XI2	CNDG2_HUMAN Condensin-2 complex subunit G2 OS=Homo sapiens GN=NCAPG2 PE=1 SV=1		
Q86XI2-2	CNDG2_HUMAN Isoform 2 of Condensin-2 complex subunit G2 OS=Homo sapiens GN=NCAPG2		
Q86XI2-2;Q86XI2	2|CNDG2_HUMAN Isoform 2 of Condensin-2 complex subunit G2 OS=Homo sapiens GN=NCAPG2;>sp|Q86XI2|CNDG2_HUMAN Conde	1.01E-05	
Q86XL3-2	ANKL2_HUMAN Isoform 2 of Ankyrin repeat and LEM domain-containing protein 2 OS=Homo sapiens GN=ANKLE2		
Q86XN6	ZN761_HUMAN Zinc finger protein 761 OS=Homo sapiens GN=ZNF761 PE=2 SV=2		
Q86XN6-2	ZN761_HUMAN Isoform 2 of Zinc finger protein 761 OS=Homo sapiens GN=ZNF761		
Q86XN6-3	ZN761_HUMAN Isoform 3 of Zinc finger protein 761 OS=Homo sapiens GN=ZNF761		
Q86XP3	DDX42_HUMAN ATP-dependent RNA helicase DDX42 OS=Homo sapiens GN=DDX42 PE=1 SV=1		214.75
Q86XP3-2	DDX42_HUMAN Isoform 2 of ATP-dependent RNA helicase DDX42 OS=Homo sapiens GN=DDX42		212.8
Q86XP3;Q86XP3-2	DDX42_HUMAN ATP-dependent RNA helicase DDX42 OS=Homo sapiens GN=DDX42 PE=1 SV=1;>sp|Q86XP3-2|DDX42_HUMAN Isofor	2.93E-60	
Q86XR2	NIBL2_HUMAN Niban-like protein 2 OS=Homo sapiens GN=FAM129C PE=1 SV=2		24.49
Q86XR2-2	NIBL2_HUMAN Isoform 2 of Niban-like protein 2 OS=Homo sapiens GN=FAM129C		24.49
Q86XR2-3	NIBL2_HUMAN Isoform 3 of Niban-like protein 2 OS=Homo sapiens GN=FAM129C		24.49
Q86XR2-5	NIBL2_HUMAN Isoform 5 of Niban-like protein 2 OS=Homo sapiens GN=FAM129C		24.49
Q86XZ4	SPAS2_HUMAN Spermatogenesis-associated serine-rich protein 2 OS=Homo sapiens GN=SPATS2 PE=1 SV=1		
Q86Y30	BAGE2_HUMAN B melanoma antigen 2 OS=Homo sapiens GN=BAGE2 PE=2 SV=1		
Q86Y33	CD20B_HUMAN Cell division cycle protein 20 homolog B OS=Homo sapiens GN=CDC20B PE=2 SV=3		
Q86Y33-2	CD20B_HUMAN Isoform 2 of Cell division cycle protein 20 homolog B OS=Homo sapiens GN=CDC20B		
Q86Y33-3	CD20B_HUMAN Isoform 3 of Cell division cycle protein 20 homolog B OS=Homo sapiens GN=CDC20B		
Q86Y33-4	CD20B_HUMAN Isoform 4 of Cell division cycle protein 20 homolog B OS=Homo sapiens GN=CDC20B		
Q86Y33-5	CD20B_HUMAN Isoform 5 of Cell division cycle protein 20 homolog B OS=Homo sapiens GN=CDC20B		
Q86Y39	NDUAB_HUMAN NADH dehydrogenase [ubiquinone] 1 alpha subcomplex subunit 11 OS=Homo sapiens GN=NDUFA11 PE=1 SV=3		61.69
Q86Y39-2	NDUAB_HUMAN Isoform 2 of NADH dehydrogenase [ubiquinone] 1 alpha subcomplex subunit 11 OS=Homo sapiens GN=NDUFA11		27.99
Q86Y39-2;Q86Y39	2|NDUAB_HUMAN Isoform 2 of NADH dehydrogenase [ubiquinone] 1 alpha subcomplex subunit 11 OS=Homo sapiens GN=NDUFA11;>	3.19E-05	
Q86Y39;Q86Y39-2	NDUAB_HUMAN NADH dehydrogenase [ubiquinone] 1 alpha subcomplex subunit 11 OS=Homo sapiens GN=NDUFA11 PE=1 SV=3;>sp	2.43E-14	
Q86Y46	K2C73_HUMAN Keratin, type II cytoskeletal 73 OS=Homo sapiens GN=KRT73 PE=1 SV=1		311.64
Q86Y46-2	K2C73_HUMAN Isoform 2 of Keratin, type II cytoskeletal 73 OS=Homo sapiens GN=KRT73		210.15
Q86Y56;Q86Y56-2	HEAT2_HUMAN HEAT repeat-containing protein 2 OS=Homo sapiens GN=HEATR2 PE=1 SV=4;>sp|Q86Y56-2|HEAT2_HUMAN Isoform	4.36E-29	
Q86Y79	PTH_HUMAN Probable peptidyl-tRNA hydrolase OS=Homo sapiens GN=PTRH1 PE=1 SV=1	4.30E-08	
Q86Y82	STX12_HUMAN Syntaxin-12 OS=Homo sapiens GN=STX12 PE=1 SV=1		294.59
Q86YB8	ERO1B_HUMAN ERO1-like protein beta OS=Homo sapiens GN=ERO1LB PE=1 SV=2		56.36
Q86YD7	F90A1_HUMAN Protein FAM90A1 OS=Homo sapiens GN=FAM90A1 PE=2 SV=3		
Q86YJ6	THNS2_HUMAN Threonine synthase-like 2 OS=Homo sapiens GN=THNSL2 PE=2 SV=3		

Q86YJ6-3	THNS2_HUMAN Isoform 3 of Threonine synthase-like 2 OS=Homo sapiens GN=THNSL2		
Q86YJ6-4	THNS2_HUMAN Isoform 4 of Threonine synthase-like 2 OS=Homo sapiens GN=THNSL2		
Q86YN1	DOPP1_HUMAN Dolichyldiphosphatase 1 OS=Homo sapiens GN=DOLPP1 PE=1 SV=1		
Q86YN6	PRGC2_HUMAN Peroxisome proliferator-activated receptor gamma coactivator 1-beta OS=Homo sapiens GN=PPARGC1B PE=1 SV=2		
Q86YN6-2	PRGC2_HUMAN Isoform 2 of Peroxisome proliferator-activated receptor gamma coactivator 1-beta OS=Homo sapiens GN=PPARGC1B		
Q86YN6-3	PRGC2_HUMAN Isoform 3 of Peroxisome proliferator-activated receptor gamma coactivator 1-beta OS=Homo sapiens GN=PPARGC1B		
Q86YN6-4	PRGC2_HUMAN Isoform 4 of Peroxisome proliferator-activated receptor gamma coactivator 1-beta OS=Homo sapiens GN=PPARGC1B		
Q86YN6-5	PRGC2_HUMAN Isoform 5 of Peroxisome proliferator-activated receptor gamma coactivator 1-beta OS=Homo sapiens GN=PPARGC1B		
Q86YP4	P66A_HUMAN Transcriptional repressor p66-alpha OS=Homo sapiens GN=GATAD2A PE=1 SV=1		
Q86YP4-2	P66A_HUMAN Isoform 2 of Transcriptional repressor p66-alpha OS=Homo sapiens GN=GATAD2A		
Q86YP4;Q86YP4-2	P66A_HUMAN Transcriptional repressor p66-alpha OS=Homo sapiens GN=GATAD2A PE=1 SV=1;>sp|Q86YP4-2|P66A_HUMAN Isoform	2.84E-06	
Q86YQ8	CPNE8_HUMAN Copine-8 OS=Homo sapiens GN=CPNE8 PE=1 SV=2		26.97
Q86YR5	GPSM1_HUMAN G-protein-signaling modulator 1 OS=Homo sapiens GN=GPSM1 PE=1 SV=2		
Q86YR5-3	GPSM1_HUMAN Isoform 3 of G-protein-signaling modulator 1 OS=Homo sapiens GN=GPSM1		
Q86YR5-4	GPSM1_HUMAN Isoform 4 of G-protein-signaling modulator 1 OS=Homo sapiens GN=GPSM1		
Q86YR5;Q86YR5-4	GPSM1_HUMAN G-protein-signaling modulator 1 OS=Homo sapiens GN=GPSM1 PE=1 SV=2;>sp|Q86YR5-4|GPSM1_HUMAN Isoform	9.58E-06	
Q86YR6	POTED_HUMAN POTE ankyrin domain family member D OS=Homo sapiens GN=POTED PE=2 SV=2		
Q86YS6	RAB43_HUMAN Ras-related protein Rab-43 OS=Homo sapiens GN=RAB43 PE=1 SV=1		97.75
Q86YV5	SG223_HUMAN Tyrosine-protein kinase SgK223 OS=Homo sapiens GN=SGK223 PE=1 SV=3	2.82E-12	
Q86YV6	MYLK4_HUMAN Myosin light chain kinase family member 4 OS=Homo sapiens GN=MYLK4 PE=1 SV=2		41.33
Q86YZ3	HORN_HUMAN Hornerin OS=Homo sapiens GN=HRNR PE=1 SV=2		46.35
Q8IU81	I2BP1_HUMAN Interferon regulatory factor 2-binding protein 1 OS=Homo sapiens GN=IRF2BP1 PE=1 SV=1	6.02E-11	
Q8IUC4	RHPN2_HUMAN Rhophilin-2 OS=Homo sapiens GN=RHPN2 PE=1 SV=1		
Q8IUC6	TCAM1_HUMAN TIR domain-containing adapter molecule 1 OS=Homo sapiens GN=TICAM1 PE=1 SV=1		
Q8IUD2	RB6I2_HUMAN ELKS/Rab6-interacting/CAST family member 1 OS=Homo sapiens GN=ERC1 PE=1 SV=1		51.3
Q8IUD2-2	RB6I2_HUMAN Isoform 2 of ELKS/Rab6-interacting/CAST family member 1 OS=Homo sapiens GN=ERC1		41.07
Q8IUD2-2;Q8IUD2;Q8IUD	2|RB6I2_HUMAN Isoform 2 of ELKS/Rab6-interacting/CAST family member 1 OS=Homo sapiens GN=ERC1;>sp|Q8IUD2|RB6I2_HUMA	9.82E-24	
Q8IUD2-3	RB6I2_HUMAN Isoform 3 of ELKS/Rab6-interacting/CAST family member 1 OS=Homo sapiens GN=ERC1		51.3
Q8IUD2-4	RB6I2_HUMAN Isoform 4 of ELKS/Rab6-interacting/CAST family member 1 OS=Homo sapiens GN=ERC1		41.07
Q8IUD2-5	RB6I2_HUMAN Isoform 5 of ELKS/Rab6-interacting/CAST family member 1 OS=Homo sapiens GN=ERC1		7.3
Q8IUE6	H2A2B_HUMAN Histone H2A type 2-B OS=Homo sapiens GN=HIST2H2AB PE=1 SV=3		104.62
Q8IUF8	MINA_HUMAN MYC-induced nuclear antigen OS=Homo sapiens GN=MINA PE=1 SV=1		55.25
Q8IUF8-2	MINA_HUMAN Isoform 2 of MYC-induced nuclear antigen OS=Homo sapiens GN=MINA		55.25
Q8IUF8-4	MINA_HUMAN Isoform 4 of MYC-induced nuclear antigen OS=Homo sapiens GN=MINA		55.25
Q8IUF8;D3DN35;Q8IUF8-	MINA_HUMAN MYC-induced nuclear antigen OS=Homo sapiens GN=MINA PE=1 SV=1;>tr|D3DN35|D3DN35_HUMAN MYC induced nuc	7.53E-10	
Q8IUG5;Q8IUG5-2	MY18B_HUMAN Myosin-XVIIIb OS=Homo sapiens GN=MYO18B PE=1 SV=1;>sp|Q8IUG5-2|MY18B_HUMAN Isoform 2 of Myosin-XVIII	8.10E-10	
Q8IUH3	RBM45_HUMAN RNA-binding protein 45 OS=Homo sapiens GN=RBM45 PE=1 SV=1		
Q8IUH3-2	RBM45_HUMAN Isoform 2 of RNA-binding protein 45 OS=Homo sapiens GN=RBM45		
Q8IUH3-3	RBM45_HUMAN Isoform 3 of RNA-binding protein 45 OS=Homo sapiens GN=RBM45		
Q8IUI8	CRLF3_HUMAN Cytokine receptor-like factor 3 OS=Homo sapiens GN=CRLF3 PE=1 SV=2	1.94E-16	
Q8IUQ0	CLVS1_HUMAN Clavesin-1 OS=Homo sapiens GN=CLVS1 PE=1 SV=1		4.91
Q8IUQ0-2	CLVS1_HUMAN Isoform 2 of Clavesin-1 OS=Homo sapiens GN=CLVS1		4.91
Q8IUR0	TPPC5_HUMAN Trafficking protein particle complex subunit 5 OS=Homo sapiens GN=TRAPPC5 PE=1 SV=1		57.75
Q8IUR6	CE041_HUMAN UPF0474 protein C5orf41 OS=Homo sapiens GN=C5orf41 PE=1 SV=2		
Q8IUR6-2	CE041_HUMAN Isoform 2 of UPF0474 protein C5orf41 OS=Homo sapiens GN=C5orf41		
Q8IUR6-3	CE041_HUMAN Isoform 3 of UPF0474 protein C5orf41 OS=Homo sapiens GN=C5orf41		
Q8IUR7	ARMC8_HUMAN Armadillo repeat-containing protein 8 OS=Homo sapiens GN=ARMC8 PE=1 SV=2		
Q8IUR7-3	ARMC8_HUMAN Isoform 3 of Armadillo repeat-containing protein 8 OS=Homo sapiens GN=ARMC8		
Q8IUX4	ABC3F_HUMAN DNA dC->dU-editing enzyme APOBEC-3F OS=Homo sapiens GN=APOBEC3F PE=1 SV=3		26.21
Q8IUX7;Q8IUX7-2	AEBP1_HUMAN Adipocyte enhancer-binding protein 1 OS=Homo sapiens GN=AEBP1 PE=1 SV=1;>sp|Q8IUX7-2|AEBP1_HUMAN Isofor	2.78E-160	
Q8IUZ5	AT2L2_HUMAN Alanine--glyoxylate aminotransferase 2-like 2 OS=Homo sapiens GN=AGXT2L2 PE=2 SV=1		
Q8IUZ5-2	AT2L2_HUMAN Isoform 2 of Alanine--glyoxylate aminotransferase 2-like 2 OS=Homo sapiens GN=AGXT2L2		
Q8IUZ5-3	AT2L2_HUMAN Isoform 3 of Alanine--glyoxylate aminotransferase 2-like 2 OS=Homo sapiens GN=AGXT2L2		
Q8IV08	PLD3_HUMAN Phospholipase D3 OS=Homo sapiens GN=PLD3 PE=1 SV=1		46.78
Q8IV33	K0825_HUMAN Uncharacterized protein KIAA0825 OS=Homo sapiens GN=KIAA0825 PE=2 SV=2		
Q8IV33-2	K0825_HUMAN Isoform 2 of Uncharacterized protein KIAA0825 OS=Homo sapiens GN=KIAA0825		
Q8IV33-3	K0825_HUMAN Isoform 3 of Uncharacterized protein KIAA0825 OS=Homo sapiens GN=KIAA0825		
Q8IV36	CQ028_HUMAN UPF0663 transmembrane protein C17orf28 OS=Homo sapiens GN=C17orf28 PE=1 SV=1		44.73
Q8IV36-2	CQ028_HUMAN Isoform 2 of UPF0663 transmembrane protein C17orf28 OS=Homo sapiens GN=C17orf28		44.73
Q8IV36-3	CQ028_HUMAN Isoform 3 of UPF0663 transmembrane protein C17orf28 OS=Homo sapiens GN=C17orf28		44.73
Q8IV36;Q8IV36-2;Q8IV36	CQ028_HUMAN UPF0663 transmembrane protein C17orf28 OS=Homo sapiens GN=C17orf28 PE=1 SV=1;>sp|Q8IV36-2|CQ028_HUMA	4.20E-39	

Q8IV48	ERI1_HUMAN 3'-5' exoribonuclease 1 OS=Homo sapiens GN=ERI1 PE=1 SV=3		27.17
Q8IV76	PASD1_HUMAN PAS domain-containing protein 1 OS=Homo sapiens GN=PASD1 PE=2 SV=1		
Q8IV76-2	PASD1_HUMAN Isoform 2 of PAS domain-containing protein 1 OS=Homo sapiens GN=PASD1		
Q8IVD9	NUDC3_HUMAN NudC domain-containing protein 3 OS=Homo sapiens GN=NUDCD3 PE=1 SV=3	2.87E-16	
Q8IVE0	CROL2_HUMAN Putative ciliary rootlet coiled-coil protein-like 2 protein OS=Homo sapiens GN=CROCCP3 PE=5 SV=1		21.61
Q8IVE3	PKHH2_HUMAN Pleckstrin homology domain-containing family H member 2 OS=Homo sapiens GN=PLEKHH2 PE=2 SV=2		63.19
Q8IVE3-2	PKHH2_HUMAN Isoform 2 of Pleckstrin homology domain-containing family H member 2 OS=Homo sapiens GN=PLEKHH2		9.67
Q8IVE3-3	PKHH2_HUMAN Isoform 3 of Pleckstrin homology domain-containing family H member 2 OS=Homo sapiens GN=PLEKHH2		59.73
Q8IVF2	AHNK2_HUMAN Protein AHNAK2 OS=Homo sapiens GN=AHNAK2 PE=1 SV=2		
Q8IVF2-2	AHNK2_HUMAN Isoform 2 of Protein AHNAK2 OS=Homo sapiens GN=AHNAK2		
Q8IVF2-3	AHNK2_HUMAN Isoform 3 of Protein AHNAK2 OS=Homo sapiens GN=AHNAK2		
Q8IVF2;Q8IVF2-3	AHNK2_HUMAN Protein AHNAK2 OS=Homo sapiens GN=AHNAK2 PE=1 SV=2;>sp|Q8IVF2-3|AHNK2_HUMAN Isoform 3 of Protein AHN	5.82E-09	
Q8IVF4	DYH10_HUMAN Dynein heavy chain 10, axonemal OS=Homo sapiens GN=DNAH10 PE=1 SV=4		
Q8IVF6	AN18A_HUMAN Ankyrin repeat domain-containing protein 18A OS=Homo sapiens GN=ANKRD18A PE=1 SV=3		
Q8IVH2	FOXP4_HUMAN Forkhead box protein P4 OS=Homo sapiens GN=FOXP4 PE=1 SV=1		
Q8IVL0	NAV3_HUMAN Neuron navigator 3 OS=Homo sapiens GN=NAV3 PE=1 SV=3		
Q8IVL0-2	NAV3_HUMAN Isoform 2 of Neuron navigator 3 OS=Homo sapiens GN=NAV3		
Q8IVL0-3	NAV3_HUMAN Isoform 3 of Neuron navigator 3 OS=Homo sapiens GN=NAV3		
Q8IVL6;Q59FY9;Q8IVL6-2	P3H3_HUMAN Prolyl 3-hydroxylase 3 OS=Homo sapiens GN=LEPREL2 PE=2 SV=1;>tr|Q59FY9|Q59FY9_HUMAN LEPREL2 protein varia	1.20E-16	
Q8IVM0	CCD50_HUMAN Coiled-coil domain-containing protein 50 OS=Homo sapiens GN=CCDC50 PE=1 SV=1		47.08
Q8IVM0-2	CCD50_HUMAN Isoform 2 of Coiled-coil domain-containing protein 50 OS=Homo sapiens GN=CCDC50		58.13
Q8IVM0-2;Q8IVM0	2|CCD50_HUMAN Isoform 2 of Coiled-coil domain-containing protein 50 OS=Homo sapiens GN=CCDC50;>sp|Q8IVM0|CCD50_HUMAN	4.43E-29	
Q8IVN8	RPESP_HUMAN RPE-spondin OS=Homo sapiens GN=RPESP PE=1 SV=2	1.28E-15	
Q8IVS8	GLCTK_HUMAN Glycerate kinase OS=Homo sapiens GN=GLYCTK PE=1 SV=1		
Q8IVS8-2	GLCTK_HUMAN Isoform 2 of Glycerate kinase OS=Homo sapiens GN=GLYCTK		
Q8IVS8-3	GLCTK_HUMAN Isoform 3 of Glycerate kinase OS=Homo sapiens GN=GLYCTK		
Q8IVS8-4	GLCTK_HUMAN Isoform 4 of Glycerate kinase OS=Homo sapiens GN=GLYCTK		
Q8IVS8-7	GLCTK_HUMAN Isoform 7 of Glycerate kinase OS=Homo sapiens GN=GLYCTK		
Q8IVT2	CS021_HUMAN Uncharacterized protein C19orf21 OS=Homo sapiens GN=C19orf21 PE=1 SV=1	2.56E-27	
Q8IW35-2;Q8IW35	2|CEP97_HUMAN Isoform 2 of Centrosomal protein of 97 kDa OS=Homo sapiens GN=CEP97;>sp|Q8IW35|CEP97_HUMAN Centrosoma	6.81E-21	
Q8IW35;Q8IW35-2	CEP97_HUMAN Centrosomal protein of 97 kDa OS=Homo sapiens GN=CEP97 PE=1 SV=1;>sp|Q8IW35-2|CEP97_HUMAN Isoform 2 of	6.61E-09	
Q8IW45-2;Q8IW45;B4DK	2|CARKD_HUMAN Isoform 2 of Carbohydrate kinase domain-containing protein OS=Homo sapiens GN=CARKD;>sp|Q8IW45|CARKD_H	2.56E-23	
Q8IWA0	WDR75_HUMAN WD repeat-containing protein 75 OS=Homo sapiens GN=WDR75 PE=1 SV=1		
Q8IWA5	CTL2_HUMAN Choline transporter-like protein 2 OS=Homo sapiens GN=SLC44A2 PE=1 SV=3		126.28
Q8IWA5-2	CTL2_HUMAN Isoform 2 of Choline transporter-like protein 2 OS=Homo sapiens GN=SLC44A2		126.28
Q8IWA5-2;Q8IWA5;Q8IW	2|CTL2_HUMAN Isoform 2 of Choline transporter-like protein 2 OS=Homo sapiens GN=SLC44A2;>sp|Q8IWA5|CTL2_HUMAN Choline tı	3.74E-12	
Q8IWA5;Q8IWA5-2;B3KN	CTL2_HUMAN Choline transporter-like protein 2 OS=Homo sapiens GN=SLC44A2 PE=1 SV=3;>sp|Q8IWA5-2|CTL2_HUMAN Isoform 2	8.31E-08	
Q8IWA6	CCD60_HUMAN Coiled-coil domain-containing protein 60 OS=Homo sapiens GN=CCDC60 PE=2 SV=2		
Q8IWB7	WDFY1_HUMAN WD repeat and FYVE domain-containing protein 1 OS=Homo sapiens GN=WDFY1 PE=1 SV=1	5.39E-22	
Q8IWC1	MA7D3_HUMAN MAP7 domain-containing protein 3 OS=Homo sapiens GN=MAP7D3 PE=1 SV=2		102.68
Q8IWC1-2	MA7D3_HUMAN Isoform 2 of MAP7 domain-containing protein 3 OS=Homo sapiens GN=MAP7D3		102.68
Q8IWC1-3	MA7D3_HUMAN Isoform 3 of MAP7 domain-containing protein 3 OS=Homo sapiens GN=MAP7D3		102.68
Q8IWC1;B4DWD2;Q8IWC	MA7D3_HUMAN MAP7 domain-containing protein 3 OS=Homo sapiens GN=MAP7D3 PE=1 SV=2;>tr|B4DWD2|B4DWD2_HUMAN cDNA	1.20E-08	
Q8IWE2	NXP20_HUMAN Protein NOXP20 OS=Homo sapiens GN=FAM114A1 PE=1 SV=2	1.45E-299	
Q8IWE4	DCNL3_HUMAN DCN1-like protein 3 OS=Homo sapiens GN=DCUN1D3 PE=2 SV=1		42.28
Q8IWJ2	GCC2_HUMAN GRIP and coiled-coil domain-containing protein 2 OS=Homo sapiens GN=GCC2 PE=1 SV=4		
Q8IWJ2-3	GCC2_HUMAN Isoform 2 of GRIP and coiled-coil domain-containing protein 2 OS=Homo sapiens GN=GCC2		
Q8IWJ2;D3DX70;B9EK47;	GCC2_HUMAN GRIP and coiled-coil domain-containing protein 2 OS=Homo sapiens GN=GCC2 PE=1 SV=4;>tr|D3DX70|D3DX70_HUM	4.54E-08	
Q8IWK6	GP125_HUMAN Probable G-protein coupled receptor 125 OS=Homo sapiens GN=GPR125 PE=1 SV=2		
Q8IWK6-3	GP125_HUMAN Isoform 3 of Probable G-protein coupled receptor 125 OS=Homo sapiens GN=GPR125		
Q8IWQ3-4	BRSK2_HUMAN Isoform 4 of BR serine/threonine-protein kinase 2 OS=Homo sapiens GN=BRSK2		17.06
Q8IWR1	TRI59_HUMAN Tripartite motif-containing protein 59 OS=Homo sapiens GN=TRIM59 PE=2 SV=1		23.69
Q8IWS0	PHF6_HUMAN PHD finger protein 6 OS=Homo sapiens GN=PHF6 PE=1 SV=1		
Q8IWS0-2	PHF6_HUMAN Isoform 2 of PHD finger protein 6 OS=Homo sapiens GN=PHF6		
Q8IWS0;Q8IWS0-2	PHF6_HUMAN PHD finger protein 6 OS=Homo sapiens GN=PHF6 PE=1 SV=1;>sp|Q8IWS0-2|PHF6_HUMAN Isoform 2 of PHD finger pr	2.81E-06	
Q8IWT6	LRC8A_HUMAN Leucine-rich repeat-containing protein 8A OS=Homo sapiens GN=LRRC8A PE=1 SV=1		31.46
Q8IWU9	TPH2_HUMAN Tryptophan 5-hydroxylase 2 OS=Homo sapiens GN=TPH2 PE=1 SV=1		
Q8IWU9-2	TPH2_HUMAN Isoform b of Tryptophan 5-hydroxylase 2 OS=Homo sapiens GN=TPH2		
Q8IWV8	UBR2_HUMAN E3 ubiquitin-protein ligase UBR2 OS=Homo sapiens GN=UBR2 PE=1 SV=1		17.27
Q8IWV8-2	UBR2_HUMAN Isoform 2 of E3 ubiquitin-protein ligase UBR2 OS=Homo sapiens GN=UBR2		
Q8IWV8-3	UBR2_HUMAN Isoform 3 of E3 ubiquitin-protein ligase UBR2 OS=Homo sapiens GN=UBR2		

Q8IWV8-4	UBR2_HUMAN Isoform 4 of E3 ubiquitin-protein ligase UBR2 OS=Homo sapiens GN=UBR2		17.27
Q8IWV8;Q8IWV8-4	UBR2_HUMAN E3 ubiquitin-protein ligase UBR2 OS=Homo sapiens GN=UBR2 PE=1 SV=1;>sp|Q8IWV8-4|UBR2_HUMAN Isoform 4 of	5.25E-09	
Q8IWW6;Q8IWW6-2;Q8IV	RHG12_HUMAN Rho GTPase-activating protein 12 OS=Homo sapiens GN=ARHGAP12 PE=1 SV=1;>sp|Q8IWW6-2|RHG12_HUMAN Iso	3.23E-12	
Q8IWX8	CHERP_HUMAN Calcium homeostasis endoplasmic reticulum protein OS=Homo sapiens GN=CHERP PE=1 SV=3		
Q8IWY4	SCUB1_HUMAN Signal peptide, CUB and EGF-like domain-containing protein 1 OS=Homo sapiens GN=SCUBE1 PE=1 SV=3		
Q8IWZ3-4;Q8IWZ3	4|ANKH1_HUMAN Isoform 4 of Ankyrin repeat and KH domain-containing protein 1 OS=Homo sapiens GN=ANKHD1;>sp|Q8IWZ3|ANK	6.58E-21	
Q8IX01	SUGP2_HUMAN SURP and G-patch domain-containing protein 2 OS=Homo sapiens GN=SUGP2 PE=1 SV=2		
Q8IX01-3	SUGP2_HUMAN Isoform 3 of SURP and G-patch domain-containing protein 2 OS=Homo sapiens GN=SUGP2		
Q8IX01-4	SUGP2_HUMAN Isoform 4 of SURP and G-patch domain-containing protein 2 OS=Homo sapiens GN=SUGP2		
Q8IX01;Q8IX01-3;Q8IX01	SUGP2_HUMAN SURP and G-patch domain-containing protein 2 OS=Homo sapiens GN=SUGP2 PE=1 SV=2;>sp|Q8IX01-3|SUGP2_HU	1.91E-14	
Q8IX12	CCAR1_HUMAN Cell division cycle and apoptosis regulator protein 1 OS=Homo sapiens GN=CCAR1 PE=1 SV=2		225.59
Q8IX12-2	CCAR1_HUMAN Isoform 2 of Cell division cycle and apoptosis regulator protein 1 OS=Homo sapiens GN=CCAR1		225.59
Q8IX12;Q8IX12-2	CCAR1_HUMAN Cell division cycle and apoptosis regulator protein 1 OS=Homo sapiens GN=CCAR1 PE=1 SV=2;>sp|Q8IX12-2|CCAR1_	1.03E-48	
Q8IX18	DHX40_HUMAN Probable ATP-dependent RNA helicase DHX40 OS=Homo sapiens GN=DHX40 PE=1 SV=2		35.68
Q8IX18-2	DHX40_HUMAN Isoform 2 of Probable ATP-dependent RNA helicase DHX40 OS=Homo sapiens GN=DHX40		35.68
Q8IX18-3	DHX40_HUMAN Isoform 3 of Probable ATP-dependent RNA helicase DHX40 OS=Homo sapiens GN=DHX40		36.32
Q8IX21	F178A_HUMAN Protein FAM178A OS=Homo sapiens GN=FAM178A PE=1 SV=2		
Q8IX90	SKA3_HUMAN Spindle and kinetochore-associated protein 3 OS=Homo sapiens GN=SKA3 PE=1 SV=2		22.86
Q8IX90-3	SKA3_HUMAN Isoform 3 of Spindle and kinetochore-associated protein 3 OS=Homo sapiens GN=SKA3		22.86
Q8IXB1	DJC10_HUMAN DnaJ homolog subfamily C member 10 OS=Homo sapiens GN=DNAJC10 PE=1 SV=2		108.12
Q8IXB1-2	DJC10_HUMAN Isoform 2 of DnaJ homolog subfamily C member 10 OS=Homo sapiens GN=DNAJC10		108.12
Q8IXB1;B3KNK5;Q8IXB1-	DJC10_HUMAN DnaJ homolog subfamily C member 10 OS=Homo sapiens GN=DNAJC10 PE=1 SV=2;>tr|B3KNK5|B3KNK5_HUMAN cD	4.20E-25	
Q8IXF0-3	NPAS3_HUMAN Isoform 3 of Neuronal PAS domain-containing protein 3 OS=Homo sapiens GN=NPAS3		
Q8IXH7	NELFD_HUMAN Negative elongation factor C/D OS=Homo sapiens GN=TH1L PE=1 SV=2		54.14
Q8IXH7-3	NELFD_HUMAN Isoform 3 of Negative elongation factor C/D OS=Homo sapiens GN=TH1L		
Q8IXH7-4	NELFD_HUMAN Isoform NELF-D of Negative elongation factor C/D OS=Homo sapiens GN=TH1L		54.14
Q8IXH7;Q8IXH7-4	NELFD_HUMAN Negative elongation factor C/D OS=Homo sapiens GN=TH1L PE=1 SV=2;>sp|Q8IXH7-4|NELFD_HUMAN Isoform NELF	2.63E-14	
Q8IXI1	MIRO2_HUMAN Mitochondrial Rho GTPase 2 OS=Homo sapiens GN=RHOT2 PE=1 SV=2		46.15
Q8IXJ6;Q8IXJ6-4;A7VJH3;	SIRT2_HUMAN NAD-dependent deacetylase sirtuin-2 OS=Homo sapiens GN=SIRT2 PE=1 SV=2;>sp|Q8IXJ6-4|SIRT2_HUMAN Isoform	6.39E-09	
Q8IXK0	PHC2_HUMAN Polyhomeotic-like protein 2 OS=Homo sapiens GN=PHC2 PE=1 SV=1		87.89
Q8IXK0-2	PHC2_HUMAN Isoform 2 of Polyhomeotic-like protein 2 OS=Homo sapiens GN=PHC2		87.89
Q8IXK0-3	PHC2_HUMAN Isoform 3 of Polyhomeotic-like protein 2 OS=Homo sapiens GN=PHC2		52.52
Q8IXK0-4	PHC2_HUMAN Isoform 4 of Polyhomeotic-like protein 2 OS=Homo sapiens GN=PHC2		87.89
Q8IXK0-5	PHC2_HUMAN Isoform 5 of Polyhomeotic-like protein 2 OS=Homo sapiens GN=PHC2		87.89
Q8IXL7	MSRB3_HUMAN Methionine-R-sulfoxide reductase B3, mitochondrial OS=Homo sapiens GN=MSRB3 PE=1 SV=1	4.75E-12	
Q8IXL7-2	2|MSRB3_HUMAN Isoform 2 of Methionine-R-sulfoxide reductase B3 OS=Homo sapiens GN=MSRB3	1.66E-06	
Q8IXM2	BAP18_HUMAN Chromatin complexes subunit BAP18 OS=Homo sapiens GN=BAP18 PE=1 SV=1		235.93
Q8IXM3	RM41_HUMAN 39S ribosomal protein L41, mitochondrial OS=Homo sapiens GN=MRPL41 PE=1 SV=1		149.76
Q8IXM6	NRM_HUMAN Nurim OS=Homo sapiens GN=NRM PE=1 SV=1		
Q8IXM6-2	NRM_HUMAN Isoform 2 of Nurim OS=Homo sapiens GN=NRM		
Q8IXN7	RIMKA_HUMAN N-acetylaspartyl-glutamate synthetase A OS=Homo sapiens GN=RIMKLA PE=2 SV=2		
Q8IXQ6;Q8IXQ6-2	PARP9_HUMAN Poly [ADP-ribose] polymerase 9 OS=Homo sapiens GN=PARP9 PE=1 SV=2;>sp|Q8IXQ6-2|PARP9_HUMAN Isoform 2 o	9.69E-28	
Q8IXS2	CCD65_HUMAN Coiled-coil domain-containing protein 65 OS=Homo sapiens GN=CCDC65 PE=1 SV=2		
Q8IXS2-2	CCD65_HUMAN Isoform 2 of Coiled-coil domain-containing protein 65 OS=Homo sapiens GN=CCDC65		
Q8IXT5	RB12B_HUMAN RNA-binding protein 12B OS=Homo sapiens GN=RBM12B PE=1 SV=2		
Q8IY17	PLPL6_HUMAN Neuropathy target esterase OS=Homo sapiens GN=PNPLA6 PE=1 SV=2		
Q8IY17-2	PLPL6_HUMAN Isoform 2 of Neuropathy target esterase OS=Homo sapiens GN=PNPLA6		
Q8IY17-3	PLPL6_HUMAN Isoform 3 of Neuropathy target esterase OS=Homo sapiens GN=PNPLA6		
Q8IY17;Q8IY17-3;Q8IY17	PLPL6_HUMAN Neuropathy target esterase OS=Homo sapiens GN=PNPLA6 PE=1 SV=2;>sp|Q8IY17-3|PLPL6_HUMAN Isoform 3 of Neu	1.04E-32	
Q8IY21	DDX60_HUMAN Probable ATP-dependent RNA helicase DDX60 OS=Homo sapiens GN=DDX60 PE=2 SV=3		33.06
Q8IY33-2;Q8IY33;Q8IY33	2|MILK2_HUMAN Isoform 2 of MICAL-like protein 2 OS=Homo sapiens GN=MICALL2;>sp|Q8IY33|MILK2_HUMAN MICAL-like protein 2	8.27E-11	
Q8IY37	DHX37_HUMAN Probable ATP-dependent RNA helicase DHX37 OS=Homo sapiens GN=DHX37 PE=1 SV=1		
Q8IY63;Q8IY63-2	AMOL1_HUMAN Angiomotin-like protein 1 OS=Homo sapiens GN=AMOTL1 PE=1 SV=1;>sp|Q8IY63-2|AMOL1_HUMAN Isoform 2 of An	3.54E-10	
Q8IY67	RAVR1_HUMAN Ribonucleoprotein PTB-binding 1 OS=Homo sapiens GN=RAVER1 PE=1 SV=1		77.07
Q8IY67-2	RAVR1_HUMAN Isoform 2 of Ribonucleoprotein PTB-binding 1 OS=Homo sapiens GN=RAVER1		123.96
Q8IY67-2;Q8IY67	2|RAVR1_HUMAN Isoform 2 of Ribonucleoprotein PTB-binding 1 OS=Homo sapiens GN=RAVER1;>sp|Q8IY67|RAVR1_HUMAN Ribonucl	4.96E-95	
Q8IY67-3	RAVR1_HUMAN Isoform 3 of Ribonucleoprotein PTB-binding 1 OS=Homo sapiens GN=RAVER1		
Q8IY81	RRMJ3_HUMAN Putative rRNA methyltransferase 3 OS=Homo sapiens GN=FTSJ3 PE=1 SV=2		126.87
Q8IY92	SLX4_HUMAN Structure-specific endonuclease subunit SLX4 OS=Homo sapiens GN=SLX4 PE=1 SV=3		
Q8IY92-2	SLX4_HUMAN Isoform 2 of Structure-specific endonuclease subunit SLX4 OS=Homo sapiens GN=SLX4		
Q8IYA2	C144C_HUMAN Coiled-coil domain-containing protein 144C OS=Homo sapiens GN=CCDC144C PE=2 SV=3		

Q8IYA6	CKP2L_HUMAN Cytoskeleton-associated protein 2-like OS=Homo sapiens GN=CKAP2L PE=1 SV=4		
Q8IYA6-2	CKP2L_HUMAN Isoform 2 of Cytoskeleton-associated protein 2-like OS=Homo sapiens GN=CKAP2L		
Q8IYB3	SRRM1_HUMAN Serine/arginine repetitive matrix protein 1 OS=Homo sapiens GN=SRRM1 PE=1 SV=2		200.06
Q8IYB3-2	SRRM1_HUMAN Isoform 2 of Serine/arginine repetitive matrix protein 1 OS=Homo sapiens GN=SRRM1		200.06
Q8IYB3;Q8IYB3-2	SRRM1_HUMAN Serine/arginine repetitive matrix protein 1 OS=Homo sapiens GN=SRRM1 PE=1 SV=2;>sp|Q8IYB3-2|SRRM1_HUMAN	1.69E-59	
Q8IYB7	DI3L2_HUMAN DIS3-like exonuclease 2 OS=Homo sapiens GN=DIS3L2 PE=1 SV=4	1.63E-72	
Q8IYB8	SUV3_HUMAN ATP-dependent RNA helicase SUPV3L1, mitochondrial OS=Homo sapiens GN=SUPV3L1 PE=1 SV=1	4.95E-05	
Q8IYD1	ERF3B_HUMAN Eukaryotic peptide chain release factor GTP-binding subunit ERF3B OS=Homo sapiens GN=GSPT2 PE=1 SV=2	3.57E-30	
Q8IYE0	CC146_HUMAN Coiled-coil domain-containing protein 146 OS=Homo sapiens GN=CCDC146 PE=2 SV=2		63.45
Q8IYE0-2	CC146_HUMAN Isoform 2 of Coiled-coil domain-containing protein 146 OS=Homo sapiens GN=CCDC146		46.45
Q8IYE1	CCD13_HUMAN Coiled-coil domain-containing protein 13 OS=Homo sapiens GN=CCDC13 PE=2 SV=2		
Q8IYF3	TEX11_HUMAN Testis-expressed sequence 11 protein OS=Homo sapiens GN=TEX11 PE=1 SV=3		28.44
Q8IYF3-2	TEX11_HUMAN Isoform 2 of Testis-expressed sequence 11 protein OS=Homo sapiens GN=TEX11		28.25
Q8IYF3-3	TEX11_HUMAN Isoform 3 of Testis-expressed sequence 11 protein OS=Homo sapiens GN=TEX11		28.44
Q8IYI6	EXOC8_HUMAN Exocyst complex component 8 OS=Homo sapiens GN=EXOC8 PE=1 SV=2		86.15
Q8IYJ1	CPNE9_HUMAN Copine-9 OS=Homo sapiens GN=CPNE9 PE=1 SV=3		
Q8IYJ1-2	CPNE9_HUMAN Isoform 2 of Copine-9 OS=Homo sapiens GN=CPNE9		
Q8IYK4	GT252_HUMAN Procollagen galactosyltransferase 2 OS=Homo sapiens GN=GLT25D2 PE=1 SV=1		
Q8IYM9;Q8IYM9-2	TRI22_HUMAN E3 ubiquitin-protein ligase TRIM22 OS=Homo sapiens GN=TRIM22 PE=1 SV=1;>sp|Q8IYM9-2|TRI22_HUMAN Isoform	5.46E-26	
Q8IYS0	GRM1C_HUMAN GRAM domain-containing protein 1C OS=Homo sapiens GN=GRAMD1C PE=2 SV=2		
Q8IYS0-2	GRM1C_HUMAN Isoform 2 of GRAM domain-containing protein 1C OS=Homo sapiens GN=GRAMD1C		
Q8IYS1	P20D2_HUMAN Peptidase M20 domain-containing protein 2 OS=Homo sapiens GN=PM20D2 PE=1 SV=2	1.88E-10	
Q8IYT4	KATL2_HUMAN Katanin p60 ATPase-containing subunit A-like 2 OS=Homo sapiens GN=KATNAL2 PE=2 SV=3		77.63
Q8IYT4-2	KATL2_HUMAN Isoform 2 of Katanin p60 ATPase-containing subunit A-like 2 OS=Homo sapiens GN=KATNAL2		77.63
Q8IYT4;Q8IYT4-2	KATL2_HUMAN Katanin p60 ATPase-containing subunit A-like 2 OS=Homo sapiens GN=KATNAL2 PE=2 SV=3;>sp|Q8IYT4-2|KATL2_HU	2.86E-09	
Q8IYT8	ULK2_HUMAN Serine/threonine-protein kinase ULK2 OS=Homo sapiens GN=ULK2 PE=1 SV=3		25.57
Q8IYU4	UBQLN_HUMAN Ubiquilin-like protein OS=Homo sapiens GN=UBQLNL PE=2 SV=3		26.12
Q8IYU4-2	UBQLN_HUMAN Isoform 2 of Ubiquilin-like protein OS=Homo sapiens GN=UBQLNL		26.12
Q8IYU8	EFHA1_HUMAN EF-hand domain-containing family member A1 OS=Homo sapiens GN=EFHA1 PE=1 SV=2	5.06E-11	
Q8IZ40	RCOR2_HUMAN REST corepressor 2 OS=Homo sapiens GN=RCOR2 PE=2 SV=2		24.43
Q8IZ83	A16A1_HUMAN Aldehyde dehydrogenase family 16 member A1 OS=Homo sapiens GN=ALDH16A1 PE=1 SV=2	3.14E-50	
Q8IZC4	RTKN2_HUMAN Rhotekin-2 OS=Homo sapiens GN=RTKN2 PE=2 SV=1		
Q8IZC4-2	RTKN2_HUMAN Isoform 2 of Rhotekin-2 OS=Homo sapiens GN=RTKN2		
Q8IZC4-3	RTKN2_HUMAN Isoform 3 of Rhotekin-2 OS=Homo sapiens GN=RTKN2		
Q8IZC6	CORA1_HUMAN Collagen alpha-1(XXVII) chain OS=Homo sapiens GN=COL27A1 PE=1 SV=1		
Q8IZC6-3	CORA1_HUMAN Isoform 3 of Collagen alpha-1(XXVII) chain OS=Homo sapiens GN=COL27A1		
Q8IZE3;Q8IZE3-2	PACE1_HUMAN Protein-associating with the carboxyl-terminal domain of ezrin OS=Homo sapiens GN=SCYL3 PE=1 SV=3;>sp|Q8IZE3	5.30E-13	
Q8IZF6	GP112_HUMAN Probable G-protein coupled receptor 112 OS=Homo sapiens GN=GPR112 PE=2 SV=2		45.88
Q8IZF6-2	GP112_HUMAN Isoform 2 of Probable G-protein coupled receptor 112 OS=Homo sapiens GN=GPR112		44.82
Q8IZF6-3	GP112_HUMAN Isoform 3 of Probable G-protein coupled receptor 112 OS=Homo sapiens GN=GPR112		5.3
Q8IZH2;Q8IZH2-2	XRN1_HUMAN 5-3 exoribonuclease 1 OS=Homo sapiens GN=XRN1 PE=1 SV=1;>sp|Q8IZH2-2|XRN1_HUMAN Isoform 2 of 5-3 exoribo	7.37E-06	
Q8IZJ1	UNC5B_HUMAN Netrin receptor UNC5B OS=Homo sapiens GN=UNC5B PE=1 SV=2		
Q8IZJ1-2	UNC5B_HUMAN Isoform 2 of Netrin receptor UNC5B OS=Homo sapiens GN=UNC5B		
Q8IZL8	PELP1_HUMAN Proline-, glutamic acid- and leucine-rich protein 1 OS=Homo sapiens GN=PELP1 PE=1 SV=2		
Q8IZL8-2	PELP1_HUMAN Isoform 2 of Proline-, glutamic acid- and leucine-rich protein 1 OS=Homo sapiens GN=PELP1		
Q8IZL8;Q8IZL8-2	PELP1_HUMAN Proline-, glutamic acid- and leucine-rich protein 1 OS=Homo sapiens GN=PELP1 PE=1 SV=2;>sp|Q8IZL8-2|PELP1_HUI	8.08E-74	
Q8IZL9	CDK20_HUMAN Cyclin-dependent kinase 20 OS=Homo sapiens GN=CDK20 PE=1 SV=1		54.71
Q8IZL9-2	CDK20_HUMAN Isoform 2 of Cyclin-dependent kinase 20 OS=Homo sapiens GN=CDK20		59.16
Q8IZL9-3	CDK20_HUMAN Isoform 3 of Cyclin-dependent kinase 20 OS=Homo sapiens GN=CDK20		61.76
Q8IZN3	ZDH14_HUMAN Probable palmitoyltransferase ZDHHC14 OS=Homo sapiens GN=ZDHHC14 PE=1 SV=1		21.77
Q8IZN3-2	ZDH14_HUMAN Isoform 2 of Probable palmitoyltransferase ZDHHC14 OS=Homo sapiens GN=ZDHHC14		21.77
Q8IZP0;Q8IZP0-9;Q8IZP0	ABI1_HUMAN Abl interactor 1 OS=Homo sapiens GN=ABI1 PE=1 SV=4;>sp|Q8IZP0-9|ABI1_HUMAN Isoform 9 of Abl interactor 1 OS=	2.71E-26	
Q8IZP2	ST134_HUMAN Putative protein FAM10A4 OS=Homo sapiens GN=ST13P4 PE=5 SV=1		123.18
Q8IZQ5	SELH_HUMAN Selenoprotein H OS=Homo sapiens GN=SELH PE=1 SV=2		129.83
Q8IZT6	ASPM_HUMAN Abnormal spindle-like microcephaly-associated protein OS=Homo sapiens GN=ASPM PE=1 SV=2		
Q8IZT6-2	ASPM_HUMAN Isoform 2 of Abnormal spindle-like microcephaly-associated protein OS=Homo sapiens GN=ASPM		
Q8IZU1	FAM9A_HUMAN Protein FAM9A OS=Homo sapiens GN=FAM9A PE=2 SV=1		
Q8IZX4	TAF1L_HUMAN Transcription initiation factor TFIID subunit 1-like OS=Homo sapiens GN=TAF1L PE=1 SV=1		
Q8MH63	LAT1N_HUMAN Putative L-type amino acid transporter 1-like protein MLAS OS=Homo sapiens GN=SLC7A5P1 PE=5 SV=1		
Q8N0S6	CENPL_HUMAN Centromere protein L OS=Homo sapiens GN=CENPL PE=1 SV=2		22.51
Q8N0S6-2	CENPL_HUMAN Isoform 2 of Centromere protein L OS=Homo sapiens GN=CENPL		22.51

Q8N0T1	CH059_HUMAN Uncharacterized protein C8orf59 OS=Homo sapiens GN=C8orf59 PE=1 SV=2		49.09
Q8N0U4	F185A_HUMAN Protein FAM185A OS=Homo sapiens GN=FAM185A PE=2 SV=3		
Q8N0U4-3	F185A_HUMAN Isoform 3 of Protein FAM185A OS=Homo sapiens GN=FAM185A		
Q8N0U8	VKORL_HUMAN Vitamin K epoxide reductase complex subunit 1-like protein 1 OS=Homo sapiens GN=VKORC1L1 PE=1 SV=2		35
Q8N0V4	LGI2_HUMAN Leucine-rich repeat LGI family member 2 OS=Homo sapiens GN=LGI2 PE=1 SV=1		27.98
Q8N0X4;Q8N0X4-2	CLYBL_HUMAN Citrate lyase subunit beta-like protein, mitochondrial OS=Homo sapiens GN=CLYBL PE=2 SV=2;>sp|Q8N0X4-2|CLYBL_	0.00024599	
Q8N0X7	SPG20_HUMAN Spartin OS=Homo sapiens GN=SPG20 PE=1 SV=1	2.30E-14	
Q8N0Y7	PGAM4_HUMAN Probable phosphoglycerate mutase 4 OS=Homo sapiens GN=PGAM4 PE=1 SV=1		149.71
Q8N0Z2	ABRA_HUMAN Actin-binding Rho-activating protein OS=Homo sapiens GN=ABRA PE=2 SV=1		
Q8N122	RPTOR_HUMAN Regulatory-associated protein of mTOR OS=Homo sapiens GN=RPTOR PE=1 SV=1	6.93E-09	
Q8N126-2;Q8N126;Q8N12	2|CADM3_HUMAN Isoform 2 of Cell adhesion molecule 3 OS=Homo sapiens GN=CADM3;>sp|Q8N126|CADM3_HUMAN Cell adhesion m	5.04E-19	
Q8N129	CNPY4_HUMAN Protein canopy homolog 4 OS=Homo sapiens GN=CNPY4 PE=2 SV=1		55.42
Q8N131	PORIM_HUMAN Porimin OS=Homo sapiens GN=TMEM123 PE=1 SV=1		
Q8N131-2	PORIM_HUMAN Isoform 2 of Porimin OS=Homo sapiens GN=TMEM123		
Q8N138	ORML3_HUMAN ORM1-like protein 3 OS=Homo sapiens GN=ORMDL3 PE=1 SV=1		
Q8N138-4	ORML3_HUMAN Isoform 2 of ORM1-like protein 3 OS=Homo sapiens GN=ORMDL3		
Q8N138;Q8N138-4	ORML3_HUMAN ORM1-like protein 3 OS=Homo sapiens GN=ORMDL3 PE=1 SV=1;>sp|Q8N138-4|ORML3_HUMAN Isoform 2 of ORM1-	1.94E-12	
Q8N140	EID3_HUMAN EP300-interacting inhibitor of differentiation 3 OS=Homo sapiens GN=EID3 PE=1 SV=1		20.08
Q8N163	K1967_HUMAN Protein KIAA1967 OS=Homo sapiens GN=KIAA1967 PE=1 SV=2		403.61
Q8N163-2	K1967_HUMAN Isoform 2 of Protein KIAA1967 OS=Homo sapiens GN=KIAA1967		403.61
Q8N163;Q8N163-2	K1967_HUMAN Protein KIAA1967 OS=Homo sapiens GN=KIAA1967 PE=1 SV=2;>sp|Q8N163-2|K1967_HUMAN Isoform 2 of Protein K	4.28E-165	
Q8N183	MIMIT_HUMAN Mimitin, mitochondrial OS=Homo sapiens GN=NDUFAF2 PE=1 SV=1		184.66
Q8N1A0	KT222_HUMAN Keratin-like protein KRT222 OS=Homo sapiens GN=KRT222 PE=2 SV=1		
Q8N1A0-2	KT222_HUMAN Isoform 2 of Keratin-like protein KRT222 OS=Homo sapiens GN=KRT222		
Q8N1B4	VPS52_HUMAN Vacuolar protein sorting-associated protein 52 homolog OS=Homo sapiens GN=VPS52 PE=1 SV=1		39.05
Q8N1F1	NC188_HUMAN Putative uncharacterized protein encoded by NCRNA00188, mitochondrial OS=Homo sapiens GN=NCRNA00188 PE=5 SV=1		
Q8N1F7	NUP93_HUMAN Nuclear pore complex protein Nup93 OS=Homo sapiens GN=NUP93 PE=1 SV=2		
Q8N1G2	MTR1_HUMAN Cap-specific mRNA (nucleoside-2'-O-)-methyltransferase 1 OS=Homo sapiens GN=FTSJD2 PE=1 SV=1		126.42
Q8N1G4	LRC47_HUMAN Leucine-rich repeat-containing protein 47 OS=Homo sapiens GN=LRRC47 PE=1 SV=1		164.33
Q8N1N4	K2C78_HUMAN Keratin, type II cytoskeletal 78 OS=Homo sapiens GN=KRT78 PE=1 SV=2		76.32
Q8N1N4-2	K2C78_HUMAN Isoform 2 of Keratin, type II cytoskeletal 78 OS=Homo sapiens GN=KRT78		76.32
Q8N1Q1	CAH13_HUMAN Carbonic anhydrase 13 OS=Homo sapiens GN=CA13 PE=1 SV=1	7.96E-11	
Q8N1S5;Q8N1S5-2	S39AB_HUMAN Zinc transporter ZIP11 OS=Homo sapiens GN=SLC39A11 PE=2 SV=3;>sp|Q8N1S5-2|S39AB_HUMAN Isoform 2 of Zin	1.51E-18	
Q8N1W1;Q8N1W1-3;Q8N:	RGNEF_HUMAN Rho-guanine nucleotide exchange factor OS=Homo sapiens GN=RGNEF PE=1 SV=3;>sp|Q8N1W1-3|RGNEF_HUMAN I	2.70E-10	
Q8N1Y9	YI025_HUMAN Putative uncharacterized protein FLJ37218 OS=Homo sapiens PE=5 SV=2		49.84
Q8N201	INT1_HUMAN Integrator complex subunit 1 OS=Homo sapiens GN=INTS1 PE=1 SV=2		
Q8N257	H2B3B_HUMAN Histone H2B type 3-B OS=Homo sapiens GN=HIST3H2BB PE=1 SV=3		715.52
Q8N264	RHG24_HUMAN Rho GTPase-activating protein 24 OS=Homo sapiens GN=ARHGAP24 PE=1 SV=2		
Q8N264-2	RHG24_HUMAN Isoform 2 of Rho GTPase-activating protein 24 OS=Homo sapiens GN=ARHGAP24		
Q8N264-3	RHG24_HUMAN Isoform 3 of Rho GTPase-activating protein 24 OS=Homo sapiens GN=ARHGAP24		
Q8N283	ANR35_HUMAN Ankyrin repeat domain-containing protein 35 OS=Homo sapiens GN=ANKRD35 PE=2 SV=2		
Q8N2F6	ARM10_HUMAN Armadillo repeat-containing protein 10 OS=Homo sapiens GN=ARMC10 PE=1 SV=1		34.37
Q8N2F6-2	ARM10_HUMAN Isoform 2 of Armadillo repeat-containing protein 10 OS=Homo sapiens GN=ARMC10		34.37
Q8N2F6-2;Q8N2F6-4;Q8N	2|ARM10_HUMAN Isoform 2 of Armadillo repeat-containing protein 10 OS=Homo sapiens GN=ARMC10;>sp|Q8N2F6-4|ARM10_HUMAN	2.43E-77	
Q8N2F6-3	ARM10_HUMAN Isoform 3 of Armadillo repeat-containing protein 10 OS=Homo sapiens GN=ARMC10		34.37
Q8N2F6-4	ARM10_HUMAN Isoform 4 of Armadillo repeat-containing protein 10 OS=Homo sapiens GN=ARMC10		34.37
Q8N2F6;Q8N2F6-2;Q8N2F	ARM10_HUMAN Armadillo repeat-containing protein 10 OS=Homo sapiens GN=ARMC10 PE=1 SV=1;>sp|Q8N2F6-2|ARM10_HUMAN Is	4.49E-18	
Q8N2G8	GHDC_HUMAN GH3 domain-containing protein OS=Homo sapiens GN=GHDC PE=1 SV=2		
Q8N2G8-2	GHDC_HUMAN Isoform 2 of GH3 domain-containing protein OS=Homo sapiens GN=GHDC		
Q8N2G8;B4DQS4;B3KVB0	GHDC_HUMAN GH3 domain-containing protein OS=Homo sapiens GN=GHDC PE=1 SV=2;>tr|B4DQS4|B4DQS4_HUMAN cDNA FLJ593	1.94E-09	
Q8N2K0	ABD12_HUMAN Monoacylglycerol lipase ABHD12 OS=Homo sapiens GN=ABHD12 PE=2 SV=2		30.6
Q8N2K0-2	ABD12_HUMAN Isoform 2 of Monoacylglycerol lipase ABHD12 OS=Homo sapiens GN=ABHD12		30.6
Q8N2K0-2;Q8N2K0;Q8N2I	2|ABD12_HUMAN Isoform 2 of Monoacylglycerol lipase ABHD12 OS=Homo sapiens GN=ABHD12;>sp|Q8N2K0|ABD12_HUMAN Monoac	1.05E-17	
Q8N2K0-3	ABD12_HUMAN Isoform 3 of Monoacylglycerol lipase ABHD12 OS=Homo sapiens GN=ABHD12		30.6
Q8N2N9	AN36B_HUMAN Ankyrin repeat domain-containing protein 36B OS=Homo sapiens GN=ANKRD36B PE=2 SV=4		
Q8N2N9-2	AN36B_HUMAN Isoform 2 of Ankyrin repeat domain-containing protein 36B OS=Homo sapiens GN=ANKRD36B		
Q8N2N9-3	AN36B_HUMAN Isoform 3 of Ankyrin repeat domain-containing protein 36B OS=Homo sapiens GN=ANKRD36B		
Q8N2N9-4	AN36B_HUMAN Isoform 4 of Ankyrin repeat domain-containing protein 36B OS=Homo sapiens GN=ANKRD36B		
Q8N2S1-3	LTBP4_HUMAN Isoform 3 of Latent-transforming growth factor beta-binding protein 4 OS=Homo sapiens GN=LTBP4		14.28
Q8N2Y8	RUSC2_HUMAN Iporin OS=Homo sapiens GN=RUSC2 PE=1 SV=3		
Q8N335	GPD1L_HUMAN Glycerol-3-phosphate dehydrogenase 1-like protein OS=Homo sapiens GN=GPD1L PE=1 SV=1	3.65E-115	

Q8N357	CB018_HUMAN Transmembrane protein C2orf18 OS=Homo sapiens GN=C2orf18 PE=1 SV=1		
Q8N371	KDM8_HUMAN Lysine-specific demethylase 8 OS=Homo sapiens GN=JMJD5 PE=1 SV=1		34.7
Q8N371-3	KDM8_HUMAN Isoform 3 of Lysine-specific demethylase 8 OS=Homo sapiens GN=JMJD5		34.7
Q8N392;Q8N392-2	RHG18_HUMAN Rho GTPase-activating protein 18 OS=Homo sapiens GN=ARHGAP18 PE=1 SV=3;>sp|Q8N392-2|RHG18_HUMAN Isof	2.14E-57	
Q8N394	TMTC2_HUMAN Transmembrane and TPR repeat-containing protein 2 OS=Homo sapiens GN=TMTC2 PE=2 SV=1		27.26
Q8N398	VW5B2_HUMAN von Willebrand factor A domain-containing protein 5B2 OS=Homo sapiens GN=VWA5B2 PE=1 SV=2		
Q8N3C0	HELC1_HUMAN Activating signal cointegrator 1 complex subunit 3 OS=Homo sapiens GN=ASCC3 PE=1 SV=3		
Q8N3D4	EH1L1_HUMAN EH domain-binding protein 1-like protein 1 OS=Homo sapiens GN=EHBP1L1 PE=1 SV=2	2.78E-09	
Q8N3F8	MILK1_HUMAN MICAL-like protein 1 OS=Homo sapiens GN=MICALL1 PE=1 SV=2		
Q8N3K9	CMYA5_HUMAN Cardiomyopathy-associated protein 5 OS=Homo sapiens GN=CMYA5 PE=1 SV=3		
Q8N3R9	MPP5_HUMAN MAGUK p55 subfamily member 5 OS=Homo sapiens GN=MPP5 PE=1 SV=3		
Q8N3R9-2	MPP5_HUMAN Isoform 2 of MAGUK p55 subfamily member 5 OS=Homo sapiens GN=MPP5		
Q8N3R9;Q8N3R9-2	MPP5_HUMAN MAGUK p55 subfamily member 5 OS=Homo sapiens GN=MPP5 PE=1 SV=3;>sp|Q8N3R9-2|MPP5_HUMAN Isoform 2 of	3.88E-28	
Q8N3T6	T132C_HUMAN Transmembrane protein 132C OS=Homo sapiens GN=TMEM132C PE=2 SV=3		23.75
Q8N3U4	STAG2_HUMAN Cohesin subunit SA-2 OS=Homo sapiens GN=STAG2 PE=1 SV=3		38.71
Q8N3U4-2	STAG2_HUMAN Isoform 2 of Cohesin subunit SA-2 OS=Homo sapiens GN=STAG2		38.71
Q8N3U4-2;Q8N3U4	2|STAG2_HUMAN Isoform 2 of Cohesin subunit SA-2 OS=Homo sapiens GN=STAG2;>sp|Q8N3U4|STAG2_HUMAN Cohesin subunit SA-	2.35E-39	
Q8N3V7	SYNPO_HUMAN Synaptopodin OS=Homo sapiens GN=SYNPO PE=1 SV=2		
Q8N3V7-2	SYNPO_HUMAN Isoform 2 of Synaptopodin OS=Homo sapiens GN=SYNPO		
Q8N3V7-3	SYNPO_HUMAN Isoform 3 of Synaptopodin OS=Homo sapiens GN=SYNPO		
Q8N3X1	FNBP4_HUMAN Formin-binding protein 4 OS=Homo sapiens GN=FNBP4 PE=1 SV=3		
Q8N3X1-2	FNBP4_HUMAN Isoform 2 of Formin-binding protein 4 OS=Homo sapiens GN=FNBP4		
Q8N3Z3	GTPB8_HUMAN GTP-binding protein 8 OS=Homo sapiens GN=GTPBP8 PE=2 SV=1		
Q8N3Z3-2	GTPB8_HUMAN Isoform 2 of GTP-binding protein 8 OS=Homo sapiens GN=GTPBP8		
Q8N414	PGBD5_HUMAN PiggyBac transposable element-derived protein 5 OS=Homo sapiens GN=PGBD5 PE=2 SV=3		
Q8N474	SFRP1_HUMAN Secreted frizzled-related protein 1 OS=Homo sapiens GN=SFRP1 PE=1 SV=1	1.55E-19	
Q8N490-2	PNKD_HUMAN Isoform 2 of Probable hydrolase PNKD OS=Homo sapiens GN=PNKD		44.82
Q8N4A0	GALT4_HUMAN Polypeptide N-acetylgalactosaminyltransferase 4 OS=Homo sapiens GN=GALNT4 PE=1 SV=2		
Q8N4C6	NIN_HUMAN Ninein OS=Homo sapiens GN=NIN PE=1 SV=4		64.42
Q8N4C6-10	NIN_HUMAN Isoform 3 of Ninein OS=Homo sapiens GN=NIN		64.42
Q8N4C6-2	NIN_HUMAN Isoform 2 of Ninein OS=Homo sapiens GN=NIN		64.42
Q8N4C6-4	NIN_HUMAN Isoform 4 of Ninein OS=Homo sapiens GN=NIN		55.89
Q8N4C6-5	NIN_HUMAN Isoform 5 of Ninein OS=Homo sapiens GN=NIN		64.42
Q8N4C6-6	NIN_HUMAN Isoform 6 of Ninein OS=Homo sapiens GN=NIN		49.4
Q8N4C6-7	NIN_HUMAN Isoform 7 of Ninein OS=Homo sapiens GN=NIN		64.42
Q8N4C6-9	NIN_HUMAN Isoform 8 of Ninein OS=Homo sapiens GN=NIN		64.42
Q8N4C8	MINK1_HUMAN Misshapen-like kinase 1 OS=Homo sapiens GN=MINK1 PE=1 SV=2		113.99
Q8N4C8-2	MINK1_HUMAN Isoform 1 of Misshapen-like kinase 1 OS=Homo sapiens GN=MINK1		113.99
Q8N4C8-3	MINK1_HUMAN Isoform 2 of Misshapen-like kinase 1 OS=Homo sapiens GN=MINK1		113.99
Q8N4C8;Q8N4C8-3;Q8N4	MINK1_HUMAN Misshapen-like kinase 1 OS=Homo sapiens GN=MINK1 PE=1 SV=2;>sp|Q8N4C8-3|MINK1_HUMAN Isoform 2 of Missh	3.76E-14	
Q8N4H5	TOM5_HUMAN Mitochondrial import receptor subunit TOM5 homolog OS=Homo sapiens GN=TOMM5 PE=1 SV=1		
Q8N4N8	KIF2B_HUMAN Kinesin-like protein KIF2B OS=Homo sapiens GN=KIF2B PE=1 SV=3		
Q8N4P3;Q8N4P3-2	MESH1_HUMAN Guanosine-3,5-bis(diphosphate) 3-pyrophosphohydrolase MESH1 OS=Homo sapiens GN=HDDC3 PE=1 SV=3;>sp|Q8N	1.07E-06	
Q8N4Q1	MIA40_HUMAN Mitochondrial intermembrane space import and assembly protein 40 OS=Homo sapiens GN=CHCHD4 PE=1 SV=1		65.05
Q8N4Q1-2	MIA40_HUMAN Isoform 2 of Mitochondrial intermembrane space import and assembly protein 40 OS=Homo sapiens GN=CHCHD4		65.05
Q8N4Q1-2;Q8N4Q1	2|MIA40_HUMAN Isoform 2 of Mitochondrial intermembrane space import and assembly protein 40 OS=Homo sapiens GN=CHCHD4;>	1.60E-46	
Q8N4V1	MMGT1_HUMAN Membrane magnesium transporter 1 OS=Homo sapiens GN=MMGT1 PE=1 SV=1		69.38
Q8N4V1-2	MMGT1_HUMAN Isoform 2 of Membrane magnesium transporter 1 OS=Homo sapiens GN=MMGT1		69.38
Q8N4V1-2;D3DWG7;Q8N4	2|MMGT1_HUMAN Isoform 2 of Membrane magnesium transporter 1 OS=Homo sapiens GN=MMGT1;>tr|D3DWG7|D3DWG7_HUMAN T	1.62E-31	
Q8N4W9	ZN808_HUMAN Zinc finger protein 808 OS=Homo sapiens GN=ZNF808 PE=2 SV=2		
Q8N4W9-2	ZN808_HUMAN Isoform 2 of Zinc finger protein 808 OS=Homo sapiens GN=ZNF808		
Q8N4X5	AF1L2_HUMAN Actin filament-associated protein 1-like 2 OS=Homo sapiens GN=AFAP1L2 PE=1 SV=1		
Q8N4X5-2	AF1L2_HUMAN Isoform 2 of Actin filament-associated protein 1-like 2 OS=Homo sapiens GN=AFAP1L2		
Q8N4X5-4	AF1L2_HUMAN Isoform 4 of Actin filament-associated protein 1-like 2 OS=Homo sapiens GN=AFAP1L2		
Q8N4X5-4;Q8N4X5;Q8N4	4|AF1L2_HUMAN Isoform 4 of Actin filament-associated protein 1-like 2 OS=Homo sapiens GN=AFAP1L2;>sp|Q8N4X5|AF1L2_HUMAN	7.94E-11	
Q8N511	TM199_HUMAN Transmembrane protein 199 OS=Homo sapiens GN=TMEM199 PE=1 SV=1		
Q8N543;Q8N543-2	OGFD1_HUMAN 2-oxoglutarate and iron-dependent oxygenase domain-containing protein 1 OS=Homo sapiens GN=OGFOD1 PE=1 SV	4.77E-19	
Q8N556	AFAP1_HUMAN Actin filament-associated protein 1 OS=Homo sapiens GN=AFAP1 PE=1 SV=2		115.99
Q8N5C6	SRBD1_HUMAN S1 RNA-binding domain-containing protein 1 OS=Homo sapiens GN=SRBD1 PE=1 SV=2		61.37
Q8N5C6-2	SRBD1_HUMAN Isoform 2 of S1 RNA-binding domain-containing protein 1 OS=Homo sapiens GN=SRBD1		2.12
Q8N5K1	CISD2_HUMAN CDGSH iron-sulfur domain-containing protein 2 OS=Homo sapiens GN=CISD2 PE=1 SV=1		316.94

Q8N5L8	CI023_HUMAN Alba-like protein C9orf23 OS=Homo sapiens GN=C9orf23 PE=1 SV=1		55.57
Q8N5M1	ATPF2_HUMAN ATP synthase mitochondrial F1 complex assembly factor 2 OS=Homo sapiens GN=ATPAF2 PE=1 SV=1	6.94E-09	
Q8N5M4	TTC9C_HUMAN Tetratricopeptide repeat protein 9C OS=Homo sapiens GN=TTC9C PE=1 SV=1	6.10E-10	
Q8N5M9	JAGN1_HUMAN Protein jagunal homolog 1 OS=Homo sapiens GN=JAGN1 PE=1 SV=1	4.83E-19	
Q8N5N7	RM50_HUMAN 39S ribosomal protein L50, mitochondrial OS=Homo sapiens GN=MRPL50 PE=1 SV=2		51.13
Q8N5S1	S2541_HUMAN Solute carrier family 25 member 41 OS=Homo sapiens GN=SLC25A41 PE=2 SV=2		2.02
Q8N5T2	TBC19_HUMAN TBC1 domain family member 19 OS=Homo sapiens GN=TBC1D19 PE=1 SV=2		
Q8N5Z5	KCD17_HUMAN BTB/POZ domain-containing protein KCTD17 OS=Homo sapiens GN=KCTD17 PE=1 SV=3		
Q8N5Z5-2	KCD17_HUMAN Isoform 2 of BTB/POZ domain-containing protein KCTD17 OS=Homo sapiens GN=KCTD17		
Q8N655	CJ012_HUMAN Uncharacterized protein C10orf12 OS=Homo sapiens GN=C10orf12 PE=1 SV=1		
Q8N668	COMD1_HUMAN COMM domain-containing protein 1 OS=Homo sapiens GN=COMMD1 PE=1 SV=1		26.4
Q8N684	CPSF7_HUMAN Cleavage and polyadenylation specificity factor subunit 7 OS=Homo sapiens GN=CPSF7 PE=1 SV=1		209.28
Q8N684-2	CPSF7_HUMAN Isoform 2 of Cleavage and polyadenylation specificity factor subunit 7 OS=Homo sapiens GN=CPSF7		209.28
Q8N684-3	CPSF7_HUMAN Isoform 3 of Cleavage and polyadenylation specificity factor subunit 7 OS=Homo sapiens GN=CPSF7		211.62
Q8N684-3;Q8N684;B4DGI	3|CPSF7_HUMAN Isoform 3 of Cleavage and polyadenylation specificity factor subunit 7 OS=Homo sapiens GN=CPSF7;>sp|Q8N684|C	9.00E-111	
Q8N693	ESX1_HUMAN Homeobox protein ESX1 OS=Homo sapiens GN=ESX1 PE=1 SV=3		
Q8N6C5	IGSF1_HUMAN Immunoglobulin superfamily member 1 OS=Homo sapiens GN=IGSF1 PE=1 SV=3		
Q8N6C5-2	IGSF1_HUMAN Isoform 2 of Immunoglobulin superfamily member 1 OS=Homo sapiens GN=IGSF1		
Q8N6H7	ARFG2_HUMAN ADP-ribosylation factor GTPase-activating protein 2 OS=Homo sapiens GN=ARFGAP2 PE=1 SV=1	1.01E-07	
Q8N6L1	KTAP2_HUMAN Keratinocyte-associated protein 2 OS=Homo sapiens GN=KRTCAP2 PE=1 SV=1		47.84
Q8N6M0	OTU6B_HUMAN OTU domain-containing protein 6B OS=Homo sapiens GN=OTUD6B PE=1 SV=1		89.04
Q8N6P7	I22R1_HUMAN Interleukin-22 receptor subunit alpha-1 OS=Homo sapiens GN=IL22RA1 PE=1 SV=1		
Q8N6T3-2;Q8N6T3;Q8N6T	2|ARFG1_HUMAN Isoform 2 of ADP-ribosylation factor GTPase-activating protein 1 OS=Homo sapiens GN=ARFGAP1;>sp|Q8N6T3|ARF	4.77E-44	
Q8N6W0	CELF5_HUMAN CUGBP Elav-like family member 5 OS=Homo sapiens GN=CELF5 PE=1 SV=1		
Q8N6W0-2	CELF5_HUMAN Isoform 2 of CUGBP Elav-like family member 5 OS=Homo sapiens GN=CELF5		
Q8N720	ZN655_HUMAN Zinc finger protein 655 OS=Homo sapiens GN=ZNF655 PE=1 SV=3		
Q8N720-3	ZN655_HUMAN Isoform 3 of Zinc finger protein 655 OS=Homo sapiens GN=ZNF655		
Q8N729	NPW_HUMAN Neuropeptide W OS=Homo sapiens GN=NPW PE=1 SV=2		
Q8N743	KI3L3_HUMAN Killer cell immunoglobulin-like receptor 3DL3 OS=Homo sapiens GN=KIR3DL3 PE=2 SV=2		
Q8N752	KC1AL_HUMAN Casein kinase I isoform alpha-like OS=Homo sapiens GN=CSNK1A1L PE=2 SV=2		27.88
Q8N766	K0090_HUMAN Uncharacterized protein KIAA0090 OS=Homo sapiens GN=KIAA0090 PE=1 SV=1		164.84
Q8N766-2	K0090_HUMAN Isoform 2 of Uncharacterized protein KIAA0090 OS=Homo sapiens GN=KIAA0090		164.84
Q8N766-3	K0090_HUMAN Isoform 3 of Uncharacterized protein KIAA0090 OS=Homo sapiens GN=KIAA0090		164.84
Q8N766-4	K0090_HUMAN Isoform 4 of Uncharacterized protein KIAA0090 OS=Homo sapiens GN=KIAA0090		164.84
Q8N766;Q8N766-2;Q8N76	K0090_HUMAN Uncharacterized protein KIAA0090 OS=Homo sapiens GN=KIAA0090 PE=1 SV=1;>sp|Q8N766-2|K0090_HUMAN Isofo	2.61E-31	
Q8N7H5	PAF1_HUMAN RNA polymerase II-associated factor 1 homolog OS=Homo sapiens GN=PAF1 PE=1 SV=2		34.95
Q8N7H5-2	PAF1_HUMAN Isoform 2 of RNA polymerase II-associated factor 1 homolog OS=Homo sapiens GN=PAF1		30.76
Q8N7H5-2;Q8N7H5	2|PAF1_HUMAN Isoform 2 of RNA polymerase II-associated factor 1 homolog OS=Homo sapiens GN=PAF1;>sp|Q8N7H5|PAF1_HUMAN	1.90E-09	
Q8N7H5;Q8N7H5-2	PAF1_HUMAN RNA polymerase II-associated factor 1 homolog OS=Homo sapiens GN=PAF1 PE=1 SV=2;>sp|Q8N7H5-2|PAF1_HUMAN	1.27E-12	
Q8N7X0	CAN7L_HUMAN Calpain-7-like protein OS=Homo sapiens GN=C6orf103 PE=2 SV=3		
Q8N7X0-2	CAN7L_HUMAN Isoform 2 of Calpain-7-like protein OS=Homo sapiens GN=C6orf103		
Q8N7X1	RMXL3_HUMAN RNA-binding motif protein, X-linked-like-3 OS=Homo sapiens GN=RBMXL3 PE=2 SV=2		74.34
Q8N7Z5	ANR31_HUMAN Ankyrin repeat domain-containing protein 31 OS=Homo sapiens GN=ANKRD31 PE=5 SV=2		
Q8N806	UBR7_HUMAN Putative E3 ubiquitin-protein ligase UBR7 OS=Homo sapiens GN=UBR7 PE=1 SV=2	1.22E-11	
Q8N823	ZN611_HUMAN Zinc finger protein 611 OS=Homo sapiens GN=ZNF611 PE=2 SV=2		
Q8N823-2	ZN611_HUMAN Isoform 2 of Zinc finger protein 611 OS=Homo sapiens GN=ZNF611		
Q8N884	M21D1_HUMAN Protein MB21D1 OS=Homo sapiens GN=MB21D1 PE=1 SV=2		
Q8N884-2	M21D1_HUMAN Isoform 2 of Protein MB21D1 OS=Homo sapiens GN=MB21D1		
Q8N8J0	PI4P1_HUMAN Putative phosphatidylinositol 4-kinase alpha-like protein P1 OS=Homo sapiens GN=PI4KAP1 PE=5 SV=1		
Q8N8L2	ZN491_HUMAN Zinc finger protein 491 OS=Homo sapiens GN=ZNF491 PE=2 SV=1		
Q8N8N7;Q8N8N7-2	PTGR2_HUMAN Prostaglandin reductase 2 OS=Homo sapiens GN=PTGR2 PE=1 SV=1;>sp|Q8N8N7-2|PTGR2_HUMAN Isoform 2 of Pro	1.87E-25	
Q8N8S7;Q8N8S7-2	ENAH_HUMAN Protein enabled homolog OS=Homo sapiens GN=ENAH PE=1 SV=2;>sp|Q8N8S7-2|ENAH_HUMAN Isoform 2 of Protein	1.92E-87	
Q8N8V2	GBP7_HUMAN Guanylate-binding protein 7 OS=Homo sapiens GN=GBP7 PE=2 SV=2		24.21
Q8N907	DAND5_HUMAN DAN domain family member 5 OS=Homo sapiens GN=DAND5 PE=2 SV=1		
Q8N954	CCD75_HUMAN Coiled-coil domain-containing protein 75 OS=Homo sapiens GN=CCDC75 PE=1 SV=3		
Q8N954-2	CCD75_HUMAN Isoform 2 of Coiled-coil domain-containing protein 75 OS=Homo sapiens GN=CCDC75		
Q8N954;Q8N954-2	CCD75_HUMAN Coiled-coil domain-containing protein 75 OS=Homo sapiens GN=CCDC75 PE=1 SV=3;>sp|Q8N954-2|CCD75_HUMAN	8.76E-12	
Q8N983	RM43_HUMAN 39S ribosomal protein L43, mitochondrial OS=Homo sapiens GN=MRPL43 PE=1 SV=1		149.05
Q8N983-2	RM43_HUMAN Isoform 2 of 39S ribosomal protein L43, mitochondrial OS=Homo sapiens GN=MRPL43		149.05
Q8N983-3	RM43_HUMAN Isoform 3 of 39S ribosomal protein L43, mitochondrial OS=Homo sapiens GN=MRPL43		149.05
Q8N983-4	RM43_HUMAN Isoform 4 of 39S ribosomal protein L43, mitochondrial OS=Homo sapiens GN=MRPL43		149.05

Q8N983-4;Q8N983;Q8N9	4|RM43_HUMAN Isoform 4 of 39S ribosomal protein L43, mitochondrial OS=Homo sapiens GN=MRPL43;>sp|Q8N983|RM43_HUMAN 3	5.15E-33	
Q8N999	CL029_HUMAN Uncharacterized protein C12orf29 OS=Homo sapiens GN=C12orf29 PE=1 SV=2		35.31
Q8N999-2	CL029_HUMAN Isoform 2 of Uncharacterized protein C12orf29 OS=Homo sapiens GN=C12orf29		
Q8N999-3	CL029_HUMAN Isoform 3 of Uncharacterized protein C12orf29 OS=Homo sapiens GN=C12orf29		35.31
Q8N999;Q8N999-3	CL029_HUMAN Uncharacterized protein C12orf29 OS=Homo sapiens GN=C12orf29 PE=1 SV=2;>sp|Q8N999-3|CL029_HUMAN Isoform	5.26E-09	
Q8N9B4	ANR42_HUMAN Ankyrin repeat domain-containing protein 42 OS=Homo sapiens GN=ANKRD42 PE=2 SV=2		
Q8N9B4-2	ANR42_HUMAN Isoform 2 of Ankyrin repeat domain-containing protein 42 OS=Homo sapiens GN=ANKRD42		
Q8N9C0	IGS22_HUMAN Immunoglobulin superfamily member 22 OS=Homo sapiens GN=IGSF22 PE=1 SV=2		
Q8N9F7;Q8N9F7-2;Q8N9F	GDPD1_HUMAN Glycerophosphodiester phosphodiesterase domain-containing protein 1 OS=Homo sapiens GN=GDPD1 PE=1 SV=2;>s	1.87E-07	
Q8N9I0	SYT2_HUMAN Synaptotagmin-2 OS=Homo sapiens GN=SYT2 PE=1 SV=2		17.45
Q8N9L1	ZIC4_HUMAN Zinc finger protein ZIC 4 OS=Homo sapiens GN=ZIC4 PE=1 SV=3		7.49
Q8N9L1-2	ZIC4_HUMAN Isoform 2 of Zinc finger protein ZIC 4 OS=Homo sapiens GN=ZIC4		7.49
Q8N9N7	LRC57_HUMAN Leucine-rich repeat-containing protein 57 OS=Homo sapiens GN=LRRC57 PE=1 SV=1		47.35
Q8N9N8	EIF1A_HUMAN Probable RNA-binding protein EIF1AD OS=Homo sapiens GN=EIF1AD PE=1 SV=1	1.12E-11	
Q8N9T8	KRI1_HUMAN Protein KRI1 homolog OS=Homo sapiens GN=KRI1 PE=1 SV=2		100.46
Q8N9T8-2	KRI1_HUMAN Isoform 2 of Protein KRI1 homolog OS=Homo sapiens GN=KRI1		100.46
Q8N9T8;Q8N9T8-2	KRI1_HUMAN Protein KRI1 homolog OS=Homo sapiens GN=KRI1 PE=1 SV=2;>sp|Q8N9T8-2|KRI1_HUMAN Isoform 2 of Protein KRI1	3.47E-56	
Q8N9V6	ANR53_HUMAN Ankyrin repeat domain-containing protein 53 OS=Homo sapiens GN=ANKRD53 PE=2 SV=2		
Q8N9V6-2	ANR53_HUMAN Isoform 2 of Ankyrin repeat domain-containing protein 53 OS=Homo sapiens GN=ANKRD53		
Q8N9Z9	ILFT1_HUMAN Intermediate filament tail domain-containing protein 1 OS=Homo sapiens GN=IFLTD1 PE=2 SV=2		36.7
Q8N9Z9-2	ILFT1_HUMAN Isoform 2 of Intermediate filament tail domain-containing protein 1 OS=Homo sapiens GN=IFLTD1		
Q8N9Z9-3	ILFT1_HUMAN Isoform 3 of Intermediate filament tail domain-containing protein 1 OS=Homo sapiens GN=IFLTD1		
Q8N9Z9-4	ILFT1_HUMAN Isoform 4 of Intermediate filament tail domain-containing protein 1 OS=Homo sapiens GN=IFLTD1		36.7
Q8N9Z9-5	ILFT1_HUMAN Isoform 5 of Intermediate filament tail domain-containing protein 1 OS=Homo sapiens GN=IFLTD1		36.7
Q8NA42	ZN383_HUMAN Zinc finger protein 383 OS=Homo sapiens GN=ZNF383 PE=2 SV=1		
Q8NAC3	I17RC_HUMAN Interleukin-17 receptor C OS=Homo sapiens GN=IL17RC PE=1 SV=2		
Q8NAC3-2	I17RC_HUMAN Isoform 2 of Interleukin-17 receptor C OS=Homo sapiens GN=IL17RC		
Q8NAC3-3	I17RC_HUMAN Isoform 3 of Interleukin-17 receptor C OS=Homo sapiens GN=IL17RC		
Q8NAC3-4	I17RC_HUMAN Isoform 4 of Interleukin-17 receptor C OS=Homo sapiens GN=IL17RC		
Q8NAF0	ZN579_HUMAN Zinc finger protein 579 OS=Homo sapiens GN=ZNF579 PE=1 SV=2		
Q8NAP1	GATS_HUMAN Putative protein GATS OS=Homo sapiens GN=GATS PE=5 SV=1		
Q8NAP3	ZBT38_HUMAN Zinc finger and BTB domain-containing protein 38 OS=Homo sapiens GN=ZBTB38 PE=1 SV=2		
Q8NAT2	TDRD5_HUMAN Tudor domain-containing protein 5 OS=Homo sapiens GN=TDRD5 PE=1 SV=3		58.52
Q8NAT2-1	TDRD5_HUMAN Isoform 1 of Tudor domain-containing protein 5 OS=Homo sapiens GN=TDRD5		58.52
Q8NAV1	PR38A_HUMAN Pre-splicing factor 38A OS=Homo sapiens GN=PRPF38A PE=1 SV=1		44.67
Q8NB15	ZN511_HUMAN Zinc finger protein 511 OS=Homo sapiens GN=ZNF511 PE=2 SV=1		50.02
Q8NB15-2	ZN511_HUMAN Isoform 2 of Zinc finger protein 511 OS=Homo sapiens GN=ZNF511		50.02
Q8NB15;Q8NB15-2	ZN511_HUMAN Zinc finger protein 511 OS=Homo sapiens GN=ZNF511 PE=2 SV=1;>sp|Q8NB15-2|ZN511_HUMAN Isoform 2 of Zinc	4.36E-15	
Q8NB16	MLKL_HUMAN Mixed lineage kinase domain-like protein OS=Homo sapiens GN=MLKL PE=1 SV=1		
Q8NB16-2	MLKL_HUMAN Isoform 2 of Mixed lineage kinase domain-like protein OS=Homo sapiens GN=MLKL		
Q8NB37	PDDC1_HUMAN Parkinson disease 7 domain-containing protein 1 OS=Homo sapiens GN=PDDC1 PE=1 SV=1		22.89
Q8NB37-2	PDDC1_HUMAN Isoform 2 of Parkinson disease 7 domain-containing protein 1 OS=Homo sapiens GN=PDDC1		33.04
Q8NB37-2;Q8NB37;B7ZK	2|PDDC1_HUMAN Isoform 2 of Parkinson disease 7 domain-containing protein 1 OS=Homo sapiens GN=PDDC1;>sp|Q8NB37|PDDC1_	3.65E-06	
Q8NB37-3	PDDC1_HUMAN Isoform 3 of Parkinson disease 7 domain-containing protein 1 OS=Homo sapiens GN=PDDC1		22.89
Q8NB90	SPAT5_HUMAN Spermatogenesis-associated protein 5 OS=Homo sapiens GN=SPATA5 PE=1 SV=3		32.04
Q8NB90-2	SPAT5_HUMAN Isoform 2 of Spermatogenesis-associated protein 5 OS=Homo sapiens GN=SPATA5		32.04
Q8NB90-3	SPAT5_HUMAN Isoform 3 of Spermatogenesis-associated protein 5 OS=Homo sapiens GN=SPATA5		32.04
Q8NB90;C9JT97;Q8NB90-	SPAT5_HUMAN Spermatogenesis-associated protein 5 OS=Homo sapiens GN=SPATA5 PE=1 SV=3;>tr|C9JT97|C9JT97_HUMAN Uncha	6.53E-11	
Q8NBD8	T229B_HUMAN Transmembrane protein 229B OS=Homo sapiens GN=TMEM229B PE=2 SV=1		
Q8NBE8	KLH23_HUMAN Kelch-like protein 23 OS=Homo sapiens GN=KLHL23 PE=2 SV=1		
Q8NBF1	GLIS1_HUMAN Zinc finger protein GLIS1 OS=Homo sapiens GN=GLIS1 PE=2 SV=2		
Q8NBF2	NHLC2_HUMAN NHL repeat-containing protein 2 OS=Homo sapiens GN=NHLRC2 PE=1 SV=1		
Q8NBF2-2	NHLC2_HUMAN Isoform 2 of NHL repeat-containing protein 2 OS=Homo sapiens GN=NHLRC2		
Q8NBF2;Q8NBF2-2	NHLC2_HUMAN NHL repeat-containing protein 2 OS=Homo sapiens GN=NHLRC2 PE=1 SV=1;>sp|Q8NBF2-2|NHLC2_HUMAN Isoform	1.74E-18	
Q8NBF6	AVL9_HUMAN Late secretory pathway protein AVL9 homolog OS=Homo sapiens GN=AVL9 PE=1 SV=1		32.61
Q8NBF6-2	AVL9_HUMAN Isoform 2 of Late secretory pathway protein AVL9 homolog OS=Homo sapiens GN=AVL9		32.61
Q8NBI5	S43A3_HUMAN Solute carrier family 43 member 3 OS=Homo sapiens GN=SLC43A3 PE=1 SV=2		109.25
Q8NBJ4;Q8NBJ4-2	GOLM1_HUMAN Golgi membrane protein 1 OS=Homo sapiens GN=GOLM1 PE=1 SV=1;>sp|Q8NBJ4-2|GOLM1_HUMAN Isoform 2 of G	1.26E-06	
Q8NBJ5	GT251_HUMAN Procollagen galactosyltransferase 1 OS=Homo sapiens GN=GLT25D1 PE=1 SV=1		666.63
Q8NBJ7	SUMF2_HUMAN Sulfatase-modifying factor 2 OS=Homo sapiens GN=SUMF2 PE=1 SV=2		295.59
Q8NBJ7-2	SUMF2_HUMAN Isoform 2 of Sulfatase-modifying factor 2 OS=Homo sapiens GN=SUMF2		219.81

Q8NBJ7-3	SUMF2_HUMAN Isoform 3 of Sulfatase-modifying factor 2 OS=Homo sapiens GN=SUMF2		215.26
Q8NBJ7-4	SUMF2_HUMAN Isoform 4 of Sulfatase-modifying factor 2 OS=Homo sapiens GN=SUMF2		75.78
Q8NBJ7-5	SUMF2_HUMAN Isoform 5 of Sulfatase-modifying factor 2 OS=Homo sapiens GN=SUMF2		156.11
Q8NBJ7;Q8NBJ7-3;Q8NBJ	SUMF2_HUMAN Sulfatase-modifying factor 2 OS=Homo sapiens GN=SUMF2 PE=1 SV=2;>sp|Q8NBJ7-3|SUMF2_HUMAN Isoform 3 of S	1.51E-23	
Q8NBL1	PGLT1_HUMAN Protein O-glucosyltransferase 1 OS=Homo sapiens GN=POGLUT1 PE=1 SV=1		
Q8NBL1;B4DJ97	KTEL1_HUMAN KTEL motif-containing protein 1 OS=Homo sapiens GN=KTELC1 PE=1 SV=1;>tr|B4DJ97|B4DJ97_HUMAN cDNA FLJ530	6.95E-05	
Q8NBM8	PCYXL_HUMAN Prenylcysteine oxidase-like OS=Homo sapiens GN=PCYOX1L PE=1 SV=2		
Q8NBN7;B2RDH1;Q8NBN7	RDH13_HUMAN Retinol dehydrogenase 13 OS=Homo sapiens GN=RDH13 PE=1 SV=2;>tr|B2RDH1|B2RDH1_HUMAN cDNA, FLJ96605,	6.00E-10	
Q8NBP0	TTC13_HUMAN Tetratricopeptide repeat protein 13 OS=Homo sapiens GN=TTC13 PE=2 SV=3		
Q8NBP0-2	TTC13_HUMAN Isoform 2 of Tetratricopeptide repeat protein 13 OS=Homo sapiens GN=TTC13		
Q8NBP7	PCSK9_HUMAN Proprotein convertase subtilisin/kexin type 9 OS=Homo sapiens GN=PCSK9 PE=1 SV=3		
Q8NBP7-2	PCSK9_HUMAN Isoform 2 of Proprotein convertase subtilisin/kexin type 9 OS=Homo sapiens GN=PCSK9		
Q8NBQ5	DHB11_HUMAN Estradiol 17-beta-dehydrogenase 11 OS=Homo sapiens GN=HSD17B11 PE=1 SV=3		345.91
Q8NBR6	FA63B_HUMAN Protein FAM63B OS=Homo sapiens GN=FAM63B PE=1 SV=2		28.28
Q8NBR6-2	FA63B_HUMAN Isoform 2 of Protein FAM63B OS=Homo sapiens GN=FAM63B		28.28
Q8NBS9	TXND5_HUMAN Thioredoxin domain-containing protein 5 OS=Homo sapiens GN=TXNDC5 PE=1 SV=2		751.91
Q8NBT2	SPC24_HUMAN Kinetochore protein Spc24 OS=Homo sapiens GN=SPC24 PE=1 SV=1	5.88E-98	
Q8NBU5	ATAD1_HUMAN ATPase family AAA domain-containing protein 1 OS=Homo sapiens GN=ATAD1 PE=1 SV=1		76.25
Q8NBU5-2	ATAD1_HUMAN Isoform 2 of ATPase family AAA domain-containing protein 1 OS=Homo sapiens GN=ATAD1		76.25
Q8NBU5;Q8NBU5-2	ATAD1_HUMAN ATPase family AAA domain-containing protein 1 OS=Homo sapiens GN=ATAD1 PE=1 SV=1;>sp|Q8NBU5-2|ATAD1_HU	1.07E-10	
Q8NBX0	SCPDH_HUMAN Probable saccharopine dehydrogenase OS=Homo sapiens GN=SCCPDH PE=1 SV=1		39.01
Q8NBZ7-2;Q8NBZ7;Q8NB	2|UXS1_HUMAN Isoform 2 of UDP-glucuronic acid decarboxylase 1 OS=Homo sapiens GN=UXS1;>sp|Q8NBZ7|UXS1_HUMAN UDP-glu	3.86E-24	
Q8NC42	RN149_HUMAN E3 ubiquitin-protein ligase RNF149 OS=Homo sapiens GN=RNF149 PE=2 SV=2		
Q8NC51	PAIRB_HUMAN Plasminogen activator inhibitor 1 RNA-binding protein OS=Homo sapiens GN=SERBP1 PE=1 SV=2		1077.31
Q8NC51-2	PAIRB_HUMAN Isoform 2 of Plasminogen activator inhibitor 1 RNA-binding protein OS=Homo sapiens GN=SERBP1		1041.65
Q8NC51-3	PAIRB_HUMAN Isoform 3 of Plasminogen activator inhibitor 1 RNA-binding protein OS=Homo sapiens GN=SERBP1		1077.31
Q8NC51-4	PAIRB_HUMAN Isoform 4 of Plasminogen activator inhibitor 1 RNA-binding protein OS=Homo sapiens GN=SERBP1		1041.65
Q8NC51;D3DQ70;Q8NC51	PAIRB_HUMAN Plasminogen activator inhibitor 1 RNA-binding protein OS=Homo sapiens GN=SERBP1 PE=1 SV=2;>tr|D3DQ70|D3DQ7	0	
Q8NC56	LEMD2_HUMAN LEM domain-containing protein 2 OS=Homo sapiens GN=LEMD2 PE=1 SV=1		
Q8NC60	CD014_HUMAN Uncharacterized protein C4orf14 OS=Homo sapiens GN=C4orf14 PE=1 SV=2	1.31E-21	
Q8NCA5	FA98A_HUMAN Protein FAM98A OS=Homo sapiens GN=FAM98A PE=1 SV=1		119.12
Q8NCA5-2	FA98A_HUMAN Isoform 2 of Protein FAM98A OS=Homo sapiens GN=FAM98A		119.12
Q8NCA5;Q8NCA5-2	FA98A_HUMAN Protein FAM98A OS=Homo sapiens GN=FAM98A PE=1 SV=1;>sp|Q8NCA5-2|FA98A_HUMAN Isoform 2 of Protein FAM9	5.38E-11	
Q8NCD3	HJURP_HUMAN Holliday junction recognition protein OS=Homo sapiens GN=HJURP PE=1 SV=2		
Q8NCD3-2	HJURP_HUMAN Isoform 2 of Holliday junction recognition protein OS=Homo sapiens GN=HJURP		
Q8NCD3-3	HJURP_HUMAN Isoform 3 of Holliday junction recognition protein OS=Homo sapiens GN=HJURP		
Q8NCG7	DGLB_HUMAN Sn1-specific diacylglycerol lipase beta OS=Homo sapiens GN=DAGLB PE=1 SV=2		23.1
Q8NCG7-2	DGLB_HUMAN Isoform 2 of Sn1-specific diacylglycerol lipase beta OS=Homo sapiens GN=DAGLB		2.73
Q8NCG7-3	DGLB_HUMAN Isoform 3 of Sn1-specific diacylglycerol lipase beta OS=Homo sapiens GN=DAGLB		20.37
Q8NCG7;Q8NCG7-2	DGLB_HUMAN Sn1-specific diacylglycerol lipase beta OS=Homo sapiens GN=DAGLB PE=1 SV=2;>sp|Q8NCG7-2|DGLB_HUMAN Isofor	5.25E-16	
Q8NCJ5	SPRY3_HUMAN SPRY domain-containing protein 3 OS=Homo sapiens GN=SPRYD3 PE=1 SV=2		26.32
Q8NCL4	GALT6_HUMAN Polypeptide N-acetylgalactosaminyltransferase 6 OS=Homo sapiens GN=GALNT6 PE=2 SV=2	5.38E-45	
Q8NCM8-2;B0I1S0;Q8NCM	2|DYHC2_HUMAN Isoform 2 of Cytoplasmic dynein 2 heavy chain 1 OS=Homo sapiens GN=DYNC2H1;>tr|B0I1S0|B0I1S0_HUMAN DYI	2.09E-06	
Q8NCN5	PDPR_HUMAN Pyruvate dehydrogenase phosphatase regulatory subunit, mitochondrial OS=Homo sapiens GN=PDPR PE=1 SV=2	3.10E-09	
Q8NCW5;Q8NCW5-2	AIBP_HUMAN Apolipoprotein A-I-binding protein OS=Homo sapiens GN=APOA1BP PE=1 SV=2;>sp|Q8NCW5-2|AIBP_HUMAN Isoform	9.27E-28	
Q8NCX0	CC150_HUMAN Coiled-coil domain-containing protein 150 OS=Homo sapiens GN=CCDC150 PE=2 SV=2		63.17
Q8NCX0-2	CC150_HUMAN Isoform 2 of Coiled-coil domain-containing protein 150 OS=Homo sapiens GN=CCDC150		22.26
Q8NCX0-3	CC150_HUMAN Isoform 3 of Coiled-coil domain-containing protein 150 OS=Homo sapiens GN=CCDC150		15.16
Q8NCX0-4	CC150_HUMAN Isoform 4 of Coiled-coil domain-containing protein 150 OS=Homo sapiens GN=CCDC150		5.8
Q8ND04-2;Q8ND04;Q8ND	2|SMG8_HUMAN Isoform 2 of Protein SMG8 OS=Homo sapiens GN=SMG8;>sp|Q8ND04|SMG8_HUMAN Protein SMG8 OS=Homo sapie	7.92E-09	
Q8ND07	CN045_HUMAN Uncharacterized protein C14orf45 OS=Homo sapiens GN=C14orf45 PE=1 SV=3	9.05E-05	
Q8ND24	RN214_HUMAN RING finger protein 214 OS=Homo sapiens GN=RNF214 PE=1 SV=2	1.24E-20	
Q8ND30	LIPB2_HUMAN Liprin-beta-2 OS=Homo sapiens GN=PPFIBP2 PE=2 SV=3	6.42E-51	
Q8ND56	LS14A_HUMAN Protein LSM14 homolog A OS=Homo sapiens GN=LSM14A PE=1 SV=3		28.61
Q8ND56-2	LS14A_HUMAN Isoform 2 of Protein LSM14 homolog A OS=Homo sapiens GN=LSM14A		28.61
Q8ND76	CCNY_HUMAN Cyclin-Y OS=Homo sapiens GN=CCNY PE=1 SV=2		38.16
Q8ND76-2	CCNY_HUMAN Isoform 2 of Cyclin-Y OS=Homo sapiens GN=CCNY		38.42
Q8ND76-3	CCNY_HUMAN Isoform 3 of Cyclin-Y OS=Homo sapiens GN=CCNY		38.16
Q8NDA8	HTR7A_HUMAN HEAT repeat-containing protein 7A OS=Homo sapiens GN=HEATR7A PE=1 SV=3		36.11
Q8NDA8-2	HTR7A_HUMAN Isoform 2 of HEAT repeat-containing protein 7A OS=Homo sapiens GN=HEATR7A		28.07
Q8NDA8-3	HTR7A_HUMAN Isoform 3 of HEAT repeat-containing protein 7A OS=Homo sapiens GN=HEATR7A		8.04

Q8NDA8-7	HTR7A_HUMAN Isoform 7 of HEAT repeat-containing protein 7A OS=Homo sapiens GN=HEATR7A		24.63
Q8NDB2	BANK1_HUMAN B-cell scaffold protein with ankyrin repeats OS=Homo sapiens GN=BANK1 PE=1 SV=3		
Q8NDB2-2	BANK1_HUMAN Isoform 2 of B-cell scaffold protein with ankyrin repeats OS=Homo sapiens GN=BANK1		
Q8NDB2-3	BANK1_HUMAN Isoform 3 of B-cell scaffold protein with ankyrin repeats OS=Homo sapiens GN=BANK1		
Q8NDB2-4	BANK1_HUMAN Isoform 4 of B-cell scaffold protein with ankyrin repeats OS=Homo sapiens GN=BANK1		
Q8NDC0	MISSL_HUMAN MAPK-interacting and spindle-stabilizing protein-like OS=Homo sapiens GN=MAPK1IP1L PE=1 SV=4	3.13E-45	
Q8NDH2	CM040_HUMAN Uncharacterized protein C13orf40 OS=Homo sapiens GN=C13orf40 PE=2 SV=2		
Q8NDH3	PEPL1_HUMAN Probable aminopeptidase NPEPL1 OS=Homo sapiens GN=NPEPL1 PE=1 SV=3		20.3
Q8NDH3-2	PEPL1_HUMAN Isoform 2 of Probable aminopeptidase NPEPL1 OS=Homo sapiens GN=NPEPL1		20.3
Q8NDH3-3	PEPL1_HUMAN Isoform 3 of Probable aminopeptidase NPEPL1 OS=Homo sapiens GN=NPEPL1		
Q8NDH3;Q8NDH3-2;Q8ND	PEPL1_HUMAN Probable aminopeptidase NPEPL1 OS=Homo sapiens GN=NPEPL1 PE=1 SV=3;>sp|Q8NDH3-2|PEPL1_HUMAN Isoform 2	6.05E-136	
Q8NDI1;Q8NDI1-2;A8K93	EHBP1_HUMAN EH domain-binding protein 1 OS=Homo sapiens GN=EHBP1 PE=1 SV=3;>sp|Q8NDI1-2|EHBP1_HUMAN Isoform 2 of E	5.56E-15	
Q8NDM7	WDR96_HUMAN WD repeat-containing protein 96 OS=Homo sapiens GN=WDR96 PE=2 SV=3		38.38
Q8NDM7-2	WDR96_HUMAN Isoform 2 of WD repeat-containing protein 96 OS=Homo sapiens GN=WDR96		38.38
Q8NDM7-3	WDR96_HUMAN Isoform 3 of WD repeat-containing protein 96 OS=Homo sapiens GN=WDR96		31.78
Q8NDM7-4	WDR96_HUMAN Isoform 4 of WD repeat-containing protein 96 OS=Homo sapiens GN=WDR96		0.38
Q8NDM7-5	WDR96_HUMAN Isoform 5 of WD repeat-containing protein 96 OS=Homo sapiens GN=WDR96		0.38
Q8NDQ6-4	ZN540_HUMAN Isoform 4 of Zinc finger protein 540 OS=Homo sapiens GN=ZNF540		
Q8NDT2	RB15B_HUMAN Putative RNA-binding protein 15B OS=Homo sapiens GN=RBM15B PE=1 SV=3		
Q8NDV3	SMC1B_HUMAN Structural maintenance of chromosomes protein 1B OS=Homo sapiens GN=SMC1B PE=1 SV=2		62.31
Q8NDV3-2	SMC1B_HUMAN Isoform 2 of Structural maintenance of chromosomes protein 1B OS=Homo sapiens GN=SMC1B		62.31
Q8NDV3-3	SMC1B_HUMAN Isoform 3 of Structural maintenance of chromosomes protein 1B OS=Homo sapiens GN=SMC1B		62.31
Q8NDW4	ZN248_HUMAN Zinc finger protein 248 OS=Homo sapiens GN=ZNF248 PE=1 SV=1		23.91
Q8NDX1	PSD4_HUMAN PH and SEC7 domain-containing protein 4 OS=Homo sapiens GN=PSD4 PE=1 SV=2		
Q8NDX1-2	PSD4_HUMAN Isoform 2 of PH and SEC7 domain-containing protein 4 OS=Homo sapiens GN=PSD4		
Q8NDX5	PHC3_HUMAN Polyhomeotic-like protein 3 OS=Homo sapiens GN=PHC3 PE=1 SV=1		
Q8NDX5-2	PHC3_HUMAN Isoform 2 of Polyhomeotic-like protein 3 OS=Homo sapiens GN=PHC3		
Q8NDX5-7	PHC3_HUMAN Isoform 7 of Polyhomeotic-like protein 3 OS=Homo sapiens GN=PHC3		
Q8NDZ4	CC058_HUMAN UPF0672 protein C3orf58 OS=Homo sapiens GN=C3orf58 PE=1 SV=1	2.99E-15	
Q8NE18	NSUN7_HUMAN Putative methyltransferase NSUN7 OS=Homo sapiens GN=NSUN7 PE=2 SV=4		
Q8NE18-2	NSUN7_HUMAN Isoform 2 of Putative methyltransferase NSUN7 OS=Homo sapiens GN=NSUN7		
Q8NE28-3	SGK71_HUMAN Isoform 3 of Protein kinase-like protein SgK071 OS=Homo sapiens GN=SGK071		
Q8NE31-3	FA13C_HUMAN Isoform 3 of Protein FAM13C OS=Homo sapiens GN=FAM13C		
Q8NE35	CPEB3_HUMAN Cytoplasmic polyadenylation element-binding protein 3 OS=Homo sapiens GN=CPEB3 PE=1 SV=2		24.82
Q8NE35-2	CPEB3_HUMAN Isoform 2 of Cytoplasmic polyadenylation element-binding protein 3 OS=Homo sapiens GN=CPEB3		24.82
Q8NE62;B4DMQ4	CHDH_HUMAN Choline dehydrogenase, mitochondrial OS=Homo sapiens GN=CHDH PE=2 SV=2;>tr|B4DMQ4|B4DMQ4_HUMAN cDNA	5.96E-19	
Q8NE63	HIPK4_HUMAN Homeodomain-interacting protein kinase 4 OS=Homo sapiens GN=HIPK4 PE=2 SV=1		
Q8NE63-2	HIPK4_HUMAN Isoform 2 of Homeodomain-interacting protein kinase 4 OS=Homo sapiens GN=HIPK4		
Q8NE71	ABCF1_HUMAN ATP-binding cassette sub-family F member 1 OS=Homo sapiens GN=ABCF1 PE=1 SV=2		280.3
Q8NE71-2	ABCF1_HUMAN Isoform 2 of ATP-binding cassette sub-family F member 1 OS=Homo sapiens GN=ABCF1		280.3
Q8NE71;Q8NE71-2	ABCF1_HUMAN ATP-binding cassette sub-family F member 1 OS=Homo sapiens GN=ABCF1 PE=1 SV=2;>sp|Q8NE71-2|ABCF1_HUMA	7.45E-136	
Q8NEA6	GLIS3_HUMAN Zinc finger protein GLIS3 OS=Homo sapiens GN=GLIS3 PE=2 SV=5		
Q8NEA6-2	GLIS3_HUMAN Isoform 2 of Zinc finger protein GLIS3 OS=Homo sapiens GN=GLIS3		
Q8NEB9	PK3C3_HUMAN Phosphatidylinositol 3-kinase catalytic subunit type 3 OS=Homo sapiens GN=PIK3C3 PE=1 SV=1	3.79E-13	
Q8NEE6	FXL13_HUMAN F-box/LRR-repeat protein 13 OS=Homo sapiens GN=FBXL13 PE=2 SV=3		
Q8NEE6-2	FXL13_HUMAN Isoform 2 of F-box/LRR-repeat protein 13 OS=Homo sapiens GN=FBXL13		
Q8NEE6-3	FXL13_HUMAN Isoform 3 of F-box/LRR-repeat protein 13 OS=Homo sapiens GN=FBXL13		
Q8NEE6-4	FXL13_HUMAN Isoform 4 of F-box/LRR-repeat protein 13 OS=Homo sapiens GN=FBXL13		
Q8NEE8	TTC16_HUMAN Tetratricopeptide repeat protein 16 OS=Homo sapiens GN=TTC16 PE=2 SV=2		
Q8NEG5	ZSWM2_HUMAN E3 ubiquitin-protein ligase ZSWIM2 OS=Homo sapiens GN=ZSWIM2 PE=1 SV=2		
Q8NEJ0	DUS18_HUMAN Dual specificity protein phosphatase 18 OS=Homo sapiens GN=DUSP18 PE=1 SV=1		13.65
Q8NEJ9	NGDN_HUMAN Neuroguidin OS=Homo sapiens GN=NGDN PE=1 SV=1		
Q8NEJ9-2	NGDN_HUMAN Isoform 2 of Neuroguidin OS=Homo sapiens GN=NGDN		
Q8NEJ9;Q8NEJ9-2	NGDN_HUMAN Neuroguidin OS=Homo sapiens GN=NGDN PE=1 SV=1;>sp|Q8NEJ9-2|NGDN_HUMAN Isoform 2 of Neuroguidin OS=Ho	3.54E-12	
Q8NEM2	SHCBP_HUMAN SHC SH2 domain-binding protein 1 OS=Homo sapiens GN=SHCBP1 PE=1 SV=3	1.52E-07	
Q8NEV4	MYO3A_HUMAN Myosin-IIIa OS=Homo sapiens GN=MYO3A PE=1 SV=2		
Q8NEW0	ZNT7_HUMAN Zinc transporter 7 OS=Homo sapiens GN=SLC30A7 PE=1 SV=1		33.66
Q8NEY1	NAV1_HUMAN Neuron navigator 1 OS=Homo sapiens GN=NAV1 PE=1 SV=2		31.67
Q8NEY1-2	NAV1_HUMAN Isoform 2 of Neuron navigator 1 OS=Homo sapiens GN=NAV1		31.67
Q8NEY1-3	NAV1_HUMAN Isoform 3 of Neuron navigator 1 OS=Homo sapiens GN=NAV1		31.67
Q8NEY1-4	NAV1_HUMAN Isoform 4 of Neuron navigator 1 OS=Homo sapiens GN=NAV1		31.67

Q8NEY1-5	NAV1_HUMAN Isoform 5 of Neuron navigator 1 OS=Homo sapiens GN=NAV1		31.67
Q8NEY1-6	NAV1_HUMAN Isoform 6 of Neuron navigator 1 OS=Homo sapiens GN=NAV1		21.63
Q8NEY1-7	NAV1_HUMAN Isoform 7 of Neuron navigator 1 OS=Homo sapiens GN=NAV1		31.67
Q8NEY1;Q8NEY1-3;Q8NEY	NAV1_HUMAN Neuron navigator 1 OS=Homo sapiens GN=NAV1 PE=1 SV=2;>sp|Q8NEY1-3|NAV1_HUMAN Isoform 3 of Neuron naviga	1.05E-13	
Q8NEY8	PPHLN_HUMAN Periphilin-1 OS=Homo sapiens GN=PPHLN1 PE=1 SV=2		
Q8NEY8-2	PPHLN_HUMAN Isoform 2 of Periphilin-1 OS=Homo sapiens GN=PPHLN1		
Q8NEY8-3	PPHLN_HUMAN Isoform 3 of Periphilin-1 OS=Homo sapiens GN=PPHLN1		
Q8NEY8-5	PPHLN_HUMAN Isoform 5 of Periphilin-1 OS=Homo sapiens GN=PPHLN1		
Q8NEY8-6	PPHLN_HUMAN Isoform 6 of Periphilin-1 OS=Homo sapiens GN=PPHLN1		
Q8NEY8-8	PPHLN_HUMAN Isoform 8 of Periphilin-1 OS=Homo sapiens GN=PPHLN1		
Q8NEZ4	MLL3_HUMAN Histone-lysine N-methyltransferase MLL3 OS=Homo sapiens GN=MLL3 PE=1 SV=3		
Q8NEZ4-2	MLL3_HUMAN Isoform 2 of Histone-lysine N-methyltransferase MLL3 OS=Homo sapiens GN=MLL3		
Q8NEZ4-3	MLL3_HUMAN Isoform 3 of Histone-lysine N-methyltransferase MLL3 OS=Homo sapiens GN=MLL3		
Q8NEZ5;Q8NEZ5-3	FBX22_HUMAN F-box only protein 22 OS=Homo sapiens GN=FBXO22 PE=1 SV=1;>sp|Q8NEZ5-3|FBX22_HUMAN Isoform 3 of F-box o	2.18E-07	
Q8NF37	PCAT1_HUMAN Lysophosphatidylcholine acyltransferase 1 OS=Homo sapiens GN=LPCAT1 PE=1 SV=2		136.23
Q8NF50	DOCK8_HUMAN Dedicator of cytokinesis protein 8 OS=Homo sapiens GN=DOCK8 PE=1 SV=3		
Q8NF50-2	DOCK8_HUMAN Isoform 2 of Dedicator of cytokinesis protein 8 OS=Homo sapiens GN=DOCK8		
Q8NF50-3	DOCK8_HUMAN Isoform 3 of Dedicator of cytokinesis protein 8 OS=Homo sapiens GN=DOCK8		
Q8NF91	SYNE1_HUMAN Nesprin-1 OS=Homo sapiens GN=SYNE1 PE=1 SV=3		272.08
Q8NF91-2	SYNE1_HUMAN Isoform 2 of Nesprin-1 OS=Homo sapiens GN=SYNE1		59.9
Q8NF91-3	SYNE1_HUMAN Isoform 3 of Nesprin-1 OS=Homo sapiens GN=SYNE1		9.3
Q8NF91-4	SYNE1_HUMAN Isoform 4 of Nesprin-1 OS=Homo sapiens GN=SYNE1		272.08
Q8NF91-5	SYNE1_HUMAN Isoform 5 of Nesprin-1 OS=Homo sapiens GN=SYNE1		77.59
Q8NF91-6	SYNE1_HUMAN Isoform 6 of Nesprin-1 OS=Homo sapiens GN=SYNE1		96.79
Q8NF91-7	SYNE1_HUMAN Isoform 7 of Nesprin-1 OS=Homo sapiens GN=SYNE1		212.18
Q8NF91-8	SYNE1_HUMAN Isoform 8 of Nesprin-1 OS=Homo sapiens GN=SYNE1		56.53
Q8NF91-9	SYNE1_HUMAN Isoform 9 of Nesprin-1 OS=Homo sapiens GN=SYNE1		
Q8NF91;Q8NF91-4;Q8NF9	SYNE1_HUMAN Nesprin-1 OS=Homo sapiens GN=SYNE1 PE=1 SV=3;>sp|Q8NF91-4|SYNE1_HUMAN Isoform 4 of Nesprin-1 OS=Hom	1.10E-29	
Q8NF99	ZN397_HUMAN Zinc finger protein 397 OS=Homo sapiens GN=ZNF397 PE=1 SV=2		
Q8NFA0	UBP32_HUMAN Ubiquitin carboxyl-terminal hydrolase 32 OS=Homo sapiens GN=USP32 PE=1 SV=1		
Q8NFC6	BOD1L_HUMAN Biorientation of chromosomes in cell division protein 1-like OS=Homo sapiens GN=BOD1L PE=1 SV=2		96.6
Q8NFD5	ARI1B_HUMAN AT-rich interactive domain-containing protein 1B OS=Homo sapiens GN=ARID1B PE=1 SV=2		
Q8NFD5-2	ARI1B_HUMAN Isoform 2 of AT-rich interactive domain-containing protein 1B OS=Homo sapiens GN=ARID1B		
Q8NFD5-3	ARI1B_HUMAN Isoform 3 of AT-rich interactive domain-containing protein 1B OS=Homo sapiens GN=ARID1B		
Q8NFD5-4	ARI1B_HUMAN Isoform 4 of AT-rich interactive domain-containing protein 1B OS=Homo sapiens GN=ARID1B		
Q8NFF5	FAD1_HUMAN FAD synthase OS=Homo sapiens GN=FLAD1 PE=1 SV=1		
Q8NFF5-2	FAD1_HUMAN Isoform 2 of FAD synthase OS=Homo sapiens GN=FLAD1		
Q8NFF5-3	FAD1_HUMAN Isoform 3 of FAD synthase OS=Homo sapiens GN=FLAD1		
Q8NFF5-4	FAD1_HUMAN Isoform 4 of FAD synthase OS=Homo sapiens GN=FLAD1		
Q8NFF5-5	FAD1_HUMAN Isoform 5 of FAD synthase OS=Homo sapiens GN=FLAD1		
Q8NFF5;Q8NFF5-2;Q8NFF	FAD1_HUMAN FAD synthase OS=Homo sapiens GN=FLAD1 PE=1 SV=1;>sp|Q8NFF5-2|FAD1_HUMAN Isoform 2 of FAD synthase OS=l	1.20E-16	
Q8NFH3	NUP43_HUMAN Nucleoporin Nup43 OS=Homo sapiens GN=NUP43 PE=1 SV=1	4.85E-11	
Q8NFH4	NUP37_HUMAN Nucleoporin Nup37 OS=Homo sapiens GN=NUP37 PE=1 SV=1		
Q8NFH5	NUP53_HUMAN Nucleoporin NUP53 OS=Homo sapiens GN=NUP35 PE=1 SV=1		
Q8NFI4	F10A5_HUMAN Putative protein FAM10A5 OS=Homo sapiens GN=ST13P5 PE=5 SV=1		40.25
Q8NFJ5	RAI3_HUMAN Retinoic acid-induced protein 3 OS=Homo sapiens GN=GPRC5A PE=1 SV=2		59.31
Q8NFP9	NBEA_HUMAN Neurobeachin OS=Homo sapiens GN=NBEA PE=1 SV=3		59.43
Q8NFP9-2	NBEA_HUMAN Isoform 2 of Neurobeachin OS=Homo sapiens GN=NBEA		18.54
Q8NFP9;P50851;P50851-2	NBEA_HUMAN Neurobeachin OS=Homo sapiens GN=NBEA PE=1 SV=3;>sp|P50851|LRBA_HUMAN Lipopolysaccharide-responsive and	1.07E-05	
Q8NFQ8	TOIP2_HUMAN Torsin-1A-interacting protein 2 OS=Homo sapiens GN=TOR1AIP2 PE=1 SV=1		
Q8NFR3	SSPTB_HUMAN Small subunit of serine palmitoyltransferase B OS=Homo sapiens GN=SSSPTB PE=1 SV=1		
Q8NFU3-4;Q8NFU3;Q8NFU	4|TSTD1_HUMAN Isoform 4 of Thiosulfate sulfurtransferase/rhodanese-like domain-containing protein 1 OS=Homo sapiens GN=TSTD1	1.97E-12	
Q8NFU7	TET1_HUMAN Methylcytosine dioxygenase TET1 OS=Homo sapiens GN=TET1 PE=1 SV=2		
Q8NFV4	ABHDB_HUMAN Abhydrolase domain-containing protein 11 OS=Homo sapiens GN=ABHD11 PE=1 SV=1		38.01
Q8NFV4-2	ABHDB_HUMAN Isoform 2 of Abhydrolase domain-containing protein 11 OS=Homo sapiens GN=ABHD11		
Q8NFV4-3	ABHDB_HUMAN Isoform 3 of Abhydrolase domain-containing protein 11 OS=Homo sapiens GN=ABHD11		
Q8NFV4-4	ABHDB_HUMAN Isoform 4 of Abhydrolase domain-containing protein 11 OS=Homo sapiens GN=ABHD11		38.01
Q8NFV4-5	ABHDB_HUMAN Isoform 5 of Abhydrolase domain-containing protein 11 OS=Homo sapiens GN=ABHD11		
Q8NFV4;Q8NFV4-4	ABHDB_HUMAN Abhydrolase domain-containing protein 11 OS=Homo sapiens GN=ABHD11 PE=1 SV=1;>sp|Q8NFV4-4|ABHDB_HUMA	1.46E-17	
Q8NFW1;Q8NFW1-2;Q8NF	COMA1_HUMAN Collagen alpha-1(XXII) chain OS=Homo sapiens GN=COL22A1 PE=1 SV=2;>sp|Q8NFW1-2|COMA1_HUMAN Isoform 2	3.92E-05	
Q8NFW8	NEUA_HUMAN N-acylneuraminate cytidylyltransferase OS=Homo sapiens GN=CMAS PE=1 SV=2		

Q8NFW8-2	NEUA_HUMAN Isoform 2 of N-acylneuraminate cytidylyltransferase OS=Homo sapiens GN=CMAS		
Q8NFW8;Q8NFW8-2	NEUA_HUMAN N-acylneuraminate cytidylyltransferase OS=Homo sapiens GN=CMAS PE=1 SV=2;>sp|Q8NFW8-2|NEUA_HUMAN Isofor	5.38E-09	
Q8NG11	TSN14_HUMAN Tetraspanin-14 OS=Homo sapiens GN=TSPAN14 PE=2 SV=1		40.88
Q8NG11-2	TSN14_HUMAN Isoform 2 of Tetraspanin-14 OS=Homo sapiens GN=TSPAN14		40.88
Q8NG11;Q8NG11-2	TSN14_HUMAN Tetraspanin-14 OS=Homo sapiens GN=TSPAN14 PE=2 SV=1;>sp|Q8NG11-2|TSN14_HUMAN Isoform 2 of Tetraspanin-	2.46E-13	
Q8NG48	LINES_HUMAN Protein Lines homolog OS=Homo sapiens GN=LINS PE=2 SV=2		
Q8NG48-2	LINES_HUMAN Isoform 2 of Protein Lines homolog OS=Homo sapiens GN=LINS		
Q8NG48-3	LINES_HUMAN Isoform 3 of Protein Lines homolog OS=Homo sapiens GN=LINS		
Q8NG57	ELOA3_HUMAN RNA polymerase II transcription factor SIII subunit A3 OS=Homo sapiens GN=TCEB3C PE=1 SV=2		
Q8NGC1	O11G2_HUMAN Olfactory receptor 11G2 OS=Homo sapiens GN=OR11G2 PE=2 SV=2		
Q8NGF0	O52B6_HUMAN Olfactory receptor 52B6 OS=Homo sapiens GN=OR52B6 PE=2 SV=3		
Q8NGI6	OR4DA_HUMAN Olfactory receptor 4D10 OS=Homo sapiens GN=OR4D10 PE=2 SV=1		
Q8NGU9	GP150_HUMAN Probable G-protein coupled receptor 150 OS=Homo sapiens GN=GPR150 PE=2 SV=1		21.37
Q8NGW1	OR6B3_HUMAN Olfactory receptor 6B3 OS=Homo sapiens GN=OR6B3 PE=2 SV=1		
Q8NGZ6	OR6F1_HUMAN Olfactory receptor 6F1 OS=Homo sapiens GN=OR6F1 PE=2 SV=1		
Q8NH61	O51F2_HUMAN Olfactory receptor 51F2 OS=Homo sapiens GN=OR51F2 PE=2 SV=2		
Q8NHG7	SVIP_HUMAN Small VCP/p97-interacting protein OS=Homo sapiens GN=SVIP PE=2 SV=1		79.18
Q8NHG8	ZNRF2_HUMAN E3 ubiquitin-protein ligase ZNRF2 OS=Homo sapiens GN=ZNRF2 PE=1 SV=1	2.01E-06	
Q8NHH9	ATLA2_HUMAN Atlastin-2 OS=Homo sapiens GN=ATL2 PE=1 SV=2		31.88
Q8NHH9-2	ATLA2_HUMAN Isoform 2 of Atlastin-2 OS=Homo sapiens GN=ATL2		31.88
Q8NHH9-3	ATLA2_HUMAN Isoform 3 of Atlastin-2 OS=Homo sapiens GN=ATL2		2.63
Q8NHH9-4	ATLA2_HUMAN Isoform 4 of Atlastin-2 OS=Homo sapiens GN=ATL2		31.88
Q8NHH9;Q8NHH9-2;B7Z1	ATLA2_HUMAN Atlastin-2 OS=Homo sapiens GN=ATL2 PE=1 SV=2;>sp|Q8NHH9-2|ATLA2_HUMAN Isoform 2 of Atlastin-2 OS=Homo s	8.89E-09	
Q8NHM4	TRY6_HUMAN Putative trypsin-6 OS=Homo sapiens GN=TRY6 PE=5 SV=1		
Q8NHP1	ARK74_HUMAN Aflatoxin B1 aldehyde reductase member 4 OS=Homo sapiens GN=AKR7L PE=2 SV=6		67.59
Q8NHP6	MSPD2_HUMAN Motile sperm domain-containing protein 2 OS=Homo sapiens GN=MOSPD2 PE=1 SV=1	1.29E-12	
Q8NHP8	PLBL2_HUMAN Putative phospholipase B-like 2 OS=Homo sapiens GN=PLBD2 PE=1 SV=2		43.31
Q8NHQ9	DDX55_HUMAN ATP-dependent RNA helicase DDX55 OS=Homo sapiens GN=DDX55 PE=1 SV=3	2.97E-14	
Q8NHS0	DNJB8_HUMAN DnaJ homolog subfamily B member 8 OS=Homo sapiens GN=DNAJB8 PE=1 SV=1		
Q8NHS9	SPT22_HUMAN Spermatogenesis-associated protein 22 OS=Homo sapiens GN=SPATA22 PE=1 SV=2		
Q8NHS9-2	SPT22_HUMAN Isoform 2 of Spermatogenesis-associated protein 22 OS=Homo sapiens GN=SPATA22		
Q8NHU6;Q8NHU6-2	TDRD7_HUMAN Tudor domain-containing protein 7 OS=Homo sapiens GN=TDRD7 PE=1 SV=2;>sp|Q8NHU6-2|TDRD7_HUMAN Isoforr	7.75E-10	
Q8NHV1	GIMA7_HUMAN GTPase IMAP family member 7 OS=Homo sapiens GN=GIMAP7 PE=2 SV=1	2.55E-09	
Q8NHW5	RLA0L_HUMAN 60S acidic ribosomal protein P0-like OS=Homo sapiens GN=RPLP0P6 PE=5 SV=1		573.66
Q8NHY3	GA2L2_HUMAN GAS2-like protein 2 OS=Homo sapiens GN=GAS2L2 PE=2 SV=1		28.85
Q8NI22	MCFD2_HUMAN Multiple coagulation factor deficiency protein 2 OS=Homo sapiens GN=MCFD2 PE=1 SV=1		63.37
Q8NI27	THOC2_HUMAN THO complex subunit 2 OS=Homo sapiens GN=THOC2 PE=1 SV=2		81.67
Q8NI27-2	THOC2_HUMAN Isoform 2 of THO complex subunit 2 OS=Homo sapiens GN=THOC2		0.99
Q8NI27;B4DKZ6;Q6P441;	THOC2_HUMAN THO complex subunit 2 OS=Homo sapiens GN=THOC2 PE=1 SV=2;>tr|B4DKZ6|B4DKZ6_HUMAN cDNA FLJ61096, hig	3.30E-09	
Q8NI29	FBX27_HUMAN F-box only protein 27 OS=Homo sapiens GN=FBXO27 PE=1 SV=2		
Q8NI36	WDR36_HUMAN WD repeat-containing protein 36 OS=Homo sapiens GN=WDR36 PE=1 SV=1		
Q8NI77	KI18A_HUMAN Kinesin-like protein KIF18A OS=Homo sapiens GN=KIF18A PE=1 SV=2		
Q8TAA1	RNS11_HUMAN Probable ribonuclease 11 OS=Homo sapiens GN=RNASE11 PE=2 SV=1		
Q8TAA3	PSA7L_HUMAN Proteasome subunit alpha type-7-like OS=Homo sapiens GN=PSMA8 PE=1 SV=3		330.79
Q8TAA3-2	PSA7L_HUMAN Isoform 2 of Proteasome subunit alpha type-7-like OS=Homo sapiens GN=PSMA8		323.84
Q8TAA3-5	PSA7L_HUMAN Isoform 3 of Proteasome subunit alpha type-7-like OS=Homo sapiens GN=PSMA8		330.79
Q8TAD4	ZNT5_HUMAN Zinc transporter 5 OS=Homo sapiens GN=SLC30A5 PE=1 SV=1		
Q8TAE8	G45IP_HUMAN Growth arrest and DNA damage-inducible proteins-interacting protein 1 OS=Homo sapiens GN=GADD45GIP1 PE=1 SV=1		
Q8TAF5	LQK1_HUMAN Putative uncharacterized protein LQK1 OS=Homo sapiens GN=LQK1 PE=2 SV=1		
Q8TAL5	CI043_HUMAN Uncharacterized protein C9orf43 OS=Homo sapiens GN=C9orf43 PE=1 SV=1		
Q8TAM1	BBS10_HUMAN Bardet-Biedl syndrome 10 protein OS=Homo sapiens GN=BBS10 PE=1 SV=2		
Q8TAP9	TTDN1_HUMAN TTD non-photosensitive 1 protein OS=Homo sapiens GN=TTDN1 PE=1 SV=1	1.41E-21	
Q8TAQ2	SMRC2_HUMAN SWI/SNF complex subunit SMARCC2 OS=Homo sapiens GN=SMARCC2 PE=1 SV=1		
Q8TAQ2-2	SMRC2_HUMAN Isoform 2 of SWI/SNF complex subunit SMARCC2 OS=Homo sapiens GN=SMARCC2		
Q8TAQ2;Q59GV3;Q59G16	SMRC2_HUMAN SWI/SNF complex subunit SMARCC2 OS=Homo sapiens GN=SMARCC2 PE=1 SV=1;>tr|Q59GV3|Q59GV3_HUMAN SW	1.61E-205	
Q8TAT5	NEIL3_HUMAN Endonuclease 8-like 3 OS=Homo sapiens GN=NEIL3 PE=1 SV=3	1.37E-11	
Q8TAT6	NPL4_HUMAN Nuclear protein localization protein 4 homolog OS=Homo sapiens GN=NPLOC4 PE=1 SV=3		148.59
Q8TAT6-2	NPL4_HUMAN Isoform 2 of Nuclear protein localization protein 4 homolog OS=Homo sapiens GN=NPLOC4		148.59
Q8TAT6-2;Q8TAT6	2|NPL4_HUMAN Isoform 2 of Nuclear protein localization protein 4 homolog OS=Homo sapiens GN=NPLOC4;>sp|Q8TAT6|NPL4_HUMA	2.32E-65	
Q8TAU0	NKX23_HUMAN Homeobox protein Nkx-2.3 OS=Homo sapiens GN=NKX2-3 PE=1 SV=2		
Q8TAV4	STML3_HUMAN Stomatin-like protein 3 OS=Homo sapiens GN=STOML3 PE=1 SV=1		17.18

Q8TB03;Q8TB03-2	CX038_HUMAN Uncharacterized protein CXorf38 OS=Homo sapiens GN=CXorf38 PE=1 SV=1;>sp|Q8TB03-2|CX038_HUMAN Isoform 2	1.61E-10	
Q8TB24	RIN3_HUMAN Ras and Rab interactor 3 OS=Homo sapiens GN=RIN3 PE=1 SV=4		15.93
Q8TB24-4	RIN3_HUMAN Isoform 4 of Ras and Rab interactor 3 OS=Homo sapiens GN=RIN3		15.93
Q8TB45	DPTOR_HUMAN DEP domain-containing mTOR-interacting protein OS=Homo sapiens GN=DEPTOR PE=1 SV=2		30.67
Q8TB61	S35B2_HUMAN Adenosine 3'-phospho 5'-phosphosulfate transporter 1 OS=Homo sapiens GN=SLC35B2 PE=1 SV=1		27.61
Q8TB61-2	S35B2_HUMAN Isoform 2 of Adenosine 3'-phospho 5'-phosphosulfate transporter 1 OS=Homo sapiens GN=SLC35B2		27.61
Q8TB61-3	S35B2_HUMAN Isoform 3 of Adenosine 3'-phospho 5'-phosphosulfate transporter 1 OS=Homo sapiens GN=SLC35B2		27.61
Q8TB61;Q8TB61-2;Q8TB6	S35B2_HUMAN Adenosine 3-phospho 5-phosphosulfate transporter 1 OS=Homo sapiens GN=SLC35B2 PE=1 SV=1;>sp|Q8TB61-2|S35	1.04E-15	
Q8TB68	PRR7_HUMAN Proline-rich protein 7 OS=Homo sapiens GN=PRR7 PE=2 SV=1		
Q8TB68-2	PRR7_HUMAN Isoform 2 of Proline-rich protein 7 OS=Homo sapiens GN=PRR7		
Q8TB72;Q8TB72-3;Q8TB7	PUM2_HUMAN Pumilio homolog 2 OS=Homo sapiens GN=PUM2 PE=1 SV=2;>sp|Q8TB72-3|PUM2_HUMAN Isoform 3 of Pumilio homol	2.10E-08	
Q8TBA6;Q8TBA6-2	GOGA5_HUMAN Golgin subfamily A member 5 OS=Homo sapiens GN=GOLGA5 PE=1 SV=3;>sp|Q8TBA6-2|GOGA5_HUMAN Isoform 2	9.75E-10	
Q8TBB1	LNX1_HUMAN E3 ubiquitin-protein ligase LNX OS=Homo sapiens GN=LNX1 PE=1 SV=1		
Q8TBB1-2	LNX1_HUMAN Isoform 2 of E3 ubiquitin-protein ligase LNX OS=Homo sapiens GN=LNX1		
Q8TBC4	UBA3_HUMAN NEDD8-activating enzyme E1 catalytic subunit OS=Homo sapiens GN=UBA3 PE=1 SV=2		52.74
Q8TBC4-2	UBA3_HUMAN Isoform 2 of NEDD8-activating enzyme E1 catalytic subunit OS=Homo sapiens GN=UBA3		52.74
Q8TBC4;Q8TBC4-2	UBA3_HUMAN NEDD8-activating enzyme E1 catalytic subunit OS=Homo sapiens GN=UBA3 PE=1 SV=2;>sp|Q8TBC4-2|UBA3_HUMAN	9.42E-24	
Q8TBE9	NANP_HUMAN N-acylneuraminate-9-phosphatase OS=Homo sapiens GN=NANP PE=1 SV=1		26.59
Q8TBF4	ZCRB1_HUMAN Zinc finger CCHC-type and RNA-binding motif-containing protein 1 OS=Homo sapiens GN=ZCRB1 PE=1 SV=2		36.38
Q8TBQ9	KISHA_HUMAN Protein kish-A OS=Homo sapiens GN=TMEM167A PE=2 SV=1		77.63
Q8TBR4	STG34_HUMAN STAG3-like protein 4 OS=Homo sapiens GN=STAG3L4 PE=2 SV=1		
Q8TBX8	PI42C_HUMAN Phosphatidylinositol-5-phosphate 4-kinase type-2 gamma OS=Homo sapiens GN=PIP4K2C PE=1 SV=3		
Q8TBY9	WDR66_HUMAN WD repeat-containing protein 66 OS=Homo sapiens GN=WDR66 PE=1 SV=2		
Q8TBY9-2	WDR66_HUMAN Isoform 2 of WD repeat-containing protein 66 OS=Homo sapiens GN=WDR66		
Q8TBY9-3	WDR66_HUMAN Isoform 3 of WD repeat-containing protein 66 OS=Homo sapiens GN=WDR66		
Q8TBZ3	WDR20_HUMAN WD repeat-containing protein 20 OS=Homo sapiens GN=WDR20 PE=1 SV=2		
Q8TBZ3-2	WDR20_HUMAN Isoform 2 of WD repeat-containing protein 20 OS=Homo sapiens GN=WDR20		
Q8TC07-2;Q8TC07	2|TBC15_HUMAN Isoform 2 of TBC1 domain family member 15 OS=Homo sapiens GN=TBC1D15;>sp|Q8TC07|TBC15_HUMAN TBC1 d	6.21E-09	
Q8TC07;Q8TC07-2	TBC15_HUMAN TBC1 domain family member 15 OS=Homo sapiens GN=TBC1D15 PE=1 SV=2;>sp|Q8TC07-2|TBC15_HUMAN Isoform	1.60E-09	
Q8TC12	RDH11_HUMAN Retinol dehydrogenase 11 OS=Homo sapiens GN=RDH11 PE=1 SV=2		369.56
Q8TC12-2	RDH11_HUMAN Isoform 2 of Retinol dehydrogenase 11 OS=Homo sapiens GN=RDH11		302.63
Q8TC12;Q8TC12-2	RDH11_HUMAN Retinol dehydrogenase 11 OS=Homo sapiens GN=RDH11 PE=1 SV=2;>sp|Q8TC12-2|RDH11_HUMAN Isoform 2 of Re	2.34E-62	
Q8TCA0;Q8TCA0-3;Q8TCA	LRC20_HUMAN Leucine-rich repeat-containing protein 20 OS=Homo sapiens GN=LRRC20 PE=2 SV=1;>sp|Q8TCA0-3|LRC20_HUMAN I	1.74E-08	
Q8TCC3	RM30_HUMAN 39S ribosomal protein L30, mitochondrial OS=Homo sapiens GN=MRPL30 PE=1 SV=1		68.02
Q8TCC3-2	RM30_HUMAN Isoform 2 of 39S ribosomal protein L30, mitochondrial OS=Homo sapiens GN=MRPL30		68.02
Q8TCC3-2;D3DVI0;Q8TCC	2|RM30_HUMAN Isoform 2 of 39S ribosomal protein L30, mitochondrial OS=Homo sapiens GN=MRPL30;>tr|D3DVI0|D3DVI0_HUMAN	4.77E-19	
Q8TCC3-3	RM30_HUMAN Isoform 3 of 39S ribosomal protein L30, mitochondrial OS=Homo sapiens GN=MRPL30		68.02
Q8TCD5	NT5C_HUMAN 5(3)-deoxyribonucleotidase, cytosolic type OS=Homo sapiens GN=NT5C PE=1 SV=2	1.58E-05	
Q8TCF1	ZFAN1_HUMAN AN1-type zinc finger protein 1 OS=Homo sapiens GN=ZFAND1 PE=1 SV=1		29.46
Q8TCF1-2	ZFAN1_HUMAN Isoform 2 of AN1-type zinc finger protein 1 OS=Homo sapiens GN=ZFAND1		29.46
Q8TCF1;Q05BD2;Q8TCF1-	ZFAN1_HUMAN AN1-type zinc finger protein 1 OS=Homo sapiens GN=ZFAND1 PE=1 SV=1;>tr|Q05BD2|Q05BD2_HUMAN ZFAND1 pro	4.41E-08	
Q8TCG1	CIP2A_HUMAN Protein CIP2A OS=Homo sapiens GN=KIAA1524 PE=1 SV=2		33.45
Q8TCG1-2	CIP2A_HUMAN Isoform 2 of Protein CIP2A OS=Homo sapiens GN=KIAA1524		33.45
Q8TCG1;Q8TCG1-2	CIP2A_HUMAN Protein CIP2A OS=Homo sapiens GN=KIAA1524 PE=1 SV=2;>sp|Q8TCG1-2|CIP2A_HUMAN Isoform 2 of Protein CIP2A	8.92E-13	
Q8TCJ2	STT3B_HUMAN Dolichyl-diphosphooligosaccharide--protein glycosyltransferase subunit STT3B OS=Homo sapiens GN=STT3B PE=1 SV=1		225.69
Q8TCS8	PNPT1_HUMAN Polyribonucleotide nucleotidyltransferase 1, mitochondrial OS=Homo sapiens GN=PNPT1 PE=1 SV=2		86.6
Q8TCT8	PSL2_HUMAN Signal peptide peptidase-like 2A OS=Homo sapiens GN=SPPL2A PE=1 SV=2		
Q8TCT9	HM13_HUMAN Minor histocompatibility antigen H13 OS=Homo sapiens GN=HM13 PE=1 SV=1		207.19
Q8TCT9-2	HM13_HUMAN Isoform 2 of Minor histocompatibility antigen H13 OS=Homo sapiens GN=HM13		176.18
Q8TCT9-4	HM13_HUMAN Isoform 4 of Minor histocompatibility antigen H13 OS=Homo sapiens GN=HM13		174.68
Q8TCT9-5	HM13_HUMAN Isoform 5 of Minor histocompatibility antigen H13 OS=Homo sapiens GN=HM13		207.19
Q8TCT9;Q8TCT9-5;E1P5L3	HM13_HUMAN Minor histocompatibility antigen H13 OS=Homo sapiens GN=HM13 PE=1 SV=1;>sp|Q8TCT9-5|HM13_HUMAN Isoform 5	4.45E-28	
Q8TCU4	ALMS1_HUMAN Alstrom syndrome protein 1 OS=Homo sapiens GN=ALMS1 PE=1 SV=3		
Q8TCU4-2	ALMS1_HUMAN Isoform 2 of Alstrom syndrome protein 1 OS=Homo sapiens GN=ALMS1		
Q8TCU4-3	ALMS1_HUMAN Isoform 3 of Alstrom syndrome protein 1 OS=Homo sapiens GN=ALMS1		
Q8TCU4;Q8TCU4-2;Q8TCU	ALMS1_HUMAN Alstrom syndrome protein 1 OS=Homo sapiens GN=ALMS1 PE=1 SV=3;>sp|Q8TCU4-2|ALMS1_HUMAN Isoform 2 of A	3.38E-17	
Q8TCU6	PREX1_HUMAN Phosphatidylinositol 3,4,5-trisphosphate-dependent Rac exchanger 1 protein OS=Homo sapiens GN=PREX1 PE=1 SV=3		25
Q8TCU6-3	PREX1_HUMAN Isoform 3 of Phosphatidylinositol 3,4,5-trisphosphate-dependent Rac exchanger 1 protein OS=Homo sapiens GN=PREX1		25
Q8TCU6;Q8TCU6-3	PREX1_HUMAN Phosphatidylinositol 3,4,5-trisphosphate-dependent Rac exchanger 1 protein OS=Homo sapiens GN=PREX1 PE=1 SV=	9.30E-11	
Q8TCZ2	C99L2_HUMAN CD99 antigen-like protein 2 OS=Homo sapiens GN=CD99L2 PE=2 SV=1		30.31
Q8TCZ2-2	C99L2_HUMAN Isoform 2 of CD99 antigen-like protein 2 OS=Homo sapiens GN=CD99L2		30.31

Q8TCZ2-3	C99L2_HUMAN Isoform 3 of CD99 antigen-like protein 2 OS=Homo sapiens GN=CD99L2		30.31
Q8TD06	AGR3_HUMAN Anterior gradient protein 3 homolog OS=Homo sapiens GN=AGR3 PE=1 SV=1		23.53
Q8TD07	N2DL4_HUMAN NKG2D ligand 4 OS=Homo sapiens GN=RAET1E PE=1 SV=1		31.18
Q8TD07-2	N2DL4_HUMAN Isoform 2 of NKG2D ligand 4 OS=Homo sapiens GN=RAET1E		31.18
Q8TD07-3	N2DL4_HUMAN Isoform 3 of NKG2D ligand 4 OS=Homo sapiens GN=RAET1E		31.18
Q8TD08;Q8TD08-3;Q8TD(	MK15_HUMAN Mitogen-activated protein kinase 15 OS=Homo sapiens GN=MAPK15 PE=1 SV=1;>sp|Q8TD08-3|MK15_HUMAN Isoforn	0.00010541	
Q8TD16-2;Q8TD16	2|BICD2_HUMAN Isoform 2 of Protein bicaudal D homolog 2 OS=Homo sapiens GN=BICD2;>sp|Q8TD16|BICD2_HUMAN Protein bicau	4.29E-05	
Q8TD19	NEK9_HUMAN Serine/threonine-protein kinase Nek9 OS=Homo sapiens GN=NEK9 PE=1 SV=2	2.98E-10	
Q8TD26	CHD6_HUMAN Chromodomain-helicase-DNA-binding protein 6 OS=Homo sapiens GN=CHD6 PE=1 SV=4		74.92
Q8TD26-3	CHD6_HUMAN Isoform 3 of Chromodomain-helicase-DNA-binding protein 6 OS=Homo sapiens GN=CHD6		16.08
Q8TD26;Q3L8U1;Q3L8U1-	CHD6_HUMAN Chromodomain-helicase-DNA-binding protein 6 OS=Homo sapiens GN=CHD6 PE=1 SV=4;>sp|Q3L8U1|CHD9_HUMAN (	1.12E-08	
Q8TD30	ALAT2_HUMAN Alanine aminotransferase 2 OS=Homo sapiens GN=GPT2 PE=1 SV=1		
Q8TD43	TRPM4_HUMAN Transient receptor potential cation channel subfamily M member 4 OS=Homo sapiens GN=TRPM4 PE=1 SV=1		
Q8TD43-2	TRPM4_HUMAN Isoform 2 of Transient receptor potential cation channel subfamily M member 4 OS=Homo sapiens GN=TRPM4		
Q8TD43-3	TRPM4_HUMAN Isoform 3 of Transient receptor potential cation channel subfamily M member 4 OS=Homo sapiens GN=TRPM4		
Q8TD47	RS4Y2_HUMAN 40S ribosomal protein S4, Y isoform 2 OS=Homo sapiens GN=RPS4Y2 PE=1 SV=3		273.32
Q8TD55	PKHO2_HUMAN Pleckstrin homology domain-containing family O member 2 OS=Homo sapiens GN=PLEKHO2 PE=1 SV=1		
Q8TD55-2	PKHO2_HUMAN Isoform 2 of Pleckstrin homology domain-containing family O member 2 OS=Homo sapiens GN=PLEKHO2		
Q8TD55;Q8TD55-2	PKHO2_HUMAN Pleckstrin homology domain-containing family O member 2 OS=Homo sapiens GN=PLEKHO2 PE=1 SV=1;>sp|Q8TD5!	1.68E-07	
Q8TDB6	DTX3L_HUMAN E3 ubiquitin-protein ligase DTX3L OS=Homo sapiens GN=DTX3L PE=1 SV=1	1.83E-13	
Q8TDD1	DDX54_HUMAN ATP-dependent RNA helicase DDX54 OS=Homo sapiens GN=DDX54 PE=1 SV=2		75.24
Q8TDD1-2	DDX54_HUMAN Isoform 2 of ATP-dependent RNA helicase DDX54 OS=Homo sapiens GN=DDX54		60.54
Q8TDD1-2;Q8TDD1	2|DDX54_HUMAN Isoform 2 of ATP-dependent RNA helicase DDX54 OS=Homo sapiens GN=DDX54;>sp|Q8TDD1|DDX54_HUMAN ATP-	1.17E-27	
Q8TDH9	MUTED_HUMAN Protein Muted homolog OS=Homo sapiens GN=MUTED PE=1 SV=1	1.79E-08	
Q8TDI0	CHD5_HUMAN Chromodomain-helicase-DNA-binding protein 5 OS=Homo sapiens GN=CHD5 PE=1 SV=1		651.5
Q8TDI8	TMC1_HUMAN Transmembrane channel-like protein 1 OS=Homo sapiens GN=TMC1 PE=1 SV=2		35.08
Q8TDJ6	DMXL2_HUMAN DmX-like protein 2 OS=Homo sapiens GN=DMXL2 PE=1 SV=2		37.63
Q8TDM6	DLG5_HUMAN Disks large homolog 5 OS=Homo sapiens GN=DLG5 PE=1 SV=4		
Q8TDM6-2	DLG5_HUMAN Isoform 2 of Disks large homolog 5 OS=Homo sapiens GN=DLG5		
Q8TDM6-3	DLG5_HUMAN Isoform 3 of Disks large homolog 5 OS=Homo sapiens GN=DLG5		
Q8TDM6-4	DLG5_HUMAN Isoform 4 of Disks large homolog 5 OS=Homo sapiens GN=DLG5		
Q8TDM6-5	DLG5_HUMAN Isoform 5 of Disks large homolog 5 OS=Homo sapiens GN=DLG5		
Q8TDN6	BRX1_HUMAN Ribosome biogenesis protein BRX1 homolog OS=Homo sapiens GN=BRIX1 PE=1 SV=2		189.93
Q8TDP1	RNH2C_HUMAN Ribonuclease H2 subunit C OS=Homo sapiens GN=RNASEH2C PE=1 SV=1		181.6
Q8TDP1-2	RNH2C_HUMAN Isoform 2 of Ribonuclease H2 subunit C OS=Homo sapiens GN=RNASEH2C		181.6
Q8TDP1;Q8TDP1-2	RNH2C_HUMAN Ribonuclease H2 subunit C OS=Homo sapiens GN=RNASEH2C PE=1 SV=1;>sp|Q8TDP1-2|RNH2C_HUMAN Isoform 2 (	4.33E-45	
Q8TDQ7;Q8TDQ7-2;Q8TD	GNPI2_HUMAN Glucosamine-6-phosphate isomerase 2 OS=Homo sapiens GN=GNPDA2 PE=1 SV=1;>sp|Q8TDQ7-2|GNPI2_HUMAN Is	5.25E-32	
Q8TDR0	MIPT3_HUMAN TRAF3-interacting protein 1 OS=Homo sapiens GN=TRAF3IP1 PE=1 SV=1		
Q8TDR0-2	MIPT3_HUMAN Isoform 2 of TRAF3-interacting protein 1 OS=Homo sapiens GN=TRAF3IP1		
Q8TDT2	GP152_HUMAN Probable G-protein coupled receptor 152 OS=Homo sapiens GN=GPR152 PE=2 SV=1		
Q8TDX5-2	ACMSD_HUMAN Isoform 2 of 2-amino-3-carboxymuconate-6-semialdehyde decarboxylase OS=Homo sapiens GN=ACMSD		
Q8TDX7	NEK7_HUMAN Serine/threonine-protein kinase Nek7 OS=Homo sapiens GN=NEK7 PE=1 SV=1	3.18E-08	
Q8TDZ2;Q8TDZ2-2	MICA1_HUMAN NEDD9-interacting protein with calponin homology and LIM domains OS=Homo sapiens GN=MICAL1 PE=1 SV=2;>sp|(	9.23E-12	
Q8TE56	ATS17_HUMAN A disintegrin and metalloproteinase with thrombospondin motifs 17 OS=Homo sapiens GN=ADAMTS17 PE=2 SV=2		29.6
Q8TE58	ATS15_HUMAN A disintegrin and metalloproteinase with thrombospondin motifs 15 OS=Homo sapiens GN=ADAMTS15 PE=2 SV=1		
Q8TE68	ES8L1_HUMAN Epidermal growth factor receptor kinase substrate 8-like protein 1 OS=Homo sapiens GN=EPS8L1 PE=1 SV=3		
Q8TE68-2	ES8L1_HUMAN Isoform 2 of Epidermal growth factor receptor kinase substrate 8-like protein 1 OS=Homo sapiens GN=EPS8L1		
Q8TE68-3	ES8L1_HUMAN Isoform 3 of Epidermal growth factor receptor kinase substrate 8-like protein 1 OS=Homo sapiens GN=EPS8L1		
Q8TE68-4	ES8L1_HUMAN Isoform 4 of Epidermal growth factor receptor kinase substrate 8-like protein 1 OS=Homo sapiens GN=EPS8L1		
Q8TE68;Q8TE68-2;Q8TE6	ES8L1_HUMAN Epidermal growth factor receptor kinase substrate 8-like protein 1 OS=Homo sapiens GN=EPS8L1 PE=1 SV=3;>sp|Q8	1.02E-13	
Q8TE73	DYH5_HUMAN Dynein heavy chain 5, axonemal OS=Homo sapiens GN=DNAH5 PE=1 SV=3		61.01
Q8TE77;Q8TE77-3;Q8TE7	SSH3_HUMAN Protein phosphatase Slingshot homolog 3 OS=Homo sapiens GN=SSH3 PE=1 SV=2;>sp|Q8TE77-3|SSH3_HUMAN Isofc	2.43E-25	
Q8TEA1	NSUN6_HUMAN Putative methyltransferase NSUN6 OS=Homo sapiens GN=NSUN6 PE=1 SV=1		
Q8TEA7	TBCK_HUMAN TBC domain-containing protein kinase-like protein OS=Homo sapiens GN=TBCK PE=1 SV=4		24.99
Q8TEA7-2	TBCK_HUMAN Isoform 2 of TBC domain-containing protein kinase-like protein OS=Homo sapiens GN=TBCK		24.99
Q8TEA7-3	TBCK_HUMAN Isoform 3 of TBC domain-containing protein kinase-like protein OS=Homo sapiens GN=TBCK		24.99
Q8TEA8	DTD1_HUMAN D-tyrosyl-tRNA(Tyr) deacylase 1 OS=Homo sapiens GN=DTD1 PE=1 SV=2		114.73
Q8TED0	UTP15_HUMAN U3 small nucleolar RNA-associated protein 15 homolog OS=Homo sapiens GN=UTP15 PE=1 SV=3		
Q8TED1	GPX8_HUMAN Probable glutathione peroxidase 8 OS=Homo sapiens GN=GPX8 PE=1 SV=2		70.91
Q8TEK3	DOT1L_HUMAN Histone-lysine N-methyltransferase, H3 lysine-79 specific OS=Homo sapiens GN=DOT1L PE=1 SV=2		
Q8TEK3-2	DOT1L_HUMAN Isoform 1 of Histone-lysine N-methyltransferase, H3 lysine-79 specific OS=Homo sapiens GN=DOT1L		

Q8TEM1	PO210_HUMAN Nuclear pore membrane glycoprotein 210 OS=Homo sapiens GN=NUP210 PE=1 SV=3		
Q8TEM1-2	PO210_HUMAN Isoform 2 of Nuclear pore membrane glycoprotein 210 OS=Homo sapiens GN=NUP210		
Q8TEM1;Q8TEM1-2	PO210_HUMAN Nuclear pore membrane glycoprotein 210 OS=Homo sapiens GN=NUP210 PE=1 SV=3;>sp|Q8TEM1-2|PO210_HUMAN	6.76E-184	
Q8TEQ6	GEMI5_HUMAN Gem-associated protein 5 OS=Homo sapiens GN=GEMIN5 PE=1 SV=3		
Q8TER5	ARH40_HUMAN Rho guanine nucleotide exchange factor 40 OS=Homo sapiens GN=ARHGEF40 PE=1 SV=3		25.94
Q8TER5-2	ARH40_HUMAN Isoform 2 of Rho guanine nucleotide exchange factor 40 OS=Homo sapiens GN=ARHGEF40		25.94
Q8TER5-3	ARH40_HUMAN Isoform 3 of Rho guanine nucleotide exchange factor 40 OS=Homo sapiens GN=ARHGEF40		30.72
Q8TER5-4	ARH40_HUMAN Isoform 4 of Rho guanine nucleotide exchange factor 40 OS=Homo sapiens GN=ARHGEF40		25.94
Q8TES7	FBF1_HUMAN Fas-binding factor 1 OS=Homo sapiens GN=FBF1 PE=1 SV=2		
Q8TES7-2	FBF1_HUMAN Isoform 2 of Fas-binding factor 1 OS=Homo sapiens GN=FBF1		
Q8TES7-3	FBF1_HUMAN Isoform 3 of Fas-binding factor 1 OS=Homo sapiens GN=FBF1		
Q8TES7-4	FBF1_HUMAN Isoform 4 of Fas-binding factor 1 OS=Homo sapiens GN=FBF1		
Q8TES7-5	FBF1_HUMAN Isoform 5 of Fas-binding factor 1 OS=Homo sapiens GN=FBF1		
Q8TES7-6	FBF1_HUMAN Isoform 6 of Fas-binding factor 1 OS=Homo sapiens GN=FBF1		
Q8TEU7;Q8TEU7-3;Q9Y4C	RPGF6_HUMAN Rap guanine nucleotide exchange factor 6 OS=Homo sapiens GN=RAPGEF6 PE=1 SV=2;>sp|Q8TEU7-3|RPGF6_HUMAI	8.59E-08	
Q8TEW0	PARD3_HUMAN Partitioning defective 3 homolog OS=Homo sapiens GN=PARD3 PE=1 SV=2		
Q8TEW0-10	PARD3_HUMAN Isoform 10 of Partitioning defective 3 homolog OS=Homo sapiens GN=PARD3		
Q8TEW0-2	PARD3_HUMAN Isoform 2 of Partitioning defective 3 homolog OS=Homo sapiens GN=PARD3		
Q8TEW0-3	PARD3_HUMAN Isoform 3 of Partitioning defective 3 homolog OS=Homo sapiens GN=PARD3		
Q8TEW0-4	PARD3_HUMAN Isoform 4 of Partitioning defective 3 homolog OS=Homo sapiens GN=PARD3		
Q8TEW0-5	PARD3_HUMAN Isoform 5 of Partitioning defective 3 homolog OS=Homo sapiens GN=PARD3		
Q8TEW0-6	PARD3_HUMAN Isoform 6 of Partitioning defective 3 homolog OS=Homo sapiens GN=PARD3		
Q8TEW0-7	PARD3_HUMAN Isoform 7 of Partitioning defective 3 homolog OS=Homo sapiens GN=PARD3		
Q8TEW0-8	PARD3_HUMAN Isoform 8 of Partitioning defective 3 homolog OS=Homo sapiens GN=PARD3		
Q8TEW0-9	PARD3_HUMAN Isoform 9 of Partitioning defective 3 homolog OS=Homo sapiens GN=PARD3		
Q8TEW8-4	PAR3L_HUMAN Isoform 4 of Partitioning defective 3 homolog B OS=Homo sapiens GN=PARD3B		
Q8TEW8;Q8TEW8-2;Q8TE	PAR3L_HUMAN Partitioning defective 3 homolog B OS=Homo sapiens GN=PARD3B PE=1 SV=2;>sp|Q8TEW8-2|PAR3L_HUMAN Isoforn	1.66E-06	
Q8TEX9	IPO4_HUMAN Importin-4 OS=Homo sapiens GN=IPO4 PE=1 SV=2		
Q8TEX9-2	IPO4_HUMAN Isoform 2 of Importin-4 OS=Homo sapiens GN=IPO4		
Q8TEX9-2;Q8TEX9	2|IPO4_HUMAN Isoform 2 of Importin-4 OS=Homo sapiens GN=IPO4;>sp|Q8TEX9|IPO4_HUMAN Importin-4 OS=Homo sapiens GN=I	3.37E-298	
Q8TF05;Q8TF05-2	PP4R1_HUMAN Serine/threonine-protein phosphatase 4 regulatory subunit 1 OS=Homo sapiens GN=PPP4R1 PE=1 SV=1;>sp|Q8TF05-	1.21E-27	
Q8TF09	DLRB2_HUMAN Dynein light chain roadblock-type 2 OS=Homo sapiens GN=DYNLRB2 PE=1 SV=1		64.64
Q8TF17	S3TC2_HUMAN SH3 domain and tetratricopeptide repeats-containing protein 2 OS=Homo sapiens GN=SH3TC2 PE=1 SV=2		
Q8TF42	UBS3B_HUMAN Ubiquitin-associated and SH3 domain-containing protein B OS=Homo sapiens GN=UBASH3B PE=1 SV=2		
Q8TF46	DI3L1_HUMAN DIS3-like exonuclease 1 OS=Homo sapiens GN=DIS3L PE=1 SV=2		
Q8TF46-2	DI3L1_HUMAN Isoform 2 of DIS3-like exonuclease 1 OS=Homo sapiens GN=DIS3L		
Q8TF46-4	DI3L1_HUMAN Isoform 4 of DIS3-like exonuclease 1 OS=Homo sapiens GN=DIS3L		
Q8TF64	GIPC3_HUMAN PDZ domain-containing protein GIPC3 OS=Homo sapiens GN=GIPC3 PE=2 SV=1		
Q8TF65	GIPC2_HUMAN PDZ domain-containing protein GIPC2 OS=Homo sapiens GN=GIPC2 PE=1 SV=1		64.62
Q8TF66	LRC15_HUMAN Leucine-rich repeat-containing protein 15 OS=Homo sapiens GN=LRRC15 PE=1 SV=2		
Q8TF66-2	LRC15_HUMAN Isoform 2 of Leucine-rich repeat-containing protein 15 OS=Homo sapiens GN=LRRC15		
Q8TF66-2;Q8TF66	2|LRC15_HUMAN Isoform 2 of Leucine-rich repeat-containing protein 15 OS=Homo sapiens GN=LRRC15;>sp|Q8TF66|LRC15_HUMAN	1.03E-109	
Q8TF72	SHRM3_HUMAN Protein Shroom3 OS=Homo sapiens GN=SHROOM3 PE=1 SV=2	1.72E-05	
Q8TF74	WIPF2_HUMAN WAS/WASL-interacting protein family member 2 OS=Homo sapiens GN=WIPF2 PE=1 SV=1		157.65
Q8TF74-2	WIPF2_HUMAN Isoform 2 of WAS/WASL-interacting protein family member 2 OS=Homo sapiens GN=WIPF2		86.26
Q8WTS6	SETD7_HUMAN Histone-lysine N-methyltransferase SETD7 OS=Homo sapiens GN=SETD7 PE=1 SV=1	1.36E-13	
Q8WTT2	NOC3L_HUMAN Nucleolar complex protein 3 homolog OS=Homo sapiens GN=NOC3L PE=1 SV=1		24.49
Q8WTV1	THAP3_HUMAN THAP domain-containing protein 3 OS=Homo sapiens GN=THAP3 PE=2 SV=1		
Q8WTV1-3	THAP3_HUMAN Isoform 2 of THAP domain-containing protein 3 OS=Homo sapiens GN=THAP3		
Q8WTW3	COG1_HUMAN Conserved oligomeric Golgi complex subunit 1 OS=Homo sapiens GN=COG1 PE=1 SV=1	2.86E-22	
Q8WU03	GLYL2_HUMAN Glycine N-acyltransferase-like protein 2 OS=Homo sapiens GN=GLYATL2 PE=2 SV=1	1.80E-10	
Q8WU39;Q8WU39-3	PERP1_HUMAN Plasma cell-induced resident endoplasmic reticulum protein OS=Homo sapiens GN=PACAP PE=1 SV=1;>sp|Q8WU39-3	1.25E-247	
Q8WU68	U2AF4_HUMAN Splicing factor U2AF 26 kDa subunit OS=Homo sapiens GN=U2AF1L4 PE=1 SV=2		44.17
Q8WU68-2	U2AF4_HUMAN Isoform 2 of Splicing factor U2AF 26 kDa subunit OS=Homo sapiens GN=U2AF1L4		44.17
Q8WU68-3	U2AF4_HUMAN Isoform 3 of Splicing factor U2AF 26 kDa subunit OS=Homo sapiens GN=U2AF1L4		44.17
Q8WU68;Q8WU68-2;Q8W	U2AF4_HUMAN Splicing factor U2AF 26 kDa subunit OS=Homo sapiens GN=U2AF1L4 PE=1 SV=2;>sp|Q8WU68-2|U2AF4_HUMAN Isof	1.84E-35	
Q8WU76	SCFD2_HUMAN Sec1 family domain-containing protein 2 OS=Homo sapiens GN=SCFD2 PE=1 SV=2		
Q8WU76-2	SCFD2_HUMAN Isoform 2 of Sec1 family domain-containing protein 2 OS=Homo sapiens GN=SCFD2		
Q8WU90	ZC3HF_HUMAN Zinc finger CCCH domain-containing protein 15 OS=Homo sapiens GN=ZC3H15 PE=1 SV=1		163.12
Q8WUA2	PPIL4_HUMAN Peptidyl-prolyl cis-trans isomerase-like 4 OS=Homo sapiens GN=PPIL4 PE=1 SV=1		23.85
Q8WUA4	TF3C2_HUMAN General transcription factor 3C polypeptide 2 OS=Homo sapiens GN=GTF3C2 PE=1 SV=2		

Q8WUA4-2	TF3C2_HUMAN Isoform 2 of General transcription factor 3C polypeptide 2 OS=Homo sapiens GN=GTF3C2		
Q8WUD1	RAB2B_HUMAN Ras-related protein Rab-2B OS=Homo sapiens GN=RAB2B PE=1 SV=1		415.83
Q8WUF5	IASPP_HUMAN RelA-associated inhibitor OS=Homo sapiens GN=PPP1R13L PE=1 SV=4	2.95E-22	
Q8WUH6	CL023_HUMAN UPF0444 transmembrane protein C12orf23 OS=Homo sapiens GN=C12orf23 PE=1 SV=1		53.73
Q8WUK0	PTPM1_HUMAN Protein-tyrosine phosphatase mitochondrial 1 OS=Homo sapiens GN=PTPMT1 PE=1 SV=1		19.1
Q8WUK0-2	PTPM1_HUMAN Isoform 2 of Protein-tyrosine phosphatase mitochondrial 1 OS=Homo sapiens GN=PTPMT1		19.1
Q8WUK0;Q8WUK0-2	PTPM1_HUMAN Protein-tyrosine phosphatase mitochondrial 1 OS=Homo sapiens GN=PTPMT1 PE=1 SV=1;>sp|Q8WUK0-2|PTPM1_HUI	4.34E-09	
Q8WUM0	NU133_HUMAN Nuclear pore complex protein Nup133 OS=Homo sapiens GN=NUP133 PE=1 SV=2		
Q8WUM4	PDC6I_HUMAN Programmed cell death 6-interacting protein OS=Homo sapiens GN=PDCD6IP PE=1 SV=1		190.43
Q8WUP2-2;Q8WUP2;Q8W	2|FBLI1_HUMAN Isoform 2 of Filamin-binding LIM protein 1 OS=Homo sapiens GN=FBLIM1;>sp|Q8WUP2|FBLI1_HUMAN Filamin-bindi	7.54E-17	
Q8WUP2;Q8WUP2-3	FBLI1_HUMAN Filamin-binding LIM protein 1 OS=Homo sapiens GN=FBLIM1 PE=1 SV=2;>sp|Q8WUP2-3|FBLI1_HUMAN Isoform 3 of I	5.27E-05	
Q8WUW1	BRK1_HUMAN Protein BRICK1 OS=Homo sapiens GN=BRK1 PE=1 SV=1		36.14
Q8WUW1-2	BRK1_HUMAN Isoform 2 of Protein BRICK1 OS=Homo sapiens GN=BRK1		36.14
Q8WUX9	CHMP7_HUMAN Charged multivesicular body protein 7 OS=Homo sapiens GN=CHMP7 PE=1 SV=1	5.34E-10	
Q8WUY1	CH055_HUMAN UPF0670 protein C8orf55 OS=Homo sapiens GN=C8orf55 PE=1 SV=2		47.08
Q8WUZ0	BCL7C_HUMAN B-cell CLL/lymphoma 7 protein family member C OS=Homo sapiens GN=BCL7C PE=1 SV=3		
Q8WUZ0-2	BCL7C_HUMAN Isoform 2 of B-cell CLL/lymphoma 7 protein family member C OS=Homo sapiens GN=BCL7C		
Q8WV22	NSE1_HUMAN Non-structural maintenance of chromosomes element 1 homolog OS=Homo sapiens GN=NSMCE1 PE=1 SV=5		24.93
Q8WV24	PHLA1_HUMAN Pleckstrin homology-like domain family A member 1 OS=Homo sapiens GN=PHLDA1 PE=1 SV=4		70.85
Q8WV28	BLNK_HUMAN B-cell linker protein OS=Homo sapiens GN=BLNK PE=1 SV=2		
Q8WV28-2	BLNK_HUMAN Isoform 2 of B-cell linker protein OS=Homo sapiens GN=BLNK		
Q8WV92	MITD1_HUMAN MIT domain-containing protein 1 OS=Homo sapiens GN=MITD1 PE=1 SV=1		
Q8WV99	ZFN2B_HUMAN AN1-type zinc finger protein 2B OS=Homo sapiens GN=ZFAND2B PE=1 SV=1		
Q8WV99-2	ZFN2B_HUMAN Isoform 2 of AN1-type zinc finger protein 2B OS=Homo sapiens GN=ZFAND2B		
Q8WVB6-2;Q8WVB6;Q8W	2|CTF18_HUMAN Isoform 2 of Chromosome transmission fidelity protein 18 homolog OS=Homo sapiens GN=CHTF18;>sp|Q8WVB6|CT	2.82E-06	
Q8WVC0	LEO1_HUMAN RNA polymerase-associated protein LEO1 OS=Homo sapiens GN=LEO1 PE=1 SV=1		32.52
Q8WVC0-2	LEO1_HUMAN Isoform 2 of RNA polymerase-associated protein LEO1 OS=Homo sapiens GN=LEO1		32.52
Q8WVC0;B4DNM0;Q8WVC	LEO1_HUMAN RNA polymerase-associated protein LEO1 OS=Homo sapiens GN=LEO1 PE=1 SV=1;>tr|B4DNM0|B4DNM0_HUMAN cDN.	7.51E-10	
Q8WVC6	DCAKD_HUMAN Dephospho-CoA kinase domain-containing protein OS=Homo sapiens GN=DCAKD PE=1 SV=1	8.48E-10	
Q8WVI0	CC078_HUMAN UPF0640 protein C3orf78 OS=Homo sapiens GN=C3orf78 PE=2 SV=2		
Q8WVJ2	NUDC2_HUMAN NudC domain-containing protein 2 OS=Homo sapiens GN=NUDCD2 PE=1 SV=1		91.38
Q8WVJ9	TWST2_HUMAN Twist-related protein 2 OS=Homo sapiens GN=TWIST2 PE=1 SV=1		
Q8WVK2	SNR27_HUMAN U4/U6.U5 small nuclear ribonucleoprotein 27 kDa protein OS=Homo sapiens GN=SNRNP27 PE=1 SV=1		40.92
Q8WVK7;P0CB44	SKA2_HUMAN Spindle and kinetochore-associated protein 2 OS=Homo sapiens GN=SKA2 PE=1 SV=1;>sp|P0CB44|SKA2L_HUMAN Pu	9.45E-06	
Q8WVM0	TFB1M_HUMAN Dimethyladenosine transferase 1, mitochondrial OS=Homo sapiens GN=TFB1M PE=1 SV=1		38.17
Q8WVM7	STAG1_HUMAN Cohesin subunit SA-1 OS=Homo sapiens GN=STAG1 PE=1 SV=3		
Q8WVM8	SCFD1_HUMAN Sec1 family domain-containing protein 1 OS=Homo sapiens GN=SCFD1 PE=1 SV=4		178.73
Q8WVQ1	CANT1_HUMAN Soluble calcium-activated nucleotidase 1 OS=Homo sapiens GN=CANT1 PE=1 SV=1		
Q8WVQ1-2	CANT1_HUMAN Isoform 2 of Soluble calcium-activated nucleotidase 1 OS=Homo sapiens GN=CANT1		
Q8WVQ1;B4DJ54;Q8WVQ	CANT1_HUMAN Soluble calcium-activated nucleotidase 1 OS=Homo sapiens GN=CANT1 PE=1 SV=1;>tr|B4DJ54|B4DJ54_HUMAN cDN	4.62E-06	
Q8WVR3	CG043_HUMAN Uncharacterized protein C7orf43 OS=Homo sapiens GN=C7orf43 PE=1 SV=2		
Q8WVR3-2	CG043_HUMAN Isoform 2 of Uncharacterized protein C7orf43 OS=Homo sapiens GN=C7orf43		
Q8WVR3-3	CG043_HUMAN Isoform 3 of Uncharacterized protein C7orf43 OS=Homo sapiens GN=C7orf43		
Q8WVV4	POF1B_HUMAN Protein POF1B OS=Homo sapiens GN=POF1B PE=1 SV=3		
Q8WVV4-1	POF1B_HUMAN Isoform 1 of Protein POF1B OS=Homo sapiens GN=POF1B		
Q8WVV4-3	POF1B_HUMAN Isoform 3 of Protein POF1B OS=Homo sapiens GN=POF1B		
Q8WVV9	HNRLL_HUMAN Heterogeneous nuclear ribonucleoprotein L-like OS=Homo sapiens GN=HNRPLL PE=1 SV=1		184.71
Q8WVV9-2	HNRLL_HUMAN Isoform 2 of Heterogeneous nuclear ribonucleoprotein L-like OS=Homo sapiens GN=HNRPLL		145.4
Q8WVV9-3	HNRLL_HUMAN Isoform 3 of Heterogeneous nuclear ribonucleoprotein L-like OS=Homo sapiens GN=HNRPLL		117.87
Q8WVV9-4	HNRLL_HUMAN Isoform 4 of Heterogeneous nuclear ribonucleoprotein L-like OS=Homo sapiens GN=HNRPLL		184.71
Q8WVV9;Q8WVV9-4	HNRLL_HUMAN Heterogeneous nuclear ribonucleoprotein L-like OS=Homo sapiens GN=HNRPLL PE=1 SV=1;>sp|Q8WVV9-4|HNRLL_H	1.10E-15	
Q8WVX9	FACR1_HUMAN Fatty acyl-CoA reductase 1 OS=Homo sapiens GN=FAR1 PE=1 SV=1		22.6
Q8WVY7	UBCP1_HUMAN Ubiquitin-like domain-containing CTD phosphatase 1 OS=Homo sapiens GN=UBLCP1 PE=1 SV=2		31.76
Q8WW01	SEN15_HUMAN tRNA-splicing endonuclease subunit Sen15 OS=Homo sapiens GN=TSEN15 PE=1 SV=1	4.43E-87	
Q8WW12	PCNP_HUMAN PEST proteolytic signal-containing nuclear protein OS=Homo sapiens GN=PCNP PE=1 SV=2		166.64
Q8WW12-2	PCNP_HUMAN Isoform 2 of PEST proteolytic signal-containing nuclear protein OS=Homo sapiens GN=PCNP		121.29
Q8WW12-3	PCNP_HUMAN Isoform 3 of PEST proteolytic signal-containing nuclear protein OS=Homo sapiens GN=PCNP		45.35
Q8WW12;Q8WW12-2	PCNP_HUMAN PEST proteolytic signal-containing nuclear protein OS=Homo sapiens GN=PCNP PE=1 SV=2;>sp|Q8WW12-2|PCNP_HUI	7.71E-49	
Q8WW22	DNJA4_HUMAN DnaJ homolog subfamily A member 4 OS=Homo sapiens GN=DNAJA4 PE=1 SV=1		30.88
Q8WW22-2	DNJA4_HUMAN Isoform 2 of DnaJ homolog subfamily A member 4 OS=Homo sapiens GN=DNAJA4		33.08
Q8WW22-2;Q8WW22	2|DNJA4_HUMAN Isoform 2 of DnaJ homolog subfamily A member 4 OS=Homo sapiens GN=DNAJA4;>sp|Q8WW22|DNJA4_HUMAN Dı	2.80E-16	

Q8WW59	SPRY4_HUMAN SPRY domain-containing protein 4 OS=Homo sapiens GN=SPRYD4 PE=1 SV=2	2.48E-09	
Q8WWA1;Q8WWA1-2	TMM40_HUMAN Transmembrane protein 40 OS=Homo sapiens GN=TMEM40 PE=1 SV=2;>sp|Q8WWA1-2|TMM40_HUMAN Isoform 2 o	9.76E-50	
Q8WWC4	CB047_HUMAN Uncharacterized protein C2orf47, mitochondrial OS=Homo sapiens GN=C2orf47 PE=1 SV=1		
Q8WWI1	LMO7_HUMAN LIM domain only protein 7 OS=Homo sapiens GN=LMO7 PE=1 SV=3		138.98
Q8WWI1-2	LMO7_HUMAN Isoform 2 of LIM domain only protein 7 OS=Homo sapiens GN=LMO7		138.98
Q8WWI1-3	LMO7_HUMAN Isoform 3 of LIM domain only protein 7 OS=Homo sapiens GN=LMO7		129.42
Q8WWI1-4	LMO7_HUMAN Isoform 4 of LIM domain only protein 7 OS=Homo sapiens GN=LMO7		138.98
Q8WWI1;Q8WWI1-2;Q8W	LMO7_HUMAN LIM domain only protein 7 OS=Homo sapiens GN=LMO7 PE=1 SV=3;>sp|Q8WWI1-2|LMO7_HUMAN Isoform 2 of LIM d	6.33E-06	
Q8WWI5	CTL1_HUMAN Choline transporter-like protein 1 OS=Homo sapiens GN=SLC44A1 PE=1 SV=1		
Q8WWI5-2	CTL1_HUMAN Isoform 2 of Choline transporter-like protein 1 OS=Homo sapiens GN=SLC44A1		
Q8WWI5-3	CTL1_HUMAN Isoform 3 of Choline transporter-like protein 1 OS=Homo sapiens GN=SLC44A1		
Q8WWI5;Q8WWI5-2;Q8W	CTL1_HUMAN Choline transporter-like protein 1 OS=Homo sapiens GN=SLC44A1 PE=1 SV=1;>sp|Q8WWI5-2|CTL1_HUMAN Isoform 2	7.75E-10	
Q8WWK9;Q8WWK9-3	CKAP2_HUMAN Cytoskeleton-associated protein 2 OS=Homo sapiens GN=CKAP2 PE=1 SV=1;>sp|Q8WWK9-3|CKAP2_HUMAN Isoform	2.04E-07	
Q8WWM7	ATX2L_HUMAN Ataxin-2-like protein OS=Homo sapiens GN=ATXN2L PE=1 SV=2		494.78
Q8WWM7-2	ATX2L_HUMAN Isoform 2 of Ataxin-2-like protein OS=Homo sapiens GN=ATXN2L		443.57
Q8WWM7-3	ATX2L_HUMAN Isoform 3 of Ataxin-2-like protein OS=Homo sapiens GN=ATXN2L		450.23
Q8WWM7-3;Q8WWM7;Q6	3|ATX2L_HUMAN Isoform 3 of Ataxin-2-like protein OS=Homo sapiens GN=ATXN2L;>sp|Q8WWM7|ATX2L_HUMAN Ataxin-2-like protei	4.61E-25	
Q8WWM7-4	ATX2L_HUMAN Isoform 4 of Ataxin-2-like protein OS=Homo sapiens GN=ATXN2L		443.57
Q8WWM7-5	ATX2L_HUMAN Isoform 5 of Ataxin-2-like protein OS=Homo sapiens GN=ATXN2L		443.57
Q8WWM7-6	ATX2L_HUMAN Isoform 6 of Ataxin-2-like protein OS=Homo sapiens GN=ATXN2L		443.57
Q8WWM7-7	ATX2L_HUMAN Isoform 7 of Ataxin-2-like protein OS=Homo sapiens GN=ATXN2L		73.39
Q8WWM9	CYGB_HUMAN Cytoglobin OS=Homo sapiens GN=CYGB PE=1 SV=1	1.68E-15	
Q8WWN8	ARAP3_HUMAN Arf-GAP with Rho-GAP domain, ANK repeat and PH domain-containing protein 3 OS=Homo sapiens GN=ARAP3 PE=1 S	1.74E-11	
Q8WWP7	GIMA1_HUMAN GTPase IMAP family member 1 OS=Homo sapiens GN=GIMAP1 PE=1 SV=1	2.90E-08	
Q8WWQ0	PHIP_HUMAN PH-interacting protein OS=Homo sapiens GN=PHIP PE=1 SV=2		
Q8WWW0;Q8WWW0-4;Q8	RASF5_HUMAN Ras association domain-containing protein 5 OS=Homo sapiens GN=RASSF5 PE=1 SV=1;>sp|Q8WWW0-4|RASF5_HUI	1.60E-06	
Q8WWX9	SELM_HUMAN Selenoprotein M OS=Homo sapiens GN=SELM PE=1 SV=3	1.26E-08	
Q8WWY3	PRP31_HUMAN U4/U6 small nuclear ribonucleoprotein Prp31 OS=Homo sapiens GN=PRPF31 PE=1 SV=2		171.77
Q8WWY3-2	PRP31_HUMAN Isoform 2 of U4/U6 small nuclear ribonucleoprotein Prp31 OS=Homo sapiens GN=PRPF31		96.09
Q8WWY3-3	PRP31_HUMAN Isoform 3 of U4/U6 small nuclear ribonucleoprotein Prp31 OS=Homo sapiens GN=PRPF31		96.09
Q8WWY3;B4DWW8;Q8WV	PRP31_HUMAN U4/U6 small nuclear ribonucleoprotein Prp31 OS=Homo sapiens GN=PRPF31 PE=1 SV=2;>tr|B4DWW8|B4DWW8_HUM	1.95E-165	
Q8WX92	NELFB_HUMAN Negative elongation factor B OS=Homo sapiens GN=COBRA1 PE=1 SV=1		111.49
Q8WX93;Q8WX93-3;Q8W	PALLD_HUMAN Palladin OS=Homo sapiens GN=PALLD PE=1 SV=3;>sp|Q8WX93-3|PALLD_HUMAN Isoform 3 of Palladin OS=Homo sap	1.09E-103	
Q8WXA3;Q8WXA3-3;Q8W	RUFY2_HUMAN RUN and FYVE domain-containing protein 2 OS=Homo sapiens GN=RUFY2 PE=1 SV=2;>sp|Q8WXA3-3|RUFY2_HUMAN	3.25E-05	
Q8WXA9-2	SREK1_HUMAN Isoform 2 of Splicing regulatory glutamine/lysine-rich protein 1 OS=Homo sapiens GN=SREK1		65.63
Q8WXA9-2;B3KRJ9;Q8WX	2|SFR12_HUMAN Isoform 2 of Splicing factor, arginine/serine-rich 12 OS=Homo sapiens GN=SFRS12;>tr|B3KRJ9|B3KRJ9_HUMAN cDI	2.87E-58	
Q8WXA9-2;Q8WXA9	2|SREK1_HUMAN Isoform 2 of Splicing regulatory glutamine/lysine-rich protein 1 OS=Homo sapiens GN=SREK1;>sp|Q8WXA9|SREK1	1.42E-12	
Q8WXB1	MT21A_HUMAN Methyltransferase-like protein 21A OS=Homo sapiens GN=METTL21A PE=2 SV=2		
Q8WXD0	RXFP2_HUMAN Relaxin receptor 2 OS=Homo sapiens GN=RXFP2 PE=1 SV=1		
Q8WXE0	CSKI2_HUMAN Caskin-2 OS=Homo sapiens GN=CASKIN2 PE=1 SV=2		
Q8WXE0-2	CSKI2_HUMAN Isoform 2 of Caskin-2 OS=Homo sapiens GN=CASKIN2		
Q8WXE1	ATRIP_HUMAN ATR-interacting protein OS=Homo sapiens GN=ATRIP PE=1 SV=1		
Q8WXE1-2	ATRIP_HUMAN Isoform 2 of ATR-interacting protein OS=Homo sapiens GN=ATRIP		
Q8WXE1-3	ATRIP_HUMAN Isoform 3 of ATR-interacting protein OS=Homo sapiens GN=ATRIP		
Q8WXE1;Q8WXE1-2	ATRIP_HUMAN ATR-interacting protein OS=Homo sapiens GN=ATRIP PE=1 SV=1;>sp|Q8WXE1-2|ATRIP_HUMAN Isoform 2 of ATR-inte	8.87E-13	
Q8WXF0	SRS12_HUMAN Serine/arginine-rich splicing factor 12 OS=Homo sapiens GN=SRSF12 PE=1 SV=1		42.13
Q8WXF1	PSPC1_HUMAN Paraspeckle component 1 OS=Homo sapiens GN=PSPC1 PE=1 SV=1		317.66
Q8WXF1-2	PSPC1_HUMAN Isoform 2 of Paraspeckle component 1 OS=Homo sapiens GN=PSPC1		317.66
Q8WXF1;B4DWI8;Q8WXF	PSPC1_HUMAN Paraspeckle component 1 OS=Homo sapiens GN=PSPC1 PE=1 SV=1;>tr|B4DWI8|B4DWI8_HUMAN cDNA FLJ57805, h	8.68E-99	
Q8WXH0	SYNE2_HUMAN Nesprin-2 OS=Homo sapiens GN=SYNE2 PE=1 SV=3		143.97
Q8WXH0-2	SYNE2_HUMAN Isoform 2 of Nesprin-2 OS=Homo sapiens GN=SYNE2		143.97
Q8WXH0-2;Q8WXH0	2|SYNE2_HUMAN Isoform 2 of Nesprin-2 OS=Homo sapiens GN=SYNE2;>sp|Q8WXH0|SYNE2_HUMAN Nesprin-2 OS=Homo sapiens G	2.11E-07	
Q8WXH0-3	SYNE2_HUMAN Isoform 3 of Nesprin-2 OS=Homo sapiens GN=SYNE2		8.4
Q8WXH0-4	SYNE2_HUMAN Isoform 4 of Nesprin-2 OS=Homo sapiens GN=SYNE2		8.4
Q8WXH0-5	SYNE2_HUMAN Isoform 5 of Nesprin-2 OS=Homo sapiens GN=SYNE2		0.51
Q8WXH0-6	SYNE2_HUMAN Isoform 6 of Nesprin-2 OS=Homo sapiens GN=SYNE2		0.51
Q8WXH0-7	SYNE2_HUMAN Isoform 7 of Nesprin-2 OS=Homo sapiens GN=SYNE2		10.3
Q8WXH0-8	SYNE2_HUMAN Isoform 8 of Nesprin-2 OS=Homo sapiens GN=SYNE2		
Q8WXH0-9	SYNE2_HUMAN Isoform 9 of Nesprin-2 OS=Homo sapiens GN=SYNE2		
Q8WXI2	CNKR2_HUMAN Connector enhancer of kinase suppressor of ras 2 OS=Homo sapiens GN=CNKSR2 PE=1 SV=1		
Q8WXI2-2	CNKR2_HUMAN Isoform 2 of Connector enhancer of kinase suppressor of ras 2 OS=Homo sapiens GN=CNKSR2		

Q8WXI9	P66B_HUMAN Transcriptional repressor p66-beta OS=Homo sapiens GN=GATAD2B PE=1 SV=1		
Q8WXR4	MYO3B_HUMAN Myosin-IIIb OS=Homo sapiens GN=MYO3B PE=2 SV=4		48.9
Q8WXR4-2	MYO3B_HUMAN Isoform 2 of Myosin-IIIb OS=Homo sapiens GN=MYO3B		48.72
Q8WXR4-3	MYO3B_HUMAN Isoform 3 of Myosin-IIIb OS=Homo sapiens GN=MYO3B		40.81
Q8WXR4-4	MYO3B_HUMAN Isoform 4 of Myosin-IIIb OS=Homo sapiens GN=MYO3B		40.99
Q8WXR4-5	MYO3B_HUMAN Isoform 5 of Myosin-IIIb OS=Homo sapiens GN=MYO3B		48.72
Q8WXR4-6	MYO3B_HUMAN Isoform 6 of Myosin-IIIb OS=Homo sapiens GN=MYO3B		48.72
Q8WXR4-7	MYO3B_HUMAN Isoform 7 of Myosin-IIIb OS=Homo sapiens GN=MYO3B		48.9
Q8WXX5	DNJC9_HUMAN DnaJ homolog subfamily C member 9 OS=Homo sapiens GN=DNAJC9 PE=1 SV=1		58.56
Q8WXX7	AUTS2_HUMAN Autism susceptibility gene 2 protein OS=Homo sapiens GN=AUTS2 PE=1 SV=1		
Q8WXX7-2	AUTS2_HUMAN Isoform Short of Autism susceptibility gene 2 protein OS=Homo sapiens GN=AUTS2		
Q8WY21-2	SORC1_HUMAN Isoform 2 of VPS10 domain-containing receptor SorCS1 OS=Homo sapiens GN=SORCS1		
Q8WY21-3	SORC1_HUMAN Isoform 3 of VPS10 domain-containing receptor SorCS1 OS=Homo sapiens GN=SORCS1		
Q8WY22	BRI3B_HUMAN BRI3-binding protein OS=Homo sapiens GN=BRI3BP PE=1 SV=1		74.98
Q8WYA1-3	BMAL2_HUMAN Isoform 3 of Aryl hydrocarbon receptor nuclear translocator-like protein 2 OS=Homo sapiens GN=ARNTL2		
Q8WYA1-4	BMAL2_HUMAN Isoform 4 of Aryl hydrocarbon receptor nuclear translocator-like protein 2 OS=Homo sapiens GN=ARNTL2		
Q8WYA1-5	BMAL2_HUMAN Isoform 5 of Aryl hydrocarbon receptor nuclear translocator-like protein 2 OS=Homo sapiens GN=ARNTL2		
Q8WYA1-6	BMAL2_HUMAN Isoform 6 of Aryl hydrocarbon receptor nuclear translocator-like protein 2 OS=Homo sapiens GN=ARNTL2		
Q8WYA1-8	BMAL2_HUMAN Isoform 8 of Aryl hydrocarbon receptor nuclear translocator-like protein 2 OS=Homo sapiens GN=ARNTL2		
Q8WYA6	CTBL1_HUMAN Beta-catenin-like protein 1 OS=Homo sapiens GN=CTNNBL1 PE=1 SV=1		92.91
Q8WYA6-2	CTBL1_HUMAN Isoform 2 of Beta-catenin-like protein 1 OS=Homo sapiens GN=CTNNBL1		90
Q8WYA6-3	CTBL1_HUMAN Isoform 3 of Beta-catenin-like protein 1 OS=Homo sapiens GN=CTNNBL1		90
Q8WYA6;Q8WYA6-2;Q8W	CTBL1_HUMAN Beta-catenin-like protein 1 OS=Homo sapiens GN=CTNNBL1 PE=1 SV=1;>sp|Q8WYA6-2|CTBL1_HUMAN Isoform 2 of	6.05E-33	
Q8WYH8	ING5_HUMAN Inhibitor of growth protein 5 OS=Homo sapiens GN=ING5 PE=1 SV=1		36.78
Q8WYH8-2	ING5_HUMAN Isoform 2 of Inhibitor of growth protein 5 OS=Homo sapiens GN=ING5		36.78
Q8WYJ6	SEPT1_HUMAN Septin-1 OS=Homo sapiens GN=SEPT1 PE=1 SV=2		55.86
Q8WYJ6-2	SEPT1_HUMAN Isoform 2 of Septin-1 OS=Homo sapiens GN=SEPT1		14.9
Q8WYP5	ELYS_HUMAN Protein ELYS OS=Homo sapiens GN=AHCTF1 PE=1 SV=3		
Q8WYP5-2	ELYS_HUMAN Isoform 2 of Protein ELYS OS=Homo sapiens GN=AHCTF1		
Q8WYP5-2;Q8WYP5	2|ELYS_HUMAN Isoform 2 of Protein ELYS OS=Homo sapiens GN=AHCTF1;>sp|Q8WYP5|ELYS_HUMAN Protein ELYS OS=Homo sapiens	1.02E-09	
Q8WZ42	TITIN_HUMAN Titin OS=Homo sapiens GN=TTN PE=1 SV=2		725.73
Q8WZ42-2	TITIN_HUMAN Isoform 2 of Titin OS=Homo sapiens GN=TTN		724.18
Q8WZ42-3	TITIN_HUMAN Isoform 3 of Titin OS=Homo sapiens GN=TTN		530.92
Q8WZ42-4	TITIN_HUMAN Isoform 4 of Titin OS=Homo sapiens GN=TTN		718.98
Q8WZ42-5	TITIN_HUMAN Isoform 5 of Titin OS=Homo sapiens GN=TTN		617.37
Q8WZ42-6	TITIN_HUMAN Isoform 6 of Titin OS=Homo sapiens GN=TTN		
Q8WZ42-7	TITIN_HUMAN Isoform 7 of Titin OS=Homo sapiens GN=TTN		718.98
Q8WZ42-7;Q8WZ42-8;Q8	7|TITIN_HUMAN Isoform 7 of Titin OS=Homo sapiens GN=TTN;>sp|Q8WZ42-8|TITIN_HUMAN Isoform 8 of Titin OS=Homo sapiens GI	9.27E-56	
Q8WZ42-8	TITIN_HUMAN Isoform 8 of Titin OS=Homo sapiens GN=TTN		732.6
Q8WZ42-8;Q8WZ42;Q8W	8|TITIN_HUMAN Isoform 8 of Titin OS=Homo sapiens GN=TTN;>sp|Q8WZ42|TITIN_HUMAN Titin OS=Homo sapiens GN=TTN PE=1 S\	1.11E-42	
Q8WZ74	CTTB2_HUMAN Cortactin-binding protein 2 OS=Homo sapiens GN=CTTNBP2 PE=1 SV=1		47.95
Q8WZA0	LZIC_HUMAN Protein LZIC OS=Homo sapiens GN=LZIC PE=1 SV=1		22.73
Q8WZA9	IRGQ_HUMAN Immunity-related GTPase family Q protein OS=Homo sapiens GN=IRGQ PE=1 SV=1	4.15E-07	
Q902F8	ENK6_HUMAN HERV-K_8p23.1 provirus ancestral Env polyprotein OS=Homo sapiens PE=1 SV=1		
Q902F9	ENK5_HUMAN HERV-K_19p13.11 provirus ancestral Env polyprotein OS=Homo sapiens PE=1 SV=1		
Q92466	DDB2_HUMAN DNA damage-binding protein 2 OS=Homo sapiens GN=DDB2 PE=1 SV=1		
Q92466-2	DDB2_HUMAN Isoform D1 of DNA damage-binding protein 2 OS=Homo sapiens GN=DDB2		
Q92466-3	DDB2_HUMAN Isoform D2 of DNA damage-binding protein 2 OS=Homo sapiens GN=DDB2		
Q92466-4	DDB2_HUMAN Isoform D3 of DNA damage-binding protein 2 OS=Homo sapiens GN=DDB2		
Q92466-5	DDB2_HUMAN Isoform D4 of DNA damage-binding protein 2 OS=Homo sapiens GN=DDB2		
Q92466;Q92466-4;Q9246	DDB2_HUMAN DNA damage-binding protein 2 OS=Homo sapiens GN=DDB2 PE=1 SV=1;>sp|Q92466-4|DDB2_HUMAN Isoform D3 of	5.09E-17	
Q92481	AP2B_HUMAN Transcription factor AP-2-beta OS=Homo sapiens GN=TFAP2B PE=1 SV=2		
Q92481-2	AP2B_HUMAN Isoform 2 of Transcription factor AP-2-beta OS=Homo sapiens GN=TFAP2B		
Q92499	DDX1_HUMAN ATP-dependent RNA helicase DDX1 OS=Homo sapiens GN=DDX1 PE=1 SV=2		634.28
Q92503-2;Q92503;Q9250	2|S14L1_HUMAN Isoform 2 of SEC14-like protein 1 OS=Homo sapiens GN=SEC14L1;>sp|Q92503|S14L1_HUMAN SEC14-like protein :	0.00024729	
Q92504	S39A7_HUMAN Zinc transporter SLC39A7 OS=Homo sapiens GN=SLC39A7 PE=1 SV=2		24.6
Q92506	DHB8_HUMAN Estradiol 17-beta-dehydrogenase 8 OS=Homo sapiens GN=HSD17B8 PE=1 SV=2	2.49E-14	
Q92508	PIEZ1_HUMAN Protein PIEZO1 OS=Homo sapiens GN=FAM38A PE=1 SV=4		50.73
Q92520	FAM3C_HUMAN Protein FAM3C OS=Homo sapiens GN=FAM3C PE=1 SV=1		722.6
Q92522	H1X_HUMAN Histone H1x OS=Homo sapiens GN=H1FX PE=1 SV=1		235.45
Q92526	TCPW_HUMAN T-complex protein 1 subunit zeta-2 OS=Homo sapiens GN=CCT6B PE=1 SV=5		58.45

Q92530	PSMF1_HUMAN Proteasome inhibitor PI31 subunit OS=Homo sapiens GN=PSMF1 PE=1 SV=2	1.50E-14	
Q92536	YLAT2_HUMAN Y+L amino acid transporter 2 OS=Homo sapiens GN=SLC7A6 PE=1 SV=3		29.19
Q92538	GBF1_HUMAN Golgi-specific brefeldin A-resistance guanine nucleotide exchange factor 1 OS=Homo sapiens GN=GBF1 PE=1 SV=2	1.89E-12	
Q92540	SMG7_HUMAN Protein SMG7 OS=Homo sapiens GN=SMG7 PE=1 SV=2		
Q92540-2	SMG7_HUMAN Isoform 2 of Protein SMG7 OS=Homo sapiens GN=SMG7		
Q92540-4	SMG7_HUMAN Isoform 4 of Protein SMG7 OS=Homo sapiens GN=SMG7		
Q92541	RTF1_HUMAN RNA polymerase-associated protein RTF1 homolog OS=Homo sapiens GN=RTF1 PE=1 SV=4	9.55E-21	
Q92542	NICA_HUMAN Nicastrin OS=Homo sapiens GN=NCSTN PE=1 SV=2		197.42
Q92542-2	NICA_HUMAN Isoform 2 of Nicastrin OS=Homo sapiens GN=NCSTN		197.42
Q92542;Q92542-2	NICA_HUMAN Nicastrin OS=Homo sapiens GN=NCSTN PE=1 SV=2;>sp|Q92542-2|NICA_HUMAN Isoform 2 of Nicastrin OS=Homo sap	9.54E-13	
Q92544	TM9S4_HUMAN Transmembrane 9 superfamily member 4 OS=Homo sapiens GN=TM9SF4 PE=1 SV=2		205.66
Q92552	RT27_HUMAN 28S ribosomal protein S27, mitochondrial OS=Homo sapiens GN=MRPS27 PE=1 SV=3		
Q92556	ELMO1_HUMAN Engulfment and cell motility protein 1 OS=Homo sapiens GN=ELMO1 PE=1 SV=2		20.39
Q92556-2	ELMO1_HUMAN Isoform 2 of Engulfment and cell motility protein 1 OS=Homo sapiens GN=ELMO1		20.39
Q92556-3	ELMO1_HUMAN Isoform 3 of Engulfment and cell motility protein 1 OS=Homo sapiens GN=ELMO1		20.39
Q92564;Q92564-2	DCNL4_HUMAN DCN1-like protein 4 OS=Homo sapiens GN=DCUN1D4 PE=1 SV=2;>sp|Q92564-2|DCNL4_HUMAN Isoform 2 of DCN1-	8.19E-06	
Q92572	AP3S1_HUMAN AP-3 complex subunit sigma-1 OS=Homo sapiens GN=AP3S1 PE=1 SV=1		62.92
Q92575	UBXN4_HUMAN UBX domain-containing protein 4 OS=Homo sapiens GN=UBXN4 PE=1 SV=2		71.49
Q92576	PHF3_HUMAN PHD finger protein 3 OS=Homo sapiens GN=PHF3 PE=1 SV=3		393.26
Q92576-2	PHF3_HUMAN Isoform 2 of PHD finger protein 3 OS=Homo sapiens GN=PHF3		393.26
Q92576;Q92576-2	PHF3_HUMAN PHD finger protein 3 OS=Homo sapiens GN=PHF3 PE=1 SV=3;>sp|Q92576-2|PHF3_HUMAN Isoform 2 of PHD finger pr	5.56E-15	
Q92597	NDRG1_HUMAN Protein NDRG1 OS=Homo sapiens GN=NDRG1 PE=1 SV=1		
Q92598	HS105_HUMAN Heat shock protein 105 kDa OS=Homo sapiens GN=HSPH1 PE=1 SV=1		44.48
Q92598-2	HS105_HUMAN Isoform Beta of Heat shock protein 105 kDa OS=Homo sapiens GN=HSPH1		44.48
Q92598-3	HS105_HUMAN Isoform 3 of Heat shock protein 105 kDa OS=Homo sapiens GN=HSPH1		44.48
Q92598;Q92598-2;Q9259	HS105_HUMAN Heat shock protein 105 kDa OS=Homo sapiens GN=HSPH1 PE=1 SV=1;>sp|Q92598-2|HS105_HUMAN Isoform Beta o	0	
Q92599	SEPT8_HUMAN Septin-8 OS=Homo sapiens GN=SEPT8 PE=1 SV=4		206.37
Q92599-2	SEPT8_HUMAN Isoform 2 of Septin-8 OS=Homo sapiens GN=SEPT8		206.37
Q92599-3	SEPT8_HUMAN Isoform 3 of Septin-8 OS=Homo sapiens GN=SEPT8		206.37
Q92599;A6NFQ9;C9J9B6;	SEPT8_HUMAN Septin-8 OS=Homo sapiens GN=SEPT8 PE=1 SV=4;>tr|A6NFQ9|A6NFQ9_HUMAN Uncharacterized protein OS=Homo s	1.16E-13	
Q92600	RCD1_HUMAN Cell differentiation protein RCD1 homolog OS=Homo sapiens GN=RQCD1 PE=1 SV=1		135.05
Q92608	DOCK2_HUMAN Dedicator of cytokinesis protein 2 OS=Homo sapiens GN=DOCK2 PE=1 SV=2		72.51
Q92608-2	DOCK2_HUMAN Isoform 2 of Dedicator of cytokinesis protein 2 OS=Homo sapiens GN=DOCK2		2.83
Q92610	ZN592_HUMAN Zinc finger protein 592 OS=Homo sapiens GN=ZNF592 PE=1 SV=2		
Q92614	MY18A_HUMAN Myosin-XVIIIa OS=Homo sapiens GN=MYO18A PE=1 SV=3		
Q92614-2	MY18A_HUMAN Isoform 2 of Myosin-XVIIIa OS=Homo sapiens GN=MYO18A		
Q92614-3	MY18A_HUMAN Isoform 3 of Myosin-XVIIIa OS=Homo sapiens GN=MYO18A		
Q92614-4	MY18A_HUMAN Isoform 4 of Myosin-XVIIIa OS=Homo sapiens GN=MYO18A		
Q92614-5	MY18A_HUMAN Isoform 5 of Myosin-XVIIIa OS=Homo sapiens GN=MYO18A		
Q92614;Q92614-4;Q9261	MY18A_HUMAN Myosin-XVIIIa OS=Homo sapiens GN=MYO18A PE=1 SV=3;>sp|Q92614-4|MY18A_HUMAN Isoform 4 of Myosin-XVIIIa	1.62E-14	
Q92615	LAR4B_HUMAN La-related protein 4B OS=Homo sapiens GN=LARP4B PE=1 SV=3		204.88
Q92616	GCN1L_HUMAN Translational activator GCN1 OS=Homo sapiens GN=GCN1L1 PE=1 SV=6		1055.21
Q92618;O15090;Q9P2Y4	ZN516_HUMAN Zinc finger protein 516 OS=Homo sapiens GN=ZNF516 PE=1 SV=1;>sp|O15090|ZN536_HUMAN Zinc finger protein 5:	1.81E-12	
Q92619	HMHA1_HUMAN Minor histocompatibility protein HA-1 OS=Homo sapiens GN=HMHA1 PE=1 SV=2	2.10E-22	
Q92620	PRP16_HUMAN Pre-splicing factor ATP-dependent RNA helicase PRP16 OS=Homo sapiens GN=DHX38 PE=1 SV=2		
Q92621	NU205_HUMAN Nuclear pore complex protein Nup205 OS=Homo sapiens GN=NUP205 PE=1 SV=3		64.17
Q92625	ANS1A_HUMAN Ankyrin repeat and SAM domain-containing protein 1A OS=Homo sapiens GN=ANKS1A PE=1 SV=4	2.56E-08	
Q92626;Q92626-2	PXDN_HUMAN Peroxidasin homolog OS=Homo sapiens GN=PXDN PE=1 SV=2;>sp|Q92626-2|PXDN_HUMAN Isoform 2 of Peroxidasin	5.62E-44	
Q92643	GPI8_HUMAN GPI-anchor transamidase OS=Homo sapiens GN=PIGK PE=1 SV=2		129.69
Q92665	RT31_HUMAN 28S ribosomal protein S31, mitochondrial OS=Homo sapiens GN=MRPS31 PE=1 SV=3		380.68
Q92667;Q92667-2	AKAP1_HUMAN A-kinase anchor protein 1, mitochondrial OS=Homo sapiens GN=AKAP1 PE=1 SV=1;>sp|Q92667-2|AKAP1_HUMAN Is	9.79E-06	
Q92685	ALG3_HUMAN Dol-P-Man:Man(5)GlcNAc(2)-PP-Dol alpha-1,3-mannosyltransferase OS=Homo sapiens GN=ALG3 PE=1 SV=1		
Q92686	NEUG_HUMAN Neurogranin OS=Homo sapiens GN=NRGN PE=1 SV=1	0.00023684	
Q92688	AN32B_HUMAN Acidic leucine-rich nuclear phosphoprotein 32 family member B OS=Homo sapiens GN=ANP32B PE=1 SV=1		598.62
Q92688-2	AN32B_HUMAN Isoform 2 of Acidic leucine-rich nuclear phosphoprotein 32 family member B OS=Homo sapiens GN=ANP32B		598.62
Q92688;Q53F35;Q92688-	AN32B_HUMAN Acidic leucine-rich nuclear phosphoprotein 32 family member B OS=Homo sapiens GN=ANP32B PE=1 SV=1;>tr|Q53F:	2.98E-60	
Q92692	PVRL2_HUMAN Poliovirus receptor-related protein 2 OS=Homo sapiens GN=PVRL2 PE=1 SV=1		143.02
Q92692-2	PVRL2_HUMAN Isoform Alpha of Poliovirus receptor-related protein 2 OS=Homo sapiens GN=PVRL2		143.02
Q92692;A8K5L5;Q92692-	PVRL2_HUMAN Poliovirus receptor-related protein 2 OS=Homo sapiens GN=PVRL2 PE=1 SV=1;>tr|A8K5L5|A8K5L5_HUMAN cDNA FLJ	5.33E-52	
Q92696	PGTA_HUMAN Geranylgeranyl transferase type-2 subunit alpha OS=Homo sapiens GN=RABGGTA PE=1 SV=2	2.18E-16	
Q92734	TFG_HUMAN Protein TFG OS=Homo sapiens GN=TFG PE=1 SV=2		

Q92736	RYR2_HUMAN Ryanodine receptor 2 OS=Homo sapiens GN=RYR2 PE=1 SV=3		
Q92736-2	RYR2_HUMAN Isoform 2 of Ryanodine receptor 2 OS=Homo sapiens GN=RYR2		
Q92743	HTRA1_HUMAN Serine protease HTRA1 OS=Homo sapiens GN=HTRA1 PE=1 SV=1	2.15E-23	
Q92747	ARC1A_HUMAN Actin-related protein 2/3 complex subunit 1A OS=Homo sapiens GN=ARPC1A PE=1 SV=2	1.43E-28	
Q92754	AP2C_HUMAN Transcription factor AP-2 gamma OS=Homo sapiens GN=TFAP2C PE=1 SV=1		
Q92759	TF2H4_HUMAN General transcription factor IIH subunit 4 OS=Homo sapiens GN=GTF2H4 PE=2 SV=1		43.09
Q92764	KRT35_HUMAN Keratin, type I cuticular Ha5 OS=Homo sapiens GN=KRT35 PE=1 SV=5		70.66
Q92769	HDAC2_HUMAN Histone deacetylase 2 OS=Homo sapiens GN=HDAC2 PE=1 SV=2		126.82
Q92783;Q92783-2	STAM1_HUMAN Signal transducing adapter molecule 1 OS=Homo sapiens GN=STAM PE=1 SV=3;>sp|Q92783-2|STAM1_HUMAN Isofo	1.13E-62	
Q92785	REQU_HUMAN Zinc finger protein ubi-d4 OS=Homo sapiens GN=DPF2 PE=1 SV=2		
Q92791;B4DVZ5	SC65_HUMAN Synaptonemal complex protein SC65 OS=Homo sapiens GN=LEPREL4 PE=1 SV=1;>tr|B4DVZ5|B4DVZ5_HUMAN cDNA	1.47E-09	
Q92793	CBP_HUMAN CREB-binding protein OS=Homo sapiens GN=CREBBP PE=1 SV=3		12.16
Q92796;Q92796-2	DLG3_HUMAN Disks large homolog 3 OS=Homo sapiens GN=DLG3 PE=1 SV=2;>sp|Q92796-2|DLG3_HUMAN Isoform 2 of Disks large	3.84E-09	
Q92797	SYMPK_HUMAN Symplekin OS=Homo sapiens GN=SYMPK PE=1 SV=2		80.43
Q92797-2	SYMPK_HUMAN Isoform 2 of Symplekin OS=Homo sapiens GN=SYMPK		69.47
Q92797-3	SYMPK_HUMAN Isoform 3 of Symplekin OS=Homo sapiens GN=SYMPK		69.47
Q92797;Q92797-2	SYMPK_HUMAN Symplekin OS=Homo sapiens GN=SYMPK PE=1 SV=2;>sp|Q92797-2|SYMPK_HUMAN Isoform 2 of Symplekin OS=Hor	4.43E-140	
Q92800	EZH1_HUMAN Histone-lysine N-methyltransferase EZH1 OS=Homo sapiens GN=EZH1 PE=1 SV=2		
Q92800-2	EZH1_HUMAN Isoform 2 of Histone-lysine N-methyltransferase EZH1 OS=Homo sapiens GN=EZH1		
Q92800-3	EZH1_HUMAN Isoform 3 of Histone-lysine N-methyltransferase EZH1 OS=Homo sapiens GN=EZH1		
Q92800-4	EZH1_HUMAN Isoform 4 of Histone-lysine N-methyltransferase EZH1 OS=Homo sapiens GN=EZH1		
Q92800-5	EZH1_HUMAN Isoform 5 of Histone-lysine N-methyltransferase EZH1 OS=Homo sapiens GN=EZH1		
Q92804	RBP56_HUMAN TATA-binding protein-associated factor 2N OS=Homo sapiens GN=TAF15 PE=1 SV=1		28.16
Q92804-2	RBP56_HUMAN Isoform Short of TATA-binding protein-associated factor 2N OS=Homo sapiens GN=TAF15		28.16
Q92804;Q92804-2	RBP56_HUMAN TATA-binding protein-associated factor 2N OS=Homo sapiens GN=TAF15 PE=1 SV=1;>sp|Q92804-2|RBP56_HUMAN Is	2.79E-124	
Q92805	GOGA1_HUMAN Golgin subfamily A member 1 OS=Homo sapiens GN=GOLGA1 PE=1 SV=3		
Q92817	EVPL_HUMAN Envoplakin OS=Homo sapiens GN=EVPL PE=1 SV=3		
Q92820	GGH_HUMAN Gamma-glutamyl hydrolase OS=Homo sapiens GN=GGH PE=1 SV=2		80.91
Q92824	PCSK5_HUMAN Proprotein convertase subtilisin/kexin type 5 OS=Homo sapiens GN=PCSK5 PE=2 SV=3		
Q92830	KAT2A_HUMAN Histone acetyltransferase KAT2A OS=Homo sapiens GN=KAT2A PE=1 SV=3		
Q92830-2	KAT2A_HUMAN Isoform 2 of Histone acetyltransferase KAT2A OS=Homo sapiens GN=KAT2A		
Q92833	JARD2_HUMAN Protein Jumonji OS=Homo sapiens GN=JARID2 PE=1 SV=2		55.17
Q92833-2	JARD2_HUMAN Isoform 2 of Protein Jumonji OS=Homo sapiens GN=JARID2		51.2
Q92834	RPGR_HUMAN X-linked retinitis pigmentosa GTPase regulator OS=Homo sapiens GN=RPGR PE=1 SV=2		
Q92834-2	RPGR_HUMAN Isoform 2 of X-linked retinitis pigmentosa GTPase regulator OS=Homo sapiens GN=RPGR		
Q92834-3	RPGR_HUMAN Isoform 3 of X-linked retinitis pigmentosa GTPase regulator OS=Homo sapiens GN=RPGR		
Q92834-4	RPGR_HUMAN Isoform 4 of X-linked retinitis pigmentosa GTPase regulator OS=Homo sapiens GN=RPGR		
Q92834-5	RPGR_HUMAN Isoform 5 of X-linked retinitis pigmentosa GTPase regulator OS=Homo sapiens GN=RPGR		
Q92835	SHIP1_HUMAN Phosphatidylinositol-3,4,5-trisphosphate 5-phosphatase 1 OS=Homo sapiens GN=INPP5D PE=1 SV=2		29.22
Q92835-2	SHIP1_HUMAN Isoform 2 of Phosphatidylinositol-3,4,5-trisphosphate 5-phosphatase 1 OS=Homo sapiens GN=INPP5D		29.22
Q92835-3	SHIP1_HUMAN Isoform 3 of Phosphatidylinositol-3,4,5-trisphosphate 5-phosphatase 1 OS=Homo sapiens GN=INPP5D		24.15
Q92835;Q92835-2;Q9283	SHIP1_HUMAN Phosphatidylinositol-3,4,5-trisphosphate 5-phosphatase 1 OS=Homo sapiens GN=INPP5D PE=1 SV=2;>sp|Q92835-2|S	4.96E-12	
Q92839	HAS1_HUMAN Hyaluronan synthase 1 OS=Homo sapiens GN=HAS1 PE=1 SV=2		
Q92841	DDX17_HUMAN Probable ATP-dependent RNA helicase DDX17 OS=Homo sapiens GN=DDX17 PE=1 SV=1		400.56
Q92841-2	DDX17_HUMAN Isoform 2 of Probable ATP-dependent RNA helicase DDX17 OS=Homo sapiens GN=DDX17		400.56
Q92841-3	DDX17_HUMAN Isoform 3 of Probable ATP-dependent RNA helicase DDX17 OS=Homo sapiens GN=DDX17		400.56
Q92841-4	DDX17_HUMAN Isoform 4 of Probable ATP-dependent RNA helicase DDX17 OS=Homo sapiens GN=DDX17		433.87
Q92841-4;Q92841-3;Q92	4|DDX17_HUMAN Isoform 4 of Probable ATP-dependent RNA helicase DDX17 OS=Homo sapiens GN=DDX17;>sp|Q92841-3|DDX17_H	0	
Q92847	GHSR_HUMAN Growth hormone secretagogue receptor type 1 OS=Homo sapiens GN=GHSR PE=1 SV=1		
Q92847-2	GHSR_HUMAN Isoform 1B of Growth hormone secretagogue receptor type 1 OS=Homo sapiens GN=GHSR		
Q92858	ATOH1_HUMAN Protein atonal homolog 1 OS=Homo sapiens GN=ATOH1 PE=2 SV=1		
Q92859	NEO1_HUMAN Neogenin OS=Homo sapiens GN=NEO1 PE=1 SV=2		
Q92859-2	NEO1_HUMAN Isoform 2 of Neogenin OS=Homo sapiens GN=NEO1		
Q92876;Q92876-2	KLK6_HUMAN Kallikrein-6 OS=Homo sapiens GN=KLK6 PE=1 SV=1;>sp|Q92876-2|KLK6_HUMAN Isoform 2 of Kallikrein-6 OS=Homo	1.64E-13	
Q92878	RAD50_HUMAN DNA repair protein RAD50 OS=Homo sapiens GN=RAD50 PE=1 SV=1		178.49
Q92878-2	RAD50_HUMAN Isoform 2 of DNA repair protein RAD50 OS=Homo sapiens GN=RAD50		178.49
Q92878-2;Q92878;Q9287	2|RAD50_HUMAN Isoform 2 of DNA repair protein RAD50 OS=Homo sapiens GN=RAD50;>sp|Q92878|RAD50_HUMAN DNA repair prot	2.73E-180	
Q92878-3	RAD50_HUMAN Isoform 3 of DNA repair protein RAD50 OS=Homo sapiens GN=RAD50		179.39
Q92879	CELF1_HUMAN CUGBP Elav-like family member 1 OS=Homo sapiens GN=CELF1 PE=1 SV=2		48.93
Q92879-2	CELF1_HUMAN Isoform 2 of CUGBP Elav-like family member 1 OS=Homo sapiens GN=CELF1		48.93
Q92879-3	CELF1_HUMAN Isoform 3 of CUGBP Elav-like family member 1 OS=Homo sapiens GN=CELF1		48.93

Q92879-4	CELF1_HUMAN Isoform 4 of CUGBP Elav-like family member 1 OS=Homo sapiens GN=CELF1		48.93
Q92879-4;D3DQS0;Q9287	4|CELF1_HUMAN Isoform 4 of CUGBP Elav-like family member 1 OS=Homo sapiens GN=CELF1;>tr|D3DQS0|D3DQS0_HUMAN CUG tri	4.65E-11	
Q92882	OSTF1_HUMAN Osteoclast-stimulating factor 1 OS=Homo sapiens GN=OSTF1 PE=1 SV=2		98.36
Q92887	MRP2_HUMAN Canalicular multispecific organic anion transporter 1 OS=Homo sapiens GN=ABCC2 PE=1 SV=3		27.99
Q92888	ARHG1_HUMAN Rho guanine nucleotide exchange factor 1 OS=Homo sapiens GN=ARHGEF1 PE=1 SV=2		56.13
Q92888-2	ARHG1_HUMAN Isoform 2 of Rho guanine nucleotide exchange factor 1 OS=Homo sapiens GN=ARHGEF1		56.13
Q92888-3	ARHG1_HUMAN Isoform 3 of Rho guanine nucleotide exchange factor 1 OS=Homo sapiens GN=ARHGEF1		56.13
Q92888-3;Q92888;Q9288	3|ARHG1_HUMAN Isoform 3 of Rho guanine nucleotide exchange factor 1 OS=Homo sapiens GN=ARHGEF1;>sp|Q92888|ARHG1_HUM	1.58E-15	
Q92889;C9J754;B4DXD8	XPF_HUMAN DNA repair endonuclease XPF OS=Homo sapiens GN=ERCC4 PE=1 SV=3;>tr|C9J754|C9J754_HUMAN Uncharacterized pr	7.46E-08	
Q92890	UFD1_HUMAN Ubiquitin fusion degradation protein 1 homolog OS=Homo sapiens GN=UFD1L PE=1 SV=3		113.82
Q92890-1	UFD1_HUMAN Isoform Long of Ubiquitin fusion degradation protein 1 homolog OS=Homo sapiens GN=UFD1L		113.82
Q92890-1;Q92890	1|UFD1_HUMAN Isoform Long of Ubiquitin fusion degradation protein 1 homolog OS=Homo sapiens GN=UFD1L;>sp|Q92890|UFD1_HU	1.01E-12	
Q92896	GSLG1_HUMAN Golgi apparatus protein 1 OS=Homo sapiens GN=GLG1 PE=1 SV=2		210.86
Q92896-2	GSLG1_HUMAN Isoform 2 of Golgi apparatus protein 1 OS=Homo sapiens GN=GLG1		210.86
Q92896-2;B7Z8Y4;D3DUJ	2|GSLG1_HUMAN Isoform 2 of Golgi apparatus protein 1 OS=Homo sapiens GN=GLG1;>tr|B7Z8Y4|B7Z8Y4_HUMAN cDNA FLJ61698,	3.05E-54	
Q92900	RENT1_HUMAN Regulator of nonsense transcripts 1 OS=Homo sapiens GN=UPF1 PE=1 SV=2		290.7
Q92900-2	RENT1_HUMAN Isoform 2 of Regulator of nonsense transcripts 1 OS=Homo sapiens GN=UPF1		290.7
Q92900;Q92900-2	RENT1_HUMAN Regulator of nonsense transcripts 1 OS=Homo sapiens GN=UPF1 PE=1 SV=2;>sp|Q92900-2|RENT1_HUMAN Isoform	2.20E-23	
Q92901	RL3L_HUMAN 60S ribosomal protein L3-like OS=Homo sapiens GN=RPL3L PE=1 SV=3		76.86
Q92905	CSN5_HUMAN COP9 signalosome complex subunit 5 OS=Homo sapiens GN=COPS5 PE=1 SV=4		188.88
Q92917	GPKOW_HUMAN G patch domain and KOW motifs-containing protein OS=Homo sapiens GN=GPKOW PE=1 SV=2		75.38
Q92918	M4K1_HUMAN Mitogen-activated protein kinase kinase kinase kinase 1 OS=Homo sapiens GN=MAP4K1 PE=1 SV=1		
Q92918-2	M4K1_HUMAN Isoform 2 of Mitogen-activated protein kinase kinase kinase kinase 1 OS=Homo sapiens GN=MAP4K1		
Q92918;Q92918-2	M4K1_HUMAN Mitogen-activated protein kinase kinase kinase kinase 1 OS=Homo sapiens GN=MAP4K1 PE=1 SV=1;>sp|Q92918-2|M4	3.08E-21	
Q92922	SMRC1_HUMAN SWI/SNF complex subunit SMARCC1 OS=Homo sapiens GN=SMARCC1 PE=1 SV=3		31.79
Q92925	SMRD2_HUMAN SWI/SNF-related matrix-associated actin-dependent regulator of chromatin subfamily D member 2 OS=Homo sapiens GN=SMARCD2 PE=1 SV=3		
Q92925-2	SMRD2_HUMAN Isoform 2 of SWI/SNF-related matrix-associated actin-dependent regulator of chromatin subfamily D member 2 OS=Homo sapiens GN=SMARCD2		
Q92925-3	SMRD2_HUMAN Isoform 3 of SWI/SNF-related matrix-associated actin-dependent regulator of chromatin subfamily D member 2 OS=Homo sapiens GN=SMARCD2		
Q92925;Q92925-2;Q9292	SMRD2_HUMAN SWI/SNF-related matrix-associated actin-dependent regulator of chromatin subfamily D member 2 OS=Homo sapiens	5.28E-69	
Q92928	RAB1C_HUMAN Putative Ras-related protein Rab-1C OS=Homo sapiens GN=RAB1C PE=5 SV=2		768.31
Q92930	RAB8B_HUMAN Ras-related protein Rab-8B OS=Homo sapiens GN=RAB8B PE=1 SV=2		524.69
Q92934	BAD_HUMAN Bcl2 antagonist of cell death OS=Homo sapiens GN=BAD PE=1 SV=3		26.53
Q92935	EXTL1_HUMAN Exostosin-like 1 OS=Homo sapiens GN=EXTL1 PE=1 SV=2		
Q92945	FUBP2_HUMAN Far upstream element-binding protein 2 OS=Homo sapiens GN=KHSRP PE=1 SV=4		681.96
Q92945-2	FUBP2_HUMAN Isoform 2 of Far upstream element-binding protein 2 OS=Homo sapiens GN=KHSRP		681.96
Q92945;Q92945-2	FUBP2_HUMAN Far upstream element-binding protein 2 OS=Homo sapiens GN=KHSRP PE=1 SV=4;>sp|Q92945-2|FUBP2_HUMAN Iso	0	
Q92947	GCDH_HUMAN Glutaryl-CoA dehydrogenase, mitochondrial OS=Homo sapiens GN=GCDH PE=1 SV=1	0.0001469	
Q92954	PRG4_HUMAN Proteoglycan 4 OS=Homo sapiens GN=PRG4 PE=1 SV=2		21.83
Q92954-2	PRG4_HUMAN Isoform B of Proteoglycan 4 OS=Homo sapiens GN=PRG4		21.83
Q92954-3	PRG4_HUMAN Isoform C of Proteoglycan 4 OS=Homo sapiens GN=PRG4		21.83
Q92954-4	PRG4_HUMAN Isoform D of Proteoglycan 4 OS=Homo sapiens GN=PRG4		21.83
Q92954-6	PRG4_HUMAN Isoform F of Proteoglycan 4 OS=Homo sapiens GN=PRG4		21.83
Q92973	TNPO1_HUMAN Transportin-1 OS=Homo sapiens GN=TNPO1 PE=1 SV=2		65.88
Q92973-2	TNPO1_HUMAN Isoform 2 of Transportin-1 OS=Homo sapiens GN=TNPO1		65.88
Q92973-3	TNPO1_HUMAN Isoform 3 of Transportin-1 OS=Homo sapiens GN=TNPO1		61.15
Q92973;Q92973-2;Q9297	TNPO1_HUMAN Transportin-1 OS=Homo sapiens GN=TNPO1 PE=1 SV=2;>sp|Q92973-2|TNPO1_HUMAN Isoform 2 of Transportin-1 O	0	
Q92974	ARHG2_HUMAN Rho guanine nucleotide exchange factor 2 OS=Homo sapiens GN=ARHGEF2 PE=1 SV=4		120.24
Q92974-2	ARHG2_HUMAN Isoform 2 of Rho guanine nucleotide exchange factor 2 OS=Homo sapiens GN=ARHGEF2		120.24
Q92974-3	ARHG2_HUMAN Isoform 3 of Rho guanine nucleotide exchange factor 2 OS=Homo sapiens GN=ARHGEF2		120.24
Q92974;Q92974-2;Q9297	ARHG2_HUMAN Rho guanine nucleotide exchange factor 2 OS=Homo sapiens GN=ARHGEF2 PE=1 SV=4;>sp|Q92974-2|ARHG2_HUM	1.12E-155	
Q92979	NEP1_HUMAN Ribosomal RNA small subunit methyltransferase NEP1 OS=Homo sapiens GN=EMG1 PE=1 SV=4		112.3
Q92993	KAT5_HUMAN Histone acetyltransferase KAT5 OS=Homo sapiens GN=KAT5 PE=1 SV=2		
Q92993-2	KAT5_HUMAN Isoform 2 of Histone acetyltransferase KAT5 OS=Homo sapiens GN=KAT5		
Q92993-3	KAT5_HUMAN Isoform 3 of Histone acetyltransferase KAT5 OS=Homo sapiens GN=KAT5		
Q92995	UBP13_HUMAN Ubiquitin carboxyl-terminal hydrolase 13 OS=Homo sapiens GN=USP13 PE=1 SV=2	1.54E-07	
Q93008	USP9X_HUMAN Probable ubiquitin carboxyl-terminal hydrolase FAF-X OS=Homo sapiens GN=USP9X PE=1 SV=3		
Q93008-1	USP9X_HUMAN Isoform 2 of Probable ubiquitin carboxyl-terminal hydrolase FAF-X OS=Homo sapiens GN=USP9X		
Q93008;Q93008-1	USP9X_HUMAN Probable ubiquitin carboxyl-terminal hydrolase FAF-X OS=Homo sapiens GN=USP9X PE=1 SV=3;>sp|Q93008-1|USP9	1.07E-110	
Q93009	UBP7_HUMAN Ubiquitin carboxyl-terminal hydrolase 7 OS=Homo sapiens GN=USP7 PE=1 SV=2		252.59
Q93034	CUL5_HUMAN Cullin-5 OS=Homo sapiens GN=CUL5 PE=1 SV=4		50.33
Q93045	STMN2_HUMAN Stathmin-2 OS=Homo sapiens GN=STMN2 PE=1 SV=3		136.47

Q93052	LPP_HUMAN Lipoma-preferred partner OS=Homo sapiens GN=LPP PE=1 SV=1	1.78E-55	
Q93063-2;Q93063	2|EXT2_HUMAN Isoform 2 of Exostosin-2 OS=Homo sapiens GN=EXT2;>sp|Q93063|EXT2_HUMAN Exostosin-2 OS=Homo sapiens GN	6.81E-05	
Q93074	MED12_HUMAN Mediator of RNA polymerase II transcription subunit 12 OS=Homo sapiens GN=MED12 PE=1 SV=4		44.74
Q93074-2	MED12_HUMAN Isoform 2 of Mediator of RNA polymerase II transcription subunit 12 OS=Homo sapiens GN=MED12		44.74
Q93074-3	MED12_HUMAN Isoform 3 of Mediator of RNA polymerase II transcription subunit 12 OS=Homo sapiens GN=MED12		44.74
Q93077	H2A1C_HUMAN Histone H2A type 1-C OS=Homo sapiens GN=HIST1H2AC PE=1 SV=3		212.14
Q93079	H2B1H_HUMAN Histone H2B type 1-H OS=Homo sapiens GN=HIST1H2BH PE=1 SV=3		708.52
Q93084	AT2A3_HUMAN Sarcoplasmic/endoplasmic reticulum calcium ATPase 3 OS=Homo sapiens GN=ATP2A3 PE=1 SV=2		239.61
Q93084-2	AT2A3_HUMAN Isoform SERCA3A of Sarcoplasmic/endoplasmic reticulum calcium ATPase 3 OS=Homo sapiens GN=ATP2A3		239.61
Q93084-3	AT2A3_HUMAN Isoform SERCA3C of Sarcoplasmic/endoplasmic reticulum calcium ATPase 3 OS=Homo sapiens GN=ATP2A3		239.61
Q93084-4	AT2A3_HUMAN Isoform SERCA3D of Sarcoplasmic/endoplasmic reticulum calcium ATPase 3 OS=Homo sapiens GN=ATP2A3		239.61
Q93084-5	AT2A3_HUMAN Isoform SERCA3E of Sarcoplasmic/endoplasmic reticulum calcium ATPase 3 OS=Homo sapiens GN=ATP2A3		239.61
Q93084-5;Q93084;Q9308	5|AT2A3_HUMAN Isoform SERCA3E of Sarcoplasmic/endoplasmic reticulum calcium ATPase 3 OS=Homo sapiens GN=ATP2A3;>sp|Q93	9.32E-191	
Q93084-6	AT2A3_HUMAN Isoform SERCA3F of Sarcoplasmic/endoplasmic reticulum calcium ATPase 3 OS=Homo sapiens GN=ATP2A3		240.59
Q93096	TP4A1_HUMAN Protein tyrosine phosphatase type IVA 1 OS=Homo sapiens GN=PTP4A1 PE=1 SV=2		100.86
Q93097	WNT2B_HUMAN Protein Wnt-2b OS=Homo sapiens GN=WNT2B PE=1 SV=2		
Q93097-2	WNT2B_HUMAN Isoform 1 of Protein Wnt-2b OS=Homo sapiens GN=WNT2B		
Q95365	1B38_HUMAN HLA class I histocompatibility antigen, B-38 alpha chain OS=Homo sapiens GN=HLA-B PE=2 SV=1		382.83
Q95604	1C17_HUMAN HLA class I histocompatibility antigen, Cw-17 alpha chain OS=Homo sapiens GN=HLA-C PE=1 SV=1		473.08
Q95IE3	2B1C_HUMAN HLA class II histocompatibility antigen, DRB1-12 beta chain OS=Homo sapiens GN=HLA-DRB1 PE=1 SV=1		0.15
Q969E2	SCAM4_HUMAN Secretory carrier-associated membrane protein 4 OS=Homo sapiens GN=SCAMP4 PE=2 SV=1		69.53
Q969E2-2	SCAM4_HUMAN Isoform 2 of Secretory carrier-associated membrane protein 4 OS=Homo sapiens GN=SCAMP4		69.53
Q969E2-3	SCAM4_HUMAN Isoform 3 of Secretory carrier-associated membrane protein 4 OS=Homo sapiens GN=SCAMP4		24.97
Q969E2;Q969E2-2;Q969E	SCAM4_HUMAN Secretory carrier-associated membrane protein 4 OS=Homo sapiens GN=SCAMP4 PE=2 SV=1;>sp|Q969E2-2|SCAM4_	1.02E-08	
Q969E4;Q5H9L2	TCAL3_HUMAN Transcription elongation factor A protein-like 3 OS=Homo sapiens GN=TCEAL3 PE=1 SV=1;>sp|Q5H9L2|TCAL5_HUMA	4.09E-14	
Q969F9	HPS3_HUMAN Hermansky-Pudlak syndrome 3 protein OS=Homo sapiens GN=HPS3 PE=1 SV=1		26.85
Q969F9-2	HPS3_HUMAN Isoform 2 of Hermansky-Pudlak syndrome 3 protein OS=Homo sapiens GN=HPS3		26.85
Q969G3	SMCE1_HUMAN SWI/SNF-related matrix-associated actin-dependent regulator of chromatin subfamily E member 1 OS=Homo sapiens GN=SMARCE1 PE=1 SV=2		
Q969G3-2	SMCE1_HUMAN Isoform 2 of SWI/SNF-related matrix-associated actin-dependent regulator of chromatin subfamily E member 1 OS=Homo sapiens GN=SMARCE1		
Q969G3;B4DGM3;Q969G3	SMCE1_HUMAN SWI/SNF-related matrix-associated actin-dependent regulator of chromatin subfamily E member 1 OS=Homo sapiens	7.66E-09	
Q969G5	PRDBP_HUMAN Protein kinase C delta-binding protein OS=Homo sapiens GN=PRKCDBP PE=1 SV=3		465.78
Q969H6	POP5_HUMAN Ribonuclease P/MRP protein subunit POP5 OS=Homo sapiens GN=POP5 PE=1 SV=1		125.85
Q969H8	CS010_HUMAN UPF0556 protein C19orf10 OS=Homo sapiens GN=C19orf10 PE=1 SV=1		332.43
Q969J2	ZKSC4_HUMAN Zinc finger protein with KRAB and SCAN domains 4 OS=Homo sapiens GN=ZKSCAN4 PE=1 SV=1		
Q969J3	L12R1_HUMAN Loss of heterozygosity 12 chromosomal region 1 protein OS=Homo sapiens GN=LOH12CR1 PE=1 SV=1		41.52
Q969L2	MAL2_HUMAN Protein MAL2 OS=Homo sapiens GN=MAL2 PE=1 SV=1		
Q969M3	YIPF5_HUMAN Protein YIPF5 OS=Homo sapiens GN=YIPF5 PE=1 SV=1		55.31
Q969M3-2	YIPF5_HUMAN Isoform 2 of Protein YIPF5 OS=Homo sapiens GN=YIPF5		37.76
Q969M3-3	YIPF5_HUMAN Isoform 3 of Protein YIPF5 OS=Homo sapiens GN=YIPF5		55.31
Q969N2	PIGT_HUMAN GPI transamidase component PIG-T OS=Homo sapiens GN=PIGT PE=1 SV=1		47.31
Q969N2-2	PIGT_HUMAN Isoform 2 of GPI transamidase component PIG-T OS=Homo sapiens GN=PIGT		47.31
Q969N2-3	PIGT_HUMAN Isoform 3 of GPI transamidase component PIG-T OS=Homo sapiens GN=PIGT		10.56
Q969N2-4	PIGT_HUMAN Isoform 4 of GPI transamidase component PIG-T OS=Homo sapiens GN=PIGT		47.31
Q969N2;Q969N2-4;Q969N	PIGT_HUMAN GPI transamidase component PIG-T OS=Homo sapiens GN=PIGT PE=1 SV=1;>sp|Q969N2-4|PIGT_HUMAN Isoform 4 of	6.31E-15	
Q969P6	TOP1M_HUMAN DNA topoisomerase I, mitochondrial OS=Homo sapiens GN=TOP1MT PE=2 SV=1		119.6
Q969Q0	RL36L_HUMAN 60S ribosomal protein L36a-like OS=Homo sapiens GN=RPL36AL PE=1 SV=3		241.36
Q969Q5	RAB24_HUMAN Ras-related protein Rab-24 OS=Homo sapiens GN=RAB24 PE=1 SV=1	4.54E-12	
Q969S3	ZN622_HUMAN Zinc finger protein 622 OS=Homo sapiens GN=ZNF622 PE=1 SV=1		
Q969T4	UB2E3_HUMAN Ubiquitin-conjugating enzyme E2 E3 OS=Homo sapiens GN=UBE2E3 PE=1 SV=1	9.30E-11	
Q969T7	5NT3L_HUMAN Cytosolic 5'-nucleotidase III-like protein OS=Homo sapiens GN=NT5C3L PE=1 SV=3		39.01
Q969T9	WBP2_HUMAN WW domain-binding protein 2 OS=Homo sapiens GN=WBP2 PE=1 SV=1		37.15
Q969U7	PSMG2_HUMAN Proteasome assembly chaperone 2 OS=Homo sapiens GN=PSMG2 PE=1 SV=1	1.14E-15	
Q969V3	NCLN_HUMAN Nicalin OS=Homo sapiens GN=NCLN PE=1 SV=2		226.4
Q969V3-2	NCLN_HUMAN Isoform 2 of Nicalin OS=Homo sapiens GN=NCLN		226.4
Q969V3;Q969V3-2	NCLN_HUMAN Nicalin OS=Homo sapiens GN=NCLN PE=1 SV=2;>sp|Q969V3-2|NCLN_HUMAN Isoform 2 of Nicalin OS=Homo sapiens	7.86E-13	
Q969X1	TMBI1_HUMAN Transmembrane BAX inhibitor motif-containing protein 1 OS=Homo sapiens GN=TMBIM1 PE=1 SV=2		74.44
Q969X5	ERGI1_HUMAN Endoplasmic reticulum-Golgi intermediate compartment protein 1 OS=Homo sapiens GN=ERGIC1 PE=1 SV=1		
Q969X5-2	ERGI1_HUMAN Isoform 2 of Endoplasmic reticulum-Golgi intermediate compartment protein 1 OS=Homo sapiens GN=ERGIC1		
Q969X5-3	ERGI1_HUMAN Isoform 3 of Endoplasmic reticulum-Golgi intermediate compartment protein 1 OS=Homo sapiens GN=ERGIC1		
Q969X5;B4E0N6;Q969X5-	ERGI1_HUMAN Endoplasmic reticulum-Golgi intermediate compartment protein 1 OS=Homo sapiens GN=ERGIC1 PE=1 SV=1;>tr|B4E	8.94E-49	
Q969X6	CIR1A_HUMAN Cirhin OS=Homo sapiens GN=CIRH1A PE=1 SV=1		

Q969X6-2	CIR1A_HUMAN Isoform 2 of Cirhin OS=Homo sapiens GN=CIRH1A		
Q969X6-3	CIR1A_HUMAN Isoform 3 of Cirhin OS=Homo sapiens GN=CIRH1A		
Q969X6;Q969X6-2	CIR1A_HUMAN Cirhin OS=Homo sapiens GN=CIRH1A PE=1 SV=1;>sp|Q969X6-2|CIR1A_HUMAN Isoform 2 of Cirhin OS=Homo sapier	6.50E-10	
Q969Y2	GTPB3_HUMAN tRNA modification GTPase GTPBP3, mitochondrial OS=Homo sapiens GN=GTPBP3 PE=2 SV=2		
Q969Y2-2	GTPB3_HUMAN Isoform 2 of tRNA modification GTPase GTPBP3, mitochondrial OS=Homo sapiens GN=GTPBP3		
Q969Y2-3	GTPB3_HUMAN Isoform 3 of tRNA modification GTPase GTPBP3, mitochondrial OS=Homo sapiens GN=GTPBP3		
Q969Z0	TBRG4_HUMAN Protein TBRG4 OS=Homo sapiens GN=TBRG4 PE=1 SV=1		58.39
Q969Z0-2	TBRG4_HUMAN Isoform 2 of Protein TBRG4 OS=Homo sapiens GN=TBRG4		58.39
Q969Z0;Q969Z0-2	TBRG4_HUMAN Protein TBRG4 OS=Homo sapiens GN=TBRG4 PE=1 SV=1;>sp|Q969Z0-2|TBRG4_HUMAN Isoform 2 of Protein TBRG4	4.90E-53	
Q96A08	H2B1A_HUMAN Histone H2B type 1-A OS=Homo sapiens GN=HIST1H2BA PE=1 SV=3		184.64
Q96A22	CK052_HUMAN Uncharacterized protein C11orf52 OS=Homo sapiens GN=C11orf52 PE=1 SV=2	8.89E-07	
Q96A23	CPNE4_HUMAN Copine-4 OS=Homo sapiens GN=CPNE4 PE=2 SV=1		
Q96A23-2	CPNE4_HUMAN Isoform 2 of Copine-4 OS=Homo sapiens GN=CPNE4		
Q96A26	F162A_HUMAN Protein FAM162A OS=Homo sapiens GN=FAM162A PE=1 SV=2		48.68
Q96A33	CCD47_HUMAN Coiled-coil domain-containing protein 47 OS=Homo sapiens GN=CCDC47 PE=1 SV=1		86.97
Q96A33-2	CCD47_HUMAN Isoform 2 of Coiled-coil domain-containing protein 47 OS=Homo sapiens GN=CCDC47		88.02
Q96A33;Q96A33-2	CCD47_HUMAN Coiled-coil domain-containing protein 47 OS=Homo sapiens GN=CCDC47 PE=1 SV=1;>sp|Q96A33-2|CCD47_HUMAN	3.28E-17	
Q96A35	RM24_HUMAN 39S ribosomal protein L24, mitochondrial OS=Homo sapiens GN=MRPL24 PE=1 SV=1		46.02
Q96A49	SYAP1_HUMAN Synapse-associated protein 1 OS=Homo sapiens GN=SYAP1 PE=1 SV=1	2.31E-09	
Q96A57	CT030_HUMAN UPF0414 transmembrane protein C20orf30 OS=Homo sapiens GN=C20orf30 PE=1 SV=1		
Q96A57-2	CT030_HUMAN Isoform 1 of UPF0414 transmembrane protein C20orf30 OS=Homo sapiens GN=C20orf30		
Q96A65	EXOC4_HUMAN Exocyst complex component 4 OS=Homo sapiens GN=EXOC4 PE=1 SV=1		64.65
Q96A72	MGN2_HUMAN Protein mago nashi homolog 2 OS=Homo sapiens GN=MAGOHB PE=1 SV=1		300.21
Q96A73	K1191_HUMAN UPF0498 protein KIAA1191 OS=Homo sapiens GN=KIAA1191 PE=1 SV=1		71.91
Q96A73-2	K1191_HUMAN Isoform 2 of UPF0498 protein KIAA1191 OS=Homo sapiens GN=KIAA1191		71.91
Q96A73-3	K1191_HUMAN Isoform 3 of UPF0498 protein KIAA1191 OS=Homo sapiens GN=KIAA1191		44.81
Q96A84	EMID1_HUMAN EMI domain-containing protein 1 OS=Homo sapiens GN=EMID1 PE=2 SV=1		
Q96A84-2	EMID1_HUMAN Isoform 2 of EMI domain-containing protein 1 OS=Homo sapiens GN=EMID1		
Q96A84-3	EMID1_HUMAN Isoform 3 of EMI domain-containing protein 1 OS=Homo sapiens GN=EMID1		
Q96AB3	ISOC2_HUMAN Isochorismatase domain-containing protein 2, mitochondrial OS=Homo sapiens GN=ISOC2 PE=1 SV=1		37.27
Q96AB3-2	ISOC2_HUMAN Isoform 2 of Isochorismatase domain-containing protein 2, mitochondrial OS=Homo sapiens GN=ISOC2		37.27
Q96AB3-2;Q96AB3;Q96AE	2|ISOC2_HUMAN Isoform 2 of Isochorismatase domain-containing protein 2, mitochondrial OS=Homo sapiens GN=ISOC2;>sp|Q96AB	3.87E-89	
Q96AB3-3	ISOC2_HUMAN Isoform 3 of Isochorismatase domain-containing protein 2, mitochondrial OS=Homo sapiens GN=ISOC2		24.86
Q96AC1	FERM2_HUMAN Fermitin family homolog 2 OS=Homo sapiens GN=FERMT2 PE=1 SV=1		345.81
Q96AC1-2	FERM2_HUMAN Isoform 2 of Fermitin family homolog 2 OS=Homo sapiens GN=FERMT2		345.81
Q96AC1;Q96AC1-2	FERM2_HUMAN Fermitin family homolog 2 OS=Homo sapiens GN=FERMT2 PE=1 SV=1;>sp|Q96AC1-2|FERM2_HUMAN Isoform 2 of Fe	2.53E-17	
Q96AE4	FUBP1_HUMAN Far upstream element-binding protein 1 OS=Homo sapiens GN=FUBP1 PE=1 SV=3		299.33
Q96AE4-2	FUBP1_HUMAN Isoform 2 of Far upstream element-binding protein 1 OS=Homo sapiens GN=FUBP1		293.93
Q96AE4-2;Q96AE4	2|FUBP1_HUMAN Isoform 2 of Far upstream element-binding protein 1 OS=Homo sapiens GN=FUBP1;>sp|Q96AE4|FUBP1_HUMAN Fa	1.66E-212	
Q96AE4;Q96AE4-2	FUBP1_HUMAN Far upstream element-binding protein 1 OS=Homo sapiens GN=FUBP1 PE=1 SV=3;>sp|Q96AE4-2|FUBP1_HUMAN Iso	0	
Q96AG4	LRC59_HUMAN Leucine-rich repeat-containing protein 59 OS=Homo sapiens GN=LRRC59 PE=1 SV=1		720.26
Q96AJ9-2;Q96AJ9	2|VTI1A_HUMAN Isoform 2 of Vesicle transport through interaction with t-SNAREs homolog 1A OS=Homo sapiens GN=VTI1A;>sp|Q96	1.94E-09	
Q96AK3	ABC3D_HUMAN Probable DNA dC->dU-editing enzyme APOBEC-3D OS=Homo sapiens GN=APOBEC3D PE=1 SV=1		16.73
Q96AP7	ESAM_HUMAN Endothelial cell-selective adhesion molecule OS=Homo sapiens GN=ESAM PE=1 SV=1		122.08
Q96AQ8	CC90A_HUMAN Coiled-coil domain-containing protein 90A, mitochondrial OS=Homo sapiens GN=CCDC90A PE=2 SV=1		
Q96AX1	VP33A_HUMAN Vacuolar protein sorting-associated protein 33A OS=Homo sapiens GN=VPS33A PE=1 SV=1		
Q96AX2	RAB37_HUMAN Ras-related protein Rab-37 OS=Homo sapiens GN=RAB37 PE=1 SV=3		70.52
Q96AX2-2	RAB37_HUMAN Isoform 2 of Ras-related protein Rab-37 OS=Homo sapiens GN=RAB37		70.52
Q96AY3	FKB10_HUMAN Peptidyl-prolyl cis-trans isomerase FKBP10 OS=Homo sapiens GN=FKBP10 PE=1 SV=1	2.04E-56	
Q96AZ6	ISG20_HUMAN Interferon-stimulated gene 20 kDa protein OS=Homo sapiens GN=ISG20 PE=1 SV=2	2.08E-19	
Q96B18	DACT3_HUMAN Dapper homolog 3 OS=Homo sapiens GN=DACT3 PE=2 SV=2		
Q96B26	EXOS8_HUMAN Exosome complex component RRP43 OS=Homo sapiens GN=EXOSC8 PE=1 SV=1		33.21
Q96B26;Q5JXM0	EXOS8_HUMAN Exosome complex exonuclease RRP43 OS=Homo sapiens GN=EXOSC8 PE=1 SV=1;>tr|Q5JXM0|Q5JXM0_HUMAN Puta	2.23E-19	
Q96B36;Q96B36-2	AKTS1_HUMAN Proline-rich AKT1 substrate 1 OS=Homo sapiens GN=AKT1S1 PE=1 SV=1;>sp|Q96B36-2|AKTS1_HUMAN Isoform 2 of	1.08E-13	
Q96B49	TOM6_HUMAN Mitochondrial import receptor subunit TOM6 homolog OS=Homo sapiens GN=TOMM6 PE=1 SV=1	3.63E-18	
Q96B54	ZN428_HUMAN Zinc finger protein 428 OS=Homo sapiens GN=ZNF428 PE=1 SV=2	1.23E-06	
Q96B97	SH3K1_HUMAN SH3 domain-containing kinase-binding protein 1 OS=Homo sapiens GN=SH3KBP1 PE=1 SV=2		139.67
Q96B97-2	SH3K1_HUMAN Isoform 2 of SH3 domain-containing kinase-binding protein 1 OS=Homo sapiens GN=SH3KBP1		139.67
Q96B97;Q96B97-2	SH3K1_HUMAN SH3 domain-containing kinase-binding protein 1 OS=Homo sapiens GN=SH3KBP1 PE=1 SV=2;>sp|Q96B97-2|SH3K1_	3.98E-10	
Q96BD8;Q96BD8-2	SKA1_HUMAN Spindle and kinetochore-associated protein 1 OS=Homo sapiens GN=SKA1 PE=1 SV=1;>sp|Q96BD8-2|SKA1_HUMAN I	3.36E-24	
Q96BF6	NACC2_HUMAN Nucleus accumbens-associated protein 2 OS=Homo sapiens GN=NACC2 PE=2 SV=1		

Q96BI1	S22AI_HUMAN Solute carrier family 22 member 18 OS=Homo sapiens GN=SLC22A18 PE=1 SV=3	8.49E-09	
Q96BJ3	AIDA_HUMAN Axin interactor, dorsalization-associated protein OS=Homo sapiens GN=AIDA PE=1 SV=1		
Q96BJ3-3	AIDA_HUMAN Isoform 3 of Axin interactor, dorsalization-associated protein OS=Homo sapiens GN=AIDA		
Q96BJ3;B7Z7D5;B7ZW30;	AIDA_HUMAN Axin interactor, dorsalization-associated protein OS=Homo sapiens GN=AIDA PE=1 SV=1;>tr|B7Z7D5|B7Z7D5_HUMAN	1.53E-17	
Q96BM9	ARL8A_HUMAN ADP-ribosylation factor-like protein 8A OS=Homo sapiens GN=ARL8A PE=1 SV=1		107.08
Q96BN8	F105B_HUMAN Protein FAM105B OS=Homo sapiens GN=FAM105B PE=1 SV=3		
Q96BP2	CHCH1_HUMAN Coiled-coil-helix-coiled-coil-helix domain-containing protein 1 OS=Homo sapiens GN=CHCHD1 PE=1 SV=1		131.44
Q96BP3	PPWD1_HUMAN Peptidylprolyl isomerase domain and WD repeat-containing protein 1 OS=Homo sapiens GN=PPWD1 PE=1 SV=1		39
Q96BR5	SELR1_HUMAN Sel1 repeat-containing protein 1 OS=Homo sapiens GN=SELRC1 PE=1 SV=2		64.47
Q96BR9	ZBT8A_HUMAN Zinc finger and BTB domain-containing protein 8A OS=Homo sapiens GN=ZBTB8A PE=1 SV=2		21.54
Q96BR9-2	ZBT8A_HUMAN Isoform 2 of Zinc finger and BTB domain-containing protein 8A OS=Homo sapiens GN=ZBTB8A		21.54
Q96BW5;Q96BW5-2	PTER_HUMAN Phosphotriesterase-related protein OS=Homo sapiens GN=PTER PE=1 SV=1;>sp|Q96BW5-2|PTER_HUMAN Isoform 2 of	5.29E-10	
Q96BW9	MMP37_HUMAN MMP37-like protein, mitochondrial OS=Homo sapiens GN=C3orf31 PE=2 SV=1	2.99E-22	
Q96BX8	MOL2A_HUMAN Mps one binder kinase activator-like 2A OS=Homo sapiens GN=MOBKL2A PE=1 SV=1	8.61E-10	
Q96BY2	MOAP1_HUMAN Modulator of apoptosis 1 OS=Homo sapiens GN=MOAP1 PE=1 SV=1		27.67
Q96BY6	DOC10_HUMAN Dedicator of cytokinesis protein 10 OS=Homo sapiens GN=DOCK10 PE=1 SV=3		
Q96BZ4	PLD4_HUMAN Phospholipase D4 OS=Homo sapiens GN=PLD4 PE=1 SV=2	4.39E-17	
Q96BZ8	LENG1_HUMAN Leukocyte receptor cluster member 1 OS=Homo sapiens GN=LENG1 PE=1 SV=1		26.12
Q96C01	F136A_HUMAN Protein FAM136A OS=Homo sapiens GN=FAM136A PE=1 SV=1		185.28
Q96C19	EFHD2_HUMAN EF-hand domain-containing protein D2 OS=Homo sapiens GN=EFHD2 PE=1 SV=1		246.45
Q96C23	GALM_HUMAN Aldose 1-epimerase OS=Homo sapiens GN=GALM PE=1 SV=1	8.20E-41	
Q96C36	P5CR2_HUMAN Pyrroline-5-carboxylate reductase 2 OS=Homo sapiens GN=PYCR2 PE=1 SV=1		225.48
Q96C57	CL043_HUMAN Uncharacterized protein C12orf43 OS=Homo sapiens GN=C12orf43 PE=1 SV=2		48.39
Q96C86	DCPS_HUMAN Scavenger mRNA-decapping enzyme DcpS OS=Homo sapiens GN=DCPS PE=1 SV=2		235.67
Q96C90	PP14B_HUMAN Protein phosphatase 1 regulatory subunit 14B OS=Homo sapiens GN=PPP1R14B PE=1 SV=3		119.14
Q96C92;Q96C92-2;Q96C9	SDCG3_HUMAN Serologically defined colon cancer antigen 3 OS=Homo sapiens GN=SDCCAG3 PE=1 SV=3;>sp|Q96C92-2|SDCG3_HU	2.92E-09	
Q96CB9	NSUN4_HUMAN Putative methyltransferase NSUN4 OS=Homo sapiens GN=NSUN4 PE=1 SV=2		
Q96CB9-2	NSUN4_HUMAN Isoform 2 of Putative methyltransferase NSUN4 OS=Homo sapiens GN=NSUN4		
Q96CB9-3	NSUN4_HUMAN Isoform 3 of Putative methyltransferase NSUN4 OS=Homo sapiens GN=NSUN4		
Q96CD2;Q96CD2-2	COAC_HUMAN Phosphopantothenoylcysteine decarboxylase OS=Homo sapiens GN=PPCDC PE=1 SV=2;>sp|Q96CD2-2|COAC_HUMAN	1.29E-08	
Q96CG8;C9JVR3;Q96CG8-	CTHR1_HUMAN Collagen triple helix repeat-containing protein 1 OS=Homo sapiens GN=CTHRC1 PE=1 SV=1;>tr|C9JVR3|C9JVR3_HUI	1.52E-29	
Q96CM8	ACSF2_HUMAN Acyl-CoA synthetase family member 2, mitochondrial OS=Homo sapiens GN=ACSF2 PE=1 SV=2		25.9
Q96CN4	EVI5L_HUMAN EVI5-like protein OS=Homo sapiens GN=EVI5L PE=1 SV=1		
Q96CN7	ISOC1_HUMAN Isochorismatase domain-containing protein 1 OS=Homo sapiens GN=ISOC1 PE=1 SV=3		
Q96CN9	GCC1_HUMAN GRIP and coiled-coil domain-containing protein 1 OS=Homo sapiens GN=GCC1 PE=1 SV=1	9.50E-07	
Q96CP2	FWCH2_HUMAN FLYWCH family member 2 OS=Homo sapiens GN=FLYWCH2 PE=1 SV=1	2.94E-11	
Q96CS2	HAUS1_HUMAN HAUS augmin-like complex subunit 1 OS=Homo sapiens GN=HAUS1 PE=1 SV=1	5.54E-12	
Q96CS3	FAF2_HUMAN FAS-associated factor 2 OS=Homo sapiens GN=FAF2 PE=1 SV=2		133.96
Q96CT7	CC124_HUMAN Coiled-coil domain-containing protein 124 OS=Homo sapiens GN=CCDC124 PE=1 SV=1		584.31
Q96CU9	FXRD1_HUMAN FAD-dependent oxidoreductase domain-containing protein 1 OS=Homo sapiens GN=FOXRED1 PE=1 SV=2		
Q96CU9-2	FXRD1_HUMAN Isoform 2 of FAD-dependent oxidoreductase domain-containing protein 1 OS=Homo sapiens GN=FOXRED1		
Q96CU9-3	FXRD1_HUMAN Isoform 3 of FAD-dependent oxidoreductase domain-containing protein 1 OS=Homo sapiens GN=FOXRED1		
Q96CV9;Q96CV9-2;Q96C\	OPTN_HUMAN Optineurin OS=Homo sapiens GN=OPTN PE=1 SV=2;>sp|Q96CV9-2|OPTN_HUMAN Isoform 2 of Optineurin OS=Homo	1.52E-21	
Q96CW1	AP2M1_HUMAN AP-2 complex subunit mu OS=Homo sapiens GN=AP2M1 PE=1 SV=2		782.26
Q96CW1-2	AP2M1_HUMAN Isoform 2 of AP-2 complex subunit mu OS=Homo sapiens GN=AP2M1		782.26
Q96CW1;D3DNT1;Q96CW	AP2M1_HUMAN AP-2 complex subunit mu OS=Homo sapiens GN=AP2M1 PE=1 SV=2;>tr|D3DNT1|D3DNT1_HUMAN Adaptor-related p	3.29E-76	
Q96CW5;Q96CW5-2	GCP3_HUMAN Gamma-tubulin complex component 3 OS=Homo sapiens GN=TUBGCP3 PE=1 SV=2;>sp|Q96CW5-2|GCP3_HUMAN Iso	2.11E-14	
Q96CX2	KCD12_HUMAN BTB/POZ domain-containing protein KCTD12 OS=Homo sapiens GN=KCTD12 PE=1 SV=1	3.37E-49	
Q96D05	CJ035_HUMAN Uncharacterized protein C10orf35 OS=Homo sapiens GN=C10orf35 PE=1 SV=1	3.18E-11	
Q96D15	RCN3_HUMAN Reticulocalbin-3 OS=Homo sapiens GN=RCN3 PE=1 SV=1	1.32E-46	
Q96D46	NMD3_HUMAN 60S ribosomal export protein NMD3 OS=Homo sapiens GN=NMD3 PE=1 SV=1		
Q96D71;Q96D71-3;Q96D7	REPS1_HUMAN RalBP1-associated Eps domain-containing protein 1 OS=Homo sapiens GN=REPS1 PE=1 SV=3;>sp|Q96D71-3|REPS1_	1.45E-05	
Q96D96	HVCN1_HUMAN Voltage-gated hydrogen channel 1 OS=Homo sapiens GN=HVCN1 PE=1 SV=1		49.78
Q96D96-2	HVCN1_HUMAN Isoform 2 of Voltage-gated hydrogen channel 1 OS=Homo sapiens GN=HVCN1		13.09
Q96D96-3	HVCN1_HUMAN Isoform 3 of Voltage-gated hydrogen channel 1 OS=Homo sapiens GN=HVCN1		49.78
Q96DA0	ZG16B_HUMAN Zymogen granule protein 16 homolog B OS=Homo sapiens GN=ZG16B PE=1 SV=3	2.86E-13	
Q96DA2	RB39B_HUMAN Ras-related protein Rab-39B OS=Homo sapiens GN=RAB39B PE=1 SV=1		61.85
Q96DA6	TIM14_HUMAN Mitochondrial import inner membrane translocase subunit TIM14 OS=Homo sapiens GN=DNAJC19 PE=1 SV=3		126.05
Q96DB5	RMD1_HUMAN Regulator of microtubule dynamics protein 1 OS=Homo sapiens GN=FAM82B PE=1 SV=1		62.25
Q96DC8;Q5W0J8;Q96DC8	ECHD3_HUMAN Enoyl-CoA hydratase domain-containing protein 3, mitochondrial OS=Homo sapiens GN=ECHDC3 PE=1 SV=2;>tr|Q5\	6.30E-20	
Q96DE0	NUD16_HUMAN U8 snoRNA-decapping enzyme OS=Homo sapiens GN=NUDT16 PE=1 SV=2		

Q96DG6	CMBL_HUMAN Carboxymethylenebutenolidase homolog OS=Homo sapiens GN=CMBL PE=1 SV=1	1.13E-17	
Q96DH6	MSI2H_HUMAN RNA-binding protein Musashi homolog 2 OS=Homo sapiens GN=MSI2 PE=1 SV=1		
Q96DH6-2	MSI2H_HUMAN Isoform 2 of RNA-binding protein Musashi homolog 2 OS=Homo sapiens GN=MSI2		
Q96DH6-3	MSI2H_HUMAN Isoform 3 of RNA-binding protein Musashi homolog 2 OS=Homo sapiens GN=MSI2		
Q96DH6;B4DHE8;Q96DH6	MSI2H_HUMAN RNA-binding protein Musashi homolog 2 OS=Homo sapiens GN=MSI2 PE=1 SV=1;>tr|B4DHE8|B4DHE8_HUMAN cDNA	1.65E-58	
Q96DI7	SNR40_HUMAN U5 small nuclear ribonucleoprotein 40 kDa protein OS=Homo sapiens GN=SNRNP40 PE=1 SV=1		
Q96DR7-3	ARHGQ_HUMAN Isoform 2 of Rho guanine nucleotide exchange factor 26 OS=Homo sapiens GN=ARHGEF26		27.14
Q96DT7	ZBT10_HUMAN Zinc finger and BTB domain-containing protein 10 OS=Homo sapiens GN=ZBTB10 PE=1 SV=2		
Q96DT7-2	ZBT10_HUMAN Isoform 2 of Zinc finger and BTB domain-containing protein 10 OS=Homo sapiens GN=ZBTB10		
Q96DT7-3	ZBT10_HUMAN Isoform 3 of Zinc finger and BTB domain-containing protein 10 OS=Homo sapiens GN=ZBTB10		
Q96DT7-4	ZBT10_HUMAN Isoform 4 of Zinc finger and BTB domain-containing protein 10 OS=Homo sapiens GN=ZBTB10		
Q96DU9	PABP5_HUMAN Polyadenylate-binding protein 5 OS=Homo sapiens GN=PABPC5 PE=1 SV=1		102.55
Q96DV4	RM38_HUMAN 39S ribosomal protein L38, mitochondrial OS=Homo sapiens GN=MRPL38 PE=1 SV=2		85.7
Q96DX7	TRI44_HUMAN Tripartite motif-containing protein 44 OS=Homo sapiens GN=TRIM44 PE=1 SV=1		
Q96DZ1-2	ERLEC_HUMAN Isoform 2 of Endoplasmic reticulum lectin 1 OS=Homo sapiens GN=ERLEC1		0.83
Q96DZ1;B5MC72;Q96DZ1	ERLEC_HUMAN Endoplasmic reticulum lectin 1 OS=Homo sapiens GN=ERLEC1 PE=1 SV=1;>tr|B5MC72|B5MC72_HUMAN Uncharacter	1.75E-06	
Q96E11	RRFM_HUMAN Ribosome-recycling factor, mitochondrial OS=Homo sapiens GN=MRRF PE=1 SV=1		166.08
Q96E11-2	RRFM_HUMAN Isoform 2 of Ribosome-recycling factor, mitochondrial OS=Homo sapiens GN=MRRF		70.97
Q96E11-3	RRFM_HUMAN Isoform 3 of Ribosome-recycling factor, mitochondrial OS=Homo sapiens GN=MRRF		166.08
Q96E11-4	RRFM_HUMAN Isoform 4 of Ribosome-recycling factor, mitochondrial OS=Homo sapiens GN=MRRF		31.71
Q96E11-5	RRFM_HUMAN Isoform 5 of Ribosome-recycling factor, mitochondrial OS=Homo sapiens GN=MRRF		31.71
Q96E11-6	RRFM_HUMAN Isoform 6 of Ribosome-recycling factor, mitochondrial OS=Homo sapiens GN=MRRF		31.71
Q96E11-7	RRFM_HUMAN Isoform 7 of Ribosome-recycling factor, mitochondrial OS=Homo sapiens GN=MRRF		31.71
Q96E11;Q96E11-3;B7Z6P	RRFM_HUMAN Ribosome-recycling factor, mitochondrial OS=Homo sapiens GN=MRRF PE=1 SV=1;>sp|Q96E11-3|RRFM_HUMAN Isofo	5.59E-51	
Q96E17	RAB3C_HUMAN Ras-related protein Rab-3C OS=Homo sapiens GN=RAB3C PE=2 SV=1		90.26
Q96E29	MTER1_HUMAN mTERF domain-containing protein 1, mitochondrial OS=Homo sapiens GN=MTERFD1 PE=1 SV=2		
Q96E29-2	MTER1_HUMAN Isoform 2 of mTERF domain-containing protein 1, mitochondrial OS=Homo sapiens GN=MTERFD1		
Q96E39	RBMXL_HUMAN Heterogeneous nuclear ribonucleoprotein G-like 1 OS=Homo sapiens GN=RBMXL1 PE=1 SV=1		226.35
Q96EB1-2;B1B1I7;B4E3W	2|ELP4_HUMAN Isoform 2 of Elongator complex protein 4 OS=Homo sapiens GN=ELP4;>tr|B1B1I7|B1B1I7_HUMAN Elongation protein	1.04E-12	
Q96EE3-1;Q96EE3	1|SEH1_HUMAN Isoform B of Nucleoporin SEH1 OS=Homo sapiens GN=SEH1L;>sp|Q96EE3|SEH1_HUMAN Nucleoporin SEH1 OS=Hor	6.08E-06	
Q96EI5-2;Q96EI5	2|TCAL4_HUMAN Isoform 2 of Transcription elongation factor A protein-like 4 OS=Homo sapiens GN=TCEAL4;>sp|Q96EI5|TCAL4_HUN	6.45E-24	
Q96EK5	KBP_HUMAN KIF1-binding protein OS=Homo sapiens GN=KIAA1279 PE=1 SV=1	5.19E-246	
Q96EK6	GNA1_HUMAN Glucosamine 6-phosphate N-acetyltransferase OS=Homo sapiens GN=GNPNAT1 PE=1 SV=1		48.76
Q96EK7;Q96EK7-2	F120B_HUMAN Constitutive coactivator of peroxisome proliferator-activated receptor gamma OS=Homo sapiens GN=FAM120B PE=1 S	2.95E-12	
Q96EL2	RT24_HUMAN 28S ribosomal protein S24, mitochondrial OS=Homo sapiens GN=MRPS24 PE=1 SV=1		31.13
Q96EL3	RM53_HUMAN 39S ribosomal protein L53, mitochondrial OS=Homo sapiens GN=MRPL53 PE=1 SV=1		160.87
Q96EN8	MOCOS_HUMAN Molybdenum cofactor sulfurase OS=Homo sapiens GN=MOCOS PE=1 SV=2		
Q96EP0	RNF31_HUMAN RING finger protein 31 OS=Homo sapiens GN=RNF31 PE=1 SV=1	7.23E-06	
Q96EP5	DAZP1_HUMAN DAZ-associated protein 1 OS=Homo sapiens GN=DAZAP1 PE=1 SV=1		70.89
Q96EP5-2	DAZP1_HUMAN Isoform 2 of DAZ-associated protein 1 OS=Homo sapiens GN=DAZAP1		70.89
Q96EP5;Q96EP5-2	DAZP1_HUMAN DAZ-associated protein 1 OS=Homo sapiens GN=DAZAP1 PE=1 SV=1;>sp|Q96EP5-2|DAZP1_HUMAN Isoform 2 of DA	1.74E-149	
Q96EQ0	SGTB_HUMAN Small glutamine-rich tetratricopeptide repeat-containing protein beta OS=Homo sapiens GN=SGTB PE=1 SV=1	0.00011412	
Q96ER3	SAAL1_HUMAN Protein SAAL1 OS=Homo sapiens GN=SAAL1 PE=1 SV=2	8.18E-14	
Q96ES7	SGF29_HUMAN SAGA-associated factor 29 homolog OS=Homo sapiens GN=CCDC101 PE=1 SV=1		
Q96EY1	DNJA3_HUMAN DnaJ homolog subfamily A member 3, mitochondrial OS=Homo sapiens GN=DNAJA3 PE=1 SV=2		
Q96EY1-2	DNJA3_HUMAN Isoform 2 of DnaJ homolog subfamily A member 3, mitochondrial OS=Homo sapiens GN=DNAJA3		
Q96EY1;Q96EY1-2	DNJA3_HUMAN DnaJ homolog subfamily A member 3, mitochondrial OS=Homo sapiens GN=DNAJA3 PE=1 SV=2;>sp|Q96EY1-2|DNJA	1.99E-08	
Q96EY4	CD043_HUMAN UPF0534 protein C4orf43 OS=Homo sapiens GN=C4orf43 PE=1 SV=2		73.85
Q96EY5;Q96EY5-3;Q96EY	F125A_HUMAN Multivesicular body subunit 12A OS=Homo sapiens GN=FAM125A PE=1 SV=1;>sp|Q96EY5-3|F125A_HUMAN Isoform	9.16E-56	
Q96EY7	PTCD3_HUMAN Pentatricopeptide repeat-containing protein 3, mitochondrial OS=Homo sapiens GN=PTCD3 PE=1 SV=3		150.68
Q96EY8	MMAB_HUMAN Cob(I)yrinic acid a,c-diamide adenosyltransferase, mitochondrial OS=Homo sapiens GN=MMAB PE=1 SV=1		66.06
Q96F07	CYFP2_HUMAN Cytoplasmic FMR1-interacting protein 2 OS=Homo sapiens GN=CYFIP2 PE=1 SV=2		94.24
Q96F07-2	CYFP2_HUMAN Isoform 2 of Cytoplasmic FMR1-interacting protein 2 OS=Homo sapiens GN=CYFIP2		94.24
Q96F07;Q96F07-2	CYFP2_HUMAN Cytoplasmic FMR1-interacting protein 2 OS=Homo sapiens GN=CYFIP2 PE=1 SV=2;>sp|Q96F07-2|CYFP2_HUMAN Isof	1.21E-50	
Q96F44	TRI11_HUMAN E3 ubiquitin-protein ligase TRIM11 OS=Homo sapiens GN=TRIM11 PE=1 SV=2		28.2
Q96F44-2	TRI11_HUMAN Isoform 2 of E3 ubiquitin-protein ligase TRIM11 OS=Homo sapiens GN=TRIM11		28.03
Q96F44-3	TRI11_HUMAN Isoform 3 of E3 ubiquitin-protein ligase TRIM11 OS=Homo sapiens GN=TRIM11		23.22
Q96F85;B8ZZB8;Q96F85-	CNRP1_HUMAN CB1 cannabinoid receptor-interacting protein 1 OS=Homo sapiens GN=CNRIP1 PE=1 SV=1;>tr|B8ZZB8|B8ZZB8_HUM	1.46E-22	
Q96F86	EDC3_HUMAN Enhancer of mRNA-decapping protein 3 OS=Homo sapiens GN=EDC3 PE=1 SV=1	1.48E-08	
Q96FA3	PELI1_HUMAN Protein pellino homolog 1 OS=Homo sapiens GN=PELI1 PE=1 SV=2		
Q96FE5	LIGO1_HUMAN Leucine-rich repeat and immunoglobulin-like domain-containing nogo receptor-interacting protein 1 OS=Homo sapiens GN=LINGO1 PE=1 SV=2		

Q96FE5-2	LIGO1_HUMAN Isoform 2 of Leucine-rich repeat and immunoglobulin-like domain-containing nogo receptor-interacting protein 1 OS=Homo sapiens GN=LINGO1		
Q96FF9	CDCA5_HUMAN Sororin OS=Homo sapiens GN=CDCA5 PE=1 SV=1		
Q96FJ0	STALP_HUMAN AMSH-like protease OS=Homo sapiens GN=STAMBPL1 PE=1 SV=2		168.59
Q96FJ0-2	STALP_HUMAN Isoform 2 of AMSH-like protease OS=Homo sapiens GN=STAMBPL1		168.59
Q96FJ0-2;B3KS69;Q96FJ0	2|STALP_HUMAN Isoform 2 of AMSH-like protease OS=Homo sapiens GN=STAMBPL1;>tr|B3KS69|B3KS69_HUMAN cDNA FLJ35627 fis	3.30E-19	
Q96FJ0;Q96FJ0-2	STALP_HUMAN AMSH-like protease OS=Homo sapiens GN=STAMBPL1 PE=1 SV=2;>sp|Q96FJ0-2|STALP_HUMAN Isoform 2 of AMSH-li	9.65E-24	
Q96FJ2	DYL2_HUMAN Dynein light chain 2, cytoplasmic OS=Homo sapiens GN=DYNLL2 PE=1 SV=1		200.19
Q96FN4	CPNE2_HUMAN Copine-2 OS=Homo sapiens GN=CPNE2 PE=1 SV=3		
Q96FN9	DTD2_HUMAN Probable D-tyrosyl-tRNA(Tyr) deacylase 2 OS=Homo sapiens GN=C14orf126 PE=2 SV=1		30.24
Q96FQ6	S10AG_HUMAN Protein S100-A16 OS=Homo sapiens GN=S100A16 PE=1 SV=1		210.5
Q96FS4	SIPA1_HUMAN Signal-induced proliferation-associated protein 1 OS=Homo sapiens GN=SIPA1 PE=1 SV=1		27.87
Q96FV2	SCRN2_HUMAN Secernin-2 OS=Homo sapiens GN=SCRN2 PE=2 SV=3	3.64E-06	
Q96FV9	THOC1_HUMAN THO complex subunit 1 OS=Homo sapiens GN=THOC1 PE=1 SV=1		
Q96FV9-2	THOC1_HUMAN Isoform 2 of THO complex subunit 1 OS=Homo sapiens GN=THOC1		
Q96FV9-3	THOC1_HUMAN Isoform 3 of THO complex subunit 1 OS=Homo sapiens GN=THOC1		
Q96FV9;Q96FV9-2	THOC1_HUMAN THO complex subunit 1 OS=Homo sapiens GN=THOC1 PE=1 SV=1;>sp|Q96FV9-2|THOC1_HUMAN Isoform 2 of THO c	1.35E-07	
Q96FW1	OTUB1_HUMAN Ubiquitin thioesterase OTUB1 OS=Homo sapiens GN=OTUB1 PE=1 SV=2		210.25
Q96FW1-2	OTUB1_HUMAN Isoform 2 of Ubiquitin thioesterase OTUB1 OS=Homo sapiens GN=OTUB1		63.46
Q96FW1;Q96FW1-2	OTUB1_HUMAN Ubiquitin thioesterase OTUB1 OS=Homo sapiens GN=OTUB1 PE=1 SV=2;>sp|Q96FW1-2|OTUB1_HUMAN Isoform 2 of	1.09E-188	
Q96FX7	TRM61_HUMAN tRNA (adenine-N(1)-)-methyltransferase catalytic subunit TRMT61A OS=Homo sapiens GN=TRMT61A PE=1 SV=1		103.95
Q96FX7-2;Q96FX7;Q96B3	2|TRM61_HUMAN Isoform 2 of tRNA (adenine-N(1)-)-methyltransferase catalytic subunit TRMT61A OS=Homo sapiens GN=TRMT61A;>	9.90E-09	
Q96FZ2	CC037_HUMAN UPF0361 protein C3orf37 OS=Homo sapiens GN=C3orf37 PE=1 SV=1	1.13E-05	
Q96FZ7	CHMP6_HUMAN Charged multivesicular body protein 6 OS=Homo sapiens GN=CHMP6 PE=1 SV=3		137.2
Q96G03	PGM2_HUMAN Phosphoglucomutase-2 OS=Homo sapiens GN=PGM2 PE=1 SV=4		41.06
Q96G21	IMP4_HUMAN U3 small nucleolar ribonucleoprotein protein IMP4 OS=Homo sapiens GN=IMP4 PE=1 SV=1		
Q96G23	LASS2_HUMAN LAG1 longevity assurance homolog 2 OS=Homo sapiens GN=LASS2 PE=1 SV=1		304.33
Q96G28	CC104_HUMAN Coiled-coil domain-containing protein 104 OS=Homo sapiens GN=CCDC104 PE=1 SV=2		
Q96G28-2	CC104_HUMAN Isoform 2 of Coiled-coil domain-containing protein 104 OS=Homo sapiens GN=CCDC104		
Q96G74	OTUD5_HUMAN OTU domain-containing protein 5 OS=Homo sapiens GN=OTUD5 PE=1 SV=1		
Q96G74-2	OTUD5_HUMAN Isoform 2 of OTU domain-containing protein 5 OS=Homo sapiens GN=OTUD5		
Q96G74-3	OTUD5_HUMAN Isoform 3 of OTU domain-containing protein 5 OS=Homo sapiens GN=OTUD5		
Q96GA3	LTV1_HUMAN Protein LTV1 homolog OS=Homo sapiens GN=LTV1 PE=1 SV=1	2.99E-06	
Q96GA7	SDSL_HUMAN Serine dehydratase-like OS=Homo sapiens GN=SDSL PE=1 SV=1	5.96E-17	
Q96GC5	RM48_HUMAN 39S ribosomal protein L48, mitochondrial OS=Homo sapiens GN=MRPL48 PE=1 SV=2		111.99
Q96GC9	VMP1_HUMAN Vacuole membrane protein 1 OS=Homo sapiens GN=VMP1 PE=1 SV=1		72.19
Q96GD0	PLPP_HUMAN Pyridoxal phosphate phosphatase OS=Homo sapiens GN=PDXP PE=1 SV=2		
Q96GD4	AURKB_HUMAN Serine/threonine-protein kinase 12 OS=Homo sapiens GN=AURKB PE=1 SV=3		
Q96GG9	DCNL1_HUMAN DCN1-like protein 1 OS=Homo sapiens GN=DCUN1D1 PE=1 SV=1		136.2
Q96GJ1;Q96GJ1-2;Q96GJ	TRM2_HUMAN tRNA (uracil-5-)-methyltransferase homolog OS=Homo sapiens GN=TRMT2B PE=2 SV=1;>sp|Q96GJ1-2|TRM2_HUMAN	8.67E-05	
Q96GK7;Q6P2I3	FAH2A_HUMAN Fumarylacetoacetate hydrolase domain-containing protein 2A OS=Homo sapiens GN=FAHD2A PE=1 SV=1;>sp|Q6P2I	3.76E-42	
Q96GM5	SMRD1_HUMAN SWI/SNF-related matrix-associated actin-dependent regulator of chromatin subfamily D member 1 OS=Homo sapiens GN=SMARCD1 PE=1 SV=2		
Q96GM5-2	SMRD1_HUMAN Isoform 2 of SWI/SNF-related matrix-associated actin-dependent regulator of chromatin subfamily D member 1 OS=Homo sapiens GN=SMARCD1		
Q96GM5;Q96GM5-2	SMRD1_HUMAN SWI/SNF-related matrix-associated actin-dependent regulator of chromatin subfamily D member 1 OS=Homo sapiens	1.00E-10	
Q96GM8	TOE1_HUMAN Target of EGR1 protein 1 OS=Homo sapiens GN=TOE1 PE=1 SV=1		
Q96GQ7	DDX27_HUMAN Probable ATP-dependent RNA helicase DDX27 OS=Homo sapiens GN=DDX27 PE=1 SV=2		
Q96GW7	PGCB_HUMAN Brevican core protein OS=Homo sapiens GN=BCAN PE=1 SV=2		
Q96GW7-2	PGCB_HUMAN Isoform 2 of Brevican core protein OS=Homo sapiens GN=BCAN		
Q96GW9	SYMM_HUMAN Methionyl-tRNA synthetase, mitochondrial OS=Homo sapiens GN=MARS2 PE=1 SV=2	6.97E-08	
Q96GX5;Q96GX5-3;Q96G	GWL_HUMAN Serine/threonine-protein kinase greatwall OS=Homo sapiens GN=MASTL PE=1 SV=1;>sp|Q96GX5-3|GWL_HUMAN Isofo	4.81E-05	
Q96GX9	MTNB_HUMAN Probable methylthioribulose-1-phosphate dehydratase OS=Homo sapiens GN=APIP PE=1 SV=1	1.58E-18	
Q96H12	CI030_HUMAN UPF0439 protein C9orf30 OS=Homo sapiens GN=C9orf30 PE=1 SV=1		32.05
Q96H15	TIMD4_HUMAN T-cell immunoglobulin and mucin domain-containing protein 4 OS=Homo sapiens GN=TIMD4 PE=1 SV=2		
Q96H20;Q96H20-2	SNF8_HUMAN Vacuolar-sorting protein SNF8 OS=Homo sapiens GN=SNF8 PE=1 SV=1;>sp|Q96H20-2|SNF8_HUMAN Isoform 2 of Vac	2.24E-08	
Q96H55	MYO19_HUMAN Myosin-XIX OS=Homo sapiens GN=MYO19 PE=2 SV=2		28.5
Q96H55-2	MYO19_HUMAN Isoform 2 of Myosin-XIX OS=Homo sapiens GN=MYO19		
Q96H55-3	MYO19_HUMAN Isoform 3 of Myosin-XIX OS=Homo sapiens GN=MYO19		
Q96H55-4	MYO19_HUMAN Isoform 4 of Myosin-XIX OS=Homo sapiens GN=MYO19		28.5
Q96H79	ZCCHL_HUMAN Zinc finger CCCH-type antiviral protein 1-like OS=Homo sapiens GN=ZC3HAV1L PE=1 SV=2		
Q96H79-2	ZCCHL_HUMAN Isoform 2 of Zinc finger CCCH-type antiviral protein 1-like OS=Homo sapiens GN=ZC3HAV1L		
Q96HA9	PX11C_HUMAN Peroxisomal membrane protein 11C OS=Homo sapiens GN=PEX11G PE=1 SV=1	1.64E-06	
Q96HC4	PDLI5_HUMAN PDZ and LIM domain protein 5 OS=Homo sapiens GN=PDLIM5 PE=1 SV=5		

Q96HC4-4	PDLI5_HUMAN Isoform 4 of PDZ and LIM domain protein 5 OS=Homo sapiens GN=PDLIM5		
Q96HC4;Q96HC4-4	PDLI5_HUMAN PDZ and LIM domain protein 5 OS=Homo sapiens GN=PDLIM5 PE=1 SV=5;>sp|Q96HC4-4|PDLI5_HUMAN Isoform 4 of	2.40E-34	
Q96HE7	ERO1A_HUMAN ERO1-like protein alpha OS=Homo sapiens GN=ERO1L PE=1 SV=2		712.63
Q96HE9	PRR11_HUMAN Proline-rich protein 11 OS=Homo sapiens GN=PRR11 PE=1 SV=1		
Q96HF1	SFRP2_HUMAN Secreted frizzled-related protein 2 OS=Homo sapiens GN=SFRP2 PE=1 SV=2	1.98E-07	
Q96HJ9	CG055_HUMAN UPF0562 protein C7orf55 OS=Homo sapiens GN=C7orf55 PE=1 SV=2	4.81E-14	
Q96HN2	SAHH3_HUMAN Putative adenosylhomocysteinase 3 OS=Homo sapiens GN=AHCYL2 PE=1 SV=1		
Q96HN2-2	SAHH3_HUMAN Isoform 2 of Putative adenosylhomocysteinase 3 OS=Homo sapiens GN=AHCYL2		
Q96HN2;Q96HN2-2	SAHH3_HUMAN Putative adenosylhomocysteinase 3 OS=Homo sapiens GN=AHCYL2 PE=1 SV=1;>sp|Q96HN2-2|SAHH3_HUMAN Isofo	5.55E-24	
Q96HQ0	ZN419_HUMAN Zinc finger protein 419 OS=Homo sapiens GN=ZNF419 PE=2 SV=2		
Q96HQ0-2	ZN419_HUMAN Isoform 2 of Zinc finger protein 419 OS=Homo sapiens GN=ZNF419		
Q96HQ2	C2AIL_HUMAN CDKN2AIP N-terminal-like protein OS=Homo sapiens GN=CDKN2AIPNL PE=1 SV=1		110.12
Q96HQ2-2	C2AIL_HUMAN Isoform 2 of CDKN2AIP N-terminal-like protein OS=Homo sapiens GN=CDKN2AIPNL		110.12
Q96HR3	MED30_HUMAN Mediator of RNA polymerase II transcription subunit 30 OS=Homo sapiens GN=MED30 PE=1 SV=1		103.77
Q96HR8	NAF1_HUMAN H/ACA ribonucleoprotein complex non-core subunit NAF1 OS=Homo sapiens GN=NAF1 PE=1 SV=2	1.17E-08	
Q96HR9	REEP6_HUMAN Receptor expression-enhancing protein 6 OS=Homo sapiens GN=REEP6 PE=1 SV=1		28.79
Q96HS1	PGAM5_HUMAN Serine/threonine-protein phosphatase PGAM5, mitochondrial OS=Homo sapiens GN=PGAM5 PE=1 SV=2		251.42
Q96HS1-2	PGAM5_HUMAN Isoform 2 of Serine/threonine-protein phosphatase PGAM5, mitochondrial OS=Homo sapiens GN=PGAM5		221.2
Q96HS1;Q96HS1-2	PGAM5_HUMAN Serine/threonine-protein phosphatase PGAM5, mitochondrial OS=Homo sapiens GN=PGAM5 PE=1 SV=2;>sp|Q96HS1	4.94E-50	
Q96HU1	SGSM3_HUMAN Small G protein signaling modulator 3 OS=Homo sapiens GN=SGSM3 PE=1 SV=1		
Q96HU1-2	SGSM3_HUMAN Isoform 2 of Small G protein signaling modulator 3 OS=Homo sapiens GN=SGSM3		
Q96HW7	INT4_HUMAN Integrator complex subunit 4 OS=Homo sapiens GN=INTS4 PE=1 SV=2		
Q96HW7-2	INT4_HUMAN Isoform 2 of Integrator complex subunit 4 OS=Homo sapiens GN=INTS4		
Q96HW7-3	INT4_HUMAN Isoform 3 of Integrator complex subunit 4 OS=Homo sapiens GN=INTS4		
Q96HY6	DDRGK_HUMAN DDRGK domain-containing protein 1 OS=Homo sapiens GN=DDRGK1 PE=1 SV=2		
Q96HY6-2	DDRGK_HUMAN Isoform 2 of DDRGK domain-containing protein 1 OS=Homo sapiens GN=DDRGK1		
Q96HY6;Q96HY6-2	DDRGK_HUMAN DDRGK domain-containing protein 1 OS=Homo sapiens GN=DDRGK1 PE=1 SV=2;>sp|Q96HY6-2|DDRGK_HUMAN Iso	3.46E-25	
Q96HY7	DHTK1_HUMAN Probable 2-oxoglutarate dehydrogenase E1 component DHKTD1, mitochondrial OS=Homo sapiens GN=DHTKD1 PE=2 SV=2		
Q96HZ4	HES6_HUMAN Transcription cofactor HES-6 OS=Homo sapiens GN=HES6 PE=1 SV=1		
Q96HZ4-2	HES6_HUMAN Isoform 2 of Transcription cofactor HES-6 OS=Homo sapiens GN=HES6		
Q96HZ4-3	HES6_HUMAN Isoform 3 of Transcription cofactor HES-6 OS=Homo sapiens GN=HES6		
Q96I15	SCLY_HUMAN Selenocysteine lyase OS=Homo sapiens GN=SCLY PE=1 SV=2		40.13
Q96I15-2	SCLY_HUMAN Isoform 2 of Selenocysteine lyase OS=Homo sapiens GN=SCLY		40.13
Q96I24	FUBP3_HUMAN Far upstream element-binding protein 3 OS=Homo sapiens GN=FUBP3 PE=1 SV=2		281.28
Q96I24-2	FUBP3_HUMAN Isoform 2 of Far upstream element-binding protein 3 OS=Homo sapiens GN=FUBP3		146.2
Q96I25	SPF45_HUMAN Splicing factor 45 OS=Homo sapiens GN=RBM17 PE=1 SV=1		
Q96I36	CL062_HUMAN Uncharacterized protein C12orf62 OS=Homo sapiens GN=C12orf62 PE=2 SV=1		
Q96I59	SYNM_HUMAN Probable asparaginyl-tRNA synthetase, mitochondrial OS=Homo sapiens GN=NARS2 PE=1 SV=3	4.67E-37	
Q96I99	SUCB2_HUMAN Succinyl-CoA ligase [GDP-forming] subunit beta, mitochondrial OS=Homo sapiens GN=SUCLG2 PE=1 SV=2		171.86
Q96IF1	JUB_HUMAN Protein ajuba OS=Homo sapiens GN=JUB PE=1 SV=1		
Q96IJ6-2;Q96IJ6	2|GMPPA_HUMAN Isoform 2 of Mannose-1-phosphate guanyltransferase alpha OS=Homo sapiens GN=GMPPA;>sp|Q96IJ6|GMPPA_HU	9.74E-14	
Q96IS3	RAX2_HUMAN Retina and anterior neural fold homeobox protein 2 OS=Homo sapiens GN=RAX2 PE=1 SV=1		
Q96IU4	ABHEB_HUMAN Abhydrolase domain-containing protein 14B OS=Homo sapiens GN=ABHD14B PE=1 SV=1		37.79
Q96IU4-2	2|ABHEB_HUMAN Isoform 2 of Abhydrolase domain-containing protein 14B OS=Homo sapiens GN=ABHD14B	1.14E-25	
Q96IU4;B4DNR3;A6NGU1	ABHEB_HUMAN Abhydrolase domain-containing protein 14B OS=Homo sapiens GN=ABHD14B PE=1 SV=1;>tr|B4DNR3|B4DNR3_HUM	2.37E-38	
Q96IX5	USMG5_HUMAN Up-regulated during skeletal muscle growth protein 5 OS=Homo sapiens GN=USMG5 PE=1 SV=1		51.27
Q96IY4	CBPB2_HUMAN Carboxypeptidase B2 OS=Homo sapiens GN=CPB2 PE=1 SV=2		21.6
Q96IY4-2	CBPB2_HUMAN Isoform 2 of Carboxypeptidase B2 OS=Homo sapiens GN=CPB2		16.61
Q96IZ0	PAWR_HUMAN PRKC apoptosis WT1 regulator protein OS=Homo sapiens GN=PAWR PE=1 SV=1		91.4
Q96IZ5	RBM41_HUMAN RNA-binding protein 41 OS=Homo sapiens GN=RBM41 PE=1 SV=2		26.07
Q96IZ5-2	RBM41_HUMAN Isoform 2 of RNA-binding protein 41 OS=Homo sapiens GN=RBM41		26.07
Q96J01	THOC3_HUMAN THO complex subunit 3 OS=Homo sapiens GN=THOC3 PE=1 SV=1		
Q96J02;Q96J02-2	ITCH_HUMAN E3 ubiquitin-protein ligase Itchy homolog OS=Homo sapiens GN=ITCH PE=1 SV=2;>sp|Q96J02-2|ITCH_HUMAN Isoforn	4.48E-47	
Q96J65	MRP9_HUMAN Multidrug resistance-associated protein 9 OS=Homo sapiens GN=ABCC12 PE=1 SV=2		
Q96J65-2	MRP9_HUMAN Isoform 2 of Multidrug resistance-associated protein 9 OS=Homo sapiens GN=ABCC12		
Q96J65-3	MRP9_HUMAN Isoform 3 of Multidrug resistance-associated protein 9 OS=Homo sapiens GN=ABCC12		
Q96J65-5	MRP9_HUMAN Isoform 5 of Multidrug resistance-associated protein 9 OS=Homo sapiens GN=ABCC12		
Q96J87	CELF6_HUMAN CUGBP Elav-like family member 6 OS=Homo sapiens GN=CELF6 PE=1 SV=1		
Q96JB1	DYH8_HUMAN Dynein heavy chain 8, axonemal OS=Homo sapiens GN=DNAH8 PE=1 SV=2		
Q96JB1-2	DYH8_HUMAN Isoform 2 of Dynein heavy chain 8, axonemal OS=Homo sapiens GN=DNAH8		
Q96JB1;Q96JB1-2	DYH8_HUMAN Dynein heavy chain 8, axonemal OS=Homo sapiens GN=DNAH8 PE=1 SV=2;>sp|Q96JB1-2|DYH8_HUMAN Isoform 2 of	5.39E-14	

Q96JB2;Q96JB2-2	COG3_HUMAN Conserved oligomeric Golgi complex subunit 3 OS=Homo sapiens GN=COG3 PE=1 SV=3;>sp|Q96JB2-2|COG3_HUMAN	4.39E-12	
Q96JB3	HIC2_HUMAN Hypermethylated in cancer 2 protein OS=Homo sapiens GN=HIC2 PE=1 SV=2		
Q96JB3-2	HIC2_HUMAN Isoform 2 of Hypermethylated in cancer 2 protein OS=Homo sapiens GN=HIC2		
Q96JB5	CK5P3_HUMAN CDK5 regulatory subunit-associated protein 3 OS=Homo sapiens GN=CDK5RAP3 PE=1 SV=2		
Q96JB5-2	CK5P3_HUMAN Isoform 2 of CDK5 regulatory subunit-associated protein 3 OS=Homo sapiens GN=CDK5RAP3		
Q96JB5-3	CK5P3_HUMAN Isoform 3 of CDK5 regulatory subunit-associated protein 3 OS=Homo sapiens GN=CDK5RAP3		
Q96JB5;Q96JB5-2	CK5P3_HUMAN CDK5 regulatory subunit-associated protein 3 OS=Homo sapiens GN=CDK5RAP3 PE=1 SV=2;>sp|Q96JB5-2|CK5P3_H	6.15E-14	
Q96JG6;Q96JG6-2	CC132_HUMAN Coiled-coil domain-containing protein 132 OS=Homo sapiens GN=CCDC132 PE=1 SV=3;>sp|Q96JG6-2|CC132_HUMA	6.32E-10	
Q96JH7	VCIP1_HUMAN Deubiquitinating protein VCIP135 OS=Homo sapiens GN=VCPIP1 PE=1 SV=2	9.68E-68	
Q96JJ3	ELMO2_HUMAN Engulfment and cell motility protein 2 OS=Homo sapiens GN=ELMO2 PE=1 SV=2		17.85
Q96JJ7	TMX3_HUMAN Protein disulfide-isomerase TMX3 OS=Homo sapiens GN=TMX3 PE=1 SV=2		146.51
Q96JJ7-2	TMX3_HUMAN Isoform 2 of Protein disulfide-isomerase TMX3 OS=Homo sapiens GN=TMX3		97.84
Q96JJ7;Q3B7X1;Q96JJ7-2	TMX3_HUMAN Protein disulfide-isomerase TMX3 OS=Homo sapiens GN=TMX3 PE=1 SV=2;>tr|Q3B7X1|Q3B7X1_HUMAN TMX3 protein	5.37E-24	
Q96JM3	ZN828_HUMAN Zinc finger protein 828 OS=Homo sapiens GN=ZNF828 PE=1 SV=2		
Q96JN2	CC136_HUMAN Coiled-coil domain-containing protein 136 OS=Homo sapiens GN=CCDC136 PE=1 SV=3		
Q96JN2-2	CC136_HUMAN Isoform 2 of Coiled-coil domain-containing protein 136 OS=Homo sapiens GN=CCDC136		
Q96JN2-3	CC136_HUMAN Isoform 3 of Coiled-coil domain-containing protein 136 OS=Homo sapiens GN=CCDC136		
Q96JN2-4	CC136_HUMAN Isoform 4 of Coiled-coil domain-containing protein 136 OS=Homo sapiens GN=CCDC136		
Q96JP0	FEM1C_HUMAN Protein fem-1 homolog C OS=Homo sapiens GN=FEM1C PE=2 SV=1		
Q96JP2	MY15B_HUMAN Putative myosin-XVB OS=Homo sapiens GN=MYO15B PE=5 SV=2		51.63
Q96JP5	ZFP91_HUMAN E3 ubiquitin-protein ligase ZFP91 OS=Homo sapiens GN=ZFP91 PE=1 SV=1		54.66
Q96JP5-2	ZFP91_HUMAN Isoform 2 of E3 ubiquitin-protein ligase ZFP91 OS=Homo sapiens GN=ZFP91		54.66
Q96JP5;Q96JP5-2	ZFP91_HUMAN E3 ubiquitin-protein ligase ZFP91 OS=Homo sapiens GN=ZFP91 PE=1 SV=1;>sp|Q96JP5-2|ZFP91_HUMAN Isoform 2 o	5.47E-10	
Q96JY6-3;D3DSR5;Q96JY	3|PDLI2_HUMAN Isoform 3 of PDZ and LIM domain protein 2 OS=Homo sapiens GN=PDLIM2;>tr|D3DSR5|D3DSR5_HUMAN PDZ and L	5.86E-16	
Q96K17	BT3L4_HUMAN Transcription factor BTF3 homolog 4 OS=Homo sapiens GN=BTF3L4 PE=1 SV=1		191.83
Q96K37	S35E1_HUMAN Solute carrier family 35 member E1 OS=Homo sapiens GN=SLC35E1 PE=1 SV=2		46.27
Q96K37-2	S35E1_HUMAN Isoform 2 of Solute carrier family 35 member E1 OS=Homo sapiens GN=SLC35E1		0.24
Q96K76	UBP47_HUMAN Ubiquitin carboxyl-terminal hydrolase 47 OS=Homo sapiens GN=USP47 PE=1 SV=3		28.09
Q96K76-2	UBP47_HUMAN Isoform 2 of Ubiquitin carboxyl-terminal hydrolase 47 OS=Homo sapiens GN=USP47		28.09
Q96K76;B3KXF5;Q96K76-	UBP47_HUMAN Ubiquitin carboxyl-terminal hydrolase 47 OS=Homo sapiens GN=USP47 PE=1 SV=3;>tr|B3KXF5|B3KXF5_HUMAN Ubiq	2.46E-13	
Q96K83	ZN521_HUMAN Zinc finger protein 521 OS=Homo sapiens GN=ZNF521 PE=1 SV=1		2
Q96KA5	CLP1L_HUMAN Cleft lip and palate transmembrane protein 1-like protein OS=Homo sapiens GN=CLPTM1L PE=1 SV=1		
Q96KA5-2	CLP1L_HUMAN Isoform 2 of Cleft lip and palate transmembrane protein 1-like protein OS=Homo sapiens GN=CLPTM1L		
Q96KB5	TOPK_HUMAN Lymphokine-activated killer T-cell-originated protein kinase OS=Homo sapiens GN=PBK PE=1 SV=3		
Q96KC8	DNJC1_HUMAN DnaJ homolog subfamily C member 1 OS=Homo sapiens GN=DNAJC1 PE=1 SV=1		105.63
Q96KG9;Q96KG9-2;Q96KG	NTKL_HUMAN N-terminal kinase-like protein OS=Homo sapiens GN=SCYL1 PE=1 SV=1;>sp|Q96KG9-2|NTKL_HUMAN Isoform 2 of N-	6.95E-95	
Q96KK5	H2A1H_HUMAN Histone H2A type 1-H OS=Homo sapiens GN=HIST1H2AH PE=1 SV=3		288.83
Q96KN1	FA84B_HUMAN Protein FAM84B OS=Homo sapiens GN=FAM84B PE=1 SV=1	5.31E-06	
Q96KP1	EXOC2_HUMAN Exocyst complex component 2 OS=Homo sapiens GN=EXOC2 PE=1 SV=1	5.61E-19	
Q96KP4;B4DV28;Q96KP4-	CNDP2_HUMAN Cytosolic non-specific dipeptidase OS=Homo sapiens GN=CNDP2 PE=1 SV=2;>tr|B4DV28|B4DV28_HUMAN cDNA FLJ5	9.23E-284	
Q96KQ7	EHMT2_HUMAN Histone-lysine N-methyltransferase EHMT2 OS=Homo sapiens GN=EHMT2 PE=1 SV=3		
Q96KQ7-2	EHMT2_HUMAN Isoform 2 of Histone-lysine N-methyltransferase EHMT2 OS=Homo sapiens GN=EHMT2		
Q96KQ7-3	EHMT2_HUMAN Isoform 3 of Histone-lysine N-methyltransferase EHMT2 OS=Homo sapiens GN=EHMT2		
Q96KQ7;Q96KQ7-2	EHMT2_HUMAN Histone-lysine N-methyltransferase EHMT2 OS=Homo sapiens GN=EHMT2 PE=1 SV=3;>sp|Q96KQ7-2|EHMT2_HUMAN	2.73E-57	
Q96KR1	ZFR_HUMAN Zinc finger RNA-binding protein OS=Homo sapiens GN=ZFR PE=1 SV=2		188.48
Q96L16	ALM1L_HUMAN Putative ALMS1-like protein OS=Homo sapiens GN=ALMS1P PE=5 SV=1		
Q96L21	RL10L_HUMAN 60S ribosomal protein L10-like OS=Homo sapiens GN=RPL10L PE=1 SV=3		307.3
Q96L46	CPNS2_HUMAN Calpain small subunit 2 OS=Homo sapiens GN=CAPNS2 PE=2 SV=2		75.18
Q96L73	NSD1_HUMAN Histone-lysine N-methyltransferase, H3 lysine-36 and H4 lysine-20 specific OS=Homo sapiens GN=NSD1 PE=1 SV=1		
Q96L73-2	NSD1_HUMAN Isoform 2 of Histone-lysine N-methyltransferase, H3 lysine-36 and H4 lysine-20 specific OS=Homo sapiens GN=NSD1		
Q96L73-3	NSD1_HUMAN Isoform 3 of Histone-lysine N-methyltransferase, H3 lysine-36 and H4 lysine-20 specific OS=Homo sapiens GN=NSD1		
Q96L91	EP400_HUMAN E1A-binding protein p400 OS=Homo sapiens GN=EP400 PE=1 SV=4		
Q96L91-2	EP400_HUMAN Isoform 2 of E1A-binding protein p400 OS=Homo sapiens GN=EP400		
Q96L91-3	EP400_HUMAN Isoform 3 of E1A-binding protein p400 OS=Homo sapiens GN=EP400		
Q96L91-4	EP400_HUMAN Isoform 4 of E1A-binding protein p400 OS=Homo sapiens GN=EP400		
Q96L91-5	EP400_HUMAN Isoform 5 of E1A-binding protein p400 OS=Homo sapiens GN=EP400		
Q96L91;Q96L91-2;Q96L9	EP400_HUMAN E1A-binding protein p400 OS=Homo sapiens GN=EP400 PE=1 SV=4;>sp|Q96L91-2|EP400_HUMAN Isoform 2 of E1A-b	5.71E-07	
Q96L92;Q96L92-3;Q96L9	SNX27_HUMAN Sorting nexin-27 OS=Homo sapiens GN=SNX27 PE=1 SV=2;>sp|Q96L92-3|SNX27_HUMAN Isoform 2 of Sorting nexin	4.55E-17	
Q96L93	KI16B_HUMAN Kinesin-like protein KIF16B OS=Homo sapiens GN=KIF16B PE=1 SV=2		
Q96L93-2	KI16B_HUMAN Isoform 2 of Kinesin-like protein KIF16B OS=Homo sapiens GN=KIF16B		
Q96L93-2;Q96L93;Q96L9	2|KI16B_HUMAN Isoform 2 of Kinesin-like protein KIF16B OS=Homo sapiens GN=KIF16B;>sp|Q96L93|KI16B_HUMAN Kinesin-like pro	2.18E-05	

Q96L93-4	KI16B_HUMAN Isoform 3 of Kinesin-like protein KIF16B OS=Homo sapiens GN=KIF16B		
Q96L93-5	KI16B_HUMAN Isoform 4 of Kinesin-like protein KIF16B OS=Homo sapiens GN=KIF16B		
Q96L93-6	KI16B_HUMAN Isoform 5 of Kinesin-like protein KIF16B OS=Homo sapiens GN=KIF16B		
Q96LA8;Q96LA8-2	ANM6_HUMAN Protein arginine N-methyltransferase 6 OS=Homo sapiens GN=PRMT6 PE=1 SV=1;>sp|Q96LA8-2|ANM6_HUMAN Isofor	1.28E-126	
Q96LB3	IFT74_HUMAN Intraflagellar transport protein 74 homolog OS=Homo sapiens GN=IFT74 PE=2 SV=1		88.69
Q96LB3-2	IFT74_HUMAN Isoform 2 of Intraflagellar transport protein 74 homolog OS=Homo sapiens GN=IFT74		67.87
Q96LD4	TRI47_HUMAN Tripartite motif-containing protein 47 OS=Homo sapiens GN=TRIM47 PE=1 SV=2		
Q96LI9	CX058_HUMAN Putative uncharacterized protein CXorf58 OS=Homo sapiens GN=CXorf58 PE=2 SV=2		
Q96LJ7	DHRS1_HUMAN Dehydrogenase/reductase SDR family member 1 OS=Homo sapiens GN=DHRS1 PE=1 SV=1	1.62E-10	
Q96LP2	FA81B_HUMAN Protein FAM81B OS=Homo sapiens GN=FAM81B PE=2 SV=3		
Q96LR5	UB2E2_HUMAN Ubiquitin-conjugating enzyme E2 E2 OS=Homo sapiens GN=UBE2E2 PE=1 SV=1	1.20E-10	
Q96LV5	IN4L1_HUMAN Integrator complex subunit 4-like protein 1 OS=Homo sapiens GN=INTS4L1 PE=2 SV=1		
Q96LX7	CCD17_HUMAN Coiled-coil domain-containing protein 17 OS=Homo sapiens GN=CCDC17 PE=2 SV=2		
Q96LX7-2	CCD17_HUMAN Isoform 3 of Coiled-coil domain-containing protein 17 OS=Homo sapiens GN=CCDC17		
Q96LX7-5	CCD17_HUMAN Isoform 2 of Coiled-coil domain-containing protein 17 OS=Homo sapiens GN=CCDC17		
Q96M02	CJ090_HUMAN Uncharacterized protein C10orf90 OS=Homo sapiens GN=C10orf90 PE=2 SV=2		
Q96M02-2	CJ090_HUMAN Isoform 2 of Uncharacterized protein C10orf90 OS=Homo sapiens GN=C10orf90		
Q96M27-2;Q96M27-3;Q96	2|PRRC1_HUMAN Isoform 2 of Protein PRRC1 OS=Homo sapiens GN=PRRC1;>sp|Q96M27-3|PRRC1_HUMAN Isoform 3 of Protein PRR	3.53E-64	
Q96M42	CU129_HUMAN Uncharacterized protein C21orf129 OS=Homo sapiens GN=C21orf129 PE=2 SV=2		31.34
Q96M86	DNHD1_HUMAN Dynein heavy chain domain-containing protein 1 OS=Homo sapiens GN=DNHD1 PE=1 SV=2		
Q96M86-4	DNHD1_HUMAN Isoform 2 of Dynein heavy chain domain-containing protein 1 OS=Homo sapiens GN=DNHD1		
Q96M86-5	DNHD1_HUMAN Isoform 3 of Dynein heavy chain domain-containing protein 1 OS=Homo sapiens GN=DNHD1		
Q96M96	FGD4_HUMAN FYVE, RhoGEF and PH domain-containing protein 4 OS=Homo sapiens GN=FGD4 PE=1 SV=2		
Q96MC2	CC164_HUMAN Coiled-coil domain-containing protein 164 OS=Homo sapiens GN=CCDC164 PE=2 SV=2		
Q96MD7	CI085_HUMAN Uncharacterized protein C9orf85 OS=Homo sapiens GN=C9orf85 PE=1 SV=1		
Q96MG7	MAGG1_HUMAN Melanoma-associated antigen G1 OS=Homo sapiens GN=NDNL2 PE=1 SV=1		
Q96MI9	CBPC4_HUMAN Cytosolic carboxypeptidase 4 OS=Homo sapiens GN=AGBL1 PE=2 SV=2		37.97
Q96MI9-2	CBPC4_HUMAN Isoform 2 of Cytosolic carboxypeptidase 4 OS=Homo sapiens GN=AGBL1		30.58
Q96MI9-3	CBPC4_HUMAN Isoform 3 of Cytosolic carboxypeptidase 4 OS=Homo sapiens GN=AGBL1		30.58
Q96MK2	FA65C_HUMAN Protein FAM65C OS=Homo sapiens GN=FAM65C PE=2 SV=4		23.58
Q96MK2-2	FA65C_HUMAN Isoform 2 of Protein FAM65C OS=Homo sapiens GN=FAM65C		23.58
Q96MM3	ZFP42_HUMAN Zinc finger protein 42 homolog OS=Homo sapiens GN=ZFP42 PE=1 SV=2		
Q96MR6	WDR65_HUMAN WD repeat-containing protein 65 OS=Homo sapiens GN=WDR65 PE=2 SV=3	7.47E-11	
Q96MT1	RN145_HUMAN RING finger protein 145 OS=Homo sapiens GN=RNF145 PE=2 SV=2		
Q96MT1-2	RN145_HUMAN Isoform 2 of RING finger protein 145 OS=Homo sapiens GN=RNF145		
Q96MT3	PRIC1_HUMAN Prickle-like protein 1 OS=Homo sapiens GN=PRICKLE1 PE=1 SV=2		
Q96MT7	WDR52_HUMAN WD repeat-containing protein 52 OS=Homo sapiens GN=WDR52 PE=1 SV=1		
Q96MT7-2	WDR52_HUMAN Isoform 2 of WD repeat-containing protein 52 OS=Homo sapiens GN=WDR52		
Q96MT7-2;Q96MT7	2|WDR52_HUMAN Isoform 2 of WD repeat-containing protein 52 OS=Homo sapiens GN=WDR52;>sp|Q96MT7|WDR52_HUMAN WD re	7.41E-09	
Q96MT8	CEP63_HUMAN Centrosomal protein of 63 kDa OS=Homo sapiens GN=CEP63 PE=1 SV=1		
Q96MT8-2	CEP63_HUMAN Isoform 2 of Centrosomal protein of 63 kDa OS=Homo sapiens GN=CEP63		
Q96MT8-3	CEP63_HUMAN Isoform 3 of Centrosomal protein of 63 kDa OS=Homo sapiens GN=CEP63		
Q96MT8-4	CEP63_HUMAN Isoform 4 of Centrosomal protein of 63 kDa OS=Homo sapiens GN=CEP63		
Q96MU7	YTDC1_HUMAN YTH domain-containing protein 1 OS=Homo sapiens GN=YTHDC1 PE=1 SV=3		46.21
Q96MU7-2	YTDC1_HUMAN Isoform 2 of YTH domain-containing protein 1 OS=Homo sapiens GN=YTHDC1		46.21
Q96MW1;Q96MW1-2	CCD43_HUMAN Coiled-coil domain-containing protein 43 OS=Homo sapiens GN=CCDC43 PE=1 SV=2;>sp|Q96MW1-2|CCD43_HUMAN	3.32E-18	
Q96MW5	COG8_HUMAN Conserved oligomeric Golgi complex subunit 8 OS=Homo sapiens GN=COG8 PE=1 SV=2	8.69E-08	
Q96MW7	TIGD1_HUMAN Tigger transposable element-derived protein 1 OS=Homo sapiens GN=TIGD1 PE=2 SV=1		28.29
Q96MX6	WDR92_HUMAN WD repeat-containing protein 92 OS=Homo sapiens GN=WDR92 PE=1 SV=1	2.39E-13	
Q96N64	PWP2A_HUMAN PWWP domain-containing protein 2A OS=Homo sapiens GN=PWWP2A PE=1 SV=2		
Q96N64-2	PWP2A_HUMAN Isoform 2 of PWWP domain-containing protein 2A OS=Homo sapiens GN=PWWP2A		
Q96N66	MBOA7_HUMAN Lysophospholipid acyltransferase 7 OS=Homo sapiens GN=MBOAT7 PE=1 SV=2		324.7
Q96N66-2	MBOA7_HUMAN Isoform 2 of Lysophospholipid acyltransferase 7 OS=Homo sapiens GN=MBOAT7		290.54
Q96N66-3	MBOA7_HUMAN Isoform 3 of Lysophospholipid acyltransferase 7 OS=Homo sapiens GN=MBOAT7		253.01
Q96N66;B4DDH8;Q96N66	MBOA7_HUMAN Lysophospholipid acyltransferase 7 OS=Homo sapiens GN=MBOAT7 PE=1 SV=2;>tr|B4DDH8|B4DDH8_HUMAN cDNA	3.37E-06	
Q96N67	DOCK7_HUMAN Dedicator of cytokinesis protein 7 OS=Homo sapiens GN=DOCK7 PE=1 SV=4		31.12
Q96N67-2	DOCK7_HUMAN Isoform 2 of Dedicator of cytokinesis protein 7 OS=Homo sapiens GN=DOCK7		31.12
Q96N67-3	DOCK7_HUMAN Isoform 3 of Dedicator of cytokinesis protein 7 OS=Homo sapiens GN=DOCK7		29.6
Q96N67-4	DOCK7_HUMAN Isoform 4 of Dedicator of cytokinesis protein 7 OS=Homo sapiens GN=DOCK7		29.6
Q96N67-5	DOCK7_HUMAN Isoform 5 of Dedicator of cytokinesis protein 7 OS=Homo sapiens GN=DOCK7		29.6
Q96N67-6	DOCK7_HUMAN Isoform 6 of Dedicator of cytokinesis protein 7 OS=Homo sapiens GN=DOCK7		31.12

Q96N67;Q96N67-6;Q96N(	DOCK7_HUMAN Dedicator of cytokinesis protein 7 OS=Homo sapiens GN=DOCK7 PE=1 SV=4;>sp|Q96N67-6|DOCK7_HUMAN Isoform	1.13E-22	
Q96N76	HUTU_HUMAN Urocanate hydratase OS=Homo sapiens GN=UROC1 PE=1 SV=1		
Q96NB2	SFXN2_HUMAN Sideroflexin-2 OS=Homo sapiens GN=SFXN2 PE=1 SV=2	2.36E-12	
Q96ND0	CR019_HUMAN Uncharacterized protein C18orf19 OS=Homo sapiens GN=C18orf19 PE=2 SV=2		69.12
Q96NE9	FRMD6_HUMAN FERM domain-containing protein 6 OS=Homo sapiens GN=FRMD6 PE=1 SV=1		56.12
Q96NE9-2	FRMD6_HUMAN Isoform 2 of FERM domain-containing protein 6 OS=Homo sapiens GN=FRMD6		56.12
Q96NE9-3	FRMD6_HUMAN Isoform 3 of FERM domain-containing protein 6 OS=Homo sapiens GN=FRMD6		56.12
Q96NJ3	ZN285_HUMAN Zinc finger protein 285 OS=Homo sapiens GN=ZNF285 PE=2 SV=2		
Q96NJ3-2	ZN285_HUMAN Isoform 2 of Zinc finger protein 285 OS=Homo sapiens GN=ZNF285		
Q96NM4	TOX2_HUMAN TOX high mobility group box family member 2 OS=Homo sapiens GN=TOX2 PE=2 SV=2		26.7
Q96NM4-2	TOX2_HUMAN Isoform 2 of TOX high mobility group box family member 2 OS=Homo sapiens GN=TOX2		26.7
Q96NW7-2	LRRC7_HUMAN Isoform 2 of Leucine-rich repeat-containing protein 7 OS=Homo sapiens GN=LRRC7		0.5
Q96NW7;Q96NW7-2	LRRC7_HUMAN Leucine-rich repeat-containing protein 7 OS=Homo sapiens GN=LRRC7 PE=1 SV=1;>sp|Q96NW7-2|LRRC7_HUMAN Is	1.60E-08	
Q96NY7	CLIC6_HUMAN Chloride intracellular channel protein 6 OS=Homo sapiens GN=CLIC6 PE=2 SV=3		67.31
Q96NY7-2	CLIC6_HUMAN Isoform A of Chloride intracellular channel protein 6 OS=Homo sapiens GN=CLIC6		67.31
Q96NY7;Q96NY7-2	CLIC6_HUMAN Chloride intracellular channel protein 6 OS=Homo sapiens GN=CLIC6 PE=2 SV=3;>sp|Q96NY7-2|CLIC6_HUMAN Isofor	8.67E-163	
Q96NZ8	WFKN1_HUMAN WAP, kazal, immunoglobulin, kunitz and NTR domain-containing protein 1 OS=Homo sapiens GN=WFIKKN1 PE=1 SV=1		
Q96P11	NSUN5_HUMAN Putative methyltransferase NSUN5 OS=Homo sapiens GN=NSUN5 PE=1 SV=2		89.2
Q96P11-2	NSUN5_HUMAN Isoform 2 of Putative methyltransferase NSUN5 OS=Homo sapiens GN=NSUN5		99.69
Q96P11-2;Q96P11	2|NSUN5_HUMAN Isoform 2 of Putative methyltransferase NSUN5 OS=Homo sapiens GN=NSUN5;>sp|Q96P11|NSUN5_HUMAN Putativ	2.64E-12	
Q96P11-3	NSUN5_HUMAN Isoform 3 of Putative methyltransferase NSUN5 OS=Homo sapiens GN=NSUN5		1.09
Q96P16	RPR1A_HUMAN Regulation of nuclear pre domain-containing protein 1A OS=Homo sapiens GN=RPRD1A PE=1 SV=1		44.69
Q96P16-2	RPR1A_HUMAN Isoform 2 of Regulation of nuclear pre domain-containing protein 1A OS=Homo sapiens GN=RPRD1A		44.69
Q96P16-3	RPR1A_HUMAN Isoform 3 of Regulation of nuclear pre domain-containing protein 1A OS=Homo sapiens GN=RPRD1A		14.76
Q96P16;Q96P16-2;Q96P1(	RPR1A_HUMAN Regulation of nuclear pre-mRNA domain-containing protein 1A OS=Homo sapiens GN=RPRD1A PE=1 SV=1;>sp|Q96P	2.34E-27	
Q96P20	NALP3_HUMAN NACHT, LRR and PYD domains-containing protein 3 OS=Homo sapiens GN=NLRP3 PE=1 SV=3		27.14
Q96P20-2	NALP3_HUMAN Isoform 1 of NACHT, LRR and PYD domains-containing protein 3 OS=Homo sapiens GN=NLRP3		22.99
Q96P20-3	NALP3_HUMAN Isoform 3 of NACHT, LRR and PYD domains-containing protein 3 OS=Homo sapiens GN=NLRP3		22.99
Q96P20-4	NALP3_HUMAN Isoform 4 of NACHT, LRR and PYD domains-containing protein 3 OS=Homo sapiens GN=NLRP3		27.14
Q96P20-5	NALP3_HUMAN Isoform 5 of NACHT, LRR and PYD domains-containing protein 3 OS=Homo sapiens GN=NLRP3		22.99
Q96P47	AGAP3_HUMAN Arf-GAP with GTPase, ANK repeat and PH domain-containing protein 3 OS=Homo sapiens GN=AGAP3 PE=1 SV=2		25.51
Q96P47-2	AGAP3_HUMAN Isoform 2 of Arf-GAP with GTPase, ANK repeat and PH domain-containing protein 3 OS=Homo sapiens GN=AGAP3		25.51
Q96P47-3	AGAP3_HUMAN Isoform 3 of Arf-GAP with GTPase, ANK repeat and PH domain-containing protein 3 OS=Homo sapiens GN=AGAP3		1.92
Q96P47-4	AGAP3_HUMAN Isoform 4 of Arf-GAP with GTPase, ANK repeat and PH domain-containing protein 3 OS=Homo sapiens GN=AGAP3		25.51
Q96P48	ARAP1_HUMAN Arf-GAP with Rho-GAP domain, ANK repeat and PH domain-containing protein 1 OS=Homo sapiens GN=ARAP1 PE=1 SV=3		26.68
Q96P48-1	ARAP1_HUMAN Isoform 1 of Arf-GAP with Rho-GAP domain, ANK repeat and PH domain-containing protein 1 OS=Homo sapiens GN=ARAP1		4.95
Q96P48-2	ARAP1_HUMAN Isoform 2 of Arf-GAP with Rho-GAP domain, ANK repeat and PH domain-containing protein 1 OS=Homo sapiens GN=ARAP1		4.95
Q96P48-3	ARAP1_HUMAN Isoform 3 of Arf-GAP with Rho-GAP domain, ANK repeat and PH domain-containing protein 1 OS=Homo sapiens GN=ARAP1		26.68
Q96P48-4	ARAP1_HUMAN Isoform 4 of Arf-GAP with Rho-GAP domain, ANK repeat and PH domain-containing protein 1 OS=Homo sapiens GN=ARAP1		4.95
Q96P48-5	ARAP1_HUMAN Isoform 5 of Arf-GAP with Rho-GAP domain, ANK repeat and PH domain-containing protein 1 OS=Homo sapiens GN=ARAP1		
Q96P48;Q96P48-3;Q96P4	ARAP1_HUMAN Arf-GAP with Rho-GAP domain, ANK repeat and PH domain-containing protein 1 OS=Homo sapiens GN=ARAP1 PE=1 S	4.06E-17	
Q96P67	GPR82_HUMAN Probable G-protein coupled receptor 82 OS=Homo sapiens GN=GPR82 PE=2 SV=1		
Q96P70	IPO9_HUMAN Importin-9 OS=Homo sapiens GN=IPO9 PE=1 SV=3		
Q96PC3	AP1S3_HUMAN AP-1 complex subunit sigma-3 OS=Homo sapiens GN=AP1S3 PE=2 SV=1		
Q96PC3-2	AP1S3_HUMAN Isoform 2 of AP-1 complex subunit sigma-3 OS=Homo sapiens GN=AP1S3		
Q96PC3-3	AP1S3_HUMAN Isoform 3 of AP-1 complex subunit sigma-3 OS=Homo sapiens GN=AP1S3		
Q96PC3-4	AP1S3_HUMAN Isoform 4 of AP-1 complex subunit sigma-3 OS=Homo sapiens GN=AP1S3		
Q96PD2-2;Q96PD2	2|DCBD2_HUMAN Isoform 2 of Discoidin, CUB and LCCL domain-containing protein 2 OS=Homo sapiens GN=DCBLD2;>sp|Q96PD2|D(	2.05E-10	
Q96PD5-2;A8K050;Q96PD	2|PGRP2_HUMAN Isoform 2 of N-acetylmuramoyl-L-alanine amidase OS=Homo sapiens GN=PGLYRP2;>tr|A8K050|A8K050_HUMAN cD	6.58E-193	
Q96PE2	ARHGH_HUMAN Rho guanine nucleotide exchange factor 17 OS=Homo sapiens GN=ARHGEF17 PE=1 SV=1		
Q96PH1	NOX5_HUMAN NADPH oxidase 5 OS=Homo sapiens GN=NOX5 PE=1 SV=1		
Q96PH1-2	NOX5_HUMAN Isoform v4 of NADPH oxidase 5 OS=Homo sapiens GN=NOX5		
Q96PH1-3	NOX5_HUMAN Isoform v1 of NADPH oxidase 5 OS=Homo sapiens GN=NOX5		
Q96PH1-4	NOX5_HUMAN Isoform v2 of NADPH oxidase 5 OS=Homo sapiens GN=NOX5		
Q96PH1-5	NOX5_HUMAN Isoform v5 of NADPH oxidase 5 OS=Homo sapiens GN=NOX5		
Q96PH1-6	NOX5_HUMAN Isoform v6 of NADPH oxidase 5 OS=Homo sapiens GN=NOX5		
Q96PK2	MACF4_HUMAN Microtubule-actin cross-linking factor 1, isoform 4 OS=Homo sapiens GN=MACF1 PE=1 SV=2		1579.58
Q96PK6	RBM14_HUMAN RNA-binding protein 14 OS=Homo sapiens GN=RBM14 PE=1 SV=2		78.39
Q96PK6-2	RBM14_HUMAN Isoform 2 of RNA-binding protein 14 OS=Homo sapiens GN=RBM14		
Q96PK6;Q96PK6-2	RBM14_HUMAN RNA-binding protein 14 OS=Homo sapiens GN=RBM14 PE=1 SV=2;>sp|Q96PK6-2|RBM14_HUMAN Isoform 2 of RNA-	3.12E-87	
Q96PN6	ADCYA_HUMAN Adenylate cyclase type 10 OS=Homo sapiens GN=ADCY10 PE=1 SV=3		57.27

Q96PN6-2	ADCYA_HUMAN Isoform 2 of Adenylate cyclase type 10 OS=Homo sapiens GN=ADCY10		57.27
Q96PN6-3	ADCYA_HUMAN Isoform 3 of Adenylate cyclase type 10 OS=Homo sapiens GN=ADCY10		0.36
Q96PN8	TSSK3_HUMAN Testis-specific serine/threonine-protein kinase 3 OS=Homo sapiens GN=TSSK3 PE=2 SV=1		
Q96PP8;Q8NF03;Q86TM5;	GBP5_HUMAN Guanylate-binding protein 5 OS=Homo sapiens GN=GBP5 PE=2 SV=1;>tr|Q8NF03|Q8NF03_HUMAN FLJ00401 protein (	1.05E-38	
Q96PP9	GBP4_HUMAN Guanylate-binding protein 4 OS=Homo sapiens GN=GBP4 PE=1 SV=2		34.08
Q96PQ0	SORC2_HUMAN VPS10 domain-containing receptor SorCS2 OS=Homo sapiens GN=SORCS2 PE=1 SV=3		66.37
Q96PU4	UHRF2_HUMAN E3 ubiquitin-protein ligase UHRF2 OS=Homo sapiens GN=UHRF2 PE=1 SV=1	7.25E-14	
Q96PU5-8;Q96PU5;Q96PU	8|NED4L_HUMAN Isoform 8 of E3 ubiquitin-protein ligase NEDD4-like OS=Homo sapiens GN=NEDD4L;>sp|Q96PU5|NED4L_HUMAN E3	1.82E-25	
Q96PU8	QKI_HUMAN Protein quaking OS=Homo sapiens GN=QKI PE=1 SV=1		144.43
Q96PU8-3	QKI_HUMAN Isoform 2 of Protein quaking OS=Homo sapiens GN=QKI		144.43
Q96PU8-4;Q96PU8-7;Q96	4|QKI_HUMAN Isoform 4 of Protein quaking OS=Homo sapiens GN=QKI;>sp|Q96PU8-7|QKI_HUMAN Isoform 7 of Protein quaking OS	2.26E-11	
Q96PU8-5	QKI_HUMAN Isoform 3 of Protein quaking OS=Homo sapiens GN=QKI		144.43
Q96PU8-6	QKI_HUMAN Isoform 4 of Protein quaking OS=Homo sapiens GN=QKI		144.43
Q96PU8-8	QKI_HUMAN Isoform 5 of Protein quaking OS=Homo sapiens GN=QKI		144.43
Q96PU8-9	QKI_HUMAN Isoform 6 of Protein quaking OS=Homo sapiens GN=QKI		144.43
Q96PU8;Q96PU8-3;Q96PU	QKI_HUMAN Protein quaking OS=Homo sapiens GN=QKI PE=1 SV=1;>sp|Q96PU8-3|QKI_HUMAN Isoform 2 of Protein quaking OS=H	3.86E-09	
Q96PV0	SYGP1_HUMAN Ras GTPase-activating protein SynGAP OS=Homo sapiens GN=SYNGAP1 PE=1 SV=4		54.19
Q96PV0-2	SYGP1_HUMAN Isoform 2 of Ras GTPase-activating protein SynGAP OS=Homo sapiens GN=SYNGAP1		54.19
Q96PV0-3	SYGP1_HUMAN Isoform 3 of Ras GTPase-activating protein SynGAP OS=Homo sapiens GN=SYNGAP1		54.19
Q96PV0-4	SYGP1_HUMAN Isoform 4 of Ras GTPase-activating protein SynGAP OS=Homo sapiens GN=SYNGAP1		54.19
Q96PV4	PNMA5_HUMAN Paraneoplastic antigen-like protein 5 OS=Homo sapiens GN=PNMA5 PE=2 SV=2		29.22
Q96PX9	PKH4B_HUMAN Pleckstrin homology domain-containing family G member 4B OS=Homo sapiens GN=PLEKHG4B PE=2 SV=4	1.48E-10	
Q96PY0	K1908_HUMAN Uncharacterized protein KIAA1908 OS=Homo sapiens GN=KIAA1908 PE=2 SV=2		
Q96PY5	FMNL2_HUMAN Formin-like protein 2 OS=Homo sapiens GN=FMNL2 PE=1 SV=3		84.5
Q96PY5-3	FMNL2_HUMAN Isoform 2 of Formin-like protein 2 OS=Homo sapiens GN=FMNL2		84.5
Q96PY5-3;Q96PY5;Q8IVF7	3|FMNL2_HUMAN Isoform 2 of Formin-like protein 2 OS=Homo sapiens GN=FMNL2;>sp|Q96PY5|FMNL2_HUMAN Formin-like protein 2	5.83E-06	
Q96PZ0	PUS7_HUMAN Pseudouridylate synthase 7 homolog OS=Homo sapiens GN=PUS7 PE=1 SV=2		37.82
Q96Q06-2;Q96Q06	2|PLIN4_HUMAN Isoform 2 of Perilipin-4 OS=Homo sapiens GN=PLIN4;>sp|Q96Q06|PLIN4_HUMAN Perilipin-4 OS=Homo sapiens GN=	1.12E-93	
Q96Q11;C9JKA2;Q96Q11-	TRNT1_HUMAN tRNA-nucleotidyltransferase 1, mitochondrial OS=Homo sapiens GN=TRNT1 PE=1 SV=2;>tr|C9JKA2|C9JKA2_HUMAN	3.23E-05	
Q96Q11;Q96Q11-2	TRNT1_HUMAN CCA tRNA nucleotidyltransferase 1, mitochondrial OS=Homo sapiens GN=TRNT1 PE=1 SV=2;>sp|Q96Q11-2|TRNT1_H	1.41E-15	
Q96Q15	SMG1_HUMAN Serine/threonine-protein kinase SMG1 OS=Homo sapiens GN=SMG1 PE=1 SV=3		50.43
Q96Q15-2	SMG1_HUMAN Isoform 2 of Serine/threonine-protein kinase SMG1 OS=Homo sapiens GN=SMG1		50.43
Q96Q15-3	SMG1_HUMAN Isoform 3 of Serine/threonine-protein kinase SMG1 OS=Homo sapiens GN=SMG1		50.43
Q96Q15-4	SMG1_HUMAN Isoform 4 of Serine/threonine-protein kinase SMG1 OS=Homo sapiens GN=SMG1		50.34
Q96Q15;Q96Q15-2;Q96Q1	SMG1_HUMAN Serine/threonine-protein kinase SMG1 OS=Homo sapiens GN=SMG1 PE=1 SV=3;>sp|Q96Q15-2|SMG1_HUMAN Isoforı	2.78E-12	
Q96Q40	CDK15_HUMAN Cyclin-dependent kinase 15 OS=Homo sapiens GN=CDK15 PE=1 SV=2		60.32
Q96Q40-2	CDK15_HUMAN Isoform 2 of Cyclin-dependent kinase 15 OS=Homo sapiens GN=CDK15		48.96
Q96Q40-3	CDK15_HUMAN Isoform 3 of Cyclin-dependent kinase 15 OS=Homo sapiens GN=CDK15		58.74
Q96Q40-4	CDK15_HUMAN Isoform 4 of Cyclin-dependent kinase 15 OS=Homo sapiens GN=CDK15		60.45
Q96Q89-2;C9J6W2;Q96Q8	2|KI20B_HUMAN Isoform 2 of Kinesin-like protein KIF20B OS=Homo sapiens GN=KIF20B;>tr|C9J6W2|C9J6W2_HUMAN Characteriz	4.05E-06	
Q96Q89-4;Q96Q89;Q96Q8	4|KI20B_HUMAN Isoform 4 of Kinesin-like protein KIF20B OS=Homo sapiens GN=KIF20B;>sp|Q96Q89|KI20B_HUMAN Kinesin-like pro	2.39E-13	
Q96QA5	GSDMA_HUMAN Gasdermin-A OS=Homo sapiens GN=GSDMA PE=2 SV=4		59.91
Q96QC0	PP1RA_HUMAN Serine/threonine-protein phosphatase 1 regulatory subunit 10 OS=Homo sapiens GN=PPP1R10 PE=1 SV=1		
Q96QE4	LR37B_HUMAN Leucine-rich repeat-containing protein 37B OS=Homo sapiens GN=LRRC37B PE=2 SV=3		
Q96QE5	TEFM_HUMAN Transcription elongation factor, mitochondrial OS=Homo sapiens GN=TEFM PE=1 SV=1		
Q96QH2	PRAM_HUMAN PML-RARA-regulated adapter molecule 1 OS=Homo sapiens GN=PRAM1 PE=1 SV=2		
Q96QH2-2	PRAM_HUMAN Isoform 2 of PML-RARA-regulated adapter molecule 1 OS=Homo sapiens GN=PRAM1		
Q96QK1	VPS35_HUMAN Vacuolar protein sorting-associated protein 35 OS=Homo sapiens GN=VPS35 PE=1 SV=2		408.16
Q96QR8	PURB_HUMAN Transcriptional activator protein Pur-beta OS=Homo sapiens GN=PURB PE=1 SV=3		124.85
Q96QS3	ARX_HUMAN Homeobox protein ARX OS=Homo sapiens GN=ARX PE=1 SV=1		
Q96QU6	1A1L1_HUMAN 1-aminocyclopropane-1-carboxylate synthase-like protein 1 OS=Homo sapiens GN=ACCS PE=2 SV=1		
Q96QV6	H2A1A_HUMAN Histone H2A type 1-A OS=Homo sapiens GN=HIST1H2AA PE=1 SV=3		145.19
Q96R06	SPAG5_HUMAN Sperm-associated antigen 5 OS=Homo sapiens GN=SPAG5 PE=1 SV=2	2.68E-23	
Q96R72	OR4K3_HUMAN Olfactory receptor 4K3 OS=Homo sapiens GN=OR4K3 PE=3 SV=3		
Q96RD7	PANX1_HUMAN Pannexin-1 OS=Homo sapiens GN=PANX1 PE=1 SV=4		
Q96RD7-2	PANX1_HUMAN Isoform 2 of Pannexin-1 OS=Homo sapiens GN=PANX1		
Q96RE7	NACC1_HUMAN Nucleus accumbens-associated protein 1 OS=Homo sapiens GN=NACC1 PE=1 SV=1	0.00024605	
Q96RK0	CIC_HUMAN Protein capicua homolog OS=Homo sapiens GN=CIC PE=1 SV=2	9.64E-10	
Q96RM1	SPR2F_HUMAN Small proline-rich protein 2F OS=Homo sapiens GN=SPRR2F PE=2 SV=1		23.4
Q96RN5	MED15_HUMAN Mediator of RNA polymerase II transcription subunit 15 OS=Homo sapiens GN=MED15 PE=1 SV=2		
Q96RN5-2	MED15_HUMAN Isoform 2 of Mediator of RNA polymerase II transcription subunit 15 OS=Homo sapiens GN=MED15		

Q96RN5-3	MED15_HUMAN Isoform 3 of Mediator of RNA polymerase II transcription subunit 15 OS=Homo sapiens GN=MED15		
Q96RP9-2;Q96RP9;C9IZ0:	2|EFGM_HUMAN Isoform 2 of Elongation factor G, mitochondrial OS=Homo sapiens GN=GFM1;>sp|Q96RP9|EFGM_HUMAN Elongation	1.09E-20	
Q96RP9;Q96RP9-2	EFGM_HUMAN Elongation factor G, mitochondrial OS=Homo sapiens GN=GFM1 PE=1 SV=2;>sp|Q96RP9-2|EFGM_HUMAN Isoform 2 c	7.29E-64	
Q96RQ3	MCCA_HUMAN Methylcrotonoyl-CoA carboxylase subunit alpha, mitochondrial OS=Homo sapiens GN=MCCC1 PE=1 SV=3		
Q96RQ9-2;Q96RQ9	2|OXLA_HUMAN Isoform 2 of L-amino-acid oxidase OS=Homo sapiens GN=IL4I1;>sp|Q96RQ9|OXLA_HUMAN L-amino-acid oxidase OS	1.49E-12	
Q96RS0	TGS1_HUMAN Trimethylguanosine synthase OS=Homo sapiens GN=TGS1 PE=1 SV=3		
Q96RS6	NUDC1_HUMAN NudC domain-containing protein 1 OS=Homo sapiens GN=NUDCD1 PE=1 SV=2		55.13
Q96RS6-2	NUDC1_HUMAN Isoform 2 of NudC domain-containing protein 1 OS=Homo sapiens GN=NUDCD1		57.35
Q96RS6-3	NUDC1_HUMAN Isoform 3 of NudC domain-containing protein 1 OS=Homo sapiens GN=NUDCD1		24.71
Q96RS6;Q96RS6-2;Q96RS	NUDC1_HUMAN NudC domain-containing protein 1 OS=Homo sapiens GN=NUDCD1 PE=1 SV=2;>sp|Q96RS6-2|NUDC1_HUMAN Isofo	0	
Q96RT1	LAP2_HUMAN Protein LAP2 OS=Homo sapiens GN=ERBB2IP PE=1 SV=2		165.28
Q96RT1-2	LAP2_HUMAN Isoform 2 of Protein LAP2 OS=Homo sapiens GN=ERBB2IP		165.28
Q96RT1-3	LAP2_HUMAN Isoform 3 of Protein LAP2 OS=Homo sapiens GN=ERBB2IP		165.28
Q96RT1-4	LAP2_HUMAN Isoform 4 of Protein LAP2 OS=Homo sapiens GN=ERBB2IP		165.28
Q96RT1-5	LAP2_HUMAN Isoform 5 of Protein LAP2 OS=Homo sapiens GN=ERBB2IP		165.28
Q96RT1-6	LAP2_HUMAN Isoform 6 of Protein LAP2 OS=Homo sapiens GN=ERBB2IP		165.28
Q96RT1-7	LAP2_HUMAN Isoform 7 of Protein LAP2 OS=Homo sapiens GN=ERBB2IP		165.28
Q96RT1;Q96RT1-2;Q96RT	LAP2_HUMAN Protein LAP2 OS=Homo sapiens GN=ERBB2IP PE=1 SV=2;>sp|Q96RT1-2|LAP2_HUMAN Isoform 2 of Protein LAP2 OS=I	8.57E-30	
Q96RT7;Q96RT7-3;Q96RT	GCP6_HUMAN Gamma-tubulin complex component 6 OS=Homo sapiens GN=TUBGCP6 PE=1 SV=3;>sp|Q96RT7-3|GCP6_HUMAN Isof	1.19E-08	
Q96RU2;Q96RU2-2	UBP28_HUMAN Ubiquitin carboxyl-terminal hydrolase 28 OS=Homo sapiens GN=USP28 PE=1 SV=1;>sp|Q96RU2-2|UBP28_HUMAN Is	2.53E-06	
Q96RU3;Q96RU3-2;B7ZL1	FNBP1_HUMAN Formin-binding protein 1 OS=Homo sapiens GN=FNBP1 PE=1 SV=2;>sp|Q96RU3-2|FNBP1_HUMAN Isoform 2 of Form	4.73E-89	
Q96RU8	TRIB1_HUMAN Tribbles homolog 1 OS=Homo sapiens GN=TRIB1 PE=1 SV=2		23.24
Q96RW7	HMCN1_HUMAN Hemicentin-1 OS=Homo sapiens GN=HMCN1 PE=1 SV=2		
Q96RW7-2	HMCN1_HUMAN Isoform 2 of Hemicentin-1 OS=Homo sapiens GN=HMCN1		
Q96S19;Q96S19-2;Q96S1	CP013_HUMAN UPF0585 protein C16orf13 OS=Homo sapiens GN=C16orf13 PE=1 SV=2;>sp|Q96S19-2|CP013_HUMAN Isoform 2 of U	1.34E-09	
Q96S44	PRPK_HUMAN TP53-regulating kinase OS=Homo sapiens GN=TP53RK PE=1 SV=2		45.67
Q96S52	PIGS_HUMAN GPI transamidase component PIG-S OS=Homo sapiens GN=PIGS PE=1 SV=3		
Q96S52-2	PIGS_HUMAN Isoform 2 of GPI transamidase component PIG-S OS=Homo sapiens GN=PIGS		
Q96S52;Q96S52-2	PIGS_HUMAN GPI transamidase component PIG-S OS=Homo sapiens GN=PIGS PE=1 SV=3;>sp|Q96S52-2|PIGS_HUMAN Isoform 2 o	3.14E-14	
Q96S55;Q96S55-2;B3KT2	WRIP1_HUMAN ATPase WRNIP1 OS=Homo sapiens GN=WRNIP1 PE=1 SV=2;>sp|Q96S55-2|WRIP1_HUMAN Isoform 2 of ATPase WRN	1.98E-48	
Q96S59;B2R8E1;Q96S59-	RANB9_HUMAN Ran-binding protein 9 OS=Homo sapiens GN=RANBP9 PE=1 SV=1;>tr|B2R8E1|B2R8E1_HUMAN cDNA, FLJ93856, Hor	1.18E-50	
Q96S66	CLCC1_HUMAN Chloride channel CLIC-like protein 1 OS=Homo sapiens GN=CLCC1 PE=1 SV=1		
Q96S66-2	CLCC1_HUMAN Isoform 2 of Chloride channel CLIC-like protein 1 OS=Homo sapiens GN=CLCC1		
Q96S66-3	CLCC1_HUMAN Isoform 3 of Chloride channel CLIC-like protein 1 OS=Homo sapiens GN=CLCC1		
Q96S66-4	CLCC1_HUMAN Isoform 4 of Chloride channel CLIC-like protein 1 OS=Homo sapiens GN=CLCC1		
Q96S66;Q96S66-2;Q96S6	CLCC1_HUMAN Chloride channel CLIC-like protein 1 OS=Homo sapiens GN=CLCC1 PE=1 SV=1;>sp|Q96S66-2|CLCC1_HUMAN Isoforn	6.58E-20	
Q96S86	HPLN3_HUMAN Hyaluronan and proteoglycan link protein 3 OS=Homo sapiens GN=HAPLN3 PE=2 SV=1	1.44E-13	
Q96S97	MYADM_HUMAN Myeloid-associated differentiation marker OS=Homo sapiens GN=MYADM PE=1 SV=2		378.31
Q96S99	PKHF1_HUMAN Pleckstrin homology domain-containing family F member 1 OS=Homo sapiens GN=PLEKHF1 PE=1 SV=3	2.03E-08	
Q96SB3	NEB2_HUMAN Neurabin-2 OS=Homo sapiens GN=PPP1R9B PE=1 SV=2	1.28E-11	
Q96SB4	SRPK1_HUMAN Serine/threonine-protein kinase SRPK1 OS=Homo sapiens GN=SRPK1 PE=1 SV=2		216.01
Q96SB8	SMC6_HUMAN Structural maintenance of chromosomes protein 6 OS=Homo sapiens GN=SMC6 PE=1 SV=2		
Q96SB8-2	SMC6_HUMAN Isoform 2 of Structural maintenance of chromosomes protein 6 OS=Homo sapiens GN=SMC6		
Q96SI9	STRBP_HUMAN Spermatid perinuclear RNA-binding protein OS=Homo sapiens GN=STRBP PE=1 SV=1		142.03
Q96SI9-2	STRBP_HUMAN Isoform 2 of Spermatid perinuclear RNA-binding protein OS=Homo sapiens GN=STRBP		142.03
Q96SI9;Q96SI9-2	STRBP_HUMAN Spermatid perinuclear RNA-binding protein OS=Homo sapiens GN=STRBP PE=1 SV=1;>sp|Q96SI9-2|STRBP_HUMAN I	2.40E-108	
Q96SL4	GPX7_HUMAN Glutathione peroxidase 7 OS=Homo sapiens GN=GPX7 PE=1 SV=1	5.49E-07	
Q96ST2	IWS1_HUMAN Protein IWS1 homolog OS=Homo sapiens GN=IWS1 PE=1 SV=2		103.59
Q96ST2-2	IWS1_HUMAN Isoform 2 of Protein IWS1 homolog OS=Homo sapiens GN=IWS1		103.59
Q96ST2-3	IWS1_HUMAN Isoform 3 of Protein IWS1 homolog OS=Homo sapiens GN=IWS1		103.59
Q96ST2;B4DL52;B4DGM5	IWS1_HUMAN Protein IWS1 homolog OS=Homo sapiens GN=IWS1 PE=1 SV=2;>tr|B4DL52|B4DL52_HUMAN cDNA FLJ54017, highly s	8.56E-07	
Q96ST3	SIN3A_HUMAN Paired amphipathic helix protein Sin3a OS=Homo sapiens GN=SIN3A PE=1 SV=2		
Q96ST8	CEP89_HUMAN Centrosomal protein of 89 kDa OS=Homo sapiens GN=CEP89 PE=1 SV=3		43.36
Q96ST8-2	CEP89_HUMAN Isoform 2 of Centrosomal protein of 89 kDa OS=Homo sapiens GN=CEP89		38.8
Q96ST8-3	CEP89_HUMAN Isoform 3 of Centrosomal protein of 89 kDa OS=Homo sapiens GN=CEP89		4.56
Q96SU4	OSBL9_HUMAN Oxysterol-binding protein-related protein 9 OS=Homo sapiens GN=OSBPL9 PE=1 SV=2		
Q96SU4-2	OSBL9_HUMAN Isoform 2 of Oxysterol-binding protein-related protein 9 OS=Homo sapiens GN=OSBPL9		
Q96SU4-3	OSBL9_HUMAN Isoform 3 of Oxysterol-binding protein-related protein 9 OS=Homo sapiens GN=OSBPL9		
Q96SU4-4	OSBL9_HUMAN Isoform 4 of Oxysterol-binding protein-related protein 9 OS=Homo sapiens GN=OSBPL9		
Q96SU4-5	OSBL9_HUMAN Isoform 5 of Oxysterol-binding protein-related protein 9 OS=Homo sapiens GN=OSBPL9		
Q96T23	RSF1_HUMAN Remodeling and spacing factor 1 OS=Homo sapiens GN=RSF1 PE=1 SV=2		37.7

Q96T23-2	RSF1_HUMAN Isoform 2 of Remodeling and spacing factor 1 OS=Homo sapiens GN=RSF1		34.83
Q96T23-3	RSF1_HUMAN Isoform 3 of Remodeling and spacing factor 1 OS=Homo sapiens GN=RSF1		4.05
Q96T23;Q96T23-2;Q96T2	RSF1_HUMAN Remodeling and spacing factor 1 OS=Homo sapiens GN=RSF1 PE=1 SV=2;>sp|Q96T23-2|RSF1_HUMAN Isoform 2 of R	3.60E-20	
Q96T25	ZIC5_HUMAN Zinc finger protein ZIC 5 OS=Homo sapiens GN=ZIC5 PE=1 SV=2		7.49
Q96T37	RBM15_HUMAN Putative RNA-binding protein 15 OS=Homo sapiens GN=RBM15 PE=1 SV=2		
Q96T37-2	RBM15_HUMAN Isoform 2 of Putative RNA-binding protein 15 OS=Homo sapiens GN=RBM15		
Q96T37-3	RBM15_HUMAN Isoform 3 of Putative RNA-binding protein 15 OS=Homo sapiens GN=RBM15		
Q96T37;Q3ZB86;Q96T37-	RBM15_HUMAN Putative RNA-binding protein 15 OS=Homo sapiens GN=RBM15 PE=1 SV=2;>tr|Q3ZB86|Q3ZB86_HUMAN RBM15 pro	2.14E-05	
Q96T51	RUFY1_HUMAN RUN and FYVE domain-containing protein 1 OS=Homo sapiens GN=RUFY1 PE=1 SV=2		29.78
Q96T51-2	RUFY1_HUMAN Isoform 2 of RUN and FYVE domain-containing protein 1 OS=Homo sapiens GN=RUFY1		29.78
Q96T51-3	RUFY1_HUMAN Isoform 3 of RUN and FYVE domain-containing protein 1 OS=Homo sapiens GN=RUFY1		29.26
Q96T51;A8MSJ1;A8K7B1;	RUFY1_HUMAN RUN and FYVE domain-containing protein 1 OS=Homo sapiens GN=RUFY1 PE=1 SV=2;>tr|A8MSJ1|A8MSJ1_HUMAN U	1.61E-58	
Q96T58	MINT_HUMAN Msx2-interacting protein OS=Homo sapiens GN=SPEN PE=1 SV=1		
Q96T60	PNKP_HUMAN Bifunctional polynucleotide phosphatase/kinase OS=Homo sapiens GN=PNKP PE=1 SV=1		
Q96T76	MMS19_HUMAN MMS19 nucleotide excision repair protein homolog OS=Homo sapiens GN=MMS19 PE=1 SV=2		13.77
Q96T76-5	MMS19_HUMAN Isoform 4 of MMS19 nucleotide excision repair protein homolog OS=Homo sapiens GN=MMS19		13.77
Q96T76-7	MMS19_HUMAN Isoform 2 of MMS19 nucleotide excision repair protein homolog OS=Homo sapiens GN=MMS19		13.77
Q96T76;Q96T76-5	MMS19_HUMAN MMS19 nucleotide excision repair protein homolog OS=Homo sapiens GN=MMS19 PE=1 SV=2;>sp|Q96T76-5|MMS19	1.82E-69	
Q96T88	UHRF1_HUMAN E3 ubiquitin-protein ligase UHRF1 OS=Homo sapiens GN=UHRF1 PE=1 SV=1		22.87
Q96TA0	PCDBI_HUMAN Putative protocadherin beta-18 OS=Homo sapiens GN=PCDHB18 PE=5 SV=1		
Q96TA1	NIBL1_HUMAN Niban-like protein 1 OS=Homo sapiens GN=FAM129B PE=1 SV=2		1074.46
Q96TA1-2;Q96TA1	2|NIBL1_HUMAN Isoform 2 of Niban-like protein 1 OS=Homo sapiens GN=FAM129B;>sp|Q96TA1|NIBL1_HUMAN Niban-like protein 1 (	7.19E-286	
Q96TA1;Q96TA1-2	NIBL1_HUMAN Niban-like protein 1 OS=Homo sapiens GN=FAM129B PE=1 SV=3;>sp|Q96TA1-2|NIBL1_HUMAN Isoform 2 of Niban-lik	7.34E-204	
Q96TA2	YMEL1_HUMAN ATP-dependent zinc metalloprotease YME1L1 OS=Homo sapiens GN=YME1L1 PE=1 SV=2		94.05
Q96TA2-2	YMEL1_HUMAN Isoform 2 of ATP-dependent zinc metalloprotease YME1L1 OS=Homo sapiens GN=YME1L1		93.6
Q96TA2;Q96TA2-2	YMEL1_HUMAN ATP-dependent zinc metalloprotease YME1L1 OS=Homo sapiens GN=YME1L1 PE=1 SV=2;>sp|Q96TA2-2|YMEL1_HUM/	5.04E-73	
Q96TC7	RMD3_HUMAN Regulator of microtubule dynamics protein 3 OS=Homo sapiens GN=FAM82A2 PE=1 SV=2	9.76E-10	
Q99062	CSF3R_HUMAN Granulocyte colony-stimulating factor receptor OS=Homo sapiens GN=CSF3R PE=1 SV=1		
Q99062-2	CSF3R_HUMAN Isoform 2 of Granulocyte colony-stimulating factor receptor OS=Homo sapiens GN=CSF3R		
Q99062-3	CSF3R_HUMAN Isoform 3 of Granulocyte colony-stimulating factor receptor OS=Homo sapiens GN=CSF3R		
Q99062-4	CSF3R_HUMAN Isoform 4 of Granulocyte colony-stimulating factor receptor OS=Homo sapiens GN=CSF3R		
Q99250	SCN2A_HUMAN Sodium channel protein type 2 subunit alpha OS=Homo sapiens GN=SCN2A PE=1 SV=3		
Q99250-2	SCN2A_HUMAN Isoform 2 of Sodium channel protein type 2 subunit alpha OS=Homo sapiens GN=SCN2A		
Q99417	MYCBP_HUMAN C-Myc-binding protein OS=Homo sapiens GN=MYCBP PE=1 SV=3		174.99
Q99426	TBCB_HUMAN Tubulin-folding cofactor B OS=Homo sapiens GN=TBCB PE=1 SV=2		71.27
Q99436	PSB7_HUMAN Proteasome subunit beta type-7 OS=Homo sapiens GN=PSMB7 PE=1 SV=1		297.72
Q99439	CNN2_HUMAN Calponin-2 OS=Homo sapiens GN=CNN2 PE=1 SV=4		203.63
Q99442	SEC62_HUMAN Translocation protein SEC62 OS=Homo sapiens GN=SEC62 PE=1 SV=1		41.09
Q99447	PCY2_HUMAN Ethanolamine-phosphate cytidylyltransferase OS=Homo sapiens GN=PCYT2 PE=1 SV=1	2.13E-43	
Q99453	PHX2B_HUMAN Paired mesoderm homeobox protein 2B OS=Homo sapiens GN=PHOX2B PE=1 SV=2		
Q99456	K1C12_HUMAN Keratin, type I cytoskeletal 12 OS=Homo sapiens GN=KRT12 PE=1 SV=1		130.52
Q99459	CDC5L_HUMAN Cell division cycle 5-like protein OS=Homo sapiens GN=CDC5L PE=1 SV=2		263.38
Q99460	PSMD1_HUMAN 26S proteasome non-ATPase regulatory subunit 1 OS=Homo sapiens GN=PSMD1 PE=1 SV=2		317.69
Q99460-2	PSMD1_HUMAN Isoform 2 of 26S proteasome non-ATPase regulatory subunit 1 OS=Homo sapiens GN=PSMD1		317.69
Q99460;Q99460-2	PSMD1_HUMAN 26S proteasome non-ATPase regulatory subunit 1 OS=Homo sapiens GN=PSMD1 PE=1 SV=2;>sp|Q99460-2|PSMD1_	0	
Q99470	SDF2_HUMAN Stromal cell-derived factor 2 OS=Homo sapiens GN=SDF2 PE=1 SV=2	8.93E-10	
Q99471	PFD5_HUMAN Prefoldin subunit 5 OS=Homo sapiens GN=PFDN5 PE=1 SV=2		335.18
Q99490;Q99490-2	AGAP2_HUMAN Arf-GAP with GTPase, ANK repeat and PH domain-containing protein 2 OS=Homo sapiens GN=AGAP2 PE=1 SV=2;>sp	4.19E-05	
Q99496	RING2_HUMAN E3 ubiquitin-protein ligase RING2 OS=Homo sapiens GN=RNF2 PE=1 SV=1		
Q99497	PARK7_HUMAN Protein DJ-1 OS=Homo sapiens GN=PARK7 PE=1 SV=2		639.76
Q99504;Q8IVX7;B4DIR7;(	EYA3_HUMAN Eyes absent homolog 3 OS=Homo sapiens GN=EYA3 PE=1 SV=3;>tr|Q8IVX7|Q8IVX7_HUMAN EYA3 protein OS=Homo	9.24E-07	
Q99523	SORT_HUMAN Sortilin OS=Homo sapiens GN=SORT1 PE=1 SV=3		
Q99536	VAT1_HUMAN Synaptic vesicle membrane protein VAT-1 homolog OS=Homo sapiens GN=VAT1 PE=1 SV=2		483.16
Q99538	LGMN_HUMAN Legumain OS=Homo sapiens GN=LGMN PE=1 SV=1	6.97E-22	
Q99541	PLIN2_HUMAN Perilipin-2 OS=Homo sapiens GN=PLIN2 PE=1 SV=2	7.74E-11	
Q99543	DNJC2_HUMAN DnaJ homolog subfamily C member 2 OS=Homo sapiens GN=DNAJC2 PE=1 SV=4		28.83
Q99543-2	DNJC2_HUMAN Isoform 2 of DnaJ homolog subfamily C member 2 OS=Homo sapiens GN=DNAJC2		28.83
Q99543;A4VCI0;Q99543-:	DNJC2_HUMAN DnaJ homolog subfamily C member 2 OS=Homo sapiens GN=DNAJC2 PE=1 SV=4;>tr|A4VCI0|A4VCI0_HUMAN DNAJC	2.68E-10	
Q99547	MPH6_HUMAN M-phase phosphoprotein 6 OS=Homo sapiens GN=MPHOSPH6 PE=1 SV=2		216.18
Q99549	MPP8_HUMAN M-phase phosphoprotein 8 OS=Homo sapiens GN=MPHOSPH8 PE=1 SV=2		
Q99549-2	MPP8_HUMAN Isoform 2 of M-phase phosphoprotein 8 OS=Homo sapiens GN=MPHOSPH8		

Q99558	M3K14_HUMAN Mitogen-activated protein kinase kinase kinase 14 OS=Homo sapiens GN=MAP3K14 PE=1 SV=2		
Q99567	NUP88_HUMAN Nuclear pore complex protein Nup88 OS=Homo sapiens GN=NUP88 PE=1 SV=2	1.63E-33	
Q99570	PI3R4_HUMAN Phosphoinositide 3-kinase regulatory subunit 4 OS=Homo sapiens GN=PIK3R4 PE=1 SV=3	9.39E-13	
Q99574	NEUS_HUMAN Neuroserpin OS=Homo sapiens GN=SERPINI1 PE=1 SV=1	2.10E-14	
Q99575	POP1_HUMAN Ribonucleases P/MRP protein subunit POP1 OS=Homo sapiens GN=POP1 PE=1 SV=2		164.02
Q99584	S10AD_HUMAN Protein S100-A13 OS=Homo sapiens GN=S100A13 PE=1 SV=1		37.89
Q99590;Q99590-2	SCAFB_HUMAN Protein SCAF11 OS=Homo sapiens GN=SCAF11 PE=1 SV=2;>sp|Q99590-2|SCAFB_HUMAN Isoform 2 of Protein SCAF	9.65E-09	
Q99594	TEAD3_HUMAN Transcriptional enhancer factor TEF-5 OS=Homo sapiens GN=TEAD3 PE=1 SV=2		22.7
Q99598	TSNAX_HUMAN Translin-associated protein X OS=Homo sapiens GN=TSNAX PE=1 SV=1		50.4
Q99611	SPS2_HUMAN Selenide, water dikinase 2 OS=Homo sapiens GN=SEPHS2 PE=1 SV=3	1.88E-06	
Q99612	KLF6_HUMAN Krueppel-like factor 6 OS=Homo sapiens GN=KLF6 PE=1 SV=3		
Q99612-2	KLF6_HUMAN Isoform 2 of Krueppel-like factor 6 OS=Homo sapiens GN=KLF6		
Q99613	EIF3C_HUMAN Eukaryotic translation initiation factor 3 subunit C OS=Homo sapiens GN=EIF3C PE=1 SV=1		396.65
Q99614	TTC1_HUMAN Tetratricopeptide repeat protein 1 OS=Homo sapiens GN=TTC1 PE=1 SV=1	1.79E-16	
Q99615	DNJC7_HUMAN DnaJ homolog subfamily C member 7 OS=Homo sapiens GN=DNAJC7 PE=1 SV=2	4.09E-28	
Q99618	CDCA3_HUMAN Cell division cycle-associated protein 3 OS=Homo sapiens GN=CDCA3 PE=1 SV=1		
Q99622	C10_HUMAN Protein C10 OS=Homo sapiens GN=C12orf57 PE=1 SV=1	3.27E-64	
Q99623	PHB2_HUMAN Prohibitin-2 OS=Homo sapiens GN=PHB2 PE=1 SV=2		685.5
Q99627	CSN8_HUMAN COP9 signalosome complex subunit 8 OS=Homo sapiens GN=COPS8 PE=1 SV=1		64.63
Q99643	C560_HUMAN Succinate dehydrogenase cytochrome b560 subunit, mitochondrial OS=Homo sapiens GN=SDHC PE=1 SV=1		109.54
Q99643-3	C560_HUMAN Isoform 3 of Succinate dehydrogenase cytochrome b560 subunit, mitochondrial OS=Homo sapiens GN=SDHC		109.54
Q99643-5	C560_HUMAN Isoform 5 of Succinate dehydrogenase cytochrome b560 subunit, mitochondrial OS=Homo sapiens GN=SDHC		109.54
Q99643;Q99643-3;Q9964	C560_HUMAN Succinate dehydrogenase cytochrome b560 subunit, mitochondrial OS=Homo sapiens GN=SDHC PE=1 SV=1;>sp|Q996	0.00016004	
Q99653	CHP1_HUMAN Calcium-binding protein p22 OS=Homo sapiens GN=CHP PE=1 SV=3		192.21
Q99661	KIF2C_HUMAN Kinesin-like protein KIF2C OS=Homo sapiens GN=KIF2C PE=1 SV=2		45.44
Q99661-2	KIF2C_HUMAN Isoform 2 of Kinesin-like protein KIF2C OS=Homo sapiens GN=KIF2C		29.61
Q99661;Q99661-2	KIF2C_HUMAN Kinesin-like protein KIF2C OS=Homo sapiens GN=KIF2C PE=1 SV=2;>sp|Q99661-2|KIF2C_HUMAN Isoform 2 of Kines	8.45E-92	
Q99665	I12R2_HUMAN Interleukin-12 receptor subunit beta-2 OS=Homo sapiens GN=IL12RB2 PE=1 SV=1		
Q99665-2	I12R2_HUMAN Isoform 2 of Interleukin-12 receptor subunit beta-2 OS=Homo sapiens GN=IL12RB2		
Q99666	RGPD5_HUMAN RANBP2-like and GRIP domain-containing protein 5/6 OS=Homo sapiens GN=RGPD5 PE=1 SV=3		117.18
Q99666-2	RGPD5_HUMAN Isoform 2 of RANBP2-like and GRIP domain-containing protein 5/6 OS=Homo sapiens GN=RGPD5		89.86
Q99666;O14715	RGPD5_HUMAN RANBP2-like and GRIP domain-containing protein 5/6 OS=Homo sapiens GN=RGPD5 PE=1 SV=3;>sp|O14715|RGPD8	2.35E-99	
Q99674	CGRE1_HUMAN Cell growth regulator with EF hand domain protein 1 OS=Homo sapiens GN=CGREF1 PE=2 SV=2	8.89E-10	
Q99698	LYST_HUMAN Lysosomal-trafficking regulator OS=Homo sapiens GN=LYST PE=1 SV=3		
Q99698-2	LYST_HUMAN Isoform 2 of Lysosomal-trafficking regulator OS=Homo sapiens GN=LYST		
Q99698-3	LYST_HUMAN Isoform 3 of Lysosomal-trafficking regulator OS=Homo sapiens GN=LYST		
Q99700;Q99700-4;Q2M2F	ATX2_HUMAN Ataxin-2 OS=Homo sapiens GN=ATXN2 PE=1 SV=2;>sp|Q99700-4|ATX2_HUMAN Isoform 4 of Ataxin-2 OS=Homo sapi	1.98E-63	
Q99707	METH_HUMAN Methionine synthase OS=Homo sapiens GN=MTR PE=1 SV=2		24.76
Q99714	HCD2_HUMAN 3-hydroxyacyl-CoA dehydrogenase type-2 OS=Homo sapiens GN=HSD17B10 PE=1 SV=3		771.84
Q99714-2	HCD2_HUMAN Isoform 2 of 3-hydroxyacyl-CoA dehydrogenase type-2 OS=Homo sapiens GN=HSD17B10		585.78
Q99714;Q99714-2	HCD2_HUMAN 3-hydroxyacyl-CoA dehydrogenase type-2 OS=Homo sapiens GN=HSD17B10 PE=1 SV=3;>sp|Q99714-2|HCD2_HUMAI	0	
Q99715;D6RGG3;Q99715-	COCA1_HUMAN Collagen alpha-1(XII) chain OS=Homo sapiens GN=COL12A1 PE=1 SV=2;>tr|D6RGG3|D6RGG3_HUMAN Uncharacteri	0	
Q99719	SEPT5_HUMAN Septin-5 OS=Homo sapiens GN=SEPT5 PE=1 SV=1		94.84
Q99720	SGMR1_HUMAN Sigma non-opioid intracellular receptor 1 OS=Homo sapiens GN=SIGMAR1 PE=1 SV=1		62.49
Q99720-2	SGMR1_HUMAN Isoform 2 of Sigma non-opioid intracellular receptor 1 OS=Homo sapiens GN=SIGMAR1		3.59
Q99720-3	SGMR1_HUMAN Isoform 3 of Sigma non-opioid intracellular receptor 1 OS=Homo sapiens GN=SIGMAR1		62.49
Q99720-4	SGMR1_HUMAN Isoform 4 of Sigma non-opioid intracellular receptor 1 OS=Homo sapiens GN=SIGMAR1		58.9
Q99720-5	SGMR1_HUMAN Isoform 5 of Sigma non-opioid intracellular receptor 1 OS=Homo sapiens GN=SIGMAR1		3.59
Q99720;Q99720-2;Q9972	SGMR1_HUMAN Sigma non-opioid intracellular receptor 1 OS=Homo sapiens GN=SIGMAR1 PE=1 SV=1;>sp|Q99720-2|SGMR1_HUMA	1.21E-05	
Q99729	ROAA_HUMAN Heterogeneous nuclear ribonucleoprotein A/B OS=Homo sapiens GN=HNRNPAB PE=1 SV=2		296.14
Q99729-2	ROAA_HUMAN Isoform 2 of Heterogeneous nuclear ribonucleoprotein A/B OS=Homo sapiens GN=HNRNPAB		390.3
Q99729-2;Q99729-3;Q997	2|ROAA_HUMAN Isoform 2 of Heterogeneous nuclear ribonucleoprotein A/B OS=Homo sapiens GN=HNRNPAB;>sp|Q99729-3|ROAA_H	8.72E-284	
Q99729-3	ROAA_HUMAN Isoform 3 of Heterogeneous nuclear ribonucleoprotein A/B OS=Homo sapiens GN=HNRNPAB		390.3
Q99729-3;Q99729-2;Q997	3|ROAA_HUMAN Isoform 3 of Heterogeneous nuclear ribonucleoprotein A/B OS=Homo sapiens GN=HNRNPAB;>sp|Q99729-2|ROAA_H	1.62E-141	
Q99729-4	ROAA_HUMAN Isoform 4 of Heterogeneous nuclear ribonucleoprotein A/B OS=Homo sapiens GN=HNRNPAB		296.14
Q99733	NP1L4_HUMAN Nucleosome assembly protein 1-like 4 OS=Homo sapiens GN=NAP1L4 PE=1 SV=1		104.97
Q99742	NPAS1_HUMAN Neuronal PAS domain-containing protein 1 OS=Homo sapiens GN=NPAS1 PE=2 SV=2		
Q99747	SNAG_HUMAN Gamma-soluble NSF attachment protein OS=Homo sapiens GN=NAPG PE=1 SV=1		51.24
Q99755	PI51A_HUMAN Phosphatidylinositol-4-phosphate 5-kinase type-1 alpha OS=Homo sapiens GN=PIP5K1A PE=1 SV=1		
Q99755-2	PI51A_HUMAN Isoform 2 of Phosphatidylinositol-4-phosphate 5-kinase type-1 alpha OS=Homo sapiens GN=PIP5K1A		
Q99755-3	PI51A_HUMAN Isoform 3 of Phosphatidylinositol-4-phosphate 5-kinase type-1 alpha OS=Homo sapiens GN=PIP5K1A		

Q99766	ATP5S_HUMAN ATP synthase subunit s, mitochondrial OS=Homo sapiens GN=ATP5S PE=1 SV=3	7.65E-08	
Q99797	MIPEP_HUMAN Mitochondrial intermediate peptidase OS=Homo sapiens GN=MIPEP PE=1 SV=2		
Q99798	ACON_HUMAN Aconitate hydratase, mitochondrial OS=Homo sapiens GN=ACO2 PE=1 SV=2		261.63
Q99805	TM9S2_HUMAN Transmembrane 9 superfamily member 2 OS=Homo sapiens GN=TM9SF2 PE=1 SV=1		140.69
Q99807;Q49A71;Q8NI45;	COQ7_HUMAN Ubiquinone biosynthesis protein COQ7 homolog OS=Homo sapiens GN=COQ7 PE=1 SV=3;>tr|Q49A71|Q49A71_HUMA	4.78E-05	
Q99808	S29A1_HUMAN Equilibrative nucleoside transporter 1 OS=Homo sapiens GN=SLC29A1 PE=1 SV=3		325.11
Q99811	PRRX2_HUMAN Paired mesoderm homeobox protein 2 OS=Homo sapiens GN=PRRX2 PE=2 SV=2		
Q99816;Q99816-2	TS101_HUMAN Tumor susceptibility gene 101 protein OS=Homo sapiens GN=TSG101 PE=1 SV=2;>sp|Q99816-2|TS101_HUMAN Isofo	6.38E-18	
Q99828	CIB1_HUMAN Calcium and integrin-binding protein 1 OS=Homo sapiens GN=CIB1 PE=1 SV=4	9.26E-14	
Q99829	CPNE1_HUMAN Copine-1 OS=Homo sapiens GN=CPNE1 PE=1 SV=1		145.08
Q99832	TCPH_HUMAN T-complex protein 1 subunit eta OS=Homo sapiens GN=CCT7 PE=1 SV=2		768.94
Q99848	EBP2_HUMAN Probable rRNA-processing protein EBP2 OS=Homo sapiens GN=EBNA1BP2 PE=1 SV=2		103.63
Q99856	ARI3A_HUMAN AT-rich interactive domain-containing protein 3A OS=Homo sapiens GN=ARID3A PE=1 SV=2	8.91E-11	
Q99865	SPI2A_HUMAN Spindlin-2A OS=Homo sapiens GN=SPIN2A PE=1 SV=3		
Q99867	TBB4Q_HUMAN Putative tubulin beta-4q chain OS=Homo sapiens GN=TUBB4Q PE=5 SV=1		465.76
Q99871-2;D3DWT9;Q998	2|HAUS7_HUMAN Isoform 2 of HAUS augmin-like complex subunit 7 OS=Homo sapiens GN=HAUS7;>tr|D3DWT9|D3DWT9_HUMAN H	1.11E-06	
Q99873	ANM1_HUMAN Protein arginine N-methyltransferase 1 OS=Homo sapiens GN=PRMT1 PE=1 SV=2		577.22
Q99873-2	ANM1_HUMAN Isoform 2 of Protein arginine N-methyltransferase 1 OS=Homo sapiens GN=PRMT1		577.22
Q99873-3	ANM1_HUMAN Isoform 3 of Protein arginine N-methyltransferase 1 OS=Homo sapiens GN=PRMT1		577.22
Q99873;B4E3C3;Q8WUW	ANM1_HUMAN Protein arginine N-methyltransferase 1 OS=Homo sapiens GN=PRMT1 PE=1 SV=2;>tr|B4E3C3|B4E3C3_HUMAN cDNA	4.61E-201	
Q99877	H2B1N_HUMAN Histone H2B type 1-N OS=Homo sapiens GN=HIST1H2BN PE=1 SV=3		705.85
Q99878	H2A1J_HUMAN Histone H2A type 1-J OS=Homo sapiens GN=HIST1H2AJ PE=1 SV=3		288.83
Q99879	H2B1M_HUMAN Histone H2B type 1-M OS=Homo sapiens GN=HIST1H2BM PE=1 SV=3		705.5
Q99880	H2B1L_HUMAN Histone H2B type 1-L OS=Homo sapiens GN=HIST1H2BL PE=1 SV=3		707.99
Q99943	PLCA_HUMAN 1-acyl-sn-glycerol-3-phosphate acyltransferase alpha OS=Homo sapiens GN=AGPAT1 PE=2 SV=2		85.49
Q99959;Q99959-2	PKP2_HUMAN Plakophilin-2 OS=Homo sapiens GN=PKP2 PE=1 SV=2;>sp|Q99959-2|PKP2_HUMAN Isoform 1 of Plakophilin-2 OS=Hor	2.59E-06	
Q99961	SH3G1_HUMAN Endophilin-A2 OS=Homo sapiens GN=SH3GL1 PE=1 SV=1	2.11E-78	
Q99983	OMD_HUMAN Osteomodulin OS=Homo sapiens GN=OMD PE=1 SV=1	4.38E-05	
Q99986	VRK1_HUMAN Serine/threonine-protein kinase VRK1 OS=Homo sapiens GN=VRK1 PE=1 SV=1		174.6
Q99996	AKAP9_HUMAN A-kinase anchor protein 9 OS=Homo sapiens GN=AKAP9 PE=1 SV=3		
Q99996-2	AKAP9_HUMAN Isoform 2 of A-kinase anchor protein 9 OS=Homo sapiens GN=AKAP9		
Q99996-3	AKAP9_HUMAN Isoform 3 of A-kinase anchor protein 9 OS=Homo sapiens GN=AKAP9		
Q99996-4	AKAP9_HUMAN Isoform 4 of A-kinase anchor protein 9 OS=Homo sapiens GN=AKAP9		
Q99996-5	AKAP9_HUMAN Isoform 5 of A-kinase anchor protein 9 OS=Homo sapiens GN=AKAP9		
Q99996-6	AKAP9_HUMAN Isoform 6 of A-kinase anchor protein 9 OS=Homo sapiens GN=AKAP9		
Q99996-6;Q99996;Q9999	6|AKAP9_HUMAN Isoform 6 of A-kinase anchor protein 9 OS=Homo sapiens GN=AKAP9;>sp|Q99996|AKAP9_HUMAN A-kinase anchor	2.49E-12	
Q9BPW4	APOL4_HUMAN Apolipoprotein L4 OS=Homo sapiens GN=APOL4 PE=2 SV=3		
Q9BPW4-2	APOL4_HUMAN Isoform 2 of Apolipoprotein L4 OS=Homo sapiens GN=APOL4		
Q9BPW4-3	APOL4_HUMAN Isoform 3 of Apolipoprotein L4 OS=Homo sapiens GN=APOL4		
Q9BPW8	NIPS1_HUMAN Protein NipSnap homolog 1 OS=Homo sapiens GN=NIPSNAP1 PE=1 SV=1		76.38
Q9BPX3	CND3_HUMAN Condensin complex subunit 3 OS=Homo sapiens GN=NCAPG PE=1 SV=1		100.72
Q9BPX5	ARP5L_HUMAN Actin-related protein 2/3 complex subunit 5-like protein OS=Homo sapiens GN=ARPC5L PE=1 SV=1		
Q9BPX6-3;Q9BPX6;Q9BP	3|MICU1_HUMAN Isoform 3 of Calcium uptake protein 1, mitochondrial OS=Homo sapiens GN=MICU1;>sp|Q9BPX6|MICU1_HUMAN Ca	2.18E-41	
Q9BPY3	F118B_HUMAN Protein FAM118B OS=Homo sapiens GN=FAM118B PE=1 SV=1	1.66E-11	
Q9BPZ2	SPI2B_HUMAN Spindlin-2B OS=Homo sapiens GN=SPIN2B PE=2 SV=1		
Q9BPZ3	PAIP2_HUMAN Polyadenylate-binding protein-interacting protein 2 OS=Homo sapiens GN=PAIP2 PE=1 SV=1	1.16E-11	
Q9BPZ7;Q9BPZ7-2;Q9BP	SIN1_HUMAN Target of rapamycin complex 2 subunit MAPKAP1 OS=Homo sapiens GN=MAPKAP1 PE=1 SV=2;>sp|Q9BPZ7-2|SIN1_HU	7.17E-09	
Q9BQ04	RBM4B_HUMAN RNA-binding protein 4B OS=Homo sapiens GN=RBM4B PE=1 SV=1		25.04
Q9BQ15	SOSB1_HUMAN SOSS complex subunit B1 OS=Homo sapiens GN=OBFC2B PE=1 SV=1		39.42
Q9BQ39	DDX50_HUMAN ATP-dependent RNA helicase DDX50 OS=Homo sapiens GN=DDX50 PE=1 SV=1		50.53
Q9BQ48	RM34_HUMAN 39S ribosomal protein L34, mitochondrial OS=Homo sapiens GN=MRPL34 PE=1 SV=1		
Q9BQ52	RNZ2_HUMAN Zinc phosphodiesterase ELAC protein 2 OS=Homo sapiens GN=ELAC2 PE=1 SV=2		
Q9BQ52-2	RNZ2_HUMAN Isoform 2 of Zinc phosphodiesterase ELAC protein 2 OS=Homo sapiens GN=ELAC2		
Q9BQ52-3	RNZ2_HUMAN Isoform 3 of Zinc phosphodiesterase ELAC protein 2 OS=Homo sapiens GN=ELAC2		
Q9BQ52;B4DT15;B4DPL9;	RNZ2_HUMAN Zinc phosphodiesterase ELAC protein 2 OS=Homo sapiens GN=ELAC2 PE=1 SV=2;>tr|B4DT15|B4DT15_HUMAN cDNA I	3.11E-50	
Q9BQ61	CS043_HUMAN Uncharacterized protein C19orf43 OS=Homo sapiens GN=C19orf43 PE=1 SV=1		
Q9BQ67	GRWD1_HUMAN Glutamate-rich WD repeat-containing protein 1 OS=Homo sapiens GN=GRWD1 PE=1 SV=1		73.69
Q9BQ69	MACD1_HUMAN MACRO domain-containing protein 1 OS=Homo sapiens GN=MACROD1 PE=1 SV=2		47.3
Q9BQ70	TCF25_HUMAN Transcription factor 25 OS=Homo sapiens GN=TCF25 PE=1 SV=1		
Q9BQ75	CC026_HUMAN Uncharacterized protein C3orf26 OS=Homo sapiens GN=C3orf26 PE=1 SV=2		66.23
Q9BQ87	TBL1Y_HUMAN F-box-like/WD repeat-containing protein TBL1Y OS=Homo sapiens GN=TBL1Y PE=2 SV=1		22.51

Q9BQ95;Q9BQ95-2	ECSIT_HUMAN Evolutionarily conserved signaling intermediate in Toll pathway, mitochondrial OS=Homo sapiens GN=ECSIT PE=1 SV=	4.70E-06	
Q9BQA1	MEP50_HUMAN Methylosome protein 50 OS=Homo sapiens GN=WDR77 PE=1 SV=1		
Q9BQB6	VKOR1_HUMAN Vitamin K epoxide reductase complex subunit 1 OS=Homo sapiens GN=VKORC1 PE=1 SV=1		29.99
Q9BQB6-2	VKOR1_HUMAN Isoform 2 of Vitamin K epoxide reductase complex subunit 1 OS=Homo sapiens GN=VKORC1		29.99
Q9BQC6	RT63_HUMAN Ribosomal protein 63, mitochondrial OS=Homo sapiens GN=MRP63 PE=2 SV=1		85.76
Q9BQD7	F173A_HUMAN Protein FAM173A OS=Homo sapiens GN=FAM173A PE=2 SV=1		23
Q9BQE3	TBA1C_HUMAN Tubulin alpha-1C chain OS=Homo sapiens GN=TUBA1C PE=1 SV=1		1122.21
Q9BQE4	SELS_HUMAN Selenoprotein S OS=Homo sapiens GN=SELS PE=1 SV=3		33.13
Q9BQE5	APOL2_HUMAN Apolipoprotein L2 OS=Homo sapiens GN=APOL2 PE=1 SV=1	5.23E-41	
Q9BQE9	BCL7B_HUMAN B-cell CLL/lymphoma 7 protein family member B OS=Homo sapiens GN=BCL7B PE=1 SV=1		
Q9BQE9-3	BCL7B_HUMAN Isoform 3 of B-cell CLL/lymphoma 7 protein family member B OS=Homo sapiens GN=BCL7B		
Q9BQG0	MBB1A_HUMAN Myb-binding protein 1A OS=Homo sapiens GN=MYBBP1A PE=1 SV=2		961.07
Q9BQG0-2	MBB1A_HUMAN Isoform 2 of Myb-binding protein 1A OS=Homo sapiens GN=MYBBP1A		961.07
Q9BQG0-2;Q9BQG0	2|MBB1A_HUMAN Isoform 2 of Myb-binding protein 1A OS=Homo sapiens GN=MYBBP1A;>sp|Q9BQG0|MBB1A_HUMAN Myb-binding p	6.12E-162	
Q9BQI0	AIF1L_HUMAN Allograft inflammatory factor 1-like OS=Homo sapiens GN=AIF1L PE=1 SV=1		
Q9BQI0-2	AIF1L_HUMAN Isoform 2 of Allograft inflammatory factor 1-like OS=Homo sapiens GN=AIF1L		
Q9BQI0-2;B7Z6K5;Q9BQI	2|AIF1L_HUMAN Isoform 2 of Allograft inflammatory factor 1-like OS=Homo sapiens GN=AIF1L;>tr|B7Z6K5|B7Z6K5_HUMAN cDNA FL	6.33E-09	
Q9BQI0-3	AIF1L_HUMAN Isoform 3 of Allograft inflammatory factor 1-like OS=Homo sapiens GN=AIF1L		
Q9BQI0-4	AIF1L_HUMAN Isoform 4 of Allograft inflammatory factor 1-like OS=Homo sapiens GN=AIF1L		
Q9BQL6;Q9BQL6-2;Q9BQI	FERM1_HUMAN Fermitin family homolog 1 OS=Homo sapiens GN=FERMT1 PE=1 SV=1;>sp|Q9BQL6-2|FERM1_HUMAN Isoform 2 of Fe	1.94E-19	
Q9BQP7	CT072_HUMAN Uncharacterized protein C20orf72 OS=Homo sapiens GN=C20orf72 PE=1 SV=1	1.40E-08	
Q9BQS8;Q9BQS8-2;Q9BQ	FYCO1_HUMAN FYVE and coiled-coil domain-containing protein 1 OS=Homo sapiens GN=FYCO1 PE=1 SV=3;>sp|Q9BQS8-2|FYCO1_H	1.67E-17	
Q9BR61	ACBD6_HUMAN Acyl-CoA-binding domain-containing protein 6 OS=Homo sapiens GN=ACBD6 PE=1 SV=1	9.27E-82	
Q9BR76	COR1B_HUMAN Coronin-1B OS=Homo sapiens GN=CORO1B PE=1 SV=1		92.55
Q9BRA2	TXD17_HUMAN Thioredoxin domain-containing protein 17 OS=Homo sapiens GN=TXNDC17 PE=1 SV=1		83.91
Q9BRF8;Q9BRF8-2	CPPED_HUMAN Calcineurin-like phosphoesterase domain-containing protein 1 OS=Homo sapiens GN=CPPED1 PE=1 SV=3;>sp|Q9BRF	1.36E-46	
Q9BRG1	VPS25_HUMAN Vacuolar protein-sorting-associated protein 25 OS=Homo sapiens GN=VPS25 PE=1 SV=1		83.45
Q9BRJ2	RM45_HUMAN 39S ribosomal protein L45, mitochondrial OS=Homo sapiens GN=MRPL45 PE=1 SV=2		95.43
Q9BRJ6	CG050_HUMAN Uncharacterized protein C7orf50 OS=Homo sapiens GN=C7orf50 PE=1 SV=1		207.83
Q9BRJ7	SDOS_HUMAN Protein syndesmos OS=Homo sapiens GN=NUDT16L1 PE=1 SV=1		72.55
Q9BRJ7-2	SDOS_HUMAN Isoform 2 of Protein syndesmos OS=Homo sapiens GN=NUDT16L1		23.8
Q9BRK5	CAB45_HUMAN 45 kDa calcium-binding protein OS=Homo sapiens GN=SDF4 PE=1 SV=1		
Q9BRK5-2	CAB45_HUMAN Isoform 2 of 45 kDa calcium-binding protein OS=Homo sapiens GN=SDF4		
Q9BRK5-3	CAB45_HUMAN Isoform 3 of 45 kDa calcium-binding protein OS=Homo sapiens GN=SDF4		
Q9BRK5-4	CAB45_HUMAN Isoform 4 of 45 kDa calcium-binding protein OS=Homo sapiens GN=SDF4		
Q9BRK5-6	CAB45_HUMAN Isoform 6 of 45 kDa calcium-binding protein OS=Homo sapiens GN=SDF4		
Q9BRL6	SRSF8_HUMAN Serine/arginine-rich splicing factor 8 OS=Homo sapiens GN=SRSF8 PE=1 SV=1		69.11
Q9BRL6-2	SRSF8_HUMAN Isoform 2 of Serine/arginine-rich splicing factor 8 OS=Homo sapiens GN=SRSF8		69.11
Q9BRL6;Q9BRL6-2;Q0113	SRSF8_HUMAN Serine/arginine-rich splicing factor 8 OS=Homo sapiens GN=SRSF8 PE=1 SV=1;>sp|Q9BRL6-2|SRSF8_HUMAN Isoforr	0.00010645	
Q9BRN9	TM2D3_HUMAN TM2 domain-containing protein 3 OS=Homo sapiens GN=TM2D3 PE=1 SV=2		
Q9BRN9-2	TM2D3_HUMAN Isoform 2 of TM2 domain-containing protein 3 OS=Homo sapiens GN=TM2D3		
Q9BRP1	PDD2L_HUMAN Programmed cell death protein 2-like OS=Homo sapiens GN=PDCD2L PE=1 SV=1	8.68E-06	
Q9BRP4	PAAF1_HUMAN Proteasomal ATPase-associated factor 1 OS=Homo sapiens GN=PAAF1 PE=1 SV=2		
Q9BRP4-2	PAAF1_HUMAN Isoform 2 of Proteasomal ATPase-associated factor 1 OS=Homo sapiens GN=PAAF1		
Q9BRP4;Q9BRP4-2	PAAF1_HUMAN Proteasomal ATPase-associated factor 1 OS=Homo sapiens GN=PAAF1 PE=1 SV=2;>sp|Q9BRP4-2|PAAF1_HUMAN Isof	2.31E-12	
Q9BRP8	WIBG_HUMAN Partner of Y14 and mago OS=Homo sapiens GN=WIBG PE=1 SV=1		114.33
Q9BRP8-2	WIBG_HUMAN Isoform 2 of Partner of Y14 and mago OS=Homo sapiens GN=WIBG		114.33
Q9BRP8;Q9BRP8-2	WIBG_HUMAN Partner of Y14 and mago OS=Homo sapiens GN=WIBG PE=1 SV=1;>sp|Q9BRP8-2|WIBG_HUMAN Isoform 2 of Partner	2.57E-140	
Q9BRQ8;C9JI05;Q9BRQ8-	AIFM2_HUMAN Apoptosis-inducing factor 2 OS=Homo sapiens GN=AIFM2 PE=2 SV=1;>tr|C9JI05|C9JI05_HUMAN Uncharacterized prc	2.66E-17	
Q9BRR6	ADPGK_HUMAN ADP-dependent glucokinase OS=Homo sapiens GN=ADPGK PE=1 SV=1		
Q9BRR6-2	ADPGK_HUMAN Isoform 2 of ADP-dependent glucokinase OS=Homo sapiens GN=ADPGK		
Q9BRR6-3	ADPGK_HUMAN Isoform 3 of ADP-dependent glucokinase OS=Homo sapiens GN=ADPGK		
Q9BRR6-4	ADPGK_HUMAN Isoform 4 of ADP-dependent glucokinase OS=Homo sapiens GN=ADPGK		
Q9BRR6-5	ADPGK_HUMAN Isoform 5 of ADP-dependent glucokinase OS=Homo sapiens GN=ADPGK		
Q9BRR6;Q9BRR6-2;Q9BRI	ADPGK_HUMAN ADP-dependent glucokinase OS=Homo sapiens GN=ADPGK PE=1 SV=1;>sp|Q9BRR6-2|ADPGK_HUMAN Isoform 2 of .	2.24E-62	
Q9BRR8	GPTC1_HUMAN G patch domain-containing protein 1 OS=Homo sapiens GN=GPATCH1 PE=1 SV=1	2.34E-08	
Q9BRS8	LARP6_HUMAN La-related protein 6 OS=Homo sapiens GN=LARP6 PE=1 SV=1		
Q9BRT2	CF125_HUMAN Uncharacterized protein C6orf125 OS=Homo sapiens GN=C6orf125 PE=1 SV=1		26.19
Q9BRT3	CQ037_HUMAN Protein C17orf37 OS=Homo sapiens GN=C17orf37 PE=1 SV=1	5.51E-07	
Q9BRT6	LLPH_HUMAN Protein LLP homolog OS=Homo sapiens GN=LLPH PE=2 SV=1		
Q9BRT9	SLD5_HUMAN DNA replication complex GINS protein SLD5 OS=Homo sapiens GN=GINS4 PE=1 SV=1	8.36E-05	

Q9BRU9	UTP23_HUMAN rRNA-processing protein UTP23 homolog OS=Homo sapiens GN=UTP23 PE=2 SV=2		
Q9BRV3	SWET1_HUMAN Sugar transporter SWEET1 OS=Homo sapiens GN=SLC50A1 PE=2 SV=1		
Q9BRV3-2	SWET1_HUMAN Isoform 2 of Sugar transporter SWEET1 OS=Homo sapiens GN=SLC50A1		
Q9BRV3-3	SWET1_HUMAN Isoform 3 of Sugar transporter SWEET1 OS=Homo sapiens GN=SLC50A1		
Q9BRX2	PELO_HUMAN Protein pelota homolog OS=Homo sapiens GN=PELO PE=1 SV=2		
Q9BRX5	PSF3_HUMAN DNA replication complex GINS protein PSF3 OS=Homo sapiens GN=GINS3 PE=1 SV=1	1.77E-06	
Q9BRX8	CJ058_HUMAN UPF0765 protein C10orf58 OS=Homo sapiens GN=C10orf58 PE=1 SV=3		212.22
Q9BRZ2	TRI56_HUMAN E3 ubiquitin-protein ligase TRIM56 OS=Homo sapiens GN=TRIM56 PE=1 SV=3	3.07E-12	
Q9BS26	ERP44_HUMAN Endoplasmic reticulum resident protein 44 OS=Homo sapiens GN=ERP44 PE=1 SV=1		391.52
Q9BS40	LXN_HUMAN Latexin OS=Homo sapiens GN=LXN PE=1 SV=2	5.07E-06	
Q9BS92	NPS3B_HUMAN Protein NipSnap homolog 3B OS=Homo sapiens GN=NIPSNAP3B PE=2 SV=1	2.79E-11	
Q9BSC4	NOL10_HUMAN Nucleolar protein 10 OS=Homo sapiens GN=NOL10 PE=1 SV=1		25.92
Q9BSC4-2	NOL10_HUMAN Isoform 2 of Nucleolar protein 10 OS=Homo sapiens GN=NOL10		25.92
Q9BSC4-3	NOL10_HUMAN Isoform 3 of Nucleolar protein 10 OS=Homo sapiens GN=NOL10		
Q9BSD7	NTPCR_HUMAN Cancer-related nucleoside-triphosphatase OS=Homo sapiens GN=NTPCR PE=1 SV=1		142.82
Q9BSH4	TACO1_HUMAN Translational activator of cytochrome c oxidase 1 OS=Homo sapiens GN=TACO1 PE=1 SV=1		86.38
Q9BSH5	HDHD3_HUMAN Haloacid dehalogenase-like hydrolase domain-containing protein 3 OS=Homo sapiens GN=HDHD3 PE=1 SV=1		
Q9BSJ2	GCP2_HUMAN Gamma-tubulin complex component 2 OS=Homo sapiens GN=TUBGCP2 PE=1 SV=2	4.65E-10	
Q9BSJ8	ESYT1_HUMAN Extended synaptotagmin-1 OS=Homo sapiens GN=ESYT1 PE=1 SV=1		817.6
Q9BSJ8-2	ESYT1_HUMAN Isoform 2 of Extended synaptotagmin-1 OS=Homo sapiens GN=ESYT1		817.6
Q9BSJ8-2;Q9BSJ8	2|ESYT1_HUMAN Isoform 2 of Extended synaptotagmin-1 OS=Homo sapiens GN=ESYT1;>sp|Q9BSJ8|ESYT1_HUMAN Extended synap	1.93E-84	
Q9BSJ8;Q9BSJ8-2	ESYT1_HUMAN Extended synaptotagmin-1 OS=Homo sapiens GN=ESYT1 PE=1 SV=1;>sp|Q9BSJ8-2|ESYT1_HUMAN Isoform 2 of Exte	3.44E-299	
Q9BSK2	S2533_HUMAN Solute carrier family 25 member 33 OS=Homo sapiens GN=SLC25A33 PE=2 SV=1		
Q9BSR8	YIPF4_HUMAN Protein YIPF4 OS=Homo sapiens GN=YIPF4 PE=1 SV=1		
Q9BSU1	CP070_HUMAN UPF0183 protein C16orf70 OS=Homo sapiens GN=C16orf70 PE=1 SV=1	2.99E-18	
Q9BSU3	NAA11_HUMAN N-alpha-acetyltransferase 11, NatA catalytic subunit OS=Homo sapiens GN=NAA11 PE=1 SV=3		12.31
Q9BSV6	SEN34_HUMAN tRNA-splicing endonuclease subunit Sen34 OS=Homo sapiens GN=TSEN34 PE=1 SV=1	2.30E-08	
Q9BSW2	EFC4B_HUMAN EF-hand calcium-binding domain-containing protein 4B OS=Homo sapiens GN=EFCAB4B PE=1 SV=1		
Q9BSW2-2	EFC4B_HUMAN Isoform 2 of EF-hand calcium-binding domain-containing protein 4B OS=Homo sapiens GN=EFCAB4B		
Q9BSY4	CHCH5_HUMAN Coiled-coil-helix-coiled-coil-helix domain-containing protein 5 OS=Homo sapiens GN=CHCHD5 PE=2 SV=1	2.09E-09	
Q9BT09	CNPY3_HUMAN Protein canopy homolog 3 OS=Homo sapiens GN=CNPY3 PE=1 SV=1		331.05
Q9BT23	LIMD2_HUMAN LIM domain-containing protein 2 OS=Homo sapiens GN=LIMD2 PE=1 SV=1	4.63E-08	
Q9BT73	PSMG3_HUMAN Proteasome assembly chaperone 3 OS=Homo sapiens GN=PSMG3 PE=1 SV=1		19.59
Q9BT78	CSN4_HUMAN COP9 signalosome complex subunit 4 OS=Homo sapiens GN=COPS4 PE=1 SV=1		504.42
Q9BT88	SYT11_HUMAN Synaptotagmin-11 OS=Homo sapiens GN=SYT11 PE=1 SV=2		
Q9BTC0	DIDO1_HUMAN Death-inducer obliterator 1 OS=Homo sapiens GN=DIDO1 PE=1 SV=5		137.53
Q9BTC0-1	DIDO1_HUMAN Isoform 1 of Death-inducer obliterator 1 OS=Homo sapiens GN=DIDO1		129.12
Q9BTC0-2	DIDO1_HUMAN Isoform 2 of Death-inducer obliterator 1 OS=Homo sapiens GN=DIDO1		10.31
Q9BTC0-3	DIDO1_HUMAN Isoform 3 of Death-inducer obliterator 1 OS=Homo sapiens GN=DIDO1		10.31
Q9BTC8	MTA3_HUMAN Metastasis-associated protein MTA3 OS=Homo sapiens GN=MTA3 PE=1 SV=2		
Q9BTC8-2	MTA3_HUMAN Isoform 2 of Metastasis-associated protein MTA3 OS=Homo sapiens GN=MTA3		
Q9BTD8	RBM42_HUMAN RNA-binding protein 42 OS=Homo sapiens GN=RBM42 PE=1 SV=1		
Q9BTD8-2	RBM42_HUMAN Isoform 2 of RNA-binding protein 42 OS=Homo sapiens GN=RBM42		
Q9BTD8-3	RBM42_HUMAN Isoform 3 of RNA-binding protein 42 OS=Homo sapiens GN=RBM42		
Q9BTD8-4	RBM42_HUMAN Isoform 4 of RNA-binding protein 42 OS=Homo sapiens GN=RBM42		
Q9BTD8;Q9BTD8-3;Q9BTI	RBM42_HUMAN RNA-binding protein 42 OS=Homo sapiens GN=RBM42 PE=1 SV=1;>sp|Q9BTD8-3|RBM42_HUMAN Isoform 3 of RNA-	7.58E-48	
Q9BTE1	DCTN5_HUMAN Dynactin subunit 5 OS=Homo sapiens GN=DCTN5 PE=1 SV=1	9.83E-15	
Q9BTE3	MCMBP_HUMAN Mini-chromosome maintenance complex-binding protein OS=Homo sapiens GN=MCMBP PE=1 SV=2		7.45
Q9BTE3-2	MCMBP_HUMAN Isoform 2 of Mini-chromosome maintenance complex-binding protein OS=Homo sapiens GN=MCMBP		31.01
Q9BTE3-2;Q9BTE3;B3KSP	2|CJ119_HUMAN Isoform 2 of UPF0557 protein C10orf119 OS=Homo sapiens GN=C10orf119;>sp|Q9BTE3|CJ119_HUMAN UPF0557 pr	4.33E-34	
Q9BTE3-2;Q9BTE3;Q9BTE	2|MCMBP_HUMAN Isoform 2 of Mini-chromosome maintenance complex-binding protein OS=Homo sapiens GN=MCMBP;>sp|Q9BTE3|I	8.07E-12	
Q9BTE3-3	MCMBP_HUMAN Isoform 3 of Mini-chromosome maintenance complex-binding protein OS=Homo sapiens GN=MCMBP		23.56
Q9BTE3;Q9BTE3-2;Q9BTE	MCMBP_HUMAN Mini-chromosome maintenance complex-binding protein OS=Homo sapiens GN=MCMBP PE=1 SV=2;>sp|Q9BTE3-2|M	1.59E-11	
Q9BTE6-2;Q9BTE6	2|AASD1_HUMAN Isoform 2 of Alanyl-tRNA editing protein Aarsd1 OS=Homo sapiens GN=AARSD1;>sp|Q9BTE6|AASD1_HUMAN Alany	5.50E-11	
Q9BTE7	DCNL5_HUMAN DCN1-like protein 5 OS=Homo sapiens GN=DCUN1D5 PE=1 SV=1		100.76
Q9BTF0	THUM2_HUMAN THUMP domain-containing protein 2 OS=Homo sapiens GN=THUMPD2 PE=2 SV=2		
Q9BTF0-2	THUM2_HUMAN Isoform 2 of THUMP domain-containing protein 2 OS=Homo sapiens GN=THUMPD2		
Q9BTL3	F103A_HUMAN Protein FAM103A1 OS=Homo sapiens GN=FAM103A1 PE=1 SV=1		54.92
Q9BTM1	H2AJ_HUMAN Histone H2A.J OS=Homo sapiens GN=H2AFJ PE=1 SV=1		288.83
Q9BTM1-2	H2AJ_HUMAN Isoform 2 of Histone H2A.J OS=Homo sapiens GN=H2AFJ		221.88
Q9BTP7	FAP24_HUMAN Fanconi anemia-associated protein of 24 kDa OS=Homo sapiens GN=FAAP24 PE=1 SV=2		

Q9BTT0	AN32E_HUMAN Acidic leucine-rich nuclear phosphoprotein 32 family member E OS=Homo sapiens GN=ANP32E PE=1 SV=1		381.27
Q9BTT4	MED10_HUMAN Mediator of RNA polymerase II transcription subunit 10 OS=Homo sapiens GN=MED10 PE=1 SV=1		
Q9BTT6	LRRC1_HUMAN Leucine-rich repeat-containing protein 1 OS=Homo sapiens GN=LRRC1 PE=1 SV=1		
Q9BTT6-2	LRRC1_HUMAN Isoform 2 of Leucine-rich repeat-containing protein 1 OS=Homo sapiens GN=LRRC1		
Q9BTU6	P4K2A_HUMAN Phosphatidylinositol 4-kinase type 2-alpha OS=Homo sapiens GN=PI4K2A PE=1 SV=1		82.73
Q9BTV4	TMM43_HUMAN Transmembrane protein 43 OS=Homo sapiens GN=TMEM43 PE=1 SV=1		286.66
Q9BTW9-4;Q9BTW9;Q9BT	4|TBCD_HUMAN Isoform 4 of Tubulin-specific chaperone D OS=Homo sapiens GN=TBCD;>sp|Q9BTW9|TBCD_HUMAN Tubulin-specific	1.01E-16	
Q9BTZ2	DHRS4_HUMAN Dehydrogenase/reductase SDR family member 4 OS=Homo sapiens GN=DHRS4 PE=1 SV=3		43.71
Q9BTZ2-2	DHRS4_HUMAN Isoform 2 of Dehydrogenase/reductase SDR family member 4 OS=Homo sapiens GN=DHRS4		
Q9BTZ2-3	DHRS4_HUMAN Isoform 3 of Dehydrogenase/reductase SDR family member 4 OS=Homo sapiens GN=DHRS4		
Q9BTZ2-4	DHRS4_HUMAN Isoform 4 of Dehydrogenase/reductase SDR family member 4 OS=Homo sapiens GN=DHRS4		43.71
Q9BTZ2-5	DHRS4_HUMAN Isoform 5 of Dehydrogenase/reductase SDR family member 4 OS=Homo sapiens GN=DHRS4		
Q9BTZ2-7	DHRS4_HUMAN Isoform 7 of Dehydrogenase/reductase SDR family member 4 OS=Homo sapiens GN=DHRS4		
Q9BTZ2;Q9BTZ2-4;E2QRL	DHRS4_HUMAN Dehydrogenase/reductase SDR family member 4 OS=Homo sapiens GN=DHRS4 PE=1 SV=3;>sp|Q9BTZ2-4|DHRS4_H	8.39E-09	
Q9BU02	THTPA_HUMAN Thiamine-triphosphatase OS=Homo sapiens GN=THTPA PE=1 SV=3	1.93E-13	
Q9BU61	NDUF3_HUMAN NADH dehydrogenase [ubiquinone] 1 alpha subcomplex assembly factor 3 OS=Homo sapiens GN=NDUFAF3 PE=1 SV=1		50.57
Q9BU76	MMTA2_HUMAN Multiple myeloma tumor-associated protein 2 OS=Homo sapiens GN=MMTAG2 PE=1 SV=1		66.78
Q9BU76-2	MMTA2_HUMAN Isoform 2 of Multiple myeloma tumor-associated protein 2 OS=Homo sapiens GN=MMTAG2		23.63
Q9BU76-3	MMTA2_HUMAN Isoform 3 of Multiple myeloma tumor-associated protein 2 OS=Homo sapiens GN=MMTAG2		23.63
Q9BU76-4	MMTA2_HUMAN Isoform 4 of Multiple myeloma tumor-associated protein 2 OS=Homo sapiens GN=MMTAG2		66.78
Q9BU76;Q9BU76-4;Q9BU	MMTA2_HUMAN Multiple myeloma tumor-associated protein 2 OS=Homo sapiens GN=MMTAG2 PE=1 SV=1;>sp|Q9BU76-4|MMTA2_HU	5.48E-12	
Q9BU89	DOHH_HUMAN Deoxyhypusine hydroxylase OS=Homo sapiens GN=DOHH PE=1 SV=1	7.73E-07	
Q9BUA3	CK084_HUMAN Uncharacterized protein C11orf84 OS=Homo sapiens GN=C11orf84 PE=1 SV=3	6.08E-07	
Q9BUB7;Q9BUB7-2	TMM70_HUMAN Transmembrane protein 70, mitochondrial OS=Homo sapiens GN=TMEM70 PE=1 SV=2;>sp|Q9BUB7-2|TMM70_HUMA	2.82E-09	
Q9BUE0	MED18_HUMAN Mediator of RNA polymerase II transcription subunit 18 OS=Homo sapiens GN=MED18 PE=1 SV=1		
Q9BUF5	TBB6_HUMAN Tubulin beta-6 chain OS=Homo sapiens GN=TUBB6 PE=1 SV=1		952.4
Q9BUH6	CI142_HUMAN Uncharacterized protein C9orf142 OS=Homo sapiens GN=C9orf142 PE=1 SV=2		115.67
Q9BUH8	BEGIN_HUMAN Brain-enriched guanylate kinase-associated protein OS=Homo sapiens GN=BEGAIN PE=1 SV=1		35.24
Q9BUJ2	HNRL1_HUMAN Heterogeneous nuclear ribonucleoprotein U-like protein 1 OS=Homo sapiens GN=HNRNPUL1 PE=1 SV=2		412.34
Q9BUJ2-2	HNRL1_HUMAN Isoform 2 of Heterogeneous nuclear ribonucleoprotein U-like protein 1 OS=Homo sapiens GN=HNRNPUL1		412.34
Q9BUJ2-3	HNRL1_HUMAN Isoform 3 of Heterogeneous nuclear ribonucleoprotein U-like protein 1 OS=Homo sapiens GN=HNRNPUL1		381.97
Q9BUJ2-4	HNRL1_HUMAN Isoform 4 of Heterogeneous nuclear ribonucleoprotein U-like protein 1 OS=Homo sapiens GN=HNRNPUL1		422.76
Q9BUJ2;Q9BUJ2-2;B7Z4B	HNRL1_HUMAN Heterogeneous nuclear ribonucleoprotein U-like protein 1 OS=Homo sapiens GN=HNRNPUL1 PE=1 SV=2;>sp|Q9BUJ2	0	
Q9BUK6	MSTO1_HUMAN Protein misato homolog 1 OS=Homo sapiens GN=MSTO1 PE=1 SV=1		
Q9BUK6-2	MSTO1_HUMAN Isoform 2 of Protein misato homolog 1 OS=Homo sapiens GN=MSTO1		
Q9BUK6-3	MSTO1_HUMAN Isoform 3 of Protein misato homolog 1 OS=Homo sapiens GN=MSTO1		
Q9BUK6-4	MSTO1_HUMAN Isoform 4 of Protein misato homolog 1 OS=Homo sapiens GN=MSTO1		
Q9BUK6-5	MSTO1_HUMAN Isoform 5 of Protein misato homolog 1 OS=Homo sapiens GN=MSTO1		
Q9BUK6-6	MSTO1_HUMAN Isoform 6 of Protein misato homolog 1 OS=Homo sapiens GN=MSTO1		
Q9BUK6-7	MSTO1_HUMAN Isoform 7 of Protein misato homolog 1 OS=Homo sapiens GN=MSTO1		
Q9BUL8	PDC10_HUMAN Programmed cell death protein 10 OS=Homo sapiens GN=PDCD10 PE=1 SV=1		155.57
Q9BUL9	RPP25_HUMAN Ribonuclease P protein subunit p25 OS=Homo sapiens GN=RPP25 PE=1 SV=1		29.9
Q9BUN8	DERL1_HUMAN Derlin-1 OS=Homo sapiens GN=DERL1 PE=1 SV=1		53.53
Q9BUN8-2	DERL1_HUMAN Isoform 2 of Derlin-1 OS=Homo sapiens GN=DERL1		53.53
Q9BUN8;Q9BUN8-2	DERL1_HUMAN Derlin-1 OS=Homo sapiens GN=DERL1 PE=1 SV=1;>sp|Q9BUN8-2|DERL1_HUMAN Isoform 2 of Derlin-1 OS=Homo sa	1.57E-65	
Q9BUP0	EFHD1_HUMAN EF-hand domain-containing protein D1 OS=Homo sapiens GN=EFHD1 PE=1 SV=1		65.38
Q9BUP3-3;D3DQY8;Q9BU	3|HTAI2_HUMAN Isoform 3 of Oxidoreductase HTATIP2 OS=Homo sapiens GN=HTATIP2;>tr|D3DQY8|D3DQY8_HUMAN HIV-1 Tat inter	7.05E-16	
Q9BUQ8	DDX23_HUMAN Probable ATP-dependent RNA helicase DDX23 OS=Homo sapiens GN=DDX23 PE=1 SV=3		
Q9BUR4	WAP53_HUMAN Telomerase Cajal body protein 1 OS=Homo sapiens GN=WRAP53 PE=1 SV=1	2.21E-10	
Q9BUR5	APOO_HUMAN Apolipoprotein O OS=Homo sapiens GN=APOO PE=1 SV=1		175.64
Q9BUR5-2	APOO_HUMAN Isoform 2 of Apolipoprotein O OS=Homo sapiens GN=APOO		175.64
Q9BUR5;Q9BUR5-2	APOO_HUMAN Apolipoprotein O OS=Homo sapiens GN=APOO PE=1 SV=1;>sp|Q9BUR5-2|APOO_HUMAN Isoform 2 of Apolipoprotein	1.01E-17	
Q9BUT1;A8K5M0;Q9BUT1	BDH2_HUMAN 3-hydroxybutyrate dehydrogenase type 2 OS=Homo sapiens GN=BDH2 PE=1 SV=2;>tr|A8K5M0|A8K5M0_HUMAN cDN	1.44E-49	
Q9BUW7	CI016_HUMAN UPF0184 protein C9orf16 OS=Homo sapiens GN=C9orf16 PE=1 SV=1		27.92
Q9BV19	CA050_HUMAN Uncharacterized protein C1orf50 OS=Homo sapiens GN=C1orf50 PE=2 SV=2	1.31E-09	
Q9BV20;Q9BV20-2	MTNA_HUMAN Methylthioribose-1-phosphate isomerase OS=Homo sapiens GN=MRI1 PE=1 SV=1;>sp|Q9BV20-2|MTNA_HUMAN Isofo	4.88E-16	
Q9BV35	SCMC3_HUMAN Calcium-binding mitochondrial carrier protein SCaMC-3 OS=Homo sapiens GN=SLC25A23 PE=1 SV=2		17.09
Q9BV35-2	SCMC3_HUMAN Isoform 2 of Calcium-binding mitochondrial carrier protein SCaMC-3 OS=Homo sapiens GN=SLC25A23		17.09
Q9BV35-3	SCMC3_HUMAN Isoform 3 of Calcium-binding mitochondrial carrier protein SCaMC-3 OS=Homo sapiens GN=SLC25A23		17.09
Q9BV35-4	SCMC3_HUMAN Isoform 4 of Calcium-binding mitochondrial carrier protein SCaMC-3 OS=Homo sapiens GN=SLC25A23		17.09
Q9BV36;Q9BV36-2	MELPH_HUMAN Melanophilin OS=Homo sapiens GN=MLPH PE=1 SV=1;>sp|Q9BV36-2|MELPH_HUMAN Isoform 2 of Melanophilin OS=	3.51E-09	

Q9BV38	WDR18_HUMAN WD repeat-containing protein 18 OS=Homo sapiens GN=WDR18 PE=1 SV=2		
Q9BV40	VAMP8_HUMAN Vesicle-associated membrane protein 8 OS=Homo sapiens GN=VAMP8 PE=1 SV=1		195.8
Q9BV44	THUM3_HUMAN THUMP domain-containing protein 3 OS=Homo sapiens GN=THUMPD3 PE=1 SV=1	1.22E-06	
Q9BV57	MTND_HUMAN 1,2-dihydroxy-3-keto-5-methylthiopentene dioxygenase OS=Homo sapiens GN=ADI1 PE=1 SV=1		44.55
Q9BV57-2	MTND_HUMAN Isoform 2 of 1,2-dihydroxy-3-keto-5-methylthiopentene dioxygenase OS=Homo sapiens GN=ADI1		10.7
Q9BV57;Q9BV57-2	MTND_HUMAN 1,2-dihydroxy-3-keto-5-methylthiopentene dioxygenase OS=Homo sapiens GN=ADI1 PE=1 SV=1;>sp|Q9BV57-2|MTN	3.41E-30	
Q9BV73;Q9BV73-2	CP250_HUMAN Centrosome-associated protein CEP250 OS=Homo sapiens GN=CEP250 PE=1 SV=2;>sp|Q9BV73-2|CP250_HUMAN Isc	2.33E-22	
Q9BV79	MECR_HUMAN Trans-2-enoyl-CoA reductase, mitochondrial OS=Homo sapiens GN=MECR PE=1 SV=2	9.82E-05	
Q9BV81	TMM93_HUMAN Transmembrane protein 93 OS=Homo sapiens GN=TMEM93 PE=1 SV=1		30.92
Q9BV86	NTM1A_HUMAN Alpha N-terminal protein methyltransferase 1A OS=Homo sapiens GN=METTL11A PE=1 SV=3		24.56
Q9BV86-2	NTM1A_HUMAN Isoform 2 of Alpha N-terminal protein methyltransferase 1A OS=Homo sapiens GN=METTL11A		24.17
Q9BV86;Q9BV86-2	NTM1A_HUMAN Alpha N-terminal protein methyltransferase 1A OS=Homo sapiens GN=METTL11A PE=1 SV=3;>sp|Q9BV86-2|NTM1A_	2.23E-13	
Q9BVA1	TBB2B_HUMAN Tubulin beta-2B chain OS=Homo sapiens GN=TUBB2B PE=1 SV=1		2168.12
Q9BVC5	ASHWN_HUMAN Ashwin OS=Homo sapiens GN=C2orf49 PE=1 SV=1		43.34
Q9BVC6	TM109_HUMAN Transmembrane protein 109 OS=Homo sapiens GN=TMEM109 PE=1 SV=1		65.28
Q9BVG4	CX026_HUMAN UPF0368 protein Cxorf26 OS=Homo sapiens GN=CXorf26 PE=1 SV=1	6.57E-36	
Q9BVG8;Q9BVG8-2	KIFC3_HUMAN Kinesin-like protein KIFC3 OS=Homo sapiens GN=KIFC3 PE=1 SV=3;>sp|Q9BVG8-2|KIFC3_HUMAN Isoform 2 of Kines	6.66E-06	
Q9BVI4	NOC4L_HUMAN Nucleolar complex protein 4 homolog OS=Homo sapiens GN=NOC4L PE=1 SV=1		
Q9BVJ6	UT14A_HUMAN U3 small nucleolar RNA-associated protein 14 homolog A OS=Homo sapiens GN=UTP14A PE=1 SV=1		
Q9BVJ6-2	UT14A_HUMAN Isoform 2 of U3 small nucleolar RNA-associated protein 14 homolog A OS=Homo sapiens GN=UTP14A		
Q9BVJ6;Q5TAP6;Q9BVJ6-2	UT14A_HUMAN U3 small nucleolar RNA-associated protein 14 homolog A OS=Homo sapiens GN=UTP14A PE=1 SV=1;>sp|Q5TAP6|UT	2.21E-56	
Q9BVJ7	DUS23_HUMAN Dual specificity protein phosphatase 23 OS=Homo sapiens GN=DUSP23 PE=1 SV=1	3.72E-06	
Q9BVK6	TMED9_HUMAN Transmembrane emp24 domain-containing protein 9 OS=Homo sapiens GN=TMED9 PE=1 SV=2		518.59
Q9BVL2	NUPL1_HUMAN Nucleoporin p58/p45 OS=Homo sapiens GN=NUPL1 PE=1 SV=1		
Q9BVL2-2	NUPL1_HUMAN Isoform 2 of Nucleoporin p58/p45 OS=Homo sapiens GN=NUPL1		
Q9BVL2;Q9BVL2-2	NUPL1_HUMAN Nucleoporin p58/p45 OS=Homo sapiens GN=NUPL1 PE=1 SV=1;>sp|Q9BVL2-2|NUPL1_HUMAN Isoform 2 of Nucleopo	1.99E-09	
Q9BVP2	GNL3_HUMAN Guanine nucleotide-binding protein-like 3 OS=Homo sapiens GN=GNL3 PE=1 SV=2		180.13
Q9BVP2-2	GNL3_HUMAN Isoform 2 of Guanine nucleotide-binding protein-like 3 OS=Homo sapiens GN=GNL3		180.13
Q9BVP2;Q9BVP2-2	GNL3_HUMAN Guanine nucleotide-binding protein-like 3 OS=Homo sapiens GN=GNL3 PE=1 SV=2;>sp|Q9BVP2-2|GNL3_HUMAN Isofc	5.17E-43	
Q9BVQ7	SPA5L_HUMAN Spermatogenesis-associated protein 5-like protein 1 OS=Homo sapiens GN=SPATA5L1 PE=1 SV=2		
Q9BVQ7-2	SPA5L_HUMAN Isoform 2 of Spermatogenesis-associated protein 5-like protein 1 OS=Homo sapiens GN=SPATA5L1		
Q9BVQ7-3	SPA5L_HUMAN Isoform 3 of Spermatogenesis-associated protein 5-like protein 1 OS=Homo sapiens GN=SPATA5L1		
Q9BVQ7;Q9BVQ7-2	SPA5L_HUMAN Spermatogenesis-associated protein 5-like protein 1 OS=Homo sapiens GN=SPATA5L1 PE=1 SV=2;>sp|Q9BVQ7-2|SP/	8.86E-07	
Q9BVT8	TMUB1_HUMAN Transmembrane and ubiquitin-like domain-containing protein 1 OS=Homo sapiens GN=TMUB1 PE=1 SV=1		112.51
Q9BVV7	TI21L_HUMAN TIM21-like protein, mitochondrial OS=Homo sapiens GN=C18orf55 PE=1 SV=1		16.98
Q9BW04;Q9BW04-2	SARG_HUMAN Specifically androgen-regulated gene protein OS=Homo sapiens GN=SARG PE=1 SV=2;>sp|Q9BW04-2|SARG_HUMAN	1.07E-14	
Q9BW19	KIFC1_HUMAN Kinesin-like protein KIFC1 OS=Homo sapiens GN=KIFC1 PE=1 SV=2		61.76
Q9BW27	NUP85_HUMAN Nuclear pore complex protein Nup85 OS=Homo sapiens GN=NUP85 PE=1 SV=1	1.28E-31	
Q9BW30	TPPP3_HUMAN Tubulin polymerization-promoting protein family member 3 OS=Homo sapiens GN=TPPP3 PE=1 SV=1	3.15E-09	
Q9BW60	ELOV1_HUMAN Elongation of very long chain fatty acids protein 1 OS=Homo sapiens GN=ELOVL1 PE=1 SV=1		77.34
Q9BW61	DDA1_HUMAN DET1- and DDB1-associated protein 1 OS=Homo sapiens GN=DDA1 PE=1 SV=1		31.59
Q9BW71	HIRP3_HUMAN HIRA-interacting protein 3 OS=Homo sapiens GN=HIRIP3 PE=1 SV=3		
Q9BW71-2	HIRP3_HUMAN Isoform 2 of HIRA-interacting protein 3 OS=Homo sapiens GN=HIRIP3		
Q9BW83	IFT27_HUMAN Intraflagellar transport protein 27 homolog OS=Homo sapiens GN=IFT27 PE=1 SV=1		
Q9BW83-2	IFT27_HUMAN Isoform 2 of Intraflagellar transport protein 27 homolog OS=Homo sapiens GN=IFT27		
Q9BW83;Q9BW83-2	IFT27_HUMAN Intraflagellar transport protein 27 homolog OS=Homo sapiens GN=IFT27 PE=1 SV=1;>sp|Q9BW83-2|IFT27_HUMAN Is	5.21E-06	
Q9BW91;Q9BW91-2	NUDT9_HUMAN ADP-ribose pyrophosphatase, mitochondrial OS=Homo sapiens GN=NUDT9 PE=1 SV=1;>sp|Q9BW91-2|NUDT9_HUM/	2.30E-35	
Q9BW92	SYTM_HUMAN Threonyl-tRNA synthetase, mitochondrial OS=Homo sapiens GN=TARS2 PE=1 SV=1	8.63E-30	
Q9BWD1	THIC_HUMAN Acetyl-CoA acetyltransferase, cytosolic OS=Homo sapiens GN=ACAT2 PE=1 SV=2		68.03
Q9BWE0	REPI1_HUMAN Replication initiator 1 OS=Homo sapiens GN=REPIN1 PE=1 SV=1	5.28E-29	
Q9BWF3	RBM4_HUMAN RNA-binding protein 4 OS=Homo sapiens GN=RBM4 PE=1 SV=1		27.01
Q9BWF3-2	RBM4_HUMAN Isoform 2 of RNA-binding protein 4 OS=Homo sapiens GN=RBM4		25.04
Q9BWF3-3	RBM4_HUMAN Isoform 3 of RNA-binding protein 4 OS=Homo sapiens GN=RBM4		25.04
Q9BWF3;Q9BQ04;Q9BWF	RBM4_HUMAN RNA-binding protein 4 OS=Homo sapiens GN=RBM4 PE=1 SV=1;>sp|Q9BQ04|RBM4B_HUMAN RNA-binding protein 4B	9.75E-10	
Q9BWH2	FUND2_HUMAN FUN14 domain-containing protein 2 OS=Homo sapiens GN=FUNDC2 PE=1 SV=2		
Q9BWH6;Q9BWH6-2	RPAP1_HUMAN RNA polymerase II-associated protein 1 OS=Homo sapiens GN=RPAP1 PE=1 SV=3;>sp|Q9BWH6-2|RPAP1_HUMAN Isc	9.47E-05	
Q9BWJ5	SF3B5_HUMAN Splicing factor 3B subunit 5 OS=Homo sapiens GN=SF3B5 PE=1 SV=1		205.85
Q9BWM7	SFXN3_HUMAN Sideroflexin-3 OS=Homo sapiens GN=SFXN3 PE=2 SV=2		176.12
Q9BWS9	CHID1_HUMAN Chitinase domain-containing protein 1 OS=Homo sapiens GN=CHID1 PE=1 SV=1		102.41
Q9BWS9-2	CHID1_HUMAN Isoform 2 of Chitinase domain-containing protein 1 OS=Homo sapiens GN=CHID1		88.33
Q9BWS9-2;Q9BWS9;Q9B\	2|CHID1_HUMAN Isoform 2 of Chitinase domain-containing protein 1 OS=Homo sapiens GN=CHID1;>sp|Q9BWS9|CHID1_HUMAN Chi	2.97E-22	

Q9BWS9-3	CHID1_HUMAN Isoform 3 of Chitinase domain-containing protein 1 OS=Homo sapiens GN=CHID1		102.41
Q9BWT6	MND1_HUMAN Meiotic nuclear division protein 1 homolog OS=Homo sapiens GN=MND1 PE=1 SV=1		59.14
Q9BWU0	NADAP_HUMAN Kanadaptin OS=Homo sapiens GN=SLC4A1AP PE=1 SV=1		66.02
Q9BWV7	TTLL2_HUMAN Probable tubulin polyglutamylase TTLL2 OS=Homo sapiens GN=TTLL2 PE=5 SV=3		
Q9BX40	LS14B_HUMAN Protein LSM14 homolog B OS=Homo sapiens GN=LSM14B PE=1 SV=1		
Q9BX40-2	LS14B_HUMAN Isoform 2 of Protein LSM14 homolog B OS=Homo sapiens GN=LSM14B		
Q9BX40;Q9BX40-2	LS14B_HUMAN Protein LSM14 homolog B OS=Homo sapiens GN=LSM14B PE=1 SV=1;>sp|Q9BX40-2|LS14B_HUMAN Isoform 2 of Pro	5.68E-12	
Q9BX63	FANCJ_HUMAN Fanconi anemia group J protein OS=Homo sapiens GN=BRIP1 PE=1 SV=1		18.19
Q9BX63-2	FANCJ_HUMAN Isoform 2 of Fanconi anemia group J protein OS=Homo sapiens GN=BRIP1		23.08
Q9BX66-10	SRBS1_HUMAN Isoform 10 of Sorbin and SH3 domain-containing protein 1 OS=Homo sapiens GN=SORBS1		
Q9BX66-12	SRBS1_HUMAN Isoform 12 of Sorbin and SH3 domain-containing protein 1 OS=Homo sapiens GN=SORBS1		
Q9BX66-4	SRBS1_HUMAN Isoform 4 of Sorbin and SH3 domain-containing protein 1 OS=Homo sapiens GN=SORBS1		
Q9BX66-5	SRBS1_HUMAN Isoform 5 of Sorbin and SH3 domain-containing protein 1 OS=Homo sapiens GN=SORBS1		
Q9BX66-6	SRBS1_HUMAN Isoform 6 of Sorbin and SH3 domain-containing protein 1 OS=Homo sapiens GN=SORBS1		
Q9BX66-8	SRBS1_HUMAN Isoform 8 of Sorbin and SH3 domain-containing protein 1 OS=Homo sapiens GN=SORBS1		
Q9BX66-9	SRBS1_HUMAN Isoform 9 of Sorbin and SH3 domain-containing protein 1 OS=Homo sapiens GN=SORBS1		
Q9BX68	HINT2_HUMAN Histidine triad nucleotide-binding protein 2, mitochondrial OS=Homo sapiens GN=HINT2 PE=1 SV=1		343.68
Q9BX97	PLVAP_HUMAN Plasmalemma vesicle-associated protein OS=Homo sapiens GN=PLVAP PE=2 SV=1		30.06
Q9BXB5	OSB10_HUMAN Oxysterol-binding protein-related protein 10 OS=Homo sapiens GN=OSBPL10 PE=1 SV=2	2.87E-09	
Q9BXD5;Q9BXD5-2;Q9BX	NPL_HUMAN N-acetylneuraminate lyase OS=Homo sapiens GN=NPL PE=2 SV=1;>sp|Q9BXD5-2|NPL_HUMAN Isoform 2 of N-acetylne	2.93E-41	
Q9BXF6	RFIP5_HUMAN Rab11 family-interacting protein 5 OS=Homo sapiens GN=RAB11FIP5 PE=1 SV=1		331.06
Q9BXI6	TB10A_HUMAN TBC1 domain family member 10A OS=Homo sapiens GN=TBC1D10A PE=1 SV=1		43.43
Q9BXJ9	NAA15_HUMAN N-alpha-acetyltransferase 15, NatA auxiliary subunit OS=Homo sapiens GN=NAA15 PE=1 SV=1		78.54
Q9BXJ9-4	NAA15_HUMAN Isoform 2 of N-alpha-acetyltransferase 15, NatA auxiliary subunit OS=Homo sapiens GN=NAA15		78.54
Q9BXJ9;Q9BXJ9-4	NAA15_HUMAN N-alpha-acetyltransferase 15, NatA auxiliary subunit OS=Homo sapiens GN=NAA15 PE=1 SV=1;>sp|Q9BXJ9-4|NAA15	3.38E-109	
Q9BXK5;Q9BXK5-2	B2L13_HUMAN Bcl-2-like protein 13 OS=Homo sapiens GN=BCL2L13 PE=1 SV=1;>sp|Q9BXK5-2|B2L13_HUMAN Isoform 1 of Bcl-2-lil	1.00E-05	
Q9BXL6	CAR14_HUMAN Caspase recruitment domain-containing protein 14 OS=Homo sapiens GN=CARD14 PE=1 SV=2		30.59
Q9BXL8	CDCA4_HUMAN Cell division cycle-associated protein 4 OS=Homo sapiens GN=CDCA4 PE=2 SV=1		34.09
Q9BXM0	PRAX_HUMAN Periaxin OS=Homo sapiens GN=PRX PE=1 SV=2		
Q9BXM0-3	PRAX_HUMAN Isoform 3 of Periaxin OS=Homo sapiens GN=PRX		
Q9BXN1	ASPN_HUMAN Asporin OS=Homo sapiens GN=ASPN PE=1 SV=2	4.72E-263	
Q9BXP2	S12A9_HUMAN Solute carrier family 12 member 9 OS=Homo sapiens GN=SLC12A9 PE=1 SV=1	1.91E-05	
Q9BXP5	SRRT_HUMAN Serrate RNA effector molecule homolog OS=Homo sapiens GN=SRRT PE=1 SV=1		337.15
Q9BXP5-2	SRRT_HUMAN Isoform 2 of Serrate RNA effector molecule homolog OS=Homo sapiens GN=SRRT		337.15
Q9BXP5-3	SRRT_HUMAN Isoform 3 of Serrate RNA effector molecule homolog OS=Homo sapiens GN=SRRT		337.15
Q9BXP5-4	SRRT_HUMAN Isoform 4 of Serrate RNA effector molecule homolog OS=Homo sapiens GN=SRRT		337.15
Q9BXP5-5	SRRT_HUMAN Isoform 5 of Serrate RNA effector molecule homolog OS=Homo sapiens GN=SRRT		337.15
Q9BXP5;Q9BXP5-3;Q9BXF	SRRT_HUMAN Serrate RNA effector molecule homolog OS=Homo sapiens GN=SRRT PE=1 SV=1;>sp|Q9BXP5-3|SRRT_HUMAN Isoforn	2.49E-147	
Q9BXP8	PAPP2_HUMAN Pappalysin-2 OS=Homo sapiens GN=PAPPA2 PE=1 SV=4		23.8
Q9BXP8-2	PAPP2_HUMAN Isoform 2 of Pappalysin-2 OS=Homo sapiens GN=PAPPA2		23.8
Q9BXR0	TGT_HUMAN Queuine tRNA-ribosyltransferase OS=Homo sapiens GN=QTRT1 PE=1 SV=3	4.94E-14	
Q9BXR5	TLR10_HUMAN Toll-like receptor 10 OS=Homo sapiens GN=TLR10 PE=1 SV=2		35.32
Q9BXS5	AP1M1_HUMAN AP-1 complex subunit mu-1 OS=Homo sapiens GN=AP1M1 PE=1 SV=3		21.93
Q9BXT4	TDRD1_HUMAN Tudor domain-containing protein 1 OS=Homo sapiens GN=TDRD1 PE=1 SV=2		
Q9BXT4-2	TDRD1_HUMAN Isoform 2 of Tudor domain-containing protein 1 OS=Homo sapiens GN=TDRD1		
Q9BXT4-3	TDRD1_HUMAN Isoform 3 of Tudor domain-containing protein 1 OS=Homo sapiens GN=TDRD1		
Q9BXT5	TEX15_HUMAN Testis-expressed sequence 15 protein OS=Homo sapiens GN=TEX15 PE=1 SV=2		
Q9BXT8	RNF17_HUMAN RING finger protein 17 OS=Homo sapiens GN=RNF17 PE=1 SV=3		
Q9BXT8-1	RNF17_HUMAN Isoform 2 of RING finger protein 17 OS=Homo sapiens GN=RNF17		
Q9BXT8-4	RNF17_HUMAN Isoform 4 of RING finger protein 17 OS=Homo sapiens GN=RNF17		
Q9BXT8-5	RNF17_HUMAN Isoform 5 of RING finger protein 17 OS=Homo sapiens GN=RNF17		
Q9BXU9	CABP8_HUMAN Calcium-binding protein 8 OS=Homo sapiens GN=CALN1 PE=2 SV=1		19.23
Q9BXV9	CN142_HUMAN Uncharacterized protein C14orf142 OS=Homo sapiens GN=C14orf142 PE=2 SV=2		21.27
Q9BXW6	OSBL1_HUMAN Oxysterol-binding protein-related protein 1 OS=Homo sapiens GN=OSBPL1A PE=1 SV=2		
Q9BXW6-2	OSBL1_HUMAN Isoform A of Oxysterol-binding protein-related protein 1 OS=Homo sapiens GN=OSBPL1A		
Q9BXW6-3	OSBL1_HUMAN Isoform C of Oxysterol-binding protein-related protein 1 OS=Homo sapiens GN=OSBPL1A		
Q9BXW7	CECR5_HUMAN Cat eye syndrome critical region protein 5 OS=Homo sapiens GN=CECR5 PE=1 SV=1		
Q9BXW7-2	CECR5_HUMAN Isoform 1 of Cat eye syndrome critical region protein 5 OS=Homo sapiens GN=CECR5		
Q9BXW7;Q9BXW7-2	CECR5_HUMAN Cat eye syndrome critical region protein 5 OS=Homo sapiens GN=CECR5 PE=1 SV=1;>sp|Q9BXW7-2|CECR5_HUMAN	2.26E-32	
Q9BXW9	FACD2_HUMAN Fanconi anemia group D2 protein OS=Homo sapiens GN=FANCD2 PE=1 SV=1		
Q9BXW9-2	FACD2_HUMAN Isoform 2 of Fanconi anemia group D2 protein OS=Homo sapiens GN=FANCD2		

Q9BXW9-3	FACD2_HUMAN Isoform 3 of Fanconi anemia group D2 protein OS=Homo sapiens GN=FANCD2		
Q9BXW9-4	FACD2_HUMAN Isoform 4 of Fanconi anemia group D2 protein OS=Homo sapiens GN=FANCD2		
Q9BXW9;Q9BXW9-2;Q9B>	FACD2_HUMAN Fanconi anemia group D2 protein OS=Homo sapiens GN=FANCD2 PE=1 SV=1;>sp|Q9BXW9-2|FACD2_HUMAN Isoform	1.08E-14	
Q9BXX2	AN30B_HUMAN Ankyrin repeat domain-containing protein 30B OS=Homo sapiens GN=ANKRD30B PE=2 SV=2		
Q9BXX2-2	AN30B_HUMAN Isoform 2 of Ankyrin repeat domain-containing protein 30B OS=Homo sapiens GN=ANKRD30B		
Q9BXX2-3	AN30B_HUMAN Isoform 3 of Ankyrin repeat domain-containing protein 30B OS=Homo sapiens GN=ANKRD30B		
Q9BXX3	AN30A_HUMAN Ankyrin repeat domain-containing protein 30A OS=Homo sapiens GN=ANKRD30A PE=1 SV=3		
Q9BXY0	MAK16_HUMAN Protein MAK16 homolog OS=Homo sapiens GN=MAK16 PE=1 SV=2		
Q9BY11	PACN1_HUMAN Protein kinase C and casein kinase substrate in neurons protein 1 OS=Homo sapiens GN=PACSIN1 PE=1 SV=1		
Q9BY32	ITPA_HUMAN Inosine triphosphate pyrophosphatase OS=Homo sapiens GN=ITPA PE=1 SV=2		52.07
Q9BY42	CT043_HUMAN UPF0549 protein C20orf43 OS=Homo sapiens GN=C20orf43 PE=1 SV=3		
Q9BY43	CHM4A_HUMAN Charged multivesicular body protein 4a OS=Homo sapiens GN=CHMP4A PE=1 SV=3	1.10E-15	
Q9BY44	EIF2A_HUMAN Eukaryotic translation initiation factor 2A OS=Homo sapiens GN=EIF2A PE=1 SV=3		235.25
Q9BY44-2	EIF2A_HUMAN Isoform 2 of Eukaryotic translation initiation factor 2A OS=Homo sapiens GN=EIF2A		147.24
Q9BY50	SC11C_HUMAN Signal peptidase complex catalytic subunit SEC11C OS=Homo sapiens GN=SEC11C PE=1 SV=3	1.67E-18	
Q9BY66	KDM5D_HUMAN Lysine-specific demethylase 5D OS=Homo sapiens GN=KDM5D PE=1 SV=2		28.1
Q9BY66-2	KDM5D_HUMAN Isoform 2 of Lysine-specific demethylase 5D OS=Homo sapiens GN=KDM5D		28.1
Q9BY77	PDIP3_HUMAN Polymerase delta-interacting protein 3 OS=Homo sapiens GN=POLDIP3 PE=1 SV=2		76.12
Q9BY77-2	PDIP3_HUMAN Isoform 2 of Polymerase delta-interacting protein 3 OS=Homo sapiens GN=POLDIP3		57.06
Q9BY77;Q9BY77-2	PDIP3_HUMAN Polymerase delta-interacting protein 3 OS=Homo sapiens GN=POLDIP3 PE=1 SV=2;>sp|Q9BY77-2|PDIP3_HUMAN Iso	1.40E-11	
Q9BYC5;Q9BYC5-2	FUT8_HUMAN Alpha-(1,6)-fucosyltransferase OS=Homo sapiens GN=FUT8 PE=1 SV=2;>sp|Q9BYC5-2|FUT8_HUMAN Isoform 2 of Alp	4.88E-06	
Q9BYC8	RM32_HUMAN 39S ribosomal protein L32, mitochondrial OS=Homo sapiens GN=MRPL32 PE=1 SV=1	5.76E-08	
Q9BYC9	RM20_HUMAN 39S ribosomal protein L20, mitochondrial OS=Homo sapiens GN=MRPL20 PE=1 SV=1		46.07
Q9BYD1	RM13_HUMAN 39S ribosomal protein L13, mitochondrial OS=Homo sapiens GN=MRPL13 PE=1 SV=1		113.28
Q9BYD2	RM09_HUMAN 39S ribosomal protein L9, mitochondrial OS=Homo sapiens GN=MRPL9 PE=1 SV=2		114.48
Q9BYD3	RM04_HUMAN 39S ribosomal protein L4, mitochondrial OS=Homo sapiens GN=MRPL4 PE=1 SV=1		113.88
Q9BYD3-2	RM04_HUMAN Isoform 2 of 39S ribosomal protein L4, mitochondrial OS=Homo sapiens GN=MRPL4		113.88
Q9BYD3;Q9BYD3-2	RM04_HUMAN 39S ribosomal protein L4, mitochondrial OS=Homo sapiens GN=MRPL4 PE=1 SV=1;>sp|Q9BYD3-2|RM04_HUMAN Iso	8.99E-09	
Q9BYD6	RM01_HUMAN 39S ribosomal protein L1, mitochondrial OS=Homo sapiens GN=MRPL1 PE=1 SV=2		207.22
Q9BYE4	SPR2G_HUMAN Small proline-rich protein 2G OS=Homo sapiens GN=SPRR2G PE=2 SV=1		23.4
Q9BYF1	ACE2_HUMAN Angiotensin-converting enzyme 2 OS=Homo sapiens GN=ACE2 PE=1 SV=2		23.28
Q9BYG3	MK67I_HUMAN MKI67 FHA domain-interacting nucleolar phosphoprotein OS=Homo sapiens GN=MKI67IP PE=1 SV=1		71.68
Q9BYG4	PAR6G_HUMAN Partitioning defective 6 homolog gamma OS=Homo sapiens GN=PARD6G PE=1 SV=1	0.00011439	
Q9BYJ4	TRI34_HUMAN Tripartite motif-containing protein 34 OS=Homo sapiens GN=TRIM34 PE=1 SV=2		28.93
Q9BYJ4-2	TRI34_HUMAN Isoform 2 of Tripartite motif-containing protein 34 OS=Homo sapiens GN=TRIM34		28.93
Q9BYJ9	YTHD1_HUMAN YTH domain family protein 1 OS=Homo sapiens GN=YTHDF1 PE=1 SV=1		59.35
Q9BYK8	PR285_HUMAN Peroxisomal proliferator-activated receptor A-interacting complex 285 kDa protein OS=Homo sapiens GN=PRIC285 PE=1 SV=6		131.22
Q9BYK8-2	PR285_HUMAN Isoform 2 of Peroxisomal proliferator-activated receptor A-interacting complex 285 kDa protein OS=Homo sapiens GN=PRIC285		125.75
Q9BYK8-4	PR285_HUMAN Isoform 3 of Peroxisomal proliferator-activated receptor A-interacting complex 285 kDa protein OS=Homo sapiens GN=PRIC285		125.75
Q9BYN0	SRXN1_HUMAN Sulfiredoxin-1 OS=Homo sapiens GN=SRXN1 PE=1 SV=2	6.23E-10	
Q9BYN8	RT26_HUMAN 28S ribosomal protein S26, mitochondrial OS=Homo sapiens GN=MRPS26 PE=1 SV=1		36.06
Q9BYP7	WNK3_HUMAN Serine/threonine-protein kinase WNK3 OS=Homo sapiens GN=WNK3 PE=1 SV=2		
Q9BYP7-2	WNK3_HUMAN Isoform 2 of Serine/threonine-protein kinase WNK3 OS=Homo sapiens GN=WNK3		
Q9BYT8	NEUL_HUMAN Neurolysin, mitochondrial OS=Homo sapiens GN=NLN PE=1 SV=1		15.07
Q9BYV8;Q9BYV8-2;Q9BYV	CEP41_HUMAN Centrosomal protein of 41 kDa OS=Homo sapiens GN=TSGA14 PE=1 SV=1;>sp|Q9BYV8-2|CEP41_HUMAN Isoform 2 c	8.70E-19	
Q9BYX2;Q9BYX2-2;Q5TBC	TBD2A_HUMAN TBC1 domain family member 2A OS=Homo sapiens GN=TBC1D2 PE=1 SV=3;>sp|Q9BYX2-2|TBD2A_HUMAN Isoform	7.64E-13	
Q9BYX4;Q9BYX4-2	IFIH1_HUMAN Interferon-induced helicase C domain-containing protein 1 OS=Homo sapiens GN=IFIH1 PE=1 SV=3;>sp|Q9BYX4-2|IF	7.54E-07	
Q9BYX7	ACTBM_HUMAN Putative beta-actin-like protein 3 OS=Homo sapiens GN=POTEKP PE=5 SV=1		343.95
Q9BYZ2	LDH6B_HUMAN L-lactate dehydrogenase A-like 6B OS=Homo sapiens GN=LDHAL6B PE=1 SV=3		123.7
Q9BZ23	PANK2_HUMAN Pantothenate kinase 2, mitochondrial OS=Homo sapiens GN=PANK2 PE=1 SV=3		19.79
Q9BZ23-3	PANK2_HUMAN Isoform 2 of Pantothenate kinase 2, mitochondrial OS=Homo sapiens GN=PANK2		2.34
Q9BZ23-4	PANK2_HUMAN Isoform 4 of Pantothenate kinase 2, mitochondrial OS=Homo sapiens GN=PANK2		19.79
Q9BZ29	DOCK9_HUMAN Dedicator of cytokinesis protein 9 OS=Homo sapiens GN=DOCK9 PE=1 SV=2		
Q9BZ29-3	DOCK9_HUMAN Isoform 3 of Dedicator of cytokinesis protein 9 OS=Homo sapiens GN=DOCK9		
Q9BZ29-4	DOCK9_HUMAN Isoform 4 of Dedicator of cytokinesis protein 9 OS=Homo sapiens GN=DOCK9		
Q9BZ29-4;Q9BZ29;Q9BZ2	4|DOCK9_HUMAN Isoform 4 of Dedicator of cytokinesis protein 9 OS=Homo sapiens GN=DOCK9;>sp|Q9BZ29|DOCK9_HUMAN Dedica	1.11E-10	
Q9BZ29-5	DOCK9_HUMAN Isoform 2 of Dedicator of cytokinesis protein 9 OS=Homo sapiens GN=DOCK9		
Q9BZ67	FRMD8_HUMAN FERM domain-containing protein 8 OS=Homo sapiens GN=FRMD8 PE=1 SV=1		
Q9BZ67-2	FRMD8_HUMAN Isoform 2 of FERM domain-containing protein 8 OS=Homo sapiens GN=FRMD8		
Q9BZ72	PITM2_HUMAN Membrane-associated phosphatidylinositol transfer protein 2 OS=Homo sapiens GN=PITPNM2 PE=1 SV=1		77.34
Q9BZ72-2	PITM2_HUMAN Isoform 2 of Membrane-associated phosphatidylinositol transfer protein 2 OS=Homo sapiens GN=PITPNM2		77.34

Q9BZ72-3	PITM2_HUMAN Isoform 3 of Membrane-associated phosphatidylinositol transfer protein 2 OS=Homo sapiens GN=PITPNM2		26.27
Q9BZA7	PC11X_HUMAN Protocadherin-11 X-linked OS=Homo sapiens GN=PCDH11X PE=2 SV=1		
Q9BZA7-2	PC11X_HUMAN Isoform 2 of Protocadherin-11 X-linked OS=Homo sapiens GN=PCDH11X		
Q9BZA7-3	PC11X_HUMAN Isoform 3 of Protocadherin-11 X-linked OS=Homo sapiens GN=PCDH11X		
Q9BZA7-4	PC11X_HUMAN Isoform 4 of Protocadherin-11 X-linked OS=Homo sapiens GN=PCDH11X		
Q9BZA7-5	PC11X_HUMAN Isoform 5 of Protocadherin-11 X-linked OS=Homo sapiens GN=PCDH11X		
Q9BZA7-6	PC11X_HUMAN Isoform 6 of Protocadherin-11 X-linked OS=Homo sapiens GN=PCDH11X		
Q9BZA7-7	PC11X_HUMAN Isoform 7 of Protocadherin-11 X-linked OS=Homo sapiens GN=PCDH11X		
Q9BZA7-8	PC11X_HUMAN Isoform 8 of Protocadherin-11 X-linked OS=Homo sapiens GN=PCDH11X		
Q9BZA8	PC11Y_HUMAN Protocadherin-11 Y-linked OS=Homo sapiens GN=PCDH11Y PE=1 SV=1		
Q9BZA8-2	PC11Y_HUMAN Isoform 2 of Protocadherin-11 Y-linked OS=Homo sapiens GN=PCDH11Y		
Q9BZA8-3	PC11Y_HUMAN Isoform 3 of Protocadherin-11 Y-linked OS=Homo sapiens GN=PCDH11Y		
Q9BZA8-4	PC11Y_HUMAN Isoform 4 of Protocadherin-11 Y-linked OS=Homo sapiens GN=PCDH11Y		
Q9BZC1	CELF4_HUMAN CUGBP Elav-like family member 4 OS=Homo sapiens GN=CELF4 PE=1 SV=1		
Q9BZC1-2	CELF4_HUMAN Isoform 2 of CUGBP Elav-like family member 4 OS=Homo sapiens GN=CELF4		
Q9BZC1-3	CELF4_HUMAN Isoform 3 of CUGBP Elav-like family member 4 OS=Homo sapiens GN=CELF4		
Q9BZC1-4	CELF4_HUMAN Isoform 4 of CUGBP Elav-like family member 4 OS=Homo sapiens GN=CELF4		
Q9BZC1-5	CELF4_HUMAN Isoform 5 of CUGBP Elav-like family member 4 OS=Homo sapiens GN=CELF4		
Q9BZD4	NUF2_HUMAN Kinetochore protein Nuf2 OS=Homo sapiens GN=NUF2 PE=1 SV=2		
Q9BZE0	GLIS2_HUMAN Zinc finger protein GLIS2 OS=Homo sapiens GN=GLIS2 PE=1 SV=2		
Q9BZE1	RM37_HUMAN 39S ribosomal protein L37, mitochondrial OS=Homo sapiens GN=MRPL37 PE=1 SV=2		21.83
Q9BZE4	NOG1_HUMAN Nucleolar GTP-binding protein 1 OS=Homo sapiens GN=GTPBP4 PE=1 SV=3		171.58
Q9BZF1	OSBL8_HUMAN Oxysterol-binding protein-related protein 8 OS=Homo sapiens GN=OSBPL8 PE=1 SV=3		105.32
Q9BZF1-2	OSBL8_HUMAN Isoform 2 of Oxysterol-binding protein-related protein 8 OS=Homo sapiens GN=OSBPL8		105.32
Q9BZF1;Q9BZF1-2	OSBL8_HUMAN Oxysterol-binding protein-related protein 8 OS=Homo sapiens GN=OSBPL8 PE=1 SV=3;>sp|Q9BZF1-2|OSBL8_HUMAI	2.32E-26	
Q9BZF2	OSBL7_HUMAN Oxysterol-binding protein-related protein 7 OS=Homo sapiens GN=OSBPL7 PE=2 SV=1		
Q9BZF9	UACA_HUMAN Uveal autoantigen with coiled-coil domains and ankyrin repeats OS=Homo sapiens GN=UACA PE=1 SV=2		77.82
Q9BZG1	RAB34_HUMAN Ras-related protein Rab-34 OS=Homo sapiens GN=RAB34 PE=1 SV=1		362.38
Q9BZG1-2	RAB34_HUMAN Isoform 2 of Ras-related protein Rab-34 OS=Homo sapiens GN=RAB34		287.26
Q9BZG1;Q9BZG1-2	RAB34_HUMAN Ras-related protein Rab-34 OS=Homo sapiens GN=RAB34 PE=1 SV=1;>sp|Q9BZG1-2|RAB34_HUMAN Isoform 2 of Ra	2.01E-27	
Q9BZH6	WDR11_HUMAN WD repeat-containing protein 11 OS=Homo sapiens GN=WDR11 PE=1 SV=1		
Q9BZJ0	CRNL1_HUMAN Crooked neck-like protein 1 OS=Homo sapiens GN=CRNKL1 PE=1 SV=4		
Q9BZJ0-2	CRNL1_HUMAN Isoform 2 of Crooked neck-like protein 1 OS=Homo sapiens GN=CRNKL1		
Q9BZJ0-3	CRNL1_HUMAN Isoform 3 of Crooked neck-like protein 1 OS=Homo sapiens GN=CRNKL1		
Q9BZJ0-4	CRNL1_HUMAN Isoform 4 of Crooked neck-like protein 1 OS=Homo sapiens GN=CRNKL1		
Q9BZJ0-5	CRNL1_HUMAN Isoform 5 of Crooked neck-like protein 1 OS=Homo sapiens GN=CRNKL1		
Q9BZJ0;Q9BZJ0-3;Q9BZJ(	CRNL1_HUMAN Crooked neck-like protein 1 OS=Homo sapiens GN=CRNKL1 PE=1 SV=4;>sp|Q9BZJ0-3|CRNL1_HUMAN Isoform 3 of C	8.70E-21	
Q9BZK3	NACP1_HUMAN Putative nascent polypeptide-associated complex subunit alpha-like protein OS=Homo sapiens GN=NACAP1 PE=5 SV=1		182.22
Q9BZK7	TBL1R_HUMAN F-box-like/WD repeat-containing protein TBL1XR1 OS=Homo sapiens GN=TBL1XR1 PE=1 SV=1	1.89E-29	
Q9BZL1	UBL5_HUMAN Ubiquitin-like protein 5 OS=Homo sapiens GN=UBL5 PE=1 SV=1		79.51
Q9BZL4	PP12C_HUMAN Protein phosphatase 1 regulatory subunit 12C OS=Homo sapiens GN=PPP1R12C PE=1 SV=1		67.27
Q9BZL4-2	PP12C_HUMAN Isoform 2 of Protein phosphatase 1 regulatory subunit 12C OS=Homo sapiens GN=PPP1R12C		23.8
Q9BZL4-3	PP12C_HUMAN Isoform 3 of Protein phosphatase 1 regulatory subunit 12C OS=Homo sapiens GN=PPP1R12C		67.27
Q9BZL4-4	PP12C_HUMAN Isoform 4 of Protein phosphatase 1 regulatory subunit 12C OS=Homo sapiens GN=PPP1R12C		67.27
Q9BZL6	KPCD2_HUMAN Serine/threonine-protein kinase D2 OS=Homo sapiens GN=PRKD2 PE=1 SV=2		
Q9BZM5	N2DL2_HUMAN NKG2D ligand 2 OS=Homo sapiens GN=ULBP2 PE=1 SV=1		
Q9BZQ8	NIBAN_HUMAN Protein Niban OS=Homo sapiens GN=FAM129A PE=1 SV=1	6.80E-22	
Q9BZV1;Q9BZV1-2	UBXN6_HUMAN UBX domain-containing protein 6 OS=Homo sapiens GN=UBXN6 PE=1 SV=1;>sp|Q9BZV1-2|UBXN6_HUMAN Isoform	5.41E-20	
Q9BZX2;B4DGD3;Q9BZX2	UCK2_HUMAN Uridine-cytidine kinase 2 OS=Homo sapiens GN=UCK2 PE=1 SV=1;>tr|B4DGD3|B4DGD3_HUMAN Uridine kinase OS=H	6.57E-08	
Q9BZZ2	SN_HUMAN Sialoadhesin OS=Homo sapiens GN=SIGLEC1 PE=1 SV=2		23.47
Q9BZZ2-2	SN_HUMAN Isoform 2 of Sialoadhesin OS=Homo sapiens GN=SIGLEC1		23.47
Q9BZZ2-3	SN_HUMAN Isoform 3 of Sialoadhesin OS=Homo sapiens GN=SIGLEC1		23.47
Q9BZZ2;Q9BZZ2-3;A5D82	SN_HUMAN Sialoadhesin OS=Homo sapiens GN=SIGLEC1 PE=1 SV=2;>sp|Q9BZZ2-3|SN_HUMAN Isoform 3 of Sialoadhesin OS=Hom	2.47E-38	
Q9BZZ5	API5_HUMAN Apoptosis inhibitor 5 OS=Homo sapiens GN=API5 PE=1 SV=2		314.32
Q9BZZ5-2	API5_HUMAN Isoform 2 of Apoptosis inhibitor 5 OS=Homo sapiens GN=API5		314.32
Q9BZZ5-2;Q9BZZ5-4;Q9B	2|API5_HUMAN Isoform 2 of Apoptosis inhibitor 5 OS=Homo sapiens GN=API5;>sp|Q9BZZ5-4|API5_HUMAN Isoform 4 of Apoptosis ir	7.91E-162	
Q9BZZ5-3	API5_HUMAN Isoform 3 of Apoptosis inhibitor 5 OS=Homo sapiens GN=API5		284.95
Q9BZZ5-4	API5_HUMAN Isoform 4 of Apoptosis inhibitor 5 OS=Homo sapiens GN=API5		314.32
Q9BZZ5;Q9BZZ5-2;Q9BZ;	API5_HUMAN Apoptosis inhibitor 5 OS=Homo sapiens GN=API5 PE=1 SV=2;>sp|Q9BZZ5-2|API5_HUMAN Isoform 2 of Apoptosis inhit	6.88E-86	
Q9C002	NMES1_HUMAN Normal mucosa of esophagus-specific gene 1 protein OS=Homo sapiens GN=NMES1 PE=2 SV=1		80.94
Q9C005	DPY30_HUMAN Protein dpy-30 homolog OS=Homo sapiens GN=DPY30 PE=1 SV=1		

Q9C035-5	TRIM5_HUMAN Isoform Epsilon of Tripartite motif-containing protein 5 OS=Homo sapiens GN=TRIM5		
Q9C037	TRIM4_HUMAN Tripartite motif-containing protein 4 OS=Homo sapiens GN=TRIM4 PE=2 SV=2		75.06
Q9C037-2	TRIM4_HUMAN Isoform Beta of Tripartite motif-containing protein 4 OS=Homo sapiens GN=TRIM4		55.14
Q9C037-3	TRIM4_HUMAN Isoform Gamma of Tripartite motif-containing protein 4 OS=Homo sapiens GN=TRIM4		48.63
Q9C075	K1C23_HUMAN Keratin, type I cytoskeletal 23 OS=Homo sapiens GN=KRT23 PE=1 SV=2		
Q9C091	GRB1L_HUMAN GREB1-like protein OS=Homo sapiens GN=GREB1L PE=2 SV=2		55.66
Q9C091-2	GRB1L_HUMAN Isoform 2 of GREB1-like protein OS=Homo sapiens GN=GREB1L		28.93
Q9C091-3	GRB1L_HUMAN Isoform 3 of GREB1-like protein OS=Homo sapiens GN=GREB1L		32.88
Q9C091-4	GRB1L_HUMAN Isoform 4 of GREB1-like protein OS=Homo sapiens GN=GREB1L		28.93
Q9C091-5	GRB1L_HUMAN Isoform 5 of GREB1-like protein OS=Homo sapiens GN=GREB1L		28.93
Q9C093	SPEF2_HUMAN Sperm flagellar protein 2 OS=Homo sapiens GN=SPEF2 PE=1 SV=2		
Q9C093-2	SPEF2_HUMAN Isoform 2 of Sperm flagellar protein 2 OS=Homo sapiens GN=SPEF2		
Q9C093-3	SPEF2_HUMAN Isoform 3 of Sperm flagellar protein 2 OS=Homo sapiens GN=SPEF2		
Q9C093;Q9C093-2;D6REZ	SPEF2_HUMAN Sperm flagellar protein 2 OS=Homo sapiens GN=SPEF2 PE=1 SV=2;>sp|Q9C093-2|SPEF2_HUMAN Isoform 2 of Sperm	4.48E-06	
Q9C098	DCLK3_HUMAN Serine/threonine-protein kinase DCLK3 OS=Homo sapiens GN=DCLK3 PE=1 SV=2		10.89
Q9C099	LRCC1_HUMAN Leucine-rich repeat and coiled-coil domain-containing protein 1 OS=Homo sapiens GN=LRRCC1 PE=2 SV=2		
Q9C099-2	LRCC1_HUMAN Isoform 2 of Leucine-rich repeat and coiled-coil domain-containing protein 1 OS=Homo sapiens GN=LRRCC1		
Q9C0A0	CNTP4_HUMAN Contactin-associated protein-like 4 OS=Homo sapiens GN=CNTNAP4 PE=1 SV=3		
Q9C0B1	FTO_HUMAN Alpha-ketoglutarate-dependent dioxygenase FTO OS=Homo sapiens GN=FTO PE=1 SV=3	4.13E-05	
Q9C0C2	TB182_HUMAN 182 kDa tankyrase-1-binding protein OS=Homo sapiens GN=TNKS1BP1 PE=1 SV=4		71.93
Q9C0C9	UBE2O_HUMAN Ubiquitin-conjugating enzyme E2 O OS=Homo sapiens GN=UBE2O PE=1 SV=3		
Q9C0D2	K1731_HUMAN Protein KIAA1731 OS=Homo sapiens GN=KIAA1731 PE=2 SV=4		
Q9C0D2-2	K1731_HUMAN Isoform 2 of Protein KIAA1731 OS=Homo sapiens GN=KIAA1731		
Q9C0D2-3	K1731_HUMAN Isoform 3 of Protein KIAA1731 OS=Homo sapiens GN=KIAA1731		
Q9C0D2-4	K1731_HUMAN Isoform 4 of Protein KIAA1731 OS=Homo sapiens GN=KIAA1731		
Q9C0D5	TANC1_HUMAN Protein TANC1 OS=Homo sapiens GN=TANC1 PE=1 SV=3		
Q9C0D5-2	TANC1_HUMAN Isoform 2 of Protein TANC1 OS=Homo sapiens GN=TANC1		
Q9C0D5;Q9C0D5-2	TANC1_HUMAN Protein TANC1 OS=Homo sapiens GN=TANC1 PE=1 SV=3;>sp|Q9C0D5-2|TANC1_HUMAN Isoform 2 of Protein TANC1	3.38E-06	
Q9C0D6	FHDC1_HUMAN FH2 domain-containing protein 1 OS=Homo sapiens GN=FHDC1 PE=2 SV=2		
Q9C0D9	EPT1_HUMAN Ethanolaminephosphotransferase 1 OS=Homo sapiens GN=EPT1 PE=1 SV=3	3.26E-08	
Q9C0E8	LNP_HUMAN Protein lunapark OS=Homo sapiens GN=LNP PE=1 SV=2		
Q9C0E8-2	LNP_HUMAN Isoform 2 of Protein lunapark OS=Homo sapiens GN=LNP		
Q9C0E8-3	LNP_HUMAN Isoform 3 of Protein lunapark OS=Homo sapiens GN=LNP		
Q9C0G0	ZN407_HUMAN Zinc finger protein 407 OS=Homo sapiens GN=ZNF407 PE=2 SV=2		
Q9C0G0-2	ZN407_HUMAN Isoform 2 of Zinc finger protein 407 OS=Homo sapiens GN=ZNF407		
Q9C0G0-3	ZN407_HUMAN Isoform 3 of Zinc finger protein 407 OS=Homo sapiens GN=ZNF407		
Q9C0G6	DYH6_HUMAN Dynein heavy chain 6, axonemal OS=Homo sapiens GN=DNAH6 PE=1 SV=3		
Q9C0G6-2	DYH6_HUMAN Isoform 2 of Dynein heavy chain 6, axonemal OS=Homo sapiens GN=DNAH6		
Q9C0G6-4	DYH6_HUMAN Isoform 4 of Dynein heavy chain 6, axonemal OS=Homo sapiens GN=DNAH6		
Q9C0G6;Q9C0G6-4	DYH6_HUMAN Dynein heavy chain 6, axonemal OS=Homo sapiens GN=DNAH6 PE=1 SV=3;>sp|Q9C0G6-4|DYH6_HUMAN Isoform 4 c	2.91E-10	
Q9C0I1;Q9C0I1-2;Q9C0I1	MTMRC_HUMAN Myotubularin-related protein 12 OS=Homo sapiens GN=MTMR12 PE=1 SV=2;>sp|Q9C0I1-2|MTMRC_HUMAN Isoform	2.50E-18	
Q9C0I9	LRC27_HUMAN Leucine-rich repeat-containing protein 27 OS=Homo sapiens GN=LRRC27 PE=2 SV=2		
Q9C0I9-2	LRC27_HUMAN Isoform 2 of Leucine-rich repeat-containing protein 27 OS=Homo sapiens GN=LRRC27		
Q9C0I9-3	LRC27_HUMAN Isoform 3 of Leucine-rich repeat-containing protein 27 OS=Homo sapiens GN=LRRC27		
Q9C0I9-4	LRC27_HUMAN Isoform 4 of Leucine-rich repeat-containing protein 27 OS=Homo sapiens GN=LRRC27		
Q9C0J8	WDR33_HUMAN WD repeat-containing protein 33 OS=Homo sapiens GN=WDR33 PE=1 SV=2		67.58
Q9C0J8-2	WDR33_HUMAN Isoform 2 of WD repeat-containing protein 33 OS=Homo sapiens GN=WDR33		2.52
Q9C0K3	ARP3C_HUMAN Actin-related protein 3C OS=Homo sapiens GN=ACTR3C PE=2 SV=1		61.16
Q9GIP4	LAT1L_HUMAN Putative L-type amino acid transporter 1-like protein IMAA OS=Homo sapiens GN=SLC7A5P2 PE=5 SV=2		
Q9GIP4-1	LAT1L_HUMAN Isoform 2 of Putative L-type amino acid transporter 1-like protein IMAA OS=Homo sapiens GN=SLC7A5P2		
Q9GIY3	2B1E_HUMAN HLA class II histocompatibility antigen, DRB1-14 beta chain OS=Homo sapiens GN=HLA-DRB1 PE=1 SV=1		0.15
Q9GZL7	WDR12_HUMAN Ribosome biogenesis protein WDR12 OS=Homo sapiens GN=WDR12 PE=1 SV=2		
Q9GZM3	RPB1B_HUMAN DNA-directed RNA polymerase II subunit RPB11-b1 OS=Homo sapiens GN=POLR2J2 PE=1 SV=1		30.42
Q9GZM3-2	RPB1B_HUMAN Isoform 2 of DNA-directed RNA polymerase II subunit RPB11-b1 OS=Homo sapiens GN=POLR2J2		3.43
Q9GZM5	YIPF3_HUMAN Protein YIPF3 OS=Homo sapiens GN=YIPF3 PE=1 SV=1		19.78
Q9GZM7	TINAL_HUMAN Tubulointerstitial nephritis antigen-like OS=Homo sapiens GN=TINAGL1 PE=1 SV=1	2.99E-88	
Q9GZM8	NDEL1_HUMAN Nuclear distribution protein nudE-like 1 OS=Homo sapiens GN=NDEL1 PE=1 SV=1		
Q9GZM8-2	NDEL1_HUMAN Isoform 2 of Nuclear distribution protein nudE-like 1 OS=Homo sapiens GN=NDEL1		
Q9GZN7	ROGDI_HUMAN Protein rogdi homolog OS=Homo sapiens GN=ROGDI PE=2 SV=1		
Q9GZN8-2;Q9GZN8	2|CT027_HUMAN Isoform 2 of UPF0687 protein C20orf27 OS=Homo sapiens GN=C20orf27;>sp|Q9GZN8|CT027_HUMAN UPF0687 pro	1.93E-18	
Q9GZP4	PITH1_HUMAN PITH domain-containing protein 1 OS=Homo sapiens GN=PITHD1 PE=1 SV=1		

Q9GZP4-2	PITH1_HUMAN Isoform 2 of PITH domain-containing protein 1 OS=Homo sapiens GN=PITHD1		
Q9GZP4;Q9GZP4-2	PITH1_HUMAN PITH domain-containing protein 1 OS=Homo sapiens GN=PITHD1 PE=1 SV=1;>sp|Q9GZP4-2|PITH1_HUMAN Isoform	8.28E-12	
Q9GZP4;Q9GZP4-2;B4DKI	CA128_HUMAN UPF0424 protein C1orf128 OS=Homo sapiens GN=C1orf128 PE=1 SV=1;>sp|Q9GZP4-2|CA128_HUMAN Isoform 2 of	3.11E-17	
Q9GZP8;Q9GZP8-2	IMUP_HUMAN Immortalization up-regulated protein OS=Homo sapiens GN=IMUP PE=1 SV=1;>sp|Q9GZP8-2|IMUP_HUMAN Isoform 2	1.31E-06	
Q9GZP9	DERL2_HUMAN Derlin-2 OS=Homo sapiens GN=DERL2 PE=1 SV=1		50.94
Q9GZQ8	MLP3B_HUMAN Microtubule-associated proteins 1A/1B light chain 3B OS=Homo sapiens GN=MAP1LC3B PE=1 SV=3		128.32
Q9GZR2	REXO4_HUMAN RNA exonuclease 4 OS=Homo sapiens GN=REXO4 PE=1 SV=2		25.88
Q9GZR7	DDX24_HUMAN ATP-dependent RNA helicase DDX24 OS=Homo sapiens GN=DDX24 PE=1 SV=1		123.41
Q9GZS1	RPA49_HUMAN DNA-directed RNA polymerase I subunit RPA49 OS=Homo sapiens GN=POLR1E PE=1 SV=2		23.99
Q9GZS1-2	RPA49_HUMAN Isoform 2 of DNA-directed RNA polymerase I subunit RPA49 OS=Homo sapiens GN=POLR1E		31.24
Q9GZS1;Q9GZS1-2	RPA49_HUMAN DNA-directed RNA polymerase I subunit RPA49 OS=Homo sapiens GN=POLR1E PE=1 SV=2;>sp|Q9GZS1-2|RPA49_HU	7.09E-15	
Q9GZS3	WDR61_HUMAN WD repeat-containing protein 61 OS=Homo sapiens GN=WDR61 PE=1 SV=1		74.11
Q9GZT3	SLIRP_HUMAN SRA stem-loop-interacting RNA-binding protein, mitochondrial OS=Homo sapiens GN=SLIRP PE=1 SV=1		363.44
Q9GZT8;Q9GZT8-2	NIF3L_HUMAN NIF3-like protein 1 OS=Homo sapiens GN=NIF3L1 PE=1 SV=2;>sp|Q9GZT8-2|NIF3L_HUMAN Isoform 2 of NIF3-like pr	2.81E-11	
Q9GZU2	PEG3_HUMAN Paternally-expressed gene 3 protein OS=Homo sapiens GN=PEG3 PE=2 SV=1		
Q9GZU2-2	PEG3_HUMAN Isoform 2 of Paternally-expressed gene 3 protein OS=Homo sapiens GN=PEG3		
Q9GZU2-3	PEG3_HUMAN Isoform 3 of Paternally-expressed gene 3 protein OS=Homo sapiens GN=PEG3		25.33
Q9GZU8	F192A_HUMAN Protein FAM192A OS=Homo sapiens GN=FAM192A PE=1 SV=1		114.59
Q9GZV4	IF5A2_HUMAN Eukaryotic translation initiation factor 5A-2 OS=Homo sapiens GN=EIF5A2 PE=1 SV=3		317.85
Q9GZX9	TWSG1_HUMAN Twisted gastrulation protein homolog 1 OS=Homo sapiens GN=TWSG1 PE=2 SV=1		47.59
Q9GZX9-2	TWSG1_HUMAN Isoform 2 of Twisted gastrulation protein homolog 1 OS=Homo sapiens GN=TWSG1		47.59
Q9GZY4	CG044_HUMAN Uncharacterized protein C7orf44 OS=Homo sapiens GN=C7orf44 PE=1 SV=1		
Q9GZZ1	NAA50_HUMAN N-alpha-acetyltransferase 50, NatE catalytic subunit OS=Homo sapiens GN=NAA50 PE=1 SV=1		153.55
Q9GZZ1-2	NAA50_HUMAN Isoform 2 of N-alpha-acetyltransferase 50, NatE catalytic subunit OS=Homo sapiens GN=NAA50		134.6
Q9GZZ1;Q9GZZ1-2	NAA50_HUMAN N-alpha-acetyltransferase 50, NatE catalytic subunit OS=Homo sapiens GN=NAA50 PE=1 SV=1;>sp|Q9GZZ1-2|NAA5	2.28E-79	
Q9GZZ9;Q9GZZ9-2	UBA5_HUMAN Ubiquitin-like modifier-activating enzyme 5 OS=Homo sapiens GN=UBA5 PE=1 SV=1;>sp|Q9GZZ9-2|UBA5_HUMAN Iso	4.44E-41	
Q9H008;Q9H008-2	LHPP_HUMAN Phospholysine phosphohistidine inorganic pyrophosphate phosphatase OS=Homo sapiens GN=LHPP PE=1 SV=2;>sp|Q	8.78E-16	
Q9H061	T126A_HUMAN Transmembrane protein 126A OS=Homo sapiens GN=TMEM126A PE=1 SV=1		
Q9H074	PAIP1_HUMAN Polyadenylate-binding protein-interacting protein 1 OS=Homo sapiens GN=PAIP1 PE=1 SV=1	8.42E-33	
Q9H074-2;A6NKV8;D6REE	2|PAIP1_HUMAN Isoform 2 of Polyadenylate-binding protein-interacting protein 1 OS=Homo sapiens GN=PAIP1;>tr|A6NKV8|A6NKV8_	6.29E-34	
Q9H074;Q9H074-2	PAIP1_HUMAN Polyadenylate-binding protein-interacting protein 1 OS=Homo sapiens GN=PAIP1 PE=1 SV=1;>sp|Q9H074-2|PAIP1_HU	1.02E-23	
Q9H078	CLPB_HUMAN Caseinolytic peptidase B protein homolog OS=Homo sapiens GN=CLPB PE=1 SV=1		33.73
Q9H078-2	CLPB_HUMAN Isoform 2 of Caseinolytic peptidase B protein homolog OS=Homo sapiens GN=CLPB		33.73
Q9H078;Q9H078-2	CLPB_HUMAN Caseinolytic peptidase B protein homolog OS=Homo sapiens GN=CLPB PE=1 SV=1;>sp|Q9H078-2|CLPB_HUMAN Isofor	6.93E-18	
Q9H082	RB33B_HUMAN Ras-related protein Rab-33B OS=Homo sapiens GN=RAB33B PE=1 SV=1		65.77
Q9H089	LSG1_HUMAN Large subunit GTPase 1 homolog OS=Homo sapiens GN=LSG1 PE=1 SV=2		21.57
Q9H098-2	F107B_HUMAN Isoform 2 of Protein FAM107B OS=Homo sapiens GN=FAM107B		20.32
Q9H098-2;Q9H098	2|F107B_HUMAN Isoform 2 of Protein FAM107B OS=Homo sapiens GN=FAM107B;>sp|Q9H098|F107B_HUMAN Protein FAM107B OS=I	1.51E-79	
Q9H0A0	NAT10_HUMAN N-acetyltransferase 10 OS=Homo sapiens GN=NAT10 PE=1 SV=2		333.63
Q9H0A8	COMD4_HUMAN COMM domain-containing protein 4 OS=Homo sapiens GN=COMMD4 PE=1 SV=1		168.53
Q9H0A8-2	COMD4_HUMAN Isoform 2 of COMM domain-containing protein 4 OS=Homo sapiens GN=COMMD4		166.45
Q9H0A8;Q9H0A8-2	COMD4_HUMAN COMM domain-containing protein 4 OS=Homo sapiens GN=COMMD4 PE=1 SV=1;>sp|Q9H0A8-2|COMD4_HUMAN Iso	2.29E-15	
Q9H0B6	KLC2_HUMAN Kinesin light chain 2 OS=Homo sapiens GN=KLC2 PE=1 SV=1	1.90E-57	
Q9H0C2	ADT4_HUMAN ADP/ATP translocase 4 OS=Homo sapiens GN=SLC25A31 PE=1 SV=1		195.76
Q9H0C8	ILKAP_HUMAN Integrin-linked kinase-associated serine/threonine phosphatase 2C OS=Homo sapiens GN=ILKAP PE=1 SV=1		83.45
Q9H0D6	XRN2_HUMAN 5'-3' exoribonuclease 2 OS=Homo sapiens GN=XRN2 PE=1 SV=1		249.4
Q9H0D6-2	XRN2_HUMAN Isoform 2 of 5'-3' exoribonuclease 2 OS=Homo sapiens GN=XRN2		161.24
Q9H0D6;Q9H0D6-2	XRN2_HUMAN 5-3 exoribonuclease 2 OS=Homo sapiens GN=XRN2 PE=1 SV=1;>sp|Q9H0D6-2|XRN2_HUMAN Isoform 2 of 5-3 exorib	4.17E-73	
Q9H0E2	TOLIP_HUMAN Toll-interacting protein OS=Homo sapiens GN=TOLLIP PE=1 SV=1		25.62
Q9H0E7	UBP44_HUMAN Ubiquitin carboxyl-terminal hydrolase 44 OS=Homo sapiens GN=USP44 PE=1 SV=2		
Q9H0F6	SHRPN_HUMAN Sharpin OS=Homo sapiens GN=SHARPIN PE=1 SV=1		
Q9H0F6-2	SHRPN_HUMAN Isoform 2 of Sharpin OS=Homo sapiens GN=SHARPIN		
Q9H0G5	NSRP1_HUMAN Nuclear speckle splicing regulatory protein 1 OS=Homo sapiens GN=NSRP1 PE=1 SV=1	1.96E-08	
Q9H0H0	INT2_HUMAN Integrator complex subunit 2 OS=Homo sapiens GN=INTS2 PE=1 SV=2		
Q9H0H5	RGAP1_HUMAN Rac GTPase-activating protein 1 OS=Homo sapiens GN=RACGAP1 PE=1 SV=1		
Q9H0I3	CC113_HUMAN Coiled-coil domain-containing protein 113 OS=Homo sapiens GN=CCDC113 PE=2 SV=1		
Q9H0L4	CSTFT_HUMAN Cleavage stimulation factor subunit 2 tau variant OS=Homo sapiens GN=CSTF2T PE=1 SV=1		90.11
Q9H0N0	RAB6C_HUMAN Ras-related protein Rab-6C OS=Homo sapiens GN=RAB6C PE=2 SV=2		91.09
Q9H0N5	PHS2_HUMAN Pterin-4-alpha-carbinolamine dehydratase 2 OS=Homo sapiens GN=PCBD2 PE=1 SV=4	1.52E-09	
Q9H0P0-1	1|5NT3_HUMAN Isoform 1 of Cytosolic 5-nucleotidase 3 OS=Homo sapiens GN=NT5C3	1.97E-17	
Q9H0P0;Q9H0P0-1;Q9H0I	5NT3_HUMAN Cytosolic 5-nucleotidase 3 OS=Homo sapiens GN=NT5C3 PE=1 SV=3;>sp|Q9H0P0-1|5NT3_HUMAN Isoform 1 of Cytos	3.89E-29	

Q9H0Q0	FA49A_HUMAN Protein FAM49A OS=Homo sapiens GN=FAM49A PE=1 SV=1		
Q9H0R4;Q9H0R4-2	HDHD2_HUMAN Haloacid dehalogenase-like hydrolase domain-containing protein 2 OS=Homo sapiens GN=HDHD2 PE=1 SV=1;>sp|Q	1.05E-14	
Q9H0S4	DDX47_HUMAN Probable ATP-dependent RNA helicase DDX47 OS=Homo sapiens GN=DDX47 PE=1 SV=1		75.14
Q9H0U3	MAGT1_HUMAN Magnesium transporter protein 1 OS=Homo sapiens GN=MAGT1 PE=1 SV=1		104.95
Q9H0U4	RAB1B_HUMAN Ras-related protein Rab-1B OS=Homo sapiens GN=RAB1B PE=1 SV=1		1125.06
Q9H0U6	RM18_HUMAN 39S ribosomal protein L18, mitochondrial OS=Homo sapiens GN=MRPL18 PE=1 SV=1		85.19
Q9H0U9	TSYL1_HUMAN Testis-specific Y-encoded-like protein 1 OS=Homo sapiens GN=TSPYL1 PE=1 SV=3		
Q9H0V9-2;Q9H0V9	2|LMA2L_HUMAN Isoform 2 of VIP36-like protein OS=Homo sapiens GN=LMAN2L;>sp|Q9H0V9|LMA2L_HUMAN VIP36-like protein OS=	2.82E-09	
Q9H0W5	CCDC8_HUMAN Coiled-coil domain-containing protein 8 OS=Homo sapiens GN=CCDC8 PE=1 SV=2		
Q9H0W8;Q9H0W8-2	SMG9_HUMAN Protein SMG9 OS=Homo sapiens GN=SMG9 PE=1 SV=1;>sp|Q9H0W8-2|SMG9_HUMAN Isoform 2 of Protein SMG9 OS	3.26E-06	
Q9H0W9;Q9H0W9-2;Q9H(	CK054_HUMAN Ester hydrolase C11orf54 OS=Homo sapiens GN=C11orf54 PE=1 SV=1;>sp|Q9H0W9-2|CK054_HUMAN Isoform 2 of E	7.84E-15	
Q9H106	SIRPD_HUMAN Signal-regulatory protein delta OS=Homo sapiens GN=SIRPD PE=2 SV=2		
Q9H115	SNAB_HUMAN Beta-soluble NSF attachment protein OS=Homo sapiens GN=NAPB PE=1 SV=2		
Q9H147	TDIF1_HUMAN Deoxynucleotidyltransferase terminal-interacting protein 1 OS=Homo sapiens GN=DNTTIP1 PE=1 SV=2		63.01
Q9H159	CAD19_HUMAN Cadherin-19 OS=Homo sapiens GN=CDH19 PE=2 SV=1	1.78E-09	
Q9H169-2;Q9H169	2|STMN4_HUMAN Isoform 2 of Stathmin-4 OS=Homo sapiens GN=STMN4;>sp|Q9H169|STMN4_HUMAN Stathmin-4 OS=Homo sapien	2.87E-11	
Q9H171	ZBP1_HUMAN Z-DNA-binding protein 1 OS=Homo sapiens GN=ZBP1 PE=1 SV=2		
Q9H171-2	ZBP1_HUMAN Isoform 2 of Z-DNA-binding protein 1 OS=Homo sapiens GN=ZBP1		
Q9H171-3	ZBP1_HUMAN Isoform 3 of Z-DNA-binding protein 1 OS=Homo sapiens GN=ZBP1		
Q9H171-4	ZBP1_HUMAN Isoform 4 of Z-DNA-binding protein 1 OS=Homo sapiens GN=ZBP1		
Q9H171-5	ZBP1_HUMAN Isoform 5 of Z-DNA-binding protein 1 OS=Homo sapiens GN=ZBP1		
Q9H173	SIL1_HUMAN Nucleotide exchange factor SIL1 OS=Homo sapiens GN=SIL1 PE=1 SV=1		57.29
Q9H1A4	APC1_HUMAN Anaphase-promoting complex subunit 1 OS=Homo sapiens GN=ANAPC1 PE=1 SV=1		
Q9H1A7	RPB1C_HUMAN DNA-directed RNA polymerase II subunit RPB11-b2 OS=Homo sapiens GN=POLR2J3 PE=3 SV=1		30.42
Q9H1C4	UN93B_HUMAN Protein unc-93 homolog B1 OS=Homo sapiens GN=UNC93B1 PE=1 SV=2		56.38
Q9H1C7	CE032_HUMAN UPF0467 protein C5orf32 OS=Homo sapiens GN=C5orf32 PE=2 SV=1		58.75
Q9H1E3	NUCKS_HUMAN Nuclear ubiquitous casein and cyclin-dependent kinases substrate OS=Homo sapiens GN=NUCKS1 PE=1 SV=1		
Q9H1E3-2	NUCKS_HUMAN Isoform 2 of Nuclear ubiquitous casein and cyclin-dependent kinases substrate OS=Homo sapiens GN=NUCKS1		
Q9H1E3;Q9H1E3-2	NUCKS_HUMAN Nuclear ubiquitous casein and cyclin-dependent kinases substrate OS=Homo sapiens GN=NUCKS1 PE=1 SV=1;>sp|Q	1.13E-214	
Q9H1E5	TMX4_HUMAN Thioredoxin-related transmembrane protein 4 OS=Homo sapiens GN=TMX4 PE=1 SV=1		
Q9H1H9;Q9H1H9-2;Q9H1	KI13A_HUMAN Kinesin-like protein KIF13A OS=Homo sapiens GN=KIF13A PE=1 SV=2;>sp|Q9H1H9-2|KI13A_HUMAN Isoform 2 of Kir	1.60E-08	
Q9H1J5	WNT8A_HUMAN Protein Wnt-8a OS=Homo sapiens GN=WNT8A PE=2 SV=2		
Q9H1J5-2	WNT8A_HUMAN Isoform 2 of Protein Wnt-8a OS=Homo sapiens GN=WNT8A		
Q9H1K1	ISCU_HUMAN Iron-sulfur cluster assembly enzyme ISCU, mitochondrial OS=Homo sapiens GN=ISCU PE=1 SV=2		37.04
Q9H1K1-2	ISCU_HUMAN Isoform 2 of Iron-sulfur cluster assembly enzyme ISCU, mitochondrial OS=Homo sapiens GN=ISCU		37.04
Q9H1K1;Q9H1K1-2	ISCU_HUMAN Iron-sulfur cluster assembly enzyme ISCU, mitochondrial OS=Homo sapiens GN=ISCU PE=1 SV=2;>sp|Q9H1K1-2|ISCU	3.87E-06	
Q9H1K4	GHC2_HUMAN Mitochondrial glutamate carrier 2 OS=Homo sapiens GN=SLC25A18 PE=2 SV=1		48.58
Q9H1K6	MESD1_HUMAN Mesoderm development candidate 1 OS=Homo sapiens GN=MESDC1 PE=1 SV=1		
Q9H1R2	DUS15_HUMAN Dual specificity protein phosphatase 15 OS=Homo sapiens GN=DUSP15 PE=1 SV=4		0.68
Q9H1R2-2	DUS15_HUMAN Isoform 2 of Dual specificity protein phosphatase 15 OS=Homo sapiens GN=DUSP15		20.56
Q9H1R2-3	DUS15_HUMAN Isoform 3 of Dual specificity protein phosphatase 15 OS=Homo sapiens GN=DUSP15		20.56
Q9H1R3	MYLK2_HUMAN Myosin light chain kinase 2, skeletal/cardiac muscle OS=Homo sapiens GN=MYLK2 PE=1 SV=3		41.33
Q9H1X3-3	DJC25_HUMAN Isoform 3 of DnaJ homolog subfamily C member 25 OS=Homo sapiens GN=DNAJC25		
Q9H1Y0	ATG5_HUMAN Autophagy protein 5 OS=Homo sapiens GN=ATG5 PE=1 SV=2		39.21
Q9H1Z8	AUGN_HUMAN Augurin OS=Homo sapiens GN=C2orf40 PE=1 SV=1		
Q9H201	EPN3_HUMAN Epsin-3 OS=Homo sapiens GN=EPN3 PE=1 SV=1		58.77
Q9H201-2	EPN3_HUMAN Isoform 2 of Epsin-3 OS=Homo sapiens GN=EPN3		58.77
Q9H201;Q9H201-2	EPN3_HUMAN Epsin-3 OS=Homo sapiens GN=EPN3 PE=1 SV=1;>sp|Q9H201-2|EPN3_HUMAN Isoform 2 of Epsin-3 OS=Homo sapiens	8.79E-10	
Q9H223	EHD4_HUMAN EH domain-containing protein 4 OS=Homo sapiens GN=EHD4 PE=1 SV=1		359.86
Q9H227	GBA3_HUMAN Cytosolic beta-glucosidase OS=Homo sapiens GN=GBA3 PE=1 SV=2		
Q9H227-2	GBA3_HUMAN Isoform 2 of Cytosolic beta-glucosidase OS=Homo sapiens GN=GBA3		
Q9H254	SPTN4_HUMAN Spectrin beta chain, brain 3 OS=Homo sapiens GN=SPTBN4 PE=1 SV=2		208.4
Q9H254-2	SPTN4_HUMAN Isoform 2 of Spectrin beta chain, brain 3 OS=Homo sapiens GN=SPTBN4		158.03
Q9H254-3	SPTN4_HUMAN Isoform 3 of Spectrin beta chain, brain 3 OS=Homo sapiens GN=SPTBN4		50.37
Q9H254-4	SPTN4_HUMAN Isoform 4 of Spectrin beta chain, brain 3 OS=Homo sapiens GN=SPTBN4		193.48
Q9H254;Q9H254-4;Q9H2!	SPTN4_HUMAN Spectrin beta chain, brain 3 OS=Homo sapiens GN=SPTBN4 PE=1 SV=2;>sp|Q9H254-4|SPTN4_HUMAN Isoform 4 of !	6.42E-22	
Q9H267	VP33B_HUMAN Vacuolar protein sorting-associated protein 33B OS=Homo sapiens GN=VPS33B PE=1 SV=2	1.09E-159	
Q9H299	SH3L3_HUMAN SH3 domain-binding glutamic acid-rich-like protein 3 OS=Homo sapiens GN=SH3BGRL3 PE=1 SV=1		106.13
Q9H2D6	TARA_HUMAN TRIO and F-actin-binding protein OS=Homo sapiens GN=TRIOBP PE=1 SV=3		91.62
Q9H2D6-2	TARA_HUMAN Isoform 3 of TRIO and F-actin-binding protein OS=Homo sapiens GN=TRIOBP		91.62
Q9H2D6-3	TARA_HUMAN Isoform 4 of TRIO and F-actin-binding protein OS=Homo sapiens GN=TRIOBP		91.62

Q9H2D6-4	TARA_HUMAN Isoform 5 of TRIO and F-actin-binding protein OS=Homo sapiens GN=TRIOBP		0.37
Q9H2D6-5	TARA_HUMAN Isoform 1 of TRIO and F-actin-binding protein OS=Homo sapiens GN=TRIOBP		91.25
Q9H2D6;Q9H2D6-2;Q9H2	TARA_HUMAN TRIO and F-actin-binding protein OS=Homo sapiens GN=TRIOBP PE=1 SV=3;>sp|Q9H2D6-2|TARA_HUMAN Isoform 3 c	1.72E-15	
Q9H2E6	SEM6A_HUMAN Semaphorin-6A OS=Homo sapiens GN=SEMA6A PE=1 SV=2		
Q9H2E6-2	SEM6A_HUMAN Isoform 2 of Semaphorin-6A OS=Homo sapiens GN=SEMA6A		
Q9H2G2	SLK_HUMAN STE20-like serine/threonine-protein kinase OS=Homo sapiens GN=SLK PE=1 SV=1	6.28E-187	
Q9H2G2-2	2|SLK_HUMAN Isoform 2 of STE20-like serine/threonine-protein kinase OS=Homo sapiens GN=SLK	2.79E-191	
Q9H2H8	PPIL3_HUMAN Peptidyl-prolyl cis-trans isomerase-like 3 OS=Homo sapiens GN=PPIL3 PE=1 SV=1		132.1
Q9H2H8-2	PPIL3_HUMAN Isoform 2 of Peptidyl-prolyl cis-trans isomerase-like 3 OS=Homo sapiens GN=PPIL3		62.27
Q9H2H8-2;Q9H2H8	2|PPIL3_HUMAN Isoform 2 of Peptidyl-prolyl cis-trans isomerase-like 3 OS=Homo sapiens GN=PPIL3;>sp|Q9H2H8|PPIL3_HUMAN Pep	9.25E-10	
Q9H2H9	S38A1_HUMAN Sodium-coupled neutral amino acid transporter 1 OS=Homo sapiens GN=SLC38A1 PE=1 SV=1		
Q9H2J4	PDCL3_HUMAN Phosducin-like protein 3 OS=Homo sapiens GN=PDCL3 PE=1 SV=1		37.94
Q9H2K0	IF3M_HUMAN Translation initiation factor IF-3, mitochondrial OS=Homo sapiens GN=MTIF3 PE=1 SV=4	1.70E-49	
Q9H2K8	TAOK3_HUMAN Serine/threonine-protein kinase TAO3 OS=Homo sapiens GN=TAOK3 PE=1 SV=2	5.24E-20	
Q9H2M9	RBGPR_HUMAN Rab3 GTPase-activating protein non-catalytic subunit OS=Homo sapiens GN=RAB3GAP2 PE=1 SV=1		
Q9H2M9-2	RBGPR_HUMAN Isoform 2 of Rab3 GTPase-activating protein non-catalytic subunit OS=Homo sapiens GN=RAB3GAP2		
Q9H2P0	ADNP_HUMAN Activity-dependent neuroprotector homeobox protein OS=Homo sapiens GN=ADNP PE=1 SV=1		
Q9H2U1	DHX36_HUMAN Probable ATP-dependent RNA helicase DHX36 OS=Homo sapiens GN=DHX36 PE=1 SV=2		65
Q9H2U1-2	DHX36_HUMAN Isoform 2 of Probable ATP-dependent RNA helicase DHX36 OS=Homo sapiens GN=DHX36		65
Q9H2U1-3	DHX36_HUMAN Isoform 3 of Probable ATP-dependent RNA helicase DHX36 OS=Homo sapiens GN=DHX36		65
Q9H2U1;Q9H2U1-2;Q9H2	DHX36_HUMAN Probable ATP-dependent RNA helicase DHX36 OS=Homo sapiens GN=DHX36 PE=1 SV=2;>sp|Q9H2U1-2|DHX36_HUM	5.92E-12	
Q9H2U2	IPYR2_HUMAN Inorganic pyrophosphatase 2, mitochondrial OS=Homo sapiens GN=PPA2 PE=1 SV=2		152.12
Q9H2U2-2	IPYR2_HUMAN Isoform 2 of Inorganic pyrophosphatase 2, mitochondrial OS=Homo sapiens GN=PPA2		152.12
Q9H2U2-2;Q9H2U2;E2QRI	2|IPYR2_HUMAN Isoform 2 of Inorganic pyrophosphatase 2, mitochondrial OS=Homo sapiens GN=PPA2;>sp|Q9H2U2|IPYR2_HUMAN I	1.74E-122	
Q9H2U2-3	IPYR2_HUMAN Isoform 3 of Inorganic pyrophosphatase 2, mitochondrial OS=Homo sapiens GN=PPA2		145.06
Q9H2U2-4	IPYR2_HUMAN Isoform 4 of Inorganic pyrophosphatase 2, mitochondrial OS=Homo sapiens GN=PPA2		64.81
Q9H2W6	RM46_HUMAN 39S ribosomal protein L46, mitochondrial OS=Homo sapiens GN=MRPL46 PE=1 SV=1		117.78
Q9H2X6	HIPK2_HUMAN Homeodomain-interacting protein kinase 2 OS=Homo sapiens GN=HIPK2 PE=1 SV=2		
Q9H2X6-2	HIPK2_HUMAN Isoform 2 of Homeodomain-interacting protein kinase 2 OS=Homo sapiens GN=HIPK2		
Q9H2X6-3	HIPK2_HUMAN Isoform 3 of Homeodomain-interacting protein kinase 2 OS=Homo sapiens GN=HIPK2		
Q9H307	PININ_HUMAN Pinin OS=Homo sapiens GN=PNN PE=1 SV=4		
Q9H307-2	PININ_HUMAN Isoform 2 of Pinin OS=Homo sapiens GN=PNN		
Q9H307;Q9H307-2	PININ_HUMAN Pinin OS=Homo sapiens GN=PNN PE=1 SV=4;>sp|Q9H307-2|PININ_HUMAN Isoform 2 of Pinin OS=Homo sapiens GN=	9.95E-95	
Q9H329	E41LB_HUMAN Band 4.1-like protein 4B OS=Homo sapiens GN=EPB41L4B PE=2 SV=2		
Q9H329-2	E41LB_HUMAN Isoform 2 of Band 4.1-like protein 4B OS=Homo sapiens GN=EPB41L4B		
Q9H330	CI005_HUMAN Transmembrane protein C9orf5 OS=Homo sapiens GN=C9orf5 PE=1 SV=2		143.03
Q9H330-2	CI005_HUMAN Isoform 2 of Transmembrane protein C9orf5 OS=Homo sapiens GN=C9orf5		131.23
Q9H330-3	CI005_HUMAN Isoform 3 of Transmembrane protein C9orf5 OS=Homo sapiens GN=C9orf5		5.63
Q9H330-4	CI005_HUMAN Isoform 4 of Transmembrane protein C9orf5 OS=Homo sapiens GN=C9orf5		114.93
Q9H330;Q9H330-2;Q9H3	CI005_HUMAN Transmembrane protein C9orf5 OS=Homo sapiens GN=C9orf5 PE=1 SV=2;>sp|Q9H330-2|CI005_HUMAN Isoform 2 of	3.77E-12	
Q9H334	FOXP1_HUMAN Forkhead box protein P1 OS=Homo sapiens GN=FOXP1 PE=1 SV=1		
Q9H334-2	FOXP1_HUMAN Isoform 2 of Forkhead box protein P1 OS=Homo sapiens GN=FOXP1		
Q9H334-3	FOXP1_HUMAN Isoform 3 of Forkhead box protein P1 OS=Homo sapiens GN=FOXP1		
Q9H334-4	FOXP1_HUMAN Isoform 4 of Forkhead box protein P1 OS=Homo sapiens GN=FOXP1		
Q9H361	PABP3_HUMAN Polyadenylate-binding protein 3 OS=Homo sapiens GN=PABPC3 PE=1 SV=2		930.66
Q9H3H3	CK068_HUMAN UPF0696 protein C11orf68 OS=Homo sapiens GN=C11orf68 PE=1 SV=1	2.26E-11	
Q9H3H3-3;Q9H3H3-2;Q9H	3|CK068_HUMAN Isoform 3 of UPF0696 protein C11orf68 OS=Homo sapiens GN=C11orf68;>sp|Q9H3H3-2|CK068_HUMAN Isoform 2	7.75E-30	
Q9H3K2	GHITM_HUMAN Growth hormone-inducible transmembrane protein OS=Homo sapiens GN=GHITM PE=1 SV=2		105.38
Q9H3K6	BOLA2_HUMAN BolA-like protein 2 OS=Homo sapiens GN=BOLA2 PE=1 SV=1		64.04
Q9H3K6-2	BOLA2_HUMAN Isoform 2 of BolA-like protein 2 OS=Homo sapiens GN=BOLA2		52.29
Q9H3K6;Q9H3K6-2	BOLA2_HUMAN BolA-like protein 2 OS=Homo sapiens GN=BOLA2 PE=1 SV=1;>sp|Q9H3K6-2|BOLA2_HUMAN Isoform 2 of BolA-like p	1.04E-217	
Q9H3M7	TXNIP_HUMAN Thioredoxin-interacting protein OS=Homo sapiens GN=TXNIP PE=1 SV=1		59.01
Q9H3N1	TMX1_HUMAN Thioredoxin-related transmembrane protein 1 OS=Homo sapiens GN=TMX1 PE=1 SV=1		411.73
Q9H3N8	HRH4_HUMAN Histamine H4 receptor OS=Homo sapiens GN=HRH4 PE=1 SV=2		22.53
Q9H3P2	NELFA_HUMAN Negative elongation factor A OS=Homo sapiens GN=WHSC2 PE=1 SV=3		30.39
Q9H3P7	GCP60_HUMAN Golgi resident protein GCP60 OS=Homo sapiens GN=ACBD3 PE=1 SV=4		
Q9H3Q1	BORG4_HUMAN Cdc42 effector protein 4 OS=Homo sapiens GN=CDC42EP4 PE=1 SV=1	2.84E-06	
Q9H3S4	TPK1_HUMAN Thiamin pyrophosphokinase 1 OS=Homo sapiens GN=TPK1 PE=1 SV=1		
Q9H3S7	PTN23_HUMAN Tyrosine-protein phosphatase non-receptor type 23 OS=Homo sapiens GN=PTPN23 PE=1 SV=1		36.97
Q9H3U1	UN45A_HUMAN Protein unc-45 homolog A OS=Homo sapiens GN=UNC45A PE=1 SV=1		
Q9H3U1-2	UN45A_HUMAN Isoform 2 of Protein unc-45 homolog A OS=Homo sapiens GN=UNC45A		

Q9H3U1-3	UN45A_HUMAN Isoform 3 of Protein unc-45 homolog A OS=Homo sapiens GN=UNC45A		
Q9H3U1;A8K6F7;Q9H3U1·	UN45A_HUMAN Protein unc-45 homolog A OS=Homo sapiens GN=UNC45A PE=1 SV=1;>tr|A8K6F7|A8K6F7_HUMAN Unc-45 homolog	8.25E-26	
Q9H3Y0	CRSPL_HUMAN Peptidase inhibitor R3HDML OS=Homo sapiens GN=R3HDML PE=1 SV=1		
Q9H3Z4	DNJC5_HUMAN DnaJ homolog subfamily C member 5 OS=Homo sapiens GN=DNAJC5 PE=1 SV=1		29.78
Q9H3Z4-2	DNJC5_HUMAN Isoform 2 of DnaJ homolog subfamily C member 5 OS=Homo sapiens GN=DNAJC5		29.78
Q9H3Z4;Q9H3Z4-2	DNJC5_HUMAN DnaJ homolog subfamily C member 5 OS=Homo sapiens GN=DNAJC5 PE=1 SV=1;>sp|Q9H3Z4-2|DNJC5_HUMAN Isof	1.47E-06	
Q9H444	CHM4B_HUMAN Charged multivesicular body protein 4b OS=Homo sapiens GN=CHMP4B PE=1 SV=1		35.46
Q9H446	RWDD1_HUMAN RWD domain-containing protein 1 OS=Homo sapiens GN=RWDD1 PE=1 SV=1	3.93E-10	
Q9H467	CUED2_HUMAN CUE domain-containing protein 2 OS=Homo sapiens GN=CUEDC2 PE=1 SV=1		28.14
Q9H477	RBSK_HUMAN Ribokinase OS=Homo sapiens GN=RBKS PE=1 SV=1		
Q9H479	FN3K_HUMAN Fructosamine-3-kinase OS=Homo sapiens GN=FN3K PE=1 SV=1		
Q9H488	OFUT1_HUMAN GDP-fucose protein O-fucosyltransferase 1 OS=Homo sapiens GN=POFUT1 PE=1 SV=1		121.22
Q9H488-2	OFUT1_HUMAN Isoform 2 of GDP-fucose protein O-fucosyltransferase 1 OS=Homo sapiens GN=POFUT1		117.76
Q9H488;Q9H488-2	OFUT1_HUMAN GDP-fucose protein O-fucosyltransferase 1 OS=Homo sapiens GN=POFUT1 PE=1 SV=1;>sp|Q9H488-2|OFUT1_HUMAN	2.98E-21	
Q9H492	MLP3A_HUMAN Microtubule-associated proteins 1A/1B light chain 3A OS=Homo sapiens GN=MAP1LC3A PE=1 SV=2		45.87
Q9H492-2	MLP3A_HUMAN Isoform 2 of Microtubule-associated proteins 1A/1B light chain 3A OS=Homo sapiens GN=MAP1LC3A		44.61
Q9H4A3-6;Q9H4A3-5;D3D	6|WNK1_HUMAN Isoform 5 of Serine/threonine-protein kinase WNK1 OS=Homo sapiens GN=WNK1;>sp|Q9H4A3-5|WNK1_HUMAN Is	2.38E-07	
Q9H4A4	AMPB_HUMAN Aminopeptidase B OS=Homo sapiens GN=RNPEP PE=1 SV=2	6.74E-103	
Q9H4A5	GLP3L_HUMAN Golgi phosphoprotein 3-like OS=Homo sapiens GN=GOLPH3L PE=1 SV=1		83.44
Q9H4A6	GOLP3_HUMAN Golgi phosphoprotein 3 OS=Homo sapiens GN=GOLPH3 PE=1 SV=1		197.25
Q9H4B7	TBB1_HUMAN Tubulin beta-1 chain OS=Homo sapiens GN=TUBB1 PE=1 SV=1		320.46
Q9H4E5	RHOJ_HUMAN Rho-related GTP-binding protein RhoJ OS=Homo sapiens GN=RHOJ PE=1 SV=1		55.7
Q9H4E5-2	RHOJ_HUMAN Isoform 2 of Rho-related GTP-binding protein RhoJ OS=Homo sapiens GN=RHOJ		55.7
Q9H4E7	DEFI6_HUMAN Differentially expressed in FDCP 6 homolog OS=Homo sapiens GN=DEF6 PE=1 SV=1	9.80E-54	
Q9H4G0	E41L1_HUMAN Band 4.1-like protein 1 OS=Homo sapiens GN=EPB41L1 PE=1 SV=2		3.07
Q9H4G0-2	E41L1_HUMAN Isoform 2 of Band 4.1-like protein 1 OS=Homo sapiens GN=EPB41L1		3.07
Q9H4G0-2;Q9H4G0-3;Q9I	2|E41L1_HUMAN Isoform 2 of Band 4.1-like protein 1 OS=Homo sapiens GN=EPB41L1;>sp|Q9H4G0-3|E41L1_HUMAN Isoform 3 of Ba	9.85E-102	
Q9H4G0-3	E41L1_HUMAN Isoform 3 of Band 4.1-like protein 1 OS=Homo sapiens GN=EPB41L1		3.07
Q9H4G0-4	E41L1_HUMAN Isoform 4 of Band 4.1-like protein 1 OS=Homo sapiens GN=EPB41L1		3.07
Q9H4G4	GAPR1_HUMAN Golgi-associated plant pathogenesis-related protein 1 OS=Homo sapiens GN=GLIPR2 PE=1 SV=3		61.6
Q9H4I0	RD21L_HUMAN Double-strand-break repair protein rad21-like protein 1 OS=Homo sapiens GN=RAD21L1 PE=2 SV=3		
Q9H4I0-2	RD21L_HUMAN Isoform 2 of Double-strand-break repair protein rad21-like protein 1 OS=Homo sapiens GN=RAD21L1		
Q9H4I2	ZHX3_HUMAN Zinc fingers and homeoboxes protein 3 OS=Homo sapiens GN=ZHX3 PE=1 SV=3		
Q9H4I8-3	SEHL2_HUMAN Isoform 3 of Serine hydrolase-like protein 2 OS=Homo sapiens GN=SERHL2		
Q9H4L4	SENP3_HUMAN Sentrin-specific protease 3 OS=Homo sapiens GN=SENP3 PE=1 SV=2		
Q9H4L5	OSBL3_HUMAN Oxysterol-binding protein-related protein 3 OS=Homo sapiens GN=OSBPL3 PE=1 SV=1		28.37
Q9H4L5-2	OSBL3_HUMAN Isoform 1b of Oxysterol-binding protein-related protein 3 OS=Homo sapiens GN=OSBPL3		28.37
Q9H4L5-3	OSBL3_HUMAN Isoform 1c of Oxysterol-binding protein-related protein 3 OS=Homo sapiens GN=OSBPL3		28.37
Q9H4L5-4	OSBL3_HUMAN Isoform 1d of Oxysterol-binding protein-related protein 3 OS=Homo sapiens GN=OSBPL3		28.37
Q9H4L5-5	OSBL3_HUMAN Isoform 2a of Oxysterol-binding protein-related protein 3 OS=Homo sapiens GN=OSBPL3		
Q9H4L5-6	OSBL3_HUMAN Isoform 2b of Oxysterol-binding protein-related protein 3 OS=Homo sapiens GN=OSBPL3		
Q9H4L5-7	OSBL3_HUMAN Isoform 2c of Oxysterol-binding protein-related protein 3 OS=Homo sapiens GN=OSBPL3		
Q9H4L5-8	OSBL3_HUMAN Isoform 2d of Oxysterol-binding protein-related protein 3 OS=Homo sapiens GN=OSBPL3		
Q9H4L5;Q9H4L5-2;Q9H4L	OSBL3_HUMAN Oxysterol-binding protein-related protein 3 OS=Homo sapiens GN=OSBPL3 PE=1 SV=1;>sp|Q9H4L5-2|OSBL3_HUMAI	1.65E-91	
Q9H4L7	SMRCD_HUMAN SWI/SNF-related matrix-associated actin-dependent regulator of chromatin subfamily A containing DEAD/H box 1 OS=Homo sapiens		51.74
Q9H4L7-2	SMRCD_HUMAN Isoform 2 of SWI/SNF-related matrix-associated actin-dependent regulator of chromatin subfamily A containing DEAD/H box 1 OS=I		51.74
Q9H4L7-2;Q9H4L7	2|SMRCD_HUMAN Isoform 2 of SWI/SNF-related matrix-associated actin-dependent regulator of chromatin subfamily A containing DE/	9.22E-06	
Q9H4M9	EHD1_HUMAN EH domain-containing protein 1 OS=Homo sapiens GN=EHD1 PE=1 SV=2		1145.06
Q9H4T2	ZSC16_HUMAN Zinc finger and SCAN domain-containing protein 16 OS=Homo sapiens GN=ZSCAN16 PE=1 SV=2		
Q9H4Z3	PCIF1_HUMAN Phosphorylated CTD-interacting factor 1 OS=Homo sapiens GN=PCIF1 PE=1 SV=1		17.76
Q9H501	ESF1_HUMAN ESF1 homolog OS=Homo sapiens GN=ESF1 PE=1 SV=1		44.39
Q9H553	ALG2_HUMAN Alpha-1,3/1,6-mannosyltransferase ALG2 OS=Homo sapiens GN=ALG2 PE=2 SV=1		
Q9H553-2	ALG2_HUMAN Isoform 2 of Alpha-1,3/1,6-mannosyltransferase ALG2 OS=Homo sapiens GN=ALG2		
Q9H553;Q9H553-2	ALG2_HUMAN Alpha-1,3/1,6-mannosyltransferase ALG2 OS=Homo sapiens GN=ALG2 PE=2 SV=1;>sp|Q9H553-2|ALG2_HUMAN Isofo	1.58E-10	
Q9H579	CT132_HUMAN Uncharacterized protein C20orf132 OS=Homo sapiens GN=C20orf132 PE=2 SV=2		
Q9H579-2	CT132_HUMAN Isoform 2 of Uncharacterized protein C20orf132 OS=Homo sapiens GN=C20orf132		34.46
Q9H583	HEAT1_HUMAN HEAT repeat-containing protein 1 OS=Homo sapiens GN=HEATR1 PE=1 SV=3		
Q9H5N1	RABE2_HUMAN Rab GTPase-binding effector protein 2 OS=Homo sapiens GN=RABEP2 PE=1 SV=2		
Q9H5N1-2	RABE2_HUMAN Isoform 2 of Rab GTPase-binding effector protein 2 OS=Homo sapiens GN=RABEP2		
Q9H5Q4	TFB2M_HUMAN Dimethyladenosine transferase 2, mitochondrial OS=Homo sapiens GN=TFB2M PE=1 SV=1	2.18E-08	
Q9H5V8	CDCP1_HUMAN CUB domain-containing protein 1 OS=Homo sapiens GN=CDCP1 PE=1 SV=3		178.16

Q9H5V8-2	CDCP1_HUMAN Isoform 2 of CUB domain-containing protein 1 OS=Homo sapiens GN=CDCP1		146.15
Q9H5V8-3	CDCP1_HUMAN Isoform 3 of CUB domain-containing protein 1 OS=Homo sapiens GN=CDCP1		75.33
Q9H5V8;Q9H5V8-2	CDCP1_HUMAN CUB domain-containing protein 1 OS=Homo sapiens GN=CDCP1 PE=1 SV=3;>sp|Q9H5V8-2|CDCP1_HUMAN Isoform	3.77E-08	
Q9H5V9	CX056_HUMAN UPF0428 protein CXorf56 OS=Homo sapiens GN=CXorf56 PE=1 SV=1	2.04E-05	
Q9H5X1	FA96A_HUMAN MIP18 family protein FAM96A OS=Homo sapiens GN=FAM96A PE=1 SV=1	7.17E-06	
Q9H6D7;Q9H6D7-4;Q9H6	HAUS4_HUMAN HAUS augmin-like complex subunit 4 OS=Homo sapiens GN=HAUS4 PE=1 SV=1;>sp|Q9H6D7-4|HAUS4_HUMAN Isof	2.55E-10	
Q9H6F5	CCD86_HUMAN Coiled-coil domain-containing protein 86 OS=Homo sapiens GN=CCDC86 PE=1 SV=1		
Q9H6K4	OPA3_HUMAN Optic atrophy 3 protein OS=Homo sapiens GN=OPA3 PE=1 SV=1		24.06
Q9H6K4-2	OPA3_HUMAN Isoform 2 of Optic atrophy 3 protein OS=Homo sapiens GN=OPA3		24.06
Q9H6P5	TASP1_HUMAN Threonine aspartase 1 OS=Homo sapiens GN=TASP1 PE=1 SV=1		
Q9H6R4	NOL6_HUMAN Nucleolar protein 6 OS=Homo sapiens GN=NOL6 PE=1 SV=2		
Q9H6R4-2	NOL6_HUMAN Isoform 2 of Nucleolar protein 6 OS=Homo sapiens GN=NOL6		
Q9H6R4-3	NOL6_HUMAN Isoform 3 of Nucleolar protein 6 OS=Homo sapiens GN=NOL6		
Q9H6R4-4	NOL6_HUMAN Isoform 4 of Nucleolar protein 6 OS=Homo sapiens GN=NOL6		
Q9H6R4;Q9H6R4-4;B4DF8	NOL6_HUMAN Nucleolar protein 6 OS=Homo sapiens GN=NOL6 PE=1 SV=2;>sp|Q9H6R4-4|NOL6_HUMAN Isoform 4 of Nucleolar prot	7.48E-12	
Q9H6S0	YTDC2_HUMAN Probable ATP-dependent RNA helicase YTHDC2 OS=Homo sapiens GN=YTHDC2 PE=1 SV=2		72.83
Q9H6S3	ES8L2_HUMAN Epidermal growth factor receptor kinase substrate 8-like protein 2 OS=Homo sapiens GN=EPS8L2 PE=1 SV=2	6.58E-18	
Q9H6T0	ESRP2_HUMAN Epithelial splicing regulatory protein 2 OS=Homo sapiens GN=ESRP2 PE=1 SV=1		37.7
Q9H6T0-2	ESRP2_HUMAN Isoform 2 of Epithelial splicing regulatory protein 2 OS=Homo sapiens GN=ESRP2		37.7
Q9H6T0;Q9H6T0-2;Q6NX0	ESRP2_HUMAN Epithelial splicing regulatory protein 2 OS=Homo sapiens GN=ESRP2 PE=1 SV=1;>sp|Q9H6T0-2|ESRP2_HUMAN Isofo	2.84E-07	
Q9H6T3;Q9H6T3-2	RPAP3_HUMAN RNA polymerase II-associated protein 3 OS=Homo sapiens GN=RPAP3 PE=1 SV=2;>sp|Q9H6T3-2|RPAP3_HUMAN Isof	1.46E-11	
Q9H6V9	CB043_HUMAN UPF0554 protein C2orf43 OS=Homo sapiens GN=C2orf43 PE=1 SV=1		
Q9H6Y2	WDR55_HUMAN WD repeat-containing protein 55 OS=Homo sapiens GN=WDR55 PE=1 SV=2	2.07E-14	
Q9H6Z4	RANB3_HUMAN Ran-binding protein 3 OS=Homo sapiens GN=RANBP3 PE=1 SV=1		
Q9H6Z4-2	RANB3_HUMAN Isoform 2 of Ran-binding protein 3 OS=Homo sapiens GN=RANBP3		
Q9H6Z4-2;Q9H6Z4;Q9H6Z	2|RANB3_HUMAN Isoform 2 of Ran-binding protein 3 OS=Homo sapiens GN=RANBP3;>sp|Q9H6Z4|RANB3_HUMAN Ran-binding prote	2.79E-43	
Q9H6Z4-3	RANB3_HUMAN Isoform 3 of Ran-binding protein 3 OS=Homo sapiens GN=RANBP3		
Q9H6Z4-4	RANB3_HUMAN Isoform 4 of Ran-binding protein 3 OS=Homo sapiens GN=RANBP3		
Q9H6Z4;Q9H6Z4-2;Q53GI	RANB3_HUMAN Ran-binding protein 3 OS=Homo sapiens GN=RANBP3 PE=1 SV=1;>sp|Q9H6Z4-2|RANB3_HUMAN Isoform 2 of Ran-b	4.26E-53	
Q9H773	DCTP1_HUMAN dCTP pyrophosphatase 1 OS=Homo sapiens GN=DCTPP1 PE=1 SV=1		
Q9H7B2	RPF2_HUMAN Ribosome production factor 2 homolog OS=Homo sapiens GN=RPF2 PE=1 SV=2		29.37
Q9H7B4;Q9H7B4-3;Q9H7I	SMYD3_HUMAN SET and MYND domain-containing protein 3 OS=Homo sapiens GN=SMYD3 PE=1 SV=4;>sp|Q9H7B4-3|SMYD3_HUMA	1.70E-08	
Q9H7C9	CK067_HUMAN UPF0366 protein C11orf67 OS=Homo sapiens GN=C11orf67 PE=1 SV=1	4.71E-08	
Q9H7D0	DOCK5_HUMAN Dedicator of cytokinesis protein 5 OS=Homo sapiens GN=DOCK5 PE=1 SV=3	1.85E-05	
Q9H7D7;Q9H7D7-2	WDR26_HUMAN WD repeat-containing protein 26 OS=Homo sapiens GN=WDR26 PE=1 SV=3;>sp|Q9H7D7-2|WDR26_HUMAN Isoform	1.94E-34	
Q9H7E9-2;Q9H7E9	2|CH033_HUMAN Isoform 2 of UPF0488 protein C8orf33 OS=Homo sapiens GN=C8orf33;>sp|Q9H7E9|CH033_HUMAN UPF0488 protei	1.39E-11	
Q9H7Z7	PGES2_HUMAN Prostaglandin E synthase 2 OS=Homo sapiens GN=PTGES2 PE=1 SV=1		88.47
Q9H814	PHAX_HUMAN Phosphorylated adapter RNA export protein OS=Homo sapiens GN=PHAX PE=1 SV=1		34.22
Q9H819	DJC18_HUMAN DnaJ homolog subfamily C member 18 OS=Homo sapiens GN=DNAJC18 PE=2 SV=1		
Q9H832;D3DTW2;B4DL66	UBE2Z_HUMAN Ubiquitin-conjugating enzyme E2 Z OS=Homo sapiens GN=UBE2Z PE=1 SV=2;>tr|D3DTW2|D3DTW2_HUMAN Ubiquit	7.81E-19	
Q9H845	ACAD9_HUMAN Acyl-CoA dehydrogenase family member 9, mitochondrial OS=Homo sapiens GN=ACAD9 PE=1 SV=1		171.31
Q9H853	TBA4B_HUMAN Putative tubulin-like protein alpha-4B OS=Homo sapiens GN=TUBA4B PE=5 SV=2		198.08
Q9H857;Q9H857-2;Q9H85	NT5D2_HUMAN 5-nucleotidase domain-containing protein 2 OS=Homo sapiens GN=NT5DC2 PE=1 SV=1;>sp|Q9H857-2|NT5D2_HUMA	4.85E-05	
Q9H871	RMD5A_HUMAN Protein RMD5 homolog A OS=Homo sapiens GN=RMND5A PE=1 SV=1		
Q9H871-2	RMD5A_HUMAN Isoform 2 of Protein RMD5 homolog A OS=Homo sapiens GN=RMND5A		
Q9H8G2	CI082_HUMAN Uncharacterized protein C9orf82 OS=Homo sapiens GN=C9orf82 PE=1 SV=2	2.42E-05	
Q9H8H0	NOL11_HUMAN Nucleolar protein 11 OS=Homo sapiens GN=NOL11 PE=1 SV=1		
Q9H8H2	DDX31_HUMAN Probable ATP-dependent RNA helicase DDX31 OS=Homo sapiens GN=DDX31 PE=1 SV=2		
Q9H8H2-2	DDX31_HUMAN Isoform 2 of Probable ATP-dependent RNA helicase DDX31 OS=Homo sapiens GN=DDX31		
Q9H8H2-3	DDX31_HUMAN Isoform 3 of Probable ATP-dependent RNA helicase DDX31 OS=Homo sapiens GN=DDX31		
Q9H8H2-4	DDX31_HUMAN Isoform 4 of Probable ATP-dependent RNA helicase DDX31 OS=Homo sapiens GN=DDX31		
Q9H8H3	MET7A_HUMAN Methyltransferase-like protein 7A OS=Homo sapiens GN=METTL7A PE=1 SV=1		
Q9H8M9	F176A_HUMAN Protein FAM176A OS=Homo sapiens GN=FAM176A PE=2 SV=1		49.96
Q9H8T0-2;Q9H8T0	2|AKTIP_HUMAN Isoform 2 of AKT-interacting protein OS=Homo sapiens GN=AKTIP;>sp|Q9H8T0|AKTIP_HUMAN AKT-interacting prot	5.65E-08	
Q9H8U3	ZFAN3_HUMAN AN1-type zinc finger protein 3 OS=Homo sapiens GN=ZFAND3 PE=1 SV=1	0.00015016	
Q9H8Y5	ANKZ1_HUMAN Ankyrin repeat and zinc finger domain-containing protein 1 OS=Homo sapiens GN=ANKZF1 PE=1 SV=1		
Q9H8Y8	GORS2_HUMAN Golgi reassembly-stacking protein 2 OS=Homo sapiens GN=GORASP2 PE=1 SV=3	2.39E-18	
Q9H900;A8K4T3;Q9H900-	ZWILC_HUMAN Protein zwilch homolog OS=Homo sapiens GN=ZWILCH PE=1 SV=2;>tr|A8K4T3|A8K4T3_HUMAN cDNA FLJ77716, hig	1.87E-11	
Q9H910	HN1L_HUMAN Hematological and neurological expressed 1-like protein OS=Homo sapiens GN=HN1L PE=1 SV=1		92.53
Q9H910-2	HN1L_HUMAN Isoform 2 of Hematological and neurological expressed 1-like protein OS=Homo sapiens GN=HN1L		92.53
Q9H910;Q9H910-2	HN1L_HUMAN Hematological and neurological expressed 1-like protein OS=Homo sapiens GN=HN1L PE=1 SV=1;>sp|Q9H910-2|HN1L	1.27E-83	

Q9H930	LY10L_HUMAN Nuclear body protein SP140-like protein OS=Homo sapiens GN=SP140L PE=2 SV=2		60.37
Q9H930-2	LY10L_HUMAN Isoform 2 of Nuclear body protein SP140-like protein OS=Homo sapiens GN=SP140L		60.37
Q9H930-3	LY10L_HUMAN Isoform 3 of Nuclear body protein SP140-like protein OS=Homo sapiens GN=SP140L		47.51
Q9H930-4	LY10L_HUMAN Isoform 4 of Nuclear body protein SP140-like protein OS=Homo sapiens GN=SP140L		60.62
Q9H930-4;Q9H930;Q9H9:	4|LY10L_HUMAN Isoform 4 of Nuclear body protein SP140-like protein OS=Homo sapiens GN=SP140L;>sp|Q9H930|LY10L_HUMAN Nu	6.15E-10	
Q9H936	GHC1_HUMAN Mitochondrial glutamate carrier 1 OS=Homo sapiens GN=SLC25A22 PE=1 SV=1		82.42
Q9H939;Q9H939-2	PPIP2_HUMAN Proline-serine-threonine phosphatase-interacting protein 2 OS=Homo sapiens GN=PSTPIP2 PE=1 SV=4;>sp|Q9H939-2	0.00018216	
Q9H943	CJ068_HUMAN Uncharacterized protein C10orf68 OS=Homo sapiens GN=C10orf68 PE=2 SV=2		
Q9H943-2	CJ068_HUMAN Isoform 2 of Uncharacterized protein C10orf68 OS=Homo sapiens GN=C10orf68		
Q9H943-3	CJ068_HUMAN Isoform 3 of Uncharacterized protein C10orf68 OS=Homo sapiens GN=C10orf68		
Q9H944	MED20_HUMAN Mediator of RNA polymerase II transcription subunit 20 OS=Homo sapiens GN=MED20 PE=1 SV=1		62.38
Q9H974	QTRD1_HUMAN Queuine tRNA-ribosyltransferase subunit QTRTD1 OS=Homo sapiens GN=QTRTD1 PE=1 SV=1	3.23E-06	
Q9H993	CF211_HUMAN UPF0364 protein C6orf211 OS=Homo sapiens GN=C6orf211 PE=1 SV=1	2.01E-280	
Q9H9A5;Q9H9A5-2;Q9H9/	CNOTA_HUMAN CCR4-NOT transcription complex subunit 10 OS=Homo sapiens GN=CNOT10 PE=1 SV=1;>sp|Q9H9A5-2|CNOTA_HUM	2.94E-13	
Q9H9A6	LRC40_HUMAN Leucine-rich repeat-containing protein 40 OS=Homo sapiens GN=LRRC40 PE=1 SV=1		43.74
Q9H9B1	EHMT1_HUMAN Histone-lysine N-methyltransferase EHMT1 OS=Homo sapiens GN=EHMT1 PE=1 SV=4		
Q9H9B1-2	EHMT1_HUMAN Isoform 2 of Histone-lysine N-methyltransferase EHMT1 OS=Homo sapiens GN=EHMT1		
Q9H9B1-3	EHMT1_HUMAN Isoform 3 of Histone-lysine N-methyltransferase EHMT1 OS=Homo sapiens GN=EHMT1		
Q9H9B1-4	EHMT1_HUMAN Isoform 4 of Histone-lysine N-methyltransferase EHMT1 OS=Homo sapiens GN=EHMT1		
Q9H9B4	SFXN1_HUMAN Sideroflexin-1 OS=Homo sapiens GN=SFXN1 PE=1 SV=4		454.07
Q9H9F9	ARP5_HUMAN Actin-related protein 5 OS=Homo sapiens GN=ACTR5 PE=1 SV=2		39.39
Q9H9H4	VP37B_HUMAN Vacuolar protein sorting-associated protein 37B OS=Homo sapiens GN=VPS37B PE=1 SV=1		186.41
Q9H9J2	RM44_HUMAN 39S ribosomal protein L44, mitochondrial OS=Homo sapiens GN=MRPL44 PE=1 SV=1		164.31
Q9H9P8	L2HDH_HUMAN L-2-hydroxyglutarate dehydrogenase, mitochondrial OS=Homo sapiens GN=L2HGDH PE=1 SV=3		
Q9H9P8-2	L2HDH_HUMAN Isoform 2 of L-2-hydroxyglutarate dehydrogenase, mitochondrial OS=Homo sapiens GN=L2HGDH		
Q9H9P8;Q9H9P8-2	L2HDH_HUMAN L-2-hydroxyglutarate dehydrogenase, mitochondrial OS=Homo sapiens GN=L2HGDH PE=1 SV=3;>sp|Q9H9P8-2|L2HI	4.64E-22	
Q9H9Q2;Q9H9Q2-2;Q9H9	CSN7B_HUMAN COP9 signalosome complex subunit 7b OS=Homo sapiens GN=COPS7B PE=1 SV=1;>sp|Q9H9Q2-2|CSN7B_HUMAN Is	5.04E-67	
Q9H9S3	S61A2_HUMAN Protein transport protein Sec61 subunit alpha isoform 2 OS=Homo sapiens GN=SEC61A2 PE=2 SV=3		67.52
Q9H9S3-2	S61A2_HUMAN Isoform 2 of Protein transport protein Sec61 subunit alpha isoform 2 OS=Homo sapiens GN=SEC61A2		74.15
Q9H9S4	CB39L_HUMAN Calcium-binding protein 39-like OS=Homo sapiens GN=CAB39L PE=1 SV=3		64.76
Q9H9T3	ELP3_HUMAN Elongator complex protein 3 OS=Homo sapiens GN=ELP3 PE=1 SV=2		
Q9H9T3-2	ELP3_HUMAN Isoform 2 of Elongator complex protein 3 OS=Homo sapiens GN=ELP3		
Q9H9T3;Q9H9T3-2;E2QRI	ELP3_HUMAN Elongator complex protein 3 OS=Homo sapiens GN=ELP3 PE=1 SV=2;>sp|Q9H9T3-2|ELP3_HUMAN Isoform 2 of Elonga	1.08E-07	
Q9H9Y2	RPF1_HUMAN Ribosome production factor 1 OS=Homo sapiens GN=RPF1 PE=1 SV=2		
Q9H9Y6	RPA2_HUMAN DNA-directed RNA polymerase I subunit RPA2 OS=Homo sapiens GN=POLR1B PE=1 SV=2		
Q9H9Y6-2	RPA2_HUMAN Isoform 2 of DNA-directed RNA polymerase I subunit RPA2 OS=Homo sapiens GN=POLR1B		
Q9H9Y6;Q9H9Y6-2	RPA2_HUMAN DNA-directed RNA polymerase I subunit RPA2 OS=Homo sapiens GN=POLR1B PE=1 SV=2;>sp|Q9H9Y6-2|RPA2_HUMAI	7.73E-19	
Q9HA26	RUN2A_HUMAN RUN domain-containing protein 2A OS=Homo sapiens GN=RUNDC2A PE=2 SV=2		
Q9HA64	KT3K_HUMAN Ketosamine-3-kinase OS=Homo sapiens GN=FN3KRP PE=1 SV=2	2.50E-11	
Q9HA77	SYCM_HUMAN Probable cysteinyl-tRNA synthetase, mitochondrial OS=Homo sapiens GN=CARS2 PE=1 SV=1		
Q9HA92	RSAD1_HUMAN Radical S-adenosyl methionine domain-containing protein 1, mitochondrial OS=Homo sapiens GN=RSAD1 PE=2 SV=2		
Q9HAB8	PPCS_HUMAN Phosphopantothenate--cysteine ligase OS=Homo sapiens GN=PPCS PE=1 SV=2	3.38E-20	
Q9HAN9	NMNA1_HUMAN Nicotinamide mononucleotide adenylyltransferase 1 OS=Homo sapiens GN=NMNAT1 PE=1 SV=1		113.37
Q9HAP6	LIN7B_HUMAN Protein lin-7 homolog B OS=Homo sapiens GN=LIN7B PE=1 SV=1		69.25
Q9HAR2	LPHN3_HUMAN Latrophilin-3 OS=Homo sapiens GN=LPHN3 PE=1 SV=2		
Q9HAR2-2	LPHN3_HUMAN Isoform 2 of Latrophilin-3 OS=Homo sapiens GN=LPHN3		
Q9HAR2-3	LPHN3_HUMAN Isoform 3 of Latrophilin-3 OS=Homo sapiens GN=LPHN3		
Q9HAS0	NJMU_HUMAN Protein Njmu-R1 OS=Homo sapiens GN=C17orf75 PE=1 SV=2	1.15E-09	
Q9HAT2;Q9HAT2-2	SIAE_HUMAN Sialate O-acetylesterase OS=Homo sapiens GN=SIAE PE=1 SV=1;>sp|Q9HAT2-2|SIAE_HUMAN Isoform 2 of Sialate O-a	1.37E-32	
Q9HAU0-4;Q9HAU0-2;Q9H	4|PKHA5_HUMAN Isoform 4 of Pleckstrin homology domain-containing family A member 5 OS=Homo sapiens GN=PLEKHA5;>sp|Q9H/	5.44E-06	
Q9HAU5	RENT2_HUMAN Regulator of nonsense transcripts 2 OS=Homo sapiens GN=UPF2 PE=1 SV=1		
Q9HAU5-2	RENT2_HUMAN Isoform 2 of Regulator of nonsense transcripts 2 OS=Homo sapiens GN=UPF2		
Q9HAU8	RNPL1_HUMAN Arginyl aminopeptidase-like 1 OS=Homo sapiens GN=RNPEPL1 PE=2 SV=2		
Q9HAV0	GBB4_HUMAN Guanine nucleotide-binding protein subunit beta-4 OS=Homo sapiens GN=GNB4 PE=1 SV=3		217.73
Q9HAV4	XPO5_HUMAN Exportin-5 OS=Homo sapiens GN=XPO5 PE=1 SV=1		324.16
Q9HAV7	GRPE1_HUMAN GrpE protein homolog 1, mitochondrial OS=Homo sapiens GN=GRPEL1 PE=1 SV=2		462.73
Q9HAW4	CLSPN_HUMAN Claspin OS=Homo sapiens GN=CLSPN PE=1 SV=3		
Q9HAW4-2	CLSPN_HUMAN Isoform 2 of Claspin OS=Homo sapiens GN=CLSPN		
Q9HAW4-3	CLSPN_HUMAN Isoform 3 of Claspin OS=Homo sapiens GN=CLSPN		
Q9HAZ2	PRD16_HUMAN PR domain zinc finger protein 16 OS=Homo sapiens GN=PRDM16 PE=1 SV=3		
Q9HAZ2-2	PRD16_HUMAN Isoform 2 of PR domain zinc finger protein 16 OS=Homo sapiens GN=PRDM16		

Q9HAZ2-3	PRD16_HUMAN Isoform 3 of PR domain zinc finger protein 16 OS=Homo sapiens GN=PRDM16		
Q9HAZ2-4	PRD16_HUMAN Isoform 4 of PR domain zinc finger protein 16 OS=Homo sapiens GN=PRDM16		
Q9HB07	MYG1_HUMAN UPF0160 protein MYG1, mitochondrial OS=Homo sapiens GN=C12orf10 PE=1 SV=2	1.25E-27	
Q9HB19	PKHA2_HUMAN Pleckstrin homology domain-containing family A member 2 OS=Homo sapiens GN=PLEKHA2 PE=1 SV=2	7.17E-08	
Q9HB40;B4DP22;Q9HB40	RISC_HUMAN Retinoid-inducible serine carboxypeptidase OS=Homo sapiens GN=SCPEP1 PE=1 SV=1;>tr|B4DP22|B4DP22_HUMAN cD	2.90E-37	
Q9HB58	SP110_HUMAN Sp110 nuclear body protein OS=Homo sapiens GN=SP110 PE=1 SV=5		19.41
Q9HB58-2	SP110_HUMAN Isoform 2 of Sp110 nuclear body protein OS=Homo sapiens GN=SP110		17.51
Q9HB58-3	SP110_HUMAN Isoform 3 of Sp110 nuclear body protein OS=Homo sapiens GN=SP110		2.15
Q9HB58-4	SP110_HUMAN Isoform 4 of Sp110 nuclear body protein OS=Homo sapiens GN=SP110		0.25
Q9HB58-5	SP110_HUMAN Isoform 5 of Sp110 nuclear body protein OS=Homo sapiens GN=SP110		2.15
Q9HB58-6	SP110_HUMAN Isoform 6 of Sp110 nuclear body protein OS=Homo sapiens GN=SP110		19.41
Q9HB71	CYBP_HUMAN Calcyclin-binding protein OS=Homo sapiens GN=CACYBP PE=1 SV=2		495.59
Q9HB71-2	CYBP_HUMAN Isoform 2 of Calcyclin-binding protein OS=Homo sapiens GN=CACYBP		22.91
Q9HB90;B4DQG4;D3DPT8	RRAGC_HUMAN Ras-related GTP-binding protein C OS=Homo sapiens GN=RRAGC PE=1 SV=1;>tr|B4DQG4|B4DQG4_HUMAN cDNA FL	9.83E-11	
Q9HBA0	TRPV4_HUMAN Transient receptor potential cation channel subfamily V member 4 OS=Homo sapiens GN=TRPV4 PE=1 SV=2		
Q9HBA0-2	TRPV4_HUMAN Isoform 2 of Transient receptor potential cation channel subfamily V member 4 OS=Homo sapiens GN=TRPV4		
Q9HBA0-3	TRPV4_HUMAN Isoform 3 of Transient receptor potential cation channel subfamily V member 4 OS=Homo sapiens GN=TRPV4		
Q9HBA0-4	TRPV4_HUMAN Isoform 4 of Transient receptor potential cation channel subfamily V member 4 OS=Homo sapiens GN=TRPV4		
Q9HBA0-5	TRPV4_HUMAN Isoform 5 of Transient receptor potential cation channel subfamily V member 4 OS=Homo sapiens GN=TRPV4		
Q9HBA0-6	TRPV4_HUMAN Isoform 6 of Transient receptor potential cation channel subfamily V member 4 OS=Homo sapiens GN=TRPV4		
Q9HBA9	FOH1B_HUMAN Putative N-acetylated-alpha-linked acidic dipeptidase OS=Homo sapiens GN=FOLH1B PE=2 SV=1		
Q9HBE1	PATZ1_HUMAN POZ-, AT hook-, and zinc finger-containing protein 1 OS=Homo sapiens GN=PATZ1 PE=1 SV=1		
Q9HBE1-2	PATZ1_HUMAN Isoform 2 of POZ-, AT hook-, and zinc finger-containing protein 1 OS=Homo sapiens GN=PATZ1		
Q9HBE1-3	PATZ1_HUMAN Isoform 3 of POZ-, AT hook-, and zinc finger-containing protein 1 OS=Homo sapiens GN=PATZ1		
Q9HBE1-4	PATZ1_HUMAN Isoform 4 of POZ-, AT hook-, and zinc finger-containing protein 1 OS=Homo sapiens GN=PATZ1		
Q9HBG4	VPP4_HUMAN V-type proton ATPase 116 kDa subunit a isoform 4 OS=Homo sapiens GN=ATP6V0A4 PE=1 SV=2		
Q9HBH5	RDH14_HUMAN Retinol dehydrogenase 14 OS=Homo sapiens GN=RDH14 PE=1 SV=1		
Q9HBI0;Q9HBI0-2;Q9HBI	PARVG_HUMAN Gamma-parvin OS=Homo sapiens GN=PARVG PE=2 SV=1;>sp|Q9HBI0-2|PARVG_HUMAN Isoform 2 of Gamma-parvir	1.22E-37	
Q9HBI1	PARVB_HUMAN Beta-parvin OS=Homo sapiens GN=PARVB PE=1 SV=1		37.37
Q9HBI1-2	PARVB_HUMAN Isoform 2 of Beta-parvin OS=Homo sapiens GN=PARVB		37.37
Q9HBI6	CP4FB_HUMAN Cytochrome P450 4F11 OS=Homo sapiens GN=CYP4F11 PE=2 SV=3	6.88E-09	
Q9HBL0	TENS1_HUMAN Tensin-1 OS=Homo sapiens GN=TNS1 PE=1 SV=2	2.89E-06	
Q9HBL7	CI046_HUMAN Transmembrane protein C9orf46 OS=Homo sapiens GN=C9orf46 PE=2 SV=1	2.32E-15	
Q9HBM0-2	VEZA_HUMAN Isoform 2 of Vezatin OS=Homo sapiens GN=VEZT		18.13
Q9HBM1	SPC25_HUMAN Kinetochore protein Spc25 OS=Homo sapiens GN=SPC25 PE=1 SV=1	4.43E-32	
Q9HBM6	TAF9B_HUMAN Transcription initiation factor TFIID subunit 9B OS=Homo sapiens GN=TAF9B PE=1 SV=1		118.03
Q9HBT7	ZN287_HUMAN Zinc finger protein 287 OS=Homo sapiens GN=ZNF287 PE=2 SV=1		
Q9HBT8	Z286A_HUMAN Zinc finger protein 286A OS=Homo sapiens GN=ZNF286A PE=2 SV=1		1.46
Q9HBX8	LGR6_HUMAN Leucine-rich repeat-containing G-protein coupled receptor 6 OS=Homo sapiens GN=LGR6 PE=1 SV=3		18.09
Q9HBX8-1	LGR6_HUMAN Isoform 1 of Leucine-rich repeat-containing G-protein coupled receptor 6 OS=Homo sapiens GN=LGR6		18.09
Q9HBX8-2	LGR6_HUMAN Isoform 2 of Leucine-rich repeat-containing G-protein coupled receptor 6 OS=Homo sapiens GN=LGR6		18.09
Q9HC07	TM165_HUMAN Transmembrane protein 165 OS=Homo sapiens GN=TMEM165 PE=1 SV=1	1.50E-66	
Q9HC16	ABC3G_HUMAN DNA dC->dU-editing enzyme APOBEC-3G OS=Homo sapiens GN=APOBEC3G PE=1 SV=1	4.10E-06	
Q9HC35	EMAL4_HUMAN Echinoderm microtubule-associated protein-like 4 OS=Homo sapiens GN=EML4 PE=1 SV=3		24.55
Q9HC36	RMTL1_HUMAN RNA methyltransferase-like protein 1 OS=Homo sapiens GN=RNMTL1 PE=1 SV=2	1.07E-12	
Q9HC38	GLOD4_HUMAN Glyoxalase domain-containing protein 4 OS=Homo sapiens GN=GLOD4 PE=1 SV=1		60.83
Q9HC38-2	GLOD4_HUMAN Isoform 2 of Glyoxalase domain-containing protein 4 OS=Homo sapiens GN=GLOD4		60.83
Q9HC38-2;Q9HC38	2|GLOD4_HUMAN Isoform 2 of Glyoxalase domain-containing protein 4 OS=Homo sapiens GN=GLOD4;>sp|Q9HC38|GLOD4_HUMAN (	3.50E-62	
Q9HC38-3	GLOD4_HUMAN Isoform 3 of Glyoxalase domain-containing protein 4 OS=Homo sapiens GN=GLOD4		10.27
Q9HC38;Q9HC38-2	GLOD4_HUMAN Glyoxalase domain-containing protein 4 OS=Homo sapiens GN=GLOD4 PE=1 SV=1;>sp|Q9HC38-2|GLOD4_HUMAN Is	3.26E-37	
Q9HC52	CBX8_HUMAN Chromobox protein homolog 8 OS=Homo sapiens GN=CBX8 PE=1 SV=3		
Q9HC62	SENP2_HUMAN Sentrin-specific protease 2 OS=Homo sapiens GN=SENP2 PE=1 SV=3		
Q9HC77	CENPJ_HUMAN Centromere protein J OS=Homo sapiens GN=CENPJ PE=1 SV=2		
Q9HC98-2;Q9HC98-4;Q9H	2|NEK6_HUMAN Isoform 2 of Serine/threonine-protein kinase Nek6 OS=Homo sapiens GN=NEK6;>sp|Q9HC98-4|NEK6_HUMAN Isofor	1.61E-08	
Q9HCC0	MCCB_HUMAN Methylcrotonoyl-CoA carboxylase beta chain, mitochondrial OS=Homo sapiens GN=MCCC2 PE=1 SV=1		47.02
Q9HCC0-2	MCCB_HUMAN Isoform 2 of Methylcrotonoyl-CoA carboxylase beta chain, mitochondrial OS=Homo sapiens GN=MCCC2		47.02
Q9HCC0;D6R932;Q9HCC0	MCCB_HUMAN Methylcrotonoyl-CoA carboxylase beta chain, mitochondrial OS=Homo sapiens GN=MCCC2 PE=1 SV=1;>tr|D6R932|D6	8.15E-181	
Q9HCD5	NCOA5_HUMAN Nuclear receptor coactivator 5 OS=Homo sapiens GN=NCOA5 PE=1 SV=2		
Q9HCE0	EPG5_HUMAN Ectopic P granules protein 5 homolog OS=Homo sapiens GN=EPG5 PE=2 SV=2		
Q9HCE0-2	EPG5_HUMAN Isoform 2 of Ectopic P granules protein 5 homolog OS=Homo sapiens GN=EPG5		
Q9HCE0;Q9HCE0-2	EPG5_HUMAN Ectopic P granules protein 5 homolog OS=Homo sapiens GN=EPG5 PE=2 SV=2;>sp|Q9HCE0-2|EPG5_HUMAN Isoform 2	1.63E-17	

Q9HCE1	MOV10_HUMAN Putative helicase MOV-10 OS=Homo sapiens GN=MOV10 PE=1 SV=2		
Q9HCE1-2	MOV10_HUMAN Isoform 2 of Putative helicase MOV-10 OS=Homo sapiens GN=MOV10		
Q9HCE1;Q9HCE1-2	MOV10_HUMAN Putative helicase MOV-10 OS=Homo sapiens GN=MOV10 PE=1 SV=2;>sp|Q9HCE1-2|MOV10_HUMAN Isoform 2 of Pu	1.26E-09	
Q9HCE3	ZN532_HUMAN Zinc finger protein 532 OS=Homo sapiens GN=ZNF532 PE=1 SV=2		
Q9HCF4	ALO17_HUMAN Protein ALO17 OS=Homo sapiens GN=KIAA1618 PE=1 SV=2		
Q9HCF4-2	ALO17_HUMAN Isoform 2 of Protein ALO17 OS=Homo sapiens GN=KIAA1618		
Q9HCF4-3	ALO17_HUMAN Isoform 3 of Protein ALO17 OS=Homo sapiens GN=KIAA1618		
Q9HCF4-3;Q9HCF4	3|ALO17_HUMAN Isoform 3 of Protein ALO17 OS=Homo sapiens GN=KIAA1618;>sp|Q9HCF4|ALO17_HUMAN Protein ALO17 OS=Hom	1.78E-29	
Q9HCF6	TRPM3_HUMAN Transient receptor potential cation channel subfamily M member 3 OS=Homo sapiens GN=TRPM3 PE=2 SV=4		16.85
Q9HCF6-11	TRPM3_HUMAN Isoform 11 of Transient receptor potential cation channel subfamily M member 3 OS=Homo sapiens GN=TRPM3		39.49
Q9HCF6-2	TRPM3_HUMAN Isoform 2 of Transient receptor potential cation channel subfamily M member 3 OS=Homo sapiens GN=TRPM3		16.85
Q9HCF6-3	TRPM3_HUMAN Isoform 3 of Transient receptor potential cation channel subfamily M member 3 OS=Homo sapiens GN=TRPM3		16.85
Q9HCF6-4	TRPM3_HUMAN Isoform 4 of Transient receptor potential cation channel subfamily M member 3 OS=Homo sapiens GN=TRPM3		16.85
Q9HCF6-5	TRPM3_HUMAN Isoform 5 of Transient receptor potential cation channel subfamily M member 3 OS=Homo sapiens GN=TRPM3		16.85
Q9HCF6-6	TRPM3_HUMAN Isoform 6 of Transient receptor potential cation channel subfamily M member 3 OS=Homo sapiens GN=TRPM3		16.85
Q9HCF6-7	TRPM3_HUMAN Isoform 7 of Transient receptor potential cation channel subfamily M member 3 OS=Homo sapiens GN=TRPM3		16.85
Q9HCF6-8	TRPM3_HUMAN Isoform 8 of Transient receptor potential cation channel subfamily M member 3 OS=Homo sapiens GN=TRPM3		16.85
Q9HCG8	CWC22_HUMAN Pre-splicing factor CWC22 homolog OS=Homo sapiens GN=CWC22 PE=1 SV=3		
Q9HCH3	CPNE5_HUMAN Copine-5 OS=Homo sapiens GN=CPNE5 PE=1 SV=2		
Q9HCI6	K1586_HUMAN Uncharacterized protein KIAA1586 OS=Homo sapiens GN=KIAA1586 PE=2 SV=2		
Q9HCI6-2	K1586_HUMAN Isoform 2 of Uncharacterized protein KIAA1586 OS=Homo sapiens GN=KIAA1586		
Q9HCJ0	TNR6C_HUMAN Trinucleotide repeat-containing gene 6C protein OS=Homo sapiens GN=TNRC6C PE=1 SV=3		
Q9HCJ0-2	TNR6C_HUMAN Isoform 2 of Trinucleotide repeat-containing gene 6C protein OS=Homo sapiens GN=TNRC6C		
Q9HCJ2	LRC4C_HUMAN Leucine-rich repeat-containing protein 4C OS=Homo sapiens GN=LRRC4C PE=1 SV=1		
Q9HCK8	CHD8_HUMAN Chromodomain-helicase-DNA-binding protein 8 OS=Homo sapiens GN=CHD8 PE=1 SV=5		76.89
Q9HCK8-2	CHD8_HUMAN Isoform 2 of Chromodomain-helicase-DNA-binding protein 8 OS=Homo sapiens GN=CHD8		76.89
Q9HCK8;Q9HCK8-2	CHD8_HUMAN Chromodomain-helicase-DNA-binding protein 8 OS=Homo sapiens GN=CHD8 PE=1 SV=5;>sp|Q9HCK8-2|CHD8_HUMA	2.16E-10	
Q9HCM4	E41L5_HUMAN Band 4.1-like protein 5 OS=Homo sapiens GN=EPB41L5 PE=1 SV=3		
Q9HCM4-2	E41L5_HUMAN Isoform 2 of Band 4.1-like protein 5 OS=Homo sapiens GN=EPB41L5		
Q9HCM4-3	E41L5_HUMAN Isoform 3 of Band 4.1-like protein 5 OS=Homo sapiens GN=EPB41L5		
Q9HCM4-4	E41L5_HUMAN Isoform 4 of Band 4.1-like protein 5 OS=Homo sapiens GN=EPB41L5		
Q9HCM4;Q9HCM4-3;Q9HC	E41L5_HUMAN Band 4.1-like protein 5 OS=Homo sapiens GN=EPB41L5 PE=1 SV=3;>sp|Q9HCM4-3|E41L5_HUMAN Isoform 3 of Banc	2.01E-08	
Q9HCN4	GPN1_HUMAN GPN-loop GTPase 1 OS=Homo sapiens GN=GPN1 PE=1 SV=1	2.04E-07	
Q9HCN8	SDF2L_HUMAN Stromal cell-derived factor 2-like protein 1 OS=Homo sapiens GN=SDF2L1 PE=1 SV=2		25.68
Q9HCQ7	RFRP_HUMAN FMRFamide-related peptides OS=Homo sapiens GN=NPVF PE=1 SV=2		
Q9HCQ7-2	RFRP_HUMAN Isoform 2 of FMRFamide-related peptides OS=Homo sapiens GN=NPVF		
Q9HCS7	SYF1_HUMAN Pre-splicing factor SYF1 OS=Homo sapiens GN=XAB2 PE=1 SV=2		
Q9HCS7;Q71SV8;Q68CN2	SYF1_HUMAN Pre-mRNA-splicing factor SYF1 OS=Homo sapiens GN=XAB2 PE=1 SV=2;>tr|Q71SV8|Q71SV8_HUMAN Crn-related prot	5.06E-07	
Q9HCU4	CELR2_HUMAN Cadherin EGF LAG seven-pass G-type receptor 2 OS=Homo sapiens GN=CELSR2 PE=1 SV=1		
Q9HCY8	S10AE_HUMAN Protein S100-A14 OS=Homo sapiens GN=S100A14 PE=1 SV=1		
Q9HD15	SRA1_HUMAN Steroid receptor RNA activator 1 OS=Homo sapiens GN=SRA1 PE=1 SV=1	3.78E-45	
Q9HD26;Q9HD26-2;Q9HD	GOPC_HUMAN Golgi-associated PDZ and coiled-coil motif-containing protein OS=Homo sapiens GN=GOPC PE=1 SV=1;>sp|Q9HD26-2	1.99E-08	
Q9HD33	RM47_HUMAN 39S ribosomal protein L47, mitochondrial OS=Homo sapiens GN=MRPL47 PE=1 SV=2		105.65
Q9HD33-2	RM47_HUMAN Isoform 2 of 39S ribosomal protein L47, mitochondrial OS=Homo sapiens GN=MRPL47		105.65
Q9HD33-3	RM47_HUMAN Isoform 3 of 39S ribosomal protein L47, mitochondrial OS=Homo sapiens GN=MRPL47		57.32
Q9HD33;Q9HD33-2	RM47_HUMAN 39S ribosomal protein L47, mitochondrial OS=Homo sapiens GN=MRPL47 PE=1 SV=2;>sp|Q9HD33-2|RM47_HUMAN Is	5.73E-14	
Q9HD34	LYRM4_HUMAN LYR motif-containing protein 4 OS=Homo sapiens GN=LYRM4 PE=1 SV=1	1.77E-18	
Q9HD42	CHM1A_HUMAN Charged multivesicular body protein 1a OS=Homo sapiens GN=CHMP1A PE=1 SV=1		32.97
Q9HD45	TM9S3_HUMAN Transmembrane 9 superfamily member 3 OS=Homo sapiens GN=TM9SF3 PE=1 SV=2		154.23
Q9HD64;Q9HD64-4	GAGD2_HUMAN G antigen family D member 2 OS=Homo sapiens GN=XAGE1A PE=2 SV=3;>sp|Q9HD64-4|GAGD2_HUMAN Isoform D	1.40E-10	
Q9HD67	MYO10_HUMAN Myosin-X OS=Homo sapiens GN=MYO10 PE=1 SV=3		
Q9HDB5	NRX3B_HUMAN Neurexin-3-beta OS=Homo sapiens GN=NRXN3 PE=1 SV=4		
Q9HDB5-2	NRX3B_HUMAN Isoform 2 of Neurexin-3-beta OS=Homo sapiens GN=NRXN3		
Q9HDB5-3	NRX3B_HUMAN Isoform 3 of Neurexin-3-beta OS=Homo sapiens GN=NRXN3		
Q9HDB5-4	NRX3B_HUMAN Isoform 4 of Neurexin-3-beta OS=Homo sapiens GN=NRXN3		
Q9HDC9	APMAP_HUMAN Adipocyte plasma membrane-associated protein OS=Homo sapiens GN=APMAP PE=1 SV=2		506.99
Q9HDC9-2	APMAP_HUMAN Isoform 2 of Adipocyte plasma membrane-associated protein OS=Homo sapiens GN=APMAP		336.2
Q9HDC9;A2A2F9;Q9HDC9	APMAP_HUMAN Adipocyte plasma membrane-associated protein OS=Homo sapiens GN=APMAP PE=1 SV=2;>tr|A2A2F9|A2A2F9_HUM	9.66E-39	
Q9NNW5	WDR6_HUMAN WD repeat-containing protein 6 OS=Homo sapiens GN=WDR6 PE=1 SV=1	5.51E-15	
Q9NNW7;D3YTF9;Q59ET7	TRXR2_HUMAN Thioredoxin reductase 2, mitochondrial OS=Homo sapiens GN=TXNRD2 PE=1 SV=3;>tr|D3YTF9|D3YTF9_HUMAN Unc	3.04E-37	
Q9NP58	ABCB6_HUMAN ATP-binding cassette sub-family B member 6, mitochondrial OS=Homo sapiens GN=ABCB6 PE=1 SV=1		

Q9NP58-4	ABCB6_HUMAN Isoform 2 of ATP-binding cassette sub-family B member 6, mitochondrial OS=Homo sapiens GN=ABCB6		
Q9NP61;B7Z4Q0;B7Z6L2;	ARFG3_HUMAN ADP-ribosylation factor GTPase-activating protein 3 OS=Homo sapiens GN=ARFGAP3 PE=1 SV=1;>tr|B7Z4Q0|B7Z4Q(	9.00E-09	
Q9NP72	RAB18_HUMAN Ras-related protein Rab-18 OS=Homo sapiens GN=RAB18 PE=1 SV=1		300.15
Q9NP73	ALG13_HUMAN UDP-N-acetylglucosamine transferase subunit ALG13 homolog OS=Homo sapiens GN=ALG13 PE=1 SV=2		29.34
Q9NP73-2	ALG13_HUMAN Isoform 2 of UDP-N-acetylglucosamine transferase subunit ALG13 homolog OS=Homo sapiens GN=ALG13		53.81
Q9NP73-3	ALG13_HUMAN Isoform 3 of UDP-N-acetylglucosamine transferase subunit ALG13 homolog OS=Homo sapiens GN=ALG13		
Q9NP73-4	ALG13_HUMAN Isoform 4 of UDP-N-acetylglucosamine transferase subunit ALG13 homolog OS=Homo sapiens GN=ALG13		
Q9NP74	PALMD_HUMAN Palmdelphin OS=Homo sapiens GN=PALMD PE=1 SV=1		29.3
Q9NP77	SSU72_HUMAN RNA polymerase II subunit A C-terminal domain phosphatase SSU72 OS=Homo sapiens GN=SSU72 PE=1 SV=1		83.03
Q9NP77-2	SSU72_HUMAN Isoform 2 of RNA polymerase II subunit A C-terminal domain phosphatase SSU72 OS=Homo sapiens GN=SSU72		83.03
Q9NP77;B4DMK6;Q5SV20	SSU72_HUMAN RNA polymerase II subunit A C-terminal domain phosphatase SSU72 OS=Homo sapiens GN=SSU72 PE=1 SV=1;>tr|B	8.03E-09	
Q9NP79	VTA1_HUMAN Vacuolar protein sorting-associated protein VTA1 homolog OS=Homo sapiens GN=VTA1 PE=1 SV=1	1.28E-16	
Q9NP81	SYSM_HUMAN Seryl-tRNA synthetase, mitochondrial OS=Homo sapiens GN=SARS2 PE=1 SV=1	2.64E-05	
Q9NP90	RAB9B_HUMAN Ras-related protein Rab-9B OS=Homo sapiens GN=RAB9B PE=1 SV=1		31.61
Q9NP92	RT30_HUMAN 28S ribosomal protein S30, mitochondrial OS=Homo sapiens GN=MRPS30 PE=1 SV=2		27.52
Q9NP97	DLRB1_HUMAN Dynein light chain roadblock-type 1 OS=Homo sapiens GN=DYNLRB1 PE=1 SV=3		169.78
Q9NPA0	CO024_HUMAN UPF0480 protein C15orf24 OS=Homo sapiens GN=C15orf24 PE=1 SV=1		58.09
Q9NPA8	ENY2_HUMAN Enhancer of yellow 2 transcription factor homolog OS=Homo sapiens GN=ENY2 PE=1 SV=1		92.67
Q9NPD3	EXOS4_HUMAN Exosome complex component RRP41 OS=Homo sapiens GN=EXOSC4 PE=1 SV=3		164.82
Q9NPD8	UBE2T_HUMAN Ubiquitin-conjugating enzyme E2 T OS=Homo sapiens GN=UBE2T PE=1 SV=1	4.71E-22	
Q9NPE3	NOP10_HUMAN H/ACA ribonucleoprotein complex subunit 3 OS=Homo sapiens GN=NOP10 PE=1 SV=1		277.37
Q9NPF4	OSGEP_HUMAN Probable tRNA threonylcarbamoyladenosine biosynthesis protein OSGEP OS=Homo sapiens GN=OSGEP PE=1 SV=1		45.85
Q9NPF5	DMAP1_HUMAN DNA methyltransferase 1-associated protein 1 OS=Homo sapiens GN=DMAP1 PE=1 SV=1		
Q9NPH2;B3KRT1;Q9NPH2	INO1_HUMAN Inositol-3-phosphate synthase 1 OS=Homo sapiens GN=ISYNA1 PE=1 SV=1;>tr|B3KRT1|B3KRT1_HUMAN cDNA FLJ348	5.41E-119	
Q9NPI1	BRD7_HUMAN Bromodomain-containing protein 7 OS=Homo sapiens GN=BRD7 PE=1 SV=1		
Q9NPI1-2	BRD7_HUMAN Isoform 2 of Bromodomain-containing protein 7 OS=Homo sapiens GN=BRD7		
Q9NPJ1	MKKS_HUMAN McKusick-Kaufman/Bardet-Biedl syndromes putative chaperonin OS=Homo sapiens GN=MKKS PE=1 SV=1		
Q9NPJ3	ACO13_HUMAN Acyl-coenzyme A thioesterase 13 OS=Homo sapiens GN=ACOT13 PE=1 SV=1		105.69
Q9NPJ6	MED4_HUMAN Mediator of RNA polymerase II transcription subunit 4 OS=Homo sapiens GN=MED4 PE=1 SV=1		
Q9NPJ8	NXT2_HUMAN NTF2-related export protein 2 OS=Homo sapiens GN=NXT2 PE=2 SV=1		
Q9NPJ8-2	NXT2_HUMAN Isoform 2 of NTF2-related export protein 2 OS=Homo sapiens GN=NXT2		
Q9NPJ8-3	NXT2_HUMAN Isoform 3 of NTF2-related export protein 2 OS=Homo sapiens GN=NXT2		
Q9NPJ8-3;Q9NPJ8-2;Q9NI	3|NXT2_HUMAN Isoform 3 of NTF2-related export protein 2 OS=Homo sapiens GN=NXT2;>sp|Q9NPJ8-2|NXT2_HUMAN Isoform 2 of N	5.06E-11	
Q9NPL8	TIDC1_HUMAN Translocase of inner mitochondrial membrane domain-containing protein 1 OS=Homo sapiens GN=TIMMDC1 PE=1 SV=	8.89E-06	
Q9NPP4	NLRC4_HUMAN NLR family CARD domain-containing protein 4 OS=Homo sapiens GN=NLRC4 PE=1 SV=2		31.36
Q9NPP4-2	NLRC4_HUMAN Isoform 2 of NLR family CARD domain-containing protein 4 OS=Homo sapiens GN=NLRC4		29.41
Q9NPP4-3	NLRC4_HUMAN Isoform 3 of NLR family CARD domain-containing protein 4 OS=Homo sapiens GN=NLRC4		1.01
Q9NPP4-4	NLRC4_HUMAN Isoform 4 of NLR family CARD domain-containing protein 4 OS=Homo sapiens GN=NLRC4		1.01
Q9NPQ8-3;Q9NPQ8;Q9NP	3|RIC8A_HUMAN Isoform 3 of Synembryn-A OS=Homo sapiens GN=RIC8A;>sp|Q9NPQ8|RIC8A_HUMAN Synembryn-A OS=Homo sap	1.09E-09	
Q9NQ29	LUC7L_HUMAN Putative RNA-binding protein Luc7-like 1 OS=Homo sapiens GN=LUC7L PE=1 SV=1		251.05
Q9NQ29-2	LUC7L_HUMAN Isoform 2 of Putative RNA-binding protein Luc7-like 1 OS=Homo sapiens GN=LUC7L		251.05
Q9NQ29-3	LUC7L_HUMAN Isoform 3 of Putative RNA-binding protein Luc7-like 1 OS=Homo sapiens GN=LUC7L		251.05
Q9NQ29;Q9NQ29-2;Q9NQ	LUC7L_HUMAN Putative RNA-binding protein Luc7-like 1 OS=Homo sapiens GN=LUC7L PE=1 SV=1;>sp|Q9NQ29-2|LUC7L_HUMAN Iso	4.67E-64	
Q9NQ38	ISK5_HUMAN Serine protease inhibitor Kazal-type 5 OS=Homo sapiens GN=SPINK5 PE=1 SV=2		28.93
Q9NQ38-2	ISK5_HUMAN Isoform short of Serine protease inhibitor Kazal-type 5 OS=Homo sapiens GN=SPINK5		28.93
Q9NQ38-3	ISK5_HUMAN Isoform long of Serine protease inhibitor Kazal-type 5 OS=Homo sapiens GN=SPINK5		28.93
Q9NQ39	RS10L_HUMAN Putative 40S ribosomal protein S10-like OS=Homo sapiens GN=RPS10P5 PE=5 SV=1		270.14
Q9NQ48	LZTL1_HUMAN Leucine zipper transcription factor-like protein 1 OS=Homo sapiens GN=LZTFL1 PE=1 SV=1	1.62E-146	
Q9NQ50	RM40_HUMAN 39S ribosomal protein L40, mitochondrial OS=Homo sapiens GN=MRPL40 PE=1 SV=1		66.18
Q9NQ55	SSF1_HUMAN Suppressor of SWI4 1 homolog OS=Homo sapiens GN=PPAN PE=1 SV=1		
Q9NQ55-2	SSF1_HUMAN Isoform 2 of Suppressor of SWI4 1 homolog OS=Homo sapiens GN=PPAN		
Q9NQ55;Q9NQ55-2	SSF1_HUMAN Suppressor of SWI4 1 homolog OS=Homo sapiens GN=PPAN PE=1 SV=1;>sp|Q9NQ55-2|SSF1_HUMAN Isoform 2 of Su	3.35E-22	
Q9NQ75	CASS4_HUMAN Cas scaffolding protein family member 4 OS=Homo sapiens GN=CASS4 PE=1 SV=2		
Q9NQ75-2	CASS4_HUMAN Isoform 2 of Cas scaffolding protein family member 4 OS=Homo sapiens GN=CASS4		
Q9NQ75-3	CASS4_HUMAN Isoform 3 of Cas scaffolding protein family member 4 OS=Homo sapiens GN=CASS4		
Q9NQ88	TIGAR_HUMAN Probable fructose-2,6-bisphosphatase TIGAR OS=Homo sapiens GN=TIGAR PE=1 SV=1	5.63E-44	
Q9NQC3	RTN4_HUMAN Reticulon-4 OS=Homo sapiens GN=RTN4 PE=1 SV=2		625.57
Q9NQC3-2	RTN4_HUMAN Isoform 2 of Reticulon-4 OS=Homo sapiens GN=RTN4		677.04
Q9NQC3-3	RTN4_HUMAN Isoform 3 of Reticulon-4 OS=Homo sapiens GN=RTN4		403.04
Q9NQC3-4	RTN4_HUMAN Isoform 4 of Reticulon-4 OS=Homo sapiens GN=RTN4		413.24
Q9NQC3-5	RTN4_HUMAN Isoform 5 of Reticulon-4 OS=Homo sapiens GN=RTN4		677.49

Q9NQC3-6	RTN4_HUMAN Isoform 6 of Reticulon-4 OS=Homo sapiens GN=RTN4		413.14
Q9NQC3;Q9NQC3-5;Q7L7	RTN4_HUMAN Reticulon-4 OS=Homo sapiens GN=RTN4 PE=1 SV=2;>sp|Q9NQC3-5|RTN4_HUMAN Isoform 5 of Reticulon-4 OS=Homo	2.16E-213	
Q9NQE9	HINT3_HUMAN Histidine triad nucleotide-binding protein 3 OS=Homo sapiens GN=HINT3 PE=1 SV=1	7.40E-10	
Q9NQG5	RPR1B_HUMAN Regulation of nuclear pre domain-containing protein 1B OS=Homo sapiens GN=RPRD1B PE=1 SV=1		333.74
Q9NQH7	XPP3_HUMAN Probable Xaa-Pro aminopeptidase 3 OS=Homo sapiens GN=XPNPEP3 PE=1 SV=1		51.48
Q9NQH7-2	XPP3_HUMAN Isoform 2 of Probable Xaa-Pro aminopeptidase 3 OS=Homo sapiens GN=XPNPEP3		40.2
Q9NQH7-3	XPP3_HUMAN Isoform 3 of Probable Xaa-Pro aminopeptidase 3 OS=Homo sapiens GN=XPNPEP3		45.86
Q9NQH7-4	XPP3_HUMAN Isoform 4 of Probable Xaa-Pro aminopeptidase 3 OS=Homo sapiens GN=XPNPEP3		51.48
Q9NQH7-5	XPP3_HUMAN Isoform 5 of Probable Xaa-Pro aminopeptidase 3 OS=Homo sapiens GN=XPNPEP3		45.86
Q9NQH7;Q9NQH7-4;Q9NQ	XPP3_HUMAN Probable Xaa-Pro aminopeptidase 3 OS=Homo sapiens GN=XPNPEP3 PE=1 SV=1;>sp|Q9NQH7-4|XPP3_HUMAN Isoform	1.60E-09	
Q9NQI0	DDX4_HUMAN Probable ATP-dependent RNA helicase DDX4 OS=Homo sapiens GN=DDX4 PE=2 SV=2		86.82
Q9NQI0-2	DDX4_HUMAN Isoform 2 of Probable ATP-dependent RNA helicase DDX4 OS=Homo sapiens GN=DDX4		86.82
Q9NQP4	PFD4_HUMAN Prefoldin subunit 4 OS=Homo sapiens GN=PFDN4 PE=1 SV=1		
Q9NQR4	NIT2_HUMAN Omega-amidase NIT2 OS=Homo sapiens GN=NIT2 PE=1 SV=1	8.59E-22	
Q9NQS1	AVEN_HUMAN Cell death regulator Aven OS=Homo sapiens GN=AVEN PE=1 SV=1		
Q9NQS7	INCE_HUMAN Inner centromere protein OS=Homo sapiens GN=INCENP PE=1 SV=3		
Q9NQS7-2	INCE_HUMAN Isoform 2 of Inner centromere protein OS=Homo sapiens GN=INCENP		
Q9NQT4	EXOS5_HUMAN Exosome complex component RRP46 OS=Homo sapiens GN=EXOSC5 PE=1 SV=1		80.11
Q9NQT4;B2RC50	EXOS5_HUMAN Exosome complex exonuclease RRP46 OS=Homo sapiens GN=EXOSC5 PE=1 SV=1;>tr|B2RC50|B2RC50_HUMAN cDNA	2.25E-43	
Q9NQT5	EXOS3_HUMAN Exosome complex component RRP40 OS=Homo sapiens GN=EXOSC3 PE=1 SV=3		243.11
Q9NQT5;A8K0K6;Q9NYS3	EXOS3_HUMAN Exosome complex exonuclease RRP40 OS=Homo sapiens GN=EXOSC3 PE=1 SV=3;>tr|A8K0K6|A8K0K6_HUMAN cDNA	3.14E-39	
Q9NQT8	KI13B_HUMAN Kinesin-like protein KIF13B OS=Homo sapiens GN=KIF13B PE=1 SV=1		
Q9NQW1	SC31B_HUMAN Protein transport protein Sec31B OS=Homo sapiens GN=SEC31B PE=1 SV=1		
Q9NQW1-2	SC31B_HUMAN Isoform 2 of Protein transport protein Sec31B OS=Homo sapiens GN=SEC31B		
Q9NQW1-4	SC31B_HUMAN Isoform 4 of Protein transport protein Sec31B OS=Homo sapiens GN=SEC31B		
Q9NQW1-5	SC31B_HUMAN Isoform 5 of Protein transport protein Sec31B OS=Homo sapiens GN=SEC31B		
Q9NQW6	ANLN_HUMAN Actin-binding protein anillin OS=Homo sapiens GN=ANLN PE=1 SV=2		103.86
Q9NQW6-2	ANLN_HUMAN Isoform 2 of Actin-binding protein anillin OS=Homo sapiens GN=ANLN		103.86
Q9NQW6;Q9NQW6-2	ANLN_HUMAN Actin-binding protein anillin OS=Homo sapiens GN=ANLN PE=1 SV=2;>sp|Q9NQW6-2|ANLN_HUMAN Isoform 2 of Actin	1.19E-113	
Q9NQW7	XPP1_HUMAN Xaa-Pro aminopeptidase 1 OS=Homo sapiens GN=XPNPEP1 PE=1 SV=3		
Q9NQW7-2	XPP1_HUMAN Isoform 2 of Xaa-Pro aminopeptidase 1 OS=Homo sapiens GN=XPNPEP1		
Q9NQW7;Q9NQW7-2	XPP1_HUMAN Xaa-Pro aminopeptidase 1 OS=Homo sapiens GN=XPNPEP1 PE=1 SV=3;>sp|Q9NQW7-2|XPP1_HUMAN Isoform 2 of Xaa	9.76E-95	
Q9NQX3-2;Q9NQX3	2|GEPH_HUMAN Isoform 2 of Gephyrin OS=Homo sapiens GN=GPHN;>sp|Q9NQX3|GEPH_HUMAN Gephyrin OS=Homo sapiens GN=G	1.33E-19	
Q9NQX4	MYO5C_HUMAN Myosin-Vc OS=Homo sapiens GN=MYO5C PE=1 SV=2		
Q9NQY0;Q9NQY0-2	BIN3_HUMAN Bridging integrator 3 OS=Homo sapiens GN=BIN3 PE=1 SV=1;>sp|Q9NQY0-2|BIN3_HUMAN Isoform 2 of Bridging integ	1.36E-13	
Q9NQZ2	SAS10_HUMAN Something about silencing protein 10 OS=Homo sapiens GN=UTP3 PE=1 SV=1		
Q9NR09	BIRC6_HUMAN Baculoviral IAP repeat-containing protein 6 OS=Homo sapiens GN=BIRC6 PE=1 SV=2		
Q9NR12	PDLI7_HUMAN PDZ and LIM domain protein 7 OS=Homo sapiens GN=PDLIM7 PE=1 SV=1		171.53
Q9NR12-2	PDLI7_HUMAN Isoform 2 of PDZ and LIM domain protein 7 OS=Homo sapiens GN=PDLIM7		97.3
Q9NR12-3	PDLI7_HUMAN Isoform 3 of PDZ and LIM domain protein 7 OS=Homo sapiens GN=PDLIM7		158.94
Q9NR12-4	PDLI7_HUMAN Isoform 4 of PDZ and LIM domain protein 7 OS=Homo sapiens GN=PDLIM7		162.05
Q9NR12-5	PDLI7_HUMAN Isoform 5 of PDZ and LIM domain protein 7 OS=Homo sapiens GN=PDLIM7		162.05
Q9NR12-6	PDLI7_HUMAN Isoform 6 of PDZ and LIM domain protein 7 OS=Homo sapiens GN=PDLIM7		162.05
Q9NR12;Q9NR12-2	PDLI7_HUMAN PDZ and LIM domain protein 7 OS=Homo sapiens GN=PDLIM7 PE=1 SV=1;>sp|Q9NR12-2|PDLI7_HUMAN Isoform 2 of	1.56E-67	
Q9NR19	ACSA_HUMAN Acetyl-coenzyme A synthetase, cytoplasmic OS=Homo sapiens GN=ACSS2 PE=1 SV=1	6.76E-10	
Q9NR22	ANM8_HUMAN Protein arginine N-methyltransferase 8 OS=Homo sapiens GN=PRMT8 PE=1 SV=2		79.99
Q9NR22-2	ANM8_HUMAN Isoform 2 of Protein arginine N-methyltransferase 8 OS=Homo sapiens GN=PRMT8		79.99
Q9NR28	DBLOH_HUMAN Diablo homolog, mitochondrial OS=Homo sapiens GN=DIABLO PE=1 SV=1		118.76
Q9NR28-2	DBLOH_HUMAN Isoform 2 of Diablo homolog, mitochondrial OS=Homo sapiens GN=DIABLO		118.76
Q9NR28;Q9NR28-2	DBLOH_HUMAN Diablo homolog, mitochondrial OS=Homo sapiens GN=DIABLO PE=1 SV=1;>sp|Q9NR28-2|DBLOH_HUMAN Isoform 2	5.42E-11	
Q9NR30	DDX21_HUMAN Nucleolar RNA helicase 2 OS=Homo sapiens GN=DDX21 PE=1 SV=5		329.42
Q9NR30-2	DDX21_HUMAN Isoform 2 of Nucleolar RNA helicase 2 OS=Homo sapiens GN=DDX21		329.42
Q9NR30;Q9NR30-2	DDX21_HUMAN Nucleolar RNA helicase 2 OS=Homo sapiens GN=DDX21 PE=1 SV=5;>sp|Q9NR30-2|DDX21_HUMAN Isoform 2 of Nuc	0	
Q9NR31	SAR1A_HUMAN GTP-binding protein SAR1a OS=Homo sapiens GN=SAR1A PE=1 SV=1		107.91
Q9NR33	DPOE4_HUMAN DNA polymerase epsilon subunit 4 OS=Homo sapiens GN=POLE4 PE=1 SV=2		60.9
Q9NR45	SIAS_HUMAN Sialic acid synthase OS=Homo sapiens GN=NANS PE=1 SV=2		247.29
Q9NR46-2;Q9NR46	2|SHLB2_HUMAN Isoform 2 of Endophilin-B2 OS=Homo sapiens GN=SH3GLB2;>sp|Q9NR46|SHLB2_HUMAN Endophilin-B2 OS=Homo	3.52E-13	
Q9NR46;Q9NR46-2	SHLB2_HUMAN Endophilin-B2 OS=Homo sapiens GN=SH3GLB2 PE=1 SV=1;>sp|Q9NR46-2|SHLB2_HUMAN Isoform 2 of Endophilin-B	6.23E-28	
Q9NR48	ASH1L_HUMAN Probable histone-lysine N-methyltransferase ASH1L OS=Homo sapiens GN=ASH1L PE=1 SV=2		42.21
Q9NR48-2	ASH1L_HUMAN Isoform 2 of Probable histone-lysine N-methyltransferase ASH1L OS=Homo sapiens GN=ASH1L		42.21
Q9NR50;Q9NR50-2;Q9NR	EI2BG_HUMAN Translation initiation factor eIF-2B subunit gamma OS=Homo sapiens GN=EIF2B3 PE=1 SV=1;>sp|Q9NR50-2|EI2BG_	2.03E-18	

Q9NR56	MBNL1_HUMAN Muscleblind-like protein 1 OS=Homo sapiens GN=MBNL1 PE=1 SV=2		
Q9NR56-2	MBNL1_HUMAN Isoform 2 of Muscleblind-like protein 1 OS=Homo sapiens GN=MBNL1		
Q9NR56-3	MBNL1_HUMAN Isoform 3 of Muscleblind-like protein 1 OS=Homo sapiens GN=MBNL1		
Q9NR56;Q5VZF2;Q9NR56	MBNL1_HUMAN Muscleblind-like protein 1 OS=Homo sapiens GN=MBNL1 PE=1 SV=2;>sp|Q5VZF2|MBNL2_HUMAN Muscleblind-like pr	1.77E-12	
Q9NR61	DLL4_HUMAN Delta-like protein 4 OS=Homo sapiens GN=DLL4 PE=1 SV=1		
Q9NR64	KLHL1_HUMAN Kelch-like protein 1 OS=Homo sapiens GN=KLHL1 PE=2 SV=1		26.56
Q9NR77	PXMP2_HUMAN Peroxisomal membrane protein 2 OS=Homo sapiens GN=PXMP2 PE=1 SV=3		15.23
Q9NR96	TLR9_HUMAN Toll-like receptor 9 OS=Homo sapiens GN=TLR9 PE=1 SV=2		
Q9NR96-2	TLR9_HUMAN Isoform 2 of Toll-like receptor 9 OS=Homo sapiens GN=TLR9		
Q9NR96-3	TLR9_HUMAN Isoform 3 of Toll-like receptor 9 OS=Homo sapiens GN=TLR9		
Q9NR96-4	TLR9_HUMAN Isoform 4 of Toll-like receptor 9 OS=Homo sapiens GN=TLR9		
Q9NR96-5	TLR9_HUMAN Isoform 5 of Toll-like receptor 9 OS=Homo sapiens GN=TLR9		
Q9NR99	MXRA5_HUMAN Matrix-remodeling-associated protein 5 OS=Homo sapiens GN=MXRA5 PE=2 SV=3		
Q9NRC6	SPTN5_HUMAN Spectrin beta chain, brain 4 OS=Homo sapiens GN=SPTBN5 PE=1 SV=1		
Q9NRD1	FBX6_HUMAN F-box only protein 6 OS=Homo sapiens GN=FBXO6 PE=1 SV=1	5.75E-11	
Q9NRD5	PICK1_HUMAN PRKCA-binding protein OS=Homo sapiens GN=PICK1 PE=1 SV=2		49.25
Q9NRF8	PYRG2_HUMAN CTP synthase 2 OS=Homo sapiens GN=CTPS2 PE=1 SV=1	1.07E-34	
Q9NRF9	DPOE3_HUMAN DNA polymerase epsilon subunit 3 OS=Homo sapiens GN=POLE3 PE=1 SV=1		130.64
Q9NRG0	CHRC1_HUMAN Chromatin accessibility complex protein 1 OS=Homo sapiens GN=CHRAC1 PE=1 SV=1		
Q9NRG7-2;Q9NRG7;Q9NR	2|D39U1_HUMAN Isoform 2 of Epimerase family protein SDR39U1 OS=Homo sapiens GN=SDR39U1;>sp|Q9NRG7|D39U1_HUMAN Epi	1.01E-09	
Q9NRG7;Q9NRG7-2;Q9NR	D39U1_HUMAN Epimerase family protein SDR39U1 OS=Homo sapiens GN=SDR39U1 PE=2 SV=2;>sp|Q9NRG7-2|D39U1_HUMAN Isof	1.85E-12	
Q9NRG9	AAAS_HUMAN Aladin OS=Homo sapiens GN=AAAS PE=1 SV=1	7.96E-10	
Q9NRH3	TBG2_HUMAN Tubulin gamma-2 chain OS=Homo sapiens GN=TUBG2 PE=1 SV=1		30.66
Q9NRI5	DISC1_HUMAN Disrupted in schizophrenia 1 protein OS=Homo sapiens GN=DISC1 PE=1 SV=3		
Q9NRI5-2	DISC1_HUMAN Isoform 2 of Disrupted in schizophrenia 1 protein OS=Homo sapiens GN=DISC1		
Q9NRI5-3	DISC1_HUMAN Isoform 3 of Disrupted in schizophrenia 1 protein OS=Homo sapiens GN=DISC1		
Q9NRI5-4	DISC1_HUMAN Isoform 4 of Disrupted in schizophrenia 1 protein OS=Homo sapiens GN=DISC1		
Q9NRK6	ABCBA_HUMAN ATP-binding cassette sub-family B member 10, mitochondrial OS=Homo sapiens GN=ABCB10 PE=1 SV=2		
Q9NRL2	BAZ1A_HUMAN Bromodomain adjacent to zinc finger domain protein 1A OS=Homo sapiens GN=BAZ1A PE=1 SV=2		34.84
Q9NRL2-2	BAZ1A_HUMAN Isoform 2 of Bromodomain adjacent to zinc finger domain protein 1A OS=Homo sapiens GN=BAZ1A		34.84
Q9NRL2;Q9NRL2-2	BAZ1A_HUMAN Bromodomain adjacent to zinc finger domain protein 1A OS=Homo sapiens GN=BAZ1A PE=1 SV=2;>sp|Q9NRL2-2|BA	0.00019025	
Q9NRL3	STRN4_HUMAN Striatin-4 OS=Homo sapiens GN=STRN4 PE=1 SV=2	5.46E-16	
Q9NRN5;Q9NRN5-2;Q9NR	OLFL3_HUMAN Olfactomedin-like protein 3 OS=Homo sapiens GN=OLFML3 PE=2 SV=1;>sp|Q9NRN5-2|OLFL3_HUMAN Isoform 2 of O	1.29E-25	
Q9NRN7	ADPPT_HUMAN L-aminoadipate-semialdehyde dehydrogenase-phosphopantetheinyl transferase OS=Homo sapiens GN=AASDHPPT PE=1 SV=2		39.08
Q9NRP0	OSTC_HUMAN Oligosaccharyltransferase complex subunit OSTC OS=Homo sapiens GN=OSTC PE=1 SV=1		68.74
Q9NRP2	CP061_HUMAN Uncharacterized protein C16orf61 OS=Homo sapiens GN=C16orf61 PE=1 SV=1		44.48
Q9NRR4	RNC_HUMAN Ribonuclease 3 OS=Homo sapiens GN=DROSHA PE=1 SV=2		
Q9NRR4-2	RNC_HUMAN Isoform 2 of Ribonuclease 3 OS=Homo sapiens GN=DROSHA		
Q9NRR4-3	RNC_HUMAN Isoform 3 of Ribonuclease 3 OS=Homo sapiens GN=DROSHA		
Q9NRR5	UBQL4_HUMAN Ubiquilin-4 OS=Homo sapiens GN=UBQLN4 PE=1 SV=2	3.60E-17	
Q9NRU3	CNNM1_HUMAN Metal transporter CNNM1 OS=Homo sapiens GN=CNNM1 PE=2 SV=3		24.81
Q9NRU3-2	CNNM1_HUMAN Isoform 2 of Metal transporter CNNM1 OS=Homo sapiens GN=CNNM1		24.81
Q9NRV9	HEBP1_HUMAN Heme-binding protein 1 OS=Homo sapiens GN=HEBP1 PE=1 SV=1		163.29
Q9NRW1	RAB6B_HUMAN Ras-related protein Rab-6B OS=Homo sapiens GN=RAB6B PE=1 SV=1		305.19
Q9NRW3	ABC3C_HUMAN Probable DNA dC->dU-editing enzyme APOBEC-3C OS=Homo sapiens GN=APOBEC3C PE=1 SV=2		98.27
Q9NRW7	VPS45_HUMAN Vacuolar protein sorting-associated protein 45 OS=Homo sapiens GN=VPS45 PE=1 SV=1		90.09
Q9NRX1	PNO1_HUMAN RNA-binding protein PNO1 OS=Homo sapiens GN=PNO1 PE=1 SV=1		49.07
Q9NRX2	RM17_HUMAN 39S ribosomal protein L17, mitochondrial OS=Homo sapiens GN=MRPL17 PE=1 SV=1		138.89
Q9NRX4	PHP14_HUMAN 14 kDa phosphohistidine phosphatase OS=Homo sapiens GN=PHPT1 PE=1 SV=1	6.33E-58	
Q9NRY2	SOSSC_HUMAN SOSS complex subunit C OS=Homo sapiens GN=SSBIP1 PE=1 SV=1		24.25
Q9NRY2-2	SOSSC_HUMAN Isoform 2 of SOSS complex subunit C OS=Homo sapiens GN=SSBIP1		24.25
Q9NRY4;Q9NRY4-2	RHG35_HUMAN Rho GTPase-activating protein 35 OS=Homo sapiens GN=ARHGAP35 PE=1 SV=2;>sp|Q9NRY4-2|RHG35_HUMAN Isof	2.29E-23	
Q9NRY5	F1142_HUMAN Protein FAM114A2 OS=Homo sapiens GN=FAM114A2 PE=1 SV=4		
Q9NRZ9	HELLS_HUMAN Lymphoid-specific helicase OS=Homo sapiens GN=HELLS PE=1 SV=1		
Q9NRZ9-2	HELLS_HUMAN Isoform 2 of Lymphoid-specific helicase OS=Homo sapiens GN=HELLS		
Q9NRZ9-3	HELLS_HUMAN Isoform 3 of Lymphoid-specific helicase OS=Homo sapiens GN=HELLS		
Q9NRZ9-4	HELLS_HUMAN Isoform 4 of Lymphoid-specific helicase OS=Homo sapiens GN=HELLS		
Q9NRZ9-5	HELLS_HUMAN Isoform 5 of Lymphoid-specific helicase OS=Homo sapiens GN=HELLS		
Q9NRZ9-6	HELLS_HUMAN Isoform 6 of Lymphoid-specific helicase OS=Homo sapiens GN=HELLS		
Q9NRZ9-7	HELLS_HUMAN Isoform 7 of Lymphoid-specific helicase OS=Homo sapiens GN=HELLS		
Q9NRZ9-8	HELLS_HUMAN Isoform 8 of Lymphoid-specific helicase OS=Homo sapiens GN=HELLS		

Q9NRZ9;Q9NRZ9-2;Q9NR	HELLS_HUMAN Lymphoid-specific helicase OS=Homo sapiens GN=HELLS PE=1 SV=1;>sp|Q9NRZ9-2|HELLS_HUMAN Isoform 2 of Lym	5.10E-10	
Q9NS23	RASF1_HUMAN Ras association domain-containing protein 1 OS=Homo sapiens GN=RASSF1 PE=1 SV=1		
Q9NS23-2	RASF1_HUMAN Isoform A of Ras association domain-containing protein 1 OS=Homo sapiens GN=RASSF1		
Q9NS23-3	RASF1_HUMAN Isoform B of Ras association domain-containing protein 1 OS=Homo sapiens GN=RASSF1		
Q9NS23-4	RASF1_HUMAN Isoform C of Ras association domain-containing protein 1 OS=Homo sapiens GN=RASSF1		
Q9NS23-5	RASF1_HUMAN Isoform E of Ras association domain-containing protein 1 OS=Homo sapiens GN=RASSF1		
Q9NS25	SPNXB_HUMAN Sperm protein associated with the nucleus on the X chromosome B/F OS=Homo sapiens GN=SPANXB1 PE=2 SV=1		30.11
Q9NS69	TOM22_HUMAN Mitochondrial import receptor subunit TOM22 homolog OS=Homo sapiens GN=TOMM22 PE=1 SV=3		176.72
Q9NS84	CHST7_HUMAN Carbohydrate sulfotransferase 7 OS=Homo sapiens GN=CHST7 PE=1 SV=2		
Q9NS86	LANC2_HUMAN LanC-like protein 2 OS=Homo sapiens GN=LANCL2 PE=1 SV=1		
Q9NS87;Q9NS87-2;Q9NS	KIF15_HUMAN Kinesin-like protein KIF15 OS=Homo sapiens GN=KIF15 PE=1 SV=1;>sp|Q9NS87-2|KIF15_HUMAN Isoform 2 of Kines	4.00E-19	
Q9NSB2	KRT84_HUMAN Keratin, type II cuticular Hb4 OS=Homo sapiens GN=KRT84 PE=1 SV=2		187.04
Q9NSB4	KRT82_HUMAN Keratin, type II cuticular Hb2 OS=Homo sapiens GN=KRT82 PE=1 SV=3		22.17
Q9NSC5	HOME3_HUMAN Homer protein homolog 3 OS=Homo sapiens GN=HOMER3 PE=1 SV=2		36.75
Q9NSC5-2	HOME3_HUMAN Isoform 2 of Homer protein homolog 3 OS=Homo sapiens GN=HOMER3		36.75
Q9NSC5;Q9NSC5-2	HOME3_HUMAN Homer protein homolog 3 OS=Homo sapiens GN=HOMER3 PE=1 SV=2;>sp|Q9NSC5-2|HOME3_HUMAN Isoform 2 of	4.41E-09	
Q9NSD9	SYFB_HUMAN Phenylalanyl-tRNA synthetase beta chain OS=Homo sapiens GN=FARSB PE=1 SV=3		565.87
Q9NSE4	SYIM_HUMAN Isoleucyl-tRNA synthetase, mitochondrial OS=Homo sapiens GN=IARS2 PE=1 SV=2		203.81
Q9NSE7	ABCCD_HUMAN Putative ATP-binding cassette sub-family C member 13 OS=Homo sapiens GN=ABCC13 PE=2 SV=2		28.97
Q9NSE7-2	ABCCD_HUMAN Isoform 2 of Putative ATP-binding cassette sub-family C member 13 OS=Homo sapiens GN=ABCC13		28.97
Q9NSE7-3	ABCCD_HUMAN Isoform 3 of Putative ATP-binding cassette sub-family C member 13 OS=Homo sapiens GN=ABCC13		28.97
Q9NSE7-4	ABCCD_HUMAN Isoform 4 of Putative ATP-binding cassette sub-family C member 13 OS=Homo sapiens GN=ABCC13		31.88
Q9NSE7-5	ABCCD_HUMAN Isoform 5 of Putative ATP-binding cassette sub-family C member 13 OS=Homo sapiens GN=ABCC13		
Q9NSI2	CU070_HUMAN Uncharacterized protein C21orf70 OS=Homo sapiens GN=C21orf70 PE=1 SV=2		
Q9NSI2-2	CU070_HUMAN Isoform B of Uncharacterized protein C21orf70 OS=Homo sapiens GN=C21orf70		
Q9NSI2;Q9NSI2-2	CU070_HUMAN Uncharacterized protein C21orf70 OS=Homo sapiens GN=C21orf70 PE=1 SV=2;>sp|Q9NSI2-2|CU070_HUMAN Isoforr	3.94E-08	
Q9NSI6	BRWD1_HUMAN Bromodomain and WD repeat-containing protein 1 OS=Homo sapiens GN=BRWD1 PE=1 SV=4		
Q9NSI6-2	BRWD1_HUMAN Isoform B of Bromodomain and WD repeat-containing protein 1 OS=Homo sapiens GN=BRWD1		
Q9NSI6-3	BRWD1_HUMAN Isoform C of Bromodomain and WD repeat-containing protein 1 OS=Homo sapiens GN=BRWD1		
Q9NSV4	DIAP3_HUMAN Protein diaphanous homolog 3 OS=Homo sapiens GN=DIAPH3 PE=1 SV=4		0.83
Q9NSV4-1	DIAP3_HUMAN Isoform 1 of Protein diaphanous homolog 3 OS=Homo sapiens GN=DIAPH3		0.83
Q9NSV4-4	DIAP3_HUMAN Isoform 4 of Protein diaphanous homolog 3 OS=Homo sapiens GN=DIAPH3		0.83
Q9NSV4-5	DIAP3_HUMAN Isoform 5 of Protein diaphanous homolog 3 OS=Homo sapiens GN=DIAPH3		0.83
Q9NSV4-6	DIAP3_HUMAN Isoform 6 of Protein diaphanous homolog 3 OS=Homo sapiens GN=DIAPH3		0.83
Q9NSV4-7	DIAP3_HUMAN Isoform 7 of Protein diaphanous homolog 3 OS=Homo sapiens GN=DIAPH3		0.83
Q9NSV4;Q9NSV4-4;Q9NS	DIAP3_HUMAN Protein diaphanous homolog 3 OS=Homo sapiens GN=DIAPH3 PE=1 SV=4;>sp|Q9NSV4-4|DIAP3_HUMAN Isoform 4 o	1.94E-67	
Q9NSY0-5	NRBP2_HUMAN Isoform 4 of Nuclear receptor-binding protein 2 OS=Homo sapiens GN=NRBP2		
Q9NSY1	BMP2K_HUMAN BMP-2-inducible protein kinase OS=Homo sapiens GN=BMP2K PE=1 SV=2	2.86E-06	
Q9NT62;Q9NT62-2	ATG3_HUMAN Ubiquitin-like-conjugating enzyme ATG3 OS=Homo sapiens GN=ATG3 PE=1 SV=1;>sp|Q9NT62-2|ATG3_HUMAN Isoforr	5.40E-15	
Q9NTI2	AT8A2_HUMAN Probable phospholipid-transporting ATPase IB OS=Homo sapiens GN=ATP8A2 PE=2 SV=2		
Q9NTI2-3	AT8A2_HUMAN Isoform 2 of Probable phospholipid-transporting ATPase IB OS=Homo sapiens GN=ATP8A2		
Q9NTI5	PDS5B_HUMAN Sister chromatid cohesion protein PDS5 homolog B OS=Homo sapiens GN=PDS5B PE=1 SV=1		61.75
Q9NTI5-2	PDS5B_HUMAN Isoform 2 of Sister chromatid cohesion protein PDS5 homolog B OS=Homo sapiens GN=PDS5B		61.75
Q9NTI5-3	PDS5B_HUMAN Isoform 3 of Sister chromatid cohesion protein PDS5 homolog B OS=Homo sapiens GN=PDS5B		30.64
Q9NTI5-4	PDS5B_HUMAN Isoform 4 of Sister chromatid cohesion protein PDS5 homolog B OS=Homo sapiens GN=PDS5B		
Q9NTI5-5	PDS5B_HUMAN Isoform 5 of Sister chromatid cohesion protein PDS5 homolog B OS=Homo sapiens GN=PDS5B		
Q9NTI5;Q9NTI5-2	PDS5B_HUMAN Sister chromatid cohesion protein PDS5 homolog B OS=Homo sapiens GN=PDS5B PE=1 SV=1;>sp|Q9NTI5-2|PDS5B_	9.89E-209	
Q9NTJ3	SMC4_HUMAN Structural maintenance of chromosomes protein 4 OS=Homo sapiens GN=SMC4 PE=1 SV=2		141.98
Q9NTJ3-2	SMC4_HUMAN Isoform 2 of Structural maintenance of chromosomes protein 4 OS=Homo sapiens GN=SMC4		129.47
Q9NTJ3;Q9NTJ3-2	SMC4_HUMAN Structural maintenance of chromosomes protein 4 OS=Homo sapiens GN=SMC4 PE=1 SV=2;>sp|Q9NTJ3-2|SMC4_HUI	1.00E-76	
Q9NTJ4	MA2C1_HUMAN Alpha-mannosidase 2C1 OS=Homo sapiens GN=MAN2C1 PE=1 SV=1		
Q9NTJ5	SAC1_HUMAN Phosphatidylinositide phosphatase SAC1 OS=Homo sapiens GN=SACM1L PE=1 SV=2		26.14
Q9NTK5	OLA1_HUMAN Obg-like ATPase 1 OS=Homo sapiens GN=OLA1 PE=1 SV=2		245.36
Q9NTK5-2	OLA1_HUMAN Isoform 2 of Obg-like ATPase 1 OS=Homo sapiens GN=OLA1		84.75
Q9NTK5-3	OLA1_HUMAN Isoform 3 of Obg-like ATPase 1 OS=Homo sapiens GN=OLA1		169.26
Q9NTK5;Q9NTK5-2	OLA1_HUMAN Obg-like ATPase 1 OS=Homo sapiens GN=OLA1 PE=1 SV=2;>sp|Q9NTK5-2|OLA1_HUMAN Isoform 2 of Obg-like ATPase	4.39E-126	
Q9NTX5;Q9NTX5-2	ECHD1_HUMAN Enoyl-CoA hydratase domain-containing protein 1 OS=Homo sapiens GN=ECHDC1 PE=1 SV=2;>sp|Q9NTX5-2|ECHD1	1.21E-32	
Q9NTZ6	RBM12_HUMAN RNA-binding protein 12 OS=Homo sapiens GN=RBM12 PE=1 SV=1		94.51
Q9NU22	MDN1_HUMAN Midasin OS=Homo sapiens GN=MDN1 PE=1 SV=2		
Q9NUB1;Q9NUB1-2	ACS2L_HUMAN Acetyl-coenzyme A synthetase 2-like, mitochondrial OS=Homo sapiens GN=ACSS1 PE=1 SV=2;>sp|Q9NUB1-2|ACS2L	7.11E-40	
Q9NUH8	TM14B_HUMAN Transmembrane protein 14B OS=Homo sapiens GN=TMEM14B PE=2 SV=1		81.8

Q9NUI1;Q4VXZ8;Q9NUI1-	DECR2_HUMAN Peroxisomal 2,4-dienoyl-CoA reductase OS=Homo sapiens GN=DECR2 PE=1 SV=1;>tr|Q4VXZ8|Q4VXZ8_HUMAN 2,4-	2.06E-09	
Q9NUJ1	ABHDA_HUMAN Abhydrolase domain-containing protein 10, mitochondrial OS=Homo sapiens GN=ABHD10 PE=1 SV=1		224.6
Q9NUJ7	PLCX1_HUMAN PI-PLC X domain-containing protein 1 OS=Homo sapiens GN=PLCXD1 PE=2 SV=1		
Q9NUL3	STAU2_HUMAN Double-stranded RNA-binding protein Staufen homolog 2 OS=Homo sapiens GN=STAU2 PE=1 SV=1		55.63
Q9NUL3-2	STAU2_HUMAN Isoform 2 of Double-stranded RNA-binding protein Staufen homolog 2 OS=Homo sapiens GN=STAU2		55.63
Q9NUL3-3	STAU2_HUMAN Isoform 3 of Double-stranded RNA-binding protein Staufen homolog 2 OS=Homo sapiens GN=STAU2		55.63
Q9NUL3-4	STAU2_HUMAN Isoform 4 of Double-stranded RNA-binding protein Staufen homolog 2 OS=Homo sapiens GN=STAU2		60.46
Q9NUL3;Q9NUL3-2;Q9NUI	STAU2_HUMAN Double-stranded RNA-binding protein Staufen homolog 2 OS=Homo sapiens GN=STAU2 PE=1 SV=1;>sp|Q9NUL3-2|S1	4.84E-16	
Q9NUM4	T106B_HUMAN Transmembrane protein 106B OS=Homo sapiens GN=TMEM106B PE=1 SV=2		53.29
Q9NUP9	LIN7C_HUMAN Protein lin-7 homolog C OS=Homo sapiens GN=LIN7C PE=1 SV=1		464.26
Q9NUQ2	PLCE_HUMAN 1-acyl-sn-glycerol-3-phosphate acyltransferase epsilon OS=Homo sapiens GN=AGPAT5 PE=1 SV=3		45.48
Q9NUQ3	TXLNG_HUMAN Gamma-taxilin OS=Homo sapiens GN=TXLNG PE=1 SV=2		103.84
Q9NUQ3-2	TXLNG_HUMAN Isoform 2 of Gamma-taxilin OS=Homo sapiens GN=TXLNG		59.1
Q9NUQ3;Q9NUQ3-2	TXLNG_HUMAN Gamma-taxilin OS=Homo sapiens GN=TXLNG PE=1 SV=2;>sp|Q9NUQ3-2|TXLNG_HUMAN Isoform 2 of Gamma-taxilin	6.55E-12	
Q9NUQ6	SPS2L_HUMAN SPATS2-like protein OS=Homo sapiens GN=SPATS2L PE=1 SV=2		174.09
Q9NUQ6-2	SPS2L_HUMAN Isoform 2 of SPATS2-like protein OS=Homo sapiens GN=SPATS2L		185.1
Q9NUQ7	UFSP2_HUMAN Ufm1-specific protease 2 OS=Homo sapiens GN=UFSP2 PE=2 SV=3	1.01E-16	
Q9NUQ8	ABCF3_HUMAN ATP-binding cassette sub-family F member 3 OS=Homo sapiens GN=ABCF3 PE=1 SV=2		
Q9NUQ8-2	ABCF3_HUMAN Isoform 2 of ATP-binding cassette sub-family F member 3 OS=Homo sapiens GN=ABCF3		
Q9NUQ8;Q9NUQ8-2	ABCF3_HUMAN ATP-binding cassette sub-family F member 3 OS=Homo sapiens GN=ABCF3 PE=1 SV=2;>sp|Q9NUQ8-2|ABCF3_HUMA	1.92E-07	
Q9NUQ9	FA49B_HUMAN Protein FAM49B OS=Homo sapiens GN=FAM49B PE=1 SV=1		144.19
Q9NUS5	CT029_HUMAN Protein C20orf29 OS=Homo sapiens GN=C20orf29 PE=1 SV=1		
Q9NUT2	ABCB8_HUMAN ATP-binding cassette sub-family B member 8, mitochondrial OS=Homo sapiens GN=ABCB8 PE=1 SV=3		
Q9NUT2-2	ABCB8_HUMAN Isoform Short of ATP-binding cassette sub-family B member 8, mitochondrial OS=Homo sapiens GN=ABCB8		
Q9NUU7;Q9UMR2;Q9UMR	DD19A_HUMAN ATP-dependent RNA helicase DDX19A OS=Homo sapiens GN=DDX19A PE=1 SV=1;>sp|Q9UMR2|DD19B_HUMAN ATP-	2.33E-62	
Q9NUV9	GIMA4_HUMAN GTPase IMAP family member 4 OS=Homo sapiens GN=GIMAP4 PE=1 SV=1	2.76E-49	
Q9NUY8;B9A6M5;Q9NUY8	TBC23_HUMAN TBC1 domain family member 23 OS=Homo sapiens GN=TBC1D23 PE=1 SV=3;>tr|B9A6M5|B9A6M5_HUMAN TBC1 dor	3.41E-18	
Q9NV06	DCA13_HUMAN DDB1- and CUL4-associated factor 13 OS=Homo sapiens GN=DCAF13 PE=1 SV=2		
Q9NV06-2	DCA13_HUMAN Isoform 2 of DDB1- and CUL4-associated factor 13 OS=Homo sapiens GN=DCAF13		
Q9NV31	IMP3_HUMAN U3 small nucleolar ribonucleoprotein protein IMP3 OS=Homo sapiens GN=IMP3 PE=1 SV=1		54.15
Q9NV35	NUD15_HUMAN Probable 7,8-dihydro-8-oxoguanine triphosphatase NUDT15 OS=Homo sapiens GN=NUDT15 PE=1 SV=1	5.43E-18	
Q9NV56	MRGBP_HUMAN MRG-binding protein OS=Homo sapiens GN=MRGBP PE=1 SV=1		111.98
Q9NV70	EXOC1_HUMAN Exocyst complex component 1 OS=Homo sapiens GN=EXOC1 PE=1 SV=4		
Q9NV70-2	EXOC1_HUMAN Isoform 2 of Exocyst complex component 1 OS=Homo sapiens GN=EXOC1		
Q9NV70;Q9NV70-2	EXOC1_HUMAN Exocyst complex component 1 OS=Homo sapiens GN=EXOC1 PE=1 SV=4;>sp|Q9NV70-2|EXOC1_HUMAN Isoform 2 c	8.37E-11	
Q9NV96	CC50A_HUMAN Cell cycle control protein 50A OS=Homo sapiens GN=TMEM30A PE=1 SV=1		50.36
Q9NV96-2	CC50A_HUMAN Isoform 2 of Cell cycle control protein 50A OS=Homo sapiens GN=TMEM30A		50.36
Q9NV96-3	CC50A_HUMAN Isoform 3 of Cell cycle control protein 50A OS=Homo sapiens GN=TMEM30A		50.36
Q9NV96;Q9NV96-2;Q9NV	CC50A_HUMAN Cell cycle control protein 50A OS=Homo sapiens GN=TMEM30A PE=1 SV=1;>sp|Q9NV96-2|CC50A_HUMAN Isoform 2	3.27E-17	
Q9NVA1	UQCC_HUMAN Ubiquinol-cytochrome c reductase complex chaperone CBP3 homolog OS=Homo sapiens GN=UQCC PE=1 SV=3		52.33
Q9NVA1-2	UQCC_HUMAN Isoform 2 of Ubiquinol-cytochrome c reductase complex chaperone CBP3 homolog OS=Homo sapiens GN=UQCC		52.33
Q9NVA1-3	UQCC_HUMAN Isoform 3 of Ubiquinol-cytochrome c reductase complex chaperone CBP3 homolog OS=Homo sapiens GN=UQCC		
Q9NVA1-4	UQCC_HUMAN Isoform 4 of Ubiquinol-cytochrome c reductase complex chaperone CBP3 homolog OS=Homo sapiens GN=UQCC		
Q9NVA1;Q9NVA1-2	UQCC_HUMAN Ubiquinol-cytochrome c reductase complex chaperone CBP3 homolog OS=Homo sapiens GN=UQCC PE=1 SV=3;>sp|Q	1.22E-09	
Q9NVA2	SEP11_HUMAN Septin-11 OS=Homo sapiens GN=SEPT11 PE=1 SV=3		485.89
Q9NVA2-2	SEP11_HUMAN Isoform 2 of Septin-11 OS=Homo sapiens GN=SEPT11		485.89
Q9NVA2-2;D6RER5;Q9NV	2|SEP11_HUMAN Isoform 2 of Septin-11 OS=Homo sapiens GN=SEPT11;>tr|D6RER5|D6RER5_HUMAN Uncharacterized protein OS=H	1.87E-298	
Q9NVA2;Q9NVA2-2	SEP11_HUMAN Septin-11 OS=Homo sapiens GN=SEPT11 PE=1 SV=3;>sp|Q9NVA2-2|SEP11_HUMAN Isoform 2 of Septin-11 OS=Hom	6.35E-50	
Q9NVD7	PARVA_HUMAN Alpha-parvin OS=Homo sapiens GN=PARVA PE=1 SV=1		363.91
Q9NVD7-2	PARVA_HUMAN Isoform 2 of Alpha-parvin OS=Homo sapiens GN=PARVA		72.14
Q9NVD7;B7Z952	PARVA_HUMAN Alpha-parvin OS=Homo sapiens GN=PARVA PE=1 SV=1;>tr|B7Z952|B7Z952_HUMAN cDNA FLJ54827, highly similar t	5.87E-93	
Q9NVE4	CCD87_HUMAN Coiled-coil domain-containing protein 87 OS=Homo sapiens GN=CCDC87 PE=2 SV=2		29.09
Q9NVE7	PANK4_HUMAN Pantothenate kinase 4 OS=Homo sapiens GN=PANK4 PE=1 SV=1	7.33E-13	
Q9NVG8;Q9NVG8-2	TBC13_HUMAN TBC1 domain family member 13 OS=Homo sapiens GN=TBC1D13 PE=1 SV=3;>sp|Q9NVG8-2|TBC13_HUMAN Isoform	2.30E-13	
Q9NVH1	DJC11_HUMAN DnaJ homolog subfamily C member 11 OS=Homo sapiens GN=DNAJC11 PE=1 SV=2		
Q9NVH1-2	DJC11_HUMAN Isoform 2 of DnaJ homolog subfamily C member 11 OS=Homo sapiens GN=DNAJC11		
Q9NVH1-3	DJC11_HUMAN Isoform 3 of DnaJ homolog subfamily C member 11 OS=Homo sapiens GN=DNAJC11		
Q9NVH1;Q9NVH1-3;Q9NV	DJC11_HUMAN DnaJ homolog subfamily C member 11 OS=Homo sapiens GN=DNAJC11 PE=1 SV=2;>sp|Q9NVH1-3|DJC11_HUMAN Is	5.39E-11	
Q9NVH2	INT7_HUMAN Integrator complex subunit 7 OS=Homo sapiens GN=INTS7 PE=1 SV=1		29.57
Q9NVH2-2	INT7_HUMAN Isoform 2 of Integrator complex subunit 7 OS=Homo sapiens GN=INTS7		29.57
Q9NVH2-3	INT7_HUMAN Isoform 3 of Integrator complex subunit 7 OS=Homo sapiens GN=INTS7		29.57

Q9NVH6;Q9NVH6-2	TMLH_HUMAN Trimethyllysine dioxygenase, mitochondrial OS=Homo sapiens GN=TMLHE PE=1 SV=1;>sp|Q9NVH6-2|TMLH_HUMAN I:	3.32E-05	
Q9NVI1	FANCI_HUMAN Fanconi anemia group I protein OS=Homo sapiens GN=FANCI PE=1 SV=4		81.81
Q9NVI1-1	FANCI_HUMAN Isoform 1 of Fanconi anemia group I protein OS=Homo sapiens GN=FANCI		81.81
Q9NVI1-2	FANCI_HUMAN Isoform 2 of Fanconi anemia group I protein OS=Homo sapiens GN=FANCI		81.81
Q9NVI1-4	FANCI_HUMAN Isoform 4 of Fanconi anemia group I protein OS=Homo sapiens GN=FANCI		3.4
Q9NVI1;Q9NVI1-1;Q9NVI	FANCI_HUMAN Fanconi anemia group I protein OS=Homo sapiens GN=FANCI PE=1 SV=4;>sp|Q9NVI1-1|FANCI_HUMAN Isoform 1 of	7.79E-120	
Q9NVI7	ATD3A_HUMAN ATPase family AAA domain-containing protein 3A OS=Homo sapiens GN=ATAD3A PE=1 SV=2		464.12
Q9NVI7-2	ATD3A_HUMAN Isoform 2 of ATPase family AAA domain-containing protein 3A OS=Homo sapiens GN=ATAD3A		565.37
Q9NVI7-2;Q9NVI7	2|ATD3A_HUMAN Isoform 2 of ATPase family AAA domain-containing protein 3A OS=Homo sapiens GN=ATAD3A;>sp|Q9NVI7|ATD3A_	5.25E-139	
Q9NVJ2	ARL8B_HUMAN ADP-ribosylation factor-like protein 8B OS=Homo sapiens GN=ARL8B PE=1 SV=1		315.84
Q9NVM6	DJC17_HUMAN DnaJ homolog subfamily C member 17 OS=Homo sapiens GN=DNAJC17 PE=1 SV=1		40.01
Q9NVM9	M89BB_HUMAN Cell cycle regulator Mat89Bb homolog OS=Homo sapiens GN=C12orf11 PE=1 SV=2	8.23E-16	
Q9NVN3	RIC8B_HUMAN Synembryn-B OS=Homo sapiens GN=RIC8B PE=1 SV=2		
Q9NVN3-1	RIC8B_HUMAN Isoform 1 of Synembryn-B OS=Homo sapiens GN=RIC8B		
Q9NVN3-1;Q9NVN3;Q9NV	1|RIC8B_HUMAN Isoform 1 of Synembryn-B OS=Homo sapiens GN=RIC8B;>sp|Q9NVN3|RIC8B_HUMAN Synembryn-B OS=Homo sap	1.20E-10	
Q9NVN3-3	RIC8B_HUMAN Isoform 3 of Synembryn-B OS=Homo sapiens GN=RIC8B		
Q9NVN3-4	RIC8B_HUMAN Isoform 4 of Synembryn-B OS=Homo sapiens GN=RIC8B		
Q9NVN8	GNL3L_HUMAN Guanine nucleotide-binding protein-like 3-like protein OS=Homo sapiens GN=GNL3L PE=1 SV=1		15.11
Q9NVP1	DDX18_HUMAN ATP-dependent RNA helicase DDX18 OS=Homo sapiens GN=DDX18 PE=1 SV=2		232.56
Q9NVP2	ASF1B_HUMAN Histone chaperone ASF1B OS=Homo sapiens GN=ASF1B PE=1 SV=1	1.52E-11	
Q9NVR0	KLH11_HUMAN Kelch-like protein 11 OS=Homo sapiens GN=KLHL11 PE=1 SV=1		
Q9NVR2	INT10_HUMAN Integrator complex subunit 10 OS=Homo sapiens GN=INTS10 PE=1 SV=2		
Q9NVR7	TBCC1_HUMAN TBCC domain-containing protein 1 OS=Homo sapiens GN=TBCCD1 PE=1 SV=1		
Q9NVS2-2;Q9NVS2	2|RT18A_HUMAN Isoform 2 of 28S ribosomal protein S18a, mitochondrial OS=Homo sapiens GN=MRPS18A;>sp|Q9NVS2|RT18A_HUM	5.23E-06	
Q9NVS9	PNPO_HUMAN Pyridoxine-5'-phosphate oxidase OS=Homo sapiens GN=PNPO PE=1 SV=1		25.56
Q9NVU7	SDA1_HUMAN Protein SDA1 homolog OS=Homo sapiens GN=SDAD1 PE=1 SV=3		137.25
Q9NVU7-2	SDA1_HUMAN Isoform 2 of Protein SDA1 homolog OS=Homo sapiens GN=SDAD1		95.9
Q9NVU7;B4DT66;Q9NVU7	SDA1_HUMAN Protein SDA1 homolog OS=Homo sapiens GN=SDAD1 PE=1 SV=3;>tr|B4DT66|B4DT66_HUMAN cDNA FLJ55367, highly	1.02E-10	
Q9NVV4-2	PAPD1_HUMAN Isoform 2 of Poly(A) RNA polymerase, mitochondrial OS=Homo sapiens GN=MTPAP		
Q9NVV4-2;D3DRX0;Q9NV	2|PAPD1_HUMAN Isoform 2 of Poly(A) RNA polymerase, mitochondrial OS=Homo sapiens GN=MTPAP;>tr|D3DRX0|D3DRX0_HUMAN P	2.21E-14	
Q9NVV5	AIG1_HUMAN Androgen-induced gene 1 protein OS=Homo sapiens GN=AIG1 PE=2 SV=2		23.41
Q9NVV5-2	AIG1_HUMAN Isoform 2 of Androgen-induced gene 1 protein OS=Homo sapiens GN=AIG1		23.41
Q9NVV5-3	AIG1_HUMAN Isoform 3 of Androgen-induced gene 1 protein OS=Homo sapiens GN=AIG1		23.41
Q9NVV5-4	AIG1_HUMAN Isoform 4 of Androgen-induced gene 1 protein OS=Homo sapiens GN=AIG1		3.07
Q9NVV5-5	AIG1_HUMAN Isoform 5 of Androgen-induced gene 1 protein OS=Homo sapiens GN=AIG1		23.41
Q9NVX0	HAUS2_HUMAN HAUS augmin-like complex subunit 2 OS=Homo sapiens GN=HAUS2 PE=1 SV=1		
Q9NVX0-3	HAUS2_HUMAN Isoform 3 of HAUS augmin-like complex subunit 2 OS=Homo sapiens GN=HAUS2		
Q9NVX0;C9JH36;Q9NVX0-	HAUS2_HUMAN HAUS augmin-like complex subunit 2 OS=Homo sapiens GN=HAUS2 PE=1 SV=1;>tr|C9JH36|C9JH36_HUMAN Unchar	3.54E-08	
Q9NVX2	NLE1_HUMAN Notchless protein homolog 1 OS=Homo sapiens GN=NLE1 PE=1 SV=4		19.14
Q9NVZ3	NECP2_HUMAN Adaptin ear-binding coat-associated protein 2 OS=Homo sapiens GN=NECAP2 PE=1 SV=1		24.87
Q9NVZ3-2	NECP2_HUMAN Isoform 2 of Adaptin ear-binding coat-associated protein 2 OS=Homo sapiens GN=NECAP2		24.87
Q9NVZ3-3	NECP2_HUMAN Isoform 3 of Adaptin ear-binding coat-associated protein 2 OS=Homo sapiens GN=NECAP2		15.77
Q9NVZ3;Q9NVZ3-2;Q9NV	NECP2_HUMAN Adaptin ear-binding coat-associated protein 2 OS=Homo sapiens GN=NECAP2 PE=1 SV=1;>sp|Q9NVZ3-2|NECP2_HUI	2.79E-11	
Q9NW08	RPC2_HUMAN DNA-directed RNA polymerase III subunit RPC2 OS=Homo sapiens GN=POLR3B PE=1 SV=2		
Q9NW13	RBM28_HUMAN RNA-binding protein 28 OS=Homo sapiens GN=RBM28 PE=1 SV=3		52.6
Q9NW64	RBM22_HUMAN Pre-mRNA-splicing factor RBM22 OS=Homo sapiens GN=RBM22 PE=1 SV=1	1.16E-09	
Q9NW68;Q9NW68-2;C9JB	BSDC1_HUMAN BSD domain-containing protein 1 OS=Homo sapiens GN=BSDC1 PE=1 SV=1;>sp|Q9NW68-2|BSDC1_HUMAN Isoform	6.38E-12	
Q9NWB6	ARGL1_HUMAN Arginine and glutamate-rich protein 1 OS=Homo sapiens GN=ARGLU1 PE=1 SV=1		
Q9NWB6-2	ARGL1_HUMAN Isoform 2 of Arginine and glutamate-rich protein 1 OS=Homo sapiens GN=ARGLU1		
Q9NWB6;Q9NWB6-2	ARGL1_HUMAN Arginine and glutamate-rich protein 1 OS=Homo sapiens GN=ARGLU1 PE=1 SV=1;>sp|Q9NWB6-2|ARGL1_HUMAN Is	5.20E-06	
Q9NWF9	RN216_HUMAN E3 ubiquitin-protein ligase RNF216 OS=Homo sapiens GN=RNF216 PE=1 SV=3		
Q9NWF9-1	RN216_HUMAN Isoform 2 of E3 ubiquitin-protein ligase RNF216 OS=Homo sapiens GN=RNF216		
Q9NWF9-3	RN216_HUMAN Isoform 3 of E3 ubiquitin-protein ligase RNF216 OS=Homo sapiens GN=RNF216		
Q9NWH9;C9IZZ3	SLTM_HUMAN SAFB-like transcription modulator OS=Homo sapiens GN=SLTM PE=1 SV=2;>tr|C9IZZ3|C9IZZ3_HUMAN Uncharacterize	3.74E-23	
Q9NWS0	PIHD1_HUMAN PIH1 domain-containing protein 1 OS=Homo sapiens GN=PIH1D1 PE=1 SV=1	5.08E-05	
Q9NWS8	RMND1_HUMAN Required for meiotic nuclear division protein 1 homolog OS=Homo sapiens GN=RMND1 PE=1 SV=2		
Q9NWS8-2	RMND1_HUMAN Isoform 2 of Required for meiotic nuclear division protein 1 homolog OS=Homo sapiens GN=RMND1		
Q9NWS8-3	RMND1_HUMAN Isoform 3 of Required for meiotic nuclear division protein 1 homolog OS=Homo sapiens GN=RMND1		
Q9NWS8;Q9NWS8-2	RMND1_HUMAN Required for meiotic nuclear division protein 1 homolog OS=Homo sapiens GN=RMND1 PE=1 SV=2;>sp|Q9NWS8-2|F	7.43E-07	
Q9NWT1	PK1IP_HUMAN p21-activated protein kinase-interacting protein 1 OS=Homo sapiens GN=PAK1IP1 PE=1 SV=2		
Q9NWU2	CT011_HUMAN Protein C20orf11 OS=Homo sapiens GN=C20orf11 PE=1 SV=1		94.76

Q9NWU5	RM22_HUMAN 39S ribosomal protein L22, mitochondrial OS=Homo sapiens GN=MRPL22 PE=1 SV=1		94.74
Q9NWU5-2	RM22_HUMAN Isoform 2 of 39S ribosomal protein L22, mitochondrial OS=Homo sapiens GN=MRPL22		63.8
Q9NWU5;A6NGJ8;Q9NWU	RM22_HUMAN 39S ribosomal protein L22, mitochondrial OS=Homo sapiens GN=MRPL22 PE=1 SV=1;>tr|A6NGJ8|A6NGJ8_HUMAN Un	6.91E-13	
Q9NWV4	CA123_HUMAN UPF0587 protein C1orf123 OS=Homo sapiens GN=C1orf123 PE=1 SV=1	5.94E-18	
Q9NWV8;C9J462;Q9NWV8	MRI40_HUMAN BRCA1-A complex subunit MERIT40 OS=Homo sapiens GN=MERIT40 PE=1 SV=1;>tr|C9J462|C9J462_HUMAN Unchara	2.44E-06	
Q9NWV8;Q9NWV8-3	BABA1_HUMAN BRISC and BRCA1-A complex member 1 OS=Homo sapiens GN=BABAM1 PE=1 SV=1;>sp|Q9NWV8-3|BABA1_HUMAN	1.39E-21	
Q9NWW9	HRSL2_HUMAN HRAS-like suppressor 2 OS=Homo sapiens GN=HRASLS2 PE=1 SV=1		
Q9NWX6	THG1_HUMAN Probable tRNA(His) guanylyltransferase OS=Homo sapiens GN=THG1L PE=1 SV=2	4.09E-13	
Q9NWZ5	UCKL1_HUMAN Uridine-cytidine kinase-like 1 OS=Homo sapiens GN=UCKL1 PE=1 SV=2		
Q9NWZ5-2	UCKL1_HUMAN Isoform 2 of Uridine-cytidine kinase-like 1 OS=Homo sapiens GN=UCKL1		
Q9NWZ5-3	UCKL1_HUMAN Isoform 3 of Uridine-cytidine kinase-like 1 OS=Homo sapiens GN=UCKL1		
Q9NX00	TM160_HUMAN Transmembrane protein 160 OS=Homo sapiens GN=TMEM160 PE=2 SV=1		22.3
Q9NX08	COMD8_HUMAN COMM domain-containing protein 8 OS=Homo sapiens GN=COMMD8 PE=1 SV=1	2.36E-08	
Q9NX14	NDUBB_HUMAN NADH dehydrogenase [ubiquinone] 1 beta subcomplex subunit 11, mitochondrial OS=Homo sapiens GN=NDUFB11 PE=1 SV=1		61.57
Q9NX14-2	NDUBB_HUMAN Isoform 2 of NADH dehydrogenase [ubiquinone] 1 beta subcomplex subunit 11, mitochondrial OS=Homo sapiens GN=NDUFB11		61.57
Q9NX14-2;Q9NX14	2|NDUBB_HUMAN Isoform 2 of NADH dehydrogenase [ubiquinone] 1 beta subcomplex subunit 11, mitochondrial OS=Homo sapiens GI	4.89E-09	
Q9NX18	SDHF2_HUMAN Succinate dehydrogenase assembly factor 2, mitochondrial OS=Homo sapiens GN=SDHAF2 PE=1 SV=1	2.73E-06	
Q9NX20	RM16_HUMAN 39S ribosomal protein L16, mitochondrial OS=Homo sapiens GN=MRPL16 PE=1 SV=1		35.4
Q9NX24	NHP2_HUMAN H/ACA ribonucleoprotein complex subunit 2 OS=Homo sapiens GN=NHP2 PE=1 SV=1		206.72
Q9NX40	OCAD1_HUMAN OCIA domain-containing protein 1 OS=Homo sapiens GN=OCIAD1 PE=1 SV=1		274.17
Q9NX40-2	OCAD1_HUMAN Isoform 2 of OCIA domain-containing protein 1 OS=Homo sapiens GN=OCIAD1		202.83
Q9NX40-3	OCAD1_HUMAN Isoform 3 of OCIA domain-containing protein 1 OS=Homo sapiens GN=OCIAD1		202.83
Q9NX40;D6RBN5;C9K030	OCAD1_HUMAN OCIA domain-containing protein 1 OS=Homo sapiens GN=OCIAD1 PE=1 SV=1;>tr|D6RBN5|D6RBN5_HUMAN Unchara	2.38E-15	
Q9NX46	ARHL2_HUMAN Poly(ADP-ribose) glycohydrolase ARH3 OS=Homo sapiens GN=ADPRHL2 PE=1 SV=1	3.84E-62	
Q9NX47	MARH5_HUMAN E3 ubiquitin-protein ligase MARCH5 OS=Homo sapiens GN=MARCH5 PE=1 SV=1	5.99E-14	
Q9NX55	HYPK_HUMAN Huntingtin-interacting protein K OS=Homo sapiens GN=HYPK PE=1 SV=2		46.51
Q9NX58	LYAR_HUMAN Cell growth-regulating nucleolar protein OS=Homo sapiens GN=LYAR PE=1 SV=2		78.33
Q9NX61	T161A_HUMAN Transmembrane protein 161A OS=Homo sapiens GN=TMEM161A PE=2 SV=1		
Q9NX62	IMPA3_HUMAN Inositol monophosphatase 3 OS=Homo sapiens GN=IMPAD1 PE=1 SV=1		
Q9NX63	CHCH3_HUMAN Coiled-coil-helix-coiled-coil-helix domain-containing protein 3, mitochondrial OS=Homo sapiens GN=CHCHD3 PE=1 SV=1		312.99
Q9NX70	MED29_HUMAN Mediator of RNA polymerase II transcription subunit 29 OS=Homo sapiens GN=MED29 PE=1 SV=1	3.14E-20	
Q9NX74	DUS2L_HUMAN tRNA-dihydrouridine synthase 2-like OS=Homo sapiens GN=DUS2L PE=1 SV=1		21.56
Q9NX76	CKLF6_HUMAN CKLF-like MARVEL transmembrane domain-containing protein 6 OS=Homo sapiens GN=CMTM6 PE=1 SV=1		29.24
Q9NXA8-2	SIRT5_HUMAN Isoform 2 of NAD-dependent deacetylase sirtuin-5 OS=Homo sapiens GN=SIRT5		13.57
Q9NXA8;Q9NXA8-2	SIRT5_HUMAN NAD-dependent deacetylase sirtuin-5 OS=Homo sapiens GN=SIRT5 PE=1 SV=2;>sp|Q9NXA8-2|SIRT5_HUMAN Isoforr	1.44E-08	
Q9NXB9	ELOV2_HUMAN Elongation of very long chain fatty acids protein 2 OS=Homo sapiens GN=ELOVL2 PE=2 SV=2		
Q9NXE4-4;Q9NXE4;Q9NXI	4|NSMA3_HUMAN Isoform 4 of Sphingomyelin phosphodiesterase 4 OS=Homo sapiens GN=SMPD4;>sp|Q9NXE4|NSMA3_HUMAN Sphi	4.96E-33	
Q9NXF1	TEX10_HUMAN Testis-expressed sequence 10 protein OS=Homo sapiens GN=TEX10 PE=1 SV=2		
Q9NXG0	CNTLN_HUMAN Centlein OS=Homo sapiens GN=CNTLN PE=2 SV=5		
Q9NXG0-2	CNTLN_HUMAN Isoform 2 of Centlein OS=Homo sapiens GN=CNTLN		
Q9NXG0-2;Q9NXG0;Q9NX	2|CNTLN_HUMAN Isoform 2 of Centlein OS=Homo sapiens GN=CNTLN;>sp|Q9NXG0|CNTLN_HUMAN Centlein OS=Homo sapiens GN=	8.68E-09	
Q9NXG0-3	CNTLN_HUMAN Isoform 3 of Centlein OS=Homo sapiens GN=CNTLN		
Q9NXG2	THUM1_HUMAN THUMP domain-containing protein 1 OS=Homo sapiens GN=THUMPD1 PE=1 SV=2		45.1
Q9NXH8	CI167_HUMAN Torsin family protein C9orf167 OS=Homo sapiens GN=C9orf167 PE=1 SV=2	9.53E-14	
Q9NXH9	TRM1_HUMAN N(2),N(2)-dimethylguanosine tRNA methyltransferase OS=Homo sapiens GN=TRMT1 PE=1 SV=1		28.82
Q9NXH9-2	TRM1_HUMAN Isoform 2 of N(2),N(2)-dimethylguanosine tRNA methyltransferase OS=Homo sapiens GN=TRMT1		28.82
Q9NXH9;Q9NXH9-2	TRM1_HUMAN N(2),N(2)-dimethylguanosine tRNA methyltransferase OS=Homo sapiens GN=TRMT1 PE=1 SV=1;>sp|Q9NXH9-2|TRM1	3.16E-09	
Q9NXR1	NDE1_HUMAN Nuclear distribution protein nudE homolog 1 OS=Homo sapiens GN=NDE1 PE=1 SV=2		
Q9NXR1-2	NDE1_HUMAN Isoform 2 of Nuclear distribution protein nudE homolog 1 OS=Homo sapiens GN=NDE1		
Q9NXR7	BRE_HUMAN BRCA1-A complex subunit BRE OS=Homo sapiens GN=BRE PE=1 SV=2		55.58
Q9NXR7-1	BRE_HUMAN Isoform 1 of BRCA1-A complex subunit BRE OS=Homo sapiens GN=BRE		55.58
Q9NXR7-1;D6W562;Q9NX	1|BRE_HUMAN Isoform 1 of BRCA1-A complex subunit BRE OS=Homo sapiens GN=BRE;>tr|D6W562|D6W562_HUMAN Brain and repr	2.80E-15	
Q9NXR7-3	BRE_HUMAN Isoform 3 of BRCA1-A complex subunit BRE OS=Homo sapiens GN=BRE		55.58
Q9NXR7-4	BRE_HUMAN Isoform 4 of BRCA1-A complex subunit BRE OS=Homo sapiens GN=BRE		55.58
Q9NXS2	QPCTL_HUMAN Glutaminyl-peptide cyclotransferase-like protein OS=Homo sapiens GN=QPCTL PE=1 SV=2		114.49
Q9NXV2	KCTD5_HUMAN BTB/POZ domain-containing protein KCTD5 OS=Homo sapiens GN=KCTD5 PE=1 SV=1	4.45E-05	
Q9NXV6	CARF_HUMAN CDKN2A-interacting protein OS=Homo sapiens GN=CDKN2AIP PE=1 SV=3		157.2
Q9NXW2	DJB12_HUMAN DnaJ homolog subfamily B member 12 OS=Homo sapiens GN=DNAJB12 PE=1 SV=4		26.32
Q9NXW2-2	DJB12_HUMAN Isoform 2 of DnaJ homolog subfamily B member 12 OS=Homo sapiens GN=DNAJB12		26.32
Q9NXW2-2;Q9NXW2	2|DJB12_HUMAN Isoform 2 of DnaJ homolog subfamily B member 12 OS=Homo sapiens GN=DNAJB12;>sp|Q9NXW2|DJB12_HUMAN	6.49E-10	
Q9NXW9	ALKB4_HUMAN Probable alpha-ketoglutarate-dependent dioxygenase ABH4 OS=Homo sapiens GN=ALKBH4 PE=1 SV=1		

Q9NY12	GAR1_HUMAN H/ACA ribonucleoprotein complex subunit 1 OS=Homo sapiens GN=GAR1 PE=1 SV=1		98.33
Q9NY12-2	GAR1_HUMAN Isoform 2 of H/ACA ribonucleoprotein complex subunit 1 OS=Homo sapiens GN=GAR1		98.33
Q9NY12;Q9NY12-2	GAR1_HUMAN H/ACA ribonucleoprotein complex subunit 1 OS=Homo sapiens GN=GAR1 PE=1 SV=1;>sp|Q9NY12-2|GAR1_HUMAN Is	1.38E-10	
Q9NY15	STAB1_HUMAN Stabilin-1 OS=Homo sapiens GN=STAB1 PE=1 SV=3	3.64E-19	
Q9NY27;Q9NY27-2;Q9NY2	PP4R2_HUMAN Serine/threonine-protein phosphatase 4 regulatory subunit 2 OS=Homo sapiens GN=PPP4R2 PE=1 SV=3;>sp|Q9NY27	6.49E-20	
Q9NY28	GALT8_HUMAN Probable polypeptide N-acetylgalactosaminyltransferase 8 OS=Homo sapiens GN=GALNT8 PE=2 SV=1		
Q9NY33	DPP3_HUMAN Dipeptidyl peptidase 3 OS=Homo sapiens GN=DPP3 PE=1 SV=2	1.13E-142	
Q9NY35;Q9NY35-2	CLDN1_HUMAN Claudin domain-containing protein 1 OS=Homo sapiens GN=CLDND1 PE=1 SV=1;>sp|Q9NY35-2|CLDN1_HUMAN Isof	3.67E-09	
Q9NY47	CA2D2_HUMAN Voltage-dependent calcium channel subunit alpha-2/delta-2 OS=Homo sapiens GN=CACNA2D2 PE=1 SV=1		
Q9NY47-2	CA2D2_HUMAN Isoform 2 of Voltage-dependent calcium channel subunit alpha-2/delta-2 OS=Homo sapiens GN=CACNA2D2		
Q9NY47-3	CA2D2_HUMAN Isoform 3 of Voltage-dependent calcium channel subunit alpha-2/delta-2 OS=Homo sapiens GN=CACNA2D2		
Q9NY47-4	CA2D2_HUMAN Isoform 4 of Voltage-dependent calcium channel subunit alpha-2/delta-2 OS=Homo sapiens GN=CACNA2D2		
Q9NY47-5	CA2D2_HUMAN Isoform 5 of Voltage-dependent calcium channel subunit alpha-2/delta-2 OS=Homo sapiens GN=CACNA2D2		
Q9NY61	AATF_HUMAN Protein AATF OS=Homo sapiens GN=AATF PE=1 SV=1		
Q9NY65	TBA8_HUMAN Tubulin alpha-8 chain OS=Homo sapiens GN=TUBA8 PE=1 SV=1		839.57
Q9NY91	SC5A4_HUMAN Low affinity sodium-glucose cotransporter OS=Homo sapiens GN=SLC5A4 PE=2 SV=1		
Q9NY93	DDX56_HUMAN Probable ATP-dependent RNA helicase DDX56 OS=Homo sapiens GN=DDX56 PE=1 SV=1		
Q9NYB0	TE2IP_HUMAN Telomeric repeat-binding factor 2-interacting protein 1 OS=Homo sapiens GN=TERF2IP PE=1 SV=1		
Q9NYB9;B4DSN1;Q9NYB9	ABI2_HUMAN Abl interactor 2 OS=Homo sapiens GN=ABI2 PE=1 SV=1;>tr|B4DSN1|B4DSN1_HUMAN cDNA FLJ59236, highly similar t	1.24E-54	
Q9NYF0	DACT1_HUMAN Dapper homolog 1 OS=Homo sapiens GN=DACT1 PE=2 SV=2		
Q9NYF8	BCLF1_HUMAN Bcl-2-associated transcription factor 1 OS=Homo sapiens GN=BCLAF1 PE=1 SV=2		
Q9NYF8-2	BCLF1_HUMAN Isoform 2 of Bcl-2-associated transcription factor 1 OS=Homo sapiens GN=BCLAF1		
Q9NYF8-3	BCLF1_HUMAN Isoform 3 of Bcl-2-associated transcription factor 1 OS=Homo sapiens GN=BCLAF1		
Q9NYF8-4	BCLF1_HUMAN Isoform 4 of Bcl-2-associated transcription factor 1 OS=Homo sapiens GN=BCLAF1		
Q9NYF8;Q9NYF8-2;Q9NYF	BCLF1_HUMAN Bcl-2-associated transcription factor 1 OS=Homo sapiens GN=BCLAF1 PE=1 SV=2;>sp|Q9NYF8-2|BCLF1_HUMAN Isof	2.87E-55	
Q9NYH9	UTP6_HUMAN U3 small nucleolar RNA-associated protein 6 homolog OS=Homo sapiens GN=UTP6 PE=1 SV=2		47.3
Q9NYK1	TLR7_HUMAN Toll-like receptor 7 OS=Homo sapiens GN=TLR7 PE=1 SV=1		
Q9NYK5	RM39_HUMAN 39S ribosomal protein L39, mitochondrial OS=Homo sapiens GN=MRPL39 PE=1 SV=3		85.32
Q9NYK5-2	RM39_HUMAN Isoform 2 of 39S ribosomal protein L39, mitochondrial OS=Homo sapiens GN=MRPL39		85.32
Q9NYK5-2;Q9NYK5	2|RM39_HUMAN Isoform 2 of 39S ribosomal protein L39, mitochondrial OS=Homo sapiens GN=MRPL39;>sp|Q9NYK5|RM39_HUMAN 3	3.68E-16	
Q9NYL2-2	2|MLTK_HUMAN Isoform 2 of Mitogen-activated protein kinase kinase kinase MLT OS=Homo sapiens GN=MLTK	3.67E-13	
Q9NYL4	FKB11_HUMAN Peptidyl-prolyl cis-trans isomerase FKBP11 OS=Homo sapiens GN=FKBP11 PE=1 SV=1		32.28
Q9NYL9	TMOD3_HUMAN Tropomodulin-3 OS=Homo sapiens GN=TMOD3 PE=1 SV=1		148.87
Q9NYP3	DONS_HUMAN Protein downstream neighbor of Son OS=Homo sapiens GN=DONSON PE=1 SV=2		
Q9NYP3-2	DONS_HUMAN Isoform 2 of Protein downstream neighbor of Son OS=Homo sapiens GN=DONSON		
Q9NYP3-3	DONS_HUMAN Isoform 3 of Protein downstream neighbor of Son OS=Homo sapiens GN=DONSON		
Q9NYP7	ELOV5_HUMAN Elongation of very long chain fatty acids protein 5 OS=Homo sapiens GN=ELOVL5 PE=1 SV=1		
Q9NYQ6	CELR1_HUMAN Cadherin EGF LAG seven-pass G-type receptor 1 OS=Homo sapiens GN=CELSR1 PE=1 SV=1	2.94E-05	
Q9NYS0	KBRS1_HUMAN NF-kappa-B inhibitor-interacting Ras-like protein 1 OS=Homo sapiens GN=NKIRAS1 PE=1 SV=1	8.31E-08	
Q9NYT0	PLEK2_HUMAN Pleckstrin-2 OS=Homo sapiens GN=PLEK2 PE=1 SV=1	2.00E-09	
Q9NYU2	UGGG1_HUMAN UDP-glucose:glycoprotein glucosyltransferase 1 OS=Homo sapiens GN=UGGT1 PE=1 SV=3		468.39
Q9NYU2-2	UGGG1_HUMAN Isoform 2 of UDP-glucose:glycoprotein glucosyltransferase 1 OS=Homo sapiens GN=UGGT1		468.39
Q9NYU2;A8KAK1;Q9NYU2	UGGG1_HUMAN UDP-glucose:glycoprotein glucosyltransferase 1 OS=Homo sapiens GN=UGGT1 PE=1 SV=3;>tr|A8KAK1|A8KAK1_HUI	1.05E-118	
Q9NYV4	CDK12_HUMAN Cyclin-dependent kinase 12 OS=Homo sapiens GN=CDK12 PE=1 SV=2		93.26
Q9NYV4-2	CDK12_HUMAN Isoform 2 of Cyclin-dependent kinase 12 OS=Homo sapiens GN=CDK12		93.26
Q9NYV4-3	CDK12_HUMAN Isoform 3 of Cyclin-dependent kinase 12 OS=Homo sapiens GN=CDK12		69.42
Q9NYV4;Q9NYV4-2;Q9NY\	CDK12_HUMAN Cyclin-dependent kinase 12 OS=Homo sapiens GN=CDK12 PE=1 SV=2;>sp|Q9NYV4-2|CDK12_HUMAN Isoform 2 of C	1.62E-05	
Q9NYV6	RRN3_HUMAN RNA polymerase I-specific transcription initiation factor RRN3 OS=Homo sapiens GN=RRN3 PE=1 SV=1		
Q9NYY8	FAKD2_HUMAN FAST kinase domain-containing protein 2 OS=Homo sapiens GN=FASTKD2 PE=1 SV=1		
Q9NYY8-2	FAKD2_HUMAN Isoform 2 of FAST kinase domain-containing protein 2 OS=Homo sapiens GN=FASTKD2		
Q9NZ01	TECR_HUMAN Trans-2,3-enoyl-CoA reductase OS=Homo sapiens GN=TECR PE=1 SV=1		291.61
Q9NZ01-2	TECR_HUMAN Isoform 2 of Trans-2,3-enoyl-CoA reductase OS=Homo sapiens GN=TECR		63.8
Q9NZ01;Q9NZ01-2	TECR_HUMAN Trans-2,3-enoyl-CoA reductase OS=Homo sapiens GN=TECR PE=1 SV=1;>sp|Q9NZ01-2|TECR_HUMAN Isoform 2 of Tra	1.34E-08	
Q9NZ08	ERAP1_HUMAN Endoplasmic reticulum aminopeptidase 1 OS=Homo sapiens GN=ERAP1 PE=1 SV=3		200.48
Q9NZ08-2	ERAP1_HUMAN Isoform 2 of Endoplasmic reticulum aminopeptidase 1 OS=Homo sapiens GN=ERAP1		200.48
Q9NZ08-2;Q9NZ08	2|ERAP1_HUMAN Isoform 2 of Endoplasmic reticulum aminopeptidase 1 OS=Homo sapiens GN=ERAP1;>sp|Q9NZ08|ERAP1_HUMAN E	4.66E-185	
Q9NZ09	UBAP1_HUMAN Ubiquitin-associated protein 1 OS=Homo sapiens GN=UBAP1 PE=1 SV=1		
Q9NZ09-2	UBAP1_HUMAN Isoform 2 of Ubiquitin-associated protein 1 OS=Homo sapiens GN=UBAP1		
Q9NZ32	ARP10_HUMAN Actin-related protein 10 OS=Homo sapiens GN=ACTR10 PE=1 SV=1	1.50E-12	
Q9NZ43	USE1_HUMAN Vesicle transport protein USE1 OS=Homo sapiens GN=USE1 PE=1 SV=2		38.98
Q9NZ43-2	USE1_HUMAN Isoform 2 of Vesicle transport protein USE1 OS=Homo sapiens GN=USE1		11.38

Q9NZ43-3	USE1_HUMAN Isoform 3 of Vesicle transport protein USE1 OS=Homo sapiens GN=USE1		11.38
Q9NZ45	CISD1_HUMAN CDGSH iron-sulfur domain-containing protein 1 OS=Homo sapiens GN=CISD1 PE=1 SV=1		137.51
Q9NZ52	GGA3_HUMAN ADP-ribosylation factor-binding protein GGA3 OS=Homo sapiens GN=GGA3 PE=1 SV=1		36.71
Q9NZ52-2	GGA3_HUMAN Isoform Short of ADP-ribosylation factor-binding protein GGA3 OS=Homo sapiens GN=GGA3		2.99
Q9NZ53	PDXL2_HUMAN Podocalyxin-like protein 2 OS=Homo sapiens GN=PODXL2 PE=1 SV=1		21.89
Q9NZ53-2	PDXL2_HUMAN Isoform 2 of Podocalyxin-like protein 2 OS=Homo sapiens GN=PODXL2		21.89
Q9NZ63	CI078_HUMAN Uncharacterized protein C9orf78 OS=Homo sapiens GN=C9orf78 PE=1 SV=1		
Q9NZ71	RTEL1_HUMAN Regulator of telomere elongation helicase 1 OS=Homo sapiens GN=RTEL1 PE=1 SV=2		55.65
Q9NZ71-2	RTEL1_HUMAN Isoform 1 of Regulator of telomere elongation helicase 1 OS=Homo sapiens GN=RTEL1		55.65
Q9NZ71-4	RTEL1_HUMAN Isoform 4 of Regulator of telomere elongation helicase 1 OS=Homo sapiens GN=RTEL1		42.77
Q9NZ71-5	RTEL1_HUMAN Isoform 5 of Regulator of telomere elongation helicase 1 OS=Homo sapiens GN=RTEL1		45.17
Q9NZ71-6	RTEL1_HUMAN Isoform 6 of Regulator of telomere elongation helicase 1 OS=Homo sapiens GN=RTEL1		55.65
Q9NZ71-7	RTEL1_HUMAN Isoform 7 of Regulator of telomere elongation helicase 1 OS=Homo sapiens GN=RTEL1		55.65
Q9NZ71-8	RTEL1_HUMAN Isoform 8 of Regulator of telomere elongation helicase 1 OS=Homo sapiens GN=RTEL1		42.77
Q9NZ71-9	RTEL1_HUMAN Isoform 9 of Regulator of telomere elongation helicase 1 OS=Homo sapiens GN=RTEL1		48.68
Q9NZA1	CLIC5_HUMAN Chloride intracellular channel protein 5 OS=Homo sapiens GN=CLIC5 PE=1 SV=3		57.09
Q9NZA1-2	CLIC5_HUMAN Isoform 1 of Chloride intracellular channel protein 5 OS=Homo sapiens GN=CLIC5		57.09
Q9NZB2	F120A_HUMAN Constitutive coactivator of PPAR-gamma-like protein 1 OS=Homo sapiens GN=FAM120A PE=1 SV=2		206.13
Q9NZB2-2	F120A_HUMAN Isoform B of Constitutive coactivator of PPAR-gamma-like protein 1 OS=Homo sapiens GN=FAM120A		64.59
Q9NZB2-4	F120A_HUMAN Isoform D of Constitutive coactivator of PPAR-gamma-like protein 1 OS=Homo sapiens GN=FAM120A		182.14
Q9NZB2-5	F120A_HUMAN Isoform E of Constitutive coactivator of PPAR-gamma-like protein 1 OS=Homo sapiens GN=FAM120A		64.59
Q9NZB2-6	F120A_HUMAN Isoform F of Constitutive coactivator of PPAR-gamma-like protein 1 OS=Homo sapiens GN=FAM120A		206.13
Q9NZB2-6;Q9NZB2;Q9NZ	6|F120A_HUMAN Isoform F of Constitutive coactivator of PPAR-gamma-like protein 1 OS=Homo sapiens GN=FAM120A;>sp|Q9NZB2|F	1.01E-18	
Q9NZD2	GLTP_HUMAN Glycolipid transfer protein OS=Homo sapiens GN=GLTP PE=1 SV=3	1.51E-41	
Q9NZF1	PLAC8_HUMAN Placenta-specific gene 8 protein OS=Homo sapiens GN=PLAC8 PE=2 SV=1	3.13E-09	
Q9NZI8	IF2B1_HUMAN Insulin-like growth factor 2 mRNA-binding protein 1 OS=Homo sapiens GN=IGF2BP1 PE=1 SV=2		30.71
Q9NZJ4;Q9NZJ4-2	SACS_HUMAN Sacsin OS=Homo sapiens GN=SACS PE=1 SV=2;>sp|Q9NZJ4-2|SACS_HUMAN Isoform 2 of Sacsin OS=Homo sapiens	8.46E-12	
Q9NZJ7	MTCH1_HUMAN Mitochondrial carrier homolog 1 OS=Homo sapiens GN=MTCH1 PE=1 SV=1		
Q9NZJ7-2	MTCH1_HUMAN Isoform 2 of Mitochondrial carrier homolog 1 OS=Homo sapiens GN=MTCH1		
Q9NZJ7-3	MTCH1_HUMAN Isoform 3 of Mitochondrial carrier homolog 1 OS=Homo sapiens GN=MTCH1		
Q9NZJ9;B2RE29;Q9NZJ9-	NUDT4_HUMAN Diphosphoinositol polyphosphate phosphohydrolase 2 OS=Homo sapiens GN=NUDT4 PE=1 SV=2;>tr|B2RE29|B2RE2	2.01E-07	
Q9NZL4	HPBP1_HUMAN Hsp70-binding protein 1 OS=Homo sapiens GN=HSPBP1 PE=1 SV=1	2.07E-33	
Q9NZL9	MAT2B_HUMAN Methionine adenosyltransferase 2 subunit beta OS=Homo sapiens GN=MAT2B PE=1 SV=1		114.44
Q9NZL9-2	MAT2B_HUMAN Isoform 2 of Methionine adenosyltransferase 2 subunit beta OS=Homo sapiens GN=MAT2B		114.44
Q9NZL9-3	MAT2B_HUMAN Isoform 3 of Methionine adenosyltransferase 2 subunit beta OS=Homo sapiens GN=MAT2B		114.44
Q9NZL9-4	MAT2B_HUMAN Isoform 4 of Methionine adenosyltransferase 2 subunit beta OS=Homo sapiens GN=MAT2B		114.44
Q9NZL9-5	MAT2B_HUMAN Isoform 5 of Methionine adenosyltransferase 2 subunit beta OS=Homo sapiens GN=MAT2B		76.35
Q9NZL9;A8K7A4;Q9NZL9-	MAT2B_HUMAN Methionine adenosyltransferase 2 subunit beta OS=Homo sapiens GN=MAT2B PE=1 SV=1;>tr|A8K7A4|A8K7A4_HUMA	1.88E-12	
Q9NZM1	MYOF_HUMAN Myoferlin OS=Homo sapiens GN=MYOF PE=1 SV=1		2947.29
Q9NZM1-2	MYOF_HUMAN Isoform 2 of Myoferlin OS=Homo sapiens GN=MYOF		2700
Q9NZM1-3	MYOF_HUMAN Isoform 3 of Myoferlin OS=Homo sapiens GN=MYOF		2784.81
Q9NZM1-4	MYOF_HUMAN Isoform 4 of Myoferlin OS=Homo sapiens GN=MYOF		273.46
Q9NZM1-5	MYOF_HUMAN Isoform 5 of Myoferlin OS=Homo sapiens GN=MYOF		2090.95
Q9NZM1-6	MYOF_HUMAN Isoform 6 of Myoferlin OS=Homo sapiens GN=MYOF		2947.29
Q9NZM1-7	MYOF_HUMAN Isoform 7 of Myoferlin OS=Homo sapiens GN=MYOF		736.46
Q9NZM1-8	MYOF_HUMAN Isoform 8 of Myoferlin OS=Homo sapiens GN=MYOF		625.51
Q9NZM1;Q9NZM1-6;C9JC	MYOF_HUMAN Myoferlin OS=Homo sapiens GN=MYOF PE=1 SV=1;>sp|Q9NZM1-6|MYOF_HUMAN Isoform 6 of Myoferlin OS=Homo sa	0	
Q9NZM5	GSCR2_HUMAN Glioma tumor suppressor candidate region gene 2 protein OS=Homo sapiens GN=GLTSCR2 PE=1 SV=2		
Q9NZN1	IRPL1_HUMAN Interleukin-1 receptor accessory protein-like 1 OS=Homo sapiens GN=IL1RAPL1 PE=1 SV=2		30.93
Q9NZN3	EHD3_HUMAN EH domain-containing protein 3 OS=Homo sapiens GN=EHD3 PE=1 SV=2		376.12
Q9NZN4	EHD2_HUMAN EH domain-containing protein 2 OS=Homo sapiens GN=EHD2 PE=1 SV=2		233.32
Q9NZN8	CNOT2_HUMAN CCR4-NOT transcription complex subunit 2 OS=Homo sapiens GN=CNOT2 PE=1 SV=1		
Q9NZN8-4	CNOT2_HUMAN Isoform 4 of CCR4-NOT transcription complex subunit 2 OS=Homo sapiens GN=CNOT2		
Q9NZN8-5	CNOT2_HUMAN Isoform 5 of CCR4-NOT transcription complex subunit 2 OS=Homo sapiens GN=CNOT2		
Q9NZQ3;Q9NZQ3-2;Q9NZ	SPN90_HUMAN NCK-interacting protein with SH3 domain OS=Homo sapiens GN=NCKIPSD PE=1 SV=1;>sp|Q9NZQ3-2|SPN90_HUMA	2.81E-09	
Q9NZQ7;Q9NZQ7-3;Q9NZ	PD1L1_HUMAN Programmed cell death 1 ligand 1 OS=Homo sapiens GN=CD274 PE=1 SV=1;>sp|Q9NZQ7-3|PD1L1_HUMAN Isoform	0.00024388	
Q9NZT1	CALL5_HUMAN Calmodulin-like protein 5 OS=Homo sapiens GN=CALML5 PE=1 SV=2	1.90E-23	
Q9NZT2;Q05BV5;Q9NZT2	OGFR_HUMAN Opioid growth factor receptor OS=Homo sapiens GN=OGFR PE=1 SV=3;>tr|Q05BV5|Q05BV5_HUMAN OGFR protein O	7.22E-43	
Q9NZU5	LMCD1_HUMAN LIM and cysteine-rich domains protein 1 OS=Homo sapiens GN=LMCD1 PE=1 SV=1	1.13E-30	
Q9NZV7	ZIM2_HUMAN Zinc finger imprinted 2 OS=Homo sapiens GN=ZIM2 PE=2 SV=1		
Q9NZW5	MPP6_HUMAN MAGUK p55 subfamily member 6 OS=Homo sapiens GN=MPP6 PE=1 SV=2	2.50E-14	

Q9NZZ3	CHMP5_HUMAN Charged multivesicular body protein 5 OS=Homo sapiens GN=CHMP5 PE=1 SV=1	2.55E-20	
Q9P000;Q9P000-2	COMD9_HUMAN COMM domain-containing protein 9 OS=Homo sapiens GN=COMMD9 PE=1 SV=2;>sp|Q9P000-2|COMD9_HUMAN Iso	7.73E-25	
Q9P003	CNIH4_HUMAN Protein cornichon homolog 4 OS=Homo sapiens GN=CNIH4 PE=1 SV=1		36.53
Q9P013	CWC15_HUMAN Protein CWC15 homolog OS=Homo sapiens GN=CWC15 PE=1 SV=2		50.53
Q9P015	RM15_HUMAN 39S ribosomal protein L15, mitochondrial OS=Homo sapiens GN=MRPL15 PE=1 SV=1		85.2
Q9P016	THYN1_HUMAN Thymocyte nuclear protein 1 OS=Homo sapiens GN=THYN1 PE=1 SV=1		283.07
Q9P016-2	THYN1_HUMAN Isoform 2 of Thymocyte nuclear protein 1 OS=Homo sapiens GN=THYN1		156.36
Q9P016;Q9P016-2	THYN1_HUMAN Thymocyte nuclear protein 1 OS=Homo sapiens GN=THYN1 PE=1 SV=1;>sp|Q9P016-2|THYN1_HUMAN Isoform 2 of T	6.91E-15	
Q9P031	TAP26_HUMAN Thyroid transcription factor 1-associated protein 26 OS=Homo sapiens GN=CCDC59 PE=1 SV=2		
Q9P032	NDUF4_HUMAN NADH dehydrogenase [ubiquinone] 1 alpha subcomplex assembly factor 4 OS=Homo sapiens GN=NDUFAF4 PE=1 SV=1		53.08
Q9P035	HACD3_HUMAN 3-hydroxyacyl-CoA dehydratase 3 OS=Homo sapiens GN=PTPLAD1 PE=1 SV=2		157.6
Q9P0I2	TM111_HUMAN Transmembrane protein 111 OS=Homo sapiens GN=TMEM111 PE=1 SV=3		136.84
Q9P0I2-2	TM111_HUMAN Isoform 2 of Transmembrane protein 111 OS=Homo sapiens GN=TMEM111		136.84
Q9P0I2;Q9P0I2-2	TM111_HUMAN Transmembrane protein 111 OS=Homo sapiens GN=TMEM111 PE=1 SV=3;>sp|Q9P0I2-2|TM111_HUMAN Isoform 2 o	1.88E-06	
Q9P0J0	NDUAD_HUMAN NADH dehydrogenase [ubiquinone] 1 alpha subcomplex subunit 13 OS=Homo sapiens GN=NDUFA13 PE=1 SV=3		127.13
Q9P0J1	PDP1_HUMAN [Pyruvate dehydrogenase [acetyl-transferring]]-phosphatase 1, mitochondrial OS=Homo sapiens GN=PDP1 PE=1 SV=3		71.8
Q9P0J7	KCMF1_HUMAN E3 ubiquitin-protein ligase KCMF1 OS=Homo sapiens GN=KCMF1 PE=1 SV=2	4.73E-10	
Q9P0K7	RAI14_HUMAN Ankycorbin OS=Homo sapiens GN=RAI14 PE=1 SV=2		25.97
Q9P0K7-2	RAI14_HUMAN Isoform 2 of Ankycorbin OS=Homo sapiens GN=RAI14		31.72
Q9P0K7-2;Q9P0K7;Q9P0K	2|RAI14_HUMAN Isoform 2 of Ankycorbin OS=Homo sapiens GN=RAI14;>sp|Q9P0K7|RAI14_HUMAN Ankycorbin OS=Homo sapiens G	9.01E-64	
Q9P0K7-3	RAI14_HUMAN Isoform 3 of Ankycorbin OS=Homo sapiens GN=RAI14		25.97
Q9P0K8	FOXJ2_HUMAN Forkhead box protein J2 OS=Homo sapiens GN=FOXJ2 PE=1 SV=1		
Q9P0K8-2	FOXJ2_HUMAN Isoform FOXJ2.S of Forkhead box protein J2 OS=Homo sapiens GN=FOXJ2		
Q9P0L0	VAPA_HUMAN Vesicle-associated membrane protein-associated protein A OS=Homo sapiens GN=VAPA PE=1 SV=3		600.64
Q9P0L0-2	VAPA_HUMAN Isoform 2 of Vesicle-associated membrane protein-associated protein A OS=Homo sapiens GN=VAPA		565.24
Q9P0L0-2;D3DUI3;Q9P0L(	2|VAPA_HUMAN Isoform 2 of Vesicle-associated membrane protein-associated protein A OS=Homo sapiens GN=VAPA;>tr|D3DUI3|D3I	1.64E-94	
Q9P0L0;Q9P0L0-2	VAPA_HUMAN Vesicle-associated membrane protein-associated protein A OS=Homo sapiens GN=VAPA PE=1 SV=3;>sp|Q9P0L0-2|VAF	3.62E-32	
Q9P0M6	H2AW_HUMAN Core histone macro-H2A.2 OS=Homo sapiens GN=H2AFY2 PE=1 SV=3		85.09
Q9P0M9	RM27_HUMAN 39S ribosomal protein L27, mitochondrial OS=Homo sapiens GN=MRPL27 PE=1 SV=1		145.05
Q9P0N8	MARH2_HUMAN E3 ubiquitin-protein ligase MARCH2 OS=Homo sapiens GN=MARCH2 PE=1 SV=1		
Q9P0N8-2	MARH2_HUMAN Isoform 2 of E3 ubiquitin-protein ligase MARCH2 OS=Homo sapiens GN=MARCH2		
Q9P0S3	ORML1_HUMAN ORM1-like protein 1 OS=Homo sapiens GN=ORMDL1 PE=2 SV=1		
Q9P0S9	TM14C_HUMAN Transmembrane protein 14C OS=Homo sapiens GN=TMEM14C PE=1 SV=1		222.67
Q9P0T7	TMEM9_HUMAN Transmembrane protein 9 OS=Homo sapiens GN=TMEM9 PE=1 SV=1		25.6
Q9P0V3	SH3B4_HUMAN SH3 domain-binding protein 4 OS=Homo sapiens GN=SH3BP4 PE=1 SV=1		
Q9P0V3-2	SH3B4_HUMAN Isoform 2 of SH3 domain-binding protein 4 OS=Homo sapiens GN=SH3BP4		
Q9P0V9	SEP10_HUMAN Septin-10 OS=Homo sapiens GN=SEPT10 PE=1 SV=2		139.78
Q9P0V9-2	SEP10_HUMAN Isoform 2 of Septin-10 OS=Homo sapiens GN=SEPT10		139.78
Q9P0V9-2;Q9P0V9;Q9P0V	2|SEP10_HUMAN Isoform 2 of Septin-10 OS=Homo sapiens GN=SEPT10;>sp|Q9P0V9|SEP10_HUMAN Septin-10 OS=Homo sapiens GI	2.67E-34	
Q9P0V9-3	SEP10_HUMAN Isoform 3 of Septin-10 OS=Homo sapiens GN=SEPT10		137.84
Q9P0X4	CAC1I_HUMAN Voltage-dependent T-type calcium channel subunit alpha-1I OS=Homo sapiens GN=CACNA1I PE=1 SV=1		
Q9P0X4-2	CAC1I_HUMAN Isoform 2 of Voltage-dependent T-type calcium channel subunit alpha-1I OS=Homo sapiens GN=CACNA1I		
Q9P0X4-3	CAC1I_HUMAN Isoform 3 of Voltage-dependent T-type calcium channel subunit alpha-1I OS=Homo sapiens GN=CACNA1I		
Q9P0X4-4	CAC1I_HUMAN Isoform 4 of Voltage-dependent T-type calcium channel subunit alpha-1I OS=Homo sapiens GN=CACNA1I		
Q9P107	GMIP_HUMAN GEM-interacting protein OS=Homo sapiens GN=GMIP PE=1 SV=2		
Q9P1F3	CF115_HUMAN Costars family protein C6orf115 OS=Homo sapiens GN=C6orf115 PE=1 SV=1	2.02E-115	
Q9P1U1	ARP3B_HUMAN Actin-related protein 3B OS=Homo sapiens GN=ACTR3B PE=1 SV=1		80.64
Q9P1U1-2	ARP3B_HUMAN Isoform 2 of Actin-related protein 3B OS=Homo sapiens GN=ACTR3B		79.4
Q9P1U1-3	ARP3B_HUMAN Isoform 3 of Actin-related protein 3B OS=Homo sapiens GN=ACTR3B		80.64
Q9P219	DAPLE_HUMAN Protein Daple OS=Homo sapiens GN=CCDC88C PE=1 SV=3		
Q9P225;Q9P225-2	DYH2_HUMAN Dynein heavy chain 2, axonemal OS=Homo sapiens GN=DNAH2 PE=1 SV=3;>sp|Q9P225-2|DYH2_HUMAN Isoform 2 o	3.01E-10	
Q9P227	RHG23_HUMAN Rho GTPase-activating protein 23 OS=Homo sapiens GN=ARHGAP23 PE=1 SV=2		33.58
Q9P227-2	RHG23_HUMAN Isoform 2 of Rho GTPase-activating protein 23 OS=Homo sapiens GN=ARHGAP23		1.68
Q9P244	LRFN1_HUMAN Leucine-rich repeat and fibronectin type III domain-containing protein 1 OS=Homo sapiens GN=LRFN1 PE=1 SV=2		
Q9P246	STIM2_HUMAN Stromal interaction molecule 2 OS=Homo sapiens GN=STIM2 PE=1 SV=2		
Q9P258	RCC2_HUMAN Protein RCC2 OS=Homo sapiens GN=RCC2 PE=1 SV=2		287.33
Q9P260	K1468_HUMAN LisH domain and HEAT repeat-containing protein KIAA1468 OS=Homo sapiens GN=KIAA1468 PE=1 SV=2		
Q9P260-2;Q9P260	2|K1468_HUMAN Isoform 2 of LisH domain and HEAT repeat-containing protein KIAA1468 OS=Homo sapiens GN=KIAA1468;>sp|Q9P2	2.50E-19	
Q9P265	DIP2B_HUMAN Disco-interacting protein 2 homolog B OS=Homo sapiens GN=DIP2B PE=1 SV=3	2.45E-10	
Q9P270	SLAI2_HUMAN SLAIN motif-containing protein 2 OS=Homo sapiens GN=SLAIN2 PE=1 SV=2		26.9
Q9P281	BAHC1_HUMAN BAH and coiled-coil domain-containing protein 1 OS=Homo sapiens GN=BAHCC1 PE=1 SV=3		

Q9P287	BCCIP_HUMAN BRCA2 and CDKN1A-interacting protein OS=Homo sapiens GN=BCCIP PE=1 SV=1		65.65
Q9P287-2	BCCIP_HUMAN Isoform 2 of BRCA2 and CDKN1A-interacting protein OS=Homo sapiens GN=BCCIP		65.65
Q9P287-2;Q9P287;B3KP4	2|BCCIP_HUMAN Isoform 2 of BRCA2 and CDKN1A-interacting protein OS=Homo sapiens GN=BCCIP;>sp|Q9P287|BCCIP_HUMAN BRC	5.52E-140	
Q9P287-3	BCCIP_HUMAN Isoform 3 of BRCA2 and CDKN1A-interacting protein OS=Homo sapiens GN=BCCIP		65.65
Q9P287;Q9P287-2;Q9P28	BCCIP_HUMAN BRCA2 and CDKN1A-interacting protein OS=Homo sapiens GN=BCCIP PE=1 SV=1;>sp|Q9P287-2|BCCIP_HUMAN Isofo	2.96E-191	
Q9P289	MST4_HUMAN Serine/threonine-protein kinase MST4 OS=Homo sapiens GN=MST4 PE=1 SV=2		105.17
Q9P289-2	MST4_HUMAN Isoform 2 of Serine/threonine-protein kinase MST4 OS=Homo sapiens GN=MST4		98.17
Q9P289-3	MST4_HUMAN Isoform 3 of Serine/threonine-protein kinase MST4 OS=Homo sapiens GN=MST4		48.85
Q9P289;Q9P289-2;Q9P28	MST4_HUMAN Serine/threonine-protein kinase MST4 OS=Homo sapiens GN=MST4 PE=1 SV=2;>sp|Q9P289-2|MST4_HUMAN Isoform	4.26E-46	
Q9P2A4	ABI3_HUMAN ABI gene family member 3 OS=Homo sapiens GN=ABI3 PE=1 SV=2		21.63
Q9P2A4-2	ABI3_HUMAN Isoform 2 of ABI gene family member 3 OS=Homo sapiens GN=ABI3		21.61
Q9P2B2	FPRP_HUMAN Prostaglandin F2 receptor negative regulator OS=Homo sapiens GN=PTGFRN PE=1 SV=2	7.65E-17	
Q9P2B4	CT2NL_HUMAN CTTNBP2 N-terminal-like protein OS=Homo sapiens GN=CTTNBP2NL PE=1 SV=2		
Q9P2D1	CHD7_HUMAN Chromodomain-helicase-DNA-binding protein 7 OS=Homo sapiens GN=CHD7 PE=1 SV=3		90.96
Q9P2D1-2	CHD7_HUMAN Isoform 2 of Chromodomain-helicase-DNA-binding protein 7 OS=Homo sapiens GN=CHD7		8.06
Q9P2D3	HTR5B_HUMAN HEAT repeat-containing protein 5B OS=Homo sapiens GN=HEATR5B PE=1 SV=2		24.52
Q9P2D3-2	HTR5B_HUMAN Isoform 2 of HEAT repeat-containing protein 5B OS=Homo sapiens GN=HEATR5B		0.79
Q9P2D3-3	HTR5B_HUMAN Isoform 3 of HEAT repeat-containing protein 5B OS=Homo sapiens GN=HEATR5B		24.52
Q9P2D8	UNC79_HUMAN Protein unc-79 homolog OS=Homo sapiens GN=KIAA1409 PE=2 SV=3		29.54
Q9P2D8-2	UNC79_HUMAN Isoform 2 of Protein unc-79 homolog OS=Homo sapiens GN=KIAA1409		29.54
Q9P2D8-3	UNC79_HUMAN Isoform 3 of Protein unc-79 homolog OS=Homo sapiens GN=KIAA1409		29.54
Q9P2E3	ZNFX1_HUMAN NFX1-type zinc finger-containing protein 1 OS=Homo sapiens GN=ZNFX1 PE=1 SV=2		
Q9P2E9	RRBP1_HUMAN Ribosome-binding protein 1 OS=Homo sapiens GN=RRBP1 PE=1 SV=4		2137.16
Q9P2E9-2	RRBP1_HUMAN Isoform 1 of Ribosome-binding protein 1 OS=Homo sapiens GN=RRBP1		2110.08
Q9P2E9-3	RRBP1_HUMAN Isoform 2 of Ribosome-binding protein 1 OS=Homo sapiens GN=RRBP1		2027.22
Q9P2E9;Q9P2E9-2;Q9P2E	RRBP1_HUMAN Ribosome-binding protein 1 OS=Homo sapiens GN=RRBP1 PE=1 SV=4;>sp|Q9P2E9-2|RRBP1_HUMAN Isoform 1 of Ri	0	
Q9P2H0	K1377_HUMAN Uncharacterized protein KIAA1377 OS=Homo sapiens GN=KIAA1377 PE=1 SV=3		
Q9P2H3	IFT80_HUMAN Intraflagellar transport protein 80 homolog OS=Homo sapiens GN=IFT80 PE=1 SV=3		
Q9P2H5	UBP35_HUMAN Ubiquitin carboxyl-terminal hydrolase 35 OS=Homo sapiens GN=USP35 PE=1 SV=3		
Q9P2H5-2	UBP35_HUMAN Isoform 2 of Ubiquitin carboxyl-terminal hydrolase 35 OS=Homo sapiens GN=USP35		
Q9P2I0	CPSF2_HUMAN Cleavage and polyadenylation specificity factor subunit 2 OS=Homo sapiens GN=CPSF2 PE=1 SV=2		72.14
Q9P2J5	SYLC_HUMAN Leucyl-tRNA synthetase, cytoplasmic OS=Homo sapiens GN=LARS PE=1 SV=2		805.75
Q9P2K1	C2D2A_HUMAN Coiled-coil and C2 domain-containing protein 2A OS=Homo sapiens GN=CC2D2A PE=1 SV=3		
Q9P2K1-2	C2D2A_HUMAN Isoform 2 of Coiled-coil and C2 domain-containing protein 2A OS=Homo sapiens GN=CC2D2A		
Q9P2K1;Q9P2K1-2	C2D2A_HUMAN Coiled-coil and C2 domain-containing protein 2A OS=Homo sapiens GN=CC2D2A PE=1 SV=3;>sp|Q9P2K1-2|C2D2A_I	9.88E-11	
Q9P2K3	RCOR3_HUMAN REST corepressor 3 OS=Homo sapiens GN=RCOR3 PE=1 SV=2		27.13
Q9P2K3-2	RCOR3_HUMAN Isoform 2 of REST corepressor 3 OS=Homo sapiens GN=RCOR3		24.43
Q9P2K3-3	RCOR3_HUMAN Isoform 3 of REST corepressor 3 OS=Homo sapiens GN=RCOR3		27.13
Q9P2K3-4	RCOR3_HUMAN Isoform 4 of REST corepressor 3 OS=Homo sapiens GN=RCOR3		24.43
Q9P2K5	MYEF2_HUMAN Myelin expression factor 2 OS=Homo sapiens GN=MYEF2 PE=1 SV=3		
Q9P2K5-2	MYEF2_HUMAN Isoform 2 of Myelin expression factor 2 OS=Homo sapiens GN=MYEF2		
Q9P2K5-3	MYEF2_HUMAN Isoform 3 of Myelin expression factor 2 OS=Homo sapiens GN=MYEF2		
Q9P2K5-4	MYEF2_HUMAN Isoform 4 of Myelin expression factor 2 OS=Homo sapiens GN=MYEF2		
Q9P2K8;Q9P2K8-2	E2AK4_HUMAN Eukaryotic translation initiation factor 2-alpha kinase 4 OS=Homo sapiens GN=EIF2AK4 PE=1 SV=3;>sp|Q9P2K8-2|E2	1.87E-12	
Q9P2M7	CING_HUMAN Cingulin OS=Homo sapiens GN=CGN PE=1 SV=2		
Q9P2M7-2	CING_HUMAN Isoform 2 of Cingulin OS=Homo sapiens GN=CGN		
Q9P2M7;Q9P2M7-2	CING_HUMAN Cingulin OS=Homo sapiens GN=CGN PE=1 SV=2;>sp|Q9P2M7-2|CING_HUMAN Isoform 2 of Cingulin OS=Homo sapien	1.75E-14	
Q9P2N5	RBM27_HUMAN RNA-binding protein 27 OS=Homo sapiens GN=RBM27 PE=1 SV=2		26.7
Q9P2P6	STAR9_HUMAN StAR-related lipid transfer protein 9 OS=Homo sapiens GN=STARD9 PE=2 SV=2	1.26E-13	
Q9P2P6-3	STAR9_HUMAN Isoform 3 of StAR-related lipid transfer protein 9 OS=Homo sapiens GN=STARD9		
Q9P2R3	ANFY1_HUMAN Ankyrin repeat and FYVE domain-containing protein 1 OS=Homo sapiens GN=ANKFY1 PE=1 SV=2		
Q9P2R3-2	ANFY1_HUMAN Isoform 2 of Ankyrin repeat and FYVE domain-containing protein 1 OS=Homo sapiens GN=ANKFY1		
Q9P2R3-2;Q9P2R3;B3KPZ	2|ANFY1_HUMAN Isoform 2 of Ankyrin repeat and FYVE domain-containing protein 1 OS=Homo sapiens GN=ANKFY1;>sp|Q9P2R3|AN	9.89E-06	
Q9P2R3-3	ANFY1_HUMAN Isoform 3 of Ankyrin repeat and FYVE domain-containing protein 1 OS=Homo sapiens GN=ANKFY1		
Q9P2R3-4	ANFY1_HUMAN Isoform 4 of Ankyrin repeat and FYVE domain-containing protein 1 OS=Homo sapiens GN=ANKFY1		
Q9P2R3-4;Q9P2R3-2;Q9P2	4|ANFY1_HUMAN Isoform 4 of Ankyrin repeat and FYVE domain-containing protein 1 OS=Homo sapiens GN=ANKFY1;>sp|Q9P2R3-2|A	2.85E-10	
Q9P2R7	SUCB1_HUMAN Succinyl-CoA ligase [ADP-forming] subunit beta, mitochondrial OS=Homo sapiens GN=SUCLA2 PE=1 SV=3		23.98
Q9P2R7-2	SUCB1_HUMAN Isoform 2 of Succinyl-CoA ligase [ADP-forming] subunit beta, mitochondrial OS=Homo sapiens GN=SUCLA2		23.98
Q9P2R7;Q9P2R7-2	SUCB1_HUMAN Succinyl-CoA ligase [ADP-forming] subunit beta, mitochondrial OS=Homo sapiens GN=SUCLA2 PE=1 SV=3;>sp|Q9P2	2.06E-69	
Q9P2S6	ANKY1_HUMAN Ankyrin repeat and MYND domain-containing protein 1 OS=Homo sapiens GN=ANKMY1 PE=2 SV=2		
Q9P2T1	GMPR2_HUMAN GMP reductase 2 OS=Homo sapiens GN=GMPR2 PE=1 SV=1		

Q9P2T1-2	GMPR2_HUMAN Isoform 2 of GMP reductase 2 OS=Homo sapiens GN=GMPR2		
Q9P2T1-2;Q9P2T1	2|GMPR2_HUMAN Isoform 2 of GMP reductase 2 OS=Homo sapiens GN=GMPR2;>sp|Q9P2T1|GMPR2_HUMAN GMP reductase 2 OS=Ho	9.90E-14	
Q9P2W9	STX18_HUMAN Syntaxin-18 OS=Homo sapiens GN=STX18 PE=1 SV=1		
Q9P2X0	DPM3_HUMAN Dolichol-phosphate mannosyltransferase subunit 3 OS=Homo sapiens GN=DPM3 PE=1 SV=2		46.92
Q9P2X0-2	DPM3_HUMAN Isoform 2 of Dolichol-phosphate mannosyltransferase subunit 3 OS=Homo sapiens GN=DPM3		46.92
Q9P2X0-2;Q9P2X0	2|DPM3_HUMAN Isoform 2 of Dolichol-phosphate mannosyltransferase subunit 3 OS=Homo sapiens GN=DPM3;>sp|Q9P2X0|DPM3_HU	6.48E-08	
Q9P2Y4	ZN219_HUMAN Zinc finger protein 219 OS=Homo sapiens GN=ZNF219 PE=1 SV=2		
Q9TNN7	1C05_HUMAN HLA class I histocompatibility antigen, Cw-5 alpha chain OS=Homo sapiens GN=HLA-C PE=2 SV=1		300.74
Q9TQE0	2B19_HUMAN HLA class II histocompatibility antigen, DRB1-9 beta chain OS=Homo sapiens GN=HLA-DRB1 PE=1 SV=1		29.42
Q9UBB4	ATX10_HUMAN Ataxin-10 OS=Homo sapiens GN=ATXN10 PE=1 SV=1		
Q9UBB5	MBD2_HUMAN Methyl-CpG-binding domain protein 2 OS=Homo sapiens GN=MBD2 PE=1 SV=1		
Q9UBB5-2	MBD2_HUMAN Isoform 2 of Methyl-CpG-binding domain protein 2 OS=Homo sapiens GN=MBD2		
Q9UBB5-3	MBD2_HUMAN Isoform 3 of Methyl-CpG-binding domain protein 2 OS=Homo sapiens GN=MBD2		
Q9UBB6-3;D3DPR9;Q9UBI	3|NCDN_HUMAN Isoform 3 of Neurochondrin OS=Homo sapiens GN=NCDN;>tr|D3DPR9|D3DPR9_HUMAN Neurochondrin, isoform CRA	2.71E-06	
Q9UBC2	EP15R_HUMAN Epidermal growth factor receptor substrate 15-like 1 OS=Homo sapiens GN=EPS15L1 PE=1 SV=1		
Q9UBC5	MYO1A_HUMAN Myosin-Ia OS=Homo sapiens GN=MYO1A PE=1 SV=1		198.78
Q9UBD5	ORC3_HUMAN Origin recognition complex subunit 3 OS=Homo sapiens GN=ORC3 PE=1 SV=1		61.04
Q9UBD5-2	ORC3_HUMAN Isoform 2 of Origin recognition complex subunit 3 OS=Homo sapiens GN=ORC3		61.04
Q9UBD5-2;Q9UBD5	2|ORC3_HUMAN Isoform 2 of Origin recognition complex subunit 3 OS=Homo sapiens GN=ORC3;>sp|Q9UBD5|ORC3_HUMAN Origin r	9.65E-17	
Q9UBD9	CLCF1_HUMAN Cardiotrophin-like cytokine factor 1 OS=Homo sapiens GN=CLCF1 PE=1 SV=1		57.25
Q9UBE0	SAE1_HUMAN SUMO-activating enzyme subunit 1 OS=Homo sapiens GN=SAE1 PE=1 SV=1		289.8
Q9UBF2	COPG2_HUMAN Coatomer subunit gamma-2 OS=Homo sapiens GN=COPG2 PE=1 SV=1		59.49
Q9UBG0	MRC2_HUMAN C-type mannose receptor 2 OS=Homo sapiens GN=MRC2 PE=1 SV=2	8.06E-48	
Q9UBI1	COMD3_HUMAN COMM domain-containing protein 3 OS=Homo sapiens GN=COMMD3 PE=1 SV=1		49.31
Q9UBI6	GBG12_HUMAN Guanine nucleotide-binding protein G(I)/G(S)/G(O) subunit gamma-12 OS=Homo sapiens GN=GNG12 PE=1 SV=3		186.98
Q9UBJ2	ABCD2_HUMAN ATP-binding cassette sub-family D member 2 OS=Homo sapiens GN=ABCD2 PE=1 SV=1		
Q9UBK7	RBL2A_HUMAN Rab-like protein 2A OS=Homo sapiens GN=RABL2A PE=2 SV=1		
Q9UBK7-3	RBL2A_HUMAN Isoform 3 of Rab-like protein 2A OS=Homo sapiens GN=RABL2A		
Q9UBK9	UXT_HUMAN Protein UXT OS=Homo sapiens GN=UXT PE=1 SV=1	9.44E-10	
Q9UBL3	ASH2L_HUMAN Set1/Ash2 histone methyltransferase complex subunit ASH2 OS=Homo sapiens GN=ASH2L PE=1 SV=1		
Q9UBL3-2	ASH2L_HUMAN Isoform 2 of Set1/Ash2 histone methyltransferase complex subunit ASH2 OS=Homo sapiens GN=ASH2L		
Q9UBL3-3	ASH2L_HUMAN Isoform 3 of Set1/Ash2 histone methyltransferase complex subunit ASH2 OS=Homo sapiens GN=ASH2L		
Q9UBL3;Q9UBL3-3;Q9UBL	ASH2L_HUMAN Set1/Ash2 histone methyltransferase complex subunit ASH2 OS=Homo sapiens GN=ASH2L PE=1 SV=1;>sp|Q9UBL3-3	9.81E-58	
Q9UBL6	CPNE7_HUMAN Copine-7 OS=Homo sapiens GN=CPNE7 PE=2 SV=1		
Q9UBL6-2	CPNE7_HUMAN Isoform 2 of Copine-7 OS=Homo sapiens GN=CPNE7		
Q9UBM7	DHCR7_HUMAN 7-dehydrocholesterol reductase OS=Homo sapiens GN=DHCR7 PE=1 SV=1		262.55
Q9UBM8	MGT4C_HUMAN Alpha-1,3-mannosyl-glycoprotein 4-beta-N-acetylglucosaminyltransferase C OS=Homo sapiens GN=MGAT4C PE=2 SV=2		
Q9UBN7	HDAC6_HUMAN Histone deacetylase 6 OS=Homo sapiens GN=HDAC6 PE=1 SV=2	3.14E-14	
Q9UBP6	TRMB_HUMAN tRNA (guanine-N(7)-)-methyltransferase OS=Homo sapiens GN=METTL1 PE=1 SV=1		52.7
Q9UBQ0	VPS29_HUMAN Vacuolar protein sorting-associated protein 29 OS=Homo sapiens GN=VPS29 PE=1 SV=1		346.99
Q9UBQ0-2	VPS29_HUMAN Isoform 2 of Vacuolar protein sorting-associated protein 29 OS=Homo sapiens GN=VPS29		346.99
Q9UBQ0-2;Q9UBQ0	2|VPS29_HUMAN Isoform 2 of Vacuolar protein sorting-associated protein 29 OS=Homo sapiens GN=VPS29;>sp|Q9UBQ0|VPS29_HUM	5.35E-14	
Q9UBQ5	EIF3K_HUMAN Eukaryotic translation initiation factor 3 subunit K OS=Homo sapiens GN=EIF3K PE=1 SV=1		473.34
Q9UBQ7	GRHPR_HUMAN Glyoxylate reductase/hydroxypyruvate reductase OS=Homo sapiens GN=GRHPR PE=1 SV=1		44.37
Q9UBR2	CATZ_HUMAN Cathepsin Z OS=Homo sapiens GN=CTSZ PE=1 SV=1		183.77
Q9UBS4	DJB11_HUMAN DnaJ homolog subfamily B member 11 OS=Homo sapiens GN=DNAJB11 PE=1 SV=1		124.28
Q9UBT2	SAE2_HUMAN SUMO-activating enzyme subunit 2 OS=Homo sapiens GN=UBA2 PE=1 SV=2		161.32
Q9UBT7;Q9UBT7-2;Q9UBT	CTNL1_HUMAN Alpha-catulin OS=Homo sapiens GN=CTNNAL1 PE=1 SV=2;>sp|Q9UBT7-2|CTNL1_HUMAN Isoform 2 of Alpha-catulin	3.18E-26	
Q9UBU8	MO4L1_HUMAN Mortality factor 4-like protein 1 OS=Homo sapiens GN=MORF4L1 PE=1 SV=2		
Q9UBU8-2	MO4L1_HUMAN Isoform 2 of Mortality factor 4-like protein 1 OS=Homo sapiens GN=MORF4L1		
Q9UBU8;Q9UBU8-2	MO4L1_HUMAN Mortality factor 4-like protein 1 OS=Homo sapiens GN=MORF4L1 PE=1 SV=2;>sp|Q9UBU8-2|MO4L1_HUMAN Isoform	1.75E-09	
Q9UBU9	NXF1_HUMAN Nuclear RNA export factor 1 OS=Homo sapiens GN=NXF1 PE=1 SV=1		89.61
Q9UBU9-2	NXF1_HUMAN Isoform 2 of Nuclear RNA export factor 1 OS=Homo sapiens GN=NXF1		39.3
Q9UBU9;Q59E96;B4E269	NXF1_HUMAN Nuclear RNA export factor 1 OS=Homo sapiens GN=NXF1 PE=1 SV=1;>tr|Q59E96|Q59E96_HUMAN Nuclear RNA expor	8.83E-75	
Q9UBV2	SE1L1_HUMAN Protein sel-1 homolog 1 OS=Homo sapiens GN=SEL1L PE=1 SV=3		
Q9UBV2-2	SE1L1_HUMAN Isoform 2 of Protein sel-1 homolog 1 OS=Homo sapiens GN=SEL1L		
Q9UBV2;Q9UBV2-2	SE1L1_HUMAN Protein sel-1 homolog 1 OS=Homo sapiens GN=SEL1L PE=1 SV=3;>sp|Q9UBV2-2|SE1L1_HUMAN Isoform 2 of Proteir	9.90E-19	
Q9UBV8	PEF1_HUMAN Peflin OS=Homo sapiens GN=PEF1 PE=1 SV=1		89.81
Q9UBW7	ZMYM2_HUMAN Zinc finger MYM-type protein 2 OS=Homo sapiens GN=ZMYM2 PE=1 SV=1		
Q9UBW7-2	ZMYM2_HUMAN Isoform 2 of Zinc finger MYM-type protein 2 OS=Homo sapiens GN=ZMYM2		
Q9UBW8	CSN7A_HUMAN COP9 signalosome complex subunit 7a OS=Homo sapiens GN=COPS7A PE=1 SV=1		307.36

Q9UBX3	DIC_HUMAN Mitochondrial dicarboxylate carrier OS=Homo sapiens GN=SLC25A10 PE=1 SV=2		162.32
Q9UBX3-2	DIC_HUMAN Isoform 2 of Mitochondrial dicarboxylate carrier OS=Homo sapiens GN=SLC25A10		162.32
Q9UBX3-2;Q9UBX3	2|DIC_HUMAN Isoform 2 of Mitochondrial dicarboxylate carrier OS=Homo sapiens GN=SLC25A10;>sp|Q9UBX3|DIC_HUMAN Mitochon	3.39E-59	
Q9UBX5	FBLN5_HUMAN Fibulin-5 OS=Homo sapiens GN=FBLN5 PE=1 SV=1	1.10E-22	
Q9UBX7	KLK11_HUMAN Kallikrein-11 OS=Homo sapiens GN=KLK11 PE=1 SV=2		
Q9UBX7-1	KLK11_HUMAN Isoform 1 of Kallikrein-11 OS=Homo sapiens GN=KLK11		
Q9UBY0	SL9A2_HUMAN Sodium/hydrogen exchanger 2 OS=Homo sapiens GN=SLC9A2 PE=2 SV=1		
Q9UDR5	AASS_HUMAN Alpha-aminoadipic semialdehyde synthase, mitochondrial OS=Homo sapiens GN=AASS PE=1 SV=1		
Q9UDT6	CLIP2_HUMAN CAP-Gly domain-containing linker protein 2 OS=Homo sapiens GN=CLIP2 PE=1 SV=1		
Q9UDT6-2	CLIP2_HUMAN Isoform 2 of CAP-Gly domain-containing linker protein 2 OS=Homo sapiens GN=CLIP2		
Q9UDW1	QCR9_HUMAN Cytochrome b-c1 complex subunit 9 OS=Homo sapiens GN=UQCR10 PE=1 SV=3		
Q9UDW1-2	QCR9_HUMAN Isoform 2 of Cytochrome b-c1 complex subunit 9 OS=Homo sapiens GN=UQCR10		
Q9UDY2	ZO2_HUMAN Tight junction protein ZO-2 OS=Homo sapiens GN=TJP2 PE=1 SV=2		98.1
Q9UDY2-2	ZO2_HUMAN Isoform A2 of Tight junction protein ZO-2 OS=Homo sapiens GN=TJP2		90.64
Q9UDY2-3	ZO2_HUMAN Isoform C1 of Tight junction protein ZO-2 OS=Homo sapiens GN=TJP2		85.01
Q9UDY2-4	ZO2_HUMAN Isoform C2 of Tight junction protein ZO-2 OS=Homo sapiens GN=TJP2		77.55
Q9UDY2-5	ZO2_HUMAN Isoform A3 of Tight junction protein ZO-2 OS=Homo sapiens GN=TJP2		60.22
Q9UDY2;A2A3H9;Q9UDY2	ZO2_HUMAN Tight junction protein ZO-2 OS=Homo sapiens GN=TJP2 PE=1 SV=2;>tr|A2A3H9|A2A3H9_HUMAN Tight junction proteir	2.13E-108	
Q9UDY4	DNJB4_HUMAN DnaJ homolog subfamily B member 4 OS=Homo sapiens GN=DNAJB4 PE=1 SV=1		80.89
Q9UDY8;Q9UDY8-2	MALT1_HUMAN Mucosa-associated lymphoid tissue lymphoma translocation protein 1 OS=Homo sapiens GN=MALT1 PE=1 SV=1;>sp|(	5.96E-11	
Q9UEE5	ST17A_HUMAN Serine/threonine-protein kinase 17A OS=Homo sapiens GN=STK17A PE=1 SV=2		
Q9UEE9;Q9UEE9-2	CFDP1_HUMAN Craniofacial development protein 1 OS=Homo sapiens GN=CFDP1 PE=1 SV=1;>sp|Q9UEE9-2|CFDP1_HUMAN Isoform	8.28E-21	
Q9UEG4	ZN629_HUMAN Zinc finger protein 629 OS=Homo sapiens GN=ZNF629 PE=2 SV=2		
Q9UET6;B3KN91;B7Z4K4	RRMJ1_HUMAN Putative ribosomal RNA methyltransferase 1 OS=Homo sapiens GN=FTSJ1 PE=1 SV=2;>tr|B3KN91|B3KN91_HUMAN (	2.53E-23	
Q9UEU0	VTI1B_HUMAN Vesicle transport through interaction with t-SNAREs homolog 1B OS=Homo sapiens GN=VTI1B PE=1 SV=3		59.82
Q9UEU0-2	VTI1B_HUMAN Isoform Short of Vesicle transport through interaction with t-SNAREs homolog 1B OS=Homo sapiens GN=VTI1B		59.82
Q9UEU0;Q9UEU0-2	VTI1B_HUMAN Vesicle transport through interaction with t-SNAREs homolog 1B OS=Homo sapiens GN=VTI1B PE=1 SV=3;>sp|Q9UEU	6.24E-08	
Q9UEW8	STK39_HUMAN STE20/SPS1-related proline-alanine-rich protein kinase OS=Homo sapiens GN=STK39 PE=1 SV=3	1.46E-12	
Q9UEY8;Q9UEY8-2	ADDG_HUMAN Gamma-adducin OS=Homo sapiens GN=ADD3 PE=1 SV=1;>sp|Q9UEY8-2|ADDG_HUMAN Isoform 1 of Gamma-adduci	6.06E-08	
Q9UF33	EPHA6_HUMAN Ephrin type-A receptor 6 OS=Homo sapiens GN=EPHA6 PE=2 SV=2		89.59
Q9UF33-2	EPHA6_HUMAN Isoform 2 of Ephrin type-A receptor 6 OS=Homo sapiens GN=EPHA6		89.59
Q9UF83	YM012_HUMAN Uncharacterized protein DKFZp434B061 OS=Homo sapiens PE=2 SV=2		
Q9UFC0	LRWD1_HUMAN Leucine-rich repeat and WD repeat-containing protein 1 OS=Homo sapiens GN=LRWD1 PE=1 SV=2		54.24
Q9UFD9	RIM3A_HUMAN RIMS-binding protein 3A OS=Homo sapiens GN=RIMBP3 PE=1 SV=3		
Q9UFF9	CNOT8_HUMAN CCR4-NOT transcription complex subunit 8 OS=Homo sapiens GN=CNOT8 PE=1 SV=1		14.59
Q9UFN0	NPS3A_HUMAN Protein NipSnap homolog 3A OS=Homo sapiens GN=NIPSNAP3A PE=1 SV=2		103.51
Q9UFW8	CGBP1_HUMAN CGG triplet repeat-binding protein 1 OS=Homo sapiens GN=CGGBP1 PE=1 SV=2		75.13
Q9UG56	PISD_HUMAN Phosphatidylserine decarboxylase proenzyme OS=Homo sapiens GN=PISD PE=2 SV=4		
Q9UG56-2	PISD_HUMAN Isoform 2 of Phosphatidylserine decarboxylase proenzyme OS=Homo sapiens GN=PISD		
Q9UG63	ABCF2_HUMAN ATP-binding cassette sub-family F member 2 OS=Homo sapiens GN=ABCF2 PE=1 SV=2		137.78
Q9UGI0	ZRAN1_HUMAN Ubiquitin thioesterase ZRANB1 OS=Homo sapiens GN=ZRANB1 PE=1 SV=2		22.69
Q9UGI8;Q9UGI8-2	TES_HUMAN Testin OS=Homo sapiens GN=TES PE=1 SV=1;>sp|Q9UGI8-2|TES_HUMAN Isoform 2 of Testin OS=Homo sapiens GN=TI	2.65E-19	
Q9UGJ0;Q9UGJ0-3;Q9UGJ	AAKG2_HUMAN 5-AMP-activated protein kinase subunit gamma-2 OS=Homo sapiens GN=PRKAG2 PE=1 SV=1;>sp|Q9UGJ0-3|AAKG2_	6.15E-12	
Q9UGM3-6;Q9UGM3-7;Q9	6|DMBT1_HUMAN Isoform 6 of Deleted in malignant brain tumors 1 protein OS=Homo sapiens GN=DMBT1;>sp|Q9UGM3-7|DMBT1_HU	1.40E-12	
Q9UGM6	SYWM_HUMAN Tryptophanyl-tRNA synthetase, mitochondrial OS=Homo sapiens GN=WARS2 PE=1 SV=1		37.97
Q9UGP8	SEC63_HUMAN Translocation protein SEC63 homolog OS=Homo sapiens GN=SEC63 PE=1 SV=2	3.82E-14	
Q9UGT4	SUSD2_HUMAN Sushi domain-containing protein 2 OS=Homo sapiens GN=SUSD2 PE=1 SV=1	9.25E-35	
Q9UGU5	HMGX4_HUMAN HMG domain-containing protein 4 OS=Homo sapiens GN=HMGXB4 PE=2 SV=2		
Q9UGV2;Q9UGV2-2;Q9UG	NDRG3_HUMAN Protein NDRG3 OS=Homo sapiens GN=NDRG3 PE=1 SV=2;>sp|Q9UGV2-2|NDRG3_HUMAN Isoform 2 of Protein NDR	1.62E-08	
Q9UH17	ABC3B_HUMAN Probable DNA dC->dU-editing enzyme APOBEC-3B OS=Homo sapiens GN=APOBEC3B PE=1 SV=1		
Q9UH17-2	ABC3B_HUMAN Isoform 2 of Probable DNA dC->dU-editing enzyme APOBEC-3B OS=Homo sapiens GN=APOBEC3B		0.07
Q9UH17;Q96AK3;Q9UH17	ABC3B_HUMAN Probable DNA dC->dU-editing enzyme APOBEC-3B OS=Homo sapiens GN=APOBEC3B PE=1 SV=1;>sp|Q96AK3|ABC3I	7.98E-06	
Q9UH62	ARMX3_HUMAN Armadillo repeat-containing X-linked protein 3 OS=Homo sapiens GN=ARMCX3 PE=1 SV=1		
Q9UH65	SWP70_HUMAN Switch-associated protein 70 OS=Homo sapiens GN=SWAP70 PE=1 SV=1	1.39E-41	
Q9UH92	MLX_HUMAN Max-like protein X OS=Homo sapiens GN=MLX PE=1 SV=2		34.88
Q9UH92-2	MLX_HUMAN Isoform Alpha of Max-like protein X OS=Homo sapiens GN=MLX		6.73
Q9UH92-3	MLX_HUMAN Isoform Beta of Max-like protein X OS=Homo sapiens GN=MLX		6.73
Q9UH99	SUN2_HUMAN SUN domain-containing protein 2 OS=Homo sapiens GN=SUN2 PE=1 SV=3		22.12
Q9UHA3	RLP24_HUMAN Probable ribosome biogenesis protein RLP24 OS=Homo sapiens GN=RSL24D1 PE=1 SV=1		32.04
Q9UHA4	LTOR3_HUMAN Ragulator complex protein LAMTOR3 OS=Homo sapiens GN=LAMTOR3 PE=1 SV=1		43.16
Q9UHB6	LIMA1_HUMAN LIM domain and actin-binding protein 1 OS=Homo sapiens GN=LIMA1 PE=1 SV=1		46.68

Q9UHB6-2	LIMA1_HUMAN Isoform Alpha of LIM domain and actin-binding protein 1 OS=Homo sapiens GN=LIMA1		46.68
Q9UHB6-3	LIMA1_HUMAN Isoform 3 of LIM domain and actin-binding protein 1 OS=Homo sapiens GN=LIMA1		46.61
Q9UHB6-4	LIMA1_HUMAN Isoform 4 of LIM domain and actin-binding protein 1 OS=Homo sapiens GN=LIMA1		46.68
Q9UHB6-4;Q9UHB6;Q9UH	4|LIMA1_HUMAN Isoform 4 of LIM domain and actin-binding protein 1 OS=Homo sapiens GN=LIMA1;>sp|Q9UHB6|LIMA1_HUMAN LIM	1.57E-91	
Q9UHB6;Q9UHB6-4;Q9UH	LIMA1_HUMAN LIM domain and actin-binding protein 1 OS=Homo sapiens GN=LIMA1 PE=1 SV=1;>sp|Q9UHB6-4|LIMA1_HUMAN Isof	2.86E-226	
Q9UHB7	AFF4_HUMAN AF4/FMR2 family member 4 OS=Homo sapiens GN=AFF4 PE=1 SV=1		
Q9UHB7-2	AFF4_HUMAN Isoform 2 of AF4/FMR2 family member 4 OS=Homo sapiens GN=AFF4		
Q9UHB7-3	AFF4_HUMAN Isoform 3 of AF4/FMR2 family member 4 OS=Homo sapiens GN=AFF4		
Q9UHB9	SRP68_HUMAN Signal recognition particle 68 kDa protein OS=Homo sapiens GN=SRP68 PE=1 SV=2		273.55
Q9UHB9-2	SRP68_HUMAN Isoform 2 of Signal recognition particle 68 kDa protein OS=Homo sapiens GN=SRP68		249.06
Q9UHB9;B3KWY7;Q9UHB	SRP68_HUMAN Signal recognition particle 68 kDa protein OS=Homo sapiens GN=SRP68 PE=1 SV=2;>tr|B3KWY7|B3KWY7_HUMAN cD	4.32E-110	
Q9UHC1	MLH3_HUMAN DNA mismatch repair protein Mlh3 OS=Homo sapiens GN=MLH3 PE=1 SV=3		
Q9UHC1-2	MLH3_HUMAN Isoform 2 of DNA mismatch repair protein Mlh3 OS=Homo sapiens GN=MLH3		
Q9UHD1;Q9UHD1-2	CHRD1_HUMAN Cysteine and histidine-rich domain-containing protein 1 OS=Homo sapiens GN=CHORDC1 PE=1 SV=2;>sp|Q9UHD1-2	9.91E-68	
Q9UHD2	TBK1_HUMAN Serine/threonine-protein kinase TBK1 OS=Homo sapiens GN=TBK1 PE=1 SV=1	4.47E-07	
Q9UHD8	SEPT9_HUMAN Septin-9 OS=Homo sapiens GN=SEPT9 PE=1 SV=2		806.8
Q9UHD8-2	SEPT9_HUMAN Isoform 2 of Septin-9 OS=Homo sapiens GN=SEPT9		787.64
Q9UHD8-3	SEPT9_HUMAN Isoform 3 of Septin-9 OS=Homo sapiens GN=SEPT9		569.84
Q9UHD8-4	SEPT9_HUMAN Isoform 4 of Septin-9 OS=Homo sapiens GN=SEPT9		465.75
Q9UHD8-5	SEPT9_HUMAN Isoform 5 of Septin-9 OS=Homo sapiens GN=SEPT9		787.64
Q9UHD8-6	SEPT9_HUMAN Isoform 6 of Septin-9 OS=Homo sapiens GN=SEPT9		366.18
Q9UHD8-7	SEPT9_HUMAN Isoform 7 of Septin-9 OS=Homo sapiens GN=SEPT9		787.64
Q9UHD8-8	SEPT9_HUMAN Isoform 8 of Septin-9 OS=Homo sapiens GN=SEPT9		473.81
Q9UHD8-9	SEPT9_HUMAN Isoform 9 of Septin-9 OS=Homo sapiens GN=SEPT9		465.75
Q9UHD8;Q9UHD8-5;Q9UH	SEPT9_HUMAN Septin-9 OS=Homo sapiens GN=SEPT9 PE=1 SV=2;>sp|Q9UHD8-5|SEPT9_HUMAN Isoform 5 of Septin-9 OS=Homo s	8.69E-123	
Q9UHD9	UBQL2_HUMAN Ubiquilin-2 OS=Homo sapiens GN=UBQLN2 PE=1 SV=2	2.04E-26	
Q9UHF7	TRPS1_HUMAN Zinc finger transcription factor Trps1 OS=Homo sapiens GN=TRPS1 PE=1 SV=2		27.37
Q9UHF7-2	TRPS1_HUMAN Isoform 2 of Zinc finger transcription factor Trps1 OS=Homo sapiens GN=TRPS1		27.37
Q9UHF7-2;Q9UHF7-3;Q9U	2|TRPS1_HUMAN Isoform 2 of Zinc finger transcription factor Trps1 OS=Homo sapiens GN=TRPS1;>sp|Q9UHF7-3|TRPS1_HUMAN Isof	7.01E-06	
Q9UHF7-3	TRPS1_HUMAN Isoform 3 of Zinc finger transcription factor Trps1 OS=Homo sapiens GN=TRPS1		27.37
Q9UHG3	PCYOX_HUMAN Prenylcysteine oxidase 1 OS=Homo sapiens GN=PCYOX1 PE=1 SV=3		193
Q9UHH9	IP6K2_HUMAN Inositol hexakisphosphate kinase 2 OS=Homo sapiens GN=IP6K2 PE=2 SV=2		
Q9UHI6	DDX20_HUMAN Probable ATP-dependent RNA helicase DDX20 OS=Homo sapiens GN=DDX20 PE=1 SV=2	1.14E-11	
Q9UHJ6	SHPK_HUMAN Sedoheptulokinase OS=Homo sapiens GN=SHPK PE=1 SV=3	3.39E-17	
Q9UHL4	DPP2_HUMAN Dipeptidyl peptidase 2 OS=Homo sapiens GN=DPP7 PE=1 SV=3	1.37E-47	
Q9UHN1	DPOG2_HUMAN DNA polymerase subunit gamma-2, mitochondrial OS=Homo sapiens GN=POLG2 PE=1 SV=1		21.56
Q9UHN6	TMEM2_HUMAN Transmembrane protein 2 OS=Homo sapiens GN=TMEM2 PE=1 SV=1		33.19
Q9UHN6-2	TMEM2_HUMAN Isoform 2 of Transmembrane protein 2 OS=Homo sapiens GN=TMEM2		33.19
Q9UHQ4	BAP29_HUMAN B-cell receptor-associated protein 29 OS=Homo sapiens GN=BCAP29 PE=1 SV=2		43.08
Q9UHQ9	NB5R1_HUMAN NADH-cytochrome b5 reductase 1 OS=Homo sapiens GN=CYB5R1 PE=1 SV=1		311.04
Q9UHR5;Q9UHR5-2	S30BP_HUMAN SAP30-binding protein OS=Homo sapiens GN=SAP30BP PE=1 SV=1;>sp|Q9UHR5-2|S30BP_HUMAN Isoform 2 of SAP3	3.98E-10	
Q9UHV9	PFD2_HUMAN Prefoldin subunit 2 OS=Homo sapiens GN=PFDN2 PE=1 SV=1		290.01
Q9UHW5-2;D4YWV1;Q9UI	2|GPN3_HUMAN Isoform 2 of GPN-loop GTPase 3 OS=Homo sapiens GN=GPN3;>tr|D4YWV1|D4YWV1_HUMAN GPN-loop GTPase 3 OS	5.07E-10	
Q9UHX1	PUF60_HUMAN Poly(U)-binding-splicing factor PUF60 OS=Homo sapiens GN=PUF60 PE=1 SV=1		439.38
Q9UHX1-2	PUF60_HUMAN Isoform 2 of Poly(U)-binding-splicing factor PUF60 OS=Homo sapiens GN=PUF60		439.38
Q9UHX1-3	PUF60_HUMAN Isoform 3 of Poly(U)-binding-splicing factor PUF60 OS=Homo sapiens GN=PUF60		434.38
Q9UHX1-4	PUF60_HUMAN Isoform 4 of Poly(U)-binding-splicing factor PUF60 OS=Homo sapiens GN=PUF60		434.38
Q9UHX1-5	PUF60_HUMAN Isoform 5 of Poly(U)-binding-splicing factor PUF60 OS=Homo sapiens GN=PUF60		434.38
Q9UHX1-5;Q9UHX1-6;Q9U	5|PUF60_HUMAN Isoform 5 of Poly(U)-binding-splicing factor PUF60 OS=Homo sapiens GN=PUF60;>sp|Q9UHX1-6|PUF60_HUMAN Isc	1.81E-126	
Q9UHX1-6	PUF60_HUMAN Isoform 6 of Poly(U)-binding-splicing factor PUF60 OS=Homo sapiens GN=PUF60		434.38
Q9UHX1;Q9UHX1-2;Q9UH	PUF60_HUMAN Poly(U)-binding-splicing factor PUF60 OS=Homo sapiens GN=PUF60 PE=1 SV=1;>sp|Q9UHX1-2|PUF60_HUMAN Isofor	8.37E-197	
Q9UHY1	NRBP_HUMAN Nuclear receptor-binding protein OS=Homo sapiens GN=NRBP1 PE=1 SV=1	1.18E-08	
Q9UHY7	ENOPH_HUMAN Enolase-phosphatase E1 OS=Homo sapiens GN=ENOPH1 PE=1 SV=1	9.99E-22	
Q9UI08-2;Q9UI08	2|EVL_HUMAN Isoform 1 of Ena/VASP-like protein OS=Homo sapiens GN=EVL;>sp|Q9UI08|EVL_HUMAN Ena/VASP-like protein OS=H	7.75E-108	
Q9UI09	NDUAC_HUMAN NADH dehydrogenase [ubiquinone] 1 alpha subcomplex subunit 12 OS=Homo sapiens GN=NDUFA12 PE=1 SV=1		135.61
Q9UI10	EI2BD_HUMAN Translation initiation factor eIF-2B subunit delta OS=Homo sapiens GN=EIF2B4 PE=1 SV=2		66.72
Q9UI10-2	EI2BD_HUMAN Isoform 2 of Translation initiation factor eIF-2B subunit delta OS=Homo sapiens GN=EIF2B4		71.51
Q9UI10-2;Q9UI10;Q9UI1	2|EI2BD_HUMAN Isoform 2 of Translation initiation factor eIF-2B subunit delta OS=Homo sapiens GN=EIF2B4;>sp|Q9UI10|EI2BD_HU	8.67E-13	
Q9UI10-3	EI2BD_HUMAN Isoform 3 of Translation initiation factor eIF-2B subunit delta OS=Homo sapiens GN=EIF2B4		66.72
Q9UI12;Q9UI12-2	VATH_HUMAN V-type proton ATPase subunit H OS=Homo sapiens GN=ATP6V1H PE=1 SV=1;>sp|Q9UI12-2|VATH_HUMAN Isoform 2 c	1.22E-07	
Q9UI14	PRAF1_HUMAN Prenylated Rab acceptor protein 1 OS=Homo sapiens GN=RABAC1 PE=1 SV=1	1.08E-23	

Q9UI15	TAGL3_HUMAN Transgelin-3 OS=Homo sapiens GN=TAGLN3 PE=1 SV=2		138.02
Q9UI17	M2GD_HUMAN Dimethylglycine dehydrogenase, mitochondrial OS=Homo sapiens GN=DMGDH PE=1 SV=2		66.22
Q9UI26-2;Q9UI26	2|IPO11_HUMAN Isoform 2 of Importin-11 OS=Homo sapiens GN=IPO11;>sp|Q9UI26|IPO11_HUMAN Importin-11 OS=Homo sapiens	9.69E-45	
Q9UI30	TR112_HUMAN tRNA methyltransferase 112 homolog OS=Homo sapiens GN=TRMT112 PE=1 SV=1		215.4
Q9UI33	SCNBA_HUMAN Sodium channel protein type 11 subunit alpha OS=Homo sapiens GN=SCN11A PE=1 SV=2		17.9
Q9UI33-2	SCNBA_HUMAN Isoform 2 of Sodium channel protein type 11 subunit alpha OS=Homo sapiens GN=SCN11A		17.9
Q9UI33-3	SCNBA_HUMAN Isoform 3 of Sodium channel protein type 11 subunit alpha OS=Homo sapiens GN=SCN11A		17.9
Q9UI46	DNAI1_HUMAN Dynein intermediate chain 1, axonemal OS=Homo sapiens GN=DNAI1 PE=1 SV=1		18.53
Q9UIA0	CYH4_HUMAN Cytohesin-4 OS=Homo sapiens GN=CYTH4 PE=1 SV=1		24.94
Q9UIA9	XPO7_HUMAN Exportin-7 OS=Homo sapiens GN=XPO7 PE=1 SV=3		20.39
Q9UID3;Q9UID3-2	FFR_HUMAN Protein fat-free homolog OS=Homo sapiens GN=FFR PE=1 SV=2;>sp|Q9UID3-2|FFR_HUMAN Isoform 2 of Protein fat-fre	9.10E-08	
Q9UIF9	BAZ2A_HUMAN Bromodomain adjacent to zinc finger domain protein 2A OS=Homo sapiens GN=BAZ2A PE=1 SV=4		39.68
Q9UIF9-2	BAZ2A_HUMAN Isoform 1 of Bromodomain adjacent to zinc finger domain protein 2A OS=Homo sapiens GN=BAZ2A		39.68
Q9UIF9-3	BAZ2A_HUMAN Isoform 3 of Bromodomain adjacent to zinc finger domain protein 2A OS=Homo sapiens GN=BAZ2A		39.68
Q9UIG0	BAZ1B_HUMAN Tyrosine-protein kinase BAZ1B OS=Homo sapiens GN=BAZ1B PE=1 SV=2		
Q9UIG0-2	BAZ1B_HUMAN Isoform 2 of Tyrosine-protein kinase BAZ1B OS=Homo sapiens GN=BAZ1B		
Q9UIG0;Q9UIG0-2	BAZ1B_HUMAN Tyrosine-protein kinase BAZ1B OS=Homo sapiens GN=BAZ1B PE=1 SV=2;>sp|Q9UIG0-2|BAZ1B_HUMAN Isoform 2 o	4.60E-130	
Q9UII2	ATIF1_HUMAN ATPase inhibitor, mitochondrial OS=Homo sapiens GN=ATPIF1 PE=1 SV=1		33.18
Q9UIJ7	KAD3_HUMAN GTP:AMP phosphotransferase, mitochondrial OS=Homo sapiens GN=AK3 PE=1 SV=4		130.57
Q9UIL1	SCOC_HUMAN Short coiled-coil protein OS=Homo sapiens GN=SCOC PE=1 SV=2		28.29
Q9UIL1-2	SCOC_HUMAN Isoform 2 of Short coiled-coil protein OS=Homo sapiens GN=SCOC		28.29
Q9UIL1-3	SCOC_HUMAN Isoform 3 of Short coiled-coil protein OS=Homo sapiens GN=SCOC		28.29
Q9UIL1-4	SCOC_HUMAN Isoform 4 of Short coiled-coil protein OS=Homo sapiens GN=SCOC		7.83
Q9UIQ6	LCAP_HUMAN Leucyl-cystinyl aminopeptidase OS=Homo sapiens GN=LNPEP PE=1 SV=3		357.93
Q9UIQ6-2	LCAP_HUMAN Isoform 2 of Leucyl-cystinyl aminopeptidase OS=Homo sapiens GN=LNPEP		357.93
Q9UIQ6-3	LCAP_HUMAN Isoform 3 of Leucyl-cystinyl aminopeptidase OS=Homo sapiens GN=LNPEP		357.93
Q9UIQ6;Q9UIQ6-2;Q9UIQ	LCAP_HUMAN Leucyl-cystinyl aminopeptidase OS=Homo sapiens GN=LNPEP PE=1 SV=3;>sp|Q9UIQ6-2|LCAP_HUMAN Isoform 2 of Le	8.16E-75	
Q9UIU6	SIX4_HUMAN Homeobox protein SIX4 OS=Homo sapiens GN=SIX4 PE=1 SV=2	4.71E-07	
Q9UIV1	CNOT7_HUMAN CCR4-NOT transcription complex subunit 7 OS=Homo sapiens GN=CNOT7 PE=1 SV=3		107.03
Q9UJ41	RABX5_HUMAN Rab5 GDP/GTP exchange factor OS=Homo sapiens GN=RABGEF1 PE=1 SV=2		
Q9UJ41-2	RABX5_HUMAN Isoform 2 of Rab5 GDP/GTP exchange factor OS=Homo sapiens GN=RABGEF1		
Q9UJ41-3	RABX5_HUMAN Isoform 3 of Rab5 GDP/GTP exchange factor OS=Homo sapiens GN=RABGEF1		
Q9UJ68	MSRA_HUMAN Mitochondrial peptide methionine sulfoxide reductase OS=Homo sapiens GN=MSRA PE=1 SV=1		
Q9UJ68-2	MSRA_HUMAN Isoform 2 of Mitochondrial peptide methionine sulfoxide reductase OS=Homo sapiens GN=MSRA		
Q9UJ68-3	MSRA_HUMAN Isoform 3 of Mitochondrial peptide methionine sulfoxide reductase OS=Homo sapiens GN=MSRA		
Q9UJ68-4	MSRA_HUMAN Isoform 4 of Mitochondrial peptide methionine sulfoxide reductase OS=Homo sapiens GN=MSRA		
Q9UJ70	NAGK_HUMAN N-acetyl-D-glucosamine kinase OS=Homo sapiens GN=NAGK PE=1 SV=4	1.82E-51	
Q9UJ98	STAG3_HUMAN Cohesin subunit SA-3 OS=Homo sapiens GN=STAG3 PE=1 SV=2		
Q9UJ98-2	STAG3_HUMAN Isoform 2 of Cohesin subunit SA-3 OS=Homo sapiens GN=STAG3		
Q9UJA5	TRM6_HUMAN tRNA (adenine-N(1)-)-methyltransferase non-catalytic subunit TRM6 OS=Homo sapiens GN=TRMT6 PE=1 SV=1		21.4
Q9UJA5-2	TRM6_HUMAN Isoform 2 of tRNA (adenine-N(1)-)-methyltransferase non-catalytic subunit TRM6 OS=Homo sapiens GN=TRMT6		21.4
Q9UJA5-3	TRM6_HUMAN Isoform 3 of tRNA (adenine-N(1)-)-methyltransferase non-catalytic subunit TRM6 OS=Homo sapiens GN=TRMT6		21.4
Q9UJA5;Q9UJA5-2;Q9UJA	TRM6_HUMAN tRNA (adenine-N(1)-)-methyltransferase non-catalytic subunit TRM6 OS=Homo sapiens GN=TRMT6 PE=1 SV=1;>sp|Q9	2.36E-35	
Q9UJC3	HOOK1_HUMAN Protein Hook homolog 1 OS=Homo sapiens GN=HOOK1 PE=1 SV=2	2.39E-23	
Q9UJF2	NGAP_HUMAN Ras GTPase-activating protein nGAP OS=Homo sapiens GN=RASAL2 PE=1 SV=2		42.96
Q9UJG1-3;D3DTG5;Q9UJG	3|MSPD1_HUMAN Isoform 3 of Motile sperm domain-containing protein 1 OS=Homo sapiens GN=MOSPD1;>tr|D3DTG5|D3DTG5_HUM	3.00E-07	
Q9UJS0	CMC2_HUMAN Calcium-binding mitochondrial carrier protein Aralar2 OS=Homo sapiens GN=SLC25A13 PE=1 SV=2		105.23
Q9UJU6-2;Q9UJU6;Q9UJU	2|DBNL_HUMAN Isoform 2 of Drebrin-like protein OS=Homo sapiens GN=DBNL;>sp|Q9UJU6|DBNL_HUMAN Drebrin-like protein OS=H	1.01E-268	
Q9UJU6;Q9UJU6-2;Q9UJU	DBNL_HUMAN Drebrin-like protein OS=Homo sapiens GN=DBNL PE=1 SV=1;>sp|Q9UJU6-2|DBNL_HUMAN Isoform 2 of Drebrin-like p	0	
Q9UJV8	PURG_HUMAN Purine-rich element-binding protein gamma OS=Homo sapiens GN=PURG PE=2 SV=1		8.57
Q9UJV8-2	PURG_HUMAN Isoform 2 of Purine-rich element-binding protein gamma OS=Homo sapiens GN=PURG		8.57
Q9UJV9	DDX41_HUMAN Probable ATP-dependent RNA helicase DDX41 OS=Homo sapiens GN=DDX41 PE=1 SV=2		
Q9UJW0	DCTN4_HUMAN Dynactin subunit 4 OS=Homo sapiens GN=DCTN4 PE=1 SV=1		
Q9UJW0-3	DCTN4_HUMAN Isoform 3 of Dynactin subunit 4 OS=Homo sapiens GN=DCTN4		
Q9UJW0-3;Q9UJW0;Q9UJ\	3|DCTN4_HUMAN Isoform 3 of Dynactin subunit 4 OS=Homo sapiens GN=DCTN4;>sp|Q9UJW0|DCTN4_HUMAN Dynactin subunit 4 OS	8.18E-24	
Q9UJX2	CDC23_HUMAN Cell division cycle protein 23 homolog OS=Homo sapiens GN=CDC23 PE=1 SV=3		
Q9UJX2-3	CDC23_HUMAN Isoform 3 of Cell division cycle protein 23 homolog OS=Homo sapiens GN=CDC23		
Q9UJX3	APC7_HUMAN Anaphase-promoting complex subunit 7 OS=Homo sapiens GN=ANAPC7 PE=1 SV=4		103.93
Q9UJX3-2	APC7_HUMAN Isoform 2 of Anaphase-promoting complex subunit 7 OS=Homo sapiens GN=ANAPC7		65.91
Q9UJX3;A5D8X2;Q4KMX6	APC7_HUMAN Anaphase-promoting complex subunit 7 OS=Homo sapiens GN=ANAPC7 PE=1 SV=4;>tr|A5D8X2|A5D8X2_HUMAN Ana	1.11E-19	
Q9UJX4	APC5_HUMAN Anaphase-promoting complex subunit 5 OS=Homo sapiens GN=ANAPC5 PE=1 SV=2		

Q9UJX5;Q9UJX5-2	APC4_HUMAN Anaphase-promoting complex subunit 4 OS=Homo sapiens GN=ANAPC4 PE=1 SV=2;>sp|Q9UJX5-2|APC4_HUMAN Isofo	9.32E-12	
Q9UJY4	GGA2_HUMAN ADP-ribosylation factor-binding protein GGA2 OS=Homo sapiens GN=GGA2 PE=1 SV=3		43.99
Q9UJY5	GGA1_HUMAN ADP-ribosylation factor-binding protein GGA1 OS=Homo sapiens GN=GGA1 PE=1 SV=1		33.72
Q9UJY5-2	GGA1_HUMAN Isoform 2 of ADP-ribosylation factor-binding protein GGA1 OS=Homo sapiens GN=GGA1		
Q9UJY5;Q9UJY5-2	GGA1_HUMAN ADP-ribosylation factor-binding protein GGA1 OS=Homo sapiens GN=GGA1 PE=1 SV=1;>sp|Q9UJY5-2|GGA1_HUMAN I	1.84E-12	
Q9UJZ1	STML2_HUMAN Stomatin-like protein 2 OS=Homo sapiens GN=STOML2 PE=1 SV=1		792.06
Q9UK22	FBX2_HUMAN F-box only protein 2 OS=Homo sapiens GN=FBXO2 PE=1 SV=2	1.76E-09	
Q9UK41	VPS28_HUMAN Vacuolar protein sorting-associated protein 28 homolog OS=Homo sapiens GN=VPS28 PE=1 SV=1	1.18E-25	
Q9UK45	LSM7_HUMAN U6 snRNA-associated Sm-like protein LSm7 OS=Homo sapiens GN=LSM7 PE=1 SV=1		101.58
Q9UK59	DBR1_HUMAN Lariat debranching enzyme OS=Homo sapiens GN=DBR1 PE=1 SV=2		38.11
Q9UK59-2	DBR1_HUMAN Isoform 2 of Lariat debranching enzyme OS=Homo sapiens GN=DBR1		0.85
Q9UK76-2;Q9UK76;Q9UK	2|HN1_HUMAN Isoform 2 of Hematological and neurological expressed 1 protein OS=Homo sapiens GN=HN1;>sp|Q9UK76|HN1_HUMA	8.76E-11	
Q9UK99	FBX3_HUMAN F-box only protein 3 OS=Homo sapiens GN=FBXO3 PE=1 SV=3		
Q9UK99-2	FBX3_HUMAN Isoform 2 of F-box only protein 3 OS=Homo sapiens GN=FBXO3		
Q9UK99-3	FBX3_HUMAN Isoform 3 of F-box only protein 3 OS=Homo sapiens GN=FBXO3		
Q9UKA9	PTBP2_HUMAN Polypyrimidine tract-binding protein 2 OS=Homo sapiens GN=PTBP2 PE=1 SV=1		61.1
Q9UKA9-2	PTBP2_HUMAN Isoform 2 of Polypyrimidine tract-binding protein 2 OS=Homo sapiens GN=PTBP2		61.1
Q9UKA9-2;Q9UKA9;Q9UK	2|PTBP2_HUMAN Isoform 2 of Polypyrimidine tract-binding protein 2 OS=Homo sapiens GN=PTBP2;>sp|Q9UKA9|PTBP2_HUMAN Polyp	6.13E-23	
Q9UKA9-3	PTBP2_HUMAN Isoform 3 of Polypyrimidine tract-binding protein 2 OS=Homo sapiens GN=PTBP2		61.1
Q9UKA9-4	PTBP2_HUMAN Isoform 4 of Polypyrimidine tract-binding protein 2 OS=Homo sapiens GN=PTBP2		61.1
Q9UKA9-5	PTBP2_HUMAN Isoform 5 of Polypyrimidine tract-binding protein 2 OS=Homo sapiens GN=PTBP2		61.1
Q9UKA9-6	PTBP2_HUMAN Isoform 6 of Polypyrimidine tract-binding protein 2 OS=Homo sapiens GN=PTBP2		61.1
Q9UKD2	MRT4_HUMAN mRNA turnover protein 4 homolog OS=Homo sapiens GN=MRTO4 PE=1 SV=2		165.44
Q9UKE5	TNIK_HUMAN TRAF2 and NCK-interacting protein kinase OS=Homo sapiens GN=TNIK PE=1 SV=1		103.98
Q9UKE5-2	TNIK_HUMAN Isoform 2 of TRAF2 and NCK-interacting protein kinase OS=Homo sapiens GN=TNIK		99.33
Q9UKE5-3	TNIK_HUMAN Isoform 3 of TRAF2 and NCK-interacting protein kinase OS=Homo sapiens GN=TNIK		100.18
Q9UKE5-4	TNIK_HUMAN Isoform 4 of TRAF2 and NCK-interacting protein kinase OS=Homo sapiens GN=TNIK		103.98
Q9UKE5-5	TNIK_HUMAN Isoform 5 of TRAF2 and NCK-interacting protein kinase OS=Homo sapiens GN=TNIK		95.53
Q9UKE5-6	TNIK_HUMAN Isoform 6 of TRAF2 and NCK-interacting protein kinase OS=Homo sapiens GN=TNIK		99.33
Q9UKE5-7	TNIK_HUMAN Isoform 7 of TRAF2 and NCK-interacting protein kinase OS=Homo sapiens GN=TNIK		100.18
Q9UKE5-8	TNIK_HUMAN Isoform 8 of TRAF2 and NCK-interacting protein kinase OS=Homo sapiens GN=TNIK		95.53
Q9UKF6	CPSF3_HUMAN Cleavage and polyadenylation specificity factor subunit 3 OS=Homo sapiens GN=CPSF3 PE=1 SV=1		
Q9UKG1;B4DQX8	DP13A_HUMAN DCC-interacting protein 13-alpha OS=Homo sapiens GN=APPL1 PE=1 SV=1;>tr|B4DQX8|B4DQX8_HUMAN cDNA FLJ5	1.21E-06	
Q9UKG9;C9J3D7;B4DT97;	OCTC_HUMAN Peroxisomal carnitine O-octanoyltransferase OS=Homo sapiens GN=CROT PE=1 SV=2;>tr|C9J3D7|C9J3D7_HUMAN Un	2.33E-06	
Q9UKH3	ENK4_HUMAN HERV-K_6q14.1 provirus ancestral Env polyprotein OS=Homo sapiens PE=1 SV=1		
Q9UKI8	TLK1_HUMAN Serine/threonine-protein kinase tousled-like 1 OS=Homo sapiens GN=TLK1 PE=1 SV=2		11.08
Q9UKI8-2	TLK1_HUMAN Isoform 2 of Serine/threonine-protein kinase tousled-like 1 OS=Homo sapiens GN=TLK1		11.08
Q9UKI8-3	TLK1_HUMAN Isoform 3 of Serine/threonine-protein kinase tousled-like 1 OS=Homo sapiens GN=TLK1		11.08
Q9UKJ0-2	PILRB_HUMAN Isoform 2 of Paired immunoglobulin-like type 2 receptor beta OS=Homo sapiens GN=PILRB		
Q9UKJ0-3	PILRB_HUMAN Isoform 3 of Paired immunoglobulin-like type 2 receptor beta OS=Homo sapiens GN=PILRB		
Q9UKJ3	GPTC8_HUMAN G patch domain-containing protein 8 OS=Homo sapiens GN=GPATCH8 PE=1 SV=2		102.72
Q9UKJ3-2	GPTC8_HUMAN Isoform 2 of G patch domain-containing protein 8 OS=Homo sapiens GN=GPATCH8		102.72
Q9UKK3	PARP4_HUMAN Poly [ADP-ribose] polymerase 4 OS=Homo sapiens GN=PARP4 PE=1 SV=3		41.39
Q9UKK6	NXT1_HUMAN NTF2-related export protein 1 OS=Homo sapiens GN=NXT1 PE=1 SV=1		
Q9UKK9	NUDT5_HUMAN ADP-sugar pyrophosphatase OS=Homo sapiens GN=NUDT5 PE=1 SV=1		18.06
Q9UKL0	RCOR1_HUMAN REST corepressor 1 OS=Homo sapiens GN=RCOR1 PE=1 SV=1		82.28
Q9UKM9	RALY_HUMAN RNA-binding protein Raly OS=Homo sapiens GN=RALY PE=1 SV=1		241.28
Q9UKM9-2	RALY_HUMAN Isoform 1 of RNA-binding protein Raly OS=Homo sapiens GN=RALY		241.28
Q9UKM9;Q9UKM9-2	RALY_HUMAN RNA-binding protein Raly OS=Homo sapiens GN=RALY PE=1 SV=1;>sp|Q9UKM9-2|RALY_HUMAN Isoform 1 of RNA-binc	1.45E-120	
Q9UKN7	MYO15_HUMAN Myosin-XV OS=Homo sapiens GN=MYO15A PE=1 SV=2		
Q9UKN8	TF3C4_HUMAN General transcription factor 3C polypeptide 4 OS=Homo sapiens GN=GTF3C4 PE=1 SV=2		57.89
Q9UKR5	ERG28_HUMAN Probable ergosterol biosynthetic protein 28 OS=Homo sapiens GN=C14orf1 PE=1 SV=1		37.97
Q9UKS6	PACN3_HUMAN Protein kinase C and casein kinase substrate in neurons protein 3 OS=Homo sapiens GN=PACSIN3 PE=1 SV=2		115.6
Q9UKU7;B7Z5W4;Q6ZWP	ACAD8_HUMAN Isobutyryl-CoA dehydrogenase, mitochondrial OS=Homo sapiens GN=ACAD8 PE=1 SV=1;>tr|B7Z5W4|B7Z5W4_HUM	4.21E-06	
Q9UKV0	HDAC9_HUMAN Histone deacetylase 9 OS=Homo sapiens GN=HDAC9 PE=1 SV=2		
Q9UKV0-2	HDAC9_HUMAN Isoform 2 of Histone deacetylase 9 OS=Homo sapiens GN=HDAC9		
Q9UKV0-3	HDAC9_HUMAN Isoform 3 of Histone deacetylase 9 OS=Homo sapiens GN=HDAC9		
Q9UKV0-4	HDAC9_HUMAN Isoform 4 of Histone deacetylase 9 OS=Homo sapiens GN=HDAC9		
Q9UKV0-5	HDAC9_HUMAN Isoform 5 of Histone deacetylase 9 OS=Homo sapiens GN=HDAC9		
Q9UKV0-6	HDAC9_HUMAN Isoform 6 of Histone deacetylase 9 OS=Homo sapiens GN=HDAC9		
Q9UKV0-7	HDAC9_HUMAN Isoform 7 of Histone deacetylase 9 OS=Homo sapiens GN=HDAC9		

Q9UKV3	ACINU_HUMAN Apoptotic chromatin condensation inducer in the nucleus OS=Homo sapiens GN=ACIN1 PE=1 SV=2		296.26
Q9UKV3-2	ACINU_HUMAN Isoform 2 of Apoptotic chromatin condensation inducer in the nucleus OS=Homo sapiens GN=ACIN1		227.54
Q9UKV3-3	ACINU_HUMAN Isoform 3 of Apoptotic chromatin condensation inducer in the nucleus OS=Homo sapiens GN=ACIN1		227.54
Q9UKV3;Q9UKV3-2;Q9UK	ACINU_HUMAN Apoptotic chromatin condensation inducer in the nucleus OS=Homo sapiens GN=ACIN1 PE=1 SV=2;>sp|Q9UKV3-2|A(	6.19E-63	
Q9UKX2	MYH2_HUMAN Myosin-2 OS=Homo sapiens GN=MYH2 PE=1 SV=1		140.79
Q9UKX3	MYH13_HUMAN Myosin-13 OS=Homo sapiens GN=MYH13 PE=1 SV=2		82.89
Q9UKX7;Q8N6V5;B4E2D3	NUP50_HUMAN Nuclear pore complex protein Nup50 OS=Homo sapiens GN=NUP50 PE=1 SV=2;>tr|Q8N6V5|Q8N6V5_HUMAN HCG19	9.14E-66	
Q9UKY1	ZHX1_HUMAN Zinc fingers and homeoboxes protein 1 OS=Homo sapiens GN=ZHX1 PE=1 SV=1		
Q9UKY3	CES1P_HUMAN Putative inactive carboxylesterase 4 OS=Homo sapiens GN=CES1P1 PE=5 SV=2		
Q9UKY7	CDV3_HUMAN Protein CDV3 homolog OS=Homo sapiens GN=CDV3 PE=1 SV=1		205.14
Q9UKY7-2	CDV3_HUMAN Isoform 2 of Protein CDV3 homolog OS=Homo sapiens GN=CDV3		133.23
Q9UKY7-3	CDV3_HUMAN Isoform 3 of Protein CDV3 homolog OS=Homo sapiens GN=CDV3		19.45
Q9UKY7;Q9UKY7-2	CDV3_HUMAN Protein CDV3 homolog OS=Homo sapiens GN=CDV3 PE=1 SV=1;>sp|Q9UKY7-2|CDV3_HUMAN Isoform 2 of Protein CD	3.34E-51	
Q9UKZ1	CB029_HUMAN UPF0760 protein C2orf29 OS=Homo sapiens GN=C2orf29 PE=1 SV=1		76.41
Q9UL01	DSE_HUMAN Dermatan-sulfate epimerase OS=Homo sapiens GN=DSE PE=1 SV=1		
Q9UL15	BAG5_HUMAN BAG family molecular chaperone regulator 5 OS=Homo sapiens GN=BAG5 PE=1 SV=1		
Q9UL15-2	BAG5_HUMAN Isoform 2 of BAG family molecular chaperone regulator 5 OS=Homo sapiens GN=BAG5		
Q9UL15-2;A8K5J7;Q9UL1!	2|BAG5_HUMAN Isoform 2 of BAG family molecular chaperone regulator 5 OS=Homo sapiens GN=BAG5;>tr|A8K5J7|A8K5J7_HUMAN	8.21E-08	
Q9UL16	CCD19_HUMAN Coiled-coil domain-containing protein 19, mitochondrial OS=Homo sapiens GN=CCDC19 PE=1 SV=2		54.85
Q9UL25	RAB21_HUMAN Ras-related protein Rab-21 OS=Homo sapiens GN=RAB21 PE=1 SV=3		614.52
Q9UL26	RB22A_HUMAN Ras-related protein Rab-22A OS=Homo sapiens GN=RAB22A PE=1 SV=2		155.27
Q9UL33	TPC2L_HUMAN Trafficking protein particle complex subunit 2-like protein OS=Homo sapiens GN=TRAPPC2L PE=1 SV=1		
Q9UL33-2	TPC2L_HUMAN Isoform 2 of Trafficking protein particle complex subunit 2-like protein OS=Homo sapiens GN=TRAPPC2L		
Q9UL33;Q9UL33-2	TPC2L_HUMAN Trafficking protein particle complex subunit 2-like protein OS=Homo sapiens GN=TRAPPC2L PE=1 SV=1;>sp|Q9UL33-2	6.68E-05	
Q9UL42	PNMA2_HUMAN Paraneoplastic antigen Ma2 OS=Homo sapiens GN=PNMA2 PE=1 SV=2		
Q9UL46	PSME2_HUMAN Proteasome activator complex subunit 2 OS=Homo sapiens GN=PSME2 PE=1 SV=4		855.68
Q9UL63	MKLN1_HUMAN Muskelin OS=Homo sapiens GN=MKLN1 PE=1 SV=2		
Q9UL63-2	MKLN1_HUMAN Isoform 2 of Muskelin OS=Homo sapiens GN=MKLN1		
Q9ULA0	DNPEP_HUMAN Aspartyl aminopeptidase OS=Homo sapiens GN=DNPEP PE=1 SV=1		49.67
Q9ULC3	RAB23_HUMAN Ras-related protein Rab-23 OS=Homo sapiens GN=RAB23 PE=1 SV=1		88.23
Q9ULC4	MCTS1_HUMAN Malignant T cell-amplified sequence 1 OS=Homo sapiens GN=MCTS1 PE=1 SV=1		535.26
Q9ULC4-2	MCTS1_HUMAN Isoform 2 of Malignant T cell-amplified sequence 1 OS=Homo sapiens GN=MCTS1		534.23
Q9ULC4-3	MCTS1_HUMAN Isoform 3 of Malignant T cell-amplified sequence 1 OS=Homo sapiens GN=MCTS1		535.26
Q9ULC4-3;Q9ULC4;Q9ULC	3|MCTS1_HUMAN Isoform 3 of Malignant T cell-amplified sequence 1 OS=Homo sapiens GN=MCTS1;>sp|Q9ULC4|MCTS1_HUMAN Mal	1.50E-164	
Q9ULC4;B4DGY2;Q9ULC4-	MCTS1_HUMAN Malignant T cell-amplified sequence 1 OS=Homo sapiens GN=MCTS1 PE=1 SV=1;>tr|B4DGY2|B4DGY2_HUMAN cDNA	3.19E-137	
Q9ULC5-3;Q9ULC5;Q9ULC	3|ACSL5_HUMAN Isoform 2 of Long-chain-fatty-acid--CoA ligase 5 OS=Homo sapiens GN=ACSL5;>sp|Q9ULC5|ACSL5_HUMAN Long-c	4.85E-12	
Q9ULD0	OGDHL_HUMAN 2-oxoglutarate dehydrogenase-like, mitochondrial OS=Homo sapiens GN=OGDHL PE=2 SV=3		34.96
Q9ULD0-2	OGDHL_HUMAN Isoform 2 of 2-oxoglutarate dehydrogenase-like, mitochondrial OS=Homo sapiens GN=OGDHL		34.96
Q9ULD0-3	OGDHL_HUMAN Isoform 3 of 2-oxoglutarate dehydrogenase-like, mitochondrial OS=Homo sapiens GN=OGDHL		34.96
Q9ULD5	ZN777_HUMAN Zinc finger protein 777 OS=Homo sapiens GN=ZNF777 PE=1 SV=2		31.41
Q9ULD5-2	ZN777_HUMAN Isoform 2 of Zinc finger protein 777 OS=Homo sapiens GN=ZNF777		31.41
Q9ULD5-3	ZN777_HUMAN Isoform 3 of Zinc finger protein 777 OS=Homo sapiens GN=ZNF777		31.41
Q9ULD9	ZN608_HUMAN Zinc finger protein 608 OS=Homo sapiens GN=ZNF608 PE=1 SV=4		
Q9ULD9-2	ZN608_HUMAN Isoform 2 of Zinc finger protein 608 OS=Homo sapiens GN=ZNF608		
Q9ULD9;Q9ULD9-2	ZN608_HUMAN Zinc finger protein 608 OS=Homo sapiens GN=ZNF608 PE=1 SV=4;>sp|Q9ULD9-2|ZN608_HUMAN Isoform 2 of Zinc f	2.78E-05	
Q9ULE0	WWC3_HUMAN Protein WWC3 OS=Homo sapiens GN=WWC3 PE=2 SV=3		
Q9ULE4	F184B_HUMAN Protein FAM184B OS=Homo sapiens GN=FAM184B PE=2 SV=3		
Q9ULE6	PALD_HUMAN Paladin OS=Homo sapiens GN=PALD PE=1 SV=3		
Q9ULG1	INO80_HUMAN DNA helicase INO80 OS=Homo sapiens GN=INO80 PE=1 SV=2		
Q9ULH0	KDIS_HUMAN Kinase D-interacting substrate of 220 kDa OS=Homo sapiens GN=KIDINS220 PE=1 SV=3		
Q9ULH0-2	KDIS_HUMAN Isoform 2 of Kinase D-interacting substrate of 220 kDa OS=Homo sapiens GN=KIDINS220		
Q9ULH0-3	KDIS_HUMAN Isoform 3 of Kinase D-interacting substrate of 220 kDa OS=Homo sapiens GN=KIDINS220		
Q9ULH0-4	KDIS_HUMAN Isoform 4 of Kinase D-interacting substrate of 220 kDa OS=Homo sapiens GN=KIDINS220		
Q9ULH1	ASAP1_HUMAN Arf-GAP with SH3 domain, ANK repeat and PH domain-containing protein 1 OS=Homo sapiens GN=ASAP1 PE=1 SV=4		6.52
Q9ULH1-2	ASAP1_HUMAN Isoform 1 of Arf-GAP with SH3 domain, ANK repeat and PH domain-containing protein 1 OS=Homo sapiens GN=ASAP1		6.52
Q9ULH1-2;Q9ULH1	2|ASAP1_HUMAN Isoform 1 of Arf-GAP with SH3 domain, ANK repeat and PH domain-containing protein 1 OS=Homo sapiens GN=ASA	1.14E-62	
Q9ULI0	ATD2B_HUMAN ATPase family AAA domain-containing protein 2B OS=Homo sapiens GN=ATAD2B PE=1 SV=3		
Q9ULI0-2	ATD2B_HUMAN Isoform 2 of ATPase family AAA domain-containing protein 2B OS=Homo sapiens GN=ATAD2B		
Q9ULJ1	ODF2L_HUMAN Outer dense fiber protein 2-like OS=Homo sapiens GN=ODF2L PE=2 SV=2		24.48
Q9ULJ1-2	ODF2L_HUMAN Isoform 2 of Outer dense fiber protein 2-like OS=Homo sapiens GN=ODF2L		24.48
Q9ULJ1-3	ODF2L_HUMAN Isoform 3 of Outer dense fiber protein 2-like OS=Homo sapiens GN=ODF2L		24.48

Q9ULJ1-4	ODF2L_HUMAN Isoform 4 of Outer dense fiber protein 2-like OS=Homo sapiens GN=ODF2L		24.48
Q9ULJ1-5	ODF2L_HUMAN Isoform 5 of Outer dense fiber protein 2-like OS=Homo sapiens GN=ODF2L		23.53
Q9ULJ3	ZN295_HUMAN Zinc finger protein 295 OS=Homo sapiens GN=ZNF295 PE=1 SV=2		
Q9ULJ3-2	ZN295_HUMAN Isoform 2 of Zinc finger protein 295 OS=Homo sapiens GN=ZNF295		
Q9ULJ7	ANR50_HUMAN Ankyrin repeat domain-containing protein 50 OS=Homo sapiens GN=ANKRD50 PE=1 SV=4		
Q9ULK2	AT7L1_HUMAN Ataxin-7-like protein 1 OS=Homo sapiens GN=ATXN7L1 PE=2 SV=3		
Q9ULK2-3	AT7L1_HUMAN Isoform 3 of Ataxin-7-like protein 1 OS=Homo sapiens GN=ATXN7L1		
Q9ULK4	MED23_HUMAN Mediator of RNA polymerase II transcription subunit 23 OS=Homo sapiens GN=MED23 PE=1 SV=2		
Q9ULK4-2	MED23_HUMAN Isoform 2 of Mediator of RNA polymerase II transcription subunit 23 OS=Homo sapiens GN=MED23		
Q9ULK4-3	MED23_HUMAN Isoform 3 of Mediator of RNA polymerase II transcription subunit 23 OS=Homo sapiens GN=MED23		
Q9ULK4-4	MED23_HUMAN Isoform 4 of Mediator of RNA polymerase II transcription subunit 23 OS=Homo sapiens GN=MED23		
Q9ULK4-5	MED23_HUMAN Isoform 5 of Mediator of RNA polymerase II transcription subunit 23 OS=Homo sapiens GN=MED23		
Q9ULK4;Q9ULK4-3;Q9ULK	MED23_HUMAN Mediator of RNA polymerase II transcription subunit 23 OS=Homo sapiens GN=MED23 PE=1 SV=2;>sp|Q9ULK4-3|ME	1.67E-10	
Q9ULL5	PRR12_HUMAN Proline-rich protein 12 OS=Homo sapiens GN=PRR12 PE=1 SV=2		83.89
Q9ULL5-2	PRR12_HUMAN Isoform 2 of Proline-rich protein 12 OS=Homo sapiens GN=PRR12		83.89
Q9ULL5-3	PRR12_HUMAN Isoform 3 of Proline-rich protein 12 OS=Homo sapiens GN=PRR12		83.89
Q9ULS6	KCNS2_HUMAN Potassium voltage-gated channel subfamily S member 2 OS=Homo sapiens GN=KCNS2 PE=2 SV=2		
Q9ULT8	HECD1_HUMAN E3 ubiquitin-protein ligase HECTD1 OS=Homo sapiens GN=HECTD1 PE=1 SV=3	4.57E-29	
Q9ULU4	PKCB1_HUMAN Protein kinase C-binding protein 1 OS=Homo sapiens GN=ZMYND8 PE=1 SV=2		
Q9ULU4-10	PKCB1_HUMAN Isoform 10 of Protein kinase C-binding protein 1 OS=Homo sapiens GN=ZMYND8		
Q9ULU4-11	PKCB1_HUMAN Isoform 11 of Protein kinase C-binding protein 1 OS=Homo sapiens GN=ZMYND8		
Q9ULU4-12	PKCB1_HUMAN Isoform 12 of Protein kinase C-binding protein 1 OS=Homo sapiens GN=ZMYND8		
Q9ULU4-13	PKCB1_HUMAN Isoform 13 of Protein kinase C-binding protein 1 OS=Homo sapiens GN=ZMYND8		
Q9ULU4-14	PKCB1_HUMAN Isoform 14 of Protein kinase C-binding protein 1 OS=Homo sapiens GN=ZMYND8		
Q9ULU4-2	PKCB1_HUMAN Isoform 2 of Protein kinase C-binding protein 1 OS=Homo sapiens GN=ZMYND8		
Q9ULU4-3	PKCB1_HUMAN Isoform 3 of Protein kinase C-binding protein 1 OS=Homo sapiens GN=ZMYND8		
Q9ULU4-4	PKCB1_HUMAN Isoform 4 of Protein kinase C-binding protein 1 OS=Homo sapiens GN=ZMYND8		
Q9ULU4-5	PKCB1_HUMAN Isoform 5 of Protein kinase C-binding protein 1 OS=Homo sapiens GN=ZMYND8		
Q9ULU4-6	PKCB1_HUMAN Isoform 6 of Protein kinase C-binding protein 1 OS=Homo sapiens GN=ZMYND8		
Q9ULU4-7	PKCB1_HUMAN Isoform 7 of Protein kinase C-binding protein 1 OS=Homo sapiens GN=ZMYND8		
Q9ULU4-7;Q9ULU4-11;Q9	7|PKCB1_HUMAN Isoform 7 of Protein kinase C-binding protein 1 OS=Homo sapiens GN=ZMYND8;>sp|Q9ULU4-11|PKCB1_HUMAN Iso	1.49E-09	
Q9ULU4-8	PKCB1_HUMAN Isoform 8 of Protein kinase C-binding protein 1 OS=Homo sapiens GN=ZMYND8		
Q9ULU4-9	PKCB1_HUMAN Isoform 9 of Protein kinase C-binding protein 1 OS=Homo sapiens GN=ZMYND8		
Q9ULV0	MYO5B_HUMAN Myosin-Vb OS=Homo sapiens GN=MYO5B PE=1 SV=3		
Q9ULV4	COR1C_HUMAN Coronin-1C OS=Homo sapiens GN=CORO1C PE=1 SV=1		943.9
Q9ULW0	TPX2_HUMAN Targeting protein for Xklp2 OS=Homo sapiens GN=TPX2 PE=1 SV=2		
Q9ULX9	MAFF_HUMAN Transcription factor MafF OS=Homo sapiens GN=MAFF PE=1 SV=2		17.23
Q9ULZ3;Q9ULZ3-2;Q9ULZ	ASC_HUMAN Apoptosis-associated speck-like protein containing a CARD OS=Homo sapiens GN=PYCARD PE=1 SV=2;>sp|Q9ULZ3-2|A	1.67E-14	
Q9UM00	TMCO1_HUMAN Transmembrane and coiled-coil domain-containing protein 1 OS=Homo sapiens GN=TMCO1 PE=1 SV=1		206.64
Q9UM00-2	TMCO1_HUMAN Isoform 2 of Transmembrane and coiled-coil domain-containing protein 1 OS=Homo sapiens GN=TMCO1		206.64
Q9UM00;B7Z591;C9JX01;	TMCO1_HUMAN Transmembrane and coiled-coil domain-containing protein 1 OS=Homo sapiens GN=TMCO1 PE=1 SV=1;>tr|B7Z591|I	1.02E-07	
Q9UM01	YLAT1_HUMAN Y+L amino acid transporter 1 OS=Homo sapiens GN=SLC7A7 PE=1 SV=2		29.19
Q9UM13	APC10_HUMAN Anaphase-promoting complex subunit 10 OS=Homo sapiens GN=ANAPC10 PE=1 SV=1		68.21
Q9UM22	EPDR1_HUMAN Mammalian ependymin-related protein 1 OS=Homo sapiens GN=EPDR1 PE=1 SV=2		220.42
Q9UM22-2	EPDR1_HUMAN Isoform 2 of Mammalian ependymin-related protein 1 OS=Homo sapiens GN=EPDR1		3.5
Q9UM54	MYO6_HUMAN Myosin-VI OS=Homo sapiens GN=MYO6 PE=1 SV=4		
Q9UM54-1	MYO6_HUMAN Isoform 1 of Myosin-VI OS=Homo sapiens GN=MYO6		
Q9UM54-2	MYO6_HUMAN Isoform 2 of Myosin-VI OS=Homo sapiens GN=MYO6		
Q9UM54-4	MYO6_HUMAN Isoform 4 of Myosin-VI OS=Homo sapiens GN=MYO6		
Q9UM54-5	MYO6_HUMAN Isoform 5 of Myosin-VI OS=Homo sapiens GN=MYO6		
Q9UM54;E1P540;Q9UM54	MYO6_HUMAN Myosin-VI OS=Homo sapiens GN=MYO6 PE=1 SV=4;>tr|E1P540|E1P540_HUMAN Myosin VI, isoform CRA_c OS=Homo	4.08E-189	
Q9UMD9	COHA1_HUMAN Collagen alpha-1(XVII) chain OS=Homo sapiens GN=COL17A1 PE=1 SV=3		
Q9UMD9-2	COHA1_HUMAN Isoform 2 of Collagen alpha-1(XVII) chain OS=Homo sapiens GN=COL17A1		
Q9UMD9;Q9UMD9-2	COHA1_HUMAN Collagen alpha-1(XVII) chain OS=Homo sapiens GN=COL17A1 PE=1 SV=3;>sp|Q9UMD9-2|COHA1_HUMAN Isoform 2	2.56E-08	
Q9UMN6	MLL4_HUMAN Histone-lysine N-methyltransferase MLL4 OS=Homo sapiens GN=WBP7 PE=1 SV=1		80.91
Q9UMN6-2	MLL4_HUMAN Isoform 2 of Histone-lysine N-methyltransferase MLL4 OS=Homo sapiens GN=WBP7		72.97
Q9UMR2;Q9NUU7;Q9UMR	DD19B_HUMAN ATP-dependent RNA helicase DDX19B OS=Homo sapiens GN=DDX19B PE=1 SV=1;>sp|Q9NUU7|DD19A_HUMAN ATP-	7.04E-29	
Q9UMS0	NFU1_HUMAN NFU1 iron-sulfur cluster scaffold homolog, mitochondrial OS=Homo sapiens GN=NFU1 PE=1 SV=2		47.8
Q9UMS0-2	NFU1_HUMAN Isoform 2 of NFU1 iron-sulfur cluster scaffold homolog, mitochondrial OS=Homo sapiens GN=NFU1		47.8
Q9UMS0-3	NFU1_HUMAN Isoform 3 of NFU1 iron-sulfur cluster scaffold homolog, mitochondrial OS=Homo sapiens GN=NFU1		47.8
Q9UMS0;C9JGG7;C9J8Q1	NFU1_HUMAN NFU1 iron-sulfur cluster scaffold homolog, mitochondrial OS=Homo sapiens GN=NFU1 PE=1 SV=2;>tr|C9JGG7|C9JGG7	2.05E-09	

Q9UMS4	PRP19_HUMAN Pre-processing factor 19 OS=Homo sapiens GN=PRPF19 PE=1 SV=1		99.89
Q9UMX0;Q9UMX0-2	UBQL1_HUMAN Ubiquilin-1 OS=Homo sapiens GN=UBQLN1 PE=1 SV=2;>sp|Q9UMX0-2|UBQL1_HUMAN Isoform 2 of Ubiquilin-1 OS=	1.02E-29	
Q9UMX5	NENF_HUMAN Neudesin OS=Homo sapiens GN=NENF PE=1 SV=1		253.85
Q9UMY1	NOL7_HUMAN Nucleolar protein 7 OS=Homo sapiens GN=NOL7 PE=1 SV=2		
Q9UMY1-2	NOL7_HUMAN Isoform 2 of Nucleolar protein 7 OS=Homo sapiens GN=NOL7		
Q9UMY1;Q9UMY1-2	NOL7_HUMAN Nucleolar protein 7 OS=Homo sapiens GN=NOL7 PE=1 SV=2;>sp|Q9UMY1-2|NOL7_HUMAN Isoform 2 of Nucleolar prot	1.85E-18	
Q9UMY4	SNX12_HUMAN Sorting nexin-12 OS=Homo sapiens GN=SNX12 PE=1 SV=3		221.81
Q9UMY4-2	SNX12_HUMAN Isoform 2 of Sorting nexin-12 OS=Homo sapiens GN=SNX12		221.81
Q9UMY4;Q3SYF1;Q9UMY4	SNX12_HUMAN Sorting nexin-12 OS=Homo sapiens GN=SNX12 PE=1 SV=3;>tr|Q3SYF1|Q3SYF1_HUMAN Sorting nexin 12 OS=Homo	1.83E-15	
Q9UN36;Q9UN36-3;Q9UN	NDRG2_HUMAN Protein NDRG2 OS=Homo sapiens GN=NDRG2 PE=1 SV=2;>sp|Q9UN36-3|NDRG2_HUMAN Isoform 3 of Protein NDR	3.07E-22	
Q9UN37	VPS4A_HUMAN Vacuolar protein sorting-associated protein 4A OS=Homo sapiens GN=VPS4A PE=1 SV=1	2.87E-10	
Q9UN67	PCDBA_HUMAN Protocadherin beta-10 OS=Homo sapiens GN=PCDHB10 PE=1 SV=1		
Q9UN86	G3BP2_HUMAN Ras GTPase-activating protein-binding protein 2 OS=Homo sapiens GN=G3BP2 PE=1 SV=2		486.82
Q9UN86-2	G3BP2_HUMAN Isoform B of Ras GTPase-activating protein-binding protein 2 OS=Homo sapiens GN=G3BP2		486.82
Q9UN86;A8K6V7;Q9UN86	G3BP2_HUMAN Ras GTPase-activating protein-binding protein 2 OS=Homo sapiens GN=G3BP2 PE=1 SV=2;>tr|A8K6V7|A8K6V7_HUM	5.25E-88	
Q9UNE7	CHIP_HUMAN E3 ubiquitin-protein ligase CHIP OS=Homo sapiens GN=STUB1 PE=1 SV=2		97.97
Q9UNE7-2	CHIP_HUMAN Isoform 2 of E3 ubiquitin-protein ligase CHIP OS=Homo sapiens GN=STUB1		97.97
Q9UNE7;Q9UNE7-2	CHIP_HUMAN E3 ubiquitin-protein ligase CHIP OS=Homo sapiens GN=STUB1 PE=1 SV=2;>sp|Q9UNE7-2|CHIP_HUMAN Isoform 2 of E	5.36E-22	
Q9UNF0	PACN2_HUMAN Protein kinase C and casein kinase substrate in neurons protein 2 OS=Homo sapiens GN=PACSIN2 PE=1 SV=2		149.21
Q9UNF0-2	PACN2_HUMAN Isoform 2 of Protein kinase C and casein kinase substrate in neurons protein 2 OS=Homo sapiens GN=PACSIN2		149.21
Q9UNF0;Q6FIA3;Q9UNF0-	PACN2_HUMAN Protein kinase C and casein kinase substrate in neurons protein 2 OS=Homo sapiens GN=PACSIN2 PE=1 SV=2;>tr|Q6	7.93E-50	
Q9UNF1;C9JMB9;Q9UNF1-	MAGD2_HUMAN Melanoma-associated antigen D2 OS=Homo sapiens GN=MAGED2 PE=1 SV=2;>tr|C9JMB9|C9JMB9_HUMAN Unchara	2.20E-90	
Q9UNH7	SNX6_HUMAN Sorting nexin-6 OS=Homo sapiens GN=SNX6 PE=1 SV=1		42.14
Q9UNI6	DUS12_HUMAN Dual specificity protein phosphatase 12 OS=Homo sapiens GN=DUSP12 PE=1 SV=1		60.47
Q9UNK0	STX8_HUMAN Syntaxin-8 OS=Homo sapiens GN=STX8 PE=1 SV=2		193.87
Q9UNL2	SSRG_HUMAN Translocon-associated protein subunit gamma OS=Homo sapiens GN=SSR3 PE=1 SV=1		84.21
Q9UNM6	PSD13_HUMAN 26S proteasome non-ATPase regulatory subunit 13 OS=Homo sapiens GN=PSMD13 PE=1 SV=2		776.02
Q9UNM6-2	PSD13_HUMAN Isoform 2 of 26S proteasome non-ATPase regulatory subunit 13 OS=Homo sapiens GN=PSMD13		701.01
Q9UNM6-2;Q9UNM6	2|PSD13_HUMAN Isoform 2 of 26S proteasome non-ATPase regulatory subunit 13 OS=Homo sapiens GN=PSMD13;>sp|Q9UNM6|PSD1	3.31E-87	
Q9UNM6;Q9UNM6-2	PSD13_HUMAN 26S proteasome non-ATPase regulatory subunit 13 OS=Homo sapiens GN=PSMD13 PE=1 SV=2;>sp|Q9UNM6-2|PSD1	2.19E-196	
Q9UNN5;Q9UNN5-2	FAF1_HUMAN FAS-associated factor 1 OS=Homo sapiens GN=FAF1 PE=1 SV=2;>sp|Q9UNN5-2|FAF1_HUMAN Isoform Short of FAS-as	1.85E-28	
Q9UNN8	EPCR_HUMAN Endothelial protein C receptor OS=Homo sapiens GN=PROCR PE=1 SV=1		
Q9UNP9	PPIE_HUMAN Peptidyl-prolyl cis-trans isomerase E OS=Homo sapiens GN=PPIE PE=1 SV=1		
Q9UNP9-2	PPIE_HUMAN Isoform B of Peptidyl-prolyl cis-trans isomerase E OS=Homo sapiens GN=PPIE		
Q9UNP9;Q9UNP9-2	PPIE_HUMAN Peptidyl-prolyl cis-trans isomerase E OS=Homo sapiens GN=PPIE PE=1 SV=1;>sp|Q9UNP9-2|PPIE_HUMAN Isoform B of	9.72E-10	
Q9UNQ2	DIMT1_HUMAN Probable dimethyladenosine transferase OS=Homo sapiens GN=DIMT1L PE=1 SV=1		27.43
Q9UNS2	CSN3_HUMAN COP9 signalosome complex subunit 3 OS=Homo sapiens GN=COPS3 PE=1 SV=3	7.43E-13	
Q9UNT1	RBL2B_HUMAN Rab-like protein 2B OS=Homo sapiens GN=RABL2B PE=2 SV=1		
Q9UNT1-3	RBL2B_HUMAN Isoform 3 of Rab-like protein 2B OS=Homo sapiens GN=RABL2B		
Q9UNU6	CP8B1_HUMAN 7-alpha-hydroxycholest-4-en-3-one 12-alpha-hydroxylase OS=Homo sapiens GN=CYP8B1 PE=2 SV=2		16.74
Q9UNW1	MINP1_HUMAN Multiple inositol polyphosphate phosphatase 1 OS=Homo sapiens GN=MINPP1 PE=1 SV=1		53.51
Q9UNW1-2	MINP1_HUMAN Isoform 2 of Multiple inositol polyphosphate phosphatase 1 OS=Homo sapiens GN=MINPP1		53.51
Q9UNW1-3	MINP1_HUMAN Isoform 3 of Multiple inositol polyphosphate phosphatase 1 OS=Homo sapiens GN=MINPP1		53.51
Q9UNW9	NOVA2_HUMAN RNA-binding protein Nova-2 OS=Homo sapiens GN=NOVA2 PE=1 SV=1	4.84E-14	
Q9UNX3	RL26L_HUMAN 60S ribosomal protein L26-like 1 OS=Homo sapiens GN=RPL26L1 PE=1 SV=1		611.98
Q9UNX4	WDR3_HUMAN WD repeat-containing protein 3 OS=Homo sapiens GN=WDR3 PE=1 SV=1		
Q9UNZ2-5;Q53FE8;Q53FF	5|NSF1C_HUMAN Isoform 3 of NSFL1 cofactor p47 OS=Homo sapiens GN=NSFL1C;>tr|Q53FE8|Q53FE8_HUMAN cDNA FLJ36526 fis, c	9.21E-95	
Q9UNZ5	L10K_HUMAN Leydig cell tumor 10 kDa protein homolog OS=Homo sapiens GN=C19orf53 PE=1 SV=1		29.71
Q9UP83-2;A8MUI4;Q9UP8	2|COG5_HUMAN Isoform 2 of Conserved oligomeric Golgi complex subunit 5 OS=Homo sapiens GN=COG5;>tr|A8MUI4|A8MUI4_HUM/	5.12E-08	
Q9UP95;Q9UP95-2;Q9UP9	S12A4_HUMAN Solute carrier family 12 member 4 OS=Homo sapiens GN=SLC12A4 PE=1 SV=2;>sp|Q9UP95-2|S12A4_HUMAN Isofor	3.99E-10	
Q9UPN3	MACF1_HUMAN Microtubule-actin cross-linking factor 1, isoforms 1/2/3/5 OS=Homo sapiens GN=MACF1 PE=1 SV=4		2048.61
Q9UPN3-2	MACF1_HUMAN Isoform 2 of Microtubule-actin cross-linking factor 1, isoforms 1/2/3/5 OS=Homo sapiens GN=MACF1		1919.02
Q9UPN3-2;Q9UPN3-3	2|MACF1_HUMAN Isoform 2 of Microtubule-actin cross-linking factor 1, isoforms 1/2/3/5 OS=Homo sapiens GN=MACF1;>sp|Q9UPN3-	0	
Q9UPN3-3	MACF1_HUMAN Isoform 3 of Microtubule-actin cross-linking factor 1, isoforms 1/2/3/5 OS=Homo sapiens GN=MACF1		1919.02
Q9UPN3-4	MACF1_HUMAN Isoform 5 of Microtubule-actin cross-linking factor 1, isoforms 1/2/3/5 OS=Homo sapiens GN=MACF1		1902.68
Q9UPN3;Q9UPN3-1;Q9UPI	MACF1_HUMAN Microtubule-actin cross-linking factor 1, isoforms 1/2/3/5 OS=Homo sapiens GN=MACF1 PE=1 SV=3;>sp|Q9UPN3-1|I	3.99E-288	
Q9UPN4	AZI1_HUMAN 5-azacytidine-induced protein 1 OS=Homo sapiens GN=AZI1 PE=1 SV=3		45.82
Q9UPN4-2	AZI1_HUMAN Isoform 2 of 5-azacytidine-induced protein 1 OS=Homo sapiens GN=AZI1		45.82
Q9UPN4-3	AZI1_HUMAN Isoform 3 of 5-azacytidine-induced protein 1 OS=Homo sapiens GN=AZI1		45.82
Q9UPN6	SCAF8_HUMAN Protein SCAF8 OS=Homo sapiens GN=SCAF8 PE=1 SV=1		62.67
Q9UPN7	PP6R1_HUMAN Serine/threonine-protein phosphatase 6 regulatory subunit 1 OS=Homo sapiens GN=PPP6R1 PE=1 SV=5	2.60E-10	

Q9UPN9	TRI33_HUMAN E3 ubiquitin-protein ligase TRIM33 OS=Homo sapiens GN=TRIM33 PE=1 SV=3		29.02
Q9UPN9-2	TRI33_HUMAN Isoform Beta of E3 ubiquitin-protein ligase TRIM33 OS=Homo sapiens GN=TRIM33		29.02
Q9UPN9;Q9UPN9-2	TRI33_HUMAN E3 ubiquitin-protein ligase TRIM33 OS=Homo sapiens GN=TRIM33 PE=1 SV=3;>sp|Q9UPN9-2|TRI33_HUMAN Isoform	1.07E-08	
Q9UPQ0	LIMC1_HUMAN LIM and calponin homology domains-containing protein 1 OS=Homo sapiens GN=LIMCH1 PE=1 SV=4		56.1
Q9UPQ0-2	LIMC1_HUMAN Isoform 2 of LIM and calponin homology domains-containing protein 1 OS=Homo sapiens GN=LIMCH1		56.1
Q9UPQ0-3	LIMC1_HUMAN Isoform 3 of LIM and calponin homology domains-containing protein 1 OS=Homo sapiens GN=LIMCH1		26.43
Q9UPQ0-4	LIMC1_HUMAN Isoform 4 of LIM and calponin homology domains-containing protein 1 OS=Homo sapiens GN=LIMCH1		56.1
Q9UPQ0-5	LIMC1_HUMAN Isoform 5 of LIM and calponin homology domains-containing protein 1 OS=Homo sapiens GN=LIMCH1		22.24
Q9UPQ0-6	LIMC1_HUMAN Isoform 6 of LIM and calponin homology domains-containing protein 1 OS=Homo sapiens GN=LIMCH1		22.77
Q9UPQ0-6;Q9UPQ0-9;Q9U	6|LIMC1_HUMAN Isoform 6 of LIM and calponin homology domains-containing protein 1 OS=Homo sapiens GN=LIMCH1;>sp|Q9UPQ0	1.48E-59	
Q9UPQ0-8	LIMC1_HUMAN Isoform 8 of LIM and calponin homology domains-containing protein 1 OS=Homo sapiens GN=LIMCH1		22.24
Q9UPQ0-9	LIMC1_HUMAN Isoform 9 of LIM and calponin homology domains-containing protein 1 OS=Homo sapiens GN=LIMCH1		22.77
Q9UPQ0;Q9UPQ0-4;Q9UP	LIMC1_HUMAN LIM and calponin homology domains-containing protein 1 OS=Homo sapiens GN=LIMCH1 PE=1 SV=4;>sp|Q9UPQ0-4|	1.35E-100	
Q9UPQ8	DOLK_HUMAN Dolichol kinase OS=Homo sapiens GN=DOLK PE=1 SV=1		
Q9UPS8	ANR26_HUMAN Ankyrin repeat domain-containing protein 26 OS=Homo sapiens GN=ANKRD26 PE=1 SV=3		
Q9UPS8-2	ANR26_HUMAN Isoform 2 of Ankyrin repeat domain-containing protein 26 OS=Homo sapiens GN=ANKRD26		
Q9UPS8-3	ANR26_HUMAN Isoform 3 of Ankyrin repeat domain-containing protein 26 OS=Homo sapiens GN=ANKRD26		
Q9UPT5	EXOC7_HUMAN Exocyst complex component 7 OS=Homo sapiens GN=EXOC7 PE=1 SV=3		46.97
Q9UPT5-1	EXOC7_HUMAN Isoform 1 of Exocyst complex component 7 OS=Homo sapiens GN=EXOC7		47.82
Q9UPT5-2	EXOC7_HUMAN Isoform 2 of Exocyst complex component 7 OS=Homo sapiens GN=EXOC7		46.97
Q9UPT5-4	EXOC7_HUMAN Isoform 4 of Exocyst complex component 7 OS=Homo sapiens GN=EXOC7		42.57
Q9UPT5-5	EXOC7_HUMAN Isoform 5 of Exocyst complex component 7 OS=Homo sapiens GN=EXOC7		46.97
Q9UPT5-6	EXOC7_HUMAN Isoform 6 of Exocyst complex component 7 OS=Homo sapiens GN=EXOC7		46.97
Q9UPT5;B5MC69;Q9UPT5-	EXOC7_HUMAN Exocyst complex component 7 OS=Homo sapiens GN=EXOC7 PE=1 SV=3;>tr|B5MC69|B5MC69_HUMAN EXOC7 splice	6.81E-15	
Q9UPT8	ZC3H4_HUMAN Zinc finger CCCH domain-containing protein 4 OS=Homo sapiens GN=ZC3H4 PE=1 SV=3		268.22
Q9UPU5	UBP24_HUMAN Ubiquitin carboxyl-terminal hydrolase 24 OS=Homo sapiens GN=USP24 PE=1 SV=3	6.58E-09	
Q9UPU9;Q9UPU9-3;Q9UP	SMAG1_HUMAN Protein Smaug homolog 1 OS=Homo sapiens GN=SAMD4A PE=1 SV=3;>sp|Q9UPU9-3|SMAG1_HUMAN Isoform 3 of	5.34E-16	
Q9UPW5	CBPC1_HUMAN Cytosolic carboxypeptidase 1 OS=Homo sapiens GN=AGTPBP1 PE=1 SV=3		
Q9UPW5-2	CBPC1_HUMAN Isoform 2 of Cytosolic carboxypeptidase 1 OS=Homo sapiens GN=AGTPBP1		
Q9UPW5-3	CBPC1_HUMAN Isoform 3 of Cytosolic carboxypeptidase 1 OS=Homo sapiens GN=AGTPBP1		
Q9UPW5-3;Q9UPW5;Q9UF	3|CBPC1_HUMAN Isoform 3 of Cytosolic carboxypeptidase 1 OS=Homo sapiens GN=AGTPBP1;>sp|Q9UPW5|CBPC1_HUMAN Cytosolic	1.55E-10	
Q9UPX6	K1024_HUMAN UPF0258 protein KIAA1024 OS=Homo sapiens GN=KIAA1024 PE=2 SV=3	2.09E-05	
Q9UPX8	SHAN2_HUMAN SH3 and multiple ankyrin repeat domains protein 2 OS=Homo sapiens GN=SHANK2 PE=1 SV=3		
Q9UPX8-3	SHAN2_HUMAN Isoform 3 of SH3 and multiple ankyrin repeat domains protein 2 OS=Homo sapiens GN=SHANK2		
Q9UPX8-3;Q9UPX8;Q9UP)	3|SHAN2_HUMAN Isoform 3 of SH3 and multiple ankyrin repeat domains protein 2 OS=Homo sapiens GN=SHANK2;>sp|Q9UPX8|SHA	1.03E-12	
Q9UPX8-4	SHAN2_HUMAN Isoform 4 of SH3 and multiple ankyrin repeat domains protein 2 OS=Homo sapiens GN=SHANK2		
Q9UPY8	MARE3_HUMAN Microtubule-associated protein RP/EB family member 3 OS=Homo sapiens GN=MAPRE3 PE=1 SV=1		26.73
Q9UPY8-2	MARE3_HUMAN Isoform 2 of Microtubule-associated protein RP/EB family member 3 OS=Homo sapiens GN=MAPRE3		26.73
Q9UPY8;Q9UPY8-2	MARE3_HUMAN Microtubule-associated protein RP/EB family member 3 OS=Homo sapiens GN=MAPRE3 PE=1 SV=1;>sp|Q9UPY8-2|M	2.49E-11	
Q9UPZ9	ICK_HUMAN Serine/threonine-protein kinase ICK OS=Homo sapiens GN=ICK PE=1 SV=1		70.37
Q9UPZ9-2	ICK_HUMAN Isoform 2 of Serine/threonine-protein kinase ICK OS=Homo sapiens GN=ICK		55.57
Q9UQ07	MOK_HUMAN MAPK/MAK/MRK overlapping kinase OS=Homo sapiens GN=RAGE PE=1 SV=1		
Q9UQ07-3	MOK_HUMAN Isoform 3 of MAPK/MAK/MRK overlapping kinase OS=Homo sapiens GN=RAGE		
Q9UQ13;Q9UQ13-2	SHOC2_HUMAN Leucine-rich repeat protein SHOC-2 OS=Homo sapiens GN=SHOC2 PE=1 SV=2;>sp|Q9UQ13-2|SHOC2_HUMAN Isofo	9.35E-08	
Q9UQ16	DYN3_HUMAN Dynamin-3 OS=Homo sapiens GN=DNM3 PE=1 SV=4		69.97
Q9UQ16-2	DYN3_HUMAN Isoform 2 of Dynamin-3 OS=Homo sapiens GN=DNM3		69.97
Q9UQ16-3	DYN3_HUMAN Isoform 3 of Dynamin-3 OS=Homo sapiens GN=DNM3		69.97
Q9UQ16-4	DYN3_HUMAN Isoform 4 of Dynamin-3 OS=Homo sapiens GN=DNM3		69.97
Q9UQ16-4;Q9UQ16;Q9UQ	4|DYN3_HUMAN Isoform 4 of Dynamin-3 OS=Homo sapiens GN=DNM3;>sp|Q9UQ16|DYN3_HUMAN Dynamin-3 OS=Homo sapiens GN	1.03E-28	
Q9UQ35	SRRM2_HUMAN Serine/arginine repetitive matrix protein 2 OS=Homo sapiens GN=SRRM2 PE=1 SV=2		754.05
Q9UQ35-2	SRRM2_HUMAN Isoform 2 of Serine/arginine repetitive matrix protein 2 OS=Homo sapiens GN=SRRM2		329.06
Q9UQ35-3	SRRM2_HUMAN Isoform 3 of Serine/arginine repetitive matrix protein 2 OS=Homo sapiens GN=SRRM2		81.8
Q9UQ35;Q9UQ35-2	SRRM2_HUMAN Serine/arginine repetitive matrix protein 2 OS=Homo sapiens GN=SRRM2 PE=1 SV=2;>sp|Q9UQ35-2|SRRM2_HUMAI	2.69E-84	
Q9UQ80	PA2G4_HUMAN Proliferation-associated protein 2G4 OS=Homo sapiens GN=PA2G4 PE=1 SV=3		946.81
Q9UQ88	CD11A_HUMAN Cyclin-dependent kinase 11A OS=Homo sapiens GN=CDK11A PE=1 SV=4		73.21
Q9UQ88-10	CD11A_HUMAN Isoform 4 of Cyclin-dependent kinase 11A OS=Homo sapiens GN=CDK11A		67.41
Q9UQ88-2	CD11A_HUMAN Isoform SV1 of Cyclin-dependent kinase 11A OS=Homo sapiens GN=CDK11A		73.21
Q9UQ88-3	CD11A_HUMAN Isoform SV2 of Cyclin-dependent kinase 11A OS=Homo sapiens GN=CDK11A		73.21
Q9UQ88-4	CD11A_HUMAN Isoform SV3 of Cyclin-dependent kinase 11A OS=Homo sapiens GN=CDK11A		73.21
Q9UQ88-5	CD11A_HUMAN Isoform SV7 of Cyclin-dependent kinase 11A OS=Homo sapiens GN=CDK11A		67.41
Q9UQ88-8	CD11A_HUMAN Isoform SV12 of Cyclin-dependent kinase 11A OS=Homo sapiens GN=CDK11A		

Q9UQ88-9	CD11A_HUMAN Isoform SV13 of Cyclin-dependent kinase 11A OS=Homo sapiens GN=CDK11A		27.54
Q9UQB3	CTND2_HUMAN Catenin delta-2 OS=Homo sapiens GN=CTNND2 PE=1 SV=3		39.82
Q9UQB3-2	CTND2_HUMAN Isoform 2 of Catenin delta-2 OS=Homo sapiens GN=CTNND2		39.82
Q9UQB8	BAIP2_HUMAN Brain-specific angiogenesis inhibitor 1-associated protein 2 OS=Homo sapiens GN=BAIAP2 PE=1 SV=1		89.54
Q9UQB8-2	BAIP2_HUMAN Isoform 2 of Brain-specific angiogenesis inhibitor 1-associated protein 2 OS=Homo sapiens GN=BAIAP2		89.54
Q9UQB8-3	BAIP2_HUMAN Isoform 3 of Brain-specific angiogenesis inhibitor 1-associated protein 2 OS=Homo sapiens GN=BAIAP2		89.54
Q9UQB8-4	BAIP2_HUMAN Isoform 4 of Brain-specific angiogenesis inhibitor 1-associated protein 2 OS=Homo sapiens GN=BAIAP2		89.54
Q9UQB8-5	BAIP2_HUMAN Isoform 5 of Brain-specific angiogenesis inhibitor 1-associated protein 2 OS=Homo sapiens GN=BAIAP2		89.54
Q9UQB8-6	BAIP2_HUMAN Isoform 6 of Brain-specific angiogenesis inhibitor 1-associated protein 2 OS=Homo sapiens GN=BAIAP2		89.54
Q9UQB8;Q9UQB8-2;Q9UQ	BAIP2_HUMAN Brain-specific angiogenesis inhibitor 1-associated protein 2 OS=Homo sapiens GN=BAIAP2 PE=1 SV=1;>sp|Q9UQB8-2	8.80E-20	
Q9UQB9	AURKC_HUMAN Serine/threonine-protein kinase 13 OS=Homo sapiens GN=AURKC PE=1 SV=1		
Q9UQB9-2	AURKC_HUMAN Isoform 2 of Serine/threonine-protein kinase 13 OS=Homo sapiens GN=AURKC		
Q9UQB9-3	AURKC_HUMAN Isoform 3 of Serine/threonine-protein kinase 13 OS=Homo sapiens GN=AURKC		
Q9UQE7	SMC3_HUMAN Structural maintenance of chromosomes protein 3 OS=Homo sapiens GN=SMC3 PE=1 SV=2		265.54
Q9UQL6	HDAC5_HUMAN Histone deacetylase 5 OS=Homo sapiens GN=HDAC5 PE=1 SV=2		
Q9UQL6-2	HDAC5_HUMAN Isoform 2 of Histone deacetylase 5 OS=Homo sapiens GN=HDAC5		
Q9UQL6-3	HDAC5_HUMAN Isoform 3 of Histone deacetylase 5 OS=Homo sapiens GN=HDAC5		
Q9UQM7	KCC2A_HUMAN Calcium/calmodulin-dependent protein kinase type II subunit alpha OS=Homo sapiens GN=CAMK2A PE=1 SV=2		5.71
Q9UQM7-2	KCC2A_HUMAN Isoform B of Calcium/calmodulin-dependent protein kinase type II subunit alpha OS=Homo sapiens GN=CAMK2A		5.71
Q9UQN3	CHM2B_HUMAN Charged multivesicular body protein 2b OS=Homo sapiens GN=CHMP2B PE=1 SV=1	9.36E-06	
Q9UQP3	TENN_HUMAN Tenascin-N OS=Homo sapiens GN=TNN PE=1 SV=2	7.78E-225	
Q9UQR1	ZN148_HUMAN Zinc finger protein 148 OS=Homo sapiens GN=ZNF148 PE=1 SV=2		
Q9Y221	NIP7_HUMAN 60S ribosome subunit biogenesis protein NIP7 homolog OS=Homo sapiens GN=NIP7 PE=1 SV=1		271.08
Q9Y221-2	NIP7_HUMAN Isoform 2 of 60S ribosome subunit biogenesis protein NIP7 homolog OS=Homo sapiens GN=NIP7		237.38
Q9Y221;B3KM49;Q9Y221-	NIP7_HUMAN 60S ribosome subunit biogenesis protein NIP7 homolog OS=Homo sapiens GN=NIP7 PE=1 SV=1;>tr|B3KM49|B3KM49_	1.14E-05	
Q9Y223-2;Q9Y223;Q9Y22	2|GLCNE_HUMAN Isoform 2 of Bifunctional UDP-N-acetylglucosamine 2-epimerase/N-acetylmannosamine kinase OS=Homo sapiens GI	2.65E-22	
Q9Y224	CN166_HUMAN UPF0568 protein C14orf166 OS=Homo sapiens GN=C14orf166 PE=1 SV=1		865.48
Q9Y230	RUVB2_HUMAN RuvB-like 2 OS=Homo sapiens GN=RUVBL2 PE=1 SV=3		308.52
Q9Y234	LIPT_HUMAN Lipoyltransferase 1, mitochondrial OS=Homo sapiens GN=LIPT1 PE=1 SV=1		
Q9Y236	OSGI2_HUMAN Oxidative stress-induced growth inhibitor 2 OS=Homo sapiens GN=OSGIN2 PE=2 SV=1		
Q9Y236-2	OSGI2_HUMAN Isoform 2 of Oxidative stress-induced growth inhibitor 2 OS=Homo sapiens GN=OSGIN2		
Q9Y237	PIN4_HUMAN Peptidyl-prolyl cis-trans isomerase NIMA-interacting 4 OS=Homo sapiens GN=PIN4 PE=1 SV=1		183.07
Q9Y237-2	PIN4_HUMAN Isoform 2 of Peptidyl-prolyl cis-trans isomerase NIMA-interacting 4 OS=Homo sapiens GN=PIN4		184.05
Q9Y237-2;Q9Y237	2|PIN4_HUMAN Isoform 2 of Peptidyl-prolyl cis-trans isomerase NIMA-interacting 4 OS=Homo sapiens GN=PIN4;>sp|Q9Y237|PIN4_H	3.90E-86	
Q9Y241	HIG1A_HUMAN HIG1 domain family member 1A OS=Homo sapiens GN=HIGD1A PE=1 SV=1	2.61E-14	
Q9Y244	POMP_HUMAN Proteasome maturation protein OS=Homo sapiens GN=POMP PE=1 SV=1	4.72E-10	
Q9Y247	FA50B_HUMAN Protein FAM50B OS=Homo sapiens GN=FAM50B PE=2 SV=1		
Q9Y248	PSF2_HUMAN DNA replication complex GINS protein PSF2 OS=Homo sapiens GN=GINS2 PE=1 SV=1		
Q9Y250	LZTS1_HUMAN Leucine zipper putative tumor suppressor 1 OS=Homo sapiens GN=LZTS1 PE=1 SV=3		28.2
Q9Y250-2	LZTS1_HUMAN Isoform 2 of Leucine zipper putative tumor suppressor 1 OS=Homo sapiens GN=LZTS1		28.2
Q9Y250-3	LZTS1_HUMAN Isoform 3 of Leucine zipper putative tumor suppressor 1 OS=Homo sapiens GN=LZTS1		28.2
Q9Y250-4	LZTS1_HUMAN Isoform 4 of Leucine zipper putative tumor suppressor 1 OS=Homo sapiens GN=LZTS1		28.2
Q9Y250-5	LZTS1_HUMAN Isoform 5 of Leucine zipper putative tumor suppressor 1 OS=Homo sapiens GN=LZTS1		25.91
Q9Y250-6	LZTS1_HUMAN Isoform 6 of Leucine zipper putative tumor suppressor 1 OS=Homo sapiens GN=LZTS1		
Q9Y250-7	LZTS1_HUMAN Isoform 7 of Leucine zipper putative tumor suppressor 1 OS=Homo sapiens GN=LZTS1		
Q9Y262	EIF3L_HUMAN Eukaryotic translation initiation factor 3 subunit L OS=Homo sapiens GN=EIF3L PE=1 SV=1		635.76
Q9Y263	PLAP_HUMAN Phospholipase A-2-activating protein OS=Homo sapiens GN=PLAA PE=1 SV=2		99.47
Q9Y265	RUVB1_HUMAN RuvB-like 1 OS=Homo sapiens GN=RUVBL1 PE=1 SV=1		352.21
Q9Y265-2	RUVB1_HUMAN Isoform 2 of RuvB-like 1 OS=Homo sapiens GN=RUVBL1		336.07
Q9Y265;Q9Y265-2	RUVB1_HUMAN RuvB-like 1 OS=Homo sapiens GN=RUVBL1 PE=1 SV=1;>sp|Q9Y265-2|RUVB1_HUMAN Isoform 2 of RuvB-like 1 OS=	0	
Q9Y266	NUDC_HUMAN Nuclear migration protein nudC OS=Homo sapiens GN=NUDC PE=1 SV=1		356.84
Q9Y274	SIA10_HUMAN Type 2 lactosamine alpha-2,3-sialyltransferase OS=Homo sapiens GN=ST3GAL6 PE=1 SV=1		
Q9Y276	BCS1_HUMAN Mitochondrial chaperone BCS1 OS=Homo sapiens GN=BCS1L PE=1 SV=1	1.43E-19	
Q9Y277	VDAC3_HUMAN Voltage-dependent anion-selective channel protein 3 OS=Homo sapiens GN=VDAC3 PE=1 SV=1		309.55
Q9Y277-2	VDAC3_HUMAN Isoform 2 of Voltage-dependent anion-selective channel protein 3 OS=Homo sapiens GN=VDAC3		300.1
Q9Y277-2;Q9Y277	2|VDAC3_HUMAN Isoform 2 of Voltage-dependent anion-selective channel protein 3 OS=Homo sapiens GN=VDAC3;>sp|Q9Y277|VDA(	3.42E-43	
Q9Y277;Q9Y277-2	VDAC3_HUMAN Voltage-dependent anion-selective channel protein 3 OS=Homo sapiens GN=VDAC3 PE=1 SV=1;>sp|Q9Y277-2|VDAC	6.45E-184	
Q9Y281	COF2_HUMAN Cofilin-2 OS=Homo sapiens GN=CFL2 PE=1 SV=1		335.65
Q9Y282-3;Q9Y282;Q9Y28	3|ERGI3_HUMAN Isoform 3 of Endoplasmic reticulum-Golgi intermediate compartment protein 3 OS=Homo sapiens GN=ERGIC3;>sp|(	1.38E-05	
Q9Y283	INVS_HUMAN Inversin OS=Homo sapiens GN=INVS PE=1 SV=2		46.68
Q9Y283-2	INVS_HUMAN Isoform 2 of Inversin OS=Homo sapiens GN=INVS		46.68

Q9Y283-3	INVS_HUMAN Isoform 3 of Inversin OS=Homo sapiens GN=INVS		9.24
Q9Y285	SYFA_HUMAN Phenylalanyl-tRNA synthetase alpha chain OS=Homo sapiens GN=FARSA PE=1 SV=3		535.92
Q9Y287	ITM2B_HUMAN Integral membrane protein 2B OS=Homo sapiens GN=ITM2B PE=1 SV=1		35.59
Q9Y291	RT33_HUMAN 28S ribosomal protein S33, mitochondrial OS=Homo sapiens GN=MRPS33 PE=1 SV=1		43.07
Q9Y294	ASF1A_HUMAN Histone chaperone ASF1A OS=Homo sapiens GN=ASF1A PE=1 SV=1		29.19
Q9Y295	DRG1_HUMAN Developmentally-regulated GTP-binding protein 1 OS=Homo sapiens GN=DRG1 PE=1 SV=1		588.81
Q9Y296;B4DF86;B4DF36;	TPPC4_HUMAN Trafficking protein particle complex subunit 4 OS=Homo sapiens GN=TRAPPC4 PE=1 SV=1;>tr|B4DF86|B4DF86_HUM/	4.11E-20	
Q9Y2A7	NCKP1_HUMAN Nck-associated protein 1 OS=Homo sapiens GN=NCKAP1 PE=1 SV=1		35.04
Q9Y2A7-2	NCKP1_HUMAN Isoform 2 of Nck-associated protein 1 OS=Homo sapiens GN=NCKAP1		35.04
Q9Y2A7-2;Q9Y2A7	2|NCKP1_HUMAN Isoform 2 of Nck-associated protein 1 OS=Homo sapiens GN=NCKAP1;>sp|Q9Y2A7|NCKP1_HUMAN Nck-associated	8.81E-43	
Q9Y2B0	CNPY2_HUMAN Protein canopy homolog 2 OS=Homo sapiens GN=CNPY2 PE=1 SV=1		440.98
Q9Y2B0-2	CNPY2_HUMAN Isoform 2 of Protein canopy homolog 2 OS=Homo sapiens GN=CNPY2		96.4
Q9Y2B0;Q9Y2B0-2	CNPY2_HUMAN Protein canopy homolog 2 OS=Homo sapiens GN=CNPY2 PE=1 SV=1;>sp|Q9Y2B0-2|CNPY2_HUMAN Isoform 2 of Prot	1.14E-10	
Q9Y2B2	PIGL_HUMAN N-acetylglucosaminyl-phosphatidylinositol de-N-acetylase OS=Homo sapiens GN=PIGL PE=2 SV=1		
Q9Y2C4;Q9Y2C4-4;Q9Y2C	EXOG_HUMAN Nuclease EXOG, mitochondrial OS=Homo sapiens GN=EXOG PE=1 SV=2;>sp|Q9Y2C4-4|EXOG_HUMAN Isoform 4 of Nu	4.70E-09	
Q9Y2C9	TLR6_HUMAN Toll-like receptor 6 OS=Homo sapiens GN=TLR6 PE=2 SV=2		
Q9Y2D4	EXC6B_HUMAN Exocyst complex component 6B OS=Homo sapiens GN=EXOC6B PE=1 SV=3		89.22
Q9Y2D4-2	EXC6B_HUMAN Isoform 2 of Exocyst complex component 6B OS=Homo sapiens GN=EXOC6B		65.63
Q9Y2D4;Q9Y2D4-2	EXC6B_HUMAN Exocyst complex component 6B OS=Homo sapiens GN=EXOC6B PE=1 SV=3;>sp|Q9Y2D4-2|EXC6B_HUMAN Isoform 2	1.28E-12	
Q9Y2D5	AKAP2_HUMAN A-kinase anchor protein 2 OS=Homo sapiens GN=AKAP2 PE=1 SV=3		130.85
Q9Y2D5-4	AKAP2_HUMAN Isoform 2 of A-kinase anchor protein 2 OS=Homo sapiens GN=AKAP2		138.1
Q9Y2D5-4;Q9Y2D5-6;Q9Y	4|AKAP2_HUMAN Isoform 2 of A-kinase anchor protein 2 OS=Homo sapiens GN=AKAP2;>sp|Q9Y2D5-6|AKAP2_HUMAN Isoform 4 of A	1.76E-67	
Q9Y2D5-5	AKAP2_HUMAN Isoform 3 of A-kinase anchor protein 2 OS=Homo sapiens GN=AKAP2		130.85
Q9Y2D5-6	AKAP2_HUMAN Isoform 4 of A-kinase anchor protein 2 OS=Homo sapiens GN=AKAP2		138.1
Q9Y2D5-7	AKAP2_HUMAN Isoform 5 of A-kinase anchor protein 2 OS=Homo sapiens GN=AKAP2		130.85
Q9Y2G5	OFUT2_HUMAN GDP-fucose protein O-fucosyltransferase 2 OS=Homo sapiens GN=POFUT2 PE=2 SV=3		49.65
Q9Y2G5-1	OFUT2_HUMAN Isoform A of GDP-fucose protein O-fucosyltransferase 2 OS=Homo sapiens GN=POFUT2		49.65
Q9Y2G5-2	OFUT2_HUMAN Isoform B of GDP-fucose protein O-fucosyltransferase 2 OS=Homo sapiens GN=POFUT2		49.65
Q9Y2G5;Q9Y2G5-1;Q9Y2G	OFUT2_HUMAN GDP-fucose protein O-fucosyltransferase 2 OS=Homo sapiens GN=POFUT2 PE=2 SV=3;>sp|Q9Y2G5-1|OFUT2_HUMAN	4.83E-19	
Q9Y2H0	DLGP4_HUMAN Disks large-associated protein 4 OS=Homo sapiens GN=DLGAP4 PE=1 SV=3		
Q9Y2H0-1	DLGP4_HUMAN Isoform 2 of Disks large-associated protein 4 OS=Homo sapiens GN=DLGAP4		
Q9Y2H0-3	DLGP4_HUMAN Isoform 3 of Disks large-associated protein 4 OS=Homo sapiens GN=DLGAP4		
Q9Y2H0;E1P5T5;Q9Y2H0-	DLGP4_HUMAN Disks large-associated protein 4 OS=Homo sapiens GN=DLGAP4 PE=1 SV=3;>tr|E1P5T5|E1P5T5_HUMAN Discs, large	1.07E-19	
Q9Y2H2	SAC2_HUMAN Phosphatidylinositide phosphatase SAC2 OS=Homo sapiens GN=INPP5F PE=1 SV=3	8.31E-10	
Q9Y2H6	FND3A_HUMAN Fibronectin type-III domain-containing protein 3A OS=Homo sapiens GN=FNDC3A PE=1 SV=4		
Q9Y2H6-2	FND3A_HUMAN Isoform 2 of Fibronectin type-III domain-containing protein 3A OS=Homo sapiens GN=FNDC3A		
Q9Y2J8	PADI2_HUMAN Protein-arginine deiminase type-2 OS=Homo sapiens GN=PADI2 PE=1 SV=2	2.00E-80	
Q9Y2K3	MYH15_HUMAN Myosin-15 OS=Homo sapiens GN=MYH15 PE=1 SV=5		
Q9Y2K6	UBP20_HUMAN Ubiquitin carboxyl-terminal hydrolase 20 OS=Homo sapiens GN=USP20 PE=1 SV=2		26.9
Q9Y2K7	KDM2A_HUMAN Lysine-specific demethylase 2A OS=Homo sapiens GN=KDM2A PE=1 SV=3		
Q9Y2K7-2	KDM2A_HUMAN Isoform 2 of Lysine-specific demethylase 2A OS=Homo sapiens GN=KDM2A		
Q9Y2K7-3	KDM2A_HUMAN Isoform 3 of Lysine-specific demethylase 2A OS=Homo sapiens GN=KDM2A		
Q9Y2L1	RRP44_HUMAN Exosome complex exonuclease RRP44 OS=Homo sapiens GN=DIS3 PE=1 SV=2		210.18
Q9Y2L1-2	RRP44_HUMAN Isoform 2 of Exosome complex exonuclease RRP44 OS=Homo sapiens GN=DIS3		162.81
Q9Y2L1;Q9Y2L1-2	RRP44_HUMAN Exosome complex exonuclease RRP44 OS=Homo sapiens GN=DIS3 PE=1 SV=2;>sp|Q9Y2L1-2|RRP44_HUMAN Isoforr	7.10E-64	
Q9Y2L5	TPPC8_HUMAN Trafficking protein particle complex subunit 8 OS=Homo sapiens GN=TRAPPC8 PE=1 SV=2		
Q9Y2L5-2	TPPC8_HUMAN Isoform 2 of Trafficking protein particle complex subunit 8 OS=Homo sapiens GN=TRAPPC8		
Q9Y2M0	FAN1_HUMAN Fanconi-associated nuclease 1 OS=Homo sapiens GN=FAN1 PE=1 SV=4		
Q9Y2M0-2	FAN1_HUMAN Isoform 2 of Fanconi-associated nuclease 1 OS=Homo sapiens GN=FAN1		
Q9Y2N7	HIF3A_HUMAN Hypoxia-inducible factor 3-alpha OS=Homo sapiens GN=HIF3A PE=1 SV=2		
Q9Y2N7-2	HIF3A_HUMAN Isoform 2 of Hypoxia-inducible factor 3-alpha OS=Homo sapiens GN=HIF3A		
Q9Y2N7-3	HIF3A_HUMAN Isoform 3 of Hypoxia-inducible factor 3-alpha OS=Homo sapiens GN=HIF3A		
Q9Y2N7-4	HIF3A_HUMAN Isoform 4 of Hypoxia-inducible factor 3-alpha OS=Homo sapiens GN=HIF3A		
Q9Y2N7-5	HIF3A_HUMAN Isoform 5 of Hypoxia-inducible factor 3-alpha OS=Homo sapiens GN=HIF3A		
Q9Y2P8	RCL1_HUMAN RNA 3'-terminal phosphate cyclase-like protein OS=Homo sapiens GN=RCL1 PE=1 SV=3		
Q9Y2Q0	AT8A1_HUMAN Probable phospholipid-transporting ATPase IA OS=Homo sapiens GN=ATP8A1 PE=1 SV=1		
Q9Y2Q0-2	AT8A1_HUMAN Isoform 2 of Probable phospholipid-transporting ATPase IA OS=Homo sapiens GN=ATP8A1		
Q9Y2Q3	GSTK1_HUMAN Glutathione S-transferase kappa 1 OS=Homo sapiens GN=GSTK1 PE=1 SV=3		417.02
Q9Y2Q3-2	GSTK1_HUMAN Isoform 2 of Glutathione S-transferase kappa 1 OS=Homo sapiens GN=GSTK1		336.43
Q9Y2Q3-2;Q6FII1;Q9Y2Q3	2|GSTK1_HUMAN Isoform 2 of Glutathione S-transferase kappa 1 OS=Homo sapiens GN=GSTK1;>tr|Q6FII1|Q6FII1_HUMAN Glutathic	1.48E-157	
Q9Y2Q3-3	GSTK1_HUMAN Isoform 3 of Glutathione S-transferase kappa 1 OS=Homo sapiens GN=GSTK1		336.43

Q9Y2Q3-4	GSTK1_HUMAN Isoform 4 of Glutathione S-transferase kappa 1 OS=Homo sapiens GN=GSTK1		314.61
Q9Y2Q5	LTOR2_HUMAN Ragulator complex protein LAMTOR2 OS=Homo sapiens GN=LAMTOR2 PE=1 SV=1		
Q9Y2Q5-2	LTOR2_HUMAN Isoform 2 of Ragulator complex protein LAMTOR2 OS=Homo sapiens GN=LAMTOR2		
Q9Y2Q5;Q9Y2Q5-2	LTOR2_HUMAN Ragulator complex protein LAMTOR2 OS=Homo sapiens GN=LAMTOR2 PE=1 SV=1;>sp|Q9Y2Q5-2|LTOR2_HUMAN Isof	1.26E-17	
Q9Y2Q5;Q9Y2Q5-2;Q5VY9	MAPIP_HUMAN Mitogen-activated protein-binding protein-interacting protein OS=Homo sapiens GN=ROBLD3 PE=1 SV=1;>sp|Q9Y2Q5	4.63E-18	
Q9Y2Q5;Q9Y2Q5-2;Q9Y2C	LTOR2_HUMAN Ragulator complex protein LAMTOR2 OS=Homo sapiens GN=LAMTOR2 PE=1 SV=1;>sp|Q9Y2Q5-2|LTOR2_HUMAN Isof	4.40E-16	
Q9Y2Q9	RT28_HUMAN 28S ribosomal protein S28, mitochondrial OS=Homo sapiens GN=MRPS28 PE=1 SV=1		128.52
Q9Y2R0	CCD56_HUMAN Coiled-coil domain-containing protein 56 OS=Homo sapiens GN=CCDC56 PE=1 SV=1		
Q9Y2R4	DDX52_HUMAN Probable ATP-dependent RNA helicase DDX52 OS=Homo sapiens GN=DDX52 PE=1 SV=3		
Q9Y2R5	RT17_HUMAN 28S ribosomal protein S17, mitochondrial OS=Homo sapiens GN=MRPS17 PE=1 SV=1		34.84
Q9Y2R9	RT07_HUMAN 28S ribosomal protein S7, mitochondrial OS=Homo sapiens GN=MRPS7 PE=1 SV=2		121.49
Q9Y2S0	RPAC2_HUMAN DNA-directed RNA polymerases I and III subunit RPAC2 OS=Homo sapiens GN=POLR1D PE=1 SV=1		75.01
Q9Y2S0-2	RPAC2_HUMAN Isoform 2 of DNA-directed RNA polymerases I and III subunit RPAC2 OS=Homo sapiens GN=POLR1D		37.09
Q9Y2S2;Q9Y2S2-2	CRYL1_HUMAN Lambda-crystallin homolog OS=Homo sapiens GN=CRYL1 PE=1 SV=3;>sp|Q9Y2S2-2|CRYL1_HUMAN Isoform 2 of Lan	3.14E-19	
Q9Y2S6	CCD72_HUMAN Coiled-coil domain-containing protein 72 OS=Homo sapiens GN=CCDC72 PE=1 SV=1		45.7
Q9Y2S7	PDIP2_HUMAN Polymerase delta-interacting protein 2 OS=Homo sapiens GN=POLDIP2 PE=1 SV=1		190.99
Q9Y2T2	AP3M1_HUMAN AP-3 complex subunit mu-1 OS=Homo sapiens GN=AP3M1 PE=1 SV=1	1.19E-31	
Q9Y2T3	GUAD_HUMAN Guanine deaminase OS=Homo sapiens GN=GDA PE=1 SV=1	7.54E-10	
Q9Y2T7	YBOX2_HUMAN Y-box-binding protein 2 OS=Homo sapiens GN=YBX2 PE=1 SV=2		227.84
Q9Y2U8	MAN1_HUMAN Inner nuclear membrane protein Man1 OS=Homo sapiens GN=LEMD3 PE=1 SV=2	0.00021367	
Q9Y2V2	CHSP1_HUMAN Calcium-regulated heat stable protein 1 OS=Homo sapiens GN=CARHSP1 PE=1 SV=2		65.23
Q9Y2W1	TR150_HUMAN Thyroid hormone receptor-associated protein 3 OS=Homo sapiens GN=THRAP3 PE=1 SV=2		176.69
Q9Y2W2	WBP11_HUMAN WW domain-binding protein 11 OS=Homo sapiens GN=WBP11 PE=1 SV=1		
Q9Y2X3	NOP58_HUMAN Nucleolar protein 58 OS=Homo sapiens GN=NOP58 PE=1 SV=1		509.83
Q9Y2X7	GIT1_HUMAN ARF GTPase-activating protein GIT1 OS=Homo sapiens GN=GIT1 PE=1 SV=2		53.64
Q9Y2X7-3	GIT1_HUMAN Isoform 3 of ARF GTPase-activating protein GIT1 OS=Homo sapiens GN=GIT1		53.64
Q9Y2X7-3;Q9Y2X7	3|GIT1_HUMAN Isoform 3 of ARF GTPase-activating protein GIT1 OS=Homo sapiens GN=GIT1;>sp|Q9Y2X7|GIT1_HUMAN ARF GTPase	2.20E-18	
Q9Y2Z0	SUGT1_HUMAN Suppressor of G2 allele of SKP1 homolog OS=Homo sapiens GN=SUGT1 PE=1 SV=3		25.35
Q9Y2Z0-2	SUGT1_HUMAN Isoform 2 of Suppressor of G2 allele of SKP1 homolog OS=Homo sapiens GN=SUGT1		25.35
Q9Y2Z0;Q9Y2Z0-2	SUGT1_HUMAN Suppressor of G2 allele of SKP1 homolog OS=Homo sapiens GN=SUGT1 PE=1 SV=3;>sp|Q9Y2Z0-2|SUGT1_HUMAN I	1.74E-64	
Q9Y2Z2-6;Q9Y2Z2;Q9Y2Z	6|MTO1_HUMAN Isoform 7 of Protein MTO1 homolog, mitochondrial OS=Homo sapiens GN=MTO1;>sp|Q9Y2Z2|MTO1_HUMAN Protein	0.00011339	
Q9Y2Z2;Q9Y2Z2-4;Q9Y2Z	MTO1_HUMAN Protein MTO1 homolog, mitochondrial OS=Homo sapiens GN=MTO1 PE=1 SV=2;>sp|Q9Y2Z2-4|MTO1_HUMAN Isoform	6.95E-05	
Q9Y2Z4	SYYM_HUMAN Tyrosyl-tRNA synthetase, mitochondrial OS=Homo sapiens GN=YARS2 PE=1 SV=2		
Q9Y2Z9	COQ6_HUMAN Ubiquinone biosynthesis monooxygenase COQ6 OS=Homo sapiens GN=COQ6 PE=1 SV=2		35.87
Q9Y303-3;Q9Y303-2;Q9Y3	3|NAGA_HUMAN Isoform 3 of Putative N-acetylglucosamine-6-phosphate deacetylase OS=Homo sapiens GN=AMDHD2;>sp|Q9Y303-2	4.46E-11	
Q9Y305	ACOT9_HUMAN Acyl-coenzyme A thioesterase 9, mitochondrial OS=Homo sapiens GN=ACOT9 PE=1 SV=2		220.33
Q9Y305-2	ACOT9_HUMAN Isoform 2 of Acyl-coenzyme A thioesterase 9, mitochondrial OS=Homo sapiens GN=ACOT9		220.33
Q9Y305-3	ACOT9_HUMAN Isoform 3 of Acyl-coenzyme A thioesterase 9, mitochondrial OS=Homo sapiens GN=ACOT9		220.33
Q9Y305-4	ACOT9_HUMAN Isoform 4 of Acyl-coenzyme A thioesterase 9, mitochondrial OS=Homo sapiens GN=ACOT9		220.33
Q9Y305-4;Q9Y305;Q9Y30	4|ACOT9_HUMAN Isoform 4 of Acyl-coenzyme A thioesterase 9, mitochondrial OS=Homo sapiens GN=ACOT9;>sp|Q9Y305|ACOT9_HU	1.97E-61	
Q9Y305;Q9Y305-2;Q9Y30	ACOT9_HUMAN Acyl-coenzyme A thioesterase 9, mitochondrial OS=Homo sapiens GN=ACOT9 PE=1 SV=2;>sp|Q9Y305-2|ACOT9_HUI	1.17E-55	
Q9Y312	CT004_HUMAN Uncharacterized protein C20orf4 OS=Homo sapiens GN=C20orf4 PE=1 SV=2	9.12E-10	
Q9Y314	NOSIP_HUMAN Nitric oxide synthase-interacting protein OS=Homo sapiens GN=NOSIP PE=1 SV=1		30.07
Q9Y315	DEOC_HUMAN Putative deoxyribose-phosphate aldolase OS=Homo sapiens GN=DERA PE=1 SV=2	7.74E-11	
Q9Y316;Q9Y316-2	MEMO1_HUMAN Protein MEMO1 OS=Homo sapiens GN=MEMO1 PE=1 SV=1;>sp|Q9Y316-2|MEMO1_HUMAN Isoform 2 of Protein MEM	3.51E-11	
Q9Y320	TMX2_HUMAN Thioredoxin-related transmembrane protein 2 OS=Homo sapiens GN=TMX2 PE=1 SV=1		40.92
Q9Y320-2	TMX2_HUMAN Isoform 2 of Thioredoxin-related transmembrane protein 2 OS=Homo sapiens GN=TMX2		37.04
Q9Y330	ZBT12_HUMAN Zinc finger and BTB domain-containing protein 12 OS=Homo sapiens GN=ZBTB12 PE=1 SV=1		
Q9Y333	LSM2_HUMAN U6 snRNA-associated Sm-like protein LSm2 OS=Homo sapiens GN=LSM2 PE=1 SV=1		365.95
Q9Y343	SNX24_HUMAN Sorting nexin-24 OS=Homo sapiens GN=SNX24 PE=1 SV=1		20.89
Q9Y343-2	SNX24_HUMAN Isoform 2 of Sorting nexin-24 OS=Homo sapiens GN=SNX24		20.89
Q9Y365	PCTL_HUMAN PCTP-like protein OS=Homo sapiens GN=STARD10 PE=2 SV=2	4.62E-51	
Q9Y371-2;Q9Y371	2|SHLB1_HUMAN Isoform 2 of Endophilin-B1 OS=Homo sapiens GN=SH3GLB1;>sp|Q9Y371|SHLB1_HUMAN Endophilin-B1 OS=Homo	2.30E-18	
Q9Y376	CAB39_HUMAN Calcium-binding protein 39 OS=Homo sapiens GN=CAB39 PE=1 SV=1		86.99
Q9Y383	LC7L2_HUMAN Putative RNA-binding protein Luc7-like 2 OS=Homo sapiens GN=LUC7L2 PE=1 SV=2		343.35
Q9Y383-2	LC7L2_HUMAN Isoform 2 of Putative RNA-binding protein Luc7-like 2 OS=Homo sapiens GN=LUC7L2		253.33
Q9Y383;Q9Y383-2	LC7L2_HUMAN Putative RNA-binding protein Luc7-like 2 OS=Homo sapiens GN=LUC7L2 PE=1 SV=2;>sp|Q9Y383-2|LC7L2_HUMAN Is	5.45E-220	
Q9Y388	RBMX2_HUMAN RNA-binding motif protein, X-linked 2 OS=Homo sapiens GN=RBMX2 PE=1 SV=2	6.32E-07	
Q9Y394	DHRS7_HUMAN Dehydrogenase/reductase SDR family member 7 OS=Homo sapiens GN=DHRS7 PE=1 SV=1		
Q9Y394-2	DHRS7_HUMAN Isoform 2 of Dehydrogenase/reductase SDR family member 7 OS=Homo sapiens GN=DHRS7		
Q9Y394;Q9Y394-2	DHRS7_HUMAN Dehydrogenase/reductase SDR family member 7 OS=Homo sapiens GN=DHRS7 PE=1 SV=1;>sp|Q9Y394-2|DHRS7_H	1.06E-09	

Q9Y399	RT02_HUMAN 28S ribosomal protein S2, mitochondrial OS=Homo sapiens GN=MRPS2 PE=1 SV=1		125.22
Q9Y3A3;B4DML0;A8K012;	MOBL3_HUMAN Mps one binder kinase activator-like 3 OS=Homo sapiens GN=MOBKL3 PE=1 SV=1;>tr|B4DML0|B4DML0_HUMAN Pre	6.99E-17	
Q9Y3A4	RRP7A_HUMAN Ribosomal RNA-processing protein 7 homolog A OS=Homo sapiens GN=RRP7A PE=1 SV=2		
Q9Y3A5	SBDS_HUMAN Ribosome maturation protein SBDS OS=Homo sapiens GN=SBDS PE=1 SV=4		218.49
Q9Y3A6	TMED5_HUMAN Transmembrane emp24 domain-containing protein 5 OS=Homo sapiens GN=TMED5 PE=1 SV=1		118.31
Q9Y3B2	EXOS1_HUMAN Exosome complex component CSL4 OS=Homo sapiens GN=EXOSC1 PE=1 SV=1		193.63
Q9Y3B2;B1AMU4;B1AMU3	EXOS1_HUMAN 3-5 exoribonuclease CSL4 homolog OS=Homo sapiens GN=EXOSC1 PE=1 SV=1;>tr|B1AMU4|B1AMU4_HUMAN Exosor	2.92E-24	
Q9Y3B3	TMED7_HUMAN Transmembrane emp24 domain-containing protein 7 OS=Homo sapiens GN=TMED7 PE=1 SV=2		204.62
Q9Y3B4	PM14_HUMAN Pre branch site protein p14 OS=Homo sapiens GN=SF3B14 PE=1 SV=1		293.33
Q9Y3B7	RM11_HUMAN 39S ribosomal protein L11, mitochondrial OS=Homo sapiens GN=MRPL11 PE=1 SV=1		425.82
Q9Y3B7-2	RM11_HUMAN Isoform 2 of 39S ribosomal protein L11, mitochondrial OS=Homo sapiens GN=MRPL11		333.03
Q9Y3B7;Q9Y3B7-2	RM11_HUMAN 39S ribosomal protein L11, mitochondrial OS=Homo sapiens GN=MRPL11 PE=1 SV=1;>sp|Q9Y3B7-2|RM11_HUMAN Is	5.59E-57	
Q9Y3B8	ORN_HUMAN Oligoribonuclease, mitochondrial OS=Homo sapiens GN=REXO2 PE=1 SV=3		184.84
Q9Y3B8-2	ORN_HUMAN Isoform 2 of Oligoribonuclease, mitochondrial OS=Homo sapiens GN=REXO2		146.78
Q9Y3B8;Q9Y3B8-2	ORN_HUMAN Oligoribonuclease, mitochondrial OS=Homo sapiens GN=REXO2 PE=1 SV=3;>sp|Q9Y3B8-2|ORN_HUMAN Isoform 2 of C	2.22E-42	
Q9Y3B9	RRP15_HUMAN RRP15-like protein OS=Homo sapiens GN=RRP15 PE=1 SV=2		
Q9Y3C0	CCD53_HUMAN WASH complex subunit CCDC53 OS=Homo sapiens GN=CCDC53 PE=1 SV=1	1.79E-18	
Q9Y3C1	NOP16_HUMAN Nucleolar protein 16 OS=Homo sapiens GN=NOP16 PE=1 SV=2		195.02
Q9Y3C4	TPRKB_HUMAN TP53RK-binding protein OS=Homo sapiens GN=TPRKB PE=1 SV=1		94.83
Q9Y3C4-2	TPRKB_HUMAN Isoform 2 of TP53RK-binding protein OS=Homo sapiens GN=TPRKB		90.29
Q9Y3C4-3	TPRKB_HUMAN Isoform 3 of TP53RK-binding protein OS=Homo sapiens GN=TPRKB		94.83
Q9Y3C4;Q9Y3C4-3	TPRKB_HUMAN TP53RK-binding protein OS=Homo sapiens GN=TPRKB PE=1 SV=1;>sp|Q9Y3C4-3|TPRKB_HUMAN Isoform 3 of TP53R	4.25E-20	
Q9Y3C6	PPIL1_HUMAN Peptidyl-prolyl cis-trans isomerase-like 1 OS=Homo sapiens GN=PPIL1 PE=1 SV=1		212.79
Q9Y3C7	MED31_HUMAN Mediator of RNA polymerase II transcription subunit 31 OS=Homo sapiens GN=MED31 PE=1 SV=1		
Q9Y3C8	UFC1_HUMAN Ubiquitin-fold modifier-conjugating enzyme 1 OS=Homo sapiens GN=UFC1 PE=1 SV=3		16.67
Q9Y3D0	MIP18_HUMAN Mitotic spindle-associated MMXD complex subunit MIP18 OS=Homo sapiens GN=FAM96B PE=1 SV=1	5.74E-06	
Q9Y3D2	MSRB2_HUMAN Methionine-R-sulfoxide reductase B2, mitochondrial OS=Homo sapiens GN=MSRB2 PE=2 SV=2		
Q9Y3D3	RT16_HUMAN 28S ribosomal protein S16, mitochondrial OS=Homo sapiens GN=MRPS16 PE=1 SV=1		65.69
Q9Y3D6	FIS1_HUMAN Mitochondrial fission 1 protein OS=Homo sapiens GN=FIS1 PE=1 SV=2		35.56
Q9Y3D7	TIM16_HUMAN Mitochondrial import inner membrane translocase subunit TIM16 OS=Homo sapiens GN=PAM16 PE=1 SV=2		63.39
Q9Y3D8	KAD6_HUMAN Adenylate kinase isoenzyme 6 OS=Homo sapiens GN=TAF9 PE=1 SV=1	1.76E-07	
Q9Y3D9	RT23_HUMAN 28S ribosomal protein S23, mitochondrial OS=Homo sapiens GN=MRPS23 PE=1 SV=2		198.57
Q9Y3E0	GOT1B_HUMAN Vesicle transport protein GOT1B OS=Homo sapiens GN=GOLT1B PE=1 SV=1		63.12
Q9Y3E1	HDGR3_HUMAN Hepatoma-derived growth factor-related protein 3 OS=Homo sapiens GN=HDGFRP3 PE=1 SV=1		
Q9Y3E5	PTH2_HUMAN Peptidyl-tRNA hydrolase 2, mitochondrial OS=Homo sapiens GN=PTRH2 PE=1 SV=1		331.17
Q9Y3E7;Q9Y3E7-2	CHMP3_HUMAN Charged multivesicular body protein 3 OS=Homo sapiens GN=VPS24 PE=1 SV=3;>sp|Q9Y3E7-2|CHMP3_HUMAN Isof	6.75E-08	
Q9Y3F4	STRAP_HUMAN Serine-threonine kinase receptor-associated protein OS=Homo sapiens GN=STRAP PE=1 SV=1		170.63
Q9Y3I0	RTCB_HUMAN tRNA-splicing ligase RtcB homolog OS=Homo sapiens GN=C22orf28 PE=1 SV=1		520.94
Q9Y3I0;B4DNA0	CV028_HUMAN UPF0027 protein C22orf28 OS=Homo sapiens GN=C22orf28 PE=1 SV=1;>tr|B4DNA0|B4DNA0_HUMAN cDNA FLJ5802	2.51E-66	
Q9Y3L3	3BP1_HUMAN SH3 domain-binding protein 1 OS=Homo sapiens GN=SH3BP1 PE=1 SV=3		
Q9Y3L3-2	3BP1_HUMAN Isoform 2 of SH3 domain-binding protein 1 OS=Homo sapiens GN=SH3BP1		
Q9Y3L3;B7Z6Y3;Q9Y3L3-2	3BP1_HUMAN SH3 domain-binding protein 1 OS=Homo sapiens GN=SH3BP1 PE=1 SV=3;>tr|B7Z6Y3|B7Z6Y3_HUMAN cDNA FLJ6163	1.18E-15	
Q9Y3L5	RAP2C_HUMAN Ras-related protein Rap-2c OS=Homo sapiens GN=RAP2C PE=1 SV=1		196.89
Q9Y3P9;Q9Y3P9-2	RBGP1_HUMAN Rab GTPase-activating protein 1 OS=Homo sapiens GN=RABGAP1 PE=1 SV=3;>sp|Q9Y3P9-2|RBGP1_HUMAN Isoform	4.79E-48	
Q9Y3Q3	TMED3_HUMAN Transmembrane emp24 domain-containing protein 3 OS=Homo sapiens GN=TMED3 PE=2 SV=1		
Q9Y3R4	NEUR2_HUMAN Sialidase-2 OS=Homo sapiens GN=NEU2 PE=1 SV=2		
Q9Y3R5	DOP2_HUMAN Protein dopey-2 OS=Homo sapiens GN=DOPEY2 PE=1 SV=5		
Q9Y3R5-2	DOP2_HUMAN Isoform 2 of Protein dopey-2 OS=Homo sapiens GN=DOPEY2		
Q9Y3R5;Q9Y3R5-2	DOP2_HUMAN Protein dopey-2 OS=Homo sapiens GN=DOPEY2 PE=1 SV=5;>sp|Q9Y3R5-2|DOP2_HUMAN Isoform 2 of Protein dopey-	1.47E-06	
Q9Y3S1-3	WNK2_HUMAN Isoform 3 of Serine/threonine-protein kinase WNK2 OS=Homo sapiens GN=WNK2		
Q9Y3T9	NOC2L_HUMAN Nucleolar complex protein 2 homolog OS=Homo sapiens GN=NOC2L PE=1 SV=4		
Q9Y3U8	RL36_HUMAN 60S ribosomal protein L36 OS=Homo sapiens GN=RPL36 PE=1 SV=3		362.34
Q9Y3V2	RWDD3_HUMAN RWD domain-containing protein 3 OS=Homo sapiens GN=RWDD3 PE=1 SV=4		20.85
Q9Y3Y2	CHTOP_HUMAN Chromatin target of PRMT1 protein OS=Homo sapiens GN=CHTOP PE=1 SV=2		89.55
Q9Y3Y2-3	CHTOP_HUMAN Isoform 2 of Chromatin target of PRMT1 protein OS=Homo sapiens GN=CHTOP		89.55
Q9Y3Y2-3;Q9Y3Y2;Q9Y3Y	3|FOP_HUMAN Isoform 2 of Friend of PRMT1 protein OS=Homo sapiens GN=C1orf77;>sp|Q9Y3Y2|FOP_HUMAN Friend of PRMT1 prote	6.84E-31	
Q9Y3Y2-4	CHTOP_HUMAN Isoform 3 of Chromatin target of PRMT1 protein OS=Homo sapiens GN=CHTOP		84.55
Q9Y3Z3	SAMH1_HUMAN SAM domain and HD domain-containing protein 1 OS=Homo sapiens GN=SAMHD1 PE=1 SV=2		197.72
Q9Y3Z3-2	SAMH1_HUMAN Isoform 2 of SAM domain and HD domain-containing protein 1 OS=Homo sapiens GN=SAMHD1		197.72
Q9Y3Z3;Q9Y3Z3-2	SAMH1_HUMAN SAM domain and HD domain-containing protein 1 OS=Homo sapiens GN=SAMHD1 PE=1 SV=2;>sp|Q9Y3Z3-2|SAMH1	4.87E-13	
Q9Y426	CU025_HUMAN C2 domain-containing protein 2 OS=Homo sapiens GN=C2CD2 PE=1 SV=2		

Q9Y426-2	CU025_HUMAN Isoform 2 of C2 domain-containing protein 2 OS=Homo sapiens GN=C2CD2		
Q9Y426-3	CU025_HUMAN Isoform 3 of C2 domain-containing protein 2 OS=Homo sapiens GN=C2CD2		
Q9Y446	PKP3_HUMAN Plakophilin-3 OS=Homo sapiens GN=PKP3 PE=1 SV=1		
Q9Y448;C9J424;B4DP18	T4AF1_HUMAN Putative TRAF4-associated factor 1 OS=Homo sapiens GN=TRAF4AF1 PE=1 SV=2;>tr|C9J424|C9J424_HUMAN Unchar	1.17E-17	
Q9Y450;Q9Y450-4	HBS1L_HUMAN HBS1-like protein OS=Homo sapiens GN=HBS1L PE=1 SV=1;>sp|Q9Y450-4|HBS1L_HUMAN Isoform 3 of HBS1-like pr	1.00E-08	
Q9Y490	TLN1_HUMAN Talin-1 OS=Homo sapiens GN=TLN1 PE=1 SV=3		2053.67
Q9Y4A5	TRRAP_HUMAN Transformation/transcription domain-associated protein OS=Homo sapiens GN=TRRAP PE=1 SV=3		140.85
Q9Y4A5-2	TRRAP_HUMAN Isoform 2 of Transformation/transcription domain-associated protein OS=Homo sapiens GN=TRRAP		140.85
Q9Y4A5;Q9Y4A5-2	TRRAP_HUMAN Transformation/transcription domain-associated protein OS=Homo sapiens GN=TRRAP PE=1 SV=3;>sp|Q9Y4A5-2|TRI	1.32E-12	
Q9Y4B5	CC165_HUMAN Coiled-coil domain-containing protein 165 OS=Homo sapiens GN=CCDC165 PE=1 SV=4		111.67
Q9Y4B5-2	CC165_HUMAN Isoform 2 of Coiled-coil domain-containing protein 165 OS=Homo sapiens GN=CCDC165		107.42
Q9Y4B5-3	CC165_HUMAN Isoform 3 of Coiled-coil domain-containing protein 165 OS=Homo sapiens GN=CCDC165		107.42
Q9Y4B5-4	CC165_HUMAN Isoform 4 of Coiled-coil domain-containing protein 165 OS=Homo sapiens GN=CCDC165		29.41
Q9Y4B5;Q9Y4B5-3;Q9Y4B	CC165_HUMAN Coiled-coil domain-containing protein 165 OS=Homo sapiens GN=CCDC165 PE=1 SV=4;>sp|Q9Y4B5-3|CC165_HUMA	2.78E-05	
Q9Y4C0	NRX3A_HUMAN Neurexin-3-alpha OS=Homo sapiens GN=NRXN3 PE=2 SV=4		
Q9Y4C0-3	NRX3A_HUMAN Isoform 3 of Neurexin-3-alpha OS=Homo sapiens GN=NRXN3		
Q9Y4C1	KDM3A_HUMAN Lysine-specific demethylase 3A OS=Homo sapiens GN=KDM3A PE=1 SV=4		6.66
Q9Y4C2;Q9Y4C2-2	F115A_HUMAN Protein FAM115A OS=Homo sapiens GN=FAM115A PE=1 SV=3;>sp|Q9Y4C2-2|F115A_HUMAN Isoform 2 of Protein FAI	6.83E-12	
Q9Y4C8	RBM19_HUMAN Probable RNA-binding protein 19 OS=Homo sapiens GN=RBM19 PE=1 SV=3		
Q9Y4D7	PLXD1_HUMAN Plexin-D1 OS=Homo sapiens GN=PLXND1 PE=1 SV=3		43.21
Q9Y4D7-2	PLXD1_HUMAN Isoform 2 of Plexin-D1 OS=Homo sapiens GN=PLXND1		18.79
Q9Y4D8	K0614_HUMAN Probable E3 ubiquitin-protein ligase C12orf51 OS=Homo sapiens GN=C12orf51 PE=1 SV=5		
Q9Y4D8-2	K0614_HUMAN Isoform 2 of Probable E3 ubiquitin-protein ligase C12orf51 OS=Homo sapiens GN=C12orf51		
Q9Y4D8-4	K0614_HUMAN Isoform 4 of Probable E3 ubiquitin-protein ligase C12orf51 OS=Homo sapiens GN=C12orf51		
Q9Y4E1	FA21C_HUMAN WASH complex subunit FAM21C OS=Homo sapiens GN=FAM21C PE=1 SV=3		35.35
Q9Y4E1-2	FA21C_HUMAN Isoform 2 of WASH complex subunit FAM21C OS=Homo sapiens GN=FAM21C		35.35
Q9Y4E1-3	FA21C_HUMAN Isoform 3 of WASH complex subunit FAM21C OS=Homo sapiens GN=FAM21C		35.35
Q9Y4E1-4	FA21C_HUMAN Isoform 4 of WASH complex subunit FAM21C OS=Homo sapiens GN=FAM21C		35.35
Q9Y4E8;Q9Y4E8-3;Q9Y4E	UBP15_HUMAN Ubiquitin carboxyl-terminal hydrolase 15 OS=Homo sapiens GN=USP15 PE=1 SV=3;>sp|Q9Y4E8-3|UBP15_HUMAN Is	2.46E-13	
Q9Y4F1-2;Q9Y4F1	2|FARP1_HUMAN Isoform 2 of FERM, RhoGEF and pleckstrin domain-containing protein 1 OS=Homo sapiens GN=FARP1;>sp|Q9Y4F1|I	5.73E-06	
Q9Y4F3	LKAP_HUMAN Limkain-b1 OS=Homo sapiens GN=LKAP PE=1 SV=5		
Q9Y4F3-3	LKAP_HUMAN Isoform 2 of Limkain-b1 OS=Homo sapiens GN=LKAP		
Q9Y4F3-4	LKAP_HUMAN Isoform 3 of Limkain-b1 OS=Homo sapiens GN=LKAP		
Q9Y4F3-5	LKAP_HUMAN Isoform 4 of Limkain-b1 OS=Homo sapiens GN=LKAP		
Q9Y4F3-6	LKAP_HUMAN Isoform 5 of Limkain-b1 OS=Homo sapiens GN=LKAP		
Q9Y4G6	TLN2_HUMAN Talin-2 OS=Homo sapiens GN=TLN2 PE=1 SV=4		204.22
Q9Y4I1	MYO5A_HUMAN Myosin-Va OS=Homo sapiens GN=MYO5A PE=1 SV=2		
Q9Y4I1-2	MYO5A_HUMAN Isoform 2 of Myosin-Va OS=Homo sapiens GN=MYO5A		
Q9Y4I1-3	MYO5A_HUMAN Isoform 3 of Myosin-Va OS=Homo sapiens GN=MYO5A		
Q9Y4I1-3;Q9Y4I1;A8CDT9	3|MYO5A_HUMAN Isoform 3 of Myosin-Va OS=Homo sapiens GN=MYO5A;>sp|Q9Y4I1|MYO5A_HUMAN Myosin-Va OS=Homo sapiens (	3.47E-07	
Q9Y4J8	DTNA_HUMAN Dystrobrevin alpha OS=Homo sapiens GN=DTNA PE=1 SV=2		
Q9Y4J8-2	DTNA_HUMAN Isoform 2 of Dystrobrevin alpha OS=Homo sapiens GN=DTNA		
Q9Y4J8-3	DTNA_HUMAN Isoform 3 of Dystrobrevin alpha OS=Homo sapiens GN=DTNA		
Q9Y4J8-4	DTNA_HUMAN Isoform 4 of Dystrobrevin alpha OS=Homo sapiens GN=DTNA		
Q9Y4J8-5	DTNA_HUMAN Isoform 5 of Dystrobrevin alpha OS=Homo sapiens GN=DTNA		
Q9Y4J8-7	DTNA_HUMAN Isoform 7 of Dystrobrevin alpha OS=Homo sapiens GN=DTNA		
Q9Y4K1	AIM1_HUMAN Absent in melanoma 1 protein OS=Homo sapiens GN=AIM1 PE=1 SV=3	3.21E-05	
Q9Y4L1	HYOU1_HUMAN Hypoxia up-regulated protein 1 OS=Homo sapiens GN=HYOU1 PE=1 SV=1		2162.51
Q9Y4P3	TBL2_HUMAN Transducin beta-like protein 2 OS=Homo sapiens GN=TBL2 PE=1 SV=1		79.84
Q9Y4P9	SPEF1_HUMAN Sperm flagellar protein 1 OS=Homo sapiens GN=SPEF1 PE=1 SV=3		
Q9Y4P9-2	SPEF1_HUMAN Isoform 2 of Sperm flagellar protein 1 OS=Homo sapiens GN=SPEF1		
Q9Y4W2	LAS1L_HUMAN Protein LAS1 homolog OS=Homo sapiens GN=LAS1L PE=1 SV=2		
Q9Y4W2-2	LAS1L_HUMAN Isoform 2 of Protein LAS1 homolog OS=Homo sapiens GN=LAS1L		
Q9Y4W2-3	LAS1L_HUMAN Isoform 3 of Protein LAS1 homolog OS=Homo sapiens GN=LAS1L		
Q9Y4W2-4	LAS1L_HUMAN Isoform 4 of Protein LAS1 homolog OS=Homo sapiens GN=LAS1L		
Q9Y4W2;Q9Y4W2-2;Q9Y4	LAS1L_HUMAN Protein LAS1 homolog OS=Homo sapiens GN=LAS1L PE=1 SV=2;>sp|Q9Y4W2-2|LAS1L_HUMAN Isoform 2 of Protein I	1.47E-09	
Q9Y4W6	AFG32_HUMAN AFG3-like protein 2 OS=Homo sapiens GN=AFG3L2 PE=1 SV=2		
Q9Y4X5;Q59FM8	ARI1_HUMAN Protein ariadne-1 homolog OS=Homo sapiens GN=ARIH1 PE=1 SV=2;>tr|Q59FM8|Q59FM8_HUMAN Ariadne-1 protein h	2.87E-05	
Q9Y4Y9	LSM5_HUMAN U6 snRNA-associated Sm-like protein LSm5 OS=Homo sapiens GN=LSM5 PE=1 SV=3	6.50E-09	
Q9Y4Z0	LSM4_HUMAN U6 snRNA-associated Sm-like protein LSm4 OS=Homo sapiens GN=LSM4 PE=1 SV=1		386.66
Q9Y508;Q9Y508-2	RN114_HUMAN RING finger protein 114 OS=Homo sapiens GN=RNF114 PE=1 SV=1;>sp|Q9Y508-2|RN114_HUMAN Isoform 2 of RIN(	5.01E-09	

Q9Y512	SAM50_HUMAN Sorting and assembly machinery component 50 homolog OS=Homo sapiens GN=SAMM50 PE=1 SV=3	4.66E-49	
Q9Y520	PRC2C_HUMAN Protein PRRC2C OS=Homo sapiens GN=PRRC2C PE=1 SV=4		761.45
Q9Y520-2	PRC2C_HUMAN Isoform 2 of Protein PRRC2C OS=Homo sapiens GN=PRRC2C		686.32
Q9Y520-3	PRC2C_HUMAN Isoform 3 of Protein PRRC2C OS=Homo sapiens GN=PRRC2C		695.38
Q9Y520-4	PRC2C_HUMAN Isoform 4 of Protein PRRC2C OS=Homo sapiens GN=PRRC2C		759.44
Q9Y520-5	PRC2C_HUMAN Isoform 5 of Protein PRRC2C OS=Homo sapiens GN=PRRC2C		761.45
Q9Y520-6	PRC2C_HUMAN Isoform 6 of Protein PRRC2C OS=Homo sapiens GN=PRRC2C		759.44
Q9Y520-7	PRC2C_HUMAN Isoform 7 of Protein PRRC2C OS=Homo sapiens GN=PRRC2C		759.05
Q9Y520-7;Q9Y520;Q9Y52	7|BA2L2_HUMAN Isoform 7 of Protein BAT2-like 2 OS=Homo sapiens GN=BAT2L2;>sp|Q9Y520|BA2L2_HUMAN Protein BAT2-like 2 OS	2.76E-15	
Q9Y520-7;Q9Y520;Q9Y52	7|PRC2C_HUMAN Isoform 7 of Protein PRRC2C OS=Homo sapiens GN=PRRC2C;>sp|Q9Y520|PRC2C_HUMAN Protein PRRC2C OS=Hom	1.52E-32	
Q9Y530	CF130_HUMAN Uncharacterized protein C6orf130 OS=Homo sapiens GN=C6orf130 PE=1 SV=2		202.18
Q9Y536	PAL4A_HUMAN Peptidylprolyl cis-trans isomerase A-like 4A/B/C OS=Homo sapiens GN=PPIAL4A PE=1 SV=1		174.74
Q9Y547	HSB11_HUMAN Heat shock protein beta-11 OS=Homo sapiens GN=HSPB11 PE=1 SV=1	2.14E-06	
Q9Y570	PPME1_HUMAN Protein phosphatase methylesterase 1 OS=Homo sapiens GN=PPME1 PE=1 SV=3		21.58
Q9Y570-2	PPME1_HUMAN Isoform 2 of Protein phosphatase methylesterase 1 OS=Homo sapiens GN=PPME1		21.58
Q9Y570-3	PPME1_HUMAN Isoform 3 of Protein phosphatase methylesterase 1 OS=Homo sapiens GN=PPME1		21.58
Q9Y570;Q9Y570-2	PPME1_HUMAN Protein phosphatase methylesterase 1 OS=Homo sapiens GN=PPME1 PE=1 SV=3;>sp|Q9Y570-2|PPME1_HUMAN Isofo	1.39E-253	
Q9Y580	RBM7_HUMAN RNA-binding protein 7 OS=Homo sapiens GN=RBM7 PE=1 SV=1		43.41
Q9Y584	TIM22_HUMAN Mitochondrial import inner membrane translocase subunit Tim22 OS=Homo sapiens GN=TIMM22 PE=1 SV=2		48.51
Q9Y592	CCD41_HUMAN Coiled-coil domain-containing protein 41 OS=Homo sapiens GN=CCDC41 PE=1 SV=2		
Q9Y592-2	CCD41_HUMAN Isoform 2 of Coiled-coil domain-containing protein 41 OS=Homo sapiens GN=CCDC41		
Q9Y5A7;Q9Y5A7-2	NUB1_HUMAN NEDD8 ultimate buster 1 OS=Homo sapiens GN=NUB1 PE=1 SV=2;>sp|Q9Y5A7-2|NUB1_HUMAN Isoform 2 of NEDD8	7.08E-17	
Q9Y5A9	YTHD2_HUMAN YTH domain family protein 2 OS=Homo sapiens GN=YTHDF2 PE=1 SV=2		130.11
Q9Y5A9-2	YTHD2_HUMAN Isoform 2 of YTH domain family protein 2 OS=Homo sapiens GN=YTHDF2		127.19
Q9Y5A9;Q9Y5A9-2	YTHD2_HUMAN YTH domain family protein 2 OS=Homo sapiens GN=YTHDF2 PE=1 SV=2;>sp|Q9Y5A9-2|YTHD2_HUMAN Isoform 2 of	4.74E-19	
Q9Y5B0	CTDP1_HUMAN RNA polymerase II subunit A C-terminal domain phosphatase OS=Homo sapiens GN=CTDP1 PE=1 SV=3		
Q9Y5B0-2	CTDP1_HUMAN Isoform 2 of RNA polymerase II subunit A C-terminal domain phosphatase OS=Homo sapiens GN=CTDP1		
Q9Y5B0-3	CTDP1_HUMAN Isoform 3 of RNA polymerase II subunit A C-terminal domain phosphatase OS=Homo sapiens GN=CTDP1		
Q9Y5B0-4	CTDP1_HUMAN Isoform 4 of RNA polymerase II subunit A C-terminal domain phosphatase OS=Homo sapiens GN=CTDP1		
Q9Y5B0;Q9Y5B0-4;Q9Y5B	CTDP1_HUMAN RNA polymerase II subunit A C-terminal domain phosphatase OS=Homo sapiens GN=CTDP1 PE=1 SV=3;>sp|Q9Y5B0	1.25E-08	
Q9Y5B6	GCFC1_HUMAN GC-rich sequence DNA-binding factor 1 OS=Homo sapiens GN=GCFC1 PE=1 SV=2		57.73
Q9Y5B6-2	GCFC1_HUMAN Isoform B of GC-rich sequence DNA-binding factor 1 OS=Homo sapiens GN=GCFC1		57.73
Q9Y5B6-3	GCFC1_HUMAN Isoform C of GC-rich sequence DNA-binding factor 1 OS=Homo sapiens GN=GCFC1		7.88
Q9Y5B6-4	GCFC1_HUMAN Isoform D of GC-rich sequence DNA-binding factor 1 OS=Homo sapiens GN=GCFC1		57.73
Q9Y5B6;Q9Y5B6-2	GCFC1_HUMAN GC-rich sequence DNA-binding factor 1 OS=Homo sapiens GN=GCFC1 PE=1 SV=2;>sp|Q9Y5B6-2|GCFC1_HUMAN Iso	1.30E-14	
Q9Y5B8;B3KSW9;B4DXC8	NDK7_HUMAN Nucleoside diphosphate kinase 7 OS=Homo sapiens GN=NME7 PE=1 SV=1;>tr|B3KSW9|B3KSW9_HUMAN Non-metast	6.15E-40	
Q9Y5B9	SP16H_HUMAN FACT complex subunit SPT16 OS=Homo sapiens GN=SUPT16H PE=1 SV=1		620.8
Q9Y5E1	PCDB9_HUMAN Protocadherin beta-9 OS=Homo sapiens GN=PCDHB9 PE=2 SV=2		
Q9Y5E2	PCDB7_HUMAN Protocadherin beta-7 OS=Homo sapiens GN=PCDHB7 PE=2 SV=1		
Q9Y5E3	PCDB6_HUMAN Protocadherin beta-6 OS=Homo sapiens GN=PCDHB6 PE=1 SV=1		
Q9Y5E4	PCDB5_HUMAN Protocadherin beta-5 OS=Homo sapiens GN=PCDHB5 PE=1 SV=1		
Q9Y5E5	PCDB4_HUMAN Protocadherin beta-4 OS=Homo sapiens GN=PCDHB4 PE=2 SV=1		
Q9Y5E6	PCDB3_HUMAN Protocadherin beta-3 OS=Homo sapiens GN=PCDHB3 PE=2 SV=1		
Q9Y5E9	PCDBE_HUMAN Protocadherin beta-14 OS=Homo sapiens GN=PCDHB14 PE=2 SV=1		
Q9Y5F1	PCDBC_HUMAN Protocadherin beta-12 OS=Homo sapiens GN=PCDHB12 PE=2 SV=1		
Q9Y5H4	PCDG1_HUMAN Protocadherin gamma-A1 OS=Homo sapiens GN=PCDHGA1 PE=2 SV=1		
Q9Y5H4-2	PCDG1_HUMAN Isoform 2 of Protocadherin gamma-A1 OS=Homo sapiens GN=PCDHGA1		
Q9Y5J1	UTP18_HUMAN U3 small nucleolar RNA-associated protein 18 homolog OS=Homo sapiens GN=UTP18 PE=1 SV=3		69.66
Q9Y5J5	PHLA3_HUMAN Pleckstrin homology-like domain family A member 3 OS=Homo sapiens GN=PHLDA3 PE=1 SV=1		13.44
Q9Y5J7	TIM9_HUMAN Mitochondrial import inner membrane translocase subunit Tim9 OS=Homo sapiens GN=TIMM9 PE=1 SV=1		33.12
Q9Y5J9	TIM8B_HUMAN Mitochondrial import inner membrane translocase subunit Tim8 B OS=Homo sapiens GN=TIMM8B PE=1 SV=1		
Q9Y5K3	PCY1B_HUMAN Choline-phosphate cytidylyltransferase B OS=Homo sapiens GN=PCYT1B PE=1 SV=1		66.84
Q9Y5K3-2	PCY1B_HUMAN Isoform 1 of Choline-phosphate cytidylyltransferase B OS=Homo sapiens GN=PCYT1B		66.84
Q9Y5K3-3	PCY1B_HUMAN Isoform 3 of Choline-phosphate cytidylyltransferase B OS=Homo sapiens GN=PCYT1B		66.84
Q9Y5K5	UCHL5_HUMAN Ubiquitin carboxyl-terminal hydrolase isozyme L5 OS=Homo sapiens GN=UCHL5 PE=1 SV=3		69.58
Q9Y5K5-2	UCHL5_HUMAN Isoform 2 of Ubiquitin carboxyl-terminal hydrolase isozyme L5 OS=Homo sapiens GN=UCHL5		69.58
Q9Y5K5-3	UCHL5_HUMAN Isoform 3 of Ubiquitin carboxyl-terminal hydrolase isozyme L5 OS=Homo sapiens GN=UCHL5		69.58
Q9Y5K5-4	UCHL5_HUMAN Isoform 4 of Ubiquitin carboxyl-terminal hydrolase isozyme L5 OS=Homo sapiens GN=UCHL5		69.58
Q9Y5K5;Q9Y5K5-3;Q9Y5K	UCHL5_HUMAN Ubiquitin carboxyl-terminal hydrolase isozyme L5 OS=Homo sapiens GN=UCHL5 PE=1 SV=3;>sp|Q9Y5K5-3|UCHL5_H	1.26E-37	
Q9Y5K6	CD2AP_HUMAN CD2-associated protein OS=Homo sapiens GN=CD2AP PE=1 SV=1		302.85
Q9Y5K8	VATD_HUMAN V-type proton ATPase subunit D OS=Homo sapiens GN=ATP6V1D PE=1 SV=1	1.51E-44	

Q9Y5L0	TNPO3_HUMAN Transportin-3 OS=Homo sapiens GN=TNPO3 PE=1 SV=3		
Q9Y5L0-1	TNPO3_HUMAN Isoform 1 of Transportin-3 OS=Homo sapiens GN=TNPO3		
Q9Y5L0-1;Q9Y5L0;Q9Y5L(	1|TNPO3_HUMAN Isoform 1 of Transportin-3 OS=Homo sapiens GN=TNPO3;>sp|Q9Y5L0|TNPO3_HUMAN Transportin-3 OS=Homo sap	9.88E-15	
Q9Y5L0-3	TNPO3_HUMAN Isoform 3 of Transportin-3 OS=Homo sapiens GN=TNPO3		
Q9Y5L0;Q9Y5L0-3;Q9Y5L(	TNPO3_HUMAN Transportin-3 OS=Homo sapiens GN=TNPO3 PE=1 SV=3;>sp|Q9Y5L0-3|TNPO3_HUMAN Isoform 3 of Transportin-3 OS	4.30E-57	
Q9Y5L4	TIM13_HUMAN Mitochondrial import inner membrane translocase subunit Tim13 OS=Homo sapiens GN=TIMM13 PE=1 SV=1		207.22
Q9Y5M8	SRPRB_HUMAN Signal recognition particle receptor subunit beta OS=Homo sapiens GN=SRPRB PE=1 SV=3		508.69
Q9Y5N5	HEMK2_HUMAN HemK methyltransferase family member 2 OS=Homo sapiens GN=N6AMT1 PE=1 SV=3		
Q9Y5N5-2	HEMK2_HUMAN Isoform 2 of HemK methyltransferase family member 2 OS=Homo sapiens GN=N6AMT1		
Q9Y5N6	ORC6_HUMAN Origin recognition complex subunit 6 OS=Homo sapiens GN=ORC6 PE=1 SV=1		
Q9Y5P6-2;Q9Y5P6	2|GMPPB_HUMAN Isoform 2 of Mannose-1-phosphate guanyltransferase beta OS=Homo sapiens GN=GMPPB;>sp|Q9Y5P6|GMPPB_HUI	5.69E-40	
Q9Y5P8	P2R3B_HUMAN Serine/threonine-protein phosphatase 2A regulatory subunit B'' subunit beta OS=Homo sapiens GN=PPP2R3B PE=1 SV=2		
Q9Y5Q8	TF3C5_HUMAN General transcription factor 3C polypeptide 5 OS=Homo sapiens GN=GTF3C5 PE=1 SV=2		30.46
Q9Y5Q8-2	TF3C5_HUMAN Isoform 2 of General transcription factor 3C polypeptide 5 OS=Homo sapiens GN=GTF3C5		3.07
Q9Y5Q8-3	TF3C5_HUMAN Isoform 3 of General transcription factor 3C polypeptide 5 OS=Homo sapiens GN=GTF3C5		30.46
Q9Y5Q9	TF3C3_HUMAN General transcription factor 3C polypeptide 3 OS=Homo sapiens GN=GTF3C3 PE=1 SV=1		
Q9Y5Q9-2	TF3C3_HUMAN Isoform 2 of General transcription factor 3C polypeptide 3 OS=Homo sapiens GN=GTF3C3		
Q9Y5Q9;Q9Y5Q9-2	TF3C3_HUMAN General transcription factor 3C polypeptide 3 OS=Homo sapiens GN=GTF3C3 PE=1 SV=1;>sp|Q9Y5Q9-2|TF3C3_HUM/	5.36E-19	
Q9Y5R2	MMP24_HUMAN Matrix metalloproteinase-24 OS=Homo sapiens GN=MMP24 PE=1 SV=1		10.09
Q9Y5R8	TPPC1_HUMAN Trafficking protein particle complex subunit 1 OS=Homo sapiens GN=TRAPPC1 PE=1 SV=1	3.42E-05	
Q9Y5S2	MRCKB_HUMAN Serine/threonine-protein kinase MRCK beta OS=Homo sapiens GN=CDC42BPB PE=1 SV=2		
Q9Y5S9	RBM8A_HUMAN RNA-binding protein 8A OS=Homo sapiens GN=RBM8A PE=1 SV=1		215.79
Q9Y5S9-2	RBM8A_HUMAN Isoform 2 of RNA-binding protein 8A OS=Homo sapiens GN=RBM8A		220.79
Q9Y5S9;Q9Y5S9-2	RBM8A_HUMAN RNA-binding protein 8A OS=Homo sapiens GN=RBM8A PE=1 SV=1;>sp|Q9Y5S9-2|RBM8A_HUMAN Isoform 2 of RNA-	6.87E-156	
Q9Y5T4	DJC15_HUMAN DnaJ homolog subfamily C member 15 OS=Homo sapiens GN=DNAJC15 PE=1 SV=2	3.13E-10	
Q9Y5T5;Q9Y5T5-2;Q9Y5T	UBP16_HUMAN Ubiquitin carboxyl-terminal hydrolase 16 OS=Homo sapiens GN=USP16 PE=1 SV=1;>sp|Q9Y5T5-2|UBP16_HUMAN Is	3.10E-06	
Q9Y5U9	IR3IP_HUMAN Immediate early response 3-interacting protein 1 OS=Homo sapiens GN=IER3IP1 PE=1 SV=1		
Q9Y5V0	ZN706_HUMAN Zinc finger protein 706 OS=Homo sapiens GN=ZNF706 PE=1 SV=1		57.1
Q9Y5V3-2;Q9Y5V3	2|MAGD1_HUMAN Isoform 2 of Melanoma-associated antigen D1 OS=Homo sapiens GN=MAGED1;>sp|Q9Y5V3|MAGD1_HUMAN Melar	5.23E-43	
Q9Y5X1	SNX9_HUMAN Sorting nexin-9 OS=Homo sapiens GN=SNX9 PE=1 SV=1	5.41E-20	
Q9Y5X2	SNX8_HUMAN Sorting nexin-8 OS=Homo sapiens GN=SNX8 PE=1 SV=1	5.74E-09	
Q9Y5X3	SNX5_HUMAN Sorting nexin-5 OS=Homo sapiens GN=SNX5 PE=1 SV=1	1.28E-34	
Q9Y5Y2	NUBP2_HUMAN Cytosolic Fe-S cluster assembly factor NUBP2 OS=Homo sapiens GN=NUBP2 PE=1 SV=1	2.60E-99	
Q9Y5Z4;Q9Y5Z4-2	HEBP2_HUMAN Heme-binding protein 2 OS=Homo sapiens GN=HEBP2 PE=1 SV=1;>sp|Q9Y5Z4-2|HEBP2_HUMAN Isoform 2 of Heme	2.96E-29	
Q9Y5Z7	HCFC2_HUMAN Host cell factor 2 OS=Homo sapiens GN=HCFC2 PE=1 SV=1		38.48
Q9Y605	MOFA1_HUMAN MORF4 family-associated protein 1 OS=Homo sapiens GN=MRFAP1 PE=1 SV=1		
Q9Y606	TRUA_HUMAN tRNA pseudouridine synthase A, mitochondrial OS=Homo sapiens GN=PUS1 PE=1 SV=3		
Q9Y606-2	TRUA_HUMAN Isoform 2 of tRNA pseudouridine synthase A, mitochondrial OS=Homo sapiens GN=PUS1		
Q9Y606;Q9Y606-2	TRUA_HUMAN tRNA pseudouridine synthase A, mitochondrial OS=Homo sapiens GN=PUS1 PE=1 SV=3;>sp|Q9Y606-2|TRUA_HUMAN	8.51E-49	
Q9Y608	LRRF2_HUMAN Leucine-rich repeat flightless-interacting protein 2 OS=Homo sapiens GN=LRRFIP2 PE=1 SV=1		224.62
Q9Y608-2	LRRF2_HUMAN Isoform 2 of Leucine-rich repeat flightless-interacting protein 2 OS=Homo sapiens GN=LRRFIP2		217.36
Q9Y608-3	LRRF2_HUMAN Isoform 3 of Leucine-rich repeat flightless-interacting protein 2 OS=Homo sapiens GN=LRRFIP2		109.5
Q9Y608-4	LRRF2_HUMAN Isoform 4 of Leucine-rich repeat flightless-interacting protein 2 OS=Homo sapiens GN=LRRFIP2		217.36
Q9Y613	FHOD1_HUMAN FH1/FH2 domain-containing protein 1 OS=Homo sapiens GN=FHOD1 PE=1 SV=3	5.35E-112	
Q9Y617	SERC_HUMAN Phosphoserine aminotransferase OS=Homo sapiens GN=PSAT1 PE=1 SV=2		190.45
Q9Y617-2	SERC_HUMAN Isoform 2 of Phosphoserine aminotransferase OS=Homo sapiens GN=PSAT1		148.49
Q9Y617;Q9Y617-2	SERC_HUMAN Phosphoserine aminotransferase OS=Homo sapiens GN=PSAT1 PE=1 SV=2;>sp|Q9Y617-2|SERC_HUMAN Isoform 2 of	3.52E-122	
Q9Y618	NCOR2_HUMAN Nuclear receptor corepressor 2 OS=Homo sapiens GN=NCOR2 PE=1 SV=2		69.76
Q9Y618-2	NCOR2_HUMAN Isoform 2 of Nuclear receptor corepressor 2 OS=Homo sapiens GN=NCOR2		2.79
Q9Y618-3	NCOR2_HUMAN Isoform 3 of Nuclear receptor corepressor 2 OS=Homo sapiens GN=NCOR2		69.76
Q9Y618-4	NCOR2_HUMAN Isoform 4 of Nuclear receptor corepressor 2 OS=Homo sapiens GN=NCOR2		69.76
Q9Y618-5	NCOR2_HUMAN Isoform 5 of Nuclear receptor corepressor 2 OS=Homo sapiens GN=NCOR2		69.76
Q9Y618;Q9Y618-3;Q9Y61	NCOR2_HUMAN Nuclear receptor corepressor 2 OS=Homo sapiens GN=NCOR2 PE=1 SV=2;>sp|Q9Y618-3|NCOR2_HUMAN Isoform 3	3.83E-13	
Q9Y619	ORNT1_HUMAN Mitochondrial ornithine transporter 1 OS=Homo sapiens GN=SLC25A15 PE=1 SV=1	1.58E-09	
Q9Y623	MYH4_HUMAN Myosin-4 OS=Homo sapiens GN=MYH4 PE=1 SV=2		93.11
Q9Y624	JAM1_HUMAN Junctional adhesion molecule A OS=Homo sapiens GN=F11R PE=1 SV=1		40.7
Q9Y625;B4E2M1	GPC6_HUMAN Glypican-6 OS=Homo sapiens GN=GPC6 PE=2 SV=1;>tr|B4E2M1|B4E2M1_HUMAN cDNA FLJ58478, highly similar to G	2.82E-10	
Q9Y639	NPTN_HUMAN Neuroplastin OS=Homo sapiens GN=NPTN PE=1 SV=2		160.76
Q9Y639-1	NPTN_HUMAN Isoform 1 of Neuroplastin OS=Homo sapiens GN=NPTN		160.76
Q9Y639-3	NPTN_HUMAN Isoform 3 of Neuroplastin OS=Homo sapiens GN=NPTN		118.57
Q9Y639-4	NPTN_HUMAN Isoform 4 of Neuroplastin OS=Homo sapiens GN=NPTN		118.57

Q9Y639-5	NPTN_HUMAN Isoform 5 of Neuroplastin OS=Homo sapiens GN=NPTN		118.57
Q9Y639;Q9Y639-1;Q9Y63	NPTN_HUMAN Neuroplastin OS=Homo sapiens GN=NPTN PE=1 SV=2;>sp|Q9Y639-1|NPTN_HUMAN Isoform 1 of Neuroplastin OS=Ho	5.62E-148	
Q9Y657	SPIN1_HUMAN Spindlin-1 OS=Homo sapiens GN=SPIN1 PE=1 SV=3		48.66
Q9Y673;Q9Y673-2	ALG5_HUMAN Dolichyl-phosphate beta-glucosyltransferase OS=Homo sapiens GN=ALG5 PE=1 SV=1;>sp|Q9Y673-2|ALG5_HUMAN Iso	3.23E-12	
Q9Y676	RT18B_HUMAN 28S ribosomal protein S18b, mitochondrial OS=Homo sapiens GN=MRPS18B PE=1 SV=1		173.61
Q9Y678	COPG_HUMAN Coatomer subunit gamma OS=Homo sapiens GN=COPG PE=1 SV=1		383.48
Q9Y679	AUP1_HUMAN Ancient ubiquitous protein 1 OS=Homo sapiens GN=AUP1 PE=1 SV=1		27.15
Q9Y679-2	AUP1_HUMAN Isoform Short of Ancient ubiquitous protein 1 OS=Homo sapiens GN=AUP1		27.15
Q9Y679-3	AUP1_HUMAN Isoform 3 of Ancient ubiquitous protein 1 OS=Homo sapiens GN=AUP1		27.15
Q9Y679;Q9Y679-2;Q9Y67	AUP1_HUMAN Ancient ubiquitous protein 1 OS=Homo sapiens GN=AUP1 PE=1 SV=1;>sp|Q9Y679-2|AUP1_HUMAN Isoform Short of A	6.30E-17	
Q9Y680;Q4ZG70;Q9Y680-	FKBP7_HUMAN Peptidyl-prolyl cis-trans isomerase FKBP7 OS=Homo sapiens GN=FKBP7 PE=1 SV=1;>tr|Q4ZG70|Q4ZG70_HUMAN Pu	4.20E-08	
Q9Y696	CLIC4_HUMAN Chloride intracellular channel protein 4 OS=Homo sapiens GN=CLIC4 PE=1 SV=4		980.15
Q9Y697	NFS1_HUMAN Cysteine desulfurase, mitochondrial OS=Homo sapiens GN=NFS1 PE=1 SV=3		
Q9Y697-2	NFS1_HUMAN Isoform Cytoplasmic of Cysteine desulfurase, mitochondrial OS=Homo sapiens GN=NFS1		
Q9Y697;Q9Y697-2	NFS1_HUMAN Cysteine desulfurase, mitochondrial OS=Homo sapiens GN=NFS1 PE=1 SV=3;>sp|Q9Y697-2|NFS1_HUMAN Isoform Cy	1.40E-12	
Q9Y6A4	CP080_HUMAN UPF0468 protein C16orf80 OS=Homo sapiens GN=C16orf80 PE=1 SV=1		235.1
Q9Y6A5	TACC3_HUMAN Transforming acidic coiled-coil-containing protein 3 OS=Homo sapiens GN=TACC3 PE=1 SV=1	1.17E-16	
Q9Y6B6	SAR1B_HUMAN GTP-binding protein SAR1b OS=Homo sapiens GN=SAR1B PE=1 SV=1		149.51
Q9Y6B7	AP4B1_HUMAN AP-4 complex subunit beta-1 OS=Homo sapiens GN=AP4B1 PE=2 SV=2	4.44E-47	
Q9Y6C2	EMIL1_HUMAN EMILIN-1 OS=Homo sapiens GN=EMILIN1 PE=1 SV=2		27.58
Q9Y6C9	MTCH2_HUMAN Mitochondrial carrier homolog 2 OS=Homo sapiens GN=MTCH2 PE=1 SV=1		136.37
Q9Y6D0	SELK_HUMAN Selenoprotein K OS=Homo sapiens GN=SELK PE=1 SV=3		67.43
Q9Y6D5	BIG2_HUMAN Brefeldin A-inhibited guanine nucleotide-exchange protein 2 OS=Homo sapiens GN=ARFGEF2 PE=1 SV=3	1.38E-16	
Q9Y6D6	BIG1_HUMAN Brefeldin A-inhibited guanine nucleotide-exchange protein 1 OS=Homo sapiens GN=ARFGEF1 PE=1 SV=2		
Q9Y6D9	MD1L1_HUMAN Mitotic spindle assembly checkpoint protein MAD1 OS=Homo sapiens GN=MAD1L1 PE=1 SV=2		47.2
Q9Y6E0	STK24_HUMAN Serine/threonine-protein kinase 24 OS=Homo sapiens GN=STK24 PE=1 SV=1		93.67
Q9Y6E0-2	STK24_HUMAN Isoform A of Serine/threonine-protein kinase 24 OS=Homo sapiens GN=STK24		93.67
Q9Y6E0;Q9Y6E0-2	STK24_HUMAN Serine/threonine-protein kinase 24 OS=Homo sapiens GN=STK24 PE=1 SV=1;>sp|Q9Y6E0-2|STK24_HUMAN Isoform	2.70E-22	
Q9Y6E2	BZW2_HUMAN Basic leucine zipper and W2 domain-containing protein 2 OS=Homo sapiens GN=BZW2 PE=1 SV=1		94
Q9Y6F6	MRVI1_HUMAN Protein MRVI1 OS=Homo sapiens GN=MRVI1 PE=1 SV=2		
Q9Y6F6-2	MRVI1_HUMAN Isoform 2 of Protein MRVI1 OS=Homo sapiens GN=MRVI1		
Q9Y6F6-3	MRVI1_HUMAN Isoform 3 of Protein MRVI1 OS=Homo sapiens GN=MRVI1		
Q9Y6F6-4	MRVI1_HUMAN Isoform 4 of Protein MRVI1 OS=Homo sapiens GN=MRVI1		
Q9Y6F6-5	MRVI1_HUMAN Isoform 5 of Protein MRVI1 OS=Homo sapiens GN=MRVI1		
Q9Y6F6-6	MRVI1_HUMAN Isoform 6 of Protein MRVI1 OS=Homo sapiens GN=MRVI1		
Q9Y6F7	CDY2_HUMAN Testis-specific chromodomain protein Y 2 OS=Homo sapiens GN=CDY2A PE=1 SV=1	8.68E-16	
Q9Y6G3	RM42_HUMAN 39S ribosomal protein L42, mitochondrial OS=Homo sapiens GN=MRPL42 PE=1 SV=1	4.26E-34	
Q9Y6G9	DC1L1_HUMAN Cytoplasmic dynein 1 light intermediate chain 1 OS=Homo sapiens GN=DYNC1LI1 PE=1 SV=3		76.93
Q9Y6H1	CHCH2_HUMAN Coiled-coil-helix-coiled-coil-helix domain-containing protein 2, mitochondrial OS=Homo sapiens GN=CHCHD2 PE=1 SV=1		40.68
Q9Y6H3	ATP23_HUMAN Mitochondrial inner membrane protease ATP23 homolog OS=Homo sapiens GN=XRCC6BP1 PE=1 SV=3		
Q9Y6I3	EPN1_HUMAN Epsin-1 OS=Homo sapiens GN=EPN1 PE=1 SV=2		210.23
Q9Y6I3-1	EPN1_HUMAN Isoform 2 of Epsin-1 OS=Homo sapiens GN=EPN1		210.23
Q9Y6I3-1;Q9Y6I3;Q9Y6I3	1|EPN1_HUMAN Isoform 2 of Epsin-1 OS=Homo sapiens GN=EPN1;>sp|Q9Y6I3|EPN1_HUMAN Epsin-1 OS=Homo sapiens GN=EPN1 P	1.43E-07	
Q9Y6I3-3	EPN1_HUMAN Isoform 3 of Epsin-1 OS=Homo sapiens GN=EPN1		210.23
Q9Y6I9	TX264_HUMAN Testis-expressed sequence 264 protein OS=Homo sapiens GN=TEX264 PE=1 SV=1		
Q9Y6J9	TAF6L_HUMAN TAF6-like RNA polymerase II p300/CBP-associated factor-associated factor 65 kDa subunit 6L OS=Homo sapiens GN=TAF6L PE=1 SV		17.29
Q9Y6K1	DNM3A_HUMAN DNA (cytosine-5)-methyltransferase 3A OS=Homo sapiens GN=DNMT3A PE=1 SV=4		
Q9Y6K1-2	DNM3A_HUMAN Isoform 2 of DNA (cytosine-5)-methyltransferase 3A OS=Homo sapiens GN=DNMT3A		
Q9Y6K1-3	DNM3A_HUMAN Isoform 3 of DNA (cytosine-5)-methyltransferase 3A OS=Homo sapiens GN=DNMT3A		
Q9Y6K5	OAS3_HUMAN 2'-5'-oligoadenylate synthase 3 OS=Homo sapiens GN=OAS3 PE=1 SV=3		30.41
Q9Y6K8;Q9Y6K8-3;Q9Y6K	KAD5_HUMAN Adenylate kinase isoenzyme 5 OS=Homo sapiens GN=AK5 PE=1 SV=2;>sp|Q9Y6K8-3|KAD5_HUMAN Isoform 3 of Ader	5.05E-10	
Q9Y6M1	IF2B2_HUMAN Insulin-like growth factor 2 mRNA-binding protein 2 OS=Homo sapiens GN=IGF2BP2 PE=1 SV=2		339.5
Q9Y6M1-1	IF2B2_HUMAN Isoform 2 of Insulin-like growth factor 2 mRNA-binding protein 2 OS=Homo sapiens GN=IGF2BP2		339.5
Q9Y6M1-3	IF2B2_HUMAN Isoform 3 of Insulin-like growth factor 2 mRNA-binding protein 2 OS=Homo sapiens GN=IGF2BP2		272.58
Q9Y6M1-4	IF2B2_HUMAN Isoform 4 of Insulin-like growth factor 2 mRNA-binding protein 2 OS=Homo sapiens GN=IGF2BP2		272.58
Q9Y6M1-5	IF2B2_HUMAN Isoform 5 of Insulin-like growth factor 2 mRNA-binding protein 2 OS=Homo sapiens GN=IGF2BP2		272.58
Q9Y6M1;Q9Y6M1-1;Q9Y6I	IF2B2_HUMAN Insulin-like growth factor 2 mRNA-binding protein 2 OS=Homo sapiens GN=IGF2BP2 PE=1 SV=2;>sp|Q9Y6M1-1|IF2B2	2.79E-19	
Q9Y6M9	NDUB9_HUMAN NADH dehydrogenase [ubiquinone] 1 beta subcomplex subunit 9 OS=Homo sapiens GN=NDUFB9 PE=1 SV=3		44.58
Q9Y6N5	SQRD_HUMAN Sulfide:quinone oxidoreductase, mitochondrial OS=Homo sapiens GN=SQRDL PE=1 SV=1		159.19
Q9Y6Q5	AP1M2_HUMAN AP-1 complex subunit mu-2 OS=Homo sapiens GN=AP1M2 PE=1 SV=4		
Q9Y6Q5-2	AP1M2_HUMAN Isoform 2 of AP-1 complex subunit mu-2 OS=Homo sapiens GN=AP1M2		

Q9Y6Q5-2;Q9Y6Q5	2|AP1M2_HUMAN Isoform 2 of AP-1 complex subunit mu-2 OS=Homo sapiens GN=AP1M2;>sp|Q9Y6Q5|AP1M2_HUMAN AP-1 complex	1.46E-118	
Q9Y6R4	M3K4_HUMAN Mitogen-activated protein kinase kinase kinase 4 OS=Homo sapiens GN=MAP3K4 PE=1 SV=2		
Q9Y6R4-2	M3K4_HUMAN Isoform 2 of Mitogen-activated protein kinase kinase kinase 4 OS=Homo sapiens GN=MAP3K4		
Q9Y6U3	ADSV_HUMAN Adseverin OS=Homo sapiens GN=SCIN PE=1 SV=4		
Q9Y6U3-3	ADSV_HUMAN Isoform 3 of Adseverin OS=Homo sapiens GN=SCIN		
Q9Y6U3;Q9Y6U3-2;Q9Y6L	ADSV_HUMAN Adseverin OS=Homo sapiens GN=SCIN PE=1 SV=4;>sp|Q9Y6U3-2|ADSV_HUMAN Isoform 2 of Adseverin OS=Homo sa	5.06E-27	
Q9Y6V0	PCLO_HUMAN Protein piccolo OS=Homo sapiens GN=PCLO PE=1 SV=4		61.32
Q9Y6V0-2	PCLO_HUMAN Isoform 2 of Protein piccolo OS=Homo sapiens GN=PCLO		57.64
Q9Y6V0-4	PCLO_HUMAN Isoform 4 of Protein piccolo OS=Homo sapiens GN=PCLO		6.49
Q9Y6V0-5	PCLO_HUMAN Isoform 5 of Protein piccolo OS=Homo sapiens GN=PCLO		61.32
Q9Y6V0-6	PCLO_HUMAN Isoform 6 of Protein piccolo OS=Homo sapiens GN=PCLO		57.64
Q9Y6V7	DDX49_HUMAN Probable ATP-dependent RNA helicase DDX49 OS=Homo sapiens GN=DDX49 PE=1 SV=1		
Q9Y6W5	WASF2_HUMAN Wiskott-Aldrich syndrome protein family member 2 OS=Homo sapiens GN=WASF2 PE=1 SV=3	1.79E-29	
Q9Y6X0	SETBP_HUMAN SET-binding protein OS=Homo sapiens GN=SETBP1 PE=1 SV=3		
Q9Y6X0-2	SETBP_HUMAN Isoform 2 of SET-binding protein OS=Homo sapiens GN=SETBP1		
Q9Y6X3	SCC4_HUMAN MAU2 chromatid cohesion factor homolog OS=Homo sapiens GN=MAU2 PE=1 SV=2		
Q9Y6X3-2	SCC4_HUMAN Isoform 2 of MAU2 chromatid cohesion factor homolog OS=Homo sapiens GN=MAU2		
Q9Y6X3-3	SCC4_HUMAN Isoform 3 of MAU2 chromatid cohesion factor homolog OS=Homo sapiens GN=MAU2		
Q9Y6X9	MORC2_HUMAN MORC family CW-type zinc finger protein 2 OS=Homo sapiens GN=MORC2 PE=1 SV=2		
Q9Y6Y0	NS1BP_HUMAN Influenza virus NS1A-binding protein OS=Homo sapiens GN=IVNS1ABP PE=1 SV=3		
Q9Y6Y8;Q9Y6Y8-2	S23IP_HUMAN SEC23-interacting protein OS=Homo sapiens GN=SEC23IP PE=1 SV=1;>sp|Q9Y6Y8-2|S23IP_HUMAN Isoform 2 of SE(	1.89E-08	
Q9Y6Z7	COL10_HUMAN Collectin-10 OS=Homo sapiens GN=COLEC10 PE=2 SV=2		
A0AVT1;B3KSS1;A0AVT1-	UBA6_HUMAN Ubiquitin-like modifier-activating enzyme 6 OS=Homo sapiens GN=UBA6 PE=1 SV=1;>tr|B3KSS1|B3KSS1_HUMAN cDN	0	
A1L0T0;Q59GP4;A1L0T0-:	ILVBL_HUMAN Acetolactate synthase-like protein OS=Homo sapiens GN=ILVBL PE=1 SV=2;>tr|Q59GP4|Q59GP4_HUMAN IlvB (Bacter	6.20E-17	
A6NCE7;Q658J6;Q9GZQ8	MP3B2_HUMAN Microtubule-associated proteins 1A/1B light chain 3 beta 2 OS=Homo sapiens GN=MAP1LC3B2 PE=2 SV=1;>tr|Q658J	1.58E-05	
A6NCE7;Q9GZQ8	MP3B2_HUMAN Microtubule-associated proteins 1A/1B light chain 3 beta 2 OS=Homo sapiens GN=MAP1LC3B2 PE=2 SV=1;>sp|Q9GZ	3.81E-07	
A6NCE7;Q9GZQ8;Q9H492	MP3B2_HUMAN Microtubule-associated proteins 1A/1B light chain 3 beta 2 OS=Homo sapiens GN=MAP1LC3B2 PE=2 SV=1;>sp|Q9GZ	4.14E-09	
A6NDU8;C9JZ41	CE051_HUMAN UPF0600 protein C5orf51 OS=Homo sapiens GN=C5orf51 PE=1 SV=1;>tr|C9JZ41|C9JZ41_HUMAN Uncharacterized pr	1.22E-08	
A6NI72;P14598;A8MVU1;I	NCF1B_HUMAN Putative neutrophil cytosol factor 1B OS=Homo sapiens GN=NCF1B PE=5 SV=2;>sp|P14598|NCF1_HUMAN Neutrophil	7.98E-06	
A6NKT7;Q7Z3J3	RGPD3_HUMAN RanBP2-like and GRIP domain-containing protein 3 OS=Homo sapiens GN=RGPD3 PE=2 SV=2;>sp|Q7Z3J3|RGPD4_H	5.57E-98	
A6NKT7;Q7Z3J3;O14715;	RGPD3_HUMAN RanBP2-like and GRIP domain-containing protein 3 OS=Homo sapiens GN=RGPD3 PE=2 SV=2;>sp|Q7Z3J3|RGPD4_H	1.02E-89	
B9A064;P0CG04	IGLL5_HUMAN Immunoglobulin lambda-like polypeptide 5 OS=Homo sapiens GN=IGLL5 PE=2 SV=2;>sp|P0CG04|LAC1_HUMAN Ig lar	0	
O00116;B7Z3Q4	ADAS_HUMAN Alkyldihydroxyacetonephosphate synthase, peroxisomal OS=Homo sapiens GN=AGPS PE=1 SV=1;>tr|B7Z3Q4|B7Z3Q4	1.50E-95	
O00139-4;O00139;O0013	4|KIF2A_HUMAN Isoform 4 of Kinesin-like protein KIF2A OS=Homo sapiens GN=KIF2A;>sp|O00139|KIF2A_HUMAN Kinesin-like protei	4.10E-10	
O00154-4;O00154;O0015	4|BACH_HUMAN Isoform 4 of Cytosolic acyl coenzyme A thioester hydrolase OS=Homo sapiens GN=ACOT7;>sp|O00154|BACH_HUMA	2.07E-98	
O00159;O00159-3;O0015	MYO1C_HUMAN Myosin-Ic OS=Homo sapiens GN=MYO1C PE=1 SV=4;>sp|O00159-3|MYO1C_HUMAN Isoform 3 of Myosin-Ic OS=Hon	2.97E-07	
O00178;B2RDW9;B0QY59	GTPB1_HUMAN GTP-binding protein 1 OS=Homo sapiens GN=GTPBP1 PE=1 SV=3;>tr|B2RDW9|B2RDW9_HUMAN cDNA, FLJ96803, hi	9.14E-26	
O00193;E3W975	SMAP_HUMAN Small acidic protein OS=Homo sapiens GN=SMAP PE=1 SV=1;>tr|E3W975|E3W975_HUMAN Uncharacterized protein C	6.12E-74	
O00217;B4DYI3;Q53G17	NDUS8_HUMAN NADH dehydrogenase [ubiquinone] iron-sulfur protein 8, mitochondrial OS=Homo sapiens GN=NDUFS8 PE=1 SV=1;>	1.32E-12	
O00232;A6NP15	PSD12_HUMAN 26S proteasome non-ATPase regulatory subunit 12 OS=Homo sapiens GN=PSMD12 PE=1 SV=3;>tr|A6NP15|A6NP15_	2.21E-235	
O00233;O00233-2;C9JR2!	PSMD9_HUMAN 26S proteasome non-ATPase regulatory subunit 9 OS=Homo sapiens GN=PSMD9 PE=1 SV=3;>sp|O00233-2|PSMD9_	8.99E-29	
O00264;Q6IB11;B7Z1L3	PGRC1_HUMAN Membrane-associated progesterone receptor component 1 OS=Homo sapiens GN=PGRMC1 PE=1 SV=3;>tr|Q6IB11|Q	1.87E-45	
O00273;Q53HN4;Q5T6G6	DFFA_HUMAN DNA fragmentation factor subunit alpha OS=Homo sapiens GN=DFFA PE=1 SV=1;>tr|Q53HN4|Q53HN4_HUMAN DNAat	4.19E-22	
O00299;Q53FB0;Q5SRT3	CLIC1_HUMAN Chloride intracellular channel protein 1 OS=Homo sapiens GN=CLIC1 PE=1 SV=4;>tr|Q53FB0|Q53FB0_HUMAN Chloric	1.02E-202	
O00391;O00391-2	QSOX1_HUMAN Sulfhydryl oxidase 1 OS=Homo sapiens GN=QSOX1 PE=1 SV=3;>sp|O00391-2|QSOX1_HUMAN Isoform 2 of Sulfhydı	3.49E-10	
O00410-3;O00410;O0041	3|IPO5_HUMAN Isoform 3 of Importin-5 OS=Homo sapiens GN=IPO5;>sp|O00410|IPO5_HUMAN Importin-5 OS=Homo sapiens GN=I	1.00E-249	
O00442-2;O00442	2|RTC1_HUMAN Isoform 2 of RNA 3-terminal phosphate cyclase OS=Homo sapiens GN=RTCD1;>sp|O00442|RTC1_HUMAN RNA 3-terr	4.57E-08	
O00469-2;O00469;B4DHC	2|PLOD2_HUMAN Isoform 2 of Procollagen-lysine,2-oxoglutarate 5-dioxygenase 2 OS=Homo sapiens GN=PLOD2;>sp|O00469|PLOD2_	9.22E-30	
O00487;Q59G89;Q53TH1;	PSDE_HUMAN 26S proteasome non-ATPase regulatory subunit 14 OS=Homo sapiens GN=PSMD14 PE=1 SV=1;>tr|Q59G89|Q59G89_I	6.50E-42	
O00505;Q5JVM9;Q53F09;	IMA3_HUMAN Importin subunit alpha-3 OS=Homo sapiens GN=KPNA3 PE=1 SV=2;>tr|Q5JVM9|Q5JVM9_HUMAN Importin alpha-3 sul	2.21E-76	
O00506;Q9P289-2	STK25_HUMAN Serine/threonine-protein kinase 25 OS=Homo sapiens GN=STK25 PE=1 SV=1;>sp|Q9P289-2|MST4_HUMAN Isoform 2	2.09E-75	
O00507;O00507-2;Q9300	USP9Y_HUMAN Probable ubiquitin carboxyl-terminal hydrolase FAF-Y OS=Homo sapiens GN=USP9Y PE=1 SV=2;>sp|O00507-2|USP9Y	7.98E-17	
O00560;O00560-3	SDCB1_HUMAN Syntenin-1 OS=Homo sapiens GN=SDCBP PE=1 SV=1;>sp|O00560-3|SDCB1_HUMAN Isoform 3 of Syntenin-1 OS=H	6.90E-23	
O00571;O15523	DDX3X_HUMAN ATP-dependent RNA helicase DDX3X OS=Homo sapiens GN=DDX3X PE=1 SV=3;>sp|O15523|DDX3Y_HUMAN ATP-de	0	
O00622;Q6FI18;B4DI61;C	CYR61_HUMAN Protein CYR61 OS=Homo sapiens GN=CYR61 PE=1 SV=1;>tr|Q6FI18|Q6FI18_HUMAN CYR61 protein OS=Homo sapie	5.75E-56	
O00625;Q6FHD2	PIR_HUMAN Pirin OS=Homo sapiens GN=PIR PE=1 SV=1;>tr|Q6FHD2|Q6FHD2_HUMAN PIR protein (Fragment) OS=Homo sapiens GN	1.55E-14	
O00764;O00764-3	PDXK_HUMAN Pyridoxal kinase OS=Homo sapiens GN=PDXK PE=1 SV=1;>sp|O00764-3|PDXK_HUMAN Isoform 3 of Pyridoxal kinase	1.54E-14	
O00764;O00764-3;O0076	PDXK_HUMAN Pyridoxal kinase OS=Homo sapiens GN=PDXK PE=1 SV=1;>sp|O00764-3|PDXK_HUMAN Isoform 3 of Pyridoxal kinase	2.85E-89	
O14493;P56747;O95484	CLD4_HUMAN Claudin-4 OS=Homo sapiens GN=CLDN4 PE=1 SV=1;>sp|P56747|CLD6_HUMAN Claudin-6 OS=Homo sapiens GN=CLD	2.19E-132	
O14548;Q6FGA0;A8MZH9	COX7R_HUMAN Cytochrome c oxidase subunit 7A-related protein, mitochondrial OS=Homo sapiens GN=COX7A2L PE=1 SV=2;>tr|Q6I	2.28E-09	

O14579;Q53HJ6;Q9UGP6;	COPE_HUMAN Coatomer subunit epsilon OS=Homo sapiens GN=COPE PE=1 SV=3;>tr|Q53HJ6|Q53HJ6_HUMAN Epsilon subunit of coa	1.82E-69
O14602;P47813	IF1AY_HUMAN Eukaryotic translation initiation factor 1A, Y-chromosomal OS=Homo sapiens GN=EIF1AY PE=1 SV=4;>sp|P47813|IF1A	2.13E-55
O14617-5;O14617;O1461	5|AP3D1_HUMAN Isoform 5 of AP-3 complex subunit delta-1 OS=Homo sapiens GN=AP3D1;>sp|O14617|AP3D1_HUMAN AP-3 comple	2.29E-19
O14618;Q8NEV0	CCS_HUMAN Copper chaperone for superoxide dismutase OS=Homo sapiens GN=CCS PE=1 SV=1;>tr|Q8NEV0|Q8NEV0_HUMAN Supe	3.84E-11
O14641;Q92997	DVL2_HUMAN Segment polarity protein dishevelled homolog DVL-2 OS=Homo sapiens GN=DVL2 PE=1 SV=1;>sp|Q92997|DVL3_HUM	4.68E-13
O14672;A0AV88;A8MY20	ADA10_HUMAN Disintegrin and metalloproteinase domain-containing protein 10 OS=Homo sapiens GN=ADAM10 PE=1 SV=1;>tr|A0AV	8.67E-57
O14734;Q6FHI2;A6NDT2;	ACOT8_HUMAN Acyl-coenzyme A thioesterase 8 OS=Homo sapiens GN=ACOT8 PE=1 SV=1;>tr|Q6FHI2|Q6FHI2_HUMAN PTE1 protein	3.79E-13
O14737;B4DE64	PDCD5_HUMAN Programmed cell death protein 5 OS=Homo sapiens GN=PDCD5 PE=1 SV=3;>tr|B4DE64|B4DE64_HUMAN cDNA FLJ5	2.28E-46
O14745;B2R6A3;B3KSV9;	NHRF1_HUMAN Na(+)/H(+) exchange regulatory cofactor NHE-RF1 OS=Homo sapiens GN=SLC9A3R1 PE=1 SV=4;>tr|B2R6A3|B2R6A	4.26E-78
O14818;O14818-2;Q8TAA	PSA7_HUMAN Proteasome subunit alpha type-7 OS=Homo sapiens GN=PSMA7 PE=1 SV=1;>sp|O14818-2|PSA7_HUMAN Isoform 2 of	0
O14818;Q05DH1;O14818-	PSA7_HUMAN Proteasome subunit alpha type-7 OS=Homo sapiens GN=PSMA7 PE=1 SV=1;>tr|Q05DH1|Q05DH1_HUMAN Proteasome	6.86E-233
O14828;Q6FHJ5;O14828-	SCAM3_HUMAN Secretory carrier-associated membrane protein 3 OS=Homo sapiens GN=SCAMP3 PE=1 SV=3;>tr|Q6FHJ5|Q6FHJ5_H	1.45E-08
O14879;Q5T765;B4DLS7	IFIT3_HUMAN Interferon-induced protein with tetratricopeptide repeats 3 OS=Homo sapiens GN=IFIT3 PE=1 SV=1;>tr|Q5T765|Q5T7	0
O14920;O14920-2	IKKB_HUMAN Inhibitor of nuclear factor kappa-B kinase subunit beta OS=Homo sapiens GN=IKBKB PE=1 SV=1;>sp|O14920-2|IKKB_	9.45E-07
O14929;Q6P594;Q8WWB	HAT1_HUMAN Histone acetyltransferase type B catalytic subunit OS=Homo sapiens GN=HAT1 PE=1 SV=1;>tr|Q6P594|Q6P594_HUMA	3.27E-201
O14949;B7Z4A1	QCR8_HUMAN Cytochrome b-c1 complex subunit 8 OS=Homo sapiens GN=UQCRQ PE=1 SV=4;>tr|B7Z4A1|B7Z4A1_HUMAN cDNA FL	4.39E-24
O14950;P19105	ML12B_HUMAN Myosin regulatory light chain 12B OS=Homo sapiens GN=MYL12B PE=1 SV=2;>sp|P19105|ML12A_HUMAN Myosin reg	0
O14950;P19105;P24844	ML12B_HUMAN Myosin regulatory light chain 12B OS=Homo sapiens GN=MYL12B PE=1 SV=2;>sp|P19105|ML12A_HUMAN Myosin reg	4.65E-286
O14964;O14964-2;B4E1E	HGS_HUMAN Hepatocyte growth factor-regulated tyrosine kinase substrate OS=Homo sapiens GN=HGS PE=1 SV=1;>sp|O14964-2|H	4.81E-19
O14974;O14974-4;O1497	MYPT1_HUMAN Protein phosphatase 1 regulatory subunit 12A OS=Homo sapiens GN=PPP1R12A PE=1 SV=1;>sp|O14974-4|MYPT1_H	0
O15031;A6QRH2;Q3LIB1;	PLXB2_HUMAN Plexin-B2 OS=Homo sapiens GN=PLXNB2 PE=1 SV=3;>tr|A6QRH2|A6QRH2_HUMAN Plexin B2 OS=Homo sapiens GN=	7.27E-49
O15144;Q53R19;Q9BXV5	ARPC2_HUMAN Actin-related protein 2/3 complex subunit 2 OS=Homo sapiens GN=ARPC2 PE=1 SV=1;>tr|Q53R19|Q53R19_HUMAN	1.08E-136
O15155;Q53XK0;Q68DU7	BET1_HUMAN BET1 homolog OS=Homo sapiens GN=BET1 PE=1 SV=1;>tr|Q53XK0|Q53XK0_HUMAN BET1 homolog (S. cerevisiae) OS	9.48E-77
O15160;O15160-2;Q96HT	RPAC1_HUMAN DNA-directed RNA polymerases I and III subunit RPAC1 OS=Homo sapiens GN=POLR1C PE=1 SV=1;>sp|O15160-2|R	3.73E-101
O15162;C9J7K9;B4DTE8;	PLS1_HUMAN Phospholipid scramblase 1 OS=Homo sapiens GN=PLSCR1 PE=1 SV=1;>tr|C9J7K9|C9J7K9_HUMAN Phospholipid scram	6.83E-35
O15212;Q5STK2	PFD6_HUMAN Prefoldin subunit 6 OS=Homo sapiens GN=PFDN6 PE=1 SV=1;>tr|Q5STK2|Q5STK2_HUMAN Prefoldin subunit 6 OS=Ho	4.00E-11
O15231-3;O15231;B8K2M	3|ZN185_HUMAN Isoform 3 of Zinc finger protein 185 OS=Homo sapiens GN=ZNF185;>sp|O15231|ZN185_HUMAN Zinc finger protein	2.38E-55
O15231-3;O15231;O1523	3|ZN185_HUMAN Isoform 3 of Zinc finger protein 185 OS=Homo sapiens GN=ZNF185;>sp|O15231|ZN185_HUMAN Zinc finger protein	2.41E-54
O15247;A6NN85;A6PVS0;	CLIC2_HUMAN Chloride intracellular channel protein 2 OS=Homo sapiens GN=CLIC2 PE=1 SV=3;>tr|A6NN85|A6NN85_HUMAN Uncha	4.60E-15
O15260;O15260-2	SURF4_HUMAN Surfeit locus protein 4 OS=Homo sapiens GN=SURF4 PE=1 SV=3;>sp|O15260-2|SURF4_HUMAN Isoform 2 of Surfeit	4.78E-28
O15269;Q6NUL7;Q59EQ4	SPTC1_HUMAN Serine palmitoyltransferase 1 OS=Homo sapiens GN=SPTLC1 PE=1 SV=1;>tr|Q6NUL7|Q6NUL7_HUMAN SPTLC1 prote	1.39E-53
O15327;D6RJC3;B7Z6T2;	INP4B_HUMAN Type II inositol-3,4-bisphosphate 4-phosphatase OS=Homo sapiens GN=INPP4B PE=2 SV=4;>tr|D6RJC3|D6RJC3_HUN	8.05E-19
O15347;Q7Z684	HMGB3_HUMAN High mobility group protein B3 OS=Homo sapiens GN=HMGB3 PE=1 SV=4;>tr|Q7Z684|Q7Z684_HUMAN Putative unc	2.34E-27
O15382;Q53EW7;B3KSI3;	BCAT2_HUMAN Branched-chain-amino-acid aminotransferase, mitochondrial OS=Homo sapiens GN=BCAT2 PE=1 SV=2;>tr|Q53EW7|	1.66E-67
O15427;Q53G91	MOT4_HUMAN Monocarboxylate transporter 4 OS=Homo sapiens GN=SLC16A3 PE=1 SV=1;>tr|Q53G91|Q53G91_HUMAN Solute carri	8.78E-14
O15446-2;O15446	2|RPA34_HUMAN Isoform 2 of DNA-directed RNA polymerase I subunit RPA34 OS=Homo sapiens GN=CD3EAP;>sp|O15446|RPA34_HU	1.14E-08
O15511;O15511-2	ARPC5_HUMAN Actin-related protein 2/3 complex subunit 5 OS=Homo sapiens GN=ARPC5 PE=1 SV=3;>sp|O15511-2|ARPC5_HUMAN	1.87E-14
O15533-3;O15533;O1553	3|TPSN_HUMAN Isoform 3 of Tapasin OS=Homo sapiens GN=TAPBP;>sp|O15533|TPSN_HUMAN Tapasin OS=Homo sapiens GN=TAPBI	1.40E-15
O15540;Q9H047	FABP7_HUMAN Fatty acid-binding protein, brain OS=Homo sapiens GN=FABP7 PE=1 SV=3;>tr|Q9H047|Q9H047_HUMAN Fatty acid bi	1.03E-152
O15551;Q75L79	CLD3_HUMAN Claudin-3 OS=Homo sapiens GN=CLDN3 PE=1 SV=1;>tr|Q75L79|Q75L79_HUMAN Claudin 3 OS=Homo sapiens GN=CL	4.71E-11
O43169;Q5HYD9	CYB5B_HUMAN Cytochrome b5 type B OS=Homo sapiens GN=CYB5B PE=1 SV=2;>tr|Q5HYD9|Q5HYD9_HUMAN Putative uncharacteri	1.07E-14
O43175;B3KSC3;Q5SZU1;	SERA_HUMAN D-3-phosphoglycerate dehydrogenase OS=Homo sapiens GN=PHGDH PE=1 SV=4;>tr|B3KSC3|B3KSC3_HUMAN cDNA	1.27E-164
O43237;B4DZP4	DC1L2_HUMAN Cytoplasmic dynein 1 light intermediate chain 2 OS=Homo sapiens GN=DYNC1LI2 PE=1 SV=1;>tr|B4DZP4|B4DZP4_H	1.19E-213
O43242;Q6IBN0;Q8N9M2;	PSMD3_HUMAN 26S proteasome non-ATPase regulatory subunit 3 OS=Homo sapiens GN=PSMD3 PE=1 SV=2;>tr|Q6IBN0|Q6IBN0_HU	1.20E-179
O43252;Q6IAX6;Q05BW9	PAPS1_HUMAN Bifunctional 3-phosphoadenosine 5-phosphosulfate synthase 1 OS=Homo sapiens GN=PAPSS1 PE=1 SV=2;>tr|Q6IAX	4.44E-52
O43290;B4DDH9	SNUT1_HUMAN U4/U6.U5 tri-snRNP-associated protein 1 OS=Homo sapiens GN=SART1 PE=1 SV=1;>tr|B4DDH9|B4DDH9_HUMAN cD	1.40E-53
O43301;B7Z2F7;B7Z2M8	HS12A_HUMAN Heat shock 70 kDa protein 12A OS=Homo sapiens GN=HSPA12A PE=1 SV=2;>tr|B7Z2F7|B7Z2F7_HUMAN cDNA FLJ5	1.08E-13
O43324;C9J1V9;D6RBD7;	MCA3_HUMAN Eukaryotic translation elongation factor 1 epsilon-1 OS=Homo sapiens GN=EEF1E1 PE=1 SV=1;>tr|C9J1V9|C9J1V9_HU	8.25E-56
O43399-3;O43399;O4339	3|TPD54_HUMAN Isoform 3 of Tumor protein D54 OS=Homo sapiens GN=TPD52L2;>sp|O43399|TPD54_HUMAN Tumor protein D54 O	1.41E-14
O43402;Q53Y03;C9JB21	CX4NB_HUMAN Neighbor of COX4 OS=Homo sapiens GN=COX4NB PE=1 SV=1;>tr|Q53Y03|Q53Y03_HUMAN COX4 neighbor, isoform	4.71E-09
O43414;O43414-2;O4341	ERI3_HUMAN ERI1 exoribonuclease 3 OS=Homo sapiens GN=ERI3 PE=1 SV=2;>sp|O43414-2|ERI3_HUMAN Isoform 2 of ERI1 exorib	6.61E-08
O43427;O43427-2;Q53F5	FIBP_HUMAN Acidic fibroblast growth factor intracellular-binding protein OS=Homo sapiens GN=FIBP PE=1 SV=3;>sp|O43427-2|FIBP	1.56E-07
O43447;Q6FH36;C9JQD4;	PPIH_HUMAN Peptidyl-prolyl cis-trans isomerase H OS=Homo sapiens GN=PPIH PE=1 SV=1;>tr|Q6FH36|Q6FH36_HUMAN Peptidyl-pro	1.06E-15
O43464;O43464-3;O4346	HTRA2_HUMAN Serine protease HTRA2, mitochondrial OS=Homo sapiens GN=HTRA2 PE=1 SV=2;>sp|O43464-3|HTRA2_HUMAN Isofo	9.55E-10
O43464;O43464-3;O4346	HTRA2_HUMAN Serine protease HTRA2, mitochondrial OS=Homo sapiens GN=HTRA2 PE=1 SV=2;>sp|O43464-3|HTRA2_HUMAN Isofo	5.87E-42
O43488;C9JSL3	ARK72_HUMAN Aflatoxin B1 aldehyde reductase member 2 OS=Homo sapiens GN=AKR7A2 PE=1 SV=3;>tr|C9JSL3|C9JSL3_HUMAN U	1.04E-68
O43488;Q8NHP1	ARK72_HUMAN Aflatoxin B1 aldehyde reductase member 2 OS=Homo sapiens GN=AKR7A2 PE=1 SV=3;>sp|Q8NHP1|ARK74_HUMAN	1.75E-66
O43491;Q68DV2;Q59FD8;	E41L2_HUMAN Band 4.1-like protein 2 OS=Homo sapiens GN=EPB41L2 PE=1 SV=1;>tr|Q68DV2|Q68DV2_HUMAN Putative uncharact	3.49E-58
O43598;A8MXA0	RCL_HUMAN Deoxyribonucleoside 5-monophosphate N-glycosidase OS=Homo sapiens GN=RCL PE=1 SV=1;>tr|A8MXA0|A8MXA0_HU	8.14E-30
O43598;O43598-2	RCL_HUMAN Deoxyribonucleoside 5-monophosphate N-glycosidase OS=Homo sapiens GN=RCL PE=1 SV=1;>sp|O43598-2|RCL_HUMA	2.53E-31
O43670;O43670-2;O4367	ZN207_HUMAN Zinc finger protein 207 OS=Homo sapiens GN=ZNF207 PE=1 SV=1;>sp|O43670-2|ZN207_HUMAN Isoform 2 of Zinc f	1.20E-07

O43676;Q6IB80;C9JKQ2	NDUB3_HUMAN NADH dehydrogenase [ubiquinone] 1 beta subcomplex subunit 3 OS=Homo sapiens GN=NDUFB3 PE=1 SV=3;>tr|Q6I	2.44E-09
O43681;B4DEE5	ASNA_HUMAN ATPase ASNA1 OS=Homo sapiens GN=ASNA1 PE=1 SV=2;>tr|B4DEE5|B4DEE5_HUMAN cDNA FLJ60230, highly similar	2.79E-79
O43707;D6PXK4;Q96BG6;	ACTN4_HUMAN Alpha-actinin-4 OS=Homo sapiens GN=ACTN4 PE=1 SV=2;>tr|D6PXK4|D6PXK4_HUMAN Alpha actinin 4 short isoform	0
O43776;B4DN60;B4DGT6	SYNC_HUMAN Asparaginyl-tRNA synthetase, cytoplasmic OS=Homo sapiens GN=NARS PE=1 SV=1;>tr|B4DN60|B4DN60_HUMAN cDN	1.51E-204
O43815;Q3B874;O43815-	STRN_HUMAN Striatin OS=Homo sapiens GN=STRN PE=1 SV=4;>tr|Q3B874|Q3B874_HUMAN STRN protein (Fragment) OS=Homo sa	5.85E-17
O43823;Q8NE02;Q9UG73	AKAP8_HUMAN A-kinase anchor protein 8 OS=Homo sapiens GN=AKAP8 PE=1 SV=1;>tr|Q8NE02|Q8NE02_HUMAN AKAP8 protein OS	1.42E-52
O43837;O43837-2;O4383	IDH3B_HUMAN Isocitrate dehydrogenase [NAD] subunit beta, mitochondrial OS=Homo sapiens GN=IDH3B PE=1 SV=2;>sp|O43837-2	3.56E-161
O43865;O43865-2;Q96HN	SAHH2_HUMAN Putative adenosylhomocysteinase 2 OS=Homo sapiens GN=AHCYL1 PE=1 SV=2;>sp|O43865-2|SAHH2_HUMAN Isofo	1.96E-10
O43920;Q6IBA0	NDUS5_HUMAN NADH dehydrogenase [ubiquinone] iron-sulfur protein 5 OS=Homo sapiens GN=NDUFS5 PE=1 SV=3;>tr|Q6IBA0|Q6I	5.80E-11
O43924;Q6IB24;C9IZ52;E	PDE6D_HUMAN Retinal rod rhodopsin-sensitive cGMP 3,5-cyclic phosphodiesterase subunit delta OS=Homo sapiens GN=PDE6D PE=1	2.75E-08
O60234;Q6IB37;Q8TDZ6	GMFG_HUMAN Glia maturation factor gamma OS=Homo sapiens GN=GMFG PE=1 SV=1;>tr|Q6IB37|Q6IB37_HUMAN GMFG protein O	1.26E-28
O60264;B4DZC0;B7ZAX9;	SMCA5_HUMAN SWI/SNF-related matrix-associated actin-dependent regulator of chromatin subfamily A member 5 OS=Homo sapiens	1.10E-14
O60313-2;O60313	2|OPA1_HUMAN Isoform 2 of Dynamin-like 120 kDa protein, mitochondrial OS=Homo sapiens GN=OPA1;>sp|O60313|OPA1_HUMAN D	4.60E-29
O60493;O60493-4;O6049	SNX3_HUMAN Sorting nexin-3 OS=Homo sapiens GN=SNX3 PE=1 SV=3;>sp|O60493-4|SNX3_HUMAN Isoform 4 of Sorting nexin-3 O	1.25E-32
O60499;Q5U8S2;O60499-	STX10_HUMAN Syntaxin-10 OS=Homo sapiens GN=STX10 PE=1 SV=1;>tr|Q5U8S2|Q5U8S2_HUMAN Syntaxin 10 OS=Homo sapiens	8.15E-16
O60502;O60502-2;O6050	NCOAT_HUMAN Bifunctional protein NCOAT OS=Homo sapiens GN=MGEA5 PE=1 SV=2;>sp|O60502-2|NCOAT_HUMAN Isoform 2 of B	1.98E-52
O60506;O60506-2;O6050	HNRPQ_HUMAN Heterogeneous nuclear ribonucleoprotein Q OS=Homo sapiens GN=SYNCRIP PE=1 SV=2;>sp|O60506-2|HNRPQ_HUM	1.20E-255
O60506;O60506-3;O6050	HNRPQ_HUMAN Heterogeneous nuclear ribonucleoprotein Q OS=Homo sapiens GN=SYNCRIP PE=1 SV=2;>sp|O60506-3|HNRPQ_HUM	0
O60568;B3KQQ3;Q9UG85	PLOD3_HUMAN Procollagen-lysine,2-oxoglutarate 5-dioxygenase 3 OS=Homo sapiens GN=PLOD3 PE=1 SV=1;>tr|B3KQQ3|B3KQQ3_	3.92E-67
O60573;Q53RG0;Q59FE1;	IF4E2_HUMAN Eukaryotic translation initiation factor 4E type 2 OS=Homo sapiens GN=EIF4E2 PE=1 SV=1;>tr|Q53RG0|Q53RG0_HUM	6.03E-07
O60610;Q6URC4;Q17RN4	DIAP1_HUMAN Protein diaphanous homolog 1 OS=Homo sapiens GN=DIAPH1 PE=1 SV=2;>tr|Q6URC4|Q6URC4_HUMAN Diaphanous	1.00E-146
O60664;O60664-3;O6066	PLIN3_HUMAN Perilipin-3 OS=Homo sapiens GN=PLIN3 PE=1 SV=3;>sp|O60664-3|PLIN3_HUMAN Isoform 3 of Perilipin-3 OS=Homo	8.17E-128
O60684;O15131	IMA7_HUMAN Importin subunit alpha-7 OS=Homo sapiens GN=KPNA6 PE=1 SV=1;>sp|O15131|IMA5_HUMAN Importin subunit alpha	1.50E-55
O60701;B3KUU2;B4DN25	UGDH_HUMAN UDP-glucose 6-dehydrogenase OS=Homo sapiens GN=UGDH PE=1 SV=1;>tr|B3KUU2|B3KUU2_HUMAN UDP-glucose d	0
O60716;O60716-2;O6071	CTND1_HUMAN Catenin delta-1 OS=Homo sapiens GN=CTNND1 PE=1 SV=1;>sp|O60716-2|CTND1_HUMAN Isoform 1AB of Catenin d	6.77E-20
O60739;Q53F41;Q6FG85	EIF1B_HUMAN Eukaryotic translation initiation factor 1b OS=Homo sapiens GN=EIF1B PE=1 SV=2;>tr|Q53F41|Q53F41_HUMAN Trans	5.05E-51
O60783;B2R4A5	RT14_HUMAN 28S ribosomal protein S14, mitochondrial OS=Homo sapiens GN=MRPS14 PE=1 SV=1;>tr|B2R4A5|B2R4A5_HUMAN cD	3.15E-38
O60784-2;O60784;O6078	2|TOM1_HUMAN Isoform 2 of Target of Myb protein 1 OS=Homo sapiens GN=TOM1;>sp|O60784|TOM1_HUMAN Target of Myb protein	1.07E-22
O60814;P57053;P58876;F	H2B1K_HUMAN Histone H2B type 1-K OS=Homo sapiens GN=HIST1H2BK PE=1 SV=3;>sp|P57053|H2BFS_HUMAN Histone H2B type	3.24E-39
O60814;P58876;P62807;(	H2B1K_HUMAN Histone H2B type 1-K OS=Homo sapiens GN=HIST1H2BK PE=1 SV=3;>sp|P58876|H2B1D_HUMAN Histone H2B type	7.52E-137
O60825;O60825-2	F262_HUMAN 6-phosphofructo-2-kinase/fructose-2,6-biphosphatase 2 OS=Homo sapiens GN=PFKFB2 PE=1 SV=2;>sp|O60825-2|F26	6.17E-10
O60826;B4DPB6;B7ZAR0;	CCD22_HUMAN Coiled-coil domain-containing protein 22 OS=Homo sapiens GN=CCDC22 PE=1 SV=1;>tr|B4DPB6|B4DPB6_HUMAN cD	3.46E-10
O60841;Q8N5A0;D3DVI5;	IF2P_HUMAN Eukaryotic translation initiation factor 5B OS=Homo sapiens GN=EIF5B PE=1 SV=4;>tr|Q8N5A0|Q8N5A0_HUMAN Eukar	3.56E-60
O60885;Q4G0X8;O60885-	BRD4_HUMAN Bromodomain-containing protein 4 OS=Homo sapiens GN=BRD4 PE=1 SV=2;>tr|Q4G0X8|Q4G0X8_HUMAN BRD4 prote	5.96E-15
O60888-2;O60888;O6088	2|CUTA_HUMAN Isoform A of Protein CutA OS=Homo sapiens GN=CUTA;>sp|O60888|CUTA_HUMAN Protein CutA OS=Homo sapiens (	4.05E-30
O75084;Q14332	FZD7_HUMAN Frizzled-7 OS=Homo sapiens GN=FZD7 PE=2 SV=2;>sp|Q14332|FZD2_HUMAN Frizzled-2 OS=Homo sapiens GN=FZD2	0.0001056
O75116;B4DRJ9;Q14DU5	ROCK2_HUMAN Rho-associated protein kinase 2 OS=Homo sapiens GN=ROCK2 PE=1 SV=4;>tr|B4DRJ9|B4DRJ9_HUMAN cDNA FLJ51	2.80E-104
O75131;Q05DL8	CPNE3_HUMAN Copine-3 OS=Homo sapiens GN=CPNE3 PE=1 SV=1;>tr|Q05DL8|Q05DL8_HUMAN CPNE3 protein (Fragment) OS=Hor	2.24E-153
O75146;Q6NXG8;B3KQW8	HIP1R_HUMAN Huntingtin-interacting protein 1-related protein OS=Homo sapiens GN=HIP1R PE=1 SV=2;>tr|Q6NXG8|Q6NXG8_HUM	1.71E-12
O75150;O75150-3;O7515	BRE1B_HUMAN E3 ubiquitin-protein ligase BRE1B OS=Homo sapiens GN=RNF40 PE=1 SV=4;>sp|O75150-3|BRE1B_HUMAN Isoform 3	1.10E-10
O75150;O75150-4;O7515	BRE1B_HUMAN E3 ubiquitin-protein ligase BRE1B OS=Homo sapiens GN=RNF40 PE=1 SV=4;>sp|O75150-4|BRE1B_HUMAN Isoform 4	4.63E-14
O75175;O75175-2	CNOT3_HUMAN CCR4-NOT transcription complex subunit 3 OS=Homo sapiens GN=CNOT3 PE=1 SV=1;>sp|O75175-2|CNOT3_HUMAN	8.39E-20
O75179;O75179-2;O7517	ANR17_HUMAN Ankyrin repeat domain-containing protein 17 OS=Homo sapiens GN=ANKRD17 PE=1 SV=3;>sp|O75179-2|ANR17_HU	6.87E-23
O75179;O75179-2;Q8IWZ	ANR17_HUMAN Ankyrin repeat domain-containing protein 17 OS=Homo sapiens GN=ANKRD17 PE=1 SV=3;>sp|O75179-2|ANR17_HU	2.42E-157
O75182;O75182-2;O7518	SIN3B_HUMAN Paired amphipathic helix protein Sin3b OS=Homo sapiens GN=SIN3B PE=1 SV=2;>sp|O75182-2|SIN3B_HUMAN Isofo	5.17E-06
O75208;B4DIV2;B4E0U3;I	COQ9_HUMAN Ubiquinone biosynthesis protein COQ9, mitochondrial OS=Homo sapiens GN=COQ9 PE=1 SV=1;>tr|B4DIV2|B4DIV2_H	1.11E-07
O75223;B3KMN7;B8ZZK2	GGCT_HUMAN Gamma-glutamylcyclotransferase OS=Homo sapiens GN=GGCT PE=1 SV=1;>tr|B3KMN7|B3KMN7_HUMAN cDNA FLJ11	9.71E-141
O75312;B4DVT8	ZPR1_HUMAN Zinc finger protein ZPR1 OS=Homo sapiens GN=ZNF259 PE=1 SV=1;>tr|B4DVT8|B4DVT8_HUMAN cDNA FLJ60173, hig	1.09E-39
O75323;C9IYJ3	NIPS2_HUMAN Protein NipSnap homolog 2 OS=Homo sapiens GN=GBAS PE=1 SV=1;>tr|C9IYJ3|C9IYJ3_HUMAN Glioblastoma amplifi	3.67E-17
O75340;Q53FC3;Q2YDC2	PDCD6_HUMAN Programmed cell death protein 6 OS=Homo sapiens GN=PDCD6 PE=1 SV=1;>tr|Q53FC3|Q53FC3_HUMAN Programme	1.54E-11
O75347;Q6FGD7;B4DT30	TBCA_HUMAN Tubulin-specific chaperone A OS=Homo sapiens GN=TBCA PE=1 SV=3;>tr|Q6FGD7|Q6FGD7_HUMAN TBCA protein OS=	1.25E-59
O75348;O95670	VATG1_HUMAN V-type proton ATPase subunit G 1 OS=Homo sapiens GN=ATP6V1G1 PE=1 SV=3;>sp|O95670|VATG2_HUMAN V-type	4.27E-62
O75348;Q6IB33;C9JRT2;(	VATG1_HUMAN V-type proton ATPase subunit G 1 OS=Homo sapiens GN=ATP6V1G1 PE=1 SV=3;>tr|Q6IB33|Q6IB33_HUMAN ATP6V1	1.11E-68
O75367;O75367-3;O7536	H2AY_HUMAN Core histone macro-H2A.1 OS=Homo sapiens GN=H2AFY PE=1 SV=4;>sp|O75367-3|H2AY_HUMAN Isoform 3 of Core h	4.19E-165
O75368;D3DTE6	SH3L1_HUMAN SH3 domain-binding glutamic acid-rich-like protein OS=Homo sapiens GN=SH3BGRL PE=1 SV=1;>tr|D3DTE6|D3DTE6	5.16E-190
O75369;Q60FE7;O75369-	FLNB_HUMAN Filamin-B OS=Homo sapiens GN=FLNB PE=1 SV=2;>tr|Q60FE7|Q60FE7_HUMAN Filamin B OS=Homo sapiens GN=FLNI	0
O75379;Q6IAZ3;O75379-	VAMP4_HUMAN Vesicle-associated membrane protein 4 OS=Homo sapiens GN=VAMP4 PE=1 SV=2;>tr|Q6IAZ3|Q6IAZ3_HUMAN VAMF	5.64E-19
O75380;Q6IBC4;D6RBT3	NDUS6_HUMAN NADH dehydrogenase [ubiquinone] iron-sulfur protein 6, mitochondrial OS=Homo sapiens GN=NDUFS6 PE=1 SV=1;>	3.91E-08
O75381;O75381-2;B7Z4Z	PEX14_HUMAN Peroxisomal membrane protein PEX14 OS=Homo sapiens GN=PEX14 PE=1 SV=1;>sp|O75381-2|PEX14_HUMAN Isofo	1.32E-25
O75390;B4DJV2;B3KTN4	CISY_HUMAN Citrate synthase, mitochondrial OS=Homo sapiens GN=CS PE=1 SV=2;>tr|B4DJV2|B4DJV2_HUMAN Citrate synthase OS	1.47E-43
O75400;O75400-2;O7540	PR40A_HUMAN Pre-mRNA-processing factor 40 homolog A OS=Homo sapiens GN=PRPF40A PE=1 SV=2;>sp|O75400-2|PR40A_HUMA	8.17E-110

O75400;O75400-3;O7540	PR40A_HUMAN Pre-mRNA-processing factor 40 homolog A OS=Homo sapiens GN=PRPF40A PE=1 SV=2;>sp|O75400-3|PR40A_HUMA	1.53E-45
O75410-2;O75410;O7541	2|TACC1_HUMAN Isoform 2 of Transforming acidic coiled-coil-containing protein 1 OS=Homo sapiens GN=TACC1;>sp|O75410|TACC1_	1.52E-10
O75414;C9J9V6;C9JQB1	NDK6_HUMAN Nucleoside diphosphate kinase 6 OS=Homo sapiens GN=NME6 PE=1 SV=3;>tr|C9J9V6|C9J9V6_HUMAN Nucleoside dip	4.89E-05
O75436;B4DLT1;A8MZ56;	VP26A_HUMAN Vacuolar protein sorting-associated protein 26A OS=Homo sapiens GN=VPS26A PE=1 SV=2;>tr|B4DLT1|B4DLT1_HUM	6.98E-62
O75477;O94905-3;O9490	ERLN1_HUMAN Erlin-1 OS=Homo sapiens GN=ERLIN1 PE=1 SV=1;>sp|O94905-3|ERLN2_HUMAN Isoform 3 of Erlin-2 OS=Homo sapi	7.46E-18
O75487;Q86SV6;B4E2C0	GPC4_HUMAN Glypican-4 OS=Homo sapiens GN=GPC4 PE=1 SV=4;>tr|Q86SV6|Q86SV6_HUMAN K-glypican OS=Homo sapiens GN=	3.10E-10
O75489;Q53FM7;Q9UF24;	NDUS3_HUMAN NADH dehydrogenase [ubiquinone] iron-sulfur protein 3, mitochondrial OS=Homo sapiens GN=NDUFS3 PE=1 SV=1;>	1.22E-26
O75494;Q53GD7;O75494	SRS10_HUMAN Serine/arginine-rich splicing factor 10 OS=Homo sapiens GN=SRSF10 PE=1 SV=1;>tr|Q53GD7|Q53GD7_HUMAN FUS	9.18E-08
O75526;Q8N7X1	HNRGT_HUMAN RNA-binding motif protein, X-linked-like-2 OS=Homo sapiens GN=RBMXL2 PE=1 SV=3;>sp|Q8N7X1|RMXL3_HUMAN	1.96E-40
O75531;B2R4V4	BAF_HUMAN Barrier-to-autointegration factor OS=Homo sapiens GN=BANF1 PE=1 SV=1;>tr|B2R4V4|B2R4V4_HUMAN cDNA, FLJ922	5.84E-72
O75533;Q7Z497	SF3B1_HUMAN Splicing factor 3B subunit 1 OS=Homo sapiens GN=SF3B1 PE=1 SV=3;>tr|Q7Z497|Q7Z497_HUMAN SF3B1 protein (F	5.04E-67
O75534;O75534-2;Q9GZ	CSDE1_HUMAN Cold shock domain-containing protein E1 OS=Homo sapiens GN=CSDE1 PE=1 SV=2;>sp|O75534-2|CSDE1_HUMAN I	3.81E-97
O75581;B7ZLD0;Q59EX8;	LRP6_HUMAN Low-density lipoprotein receptor-related protein 6 OS=Homo sapiens GN=LRP6 PE=1 SV=2;>tr|B7ZLD0|B7ZLD0_HUMA	2.38E-06
O75676;O75676-2;A0PJF	KS6A4_HUMAN Ribosomal protein S6 kinase alpha-4 OS=Homo sapiens GN=RPS6KA4 PE=1 SV=1;>sp|O75676-2|KS6A4_HUMAN Iso	1.46E-18
O75694;O75694-2	NU155_HUMAN Nuclear pore complex protein Nup155 OS=Homo sapiens GN=NUP155 PE=1 SV=1;>sp|O75694-2|NU155_HUMAN Iso	1.04E-131
O75787;B7Z4S4;B7Z9I3;	RENR_HUMAN Renin receptor OS=Homo sapiens GN=ATP6AP2 PE=1 SV=2;>tr|B7Z4S4|B7Z4S4_HUMAN cDNA FLJ52567, highly simil	1.41E-16
O75792;Q6IBG3;Q6UQL6	RNH2A_HUMAN Ribonuclease H2 subunit A OS=Homo sapiens GN=RNASEH2A PE=1 SV=2;>tr|Q6IBG3|Q6IBG3_HUMAN Ribonuclease	7.42E-46
O75818;O75818-2;Q5VX9	RPP40_HUMAN Ribonuclease P protein subunit p40 OS=Homo sapiens GN=RPP40 PE=1 SV=3;>sp|O75818-2|RPP40_HUMAN Isoform	2.04E-10
O75821;Q6IAM0;A8K5K5	EIF3G_HUMAN Eukaryotic translation initiation factor 3 subunit G OS=Homo sapiens GN=EIF3G PE=1 SV=2;>tr|Q6IAM0|Q6IAM0_HU	4.87E-120
O75822;B4DUI3	EIF3J_HUMAN Eukaryotic translation initiation factor 3 subunit J OS=Homo sapiens GN=EIF3J PE=1 SV=2;>tr|B4DUI3|B4DUI3_HUMA	4.37E-49
O75828;Q53F60	CBR3_HUMAN Carbonyl reductase [NADPH] 3 OS=Homo sapiens GN=CBR3 PE=1 SV=3;>tr|Q53F60|Q53F60_HUMAN Carbonyl reduct	4.19E-58
O75832;Q5U0B2;B1AJY7;	PSD10_HUMAN 26S proteasome non-ATPase regulatory subunit 10 OS=Homo sapiens GN=PSMD10 PE=1 SV=1;>tr|Q5U0B2|Q5U0B2_	3.16E-34
O75909-4;O75909;O7590	4|CCNK_HUMAN Isoform 4 of Cyclin-K OS=Homo sapiens GN=CCNK;>sp|O75909|CCNK_HUMAN Cyclin-K OS=Homo sapiens GN=CC	7.93E-10
O75935;O75935-3;O7593	DCTN3_HUMAN Dynactin subunit 3 OS=Homo sapiens GN=DCTN3 PE=1 SV=1;>sp|O75935-3|DCTN3_HUMAN Isoform 3 of Dynactin	2.38E-18
O75947;O75947-2;A0PJH	ATP5H_HUMAN ATP synthase subunit d, mitochondrial OS=Homo sapiens GN=ATP5H PE=1 SV=3;>sp|O75947-2|ATP5H_HUMAN Isofo	4.71E-75
O75955;Q53HQ0;Q5ST80	FLOT1_HUMAN Flotillin-1 OS=Homo sapiens GN=FLOT1 PE=1 SV=3;>tr|Q53HQ0|Q53HQ0_HUMAN Flotillin 1 variant (Fragment) OS=	5.49E-62
O75962;O75962-4;O7596	TRIO_HUMAN Triple functional domain protein OS=Homo sapiens GN=TRIO PE=1 SV=2;>sp|O75962-4|TRIO_HUMAN Isoform 4 of Tri	2.54E-11
O75962;O75962-4;O7596	TRIO_HUMAN Triple functional domain protein OS=Homo sapiens GN=TRIO PE=1 SV=2;>sp|O75962-4|TRIO_HUMAN Isoform 4 of Tri	1.08E-42
O75976;B7Z7T9;B7ZAU4;	CBPD_HUMAN Carboxypeptidase D OS=Homo sapiens GN=CPD PE=1 SV=2;>tr|B7Z7T9|B7Z7T9_HUMAN cDNA FLJ50395, highly simi	1.09E-44
O76031;Q9H072	CLPX_HUMAN ATP-dependent Clp protease ATP-binding subunit clpX-like, mitochondrial OS=Homo sapiens GN=CLPX PE=1 SV=2;>tr|	8.08E-13
O76070;Q6FHG5;A9XXE1	SYUG_HUMAN Gamma-synuclein OS=Homo sapiens GN=SNCG PE=1 SV=2;>tr|Q6FHG5|Q6FHG5_HUMAN SNCG protein OS=Homo sa	3.69E-176
O76071;B4DRZ6;B2RDJ6	CIAO1_HUMAN Probable cytosolic iron-sulfur protein assembly protein CIAO1 OS=Homo sapiens GN=CIAO1 PE=1 SV=1;>tr|B4DRZ6|	6.05E-35
O76094;Q71V07;A0PJJ5;	SRP72_HUMAN Signal recognition particle 72 kDa protein OS=Homo sapiens GN=SRP72 PE=1 SV=3;>tr|Q71V07|Q71V07_HUMAN Pu	0
O94763;Q6NX55;O94763	RMP_HUMAN Unconventional prefoldin RPB5 interactor OS=Homo sapiens GN=RMP PE=1 SV=3;>tr|Q6NX55|Q6NX55_HUMAN Chromo	1.07E-08
O94776;Q68DB1	MTA2_HUMAN Metastasis-associated protein MTA2 OS=Homo sapiens GN=MTA2 PE=1 SV=1;>tr|Q68DB1|Q68DB1_HUMAN Putative u	1.30E-193
O94832;Q7Z3N6	MYO1D_HUMAN Myosin-Id OS=Homo sapiens GN=MYO1D PE=1 SV=2;>tr|Q7Z3N6|Q7Z3N6_HUMAN Putative uncharacterized protein	1.66E-22
O94855-2;O94855	2|SC24D_HUMAN Isoform 2 of Protein transport protein Sec24D OS=Homo sapiens GN=SEC24D;>sp|O94855|SC24D_HUMAN Protein	3.74E-10
O94874;O94874-2;O9487	UFL1_HUMAN E3 UFM1-protein ligase 1 OS=Homo sapiens GN=KIAA0776 PE=1 SV=2;>sp|O94874-2|UFL1_HUMAN Isoform 2 of E3 U	5.54E-28
O94925-3;O94925	3|GLSK_HUMAN Isoform 3 of Glutaminase kidney isoform, mitochondrial OS=Homo sapiens GN=GLS;>sp|O94925|GLSK_HUMAN Glut	7.41E-28
O94973-2;O94973;O9497	2|AP2A2_HUMAN Isoform 2 of AP-2 complex subunit alpha-2 OS=Homo sapiens GN=AP2A2;>sp|O94973|AP2A2_HUMAN AP-2 comple	6.27E-27
O95155;O95155-2;O9515	UBE4B_HUMAN Ubiquitin conjugation factor E4 B OS=Homo sapiens GN=UBE4B PE=1 SV=1;>sp|O95155-2|UBE4B_HUMAN Isoform 2	9.07E-17
O95168;B2RUY3;C9JXQ9	NDUB4_HUMAN NADH dehydrogenase [ubiquinone] 1 beta subcomplex subunit 4 OS=Homo sapiens GN=NDUFB4 PE=1 SV=3;>tr|B2	1.17E-25
O95169;Q5W143;Q5W14	NDUB8_HUMAN NADH dehydrogenase [ubiquinone] 1 beta subcomplex subunit 8, mitochondrial OS=Homo sapiens GN=NDUFB8 PE=1	3.26E-12
O95229;A6NNV6;B4DP12;	ZWINT_HUMAN ZW10 interactor OS=Homo sapiens GN=ZWINT PE=1 SV=2;>tr|A6NNV6|A6NNV6_HUMAN Uncharacterized protein O	1.36E-07
O95239;O95239-2;Q2VIQ	KIF4A_HUMAN Chromosome-associated kinesin KIF4A OS=Homo sapiens GN=KIF4A PE=1 SV=3;>sp|O95239-2|KIF4A_HUMAN Isofor	6.26E-31
O95297;O95297-2;O9529	MPZL1_HUMAN Myelin protein zero-like protein 1 OS=Homo sapiens GN=MPZL1 PE=1 SV=1;>sp|O95297-2|MPZL1_HUMAN Isoform 2	0.00012997
O95298;Q549M5	NDUC2_HUMAN NADH dehydrogenase [ubiquinone] 1 subunit C2 OS=Homo sapiens GN=NDUFC2 PE=1 SV=1;>tr|Q549M5|Q549M5_	8.47E-06
O95340-2;O95340;Q5TB5	2|PAPS2_HUMAN Isoform B of Bifunctional 3-phosphoadenosine 5-phosphosulfate synthase 2 OS=Homo sapiens GN=PAPSS2;>sp|O9	9.55E-88
O95372;Q5QPQ2;Q5QPQ3	LYPA2_HUMAN Acyl-protein thioesterase 2 OS=Homo sapiens GN=LYPLA2 PE=1 SV=1;>tr|Q5QPQ2|Q5QPQ2_HUMAN Lysophospholipa	2.12E-16
O95373;B3KQG6	IPO7_HUMAN Importin-7 OS=Homo sapiens GN=IPO7 PE=1 SV=1;>tr|B3KQG6|B3KQG6_HUMAN cDNA FLJ90427 fis, clone NT2RP300	8.31E-130
O95433;B4DUR9	AHSA1_HUMAN Activator of 90 kDa heat shock protein ATPase homolog 1 OS=Homo sapiens GN=AHSA1 PE=1 SV=1;>tr|B4DUR9|B4	1.86E-43
O95445;Q5SRP5;Q5SRP4	APOM_HUMAN Apolipoprotein M OS=Homo sapiens GN=APOM PE=1 SV=2;>tr|Q5SRP5|Q5SRP5_HUMAN Apolipoprotein M OS=Homo	6.92E-07
O95466-2;O95466;D2DG	2|FMNL_HUMAN Isoform 2 of Formin-like protein 1 OS=Homo sapiens GN=FMNL1;>sp|O95466|FMNL_HUMAN Formin-like protein 1 O	3.34E-08
O95478;Q5J7U2	NSA2_HUMAN Ribosome biogenesis protein NSA2 homolog OS=Homo sapiens GN=NSA2 PE=1 SV=1;>tr|Q5J7U2|Q5J7U2_HUMAN TG	1.08E-07
O95486;B4E205	SC24A_HUMAN Protein transport protein Sec24A OS=Homo sapiens GN=SEC24A PE=1 SV=2;>tr|B4E205|B4E205_HUMAN cDNA FLJ6	5.02E-30
O95486;O95486-2	SC24A_HUMAN Protein transport protein Sec24A OS=Homo sapiens GN=SEC24A PE=1 SV=2;>sp|O95486-2|SC24A_HUMAN Isoform	9.65E-13
O95571;B2RCZ7	ETHE1_HUMAN Protein ETHE1, mitochondrial OS=Homo sapiens GN=ETHE1 PE=1 SV=2;>tr|B2RCZ7|B2RCZ7_HUMAN Ethylmalonic e	4.72E-16
O95721;C9JAF7	SNP29_HUMAN Synaptosomal-associated protein 29 OS=Homo sapiens GN=SNAP29 PE=1 SV=1;>tr|C9JAF7|C9JAF7_HUMAN Synapt	2.69E-27
O95747;C9JIG9	OXSR1_HUMAN Serine/threonine-protein kinase OSR1 OS=Homo sapiens GN=OXSR1 PE=1 SV=1;>tr|C9JIG9|C9JIG9_HUMAN Unchar	6.71E-133
O95749;C9J7M1;C9J6G3	GGPPS_HUMAN Geranylgeranyl pyrophosphate synthase OS=Homo sapiens GN=GGPS1 PE=1 SV=1;>tr|C9J7M1|C9J7M1_HUMAN Unc	2.31E-18
O95782;O95782-2;Q8N9K	AP2A1_HUMAN AP-2 complex subunit alpha-1 OS=Homo sapiens GN=AP2A1 PE=1 SV=3;>sp|O95782-2|AP2A1_HUMAN Isoform B of	8.72E-106

O95786;O95786-2;B3KW\	DDX58_HUMAN Probable ATP-dependent RNA helicase DDX58 OS=Homo sapiens GN=DDX58 PE=1 SV=2;>sp|O95786-2|DDX58_HUM	2.61E-85
O95817;Q53GY1;C9JFK9	BAG3_HUMAN BAG family molecular chaperone regulator 3 OS=Homo sapiens GN=BAG3 PE=1 SV=3;>tr|Q53GY1|Q53GY1_HUMAN B	3.29E-09
O95825;A6NMA8;A6NHJ8;	QORL1_HUMAN Quinone oxidoreductase-like protein 1 OS=Homo sapiens GN=CRYZL1 PE=1 SV=2;>tr|A6NMA8|A6NMA8_HUMAN Unc	1.66E-12
O95831;O95831-3;O9583	AIFM1_HUMAN Apoptosis-inducing factor 1, mitochondrial OS=Homo sapiens GN=AIFM1 PE=1 SV=1;>sp|O95831-3|AIFM1_HUMAN Is	8.36E-63
O95833;Q6FGP1;Q5SQ17	CLIC3_HUMAN Chloride intracellular channel protein 3 OS=Homo sapiens GN=CLIC3 PE=1 SV=2;>tr|Q6FGP1|Q6FGP1_HUMAN CLIC3	1.56E-22
O95861-2;O95861	2|BPNT1_HUMAN Isoform 2 of 3(2),5-bisphosphate nucleotidase 1 OS=Homo sapiens GN=BPNT1;>sp|O95861|BPNT1_HUMAN 3(2),5-	9.31E-15
O95864;O95864-3;O9586	FADS2_HUMAN Fatty acid desaturase 2 OS=Homo sapiens GN=FADS2 PE=1 SV=1;>sp|O95864-3|FADS2_HUMAN Isoform 3 of Fatty a	2.12E-09
O95865;Q5SRR8;Q5SSV3	DDAH2_HUMAN N(G),N(G)-dimethylarginine dimethylaminohydrolase 2 OS=Homo sapiens GN=DDAH2 PE=1 SV=1;>tr|Q5SRR8|Q5SF	1.41E-112
O95999;Q5VUF1;A2TDT2	BCL10_HUMAN B-cell lymphoma/leukemia 10 OS=Homo sapiens GN=BCL10 PE=1 SV=1;>tr|Q5VUF1|Q5VUF1_HUMAN B-cell CLL/lym	2.40E-16
O96011;B4DXH9	PX11B_HUMAN Peroxisomal membrane protein 11B OS=Homo sapiens GN=PEX11B PE=1 SV=1;>tr|B4DXH9|B4DXH9_HUMAN cDNA F	7.08E-05
O96013;O96013-4;O9601	PAK4_HUMAN Serine/threonine-protein kinase PAK 4 OS=Homo sapiens GN=PAK4 PE=1 SV=1;>sp|O96013-4|PAK4_HUMAN Isoform	4.30E-06
O96019;Q53FS0;Q6FI97;I	ACL6A_HUMAN Actin-like protein 6A OS=Homo sapiens GN=ACTL6A PE=1 SV=1;>tr|Q53FS0|Q53FS0_HUMAN Actin-like 6A isoform 1	6.98E-63
P00167;Q59F44;P00167-2	CYB5_HUMAN Cytochrome b5 OS=Homo sapiens GN=CYB5A PE=1 SV=2;>tr|Q59F44|Q59F44_HUMAN Cytochrome b-5 isoform 1 vari	3.04E-31
P00325;P07327	ADH1B_HUMAN Alcohol dehydrogenase 1B OS=Homo sapiens GN=ADH1B PE=1 SV=2;>sp|P07327|ADH1A_HUMAN Alcohol dehydroge	1.13E-254
P00352;B4DDF8;B4DXX3;	AL1A1_HUMAN Retinal dehydrogenase 1 OS=Homo sapiens GN=ALDH1A1 PE=1 SV=2;>tr|B4DDF8|B4DDF8_HUMAN cDNA FLJ51786,	0
P00367;B4DMF5;Q53GW3	DHE3_HUMAN Glutamate dehydrogenase 1, mitochondrial OS=Homo sapiens GN=GLUD1 PE=1 SV=2;>tr|B4DMF5|B4DMF5_HUMAN c	4.12E-258
P00367;P49448	DHE3_HUMAN Glutamate dehydrogenase 1, mitochondrial OS=Homo sapiens GN=GLUD1 PE=1 SV=2;>sp|P49448|DHE4_HUMAN Glut	4.56E-181
P00374;Q86XF0	DYR_HUMAN Dihydrofolate reductase OS=Homo sapiens GN=DHFR PE=1 SV=2;>sp|Q86XF0|DYRL1_HUMAN Dihydrofolate reductase-	1.01E-16
P00488;B2R6V9;B4E2L8	F13A_HUMAN Coagulation factor XIII A chain OS=Homo sapiens GN=F13A1 PE=1 SV=4;>tr|B2R6V9|B2R6V9_HUMAN cDNA, FLJ9314	2.65E-260
P00492;Q6LET3;A8MSU4	HPRT_HUMAN Hypoxanthine-guanine phosphoribosyltransferase OS=Homo sapiens GN=HPRT1 PE=1 SV=2;>tr|Q6LET3|Q6LET3_HUM	8.44E-81
P00505;B3KUZ8;A8K482;I	AATM_HUMAN Aspartate aminotransferase, mitochondrial OS=Homo sapiens GN=GOT2 PE=1 SV=3;>tr|B3KUZ8|B3KUZ8_HUMAN Asp	1.88E-197
P00734;B4DDT3;C9JV37	THRB_HUMAN Prothrombin OS=Homo sapiens GN=F2 PE=1 SV=2;>tr|B4DDT3|B4DDT3_HUMAN cDNA FLJ54622, highly similar to Pro	0
P00738;Q0VAC5;Q6NSB4;	HPT_HUMAN Haptoglobin OS=Homo sapiens GN=HP PE=1 SV=1;>tr|Q0VAC5|Q0VAC5_HUMAN HP protein OS=Homo sapiens GN=HP	0
P00747;B2R7F8;B4DPH4	PLMN_HUMAN Plasminogen OS=Homo sapiens GN=PLG PE=1 SV=2;>tr|B2R7F8|B2R7F8_HUMAN cDNA, FLJ93426, highly similar to H	2.66E-63
P00748;Q8IZZ5	FA12_HUMAN Coagulation factor XII OS=Homo sapiens GN=F12 PE=1 SV=3;>tr|Q8IZZ5|Q8IZZ5_HUMAN Coagulation factor XII-Mie	5.90E-98
P00966;Q5T6L4;A8KAP9;(	ASSY_HUMAN Argininosuccinate synthase OS=Homo sapiens GN=ASS1 PE=1 SV=2;>tr|Q5T6L4|Q5T6L4_HUMAN Argininosuccinate sy	3.74E-43
P01009;P01009-2;P01009	A1AT_HUMAN Alpha-1-antitrypsin OS=Homo sapiens GN=SERPINA1 PE=1 SV=3;>sp|P01009-2|A1AT_HUMAN Isoform 2 of Alpha-1-ar	0
P01024;B4E216	CO3_HUMAN Complement C3 OS=Homo sapiens GN=C3 PE=1 SV=2;>tr|B4E216|B4E216_HUMAN cDNA FLJ57339, highly similar to C	0
P01031;Q59GS8	CO5_HUMAN Complement C5 OS=Homo sapiens GN=C5 PE=1 SV=4;>tr|Q59GS8|Q59GS8_HUMAN Complement component 5 variant	8.90E-09
P01040;Q6IB90;C9J0E4	CYTA_HUMAN Cystatin-A OS=Homo sapiens GN=CSTA PE=1 SV=1;>tr|Q6IB90|Q6IB90_HUMAN CSTA protein OS=Homo sapiens GN=	2.08E-21
P01112;Q9BR65;Q92468	RASH_HUMAN GTPase HRas OS=Homo sapiens GN=HRAS PE=1 SV=1;>tr|Q9BR65|Q9BR65_HUMAN V-Ha-ras Harvey rat sarcoma vira	8.55E-41
P01116-2;P01116;P01111	2|RASK_HUMAN Isoform 2B of GTPase KRas OS=Homo sapiens GN=KRAS;>sp|P01116|RASK_HUMAN GTPase KRas OS=Homo sapiens	2.72E-45
P01591;D6RD17;D6RHJ6	IGJ_HUMAN Immunoglobulin J chain OS=Homo sapiens GN=IGJ PE=1 SV=4;>tr|D6RD17|D6RD17_HUMAN Uncharacterized protein O	4.49E-45
P01598;P01596	KV106_HUMAN Ig kappa chain V-I region EU OS=Homo sapiens PE=1 SV=1;>sp|P01596|KV104_HUMAN Ig kappa chain V-I region CA	1.39E-129
P01620;P01623;A2NB46;I	KV302_HUMAN Ig kappa chain V-III region SIE OS=Homo sapiens PE=1 SV=1;>sp|P01623|KV305_HUMAN Ig kappa chain V-III regio	6.49E-202
P01620;P01623;P01622;P	KV302_HUMAN Ig kappa chain V-III region SIE OS=Homo sapiens PE=1 SV=1;>sp|P01623|KV305_HUMAN Ig kappa chain V-III regio	4.78E-21
P01620;P01623;P04206	KV302_HUMAN Ig kappa chain V-III region SIE OS=Homo sapiens PE=1 SV=1;>sp|P01623|KV305_HUMAN Ig kappa chain V-III regio	0
P01779;P01770	HV318_HUMAN Ig heavy chain V-III region TUR OS=Homo sapiens PE=1 SV=1;>sp|P01770|HV309_HUMAN Ig heavy chain V-III regio	5.86E-244
P01857;P01860;P01861;P	IGHG1_HUMAN Ig gamma-1 chain C region OS=Homo sapiens GN=IGHG1 PE=1 SV=1;>sp|P01860|IGHG3_HUMAN Ig gamma-3 chai	1.06E-09
P01871-2;P01871;P04220	2|IGHM_HUMAN Isoform 2 of Ig mu chain C region OS=Homo sapiens GN=IGHM;>sp|P01871|IGHM_HUMAN Ig mu chain C region OS	0
P01876;P01877	IGHA1_HUMAN Ig alpha-1 chain C region OS=Homo sapiens GN=IGHA1 PE=1 SV=2;>sp|P01877|IGHA2_HUMAN Ig alpha-2 chain C r	1.45E-22
P01889;Q31610	1B07_HUMAN HLA class I histocompatibility antigen, B-7 alpha chain OS=Homo sapiens GN=HLA-B PE=1 SV=3;>sp|Q31610|1B81_HU	6.75E-97
P01891;P16189;P30512;P	1A68_HUMAN HLA class I histocompatibility antigen, A-68 alpha chain OS=Homo sapiens GN=HLA-A PE=1 SV=4;>sp|P16189|1A31_H	7.45E-185
P01909;Q5Y7C6;Q5Y7D2;	DQA1_HUMAN HLA class II histocompatibility antigen, DQ alpha 1 chain OS=Homo sapiens GN=HLA-DQA1 PE=1 SV=1;>tr|Q5Y7C6|Q	3.77E-14
P02144;B0QYF8;B4DUI1;(	MYG_HUMAN Myoglobin OS=Homo sapiens GN=MB PE=1 SV=2;>tr|B0QYF8|B0QYF8_HUMAN Myoglobin (Fragment) OS=Homo sapier	7.31E-12
P02452;Q6LAN8	CO1A1_HUMAN Collagen alpha-1(I) chain OS=Homo sapiens GN=COL1A1 PE=1 SV=5;>tr|Q6LAN8|Q6LAN8_HUMAN Collagen type I a	3.52E-152
P02462;P02462-2;Q59F15	CO4A1_HUMAN Collagen alpha-1(IV) chain OS=Homo sapiens GN=COL4A1 PE=1 SV=3;>sp|P02462-2|CO4A1_HUMAN Isoform 2 of C	3.49E-43
P02511;C3VMY8;B4DIA6	CRYAB_HUMAN Alpha-crystallin B chain OS=Homo sapiens GN=CRYAB PE=1 SV=2;>tr|C3VMY8|C3VMY8_HUMAN Alpha B crystallin OS	9.20E-47
P02545;P02545-3	LMNA_HUMAN Prelamin-A/C OS=Homo sapiens GN=LMNA PE=1 SV=1;>sp|P02545-3|LMNA_HUMAN Isoform ADelta10 of Prelamin-A/	0
P02545;P02545-3;P02545	LMNA_HUMAN Prelamin-A/C OS=Homo sapiens GN=LMNA PE=1 SV=1;>sp|P02545-3|LMNA_HUMAN Isoform ADelta10 of Prelamin-A/	0
P02545;Q5I6Y6;Q6UYC3;(	LMNA_HUMAN Prelamin-A/C OS=Homo sapiens GN=LMNA PE=1 SV=1;>tr|Q5I6Y6|Q5I6Y6_HUMAN Lamin A/C transcript variant 1 OS	0
P02649;Q6LBZ1	APOE_HUMAN Apolipoprotein E OS=Homo sapiens GN=APOE PE=1 SV=1;>tr|Q6LBZ1|Q6LBZ1_HUMAN MRNA for apolipoprotein E (ap	0
P02675;Q32Q65;B4E1D3;	FIBB_HUMAN Fibrinogen beta chain OS=Homo sapiens GN=FGB PE=1 SV=2;>tr|Q32Q65|Q32Q65_HUMAN Fibrinogen beta chain OS=	0
P02746;D6R934;Q5T960;I	C1QB_HUMAN Complement C1q subcomponent subunit B OS=Homo sapiens GN=C1QB PE=1 SV=3;>tr|D6R934|D6R934_HUMAN Unc	1.38E-47
P02749;D9IWP9;B4DPN0	APOH_HUMAN Beta-2-glycoprotein 1 OS=Homo sapiens GN=APOH PE=1 SV=3;>tr|D9IWP9|D9IWP9_HUMAN Beta-2-glycoprotein I (F	0
P02751;P02751-3;P02751	FINC_HUMAN Fibronectin OS=Homo sapiens GN=FN1 PE=1 SV=4;>sp|P02751-3|FINC_HUMAN Isoform 3 of Fibronectin OS=Homo sa	0
P02760;B7Z8R6	AMBP_HUMAN Protein AMBP OS=Homo sapiens GN=AMBP PE=1 SV=1;>tr|B7Z8R6|B7Z8R6_HUMAN cDNA FLJ51445, highly similar to	4.20E-84
P02763;Q5T539	A1AG1_HUMAN Alpha-1-acid glycoprotein 1 OS=Homo sapiens GN=ORM1 PE=1 SV=1;>tr|Q5T539|Q5T539_HUMAN Orosomucoid 1 O	3.41E-196
P02787;Q06AH7	TRFE_HUMAN Serotransferrin OS=Homo sapiens GN=TF PE=1 SV=3;>tr|Q06AH7|Q06AH7_HUMAN Transferrin OS=Homo sapiens GN=	0
P02790;Q9BS19	HEMO_HUMAN Hemopexin OS=Homo sapiens GN=HPX PE=1 SV=2;>tr|Q9BS19|Q9BS19_HUMAN HPX protein OS=Homo sapiens PE=	0
P04003;Q5VVQ8;B4E1D8;	C4BPA_HUMAN C4b-binding protein alpha chain OS=Homo sapiens GN=C4BPA PE=1 SV=2;>tr|Q5VVQ8|Q5VVQ8_HUMAN Complemer	1.61E-168

P04062;P04062-2;P04062	GLCM_HUMAN Glucosylceramidase OS=Homo sapiens GN=GBA PE=1 SV=3;>sp|P04062-2|GLCM_HUMAN Isoform Short of Glucosylce	1.14E-16
P04080;Q76LA1	CYTB_HUMAN Cystatin-B OS=Homo sapiens GN=CSTB PE=1 SV=2;>tr|Q76LA1|Q76LA1_HUMAN CSTB protein OS=Homo sapiens GN=	8.40E-63
P04083;Q5TZZ9;B5BU38;	ANXA1_HUMAN Annexin A1 OS=Homo sapiens GN=ANXA1 PE=1 SV=2;>tr|Q5TZZ9|Q5TZZ9_HUMAN ANXA1 protein OS=Homo sapien	0
P04150-3;P04150;P04150	3|GCR_HUMAN Isoform Alpha-2 of Glucocorticoid receptor OS=Homo sapiens GN=NR3C1;>sp|P04150|GCR_HUMAN Glucocorticoid rec	0.00018095
P04207;Q9UL85;P04434;P	KV308_HUMAN Ig kappa chain V-III region CLL OS=Homo sapiens PE=1 SV=2;>tr|Q9UL85|Q9UL85_HUMAN Myosin-reactive immuno	6.28E-117
P04217;P04217-2;B3KP79	A1BG_HUMAN Alpha-1B-glycoprotein OS=Homo sapiens GN=A1BG PE=1 SV=4;>sp|P04217-2|A1BG_HUMAN Isoform 2 of Alpha-1B-g	0
P04229;Q30154	2B11_HUMAN HLA class II histocompatibility antigen, DRB1-1 beta chain OS=Homo sapiens GN=HLA-DRB1 PE=1 SV=2;>sp|Q30154|	4.84E-104
P04350;B4DE77;B4DJ43;B	TBB4_HUMAN Tubulin beta-4 chain OS=Homo sapiens GN=TUBB4 PE=1 SV=2;>tr|B4DE77|B4DE77_HUMAN cDNA FLJ55189, highly s	0
P04406;Q2TSD0;Q0QET7;	G3P_HUMAN Glyceraldehyde-3-phosphate dehydrogenase OS=Homo sapiens GN=GAPDH PE=1 SV=3;>tr|Q2TSD0|Q2TSD0_HUMAN G	3.87E-275
P04439;P30443;P30455	1A03_HUMAN HLA class I histocompatibility antigen, A-3 alpha chain OS=Homo sapiens GN=HLA-A PE=1 SV=2;>sp|P30443|1A01_HU	1.18E-236
P04626;P04626-4;P04626	ERBB2_HUMAN Receptor tyrosine-protein kinase erbB-2 OS=Homo sapiens GN=ERBB2 PE=1 SV=1;>sp|P04626-4|ERBB2_HUMAN Iso	5.47E-57
P04792;B4DL87	HSPB1_HUMAN Heat shock protein beta-1 OS=Homo sapiens GN=HSPB1 PE=1 SV=2;>tr|B4DL87|B4DL87_HUMAN cDNA FLJ52243, h	1.64E-129
P04899;P04899-2;P63096	GNAI2_HUMAN Guanine nucleotide-binding protein G(i) subunit alpha-2 OS=Homo sapiens GN=GNAI2 PE=1 SV=3;>sp|P04899-2|GN	5.52E-138
P04899;Q96C71;B3KTZ0;	GNAI2_HUMAN Guanine nucleotide-binding protein G(i) subunit alpha-2 OS=Homo sapiens GN=GNAI2 PE=1 SV=3;>tr|Q96C71|Q96C	7.93E-160
P04908;Q08AJ9;Q7L7L0;Q	H2A1B_HUMAN Histone H2A type 1-B/E OS=Homo sapiens GN=HIST1H2AB PE=1 SV=2;>tr|Q08AJ9|Q08AJ9_HUMAN Histone H2A OS	1.30E-97
P04908;Q7L7L0;Q93077	H2A1B_HUMAN Histone H2A type 1-B/E OS=Homo sapiens GN=HIST1H2AB PE=1 SV=2;>sp|Q7L7L0|H2A3_HUMAN Histone H2A type	6.31E-150
P05023;P05023-2;P13637	AT1A1_HUMAN Sodium/potassium-transporting ATPase subunit alpha-1 OS=Homo sapiens GN=ATP1A1 PE=1 SV=1;>sp|P05023-2|AT	1.51E-51
P05091;Q53FB6;B4DW54;	ALDH2_HUMAN Aldehyde dehydrogenase, mitochondrial OS=Homo sapiens GN=ALDH2 PE=1 SV=2;>tr|Q53FB6|Q53FB6_HUMAN Mito	0
P05156;B4DRF2;A8K3L0;Q	CFAI_HUMAN Complement factor I OS=Homo sapiens GN=CFI PE=1 SV=2;>tr|B4DRF2|B4DRF2_HUMAN cDNA FLJ58124, highly simil	1.91E-53
P05198;Q53XC0	IF2A_HUMAN Eukaryotic translation initiation factor 2 subunit 1 OS=Homo sapiens GN=EIF2S1 PE=1 SV=3;>tr|Q53XC0|Q53XC0_HUN	2.19E-145
P05204;Q15651;Q15651-2	HMGN2_HUMAN Non-histone chromosomal protein HMG-17 OS=Homo sapiens GN=HMGN2 PE=1 SV=3;>sp|Q15651|HMGN3_HUMAN	1.28E-08
P05362;Q5NKV8	ICAM1_HUMAN Intercellular adhesion molecule 1 OS=Homo sapiens GN=ICAM1 PE=1 SV=2;>tr|Q5NKV8|Q5NKV8_HUMAN Intercellul	3.26E-38
P05386;Q6FG99;Q6ICQ4;(	RLA1_HUMAN 60S acidic ribosomal protein P1 OS=Homo sapiens GN=RPLP1 PE=1 SV=1;>tr|Q6FG99|Q6FG99_HUMAN RPLP1 protein	4.63E-61
P05387;Q6FG96	RLA2_HUMAN 60S acidic ribosomal protein P2 OS=Homo sapiens GN=RPLP2 PE=1 SV=1;>tr|Q6FG96|Q6FG96_HUMAN RPLP2 protein	1.71E-146
P05388;Q8NHW5	RLA0_HUMAN 60S acidic ribosomal protein P0 OS=Homo sapiens GN=RPLP0 PE=1 SV=1;>sp|Q8NHW5|RLA0L_HUMAN 60S acidic ribo	0
P05412;Q6FHM7;Q6FHK0	JUN_HUMAN Transcription factor AP-1 OS=Homo sapiens GN=JUN PE=1 SV=2;>tr|Q6FHM7|Q6FHM7_HUMAN JUN protein OS=Homo s	9.83E-10
P05413;Q6IBD7	FABPH_HUMAN Fatty acid-binding protein, heart OS=Homo sapiens GN=FABP3 PE=1 SV=4;>tr|Q6IBD7|Q6IBD7_HUMAN FABP3 protei	1.32E-13
P05455;B5BUB5;Q14730	LA_HUMAN Lupus La protein OS=Homo sapiens GN=SSB PE=1 SV=2;>tr|B5BUB5|B5BUB5_HUMAN Autoantigen La (Fragment) OS=H	7.78E-228
P05997;B4DNJ0	CO5A2_HUMAN Collagen alpha-2(V) chain OS=Homo sapiens GN=COL5A2 PE=1 SV=3;>tr|B4DNJ0|B4DNJ0_HUMAN cDNA FLJ60734,	1.39E-12
P06132;Q53ZP6;Q5T450;(	DCUP_HUMAN Uroporphyrinogen decarboxylase OS=Homo sapiens GN=UROD PE=1 SV=2;>tr|Q53ZP6|Q53ZP6_HUMAN Uroporphyrin	5.18E-18
P06280;Q53HF3;Q53Y83;(	AGAL_HUMAN Alpha-galactosidase A OS=Homo sapiens GN=GLA PE=1 SV=1;>tr|Q53HF3|Q53HF3_HUMAN Galactosidase, alpha varia	3.90E-31
P06401;Q8TDS3;P06401-2	PRGR_HUMAN Progesterone receptor OS=Homo sapiens GN=PGR PE=1 SV=4;>tr|Q8TDS3|Q8TDS3_HUMAN Delta 4 progesterone rec	3.57E-05
P06454;P06454-2;O60271	PTMA_HUMAN Prothymosin alpha OS=Homo sapiens GN=PTMA PE=1 SV=2;>sp|P06454-2|PTMA_HUMAN Isoform 2 of Prothymosin al	3.29E-184
P06454;P06454-2;Q1520	PTMA_HUMAN Prothymosin alpha OS=Homo sapiens GN=PTMA PE=1 SV=2;>sp|P06454-2|PTMA_HUMAN Isoform 2 of Prothymosin al	2.98E-51
P06576;Q0QEN7	ATPB_HUMAN ATP synthase subunit beta, mitochondrial OS=Homo sapiens GN=ATP5B PE=1 SV=3;>tr|Q0QEN7|Q0QEN7_HUMAN ATP	0
P06727;Q13784	APOA4_HUMAN Apolipoprotein A-IV OS=Homo sapiens GN=APOA4 PE=1 SV=3;>tr|Q13784|Q13784_HUMAN APOA4 protein (Fragmen	0
P06733;P06733-2;E2DRY6	ENOA_HUMAN Alpha-enolase OS=Homo sapiens GN=ENO1 PE=1 SV=2;>sp|P06733-2|ENOA_HUMAN Isoform MBP-1 of Alpha-enolase	0
P06737;B4DUB7;B2R825	PYGL_HUMAN Glycogen phosphorylase, liver form OS=Homo sapiens GN=PYGL PE=1 SV=4;>tr|B4DUB7|B4DUB7_HUMAN Phosphoryl	0
P06899;P23527;P33778;Q	H2B1J_HUMAN Histone H2B type 1-J OS=Homo sapiens GN=HIST1H2BJ PE=1 SV=3;>sp|P23527|H2B1O_HUMAN Histone H2B type 1	4.11E-129
P07195;Q5U077;A8MW50	LDHB_HUMAN L-lactate dehydrogenase B chain OS=Homo sapiens GN=LDHB PE=1 SV=2;>tr|Q5U077|Q5U077_HUMAN L-lactate dehy	2.92E-226
P07197;P07196	NFM_HUMAN Neurofilament medium polypeptide OS=Homo sapiens GN=NEFM PE=1 SV=3;>sp|P07196|NFL_HUMAN Neurofilament li	8.51E-13
P07203;Q6NSD4	GPX1_HUMAN Glutathione peroxidase 1 OS=Homo sapiens GN=GPX1 PE=1 SV=4;>tr|Q6NSD4|Q6NSD4_HUMAN Glutathione peroxida	4.96E-29
P07237;B4DNL5;B4DUA5;	PDIA1_HUMAN Protein disulfide-isomerase OS=Homo sapiens GN=P4HB PE=1 SV=3;>tr|B4DNL5|B4DNL5_HUMAN cDNA FLJ59361, hi	0
P07305;B4DRD6	H10_HUMAN Histone H1.0 OS=Homo sapiens GN=H1F0 PE=1 SV=3;>tr|B4DRD6|B4DRD6_HUMAN cDNA FLJ54804, highly similar to H	1.45E-39
P07339;C9JH19;C9J9G3	CATD_HUMAN Cathepsin D OS=Homo sapiens GN=CTSD PE=1 SV=1;>tr|C9JH19|C9JH19_HUMAN Uncharacterized protein OS=Homo	2.49E-161
P07355-2;P07355;A6NMY(	2|ANXA2_HUMAN Isoform 2 of Annexin A2 OS=Homo sapiens GN=ANXA2;>sp|P07355|ANXA2_HUMAN Annexin A2 OS=Homo sapiens	0
P07360;Q5SQ08	CO8G_HUMAN Complement component C8 gamma chain OS=Homo sapiens GN=C8G PE=1 SV=3;>tr|Q5SQ08|Q5SQ08_HUMAN Com	1.54E-14
P07384;B2RDI5;A8MSM1;	CAN1_HUMAN Calpain-1 catalytic subunit OS=Homo sapiens GN=CAPN1 PE=1 SV=1;>tr|B2RDI5|B2RDI5_HUMAN cDNA, FLJ96627, h	5.01E-163
P07437;Q5SU16;Q5JP53;I	TBB5_HUMAN Tubulin beta chain OS=Homo sapiens GN=TUBB PE=1 SV=2;>tr|Q5SU16|Q5SU16_HUMAN Beta 5-tubulin OS=Homo sa	0
P07585;Q6FH10;P07585-4	PGS2_HUMAN Decorin OS=Homo sapiens GN=DCN PE=1 SV=1;>tr|Q6FH10|Q6FH10_HUMAN DCN protein OS=Homo sapiens GN=DC	1.95E-280
P07686;Q5URX0	HEXB_HUMAN Beta-hexosaminidase subunit beta OS=Homo sapiens GN=HEXB PE=1 SV=3;>tr|Q5URX0|Q5URX0_HUMAN ENC-1AS O	5.94E-76
P07737;Q53Y44	PROF1_HUMAN Profilin-1 OS=Homo sapiens GN=PFN1 PE=1 SV=2;>tr|Q53Y44|Q53Y44_HUMAN Profilin OS=Homo sapiens GN=PFN1	3.99E-289
P07814;B4DKX5;Q3KQZ8	SYEP_HUMAN Bifunctional aminoacyl-tRNA synthetase OS=Homo sapiens GN=EPRS PE=1 SV=5;>tr|B4DKX5|B4DKX5_HUMAN cDNA F	0
P07858;B3KUJ8;B4DL49;E	CATB_HUMAN Cathepsin B OS=Homo sapiens GN=CTSB PE=1 SV=3;>tr|B3KUJ8|B3KUJ8_HUMAN cDNA FLJ40065 fis, clone TESOP20	4.36E-86
P07900-2;P07900;Q2VPJ6	2|HS90A_HUMAN Isoform 2 of Heat shock protein HSP 90-alpha OS=Homo sapiens GN=HSP90AA1;>sp|P07900|HS90A_HUMAN Heat	0
P07942;Q8TAS6	LAMB1_HUMAN Laminin subunit beta-1 OS=Homo sapiens GN=LAMB1 PE=1 SV=2;>tr|Q8TAS6|Q8TAS6_HUMAN LAMB1 protein (Frag	2.86E-25
P07947;P06241;P06241-2	YES_HUMAN Tyrosine-protein kinase Yes OS=Homo sapiens GN=YES1 PE=1 SV=3;>sp|P06241|FYN_HUMAN Tyrosine-protein kinase F	9.07E-12
P07948;P07948-2;P08631	LYN_HUMAN Tyrosine-protein kinase Lyn OS=Homo sapiens GN=LYN PE=1 SV=3;>sp|P07948-2|LYN_HUMAN Isoform LYN B of Tyrosin	4.83E-17
P08134;Q5JR08;Q5JR05;(	RHOC_HUMAN Rho-related GTP-binding protein RhoC OS=Homo sapiens GN=RHOC PE=1 SV=1;>tr|Q5JR08|Q5JR08_HUMAN Ras hon	4.05E-68
P08236;P08236-2	BGLR_HUMAN Beta-glucuronidase OS=Homo sapiens GN=GUSB PE=1 SV=2;>sp|P08236-2|BGLR_HUMAN Isoform Short of Beta-gluc	2.85E-32
P08238;B4DGL0;B4DMA2	HS90B_HUMAN Heat shock protein HSP 90-beta OS=Homo sapiens GN=HSP90AB1 PE=1 SV=4;>tr|B4DGL0|B4DGL0_HUMAN cDNA FL	0

<table>
<tr><th></th><th></th><th></th></tr>
<tr><td>P08238;Q58FF7</td><td>HS90B_HUMAN Heat shock protein HSP 90-beta OS=Homo sapiens GN=HSP90AB1 PE=1 SV=4;>sp|Q58FF7|H90B3_HUMAN Putative</td><td>0</td></tr>
<tr><td>P08240;Q6IAX9;B4E0H3</td><td>SRPR_HUMAN Signal recognition particle receptor subunit alpha OS=Homo sapiens GN=SRPR PE=1 SV=2;>tr|Q6IAX9|Q6IAX9_HUMA</td><td>5.93E-50</td></tr>
<tr><td>P08253;B4DWH3</td><td>MMP2_HUMAN 72 kDa type IV collagenase OS=Homo sapiens GN=MMP2 PE=1 SV=2;>tr|B4DWH3|B4DWH3_HUMAN cDNA FLJ59773,</td><td>2.05E-55</td></tr>
<tr><td>P08572;B4DH43</td><td>CO4A2_HUMAN Collagen alpha-2(IV) chain OS=Homo sapiens GN=COL4A2 PE=1 SV=4;>tr|B4DH43|B4DH43_HUMAN cDNA FLJ5643</td><td>2.47E-08</td></tr>
<tr><td>P08574;Q8TBT6</td><td>CY1_HUMAN Cytochrome c1, heme protein, mitochondrial OS=Homo sapiens GN=CYC1 PE=1 SV=3;>tr|Q8TBT6|Q8TBT6_HUMAN Put</td><td>2.37E-20</td></tr>
<tr><td>P08581-2;P08581</td><td>2|MET_HUMAN Isoform 2 of Hepatocyte growth factor receptor OS=Homo sapiens GN=MET;>sp|P08581|MET_HUMAN Hepatocyte gro</td><td>1.80E-15</td></tr>
<tr><td>P08590;P05976;P05976-2</td><td>MYL3_HUMAN Myosin light chain 3 OS=Homo sapiens GN=MYL3 PE=1 SV=3;>sp|P05976|MYL1_HUMAN Myosin light chain 1/3, skelet</td><td>2.93E-09</td></tr>
<tr><td>P08603;A8K5T0;Q14006</td><td>CFAH_HUMAN Complement factor H OS=Homo sapiens GN=CFH PE=1 SV=4;>tr|A8K5T0|A8K5T0_HUMAN cDNA FLJ75416, highly sim</td><td>1.96E-162</td></tr>
<tr><td>P08621;P08621-2;P08621</td><td>RU17_HUMAN U1 small nuclear ribonucleoprotein 70 kDa OS=Homo sapiens GN=SNRNP70 PE=1 SV=2;>sp|P08621-2|RU17_HUMAN</td><td>3.40E-77</td></tr>
<tr><td>P08637;C9JC71</td><td>FCG3A_HUMAN Low affinity immunoglobulin gamma Fc region receptor III-A OS=Homo sapiens GN=FCGR3A PE=2 SV=2;>tr|C9JC71|</td><td>7.11E-06</td></tr>
<tr><td>P08697;B4E1B7;C9JMH6;</td><td>A2AP_HUMAN Alpha-2-antiplasmin OS=Homo sapiens GN=SERPINF2 PE=1 SV=3;>tr|B4E1B7|B4E1B7_HUMAN cDNA FLJ50942, highl</td><td>8.20E-115</td></tr>
<tr><td>P08708;P0CW22</td><td>RS17_HUMAN 40S ribosomal protein S17 OS=Homo sapiens GN=RPS17 PE=1 SV=2;>sp|P0CW22|RS17L_HUMAN 40S ribosomal prot</td><td>8.97E-278</td></tr>
<tr><td>P08727;C9JM50</td><td>K1C19_HUMAN Keratin, type I cytoskeletal 19 OS=Homo sapiens GN=KRT19 PE=1 SV=4;>tr|C9JM50|C9JM50_HUMAN Uncharacterize</td><td>0</td></tr>
<tr><td>P08754;Q5TZX1</td><td>GNAI3_HUMAN Guanine nucleotide-binding protein G(k) subunit alpha OS=Homo sapiens GN=GNAI3 PE=1 SV=3;>tr|Q5TZX1|Q5TZX</td><td>3.38E-89</td></tr>
<tr><td>P08865;C9J9K3;A6NE09;</td><td>RSSA_HUMAN 40S ribosomal protein SA OS=Homo sapiens GN=RPSA PE=1 SV=4;>tr|C9J9K3|C9J9K3_HUMAN Uncharacterized prote</td><td>2.16E-270</td></tr>
<tr><td>P09104;Q6FHV6;A8K3B0;</td><td>ENOG_HUMAN Gamma-enolase OS=Homo sapiens GN=ENO2 PE=1 SV=3;>tr|Q6FHV6|Q6FHV6_HUMAN Enolase OS=Homo sapiens G</td><td>0</td></tr>
<tr><td>P09110;C9JDE9;B4DEN6;</td><td>THIK_HUMAN 3-ketoacyl-CoA thiolase, peroxisomal OS=Homo sapiens GN=ACAA1 PE=1 SV=2;>tr|C9JDE9|C9JDE9_HUMAN Uncharac</td><td>7.29E-100</td></tr>
<tr><td>P09211;A8MX94</td><td>GSTP1_HUMAN Glutathione S-transferase P OS=Homo sapiens GN=GSTP1 PE=1 SV=2;>tr|A8MX94|A8MX94_HUMAN Uncharacterized</td><td>6.41E-216</td></tr>
<tr><td>P09417;B3KW71;D6RGG7</td><td>DHPR_HUMAN Dihydropteridine reductase OS=Homo sapiens GN=QDPR PE=1 SV=2;>tr|B3KW71|B3KW71_HUMAN Quinoid dihydropt</td><td>1.80E-179</td></tr>
<tr><td>P09429;B2RPK0</td><td>HMGB1_HUMAN High mobility group protein B1 OS=Homo sapiens GN=HMGB1 PE=1 SV=3;>sp|B2RPK0|HGB1A_HUMAN Putative hig</td><td>2.17E-148</td></tr>
<tr><td>P09429;Q59GW1;B7Z965;</td><td>HMGB1_HUMAN High mobility group protein B1 OS=Homo sapiens GN=HMGB1 PE=1 SV=3;>tr|Q59GW1|Q59GW1_HUMAN High-mob</td><td>9.90E-174</td></tr>
<tr><td>P09488;P09488-2;B9ZVX</td><td>GSTM1_HUMAN Glutathione S-transferase Mu 1 OS=Homo sapiens GN=GSTM1 PE=1 SV=3;>sp|P09488-2|GSTM1_HUMAN Isoform 2</td><td>6.42E-96</td></tr>
<tr><td>P09488;P09488-2;Q0301</td><td>GSTM1_HUMAN Glutathione S-transferase Mu 1 OS=Homo sapiens GN=GSTM1 PE=1 SV=3;>sp|P09488-2|GSTM1_HUMAN Isoform 2</td><td>1.43E-74</td></tr>
<tr><td>P09493-3;P09493-4;P094</td><td>3|TPM1_HUMAN Isoform 3 of Tropomyosin alpha-1 chain OS=Homo sapiens GN=TPM1;>sp|P09493-4|TPM1_HUMAN Isoform 4 of Trop</td><td>0</td></tr>
<tr><td>P09493-3;P09493-4;P094</td><td>3|TPM1_HUMAN Isoform 3 of Tropomyosin alpha-1 chain OS=Homo sapiens GN=TPM1;>sp|P09493-4|TPM1_HUMAN Isoform 4 of Trop</td><td>0</td></tr>
<tr><td>P09496;P09496-3;P09496</td><td>CLCA_HUMAN Clathrin light chain A OS=Homo sapiens GN=CLTA PE=1 SV=1;>sp|P09496-3|CLCA_HUMAN Isoform 3 of Clathrin light</td><td>2.63E-09</td></tr>
<tr><td>P09497;P09497-2;D6RJD1</td><td>CLCB_HUMAN Clathrin light chain B OS=Homo sapiens GN=CLTB PE=1 SV=1;>sp|P09497-2|CLCB_HUMAN Isoform Non-brain of Clath</td><td>1.36E-17</td></tr>
<tr><td>P09543;P09543-2;B4DI06</td><td>CN37_HUMAN 2,3-cyclic-nucleotide 3-phosphodiesterase OS=Homo sapiens GN=CNP PE=1 SV=2;>sp|P09543-2|CN37_HUMAN Isofor</td><td>1.91E-86</td></tr>
<tr><td>P09622;B4DFL1;B4DMK9;</td><td>DLDH_HUMAN Dihydrolipoyl dehydrogenase, mitochondrial OS=Homo sapiens GN=DLD PE=1 SV=2;>tr|B4DFL1|B4DFL1_HUMAN Dihy</td><td>1.02E-212</td></tr>
<tr><td>P09651;P09651-2;Q6IPF2</td><td>ROA1_HUMAN Heterogeneous nuclear ribonucleoprotein A1 OS=Homo sapiens GN=HNRNPA1 PE=1 SV=5;>sp|P09651-2|ROA1_HUMA</td><td>3.70E-147</td></tr>
<tr><td>P09661;Q53G21;Q53G61;</td><td>RU2A_HUMAN U2 small nuclear ribonucleoprotein A OS=Homo sapiens GN=SNRPA1 PE=1 SV=2;>tr|Q53G21|Q53G21_HUMAN Small</td><td>2.17E-45</td></tr>
<tr><td>P09874;B2R5W3;B4E0E1</td><td>PARP1_HUMAN Poly [ADP-ribose] polymerase 1 OS=Homo sapiens GN=PARP1 PE=1 SV=4;>tr|B2R5W3|B2R5W3_HUMAN cDNA, FLJ9</td><td>6.41E-198</td></tr>
<tr><td>P09914;Q5T7J1;D3DR31;</td><td>IFIT1_HUMAN Interferon-induced protein with tetratricopeptide repeats 1 OS=Homo sapiens GN=IFIT1 PE=1 SV=2;>tr|Q5T7J1|Q5T7</td><td>3.63E-269</td></tr>
<tr><td>P09960;P09960-2</td><td>LKHA4_HUMAN Leukotriene A-4 hydrolase OS=Homo sapiens GN=LTA4H PE=1 SV=2;>sp|P09960-2|LKHA4_HUMAN Isoform 2 of Leu</td><td>2.30E-166</td></tr>
<tr><td>P09960;P09960-2;P09960</td><td>LKHA4_HUMAN Leukotriene A-4 hydrolase OS=Homo sapiens GN=LTA4H PE=1 SV=2;>sp|P09960-2|LKHA4_HUMAN Isoform 2 of Leu</td><td>7.34E-212</td></tr>
<tr><td>P0C0S5;Q71UI9</td><td>H2AZ_HUMAN Histone H2A.Z OS=Homo sapiens GN=H2AFZ PE=1 SV=2;>sp|Q71UI9|H2AV_HUMAN Histone H2A.V OS=Homo sapien</td><td>1.79E-121</td></tr>
<tr><td>P0C0S5;Q71UI9;C9J0D1;</td><td>H2AZ_HUMAN Histone H2A.Z OS=Homo sapiens GN=H2AFZ PE=1 SV=2;>sp|Q71UI9|H2AV_HUMAN Histone H2A.V OS=Homo sapien</td><td>1.81E-19</td></tr>
<tr><td>P0C0S8;P20671;Q6FI13;</td><td>H2A1_HUMAN Histone H2A type 1 OS=Homo sapiens GN=HIST1H2AG PE=1 SV=2;>sp|P20671|H2A1D_HUMAN Histone H2A type 1-D</td><td>2.34E-144</td></tr>
<tr><td>P0C0S8;P20671;Q96KK5;</td><td>H2A1_HUMAN Histone H2A type 1 OS=Homo sapiens GN=HIST1H2AG PE=1 SV=2;>sp|P20671|H2A1D_HUMAN Histone H2A type 1-D</td><td>6.10E-128</td></tr>
<tr><td>P0C7P4;P47985</td><td>UCRIL_HUMAN Putative cytochrome b-c1 complex subunit Rieske-like protein 1 OS=Homo sapiens GN=UQCRFSL1 PE=5 SV=1;>sp|P4</td><td>9.31E-61</td></tr>
<tr><td>P0CF74;P0CG05;P0CG06;</td><td>LAC6_HUMAN Ig lambda-6 chain C region OS=Homo sapiens GN=IGLC6 PE=4 SV=1;>sp|P0CG05|LAC2_HUMAN Ig lambda-2 chain C</td><td>1.25E-11</td></tr>
<tr><td>P0CG05;P0CG06;P0CF74</td><td>LAC2_HUMAN Ig lambda-2 chain C regions OS=Homo sapiens GN=IGLC2 PE=1 SV=1;>sp|P0CG06|LAC3_HUMAN Ig lambda-3 chain</td><td>1.13E-242</td></tr>
<tr><td>P0CG05;P0CG06;P0CF74;</td><td>LAC2_HUMAN Ig lambda-2 chain C regions OS=Homo sapiens GN=IGLC2 PE=1 SV=1;>sp|P0CG06|LAC3_HUMAN Ig lambda-3 chain</td><td>0</td></tr>
<tr><td>P0CG29;P0CG30</td><td>GST2_HUMAN Glutathione S-transferase theta-2 OS=Homo sapiens GN=GSTT2 PE=1 SV=1;>sp|P0CG30|GSTT2_HUMAN Glutathione</td><td>5.02E-15</td></tr>
<tr><td>P10155;P10155-2</td><td>RO60_HUMAN 60 kDa SS-A/Ro ribonucleoprotein OS=Homo sapiens GN=TROVE2 PE=1 SV=2;>sp|P10155-2|RO60_HUMAN Isoform S</td><td>6.85E-37</td></tr>
<tr><td>P10155;Q5LJ99;Q86WL3;</td><td>RO60_HUMAN 60 kDa SS-A/Ro ribonucleoprotein OS=Homo sapiens GN=TROVE2 PE=1 SV=2;>tr|Q5LJ99|Q5LJ99_HUMAN TROVE do</td><td>2.00E-56</td></tr>
<tr><td>P10319;P18465</td><td>1B58_HUMAN HLA class I histocompatibility antigen, B-58 alpha chain OS=Homo sapiens GN=HLA-B PE=2 SV=1;>sp|P18465|1B57_</td><td>4.31E-139</td></tr>
<tr><td>P10319;P18465;P30484</td><td>1B58_HUMAN HLA class I histocompatibility antigen, B-58 alpha chain OS=Homo sapiens GN=HLA-B PE=2 SV=1;>sp|P18465|1B57_</td><td>9.30E-201</td></tr>
<tr><td>P10412;P16402</td><td>H14_HUMAN Histone H1.4 OS=Homo sapiens GN=HIST1H1E PE=1 SV=2;>sp|P16402|H13_HUMAN Histone H1.3 OS=Homo sapiens</td><td>1.91E-130</td></tr>
<tr><td>P10515;Q86YI5;B4DJX1;</td><td>ODP2_HUMAN Dihydrolipoyllysine-residue acetyltransferase component of pyruvate dehydrogenase complex, mitochondrial OS=Homo</td><td>1.30E-82</td></tr>
<tr><td>P10599;B1ALW1;O60744</td><td>THIO_HUMAN Thioredoxin OS=Homo sapiens GN=TXN PE=1 SV=3;>tr|B1ALW1|B1ALW1_HUMAN Thioredoxin OS=Homo sapiens GN=</td><td>2.06E-91</td></tr>
<tr><td>P10606;Q53YB7;Q6FHM4;</td><td>COX5B_HUMAN Cytochrome c oxidase subunit 5B, mitochondrial OS=Homo sapiens GN=COX5B PE=1 SV=2;>tr|Q53YB7|Q53YB7_HU</td><td>4.59E-18</td></tr>
<tr><td>P10620;Q6LET6</td><td>MGST1_HUMAN Microsomal glutathione S-transferase 1 OS=Homo sapiens GN=MGST1 PE=1 SV=1;>tr|Q6LET6|Q6LET6_HUMAN MGS</td><td>8.41E-34</td></tr>
<tr><td>P10809;B3GQS7;B7Z597;</td><td>CH60_HUMAN 60 kDa heat shock protein, mitochondrial OS=Homo sapiens GN=HSPD1 PE=1 SV=2;>tr|B3GQS7|B3GQS7_HUMAN Mit</td><td>0</td></tr>
<tr><td>P10909-2;P10909;B3KSE</td><td>2|CLUS_HUMAN Isoform 2 of Clusterin OS=Homo sapiens GN=CLU;>sp|P10909|CLUS_HUMAN Clusterin OS=Homo sapiens GN=CLU</td><td>1.55E-219</td></tr>
<tr><td>P11021;Q2KHP4</td><td>GRP78_HUMAN 78 kDa glucose-regulated protein OS=Homo sapiens GN=HSPA5 PE=1 SV=2;>tr|Q2KHP4|Q2KHP4_HUMAN HSPA5 pro</td><td>0</td></tr>
<tr><td>P11142;Q53GZ6;Q96IS6;</td><td>HSP7C_HUMAN Heat shock cognate 71 kDa protein OS=Homo sapiens GN=HSPA8 PE=1 SV=1;>tr|Q53GZ6|Q53GZ6_HUMAN Heat sh</td><td>0</td></tr>
<tr><td>P11177;P11177-2</td><td>ODPB_HUMAN Pyruvate dehydrogenase E1 component subunit beta, mitochondrial OS=Homo sapiens GN=PDHB PE=1 SV=3;>sp|P11</td><td>4.53E-123</td></tr>
<tr><td>P11182;B4E1Q7;Q5VVL7</td><td>ODB2_HUMAN Lipoamide acyltransferase component of branched-chain alpha-keto acid dehydrogenase complex, mitochondrial OS=H</td><td>2.15E-22</td></tr>
<tr><td>P11215;Q4VAK1;B3KXM6</td><td>ITAM_HUMAN Integrin alpha-M OS=Homo sapiens GN=ITGAM PE=1 SV=2;>tr|Q4VAK1|Q4VAK1_HUMAN Integrin, alpha M (Compleme</td><td>1.37E-08</td></tr>
<tr><td>P11216;B4DSD8</td><td>PYGB_HUMAN Glycogen phosphorylase, brain form OS=Homo sapiens GN=PYGB PE=1 SV=5;>tr|B4DSD8|B4DSD8_HUMAN Phosphor</td><td>0</td></tr>
</table>

P11277-2;P11277;P11277	2|SPTB1_HUMAN Isoform 2 of Spectrin beta chain, erythrocyte OS=Homo sapiens GN=SPTB;>sp|P11277|SPTB1_HUMAN Spectrin bet	7.22E-30
P11279;B3KRY3;B4DWL3	LAMP1_HUMAN Lysosome-associated membrane glycoprotein 1 OS=Homo sapiens GN=LAMP1 PE=1 SV=3;>tr|B3KRY3|B3KRY3_HUM	4.82E-65
P11413-2;P11413-3;P114	2|G6PD_HUMAN Isoform Long of Glucose-6-phosphate 1-dehydrogenase OS=Homo sapiens GN=G6PD;>sp|P11413-3|G6PD_HUMAN I	3.64E-55
P11586;B7Z809	C1TC_HUMAN C-1-tetrahydrofolate synthase, cytoplasmic OS=Homo sapiens GN=MTHFD1 PE=1 SV=3;>tr|B7Z809|B7Z809_HUMAN c	3.23E-216
P11717;Q59EZ3	MPRI_HUMAN Cation-independent mannose-6-phosphate receptor OS=Homo sapiens GN=IGF2R PE=1 SV=3;>tr|Q59EZ3|Q59EZ3_HU	7.51E-78
P11766;Q5U043;Q6FI45;(	ADHX_HUMAN Alcohol dehydrogenase class-3 OS=Homo sapiens GN=ADH5 PE=1 SV=4;>tr|Q5U043|Q5U043_HUMAN Alcohol dehydr	1.44E-138
P11908-2;P11908	2|PRPS2_HUMAN Isoform 2 of Ribose-phosphate pyrophosphokinase 2 OS=Homo sapiens GN=PRPS2;>sp|P11908|PRPS2_HUMAN Rib	3.57E-53
P11940;P11940-2	PABP1_HUMAN Polyadenylate-binding protein 1 OS=Homo sapiens GN=PABPC1 PE=1 SV=2;>sp|P11940-2|PABP1_HUMAN Isoform 2	0
P11940;P11940-2;Q9H36	PABP1_HUMAN Polyadenylate-binding protein 1 OS=Homo sapiens GN=PABPC1 PE=1 SV=2;>sp|P11940-2|PABP1_HUMAN Isoform 2	0
P12081;D6REN6;B4DY73;	SYHC_HUMAN Histidyl-tRNA synthetase, cytoplasmic OS=Homo sapiens GN=HARS PE=1 SV=2;>tr|D6REN6|D6REN6_HUMAN Uncharа	2.07E-146
P12110;Q9BUM6;P12110-	CO6A2_HUMAN Collagen alpha-2(VI) chain OS=Homo sapiens GN=COL6A2 PE=1 SV=4;>tr|Q9BUM6|Q9BUM6_HUMAN COL6A2 protei	0
P12270;Q99968	TPR_HUMAN Nucleoprotein TPR OS=Homo sapiens GN=TPR PE=1 SV=3;>tr|Q99968|Q99968_HUMAN Tpr OS=Homo sapiens GN=tpr	1.95E-157
P12277;B4DP56	KCRB_HUMAN Creatine kinase B-type OS=Homo sapiens GN=CKB PE=1 SV=1;>tr|B4DP56|B4DP56_HUMAN cDNA FLJ52237, highly s	7.25E-82
P12429;D6RA82	ANXA3_HUMAN Annexin A3 OS=Homo sapiens GN=ANXA3 PE=1 SV=3;>tr|D6RA82|D6RA82_HUMAN Uncharacterized protein OS=Ho	8.39E-176
P12532;P12532-2;C9JSQ1	KCRU_HUMAN Creatine kinase U-type, mitochondrial OS=Homo sapiens GN=CKMT1A PE=1 SV=1;>sp|P12532-2|KCRU_HUMAN Isofor	1.39E-286
P12931-2;P12931	2|SRC_HUMAN Isoform 2 of Proto-oncogene tyrosine-protein kinase Src OS=Homo sapiens GN=SRC;>sp|P12931|SRC_HUMAN Proto-	8.84E-10
P12931-2;P12931;P07947	2|SRC_HUMAN Isoform 2 of Proto-oncogene tyrosine-protein kinase Src OS=Homo sapiens GN=SRC;>sp|P12931|SRC_HUMAN Proto-	1.99E-21
P12956;B1AHC9;B2RDN9;	XRCC6_HUMAN X-ray repair cross-complementing protein 6 OS=Homo sapiens GN=XRCC6 PE=1 SV=2;>tr|B1AHC9|B1AHC9_HUMAN	0
P13010;Q53T09	XRCC5_HUMAN X-ray repair cross-complementing protein 5 OS=Homo sapiens GN=XRCC5 PE=1 SV=3;>tr|Q53T09|Q53T09_HUMAN	0
P13284;Q8NEI4;Q8WU77;	GILT_HUMAN Gamma-interferon-inducible lysosomal thiol reductase OS=Homo sapiens GN=IFI30 PE=1 SV=2;>tr|Q8NEI4|Q8NEI4_HU	2.71E-33
P13473;Q6Q3G8;D3DTF0;	LAMP2_HUMAN Lysosome-associated membrane glycoprotein 2 OS=Homo sapiens GN=LAMP2 PE=1 SV=2;>tr|Q6Q3G8|Q6Q3G8_HUM	1.98E-13
P13489;Q8IZK8;Q9BQ80;	RINI_HUMAN Ribonuclease inhibitor OS=Homo sapiens GN=RNH1 PE=1 SV=2;>tr|Q8IZK8|Q8IZK8_HUMAN Ribonuclease/angiogenin	1.99E-128
P13674-2;Q5VSQ6;C9JL1	2|P4HA1_HUMAN Isoform 2 of Prolyl 4-hydroxylase subunit alpha-1 OS=Homo sapiens GN=P4HA1;>tr|Q5VSQ6|Q5VSQ6_HUMAN Pro	3.05E-189
P13716;P13716-2	HEM2_HUMAN Delta-aminolevulinic acid dehydratase OS=Homo sapiens GN=ALAD PE=1 SV=1;>sp|P13716-2|HEM2_HUMAN Isoform	1.83E-16
P13762;Q2LIR4;Q30152;(	DRB4_HUMAN HLA class II histocompatibility antigen, DR beta 4 chain OS=Homo sapiens GN=HLA-DRB4 PE=1 SV=2;>tr|Q2LIR4|Q2L	4.71E-10
P13796;Q53FI1;Q59GX5	PLSL_HUMAN Plastin-2 OS=Homo sapiens GN=LCP1 PE=1 SV=6;>tr|Q53FI1|Q53FI1_HUMAN L-plastin variant (Fragment) OS=Homo	0
P13804;Q53XN3;B4DT43	ETFA_HUMAN Electron transfer flavoprotein subunit alpha, mitochondrial OS=Homo sapiens GN=ETFA PE=1 SV=1;>tr|Q53XN3|Q53XN	3.55E-234
P13928;Q5T2P8	ANXA8_HUMAN Annexin A8 OS=Homo sapiens GN=ANXA8 PE=1 SV=3;>sp|Q5T2P8|AXA81_HUMAN Annexin A8-like protein 1 OS=Ho	0
P13929;P13929-3;P13929	ENOB_HUMAN Beta-enolase OS=Homo sapiens GN=ENO3 PE=1 SV=4;>sp|P13929-3|ENOB_HUMAN Isoform 3 of Beta-enolase OS=H	2.60E-156
P13987;Q6FHM9	CD59_HUMAN CD59 glycoprotein OS=Homo sapiens GN=CD59 PE=1 SV=1;>tr|Q6FHM9|Q6FHM9_HUMAN CD59 antigen, complement	2.42E-19
P13995;B2R7W0;Q7Z650;	MTDC_HUMAN Bifunctional methylenetetrahydrofolate dehydrogenase/cyclohydrolase, mitochondrial OS=Homo sapiens GN=MTHFD2 P	3.39E-15
P14317;B4DQ92	HCLS1_HUMAN Hematopoietic lineage cell-specific protein OS=Homo sapiens GN=HCLS1 PE=1 SV=3;>tr|B4DQ92|B4DQ92_HUMAN c	1.19E-177
P14618;P14618-2	KPYM_HUMAN Pyruvate kinase isozymes M1/M2 OS=Homo sapiens GN=PKM2 PE=1 SV=4;>sp|P14618-2|KPYM_HUMAN Isoform M1 o	0
P14625;Q5CAQ5;B4DU71;	ENPL_HUMAN Endoplasmin OS=Homo sapiens GN=HSP90B1 PE=1 SV=1;>tr|Q5CAQ5|Q5CAQ5_HUMAN Tumor rejection antigen (Gp9	0
P14678-3;P14678;P14678	3|RSMB_HUMAN Isoform SM-B1 of Small nuclear ribonucleoprotein-associated proteins B and B OS=Homo sapiens GN=SNRPB;>sp|P1	2.22E-32
P14678-3;P14678;P63162	3|RSMB_HUMAN Isoform SM-B1 of Small nuclear ribonucleoprotein-associated proteins B and B OS=Homo sapiens GN=SNRPB;>sp|P1	2.06E-25
P14780;B7Z507;B7Z747;I	MMP9_HUMAN Matrix metalloproteinase-9 OS=Homo sapiens GN=MMP9 PE=1 SV=3;>tr|B7Z507|B7Z507_HUMAN cDNA FLJ51036, hi	2.38E-45
P14866;Q6NTA2;B2R959;	HNRPL_HUMAN Heterogeneous nuclear ribonucleoprotein L OS=Homo sapiens GN=HNRNPL PE=1 SV=2;>tr|Q6NTA2|Q6NTA2_HUMAN	0
P14927;B2R4A2;B7Z2R2	QCR7_HUMAN Cytochrome b-c1 complex subunit 7 OS=Homo sapiens GN=UQCRB PE=1 SV=2;>tr|B2R4A2|B2R4A2_HUMAN cDNA, FI	1.16E-72
P15121;Q59EL5	ALDR_HUMAN Aldose reductase OS=Homo sapiens GN=AKR1B1 PE=1 SV=3;>tr|Q59EL5|Q59EL5_HUMAN Aldo-keto reductase family	1.11E-44
P15151;P15151-4;P15151	PVR_HUMAN Poliovirus receptor OS=Homo sapiens GN=PVR PE=1 SV=2;>sp|P15151-4|PVR_HUMAN Isoform Delta of Poliovirus recep	5.96E-50
P15153;B1AH78;B1AH80;	RAC2_HUMAN Ras-related C3 botulinum toxin substrate 2 OS=Homo sapiens GN=RAC2 PE=1 SV=1;>tr|B1AH78|B1AH78_HUMAN Ras	5.54E-43
P15374;Q5TBK7	UCHL3_HUMAN Ubiquitin carboxyl-terminal hydrolase isozyme L3 OS=Homo sapiens GN=UCHL3 PE=1 SV=1;>tr|Q5TBK7|Q5TBK7_HU	6.88E-131
P15559;B7ZAD1;Q53G81;	NQO1_HUMAN NAD(P)H dehydrogenase [quinone] 1 OS=Homo sapiens GN=NQO1 PE=1 SV=1;>tr|B7ZAD1|B7ZAD1_HUMAN NAD(P)I	1.24E-75
P15924;P15924-2	DESP_HUMAN Desmoplakin OS=Homo sapiens GN=DSP PE=1 SV=3;>sp|P15924-2|DESP_HUMAN Isoform DPII of Desmoplakin OS=H	3.30E-11
P15927-3;P15927-2;P159	3|RFA2_HUMAN Isoform 3 of Replication protein A 32 kDa subunit OS=Homo sapiens GN=RPA2;>sp|P15927-2|RFA2_HUMAN Isoform	1.20E-36
P16104;Q96QV6	H2AX_HUMAN Histone H2A.x OS=Homo sapiens GN=H2AFX PE=1 SV=2;>sp|Q96QV6|H2A1A_HUMAN Histone H2A type 1-A OS=Hom	6.20E-225
P16144;P16144-3;P16144	ITB4_HUMAN Integrin beta-4 OS=Homo sapiens GN=ITGB4 PE=1 SV=5;>sp|P16144-3|ITB4_HUMAN Isoform Beta-4B of Integrin bet	5.53E-71
P16152;O75828	CBR1_HUMAN Carbonyl reductase [NADPH] 1 OS=Homo sapiens GN=CBR1 PE=1 SV=3;>sp|O75828|CBR3_HUMAN Carbonyl reducta	3.92E-136
P16615;P16615-4;P16615	AT2A2_HUMAN Sarcoplasmic/endoplasmic reticulum calcium ATPase 2 OS=Homo sapiens GN=ATP2A2 PE=1 SV=1;>sp|P16615-4|AT2	0
P16989;P16989-3;P16989	DBPA_HUMAN DNA-binding protein A OS=Homo sapiens GN=CSDA PE=1 SV=4;>sp|P16989-3|DBPA_HUMAN Isoform 3 of DNA-bindin	0
P17050;B8ZZ36;B8ZZ35	NAGAB_HUMAN Alpha-N-acetylgalactosaminidase OS=Homo sapiens GN=NAGA PE=1 SV=2;>tr|B8ZZ36|B8ZZ36_HUMAN N-acetylgal	1.03E-13
P17066;P48741	HSP76_HUMAN Heat shock 70 kDa protein 6 OS=Homo sapiens GN=HSPA6 PE=1 SV=2;>sp|P48741|HSP77_HUMAN Putative heat sho	0
P17096-3;P17096;P17096	3|HMGA1_HUMAN Isoform HMG-R of High mobility group protein HMG-I/HMG-Y OS=Homo sapiens GN=HMGA1;>sp|P17096|HMGA1_I	6.48E-15
P17152;B7Z587	TMM11_HUMAN Transmembrane protein 11 OS=Homo sapiens GN=TMEM11 PE=1 SV=1;>tr|B7Z587|B7Z587_HUMAN cDNA FLJ5127	2.95E-06
P17174;B7Z7E9;Q2TU84;I	AATC_HUMAN Aspartate aminotransferase, cytoplasmic OS=Homo sapiens GN=GOT1 PE=1 SV=3;>tr|B7Z7E9|B7Z7E9_HUMAN Aspart	5.77E-265
P17252;P05771-2;P05771	KPCA_HUMAN Protein kinase C alpha type OS=Homo sapiens GN=PRKCA PE=1 SV=4;>sp|P05771-2|KPCB_HUMAN Isoform Beta-II of	2.67E-37
P17612;P17612-2	KAPCA_HUMAN cAMP-dependent protein kinase catalytic subunit alpha OS=Homo sapiens GN=PRKACA PE=1 SV=2;>sp|P17612-2|KA	1.13E-47
P17612;P17612-2;P22694	KAPCA_HUMAN cAMP-dependent protein kinase catalytic subunit alpha OS=Homo sapiens GN=PRKACA PE=1 SV=2;>sp|P17612-2|KA	1.19E-43
P17858;P17858-2;Q7L2M	K6PL_HUMAN 6-phosphofructokinase, liver type OS=Homo sapiens GN=PFKL PE=1 SV=6;>sp|P17858-2|K6PL_HUMAN Isoform 2 of 6	0
P18074;A8MX75;Q2TB79;	ERCC2_HUMAN TFIIH basal transcription factor complex helicase XPD subunit OS=Homo sapiens GN=ERCC2 PE=1 SV=1;>tr|A8MX75	3.21E-06

<table>
<tr><td>P18077;C9K025</td><td>RL35A_HUMAN 60S ribosomal protein L35a OS=Homo sapiens GN=RPL35A PE=1 SV=2;>tr|C9K025|C9K025_HUMAN Uncharacterized</td><td>1.04E-49</td></tr>
<tr><td>P18084;D3DNA1</td><td>ITB5_HUMAN Integrin beta-5 OS=Homo sapiens GN=ITGB5 PE=1 SV=1;>tr|D3DNA1|D3DNA1_HUMAN Integrin beta OS=Homo sapie</td><td>1.55E-14</td></tr>
<tr><td>P18085;C9JPM4;C9JAK5</td><td>ARF4_HUMAN ADP-ribosylation factor 4 OS=Homo sapiens GN=ARF4 PE=1 SV=3;>tr|C9JPM4|C9JPM4_HUMAN Uncharacterized protei</td><td>6.79E-131</td></tr>
<tr><td>P18124;A8MUD9;O95036</td><td>RL7_HUMAN 60S ribosomal protein L7 OS=Homo sapiens GN=RPL7 PE=1 SV=1;>tr|A8MUD9|A8MUD9_HUMAN Uncharacterized prote</td><td>1.34E-97</td></tr>
<tr><td>P18206;P18206-2;B3KXA2</td><td>VINC_HUMAN Vinculin OS=Homo sapiens GN=VCL PE=1 SV=4;>sp|P18206-2|VINC_HUMAN Isoform 1 of Vinculin OS=Homo sapiens</td><td>0</td></tr>
<tr><td>P18428;Q8TCF0</td><td>LBP_HUMAN Lipopolysaccharide-binding protein OS=Homo sapiens GN=LBP PE=1 SV=3;>tr|Q8TCF0|Q8TCF0_HUMAN LBP protein OS</td><td>2.42E-16</td></tr>
<tr><td>P18440;Q09LL5;Q6U7I7;C</td><td>ARY1_HUMAN Arylamine N-acetyltransferase 1 OS=Homo sapiens GN=NAT1 PE=1 SV=2;>tr|Q09LL5|Q09LL5_HUMAN NAT1 (Fragmen</td><td>4.46E-113</td></tr>
<tr><td>P18621;B4E3C2;B5ME31</td><td>RL17_HUMAN 60S ribosomal protein L17 OS=Homo sapiens GN=RPL17 PE=1 SV=3;>tr|B4E3C2|B4E3C2_HUMAN cDNA FLJ52184, hig</td><td>2.58E-164</td></tr>
<tr><td>P18669;Q53G35;Q6FHU2;</td><td>PGAM1_HUMAN Phosphoglycerate mutase 1 OS=Homo sapiens GN=PGAM1 PE=1 SV=2;>tr|Q53G35|Q53G35_HUMAN Phosphoglycera</td><td>1.59E-157</td></tr>
<tr><td>P18858;B4DTU4;B4DM52;</td><td>DNLI1_HUMAN DNA ligase 1 OS=Homo sapiens GN=LIG1 PE=1 SV=1;>tr|B4DTU4|B4DTU4_HUMAN DNA ligase OS=Homo sapiens PE</td><td>4.87E-18</td></tr>
<tr><td>P19338;B3KTP9;Q6ZS99;I</td><td>NUCL_HUMAN Nucleolin OS=Homo sapiens GN=NCL PE=1 SV=3;>tr|B3KTP9|B3KTP9_HUMAN cDNA FLJ38578 fis, clone HCHON20076</td><td>0</td></tr>
<tr><td>P19652;Q5T538;Q6IB74;E</td><td>A1AG2_HUMAN Alpha-1-acid glycoprotein 2 OS=Homo sapiens GN=ORM2 PE=1 SV=2;>tr|Q5T538|Q5T538_HUMAN Orosomucoid 2 O</td><td>3.17E-182</td></tr>
<tr><td>P19827;B7Z549;B7Z539</td><td>ITIH1_HUMAN Inter-alpha-trypsin inhibitor heavy chain H1 OS=Homo sapiens GN=ITIH1 PE=1 SV=3;>tr|B7Z549|B7Z549_HUMAN cD</td><td>0</td></tr>
<tr><td>P20036;Q5STP1;A2ABA5;</td><td>DPA1_HUMAN HLA class II histocompatibility antigen, DP alpha 1 chain OS=Homo sapiens GN=HLA-DPA1 PE=1 SV=1;>tr|Q5STP1|Q5</td><td>1.20E-14</td></tr>
<tr><td>P20039;Q95IE3</td><td>2B1B_HUMAN HLA class II histocompatibility antigen, DRB1-11 beta chain OS=Homo sapiens GN=HLA-DRB1 PE=1 SV=1;>sp|Q95IE3</td><td>1.05E-118</td></tr>
<tr><td>P20042;A6NK07</td><td>IF2B_HUMAN Eukaryotic translation initiation factor 2 subunit 2 OS=Homo sapiens GN=EIF2S2 PE=1 SV=2;>sp|A6NK07|IF2BL_HUMA</td><td>7.33E-127</td></tr>
<tr><td>P20042;Q6IBR8;B5BU01;</td><td>IF2B_HUMAN Eukaryotic translation initiation factor 2 subunit 2 OS=Homo sapiens GN=EIF2S2 PE=1 SV=2;>tr|Q6IBR8|Q6IBR8_HUM</td><td>1.36E-292</td></tr>
<tr><td>P20132;Q8WW81</td><td>SDHL_HUMAN L-serine dehydratase/L-threonine deaminase OS=Homo sapiens GN=SDS PE=1 SV=2;>tr|Q8WW81|Q8WW81_HUMAN</td><td>4.89E-05</td></tr>
<tr><td>P20226;Q32MN7;Q5U0C8</td><td>TBP_HUMAN TATA-box-binding protein OS=Homo sapiens GN=TBP PE=1 SV=2;>tr|Q32MN7|Q32MN7_HUMAN TATA box binding protei</td><td>7.22E-06</td></tr>
<tr><td>P20339;Q6FI44;B4DJA5</td><td>RAB5A_HUMAN Ras-related protein Rab-5A OS=Homo sapiens GN=RAB5A PE=1 SV=2;>tr|Q6FI44|Q6FI44_HUMAN Cervical cancer or</td><td>2.14E-28</td></tr>
<tr><td>P20618;Q53FT8;Q59GN1</td><td>PSB1_HUMAN Proteasome subunit beta type-1 OS=Homo sapiens GN=PSMB1 PE=1 SV=2;>tr|Q53FT8|Q53FT8_HUMAN Proteasome b</td><td>2.82E-142</td></tr>
<tr><td>P20700;Q6DC98</td><td>LMNB1_HUMAN Lamin-B1 OS=Homo sapiens GN=LMNB1 PE=1 SV=2;>tr|Q6DC98|Q6DC98_HUMAN LMNB1 protein (Fragment) OS=H</td><td>0</td></tr>
<tr><td>P20810-6;P20810-7;P208</td><td>6|ICAL_HUMAN Isoform 6 of Calpastatin OS=Homo sapiens GN=CAST;>sp|P20810-7|ICAL_HUMAN Isoform 7 of Calpastatin OS=Hom</td><td>2.21E-106</td></tr>
<tr><td>P20810-6;P20810-7;P208</td><td>6|ICAL_HUMAN Isoform 6 of Calpastatin OS=Homo sapiens GN=CAST;>sp|P20810-7|ICAL_HUMAN Isoform 7 of Calpastatin OS=Hom</td><td>1.88E-17</td></tr>
<tr><td>P20936;P20936-2</td><td>RASA1_HUMAN Ras GTPase-activating protein 1 OS=Homo sapiens GN=RASA1 PE=1 SV=1;>sp|P20936-2|RASA1_HUMAN Isoform 2</td><td>2.02E-188</td></tr>
<tr><td>P21266;Q6FGJ9;Q59EJ5;</td><td>GSTM3_HUMAN Glutathione S-transferase Mu 3 OS=Homo sapiens GN=GSTM3 PE=1 SV=3;>tr|Q6FGJ9|Q6FGJ9_HUMAN GSTM3 prote</td><td>8.51E-123</td></tr>
<tr><td>P21281;P15313</td><td>VATB2_HUMAN V-type proton ATPase subunit B, brain isoform OS=Homo sapiens GN=ATP6V1B2 PE=1 SV=3;>sp|P15313|VATB1_HUM</td><td>8.63E-33</td></tr>
<tr><td>P21283;A8MQ60;B7Z593</td><td>VATC1_HUMAN V-type proton ATPase subunit C 1 OS=Homo sapiens GN=ATP6V1C1 PE=1 SV=4;>tr|A8MQ60|A8MQ60_HUMAN Uncha</td><td>8.60E-68</td></tr>
<tr><td>P21291;Q5U0J2;B4DY28;</td><td>CSRP1_HUMAN Cysteine and glycine-rich protein 1 OS=Homo sapiens GN=CSRP1 PE=1 SV=3;>tr|Q5U0J2|Q5U0J2_HUMAN Cysteine</td><td>7.99E-217</td></tr>
<tr><td>P21333;P21333-2;Q5HY5</td><td>FLNA_HUMAN Filamin-A OS=Homo sapiens GN=FLNA PE=1 SV=4;>sp|P21333-2|FLNA_HUMAN Isoform 2 of Filamin-A OS=Homo sapi</td><td>0</td></tr>
<tr><td>P21397;Q49A63;Q53EZ0;</td><td>AOFA_HUMAN Amine oxidase [flavin-containing] A OS=Homo sapiens GN=MAOA PE=1 SV=1;>tr|Q49A63|Q49A63_HUMAN Monoamin</td><td>1.26E-15</td></tr>
<tr><td>P21579;Q8N9I0</td><td>SYT1_HUMAN Synaptotagmin-1 OS=Homo sapiens GN=SYT1 PE=1 SV=1;>sp|Q8N9I0|SYT2_HUMAN Synaptotagmin-2 OS=Homo sap</td><td>1.13E-11</td></tr>
<tr><td>P21695;B2R6C0</td><td>GPDA_HUMAN Glycerol-3-phosphate dehydrogenase [NAD+], cytoplasmic OS=Homo sapiens GN=GPD1 PE=1 SV=4;>tr|B2R6C0|B2R</td><td>6.97E-116</td></tr>
<tr><td>P21741;Q2LEK4</td><td>MK_HUMAN Midkine OS=Homo sapiens GN=MDK PE=1 SV=1;>tr|Q2LEK4|Q2LEK4_HUMAN Mutant truncated midkine A OS=Homo sa</td><td>3.36E-09</td></tr>
<tr><td>P21912;Q0QEY7</td><td>DHSB_HUMAN Succinate dehydrogenase [ubiquinone] iron-sulfur subunit, mitochondrial OS=Homo sapiens GN=SDHB PE=1 SV=3;>t</td><td>1.01E-22</td></tr>
<tr><td>P22059;B4DR25</td><td>OSBP1_HUMAN Oxysterol-binding protein 1 OS=Homo sapiens GN=OSBP PE=1 SV=1;>tr|B4DR25|B4DR25_HUMAN Oxysterol-binding</td><td>3.36E-37</td></tr>
<tr><td>P22061-2;P22061;B7Z972</td><td>2|PIMT_HUMAN Isoform 2 of Protein-L-isoaspartate(D-aspartate) O-methyltransferase OS=Homo sapiens GN=PCMT1;>sp|P22061|PI</td><td>1.11E-28</td></tr>
<tr><td>P22102;P22102-2</td><td>PUR2_HUMAN Trifunctional purine biosynthetic protein adenosine-3 OS=Homo sapiens GN=GART PE=1 SV=1;>sp|P22102-2|PUR2_H</td><td>0</td></tr>
<tr><td>P22234;Q68CQ5;B4DGX3;</td><td>PUR6_HUMAN Multifunctional protein ADE2 OS=Homo sapiens GN=PAICS PE=1 SV=3;>tr|Q68CQ5|Q68CQ5_HUMAN Putative unchara</td><td>0</td></tr>
<tr><td>P22314;B4DDE4</td><td>UBA1_HUMAN Ubiquitin-like modifier-activating enzyme 1 OS=Homo sapiens GN=UBA1 PE=1 SV=3;>tr|B4DDE4|B4DDE4_HUMAN cD</td><td>0</td></tr>
<tr><td>P22392-2;P22392;O60361</td><td>2|NDKB_HUMAN Isoform 3 of Nucleoside diphosphate kinase B OS=Homo sapiens GN=NME2;>sp|P22392|NDKB_HUMAN Nucleoside</td><td>2.42E-193</td></tr>
<tr><td>P22413;B2RBY8</td><td>ENPP1_HUMAN Ectonucleotide pyrophosphatase/phosphodiesterase family member 1 OS=Homo sapiens GN=ENPP1 PE=1 SV=2;>tr|B</td><td>3.50E-08</td></tr>
<tr><td>P22528;P35321</td><td>SPR1B_HUMAN Cornifin-B OS=Homo sapiens GN=SPRR1B PE=1 SV=2;>sp|P35321|SPR1A_HUMAN Cornifin-A OS=Homo sapiens GN=</td><td>3.83E-98</td></tr>
<tr><td>P22626;P22626-2;B8ZZ37</td><td>ROA2_HUMAN Heterogeneous nuclear ribonucleoproteins A2/B1 OS=Homo sapiens GN=HNRNPA2B1 PE=1 SV=2;>sp|P22626-2|ROA2</td><td>1.19E-168</td></tr>
<tr><td>P22676;A6NER6;A8MY71</td><td>CALB2_HUMAN Calretinin OS=Homo sapiens GN=CALB2 PE=1 SV=2;>tr|A6NER6|A6NER6_HUMAN Calbindin 2, 29kDa (Calretinin), iso</td><td>1.46E-18</td></tr>
<tr><td>P23141-2;P23141;B7Z795</td><td>2|EST1_HUMAN Isoform 2 of Liver carboxylesterase 1 OS=Homo sapiens GN=CES1;>sp|P23141|EST1_HUMAN Liver carboxylesterase</td><td>1.80E-158</td></tr>
<tr><td>P23142;P23142-3;Q8NBH</td><td>FBLN1_HUMAN Fibulin-1 OS=Homo sapiens GN=FBLN1 PE=1 SV=4;>sp|P23142-3|FBLN1_HUMAN Isoform B of Fibulin-1 OS=Homo s</td><td>0</td></tr>
<tr><td>P23193;P23193-2</td><td>TCEA1_HUMAN Transcription elongation factor A protein 1 OS=Homo sapiens GN=TCEA1 PE=1 SV=2;>sp|P23193-2|TCEA1_HUMAN I</td><td>5.87E-96</td></tr>
<tr><td>P23229;P23229-6;P23229</td><td>ITA6_HUMAN Integrin alpha-6 OS=Homo sapiens GN=ITGA6 PE=1 SV=4;>sp|P23229-6|ITA6_HUMAN Isoform Alpha-6X1X2A of Integ</td><td>3.35E-244</td></tr>
<tr><td>P23246;Q86VG2;Q9BSV4;</td><td>SFPQ_HUMAN Splicing factor, proline- and glutamine-rich OS=Homo sapiens GN=SFPQ PE=1 SV=2;>tr|Q86VG2|Q86VG2_HUMAN Spli</td><td>2.28E-184</td></tr>
<tr><td>P23258;Q9NRH3</td><td>TBG1_HUMAN Tubulin gamma-1 chain OS=Homo sapiens GN=TUBG1 PE=1 SV=2;>sp|Q9NRH3|TBG2_HUMAN Tubulin gamma-2 chain</td><td>1.63E-17</td></tr>
<tr><td>P23368;B2R8J2;Q9BWL6</td><td>MAOM_HUMAN NAD-dependent malic enzyme, mitochondrial OS=Homo sapiens GN=ME2 PE=1 SV=1;>tr|B2R8J2|B2R8J2_HUMAN Ma</td><td>1.28E-75</td></tr>
<tr><td>P23396;Q53G83;Q9NQS8;</td><td>RS3_HUMAN 40S ribosomal protein S3 OS=Homo sapiens GN=RPS3 PE=1 SV=2;>tr|Q53G83|Q53G83_HUMAN Ribosomal protein S3</td><td>3.98E-154</td></tr>
<tr><td>P23434;Q53XL7;Q6IAT2;C</td><td>GCSH_HUMAN Glycine cleavage system H protein, mitochondrial OS=Homo sapiens GN=GCSH PE=1 SV=2;>tr|Q53XL7|Q53XL7_HUM</td><td>2.67E-77</td></tr>
<tr><td>P23497;Q53TD0;P23497-</td><td>SP100_HUMAN Nuclear autoantigen Sp-100 OS=Homo sapiens GN=SP100 PE=1 SV=3;>tr|Q53TD0|Q53TD0_HUMAN Putative unchara</td><td>4.10E-56</td></tr>
<tr><td>P23528;B4E112</td><td>COF1_HUMAN Cofilin-1 OS=Homo sapiens GN=CFL1 PE=1 SV=3;>tr|B4E112|B4E112_HUMAN cDNA FLJ51435, moderately similar to</td><td>0</td></tr>
<tr><td>P23634;P23634-8;P23634</td><td>AT2B4_HUMAN Plasma membrane calcium-transporting ATPase 4 OS=Homo sapiens GN=ATP2B4 PE=1 SV=2;>sp|P23634-8|AT2B4_H</td><td>5.77E-105</td></tr>
<tr><td>P23743;B4E0C6;Q3ZE25;</td><td>DGKA_HUMAN Diacylglycerol kinase alpha OS=Homo sapiens GN=DGKA PE=1 SV=3;>tr|B4E0C6|B4E0C6_HUMAN cDNA FLJ58783, hi</td><td>2.01E-57</td></tr>
<tr><td>P23743;P23743-2</td><td>DGKA_HUMAN Diacylglycerol kinase alpha OS=Homo sapiens GN=DGKA PE=1 SV=3;>sp|P23743-2|DGKA_HUMAN Isoform 2 of Diacy</td><td>2.59E-46</td></tr>
<tr><td>P23786;Q53G79</td><td>CPT2_HUMAN Carnitine O-palmitoyltransferase 2, mitochondrial OS=Homo sapiens GN=CPT2 PE=1 SV=2;>tr|Q53G79|Q53G79_HUM</td><td>3.80E-54</td></tr>
<tr><td>P23919;Q53F55;Q6FGU2;</td><td>KTHY_HUMAN Thymidylate kinase OS=Homo sapiens GN=DTYMK PE=1 SV=4;>tr|Q53F55|Q53F55_HUMAN Deoxythymidylate kinase</td><td>1.32E-53</td></tr>
</table>

P23921;Q53GZ5;B4DNN4;	RIR1_HUMAN Ribonucleoside-diphosphate reductase large subunit OS=Homo sapiens GN=RRM1 PE=1 SV=1;>tr|Q53GZ5|Q53GZ5_HU	3.17E-68
P23946;Q3SY37	CMA1_HUMAN Chymase OS=Homo sapiens GN=CMA1 PE=1 SV=1;>tr|Q3SY37|Q3SY37_HUMAN CMA1 protein OS=Homo sapiens GN=	4.16E-39
P24468;P10589	COT2_HUMAN COUP transcription factor 2 OS=Homo sapiens GN=NR2F2 PE=1 SV=1;>sp|P10589|COT1_HUMAN COUP transcription fa	9.53E-22
P24534;A4D1M6	EF1B_HUMAN Elongation factor 1-beta OS=Homo sapiens GN=EEF1B2 PE=1 SV=3;>tr|A4D1M6|A4D1M6_HUMAN HCG19809 OS=Hon	5.56E-120
P24666;P24666-3	PPAC_HUMAN Low molecular weight phosphotyrosine protein phosphatase OS=Homo sapiens GN=ACP1 PE=1 SV=3;>sp|P24666-3|PP	1.24E-30
P24821;P24821-3;P24821	TENA_HUMAN Tenascin OS=Homo sapiens GN=TNC PE=1 SV=3;>sp|P24821-3|TENA_HUMAN Isoform 3 of Tenascin OS=Homo sapien	0
P24844;O14950;P19105	MYL9_HUMAN Myosin regulatory light polypeptide 9 OS=Homo sapiens GN=MYL9 PE=1 SV=4;>sp|O14950|ML12B_HUMAN Myosin reg	3.08E-261
P24928;B4E3V7	RPB1_HUMAN DNA-directed RNA polymerase II subunit RPB1 OS=Homo sapiens GN=POLR2A PE=1 SV=2;>tr|B4E3V7|B4E3V7_HUMA	5.84E-05
P25325;Q6FHN9;B3KP52;	THTM_HUMAN 3-mercaptopyruvate sulfurtransferase OS=Homo sapiens GN=MPST PE=1 SV=3;>tr|Q6FHN9|Q6FHN9_HUMAN MPST pr	1.85E-14
P25490;O15391	TYY1_HUMAN Transcriptional repressor protein YY1 OS=Homo sapiens GN=YY1 PE=1 SV=2;>sp|O15391|TYY2_HUMAN Transcription f	1.70E-07
P25685;Q6FHS4;Q6FI51;E	DNJB1_HUMAN DnaJ homolog subfamily B member 1 OS=Homo sapiens GN=DNAJB1 PE=1 SV=4;>tr|Q6FHS4|Q6FHS4_HUMAN DNAJ	2.24E-105
P25705;A8K092;B4DY56	ATPA_HUMAN ATP synthase subunit alpha, mitochondrial OS=Homo sapiens GN=ATP5A1 PE=1 SV=1;>tr|A8K092|A8K092_HUMAN AT	0
P25786-2;P25786;Q53YE8	2|PSA1_HUMAN Isoform Long of Proteasome subunit alpha type-1 OS=Homo sapiens GN=PSMA1;>sp|P25786|PSA1_HUMAN Proteasc	7.70E-98
P25787;Q53GF5	PSA2_HUMAN Proteasome subunit alpha type-2 OS=Homo sapiens GN=PSMA2 PE=1 SV=2;>tr|Q53GF5|Q53GF5_HUMAN Proteasome	4.96E-63
P25788;Q6IB71;P25788-2	PSA3_HUMAN Proteasome subunit alpha type-3 OS=Homo sapiens GN=PSMA3 PE=1 SV=2;>tr|Q6IB71|Q6IB71_HUMAN Proteasome s	2.96E-41
P25815;Q5J7W2	S100P_HUMAN Protein S100-P OS=Homo sapiens GN=S100P PE=1 SV=2;>tr|Q5J7W2|Q5J7W2_HUMAN Migration-inducing gene 9 pro	1.05E-32
P26038;Q6PJT4	MOES_HUMAN Moesin OS=Homo sapiens GN=MSN PE=1 SV=3;>tr|Q6PJT4|Q6PJT4_HUMAN MSN protein (Fragment) OS=Homo sapie	0
P26196;B2R858;Q8IV96	DDX6_HUMAN Probable ATP-dependent RNA helicase DDX6 OS=Homo sapiens GN=DDX6 PE=1 SV=2;>tr|B2R858|B2R858_HUMAN cD	1.10E-96
P26358-2;P26358;Q59FP7	2|DNMT1_HUMAN Isoform 2 of DNA (cytosine-5)-methyltransferase 1 OS=Homo sapiens GN=DNMT1;>sp|P26358|DNMT1_HUMAN DN	4.85E-57
P26368;P26368-2;B5BU25	U2AF2_HUMAN Splicing factor U2AF 65 kDa subunit OS=Homo sapiens GN=U2AF2 PE=1 SV=4;>sp|P26368-2|U2AF2_HUMAN Isoform	0
P26440;Q53XZ9;B3KVI7	IVD_HUMAN Isovaleryl-CoA dehydrogenase, mitochondrial OS=Homo sapiens GN=IVD PE=1 SV=1;>tr|Q53XZ9|Q53XZ9_HUMAN Isov	3.06E-39
P26639;Q53GX7;Q5M7Z9;	SYTC_HUMAN Threonyl-tRNA synthetase, cytoplasmic OS=Homo sapiens GN=TARS PE=1 SV=3;>tr|Q53GX7|Q53GX7_HUMAN Threon	0
P26641;Q53YD7;B4DTG2;	EF1G_HUMAN Elongation factor 1-gamma OS=Homo sapiens GN=EEF1G PE=1 SV=3;>tr|Q53YD7|Q53YD7_HUMAN EEF1G protein OS	0
P26885;Q53XJ5	FKBP2_HUMAN Peptidyl-prolyl cis-trans isomerase FKBP2 OS=Homo sapiens GN=FKBP2 PE=1 SV=2;>tr|Q53XJ5|Q53XJ5_HUMAN FK5	9.30E-71
P27105;B4DZK8;B4E2V5	STOM_HUMAN Erythrocyte band 7 integral membrane protein OS=Homo sapiens GN=STOM PE=1 SV=3;>tr|B4DZK8|B4DZK8_HUMAN	8.31E-215
P27169;B4DX19	PON1_HUMAN Serum paraoxonase/arylesterase 1 OS=Homo sapiens GN=PON1 PE=1 SV=3;>tr|B4DX19|B4DX19_HUMAN cDNA FLJ57	5.77E-84
P27361;Q8NHX1;Q7Z3H5;	MK03_HUMAN Mitogen-activated protein kinase 3 OS=Homo sapiens GN=MAPK3 PE=1 SV=4;>tr|Q8NHX1|Q8NHX1_HUMAN Extracellu	7.14E-40
P27635;Q96L21	RL10_HUMAN 60S ribosomal protein L10 OS=Homo sapiens GN=RPL10 PE=1 SV=4;>sp|Q96L21|RL10L_HUMAN 60S ribosomal protei	2.54E-175
P27695;Q5TZP7	APEX1_HUMAN DNA-(apurinic or apyrimidinic site) lyase OS=Homo sapiens GN=APEX1 PE=1 SV=2;>tr|Q5TZP7|Q5TZP7_HUMAN APE	8.64E-83
P27707;D6RFG8;D6RCP9	DCK_HUMAN Deoxycytidine kinase OS=Homo sapiens GN=DCK PE=1 SV=1;>tr|D6RFG8|D6RFG8_HUMAN Uncharacterized protein OS	4.97E-10
P27797;Q53G71;B4DHR1;	CALR_HUMAN Calreticulin OS=Homo sapiens GN=CALR PE=1 SV=1;>tr|Q53G71|Q53G71_HUMAN Calreticulin variant (Fragment) OS=	0
P28062;Q5QNR8;P28062-	PSB8_HUMAN Proteasome subunit beta type-8 OS=Homo sapiens GN=PSMB8 PE=1 SV=2;>tr|Q5QNR8|Q5QNR8_HUMAN Proteasome	2.81E-19
P28065;Q5JNW4;P28065-	PSB9_HUMAN Proteasome subunit beta type-9 OS=Homo sapiens GN=PSMB9 PE=1 SV=2;>tr|Q5JNW4|Q5JNW4_HUMAN Proteasome	7.88E-09
P28066;Q5U0A0;B4E2V4	PSA5_HUMAN Proteasome subunit alpha type-5 OS=Homo sapiens GN=PSMA5 PE=1 SV=3;>tr|Q5U0A0|Q5U0A0_HUMAN Proteasome	4.85E-126
P28070;B4DFL3	PSB4_HUMAN Proteasome subunit beta type-4 OS=Homo sapiens GN=PSMB4 PE=1 SV=4;>tr|B4DFL3|B4DFL3_HUMAN cDNA FLJ5666	3.13E-106
P28072;Q6IAT9	PSB6_HUMAN Proteasome subunit beta type-6 OS=Homo sapiens GN=PSMB6 PE=1 SV=4;>tr|Q6IAT9|Q6IAT9_HUMAN Proteasome su	2.52E-11
P28161;Q0D2I8;A6NGW8;	GSTM2_HUMAN Glutathione S-transferase Mu 2 OS=Homo sapiens GN=GSTM2 PE=1 SV=2;>tr|Q0D2I8|Q0D2I8_HUMAN Glutathione	2.89E-84
P28347;Q15562;Q99594;(	TEAD1_HUMAN Transcriptional enhancer factor TEF-1 OS=Homo sapiens GN=TEAD1 PE=1 SV=2;>sp|Q15562|TEAD2_HUMAN Transcr	1.18E-10
P28482;P27361	MK01_HUMAN Mitogen-activated protein kinase 1 OS=Homo sapiens GN=MAPK1 PE=1 SV=3;>sp|P27361|MK03_HUMAN Mitogen-acti	1.39E-25
P28482;Q1HBJ4;B4DHN0;	MK01_HUMAN Mitogen-activated protein kinase 1 OS=Homo sapiens GN=MAPK1 PE=1 SV=3;>tr|Q1HBJ4|Q1HBJ4_HUMAN Mitogen-ac	1.19E-81
P28676;B7Z322;C9JGR8	GRAN_HUMAN Grancalcin OS=Homo sapiens GN=GCA PE=1 SV=2;>tr|B7Z322|B7Z322_HUMAN cDNA FLJ52146, highly similar to Gra	5.85E-25
P28838;P28838-2;B4DQG	AMPL_HUMAN Cytosol aminopeptidase OS=Homo sapiens GN=LAP3 PE=1 SV=3;>sp|P28838-2|AMPL_HUMAN Isoform 2 of Cytosol am	0
P28907;P28907-2	CD38_HUMAN ADP-ribosyl cyclase 1 OS=Homo sapiens GN=CD38 PE=1 SV=2;>sp|P28907-2|CD38_HUMAN Isoform 2 of ADP-ribosyl	2.64E-55
P29034;Q5RHS7	S10A2_HUMAN Protein S100-A2 OS=Homo sapiens GN=S100A2 PE=1 SV=3;>tr|Q5RHS7|Q5RHS7_HUMAN S100 calcium binding prot	2.19E-11
P29083;Q53F88;C9IYL4	T2EA_HUMAN General transcription factor IIE subunit 1 OS=Homo sapiens GN=GTF2E1 PE=1 SV=2;>tr|Q53F88|Q53F88_HUMAN Gen	4.50E-05
P29317;B4DL04	EPHA2_HUMAN Ephrin type-A receptor 2 OS=Homo sapiens GN=EPHA2 PE=1 SV=2;>tr|B4DL04|B4DL04_HUMAN cDNA FLJ51229, hig	0
P29353-6;P29353;P29353	6|SHC1_HUMAN Isoform 6 of SHC-transforming protein 1 OS=Homo sapiens GN=SHC1;>sp|P29353|SHC1_HUMAN SHC-transforming	1.60E-06
P29373;Q5SYZ4	RABP2_HUMAN Cellular retinoic acid-binding protein 2 OS=Homo sapiens GN=CRABP2 PE=1 SV=2;>tr|Q5SYZ4|Q5SYZ4_HUMAN Cellu	3.11E-274
P29728;P29728-2;A8KA41	OAS2_HUMAN 2-5-oligoadenylate synthase 2 OS=Homo sapiens GN=OAS2 PE=1 SV=3;>sp|P29728-2|OAS2_HUMAN Isoform p69 of 2	8.76E-227
P29992;Q59FM5	GNA11_HUMAN Guanine nucleotide-binding protein subunit alpha-11 OS=Homo sapiens GN=GNA11 PE=1 SV=2;>tr|Q59FM5|Q59FM5	8.48E-88
P30038;D2D4A3;B4DGE4	AL4A1_HUMAN Delta-1-pyrroline-5-carboxylate dehydrogenase, mitochondrial OS=Homo sapiens GN=ALDH4A1 PE=1 SV=3;>tr|D2D4	4.27E-90
P30044;P30044-2;A6NG0(	PRDX5_HUMAN Peroxiredoxin-5, mitochondrial OS=Homo sapiens GN=PRDX5 PE=1 SV=4;>sp|P30044-2|PRDX5_HUMAN Isoform Cyt	8.60E-102
P30046;A6NHG4	DOPD_HUMAN D-dopachrome decarboxylase OS=Homo sapiens GN=DDT PE=1 SV=3;>sp|A6NHG4|DDTL_HUMAN D-dopachrome dec	8.93E-42
P30046;Q53Y51;B4E259;E	DOPD_HUMAN D-dopachrome decarboxylase OS=Homo sapiens GN=DDT PE=1 SV=3;>tr|Q53Y51|Q53Y51_HUMAN D-dopachrome tau	4.00E-14
P30101;B3KQT9;B3KQT2;	PDIA3_HUMAN Protein disulfide-isomerase A3 OS=Homo sapiens GN=PDIA3 PE=1 SV=4;>tr|B3KQT9|B3KQT9_HUMAN cDNA PSEC017	0
P30154-2;P30154	2|2AAB_HUMAN Isoform 2 of Serine/threonine-protein phosphatase 2A 65 kDa regulatory subunit A beta isoform OS=Homo sapiens G	1.79E-15
P30260;B4DL80	CDC27_HUMAN Cell division cycle protein 27 homolog OS=Homo sapiens GN=CDC27 PE=1 SV=2;>tr|B4DL80|B4DL80_HUMAN cDNA	7.58E-07
P30419;Q96HI4;P30419-2	NMT1_HUMAN Glycylpeptide N-tetradecanoyltransferase 1 OS=Homo sapiens GN=NMT1 PE=1 SV=2;>tr|Q96HI4|Q96HI4_HUMAN Gly	6.00E-296
P30443;P30455	1A01_HUMAN HLA class I histocompatibility antigen, A-1 alpha chain OS=Homo sapiens GN=HLA-A PE=1 SV=1;>sp|P30455|1A36_HU	3.04E-171
P30453;P18462;P30450;P	1A34_HUMAN HLA class I histocompatibility antigen, A-34 alpha chain OS=Homo sapiens GN=HLA-A PE=1 SV=1;>sp|P18462|1A25_H	0
P30480;P30462	1B42_HUMAN HLA class I histocompatibility antigen, B-42 alpha chain OS=Homo sapiens GN=HLA-B PE=1 SV=1;>sp|P30462|1B14_H	2.11E-99

P30483;A9QUT8;Q6IVJ2;[	1B45_HUMAN HLA class I histocompatibility antigen, B-45 alpha chain OS=Homo sapiens GN=HLA-B PE=2 SV=1;>tr|A9QUT8|A9QUT	4.92E-66
P30483;P30487;P30488	1B45_HUMAN HLA class I histocompatibility antigen, B-45 alpha chain OS=Homo sapiens GN=HLA-B PE=2 SV=1;>sp|P30487|1B49_H	0
P30486;Q31610	1B48_HUMAN HLA class I histocompatibility antigen, B-48 alpha chain OS=Homo sapiens GN=HLA-B PE=1 SV=1;>sp|Q31610|1B81_	3.43E-98
P30487;P30488	1B49_HUMAN HLA class I histocompatibility antigen, B-49 alpha chain OS=Homo sapiens GN=HLA-B PE=2 SV=2;>sp|P30488|1B50_	1.74E-159
P30491;P30685;P18464;P	1B53_HUMAN HLA class I histocompatibility antigen, B-53 alpha chain OS=Homo sapiens GN=HLA-B PE=1 SV=1;>sp|P30685|1B35_	2.17E-99
P30508;P30510;Q07000;F	1C12_HUMAN HLA class I histocompatibility antigen, Cw-12 alpha chain OS=Homo sapiens GN=HLA-C PE=1 SV=2;>sp|P30510|1C14_	3.21E-93
P30508;Q07000;Q29960;I	1C12_HUMAN HLA class I histocompatibility antigen, Cw-12 alpha chain OS=Homo sapiens GN=HLA-C PE=1 SV=2;>sp|Q07000|1C15	0
P30511-3;P30511;P30511	3|HLAF_HUMAN Isoform 3 of HLA class I histocompatibility antigen, alpha chain F OS=Homo sapiens GN=HLA-F;>sp|P30511|HLAF_HU	1.56E-08
P30520;B4E1L0	PURA2_HUMAN Adenylosuccinate synthetase isozyme 2 OS=Homo sapiens GN=ADSS PE=1 SV=3;>tr|B4E1L0|B4E1L0_HUMAN Adeny	2.94E-63
P30566;Q71UA4;B4DP46;	PUR8_HUMAN Adenylosuccinate lyase OS=Homo sapiens GN=ADSL PE=1 SV=2;>tr|Q71UA4|Q71UA4_HUMAN Adenylosuccinate lyase	6.22E-34
P30740;B4E3A8;B4DNT0	ILEU_HUMAN Leukocyte elastase inhibitor OS=Homo sapiens GN=SERPINB1 PE=1 SV=1;>tr|B4E3A8|B4E3A8_HUMAN cDNA FLJ5396	7.43E-172
P30876;B4DHJ3;C9J2Y9;E	RPB2_HUMAN DNA-directed RNA polymerase II subunit RPB2 OS=Homo sapiens GN=POLR2B PE=1 SV=1;>tr|B4DHJ3|B4DHJ3_HUMA	4.90E-43
P31025;Q5VSP4	LCN1_HUMAN Lipocalin-1 OS=Homo sapiens GN=LCN1 PE=1 SV=1;>sp|Q5VSP4|LC1L1_HUMAN Putative lipocalin 1-like protein 1 OS	1.59E-14
P31040;B4DJ60;D6RFM5;	DHSA_HUMAN Succinate dehydrogenase [ubiquinone] flavoprotein subunit, mitochondrial OS=Homo sapiens GN=SDHA PE=1 SV=2;>	2.41E-266
P31146;Q59G88	COR1A_HUMAN Coronin-1A OS=Homo sapiens GN=CORO1A PE=1 SV=4;>tr|Q59G88|Q59G88_HUMAN Putative uncharacterized prote	1.15E-233
P31150;B4DHX4;B4DH24	GDIA_HUMAN Rab GDP dissociation inhibitor alpha OS=Homo sapiens GN=GDI1 PE=1 SV=2;>tr|B4DHX4|B4DHX4_HUMAN cDNA FLJ5	0
P31323;Q6DHZ2;B3KY43;	KAP3_HUMAN cAMP-dependent protein kinase type II-beta regulatory subunit OS=Homo sapiens GN=PRKAR2B PE=1 SV=3;>tr|Q6DH	1.73E-14
P31689;Q5T7Q0;Q86TL9	DNJA1_HUMAN DnaJ homolog subfamily A member 1 OS=Homo sapiens GN=DNAJA1 PE=1 SV=2;>tr|Q5T7Q0|Q5T7Q0_HUMAN DnaJ	1.72E-117
P31751;Q0VAN0;B4DG79;	AKT2_HUMAN RAC-beta serine/threonine-protein kinase OS=Homo sapiens GN=AKT2 PE=1 SV=2;>tr|Q0VAN0|Q0VAN0_HUMAN AKT	6.68E-06
P31930;B4DUL5	QCR1_HUMAN Cytochrome b-c1 complex subunit 1, mitochondrial OS=Homo sapiens GN=UQCRC1 PE=1 SV=3;>tr|B4DUL5|B4DUL5_	2.67E-107
P31937;Q546Z2	3HIDH_HUMAN 3-hydroxyisobutyrate dehydrogenase, mitochondrial OS=Homo sapiens GN=HIBADH PE=1 SV=2;>tr|Q546Z2|Q546Z	2.82E-131
P31949;B2R5H0	S10AB_HUMAN Protein S100-A11 OS=Homo sapiens GN=S100A11 PE=1 SV=2;>tr|B2R5H0|B2R5H0_HUMAN cDNA, FLJ92471, highly	7.30E-125
P32119;B4DF70;A6NIW5	PRDX2_HUMAN Peroxiredoxin-2 OS=Homo sapiens GN=PRDX2 PE=1 SV=5;>tr|B4DF70|B4DF70_HUMAN cDNA FLJ60461, highly simil	6.43E-60
P32456;Q8TCE5	GBP2_HUMAN Interferon-induced guanylate-binding protein 2 OS=Homo sapiens GN=GBP2 PE=2 SV=3;>tr|Q8TCE5|Q8TCE5_HUMAN	2.47E-28
P32929;P32929-2	CGL_HUMAN Cystathionine gamma-lyase OS=Homo sapiens GN=CTH PE=1 SV=3;>sp|P32929-2|CGL_HUMAN Isoform 2 of Cystathio	2.44E-12
P32969;Q53Z07;D6RAN4;	RL9_HUMAN 60S ribosomal protein L9 OS=Homo sapiens GN=RPL9 PE=1 SV=1;>tr|Q53Z07|Q53Z07_HUMAN NPC-A-16 OS=Homo sa	4.60E-82
P33316;P33316-3;P33316	DUT_HUMAN Deoxyuridine 5-triphosphate nucleotidohydrolase, mitochondrial OS=Homo sapiens GN=DUT PE=1 SV=3;>sp|P33316-3|	4.12E-27
P34897;Q53ET4;B7Z9F1;(	GLYM_HUMAN Serine hydroxymethyltransferase, mitochondrial OS=Homo sapiens GN=SHMT2 PE=1 SV=3;>tr|Q53ET4|Q53ET4_HUM	2.75E-252
P34949;P34949-2;B4DW5	MPI_HUMAN Mannose-6-phosphate isomerase OS=Homo sapiens GN=MPI PE=1 SV=2;>sp|P34949-2|MPI_HUMAN Isoform 2 of Mann	7.91E-49
P35219;B4DFA0	CAH8_HUMAN Carbonic anhydrase-related protein OS=Homo sapiens GN=CA8 PE=1 SV=3;>tr|B4DFA0|B4DFA0_HUMAN cDNA FLJ57	1.73E-06
P35221;P35221-2	CTNA1_HUMAN Catenin alpha-1 OS=Homo sapiens GN=CTNNA1 PE=1 SV=1;>sp|P35221-2|CTNA1_HUMAN Isoform 2 of Catenin alph	1.48E-225
P35237;Q8IXH2;Q5TD02	SPB6_HUMAN Serpin B6 OS=Homo sapiens GN=SERPINB6 PE=1 SV=3;>tr|Q8IXH2|Q8IXH2_HUMAN MSTP057 OS=Homo sapiens PE=	1.71E-252
P35251;P35251-2;Q1475(	RFC1_HUMAN Replication factor C subunit 1 OS=Homo sapiens GN=RFC1 PE=1 SV=4;>sp|P35251-2|RFC1_HUMAN Isoform 2 of Repli	9.60E-15
P35268;Q7Z4W8	RL22_HUMAN 60S ribosomal protein L22 OS=Homo sapiens GN=RPL22 PE=1 SV=2;>tr|Q7Z4W8|Q7Z4W8_HUMAN Heparin-binding p	6.57E-153
P35269;Q6IBK5;B4DXY2;I	T2FA_HUMAN General transcription factor IIF subunit 1 OS=Homo sapiens GN=GTF2F1 PE=1 SV=2;>tr|Q6IBK5|Q6IBK5_HUMAN GTF	5.00E-56
P35442;Q6MZL6	TSP2_HUMAN Thrombospondin-2 OS=Homo sapiens GN=THBS2 PE=1 SV=2;>tr|Q6MZL6|Q6MZL6_HUMAN Putative uncharacterized p	1.40E-158
P35520-2;P35520	2|CBS_HUMAN Isoform 2 of Cystathionine beta-synthase OS=Homo sapiens GN=CBS;>sp|P35520|CBS_HUMAN Cystathionine beta-sy	8.27E-12
P35579;Q60FE2;Q86XU5;	MYH9_HUMAN Myosin-9 OS=Homo sapiens GN=MYH9 PE=1 SV=4;>tr|Q60FE2|Q60FE2_HUMAN MYH9 variant protein OS=Homo sapi	0
P35580-3;P35580;P35580	3|MYH10_HUMAN Isoform 3 of Myosin-10 OS=Homo sapiens GN=MYH10;>sp|P35580|MYH10_HUMAN Myosin-10 OS=Homo sapiens (	0
P35606;B4DZI8	COPB2_HUMAN Coatomer subunit beta OS=Homo sapiens GN=COPB2 PE=1 SV=2;>tr|B4DZI8|B4DZI8_HUMAN Coatomer protein con	4.40E-51
P35613;P35613-2;Q54A5	BASI_HUMAN Basigin OS=Homo sapiens GN=BSG PE=1 SV=2;>sp|P35613-2|BASI_HUMAN Isoform 2 of Basigin OS=Homo sapiens G	1.52E-215
P35637;Q6IBQ5;Q8TBR3;	FUS_HUMAN RNA-binding protein FUS OS=Homo sapiens GN=FUS PE=1 SV=1;>tr|Q6IBQ5|Q6IBQ5_HUMAN FUS protein OS=Homo s	2.17E-156
P35659;B4DNW3;B4DFG0	DEK_HUMAN Protein DEK OS=Homo sapiens GN=DEK PE=1 SV=1;>tr|B4DNW3|B4DNW3_HUMAN cDNA FLJ51914, highly similar to P	2.85E-206
P35813;P35813-2	PPM1A_HUMAN Protein phosphatase 1A OS=Homo sapiens GN=PPM1A PE=1 SV=1;>sp|P35813-2|PPM1A_HUMAN Isoform Alpha-2 of	5.99E-130
P35914;Q6IBC0;B1AK13;/	HMGCL_HUMAN Hydroxymethylglutaryl-CoA lyase, mitochondrial OS=Homo sapiens GN=HMGCL PE=1 SV=2;>tr|Q6IBC0|Q6IBC0_HU	1.56E-38
P35998;Q75L23;B7Z5E2	PRS7_HUMAN 26S protease regulatory subunit 7 OS=Homo sapiens GN=PSMC2 PE=1 SV=3;>tr|Q75L23|Q75L23_HUMAN Putative un	3.79E-134
P36404;Q53YD8;B4DGG0	ARL2_HUMAN ADP-ribosylation factor-like protein 2 OS=Homo sapiens GN=ARL2 PE=1 SV=4;>tr|Q53YD8|Q53YD8_HUMAN ADP-ribos	1.57E-45
P36507;B3KS97	MP2K2_HUMAN Dual specificity mitogen-activated protein kinase kinase 2 OS=Homo sapiens GN=MAP2K2 PE=1 SV=1;>tr|B3KS97|B	7.82E-61
P36542;Q6I9V2;Q8TAS0;F	ATPG_HUMAN ATP synthase subunit gamma, mitochondrial OS=Homo sapiens GN=ATP5C1 PE=1 SV=1;>tr|Q6I9V2|Q6I9V2_HUMAN	2.00E-126
P36543;Q53Y06;A8MUE4;	VATE1_HUMAN V-type proton ATPase subunit E 1 OS=Homo sapiens GN=ATP6V1E1 PE=1 SV=1;>tr|Q53Y06|Q53Y06_HUMAN ATPase,	4.31E-33
P36915;P36915-2	GNL1_HUMAN Guanine nucleotide-binding protein-like 1 OS=Homo sapiens GN=GNL1 PE=1 SV=2;>sp|P36915-2|GNL1_HUMAN Isofo	1.74E-12
P36952;P36952-2	SPB5_HUMAN Serpin B5 OS=Homo sapiens GN=SERPINB5 PE=1 SV=2;>sp|P36952-2|SPB5_HUMAN Isoform 2 of Serpin B5 OS=Hom	1.14E-182
P36957;Q6IBS5;B7Z5W8;	ODO2_HUMAN Dihydrolipoyllysine-residue succinyltransferase component of 2-oxoglutarate dehydrogenase complex, mitochondrial OS	1.15E-46
P36969;Q6PI42;P36969-2	GPX4_HUMAN Phospholipid hydroperoxide glutathione peroxidase, mitochondrial OS=Homo sapiens GN=GPX4 PE=1 SV=3;>tr|Q6PI42	3.71E-37
P37235;P84074	HPCL1_HUMAN Hippocalcin-like protein 1 OS=Homo sapiens GN=HPCAL1 PE=1 SV=3;>sp|P84074|HPCA_HUMAN Neuron-specific calc	4.92E-138
P37235;Q6FGY1;Q68CP5;	HPCL1_HUMAN Hippocalcin-like protein 1 OS=Homo sapiens GN=HPCAL1 PE=1 SV=3;>tr|Q6FGY1|Q6FGY1_HUMAN HPCAL1 protein C	1.73E-66
P37268;Q6IAX1;B4DJE5;E	FDFT_HUMAN Squalene synthase OS=Homo sapiens GN=FDFT1 PE=1 SV=1;>tr|Q6IAX1|Q6IAX1_HUMAN FDFT1 protein OS=Homo sa	1.77E-10
P38159;Q96E39	HNRPG_HUMAN Heterogeneous nuclear ribonucleoprotein G OS=Homo sapiens GN=RBMX PE=1 SV=3;>sp|Q96E39|RBMXL_HUMAN H	1.70E-128
P38646;B7Z4V2;Q8N1C8;	GRP75_HUMAN Stress-70 protein, mitochondrial OS=Homo sapiens GN=HSPA9 PE=1 SV=2;>tr|B7Z4V2|B7Z4V2_HUMAN cDNA FLJ51	0
P39023;Q96QL0;Q8TBW1;	RL3_HUMAN 60S ribosomal protein L3 OS=Homo sapiens GN=RPL3 PE=1 SV=2;>tr|Q96QL0|Q96QL0_HUMAN Ribosomal protein L3 O	4.23E-175
P39060;P39060-1;P39060	COIA1_HUMAN Collagen alpha-1(XVIII) chain OS=Homo sapiens GN=COL18A1 PE=1 SV=5;>sp|P39060-1|COIA1_HUMAN Isoform 2 (	4.84E-189

<table>
<tr><td>P39656;B4DLI2;B4DJE3</td><td>OST48_HUMAN Dolichyl-diphosphooligosaccharide--protein glycosyltransferase 48 kDa subunit OS=Homo sapiens GN=DDOST PE=1 S</td><td>3.28E-119</td></tr>
<tr><td>P39687;Q08AJ6;Q6PKH8;</td><td>AN32A_HUMAN Acidic leucine-rich nuclear phosphoprotein 32 family member A OS=Homo sapiens GN=ANP32A PE=1 SV=1;>tr|Q08A</td><td>9.70E-45</td></tr>
<tr><td>P39748;Q6FHX6;B4DWZ4</td><td>FEN1_HUMAN Flap endonuclease 1 OS=Homo sapiens GN=FEN1 PE=1 SV=1;>tr|Q6FHX6|Q6FHX6_HUMAN FEN1 protein OS=Homo sa</td><td>2.53E-270</td></tr>
<tr><td>P40123;Q9BYW9;B7Z4M3</td><td>CAP2_HUMAN Adenylyl cyclase-associated protein 2 OS=Homo sapiens GN=CAP2 PE=1 SV=1;>tr|Q9BYW9|Q9BYW9_HUMAN Adenyly</td><td>5.68E-60</td></tr>
<tr><td>P40261;Q6FH49</td><td>NNMT_HUMAN Nicotinamide N-methyltransferase OS=Homo sapiens GN=NNMT PE=1 SV=1;>tr|Q6FH49|Q6FH49_HUMAN NNMT prote</td><td>2.04E-175</td></tr>
<tr><td>P40306;Q6IB22</td><td>PSB10_HUMAN Proteasome subunit beta type-10 OS=Homo sapiens GN=PSMB10 PE=1 SV=1;>tr|Q6IB22|Q6IB22_HUMAN Proteasom</td><td>4.55E-12</td></tr>
<tr><td>P40429;Q5QTS3;Q9BSQ6;</td><td>RL13A_HUMAN 60S ribosomal protein L13a OS=Homo sapiens GN=RPL13A PE=1 SV=2;>tr|Q5QTS3|Q5QTS3_HUMAN FWP004 OS=H</td><td>1.18E-59</td></tr>
<tr><td>P40429;Q6NVV1</td><td>RL13A_HUMAN 60S ribosomal protein L13a OS=Homo sapiens GN=RPL13A PE=1 SV=2;>sp|Q6NVV1|R13AX_HUMAN Putative 60S rib</td><td>2.31E-95</td></tr>
<tr><td>P40616;B4DWW1;B4DR16</td><td>ARL1_HUMAN ADP-ribosylation factor-like protein 1 OS=Homo sapiens GN=ARL1 PE=1 SV=1;>tr|B4DWW1|B4DWW1_HUMAN cDNA F</td><td>7.89E-20</td></tr>
<tr><td>P40763;P40763-2;A8K7B8</td><td>STAT3_HUMAN Signal transducer and activator of transcription 3 OS=Homo sapiens GN=STAT3 PE=1 SV=2;>sp|P40763-2|STAT3_HU</td><td>0</td></tr>
<tr><td>P40926;Q6FHZ0;Q75MT9;</td><td>MDHM_HUMAN Malate dehydrogenase, mitochondrial OS=Homo sapiens GN=MDH2 PE=1 SV=3;>tr|Q6FHZ0|Q6FHZ0_HUMAN Malate</td><td>1.72E-246</td></tr>
<tr><td>P40938;B4DKE6;C9JU95;</td><td>RFC3_HUMAN Replication factor C subunit 3 OS=Homo sapiens GN=RFC3 PE=1 SV=2;>tr|B4DKE6|B4DKE6_HUMAN cDNA FLJ60629,</td><td>5.17E-34</td></tr>
<tr><td>P40939;B4DRH6;B4DDZ5;</td><td>ECHA_HUMAN Trifunctional enzyme subunit alpha, mitochondrial OS=Homo sapiens GN=HADHA PE=1 SV=2;>tr|B4DRH6|B4DRH6_HU</td><td>0</td></tr>
<tr><td>P41091;Q2VIR3;Q2VIR3-2</td><td>IF2G_HUMAN Eukaryotic translation initiation factor 2 subunit 3 OS=Homo sapiens GN=EIF2S3 PE=1 SV=3;>sp|Q2VIR3|IF2GL_HUMA</td><td>0</td></tr>
<tr><td>P41214;P41214-2</td><td>EIF2D_HUMAN Eukaryotic translation initiation factor 2D OS=Homo sapiens GN=EIF2D PE=1 SV=3;>sp|P41214-2|EIF2D_HUMAN Isof</td><td>7.25E-14</td></tr>
<tr><td>P41227;A6NM98;A8MWP7</td><td>NAA10_HUMAN N-alpha-acetyltransferase 10, NatA catalytic subunit OS=Homo sapiens GN=NAA10 PE=1 SV=1;>tr|A6NM98|A6NM98</td><td>5.99E-47</td></tr>
<tr><td>P41227;Q9BSU3</td><td>NAA10_HUMAN N-alpha-acetyltransferase 10, NatA catalytic subunit OS=Homo sapiens GN=NAA10 PE=1 SV=1;>sp|Q9BSU3|NAA11_</td><td>2.14E-10</td></tr>
<tr><td>P41567;Q6IAV3</td><td>EIF1_HUMAN Eukaryotic translation initiation factor 1 OS=Homo sapiens GN=EIF1 PE=1 SV=1;>tr|Q6IAV3|Q6IAV3_HUMAN Eukaryoti</td><td>1.32E-83</td></tr>
<tr><td>P42226;P42226-2</td><td>STAT6_HUMAN Signal transducer and activator of transcription 6 OS=Homo sapiens GN=STAT6 PE=1 SV=1;>sp|P42226-2|STAT6_HU</td><td>9.47E-14</td></tr>
<tr><td>P42229;P51692</td><td>STA5A_HUMAN Signal transducer and activator of transcription 5A OS=Homo sapiens GN=STAT5A PE=1 SV=1;>sp|P51692|STA5B_HU</td><td>2.90E-10</td></tr>
<tr><td>P42229;Q8WWS9;Q59GY7</td><td>STA5A_HUMAN Signal transducer and activator of transcription 5A OS=Homo sapiens GN=STAT5A PE=1 SV=1;>tr|Q8WWS9|Q8WWS</td><td>2.57E-32</td></tr>
<tr><td>P42677;Q5T4L6;Q5T4L4;</td><td>RS27_HUMAN 40S ribosomal protein S27 OS=Homo sapiens GN=RPS27 PE=1 SV=3;>tr|Q5T4L6|Q5T4L6_HUMAN Ribosomal protein</td><td>2.26E-32</td></tr>
<tr><td>P42677;Q71UM5</td><td>RS27_HUMAN 40S ribosomal protein S27 OS=Homo sapiens GN=RPS27 PE=1 SV=3;>sp|Q71UM5|RS27L_HUMAN 40S ribosomal prot</td><td>4.78E-50</td></tr>
<tr><td>P42704;B4DSR0</td><td>LPPRC_HUMAN Leucine-rich PPR motif-containing protein, mitochondrial OS=Homo sapiens GN=LRPPRC PE=1 SV=3;>tr|B4DSR0|B4D</td><td>0</td></tr>
<tr><td>P42766;A4D2M5</td><td>RL35_HUMAN 60S ribosomal protein L35 OS=Homo sapiens GN=RPL35 PE=1 SV=2;>tr|A4D2M5|A4D2M5_HUMAN Similar to 60S ribo</td><td>4.24E-68</td></tr>
<tr><td>P42771;P42771-3;P42771</td><td>CD2A1_HUMAN Cyclin-dependent kinase inhibitor 2A, isoforms 1/2/3 OS=Homo sapiens GN=CDKN2A PE=1 SV=2;>sp|P42771-3|CD2</td><td>5.46E-14</td></tr>
<tr><td>P42892;P42892-4;P42892</td><td>ECE1_HUMAN Endothelin-converting enzyme 1 OS=Homo sapiens GN=ECE1 PE=1 SV=2;>sp|P42892-4|ECE1_HUMAN Isoform D of E</td><td>8.42E-76</td></tr>
<tr><td>P43155;P43155-2;P43155</td><td>CACP_HUMAN Carnitine O-acetyltransferase OS=Homo sapiens GN=CRAT PE=1 SV=5;>sp|P43155-2|CACP_HUMAN Isoform 2 of Carn</td><td>7.57E-42</td></tr>
<tr><td>P43246;Q53FK0;Q53GS1;</td><td>MSH2_HUMAN DNA mismatch repair protein Msh2 OS=Homo sapiens GN=MSH2 PE=1 SV=1;>tr|Q53FK0|Q53FK0_HUMAN MutS hom</td><td>1.16E-96</td></tr>
<tr><td>P43304;Q53T76;A8K1Z2;</td><td>GPDM_HUMAN Glycerol-3-phosphate dehydrogenase, mitochondrial OS=Homo sapiens GN=GPD2 PE=1 SV=3;>tr|Q53T76|Q53T76_H</td><td>6.08E-110</td></tr>
<tr><td>P43487;C9JXG8;Q53EY3;</td><td>RANG_HUMAN Ran-specific GTPase-activating protein OS=Homo sapiens GN=RANBP1 PE=1 SV=1;>tr|C9JXG8|C9JXG8_HUMAN Uncha</td><td>4.25E-64</td></tr>
<tr><td>P43490;Q5SYT8</td><td>NAMPT_HUMAN Nicotinamide phosphoribosyltransferase OS=Homo sapiens GN=NAMPT PE=1 SV=1;>tr|Q5SYT8|Q5SYT8_HUMAN Nov</td><td>0</td></tr>
<tr><td>P43897-2;P43897;B4DHY</td><td>2|EFTS_HUMAN Isoform 2 of Elongation factor Ts, mitochondrial OS=Homo sapiens GN=TSFM;>sp|P43897|EFTS_HUMAN Elongation f</td><td>2.98E-41</td></tr>
<tr><td>P45954;Q5SQN6;B4DQ51</td><td>ACDSB_HUMAN Short/branched chain specific acyl-CoA dehydrogenase, mitochondrial OS=Homo sapiens GN=ACADSB PE=1 SV=1;>t</td><td>6.85E-221</td></tr>
<tr><td>P45973;Q6I9T7</td><td>CBX5_HUMAN Chromobox protein homolog 5 OS=Homo sapiens GN=CBX5 PE=1 SV=1;>tr|Q6I9T7|Q6I9T7_HUMAN CBX5 protein OS</td><td>3.45E-25</td></tr>
<tr><td>P45985-2;P45985</td><td>2|MP2K4_HUMAN Isoform 2 of Dual specificity mitogen-activated protein kinase kinase 4 OS=Homo sapiens GN=MAP2K4;>sp|P45985</td><td>1.72E-08</td></tr>
<tr><td>P46060;B4DY16;B4E0U0</td><td>RAGP1_HUMAN Ran GTPase-activating protein 1 OS=Homo sapiens GN=RANGAP1 PE=1 SV=1;>tr|B4DY16|B4DY16_HUMAN cDNA FL</td><td>0</td></tr>
<tr><td>P46459;B7Z5J7;B4DSK5;</td><td>NSF_HUMAN Vesicle-fusing ATPase OS=Homo sapiens GN=NSF PE=1 SV=3;>tr|B7Z5J7|B7Z5J7_HUMAN cDNA FLJ58682, highly simila</td><td>1.66E-111</td></tr>
<tr><td>P46734-3;P46734;P46734</td><td>3|MP2K3_HUMAN Isoform 2 of Dual specificity mitogen-activated protein kinase kinase 3 OS=Homo sapiens GN=MAP2K3;>sp|P46734</td><td>2.24E-14</td></tr>
<tr><td>P46776;Q6NZ52;Q9BQQ5</td><td>RL27A_HUMAN 60S ribosomal protein L27a OS=Homo sapiens GN=RPL27A PE=1 SV=2;>tr|Q6NZ52|Q6NZ52_HUMAN Ribosomal prot</td><td>5.42E-74</td></tr>
<tr><td>P46779;B4DEP9;Q59F34;</td><td>RL28_HUMAN 60S ribosomal protein L28 OS=Homo sapiens GN=RPL28 PE=1 SV=3;>tr|B4DEP9|B4DEP9_HUMAN cDNA FLJ57954, hig</td><td>3.18E-53</td></tr>
<tr><td>P46782;Q53G25</td><td>RS5_HUMAN 40S ribosomal protein S5 OS=Homo sapiens GN=RPS5 PE=1 SV=4;>tr|Q53G25|Q53G25_HUMAN Ribosomal protein S5</td><td>3.19E-138</td></tr>
<tr><td>P46783;Q9NQ39</td><td>RS10_HUMAN 40S ribosomal protein S10 OS=Homo sapiens GN=RPS10 PE=1 SV=1;>sp|Q9NQ39|RS10L_HUMAN Putative 40S riboso</td><td>7.15E-97</td></tr>
<tr><td>P46821;A2BDK6;Q5H9P1</td><td>MAP1B_HUMAN Microtubule-associated protein 1B OS=Homo sapiens GN=MAP1B PE=1 SV=2;>tr|A2BDK6|A2BDK6_HUMAN Microtub</td><td>0</td></tr>
<tr><td>P46937;P46937-2;P46937</td><td>YAP1_HUMAN Yorkie homolog OS=Homo sapiens GN=YAP1 PE=1 SV=2;>sp|P46937-2|YAP1_HUMAN Isoform 2 of Yorkie homolog OS</td><td>2.45E-15</td></tr>
<tr><td>P46977;B4DJ24</td><td>STT3A_HUMAN Dolichyl-diphosphooligosaccharide--protein glycosyltransferase subunit STT3A OS=Homo sapiens GN=STT3A PE=1 SV</td><td>6.87E-13</td></tr>
<tr><td>P47813;O14602</td><td>IF1AX_HUMAN Eukaryotic translation initiation factor 1A, X-chromosomal OS=Homo sapiens GN=EIF1AX PE=1 SV=2;>sp|O14602|IF1</td><td>9.86E-83</td></tr>
<tr><td>P47813;O14602;A6NJH9</td><td>IF1AX_HUMAN Eukaryotic translation initiation factor 1A, X-chromosomal OS=Homo sapiens GN=EIF1AX PE=1 SV=2;>sp|O14602|IF1</td><td>3.92E-139</td></tr>
<tr><td>P47897;Q53HS0;A8K3A8;</td><td>SYQ_HUMAN Glutaminyl-tRNA synthetase OS=Homo sapiens GN=QARS PE=1 SV=1;>tr|Q53HS0|Q53HS0_HUMAN Glutaminyl-tRNA s</td><td>0</td></tr>
<tr><td>P47985;P0C7P4</td><td>UCRI_HUMAN Cytochrome b-c1 complex subunit Rieske, mitochondrial OS=Homo sapiens GN=UQCRFS1 PE=1 SV=2;>sp|P0C7P4|UC</td><td>3.67E-52</td></tr>
<tr><td>P48047;Q53HH2</td><td>ATPO_HUMAN ATP synthase subunit O, mitochondrial OS=Homo sapiens GN=ATP5O PE=1 SV=1;>tr|Q53HH2|Q53HH2_HUMAN Mitoch</td><td>3.66E-114</td></tr>
<tr><td>P48059;Q7Z4I7-2;Q7Z4I7</td><td>LIMS1_HUMAN LIM and senescent cell antigen-like-containing domain protein 1 OS=Homo sapiens GN=LIMS1 PE=1 SV=4;>sp|Q7Z4I</td><td>3.04E-29</td></tr>
<tr><td>P48556;Q5U0B3;B4E2Y4</td><td>PSMD8_HUMAN 26S proteasome non-ATPase regulatory subunit 8 OS=Homo sapiens GN=PSMD8 PE=1 SV=2;>tr|Q5U0B3|Q5U0B3_H</td><td>1.59E-29</td></tr>
<tr><td>P48643;Q9BU08;B7ZAR1;</td><td>TCPE_HUMAN T-complex protein 1 subunit epsilon OS=Homo sapiens GN=CCT5 PE=1 SV=1;>tr|Q9BU08|Q9BU08_HUMAN Putative ur</td><td>0</td></tr>
<tr><td>P48729-2;P48729;Q8N75</td><td>2|KC1A_HUMAN Isoform 2 of Casein kinase I isoform alpha OS=Homo sapiens GN=CSNK1A1;>sp|P48729|KC1A_HUMAN Casein kinas</td><td>1.05E-10</td></tr>
<tr><td>P48735;Q53GL5;B4DFL2;</td><td>IDHP_HUMAN Isocitrate dehydrogenase [NADP], mitochondrial OS=Homo sapiens GN=IDH2 PE=1 SV=2;>tr|Q53GL5|Q53GL5_HUMA</td><td>0</td></tr>
<tr><td>P48960;P48960-3;P48960</td><td>CD97_HUMAN CD97 antigen OS=Homo sapiens GN=CD97 PE=1 SV=4;>sp|P48960-3|CD97_HUMAN Isoform 3 of CD97 antigen OS=</td><td>1.59E-07</td></tr>
<tr><td>P49023-3;P49023;P49023</td><td>3|PAXI_HUMAN Isoform Gamma of Paxillin OS=Homo sapiens GN=PXN;>sp|P49023|PAXI_HUMAN Paxillin OS=Homo sapiens GN=PX</td><td>8.26E-45</td></tr>
<tr><td>P49137;P49137-2;Q1664</td><td>MAPK2_HUMAN MAP kinase-activated protein kinase 2 OS=Homo sapiens GN=MAPKAPK2 PE=1 SV=1;>sp|P49137-2|MAPK2_HUMAN</td><td>3.38E-39</td></tr>
<tr><td>P49137;Q16644;P49137-</td><td>MAPK2_HUMAN MAP kinase-activated protein kinase 2 OS=Homo sapiens GN=MAPKAPK2 PE=1 SV=1;>sp|Q16644|MAPK3_HUMAN M</td><td>4.07E-09</td></tr>
<tr><td>P49321-3;P49321</td><td>3|NASP_HUMAN Isoform 3 of Nuclear autoantigenic sperm protein OS=Homo sapiens GN=NASP;>sp|P49321|NASP_HUMAN Nuclear a</td><td>1.69E-255</td></tr>
</table>

P49321-3;P49321;P49321	3|NASP_HUMAN Isoform 3 of Nuclear autoantigenic sperm protein OS=Homo sapiens GN=NASP;>sp|P49321|NASP_HUMAN Nuclear a	0
P49366;P49366-2;Q5J8M!	DHYS_HUMAN Deoxyhypusine synthase OS=Homo sapiens GN=DHPS PE=1 SV=1;>sp|P49366-2|DHYS_HUMAN Isoform Short of Deo	1.24E-159
P49406;A8K5D5;B4DIG4	RM19_HUMAN 39S ribosomal protein L19, mitochondrial OS=Homo sapiens GN=MRPL19 PE=1 SV=2;>tr|A8K5D5|A8K5D5_HUMAN cD	5.78E-34
P49419;P49419-2;B4DMA	AL7A1_HUMAN Alpha-aminoadipic semialdehyde dehydrogenase OS=Homo sapiens GN=ALDH7A1 PE=1 SV=5;>sp|P49419-2|AL7A1_	5.67E-203
P49419;P49419-2;P49419	AL7A1_HUMAN Alpha-aminoadipic semialdehyde dehydrogenase OS=Homo sapiens GN=ALDH7A1 PE=1 SV=5;>sp|P49419-2|AL7A1_	1.15E-254
P49585;Q9Y5K3;Q9Y5K3-	PCY1A_HUMAN Choline-phosphate cytidylyltransferase A OS=Homo sapiens GN=PCYT1A PE=1 SV=2;>sp|Q9Y5K3|PCY1B_HUMAN Cho	4.27E-26
P49588;B4DR45	SYAC_HUMAN Alanyl-tRNA synthetase, cytoplasmic OS=Homo sapiens GN=AARS PE=1 SV=2;>tr|B4DR45|B4DR45_HUMAN cDNA FLJ	3.68E-161
P49593;Q6IPC0;A0M8Q2;	PPM1F_HUMAN Protein phosphatase 1F OS=Homo sapiens GN=PPM1F PE=1 SV=3;>tr|Q6IPC0|Q6IPC0_HUMAN Protein phosphatase 1	1.38E-08
P49747;B4DN90;B4DKJ3;	COMP_HUMAN Cartilage oligomeric matrix protein OS=Homo sapiens GN=COMP PE=1 SV=2;>tr|B4DN90|B4DN90_HUMAN cDNA FLJ5	8.20E-196
P49753;Q86TX2;P49753-2	ACOT2_HUMAN Acyl-coenzyme A thioesterase 2, mitochondrial OS=Homo sapiens GN=ACOT2 PE=1 SV=6;>sp|Q86TX2|ACOT1_HUMA	1.01E-33
P49757;P49757-3;P49757	NUMB_HUMAN Protein numb homolog OS=Homo sapiens GN=NUMB PE=1 SV=2;>sp|P49757-3|NUMB_HUMAN Isoform 3 of Protein n	1.41E-06
P49770;Q53XC2;Q9BPX4	EI2BB_HUMAN Translation initiation factor eIF-2B subunit beta OS=Homo sapiens GN=EIF2B2 PE=1 SV=3;>tr|Q53XC2|Q53XC2_HUM	9.67E-95
P49773;A4D2H4;D6RD60;	HINT1_HUMAN Histidine triad nucleotide-binding protein 1 OS=Homo sapiens GN=HINT1 PE=1 SV=2;>tr|A4D2H4|A4D2H4_HUMAN S	5.78E-28
P49792;A6NKT7;Q7Z3J3;(	RBP2_HUMAN E3 SUMO-protein ligase RanBP2 OS=Homo sapiens GN=RANBP2 PE=1 SV=2;>sp|A6NKT7|RGPD3_HUMAN RanBP2-like	9.37E-58
P49821;P49821-2;Q53G7(	NDUV1_HUMAN NADH dehydrogenase [ubiquinone] flavoprotein 1, mitochondrial OS=Homo sapiens GN=NDUFV1 PE=1 SV=4;>sp|P4	1.95E-68
P49961-3	ENTP1_HUMAN Isoform Placental II of Ectonucleoside triphosphate diphosphohydrolase 1 OS=Homo sapiens GN=ENTPD1	
P50135;B4DWC1	HNMT_HUMAN Histamine N-methyltransferase OS=Homo sapiens GN=HNMT PE=1 SV=1;>tr|B4DWC1|B4DWC1_HUMAN cDNA FLJ513	5.89E-48
P50224;P50225-2;P50224	ST1A3_HUMAN Sulfotransferase 1A3/1A4 OS=Homo sapiens GN=SULT1A3 PE=1 SV=1;>sp|P50225-2|ST1A1_HUMAN Isoform 2 of Su	1.01E-18
P50402;Q6FI02;Q5HY57	EMD_HUMAN Emerin OS=Homo sapiens GN=EMD PE=1 SV=1;>tr|Q6FI02|Q6FI02_HUMAN EMD protein OS=Homo sapiens GN=EMD F	6.18E-38
P50453;Q6N0A8	SPB9_HUMAN Serpin B9 OS=Homo sapiens GN=SERPINB9 PE=1 SV=1;>tr|Q6N0A8|Q6N0A8_HUMAN Putative uncharacterized protei	3.58E-92
P50454;A8K259;B4DN87	SERPH_HUMAN Serpin H1 OS=Homo sapiens GN=SERPINH1 PE=1 SV=2;>tr|A8K259|A8K259_HUMAN cDNA FLJ78501, highly similar	0
P50479;P50479-2	PDLI4_HUMAN PDZ and LIM domain protein 4 OS=Homo sapiens GN=PDLIM4 PE=1 SV=2;>sp|P50479-2|PDLI4_HUMAN Isoform 2 of	1.72E-16
P50502;Q2TU77;B4E0U6;(	F10A1_HUMAN Hsc70-interacting protein OS=Homo sapiens GN=ST13 PE=1 SV=2;>tr|Q2TU77|Q2TU77_HUMAN Aging-associated pro	1.10E-198
P50502;Q8NFI4;Q8IZP2	F10A1_HUMAN Hsc70-interacting protein OS=Homo sapiens GN=ST13 PE=1 SV=2;>sp|Q8NFI4|F10A5_HUMAN Putative protein FAM1	0
P50579;B4DUX5;Q8NB11;	AMPM2_HUMAN Methionine aminopeptidase 2 OS=Homo sapiens GN=METAP2 PE=1 SV=1;>tr|B4DUX5|B4DUX5_HUMAN Methionine a	1.27E-27
P50897;Q6FGQ4;Q5T0S6;	PPT1_HUMAN Palmitoyl-protein thioesterase 1 OS=Homo sapiens GN=PPT1 PE=1 SV=1;>tr|Q6FGQ4|Q6FGQ4_HUMAN PPT1 protein (F	1.61E-20
P50990;Q53HU0;B4DEM7;	TCPQ_HUMAN T-complex protein 1 subunit theta OS=Homo sapiens GN=CCT8 PE=1 SV=4;>tr|Q53HU0|Q53HU0_HUMAN Chaperonin	0
P50991;A8K3C3;B7Z8B1;I	TCPD_HUMAN T-complex protein 1 subunit delta OS=Homo sapiens GN=CCT4 PE=1 SV=4;>tr|A8K3C3|A8K3C3_HUMAN T-complex pr	0
P51003;P51003-2;Q9NRJ!	PAPOA_HUMAN Poly(A) polymerase alpha OS=Homo sapiens GN=PAPOLA PE=1 SV=4;>sp|P51003-2|PAPOA_HUMAN Isoform 2 of Poly	1.75E-33
P51114;P51114-2;P51114	FXR1_HUMAN Fragile X mental retardation syndrome-related protein 1 OS=Homo sapiens GN=FXR1 PE=1 SV=3;>sp|P51114-2|FXR1_	1.38E-93
P51149;B4DPH9;C9J8S3;(	RAB7A_HUMAN Ras-related protein Rab-7a OS=Homo sapiens GN=RAB7A PE=1 SV=1;>tr|B4DPH9|B4DPH9_HUMAN cDNA FLJ55895,	2.76E-112
P51151;Q9NP90	RAB9A_HUMAN Ras-related protein Rab-9A OS=Homo sapiens GN=RAB9A PE=1 SV=1;>sp|Q9NP90|RAB9B_HUMAN Ras-related prote	1.39E-29
P51153;Q96E17	RAB13_HUMAN Ras-related protein Rab-13 OS=Homo sapiens GN=RAB13 PE=1 SV=1;>sp|Q96E17|RAB3C_HUMAN Ras-related prote	2.57E-19
P51398;B4DY62;B4DP59	RT29_HUMAN 28S ribosomal protein S29, mitochondrial OS=Homo sapiens GN=DAP3 PE=1 SV=1;>tr|B4DY62|B4DY62_HUMAN cDNA	2.63E-49
P51553;D3DWV9;O15384	IDH3G_HUMAN Isocitrate dehydrogenase [NAD] subunit gamma, mitochondrial OS=Homo sapiens GN=IDH3G PE=1 SV=1;>tr|D3DW\	4.22E-20
P51580;Q9BS45;Q6I929	TPMT_HUMAN Thiopurine S-methyltransferase OS=Homo sapiens GN=TPMT PE=1 SV=1;>tr|Q9BS45|Q9BS45_HUMAN Thiopurine S-m	1.39E-08
P51606;P51606-2	RENBP_HUMAN N-acylglucosamine 2-epimerase OS=Homo sapiens GN=RENBP PE=1 SV=2;>sp|P51606-2|RENBP_HUMAN Isoform 2 c	2.82E-10
P51608-2;P51608	2|MECP2_HUMAN Isoform B of Methyl-CpG-binding protein 2 OS=Homo sapiens GN=MECP2;>sp|P51608|MECP2_HUMAN Methyl-CpG-	1.63E-05
P51812;B7ZB17;B4DG22;	KS6A3_HUMAN Ribosomal protein S6 kinase alpha-3 OS=Homo sapiens GN=RPS6KA3 PE=1 SV=1;>tr|B7ZB17|B7ZB17_HUMAN cDN/	5.28E-35
P51812;Q15349-2;Q1541!	KS6A3_HUMAN Ribosomal protein S6 kinase alpha-3 OS=Homo sapiens GN=RPS6KA3 PE=1 SV=1;>sp|Q15349-2|KS6A2_HUMAN Iso	1.03E-10
P51884;Q53FV4	LUM_HUMAN Lumican OS=Homo sapiens GN=LUM PE=1 SV=2;>tr|Q53FV4|Q53FV4_HUMAN Lumican variant (Fragment) OS=Homo s	0
P51888;Q6FG38;Q6FHG6;	PRELP_HUMAN Prolargin OS=Homo sapiens GN=PRELP PE=1 SV=1;>tr|Q6FG38|Q6FG38_HUMAN PRELP protein OS=Homo sapiens GI	0
P51911;Q53FP8;B4DUX6;	CNN1_HUMAN Calponin-1 OS=Homo sapiens GN=CNN1 PE=1 SV=2;>tr|Q53FP8|Q53FP8_HUMAN Calponin 1, basic, smooth muscle va	1.90E-125
P51965;Q969T4	UB2E1_HUMAN Ubiquitin-conjugating enzyme E2 E1 OS=Homo sapiens GN=UBE2E1 PE=1 SV=1;>sp|Q969T4|UB2E3_HUMAN Ubiquit	1.71E-10
P51965;Q96LR5	UB2E1_HUMAN Ubiquitin-conjugating enzyme E2 E1 OS=Homo sapiens GN=UBE2E1 PE=1 SV=1;>sp|Q96LR5|UB2E2_HUMAN Ubiquit	1.78E-31
P52272;P52272-2;Q59ES(	HNRPM_HUMAN Heterogeneous nuclear ribonucleoprotein M OS=Homo sapiens GN=HNRNPM PE=1 SV=3;>sp|P52272-2|HNRPM_HUM	1.61E-260
P52298;P52298-2;Q7Z3W	NCBP2_HUMAN Nuclear cap-binding protein subunit 2 OS=Homo sapiens GN=NCBP2 PE=1 SV=1;>sp|P52298-2|NCBP2_HUMAN Isofo	4.57E-12
P52435;Q9GZM3-2;Q9GZI	RPB11_HUMAN DNA-directed RNA polymerase II subunit RPB11-a OS=Homo sapiens GN=POLR2J PE=1 SV=1;>sp|Q9GZM3-2|RPB1B_	8.64E-06
P52435;Q9GZM3;Q9GZM3	RPB11_HUMAN DNA-directed RNA polymerase II subunit RPB11-a OS=Homo sapiens GN=POLR2J PE=1 SV=1;>sp|Q9GZM3|RPB1B_H	2.86E-09
P52565;A8MXW0;B4DUV9	GDIR1_HUMAN Rho GDP-dissociation inhibitor 1 OS=Homo sapiens GN=ARHGDIA PE=1 SV=3;>tr|A8MXW0|A8MXW0_HUMAN Unchar	6.45E-59
P52597;B4DKS8	HNRPF_HUMAN Heterogeneous nuclear ribonucleoprotein F OS=Homo sapiens GN=HNRNPF PE=1 SV=3;>tr|B4DKS8|B4DKS8_HUMAN	1.93E-281
P52630;P52630-2	STAT2_HUMAN Signal transducer and activator of transcription 2 OS=Homo sapiens GN=STAT2 PE=1 SV=1;>sp|P52630-2|STAT2_HUI	3.61E-22
P52701;P52701-2	MSH6_HUMAN DNA mismatch repair protein Msh6 OS=Homo sapiens GN=MSH6 PE=1 SV=2;>sp|P52701-2|MSH6_HUMAN Isoform G1	7.90E-190
P52732;B2RAM6	KIF11_HUMAN Kinesin-like protein KIF11 OS=Homo sapiens GN=KIF11 PE=1 SV=2;>tr|B2RAM6|B2RAM6_HUMAN cDNA, FLJ95005, h	2.12E-59
P52758;Q6IBG0	UK114_HUMAN Ribonuclease UK114 OS=Homo sapiens GN=HRSP12 PE=1 SV=1;>tr|Q6IBG0|Q6IBG0_HUMAN Heat-responsive protei	1.48E-137
P52788;P52788-2	SPSY_HUMAN Spermine synthase OS=Homo sapiens GN=SMS PE=1 SV=2;>sp|P52788-2|SPSY_HUMAN Isoform 2 of Spermine synth	5.11E-81
P52788;P52788-2;B4DE4(	SPSY_HUMAN Spermine synthase OS=Homo sapiens GN=SMS PE=1 SV=2;>sp|P52788-2|SPSY_HUMAN Isoform 2 of Spermine synth	7.47E-184
P52888;Q96CV8;Q9BW75	THOP1_HUMAN Thimet oligopeptidase OS=Homo sapiens GN=THOP1 PE=1 SV=2;>tr|Q96CV8|Q96CV8_HUMAN Thimet oligopeptidase	1.91E-127
P52895;B4DK69;Q59GU2;	AK1C2_HUMAN Aldo-keto reductase family 1 member C2 OS=Homo sapiens GN=AKR1C2 PE=1 SV=3;>tr|B4DK69|B4DK69_HUMAN c	1.27E-19
P52907;A8K0T9	CAZA1_HUMAN F-actin-capping protein subunit alpha-1 OS=Homo sapiens GN=CAPZA1 PE=1 SV=3;>tr|A8K0T9|A8K0T9_HUMAN cDN	2.76E-188
P52943;Q53FN1;B7Z6C0	CRIP2_HUMAN Cysteine-rich protein 2 OS=Homo sapiens GN=CRIP2 PE=1 SV=1;>tr|Q53FN1|Q53FN1_HUMAN Cysteine-rich protein 2	2.36E-83

P53367-2;Q2M2X4;B4E27	2|ARFP1_HUMAN Isoform A of Arfaptin-1 OS=Homo sapiens GN=ARFIP1;>tr|Q2M2X4|Q2M2X4_HUMAN ADP-ribosylation factor intera	5.27E-56
P53582;D6RBD2;D6RF24	AMPM1_HUMAN Methionine aminopeptidase 1 OS=Homo sapiens GN=METAP1 PE=1 SV=2;>tr|D6RBD2|D6RBD2_HUMAN Uncharacter	3.00E-27
P53597;A8K4W7;Q6IAL5;	SUCA_HUMAN Succinyl-CoA ligase [GDP-forming] subunit alpha, mitochondrial OS=Homo sapiens GN=SUCLG1 PE=1 SV=4;>tr|A8K4	7.11E-64
P53602;Q59G80	MVD1_HUMAN Diphosphomevalonate decarboxylase OS=Homo sapiens GN=MVD PE=1 SV=1;>tr|Q59G80|Q59G80_HUMAN Diphosph	3.32E-06
P53611;Q6IB63;Q59GT6;	PGTB2_HUMAN Geranylgeranyl transferase type-2 subunit beta OS=Homo sapiens GN=RABGGTB PE=1 SV=2;>tr|Q6IB63|Q6IB63_HU	9.10E-20
P53634;P53634-2	CATC_HUMAN Dipeptidyl peptidase 1 OS=Homo sapiens GN=CTSC PE=1 SV=2;>sp|P53634-2|CATC_HUMAN Isoform 2 of Dipeptidyl p	6.22E-20
P53701;Q68D50	CCHL_HUMAN Cytochrome c-type heme lyase OS=Homo sapiens GN=HCCS PE=1 SV=1;>tr|Q68D50|Q68D50_HUMAN Putative uncha	2.12E-28
P53990-4;P53990;P53990	4|IST1_HUMAN Isoform 4 of IST1 homolog OS=Homo sapiens GN=KIAA0174;>sp|P53990|IST1_HUMAN IST1 homolog OS=Homo sap	4.60E-10
P53992;B4DSN3	SC24C_HUMAN Protein transport protein Sec24C OS=Homo sapiens GN=SEC24C PE=1 SV=3;>tr|B4DSN3|B4DSN3_HUMAN cDNA FLJ	3.32E-50
P54136;P54136-2;B4DXW	SYRC_HUMAN Arginyl-tRNA synthetase, cytoplasmic OS=Homo sapiens GN=RARS PE=1 SV=2;>sp|P54136-2|SYRC_HUMAN Isoform N	0
P54652;B3KUS2	HSP72_HUMAN Heat shock-related 70 kDa protein 2 OS=Homo sapiens GN=HSPA2 PE=1 SV=1;>tr|B3KUS2|B3KUS2_HUMAN cDNA F	0
P54687;Q68DQ7;B7Z2M5;	BCAT1_HUMAN Branched-chain-amino-acid aminotransferase, cytosolic OS=Homo sapiens GN=BCAT1 PE=1 SV=3;>tr|Q68DQ7|Q68D	1.05E-27
P54709;D3DNF9	AT1B3_HUMAN Sodium/potassium-transporting ATPase subunit beta-3 OS=Homo sapiens GN=ATP1B3 PE=1 SV=1;>tr|D3DNF9|D3DN	2.93E-37
P54727;Q53F10;B4DEA3;	RD23B_HUMAN UV excision repair protein RAD23 homolog B OS=Homo sapiens GN=RAD23B PE=1 SV=1;>tr|Q53F10|Q53F10_HUMA	7.60E-60
P54868;Q6IBF4;B7Z8R3;	HMCS2_HUMAN Hydroxymethylglutaryl-CoA synthase, mitochondrial OS=Homo sapiens GN=HMGCS2 PE=1 SV=1;>tr|Q6IBF4|Q6IBF4	1.10E-29
P55010;Q6IBU0;Q05DF3;	IF5_HUMAN Eukaryotic translation initiation factor 5 OS=Homo sapiens GN=EIF5 PE=1 SV=2;>tr|Q6IBU0|Q6IBU0_HUMAN EIF5 prote	1.45E-129
P55011;Q53ZR1;B7ZM24;	S12A2_HUMAN Solute carrier family 12 member 2 OS=Homo sapiens GN=SLC12A2 PE=1 SV=1;>tr|Q53ZR1|Q53ZR1_HUMAN Bumeta	1.50E-06
P55036;Q5VWC4;D3DV16	PSMD4_HUMAN 26S proteasome non-ATPase regulatory subunit 4 OS=Homo sapiens GN=PSMD4 PE=1 SV=1;>tr|Q5VWC4|Q5VWC4_	0
P55039;Q53Y50;A8MZF9	DRG2_HUMAN Developmentally-regulated GTP-binding protein 2 OS=Homo sapiens GN=DRG2 PE=1 SV=1;>tr|Q53Y50|Q53Y50_HUM	1.71E-40
P55072;Q96IF9;Q0IIN5;Q	TERA_HUMAN Transitional endoplasmic reticulum ATPase OS=Homo sapiens GN=VCP PE=1 SV=4;>tr|Q96IF9|Q96IF9_HUMAN VCP pr	0
P55084;B4E2W0;B4DY96;	ECHB_HUMAN Trifunctional enzyme subunit beta, mitochondrial OS=Homo sapiens GN=HADHB PE=1 SV=3;>tr|B4E2W0|B4E2W0_HU	1.28E-141
P55196-5;P55196;P55196	5|AFAD_HUMAN Isoform 5 of Afadin OS=Homo sapiens GN=MLLT4;>sp|P55196|AFAD_HUMAN Afadin OS=Homo sapiens GN=MLLT4 P	5.78E-78
P55209;B3KNT8;B7Z2V4;	NP1L1_HUMAN Nucleosome assembly protein 1-like 1 OS=Homo sapiens GN=NAP1L1 PE=1 SV=1;>tr|B3KNT8|B3KNT8_HUMAN cDNA	0
P55212;D6RHU3;P55212-	CASP6_HUMAN Caspase-6 OS=Homo sapiens GN=CASP6 PE=1 SV=2;>tr|D6RHU3|D6RHU3_HUMAN Uncharacterized protein OS=Hon	2.34E-17
P55212;P55212-2	CASP6_HUMAN Caspase-6 OS=Homo sapiens GN=CASP6 PE=1 SV=2;>sp|P55212-2|CASP6_HUMAN Isoform Beta of Caspase-6 OS=H	2.07E-26
P55263;P55263-2;Q5JQ1	ADK_HUMAN Adenosine kinase OS=Homo sapiens GN=ADK PE=1 SV=2;>sp|P55263-2|ADK_HUMAN Isoform Short of Adenosine kina	5.91E-137
P55265-4;P55265;P55265	4|DSRAD_HUMAN Isoform 4 of Double-stranded RNA-specific adenosine deaminase OS=Homo sapiens GN=ADAR;>sp|P55265|DSRAD	0
P55327-3;P55327;P55327	3|TPD52_HUMAN Isoform 3 of Tumor protein D52 OS=Homo sapiens GN=TPD52;>sp|P55327|TPD52_HUMAN Tumor protein D52 OS=	1.13E-207
P55786;B7Z899;B7Z463;	PSA_HUMAN Puromycin-sensitive aminopeptidase OS=Homo sapiens GN=NPEPPS PE=1 SV=2;>tr|B7Z899|B7Z899_HUMAN cDNA FLJ	5.23E-135
P55795;B4DFK9;B4DP00	HNRH2_HUMAN Heterogeneous nuclear ribonucleoprotein H2 OS=Homo sapiens GN=HNRNPH2 PE=1 SV=1;>tr|B4DFK9|B4DFK9_HUN	6.95E-157
P55809;B2R5V2;Q6IAV5	SCOT1_HUMAN Succinyl-CoA:3-ketoacid-coenzyme A transferase 1, mitochondrial OS=Homo sapiens GN=OXCT1 PE=1 SV=1;>tr|B2R	4.74E-33
P55854;Q6EEV6	SUMO3_HUMAN Small ubiquitin-related modifier 3 OS=Homo sapiens GN=SUMO3 PE=1 SV=2;>sp|Q6EEV6|SUMO4_HUMAN Small ub	1.38E-33
P55884-2;P55884;A4D21	2|EIF3B_HUMAN Isoform 2 of Eukaryotic translation initiation factor 3 subunit B OS=Homo sapiens GN=EIF3B;>sp|P55884|EIF3B_HU	3.13E-113
P56199;B4DTY8	ITA1_HUMAN Integrin alpha-1 OS=Homo sapiens GN=ITGA1 PE=1 SV=2;>tr|B4DTY8|B4DTY8_HUMAN cDNA FLJ61587, highly similar	9.93E-07
P56537;B7ZBG9	IF6_HUMAN Eukaryotic translation initiation factor 6 OS=Homo sapiens GN=EIF6 PE=1 SV=1;>tr|B7ZBG9|B7ZBG9_HUMAN Eukaryoti	1.38E-83
P56545-2;Q5SQP8;Q8IY4	2|CTBP2_HUMAN Isoform 2 of C-terminal-binding protein 2 OS=Homo sapiens GN=CTBP2;>tr|Q5SQP8|Q5SQP8_HUMAN C-terminal b	8.15E-28
P57737;P57737-2	CORO7_HUMAN Coronin-7 OS=Homo sapiens GN=CORO7 PE=1 SV=2;>sp|P57737-2|CORO7_HUMAN Isoform 2 of Coronin-7 OS=Hor	1.20E-43
P57740;B4DZ67;Q6PJE1;	NU107_HUMAN Nuclear pore complex protein Nup107 OS=Homo sapiens GN=NUP107 PE=1 SV=1;>tr|B4DZ67|B4DZ67_HUMAN cDN	3.45E-32
P58546;Q69YG1;C9JL85	MTPN_HUMAN Myotrophin OS=Homo sapiens GN=MTPN PE=1 SV=2;>tr|Q69YG1|Q69YG1_HUMAN Myotrophin OS=Homo sapiens GN	3.08E-149
P59665;P59666;Q6EZE9;	DEF1_HUMAN Neutrophil defensin 1 OS=Homo sapiens GN=DEFA1 PE=1 SV=1;>sp|P59666|DEF3_HUMAN Neutrophil defensin 3 OS=	5.70E-07
P60228;Q6IAX5;B2R806;	EIF3E_HUMAN Eukaryotic translation initiation factor 3 subunit E OS=Homo sapiens GN=EIF3E PE=1 SV=1;>tr|Q6IAX5|Q6IAX5_HUM	5.79E-166
P60468;Q53FA5	SC61B_HUMAN Protein transport protein Sec61 subunit beta OS=Homo sapiens GN=SEC61B PE=1 SV=2;>tr|Q53FA5|Q53FA5_HUMAN	1.21E-06
P60510;Q5U086	PP4C_HUMAN Serine/threonine-protein phosphatase 4 catalytic subunit OS=Homo sapiens GN=PPP4C PE=1 SV=1;>tr|Q5U086|Q5U08	2.24E-06
P60900;B4DXJ9	PSA6_HUMAN Proteasome subunit alpha type-6 OS=Homo sapiens GN=PSMA6 PE=1 SV=1;>tr|B4DXJ9|B4DXJ9_HUMAN Proteasome	4.39E-73
P60981;B4DYA6;B7Z9M9	DEST_HUMAN Destrin OS=Homo sapiens GN=DSTN PE=1 SV=3;>tr|B4DYA6|B4DYA6_HUMAN Destrin (Actin depolymerizing factor), i	9.96E-69
P61006;B4DEK7	RAB8A_HUMAN Ras-related protein Rab-8A OS=Homo sapiens GN=RAB8A PE=1 SV=1;>tr|B4DEK7|B4DEK7_HUMAN cDNA FLJ53028,	3.96E-121
P61011;B4DDS7;B4DUW6	SRP54_HUMAN Signal recognition particle 54 kDa protein OS=Homo sapiens GN=SRP54 PE=1 SV=1;>tr|B4DDS7|B4DDS7_HUMAN cD	2.64E-178
P61019;B4DMQ5;D3DS24	RAB2A_HUMAN Ras-related protein Rab-2A OS=Homo sapiens GN=RAB2A PE=1 SV=1;>tr|B4DMQ5|B4DMQ5_HUMAN cDNA FLJ5199	3.50E-178
P61019;Q8WUD1	RAB2A_HUMAN Ras-related protein Rab-2A OS=Homo sapiens GN=RAB2A PE=1 SV=1;>sp|Q8WUD1|RAB2B_HUMAN Ras-related prot	0
P61020;Q6FI54;B4DKD7	RAB5B_HUMAN Ras-related protein Rab-5B OS=Homo sapiens GN=RAB5B PE=1 SV=1;>tr|Q6FI54|Q6FI54_HUMAN RAB5B protein OS	1.45E-88
P61077-3;P61077-2;P610	3|UB2D3_HUMAN Isoform 3 of Ubiquitin-conjugating enzyme E2 D3 OS=Homo sapiens GN=UBE2D3;>sp|P61077-2|UB2D3_HUMAN Is	2.22E-11
P61086;P61086-2	UBE2K_HUMAN Ubiquitin-conjugating enzyme E2 K OS=Homo sapiens GN=UBE2K PE=1 SV=3;>sp|P61086-2|UBE2K_HUMAN Isoform	2.97E-08
P61088;Q5JXB2	UBE2N_HUMAN Ubiquitin-conjugating enzyme E2 N OS=Homo sapiens GN=UBE2N PE=1 SV=1;>sp|Q5JXB2|UE2NL_HUMAN Putative u	1.66E-147
P61158;Q53QM2;B4DXW1	ARP3_HUMAN Actin-related protein 3 OS=Homo sapiens GN=ACTR3 PE=1 SV=3;>tr|Q53QM2|Q53QM2_HUMAN ARP3 actin-related pr	1.99E-248
P61163;B4DM97;B4DXP9;	ACTZ_HUMAN Alpha-centractin OS=Homo sapiens GN=ACTR1A PE=1 SV=1;>tr|B4DM97|B4DM97_HUMAN cDNA FLJ55002, highly sim	8.69E-147
P61163;P42025	ACTZ_HUMAN Alpha-centractin OS=Homo sapiens GN=ACTR1A PE=1 SV=1;>sp|P42025|ACTY_HUMAN Beta-centractin OS=Homo sap	1.02E-188
P61221;D6R9I9	ABCE1_HUMAN ATP-binding cassette sub-family E member 1 OS=Homo sapiens GN=ABCE1 PE=1 SV=1;>tr|D6R9I9|D6R9I9_HUMAN	1.10E-290
P61224;A6NIZ1	RAP1B_HUMAN Ras-related protein Rap-1b OS=Homo sapiens GN=RAP1B PE=1 SV=1;>sp|A6NIZ1|RP1BL_HUMAN Ras-related protei	1.12E-86
P61224;B4DQI8;Q9BXV4;	RAP1B_HUMAN Ras-related protein Rap-1b OS=Homo sapiens GN=RAP1B PE=1 SV=1;>tr|B4DQI8|B4DQI8_HUMAN cDNA FLJ51696,	1.44E-26
P61225;Q5JQ44	RAP2B_HUMAN Ras-related protein Rap-2b OS=Homo sapiens GN=RAP2B PE=1 SV=1;>tr|Q5JQ44|Q5JQ44_HUMAN Putative uncharac	2.32E-09
P61289-2;P61289;Q6FHK	2|PSME3_HUMAN Isoform 2 of Proteasome activator complex subunit 3 OS=Homo sapiens GN=PSME3;>sp|P61289|PSME3_HUMAN Pr	2.46E-50

P61313;Q5U0C0;B4DLP4	RL15_HUMAN 60S ribosomal protein L15 OS=Homo sapiens GN=RPL15 PE=1 SV=2;>tr|Q5U0C0|Q5U0C0_HUMAN Ribosomal protein I	4.30E-81
P61457;Q6FGB3;Q6LEE0;(	PHS_HUMAN Pterin-4-alpha-carbinolamine dehydratase OS=Homo sapiens GN=PCBD1 PE=1 SV=2;>tr|Q6FGB3|Q6FGB3_HUMAN PCB	2.60E-200
P61513;Q6P4E4;C9J4Z3	RL37A_HUMAN 60S ribosomal protein L37a OS=Homo sapiens GN=RPL37A PE=1 SV=2;>tr|Q6P4E4|Q6P4E4_HUMAN RPL37A protein	8.93E-81
P61586;B4DKN9;C9JX21;(	RHOA_HUMAN Transforming protein RhoA OS=Homo sapiens GN=RHOA PE=1 SV=1;>tr|B4DKN9|B4DKN9_HUMAN cDNA FLJ57740, h	1.88E-76
P61586;P08134	RHOA_HUMAN Transforming protein RhoA OS=Homo sapiens GN=RHOA PE=1 SV=1;>sp|P08134|RHOC_HUMAN Rho-related GTP-binc	0
P61599;A8MZB2;A6NHA3	NAA20_HUMAN N-alpha-acetyltransferase 20, NatB catalytic subunit OS=Homo sapiens GN=NAA20 PE=1 SV=1;>tr|A8MZB2|A8MZB2_	2.05E-06
P61604;B8ZZL8;Q9UNM1	CH10_HUMAN 10 kDa heat shock protein, mitochondrial OS=Homo sapiens GN=HSPE1 PE=1 SV=2;>tr|B8ZZL8|B8ZZL8_HUMAN Heat	1.71E-30
P61619;Q9H9S3;Q9H9S3-	S61A1_HUMAN Protein transport protein Sec61 subunit alpha isoform 1 OS=Homo sapiens GN=SEC61A1 PE=1 SV=2;>sp|Q9H9S3|S6	2.48E-12
P61764-2;P61764;Q68CM	2|STXB1_HUMAN Isoform 2 of Syntaxin-binding protein 1 OS=Homo sapiens GN=STXBP1;>sp|P61764|STXB1_HUMAN Syntaxin-bindi	1.15E-69
P61812-2;P61812	2|TGFB2_HUMAN Isoform B of Transforming growth factor beta-2 OS=Homo sapiens GN=TGFB2;>sp|P61812|TGFB2_HUMAN Transfor	1.24E-24
P61923;Q53FU3;B4DDX8	COPZ1_HUMAN Coatomer subunit zeta-1 OS=Homo sapiens GN=COPZ1 PE=1 SV=1;>tr|Q53FU3|Q53FU3_HUMAN Coatomer protein c	1.20E-103
P61956;P55854;Q6EEV6	SUMO2_HUMAN Small ubiquitin-related modifier 2 OS=Homo sapiens GN=SUMO2 PE=1 SV=2;>sp|P55854|SUMO3_HUMAN Small ubi	1.02E-42
P61956;Q6EEV6	SUMO2_HUMAN Small ubiquitin-related modifier 2 OS=Homo sapiens GN=SUMO2 PE=1 SV=2;>sp|Q6EEV6|SUMO4_HUMAN Small ub	1.88E-46
P61978-2;Q6IBN1;P6197	2|HNRPK_HUMAN Isoform 2 of Heterogeneous nuclear ribonucleoprotein K OS=Homo sapiens GN=HNRNPK;>tr|Q6IBN1|Q6IBN1_HUM	0
P61978-3;P61978-2;P619	3|HNRPK_HUMAN Isoform 3 of Heterogeneous nuclear ribonucleoprotein K OS=Homo sapiens GN=HNRNPK;>sp|P61978-2|HNRPK_HU	0
P61981;B3KNB4;B4DE78;	1433G_HUMAN 14-3-3 protein gamma OS=Homo sapiens GN=YWHAG PE=1 SV=2;>tr|B3KNB4|B3KNB4_HUMAN cDNA FLJ14168 fis,	3.75E-291
P62081;Q57Z92;B5MCP9	RS7_HUMAN 40S ribosomal protein S7 OS=Homo sapiens GN=RPS7 PE=1 SV=1;>tr|Q57Z92|Q57Z92_HUMAN Putative uncharacteriz	1.93E-62
P62136;P36873-2;P36873	PP1A_HUMAN Serine/threonine-protein phosphatase PP1-alpha catalytic subunit OS=Homo sapiens GN=PPP1CA PE=1 SV=1;>sp|P368	0
P62191;Q53HB3;Q53XL8;	PRS4_HUMAN 26S protease regulatory subunit 4 OS=Homo sapiens GN=PSMC1 PE=1 SV=1;>tr|Q53HB3|Q53HB3_HUMAN Proteasom	0
P62195;A8K3Z3;Q59GS3	PRS8_HUMAN 26S protease regulatory subunit 8 OS=Homo sapiens GN=PSMC5 PE=1 SV=1;>tr|A8K3Z3|A8K3Z3_HUMAN Proteasome	1.76E-197
P62241;Q5JR94;Q5JR95	RS8_HUMAN 40S ribosomal protein S8 OS=Homo sapiens GN=RPS8 PE=1 SV=2;>tr|Q5JR94|Q5JR94_HUMAN 40S ribosomal protein S	1.16E-202
P62269;Q5SUJ3	RS18_HUMAN 40S ribosomal protein S18 OS=Homo sapiens GN=RPS18 PE=1 SV=3;>tr|Q5SUJ3|Q5SUJ3_HUMAN Ribosomal protein S	4.15E-97
P62304;Q5VYJ4	RUXE_HUMAN Small nuclear ribonucleoprotein E OS=Homo sapiens GN=SNRPE PE=1 SV=1;>sp|Q5VYJ4|RUEL1_HUMAN Putative sma	4.25E-84
P62304;Q5VYJ4;A6NHK2	RUXE_HUMAN Small nuclear ribonucleoprotein E OS=Homo sapiens GN=SNRPE PE=1 SV=1;>sp|Q5VYJ4|RUEL1_HUMAN Putative sma	8.19E-15
P62312;Q4W5J5	LSM6_HUMAN U6 snRNA-associated Sm-like protein LSm6 OS=Homo sapiens GN=LSM6 PE=1 SV=1;>tr|Q4W5J5|Q4W5J5_HUMAN LS	5.57E-05
P62314;Q7Z5A3	SMD1_HUMAN Small nuclear ribonucleoprotein Sm D1 OS=Homo sapiens GN=SNRPD1 PE=1 SV=1;>tr|Q7Z5A3|Q7Z5A3_HUMAN Sma	2.08E-124
P62318;B4DJP7	SMD3_HUMAN Small nuclear ribonucleoprotein Sm D3 OS=Homo sapiens GN=SNRPD3 PE=1 SV=1;>tr|B4DJP7|B4DJP7_HUMAN cDNA	1.36E-09
P62330;Q5U025;Q6FGZ2;	ARF6_HUMAN ADP-ribosylation factor 6 OS=Homo sapiens GN=ARF6 PE=1 SV=2;>tr|Q5U025|Q5U025_HUMAN ADP-ribosylation facto	2.86E-48
P62341;B4DTQ5;Q6IAK0	SELT_HUMAN Selenoprotein T OS=Homo sapiens GN=SELT PE=2 SV=2;>tr|B4DTQ5|B4DTQ5_HUMAN cDNA FLJ60886, highly similar t	2.48E-05
P62424;Q5T8U4;Q5T8U2;	RL7A_HUMAN 60S ribosomal protein L7a OS=Homo sapiens GN=RPL7A PE=1 SV=2;>tr|Q5T8U4|Q5T8U4_HUMAN Ribosomal protein L	1.57E-200
P62491;B4DQU5	RB11A_HUMAN Ras-related protein Rab-11A OS=Homo sapiens GN=RAB11A PE=1 SV=3;>tr|B4DQU5|B4DQU5_HUMAN cDNA FLJ571	1.84E-31
P62701;P22090;Q8TD47	RS4X_HUMAN 40S ribosomal protein S4, X isoform OS=Homo sapiens GN=RPS4X PE=1 SV=2;>sp|P22090|RS4Y1_HUMAN 40S ribosc	0
P62701;Q8TD47;P22090	RS4X_HUMAN 40S ribosomal protein S4, X isoform OS=Homo sapiens GN=RPS4X PE=1 SV=2;>sp|Q8TD47|RS4Y2_HUMAN 40S ribos	0
P62736;P63267	ACTA_HUMAN Actin, aortic smooth muscle OS=Homo sapiens GN=ACTA2 PE=1 SV=1;>sp|P63267|ACTH_HUMAN Actin, gamma-enter	0
P62750;A8MUS3;A8MXA8	RL23A_HUMAN 60S ribosomal protein L23a OS=Homo sapiens GN=RPL23A PE=1 SV=1;>tr|A8MUS3|A8MUS3_HUMAN Ribosomal prot	1.36E-49
P62820;Q5U0I6;B7Z8M7;I	RAB1A_HUMAN Ras-related protein Rab-1A OS=Homo sapiens GN=RAB1A PE=1 SV=3;>tr|Q5U0I6|Q5U0I6_HUMAN RAB1A protein OS	8.47E-266
P62829;Q9BTQ7;C9JD32;I	RL23_HUMAN 60S ribosomal protein L23 OS=Homo sapiens GN=RPL23 PE=1 SV=1;>tr|Q9BTQ7|Q9BTQ7_HUMAN Similar to ribosoma	2.19E-114
P62847;Q5T0P7;P62847-3	RS24_HUMAN 40S ribosomal protein S24 OS=Homo sapiens GN=RPS24 PE=1 SV=1;>tr|Q5T0P7|Q5T0P7_HUMAN 40S ribosomal prot	3.40E-63
P62854;Q5JNZ5	RS26_HUMAN 40S ribosomal protein S26 OS=Homo sapiens GN=RPS26 PE=1 SV=3;>sp|Q5JNZ5|RS26L_HUMAN Putative 40S ribosor	1.83E-86
P62854;Q5JVH5;Q5JNZ5;(	RS26_HUMAN 40S ribosomal protein S26 OS=Homo sapiens GN=RPS26 PE=1 SV=3;>tr|Q5JVH5|Q5JVH5_HUMAN Ribosomal protein :	1.04E-27
P62879;Q6FHM2;C9JXA5;	GBB2_HUMAN Guanine nucleotide-binding protein G(I)/G(S)/G(T) subunit beta-2 OS=Homo sapiens GN=GNB2 PE=1 SV=3;>tr|Q6FHI	1.35E-116
P62906;Q1JQ76	RL10A_HUMAN 60S ribosomal protein L10a OS=Homo sapiens GN=RPL10A PE=1 SV=2;>tr|Q1JQ76|Q1JQ76_HUMAN Ribosomal prote	4.97E-108
P62913;Q5VVD0;P62913-	RL11_HUMAN 60S ribosomal protein L11 OS=Homo sapiens GN=RPL11 PE=1 SV=2;>tr|Q5VVD0|Q5VVD0_HUMAN Ribosomal protein	1.47E-83
P62979;P0CG48;P0CG47;I	RS27A_HUMAN Ubiquitin-40S ribosomal protein S27a OS=Homo sapiens GN=RPS27A PE=1 SV=2;>sp|P0CG48|UBC_HUMAN Polyubiq	4.49E-230
P63000-2;P63000;P60763	2|RAC1_HUMAN Isoform B of Ras-related C3 botulinum toxin substrate 1 OS=Homo sapiens GN=RAC1;>sp|P63000|RAC1_HUMAN Ras	4.00E-63
P63027;Q15836;P23763;I	VAMP2_HUMAN Vesicle-associated membrane protein 2 OS=Homo sapiens GN=VAMP2 PE=1 SV=3;>sp|Q15836|VAMP3_HUMAN Vesic	3.69E-155
P63027;Q15836;Q6FGG2;	VAMP2_HUMAN Vesicle-associated membrane protein 2 OS=Homo sapiens GN=VAMP2 PE=1 SV=3;>sp|Q15836|VAMP3_HUMAN Vesic	1.86E-195
P63096;P09471;P09471-2	GNAI1_HUMAN Guanine nucleotide-binding protein G(i) subunit alpha-1 OS=Homo sapiens GN=GNAI1 PE=1 SV=2;>sp|P09471|GNAC	3.60E-41
P63098;Q96LZ3	CANB1_HUMAN Calcineurin subunit B type 1 OS=Homo sapiens GN=PPP3R1 PE=1 SV=2;>sp|Q96LZ3|CANB2_HUMAN Calcineurin sub	8.11E-12
P63167;Q6FGH9	DYL1_HUMAN Dynein light chain 1, cytoplasmic OS=Homo sapiens GN=DYNLL1 PE=1 SV=1;>tr|Q6FGH9|Q6FGH9_HUMAN DNCL1 pro	1.77E-21
P63167;Q96FJ2	DYL1_HUMAN Dynein light chain 1, cytoplasmic OS=Homo sapiens GN=DYNLL1 PE=1 SV=1;>sp|Q96FJ2|DYL2_HUMAN Dynein light cl	1.36E-119
P63172;Q5VTU4;Q5VTU3	DYLT1_HUMAN Dynein light chain Tctex-type 1 OS=Homo sapiens GN=DYNLT1 PE=1 SV=1;>tr|Q5VTU4|Q5VTU4_HUMAN Dynein, ligh	1.87E-177
P63241-2;P63241;Q6IS14	2|IF5A1_HUMAN Isoform 2 of Eukaryotic translation initiation factor 5A-1 OS=Homo sapiens GN=EIF5A;>sp|P63241|IF5A1_HUMAN Eu	0
P63261;B4E3A4;B4DVQ0	ACTG_HUMAN Actin, cytoplasmic 2 OS=Homo sapiens GN=ACTG1 PE=1 SV=1;>tr|B4E3A4|B4E3A4_HUMAN cDNA FLJ57283, highly si	0
P63313;Q596K9	TYB10_HUMAN Thymosin beta-10 OS=Homo sapiens GN=TMSB10 PE=1 SV=2;>tr|Q596K9|Q596K9_HUMAN Migration-inducing prote	3.76E-12
P67775;P62714	PP2AA_HUMAN Serine/threonine-protein phosphatase 2A catalytic subunit alpha isoform OS=Homo sapiens GN=PPP2CA PE=1 SV=1;>	2.48E-262
P67809;Q7KZ24;Q05D43;	YBOX1_HUMAN Nuclease-sensitive element-binding protein 1 OS=Homo sapiens GN=YBX1 PE=1 SV=3;>tr|Q7KZ24|Q7KZ24_HUMAN	0
P67936;P67936-2	TPM4_HUMAN Tropomyosin alpha-4 chain OS=Homo sapiens GN=TPM4 PE=1 SV=3;>sp|P67936-2|TPM4_HUMAN Isoform 2 of Tropom	8.33E-173
P68032;P68133;P62736;P	ACTC_HUMAN Actin, alpha cardiac muscle 1 OS=Homo sapiens GN=ACTC1 PE=1 SV=1;>sp|P68133|ACTS_HUMAN Actin, alpha skelet	0
P68104;Q53HM9;Q53HR5	EF1A1_HUMAN Elongation factor 1-alpha 1 OS=Homo sapiens GN=EEF1A1 PE=1 SV=1;>tr|Q53HM9|Q53HM9_HUMAN Elongation facto	0
P68104;Q5VTE0	EF1A1_HUMAN Elongation factor 1-alpha 1 OS=Homo sapiens GN=EEF1A1 PE=1 SV=1;>sp|Q5VTE0|EF1A3_HUMAN Putative elongatio	0

P68366;A8MUB1	TBA4A_HUMAN Tubulin alpha-4A chain OS=Homo sapiens GN=TUBA4A PE=1 SV=1;>tr|A8MUB1|A8MUB1_HUMAN Tubulin, alpha 1 (Te	1.38E-258
P68371;Q8IZ29;Q8N6N5;I	TBB2C_HUMAN Tubulin beta-2C chain OS=Homo sapiens GN=TUBB2C PE=1 SV=1;>tr|Q8IZ29|Q8IZ29_HUMAN Tubulin, beta 2C OS=	0
P68402;Q6IBR6;A8DPS7;/	PA1B2_HUMAN Platelet-activating factor acetylhydrolase IB subunit beta OS=Homo sapiens GN=PAFAH1B2 PE=1 SV=1;>tr|Q6IBR6|Q	5.47E-11
P68431;P84243;Q16695;(	H31_HUMAN Histone H3.1 OS=Homo sapiens GN=HIST1H3A PE=1 SV=2;>sp|P84243|H33_HUMAN Histone H3.3 OS=Homo sapiens (	1.71E-15
P68431;Q16695;Q6NXT2	H31_HUMAN Histone H3.1 OS=Homo sapiens GN=HIST1H3A PE=1 SV=2;>sp|Q16695|H31T_HUMAN Histone H3.1t OS=Homo sapiens	6.98E-258
P68431;Q16695;Q71DI3;I	H31_HUMAN Histone H3.1 OS=Homo sapiens GN=HIST1H3A PE=1 SV=2;>sp|Q16695|H31T_HUMAN Histone H3.1t OS=Homo sapiens	3.42E-05
P78330;Q53EY1;C9JBI3	SERB_HUMAN Phosphoserine phosphatase OS=Homo sapiens GN=PSPH PE=1 SV=2;>tr|Q53EY1|Q53EY1_HUMAN Phosphoserine phos	5.99E-30
P78347;P78347-3;P78347	GTF2I_HUMAN General transcription factor II-I OS=Homo sapiens GN=GTF2I PE=1 SV=2;>sp|P78347-3|GTF2I_HUMAN Isoform 3 of (	0
P78356;P78356-2	PI42B_HUMAN Phosphatidylinositol-5-phosphate 4-kinase type-2 beta OS=Homo sapiens GN=PIP4K2B PE=1 SV=1;>sp|P78356-2|PI4	5.44E-09
P78362-2;P78362;Q96SB	2|SRPK2_HUMAN Isoform 2 of Serine/threonine-protein kinase SRPK2 OS=Homo sapiens GN=SRPK2;>sp|P78362|SRPK2_HUMAN Ser	7.06E-10
P78559-2;P78559;A8K4T7	2|MAP1A_HUMAN Isoform 2 of Microtubule-associated protein 1A OS=Homo sapiens GN=MAP1A;>sp|P78559|MAP1A_HUMAN Microtul	1.99E-08
P79483;O19507;Q30131;I	DRB3_HUMAN HLA class II histocompatibility antigen, DR beta 3 chain OS=Homo sapiens GN=HLA-DRB3 PE=1 SV=1;>tr|O19507|O1	3.40E-19
P82094-2;P82094;B4DQY	2|TMF1_HUMAN Isoform 2 of TATA element modulatory factor OS=Homo sapiens GN=TMF1;>sp|P82094|TMF1_HUMAN TATA element	1.71E-07
P82650;A8K9Y7;Q8NBL6;(	RT22_HUMAN 28S ribosomal protein S22, mitochondrial OS=Homo sapiens GN=MRPS22 PE=1 SV=1;>tr|A8K9Y7|A8K9Y7_HUMAN cD	3.86E-47
P82664;B4DP77;Q9BZS5	RT10_HUMAN 28S ribosomal protein S10, mitochondrial OS=Homo sapiens GN=MRPS10 PE=1 SV=2;>tr|B4DP77|B4DP77_HUMAN cD	1.16E-06
P82673;P82673-2	RT35_HUMAN 28S ribosomal protein S35, mitochondrial OS=Homo sapiens GN=MRPS35 PE=1 SV=1;>sp|P82673-2|RT35_HUMAN Iso	6.03E-42
P82675;P82675-2	RT05_HUMAN 28S ribosomal protein S5, mitochondrial OS=Homo sapiens GN=MRPS5 PE=1 SV=2;>sp|P82675-2|RT05_HUMAN Isofor	7.83E-10
P82912;P82912-2;P82912	RT11_HUMAN 28S ribosomal protein S11, mitochondrial OS=Homo sapiens GN=MRPS11 PE=1 SV=2;>sp|P82912-2|RT11_HUMAN Iso	3.80E-08
P82912;Q53FX9;Q53GJ8;I	RT11_HUMAN 28S ribosomal protein S11, mitochondrial OS=Homo sapiens GN=MRPS11 PE=1 SV=2;>tr|Q53FX9|Q53FX9_HUMAN Mit	2.30E-14
P82914;Q59EA6;D3DPS9;	RT15_HUMAN 28S ribosomal protein S15, mitochondrial OS=Homo sapiens GN=MRPS15 PE=1 SV=1;>tr|Q59EA6|Q59EA6_HUMAN Mi	1.00E-19
P82921;Q5TB11	RT21_HUMAN 28S ribosomal protein S21, mitochondrial OS=Homo sapiens GN=MRPS21 PE=1 SV=2;>tr|Q5TB11|Q5TB11_HUMAN Mi	5.43E-54
P82933;Q86WV4	RT09_HUMAN 28S ribosomal protein S9, mitochondrial OS=Homo sapiens GN=MRPS9 PE=1 SV=2;>tr|Q86WV4|Q86WV4_HUMAN MRI	6.44E-121
P82979;Q567R9;A4D286	SARNP_HUMAN SAP domain-containing ribonucleoprotein OS=Homo sapiens GN=SARNP PE=1 SV=3;>tr|Q567R9|Q567R9_HUMAN CI	1.17E-40
P83731;C9JNW5;C9JXB8	RL24_HUMAN 60S ribosomal protein L24 OS=Homo sapiens GN=RPL24 PE=1 SV=1;>tr|C9JNW5|C9JNW5_HUMAN Ribosomal protein	4.26E-57
P83916;Q6IBN6;B5MD17;	CBX1_HUMAN Chromobox protein homolog 1 OS=Homo sapiens GN=CBX1 PE=1 SV=1;>tr|Q6IBN6|Q6IBN6_HUMAN CBX1 protein OS	9.55E-264
P84077;P61204	ARF1_HUMAN ADP-ribosylation factor 1 OS=Homo sapiens GN=ARF1 PE=1 SV=2;>sp|P61204|ARF3_HUMAN ADP-ribosylation factor 3	0
P84077;P61204;B4DEB9;I	ARF1_HUMAN ADP-ribosylation factor 1 OS=Homo sapiens GN=ARF1 PE=1 SV=2;>sp|P61204|ARF3_HUMAN ADP-ribosylation factor 3	3.31E-236
P84095;Q6ICQ8	RHOG_HUMAN Rho-related GTP-binding protein RhoG OS=Homo sapiens GN=RHOG PE=1 SV=1;>tr|Q6ICQ8|Q6ICQ8_HUMAN ARHG p	1.02E-99
P84098;Q53G49;Q8IWR8	RL19_HUMAN 60S ribosomal protein L19 OS=Homo sapiens GN=RPL19 PE=1 SV=1;>tr|Q53G49|Q53G49_HUMAN Ribosomal protein I	1.20E-167
P85037;P85037-2	FOXK1_HUMAN Forkhead box protein K1 OS=Homo sapiens GN=FOXK1 PE=1 SV=1;>sp|P85037-2|FOXK1_HUMAN Isoform 2 of Forkh	1.09E-23
P98160;Q59EG0	PGBM_HUMAN Basement membrane-specific heparan sulfate proteoglycan core protein OS=Homo sapiens GN=HSPG2 PE=1 SV=4;>tr	0
P99999;C9JFR7	CYC_HUMAN Cytochrome c OS=Homo sapiens GN=CYCS PE=1 SV=2;>tr|C9JFR7|C9JFR7_HUMAN Uncharacterized protein OS=Homo	8.87E-212
Q00059;Q6LES8;A8MRB2	TFAM_HUMAN Transcription factor A, mitochondrial OS=Homo sapiens GN=TFAM PE=1 SV=1;>tr|Q6LES8|Q6LES8_HUMAN TFAM prote	4.08E-33
Q00325-2;Q53HC3;Q8NCI	2|MPCP_HUMAN Isoform B of Phosphate carrier protein, mitochondrial OS=Homo sapiens GN=SLC25A3;>tr|Q53HC3|Q53HC3_HUMAN	3.41E-25
Q00403;B1APE2;B1APE1	TF2B_HUMAN Transcription initiation factor IIB OS=Homo sapiens GN=GTF2B PE=1 SV=1;>tr|B1APE2|B1APE2_HUMAN General transc	5.63E-14
Q00577;Q56A79;Q2NLC9;	PURA_HUMAN Transcriptional activator protein Pur-alpha OS=Homo sapiens GN=PURA PE=1 SV=2;>tr|Q56A79|Q56A79_HUMAN Purin	8.62E-27
Q00613;Q53HT3;Q00613-	HSF1_HUMAN Heat shock factor protein 1 OS=Homo sapiens GN=HSF1 PE=1 SV=1;>tr|Q53HT3|Q53HT3_HUMAN Heat shock transcri	6.52E-10
Q00688;Q53GD8	FKBP3_HUMAN Peptidyl-prolyl cis-trans isomerase FKBP3 OS=Homo sapiens GN=FKBP3 PE=1 SV=1;>tr|Q53GD8|Q53GD8_HUMAN FK	1.52E-75
Q00796;B4DKI2	DHSO_HUMAN Sorbitol dehydrogenase OS=Homo sapiens GN=SORD PE=1 SV=4;>tr|B4DKI2|B4DKI2_HUMAN cDNA FLJ60282, highly	2.37E-81
Q00839;Q00839-2	HNRPU_HUMAN Heterogeneous nuclear ribonucleoprotein U OS=Homo sapiens GN=HNRNPU PE=1 SV=6;>sp|Q00839-2|HNRPU_HUM	0
Q008S8;B7Z944	ECT2L_HUMAN Epithelial cell-transforming sequence 2 oncogene-like OS=Homo sapiens GN=ECT2L PE=2 SV=2;>tr|B7Z944|B7Z944_	1.89E-06
Q01081;Q8WU68;Q8WU6	U2AF1_HUMAN Splicing factor U2AF 35 kDa subunit OS=Homo sapiens GN=U2AF1 PE=1 SV=3;>sp|Q8WU68|U2AF4_HUMAN Splicing	4.84E-73
Q01082;Q01082-2;Q0108	SPTB2_HUMAN Spectrin beta chain, brain 1 OS=Homo sapiens GN=SPTBN1 PE=1 SV=2;>sp|Q01082-2|SPTB2_HUMAN Isoform Short	0
Q01130;Q9BRL6;Q9BRL6-	SRSF2_HUMAN Serine/arginine-rich splicing factor 2 OS=Homo sapiens GN=SRSF2 PE=1 SV=4;>sp|Q9BRL6|SRSF8_HUMAN Serine/a	1.08E-116
Q01469;A8MTQ5	FABP5_HUMAN Fatty acid-binding protein, epidermal OS=Homo sapiens GN=FABP5 PE=1 SV=3;>tr|A8MTQ5|A8MTQ5_HUMAN Unchar	1.69E-53
Q01581;Q5XJ04;Q8N995	HMCS1_HUMAN Hydroxymethylglutaryl-CoA synthase, cytoplasmic OS=Homo sapiens GN=HMGCS1 PE=1 SV=2;>tr|Q5XJ04|Q5XJ04_	3.78E-16
Q01628;Q01629;P13164	IFM3_HUMAN Interferon-induced transmembrane protein 3 OS=Homo sapiens GN=IFITM3 PE=1 SV=2;>sp|Q01629|IFM2_HUMAN Int	1.32E-157
Q01650;Q8IV97	LAT1_HUMAN Large neutral amino acids transporter small subunit 1 OS=Homo sapiens GN=SLC7A5 PE=1 SV=2;>tr|Q8IV97|Q8IV97_	9.26E-88
Q01658;Q53F47;Q658N3	NC2B_HUMAN Protein Dr1 OS=Homo sapiens GN=DR1 PE=1 SV=1;>tr|Q53F47|Q53F47_HUMAN Down-regulator of transcription 1 va	1.27E-05
Q01780;Q01780-2;B4DKG	EXOSX_HUMAN Exosome component 10 OS=Homo sapiens GN=EXOSC10 PE=1 SV=2;>sp|Q01780-2|EXOSX_HUMAN Isoform 2 of Ex	6.85E-39
Q01804;Q7RTX8;Q01804-	OTUD4_HUMAN OTU domain-containing protein 4 OS=Homo sapiens GN=OTUD4 PE=1 SV=3;>sp|Q7RTX8|HIN1L_HUMAN Putative HI	2.32E-07
Q01813;Q5VSR7;B3KS15;	K6PP_HUMAN 6-phosphofructokinase type C OS=Homo sapiens GN=PFKP PE=1 SV=2;>tr|Q5VSR7|Q5VSR7_HUMAN Phosphofructokin	0
Q01970;Q8N1A4	PLCB3_HUMAN 1-phosphatidylinositol-4,5-bisphosphate phosphodiesterase beta-3 OS=Homo sapiens GN=PLCB3 PE=1 SV=2;>tr|Q8N	2.16E-11
Q01995;Q5U0D2;Q6FI52;	TAGL_HUMAN Transgelin OS=Homo sapiens GN=TAGLN PE=1 SV=4;>tr|Q5U0D2|Q5U0D2_HUMAN Putative uncharacterized protein D	1.44E-267
Q02083;Q02083-2;Q0208	NAAA_HUMAN N-acylethanolamine-hydrolyzing acid amidase OS=Homo sapiens GN=NAAA PE=1 SV=3;>sp|Q02083-2|NAAA_HUMAN	3.67E-07
Q02252;Q53H94;Q53FN8;	MMSA_HUMAN Methylmalonate-semialdehyde dehydrogenase [acylating], mitochondrial OS=Homo sapiens GN=ALDH6A1 PE=1 SV=2	8.59E-140
Q02750;Q02750-2;P3650	MP2K1_HUMAN Dual specificity mitogen-activated protein kinase kinase 1 OS=Homo sapiens GN=MAP2K1 PE=1 SV=2;>sp|Q02750-2	4.76E-10
Q02880;Q02880-2;Q71UI	TOP2B_HUMAN DNA topoisomerase 2-beta OS=Homo sapiens GN=TOP2B PE=1 SV=3;>sp|Q02880-2|TOP2B_HUMAN Isoform Beta-1	8.74E-17
Q02952;Q02952-2;Q0295	AKA12_HUMAN A-kinase anchor protein 12 OS=Homo sapiens GN=AKAP12 PE=1 SV=4;>sp|Q02952-2|AKA12_HUMAN Isoform 2 of A	2.65E-70
Q02978;Q6IBH0	M2OM_HUMAN Mitochondrial 2-oxoglutarate/malate carrier protein OS=Homo sapiens GN=SLC25A11 PE=1 SV=3;>tr|Q6IBH0|Q6IBH	3.09E-75
Q03013;Q03013-2;A6NNT	GSTM4_HUMAN Glutathione S-transferase Mu 4 OS=Homo sapiens GN=GSTM4 PE=1 SV=3;>sp|Q03013-2|GSTM4_HUMAN Isoform 2	1.56E-89

<table>
<tr><td>Q03113;Q5PPR5;B3KXS2;</td><td>GNA12_HUMAN Guanine nucleotide-binding protein subunit alpha-12 OS=Homo sapiens GN=GNA12 PE=1 SV=4;>tr|Q5PPR5|Q5PPR5</td><td>1.81E-17</td></tr>
<tr><td>Q03591;B1AKG0</td><td>FHR1_HUMAN Complement factor H-related protein 1 OS=Homo sapiens GN=CFHR1 PE=1 SV=2;>tr|B1AKG0|B1AKG0_HUMAN Comp</td><td>1.62E-61</td></tr>
<tr><td>Q04323-2;Q04323;B4E0P</td><td>2|UBXN1_HUMAN Isoform 2 of UBX domain-containing protein 1 OS=Homo sapiens GN=UBXN1;>sp|Q04323|UBXN1_HUMAN UBX do</td><td>2.06E-19</td></tr>
<tr><td>Q04721;P46531</td><td>NOTC2_HUMAN Neurogenic locus notch homolog protein 2 OS=Homo sapiens GN=NOTCH2 PE=1 SV=3;>sp|P46531|NOTC1_HUMAN</td><td>3.37E-07</td></tr>
<tr><td>Q04721;Q96JU8;Q9UFD5;</td><td>NOTC2_HUMAN Neurogenic locus notch homolog protein 2 OS=Homo sapiens GN=NOTCH2 PE=1 SV=3;>tr|Q96JU8|Q96JU8_HUMAN</td><td>3.74E-06</td></tr>
<tr><td>Q04726;Q04726-4;Q6PI5</td><td>TLE3_HUMAN Transducin-like enhancer protein 3 OS=Homo sapiens GN=TLE3 PE=1 SV=2;>sp|Q04726-4|TLE3_HUMAN Isoform 4 of</td><td>6.61E-09</td></tr>
<tr><td>Q04826;P30485;P30461</td><td>1B40_HUMAN HLA class I histocompatibility antigen, B-40 alpha chain OS=Homo sapiens GN=HLA-B PE=1 SV=1;>sp|P30485|1B47_</td><td>0</td></tr>
<tr><td>Q04917;B2R6N6;Q9H4N8</td><td>1433F_HUMAN 14-3-3 protein eta OS=Homo sapiens GN=YWHAH PE=1 SV=4;>tr|B2R6N6|B2R6N6_HUMAN cDNA, FLJ93036, highly</td><td>6.75E-152</td></tr>
<tr><td>Q05048;Q5QPD8;B4DDG3</td><td>CSTF1_HUMAN Cleavage stimulation factor subunit 1 OS=Homo sapiens GN=CSTF1 PE=1 SV=1;>tr|Q5QPD8|Q5QPD8_HUMAN Cleava</td><td>1.08E-21</td></tr>
<tr><td>Q05519;Q8IWE6;Q6PJB9;</td><td>SRS11_HUMAN Serine/arginine-rich splicing factor 11 OS=Homo sapiens GN=SRSF11 PE=1 SV=1;>tr|Q8IWE6|Q8IWE6_HUMAN Splic</td><td>5.18E-22</td></tr>
<tr><td>Q05655;B4DFV1</td><td>KPCD_HUMAN Protein kinase C delta type OS=Homo sapiens GN=PRKCD PE=1 SV=2;>tr|B4DFV1|B4DFV1_HUMAN cDNA FLJ57052, h</td><td>9.73E-49</td></tr>
<tr><td>Q05682-5;Q05682-3;Q05</td><td>5|CALD1_HUMAN Isoform 5 of Caldesmon OS=Homo sapiens GN=CALD1;>sp|Q05682-3|CALD1_HUMAN Isoform 3 of Caldesmon OS=</td><td>1.59E-243</td></tr>
<tr><td>Q06265-2;Q06265;Q0626</td><td>2|EXOS9_HUMAN Isoform 2 of Exosome complex component RRP45 OS=Homo sapiens GN=EXOSC9;>sp|Q06265|EXOS9_HUMAN Exo</td><td>3.56E-35</td></tr>
<tr><td>Q06323;Q6IBM2;A6NJG9;</td><td>PSME1_HUMAN Proteasome activator complex subunit 1 OS=Homo sapiens GN=PSME1 PE=1 SV=1;>tr|Q6IBM2|Q6IBM2_HUMAN PS</td><td>4.55E-260</td></tr>
<tr><td>Q06330;Q06330-6;Q0633</td><td>SUH_HUMAN Recombining binding protein suppressor of hairless OS=Homo sapiens GN=RBPJ PE=1 SV=3;>sp|Q06330-6|SUH_HUMA</td><td>1.97E-06</td></tr>
<tr><td>Q06828;B4E1J3;B3KS64;</td><td>FMOD_HUMAN Fibromodulin OS=Homo sapiens GN=FMOD PE=1 SV=2;>tr|B4E1J3|B4E1J3_HUMAN cDNA FLJ53615, highly similar to</td><td>9.24E-89</td></tr>
<tr><td>Q07020;Q0QEW2;B4DDY</td><td>RL18_HUMAN 60S ribosomal protein L18 OS=Homo sapiens GN=RPL18 PE=1 SV=2;>tr|Q0QEW2|Q0QEW2_HUMAN Ribosomal protein</td><td>3.68E-65</td></tr>
<tr><td>Q07065;Q07065-2;B3KVX</td><td>CKAP4_HUMAN Cytoskeleton-associated protein 4 OS=Homo sapiens GN=CKAP4 PE=1 SV=2;>sp|Q07065-2|CKAP4_HUMAN Isoform</td><td>0</td></tr>
<tr><td>Q07866-9;Q07866-4;Q7R</td><td>9|KLC1_HUMAN Isoform I of Kinesin light chain 1 OS=Homo sapiens GN=KLC1;>sp|Q07866-4|KLC1_HUMAN Isoform J of Kinesin light</td><td>2.21E-78</td></tr>
<tr><td>Q08379;Q08379-2</td><td>GOGA2_HUMAN Golgin subfamily A member 2 OS=Homo sapiens GN=GOLGA2 PE=1 SV=3;>sp|Q08379-2|GOGA2_HUMAN Isoform 2</td><td>2.77E-103</td></tr>
<tr><td>Q08380;B4DVE1;B4DDG4</td><td>LG3BP_HUMAN Galectin-3-binding protein OS=Homo sapiens GN=LGALS3BP PE=1 SV=1;>tr|B4DVE1|B4DVE1_HUMAN cDNA FLJ5347</td><td>2.58E-174</td></tr>
<tr><td>Q08431;Q08431-3</td><td>MFGM_HUMAN Lactadherin OS=Homo sapiens GN=MFGE8 PE=1 SV=2;>sp|Q08431-3|MFGM_HUMAN Isoform 3 of Lactadherin OS=H</td><td>1.89E-10</td></tr>
<tr><td>Q08752;Q6FGM6</td><td>PPID_HUMAN Peptidyl-prolyl cis-trans isomerase D OS=Homo sapiens GN=PPID PE=1 SV=3;>tr|Q6FGM6|Q6FGM6_HUMAN PPID prot</td><td>1.75E-61</td></tr>
<tr><td>Q08AM6;B4DMP4;Q9NW5</td><td>VAC14_HUMAN Protein VAC14 homolog OS=Homo sapiens GN=VAC14 PE=1 SV=1;>tr|B4DMP4|B4DMP4_HUMAN cDNA FLJ53136, hig</td><td>5.67E-27</td></tr>
<tr><td>Q09028;Q09028-3;Q0902</td><td>RBBP4_HUMAN Histone-binding protein RBBP4 OS=Homo sapiens GN=RBBP4 PE=1 SV=3;>sp|Q09028-3|RBBP4_HUMAN Isoform 3 o</td><td>8.03E-227</td></tr>
<tr><td>Q09666;B5MEH8</td><td>AHNK_HUMAN Neuroblast differentiation-associated protein AHNAK OS=Homo sapiens GN=AHNAK PE=1 SV=2;>tr|B5MEH8|B5MEH8_</td><td>0</td></tr>
<tr><td>Q0JRZ9;Q0JRZ9-2</td><td>FCHO2_HUMAN FCH domain only protein 2 OS=Homo sapiens GN=FCHO2 PE=1 SV=2;>sp|Q0JRZ9-2|FCHO2_HUMAN Isoform 2 of FC</td><td>3.27E-09</td></tr>
<tr><td>Q10471;B7Z6K2;B7Z8V8</td><td>GALT2_HUMAN Polypeptide N-acetylgalactosaminyltransferase 2 OS=Homo sapiens GN=GALNT2 PE=1 SV=1;>tr|B7Z6K2|B7Z6K2_HU</td><td>6.58E-63</td></tr>
<tr><td>Q10713;Q5SXM9;B4DKL3</td><td>MPPA_HUMAN Mitochondrial-processing peptidase subunit alpha OS=Homo sapiens GN=PMPCA PE=1 SV=2;>tr|Q5SXM9|Q5SXM9_HU</td><td>5.44E-24</td></tr>
<tr><td>Q12849;B3KW50;B7Z3R7</td><td>GRSF1_HUMAN G-rich sequence factor 1 OS=Homo sapiens GN=GRSF1 PE=1 SV=3;>tr|B3KW50|B3KW50_HUMAN cDNA FLJ42130 fi</td><td>3.86E-67</td></tr>
<tr><td>Q12882;Q32NB0;Q96HL6</td><td>DPYD_HUMAN Dihydropyrimidine dehydrogenase [NADP+] OS=Homo sapiens GN=DPYD PE=1 SV=2;>tr|Q32NB0|Q32NB0_HUMAN D</td><td>9.01E-08</td></tr>
<tr><td>Q12888-2;Q12888;A6NN</td><td>2|TP53B_HUMAN Isoform 2 of Tumor suppressor p53-binding protein 1 OS=Homo sapiens GN=TP53BP1;>sp|Q12888|TP53B_HUMAN</td><td>6.57E-06</td></tr>
<tr><td>Q12904;B4DNK3</td><td>AIMP1_HUMAN Aminoacyl tRNA synthase complex-interacting multifunctional protein 1 OS=Homo sapiens GN=AIMP1 PE=1 SV=2;>tr</td><td>8.95E-134</td></tr>
<tr><td>Q12905;Q53FG3;B4DY09</td><td>ILF2_HUMAN Interleukin enhancer-binding factor 2 OS=Homo sapiens GN=ILF2 PE=1 SV=2;>tr|Q53FG3|Q53FG3_HUMAN Interleukin</td><td>4.46E-141</td></tr>
<tr><td>Q12906;Q12906-3;Q1290</td><td>ILF3_HUMAN Interleukin enhancer-binding factor 3 OS=Homo sapiens GN=ILF3 PE=1 SV=3;>sp|Q12906-3|ILF3_HUMAN Isoform 3 o</td><td>1.50E-270</td></tr>
<tr><td>Q12906;Q12906-3;Q1290</td><td>ILF3_HUMAN Interleukin enhancer-binding factor 3 OS=Homo sapiens GN=ILF3 PE=1 SV=3;>sp|Q12906-3|ILF3_HUMAN Isoform 3 o</td><td>2.49E-170</td></tr>
<tr><td>Q12972;Q561W4;Q5TEJ4;</td><td>PP1R8_HUMAN Nuclear inhibitor of protein phosphatase 1 OS=Homo sapiens GN=PPP1R8 PE=1 SV=2;>tr|Q561W4|Q561W4_HUMAN</td><td>1.42E-07</td></tr>
<tr><td>Q12974;Q12974-2;Q9309</td><td>TP4A2_HUMAN Protein tyrosine phosphatase type IVA 2 OS=Homo sapiens GN=PTP4A2 PE=1 SV=1;>sp|Q12974-2|TP4A2_HUMAN Is</td><td>1.37E-22</td></tr>
<tr><td>Q13045;B4DIX0;B4DIL0;</td><td>FLII_HUMAN Protein flightless-1 homolog OS=Homo sapiens GN=FLII PE=1 SV=2;>tr|B4DIX0|B4DIX0_HUMAN cDNA FLJ50778, highl</td><td>1.91E-97</td></tr>
<tr><td>Q13148-2;Q13148</td><td>2|TADBP_HUMAN Isoform 2 of TAR DNA-binding protein 43 OS=Homo sapiens GN=TARDBP;>sp|Q13148|TADBP_HUMAN TAR DNA-bin</td><td>8.77E-249</td></tr>
<tr><td>Q13177;A8K5M4</td><td>PAK2_HUMAN Serine/threonine-protein kinase PAK 2 OS=Homo sapiens GN=PAK2 PE=1 SV=3;>tr|A8K5M4|A8K5M4_HUMAN cDNA FL</td><td>1.71E-293</td></tr>
<tr><td>Q13228;Q13228-2</td><td>SBP1_HUMAN Selenium-binding protein 1 OS=Homo sapiens GN=SELENBP1 PE=1 SV=2;>sp|Q13228-2|SBP1_HUMAN Isoform 2 of S</td><td>0</td></tr>
<tr><td>Q13232;Q9NUF9</td><td>NDK3_HUMAN Nucleoside diphosphate kinase 3 OS=Homo sapiens GN=NME3 PE=1 SV=2;>tr|Q9NUF9|Q9NUF9_HUMAN Nucleoside d</td><td>2.67E-53</td></tr>
<tr><td>Q13242;A8K3M9</td><td>SRSF9_HUMAN Serine/arginine-rich splicing factor 9 OS=Homo sapiens GN=SRSF9 PE=1 SV=1;>tr|A8K3M9|A8K3M9_HUMAN cDNA F</td><td>5.46E-12</td></tr>
<tr><td>Q13243;Q13243-3;Q1324</td><td>SRSF5_HUMAN Serine/arginine-rich splicing factor 5 OS=Homo sapiens GN=SRSF5 PE=1 SV=1;>sp|Q13243-3|SRSF5_HUMAN Isofor</td><td>1.38E-29</td></tr>
<tr><td>Q13277;Q53YE2;B4DME0;</td><td>STX3_HUMAN Syntaxin-3 OS=Homo sapiens GN=STX3 PE=1 SV=3;>tr|Q53YE2|Q53YE2_HUMAN Syntaxin 3A OS=Homo sapiens GN=</td><td>2.64E-06</td></tr>
<tr><td>Q13283;Q5U0Q1;Q6FI03;</td><td>G3BP1_HUMAN Ras GTPase-activating protein-binding protein 1 OS=Homo sapiens GN=G3BP1 PE=1 SV=1;>tr|Q5U0Q1|Q5U0Q1_HU</td><td>4.76E-264</td></tr>
<tr><td>Q13330;Q59FW1;Q13330</td><td>MTA1_HUMAN Metastasis-associated protein MTA1 OS=Homo sapiens GN=MTA1 PE=1 SV=2;>tr|Q59FW1|Q59FW1_HUMAN Metastasi</td><td>2.20E-20</td></tr>
<tr><td>Q13347;Q5U0F4;Q53HU7</td><td>EIF3I_HUMAN Eukaryotic translation initiation factor 3 subunit I OS=Homo sapiens GN=EIF3I PE=1 SV=1;>tr|Q5U0F4|Q5U0F4_HUM</td><td>9.66E-86</td></tr>
<tr><td>Q13363;Q7Z2Q5;Q4KMQ</td><td>CTBP1_HUMAN C-terminal-binding protein 1 OS=Homo sapiens GN=CTBP1 PE=1 SV=2;>tr|Q7Z2Q5|Q7Z2Q5_HUMAN C-terminal bind</td><td>7.18E-24</td></tr>
<tr><td>Q13405;Q59GE9</td><td>RM49_HUMAN 39S ribosomal protein L49, mitochondrial OS=Homo sapiens GN=MRPL49 PE=1 SV=1;>tr|Q59GE9|Q59GE9_HUMAN M</td><td>1.73E-17</td></tr>
<tr><td>Q13416;B4DYU9</td><td>ORC2_HUMAN Origin recognition complex subunit 2 OS=Homo sapiens GN=ORC2L PE=1 SV=2;>tr|B4DYU9|B4DYU9_HUMAN cDNA F</td><td>1.00E-10</td></tr>
<tr><td>Q13423;Q2TB59;D6RAI5;</td><td>NNTM_HUMAN NAD(P) transhydrogenase, mitochondrial OS=Homo sapiens GN=NNT PE=1 SV=3;>tr|Q2TB59|Q2TB59_HUMAN Nicoti</td><td>1.89E-37</td></tr>
<tr><td>Q13428-4;Q13428-3;Q13</td><td>4|TCOF_HUMAN Isoform 4 of Treacle protein OS=Homo sapiens GN=TCOF1;>sp|Q13428-3|TCOF_HUMAN Isoform 3 of Treacle protein</td><td>3.16E-67</td></tr>
<tr><td>Q13435;Q7Z3K9</td><td>SF3B2_HUMAN Splicing factor 3B subunit 2 OS=Homo sapiens GN=SF3B2 PE=1 SV=2;>tr|Q7Z3K9|Q7Z3K9_HUMAN Putative unchara</td><td>1.12E-181</td></tr>
<tr><td>Q13443;Q13443-2</td><td>ADAM9_HUMAN Disintegrin and metalloproteinase domain-containing protein 9 OS=Homo sapiens GN=ADAM9 PE=1 SV=1;>sp|Q134</td><td>8.48E-18</td></tr>
<tr><td>Q13451;Q2TA84;Q53GX4;</td><td>FKBP5_HUMAN Peptidyl-prolyl cis-trans isomerase FKBP5 OS=Homo sapiens GN=FKBP5 PE=1 SV=2;>tr|Q2TA84|Q2TA84_HUMAN FK</td><td>3.50E-204</td></tr>
<tr><td>Q13501;Q13501-2;B4E3V</td><td>SQSTM_HUMAN Sequestosome-1 OS=Homo sapiens GN=SQSTM1 PE=1 SV=1;>sp|Q13501-2|SQSTM_HUMAN Isoform 2 of Sequesto</td><td>8.45E-213</td></tr>
<tr><td>Q13505;Q13505-3</td><td>MTX1_HUMAN Metaxin-1 OS=Homo sapiens GN=MTX1 PE=1 SV=2;>sp|Q13505-3|MTX1_HUMAN Isoform 3 of Metaxin-1 OS=Homo s</td><td>3.33E-15</td></tr>
<tr><td>Q13526;B3KUM4</td><td>PIN1_HUMAN Peptidyl-prolyl cis-trans isomerase NIMA-interacting 1 OS=Homo sapiens GN=PIN1 PE=1 SV=1;>tr|B3KUM4|B3KUM4_</td><td>6.33E-144</td></tr>
<tr><td>Q13546;Q13546-2</td><td>RIPK1_HUMAN Receptor-interacting serine/threonine-protein kinase 1 OS=Homo sapiens GN=RIPK1 PE=1 SV=3;>sp|Q13546-2|RIPK</td><td>1.88E-43</td></tr>
</table>

Q13547;Q6IT96;B5BU61;(	HDAC1_HUMAN Histone deacetylase 1 OS=Homo sapiens GN=HDAC1 PE=1 SV=1;>tr|Q6IT96|Q6IT96_HUMAN HDAC1 protein OS=Hc	1.65E-68
Q13576;Q13576-2	IQGA2_HUMAN Ras GTPase-activating-like protein IQGAP2 OS=Homo sapiens GN=IQGAP2 PE=1 SV=4;>sp|Q13576-2|IQGA2_HUMAN	2.35E-119
Q13596;Q53GY8;Q53HL9;	SNX1_HUMAN Sorting nexin-1 OS=Homo sapiens GN=SNX1 PE=1 SV=3;>tr|Q53GY8|Q53GY8_HUMAN Sorting nexin 1 isoform a varia	2.43E-131
Q13610;Q53FW9;Q9BV37	PWP1_HUMAN Periodic tryptophan protein 1 homolog OS=Homo sapiens GN=PWP1 PE=1 SV=1;>tr|Q53FW9|Q53FW9_HUMAN Nuclea	5.52E-06
Q13618;Q53S54;Q13618-	CUL3_HUMAN Cullin-3 OS=Homo sapiens GN=CUL3 PE=1 SV=2;>tr|Q53S54|Q53S54_HUMAN Putative uncharacterized protein CUL3	2.56E-77
Q13619;Q13619-2	CUL4A_HUMAN Cullin-4A OS=Homo sapiens GN=CUL4A PE=1 SV=3;>sp|Q13619-2|CUL4A_HUMAN Isoform 2 of Cullin-4A OS=Homo	3.17E-104
Q13620;Q13620-1;Q1362	CUL4B_HUMAN Cullin-4B OS=Homo sapiens GN=CUL4B PE=1 SV=4;>sp|Q13620-1|CUL4B_HUMAN Isoform 2 of Cullin-4B OS=Homo	5.97E-23
Q13724;Q58F09;C9J8D4;/	MOGS_HUMAN Mannosyl-oligosaccharide glucosidase OS=Homo sapiens GN=MOGS PE=1 SV=5;>tr|Q58F09|Q58F09_HUMAN Glucosi	2.00E-82
Q13740;Q13740-2	CD166_HUMAN CD166 antigen OS=Homo sapiens GN=ALCAM PE=1 SV=2;>sp|Q13740-2|CD166_HUMAN Isoform 2 of CD166 antiger	2.12E-267
Q13813;Q13813-3;Q1381	SPTA2_HUMAN Spectrin alpha chain, brain OS=Homo sapiens GN=SPTAN1 PE=1 SV=3;>sp|Q13813-3|SPTA2_HUMAN Isoform 3 of Sp	0
Q13867;B4DXF3	BLMH_HUMAN Bleomycin hydrolase OS=Homo sapiens GN=BLMH PE=1 SV=1;>tr|B4DXF3|B4DXF3_HUMAN cDNA FLJ57650, highly si	5.31E-45
Q13885;B2R6L0	TBB2A_HUMAN Tubulin beta-2A chain OS=Homo sapiens GN=TUBB2A PE=1 SV=1;>tr|B2R6L0|B2R6L0_HUMAN cDNA, FLJ93005, high	0
Q13885;Q9BVA1	TBB2A_HUMAN Tubulin beta-2A chain OS=Homo sapiens GN=TUBB2A PE=1 SV=1;>sp|Q9BVA1|TBB2B_HUMAN Tubulin beta-2B chain	0
Q13895;Q5T8J2	BYST_HUMAN Bystin OS=Homo sapiens GN=BYSL PE=1 SV=3;>tr|Q5T8J2|Q5T8J2_HUMAN Bystin-like OS=Homo sapiens GN=BYSL P	8.32E-42
Q13907-2;Q13907;C9JKM	2|IDI1_HUMAN Isoform 2 of Isopentenyl-diphosphate Delta-isomerase 1 OS=Homo sapiens GN=IDI1;>sp|Q13907|IDI1_HUMAN Isope	5.11E-34
Q13951;Q13951-2	PEBB_HUMAN Core-binding factor subunit beta OS=Homo sapiens GN=CBFB PE=1 SV=2;>sp|Q13951-2|PEBB_HUMAN Isoform 2 of C	4.74E-10
Q14005;Q14005-2;Q1400	IL16_HUMAN Pro-interleukin-16 OS=Homo sapiens GN=IL16 PE=1 SV=4;>sp|Q14005-2|IL16_HUMAN Isoform 2 of Pro-interleukin-16	2.63E-17
Q14011;Q53XX5;D6W5Y5	CIRBP_HUMAN Cold-inducible RNA-binding protein OS=Homo sapiens GN=CIRBP PE=1 SV=1;>tr|Q53XX5|Q53XX5_HUMAN Cold induc	7.51E-88
Q14103-4;Q14103-2;Q14	4|HNRPD_HUMAN Isoform 4 of Heterogeneous nuclear ribonucleoprotein D0 OS=Homo sapiens GN=HNRNPD;>sp|Q14103-2|HNRPD_I	7.98E-129
Q14103;Q14103-3	HNRPD_HUMAN Heterogeneous nuclear ribonucleoprotein D0 OS=Homo sapiens GN=HNRNPD PE=1 SV=1;>sp|Q14103-3|HNRPD_HUI	0
Q14108;Q53Y63;B4DKD8	SCRB2_HUMAN Lysosome membrane protein 2 OS=Homo sapiens GN=SCARB2 PE=1 SV=2;>tr|Q53Y63|Q53Y63_HUMAN Scavenger r	2.87E-57
Q14112;Q8IV28;Q14112-:	NID2_HUMAN Nidogen-2 OS=Homo sapiens GN=NID2 PE=1 SV=3;>tr|Q8IV28|Q8IV28_HUMAN NID2 protein OS=Homo sapiens GN=	2.31E-18
Q14139-2;Q14139;B4DK3	2|UBE4A_HUMAN Isoform 2 of Ubiquitin conjugation factor E4 A OS=Homo sapiens GN=UBE4A;>sp|Q14139|UBE4A_HUMAN Ubiquitin	1.44E-23
Q14141;Q14141-4;Q1414	SEPT6_HUMAN Septin-6 OS=Homo sapiens GN=SEPT6 PE=1 SV=4;>sp|Q14141-4|SEPT6_HUMAN Isoform V of Septin-6 OS=Homo sa	7.29E-32
Q14152;Q24JU4;B4DYS1;	EIF3A_HUMAN Eukaryotic translation initiation factor 3 subunit A OS=Homo sapiens GN=EIF3A PE=1 SV=1;>tr|Q24JU4|Q24JU4_HUM	1.21E-159
Q14157;Q14157-3;Q1415	UBP2L_HUMAN Ubiquitin-associated protein 2-like OS=Homo sapiens GN=UBAP2L PE=1 SV=2;>sp|Q14157-3|UBP2L_HUMAN Isoform	1.11E-139
Q14160-3;Q14160;Q1416	3|SCRIB_HUMAN Isoform 3 of Protein scribble homolog OS=Homo sapiens GN=SCRIB;>sp|Q14160|SCRIB_HUMAN Protein scribble hc	1.46E-56
Q14166;B1AH89	TTL12_HUMAN Tubulin--tyrosine ligase-like protein 12 OS=Homo sapiens GN=TTLL12 PE=1 SV=2;>tr|B1AH89|B1AH89_HUMAN Tubul	5.89E-69
Q14240-2;Q14240	2|IF4A2_HUMAN Isoform 2 of Eukaryotic initiation factor 4A-II OS=Homo sapiens GN=EIF4A2;>sp|Q14240|IF4A2_HUMAN Eukaryotic	8.77E-273
Q14247;Q53HG7;Q96H99	SRC8_HUMAN Src substrate cortactin OS=Homo sapiens GN=CTTN PE=1 SV=2;>tr|Q53HG7|Q53HG7_HUMAN Cortactin isoform a var	0
Q14254;Q9BTI6;Q6FG43	FLOT2_HUMAN Flotillin-2 OS=Homo sapiens GN=FLOT2 PE=1 SV=2;>tr|Q9BTI6|Q9BTI6_HUMAN FLOT2 protein OS=Homo sapiens GN	5.04E-14
Q14315;Q14315-2;Q59H9	FLNC_HUMAN Filamin-C OS=Homo sapiens GN=FLNC PE=1 SV=3;>sp|Q14315-2|FLNC_HUMAN Isoform 2 of Filamin-C OS=Homo sap	0
Q14318-2;Q14318;B2R8G	2|FKBP8_HUMAN Isoform 2 of Peptidyl-prolyl cis-trans isomerase FKBP8 OS=Homo sapiens GN=FKBP8;>sp|Q14318|FKBP8_HUMAN P	6.08E-144
Q14320;B0S8I6	FA50A_HUMAN Protein FAM50A OS=Homo sapiens GN=FAM50A PE=1 SV=2;>tr|B0S8I6|B0S8I6_HUMAN Family with sequence similar	1.15E-29
Q14344;B4DWV9;B7Z7R0	GNA13_HUMAN Guanine nucleotide-binding protein subunit alpha-13 OS=Homo sapiens GN=GNA13 PE=1 SV=2;>tr|B4DWV9|B4DWV	1.37E-37
Q14344;Q03113	GNA13_HUMAN Guanine nucleotide-binding protein subunit alpha-13 OS=Homo sapiens GN=GNA13 PE=1 SV=2;>sp|Q03113|GNA12_	6.72E-13
Q14376;Q38G75;B3KQ39;	GALE_HUMAN UDP-glucose 4-epimerase OS=Homo sapiens GN=GALE PE=1 SV=2;>tr|Q38G75|Q38G75_HUMAN UDP galactose 4-epi	6.95E-29
Q14520;B7Z8U5	HABP2_HUMAN Hyaluronan-binding protein 2 OS=Homo sapiens GN=HABP2 PE=1 SV=1;>tr|B7Z8U5|B7Z8U5_HUMAN cDNA FLJ5160	2.62E-07
Q14558-2;Q14558;B4DP3	2|KPRA_HUMAN Isoform 2 of Phosphoribosyl pyrophosphate synthase-associated protein 1 OS=Homo sapiens GN=PRPSAP1;>sp|Q14	3.52E-57
Q14566;Q4ZG57;B4DRF6	MCM6_HUMAN DNA replication licensing factor MCM6 OS=Homo sapiens GN=MCM6 PE=1 SV=1;>tr|Q4ZG57|Q4ZG57_HUMAN Putativ	1.18E-120
Q14671;Q14671-2;Q8TB7	PUM1_HUMAN Pumilio homolog 1 OS=Homo sapiens GN=PUM1 PE=1 SV=3;>sp|Q14671-2|PUM1_HUMAN Isoform 2 of Pumilio homol	6.41E-12
Q14683;Q6P2R1;A8K7A6;	SMC1A_HUMAN Structural maintenance of chromosomes protein 1A OS=Homo sapiens GN=SMC1A PE=1 SV=2;>tr|Q6P2R1|Q6P2R1_	1.29E-47
Q14684;Q14684-2	RRP1B_HUMAN Ribosomal RNA processing protein 1 homolog B OS=Homo sapiens GN=RRP1B PE=1 SV=3;>sp|Q14684-2|RRP1B_HUI	7.11E-12
Q14691;B4DGH0	PSF1_HUMAN DNA replication complex GINS protein PSF1 OS=Homo sapiens GN=GINS1 PE=1 SV=1;>tr|B4DGH0|B4DGH0_HUMAN c	6.27E-19
Q14694-2;Q14694;Q1469	2|UBP10_HUMAN Isoform 2 of Ubiquitin carboxyl-terminal hydrolase 10 OS=Homo sapiens GN=USP10;>sp|Q14694|UBP10_HUMAN U	1.42E-16
Q14739;C9JXK0;B4E0Z3	LBR_HUMAN Lamin-B receptor OS=Homo sapiens GN=LBR PE=1 SV=2;>tr|C9JXK0|C9JXK0_HUMAN Uncharacterized protein OS=Hom	4.04E-07
Q14764;B4DDR2;B4DXN0	MVP_HUMAN Major vault protein OS=Homo sapiens GN=MVP PE=1 SV=4;>tr|B4DDR2|B4DDR2_HUMAN cDNA FLJ54588, highly simila	1.24E-168
Q14767;Q6AZ94;Q59EE6	LTBP2_HUMAN Latent-transforming growth factor beta-binding protein 2 OS=Homo sapiens GN=LTBP2 PE=1 SV=3;>tr|Q6AZ94|Q6AZ	2.49E-10
Q14847;Q14847-2;C9JVR	LASP1_HUMAN LIM and SH3 domain protein 1 OS=Homo sapiens GN=LASP1 PE=1 SV=2;>sp|Q14847-2|LASP1_HUMAN Isoform 2 of	1.32E-109
Q14914;B4DPK3;Q5JVP3	PTGR1_HUMAN Prostaglandin reductase 1 OS=Homo sapiens GN=PTGR1 PE=1 SV=2;>tr|B4DPK3|B4DPK3_HUMAN cDNA FLJ59730, h	3.55E-55
Q14978-2;Q14978-3;Q14	2|NOLC1_HUMAN Isoform Beta of Nucleolar and coiled-body phosphoprotein 1 OS=Homo sapiens GN=NOLC1;>sp|Q14978-3|NOLC1_	1.55E-44
Q14978-2;Q14978;Q1497	2|NOLC1_HUMAN Isoform Beta of Nucleolar and coiled-body phosphoprotein 1 OS=Homo sapiens GN=NOLC1;>sp|Q14978|NOLC1_HU	2.09E-84
Q14997;Q14997-2;Q1499	PSME4_HUMAN Proteasome activator complex subunit 4 OS=Homo sapiens GN=PSME4 PE=1 SV=2;>sp|Q14997-2|PSME4_HUMAN Is	2.50E-08
Q15005;B4DDB9	SPCS2_HUMAN Signal peptidase complex subunit 2 OS=Homo sapiens GN=SPCS2 PE=1 SV=3;>tr|B4DDB9|B4DDB9_HUMAN cDNA FL	1.31E-41
Q15008;C9IZE4;Q6UV22;	PSMD6_HUMAN 26S proteasome non-ATPase regulatory subunit 6 OS=Homo sapiens GN=PSMD6 PE=1 SV=1;>tr|C9IZE4|C9IZE4_HU	1.77E-86
Q15019-2;Q15019;B5MC	2|SEPT2_HUMAN Isoform 2 of Septin-2 OS=Homo sapiens GN=SEPT2;>sp|Q15019|SEPT2_HUMAN Septin-2 OS=Homo sapiens GN=S	2.25E-204
Q15020;Q15020-2	SART3_HUMAN Squamous cell carcinoma antigen recognized by T-cells 3 OS=Homo sapiens GN=SART3 PE=1 SV=1;>sp|Q15020-2|S/	7.44E-91
Q15046;Q15046-2	SYK_HUMAN Lysyl-tRNA synthetase OS=Homo sapiens GN=KARS PE=1 SV=3;>sp|Q15046-2|SYK_HUMAN Isoform Mitochondrial of Ly	1.01E-177
Q15084-2;Q15084	2|PDIA6_HUMAN Isoform 2 of Protein disulfide-isomerase A6 OS=Homo sapiens GN=PDIA6;>sp|Q15084|PDIA6_HUMAN Protein disulf	0
Q15113;A4D2D2;B4DPJ4	PCOC1_HUMAN Procollagen C-endopeptidase enhancer 1 OS=Homo sapiens GN=PCOLCE PE=1 SV=2;>tr|A4D2D2|A4D2D2_HUMAN P	4.67E-57
Q15124;Q15124-2;A6NIQ	PGM5_HUMAN Phosphoglucomutase-like protein 5 OS=Homo sapiens GN=PGM5 PE=1 SV=2;>sp|Q15124-2|PGM5_HUMAN Isoform 2	1.31E-10

Q15126;Q6FGV9	PMVK_HUMAN Phosphomevalonate kinase OS=Homo sapiens GN=PMVK PE=1 SV=3;>tr|Q6FGV9|Q6FGV9_HUMAN PMVK protein (Frag	7.38E-10
Q15149;Q15149-2;Q1514	PLEC_HUMAN Plectin OS=Homo sapiens GN=PLEC PE=1 SV=3;>sp|Q15149-2|PLEC_HUMAN Isoform 2 of Plectin OS=Homo sapiens G	0
Q15181;Q5SQT6	IPYR_HUMAN Inorganic pyrophosphatase OS=Homo sapiens GN=PPA1 PE=1 SV=2;>tr|Q5SQT6|Q5SQT6_HUMAN Pyrophosphatase (In	3.49E-205
Q15257;Q15257-2;Q1525	PTPA_HUMAN Serine/threonine-protein phosphatase 2A activator OS=Homo sapiens GN=PPP2R4 PE=1 SV=3;>sp|Q15257-2|PTPA_HU	1.91E-19
Q15257;Q15257-2;Q1525	PTPA_HUMAN Serine/threonine-protein phosphatase 2A activator OS=Homo sapiens GN=PPP2R4 PE=1 SV=3;>sp|Q15257-2|PTPA_HU	2.37E-22
Q15276;Q15276-2;B4DMI	RABE1_HUMAN Rab GTPase-binding effector protein 1 OS=Homo sapiens GN=RABEP1 PE=1 SV=2;>sp|Q15276-2|RABE1_HUMAN Isof	1.06E-52
Q15363;Q6FHT8;B4DP27;	TMED2_HUMAN Transmembrane emp24 domain-containing protein 2 OS=Homo sapiens GN=TMED2 PE=1 SV=1;>tr|Q6FHT8|Q6FHT8_	1.88E-39
Q15365;Q53SS8	PCBP1_HUMAN Poly(rC)-binding protein 1 OS=Homo sapiens GN=PCBP1 PE=1 SV=2;>tr|Q53SS8|Q53SS8_HUMAN Poly(RC) binding p	1.46E-117
Q15386;Q15386-2	UBE3C_HUMAN Ubiquitin-protein ligase E3C OS=Homo sapiens GN=UBE3C PE=1 SV=3;>sp|Q15386-2|UBE3C_HUMAN Isoform 2 of U	1.70E-32
Q15386;Q15386-2;Q1538	UBE3C_HUMAN Ubiquitin-protein ligase E3C OS=Homo sapiens GN=UBE3C PE=1 SV=3;>sp|Q15386-2|UBE3C_HUMAN Isoform 2 of U	1.80E-33
Q15417;Q6FHA7;B4DP09;	CNN3_HUMAN Calponin-3 OS=Homo sapiens GN=CNN3 PE=1 SV=1;>tr|Q6FHA7|Q6FHA7_HUMAN CNN3 protein (Fragment) OS=Hom	0
Q15424;Q14151	SAFB1_HUMAN Scaffold attachment factor B1 OS=Homo sapiens GN=SAFB PE=1 SV=4;>sp|Q14151|SAFB2_HUMAN Scaffold attachm	2.92E-44
Q15435;Q15435-2;Q1543	PP1R7_HUMAN Protein phosphatase 1 regulatory subunit 7 OS=Homo sapiens GN=PPP1R7 PE=1 SV=1;>sp|Q15435-2|PP1R7_HUMAN	3.91E-123
Q15436;B4DF60;B3KXI2;I	SC23A_HUMAN Protein transport protein Sec23A OS=Homo sapiens GN=SEC23A PE=1 SV=2;>tr|B4DF60|B4DF60_HUMAN cDNA FLJ5	4.61E-61
Q15459;B4E091	SF3A1_HUMAN Splicing factor 3A subunit 1 OS=Homo sapiens GN=SF3A1 PE=1 SV=1;>tr|B4E091|B4E091_HUMAN cDNA FLJ55438, I	2.01E-106
Q15555;Q15555-2	MARE2_HUMAN Microtubule-associated protein RP/EB family member 2 OS=Homo sapiens GN=MAPRE2 PE=1 SV=1;>sp|Q15555-2|M	8.40E-08
Q15628;B3KQZ9	TRADD_HUMAN Tumor necrosis factor receptor type 1-associated DEATH domain protein OS=Homo sapiens GN=TRADD PE=1 SV=2;>	1.75E-09
Q15631;B7Z5D9;B3KRM8	TSN_HUMAN Translin OS=Homo sapiens GN=TSN PE=1 SV=1;>tr|B7Z5D9|B7Z5D9_HUMAN cDNA FLJ59957, highly similar to Translin	5.41E-21
Q15637;Q15637-2;Q1563	SF01_HUMAN Splicing factor 1 OS=Homo sapiens GN=SF1 PE=1 SV=4;>sp|Q15637-2|SF01_HUMAN Isoform 2 of Splicing factor 1 OS	8.21E-51
Q15642;Q15642-3;Q1564	CIP4_HUMAN Cdc42-interacting protein 4 OS=Homo sapiens GN=TRIP10 PE=1 SV=3;>sp|Q15642-3|CIP4_HUMAN Isoform 3 of Cdc42	2.63E-60
Q15648;Q15648-3	MED1_HUMAN Mediator of RNA polymerase II transcription subunit 1 OS=Homo sapiens GN=MED1 PE=1 SV=4;>sp|Q15648-3|MED1_	8.44E-12
Q15651;Q15651-2	HMGN3_HUMAN High mobility group nucleosome-binding domain-containing protein 3 OS=Homo sapiens GN=HMGN3 PE=1 SV=2;>sp	9.46E-10
Q15654;Q68D94;Q71V88	TRIP6_HUMAN Thyroid receptor-interacting protein 6 OS=Homo sapiens GN=TRIP6 PE=1 SV=3;>tr|Q68D94|Q68D94_HUMAN Putative	2.35E-13
Q15758;Q71UA6;B7ZB81;	AAAT_HUMAN Neutral amino acid transporter B(0) OS=Homo sapiens GN=SLC1A5 PE=1 SV=2;>tr|Q71UA6|Q71UA6_HUMAN Neutral a	2.31E-161
Q15785;B4DXU3	TOM34_HUMAN Mitochondrial import receptor subunit TOM34 OS=Homo sapiens GN=TOMM34 PE=1 SV=2;>tr|B4DXU3|B4DXU3_HUM	2.74E-185
Q15796;Q53XR6;Q15796-	SMAD2_HUMAN Mothers against decapentaplegic homolog 2 OS=Homo sapiens GN=SMAD2 PE=1 SV=1;>tr|Q53XR6|Q53XR6_HUMAN	5.55E-08
Q15819;Q13404-1;Q1340	UB2V2_HUMAN Ubiquitin-conjugating enzyme E2 variant 2 OS=Homo sapiens GN=UBE2V2 PE=1 SV=4;>sp|Q13404-1|UB2V1_HUMAN	4.81E-45
Q15904;Q8NBT4;Q9H0C7	VAS1_HUMAN V-type proton ATPase subunit S1 OS=Homo sapiens GN=ATP6AP1 PE=1 SV=2;>tr|Q8NBT4|Q8NBT4_HUMAN cDNA FLJ9	8.26E-42
Q15907;P62491	RB11B_HUMAN Ras-related protein Rab-11B OS=Homo sapiens GN=RAB11B PE=1 SV=4;>sp|P62491|RB11A_HUMAN Ras-related pro	3.22E-54
Q15942;B4DY85;Q28617;	ZYX_HUMAN Zyxin OS=Homo sapiens GN=ZYX PE=1 SV=1;>tr|B4DY85|B4DY85_HUMAN cDNA FLJ61463, highly similar to Zyxin OS=	2.54E-118
Q16181;Q16181-2;A4GYY	SEPT7_HUMAN Septin-7 OS=Homo sapiens GN=SEPT7 PE=1 SV=2;>sp|Q16181-2|SEPT7_HUMAN Isoform 2 of Septin-7 OS=Homo sa	3.04E-233
Q16204;Q05CP8	CCDC6_HUMAN Coiled-coil domain-containing protein 6 OS=Homo sapiens GN=CCDC6 PE=1 SV=2;>tr|Q05CP8|Q05CP8_HUMAN CCDC	8.03E-74
Q16270;B4E1N2;B7Z9W7	IBP7_HUMAN Insulin-like growth factor-binding protein 7 OS=Homo sapiens GN=IGFBP7 PE=1 SV=1;>tr|B4E1N2|B4E1N2_HUMAN cD	4.91E-32
Q16352;Q59EM6;B4DE66	AINX_HUMAN Alpha-internexin OS=Homo sapiens GN=INA PE=1 SV=2;>tr|Q59EM6|Q59EM6_HUMAN Internexin neuronal intermedia	3.92E-15
Q16363;Q16363-2;E1P55	LAMA4_HUMAN Laminin subunit alpha-4 OS=Homo sapiens GN=LAMA4 PE=1 SV=4;>sp|Q16363-2|LAMA4_HUMAN Isoform 2 of Lami	1.86E-63
Q16394;D7RTA7	EXT1_HUMAN Exostosin-1 OS=Homo sapiens GN=EXT1 PE=1 SV=2;>tr|D7RTA7|D7RTA7_HUMAN Multiple exostoses 1 (Fragment) OS	2.25E-06
Q16512-2;Q16512;Q1552	2|PKN1_HUMAN Isoform 2 of Serine/threonine-protein kinase N1 OS=Homo sapiens GN=PKN1;>sp|Q16512|PKN1_HUMAN Serine/thre	2.79E-11
Q16531;B7Z2A1;B4DSA8	DDB1_HUMAN DNA damage-binding protein 1 OS=Homo sapiens GN=DDB1 PE=1 SV=1;>tr|B7Z2A1|B7Z2A1_HUMAN cDNA FLJ56068	2.08E-24
Q16539;Q16539-2;Q1653	MK14_HUMAN Mitogen-activated protein kinase 14 OS=Homo sapiens GN=MAPK14 PE=1 SV=3;>sp|Q16539-2|MK14_HUMAN Isoform	8.48E-36
Q16543;Q6FG59	CDC37_HUMAN Hsp90 co-chaperone Cdc37 OS=Homo sapiens GN=CDC37 PE=1 SV=1;>tr|Q6FG59|Q6FG59_HUMAN CDC37 protein (	2.62E-182
Q16576;Q6FHQ0;Q5JP00;	RBBP7_HUMAN Histone-binding protein RBBP7 OS=Homo sapiens GN=RBBP7 PE=1 SV=1;>tr|Q6FHQ0|Q6FHQ0_HUMAN RBBP7 prote	5.07E-202
Q16595;Q16595-2	FRDA_HUMAN Frataxin, mitochondrial OS=Homo sapiens GN=FXN PE=1 SV=2;>sp|Q16595-2|FRDA_HUMAN Isoform 2 of Frataxin, m	3.58E-06
Q16610-4;Q16610;Q1661	4|ECM1_HUMAN Isoform 4 of Extracellular matrix protein 1 OS=Homo sapiens GN=ECM1;>sp|Q16610|ECM1_HUMAN Extracellular ma	1.20E-09
Q16629;Q564D3;C9JAB2;	SRSF7_HUMAN Serine/arginine-rich splicing factor 7 OS=Homo sapiens GN=SRSF7 PE=1 SV=1;>tr|Q564D3|Q564D3_HUMAN Aging-a	2.31E-22
Q16630-2;Q16630;Q1663	2|CPSF6_HUMAN Isoform 2 of Cleavage and polyadenylation specificity factor subunit 6 OS=Homo sapiens GN=CPSF6;>sp|Q16630|CI	4.77E-228
Q16666;Q16666-2;Q1666	IF16_HUMAN Gamma-interferon-inducible protein 16 OS=Homo sapiens GN=IFI16 PE=1 SV=3;>sp|Q16666-2|IF16_HUMAN Isoform 2	0
Q16698;B7Z6B8	DECR_HUMAN 2,4-dienoyl-CoA reductase, mitochondrial OS=Homo sapiens GN=DECR1 PE=1 SV=1;>tr|B7Z6B8|B7Z6B8_HUMAN cDN	8.55E-83
Q16718;Q5H9R2;C9IZN5	NDUA5_HUMAN NADH dehydrogenase [ubiquinone] 1 alpha subcomplex subunit 5 OS=Homo sapiens GN=NDUFA5 PE=1 SV=3;>tr|Q5	3.36E-85
Q16762;Q53EW8;B4DVZ4	THTR_HUMAN Thiosulfate sulfurtransferase OS=Homo sapiens GN=TST PE=1 SV=4;>tr|Q53EW8|Q53EW8_HUMAN Thiosulfate sulfurt	1.45E-24
Q16763;Q2QD04;Q6NXQ4	UBE2S_HUMAN Ubiquitin-conjugating enzyme E2 S OS=Homo sapiens GN=UBE2S PE=1 SV=2;>tr|Q2QD04|Q2QD04_HUMAN Ubiquiti	1.90E-18
Q16775;Q16775-2	GLO2_HUMAN Hydroxyacylglutathione hydrolase, mitochondrial OS=Homo sapiens GN=HAGH PE=1 SV=2;>sp|Q16775-2|GLO2_HUMA	5.81E-18
Q16795;Q9BTT5;A8K4V2	NDUA9_HUMAN NADH dehydrogenase [ubiquinone] 1 alpha subcomplex subunit 9, mitochondrial OS=Homo sapiens GN=NDUFA9 PE=	1.94E-26
Q16822;Q6IB91;B4DW73;	PCKGM_HUMAN Phosphoenolpyruvate carboxykinase [GTP], mitochondrial OS=Homo sapiens GN=PCK2 PE=1 SV=3;>tr|Q6IB91|Q6IB	3.04E-165
Q16836-2;Q16836	2|HCDH_HUMAN Isoform 2 of Hydroxyacyl-coenzyme A dehydrogenase, mitochondrial OS=Homo sapiens GN=HADH;>sp|Q16836|HCI	1.49E-213
Q16850;Q16850-2	CP51A_HUMAN Lanosterol 14-alpha demethylase OS=Homo sapiens GN=CYP51A1 PE=1 SV=3;>sp|Q16850-2|CP51A_HUMAN Isoform	1.73E-38
Q16881;Q16881-3;Q1688	TRXR1_HUMAN Thioredoxin reductase 1, cytoplasmic OS=Homo sapiens GN=TXNRD1 PE=1 SV=3;>sp|Q16881-3|TRXR1_HUMAN Isof	1.91E-221
Q16881;Q16881-6;Q1688	TRXR1_HUMAN Thioredoxin reductase 1, cytoplasmic OS=Homo sapiens GN=TXNRD1 PE=1 SV=3;>sp|Q16881-6|TRXR1_HUMAN Isof	1.50E-106
Q27J81;Q27J81-2;A8MSL	INF2_HUMAN Inverted formin-2 OS=Homo sapiens GN=INF2 PE=1 SV=2;>sp|Q27J81-2|INF2_HUMAN Isoform 2 of Inverted formin-2	5.95E-20
Q29963;Q29865	1C06_HUMAN HLA class I histocompatibility antigen, Cw-6 alpha chain OS=Homo sapiens GN=HLA-C PE=1 SV=2;>sp|Q29865|1C18_	0
Q29RF7;Q96DB6;B3KMN2	PDS5A_HUMAN Sister chromatid cohesion protein PDS5 homolog A OS=Homo sapiens GN=PDS5A PE=1 SV=1;>tr|Q96DB6|Q96DB6_I	2.79E-63
Q2LD37;Q2LD37-4;Q2LD3	K1109_HUMAN Uncharacterized protein KIAA1109 OS=Homo sapiens GN=KIAA1109 PE=1 SV=2;>sp|Q2LD37-4|K1109_HUMAN Isofo	9.12E-10

Q2M2I8;Q2M2I8-2	AAK1_HUMAN AP2-associated protein kinase 1 OS=Homo sapiens GN=AAK1 PE=1 SV=3;>sp|Q2M2I8-2|AAK1_HUMAN Isoform 2 of A	2.93E-08
Q2TAA2;Q05D21;Q6PCA9;	IAH1_HUMAN Isoamyl acetate-hydrolyzing esterase 1 homolog OS=Homo sapiens GN=IAH1 PE=1 SV=1;>tr|Q05D21|Q05D21_HUMAI	5.33E-06
Q32P28;Q32P28-3;B4DNN	P3H1_HUMAN Prolyl 3-hydroxylase 1 OS=Homo sapiens GN=LEPRE1 PE=1 SV=2;>sp|Q32P28-3|P3H1_HUMAN Isoform 3 of Prolyl 3-h	5.97E-156
Q3KQU3;Q3KQU3-4;Q3KQ	MA7D1_HUMAN MAP7 domain-containing protein 1 OS=Homo sapiens GN=MAP7D1 PE=1 SV=1;>sp|Q3KQU3-4|MA7D1_HUMAN Isofo	1.66E-16
Q3KQV9;Q3KQV9-2	UAP1L_HUMAN UDP-N-acetylhexosamine pyrophosphorylase-like protein 1 OS=Homo sapiens GN=UAP1L1 PE=2 SV=2;>sp|Q3KQV9-2	2.57E-08
Q3LXA3;Q2L9C1;Q9UFX5	DHAK_HUMAN Bifunctional ATP-dependent dihydroxyacetone kinase/FAD-AMP lyase (cyclizing) OS=Homo sapiens GN=DAK PE=1 SV=	1.27E-289
Q3MHD2-2;Q3MHD2;B4DU	2|LSM12_HUMAN Isoform 2 of Protein LSM12 homolog OS=Homo sapiens GN=LSM12;>sp|Q3MHD2|LSM12_HUMAN Protein LSM12 hc	1.01E-34
Q3V6T2;Q3V6T2-3;Q3V6T	GRDN_HUMAN Girdin OS=Homo sapiens GN=CCDC88A PE=1 SV=2;>sp|Q3V6T2-3|GRDN_HUMAN Isoform 3 of Girdin OS=Homo sapi	1.62E-06
Q3ZCM7;Q5SQY0;C9JAA5	TBB8_HUMAN Tubulin beta-8 chain OS=Homo sapiens GN=TUBB8 PE=1 SV=2;>tr|Q5SQY0|Q5SQY0_HUMAN Novel protein similar to l	8.16E-162
Q3ZCM7;Q99867	TBB8_HUMAN Tubulin beta-8 chain OS=Homo sapiens GN=TUBB8 PE=1 SV=2;>sp|Q99867|TBB4Q_HUMAN Putative tubulin beta-4q c	4.61E-282
Q4G0J3;D6RFF0	LARP7_HUMAN La-related protein 7 OS=Homo sapiens GN=LARP7 PE=1 SV=1;>tr|D6RFF0|D6RFF0_HUMAN Uncharacterized protein (	1.58E-18
Q4V328;Q4V328-4;Q4V32	GRAP1_HUMAN GRIP1-associated protein 1 OS=Homo sapiens GN=GRIPAP1 PE=1 SV=1;>sp|Q4V328-4|GRAP1_HUMAN Isoform 4 of	1.90E-09
Q4VC31;C9JQ41	CCD58_HUMAN Coiled-coil domain-containing protein 58 OS=Homo sapiens GN=CCDC58 PE=1 SV=1;>tr|C9JQ41|C9JQ41_HUMAN Ur	5.04E-66
Q53FV1;Q9P0S3	ORML2_HUMAN ORM1-like protein 2 OS=Homo sapiens GN=ORMDL2 PE=2 SV=2;>sp|Q9P0S3|ORML1_HUMAN ORM1-like protein 1 O	7.68E-09
Q567U6;A8MQC0	CCD93_HUMAN Coiled-coil domain-containing protein 93 OS=Homo sapiens GN=CCDC93 PE=1 SV=2;>tr|A8MQC0|A8MQC0_HUMAN l	4.27E-13
Q5EBL8-2;Q5EBL8	2|PDZ11_HUMAN Isoform 2 of PDZ domain-containing protein 11 OS=Homo sapiens GN=PDZD11;>sp|Q5EBL8|PDZ11_HUMAN PDZ d	5.91E-37
Q5HYI8;C9JXM3	RABL3_HUMAN Rab-like protein 3 OS=Homo sapiens GN=RABL3 PE=1 SV=1;>tr|C9JXM3|C9JXM3_HUMAN Uncharacterized protein OS	1.65E-06
Q5JPE7;Q15155;P69849;(	NOMO2_HUMAN Nodal modulator 2 OS=Homo sapiens GN=NOMO2 PE=1 SV=1;>sp|Q15155|NOMO1_HUMAN Nodal modulator 1 OS=	2.91E-28
Q5JRA6;Q5JRA6-2;Q5JRA(	MIA3_HUMAN Melanoma inhibitory activity protein 3 OS=Homo sapiens GN=MIA3 PE=1 SV=1;>sp|Q5JRA6-2|MIA3_HUMAN Isoform 2	2.59E-15
Q5JSH3;Q5JSH3-2;Q5JSH	WDR44_HUMAN WD repeat-containing protein 44 OS=Homo sapiens GN=WDR44 PE=1 SV=1;>sp|Q5JSH3-2|WDR44_HUMAN Isoform	2.24E-09
Q5JVF3-4;Q5JVF3;Q5JVF3	4|PCID2_HUMAN Isoform 4 of PCI domain-containing protein 2 OS=Homo sapiens GN=PCID2;>sp|Q5JVF3|PCID2_HUMAN PCI domair	2.11E-07
Q5JVF3-4;Q5JVF3;Q5JVF3	4|PCID2_HUMAN Isoform 4 of PCI domain-containing protein 2 OS=Homo sapiens GN=PCID2;>sp|Q5JVF3|PCID2_HUMAN PCI domair	5.95E-17
Q5M775;Q5M775-4;Q5M7	CYTSB_HUMAN Cytospin-B OS=Homo sapiens GN=SPECC1 PE=1 SV=1;>sp|Q5M775-4|CYTSB_HUMAN Isoform 4 of Cytospin-B OS=H	1.92E-07
Q5R3I4;A9UJP8;B4DUE0	TTC38_HUMAN Tetratricopeptide repeat protein 38 OS=Homo sapiens GN=TTC38 PE=1 SV=1;>tr|A9UJP8|A9UJP8_HUMAN Novel prote	1.12E-40
Q5RI15-2;Q5RI15	2|FA36A_HUMAN Isoform 2 of Protein FAM36A OS=Homo sapiens GN=FAM36A;>sp|Q5RI15|FA36A_HUMAN Protein FAM36A OS=Hom	2.20E-15
Q5SRD1;O14925;B4DI18;	TI23B_HUMAN Putative mitochondrial import inner membrane translocase subunit Tim23B OS=Homo sapiens GN=TIMM23B PE=5 SV=	7.36E-24
Q5SRE7-3;Q5SRE7;Q5SRE	3|PHYD1_HUMAN Isoform 3 of Phytanoyl-CoA dioxygenase domain-containing protein 1 OS=Homo sapiens GN=PHYHD1;>sp|Q5SRE7	4.00E-10
Q5SW79;Q5SW79-3;Q5SV	CE170_HUMAN Centrosomal protein of 170 kDa OS=Homo sapiens GN=CEP170 PE=1 SV=1;>sp|Q5SW79-3|CE170_HUMAN Isoform 3	3.00E-52
Q5T1J5;Q9Y6H1	CHCH9_HUMAN Putative coiled-coil-helix-coiled-coil-helix domain-containing protein CHCHD2P9, mitochondrial OS=Homo sapiens GN=	9.78E-53
Q5T280;Q9P041	CI114_HUMAN Uncharacterized protein C9orf114 OS=Homo sapiens GN=C9orf114 PE=1 SV=3;>tr|Q9P041|Q9P041_HUMAN HSPC10	1.91E-07
Q5T653;C9IY40	RM02_HUMAN 39S ribosomal protein L2, mitochondrial OS=Homo sapiens GN=MRPL2 PE=1 SV=2;>tr|C9IY40|C9IY40_HUMAN Uncha	5.06E-22
Q5T6F2;B4DH66;B4DRB6;	UBAP2_HUMAN Ubiquitin-associated protein 2 OS=Homo sapiens GN=UBAP2 PE=1 SV=1;>tr|B4DH66|B4DH66_HUMAN cDNA FLJ595(	1.33E-19
Q5T6V5;Q5T6V7	CI064_HUMAN UPF0553 protein C9orf64 OS=Homo sapiens GN=C9orf64 PE=1 SV=1;>tr|Q5T6V7|Q5T6V7_HUMAN Chromosome 9 op	5.13E-33
Q5T8D3;Q5T8D3-3;Q5T8I	ACBD5_HUMAN Acyl-CoA-binding domain-containing protein 5 OS=Homo sapiens GN=ACBD5 PE=1 SV=1;>sp|Q5T8D3-3|ACBD5_HUI	2.68E-06
Q5TFE4;A8K2Z3;Q5QPD0;	NT5D1_HUMAN 5-nucleotidase domain-containing protein 1 OS=Homo sapiens GN=NT5DC1 PE=1 SV=1;>tr|A8K2Z3|A8K2Z3_HUMAN	5.95E-39
Q5VT52;Q5VT52-3;Q5VT5	RPRD2_HUMAN Regulation of nuclear pre-mRNA domain-containing protein 2 OS=Homo sapiens GN=RPRD2 PE=1 SV=1;>sp|Q5VT52	2.35E-10
Q5VT79;Q5VT79-2	AXA82_HUMAN Annexin A8-like protein 2 OS=Homo sapiens GN=ANXA8L2 PE=2 SV=1;>sp|Q5VT79-2|AXA82_HUMAN Isoform 2 of A	0
Q5VTR2;Q05DC0;A0PJJ6	BRE1A_HUMAN E3 ubiquitin-protein ligase BRE1A OS=Homo sapiens GN=RNF20 PE=1 SV=2;>tr|Q05DC0|Q05DC0_HUMAN RNF20 pro	3.67E-08
Q5VW32;B7Z9G5;B7Z1V1	BROX_HUMAN BRO1 domain-containing protein BROX OS=Homo sapiens GN=BROX PE=1 SV=1;>tr|B7Z9G5|B7Z9G5_HUMAN C1orf5	5.13E-130
Q5VYK3;B3KXF2	ECM29_HUMAN Proteasome-associated protein ECM29 homolog OS=Homo sapiens GN=ECM29 PE=1 SV=2;>tr|B3KXF2|B3KXF2_HUM	2.95E-81
Q5Y7A7;P01912	2B1D_HUMAN HLA class II histocompatibility antigen, DRB1-13 beta chain OS=Homo sapiens GN=HLA-DRB1 PE=1 SV=1;>sp|P01912	8.73E-55
Q63HR2-4;Q63HR2;Q63HI	4|TENC1_HUMAN Isoform 4 of Tensin-like C1 domain-containing phosphatase OS=Homo sapiens GN=TENC1;>sp|Q63HR2|TENC1_HU	1.07E-06
Q641Q2;Q641Q2-2;Q9Y4E	FA21A_HUMAN WASH complex subunit FAM21A OS=Homo sapiens GN=FAM21A PE=1 SV=3;>sp|Q641Q2-2|FA21A_HUMAN Isoform 2	2.99E-50
Q66LE6;A8KAK0	2ABD_HUMAN Serine/threonine-protein phosphatase 2A 55 kDa regulatory subunit B delta isoform OS=Homo sapiens GN=PPP2R2D PI	3.53E-26
Q66PJ3-2;Q8TEM4;Q66PJ	2|AR6P4_HUMAN Isoform 2 of ADP-ribosylation factor-like protein 6-interacting protein 4 OS=Homo sapiens GN=ARL6IP4;>tr|Q8TEM	3.69E-21
Q68CZ2;Q63HR2-5;Q68C	TENS3_HUMAN Tensin-3 OS=Homo sapiens GN=TNS3 PE=1 SV=2;>sp|Q63HR2-5|TENC1_HUMAN Isoform 5 of Tensin-like C1 domain	5.16E-22
Q68CZ2;Q68CZ2-2	TENS3_HUMAN Tensin-3 OS=Homo sapiens GN=TNS3 PE=1 SV=2;>sp|Q68CZ2-2|TENS3_HUMAN Isoform 2 of Tensin-3 OS=Homo sa	7.57E-21
Q68EM7;Q68EM7-5;Q68EI	RHG17_HUMAN Rho GTPase-activating protein 17 OS=Homo sapiens GN=ARHGAP17 PE=1 SV=1;>sp|Q68EM7-5|RHG17_HUMAN Isof	4.25E-54
Q6AI08;Q0VD96	HEAT6_HUMAN HEAT repeat-containing protein 6 OS=Homo sapiens GN=HEATR6 PE=1 SV=1;>tr|Q0VD96|Q0VD96_HUMAN HEATR6 p	4.60E-42
Q6DD88;B4DXC4;C9JUU1	ATLA3_HUMAN Atlastin-3 OS=Homo sapiens GN=ATL3 PE=1 SV=1;>tr|B4DXC4|B4DXC4_HUMAN cDNA FLJ58636, moderately similar	0
Q6DKI1;B7Z652;B7Z4G0;	RL7L_HUMAN 60S ribosomal protein L7-like 1 OS=Homo sapiens GN=RPL7L1 PE=1 SV=1;>tr|B7Z652|B7Z652_HUMAN cDNA FLJ5085	1.52E-09
Q6DKK2;B4DN65;A8MZ52	TTC19_HUMAN Tetratricopeptide repeat protein 19 OS=Homo sapiens GN=TTC19 PE=1 SV=3;>tr|B4DN65|B4DN65_HUMAN Tetratrico	3.99E-10
Q6DN03;Q6DRA6	H2B2C_HUMAN Putative histone H2B type 2-C OS=Homo sapiens GN=HIST2H2BC PE=5 SV=3;>sp|Q6DRA6|H2B2D_HUMAN Putative	1.57E-68
Q6FHJ7;B4E1T4	SFRP4_HUMAN Secreted frizzled-related protein 4 OS=Homo sapiens GN=SFRP4 PE=1 SV=2;>tr|B4E1T4|B4E1T4_HUMAN cDNA FLJ5	2.35E-16
Q6FI13;Q16777;P0C0S8;F	H2A2A_HUMAN Histone H2A type 2-A OS=Homo sapiens GN=HIST2H2AA3 PE=1 SV=3;>sp|Q16777|H2A2C_HUMAN Histone H2A type	3.56E-249
Q6IBS0;D6RG15	TWF2_HUMAN Twinfilin-2 OS=Homo sapiens GN=TWF2 PE=1 SV=2;>tr|D6RG15|D6RG15_HUMAN Uncharacterized protein OS=Homo	5.72E-78
Q6IN85;Q6IN85-2;Q6IN8!	P4R3A_HUMAN Serine/threonine-protein phosphatase 4 regulatory subunit 3A OS=Homo sapiens GN=SMEK1 PE=1 SV=1;>sp|Q6IN8!	2.16E-08
Q6NXE6;Q6NXE6-2;B4E1N	ARMC6_HUMAN Armadillo repeat-containing protein 6 OS=Homo sapiens GN=ARMC6 PE=1 SV=2;>sp|Q6NXE6-2|ARMC6_HUMAN Isof	3.33E-92
Q6NZ67;Q6P582	MZT2B_HUMAN Mitotic-spindle organizing protein 2B OS=Homo sapiens GN=MZT2B PE=1 SV=1;>sp|Q6P582|MZT2A_HUMAN Mitotic-	9.48E-05
Q6NZI2;Q6NZI2-2	PTRF_HUMAN Polymerase I and transcript release factor OS=Homo sapiens GN=PTRF PE=1 SV=1;>sp|Q6NZI2-2|PTRF_HUMAN Isofor	0
Q6NZI2;Q6NZI2-2;Q6NZI	PTRF_HUMAN Polymerase I and transcript release factor OS=Homo sapiens GN=PTRF PE=1 SV=1;>sp|Q6NZI2-2|PTRF_HUMAN Isofor	0

Q6P179;Q6P179-3;Q6P17	ERAP2_HUMAN Endoplasmic reticulum aminopeptidase 2 OS=Homo sapiens GN=ERAP2 PE=1 SV=2;>sp|Q6P179-3|ERAP2_HUMAN Is	1.47E-23
Q6P1J9;B4DV47	CDC73_HUMAN Parafibromin OS=Homo sapiens GN=CDC73 PE=1 SV=1;>tr|B4DV47|B4DV47_HUMAN cDNA FLJ57893, highly similar	6.08E-30
Q6P1N0;Q6P1N0-2;Q6P1N	C2D1A_HUMAN Coiled-coil and C2 domain-containing protein 1A OS=Homo sapiens GN=CC2D1A PE=1 SV=1;>sp|Q6P1N0-2|C2D1A_	1.06E-05
Q6P1Q9;Q6P1Q9-2;B4DS	MTL2B_HUMAN Methyltransferase-like protein 2B OS=Homo sapiens GN=METTL2B PE=1 SV=3;>sp|Q6P1Q9-2|MTL2B_HUMAN Isofor	5.40E-09
Q6P3X3;B4DRC7	TTC27_HUMAN Tetratricopeptide repeat protein 27 OS=Homo sapiens GN=TTC27 PE=1 SV=1;>tr|B4DRC7|B4DRC7_HUMAN cDNA FLJ	7.61E-35
Q6P6C2;Q6P6C2-2	ALKB5_HUMAN Probable alpha-ketoglutarate-dependent dioxygenase ABH5 OS=Homo sapiens GN=ALKBH5 PE=1 SV=1;>sp|Q6P6C2-	1.82E-40
Q6P996;B4DR55;B4DHL7;	PDXD1_HUMAN Pyridoxal-dependent decarboxylase domain-containing protein 1 OS=Homo sapiens GN=PDXDC1 PE=1 SV=2;>tr|B4D	1.96E-248
Q6S8J3;P0CG38;P0CG39;	POTEE_HUMAN POTE ankyrin domain family member E OS=Homo sapiens GN=POTEE PE=1 SV=3;>sp|P0CG38|POTEI_HUMAN POTE a	0
Q6S8J3;Q9BYX7;P0CG38	POTEE_HUMAN POTE ankyrin domain family member E OS=Homo sapiens GN=POTEE PE=1 SV=3;>sp|Q9BYX7|ACTBM_HUMAN Putat	1.73E-127
Q6UN15;Q6UN15-3;Q6UN	FIP1_HUMAN Pre-mRNA 3-end-processing factor FIP1 OS=Homo sapiens GN=FIP1L1 PE=1 SV=1;>sp|Q6UN15-3|FIP1_HUMAN Isofor	1.32E-49
Q6UWP2;Q6UWP2-2;A8M	DHR11_HUMAN Dehydrogenase/reductase SDR family member 11 OS=Homo sapiens GN=DHRS11 PE=1 SV=1;>sp|Q6UWP2-2|DHR1	9.14E-13
Q6UWZ7;D6REL5;D6RAN	F175A_HUMAN BRCA1-A complex subunit Abraxas OS=Homo sapiens GN=FAM175A PE=1 SV=2;>tr|D6REL5|D6REL5_HUMAN Unchar	1.15E-07
Q6UX04;Q6UX04-2;D6RE	CWC27_HUMAN Peptidyl-prolyl cis-trans isomerase CWC27 homolog OS=Homo sapiens GN=CWC27 PE=1 SV=1;>sp|Q6UX04-2|CWC	2.05E-30
Q6UX71;Q6UX71-2	PXDC2_HUMAN Plexin domain-containing protein 2 OS=Homo sapiens GN=PLXDC2 PE=1 SV=1;>sp|Q6UX71-2|PXDC2_HUMAN Isofor	1.79E-07
Q6UXG2;Q6UXG2-4;Q6UX	K1324_HUMAN UPF0577 protein KIAA1324 OS=Homo sapiens GN=KIAA1324 PE=2 SV=2;>sp|Q6UXG2-4|K1324_HUMAN Isoform 4 o	3.05E-37
Q6VY07-2;Q6VY07;B4DF7	2|PACS1_HUMAN Isoform 2 of Phosphofurin acidic cluster sorting protein 1 OS=Homo sapiens GN=PACS1;>sp|Q6VY07|PACS1_HUMA	3.71E-11
Q6WCQ1-2;Q6WCQ1;Q6W	2|MPRIP_HUMAN Isoform 2 of Myosin phosphatase Rho-interacting protein OS=Homo sapiens GN=MPRIP;>sp|Q6WCQ1|MPRIP_HUMA	9.01E-21
Q6WKZ4;Q6WKZ4-3;Q6W	RFIP1_HUMAN Rab11 family-interacting protein 1 OS=Homo sapiens GN=RAB11FIP1 PE=1 SV=2;>sp|Q6WKZ4-3|RFIP1_HUMAN Isof	1.73E-34
Q6XQN6;Q6XQN6-3;Q8W	PNCB_HUMAN Nicotinate phosphoribosyltransferase OS=Homo sapiens GN=NAPRT1 PE=1 SV=2;>sp|Q6XQN6-3|PNCB_HUMAN Isofor	3.53E-27
Q6Y7W6-3;Q6Y7W6	3|PERQ2_HUMAN Isoform 2 of PERQ amino acid-rich with GYF domain-containing protein 2 OS=Homo sapiens GN=GIGYF2;>sp|Q6Y7	5.61E-34
Q6YN16;Q6YN16-2	HSDL2_HUMAN Hydroxysteroid dehydrogenase-like protein 2 OS=Homo sapiens GN=HSDL2 PE=1 SV=1;>sp|Q6YN16-2|HSDL2_HUM	1.50E-20
Q6YP21;Q6YP21-2	KAT3_HUMAN Kynurenine--oxoglutarate transaminase 3 OS=Homo sapiens GN=CCBL2 PE=1 SV=1;>sp|Q6YP21-2|KAT3_HUMAN Isof	8.50E-11
Q6ZMI0;Q6ZMI0-2;Q6ZM	KLRAQ_HUMAN KLRAQ motif-containing protein 1 OS=Homo sapiens GN=KLRAQ1 PE=1 SV=1;>sp|Q6ZMI0-2|KLRAQ_HUMAN Isofor	1.43E-05
Q6ZRP7;B3KY64;Q5TB38	QSOX2_HUMAN Sulfhydryl oxidase 2 OS=Homo sapiens GN=QSOX2 PE=1 SV=3;>tr|B3KY64|B3KY64_HUMAN cDNA FLJ44717 fis, clo	1.98E-31
Q6ZSZ5;Q6ZSZ5-3;Q6ZS	ARHGI_HUMAN Rho guanine nucleotide exchange factor 18 OS=Homo sapiens GN=ARHGEF18 PE=1 SV=3;>sp|Q6ZSZ5-3|ARHGI_HU	5.85E-15
Q70E73;Q70E73-3;Q70E7	RAPH1_HUMAN Ras-associated and pleckstrin homology domains-containing protein 1 OS=Homo sapiens GN=RAPH1 PE=1 SV=3;>sp	8.24E-131
Q70J99-3;Q70J99;B4DTQ	3|UN13D_HUMAN Isoform 3 of Protein unc-13 homolog D OS=Homo sapiens GN=UNC13D;>sp|Q70J99|UN13D_HUMAN Protein unc-1	6.01E-07
Q70UQ0;Q70UQ0-2	IKIP_HUMAN Inhibitor of nuclear factor kappa-B kinase-interacting protein OS=Homo sapiens GN=IKBIP PE=1 SV=1;>sp|Q70UQ0-2|I	1.50E-112
Q71U36;B4DQK4;Q9UQM	TBA1A_HUMAN Tubulin alpha-1A chain OS=Homo sapiens GN=TUBA1A PE=1 SV=1;>tr|B4DQK4|B4DQK4_HUMAN cDNA FLJ53743, hi	0
Q71U36;Q13748;Q13748-	TBA1A_HUMAN Tubulin alpha-1A chain OS=Homo sapiens GN=TUBA1A PE=1 SV=1;>sp|Q13748|TBA3C_HUMAN Tubulin alpha-3C/D c	0
Q71U36;Q13748;Q6PEY2;	TBA1A_HUMAN Tubulin alpha-1A chain OS=Homo sapiens GN=TUBA1A PE=1 SV=1;>sp|Q13748|TBA3C_HUMAN Tubulin alpha-3C/D c	0
Q7L0Y3;C9JVB6	MRRP1_HUMAN Mitochondrial ribonuclease P protein 1 OS=Homo sapiens GN=RG9MTD1 PE=1 SV=2;>tr|C9JVB6|C9JVB6_HUMAN Un	9.26E-42
Q7L273;B4DPW0	KCTD9_HUMAN BTB/POZ domain-containing protein KCTD9 OS=Homo sapiens GN=KCTD9 PE=2 SV=1;>tr|B4DPW0|B4DPW0_HUMAN	8.02E-10
Q7L2E3-2;Q7L2E3;Q7L2E	2|DHX30_HUMAN Isoform 2 of Putative ATP-dependent RNA helicase DHX30 OS=Homo sapiens GN=DHX30;>sp|Q7L2E3|DHX30_HUM	2.02E-26
Q7L2H7;B4E2Q4	EIF3M_HUMAN Eukaryotic translation initiation factor 3 subunit M OS=Homo sapiens GN=EIF3M PE=1 SV=1;>tr|B4E2Q4|B4E2Q4_HU	2.02E-214
Q7L5D6;Q7L5D6-2;C9JHN	GET4_HUMAN Golgi to ER traffic protein 4 homolog OS=Homo sapiens GN=GET4 PE=1 SV=1;>sp|Q7L5D6-2|GET4_HUMAN Isoform 2	2.21E-25
Q7L5N1;B2R514	CSN6_HUMAN COP9 signalosome complex subunit 6 OS=Homo sapiens GN=COPS6 PE=1 SV=1;>tr|B2R514|B2R514_HUMAN cDNA, F	2.61E-31
Q7LBR1;B2RA72	CHM1B_HUMAN Charged multivesicular body protein 1b OS=Homo sapiens GN=CHMP1B PE=1 SV=1;>tr|B2RA72|B2RA72_HUMAN cD	1.54E-06
Q7LG56;Q7LG56-5	RIR2B_HUMAN Ribonucleoside-diphosphate reductase subunit M2 B OS=Homo sapiens GN=RRM2B PE=1 SV=1;>sp|Q7LG56-5|RIR2B	1.36E-10
Q7RTV5;Q5JU03;B7ZKK1	CI021_HUMAN UPF0308 protein C9orf21 OS=Homo sapiens GN=C9orf21 PE=2 SV=1;>tr|Q5JU03|Q5JU03_HUMAN Novel protein (Fra	5.43E-05
Q7Z2W4;Q7Z2W4-2;Q7Z2	ZCCHV_HUMAN Zinc finger CCCH-type antiviral protein 1 OS=Homo sapiens GN=ZC3HAV1 PE=1 SV=3;>sp|Q7Z2W4-2|ZCCHV_HUMA	2.09E-22
Q7Z3E5;Q7Z3E5-2	ARMC9_HUMAN LisH domain-containing protein ARMC9 OS=Homo sapiens GN=ARMC9 PE=2 SV=2;>sp|Q7Z3E5-2|ARMC9_HUMAN Is	7.20E-28
Q7Z460;Q7Z460-3;Q7Z46	CLAP1_HUMAN CLIP-associating protein 1 OS=Homo sapiens GN=CLASP1 PE=1 SV=1;>sp|Q7Z460-3|CLAP1_HUMAN Isoform 3 of CL	1.76E-11
Q7Z4G1-2;Q7Z4G1;B0QZ	2|COMD6_HUMAN Isoform 2 of COMM domain-containing protein 6 OS=Homo sapiens GN=COMMD6;>sp|Q7Z4G1|COMD6_HUMAN C	1.04E-08
Q7Z4H8;Q7Z4H8-2;Q7Z4	KDEL2_HUMAN KDEL motif-containing protein 2 OS=Homo sapiens GN=KDELC2 PE=1 SV=2;>sp|Q7Z4H8-2|KDEL2_HUMAN Isoform 2	2.13E-18
Q7Z4Q2;Q7Z4Q2-2;Q7Z4	HEAT3_HUMAN HEAT repeat-containing protein 3 OS=Homo sapiens GN=HEATR3 PE=1 SV=2;>sp|Q7Z4Q2-2|HEAT3_HUMAN Isoform	1.24E-08
Q7Z739;Q9BYJ9	YTHD3_HUMAN YTH domain family protein 3 OS=Homo sapiens GN=YTHDF3 PE=1 SV=1;>sp|Q9BYJ9|YTHD1_HUMAN YTH domain fa	1.36E-15
Q7Z7H5;Q7Z7H5-2;Q7Z7	TMED4_HUMAN Transmembrane emp24 domain-containing protein 4 OS=Homo sapiens GN=TMED4 PE=1 SV=1;>sp|Q7Z7H5-2|TMED	1.71E-45
Q86SF2;Q4W5F7	GALT7_HUMAN N-acetylgalactosaminyltransferase 7 OS=Homo sapiens GN=GALNT7 PE=1 SV=1;>tr|Q4W5F7|Q4W5F7_HUMAN Putati	3.48E-09
Q86SZ2;Q86SZ2-2	TPC6B_HUMAN Trafficking protein particle complex subunit 6B OS=Homo sapiens GN=TRAPPC6B PE=1 SV=1;>sp|Q86SZ2-2|TPC6B_	6.07E-14
Q86TB9;Q86TB9-4;Q86TB	PATL1_HUMAN Protein PAT1 homolog 1 OS=Homo sapiens GN=PATL1 PE=1 SV=2;>sp|Q86TB9-4|PATL1_HUMAN Isoform 4 of Protein	2.55E-21
Q86TG7;Q86TG7-2	PEG10_HUMAN Retrotransposon-derived protein PEG10 OS=Homo sapiens GN=PEG10 PE=1 SV=2;>sp|Q86TG7-2|PEG10_HUMAN Iso	8.86E-26
Q86TI2-2;Q86TI2;Q86TI2	2|DPP9_HUMAN Isoform 2 of Dipeptidyl peptidase 9 OS=Homo sapiens GN=DPP9;>sp|Q86TI2|DPP9_HUMAN Dipeptidyl peptidase 9 O	4.48E-06
Q86UP2;Q86UP2-2	KTN1_HUMAN Kinectin OS=Homo sapiens GN=KTN1 PE=1 SV=1;>sp|Q86UP2-2|KTN1_HUMAN Isoform 2 of Kinectin OS=Homo sapie	1.78E-42
Q86V21;Q86V21-2;Q9H9	AACS_HUMAN Acetoacetyl-CoA synthetase OS=Homo sapiens GN=AACS PE=1 SV=1;>sp|Q86V21-2|AACS_HUMAN Isoform 2 of Acet	1.31E-06
Q86W42;Q86W42-3;Q86W	THOC6_HUMAN THO complex subunit 6 homolog OS=Homo sapiens GN=THOC6 PE=1 SV=1;>sp|Q86W42-3|THOC6_HUMAN Isoform	9.41E-13
Q86X76-3;Q86X76;Q86X7	3|NIT1_HUMAN Isoform 4 of Nitrilase homolog 1 OS=Homo sapiens GN=NIT1;>sp|Q86X76|NIT1_HUMAN Nitrilase homolog 1 OS=Ho	1.39E-13
Q86XP3;Q86XP3-2;B3KX2	DDX42_HUMAN ATP-dependent RNA helicase DDX42 OS=Homo sapiens GN=DDX42 PE=1 SV=1;>sp|Q86XP3-2|DDX42_HUMAN Isofo	8.29E-48
Q86Y56;Q86Y56-2;Q86Y5	HEAT2_HUMAN HEAT repeat-containing protein 2 OS=Homo sapiens GN=HEATR2 PE=1 SV=4;>sp|Q86Y56-2|HEAT2_HUMAN Isoform	1.99E-20
Q86Y56;Q86Y56-3;Q86Y5	HEAT2_HUMAN HEAT repeat-containing protein 2 OS=Homo sapiens GN=HEATR2 PE=1 SV=4;>sp|Q86Y56-3|HEAT2_HUMAN Isoform	3.52E-19
Q86YS6;A6NDJ8	RAB43_HUMAN Ras-related protein Rab-43 OS=Homo sapiens GN=RAB43 PE=1 SV=1;>sp|A6NDJ8|RB43L_HUMAN Putative Rab-43-li	8.44E-09

Q8IUE6;A2RUA4	H2A2B_HUMAN Histone H2A type 2-B OS=Homo sapiens GN=HIST2H2AB PE=1 SV=3;>tr|A2RUA4|A2RUA4_HUMAN Histone H2A OS=	5.92E-37
Q8IUF8;Q8IUF8-4;Q8IUF8	MINA_HUMAN MYC-induced nuclear antigen OS=Homo sapiens GN=MINA PE=1 SV=1;>sp|Q8IUF8-4|MINA_HUMAN Isoform 4 of MYC-	2.61E-09
Q8IUX7;Q8IUX7-2;B4DJB:	AEBP1_HUMAN Adipocyte enhancer-binding protein 1 OS=Homo sapiens GN=AEBP1 PE=1 SV=1;>sp|Q8IUX7-2|AEBP1_HUMAN Isofor	9.76E-63
Q8IVF2;Q8IVF2-3;Q8IVF2	AHNK2_HUMAN Protein AHNAK2 OS=Homo sapiens GN=AHNAK2 PE=1 SV=2;>sp|Q8IVF2-3|AHNK2_HUMAN Isoform 3 of Protein AHN	7.28E-08
Q8IVL6;Q8IVL6-2	P3H3_HUMAN Prolyl 3-hydroxylase 3 OS=Homo sapiens GN=LEPREL2 PE=2 SV=1;>sp|Q8IVL6-2|P3H3_HUMAN Isoform 2 of Prolyl 3-	2.92E-14
Q8IW45-2;Q8IW45;Q8IW4	2|CARKD_HUMAN Isoform 2 of Carbohydrate kinase domain-containing protein OS=Homo sapiens GN=CARKD;>sp|Q8IW45|CARKD_H	2.20E-17
Q8IWC1;Q8IWC1-3;Q8IW	MA7D3_HUMAN MAP7 domain-containing protein 3 OS=Homo sapiens GN=MAP7D3 PE=1 SV=2;>sp|Q8IWC1-3|MA7D3_HUMAN Isofo	4.03E-23
Q8IWE2;B4E1Z8;Q6MZV4	NXP20_HUMAN Protein NOXP20 OS=Homo sapiens GN=FAM114A1 PE=1 SV=2;>tr|B4E1Z8|B4E1Z8_HUMAN cDNA FLJ60392 OS=Hon	0
Q8IWX8;B4DPM4;B4DL82	CHERP_HUMAN Calcium homeostasis endoplasmic reticulum protein OS=Homo sapiens GN=CHERP PE=1 SV=3;>tr|B4DPM4|B4DPM4_	4.86E-10
Q8IXB1;Q8IXB1-2	DJC10_HUMAN DnaJ homolog subfamily C member 10 OS=Homo sapiens GN=DNAJC10 PE=1 SV=2;>sp|Q8IXB1-2|DJC10_HUMAN Is	7.40E-22
Q8IXI1;Q8IXI1-2;Q8IXI2-	MIRO2_HUMAN Mitochondrial Rho GTPase 2 OS=Homo sapiens GN=RHOT2 PE=1 SV=2;>sp|Q8IXI1-2|MIRO2_HUMAN Isoform 2 of Mi	7.88E-94
Q8IXI1;Q8IXI1-2;Q8IXI2-	MIRO2_HUMAN Mitochondrial Rho GTPase 2 OS=Homo sapiens GN=RHOT2 PE=1 SV=2;>sp|Q8IXI1-2|MIRO2_HUMAN Isoform 2 of Mi	1.51E-96
Q8IXQ6;Q8IXQ6-2;B7Z5L	PARP9_HUMAN Poly [ADP-ribose] polymerase 9 OS=Homo sapiens GN=PARP9 PE=1 SV=2;>sp|Q8IXQ6-2|PARP9_HUMAN Isoform 2 o	1.73E-20
Q8IXT5;B9ZVT1	RB12B_HUMAN RNA-binding protein 12B OS=Homo sapiens GN=RBM12B PE=1 SV=2;>tr|B9ZVT1|B9ZVT1_HUMAN Uncharacterized p	1.64E-06
Q8IY21;Q6MZF7	DDX60_HUMAN Probable ATP-dependent RNA helicase DDX60 OS=Homo sapiens GN=DDX60 PE=2 SV=3;>tr|Q6MZF7|Q6MZF7_HUM/	4.82E-05
Q8IY81;B4DKU3	RRMJ3_HUMAN Putative rRNA methyltransferase 3 OS=Homo sapiens GN=FTSJ3 PE=1 SV=2;>tr|B4DKU3|B4DKU3_HUMAN cDNA FLJ	3.48E-22
Q8IYT8;B3KUZ9	ULK2_HUMAN Serine/threonine-protein kinase ULK2 OS=Homo sapiens GN=ULK2 PE=1 SV=3;>tr|B3KUZ9|B3KUZ9_HUMAN cDNA FL	6.02E-07
Q8IZ83;B4DLQ1;B4DQ15;	A16A1_HUMAN Aldehyde dehydrogenase family 16 member A1 OS=Homo sapiens GN=ALDH16A1 PE=1 SV=2;>tr|B4DLQ1|B4DLQ1_	7.67E-19
Q8N1F7;A8K897;B3KPQ8	NUP93_HUMAN Nuclear pore complex protein Nup93 OS=Homo sapiens GN=NUP93 PE=1 SV=2;>tr|A8K897|A8K897_HUMAN cDNA Fl	1.15E-165
Q8N2G8;Q8N2G8-2	GHDC_HUMAN GH3 domain-containing protein OS=Homo sapiens GN=GHDC PE=1 SV=2;>sp|Q8N2G8-2|GHDC_HUMAN Isoform 2 of	3.44E-10
Q8N335;C9JM46;C9JFA7;(	GPD1L_HUMAN Glycerol-3-phosphate dehydrogenase 1-like protein OS=Homo sapiens GN=GPD1L PE=1 SV=1;>tr|C9JM46|C9JM46_H	3.00E-52
Q8N474;A8KAM5;Q05DS5	SFRP1_HUMAN Secreted frizzled-related protein 1 OS=Homo sapiens GN=SFRP1 PE=1 SV=1;>tr|A8KAM5|A8KAM5_HUMAN cDNA FLJ	6.36E-14
Q8N4V1-2;Q8N4V1	2|MMGT1_HUMAN Isoform 2 of Membrane magnesium transporter 1 OS=Homo sapiens GN=MMGT1;>sp|Q8N4V1|MMGT1_HUMAN Me	1.55E-29
Q8N5K1;D6RCF4	CISD2_HUMAN CDGSH iron-sulfur domain-containing protein 2 OS=Homo sapiens GN=CISD2 PE=1 SV=1;>tr|D6RCF4|D6RCF4_HUM/	4.09E-30
Q8N5M1;C9J2Q2;B7Z3V0	ATPF2_HUMAN ATP synthase mitochondrial F1 complex assembly factor 2 OS=Homo sapiens GN=ATPAF2 PE=1 SV=1;>tr|C9J2Q2|C9J	1.73E-09
Q8N5M9;Q9BY51	JAGN1_HUMAN Protein jagunal homolog 1 OS=Homo sapiens GN=JAGN1 PE=1 SV=1;>tr|Q9BY51|Q9BY51_HUMAN GL009 OS=Homo	8.73E-07
Q8N684-3;Q8N684;Q8N6	3|CPSF7_HUMAN Isoform 3 of Cleavage and polyadenylation specificity factor subunit 7 OS=Homo sapiens GN=CPSF7;>sp|Q8N684|C	5.04E-49
Q8N6M0;B4DEY0	OTU6B_HUMAN OTU domain-containing protein 6B OS=Homo sapiens GN=OTUD6B PE=1 SV=1;>tr|B4DEY0|B4DEY0_HUMAN cDNA Fl	1.63E-07
Q8N7H5;Q8N7H5-2;E2QR	PAF1_HUMAN RNA polymerase II-associated factor 1 homolog OS=Homo sapiens GN=PAF1 PE=1 SV=2;>sp|Q8N7H5-2|PAF1_HUMAN	1.68E-27
Q8N8S7;Q8N8S7-2;D0PQ	ENAH_HUMAN Protein enabled homolog OS=Homo sapiens GN=ENAH PE=1 SV=2;>sp|Q8N8S7-2|ENAH_HUMAN Isoform 2 of Protein	5.54E-102
Q8NB90;Q8NB90-2	SPAT5_HUMAN Spermatogenesis-associated protein 5 OS=Homo sapiens GN=SPATA5 PE=1 SV=3;>sp|Q8NB90-2|SPAT5_HUMAN Isof	6.59E-09
Q8NB90;Q8NB90-2;Q8NB	SPAT5_HUMAN Spermatogenesis-associated protein 5 OS=Homo sapiens GN=SPATA5 PE=1 SV=3;>sp|Q8NB90-2|SPAT5_HUMAN Isof	7.55E-16
Q8NBN7;Q8NBN7-2	RDH13_HUMAN Retinol dehydrogenase 13 OS=Homo sapiens GN=RDH13 PE=1 SV=2;>sp|Q8NBN7-2|RDH13_HUMAN Isoform 2 of Re	8.32E-11
Q8NBQ5;D6RCD0	DHB11_HUMAN Estradiol 17-beta-dehydrogenase 11 OS=Homo sapiens GN=HSD17B11 PE=1 SV=3;>tr|D6RCD0|D6RCD0_HUMAN Ur	1.09E-59
Q8NBS9;Q658S9;Q6EHZ3	TXND5_HUMAN Thioredoxin domain-containing protein 5 OS=Homo sapiens GN=TXNDC5 PE=1 SV=2;>tr|Q658S9|Q658S9_HUMAN P	5.23E-289
Q8NBT2;C9JGC4;B4E086;	SPC24_HUMAN Kinetochore protein Spc24 OS=Homo sapiens GN=SPC24 PE=1 SV=1;>tr|C9JGC4|C9JGC4_HUMAN Uncharacterized p	8.18E-89
Q8NC51;Q8NC51-2;Q8NC	PAIRB_HUMAN Plasminogen activator inhibitor 1 RNA-binding protein OS=Homo sapiens GN=SERBP1 PE=1 SV=2;>sp|Q8NC51-2|PAII	0
Q8NC51;Q8NC51-3;Q8NC	PAIRB_HUMAN Plasminogen activator inhibitor 1 RNA-binding protein OS=Homo sapiens GN=SERBP1 PE=1 SV=2;>sp|Q8NC51-3|PAII	0
Q8NC56;A8MS91;B4DVH5	LEMD2_HUMAN LEM domain-containing protein 2 OS=Homo sapiens GN=LEMD2 PE=1 SV=1;>tr|A8MS91|A8MS91_HUMAN Uncharact	1.83E-40
Q8NCA5;Q8NCA5-2;Q05C	FA98A_HUMAN Protein FAM98A OS=Homo sapiens GN=FAM98A PE=1 SV=1;>sp|Q8NCA5-2|FA98A_HUMAN Isoform 2 of Protein FAM	5.49E-05
Q8NCM8-2;Q8NCM8	2|DYHC2_HUMAN Isoform 2 of Cytoplasmic dynein 2 heavy chain 1 OS=Homo sapiens GN=DYNC2H1;>sp|Q8NCM8|DYHC2_HUMAN C	5.36E-08
Q8NCN5;B4DZL5;B3KU99	PDPR_HUMAN Pyruvate dehydrogenase phosphatase regulatory subunit, mitochondrial OS=Homo sapiens GN=PDPR PE=1 SV=2;>tr|E	5.70E-09
Q8NDI1;Q8NDI1-2;Q8NDI	EHBP1_HUMAN EH domain-binding protein 1 OS=Homo sapiens GN=EHBP1 PE=1 SV=3;>sp|Q8NDI1-2|EHBP1_HUMAN Isoform 2 of E	4.36E-11
Q8NEZ5;Q8NEZ5-3;Q8NE	FBX22_HUMAN F-box only protein 22 OS=Homo sapiens GN=FBXO22 PE=1 SV=1;>sp|Q8NEZ5-3|FBX22_HUMAN Isoform 3 of F-box c	1.36E-09
Q8NF37;D3DTC2	PCAT1_HUMAN Lysophosphatidylcholine acyltransferase 1 OS=Homo sapiens GN=LPCAT1 PE=1 SV=2;>tr|D3DTC2|D3DTC2_HUMAN A	9.99E-07
Q8NF91;Q8NF91-4;Q8NF9	SYNE1_HUMAN Nesprin-1 OS=Homo sapiens GN=SYNE1 PE=1 SV=3;>sp|Q8NF91-4|SYNE1_HUMAN Isoform 4 of Nesprin-1 OS=Hom	2.08E-27
Q8NFA0;P35125-2	UBP32_HUMAN Ubiquitin carboxyl-terminal hydrolase 32 OS=Homo sapiens GN=USP32 PE=1 SV=1;>sp|P35125-2|UBP6_HUMAN Isof	7.02E-09
Q8NFV4;Q8NFV4-4;D3DX	ABHDB_HUMAN Abhydrolase domain-containing protein 11 OS=Homo sapiens GN=ABHD11 PE=1 SV=1;>sp|Q8NFV4-4|ABHDB_HUM/	6.22E-18
Q8NHG8;Q8NEX1	ZNRF2_HUMAN E3 ubiquitin-protein ligase ZNRF2 OS=Homo sapiens GN=ZNRF2 PE=1 SV=1;>tr|Q8NEX1|Q8NEX1_HUMAN Ells2 (Frag	5.68E-07
Q8NHH9;Q8NHH9-2;Q8NH	ATLA2_HUMAN Atlastin-2 OS=Homo sapiens GN=ATL2 PE=1 SV=2;>sp|Q8NHH9-2|ATLA2_HUMAN Isoform 2 of Atlastin-2 OS=Homo s	0.00011348
Q8NHH9;Q8NHH9-3;Q8NH	ATLA2_HUMAN Atlastin-2 OS=Homo sapiens GN=ATL2 PE=1 SV=2;>sp|Q8NHH9-3|ATLA2_HUMAN Isoform 3 of Atlastin-2 OS=Homo s	2.68E-19
Q8NHP6;Q8NHP6-2	MSPD2_HUMAN Motile sperm domain-containing protein 2 OS=Homo sapiens GN=MOSPD2 PE=1 SV=1;>sp|Q8NHP6-2|MSPD2_HUM/	6.21E-08
Q8NHQ9;B4DVE4	DDX55_HUMAN ATP-dependent RNA helicase DDX55 OS=Homo sapiens GN=DDX55 PE=1 SV=3;>tr|B4DVE4|B4DVE4_HUMAN cDNA F	2.26E-12
Q8TAE8;Q7LAX7	G45IP_HUMAN Growth arrest and DNA damage-inducible proteins-interacting protein 1 OS=Homo sapiens GN=GADD45GIP1 PE=1 SV	3.25E-21
Q8TAQ2;Q8TAQ2-2	SMRC2_HUMAN SWI/SNF complex subunit SMARCC2 OS=Homo sapiens GN=SMARCC2 PE=1 SV=1;>sp|Q8TAQ2-2|SMRC2_HUMAN Is	6.31E-208
Q8TAT6-2;Q8TAT6;B4DG8	2|NPL4_HUMAN Isoform 2 of Nuclear protein localization protein 4 homolog OS=Homo sapiens GN=NPLOC4;>sp|Q8TAT6|NPL4_HUM/	1.10E-117
Q8TBX8;B4DY44;B4DM11	PI42C_HUMAN Phosphatidylinositol-5-phosphate 4-kinase type-2 gamma OS=Homo sapiens GN=PIP4K2C PE=1 SV=3;>tr|B4DY44|B4	1.34E-08
Q8TC07-2;Q8TC07;B4DM	2|TBC15_HUMAN Isoform 2 of TBC1 domain family member 15 OS=Homo sapiens GN=TBC1D15;>sp|Q8TC07|TBC15_HUMAN TBC1 d	1.35E-11
Q8TC12;Q8TC12-2;B4DD	RDH11_HUMAN Retinol dehydrogenase 11 OS=Homo sapiens GN=RDH11 PE=1 SV=2;>sp|Q8TC12-2|RDH11_HUMAN Isoform 2 of Re	1.48E-46
Q8TCC3-2;Q8TCC3;Q8TC	2|RM30_HUMAN Isoform 2 of 39S ribosomal protein L30, mitochondrial OS=Homo sapiens GN=MRPL30;>sp|Q8TCC3|RM30_HUMAN 3	9.48E-11
Q8TCT9;Q8TCT9-5;Q8TCT	HM13_HUMAN Minor histocompatibility antigen H13 OS=Homo sapiens GN=HM13 PE=1 SV=1;>sp|Q8TCT9-5|HM13_HUMAN Isoform	1.54E-39

Q8TD06;B5MC62	AGR3_HUMAN Anterior gradient protein 3 homolog OS=Homo sapiens GN=AGR3 PE=1 SV=1;>tr|B5MC62|B5MC62_HUMAN Uncharact	1.89E-158
Q8TD30;Q8TD30-2	ALAT2_HUMAN Alanine aminotransferase 2 OS=Homo sapiens GN=GPT2 PE=1 SV=1;>sp|Q8TD30-2|ALAT2_HUMAN Isoform 2 of Alan	2.28E-23
Q8TD55;Q8TD55-2;B3KTV	PKHO2_HUMAN Pleckstrin homology domain-containing family O member 2 OS=Homo sapiens GN=PLEKHO2 PE=1 SV=1;>sp|Q8TD5	7.44E-11
Q8TDN6;A0JLQ5;B4E0B8	BRX1_HUMAN Ribosome biogenesis protein BRX1 homolog OS=Homo sapiens GN=BRIX1 PE=1 SV=2;>tr|A0JLQ5|A0JLQ5_HUMAN BX	5.88E-100
Q8TDP1;Q8TDP1-2;B4DM	RNH2C_HUMAN Ribonuclease H2 subunit C OS=Homo sapiens GN=RNASEH2C PE=1 SV=1;>sp|Q8TDP1-2|RNH2C_HUMAN Isoform 2	3.37E-05
Q8TDQ7;Q8TDQ7-3;Q8TD	GNPI2_HUMAN Glucosamine-6-phosphate isomerase 2 OS=Homo sapiens GN=GNPDA2 PE=1 SV=1;>sp|Q8TDQ7-3|GNPI2_HUMAN Is	4.10E-276
Q8TDX7;Q8TDX7-2	NEK7_HUMAN Serine/threonine-protein kinase Nek7 OS=Homo sapiens GN=NEK7 PE=1 SV=1;>sp|Q8TDX7-2|NEK7_HUMAN Isoform	5.98E-11
Q8TED1;B4DPY0	GPX8_HUMAN Probable glutathione peroxidase 8 OS=Homo sapiens GN=GPX8 PE=1 SV=2;>tr|B4DPY0|B4DPY0_HUMAN Glutathione	3.30E-10
Q8TEQ6;B7ZLC9;Q58EZ8	GEMI5_HUMAN Gem-associated protein 5 OS=Homo sapiens GN=GEMIN5 PE=1 SV=3;>tr|B7ZLC9|B7ZLC9_HUMAN GEMIN5 protein	2.23E-30
Q8WTS6;D6RJA0	SETD7_HUMAN Histone-lysine N-methyltransferase SETD7 OS=Homo sapiens GN=SETD7 PE=1 SV=1;>tr|D6RJA0|D6RJA0_HUMAN U	1.30E-12
Q8WTW3;Q4G0L8;B3KUY	COG1_HUMAN Conserved oligomeric Golgi complex subunit 1 OS=Homo sapiens GN=COG1 PE=1 SV=1;>tr|Q4G0L8|Q4G0L8_HUMAN	4.89E-25
Q8WUA2;Q6P392;Q32Q8	PPIL4_HUMAN Peptidyl-prolyl cis-trans isomerase-like 4 OS=Homo sapiens GN=PPIL4 PE=1 SV=1;>tr|Q6P392|Q6P392_HUMAN PPIL4	7.28E-07
Q8WUF5;Q6ZNZ8	IASPP_HUMAN RelA-associated inhibitor OS=Homo sapiens GN=PPP1R13L PE=1 SV=4;>tr|Q6ZNZ8|Q6ZNZ8_HUMAN cDNA FLJ26816	9.81E-23
Q8WUH6;B3KMF8	CL023_HUMAN UPF0444 transmembrane protein C12orf23 OS=Homo sapiens GN=C12orf23 PE=1 SV=1;>tr|B3KMF8|B3KMF8_HUMA	1.27E-54
Q8WUM0;B4E206;B4E3T7	NU133_HUMAN Nuclear pore complex protein Nup133 OS=Homo sapiens GN=NUP133 PE=1 SV=2;>tr|B4E206|B4E206_HUMAN cDNA	1.89E-19
Q8WV24;A2BDE7;B4E2D4	PHLA1_HUMAN Pleckstrin homology-like domain family A member 1 OS=Homo sapiens GN=PHLDA1 PE=1 SV=4;>tr|A2BDE7|A2BDE7	6.67E-08
Q8WV92;B8ZZL5	MITD1_HUMAN MIT domain-containing protein 1 OS=Homo sapiens GN=MITD1 PE=1 SV=1;>tr|B8ZZL5|B8ZZL5_HUMAN Uncharacter	1.68E-08
Q8WVC0;Q8WVC0-2	LEO1_HUMAN RNA polymerase-associated protein LEO1 OS=Homo sapiens GN=LEO1 PE=1 SV=1;>sp|Q8WVC0-2|LEO1_HUMAN Isof	2.43E-06
Q8WVQ1;Q8WVQ1-2	CANT1_HUMAN Soluble calcium-activated nucleotidase 1 OS=Homo sapiens GN=CANT1 PE=1 SV=1;>sp|Q8WVQ1-2|CANT1_HUMAN I	1.88E-05
Q8WW59;B4DUC9	SPRY4_HUMAN SPRY domain-containing protein 4 OS=Homo sapiens GN=SPRYD4 PE=1 SV=2;>tr|B4DUC9|B4DUC9_HUMAN cDNA FL	2.77E-16
Q8WWM7-3;Q8WWM7;Q8	3|ATX2L_HUMAN Isoform 3 of Ataxin-2-like protein OS=Homo sapiens GN=ATXN2L;>sp|Q8WWM7|ATX2L_HUMAN Ataxin-2-like protei	4.81E-46
Q8WWY3;Q8WWY3-2	PRP31_HUMAN U4/U6 small nuclear ribonucleoprotein Prp31 OS=Homo sapiens GN=PRPF31 PE=1 SV=2;>sp|Q8WWY3-2|PRP31_HUM	2.72E-56
Q8WWY3;Q8WWY3-2;Q8V	PRP31_HUMAN U4/U6 small nuclear ribonucleoprotein Prp31 OS=Homo sapiens GN=PRPF31 PE=1 SV=2;>sp|Q8WWY3-2|PRP31_HUM	4.34E-56
Q8WX93;Q8WX93-5;Q8W	PALLD_HUMAN Palladin OS=Homo sapiens GN=PALLD PE=1 SV=3;>sp|Q8WX93-5|PALLD_HUMAN Isoform 5 of Palladin OS=Homo sa	3.82E-61
Q8WXF1;Q8WXF1-2	PSPC1_HUMAN Paraspeckle component 1 OS=Homo sapiens GN=PSPC1 PE=1 SV=1;>sp|Q8WXF1-2|PSPC1_HUMAN Isoform 2 of Para	5.80E-71
Q8WXH0-2;Q8WXH0;D4Y	2|SYNE2_HUMAN Isoform 2 of Nesprin-2 OS=Homo sapiens GN=SYNE2;>sp|Q8WXH0|SYNE2_HUMAN Nesprin-2 OS=Homo sapiens G	3.64E-10
Q8WXI9;Q86YP4;Q86YP4-	P66B_HUMAN Transcriptional repressor p66-beta OS=Homo sapiens GN=GATAD2B PE=1 SV=1;>sp|Q86YP4|P66A_HUMAN Transcripti	4.96E-42
Q92520;C9JMN4;C9JP35	FAM3C_HUMAN Protein FAM3C OS=Homo sapiens GN=FAM3C PE=1 SV=1;>tr|C9JMN4|C9JMN4_HUMAN Uncharacterized protein OS=	3.55E-178
Q92538;Q149P0;Q149P1	GBF1_HUMAN Golgi-specific brefeldin A-resistance guanine nucleotide exchange factor 1 OS=Homo sapiens GN=GBF1 PE=1 SV=2;>tr	8.37E-24
Q92544;B4DKC1;B4DH88	TM9S4_HUMAN Transmembrane 9 superfamily member 4 OS=Homo sapiens GN=TM9SF4 PE=1 SV=2;>tr|B4DKC1|B4DKC1_HUMAN	1.76E-08
Q92575;B4E240;B3KTD5	UBXN4_HUMAN UBX domain-containing protein 4 OS=Homo sapiens GN=UBXN4 PE=1 SV=2;>tr|B4E240|B4E240_HUMAN cDNA FLJ5	3.66E-16
Q92598;Q92598-3;Q9259	HS105_HUMAN Heat shock protein 105 kDa OS=Homo sapiens GN=HSPH1 PE=1 SV=1;>sp|Q92598-3|HS105_HUMAN Isoform 3 of H	4.31E-288
Q92599;Q92599-2;Q9259	SEPT8_HUMAN Septin-8 OS=Homo sapiens GN=SEPT8 PE=1 SV=4;>sp|Q92599-2|SEPT8_HUMAN Isoform 2 of Septin-8 OS=Homo sa	7.30E-14
Q92621;B4DE72;Q6P486;	NU205_HUMAN Nuclear pore complex protein Nup205 OS=Homo sapiens GN=NUP205 PE=1 SV=3;>tr|B4DE72|B4DE72_HUMAN cDN	1.46E-41
Q92643;A6NEM5;B4E2M3	GPI8_HUMAN GPI-anchor transamidase OS=Homo sapiens GN=PIGK PE=1 SV=2;>tr|A6NEM5|A6NEM5_HUMAN Uncharacterized prote	3.90E-26
Q92688;Q92688-2	AN32B_HUMAN Acidic leucine-rich nuclear phosphoprotein 32 family member B OS=Homo sapiens GN=ANP32B PE=1 SV=1;>sp|Q926	4.23E-114
Q92692;Q92692-2	PVRL2_HUMAN Poliovirus receptor-related protein 2 OS=Homo sapiens GN=PVRL2 PE=1 SV=1;>sp|Q92692-2|PVRL2_HUMAN Isoform	7.56E-30
Q92879-4;Q92879;Q9287	4|CELF1_HUMAN Isoform 4 of CUGBP Elav-like family member 1 OS=Homo sapiens GN=CELF1;>sp|Q92879|CELF1_HUMAN CUGBP El	5.30E-25
Q92896-2;Q92896	2|GSLG1_HUMAN Isoform 2 of Golgi apparatus protein 1 OS=Homo sapiens GN=GLG1;>sp|Q92896|GSLG1_HUMAN Golgi apparatus p	2.19E-29
Q92905;Q59GH5	CSN5_HUMAN COP9 signalosome complex subunit 5 OS=Homo sapiens GN=COPS5 PE=1 SV=4;>tr|Q59GH5|Q59GH5_HUMAN COP9	1.50E-67
Q92925;Q96GM5;Q92925	SMRD2_HUMAN SWI/SNF-related matrix-associated actin-dependent regulator of chromatin subfamily D member 2 OS=Homo sapiens	3.73E-06
Q92930;B4DMS1	RAB8B_HUMAN Ras-related protein Rab-8B OS=Homo sapiens GN=RAB8B PE=1 SV=2;>tr|B4DMS1|B4DMS1_HUMAN cDNA FLJ52970	3.35E-33
Q92947;B4DK85;Q92947-	GCDH_HUMAN Glutaryl-CoA dehydrogenase, mitochondrial OS=Homo sapiens GN=GCDH PE=1 SV=1;>tr|B4DK85|B4DK85_HUMAN c	3.76E-11
Q92947;Q92947-2	GCDH_HUMAN Glutaryl-CoA dehydrogenase, mitochondrial OS=Homo sapiens GN=GCDH PE=1 SV=1;>sp|Q92947-2|GCDH_HUMAN I	1.51E-49
Q93008;Q93008-1;O0050	USP9X_HUMAN Probable ubiquitin carboxyl-terminal hydrolase FAF-X OS=Homo sapiens GN=USP9X PE=1 SV=3;>sp|Q93008-1|USP9	6.97E-10
Q93009;Q6U8A4;B7Z815;	UBP7_HUMAN Ubiquitin carboxyl-terminal hydrolase 7 OS=Homo sapiens GN=USP7 PE=1 SV=2;>tr|Q6U8A4|Q6U8A4_HUMAN Ubiqui	1.94E-72
Q93045;B7Z4N6	STMN2_HUMAN Stathmin-2 OS=Homo sapiens GN=STMN2 PE=1 SV=3;>tr|B7Z4N6|B7Z4N6_HUMAN Stathmin OS=Homo sapiens PE	2.90E-28
Q969G3;Q969G3-2	SMCE1_HUMAN SWI/SNF-related matrix-associated actin-dependent regulator of chromatin subfamily E member 1 OS=Homo sapiens	2.08E-12
Q969Q5;Q8WY67;A8MSV	RAB24_HUMAN Ras-related protein Rab-24 OS=Homo sapiens GN=RAB24 PE=1 SV=1;>tr|Q8WY67|Q8WY67_HUMAN PP4748 OS=Ho	1.86E-17
Q969T9;B4DDJ2;Q7Z511;	WBP2_HUMAN WW domain-binding protein 2 OS=Homo sapiens GN=WBP2 PE=1 SV=1;>tr|B4DDJ2|B4DDJ2_HUMAN cDNA FLJ59459	5.27E-13
Q969U7;Q9P1R6;Q9NRV1	PSMG2_HUMAN Proteasome assembly chaperone 2 OS=Homo sapiens GN=PSMG2 PE=1 SV=1;>tr|Q9P1R6|Q9P1R6_HUMAN HDCMC	1.62E-10
Q969X5;Q969X5-2	ERGI1_HUMAN Endoplasmic reticulum-Golgi intermediate compartment protein 1 OS=Homo sapiens GN=ERGIC1 PE=1 SV=1;>sp|Q9	3.97E-27
Q969X6;Q969X6-3;Q969X	CIR1A_HUMAN Cirhin OS=Homo sapiens GN=CIRH1A PE=1 SV=1;>sp|Q969X6-3|CIR1A_HUMAN Isoform 3 of Cirhin OS=Homo sapie	1.82E-10
Q96A26;C9JYF2;B4DF97	F162A_HUMAN Protein FAM162A OS=Homo sapiens GN=FAM162A PE=1 SV=2;>tr|C9JYF2|C9JYF2_HUMAN Uncharacterized protein O	5.32E-39
Q96A72;P61326	MGN2_HUMAN Protein mago nashi homolog 2 OS=Homo sapiens GN=MAGOHB PE=1 SV=1;>sp|P61326|MGN_HUMAN Protein mago	4.48E-43
Q96A72;P61326;A6NEC0;	MGN2_HUMAN Protein mago nashi homolog 2 OS=Homo sapiens GN=MAGOHB PE=1 SV=1;>sp|P61326|MGN_HUMAN Protein mago	6.33E-51
Q96AY3;Q658U4;Q6ZME7	FKB10_HUMAN Peptidyl-prolyl cis-trans isomerase FKBP10 OS=Homo sapiens GN=FKBP10 PE=1 SV=1;>tr|Q658U4|Q658U4_HUMAN	4.76E-29
Q96AZ6;Q96AZ6-2	ISG20_HUMAN Interferon-stimulated gene 20 kDa protein OS=Homo sapiens GN=ISG20 PE=1 SV=2;>sp|Q96AZ6-2|ISG20_HUMAN I	9.85E-11
Q96BJ3;Q96BJ3-3	AIDA_HUMAN Axin interactor, dorsalization-associated protein OS=Homo sapiens GN=AIDA PE=1 SV=1;>sp|Q96BJ3-3|AIDA_HUMAN	2.97E-13
Q96BM9;B2R7U5	ARL8A_HUMAN ADP-ribosylation factor-like protein 8A OS=Homo sapiens GN=ARL8A PE=1 SV=1;>tr|B2R7U5|B2R7U5_HUMAN cDNA,	1.71E-16
Q96BP3;B4DT22;B4DP34;	PPWD1_HUMAN Peptidylprolyl isomerase domain and WD repeat-containing protein 1 OS=Homo sapiens GN=PPWD1 PE=1 SV=1;>tr|	3.06E-12

Q96BX8;Q6AI16	MOL2A_HUMAN Mps one binder kinase activator-like 2A OS=Homo sapiens GN=MOBKL2A PE=1 SV=1;>tr|Q6AI16|Q6AI16_HUMAN Pu	3.84E-06
Q96BY6;Q96BY6-2	DOC10_HUMAN Dedicator of cytokinesis protein 10 OS=Homo sapiens GN=DOCK10 PE=1 SV=3;>sp|Q96BY6-2|DOC10_HUMAN Isofo	4.30E-18
Q96C01;B0AZT6;C9J2Y4	F136A_HUMAN Protein FAM136A OS=Homo sapiens GN=FAM136A PE=1 SV=1;>tr|B0AZT6|B0AZT6_HUMAN cDNA, FLJ79526 OS=Hor	5.96E-09
Q96C19;Q5JYW8;C9JTI6	EFHD2_HUMAN EF-hand domain-containing protein D2 OS=Homo sapiens GN=EFHD2 PE=1 SV=1;>tr|Q5JYW8|Q5JYW8_HUMAN EF-h	4.54E-40
Q96C19;Q9BUP0	EFHD2_HUMAN EF-hand domain-containing protein D2 OS=Homo sapiens GN=EFHD2 PE=1 SV=1;>sp|Q9BUP0|EFHD1_HUMAN EF-ha	3.04E-08
Q96C36;Q4W8W1;B3KMB	P5CR2_HUMAN Pyrroline-5-carboxylate reductase 2 OS=Homo sapiens GN=PYCR2 PE=1 SV=1;>tr|Q4W8W1|Q4W8W1_HUMAN Pyrrol	6.65E-67
Q96C86;Q53G42	DCPS_HUMAN Scavenger mRNA-decapping enzyme DcpS OS=Homo sapiens GN=DCPS PE=1 SV=2;>tr|Q53G42|Q53G42_HUMAN mR	1.92E-105
Q96CG8;Q96CG8-2	CTHR1_HUMAN Collagen triple helix repeat-containing protein 1 OS=Homo sapiens GN=CTHRC1 PE=1 SV=1;>sp|Q96CG8-2|CTHR1_H	3.84E-33
Q96CN7;D6RE73;D6RGE2	ISOC1_HUMAN Isochorismatase domain-containing protein 1 OS=Homo sapiens GN=ISOC1 PE=1 SV=3;>tr|D6RE73|D6RE73_HUMAN	5.15E-45
Q96CS2;Q96CS2-2	HAUS1_HUMAN HAUS augmin-like complex subunit 1 OS=Homo sapiens GN=HAUS1 PE=1 SV=1;>sp|Q96CS2-2|HAUS1_HUMAN Isofo	5.86E-08
Q96CS3;B4E2M8	FAF2_HUMAN FAS-associated factor 2 OS=Homo sapiens GN=FAF2 PE=1 SV=2;>tr|B4E2M8|B4E2M8_HUMAN cDNA FLJ61076, highly	1.68E-19
Q96CW1;Q96CW1-2	AP2M1_HUMAN AP-2 complex subunit mu OS=Homo sapiens GN=AP2M1 PE=1 SV=2;>sp|Q96CW1-2|AP2M1_HUMAN Isoform 2 of AP	3.62E-64
Q96DA0;C3PTT6	ZG16B_HUMAN Zymogen granule protein 16 homolog B OS=Homo sapiens GN=ZG16B PE=1 SV=3;>tr|C3PTT6|C3PTT6_HUMAN Panc	1.19E-09
Q96DB5;B4DZW6;B4DNF5	RMD1_HUMAN Regulator of microtubule dynamics protein 1 OS=Homo sapiens GN=FAM82B PE=1 SV=1;>tr|B4DZW6|B4DZW6_HUMA	2.23E-60
Q96DC8;Q96DC8-2	ECHD3_HUMAN Enoyl-CoA hydratase domain-containing protein 3, mitochondrial OS=Homo sapiens GN=ECHDC3 PE=1 SV=2;>sp|Q9	1.79E-08
Q96DH6;Q96DH6-2	MSI2H_HUMAN RNA-binding protein Musashi homolog 2 OS=Homo sapiens GN=MSI2 PE=1 SV=1;>sp|Q96DH6-2|MSI2H_HUMAN Isof	1.11E-16
Q96DH6;Q96DH6-2;Q96D	MSI2H_HUMAN RNA-binding protein Musashi homolog 2 OS=Homo sapiens GN=MSI2 PE=1 SV=1;>sp|Q96DH6-2|MSI2H_HUMAN Isof	2.75E-101
Q96DV4;B2R894;B3KN96	RM38_HUMAN 39S ribosomal protein L38, mitochondrial OS=Homo sapiens GN=MRPL38 PE=1 SV=2;>tr|B2R894|B2R894_HUMAN Mit	4.12E-07
Q96E11;Q96E11-3;Q96E1	RRFM_HUMAN Ribosome-recycling factor, mitochondrial OS=Homo sapiens GN=MRRF PE=1 SV=1;>sp|Q96E11-3|RRFM_HUMAN Isofo	8.34E-43
Q96EB1-2;Q96EB1	2|ELP4_HUMAN Isoform 2 of Elongator complex protein 4 OS=Homo sapiens GN=ELP4;>sp|Q96EB1|ELP4_HUMAN Elongator complex	4.84E-10
Q96EL3;B9A051	RM53_HUMAN 39S ribosomal protein L53, mitochondrial OS=Homo sapiens GN=MRPL53 PE=1 SV=1;>tr|B9A051|B9A051_HUMAN Un	6.48E-07
Q96EP5;Q96EP5-2;Q5IRN	DAZP1_HUMAN DAZ-associated protein 1 OS=Homo sapiens GN=DAZAP1 PE=1 SV=1;>sp|Q96EP5-2|DAZP1_HUMAN Isoform 2 of DA	7.55E-37
Q96EQ0;D6RFW1	SGTB_HUMAN Small glutamine-rich tetratricopeptide repeat-containing protein beta OS=Homo sapiens GN=SGTB PE=1 SV=1;>tr|D6I	9.03E-09
Q96ER3;B3KVU5	SAAL1_HUMAN Protein SAAL1 OS=Homo sapiens GN=SAAL1 PE=1 SV=2;>tr|B3KVU5|B3KVU5_HUMAN cDNA FLJ41463 fis, clone BRS	5.01E-15
Q96EY4;D6RA57;B7Z504	CD043_HUMAN UPF0534 protein C4orf43 OS=Homo sapiens GN=C4orf43 PE=1 SV=2;>tr|D6RA57|D6RA57_HUMAN Uncharacterized	5.55E-13
Q96EY7;Q96EY7-2	PTCD3_HUMAN Pentatricopeptide repeat-containing protein 3, mitochondrial OS=Homo sapiens GN=PTCD3 PE=1 SV=3;>sp|Q96EY7-2	2.21E-13
Q96FJ0-2;Q96FJ0	2|STALP_HUMAN Isoform 2 of AMSH-like protease OS=Homo sapiens GN=STAMBPL1;>sp|Q96FJ0|STALP_HUMAN AMSH-like protease	3.10E-62
Q96FN4;Q9UBL6;Q9HCH3	CPNE2_HUMAN Copine-2 OS=Homo sapiens GN=CPNE2 PE=1 SV=3;>sp|Q9UBL6|CPNE7_HUMAN Copine-7 OS=Homo sapiens GN=CF	1.11E-21
Q96FV2;Q9BU04;B7Z8S7	SCRN2_HUMAN Secernin-2 OS=Homo sapiens GN=SCRN2 PE=2 SV=3;>tr|Q9BU04|Q9BU04_HUMAN Ses2 protein (Fragment) OS=Hc	1.17E-19
Q96G03;B4DN40	PGM2_HUMAN Phosphoglucomutase-2 OS=Homo sapiens GN=PGM2 PE=1 SV=4;>tr|B4DN40|B4DN40_HUMAN cDNA FLJ54368, highly	8.77E-222
Q96GA3;B4DTT8	LTV1_HUMAN Protein LTV1 homolog OS=Homo sapiens GN=LTV1 PE=1 SV=1;>tr|B4DTT8|B4DTT8_HUMAN cDNA FLJ61396 OS=Homo	2.98E-51
Q96GC5;B4DN34	RM48_HUMAN 39S ribosomal protein L48, mitochondrial OS=Homo sapiens GN=MRPL48 PE=1 SV=2;>tr|B4DN34|B4DN34_HUMAN cD	7.72E-76
Q96GG9;C9JVE2;B4DM76	DCNL1_HUMAN DCN1-like protein 1 OS=Homo sapiens GN=DCUN1D1 PE=1 SV=1;>tr|C9JVE2|C9JVE2_HUMAN DCN1, defective in cul	1.59E-39
Q96GM8;B4DP23	TOE1_HUMAN Target of EGR1 protein 1 OS=Homo sapiens GN=TOE1 PE=1 SV=1;>tr|B4DP23|B4DP23_HUMAN cDNA FLJ57546, highl	2.02E-05
Q96GQ7;B3GQE6;B7Z6D5	DDX27_HUMAN Probable ATP-dependent RNA helicase DDX27 OS=Homo sapiens GN=DDX27 PE=1 SV=2;>tr|B3GQE6|B3GQE6_HUMA	1.12E-07
Q96H20;Q96H20-2;D6RFY	SNF8_HUMAN Vacuolar-sorting protein SNF8 OS=Homo sapiens GN=SNF8 PE=1 SV=1;>sp|Q96H20-2|SNF8_HUMAN Isoform 2 of Vac	2.01E-10
Q96HF1;B3KSM5	SFRP2_HUMAN Secreted frizzled-related protein 2 OS=Homo sapiens GN=SFRP2 PE=1 SV=2;>tr|B3KSM5|B3KSM5_HUMAN cDNA FLJ	8.18E-08
Q96I99;Q3ZCW5;C9JVT2;	SUCB2_HUMAN Succinyl-CoA ligase [GDP-forming] subunit beta, mitochondrial OS=Homo sapiens GN=SUCLG2 PE=1 SV=2;>tr|Q3ZC	6.65E-199
Q96J01;Q6NZ53;D6RGZ2	THOC3_HUMAN THO complex subunit 3 OS=Homo sapiens GN=THOC3 PE=1 SV=1;>tr|Q6NZ53|Q6NZ53_HUMAN THOC3 protein OS=	2.51E-18
Q96JB2;Q96JB2-2;B4DH7	COG3_HUMAN Conserved oligomeric Golgi complex subunit 3 OS=Homo sapiens GN=COG3 PE=1 SV=3;>sp|Q96JB2-2|COG3_HUMAN	2.87E-79
Q96JB5;Q96JB5-2;Q96JB	CK5P3_HUMAN CDK5 regulatory subunit-associated protein 3 OS=Homo sapiens GN=CDK5RAP3 PE=1 SV=2;>sp|Q96JB5-2|CK5P3_H	1.91E-07
Q96JH7;B4DM84	VCIP1_HUMAN Deubiquitinating protein VCIP135 OS=Homo sapiens GN=VCPIP1 PE=1 SV=2;>tr|B4DM84|B4DM84_HUMAN cDNA FLJ	2.40E-32
Q96JJ7;Q96JJ7-2	TMX3_HUMAN Protein disulfide-isomerase TMX3 OS=Homo sapiens GN=TMX3 PE=1 SV=2;>sp|Q96JJ7-2|TMX3_HUMAN Isoform 2 of I	7.58E-41
Q96JY6-3;Q96JY6;Q96JY6	3|PDLI2_HUMAN Isoform 3 of PDZ and LIM domain protein 2 OS=Homo sapiens GN=PDLIM2;>sp|Q96JY6|PDLI2_HUMAN PDZ and LIM	5.21E-15
Q96K76;Q96K76-2	UBP47_HUMAN Ubiquitin carboxyl-terminal hydrolase 47 OS=Homo sapiens GN=USP47 PE=1 SV=3;>sp|Q96K76-2|UBP47_HUMAN Is	4.00E-15
Q96KC8;Q96NY3;A4VCI1	DNJC1_HUMAN DnaJ homolog subfamily C member 1 OS=Homo sapiens GN=DNAJC1 PE=1 SV=1;>tr|Q96NY3|Q96NY3_HUMAN DnaJ	7.93E-11
Q96KP4;Q96KP4-2	CNDP2_HUMAN Cytosolic non-specific dipeptidase OS=Homo sapiens GN=CNDP2 PE=1 SV=2;>sp|Q96KP4-2|CNDP2_HUMAN Isoform	1.74E-158
Q96N66;Q96N66-2;Q96N	MBOA7_HUMAN Lysophospholipid acyltransferase 7 OS=Homo sapiens GN=MBOAT7 PE=1 SV=2;>sp|Q96N66-2|MBOA7_HUMAN Isofc	4.93E-65
Q96NY7;Q96NY7-2;Q9NZ	CLIC6_HUMAN Chloride intracellular channel protein 6 OS=Homo sapiens GN=CLIC6 PE=2 SV=3;>sp|Q96NY7-2|CLIC6_HUMAN Isofor	1.02E-16
Q96PD5-2;Q96PD5	2|PGRP2_HUMAN Isoform 2 of N-acetylmuramoyl-L-alanine amidase OS=Homo sapiens GN=PGLYRP2;>sp|Q96PD5|PGRP2_HUMAN N-	8.07E-165
Q96PK6;Q96PK6-2;B0LM4	RBM14_HUMAN RNA-binding protein 14 OS=Homo sapiens GN=RBM14 PE=1 SV=2;>sp|Q96PK6-2|RBM14_HUMAN Isoform 2 of RNA-	5.29E-106
Q96RQ3;Q68D27;B4DH16	MCCA_HUMAN Methylcrotonoyl-CoA carboxylase subunit alpha, mitochondrial OS=Homo sapiens GN=MCCC1 PE=1 SV=3;>tr|Q68D27	5.06E-53
Q96RU3;Q96RU3-2;Q96RU	FNBP1_HUMAN Formin-binding protein 1 OS=Homo sapiens GN=FNBP1 PE=1 SV=2;>sp|Q96RU3-2|FNBP1_HUMAN Isoform 2 of Form	5.97E-129
Q96S55;Q96S55-2;Q96S5	WRIP1_HUMAN ATPase WRNIP1 OS=Homo sapiens GN=WRNIP1 PE=1 SV=2;>sp|Q96S55-2|WRIP1_HUMAN Isoform 2 of ATPase WRN	5.08E-24
Q96S55;Q96S55-2;Q96S5	WRIP1_HUMAN ATPase WRNIP1 OS=Homo sapiens GN=WRNIP1 PE=1 SV=2;>sp|Q96S55-2|WRIP1_HUMAN Isoform 2 of ATPase WRN	3.63E-29
Q96S59;Q96S59-2	RANB9_HUMAN Ran-binding protein 9 OS=Homo sapiens GN=RANBP9 PE=1 SV=1;>sp|Q96S59-2|RANB9_HUMAN Isoform 2 of Ran-b	3.72E-87
Q96S97;B4DG42;C9JC07;	MYADM_HUMAN Myeloid-associated differentiation marker OS=Homo sapiens GN=MYADM PE=1 SV=2;>tr|B4DG42|B4DG42_HUMAN c	5.53E-23
Q96ST2;Q96ST2-3;Q96ST	IWS1_HUMAN Protein IWS1 homolog OS=Homo sapiens GN=IWS1 PE=1 SV=2;>sp|Q96ST2-3|IWS1_HUMAN Isoform 3 of Protein IW	4.49E-20
Q96T37;Q96T37-3;Q96T3	RBM15_HUMAN Putative RNA-binding protein 15 OS=Homo sapiens GN=RBM15 PE=1 SV=2;>sp|Q96T37-3|RBM15_HUMAN Isoform 3	9.13E-10
Q96T51;Q96T51-2;Q96T5	RUFY1_HUMAN RUN and FYVE domain-containing protein 1 OS=Homo sapiens GN=RUFY1 PE=1 SV=2;>sp|Q96T51-2|RUFY1_HUMAN	3.82E-53
Q96T60;Q9BUL2	PNKP_HUMAN Bifunctional polynucleotide phosphatase/kinase OS=Homo sapiens GN=PNKP PE=1 SV=1;>tr|Q9BUL2|Q9BUL2_HUMAN	3.57E-24

Q96TC7;B3KUI4;Q96TC7-	RMD3_HUMAN Regulator of microtubule dynamics protein 3 OS=Homo sapiens GN=FAM82A2 PE=1 SV=2;>tr|B3KUI4|B3KUI4_HUMAN	1.44E-16
Q99426;Q6FGY5;A8MVD5	TBCB_HUMAN Tubulin-folding cofactor B OS=Homo sapiens GN=TBCB PE=1 SV=2;>tr|Q6FGY5|Q6FGY5_HUMAN CKAP1 protein OS=H	1.18E-24
Q99447;B7Z4W6;B7ZAS0	PCY2_HUMAN Ethanolamine-phosphate cytidylyltransferase OS=Homo sapiens GN=PCYT2 PE=1 SV=1;>tr|B7Z4W6|B7Z4W6_HUMAN	4.82E-57
Q99471;Q9C083	PFD5_HUMAN Prefoldin subunit 5 OS=Homo sapiens GN=PFDN5 PE=1 SV=2;>tr|Q9C083|Q9C083_HUMAN MM-1 gamma OS=Homo s	1.14E-172
Q99536;B4DPX4;B3KUF8;	VAT1_HUMAN Synaptic vesicle membrane protein VAT-1 homolog OS=Homo sapiens GN=VAT1 PE=1 SV=2;>tr|B4DPX4|B4DPX4_HUM	1.12E-233
Q99543;Q99543-2	DNJC2_HUMAN DnaJ homolog subfamily C member 2 OS=Homo sapiens GN=DNAJC2 PE=1 SV=4;>sp|Q99543-2|DNJC2_HUMAN Isof	2.06E-16
Q99584;D3DV53	S10AD_HUMAN Protein S100-A13 OS=Homo sapiens GN=S100A13 PE=1 SV=1;>tr|D3DV53|D3DV53_HUMAN S100 calcium binding p	2.92E-21
Q99615;Q59EH7;A8K4T2;	DNJC7_HUMAN DnaJ homolog subfamily C member 7 OS=Homo sapiens GN=DNAJC7 PE=1 SV=2;>tr|Q59EH7|Q59EH7_HUMAN DnaJ	9.04E-176
Q99623;B4DP75;B4DW05	PHB2_HUMAN Prohibitin-2 OS=Homo sapiens GN=PHB2 PE=1 SV=2;>tr|B4DP75|B4DP75_HUMAN cDNA FLJ56579, highly similar to P	2.03E-95
Q99661;Q99661-2;A8K3S	KIF2C_HUMAN Kinesin-like protein KIF2C OS=Homo sapiens GN=KIF2C PE=1 SV=2;>sp|Q99661-2|KIF2C_HUMAN Isoform 2 of Kines	1.33E-93
Q99700;Q99700-4;Q9970	ATX2_HUMAN Ataxin-2 OS=Homo sapiens GN=ATXN2 PE=1 SV=2;>sp|Q99700-4|ATX2_HUMAN Isoform 4 of Ataxin-2 OS=Homo sapi	2.94E-23
Q99715;Q99715-4;Q9971	COCA1_HUMAN Collagen alpha-1(XII) chain OS=Homo sapiens GN=COL12A1 PE=1 SV=2;>sp|Q99715-4|COCA1_HUMAN Isoform 4 o	0
Q99805;B3KSG9	TM9S2_HUMAN Transmembrane 9 superfamily member 2 OS=Homo sapiens GN=TM9SF2 PE=1 SV=1;>tr|B3KSG9|B3KSG9_HUMAN c	8.47E-18
Q99807;Q99807-2	COQ7_HUMAN Ubiquinone biosynthesis protein COQ7 homolog OS=Homo sapiens GN=COQ7 PE=1 SV=3;>sp|Q99807-2|COQ7_HUMA	1.53E-06
Q99816;Q99816-2;D3DQ\	TS101_HUMAN Tumor susceptibility gene 101 protein OS=Homo sapiens GN=TSG101 PE=1 SV=2;>sp|Q99816-2|TS101_HUMAN Isofo	4.85E-21
Q99873;Q99873-2;Q9987	ANM1_HUMAN Protein arginine N-methyltransferase 1 OS=Homo sapiens GN=PRMT1 PE=1 SV=2;>sp|Q99873-2|ANM1_HUMAN Isofor	1.85E-218
Q9BPX3;Q6NUR1	CND3_HUMAN Condensin complex subunit 3 OS=Homo sapiens GN=NCAPG PE=1 SV=1;>tr|Q6NUR1|Q6NUR1_HUMAN Non-SMC conc	1.34E-105
Q9BQ39;B4DW97;B4DED6	DDX50_HUMAN ATP-dependent RNA helicase DDX50 OS=Homo sapiens GN=DDX50 PE=1 SV=1;>tr|B4DW97|B4DW97_HUMAN cDNA	9.83E-68
Q9BQ52;Q9BQ52-2	RNZ2_HUMAN Zinc phosphodiesterase ELAC protein 2 OS=Homo sapiens GN=ELAC2 PE=1 SV=2;>sp|Q9BQ52-2|RNZ2_HUMAN Isofor	2.17E-08
Q9BQ52;Q9BQ52-3	RNZ2_HUMAN Zinc phosphodiesterase ELAC protein 2 OS=Homo sapiens GN=ELAC2 PE=1 SV=2;>sp|Q9BQ52-3|RNZ2_HUMAN Isofor	3.95E-48
Q9BQ52;Q9BQ52-3;Q9BQ	RNZ2_HUMAN Zinc phosphodiesterase ELAC protein 2 OS=Homo sapiens GN=ELAC2 PE=1 SV=2;>sp|Q9BQ52-3|RNZ2_HUMAN Isofor	2.94E-41
Q9BQA1;Q5QNY9;B4DP38	MEP50_HUMAN Methylosome protein 50 OS=Homo sapiens GN=WDR77 PE=1 SV=1;>tr|Q5QNY9|Q5QNY9_HUMAN WD repeat domair	9.42E-14
Q9BQE3;B7Z1K5;Q53GA7	TBA1C_HUMAN Tubulin alpha-1C chain OS=Homo sapiens GN=TUBA1C PE=1 SV=1;>tr|B7Z1K5|B7Z1K5_HUMAN cDNA FLJ55956, hig	0
Q9BQI0-2;Q9BQI0;Q9BQI	2|AIF1L_HUMAN Isoform 2 of Allograft inflammatory factor 1-like OS=Homo sapiens GN=AIF1L;>sp|Q9BQI0|AIF1L_HUMAN Allograft i	9.91E-07
Q9BR76;Q9NSK3	COR1B_HUMAN Coronin-1B OS=Homo sapiens GN=CORO1B PE=1 SV=1;>tr|Q9NSK3|Q9NSK3_HUMAN Putative uncharacterized prot	4.71E-43
Q9BRJ6;C9JQV0	CG050_HUMAN Uncharacterized protein C7orf50 OS=Homo sapiens GN=C7orf50 PE=1 SV=1;>tr|C9JQV0|C9JQV0_HUMAN Uncharacte	3.71E-87
Q9BRQ8;Q9BRQ8-2	AIFM2_HUMAN Apoptosis-inducing factor 2 OS=Homo sapiens GN=AIFM2 PE=1 SV=1;>sp|Q9BRQ8-2|AIFM2_HUMAN Isoform 2 of Ap	3.24E-14
Q9BRT2;Q5TAQ0	CF125_HUMAN Uncharacterized protein C6orf125 OS=Homo sapiens GN=C6orf125 PE=1 SV=1;>tr|Q5TAQ0|Q5TAQ0_HUMAN OTTHUN	4.93E-30
Q9BRX5;Q9BRX5-2	PSF3_HUMAN DNA replication complex GINS protein PSF3 OS=Homo sapiens GN=GINS3 PE=1 SV=1;>sp|Q9BRX5-2|PSF3_HUMAN Is	3.86E-09
Q9BRX8;A6NDF1	CJ058_HUMAN UPF0765 protein C10orf58 OS=Homo sapiens GN=C10orf58 PE=1 SV=3;>tr|A6NDF1|A6NDF1_HUMAN Uncharacterized	4.82E-56
Q9BSH4;Q8N9Q7	TACO1_HUMAN Translational activator of cytochrome c oxidase 1 OS=Homo sapiens GN=TACO1 PE=1 SV=1;>tr|Q8N9Q7|Q8N9Q7_HU	6.33E-13
Q9BTE1;B4DS97	DCTN5_HUMAN Dynactin subunit 5 OS=Homo sapiens GN=DCTN5 PE=1 SV=1;>tr|B4DS97|B4DS97_HUMAN cDNA FLJ57579, highly s	3.06E-06
Q9BTT0;Q53F20;Q5TB20;	AN32E_HUMAN Acidic leucine-rich nuclear phosphoprotein 32 family member E OS=Homo sapiens GN=ANP32E PE=1 SV=1;>tr|Q53F2	6.06E-190
Q9BTV4;Q8TEP9	TMM43_HUMAN Transmembrane protein 43 OS=Homo sapiens GN=TMEM43 PE=1 SV=1;>tr|Q8TEP9|Q8TEP9_HUMAN FLJ00144 prote	8.09E-16
Q9BTZ2;Q9BTZ2-4;P0CG2	DHRS4_HUMAN Dehydrogenase/reductase SDR family member 4 OS=Homo sapiens GN=DHRS4 PE=1 SV=3;>sp|Q9BTZ2-4|DHRS4_I	2.19E-05
Q9BUF5;B4DP54;B3KS31;	TBB6_HUMAN Tubulin beta-6 chain OS=Homo sapiens GN=TUBB6 PE=1 SV=1;>tr|B4DP54|B4DP54_HUMAN cDNA FLJ52712, highly si	0
Q9BUJ2;Q9BUJ2-2;Q9BUJ	HNRL1_HUMAN Heterogeneous nuclear ribonucleoprotein U-like protein 1 OS=Homo sapiens GN=HNRNPUL1 PE=1 SV=2;>sp|Q9BUJ2	3.19E-207
Q9BUL8;C9J5C3;C9J6F3;C	PDC10_HUMAN Programmed cell death protein 10 OS=Homo sapiens GN=PDCD10 PE=1 SV=1;>tr|C9J5C3|C9J5C3_HUMAN Uncharac	2.22E-19
Q9BUP0;B4DEE0;Q53SA2;	EFHD1_HUMAN EF-hand domain-containing protein D1 OS=Homo sapiens GN=EFHD1 PE=1 SV=1;>tr|B4DEE0|B4DEE0_HUMAN cDNA	3.19E-42
Q9BUP3-3;Q9BUP3	3|HTAI2_HUMAN Isoform 3 of Oxidoreductase HTATIP2 OS=Homo sapiens GN=HTATIP2;>sp|Q9BUP3|HTAI2_HUMAN Oxidoreductase I	6.91E-11
Q9BUR5;Q9BUR5-2;C9J57	APOO_HUMAN Apolipoprotein O OS=Homo sapiens GN=APOO PE=1 SV=1;>sp|Q9BUR5-2|APOO_HUMAN Isoform 2 of Apolipoprotein	2.31E-27
Q9BUT1;Q9BUT1-2	BDH2_HUMAN 3-hydroxybutyrate dehydrogenase type 2 OS=Homo sapiens GN=BDH2 PE=1 SV=2;>sp|Q9BUT1-2|BDH2_HUMAN Isof	1.38E-59
Q9BV40;C9JXZ5	VAMP8_HUMAN Vesicle-associated membrane protein 8 OS=Homo sapiens GN=VAMP8 PE=1 SV=1;>tr|C9JXZ5|C9JXZ5_HUMAN Uncha	4.82E-78
Q9BVA1;O43209;B7Z4N1;	TBB2B_HUMAN Tubulin beta-2B chain OS=Homo sapiens GN=TUBB2B PE=1 SV=1;>tr|O43209|O43209_HUMAN Homo sapiens clone	0
Q9BVG4;A6NDF3;Q9P0N4	CX026_HUMAN UPF0368 protein Cxorf26 OS=Homo sapiens GN=CXorf26 PE=1 SV=1;>tr|A6NDF3|A6NDF3_HUMAN Uncharacterized p	1.51E-42
Q9BVK6;B2R8A2;B4DPP4	TMED9_HUMAN Transmembrane emp24 domain-containing protein 9 OS=Homo sapiens GN=TMED9 PE=1 SV=2;>tr|B2R8A2|B2R8A2	1.52E-55
Q9BW19;B4E063;B4DMA8	KIFC1_HUMAN Kinesin-like protein KIFC1 OS=Homo sapiens GN=KIFC1 PE=1 SV=2;>tr|B4E063|B4E063_HUMAN cDNA FLJ54292, hig	1.18E-42
Q9BW27;B4DMQ3;B4DPW	NUP85_HUMAN Nuclear pore complex protein Nup85 OS=Homo sapiens GN=NUP85 PE=1 SV=1;>tr|B4DMQ3|B4DMQ3_HUMAN cDNA	1.14E-42
Q9BWU0;B4DV74	NADAP_HUMAN Kanadaptin OS=Homo sapiens GN=SLC4A1AP PE=1 SV=1;>tr|B4DV74|B4DV74_HUMAN cDNA FLJ53855, highly simil	2.05E-14
Q9BX40;Q9BX40-2;Q5TBF	LS14B_HUMAN Protein LSM14 homolog B OS=Homo sapiens GN=LSM14B PE=1 SV=1;>sp|Q9BX40-2|LS14B_HUMAN Isoform 2 of Pro	3.43E-12
Q9BX40;Q9BX40-2;Q9BX	LS14B_HUMAN Protein LSM14 homolog B OS=Homo sapiens GN=LSM14B PE=1 SV=1;>sp|Q9BX40-2|LS14B_HUMAN Isoform 2 of Pro	1.47E-33
Q9BX68;Q8WYJ5;Q53HJ8	HINT2_HUMAN Histidine triad nucleotide-binding protein 2, mitochondrial OS=Homo sapiens GN=HINT2 PE=1 SV=1;>tr|Q8WYJ5|Q8W	1.66E-38
Q9BXD5;Q9BXD5-2;Q9BX	NPL_HUMAN N-acetylneuraminate lyase OS=Homo sapiens GN=NPL PE=2 SV=1;>sp|Q9BXD5-2|NPL_HUMAN Isoform 2 of N-acetylne	5.77E-31
Q9BXF6;Q2Z1P3	RFIP5_HUMAN Rab11 family-interacting protein 5 OS=Homo sapiens GN=RAB11FIP5 PE=1 SV=1;>tr|Q2Z1P3|Q2Z1P3_HUMAN Putativ	1.16E-13
Q9BXK5;Q9BXK5-2;Q86T6	B2L13_HUMAN Bcl-2-like protein 13 OS=Homo sapiens GN=BCL2L13 PE=1 SV=1;>sp|Q9BXK5-2|B2L13_HUMAN Isoform 1 of Bcl-2-lil	4.28E-08
Q9BY32;A2A2N2	ITPA_HUMAN Inosine triphosphate pyrophosphatase OS=Homo sapiens GN=ITPA PE=1 SV=2;>tr|A2A2N2|A2A2N2_HUMAN Inosine tr	1.32E-67
Q9BY50;B4DI03	SC11C_HUMAN Signal peptidase complex catalytic subunit SEC11C OS=Homo sapiens GN=SEC11C PE=1 SV=3;>tr|B4DI03|B4DI03_I	1.85E-19
Q9BYC9;B7Z746	RM20_HUMAN 39S ribosomal protein L20, mitochondrial OS=Homo sapiens GN=MRPL20 PE=1 SV=1;>tr|B7Z746|B7Z746_HUMAN cD	7.25E-18
Q9BYD2;Q5SZR1	RM09_HUMAN 39S ribosomal protein L9, mitochondrial OS=Homo sapiens GN=MRPL9 PE=1 SV=2;>tr|Q5SZR1|Q5SZR1_HUMAN Mito	6.63E-18
Q9BYD6;A0PJ79	RM01_HUMAN 39S ribosomal protein L1, mitochondrial OS=Homo sapiens GN=MRPL1 PE=1 SV=2;>tr|A0PJ79|A0PJ79_HUMAN MRPL1	2.01E-43
Q9BYG3;C9J808;C9J6C5	MK67I_HUMAN MKI67 FHA domain-interacting nucleolar phosphoprotein OS=Homo sapiens GN=MKI67IP PE=1 SV=1;>tr|C9J808|C9J	5.88E-22

Q9BYX2;Q9BYX2-2;Q9BY	TBD2A_HUMAN TBC1 domain family member 2A OS=Homo sapiens GN=TBC1D2 PE=1 SV=3;>sp|Q9BYX2-2|TBD2A_HUMAN Isoform	1.89E-16
Q9BZE1;A6NHR2;Q6TGK8	RM37_HUMAN 39S ribosomal protein L37, mitochondrial OS=Homo sapiens GN=MRPL37 PE=1 SV=2;>tr|A6NHR2|A6NHR2_HUMAN U	9.11E-85
Q9BZK7;O60907;O60907-	TBL1R_HUMAN F-box-like/WD repeat-containing protein TBL1XR1 OS=Homo sapiens GN=TBL1XR1 PE=1 SV=1;>sp|O60907|TBL1X_	5.38E-11
Q9BZM5;B4DSS7;Q8IZX9	N2DL2_HUMAN NKG2D ligand 2 OS=Homo sapiens GN=ULBP2 PE=1 SV=1;>tr|B4DSS7|B4DSS7_HUMAN cDNA FLJ56582, highly simi	3.85E-05
Q9BZX2;Q9BZX2-2	UCK2_HUMAN Uridine-cytidine kinase 2 OS=Homo sapiens GN=UCK2 PE=1 SV=1;>sp|Q9BZX2-2|UCK2_HUMAN Isoform 2 of Uridine-	3.97E-11
Q9BZZ2;Q9BZZ2-3;Q9BZ	SN_HUMAN Sialoadhesin OS=Homo sapiens GN=SIGLEC1 PE=1 SV=2;>sp|Q9BZZ2-3|SN_HUMAN Isoform 3 of Sialoadhesin OS=Hom	4.64E-11
Q9C0B1;Q9C0B1-2;Q9C0	FTO_HUMAN Alpha-ketoglutarate-dependent dioxygenase FTO OS=Homo sapiens GN=FTO PE=1 SV=3;>sp|Q9C0B1-2|FTO_HUMAN Is	2.19E-15
Q9C0C2;Q9C0C2-2	TB182_HUMAN 182 kDa tankyrase-1-binding protein OS=Homo sapiens GN=TNKS1BP1 PE=1 SV=4;>sp|Q9C0C2-2|TB182_HUMAN Is	8.96E-154
Q9GZR7;Q4V9L5;B4DKV2	DDX24_HUMAN ATP-dependent RNA helicase DDX24 OS=Homo sapiens GN=DDX24 PE=1 SV=1;>tr|Q4V9L5|Q4V9L5_HUMAN DDX24	7.34E-28
Q9GZS1;Q9GZS1-2;B4E0	RPA49_HUMAN DNA-directed RNA polymerase I subunit RPA49 OS=Homo sapiens GN=POLR1E PE=1 SV=2;>sp|Q9GZS1-2|RPA49_HU	1.41E-13
Q9GZT8;Q9GZT8-2;Q658	NIF3L_HUMAN NIF3-like protein 1 OS=Homo sapiens GN=NIF3L1 PE=1 SV=2;>sp|Q9GZT8-2|NIF3L_HUMAN Isoform 2 of NIF3-like pr	2.22E-18
Q9GZU8;Q2VIL1;A8MQ36	F192A_HUMAN Protein FAM192A OS=Homo sapiens GN=FAM192A PE=1 SV=1;>tr|Q2VIL1|Q2VIL1_HUMAN RcNIP30 (Fragment) OS=	1.58E-06
Q9H078;Q9H078-2;B4DX	CLPB_HUMAN Caseinolytic peptidase B protein homolog OS=Homo sapiens GN=CLPB PE=1 SV=1;>sp|Q9H078-2|CLPB_HUMAN Isofor	5.20E-36
Q9H0A8;Q9H0A8-2;B4DE	COMD4_HUMAN COMM domain-containing protein 4 OS=Homo sapiens GN=COMMD4 PE=1 SV=1;>sp|Q9H0A8-2|COMD4_HUMAN Iso	9.43E-21
Q9H0B6;A8MXL7;A8MZ87	KLC2_HUMAN Kinesin light chain 2 OS=Homo sapiens GN=KLC2 PE=1 SV=1;>tr|A8MXL7|A8MXL7_HUMAN Uncharacterized protein O	5.13E-80
Q9H0P0;Q9H0P0-2;Q9H0	5NT3_HUMAN Cytosolic 5-nucleotidase 3 OS=Homo sapiens GN=NT5C3 PE=1 SV=3;>sp|Q9H0P0-2|5NT3_HUMAN Isoform 3 of Cytos	4.75E-14
Q9H0S4;Q9H4E3;Q53GJ1	DDX47_HUMAN Probable ATP-dependent RNA helicase DDX47 OS=Homo sapiens GN=DDX47 PE=1 SV=1;>tr|Q9H4E3|Q9H4E3_HUMA	1.08E-30
Q9H0U4;Q6FIG4;Q92928	RAB1B_HUMAN Ras-related protein Rab-1B OS=Homo sapiens GN=RAB1B PE=1 SV=1;>tr|Q6FIG4|Q6FIG4_HUMAN RAB1B protein O	5.23E-249
Q9H0U4;Q92928	RAB1B_HUMAN Ras-related protein Rab-1B OS=Homo sapiens GN=RAB1B PE=1 SV=1;>sp|Q92928|RAB1C_HUMAN Putative Ras-rela	0
Q9H0W9;Q9H0W9-2;Q9H	CK054_HUMAN Ester hydrolase C11orf54 OS=Homo sapiens GN=C11orf54 PE=1 SV=1;>sp|Q9H0W9-2|CK054_HUMAN Isoform 2 of E	2.07E-53
Q9H147;C9J7R7;C9J4E2;	TDIF1_HUMAN Deoxynucleotidyltransferase terminal-interacting protein 1 OS=Homo sapiens GN=DNTTIP1 PE=1 SV=2;>tr|C9J7R7|C	1.42E-20
Q9H1C4;Q05BS6;Q58F16	UN93B_HUMAN Protein unc-93 homolog B1 OS=Homo sapiens GN=UNC93B1 PE=1 SV=2;>tr|Q05BS6|Q05BS6_HUMAN UNC93B1 pro	3.36E-06
Q9H201;Q9H201-2;D6RE	EPN3_HUMAN Epsin-3 OS=Homo sapiens GN=EPN3 PE=1 SV=1;>sp|Q9H201-2|EPN3_HUMAN Isoform 2 of Epsin-3 OS=Homo sapien	9.28E-11
Q9H2G2-2;Q9H2G2	2|SLK_HUMAN Isoform 2 of STE20-like serine/threonine-protein kinase OS=Homo sapiens GN=SLK;>sp|Q9H2G2|SLK_HUMAN STE20-	6.70E-114
Q9H2H8-2;Q9H2H8;B8ZZ	2|PPIL3_HUMAN Isoform 2 of Peptidyl-prolyl cis-trans isomerase-like 3 OS=Homo sapiens GN=PPIL3;>sp|Q9H2H8|PPIL3_HUMAN Pep	3.57E-24
Q9H2J4;B7Z6S0	PDCL3_HUMAN Phosducin-like protein 3 OS=Homo sapiens GN=PDCL3 PE=1 SV=1;>tr|B7Z6S0|B7Z6S0_HUMAN cDNA FLJ51322, hig	5.12E-28
Q9H2U1;Q9H2U1-3;Q9H2	DHX36_HUMAN Probable ATP-dependent RNA helicase DHX36 OS=Homo sapiens GN=DHX36 PE=1 SV=2;>sp|Q9H2U1-3|DHX36_HUM	1.88E-17
Q9H2U2-2;Q9H2U2;Q9H2	2|IPYR2_HUMAN Isoform 2 of Inorganic pyrophosphatase 2, mitochondrial OS=Homo sapiens GN=PPA2;>sp|Q9H2U2|IPYR2_HUMAN	1.39E-82
Q9H330;Q9H330-2;Q9H3	CI005_HUMAN Transmembrane protein C9orf5 OS=Homo sapiens GN=C9orf5 PE=1 SV=2;>sp|Q9H330-2|CI005_HUMAN Isoform 2 of	8.47E-09
Q9H3N1;B4DZX7	TMX1_HUMAN Thioredoxin-related transmembrane protein 1 OS=Homo sapiens GN=TMX1 PE=1 SV=1;>tr|B4DZX7|B4DZX7_HUMAN	1.77E-52
Q9H3P2;Q9H3P2-7	NELFA_HUMAN Negative elongation factor A OS=Homo sapiens GN=WHSC2 PE=1 SV=3;>sp|Q9H3P2-7|NELFA_HUMAN Isoform 2 of N	5.74E-14
Q9H3S7;B4DJ12;B4DST5	PTN23_HUMAN Tyrosine-protein phosphatase non-receptor type 23 OS=Homo sapiens GN=PTPN23 PE=1 SV=1;>tr|B4DJ12|B4DJ12_	2.62E-35
Q9H3U1;Q9H3U1-2	UN45A_HUMAN Protein unc-45 homolog A OS=Homo sapiens GN=UNC45A PE=1 SV=1;>sp|Q9H3U1-2|UN45A_HUMAN Isoform 2 of P	7.51E-10
Q9H446;A8MT24	RWDD1_HUMAN RWD domain-containing protein 1 OS=Homo sapiens GN=RWDD1 PE=1 SV=1;>tr|A8MT24|A8MT24_HUMAN RWD do	1.94E-07
Q9H477;B4DV96	RBSK_HUMAN Ribokinase OS=Homo sapiens GN=RBKS PE=1 SV=1;>tr|B4DV96|B4DV96_HUMAN cDNA FLJ60169, highly similar to R	9.91E-07
Q9H479;B4DH12	FN3K_HUMAN Fructosamine-3-kinase OS=Homo sapiens GN=FN3K PE=1 SV=1;>tr|B4DH12|B4DH12_HUMAN cDNA FLJ60514, highly	4.96E-13
Q9H4A3-6;Q9H4A3-5;Q9	6|WNK1_HUMAN Isoform 5 of Serine/threonine-protein kinase WNK1 OS=Homo sapiens GN=WNK1;>sp|Q9H4A3-5|WNK1_HUMAN Is	3.63E-23
Q9H4A4;Q7RU04;A6NKB8	AMPB_HUMAN Aminopeptidase B OS=Homo sapiens GN=RNPEP PE=1 SV=2;>tr|Q7RU04|Q7RU04_HUMAN Aminopeptidase B OS=Ho	4.47E-174
Q9H4A6;B4DWL6;B3KQ71	GOLP3_HUMAN Golgi phosphoprotein 3 OS=Homo sapiens GN=GOLPH3 PE=1 SV=1;>tr|B4DWL6|B4DWL6_HUMAN cDNA FLJ55806, h	2.00E-26
Q9H6S3;B7ZKL3;Q9H6S3	ES8L2_HUMAN Epidermal growth factor receptor kinase substrate 8-like protein 2 OS=Homo sapiens GN=EPS8L2 PE=1 SV=2;>tr|B7Z	1.80E-173
Q9H6S3;Q9H6S3-2	ES8L2_HUMAN Epidermal growth factor receptor kinase substrate 8-like protein 2 OS=Homo sapiens GN=EPS8L2 PE=1 SV=2;>sp|Q9	5.03E-144
Q9H6T3;Q9H6T3-2;B4DR	RPAP3_HUMAN RNA polymerase II-associated protein 3 OS=Homo sapiens GN=RPAP3 PE=1 SV=2;>sp|Q9H6T3-2|RPAP3_HUMAN Isof	2.09E-12
Q9H6Z4;Q9H6Z4-2;Q9H6	RANB3_HUMAN Ran-binding protein 3 OS=Homo sapiens GN=RANBP3 PE=1 SV=1;>sp|Q9H6Z4-2|RANB3_HUMAN Isoform 2 of Ran-b	1.45E-20
Q9H6Z4;Q9H6Z4-2;Q9H6	RANB3_HUMAN Ran-binding protein 3 OS=Homo sapiens GN=RANBP3 PE=1 SV=1;>sp|Q9H6Z4-2|RANB3_HUMAN Isoform 2 of Ran-b	1.37E-16
Q9H7C9;C9JH31;Q9H7C9	CK067_HUMAN UPF0366 protein C11orf67 OS=Homo sapiens GN=C11orf67 PE=1 SV=1;>tr|C9JH31|C9JH31_HUMAN Uncharacterized	1.66E-06
Q9H7D7;Q9H7D7-2;B4DR	WDR26_HUMAN WD repeat-containing protein 26 OS=Homo sapiens GN=WDR26 PE=1 SV=3;>sp|Q9H7D7-2|WDR26_HUMAN Isoform	2.13E-40
Q9H832;Q9H832-2	UBE2Z_HUMAN Ubiquitin-conjugating enzyme E2 Z OS=Homo sapiens GN=UBE2Z PE=1 SV=2;>sp|Q9H832-2|UBE2Z_HUMAN Isoforn	1.42E-09
Q9H8H3;B4DDW1	MET7A_HUMAN Methyltransferase-like protein 7A OS=Homo sapiens GN=METTL7A PE=1 SV=1;>tr|B4DDW1|B4DDW1_HUMAN cDNA	5.14E-65
Q9H8U3;E2QRF5;Q5JVV6	ZFAN3_HUMAN AN1-type zinc finger protein 3 OS=Homo sapiens GN=ZFAND3 PE=1 SV=1;>tr|E2QRF5|E2QRF5_HUMAN Uncharacteri	7.11E-06
Q9H8Y8;Q9H8Y8-2	GORS2_HUMAN Golgi reassembly-stacking protein 2 OS=Homo sapiens GN=GORASP2 PE=1 SV=3;>sp|Q9H8Y8-2|GORS2_HUMAN Is	1.39E-159
Q9H900;Q9H900-2	ZWILC_HUMAN Protein zwilch homolog OS=Homo sapiens GN=ZWILCH PE=1 SV=2;>sp|Q9H900-2|ZWILC_HUMAN Isoform 2 of Prote	6.18E-07
Q9H936;C9J1H6	GHC1_HUMAN Mitochondrial glutamate carrier 1 OS=Homo sapiens GN=SLC25A22 PE=1 SV=1;>tr|C9J1H6|C9J1H6_HUMAN Uncharac	1.98E-13
Q9H944;B3KUJ9;B7ZBQ3	MED20_HUMAN Mediator of RNA polymerase II transcription subunit 20 OS=Homo sapiens GN=MED20 PE=1 SV=1;>tr|B3KUJ9|B3KU	5.21E-39
Q9H974;B7Z472;B7Z5R2	QTRD1_HUMAN Queuine tRNA-ribosyltransferase subunit QTRTD1 OS=Homo sapiens GN=QTRTD1 PE=1 SV=1;>tr|B7Z472|B7Z472_H	2.33E-08
Q9H9Q2;Q9H9Q2-3;Q9H9	CSN7B_HUMAN COP9 signalosome complex subunit 7b OS=Homo sapiens GN=COPS7B PE=1 SV=1;>sp|Q9H9Q2-3|CSN7B_HUMAN I	3.99E-31
Q9H9S4;Q5TAW7;B7ZBJ4	CB39L_HUMAN Calcium-binding protein 39-like OS=Homo sapiens GN=CAB39L PE=1 SV=3;>tr|Q5TAW7|Q5TAW7_HUMAN Calcium bi	2.95E-07
Q9HAB8;Q5VVM0	PPCS_HUMAN Phosphopantothenate--cysteine ligase OS=Homo sapiens GN=PPCS PE=1 SV=2;>tr|Q5VVM0|Q5VVM0_HUMAN Phosph	2.32E-15
Q9HAV7;B4DWV5	GRPE1_HUMAN GrpE protein homolog 1, mitochondrial OS=Homo sapiens GN=GRPEL1 PE=1 SV=2;>tr|B4DWV5|B4DWV5_HUMAN Gr	2.14E-167
Q9HB40;Q9HB40-2	RISC_HUMAN Retinoid-inducible serine carboxypeptidase OS=Homo sapiens GN=SCPEP1 PE=1 SV=1;>sp|Q9HB40-2|RISC_HUMAN Is	3.06E-15
Q9HB90;Q9NQL2;Q9NQL2	RRAGC_HUMAN Ras-related GTP-binding protein C OS=Homo sapiens GN=RRAGC PE=1 SV=1;>sp|Q9NQL2|RRAGD_HUMAN Ras-relat	1.50E-05
Q9HC38-2;Q9HC38;Q9HC	2|GLOD4_HUMAN Isoform 2 of Glyoxalase domain-containing protein 4 OS=Homo sapiens GN=GLOD4;>sp|Q9HC38|GLOD4_HUMAN	1.87E-164

Q9HCC0;Q9HCC0-2	MCCB_HUMAN Methylcrotonoyl-CoA carboxylase beta chain, mitochondrial OS=Homo sapiens GN=MCCC2 PE=1 SV=1;>sp|Q9HCC0-2	8.33E-82
Q9HCF4-3;Q9HCF4;Q9HCI	3|ALO17_HUMAN Isoform 3 of Protein ALO17 OS=Homo sapiens GN=KIAA1618;>sp|Q9HCF4|ALO17_HUMAN Protein ALO17 OS=Hom	5.18E-24
Q9HD33;Q9HD33-2;Q9HD	RM47_HUMAN 39S ribosomal protein L47, mitochondrial OS=Homo sapiens GN=MRPL47 PE=1 SV=2;>sp|Q9HD33-2|RM47_HUMAN Is	2.01E-15
Q9HD42;B4DXN3	CHM1A_HUMAN Charged multivesicular body protein 1a OS=Homo sapiens GN=CHMP1A PE=1 SV=1;>tr|B4DXN3|B4DXN3_HUMAN cD	1.09E-13
Q9HDC9;Q9HDC9-2	APMAP_HUMAN Adipocyte plasma membrane-associated protein OS=Homo sapiens GN=APMAP PE=1 SV=2;>sp|Q9HDC9-2|APMAP_H	1.03E-45
Q9NNW7;Q9NNW7-2;Q9N	TRXR2_HUMAN Thioredoxin reductase 2, mitochondrial OS=Homo sapiens GN=TXNRD2 PE=1 SV=3;>sp|Q9NNW7-2|TRXR2_HUMAN I	7.78E-17
Q9NP77;Q9NP77-2	SSU72_HUMAN RNA polymerase II subunit A C-terminal domain phosphatase SSU72 OS=Homo sapiens GN=SSU72 PE=1 SV=1;>sp|	2.59E-07
Q9NP97;Q8TF09;Q9NP97-	DLRB1_HUMAN Dynein light chain roadblock-type 1 OS=Homo sapiens GN=DYNLRB1 PE=1 SV=3;>sp|Q8TF09|DLRB2_HUMAN Dynein	3.86E-06
Q9NPH2;Q9NPH2-2	INO1_HUMAN Inositol-3-phosphate synthase 1 OS=Homo sapiens GN=ISYNA1 PE=1 SV=1;>sp|Q9NPH2-2|INO1_HUMAN Isoform 2 o	1.05E-100
Q9NPJ6;Q5T911;B4DX67	MED4_HUMAN Mediator of RNA polymerase II transcription subunit 4 OS=Homo sapiens GN=MED4 PE=1 SV=1;>tr|Q5T911|Q5T911_	3.78E-07
Q9NQ29;Q9NQ29-3;Q9NQ	LUC7L_HUMAN Putative RNA-binding protein Luc7-like 1 OS=Homo sapiens GN=LUC7L PE=1 SV=1;>sp|Q9NQ29-3|LUC7L_HUMAN Is	1.83E-57
Q9NQ48;B3KSI9;B4E0K7	LZTL1_HUMAN Leucine zipper transcription factor-like protein 1 OS=Homo sapiens GN=LZTFL1 PE=1 SV=1;>tr|B3KSI9|B3KSI9_HUM	5.59E-127
Q9NQC3;Q9NQC3-5;Q9NQ	RTN4_HUMAN Reticulon-4 OS=Homo sapiens GN=RTN4 PE=1 SV=2;>sp|Q9NQC3-5|RTN4_HUMAN Isoform 5 of Reticulon-4 OS=Hom	6.49E-220
Q9NQC3;Q9NQC3-5;Q9NQ	RTN4_HUMAN Reticulon-4 OS=Homo sapiens GN=RTN4 PE=1 SV=2;>sp|Q9NQC3-5|RTN4_HUMAN Isoform 5 of Reticulon-4 OS=Hom	4.30E-89
Q9NQG5;A2A2M0	RPR1B_HUMAN Regulation of nuclear pre-mRNA domain-containing protein 1B OS=Homo sapiens GN=RPRD1B PE=1 SV=1;>tr|A2A2M	5.87E-121
Q9NQW6;Q9NQW6-2;A8K	ANLN_HUMAN Actin-binding protein anillin OS=Homo sapiens GN=ANLN PE=1 SV=2;>sp|Q9NQW6-2|ANLN_HUMAN Isoform 2 of Acti	1.91E-46
Q9NR12;Q9NR12-2;Q9NR	PDLI7_HUMAN PDZ and LIM domain protein 7 OS=Homo sapiens GN=PDLIM7 PE=1 SV=1;>sp|Q9NR12-2|PDLI7_HUMAN Isoform 2 of	7.91E-23
Q9NR56;Q96P92;Q5VZF2;	MBNL1_HUMAN Muscleblind-like protein 1 OS=Homo sapiens GN=MBNL1 PE=1 SV=2;>tr|Q96P92|Q96P92_HUMAN MBNL protein OS=	2.09E-18
Q9NR56;Q9NR56-2;Q9NR	MBNL1_HUMAN Muscleblind-like protein 1 OS=Homo sapiens GN=MBNL1 PE=1 SV=2;>sp|Q9NR56-2|MBNL1_HUMAN Isoform 2 of Mu	2.63E-17
Q9NRX4;Q6FIE5;B1AMX0	PHP14_HUMAN 14 kDa phosphohistidine phosphatase OS=Homo sapiens GN=PHPT1 PE=1 SV=1;>tr|Q6FIE5|Q6FIE5_HUMAN PHP14 p	8.83E-96
Q9NRX4;Q9NRX4-2	PHP14_HUMAN 14 kDa phosphohistidine phosphatase OS=Homo sapiens GN=PHPT1 PE=1 SV=1;>sp|Q9NRX4-2|PHP14_HUMAN Isofo	4.37E-15
Q9NRY5;B4E1J2;B4DQT0	F1142_HUMAN Protein FAM114A2 OS=Homo sapiens GN=FAM114A2 PE=1 SV=4;>tr|B4E1J2|B4E1J2_HUMAN cDNA FLJ54229 OS=Ho	1.77E-22
Q9NSD9;Q9BR63;A8K666	SYFB_HUMAN Phenylalanyl-tRNA synthetase beta chain OS=Homo sapiens GN=FARSB PE=1 SV=3;>tr|Q9BR63|Q9BR63_HUMAN FARS	1.12E-123
Q9NSE4;A8K5W7	SYIM_HUMAN Isoleucyl-tRNA synthetase, mitochondrial OS=Homo sapiens GN=IARS2 PE=1 SV=2;>tr|A8K5W7|A8K5W7_HUMAN cDN	4.95E-75
Q9NSI2;Q9NSI2-2;C9JJU7	CU070_HUMAN Uncharacterized protein C21orf70 OS=Homo sapiens GN=C21orf70 PE=1 SV=2;>sp|Q9NSI2-2|CU070_HUMAN Isofor	3.16E-10
Q9NTJ5;B4DK71;B4DVV3;	SAC1_HUMAN Phosphatidylinositide phosphatase SAC1 OS=Homo sapiens GN=SACM1L PE=1 SV=2;>tr|B4DK71|B4DK71_HUMAN cDN	8.99E-10
Q9NTK5;Q9NTK5-2;Q9NT	OLA1_HUMAN Obg-like ATPase 1 OS=Homo sapiens GN=OLA1 PE=1 SV=2;>sp|Q9NTK5-2|OLA1_HUMAN Isoform 2 of Obg-like ATPas	3.09E-203
Q9NTX5;Q9NTX5-2;Q9NZ	ECHD1_HUMAN Enoyl-CoA hydratase domain-containing protein 1 OS=Homo sapiens GN=ECHDC1 PE=1 SV=2;>sp|Q9NTX5-2|ECHD1	6.89E-41
Q9NUI1;Q9NUI1-3	DECR2_HUMAN Peroxisomal 2,4-dienoyl-CoA reductase OS=Homo sapiens GN=DECR2 PE=1 SV=1;>sp|Q9NUI1-3|DECR2_HUMAN Iso	1.09E-08
Q9NUQ7;B3KRI4	UFSP2_HUMAN Ufm1-specific protease 2 OS=Homo sapiens GN=UFSP2 PE=2 SV=3;>tr|B3KRI4|B3KRI4_HUMAN cDNA FLJ34316 fis,	7.31E-22
Q9NUQ8;Q9NUQ8-2;B4DR	ABCF3_HUMAN ATP-binding cassette sub-family F member 3 OS=Homo sapiens GN=ABCF3 PE=1 SV=2;>sp|Q9NUQ8-2|ABCF3_HUMA	7.83E-73
Q9NUQ9;Q68D08	FA49B_HUMAN Protein FAM49B OS=Homo sapiens GN=FAM49B PE=1 SV=1;>tr|Q68D08|Q68D08_HUMAN Putative uncharacterized p	1.37E-84
Q9NUV9;B4DWA5;B4DWN	GIMA4_HUMAN GTPase IMAP family member 4 OS=Homo sapiens GN=GIMAP4 PE=1 SV=1;>tr|B4DWA5|B4DWA5_HUMAN cDNA FLJ5	1.53E-91
Q9NUY8;Q9NUY8-2	TBC23_HUMAN TBC1 domain family member 23 OS=Homo sapiens GN=TBC1D23 PE=1 SV=3;>sp|Q9NUY8-2|TBC23_HUMAN Isoform	6.76E-16
Q9NVA1;Q9NVA1-2;Q9NV	UQCC_HUMAN Ubiquinol-cytochrome c reductase complex chaperone CBP3 homolog OS=Homo sapiens GN=UQCC PE=1 SV=3;>sp|	1.16E-13
Q9NVA1;Q9NVA1-4;Q9NV	UQCC_HUMAN Ubiquinol-cytochrome c reductase complex chaperone CBP3 homolog OS=Homo sapiens GN=UQCC PE=1 SV=3;>sp|	1.19E-35
Q9NVA2-2;Q9NVA2	2|SEP11_HUMAN Isoform 2 of Septin-11 OS=Homo sapiens GN=SEPT11;>sp|Q9NVA2|SEP11_HUMAN Septin-11 OS=Homo sapiens G	4.37E-296
Q9NVI7-2;Q9NVI7;Q5T9A	2|ATD3A_HUMAN Isoform 2 of ATPase family AAA domain-containing protein 3A OS=Homo sapiens GN=ATAD3A;>sp|Q9NVI7|ATD3A_	6.07E-37
Q9NVJ2;Q96BM9	ARL8B_HUMAN ADP-ribosylation factor-like protein 8B OS=Homo sapiens GN=ARL8B PE=1 SV=1;>sp|Q96BM9|ARL8A_HUMAN ADP-ri	2.62E-17
Q9NVN8;A8MX10;Q05DU1	GNL3L_HUMAN Guanine nucleotide-binding protein-like 3-like protein OS=Homo sapiens GN=GNL3L PE=1 SV=1;>tr|A8MX10|A8MX10	3.24E-05
Q9NVP1;Q4ZG72;Q8N254	DDX18_HUMAN ATP-dependent RNA helicase DDX18 OS=Homo sapiens GN=DDX18 PE=1 SV=2;>tr|Q4ZG72|Q4ZG72_HUMAN Putati	6.69E-45
Q9NVU7;Q9NVU7-2	SDA1_HUMAN Protein SDA1 homolog OS=Homo sapiens GN=SDAD1 PE=1 SV=3;>sp|Q9NVU7-2|SDA1_HUMAN Isoform 2 of Protein S	1.79E-16
Q9NVV4-2;Q9NVV4	2|PAPD1_HUMAN Isoform 2 of Poly(A) RNA polymerase, mitochondrial OS=Homo sapiens GN=MTPAP;>sp|Q9NVV4|PAPD1_HUMAN Po	9.17E-08
Q9NVX0;Q9NVX0-3;Q9NV	HAUS2_HUMAN HAUS augmin-like complex subunit 2 OS=Homo sapiens GN=HAUS2 PE=1 SV=1;>sp|Q9NVX0-3|HAUS2_HUMAN Isof	4.38E-06
Q9NW64;Q9NW64-2	RBM22_HUMAN Pre-mRNA-splicing factor RBM22 OS=Homo sapiens GN=RBM22 PE=1 SV=1;>sp|Q9NW64-2|RBM22_HUMAN Isoform	9.60E-08
Q9NWS0;B4DGN7	PIHD1_HUMAN PIH1 domain-containing protein 1 OS=Homo sapiens GN=PIH1D1 PE=1 SV=1;>tr|B4DGN7|B4DGN7_HUMAN cDNA FL	1.58E-05
Q9NWS8;Q9NWS8-2;Q5S	RMND1_HUMAN Required for meiotic nuclear division protein 1 homolog OS=Homo sapiens GN=RMND1 PE=1 SV=2;>sp|Q9NWS8-2|	2.70E-07
Q9NWU5;Q9NWU5-2	RM22_HUMAN 39S ribosomal protein L22, mitochondrial OS=Homo sapiens GN=MRPL22 PE=1 SV=1;>sp|Q9NWU5-2|RM22_HUMAN I	1.96E-07
Q9NWV4;D3DQ38	CA123_HUMAN UPF0587 protein C1orf123 OS=Homo sapiens GN=C1orf123 PE=1 SV=1;>tr|D3DQ38|D3DQ38_HUMAN Chromosome	3.27E-15
Q9NX24;D6RCB9;D6RC52	NHP2_HUMAN H/ACA ribonucleoprotein complex subunit 2 OS=Homo sapiens GN=NHP2 PE=1 SV=1;>tr|D6RCB9|D6RCB9_HUMAN Ur	1.40E-49
Q9NX40;Q9NX40-2;Q9NX	OCAD1_HUMAN OCIA domain-containing protein 1 OS=Homo sapiens GN=OCIAD1 PE=1 SV=1;>sp|Q9NX40-2|OCAD1_HUMAN Isofor	2.85E-47
Q9NX46;B4DHV5	ARHL2_HUMAN Poly(ADP-ribose) glycohydrolase ARH3 OS=Homo sapiens GN=ADPRHL2 PE=1 SV=1;>tr|B4DHV5|B4DHV5_HUMAN cD	9.77E-55
Q9NX55;Q9NX55-3	HYPK_HUMAN Huntingtin-interacting protein K OS=Homo sapiens GN=HYPK PE=1 SV=2;>sp|Q9NX55-3|HYPK_HUMAN Isoform 3 of H	6.90E-09
Q9NXF1;B4DYV2;B4DQR0	TEX10_HUMAN Testis-expressed sequence 10 protein OS=Homo sapiens GN=TEX10 PE=1 SV=2;>tr|B4DYV2|B4DYV2_HUMAN cDNA	3.69E-05
Q9NXR7-1;Q9NXR7;Q9NX	1|BRE_HUMAN Isoform 1 of BRCA1-A complex subunit BRE OS=Homo sapiens GN=BRE;>sp|Q9NXR7|BRE_HUMAN BRCA1-A complex	1.31E-20
Q9NXV6;B3KTW3	CARF_HUMAN CDKN2A-interacting protein OS=Homo sapiens GN=CDKN2AIP PE=1 SV=2;>tr|B3KTW3|B3KTW3_HUMAN cDNA FLJ388	1.10E-66
Q9NY12;Q9NY12-2;D6RC	GAR1_HUMAN H/ACA ribonucleoprotein complex subunit 1 OS=Homo sapiens GN=GAR1 PE=1 SV=1;>sp|Q9NY12-2|GAR1_HUMAN Is	5.78E-11
Q9NY27;Q9NY27-3;Q9NY	PP4R2_HUMAN Serine/threonine-protein phosphatase 4 regulatory subunit 2 OS=Homo sapiens GN=PPP4R2 PE=1 SV=3;>sp|Q9NY27	2.39E-37
Q9NYB9;Q9NYB9-4;Q9NY	ABI2_HUMAN Abl interactor 2 OS=Homo sapiens GN=ABI2 PE=1 SV=1;>sp|Q9NYB9-4|ABI2_HUMAN Isoform 4 of Abl interactor 2 OS	1.06E-07
Q9NYL2-2;Q9NYL2;Q9NYL	2|MLTK_HUMAN Isoform 2 of Mitogen-activated protein kinase kinase kinase MLT OS=Homo sapiens GN=MLTK;>sp|Q9NYL2|MLTK_HU	7.24E-10
Q9NYL4;B4DWB7;Q86SR8	FKB11_HUMAN Peptidyl-prolyl cis-trans isomerase FKBP11 OS=Homo sapiens GN=FKBP11 PE=1 SV=1;>tr|B4DWB7|B4DWB7_HUMAN	8.70E-07

Q9NYU2;Q9NYU2-2	UGGG1_HUMAN UDP-glucose:glycoprotein glucosyltransferase 1 OS=Homo sapiens GN=UGGT1 PE=1 SV=3;>sp|Q9NYU2-2|UGGG1_H	0
Q9NZ08-2;Q9NZ08;A8K6I	2|ERAP1_HUMAN Isoform 2 of Endoplasmic reticulum aminopeptidase 1 OS=Homo sapiens GN=ERAP1;>sp|Q9NZ08|ERAP1_HUMAN E	4.26E-53
Q9NZ63;Q6GVN4;Q9P0Q6	CI078_HUMAN Uncharacterized protein C9orf78 OS=Homo sapiens GN=C9orf78 PE=1 SV=1;>tr|Q6GVN4|Q6GVN4_HUMAN Hepatocel	7.63E-10
Q9NZJ9;Q9NZJ9-2;Q8NFP	NUDT4_HUMAN Diphosphoinositol polyphosphate phosphohydrolase 2 OS=Homo sapiens GN=NUDT4 PE=1 SV=2;>sp|Q9NZJ9-2|NUD	4.36E-07
Q9NZL9;Q9NZL9-2;Q9NZL	MAT2B_HUMAN Methionine adenosyltransferase 2 subunit beta OS=Homo sapiens GN=MAT2B PE=1 SV=1;>sp|Q9NZL9-2|MAT2B_HU	2.62E-67
Q9NZM1;Q9NZM1-6;Q9NZ	MYOF_HUMAN Myoferlin OS=Homo sapiens GN=MYOF PE=1 SV=1;>sp|Q9NZM1-6|MYOF_HUMAN Isoform 6 of Myoferlin OS=Homo sa	0
Q9NZN4;B4DLA1;Q8NCJ3	EHD2_HUMAN EH domain-containing protein 2 OS=Homo sapiens GN=EHD2 PE=1 SV=2;>tr|B4DLA1|B4DLA1_HUMAN cDNA FLJ5345	3.56E-144
Q9NZT2;Q9NZT2-2	OGFR_HUMAN Opioid growth factor receptor OS=Homo sapiens GN=OGFR PE=1 SV=3;>sp|Q9NZT2-2|OGFR_HUMAN Isoform 2 of Op	3.70E-79
Q9NZU5;B4DG80;B4DEY6	LMCD1_HUMAN LIM and cysteine-rich domains protein 1 OS=Homo sapiens GN=LMCD1 PE=1 SV=1;>tr|B4DG80|B4DG80_HUMAN cD	2.10E-36
Q9NZZ3;B4DIR6	CHMP5_HUMAN Charged multivesicular body protein 5 OS=Homo sapiens GN=CHMP5 PE=1 SV=1;>tr|B4DIR6|B4DIR6_HUMAN cDNA	4.58E-29
Q9P0K7-2;Q9P0K7;Q9P0K	2|RAI14_HUMAN Isoform 2 of Ankycorbin OS=Homo sapiens GN=RAI14;>sp|Q9P0K7|RAI14_HUMAN Ankycorbin OS=Homo sapiens G	1.89E-32
Q9P0L0-2;Q9P0L0	2|VAPA_HUMAN Isoform 2 of Vesicle-associated membrane protein-associated protein A OS=Homo sapiens GN=VAPA;>sp|Q9P0L0|VA	1.65E-91
Q9P0M9;D6RB99;D6RAN8	RM27_HUMAN 39S ribosomal protein L27, mitochondrial OS=Homo sapiens GN=MRPL27 PE=1 SV=2;>tr|D6RB99|D6RB99_HUMAN Ur	2.12E-08
Q9P258;A5PLK7	RCC2_HUMAN Protein RCC2 OS=Homo sapiens GN=RCC2 PE=1 SV=2;>tr|A5PLK7|A5PLK7_HUMAN RCC2 protein (Fragment) OS=Hor	6.69E-82
Q9P287-2;Q9P287;Q9P28	2|BCCIP_HUMAN Isoform 2 of BRCA2 and CDKN1A-interacting protein OS=Homo sapiens GN=BCCIP;>sp|Q9P287|BCCIP_HUMAN BRC	1.41E-86
Q9P289;Q9P289-3	MST4_HUMAN Serine/threonine-protein kinase MST4 OS=Homo sapiens GN=MST4 PE=1 SV=2;>sp|Q9P289-3|MST4_HUMAN Isoform	4.10E-63
Q9P2I0;B3KN45;B3KMI0	CPSF2_HUMAN Cleavage and polyadenylation specificity factor subunit 2 OS=Homo sapiens GN=CPSF2 PE=1 SV=2;>tr|B3KN45|B3KN	3.02E-27
Q9P2N5;B3KY61;Q05DK9	RBM27_HUMAN RNA-binding protein 27 OS=Homo sapiens GN=RBM27 PE=1 SV=2;>tr|B3KY61|B3KY61_HUMAN cDNA FLJ16787 fis,	4.61E-07
Q9P2W9;D6RF48;D6RD65	STX18_HUMAN Syntaxin-18 OS=Homo sapiens GN=STX18 PE=1 SV=1;>tr|D6RF48|D6RF48_HUMAN Uncharacterized protein OS=Hor	8.62E-07
Q9TQE0;P13761	2B19_HUMAN HLA class II histocompatibility antigen, DRB1-9 beta chain OS=Homo sapiens GN=HLA-DRB1 PE=1 SV=1;>sp|P13761|	7.13E-78
Q9UBB4;B4DG05;Q7Z524	ATX10_HUMAN Ataxin-10 OS=Homo sapiens GN=ATXN10 PE=1 SV=1;>tr|B4DG05|B4DG05_HUMAN cDNA FLJ54600, highly similar to	6.02E-162
Q9UBB6-3;Q9UBB6;Q9UB	3|NCDN_HUMAN Isoform 3 of Neurochondrin OS=Homo sapiens GN=NCDN;>sp|Q9UBB6|NCDN_HUMAN Neurochondrin OS=Homo sap	1.27E-08
Q9UBE0;B3KMQ2;B3KNJ4	SAE1_HUMAN SUMO-activating enzyme subunit 1 OS=Homo sapiens GN=SAE1 PE=1 SV=1;>tr|B3KMQ2|B3KMQ2_HUMAN cDNA FLJ1	4.30E-52
Q9UBF2;Q9H9B7;B3KND4	COPG2_HUMAN Coatomer subunit gamma-2 OS=Homo sapiens GN=COPG2 PE=1 SV=1;>tr|Q9H9B7|Q9H9B7_HUMAN cDNA FLJ1287	1.03E-102
Q9UBK9;B1AJQ0	UXT_HUMAN Protein UXT OS=Homo sapiens GN=UXT PE=1 SV=1;>tr|B1AJQ0|B1AJQ0_HUMAN Ubiquitously-expressed transcript OS=	3.69E-14
Q9UBQ0-2;Q9UBQ0;Q5JP	2|VPS29_HUMAN Isoform 2 of Vacuolar protein sorting-associated protein 29 OS=Homo sapiens GN=VPS29;>sp|Q9UBQ0|VPS29_HUN	5.01E-22
Q9UBQ5;B7ZAM9;B4DVD7	EIF3K_HUMAN Eukaryotic translation initiation factor 3 subunit K OS=Homo sapiens GN=EIF3K PE=1 SV=1;>tr|B7ZAM9|B7ZAM9_HU	3.75E-193
Q9UBR2;Q5U000	CATZ_HUMAN Cathepsin Z OS=Homo sapiens GN=CTSZ PE=1 SV=1;>tr|Q5U000|Q5U000_HUMAN Cathepsin Z OS=Homo sapiens PE	3.77E-30
Q9UBS4;B3KW63	DJB11_HUMAN DnaJ homolog subfamily B member 11 OS=Homo sapiens GN=DNAJB11 PE=1 SV=1;>tr|B3KW63|B3KW63_HUMAN cD	5.84E-60
Q9UBU9;Q9UBU9-2	NXF1_HUMAN Nuclear RNA export factor 1 OS=Homo sapiens GN=NXF1 PE=1 SV=1;>sp|Q9UBU9-2|NXF1_HUMAN Isoform 2 of Nucle	3.78E-98
Q9UDY2;Q9UDY2-3;Q9UD	ZO2_HUMAN Tight junction protein ZO-2 OS=Homo sapiens GN=TJP2 PE=1 SV=2;>sp|Q9UDY2-3|ZO2_HUMAN Isoform C1 of Tight ju	1.94E-288
Q9UET6;Q9UET6-2	RRMJ1_HUMAN Putative ribosomal RNA methyltransferase 1 OS=Homo sapiens GN=FTSJ1 PE=1 SV=2;>sp|Q9UET6-2|RRMJ1_HUMAN	3.44E-12
Q9UEW8;Q6E0B2	STK39_HUMAN STE20/SPS1-related proline-alanine-rich protein kinase OS=Homo sapiens GN=STK39 PE=1 SV=3;>tr|Q6E0B2|Q6E0E	4.10E-26
Q9UFN0;B4DW81	NPS3A_HUMAN Protein NipSnap homolog 3A OS=Homo sapiens GN=NIPSNAP3A PE=1 SV=2;>tr|B4DW81|B4DW81_HUMAN cDNA FLJ	8.66E-33
Q9UGM6;B7Z6G7;B7Z5X7	SYWM_HUMAN Tryptophanyl-tRNA synthetase, mitochondrial OS=Homo sapiens GN=WARS2 PE=1 SV=1;>tr|B7Z6G7|B7Z6G7_HUMA	2.29E-10
Q9UH65;D3DQV1;B3KUB9	SWP70_HUMAN Switch-associated protein 70 OS=Homo sapiens GN=SWAP70 PE=1 SV=1;>tr|D3DQV1|D3DQV1_HUMAN SWAP-70 pr	1.82E-72
Q9UHB9;Q9UHB9-2	SRP68_HUMAN Signal recognition particle 68 kDa protein OS=Homo sapiens GN=SRP68 PE=1 SV=2;>sp|Q9UHB9-2|SRP68_HUMAN I	4.25E-63
Q9UHD9;B4DZF1;B4DM19	UBQL2_HUMAN Ubiquilin-2 OS=Homo sapiens GN=UBQLN2 PE=1 SV=2;>tr|B4DZF1|B4DZF1_HUMAN cDNA FLJ56541, highly similar t	1.03E-59
Q9UHG3;B7Z8A2;B7Z9P8	PCYOX_HUMAN Prenylcysteine oxidase 1 OS=Homo sapiens GN=PCYOX1 PE=1 SV=3;>tr|B7Z8A2|B7Z8A2_HUMAN cDNA FLJ51671, h	3.64E-88
Q9UHL4;Q59EM4	DPP2_HUMAN Dipeptidyl peptidase 2 OS=Homo sapiens GN=DPP7 PE=1 SV=3;>tr|Q59EM4|Q59EM4_HUMAN Dipeptidyl-peptidase II	1.37E-39
Q9UHW5-2;Q9UHW5	2|GPN3_HUMAN Isoform 2 of GPN-loop GTPase 3 OS=Homo sapiens GN=GPN3;>sp|Q9UHW5|GPN3_HUMAN GPN-loop GTPase 3 OS=	4.82E-08
Q9UHY7;Q7Z508;D6RA00	ENOPH_HUMAN Enolase-phosphatase E1 OS=Homo sapiens GN=ENOPH1 PE=1 SV=1;>tr|Q7Z508|Q7Z508_HUMAN MasA OS=Homo s	8.30E-16
Q9UHY7;Q9UHY7-2	ENOPH_HUMAN Enolase-phosphatase E1 OS=Homo sapiens GN=ENOPH1 PE=1 SV=1;>sp|Q9UHY7-2|ENOPH_HUMAN Isoform 2 of En	1.75E-08
Q9UI08-2;Q9UI08;B4DW5	2|EVL_HUMAN Isoform 1 of Ena/VASP-like protein OS=Homo sapiens GN=EVL;>sp|Q9UI08|EVL_HUMAN Ena/VASP-like protein OS=H	2.46E-109
Q9UI09;Q53HG1	NDUAC_HUMAN NADH dehydrogenase [ubiquinone] 1 alpha subcomplex subunit 12 OS=Homo sapiens GN=NDUFA12 PE=1 SV=1;>tr|	2.42E-16
Q9UJU6;Q9UJU6-3;Q9UJU	DBNL_HUMAN Drebrin-like protein OS=Homo sapiens GN=DBNL PE=1 SV=1;>sp|Q9UJU6-3|DBNL_HUMAN Isoform 3 of Drebrin-like p	1.97E-250
Q9UJX3;Q9UJX3-2	APC7_HUMAN Anaphase-promoting complex subunit 7 OS=Homo sapiens GN=ANAPC7 PE=1 SV=4;>sp|Q9UJX3-2|APC7_HUMAN Isof	2.03E-12
Q9UKX2;P12882	MYH2_HUMAN Myosin-2 OS=Homo sapiens GN=MYH2 PE=1 SV=1;>sp|P12882|MYH1_HUMAN Myosin-1 OS=Homo sapiens GN=MYH1	3.85E-09
Q9UKX2;P12882;P11055;	MYH2_HUMAN Myosin-2 OS=Homo sapiens GN=MYH2 PE=1 SV=1;>sp|P12882|MYH1_HUMAN Myosin-1 OS=Homo sapiens GN=MYH1	2.11E-23
Q9UKX7;Q9UKX7-2	NUP50_HUMAN Nuclear pore complex protein Nup50 OS=Homo sapiens GN=NUP50 PE=1 SV=2;>sp|Q9UKX7-2|NUP50_HUMAN Isofo	5.91E-36
Q9UKY7;Q9UKY7-2;Q9UK	CDV3_HUMAN Protein CDV3 homolog OS=Homo sapiens GN=CDV3 PE=1 SV=1;>sp|Q9UKY7-2|CDV3_HUMAN Isoform 2 of Protein CD	1.18E-10
Q9UKZ1;B3KNB0	CB029_HUMAN UPF0760 protein C2orf29 OS=Homo sapiens GN=C2orf29 PE=1 SV=1;>tr|B3KNB0|B3KNB0_HUMAN cDNA FLJ14159 f	2.80E-08
Q9UL15-2;Q9UL15	2|BAG5_HUMAN Isoform 2 of BAG family molecular chaperone regulator 5 OS=Homo sapiens GN=BAG5;>sp|Q9UL15|BAG5_HUMAN E	9.92E-16
Q9UL33;Q9UL33-2;C9JDE	TPC2L_HUMAN Trafficking protein particle complex subunit 2-like protein OS=Homo sapiens GN=TRAPPC2L PE=1 SV=1;>sp|Q9UL33-2	1.98E-05
Q9ULC4;Q9ULC4-3;Q9ULC	MCTS1_HUMAN Malignant T cell-amplified sequence 1 OS=Homo sapiens GN=MCTS1 PE=1 SV=1;>sp|Q9ULC4-3|MCTS1_HUMAN Isof	7.85E-135
Q9ULV0;Q9H6Y6;B4DXH5	MYO5B_HUMAN Myosin-Vb OS=Homo sapiens GN=MYO5B PE=1 SV=3;>tr|Q9H6Y6|Q9H6Y6_HUMAN cDNA: FLJ21683 fis, clone COL0	8.48E-10
Q9ULX9;B4DV49	MAFF_HUMAN Transcription factor MafF OS=Homo sapiens GN=MAFF PE=1 SV=2;>tr|B4DV49|B4DV49_HUMAN cDNA FLJ57607, highl	4.01E-05
Q9ULZ3;Q9ULZ3-3;Q9ULZ	ASC_HUMAN Apoptosis-associated speck-like protein containing a CARD OS=Homo sapiens GN=PYCARD PE=1 SV=2;>sp|Q9ULZ3-3|A	1.41E-27
Q9UM00;Q9UM00-2	TMCO1_HUMAN Transmembrane and coiled-coil domain-containing protein 1 OS=Homo sapiens GN=TMCO1 PE=1 SV=1;>sp|Q9UM00	3.12E-12
Q9UM54;Q9UM54-1;Q9UM	MYO6_HUMAN Myosin-VI OS=Homo sapiens GN=MYO6 PE=1 SV=4;>sp|Q9UM54-1|MYO6_HUMAN Isoform 1 of Myosin-VI OS=Homo	3.16E-141
Q9UMS0;Q9UMS0-3;Q9UM	NFU1_HUMAN NFU1 iron-sulfur cluster scaffold homolog, mitochondrial OS=Homo sapiens GN=NFU1 PE=1 SV=2;>sp|Q9UMS0-3|NFU	5.40E-06

Q9UMY4;Q9UMY4-2	SNX12_HUMAN Sorting nexin-12 OS=Homo sapiens GN=SNX12 PE=1 SV=3;>sp|Q9UMY4-2|SNX12_HUMAN Isoform 2 of Sorting nexi	1.40E-14
Q9UN36;Q9UN36-3;Q9UN	NDRG2_HUMAN Protein NDRG2 OS=Homo sapiens GN=NDRG2 PE=1 SV=2;>sp|Q9UN36-3|NDRG2_HUMAN Isoform 3 of Protein NDR	5.89E-10
Q9UN37;Q9UF30;Q9NXK5	VPS4A_HUMAN Vacuolar protein sorting-associated protein 4A OS=Homo sapiens GN=VPS4A PE=1 SV=1;>tr|Q9UF30|Q9UF30_HUMA	2.86E-13
Q9UN86;Q9UN86-2	G3BP2_HUMAN Ras GTPase-activating protein-binding protein 2 OS=Homo sapiens GN=G3BP2 PE=1 SV=2;>sp|Q9UN86-2|G3BP2_HU	3.94E-76
Q9UNF0;Q9UNF0-2	PACN2_HUMAN Protein kinase C and casein kinase substrate in neurons protein 2 OS=Homo sapiens GN=PACSIN2 PE=1 SV=2;>sp|Q	1.17E-24
Q9UNF1;Q9UNF1-2	MAGD2_HUMAN Melanoma-associated antigen D2 OS=Homo sapiens GN=MAGED2 PE=1 SV=2;>sp|Q9UNF1-2|MAGD2_HUMAN Isofor	6.77E-107
Q9UNQ2;A8K9K8;B4DRY2	DIMT1_HUMAN Probable dimethyladenosine transferase OS=Homo sapiens GN=DIMT1L PE=1 SV=1;>tr|A8K9K8|A8K9K8_HUMAN cDN	5.07E-33
Q9UNS2;B4DY81;B4DN01	CSN3_HUMAN COP9 signalosome complex subunit 3 OS=Homo sapiens GN=COPS3 PE=1 SV=3;>tr|B4DY81|B4DY81_HUMAN cDNA Fl	1.40E-64
Q9UNZ2-5;Q9UNZ2;Q9UN	5|NSF1C_HUMAN Isoform 3 of NSFL1 cofactor p47 OS=Homo sapiens GN=NSFL1C;>sp|Q9UNZ2|NSF1C_HUMAN NSFL1 cofactor p47 (	9.26E-28
Q9UP83-2;Q9UP83-3;Q9U	2|COG5_HUMAN Isoform 2 of Conserved oligomeric Golgi complex subunit 5 OS=Homo sapiens GN=COG5;>sp|Q9UP83-3|COG5_HUM	2.16E-08
Q9UPN3;Q9UPN3-4	MACF1_HUMAN Microtubule-actin cross-linking factor 1, isoforms 1/2/3/5 OS=Homo sapiens GN=MACF1 PE=1 SV=4;>sp|Q9UPN3-4|I	0
Q9UPN7;B4DLT9	PP6R1_HUMAN Serine/threonine-protein phosphatase 6 regulatory subunit 1 OS=Homo sapiens GN=PPP6R1 PE=1 SV=5;>tr|B4DLT9|	1.54E-08
Q9UPT5;Q9UPT5-6;Q9UPT	EXOC7_HUMAN Exocyst complex component 7 OS=Homo sapiens GN=EXOC7 PE=1 SV=3;>sp|Q9UPT5-6|EXOC7_HUMAN Isoform 6 o	1.31E-10
Q9UQ35;Q9UQ35-2;Q9UQ	SRRM2_HUMAN Serine/arginine repetitive matrix protein 2 OS=Homo sapiens GN=SRRM2 PE=1 SV=2;>sp|Q9UQ35-2|SRRM2_HUMAI	1.99E-08
Q9UQ80;Q6PIN5;A8K6Y1;	PA2G4_HUMAN Proliferation-associated protein 2G4 OS=Homo sapiens GN=PA2G4 PE=1 SV=3;>tr|Q6PIN5|Q6PIN5_HUMAN PA2G4 pi	0
Q9Y221;Q9Y221-2	NIP7_HUMAN 60S ribosome subunit biogenesis protein NIP7 homolog OS=Homo sapiens GN=NIP7 PE=1 SV=1;>sp|Q9Y221-2|NIP7_H	8.09E-11
Q9Y230;B3KNL2;B3KQ59	RUVB2_HUMAN RuvB-like 2 OS=Homo sapiens GN=RUVBL2 PE=1 SV=3;>tr|B3KNL2|B3KNL2_HUMAN RuvB-like 2 (E. coli), isoform Cl	2.81E-291
Q9Y237-2;Q9Y237;Q5HYW	2|PIN4_HUMAN Isoform 2 of Peptidyl-prolyl cis-trans isomerase NIMA-interacting 4 OS=Homo sapiens GN=PIN4;>sp|Q9Y237|PIN4_H	3.15E-74
Q9Y266;Q9H2R7	NUDC_HUMAN Nuclear migration protein nudC OS=Homo sapiens GN=NUDC PE=1 SV=1;>tr|Q9H2R7|Q9H2R7_HUMAN NPD011 OS=l	0
Q9Y295;Q9UFA5	DRG1_HUMAN Developmentally-regulated GTP-binding protein 1 OS=Homo sapiens GN=DRG1 PE=1 SV=1;>tr|Q9UFA5|Q9UFA5_HUM	2.88E-107
Q9Y2A7-2;Q9Y2A7;B3KMK	2|NCKP1_HUMAN Isoform 2 of Nck-associated protein 1 OS=Homo sapiens GN=NCKAP1;>sp|Q9Y2A7|NCKP1_HUMAN Nck-associated	7.27E-12
Q9Y2D5-4;Q9Y2D5-7;Q9Y	4|AKAP2_HUMAN Isoform 2 of A-kinase anchor protein 2 OS=Homo sapiens GN=AKAP2;>sp|Q9Y2D5-7|AKAP2_HUMAN Isoform 5 of A	5.48E-27
Q9Y2H0;Q9Y2H0-1;Q9Y2H	DLGP4_HUMAN Disks large-associated protein 4 OS=Homo sapiens GN=DLGAP4 PE=1 SV=3;>sp|Q9Y2H0-1|DLGP4_HUMAN Isoform 2	1.34E-12
Q9Y2J8;B4DIU3;Q96DA7	PADI2_HUMAN Protein-arginine deiminase type-2 OS=Homo sapiens GN=PADI2 PE=1 SV=2;>tr|B4DIU3|B4DIU3_HUMAN cDNA FLJ61	5.14E-72
Q9Y2K3;B3KP05;REV__Q8	MYH15_HUMAN Myosin-15 OS=Homo sapiens GN=MYH15 PE=1 SV=5;>tr|B3KP05|B3KP05_HUMAN cDNA FLJ30874 fis, clone FEBRA2	1.41E-06
Q9Y2Q3-2;Q9Y2Q3;Q9Y2C	2|GSTK1_HUMAN Isoform 2 of Glutathione S-transferase kappa 1 OS=Homo sapiens GN=GSTK1;>sp|Q9Y2Q3|GSTK1_HUMAN Glutath	4.90E-101
Q9Y2R4;Q59H21;A8MTP9;	DDX52_HUMAN Probable ATP-dependent RNA helicase DDX52 OS=Homo sapiens GN=DDX52 PE=1 SV=3;>tr|Q59H21|Q59H21_HUMA	6.84E-08
Q9Y2S7;B4DEM9	PDIP2_HUMAN Polymerase delta-interacting protein 2 OS=Homo sapiens GN=POLDIP2 PE=1 SV=1;>tr|B4DEM9|B4DEM9_HUMAN cDN	2.24E-32
Q9Y2T2;B4DRN6;Q8NDP0	AP3M1_HUMAN AP-3 complex subunit mu-1 OS=Homo sapiens GN=AP3M1 PE=1 SV=1;>tr|B4DRN6|B4DRN6_HUMAN cDNA FLJ5128	4.36E-38
Q9Y2X3;B3KN82	NOP58_HUMAN Nucleolar protein 58 OS=Homo sapiens GN=NOP58 PE=1 SV=1;>tr|B3KN82|B3KN82_HUMAN cDNA FLJ13913 fis, clor	5.51E-152
Q9Y365;B3KTJ1	PCTL_HUMAN PCTP-like protein OS=Homo sapiens GN=STARD10 PE=2 SV=2;>tr|B3KTJ1|B3KTJ1_HUMAN cDNA FLJ38349 fis, clone F	3.54E-48
Q9Y371-2;Q9Y371;B4E18	2|SHLB1_HUMAN Isoform 2 of Endophilin-B1 OS=Homo sapiens GN=SH3GLB1;>sp|Q9Y371|SHLB1_HUMAN Endophilin-B1 OS=Homo	2.01E-23
Q9Y388;Q9Y6G0;Q0VGM7	RBMX2_HUMAN RNA-binding motif protein, X-linked 2 OS=Homo sapiens GN=RBMX2 PE=1 SV=2;>tr|Q9Y6G0|Q9Y6G0_HUMAN RNA-l	4.36E-07
Q9Y399;Q5T8A0	RT02_HUMAN 28S ribosomal protein S2, mitochondrial OS=Homo sapiens GN=MRPS2 PE=1 SV=1;>tr|Q5T8A0|Q5T8A0_HUMAN Mitoc	1.15E-16
Q9Y3A3;Q9Y3A3-3;Q9Y3A	MOBL3_HUMAN Mps one binder kinase activator-like 3 OS=Homo sapiens GN=MOBKL3 PE=1 SV=1;>sp|Q9Y3A3-3|MOBL3_HUMAN Is	5.05E-10
Q9Y3B9;A8K201	RRP15_HUMAN RRP15-like protein OS=Homo sapiens GN=RRP15 PE=1 SV=2;>tr|A8K201|A8K201_HUMAN cDNA FLJ75605, highly sir	6.74E-08
Q9Y3C4;Q9Y3C4-3;Q9Y3C	TPRKB_HUMAN TP53RK-binding protein OS=Homo sapiens GN=TPRKB PE=1 SV=1;>sp|Q9Y3C4-3|TPRKB_HUMAN Isoform 3 of TP53R	5.39E-12
Q9Y3D3;B4E032;A6ND22	RT16_HUMAN 28S ribosomal protein S16, mitochondrial OS=Homo sapiens GN=MRPS16 PE=1 SV=1;>tr|B4E032|B4E032_HUMAN cDI	6.84E-17
Q9Y3D8;Q9Y3D8-2	KAD6_HUMAN Adenylate kinase isoenzyme 6 OS=Homo sapiens GN=TAF9 PE=1 SV=1;>sp|Q9Y3D8-2|KAD6_HUMAN Isoform 3 of Ade	6.37E-12
Q9Y3D8;Q9Y3D8-2;D6RDI	KAD6_HUMAN Adenylate kinase isoenzyme 6 OS=Homo sapiens GN=TAF9 PE=1 SV=1;>sp|Q9Y3D8-2|KAD6_HUMAN Isoform 3 of Ade	1.85E-08
Q9Y3L3;Q9Y3L3-2	3BP1_HUMAN SH3 domain-binding protein 1 OS=Homo sapiens GN=SH3BP1 PE=1 SV=3;>sp|Q9Y3L3-2|3BP1_HUMAN Isoform 2 of S	1.61E-16
Q9Y3Q3;Q9UMB6	TMED3_HUMAN Transmembrane emp24 domain-containing protein 3 OS=Homo sapiens GN=TMED3 PE=2 SV=1;>tr|Q9UMB6|Q9UMB	1.11E-05
Q9Y448;Q9Y448-2	T4AF1_HUMAN Putative TRAF4-associated factor 1 OS=Homo sapiens GN=TRAF4AF1 PE=1 SV=2;>sp|Q9Y448-2|T4AF1_HUMAN Isofo	4.00E-14
Q9Y448;Q9Y448-2;Q9Y44	T4AF1_HUMAN Putative TRAF4-associated factor 1 OS=Homo sapiens GN=TRAF4AF1 PE=1 SV=2;>sp|Q9Y448-2|T4AF1_HUMAN Isofo	1.81E-15
Q9Y490;Q5TCU6	TLN1_HUMAN Talin-1 OS=Homo sapiens GN=TLN1 PE=1 SV=3;>tr|Q5TCU6|Q5TCU6_HUMAN Talin 1 OS=Homo sapiens GN=TLN1 PE=	0
Q9Y4F1-2;Q9Y4F1;C9JME	2|FARP1_HUMAN Isoform 2 of FERM, RhoGEF and pleckstrin domain-containing protein 1 OS=Homo sapiens GN=FARP1;>sp|Q9Y4F1|I	1.66E-06
Q9Y4I1-3;Q9Y4I1;Q9Y4I1	3|MYO5A_HUMAN Isoform 3 of Myosin-Va OS=Homo sapiens GN=MYO5A;>sp|Q9Y4I1|MYO5A_HUMAN Myosin-Va OS=Homo sapiens (	6.46E-07
Q9Y4Y9;B8ZZF8	LSM5_HUMAN U6 snRNA-associated Sm-like protein LSm5 OS=Homo sapiens GN=LSM5 PE=1 SV=3;>tr|B8ZZF8|B8ZZF8_HUMAN Unc	2.13E-05
Q9Y4Y9;Q9Y4Y9-2	LSM5_HUMAN U6 snRNA-associated Sm-like protein LSm5 OS=Homo sapiens GN=LSM5 PE=1 SV=3;>sp|Q9Y4Y9-2|LSM5_HUMAN Isc	1.32E-74
Q9Y530;C9JNE2;C9JXC3;(	CF130_HUMAN Uncharacterized protein C6orf130 OS=Homo sapiens GN=C6orf130 PE=1 SV=2;>tr|C9JNE2|C9JNE2_HUMAN Uncharac	4.89E-17
Q9Y536;A2BFH1	PAL4A_HUMAN Peptidylprolyl cis-trans isomerase A-like 4A/B/C OS=Homo sapiens GN=PPIAL4A PE=1 SV=1;>sp|A2BFH1|PAL4G_HUM	2.19E-15
Q9Y570;Q9Y570-2;Q9Y57	PPME1_HUMAN Protein phosphatase methylesterase 1 OS=Homo sapiens GN=PPME1 PE=1 SV=3;>sp|Q9Y570-2|PPME1_HUMAN Isofo	2.02E-16
Q9Y5B8;Q9Y5B8-2	NDK7_HUMAN Nucleoside diphosphate kinase 7 OS=Homo sapiens GN=NME7 PE=1 SV=1;>sp|Q9Y5B8-2|NDK7_HUMAN Isoform 2 of	4.16E-18
Q9Y613;B4DVN5	FHOD1_HUMAN FH1/FH2 domain-containing protein 1 OS=Homo sapiens GN=FHOD1 PE=1 SV=3;>tr|B4DVN5|B4DVN5_HUMAN cDNA	3.95E-06
Q9Y623;P11055;Q9UKX3;	MYH4_HUMAN Myosin-4 OS=Homo sapiens GN=MYH4 PE=1 SV=2;>sp|P11055|MYH3_HUMAN Myosin-3 OS=Homo sapiens GN=MYH3	3.37E-09
Q9Y623;Q9UKX2;P12882	MYH4_HUMAN Myosin-4 OS=Homo sapiens GN=MYH4 PE=1 SV=2;>sp|Q9UKX2|MYH2_HUMAN Myosin-2 OS=Homo sapiens GN=MYH	8.78E-10
Q9Y625;O75487	GPC6_HUMAN Glypican-6 OS=Homo sapiens GN=GPC6 PE=2 SV=1;>sp|O75487|GPC4_HUMAN Glypican-4 OS=Homo sapiens GN=GF	6.08E-07
Q9Y639;Q9Y639-5;Q9Y63	NPTN_HUMAN Neuroplastin OS=Homo sapiens GN=NPTN PE=1 SV=2;>sp|Q9Y639-5|NPTN_HUMAN Isoform 5 of Neuroplastin OS=Ho	5.50E-15
Q9Y639;Q9Y639-5;Q9Y63	NPTN_HUMAN Neuroplastin OS=Homo sapiens GN=NPTN PE=1 SV=2;>sp|Q9Y639-5|NPTN_HUMAN Isoform 5 of Neuroplastin OS=Ho	5.74E-16
Q9Y657;Q5JUX0	SPIN1_HUMAN Spindlin-1 OS=Homo sapiens GN=SPIN1 PE=1 SV=3;>sp|Q5JUX0|SPIN3_HUMAN Spindlin-3 OS=Homo sapiens GN=S	1.05E-07
Q9Y680;Q9Y680-2;Q9Y68	FKBP7_HUMAN Peptidyl-prolyl cis-trans isomerase FKBP7 OS=Homo sapiens GN=FKBP7 PE=1 SV=1;>sp|Q9Y680-2|FKBP7_HUMAN Is	8.68E-09

Q9Y6A4;B4DN94	CP080_HUMAN UPF0468 protein C16orf80 OS=Homo sapiens GN=C16orf80 PE=1 SV=1;>tr|B4DN94|B4DN94_HUMAN cDNA FLJ5884	3.05E-28
Q9Y6C9;Q53G34	MTCH2_HUMAN Mitochondrial carrier homolog 2 OS=Homo sapiens GN=MTCH2 PE=1 SV=1;>tr|Q53G34|Q53G34_HUMAN Mitochondr	1.00E-121
Q9Y6D5;Q9Y6D6;Q59FY5;	BIG2_HUMAN Brefeldin A-inhibited guanine nucleotide-exchange protein 2 OS=Homo sapiens GN=ARFGEF2 PE=1 SV=3;>sp|Q9Y6D6|	9.60E-08
Q9Y6G9;Q6MZE7;B3KM42	DC1L1_HUMAN Cytoplasmic dynein 1 light intermediate chain 1 OS=Homo sapiens GN=DYNC1LI1 PE=1 SV=3;>tr|Q6MZE7|Q6MZE7_	2.15E-145
Q9Y6H1;Q5T1J5	CHCH2_HUMAN Coiled-coil-helix-coiled-coil-helix domain-containing protein 2, mitochondrial OS=Homo sapiens GN=CHCHD2 PE=1 S\	1.06E-13
Q9Y6M9;B7Z7N1;Q5JVG7	NDUB9_HUMAN NADH dehydrogenase [ubiquinone] 1 beta subcomplex subunit 9 OS=Homo sapiens GN=NDUFB9 PE=1 SV=3;>tr|B7Z	1.35E-51
Q9Y6W5;B4DZN0;Q8IV90	WASF2_HUMAN Wiskott-Aldrich syndrome protein family member 2 OS=Homo sapiens GN=WASF2 PE=1 SV=3;>tr|B4DZN0|B4DZN0_	3.79E-42
Q9Y6W5;Q9UPY6	WASF2_HUMAN Wiskott-Aldrich syndrome protein family member 2 OS=Homo sapiens GN=WASF2 PE=1 SV=3;>sp|Q9UPY6|WASF3_	5.17E-23
