# Supplementary material for: Proteomic analysis of breast tumors confirms the mRNA intrinsic molecular subtypes using different classifiers: a large-scale analysis of fresh frozen tissue samples
Source: Breast Cancer Res. 2016 Jun 29;18:69. doi: 10.1186/s13058-016-0732-2 (PMC4928264; doi:10.1186/s13058-016-0732-2)
Supplement: Additional file 4: Table S4. — The selected reaction monitoring (SRM) assays set up after refinement, containing 190 proteins represented by 253 peptides. The protein names, peptide sequences, Q1/Q3 transitions, collision energy, and fragment being monitored are given. (PDF 297 kb) [file 13058_2016_732_MOESM4_ESM.pdf]

Protein Name	Peptide	Begin Pos	End Pos	Q1 m/z	Q3 m/z	Collision energy (V)	Fragment
sp|O00231|PSD11_HUMAN	EQSILELGSLLAK	46	58	700.903399	588.371538	23.9	y6
sp|O00231|PSD11_HUMAN	EQSILELGSLLAK	46	58	700.903399	830.498195	23.9	y8
sp|O00231|PSD11_HUMAN	EQSILELGSLLAK	46	58	700.903399	943.582259	23.9	y9
sp|O00231|PSD11_HUMAN	EQSILELGSLLAK	46	58	700.903399	1143.698351	23.9	y11
sp|O00299|CLIC1_HUMAN	NSNPALNDNLEK	119	130	664.82568	732.352259	22.8	y6
sp|O00299|CLIC1_HUMAN	NSNPALNDNLEK	119	130	664.82568	845.436323	22.8	y7
sp|O00299|CLIC1_HUMAN	NSNPALNDNLEK	119	130	664.82568	1013.526201	22.8	y9
sp|O00299|CLIC1_HUMAN	NSNPALNDNLEK	119	130	664.82568	1127.569128	22.8	y10
sp|O00303|EIF3F_HUMAN	YAYYDTER	238	245	540.735271	683.299495	19.1	y5
sp|O00303|EIF3F_HUMAN	YAYYDTER	238	245	540.735271	846.362824	19.1	y6
sp|O00303|EIF3F_HUMAN	YAYYDTER	238	245	540.735271	917.399937	19.1	y7
sp|O14745|NHRF1_HUMAN	LLVVDPETDEQLQK	87	100	813.932885	1087.526595	27.3	y9
sp|O14745|NHRF1_HUMAN	LLVVDPETDEQLQK	87	100	813.932885	1202.553538	27.3	y10
sp|O14745|NHRF1_HUMAN	LLVVDPETDEQLQK	87	100	813.932885	1301.621952	27.3	y11
sp|O14745|NHRF1_HUMAN	LVEPGSPAEK	40	49	513.776939	444.245275	18.3	y4
sp|O14745|NHRF1_HUMAN	LVEPGSPAEK	40	49	513.776939	588.298767	18.3	y6
sp|O14745|NHRF1_HUMAN	LVEPGSPAEK	40	49	513.776939	685.351531	18.3	y7
sp|O14745|NHRF1_HUMAN	LVEPGSPAEK	40	49	513.776939	814.394124	18.3	y8
sp|O14818|PSA7_HUMAN	ALLEVVQSGGK	193	203	550.819138	348.18776	19.4	y4
sp|O14818|PSA7_HUMAN	ALLEVVQSGGK	193	203	550.819138	575.314751	19.4	y6
sp|O14818|PSA7_HUMAN	ALLEVVQSGGK	193	203	550.819138	674.383165	19.4	y7
sp|O14818|PSA7_HUMAN	ALLEVVQSGGK	193	203	550.819138	803.425758	19.4	y8
sp|O14879|IFIT3_HUMAN	EAEGEQFVEEALEK	222	235	804.3754	718.361761	27	y6
sp|O14879|IFIT3_HUMAN	EAEGEQFVEEALEK	222	235	804.3754	817.430175	27	y7
sp|O14879|IFIT3_HUMAN	EAEGEQFVEEALEK	222	235	804.3754	964.498589	27	y8
sp|O14879|IFIT3_HUMAN	EAEGEQFVEEALEK	222	235	804.3754	1092.557166	27	y9
sp|O14879|IFIT3_HUMAN	VC[+57.0]NQIEFLNTEFK	37	49	821.400698	751.398481	27.5	y6
sp|O14879|IFIT3_HUMAN	VC[+57.0]NQIEFLNTEFK	37	49	821.400698	898.466895	27.5	y7
sp|O14879|IFIT3_HUMAN	VC[+57.0]NQIEFLNTEFK	37	49	821.400698	1027.509488	27.5	y8
sp|O14879|IFIT3_HUMAN	VC[+57.0]NQIEFLNTEFK	146	154	821.400698	1140.593552	27.5	y9
sp|O43175|SERA_HUMAN	TLGILGLGR	35	43	450.287277	515.355159	16.4	c5
sp|O43175|SERA_HUMAN	TLGILGLGR	35	43	450.287277	685.435535	16.4	y7
sp|O43175|SERA_HUMAN	TLGILGLGR	35	43	450.287277	685.460687	16.4	c7
sp|O43175|SERA_HUMAN	TLGILGLGR	146	154	450.287277	402.245943	16.4	y4
sp|O43175|SERA_HUMAN	TLGILGLGR	146	154	450.287277	515.330007	16.4	y5
sp|O60814|H2B1K_HUMAN	ESYSVYVYK	776	788	569.276772	310.176132	20	y2
sp|O60814|H2B1K_HUMAN	ESYSVYVYK	776	788	569.276772	572.307875	20	y4
sp|O60814|H2B1K_HUMAN	ESYSVYVYK	776	788	569.276772	671.376289	20	y5
sp|O60814|H2B1K_HUMAN	ESYSVYVYK	776	788	569.276772	758.408317	20	y6
sp|O75400|PR40A_HUMAN	EPAFEDITLESER	328	341	768.364835	520.236166	26	y4
sp|O75400|PR40A_HUMAN	EPAFEDITLESER	328	341	768.364835	734.367909	26	y6
sp|O75400|PR40A_HUMAN	EPAFEDITLESER	328	341	768.364835	962.478916	26	y8
sp|O75400|PR40A_HUMAN	EPAFEDITLESER	328	341	768.364835	1091.521509	26	y9
sp|O75477|ERLN1_HUMAN	EALEPSGENVIQNK	97	104	764.386102	844.452307	25.8	y7
sp|O75477|ERLN1_HUMAN	EALEPSGENVIQNK	97	104	764.386102	901.473771	25.8	y8
sp|O75477|ERLN1_HUMAN	EALEPSGENVIQNK	97	104	764.386102	988.505799	25.8	y9
sp|O75477|ERLN1_HUMAN	EALEPSGENVIQNK	97	104	764.386102	1085.558563	25.8	y10
sp|O75608|LYPA1_HUMAN	ALIDQEVK	97	104	458.260925	147.112804	16.7	y1
sp|O75608|LYPA1_HUMAN	ALIDQEVK	97	104	458.260925	503.282388	16.7	y4
sp|O75608|LYPA1_HUMAN	ALIDQEVK	97	104	458.260925	618.309331	16.7	y5
sp|O75608|LYPA1_HUMAN	ALIDQEVK	97	104	458.260925	731.393395	16.7	y6
sp|O75947|ATP5H_HUMAN	AGLVDDFEK	63	71	497.248014	538.250754	17.8	y4
sp|O75947|ATP5H_HUMAN	AGLVDDFEK	63	71	497.248014	653.277697	17.8	y5
sp|O75947|ATP5H_HUMAN	AGLVDDFEK	63	71	497.248014	752.346111	17.8	y6
sp|O75947|ATP5H_HUMAN	AGLVDDFEK	63	71	497.248014	865.430175	17.8	y7
sp|O75964|ATP5L_HUMAN	LATFWYYAK	26	34	581.800217	544.276575	20.4	y4
sp|O75964|ATP5L_HUMAN	LATFWYYAK	26	34	581.800217	730.355888	20.4	y5
sp|O75964|ATP5L_HUMAN	LATFWYYAK	26	34	581.800217	877.424302	20.4	y6
sp|O75964|ATP5L_HUMAN	LATFWYYAK	26	34	581.800217	978.47198	20.4	y7
sp|O94855-2|SC24D_HUMAN	ILFQPQTNVYDSLAK	608	622	868.964519	418.26601	29	y4
sp|O94855-2|SC24D_HUMAN	ILFQPQTNVYDSLAK	608	622	868.964519	909.467623	29	y8
sp|O94855-2|SC24D_HUMAN	ILFQPQTNVYDSLAK	608	622	868.964519	1010.515302	29	y9
sp|O94855-2|SC24D_HUMAN	ILFQPQTNVYDSLAK	608	622	868.964519	1235.626643	29	y11

sp|O94925-3|GLSK_HUMAN	DGPGETDAFGNSEGK	106	120	740.812967	591.27328	25.1	y6
sp|O94925-3|GLSK_HUMAN	DGPGETDAFGNSEGK	106	120	740.812967	809.378808	25.1	y8
sp|O94925-3|GLSK_HUMAN	DGPGETDAFGNSEGK	106	120	740.812967	924.405751	25.1	y9
sp|O94925-3|GLSK_HUMAN	DGPGETDAFGNSEGK	106	120	740.812967	1025.45343	25.1	y10
sp|O95154|ARK73_HUMAN	FQLETSLK	92	99	483.26875	448.276575	17.4	y4
sp|O95154|ARK73_HUMAN	FQLETSLK	92	99	483.26875	577.319168	17.4	y5
sp|O95154|ARK73_HUMAN	FQLETSLK	92	99	483.26875	690.403232	17.4	y6
sp|O95154|ARK73_HUMAN	FQLETSLK	92	99	483.26875	818.461809	17.4	y7
sp|O95163|ELP1_HUMAN	SVVLQLADGQIFK	567	579	709.406109	592.345323	24.2	y5
sp|O95163|ELP1_HUMAN	SVVLQLADGQIFK	567	579	709.406109	778.40938	24.2	y7
sp|O95163|ELP1_HUMAN	SVVLQLADGQIFK	567	579	709.406109	891.493444	24.2	y8
sp|O95163|ELP1_HUMAN	SVVLQLADGQIFK	567	579	709.406109	1019.552021	24.2	y9
sp|P00325|ADH1B_HUMAN	AAVLWEVK	12	19	458.268553	375.223811	16.7	y3
sp|P00325|ADH1B_HUMAN	AAVLWEVK	12	19	458.268553	561.303124	16.7	y4
sp|P00325|ADH1B_HUMAN	AAVLWEVK	12	19	458.268553	674.387188	16.7	y5
sp|P00325|ADH1B_HUMAN	AAVLWEVK	12	19	458.268553	773.455602	16.7	y6
sp|P00325|ADH1B_HUMAN	MVAVGIC[+57.0]R	40	47	453.238415	505.255128	16.5	y4
sp|P00325|ADH1B_HUMAN	MVAVGIC[+57.0]R	40	47	453.238415	604.323542	16.5	y5
sp|P00325|ADH1B_HUMAN	MVAVGIC[+57.0]R	40	47	453.238415	675.360656	16.5	y6
sp|P02511|CRYAB_HUMAN	FSVNLDVK	74	81	461.255642	246.181218	16.7	y2
sp|P02511|CRYAB_HUMAN	FSVNLDVK	74	81	461.255642	588.335152	16.7	y5
sp|P02511|CRYAB_HUMAN	FSVNLDVK	74	81	461.255642	687.403566	16.7	y6
sp|P02511|CRYAB_HUMAN	FSVNLDVK	74	81	461.255642	774.435595	16.7	y7
sp|P02533|K1C14_HUMAN	DAEEWFFTK	300	308	586.766571	542.29731	20.5	y4
sp|P02533|K1C14_HUMAN	DAEEWFFTK	300	308	586.766571	728.376623	20.5	y5
sp|P02533|K1C14_HUMAN	DAEEWFFTK	300	308	586.766571	857.419216	20.5	y6
sp|P02533|K1C14_HUMAN	DAEEWFFTK	300	308	586.766571	986.461809	20.5	y7
sp|P02538|K2C6A_HUMAN	AIGGGLSSVGGGSSTIK	533	549	724.391187	649.351531	24.6	y7
sp|P02538|K2C6A_HUMAN	AIGGGLSSVGGGSSTIK	533	549	724.391187	706.372994	24.6	y8
sp|P02538|K2C6A_HUMAN	AIGGGLSSVGGGSSTIK	533	549	724.391187	892.473437	24.6	y10
sp|P02538|K2C6A_HUMAN	AIGGGLSSVGGGSSTIK	533	549	724.391187	979.505465	24.6	y11
sp|P02743|SAMP_HUMAN	AYSLFSYNTQGR	64	75	703.83859	575.289599	24	y5
sp|P02743|SAMP_HUMAN	AYSLFSYNTQGR	64	75	703.83859	738.352928	24	y6
sp|P02743|SAMP_HUMAN	AYSLFSYNTQGR	64	75	703.83859	825.384956	24	y7
sp|P02743|SAMP_HUMAN	AYSLFSYNTQGR	64	75	703.83859	972.45337	24	y8
sp|P02743|SAMP_HUMAN	VGEYSLYIGR	86	95	578.803488	508.287808	20.3	y4
sp|P02743|SAMP_HUMAN	VGEYSLYIGR	86	95	578.803488	621.371872	20.3	y5
sp|P02743|SAMP_HUMAN	VGEYSLYIGR	86	95	578.803488	708.403901	20.3	y6
sp|P02743|SAMP_HUMAN	VGEYSLYIGR	86	95	578.803488	871.467229	20.3	y7
sp|P02788|TRFL_HUMAN	GGSFQLNELQGLK	119	131	695.869891	687.403566	23.8	y6
sp|P02788|TRFL_HUMAN	GGSFQLNELQGLK	119	131	695.869891	801.446494	23.8	y7
sp|P02788|TRFL_HUMAN	GGSFQLNELQGLK	119	131	695.869891	914.530558	23.8	y8
sp|P02788|TRFL_HUMAN	GGSFQLNELQGLK	119	131	695.869891	1042.589135	23.8	y9
sp|P02788|TRFL_HUMAN	YLGPQYVAGITNLK	636	649	768.924666	475.287474	26	y4
sp|P02788|TRFL_HUMAN	YLGPQYVAGITNLK	636	649	768.924666	645.393001	26	y6
sp|P02788|TRFL_HUMAN	YLGPQYVAGITNLK	636	649	768.924666	716.430115	26	y7
sp|P02788|TRFL_HUMAN	YLGPQYVAGITNLK	636	649	768.924666	1260.694663	26	y12
sp|P04075|ALDOA_HUMAN	ALQASALK	304	311	401.245078	418.26601	14.9	y4
sp|P04075|ALDOA_HUMAN	ALQASALK	304	311	401.245078	489.303124	14.9	y5
sp|P04075|ALDOA_HUMAN	ALQASALK	304	311	401.245078	617.361701	14.9	y6
sp|P04075|ALDOA_HUMAN	GILAADESTGSIAK	28	41	666.853906	475.287474	22.9	y5
sp|P04075|ALDOA_HUMAN	GILAADESTGSIAK	28	41	666.853906	907.436717	22.9	y9
sp|P04075|ALDOA_HUMAN	GILAADESTGSIAK	28	41	666.853906	978.473831	22.9	y10
sp|P04075|ALDOA_HUMAN	GILAADESTGSIAK	28	41	666.853906	1049.510944	22.9	y11
sp|P04275|VWF_HUMAN	YAGSQVASTSEVLK	1348	1361	720.372463	763.41961	24.5	y7
sp|P04275|VWF_HUMAN	YAGSQVASTSEVLK	1348	1361	720.372463	834.456724	24.5	y8
sp|P04275|VWF_HUMAN	YAGSQVASTSEVLK	1348	1361	720.372463	933.525138	24.5	y9
sp|P04275|VWF_HUMAN	YAGSQVASTSEVLK	1348	1361	720.372463	1205.637208	24.5	y12
sp|P04275|VWF_HUMAN	YTLFQIFSK	1362	1370	573.813324	381.213246	20.1	y3
sp|P04275|VWF_HUMAN	YTLFQIFSK	1362	1370	573.813324	622.355888	20.1	y5
sp|P04275|VWF_HUMAN	YTLFQIFSK	1362	1370	573.813324	769.424302	20.1	y6
sp|P04275|VWF_HUMAN	YTLFQIFSK	1362	1370	573.813324	882.508366	20.1	y7
sp|P04350|TBB4A_HUMAN	YLTVAAVFR	309	317	520.300384	492.292893	18.5	y4
sp|P04350|TBB4A_HUMAN	YLTVAAVFR	309	317	520.300384	563.330007	18.5	y5
sp|P04350|TBB4A_HUMAN	YLTVAAVFR	309	317	520.300384	662.398421	18.5	y6

sp|P04350|TBB4A_HUMAN	YLTVAAVFR	309	317	520.300384	763.4461	18.5	y7
sp|P04792|HSPB1_HUMAN	AQLGGPEAAK	188	197	471.256174	289.187031	17	y3
sp|P04792|HSPB1_HUMAN	AQLGGPEAAK	188	197	471.256174	572.303852	17	y6
sp|P04792|HSPB1_HUMAN	AQLGGPEAAK	188	197	471.256174	629.325316	17	y7
sp|P04792|HSPB1_HUMAN	AQLGGPEAAK	188	197	471.256174	742.40938	17	y8
sp|P04792|HSPB1_HUMAN	LFDQAFGLPR	27	36	582.314023	272.171716	20.4	y2
sp|P04792|HSPB1_HUMAN	LFDQAFGLPR	27	36	582.314023	442.277243	20.4	y4
sp|P04792|HSPB1_HUMAN	LFDQAFGLPR	27	36	582.314023	660.382771	20.4	y6
sp|P04792|HSPB1_HUMAN	LFDQAFGLPR	27	36	582.314023	903.468292	20.4	y8
sp|P04839|CY24B_HUMAN	LLGSALALAR	44	53	492.813659	359.24013	17.7	y3
sp|P04839|CY24B_HUMAN	LLGSALALAR	44	53	492.813659	430.277243	17.7	y4
sp|P04839|CY24B_HUMAN	LLGSALALAR	44	53	492.813659	543.361307	17.7	y5
sp|P04839|CY24B_HUMAN	LLGSALALAR	44	53	492.813659	758.451913	17.7	y8
sp|P04839|CY24B_HUMAN	QSISNSESGPR	548	558	581.278366	416.225208	20.3	y4
sp|P04839|CY24B_HUMAN	QSISNSESGPR	548	558	581.278366	545.267801	20.3	y5
sp|P04839|CY24B_HUMAN	QSISNSESGPR	548	558	581.278366	632.299829	20.3	y6
sp|P04839|CY24B_HUMAN	QSISNSESGPR	548	558	581.278366	946.458849	20.3	y9
sp|P05090|APOD_HUMAN	NILTSNNIDVK	164	174	615.838059	702.37808	21.4	y6
sp|P05090|APOD_HUMAN	NILTSNNIDVK	164	174	615.838059	789.410108	21.4	y7
sp|P05090|APOD_HUMAN	NILTSNNIDVK	164	174	615.838059	890.457787	21.4	y8
sp|P05090|APOD_HUMAN	NILTSNNIDVK	164	174	615.838059	1003.541851	21.4	y9
sp|P05109|S10A8_HUMAN	ALNSIIDVYHK	7	17	636.850969	546.303458	22	y4
sp|P05109|S10A8_HUMAN	ALNSIIDVYHK	7	17	636.850969	661.330401	22	y5
sp|P05109|S10A8_HUMAN	ALNSIIDVYHK	7	17	636.850969	774.414465	22	y6
sp|P05109|S10A8_HUMAN	ALNSIIDVYHK	7	17	636.850969	887.498529	22	y7
sp|P05783|K1C18_HUMAN	LEAEIATYR	372	380	533.282388	510.267073	18.9	y4
sp|P05783|K1C18_HUMAN	LEAEIATYR	372	380	533.282388	623.351137	18.9	y5
sp|P05783|K1C18_HUMAN	LEAEIATYR	372	380	533.282388	752.39373	18.9	y6
sp|P05783|K1C18_HUMAN	LEAEIATYR	372	380	533.282388	823.430844	18.9	y7
sp|P05787|K2C8_HUMAN	ASLEAAIADAEQR	328	340	672.84133	618.284179	23.1	y5
sp|P05787|K2C8_HUMAN	ASLEAAIADAEQR	328	340	672.84133	689.321293	23.1	y6
sp|P05787|K2C8_HUMAN	ASLEAAIADAEQR	328	340	672.84133	727.398481	23.1	b8
sp|P05787|K2C8_HUMAN	ASLEAAIADAEQR	328	340	672.84133	802.405357	23.1	y7
sp|P05787|K2C8_HUMAN	LSELEAALQR	352	361	565.31422	558.335821	19.9	y5
sp|P05787|K2C8_HUMAN	LSELEAALQR	352	361	565.31422	687.378414	19.9	y6
sp|P05787|K2C8_HUMAN	LSELEAALQR	352	361	565.31422	800.462478	19.9	y7
sp|P05787|K2C8_HUMAN	LSELEAALQR	352	361	565.31422	929.505071	19.9	y8
sp|P06730|IF4E_HUMAN	IAIWTTEC[+57.0]ENR	162	172	696.832451	578.235121	23.8	y4
sp|P06730|IF4E_HUMAN	IAIWTTEC[+57.0]ENR	162	172	696.832451	808.325392	23.8	y6
sp|P06730|IF4E_HUMAN	IAIWTTEC[+57.0]ENR	162	172	696.832451	909.373071	23.8	y7
sp|P06730|IF4E_HUMAN	IAIWTTEC[+57.0]ENR	162	172	696.832451	1095.452384	23.8	y8
sp|P07602|SAP_HUMAN	GC[+57.0]SFLPDPYQK	438	448	656.305538	535.287474	22.6	y4
sp|P07602|SAP_HUMAN	GC[+57.0]SFLPDPYQK	438	448	656.305538	747.367181	22.6	y6
sp|P07602|SAP_HUMAN	GC[+57.0]SFLPDPYQK	438	448	656.305538	860.451245	22.6	y7
sp|P07602|SAP_HUMAN	GC[+57.0]SFLPDPYQK	438	448	656.305538	1094.551687	22.6	y9
sp|P07864|LDHC_HUMAN	VIGSGC[+57.0]NLDSAR	157	168	624.803693	448.215037	21.6	y4
sp|P07864|LDHC_HUMAN	VIGSGC[+57.0]NLDSAR	157	168	624.803693	675.342029	21.6	y6
sp|P07864|LDHC_HUMAN	VIGSGC[+57.0]NLDSAR	157	168	624.803693	892.394141	21.6	y8
sp|P07864|LDHC_HUMAN	VIGSGC[+57.0]NLDSAR	157	168	624.803693	1036.447633	21.6	y10
sp|P07942|LAMB1_HUMAN	ELAEQLEFIK	77	84	610.332078	977.530223	21.2	y8
sp|P07942|LAMB1_HUMAN	ELAEQLEFIK	1300	1309	610.332078	536.307875	21.2	y4
sp|P07942|LAMB1_HUMAN	ELAEQLEFIK	1300	1309	610.332078	649.391939	21.2	y5
sp|P07942|LAMB1_HUMAN	ELAEQLEFIK	1300	1309	610.332078	777.450516	21.2	y6
sp|P08294|SODE_HUMAN	VTGVVLFR	77	84	445.776545	435.27143	16.3	y3
sp|P08294|SODE_HUMAN	VTGVVLFR	77	84	445.776545	534.339844	16.3	y4
sp|P08294|SODE_HUMAN	VTGVVLFR	77	84	445.776545	690.429721	16.3	y6
sp|P08294|SODE_HUMAN	VTGVVLFR	77	84	445.776545	791.4774	16.3	y7
sp|P08572|CO4A2_HUMAN	SVSIGYLLVK	1484	1493	539.828974	635.412674	19.1	y5
sp|P08572|CO4A2_HUMAN	SVSIGYLLVK	1484	1493	539.828974	692.434138	19.1	y6
sp|P08572|CO4A2_HUMAN	SVSIGYLLVK	1484	1493	539.828974	805.518202	19.1	y7
sp|P08572|CO4A2_HUMAN	SVSIGYLLVK	1484	1493	539.828974	892.55023	19.1	y8
sp|P08727|K1C19_HUMAN	FGPGVAFR	25	32	425.732137	393.22448	15.7	y3
sp|P08727|K1C19_HUMAN	FGPGVAFR	25	32	425.732137	492.292893	15.7	y4
sp|P08727|K1C19_HUMAN	FGPGVAFR	25	32	425.732137	549.314357	15.7	y5
sp|P08727|K1C19_HUMAN	FGPGVAFR	25	32	425.732137	646.367121	15.7	y6

sp|P08727|K1C19_HUMAN	ILGATIENSR	140	149	537.301112	505.236501	19	y4
sp|P08727|K1C19_HUMAN	ILGATIENSR	140	149	537.301112	618.320565	19	y5
sp|P08727|K1C19_HUMAN	ILGATIENSR	140	149	537.301112	719.368243	19	y6
sp|P08727|K1C19_HUMAN	ILGATIENSR	140	149	537.301112	847.426821	19	y8
sp|P08779|K1C16_HUMAN	EVFTSSSSSSSR	441	452	630.788763	610.279094	21.8	y6
sp|P08779|K1C16_HUMAN	EVFTSSSSSSSR	441	452	630.788763	697.311122	21.8	y7
sp|P08779|K1C16_HUMAN	EVFTSSSSSSSR	441	452	630.788763	784.343151	21.8	y8
sp|P08779|K1C16_HUMAN	EVFTSSSSSSSR	441	452	630.788763	885.390829	21.8	y9
sp|P08865|RSSA_HUMAN	LLVVTDPR	120	127	456.779285	488.246337	16.6	y4
sp|P08865|RSSA_HUMAN	LLVVTDPR	120	127	456.779285	587.314751	16.6	y5
sp|P08865|RSSA_HUMAN	LLVVTDPR	120	127	456.779285	686.383165	16.6	y6
sp|P09455|RET1_HUMAN	EFEEDLTGIDDR	69	80	719.82026	405.172838	24.5	y3
sp|P09455|RET1_HUMAN	EFEEDLTGIDDR	69	80	719.82026	575.278366	24.5	y5
sp|P09455|RET1_HUMAN	EFEEDLTGIDDR	69	80	719.82026	676.326044	24.5	y6
sp|P09455|RET1_HUMAN	EFEEDLTGIDDR	69	80	719.82026	904.437051	24.5	y8
sp|P09455|RET1_HUMAN	IANLLKPDK	32	40	506.313492	359.192511	18.1	y3
sp|P09455|RET1_HUMAN	IANLLKPDK	32	40	506.313492	487.287474	18.1	y4
sp|P09455|RET1_HUMAN	IANLLKPDK	32	40	506.313492	600.371538	18.1	y5
sp|P09455|RET1_HUMAN	IANLLKPDK	32	40	506.313492	827.498529	18.1	y7
sp|P09488|GSTM1_HUMAN	LYSEFLGK	136	143	478.758018	464.286745	17.3	y4
sp|P09488|GSTM1_HUMAN	LYSEFLGK	136	143	478.758018	593.329339	17.3	y5
sp|P09488|GSTM1_HUMAN	LYSEFLGK	136	143	478.758018	680.361367	17.3	y6
sp|P09488|GSTM1_HUMAN	LYSEFLGK	136	143	478.758018	843.424696	17.3	y7
sp|P09488|GSTM1_HUMAN	YLEELPEK	126	133	510.76604	486.292225	18.2	y4
sp|P09488|GSTM1_HUMAN	YLEELPEK	126	133	510.76604	615.334818	18.2	y5
sp|P09488|GSTM1_HUMAN	YLEELPEK	126	133	510.76604	744.377411	18.2	y6
sp|P09488|GSTM1_HUMAN	YLEELPEK	126	133	510.76604	857.461475	18.2	y7
sp|P09493-6|TPM1_HUMAN	AEDSLLAAEEAAAK	268	283	694.848821	489.266738	23.8	y5
sp|P09493-6|TPM1_HUMAN	AEDSLLAAEEAAAK	268	283	694.848821	618.309331	23.8	y6
sp|P09493-6|TPM1_HUMAN	AEDSLLAAEEAAAK	268	283	694.848821	760.383559	23.8	y8
sp|P09493-6|TPM1_HUMAN	AEDSLLAAEEAAAK	268	283	694.848821	873.467623	23.8	y9
sp|P09493-6|TPM1_HUMAN	VLEELHK	61	67	434.25036	397.25578	15.9	y3
sp|P09493-6|TPM1_HUMAN	VLEELHK	61	67	434.25036	526.298373	15.9	y4
sp|P09493-6|TPM1_HUMAN	VLEELHK	61	67	434.25036	655.340966	15.9	y5
sp|P09493-6|TPM1_HUMAN	VLEELHK	61	67	434.25036	768.42503	15.9	y6
sp|P09874|PARP1_HUMAN	TTNFAGILSQGLR	865	877	689.377883	560.315085	23.6	y5
sp|P09874|PARP1_HUMAN	TTNFAGILSQGLR	865	877	689.377883	673.399149	23.6	y6
sp|P09874|PARP1_HUMAN	TTNFAGILSQGLR	865	877	689.377883	843.504677	23.6	y8
sp|P09874|PARP1_HUMAN	TTNFAGILSQGLR	865	877	689.377883	914.541791	23.6	y9
sp|P09874|PARP1_HUMAN	VVSEDFLQDVSASTK	452	466	812.906867	493.261653	27.3	y5
sp|P09874|PARP1_HUMAN	VVSEDFLQDVSASTK	452	466	812.906867	707.35701	27.3	y7
sp|P09874|PARP1_HUMAN	VVSEDFLQDVSASTK	452	466	812.906867	835.415587	27.3	y8
sp|P09874|PARP1_HUMAN	VVSEDFLQDVSASTK	452	466	812.906867	948.499651	27.3	y9
sp|P0C0S5|H2AZ_HUMAN	ATIAGGGVIPHIHK	102	115	685.898785	631.367455	23.5	y5
sp|P0C0S5|H2AZ_HUMAN	ATIAGGGVIPHIHK	102	115	685.898785	744.451519	23.5	y6
sp|P0C0S5|H2AZ_HUMAN	ATIAGGGVIPHIHK	102	115	685.898785	957.562861	23.5	y9
sp|P0C0S5|H2AZ_HUMAN	ATIAGGGVIPHIHK	102	115	685.898785	1014.584325	23.5	y10
sp|P0C0S5|H2AZ_HUMAN	GDEELDSLIK	92	101	559.782418	460.31296	19.7	y4
sp|P0C0S5|H2AZ_HUMAN	GDEELDSLIK	92	101	559.782418	575.339903	19.7	y5
sp|P0C0S5|H2AZ_HUMAN	GDEELDSLIK	92	101	559.782418	688.423967	19.7	y6
sp|P0C0S5|H2AZ_HUMAN	GDEELDSLIK	92	101	559.782418	817.46656	19.7	y7
sp|P0CB38|PAB4L_HUMAN	NLDDTIDDEK	298	307	589.26459	506.209283	20.6	y4
sp|P0CB38|PAB4L_HUMAN	NLDDTIDDEK	298	307	589.26459	720.341026	20.6	y6
sp|P0CB38|PAB4L_HUMAN	NLDDTIDDEK	298	307	589.26459	835.367969	20.6	y7
sp|P0CB38|PAB4L_HUMAN	NLDDTIDDEK	298	307	589.26459	950.394912	20.6	y8
sp|P11047|LAMC1_HUMAN	NTIEETGNLAEQAR	1121	1134	773.378808	503.257236	26.1	y4
sp|P11047|LAMC1_HUMAN	NTIEETGNLAEQAR	1121	1134	773.378808	574.29435	26.1	y5
sp|P11047|LAMC1_HUMAN	NTIEETGNLAEQAR	1121	1134	773.378808	858.442805	26.1	y8
sp|P11047|LAMC1_HUMAN	NTIEETGNLAEQAR	1121	1134	773.378808	1217.57567	26.1	y11
sp|P11277-2|SPTB1_HUMAN	ETWLSENQR	434	442	581.778001	417.220457	20.4	y3
sp|P11277-2|SPTB1_HUMAN	ETWLSENQR	434	442	581.778001	546.26305	20.4	y4
sp|P11277-2|SPTB1_HUMAN	ETWLSENQR	434	442	581.778001	633.295078	20.4	y5
sp|P11277-2|SPTB1_HUMAN	ETWLSENQR	434	442	581.778001	746.379142	20.4	y6
sp|P11387|TOP1_HUMAN	AEEVATFFAK	252	261	556.784764	613.334424	19.6	y5
sp|P11387|TOP1_HUMAN	AEEVATFFAK	252	261	556.784764	684.371538	19.6	y6

sp|P11387|TOP1_HUMAN	AEEVATFFAK	252	261	556.784764	783.439952	19.6	y7
sp|P11387|TOP1_HUMAN	AEEVATFFAK	252	261	556.784764	912.482545	19.6	y8
sp|P12273|PIP_HUMAN	YTAC[+57.0]LC[+57.0]DDNPK	85	95	678.78157	748.29303	23.3	y6
sp|P12273|PIP_HUMAN	YTAC[+57.0]LC[+57.0]DDNPK	85	95	678.78157	861.377094	23.3	y7
sp|P12273|PIP_HUMAN	YTAC[+57.0]LC[+57.0]DDNPK	85	95	678.78157	1021.407742	23.3	y8
sp|P12273|PIP_HUMAN	YTAC[+57.0]LC[+57.0]DDNPK	85	95	678.78157	1092.444856	23.3	y9
sp|P13489|RINI_HUMAN	LDDC[+57.0]GLTEAR	34	43	575.26387	476.246337	20.2	y4
sp|P13489|RINI_HUMAN	LDDC[+57.0]GLTEAR	34	43	575.26387	646.351865	20.2	y6
sp|P13489|RINI_HUMAN	LDDC[+57.0]GLTEAR	34	43	575.26387	921.409457	20.2	y8
sp|P13647|K2C5_HUMAN	LAELEEALQK	431	440	572.316428	588.335152	20.1	y5
sp|P13647|K2C5_HUMAN	LAELEEALQK	431	440	572.316428	717.377745	20.1	y6
sp|P13647|K2C5_HUMAN	LAELEEALQK	431	440	572.316428	830.461809	20.1	y7
sp|P13647|K2C5_HUMAN	LAELEEALQK	431	440	572.316428	959.504403	20.1	y8
sp|P13647|K2C5_HUMAN	VSLAGAC[+57.0]GVGGYGSR	48	62	705.84335	596.2787	24.1	y6
sp|P13647|K2C5_HUMAN	VSLAGAC[+57.0]GVGGYGSR	48	62	705.84335	752.368578	24.1	y8
sp|P13647|K2C5_HUMAN	VSLAGAC[+57.0]GVGGYGSR	48	62	705.84335	912.399226	24.1	y9
sp|P13647|K2C5_HUMAN	VSLAGAC[+57.0]GVGGYGSR	48	62	705.84335	1040.457804	24.1	y11
sp|P14543|NID1_HUMAN	EYTVTEPER	587	595	562.266935	401.214309	19.8	y3
sp|P14543|NID1_HUMAN	EYTVTEPER	587	595	562.266935	530.256902	19.8	y4
sp|P14543|NID1_HUMAN	EYTVTEPER	587	595	562.266935	631.30458	19.8	y5
sp|P14543|NID1_HUMAN	EYTVTEPER	587	595	562.266935	831.420673	19.8	y7
sp|P15086|CBPB1_HUMAN	LGENNAELNALAK	319	331	678.859523	516.314023	23.3	y5
sp|P15086|CBPB1_HUMAN	LGENNAELNALAK	319	331	678.859523	629.398087	23.3	y6
sp|P15086|CBPB1_HUMAN	LGENNAELNALAK	319	331	678.859523	758.44068	23.3	y7
sp|P15086|CBPB1_HUMAN	LGENNAELNALAK	319	331	678.859523	829.477794	23.3	y8
sp|P15104|GLNA_HUMAN	LVLC[+57.0]EVFK	95	102	504.283346	522.292225	18	y4
sp|P15104|GLNA_HUMAN	LVLC[+57.0]EVFK	95	102	504.283346	682.322873	18	y5
sp|P15104|GLNA_HUMAN	LVLC[+57.0]EVFK	95	102	504.283346	795.406937	18	y6
sp|P15104|GLNA_HUMAN	LVLC[+57.0]EVFK	95	102	504.283346	894.475351	18	y7
sp|P15104|GLNA_HUMAN	YIEEAIEK	268	275	497.758215	460.276575	17.8	y4
sp|P15104|GLNA_HUMAN	YIEEAIEK	268	275	497.758215	589.319168	17.8	y5
sp|P15104|GLNA_HUMAN	YIEEAIEK	268	275	497.758215	718.361761	17.8	y6
sp|P15104|GLNA_HUMAN	YIEEAIEK	268	275	497.758215	831.445825	17.8	y7
sp|P15498|VAV_HUMAN	AEAEQNWWEGR	629	639	688.304915	547.262322	23.6	y4
sp|P15498|VAV_HUMAN	AEAEQNWWEGR	629	639	688.304915	733.341635	23.6	y5
sp|P15498|VAV_HUMAN	AEAEQNWWEGR	629	639	688.304915	847.384562	23.6	y6
sp|P15498|VAV_HUMAN	AEAEQNWWEGR	629	639	688.304915	975.44314	23.6	y7
sp|P15559|NQO1_HUMAN	IQILEGWK	201	208	493.78711	390.21358	17.7	y3
sp|P15559|NQO1_HUMAN	IQILEGWK	201	208	493.78711	519.256174	17.7	y4
sp|P15559|NQO1_HUMAN	IQILEGWK	201	208	493.78711	632.340238	17.7	y5
sp|P15559|NQO1_HUMAN	IQILEGWK	201	208	493.78711	745.424302	17.7	y6
sp|P17661|DESM_HUMAN	DNLLDDLQR	180	188	551.280377	531.288536	19.4	y4
sp|P17661|DESM_HUMAN	DNLLDDLQR	180	188	551.280377	646.315479	19.4	y5
sp|P17661|DESM_HUMAN	DNLLDDLQR	180	188	551.280377	759.399543	19.4	y6
sp|P17661|DESM_HUMAN	DNLLDDLQR	180	188	551.280377	872.483607	19.4	y7
sp|P17661|DESM_HUMAN	FASEASGYQDNIAR	355	368	764.854969	716.368578	25.9	y6
sp|P17661|DESM_HUMAN	FASEASGYQDNIAR	355	368	764.854969	936.45337	25.9	y8
sp|P17661|DESM_HUMAN	FASEASGYQDNIAR	355	368	764.854969	1023.485398	25.9	y9
sp|P17661|DESM_HUMAN	FASEASGYQDNIAR	355	368	764.854969	1094.522512	25.9	y10
sp|P18440|ARY1_HUMAN	NYIVDAGFGR	117	126	556.28018	436.230293	19.6	y4
sp|P18440|ARY1_HUMAN	NYIVDAGFGR	117	126	556.28018	622.29435	19.6	y6
sp|P18440|ARY1_HUMAN	NYIVDAGFGR	117	126	556.28018	721.362764	19.6	y7
sp|P18440|ARY1_HUMAN	NYIVDAGFGR	117	126	556.28018	834.446828	19.6	y8
sp|P19012|K1C15_HUMAN	AGLENSLAETEC[+57.0]R	343	355	725.335555	565.239872	24.7	y4
sp|P19012|K1C15_HUMAN	AGLENSLAETEC[+57.0]R	343	355	725.335555	694.282465	24.7	y5
sp|P19012|K1C15_HUMAN	AGLENSLAETEC[+57.0]R	343	355	725.335555	765.319579	24.7	y6
sp|P19012|K1C15_HUMAN	AGLENSLAETEC[+57.0]R	343	355	725.335555	878.403643	24.7	y7
sp|P19404|NDUV2_HUMAN	AAAVLPVLDLAQR	75	87	668.900994	602.32565	23	y5
sp|P19404|NDUV2_HUMAN	AAAVLPVLDLAQR	75	87	668.900994	911.530892	23	y8
sp|P19404|NDUV2_HUMAN	AAAVLPVLDLAQR	75	87	668.900994	1024.614956	23	y9
sp|P19404|NDUV2_HUMAN	AAAVLPVLDLAQR	75	87	668.900994	1123.68337	23	y10
sp|P20774|MIME_HUMAN	ESAYLYAR	119	126	486.742899	409.219394	17.5	y3
sp|P20774|MIME_HUMAN	ESAYLYAR	119	126	486.742899	522.303458	17.5	y4
sp|P20774|MIME_HUMAN	ESAYLYAR	119	126	486.742899	685.366787	17.5	y5
sp|P20774|MIME_HUMAN	ESAYLYAR	119	126	486.742899	756.403901	17.5	y6

sp|P21266|GSTM3_HUMAN	IAAYLQSDQFC[+57.0]K	197	208	722.350476	784.329415	24.6	y6
sp|P21266|GSTM3_HUMAN	IAAYLQSDQFC[+57.0]K	197	208	722.350476	912.387993	24.6	y7
sp|P21266|GSTM3_HUMAN	IAAYLQSDQFC[+57.0]K	197	208	722.350476	1025.472057	24.6	y8
sp|P21266|GSTM3_HUMAN	IAAYLQSDQFC[+57.0]K	197	208	722.350476	1188.535385	24.6	y9
sp|P22105|TENX_HUMAN	ILISGLEPSTPYR	3606	3618	723.403566	435.235044	24.6	y3
sp|P22105|TENX_HUMAN	ILISGLEPSTPYR	3606	3618	723.403566	849.410108	24.6	y7
sp|P22105|TENX_HUMAN	ILISGLEPSTPYR	3606	3618	723.403566	1019.515636	24.6	y9
sp|P22105|TENX_HUMAN	ILISGLEPSTPYR	3606	3618	723.403566	1106.547664	24.6	y10
sp|P22352|GPX3_HUMAN	QEPGENSEILPTLK	106	119	777.904127	571.381374	26.2	y5
sp|P22352|GPX3_HUMAN	QEPGENSEILPTLK	106	119	777.904127	900.54006	26.2	y8
sp|P22352|GPX3_HUMAN	QEPGENSEILPTLK	106	119	777.904127	1014.582987	26.2	y9
sp|P22352|GPX3_HUMAN	QEPGENSEILPTLK	106	119	777.904127	1297.699808	26.2	y12
sp|P23470|PTPRG_HUMAN	DDYFVSGAGLPGR	129	141	677.325316	329.193179	23.2	y3
sp|P23470|PTPRG_HUMAN	DDYFVSGAGLPGR	129	141	677.325316	442.277243	23.2	y4
sp|P23470|PTPRG_HUMAN	DDYFVSGAGLPGR	129	141	677.325316	499.298707	23.2	y5
sp|P23470|PTPRG_HUMAN	DDYFVSGAGLPGR	129	141	677.325316	714.389313	23.2	y8
sp|P24821-4|TENA_HUMAN	VSIYGVIR	1414	1421	453.774002	444.292893	16.5	y4
sp|P24821-4|TENA_HUMAN	VSIYGVIR	1414	1421	453.774002	607.356222	16.5	y5
sp|P24821-4|TENA_HUMAN	VSIYGVIR	1414	1421	453.774002	720.440286	16.5	y6
sp|P29590|PML_HUMAN	C[+57.0]FEAHQWFLK	150	159	683.324065	858.462084	23.4	y6
sp|P29590|PML_HUMAN	C[+57.0]FEAHQWFLK	150	159	683.324065	929.499198	23.4	y7
sp|P29590|PML_HUMAN	C[+57.0]FEAHQWFLK	150	159	683.324065	1058.541791	23.4	y8
sp|P29590|PML_HUMAN	C[+57.0]FEAHQWFLK	1414	1421	683.324065	721.403172	23.4	y5
sp|P29692-2|EF1D_HUMAN	GVVQELQQAISK	95	106	650.366984	418.26601	22.4	y4
sp|P29692-2|EF1D_HUMAN	GVVQELQQAISK	95	106	650.366984	546.324588	22.4	y5
sp|P29692-2|EF1D_HUMAN	GVVQELQQAISK	95	106	650.366984	787.467229	22.4	y7
sp|P29692-2|EF1D_HUMAN	GVVQELQQAISK	95	106	650.366984	916.509822	22.4	y8
sp|P29692-2|EF1D_HUMAN	SIQLDGLVWGASK	219	231	687.374809	362.20341	23.5	y4
sp|P29692-2|EF1D_HUMAN	SIQLDGLVWGASK	219	231	687.374809	548.282723	23.5	y5
sp|P29692-2|EF1D_HUMAN	SIQLDGLVWGASK	219	231	687.374809	647.351137	23.5	y6
sp|P29692-2|EF1D_HUMAN	SIQLDGLVWGASK	219	231	687.374809	932.483607	23.5	y9
sp|P29762|RABP1_HUMAN	VGEGFEEETVDGR	67	79	712.320427	547.283451	24.3	y5
sp|P29762|RABP1_HUMAN	VGEGFEEETVDGR	67	79	712.320427	805.368637	24.3	y7
sp|P29762|RABP1_HUMAN	VGEGFEEETVDGR	67	79	712.320427	934.41123	24.3	y8
sp|P29762|RABP1_HUMAN	VGEGFEEETVDGR	67	79	712.320427	1138.501108	24.3	y10
sp|P30040|ERP29_HUMAN	DGDFENPVPYTGAVK	122	136	804.880652	735.403566	27.1	y7
sp|P30040|ERP29_HUMAN	DGDFENPVPYTGAVK	122	136	804.880652	931.524744	27.1	y9
sp|P30040|ERP29_HUMAN	DGDFENPVPYTGAVK	122	136	804.880652	1045.567671	27.1	y10
sp|P30040|ERP29_HUMAN	DGDFENPVPYTGAVK	122	136	804.880652	1174.610265	27.1	y11
sp|P30042|ES1_HUMAN	NVLTESAR	103	110	445.240524	333.188094	16.3	y3
sp|P30042|ES1_HUMAN	NVLTESAR	103	110	445.240524	462.230687	16.3	y4
sp|P30042|ES1_HUMAN	NVLTESAR	103	110	445.240524	563.278366	16.3	y5
sp|P30042|ES1_HUMAN	NVLTESAR	103	110	445.240524	676.36243	16.3	y6
sp|P31949|S10AB_HUMAN	DGYNYTLSK	27	35	530.750921	347.228896	18.8	y3
sp|P31949|S10AB_HUMAN	DGYNYTLSK	27	35	530.750921	448.276575	18.8	y4
sp|P31949|S10AB_HUMAN	DGYNYTLSK	27	35	530.750921	611.339903	18.8	y5
sp|P31949|S10AB_HUMAN	DGYNYTLSK	27	35	530.750921	725.382831	18.8	y6
sp|P32119|PRDX2_HUMAN	LSEDYGVLK	110	118	512.271489	579.350074	18.3	y5
sp|P32119|PRDX2_HUMAN	LSEDYGVLK	110	118	512.271489	694.377017	18.3	y6
sp|P32119|PRDX2_HUMAN	LSEDYGVLK	110	118	512.271489	823.41961	18.3	y7
sp|P32119|PRDX2_HUMAN	LSEDYGVLK	110	118	512.271489	910.451639	18.3	y8
sp|P32119|PRDX2_HUMAN	TDEGIAYR	119	126	462.724706	338.18228	16.8	y2
sp|P32119|PRDX2_HUMAN	TDEGIAYR	119	126	462.724706	409.219394	16.8	y3
sp|P32119|PRDX2_HUMAN	TDEGIAYR	119	126	462.724706	579.324922	16.8	y5
sp|P32119|PRDX2_HUMAN	TDEGIAYR	119	126	462.724706	708.367515	16.8	y6
sp|P33241|LSP1_HUMAN	WETGEVQAQSAAK	266	278	702.84133	575.314751	24	y6
sp|P33241|LSP1_HUMAN	WETGEVQAQSAAK	266	278	702.84133	802.441743	24	y8
sp|P33241|LSP1_HUMAN	WETGEVQAQSAAK	266	278	702.84133	988.505799	24	y10
sp|P33241|LSP1_HUMAN	WETGEVQAQSAAK	266	278	702.84133	1089.553478	24	y11
sp|P34896|GLYC_HUMAN	ALSEALTELGYK	336	347	647.848092	609.324253	22.3	y5
sp|P34896|GLYC_HUMAN	ALSEALTELGYK	336	347	647.848092	710.371932	22.3	y6
sp|P34896|GLYC_HUMAN	ALSEALTELGYK	336	347	647.848092	823.455996	22.3	y7
sp|P34896|GLYC_HUMAN	ALSEALTELGYK	336	347	647.848092	1110.567731	22.3	y10
sp|P35573|GDE_HUMAN	SGGGYIVVDPILR	101	113	673.377351	613.366787	23.1	y5
sp|P35573|GDE_HUMAN	SGGGYIVVDPILR	101	113	673.377351	712.435201	23.1	y6

sp|P35573|GDE_HUMAN	SGGGYIVVDPILR	101	113	673.377351	811.503615	23.1	y7
sp|P35573|GDE_HUMAN	SGGGYIVVDPILR	101	113	673.377351	924.587679	23.1	y8
sp|P35908|K22E_HUMAN	LNDLEEALQQAK	441	452	686.359356	474.267073	23.5	y4
sp|P35908|K22E_HUMAN	LNDLEEALQQAK	441	452	686.359356	658.38825	23.5	y6
sp|P35908|K22E_HUMAN	LNDLEEALQQAK	441	452	686.359356	787.430844	23.5	y7
sp|P35908|K22E_HUMAN	LNDLEEALQQAK	441	452	686.359356	916.473437	23.5	y8
sp|P36551|HEM6_HUMAN	AVVPSYIPLVK	359	369	593.365724	456.318046	20.7	y4
sp|P36551|HEM6_HUMAN	AVVPSYIPLVK	359	369	593.365724	569.40211	20.7	y5
sp|P36551|HEM6_HUMAN	AVVPSYIPLVK	359	369	593.365724	916.55023	20.7	y8
sp|P36551|HEM6_HUMAN	AVVPSYIPLVK	359	369	593.365724	1015.618644	20.7	y9
sp|P36551|HEM6_HUMAN	EAEILEVLR	438	446	536.305863	387.27143	19	y3
sp|P36551|HEM6_HUMAN	EAEILEVLR	438	446	536.305863	516.314023	19	y4
sp|P36551|HEM6_HUMAN	EAEILEVLR	438	446	536.305863	629.398087	19	y5
sp|P36551|HEM6_HUMAN	EAEILEVLR	438	446	536.305863	742.482151	19	y6
sp|P38606|VATA_HUMAN	TVISQSLSK	256	264	481.779482	347.228896	17.4	y3
sp|P38606|VATA_HUMAN	TVISQSLSK	256	264	481.779482	562.319502	17.4	y5
sp|P38606|VATA_HUMAN	TVISQSLSK	256	264	481.779482	649.351531	17.4	y6
sp|P38606|VATA_HUMAN	TVISQSLSK	256	264	481.779482	762.435595	17.4	y7
sp|P39019|RS19_HUMAN	IAGQVAAANK	133	142	471.771991	332.192845	17.1	y3
sp|P39019|RS19_HUMAN	IAGQVAAANK	133	142	471.771991	403.229959	17.1	y4
sp|P39019|RS19_HUMAN	IAGQVAAANK	133	142	471.771991	573.335487	17.1	y6
sp|P39019|RS19_HUMAN	IAGQVAAANK	133	142	471.771991	758.415528	17.1	y8
sp|P39019|RS19_HUMAN	VLQALEGLK	102	110	485.800217	317.218332	17.5	y3
sp|P39019|RS19_HUMAN	VLQALEGLK	102	110	485.800217	559.344989	17.5	y5
sp|P39019|RS19_HUMAN	VLQALEGLK	102	110	485.800217	630.382102	17.5	y6
sp|P39019|RS19_HUMAN	VLQALEGLK	102	110	485.800217	758.44068	17.5	y7
sp|P41223|BUD31_HUMAN	DTNFGTNC[+57.0]IC[+57.0]R	109	119	679.284811	608.264313	23.3	y4
sp|P41223|BUD31_HUMAN	DTNFGTNC[+57.0]IC[+57.0]R	109	119	679.284811	722.30724	23.3	y5
sp|P41223|BUD31_HUMAN	DTNFGTNC[+57.0]IC[+57.0]R	109	119	679.284811	880.376382	23.3	y7
sp|P41223|BUD31_HUMAN	DTNFGTNC[+57.0]IC[+57.0]R	109	119	679.284811	1027.444796	23.3	y8
sp|P41227|NAA10_HUMAN	DLSEVSETTESTDVK	210	224	820.380879	880.425818	27.5	y8
sp|P41227|NAA10_HUMAN	DLSEVSETTESTDVK	210	224	820.380879	1009.468411	27.5	y9
sp|P41227|NAA10_HUMAN	DLSEVSETTESTDVK	210	224	820.380879	1096.500439	27.5	y10
sp|P41227|NAA10_HUMAN	DLSEVSETTESTDVK	210	224	820.380879	1195.568853	27.5	y11
sp|P42126|ECI1_HUMAN	AVQELWLR	126	133	507.790184	474.282329	18.1	y3
sp|P42126|ECI1_HUMAN	AVQELWLR	126	133	507.790184	587.366393	18.1	y4
sp|P42126|ECI1_HUMAN	AVQELWLR	126	133	507.790184	716.408986	18.1	y5
sp|P42126|ECI1_HUMAN	AVQELWLR	126	133	507.790184	844.467563	18.1	y6
sp|P46781|RS9_HUMAN	IGVLDEGK	83	90	415.734543	448.203804	15.4	y4
sp|P46781|RS9_HUMAN	IGVLDEGK	83	90	415.734543	561.287868	15.4	y5
sp|P46781|RS9_HUMAN	IGVLDEGK	83	90	415.734543	660.356282	15.4	y6
sp|P46781|RS9_HUMAN	IGVLDEGK	83	90	415.734543	717.377745	15.4	y7
sp|P46781|RS9_HUMAN	LFEGNALLR	70	78	516.795466	401.28708	18.4	y3
sp|P46781|RS9_HUMAN	LFEGNALLR	70	78	516.795466	586.367121	18.4	y5
sp|P46781|RS9_HUMAN	LFEGNALLR	70	78	516.795466	643.388585	18.4	y6
sp|P46781|RS9_HUMAN	LFEGNALLR	70	78	516.795466	772.431178	18.4	y7
sp|P46977|STT3A_HUMAN	ENDYYTPTGEFR	613	624	746.32297	706.351865	25.3	y6
sp|P46977|STT3A_HUMAN	ENDYYTPTGEFR	613	624	746.32297	807.399543	25.3	y7
sp|P46977|STT3A_HUMAN	ENDYYTPTGEFR	613	624	746.32297	970.462872	25.3	y8
sp|P46977|STT3A_HUMAN	ENDYYTPTGEFR	613	624	746.32297	1133.526201	25.3	y9
sp|P46977|STT3A_HUMAN	FGQVYTEAK	646	654	521.763831	448.240189	18.6	y4
sp|P46977|STT3A_HUMAN	FGQVYTEAK	646	654	521.763831	611.303518	18.6	y5
sp|P46977|STT3A_HUMAN	FGQVYTEAK	646	654	521.763831	710.371932	18.6	y6
sp|P46977|STT3A_HUMAN	FGQVYTEAK	646	654	521.763831	838.430509	18.6	y7
sp|P47756-2|CAPZB_HUMAN	STLNEIYFGK	225	234	586.303321	514.26601	20.5	y4
sp|P47756-2|CAPZB_HUMAN	STLNEIYFGK	225	234	586.303321	627.350074	20.5	y5
sp|P47756-2|CAPZB_HUMAN	STLNEIYFGK	225	234	586.303321	870.435595	20.5	y7
sp|P47756-2|CAPZB_HUMAN	STLNEIYFGK	225	234	586.303321	983.519659	20.5	y8
sp|P49321-3|NASP_HUMAN	SIEVIENR	609	616	480.261456	418.204472	17.3	y3
sp|P49321-3|NASP_HUMAN	SIEVIENR	609	616	480.261456	531.288536	17.3	y4
sp|P49321-3|NASP_HUMAN	SIEVIENR	609	616	480.261456	630.35695	17.3	y5
sp|P49321-3|NASP_HUMAN	SIEVIENR	609	616	480.261456	759.399543	17.3	y6
sp|P49747|COMP_HUMAN	ELQETNAALQDVR	36	48	743.878444	517.272886	25.2	y4
sp|P49747|COMP_HUMAN	ELQETNAALQDVR	36	48	743.878444	630.35695	25.2	y5
sp|P49747|COMP_HUMAN	ELQETNAALQDVR	36	48	743.878444	701.394064	25.2	y6

sp|P49747|COMP_HUMAN	ELQETNAALQDVR	36	48	743.878444	772.431178	25.2	y7
sp|P50238|CRIP1_HUMAN	EVYFAER	9	15	457.224342	304.161545	16.6	y2
sp|P50238|CRIP1_HUMAN	EVYFAER	9	15	457.224342	375.198659	16.6	y3
sp|P50238|CRIP1_HUMAN	EVYFAER	9	15	457.224342	522.267073	16.6	y4
sp|P50238|CRIP1_HUMAN	EVYFAER	9	15	457.224342	685.330401	16.6	y5
sp|P50552|VASP_HUMAN	DESANQEEPEAR	286	297	687.792034	472.251423	23.5	y4
sp|P50552|VASP_HUMAN	DESANQEEPEAR	286	297	687.792034	601.294016	23.5	y5
sp|P50552|VASP_HUMAN	DESANQEEPEAR	286	297	687.792034	730.336609	23.5	y6
sp|P50552|VASP_HUMAN	DESANQEEPEAR	286	297	687.792034	972.438114	23.5	y8
sp|P51553|IDH3G_HUMAN	DIDILIVR	159	166	478.792392	387.27143	17.3	y3
sp|P51553|IDH3G_HUMAN	DIDILIVR	159	166	478.792392	500.355494	17.3	y4
sp|P51553|IDH3G_HUMAN	DIDILIVR	159	166	478.792392	613.439558	17.3	y5
sp|P51553|IDH3G_HUMAN	DIDILIVR	159	166	478.792392	728.466501	17.3	y6
sp|P51553|IDH3G_HUMAN	LGDGLFLQC[+57.0]C[+57.0]R	226	236	669.818289	495.180249	23	y3
sp|P51553|IDH3G_HUMAN	LGDGLFLQC[+57.0]C[+57.0]R	226	236	669.818289	623.238826	23	y4
sp|P51553|IDH3G_HUMAN	LGDGLFLQC[+57.0]C[+57.0]R	226	236	669.818289	736.32289	23	y5
sp|P51553|IDH3G_HUMAN	LGDGLFLQC[+57.0]C[+57.0]R	226	236	669.818289	883.391304	23	y6
sp|P51809|VAMP7_HUMAN	AFNFLNEIK	74	82	548.295299	503.282388	19.4	y4
sp|P51809|VAMP7_HUMAN	AFNFLNEIK	74	82	548.295299	616.366452	19.4	y5
sp|P51809|VAMP7_HUMAN	AFNFLNEIK	74	82	548.295299	763.434866	19.4	y6
sp|P51809|VAMP7_HUMAN	AFNFLNEIK	125	136	548.295299	877.477794	19.4	y7
sp|P51809|VAMP7_HUMAN	VMETQAQVDELK	125	136	695.847767	504.266404	23.8	y4
sp|P51809|VAMP7_HUMAN	VMETQAQVDELK	125	136	695.847767	802.430509	23.8	y7
sp|P51809|VAMP7_HUMAN	VMETQAQVDELK	125	136	695.847767	1031.536765	23.8	y9
sp|P51809|VAMP7_HUMAN	VMETQAQVDELK	125	136	695.847767	1160.579358	23.8	y10
sp|P51911|CNN1_HUMAN	EWIEGVTGR	35	43	523.766905	333.188094	18.6	y3
sp|P51911|CNN1_HUMAN	EWIEGVTGR	35	43	523.766905	432.256508	18.6	y4
sp|P51911|CNN1_HUMAN	EWIEGVTGR	35	43	523.766905	489.277972	18.6	y5
sp|P51911|CNN1_HUMAN	EWIEGVTGR	35	43	523.766905	618.320565	18.6	y6
sp|P52434|RPAB3_HUMAN	IEGDETSTEAATR	98	110	690.317885	648.33113	23.6	y6
sp|P52434|RPAB3_HUMAN	IEGDETSTEAATR	98	110	690.317885	735.363158	23.6	y7
sp|P52434|RPAB3_HUMAN	IEGDETSTEAATR	98	110	690.317885	836.410836	23.6	y8
sp|P52434|RPAB3_HUMAN	IEGDETSTEAATR	98	110	690.317885	1137.501836	23.6	y11
sp|P53999|TCP4_HUMAN	EQISDIDDAVR	114	124	630.806956	575.278366	21.8	y5
sp|P53999|TCP4_HUMAN	EQISDIDDAVR	114	124	630.806956	688.36243	21.8	y6
sp|P53999|TCP4_HUMAN	EQISDIDDAVR	114	124	630.806956	803.389373	21.8	y7
sp|P53999|TCP4_HUMAN	EQISDIDDAVR	114	124	630.806956	890.421401	21.8	y8
sp|P55268|LAMB2_HUMAN	LGIVQGIVGAR	620	629	541.837665	799.478462	19.2	y8
sp|P55268|LAMB2_HUMAN	LGIVQGIVGAR	1237	1247	541.837665	402.245943	19.2	y4
sp|P55268|LAMB2_HUMAN	LGIVQGIVGAR	1237	1247	541.837665	572.351471	19.2	y6
sp|P55268|LAMB2_HUMAN	LGIVQGIVGAR	1237	1247	541.837665	700.410049	19.2	y7
sp|P57737|CORO7_HUMAN	IWDLQAGADR	620	629	572.790912	418.204472	20.1	y4
sp|P57737|CORO7_HUMAN	IWDLQAGADR	620	629	572.790912	489.241586	20.1	y5
sp|P57737|CORO7_HUMAN	IWDLQAGADR	620	629	572.790912	617.300164	20.1	y6
sp|P57737|CORO7_HUMAN	IWDLQAGADR	620	629	572.790912	845.411171	20.1	y8
sp|P57737|CORO7_HUMAN	SLQSLLGPSSK	461	471	558.816595	418.229624	19.7	y4
sp|P57737|CORO7_HUMAN	SLQSLLGPSSK	461	471	558.816595	475.251088	19.7	y5
sp|P57737|CORO7_HUMAN	SLQSLLGPSSK	461	471	558.816595	588.335152	19.7	y6
sp|P57737|CORO7_HUMAN	SLQSLLGPSSK	461	471	558.816595	788.451245	19.7	y8
sp|P58107|EPIPL_HUMAN	AEIIDQDLYER	574	584	682.838256	938.421401	23.4	y7
sp|P58107|EPIPL_HUMAN	AEIIDQDLYER	574	584	682.838256	1051.505465	23.4	y8
sp|P58107|EPIPL_HUMAN	AEIIDQDLYER	574	584	682.838256	1164.589529	23.4	y9
sp|P58107|EPIPL_HUMAN	ALQQGLVGLELK	97	108	634.88227	559.344989	22	y5
sp|P58107|EPIPL_HUMAN	ALQQGLVGLELK	97	108	634.88227	658.413403	22	y6
sp|P58107|EPIPL_HUMAN	ALQQGLVGLELK	97	108	634.88227	828.51893	22	y8
sp|P58107|EPIPL_HUMAN	ALQQGLVGLELK	97	108	634.88227	956.577508	22	y9
sp|P59665|DEF1_HUMAN	YGTC[+57.0]IYQGR	79	87	559.25839	360.198993	19.7	y3
sp|P59665|DEF1_HUMAN	YGTC[+57.0]IYQGR	79	87	559.25839	523.262322	19.7	y4
sp|P59665|DEF1_HUMAN	YGTC[+57.0]IYQGR	79	87	559.25839	636.346386	19.7	y5
sp|P59665|DEF1_HUMAN	YGTC[+57.0]IYQGR	79	87	559.25839	796.377034	19.7	y6
sp|P60866|RS20_HUMAN	LIDLHSPSEIVK	87	98	675.885009	759.424696	23.2	y7
sp|P60866|RS20_HUMAN	LIDLHSPSEIVK	87	98	675.885009	896.483607	23.2	y8
sp|P60866|RS20_HUMAN	LIDLHSPSEIVK	87	98	675.885009	1009.567671	23.2	y9
sp|P60866|RS20_HUMAN	LIDLHSPSEIVK	87	98	675.885009	1124.594615	23.2	y10
sp|P60903|S10AA_HUMAN	EFPGFLENQK	37	46	604.800945	389.214309	21	y3

sp|P60903|S10AA_HUMAN	EFPGFLENQK	37	46	604.800945	518.256902	21	y4
sp|P60903|S10AA_HUMAN	EFPGFLENQK	37	46	604.800945	631.340966	21	y5
sp|P60903|S10AA_HUMAN	EFPGFLENQK	37	46	604.800945	932.483607	21	y8
sp|P61077-3|UB2D3_HUMAN	SQWSPALTISK	90	100	609.32987	448.276575	21.2	y4
sp|P61077-3|UB2D3_HUMAN	SQWSPALTISK	90	100	609.32987	729.450516	21.2	y7
sp|P61077-3|UB2D3_HUMAN	SQWSPALTISK	90	100	609.32987	816.482545	21.2	y8
sp|P61077-3|UB2D3_HUMAN	SQWSPALTISK	90	100	609.32987	1002.561858	21.2	y9
sp|P61604|CH10_HUMAN	GGEIQPVSVK	56	65	507.284931	333.213246	18.1	y3
sp|P61604|CH10_HUMAN	GGEIQPVSVK	56	65	507.284931	529.334424	18.1	y5
sp|P61604|CH10_HUMAN	GGEIQPVSVK	56	65	507.284931	657.393001	18.1	y6
sp|P61604|CH10_HUMAN	GGEIQPVSVK	56	65	507.284931	770.477066	18.1	y7
sp|P61604|CH10_HUMAN	VLLPEYGGTK	70	79	538.802957	525.266738	19.1	y5
sp|P61604|CH10_HUMAN	VLLPEYGGTK	70	79	538.802957	751.362095	19.1	y7
sp|P61604|CH10_HUMAN	VLLPEYGGTK	70	79	538.802957	864.446159	19.1	y8
sp|P61604|CH10_HUMAN	VLLPEYGGTK	70	79	538.802957	977.530223	19.1	y9
sp|P61769|B2MG_HUMAN	VEHSDLSFSK	68	77	574.782753	581.329339	20.1	y5
sp|P61769|B2MG_HUMAN	VEHSDLSFSK	68	77	574.782753	696.356282	20.1	y6
sp|P61769|B2MG_HUMAN	VEHSDLSFSK	68	77	574.782753	783.38831	20.1	y7
sp|P61769|B2MG_HUMAN	VEHSDLSFSK	68	77	574.782753	920.447222	20.1	y8
sp|P61981|1433G_HUMAN	YLAEVATGEK	132	141	540.782221	505.261653	19.1	y5
sp|P61981|1433G_HUMAN	YLAEVATGEK	132	141	540.782221	604.330067	19.1	y6
sp|P61981|1433G_HUMAN	YLAEVATGEK	132	141	540.782221	733.37266	19.1	y7
sp|P61981|1433G_HUMAN	YLAEVATGEK	132	141	540.782221	804.409774	19.1	y8
sp|P62424|RL7A_HUMAN	AGVNTVTTLVENK	137	149	673.369723	489.266738	23.1	y4
sp|P62424|RL7A_HUMAN	AGVNTVTTLVENK	137	149	673.369723	703.398481	23.1	y6
sp|P62424|RL7A_HUMAN	AGVNTVTTLVENK	137	149	673.369723	804.446159	23.1	y7
sp|P62424|RL7A_HUMAN	AGVNTVTTLVENK	137	149	673.369723	903.514573	23.1	y8
sp|P62750|RL23A_HUMAN	LAPDYDALDVANK	139	151	702.853906	546.288202	24	y5
sp|P62750|RL23A_HUMAN	LAPDYDALDVANK	139	151	702.853906	730.40938	24	y7
sp|P62750|RL23A_HUMAN	LAPDYDALDVANK	139	151	702.853906	1008.499651	24	y9
sp|P62750|RL23A_HUMAN	LAPDYDALDVANK	139	151	702.853906	1220.579358	24	y11
sp|P62829|RL23_HUMAN	NLYIISVK	35	42	475.289485	333.213246	17.2	y3
sp|P62829|RL23_HUMAN	NLYIISVK	35	42	475.289485	446.29731	17.2	y4
sp|P62829|RL23_HUMAN	NLYIISVK	35	42	475.289485	559.381374	17.2	y5
sp|P62829|RL23_HUMAN	NLYIISVK	35	42	475.289485	722.444703	17.2	y6
sp|P62834|RAP1A_HUMAN	LVVLGSGGVGK	104	116	493.305666	504.277637	17.7	y6
sp|P62834|RAP1A_HUMAN	LVVLGSGGVGK	104	116	493.305666	561.299101	17.7	y7
sp|P62834|RAP1A_HUMAN	LVVLGSGGVGK	104	116	493.305666	674.383165	17.7	y8
sp|P62834|RAP1A_HUMAN	LVVLGSGGVGK	104	116	493.305666	773.451579	17.7	y9
sp|P62851|RS25_HUMAN	AALQELLSK	85	93	486.789849	460.31296	17.5	y4
sp|P62851|RS25_HUMAN	AALQELLSK	85	93	486.789849	589.355553	17.5	y5
sp|P62851|RS25_HUMAN	AALQELLSK	85	93	486.789849	717.414131	17.5	y6
sp|P62851|RS25_HUMAN	AALQELLSK	85	93	486.789849	830.498195	17.5	y7
sp|P62851|RS25_HUMAN	LNNLVLFDK	43	51	538.310949	409.208161	19.1	y3
sp|P62851|RS25_HUMAN	LNNLVLFDK	43	51	538.310949	522.292225	19.1	y4
sp|P62851|RS25_HUMAN	LNNLVLFDK	43	51	538.310949	621.360639	19.1	y5
sp|P62851|RS25_HUMAN	LNNLVLFDK	43	51	538.310949	848.48763	19.1	y7
sp|P62888|RL30_HUMAN	LVILANNC[+57.0]PALR	44	55	677.387196	456.292893	23.2	y4
sp|P62888|RL30_HUMAN	LVILANNC[+57.0]PALR	44	55	677.387196	730.366469	23.2	y6
sp|P62888|RL30_HUMAN	LVILANNC[+57.0]PALR	44	55	677.387196	844.409397	23.2	y7
sp|P62888|RL30_HUMAN	LVILANNC[+57.0]PALR	44	55	677.387196	915.446511	23.2	y8
sp|P62888|RL30_HUMAN	SEIEYYAMLAK	57	67	659.323396	696.374909	22.7	y6
sp|P62888|RL30_HUMAN	SEIEYYAMLAK	57	67	659.323396	859.438237	22.7	y7
sp|P62888|RL30_HUMAN	SEIEYYAMLAK	57	67	659.323396	988.48083	22.7	y8
sp|P62888|RL30_HUMAN	SEIEYYAMLAK	57	67	659.323396	1101.564894	22.7	y9
sp|P67809|YBOX1_HUMAN	GAEAANVTGPGGVPVQGSK	118	136	848.436657	615.346051	28.4	y6
sp|P67809|YBOX1_HUMAN	GAEAANVTGPGGVPVQGSK	118	136	848.436657	925.510157	28.4	y10
sp|P67809|YBOX1_HUMAN	GAEAANVTGPGGVPVQGSK	118	136	848.436657	982.53162	28.4	y11
sp|P67809|YBOX1_HUMAN	GAEAANVTGPGGVPVQGSK	118	136	848.436657	1083.579299	28.4	y12
sp|P67809|YBOX1_HUMAN	NEGSESAPEGQAQQR	170	184	794.353322	687.353262	26.7	y6
sp|P67809|YBOX1_HUMAN	NEGSESAPEGQAQQR	170	184	794.353322	913.448619	26.7	y8
sp|P67809|YBOX1_HUMAN	NEGSESAPEGQAQQR	170	184	794.353322	984.485733	26.7	y9
sp|P67809|YBOX1_HUMAN	NEGSESAPEGQAQQR	170	184	794.353322	1071.517761	26.7	y10
sp|P68431|H31_HUMAN	STELLIR	57	63	416.25036	401.28708	15.4	y3
sp|P68431|H31_HUMAN	STELLIR	57	63	416.25036	514.371144	15.4	y4

sp|P68431|H31_HUMAN	STELLIR	57	63	416.25036	544.297704	15.4	b5
sp|P78347|GTF2I_HUMAN	IIQVGNR	577	583	400.242869	346.183343	14.9	y3
sp|P78347|GTF2I_HUMAN	IIQVGNR	577	583	400.242869	445.251757	14.9	y4
sp|P78347|GTF2I_HUMAN	IIQVGNR	577	583	400.242869	573.310334	14.9	y5
sp|P78347|GTF2I_HUMAN	IIQVGNR	577	583	400.242869	686.394398	14.9	y6
sp|P80217-2|IN35_HUMAN	VAEQVLQQK	89	97	521.798206	403.229959	18.6	y3
sp|P80217-2|IN35_HUMAN	VAEQVLQQK	89	97	521.798206	516.314023	18.6	y4
sp|P80217-2|IN35_HUMAN	VAEQVLQQK	89	97	521.798206	615.382437	18.6	y5
sp|P80217-2|IN35_HUMAN	VAEQVLQQK	89	97	521.798206	743.441014	18.6	y6
sp|P80303|NUCB2_HUMAN	LVTLEEFLK	311	319	546.320982	407.265282	19.3	y3
sp|P80303|NUCB2_HUMAN	LVTLEEFLK	311	319	546.320982	665.350468	19.3	y5
sp|P80303|NUCB2_HUMAN	LVTLEEFLK	311	319	546.320982	778.434532	19.3	y6
sp|P80303|NUCB2_HUMAN	LVTLEEFLK	311	319	546.320982	879.482211	19.3	y7
sp|Q00325-2|MPCP_HUMAN	FGFYEVFK	137	144	518.76056	393.249632	18.5	y3
sp|Q00325-2|MPCP_HUMAN	FGFYEVFK	137	144	518.76056	685.355553	18.5	y5
sp|Q00325-2|MPCP_HUMAN	FGFYEVFK	137	144	518.76056	832.423967	18.5	y6
sp|Q00325-2|MPCP_HUMAN	FGFYEVFK	137	144	518.76056	889.445431	18.5	y7
sp|Q00325-2|MPCP_HUMAN	IQTQPGYANTLR	189	200	681.362233	794.415528	23.3	y7
sp|Q00325-2|MPCP_HUMAN	IQTQPGYANTLR	189	200	681.362233	891.468292	23.3	y8
sp|Q00325-2|MPCP_HUMAN	IQTQPGYANTLR	189	200	681.362233	1019.526869	23.3	y9
sp|Q00325-2|MPCP_HUMAN	IQTQPGYANTLR	189	200	681.362233	1120.574548	23.3	y10
sp|Q01844|EWS_HUMAN	AAVEWFDGK	424	432	511.750724	466.229624	18.3	y4
sp|Q01844|EWS_HUMAN	AAVEWFDGK	424	432	511.750724	652.308937	18.3	y5
sp|Q01844|EWS_HUMAN	AAVEWFDGK	424	432	511.750724	781.351531	18.3	y6
sp|Q01844|EWS_HUMAN	AAVEWFDGK	424	432	511.750724	880.419945	18.3	y7
sp|Q01844|EWS_HUMAN	GDATVSYEDPPTAK	410	423	725.838453	513.303124	24.7	y5
sp|Q01844|EWS_HUMAN	GDATVSYEDPPTAK	410	423	725.838453	628.330067	24.7	y6
sp|Q01844|EWS_HUMAN	GDATVSYEDPPTAK	410	423	725.838453	920.435989	24.7	y8
sp|Q01844|EWS_HUMAN	GDATVSYEDPPTAK	410	423	725.838453	1007.468017	24.7	y9
sp|Q02224|CENPE_HUMAN	NDLDEFEALER	531	541	675.812238	488.282723	23.2	y4
sp|Q02224|CENPE_HUMAN	NDLDEFEALER	531	541	675.812238	617.325316	23.2	y5
sp|Q02224|CENPE_HUMAN	NDLDEFEALER	531	541	675.812238	764.39373	23.2	y6
sp|Q02224|CENPE_HUMAN	NDLDEFEALER	531	541	675.812238	1008.463266	23.2	y8
sp|Q02224|CENPE_HUMAN	SLFILGQVIK	260	269	559.352617	544.345323	19.7	y5
sp|Q02224|CENPE_HUMAN	SLFILGQVIK	260	269	559.352617	657.429387	19.7	y6
sp|Q02224|CENPE_HUMAN	SLFILGQVIK	260	269	559.352617	770.513451	19.7	y7
sp|Q02224|CENPE_HUMAN	SLFILGQVIK	260	269	559.352617	917.581865	19.7	y8
sp|Q02252|MMSA_HUMAN	TLADAEGDVFR	129	139	597.293484	421.25578	20.8	y3
sp|Q02252|MMSA_HUMAN	TLADAEGDVFR	129	139	597.293484	722.34678	20.8	y6
sp|Q02252|MMSA_HUMAN	TLADAEGDVFR	129	139	597.293484	793.383893	20.8	y7
sp|Q02252|MMSA_HUMAN	TLADAEGDVFR	129	139	597.293484	979.44795	20.8	y9
sp|Q03518|TAP1_HUMAN	SFANEEGEAQK	438	448	605.272749	532.272552	21.1	y5
sp|Q03518|TAP1_HUMAN	SFANEEGEAQK	438	448	605.272749	661.315145	21.1	y6
sp|Q03518|TAP1_HUMAN	SFANEEGEAQK	438	448	605.272749	904.400666	21.1	y8
sp|Q03518|TAP1_HUMAN	SFANEEGEAQK	438	448	605.272749	975.43778	21.1	y9
sp|Q04695|K1C17_HUMAN	DAEDWFFSK	269	277	572.750921	528.28166	20.1	y4
sp|Q04695|K1C17_HUMAN	DAEDWFFSK	269	277	572.750921	714.360973	20.1	y5
sp|Q04695|K1C17_HUMAN	DAEDWFFSK	269	277	572.750921	829.387916	20.1	y6
sp|Q04695|K1C17_HUMAN	DAEDWFFSK	269	277	572.750921	958.430509	20.1	y7
sp|Q04917|1433F_HUMAN	NC[+57.0]NDFQYESK	110	119	652.764234	526.250754	22.5	y4
sp|Q04917|1433F_HUMAN	NC[+57.0]NDFQYESK	110	119	652.764234	801.377745	22.5	y6
sp|Q04917|1433F_HUMAN	NC[+57.0]NDFQYESK	110	119	652.764234	916.404688	22.5	y7
sp|Q04917|1433F_HUMAN	NC[+57.0]NDFQYESK	110	119	652.764234	1030.447616	22.5	y8
sp|Q07507|DERM_HUMAN	YFESVLDR	118	125	514.756006	403.229959	18.3	y3
sp|Q07507|DERM_HUMAN	YFESVLDR	118	125	514.756006	502.298373	18.3	y4
sp|Q07507|DERM_HUMAN	YFESVLDR	118	125	514.756006	589.330401	18.3	y5
sp|Q07507|DERM_HUMAN	YFESVLDR	118	125	514.756006	718.372994	18.3	y6
sp|Q07954|LRP1_HUMAN	AALSGANVLTLIEK	2360	2373	700.411391	389.239461	23.9	y3
sp|Q07954|LRP1_HUMAN	AALSGANVLTLIEK	2360	2373	700.411391	502.323525	23.9	y4
sp|Q07954|LRP1_HUMAN	AALSGANVLTLIEK	2360	2373	700.411391	716.455267	23.9	y6
sp|Q07954|LRP1_HUMAN	AALSGANVLTLIEK	2360	2373	700.411391	1144.657215	23.9	y11
sp|Q08380|LG3BP_HUMAN	SQLVYQSR	427	434	490.761623	390.209558	17.6	y3
sp|Q08380|LG3BP_HUMAN	SQLVYQSR	427	434	490.761623	553.272886	17.6	y4
sp|Q08380|LG3BP_HUMAN	SQLVYQSR	427	434	490.761623	652.3413	17.6	y5
sp|Q08380|LG3BP_HUMAN	SQLVYQSR	427	434	490.761623	765.425364	17.6	y6

sp|Q12846|STX4_HUMAN	NILSSADYVER	243	253	633.819866	681.32023	21.9	y5
sp|Q12846|STX4_HUMAN	NILSSADYVER	243	253	633.819866	839.389373	21.9	y7
sp|Q12846|STX4_HUMAN	NILSSADYVER	243	253	633.819866	926.421401	21.9	y8
sp|Q12846|STX4_HUMAN	NILSSADYVER	243	253	633.819866	1039.505465	21.9	y9
sp|Q13011|ECH1_HUMAN	YQETFNVIER	148	157	649.822409	630.35695	22.4	y5
sp|Q13011|ECH1_HUMAN	YQETFNVIER	148	157	649.822409	777.425364	22.4	y6
sp|Q13011|ECH1_HUMAN	YQETFNVIER	148	157	649.822409	878.473043	22.4	y7
sp|Q13011|ECH1_HUMAN	YQETFNVIER	148	157	649.822409	1007.515636	22.4	y8
sp|Q13547|HDAC1_HUMAN	LGC[+57.0]FNLTIK	270	278	533.291702	588.371538	18.9	y5
sp|Q13547|HDAC1_HUMAN	LGC[+57.0]FNLTIK	270	278	533.291702	735.439952	18.9	y6
sp|Q13547|HDAC1_HUMAN	LGC[+57.0]FNLTIK	270	278	533.291702	895.4706	18.9	y7
sp|Q13547|HDAC1_HUMAN	LGC[+57.0]FNLTIK	270	278	533.291702	952.492064	18.9	y8
sp|Q13547|HDAC1_HUMAN	YYAVNYPLR	220	228	579.800748	548.319108	20.3	y4
sp|Q13547|HDAC1_HUMAN	YYAVNYPLR	220	228	579.800748	662.362036	20.3	y5
sp|Q13547|HDAC1_HUMAN	YYAVNYPLR	220	228	579.800748	761.43045	20.3	y6
sp|Q13547|HDAC1_HUMAN	YYAVNYPLR	220	228	579.800748	832.467563	20.3	y7
sp|Q13610|PWP1_HUMAN	NSSISGPFGSR	482	492	554.772719	620.315085	19.5	y6
sp|Q13610|PWP1_HUMAN	NSSISGPFGSR	482	492	554.772719	707.347114	19.5	y7
sp|Q13610|PWP1_HUMAN	NSSISGPFGSR	482	492	554.772719	820.431178	19.5	y8
sp|Q13610|PWP1_HUMAN	NSSISGPFGSR	482	492	554.772719	907.463206	19.5	y9
sp|Q13642-1|FHL1_HUMAN	AIVAGDQNVEYK	106	117	653.835516	439.218725	22.5	y3
sp|Q13642-1|FHL1_HUMAN	AIVAGDQNVEYK	106	117	653.835516	952.437051	22.5	y8
sp|Q13642-1|FHL1_HUMAN	AIVAGDQNVEYK	106	117	653.835516	1023.474165	22.5	y9
sp|Q13642-1|FHL1_HUMAN	AIVAGDQNVEYK	106	117	653.835516	1122.542579	22.5	y10
sp|Q13642-1|FHL1_HUMAN	QVIGTGSFFPK	133	143	590.821681	625.334424	20.6	y5
sp|Q13642-1|FHL1_HUMAN	QVIGTGSFFPK	133	143	590.821681	682.355888	20.6	y6
sp|Q13642-1|FHL1_HUMAN	QVIGTGSFFPK	133	143	590.821681	840.42503	20.6	y8
sp|Q13642-1|FHL1_HUMAN	QVIGTGSFFPK	133	143	590.821681	953.509094	20.6	y9
sp|Q14108|SCRB2_HUMAN	VEEVGPYTYR	82	91	606.798403	699.346051	21.1	y5
sp|Q14108|SCRB2_HUMAN	VEEVGPYTYR	82	91	606.798403	756.367515	21.1	y6
sp|Q14108|SCRB2_HUMAN	VEEVGPYTYR	82	91	606.798403	855.435929	21.1	y7
sp|Q14108|SCRB2_HUMAN	VEEVGPYTYR	82	91	606.798403	984.478522	21.1	y8
sp|Q14956|GPNMB_HUMAN	AYVPIAQVK	218	226	494.794935	374.239795	17.7	y3
sp|Q14956|GPNMB_HUMAN	AYVPIAQVK	218	226	494.794935	445.276909	17.7	y4
sp|Q14956|GPNMB_HUMAN	AYVPIAQVK	218	226	494.794935	655.413737	17.7	y6
sp|Q14956|GPNMB_HUMAN	AYVPIAQVK	218	226	494.794935	754.482151	17.7	y7
sp|Q15102|PA1B3_HUMAN	AIVQLVNER	118	126	521.306198	418.204472	18.5	y3
sp|Q15102|PA1B3_HUMAN	AIVQLVNER	118	126	521.306198	630.35695	18.5	y5
sp|Q15102|PA1B3_HUMAN	AIVQLVNER	118	126	521.306198	758.415528	18.5	y6
sp|Q15102|PA1B3_HUMAN	AIVQLVNER	118	126	521.306198	857.483942	18.5	y7
sp|Q15661|TRYB1_HUMAN	DSC[+57.0]QGDSGGPLVC[+57.0]K	217	230	740.313765	406.211866	25.1	y3
sp|Q15661|TRYB1_HUMAN	DSC[+57.0]QGDSGGPLVC[+57.0]K	217	230	740.313765	730.391622	25.1	y7
sp|Q15661|TRYB1_HUMAN	DSC[+57.0]QGDSGGPLVC[+57.0]K	217	230	740.313765	817.42365	25.1	y8
sp|Q15661|TRYB1_HUMAN	DSC[+57.0]QGDSGGPLVC[+57.0]K	217	230	740.313765	989.472057	25.1	y10
sp|Q15661|TRYB1_HUMAN	YHLGAYTGDDVR	191	202	683.82294	662.310394	23.4	y6
sp|Q15661|TRYB1_HUMAN	YHLGAYTGDDVR	191	202	683.82294	825.373723	23.4	y7
sp|Q15661|TRYB1_HUMAN	YHLGAYTGDDVR	191	202	683.82294	953.4323	23.4	y9
sp|Q15661|TRYB1_HUMAN	YHLGAYTGDDVR	191	202	683.82294	1066.516364	23.4	y10
sp|Q15811|ITSN1_HUMAN	VEGLQAQALYPWR	912	924	765.906807	458.251029	25.9	y3
sp|Q15811|ITSN1_HUMAN	VEGLQAQALYPWR	912	924	765.906807	621.314357	25.9	y4
sp|Q15811|ITSN1_HUMAN	VEGLQAQALYPWR	912	924	765.906807	734.398421	25.9	y5
sp|Q15811|ITSN1_HUMAN	VEGLQAQALYPWR	912	924	765.906807	1004.531226	25.9	y8
sp|Q16853|AOC3_HUMAN	YQLAVTQR	629	636	489.771991	404.225208	17.6	y3
sp|Q16853|AOC3_HUMAN	YQLAVTQR	629	636	489.771991	503.293622	17.6	y4
sp|Q16853|AOC3_HUMAN	YQLAVTQR	629	636	489.771991	574.330736	17.6	y5
sp|Q16853|AOC3_HUMAN	YQLAVTQR	629	636	489.771991	687.4148	17.6	y6
sp|Q2TBA0|KBTB5_HUMAN	LVLAAC[+57.0]SPYFR	48	58	648.84209	669.335487	22.4	y5
sp|Q2TBA0|KBTB5_HUMAN	LVLAAC[+57.0]SPYFR	48	58	648.84209	829.366135	22.4	y6
sp|Q2TBA0|KBTB5_HUMAN	LVLAAC[+57.0]SPYFR	48	58	648.84209	900.403249	22.4	y7
sp|Q2TBA0|KBTB5_HUMAN	LVLAAC[+57.0]SPYFR	48	58	648.84209	971.440363	22.4	y8
sp|Q562R1|ACTBL_HUMAN	SYELPDGQVITIGNER	239	254	895.949597	475.225936	29.8	y4
sp|Q562R1|ACTBL_HUMAN	SYELPDGQVITIGNER	239	254	895.949597	689.357679	29.8	y6
sp|Q562R1|ACTBL_HUMAN	SYELPDGQVITIGNER	239	254	895.949597	802.441743	29.8	y7
sp|Q562R1|ACTBL_HUMAN	SYELPDGQVITIGNER	239	254	895.949597	1298.669905	29.8	y12
sp|Q5T0N5|FBP1L_HUMAN	ESPEGSYTDDANQEVR	499	514	898.882109	831.395521	29.9	y7

sp|Q5T0N5|FBP1L_HUMAN	ESPEGSYTDDANQEVR	499	514	898.882109	946.422464	29.9	y8
sp|Q5T0N5|FBP1L_HUMAN	ESPEGSYTDDANQEVR	499	514	898.882109	1047.470142	29.9	y9
sp|Q5T0N5|FBP1L_HUMAN	ESPEGSYTDDANQEVR	499	514	898.882109	1210.533471	29.9	y10
sp|Q6P996|PDXD1_HUMAN	LLEEGVLR	631	638	464.776742	444.292893	16.8	y4
sp|Q6P996|PDXD1_HUMAN	LLEEGVLR	631	638	464.776742	573.335487	16.8	y5
sp|Q6P996|PDXD1_HUMAN	LLEEGVLR	631	638	464.776742	702.37808	16.8	y6
sp|Q6P996|PDXD1_HUMAN	LLEEGVLR	631	638	464.776742	815.462144	16.8	y7
sp|Q6PCE3|PGM2L_HUMAN	YVPTPFVPYAVQK	150	162	754.911027	705.393001	25.6	y6
sp|Q6PCE3|PGM2L_HUMAN	YVPTPFVPYAVQK	150	162	754.911027	804.461415	25.6	y7
sp|Q6PCE3|PGM2L_HUMAN	YVPTPFVPYAVQK	150	162	754.911027	1048.582593	25.6	y9
sp|Q6PCE3|PGM2L_HUMAN	YVPTPFVPYAVQK	150	162	754.911027	1246.683036	25.6	y11
sp|Q86U86|PB1_HUMAN	SYYKPDSPEYK	124	134	688.822075	536.271489	23.6	y4
sp|Q86U86|PB1_HUMAN	SYYKPDSPEYK	124	134	688.822075	623.303518	23.6	y5
sp|Q86U86|PB1_HUMAN	SYYKPDSPEYK	124	134	688.822075	835.383225	23.6	y7
sp|Q86U86|PB1_HUMAN	SYYKPDSPEYK	124	134	688.822075	963.478188	23.6	y8
sp|Q86UX7|URP2_HUMAN	VVLAGGVAPALFR	166	178	635.387522	603.361307	22	y5
sp|Q86UX7|URP2_HUMAN	VVLAGGVAPALFR	166	178	635.387522	674.398421	22	y6
sp|Q86UX7|URP2_HUMAN	VVLAGGVAPALFR	166	178	635.387522	830.488299	22	y8
sp|Q86UX7|URP2_HUMAN	VVLAGGVAPALFR	166	178	635.387522	958.546876	22	y10
sp|Q86VB7-2|C163A_HUMAN	TSYQVYSK	421	428	488.242732	397.208161	17.6	y3
sp|Q86VB7-2|C163A_HUMAN	TSYQVYSK	421	428	488.242732	496.276575	17.6	y4
sp|Q86VB7-2|C163A_HUMAN	TSYQVYSK	421	428	488.242732	624.335152	17.6	y5
sp|Q86VB7-2|C163A_HUMAN	TSYQVYSK	421	428	488.242732	787.398481	17.6	y6
sp|Q86Y39-2|NDUAB_HUMAN	VTLNPPGTFLEGVAK	40	54	771.929948	763.434866	26.1	y7
sp|Q86Y39-2|NDUAB_HUMAN	VTLNPPGTFLEGVAK	40	54	771.929948	1018.556772	26.1	y10
sp|Q86Y39-2|NDUAB_HUMAN	VTLNPPGTFLEGVAK	40	54	771.929948	1115.609536	26.1	y11
sp|Q86Y39-2|NDUAB_HUMAN	VTLNPPGTFLEGVAK	40	54	771.929948	1229.652464	26.1	y12
sp|Q8IUX7|AEBP1_HUMAN	VPNNNLPIPER	721	731	631.846218	401.214309	21.9	y3
sp|Q8IUX7|AEBP1_HUMAN	VPNNNLPIPER	721	731	631.846218	611.351137	21.9	y5
sp|Q8IUX7|AEBP1_HUMAN	VPNNNLPIPER	721	731	631.846218	724.435201	21.9	y6
sp|Q8IUX7|AEBP1_HUMAN	VPNNNLPIPER	721	731	631.846218	1066.563983	21.9	y9
sp|Q8IYB3|SRRM1_HUMAN	EPSVQEATSTSDILK	231	245	802.904324	763.41961	27	y7
sp|Q8IYB3|SRRM1_HUMAN	EPSVQEATSTSDILK	231	245	802.904324	864.467289	27	y8
sp|Q8IYB3|SRRM1_HUMAN	EPSVQEATSTSDILK	231	245	802.904324	935.504403	27	y9
sp|Q8IYB3|SRRM1_HUMAN	EPSVQEATSTSDILK	231	245	802.904324	1192.605573	27	y11
sp|Q8IYB3|SRRM1_HUMAN	VNLEVIKPWITK	448	457	720.434669	644.376623	24.5	y5
sp|Q8IYB3|SRRM1_HUMAN	VNLEVIKPWITK	448	457	720.434669	772.471586	24.5	y6
sp|Q8IYB3|SRRM1_HUMAN	VNLEVIKPWITK	448	457	720.434669	885.55565	24.5	y7
sp|Q8N7H5|PAF1_HUMAN	LLEEEIQAPTSSK	106	118	722.880121	519.277303	24.6	y5
sp|Q8N7H5|PAF1_HUMAN	LLEEEIQAPTSSK	106	118	722.880121	590.314417	24.6	y6
sp|Q8N7H5|PAF1_HUMAN	LLEEEIQAPTSSK	106	118	722.880121	831.457058	24.6	y8
sp|Q8N7H5|PAF1_HUMAN	LLEEEIQAPTSSK	106	118	722.880121	1089.542245	24.6	y10
sp|Q8N7H5|PAF1_HUMAN	TEYISTEFNR	133	142	630.298767	436.230293	21.8	y3
sp|Q8N7H5|PAF1_HUMAN	TEYISTEFNR	133	142	630.298767	753.352593	21.8	y6
sp|Q8N7H5|PAF1_HUMAN	TEYISTEFNR	133	142	630.298767	866.436657	21.8	y7
sp|Q8N7H5|PAF1_HUMAN	TEYISTEFNR	133	142	630.298767	1029.499986	21.8	y8
sp|Q8NC51|PAIRB_HUMAN	SAAQAAAQTNSNAAGK	52	67	730.858043	762.374057	24.8	y8
sp|Q8NC51|PAIRB_HUMAN	SAAQAAAQTNSNAAGK	52	67	730.858043	961.469748	24.8	y10
sp|Q8NC51|PAIRB_HUMAN	SAAQAAAQTNSNAAGK	52	67	730.858043	1001.464663	24.8	b11
sp|Q8NC51|PAIRB_HUMAN	SAAQAAAQTNSNAAGK	52	67	730.858043	1032.506862	24.8	y11
sp|Q8TD06|AGR3_HUMAN	DLPLLIENMK	146	155	593.331024	521.238809	20.7	y4
sp|Q8TD06|AGR3_HUMAN	DLPLLIENMK	146	155	593.331024	634.322873	20.7	y5
sp|Q8TD06|AGR3_HUMAN	DLPLLIENMK	146	155	593.331024	747.406937	20.7	y6
sp|Q8TD06|AGR3_HUMAN	DLPLLIENMK	146	155	593.331024	957.543765	20.7	y8
sp|Q8TD06|AGR3_HUMAN	VFAQNEEIQEMAQNK	79	93	889.922525	460.251423	29.6	y4
sp|Q8TD06|AGR3_HUMAN	VFAQNEEIQEMAQNK	79	93	889.922525	720.3345	29.6	y6
sp|Q8TD06|AGR3_HUMAN	VFAQNEEIQEMAQNK	79	93	889.922525	848.393078	29.6	y7
sp|Q8TD06|AGR3_HUMAN	VFAQNEEIQEMAQNK	79	93	889.922525	1090.519735	29.6	y9
sp|Q8WXX0|DYH7_HUMAN	SVLTAAGNLK	1506	1515	487.287474	431.261259	17.5	y4
sp|Q8WXX0|DYH7_HUMAN	SVLTAAGNLK	1506	1515	487.287474	502.298373	17.5	y5
sp|Q8WXX0|DYH7_HUMAN	SVLTAAGNLK	1506	1515	487.287474	674.383165	17.5	y7
sp|Q8WXX0|DYH7_HUMAN	SVLTAAGNLK	1506	1515	487.287474	787.467229	17.5	y8

sp|Q8WYJ6|SEPT1_HUMAN	VNIIPVIGK	162	170	476.813127	513.339509	17.2	y5
sp|Q8WYJ6|SEPT1_HUMAN	VNIIPVIGK	162	170	476.813127	626.423573	17.2	y6
sp|Q8WYJ6|SEPT1_HUMAN	VNIIPVIGK	162	170	476.813127	739.507637	17.2	y7
sp|Q8WYJ6|SEPT1_HUMAN	VNIIPVIGK	162	170	476.813127	853.550565	17.2	y8
sp|Q92598|HS105_HUMAN	AFNDPFIQK	73	81	540.279649	632.376623	19.1	y5
sp|Q92598|HS105_HUMAN	AFNDPFIQK	73	81	540.279649	747.403566	19.1	y6
sp|Q92598|HS105_HUMAN	AFNDPFIQK	73	81	540.279649	805.387916	19.1	b7
sp|Q92598|HS105_HUMAN	AFNDPFIQK	73	81	540.279649	861.446494	19.1	y7
sp|Q92804|RBP56_HUMAN	AAIDWFDGK	297	305	511.750724	466.229624	18.3	y4
sp|Q92804|RBP56_HUMAN	AAIDWFDGK	297	305	511.750724	652.308937	18.3	y5
sp|Q92804|RBP56_HUMAN	AAIDWFDGK	297	305	511.750724	767.335881	18.3	y6
sp|Q92804|RBP56_HUMAN	AAIDWFDGK	297	305	511.750724	880.419945	18.3	y7
sp|Q92804|RBP56_HUMAN	GEATVSFDDPPSAK	283	296	710.833171	499.287474	24.2	y5
sp|Q92804|RBP56_HUMAN	GEATVSFDDPPSAK	283	296	710.833171	876.409774	24.2	y8
sp|Q92804|RBP56_HUMAN	GEATVSFDDPPSAK	283	296	710.833171	963.441802	24.2	y9
sp|Q92804|RBP56_HUMAN	GEATVSFDDPPSAK	283	296	710.833171	1062.510216	24.2	y10
sp|Q96AE4|FUBP1_HUMAN	IGGDAGTSLNSNDYGYGGQK	44	63	987.445408	609.299101	32.5	y6
sp|Q96AE4|FUBP1_HUMAN	IGGDAGTSLNSNDYGYGGQK	44	63	987.445408	772.36243	32.5	y7
sp|Q96AE4|FUBP1_HUMAN	IGGDAGTSLNSNDYGYGGQK	44	63	987.445408	1088.464329	32.5	y10
sp|Q96AE4|FUBP1_HUMAN	IGGDAGTSLNSNDYGYGGQK	44	63	987.445408	1202.507256	32.5	y11
sp|Q96FZ2|CC037_HUMAN	GTAGLLEQWLK	328	338	608.340238	446.276181	21.2	y3
sp|Q96FZ2|CC037_HUMAN	GTAGLLEQWLK	328	338	608.340238	574.334758	21.2	y4
sp|Q96FZ2|CC037_HUMAN	GTAGLLEQWLK	328	338	608.340238	703.377351	21.2	y5
sp|Q96FZ2|CC037_HUMAN	GTAGLLEQWLK	328	338	608.340238	816.461415	21.2	y6
sp|Q96G03|PGM2_HUMAN	IVLANDPDADR	316	326	599.806759	573.262716	20.9	y5
sp|Q96G03|PGM2_HUMAN	IVLANDPDADR	316	326	599.806759	802.332586	20.9	y7
sp|Q96G03|PGM2_HUMAN	IVLANDPDADR	316	326	599.806759	873.3697	20.9	y8
sp|Q96G03|PGM2_HUMAN	IVLANDPDADR	316	326	599.806759	986.453764	20.9	y9
sp|Q96NY7|CLIC6_HUMAN	VGDGPQQEPGEDEER	358	372	821.352987	548.231081	27.5	y4
sp|Q96NY7|CLIC6_HUMAN	VGDGPQQEPGEDEER	358	372	821.352987	831.347902	27.5	y7
sp|Q96NY7|CLIC6_HUMAN	VGDGPQQEPGEDEER	358	372	821.352987	960.390495	27.5	y8
sp|Q99747|SNAG_HUMAN	LGLSLVVPGGGIK	268	280	605.381905	528.314023	21.1	y6
sp|Q99747|SNAG_HUMAN	LGLSLVVPGGGIK	268	280	605.381905	627.382437	21.1	y7
sp|Q99747|SNAG_HUMAN	LGLSLVVPGGGIK	268	280	605.381905	726.450851	21.1	y8
sp|Q99747|SNAG_HUMAN	LGLSLVVPGGGIK	268	280	605.381905	926.566943	21.1	y10
sp|Q99747|SNAG_HUMAN	LIENVDPEK	123	131	528.782221	373.208161	18.8	y3
sp|Q99747|SNAG_HUMAN	LIENVDPEK	123	131	528.782221	488.235104	18.8	y4
sp|Q99747|SNAG_HUMAN	LIENVDPEK	123	131	528.782221	701.346445	18.8	y6
sp|Q99747|SNAG_HUMAN	LIENVDPEK	123	131	528.782221	830.389038	18.8	y7
sp|Q9BR76|COR1B_HUMAN	DADPILISLR	383	392	556.819138	375.235044	19.6	y3
sp|Q9BR76|COR1B_HUMAN	DADPILISLR	383	392	556.819138	488.319108	19.6	y4
sp|Q9BR76|COR1B_HUMAN	DADPILISLR	383	392	556.819138	601.403172	19.6	y5
sp|Q9BR76|COR1B_HUMAN	DADPILISLR	383	392	556.819138	811.54	19.6	y7
sp|Q9BR76|COR1B_HUMAN	VTWDSTFC[+57.0]AVNPK	33	45	762.861208	358.208495	25.8	y3
sp|Q9BR76|COR1B_HUMAN	VTWDSTFC[+57.0]AVNPK	33	45	762.861208	457.276909	25.8	y4
sp|Q9BR76|COR1B_HUMAN	VTWDSTFC[+57.0]AVNPK	33	45	762.861208	688.344671	25.8	y6
sp|Q9BR76|COR1B_HUMAN	VTWDSTFC[+57.0]AVNPK	33	45	762.861208	835.413085	25.8	y7
sp|Q9BRJ2|RM45_HUMAN	QLTNPYGSWR	250	259	611.304186	765.367849	21.2	y6
sp|Q9BRJ2|RM45_HUMAN	QLTNPYGSWR	250	259	611.304186	879.410777	21.2	y7
sp|Q9BRJ2|RM45_HUMAN	QLTNPYGSWR	250	259	611.304186	980.458455	21.2	y8
sp|Q9BRX2|PELO_HUMAN	FYEQVVQAIQR	184	194	690.867151	487.298707	23.6	y4
sp|Q9BRX2|PELO_HUMAN	FYEQVVQAIQR	184	194	690.867151	615.357285	23.6	y5
sp|Q9BRX2|PELO_HUMAN	FYEQVVQAIQR	184	194	690.867151	714.425699	23.6	y6
sp|Q9BRX2|PELO_HUMAN	VQTESSTGSVGSNR	46	59	704.836776	433.215371	24.1	y4
sp|Q9BRX2|PELO_HUMAN	VQTESSTGSVGSNR	46	59	704.836776	532.283785	24.1	y5
sp|Q9BRX2|PELO_HUMAN	VQTESSTGSVGSNR	46	59	704.836776	951.449013	24.1	y10
sp|Q9BTT0|AN32E_HUMAN	DLSTVEALQNLK	101	112	665.864274	502.298373	22.9	y4
sp|Q9BTT0|AN32E_HUMAN	DLSTVEALQNLK	101	112	665.864274	615.382437	22.9	y5
sp|Q9BTT0|AN32E_HUMAN	DLSTVEALQNLK	101	112	665.864274	686.419551	22.9	y6
sp|Q9BTT0|AN32E_HUMAN	DLSTVEALQNLK	101	112	665.864274	815.462144	22.9	y7
sp|Q9BVK6|TMED9_HUMAN	QLVEQVEQIQK	169	179	671.372266	516.314023	23	y4
sp|Q9BVK6|TMED9_HUMAN	QLVEQVEQIQK	169	179	671.372266	645.356616	23	y5
sp|Q9BVK6|TMED9_HUMAN	QLVEQVEQIQK	169	179	671.372266	744.42503	23	y6
sp|Q9BVK6|TMED9_HUMAN	QLVEQVEQIQK	169	179	671.372266	1001.526201	23	y8
sp|Q9BXS5|AP1M1_HUMAN	HNNLYLVATSK	61	71	630.340769	781.445431	21.8	y7

sp|Q9BXS5|AP1M1_HUMAN	HNNLYLVATSK	61	71	630.340769	894.529495	21.8	y8
sp|Q9BXS5|AP1M1_HUMAN	HNNLYLVATSK	61	71	630.340769	1008.572423	21.8	y9
sp|Q9BXS5|AP1M1_HUMAN	HNNLYLVATSK	61	71	630.340769	1122.61535	21.8	y10
sp|Q9BYX2|TBD2A_HUMAN	WAALGDLVPSAELK	605	618	735.403566	644.361367	25	y6
sp|Q9BYX2|TBD2A_HUMAN	WAALGDLVPSAELK	605	618	735.403566	1028.562252	25	y10
sp|Q9BYX2|TBD2A_HUMAN	WAALGDLVPSAELK	605	618	735.403566	1141.646316	25	y11
sp|Q9BYX2|TBD2A_HUMAN	WAALGDLVPSAELK	605	618	735.403566	1212.68343	25	y12
sp|Q9GZM7|TINAL_HUMAN	ITGWGEETLPDGR	403	415	715.849155	444.220122	24.4	y4
sp|Q9GZM7|TINAL_HUMAN	ITGWGEETLPDGR	403	415	715.849155	557.304186	24.4	y5
sp|Q9GZM7|TINAL_HUMAN	ITGWGEETLPDGR	403	415	715.849155	658.351865	24.4	y6
sp|Q9GZM7|TINAL_HUMAN	ITGWGEETLPDGR	403	415	715.849155	973.458515	24.4	y9
sp|Q9H2U2-2|IPYR2_HUMAN	AFALEVIK	261	268	445.770928	359.265282	16.3	y3
sp|Q9H2U2-2|IPYR2_HUMAN	AFALEVIK	261	268	445.770928	488.307875	16.3	y4
sp|Q9H2U2-2|IPYR2_HUMAN	AFALEVIK	261	268	445.770928	601.391939	16.3	y5
sp|Q9H2U2-2|IPYR2_HUMAN	AFALEVIK	261	268	445.770928	672.429053	16.3	y6
sp|Q9H2U2-2|IPYR2_HUMAN	SLVESVSSSPNK	308	319	617.319699	445.240524	21.4	y4
sp|Q9H2U2-2|IPYR2_HUMAN	SLVESVSSSPNK	308	319	617.319699	805.405023	21.4	y8
sp|Q9H2U2-2|IPYR2_HUMAN	SLVESVSSSPNK	308	319	617.319699	934.447616	21.4	y9
sp|Q9H2U2-2|IPYR2_HUMAN	SLVESVSSSPNK	308	319	617.319699	1033.51603	21.4	y10
sp|Q9H3U1|UN45A_HUMAN	ALIPLALEGTDVGQTK	691	706	813.45907	748.383559	27.3	y7
sp|Q9H3U1|UN45A_HUMAN	ALIPLALEGTDVGQTK	691	706	813.45907	805.405023	27.3	y8
sp|Q9H3U1|UN45A_HUMAN	ALIPLALEGTDVGQTK	691	706	813.45907	1047.53168	27.3	y10
sp|Q9H3U1|UN45A_HUMAN	ALIPLALEGTDVGQTK	691	706	813.45907	1328.705622	27.3	y13
sp|Q9H3U1|UN45A_HUMAN	DNALTLLIK	312	320	500.805499	486.364996	17.9	y4
sp|Q9H3U1|UN45A_HUMAN	DNALTLLIK	312	320	500.805499	587.412674	17.9	y5
sp|Q9H3U1|UN45A_HUMAN	DNALTLLIK	312	320	500.805499	700.496738	17.9	y6
sp|Q9H3U1|UN45A_HUMAN	DNALTLLIK	312	320	500.805499	771.533852	17.9	y7
sp|Q9H4A3-6|WNK1_HUMAN	QQVEQSSASQTGIK	590	603	745.875901	633.356616	25.3	y6
sp|Q9H4A3-6|WNK1_HUMAN	QQVEQSSASQTGIK	590	603	745.875901	791.425758	25.3	y8
sp|Q9H4A3-6|WNK1_HUMAN	QQVEQSSASQTGIK	590	603	745.875901	878.457787	25.3	y9
sp|Q9H4A3-6|WNK1_HUMAN	QQVEQSSASQTGIK	590	603	745.875901	1135.558957	25.3	y11
sp|Q9H4A3-6|WNK1_HUMAN	VELAEEDDGEK	486	496	617.277697	692.27334	21.4	y6
sp|Q9H4A3-6|WNK1_HUMAN	VELAEEDDGEK	486	496	617.277697	821.315933	21.4	y7
sp|Q9H4A3-6|WNK1_HUMAN	VELAEEDDGEK	486	496	617.277697	892.353047	21.4	y8
sp|Q9H4A3-6|WNK1_HUMAN	VELAEEDDGEK	486	496	617.277697	1005.437111	21.4	y9
sp|Q9H4M9|EHD1_HUMAN	ELVNNLGEIYQK	329	340	710.377548	737.382831	24.2	y6
sp|Q9H4M9|EHD1_HUMAN	ELVNNLGEIYQK	329	340	710.377548	850.466895	24.2	y7
sp|Q9H4M9|EHD1_HUMAN	ELVNNLGEIYQK	329	340	710.377548	964.509822	24.2	y8
sp|Q9H4M9|EHD1_HUMAN	ELVNNLGEIYQK	329	340	710.377548	1078.55275	24.2	y9
sp|Q9H4M9|EHD1_HUMAN	LFEAEEQDLFK	269	279	684.837725	779.393395	23.5	y6
sp|Q9H4M9|EHD1_HUMAN	LFEAEEQDLFK	269	279	684.837725	908.435989	23.5	y7
sp|Q9H4M9|EHD1_HUMAN	LFEAEEQDLFK	269	279	684.837725	979.473102	23.5	y8
sp|Q9H4M9|EHD1_HUMAN	LFEAEEQDLFK	269	279	684.837725	1108.515696	23.5	y9
sp|Q9H993|CF211_HUMAN	LLQISLWGNK	183	192	586.345323	504.256508	20.5	y4
sp|Q9H993|CF211_HUMAN	LLQISLWGNK	183	192	586.345323	704.3726	20.5	y6
sp|Q9H993|CF211_HUMAN	LLQISLWGNK	183	192	586.345323	817.456664	20.5	y7
sp|Q9H993|CF211_HUMAN	LLQISLWGNK	183	192	586.345323	945.515242	20.5	y8
sp|Q9HB71|CYBP_HUMAN	ISNYGWDQSDK	74	84	656.793849	592.257296	22.6	y5
sp|Q9HB71|CYBP_HUMAN	ISNYGWDQSDK	74	84	656.793849	835.358073	22.6	y7
sp|Q9HB71|CYBP_HUMAN	ISNYGWDQSDK	74	84	656.793849	998.421401	22.6	y8
sp|Q9HB71|CYBP_HUMAN	ISNYGWDQSDK	74	84	656.793849	1112.464329	22.6	y9
sp|Q9HC35|EMAL4_HUMAN	WFVLDAETR	656	664	568.790381	476.246337	20	y4
sp|Q9HC35|EMAL4_HUMAN	WFVLDAETR	656	664	568.790381	591.27328	20	y5
sp|Q9HC35|EMAL4_HUMAN	WFVLDAETR	656	664	568.790381	704.357344	20	y6
sp|Q9HC35|EMAL4_HUMAN	WFVLDAETR	656	664	568.790381	803.425758	20	y7
sp|Q9P2P6|STAR9_HUMAN	DSVLTWLLK	337	345	537.813324	559.360245	19	y4
sp|Q9P2P6|STAR9_HUMAN	DSVLTWLLK	337	345	537.813324	660.407923	19	y5
sp|Q9P2P6|STAR9_HUMAN	DSVLTWLLK	337	345	537.813324	773.491987	19	y6

sp|Q9P2P6|STAR9_HUMAN	DSVLTWLLK	337	345	537.813324	872.560401	19	y7
sp|Q9UBC3|DNM3B_HUMAN	EFGIGDLVWGK	221	231	610.819138	390.21358	21.2	y3
sp|Q9UBC3|DNM3B_HUMAN	EFGIGDLVWGK	221	231	610.819138	489.281994	21.2	y4
sp|Q9UBC3|DNM3B_HUMAN	EFGIGDLVWGK	221	231	610.819138	774.414465	21.2	y7
sp|Q9UBC3|DNM3B_HUMAN	EFGIGDLVWGK	221	231	610.819138	944.519993	21.2	y9
sp|Q9UBC3|DNM3B_HUMAN	WVQWFGDGK	259	267	561.771991	376.182674	19.8	y4
sp|Q9UBC3|DNM3B_HUMAN	WVQWFGDGK	259	267	561.771991	523.251088	19.8	y5
sp|Q9UBC3|DNM3B_HUMAN	WVQWFGDGK	259	267	561.771991	709.330401	19.8	y6
sp|Q9UBC3|DNM3B_HUMAN	WVQWFGDGK	259	267	561.771991	837.388979	19.8	y7
sp|Q9UHD1|CHRD1_HUMAN	AEPMQWASLELPAAK	306	320	821.41889	386.239795	27.5	y4
sp|Q9UHD1|CHRD1_HUMAN	AEPMQWASLELPAAK	306	320	821.41889	628.366452	27.5	y6
sp|Q9UHD1|CHRD1_HUMAN	AEPMQWASLELPAAK	306	320	821.41889	741.450516	27.5	y7
sp|Q9UHD1|CHRD1_HUMAN	AEPMQWASLELPAAK	306	320	821.41889	899.519659	27.5	y9
sp|Q9UI08-2|EVL_HUMAN	AASQPPNSSEAGR	310	322	636.302372	720.327107	22	y7
sp|Q9UI08-2|EVL_HUMAN	AASQPPNSSEAGR	310	322	636.302372	817.379871	22	y8
sp|Q9UI08-2|EVL_HUMAN	AASQPPNSSEAGR	310	322	636.302372	914.432635	22	y9
sp|Q9UI08-2|EVL_HUMAN	SNSVEKPVSSILSR	330	343	501.610616	375.235044	21.3	y3
sp|Q9UI08-2|EVL_HUMAN	SNSVEKPVSSILSR	330	343	501.610616	488.319108	21.3	y4
sp|Q9UI08-2|EVL_HUMAN	SNSVEKPVSSILSR	330	343	501.610616	575.351137	21.3	y5
sp|Q9UI08-2|EVL_HUMAN	SNSVEKPVSSILSR	330	343	501.610616	662.383165	21.3	y6
sp|Q9UL46|PSME2_HUMAN	DEAAYGELR	194	202	512.240721	474.267073	18.3	y4
sp|Q9UL46|PSME2_HUMAN	DEAAYGELR	194	202	512.240721	637.330401	18.3	y5
sp|Q9UL46|PSME2_HUMAN	DEAAYGELR	194	202	512.240721	708.367515	18.3	y6
sp|Q9UL46|PSME2_HUMAN	DEAAYGELR	194	202	512.240721	779.404629	18.3	y7
sp|Q9ULH1-2|ASAP1_HUMAN	GEQSAGENSLEDLTK	424	438	789.368106	476.271489	26.6	y4
sp|Q9ULH1-2|ASAP1_HUMAN	GEQSAGENSLEDLTK	424	438	789.368106	919.473102	26.6	y8
sp|Q9ULH1-2|ASAP1_HUMAN	GEQSAGENSLEDLTK	424	438	789.368106	1105.537159	26.6	y10
sp|Q9ULH1-2|ASAP1_HUMAN	GEQSAGENSLEDLTK	424	438	789.368106	1176.574273	26.6	y11
sp|Q9Y2J8|PADI2_HUMAN	ELLGPDFGYVTR	383	394	683.853709	595.319837	23.4	y5
sp|Q9Y2J8|PADI2_HUMAN	ELLGPDFGYVTR	383	394	683.853709	742.38825	23.4	y6
sp|Q9Y2J8|PADI2_HUMAN	ELLGPDFGYVTR	383	394	683.853709	954.467957	23.4	y8
sp|Q9Y2J8|PADI2_HUMAN	ELLGPDFGYVTR	383	394	683.853709	1011.489421	23.4	y9
sp|Q9Y305-4|ACOT9_HUMAN	VHSEVASLQEK	372	382	613.822409	604.330067	21.3	y5
sp|Q9Y305-4|ACOT9_HUMAN	VHSEVASLQEK	372	382	613.822409	675.367181	21.3	y6
sp|Q9Y305-4|ACOT9_HUMAN	VHSEVASLQEK	372	382	613.822409	903.478188	21.3	y8
sp|Q9Y305-4|ACOT9_HUMAN	VHSEVASLQEK	372	382	613.822409	990.510216	21.3	y9
sp|Q9Y305-4|ACOT9_HUMAN	YLTVQNTVR	103	111	547.303655	489.277972	19.3	y4
sp|Q9Y305-4|ACOT9_HUMAN	YLTVQNTVR	103	111	547.303655	617.336549	19.3	y5
sp|Q9Y305-4|ACOT9_HUMAN	YLTVQNTVR	103	111	547.303655	716.404963	19.3	y6
sp|Q9Y305-4|ACOT9_HUMAN	YLTVQNTVR	103	111	547.303655	817.452642	19.3	y7
sp|Q9Y3B7|RM11_HUMAN	IGQPTVSYFLK	81	91	626.850438	570.32861	21.7	y4
sp|Q9Y3B7|RM11_HUMAN	IGQPTVSYFLK	81	91	626.850438	657.360639	21.7	y5
sp|Q9Y3B7|RM11_HUMAN	IGQPTVSYFLK	81	91	626.850438	954.529495	21.7	y8
sp|Q9Y3B7|RM11_HUMAN	IGQPTVSYFLK	81	91	626.850438	1139.609536	21.7	y10
sp|Q9Y3B8|ORN_HUMAN	ALDDISESIK	195	204	545.784961	563.303518	19.3	y5
sp|Q9Y3B8|ORN_HUMAN	ALDDISESIK	195	204	545.784961	676.387582	19.3	y6
sp|Q9Y3B8|ORN_HUMAN	ALDDISESIK	195	204	545.784961	791.414525	19.3	y7
sp|Q9Y3B8|ORN_HUMAN	ALDDISESIK	195	204	545.784961	906.441468	19.3	y8
sp|Q9Y520-7|PRC2C_HUMAN	ISAVESQPSR	911	920	537.28292	359.203744	19	y3
sp|Q9Y520-7|PRC2C_HUMAN	ISAVESQPSR	911	920	537.28292	574.29435	19	y5
sp|Q9Y520-7|PRC2C_HUMAN	ISAVESQPSR	911	920	537.28292	703.336943	19	y6
sp|Q9Y520-7|PRC2C_HUMAN	ISAVESQPSR	911	920	537.28292	873.442471	19	y8
sp|Q9Y520-7|PRC2C_HUMAN	QQVADEDEIWK	437	447	680.822606	819.38831	23.3	y6
sp|Q9Y520-7|PRC2C_HUMAN	QQVADEDEIWK	437	447	680.822606	934.415253	23.3	y7
sp|Q9Y520-7|PRC2C_HUMAN	QQVADEDEIWK	437	447	680.822606	1005.452367	23.3	y8

sp|Q9Y520-7|PRC2C_HUMAN	QQVADEDEIWK	437	447	680.822606	1104.520781	23.3	y9
sp|Q9Y6K5|OAS3_HUMAN	AQLEAC[+57.0]QQER	844	853	616.788043	720.309348	21.4	y5
sp|Q9Y6K5|OAS3_HUMAN	AQLEAC[+57.0]QQER	844	853	616.788043	791.346462	21.4	y6
sp|Q9Y6K5|OAS3_HUMAN	AQLEAC[+57.0]QQER	844	853	616.788043	920.389055	21.4	y7
sp|Q9Y6K5|OAS3_HUMAN	AQLEAC[+57.0]QQER	844	853	616.788043	1033.473119	21.4	y8
sp|Q9Y6K5|OAS3_HUMAN	FISEFLQPNR	757	766	625.830037	386.214643	21.7	y3
sp|Q9Y6K5|OAS3_HUMAN	FISEFLQPNR	757	766	625.830037	514.273221	21.7	y4
sp|Q9Y6K5|OAS3_HUMAN	FISEFLQPNR	757	766	625.830037	774.425699	21.7	y6
sp|Q9Y6K5|OAS3_HUMAN	FISEFLQPNR	757	766	625.830037	990.50032	21.7	y8
