# Supplementary material for: Proteomic analysis of breast tumors confirms the mRNA intrinsic molecular subtypes using different classifiers: a large-scale analysis of fresh frozen tissue samples
Source: Breast Cancer Res. 2016 Jun 29;18:69. doi: 10.1186/s13058-016-0732-2 (PMC4928264; doi:10.1186/s13058-016-0732-2)
Supplement: Additional file 5: Table S5. — The clinical data for the tumors selected for selected reaction monitoring with sample type, BRCA1 methylation status, ER, and PgR status, and the RNA classifications. (PDF 28 kb) [file 13058_2016_732_MOESM5_ESM.pdf]

Tumors from original dataset analyzed on SRM

Cancer No	HBC sample type	BRCA1 promotor methylation	ER_status	PgR_status	Expressions data ->	Sörlie subtype classified	Best Hu	Best PAM50
17613	brca1	negative	-	-	Expressions data ->	Basal	Basal	Basal
11472	sporadic	negative	er_neg	pgr_neg	Expressions data ->	Basal	Basal	Basal
8706	sporadic	negative	er_neg	pgr_neg	Expressions data ->	Basal	Basal	Basal
9833	sporadic	negative	er_neg	pgr_neg	Expressions data ->	Basal	Basal	Basal
5696	sporadic	positive	er_neg	pgr_neg	Expressions data ->	Basal	Basal	Basal
9774	sporadic	-	er_neg	pgr_neg	Expressions data ->	Basal	Basal	Basal
9877	sporadic	negative	er_neg	pgr_neg	Expressions data ->	Basal	Basal	Basal
8196	brca1	negative	er_neg	pgr_neg	Expressions data ->	Basal	Basal	Basal
11283	brca1	negative	er_neg	pgr_neg	Expressions data ->	Basal	Basal	Basal
7596	sporadic	negative	er_neg	pgr_neg	Expressions data ->	ERBB2	Basal	HER2enriched
10656	sporadic	-	er_neg	pgr_neg	Expressions data ->	ERBB2	HER2enriched	HER2enriched
11697	brcax	-	er_neg	pgr_neg	Expressions data ->	ERBB2	HER2enriched	HER2enriched
10785	brcax	-	er_neg	pgr_neg	Expressions data ->	ERBB2	HER2enriched	HER2enriched
15752	brcax	-	er_neg	pgr_neg	Expressions data ->	ERBB2	HER2enriched	Normal
10413	sporadic	-	er_pos	pgr_pos	Expressions data ->	Luminal-A	LumA	LumA
11462	sporadic	-	er_pos	pgr_pos	Expressions data ->	Luminal-A	LumA	LumA
7650	brcax	-	er_neg	pgr_neg	Expressions data ->	Luminal-A	LumA	LumA
15478	brcax	-	er_pos	pgr_pos	Expressions data ->	Luminal-A	LumA	LumA
15765	brcax	-	er_pos	pgr_neg	Expressions data ->	Luminal-A	LumA	LumA
10533	sporadic	-	er_pos	pgr_pos	Expressions data ->	Luminal-A	LumA	LumA
10017	sporadic	-	er_pos	pgr_pos	Expressions data ->	Luminal-A	Unclassified	Unclassified
13644	brcax	-	er_pos	pgr_neg	Expressions data ->	Luminal-B	Unclassified	HER2enriched
7223	brcax	negative	er_pos	pgr_neg	Expressions data ->	Luminal-B	LumB	LumB
7219	sporadic	negative	er_pos	pgr_pos	Expressions data ->	Luminal-B	Unclassified	LumB

Tumors from independent dataset analyzed on SRM

4404	sporadic	-	er_pos	pgr_pos	Expressions data ->	unclassified	unclassified	unclassified
4723	sporadic	-	er_neg	pgr_neg	Expressions data ->	Normal	Normal	Normal

6009	sporadic	-	er_pos	pgr_pos	Expressions data ->	LumA	LumA	LumB
6013	sporadic	negative	er_pos	pgr_pos	Expressions data ->	unclassified	LumB	LumB
6029	sporadic	negative	er_neg	pgr_neg	Expressions data ->	ERBB2	Basal	ERBB2
6176	sporadic	negative	er_pos	pgr_pos	Expressions data ->	LumB	LumB	LumB
6191	sporadic	positive	er_neg	pgr_neg	Expressions data ->	Basal	Basal	Basal
6514	sporadic	-	er_neg	pgr_neg	Expressions data ->	Normal	Normal	Normal
6877	sporadic	-	er_pos	pgr_pos	Expressions data ->	unclassified	unclassified	unclassified
7940	sporadic	-	er_pos	pgr_pos	Expressions data ->	Normal	Normal	Normal
8613	sporadic	negative	er_pos	pgr_pos	Expressions data ->	LumA	LumA	unclassified
9317	sporadic	-	er_pos	pgr_pos	Expressions data ->	unclassified	LumA	Normal
9322	sporadic	positive	er_neg	pgr_neg	Expressions data ->	Basal	Basal	Basal
9340	sporadic	-	er_pos	pgr_pos	Expressions data ->	LumA	LumB	LumB
9460	sporadic	negative	er_pos	pgr_pos	Expressions data ->	ERBB2	Basal	LumB
6127	sporadic	-	er_pos	pgr_pos	Expressions data ->	Normal	Normal	Normal
7286	sporadic	-	er_pos	pgr_pos	Expressions data ->	unclassified	LumA	unclassified
