# Supplementary material for: Proteomic analysis of breast tumors confirms the mRNA intrinsic molecular subtypes using different classifiers: a large-scale analysis of fresh frozen tissue samples
Source: Breast Cancer Res. 2016 Jun 29;18:69. doi: 10.1186/s13058-016-0732-2 (PMC4928264; doi:10.1186/s13058-016-0732-2)
Supplement: Additional file 6: Figure S1. — The ROC area curves for all classifiers and pairwise comparisons between each of the three gene classifications (Sörlie, PAM50, and Hu) and SRM-based peptide assays for each of the five subclasses; basal, normal, Her2, luminal A and luminal B. (PDF 178 kb) [file 13058_2016_732_MOESM6_ESM.pdf]

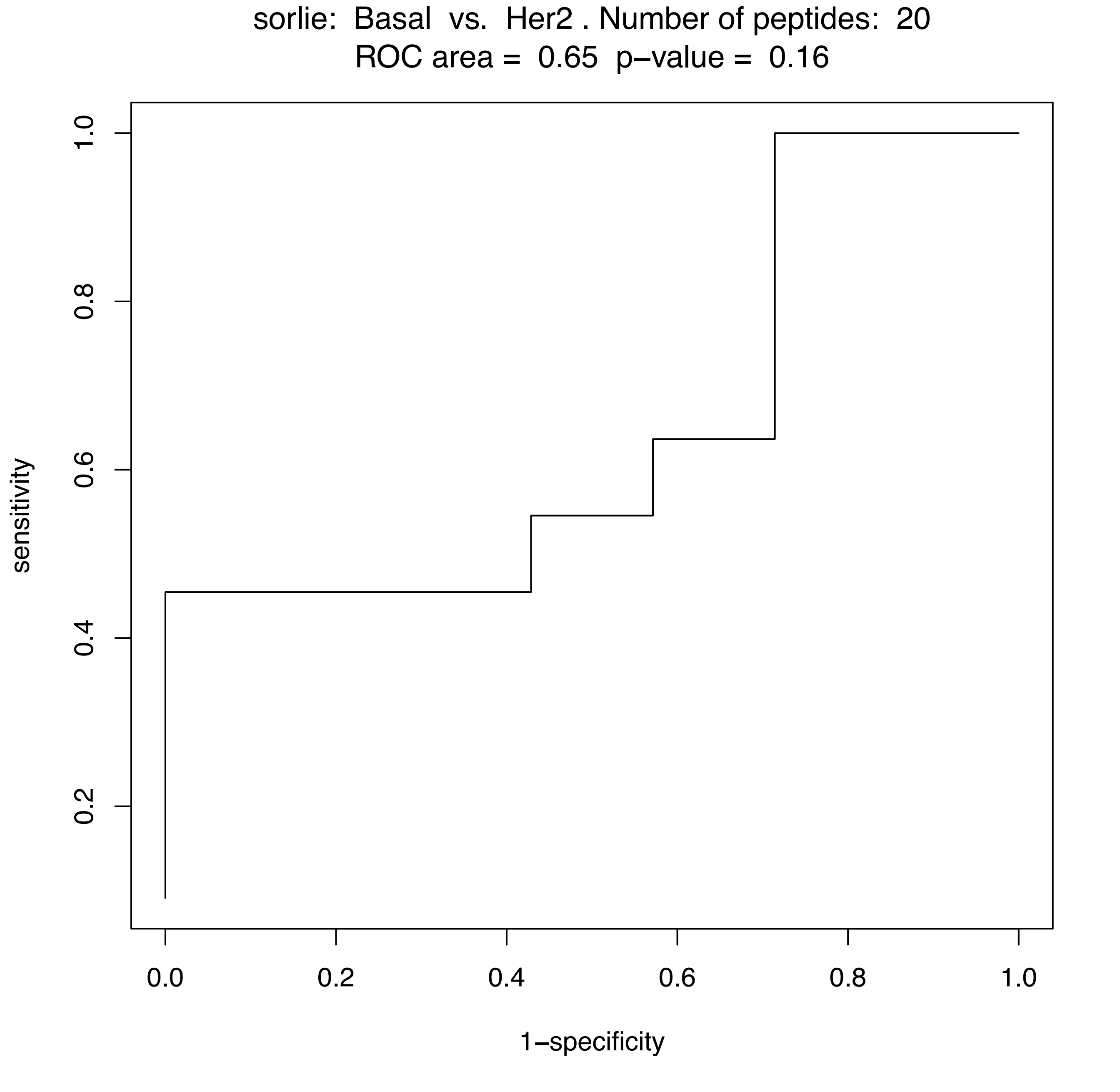
sorlie: Basal vs. Her2 . Number of peptides: 20
ROC area = 0.65 p−value = 0.16
sensitivity
1−specificity
0.0
0.2
0.4
0.6
0.8
1.0

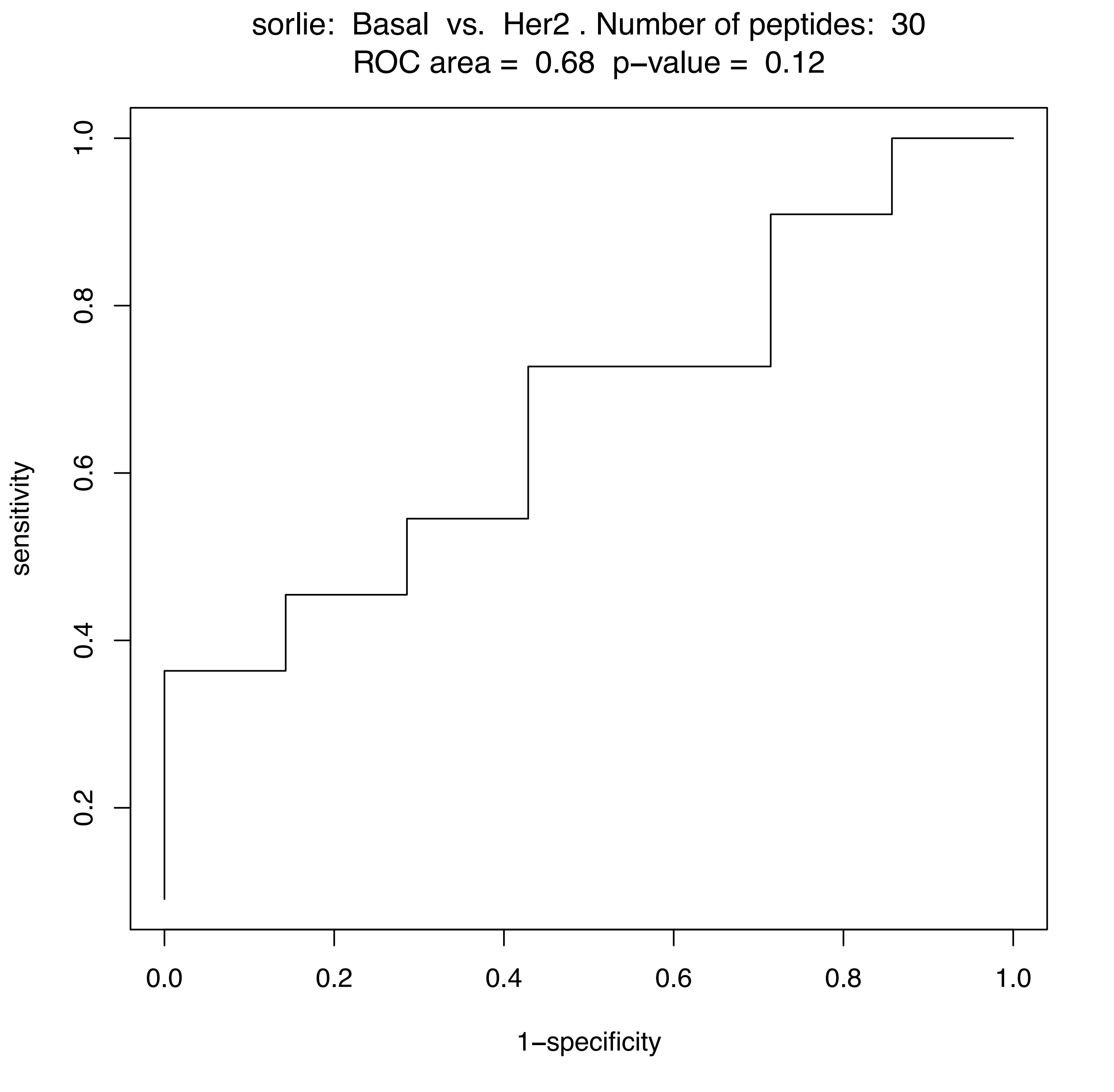
sorlie: Basal vs. Her2 . Number of peptides: 30
ROC area = 0.68 p−value = 0.12
sensitivity
1−specificity
0.2
0.4
0.6
0.8
1.0
0.0
0.2
0.4
0.6
0.8
1.0

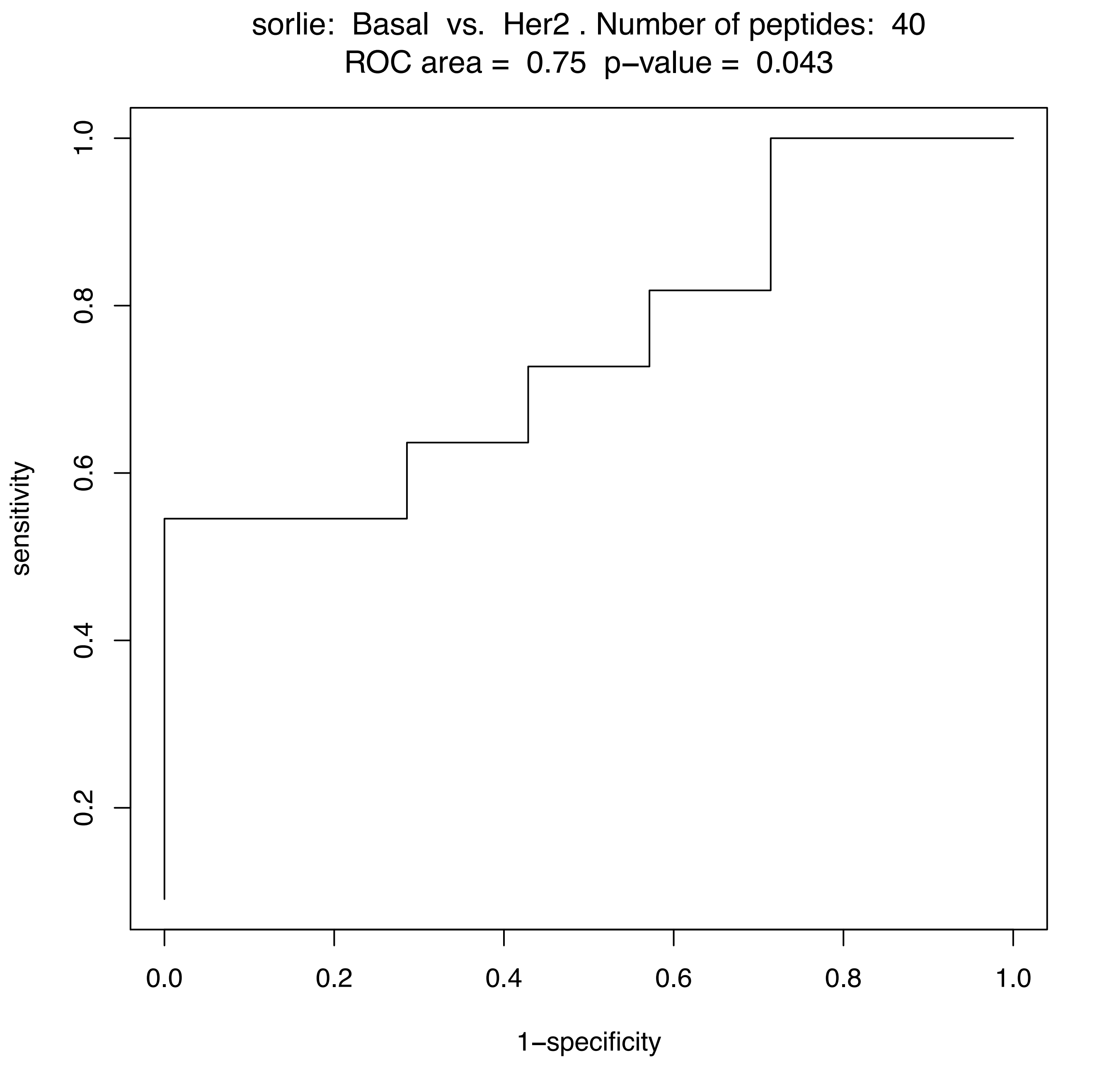

sorlie: Basal vs. Her2 . Number of peptides: 40
ROC area = 0.75 p−value = 0.043
sensitivity
0.2
0.4
0.6
0.8
1.0
0.0
0.2
0.4
0.6
0.8
1.0
1−specificity

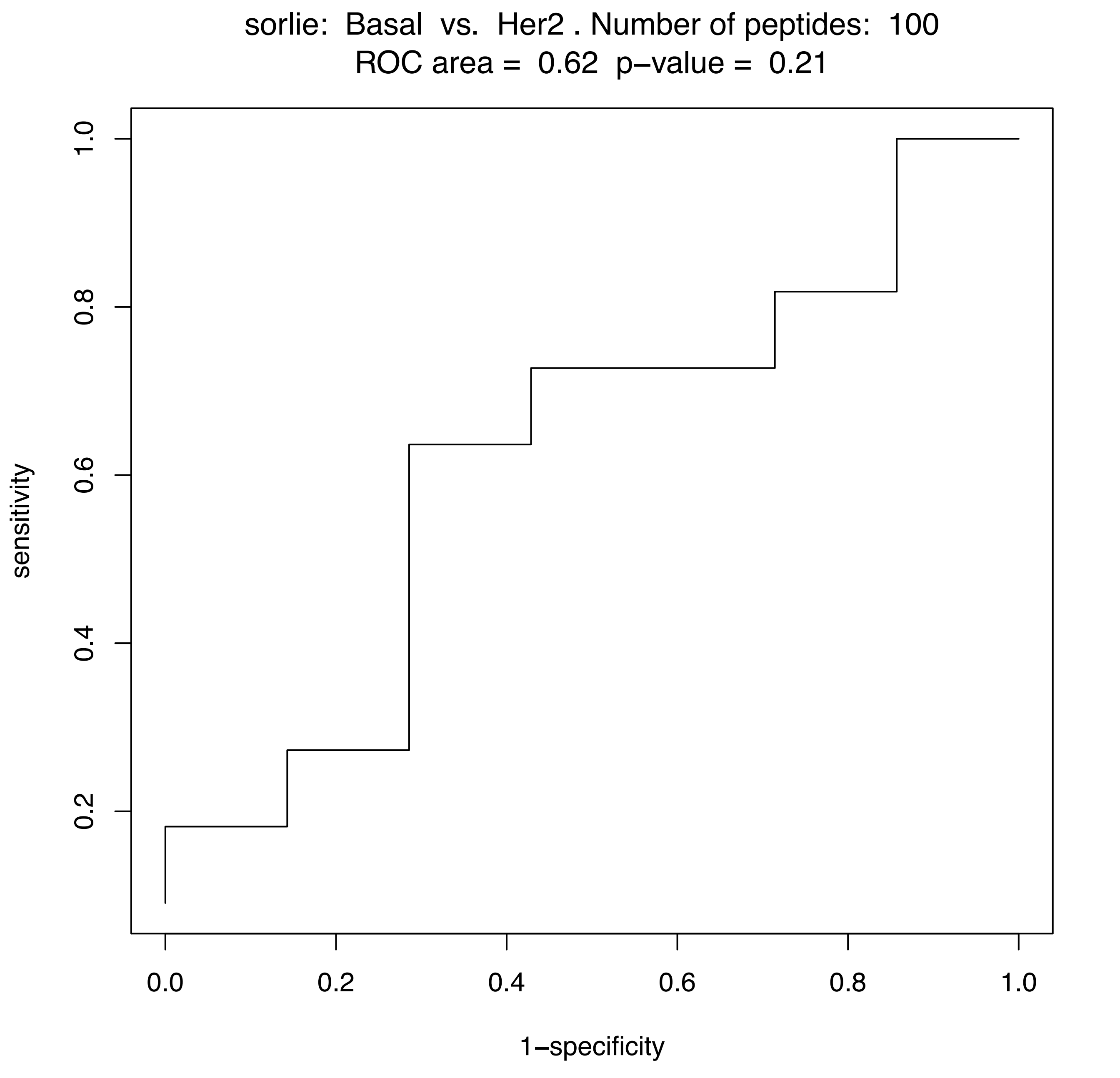
sorlie: Basal vs. Her2 . Number of peptides: 100
ROC area = 0.62 p−value = 0.21
sensitivity
1−specificity
0.0
0.2
0.4
0.6
0.8
1.0
0.2
0.4
0.6
0.8
1.0

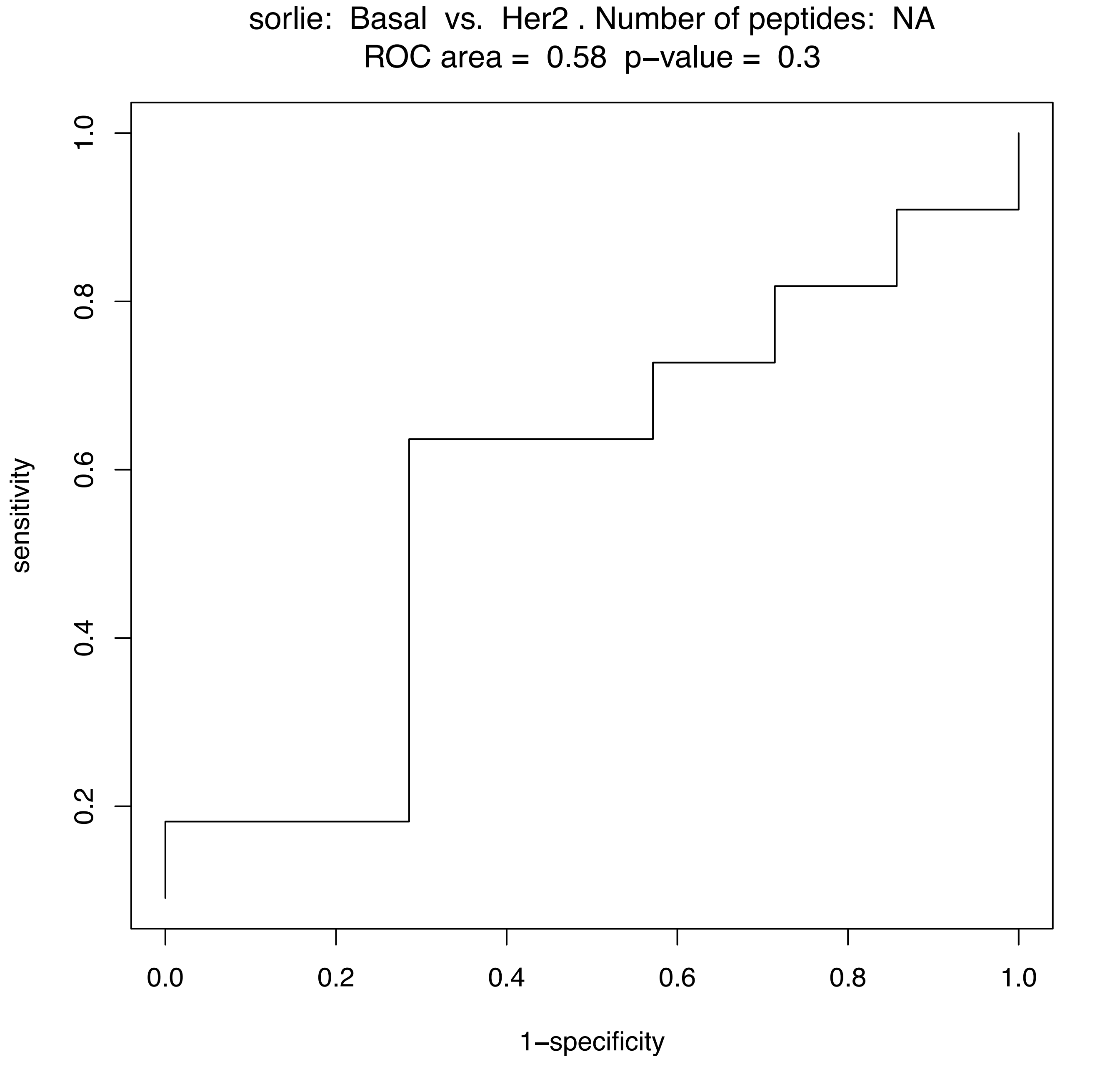
sorlie: Basal vs. Her2 . Number of peptides: NA
ROC area = 0.58 p−value = 0.3
sensitivity
1−specificity
0.2
0.4
0.6
0.8
1.0
0.0
0.2
0.4
0.6
0.8
1.0

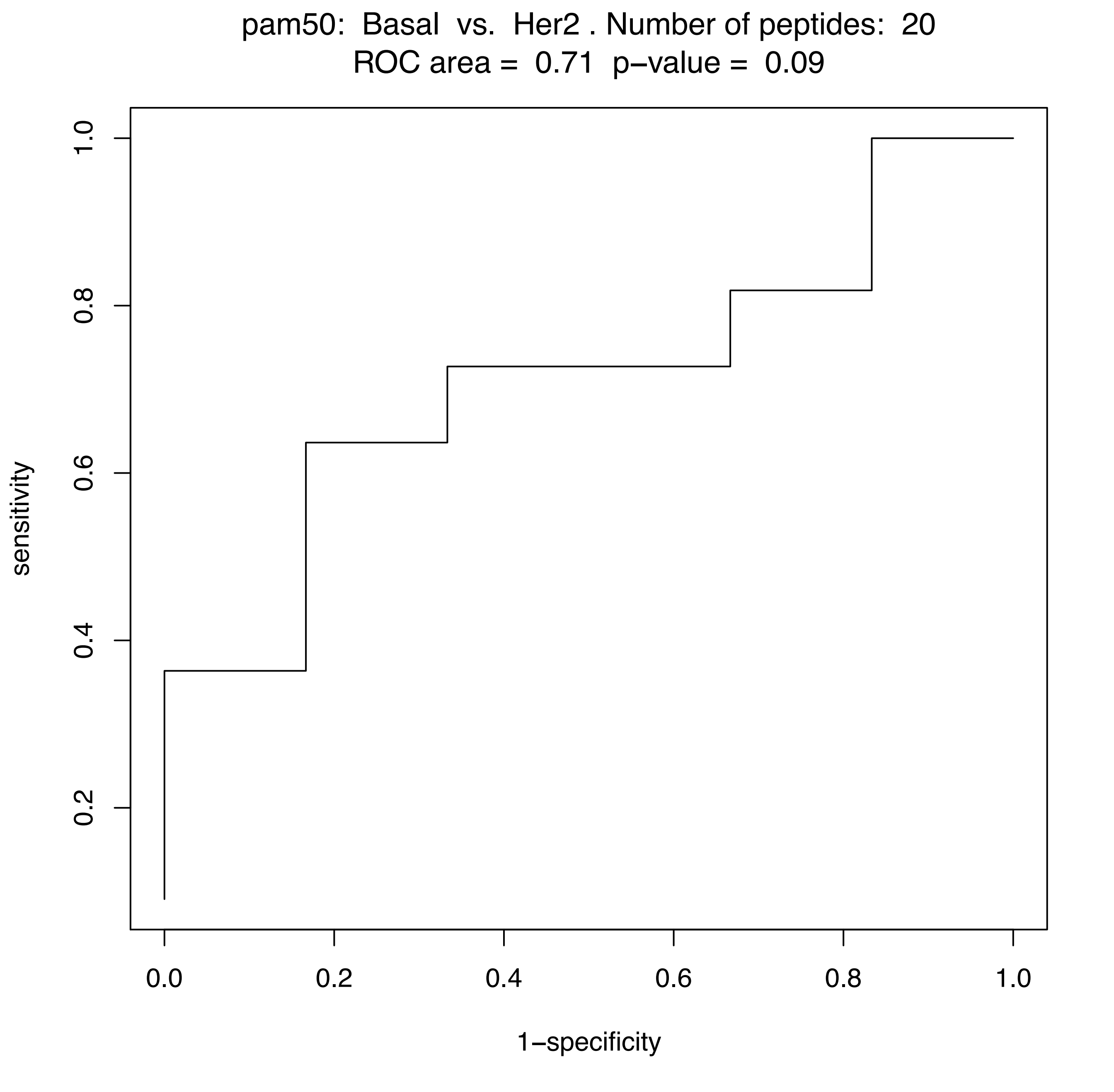
pam50: Basal vs. Her2 . Number of peptides: 20
ROC area = 0.71 p−value = 0.09
sensitivity
1−specificity
0.2
0.4
0.6
0.8
1.0
0.0
0.2
0.4
0.6
0.8
1.0

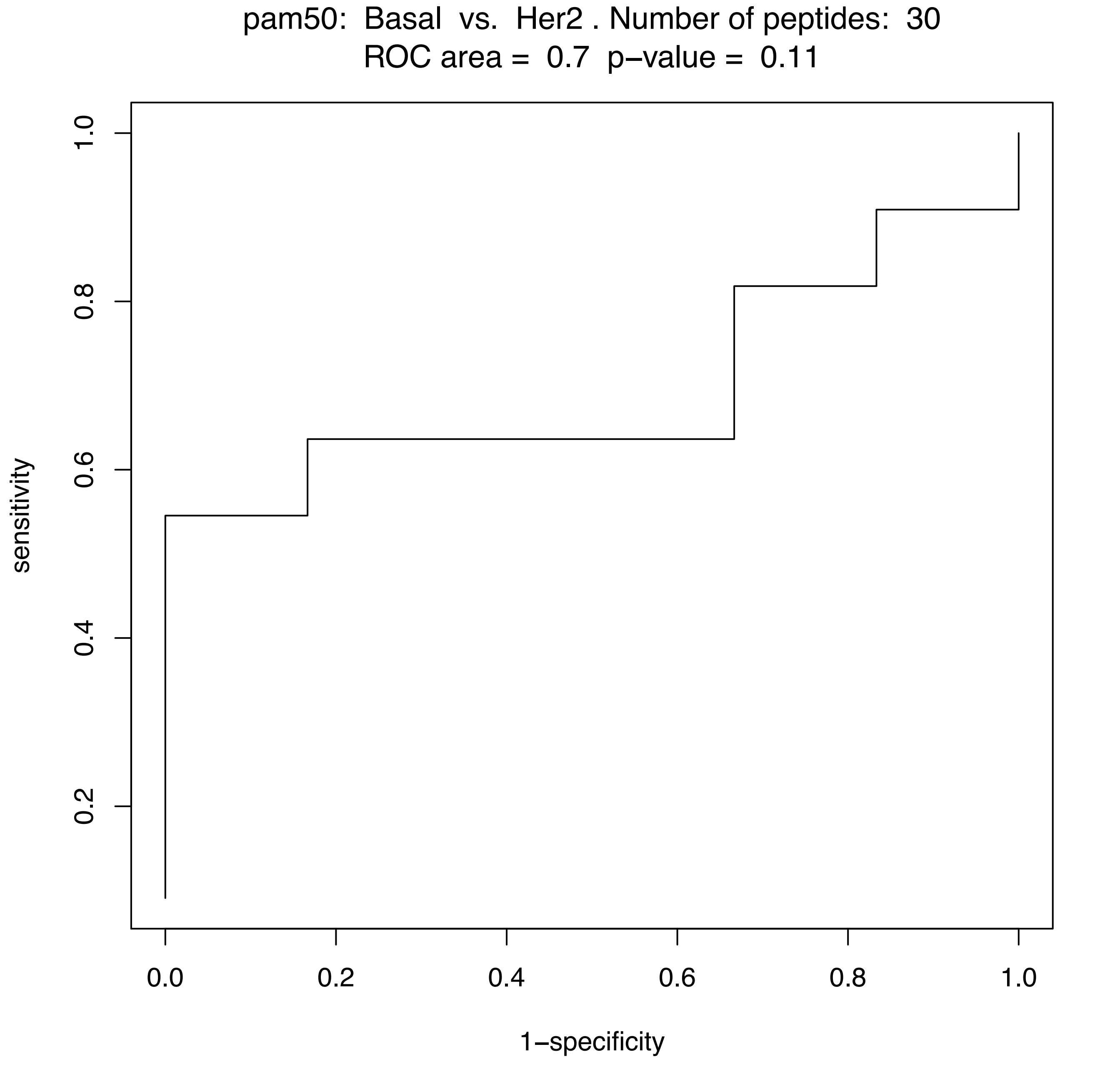
pam50: Basal vs. Her2 . Number of peptides: 30
ROC area = 0.7 p−value = 0.11
sensitivity
1−specificity
0.2
0.4
0.6
0.8
1.0
0.0
0.2
0.4
0.6
0.8
1.0

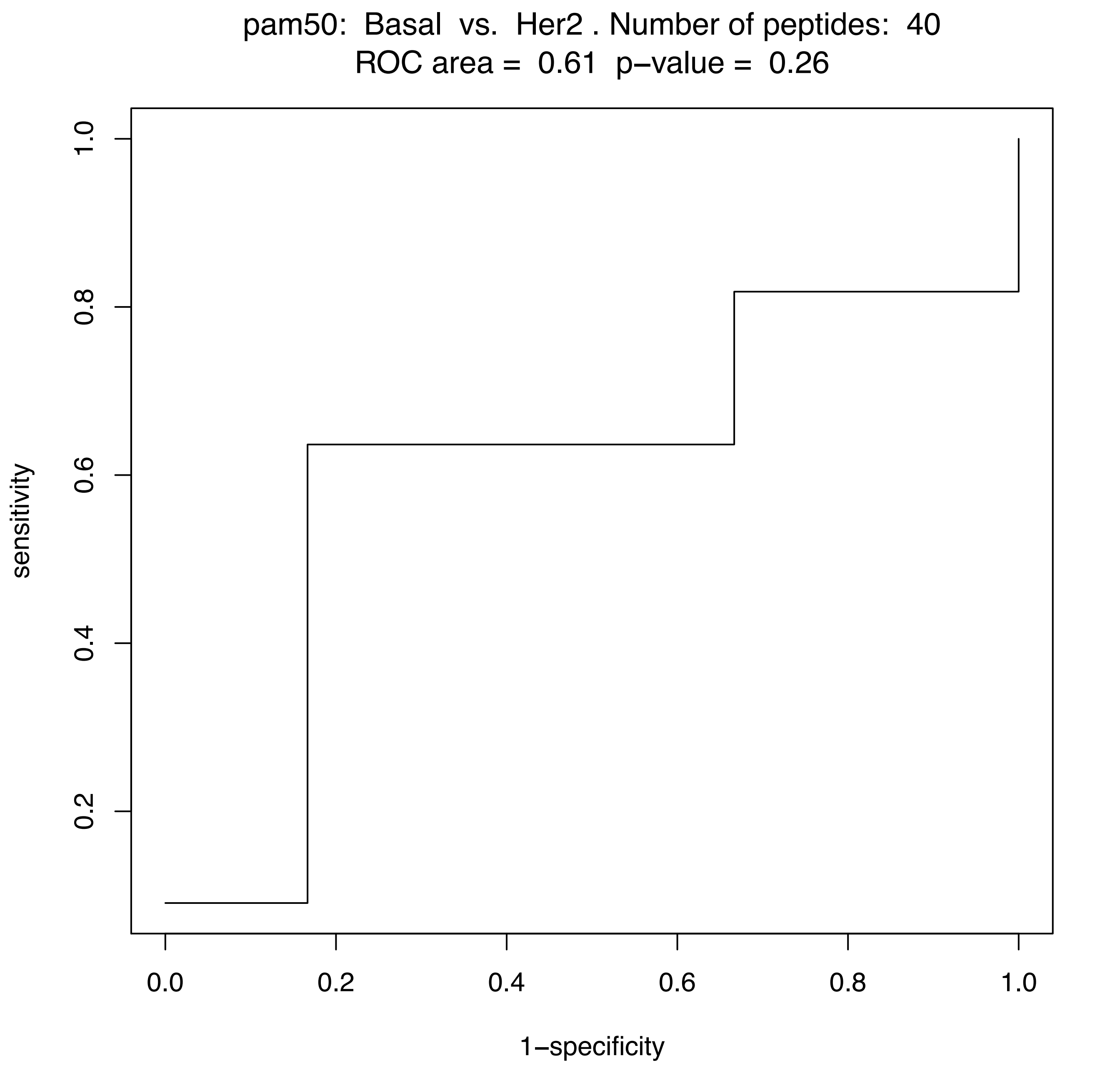
pam50: Basal vs. Her2 . Number of peptides: 40
ROC area = 0.61 p−value = 0.26
sensitivity
1−specificity
0.0
0.2
0.4
0.6
0.8
1.0

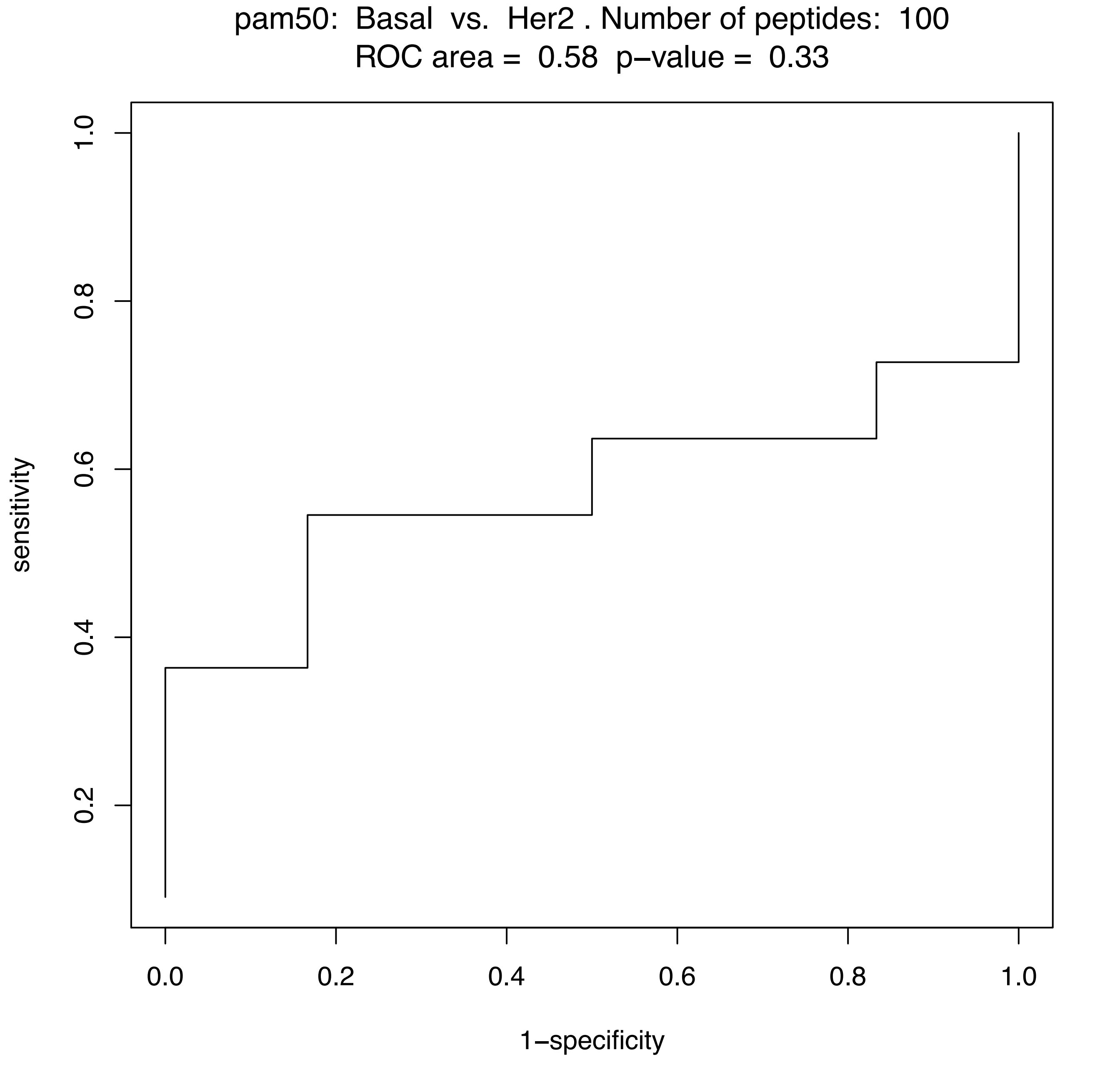
pam50: Basal vs. Her2 . Number of peptides: 100
ROC area = 0.58 p−value = 0.33
sensitivity
1−specificity
0.0
0.2
0.4
0.6
0.8
1.0

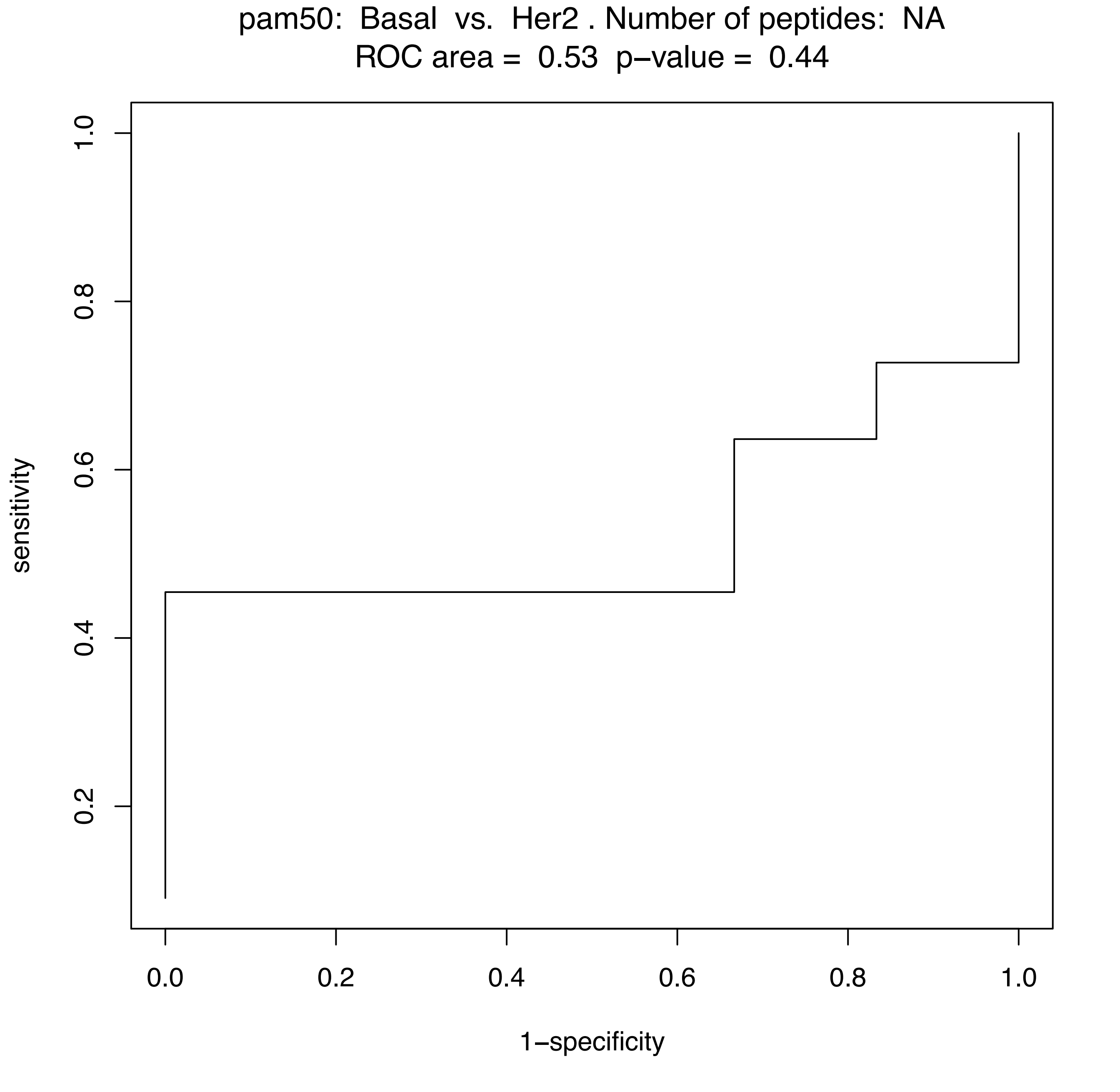
pam50: Basal vs. Her2 . Number of peptides: NA
ROC area = 0.53 p−value = 0.44
sensitivity
1−specificity
0.0
0.2
0.4
0.6
0.8
1.0
0.2
0.4
0.6
0.8
1.0

hu: Basal vs. Her2 . Number of peptides: 20
ROC area = 0.66 p−value = 0.19

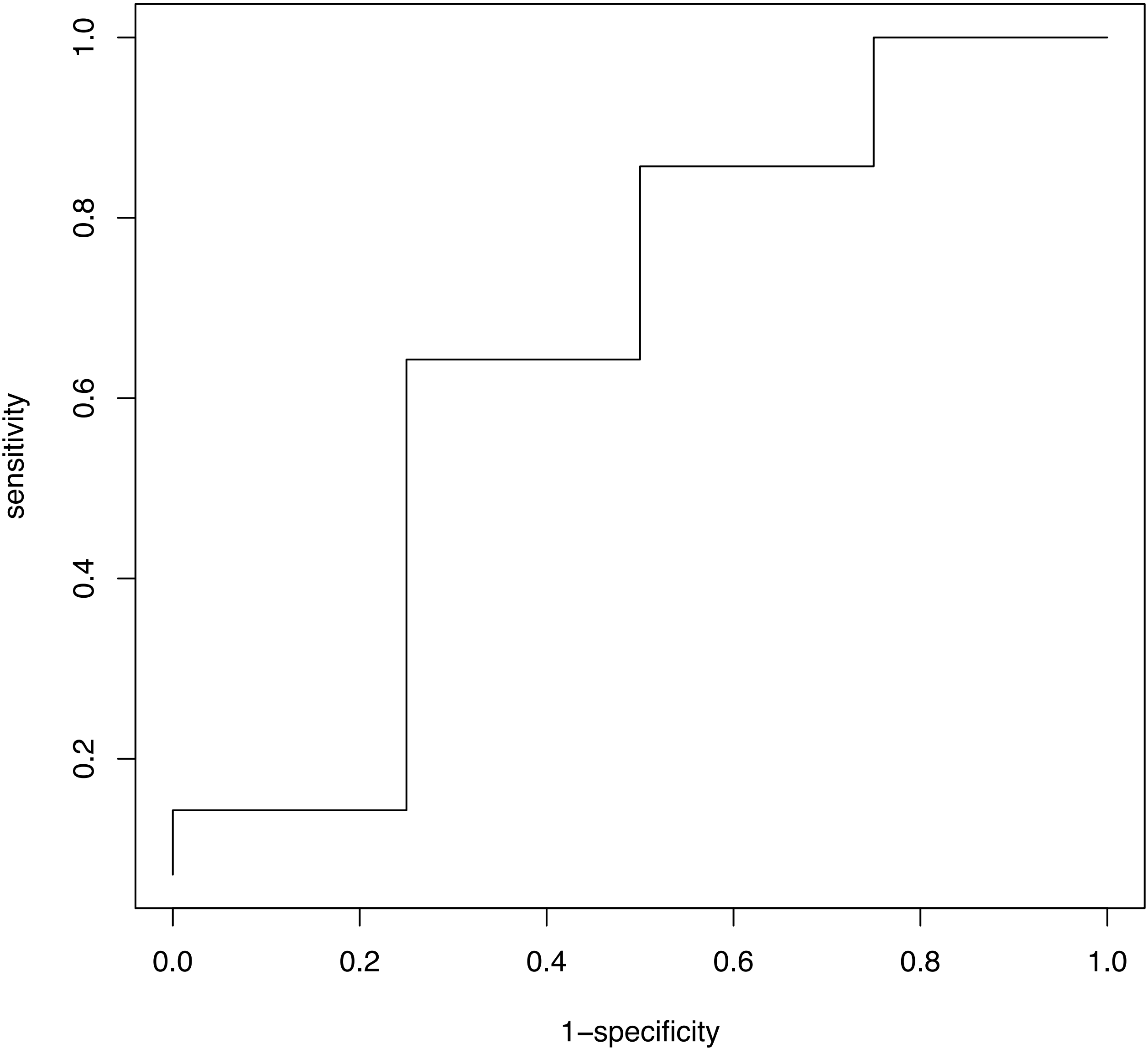

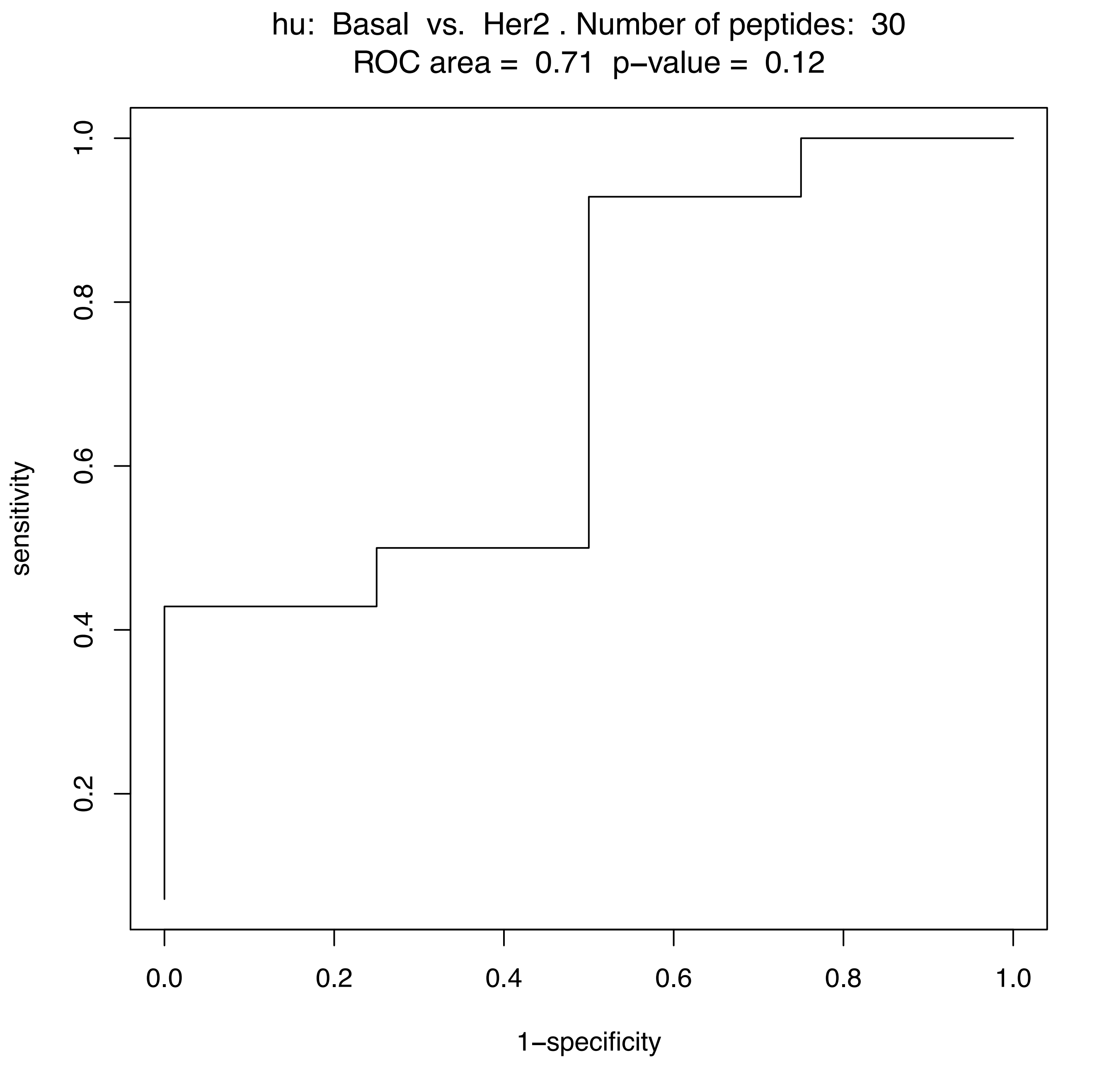
hu: Basal vs. Her2 . Number of peptides: 30
ROC area = 0.71 p−value = 0.12
sensitivity
1.0
0.8
0.6
0.4
0.2
0.0
0.2
0.4
0.6
0.8
1.0
1−specificity

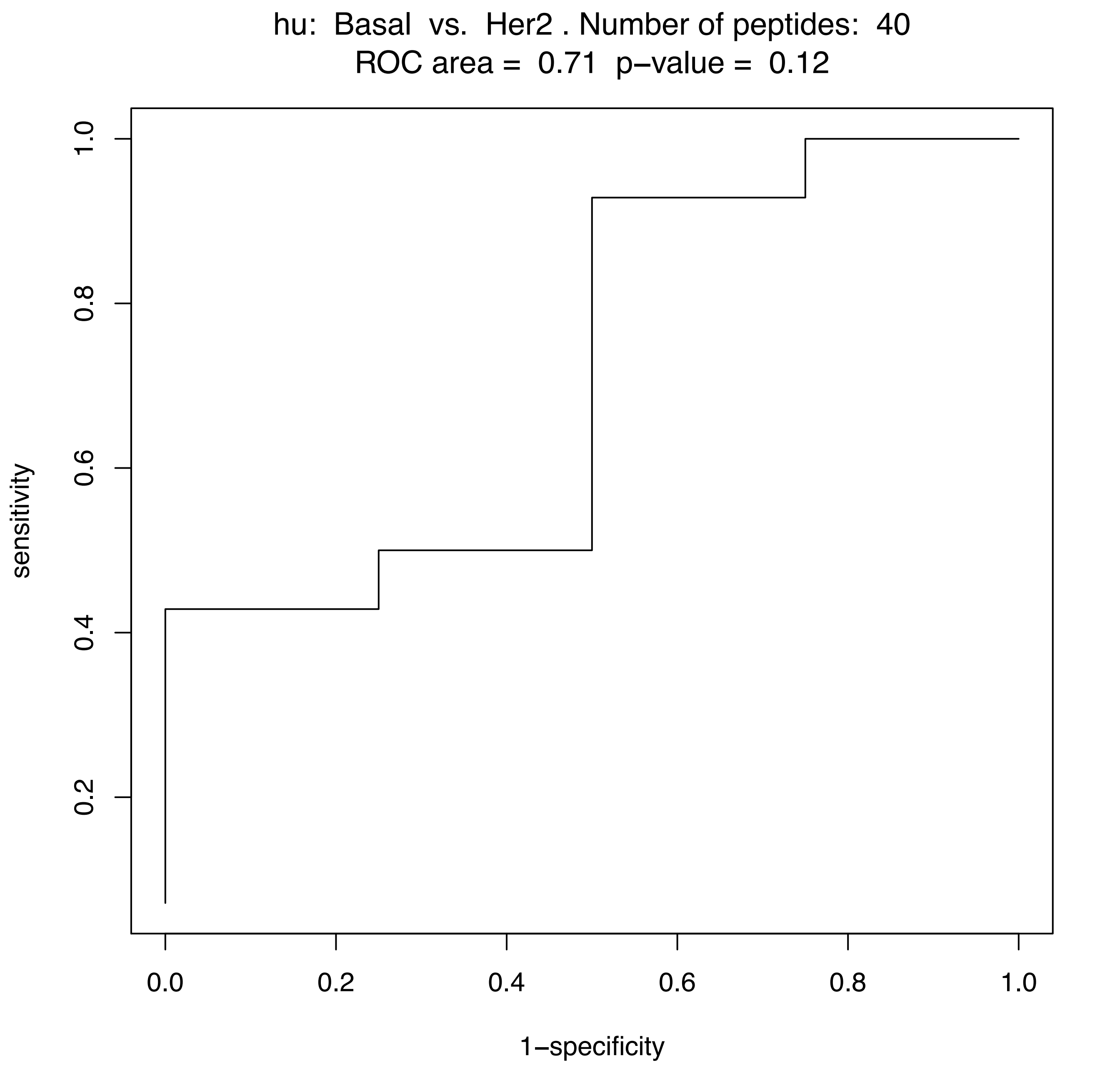
hu: Basal vs. Her2 . Number of peptides: 40
ROC area = 0.71 p−value = 0.12
sensitivity
1.0
0.8
0.6
0.4
0.2
0.0
0.2
0.4
0.6
0.8
1.0
1−specificity

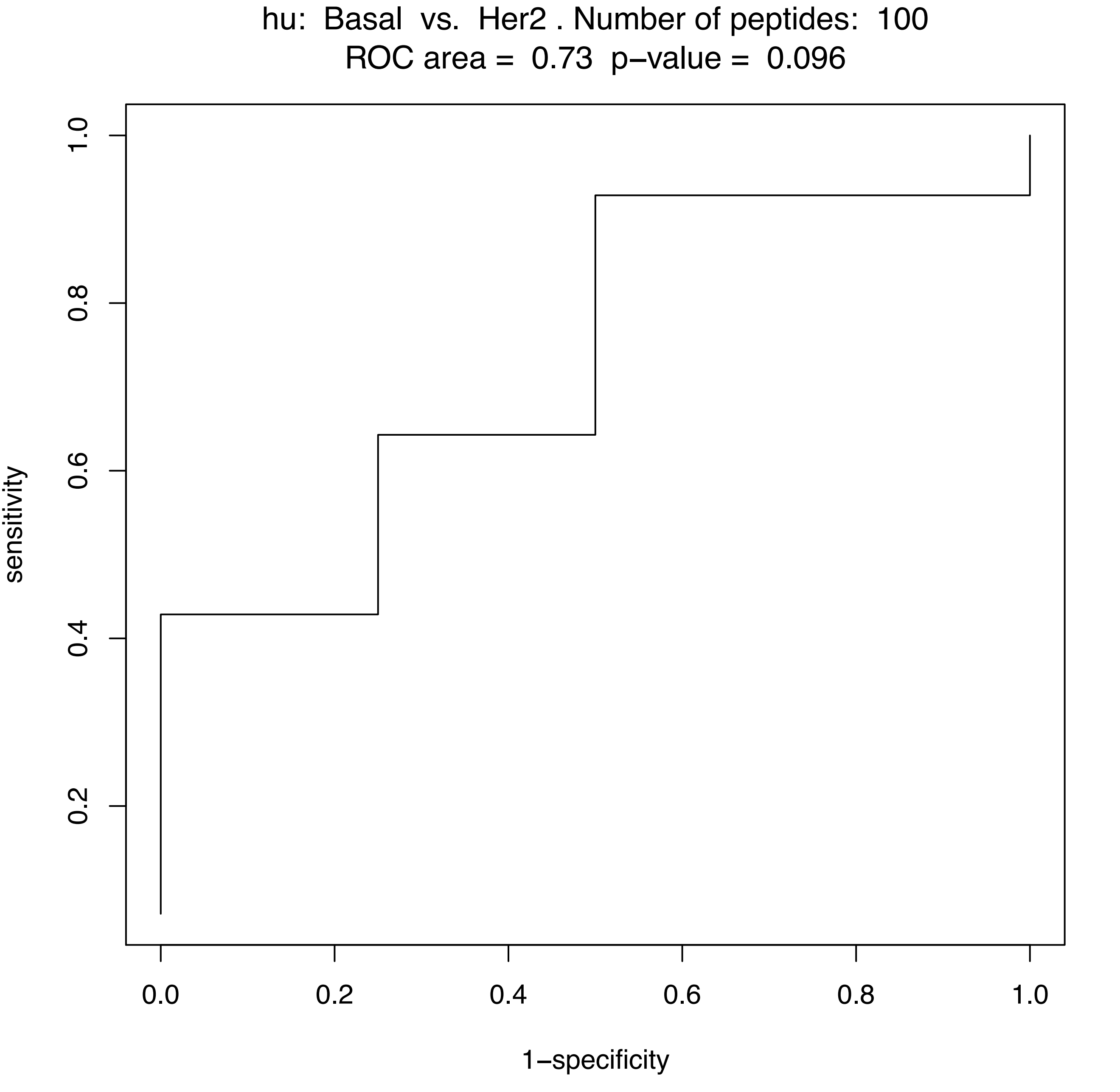
hu: Basal vs. Her2 . Number of peptides: 100
ROC area = 0.73 p−value = 0.096
sensitivity
0.2
0.4
0.6
0.8
1.0
0.0
0.2
0.4
0.6
0.8
1.0
1−specificity

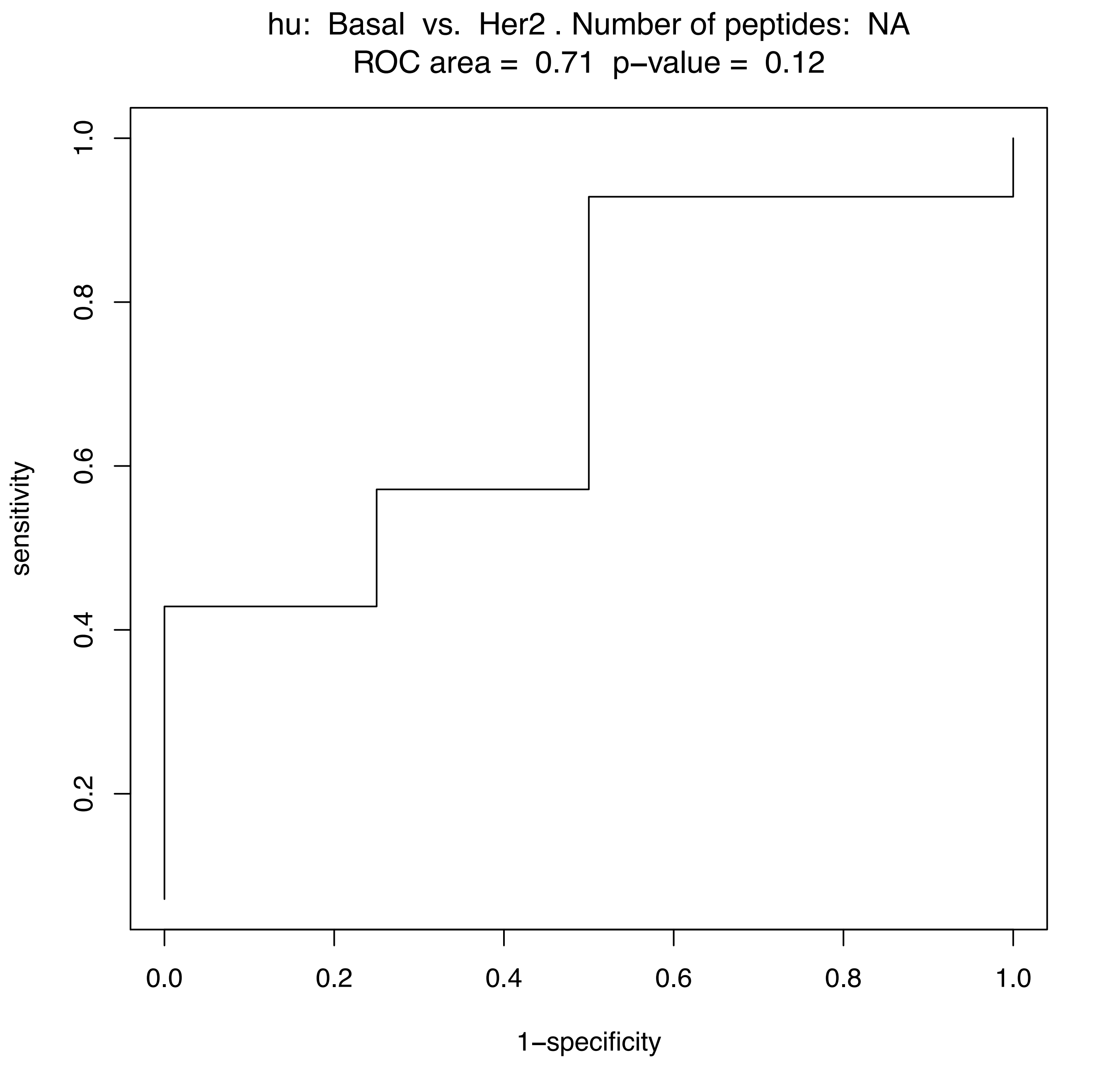
hu: Basal vs. Her2 . Number of peptides: NA
ROC area = 0.71 p−value = 0.12
sensitivity
1.0
0.8
0.6
0.4
0.2
0.0
0.2
0.4
0.6
0.8
1.0
1−specificity

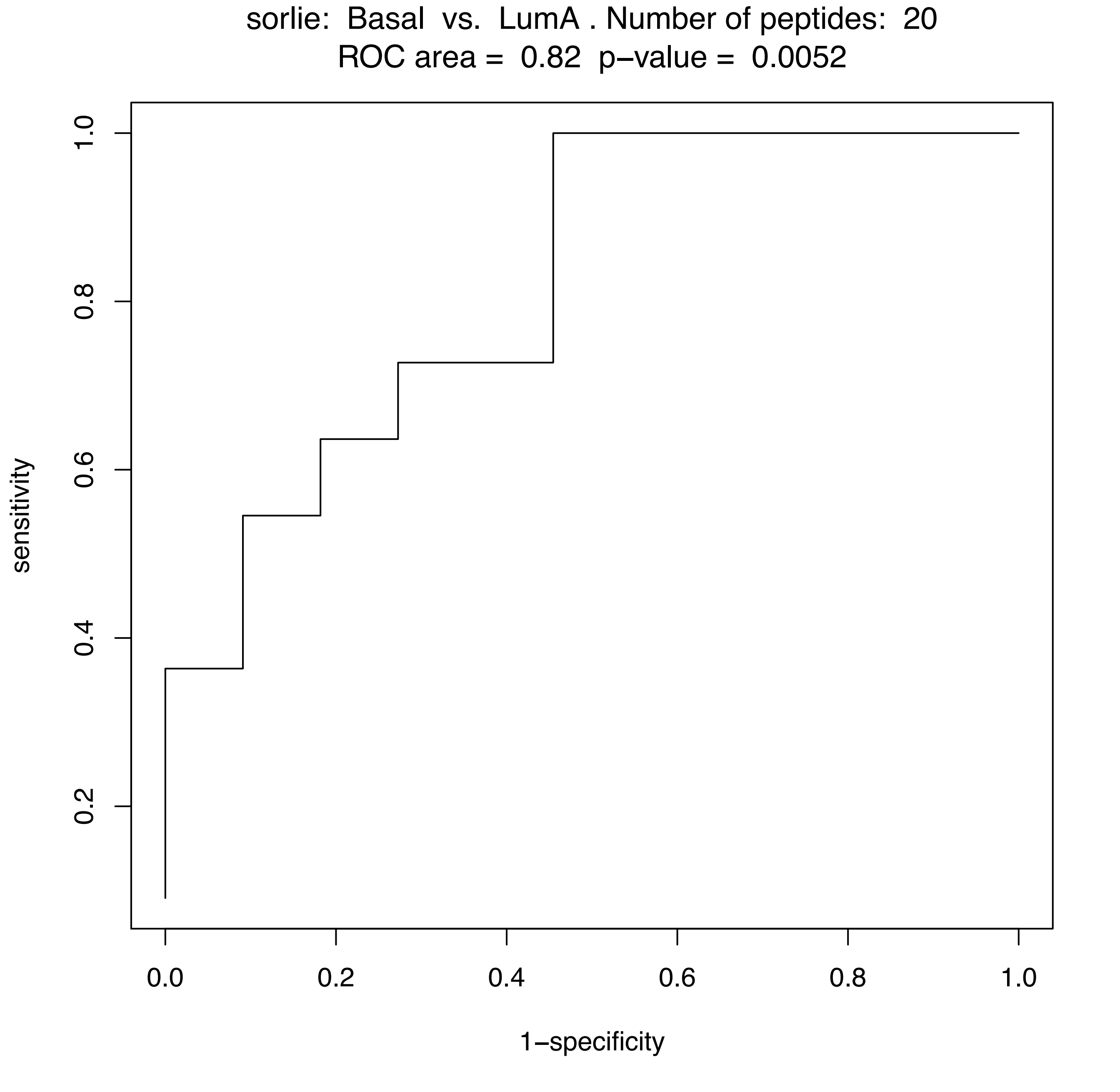
sorlie: Basal vs. LumA . Number of peptides: 20
ROC area = 0.82 p−value = 0.0052
1.0
0.8
0.6
0.4
0.2
sensitivity
0.0
0.2
0.4
0.6
0.8
1.0
1−specificity

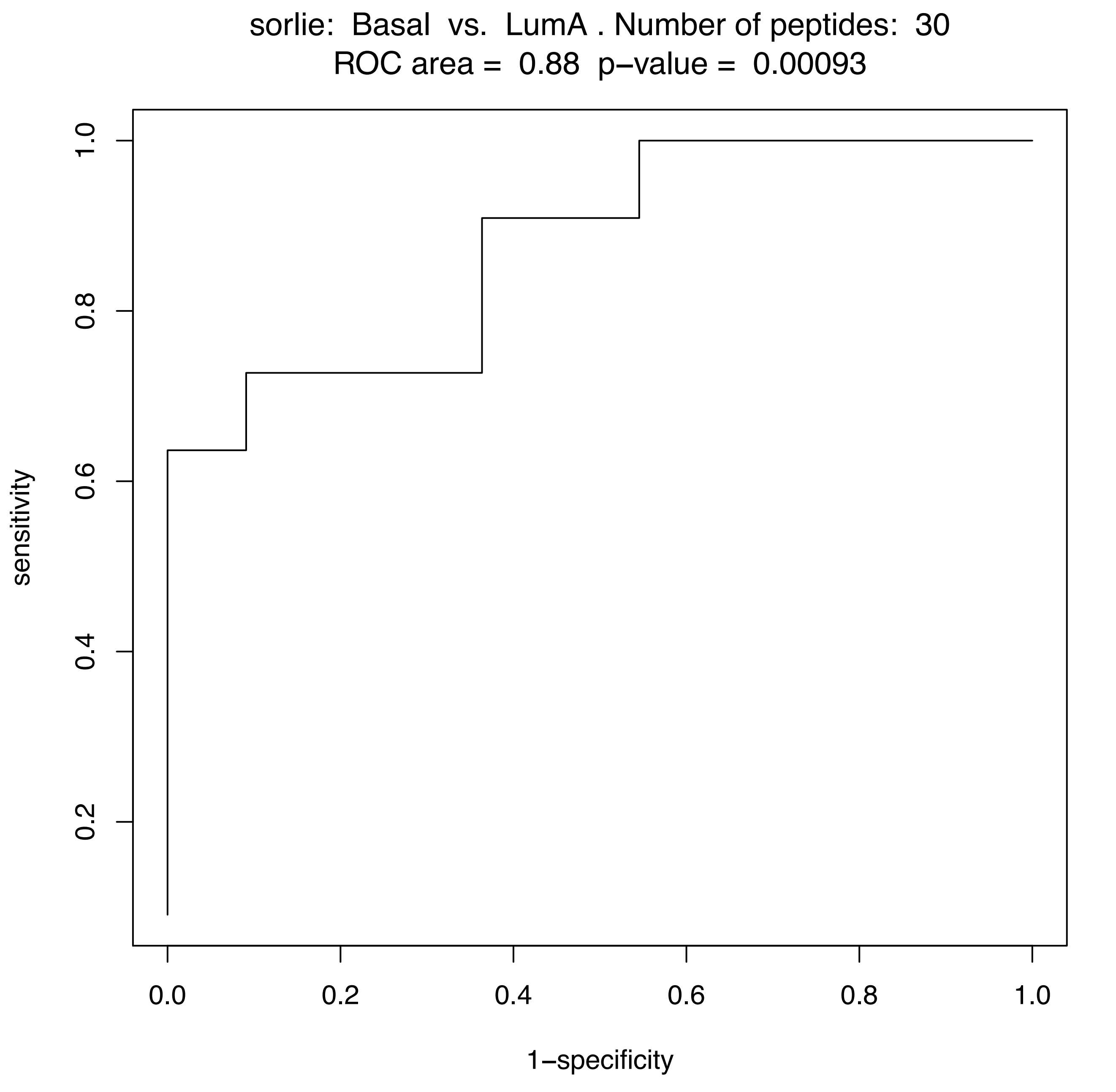
sorlie: Basal vs. LumA . Number of peptides: 30
ROC area = 0.88 p−value = 0.00093
sensitivity
1.0
0.8
0.6
0.4
0.2
0.0
0.2
0.4
0.6
0.8
1.0
1−specificity

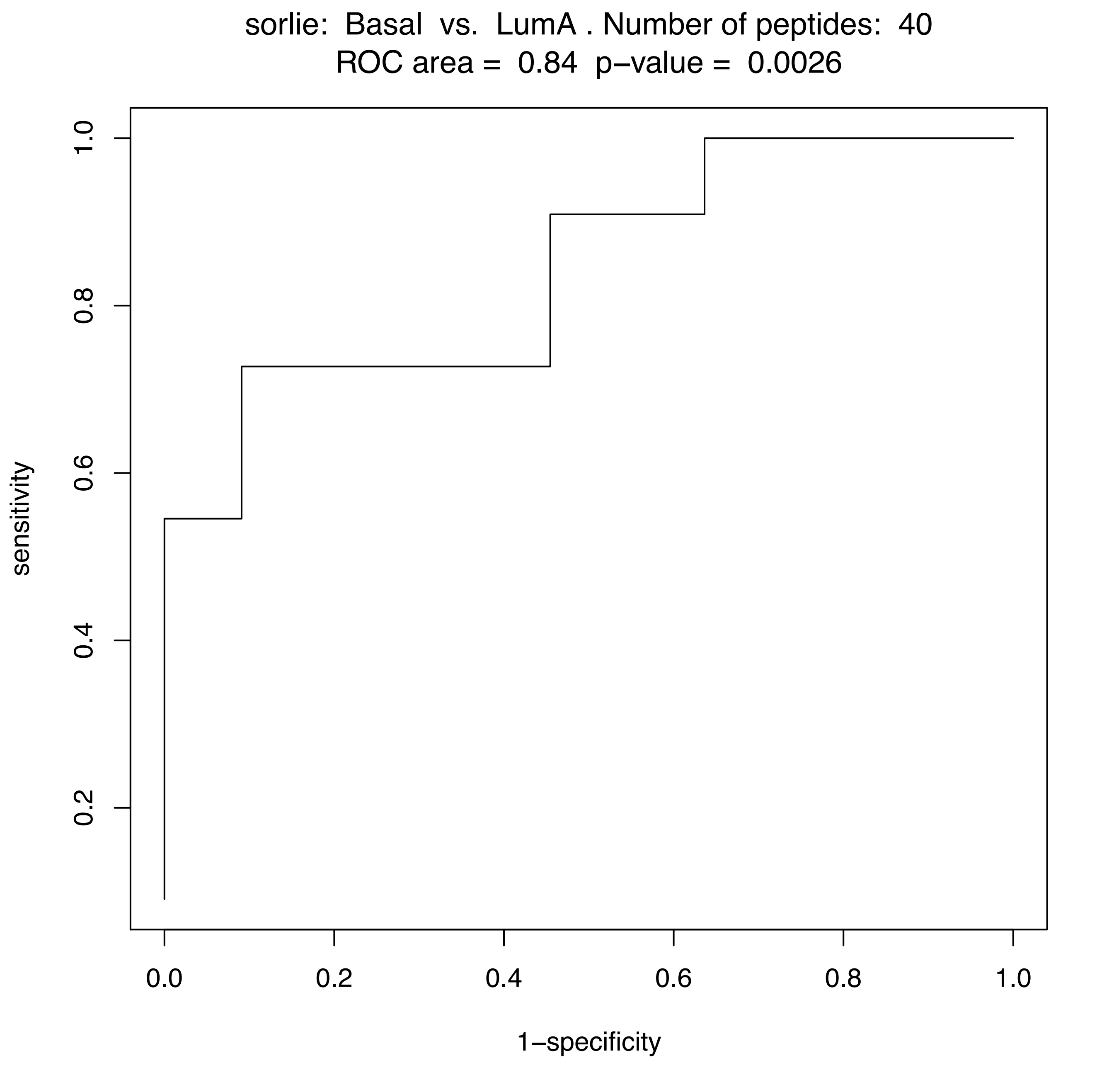
sorlie: Basal vs. LumA . Number of peptides: 40
ROC area = 0.84 p−value = 0.0026
sensitivity
1−specificity
0.0
0.2
0.4
0.6
0.8
1.0
0.2
0.4
0.6
0.8
1.0

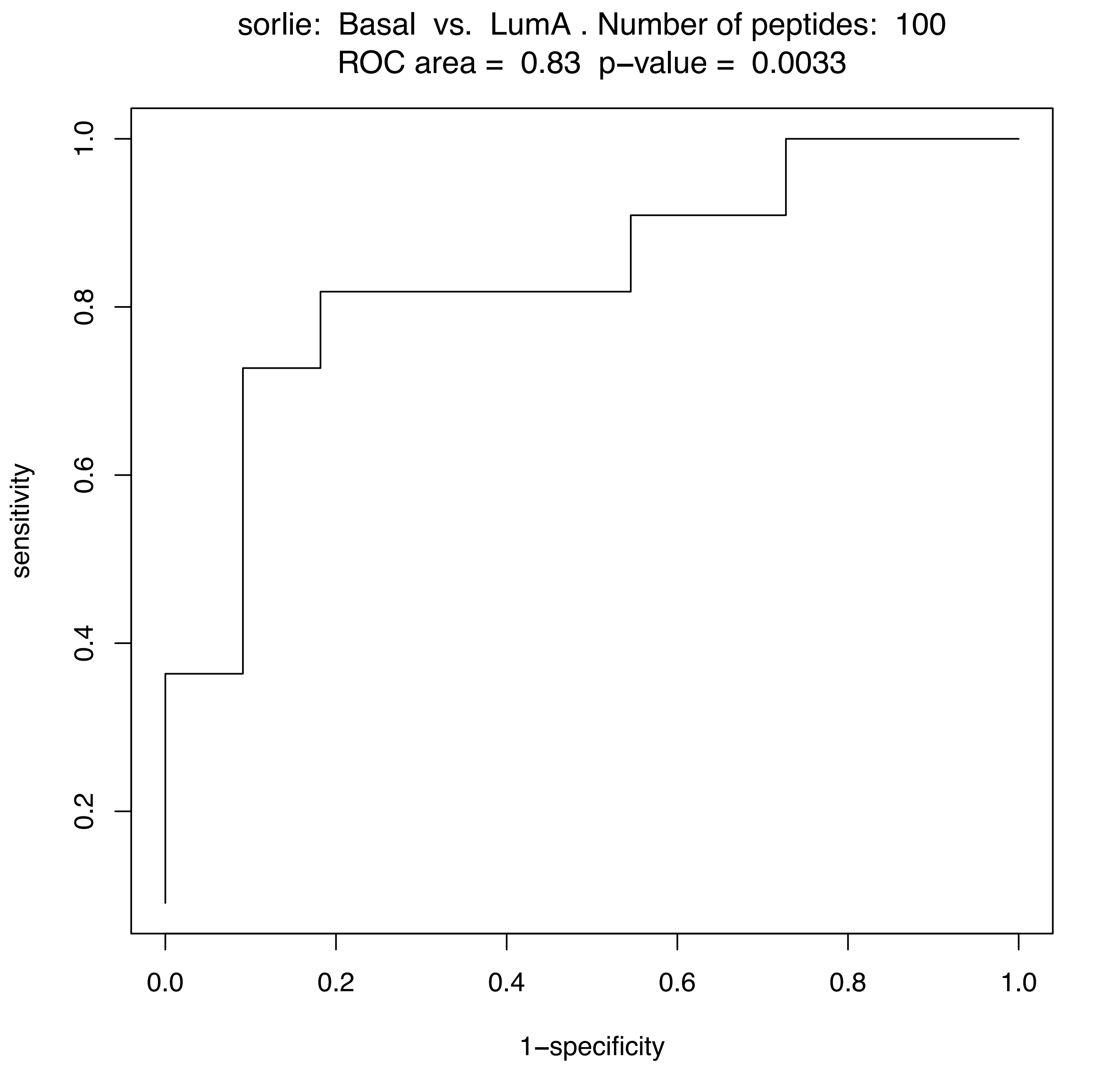
sorlie: Basal vs. LumA . Number of peptides: 100
ROC area = 0.83 p−value = 0.0033
sensitivity
1−specificity
0.0
0.2
0.4
0.6
0.8
1.0

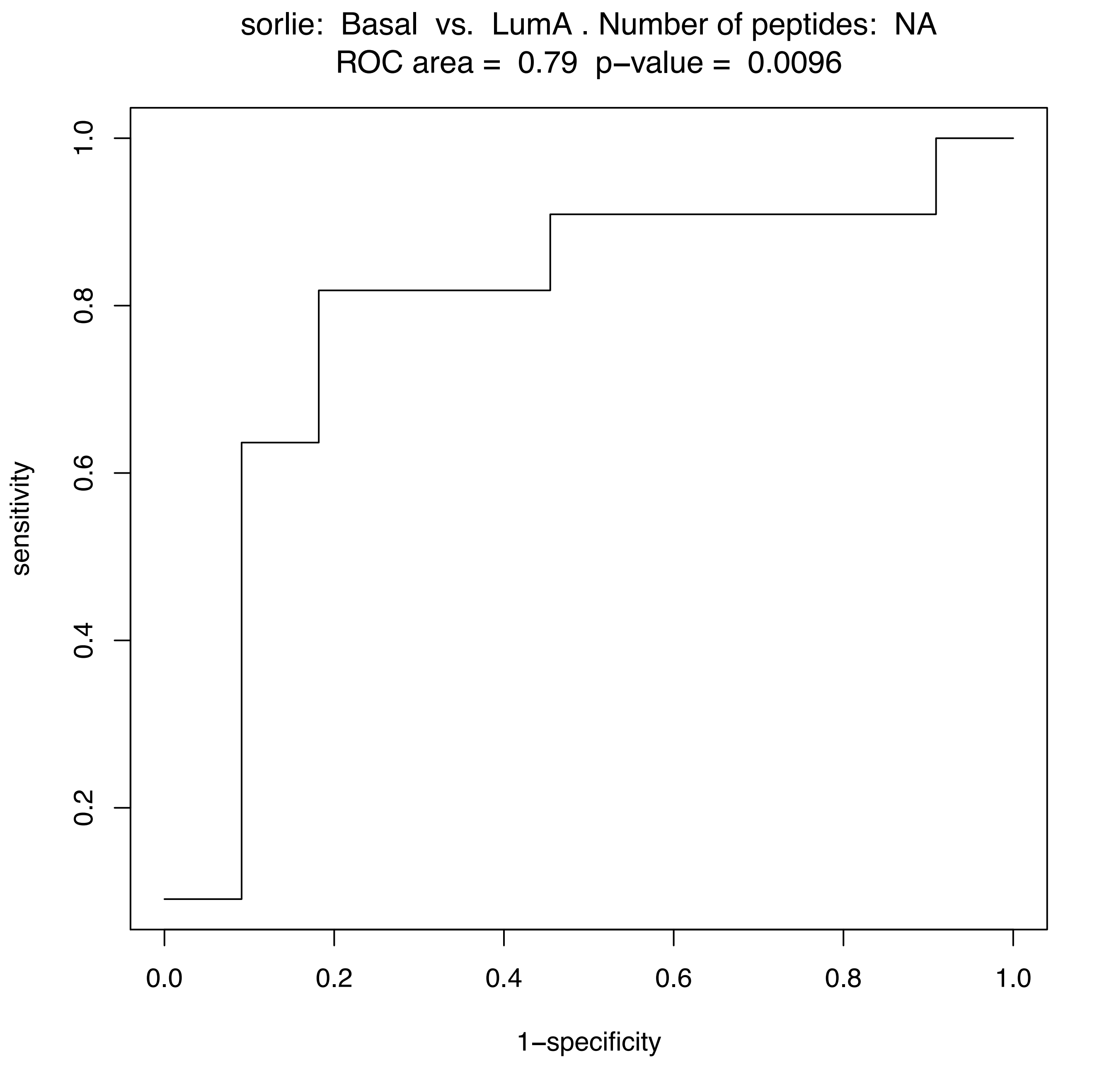

sorlie: Basal vs. LumA . Number of peptides: NA
ROC area = 0.79 p−value = 0.0096
sensitivity
1−specificity
0.2
0.4
0.6
0.8
1.0
0.0
0.2
0.4
0.6
0.8
1.0

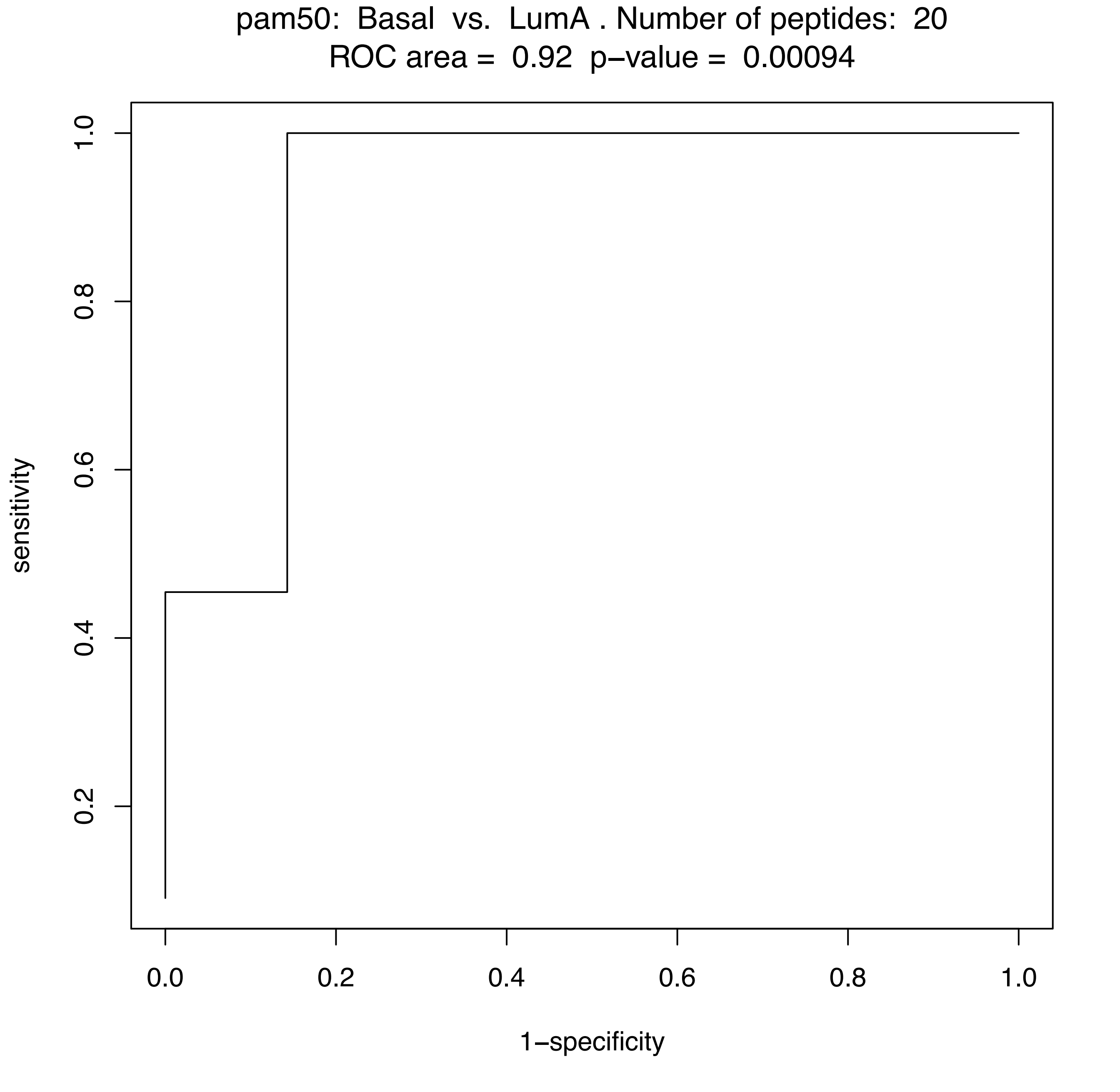

pam50: Basal vs. LumA . Number of peptides: 20
ROC area = 0.92 p−value = 0.00094
sensitivity
1−specificity
0.0
0.2
0.4
0.6
0.8
1.0

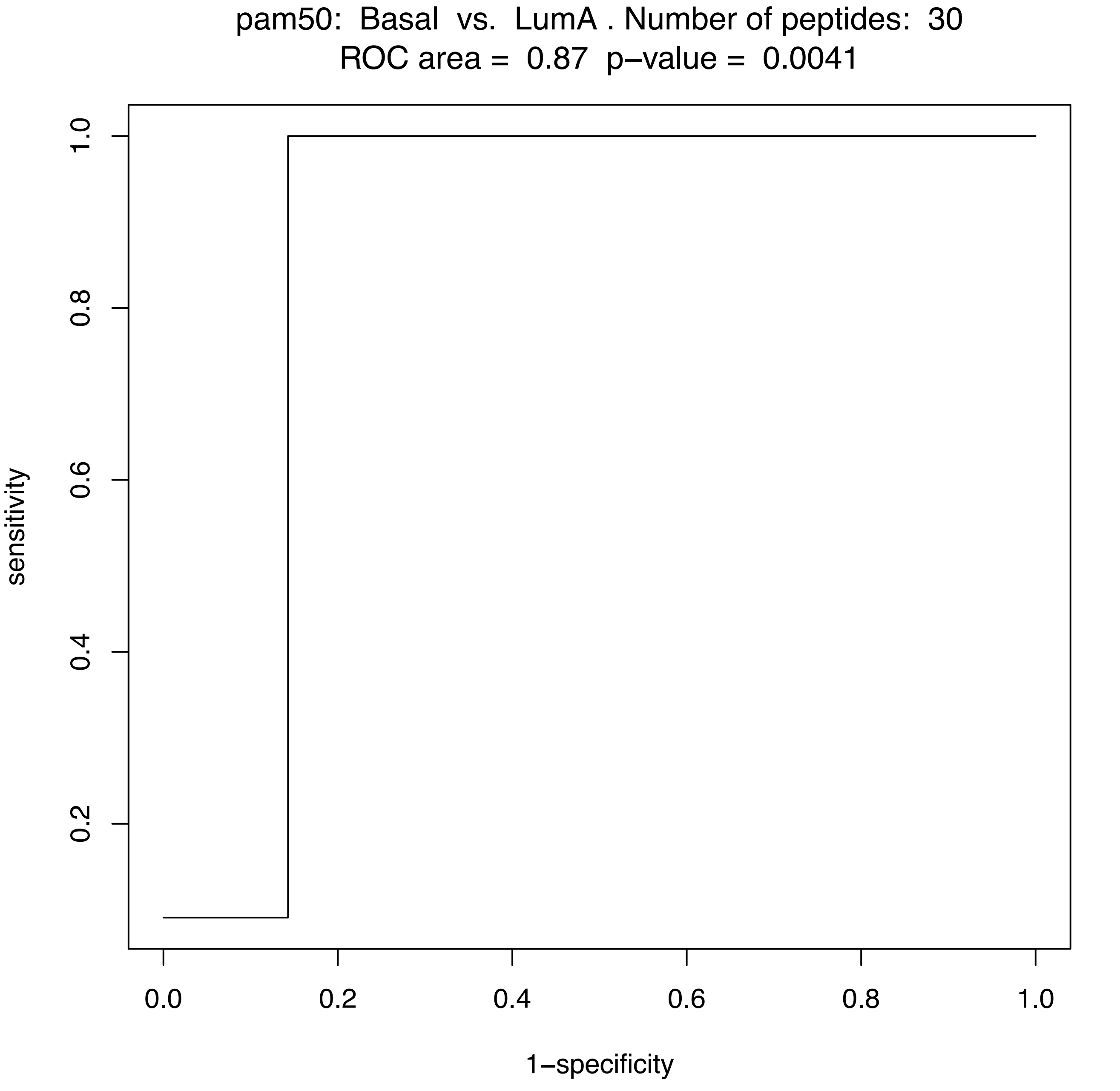
pam50: Basal vs. LumA . Number of peptides: 30
ROC area = 0.87 p−value = 0.0041
sensitivity
1−specificity
0.2
0.4
0.6
0.8
1.0
0.0

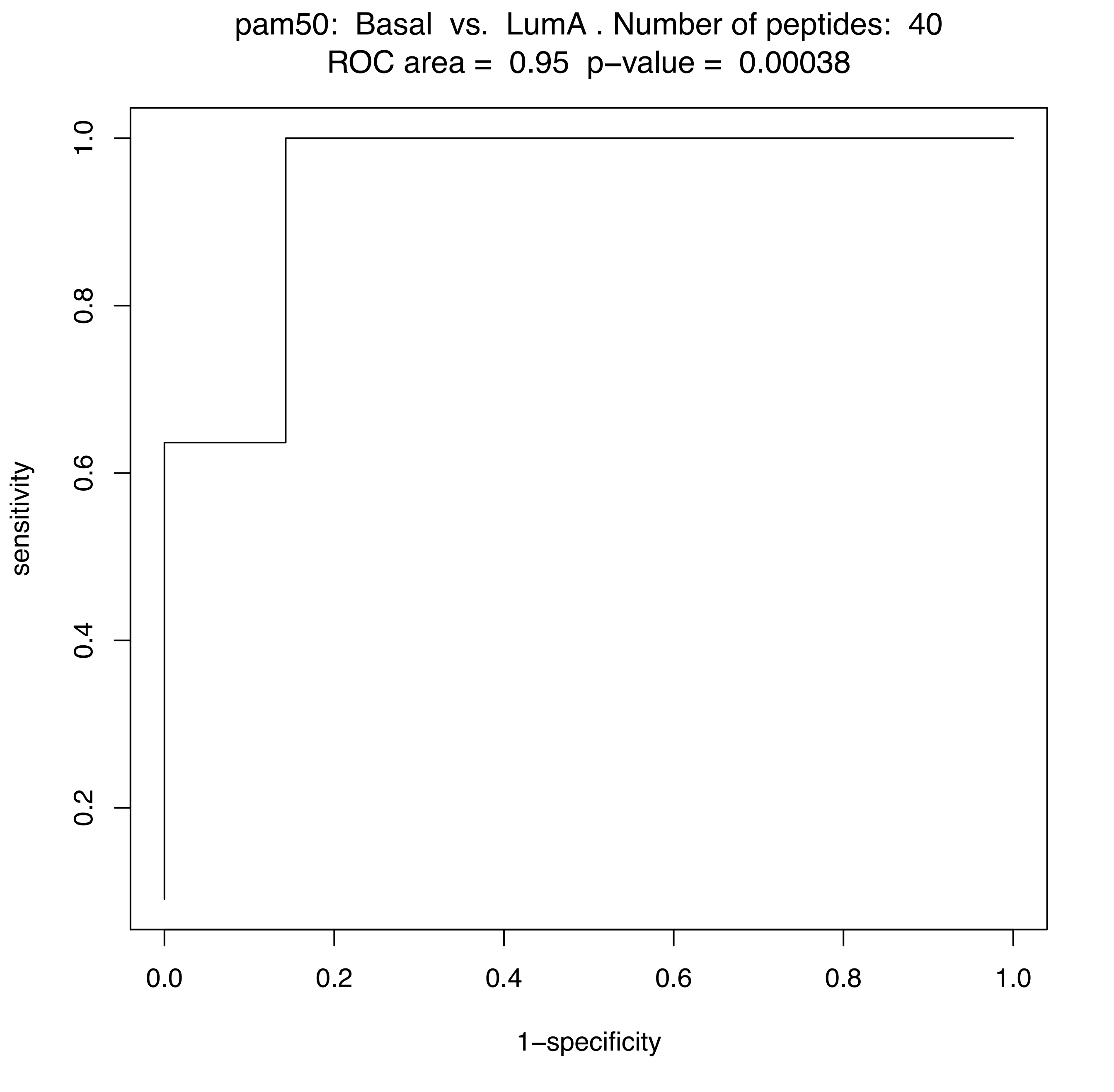
pam50: Basal vs. LumA . Number of peptides: 40
ROC area = 0.95 p−value = 0.00038
sensitivity
1−specificity
0.0
0.2
0.4
0.6
0.8
1.0

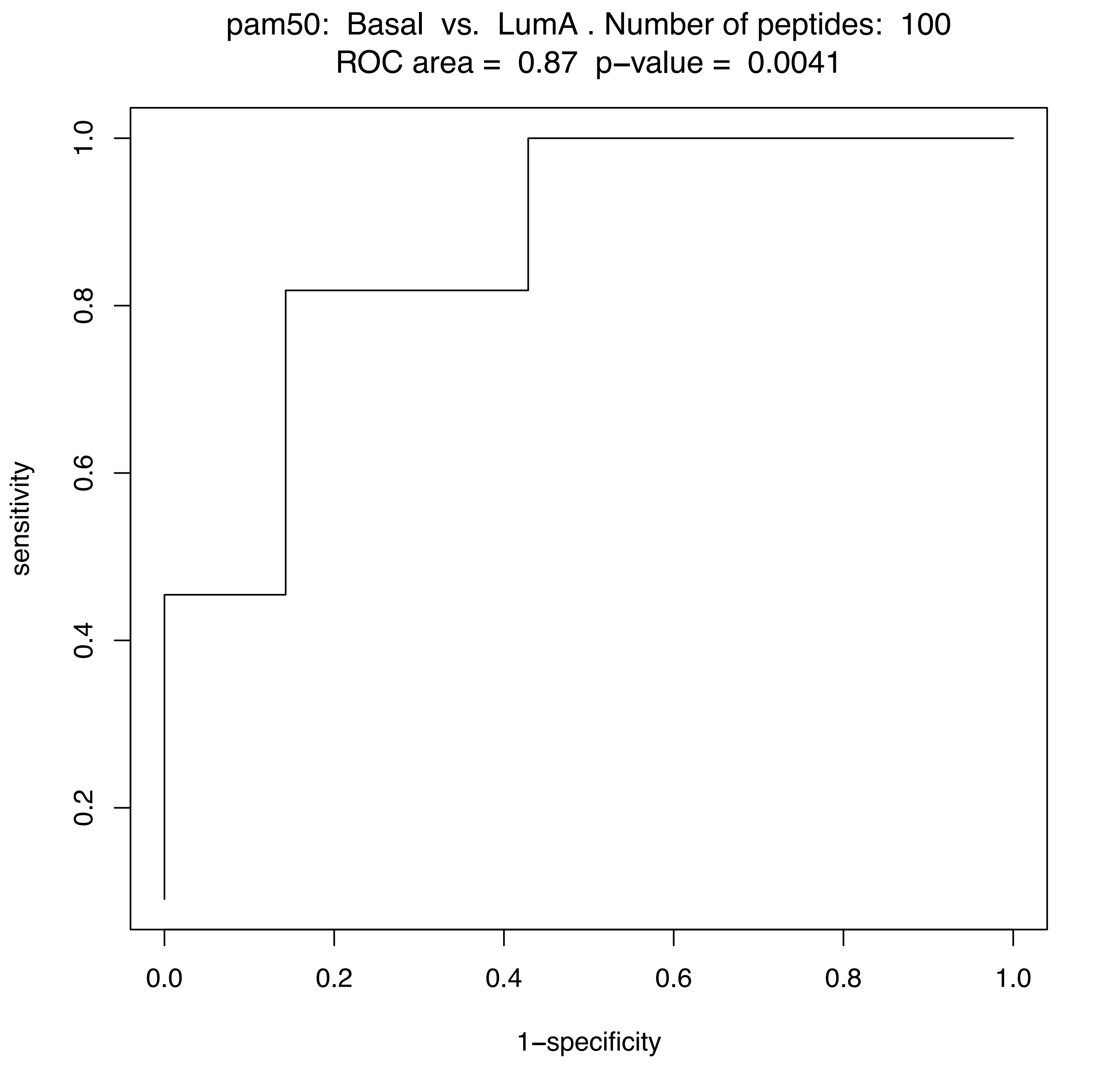
pam50: Basal vs. LumA . Number of peptides: 100
ROC area = 0.87 p−value = 0.0041
sensitivity
1−specificity
0.0
0.2
0.4
0.6
0.8
1.0
0.2
0.4
0.6
0.8
1.0

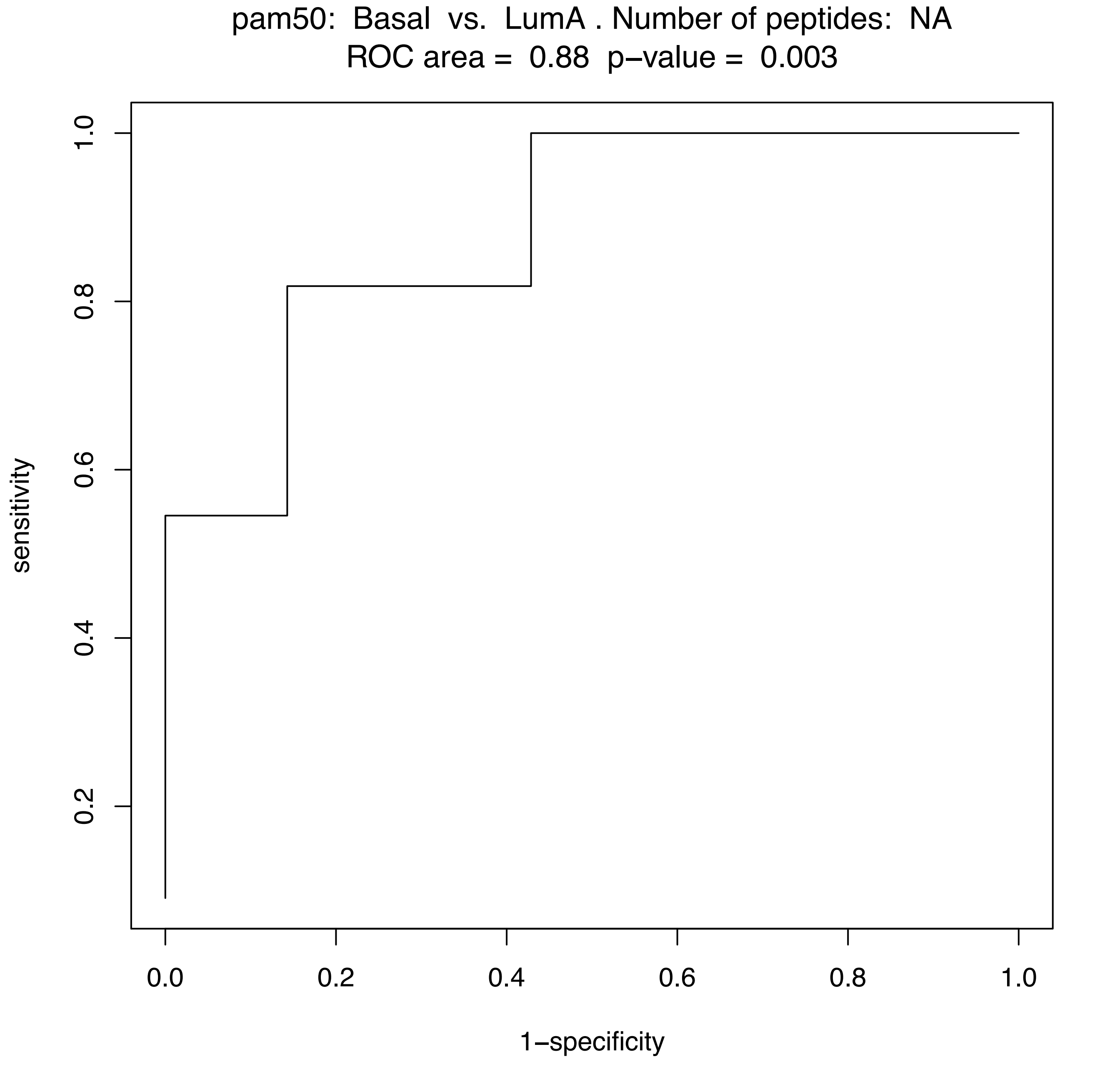
pam50: Basal vs. LumA . Number of peptides: NA
ROC area = 0.88 p−value = 0.003
sensitivity
1.0
0.8
0.6
0.4
0.2
0.0
0.2
0.4
0.6
0.8
1.0
1−specificity

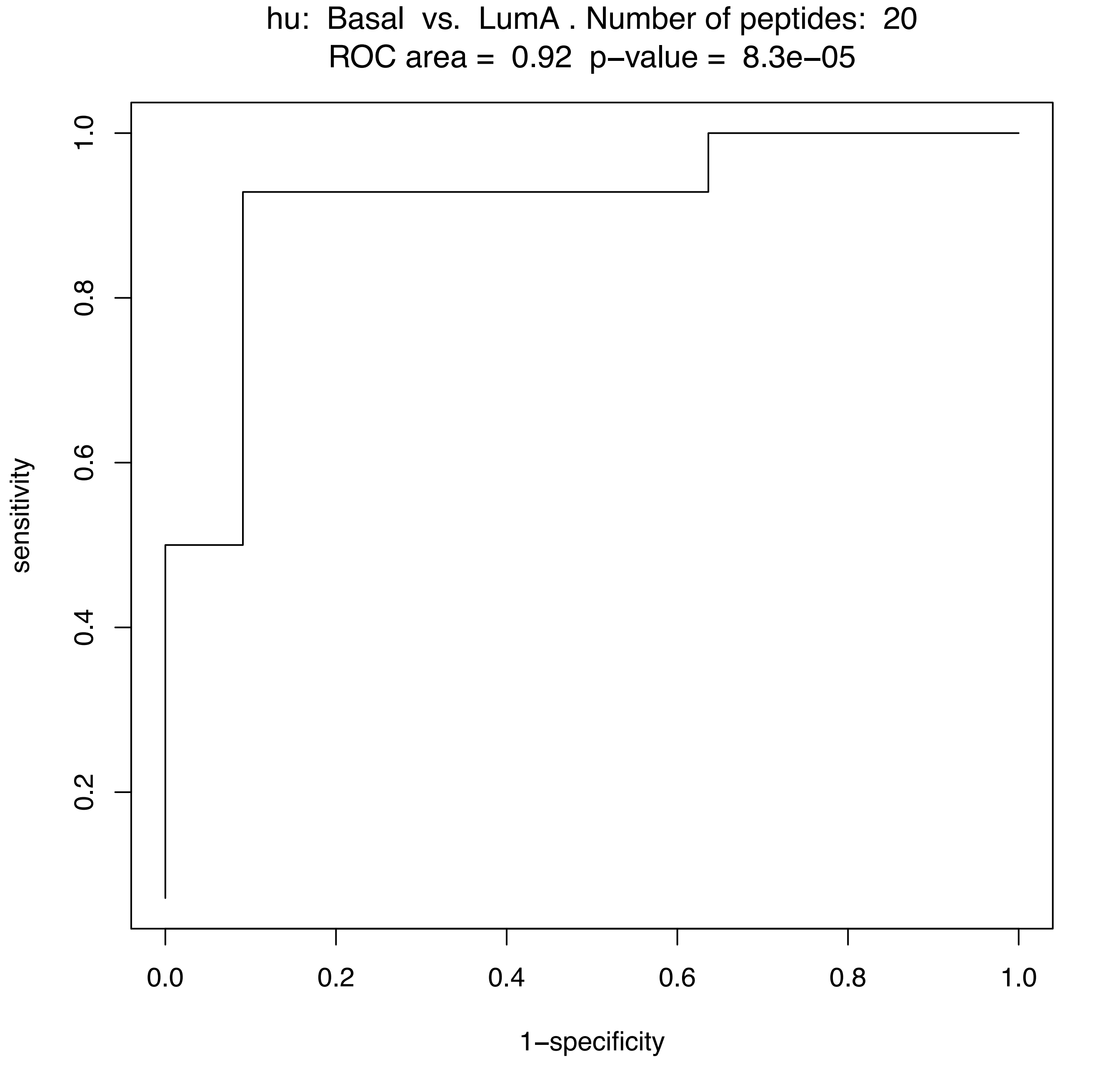
hu: Basal vs. LumA . Number of peptides: 20
ROC area = 0.92 p−value = 8.3e−05
sensitivity
0.2
0.4
0.6
0.8
1.0
0.0
0.2
0.4
0.6
0.8
1.0
1−specificity

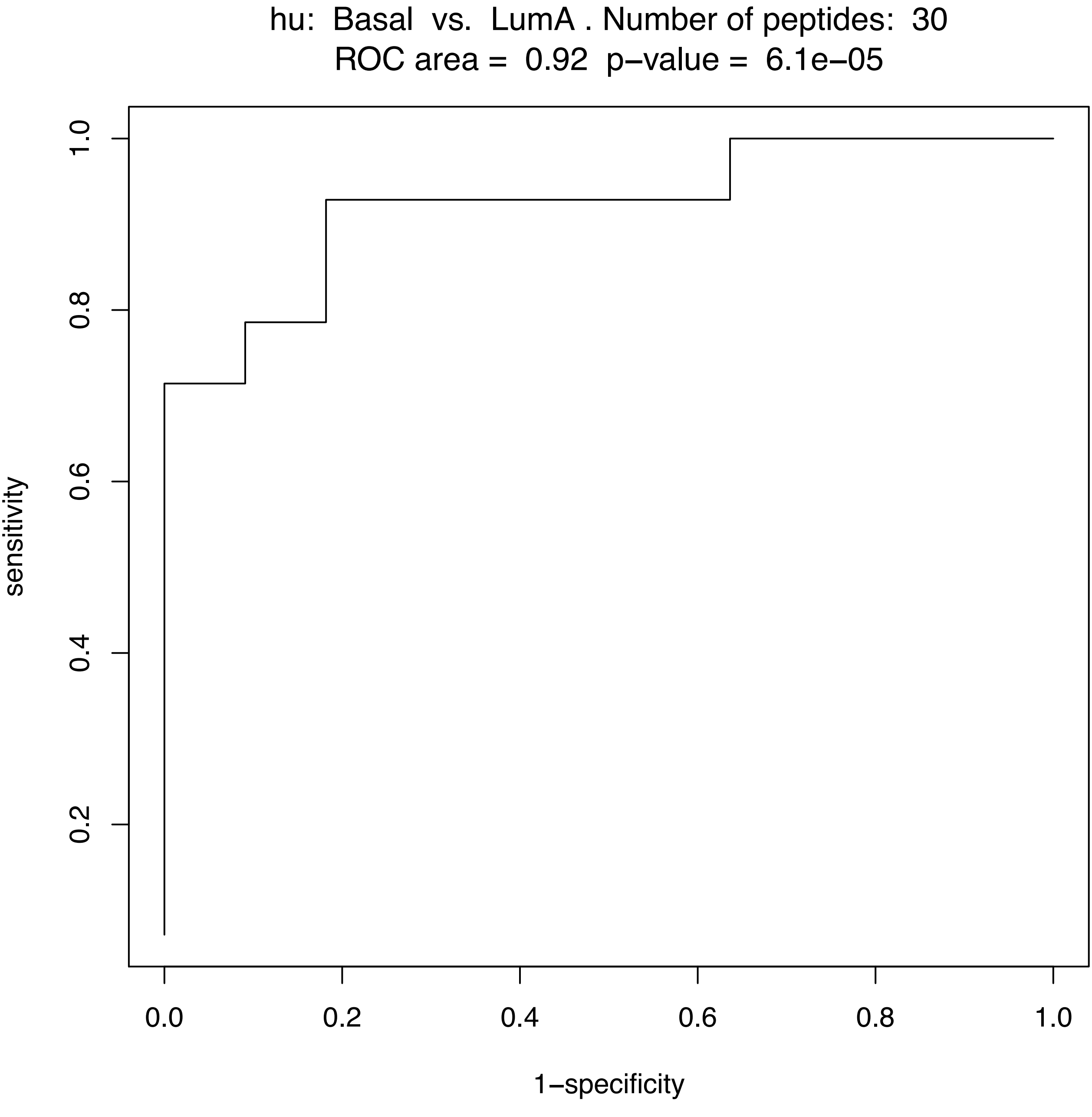
hu: Basal vs. LumA . Number of peptides: 30
ROC area = 0.92 p−value = 6.1e−05
sensitivity
1.0
0.8
0.6
0.4
0.2
0.0
0.2
0.4
0.6
0.8
1.0
1−specificity

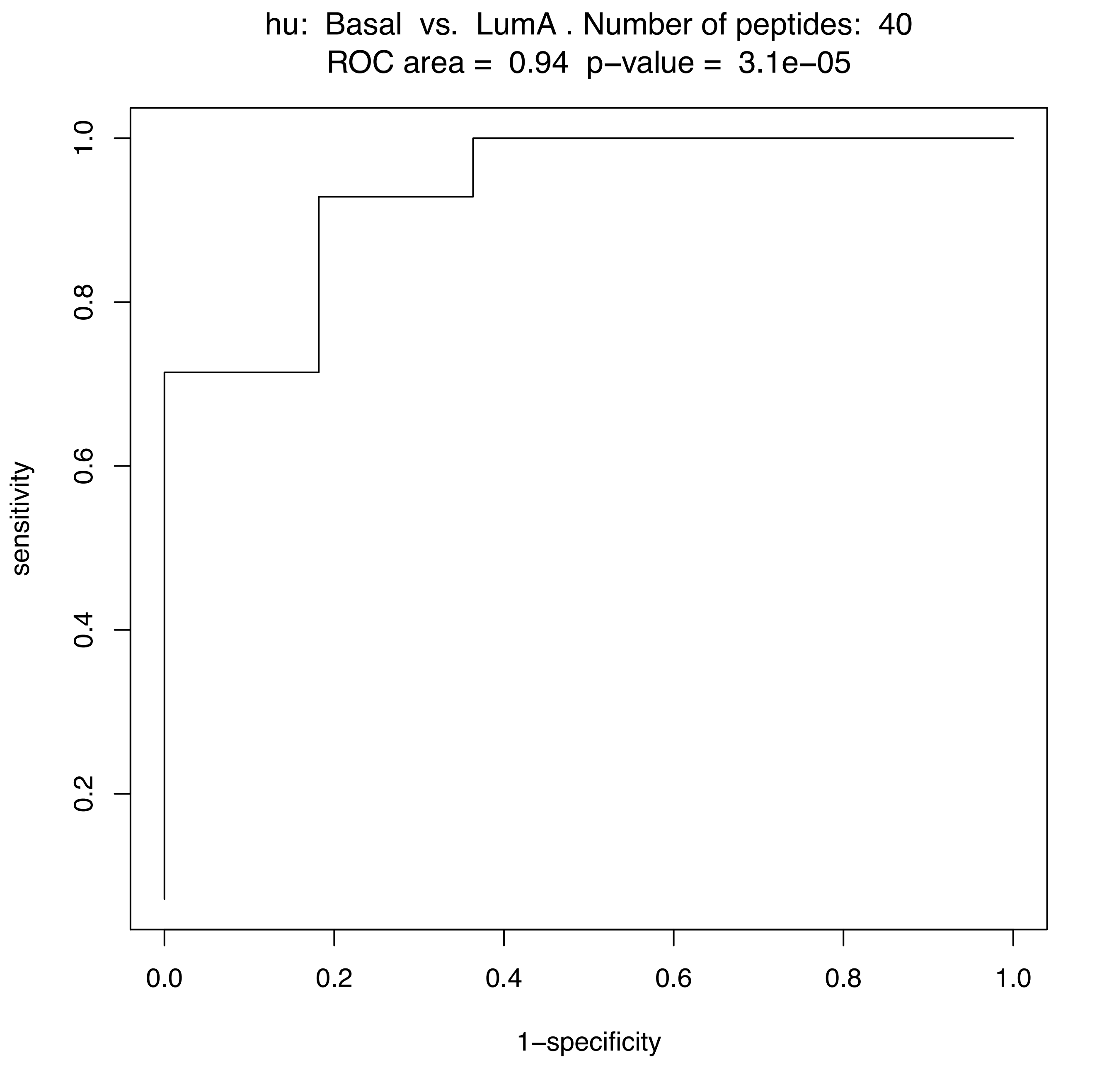
hu: Basal vs. LumA . Number of peptides: 40
ROC area = 0.94 p−value = 3.1e−05
sensitivity
1−specificity
0.0
0.2
0.4
0.6
0.8
1.0

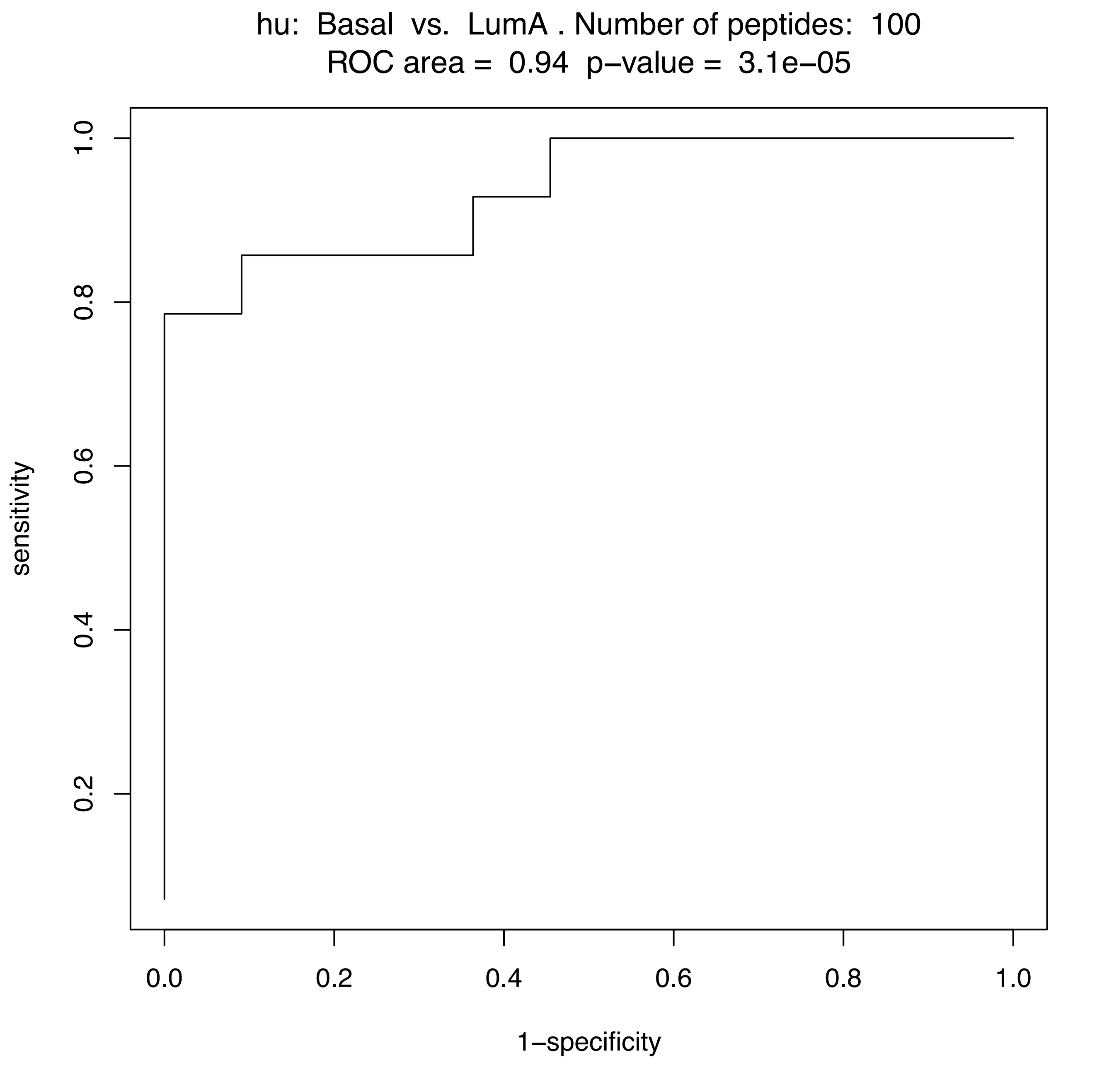
hu: Basal vs. LumA . Number of peptides: 100
ROC area = 0.94 p−value = 3.1e−05
sensitivity
1−specificity
0.2
0.4
0.6
0.8
1.0
0.0
0.2
0.4
0.6
0.8
1.0

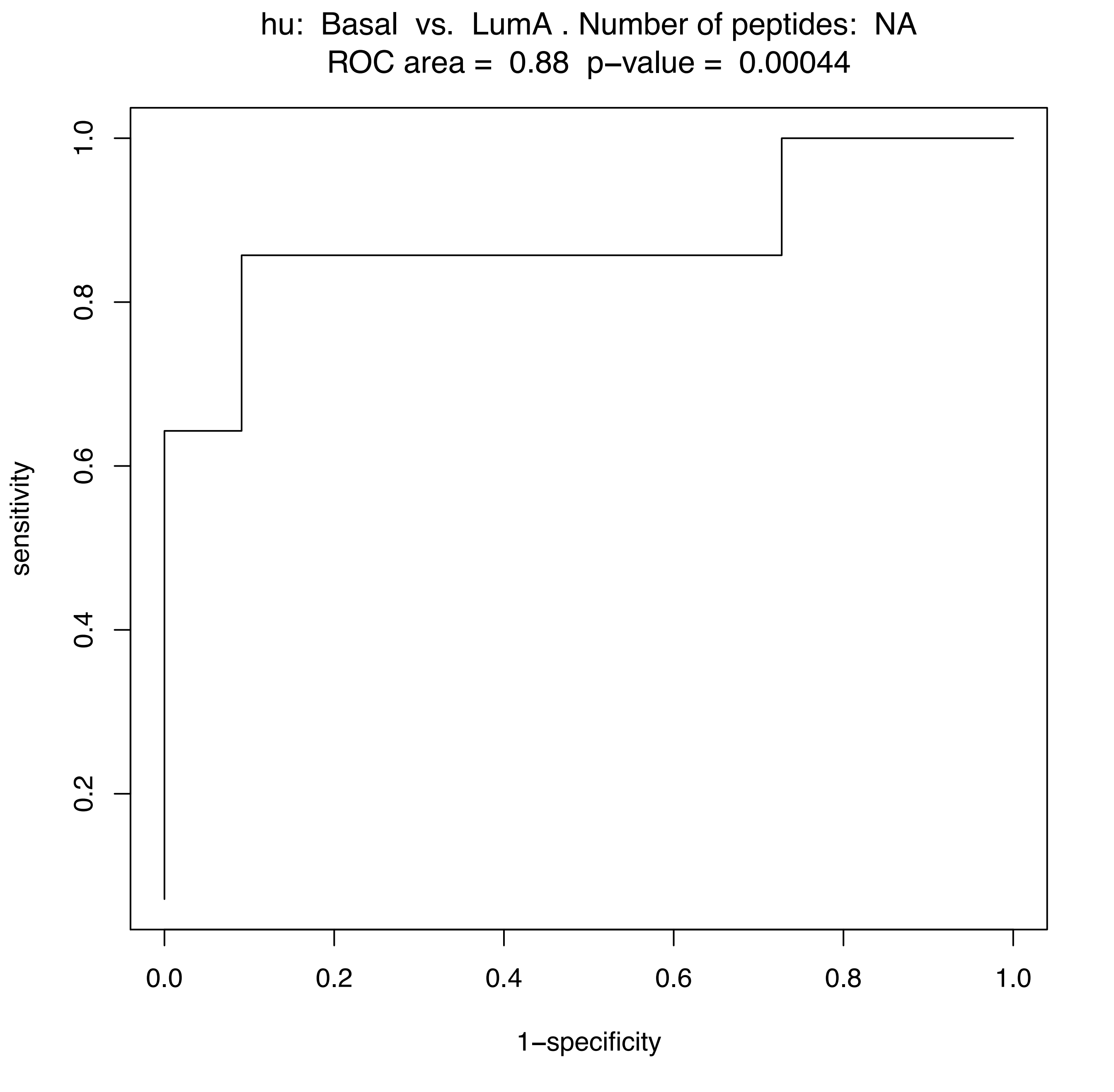
hu: Basal vs. LumA . Number of peptides: NA
ROC area = 0.88 p−value = 0.00044
sensitivity
1.0
0.8
0.6
0.4
0.2
0.0
0.2
0.4
0.6
0.8
1.0
1−specificity

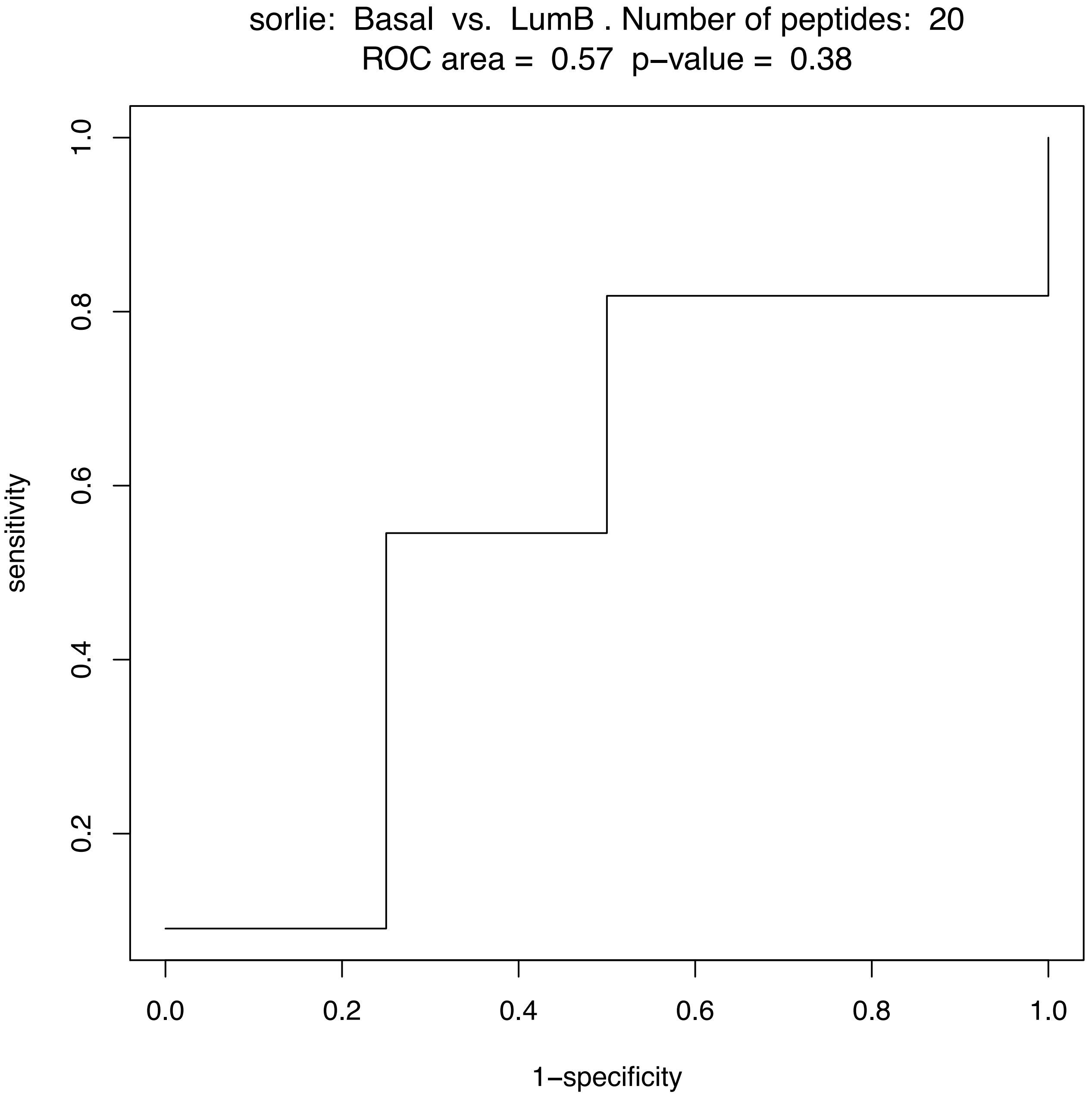
sorlie: Basal vs. LumB . Number of peptides: 20
ROC area = 0.57 p−value = 0.38
sensitivity
0.2
0.4
0.6
0.8
1.0
0.0
0.2
0.4
0.6
0.8
1.0
1−specificity

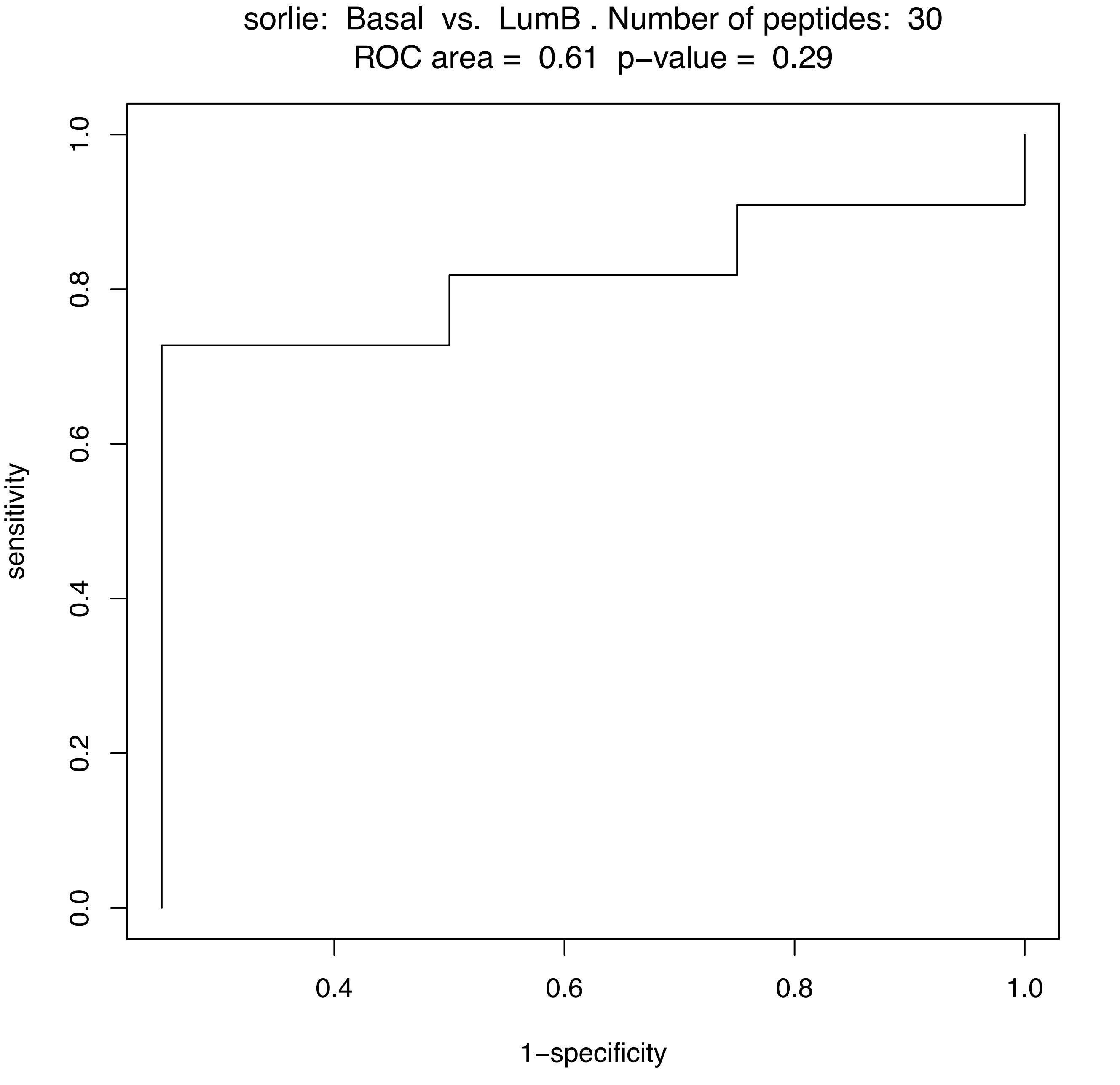
sorlie: Basal vs. LumB . Number of peptides: 30
ROC area = 0.61 p−value = 0.29
sensitivity
1−specificity
0.0
0.2
0.4
0.6
0.8
1.0
0.4
0.6
0.8
1.0

sorlie: Basal vs. LumB . Number of peptides: 40
ROC area = 0.59 p−value = 0.33

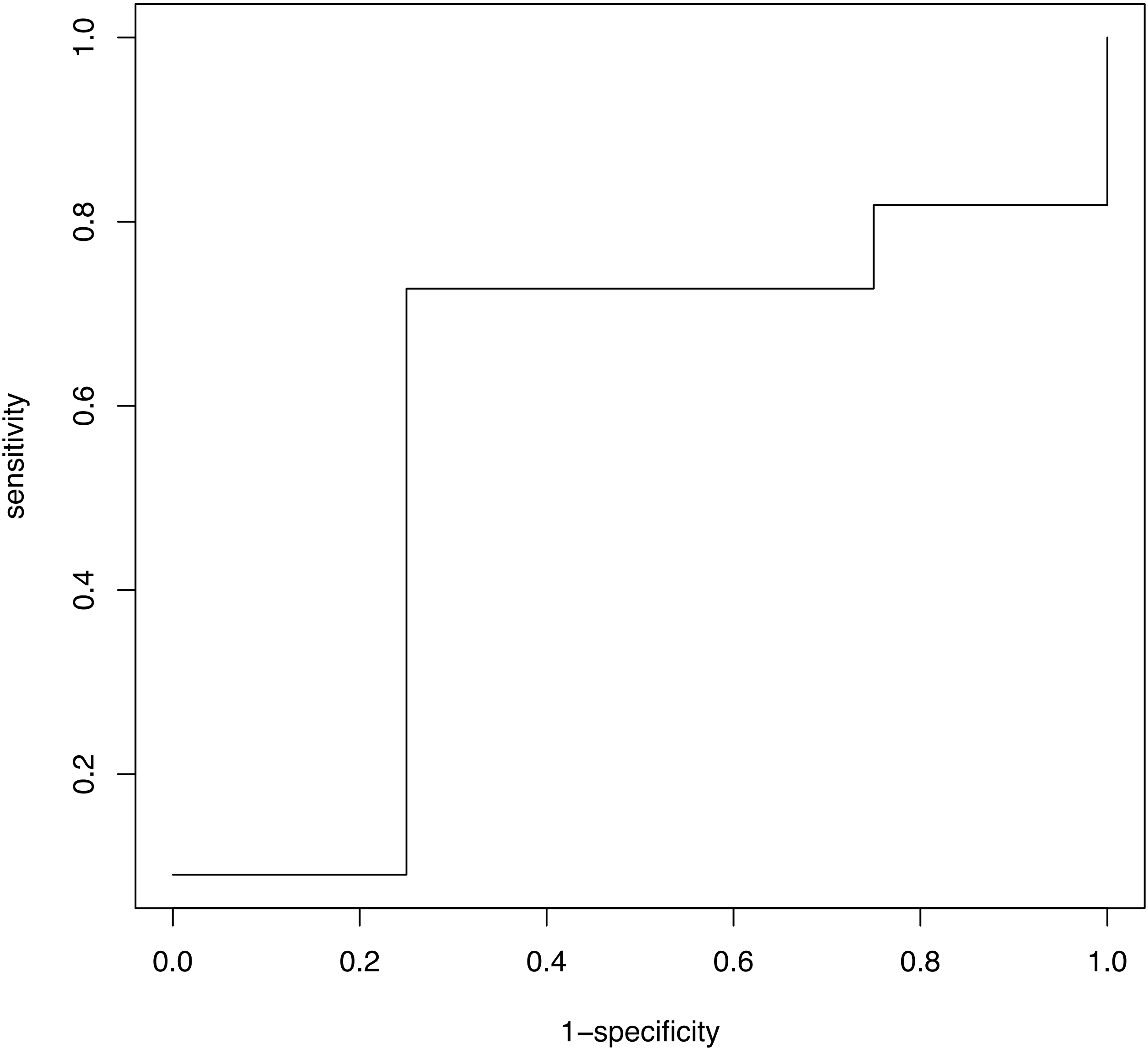

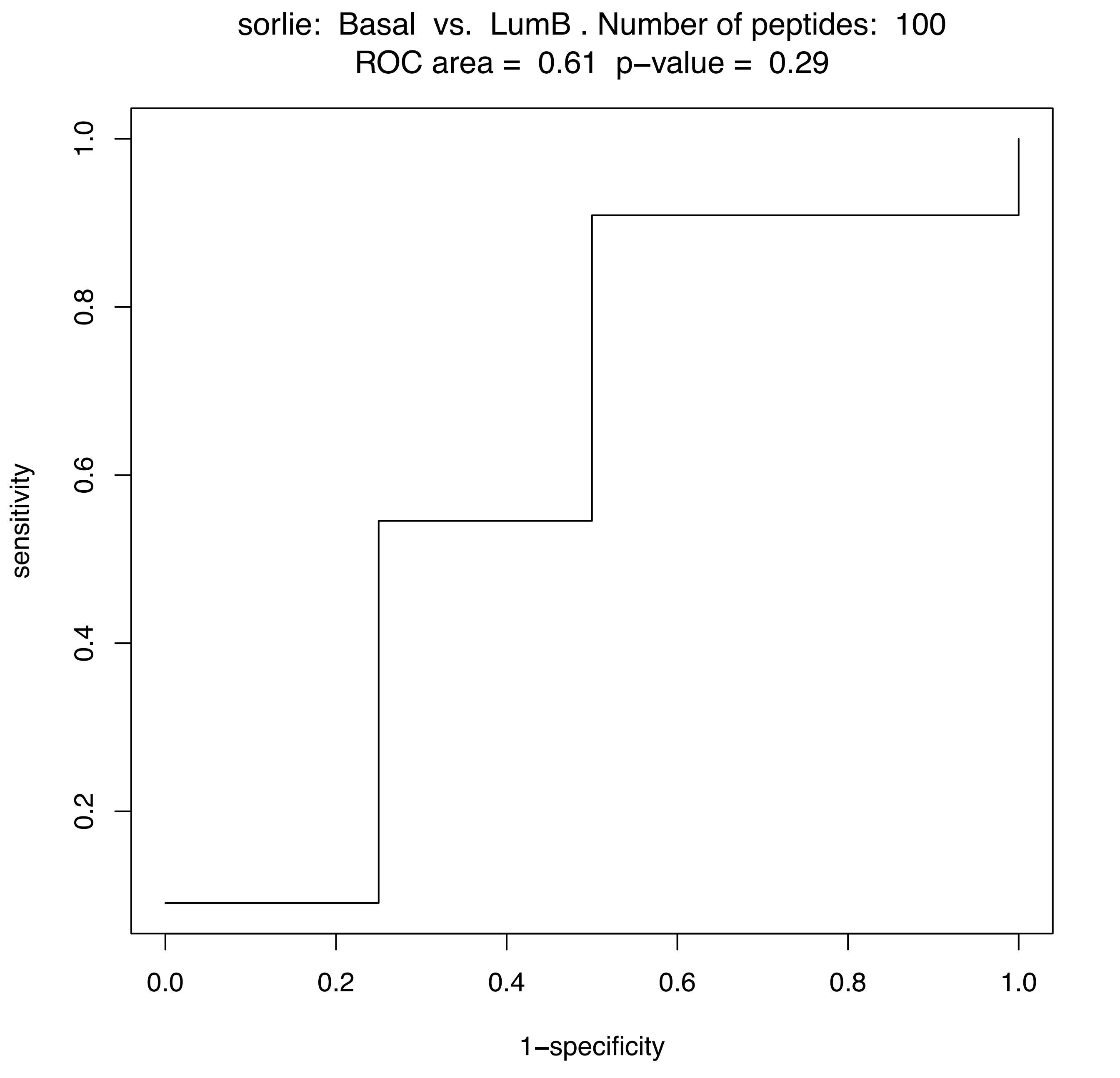
sorlie: Basal vs. LumB . Number of peptides: 100
ROC area = 0.61 p−value = 0.29
sensitivity
1−specificity
0.2
0.4
0.6
0.8
1.0
0.0
0.2
0.4
0.6
0.8
1.0

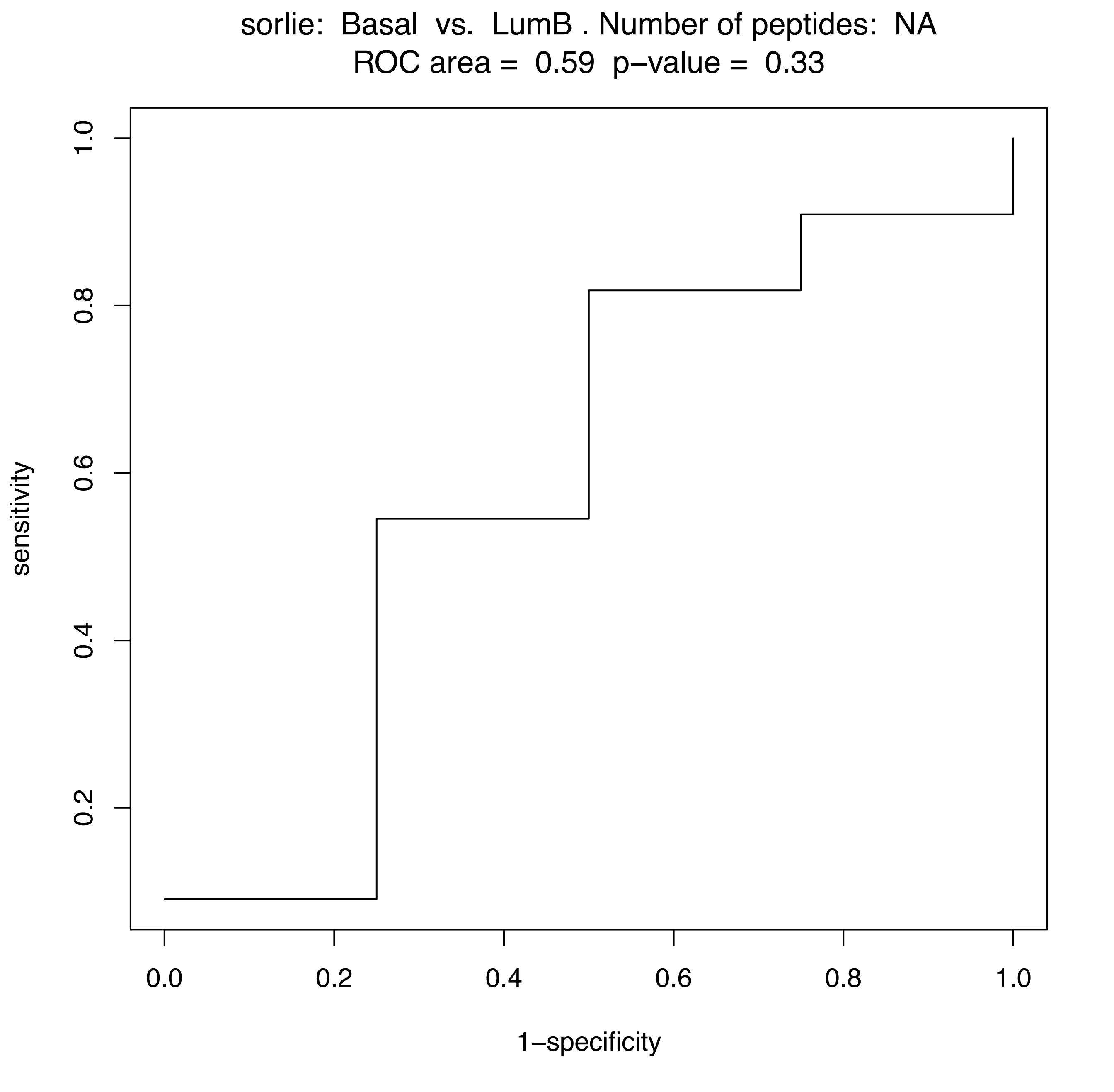
sorlie: Basal vs. LumB . Number of peptides: NA
ROC area = 0.59 p−value = 0.33
sensitivity
1−specificity
0.0
0.2
0.4
0.6
0.8
1.0

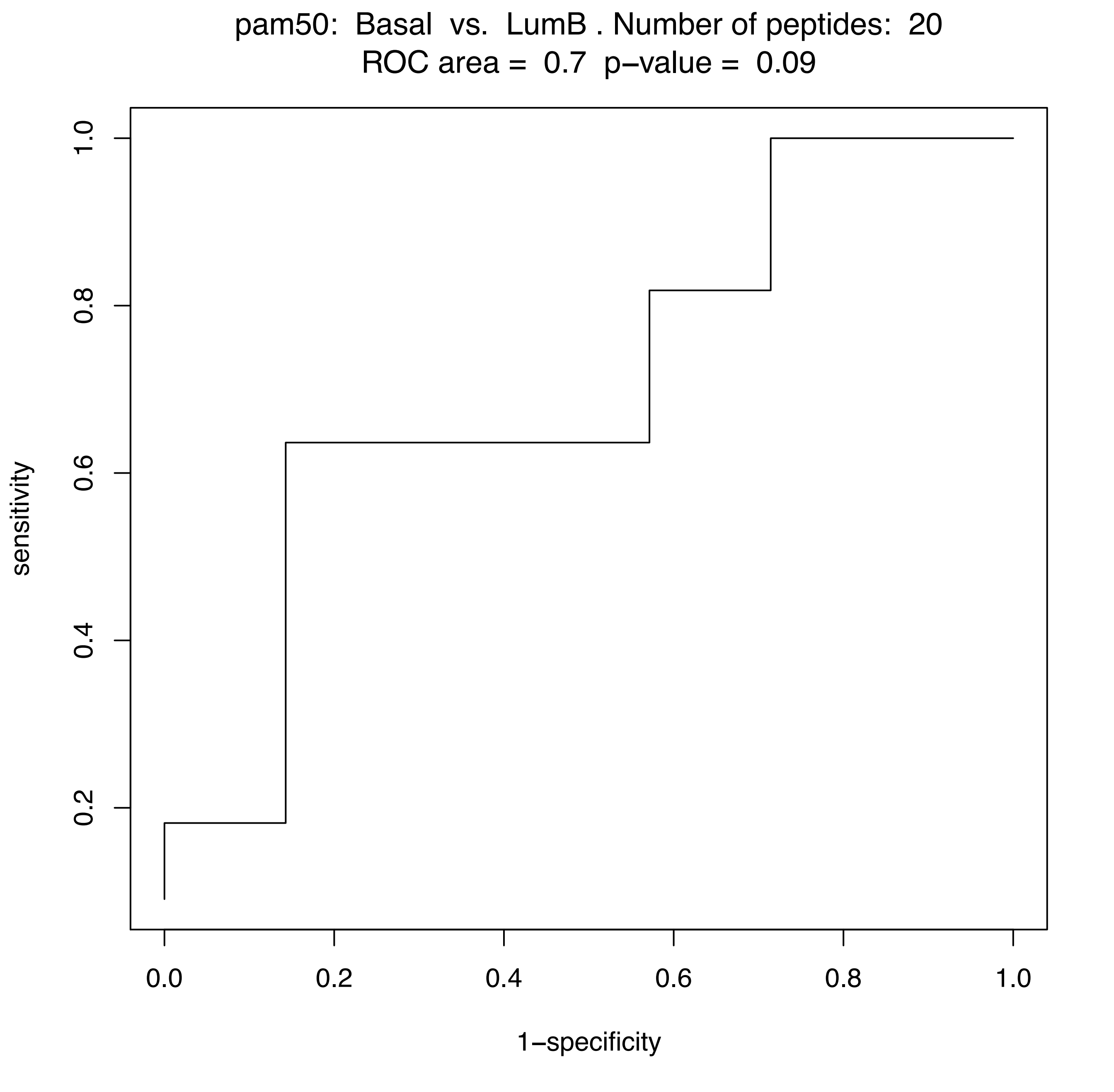
pam50: Basal vs. LumB . Number of peptides: 20
ROC area = 0.7 p−value = 0.09
sensitivity
1−specificity
0.2
0.4
0.6
0.8
1.0
0.0
0.2
0.4
0.6
0.8
1.0

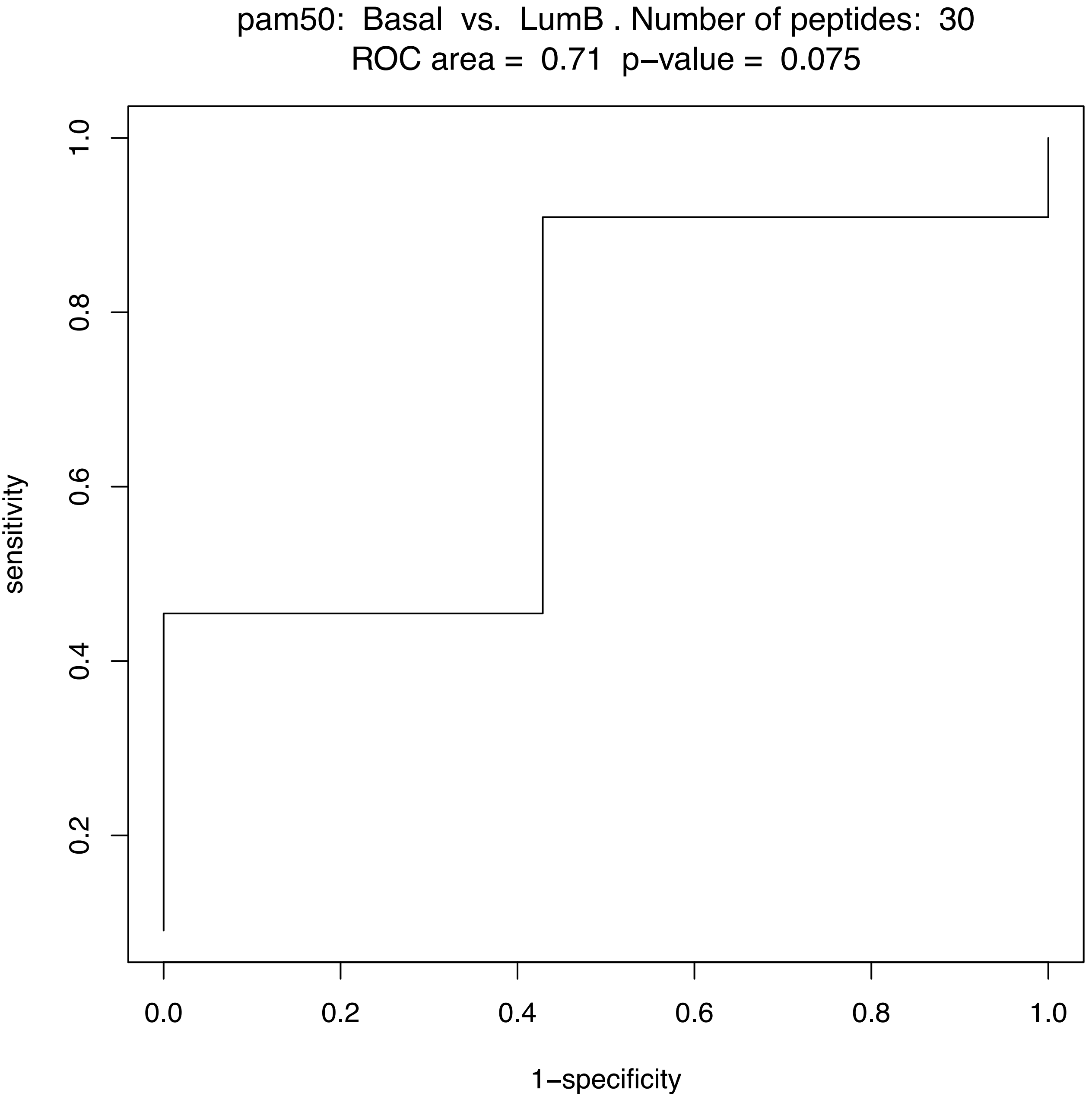
pam50: Basal vs. LumB . Number of peptides: 30
ROC area = 0.71 p−value = 0.075
sensitivity
1−specificity
0.0
0.2
0.4
0.6
0.8
1.0

pam50: Basal vs. LumB . Number of peptides: 40
ROC area = 0.73 p−value = 0.063

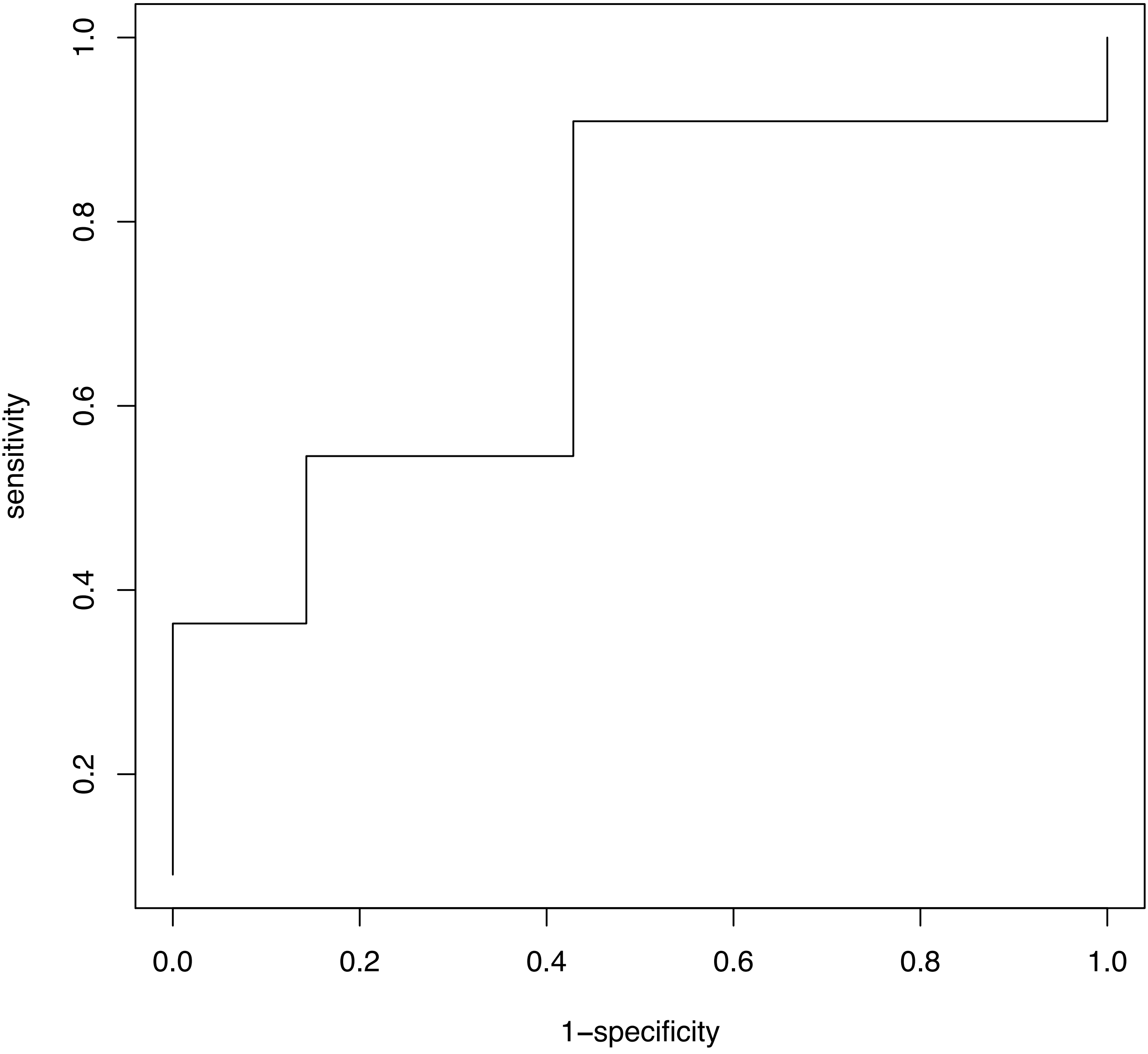

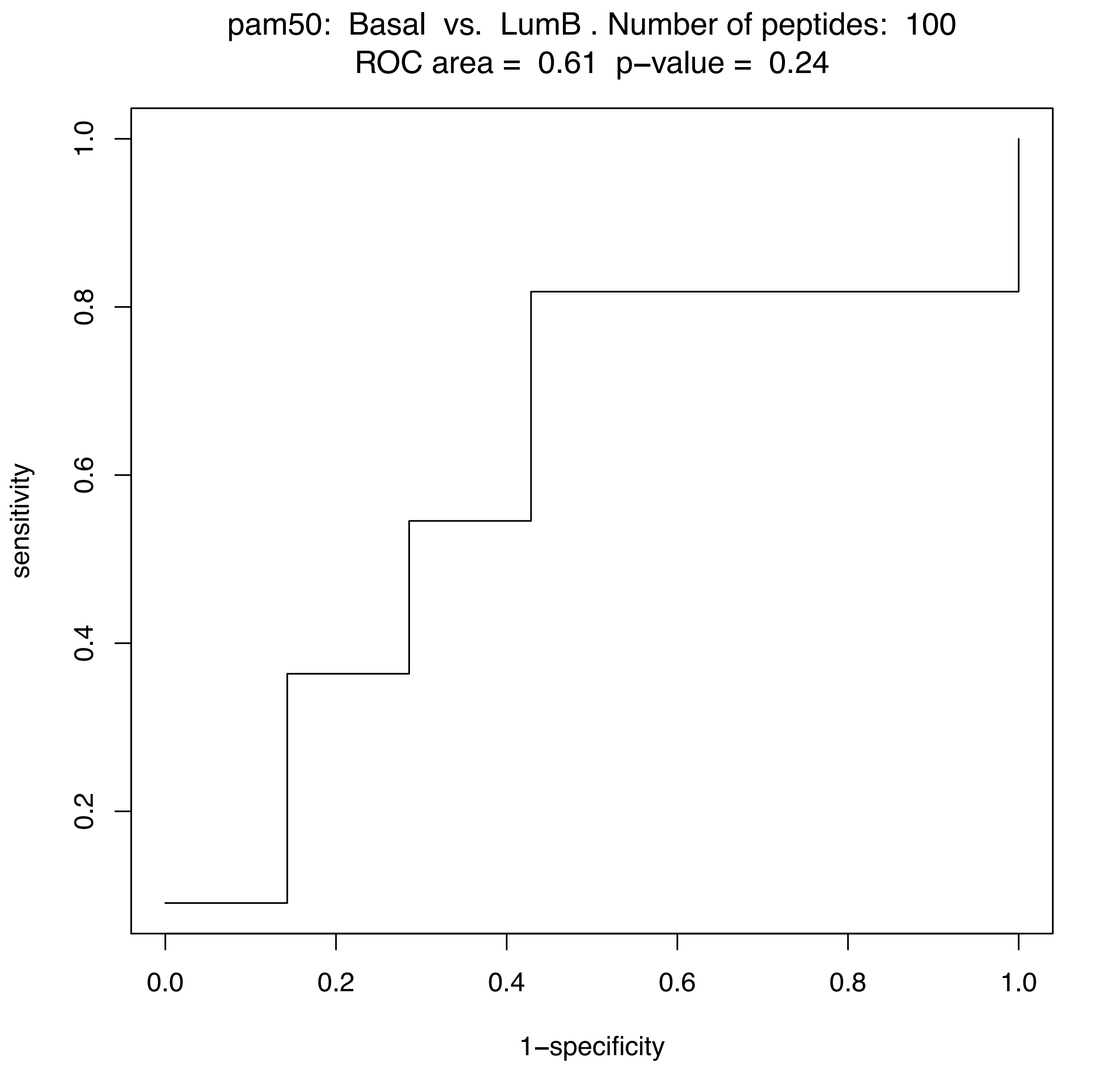
pam50: Basal vs. LumB . Number of peptides: 100
ROC area = 0.61 p−value = 0.24
sensitivity
0.2
0.4
0.6
0.8
1.0
0.0
0.2
0.4
0.6
0.8
1.0
1−specificity

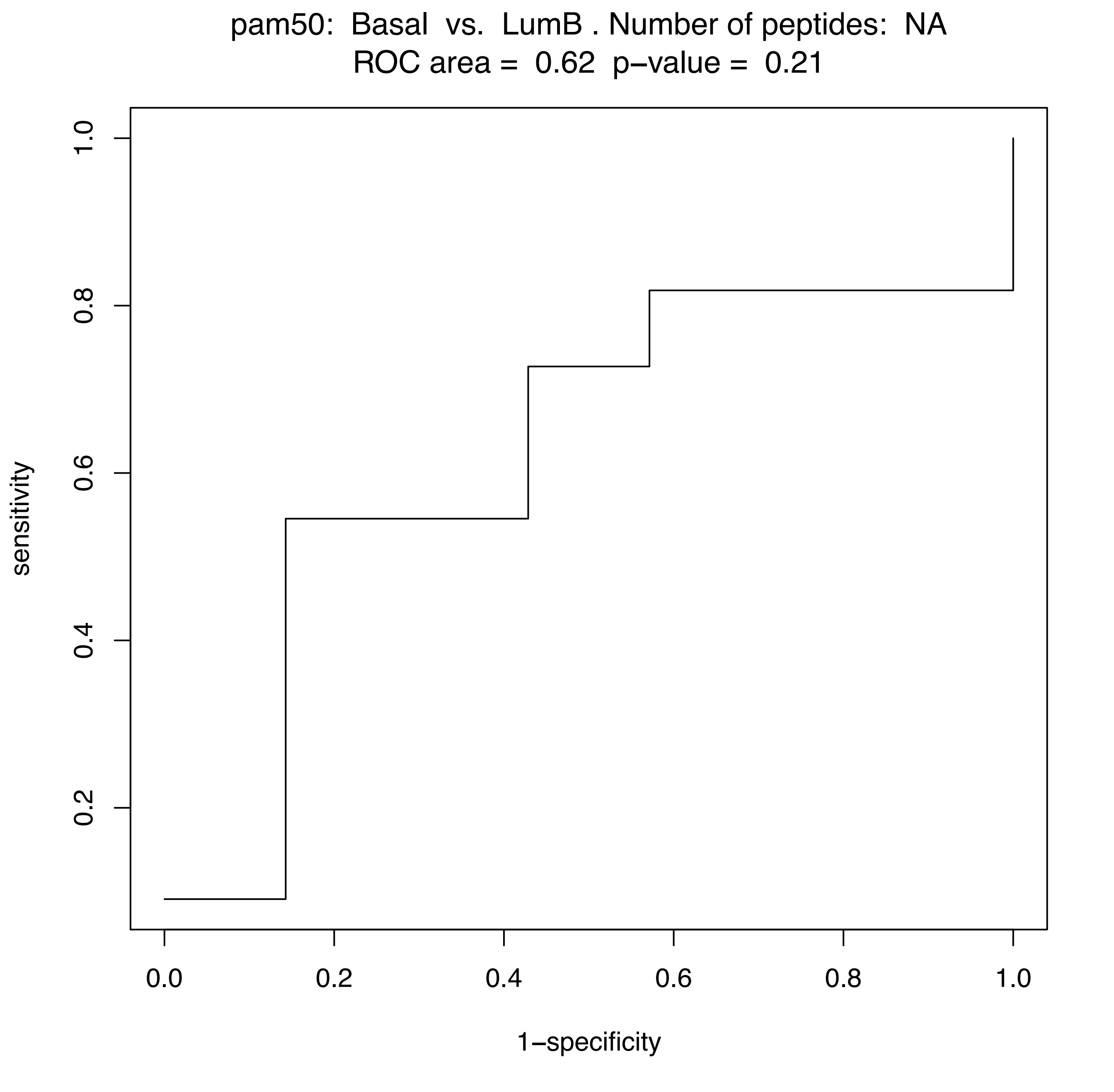
pam50: Basal vs. LumB . Number of peptides: NA
ROC area = 0.62 p−value = 0.21
sensitivity
1−specificity
0.0
0.2
0.4
0.6
0.8
1.0
0.2
0.4
0.6
0.8
1.0

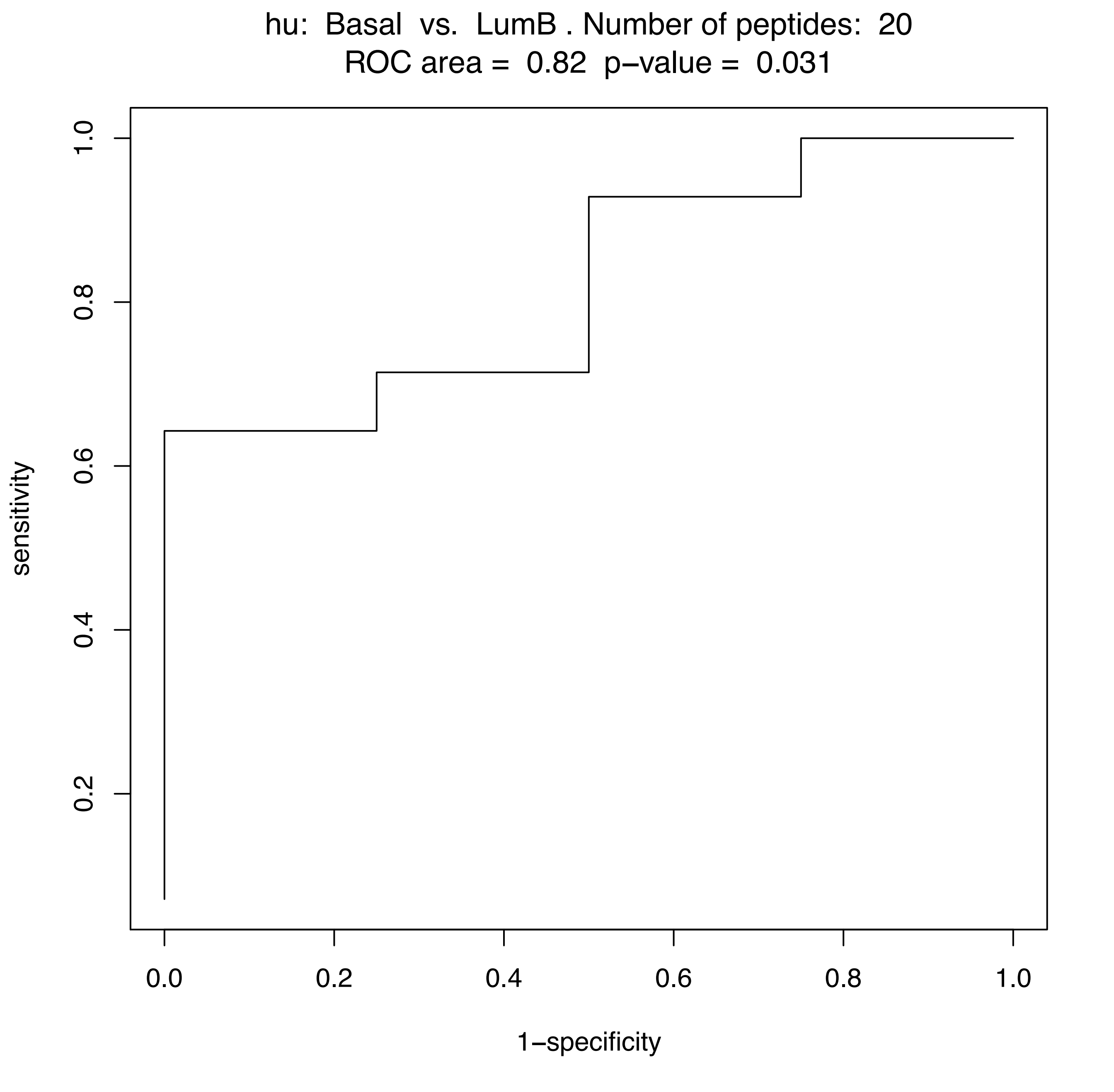
hu: Basal vs. LumB . Number of peptides: 20
ROC area = 0.82 p−value = 0.031
sensitivity
1−specificity
0.0
0.2
0.4
0.6
0.8
1.0
0.2
0.4
0.6
0.8
1.0

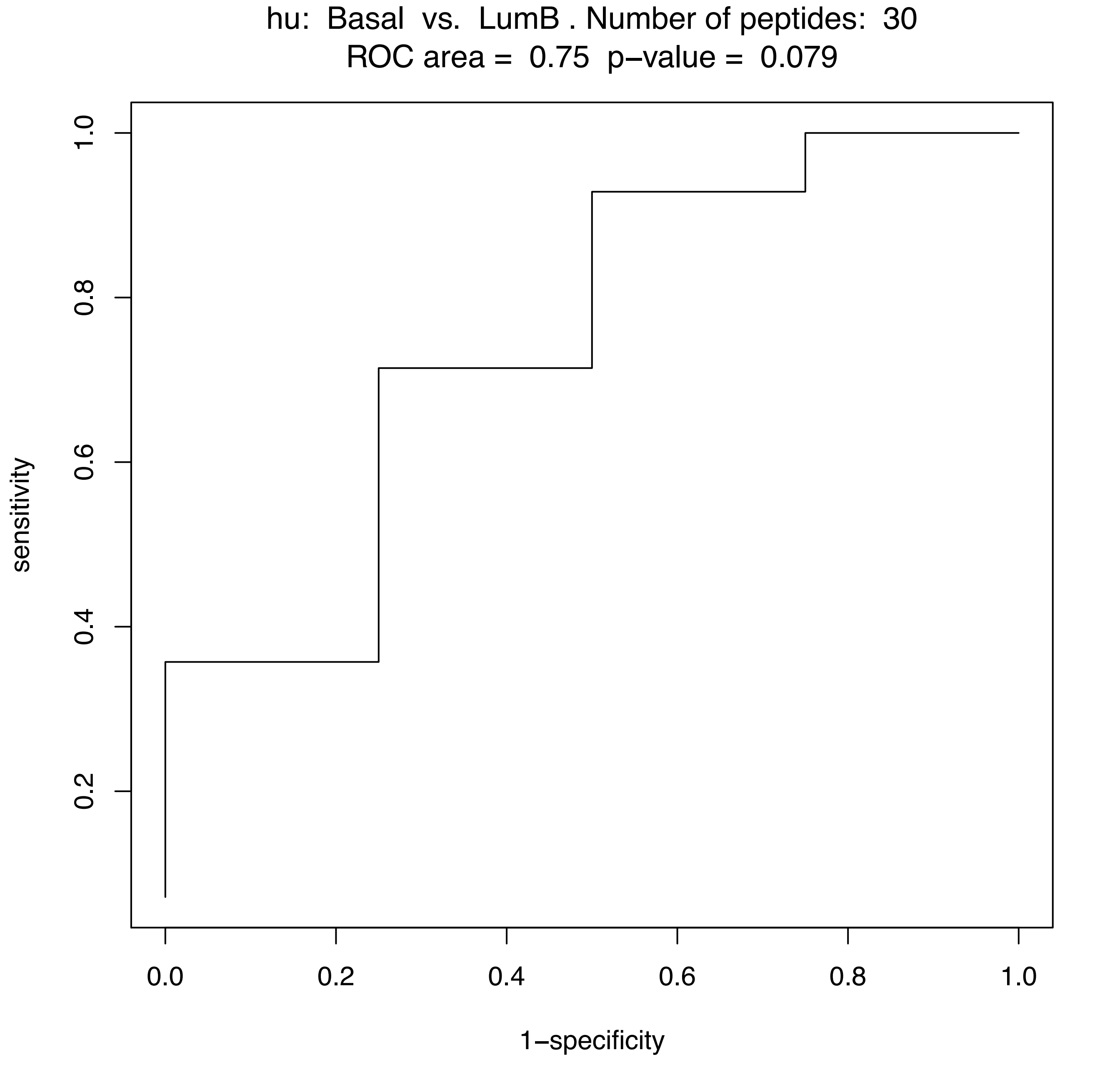
hu: Basal vs. LumB . Number of peptides: 30
ROC area = 0.75 p−value = 0.079
sensitivity
1−specificity
0.0
0.2
0.4
0.6
0.8
1.0
0.2
0.4
0.6
0.8
1.0

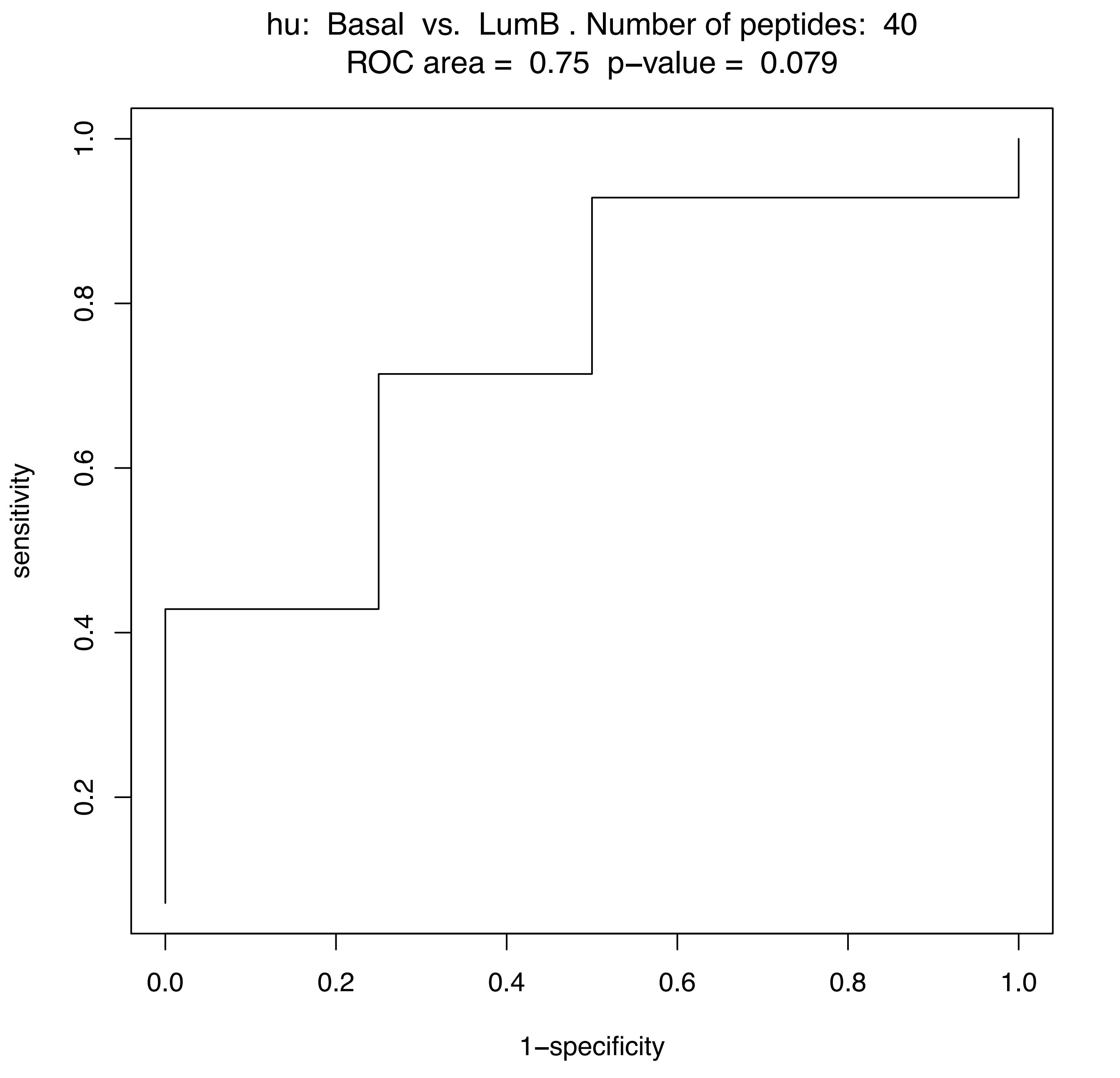

hu: Basal vs. LumB . Number of peptides: 40
ROC area = 0.75 p−value = 0.079
sensitivity
1−specificity
0.0
0.2
0.4
0.6
0.8
1.0

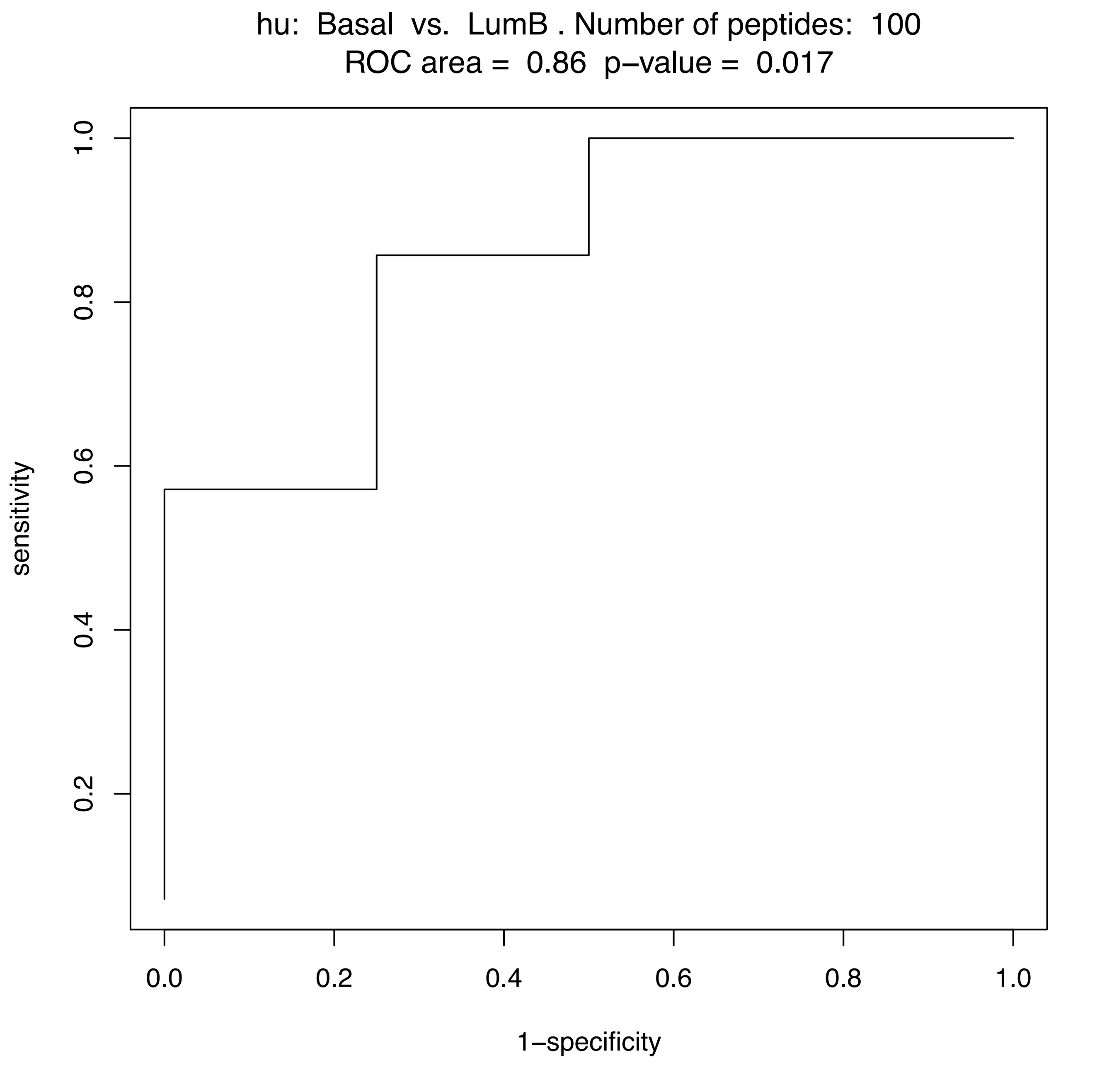
hu: Basal vs. LumB . Number of peptides: 100
ROC area = 0.86 p−value = 0.017
sensitivity
1−specificity
0.0
0.2
0.4
0.6
0.8
1.0
0.2
0.4
0.6
0.8
1.0

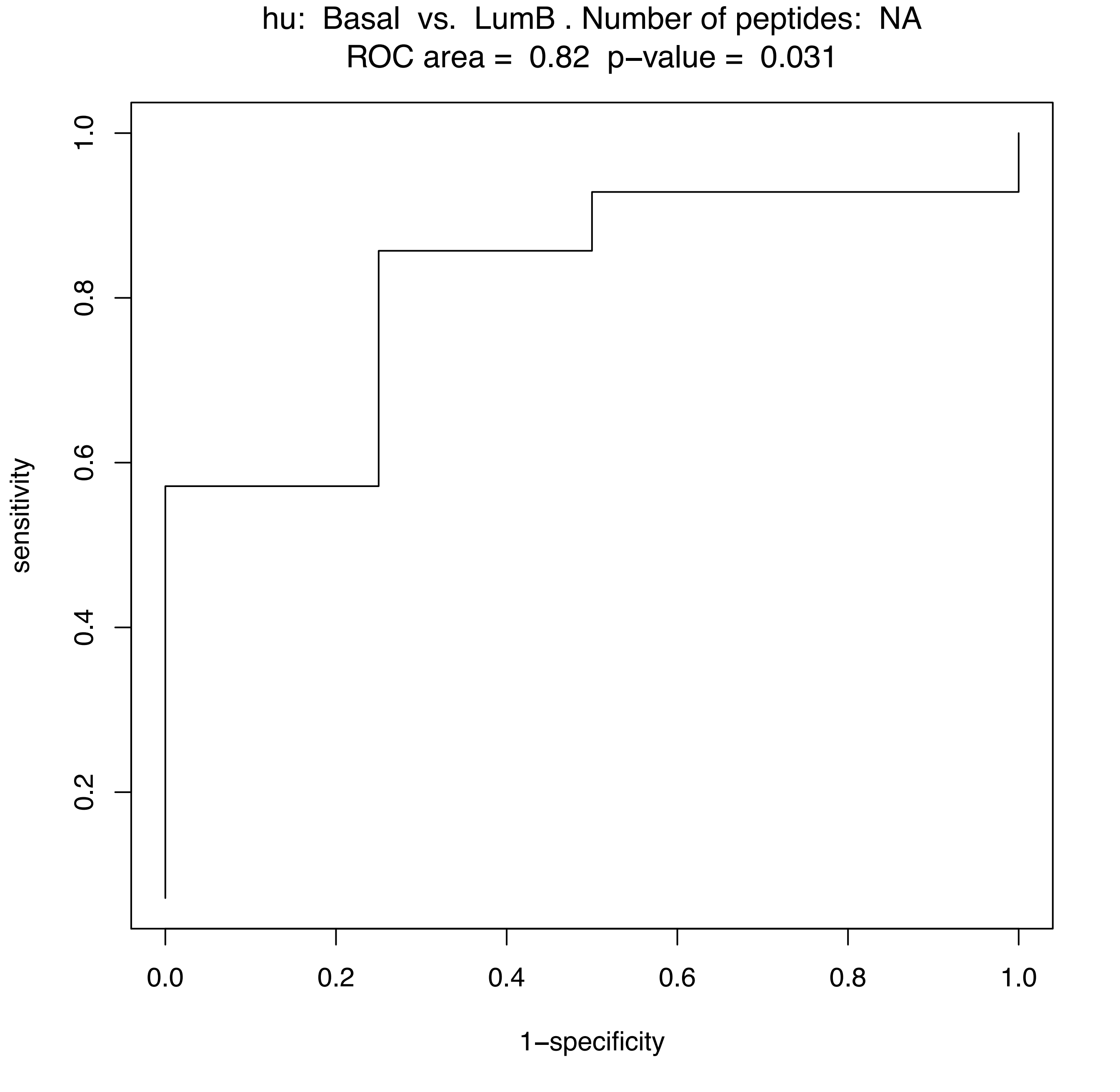
hu: Basal vs. LumB . Number of peptides: NA
ROC area = 0.82 p−value = 0.031
sensitivity
1−specificity
0.0
0.2
0.4
0.6
0.8
1.0
0.2
0.4
0.6
0.8
1.0

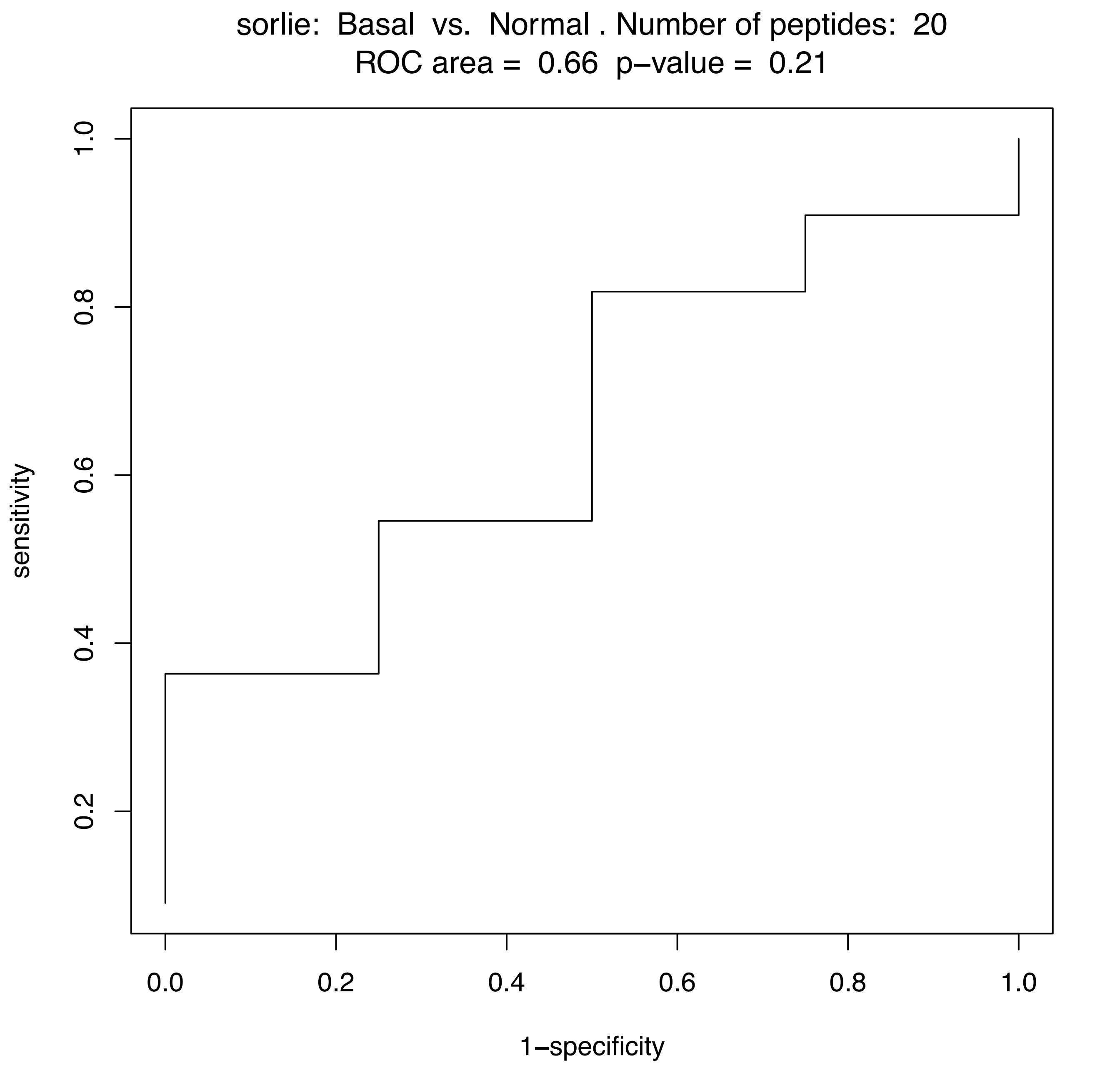
sorlie: Basal vs. Normal . Number of peptides: 20
ROC area = 0.66 p−value = 0.21
sensitivity
0.2
0.4
0.6
0.8
1.0
0.0
0.2
0.4
0.6
0.8
1.0
1−specificity

sorlie: Basal vs. Normal . Number of peptides: 30
ROC area = 0.52 p−value = 0.47

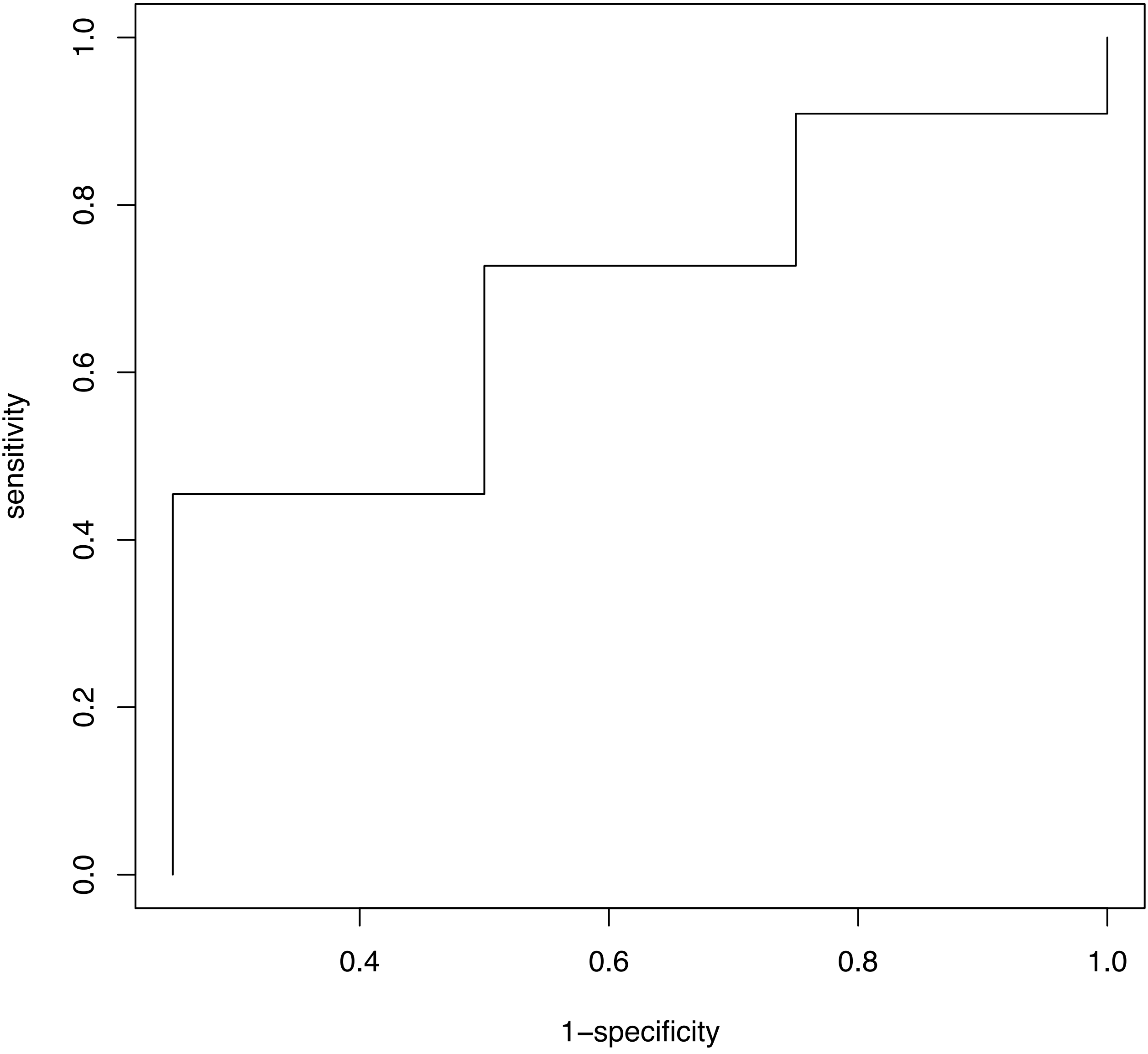

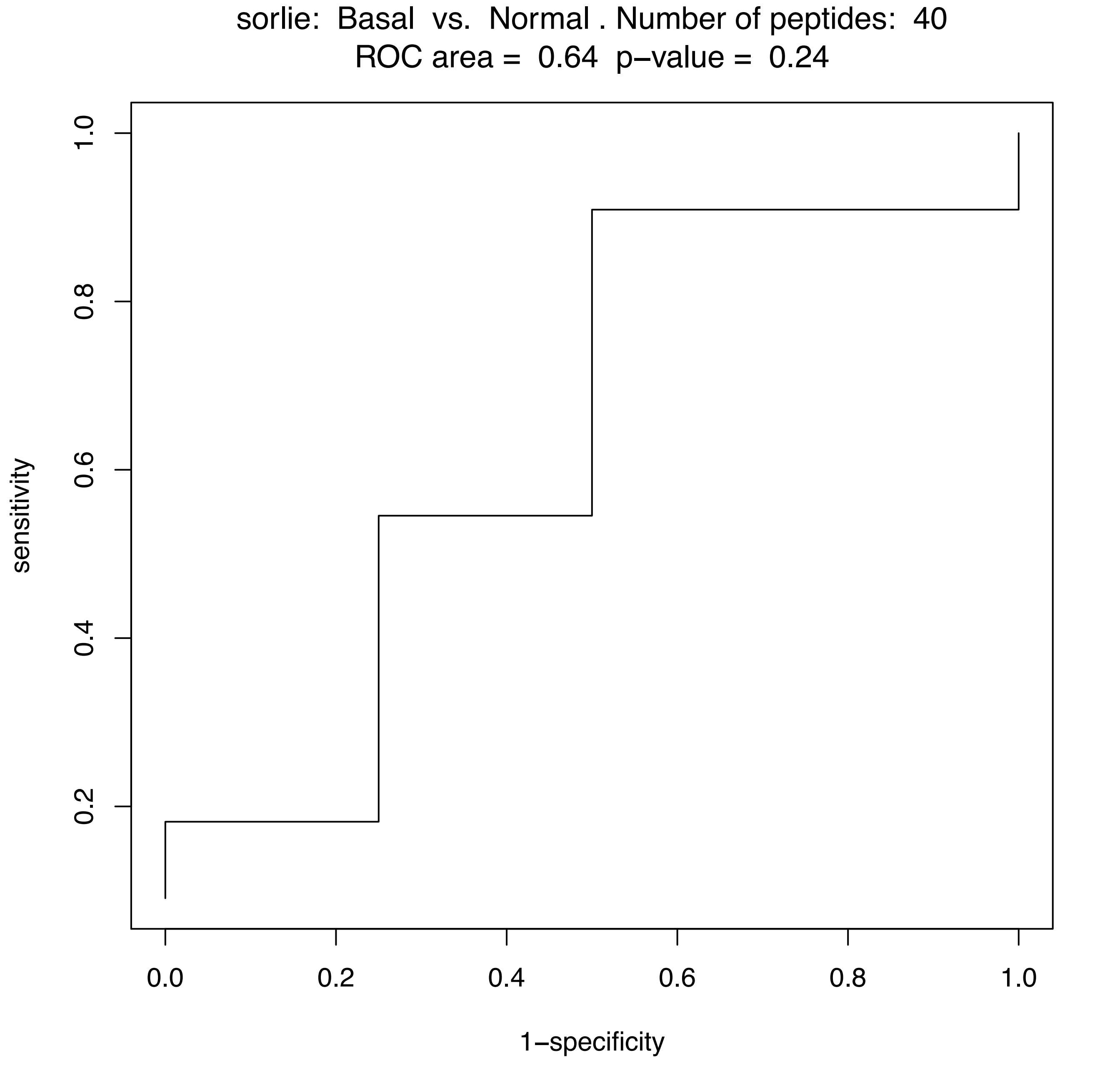

sorlie: Basal vs. Normal . Number of peptides: 40
ROC area = 0.64 p−value = 0.24
sensitivity
1−specificity
0.2
0.4
0.6
0.8
1.0
0.0
0.2
0.4
0.6
0.8
1.0

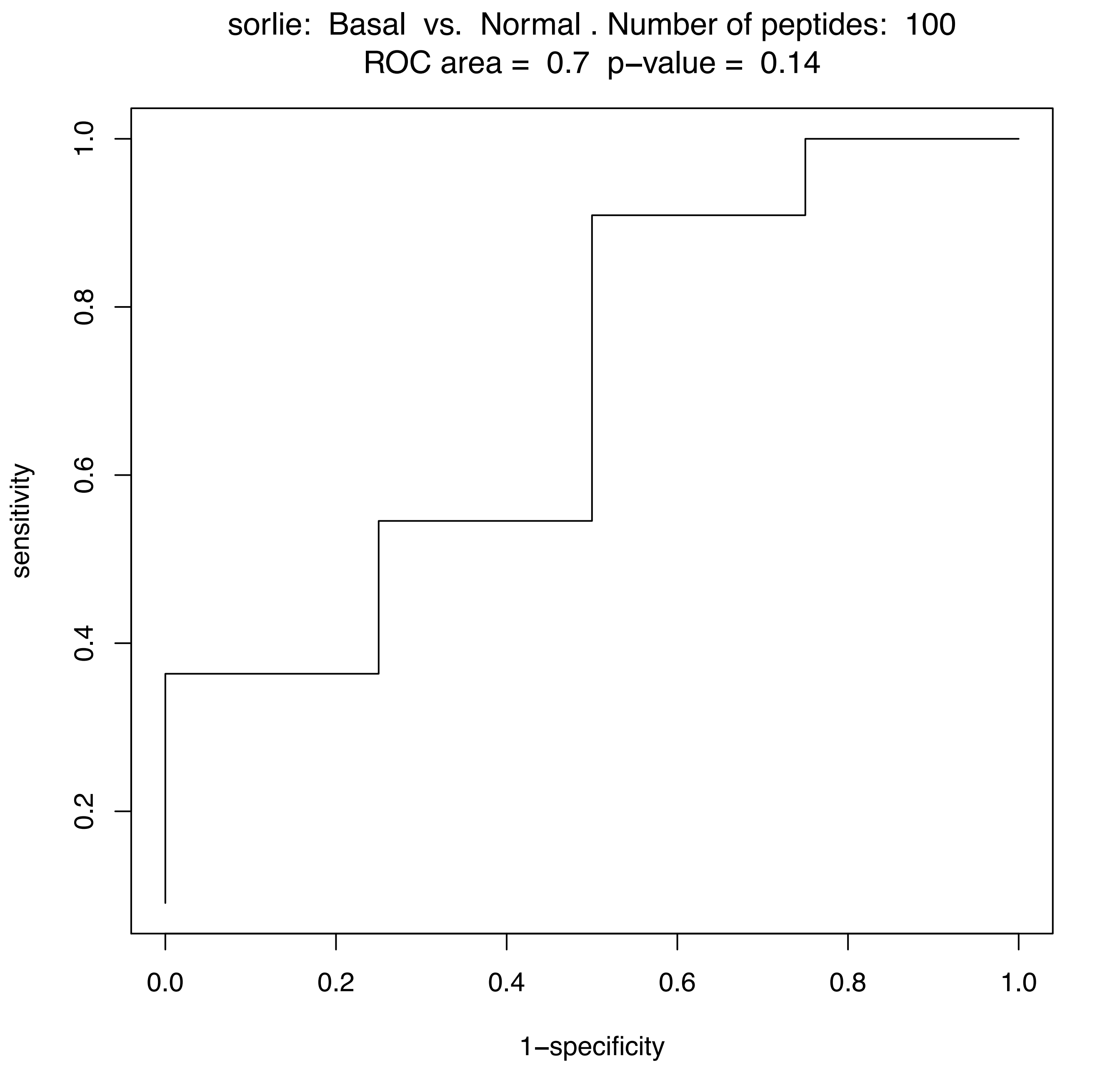

sorlie: Basal vs. Normal . Number of peptides: 100
ROC area = 0.7 p−value = 0.14
sensitivity
0.2
0.4
0.6
0.8
1.0
0.0
0.2
0.4
0.6
0.8
1.0
1−specificity

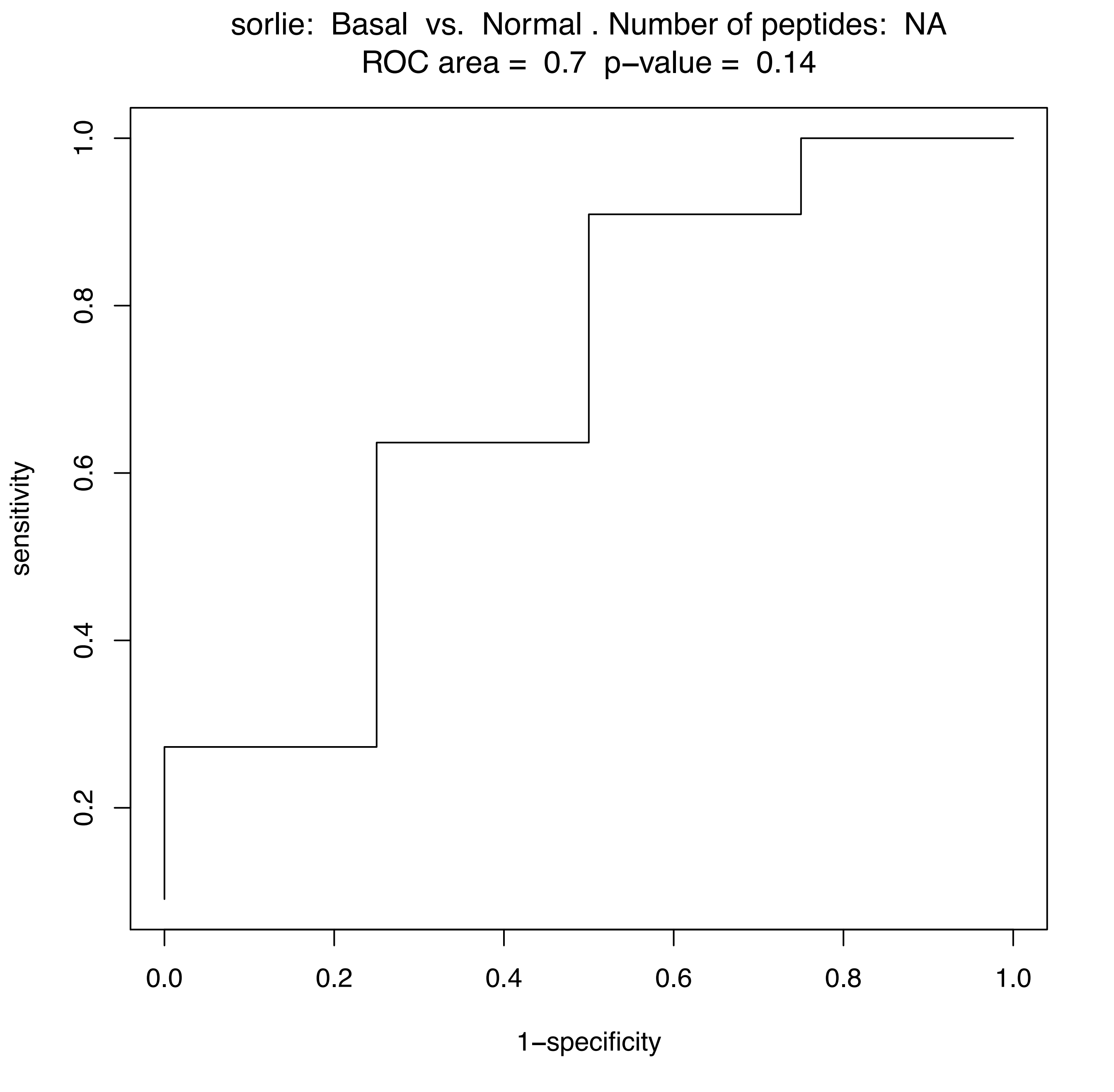
sorlie: Basal vs. Normal . Number of peptides: NA
ROC area = 0.7 p−value = 0.14
sensitivity
1−specificity
0.2
0.4
0.6
0.8
1.0
0.0
0.2
0.4
0.6
0.8
1.0

pam50: Basal vs. Normal . Number of peptides: 20
ROC area = 0.73 p−value = 0.074

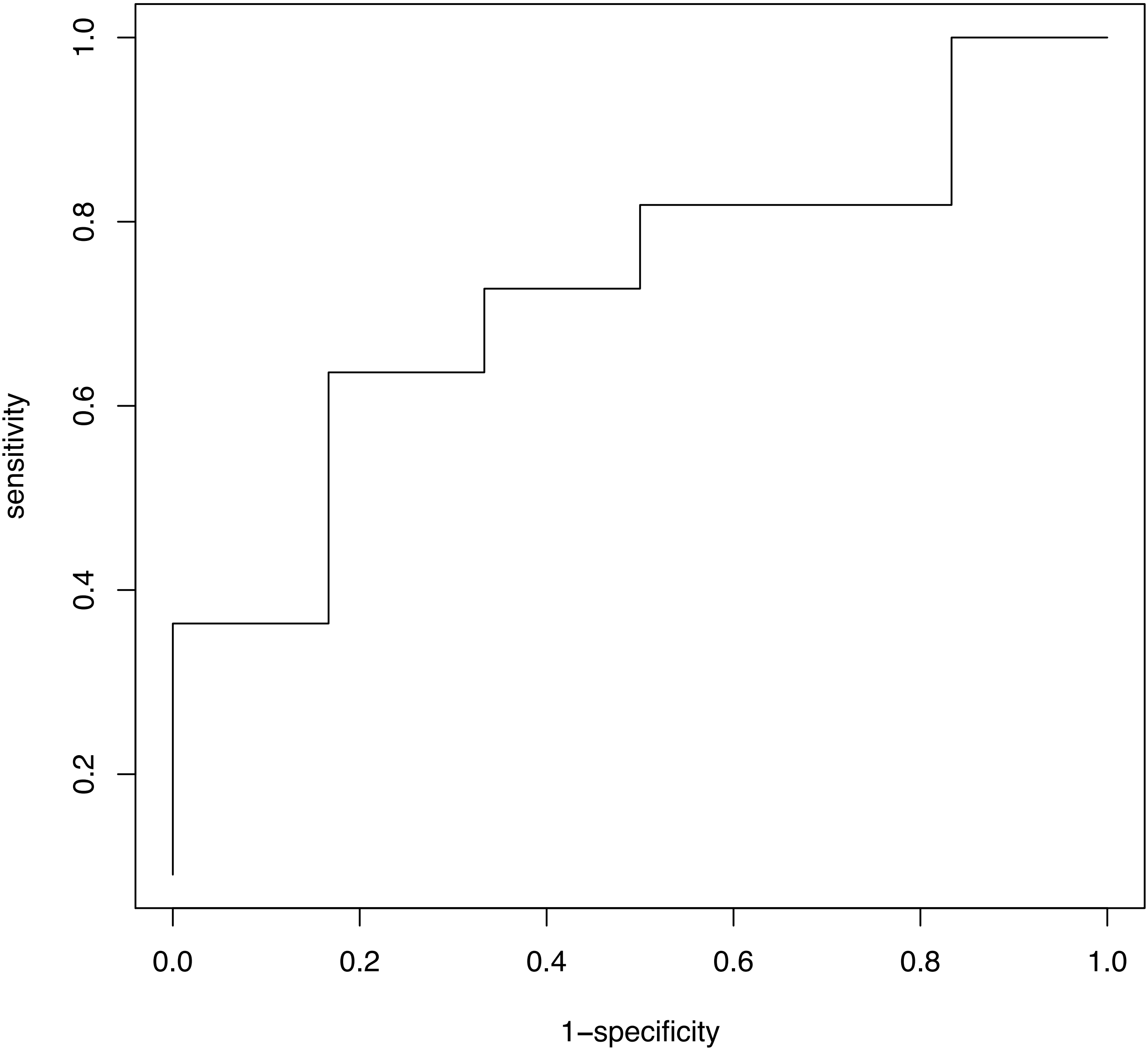

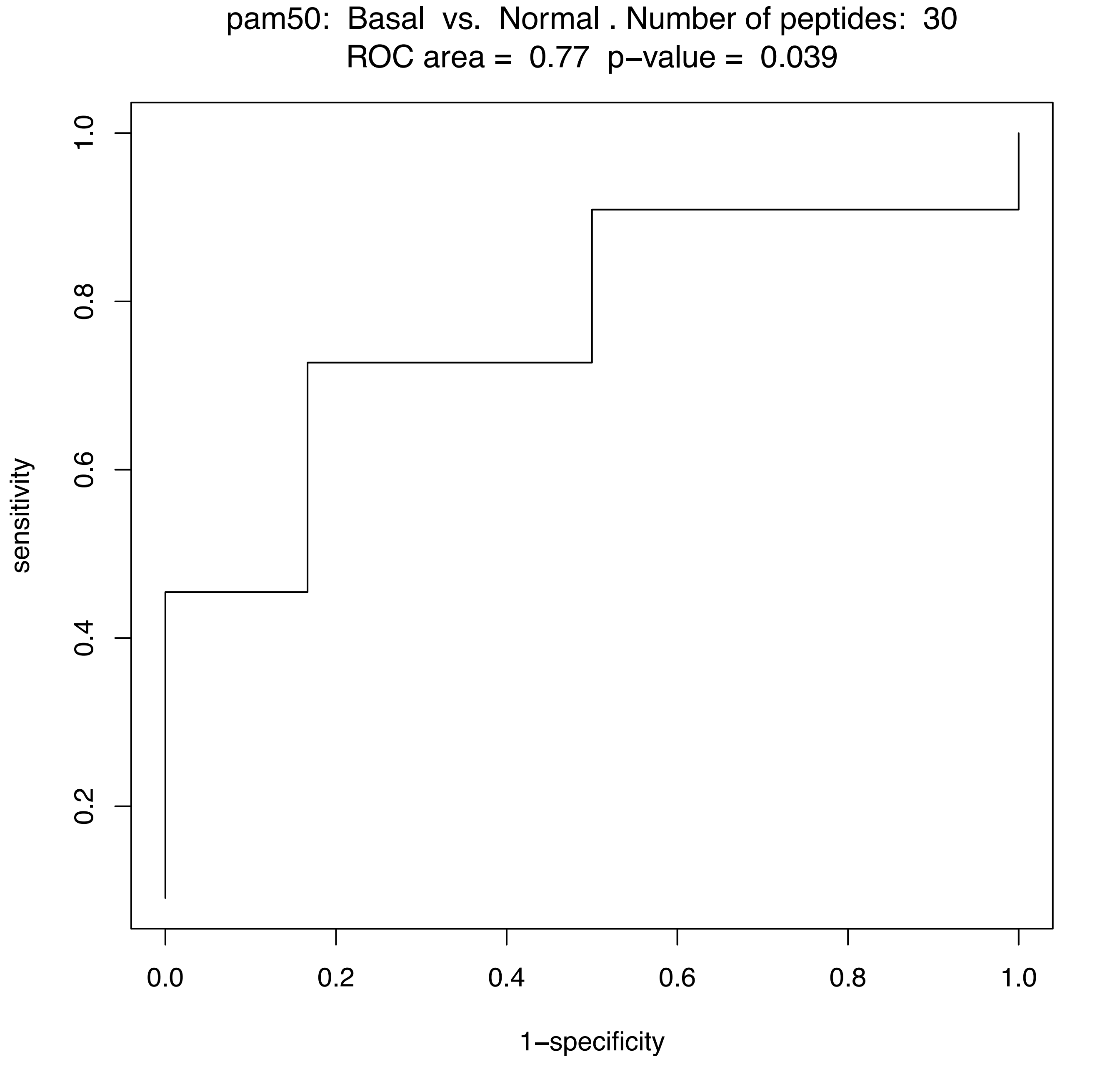
pam50: Basal vs. Normal . Number of peptides: 30
ROC area = 0.77 p−value = 0.039
sensitivity
1−specificity
0.0
0.2
0.4
0.6
0.8
1.0
0.2
0.4
0.6
0.8
1.0

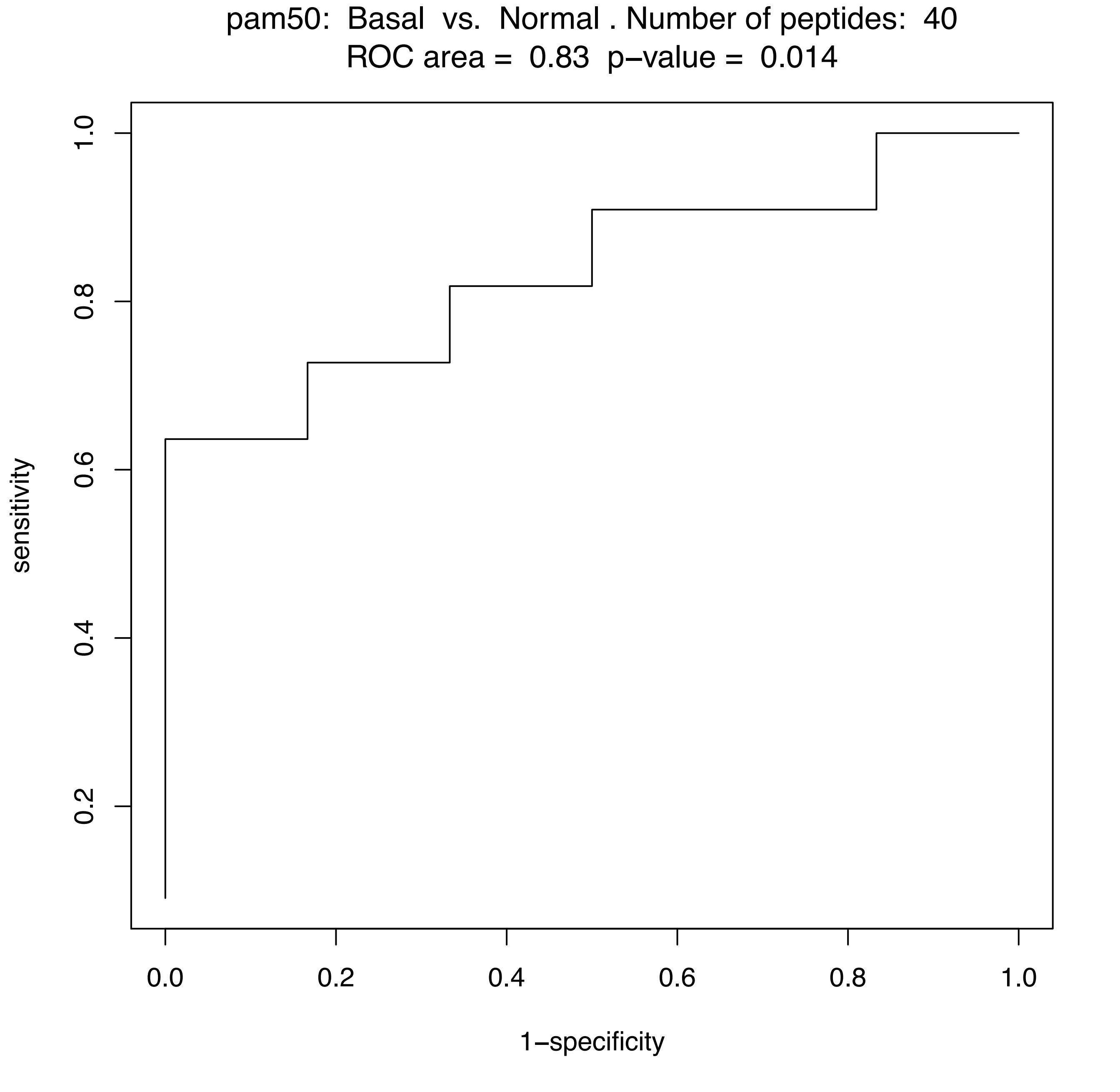
pam50: Basal vs. Normal . Number of peptides: 40
ROC area = 0.83 p−value = 0.014
sensitivity
1−specificity
0.0
0.2
0.4
0.6
0.8
1.0

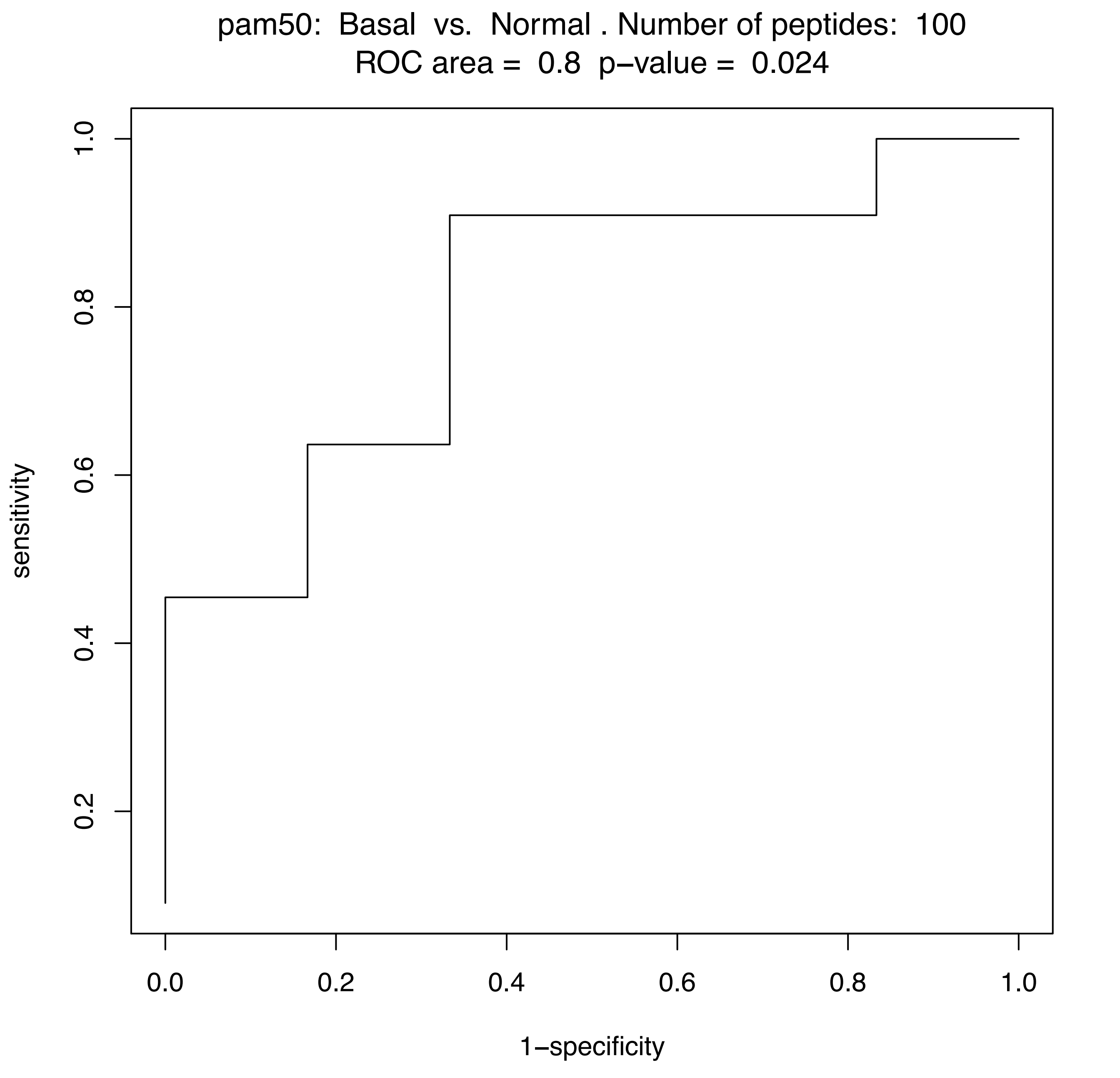
pam50: Basal vs. Normal . Number of peptides: 100
ROC area = 0.8 p−value = 0.024
sensitivity
1−specificity
0.0
0.2
0.4
0.6
0.8
1.0

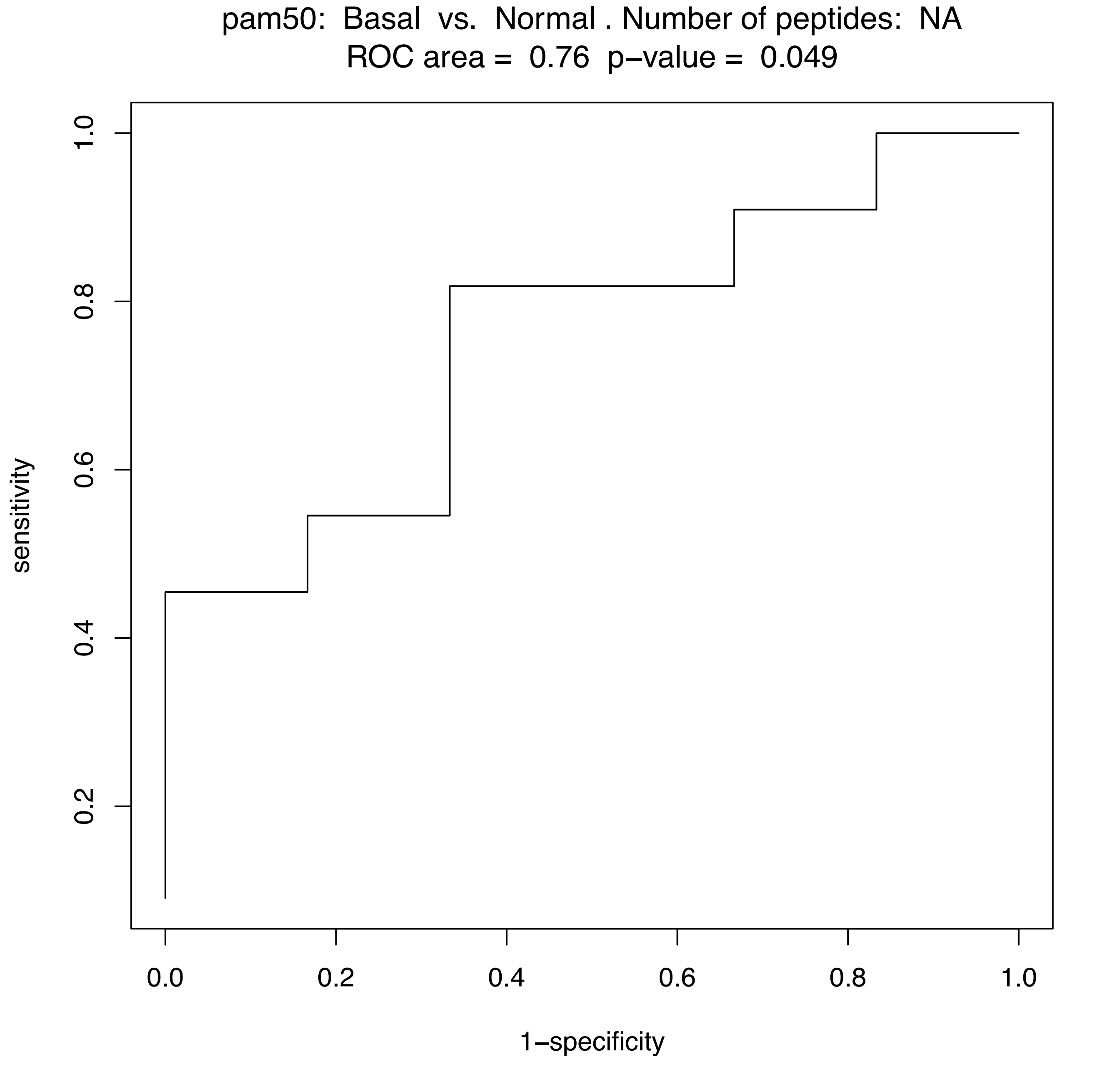
pam50: Basal vs. Normal . Number of peptides: NA
ROC area = 0.76 p−value = 0.049
sensitivity
1−specificity
0.2
0.4
0.6
0.8
1.0
0.0
0.2
0.4
0.6
0.8
1.0

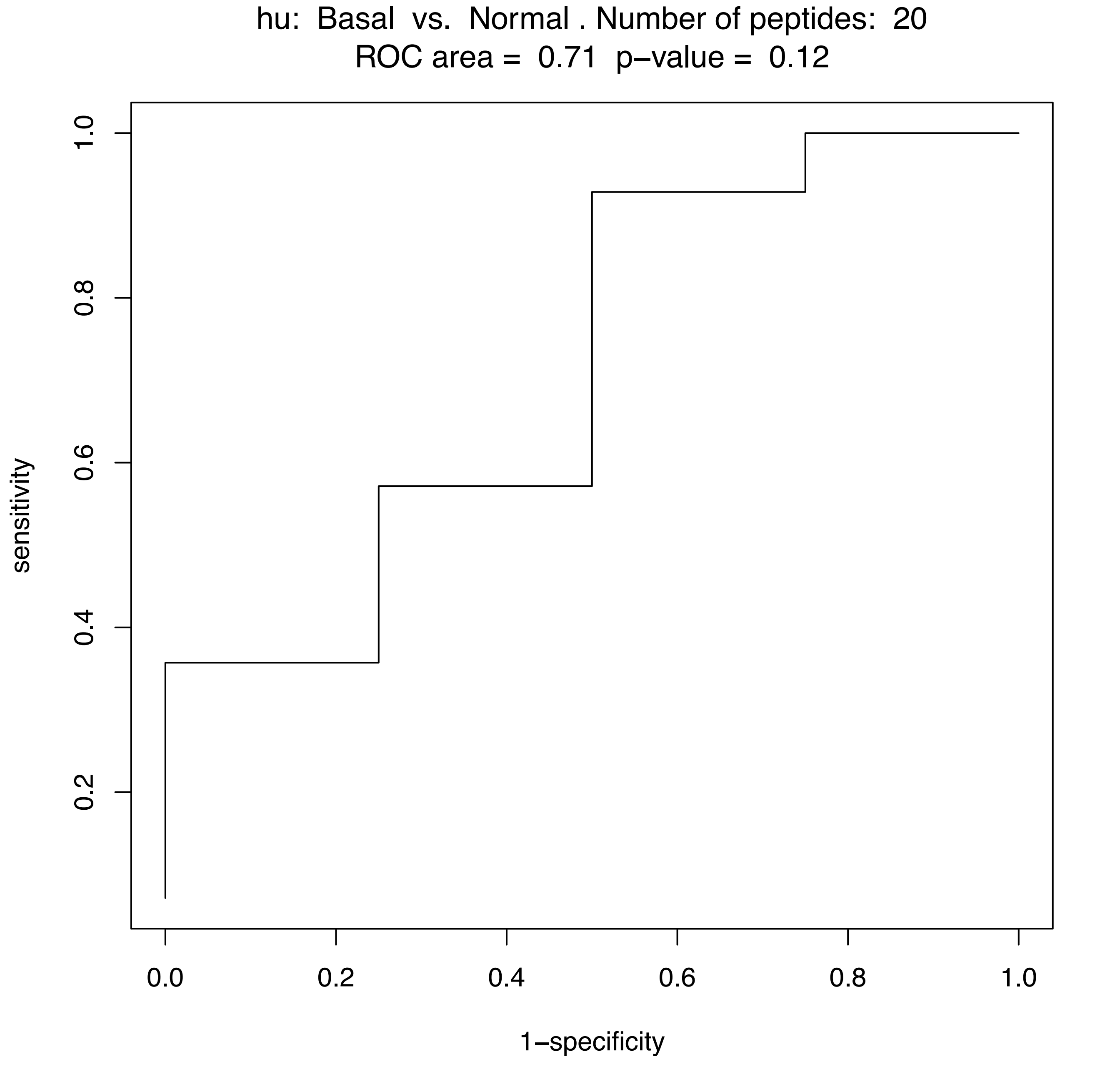

hu: Basal vs. Normal . Number of peptides: 20
ROC area = 0.71 p−value = 0.12
sensitivity
1.0
0.8
0.6
0.4
0.2
0.0
0.2
0.4
0.6
0.8
1.0
1−specificity

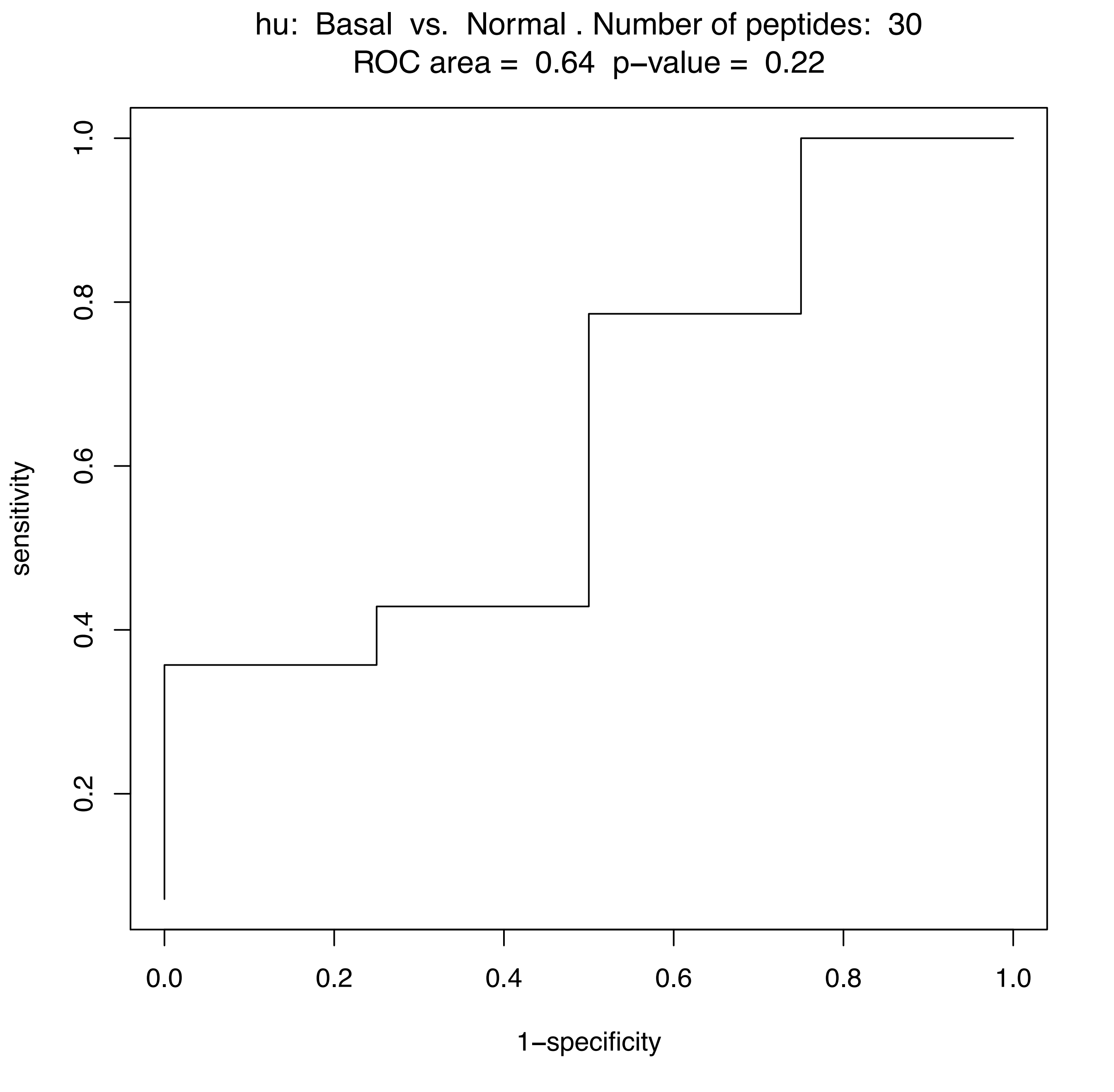
hu: Basal vs. Normal . Number of peptides: 30
ROC area = 0.64 p−value = 0.22
sensitivity
1−specificity
0.2
0.4
0.6
0.8
1.0
0.0
0.2
0.4
0.6
0.8
1.0

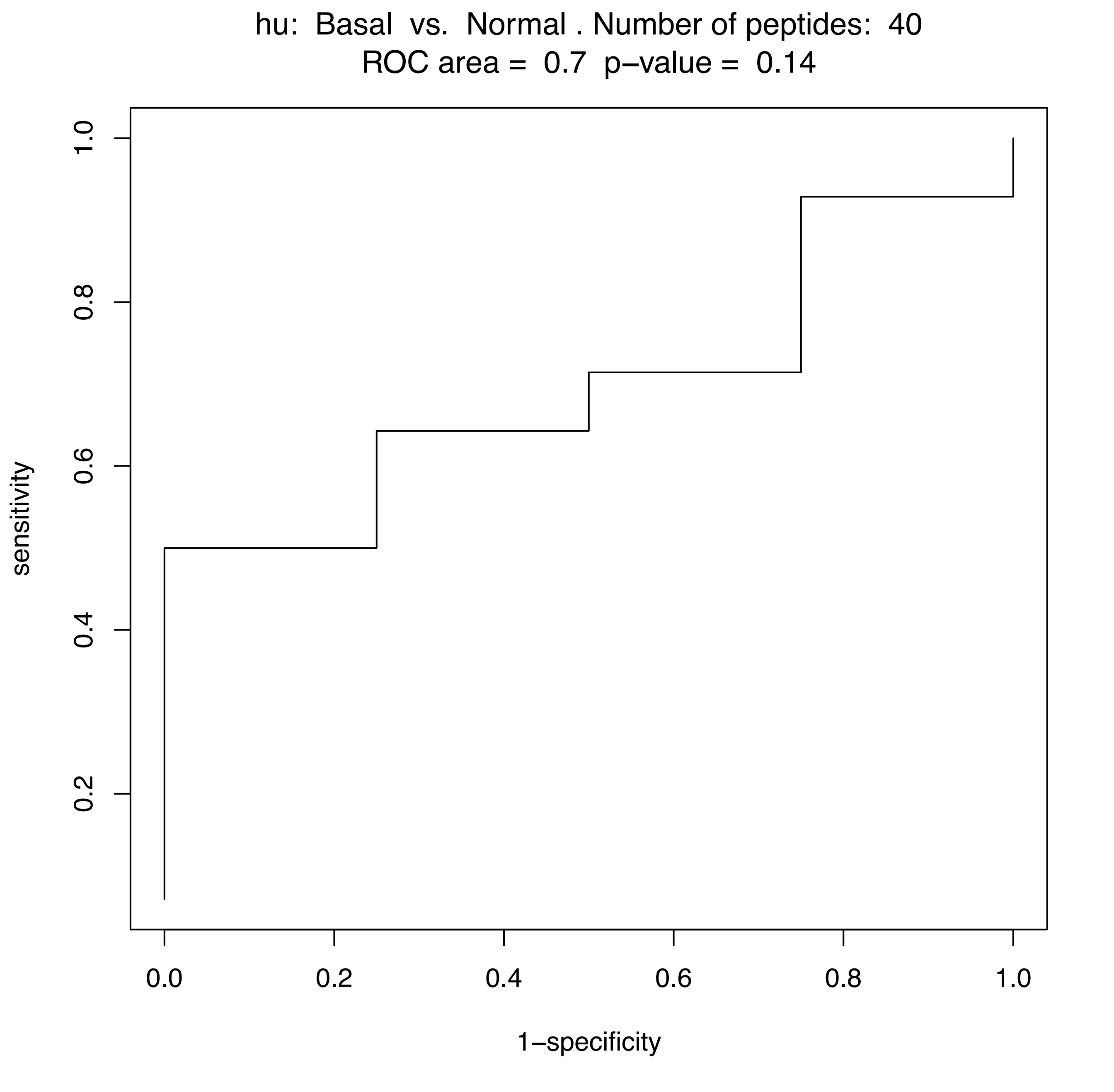
hu: Basal vs. Normal . Number of peptides: 40
ROC area = 0.7 p−value = 0.14
sensitivity
1−specificity
0.0
0.2
0.4
0.6
0.8
1.0
0.2
0.4
0.6
0.8
1.0

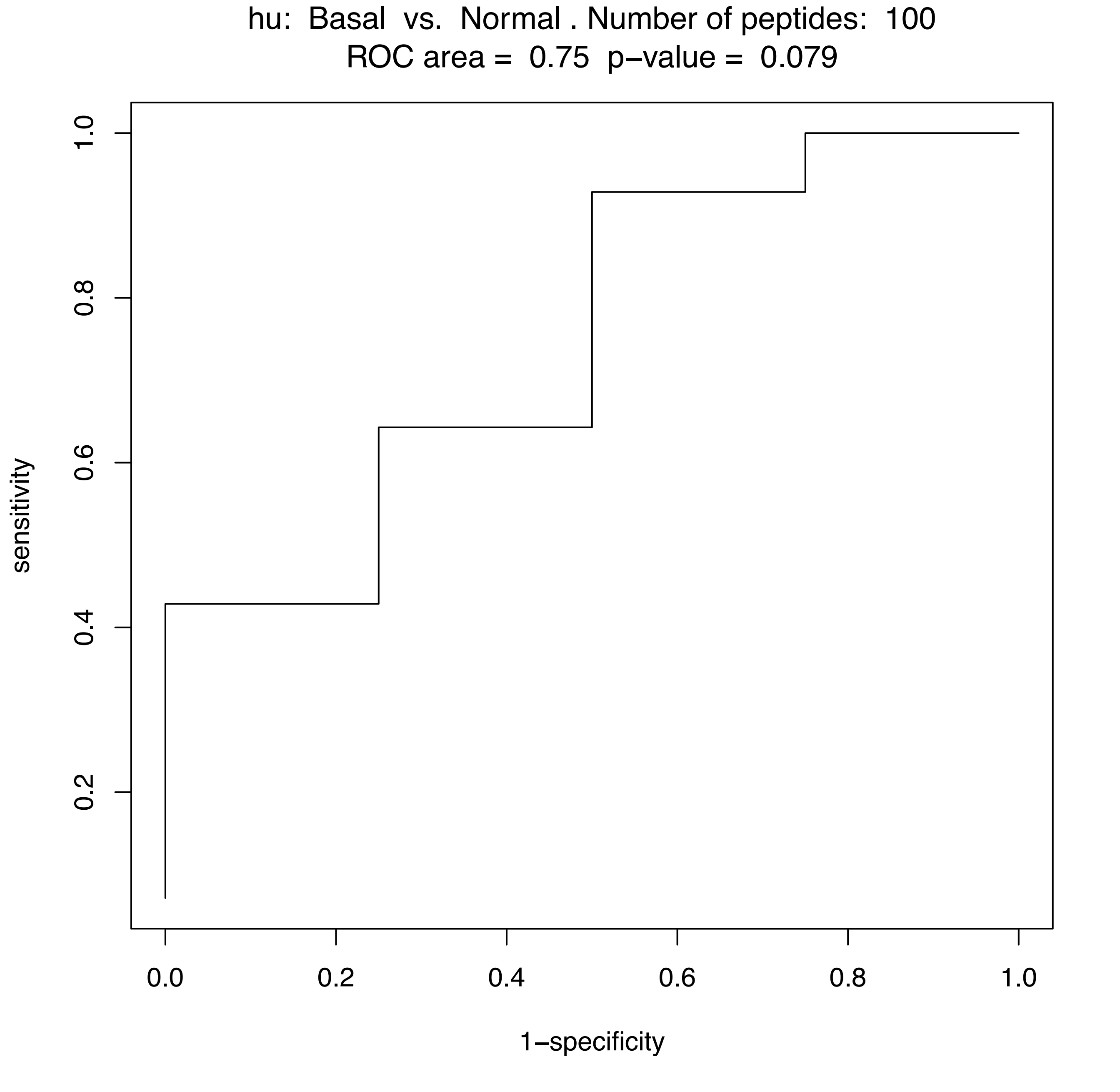
hu: Basal vs. Normal . Number of peptides: 100
ROC area = 0.75 p−value = 0.079
sensitivity
1−specificity
0.0
0.2
0.4
0.6
0.8
1.0
0.2
0.4
0.6
0.8
1.0

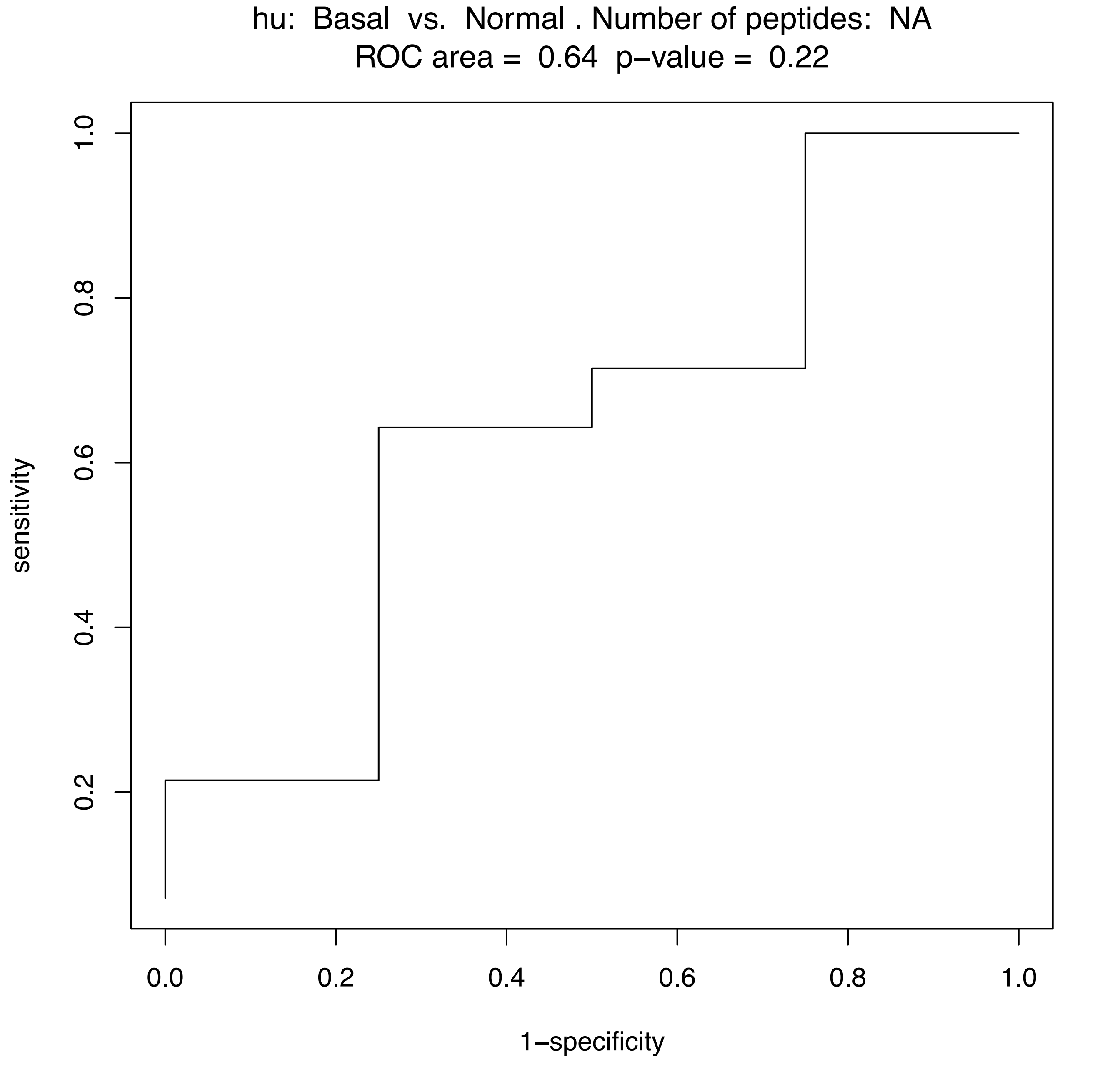
hu: Basal vs. Normal . Number of peptides: NA
ROC area = 0.64 p−value = 0.22
sensitivity
1−specificity
0.0
0.2
0.4
0.6
0.8
1.0

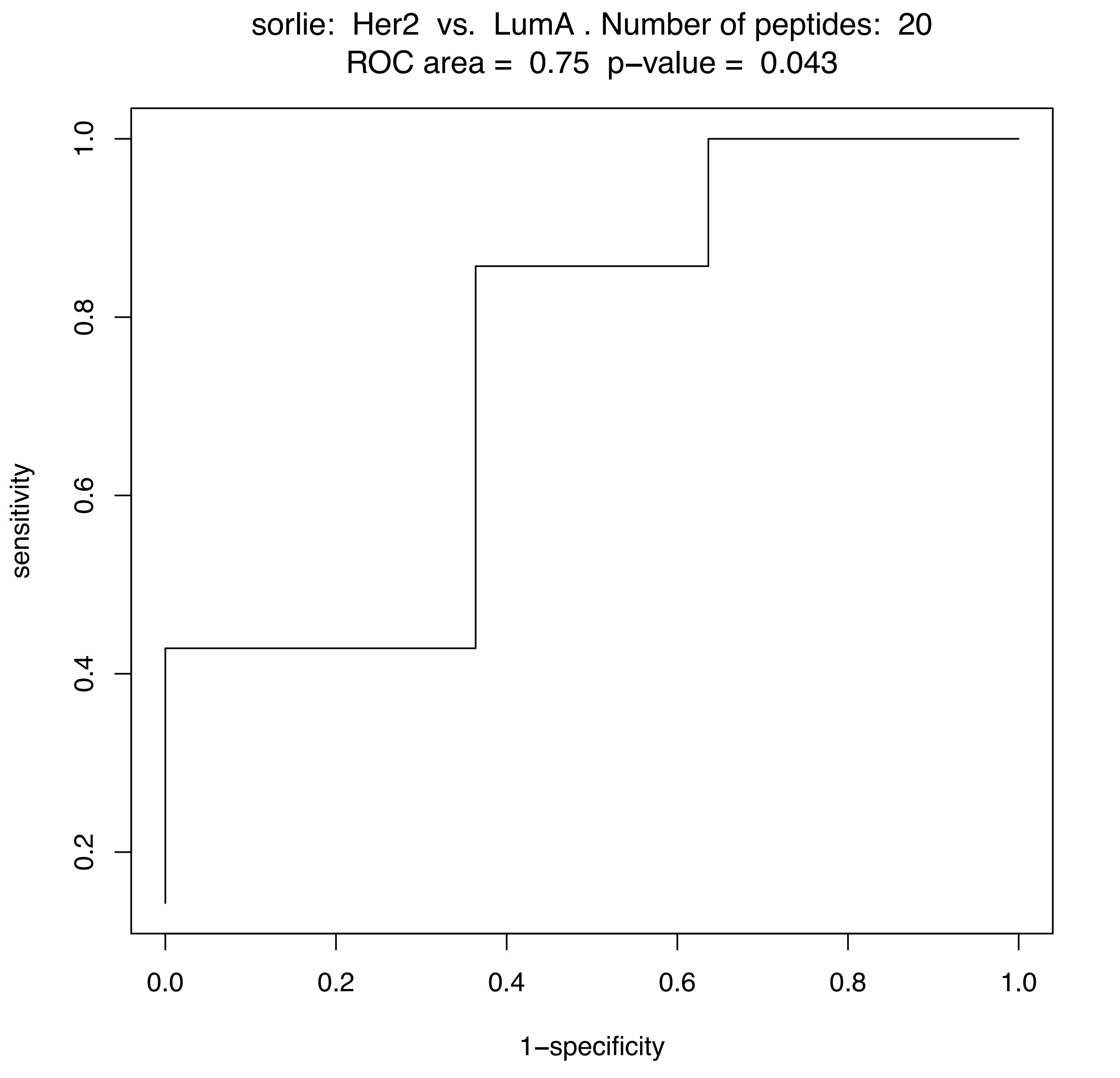
sorlie: Her2 vs. LumA . Number of peptides: 20
ROC area = 0.75 p−value = 0.043
sensitivity
0.2
0.4
0.6
0.8
1.0
0.0
0.2
0.4
0.6
0.8
1.0
1−specificity

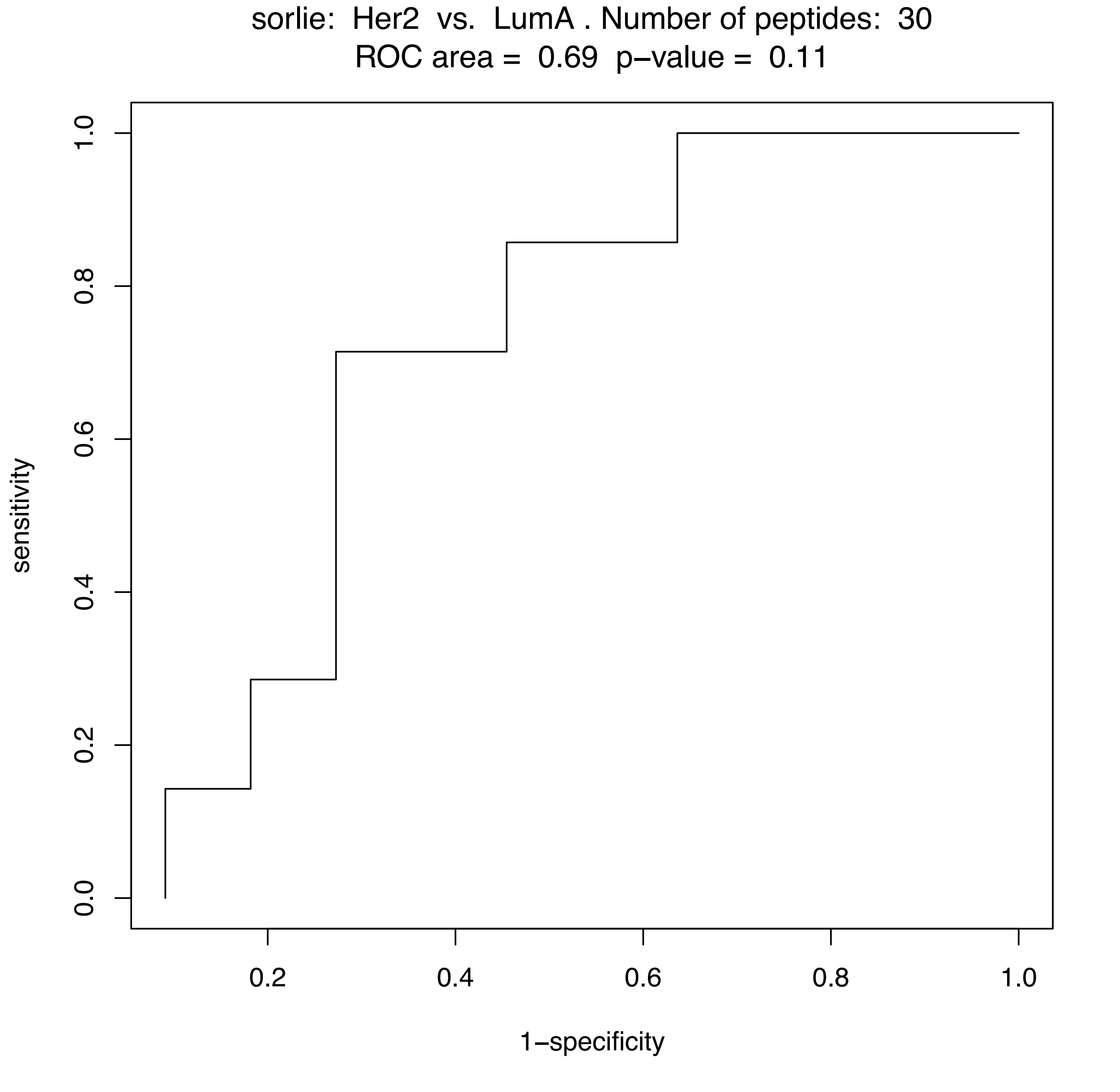
sorlie: Her2 vs. LumA . Number of peptides: 30
ROC area = 0.69 p−value = 0.11
sensitivity
1−specificity
0.0
0.2
0.4
0.6
0.8
1.0
0.2
0.4
0.6
0.8
1.0

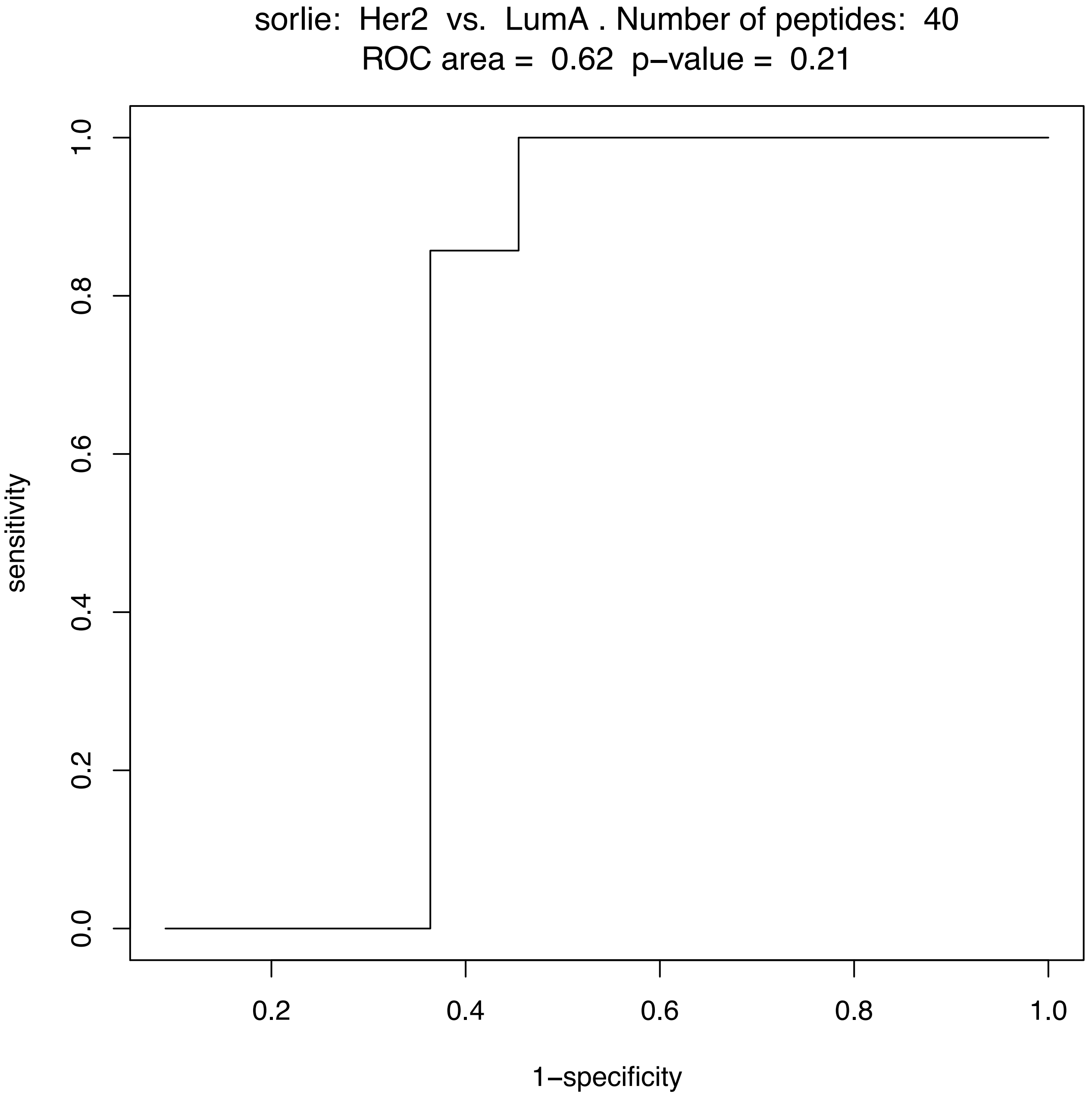
sorlie: Her2 vs. LumA . Number of peptides: 40
ROC area = 0.62 p−value = 0.21
sensitivity
0.0
0.2
0.4
0.6
0.8
1.0
0.2
0.4
0.6
0.8
1.0
1−specificity

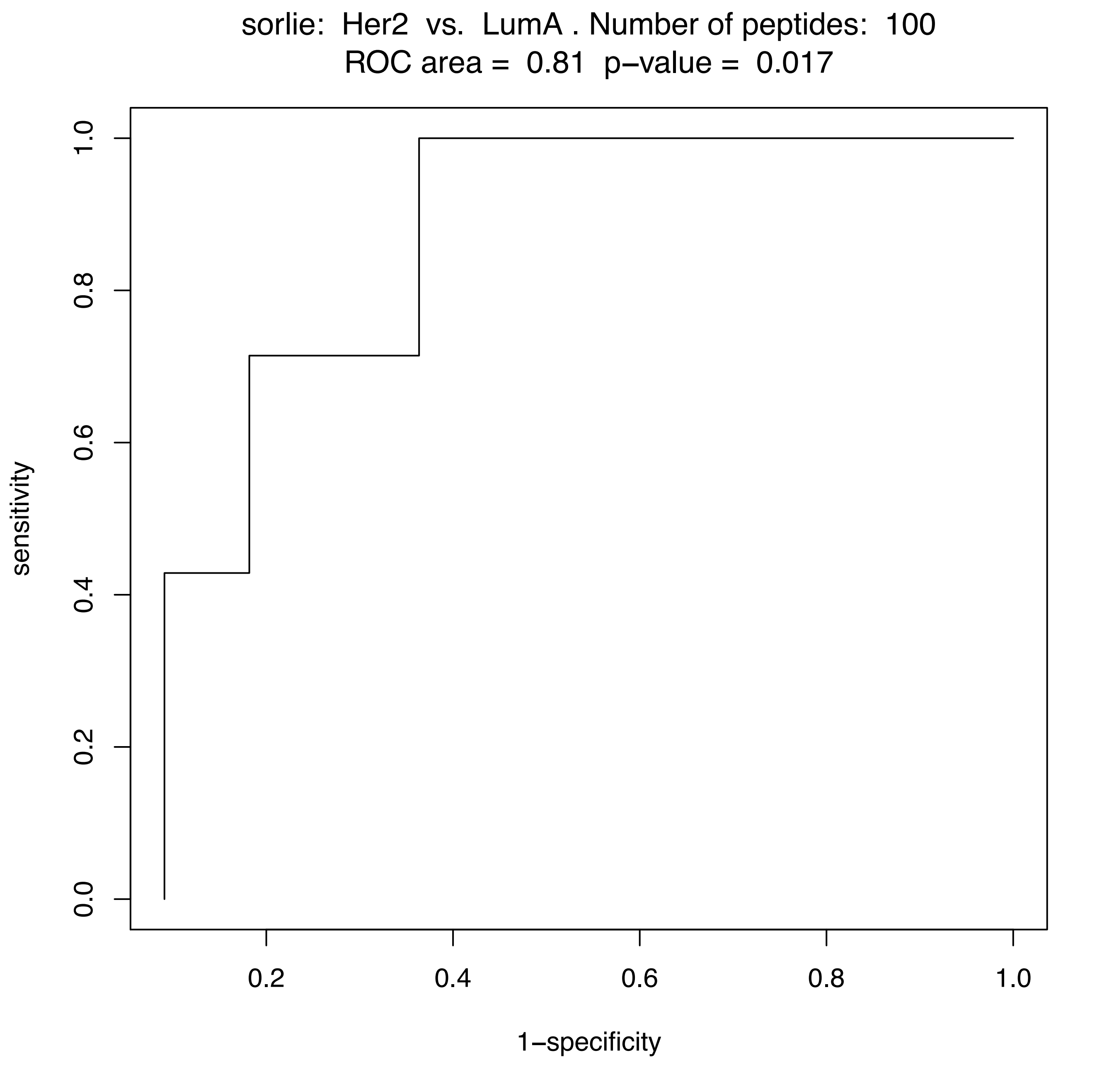
sorlie: Her2 vs. LumA . Number of peptides: 100
ROC area = 0.81 p−value = 0.017
sensitivity
1−specificity
0.0
0.2
0.4
0.6
0.8
1.0
0.2
0.4
0.6
0.8
1.0

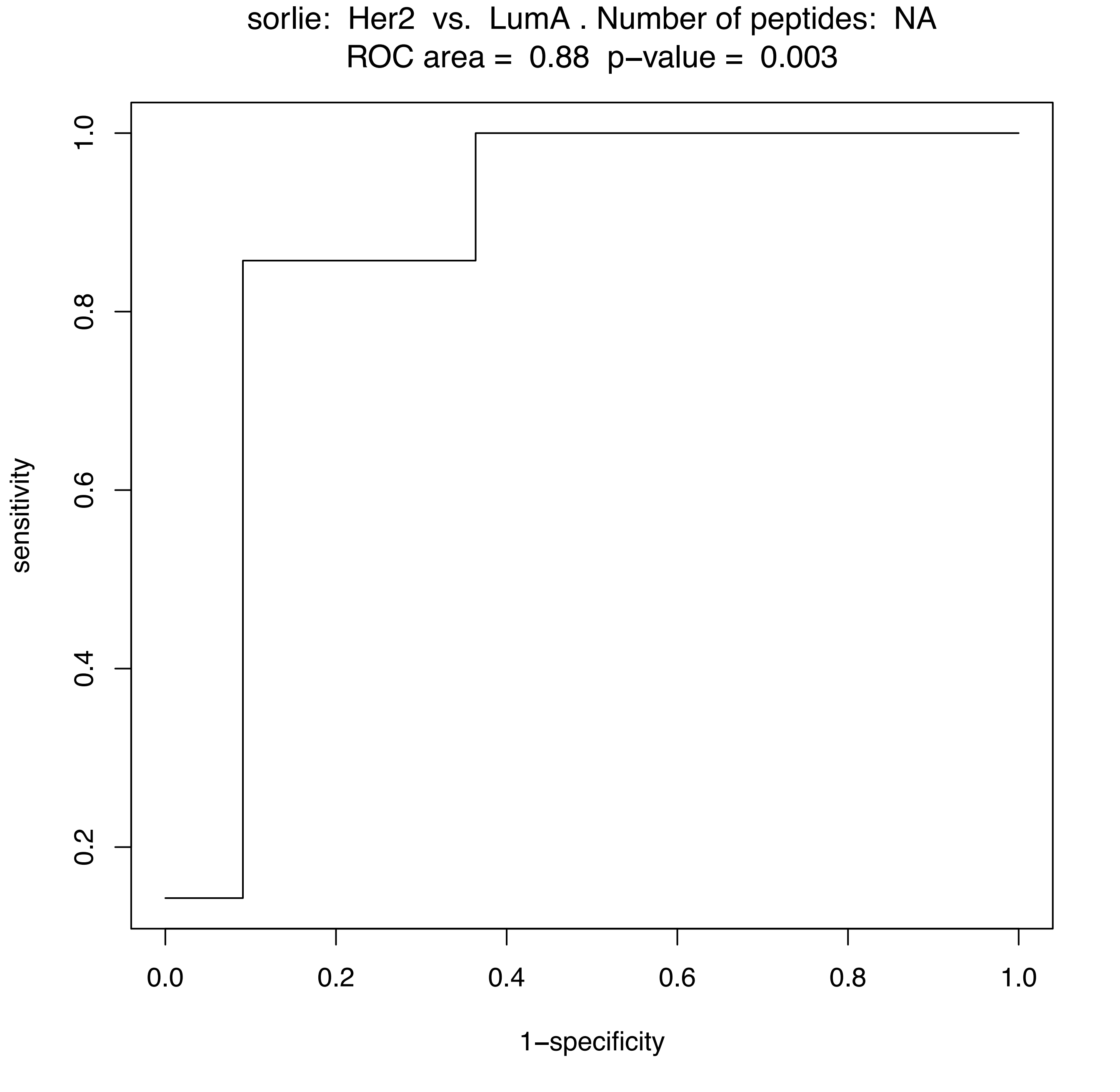
sorlie: Her2 vs. LumA . Number of peptides: NA
ROC area = 0.88 p−value = 0.003
sensitivity
1−specificity
0.2
0.4
0.6
0.8
1.0
0.0
0.2
0.4
0.6
0.8
1.0

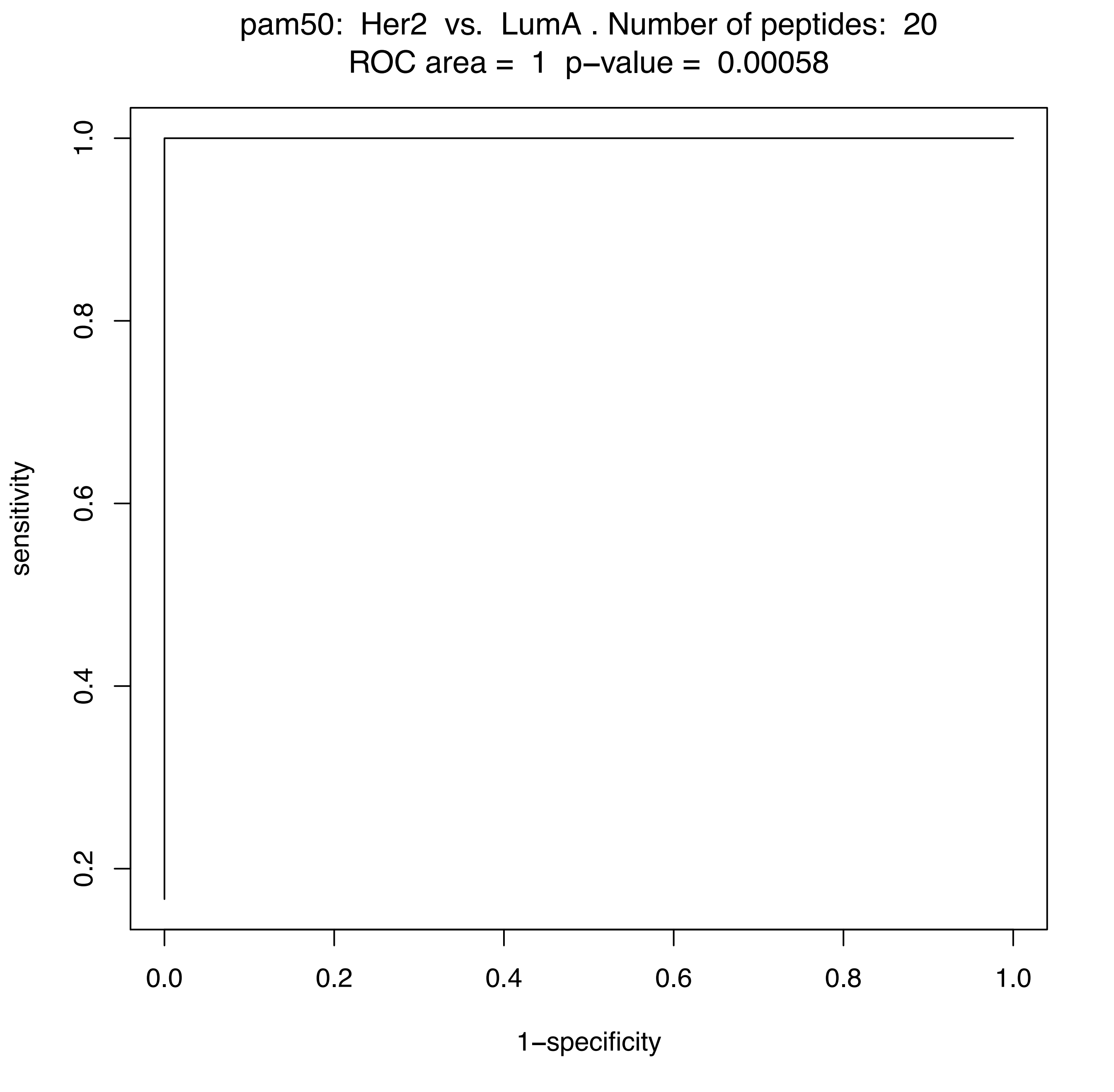

pam50: Her2 vs. LumA . Number of peptides: 20
ROC area = 1 p−value = 0.00058
sensitivity
1−specificity
0.2
0.4
0.6
0.8
1.0
0.0
0.2
0.4
0.6
0.8
1.0

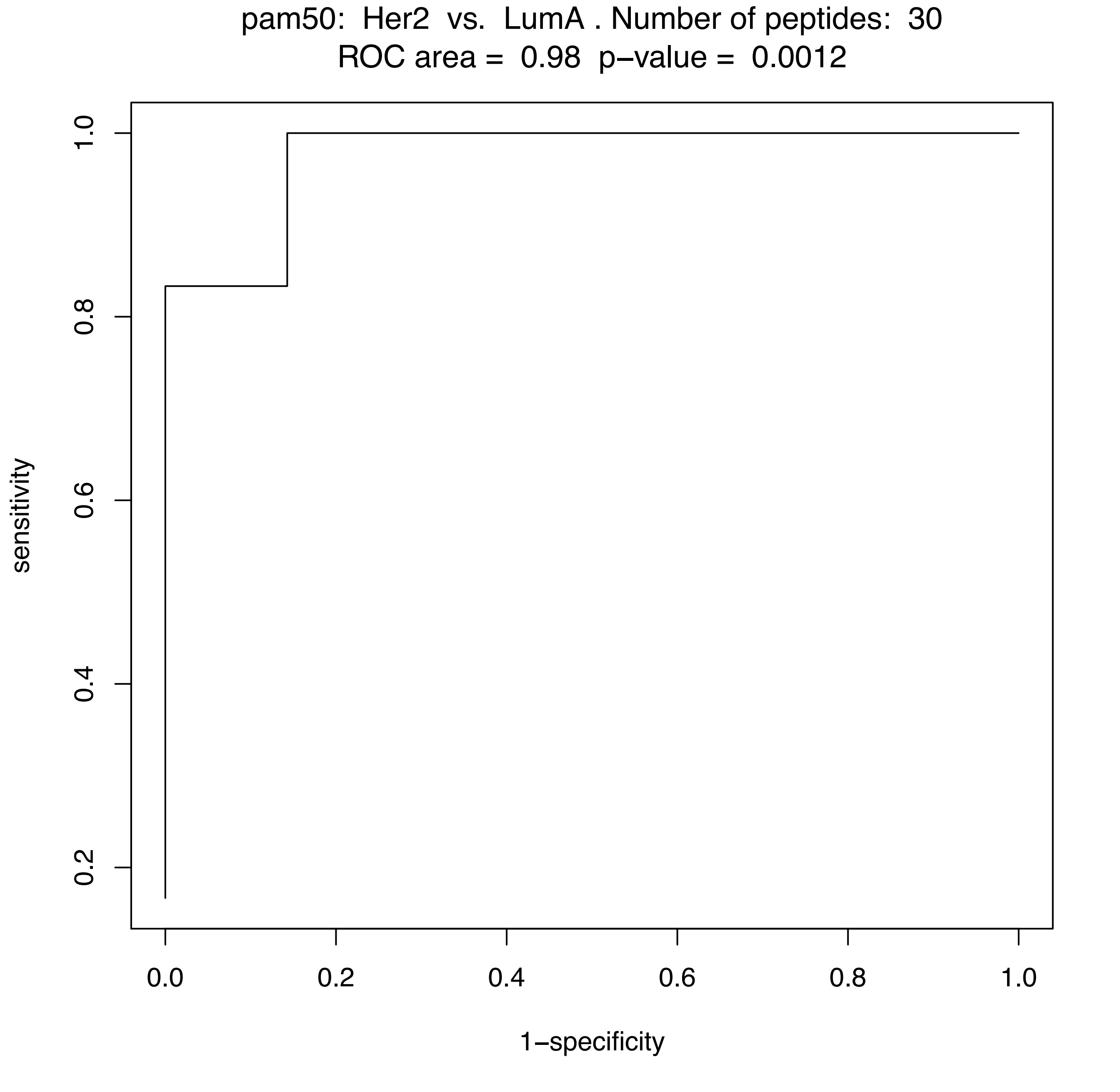
pam50: Her2 vs. LumA . Number of peptides: 30
ROC area = 0.98 p−value = 0.0012
sensitivity
1.0
0.8
0.6
0.4
0.2
0.0
0.2
0.4
0.6
0.8
1.0
1−specificity

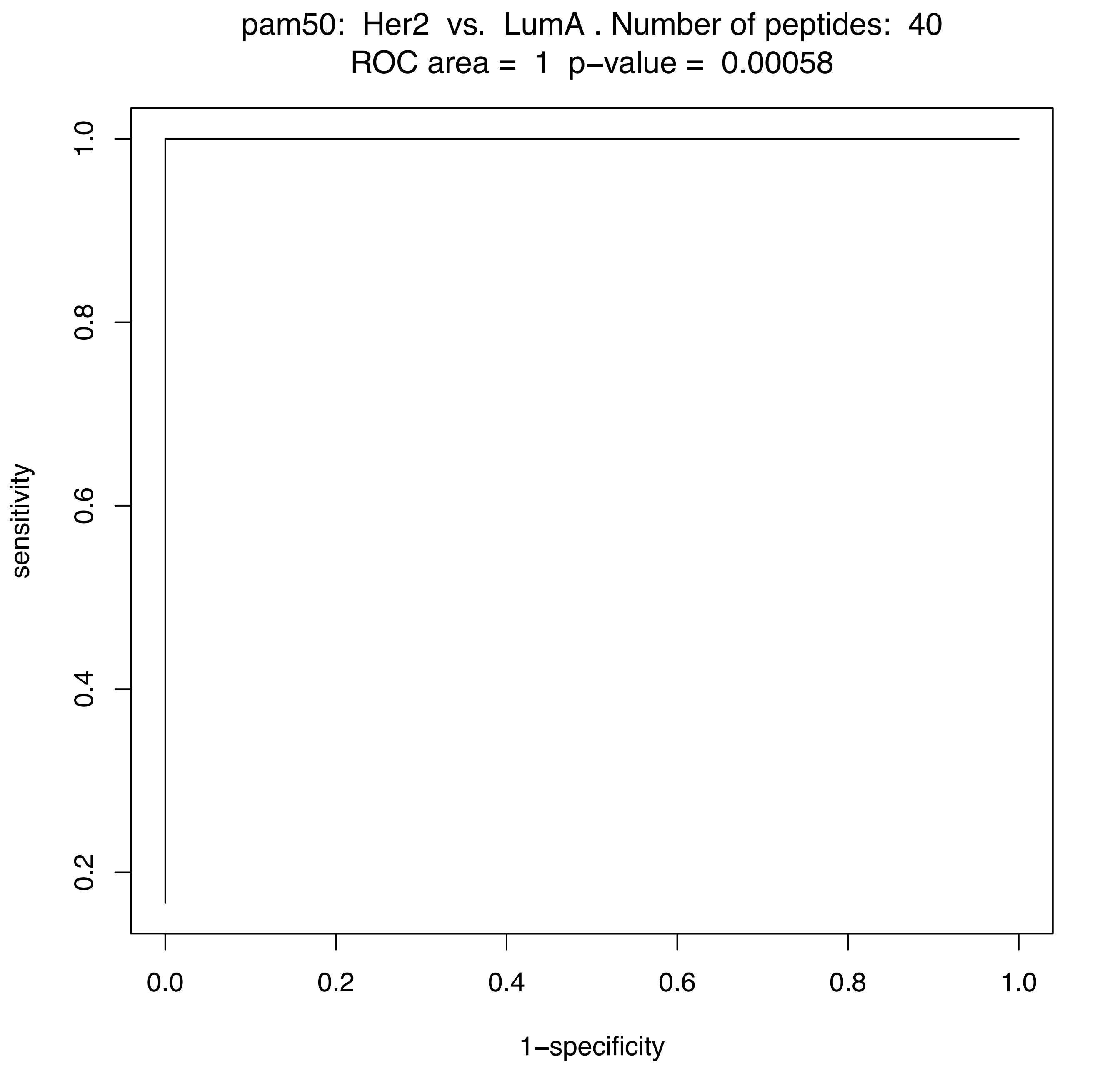
pam50: Her2 vs. LumA . Number of peptides: 40
ROC area = 1 p−value = 0.00058
sensitivity
1.0
0.8
0.6
0.4
0.2
0.0
0.2
0.4
0.6
0.8
1.0
1−specificity

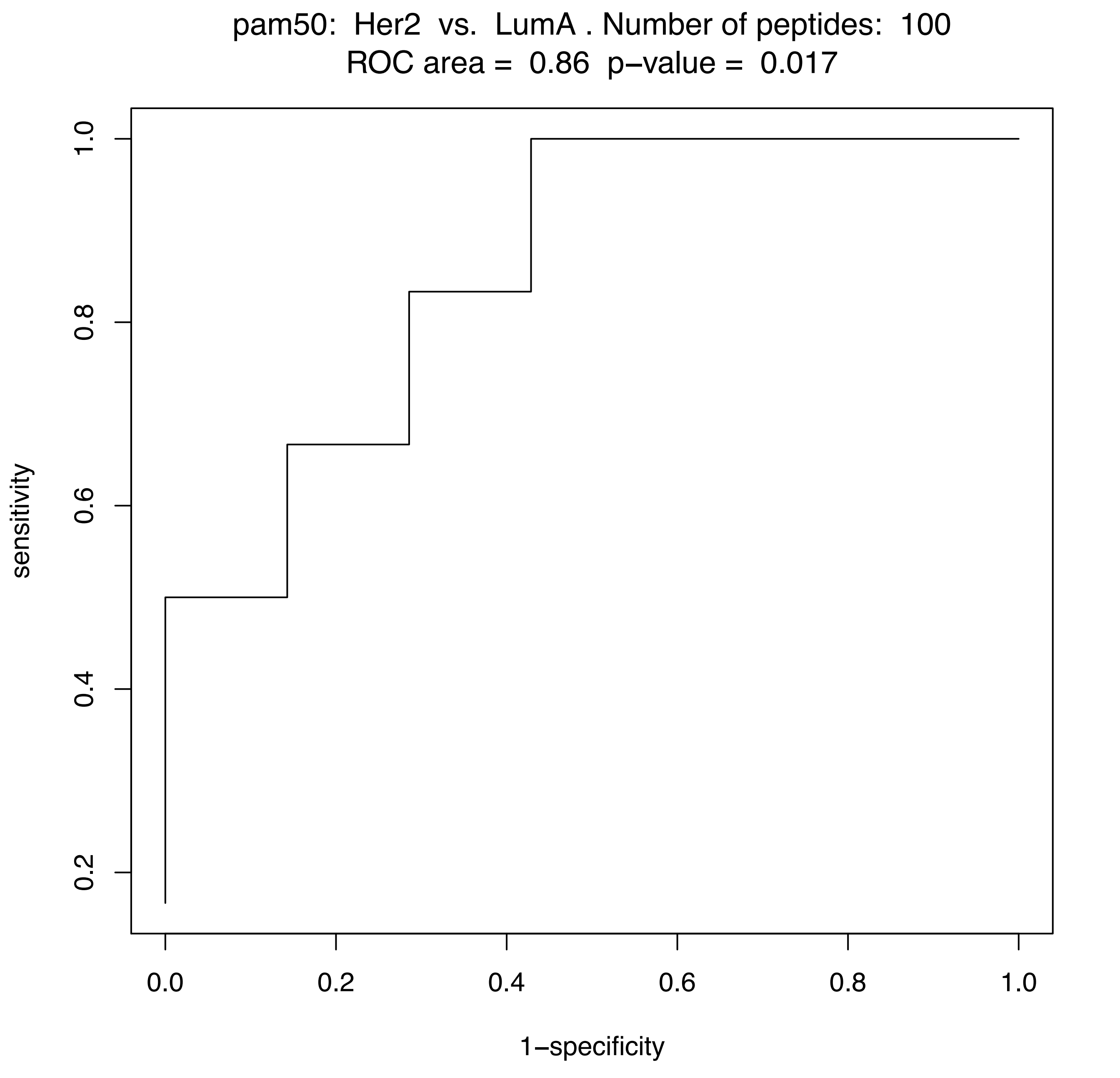
pam50: Her2 vs. LumA . Number of peptides: 100
ROC area = 0.86 p−value = 0.017
sensitivity
1−specificity
0.2
0.4
0.6
0.8
1.0
0.0
0.2
0.4
0.6
0.8
1.0

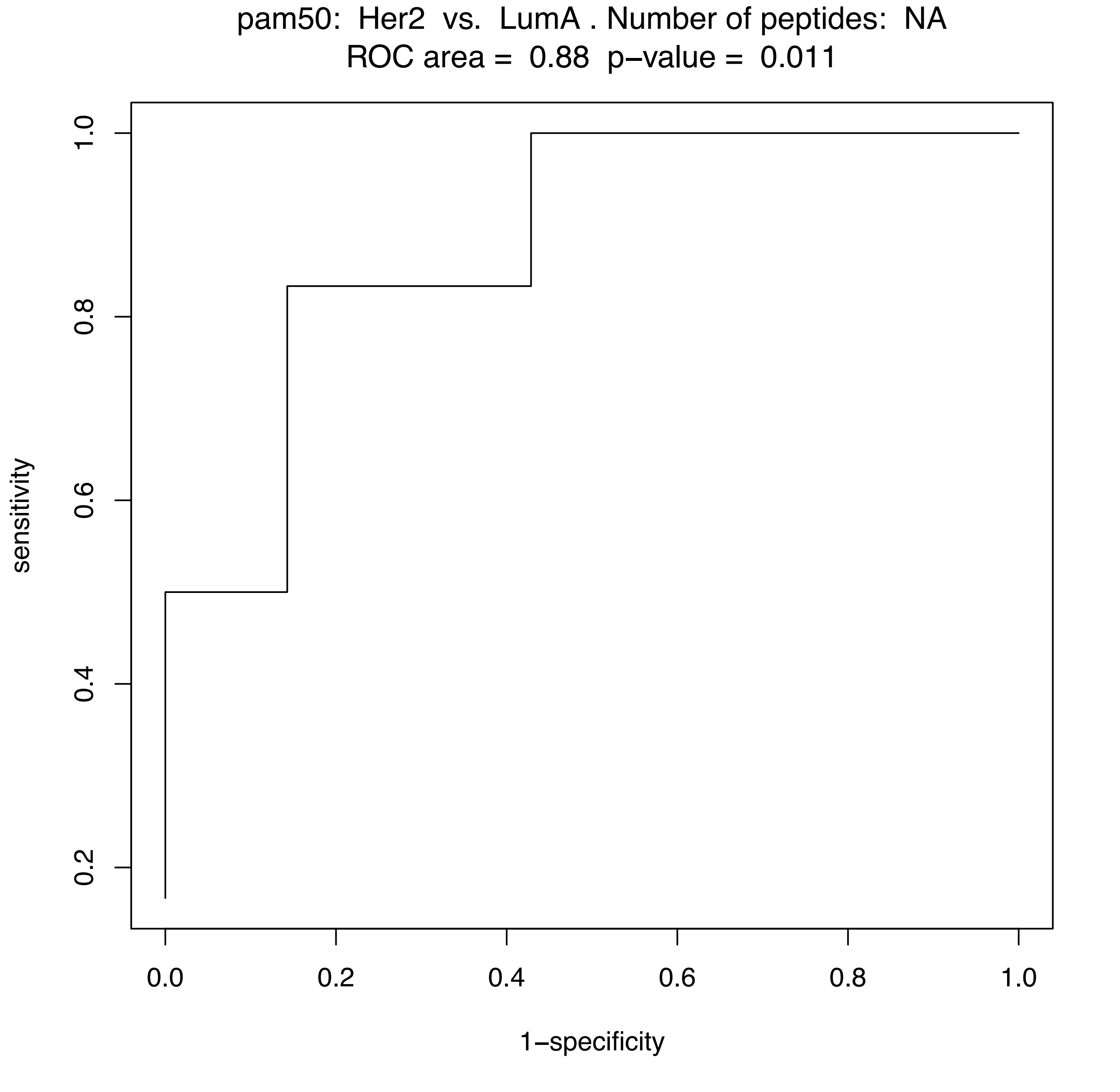
pam50: Her2 vs. LumA . Number of peptides: NA
ROC area = 0.88 p−value = 0.011
sensitivity
1−specificity
0.2
0.4
0.6
0.8
1.0
0.0
0.2
0.4
0.6
0.8
1.0

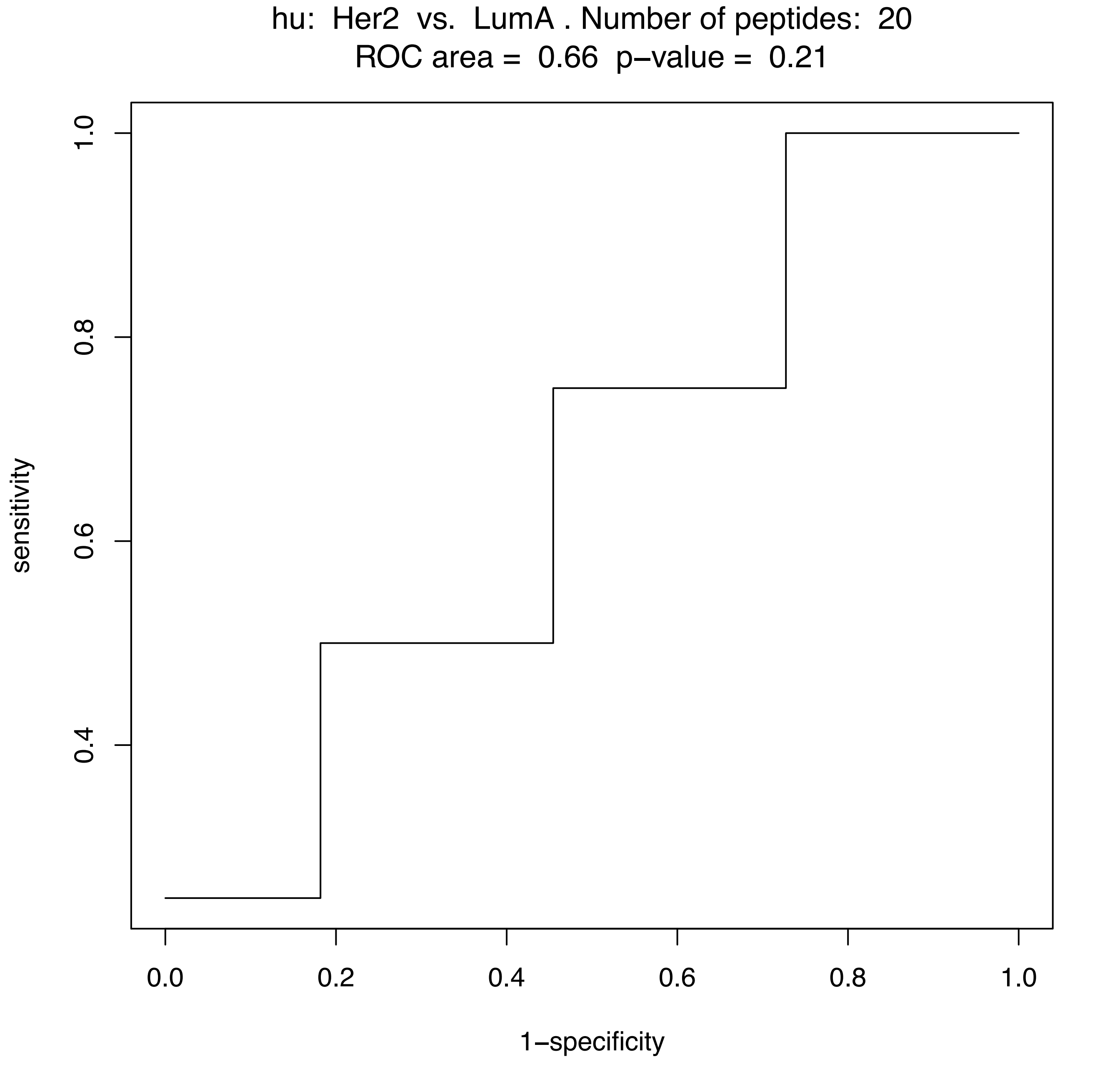
hu: Her2 vs. LumA . Number of peptides: 20
ROC area = 0.66 p−value = 0.21
sensitivity
1.0
0.8
0.6
0.4
0.0
0.2
0.4
0.6
0.8
1.0
1−specificity

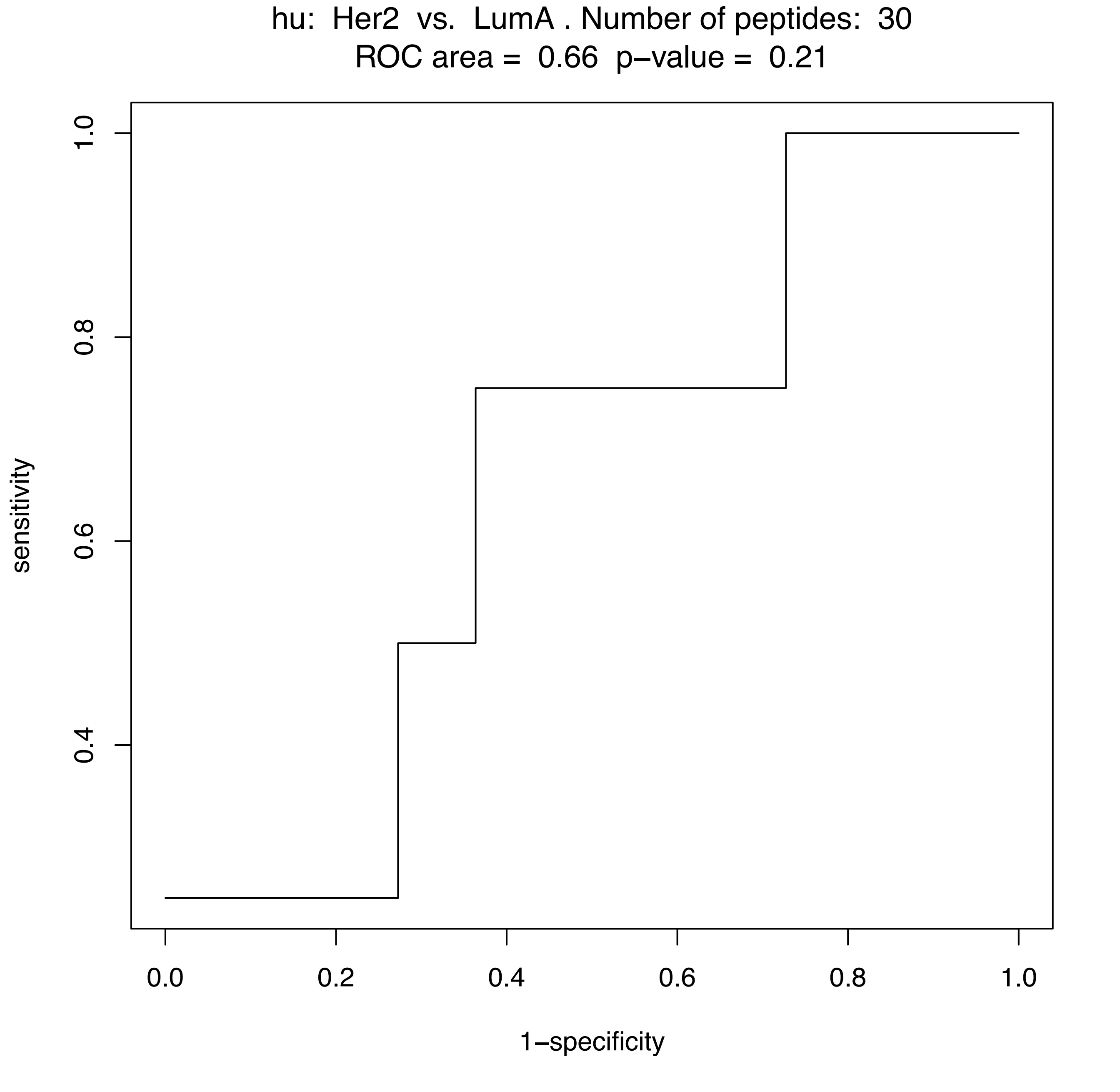
hu: Her2 vs. LumA . Number of peptides: 30
ROC area = 0.66 p−value = 0.21
sensitivity
1−specificity
0.4
0.6
0.8
1.0
0.0
0.2
0.4
0.6
0.8
1.0

hu: Her2 vs. LumA . Number of peptides: 40
ROC area = 0.7 p−value = 0.14

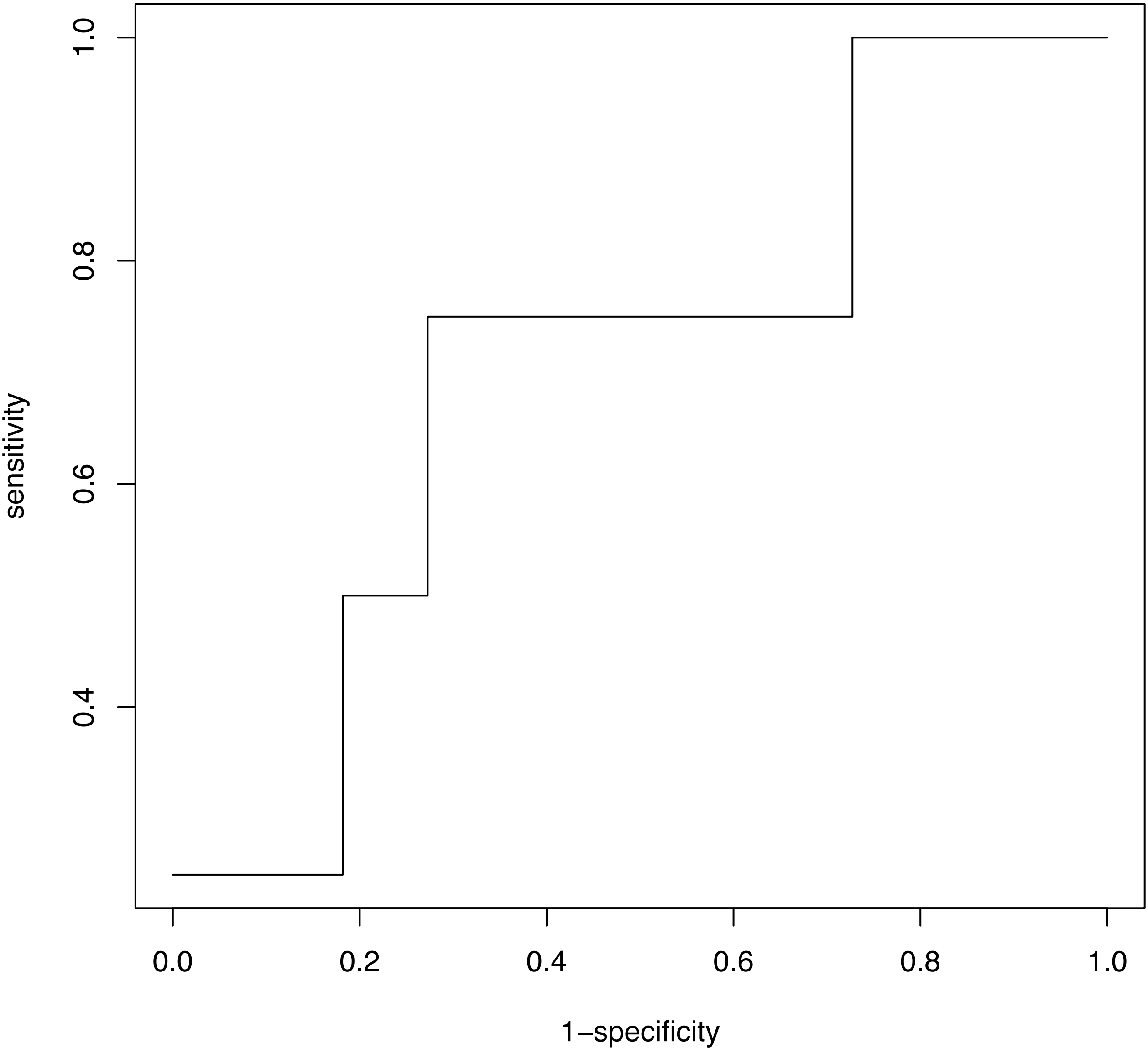

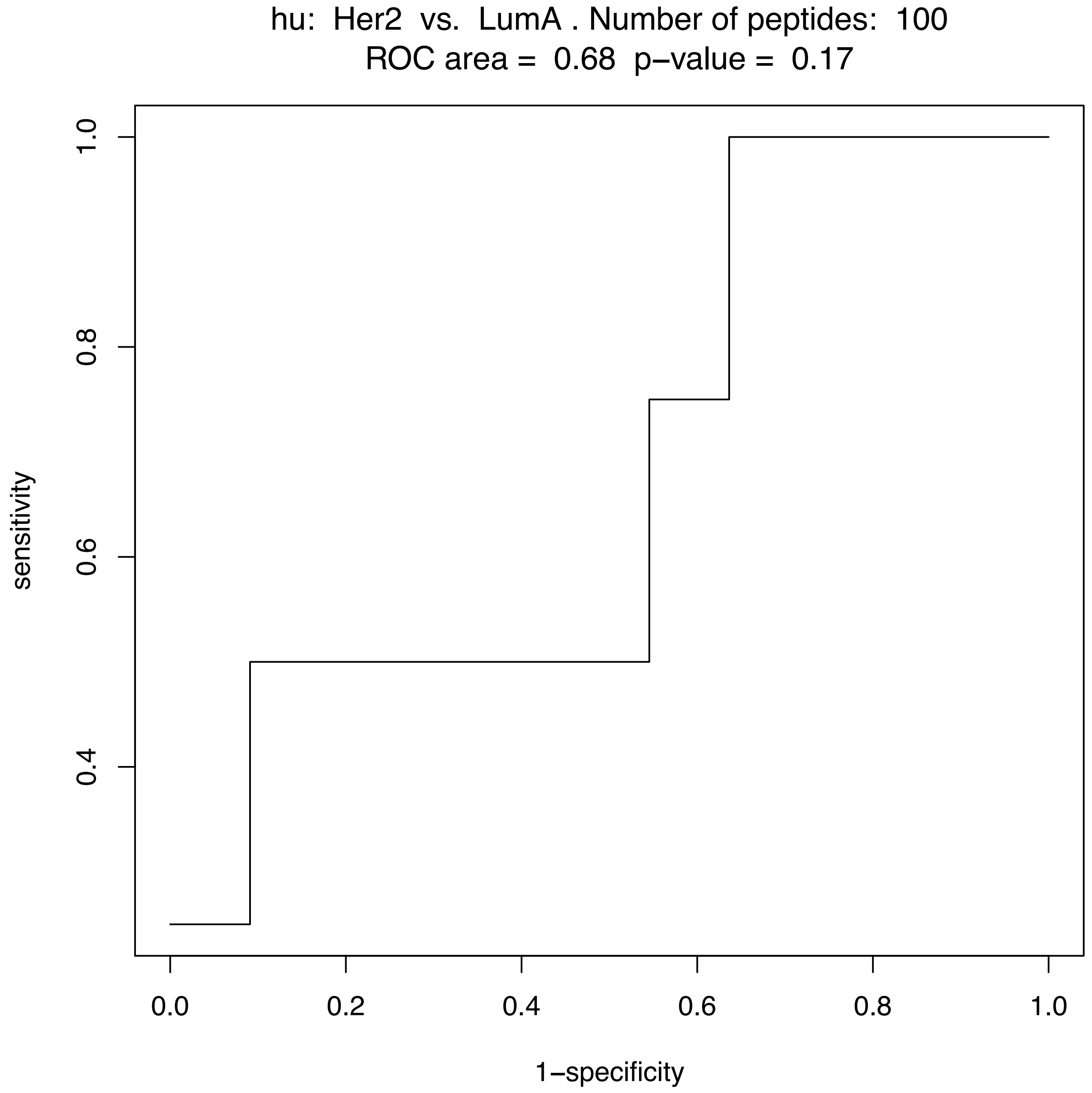
hu: Her2 vs. LumA . Number of peptides: 100
ROC area = 0.68 p−value = 0.17
sensitivity
0.4
0.6
0.8
1.0
0.0
0.2
0.4
0.6
0.8
1.0
1−specificity

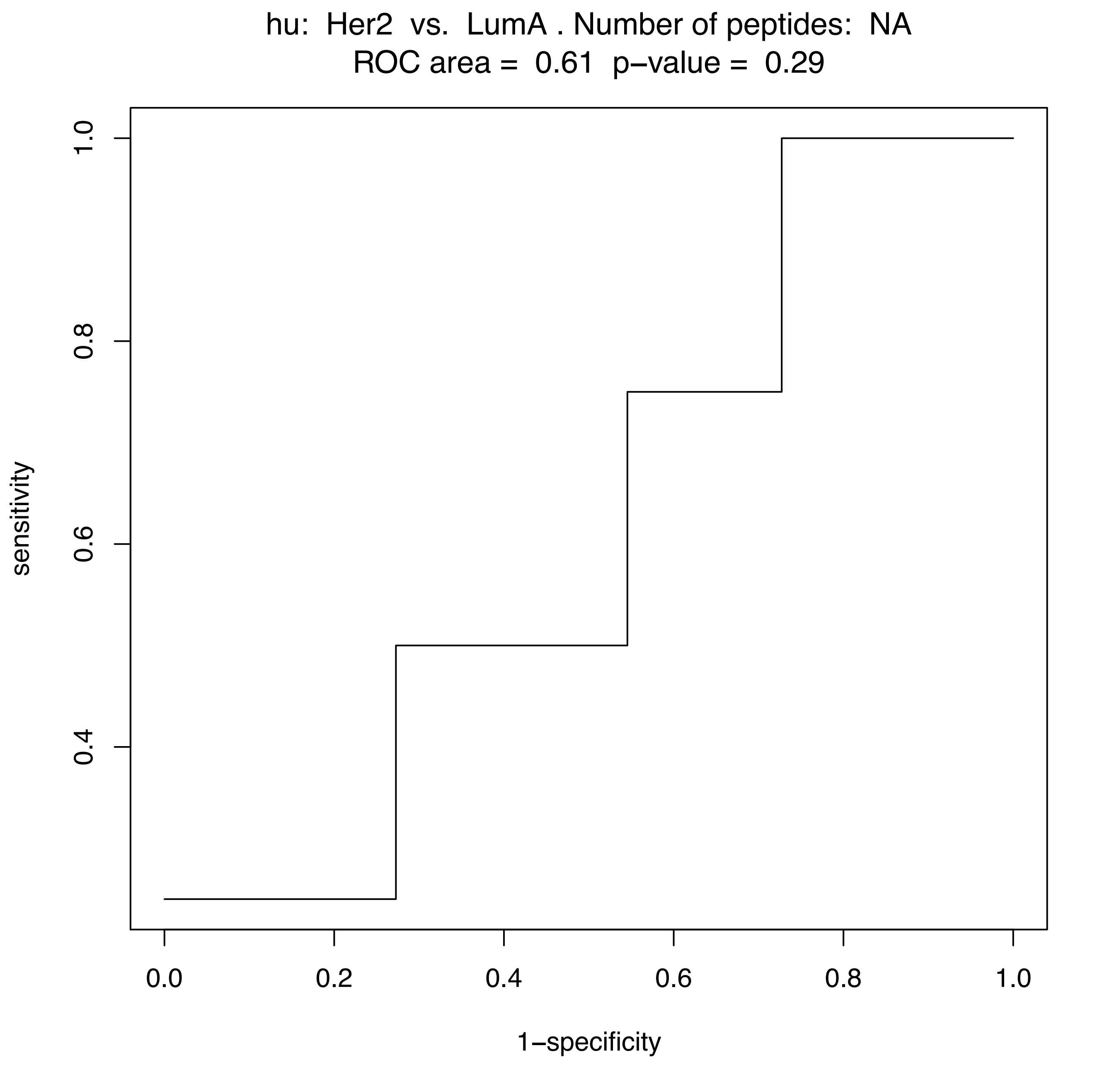
hu: Her2 vs. LumA . Number of peptides: NA
ROC area = 0.61 p−value = 0.29
sensitivity
0.4
0.6
0.8
1.0
0.0
0.2
0.4
0.6
0.8
1.0
1−specificity

sorlie: Her2 vs. LumB . Number of peptides: 20
ROC area = 0.21 p−value = 0.95

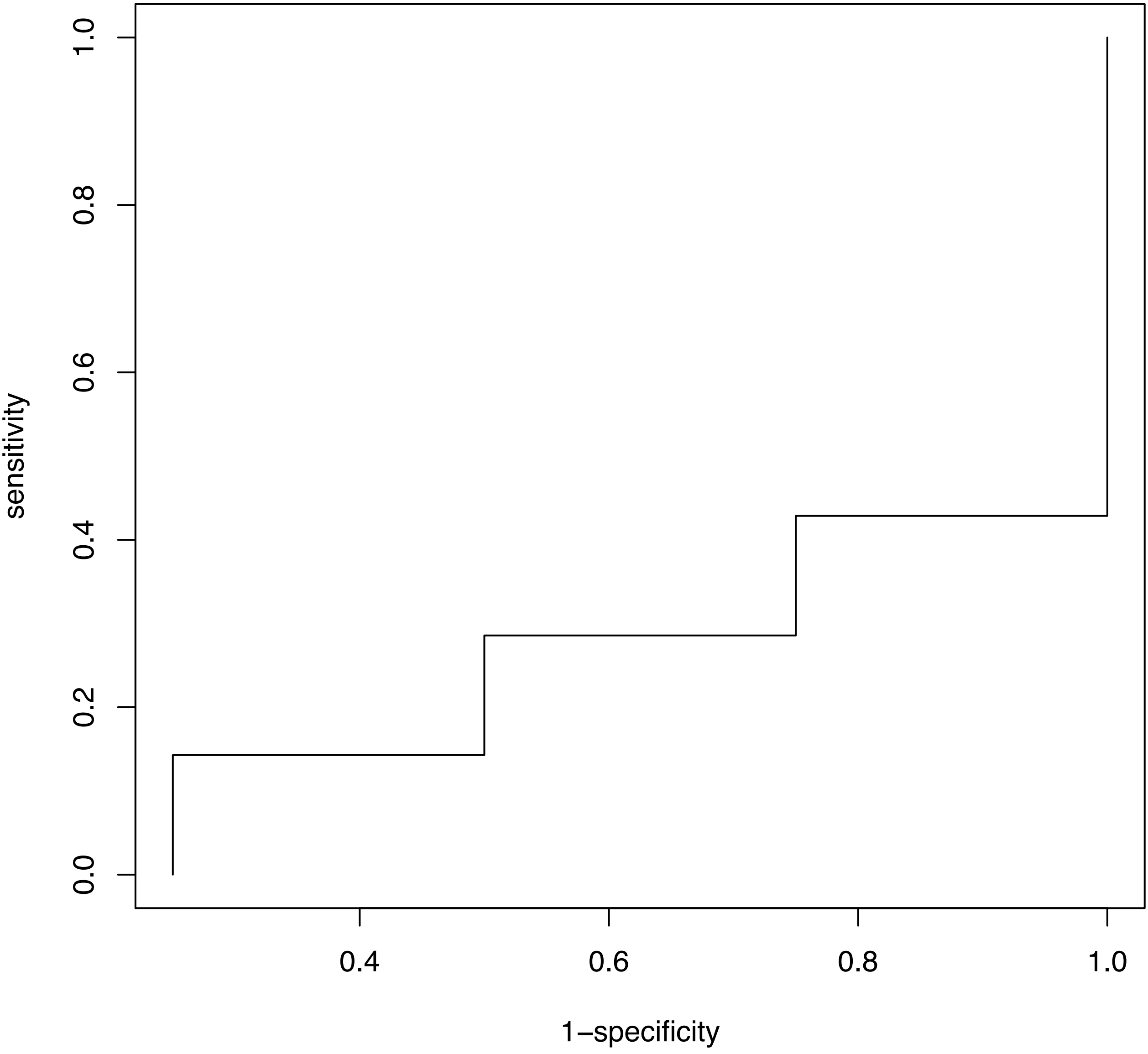

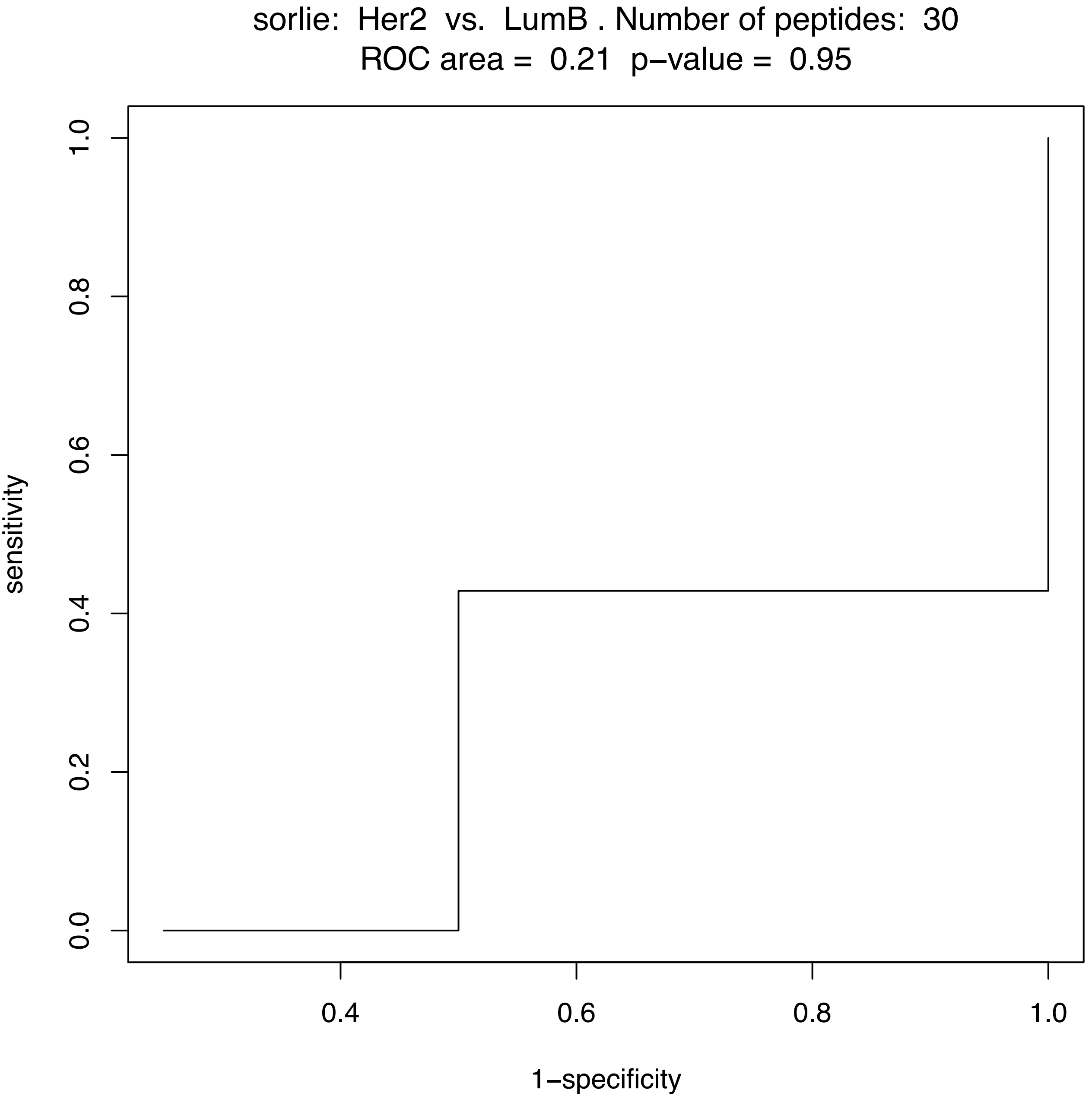
sorlie: Her2 vs. LumB . Number of peptides: 30
ROC area = 0.21 p−value = 0.95
sensitivity
1−specificity
0.0
0.2
0.4
0.6
0.8
1.0
0.4
0.6
0.8
1.0

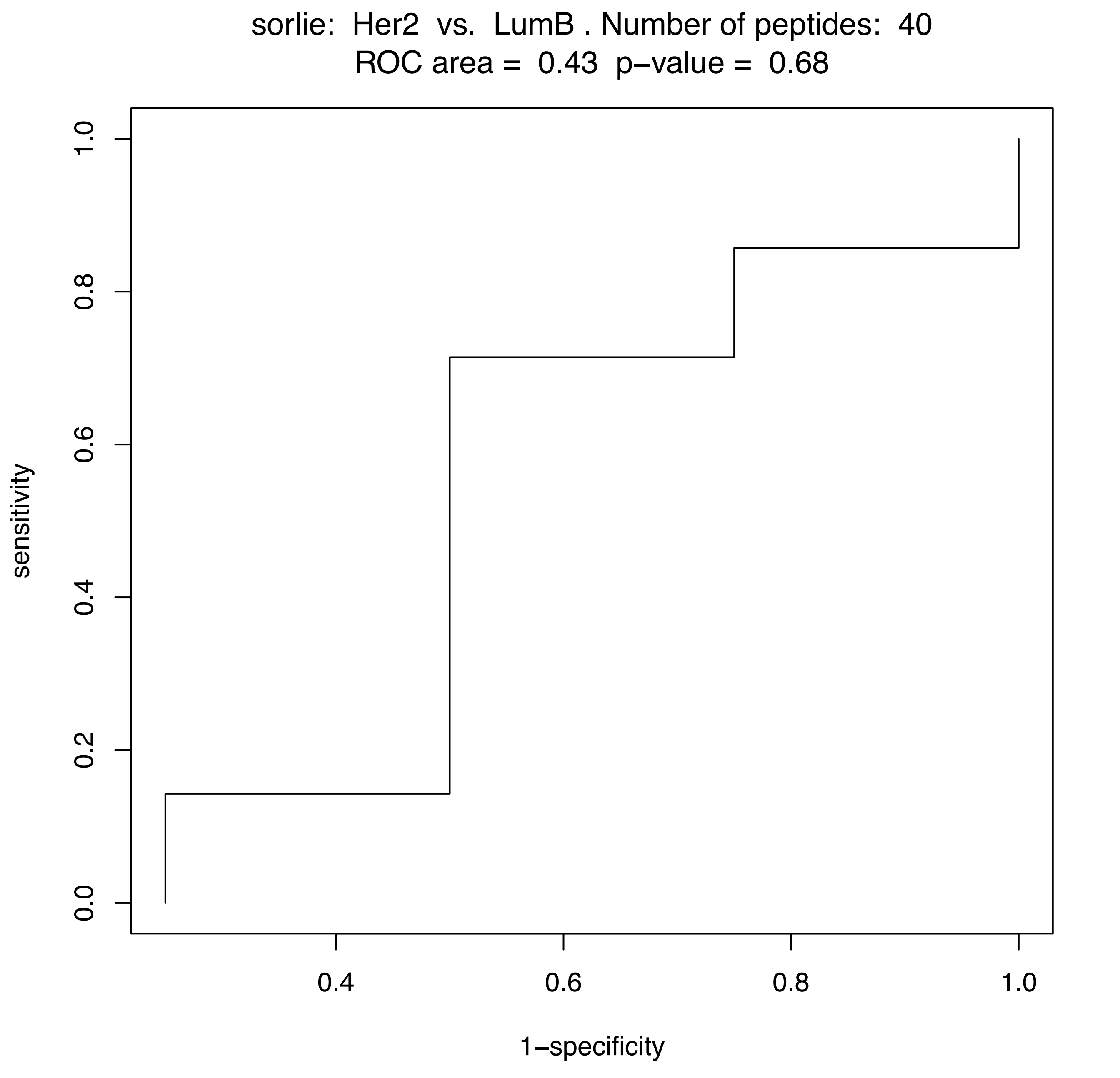

sorlie: Her2 vs. LumB . Number of peptides: 40
ROC area = 0.43 p−value = 0.68
1.0
0.8
0.6
0.4
0.2
0.0
sensitivity
0.4
0.6
0.8
1.0
1−specificity

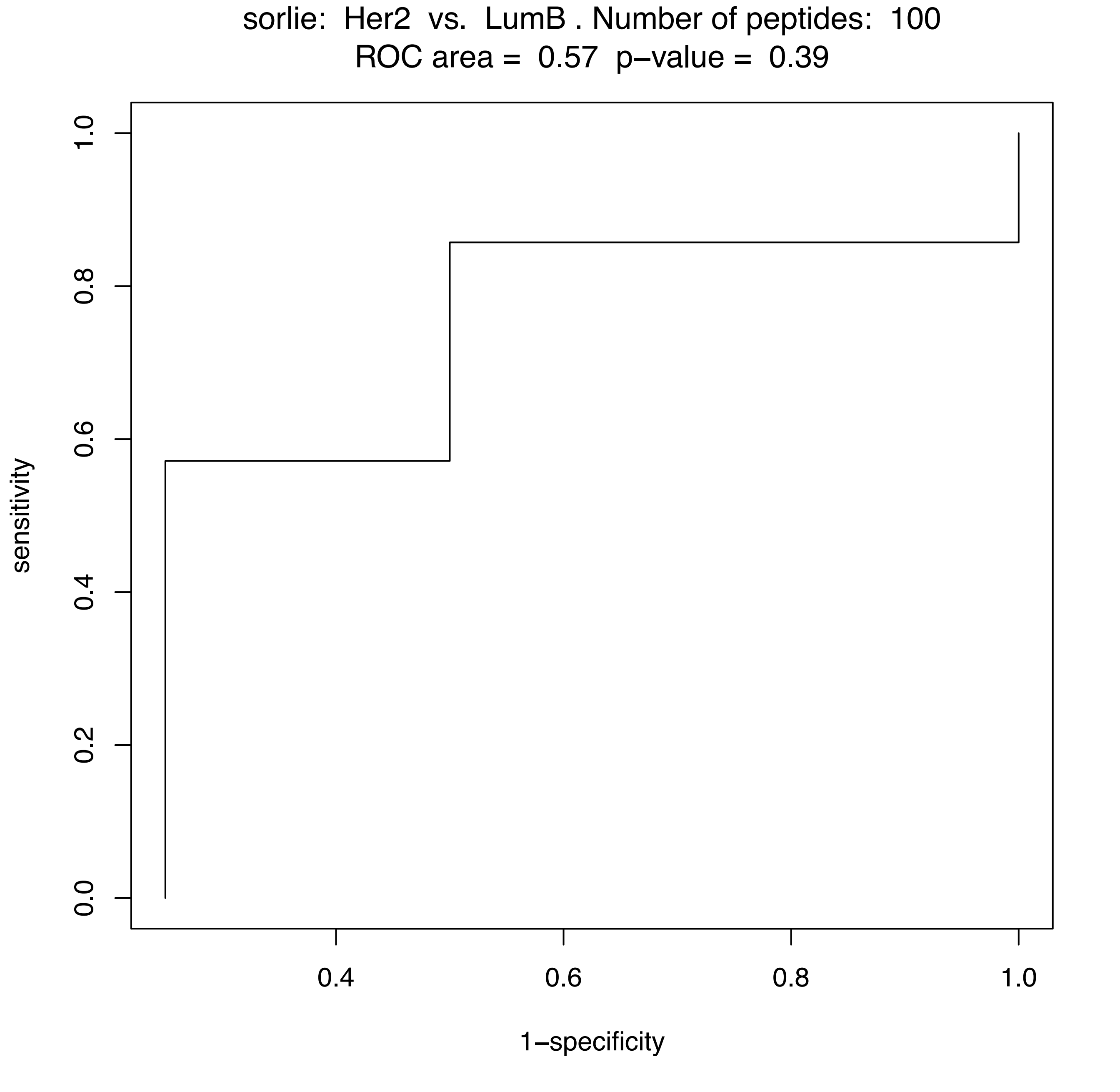

sorlie: Her2 vs. LumB . Number of peptides: 100
ROC area = 0.57 p−value = 0.39
sensitivity
1−specificity
0.0
0.2
0.4
0.6
0.8
1.0
0.4
0.6
0.8
1.0

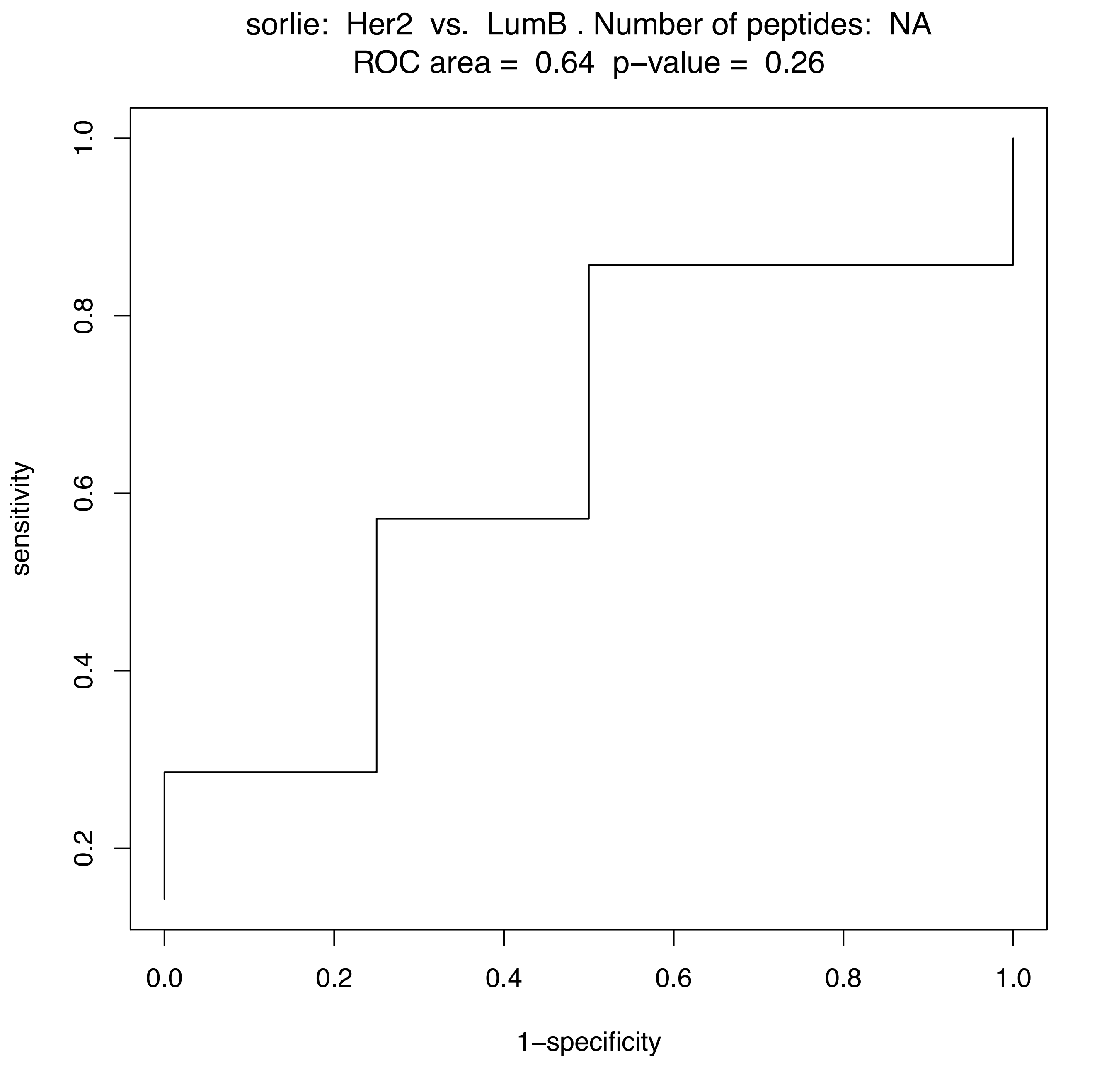
sorlie: Her2 vs. LumB . Number of peptides: NA
ROC area = 0.64 p−value = 0.26
sensitivity
1.0
0.8
0.6
0.4
0.2
0.0
0.2
0.4
0.6
0.8
1.0
1−specificity

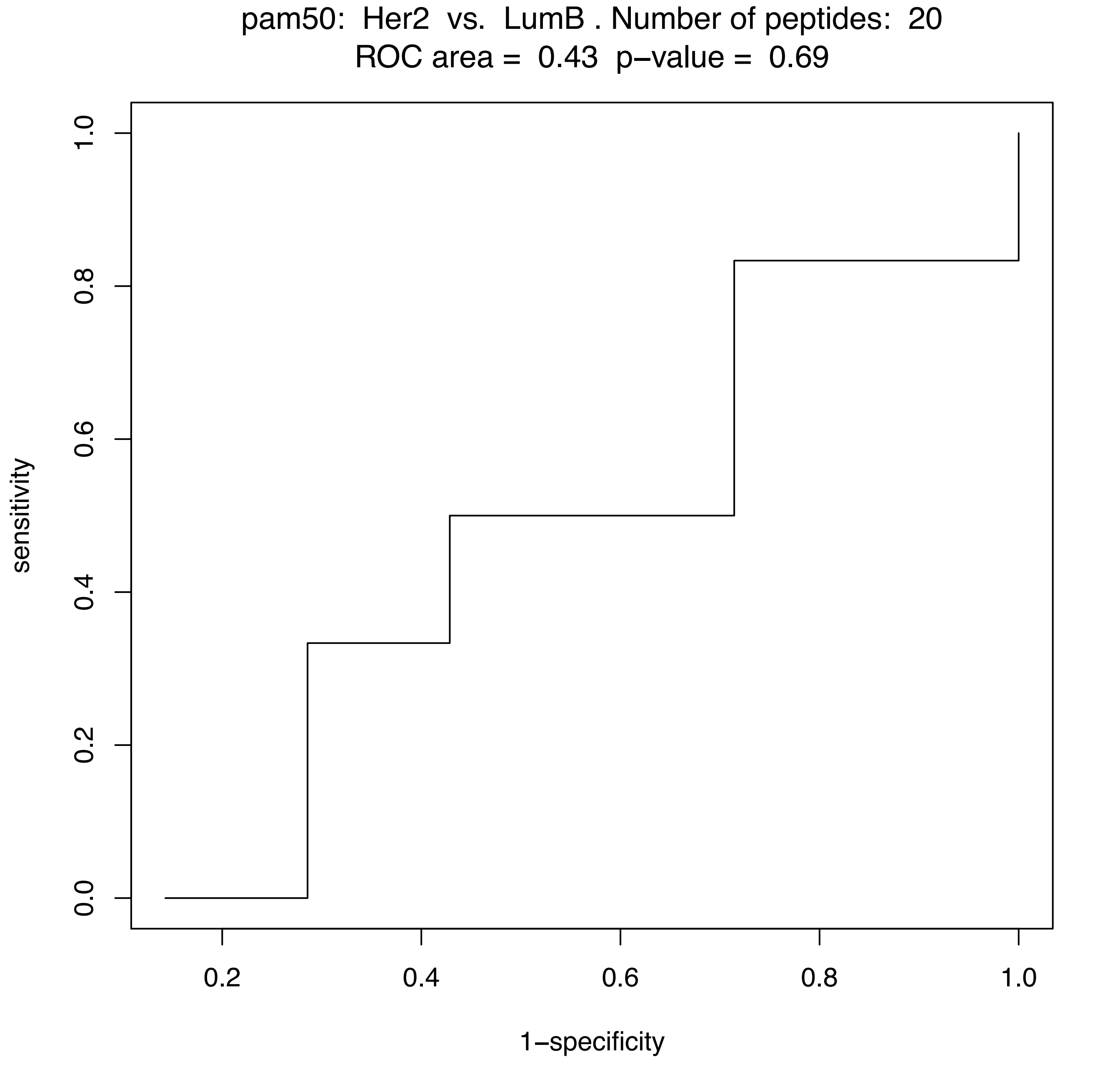
pam50: Her2 vs. LumB . Number of peptides: 20
ROC area = 0.43 p−value = 0.69
sensitivity
1−specificity
0.0
0.2
0.4
0.6
0.8
1.0
0.2
0.4
0.6
0.8
1.0

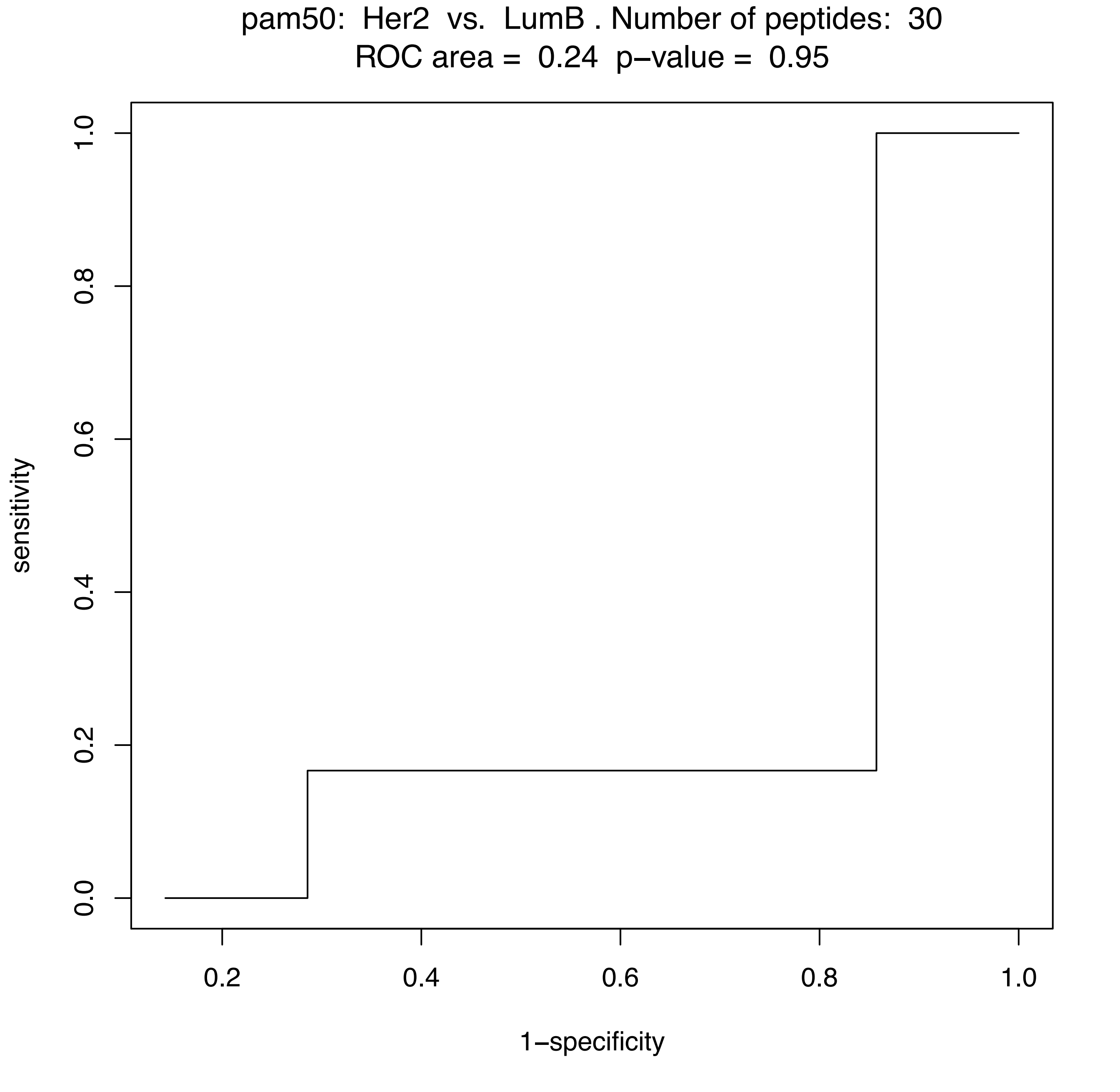
pam50: Her2 vs. LumB . Number of peptides: 30
ROC area = 0.24 p−value = 0.95
sensitivity
1−specificity
0.0
0.2
0.4
0.6
0.8
1.0
0.2
0.4
0.6
0.8
1.0

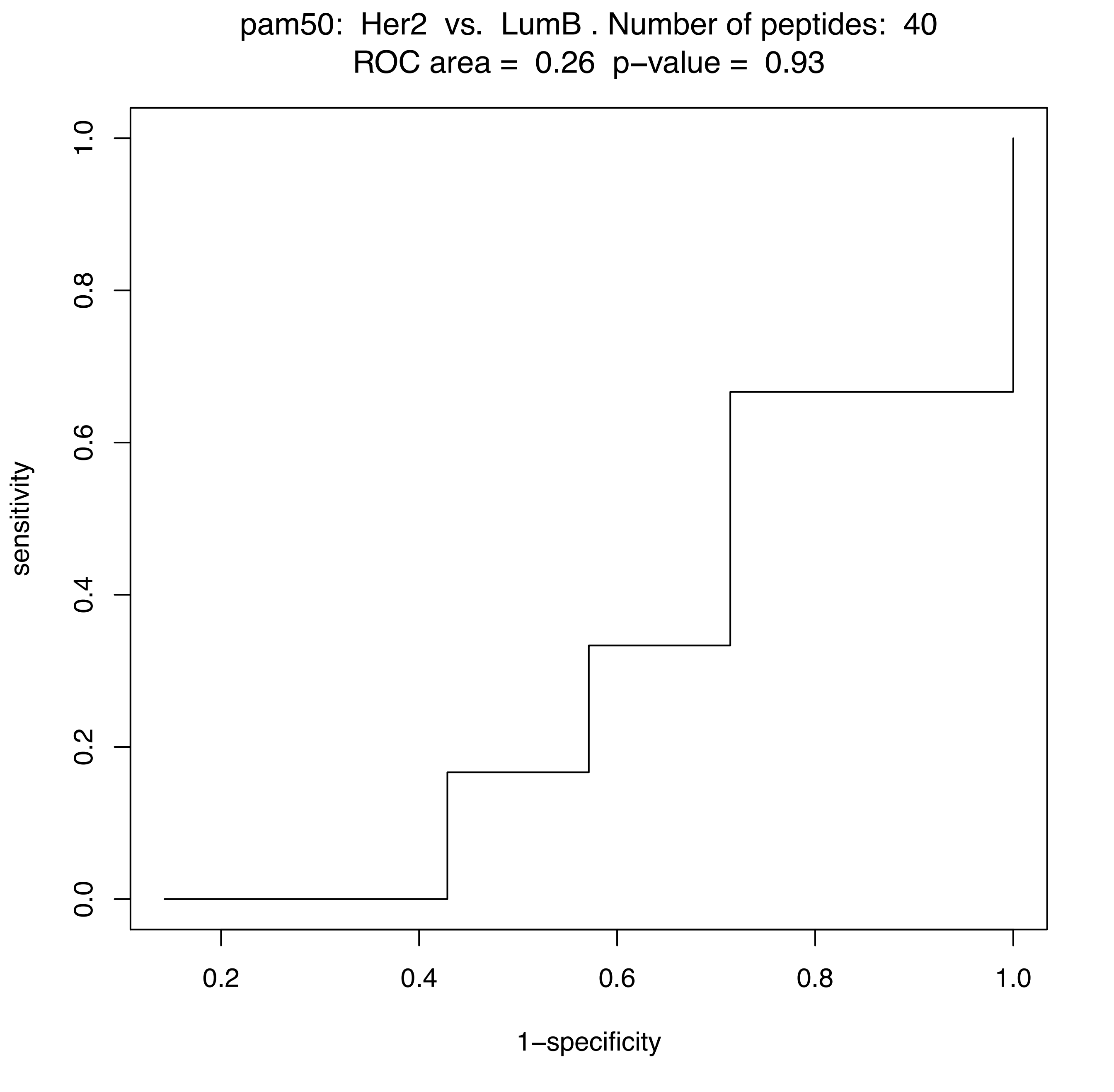
pam50: Her2 vs. LumB . Number of peptides: 40
ROC area = 0.26 p−value = 0.93
sensitivity
1−specificity
0.0
0.2
0.4
0.6
0.8
1.0
0.2
0.4
0.6
0.8
1.0

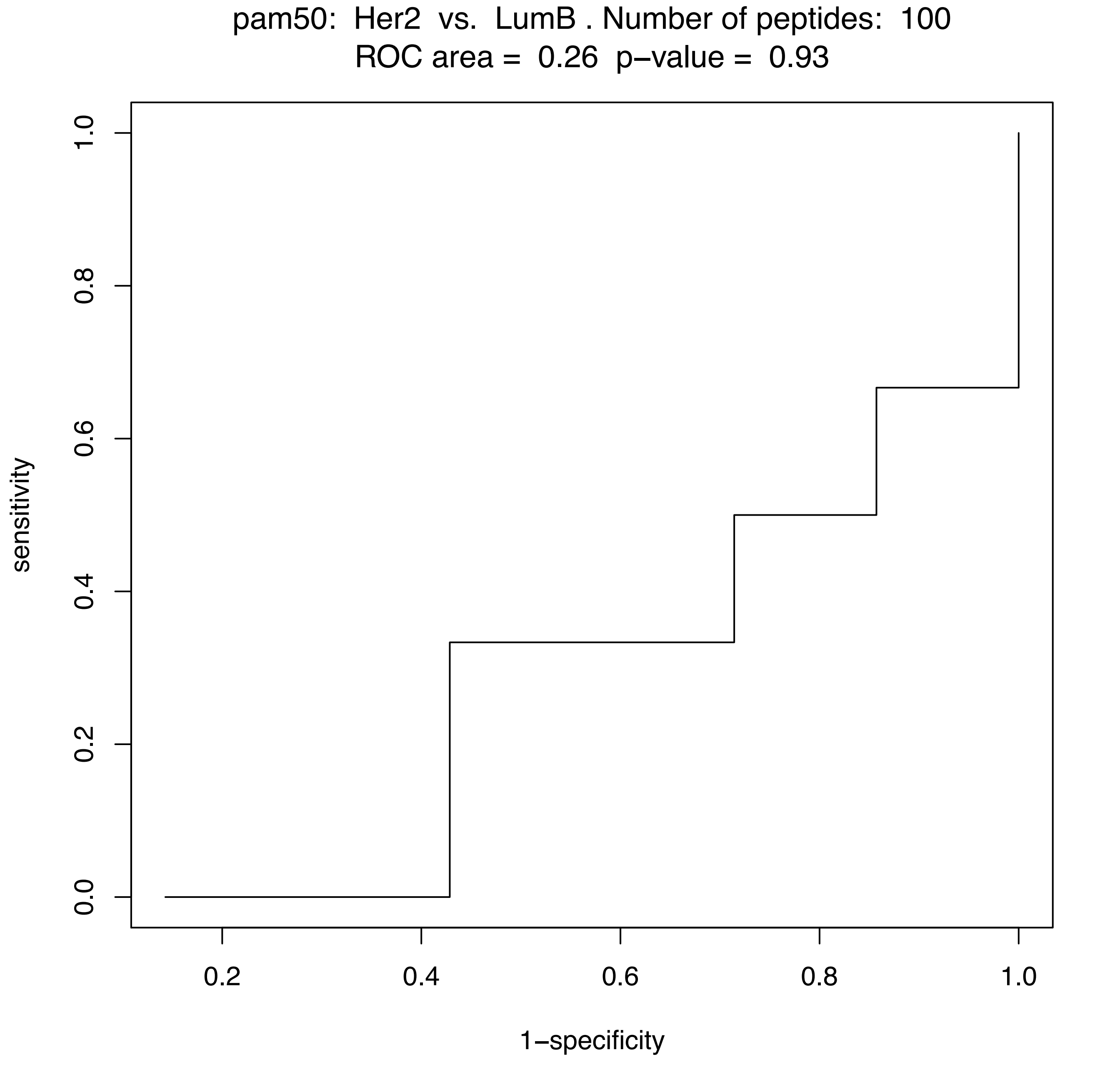
pam50: Her2 vs. LumB . Number of peptides: 100
ROC area = 0.26 p−value = 0.93
sensitivity
1−specificity
0.0
0.2
0.4
0.6
0.8
1.0
0.2
0.4
0.6
0.8
1.0

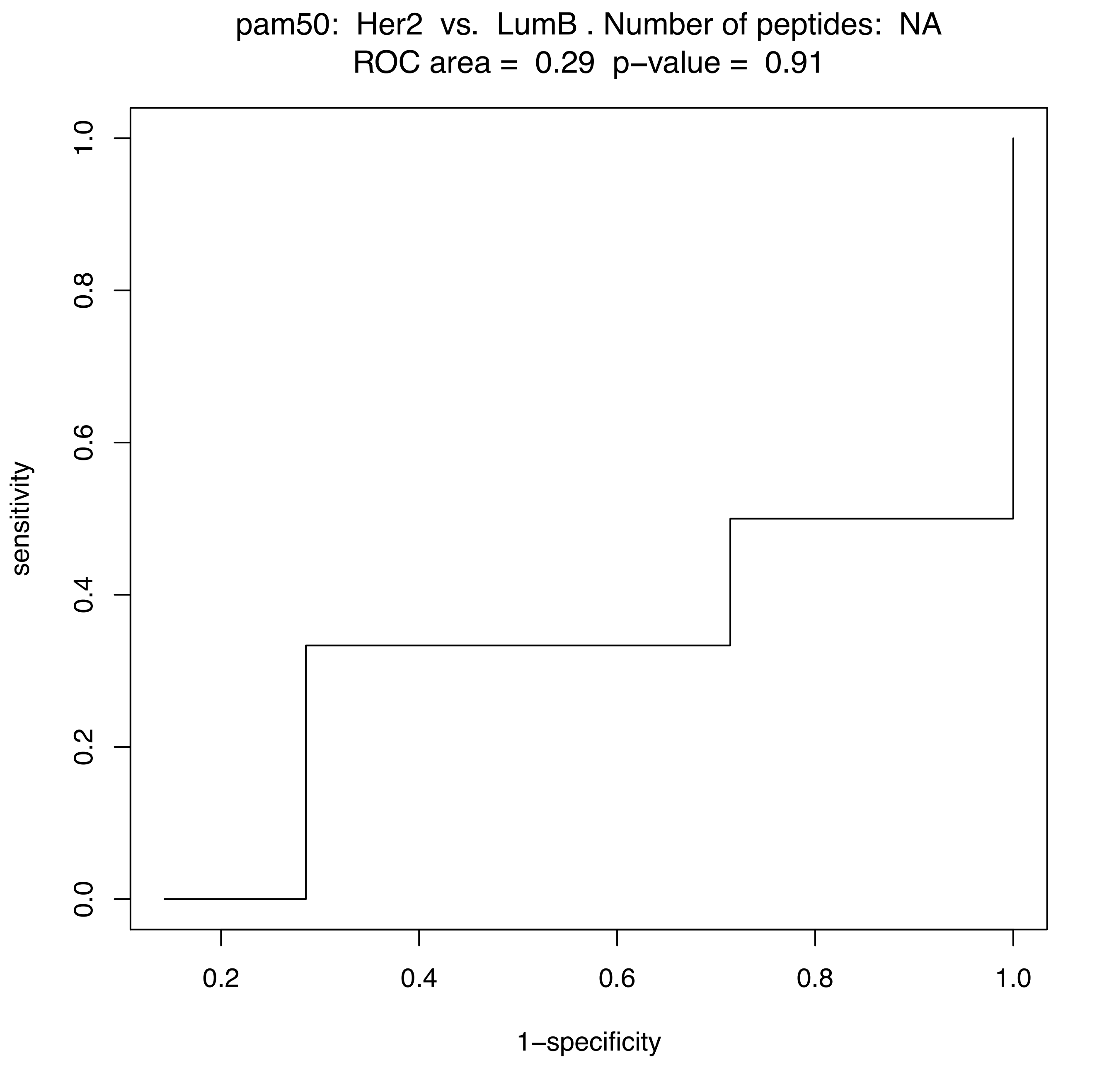
pam50: Her2 vs. LumB . Number of peptides: NA
ROC area = 0.29 p−value = 0.91
sensitivity
1−specificity
0.0
0.2
0.4
0.6
0.8
1.0
0.2
0.4
0.6
0.8
1.0

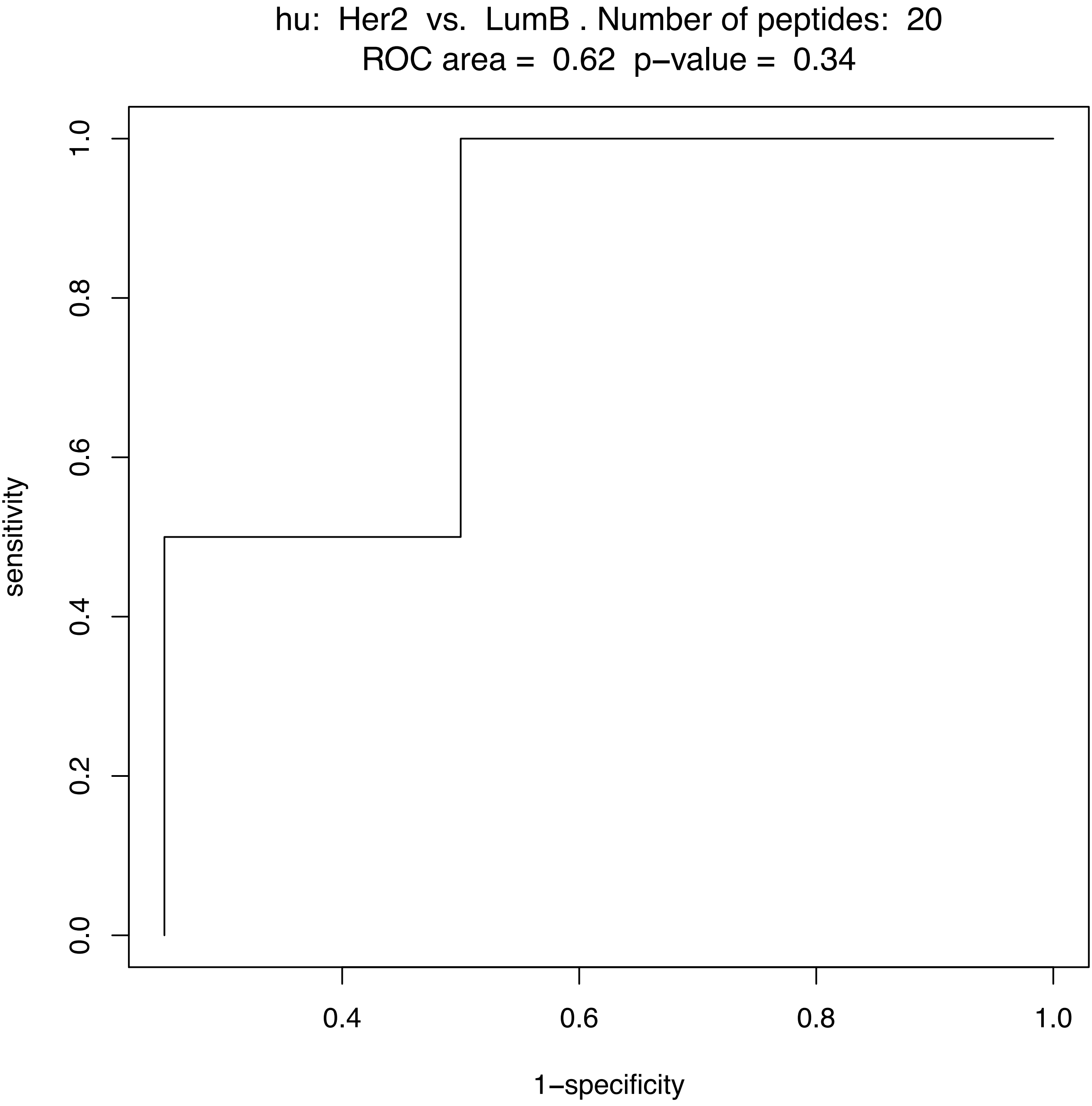

hu: Her2 vs. LumB . Number of peptides: 20
ROC area = 0.62 p−value = 0.34
sensitivity
0.0
0.2
0.4
0.6
0.8
1.0
0.4
0.6
0.8
1.0
1−specificity

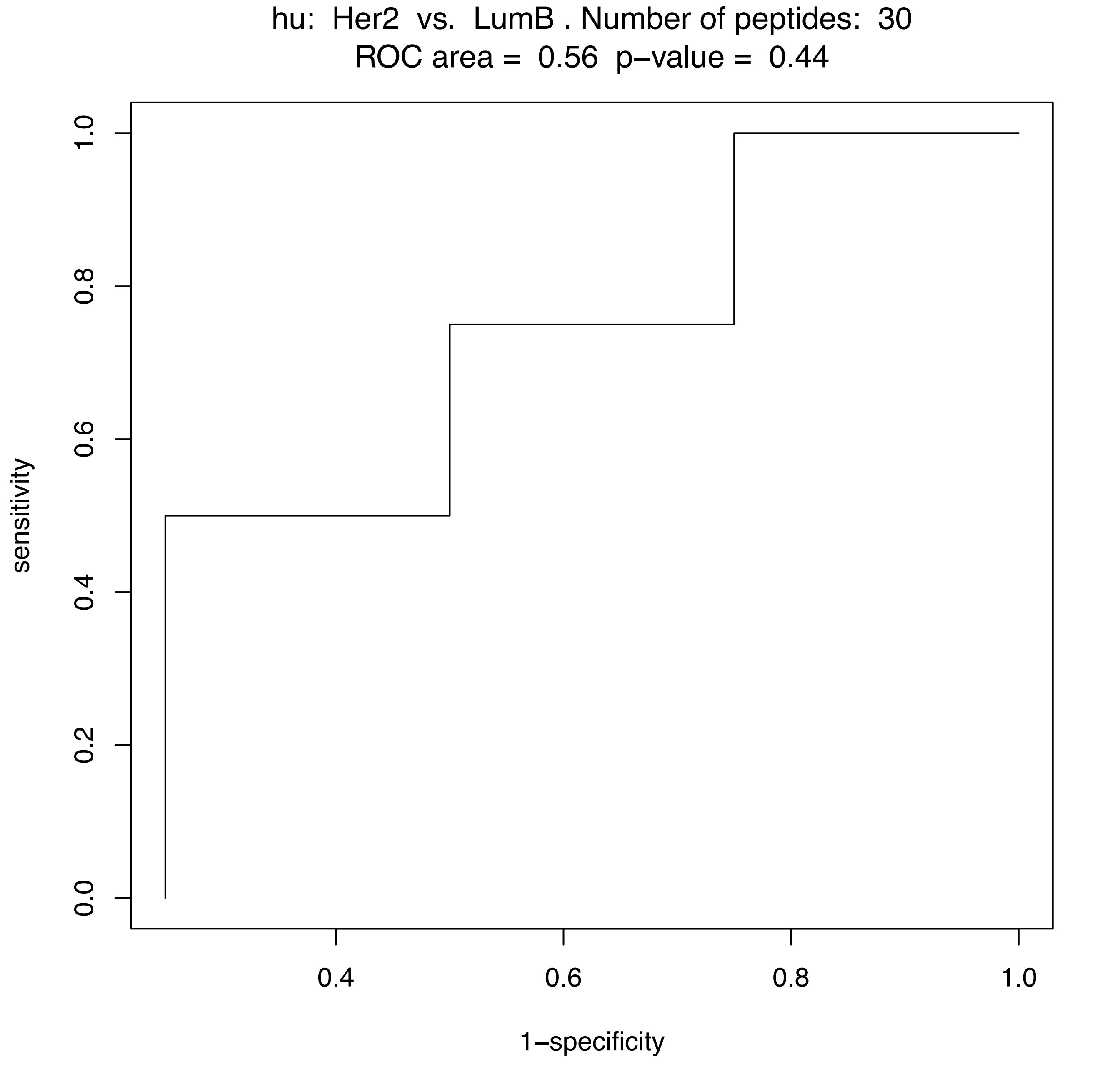
hu: Her2 vs. LumB . Number of peptides: 30
ROC area = 0.56 p−value = 0.44
sensitivity
0.0
0.2
0.4
0.6
0.8
1.0
0.4
0.6
0.8
1.0
1−specificity

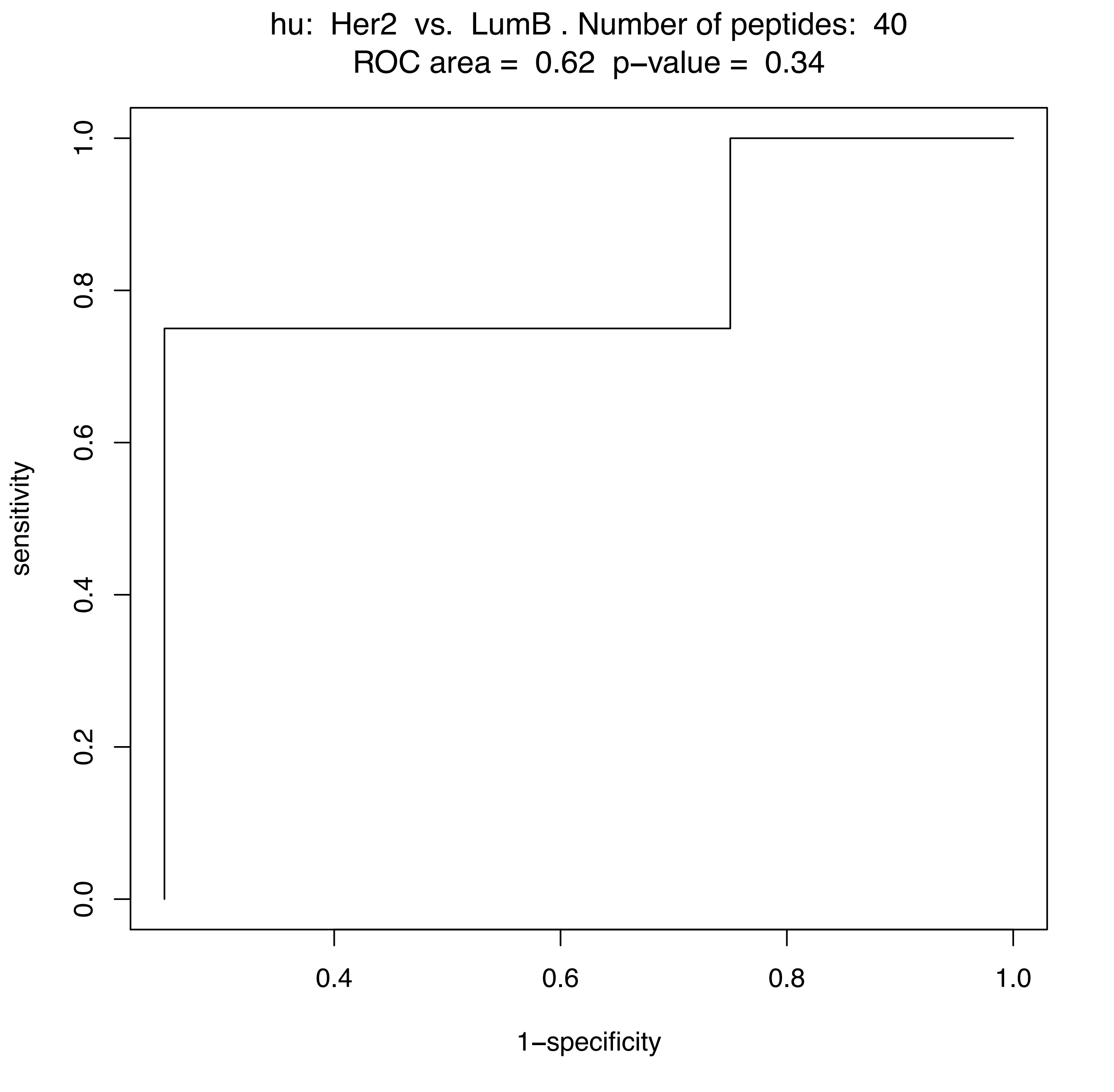
hu: Her2 vs. LumB . Number of peptides: 40
ROC area = 0.62 p−value = 0.34
sensitivity
0.0
0.2
0.4
0.6
0.8
1.0
0.4
0.6
0.8
1.0
1−specificity

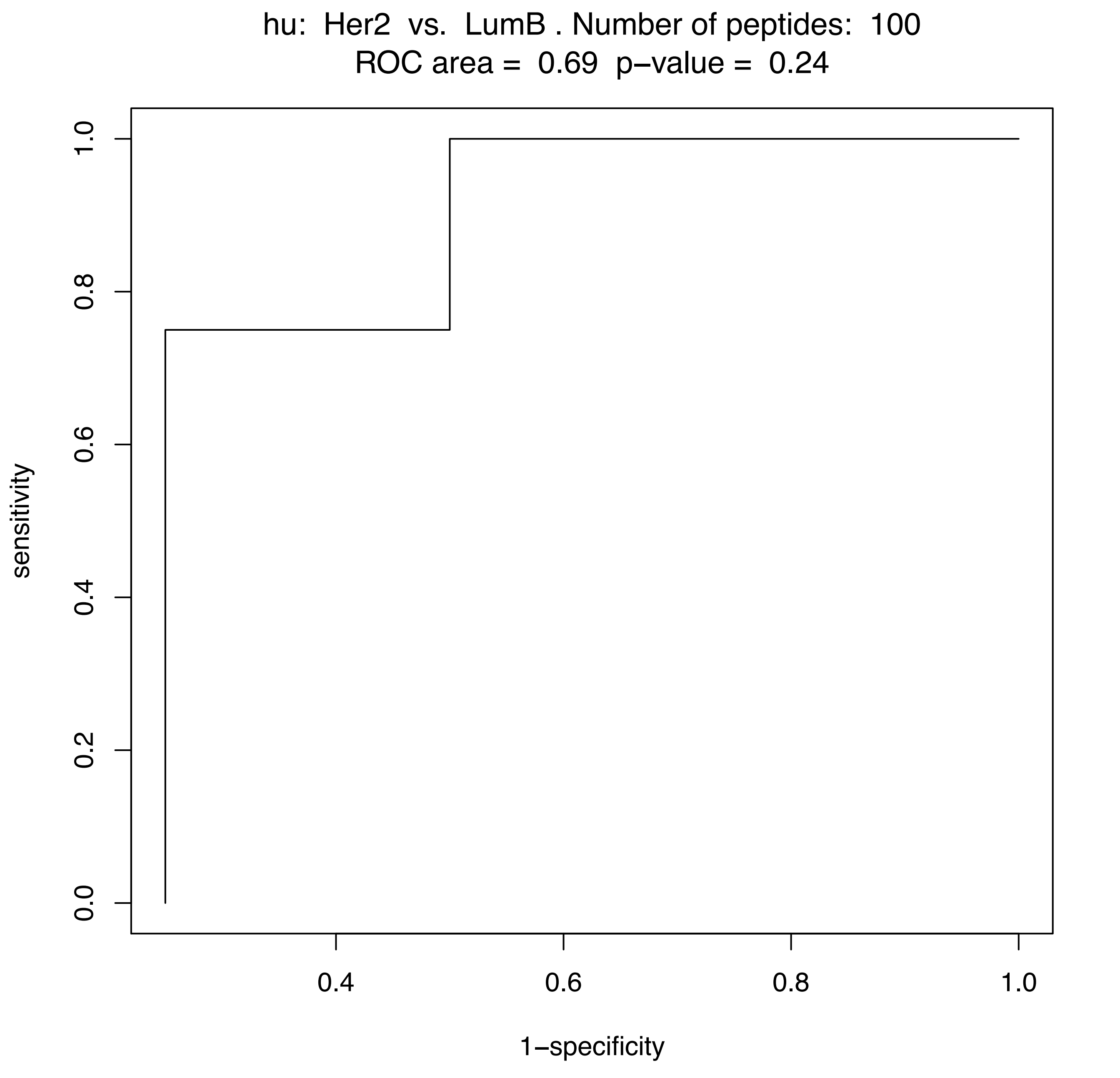
hu: Her2 vs. LumB . Number of peptides: 100
ROC area = 0.69 p−value = 0.24
sensitivity
1−specificity
0.0
0.2
0.4
0.6
0.8
1.0
0.4
0.6
0.8
1.0

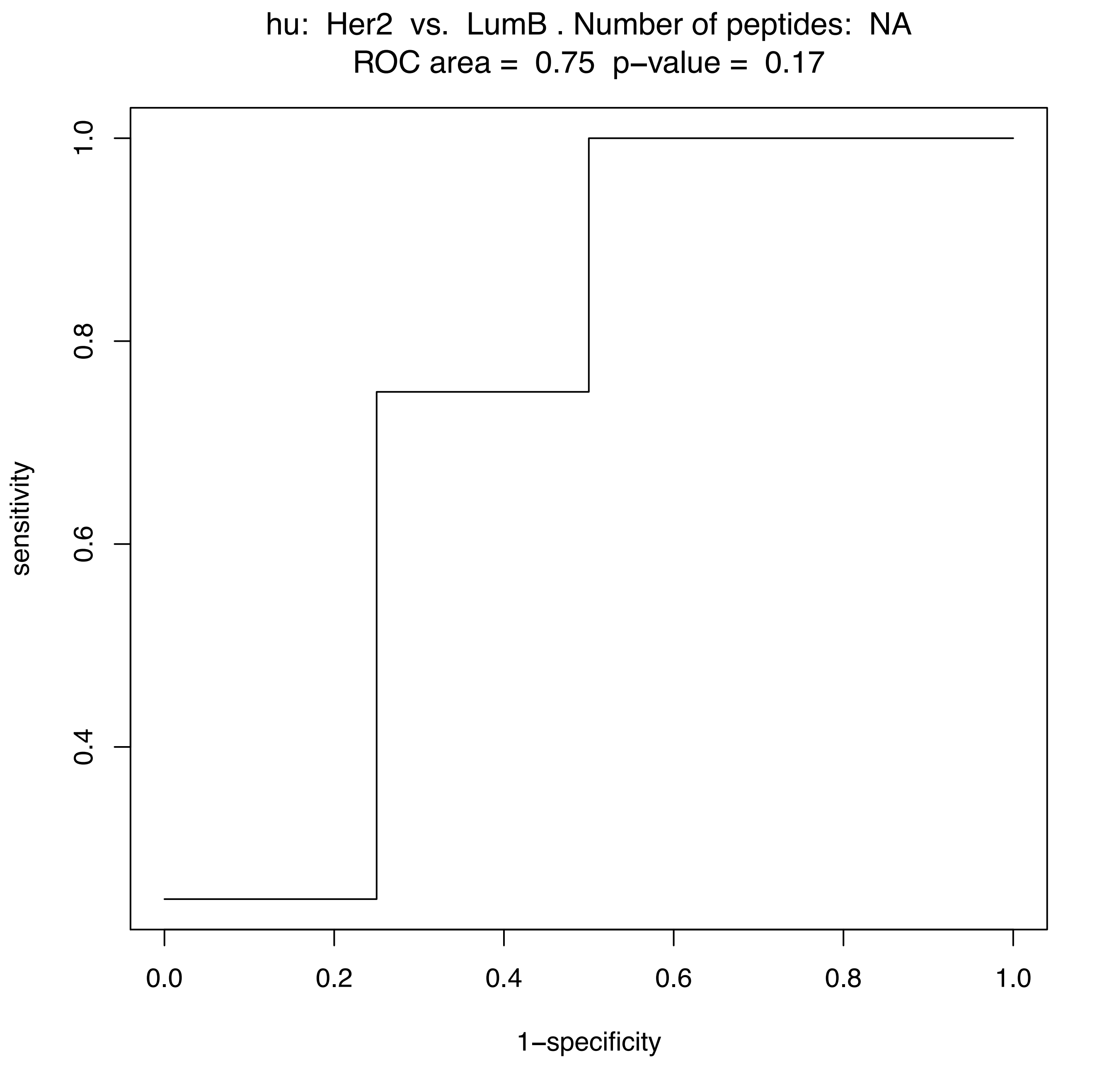
hu: Her2 vs. LumB . Number of peptides: NA
ROC area = 0.75 p−value = 0.17
sensitivity
0.4
0.6
0.8
1.0
0.0
0.2
0.4
0.6
0.8
1.0
1−specificity

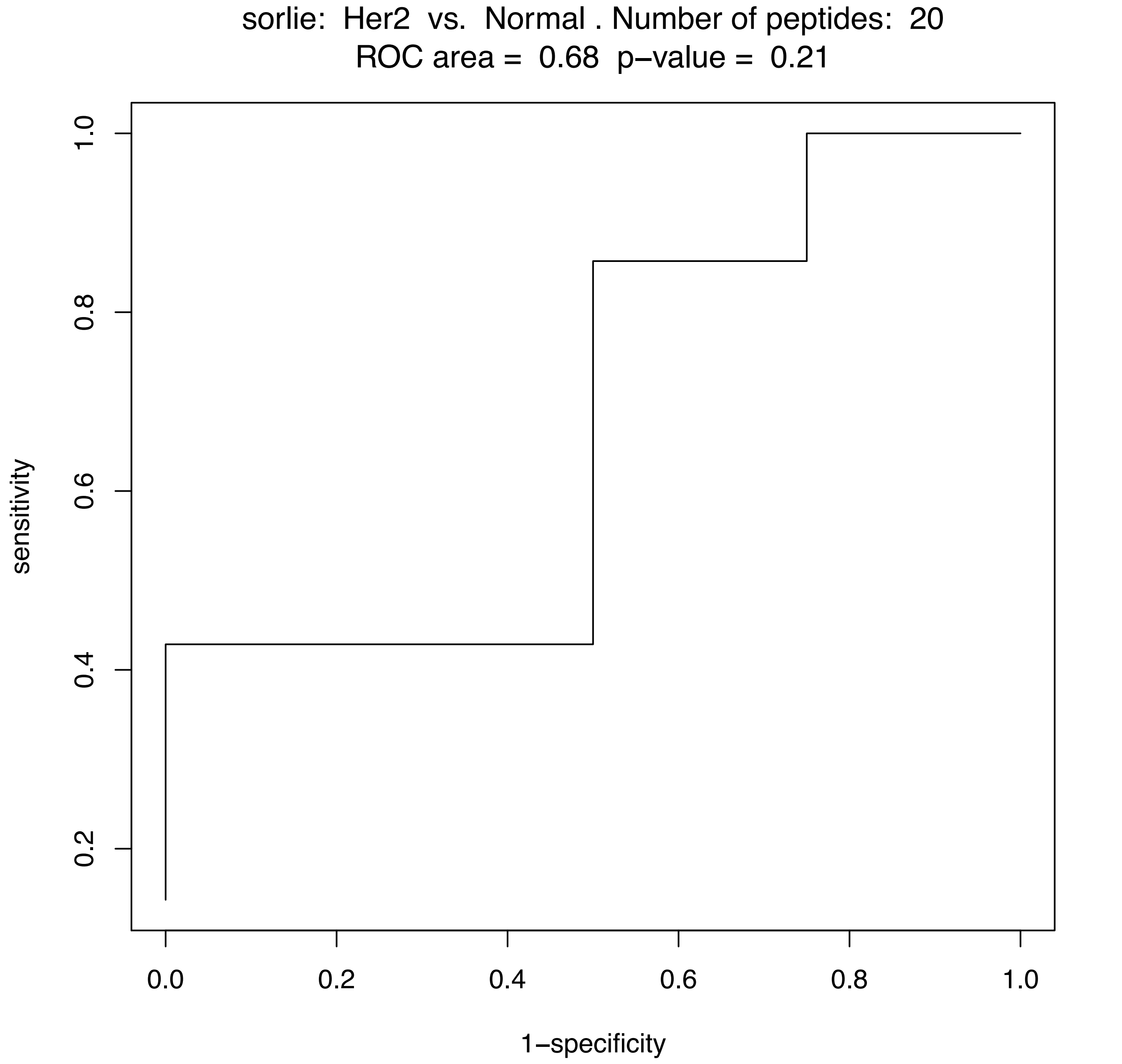
sorlie: Her2 vs. Normal . Number of peptides: 20
ROC area = 0.68 p−value = 0.21
sensitivity
0.2
0.4
0.6
0.8
1.0
0.0
0.2
0.4
0.6
0.8
1.0
1−specificity

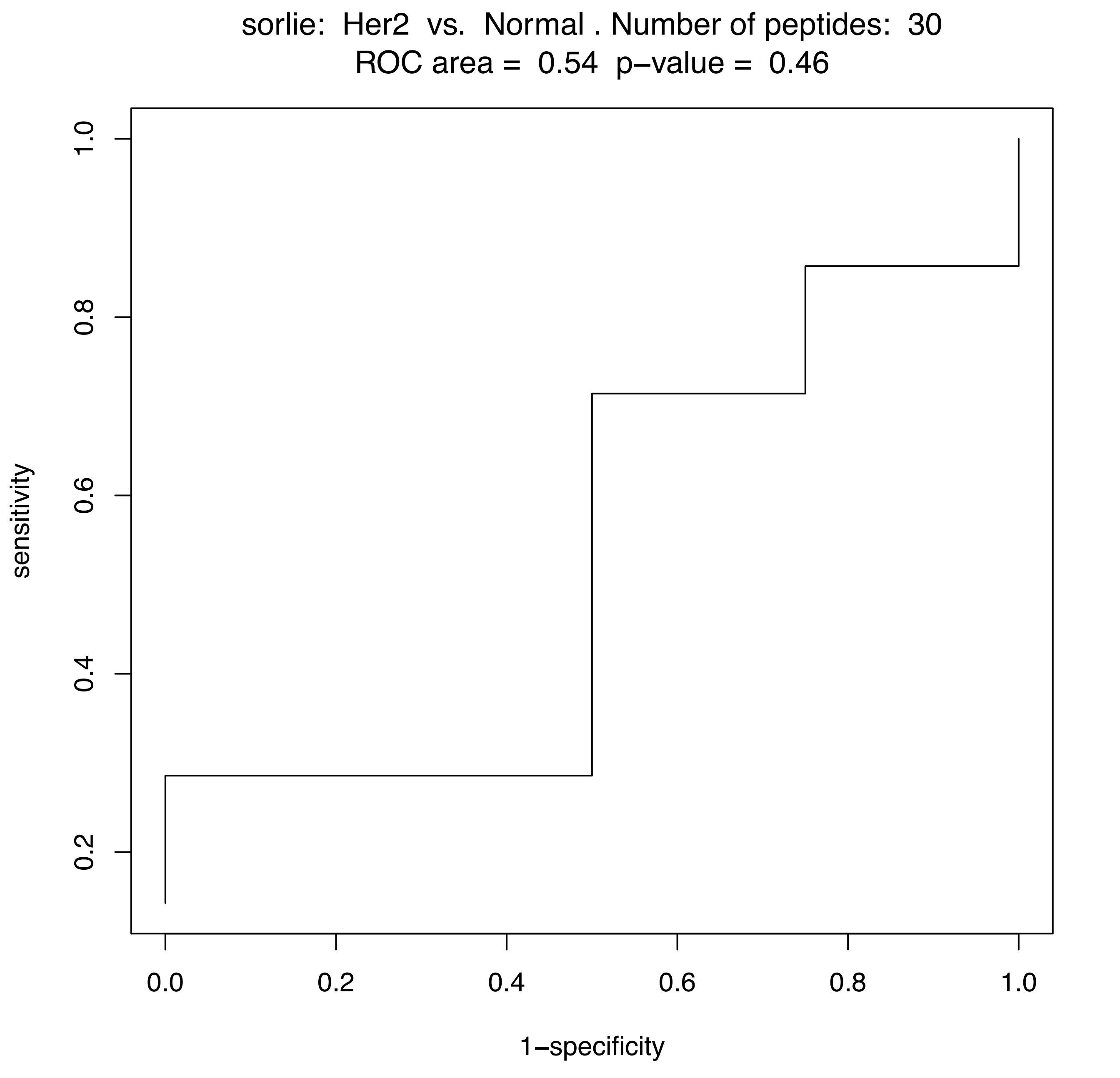
sorlie: Her2 vs. Normal . Number of peptides: 30
ROC area = 0.54 p−value = 0.46
sensitivity
0.2
0.4
0.6
0.8
1.0
0.0
0.2
0.4
0.6
0.8
1.0
1−specificity

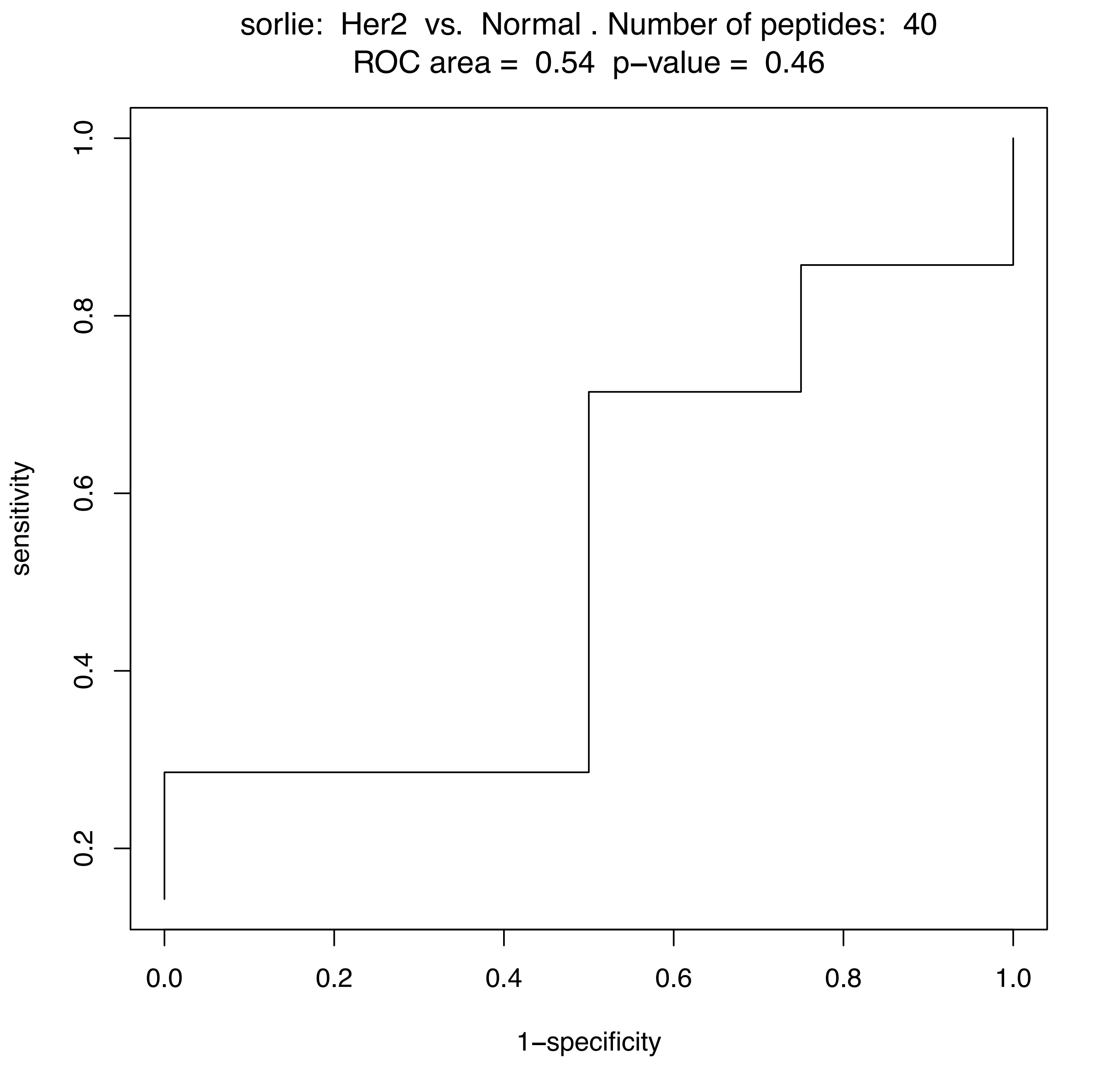
sorlie: Her2 vs. Normal . Number of peptides: 40
ROC area = 0.54 p−value = 0.46
sensitivity
0.2
0.4
0.6
0.8
1.0
0.0
0.2
0.4
0.6
0.8
1.0
1−specificity

sorlie: Her2 vs. Normal . Number of peptides: 100
ROC area = 0.43 p−value = 0.68

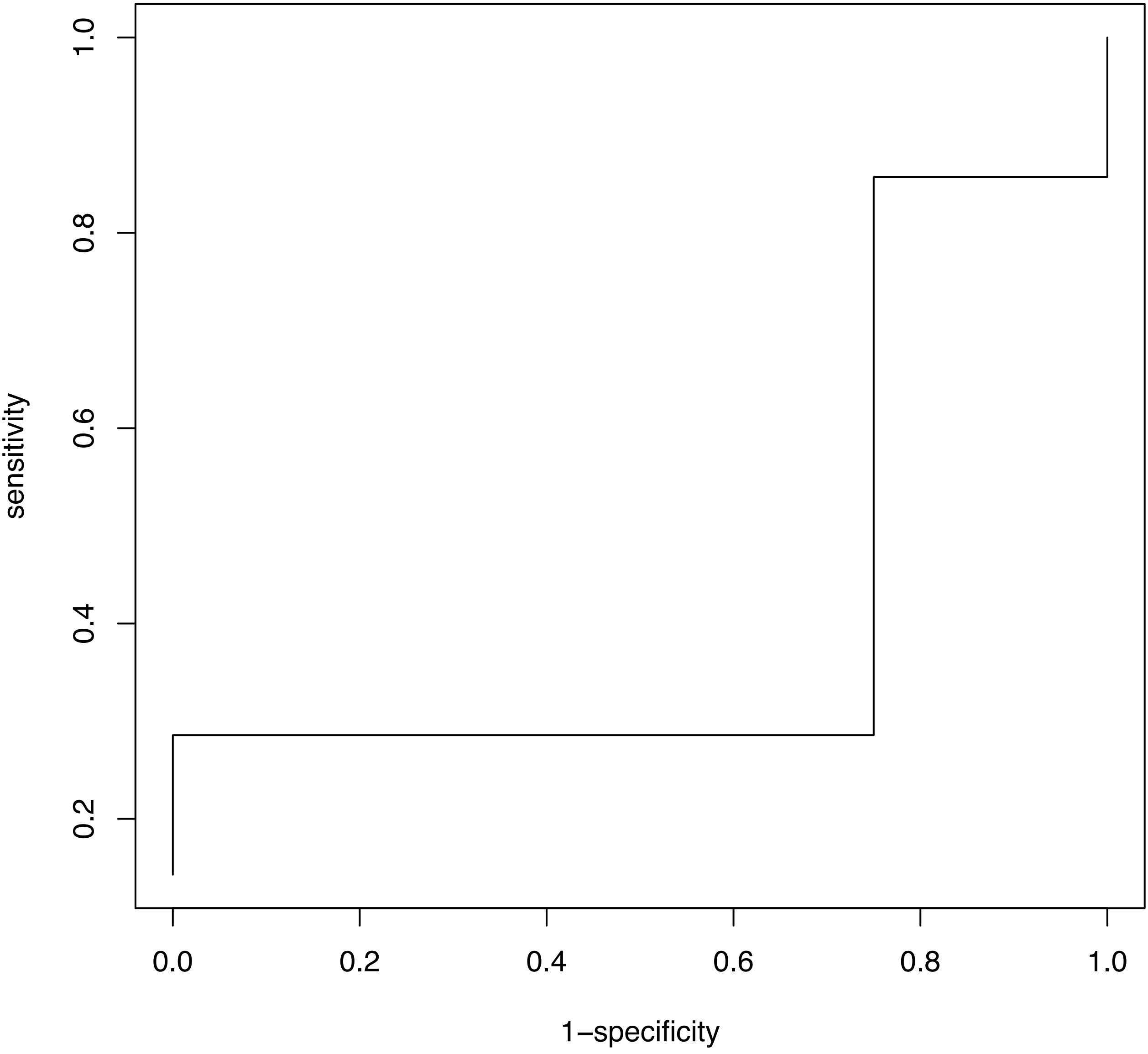

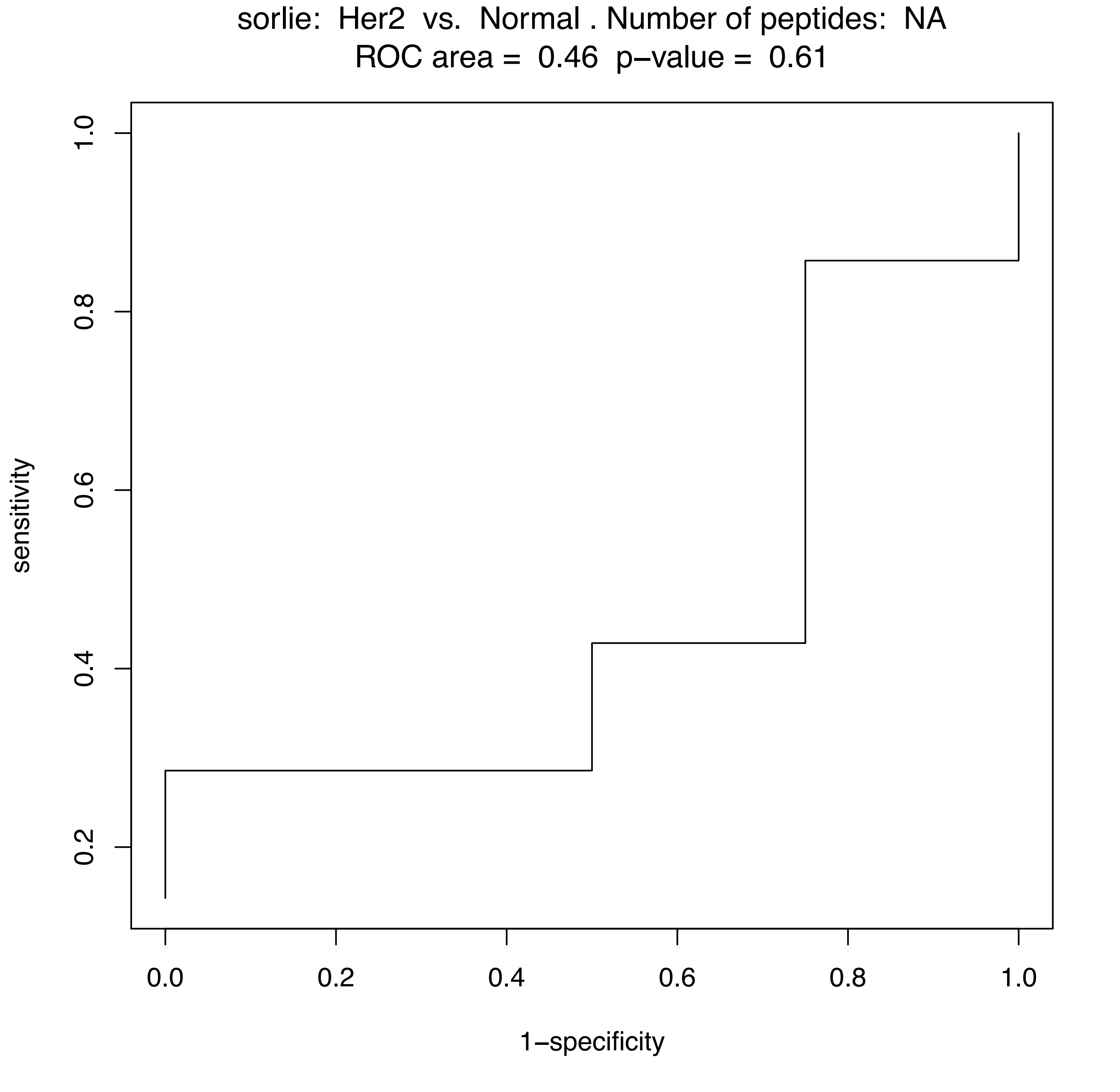
sorlie: Her2 vs. Normal . Number of peptides: NA
ROC area = 0.46 p−value = 0.61
sensitivity
1−specificity
0.2
0.4
0.6
0.8
1.0
0.0

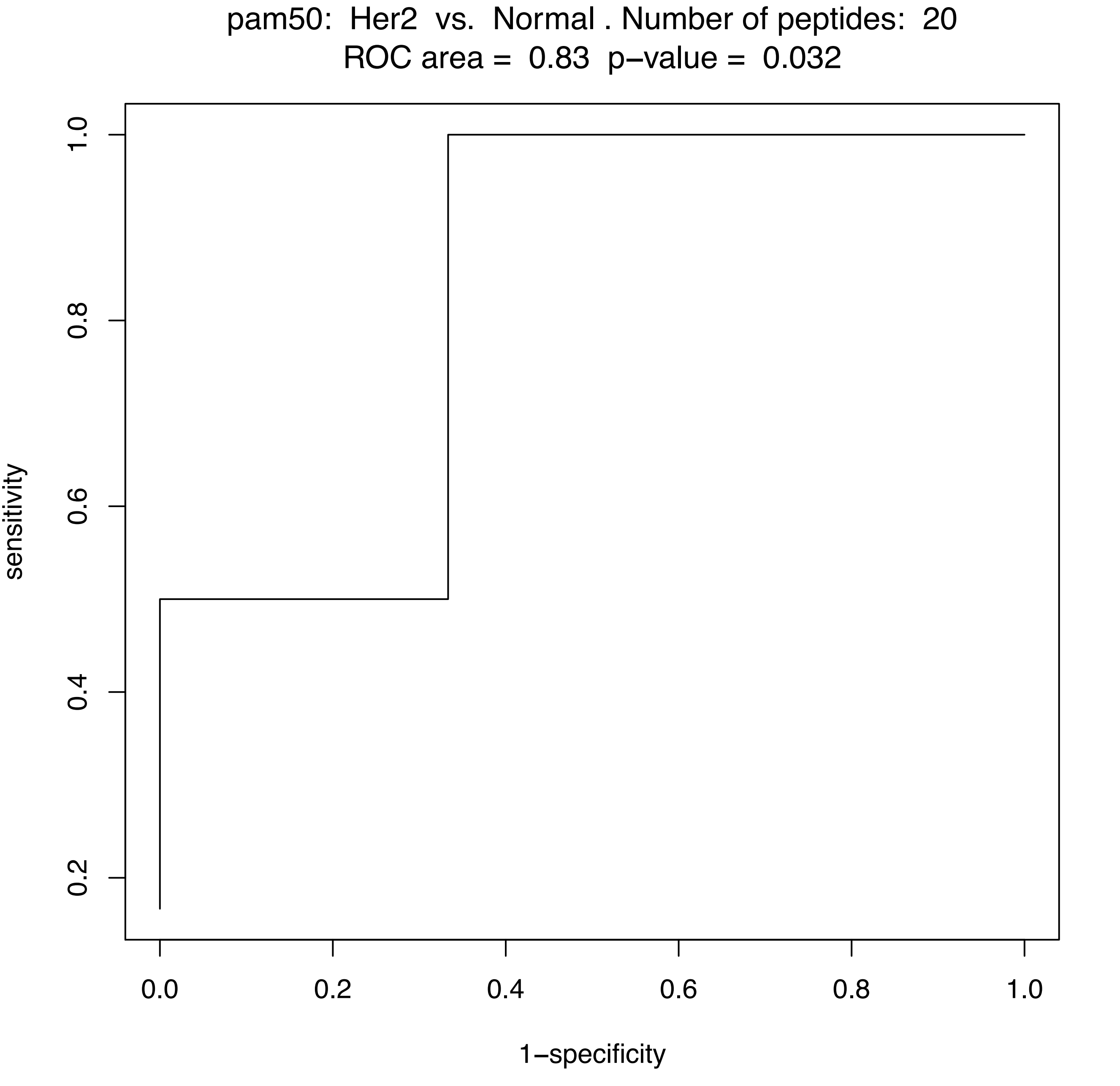
pam50: Her2 vs. Normal . Number of peptides: 20
ROC area = 0.83 p−value = 0.032
sensitivity
0.2
0.4
0.6
0.8
1.0
0.0
0.2
0.4
0.6
0.8
1.0
1−specificity

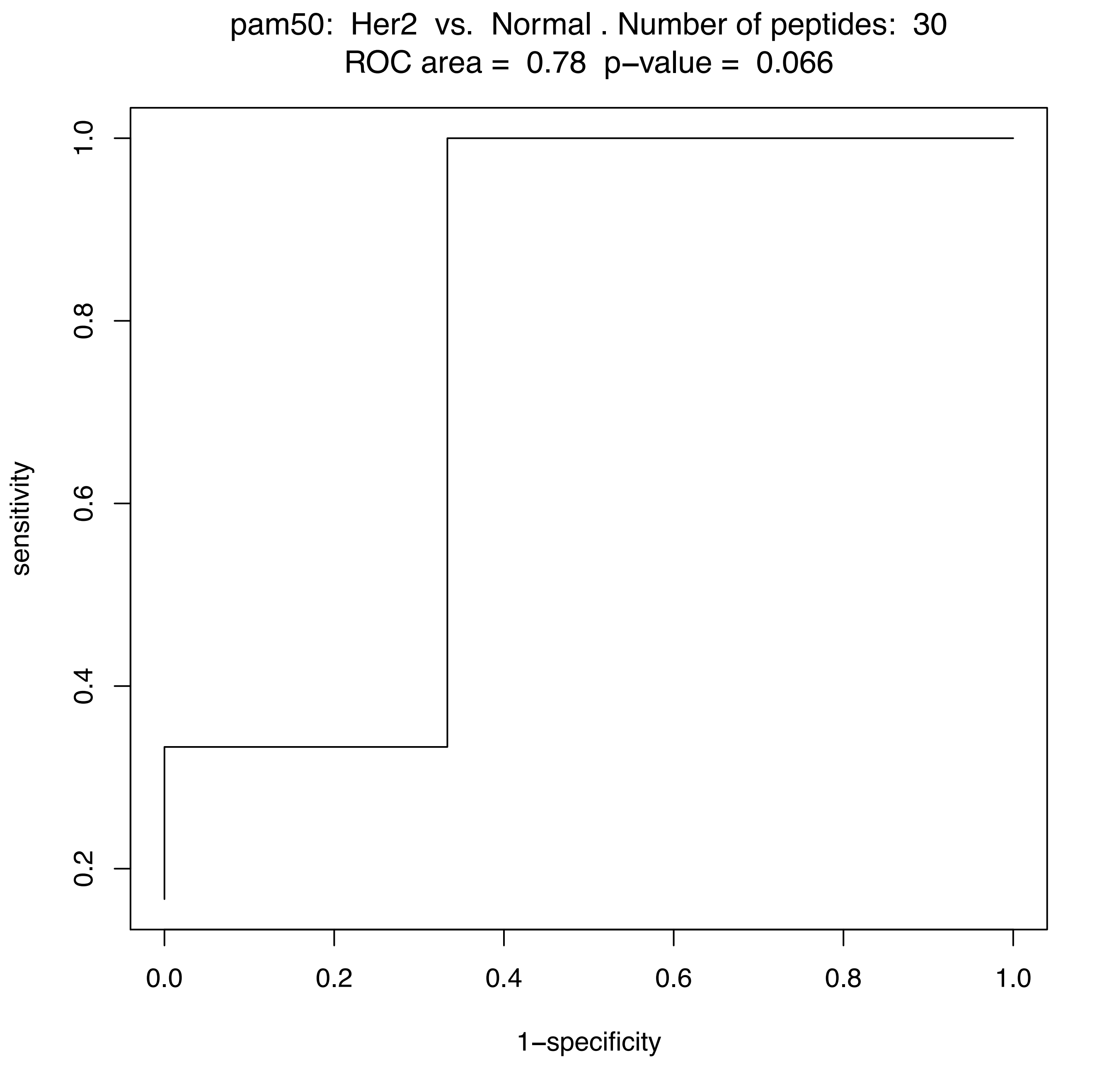
pam50: Her2 vs. Normal . Number of peptides: 30
ROC area = 0.78 p−value = 0.066
sensitivity
1.0
0.8
0.6
0.4
0.2
0.0
0.2
0.4
0.6
0.8
1.0
1−specificity

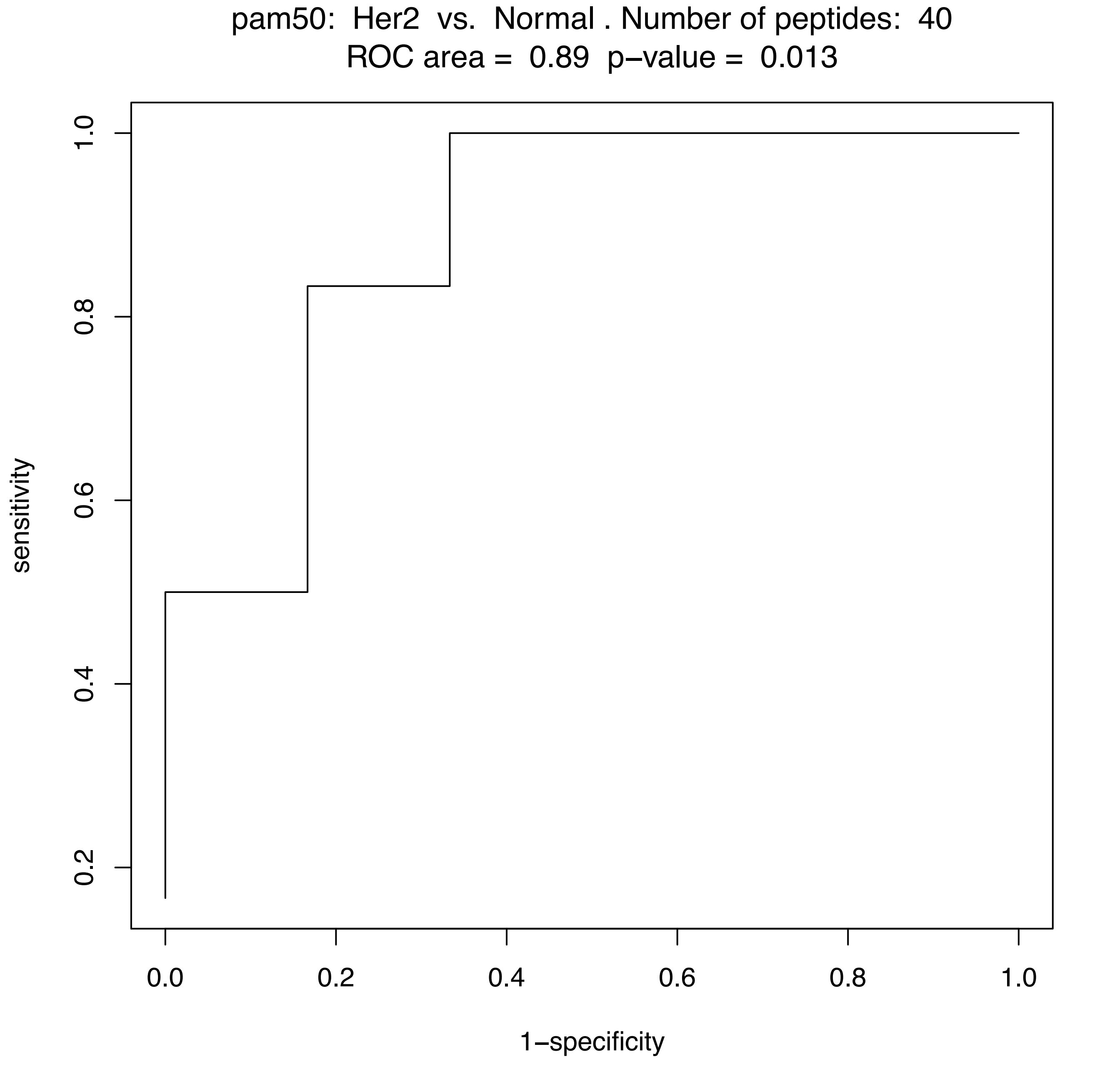
pam50: Her2 vs. Normal . Number of peptides: 40
ROC area = 0.89 p−value = 0.013
sensitivity
1.0
0.8
0.6
0.4
0.2
0.0
0.2
0.4
0.6
0.8
1.0
1−specificity

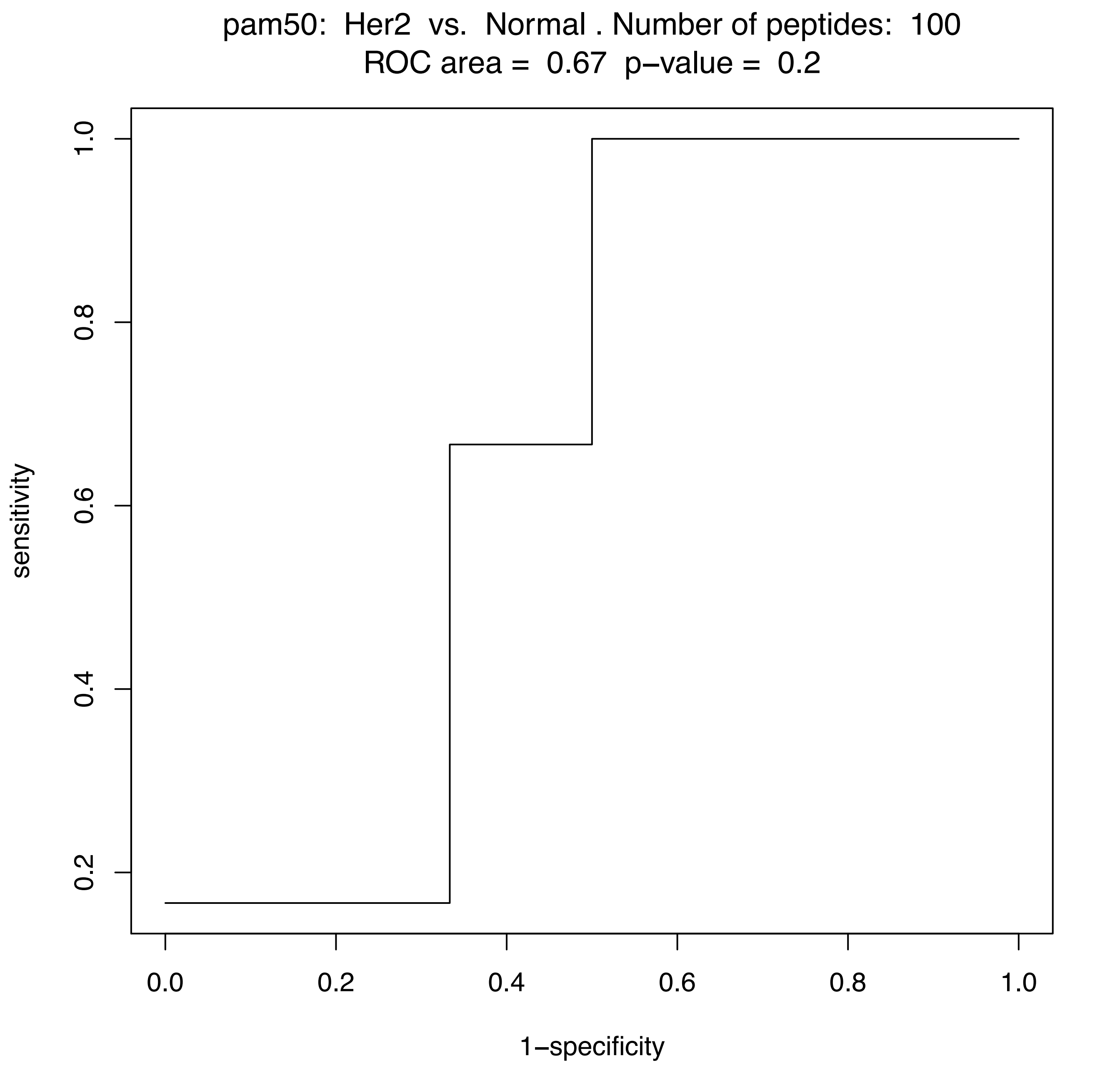
pam50: Her2 vs. Normal . Number of peptides: 100
ROC area = 0.67 p−value = 0.2
1.0
0.8
0.6
0.4
0.2
sensitivity
0.0
0.2
0.4
0.6
0.8
1.0
1−specificity

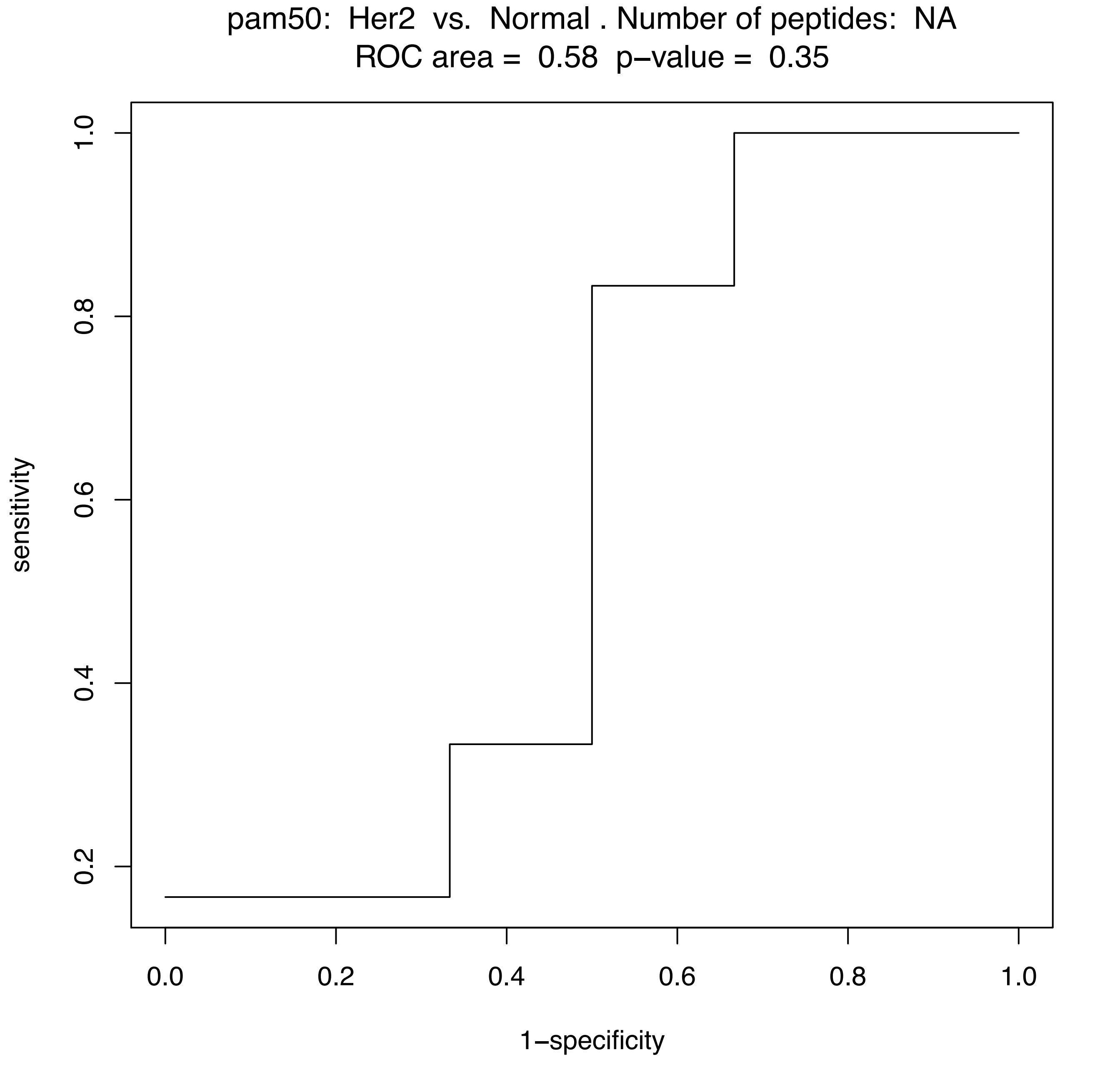
pam50: Her2 vs. Normal . Number of peptides: NA
ROC area = 0.58 p−value = 0.35
sensitivity
1.0
0.8
0.6
0.4
0.2
0.0
0.2
0.4
0.6
0.8
1.0
1−specificity

hu: Her2 vs. Normal . Number of peptides: 20
ROC area = 0.44 p−value = 0.66

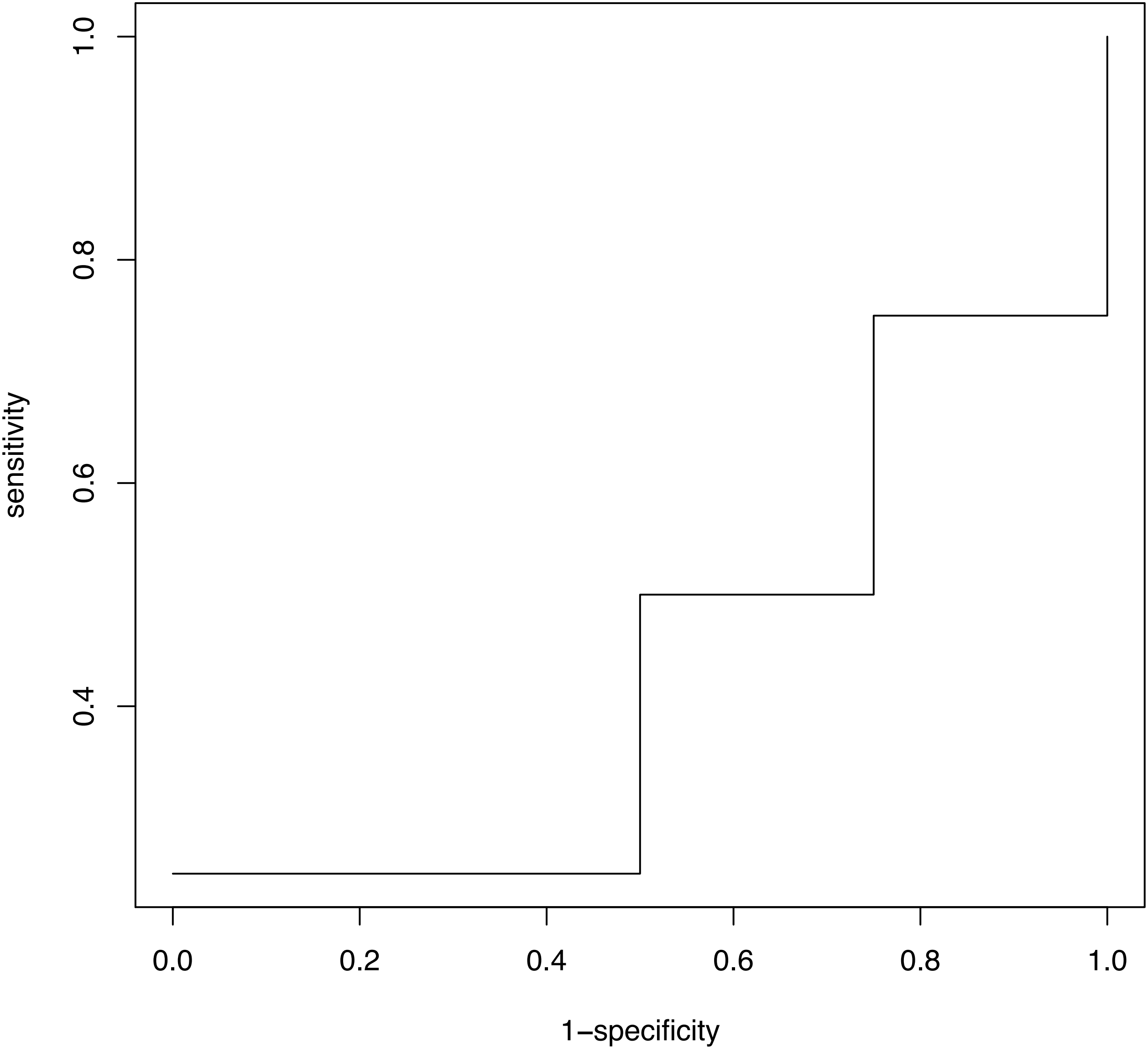

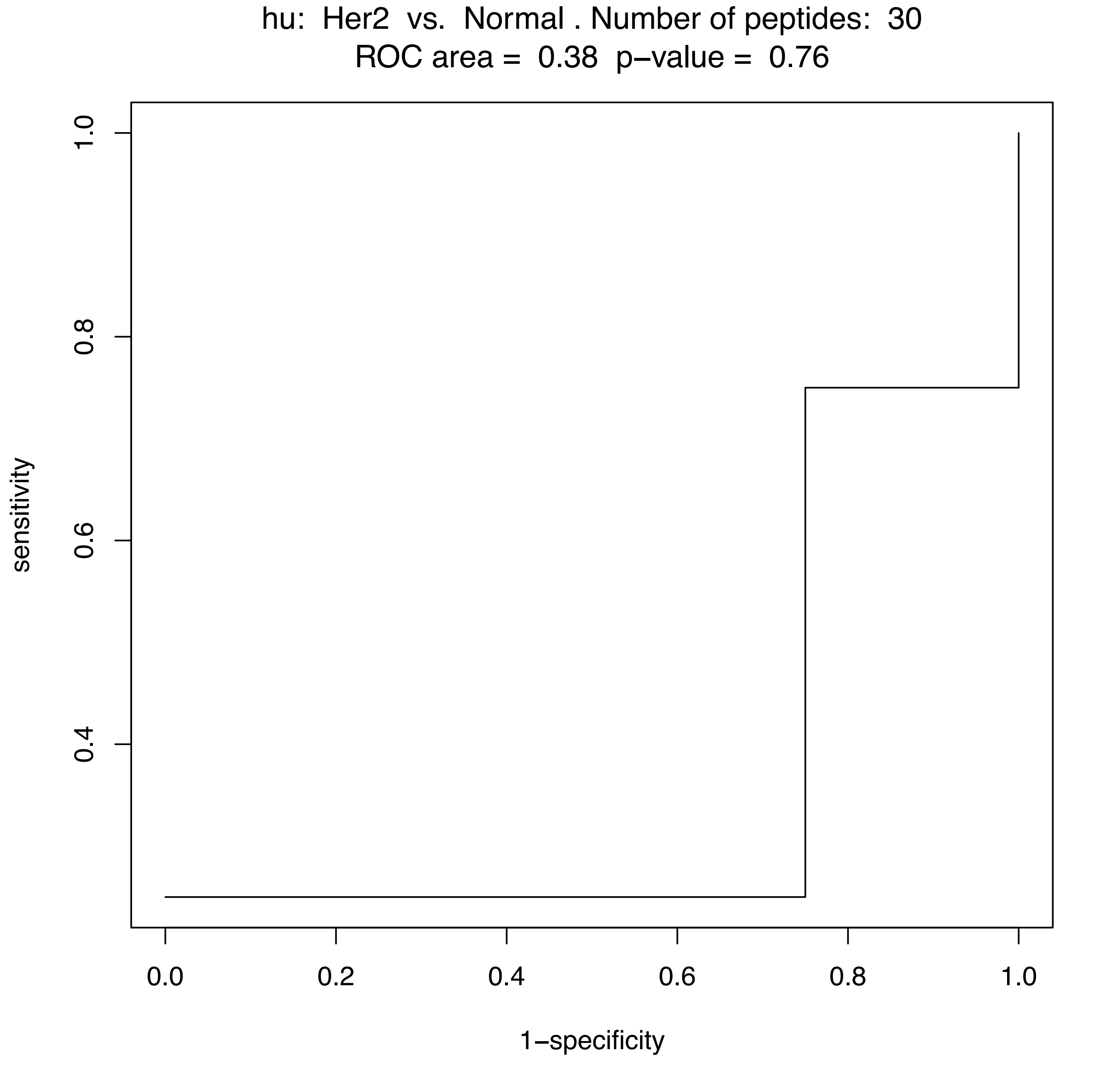

hu: Her2 vs. Normal . Number of peptides: 30
ROC area = 0.38 p−value = 0.76
sensitivity
1.0
0.8
0.6
0.4
0.0
0.2
0.4
0.6
0.8
1.0
1−specificity

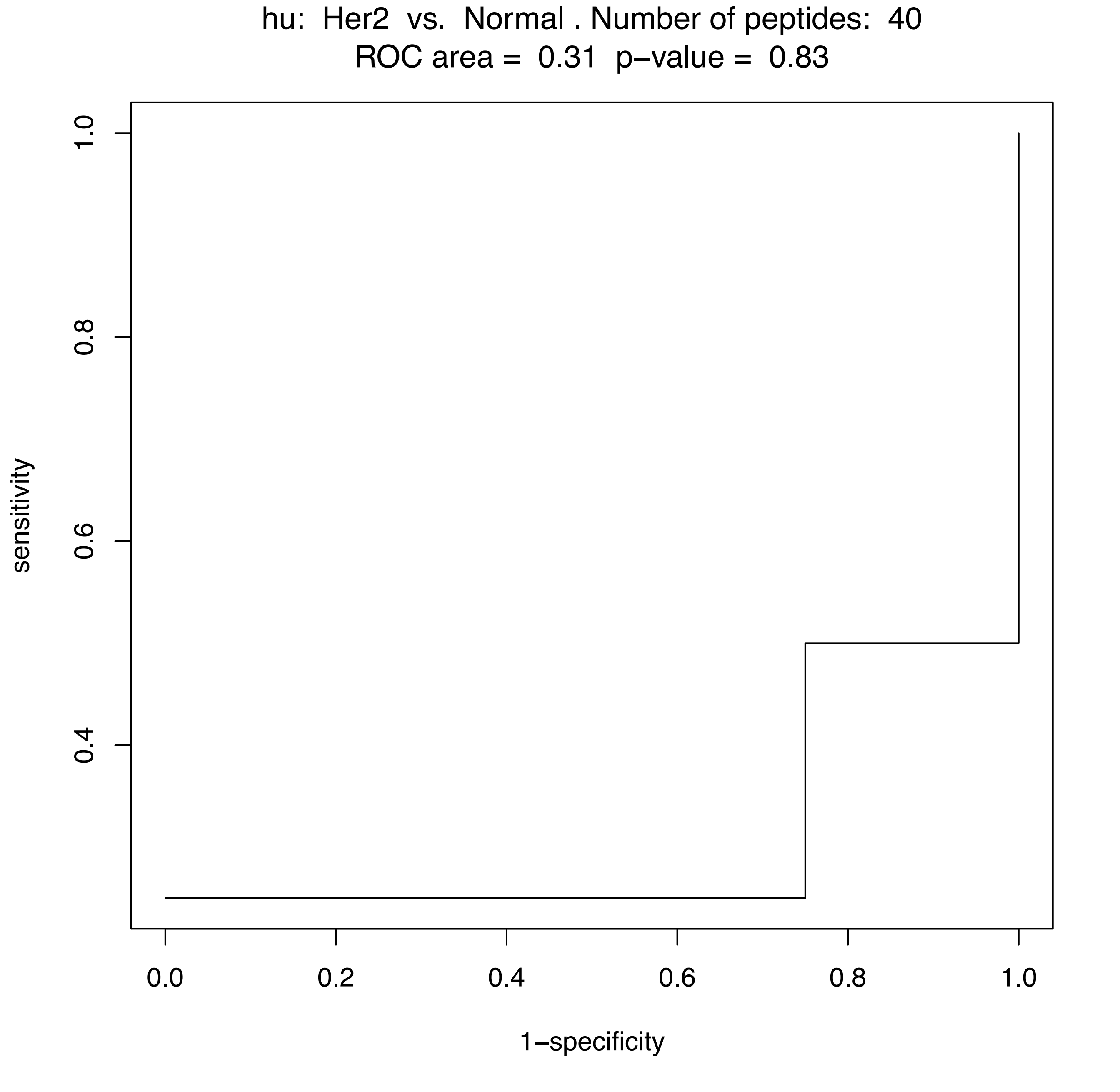
hu: Her2 vs. Normal . Number of peptides: 40
ROC area = 0.31 p−value = 0.83
sensitivity
1.0
0.8
0.6
0.4
0.0
0.2
0.4
0.6
0.8
1.0
1−specificity

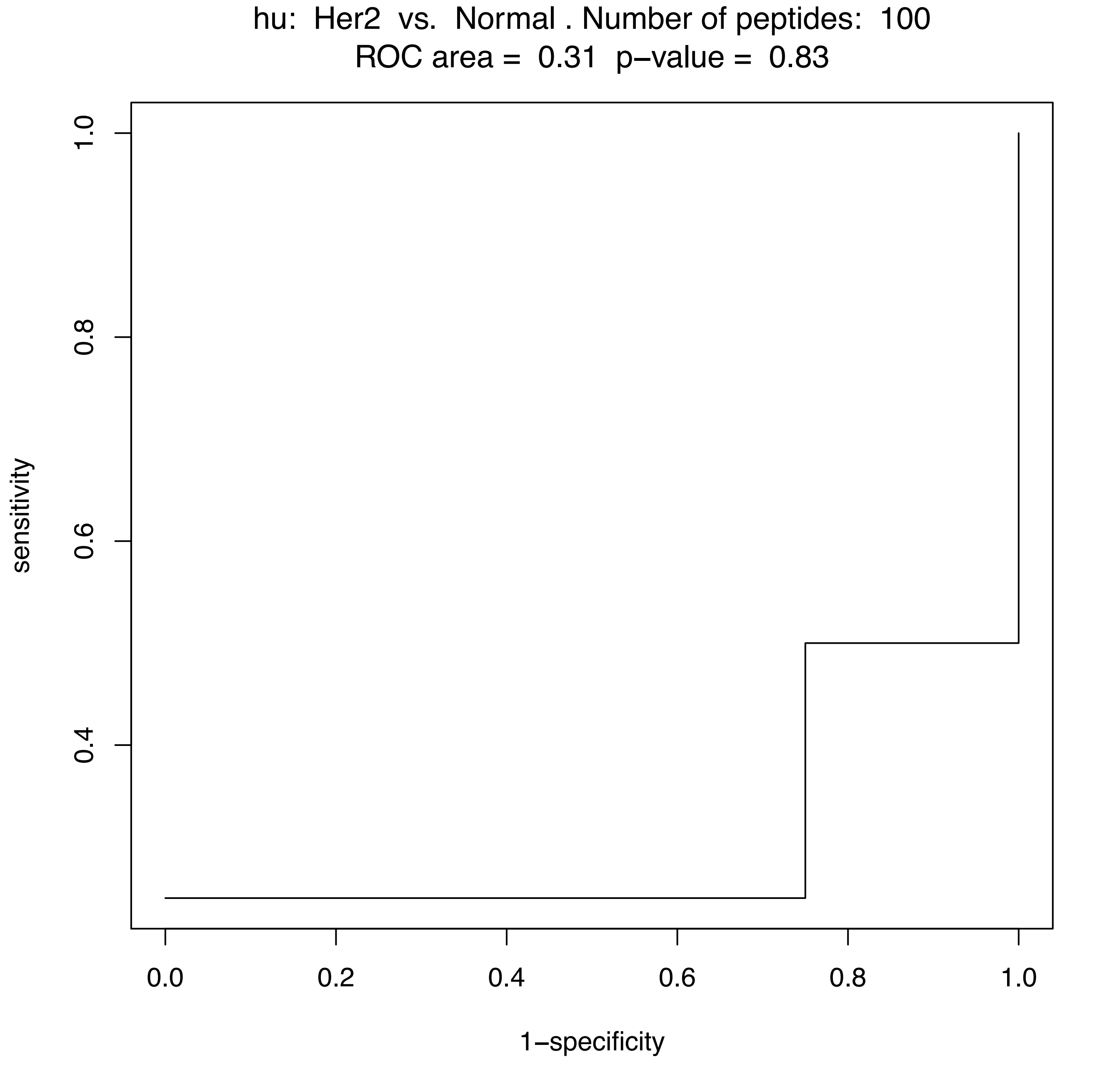
hu: Her2 vs. Normal . Number of peptides: 100
ROC area = 0.31 p−value = 0.83
sensitivity
1.0
0.8
0.6
0.4
1−specificity
0.0
0.2
0.4
0.6
0.8
1.0

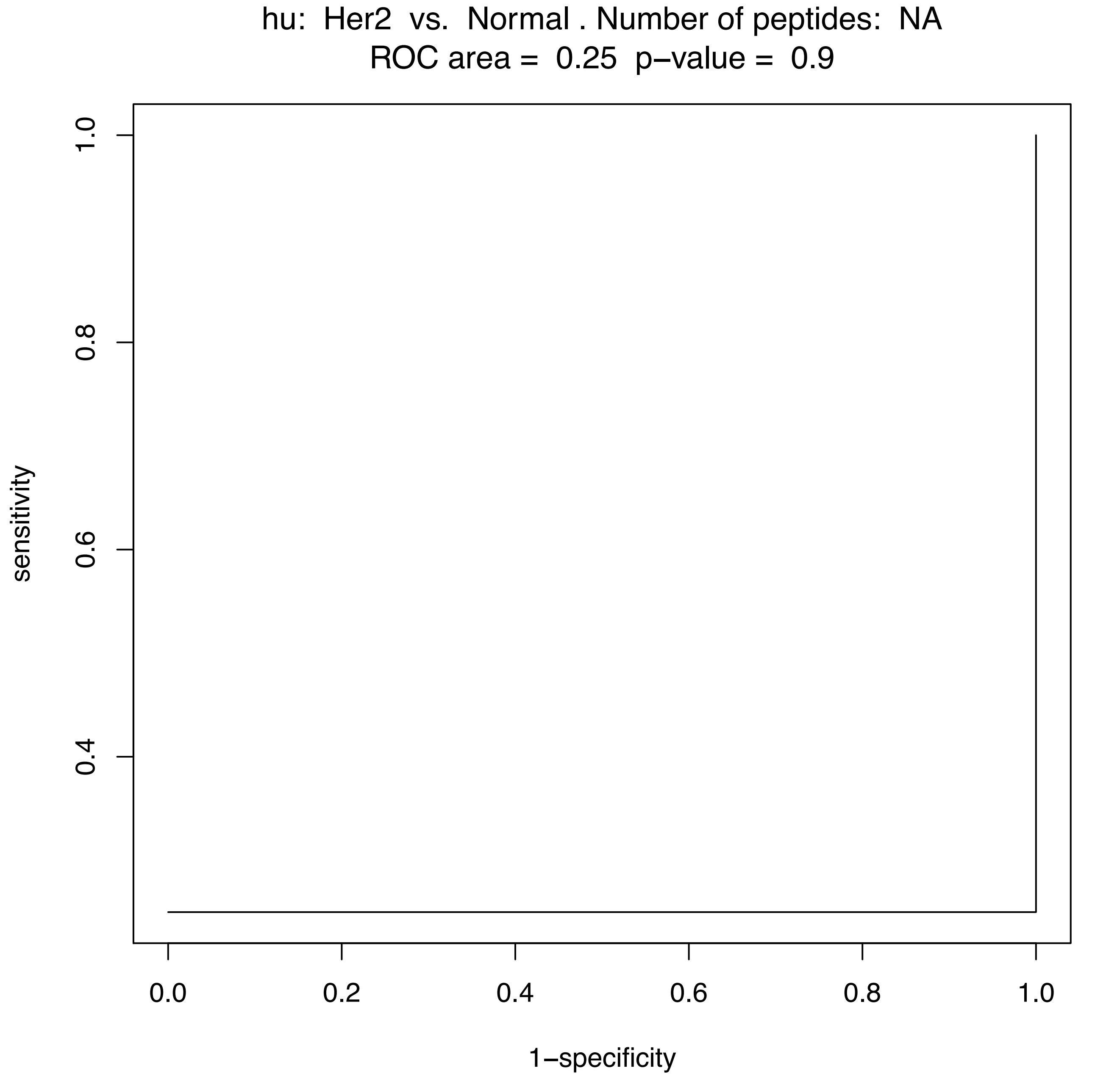
hu: Her2 vs. Normal . Number of peptides: NA
ROC area = 0.25 p−value = 0.9
sensitivity
1−specificity
0.4
0.6
0.8
1.0
0.0
0.2
0.4
0.6
0.8
1.0

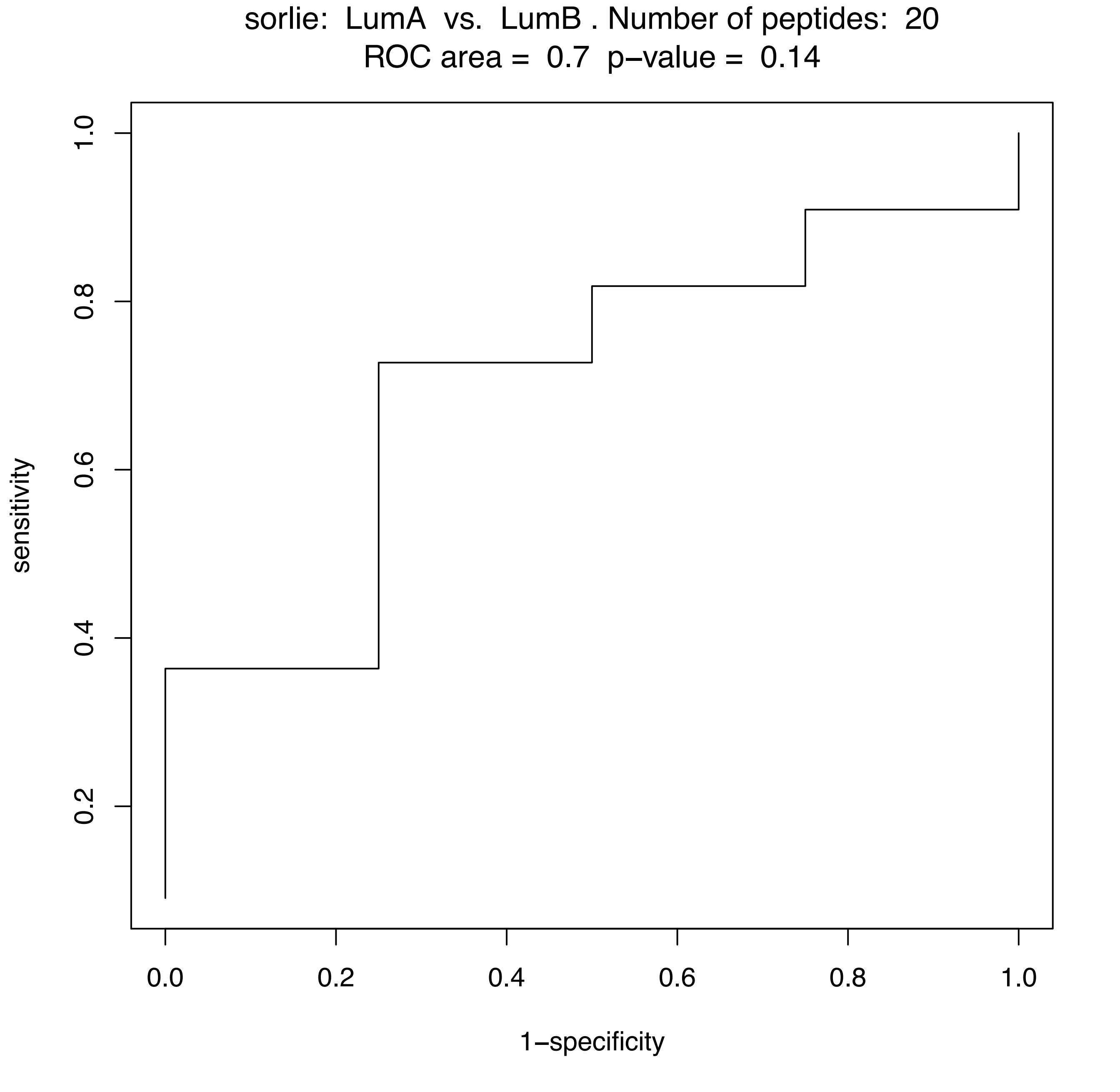
sorlie: LumA vs. LumB . Number of peptides: 20
ROC area = 0.7 p−value = 0.14
sensitivity
0.2
0.4
0.6
0.8
1.0
0.0
0.2
0.4
0.6
0.8
1.0
1−specificity

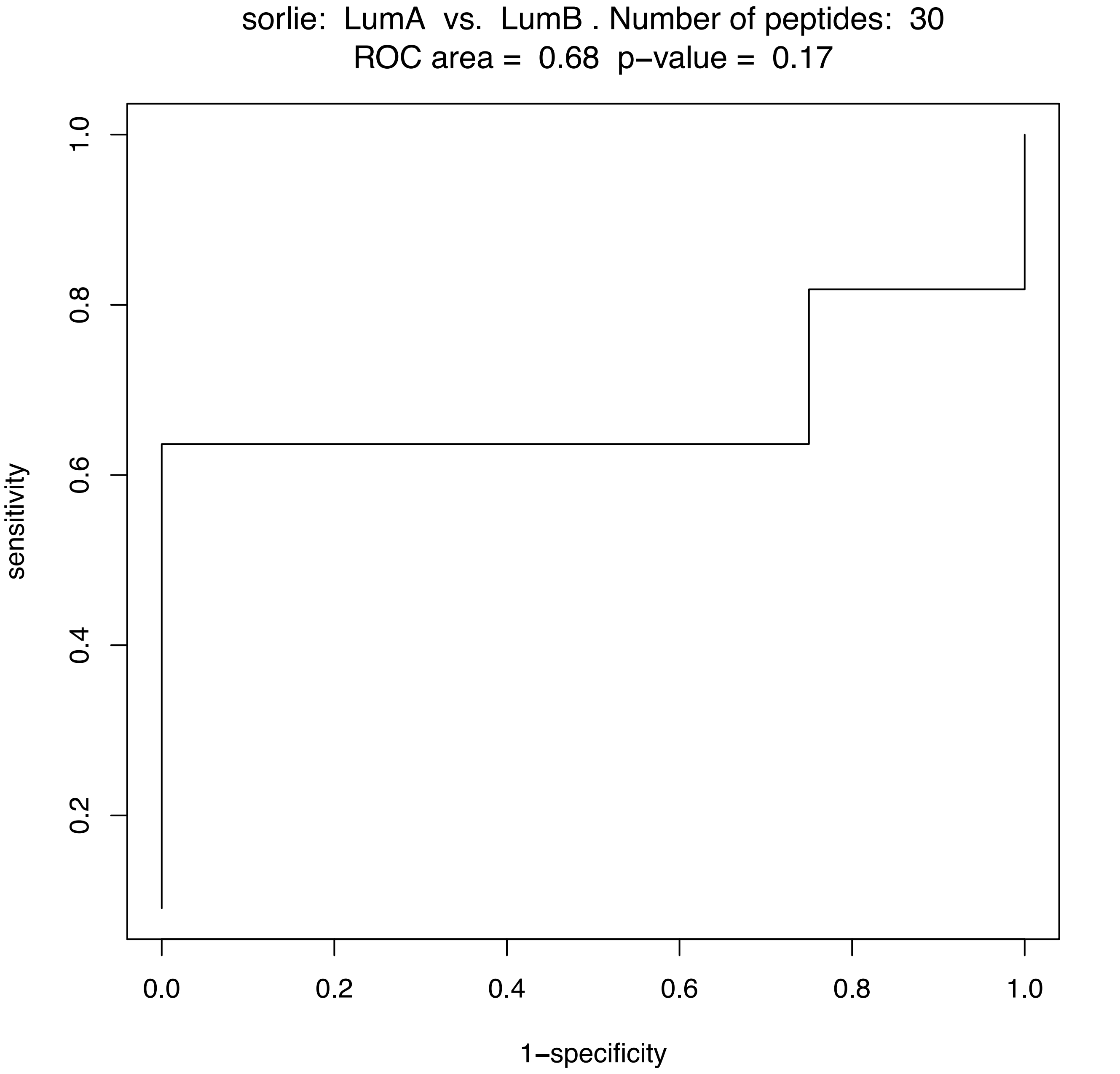
sorlie: LumA vs. LumB . Number of peptides: 30
ROC area = 0.68 p−value = 0.17
sensitivity
1−specificity
0.0
0.2
0.4
0.6
0.8
1.0

sorlie: LumA vs. LumB . Number of peptides: 40
ROC area = 0.73 p−value = 0.11

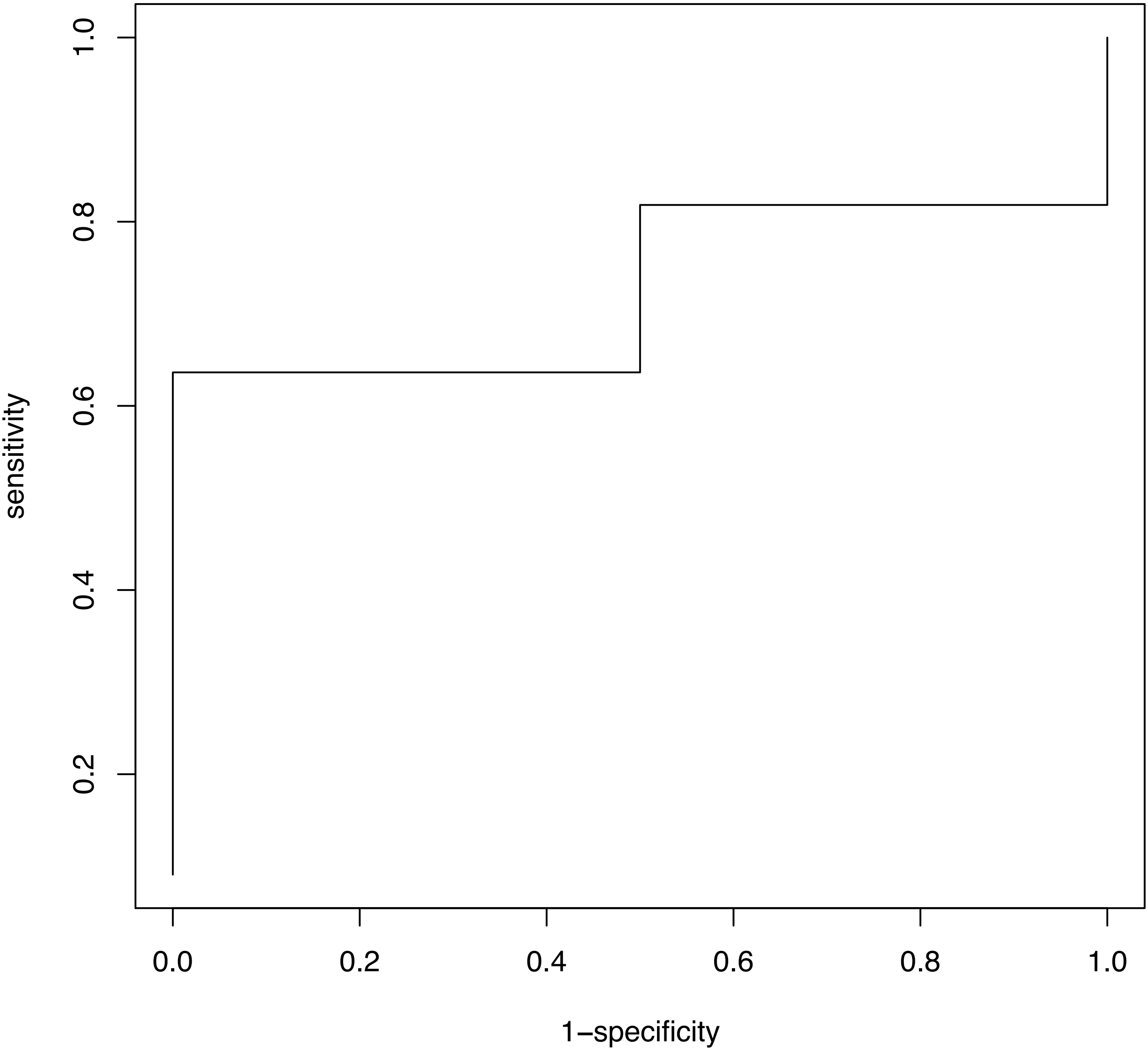

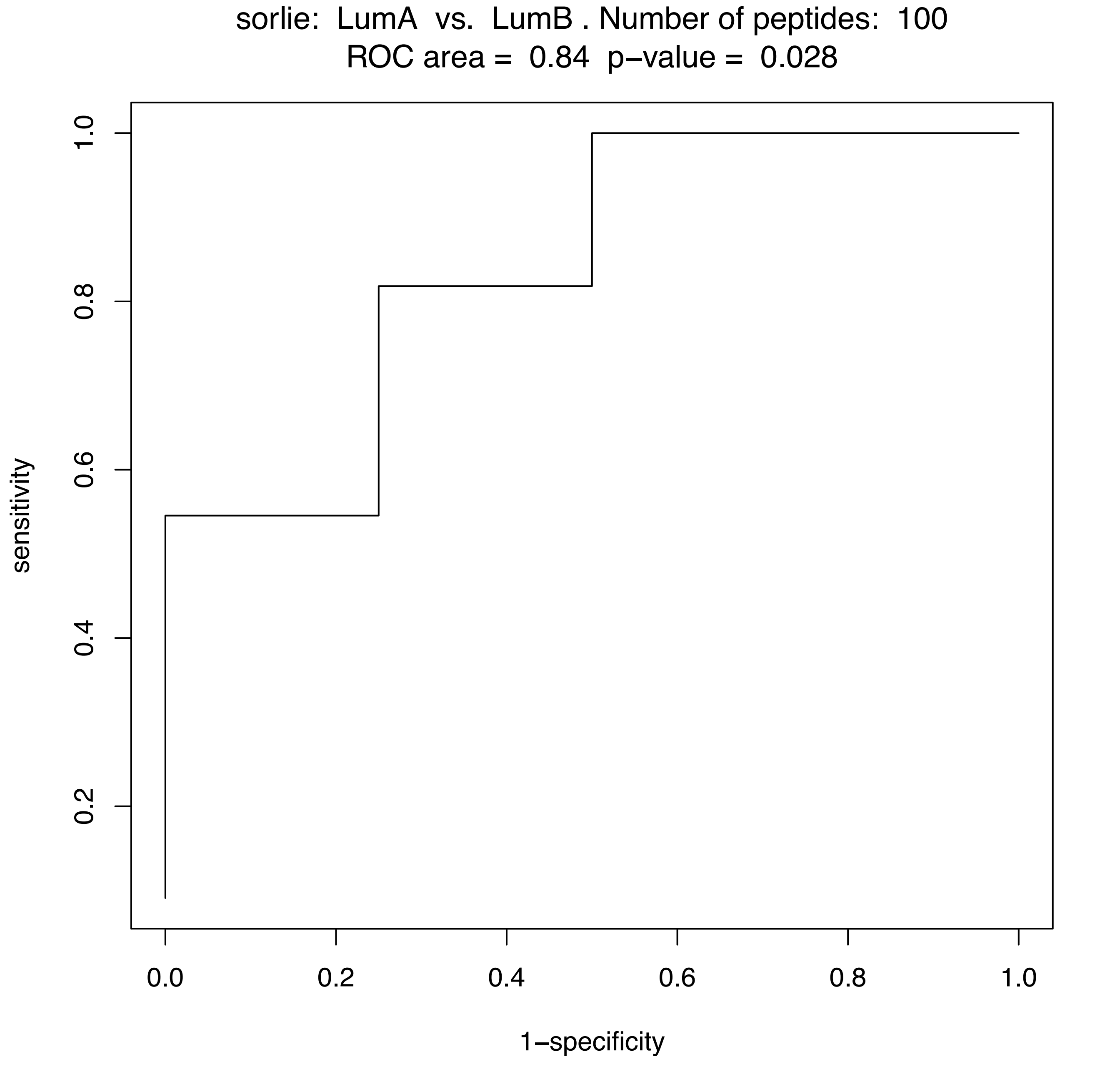
sorlie: LumA vs. LumB . Number of peptides: 100
ROC area = 0.84 p−value = 0.028
sensitivity
0.2
0.4
0.6
0.8
1.0
0.0
0.2
0.4
0.6
0.8
1.0
1−specificity

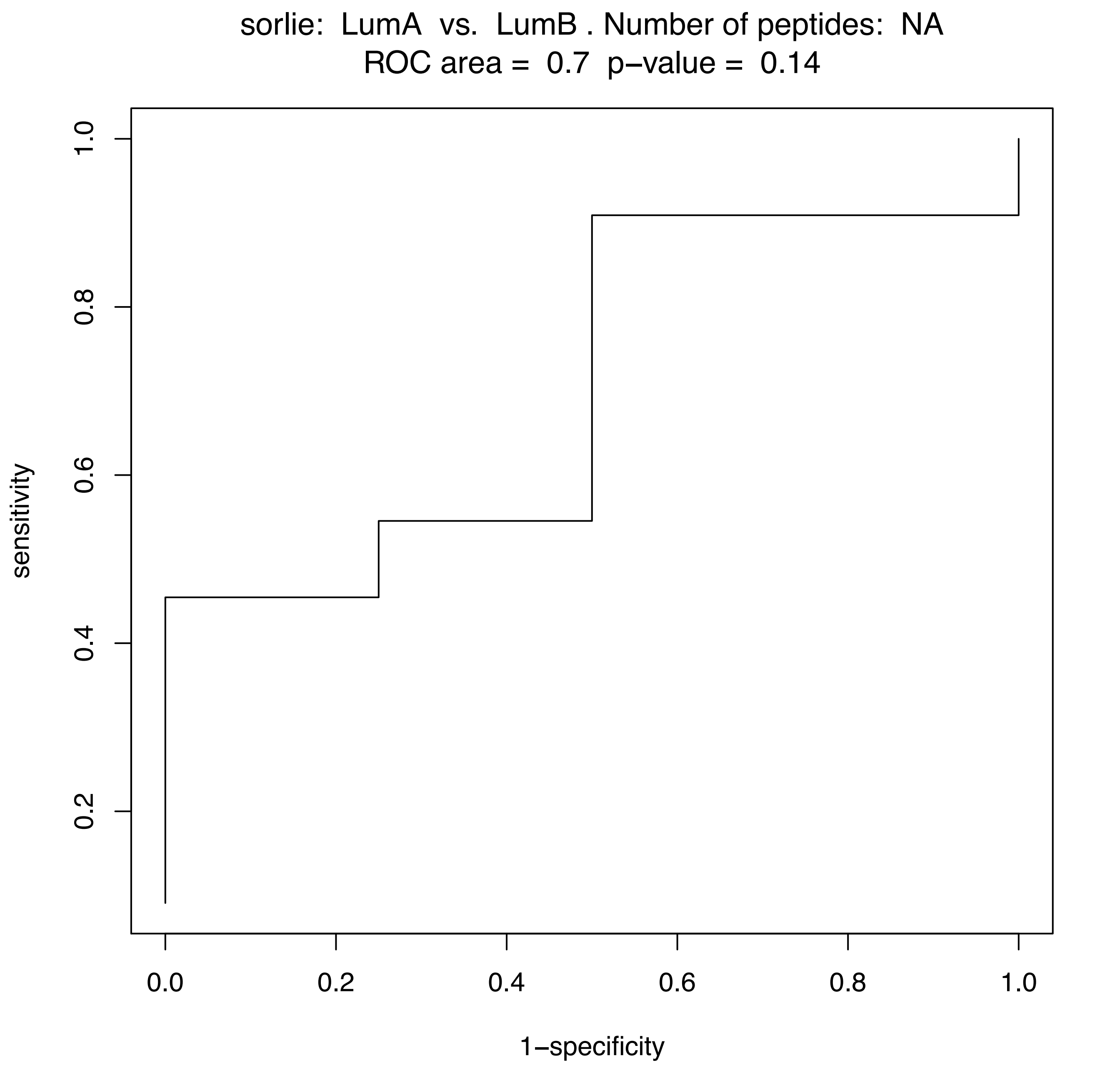
sorlie: LumA vs. LumB . Number of peptides: NA
ROC area = 0.7 p−value = 0.14
sensitivity
1−specificity
0.2
0.4
0.6
0.8
1.0
0.0
0.2
0.4
0.6
0.8
1.0

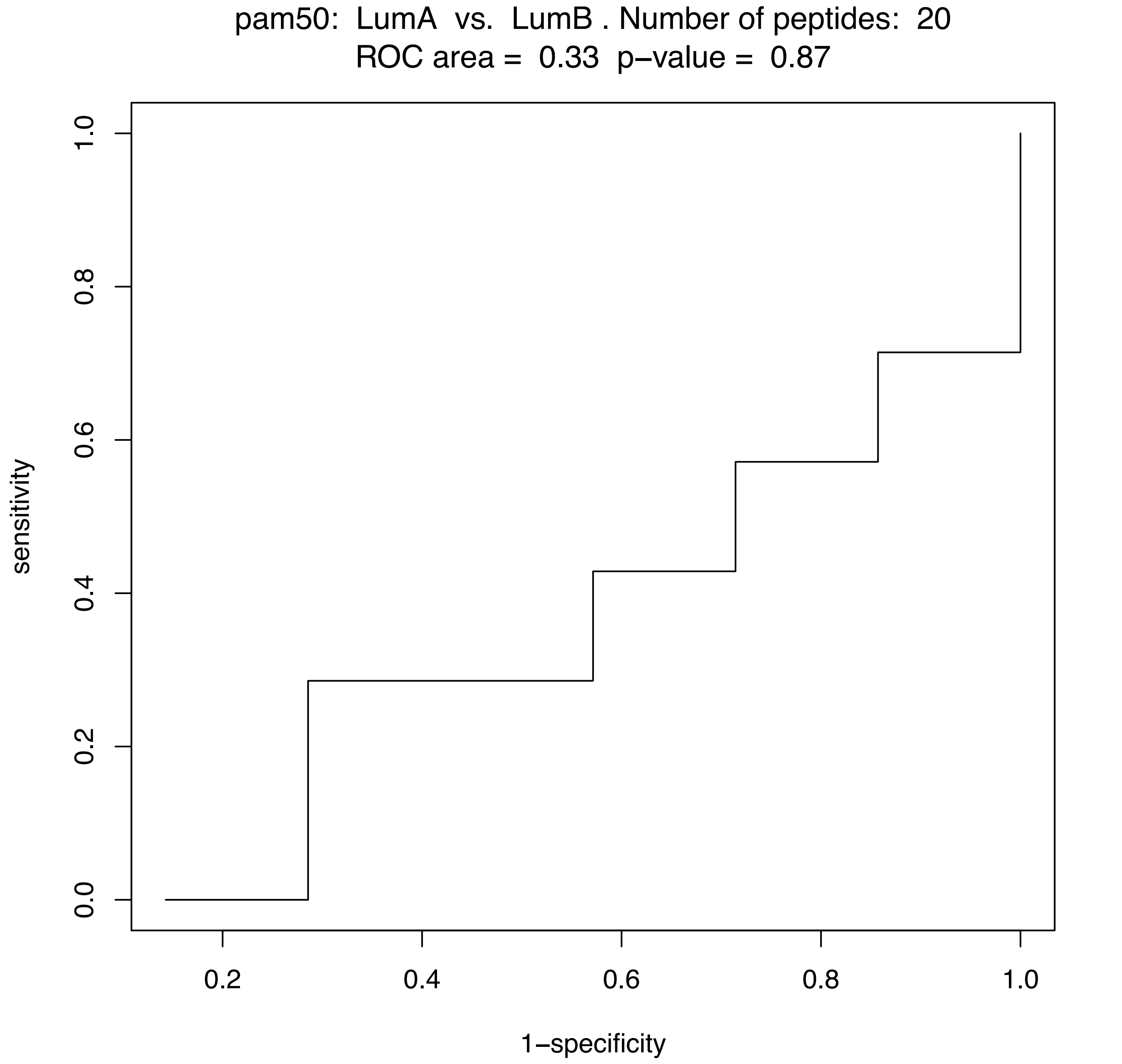
pam50: LumA vs. LumB . Number of peptides: 20
ROC area = 0.33 p−value = 0.87
sensitivity
1−specificity
0.0
0.2
0.4
0.6
0.8
1.0
0.2
0.4
0.6
0.8
1.0

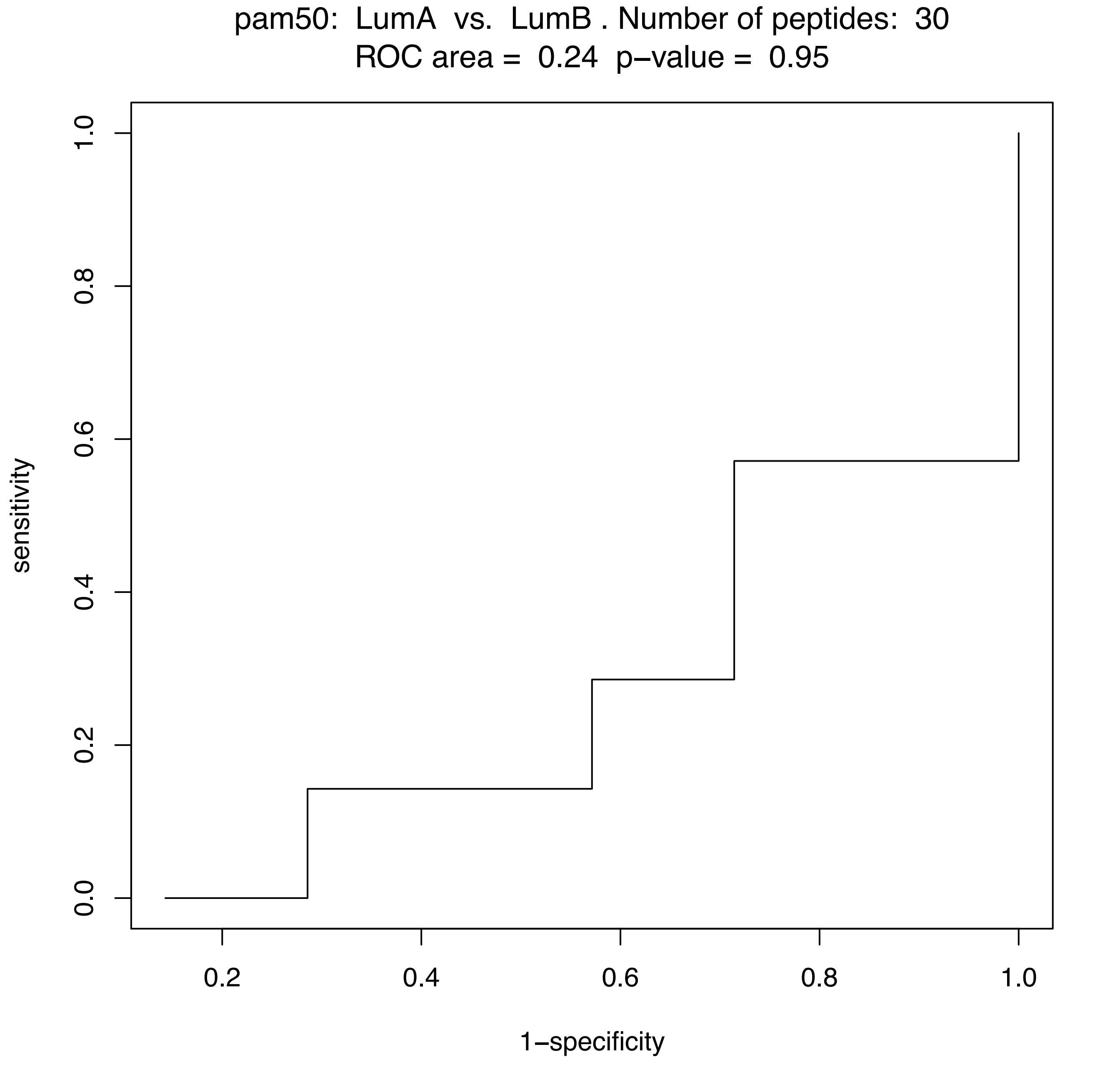
pam50: LumA vs. LumB . Number of peptides: 30
ROC area = 0.24 p−value = 0.95
sensitivity
0.0
0.2
0.4
0.6
0.8
1.0
0.2
0.4
0.6
0.8
1.0
1−specificity

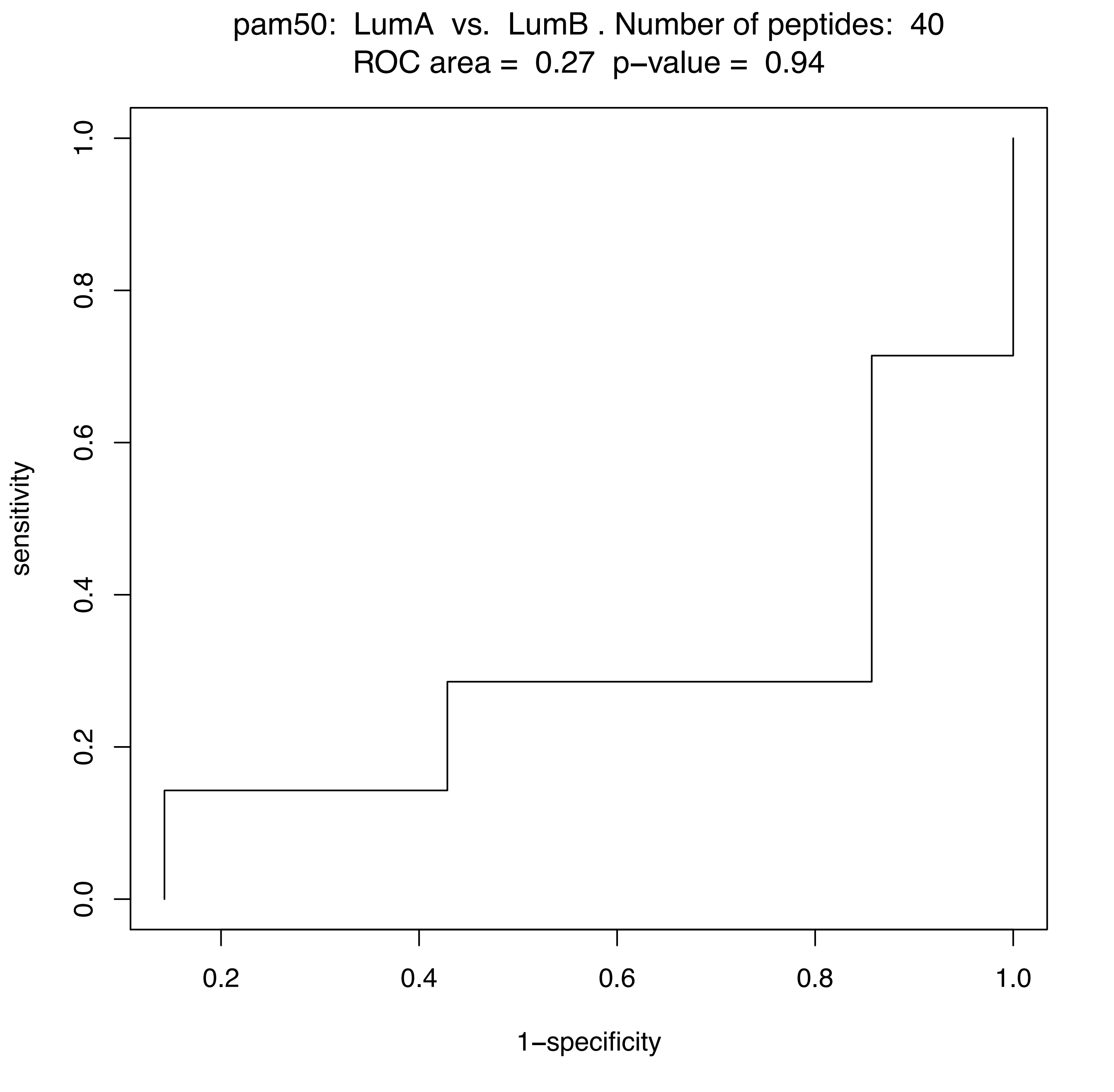
pam50: LumA vs. LumB . Number of peptides: 40
ROC area = 0.27 p−value = 0.94
sensitivity
1−specificity
0.0
0.2
0.4
0.6
0.8
1.0
0.2
0.4
0.6
0.8
1.0

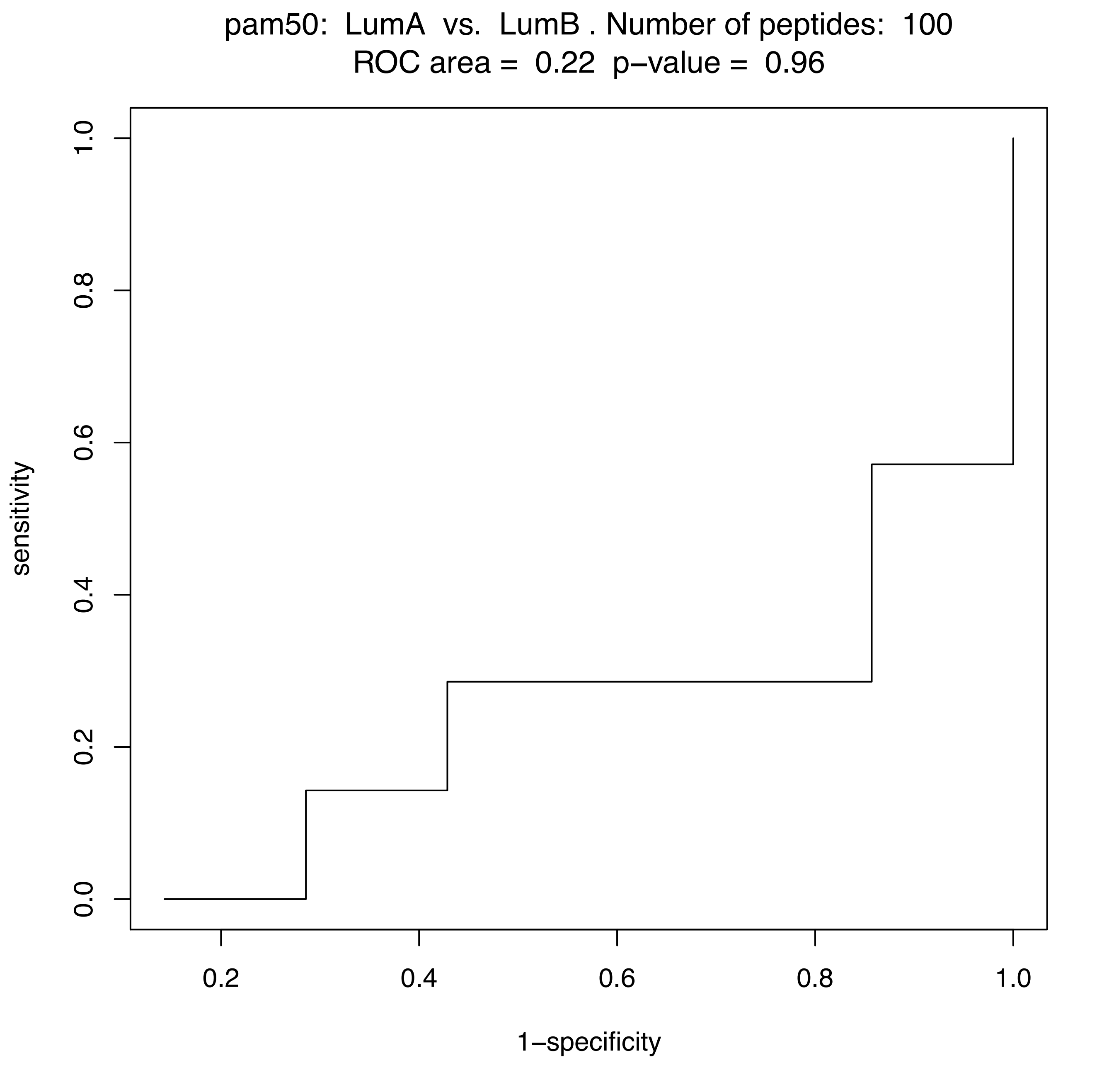
pam50: LumA vs. LumB . Number of peptides: 100
ROC area = 0.22 p−value = 0.96
sensitivity
1−specificity
0.0
0.2
0.4
0.6
0.8
1.0
0.2
0.4
0.6
0.8
1.0

pam50: LumA vs. LumB . Number of peptides: NA
ROC area = 0.27 p−value = 0.94

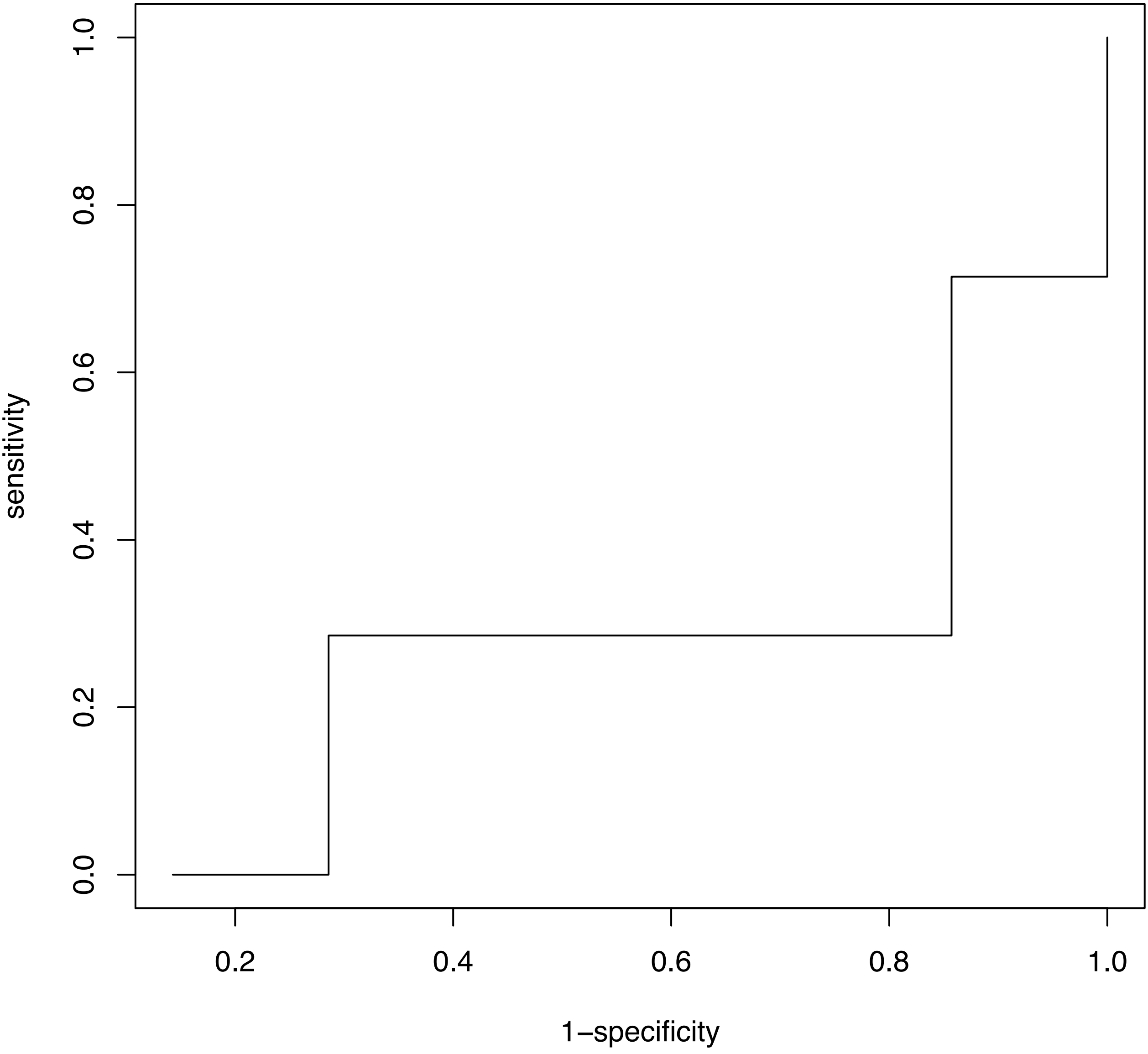

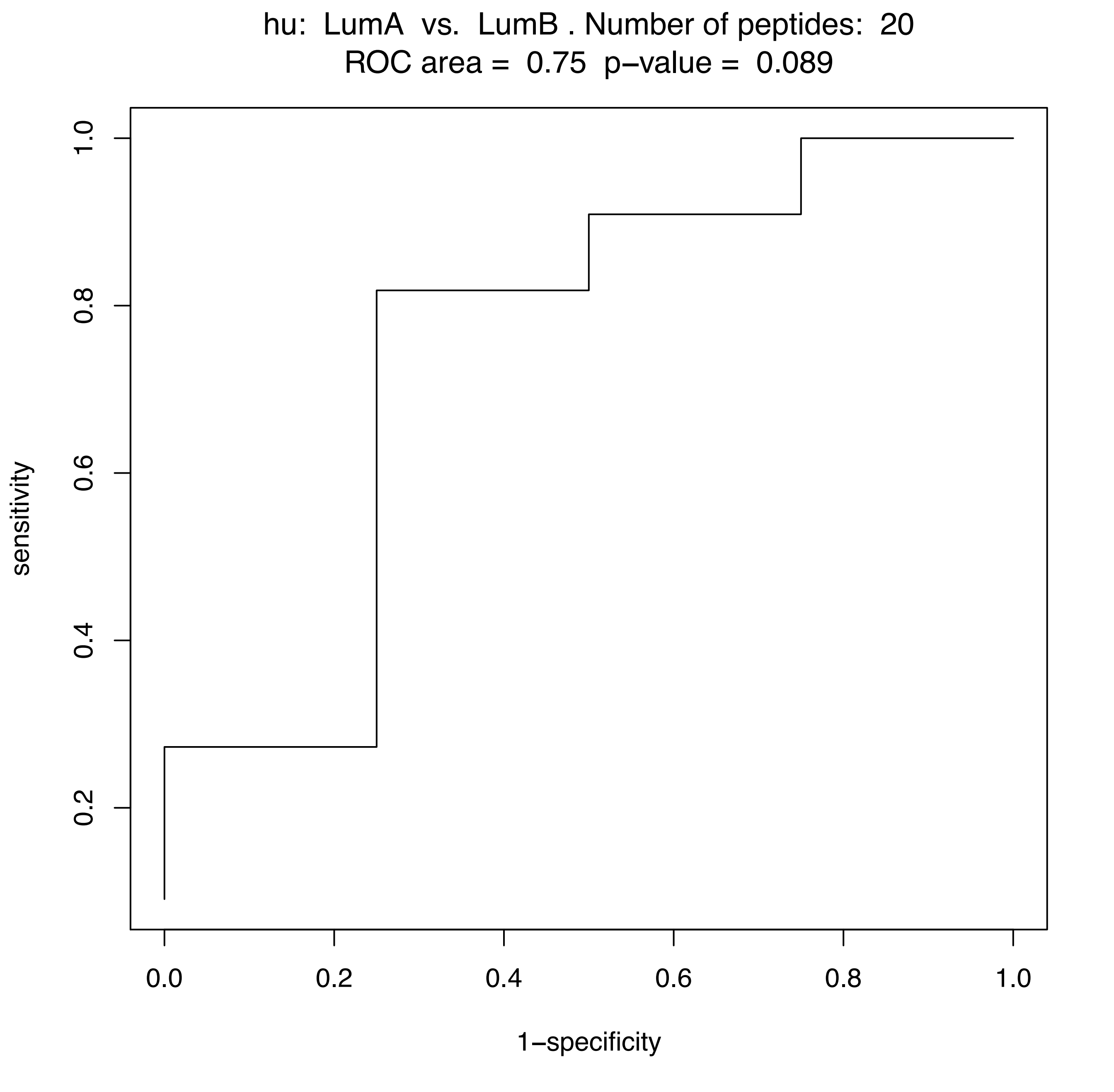

hu: LumA vs. LumB . Number of peptides: 20
ROC area = 0.75 p−value = 0.089
sensitivity
1−specificity
0.2
0.4
0.6
0.8
1.0
0.0

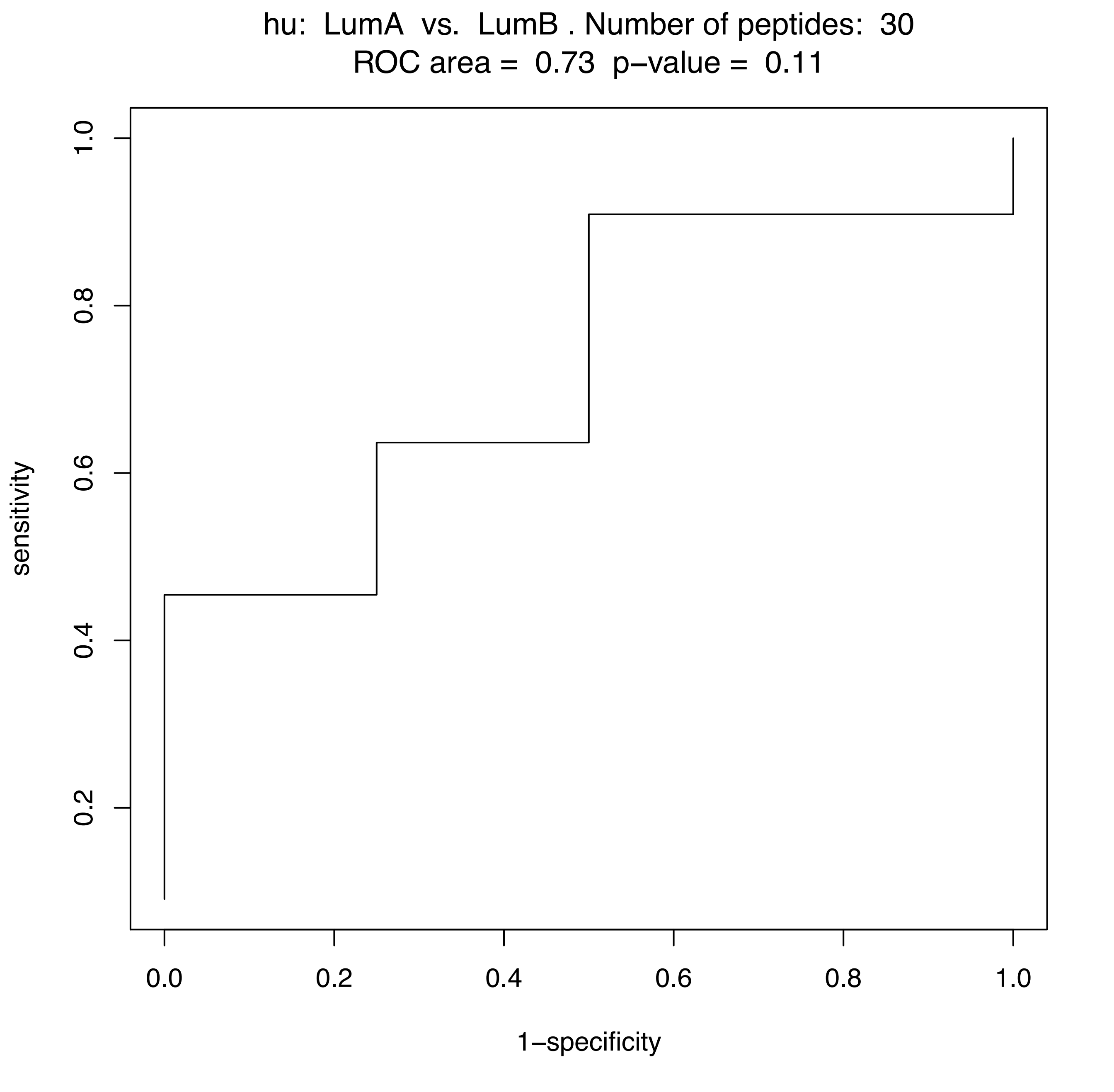
hu: LumA vs. LumB . Number of peptides: 30
ROC area = 0.73 p−value = 0.11
sensitivity
1−specificity
0.0
0.2
0.4
0.6
0.8
1.0

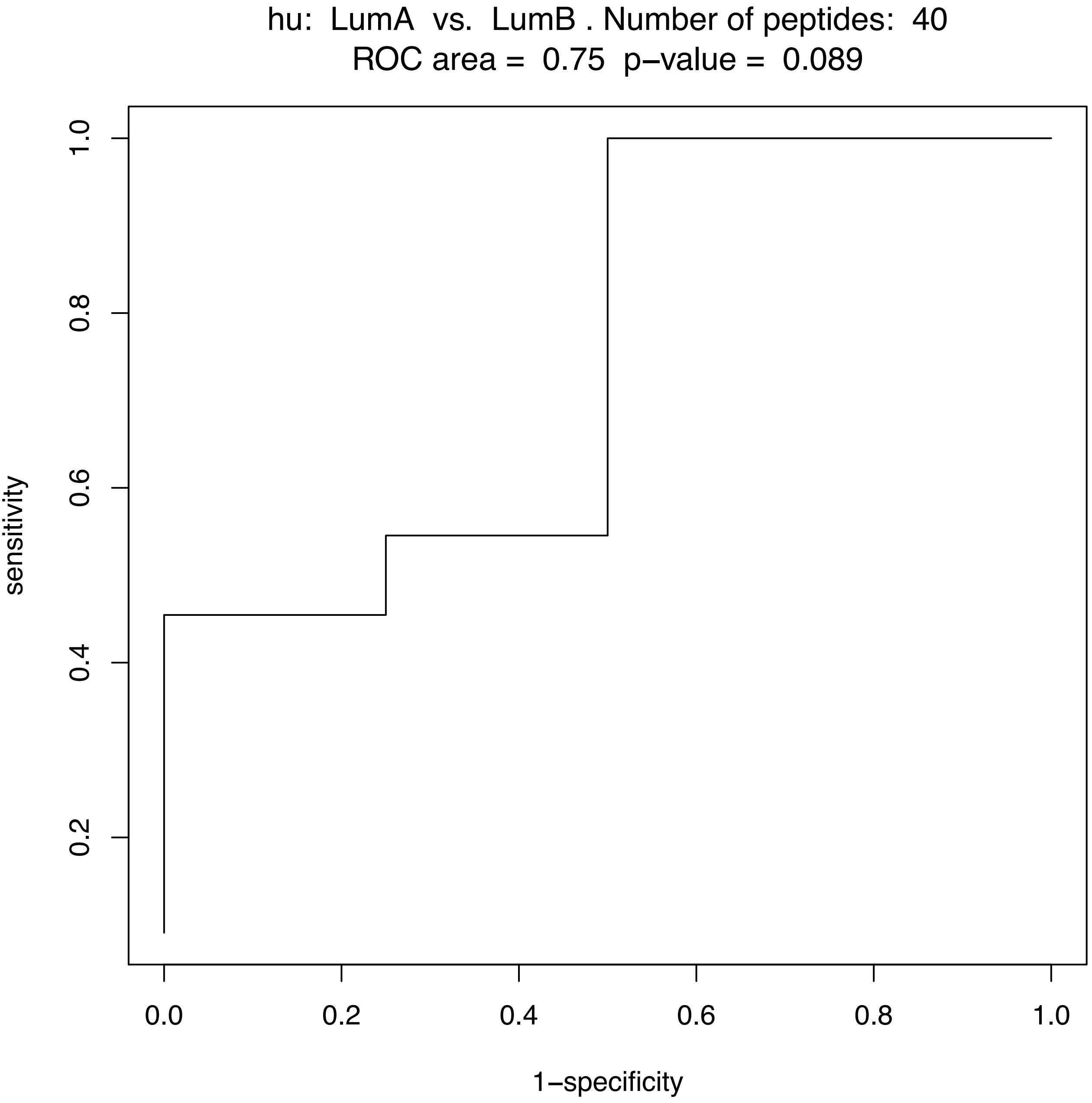
hu: LumA vs. LumB . Number of peptides: 40
ROC area = 0.75 p−value = 0.089
sensitivity
1−specificity
0.0
0.2
0.4
0.6
0.8
1.0
0.2
0.4
0.6
0.8
1.0

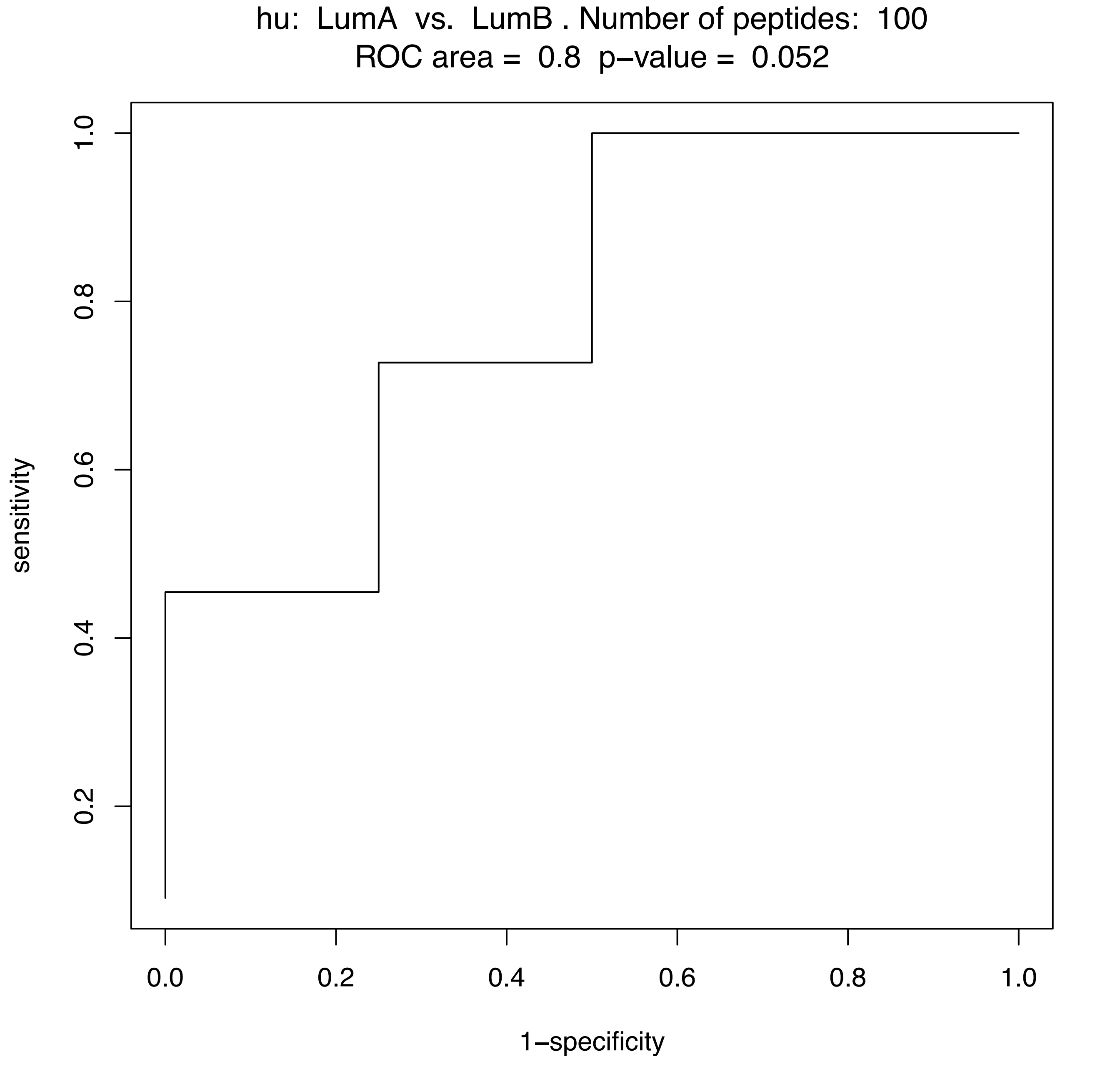
hu: LumA vs. LumB . Number of peptides: 100
ROC area = 0.8 p−value = 0.052
sensitivity
1−specificity
0.0
0.2
0.4
0.6
0.8
1.0
0.2
0.4
0.6
0.8
1.0

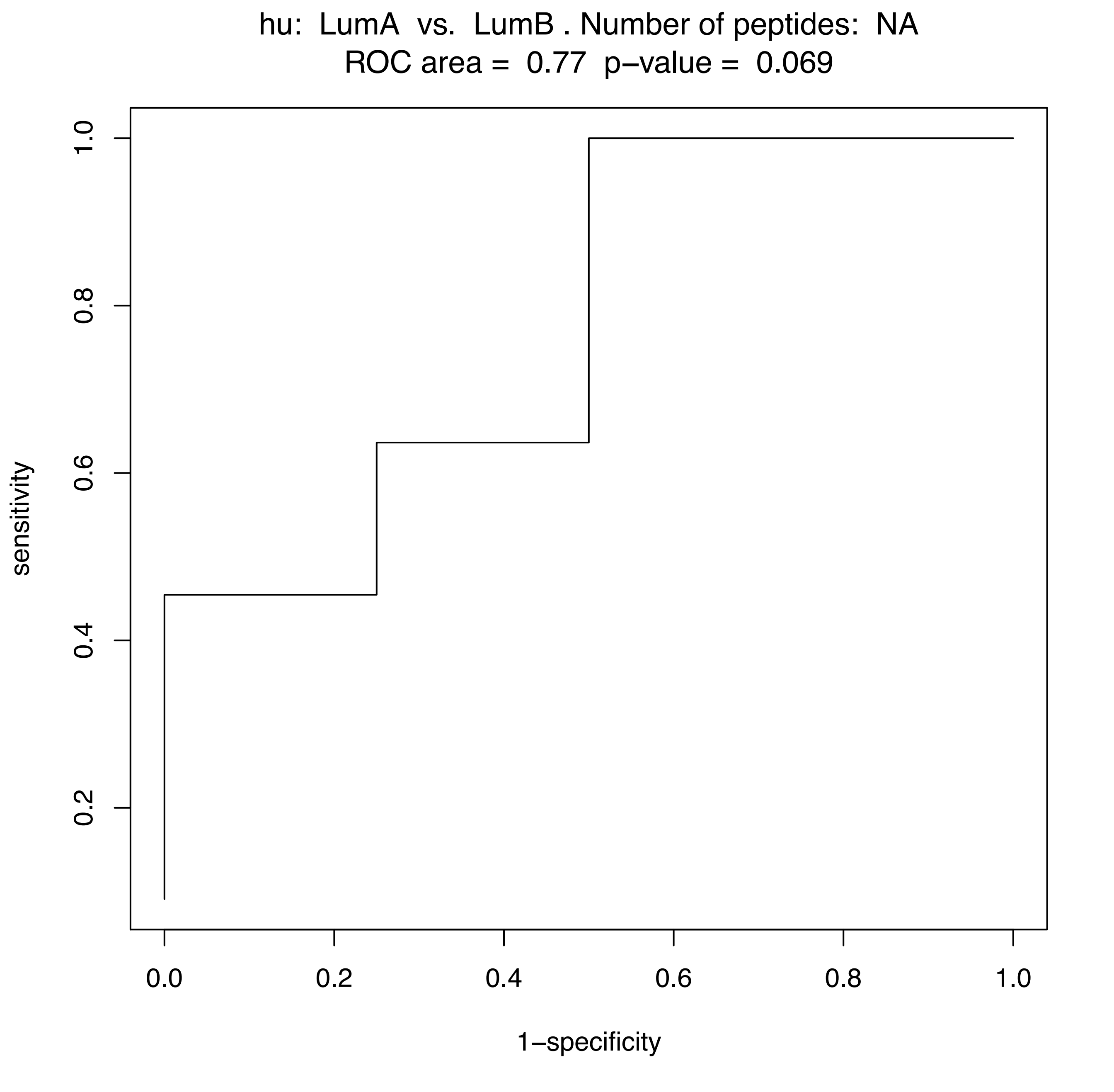
hu: LumA vs. LumB . Number of peptides: NA
ROC area = 0.77 p−value = 0.069
sensitivity
1.0
0.8
0.6
0.4
0.2
0.0
0.2
0.4
0.6
0.8
1.0
1−specificity

sorlie: LumA vs. Normal . Number of peptides: 20
ROC area = 0.5 p−value = 0.53

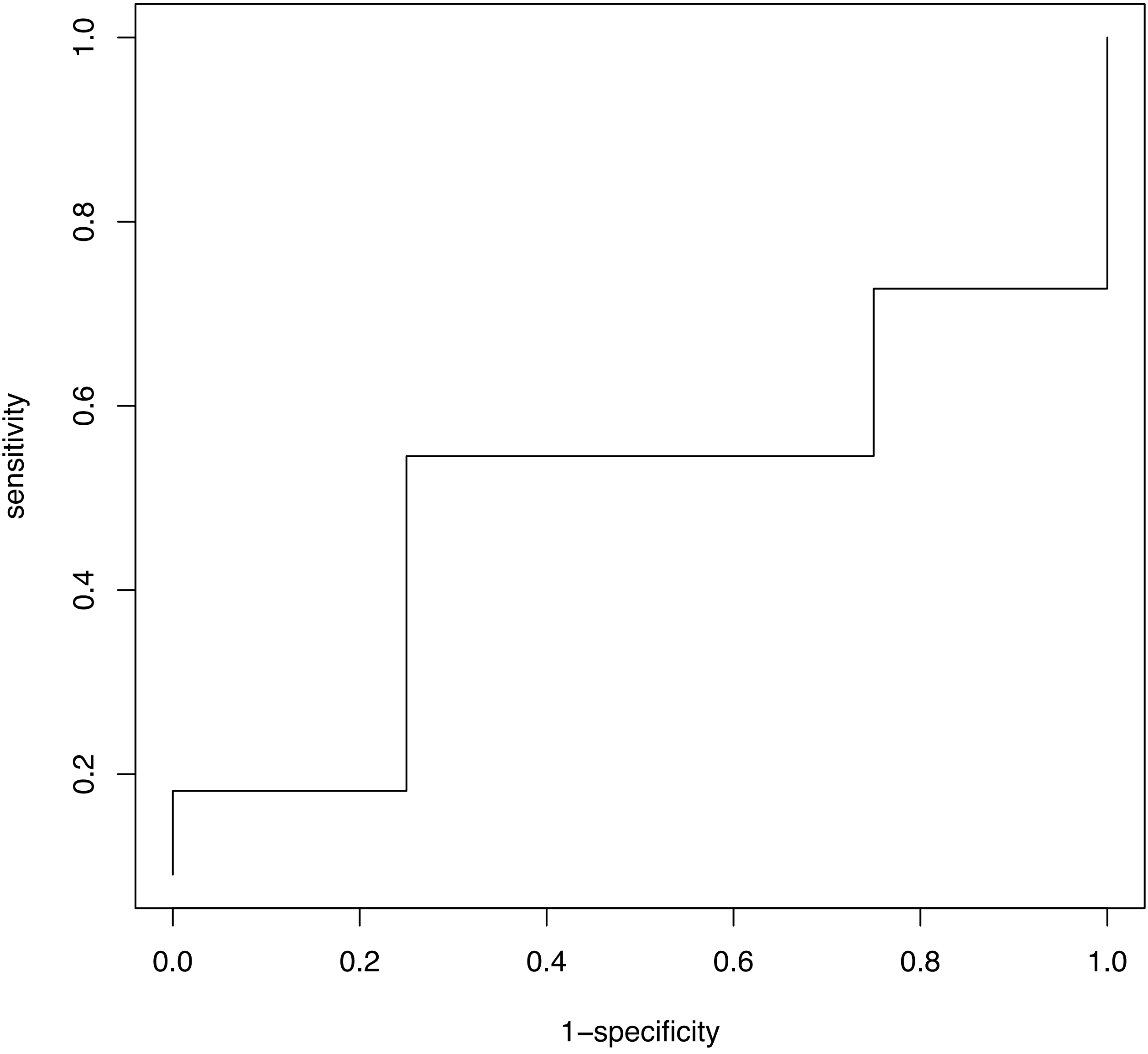

sorlie: LumA vs. Normal . Number of peptides: 30
ROC area = 0.59 p−value = 0.33

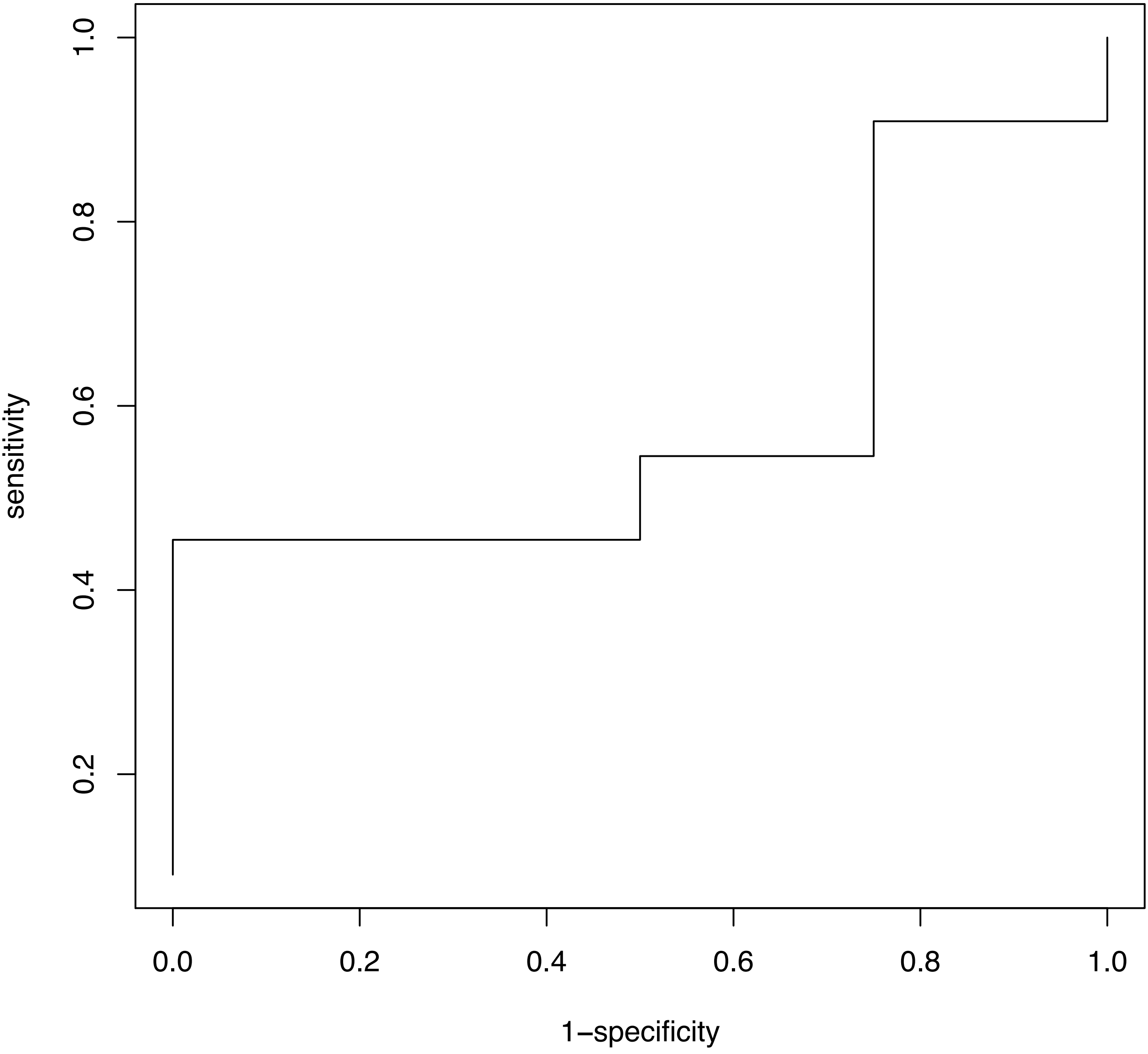

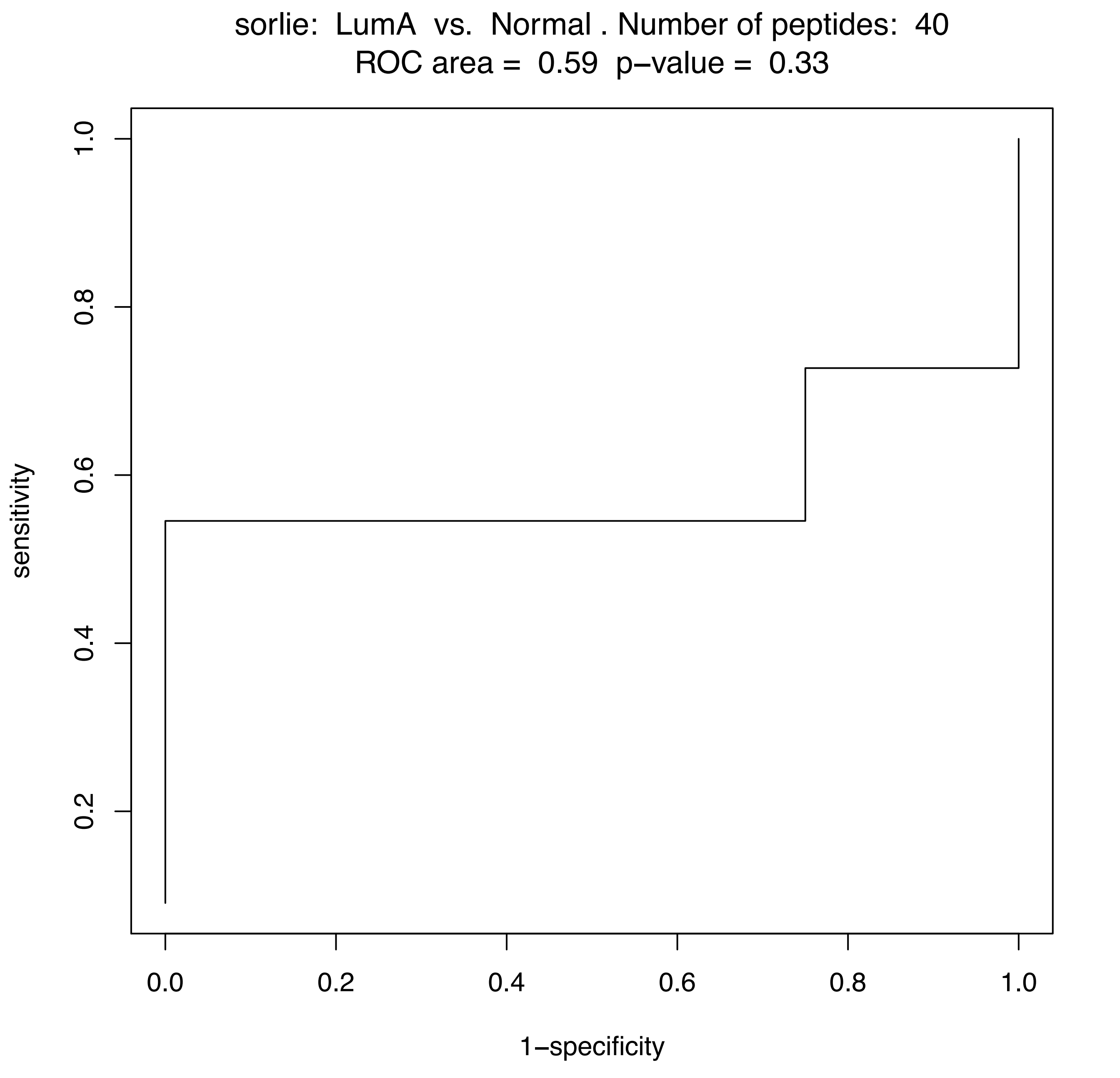
sorlie: LumA vs. Normal . Number of peptides: 40
ROC area = 0.59 p−value = 0.33
sensitivity
1−specificity
0.0
0.2
0.4
0.6
0.8
1.0

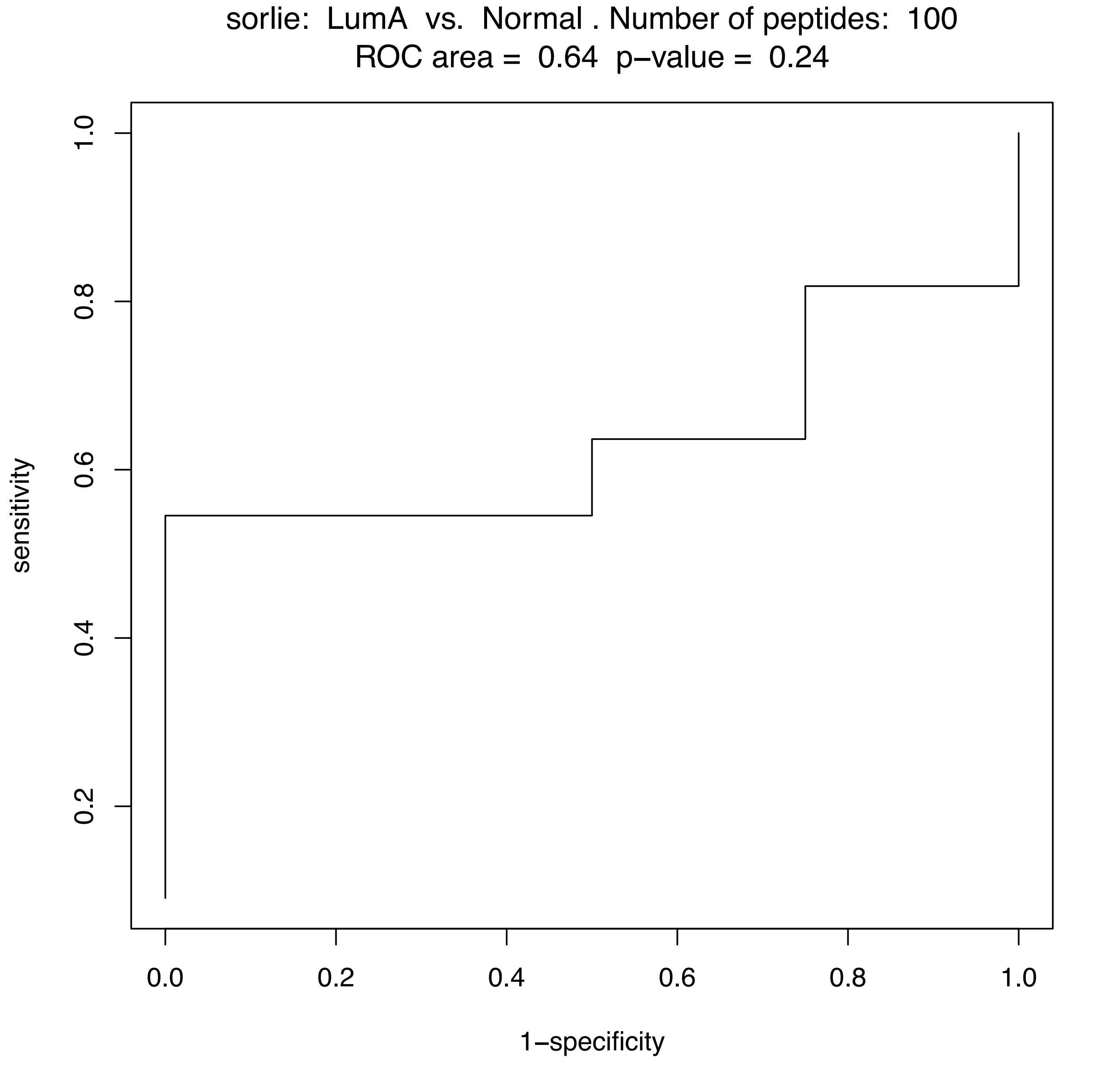
sorlie: LumA vs. Normal . Number of peptides: 100
ROC area = 0.64 p−value = 0.24
sensitivity
1−specificity
0.0
0.2
0.4
0.6
0.8
1.0

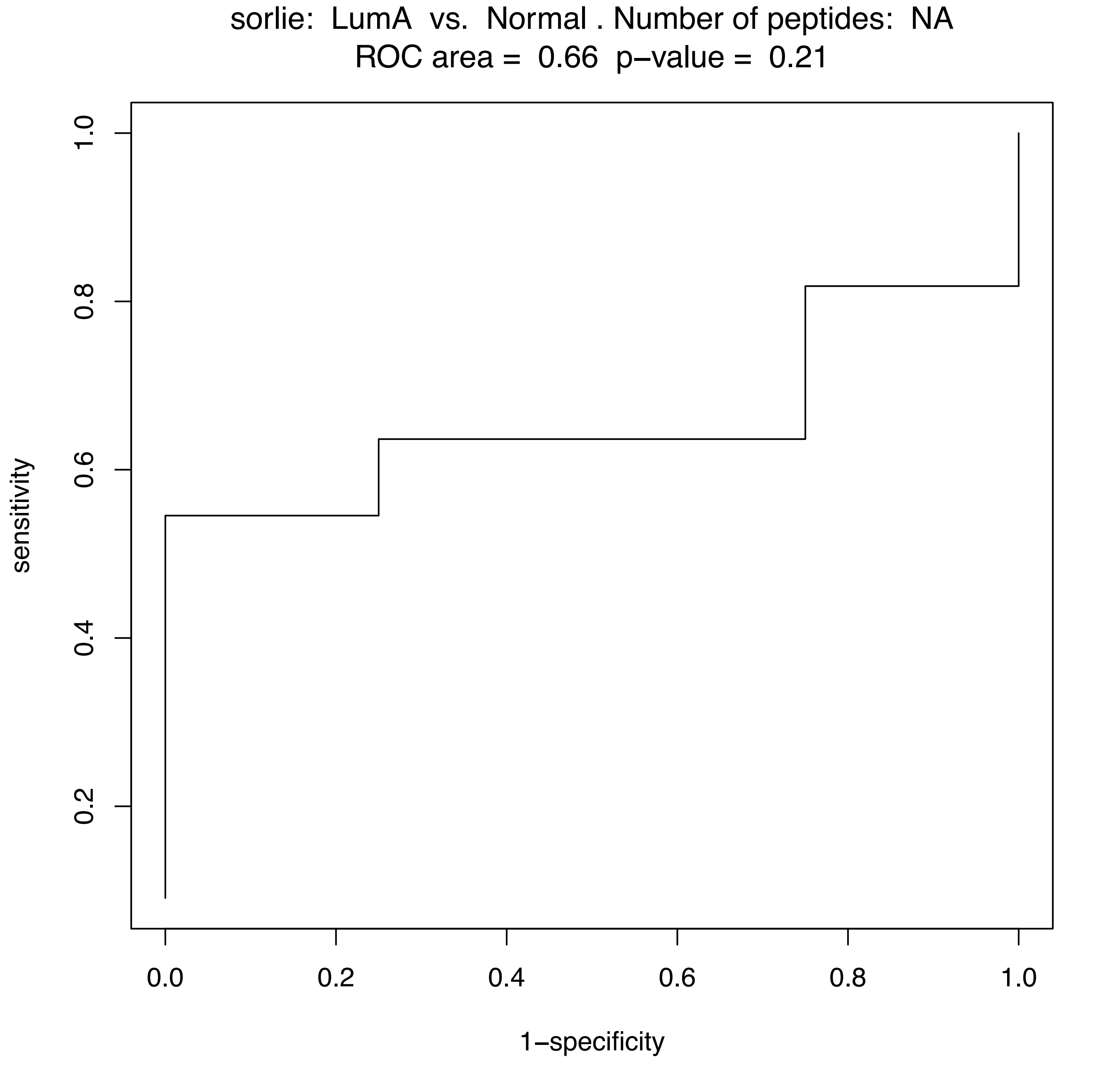
sorlie: LumA vs. Normal . Number of peptides: NA
ROC area = 0.66 p−value = 0.21
sensitivity
0.2
0.4
0.6
0.8
1.0
0.0
0.2
0.4
0.6
0.8
1.0
1−specificity

pam50: LumA vs. Normal . Number of peptides: 20
ROC area = 0.52 p−value = 0.47

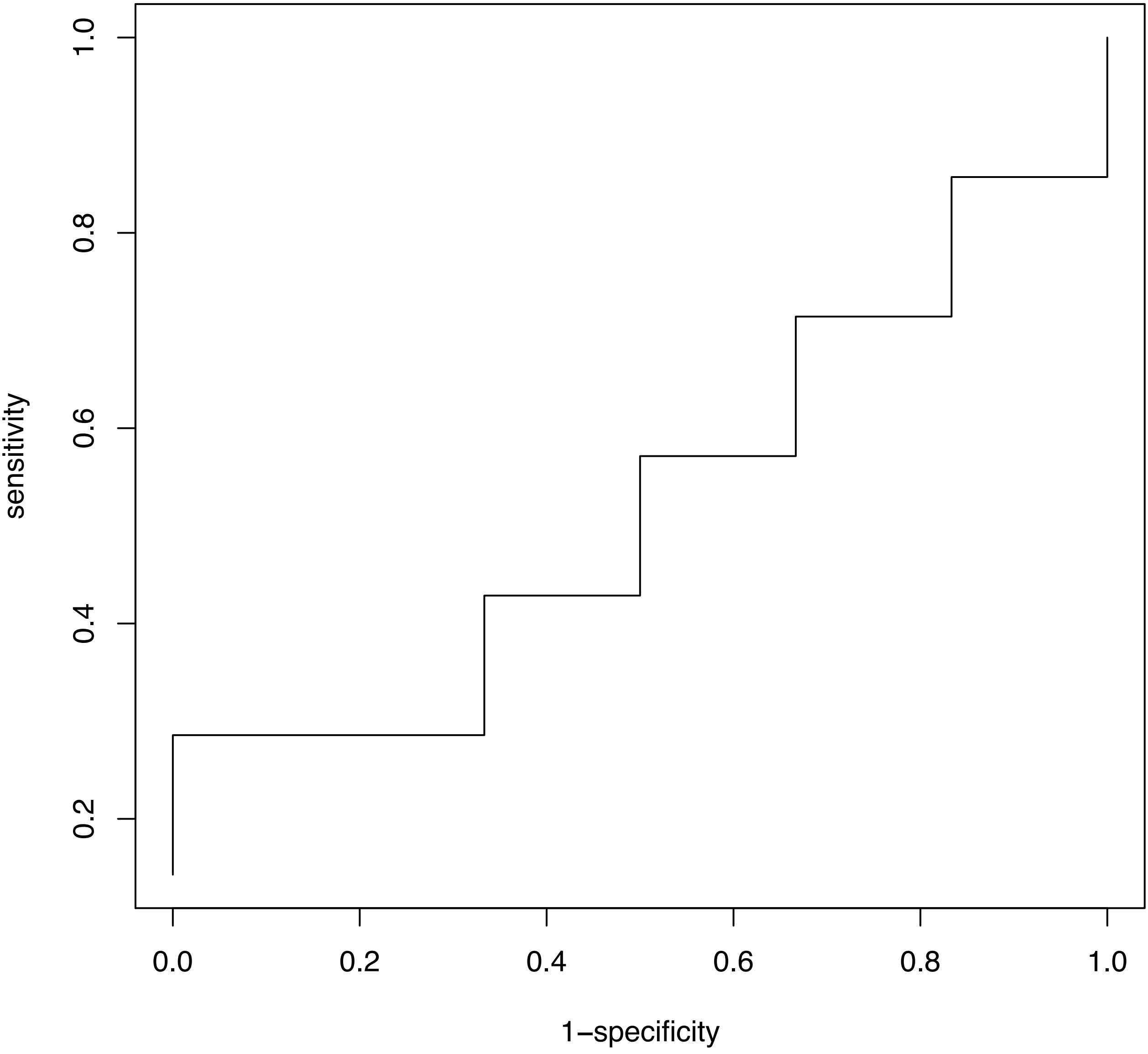

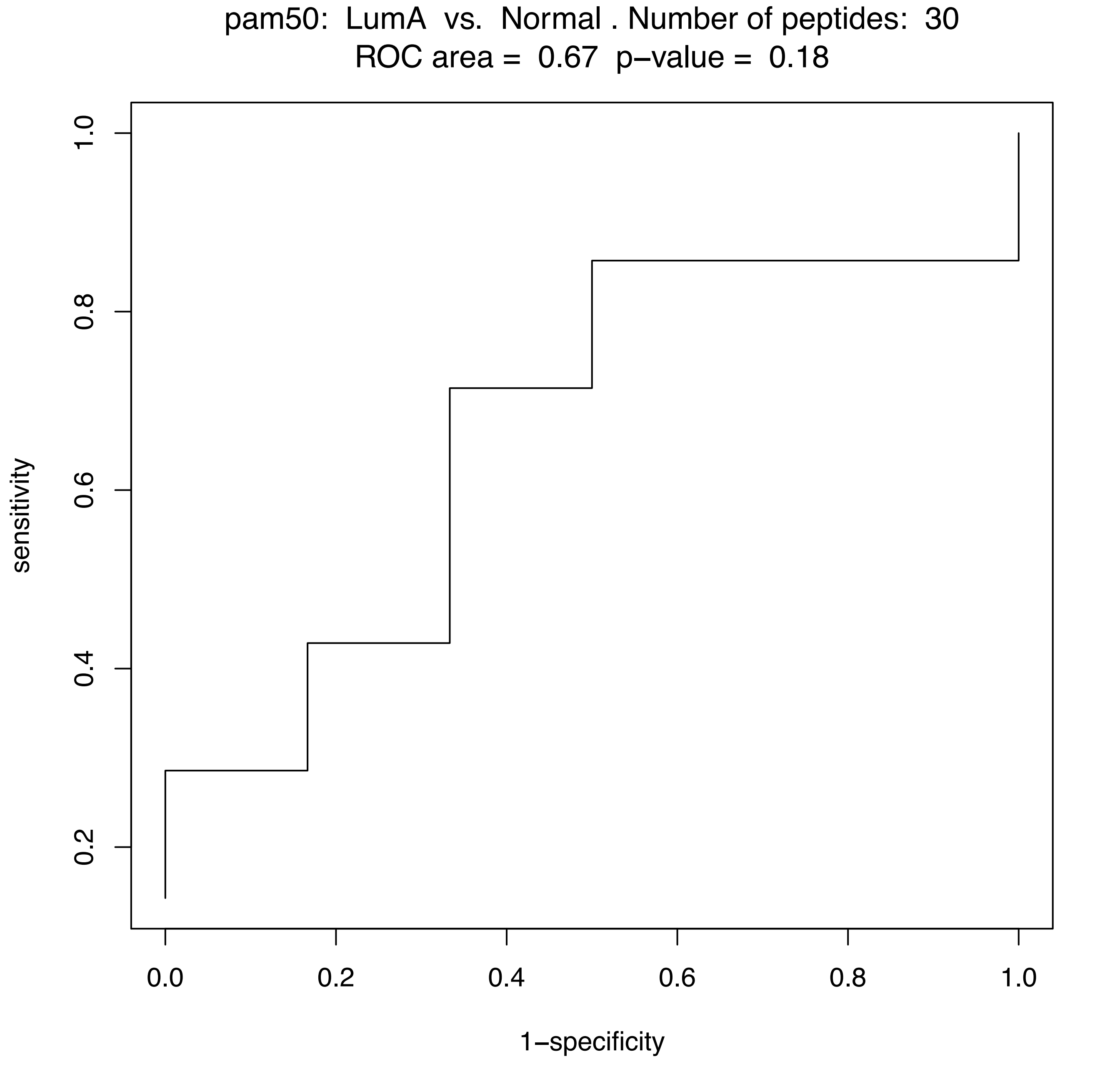
pam50: LumA vs. Normal . Number of peptides: 30
ROC area = 0.67 p−value = 0.18
sensitivity
1.0
0.8
0.6
0.4
0.2
0.0
0.2
0.4
0.6
0.8
1.0
1−specificity

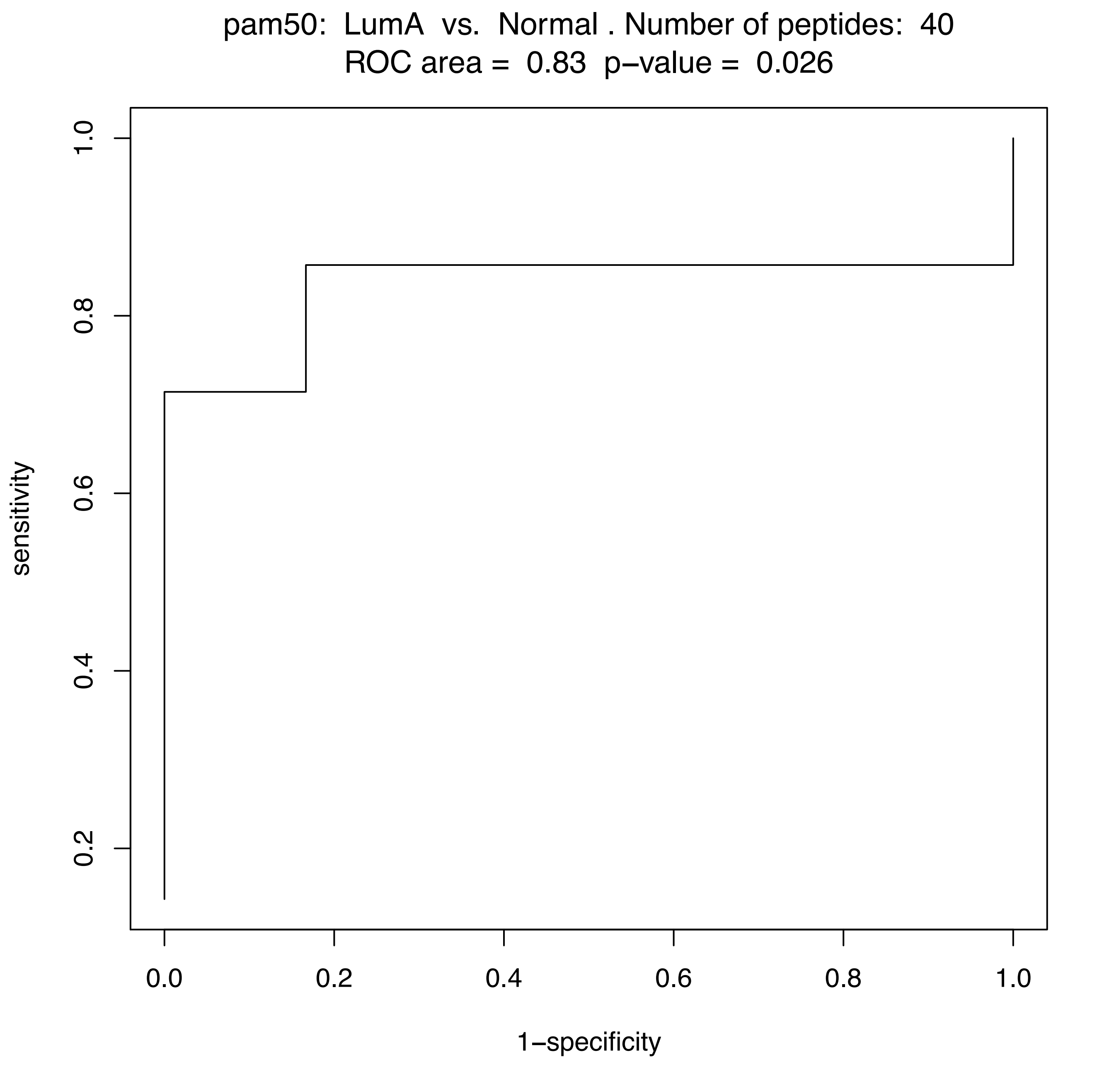
pam50: LumA vs. Normal . Number of peptides: 40
ROC area = 0.83 p−value = 0.026
sensitivity
1.0
0.8
0.6
0.4
0.2
0.0
0.2
0.4
0.6
0.8
1.0
1−specificity

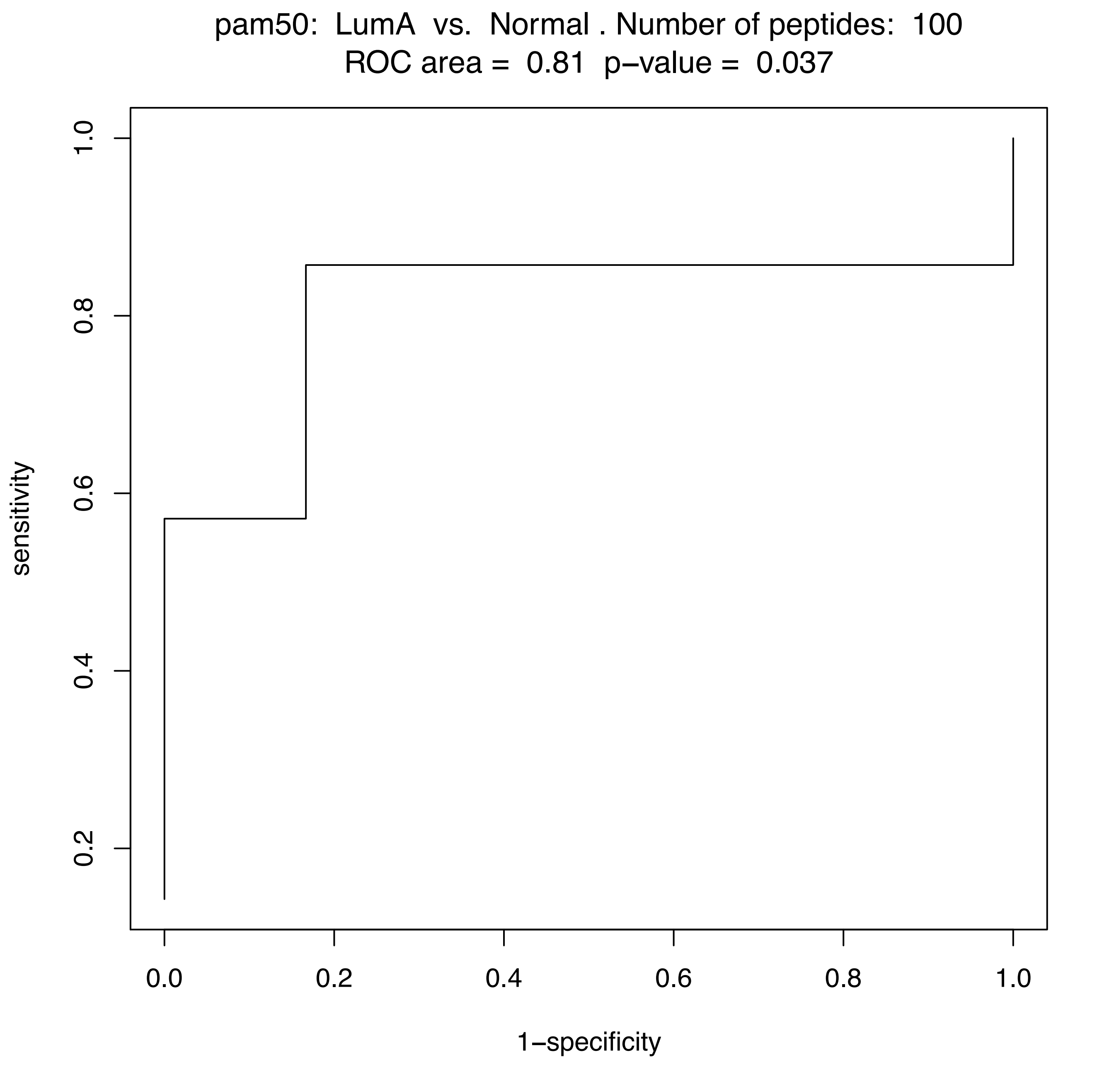

pam50: LumA vs. Normal . Number of peptides: 100
ROC area = 0.81 p−value = 0.037
sensitivity
1.0
0.8
0.6
0.4
0.2
0.0
0.2
0.4
0.6
0.8
1.0
1−specificity

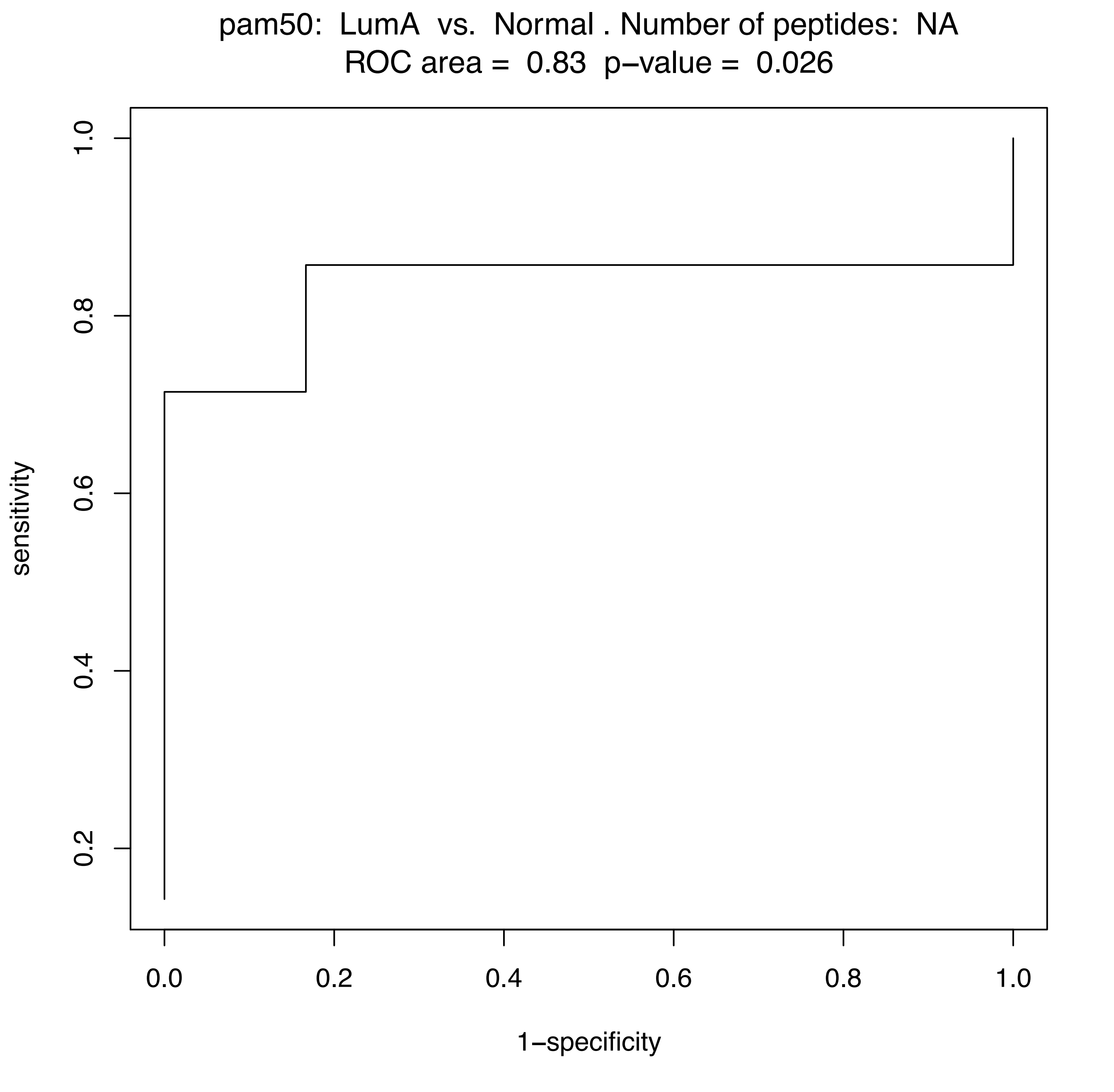
pam50: LumA vs. Normal . Number of peptides: NA
ROC area = 0.83 p−value = 0.026
sensitivity
1.0
0.8
0.6
0.4
0.2
0.0
0.2
0.4
0.6
0.8
1.0
1−specificity

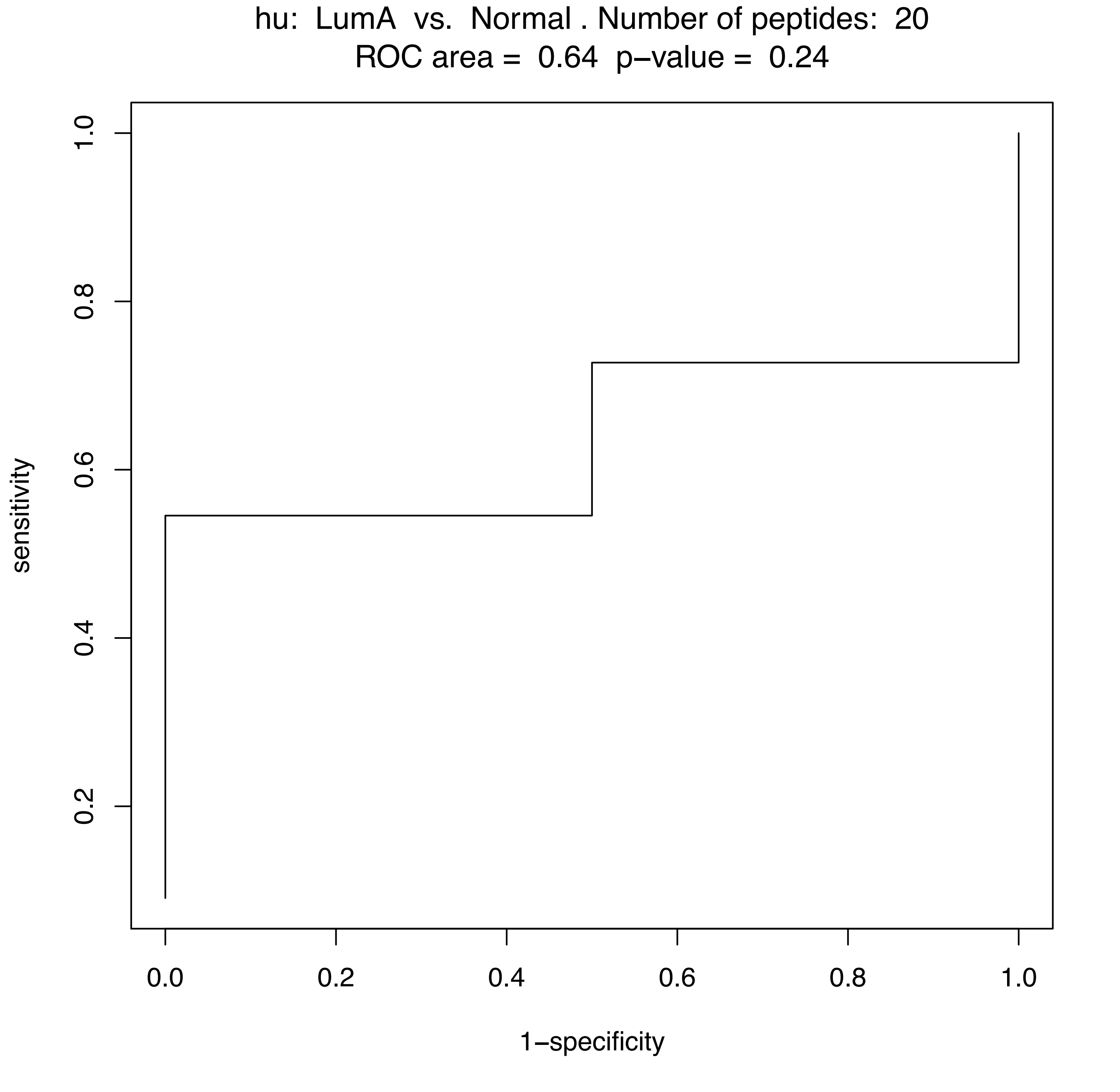
hu: LumA vs. Normal . Number of peptides: 20
ROC area = 0.64 p−value = 0.24
sensitivity
1.0
0.8
0.6
0.4
0.2
0.0
0.2
0.4
0.6
0.8
1.0
1−specificity

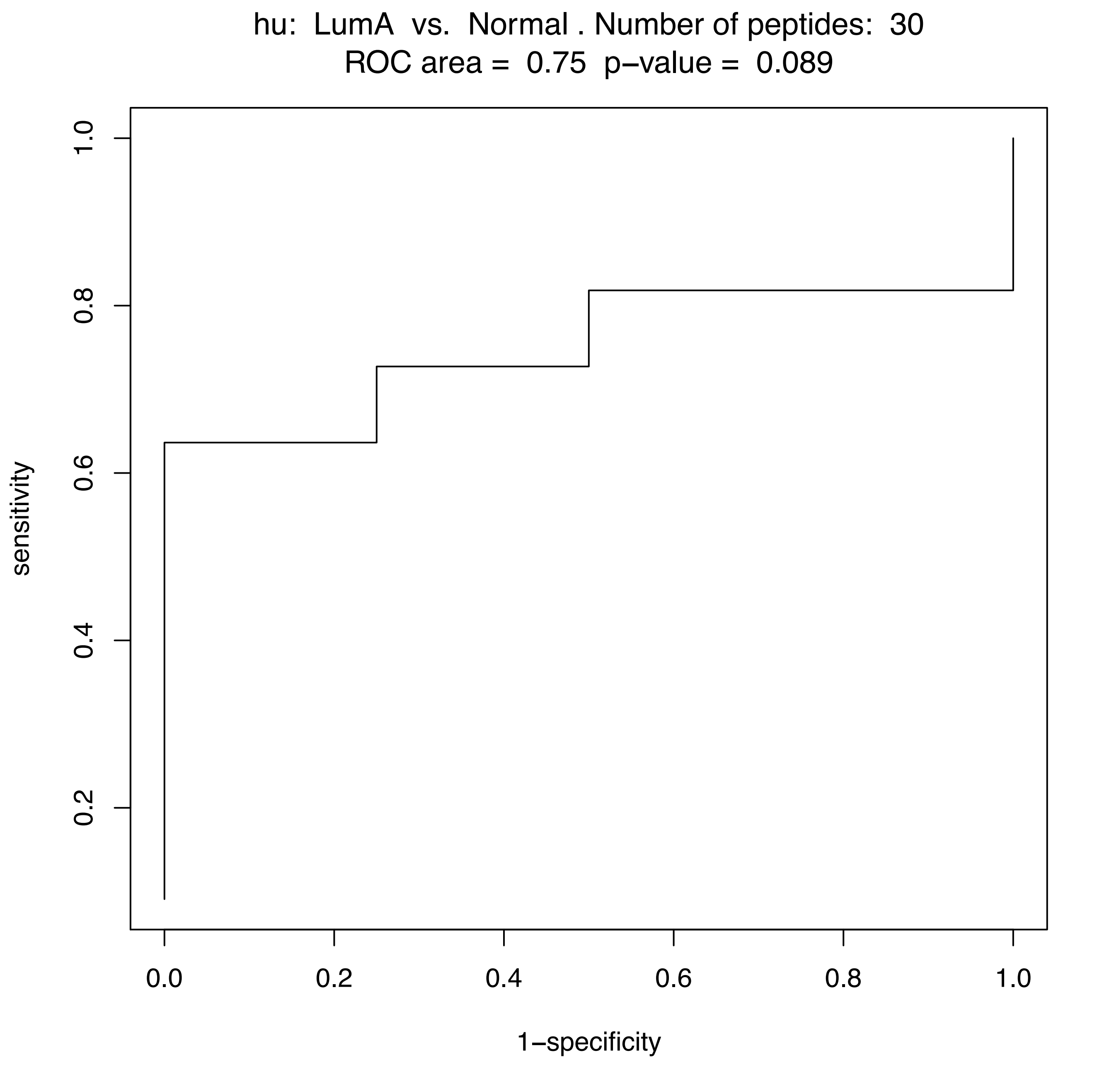
hu: LumA vs. Normal . Number of peptides: 30
ROC area = 0.75 p−value = 0.089
sensitivity
1−specificity
0.0
0.2
0.4
0.6
0.8
1.0
0.2
0.4
0.6
0.8
1.0

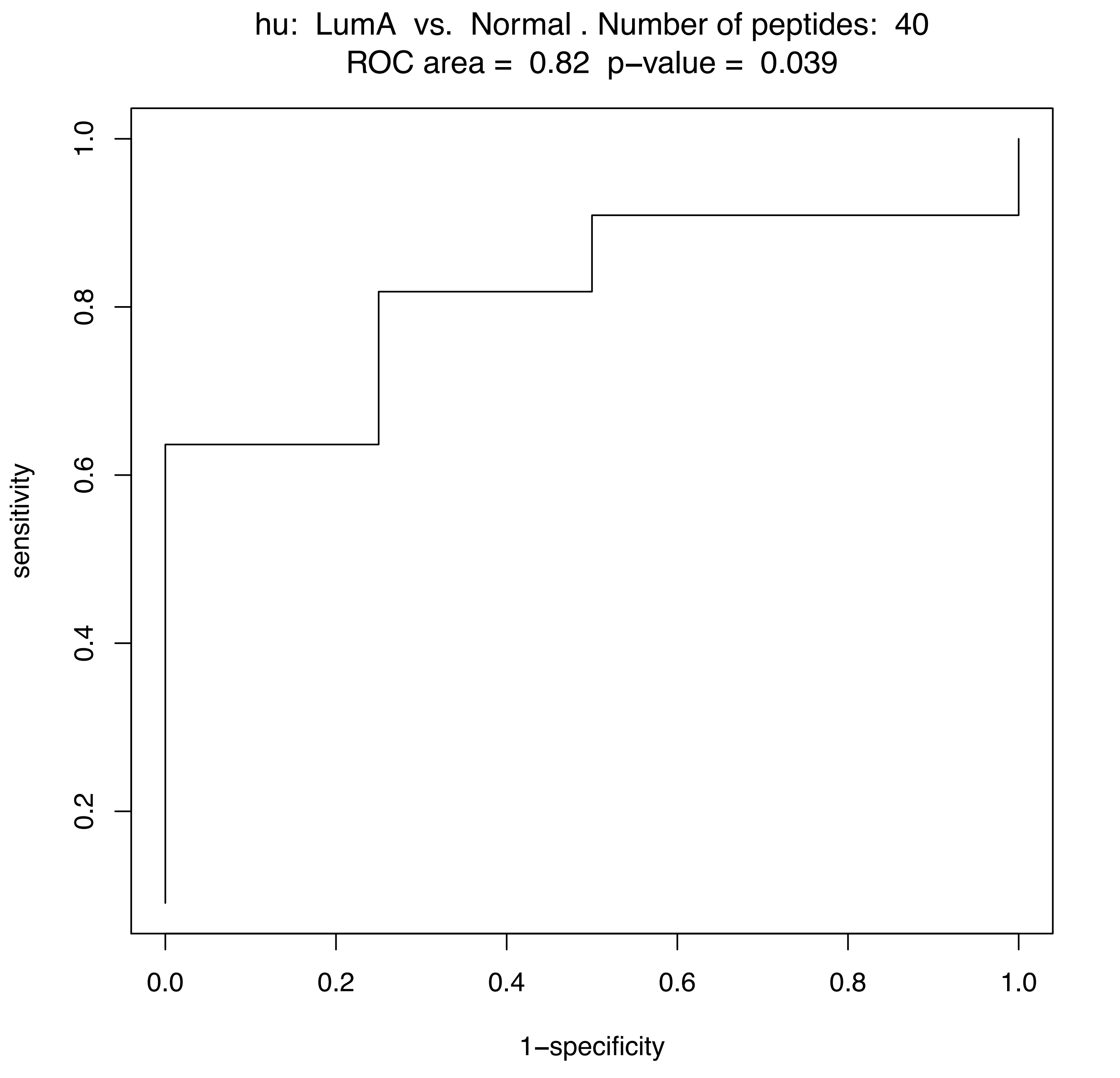
hu: LumA vs. Normal . Number of peptides: 40
ROC area = 0.82 p−value = 0.039
sensitivity
1−specificity
0.0
0.2
0.4
0.6
0.8
1.0

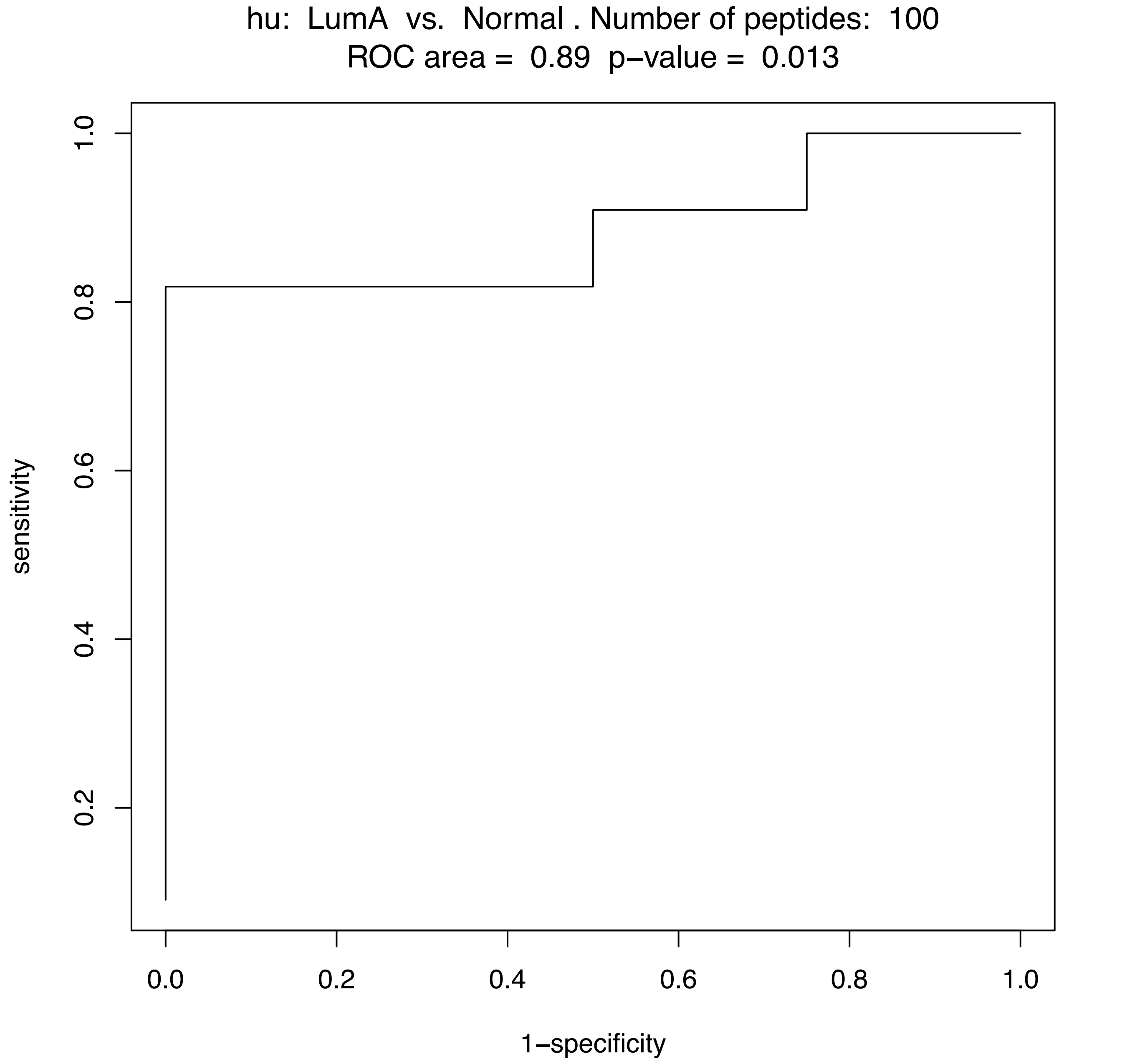
hu: LumA vs. Normal . Number of peptides: 100
ROC area = 0.89 p−value = 0.013
sensitivity
1−specificity
0.0
0.2
0.4
0.6
0.8
1.0
0.2
0.4
0.6
0.8
1.0

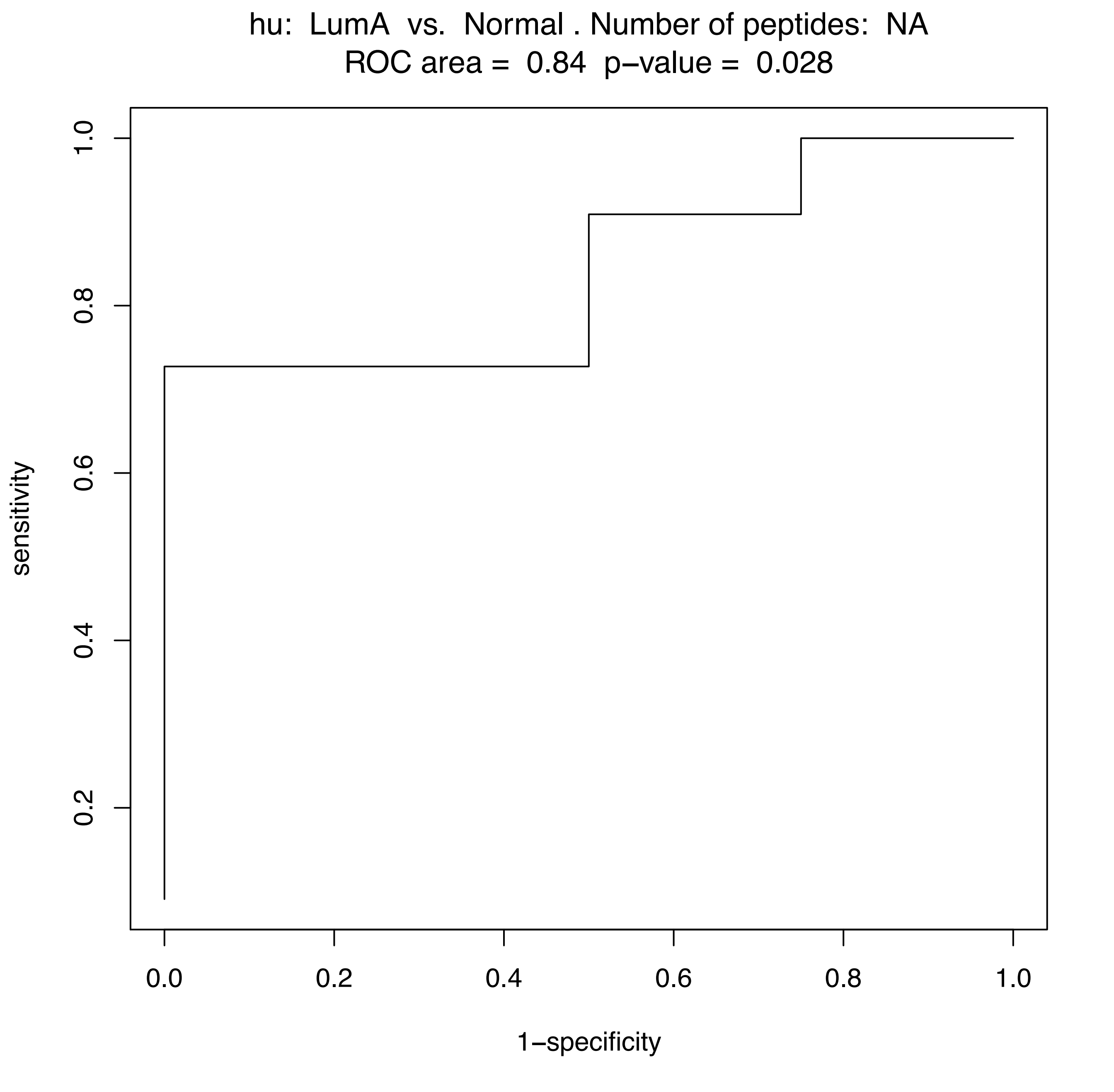
hu: LumA vs. Normal . Number of peptides: NA
ROC area = 0.84 p−value = 0.028
sensitivity
0.2
0.4
0.6
0.8
1.0
0.0
0.2
0.4
0.6
0.8
1.0
1−specificity

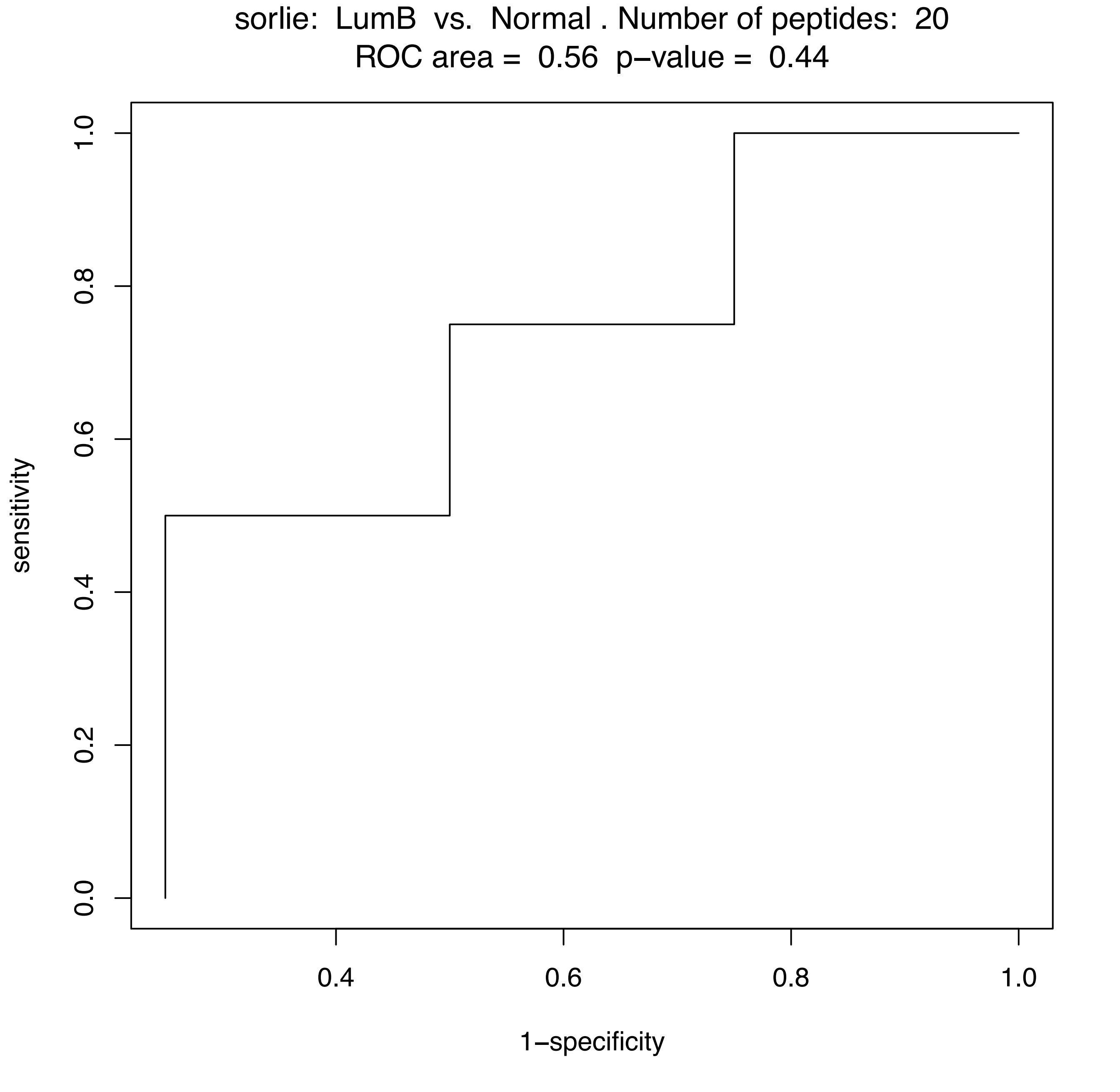

sorlie: LumB vs. Normal . Number of peptides: 20
ROC area = 0.56 p−value = 0.44
sensitivity
1−specificity
0.0
0.2
0.4
0.6
0.8
1.0
0.4
0.6
0.8
1.0

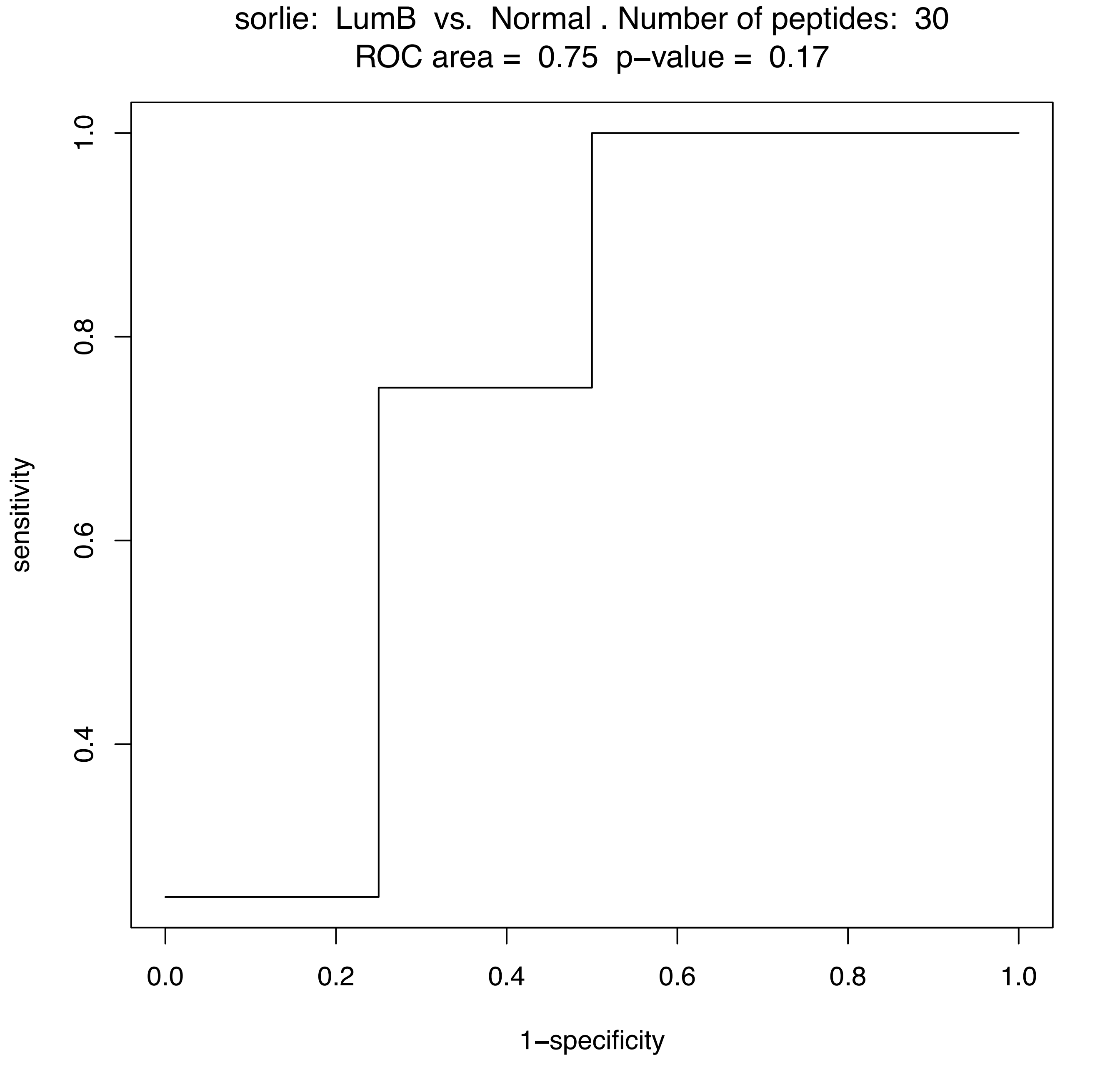
sorlie: LumB vs. Normal . Number of peptides: 30
ROC area = 0.75 p−value = 0.17
sensitivity
1.0
0.8
0.6
0.4
0.0
0.2
0.4
0.6
0.8
1.0
1−specificity

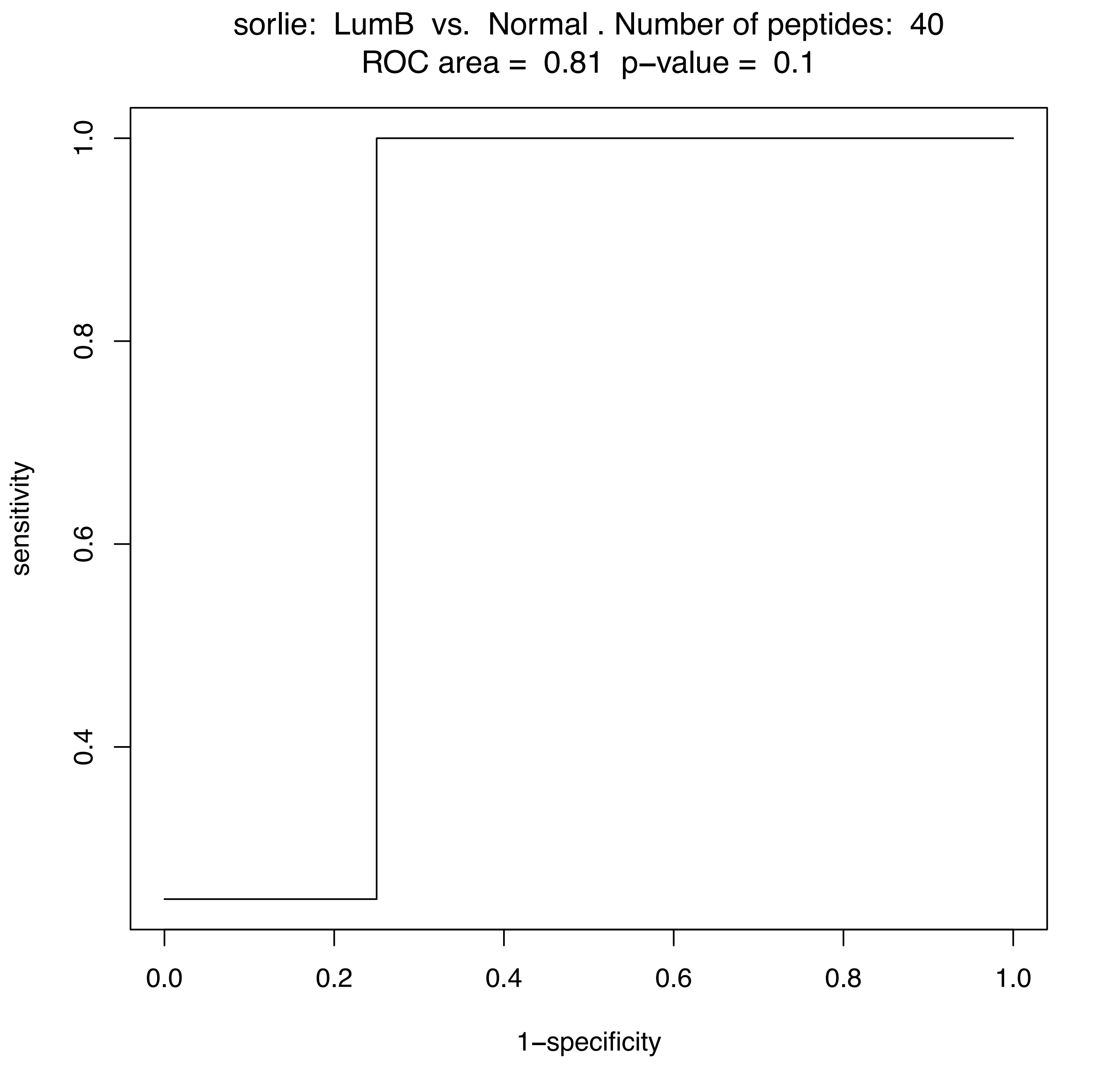

sorlie: LumB vs. Normal . Number of peptides: 40
ROC area = 0.81 p−value = 0.1
sensitivity
1.0
0.8
0.6
0.4
0.0
0.2
0.4
0.6
0.8
1.0
1−specificity

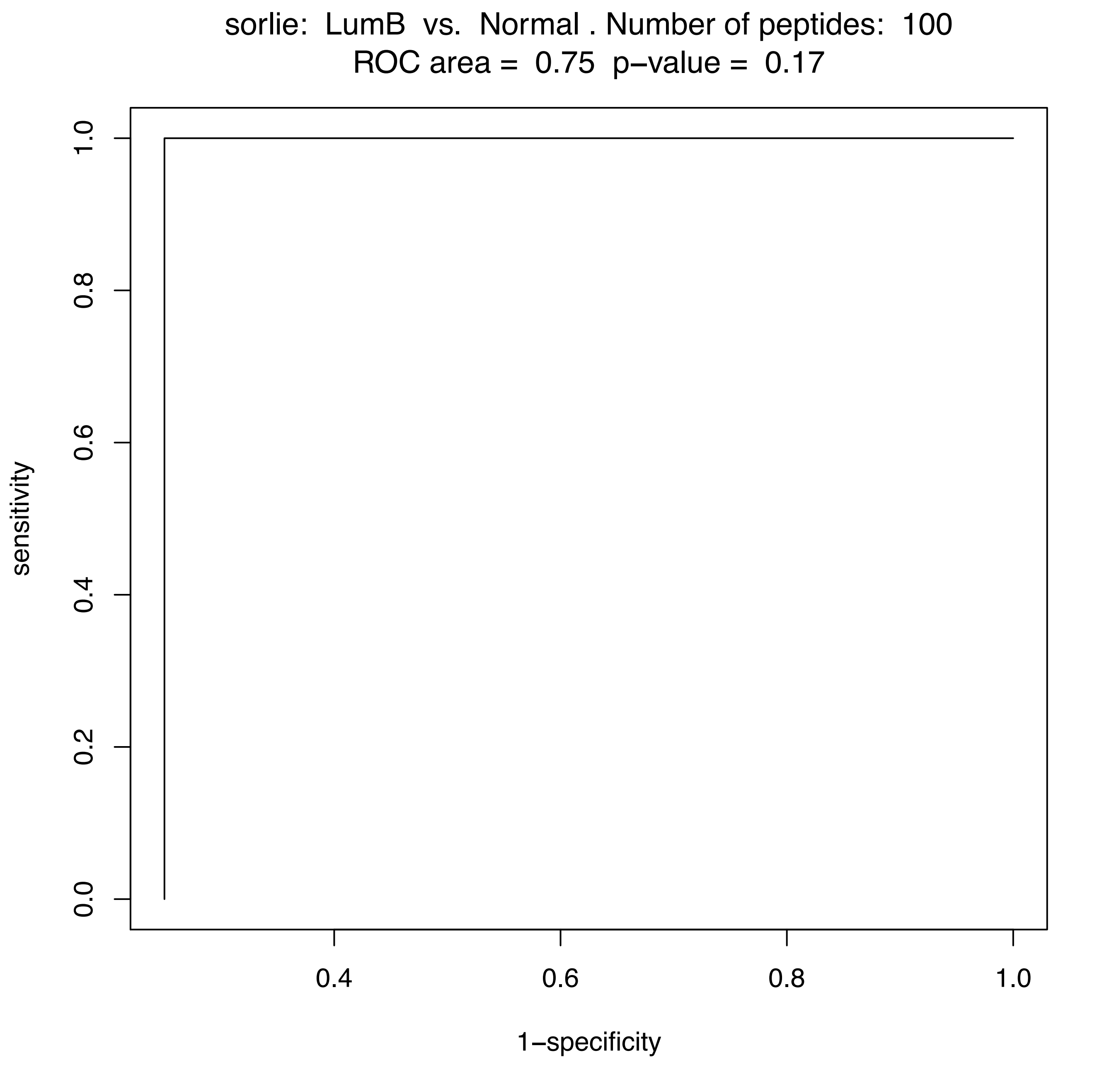
sorlie: LumB vs. Normal . Number of peptides: 100
ROC area = 0.75 p−value = 0.17
sensitivity
1−specificity
0.0
0.2
0.4
0.6
0.8
1.0
0.4
0.6
0.8
1.0

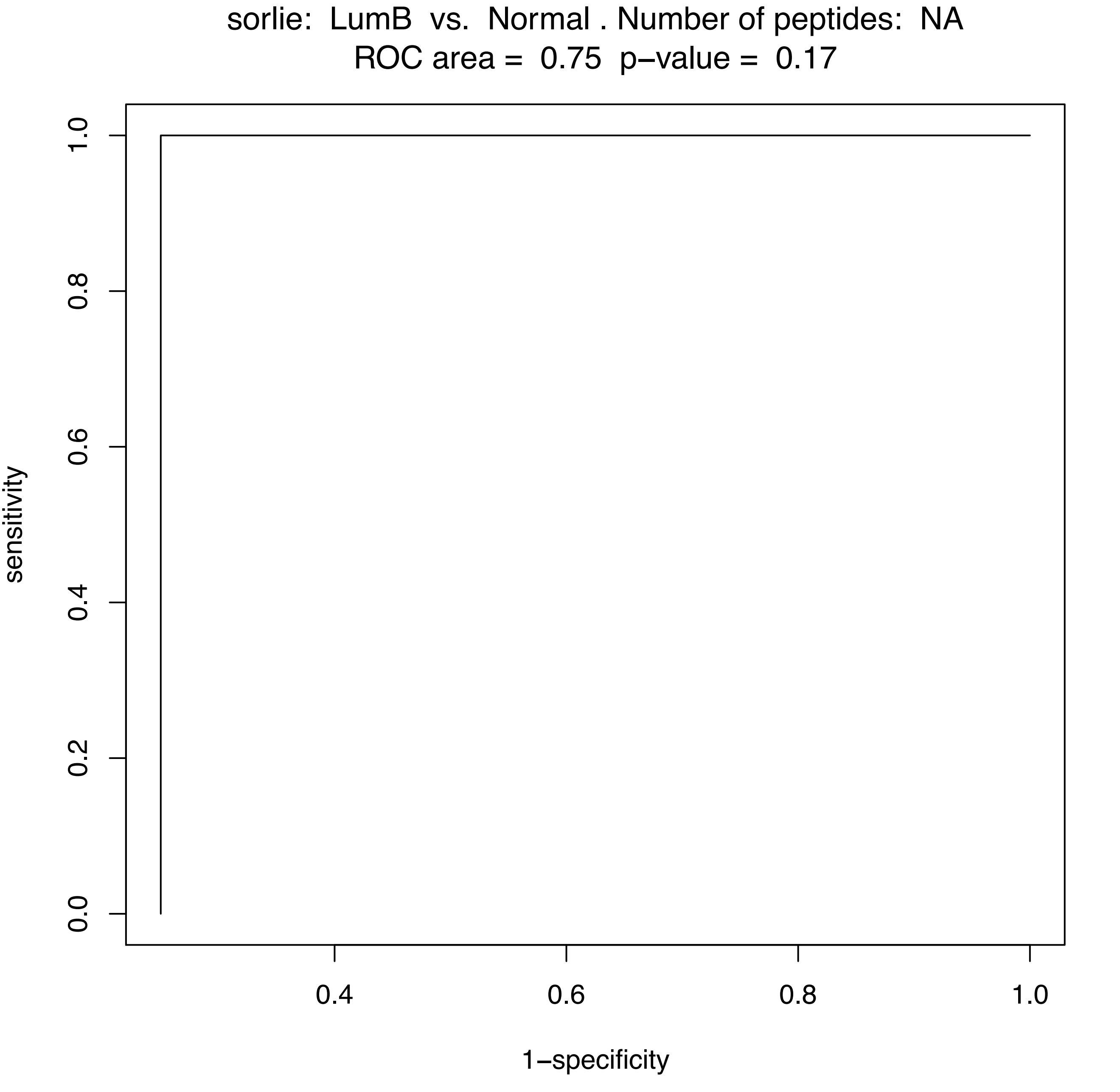
sorlie: LumB vs. Normal . Number of peptides: NA
ROC area = 0.75 p−value = 0.17
0.0
0.2
0.4
0.6
0.8
1.0
sensitivity
0.4
0.6
0.8
1.0
1−specificity

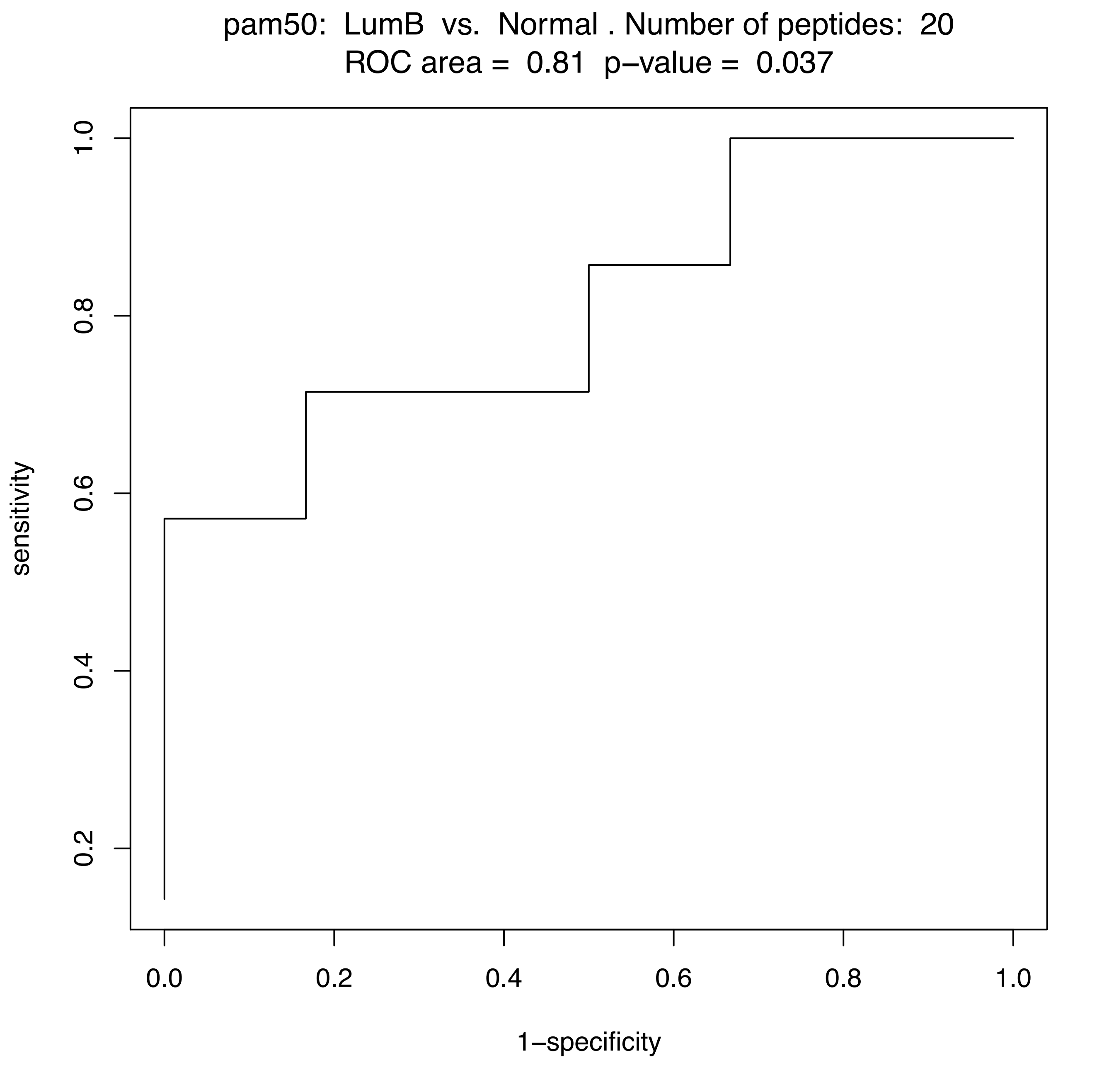
pam50: LumB vs. Normal . Number of peptides: 20
ROC area = 0.81 p−value = 0.037
sensitivity
1−specificity
0.2
0.4
0.6
0.8
1.0
0.0
0.2
0.4
0.6
0.8
1.0

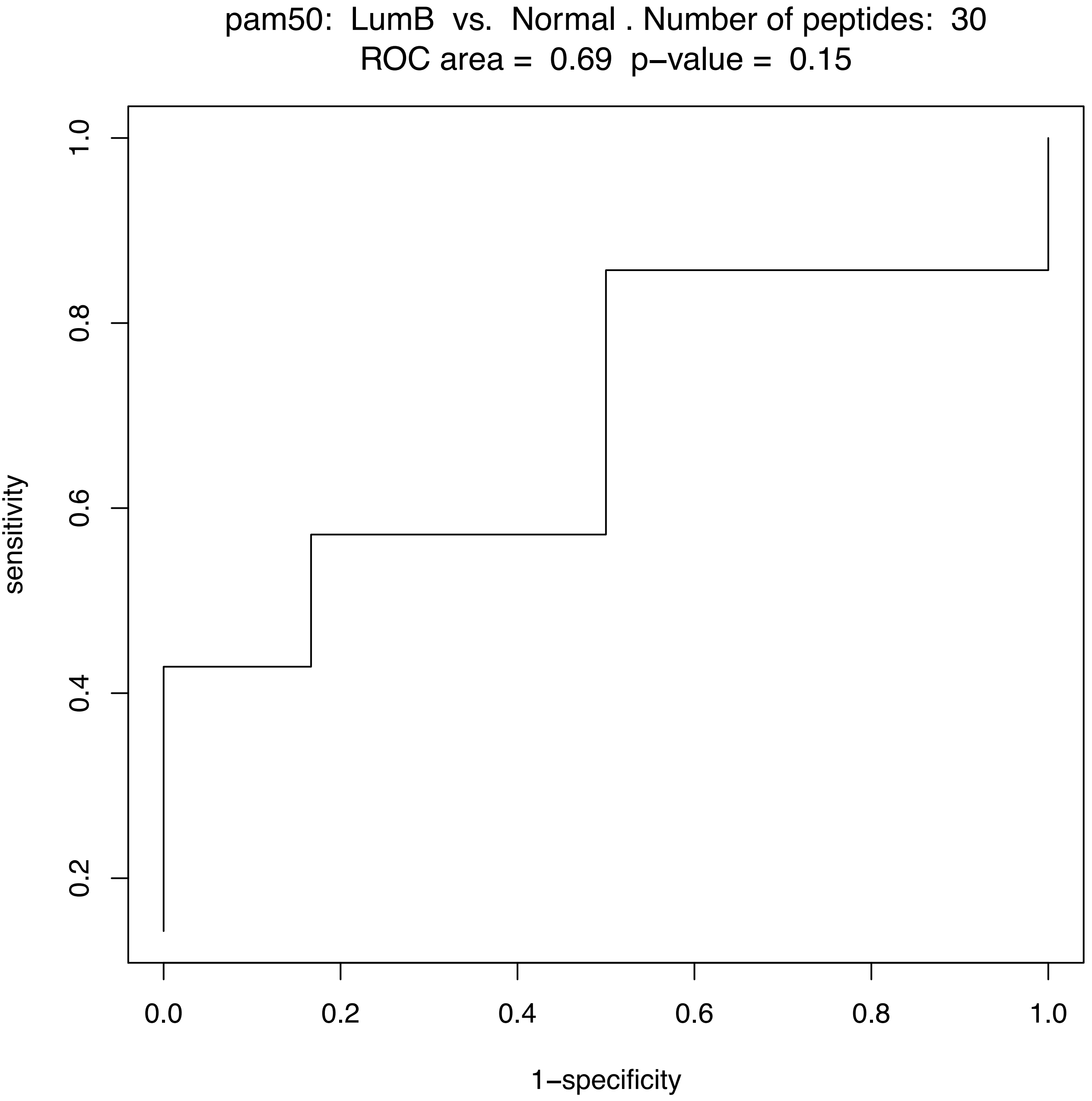

pam50: LumB vs. Normal . Number of peptides: 30
ROC area = 0.69 p−value = 0.15
sensitivity
0.2
0.4
0.6
0.8
1.0
0.0
0.2
0.4
0.6
0.8
1.0
1−specificity

pam50: LumB vs. Normal . Number of peptides: 40
ROC area = 0.81 p−value = 0.037

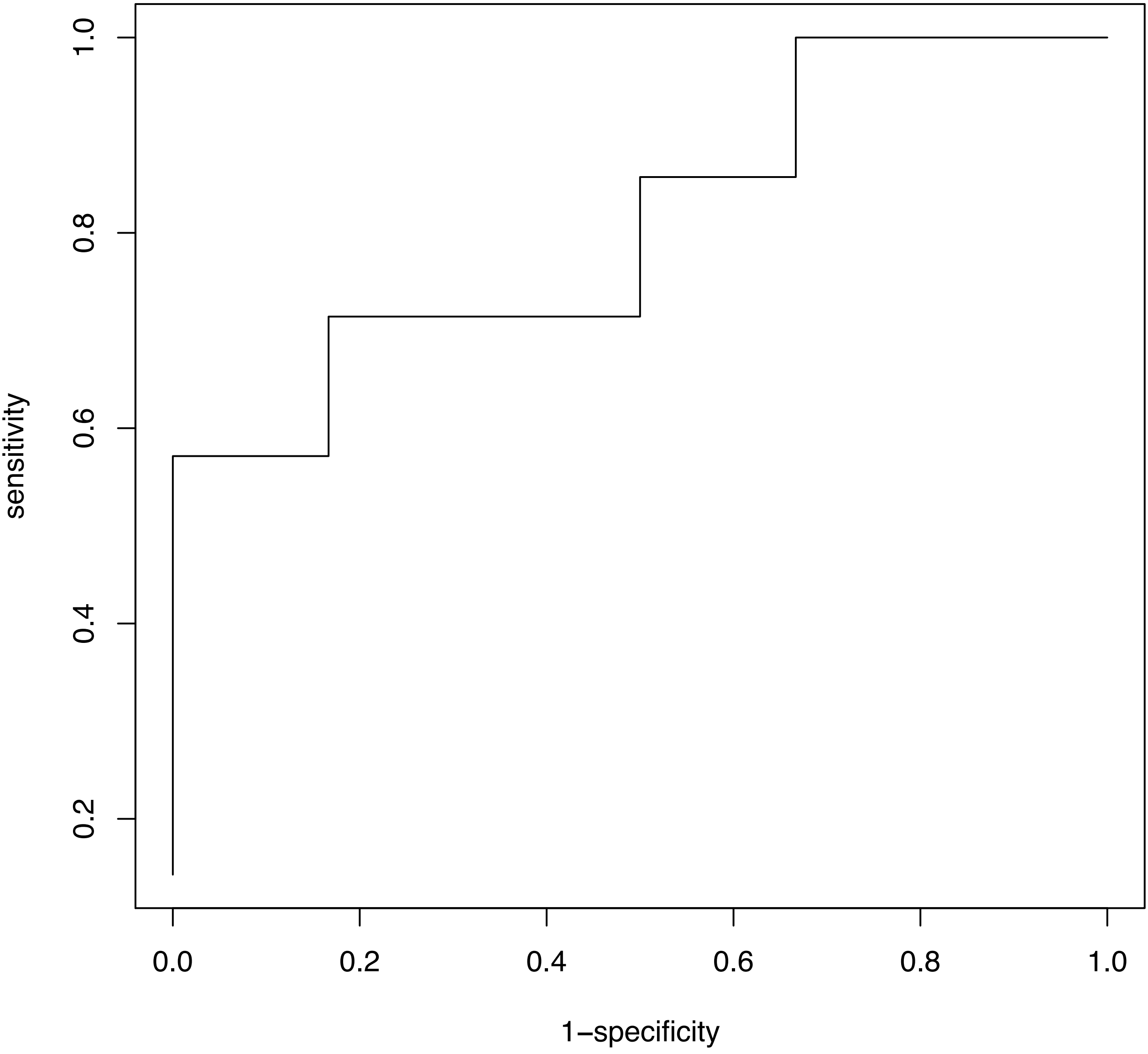

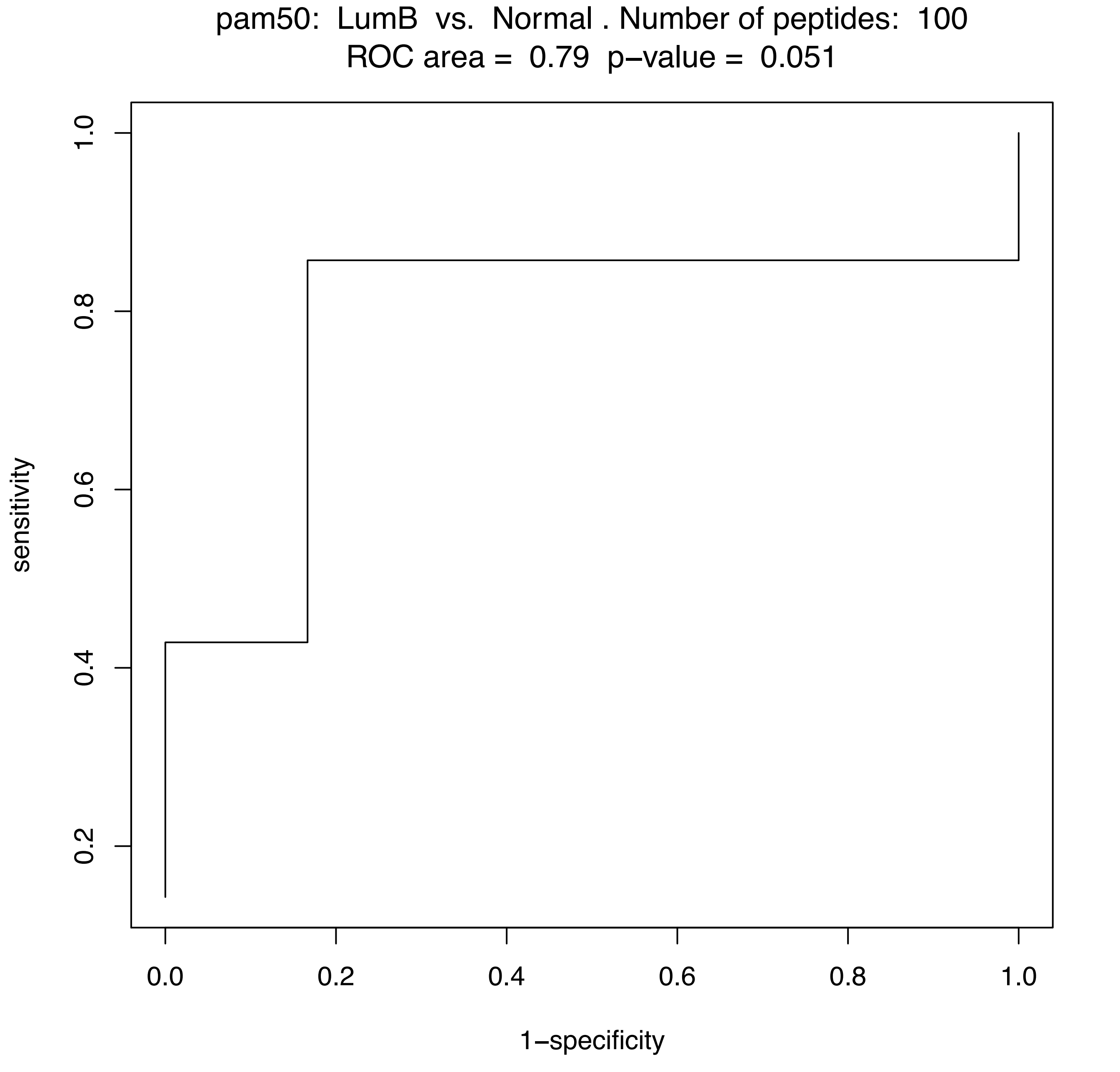
pam50: LumB vs. Normal . Number of peptides: 100
ROC area = 0.79 p−value = 0.051
sensitivity
1−specificity
0.2
0.4
0.6
0.8
1.0
0.0
0.2
0.4
0.6
0.8
1.0

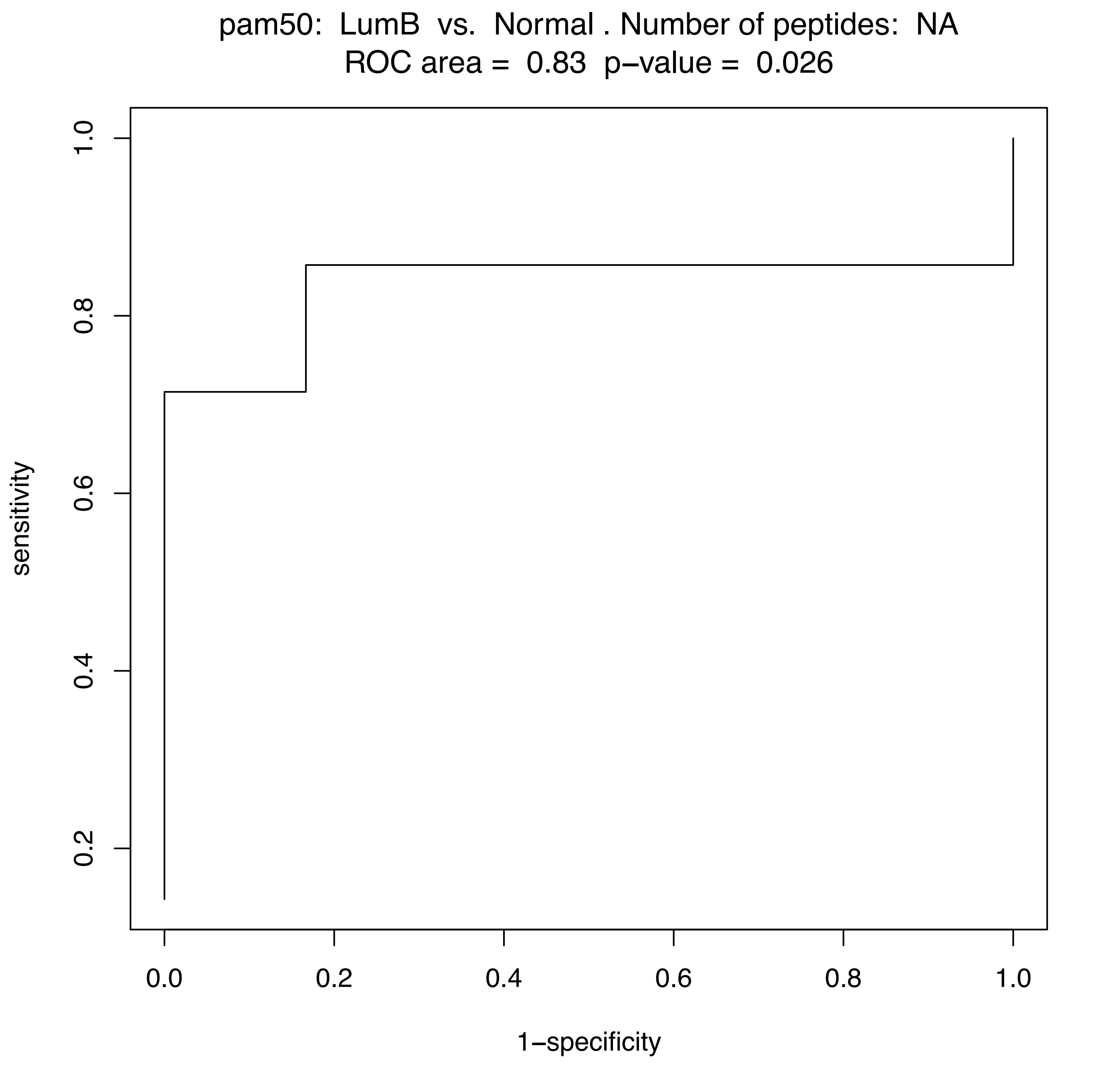
pam50: LumB vs. Normal . Number of peptides: NA
ROC area = 0.83 p−value = 0.026
sensitivity
1−specificity
0.0
0.2
0.4
0.6
0.8
1.0
0.2
0.4
0.6
0.8
1.0

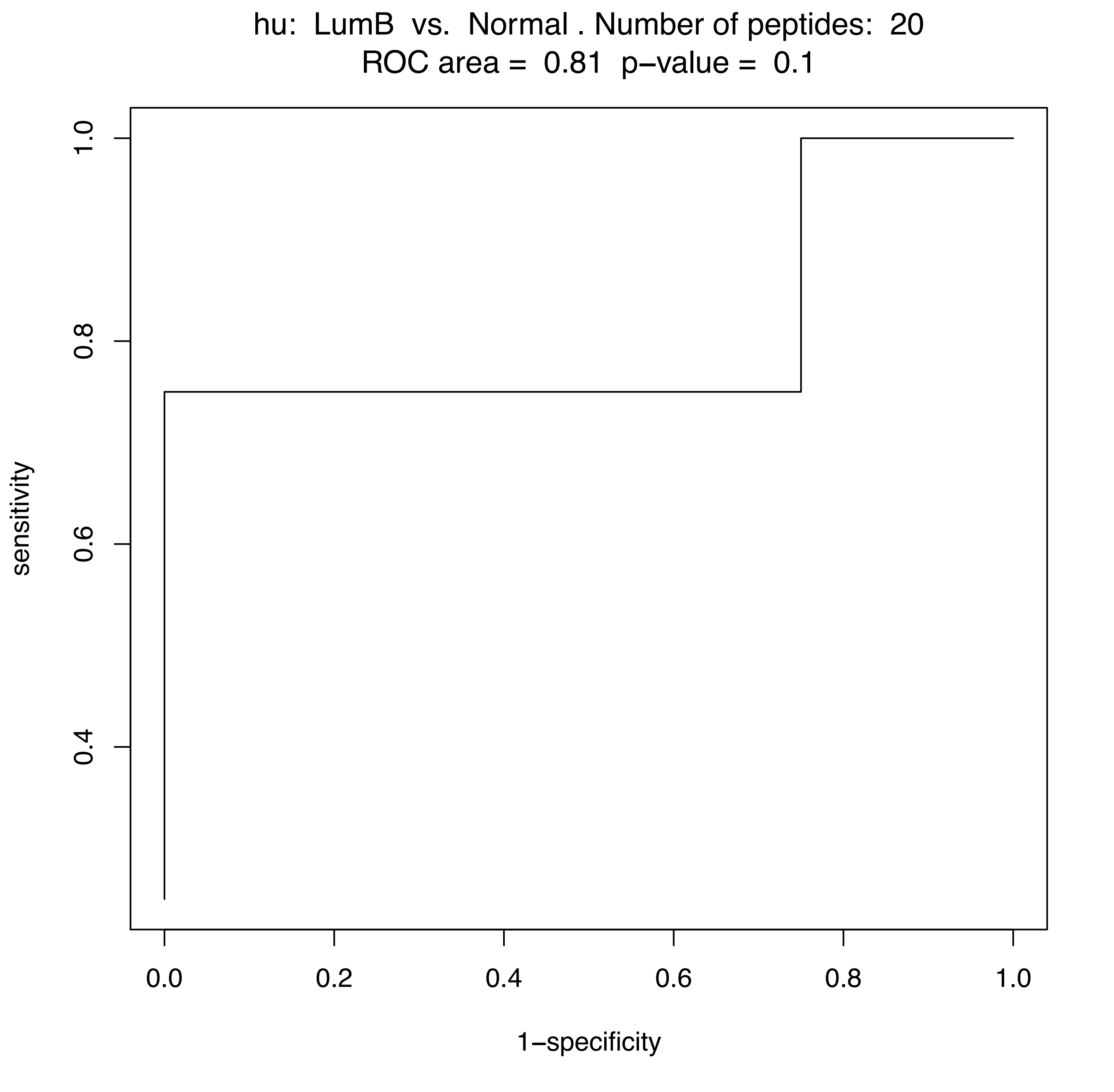
hu: LumB vs. Normal . Number of peptides: 20
ROC area = 0.81 p−value = 0.1
sensitivity
1−specificity
0.0
0.2
0.4
0.6
0.8
1.0
0.4
0.6
0.8
1.0

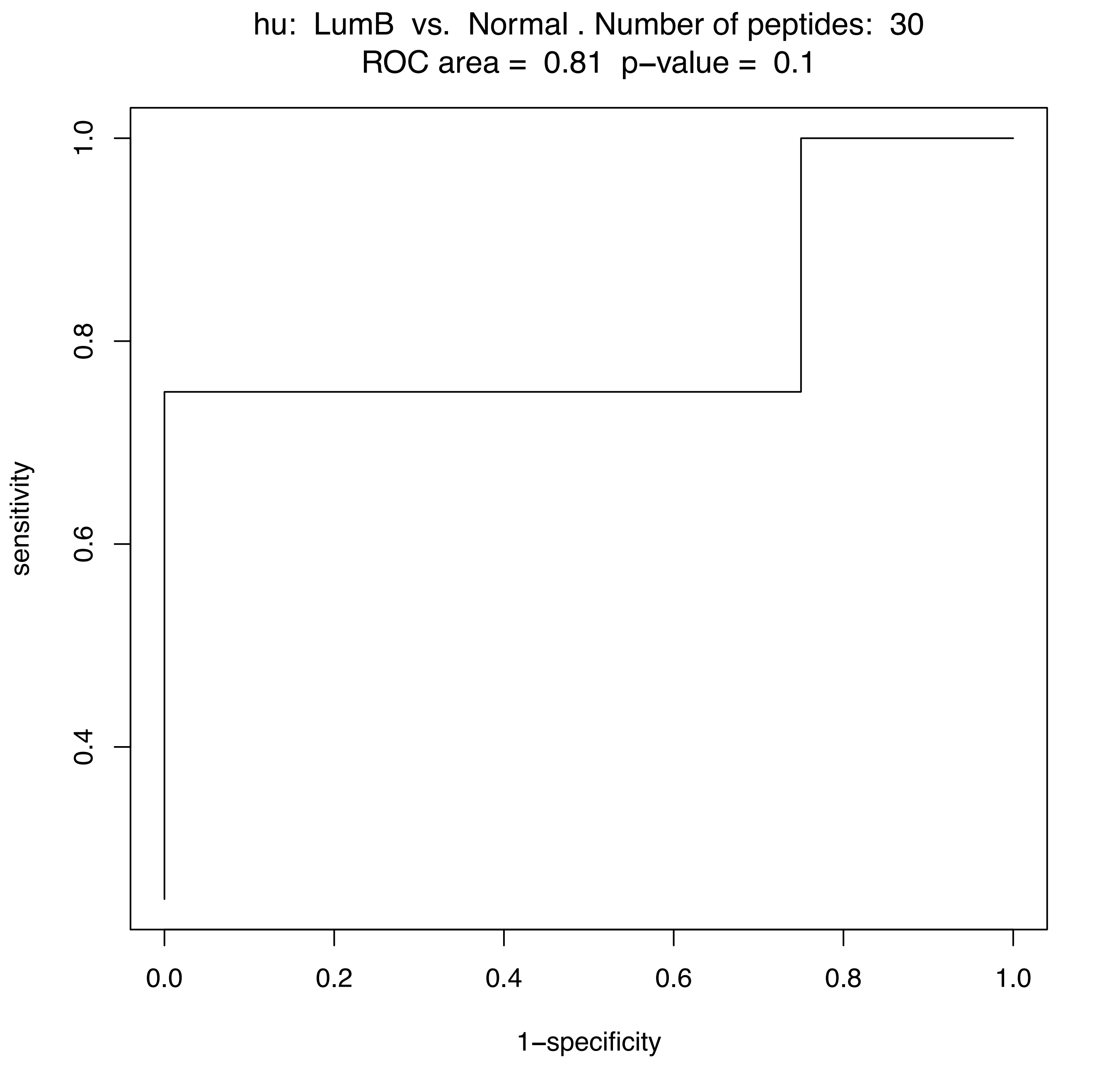
hu: LumB vs. Normal . Number of peptides: 30
ROC area = 0.81 p−value = 0.1
sensitivity
1.0
0.8
0.6
0.4
0.0
0.2
0.4
0.6
0.8
1.0
1−specificity

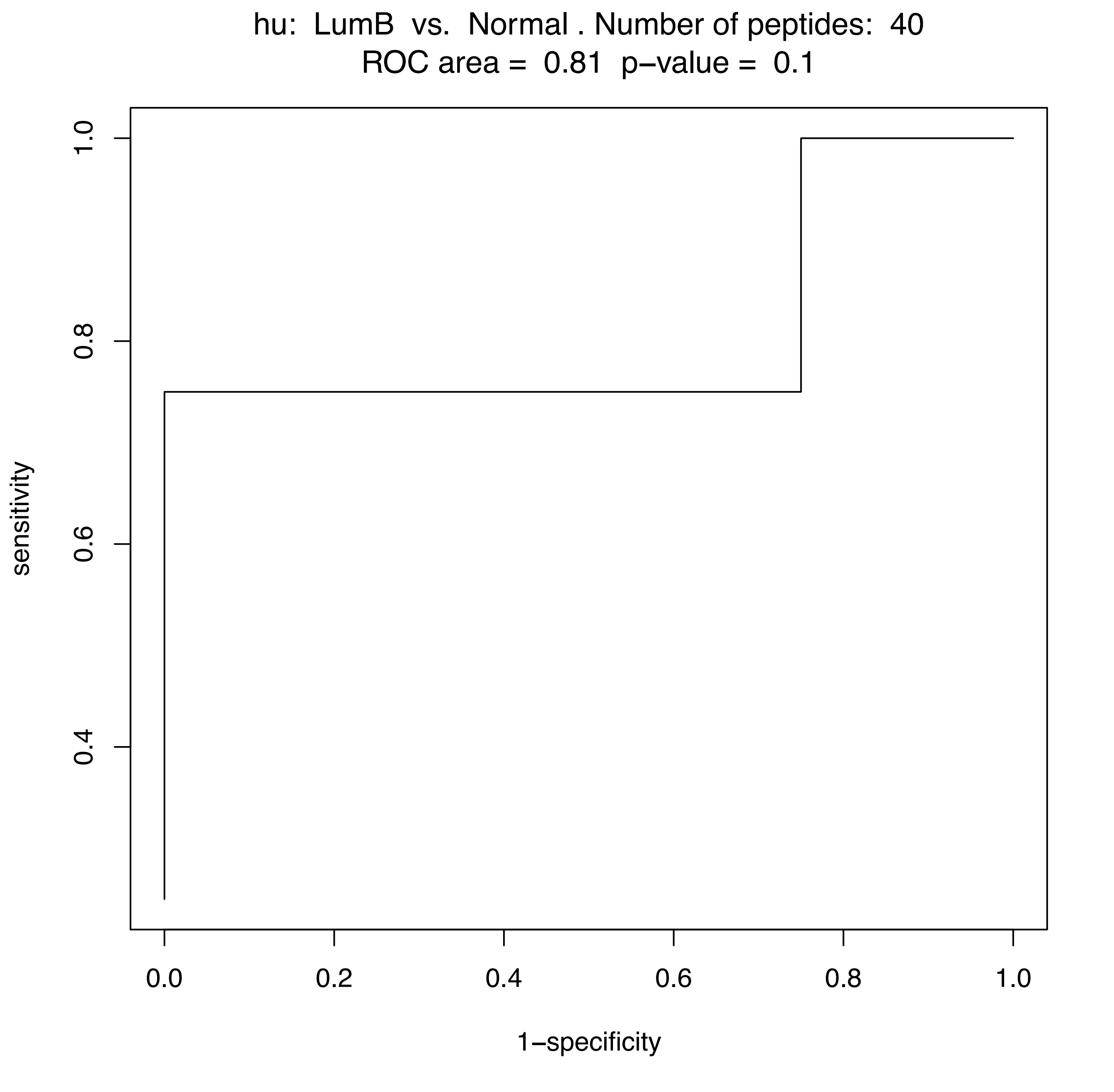
hu: LumB vs. Normal . Number of peptides: 40
ROC area = 0.81 p−value = 0.1
sensitivity
1−specificity
0.4
0.6
0.8
1.0
0.0
0.2
0.4
0.6
0.8
1.0

hu: LumB vs. Normal . Number of peptides: 100
ROC area = 0.75 p−value = 0.17

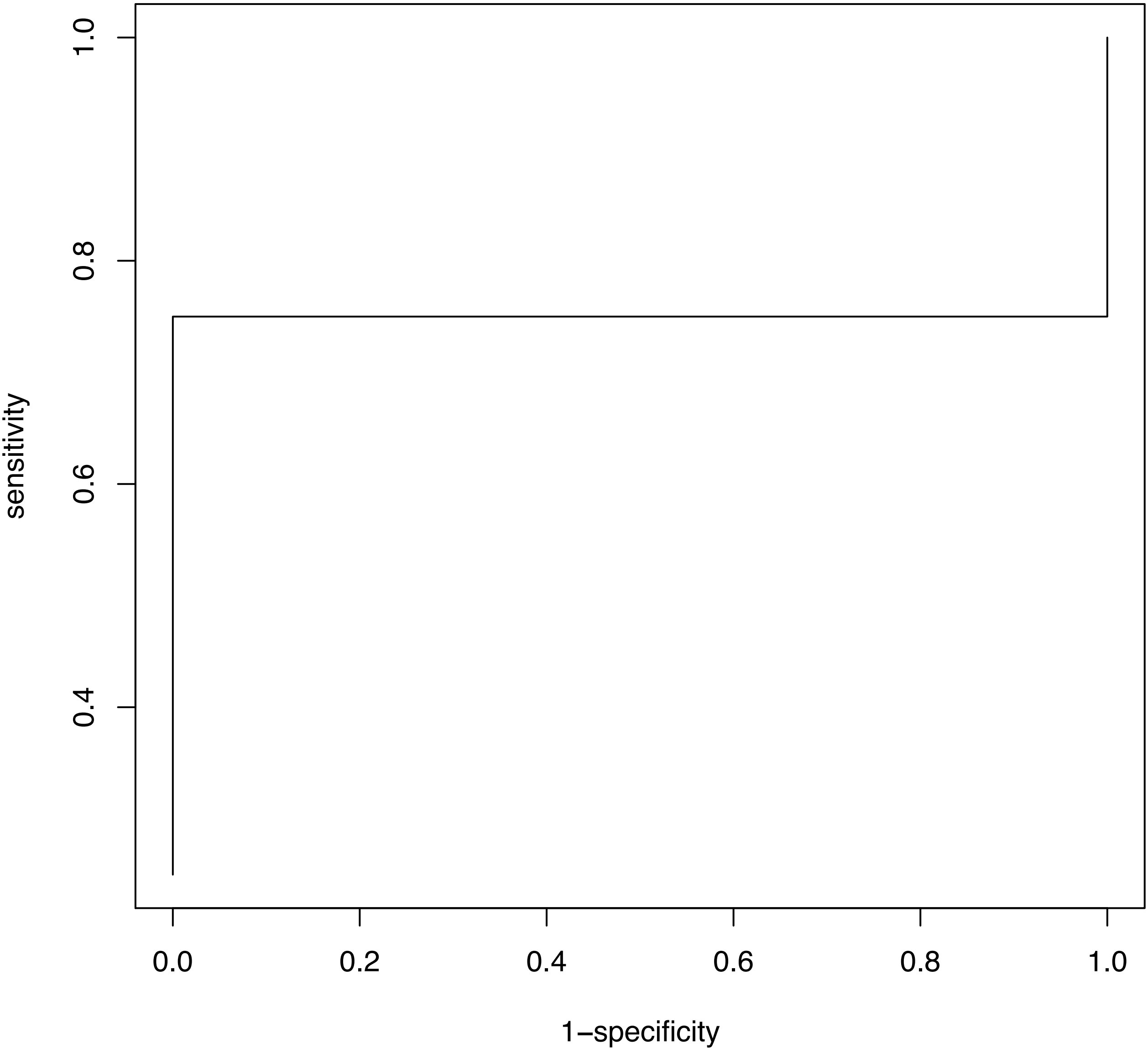

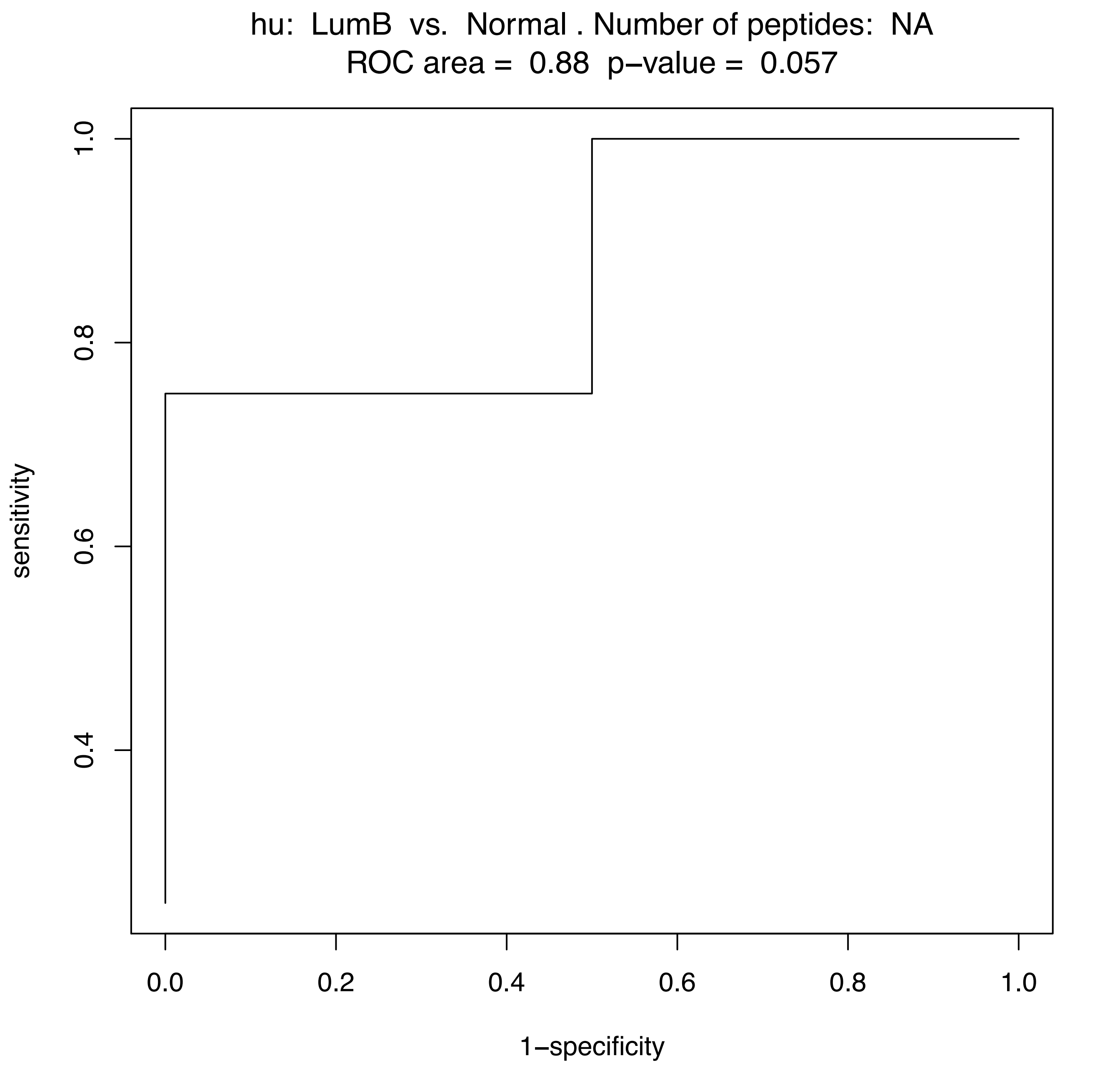
hu: LumB vs. Normal . Number of peptides: NA
ROC area = 0.88 p−value = 0.057
sensitivity
1.0
0.8
0.6
0.4
0.0
0.2
0.4
0.6
0.8
1.0
1−specificity
